# Proteomic and Phospho-Proteomic Profile of Human Platelets in Basal, Resting State: Insights into Integrin Signaling

**DOI:** 10.1371/journal.pone.0007627

**Published:** 2009-10-27

**Authors:** Amir H. Qureshi, Vineet Chaoji, Dony Maiguel, Mohd Hafeez Faridi, Constantinos J. Barth, Saeed M. Salem, Mudita Singhal, Darren Stoub, Bryan Krastins, Mitsunori Ogihara, Mohammed J. Zaki, Vineet Gupta

**Affiliations:** 1 Nephrology Division, Massachusetts General Hospital, Harvard Medical School, Boston, Massachusetts, United States of America; 2 Division of Nephrology and Hypertension, Department of Medicine, University of Miami, Miami, Florida, United States of America; 3 Department of Computer Science, Rensselaer Polytechnic Institute, Troy, New York, United States of America; 4 Computational Biology and Bioinformatics Group, Computational and Informational Sciences Directorate, Pacific Northwest National Laboratory, Richland, Washington, United States of America; 5 Department of Chemistry, Rollins College, Winter Park, Orlando, Florida, United States of America; 6 Thermo-Fisher BRIMS Center, Cambridge, Massachusetts, United States of America; 7 Department of Computer Science, University of Miami, Miami, Florida, United States of America; University of California Los Angeles, United States of America

## Abstract

During atherogenesis and vascular inflammation quiescent platelets are activated to increase the surface expression and ligand affinity of the integrin αIIbβ3 via inside-out signaling. Diverse signals such as thrombin, ADP and epinephrine transduce signals through their respective GPCRs to activate protein kinases that ultimately lead to the phosphorylation of the cytoplasmic tail of the integrin αIIbβ3 and augment its function. The signaling pathways that transmit signals from the GPCR to the cytosolic domain of the integrin are not well defined. In an effort to better understand these pathways, we employed a combination of proteomic profiling and computational analyses of isolated human platelets. We analyzed ten independent human samples and identified a total of 1507 unique proteins in platelets. This is the most comprehensive platelet proteome assembled to date and includes 190 membrane-associated and 262 phosphorylated proteins, which were identified via independent proteomic and phospho-proteomic profiling. We used this proteomic dataset to create a platelet protein-protein interaction (PPI) network and applied novel contextual information about the phosphorylation step to introduce limited directionality in the PPI graph. This newly developed contextual PPI network computationally recapitulated an integrin signaling pathway. Most importantly, our approach not only provided insights into the mechanism of integrin αIIbβ3 activation in resting platelets but also provides an improved model for analysis and discovery of PPI dynamics and signaling pathways in the future.

## Introduction

Platelets are key initiators of hemostatic mechanisms that repair injury to the vasculature. Platelets also play a central role in cardiovascular diseases, cancer, and stroke, which account for the major mortality and morbidity in the United States [1], [2]. Additionally, platelets modulate inflammatory pathways to initiate, sustain and accelerate a number of inflammatory diseases, such as atherosclerosis [3].

Platelets are enucleate cells that are characteristically small and discoidal in resting state and normally circulate at levels of approximately 150−400×10^9^/L in blood [4]. Platelets rely on integrin αIIbβ3 (also known as glycoprotein GPIIb/IIIa) to perform their primary biological function, which is to help seal and repair the circulatory system after vascular injury [5]. Defects in platelet function, such as impaired adhesion or aggregation, are also primarily mediated by the integrin αIIbβ3. A number of controls, both internal and external, keep the platelets in a resting state during circulation and prevent intracellular signals from inappropriately activating the integrins [6], through the tight regulation of the cytosolic Ca^2+^ concentration, the activity of intracellular phosphatases that limit signaling through kinase-dependent pathways and the presence of extracellular ADPases that hydrolyze released ADP.

Upon a break in the integrity of the vascular endothelial cell lining, the underlying collagen fibrils of the extracellular matrix (ECM) are exposed to and interact with the circulating platelets, which leads to platelet adhesion to collagen, via the platelet collagen receptor integrin α2β1 (also known as glycoprotein (GPIa/IIa)). In addition, the interaction provides the platelets with a strong activation signal, which induces the platelets to change shape, to spread along the collagen fibrils and to secrete thromboxane A2 and ADP into the circulation, and to induce conformational changes in the abundant second platelet integrin αIIbβ3. Normally present in an inactive conformation, integrin activation facilitates the binding of circulating coagulation protein fibrinogen (a process referred to as “inside-out signaling” [6]). Simultaneous binding of two integrin αIIbβ3 receptors by fibrinogen initiates the process of platelet aggregation [7]. Subsequently, a series of platelet intracellular signaling events are initiated and propagated, including activation of the various tyrosine and serine/threonine kinases and the protein phosphatases (so called “outside-in” integrin signaling). Since each platelet has ∼80,000 copies of integrin αIIbβ3 on its surface [8], very large aggregates of platelets can rapidly assemble at the site of platelet activation. A cross-linked fibrin clot ultimately stabilizes the growing platelet aggregate.

Detailed molecular, cellular, animal and human studies have provided incredible insights into the structure and function of platelets, both under normal physiologic conditions as well as in a variety of disease states [5], however, the molecular mechanisms of integrin activation and the identities of proteins involved in the signaling pathways leading to a variety of platelet responses *in vivo* are yet to be fully characterized. A key first step in mapping out such interactions is the cataloging of various components that make up the platelets as well as identification of post-translationally modified proteins, such as the phosphorylated proteins. Since platelets are readily available, are easily isolated in relatively large numbers, lack nuclei and genomic DNA, and have a limited RNA pool, proteomic techniques are ideally suited for the analysis of platelets. Indeed, several proteomic analysis techniques have taken a lead in identifying the proteomic content of platelets, as reported in several exciting publications (reviewed in [9], [10]). Despite these efforts, a number of proteomic components of the human platelets remain to be identified, especially notable when considering that the human platelet proteome has been predicted to contain approx. 2000–3000 unique proteins [11].

The platelet phospho-proteome has also been investigated by a number of investigators, including studies on platelets in its basal state as well as upon activation by the platelet agonists [12], [13], [14], [15], [16], [17], [18]. A recent study by Zahedi *et al*. cataloging the platelet phosphoproteins using platelet rich plasma identified 270 phosphorylated proteins in the resting platelets [17]. Researchers have also identified several proteins that change their phosphorylation state during platelet activation [13], [14], [15], [18], [19].

A recurring theme in all platelet proteomics reports is the need for multiple complementary proteomic profiling techniques and the analyses of multiple independent samples to obtain a high-confidence proteomic profile from such a complex cellular system [20]. Here, we present a comprehensive proteomic profile of human platelets from ten independent platelet samples using ten individual proteomic analyses and a total of 140 1D SDS PAGE gel slices. Additionally, we present phospho-proteomic profile of platelets using four separate samples and a proteomic profile of the platelet membrane fractions. In total, we identified 1507 unique proteins in the human platelets. This is, by far, the largest platelet proteomic dataset yet assembled from a single set of studies. We also present a contextual platelet protein-protein interaction (PPI) network created advanced bioinformatic approaches on this comprehensive platelet proteomic dataset and. Our analysis shows that computational models of the platelet interactome represents an excellent starting point for studying the protein signaling pathways.

## Results and Discussion

### Identification of Platelet Proteins using Multiple Samples

**Figure 1:**
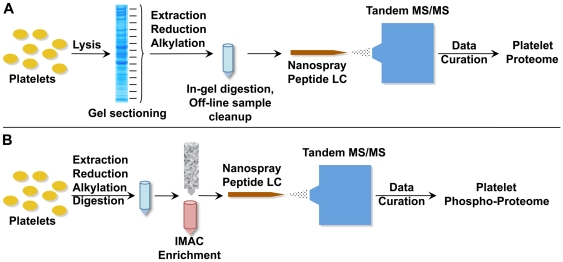
Schematic workflow of LC-MS/MS based platelet proteomic profiling. A. Workflow used in the proteomic analysis of human platelets. Isolated platelets were lysed and the extracted proteins were size-fractionated using 1D-SDS PAGE. The coomassie-stained gel lanes were cut in 14–16 equally sized sections, and in-gel digested with trypsin. Subsequently, extracted peptide mixture from each gel slice was independently analyzed using LC-MS/MS to obtain a list of unique platelet proteins. B. Workflow used in the phospho-proteomic analysis of platelets. Isolated platelets were lysed and the extracted total lysate was digested in-solution with trypsin. The trypsinized samples were enriched for phospho-peptides using an IMAC column and the enriched peptide mixtures were analyzed using LC-MS/MS to obtain a list of unique platelet phospho-proteins.

**Figure 2:**
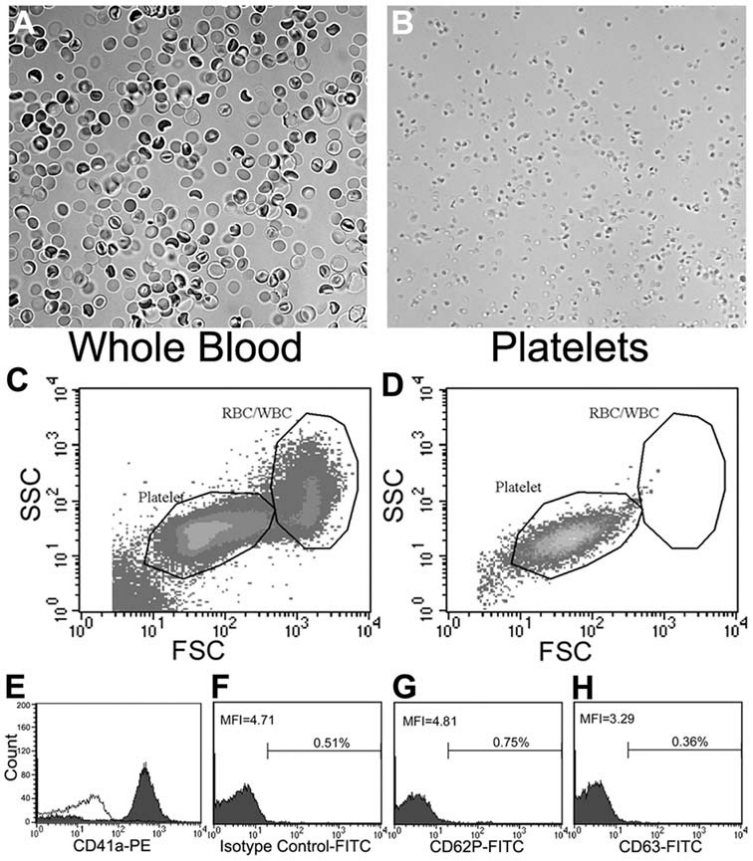
Analysis of platelet quality using light microscopy and flow cytometry. A, B. Light microscopy images of the anti-coagulated whole blood sample (A) and the isolated platelets (B) at 40× magnification. Red blood cells (RBCs) are clearly visible in the anti-coagulated whole blood samples (A), whereas no detectable RBCs were seen in the isolated platelet samples (B). C–E. Flow cytometric analyses of anti-coagulated whole blood (C) and the isolated platelets (D, E). Forward- and side-scatter density plots show that RBC/WBC and platelets populations are clearly distinguishable based on their respective light scatter patterns. E. Flow cytometric analysis of isolated platelets upon staining with anti-CD41a mAb (filled histogram) as compared to an isotype control (open histogram). F–H. Flow cytometric analyses of the isolated platelets showing that the platelets are in a quiescent state. The CD41a+ platelets were further analyzed for markers of platelet activation using antibody against CD62P (P-selectin) (G) or against CD63 (type III lysosomal glycoprotein) (H), both of which showed no increase as compared to an isotype control antibody (F). The determined percent positive events (Vs isotype control) and MFI are indicated in each histogram.

The proteomic composition of isolated platelets in a resting state was obtained via the workflow strategy detailed in Figure 1A. In order to increase the confidence associated with proteomic profiling and to catalog a large fraction of platelet proteins, ten independent platelet samples were analyzed. Additionally, the platelets were isolated from two different types of sources: from platelet rich plasma (PRP) that was obtained from a local blood bank and from fresh whole blood collected from healthy subjects. Platelets from whole blood were isolated using an optimized protocol using centrifugal separation in an acid-citrate-dextrose (ACD) buffer, which uses citrate as an anticoagulant. Purity of the isolated platelets was assessed by light microscopy and flow cytometry, which showed that the platelets isolated from whole blood were devoid of any red blood cells (RBC) or leukocytes. The presence of WBC/leukocytes was estimated to be <0.1% of platelet population (Figure 2A–D).

To verify that the isolated platelets were in the basally resting state, the cells were analyzed using flow cytometry. Since activated platelets express P-selectin (CD62P) and CD63 (type III lysosomal glycoprotein) on the platelet cell surface, fluorescently tagged anti-P-selectin and anti-CD63 antibodies were used to count activated platelets [21], [22]. These markers were completely absent on the surface of the freshly isolated platelets (which stained positively for the known platelet marker CD41), indicating that the isolated platelets are indeed in the resting state (Figure 2E–F). Furthermore, activation of the isolated platelets with thrombin, a potent agonist of platelets, showed a clear increase in the percent positive events (>70%) and >2-fold change in the MFI values for both markers, with no concomitant change in the isotype control (data not shown), as expected based on published literature [23].

**Figure 3:**
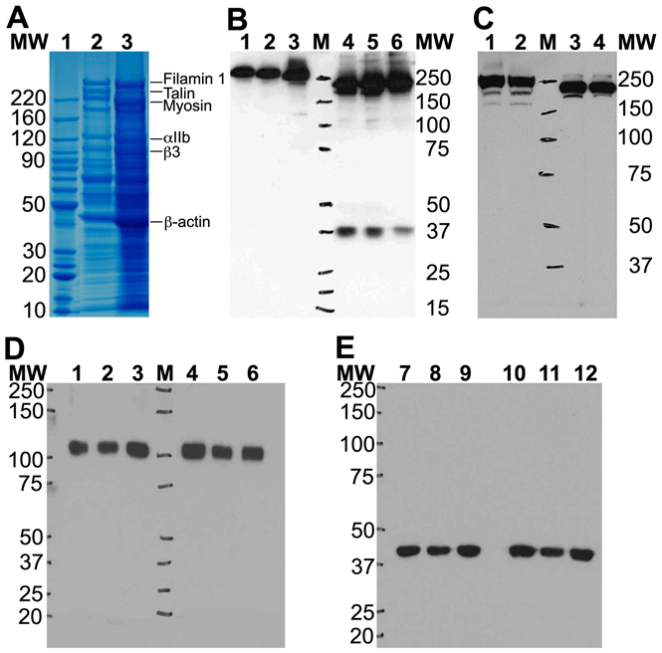
Platelets stored without citrate buffer show Talin degradation upon thawing. A. Coomassie stained 4–12% Bis-Tris SDS-PAGE gel shows selective degradation of Talin in lysates from platelet pellets stored in the absence of citrate buffer (Lane 3) Vs no degradation in the presence of citrate (Lane 2). B. Immuno-blotting with anti-Talin antibody against the Talin N-terminal rod region (antibody 8D4, [27]) shows that Talin is degraded to smaller fragments in three independent platelet samples stored in the absence of citrate buffer (Lanes 4–6) as compared to three independent platelet samples stored in the presence of citrate (lanes 1–3). Notice the slight reduction in the MW of full-length Talin band in lanes 4–6 as well as the presence of ∼37 kDa fragments in lanes 4–6. C. Similarly, immuno-blotting with anti-Talin antibody against the Talin C-terminal region (antibody C20) also shows that Talin is degraded to a smaller MW species in two independent platelet samples stored in the absence of citrate buffer (Lanes 3–4) as compared to two independent platelet samples stored in the presence of citrate (lanes 1–2). Protein MW markers are as labeled. D. Immuno-blotting with the anti-integrin β3 mAb shows no degradation of integrin β3 upon thawing of stored platelet pellets. Platelet lysates from three independent platelet samples stored either in the presence of citrate (lanes 1–3) or absence of citrate (lanes 4–6) were thawed and analyzed by 4–12% Bis-Tris 1D SDS PAGE followed by western blotting. Protein MW markers are as labeled. E. Immuno-blotting with the anti-β-actin mAb shows no β-actin degradation upon thawing of stored platelet pellets. Platelet lysates from three independent platelet samples stored either in the presence of citrate (lanes 1–3) or absence of citrate (lanes 4–6) were thawed and analyzed by 4–12% Bis-Tris 1D SDS PAGE followed by western blotting. Protein MW markers are as labeled.

We found that the conditions of platelet storage and thawing had a significant effect on Talin stability in human platelets. Platelet activation is known to leads to a rapid cleavage of signaling proteins, such as Talin [24], [25], [26]. Interestingly, the platelets stored as platelet pellets (no ACD buffer), after isolation using the ACD buffer, showed a dramatic loss of full-length Talin upon thawing/lysis in the presence of SDS-containing lysis buffer (Figure 3). The loss of full-length Talin was rapid and was readily detectable in the coomassie stained SDS PAGE gel (Figure 3A). We validated the degradation of Talin in isolated platelets by running the platelet lysates on 1D SDS PAGE followed by immunoblotting with two different anti-Talin antibodies (Figure 3B–C). Unexpectedly, western blotting with the anti-Talin antibody 8D4 showed the presence of a ∼37 kDa (Figure 3B), which seems to be slightly smaller than the ∼47 kDa N-terminal fragment generated as a result of the Talin cleavage by calpain upon platelet activation [26], [27]. Additionally, the mAb 8D4 has been shown to not recognize the calpain-cleaved 47 kDa N-terminal fragment of Talin, as the mAb 8D4 epitope lies in the Talin rod region (residues 482–636) [27]. It also suggests that this ∼37 kDa fragment retains at least part of the Talin sequence in the region 482–636. This is confirmed by the fact that the anti-Talin C-terminal antibody C-20 did not detect this smaller ∼37 kDa fragment, even though it showed a reduction in the total size of Talin in these platelet samples (Figure 3C). Future work will determine the identity and the significance of this novel fragment of Talin. Platelets also express very high levels of the integrin αIIbβ3 and actin. 1D-SDS PAGE followed by immunoblotting with mAbs against the integrin β3 or β-actin showed no degradation of these proteins (Figure 3D–E), suggesting that the Talin was selectively cleaved during the thawing/lysis of stored platelet pellets.

**Figure 4:**
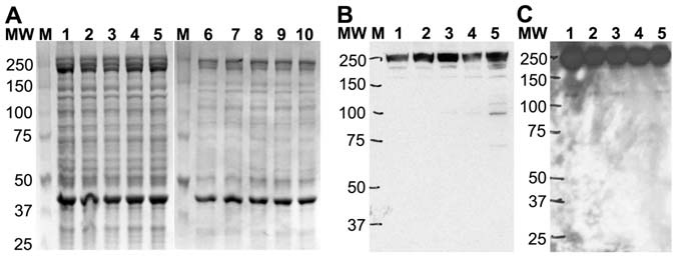
Talin is protectected from degradation during platelet thawing/lysis when stored in the presence of citrate. A. SDS-PAGE analysis of ten independent isolated platelet samples. Platelet lysates from five individual platelet samples isolated from fresh whole blood from healthy subjects (lanes 1–5) and five individual platelets samples obtained from the platelet rich plasma (PRP) (from blood bank) (lanes 6–10) were analyzed using 4–12% Bis-Tris SDS-PAGE. Images of the coomassie stained gels show that all ten samples have a very similar protein expression pattern and no noticeable Talin degradation (when compared with lane 3, Figure 3A). Lane M = Protein MW markers. B–C. Western Blots show no Talin degradation in the presence of citrate in ten platelet samples. Platelet lysates from five individual platelet samples isolated from fresh whole blood (B) and five individual samples from platelets isolated from PRP (C) were analyzed by 4–12% Bis-Tris SDS-PAGE followed by immuno-blotting with anti-Talin antibody C-20. All ten samples show a single band for Talin with minimal degradation. Protein MW markers are as labeled.

In order to limit protein degradation, the isolated platelets were stored at −80°C as a suspension in the citrate buffer, rather than as pellets. Analysis of the protein lysate using 1D SDS PAGE showed very similar protein expression pattern in all ten platelet samples upon staining with coomassie blue (Figure 4A) and no loss of the full-length Talin band (Figure 4B–C) as compared to the selective Talin degradation seen earlier. Western blot analysis of five platelet samples obtained from fresh whole blood and five platelet samples obtained from PRP with the C-terminal anti-Talin antibody showed little Talin degradation in any sample, suggesting that our platelet isolation and storage protocol preserved the platelet proteomic integrity.

Using the refined preparation and storage protocols, a total of 1451 unique proteins were unambiguously identified in resting platelets and cataloged according to the appropriate refseq IDs (a complete list of all identified proteins is shown in Table S1). Figure S1 shows an example MS/MS spectrum of MH^+^ ion of a peptide from integrin αIIβ3, identifying it in the mixture. A complete list of all identified peptides from each of the ten analyses is shown in Table S2. Approx. 956 proteins were identified based on more than one peptide hit. As this proteomic profiling methodology is biased towards the detection of proteins with higher abundance, not surprisingly, only 919 of identified proteins were found to be present in more than one sample and, on average, overlap between the proteomic profile of any two datasets was approximately 63%, consistent with the other platelet protein studies using sample replicates [28], [29]. When peptide mixtures from a single sample was analyzed using LC-MS/MS multiple times (>50 individual runs), the overlap between the proteins identified from different runs of the same sample was also approx. 60% (data not shown), suggesting that the level of overlapping protein identifications between two different samples is similar to the level of overlap obtained when the same sample is analyzed multiple times. Additionally, only a small fraction of proteins identified here were highly expressed, as judged by their relative abundance, and a majority of the protein signatures were from low level expressors. Furthermore, approximately 500–800 unique proteins were identified from a single dataset. This suggests that at the current level of sensitivity, the detection of proteins expressed at low levels greatly benefits from analyzing a large number of replicates. Among the identified proteins, the high expressors (based on the peptide count) include FLNA (Filamin A), TLN (Talin), MYH9 (Myosin, non-muscle), THBS1 (Thrombospondin), ITGA2B (integrin alphaIIb) and ITGB3 (integrin beta3), all previously known to be present in the platelets.

**Figure 5:**
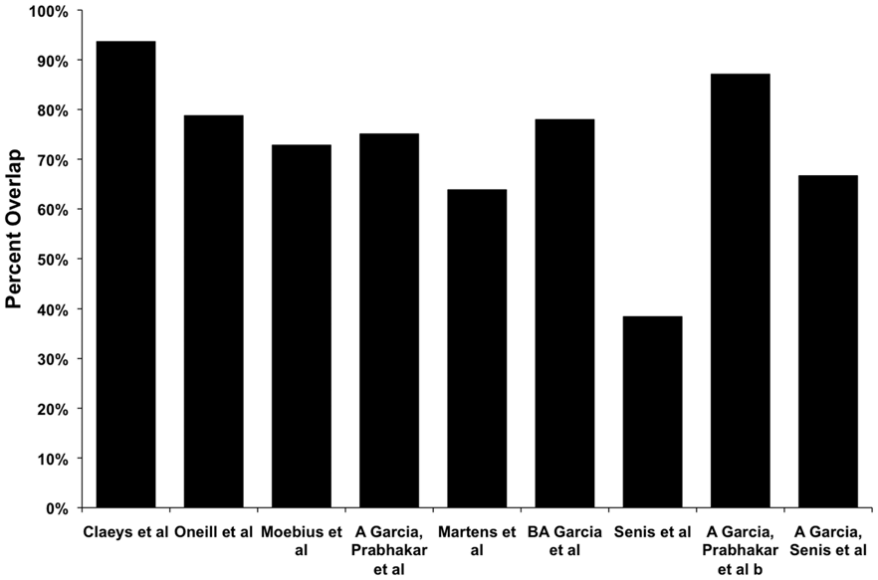
Comparison of the list of identified platelet proteins with platelet proteins described in previous studies. A bar-graph showing overlap between the protein list generated in the current study and the proteins identified in some of the previously published studies [31], [32], [33], [34], [35], [36], [37]. The percent overlap between the proteins identified in each of the prior studies and the current study is plotted along the y-axis and the prior studies are shown along the x-axis.

A comparison of the comprehensive platelet proteome for overlap with some of the previously published data from the platelet proteomic profiling studies showed >75% overlap between most of the published studies and our dataset (Figure 5) [30], [31], [32], [33], [34], [35], [36], [37], although the overlap between any two published studies was low (analysis not shown). As a result, we are confident that the proteins identified in the present study are from the human platelets. Proteins typically associated with RBCs (such as α and β globin or spectrin) were not detected, further verifying that the contamination from these cells in the isolated platelets was minimal.

### Membrane-associated Platelet Proteins

**Figure 6:**
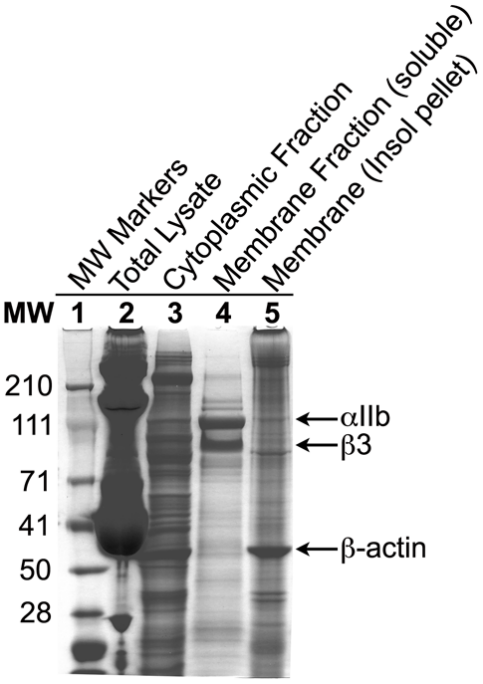
1D SDS-PAGE analysis of purified platelet membrane fraction. Platelet membrane was prepared as described in text. All fractions were analyzed using a 4–12% Tris-acetate pre-cast SDS-PAGE gel. Image of the coomassie stained gel shows that membrane fraction is highly enriched for membrane-associated proteins, such as integrin αIIb and β3 chains (arrows). Lane 1 = Molecular Weight markers (SeeBlue Plus2 from Invitrogen), Lane 2 = total platelet lysate, Lane 3 = cytoplasmic fraction after fractionation of platelet lysate on a 40% sucrose gradient, Lane 4 = extracted membrane fraction, Lane 5 = insoluble pellet after membrane fraction extraction.

In order to determine the platelet membrane proteomic content, we purified the platelet membrane associated proteins from one of the analyzed platelet sample using published protocols [38]. Figure 6 shows that known membrane-associated platelet proteins, such as integrin chains αIIb and β3, are selectively enriched in the isolated membrane fraction (lane 4), as compared to the low levels of cytosolic actin in this fraction, signifying that the membrane-fraction isolated here largely retained membranous proteins while selecting out a majority of the cytosolic proteins. For the proteomic profiling step, the purified platelet membrane fraction was pre-fractionated using 1D SDS-PAGE and processed according to the workflow shown in Figure 1A. A total of 182 unique platelet proteins from 577 unique peptides (from a total of 1089 peptide identifications) were unambiguously identified as associated with the platelet membrane fraction (as cataloged by their uniprot IDs). Representative platelet membrane proteins include well known proteins, such as integrin αIIβ3 heterodimer (ITGA2B and ITB3), GP1B, JAM-A and G6B among others. Figure S2 shows an example MS/MS spectrum of MH^+^ ion of a peptide from integrin β3, identifying it in the mixture. A complete list of all identified proteins is shown in Tables S3 and S4. Comparison with proteins identified using the whole platelets showed that >90% of the membrane proteins were co-identified in the whole platelet analyses, suggesting that our use of ten independent biological samples in the whole platelet proteomics sufficiently captured a majority of the platelet proteins.

A comparison of this platelet membrane proteome for overlap with two of the previously published proteomic profiling studies with the platelet membrane fractions showed that there were eighty-eight proteins in common between our dataset and that from Moebius *et al*. [33], which found 296 unique proteins in the platelet membranes, and twenty-eight proteins were in common with the dataset from Senis *et al.* [37]. Sixty-two proteins were reported to be common between these two previously published studies [37].

### Phospho-proteomic Profile of Platelets in Resting State

**Figure 7:**
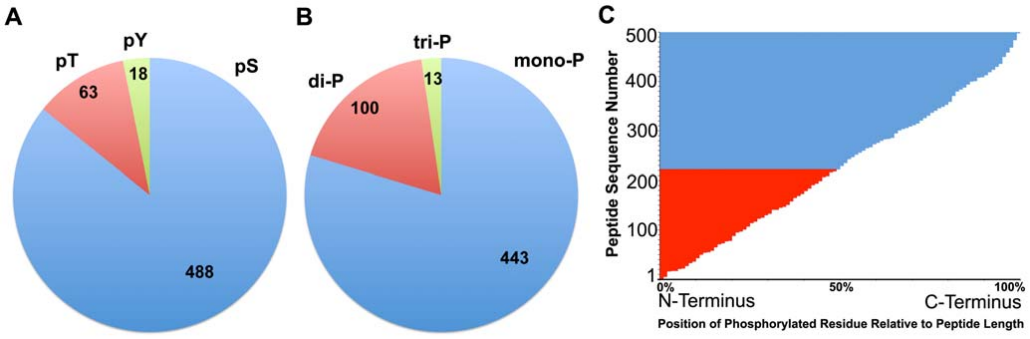
Analysis of Protein phosphorylation-site distribution. A. A pie chart showing the distribution of the three types of phosphorylation sites, pSer (pS), pThr (pT) and pTyr pY), across all of the unique phospho-peptides identified in this study. B. A pie chart showing the distribution of number of phospho-sites per identified peptide across all of the unique phospho-peptides identified in this study. C. Distribution of phospho-sites relative to protein length. A graph showing the position of the identified phosphorylated residue relative to the total protein length as a percentage for all of the identified phospho-proteins. Each phospho-peptide sequence was queried against its mapped protein sequence from the Uniprot database and the position of the phosphorylated residue in the sequence was determined. Relative position percentage of each phospho-site was calculated by dividing the determined position number for the phosphorylated residue with the total length of the mapped protein and multiplying the resulting fraction by 100. The relative position percentage of each peptide was graphed using a horizontal bar graph, with the calculated relative position percentage values on the x-axis and each of the phospho-peptide on the y-axis. Proteins where the phosho-site is in the N-terminal half (less than 50% relative position, x-axis) are colored red and the rest are colored blue.

The phospho-proteomic profile was determined using four independent platelet samples. Unlike a recent study using only one type of the platelet sample [17], we decided to include multiple samples from two different sources: two platelet samples isolated from fresh whole blood and two samples from the platelets isolated from PRP in our analyses. Figure 1B shows a schematic of immobilized metal affinity column (IMAC) based proteomic analysis setup used in our studies (reviewed in [39]). Here, we unambiguously identified a total of 262 unique platelet proteins (as cataloged by their uniprot IDs) as phosphorylated proteins in the basal, resting state from 569 unique phospho-peptides (from a total of 1300 phospho-peptide identifications). A complete list of all identified phospho-proteins is shown in Table S5. Additionally, as acidic peptides are also known to co-elute with the phosphopeptides from the positively charged IMAC columns [40], [41], we also identified a total of 104 additional unique non-phosphorylated proteins (based on their uniprot IDs, as shown as a list in Table S6) from these four peptide mixtures. In total, these combined 366 protein identifications were based on 1089 unique peptide hits from a total of 3051 peptide hits. Of the 569 unique phospho-peptide identifications, 488 peptides were identified to be mono-phosphorylated, 63 doubly phosphorylated and 18 triply phosphorylated peptides (Figure 7A). Additionally, analysis of the phospho-peptides showed that 443 were phosphorylated at the serine sites (pSer), 100 were phosphorylated at the threonines (pThr) and 13 were phosphorylated at the tyrosines (pTyr) (Figure 7B), providing a ratio of approximately 42∶1 pSer and pThr to pTyr in the present study, which is similar to results from phosphoproteomic studies in other cells [42], but is 2-fold higher than the report from a recent phospho-proteomic study by Zahedi *et al.*, which found a higher number of pTyr by focusing on the pTyr-specific precursor ion-scanning [17]. A comparison with this platelet phospho-proteome showed that 97 of the phospho-proteins identified here were also identified in this published dataset [17].

Next, the site of protein phosphorylation was qualitatively investigated by analyzing the position of the phosphorylated residue relative to the total length of the phosho-protein using >500 identified unique phosphopeptides from our analyses. For this analysis, we determined the residue number for the phosphorylated residue (pSer, pThr or pTyr) and divided that by the total length of the protein to generate it's fractional position relative to the length of each protein (where fractional position value of 0 means that the phosphorylated residue is the extreme N-terminal residue, and a fractional position value of 1 means that the phosphorylated residue is the extreme C-terminal residue). We converted the fractional positional value into a percentage number (by multiplying with 100) and generated a graph (shown in Figure 7C) displaying each peptide (Y-axis) and its fractional position (X-axis). The graph shows that a small majority of phosphorylation sites were present towards the C-terminal end of the platelet proteins (275 (blue bars) Vs 225 N-terminal peptides (red bars), Figure 7C). It is conceivable that this distribution could change with a much more comprehensive analysis in the future. However, other studies have also reported a preference for the protein phosphorylation to occur at the C-terminus [43]. Additionally, a sharp increase in the slope of this curve near the extreme C-terminus (between 90% and 100%) suggests that the extreme C-terminus is a slightly preferred phosphorylation site, similar to previous phosphosite analyses of liver proteins [43].

### Determination of Phosphorylated Sequence Motifs

**Figure 8:**
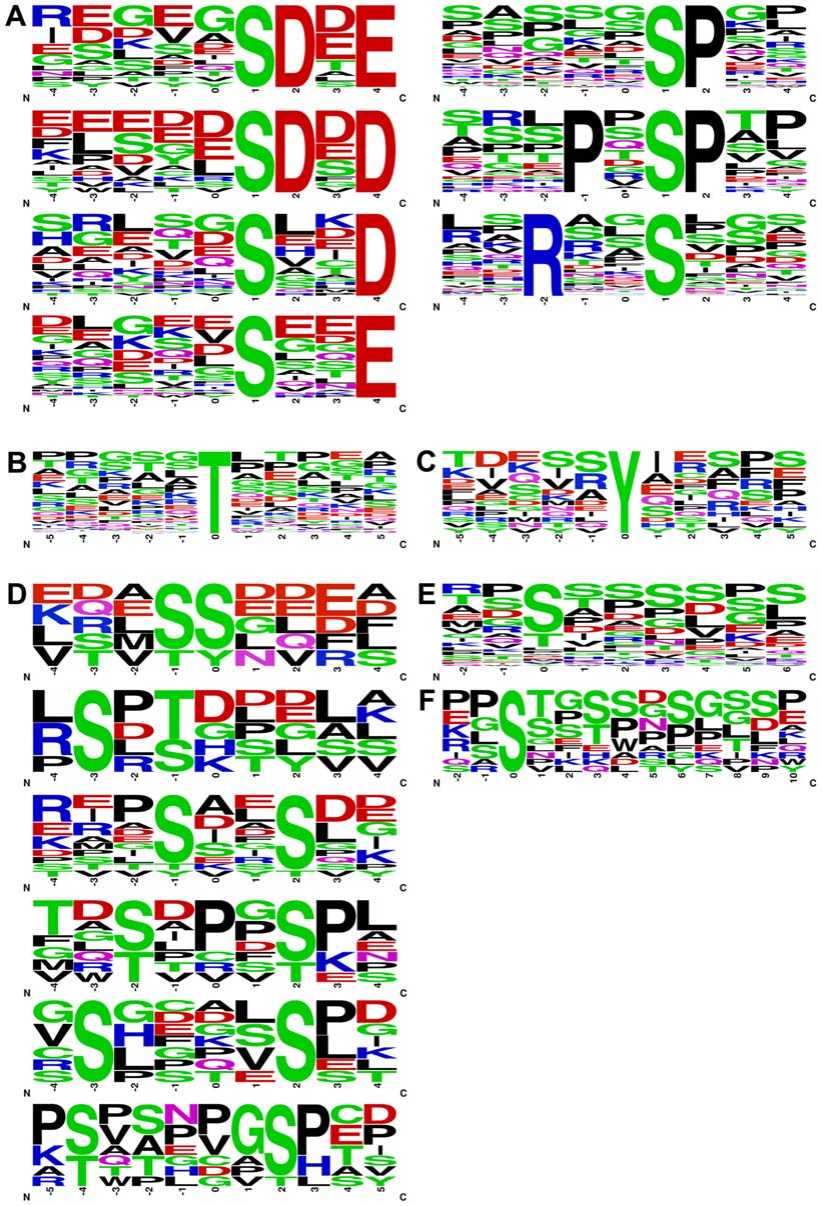
Computational identification of sequence motifs in phospho-peptides. A–C. Phospho-peptides containing single phosphorylation at (A) Ser, (B) Thr or (C) Tyr were analyzed for the presence of sequence motifs using the Motif-X algorithm [44] as described in the *Materials and Methods* section. Sequence logos for Motif-X aligned peptide sequences were generated using weblogo [45] and are shown above for each of the three types of phospho-peptides identified in this study. D–F. Identification of sequence motifs in multiply phosphorylated peptides. Phospho-peptides containing di- and tri-phospho residues were analyzed for the presence of sequence motifs by manual alignment as described in the *Materials and Methods* section. Sequence logos for the aligned peptide sequences were generated using weblogo [45] and are shown above for the (D) di-phosphopeptides with varying number of residues in between the two phosphor-sites. A composite sequence logo using all of the (E) the di-phosphopeptides and (F) the tri-phosphopeptides shows the relative preference for the second and the third phosphorylation site in these multi-phosphorylated peptides.

The phospho-peptide sequences from each of the three categories (pSer, pThr and pTyr) were analyzed for the presence or enrichment of any particular sequence motifs [43]. We aligned the unique peptides in each of the three categories around the phosphorylated residues and analyzed the sequences using Motif-X algorithm (http://motif-x.med.harvard.edu) [44]. Aligned sequences were used to generate peptide sequence representations as logos using web logo (http://weblogo.berkeley.edu) [45]. Resulting logos are shown in Figure 8A–C. Since there are far fewer phospho-peptides containing pThr and pTyr as compared to pSer, no motifs were found for pThr (Figure 8B) and pTyr (Figure 8C). Some of the over-represented pSer motifs that were identified include SDxD, SDxE, SxxD, SxxE, SP, PxSP, and RxxS (Figure 8A).

Using the Phosphomotif finder, we identified potential kinases associated with some of the identified motifs [46]. We suggest that motifs SDxD, SDxE and SxxD/E are associated with casein kinase II; SP and PxSP is associated with GSK3, ERK1, ERK2 and RxxS is associated with the kinases such as Protein Kinase A (PKA), Calmodulin (CaM) Kinase family and Akt.

The di- and tri-phosphorylated peptide sequences were manually aligned to identify any motifs represented in the di- and tri-phosphorylated phospho-peptides in our dataset. As the number of these multi-phosphorylated peptides is low, we did not perform any statistical analysis on the over-representation of any of the motifs in our dataset. The manually aligned sequences were converted into logo representations using weblogo and the results are shown in Figure 8D–F, which indicates that di-phosphorylated peptides contain a varying number of intervening residues (anywhere from 0 to 5 amino acids) in between the two phosphorylated Ser/Thr residues (Figure 8D). The composite alignment of di-phosphorylated peptides (Figure 8E) shows that the +3 and the +1 positions relative to the first phospho-site are the most preferred second-phosphorylation sites. Analysis of tri-phosphorylated peptides shows that they also contain a varying number of intervening residues (anywhere from 0 to 8 amino acids) in between the three phosphorylated Ser/Thr residues, as shown in a composite alignment of the tri-phosphorylated peptides (Figure 8F). Additionally, it shows that the +3 and +6 positions in the sequence relative to the first phospho-site are the most preferred multi-phosphorylation sites. Finally, we also found a number of di- and tri-phosphorylated peptides to be represented as mono-phosphorylated peptides in the database, where only one of the two sites in the peptide sequence was phosphorylated. This is not unexpected, as protein phosphorylation typically involves a sequential mono-phosphorylation reaction. It is also known that many mono-phosphorylated sequences can enhance the rate of subsequent second/multi-phosphorylation of the same protein.

### Classification of Identified Platelet Proteins

**Figure 9:**
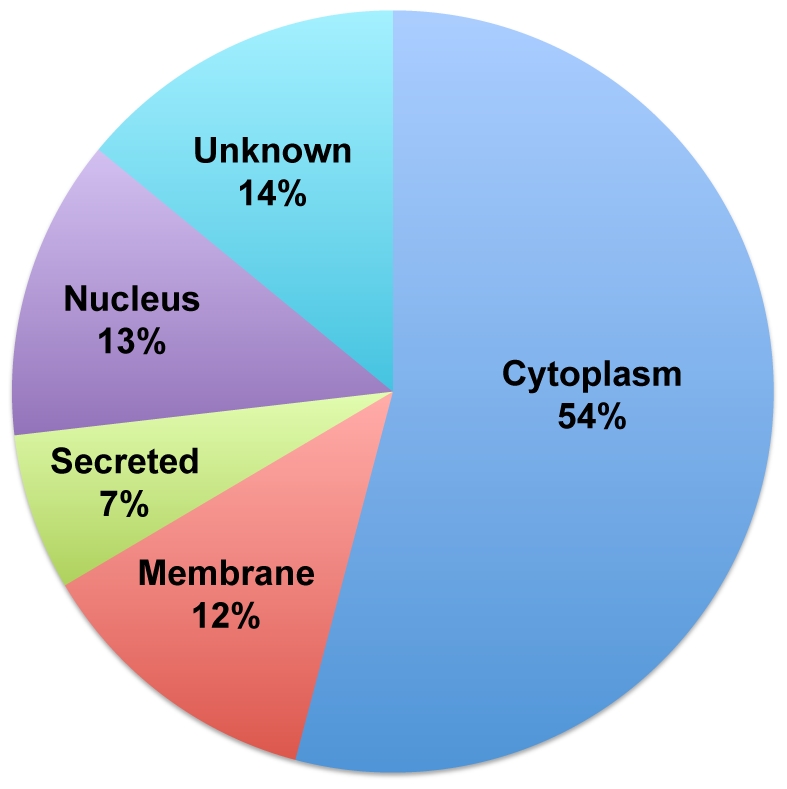
Sub-cellular localization of the identified platelet proteins. The complete list of 1507 identified platelet proteins from this study was used in determining the subcellular localization of the identified proteins using the IPA software ([47], see text). This figure shows the results for each of the identified categories as a pie chart. Each category and the relative percentage of total proteins present in that category is shown in the graph.

We combined all of the identified proteins from the three different types of proteomic analyses on multiple independent platelet samples and converted the refseq IDs to Uniprot IDs to obtain a comprehensive list of proteins from our study, resulting in a list of 1507 unique proteins in the resting platelets (as identified by their Uniprot IDs), shown in the Table S7. Using the Ingenuity Pathway Analysis (IPA [47]) software the sub-cellular localization of the identified proteins was identified. We find that 54% (∼813 proteins) are localized in the cell cytoplasm, 12% in the membrane, 7% are secreted (likely from various intra-cellular granules), 13% show mapping as nuclear proteins and the remaining 14% did not have any sub-cellular localization information available in this database (Figure 9). The analysis also revealed the presence of 81 protein kinases, 33 protein phosphatases, 55 peptidases and 369 other enzymes. Functional analysis of the proteins using IPA software (Figure S3) showed that the over-represented cellular and biological functions associated with this set of proteins include cell movement, inflammatory and immune response and hematological function–all the functions that are commonly associated with the platelets. Not surprisingly, IPA analysis also showed that the disease pathways over-represented in this protein set include hematological and inflammatory diseases (Figure S4).

### Protein-protein Interaction Network of Platelet Proteins

Placing proteins in computational interaction networks has been successfully used to not only identify biological function of individual proteins, but has also provided new insights into the functional networks of proteins in a cellular context (see [48], [49], [50], [51], [52], [53]). Recently, PPI networks have been applied to the available platelet data [11]. In order to gain deeper biological insights from this platelet proteomic dataset, we generated a comprehensive platelet protein-protein interaction (PPI) network using our proteomic dataset. First, we determined the known interactions between any set of two proteins on our list using the PPI data from the publicly available HPRD database, an aggregator of many different sources of experimentally observed direct protein-protein interaction data [54]. This yielded a base network of 2194 interactions among the 870 of all 1507 identified proteins (remaining proteins showed no interactions in HPRD database). We visualized the PPI network using Cytoscape [55]. This base network is shown in Figure S5. Each non-phosphorylated protein is represented by a solid gray colored dot (node). The phosphorylated proteins identified in this study are shown as red dots. Interaction between two proteins is represented by a line (edge) connecting two nodes.

**Figure 10:**
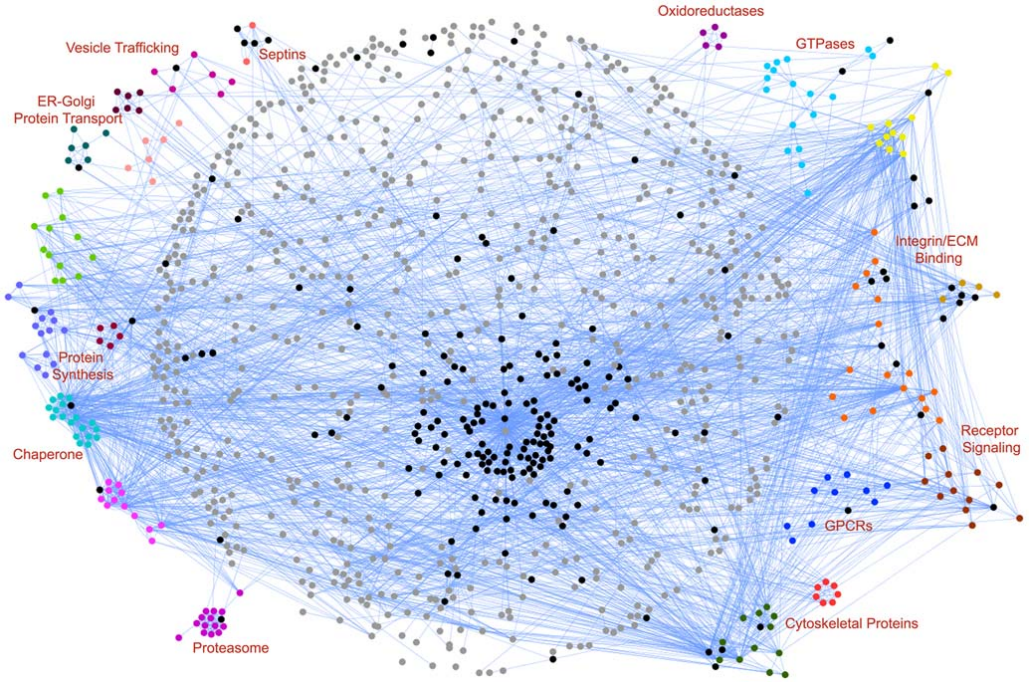
Platelet Protein-Protein Interaction (PPI) network. A graph showing the platelet PPI network. Literature curated interactions between any set of two platelet were identified using the publicly available HPRD database [54] and graphed to generate a PPI network. Each platelet protein is shown as a colored dot and interaction between any two proteins is shown as a blue colored edge. Black dots represent phospho-proteins identified in this study. The PPI network was visualized using Cytoscape [55] and proteins showing high degree of interactions were clustered using the MCODE plugin [57]. Protein nodes that were found to be clustered based on degree of interactions are grouped and are shown in a single color. Biological function of protein clusters was determined using Uniprot database and is shown above.

Next, we expanded our base PPI network as follows. First, we added potential new interactions using the phospho-peptides indentified. Phosphomotif finder database contains a literature curated mapping of the kinase binding and phosphorylation motifs [46]. Using the kinases present in our dataset and the Phosphomotif finder, we defined the likely phospho-peptide sequences that the kinases in our dataset would phosphorylate. We added these additional 297 predicted interactions to our PPI network. Second, as a goal of the present study is to create a computational framework (based on the proteomics data) for gaining deeper insights into integrin signaling pathways and be able to make testable predictions about them, we incorporated interactions from a recently described integrin adhesome network into our dataset [56]. The adhesome components are highly conserved among a variety of cell types and provide a comprehensive dataset for understanding integrin-related pathways in the PPI networks. The adhesome network contains 156 protein components and 690 interactions among these components, derived from published experimental studies. Inclusion of these two sets of data into our PPI network resulted in a platelet PPI network consisting of 1034 protein components and 2993 interactions among them. Finally, in order to obtain insights into the functional groups present in this network, we used the MCODE plugin [57] to cluster the PPI network. The resulting network is shown in Figure 10, where each protein is presented as a colored node (the phospho-proteins are shown as black colored nodes) and a blue colored edge represents interaction between any two proteins. Remarkably, this PPI network showed enrichment of several related proteins into expected functional groups. Proteins constituting a cluster are represented by a single color (except for phospho-proteins, which are black). This clustering of related proteins into expected functional groups was achieved in the absence of any other applied constrains (such as the use of Gene Ontologies to bring together related groups of proteins). However, analysis of the PPI network showed that the average shortest path length (i.e.; average length of a shortest path between a given node (protein) and any other node in the network) is only 3.5. The network analysis also showed that the network is highly connected and that the proteins have, on average, 5.8 neighbors. Such short path lengths and high connectivity make it difficult to identify signaling pathways using these kinds of PPI networks.

### Generation of a Directed Graph using phospho-proteome data: Insights into Integrin Signaling

Changes in the platelet functional state are known to dynamically regulate protein-protein interactions, thereby changing the signaling pathways and thus regulating platelet function. For example, activation of integrin αIIbβ3 leads to a stable adhesion of circulating platelets and further changes in the cell shape. However, typical PPI networks, as we developed in Figure 10, do not provide any detailed insights into the signaling pathways. One reason is that the available databases, such as HPRD, contain protein-protein interaction information from a variety of cell types and under a variety of conditions. However, protein-protein interactions are highly dynamic and change based on the local context within a cell. In order to address this weakness in the current PPI networks, we developed a novel method by incorporating additional available “contextual” information. This contextual information came in the form of mappings for a) the phospho-proteins (the phospho-proteins identified in our analyses above were labeled as such in the PPI network), b) the protein kinases and c) the protein phosphatases (the protein kinases and protein phosphatases identified were also labeled as such). This “contextual” information was used to convert some of the non-directional edges into directional edges as follows: we converted a non-directional edge between two protein partners in our PPI network into a directed edge from a kinase to a phospho-protein, if the linkage satisfied the condition that the identified kinase is likely to phosphorylate the phospho-protein at the identified phospho-peptide sequence as predicted by Phosphomotif finder [46]. Additionally, if the phospho-protein was connected with a protein phosphatase, we converted that edge into a directed edge between the phospho-protein and the phosphatase, as the currently available algorithms for predicting the consensus protein phosphatase site in a phospho-protein are quite weak (which is a shortcoming of this and other similar studies). As a result, we established a limited directionality in our PPI network to develop this contextual PPI network, which can be utilized in identifying signaling pathways in the future.

**Figure 11:**
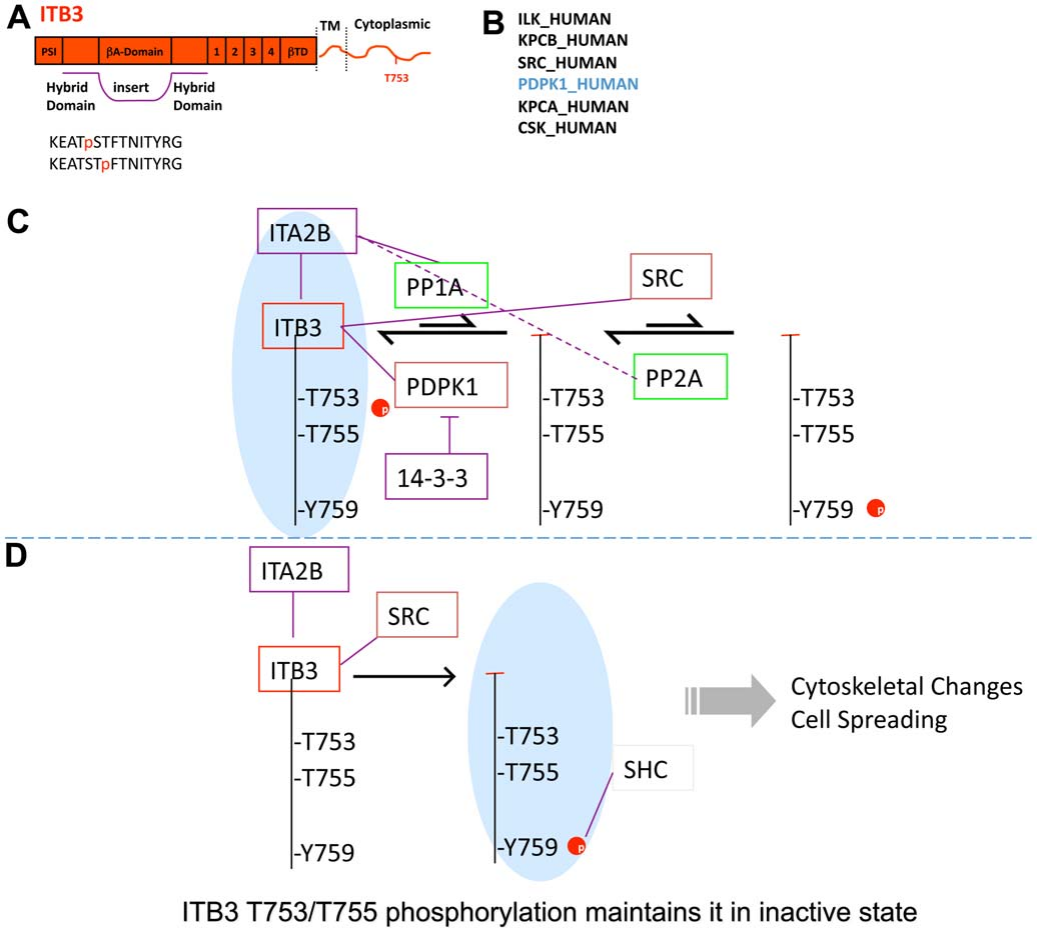
Contextual PPI network based computational recapitulation of integrin activation pathway. A. A cartoon representation of the domain structure of integrin beta3 chain, with majority of the domains labeled. Phoshorylated threonine residues identified in the current study are shown at the bottom. B. Names of six kinases that were found to show a direct interaction with ITB3 in the platelet PPI network. C. Signaling pathways showing integrin ITA2B and ITB3 and selected interacting protein kinases and phosphatases that are present in our platelet PPI network. ITB3 interacts with ITA2B (α-chain of the integrin heterodimer) and is found phosphorylated at threonine residues T753 and T755 in the resting platelets. Phosphomotif finder [46] mapping suggests that PDPK1 (3-phosphoinositide dependent protein kinase-1) is the likely kinase that phosphorylates ITB3 at this site. Literature mining also suggests that PDPK1 interacting protein 14-3-3 inhibits this kinase [58], [59], [60], [61]. Threonine phosphorylation of ITB3 maintains it in the inactive state. ITA2B interacting protein phosphatase PP1A and/or PP2A likely dephosphorylates this site and leads to generation of non-phosphorylated ITB3 [63]. D. In activated platelets, the non-phosphorylated ITB3 is a substrate for SRC kinase. HPRD database shows that ITB3 can be phosphorylated at residue Y759 [61], [62]. SRC phosphorylation of ITB3 leads to binding of other proteins, such as SHC and others [64], outside-in signaling by the integrin and initiation/augmentation of platelet activation [62], [64].

Finally, we used the newly developed contextual PPI network to determine if it would provide any signaling insights using purely computational means. We focused on integrin β3 (ITB3), the beta chain of the integrin αIIbβ3 heterodimer and recapitulated a known pathway as a model. ITB3 was identified as a phospho-protein in our phospho-proteomic analyses and the sequence of the phospho-peptides is shown in Figure 11A. Our PPI network showed that there were six potential protein kinases that were directly connected to this phospho-protein (Figure 11B). However, cross-mapping of these six potential kinases and the sequence of the ITB3 phospho-peptide using Phosphomotif finder [46] suggested that only one, PDPK1 (3-phosphoinositide dependent protein kinase-1), was capable of phosphorylating ITB3 at the threonine residues in this sequence. Indeed, literature mining confirmed that PDPK1 phosphorylates ITB3 and is responsible for maintaining this integrin in the inactive state [58], [59], [60], [61]. Furthermore, HPRD database shows that ITB3 is tyrosine phosphorylated at Y759, upon outside-in integrin activation, by SRC kinase [61], [62]. This lead to a computational recapitulation of a known mechanism for integrin ITB3 activation and signaling (which has been previously described using *in vitro* methods [58], [59], [60], [61], [62], [63], [64]) as follows: Threonine phosphorylation of ITB3 maintains it in the inactive state in resting platelets. Dephosphorylation, likely by protein phosphatase PP1A and/or PP2A leads to generation of non-phosphorylated ITB3 (Figure 11C–D) [63]. This non-phosphorylated (but not the threonine phosphorylated) ITB3 is subsequently phosphorylated at a tyrosine residue by the protein tyrosine kinase SRC [62]. SRC phosphorylation of ITB3 leads to binding of adaptor SHC and others [64], outside-in signaling by the integrin and initiation/augmentation of platelet activation [62], [64]. Thus, this simplification of platelet PPI network by incorporation of available contextual information rapidly confirmed an integrin activation and signaling pathway and, therefore, can be used to provide insights into this and other pathways. We believe that this model of contextual PPI network will serve as a new model for improving the biological significance and the predictive powers of the current PPI networks and may provide insights into their dynamics.

In summary, by using a number of individual platelet samples and three different proteomic profiling techniques, we have identified 1507 unique proteins as constituents of human platelets in its basal, resting state. The platelet proteomic dataset includes 190 membrane-associated proteins and 262 phospho-proteins. The identified platelet proteins were used to generate a comprehensive platelet protein-protein interaction network that computationally recapitulated known integrin pathways and can be used as a model for studying the dynamics of PPI and protein signaling pathways in human platelets and other cells in the future.

## Materials and Methods

### Reagents and antibodies

All biochemical reagents were from Sigma (St. Louis, MO), Invitrogen (San Diego, CA) or Fisher Scientific, unless otherwise specified. Antibodies were purchased from commercial sources as indicated: the anti-Talin mAb 8D4 was from Sigma (St. Louis, MO), the goat anti-Talin antibody (C20), rabbit anti integrin β3 (H-96, sc-14009), goat anti-rabbit-HRP and anti-goat-HRP were from SantaCruz Bio (Santa Cruz, CA), the rabbit anti-mouse-HRP and anti-β-actin (N350) were from Amersham (Piscataway, NJ), and anti-CD41a-PE (557297), anti-CD62P-FITC (550866), anti-CD63-FITC (550759), anti-glycophorin A-FITC (559943) and the IgG1 isotype control mAb (557273) were from BD Pharmingen (San Diego, CA).

### Platelet isolation

Fresh whole blood was collected from five healthy subjects under an IRB approved protocol. Five independent platelet rich plasma (PRP) samples (day 6) were obtained from a local blood bank. The platelets were isolated from each of these samples with a slight modification of the published protocols [31]. Briefly, 10 mL human whole blood was collected by venipuncture and mixed immediately with 1/9^th^ volume of acid-citrate-dextrose solution (ACD, 75 mM trisodium citrate, 124 mM dextrose, and 38 mM citric acid). Room temperature centrifugation of the above citrated blood at 200×*g* for 10 min was used to remove red blood cells (RBC) and leukocytes and to obtain platelet rich suspension. In order to avoid any contamination from the buffy coat, ∼0.5 mL of the platelet-rich suspension above the buffy coat layer was left behind in the centrifugation tube. Any residual RBC and leukocytes were removed from the platelet-rich suspension and the PRP by re-centrifugation at 200×*g* for 10 min at room temperature. The platelets were pelleted by room temperature centrifugation of this suspension at 1200×*g* for 15 min. The platelet pellet was washed by gentle re-suspension in citrate wash buffer (11 mM glucose, 128 mM NaCl, 4.3 mM NaH_2_PO_4_, 7.5 mM Na_2_HPO_4_, 4.8 mM sodium citrate, 2.4 mM citric acid, pH 6.5) and pelleted by centrifugation at 1200×*g* for 10 min at room temperature to isolate the pure platelets as pellets. Some of the platelet samples were stored as platelet pellets at this stage. As described in the text of the article, thawing/lysis of these platelet pellets showed a selective cleavage of Talin in these samples. For the proteomic profiling studies described in the text, the platelet pellets were re-suspended in 0.3 mL of the citrate wash buffer immediately following the centrifugation step with the citrate wash buffer and were stored in 75 µL aliquots at −80°C. For the proteomic profiling studies, each platelet aliquot (75 µL) was lysed by adding 25 µL of 4X SDS-PAGE loading buffer (2% SDS in 100 mM ammonium bicarbonate, 10 mM DTT, pH 8.6) and boiling at 95°C for 5 min. Lysed platelet samples were kept frozen at −80°C until proteomic profiling by 1D SDS-PAGE and LC-MS/MS.

### Isolation of platelet membrane-associated proteins

Membrane-associated proteins were obtained from isolated platelets using a slight modification of published protocols [38]. Briefly, isolated platelets were resuspended in 9 mL TBS buffer (25 mM Tris.Cl pH 7.2, 150 mM NaCl) containing protease inhibitors (5 mM diisopropyl fluorophosphate (DFP), 25 uM Leupeptin, 5 uM Pepstatin and 10 uM Phosphoramidon). The platelet suspension was homogenized, on ice, by 10 strokes of a Dounce homogenizer and kept on ice for an additional 10 min. The platelet membranes were disrupted by passing through a French press at 1000 psi. Lysate was carefully layered on top of a 40% sucrose (in TBS) cushion (3 mL) and centrifuged at 26,000 rpm for 4 h at 4°C in ultracentrifuge using SW41 rotor (Beckman-Coulter, CA). The top-layer above the interface was saved as the cytoplasmic fraction. The crude membrane fraction was collected from the gradient interface. Membrane fraction was washed by re-suspension in 10 mL Tris buffer (20 mM Tris.Cl pH 7.2, 1 mM CaCl_2_ and 0.5 mM MgCl_2_) containing protease inhibitors and was subsequently centrifuged at 26,000 rpm for 1 h at 4°C. The supernatant was discarded and the pelleted membrane fraction was re-suspended in n-octyl-beta-D-glucopyranoside (NOG, from Anatrace, OH)/Guanidinium buffer (8 M Guanidinium.HCl, 100 mM ammonium bicarbonate, 2% NOG, 10 mM DTT, pH 8.6) to extract membrane associated proteins. Any insoluble membranous debris was removed by centrifugation at 26,000 rpm for 1 h at 4°C and the membrane-associated proteins were collected from the supernatant. Subsequently, Guanidinium.HCl was removed from this protein solution by dialysis and the membrane-associate proteins were analyzed by 1D SDS PAGE followed by LC-MS/MS as described below.

### Proteomic profiling of platelets

(A) All proteomic and phospho-proteomic profiling assays and peptide identifications were performed at the Proteomics Core of the Harvard Partners Center for Genetics and Genomics (HPCGG). *A. Protein pre-fractionation and digestion*. Platelet proteins were separated in 1D using 4–12% SDS-PAGE precast gels (Invitrogen, CA) at 150 V and stained with Coomassie blue according to standard protocols. For an extensive mass spectrometric interrogation of the platelet proteome, the gel lane containing the sample was cut into 12–14 equally sized sections irrespective of staining intensity and in-gel digestion of the bands was performed according to published protocols [34], [65]. Briefly, gels were imaged with a Kodak DC280 Digital Camera fitted with a +10 Macro lens. Images were processed using Adobe Photoshop and printed out. Gels were cut with a clean razor blade into 12–14 gel slices each while marking positions of the cuts for each slice. Gel sections were placed into 2 mL Axygen tubes and destained with two washes of aqueous solution containing 50% methanol and 5% acetic acid. Subsequently, the destain solution was removed and the gel pieces were rinsed with ammonium bicarbonate. The gel slices were next reduced and alkylated using 10 mM dithiothreitol (DTT) and 55 mM iodoacetamide, respectively, for 1 h at room temperature in the dark. The gel pieces were then rinsed with three alternating washes of ammonium bicarbonate and acetonitrile. The final acetonitrile wash was removed just prior to digestion and the gel slices dried for 30 min in a speed vac (Thermo Savant SC280). The tubes containing the dried gel pieces were placed on ice and 25 µL of sequencing grade porcine trypsin (Promega, Madison, WI) at a concentration of 5.5 µg/mL in 50 mM ammonium bicarbonate was added to each sample. The gel pieces were allowed to swell for 15 minutes on ice after which excess trypsin solution was removed and an additional 25 µL of 50 mM ammonium bicarbonate was added to each tube. The tubes were then capped and incubated for 16 h at 37°C. Peptide were extracted with 2 washes of 75 µL of 50 mM ammonium bicarbonate and two washes of 75 µL of aqueous solution containing 50% acetonitrile and 0.1% formic acid. All extracts were frozen at −80°C and lyophilized to dryness in a speed vac at <10mTorr. The lyophilate was re-dissolved in 24 µL of aqueous solution containing 5% acetonitrile and 0.1% formic acid for LC-MS/MS mass spectrometry analysis. A total of 152 gel slices were used in this study. (B) Mass spectrometry using nanospray LC-MS/MS. Trypsin-treated samples were analyzed using a LCQ DECA XP plus ProteomeX workstation. 10 µL of each reconstituted sample was injected with a Famos Autosampler while the separation was done on a 75 µm i.d.×18 cm column packed with C18 media running at a 235 nL a minute flow rate provided from a Surveyor MS pump with a flow splitter with a gradient of 5–60% water 0.1% formic acid, acetonitrile 0.1% formic acid over the course of 180 min (4 h run). In between each set of samples two standards of a 5 Angio mix peptides (Michrom Bioresources, Inc., Auburn, CA) were run to ascertain column performance, and observe any potential carryover that might have occurred. The LCQ was run in a top five configuration, with one MS scans and five MS/MS scans. Dynamic exclusion was set to 1 with a limit of 30 seconds. (C) Peptide identifications. Peptide identifications (ID's) were made using the TurboSequest program through the Bioworks Browser 3.2 (Thermo Electron, San Jose, CA). Sequential database searches were made using the RefSeq Human Protein Database from NCBI (National Center for Biotechnology Information, Bethesda, MD; release 7) [66] using differential carbamidomethyl modified cysteines and oxidized methionines, followed by further searches using differential modifications. Secondary searches were performed with Sequest using RefSeq Human Gnomon predicted protein database and a reversed database generated using the db_reverse Perl script [67], to minimize false sequence detections (to <5%). In this fashion known and theoretical protein hits can be found without compromising the statistical relevance of all the data. Peptide score cutoff values were chosen at Xcorr (cross correlation) of 1.8 for singly charged ions, 2.5 for doubly charged ions, and 3.0 for triply charged ions, along with deltaCN (delta correlation) values of 0.1, and RSP (Ranking of the primary score) values of 1. Additionally, a restriction that all peptides must be fully tryptic was placed on the data. The cross correlation values chosen for each peptide assured a high confidence match for the different charge states, while the deltaCN cutoff insured the uniqueness of the peptide hit. The RSP value of 1 ensured that the peptide matched the top hit in the preliminary scoring and that the .dta peptide fragment file only matched to one protein hit. (D) Phospho-proteomic profiling of platelets. (A) Protein digestion. Platelet samples were prepared for phospho-proteomic profiling using a slight modification of published protocols [43]. Platelets were lysed in an aqueous solution containing 8 M guanidinium hydrochloride (GuHCl), 100 mM ammonium bicarbonate, 10 mM DTT and 5% n-propanol. The protein mixture was reduced and alkylated using 10 mM DTT and 45 mM iodoacetamide, respectively, for 1 h at room temperature in the dark. The solution was diluted 8-fold into an aqueous buffer with a final concentration of 25 mM Tris-HCl, pH 8.3 and 1 mM CaCl_2_ and digested with 5.5 µg/mL of sequencing-grade modified trypsin (Promega, Madison, WI) (enzyme/substrate ratio of 1∶250) for 16 h at 37°C. Digestion reaction was stopped by the addition of TFA to 0.4%. Peptides were desalted on a C18 Sep-Pak cartridge (Waters, Milford, MA) and the eluate was dried in a Speedvac and stored at −80°C. (B) Enrichment of phospho-peptides using IMAC. Platelet peptide mixtures were enriched for phospho-peptides using a slight modification of published protocols [43]. The lyophilysed platelet peptide mixture was re-dissolved in 100 µL of aqueous wash buffer (AWB) containing 30% acetonitrile and 250 mM acetic acid. 15 µL Fe(III)-loaded IMAC slurry (50% beads) (Phos-Select iron affinity gel, SIGMA), pre-equilibrated with the same buffer, was added to the peptide solution. Next, the samples were incubated with vigorous shaking for 90 min at room temperature. Subsequently, the IMAC beads were washed three times with 350 µL of AWB. Peptides were eluted from the IMAC beads twice by adding 20 µL of elution buffer (50 mM KH_2_PO_4_/NH_3_, pH 10.0) to the beads and incubating at room temperature for 15 min. Eluates were acidified with 20 µL of 5% formic acid, dried in a Speedvac, desalted using stage-tips and stored at −80°C. (C) Mass spectrometry using nanospray LC-MS/MS. IMAC-isolated samples were analyzed using a LTQ-FT ion-trap mass-spectrometer (Thermo Electron, San Jose, CA). The lyophilate from IMAC-columns was re-dissolved in 24 µL of aqueous solution containing 5% acetonitrile and 0.1% formic acid. 10 µL of each reconstituted sample was injected with a Famos Autosampler while the separation was done on a 75 µm i.d.×18 cm column packed with C18 resin (Michrom Bioresources, Inc., Auburn, CA) running at a 235 nL a minute flow rate provided from a Surveyor MS pump with a flow splitter with a gradient of 5–60% water 0.1% formic acid, acetonitrile 0.1% formic acid over the course of 100 min with a total run length of 150 min. The LTQ-FT was run in a top four configuration at 200 K resolution. For each cycle, one full MS full scan - (*m*/*z* 350–1800) was acquired in the ion-trap (MS scan), followed by MS/MS scans (MS^2^ scans) - on the four most abundant precursor ions. Dynamic exclusion was set to 1 with a limit of 30 seconds. Charge-state screening was used to reject singly charged ions. A third scan (MS^3^ scan) was automatically acquired for the most intense peak in the MS^2^ spectrum with a neutral loss trigger set at masses 98, 49 and 32.7 Da. (D) Phospho-peptide identifications. First, full scan data was analyzed using DeCyder MS software. Phospho-peptide identifications and phosphorylation site localizations were made usingthe TurboSequest program through the Bioworks Browser 3.2 (Thermo Electron, San Jose, CA) in a manner similar to the methods described for peptide IDs above.

### Consensus phospho-motif discovery

Consensus peptide sequence motifs near phosphorylation sites were determined as described in the literature using the Motif-X program (http://motif-x.med.harvard.edu) [45]. For pSer, the peptide sequences were restricted to 9 amino acids in length for alignment. The significance threshold was set to p<10^−3^. The minimum number of motif occurrences was set to 20. For pThr and pTyr, the peptide sequences were extended to 11 amino acids and the significance threshold was set to p<10^−3^. The minimum number of motif occurrences was also lowered and was set to 5. Sequence logos were generated with Weblogo at http://weblogo.berkeley.edu [45]. The di- and tri-phosphopeptide sequences were aligned manually and their logos were generated as described above.

### Flow cytometry based analysis of platelets

Flow cytometry was performed on a Becton Dickinson FACScan and analyzed with Cellquest software (Becton Dickinson, Palo Alto, CA) according to published protocols [21], [22]. Briefly, the platelet and RBC/erythrocyte populations in purified platelets or anti-coagulated whole blood were identified by their forward and side light scatter characteristics and a gate placed around each of the two cell types. The two samples were also stained with the phycoerythrin (PE)-conjugated anti-CD41a (GPIIb) and fluorescein isothiocyanate (FITC)-anti-CD235a (Glycophorin A) to verify placement of the correct forward and side scatter of platelets and RBCs/erythrocytes, respectively. Both antibodies, and their corresponding isotype controls were from BD Biosciences (San Diego, CA). Resting platelets and platelets activated by thrombin (from BD Biosciences) treatment were analyzed by staining with anti-CD41a-PE and with either anti-CD62P-FITC or anti-CD63-FITC mAbs to confirm whether the platelets are in the resting or an activated state.

### Light microscopy

Anticoagulated whole blood and washed platelets were fixed with 2% formaldehyde. A drop of diluted fixed samples was applied to a regular glass slide and incubated for 10 minutes under humidified condition. After mounting cover-slips, light micrographs were taken at 40× magnification (Nikon, Eclipse E800) using CCD camera (Hamamatsu, model 742-95). A minimum of 8 random images were taken per slide.

### Western blot analyses

Platelet samples were separated by SDS-PAGE using a 4–12% gradient Bis-Tris gels (Invitrogen, CA USA) under reducing conditions and electroblotted onto PVDF membranes (Bio-Rad Laboratories, CA). After blocking with 10% nonfat milk in 25 mM Tris-HCl, pH 7.4, 137 mM NaCl, 2.7 mM KCl (TBS, Boston Bioproducts, MA), the membrane was incubated with a primary antibody (as described in *Reagents and antibodies* section). Detection of proteins was performed using an appropriate horseradish peroxidase (HRP) linked secondary antibody and SuperSignal® Chemiluminescent kit (Pierce Chemical Company, Milwaukee, WI). The luminescent signal was detected using BioMax x-ray films (Eastman Kodak Company, NY USA).

## Supporting Information

Figure S1 An example MS/MS spectrum of MH+ ion of an identifying peptide from integrin alphaIIb in a platelet lysate. An MS/MS spectrum recorded at MH+ 2827.05 corresponding to a peptide from the integrin subunit alphaIIb (R.GAVDIDDNGYPDLIVGAYGANQVAVYR.A). Fragment ions of type b and y are labeled.
(1.60 MB TIF)

Figure S2 An example MS/MS spectrum of MH+ ion of an identifying peptide from integrin beta3 in platelet membrane sample. An MS/MS spectrum recorded at MH+ 1421.58 corresponding to a peptide from the integrin subunit beta3 (R.AKWDTANNPLYK.E). Fragment ions of type b and y are labeled.
(1.24 MB TIF)

Figure S3 Cellular and biological functions of the platelet proteome. A bar graph showing the cellular and biological functions over-represented in the identified platelet proteome, as determined by the IPA software. The y-axis shows the -log (p-value) associated with the predicted functional enrichment.
(0.33 MB TIF)

Figure S4 Disease pathways represented by the platelet proteome. A bar graph showing the disease pathways over-represented in the identified platelet proteome, as determined by the IPA software. The y-axis shows the -log (p-value) associated with the predicted pathway enrichment.
(0.31 MB TIF)

Figure S5 Platelet Protein-Protein Interaction (PPI) network. A graph showing the platelet PPI network. Literature curated interactions between any set of two platelet were identified using the publicly available HPRD database [54] and graphed using Cytoscape [55] to generate a PPI network. Each platelet protein is shown as a colored dot and interaction between any two proteins is shown as a blue colored edge. Red dots represent phospho-proteins identified in this study and gray dots represent the remaining non-phosphorylated proteins.
(5.52 MB TIF)

Table S1 A comprehensive list of identified platelet proteins (from 10 independent samples). Protein refseq IDs, genbank IDs and protein names are shown.
(0.16 MB PDF)

Table S2 Ten lists of proteins and the identifying peptides from proteomic profiling of ten individual platelet samples. Protein identification is based on its refseq ID. Definition of additional terms is as follows: P (pro) is the protein probability (normalized to 1); P (pep) is the peptide probability (normalized to 1); Sf score is the quality of the match in a TurboSEQUEST search. The protein Sf score is the sum of peptide Sf scores for all the peptides associated with that protein; consensus Score is the quality of the match in a TurboSEQUEST search.
(9.66 MB PDF)

Table S3 A list of all membrane-fraction associated proteins identified in this study. Protein uniprot name, Gene name, Uniprot accession number, Protein name, Gene ontology classification, Predicted sub-cellular localization and Protein family are shown based on its description in the Uniprot database (www.uniprot.org).
(0.13 MB PDF)

Table S4 A list of 190 unique proteins and the identifying peptides from proteomic profiling of the platelet membrane fraction. Protein identification is based on its Refseq ID and the definition of additional terms is the same as described for Table S2A.
(0.19 MB PDF)

Table S5 A list of 262 unique phosphorylated proteins identified using four independent samples in this study. Protein uniprot name, Gene name, Uniprot accession number, Protein name, Gene ontology classification, Predicted sub-cellular localization and Protein family are shown based on its descrition in the Uniprot database (www.uniprot.org).
(0.15 MB PDF)

Table S6 A list of all non-phosphorylated proteins identified during phospho-proteomic profiling of platelets. Protein uniprot name, Gene name, Uniprot accession number, Protein name, Gene ontology classification, Predicted sub-cellular localization and Protein family are shown based on its descrition in the Uniprot database (www.uniprot.org).
(0.09 MB PDF)

Table S7 A combined list of 1507 platelet proteins identified in this study. Protein uniprot name, Gene name, Uniprot accession number, Protein name, Gene ontology classification, Predicted sub-cellular localization and Protein family are shown based on its descrition in the Uniprot database (www.uniprot.org). Comments column describes whether the protein was identified as associated with the membrane fraction or as a phospho-protein in this study. Additional columns describe if the protein is known to be phosphorylated in literature databases. The function column tags known protein kinases and phosphatases in our dataset.
(1.13 MB PDF)
