# Supplementary figures and images for: Proteomic and Phospho-Proteomic Profile of Human Platelets in Basal, Resting State: Insights into Integrin Signaling
Source: PLoS One. 2009 Oct 27;4(10):e7627. doi: 10.1371/journal.pone.0007627 (PMC2762604; doi:10.1371/journal.pone.0007627)

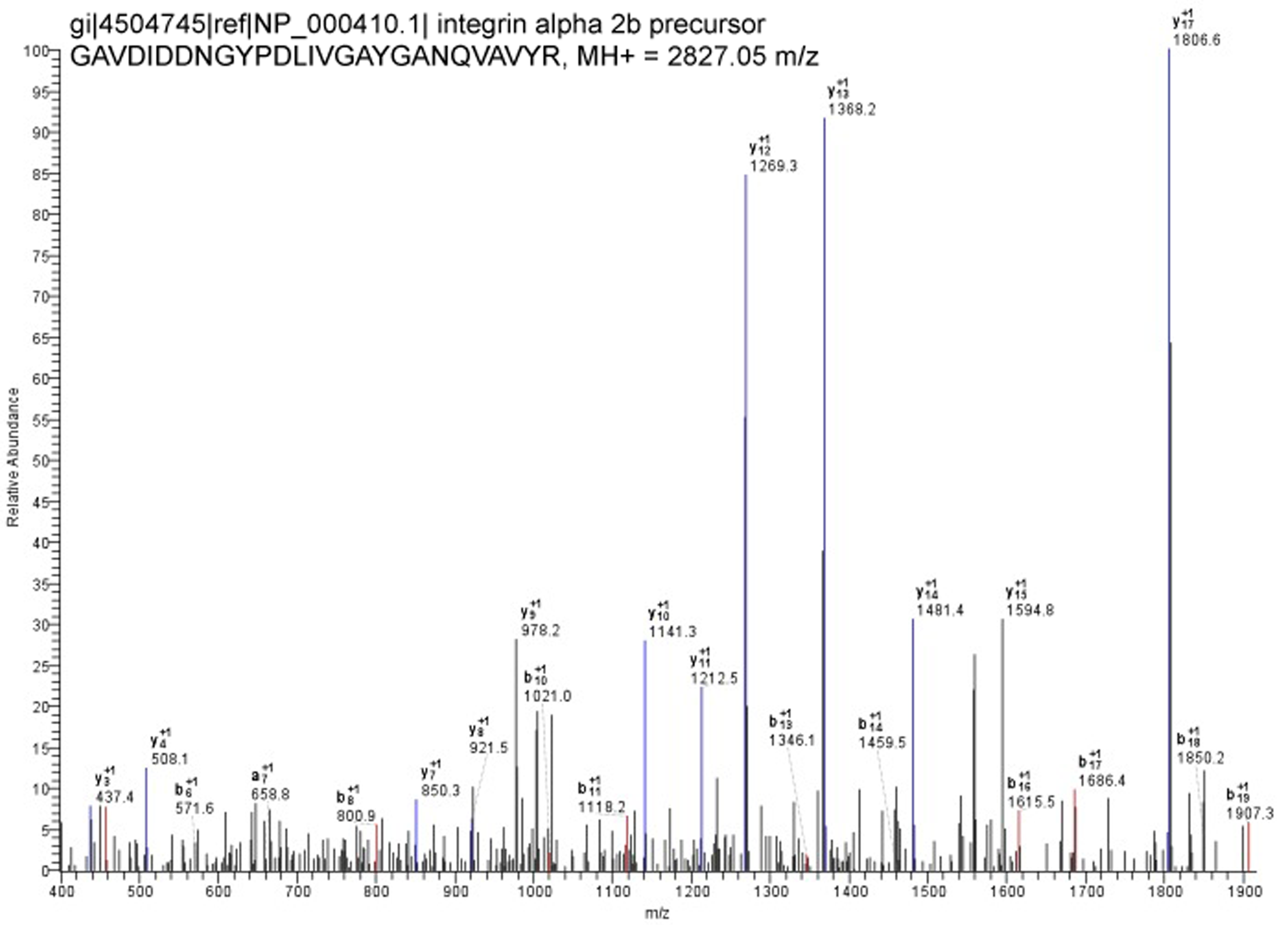

Supplement: Figure S1 — An example MS/MS spectrum of MH+ ion of an identifying peptide from integrin alphaIIb in a platelet lysate. An MS/MS spectrum recorded at MH+ 2827.05 corresponding to a peptide from the integrin subunit alphaIIb (R.GAVDIDDNGYPDLIVGAYGANQVAVYR.A). Fragment ions of type b and y are labeled. (1.60 MB TIF) [file pone.0007627.s001.tif]

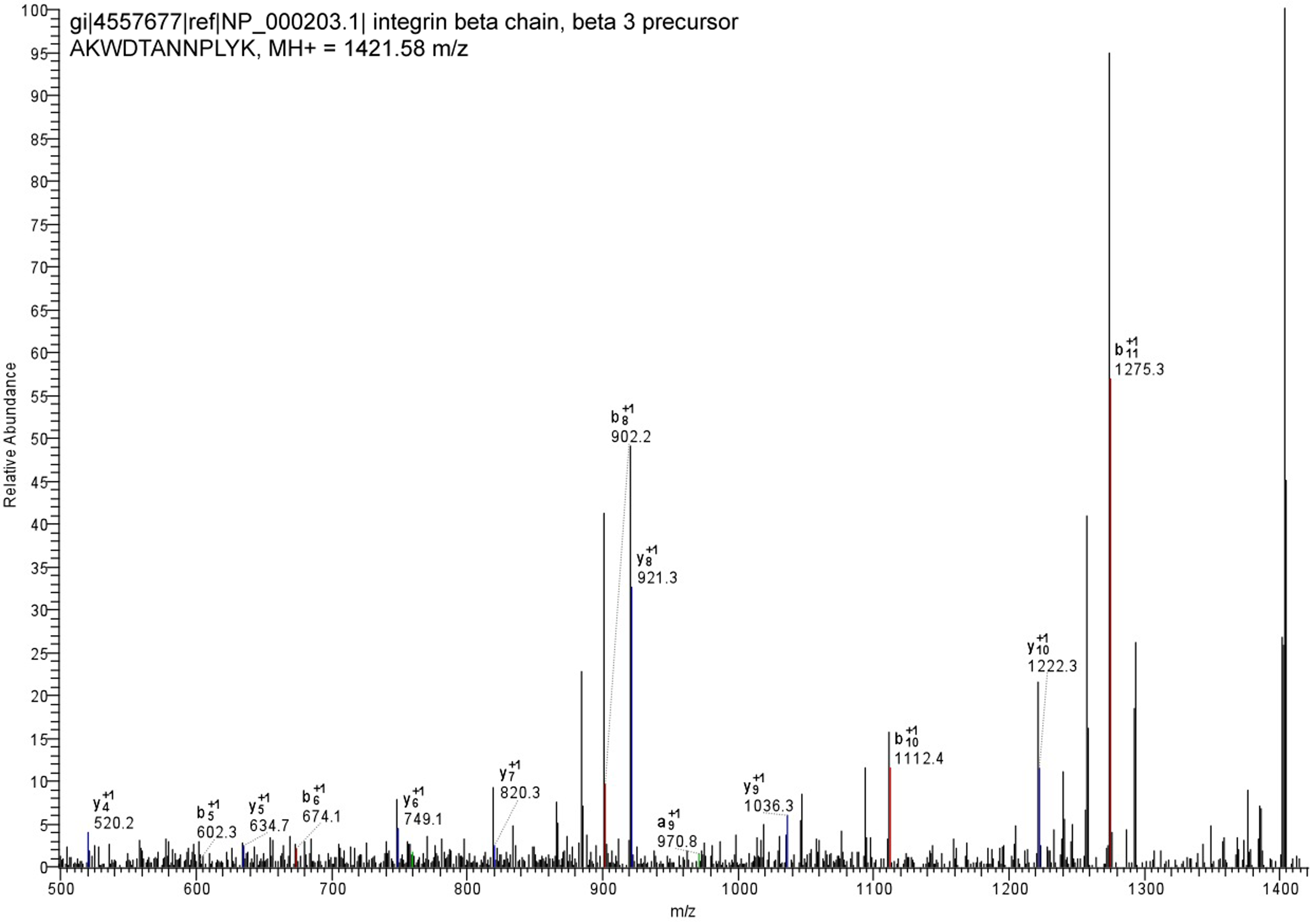

Supplement: Figure S2 — An example MS/MS spectrum of MH+ ion of an identifying peptide from integrin beta3 in platelet membrane sample. An MS/MS spectrum recorded at MH+ 1421.58 corresponding to a peptide from the integrin subunit beta3 (R.AKWDTANNPLYK.E). Fragment ions of type b and y are labeled. (1.24 MB TIF) [file pone.0007627.s002.tif]

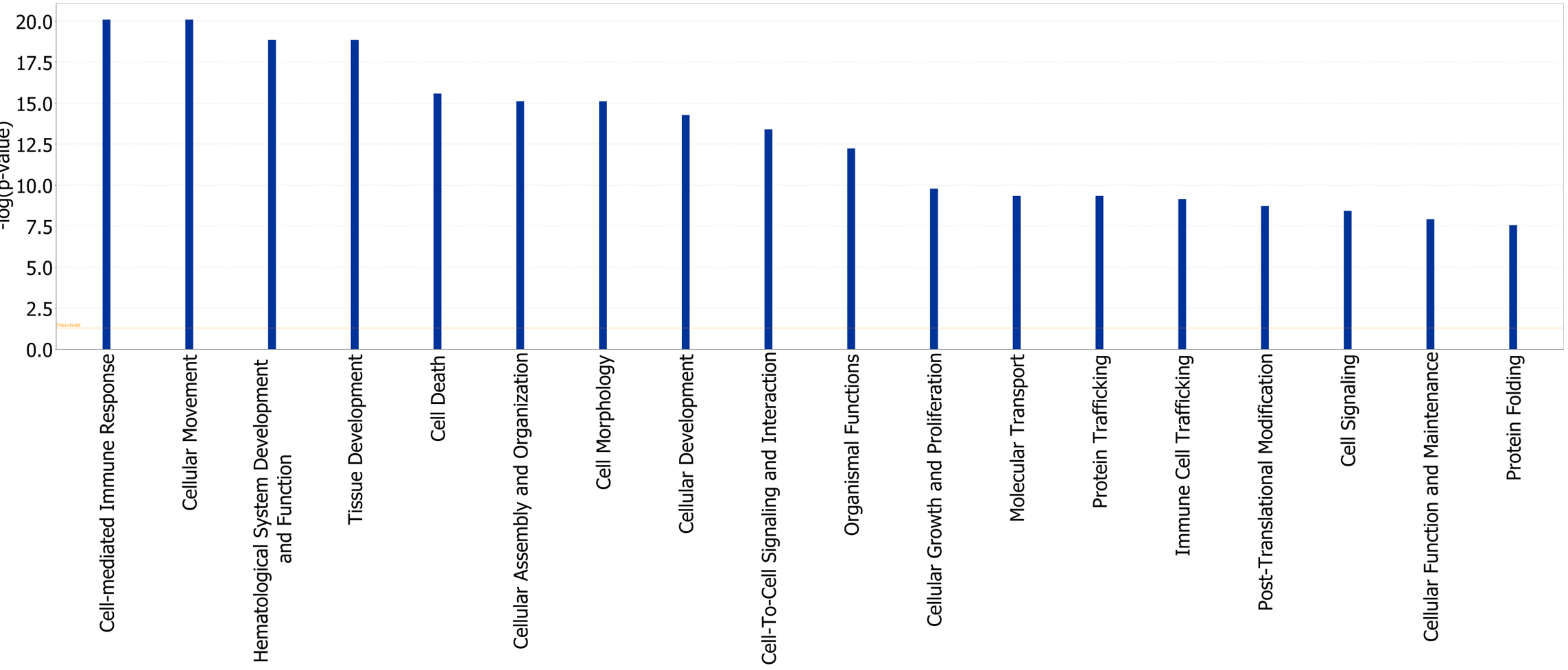

Supplement: Figure S3 — Cellular and biological functions of the platelet proteome. A bar graph showing the cellular and biological functions over-represented in the identified platelet proteome, as determined by the IPA software. The y-axis shows the -log (p-value) associated with the predicted functional enrichment. (0.33 MB TIF) [file pone.0007627.s003.tif]

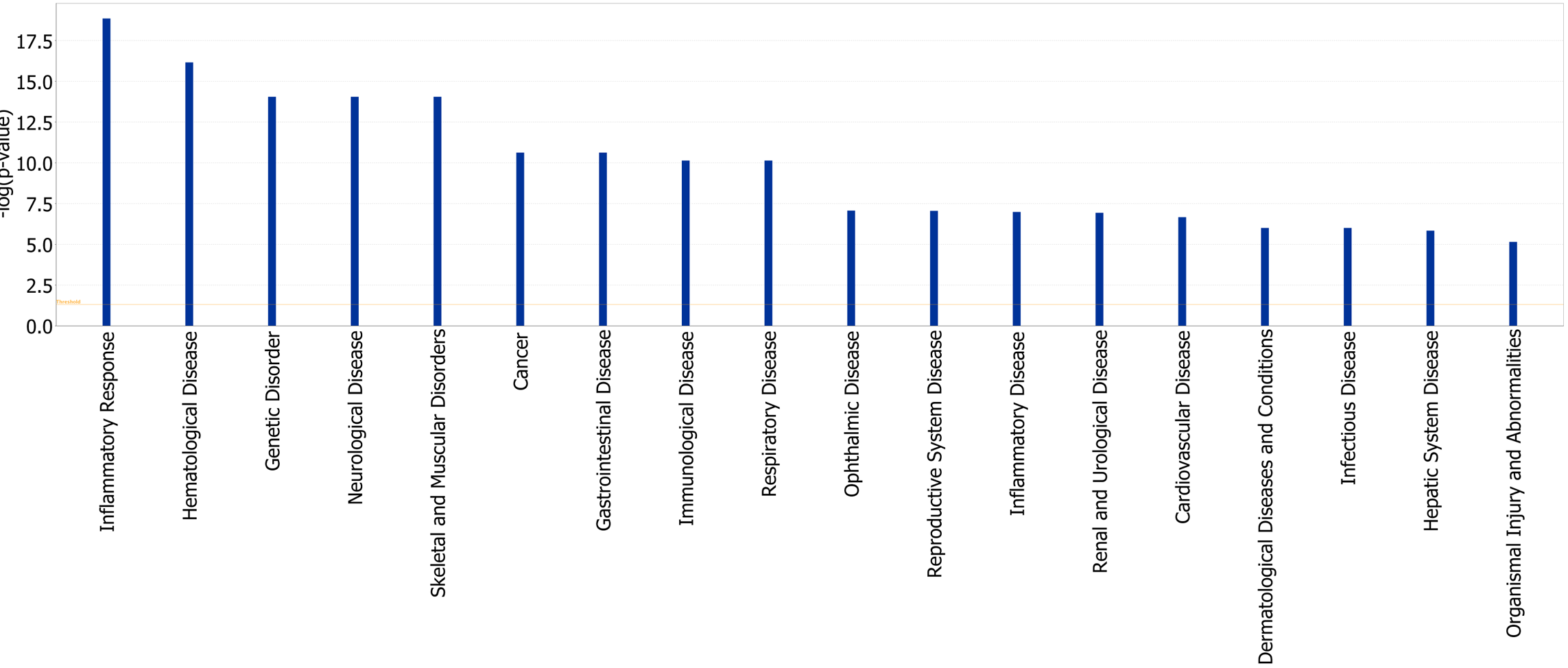

Supplement: Figure S4 — Disease pathways represented by the platelet proteome. A bar graph showing the disease pathways over-represented in the identified platelet proteome, as determined by the IPA software. The y-axis shows the -log (p-value) associated with the predicted pathway enrichment. (0.31 MB TIF) [file pone.0007627.s004.tif]

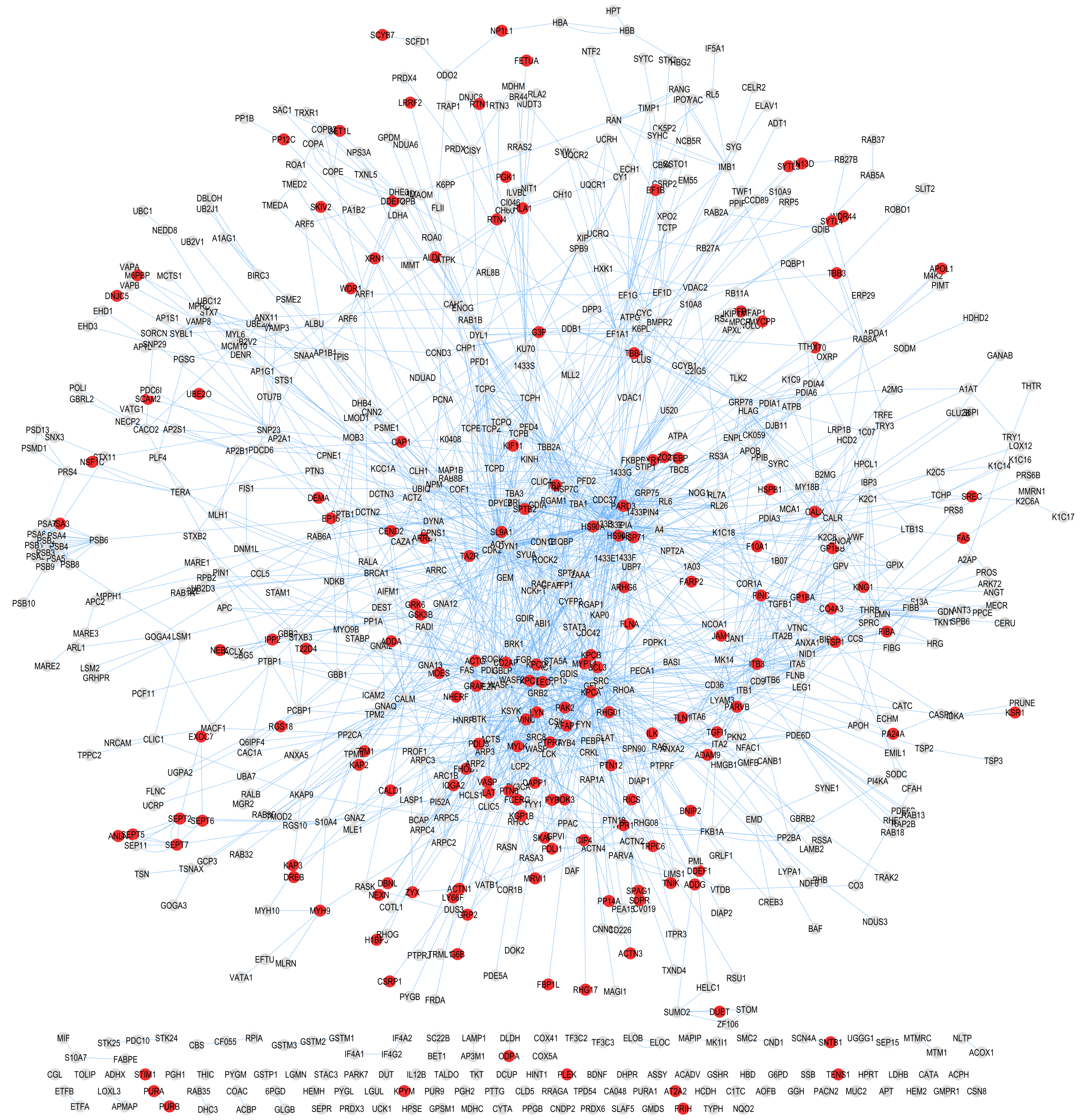

Supplement: Figure S5 — Platelet Protein-Protein Interaction (PPI) network. A graph showing the platelet PPI network. Literature curated interactions between any set of two platelet were identified using the publicly available HPRD database [54] and graphed using Cytoscape [55] to generate a PPI network. Each platelet protein is shown as a colored dot and interaction between any two proteins is shown as a blue colored edge. Red dots represent phospho-proteins identified in this study and gray dots represent the remaining non-phosphorylated proteins. (5.52 MB TIF) [file pone.0007627.s005.tif]
