# Supplementary material for: Proteomic and Phospho-Proteomic Profile of Human Platelets in Basal, Resting State: Insights into Integrin Signaling
Source: PLoS One. 2009 Oct 27;4(10):e7627. doi: 10.1371/journal.pone.0007627 (PMC2762604; doi:10.1371/journal.pone.0007627)
Supplement: Table S1 — A comprehensive list of identified platelet proteins (from 10 independent samples). Protein refseq IDs, genbank IDs and protein names are shown. (0.16 MB PDF) [file pone.0007627.s006.pdf]

	Refseq ID	gi Number	Protein Name/Description
1	NP_000005	4557225	alpha 2 macroglobulin precursor
2	NP_000009	4557235	acyl-Coenzyme A dehydrogenase, very long chain precursor
3	NP_000022	4557297	delta-aminolevulinic acid dehydratase; porphobilinogen synthase; aminol
4	NP_000025	4557305	aldolase A; fructose-bisphosphate aldolase; Aldolase A, fructose-bispho
5	NP_000029	4557319	adenomatosis polyposis coli
6	NP_000030	4557321	apolipoprotein A-I precursor
7	NP_000031	4557323	apolipoprotein C-III precursor
8	NP_000033	4557327	beta-2-glycoprotein I precursor
9	NP_000052	4557377	Bruton agammaglobulinemia tyrosine kinase
10	NP_000055	4557385	complement component 3 precursor; acylation-stimulating protein cleavag
11	NP_000058	4557395	carbonic anhydrase II; carbonate dehydratase II; carbonic dehydratase;
12	NP_000062	4557415	cystathionine-beta-synthase; serine sulfhydrase; beta-thionase; methylc
13	NP_000063	4557419	CD36 antigen (collagen type I receptor, thrombospondin receptor); CD36
14	NP_000087	4557485	ceruloplasmin (ferroxidase); Ceruloplasmin
15	NP_000091	4503117	cystatin B; stefin B; liver thiol proteinase inhibitor; CPI-B
16	NP_000099	4557525	dihydrolipoamide dehydrogenase precursor; E3 component of pyruvate dehy
17	NP_000108	4557553	emerin
18	NP_000117	4503607	electron transfer flavoprotein, alpha polypeptide; electron-transferrin
19	NP_000120	4503631	coagulation factor XIII A1 subunit precursor; Coagulation factor XIII,
20	NP_000121	4503643	coagulation factor V precursor; labile factor; factor V Leiden
21	NP_000131	4557593	ferrochelatase
22	NP_000135	4503785	frataxin; Friedreich ataxia (frataxin)
23	NP_000149	4557619	glucan (1,4-alpha-), branching enzyme 1 (glycogen branching enzyme); Gl
24	NP_000164	4504071	platelet glycoprotein Ib alpha polypeptide precursor
25	NP_000165	4504077	glycoprotein IX (platelet)
26	NP_000166	18201905	glucose phosphate isomerase; neuroleukin; glucose-6-phosphate isomeras
27	NP_000168	4504165	gelsolin (amyloidosis, Finnish type); Gelsolin
28	NP_000173	20127408	hydroxyacyl dehydrogenase, subunit A; trifunctional protein, alpha sub
29	NP_000175	6715607	G-gamma globin
30	NP_000177	4504375	H factor 1 (complement); H factor-1 (complement)
31	NP_000179	4504391	hexokinase 1 isoform HKI; brain form hexokinase
32	NP_000180	15553127	hexokinase 2; hexokinase-2, muscle
33	NP_000185	4504483	hypoxanthine phosphoribosyltransferase 1
34	NP_000201	4557675	integrin alpha chain, alpha 6
35	NP_000203	4557677	integrin beta chain, beta 3 precursor; platelet glycoprotein IIIa precu
36	NP_000217	4557705	keratin 9
37	NP_000230	4557894	lysozyme precursor
38	NP_000243	4557896	myotubularin
39	NP_000248	4557773	myosin, heavy polypeptide 7, cardiac muscle, beta
40	NP_000260	4557797	non-metastatic cells 1 protein
41	NP_000261	4557801	purine nucleoside phosphorylase
42	NP_000274	4505669	phosphodiesterase 6B, cGMP-specific, rod, beta
43	NP_000281	4505755	phosphoglycerate mutase 2 (muscle); Phosphoglycerate mutase, muscle for
44	NP_000282	4505763	phosphoglycerate kinase 1
45	NP_000286	21361198	serine (or cysteine) proteinase inhibitor, clade A (alpha-1 antiprotei
46	NP_000289	10835121	pyruvate kinase, liver and RBC; Pyruvate kinase, liver and RBC type
47	NP_000292	4505881	plasminogen
48	NP_000299	4505989	protective protein for beta-galactosidase; Protective protein for beta-
49	NP_000304	4506117	protein S (alpha); Protein S, alpha
50	NP_000311	4506359	quinoid dihydropteridine reductase; dihydropteridine reductase
51	NP_000325	4506807	sodium channel, voltage-gated, type IV, alpha; hyperkalemic periodic pa
52	NP_000336	4507109	alpha-synuclein isoform NACP140; non A4 component of amyloid precursor
53	NP_000338	27413156	spectrin, beta, erythrocytic (includes spherocytosis, clinical type I)
54	NP_000353	4507513	tissue inhibitor of metalloproteinase 3; Tissue inhibitor of metallopro
55	NP_000356	4507645	triosephosphate isomerase 1
56	NP_000357	27597085	tropomyosin 1 (alpha)
57	NP_000362	4507725	transthyretin (prealbumin, amyloidosis type I); Transthyretin (prealbum
58	NP_000365	9845522	uroporphyrinogen decarboxylase; uroporphyrinogen III decarboxylase
59	NP_000368	4507909	Wiskott-Aldrich syndrome protein; Wiskott-Aldrich syndrome (eczema-thro
60	NP_000375	4502153	apolipoprotein B precursor; apoB-100; apoB-48

	Refseq ID	gi Number	Protein Name/Description
**61**	NP_000382	5729770	ceroid-lipofuscinosis, neuronal 2, late infantile (Jansky-Bielschowsky
**62**	NP_000389	4503327	cytochrome b5 reductase membrane-bound isoform; diaphorase (NADH)
**63**	NP_000393	21614520	glucose-6-phosphate dehydrogenase
**64**	NP_000398	4504073	glycoprotein Ib beta polypeptide precursor
**65**	NP_000399	4504085	glycerol-3-phosphate dehydrogenase 2 (mitochondrial)
**66**	NP_000403	4504489	histidine-rich glycoprotein precursor; histidine-proline rich glycoprot
**67**	NP_000405	4504505	hydroxysteroid (17-beta) dehydrogenase 4
**68**	NP_000410	4504745	integrin alpha 2b precursor
**69**	NP_000412	4557697	keratin 10; Keratin-10
**70**	NP_000413	4557701	keratin 17
**71**	NP_000414	4557703	keratin 2a
**72**	NP_000415	4557890	keratin 5; Keratin-5; 58 kda cytokeratin; keratin, type II cytoskeletal
**73**	NP_000433	21314617	platelet/endothelial cell adhesion molecule (CD31 antigen); platelet/e
**74**	NP_000436	4505877	plectin 1, intermediate filament binding protein 500kDa; plectin 1, int
**75**	NP_000445	4507149	superoxide dismutase 1, soluble (amyotrophic lateral sclerosis 1 (adult
**76**	NP_000467	4502011	adenylate kinase 1
**77**	NP_000468	4502027	albumin precursor; PRO0883 protein
**78**	NP_000475	4502167	amyloid beta (A4) precursor protein (protease nexin-II, Alzheimer disea
**79**	NP_000476	4502171	adenine phosphoribosyltransferase; AMP pyrophosphorylase; AMP diphospho
**80**	NP_000479	4502261	serine (or cysteine) proteinase inhibitor, clade C (antithrombin), memb
**81**	NP_000497	4503635	coagulation factor II precursor; prothrombin
**82**	NP_000499	4503689	fibrinogen, alpha chain isoform alpha-E preproprotein
**83**	NP_000500	4503715	fibrinogen, gamma chain isoform gamma-A precursor
**84**	NP_000508	4504345	alpha 2 globin
**85**	NP_000509	4504349	beta globin
**86**	NP_000510	4504351	delta globin
**87**	NP_000517	15431310	keratin 14; cytokeratin 14
**88**	NP_000520	4505127	melanocortin 2 receptor; Melanocortin-2 receptor (ACTH receptor); melan
**89**	NP_000543	4507907	von Willebrand factor precursor; Coagulation factor VIII VWF (von Wille
**90**	NP_000552	23065544	glutathione S-transferase M1 isoform 1; HB subunit 4; glutathione S-al
**91**	NP_000565	10835143	decay accelerating factor for complement (CD55, Cromer blood group sys
**92**	NP_000572	10834976	glutathione peroxidase 1
**93**	NP_000574	9845255	group-specific component (vitamin D binding protein); hDBP
**94**	NP_000583	4502501	complement component 4B proprotein
**95**	NP_000589	4504617	insulin-like growth factor binding protein 3
**96**	NP_000598	9257232	orosomucoid 1 precursor; Orosomucoid-1 (alpha-1-acid glycoprotein-1); a
**97**	NP_000604	11321561	hemopexin
**98**	NP_000618	4557731	latent transforming growth factor beta binding protein 1 precursor
**99**	NP_000627	10835187	superoxide dismutase 2, mitochondrial
**100**	NP_000628	10835189	glutathione reductase
**101**	NP_000629	18201911	vitronectin precursor; serum spreading factor; somatomedin B; compleme
**102**	NP_000631	10834992	interleukin 13 receptor, alpha 2 precursor; interleukin 13 binding pro
**103**	NP_000651	10863873	transforming growth factor, beta 1 (Camurati-Engelmann disease); trans
**104**	NP_000662	11496891	class III alcohol dehydrogenase 5 chi subunit; Alcohol dehydrogenase (
**105**	NP_000688	4502051	arachidonate 12-lipoxygenase; 12(S)-lipoxygenase
**106**	NP_000691	4502101	annexin I; annexin I (lipocortin I); lipocortin I
**107**	NP_000703	4502417	biliverdin reductase A
**108**	NP_000704	4502419	biliverdin reductase B (flavin reductase (NADPH)); flavin reductase (NA
**109**	NP_000745	4502969	catechol-O-methyltransferase isoform MB-COMT
**110**	NP_000746	21618331	carnitine acetyltransferase isoform 1 precursor
**111**	NP_000792	4503725	FK506-binding protein 1A; FK506-binding protein 1; FK506-binding protei
**112**	NP_000806	4557609	gamma-aminobutyric acid (GABA) A receptor, delta
**113**	NP_000830	4504137	glutamate receptor, metabotropic 2 precursor
**114**	NP_000839	4504175	glutathione S-transferase M2; glutathione S-transferase 4; GST, muscle;
**115**	NP_000840	23065552	glutathione S-transferase M3; glutathione S-transferase, Mu-3; brain G
**116**	NP_000843	4504183	glutathione transferase; deafness, X-linked 7; fatty acid ethyl ester s
**117**	NP_000847	4504213	guanylate cyclase 1, soluble, alpha 3
**118**	NP_000848	4504215	guanylate cyclase 1, soluble, beta 3
**119**	NP_000849	4504221	guanylate kinase 1
**120**	NP_000864	4504557	intercellular adhesion molecule 2 precursor

	Refseq ID	gi Number	Protein Name/Description
**121**	NP_000879	9625002	integrin, beta 6
**122**	NP_000889	4505093	monoamine oxidase B
**123**	NP_000895	4505417	NAD(P)H dehydrogenase, quinone 2; NAD(P)H menadione oxidoreductase-1, d
**124**	NP_000909	20070125	prolyl 4-hydroxylase, beta subunit; v-erb-a avian erythroblastic leuke
**125**	NP_000925	11386143	alpha-2-plasmin inhibitor; alpha-2-antiplasmin
**126**	NP_000929	4505941	DNA directed RNA polymerase II polypeptide B; polymerase (RNA) II (DNA
**127**	NP_000933	4758950	peptidylprolyl isomerase B (cyclophilin B)
**128**	NP_000935	6715568	protein phosphatase 3 (formerly 2B), catalytic subunit, alpha isoform (
**129**	NP_000936	4506025	protein phosphatase 3, regulatory subunit B, alpha isoform 1; protein p
**130**	NP_000953	18104967	prostaglandin-endoperoxide synthase 1 isoform 1 precursor; prostagland
**131**	NP_000954	4506265	prostaglandin-endoperoxide synthase 2 precursor; prostaglandin G/H synt
**132**	NP_000960	14591909	ribosomal protein L5; 60S ribosomal protein L5
**133**	NP_000963	4506661	ribosomal protein L7a; 60S ribosomal protein L7a; surfeit 3; surfeit lo
**134**	NP_000995	4506671	ribosomal protein P2; 60S acidic ribosomal protein P2; acidic ribosomal
**135**	NP_000997	4506723	ribosomal protein S3a; 40S ribosomal protein S3a; v-fos transformation
**136**	NP_001007	14277700	ribosomal protein S12; 40S ribosomal protein S12
**137**	NP_001045	4507303	sulfotransferase family, cytosolic, 1A, phenol-preferring, member 2; th
**138**	NP_001052	4507383	thromboxane A synthase 1 (platelet, cytochrome P450, family 5, subfamil
**139**	NP_001054	4557871	transferrin
**140**	NP_001055	4507521	transketolase
**141**	NP_001060	4507729	tubulin, beta polypeptide
**142**	NP_001074	4505667	phosphodiesterase 5A isoform 1; cGMP-binding cGMP-specific 3',5'-cyclic
**143**	NP_001087	4501865	ATP citrate lyase
**144**	NP_001089	4501867	aconitase 2
**145**	NP_001091	4501881	alpha 1 actin precursor; alpha skeletal muscle actin
**146**	NP_001092	4501885	beta actin; beta cytoskeletal actin
**147**	NP_001093	4501891	actinin, alpha 1
**148**	NP_001094	4501893	actinin, alpha 2
**149**	NP_001095	4557241	skeletal muscle specific actinin, alpha 3
**150**	NP_001101	4557251	a disintegrin and metalloprotease domain 10
**151**	NP_001110	29826319	adducin 1 (alpha) isoform a; erythrocyte adducin alpha subunit
**152**	NP_001118	22027651	adaptor-related protein complex 1 beta 1 subunit isoform a; beta-adapt
**153**	NP_001119	18104998	adaptor-related protein complex 1, gamma 1 subunit; gamma adaptin; cla
**154**	NP_001142	4502097	solute carrier family 25 (mitochondrial carrier; adenine nucleotide tra
**155**	NP_001143	4502099	solute carrier family 25 (mitochondrial carrier; adenine nucleotide tra
**156**	NP_001145	4502107	annexin V; endonexin II; anchorin CII; lipocortin V; placental anticoag
**157**	NP_001148	4557317	annexin A11; annexin XI; autoantigen, 56-kD; calcyclin-associated annex
**158**	NP_001152	4502125	nudix-type motif 2; diadenosine 5',5''-P1,P4-tetraphosphate pyrophospho
**159**	NP_001156	4502139	baculoviral IAP repeat-containing protein 3; apoptosis inhibitor 2; TNF
**160**	NP_001166	10835002	Rho GDP dissociation inhibitor (GDI) beta; Ly-GDI
**161**	NP_001168	4502227	ADP-ribosylation factor-like 1
**162**	NP_001187	4557361	BH3 interacting domain death agonist; BH3-interacting domain death agon
**163**	NP_001195	15451916	bone morphogenetic protein receptor, type II isoform 1 precursor; type
**164**	NP_001203	4502491	complement component 1, q subcomponent binding protein precursor; hyalu
**165**	NP_001227	4502601	carbonyl reductase 3; carbonyl reductase (NADPH) 3
**166**	NP_001242	4557435	CD68 antigen; Macrophage antigen CD68 (microsialin); macrosialin; scave
**167**	NP_001272	4502849	cytoskeleton-associated protein 1
**168**	NP_001273	4557469	adaptor-related protein complex 2, beta 1 subunit; adaptin, beta 2 (bet
**169**	NP_001274	4557471	adaptor-related protein complex 1, sigma 1 subunit isoform 1; clathrin-
**170**	NP_001275	4502861	adaptor-related protein complex 3, sigma 1 subunit; clathrin-associated
**171**	NP_001279	14251209	chloride intracellular channel 1; p64CLCP; chloride channel ABP
**172**	NP_001306	4503069	mitogen-activated protein kinase 14 isoform 1; cytokine suppressive ant
**173**	NP_001307	29029559	CSE1 chromosome segregation 1-like protein isoform a; cellular apoptos
**174**	NP_001312	4503101	cysteine and glycine-rich protein 2; LIM domain only 5, smooth muscle;
**175**	NP_001313	4503105	cystatin SA precursor; cystatin 2; cystatin S5; salivary cysteine (thio
**176**	NP_001329	4503173	coxsackie virus and adenovirus receptor; 46 kD coxsackievirus and adeno
**177**	NP_001335	4503253	defender against cell death 1
**178**	NP_001346	4503291	D-dopachrome tautomerase
**179**	NP_001350	4503301	2,4-dienoyl CoA reductase 1 precursor; 4-enoyl-CoA reductase
**180**	NP_001377	4503377	dihydropyrimidinase-like 2; collapsin response mediator protein hCRMP-2

	Refseq ID	gi Number	Protein Name/Description
**181**	NP_001388	4503443	endothelin converting enzyme 1
**182**	NP_001389	4503447	peroxisomal enoyl-coenzyme A hydratase-like protein; delta3,5-delta2,4-
**183**	NP_001395	4503481	eukaryotic translation elongation factor 1 gamma; elongation factor 1-g
**184**	NP_001399	13325064	cadherin EGF LAG seven-pass G-type receptor 2; EGF-like-domain, multip
**185**	NP_001406	4503507	eukaryotic translation initiation factor 2, subunit 3 gamma, 52kDa; euk
**186**	NP_001407	4503529	eukaryotic translation initiation factor 4A, isoform 1
**187**	NP_001409	4503539	eukaryotic translation initiation factor 4 gamma, 2; Eukaryotic transla
**188**	NP_001410	4503551	ELAV (embryonic lethal, abnormal vision, Drosophila)-like 1 (Hu antigen
**189**	NP_001419	4503571	enolase 1; phosphopyruvate hydratase; MYC promoter-binding protein 1; n
**190**	NP_001427	12056465	fibrillarin; 34-kD nucleolar scleroderma antigen; RNA, U3 small nucleo
**191**	NP_001435	4557581	fatty acid binding protein 5 (psoriasis-associated); E-FABP
**192**	NP_001440	21361122	four and a half LIM domains 1; Four-and-a-half LIM domains 1
**193**	NP_001447	4503745	filamin 1 (actin-binding protein-280); filamin 1; filamin A, alpha (act
**194**	NP_001448	4503747	filamin B, beta (actin binding protein 278); beta filamin; filamin 1 (a
**195**	NP_001449	4557597	gamma filamin; filamin 2; filamin C, gamma (actin-binding protein-280);
**196**	NP_001456	4503821	FYN binding protein (FYB-120/130); FYN-binding protein (FYB-120/130)
**197**	NP_001484	4503971	GDP dissociation inhibitor 1; mental retardation, X-linked 41; mental r
**198**	NP_001485	6598323	GDP dissociation inhibitor 2; rab GDP-dissociation inhibitor, beta
**199**	NP_001487	22035694	GDNF family receptor alpha 3 preproprotein; glial cell line-derived ne
**200**	NP_001512	4504205	general transcription factor IIIC, polypeptide 2, beta 110kDa; general
**201**	NP_001531	4504517	heat shock 27kDa protein 1; heat shock 27kD protein 1
**202**	NP_001596	4501841	alanyl-tRNA synthetase
**203**	NP_001600	4501859	acyl-Coenzyme A dehydrogenase, short/branched chain precursor
**204**	NP_001606	4501889	actin, gamma 2 propeptide; actin, alpha-3
**205**	NP_001616	4502013	adenylate kinase 2 isoform a; adenylate kinase, mitochondrial; adenylat
**206**	NP_001631	23510451	N-acylaminoacyl-peptide hydrolase; oxidized protein hydrolase; acylami
**207**	NP_001649	4502201	ADP-ribosylation factor 1
**208**	NP_001651	4502205	ADP-ribosylation factor 4
**209**	NP_001653	4502209	ADP-ribosylation factor 5
**210**	NP_001654	4502211	ADP-ribosylation factor 6
**211**	NP_001655	10835049	ras homolog gene family, member A; Aplysia ras-related homolog 12; Rho
**212**	NP_001656	4502219	ras homolog gene family, member G (rho G); RhoG
**213**	NP_001672	4502285	ATPase, Ca++ transporting, cardiac muscle, slow twitch 2 isoform 2; ATP
**214**	NP_001677	4502295	ATP synthase, H+ transporting, mitochondrial F1 complex, beta polypepti
**215**	NP_001678	4502297	ATP synthase, H+ transporting, mitochondrial F1 complex, delta subunit
**216**	NP_001679	21361565	ATP synthase, H+ transporting, mitochondrial F0 complex, subunit b, is
**217**	NP_001681	19913424	ATPase, H+ transporting, lysosomal 70kD, V1 subunit A, isoform 1; V-AT
**218**	NP_001683	19913426	ATPase, H+ transporting, lysosomal 56/58kD, V1 subunit B, isoform 1; A
**219**	NP_001688	4502303	mitochondrial ATP synthase, O subunit; ATP synthase, H+ transporting, m
**220**	NP_001700	25306267	brain-derived neurotrophic factor isoform a preproprotein
**221**	NP_001719	4502459	basigin; OK blood group; emmprin; collagenase stimulatory factor; M6 an
**222**	NP_001723	4502475	butyrophilin, subfamily 1, member A1
**223**	NP_001734	4502549	calmodulin 2 (phosphorylase kinase, delta); phosphorylase kinase delta
**224**	NP_001737	10716563	calnexin
**225**	NP_001740	4502565	calpain, small subunit 1; calpain, small polypeptide; calcium-dependent
**226**	NP_001743	4557014	catalase
**227**	NP_001748	4502599	carbonyl reductase 1; carbonyl reductase (NADPH); carbonyl reductase (N
**228**	NP_001753	4502643	chaperonin containing TCP1, subunit 6A (zeta 1); chaperonin containing
**229**	NP_001760	4502693	CD9 antigen; motility related protein; leukocyte antigen MIC3; antigen
**230**	NP_001779	4502695	cell division cycle 10; cell division cycle 10 (homolog to CDC10 of S.c
**231**	NP_001782	4757952	cell division cycle 42 isoform 1; cell division cycle 42 (GTP-binding p
**232**	NP_001785	14589893	cadherin 4, type 1 preproprotein; cadherin 4, R-cadherin (retinal); R-
**233**	NP_001805	4503141	cathepsin C isoform a preproprotein; dipeptidyl-peptidase I; dipeptidyl
**234**	NP_001822	4502905	clusterin; complement-associated protein SP-40
**235**	NP_001830	4502923	calponin 3; calponin, acidic
**236**	NP_001852	4502981	cytochrome c oxidase subunit IV isoform 1 precursor; cytochrome c oxida
**237**	NP_001853	17017988	cytochrome c oxidase subunit Vb precursor; cytochrome c oxidase polype
**238**	NP_001854	4502985	cytochrome c oxidase subunit VIb; human cytochrome oxidase subunit VIb
**239**	NP_001856	4502989	cytochrome c oxidase subunit VIIa polypeptide 2 (liver) precursor; hepa
**240**	NP_001858	4502993	cytochrome c oxidase subunit VIIc precursor; cytochrome-c oxidase chain

	Refseq ID	gi Number	Protein Name/Description
**241**	NP_001889	19882251	cystatin SN precursor; cystatin 1; cystatin SA-I; cysteine proteinase
**242**	NP_001890	4503109	cystatin S precursor; cystatin 4
**243**	NP_001893	21361334	cystathionase isoform 1; homoserine deaminase; homoserine dehydratase;
**244**	NP_001907	21359867	cytochrome c-1
**245**	NP_001914	13435359	damage-specific DNA binding protein 1; damage-specific DNA binding pro
**246**	NP_001924	19923748	dihydrolipoamide S-succinyltransferase (E2 component of 2-oxo-glutarat
**247**	NP_001939	4503423	dUTP pyrophosphatase
**248**	NP_001944	4503445	endothelial cell growth factor 1 (platelet-derived); thymidine phosphor
**249**	NP_001950	4503477	eukaryotic translation elongation factor 1 beta 2; eukaryotic translati
**250**	NP_001951	25453472	eukaryotic translation elongation factor 1 delta isoform 2; guanine nu
**251**	NP_001958	4503531	eukaryotic translation initiation factor 4A, isoform 2
**252**	NP_001961	4503545	eukaryotic translation initiation factor 5A; eIF5AI; eIF5A
**253**	NP_001966	5803011	enolase 2; enolase-2, gamma, neuronal; neurone-specific enolase; neuron
**254**	NP_001967	4503573	enolase 3; enolase-3, beta, muscle; muscle specific enolase; beta enola
**255**	NP_001976	4503609	electron-transfer-flavoprotein, beta polypeptide; electron-transferring
**256**	NP_002009	4503743	flightless I homolog
**257**	NP_002017	16933542	fibronectin 1 isoform 1 preproprotein; cold-insoluble globulin
**258**	NP_002028	4503823	protein-tyrosine kinase fyn isoform a; proto-oncogene tyrosine-protein
**259**	NP_002037	7669492	glyceraldehyde-3-phosphate dehydrogenase
**260**	NP_002038	6996010	glycyl-tRNA synthetase; GlyRS; glycine tRNA ligase
**261**	NP_002046	4503979	glial fibrillary acidic protein
**262**	NP_002061	4504041	guanine nucleotide binding protein (G protein), alpha inhibiting activi
**263**	NP_002063	4504045	guanine nucleotide binding protein (G protein), q polypeptide
**264**	NP_002064	4504051	guanine nucleotide binding protein, alpha z polypeptide; transducin alp
**265**	NP_002065	11321585	guanine nucleotide-binding protein, beta-1 subunit; G protein, beta-1
**266**	NP_002067	4504061	glucosamine (N-acetyl)-6-sulfatase precursor; N-acetylglucosamine-6-sul
**267**	NP_002069	6715600	golgi autoantigen, golgin subfamily a, 4; golgin 245; golgin-240; p230
**268**	NP_002076	4504107	glutathione peroxidase 4; phospholipid hydroperoxidase; sperm nucleus g
**269**	NP_002077	4504111	growth factor receptor-bound protein 2
**270**	NP_002100	6996014	histidyl-tRNA synthetase; histidine-tRNA ligase; HisRS; histidine trans
**271**	NP_002107	24797067	major histocompatibility complex, class I, A precursor; HLA-A1 class I
**272**	NP_002108	19557677	major histocompatibility complex, class I, C precursor; HLA class I hi
**273**	NP_002118	4504415	major histocompatibility complex, class I, G precursor; HLA-G histocomp
**274**	NP_002128	4504447	heterogeneous nuclear ribonucleoprotein A2/B1 isoform A2; heterogeneous
**275**	NP_002131	14165439	heterogeneous nuclear ribonucleoprotein K isoform a; dC-stretch bindin
**276**	NP_002134	19913446	hippocalcin; neuron specific calcium-binding protein hippocalcin
**277**	NP_002140	19913441	hippocalcin-like 1; visinin-like protein 3; calcium-binding protein BD
**278**	NP_002143	4504487	histidine-rich calcium-binding protein precursor; histidine-rich calciu
**279**	NP_002146	4504515	heat shock 70kDa protein 6 (HSP70B'); heat shock 70kD protein 6 (HSP70B
**280**	NP_002147	4504521	heat shock 60kDa protein 1 (chaperonin); mitochondrial matrix protein P
**281**	NP_002148	4504523	heat shock 10kDa protein 1 (chaperonin 10); heat shock 10kD protein 1 (
**282**	NP_002159	28178832	isocitrate dehydrogenase 2 (NADP+), mitochondrial precursor; isocitrat
**283**	NP_002178	24497438	interleukin 12B precursor; natural killer cell stimulatory factor-2; i
**284**	NP_002194	4504743	integrin alpha 2 precursor; Integrin, alpha-2 (CD49B; alpha-2 subunit o
**285**	NP_002196	4504751	integrin alpha 5 precursor; fibronectin receptor, alpha subunit; very l
**286**	NP_002202	19743813	integrin beta 1 isoform 1A precursor; integrin VLA-4 beta subunit; fib
**287**	NP_002214	4504793	inositol 1,4,5-triphosphate receptor, type 2
**288**	NP_002215	4504795	inositol 1,4,5-triphosphate receptor, type 3
**289**	NP_002256	19923142	karyopherin beta 1; nuclear factor p97; importin 90; importin beta-1 s
**290**	NP_002268	14917115	type I hair keratin 1; Ha-1; hard keratin, type I, 1; keratin, hair, a
**291**	NP_002272	4504931	keratin, hair, basic, 1; hard keratin, type II, 1
**292**	NP_002283	9845496	laminin, beta 2 precursor; laminin S
**293**	NP_002286	9845502	laminin receptor 1; Laminin receptor-1 (67kD); laminin receptor 1 (67kD
**294**	NP_002291	4557032	lactate dehydrogenase B
**295**	NP_002292	4504973	lactate dehydrogenase C
**296**	NP_002296	4504981	beta-galactosidase binding lectin precursor; Lectin, galactose-binding,
**297**	NP_002346	4505061	cation-dependent mannose-6-phosphate receptor precursor; Mr 46,000 Man6
**298**	NP_002387	4505145	malic enzyme 2, NAD(+)-dependent, mitochondrial; Malic enzyme, mitochon
**299**	NP_002406	4505185	macrophage migration inhibitory factor (glycosylation-inhibiting factor
**300**	NP_002427	4505237	palmitoylated membrane protein 1; membrane protein, palmitoylated 1 (55

	Refseq ID	gi Number	Protein Name/Description
**301**	NP_002435	4505257	moesin
**302**	NP_002442	4505273	5'-methylthioadenosine phosphorylase
**303**	NP_002443	21361346	nudix (nucleoside diphosphate linked moiety X)-type motif 1; mutT (E.
**304**	NP_002463	4505301	myosin, heavy polypeptide 8, skeletal muscle, perinatal
**305**	NP_002464	12667788	myosin, heavy polypeptide 9, non-muscle
**306**	NP_002465	13124879	smooth muscle myosin heavy chain 11 isoform SM1
**307**	NP_002466	4505303	myosin alkali light chain 1 slow a; myosin light chain 1, slow-twitch m
**308**	NP_002467	4557038	atrial/embryonic alkali myosin light chain; myosin, atrial/fetal muscle
**309**	NP_002480	4505357	NADH dehydrogenase (ubiquinone) 1 alpha subcomplex, 4, 9kDa; NADH dehyd
**310**	NP_002481	4505359	NADH dehydrogenase (ubiquinone) 1 alpha subcomplex, 6, 14kDa; NADH dehy
**311**	NP_002486	4505369	NADH dehydrogenase (ubiquinone) Fe-S protein 4, 18kDa (NADH-coenzyme Q
**312**	NP_002499	4505395	nidogen (enactin); Nidogen; nidogen (entactin)
**313**	NP_002503	4505409	non-metastatic cells 2, protein (NM23B) expressed in; Non-metastatic ce
**314**	NP_002511	10835063	nucleophosmin (nucleolar phosphoprotein B23, numatrin); Nucleophosmin
**315**	NP_002515	4505451	neuroblastoma RAS viral (v-ras) oncogene homolog; v-ras neuroblastoma R
**316**	NP_002558	4505621	prostatic binding protein; phosphatidylethanolamine binding protein
**317**	NP_002563	4505585	platelet-activating factor acetylhydrolase, isoform Ib, beta subunit 30
**318**	NP_002565	4505591	peroxiredoxin 1; natural killer-enhancing factor A; proliferation-assoc
**319**	NP_002568	4505599	p21-activated kinase 2; novel serine kinase; hPAK65
**320**	NP_002578	27754771	protocadherin 1 isoform 1 precursor; protocadherin 42; cadherin-like p
**321**	NP_002583	4505641	proliferating cell nuclear antigen
**322**	NP_002592	4505671	phosphodiesterase 6D, cGMP-specific, rod, delta
**323**	NP_002604	4505695	3-phosphoinositide dependent protein kinase-1; PkB kinase
**324**	NP_002610	4505733	platelet factor 4 (chemokine (C-X-C motif) ligand 4); platelet factor 4
**325**	NP_002611	4505735	platelet factor 4 variant 1; Platelet factor 4, variant 1 (PF4-like)
**326**	NP_002613	21536449	prefoldin 1; prefoldin subunit 1
**327**	NP_002614	12408677	prefoldin 4
**328**	NP_002617	21361070	phosphofructokinase, liver; Phosphofructokinase, liver type; human liv
**329**	NP_002618	11321601	phosphofructokinase, platelet; Phosphofructokinase, platelet type
**330**	NP_002620	4505753	phosphoglycerate mutase 1 (brain); Phosphoglycerate mutase A, nonmuscle
**331**	NP_002622	4505759	phosphogluconate dehydrogenase; 6-phosphogluconate dehydrogenase
**332**	NP_002624	21361621	phosphoglucomutase 1
**333**	NP_002625	4505773	prohibitin
**334**	NP_002626	4505775	phosphate carrier precursor isoform 1b; phosphate carrier, mitochondria
**335**	NP_002643	4505821	prolactin-induced protein; prolactin-inducible protein
**336**	NP_002645	4505839	pyruvate kinase, muscle; Pyruvate kinase-3; Thyroid hormone-binding pro
**337**	NP_002655	4505879	pleckstrin; p47
**338**	NP_002666	4505903	promyelocytic leukemia protein isoform 6; promyelocytic leukemia, induc
**339**	NP_002679	9945439	peanut-like 1; septin HCDCREL-1
**340**	NP_002695	4505981	pro-platelet basic protein (includes platelet basic protein, bet; Pro-p
**341**	NP_002700	4506005	protein phosphatase 1, catalytic subunit, beta isoform
**342**	NP_002717	20149545	prolyl endopeptidase
**343**	NP_002718	4506045	proteoglycan 1, secretory granule; Proteoglycan 1, secretory granule (p
**344**	NP_002722	4506057	protein kinase, cAMP-dependent, catalytic, beta
**345**	NP_002725	4506063	protein kinase, cAMP-dependent, regulatory, type I, alpha; tissue-speci
**346**	NP_002727	4506065	protein kinase, cAMP-dependent, regulatory, type II, beta
**347**	NP_002729	20127450	protein kinase C, beta 1; Protein kinase C, beta 1 polypeptide
**348**	NP_002734	4506077	protein kinase C substrate 80K-H; glucosidase II, beta subunit; AGE-bin
**349**	NP_002760	4506145	protease, serine, 1 preproprotein; cationic trypsinogen; trypsinogen A;
**350**	NP_002762	21536452	mesotrypsin preproprotein; trypsin 4, brain; protease, serine, 4; meso
**351**	NP_002778	4506181	proteasome alpha 2 subunit; proteasome subunit HC3; proteasome componen
**352**	NP_002779	4506183	proteasome alpha 3 subunit isoform 1; proteasome subunit C8; macropain
**353**	NP_002780	4506185	proteasome alpha 4 subunit; proteasome component C9; proteasome subunit
**354**	NP_002781	23110942	proteasome alpha 5 subunit; proteasome component 5; macropain subunit
**355**	NP_002782	23110944	proteasome alpha 6 subunit; prosomal P27K protein; proteasome subunit
**356**	NP_002783	4506189	proteasome alpha 7 subunit isoform 1; proteasome subunit RC6-1; proteas
**357**	NP_002784	4506193	proteasome beta 1 subunit; proteasome subunit HC5; proteasome component
**358**	NP_002785	4506195	proteasome beta 2 subunit; proteasome subunit, beta type, 2; macropain
**359**	NP_002786	22538465	proteasome beta 3 subunit; proteasome theta chain; proteasome chain 13
**360**	NP_002787	22538467	proteasome beta 4 subunit; proteasome subunit, beta type, 4; proteasom

	Refseq ID	gi Number	Protein Name/Description
**361**	NP_002789	23110925	proteasome beta 6 subunit; proteasome subunit Y; proteasome subunit be
**362**	NP_002791	4506205	proteasome beta 9 subunit isoform 1 proprotein; proteasome subunit, bet
**363**	NP_002792	4506191	proteasome beta 10 subunit proprotein; proteasome subunit MECL1; protea
**364**	NP_002793	24430151	proteasome 26S ATPase subunit 1; proteasome 26S subunit, ATPase, 1; 26
**365**	NP_002796	24497435	proteasome 26S ATPase subunit 5; thyroid receptor interactor 1; protea
**366**	NP_002798	25777600	proteasome 26S non-ATPase subunit 1; 26S proteasome non-ATPase regulat
**367**	NP_002808	28872728	proteasome 26S non-ATPase subunit 13 isoform 1; 26S proteasome subunit
**368**	NP_002809	4506237	proteasome (prosome, macropain) activator subunit 2 (PA28 beta); Protea
**369**	NP_002810	4506243	polypyrimidine tract-binding protein 1 isoform a; RNA-binding protein;
**370**	NP_002822	18104989	protein tyrosine phosphatase, non-receptor type 6 isoform 1; protein-t
**371**	NP_002826	18375652	protein tyrosine phosphatase, non-receptor type 12; protein-tyrosine p
**372**	NP_002831	4506311	protein tyrosine phosphatase, receptor type, F isoform 1 precursor; pro
**373**	NP_002834	18860900	protein tyrosine phosphatase, receptor type, J precursor; protein tyro
**374**	NP_002853	21361370	phosphorylase, glycogen; brain
**375**	NP_002854	4506353	phosphorylase, glycogen; liver (Hers disease, glycogen storage disease
**376**	NP_002856	4506365	RAB2, member RAS oncogene family
**377**	NP_002858	19923750	RAB3B, member RAS oncogene family; Brain antigen RAB3B
**378**	NP_002860	19923231	RAB6A, member RAS oncogene family; Oncogene RAB6; RAB6, member RAS onc
**379**	NP_002861	4506363	RAB13, member RAS oncogene family; RAS-associated protein RAB13
**380**	NP_002863	4506381	ras-related C3 botulinum toxin substrate 2; Ras-related C3 botulinum to
**381**	NP_002872	4506405	v-ral simian leukemia viral oncogene homolog B; RAS-like protein B; GTP
**382**	NP_002873	4506407	RAN binding protein 1
**383**	NP_002875	4506413	RAP1A, member of RAS oncogene family; RAS-related protein RAP1A
**384**	NP_002877	4506417	RAP2B, member of RAS oncogene family
**385**	NP_002878	15149476	arginyl-tRNA synthetase
**386**	NP_002880	4506427	retinoic acid receptor responder (tazarotene induced) 2
**387**	NP_002897	4506467	radixin
**388**	NP_002916	4506505	regulator of G-protein signaling 10
**389**	NP_002930	21361547	ribonuclease/angiogenin inhibitor; Placental ribonuclease inhibitor
**390**	NP_002932	4506569	roundabout 1 isoform a; roundabout 1; axon guidance receptor
**391**	NP_002941	4506675	ribophorin I
**392**	NP_002942	4506677	ribophorin II
**393**	NP_002945	4506713	ubiquitin and ribosomal protein S27a precursor; ubiquitin carboxyl exte
**394**	NP_002952	4506765	S100 calcium-binding protein A4; 18A2; 42A; S100 calcium-binding protei
**395**	NP_002954	4506769	S100 calcium-binding protein A7; psoriasin 1
**396**	NP_002955	21614544	S100 calcium-binding protein A8; cystic fibrosis antigen; calgranulin
**397**	NP_002956	4506773	S100 calcium-binding protein A9; calgranulin B
**398**	NP_002970	19923233	sterol carrier protein 2
**399**	NP_002976	22538814	small inducible cytokine A5 precursor; T-cell specific protein p288; T
**400**	NP_002996	4506877	selectin P precursor; Selectin P (granulocyte membrane protein, 140kD;
**401**	NP_003013	4506925	SH3 domain binding glutamic acid-rich protein like; SH3-binding domain
**402**	NP_003043	4506985	solute carrier family 34 (sodium phosphate), member 1; solute carrier f
**403**	NP_003096	4507157	sortilin-related receptor containing LDLR class A repeats preproprotein
**404**	NP_003109	4507171	secreted protein, acidic, cysteine-rich (osteonectin); Osteonectin (sec
**405**	NP_003118	4507191	spectrin, alpha, non-erythrocytic 1 (alpha-fodrin)
**406**	NP_003119	4507195	spectrin, beta, non-erythrocytic 1; Spectrin, beta, nonerythrocytic-1 (
**407**	NP_003121	4507207	sorcin; Sorcin (class 4 gene)
**408**	NP_003134	4507231	single-stranded DNA binding protein; single-stranded DNA-binding protei
**409**	NP_003141	21618338	signal transducer and activator of transcription 3 isoform 2; acute-ph
**410**	NP_003143	21618342	signal transducer and activator of transcription 5A; signal transducer
**411**	NP_003147	21070997	stromal interaction molecule 1 precursor
**412**	NP_003157	10835035	sulfotransferase family, cytosolic, 1A, phenol-preferring, member 3; t
**413**	NP_003168	21361553	spleen tyrosine kinase
**414**	NP_003182	4507367	threonyl-tRNA synthetase; threonine--tRNA ligase
**415**	NP_003237	4507485	thrombospondin 1
**416**	NP_003238	4507487	thrombospondin 2
**417**	NP_003245	4507509	tissue inhibitor of metalloproteinase 1 precursor; Erythroid-potentiati
**418**	NP_003268	4502879	transmembrane protein claudin 5; androgen withdrawal and apoptosis indu
**419**	NP_003279	4507643	tumor protein D52-like 2; hD54
**420**	NP_003280	4507649	tropomyosin 2 (beta)

	Refseq ID	gi Number	Protein Name/Description
**421**	NP_003281	4507651	tropomyosin 4
**422**	NP_003282	4507657	tripeptidyl peptidase II
**423**	NP_003286	4507669	tumor protein, translationally-controlled 1; fortilin; histamine-releas
**424**	NP_003290	4507677	tumor rejection antigen (gp96) 1; Tumor rejection antigen-1 (gp96); glu
**425**	NP_003303	17402865	thiosulfate sulfurtransferase; rhodanese
**426**	NP_003312	21359837	Tu translation elongation factor, mitochondrial
**427**	NP_003320	4507745	thioredoxin
**428**	NP_003321	4507747	thioredoxin reductase 1
**429**	NP_003325	23510338	ubiquitin-activating enzyme E1; A1S9T and BN75 temperature sensitivity
**430**	NP_003326	4507767	ubiquitin-activating enzyme E1-like; Ubiquitin-activating enzyme-2; ubi
**431**	NP_003331	4507777	ubiquitin-conjugating enzyme E2D 3 (UBC4/5 homolog, yeast); UbcH5C; ubi
**432**	NP_003335	4507783	ubiquitin-conjugating enzyme E2H (UBC8 homolog, yeast); ubiquitin-conju
**433**	NP_003338	4507789	ubiquitin-conjugating enzyme E2L 3
**434**	NP_003339	4507793	ubiquitin-conjugating enzyme E2N (UBC13 homolog, yeast); ubiquitin-conj
**435**	NP_003340	4507795	ubiquitin-conjugating enzyme E2 variant 1 isoform b; DNA-binding protei
**436**	NP_003341	4507797	ubiquitin-conjugating enzyme E2 variant 2; 1 alpha,25-dihydroxyvitamin
**437**	NP_003356	4507841	ubiquinol-cytochrome c reductase core protein I
**438**	NP_003361	4507869	vasodilator-stimulated phosphoprotein
**439**	NP_003364	4507877	vinculin isoform VCL
**440**	NP_003365	4507879	voltage-dependent anion channel 1
**441**	NP_003370	21614499	villin 2; Villin-2; cytovillin
**442**	NP_003384	4507931	wingless-type MMTV integration site family, member 8B precursor
**443**	NP_003394	4507955	YY1 transcription factor
**444**	NP_003395	4507949	tyrosine 3-monooxygenase/tryptophan 5-monooxygenase activation protein,
**445**	NP_003396	4507951	tyrosine 3/tryptophan 5 -monooxygenase activation protein, eta polypept
**446**	NP_003397	4507953	tyrosine 3/tryptophan 5 -monooxygenase activation protein, zeta polypep
**447**	NP_003452	4508047	zyxin
**448**	NP_003460	4506801	secretogranin II precursor; Chromogranin C (secretogranin II); secreton
**449**	NP_003464	4507249	signal transducing adaptor molecule 1; signal transducing adaptor molec
**450**	NP_003470	4506285	protein tyrosine phosphatase type IVA, member 2 isoform 1; protein tyro
**451**	NP_003472	4507855	Ubiquitin isopeptidase T; Ubiquitin-specific protease-5 (ubiquitin isop
**452**	NP_003473	4505197	myeloid/lymphoid or mixed-lineage leukemia 2; ALL1-related gene
**453**	NP_003555	4507357	transgelin 2; SM22-alpha homolog
**454**	NP_003560	4507295	syntaxin 7
**455**	NP_003565	20070156	vessicle-associated membrane protein (VAMP)-associated protein of 33 k
**456**	NP_003567	20070158	serine/threonine kinase 24 (STE20 homolog, yeast); STE20-like kinase 3
**457**	NP_003602	4503179	oral-facial-digital syndrome 1; chromosome X open reading frame 5; oral
**458**	NP_003647	4502553	calcium/calmodulin-dependent protein kinase I
**459**	NP_003650	4501993	alkylglycerone phosphate synthase precursor
**460**	NP_003669	19923178	chromosome 22 open reading frame 19; gene from NF2/meningioma region o
**461**	NP_003671	4507947	tyrosyl-tRNA synthetase; tyrosyl-tRNA synthetase; tyrosyl tRNA ligase
**462**	NP_003680	4502021	aldo-keto reductase family 7, member A2 (aflatoxin aldehyde reductase);
**463**	NP_003734	22538455	nuclear receptor coactivator 1 isoform 1
**464**	NP_003737	4505813	cytoplasmic dynein light polypeptide; dynein, cytoplasmic, light chain
**465**	NP_003752	14043026	vesicle-associated membrane protein 8; endobrevin
**466**	NP_003755	4507287	syntaxin 11
**467**	NP_003759	4505705	phosphoprotein enriched in astrocytes 15; Phosphoprotein enriched in as
**468**	NP_003765	4503901	polypeptide N-acetylgalactosaminyltransferase 4; UDP-N-acetyl-alpha-D-g
**469**	NP_003786	4507143	sorting nexin 3 isoform a; sorting nexin 3A
**470**	NP_003816	18765729	synaptosomal-associated protein 23 isoform SNAP23A; synaptosomal-assoc
**471**	NP_003818	4505329	N-ethylmaleimide-sensitive factor attachment protein, alpha; alpha-SNAP
**472**	NP_003840	11321581	succinate-CoA ligase, GDP-forming, alpha subunit
**473**	NP_003851	4502389	barrier to autointegration factor 1; breakpoint cluster region protein,
**474**	NP_003865	4502687	CD84 antigen (leukocyte antigen); leukocyte antigen CD84
**475**	NP_003869	4503987	gamma-glutamyl hydrolase precursor; conjugase; folylpolygammaglutamyl h
**476**	NP_003878	4502249	development- and differentiation-enhancing factor 2; PYK2 C terminus-as
**477**	NP_003906	4503013	copine I
**478**	NP_003907	22027655	adaptor-related protein complex 1 sigma 2 subunit; clathrin adaptor co
**479**	NP_003921	16753212	src family associated phosphoprotein 2; src kinase-associated phosphop
**480**	NP_003922	4507913	WAS protein family, member 1; WASP family Verprolin-homologous protein;

	Refseq ID	gi Number	Protein Name/Description
**481**	NP_003928	4504937	kynureninase (L-kynurenine hydrolase); l-kynurenine hydrolase
**482**	NP_003965	4503359	docking protein 2, 56kD
**483**	NP_004026	4757712	acyl-Coenzyme A oxidase isoform a; acyl-coenzyme A oxidase 1
**484**	NP_004028	21264318	adenosine monophosphate deaminase 2 (isoform L)
**485**	NP_004030	4757756	annexin A2; annexin II; annexin II (lipocortin II); calpactin I, heavy
**486**	NP_004032	10880136	arrestin beta 1 isoform A
**487**	NP_004035	20127454	5-aminoimidazole-4-carboxamide ribonucleotide formyltransferase/IMP cy
**488**	NP_004037	4757810	ATP synthase, H+ transporting, mitochondrial F1 complex, alpha subunit,
**489**	NP_004039	4757826	beta-2-microglobulin
**490**	NP_004060	4757996	adaptor-related protein complex 2, sigma 1 subunit isoform AP17; clathr
**491**	NP_004068	4758076	citrate synthase precursor; Citrate synthase, mitochondrial
**492**	NP_004069	4758086	cysteine and glycine-rich protein 1; cysteine-rich protein; LIM-domain
**493**	NP_004073	13259510	dynactin 1 isoform 1; dynactin 1 (p150, Glued (Drosophila) homolog); p
**494**	NP_004081	4758208	dual specificity phosphatase 3; vaccinia virus phosphatase VH1-related;
**495**	NP_004083	12707570	mitochondrial short-chain enoyl-coenzyme A hydratase 1 precursor
**496**	NP_004090	21361755	stomatin; erythrocyte membrane protein band 7.2 (stomatin)
**497**	NP_004095	21618359	fatty acid synthase
**498**	NP_004108	4758384	FK506 binding protein 5; FK506-binding protein 5; 51 kDa FK506-binding
**499**	NP_004115	4758442	glia maturation factor, beta
**500**	NP_004125	24234688	heat shock 70kDa protein 9B precursor; heat shock 70kD protein 9; stre
**501**	NP_004136	4758750	myosin IXB
**502**	NP_004146	4758906	serine (or cysteine) proteinase inhibitor, clade B (ovalbumin), member
**503**	NP_004150	4758970	proteasome beta 8 subunit isoform E1 proprotein; proteasome subunit, be
**504**	NP_004152	4758988	RAB1A, member RAS oncogene family; RAB1, member RAS oncogene family
**505**	NP_004153	19923262	RAB5A, member RAS oncogene family; RAS-associated protein RAB5A
**506**	NP_004154	5729997	RAB27B, member RAS oncogene family
**507**	NP_004159	4759080	succinate dehydrogenase complex, subunit A, flavoprotein precursor; suc
**508**	NP_004160	22547186	serine hydroxymethyltransferase 1 (soluble) isoform 1; cytoplasmic ser
**509**	NP_004175	4759316	tryptophanyl-tRNA synthetase; interferon-induced protein 53
**510**	NP_004199	4757732	programmed cell death 8 isoform 1; apoptosis-inducing factor
**511**	NP_004209	4758986	RAB11B, member RAS oncogene family; RAB11B, member of RAS oncogene fami
**512**	NP_004243	4759140	solute carrier family 9 (sodium/hydrogen exchanger), isoform 3 regulato
**513**	NP_004246	4758038	cytochrome c oxidase subunit Va precursor; cytochrome c oxidase polypep
**514**	NP_004252	4759096	15 kDa selenoprotein
**515**	NP_004281	4757762	ring finger protein 14; androgen receptor associated protein 54
**516**	NP_004291	4757714	acid phosphatase 1 isoform c; acid phosphatase of erythrocyte; red cell
**517**	NP_004299	4757766	Rho GTPase activating protein 1; RhoGAP; p50rhoGAP; CDC42 GTPase-activa
**518**	NP_004300	4757768	Rho GDP dissociation inhibitor (GDI) alpha
**519**	NP_004303	4757776	arrestin 3, retinal (X-arrestin); Arrestin 3, retinal
**520**	NP_004306	4757786	N-acylsphingosine amidohydrolase (acid ceramidase) 1; N-acylsphingosine
**521**	NP_004321	4757856	BCL2/adenovirus E1B 19kD interacting protein 2; BCL2/adenovirus E1B 19k
**522**	NP_004330	4757886	pituitary tumor-transforming protein 1-interacting protein precursor; c
**523**	NP_004333	4826657	caldesmon 1 isoform 3; H-CAD; L-CAD
**524**	NP_004334	4757900	calreticulin precursor; Sicca syndrome antigen A (autoantigen Ro; calre
**525**	NP_004359	4758018	calponin 2; Calonin 2
**526**	NP_004362	4758030	coatomer protein complex, subunit alpha; alpha coat protein; xenin
**527**	NP_004365	4758040	cytochrome c oxidase subunit VIc proprotein; cytochrome c oxidase subun
**528**	NP_004374	4758078	c-src tyrosine kinase
**529**	NP_004380	7656999	catenin (cadherin-associated protein), alpha 2; Catenin, alpha-2 (cadhe
**530**	NP_004386	18426915	drebrin 1 isoform a; drebrin E; drebrin-1; drebrin E2
**531**	NP_004395	4758158	neural precursor cell expressed, developmentally down-regulated 5
**532**	NP_004399	4758182	dynamin 1; dynamin; dynamin1; Dynamin-1
**533**	NP_004448	4758330	long-chain fatty-acid-Coenzyme A ligase 3; lignoceroyl-CoA synthase
**534**	NP_004451	16933540	fibroblast activation protein, alpha subunit; integral membrane serine
**535**	NP_004461	17149842	FK506-binding protein 2 precursor; FK506-binding protein 2 (13kD); pep
**536**	NP_004479	4758460	glycoprotein V (platelet)
**537**	NP_004482	25121940	glucocorticoid receptor DNA binding factor 1 isoform b
**538**	NP_004484	4758504	hydroxyacyl-Coenzyme A dehydrogenase, type II
**539**	NP_004508	4758606	integrin-linked kinase
**540**	NP_004511	4758644	kinesin heavy chain member 2; Kinesin, heavy chain, 2

	Refseq ID	gi Number	Protein Name/Description
**541**	NP_004512	4758648	kinesin family member 5B; kinesin 1 (110-120kD)
**542**	NP_004519	4758714	microsomal glutathione S-transferase 3; microsomal glutathione S-transf
**543**	NP_004528	4758756	nucleosome assembly protein 1-like 1; HSP22-like protein interacting pr
**544**	NP_004538	6041669	NADH dehydrogenase (ubiquinone) 1 beta subcomplex, 4, 15kDa; NADH dehyd
**545**	NP_004540	4758784	NADH dehydrogenase (ubiquinone) 1, subcomplex unknown, 2, 14.5kDa; NADH
**546**	NP_004542	4758788	NADH dehydrogenase (ubiquinone) Fe-S protein 3, 30kDa (NADH-coenzyme Q
**547**	NP_004543	4758790	NADH dehydrogenase (ubiquinone) Fe-S protein 5, 15kDa (NADH-coenzyme Q
**548**	NP_004544	4758792	NADH dehydrogenase (ubiquinone) Fe-S protein 6, 13kDa (NADH-coenzyme Q
**549**	NP_004559	28077085	serine (or cysteine) proteinase inhibitor, clade B (ovalbumin), member
**550**	NP_004570	22035600	mitogen-activated protein kinase kinase kinase kinase 2; Rab8 interact
**551**	NP_004571	19923264	RAB27A, member RAS oncogene family
**552**	NP_004598	4759212	beta-tubulin cofactor A
**553**	NP_004613	4759270	translin; recombination hotspot associated factor; recombination hotspo
**554**	NP_004640	5031691	chromosome 21 open reading frame 33; human HES1 protein, homolog to E.c
**555**	NP_004648	4759082	serum deprivation response protein; serum deprivation response; phospha
**556**	NP_004654	4758984	RAB11A, member RAS oncogene family; RAB 11A, member oncogene family
**557**	NP_004684	4758618	cytokeratin type II
**558**	NP_004691	26638653	potassium voltage-gated channel KQT-like protein 4 isoform a; potassiu
**559**	NP_004709	18105037	cytochrome c oxidase subunit VIIa polypeptide 2 like; estrogen recepto
**560**	NP_004725	4758128	doublecortin and CaM kinase-like 1; doublecortin-like kinase
**561**	NP_004729	4759302	VAMP (vesicle-associated membrane protein)-associated protein B and C;
**562**	NP_004733	9257195	BAI1-associated protein 1; WW domain-containing protein 3; atrophin-1 i
**563**	NP_004748	4758266	small inducible cytokine subfamily E, member 1; endothelial monocyte-ac
**564**	NP_004757	4758032	coatomer protein complex, subunit beta 2 (beta prime); coatomer binding
**565**	NP_004772	4759300	vesicle-associated membrane protein 3 (cellubrevin); vesicle-associated
**566**	NP_004777	4759274	thioredoxin-like, 32kDa; thioredoxin-related 32 kDa protein; thioredoxi
**567**	NP_004778	4759146	slit homolog 2
**568**	NP_004800	20149563	stomatin (EPB72)-like 1; stomatin-like 1; stomatin (EBP72)-like 1
**569**	NP_004801	4758476	GRB2-related adaptor protein 2; growth factor receptor-bound protein 2-
**570**	NP_004808	4759342	tight junction protein 2 (zona occludens 2); Friedreich ataxia region g
**571**	NP_004823	4758484	glutathione-S-transferase like; glutathione transferase omega
**572**	NP_004841	4759044	Rho-associated, coiled-coil containing protein kinase 2
**573**	NP_004850	4758012	clathrin heavy chain; clathrin, heavy polypeptide-like 2
**574**	NP_004868	4758440	glia maturation factor, gamma
**575**	NP_004879	4757818	ATPase, H+ transporting, lysosomal, V1 subunit G isoform 1; vacuolar H(
**576**	NP_004880	4757812	ATP synthase, H+ transporting, mitochondrial F0 complex, subunit f, iso
**577**	NP_004883	4759086	vesicle trafficking protein sec22b
**578**	NP_004896	4758638	peroxiredoxin 6; antioxidant protein 2; non-selenium glutathione peroxi
**579**	NP_004902	4758304	protein disulfide isomerase related protein (calcium-binding protein, i
**580**	NP_004915	12025678	actinin, alpha 4
**581**	NP_004921	4826659	F-actin capping protein beta subunit; Cap Z
**582**	NP_004964	11863152	jumonji; jumonji (mouse) homolog
**583**	NP_004976	15718761	c-K-ras2 protein isoform b; Kirsten rat sarcoma-2 viral (v-Ki-ras2) on
**584**	NP_004978	13518026	LIM and senescent cell antigen-like domains 1
**585**	NP_004991	4826848	NADH dehydrogenase (ubiquinone) 1 alpha subcomplex, 5; NADH dehydrogena
**586**	NP_004993	6681764	NADH dehydrogenase (ubiquinone) 1 alpha subcomplex, 9, 39kDa; NADH dehy
**587**	NP_005001	4826864	neuronal cell adhesion molecule; Bravo
**588**	NP_005007	14141168	poly(rC)-binding protein 2 isoform a; poly(rC)-binding protein 2; alph
**589**	NP_005013	4826898	profilin 1; profilin-1
**590**	NP_005019	6857820	phosphatidylinositol-4-phosphate 5-kinase type II alpha; 1-phosphatidyl
**591**	NP_005092	4826774	interferon, alpha-inducible protein (clone IFI-15K); interferon-stimula
**592**	NP_005103	4827056	WD repeat-containing protein 1 isoform 2
**593**	NP_005106	19913412	major vault protein
**594**	NP_005116	4826665	copper chaperone for superoxide dismutase
**595**	NP_005130	4826643	annexin A3; Annexin III (lipocortin III); annexin III (lipocortin III,
**596**	NP_005132	11761631	fibrinogen, beta chain preproprotein
**597**	NP_005134	4826762	haptoglobin
**598**	NP_005142	4827050	ubiquitin specific protease 14
**599**	NP_005152	4885057	angiotensin II receptor-like 1; angiotensin receptor-like 1
**600**	NP_005156	4885063	aldolase C, fructose-bisphosphate; Aldolase C, fructose-bisphosphatase

	Refseq ID	gi Number	Protein Name/Description
**601**	NP_005164	28373103	sarco/endoplasmic reticulum Ca2+ -ATPase isoform a; ATPase, Ca(2+)-tra
**602**	NP_005165	4885079	ATP synthase, H+ transporting, mitochondrial F1 complex, gamma polypept
**603**	NP_005177	12408656	calpain 1, large subunit; calpain, large polypeptide L1; calcium-activ
**604**	NP_005198	4885153	v-crk sarcoma virus CT10 oncogene homolog (avian)-like; v-crk avian sar
**605**	NP_005204	4885165	cystatin A; stefin A; cystatin AS
**606**	NP_005210	4885183	diaphanous 1; deafness, autosomal dominant 1; hDia1
**607**	NP_005222	20357552	cortactin isoform a; oncogene EMS1
**608**	NP_005239	4885235	Gardner-Rasheed feline sarcoma viral (v-fgr) oncogene homolog; Gardner-
**609**	NP_005252	4885263	GTP binding protein overexpressed in skeletal muscle; GTP-binding prote
**610**	NP_005262	4885281	glutamate dehydrogenase 1
**611**	NP_005263	20330805	guanine nucleotide binding protein, alpha transducing activity polypep
**612**	NP_005264	20357529	guanine nucleotide-binding protein, beta-2 subunit; G protein, beta-2
**613**	NP_005265	4885287	guanine nucleotide binding protein (G protein), gamma 5
**614**	NP_005304	21361657	glucose regulated protein, 58kDa; glucose regulated protein, 58kD
**615**	NP_005318	4885387	L-3-hydroxyacyl-Coenzyme A dehydrogenase, short chain; L-3-hydroxyacyl-
**616**	NP_005326	4885405	hematopoietic cell-specific Lyn substrate 1
**617**	NP_005330	4885417	huntingtin interacting protein 2; ubiquitin-conjugating enzyme E2-25 KD
**618**	NP_005331	4885413	histidine triad nucleotide binding protein 1; Histidine triad nucleotid
**619**	NP_005334	4885425	v-Ha-ras Harvey rat sarcoma viral oncogene homolog; H-RasIDX
**620**	NP_005336	5123454	heat shock 70kDa protein 1A; heat shock 70kD protein 1A; heat shock-ind
**621**	NP_005338	16507237	heat shock 70kDa protein 5 (glucose-regulated protein, 78kDa); BiP; he
**622**	NP_005339	13129150	heat shock 90kDa protein 1, alpha; heat shock 90kD protein 1, alpha
**623**	NP_005347	20428652	lymphocyte-specific protein tyrosine kinase; oncogene LCK; membrane as
**624**	NP_005361	16933567	mel transforming oncogene; ras-associated protein RAB8
**625**	NP_005380	4885539	protein-L-isoaspartate (D-aspartate) O-methyltransferase
**626**	NP_005393	4885569	v-ral simian leukemia viral oncogene homolog A (ras related); RAS-like
**627**	NP_005397	4885583	Rho-associated, coiled-coil containing protein kinase 1; p160ROCK; p160
**628**	NP_005408	4885609	v-src sarcoma (Schmidt-Ruppin A-2) viral oncogene homolog; Protooncogen
**629**	NP_005427	4885173	DNA segment on chromosome 10 (unique) 170; DNA segment, single copy, pr
**630**	NP_005442	11496885	enigma protein; LIM domain protein
**631**	NP_005454	4885423	heterogeneous nuclear ribonucleoprotein D-like; A+U-rich element RNA bi
**632**	NP_005498	5031635	cofilin 1 (non-muscle)
**633**	NP_005505	17986001	major histocompatibility complex, class I, B; HLA class I histocompati
**634**	NP_005518	27436929	heat shock 70kDa protein 1-like; heat shock 70kD protein-like 1
**635**	NP_005545	5031839	keratin 6A; Keratin-6A; keratin, epidermal type II, K6A; cytokeratin 6A
**636**	NP_005548	24430192	keratin 16; keratin, type I cytoskeletal 16; cytokeratin 16
**637**	NP_005552	7669501	lysosomal-associated membrane protein 1
**638**	NP_005556	5031855	lymphocyte cytosolic protein 2; SH2 domain-containing leukocyte protein
**639**	NP_005557	5031857	lactate dehydrogenase A
**640**	NP_005585	5031931	nascent-polypeptide-associated complex alpha polypeptide
**641**	NP_005591	5031947	nitrilase 1
**642**	NP_005596	21361290	protein phosphatase 3 (formerly 2B), catalytic subunit, gamma isoform
**643**	NP_005597	21914881	legumain; protease, cysteine, 1 (legumain)
**644**	NP_005600	5032009	glycogen phosphorylase; phosphorylase, glycogen, muscle; myophosphoryla
**645**	NP_005605	5032041	Ras homolog enriched in brain 2
**646**	NP_005629	5032137	synaptobrevin-like 1
**647**	NP_005639	5032161	elongin C; transcription elongation factor B (SIII), polypeptide 1 (15k
**648**	NP_005653	25188179	voltage-dependent anion channel 3
**649**	NP_005681	5031677	dynamin 1-like protein isoform 3; dynamin-like protein
**650**	NP_005688	5730031	secretory carrier membrane protein 2
**651**	NP_005691	18491024	dipeptidylpeptidase III isoform 1; dipeptidylpeptidase III
**652**	NP_005701	5031957	polyglutamine binding protein 1; nuclear protein containing WW domain 3
**653**	NP_005708	5031593	actin related protein 2/3 complex subunit 5; Arp2/3 protein complex sub
**654**	NP_005709	5031595	actin related protein 2/3 complex subunit 4; Arp2/3 protein complex sub
**655**	NP_005710	5031597	actin related protein 2/3 complex subunit 3; ARP2/3 protein complex sub
**656**	NP_005711	5031601	actin related protein 2/3 complex subunit 1B; ARP2/3 protein complex su
**657**	NP_005712	5031573	ARP3 actin-related protein 3 homolog; ARP3 (actin-related protein 3, ye
**658**	NP_005713	5031571	actin-related protein 2; ARP2 (actin-related protein 2, yeast) homolog
**659**	NP_005720	5031987	peptidylprolyl isomerase F (cyclophilin F)
**660**	NP_005722	5031599	actin related protein 2/3 complex subunit 2; ARP2/3 protein complex sub

	Refseq ID	gi Number	Protein Name/Description
**661**	NP_005727	5031569	ARP1 actin-related protein 1 homolog A, centractin alpha; ARP1 (actin-r
**662**	NP_005733	5031973	protein disulfide isomerase-related protein
**663**	NP_005742	22538387	A kinase anchor protein 9 isoform 2; yotiao; A-kinase anchoring protei
**664**	NP_005787	5031985	nuclear transport factor 2; placental protein 15
**665**	NP_005800	5902726	peroxiredoxin 2; thioredoxin-dependent peroxide reductase 1 (thiol-spec
**666**	NP_005808	20127486	cargo selection protein (mannose 6 phosphate receptor binding pr
**667**	NP_005816	20149570	RAS guanyl releasing protein 2 isoform 1; calcium and diacylglycerol-r
**668**	NP_005822	5031937	nuclear domain 10 protein
**669**	NP_005850	5032007	purine-rich element binding protein A; purine-rich single-stranded DNA-
**670**	NP_005859	5031611	BET1 homolog; Golgi vesicular membrane trafficking protein p18; Bet1p h
**671**	NP_005882	5174389	acetyl-Coenzyme A acetyltransferase 2; acetoacetyl Coenzyme A thiolase
**672**	NP_005886	5174441	Golgi autoantigen, golgin subfamily a, 3; Golgin-160; male enhanced ant
**673**	NP_005887	28178825	isocitrate dehydrogenase 1 (NADP+), soluble; oxalosuccinate decarboxyl
**674**	NP_005900	5174525	microtubule-associated protein 1B isoform 1
**675**	NP_005908	5174539	cytosolic malate dehydrogenase; soluble malate dehydrogenase
**676**	NP_005909	21735621	mitochondrial malate dehydrogenase precursor
**677**	NP_005917	5174553	microfibrillar-associated protein 1
**678**	NP_005947	13699868	methylenetetrahydrofolate dehydrogenase (NADP+ dependent), methenyltet
**679**	NP_005956	16950601	myosin light chain kinase isoform 6; myosin light chain kinase
**680**	NP_005960	5174613	nucleosome assembly protein 1-like 4; nucleosome assembly protein 2
**681**	NP_005989	5174727	chaperonin containing TCP1, subunit 3 (gamma); TCP1 (t-complex-1) ring
**682**	NP_005990	5174731	translin-associated factor X; translin-like protein
**683**	NP_005991	17921989	tubulin, alpha 1; testis-specific alpha tubulin; tubulin alpha-1 chain
**684**	NP_005995	5174745	ubiquinol-cytochrome c reductase hinge protein
**685**	NP_006000	17986283	tubulin, alpha 3; tubulin, alpha, brain-specific; hum-a-tub1; hum-a-tu
**686**	NP_006001	5174393	arginine-rich, mutated in early stage tumors; arginine-rich protein
**687**	NP_006026	16357474	CDC42-binding protein kinase beta; CDC42-binding protein kinase beta (
**688**	NP_006077	5174737	tubulin, beta, 4
**689**	NP_006078	21361322	tubulin, beta, 5
**690**	NP_006085	5174427	deleted in liver cancer 1; ortholog of mouse Arhgap7; START domain cont
**691**	NP_006088	29568111	myosin, light polypeptide 9, regulatory; myosin regulatory light chain
**692**	NP_006089	5174447	guanine nucleotide binding protein (G protein), beta polypeptide 2-like
**693**	NP_006091	5174697	alpha2,3-sialyltransferase VI
**694**	NP_006112	17318569	keratin 1; Keratin-1; cytokeratin 1; hair alpha protein
**695**	NP_006126	5453597	F-actin capping protein alpha-1 subunit; Cap Z
**696**	NP_006127	5453599	capping protein (actin filament) muscle Z-line, alpha 2; F-actin cappin
**697**	NP_006139	5453710	LIM and SH3 protein 1; Lasp-1
**698**	NP_006147	5453760	neural precursor cell expressed, developmentally down-regulated 8
**699**	NP_006187	5453854	poly(rC) binding protein 1; heterogenous nuclear ribonucleoprotein X; p
**700**	NP_006207	24307907	plasminogen activator inhibitor type 1, member 2; protease inhibitor 7
**701**	NP_006209	5453892	phosphoinositide-3-kinase, catalytic, alpha polypeptide; phosphatidylin
**702**	NP_006212	5453898	protein (peptidyl-prolyl cis/trans isomerase) NIMA-interacting 1; dod;
**703**	NP_006214	5453902	protein (peptidyl-prolyl cis/trans isomerase) NIMA-interacting, 4 (parv
**704**	NP_006245	5453970	protein kinase C, delta
**705**	NP_006247	5453974	protein kinase C-like 2
**706**	NP_006249	10835242	protein kinase, cGMP-dependent, type I; Protein kinase, cGMP-dependent
**707**	NP_006254	5453990	proteasome (prosome, macropain) activator subunit 1 (PA28 alpha); Prote
**708**	NP_006261	5454028	related RAS viral (r-ras) oncogene homolog; Oncogene RRAS
**709**	NP_006271	5454090	signal sequence receptor, delta; translocon-associated protein delta
**710**	NP_006280	16753233	talin 1
**711**	NP_006285	5454152	ubiquinol-cytochrome c reductase binding protein
**712**	NP_006300	5453726	leucine rich repeat (in FLII) interacting protein 2
**713**	NP_006301	15451907	aminopeptidase puromycin sensitive; puromycin-sensitive aminopeptidase
**714**	NP_006311	5453916	progesterone membrane binding protein
**715**	NP_006316	5453555	ras-related nuclear protein
**716**	NP_006321	5453722	lysophospholipase I; lysophospholipase 1; lysophospholipid-specific lys
**717**	NP_006347	5453559	ATP synthase, H+ transporting, mitochondrial F0 complex, subunit d; ATP
**718**	NP_006358	5453595	adenylyl cyclase-associated protein
**719**	NP_006359	22219462	cAMP responsive element binding protein 3; luman; cyclic AMP response
**720**	NP_006365	21361358	serine/threonine kinase 25 (STE20 homolog, yeast); sterile 20 (oxidant

	Refseq ID	gi Number	Protein Name/Description
**721**	NP_006368	5454148	UNC13 (C. elegans)-like; homolog of rat Munc13 (diacylglycerol-binding)
**722**	NP_006380	5453832	oxygen regulated protein precursor; oxygen regulated protein (150kD)
**723**	NP_006382	5453998	importin 7; RAN-binding protein 7
**724**	NP_006391	5453629	dynactin 2; dynactin complex 50 kD subunit; dynamitin; 50 kD dynein-ass
**725**	NP_006393	5454170	hepatitis B virus x-interacting protein; hepatitis B virus x-interactin
**726**	NP_006397	5453549	thioredoxin peroxidase; thioredoxin peroxidase (antioxidant enzyme)
**727**	NP_006398	5453704	cytoskeleton related vitamin A responsive protein; glutamate transporte
**728**	NP_006420	5453607	chaperonin containing TCP1, subunit 7 (eta); chaperonin containing t-co
**729**	NP_006421	5453605	chaperonin containing TCP1, subunit 4 (delta); chaperonin containing t-
**730**	NP_006422	5453603	chaperonin containing TCP1, subunit 2 (beta); chaperonin containing t-c
**731**	NP_006435	5453591	SMC2 structural maintenance of chromosomes 2-like 1; structural mainten
**732**	NP_006448	5453714	LIM protein (similar to rat protein kinase C-binding enigma)
**733**	NP_006454	5453545	associated molecule with the SH3 domain of STAM
**734**	NP_006462	5453740	myosin regulatory light chain MRCL3; myosin, light polypeptide, regulat
**735**	NP_006467	29568101	ATP synthase, H+ transporting, mitochondrial F0 complex, subunit g; AT
**736**	NP_006494	5729991	proteasome 26S ATPase subunit 4 isoform 1; protease 26S subunit 6; Tat-
**737**	NP_006504	16306548	seryl-tRNA synthetase; serine-tRNA ligase
**738**	NP_006514	5730118	X-prolyl aminopeptidase (aminopeptidase P) 1, soluble; X-prolyl aminope
**739**	NP_006547	5729980	phosphomevalonate kinase
**740**	NP_006557	5729808	adhesion glycoprotein; platelet and T cell activation antigen 1; DNAX a
**741**	NP_006561	5729999	Ras-related GTP-binding protein RAGA
**742**	NP_006563	24111250	guanine nucleotide binding protein (G protein), alpha 13
**743**	NP_006576	6005727	chaperonin containing TCP1, subunit 8 (theta); T-complex protein 1, the
**744**	NP_006588	5729877	heat shock 70kDa protein 8 isoform 1; heat shock cognate protein, 71-kD
**745**	NP_006592	23308579	unactive progesterone receptor, 23 kD; likely ortholog of mouse telome
**746**	NP_006624	5729887	IQ motif containing GTPase activating protein 2
**747**	NP_006656	5729873	heparanase; heparanase-1
**748**	NP_006658	5729875	progesterone receptor membrane component 1; progesterone binding protei
**749**	NP_006694	5729804	nudix (nucleoside diphosphate linked moiety X)-type motif 3; diphosphoi
**750**	NP_006699	5729842	glyoxalase I; lactoyl glutathione lyase; lactoylglutathione lyase
**751**	NP_006701	5729779	COP9 homolog
**752**	NP_006717	16904381	LPS-responsive vesicle trafficking, beach and anchor containing; vesic
**753**	NP_006720	5803003	diaphanous 2 isoform 156
**754**	NP_006746	5803187	transaldolase 1; dihydroxyacetone transferase; glycerone transferase
**755**	NP_006750	13027638	UDP-glucose pyrophosphorylase 2; UTP-glucose-1-phosphate uridyltransfe
**756**	NP_006752	5803225	tyrosine 3/tryptophan 5 -monooxygenase activation protein, epsilon poly
**757**	NP_006780	5802978	apolipoprotein B mRNA editing enzyme, catalytic polypeptide-like 2; apo
**758**	NP_006784	5802974	peroxiredoxin 3; antioxidant protein 1; thioredoxin-dependent peroxide
**759**	NP_006786	5803009	EH-domain containing 1; homolog of Drosophila past; EH domain containin
**760**	NP_006793	5803167	splicing factor 3a, subunit 3, 60kDa; pre-mRNA splicing factor SF3a (60
**761**	NP_006796	5803036	heterogeneous nuclear ribonucleoprotein A0; hnRNA binding protein
**762**	NP_006801	5803121	for protein disulfide isomerase-related
**763**	NP_006806	5803149	coated vesicle membrane protein
**764**	NP_006808	5803013	endoplasmic reticulum protein 29 precursor; endoplasmic reticulum lumen
**765**	NP_006810	5803181	stress-induced-phosphoprotein 1 (Hsp70/Hsp90-organizing protein)
**766**	NP_006817	5803227	tyrosine 3/tryptophan 5 -monooxygenase activation protein, theta polype
**767**	NP_006818	5803201	transmembrane trafficking protein
**768**	NP_006819	24307917	RNA helicase family
**769**	NP_006821	5803217	ubiquinol-cytochrome c reductase (6.4kD) subunit
**770**	NP_006825	5803133	RAB32, member RAS oncogene family
**771**	NP_006830	5803115	inner membrane protein, mitochondrial (mitofilin); motor protein
**772**	NP_006843	11140819	tousled-like kinase 2; serine/threonine kinase; tousled-like kinase
**773**	NP_006852	5803135	RAB35, member RAS oncogene family; ras-related protein rab-1c (GTP-bind
**774**	NP_006861	5802966	destrin (actin depolymerizing factor); destrin
**775**	NP_006868	11321611	guanosine monophosphate reductase; guanine monophosphate reductase
**776**	NP_008839	9845511	ras-related C3 botulinum toxin substrate 1 isoform Rac1; rho family, sm
**777**	NP_008862	5902090	solute carrier family 2 (facilitated glucose transporter), member 3; GL
**778**	NP_008868	21361388	SMT3 suppressor of mif two 3 homolog 2; SMT3 (suppressor of mif two 3,
**779**	NP_008878	5902124	signal recognition particle 72kDa; signal recognition particle 72kD
**780**	NP_008880	5902128	syntaxin binding protein 2; Hunc18b

	Refseq ID	gi Number	Protein Name/Description
**781**	NP_008921	11386183	WAS protein family, member 2; suppressor of cyclic-AMP receptor (WASP-
**782**	NP_008939	5902016	reticulon 4; neuroendocrine-specific protein C like (foocen)
**783**	NP_008977	5901944	elastin microfibril interface located protein; TNF? elastin microfibril
**784**	NP_008994	5902154	vascular Rab-GAP/TBC-containing
**785**	NP_008996	5901922	CDC37 homolog
**786**	NP_009005	5902134	coronin, actin binding protein, 1A; coronin, actin-binding, 1A; coronin
**787**	NP_009031	6005717	ATP synthase, H+ transporting, mitochondrial F0 complex, subunit e; ATP
**788**	NP_009039	6005890	elongin B; transcription elongation factor B (SIII), polypeptide 2 (18k
**789**	NP_009043	6005902	thrombospondin 3
**790**	NP_009057	6005942	valosin-containing protein; yeast Cdc48p homolog; transitional endoplas
**791**	NP_009098	6005978	zinc finger protein 258
**792**	NP_009126	6005848	polymerase (DNA directed) iota; RAD30 (S. cerevisiae) homolog B; polyme
**793**	NP_009148	20127517	programmed cell death 10; apoptosis-related protein 15
**794**	NP_009160	6005826	protein kinase C and casein kinase substrate in neurons 2; pacsin 2
**795**	NP_009165	6005745	dynactin 3 isoform 1; dynactin light chain
**796**	NP_009167	6005731	calcium binding protein P22; SLC9A1 binding protein; calcineurin homolo
**797**	NP_009168	21361374	SP140 nuclear body protein; nuclear body protein Sp140
**798**	NP_009175	6005802	proline rich 4 (lacrimal); lacrimal proline rich protein
**799**	NP_009177	21359896	kelch-like 2, Mayven; mayven; kelch (Drosophila)-like 2 (Mayven)
**800**	NP_009191	9966764	lysophospholipase II; acyl-protein thioesterase
**801**	NP_009193	6005749	RNA-binding protein regulatory subunit; oncogene DJ1
**802**	NP_009194	6005735	coatomer protein complex, subunit epsilon
**803**	NP_009200	6005886	syntaxin binding protein 3; syntaxin 4 binding protein
**804**	NP_009204	6005854	repressor of estrogen receptor activity; B-cell associated protein
**805**	NP_009214	6005786	monoglyceride lipase; lysophospholipase-like
**806**	NP_009215	6005846	PTK9L protein tyrosine kinase 9-like (A6-related protein); protein tyro
**807**	NP_009216	6005768	GABA(A) receptor-associated protein-like 2; ganglioside expression fact
**808**	NP_009225	6552299	breast cancer 1, early onset; breast-ovarian cancer, included
**809**	NP_031377	6681259	multimerin
**810**	NP_031379	6680033	guanine nucleotide binding protein (G protein) alpha 12
**811**	NP_031381	20149594	heat shock 90kDa protein 1, beta; heat shock 90kD protein 1, beta; Hea
**812**	NP_031387	6679056	nidogen 2 (osteonidogen); nidogen 2
**813**	NP_031394	12545410	RAS p21 protein activator 3; GTPase activating protein III; Ins(1,3,4,
**814**	NP_036205	24307939	chaperonin containing TCP1, subunit 5 (epsilon)
**815**	NP_036208	6912322	crumbs homolog 1
**816**	NP_036218	6912398	general transcription factor IIIC, polypeptide 3, 102kDa; general trans
**817**	NP_036220	6912586	6-phosphogluconolactonase
**818**	NP_036222	15011904	microfilament and actin filament cross-linker protein isoform a; 620 k
**819**	NP_036226	6912238	peroxiredoxin 5; antioxidant enzyme B166
**820**	NP_036227	6912240	adaptor-related protein complex 3, mu 1 subunit; mu-adaptin 3A; AP-3 ad
**821**	NP_036256	6912304	cysteine and histidine-rich domain (CHORD)-containing, zinc binding pro
**822**	NP_036266	6912324	leiomodin 1 (smooth muscle); thyroid and eye muscle autoantigen D1 (64k
**823**	NP_036277	6912340	deoxythymidylate kinase (thymidylate kinase); thymidylate kinase
**824**	NP_036335	6912396	glyoxylate reductase/hydroxypyruvate reductase
**825**	NP_036357	6912540	nucleotide binding protein 2 (MinD homolog, E. coli); nucleotide bindin
**826**	NP_036372	6912662	sirtuin 4; sir2-like 4; sirtuin type 4; sirtuin silent mating type info
**827**	NP_036382	21361416	related RAS viral (r-ras) oncogene homolog 2; oncogene TC21
**828**	NP_036457	6912494	microtubule-associated protein, RP/EB family, member 1; ADENOMATOUS POL
**829**	NP_036458	10800412	microtubule-associated protein, RP/EB family, member 3; APC binding pr
**830**	NP_036463	6912516	methionine sulfoxide reductase A; peptide met (O) reductase
**831**	NP_036473	6912532	G protein-binding protein CRFG; GTP-binding protein
**832**	NP_036475	6912536	nicotinamide nucleotide transhydrogenase
**833**	NP_036515	21361409	osteoclast stimulating factor 1
**834**	NP_036526	12408675	prefoldin 2
**835**	NP_036557	6912638	ras suppressor protein 1
**836**	NP_036592	6912714	translocase of inner mitochondrial membrane 9 homolog; translocase of i
**837**	NP_036610	24430132	WW domain binding protein 2
**838**	NP_036611	21464101	tyrosine 3-monooxygenase/tryptophan 5-monooxygenase activation protein
**839**	NP_037364	7019485	programmed cell death 6; apoptosis-linked gene 2
**840**	NP_037373	7019375	formin homology 2 domain containing 1; FH1/FH2 domain-containing protei

	Refseq ID	gi Number	Protein Name/Description
**841**	NP_037391	10047092	neuronal protein
**842**	NP_037409	21361397	Rac GTPase activating protein 1; GTPase activating protein
**843**	NP_037459	20127528	parvin, beta; CGI-56 protein; beta-parvin
**844**	NP_037473	24431969	hypothetical protein PTD004; homologous yeast-44.2 protein
**845**	NP_037478	23111030	sorting nexin 12
**846**	NP_037481	7019545	secreted protein of unknown function
**847**	NP_037506	22027538	programmed cell death 6 interacting protein; ALG-2 interacting protein
**848**	NP_038464	7305303	NCK-associated protein 1
**849**	NP_038470	7305503	stomatin (EPB72)-like 2; stomatin-like protein 2; stomatin-like 2
**850**	NP_038479	7305053	myoferlin isoform a; fer-1-like 3
**851**	NP_039234	7330335	chloride intracellular channel 4; chloride intracellular channel 4 like
**852**	NP_039268	7524354	dimethylarginine dimethylaminohydrolase 2; dimethylarginine dimethylami
**853**	NP_054702	7770075	tachykinin 1 isoform alpha precursor; neurokinin A; neurokinin alpha; t
**854**	NP_054733	24307975	U5 snRNP-specific protein, 200-KD
**855**	NP_054735	7662338	suppressor of actin 1
**856**	NP_054736	7661728	HSPC003 protein
**857**	NP_054738	7661732	HSPC009 protein
**858**	NP_054752	13378141	DKFZP586A0522 protein
**859**	NP_054756	7662518	NTT5 protein
**860**	NP_054775	7661620	DKFZP564K247 protein
**861**	NP_054778	7662651	RGC32 protein
**862**	NP_054779	7662502	MCT-1 protein
**863**	NP_054782	21361670	src homology 3 domain-containing protein HIP-55; HIP-55 protein
**864**	NP_054891	24475861	phosphohistidine phosphatase; sex-regulated protein janus-a
**865**	NP_054900	7661818	HSPC159 protein
**866**	NP_055018	19913414	adaptor-related protein complex 2, alpha 1 subunit isoform 1; adaptin,
**867**	NP_055040	21361399	protein phosphatase 2 (formerly 2A), regulatory subunit A (PR 65), alp
**868**	NP_055067	7657585	solute carrier family 25 (mitochondrial carrier; ornithine transporter)
**869**	NP_055070	7657176	transmembrane protein 4
**870**	NP_055083	10346135	microtubule-associated protein, RP/EB family, member 2; T-cell activat
**871**	NP_055093	7656894	heat shock protein (hsp110 family)
**872**	NP_055140	7656991	coronin, actin binding protein, 1C; coronin, actin-binding protein, 1C;
**873**	NP_055165	7657124	TNF-induced protein
**874**	NP_055182	21361427	hypothetical protein, estradiol-induced
**875**	NP_055184	18375655	protein tyrosine phosphatase, non-receptor type 18; brain-derived phos
**876**	NP_055191	24307955	p53 inducible protein
**877**	NP_055205	7657431	EBNA-2 co-activator (100kD)
**878**	NP_055210	7657007	dual adaptor of phosphotyrosine and 3-phosphoinositides
**879**	NP_055217	7657486	low molecular mass ubiquinone-binding protein
**880**	NP_055227	7656952	calcyclin binding protein
**881**	NP_055243	10092691	tight junction protein 3 (zona occludens 3)
**882**	NP_055277	7657313	Lsm1 protein
**883**	NP_055309	21702742	trinucleotide repeat containing 6; EDIE; GW182 autoantigen
**884**	NP_055323	22907039	apolipoprotein B mRNA editing enzyme, catalytic polypeptide-like 3C
**885**	NP_055362	7657649	tropomodulin 3 (ubiquitous)
**886**	NP_055363	7657647	tropomodulin 2 (neuronal)
**887**	NP_055378	7657548	spondyloepiphyseal dysplasia, late; sedlin
**888**	NP_055415	7657056	EH-domain containing 3; EH domain containing 3
**889**	NP_055419	11993943	Tax interaction protein 1
**890**	NP_055423	24307969	cytoplasmic FMR1 interacting protein 1; selective hybridizing clone (m
**891**	NP_055425	21361456	alpha glucosidase II alpha subunit; likely ortholog of mouse G2an alph
**892**	NP_055517	7662096	KIAA0408 gene product
**893**	NP_055545	7661948	KIAA0152 gene product
**894**	NP_055644	7662318	Prp5-like DEAD-box protein
**895**	NP_055793	18379346	VPS10 domain receptor protein SORCS 3
**896**	NP_055814	7661922	RAB21, member RAS oncogene family
**897**	NP_055864	13027380	amyotrophic lateral sclerosis 2 (juvenile) chromosome region, candidat
**898**	NP_055874	22035665	talin 2
**899**	NP_055944	21361478	septin 6 isoform B; septin 2
**900**	NP_055987	28872861	HBxAg transactivated protein 2

	Refseq ID	gi Number	Protein Name/Description
**901**	NP_056015	22094121	KIAA0648 protein
**902**	NP_056034	24308035	KIAA1067 protein
**903**	NP_056057	16975484	centaurin delta 2 isoform b; ARF-GAP, RHO-GAP, ankyrin repeat, and ple
**904**	NP_056078	19745148	rabconnectin-3
**905**	NP_056107	14149680	KIAA0747 protein
**906**	NP_056202	7661624	preimplantation protein 3; likely ortholog of preimplantation protein 3
**907**	NP_056230	7661602	DKFZP564B167 protein
**908**	NP_056284	22267436	DKFZp564D177 protein
**909**	NP_056286	21361470	DKFZP566O1646 protein
**910**	NP_056348	20149621	DKFZP586B1621 protein
**911**	NP_056461	7661678	RAP1B, member of RAS oncogene family; K-REV; RAS-related protein RAP1B
**912**	NP_056505	24308123	serine/threonine kinase 36 (fused homolog, Drosophila); serine/threoni
**913**	NP_057001	7705704	glutathione S-transferase subunit 13 homolog
**914**	NP_057005	7706244	divalent cation tolerant protein CUTA
**915**	NP_057049	21361822	cell death-regulatory protein GRIM19; CGI-39 protein
**916**	NP_057070	27764873	cytokine receptor-like factor 3; cytokine receptor-like molecule 9; cy
**917**	NP_057071	20336761	heme binding protein 1
**918**	NP_057086	7705767	CGI-49 protein
**919**	NP_057095	7705777	CGI-63 protein
**920**	NP_057105	7706312	non-canonical ubquitin conjugating enzyme 1
**921**	NP_057107	7706314	CGI-77 protein
**922**	NP_057110	20070272	androgen-regulated short-chain dehydrogenase/reductase 1; prostate sho
**923**	NP_057132	7705614	CGI-111 protein
**924**	NP_057152	7705632	CGI-135 protein
**925**	NP_057153	27363461	mitochondria-associated GM-CSF signaling molecule
**926**	NP_057158	7705638	CGI-143 protein
**927**	NP_057161	7706351	CGI-147 protein
**928**	NP_057181	7705819	HSPC039 protein
**929**	NP_057187	7705827	GTP-binding protein Sara
**930**	NP_057190	7706371	vesicle transport-related protein
**931**	NP_057215	7705849	ras-related GTP-binding protein RAB10; ras-related GTP-binding protein
**932**	NP_057226	7705855	steroid dehydrogenase homolog; likely ortholog of mouse Kik1 steroid de
**933**	NP_057227	20149635	p47 protein
**934**	NP_057271	7705296	bridging integrator 2; bridging integrator-2; breast cancer associated
**935**	NP_057293	7706429	unknown
**936**	NP_057366	19923437	adenylate kinase 3 alpha like
**937**	NP_057376	7706485	tumor necrosis factor type 1 receptor associated protein
**938**	NP_057390	7706495	DnaJ (Hsp40) homolog, subfamily B, member 11; ER-associated DNAJ; ER-as
**939**	NP_057392	7706497	UMP-CMP kinase
**940**	NP_057406	19923483	GTPase Rab14
**941**	NP_057447	19923457	glycoprotein VI (platelet); platelet glycoprotein VI
**942**	NP_057454	7705963	RAB9-like protein
**943**	NP_057526	20149637	type 1 tumor necrosis factor receptor shedding aminopeptidase regulato
**944**	NP_057535	7705369	coatomer protein complex, subunit beta; beta coat protein
**945**	NP_057537	7705266	SH3 protein interacting with Nck, 90 kDa; SH3 protein; SH3 protein inte
**946**	NP_057546	7705501	hypothetical protein HSPC194
**947**	NP_057569	21361741	hypothetical protein HSPC228; My012 protein
**948**	NP_057614	7706563	RAB-8b protein
**949**	NP_057661	7706675	RAB6B, member RAS oncogene family; small GTPase RAB6B
**950**	NP_057701	7705300	hypothetical protein BM-002
**951**	NP_057736	11072091	Crn, crooked neck-like 1; CGI-201 protein; crooked neck protein (crn);
**952**	NP_058625	8393147	chloride intracellular channel 5
**953**	NP_058642	8393638	F11 receptor isoform a precursor; platelet F11 receptor; platelet adhes
**954**	NP_059118	8393159	calmodulin-like skin protein
**955**	NP_059139	8393120	polymerase (DNA directed), epsilon 3 (p17 subunit); histone fold protei
**956**	NP_059830	9257257	WD repeat-containing protein 1 isoform 1
**957**	NP_059980	22129786	gp25L2 protein
**958**	NP_060035	24308149	hypothetical protein DKFZp434F2322
**959**	NP_060055	8923733	sugar transporter (SLC2A6 gene)
**960**	NP_060140	8923114	ubiquitin-specific protease otubain 1

	Refseq ID	gi Number	Protein Name/Description
**961**	NP_060193	8923221	hypothetical protein FLJ20245
**962**	NP_060227	8923289	hypothetical protein FLJ20307
**963**	NP_060357	8923541	hypothetical protein FLJ20580
**964**	NP_060377	8923579	hypothetical protein FLJ20625
**965**	NP_060378	8923582	hypothetical protein FLJ20626
**966**	NP_060502	8922297	LUC7-like; sarcoplasmic reticulum protein LUC7B1
**967**	NP_060521	21361687	hypothetical protein FLJ10300
**968**	NP_060560	8922416	hypothetical protein FLJ10420
**969**	NP_060605	8922511	mitochondrial ribosomal protein S18A; mitochondrial ribosomal protein S
**970**	NP_060653	8922599	hypothetical protein FLJ10701
**971**	NP_060654	8922601	hypothetical protein FLJ10702
**972**	NP_060676	17999541	vacuolar protein sorting 35; maternal-embryonic 3
**973**	NP_060677	8922648	hypothetical protein FLJ10759
**974**	NP_060691	8922671	chromosome 2 open reading frame 6
**975**	NP_060692	8922673	parvin, alpha; alpha-parvin; actopaxin
**976**	NP_060705	8922699	hypothetical protein FLJ10830
**977**	NP_060713	8922712	hypothetical protein FLJ10849
**978**	NP_060719	28872786	CDK5 regulatory subunit associated protein 2; CDK5 activator-binding p
**979**	NP_060735	8922754	hypothetical protein FLJ10901
**980**	NP_060760	8922804	hypothetical protein FLJ10983
**981**	NP_060776	8922835	hypothetical protein FLJ11036
**982**	NP_060830	8922940	chromosome X open reading frame 15
**983**	NP_060835	8922950	hypothetical protein FLJ11222
**984**	NP_060849	8922974	hypothetical protein FLJ11280
**985**	NP_060899	21281669	transcription factor-like nuclear regulator; putative transcription re
**986**	NP_060918	21361794	TIP120 protein
**987**	NP_060932	27544939	uncharacterized hematopoietic stem/progenitor cells protein MDS027
**988**	NP_060935	8923932	uncharacterized hematopoietic stem/progenitor cells protein MDS030
**989**	NP_060943	8923812	uncharacterized hypothalamus protein HT012
**990**	NP_060988	19923534	MCM10 minichromosome maintenance deficient 10; homolog of yeast MCM10
**991**	NP_061027	9055270	low density lipoprotein-related protein 1B (deleted in tumors); low den
**992**	NP_061138	18105058	vacuolar protein sorting 33B (yeast homolog))
**993**	NP_061141	29725607	smooth muscle cell associated protein-1
**994**	NP_061158	8923904	bridging integrator 3
**995**	NP_061166	8922122	elaC homolog 1
**996**	NP_061174	24308179	hypothetical protein DKFZp547A023
**997**	NP_061720	17864092	axonemal dynein heavy chain 7
**998**	NP_061816	9507215	tubulin, alpha-like 2; tubulin, alpha 8
**999**	NP_061820	11128019	cytochrome c
**1000**	NP_061846	19424120	dolichyl-phosphate mannosyltransferase polypeptide 3 isoform 1; prosti
**1001**	NP_061882	21361619	TOLLIP protein; Toll-interacting protein
**1002**	NP_061907	9506667	ras homolog gene family, member F
**1003**	NP_061934	27477132	phosphatidylinositol-3 phosphate 3-phosphatase adaptor subunit; 3-phos
**1004**	NP_061969	19923499	GTP binding protein 2
**1005**	NP_062826	21361827	putative methyltransferase M6A; putative methyltransferase
**1006**	NP_063940	9845297	second mitochondria-derived activator of caspase isoform Smac-alpha, pr
**1007**	NP_064424	29788766	tubulin, beta polypeptide 4, member Q
**1008**	NP_064425	9910554	solute carrier family 2 (facilitated glucose transporter), member 9; SL
**1009**	NP_064505	9910280	UDP-glucose ceramide glucosyltransferase-like 1; UDP-glucose:glycoprote
**1010**	NP_064535	9910542	SAR1a gene homolog 1; SAR1 protein
**1011**	NP_064542	9910470	otoraplin precursor; fibrocyte-derived protein; melanoma inhibitory act
**1012**	NP_064578	19923559	EMSY protein
**1013**	NP_064590	9910156	zinc finger protein Cezanne
**1014**	NP_065089	9966825	HEF like Protein
**1015**	NP_065090	9966827	PEST-containing nuclear protein
**1016**	NP_065123	9966867	eIF-5A2 protein; eIF5AII
**1017**	NP_065174	14149734	coronin, actin binding protein, 1B
**1018**	NP_065176	27262622	neuron navigator 1; neuron navigator-1; pore membrane and/or filament
**1019**	NP_065392	24308201	chromosome 20 open reading frame 3; chromosome 20open reading frame 3
**1020**	NP_065393	24431935	reticulon 4; neuroendocrine-specific protein C like (foocen)

	Refseq ID	gi Number	Protein Name/Description
**1021**	NP_065438	10140853	diazepam binding inhibitor; GABA receptor modulator; endozepine; acyl
**1022**	NP_065691	10190678	RP42 homolog; squamous cell carcinoma-related oncogene
**1023**	NP_065765	29789112	KIAA1198 protein
**1024**	NP_065815	18079216	CASK-interacting protein 1
**1025**	NP_065871	19882229	P-Rex1
**1026**	NP_066272	13994151	PDZ and LIM domain 1 (elfin); carboxy terminal LIM domain protein 1
**1027**	NP_066283	10337595	protein phosphatase 1A isoform 1; protein phosphatase 2C alpha isoform
**1028**	NP_066299	17986258	smooth muscle and non-muscle myosin alkali light chain isoform 1
**1029**	NP_066301	11321640	basic beta 1 syntrophin; 59-DAP; syntrophin, beta 1; syntrophin, beta
**1030**	NP_066932	11056061	thymosin, beta 4
**1031**	NP_066949	13489091	3-mercaptopyruvate sulfurtransferase
**1032**	NP_066953	10863927	peptidylprolyl isomerase A (cyclophilin A)
**1033**	NP_066954	29725611	regulatory subunit PR 53 of protein phosphatase 2A isoform b; phosphot
**1034**	NP_066967	16945970	PC4 and SFRS1 interacting protein 1; transcriptional coactivator p52/p
**1035**	NP_066972	21624607	coactosin-like 1
**1036**	NP_067000	10863977	LSM2 homolog, U6 small nuclear RNA associated; U6 snRNA-associated Sm-
**1037**	NP_067022	10864011	sulfide dehydrogenase like; sulfide dehydrogenase like (yeast)
**1038**	NP_067033	10864021	MUM2 protein
**1039**	NP_067045	24308263	TcD37 homolog; prune
**1040**	NP_067058	10864047	epidermal growth factor receptor substrate EPS15R
**1041**	NP_067069	10864055	megakaryocyte-enhanced gene transcript 1 protein; lymphocyte antigen-6
**1042**	NP_067075	10880989	RAB18, member RAS oncogene family; RAB18 small GTPase
**1043**	NP_068557	11140817	retinoic acid induced 2
**1044**	NP_068595	21361900	hypothetical protein MDS018
**1045**	NP_068711	12548785	gamma-aminobutyric acid (GABA) A receptor, beta 2 isoform 1
**1046**	NP_068805	11496277	mitogen-activated protein kinase kinase 1 interacting protein 1; MEK p
**1047**	NP_071349	11545777	likely ortholog of mouse ubiquitin-conjugating enzyme E2-230K
**1048**	NP_071357	11545789	hypothetical protein FLJ22794; KIAA1895 protein
**1049**	NP_071441	11545906	fructosamine-3-kinase
**1050**	NP_071496	11559923	eukaryotic translation initiation factor 4H isoform 1
**1051**	NP_071918	11968023	zinc finger protein 106
**1052**	NP_071930	11968039	hypothetical protein FLJ22405
**1053**	NP_071934	11968045	hypothetical protein FLJ22056
**1054**	NP_073565	27475985	neurogenic differentiation 6
**1055**	NP_073728	12707562	period 2 isoform 2; period, Drosophila, homolog of, 2; period circadia
**1056**	NP_075049	12597635	B-cell CLL/lymphoma 11B isoform 2; B-cell lymphoma/leukaemia 11B; zinc
**1057**	NP_075408	13259508	dynactin 1 isoform 2; dynactin 1 (p150, Glued (Drosophila) homolog); p
**1058**	NP_076933	13128974	hypothetical protein MGC3265
**1059**	NP_076956	13129018	hypothetical protein MGC3077
**1060**	NP_076982	21359955	SECIS binding protein 2
**1061**	NP_078975	13375985	hypothetical protein FLJ14007
**1062**	NP_078982	24432013	hypothetical protein FLJ13479
**1063**	NP_079079	13376181	hypothetical protein FLJ21665
**1064**	NP_079082	13376187	hypothetical protein FLJ23554
**1065**	NP_079131	13376282	hypothetical protein FLJ12785
**1066**	NP_079199	27477134	nucleoporin 210; nuclear pore membrane glycoprotein 210; gp210
**1067**	NP_079209	21281689	hypothetical protein FLJ12056
**1068**	NP_079295	13376539	hypothetical protein FLJ13940
**1069**	NP_079356	23308567	asparaginase-like 1 protein
**1070**	NP_079390	13376689	hypothetical protein FLJ13615
**1071**	NP_079413	13376719	hypothetical protein FLJ21439
**1072**	NP_079434	22095371	RUN and FYVE domain-containing 1
**1073**	NP_079536	19913373	G6B protein isoform G6b-A precursor; G6B protein; immunoglobulin recep
**1074**	NP_085056	13385594	cytochrome b5 outer mitochondrial membrane precursor; type 2 cyt-b5
**1075**	NP_109591	13489087	serine (or cysteine) proteinase inhibitor, clade B (ovalbumin), member
**1076**	NP_110400	13562114	beta tubulin 1, class VI
**1077**	NP_112186	21359960	hypothetical protein PRTD-NY3
**1078**	NP_112243	13569962	RAB1B, member RAS oncogene family; small GTP-binding protein
**1079**	NP_112484	13654272	hypothetical protein HT036
**1080**	NP_112567	13775186	ring finger protein 17 isoform long

	Refseq ID	gi Number	Protein Name/Description
**1081**	NP_112576	13775198	SH3 domain binding glutamic acid-rich protein like 3; SH3BGRL3-like pr
**1082**	NP_112585	23200010	Williams Beuren syndrome chromosome region 21 isoform 4
**1083**	NP_112586	13786129	RAB33B, member RAS oncogene family; likely ortholog of mouse RAB33b
**1084**	NP_113620	13899253	uridine-cytidine kinase 1
**1085**	NP_113646	13899297	B aggressive lymphoma gene
**1086**	NP_113657	13899317	SH3 domain binding glutamic acid-rich protein like 2
**1087**	NP_113659	28626504	UNC-112 related protein 2
**1088**	NP_114159	13994351	MSTP043 protein
**1089**	NP_114420	14042953	FKSG17
**1090**	NP_115492	14149767	hypothetical protein MGC2599 similar to katanin p60 subunit A 1 2599
**1091**	NP_115500	14149777	hypothetical protein DKFZp564D1378
**1092**	NP_115523	14149817	hypothetical protein DKFZp434D0127
**1093**	NP_115676	14150056	hypothetical protein MGC10854
**1094**	NP_115706	14161692	calpain small subunit 2
**1095**	NP_115712	14150122	hypothetical protein MGC14799
**1096**	NP_115729	14150155	hypothetical protein MGC10540
**1097**	NP_115744	21362062	leucine zipper and CTNNBIP1 domain containing
**1098**	NP_115914	14210536	similar to chicken tubulin beta 5
**1099**	NP_115951	14211867	group XIII secreted phospholipase A2
**1100**	NP_115956	24432022	testis-specific protein NYD-TSP1
**1101**	NP_115982	14211923	PKCI-1-related HIT protein
**1102**	NP_115992	14211943	lysyl oxidase-like 3
**1103**	NP_115997	20373153	myosin XVIIIB; myosin 18B
**1104**	NP_116093	14389309	tubulin alpha 6
**1105**	NP_116120	14249348	hypothetical protein MGC14353
**1106**	NP_116139	14249382	hypothetical protein MGC15429
**1107**	NP_116189	14249478	hypothetical protein FLJ14525
**1108**	NP_116214	14249526	zinc finger protein 382; multiple-zinc-finger-Kruppel-associated box p
**1109**	NP_116235	21361884	RAB2B
**1110**	NP_116262	24497612	nm23-phosphorylated unknown substrate; SH3 domain-containing 70 kDa pr
**1111**	NP_149062	23097308	nesprin 1 isoform longest; synaptic nuclei expressed gene 1; nesprin 1
**1112**	NP_150094	15100168	CUB and Sushi multiple domains 1
**1113**	NP_150281	15147250	protein phosphatase 1, regulatory (inhibitor) subunit 14A; 17-kDa PKC-
**1114**	NP_258259	15618995	keratin 6 irs
**1115**	NP_258412	15626999	inosine triphosphatase; Inosine triphosphatase-A
**1116**	NP_277050	15723376	MacGAP protein
**1117**	NP_439892	16936530	cyclin-dependent kinase 2 isoform 2; cdc2-related protein kinase; cell
**1118**	NP_443099	24119274	voltage gated channel like 1; four repeat voltage-gated ion channel
**1119**	NP_443114	16418391	zinc finger, imprinted 3
**1120**	NP_443195	16418461	mitochondrial topoisomerase I
**1121**	NP_443728	28461290	TRALPUSH; no opposite paired repeat protein
**1122**	NP_444253	16950611	myosin light chain kinase isoform 1; myosin light chain kinase
**1123**	NP_444513	16751921	dermcidin precursor; AIDD protein
**1124**	NP_524144	17986273	fast skeletal myosin alkali light chain 1 isoform 1f; A1 catalytic; A2
**1125**	NP_524147	17986260	smooth muscle and non-muscle myosin alkali light chain isoform 2
**1126**	NP_542408	18087855	dynein light chain 2
**1127**	NP_542775	18152767	synaptotagmin-like 4 (granuphilin-a)
**1128**	NP_542937	18375611	dachshund homolog isoform a
**1129**	NP_543010	21361785	histidyl-tRNA synthetase 2; histidyl-tRNA synthetase; bA379J5.3; chrom
**1130**	NP_543152	18254478	dual specificity phosphatase 19; protein phosphatase; stress-activated
**1131**	NP_570138	18640750	regulator of G-protein signalling 18; regulator of G-protein signaling
**1132**	NP_570602	21071030	alpha 1B-glycoprotein
**1133**	NP_570967	18702323	dynein, cytoplasmic, light polypeptide 2B; dynein light chain 2B; bith
**1134**	NP_596870	20143916	titin isoform novex-3; connectin; CMH9, included; cardiomyopathy, dila
**1135**	NP_597702	20143964	glutamate receptor, ionotropic, N-methyl-D-aspartate 3A
**1136**	NP_612366	24308400	hypothetical protein BC010682
**1137**	NP_612460	19923981	hypothetical protein BC013151
**1138**	NP_612469	19923993	chemokine-like factor super family 5
**1139**	NP_620061	20270259	phosphoglycerate kinase 2; phosphoglycerate kinase 1, pseudogene 2
**1140**	NP_620124	20270303	mitochondrial Rho 2

	Refseq ID	gi Number	Protein Name/Description
**1141**	NP_620150	20270343	hypothetical protein BC015408
**1142**	NP_620411	20452464	similar to endothelial cell-selective adhesion molecule; likely orthol
**1143**	NP_620688	21265064	a disintegrin-like and metalloprotease (reprolysin type) with thrombos
**1144**	NP_631960	21245108	TGFB-induced factor 2-like, Y-linked
**1145**	NP_653164	21389337	ribose 5-phosphate isomerase A (ribose 5-phosphate epimerase); RIBOSE
**1146**	NP_653174	21389339	likely ortholog of rat F-actin binding protein nexilin
**1147**	NP_653263	21389549	hypothetical protein MGC26605
**1148**	NP_653321	21389505	hypothetical protein FLJ31564
**1149**	NP_659415	21450665	hypothetical protein FLJ32745
**1150**	NP_659447	21450721	hypothetical protein MGC26778
**1151**	NP_659501	21450838	hypothetical protein MGC2793
**1152**	NP_660202	21624643	similar to CG3714 gene product
**1153**	NP_660295	21687060	similar to common salivary protein 1
**1154**	NP_665807	21956645	myotrophin; granule cell differentiation protein
**1155**	NP_689476	22748619	tropomyosin 3
**1156**	NP_689522	22748683	hypothetical protein FLJ35564
**1157**	NP_689541	22748717	hypothetical protein FLJ38602
**1158**	NP_689584	22748799	hypothetical protein MGC26818
**1159**	NP_689590	22748811	hypothetical protein MGC34837
**1160**	NP_689598	22748825	hypothetical protein FLJ31438
**1161**	NP_689616	22748857	hypothetical protein FLJ39155
**1162**	NP_689628	22748879	hypothetical protein FLJ32642
**1163**	NP_689663	22748945	hypothetical protein MGC26690
**1164**	NP_689761	22749143	hypothetical protein FLJ25333
**1165**	NP_689822	22749247	hypothetical protein FLJ32001
**1166**	NP_689936	22749429	hypothetical protein FLJ38159
**1167**	NP_689973	22749489	hypothetical protein FLJ30934
**1168**	NP_689979	22749499	hypothetical protein MGC40107
**1169**	NP_690007	23065535	hypoxia-inducible factor-3 alpha isoform a; inhibitory PAS domain prot
**1170**	NP_690870	23097264	hypothetical protein DKFZp761P1121
**1171**	NP_694968	23397516	immune associated nucleotide protein
**1172**	NP_694978	23397534	hypothetical protein MGC45491
**1173**	NP_695023	23463289	family with sequence similarity 10, member A5
**1174**	NP_699160	23503239	hypothetical protein MGC10204
**1175**	NP_699179	24158492	F-box only protein 29; F-box protein Fbx29
**1176**	NP_705691	24111244	MAM domain containing glycosylphosphatidylinositol anchor 1; glycosyl-
**1177**	NP_705840	23957682	heparan sulfate 3-OST-5
**1178**	NP_705935	24119203	tropomyosin 3
**1179**	NP_722516	24429572	TBP-associated factor RNA polymerase 1-like
**1180**	NP_775771	28466991	tau-tubulin kinase
**1181**	NP_776155	28603830	hypothetical protein MGC51029
**1182**	NP_777550	28376664	hypothetical protein LOC93550
**1183**	NP_777604	28372565	hypothetical protein LOC283629
**1184**	NP_778250	28376654	hypothetical protein INM01
**1185**	NP_783313	28269681	NADH-ubiquinone oxidoreductase subunit B14.7
**1186**	NP_783865	28376635	Rab37-like
**1187**	NP_786886	28395033	ras homolog gene family, member C; Aplysia RAS-related homolog 9 (onco
**1188**	NP_787068	28557683	hypothetical protein FLJ38451
**1189**	NP_787082	28557709	hypothetical protein LOC221823
**1190**	NP_789797	28827809	nucleoside diphosphate kinase B-like
**1191**	NP_803875	29150259	fibrocystin L; polycystic kidney and hepatic disease-like 1
**1192**	XP_015334	27478053	similar to heat shock 70kD protein binding protein; progesterone recep
**1193**	XP_016144	14745898	similar to tyrosine 3-monooxygenase/tryptophan 5-monooxygenase activat
**1194**	XP_016625	13637631	similar to voltage-dependent anion channel isoform 2
**1195**	XP_017966	17444699	similar to Reticulon protein 3 (Neuroendocrine-specific protein-like 2
**1196**	XP_028810	27500963	similar to KIAA1755 protein
**1197**	XP_028966	18591697	similar to DnaJ homolog subfamily C member 5 (Cysteine string protein)
**1198**	XP_029179	27498357	similar to filamin-interacting protein L-Filip; filamin-interacting pr
**1199**	XP_031223	20536114	similar to Hypothetical protein KIAA1034
**1200**	XP_031992	27498919	similar to RRP5 protein homolog

	Refseq ID	gi Number	Protein Name/Description
**1201**	XP_032693	14779875	similar to KIAA0420
**1202**	XP_033004	14780596	hypothetical protein BC005107
**1203**	XP_033371	22048001	similar to DKFZP564O092 protein
**1204**	XP_033811	20553919	similar to hypothetical protein FLJ14775
**1205**	XP_035037	22065231	similar to MEGF7
**1206**	XP_035527	16160851	similar to KIAA1370 protein
**1207**	XP_035638	15306356	similar to interleukin 25; lymphocyte antigen 6 complex, locus E ligan
**1208**	XP_036408	22045984	similar to KIAA1228 protein
**1209**	XP_037574	14765644	similar to PTPL1-associated RhoGAP 1
**1210**	XP_037817	30148409	similar to hypothetical protein FLJ20035
**1211**	XP_038288	27480017	similar to The KIAA0191 gene is expressed ubiquitously.~The KIAA0191 p
**1212**	XP_039259	29732431	similar to modifier of cell adhesion
**1213**	XP_042500	14724206	similar to ribosomal protein S2; 40S ribosomal protein S2
**1214**	XP_045792	27499681	similar to RIKEN cDNA G431004K08
**1215**	XP_047659	22048785	similar to KIAA0532 protein
**1216**	XP_050793	27479431	similar to KIAA1393 protein
**1217**	XP_051091	27499555	similar to SF21 protein
**1218**	XP_051854	14749486	similar to Mesoderm development candidate 2
**1219**	XP_053177	20535366	similar to Tubulin alpha-3/alpha-7 chain (Alpha-tubulin 3/7)
**1220**	XP_058577	27499572	similar to chromosome 11 open reading frame 25
**1221**	XP_058957	17482508	similar to QIL1
**1222**	XP_059066	22041780	similar to 14-3-3 protein sigma (Stratifin) (Epithelial cell marker pr
**1223**	XP_059067	17438583	similar to nucleophosmin (nucleolar phosphoprotein B23, numatrin); Nuc
**1224**	XP_059465	17445600	similar to ribosomal protein S15
**1225**	XP_059776	17464200	similar to FK506-binding protein 1A; FK506-binding protein 1; FK506-bi
**1226**	XP_060104	30147721	similar to RIKEN cDNA 5430400H23
**1227**	XP_060316	29728979	similar to Olfactory receptor 2T1 (Olfactory receptor 1-25) (OR1-25)
**1228**	XP_060320	30147184	similar to ATP-dependent DNA helicase II, 70 kDa subunit (Lupus Ku aut
**1229**	XP_060398	17440821	similar to peptidylprolyl isomerase A (cyclophilin A)
**1230**	XP_060887	17450333	similar to peptidyl-Pro cis trans isomerase
**1231**	XP_061930	17473323	similar to Homeobox protein DBX1
**1232**	XP_062437	30156394	similar to Keratin, type I cytoskeletal 18 (Cytokeratin 18) (K18) (CK
**1233**	XP_062669	27499559	similar to lactate dehydrogenase A -like
**1234**	XP_062849	17475428	similar to Proteasome subunit alpha type 6 (Proteasome iota chain) (Ma
**1235**	XP_065237	22042381	similar to FKSG30
**1236**	XP_066916	17486695	similar to cytosolic malate dehydrogenase
**1237**	XP_067176	29730275	similar to peptidylprolyl isomerase A (cyclophilin A)
**1238**	XP_067423	17439599	similar to destrin - pig
**1239**	XP_067503	17440554	similar to peptidyl-Pro cis trans isomerase
**1240**	XP_067504	20535081	similar to Cop-coated vesicle membrane protein p24 precursor (p24A)
**1241**	XP_068464	29737116	similar to 60S ribosomal protein L19
**1242**	XP_069253	17463729	similar to prohibitin
**1243**	XP_069750	17448269	similar to GDP dissociation inhibitor isoform 2; GDI-2
**1244**	XP_070805	30149697	similar to prohibitin
**1245**	XP_071133	17452829	similar to ATP synthase, H+ transporting, mitochondrial F0 complex, su
**1246**	XP_084467	29739581	similar to eukaryotic initiation factor 5A
**1247**	XP_084514	27484943	similar to heat shock 90kDa protein 1, alpha; heat shock 90kD protein
**1248**	XP_084610	22065448	similar to tubulin alpha-1 chain - Chinese hamster
**1249**	XP_085123	29736026	similar to Ras-related protein Rab-15
**1250**	XP_085748	18588342	similar to KIAA1039 protein
**1251**	XP_086001	29741322	similar to placental protein 13; galectin-13
**1252**	XP_086916	18543899	similar to Phosphatidylethanolamine-binding protein (PEBP) (Prostatic
**1253**	XP_086931	20532885	similar to epsilon isoform of 14-3-3 protein
**1254**	XP_087062	18551340	similar to 60S acidic ribosomal protein P1
**1255**	XP_087572	18558709	similar to bA291L22.2 (similar to CDC10 (cell division cycle 10, S. ce
**1256**	XP_087939	22053085	similar to tubulin, beta 5
**1257**	XP_088293	22060072	similar to cytochrome c
**1258**	XP_088391	20539300	similar to tropomyosin 4
**1259**	XP_088393	18571222	similar to destrin - pig
**1260**	XP_088476	18572267	similar to thioredoxin domain containing 4; endoplasmic reticulum resi

	Refseq ID	gi Number	Protein Name/Description
**1261**	XP_089309	18600882	similar to Cyclophilin-LC
**1262**	XP_095590	18570595	similar to myosin regulatory light chain
**1263**	XP_096852	22054400	hypothetical protein XP_096852
**1264**	XP_097006	18585927	similar to MUSP1
**1265**	XP_097771	27485697	similar to hypothetical protein
**1266**	XP_113714	22059372	similar to Density-regulated protein (DRP) (DRP1 protein) (Smooth musc
**1267**	XP_113950	27500274	similar to Munc13-4 protein
**1268**	XP_114482	20547107	similar to HEAT SHOCK 70 KDA PROTEIN 4 (HEAT SHOCK 70-RELATED PROTEIN
**1269**	XP_114617	22059949	similar to tubulin, beta 5
**1270**	XP_115639	20546215	similar to leukocyte immunoglobulin-like receptor, subfamily B (with T
**1271**	XP_115812	20536967	similar to Latent transforming growth factor beta binding protein 1L p
**1272**	XP_115995	20470897	similar to protease (prosome, macropain) 26S subunit, ATPase 1
**1273**	XP_116321	20540570	similar to 60 kDa heat shock protein, mitochondrial precursor (Hsp60)
**1274**	XP_116396	20543399	similar to peptidylprolyl isomerase A (cyclophilin A)
**1275**	XP_117198	27481006	hypothetical protein XP_117198
**1276**	XP_166112	27482697	similar to KIAA1217 protein
**1277**	XP_166241	20483052	similar to PTD015 protein
**1278**	XP_166266	20547663	similar to Esterase D
**1279**	XP_166506	22045787	similar to Elongation factor 1-alpha 1 (EF-1-alpha-1) (Elongation fact
**1280**	XP_166540	20540302	similar to Heat shock cognate 71 kDa protein
**1281**	XP_167021	20551618	similar to calponin 2; Calonin 2
**1282**	XP_170195	30151001	similar to ADP,ATP carrier protein, fibroblast isoform (ADP/ATP transl
**1283**	XP_171081	22043610	similar to ras-related C3 botulinum toxin substrate 1 isoform Rac1; rh
**1284**	XP_171113	30153852	similar to Moesin (Membrane-organizing extension spike protein)
**1285**	XP_171447	22063113	similar to elongin B; transcription elongation factor B (SIII), polype
**1286**	XP_208062	27485813	similar to ARP2/3 complex 21 kDa subunit (P21-ARC) (Actin-related prot
**1287**	XP_208162	27483766	similar to PTK9 protein tyrosine kinase 9; protein tyrosine kinase 9
**1288**	XP_208215	27480104	similar to triosephosphate isomerase 1
**1289**	XP_208217	27481160	similar to elongation factor 1 alpha
**1290**	XP_208225	27480253	similar to hypothetical protein FLJ20420
**1291**	XP_208234	27481323	similar to unactive progesterone receptor, 23 kD; likely ortholog of m
**1292**	XP_208238	27480842	similar to bA92K2.2 (similar to ubiquitin)
**1293**	XP_208313	27477800	similar to tropomyosin 4
**1294**	XP_208318	30147468	similar to hypothetical protein
**1295**	XP_208411	27478749	similar to L-lactate dehydrogenase A chain (LDH-A) (LDH muscle subunit
**1296**	XP_208709	27482573	similar to Ubiquitin-conjugating enzyme E2-18 kDa UbcH7 (Ubiquitin-pro
**1297**	XP_208746	27479531	similar to golgi complex associated protein 1; golgi resident protein
**1298**	XP_208872	27500008	similar to tropomyosin 3
**1299**	XP_208901	27500115	similar to ubiquitin-conjugating enzyme E2M; UBC12 homolog, yeast
**1300**	XP_209234	29727772	similar to hypothetical protein
**1301**	XP_209737	27498267	similar to tyrosine 3/tryptophan 5 -monooxygenase activation protein,
**1302**	XP_210183	30158009	similar to 60S ribosomal protein L7a (Surfeit locus protein 3) (PLA-X
**1303**	XP_210184	30158216	similar to hypothetical protein BC015353
**1304**	XP_210460	27501260	similar to Glyceraldehyde 3-phosphate dehydrogenase, liver (GAPDH)
**1305**	XP_210540	30149460	similar to Heat shock protein HSP 90-alpha (HSP 86)
**1306**	XP_210550	30147105	similar to Chain , Aldose Reductase (E.C.1.1.1.21) Mutant With Tyr 48
**1307**	XP_210576	30149984	similar to RIKEN cDNA 4921517D21
**1308**	XP_210664	27484737	similar to MRDS1 protein
**1309**	XP_210750	27478732	similar to ATP synthase, H+ transporting, mitochondrial F0 complex, su
**1310**	XP_212118	27479210	hypothetical protein XP_212118
**1311**	XP_212565	27498332	similar to tubulin, beta 5
**1312**	XP_290546	29745994	similar to KIAA0830 protein
**1313**	XP_290702	29747201	similar to My016 protein
**1314**	XP_290742	29737976	similar to Phosphoribosylformylglycinamidine synthase (FGAM synthase)
**1315**	XP_290747	29737978	similar to Myosin heavy chain, nonmuscle type B (Cellular myosin heavy
**1316**	XP_290872	29727204	similar to Pyruvate kinase, M2 isozyme
**1317**	XP_290989	29728414	similar to myosin-I, Myr 1c (alternatively spliced) - rat
**1318**	XP_290995	29729345	similar to speckle-type POZ protein
**1319**	XP_291064	29731000	similar to KIAA0540 protein
**1320**	XP_291117	29734141	similar to succinate dehydrogenase flavoprotein subunit

	Refseq ID	gi Number	Protein Name/Description
**1321**	XP_291183	29742410	similar to TREM-like transcript 1
**1322**	XP_291269	29734552	similar to KIAA1875 protein
**1323**	XP_291384	29727565	similar to Cytochrome c, somatic
**1324**	XP_291446	30149221	similar to Heat shock cognate 71 kDa protein
**1325**	XP_291459	29729329	similar to Cofilin, non-muscle isoform (18 kDa phosphoprotein) (P18)
**1326**	XP_291482	29730023	similar to 78 kDa glucose-regulated protein precursor (GRP 78) (Immuno
**1327**	XP_291501	30148728	similar to Heterogeneous nuclear ribonucleoprotein A1 (Helix-destabili
**1328**	XP_291520	30148978	similar to Keratin, type I cytoskeletal 18 (Cytokeratin 18) (K18) (CK
**1329**	XP_291550	29732170	similar to Heat shock protein HSP 90-alpha (HSP 86)
**1330**	XP_291573	30149784	similar to elongation factor 1 alpha
**1331**	XP_291587	29732725	similar to Endoplasmin precursor (94 kDa glucose-regulated protein) (G
**1332**	XP_291603	29732955	similar to glyceraldehyde 3-phosphate dehydrogenase
**1333**	XP_291660	29738713	similar to RIKEN cDNA 5730509K17 gene
**1334**	XP_291710	29739273	similar to Ras suppressor protein 1 (Rsu-1) (RSP-1)
**1335**	XP_291724	29739720	similar to 60S RIBOSOMAL PROTEIN L26
**1336**	XP_291762	29741138	similar to hypothetical protein MGC33214
**1337**	XP_291768	29740995	similar to Proteasome activator complex subunit 2 (Proteasome activato
**1338**	XP_292125	29745118	similar to submaxillary apomucin
**1339**	XP_292133	30157216	similar to genethonin 1
**1340**	XP_292188	29738504	similar to ribosomal protein L31
**1341**	XP_292230	29736201	similar to DnaJ homolog subfamily C member 8 (Splicing protein spf31)
**1342**	XP_292459	29741994	similar to alpha tubulin
**1343**	XP_292513	30158844	similar to KIAA1879 protein
**1344**	XP_292801	30155465	similar to RIKEN cDNA 2310076L09
**1345**	XP_292814	30155919	similar to zinc finger protein 433
**1346**	XP_292963	29729616	similar to peptidylprolyl isomerase A (cyclophilin A)
**1347**	XP_292964	30150271	similar to Heat shock protein HSP 90-beta (HSP 84) (Tumor specific tra
**1348**	XP_292982	30148842	similar to FKSG30
**1349**	XP_292989	30148924	similar to SOCS box protein ASB-18
**1350**	XP_293007	30147857	similar to peptidylprolyl isomerase A (cyclophilin A)
**1351**	XP_293010	30150299	similar to keratin 8, type II cytoskeletal - human
**1352**	XP_293011	29731062	hypothetical protein XP_293011
**1353**	XP_293023	29731325	similar to peptidyl-Pro cis trans isomerase
**1354**	XP_293027	29731460	similar to ADP,ATP carrier protein, fibroblast isoform (ADP/ATP transl
**1355**	XP_293042	29731857	similar to ribosomal protein S12; 40S ribosomal protein S12
**1356**	XP_293066	30150448	similar to Stress-70 protein, mitochondrial precursor (75 kDa glucose
**1357**	XP_293276	29743324	similar to Heat shock 27 kDa protein (HSP 27) (Stress-responsive prote
**1358**	XP_293277	30156347	similar to keratin 8; cytokeratin 8; keratin, type II cytoskeletal 8
**1359**	XP_293394	30157691	similar to Nucleolar phosphoprotein p130 (Nucleolar 130 kDa protein) (
**1360**	XP_293411	30157848	similar to Keratin, type I cytoskeletal 18 (Cytokeratin 18) (K18) (CK
**1361**	XP_293563	29729703	similar to huntingtin-associated protein interacting protein (duo)
**1362**	XP_293567	29729857	similar to 60 kDa heat shock protein, mitochondrial precursor (Hsp60)
**1363**	XP_293602	30148456	similar to glutathione-S-transferase like; glutathione transferase ome
**1364**	XP_293610	30148492	similar to Heat shock cognate 71 kDa protein
**1365**	XP_293669	30147623	similar to actinin, alpha 4
**1366**	XP_293672	30149327	similar to ebiP7687
**1367**	XP_293689	29728393	similar to Heterogeneous nuclear ribonucleoprotein A1 (Helix-destabili
**1368**	XP_293716	30148644	similar to Heat shock protein HSP 90-alpha (HSP 86)
**1369**	XP_293759	29729950	similar to Heat shock protein HSP 90-alpha (HSP 86)
**1370**	XP_293762	30147536	similar to bridging integrator 2; bridging integrator-2; breast cancer
**1371**	XP_293786	29730632	similar to RAN, member RAS oncogene family
**1372**	XP_293814	29733950	similar to Keratin, type I cytoskeletal 18 (Cytokeratin 18) (K18) (CK
**1373**	XP_293924	29736622	similar to cytoplasmic beta-actin
**1374**	XP_293925	30153515	similar to phosphoglycerate mutase (EC 5.4.2.1) B chain - rat
**1375**	XP_293932	30153966	similar to hypothetical protein BC013073
**1376**	XP_293967	30154095	similar to gamma actin-like protein
**1377**	XP_294006	29739002	similar to 60S ribosomal protein L6 (TAX-responsive enhancer element b
**1378**	XP_294015	29739414	similar to Glyceraldehyde 3-phosphate dehydrogenase, liver (GAPDH)
**1379**	XP_294035	30160191	similar to Pyruvate kinase, M1 isozyme (Pyruvate kinase muscle isozyme
**1380**	XP_294045	29740794	similar to Translationally controlled tumor protein (TCTP) (p23) (Hist

	Refseq ID	gi Number	Protein Name/Description
**1381**	XP_294070	29741246	similar to Glyceraldehyde 3-phosphate dehydrogenase, liver (GAPDH)
**1382**	XP_294099	29742827	similar to myosin:SUBUNIT=regulatory light chain
**1383**	XP_294103	29742841	similar to 60S ribosomal protein L35
**1384**	XP_294126	30155280	similar to Keratin, type I cytoskeletal 18 (Cytokeratin 18) (K18) (CK
**1385**	XP_294215	29734916	similar to argininosuccinate synthetase
**1386**	XP_294234	30152366	similar to chromosome condensation-related SMC-associated protein 1
**1387**	XP_294438	30150422	similar to ribosomal protein L5; 60S ribosomal protein L5
**1388**	XP_294519	29732043	similar to chromosome 15 open reading frame 2
**1389**	XP_294531	30151752	similar to seven transmembrane helix receptor
**1390**	XP_294548	29733417	similar to hypothetical protein FLJ90396
**1391**	XP_294951	29729918	hypothetical protein XP_294951
**1392**	XP_295854	29741003	hypothetical protein XP_295854
**1393**	XP_296036	29746056	hypothetical protein XP_296036
**1394**	XP_296484	29736279	hypothetical protein XP_296484
**1395**	XP_296644	29740531	hypothetical protein XP_296644
**1396**	XP_296859	30159103	hypothetical protein XP_296859
**1397**	XP_297088	29743107	hypothetical protein XP_297088
**1398**	XP_297109	29743501	hypothetical protein XP_297109
**1399**	XP_297123	29743626	hypothetical protein XP_297123
**1400**	XP_297463	29730163	hypothetical protein XP_297463
**1401**	XP_297644	29732344	hypothetical protein XP_297644
**1402**	XP_297653	29732368	hypothetical protein XP_297653
**1403**	XP_297707	29732788	hypothetical protein XP_297707
**1404**	XP_297980	30157526	hypothetical protein XP_297980
**1405**	XP_298144	29727317	hypothetical protein XP_298144
**1406**	XP_298422	29727249	hypothetical protein XP_298422
**1407**	XP_298663	29730354	hypothetical protein XP_298663
**1408**	XP_299053	30154099	similar to High mobility group protein 1 (HMG-1) (Amphoterin) (Heparin
**1409**	XP_299177	29740132	hypothetical protein XP_299177
**1410**	XP_299313	30155257	hypothetical protein XP_299313
**1411**	XP_299768	29734623	hypothetical protein XP_299768
**1412**	XP_299876	29735989	hypothetical protein XP_299876
**1413**	XP_300550	30157434	similar to cDNA sequence, BC023835
**1414**	XP_300862	30150022	similar to beta-tubulin 4Q
**1415**	XP_301130	30148988	similar to Zinc finger protein 132
**1416**	XP_301154	30149555	similar to heat shock protein 86
**1417**	XP_301187	30151108	similar to actin beta, cytoskeletal - Kenyan clawed frog
**1418**	XP_301313	30156575	similar to caspase 1 isoform alpha precursor; interleukin 1-beta conve
**1419**	XP_301321	30156971	similar to Tubulin alpha-4 chain (Alpha-tubulin 4)
**1420**	XP_301350	30158159	similar to 60S RIBOSOMAL PROTEIN L34
**1421**	XP_301421	30158489	similar to Chloride intracellular channel protein 1 (Nuclear chloride
**1422**	XP_301425	30152949	similar to Heat shock protein HSP 90-beta (HSP 84) (HSP 90)
**1423**	XP_301443	30153769	similar to eukaryotic translation initiation factor 4A1; initiation fa
**1424**	XP_301485	30153076	similar to ubiquitin-conjugating enzyme E2N; 1500026J17Rik
**1425**	XP_301494	30152971	similar to RIKEN cDNA C730036D15
**1426**	XP_301540	30154839	similar to keratin 8; cytokeratin 8; keratin, type II cytoskeletal 8
**1427**	XP_301571	30156116	similar to Dual specificity protein phosphatase 3 (Dual specificity pr
**1428**	XP_301583	30158852	similar to actin CA15 - sea squirt (Styela clava) (fragments)
**1429**	XP_301585	30158950	similar to natural killer cell transcript 4
**1430**	XP_301652	30156349	similar to Eukaryotic initiation factor 4A-II (eIF-4A-II) (eIF4A-II)
**1431**	XP_301676	30157049	similar to G1/S-specific cyclin D3
**1432**	XP_301750	30147736	similar to Heterogeneous nuclear ribonucleoprotein A1 (Helix-destabili
**1433**	XP_301830	30150354	similar to Heterogeneous nuclear ribonucleoprotein A1 (Helix-destabili
**1434**	XP_301845	30157774	similar to Kunitz-type protease inhibitor 3 (HKIB9)
**1435**	XP_301899	30159310	similar to beta-actin
**1436**	XP_302052	30148500	similar to cytoplasmic actin
**1437**	XP_302053	30148290	similar to transrationally controlled tumor protein
**1438**	XP_302059	30148346	similar to MLRQ subunit of the NADH: ubiquinone oxidoreductase complex
**1439**	XP_302138	30149013	similar to alpha tubulin
**1440**	XP_302246	30159830	similar to HSPC280

	Refseq ID	gi Number	Protein Name/Description
**1441**	XP_303117	30148125	hypothetical protein XP_303117
**1442**	XP_303366	30155338	hypothetical protein XP_303366
**1443**	XP_304146	30156276	hypothetical protein XP_304146
**1444**	XP_304295	30147267	hypothetical protein XP_304295
**1445**	XP_304825	30147562	hypothetical protein XP_304825
**1446**	XP_305433	30155210	hypothetical protein XP_305433
**1447**	XP_305444	30155531	hypothetical protein XP_305444
**1448**	XP_305530	30152324	hypothetical protein XP_305530
**1449**	XP_305800	30152584	hypothetical protein XP_305800
**1450**	XP_305804	30149452	hypothetical protein XP_305804
**1451**	XP_305873	30150883	hypothetical protein XP_305873
