# Supplementary material for: Proteomic and Phospho-Proteomic Profile of Human Platelets in Basal, Resting State: Insights into Integrin Signaling
Source: PLoS One. 2009 Oct 27;4(10):e7627. doi: 10.1371/journal.pone.0007627 (PMC2762604; doi:10.1371/journal.pone.0007627)
Supplement: Table S3 — A list of all membrane-fraction associated proteins identified in this study. Protein uniprot name, Gene name, Uniprot accession number, Protein name, Gene ontology classification, Predicted sub-cellular localization and Protein family are shown based on its description in the Uniprot database (www.uniprot.org). (0.13 MB PDF) [file pone.0007627.s008.pdf]

	Entry name	Gene names	Uniprot Accession	Protein names	Gene Ontology	Subcellular Localization	Protein family
1	1433B_HUMAN	YWHAB	P31946	14-3-3 protein beta/alpha (Protein kinase C inhibitor protein 1) (KCIP-1) (Protein 1054) [Cleaved into: 14-3-3 protein beta/alpha, N-terminally processed]	Ras protein signal transduction; activation of pro-apoptotic gene products; centrosome; cytosol; enzyme binding; melanosome; negative regulation of protein amino acid dephosphorylation; nucleus; perinuclear region of cytoplasm; positive regulation of catalytic activity; protein domain specific binding	Cytoplasm. Melanosome.	14-3-3 family
2	1433E_HUMAN	YWHAE	P62258	14-3-3 protein epsilon (14-3-3E)	enzyme binding; interspecies interaction between organisms; intracellular signaling cascade; melanosome	Cytoplasm. Melanosome.	14-3-3 family
3	1433G_HUMAN	YWHAG	P61981	14-3-3 protein gamma (Protein kinase C inhibitor protein 1) (KCIP-1) [Cleaved into: 14-3-3 protein gamma, N-terminally processed]	cytoplasm; insulin-like growth factor receptor binding; negative regulation of protein kinase activity; protein kinase C binding; protein kinase C inhibitor activity; regulation of neuron differentiation; regulation of signal transduction; regulation of synaptic plasticity	Cytoplasm.	14-3-3 family
4	1433Z_HUMAN	YWHAZ	P63104	14-3-3 protein zeta/delta (Protein kinase C inhibitor protein 1) (KCIP-1)	anti-apoptosis; melanosome; signal transduction; transcription factor binding	Cytoplasm. Melanosome.	14-3-3 family
5	1A03_HUMAN	HLA-A (HLAA)	P04439	HLA class I histocompatibility antigen, A-3 alpha chain (MHC class I antigen A*3)	MHC class I protein complex; MHC class I receptor activity; antigen processing and presentation of peptide antigen via MHC class I; immune response; integral to plasma membrane; interspecies interaction between organisms; protein binding	Membrane; Single-pass type I membrane protein.	MHC class I family
6	1C07_HUMAN	HLA-C (HLAC)	P10321	HLA class I histocompatibility antigen, Cw-7 alpha chain (MHC class I antigen Cw*7)	MHC class I protein complex; antigen processing and presentation of peptide antigen via MHC class I; immune response; integral to membrane; interspecies interaction between organisms	Membrane; Single-pass type I membrane protein.	MHC class I family
7	A4_HUMAN	APP (A4) (AD1)	P05067	Amyloid beta A4 protein (Alzheimer disease amyloid protein) (ABPP) (APPI) (APP) (PreA4) (Cerebral vascular amyloid peptide) (CVAP) (Protease nexin-II) (PN-II) [Cleaved into: Soluble APP-alpha (S-APP-alpha); Soluble APP-beta (S-APP-beta); C99; Beta-amyloid protein 42 (Beta-APP42); Beta-amyloid protein 40 (Beta-APP40); C83; P3(42); P3(40); Gamma-secretase C-terminal fragment 59 (Gamma-CTF(59)) (Amyloid intracellular domain 59) (AICD-59) (AID(59)); Gamma-secretase C-terminal fragment 57 (Gamma-CTF(57)) (Amyloid intracellular domain 57) (AICD-57) (AID(57)); Gamma-secretase C-terminal fragment 50 (Gamma-CTF(50)) (Amyloid intracellular domain 50) (AICD-50) (AID(50)); C31]	Golgi apparatus; Notch signaling pathway; acetylcholine receptor binding; apoptosis; cell adhesion; cell surface; cellular copper ion homeostasis; coated pit; copper ion binding; endocytosis; extracellular region; heparin binding; identical protein binding; integral to plasma membrane; iron ion binding; neuromuscular process; platelet alpha granule lumen; serine-type endopeptidase inhibitor activity; zinc ion binding	Membrane; Single-pass type I membrane protein. Membrane , clathrin-coated pit.	APP family
8	ABI1_HUMAN	ABI1 (SSH3BP1)	Q8IZP0	Abl interactor 1 (Abelson interactor 1) (Abi-1) (Spectrin SH3 domain-binding protein 1) (Eps8 SH3 domain-binding protein) (Eps8-binding protein) (e3B1) (Nap1-binding protein) (Nap1BP) (Abl-binding protein 4) (AblBP4)	cell junction; cytoskeletal protein binding; cytoskeleton; cytosol; endoplasmic reticulum; filopodium; growth cone; lamellipodium; negative regulation of cell proliferation; nucleus; peptidyl-tyrosine phosphorylation; soluble fraction; synapse; synaptosome; transmembrane receptor protein tyrosine kinase signaling pathway	Cytoplasm. Nucleus. Cell projection , lamellipodium. Cell projection , filopodium. Cell projection , growth cone. Cell junction , synapse , synaptosome. Cytoplasm , cytoskeleton.	ABI family
9	ACOX1_HUMAN	ACOX1 (ACOX)	Q15067	Peroxisomal acyl-coenzyme A oxidase 1 (AOX) (EC 1.3.3.6) (Palmitoyl-CoA oxidase) (Straight-chain acyl-CoA oxidase) (SCOX)	FAD binding; acyl-CoA dehydrogenase activity; acyl-CoA oxidase activity; electron carrier activity; fatty acid beta-oxidation; generation of precursor metabolites and energy; oxidation reduction; prostaglandin metabolic process; protein N-terminus binding	Peroxisome.	Acyl-CoA oxidase family
10	ACTB_HUMAN	ACTB	P60709	Actin, cytoplasmic 1 (Beta-actin) [Cleaved into: Actin, cytoplasmic 1, N-terminally processed]	ATP binding; NuA4 histone acetyltransferase complex; cell motion; histone methyltransferase complex; nitric-oxide synthase binding; sensory perception of sound; structural constituent of cytoskeleton	Cytoplasm , cytoskeleton.	Actin family
11	ACTN1_HUMAN	ACTN1	P12814	Alpha-actinin-1 (Alpha-actinin cytoskeletal isoform) (Non-muscle alpha-actinin-1) (F-actin cross-linking protein)	Z disc; actin binding; calcium ion binding; cytoskeleton; focal adhesion; focal adhesion formation; integrin binding; negative regulation of cell motion; nucleolus; pseudopodium; regulation of apoptosis; vinculin binding	Cytoplasm , cytoskeleton. Cytoplasm , myofibril , sarcomere , Z-disk.	Alpha-actinin family

	Entry name	Gene names	Uniprot Accession	Protein names	Gene Ontology	Subcellular Localization	Protein family
**12**	ACTN2_HUMAN	ACTN2	P35609	Alpha-actinin-2 (Alpha-actinin skeletal muscle isoform 2) (F-actin cross-linking protein)	FATZ 1 binding; Z disc; ZASP binding; actin binding; actin filament; calcium ion binding; dendritic spine; filopodium; focal adhesion; focal adhesion formation; identical protein binding; integrin binding; microspike assembly; nucleolus; protein dimerization activity; protein homotetramerization; pseudopodium; regulation of apoptosis; structural constituent of muscle; titin Z domain binding; titin binding	Cytoplasm , myofibril , sarcomere , Z-disk.	Alpha-actinin family
**13**	ACTN4_HUMAN	ACTN4	O43707	Alpha-actinin-4 (Non-muscle alpha-actinin 4) (F-actin cross-linking protein)	actin filament binding; calcium ion binding; integrin binding; nucleolus; nucleoside binding; perinuclear region of cytoplasm; positive regulation of cell motion; positive regulation of sodium:hydrogen antiporter activity; protein complex; protein homodimerization activity; pseudopodium; regulation of apoptosis	Nucleus. Cytoplasm.	Alpha-actinin family
**14**	ACTS_HUMAN	ACTA1 (ACTA)	P68133	Actin, alpha skeletal muscle (Alpha-actin-1)	ADP binding; ATP binding; actin filament; muscle contraction; muscle thin filament assembly; myosin binding; skeletal muscle fiber development; stress fiber; striated muscle thin filament; structural constituent of cytoskeleton	Cytoplasm , cytoskeleton.	Actin family
**15**	ACTZ_HUMAN	ACTR1A (CTRN1)	P61163	Alpha-centractin (Centractin) (Centrosome-associated actin homolog) (Actin-RPV) (ARP1)	ATP binding; centrosome; dynactin complex; protein binding; vesicle-mediated transport	Cytoplasm , cytoskeleton. Centrosome.	Actin family, ARP1 subfamily
**16**	ADA10_HUMAN	ADAM10 (KUZ) (MADM)	O14672	Disintegrin and metalloproteinase domain-containing protein 10 (ADAM 10) (EC 3.4.24.81) (Mammalian disintegrin-metalloprotease) (Kuzbanian protein homolog) (CDw156) (CD antigen CD156c)	Golgi-associated vesicle; Notch signaling pathway; PMA-inducible membrane protein ectodomain proteolysis; SH3 domain binding; cell surface; cell-cell signaling; constitutive protein ectodomain proteolysis; endomembrane system; in utero embryonic development; integral to membrane; integrin binding; integrin-mediated signaling pathway; metalloendopeptidase activity; monocyte activation; negative regulation of cell adhesion; nucleus; plasma membrane; positive regulation of T cell chemotaxis; positive regulation of cell growth; positive regulation of cell proliferation; protein amino acid phosphorylation; protein homodimerization activity; protein kinase binding; response to tumor necrosis factor; zinc ion binding	Cell membrane; Single-pass type I membrane protein. Endomembrane system; Single-pass type I membrane protein.	
**17**	ADT1_HUMAN	SLC25A4 (ANT1)	P12235	ADP/ATP translocase 1 (Adenine nucleotide translocator 1) (ANT 1) (ADP,ATP carrier protein 1) (Solute carrier family 25 member 4) (ADP,ATP carrier protein, heart/skeletal muscle isoform T1)	adenine transmembrane transporter activity; generation of precursor metabolites and energy; integral to plasma membrane; interspecies interaction between organisms; mitochondrial genome maintenance; mitochondrial inner membrane; protein binding	Mitochondrion inner membrane; Multi-pass membrane protein.	Mitochondrial carrier family
**18**	ALDOA_HUMAN	ALDOA (ALDA)	P04075	Fructose-bisphosphate aldolase A (EC 4.1.2.13) (Muscle-type aldolase) (Lung cancer antigen NY-LU-1)	ATP biosynthetic process; I band; actin binding; actin cytoskeleton; actin filament organization; extracellular vesicular exosome; fructose 1,6-bisphosphate metabolic process; fructose binding; fructose-bisphosphate aldolase activity; glycolysis; identical protein binding; muscle maintenance; nucleus; regulation of cell shape; striated muscle contraction; tubulin binding		Class I fructose-bisphosphate aldolase family
**19**	ANXA5_HUMAN	ANXA5 (ANX5) (ENX2) (PP4)	P08758	Annexin A5 (Annexin-5) (Annexin V) (Lipocortin V) (Endonexin II) (Calphobindin I) (CBP-I) (Placental anticoagulant protein I) (PAP-I) (Placental anticoagulant protein 4) (PP4) (Thromboplastin inhibitor) (Vascular anticoagulant-alpha) (VAC-alpha) (Anchorin CII)	anti-apoptosis; blood coagulation; calcium ion binding; calcium-dependent phospholipid binding; cytoplasm; negative regulation of coagulation; phospholipase inhibitor activity; protein binding; signal transduction		Annexin family

	Entry name	Gene names	Uniprot Accession	Protein names	Gene Ontology	Subcellular Localization	Protein family
**20**	AP1G1_HUMAN	AP1G1 (ADTG) (CLAPG1)	O43747	AP-1 complex subunit gamma-1 (Adapter-related protein complex 1 subunit gamma-1) (Adaptor protein complex AP-1 subunit gamma 1) (Golgi adaptor HA1/AP1 adaptin subunit gamma-1) (Gamma1-adaptin) (Clathrin assembly protein complex 1 gamma-1 large chain)	Golgi apparatus part; clathrin adaptor complex; clathrin coated vesicle membrane; cytosol; endocytosis; intracellular protein transport; protein binding; protein transporter activity	Golgi apparatus. Cytoplasmic vesicle , clathrin-coated vesicle membrane; Peripheral membrane protein; Cytoplasmic side.	Adaptor complexes large subunit family
**21**	ARF1_HUMAN	ARF1	P84077	ADP-ribosylation factor 1	COPI coating of Golgi vesicle; GTP binding; GTPase activity; Golgi membrane; cytosol; plasma membrane; protein binding; protein transport; receptor signaling protein activity; retrograde vesicle-mediated transport, Golgi to ER; small GTPase mediated signal transduction	Golgi apparatus.	Small GTPase superfamily, Arf family
**22**	ARMET_HUMAN	ARMET (ARP)	P55145	Protein ARMET (Mesencephalic astrocyte-derived neurotrophic factor) (MANF) (Arginine-rich protein)	extracellular region; growth factor activity	Secreted.	ARMET family
**23**	ARP2_HUMAN	ACTR2 (ARP2)	P61160	Actin-related protein 2 (Actin-like protein 2)	ATP binding; Arp2/3 protein complex; actin binding; cell motion; cell projection; cytoplasm	Cytoplasm , cytoskeleton. Cell projection.	Actin family, ARP2 subfamily
**24**	ARP3_HUMAN	ACTR3 (ARP3)	P61158	Actin-related protein 3 (Actin-like protein 3)	ATP binding; Arp2/3 protein complex; actin binding; cell motion; cytoplasm; regulation of actin filament polymerization	Cytoplasm , cytoskeleton. Cell projection.	Actin family, ARP3 subfamily
**25**	ARPC2_HUMAN	ARPC2 (ARC34) (PRO2446)	O15144	Actin-related protein 2/3 complex subunit 2 (Arp2/3 complex 34 kDa subunit) (p34-ARC)	Arp2/3 protein complex; actin binding; cell motion; cell projection; cytoplasm; structural constituent of cytoskeleton	Cytoplasm , cytoskeleton. Cell projection.	ARPC2 family
**26**	ARPC5_HUMAN	ARPC5 (ARC16)	O15511	Actin-related protein 2/3 complex subunit 5 (Arp2/3 complex 16 kDa subunit) (p16-ARC)	Arp2/3 protein complex; actin binding; actin cytoskeleton organization; cell motion; cell projection; cytoplasm; regulation of actin filament polymerization; structural constituent of cytoskeleton	Cytoplasm , cytoskeleton. Cell projection.	ARPC5 family
**27**	AT2A1_HUMAN	ATP2A1	O14983	Sarcoplasmic/endoplasmic reticulum calcium ATPase 1 (SERCA1) (EC 3.6.3.8) (Calcium pump 1) (Calcium-transporting ATPase sarcoplasmic reticulum type, fast twitch skeletal muscle isoform) (SR Ca(2+)-ATPase 1) (Endoplasmic reticulum class 1/2 Ca(2+) ATPase)	ATP binding; ATP biosynthetic process; calcium ion binding; calcium ion transport; calcium-transporting ATPase activity; integral to membrane; magnesium ion binding; positive regulation of fast-twitch skeletal muscle fiber contraction; protein binding; sarcoplasmic reticulum membrane	Endoplasmic reticulum membrane; Multi-pass membrane protein. Sarcoplasmic reticulum membrane; Multi-pass membrane protein.	Cation transport ATPase (P-type) family, Type IIA subfamily
**28**	AT2A2_HUMAN	ATP2A2 (ATP2B)	P16615	Sarcoplasmic/endoplasmic reticulum calcium ATPase 2 (SERCA2) (EC 3.6.3.8) (Calcium pump 2) (Calcium-transporting ATPase sarcoplasmic reticulum type, slow twitch skeletal muscle isoform) (SR Ca(2+)-ATPase 2) (Endoplasmic reticulum class 1/2 Ca(2+) ATPase)	ATP binding; ATP biosynthetic process; S100 alpha binding; calcium ion binding; calcium-transporting ATPase activity; cell adhesion; epidermis development; integral to plasma membrane; magnesium ion binding; microsome; sarcoplasmic reticulum calcium ion transport; sarcoplasmic reticulum membrane	Endoplasmic reticulum membrane; Multi-pass membrane protein. Sarcoplasmic reticulum membrane; Multi-pass membrane protein.	Cation transport ATPase (P-type) family, Type IIA subfamily
**29**	AT2A3_HUMAN	ATP2A3	Q93084	Sarcoplasmic/endoplasmic reticulum calcium ATPase 3 (SERCA3) (EC 3.6.3.8) (Calcium pump 3) (SR Ca(2+)-ATPase 3)	ATP binding; ATP biosynthetic process; calcium ion binding; calcium ion transport; calcium-transporting ATPase activity; integral to membrane; magnesium ion binding; nuclear membrane; protein binding; sarcoplasmic reticulum membrane	Nucleus membrane; Multi-pass membrane protein. Endoplasmic reticulum membrane; Multi-pass membrane protein. Sarcoplasmic reticulum membrane; Multi-pass membrane protein.	Cation transport ATPase (P-type) family, Type IIA subfamily
**30**	ATD3A_HUMAN	ATAD3A	Q9NVI7	ATPase family AAA domain-containing protein 3A	ATP binding; nucleoside-triphosphatase activity; protein binding		AAA ATPase family
**31**	ATP5H_HUMAN	ATP5H (My032)	O75947	ATP synthase subunit d, mitochondrial	ATP synthesis coupled proton transport; hydrogen ion transmembrane transporter activity; mitochondrial proton-transporting ATP synthase complex, coupling factor F(o)	Mitochondrion. Mitochondrion inner membrane.	ATPase d subunit family
**32**	ATPB_HUMAN	ATP5B (ATPMB) (ATPSB)	P06576	ATP synthase subunit beta, mitochondrial (EC 3.6.3.14)	ATP binding; ATP synthesis coupled proton transport; MHC class I protein binding; angiogenesis; cell surface; eukaryotic cell surface binding; hydrogen ion transporting ATP synthase activity, rotational mechanism; hydrogen-exporting ATPase activity, phosphorylative mechanism; mitochondrial nucleoid; mitochondrial proton-transporting ATP synthase, catalytic core; plasma membrane; proton-transporting ATPase activity, rotational mechanism; regulation of intracellular pH	Mitochondrion. Mitochondrion inner membrane.	ATPase alpha/beta chains family

	Entry name	Gene names	Uniprot Accession	Protein names	Gene Ontology	Subcellular Localization	Protein family
**33**	ATPK_HUMAN	ATP5J2 (ATP5JL)	P56134	ATP synthase subunit f, mitochondrial	ATP biosynthetic process; hydrogen ion transmembrane transporter activity; mitochondrial proton-transporting ATP synthase complex; proton transport; proton-transporting ATP synthase complex, coupling factor F(o)	Mitochondrion. Mitochondrion inner membrane.	ATPase F chain family
**34**	ATPO_HUMAN	ATP5O (ATPO)	P48047	ATP synthase subunit O, mitochondrial (Oligomycin sensitivity conferral protein) (OSCP)	ATP catabolic process; drug binding; hydrogen ion transporting ATP synthase activity, rotational mechanism; mitochondrial ATP synthesis coupled proton transport; mitochondrial proton-transporting ATP synthase complex; plasma membrane; proton-transporting ATP synthase complex, catalytic core F(1); proton-transporting ATPase activity, rotational mechanism	Mitochondrion. Mitochondrion inner membrane.	ATPase delta chain family
**35**	B2MG_HUMAN	B2M (CDABP0092) (HDCMA22P)	P61769	Beta-2-microglobulin [Cleaved into: Beta-2-microglobulin form pI 5.3]	Golgi membrane; MHC class I protein complex; antigen processing and presentation of peptide antigen via MHC class I; early endosome membrane; extracellular region; immune response; protein binding	Secreted.	Beta-2-microglobulin family
**36**	BET1_HUMAN	BET1	O15155	BET1 homolog (hBET1) (Golgi vesicular membrane-trafficking protein p18)	ER to Golgi vesicle-mediated transport; Golgi membrane; endoplasmic reticulum membrane; integral to membrane; protein binding; protein transport	Golgi apparatus membrane; Single-pass type IV membrane protein. Endoplasmic reticulum membrane; Single-pass type IV membrane protein.	BET1 family
**37**	C109A_HUMAN	CCDC109A (C10orf42)	Q8NE86	Coiled-coil domain-containing protein 109A	integral to membrane	Membrane; Multi-pass membrane protein.	CCDC109 family
**38**	CALM_HUMAN	CALM1 (CALM) (CAM) (CAM1); CALM2 (CAM2) (CAMB); CALM3 (CALML2) (CAM3) (CAMC) (CAMIII)	P62158	Calmodulin (CaM)	G-protein coupled receptor protein signaling pathway; N-terminal myristoylation domain binding; calcium ion binding; centrosome; cytosol; negative regulation of ryanodine-sensitive calcium-release channel activity; plasma membrane; positive regulation of ryanodine-sensitive calcium-release channel activity; protein domain specific binding; regulation of cytokinesis; regulation of release of sequestered calcium ion into cytosol by sarcoplasmic reticulum; response to calcium ion; spindle microtubule; spindle pole; titin binding	Spindle.	Calmodulin family
**39**	CALX_HUMAN	CANX	P27824	Calnexin (Major histocompatibility complex class I antigen-binding protein p88) (p90) (IP90)	calcium ion binding; endoplasmic reticulum membrane; integral to membrane; melanosome; protein folding; protein secretion; sugar binding; unfolded protein binding	Endoplasmic reticulum membrane; Single-pass type I membrane protein. Melanosome.	Calreticulin family
**40**	CAN1_HUMAN	CAPN1 (CANPL1) (PIG30)	P07384	Calpain-1 catalytic subunit (EC 3.4.22.52) (Calpain-1 large subunit) (Calcium-activated neutral proteinase 1) (CANP 1) (Calpain mu-type) (muCANP) (Micromolar-calpain) (Cell proliferation-inducing gene 30 protein)	calcium ion binding; calcium-dependent cysteine-type endopeptidase activity; cytoplasm; plasma membrane; positive regulation of cell proliferation; protein binding; proteolysis	Cytoplasm. Cell membrane.	Peptidase C2 family
**41**	CAZA1_HUMAN	CAPZA1	P52907	F-actin-capping protein subunit alpha-1 (CapZ alpha-1)	F-actin capping protein complex; actin binding; actin cytoskeleton organization; barbed-end actin filament capping; cell motion; protein complex assembly		F-actin-capping protein alpha subunit family
**42**	CD36_HUMAN	CD36 (GP3B) (GP4)	P16671	Platelet glycoprotein 4 (Platelet glycoprotein IV) (GPIV) (Glycoprotein IIIb) (GPIIIB) (Leukocyte differentiation antigen CD36) (PAS IV) (PAS-4) (Platelet collagen receptor) (Fatty acid translocase) (FAT) (Thrombospondin receptor) (CD antigen CD36)	Golgi apparatus; blood coagulation; cGMP-mediated signaling; cell adhesion; cell surface; cholesterol transport; integral to plasma membrane; lipid binding; lipid metabolic process; lipid storage; lipoprotein catabolic process; lipoprotein particle clearance; lipoprotein transport; low-density lipoprotein receptor activity; membrane fraction; nitric oxide mediated signal transduction; plasma membrane long-chain fatty acid transport; platelet alpha granule membrane; positive regulation of cell-matrix adhesion; positive regulation of foam cell differentiation; thrombospondin receptor activity; transforming growth factor beta binding	Membrane; Multi-pass membrane protein.	CD36 family

	Entry name	Gene names	Uniprot Accession	Protein names	Gene Ontology	Subcellular Localization	Protein family
**43**	CD68_HUMAN	CD68	P34810	Macrosialin (Gp110) (CD antigen CD68)	endosome; integral to membrane; lysosomal membrane; membrane fraction; plasma membrane	Cell membrane; Single-pass type I membrane protein. Endosome membrane; Single-pass type I membrane protein. Lysosome membrane; Single-pass type I membrane protein.	LAMP family
**44**	CD9_HUMAN	CD9 (MIC3) (TSPAN29) (GIG2)	P21926	CD9 antigen (p24) (Leukocyte antigen MIC3) (Motility-related protein) (MRP-1) (Tetraspanin-29) (Tspan-29) (5H9 antigen) (Cell growth-inhibiting gene 2 protein) (CD antigen CD9)	cell adhesion; cell motion; fusion of sperm to egg plasma membrane; integral to plasma membrane; paranodal junction assembly; platelet activation; platelet alpha granule membrane; protein binding	Membrane; Multi-pass membrane protein.	Tetraspanin (TM4SF) family
**45**	CK059_HUMAN	C11orf59 (PP7157)	Q6IAA8	UPF0404 protein C11orf59			UPF0404 family
**46**	CLH1_HUMAN	CLTC (CLH17) (CLTCL2) (KIAA0034)	Q00610	Clathrin heavy chain 1 (CLH-17)	clathrin coat of coated pit; clathrin coat of trans-Golgi network vesicle; intracellular protein transport; melanosome; protein binding; structural molecule activity; vesicle-mediated transport	Cytoplasmic vesicle membrane; Peripheral membrane protein; Cytoplasmic side. Membrane , coated pit; Peripheral membrane protein; Cytoplasmic side. Melanosome.	Clathrin heavy chain family
**47**	CLUS_HUMAN	CLU (APOJ) (CLI) (KUB1) (AAG4)	P10909	Clusterin (Complement-associated protein SP-40,40) (Complement cytolysis inhibitor) (CLI) (NA1/NA2) (Apolipoprotein J) (Apo-J) (Testosterone-repressed prostate message 2) (TRPM-2) (Ku70-binding protein 1) (Aging-associated gene 4 protein) [Cleaved into: Clusterin beta chain (ApoJalpha) (Complement cytolysis inhibitor a chain); Clusterin alpha chain (ApoJbeta) (Complement cytolysis inhibitor b chain)]	apoptosis; complement activation, classical pathway; innate immune response; lipid metabolic process; protein binding; reverse cholesterol transport; spherical high-density lipoprotein particle	Secreted.	Clusterin family
**48**	CMC2_HUMAN	SLC25A13 (ARALAR2)	Q9UJS0	Calcium-binding mitochondrial carrier protein Aralar2 (Mitochondrial aspartate glutamate carrier 2) (Solute carrier family 25 member 13) (Citrin)	ATP biosynthetic process; L-aspartate transmembrane transporter activity; L-glutamate transmembrane transporter activity; L-glutamate transport; aspartate transport; calcium ion binding; integral to plasma membrane; malate-aspartate shuttle; mitochondrial inner membrane; response to calcium ion	Mitochondrion inner membrane; Multi-pass membrane protein.	Mitochondrial carrier family
**49**	COF1_HUMAN	CFL1 (CFL)	P23528	Cofilin-1 (Cofilin, non-muscle isoform) (18 kDa phosphoprotein) (p18)	Rho protein signal transduction; actin binding; anti-apoptosis; nuclear matrix	Nucleus matrix. Cytoplasm , cytoskeleton.	Actin-binding proteins ADF family
**50**	COR1B_HUMAN	CORO1B	Q9BR76	Coronin-1B (Coronin-2)	actin binding; cytoplasm; cytoskeleton	Cytoplasm , cytoskeleton.	WD repeat coronin family
**51**	CPNS1_HUMAN	CAPNS1 (CAPN4) (CAPNS)	P04632	Calpain small subunit 1 (CSS1) (Calcium-dependent protease small subunit 1) (Calcium-dependent protease small subunit) (CDPS) (Calpain regulatory subunit) (Calcium-activated neutral proteinase small subunit) (CANP small subunit)	calcium ion binding; calcium-dependent cysteine-type endopeptidase activity; cytoplasm; nucleus; plasma membrane; positive regulation of cell proliferation; protein binding	Cytoplasm. Cell membrane.	
**52**	CSRP1_HUMAN	CSRP1 (CSRP) (CYRP)	P21291	Cysteine and glycine-rich protein 1 (Cysteine-rich protein 1) (CRP1) (CRP)	nucleus; zinc ion binding	Nucleus.	
**53**	CX7A2_HUMAN	COX7A2 (COX7AL)	P14406	Cytochrome c oxidase polypeptide 7A2, mitochondrial (Cytochrome c oxidase polypeptide VIIa-liver/heart) (Cytochrome c oxidase subunit VIIa-L) (VIIaL)	cytochrome-c oxidase activity; electron carrier activity; mitochondrial respiratory chain	Mitochondrion inner membrane.	Cytochrome c oxidase VIIa family
**54**	CXCL7_HUMAN	PPBP (CTAP3) (CXCL7) (SCYB7) (TGB1) (THBGB1)	P02775	Platelet basic protein (PBP) (C-X-C motif chemokine 7) (Small-inducible cytokine B7) (Leukocyte-derived growth factor) (LDGF) (Macrophage-derived growth factor) (MDGF) [Cleaved into: Connective tissue-activating peptide III (CTAP-III) (Low-affinity platelet factor IV) (LA-PF4); TC-2; Connective tissue-activating peptide III(1-81) (CTAP-III(1-81)); Beta-thromboglobulin (Beta-TG); Neutrophil-activating peptide 2(74) (NAP-2(74)); Neutrophil-activating peptide 2(73) (NAP-2(73)); Neutrophil-activating peptide 2 (NAP-2); TC-1; Neutrophil-activating peptide 2(1-66) (NAP-2(1-66)); Neutrophil-activating peptide 2(1-63) (NAP-2(1-63))]	cell proliferation; chemokine activity; chemotaxis; defense response to bacterium; extracellular space; glucose transmembrane transporter activity; glucose transport; growth factor activity; immune response; platelet alpha granule lumen	Secreted.	Intercrine alpha (chemokine CxC) family
**55**	CYFP1_HUMAN	CYFIP1 (KIAA0068)	Q7L576	Cytoplasmic FMR1-interacting protein 1 (Specifically Rac1-associated protein 1) (Sra-1) (p140sra-1)	Rac GTPase binding; actin filament binding; axon extension; cell junction; lamellipodium; lamellipodium assembly; perinuclear region of cytoplasm; regulation of cell shape; ruffle; ruffle organization; synapse; synaptosome	Cytoplasm , perinuclear region. Cell projection , lamellipodium. Cell projection , ruffle. Cell junction , synapse , synaptosome.	CYFIP family
**56**	DCLK1_HUMAN	DCLK1 (DCAMKL1) (DCDC3A) (KIAA0369)	O15075	Serine/threonine-protein kinase DCLK1 (EC 2.7.11.1) (Doublecortin-like and CAM kinase-like 1) (Doublecortin-like kinase 1)	ATP binding; cell differentiation; central nervous system development; endosome transport; integral to plasma membrane; intracellular signaling cascade; protein amino acid phosphorylation; protein binding; protein serine/threonine kinase activity; receptor signaling protein activity		Protein kinase superfamily, CAMK Ser/Thr protein kinase family, CaMK subfamily

	Entry name	Gene names	Uniprot Accession	Protein names	Gene Ontology	Subcellular Localization	Protein family
**57**	DECR_HUMAN	DECR1 (DECR)	Q16698	2,4-dienoyl-CoA reductase, mitochondrial (EC 1.3.1.34) (2,4-dienoyl-CoA reductase [NADPH]) (4-enoyl-CoA reductase [NADPH])	2,4-dienoyl-CoA reductase (NADPH) activity; NADPH binding; fatty acid beta-oxidation; mitochondrion; oxidation reduction; protein homotetramerization	Mitochondrion.	Short-chain dehydrogenases/reductases (SDR) family, 2,4-dienoyl-CoA reductase subfamily
**58**	DHB12_HUMAN	HSD17B12	Q53GQ0	Estradiol 17-beta-dehydrogenase 12 (EC 1.1.1.62) (17-beta-hydroxysteroid dehydrogenase 12) (17-beta-HSD 12) (3-ketoacyl-CoA reductase) (KAR) (EC 1.3.1.-)	endoplasmic reticulum membrane; estradiol 17-beta-dehydrogenase activity; integral to membrane; oxidation reduction; steroid biosynthetic process	Endoplasmic reticulum membrane; Multi-pass membrane protein.	Short-chain dehydrogenases/reductases (SDR) family, 17-beta-HSD 3 subfamily
**59**	DHSA_HUMAN	SDHA (SDH2) (SDHF)	P31040	Succinate dehydrogenase [ubiquinone] flavoprotein subunit, mitochondrial (EC 1.3.5.1) (Flavoprotein subunit of complex II) (Fp)	FAD binding; electron carrier activity; mitochondrial respiratory chain complex II; nervous system development; protein binding; respiratory electron transport chain; succinate dehydrogenase (ubiquinone) activity; succinate metabolic process; transport; tricarboxylic acid cycle	Mitochondrion inner membrane; Peripheral membrane protein; Matrix side.	FAD-dependent oxidoreductase 2 family, FRD/SDH subfamily
**60**	EHD3_HUMAN	EHD3 (EHD2) (PAST3)	Q9NZN3	EH domain-containing protein 3	ATP binding; GTP binding; GTPase activity; calcium ion binding; nucleic acid binding; nucleus; plasma membrane; recycling endosome membrane	Cell membrane; Peripheral membrane protein; Cytoplasmic side. Endosome membrane; Peripheral membrane protein. Recycling endosome membrane; Peripheral membrane protein.	
**61**	ESAM_HUMAN	ESAM (UNQ220/PRO246)	Q96AP7	Endothelial cell-selective adhesion molecule	adherens junction; integral to membrane; protein binding; tight junction	Cell junction , adherens junction. Cell junction , tight junction. Cell membrane; Single-pass type I membrane protein.	
**62**	F13A_HUMAN	F13A1 (F13A)	P00488	Coagulation factor XIII A chain (Coagulation factor XIIIa) (EC 2.3.2.13) (Protein-glutamine gamma-glutamyltransferase A chain) (Transglutaminase A chain)	acyltransferase activity; blood coagulation; calcium ion binding; cytoplasm; extracellular region; protein-glutamine gamma-glutamyltransferase activity	Cytoplasm. Secreted.	Transglutaminase superfamily, Transglutaminase family
**63**	FHL1_HUMAN	FHL1 (SLIM1)	Q13642	Four and a half LIM domains protein 1 (FHL-1) (Skeletal muscle LIM-protein 1) (SLIM 1) (SLIM)	cell differentiation; cell growth; cytosol; muscle organ development; nucleus; organ morphogenesis; zinc ion binding	Cytoplasm. Cytoplasm. Nucleus. Nucleus. Cytoplasm , cytosol.	
**64**	FIBA_HUMAN	FGA	P02671	Fibrinogen alpha chain [Cleaved into: Fibrinopeptide A]	eukaryotic cell surface binding; external side of plasma membrane; fibrinogen complex; platelet activation; platelet alpha granule lumen; protein binding, bridging; protein polymerization; receptor binding; response to calcium ion; signal transduction	Secreted.	
**65**	FIBB_HUMAN	FGB	P02675	Fibrinogen beta chain [Cleaved into: Fibrinopeptide B]	eukaryotic cell surface binding; external side of plasma membrane; fibrinogen complex; nucleus; platelet activation; platelet alpha granule lumen; protein binding, bridging; protein polymerization; receptor binding; response to calcium ion; signal transduction; soluble fraction	Secreted.	
**66**	FIBG_HUMAN	FGG (PRO2061)	P02679	Fibrinogen gamma chain	calcium ion binding; eukaryotic cell surface binding; external side of plasma membrane; fibrinogen complex; platelet activation; platelet alpha granule lumen; protein binding, bridging; protein polymerization; receptor binding; response to calcium ion; signal transduction	Secreted.	
**67**	FINC_HUMAN	FN1 (FN)	P02751	Fibronectin (FN) (Cold-insoluble globulin) (CIG) [Cleaved into: Ugl-Y1; Ugl-Y2; Ugl-Y3]	ER-Golgi intermediate compartment; acute-phase response; cell migration; collagen binding; extracellular matrix structural constituent; fibrinogen complex; heparin binding; peptide cross-linking; platelet alpha granule lumen; proteinaceous extracellular matrix; regulation of cell shape; substrate adhesion-dependent cell spreading; transmembrane receptor protein tyrosine kinase signaling pathway	Secreted , extracellular space , extracellular matrix.	

	Entry name	Gene names	Uniprot Accession	Protein names	Gene Ontology	Subcellular Localization	Protein family
**68**	FLNA_HUMAN	FLNA (FLN) (FLN1)	P21333	Filamin-A (Alpha-filamin) (Filamin-1) (Endothelial actin-binding protein) (Actin-binding protein 280) (ABP-280) (Non-muscle filamin)	Fc-gamma receptor I complex binding; GTP-Ral binding; Rac GTPase binding; actin crosslink formation; actin cytoskeleton; actin cytoskeleton reorganization; actin filament binding; cell cortex; cytoplasmic sequestering of protein; establishment of protein localization; glycoprotein binding; inhibition of adenylate cyclase activity by dopamine receptor signaling pathway; negative regulation of protein catabolic process; negative regulation of transcription factor activity; nucleus; plasma membrane; positive regulation of I-kappaB kinase/NF-kappaB cascade; positive regulation of transcription factor import into nucleus; protein homodimerization activity; protein localization at cell surface; protein stabilization; receptor clustering; signal transducer activity; transcription factor binding	Cytoplasm , cell cortex. Cytoplasm , cytoskeleton.	Filamin family
**69**	FYN_HUMAN	FYN	P06241	Proto-oncogene tyrosine-protein kinase Fyn (EC 2.7.10.2) (p59-Fyn) (Protooncogene Syn) (SLK)	ATP binding; T cell receptor signaling pathway; calcium ion transport; cytosol; endosome; feeding behavior; glycoprotein binding; identical protein binding; interspecies interaction between organisms; learning; manganese ion binding; multicellular organismal development; non-membrane spanning protein tyrosine kinase activity; plasma membrane; protein amino acid phosphorylation; protein kinase cascade	Cell membrane.	Protein kinase superfamily, Tyr protein kinase family, SRC subfamily
**70**	G3P_HUMAN	GAPDH (GAPD) (CDABP0047) (OK/SW-cl.12)	P04406	Glyceraldehyde-3-phosphate dehydrogenase (GAPDH) (EC 1.2.1.12)	NAD or NADH binding; glyceraldehyde-3-phosphate dehydrogenase (phosphorylating) activity; glycolysis; membrane; oxidation reduction; perinuclear region of cytoplasm; protein binding	Cytoplasm. Cytoplasm , perinuclear region. Membrane.	Glyceraldehyde-3-phosphate dehydrogenase family
**71**	G6B_HUMAN	G6B (C6orf25)	O95866	Protein G6b	Golgi apparatus; endoplasmic reticulum; heparin binding; integral to membrane; plasma membrane; receptor activity	Endoplasmic reticulum. Golgi apparatus. Endoplasmic reticulum. Golgi apparatus. Cell membrane; Single-pass type I membrane protein. Cell membrane; Single-pass type I membrane protein.	
**72**	G6PD_HUMAN	G6PD	P11413	Glucose-6-phosphate 1-dehydrogenase (G6PD) (EC 1.1.1.49)	NADP or NADPH binding; cellular response to oxidative stress; centrosome; cholesterol biosynthetic process; cytokine production; cytosol; erythrocyte maturation; glucose 6-phosphate utilization; glucose binding; glucose-6-phosphate dehydrogenase activity; glutathione metabolic process; internal side of plasma membrane; intracellular membrane-bounded organelle; negative regulation of protein amino acid glutathionylation; oxidation reduction; pentose-phosphate shunt, oxidative branch; protein homodimerization activity; ribose phosphate biosynthetic process		Glucose-6-phosphate dehydrogenase family
**73**	GBB1_HUMAN	GNB1	P62873	Guanine nucleotide-binding protein G(I)/G(S)/G(T) subunit beta-1 (Transducin beta chain 1)	GTPase activity; Ras protein signal transduction; hormone-mediated signaling; muscarinic acetylcholine receptor signaling pathway; signal transducer activity		WD repeat G protein beta family
**74**	GELS_HUMAN	GSN	P06396	Gelsolin (Actin-depolymerizing factor) (ADF) (Brevin) (AGEL)	actin binding; actin cytoskeleton; actin filament polymerization; actin filament severing; barbed-end actin filament capping; calcium ion binding; cytosol; extracellular region	Cytoplasm , cytoskeleton. Secreted.	Villin/gelsolin family
**75**	GNA12_HUMAN	GNA12	Q03113	Guanine nucleotide-binding protein subunit alpha-12 (G-protein subunit alpha-12) (G alpha-12)	G-protein coupled receptor protein signaling pathway; GTP binding; GTPase activity; blood coagulation; plasma membrane; protein binding; signal transducer activity	Membrane; Lipid-anchor.	G-alpha family, G(12) subfamily

	Entry name	Gene names	Uniprot Accession	Protein names	Gene Ontology	Subcellular Localization	Protein family
**76**	GNAI2_HUMAN	GNAI2 (GNAI2B)	P04899	Guanine nucleotide-binding protein G(i), alpha-2 subunit (Adenylate cyclase-inhibiting G alpha protein)	GTP binding; GTPase activity; protein binding; response to nutrient; signal transducer activity		G-alpha family, G(i/o/t/z) subfamily
**77**	GP1BB_HUMAN	GP1BB	P13224	Platelet glycoprotein Ib beta chain (GP-Ib beta) (GPIb-beta) (GPIbB) (Antigen CD42b-beta) (CD antigen CD42c)	cell adhesion; cell surface receptor linked signal transduction; integral to plasma membrane; platelet activation; platelet alpha granule membrane; protein binding; transmembrane receptor activity	Membrane; Single-pass type I membrane protein.	
**78**	GPDM_HUMAN	GPD2	P43304	Glycerol-3-phosphate dehydrogenase, mitochondrial (GPDH-M) (GPD-M) (EC 1.1.5.3) (mtGPD)	calcium ion binding; glycerol-3-phosphate dehydrogenase activity; glycerol-3-phosphate dehydrogenase complex; oxidation reduction	Mitochondrion.	FAD-dependent glycerol-3-phosphate dehydrogenase family
**79**	GPV_HUMAN	GP5	P40197	Platelet glycoprotein V (GPV) (CD antigen CD42D)	blood coagulation; cell adhesion; integral to plasma membrane; platelet alpha granule membrane; protein binding	Membrane; Single-pass type I membrane protein.	
**80**	GSH0_HUMAN	GCLM (GLCLR)	P48507	Glutamate--cysteine ligase regulatory subunit (Gamma-glutamylcysteine synthetase regulatory subunit) (Gamma-ECS regulatory subunit) (GCS light chain) (Glutamate--cysteine ligase modifier subunit)	cytosol; glutamate metabolic process; glutamate-cysteine ligase catalytic subunit binding; glutathione biosynthetic process; oxidation reduction; oxidoreductase activity; regulation of blood vessel size; response to drug; response to oxidative stress; soluble fraction		Aldo/keto reductase family, Glutamate--cysteine ligase light chain subfamily
**81**	GTR3_HUMAN	SLC2A3 (GLUT3)	P11169	Solute carrier family 2, facilitated glucose transporter member 3 (Glucose transporter type 3, brain) (GLUT-3)	carbohydrate metabolic process; cytoplasm; glucose transmembrane transporter activity; glucose transport; integral to membrane; plasma membrane; sugar:hydrogen symporter activity; transmembrane transport	Membrane; Multi-pass membrane protein.	Major facilitator superfamily, Sugar transporter (TC 2.A.1.1) family, Glucose transporter subfamily
**82**	HPCA_HUMAN	HPCA (BDR2)	P84074	Neuron-specific calcium-binding protein hippocalcin (Calcium-binding protein BDR-2)	actin binding; calcium ion binding		Recoverin family
**83**	HSP7C_HUMAN	HSPA8 (HSC70) (HSP73) (HSPA10)	P11142	Heat shock cognate 71 kDa protein (Heat shock 70 kDa protein 8)	ATP binding; ATPase activity, coupled; cell surface; melanosome; protein binding; protein folding; response to unfolded protein	Cytoplasm. Melanosome.	Heat shock protein 70 family
**84**	HSPB1_HUMAN	HSPB1 (HSP27) (HSP28)	P04792	Heat shock protein beta-1 (HspB1) (Heat shock 27 kDa protein) (HSP 27) (Stress-responsive protein 27) (SRP27) (Estrogen-regulated 24 kDa protein) (28 kDa heat shock protein)	anti-apoptosis; cell death; cell motion; cell surface; cytoplasm; identical protein binding; nucleus; regulation of translational initiation; response to heat; response to unfolded protein; spindle	Cytoplasm. Nucleus. Spindle.	Small heat shock protein (HSP20) family
**85**	HXK1_HUMAN	HK1	P19367	Hexokinase-1 (EC 2.7.1.1) (Hexokinase type I) (HK I) (Brain form hexokinase)	ATP binding; cytosol; glycolysis; hexokinase activity; mitochondrial outer membrane	Mitochondrion outer membrane.	Hexokinase family
**86**	HXK2_HUMAN	HK2	P52789	Hexokinase-2 (EC 2.7.1.1) (Hexokinase type II) (HK II) (Muscle form hexokinase)	ATP binding; cytosol; glycolysis; hexokinase activity; mitochondrial outer membrane	Mitochondrion outer membrane.	Hexokinase family
**87**	IF5A1_HUMAN	EIF5A	P63241	Eukaryotic translation initiation factor 5A-1 (eIF-5A-1) (eIF-5A1) (Eukaryotic initiation factor 5A isoform 1) (eIF-5A) (eIF-4D) (Rev-binding factor)	U6 snRNA binding; annulate lamellae; cell proliferation; cytosol; induction of apoptosis; mRNA export from nucleus; nuclear pore; peptidyl-lysine modification to hypusine; positive regulation of translational initiation; protein N-terminus binding; protein export from nucleus; translation initiation factor activity; translational initiation; viral genome replication	Nucleus.	EIF-5A family
**88**	ILVBL_HUMAN	ILVBL (AHAS)	A1L0T0	Acetolactate synthase-like protein (EC 2.2.1.-) (IlvB-like protein)	integral to membrane; magnesium ion binding; thiamin pyrophosphate binding; transferase activity	Membrane; Single-pass membrane protein.	TPP enzyme family
**89**	IQGA2_HUMAN	IQGAP2	Q13576	Ras GTPase-activating-like protein IQGAP2	GTPase inhibitor activity; Ras GTPase activator activity; actin binding; actin cytoskeleton; calmodulin binding; regulation of small GTPase mediated signal transduction; small GTPase mediated signal transduction		
**90**	ITA2_HUMAN	ITGA2 (CD49B)	P17301	Integrin alpha-2 (Platelet membrane glycoprotein Ia) (GPIa) (Collagen receptor) (VLA-2 alpha chain) (CD49 antigen-like family member B) (CD antigen CD49b)	blood coagulation; calcium ion binding; cell-matrix adhesion; collagen binding; identical protein binding; integrin complex; integrin-mediated signaling pathway; interspecies interaction between organisms; magnesium ion binding; organ morphogenesis; receptor activity	Membrane; Single-pass type I membrane protein.	Integrin alpha chain family
**91**	ITA2B_HUMAN	ITGA2B (GP2B) (ITGAB)	P08514	Integrin alpha-IIb (Platelet membrane glycoprotein IIb) (GPalpha IIb) (GPIIb) (CD antigen CD41) [Cleaved into: Integrin alpha-IIb heavy chain; Integrin alpha-IIb light chain, form 1; Integrin alpha-IIb light chain, form 2]	calcium ion binding; cell adhesion; identical protein binding; integrin complex; integrin-mediated signaling pathway; platelet alpha granule membrane; receptor activity	Membrane; Single-pass type I membrane protein.	Integrin alpha chain family

	Entry name	Gene names	Uniprot Accession	Protein names	Gene Ontology	Subcellular Localization	Protein family
**92**	ITB1_HUMAN	ITGB1 (FNRB) (MDF2) (MSK12)	P05556	Integrin beta-1 (Fibronectin receptor subunit beta) (Integrin VLA-4 subunit beta) (CD antigen CD29)	B cell differentiation; cell surface; cell-cell adhesion mediated by integrin; cell-matrix adhesion; cellular defense response; homophilic cell adhesion; identical protein binding; integrin complex; integrin-mediated signaling pathway; interspecies interaction between organisms; leukocyte adhesion; melanosome; neuromuscular junction; protein heterodimerization activity; receptor activity; ruffle; sarcolemma	Cell membrane; Single-pass type I membrane protein. Melanosome.	Integrin beta chain family
**93**	ITB3_HUMAN	ITGB3 (GP3A)	P05106	Integrin beta-3 (Platelet membrane glycoprotein IIIa) (GPIIIa) (CD antigen CD61)	blood coagulation; cell-matrix adhesion; identical protein binding; integrin complex; integrin-mediated signaling pathway; interspecies interaction between organisms; negative regulation of foam cell differentiation; negative regulation of lipid storage; negative regulation of lipid transport; negative regulation of lipoprotein metabolic process; negative regulation of low-density lipoprotein receptor biosynthetic process; platelet alpha granule membrane; receptor activity	Membrane; Single-pass type I membrane protein.	Integrin beta chain family
**94**	JAM1_HUMAN	F11R (JAM1) (JCAM) (UNQ264/PRO301)	Q9Y624	Junctional adhesion molecule A (JAM-A) (Junctional adhesion molecule 1) (JAM-1) (Platelet adhesion molecule 1) (PAM-1) (Platelet F11 receptor) (CD antigen CD321)	inflammatory response; integral to membrane; interspecies interaction between organisms; tight junction	Cell junction , tight junction. Cell membrane; Single-pass type I membrane protein.	Immunoglobulin superfamily
**95**	JAM3_HUMAN	JAM3 (UNQ859/PRO1868)	Q9BX67	Junctional adhesion molecule C (JAM-C) (Junctional adhesion molecule 3) (JAM-3) (JAM-2)	integral to membrane; plasma membrane	Cell membrane; Single-pass type I membrane protein.	Immunoglobulin superfamily
**96**	K6PL_HUMAN	PFKL	P17858	6-phosphofructokinase, liver type (EC 2.7.1.11) (Phosphofructokinase 1) (Phosphohexokinase) (Phosphofructo-1-kinase isozyme B) (PFK-B)	6-phosphofructokinase activity; 6-phosphofructokinase complex; ATP binding; fructose 6-phosphate metabolic process; fructose-6-phosphate binding; glycolysis; identical protein binding; kinase binding; magnesium ion binding; protein oligomerization		Phosphofructokinase family, Two domains subfamily
**97**	K6PP_HUMAN	PFKP (PFKF)	Q01813	6-phosphofructokinase type C (EC 2.7.1.11) (Phosphofructokinase 1) (Phosphohexokinase) (Phosphofructo-1-kinase isozyme C) (PFK-C) (6-phosphofructokinase, platelet type)	6-phosphofructokinase activity; 6-phosphofructokinase complex; ATP binding; glycolysis; magnesium ion binding; protein binding		Phosphofructokinase family, Two domains subfamily
**98**	LIMS1_HUMAN	LIMS1 (PINCH) (PINCH1)	P48059	LIM and senescent cell antigen-like-containing domain protein 1 (Particularly interesting new Cys-His protein 1) (PINCH-1) (Renal carcinoma antigen NY-REN-48)	cell aging; focal adhesion; protein binding; zinc ion binding	Cell junction , focal adhesion. Cell membrane; Peripheral membrane protein; Cytoplasmic side.	
**99**	LOX12_HUMAN	ALOX12 (LOG12)	P18054	Arachidonate 12-lipoxygenase, 12S-type (12-LOX) (EC 1.13.11.31) (Platelet-type lipoxygenase 12)	anti-apoptosis; arachidonate 12-lipoxygenase activity; cell motion; cytosol; fatty acid oxidation; hepoxilin-epoxide hydrolase activity; iron ion binding; leukotriene biosynthetic process; lipoxygenase activity; oxidation reduction; positive regulation of cell adhesion; positive regulation of cell growth; positive regulation of cell proliferation; protein binding; sarcolemma; superoxide release	Cytoplasm.	Lipoxygenase family
**100**	LRBA_HUMAN	LRBA (BGL) (CDC4L) (LBA)	P50851	Lipopolysaccharide-responsive and beige-like anchor protein (CDC4-like protein) (Beige-like protein)	protein binding		
**101**	LYAM3_HUMAN	SELP (GMRP) (GRMP)	P16109	P-selectin (Granule membrane protein 140) (GMP-140) (PADGEM) (Leukocyte-endothelial cell adhesion molecule 3) (LECAM3) (CD62 antigen-like family member P) (CD antigen CD62P)	defense response to Gram-negative bacterium; external side of plasma membrane; extracellular space; fucose binding; glycosphingolipid binding; heparin binding; integral to plasma membrane; leukocyte adhesion; lipopolysaccharide binding; membrane fraction; oligosaccharide binding; platelet alpha granule membrane; positive regulation of platelet activation; protein binding; response to lipopolysaccharide; sialic acid binding; soluble fraction	Membrane; Single-pass type I membrane protein.	Selectin/LECAM family
**102**	MARE2_HUMAN	MAPRE2 (RP1)	Q15555	Microtubule-associated protein RP/EB family member 2 (APC-binding protein EB2) (End-binding protein 2) (EB2)	cell division; cell proliferation; cytoplasm; microtubule; microtubule binding; mitosis; signal transduction	Cytoplasm.	MAPRE family

	Entry name	Gene names	Uniprot Accession	Protein names	Gene Ontology	Subcellular Localization	Protein family
**103**	MDHM_HUMAN	MDH2	P40926	Malate dehydrogenase, mitochondrial (EC 1.1.1.37)	L-malate dehydrogenase activity; binding; glycolysis; malate metabolic process; mitochondrial matrix; oxidation reduction; tricarboxylic acid cycle	Mitochondrion matrix.	LDH/MDH superfamily, MDH type 1 family
**104**	MGLL_HUMAN	MGLL	Q99685	Monoglyceride lipase (MGL) (EC 3.1.1.23) (Lysophospholipase homolog) (Lysophospholipase-like) (HU-K5)	acylglycerol lipase activity; inflammatory response; lipid metabolic process; lysophospholipase activity		AB hydrolase superfamily, Monoacylglycerol lipase family
**105**	ML12A_HUMAN	MYL12A (MLCB) (MRLC3) (RLC)	P19105	Myosin regulatory light chain 12A (Myosin regulatory light chain MRLC3) (Myosin regulatory light chain 2, nonsarcomeric) (Myosin RLC) (MLC-2B)	calcium ion binding; motor activity; myosin complex; protein binding		
**106**	MLE1_HUMAN	MYL1	P05976	Myosin light chain 1, skeletal muscle isoform (MLC1F) (A1 catalytic) (Alkali myosin light chain 1)	calcium ion binding; motor activity; muscle filament sliding; muscle myosin complex; muscle organ development; sarcomere; structural constituent of muscle		
**107**	MMRN1_HUMAN	MMRN1 (ECM) (EMILIN4) (GPIA*) (MMRN)	Q13201	Multimerin-1 (Endothelial cell multimerin) (EMILIN-4) (Elastin microfibril interface located protein 4) (Elastin microfibril interfacer 4) [Cleaved into: Platelet glycoprotein Ia*; 155 kDa platelet multimerin (p-155) (p155)]	blood coagulation; cell adhesion; extracellular region; platelet alpha granule lumen	Secreted.	
**108**	MPKS1_HUMAN	MAPKSP1 (MAP2K1IP1) (PRO2783)	Q9UHA4	Mitogen-activated protein kinase scaffold protein 1 (Mitogen-activated protein kinase kinase 1-interacting protein 1) (MEK-binding partner 1) (Mp1)	late endosome membrane; protein binding	Late endosome membrane; Peripheral membrane protein; Cytoplasmic side.	MAPKSP1 family
**109**	MYH11_HUMAN	MYH11 (KIAA0866)	P35749	Myosin-11 (Myosin heavy chain 11) (Myosin heavy chain, smooth muscle isoform) (SMMHC)	ATP binding; actin binding; calmodulin binding; cardiac muscle fiber development; elastic fiber assembly; melanosome; motor activity; muscle thick filament assembly; smooth muscle contraction; striated muscle contraction; striated muscle thick filament; structural constituent of muscle	Melanosome.	
**110**	MYH9_HUMAN	MYH9	P35579	Myosin-9 (Myosin heavy chain 9) (Myosin heavy chain, non-muscle IIa) (Non-muscle myosin heavy chain IIa) (NMMHC II-a) (NMMHC-IIA) (Cellular myosin heavy chain, type A) (Non-muscle myosin heavy chain A) (NMMHC-A)	ADP binding; ATP binding; actin cytoskeleton reorganization; actin filament binding; actin filament-based movement; actin-dependent ATPase activity; angiogenesis; blood vessel endothelial cell migration; calmodulin binding; cleavage furrow; contractile ring; cytokinesis; cytosol; integrin-mediated signaling pathway; leukocyte migration; membrane protein ectodomain proteolysis; microfilament motor activity; monocyte differentiation; myosin complex; nucleus; platelet formation; protein anchor; protein homodimerization activity; protein transport; regulation of cell shape; ruffle; sensory perception of sound; stress fiber; uropod		
**111**	MYL6_HUMAN	MYL6	P60660	Myosin light polypeptide 6 (Smooth muscle and nonmuscle myosin light chain alkali 6) (Myosin light chain alkali 3) (Myosin light chain 3) (MLC-3) (LC17)	actin-dependent ATPase activity; calcium ion binding; motor activity; muscle filament sliding; skeletal muscle tissue development; structural constituent of muscle; unconventional myosin complex		
**112**	MYL6B_HUMAN	MYL6B (MLC1SA)	P14649	Myosin light chain 6B (Smooth muscle and nonmuscle myosin light chain alkali 6B) (Myosin light chain 1 slow-twitch muscle A isoform) (MLC1sa)	calcium ion binding; motor activity; muscle filament sliding; muscle myosin complex; protein binding; skeletal muscle tissue development; structural constituent of muscle; unconventional myosin complex		
**113**	MYL9_HUMAN	MYL9 (MLC2) (MRLC1) (MYRL2)	P24844	Myosin regulatory light polypeptide 9 (Myosin regulatory light chain 9) (Myosin regulatory light chain MRLC1) (Myosin regulatory light chain 2, smooth muscle isoform) (Myosin RLC) (MLC-2C) (LC20)	calcium ion binding; motor activity; muscle myosin complex; regulation of muscle contraction; structural constituent of muscle		
**114**	NB5R3_HUMAN	CYB5R3 (DIA1)	P00387	NADH-cytochrome b5 reductase 3 (Cytochrome b5 reductase) (B5R) (EC 1.6.2.2) (Diaphorase-1) [Cleaved into: NADH-cytochrome b5 reductase 3 membrane-bound form; NADH-cytochrome b5 reductase 3 soluble form]	blood circulation; cholesterol biosynthetic process; cytochrome-b5 reductase activity; electron carrier activity; endoplasmic reticulum membrane; hemoglobin complex; mitochondrial outer membrane; oxidation reduction	Endoplasmic reticulum membrane; Lipid-anchor; Cytoplasmic side. Mitochondrion outer membrane; Lipid-anchor; Cytoplasmic side. Cytoplasm.	Flavoprotein pyridine nucleotide cytochrome reductase family
**115**	NDUB8_HUMAN	NDUFB8	O95169	NADH dehydrogenase [ubiquinone] 1 beta subcomplex subunit 8, mitochondrial (NADH-ubiquinone oxidoreductase ASHI subunit) (Complex I-ASHI) (CI-ASHI)	NADH dehydrogenase (ubiquinone) activity; endoplasmic reticulum; integral to membrane; mitochondrial electron transport, NADH to ubiquinone; mitochondrial respiratory chain complex I; transport	Mitochondrion inner membrane; Single-pass membrane protein; Matrix side.	Complex I NDUFB8 subunit family

	Entry name	Gene names	Uniprot Accession	Protein names	Gene Ontology	Subcellular Localization	Protein family
**116**	NNTM_HUMAN	NNT	Q13423	NAD(P) transhydrogenase, mitochondrial (EC 1.6.1.2) (Nicotinamide nucleotide transhydrogenase) (Pyridine nucleotide transhydrogenase)	NAD or NADH binding; NAD(P)+ transhydrogenase (AB-specific) activity; NAD(P)+ transhydrogenase (B-specific) activity; NADP or NADPH binding; electron carrier activity; integral to membrane; mitochondrial respiratory chain; oxidation reduction; proton transport; tricarboxylic acid cycle	Mitochondrion inner membrane; Multi-pass membrane protein; Matrix side.	AlaDH/PNT family; PNT beta subunit family
**117**	PARVA_HUMAN	PARVA (MXRA2)	Q9NVD7	Alpha-parvin (Calponin-like integrin-linked kinase-binding protein) (CH-ILKBP) (Matrix-remodeling-associated protein 2) (Actopaxin)	actin binding; cell adhesion; cytoplasm; cytoskeleton; focal adhesion	Cell junction , focal adhesion. Cell membrane; Peripheral membrane protein; Cytoplasmic side. Cytoplasm , cytoskeleton.	Parvin family
**118**	PARVB_HUMAN	PARVB (CGI-56)	Q9HBI1	Beta-parvin (Affixin)	actin binding; cell adhesion; cytoplasm; cytoskeleton; focal adhesion	Cell junction , focal adhesion. Cell membrane; Peripheral membrane protein; Cytoplasmic side. Cytoplasm , cytoskeleton.	Parvin family
**119**	PCF11_HUMAN	PCF11 (KIAA0824)	O94913	Pre-mRNA cleavage complex 2 protein Pcf11 (Pre-mRNA cleavage complex II protein Pcf11)	mRNA cleavage; mRNA cleavage factor complex; nuclear mRNA splicing, via spliceosome	Nucleus.	
**120**	PCYXL_HUMAN	PCYOX1L (PSEC0105)	Q8NBM8	Prenylcysteine oxidase-like (EC 1.8.3.-)	extracellular region; oxidation reduction; oxidoreductase activity, acting on sulfur group of donors, oxygen as acceptor; prenylcysteine catabolic process	Secreted.	Prenylcysteine oxidase family
**121**	PDIA3_HUMAN	PDIA3 (ERP57) (ERP60) (GRP58)	P30101	Protein disulfide-isomerase A3 (EC 5.3.4.1) (Disulfide isomerase ER-60) (ERp60) (58 kDa microsomal protein) (p58) (ERp57) (58 kDa glucose-regulated protein)	cell redox homeostasis; cysteine-type endopeptidase activity; endoplasmic reticulum; endoplasmic reticulum lumen; melanosome; phospholipase C activity; protein binding; protein disulfide isomerase activity; protein import into nucleus; protein retention in ER lumen; signal transduction	Endoplasmic reticulum lumen. Melanosome.	Protein disulfide isomerase family
**122**	PDIA6_HUMAN	PDIA6 (TXNDC7)	Q15084	Protein disulfide-isomerase A6 (EC 5.3.4.1) (Protein disulfide isomerase P5) (Thioredoxin domain-containing protein 7)	ER-Golgi intermediate compartment; cell redox homeostasis; endoplasmic reticulum; endoplasmic reticulum lumen; melanosome; protein binding; protein disulfide isomerase activity; protein folding	Endoplasmic reticulum lumen. Melanosome.	Protein disulfide isomerase family
**123**	PECA1_HUMAN	PECAM1	P16284	Platelet endothelial cell adhesion molecule (PECAM-1) (EndoCAM) (GPIIA') (CD antigen CD31)	cell adhesion; cell recognition; extracellular space; integral to membrane; plasma membrane; platelet alpha granule membrane; protein binding; signal transduction	Membrane; Single-pass type I membrane protein.	
**124**	PGH1_HUMAN	PTGS1 (COX1)	P23219	Prostaglandin G/H synthase 1 (EC 1.14.99.1) (Cyclooxygenase-1) (COX-1) (Prostaglandin-endoperoxide synthase 1) (Prostaglandin H2 synthase 1) (PGH synthase 1) (PGHS-1) (PHS 1)	Golgi apparatus; endoplasmic reticulum membrane; heme binding; microsome; nucleus; oxidation reduction; oxidoreductase activity, acting on single donors with incorporation of molecular oxygen, incorporation of two atoms of oxygen; peroxidase activity; plasma membrane; prostaglandin biosynthetic process; prostaglandin-endoperoxide synthase activity; regulation of blood pressure; response to oxidative stress	Microsome membrane; Peripheral membrane protein. Endoplasmic reticulum membrane; Peripheral membrane protein.	Prostaglandin G/H synthase family
**125**	PHF2_HUMAN	PHF2 (KIAA0662)	O75151	PHD finger protein 2 (GRC5)	nucleus; protein binding; zinc ion binding	Nucleus.	
**126**	PI4KA_HUMAN	PI4KA (PIK4) (PIK4CA)	P42356	Phosphatidylinositol 4-kinase alpha (PI4-kinase alpha) (PtdIns-4-kinase alpha) (PI4K-alpha) (EC 2.7.1.67)	1-phosphatidylinositol 4-kinase activity; ATP binding; Golgi-associated vesicle; phosphatidylinositol biosynthetic process; phosphoinositide phosphorylation; phosphoinositide-mediated signaling; protein binding; synaptic transmission		PI3/PI4-kinase family, Type III PI4K subfamily

	Entry name	Gene names	Uniprot Accession	Protein names	Gene Ontology	Subcellular Localization	Protein family
**127**	PLEK_HUMAN	PLEK (P47)	P08567	Pleckstrin (Platelet p47 protein)	actin cytoskeleton reorganization; cortical actin cytoskeleton organization; cytoplasm; hemopoietic progenitor cell differentiation; inhibition of phospholipase C activity involved in G-protein coupled receptor signaling pathway; integrin-mediated signaling pathway; membrane fraction; negative regulation of calcium-mediated signaling; negative regulation of inositol phosphate biosynthetic process; phosphate binding; phosphatidylinositol-3,4-bisphosphate binding; phosphoinositide metabolic process; platelet aggregation; positive regulation of actin filament bundle formation; positive regulation of actin filament depolymerization; positive regulation of inositol-polyphosphate 5-phosphatase activity; positive regulation of integrin activation; positive regulation of platelet activation; protein homodimerization activity; protein kinase C binding; protein kinase C signaling cascade; protein secretion by platelet; regulation of cell		
**128**	PLF4_HUMAN	PF4 (CXCL4) (SCYB4)	P02776	Platelet factor 4 (PF-4) (C-X-C motif chemokine 4) (Oncostatin-A) (Iroplact) [Cleaved into: Platelet factor 4, short form]	chemokine activity; cytokine-mediated signaling pathway; extracellular space; heparin binding; immune response; leukocyte chemotaxis; negative regulation of MHC class II biosynthetic process; negative regulation of angiogenesis; negative regulation of apoptosis; negative regulation of cytolysis; negative regulation of megakaryocyte differentiation; platelet activation; platelet alpha granule lumen; positive regulation of foam cell differentiation; positive regulation of gene expression; positive regulation of tumor necrosis factor production	Secreted.	Intercrine alpha (chemokine CxC) family
**129**	PP1B_HUMAN	PPP1CB	P62140	Serine/threonine-protein phosphatase PP1-beta catalytic subunit (PP-1B) (EC 3.1.3.16)	cell cycle; cell division; cytoplasm; glycogen metabolic process; histone methyltransferase complex; iron ion binding; manganese ion binding; protein binding	Cytoplasm.	PPP phosphatase family, PP-1 subfamily
**130**	PPIA_HUMAN	PPIA (CYPA)	P62937	Peptidyl-prolyl cis-trans isomerase A (PPIase A) (Rotamase A) (EC 5.2.1.8) (Cyclophilin A) (Cyclosporin A-binding protein)	cytosol; extracellular region; initiation of viral infection; interspecies interaction between organisms; nucleus; peptide binding; peptidyl-prolyl cis-trans isomerase activity; protein folding; provirus integration; regulation of viral genome replication; unfolded protein binding; virion binding	Cytoplasm.	Cyclophilin-type PPIase family, PPIase A subfamily
**131**	PPIB_HUMAN	PPIB (CYPB)	P23284	Peptidyl-prolyl cis-trans isomerase B (PPIase) (Rotamase) (EC 5.2.1.8) (Cyclophilin B) (S-cyclophilin) (SCYLP) (CYP-S1)	endoplasmic reticulum; endoplasmic reticulum lumen; melanosome; peptide binding; peptidyl-prolyl cis-trans isomerase activity; protein folding; unfolded protein binding	Endoplasmic reticulum lumen. Melanosome.	Cyclophilin-type PPIase family, PPIase B subfamily
**132**	PPIF_HUMAN	PPIF (CYP3)	P30405	Peptidyl-prolyl cis-trans isomerase, mitochondrial (PPIase) (Rotamase) (EC 5.2.1.8) (Cyclophilin F)	membrane fraction; mitochondrial matrix; peptide binding; peptidyl-prolyl cis-trans isomerase activity; protein folding	Mitochondrion matrix.	Cyclophilin-type PPIase family
**133**	PRAF3_HUMAN	ARL6IP5 (DERP11) (JWA) (PRA2) (PRAF3) (HSPC127)	O75915	PRA1 family protein 3 (ADP-ribosylation factor-like protein 6-interacting protein 5) (ARL-6-interacting protein 5) (Aip-5) (Glutamate transporter EAAC1-interacting protein) (GTRAP3-18) (Prenylated Rab acceptor protein 2) (Protein JWa) (Dermal papilla-derived protein 11) (JM5) (Putative MAPK-activating protein PM27) (Cytoskeleton-related vitamin A-responsive protein)	L-glutamate transport; endoplasmic reticulum membrane; integral to membrane; protein binding	Endoplasmic reticulum membrane; Multi-pass membrane protein. Cytoplasm.	PRA1 family
**134**	PROF1_HUMAN	PFN1	P07737	Profilin-1 (Profilin I)	actin binding; actin cytoskeleton; actin cytoskeleton organization; cytoplasm	Cytoplasm , cytoskeleton.	Profilin family

	Entry name	Gene names	Uniprot Accession	Protein names	Gene Ontology	Subcellular Localization	Protein family
**135**	PURA_HUMAN	PURA (PUR1)	Q00577	Transcriptional activator protein Pur-alpha (Purine-rich single-stranded DNA-binding protein alpha)	DNA replication factor A complex; DNA replication initiation; DNA unwinding during replication; RNA polymerase II transcription factor activity, enhancer binding; double-stranded telomeric DNA binding; nuclear chromosome, telomeric region; single-stranded DNA binding; transcription; transcription factor binding	Nucleus.	PUR DNA-binding protein family
**136**	QCR1_HUMAN	UQCRC1	P31930	Cytochrome b-c1 complex subunit 1, mitochondrial (Ubiquinol-cytochrome-c reductase complex core protein 1) (Core protein I) (Complex III subunit 1)	aerobic respiration; metalloendopeptidase activity; mitochondrial respiratory chain; protein binding; proteolysis; transport; ubiquinol-cytochrome-c reductase activity; zinc ion binding	Mitochondrion inner membrane.	Peptidase M16 family, UQCRC1/QCR1 subfamily
**137**	QCR2_HUMAN	UQCRC2	P22695	Cytochrome b-c1 complex subunit 2, mitochondrial (Ubiquinol-cytochrome-c reductase complex core protein 2) (Core protein II) (Complex III subunit 2)	aerobic respiration; electron transport chain; metalloendopeptidase activity; mitochondrial inner membrane; nucleus; oxidative phosphorylation; protein binding; proteolysis; respiratory chain; transport; zinc ion binding	Mitochondrion inner membrane; Peripheral membrane protein; Matrix side.	Peptidase M16 family, UQCRC2/QCR2 subfamily
**138**	RAP1A_HUMAN	RAP1A (KREV1)	P62834	Ras-related protein Rap-1A (GTP-binding protein smg-p21A) (Ras-related protein Krev-1) (C21KG) (G-22K)	GTP binding; GTPase activity; cell cycle; cytosol; negative regulation of cell cycle; plasma membrane	Cell membrane; Lipid-anchor.	Small GTPase superfamily, Ras family
**139**	RASA3_HUMAN	RASA3	Q14644	Ras GTPase-activating protein 3 (GAP1(IP4BP)) (Ins P4-binding protein)	GTPase activator activity; calcium-release channel activity; intracellular; intracellular signaling cascade; intrinsic to internal side of plasma membrane; regulation of small GTPase mediated signal transduction; zinc ion binding	Cell membrane.	
**140**	RB11A_HUMAN	RAB11A (RAB11)	P62491	Ras-related protein Rab-11A (Rab-11) (YL8)	GTP binding; GTPase activity; plasma membrane; plasma membrane to endosome transport; recycling endosome membrane; small GTPase mediated signal transduction; syntaxin binding; trans-Golgi network; transporter activity	Cell membrane; Peripheral membrane protein. Recycling endosome membrane; Peripheral membrane protein.	Small GTPase superfamily, Rab family
**141**	RB27B_HUMAN	RAB27B	O00194	Ras-related protein Rab-27B (C25KG)	GTP binding; GTPase activity; membrane; protein transport; small GTPase mediated signal transduction	Membrane; Lipid-anchor.	Small GTPase superfamily, Rab family
**142**	RGS18_HUMAN	RGS18 (RGS13)	Q9NS28	Regulator of G-protein signaling 18 (RGS18)	cytoplasm; negative regulation of signal transduction; signal transducer activity	Cytoplasm.	
**143**	RHOA_HUMAN	RHOA (ARH12) (ARHA) (RHO12)	P61586	Transforming protein RhoA (H12)	GTP binding; GTPase activity; Rho protein signal transduction; cytoplasm; cytoskeleton; magnesium ion binding; myosin binding; plasma membrane; positive regulation of I-kappaB kinase/NF-kappaB cascade; positive regulation of NF-kappaB import into nucleus; positive regulation of neuron differentiation; positive regulation of stress fiber formation	Cell membrane; Lipid-anchor; Cytoplasmic side. Cytoplasm , cytoskeleton.	Small GTPase superfamily, Rho family
**144**	RHOC_HUMAN	RHOC (ARH9) (ARHC)	P08134	Rho-related GTP-binding protein RhoC (H9)	GTP binding; GTPase activity; intracellular; plasma membrane; positive regulation of I-kappaB kinase/NF-kappaB cascade; protein binding; signal transducer activity; small GTPase mediated signal transduction	Cell membrane; Lipid-anchor; Cytoplasmic side.	Small GTPase superfamily, Rho family
**145**	RLA2_HUMAN	RPLP2 (D11S2243E) (RPP2)	P05387	60S acidic ribosomal protein P2 (Renal carcinoma antigen NY-REN-44)	RNA binding; cytosolic large ribosomal subunit; structural constituent of ribosome; translational elongation		Ribosomal protein L12P family
**146**	RPN1_HUMAN	RPN1	P04843	Dolichyl-diphosphooligosaccharide--protein glycosyltransferase subunit 1 (EC 2.4.1.119) (Dolichyl-diphosphooligosaccharide--protein glycosyltransferase 67 kDa subunit) (Ribophorin-1) (Ribophorin I) (RPN-I)	dolichyl-diphosphooligosaccharide-protein glycotransferase activity; integral to membrane; melanosome; oligosaccharyltransferase complex; protein amino acid N-linked glycosylation via asparagine; protein binding; rough endoplasmic reticulum membrane	Endoplasmic reticulum membrane; Single-pass type I membrane protein. Melanosome.	OST1 family
**147**	RPN2_HUMAN	RPN2	P04844	Dolichyl-diphosphooligosaccharide--protein glycosyltransferase subunit 2 (EC 2.4.1.119) (Dolichyl-diphosphooligosaccharide--protein glycosyltransferase 63 kDa subunit) (Ribophorin-2) (Ribophorin II) (RPN-II) (RIBIIR)	dolichyl-diphosphooligosaccharide-protein glycotransferase activity; integral to membrane; oligosaccharyltransferase complex; protein amino acid N-linked glycosylation via asparagine; protein binding	Endoplasmic reticulum membrane; Multi-pass membrane protein.	SWP1 family
**148**	RSU1_HUMAN	RSU1 (RSP1)	Q15404	Ras suppressor protein 1 (Rsu-1) (RSP-1)	protein binding; signal transduction		

	Entry name	Gene names	Uniprot Accession	Protein names	Gene Ontology	Subcellular Localization	Protein family
**149**	RTN4_HUMAN	RTN4 (KIAA0886) (NOGO) (My043) (SP1507)	Q9NQC3	Reticulon-4 (Neurite outgrowth inhibitor) (Nogo protein) (Foocen) (Neuroendocrine-specific protein) (NSP) (Neuroendocrine-specific protein C homolog) (RTN-x) (Reticulon-5)	apoptosis; integral to endoplasmic reticulum membrane; negative regulation of anti-apoptosis; negative regulation of axon extension; nuclear envelope; protein binding; regulation of apoptosis; regulation of cell migration	Endoplasmic reticulum membrane; Multi-pass membrane protein.	
**150**	SC22B_HUMAN	SEC22B (SEC22L1)	O75396	Vesicle-trafficking protein SEC22b (SEC22 vesicle-trafficking protein homolog B) (SEC22 vesicle-trafficking protein-like 1) (ERS24) (ERS 24)	ER to Golgi vesicle-mediated transport; ER-Golgi intermediate compartment membrane; Golgi membrane; endoplasmic reticulum membrane; integral to membrane; melanosome; protein binding; protein transport	Endoplasmic reticulum-Golgi intermediate compartment membrane; Single-pass type IV membrane protein. Golgi apparatus membrane; Single-pass type IV membrane protein. Endoplasmic reticulum membrane; Single-pass type IV membrane protein. Melanosome.	Synaptobrevin family
**151**	SCAM2_HUMAN	SCAMP2	O15127	Secretory carrier-associated membrane protein 2 (Secretory carrier membrane protein 2)	integral to membrane; post-Golgi vesicle-mediated transport; protein binding; protein transport; recycling endosome membrane; trans-Golgi network membrane	Golgi apparatus , trans-Golgi network membrane; Multi-pass membrane protein. Recycling endosome membrane; Multi-pass membrane protein.	SCAMP family
**152**	SCPDH_HUMAN	SCCPDH (CGI-49)	Q8NBX0	Probable saccharopine dehydrogenase (EC 1.5.1.9)	binding; oxidation reduction; saccharopine dehydrogenase (NAD+, L-glutamate-forming) activity		Saccharopine dehydrogenase family
**153**	SDPR_HUMAN	SDPR	O95810	Serum deprivation-response protein (Phosphatidylserine-binding protein) (PS-p68)	caveola; cytosol; phosphatidylserine binding; protein binding	Cytoplasm , cytosol. Membrane , caveola.	PTRF/SDPR family
**154**	SEP11_HUMAN	11-Sep	Q9NVA2	Septin-11	GTP binding; cell cycle; cell division; nucleus; protein binding; protein heterooligomerization; septin complex; stress fiber		Septin family
**155**	SEPT6_HUMAN	SEPT6 (KIAA0128) (SEP2)	Q14141	Septin-6	GTP binding; cell cycle; cytokinesis; nucleus; protein binding; septin complex		Septin family
**156**	SND1_HUMAN	SND1 (TDRD11)	Q7KZF4	Staphylococcal nuclease domain-containing protein 1 (p100 co-activator) (100 kDa coactivator) (EBNA2 coactivator p100) (Tudor domain-containing protein 11)	Golgi apparatus; RNA interference; RNA-induced silencing complex; interspecies interaction between organisms; melanosome; nuclease activity; nucleic acid binding; nucleus; regulation of transcription, DNA-dependent; transcription; transcription cofactor activity	Cytoplasm. Nucleus. Melanosome.	
**157**	SNP29_HUMAN	SNAP29	O95721	Synaptosomal-associated protein 29 (SNAP-29) (Vesicle-membrane fusion protein SNAP-29) (Soluble 29 kDa NSF attachment protein)	SNAP receptor activity; cell junction; cytoplasm; exocytosis; membrane fusion; protein transport; synapse; synaptosome; vesicle targeting	Cytoplasm. Membrane; Peripheral membrane protein. Cell junction , synapse , synaptosome.	SNAP-25 family
**158**	SPCS1_HUMAN	SPCS1 (SPC12) (HSPC033)	Q9Y6A9	Signal peptidase complex subunit 1 (EC 3.4.-.-) (Microsomal signal peptidase 12 kDa subunit) (SPase 12 kDa subunit)	integral to endoplasmic reticulum membrane; microsome; peptidase activity; proteolysis; signal peptidase complex; signal peptide processing	Microsome membrane; Multi-pass membrane protein. Endoplasmic reticulum membrane; Multi-pass membrane protein.	SPCS1 family
**159**	SSBP_HUMAN	SSBP1 (SSBP)	Q04837	Single-stranded DNA-binding protein, mitochondrial (Mt-SSB) (MtSSB) (PWP1-interacting protein 17)	DNA replication; mitochondrial nucleoid; single-stranded DNA binding	Mitochondrion.	
**160**	STOM_HUMAN	STOM (BND7) (EPB72)	P27105	Erythrocyte band 7 integral membrane protein (Stomatin) (Protein 7.2b)	cytoskeleton; integral to plasma membrane; melanosome; membrane raft; protein binding; protein homooligomerization	Cell membrane; Single-pass membrane protein; Cytoplasmic side. Cell membrane; Lipid-anchor; Cytoplasmic side. Melanosome.	Band 7/mec-2 family
**161**	STXB2_HUMAN	STXBP2 (UNC18B)	Q15833	Syntaxin-binding protein 2 (Unc-18 homolog 2) (Unc18-2) (Unc-18B)	intracellular protein transport; syntaxin-2 binding; syntaxin-3 binding; vesicle docking during exocytosis		STXBP/unc-18/SEC1 family
**162**	TAGL2_HUMAN	TAGLN2 (KIAA0120) (CDABP0035)	P37802	Transgelin-2 (SM22-alpha homolog)	cytoskeleton; muscle organ development; nuclear membrane; plasma membrane; protein binding		Calponin family
**163**	TBA4A_HUMAN	TUBA4A (TUBA1)	P68366	Tubulin alpha-4A chain (Tubulin alpha-1 chain) (Alpha-tubulin 1) (Testis-specific alpha-tubulin) (Tubulin H2-alpha)	GTP binding; GTPase activity; microtubule; microtubule-based movement; protein binding; protein complex; protein polymerization; structural molecule activity		Tubulin family
**164**	TBB1_HUMAN	TUBB1	Q9H4B7	Tubulin beta-1 chain	GTP binding; GTPase activity; microtubule; microtubule-based movement; protein complex; protein polymerization		Tubulin family
**165**	TBB2A_HUMAN	TUBB2A (TUBB2)	Q13885	Tubulin beta-2A chain	GTP binding; GTPase activity; microtubule; microtubule-based movement; protein binding; protein complex; protein polymerization; structural molecule activity		Tubulin family

	Entry name	Gene names	Uniprot Accession	Protein names	Gene Ontology	Subcellular Localization	Protein family
**166**	TCPG_HUMAN	CCT3 (CCTG) (TRIC5)	P49368	T-complex protein 1 subunit gamma (TCP-1-gamma) (CCT-gamma) (hTRiC5)	ATP binding; cytoplasm; cytoskeleton; nucleus; plasma membrane; protein folding; unfolded protein binding	Cytoplasm.	TCP-1 chaperonin family
**167**	TLN1_HUMAN	TLN1 (KIAA1027) (TLN)	Q9Y490	Talin-1	LIM domain binding; actin binding; cell motion; cell-cell junction; cell-cell junction assembly; cytoplasm; cytoskeletal anchoring at plasma membrane; cytoskeleton; focal adhesion; intracellular membrane-bounded organelle; ruffle membrane; structural constituent of cytoskeleton; vinculin binding	Cell projection , ruffle membrane; Peripheral membrane protein; Cytoplasmic side. Cytoplasm , cytoskeleton.	
**168**	TMEDA_HUMAN	TMED10 (TMP21)	P49755	Transmembrane emp24 domain-containing protein 10 (21 kDa transmembrane-trafficking protein) (Transmembrane protein Tmp21) (Tmp-21-I) (p24delta) (S31III125) (S31I125)	ER-Golgi intermediate compartment; Golgi membrane; cis-Golgi network; integral to membrane; melanosome; microsome; protein binding; protein transport; regulated secretory pathway; vesicle targeting, to, from or within Golgi; zymogen granule membrane	Golgi apparatus membrane; Single-pass type I membrane protein; Lumenal side. Melanosome.	EMP24/GP25L family
**169**	TPM1_HUMAN	TPM1 (C15orf13) (TMSA)	P09493	Tropomyosin alpha-1 chain (Tropomyosin-1) (Alpha-tropomyosin)	actin binding; bleb; cardiac muscle contraction; cell motion; cellular response to reactive oxygen species; muscle filament sliding; muscle thin filament tropomyosin; negative regulation of cell migration; positive regulation of ATPase activity; positive regulation of cell adhesion; positive regulation of heart rate by epinephrine; positive regulation of stress fiber formation; regulation of muscle contraction; ruffle membrane; ruffle organization; sarcomere organization; stress fiber; structural constituent of cytoskeleton; structural constituent of muscle; ventricular cardiac muscle morphogenesis; wound healing	Cytoplasm , cytoskeleton.	Tropomyosin family
**170**	TPM3_HUMAN	TPM3	P06753	Tropomyosin alpha-3 chain (Tropomyosin-3) (Gamma-tropomyosin) (Tropomyosin-5) (hTM5)	actin binding; cell motion; muscle thin filament tropomyosin; regulation of muscle contraction	Cytoplasm , cytoskeleton.	Tropomyosin family
**171**	TPM4_HUMAN	TPM4	P67936	Tropomyosin alpha-4 chain (Tropomyosin-4) (TM30p1)	actin binding; calcium ion binding; cell motion; muscle thin filament tropomyosin; structural constituent of muscle	Cytoplasm , cytoskeleton.	Tropomyosin family
**172**	TSP1_HUMAN	THBS1 (TSP) (TSP1)	P07996	Thrombospondin-1	activation of MAPK activity; anti-apoptosis; calcium ion binding; cell adhesion; cell cycle arrest; cell migration; cellular response to heat; chronic inflammatory response; collagen V binding; engulfment of apoptotic cell; eukaryotic cell surface binding; external side of plasma membrane; extracellular matrix; fibrinogen binding; fibrinogen complex; fibroblast growth factor 2 binding; fibronectin binding; heparin binding; identical protein binding; immune response; integrin binding; laminin binding; low-density lipoprotein binding; negative regulation of angiogenesis; negative regulation of antigen processing and presentation of peptide or polysaccharide antigen via MHC class II; negative regulation of blood vessel endothelial cell migration; negative regulation of cGMP-mediated signaling; negative regulation of dendritic cell antigen processing and presentation; negative regulation of endothelial cell proliferation; negative regulation of fibrinolysis; [illegible]		Thrombospondin family
**173**	TSP3_HUMAN	THBS3 (TSP3)	P49746	Thrombospondin-3	calcium ion binding; cell-matrix adhesion; extracellular region; platelet alpha granule lumen; protein binding; structural molecule activity		Thrombospondin family
**174**	TWF2_HUMAN	TWF2 (PTK9L) (MSTP011)	Q6IBS0	Twinfilin-2 (Twinfilin-1-like protein) (A6-related protein) (hA6RP) (Protein tyrosine kinase 9-like)	ATP binding; actin binding; cytoskeleton; perinuclear region of cytoplasm	Cytoplasm , cytoskeleton. Cytoplasm , perinuclear region.	Actin-binding proteins ADF family, Twinfilin subfamily

	Entry name	Gene names	Uniprot Accession	Protein names	Gene Ontology	Subcellular Localization	Protein family
**175**	UBP7_HUMAN	USP7 (HAUSP)	Q93009	Ubiquitin carboxyl-terminal hydrolase 7 (EC 3.1.2.15) (Ubiquitin thioesterase 7) (Ubiquitin-specific-processing protease 7) (Deubiquitinating enzyme 7) (Herpesvirus-associated ubiquitin-specific protease)	cysteine-type endopeptidase activity; interspecies interaction between organisms; nucleus; protein C-terminus binding; protein deubiquitination; ubiquitin thiolesterase activity; ubiquitin-dependent protein catabolic process; ubiquitin-specific protease activity	Nucleus.	Peptidase C19 family
**176**	UBS3B_HUMAN	UBASH3B (KIAA1959) (STS1)	Q8TF42	Ubiquitin-associated and SH3 domain-containing protein B (Suppressor of T-cell receptor signaling 1) (STS-1) (Cbl-interacting protein p70)	cytoplasm; nucleus	Cytoplasm. Nucleus.	
**177**	URP2_HUMAN	FERMT3 (KIND3) (MIG2B) (URP2)	Q86UX7	Fermitin family homolog 3 (Unc-112-related protein 2) (Kindlin-3) (MIG2-like protein)	cell adhesion; cytoplasm; plasma membrane; protein binding	Cytoplasm. Cell membrane; Peripheral membrane protein; Cytoplasmic side.	Kindlin family
**178**	VDAC1_HUMAN	VDAC1 (VDAC)	P21796	Voltage-dependent anion-selective channel protein 1 (VDAC-1) (hVDAC1) (Outer mitochondrial membrane protein porin 1) (Plasmalemmal porin) (Porin 31HL) (Porin 31HM)	anion transport; apoptosis; interspecies interaction between organisms; mitochondrial nucleoid; mitochondrial outer membrane; plasma membrane; pore complex; protein binding; voltage-gated anion channel activity	Mitochondrion outer membrane. Cell membrane.	Eukaryotic mitochondrial porin family
**179**	VDAC3_HUMAN	VDAC3	Q9Y277	Voltage-dependent anion-selective channel protein 3 (VDAC-3) (hVDAC3) (Outer mitochondrial membrane protein porin 3)	adenine transport; anion transport; integral to membrane; mitochondrial outer membrane; nucleotide binding; protein binding; voltage-gated anion channel activity	Mitochondrion outer membrane.	Eukaryotic mitochondrial porin family
**180**	VINC_HUMAN	VCL	P18206	Vinculin (Metavinculin)	actin binding; actin cytoskeleton; alpha-catenin binding; apical junction assembly; cell adhesion; cell motion; cell-cell junction; costamere; focal adhesion; lamellipodium assembly; negative regulation of cell migration; oxidoreductase activity; protein complex; structural molecule activity	Cytoplasm , cytoskeleton. Cell junction , adherens junction. Cell membrane; Peripheral membrane protein; Cytoplasmic side.	Vinculin/alpha-catenin family
**181**	VWF_HUMAN	VWF (F8VWF)	P04275	von Willebrand factor (vWF) [Cleaved into: von Willebrand antigen 2 (von Willebrand antigen II)]	Weibel-Palade body; cell-substrate adhesion; chaperone binding; collagen binding; endoplasmic reticulum; extracellular space; glycoprotein binding; immunoglobulin binding; integrin binding; nucleolus; platelet activation; platelet alpha granule; protease binding; protein N-terminus binding; protein homodimerization activity; protein homooligomerization; proteinaceous extracellular matrix	Secreted. Secreted , extracellular space , extracellular matrix.	
**182**	WASF2_HUMAN	WASF2 (WAVE2)	Q9Y6W5	Wiskott-Aldrich syndrome protein family member 2 (WASP family protein member 2) (Protein WAVE-2) (Verprolin homology domain-containing protein 2)	G-protein signaling, coupled to cAMP nucleotide second messenger; actin binding; actin cytoskeleton; actin cytoskeleton organization; cytoplasm; lamellipodium	Cytoplasm , cytoskeleton. Cell projection , lamellipodium.	SCAR/WAVE family
