# Supplementary material for: Proteomic and Phospho-Proteomic Profile of Human Platelets in Basal, Resting State: Insights into Integrin Signaling
Source: PLoS One. 2009 Oct 27;4(10):e7627. doi: 10.1371/journal.pone.0007627 (PMC2762604; doi:10.1371/journal.pone.0007627)
Supplement: Table S4 — A list of 190 unique proteins and the identifying peptides from proteomic profiling of the platelet membrane fraction. Protein identification is based on its Refseq ID and the definition of additional terms is the same as described for Table S2A. (0.19 MB PDF) [file pone.0007627.s009.pdf]

Reference<br>File, Scan(s)	Peptide	MH+	z	P (pro)<br>P (pep)	Sf<br>Sf	Score<br>XC	Coverage	MW<br>Sp
gi|16753233|ref|NP_006280.2| talin 1 [Homo sapiens]				2.78E-15	35.56	430.32	25.70	269665.3
PM-3, 2187	R.AAM*EPIVISAK.T	1146.38260	1	2.29E-04	0.11	1.99	-	194.9
PM-1, 6479	K.AQEACGPLEMDSALSVVQNLEK.D	2390.67685	2	4.29E-06	0.93	3.95	-	1327.0
PM-4, 6309	K.AQEACGPLEMDSALSVVQNLEK.D	2390.67685	2	6.12E-10	0.92	4.37	-	944.6
PM-4, 2250 - 2252	K.ASAGPQPLLVQSCK.A	1456.69283	1	1.32E-04	0.19	2.17	-	338.0
PM-2, 3867 - 3906	K.ASVPTIQDQASAMQLSQCAK.N	2135.40852	2	6.98E-06	0.88	3.61	-	742.0
PM-3, 5303 - 5359	R.AVTDSINQLITMCTQQAPGQK.E	2305.61832	2	2.26E-04	0.68	2.93	-	526.7
PM-1, 5621	R.AVTDSINQLITMCTQQAPGQK.E	2305.61832	2	3.35E-06	0.87	3.93	-	650.3
PM-1, 5237 - 5239	R.AVTDSINQLITM*CTQQAPGQK.E	2321.61772	2	1.57E-04	0.87	3.98	-	555.5
PM-5, 5326 - 5353	R.AVTDSINQLITMCTQQAPGQK.E	2305.61832	2	1.05E-05	0.46	3.08	-	322.1
PM-8, 4588	K.DHFGLEGDEESTMLEDSVSPK.K	2323.43307	2	2.18E-04	0.93	3.94	-	1485.3
PM-1, 5491	R.DKAPGQLECETAIAALNSCLR.D	2318.61684	2	1.90E-09	0.92	3.71	-	1095.1
PM-8, 5136	R.DKAPGQLECETAIAALNSCLR.D	2318.61684	2	8.25E-04	0.41	2.52	-	425.3
PM-4, 4373	R.DKAPGQLECETAIAALNSCLR.D	2318.61684	2	2.02E-04	0.53	2.72	-	484.7
PM-4, 5041	R.DKAPGQLECETAIAALNSCLR.D	2318.61684	2	4.09E-09	0.89	3.83	-	772.0
PM-2, 5109	R.DKAPGQLECETAIAALNSCLR.D	2318.61684	2	9.50E-12	0.91	4.27	-	879.2
PM-1, 4801	R.DKAPGQLECETAIAALNSCLR.D	2318.61684	2	2.78E-15	0.93	4.13	-	943.8
PM-3, 5104	R.DKAPGQLECETAIAALNSCLR.D	2318.61684	2	9.28E-06	0.82	3.28	-	694.1
PM-5, 2210 - 2218	K.EAAYHPEVAPDVR.L	1454.56910	1	2.78E-05	0.68	2.57	-	553.8
PM-9, 5755 - 5823	K.EADESLNFEEQILEAAK.S	1937.05072	2	3.77E-04	0.85	3.87	-	870.1
PM-8, 5760 - 5827	K.EADESLNFEEQILEAAK.S	1937.05072	2	8.87E-07	0.94	4.02	-	1395.7
PM-4, 5862	K.EADESLNFEEQILEAAK.S	1937.05072	2	4.53E-06	0.74	3.22	-	742.3
PM-2, 5877 - 5881	K.EADESLNFEEQILEAAK.S	1937.05072	2	3.06E-04	0.94	4.18	-	1597.0
PM-1, 6128 - 6205	K.EADESLNFEEQILEAAK.S	1937.05072	2	5.27E-09	0.97	5.34	-	1868.6
PM-3, 6480	R.ECANGYLELLDHVLLTLQKPSPELK.Q	2882.32426	3	8.40E-04	0.84	4.03	-	848.6
PM-1, 6629	R.EGISQEALHTQM*LTAVQEISHLIEPLANAAR.A	3388.79744	3	6.72E-04	0.76	3.26	-	613.8
PM-3, 6527 - 6529	R.EGISQEALHTQMLTAVQEISHLIEPLANAAR.A	3372.79804	3	4.53E-11	0.96	5.87	-	1364.5
PM-2, 6530	R.EGISQEALHTQM*LTAVQEISHLIEPLANAAR.A	3388.79744	3	7.43E-04	0.82	4.00	-	695.8
PM-3, 6447 - 6448	R.EGISQEALHTQM*LTAVQEISHLIEPLANAAR.A	3388.79744	3	1.04E-08	0.91	4.83	-	880.2
PM-4, 6488	R.EGISQEALHTQMLTAVQEISHLIEPLANAAR.A	3372.79804	3	5.43E-06	0.87	4.24	-	954.9
PM-9, 5937	K.FFYSDQNVDSRDPVQLNLLYVQAR.D	2889.17074	3	1.48E-04	0.95	4.02	-	1842.3
PM-5, 6278	R.FGQDFSTFLEAGVEMAGQAPSQEDR.A	2718.89325	2	6.82E-08	0.90	4.01	-	778.7
PM-6, 6199	R.GSQAQPDSPSAQLALIAASQSFLQPGGK.M	2756.02103	2	8.12E-05	0.61	3.35	-	314.4
PM-3, 6355	R.GSQAQPDSPSAQLALIAASQSFLQPGGK.M	2756.02103	2	3.32E-06	0.89	4.51	-	606.8
PM-1, 6476	R.GSQAQPDSPSAQLALIAASQSFLQPGGK.M	2756.02103	2	1.38E-08	0.97	5.47	-	1270.6
PM-5, 6224	R.GSQAQPDSPSAQLALIAASQSFLQPGGK.M	2756.02103	2	3.59E-04	0.93	4.42	-	825.9
PM-4, 6412	R.GVAALTSDPAVQAIVLDTASDVLDK.A	2470.75835	2	2.43E-07	0.95	6.01	-	735.4
PM-1, 6559 - 6617	R.GVAALTSDPAVQAIVLDTASDVLDK.A	2470.75835	2	3.18E-06	0.97	6.29	-	994.9
PM-2, 6529	R.GVAALTSDPAVQAIVLDTASDVLDK.A	2470.75835	2	8.11E-07	0.93	4.98	-	603.1
PM-5, 6361	R.GVGAAATAVTQALNELLQHVK.A	2092.38495	2	5.31E-04	0.32	2.82	-	206.9
PM-4, 6462 - 6465	R.GVGAAATAVTQALNELLQHVK.A	2092.38495	2	7.61E-06	0.91	4.41	-	736.5
PM-1, 6204	R.IPEAPAGPPSDFGLFLSDDDPK.K	2286.47890	2	1.13E-04	0.56	3.18	-	440.2
PM-11, 5461 - 5516	R.IPEAPAGPPSDFGLFLSDDDPKK.G	2414.65181	3	1.68E-04	0.85	3.40	-	1067.0
PM-2, 2886 - 2897	R.LASEAKPAAVAAENEEIGSHIK.H	2236.46779	3	1.71E-04	0.91	4.30	-	1063.8
PM-4, 2716 - 2721	R.LASEAKPAAVAAENEEIGSHIK.H	2236.46779	3	4.75E-04	0.86	3.62	-	1106.4
PM-1, 3267	R.LASEAKPAAVAAENEEIGSHIK.H	2236.46779	3	8.11E-08	0.97	5.98	-	1934.1
PM-3, 2799	R.LASEAKPAAVAAENEEIGSHIK.H	2236.46779	3	3.98E-04	0.91	3.51	-	1623.1
PM-4, 5388	K.LGAASLGAEDPETQVVLINAVK.D	2196.48671	2	5.20E-04	0.48	2.52	-	387.6
PM-11, 5393	K.LGAASLGAEDPETQVVLINAVK.D	2196.48671	2	9.94E-05	0.65	2.51	-	686.4
PM-4, 5168 - 5224	K.LGAASLGAEDPETQVVLINAVK.D	2196.48671	2	1.50E-07	0.90	4.14	-	906.6
PM-3, 5036 - 5056	K.LGAASLGAEDPETQVVLINAVK.D	2196.48671	2	4.87E-05	0.60	2.87	-	587.1
PM-10, 5337 - 5339	K.LGAASLGAEDPETQVVLINAVK.D	2196.48671	2	2.57E-04	0.87	3.38	-	907.8
PM-3, 5235	K.LGAASLGAEDPETQVVLINAVK.D	2196.48671	2	6.90E-04	0.88	3.66	-	826.0
PM-5, 5310 - 5390	K.LGAASLGAEDPETQVVLINAVK.D	2196.48671	2	9.28E-04	0.89	3.54	-	937.6
PM-9, 5200 - 5271	K.LGAASLGAEDPETQVVLINAVK.D	2196.48671	2	9.94E-06	0.94	4.66	-	882.1
PM-8, 5243 - 5251	K.LGAASLGAEDPETQVVLINAVK.D	2196.48671	2	1.24E-05	0.77	3.35	-	584.0
PM-4, 4984 - 4992	K.LGAASLGAEDPETQVVLINAVK.D	2196.48671	2	2.39E-06	0.93	4.62	-	774.9
PM-5, 5096 - 5114	K.LGAASLGAEDPETQVVLINAVK.D	2196.48671	2	3.57E-04	0.92	4.24	-	991.3
PM-1, 5721 - 5780	K.LGAASLGAEDPETQVVLINAVK.D	2196.48671	2	7.21E-05	0.82	3.74	-	505.0
PM-5, 5277 - 5346	K.LGAASLGAEDPETQVVLINAVK.D	2196.48671	2	2.57E-04	0.92	4.12	-	941.1
PM-2, 5247 - 5307	K.LGAASLGAEDPETQVVLINAVK.D	2196.48671	2	5.66E-08	0.95	4.57	-	1092.8
PM-1, 5587 - 5588	K.LGAASLGAEDPETQVVLINAVK.D	2196.48671	2	8.60E-04	0.94	4.74	-	975.9
PM-6, 4863 - 4868	K.LGAASLGAEDPETQVVLINAVK.D	2196.48671	2	1.29E-04	0.80	2.90	-	690.0
PM-8, 3344 - 3404	K.LHTDDELNWLDHGR.T	1721.81153	2	3.37E-06	0.93	4.29	-	906.9
PM-6, 3170 - 3227	K.LHTDDELNWLDHGR.T	1721.81153	2	1.15E-04	0.92	3.81	-	886.5
PM-11, 5260 - 5283	K.LLAALLEDEGGSGRPLLQAAK.G	2123.43896	2	2.66E-08	0.97	5.34	-	1211.8
PM-8, 5008 - 5026	K.LLAALLEDEGGSGRPLLQAAK.G	2123.43896	2	4.70E-06	0.76	3.80	-	613.5
PM-4, 4864	K.LLAALLEDEGGSGRPLLQAAK.G	2123.43896	2	4.59E-05	0.69	3.45	-	575.1
PM-1, 5424	K.LLAALLEDEGGSGRPLLQAAK.G	2123.43896	2	1.27E-04	0.89	3.59	-	950.1
PM-2, 4978 - 5017	K.LLAALLEDEGGSGRPLLQAAK.G	2123.43896	2	7.92E-04	0.96	5.36	-	847.2
PM-3, 3753	R.MVAAATNNLCEAANAAVQGHASQEK.L	2557.80618	2	3.40E-04	0.56	2.98	-	277.0
PM-3, 2319	K.PAAVAAENEEIGSHIK.H	1636.78798	2	5.80E-06	0.96	4.71	-	1288.4
PM-2, 2390	K.PAAVAAENEEIGSHIK.H	1636.78798	2	1.09E-05	0.98	5.56	-	2382.7
PM-2, 6549	R.QNLLQAAGNVGQASGELLQQIGESDTDPHFQDALMQLAK.A	4138.52644	3	3.02E-08	0.94	5.94	-	669.3
PM-3, 6471	R.QNLLQAAGNVGQASGELLQQIGESDTDPHFQDALMQLAK.A	4138.52644	3	7.75E-11	0.96	6.35	-	828.8
PM-2, 6534	R.QNLLQAAGNVGQASGELLQQIGESDTDPHFQDALM*QLAK.A	4154.52584	3	1.12E-07	0.83	4.15	-	479.7
PM-3, 6455	R.QNLLQAAGNVGQASGELLQQIGESDTDPHFQDALM*QLAK.A	4154.52584	3	2.48E-05	0.82	4.43	-	509.0
PM-4, 6436	R.QNLLQAAGNVGQASGELLQQIGESDTDPHFQDALMQLAK.A	4138.52644	3	2.31E-07	0.92	4.66	-	905.8
PM-3, 5589	K.RVAGSVTELIQAAEAMK.G	1775.06387	2	5.64E-05	0.90	3.65	-	1013.6
PM-5, 5728	K.RVAGSVTELIQAAEAMK.G	1775.06387	2	1.35E-04	0.98	5.13	-	2318.6
PM-3, 4117	K.SKDHFGLEGDEESTMLEDSVSPK.K	2538.68364	2	1.56E-04	0.83	3.32	-	725.7
PM-2, 4185	K.SKDHFGLEGDEESTMLEDSVSPK.K	2538.68364	2	1.09E-04	0.85	3.56	-	947.6
PM-2, 5010 - 5015	K.SNTSPEELGPLANQLTSDYGR.L	2250.36505	2	3.55E-05	0.56	3.02	-	322.9
PM-1, 5340 - 5399	K.SNTSPEELGPLANQLTSDYGR.L	2250.36505	2	4.12E-05	0.74	3.34	-	349.9
PM-9, 4017 - 4072	K.TKEVIQEWNLTNIK.R	1716.95938	2	1.14E-06	0.91	3.48	-	1330.7
PM-2, 2866 - 2873	K.TLSHPQQMALLDQTK.T	1711.96464	2	1.53E-05	0.66	3.03	-	419.3
PM-1, 6439	R.VAGSVTELIQAAEAMK.G	1618.87752	2	6.89E-07	0.97	5.15	-	1501.1
PM-1, 5469 - 5471	R.VAGSVTELIQAAEAM*K.G	1634.87692	2	2.90E-05	0.76	3.07	-	667.6
PM-5, 5693 - 5698	K.VGAIPANALDDGQWSQGLISAAR.M	2311.53959	2	2.42E-04	0.81	3.54	-	338.1
PM-1, 6227	K.VGAIPANALDDGQWSQGLISAAR.M	2311.53959	2	3.95E-04	0.84	3.52	-	505.2
PM-1, 6111	K.VGAIPANALDDGQWSQGLISAAR.M	2311.53959	2	1.18E-09	0.93	4.43	-	755.2
PM-10, 5831	K.VGAIPANALDDGQWSQGLISAAR.M	2311.53959	2	5.02E-05	0.68	2.89	-	359.2
PM-5, 5776	K.VGAIPANALDDGQWSQGLISAAR.M	2311.53959	2	1.98E-06	0.89	3.90	-	613.0
PM-9, 5673 - 5748	K.VGAIPANALDDGQWSQGLISAAR.M	2311.53959	2	3.15E-09	0.94	4.23	-	1203.9
PM-4, 5653 - 5729	K.VGAIPANALDDGQWSQGLISAAR.M	2311.53959	2	4.74E-07	0.93	4.68	-	817.3
PM-1, 6021	K.VGAIPANALDDGQWSQGLISAAR.M	2311.53959	2	5.67E-07	0.91	3.89	-	544.7
PM-3, 5692 - 5751	K.VGAIPANALDDGQWSQGLISAAR.M	2311.53959	2	1.91E-09	0.95	5.05	-	763.6
PM-2, 5790 - 5862	K.VGAIPANALDDGQWSQGLISAAR.M	2311.53959	2	9.00E-06	0.92	4.90	-	587.8
PM-5, 3470 - 3472	K.VGDDPAVWQLK.N	1228.37879	2	2.20E-04	0.93	4.08	-	1073.3
PM-1, 3811 - 3812	K.VGDDPAVWQLK.N	1228.37879	2	1.74E-08	0.92	3.72	-	859.2
PM-3, 2757	K.VLGEAMTGISQNAK.N	1419.62961	1	4.16E-05	0.84	3.46	-	469.5
PM-1, 4435	K.VM*VTNVTSLLK.T	1221.49262	2	1.38E-04	0.84	3.44	-	751.7
PM-2, 2239	K.VSHVLAALQAGNR.G	1336.52525	1	5.33E-05	0.50	2.42	-	438.8
PM-5, 2417 - 2420	K.VSHVLAALQAGNR.G	1336.52525	1	9.01E-04	0.67	2.92	-	397.2
PM-1, 6496	K.VSQMAQYFEPLTLAAVGAASK.T	2183.51313	2	1.88E-05	0.89	4.52	-	489.4

PM-2, 6441	K.VSQMAQYFEPLTLAAVGAASK.T	2183.51313	2	1.71E-05	0.95	5.14	-	901.0
PM-1, 6095	K.VSQM*AQYFEPLTLAAVGAASK.T	2199.51253	2	4.69E-04	0.93	4.19	-	1096.1
PM-5, 6169 - 6242	K.VSQMAQYFEPLTLAAVGAASK.T	2183.51313	2	1.09E-04	0.89	4.04	-	582.0
PM-5, 5732 - 5733	K.VSQM*AQYFEPLTLAAVGAASK.T	2199.51253	2	9.66E-05	0.88	4.23	-	553.5
PM-9, 6227	K.VSQMAQYFEPLTLAAVGAASK.T	2183.51313	2	8.39E-05	0.96	4.90	-	1161.2
PM-3, 5669 - 5687	K.VSQM*AQYFEPLTLAAVGAASK.T	2199.51253	2	2.34E-04	0.88	4.04	-	727.8
PM-3, 6371 - 6375	K.VSQMAQYFEPLTLAAVGAASK.T	2183.51313	2	1.33E-05	0.93	4.74	-	794.2
PM-4, 6330 - 6337	K.VSQMAQYFEPLTLAAVGAASK.T	2183.51313	2	7.78E-05	0.94	4.58	-	931.6
PM-6, 6214 - 6215	K.VSQMAQYFEPLTLAAVGAASK.T	2183.51313	2	1.28E-04	0.93	4.46	-	812.3
PM-4, 4094 - 4152	K.VVAPTISSPVCQEQLVEAGR.L	2141.43439	2	6.36E-04	0.81	3.61	-	598.7
PM-3, 4221 - 4231	K.VVAPTISSPVCQEQLVEAGR.L	2141.43439	2	1.59E-04	0.84	3.78	-	488.6
PM-8, 4170	K.VVAPTISSPVCQEQLVEAGR.L	2141.43439	2	8.87E-04	0.44	2.88	-	143.0
PM-2, 4261	K.VVAPTISSPVCQEQLVEAGR.L	2141.43439	2	5.59E-05	0.31	2.54	-	233.2
PM-5, 4174 - 4232	K.VVAPTISSPVCQEQLVEAGR.L	2141.43439	2	3.40E-05	0.84	3.88	-	479.4
gi|4503689|ref|NP_000499.1| fibrinogen, alpha chain isoform alpha-E preproprotein [Homo sapiens] [M				2.83E-14	7.47	100.25	19.60	94972.5
PM-6, 5208 - 5279	K.DSDWPFCSDEDWNYK.C	1965.00129	2	7.73E-04	0.83	3.27	-	462.1
PM-10, 5407 - 5479	K.DSDWPFCSDEDWNYK.C	1965.00129	2	6.04E-07	0.81	3.13	-	586.9
PM-7, 5137 - 5203	K.DSDWPFCSDEDWNYK.C	1965.00129	2	5.60E-06	0.84	3.40	-	426.0
PM-11, 5484	K.DSDWPFCSDEDWNYK.C	1965.00129	2	7.09E-05	0.82	3.19	-	545.2
PM-5, 5697	K.DSHSLTTNIMEILR.G	1630.84839	2	2.82E-05	0.71	2.88	-	533.7
PM-1, 6048 - 6051	K.DSHSLTTNIMEILR.G	1630.84839	2	2.06E-04	0.96	4.68	-	1195.3
PM-7, 5237	K.DSHSLTTNIMEILR.G	1630.84839	2	3.01E-04	0.88	3.16	-	978.3
PM-8, 5534 - 5591	K.DSHSLTTNIMEILR.G	1630.84839	2	9.44E-05	0.81	3.66	-	590.8
PM-8, 2278 - 2352	R.EVDLKDYEDQQK.Q	1510.58460	2	5.87E-06	0.75	3.04	-	610.2
PM-1, 2631	R.EVDLKDYEDQQK.Q	1510.58460	2	4.63E-07	0.76	2.75	-	617.5
PM-3, 2285	R.MELERPGGNEITR.G	1502.67870	2	6.64E-04	0.75	3.48	-	464.9
PM-7, 2796	R.NPGSSGTGGTATWKPGSSGPGSTGSWNSGSSGTGSTGNQNPGSPR.P	4123.15285	3	3.41E-06	0.72	3.66	-	791.7
PM-1, 3396 - 3469	R.NPGSSGTGGTATWKPGSSGPGSTGSWNSGSSGTGSTGNQNPGSPR.P	4123.15285	3	5.19E-05	0.85	3.94	-	845.4
PM-2, 1713	R.NPSSAGSWNSGSSGPGSTGNR.N	1964.94443	2	1.28E-07	0.66	2.59	-	576.2
PM-7, 1705	R.NPSSAGSWNSGSSGPGSTGNR.N	1964.94443	2	1.51E-05	0.91	3.82	-	980.5
PM-7, 1803	R.NPSSAGSWNSGSSGPGSTGNR.N	1964.94443	2	2.83E-14	0.93	4.51	-	687.5
PM-5, 1941 - 2004	R.NPSSAGSWNSGSSGPGSTGNR.N	1964.94443	2	3.84E-07	0.96	4.38	-	1458.5
PM-8, 2000	R.NPSSAGSWNSGSSGPGSTGNR.N	1964.94443	2	1.40E-07	0.58	3.03	-	313.4
PM-3, 1784	R.NPSSAGSWNSGSSGPGSTGNR.N	1964.94443	2	1.17E-04	0.71	3.28	-	467.6
PM-6, 1679	R.NPSSAGSWNSGSSGPGSTGNR.N	1964.94443	2	2.61E-05	0.75	2.68	-	718.4
PM-8, 1886	R.NPSSAGSWNSGSSGPGSTGNR.N	1964.94443	2	9.85E-06	0.96	4.90	-	1121.5
PM-1, 4872 - 4931	R.PNNPDWGTFEEVSGNVSPGTR.R	2261.34962	2	3.67E-05	0.90	4.30	-	794.9
PM-3, 6476	K.TFPGFFSPMLGEFVSETESR.G	2266.51432	2	2.52E-04	0.67	3.00	-	404.0
PM-1, 6583 - 6589	K.TFPGFFSPMLGEFVSETESR.G	2266.51432	2	1.92E-05	0.70	3.39	-	282.2
PM-1, 6515	K.TFPGFFSPM*LGEFVSETESR.G	2282.51372	2	6.46E-04	0.55	2.73	-	183.0
PM-9, 1997	K.VQHIQLLQK.N	1107.33106	1	2.04E-04	0.30	2.36	-	431.0
PM-3, 1944	K.VQHIQLLQK.N	1107.33106	1	5.29E-04	0.69	2.49	-	875.4
PM-8, 2022	K.VQHIQLLQK.N	1107.33106	1	1.84E-05	0.64	2.92	-	575.6
gi|4503745|ref|NP_001447.1| filamin 1 (actin-binding protein-280); filamin 1; filamin A, alpha (act				2.42E-12	44.71	540.29	31.80	280757.4
PM-1, 6231 - 6291	R.ALGALVDSCAPGLCPDWDSWDASKPVTNAR.E	3231.56477	3	6.65E-05	0.83	4.28	-	405.9
PM-1, 6097 - 6175	R.ALGALVDSCAPGLCPDWDSWDASKPVTNAR.E	3231.56477	3	8.21E-04	0.75	3.59	-	392.4
PM-9, 5900 - 5905	R.ALGALVDSCAPGLCPDWDSWDASKPVTNAR.E	3231.56477	3	3.48E-06	0.72	3.17	-	534.3
PM-2, 5913 - 5973	R.ALGALVDSCAPGLCPDWDSWDASKPVTNAR.E	3231.56477	3	5.51E-05	0.93	5.01	-	792.9
PM-5, 5940	R.ALGALVDSCAPGLCPDWDSWDASKPVTNAR.E	3231.56477	3	5.85E-08	0.90	4.56	-	712.3
PM-2, 5998 - 6061	R.ALGALVDSCAPGLCPDWDSWDASKPVTNAR.E	3231.56477	3	1.44E-10	0.94	5.68	-	703.1
PM-2, 2970	R.ANLPQSFQVDTSK.A	1435.56409	1	1.92E-05	0.76	3.37	-	424.4
PM-1, 3325 - 3383	R.ANLPQSFQVDTSK.A	1435.56409	1	6.74E-09	0.57	2.87	-	376.3
PM-4, 3142	R.AWGPGLEGGVVGK.S	1227.39403	1	8.62E-05	0.39	2.21	-	533.4
PM-3, 3208	R.AWGPGLEGGVVGK.S	1227.39403	2	2.86E-05	0.80	3.08	-	611.0
PM-1, 3663	R.AWGPGLEGGVVGK.S	1227.39403	2	5.91E-05	0.89	4.03	-	708.3
PM-2, 3254	R.AWGPGLEGGVVGK.S	1227.39403	2	4.09E-07	0.92	3.31	-	1042.7
PM-2, 5081	R.CSYQPTMEGVHTVHVTFAGVPIPR.S	2685.07753	2	1.22E-06	0.56	2.59	-	354.9
PM-8, 3764	R.DAEMPATEKDLAEDAPWKK.I	2146.36338	2	2.51E-05	0.85	3.08	-	1173.6
PM-3, 4035 - 4100	K.DAGEGLLAVQITDPEGKPK.K	1939.15612	2	3.03E-05	0.91	4.07	-	1143.0
PM-2, 4263 - 4302	K.DAGEGLLAVQITDPEGKPK.K	1939.15612	2	2.67E-05	0.82	3.45	-	913.8
PM-1, 4457	K.DAGEGLLAVQITDPEGKPK.K	1939.15612	3	2.04E-07	0.88	3.70	-	1142.6
PM-8, 4059 - 4062	K.DAGEGLLAVQITDPEGKPK.K	1939.15612	2	4.82E-06	0.90	3.99	-	1064.7
PM-6, 3838	K.DAGEGLLAVQITDPEGKPK.K	1939.15612	2	1.28E-04	0.76	3.34	-	668.8
PM-2, 3947 - 4013	K.DAGEGLLAVQITDPEGKPK.K	1939.15612	2	1.10E-04	0.88	4.09	-	894.1
PM-9, 4036	K.DAGEGLLAVQITDPEGKPK.K	1939.15612	2	7.16E-07	0.89	4.03	-	792.2
PM-10, 4175	K.DAGEGLLAVQITDPEGKPK.K	1939.15612	2	6.24E-04	0.70	3.50	-	687.0
PM-1, 4712	K.DGSCGVAYVVQEPGDYEVSVK.F	2259.43510	2	6.02E-05	0.86	4.29	-	494.5
PM-11, 2232 - 2233	K.DKGEYTLVVK.W	1152.32223	2	2.30E-05	0.76	2.78	-	749.9
PM-2, 2741	R.DVDIIDHHDNTYTVK.Y	1785.89230	2	9.04E-06	0.91	3.49	-	908.6
PM-2, 2829	R.DVDIIDHHDNTYTVK.Y	1785.89230	2	1.58E-07	0.92	3.75	-	918.6
PM-3, 2745	R.DVDIIDHHDNTYTVK.Y	1785.89230	2	2.32E-06	0.89	3.61	-	786.8
PM-3, 2625 - 2683	R.DVDIIDHHDNTYTVK.Y	1785.89230	2	2.92E-06	0.94	4.20	-	1020.8
PM-4, 2962	R.EAGAGGLAIAVEGPSK.A	1427.58510	1	7.93E-05	0.88	3.95	-	496.3
PM-1, 2636	R.EATTEFSVDAR.A	1226.27493	2	9.85E-05	0.65	2.63	-	547.1
PM-3, 6188 - 6259	R.EEGPYEVEVTYDGVPVPGSPFPLEAVAPTKPSK.V	3487.85286	3	1.71E-06	0.93	5.17	-	754.6
PM-1, 6431 - 6497	R.EEGPYEVEVTYDGVPVPGSPFPLEAVAPTKPSK.V	3487.85286	3	6.22E-05	0.73	3.26	-	705.2
PM-2, 6226 - 6279	R.EEGPYEVEVTYDGVPVPGSPFPLEAVAPTKPSK.V	3487.85286	3	1.75E-05	0.81	3.72	-	612.0
PM-2, 6337 - 6399	R.EEGPYEVEVTYDGVPVPGSPFPLEAVAPTKPSK.V	3487.85286	3	8.85E-07	0.67	3.68	-	425.8
PM-3, 6320	R.EEGPYEVEVTYDGVPVPGSPFPLEAVAPTKPSK.V	3487.85286	3	2.21E-05	0.85	3.87	-	616.3
PM-5, 6166 - 6168	R.EEGPYEVEVTYDGVPVPGSPFPLEAVAPTKPSK.V	3487.85286	3	1.66E-09	0.94	5.22	-	1002.9
PM-1, 5180 - 5235	R.EGPYSISVLYGDEEVPR.S	1911.05809	2	1.17E-05	0.82	3.47	-	855.7
PM-10, 5536	R.FGGEHVPNSPFQVTALAGDQPSVQPPLR.S	2947.25342	3	5.28E-04	0.77	3.38	-	600.6
PM-5, 5416	R.FGGEHVPNSPFQVTALAGDQPSVQPPLR.S	2947.25342	2	1.49E-05	0.97	5.66	-	1214.1
PM-8, 5376	R.FGGEHVPNSPFQVTALAGDQPSVQPPLR.S	2947.25342	2	2.57E-06	0.93	4.25	-	933.4
PM-2, 5383 - 5450	R.FGGEHVPNSPFQVTALAGDQPSVQPPLR.S	2947.25342	3	1.01E-10	0.95	5.52	-	1053.3
PM-5, 5381 - 5440	R.FGGEHVPNSPFQVTALAGDQPSVQPPLR.S	2947.25342	3	3.27E-07	0.94	5.56	-	732.2
PM-4, 5274	R.FGGEHVPNSPFQVTALAGDQPSVQPPLR.S	2947.25342	2	2.13E-07	0.96	5.59	-	933.1
PM-1, 5740 - 5797	R.FGGEHVPNSPFQVTALAGDQPSVQPPLR.S	2947.25342	2	2.14E-06	0.96	5.62	-	942.9
PM-2, 5406 - 5465	R.FGGEHVPNSPFQVTALAGDQPSVQPPLR.S	2947.25342	2	3.20E-09	0.97	5.56	-	1125.4
PM-3, 5356 - 5417	R.FGGEHVPNSPFQVTALAGDQPSVQPPLR.S	2947.25342	2	6.48E-07	0.94	4.43	-	1020.5
PM-1, 3849	K.FNGTHIPGSPFK.I	1302.46259	2	1.29E-05	0.76	2.74	-	702.5
PM-1, 3025	R.FVPAEMGTHTVSVK.Y	1503.74821	2	1.00E-04	0.89	3.57	-	553.3
PM-1, 3465 - 3472	K.GKLDVQFSGLTK.G	1293.49392	2	1.29E-05	0.87	4.23	-	639.9
PM-1, 5096 - 5163	K.GLVEPVDVVDNADGTQTVNYVPSR.E	2545.74387	2	3.40E-04	0.94	4.78	-	1023.3
PM-2, 4881 - 4941	K.GLVEPVDVVDNADGTQTVNYVPSR.E	2545.74387	2	3.31E-06	0.92	4.56	-	599.4
PM-3, 4789	K.HTAM*VSWGGVSIPNSPFR.V	1960.20541	2	6.95E-06	0.95	4.45	-	1138.3
PM-1, 5313	K.HTAM*VSWGGVSIPNSPFR.V	1960.20541	3	3.62E-04	0.83	3.83	-	950.6
PM-5, 4973	K.HTAM*VSWGGVSIPNSPFR.V	1960.20541	2	1.03E-08	0.96	4.61	-	1307.0
PM-1, 5673 - 5677	K.HTAMVSWGGVSIPNSPFR.V	1944.20601	2	5.02E-06	0.97	5.46	-	1164.0
PM-3, 5225 - 5233	K.HTAMVSWGGVSIPNSPFR.V	1944.20601	2	1.61E-04	0.96	4.37	-	1430.3
PM-2, 4913	K.HTAM*VSWGGVSIPNSPFR.V	1960.20541	2	3.58E-04	0.92	4.50	-	810.6
PM-2, 5165 - 5227	K.HTAMVSWGGVSIPNSPFR.V	1944.20601	2	3.28E-05	0.96	5.31	-	996.8
PM-4, 4765	K.HTAM*VSWGGVSIPNSPFR.V	1960.20541	2	5.80E-04	0.94	4.55	-	995.4
PM-3, 3913	R.IANLQTDLSDGLR.L	1416.56231	1	2.18E-05	0.58	2.79	-	311.2
PM-4, 1985	K.IVGPSGAAVPCK.V	1156.38050	1	3.35E-04	0.54	2.24	-	577.0
PM-2, 2057	K.IVGPSGAAVPCK.V	1156.38050	2	5.53E-05	0.91	3.77	-	831.1
PM-1, 2427	K.IVGPSGAAVPCK.V	1156.38050	2	1.69E-06	0.91	3.21	-	1082.0

PM-2, 3726	K.IVGPSGAAVPCKVEPGLGADNSVVR.F	2450.79998	2	8.92E-04	0.70	3.15	-	286.8
PM-3, 3703	K.IVGPSGAAVPCKVEPGLGADNSVVR.F	2450.79998	3	3.35E-04	0.82	3.68	-	793.9
PM-1, 4108 - 4109	K.IVGPSGAAVPCKVEPGLGADNSVVR.F	2450.79998	3	8.60E-05	0.95	5.80	-	1019.3
PM-2, 3722 - 3737	K.IVGPSGAAVPCKVEPGLGADNSVVR.F	2450.79998	3	1.69E-06	0.95	4.75	-	1422.3
PM-1, 4267	R.KDGSCGVAYVVQEPGDYEVSVK.F	2387.60802	2	3.28E-04	0.78	3.62	-	350.3
PM-1, 3475 - 3476	K.KTHIQDNHDGTYTVAYVPDVTGR.Y	2588.77367	2	4.65E-04	0.84	3.78	-	735.5
PM-1, 4936 - 4992	K.LPQLPITNFSR.D	1286.50461	2	7.04E-05	0.92	3.60	-	792.0
PM-3, 5135 - 5196	K.LQVEPAVDTSGVQCYGPGIEGQGVFR.E	2765.05117	2	7.14E-07	0.95	5.03	-	894.8
PM-5, 5156	K.LQVEPAVDTSGVQCYGPGIEGQGVFR.E	2765.05117	2	4.54E-07	0.69	3.15	-	375.5
PM-4, 5070 - 5108	K.LQVEPAVDTSGVQCYGPGIEGQGVFR.E	2765.05117	2	4.22E-07	0.95	5.01	-	959.2
PM-11, 5251 - 5252	K.LQVEPAVDTSGVQCYGPGIEGQGVFR.E	2765.05117	2	5.85E-07	0.88	3.97	-	792.8
PM-6, 4954	K.LQVEPAVDTSGVQCYGPGIEGQGVFR.E	2765.05117	2	6.20E-09	0.88	3.97	-	575.6
PM-8, 5132 - 5143	K.LQVEPAVDTSGVQCYGPGIEGQGVFR.E	2765.05117	2	2.49E-07	0.94	4.97	-	802.8
PM-9, 5144	K.LQVEPAVDTSGVQCYGPGIEGQGVFR.E	2765.05117	2	3.86E-07	0.86	3.95	-	436.7
PM-1, 5495 - 5544	K.LQVEPAVDTSGVQCYGPGIEGQGVFR.E	2765.05117	2	3.71E-07	0.95	5.33	-	783.4
PM-4, 3642 - 3710	R.LVSNHSLHETSSVFVDSLTK.A	2201.42190	2	2.36E-04	0.95	4.55	-	1265.8
PM-1, 4560	R.LVSNHSLHETSSVFVDSLTK.A	2201.42190	2	9.01E-10	0.95	4.46	-	1366.8
PM-4, 4028 - 4058	R.LVSNHSLHETSSVFVDSLTK.A	2201.42190	2	4.19E-07	0.89	4.24	-	793.5
PM-2, 4214	R.LVSNHSLHETSSVFVDSLTK.A	2201.42190	2	7.96E-04	0.68	2.96	-	334.1
PM-1, 4244 - 4251	R.LVSNHSLHETSSVFVDSLTK.A	2201.42190	2	3.21E-05	0.92	4.62	-	792.8
PM-1, 4249 - 4260	R.LVSNHSLHETSSVFVDSLTK.A	2201.42190	3	1.09E-04	0.90	3.06	-	1636.3
PM-2, 2914	R.NGHVGISFVPK.E	1155.33104	1	4.65E-07	0.85	3.22	-	619.1
PM-3, 2831	R.NGHVGISFVPK.E	1155.33104	1	5.30E-04	0.35	2.39	-	258.0
PM-1, 3303	R.NGHVGISFVPK.E	1155.33104	1	4.16E-05	0.69	2.53	-	461.9
PM-4, 4580 - 4589	K.NGQHVASSPIPVVISQSEIGDASR.V	2449.66256	3	3.83E-06	0.96	4.96	-	1960.9
PM-1, 4996	K.NGQHVASSPIPVVISQSEIGDASR.V	2449.66256	2	2.82E-06	0.74	2.57	-	722.5
PM-2, 6646 - 6706	K.SADFVVEAIGDDVGTLGFSVEGPSQAK.I	2696.90399	2	6.15E-08	0.94	4.44	-	1332.5
PM-10, 3631	R.SAGQGEVLVYVEDPAGHQEEAK.V	2314.45074	2	8.35E-05	0.77	3.69	-	495.3
PM-11, 3740	R.SAGQGEVLVYVEDPAGHQEEAK.V	2314.45074	2	1.79E-05	0.84	3.42	-	563.6
PM-2, 3569 - 3638	R.SAGQGEVLVYVEDPAGHQEEAK.V	2314.45074	2	1.09E-06	0.82	3.91	-	483.4
PM-6, 3395 - 3398	R.SAGQGEVLVYVEDPAGHQEEAK.V	2314.45074	2	7.98E-05	0.90	3.93	-	743.9
PM-4, 3540 - 3609	R.SAGQGEVLVYVEDPAGHQEEAK.V	2314.45074	2	1.81E-07	0.71	3.15	-	472.5
PM-2, 4215 - 4285	R.SAGQGEVLVYVEDPAGHQEEAK.V	2314.45074	2	4.69E-04	0.44	2.59	-	349.3
PM-4, 3426	R.SAGQGEVLVYVEDPAGHQEEAK.V	2314.45074	2	4.42E-06	0.75	3.59	-	446.1
PM-4, 3418	R.SAGQGEVLVYVEDPAGHQEEAK.V	2314.45074	2	8.49E-05	0.85	3.88	-	559.7
PM-7, 3427	R.SAGQGEVLVYVEDPAGHQEEAK.V	2314.45074	2	1.07E-04	0.86	3.58	-	683.0
PM-3, 3517 - 3540	R.SAGQGEVLVYVEDPAGHQEEAK.V	2314.45074	2	1.71E-05	0.86	3.93	-	635.0
PM-3, 3601	R.SAGQGEVLVYVEDPAGHQEEAK.V	2314.45074	2	5.15E-06	0.82	3.41	-	558.6
PM-3, 3687 - 3720	R.SAGQGEVLVYVEDPAGHQEEAK.V	2314.45074	2	3.27E-04	0.89	3.97	-	656.9
PM-1, 3951 - 4007	R.SAGQGEVLVYVEDPAGHQEEAK.V	2314.45074	2	1.97E-07	0.93	4.90	-	748.3
PM-3, 3860	R.SAGQGEVLVYVEDPAGHQEEAK.V	2314.45074	2	3.61E-04	0.30	2.52	-	203.2
PM-1, 4064	R.SAGQGEVLVYVEDPAGHQEEAK.V	2314.45074	2	8.65E-07	0.76	3.41	-	458.0
PM-2, 3010	K.SPFEVYVDK.S	1084.20321	1	2.64E-04	0.89	3.25	-	931.5
PM-1, 3349	K.SPFEVYVDK.S	1084.20321	1	2.53E-06	0.85	3.14	-	979.5
PM-5, 3060	K.SPFEVYVDK.S	1084.20321	1	1.93E-05	0.54	2.42	-	655.0
PM-1, 3348	K.SPFEVYVDK.S	1084.20321	2	5.14E-05	0.92	3.36	-	983.7
PM-3, 2941	K.SPFEVYVDK.S	1084.20321	1	8.55E-05	0.87	3.08	-	1041.8
PM-1, 3001 - 3075	R.SPYTVTVGQACNPSACR.A	1869.07248	2	4.96E-04	0.40	2.59	-	239.4
PM-2, 2441	R.TGVELGKPTHFTVNAK.A	1699.93205	3	3.81E-04	0.66	3.12	-	641.7
PM-5, 3008	R.VANPSGNLTETYVQDR.G	1764.87462	2	2.47E-06	0.86	3.39	-	713.4
PM-2, 2971 - 3033	R.VANPSGNLTETYVQDR.G	1764.87462	2	2.42E-12	0.95	5.24	-	831.7
PM-1, 3457	R.VANPSGNLTETYVQDR.G	1764.87462	2	1.31E-07	0.92	3.99	-	778.5
PM-1, 3291 - 3351	R.VANPSGNLTETYVQDR.G	1764.87462	2	1.88E-10	0.96	4.73	-	1093.7
PM-10, 3051 - 3055	R.VANPSGNLTETYVQDR.G	1764.87462	2	1.84E-11	0.84	3.73	-	519.4
PM-9, 2991 - 2992	R.VANPSGNLTETYVQDR.G	1764.87462	2	9.91E-06	0.61	2.82	-	484.4
PM-3, 2920 - 2960	R.VANPSGNLTETYVQDR.G	1764.87462	2	2.41E-04	0.83	3.32	-	592.7
PM-4, 3040	K.VDINTEDLEDGTCR.V	1637.70803	2	2.58E-05	0.94	3.73	-	1424.5
PM-2, 3129	K.VDINTEDLEDGTCR.V	1637.70803	2	6.67E-07	0.81	3.09	-	807.8
PM-5, 3120	K.VDINTEDLEDGTCR.V	1637.70803	2	6.04E-05	0.92	3.87	-	1092.9
PM-1, 3419	K.VDINTEDLEDGTCR.V	1637.70803	2	1.46E-04	0.90	3.39	-	1152.2
PM-2, 2209	K.VDVGKDQEFTVK.S	1365.51377	1	6.20E-04	0.45	3.02	-	279.4
PM-3, 2179	K.VDVGKDQEFTVK.S	1365.51377	1	7.58E-04	0.51	3.22	-	409.3
PM-1, 2591	K.VDVGKDQEFTVK.S	1365.51377	2	2.76E-07	0.93	4.09	-	787.5
PM-2, 2401	K.VEPGLGADNSVVR.F	1313.44209	2	8.81E-08	0.72	2.87	-	235.0
PM-3, 2348	K.VEPGLGADNSVVR.F	1313.44209	2	1.89E-07	0.76	2.63	-	290.7
PM-2, 6153 - 6209	K.VEYTPYEEGLHSVDVTYDGSPVPSSPFQVPVTEGCDPSR.V	4299.59014	3	6.60E-05	0.93	5.73	-	504.4
PM-9, 6073	K.VEYTPYEEGLHSVDVTYDGSPVPSSPFQVPVTEGCDPSR.V	4299.59014	3	1.48E-04	0.87	4.78	-	526.5
PM-2, 6542	K.VGSAADIPINISETDLSLLTATVVPPSGR.E	2895.25495	2	9.74E-07	0.86	4.04	-	533.3
PM-3, 6460	K.VGSAADIPINISETDLSLLTATVVPPSGR.E	2895.25495	2	1.02E-07	0.91	4.65	-	534.8
PM-7, 6370	K.VGSAADIPINISETDLSLLTATVVPPSGR.E	2895.25495	2	5.18E-05	0.83	3.54	-	640.9
PM-4, 6424	K.VGSAADIPINISETDLSLLTATVVPPSGR.E	2895.25495	2	1.52E-07	0.94	5.17	-	807.8
PM-2, 6533	K.VGSAADIPINISETDLSLLTATVVPPSGREEPCLLK.R	3765.28414	3	2.57E-07	0.62	3.38	-	183.6
PM-5, 3949	K.VHSPSGALEECYVTEIDQDK.Y	2278.43842	2	7.72E-06	0.64	2.55	-	515.7
PM-1, 4308 - 4384	K.VHSPSGALEECYVTEIDQDK.Y	2278.43842	2	2.41E-05	0.96	4.89	-	1603.4
PM-4, 3748 - 3805	K.VHSPSGALEECYVTEIDQDK.Y	2278.43842	2	1.89E-04	0.54	3.05	-	450.1
PM-5, 4346	K.VHSPSGALEECYVTEIDQDKYAVR.F	2768.00869	2	2.47E-06	0.71	3.29	-	706.7
PM-2, 4290 - 4299	K.VHSPSGALEECYVTEIDQDKYAVR.F	2768.00869	2	5.51E-08	0.92	4.35	-	963.7
PM-3, 4265	K.VHSPSGALEECYVTEIDQDKYAVR.F	2768.00869	2	9.33E-10	0.91	4.35	-	892.5
PM-4, 4148	K.VHSPSGALEECYVTEIDQDKYAVR.F	2768.00869	2	6.60E-09	0.92	4.25	-	1075.9
PM-1, 3737	K.VNQPASFAVSLNGAK.G	1503.68480	2	8.12E-07	0.82	3.14	-	631.1
PM-3, 3368	K.VNQPASFAVSLNGAK.G	1503.68480	2	3.13E-04	0.76	2.80	-	524.5
PM-2, 3429 - 3430	K.VNQPASFAVSLNGAK.G	1503.68480	2	9.33E-07	0.88	3.78	-	622.1
PM-6, 3258 - 3327	K.VNQPASFAVSLNGAK.G	1503.68480	2	2.98E-05	0.81	3.11	-	530.8
PM-1, 3832	K.VNQPASFAVSLNGAK.G	1503.68480	2	8.31E-08	0.70	2.98	-	391.9
PM-1, 3943 - 3988	K.VNQPASFAVSLNGAK.G	1503.68480	2	1.62E-05	0.64	2.81	-	330.1
PM-5, 3549 - 3550	R.VQVQDNEGCPVEALVK.D	1786.00013	2	1.25E-04	0.94	4.03	-	1268.8
PM-1, 3931 - 4005	R.VQVQDNEGCPVEALVK.D	1786.00013	2	7.95E-10	0.97	5.38	-	1737.6
PM-2, 3514 - 3554	R.VQVQDNEGCPVEALVK.D	1786.00013	2	8.52E-05	0.94	4.10	-	1240.7
PM-4, 3396 - 3456	R.VQVQDNEGCPVEALVK.D	1786.00013	2	4.91E-04	0.83	3.26	-	1049.8
PM-6, 3366	R.VQVQDNEGCPVEALVK.D	1786.00013	2	3.15E-06	0.93	3.22	-	1444.6
PM-3, 3475	R.VQVQDNEGCPVEALVK.D	1786.00013	2	4.48E-04	0.95	4.28	-	1218.2
PM-4, 4320	R.VSGQGLHEGHTFEPAEFIIDTR.D	2441.64058	2	7.97E-05	0.94	4.04	-	1261.7
PM-1, 3207 - 3208	K.VTVLFAGQHIAK.S	1284.53185	2	4.14E-04	0.91	4.09	-	784.4
PM-2, 2778	K.VTVLFAGQHIAK.S	1284.53185	2	6.82E-04	0.80	3.80	-	503.3
PM-1, 5136	R.VTYTPM*APGSYLISIK.Y	1758.07165	2	3.88E-05	0.75	2.80	-	444.9
PM-2, 2958	K.YGGPYHIGGSPFK.A	1380.53182	2	4.72E-09	0.97	4.08	-	1973.2
PM-4, 2817	K.YGGPYHIGGSPFK.A	1380.53182	1	5.75E-06	0.91	4.12	-	594.7
PM-2, 2729	K.YGGQPVPNFPSK.L	1291.43658	1	1.92E-04	0.27	2.35	-	270.4
PM-1, 3029	K.YGGQPVPNFPSK.L	1291.43658	2	8.78E-05	0.93	3.76	-	596.2
PM-1, 2859	K.YNEQHVPGSPFTAR.V	1603.71978	2	1.87E-04	0.66	2.66	-	329.2
PM-1, 4588 - 4647	K.YTPVQQGPVGVNVTYGGDPIPK.S	2287.55640	2	2.18E-07	0.88	4.20	-	489.1
PM-6, 4166	K.YTPVQQGPVGVNVTYGGDPIPK.S	2287.55640	2	8.24E-06	0.58	2.98	-	471.1
PM-1, 4703 - 4781	K.YTPVQQGPVGVNVTYGGDPIPK.S	2287.55640	2	5.73E-05	0.88	3.88	-	590.0
PM-3, 4383 - 4443	K.YTPVQQGPVGVNVTYGGDPIPK.S	2287.55640	2	1.56E-07	0.75	2.89	-	647.6
PM-5, 4332 - 4388	K.YTPVQQGPVGVNVTYGGDPIPK.S	2287.55640	2	1.14E-07	0.73	2.80	-	678.3
PM-2, 4337 - 4409	K.YTPVQQGPVGVNVTYGGDPIPK.S	2287.55640	2	1.19E-05	0.76	3.35	-	420.8
PM-2, 6370	R.YWPQEAGEYAVHVLCNSEDIR.L	2537.74838	2	1.45E-10	0.91	4.01	-	628.9

PM-1, 6469	R.YWPQEAGEYAVHVLCNSEDIR.L	2537.74838	2	5.81E-07	0.78	2.91	-	699.8
PM-3, 6009 - 6020	R.YWPQEAGEYAVHVLCNSEDIR.L	2537.74838	2	7.59E-07	0.72	2.88	-	602.6
PM-4, 5957	R.YWPQEAGEYAVHVLCNSEDIR.L	2537.74838	2	2.51E-05	0.66	2.51	-	622.5
PM-4, 6270 - 6285	R.YWPQEAGEYAVHVLCNSEDIR.L	2537.74838	2	3.76E-06	0.87	3.33	-	815.6
PM-3, 6303	R.YWPQEAGEYAVHVLCNSEDIR.L	2537.74838	2	5.69E-06	0.89	3.94	-	669.1
gi|12667788|ref|NP_002464.1| myosin, heavy polypeptide 9, non-muscle [Homo sapiens]				3.46E-12	35.78	420.32	28.00	226529.8
PM-1, 3757	K.ALELDSNLYR.I	1194.31920	2	3.60E-04	0.93	3.46	-	1286.2
PM-1, 6435 - 6453	R.ALEQQVEEM*KTQLEELEDELQATEDAK.L	3165.38394	3	5.66E-05	0.87	3.96	-	702.9
PM-3, 6384 - 6451	R.ALEQQVEEMKTQLEELEDELQATEDAK.L	3149.38454	3	2.72E-05	0.94	4.73	-	1428.9
PM-2, 6531	R.ALEQQVEEMKTQLEELEDELQATEDAK.L	3149.38454	3	1.53E-05	0.88	4.13	-	1047.6
PM-3, 4903	K.ANLQIDQINTDLNLER.S	1871.04187	2	2.94E-05	0.92	4.04	-	795.6
PM-3, 6379	K.DFSALESQLQDTQELLQEENR.Q	2494.61059	2	9.43E-06	0.97	5.99	-	1361.7
PM-4, 6340	K.DFSALESQLQDTQELLQEENR.Q	2494.61059	2	1.45E-05	0.97	5.75	-	1409.4
PM-2, 6457	K.DFSALESQLQDTQELLQEENR.Q	2494.61059	2	4.16E-06	0.97	5.79	-	1293.8
PM-3, 5556 - 5629	R.ELESQISELQEDLESER.A	2035.10956	2	9.45E-04	0.42	2.67	-	381.1
PM-1, 4455 - 4528	K.EQADFAIEALAK.A	1306.44632	2	4.19E-04	0.81	2.91	-	596.1
PM-3, 2383	K.HEAMITDLEER.L	1344.47646	1	4.61E-04	0.17	2.16	-	390.6
PM-1, 3295	R.HEM*PPHIYAITDTAYR.S	1932.14884	2	2.00E-04	0.80	2.62	-	729.5
PM-2, 2905 - 2913	R.HEM*PPHIYAITDTAYR.S	1932.14884	2	1.08E-04	0.92	3.82	-	778.8
PM-3, 5319	R.IAQLEEELEEEQGNTELINDRLK.K	2714.92085	2	6.81E-07	0.88	3.77	-	909.4
PM-5, 5666	K.IRELESQISELQEDLESER.A	2304.45416	2	1.17E-04	0.91	3.79	-	963.8
PM-4, 5544 - 5608	K.IRELESQISELQEDLESER.A	2304.45416	2	2.33E-05	0.97	5.23	-	1230.6
PM-3, 5567 - 5631	K.IRELESQISELQEDLESER.A	2304.45416	2	8.36E-05	0.98	6.24	-	1047.8
PM-3, 5651	K.IRELESQISELQEDLESER.A	2304.45416	2	4.35E-06	0.90	3.69	-	732.7
PM-3, 3896 - 3933	R.KKLEMDLKDLEAHIDSANK.N	2199.51410	2	1.76E-05	0.94	4.27	-	1028.3
PM-2, 2899 - 2927	K.KLEEEQIILEDQNCK.L	1890.10512	2	4.77E-04	0.69	3.02	-	824.9
PM-8, 6243	R.KLEGDSTDLSDQIAELQAQIAELK.M	2616.86032	2	9.64E-05	0.93	4.22	-	1139.5
PM-1, 6524	R.KLEGDSTDLSDQIAELQAQIAELK.M	2616.86032	2	1.11E-07	0.97	5.67	-	1385.2
PM-3, 6403	R.KLEGDSTDLSDQIAELQAQIAELK.M	2616.86032	2	5.40E-09	0.96	5.50	-	1368.2
PM-2, 6485	R.KLEGDSTDLSDQIAELQAQIAELK.M	2616.86032	2	7.67E-07	0.97	6.32	-	1337.0
PM-4, 6368	R.KLEGDSTDLSDQIAELQAQIAELK.M	2616.86032	2	7.83E-07	0.94	4.84	-	1113.4
PM-5, 2796	K.KMEDSVGCLETAEEVKR.K	1982.22605	2	9.10E-08	0.91	4.10	-	1085.7
PM-3, 4983 - 5039	K.KMQQNIQELEEQLEEEESAR.Q	2462.63354	2	2.49E-04	0.85	3.70	-	718.6
PM-1, 5461	K.KMQQNIQELEEQLEEEESAR.Q	2462.63354	2	4.48E-05	0.83	3.63	-	665.1
PM-5, 3416 - 3448	K.KQELEEICHDLEAR.V	1770.94556	2	3.87E-04	0.96	4.57	-	1635.8
PM-2, 3270	K.LEEEQIILEDQNCK.L	1761.93220	2	6.90E-05	0.87	3.30	-	885.3
PM-3, 6439	K.LEGDSTDLSDQIAELQAQIAELK.M	2488.68740	2	6.89E-07	0.97	5.65	-	1212.8
PM-2, 6517 - 6518	K.LEGDSTDLSDQIAELQAQIAELK.M	2488.68740	2	7.39E-07	0.92	4.67	-	788.8
PM-3, 5795	R.LQQELDDLLVDLDHQR.Q	1951.12699	2	1.25E-10	0.97	5.00	-	2022.3
PM-8, 5863	R.LQQELDDLLVDLDHQR.Q	1951.12699	2	2.33E-08	0.94	4.56	-	1151.0
PM-1, 6285 - 6323	R.LQQELDDLLVDLDHQR.Q	1951.12699	2	3.63E-07	0.95	4.65	-	1309.3
PM-3, 5775 - 5856	R.LQQELDDLLVDLDHQR.Q	1951.12699	2	7.78E-09	0.98	6.04	-	2376.0
PM-5, 5889 - 5944	R.LQQELDDLLVDLDHQR.Q	1951.12699	2	4.78E-09	0.96	5.34	-	1141.5
PM-1, 6284	R.LQQELDDLLVDLDHQR.Q	1951.12699	3	1.95E-05	0.79	3.58	-	662.7
PM-4, 5726 - 5793	R.LQQELDDLLVDLDHQR.Q	1951.12699	2	2.56E-06	0.96	4.76	-	1648.2
PM-2, 5937 - 5977	R.LQQELDDLLVDLDHQR.Q	1951.12699	2	3.46E-12	0.97	5.45	-	1560.4
PM-2, 5789 - 5853	K.LQVELDNVTGLLSQSDSK.S	1947.13342	2	2.29E-06	0.95	5.06	-	1046.4
PM-3, 5903 - 5961	K.LQVELDNVTGLLSQSDSK.S	1947.13342	2	1.80E-06	0.97	5.29	-	1912.6
PM-2, 5658 - 5730	K.LQVELDNVTGLLSQSDSK.S	1947.13342	2	4.69E-07	0.97	5.45	-	1670.6
PM-2, 6074	K.LQVELDNVTGLLSQSDSK.S	1947.13342	2	6.58E-04	0.94	4.64	-	871.4
PM-3, 6024 - 6073	K.LQVELDNVTGLLSQSDSK.S	1947.13342	2	4.16E-06	0.94	4.94	-	757.4
PM-4, 5977 - 6052	K.LQVELDNVTGLLSQSDSK.S	1947.13342	2	1.62E-05	0.86	3.89	-	760.2
PM-3, 6133 - 6187	K.LQVELDNVTGLLSQSDSK.S	1947.13342	2	5.26E-04	0.68	3.42	-	622.3
PM-1, 6256	K.LQVELDNVTGLLSQSDSK.S	1947.13342	2	8.59E-06	0.93	4.65	-	696.4
PM-1, 5545	K.LQVELDNVTGLLSQSDSK.S	1947.13342	2	3.25E-05	0.80	3.03	-	755.7
PM-1, 5912 - 5967	K.LQVELDNVTGLLSQSDSK.S	1947.13342	2	6.02E-07	0.97	5.61	-	1453.9
PM-6, 5370	K.LQVELDNVTGLLSQSDSK.S	1947.13342	2	4.89E-06	0.78	3.04	-	616.6
PM-4, 5536 - 5610	K.LQVELDNVTGLLSQSDSK.S	1947.13342	2	4.00E-07	0.98	5.46	-	1865.4
PM-3, 5767 - 5845	K.LQVELDNVTGLLSQSDSK.S	1947.13342	2	2.42E-05	0.83	3.31	-	795.2
PM-1, 6023 - 6029	K.LQVELDNVTGLLSQSDSK.S	1947.13342	2	2.51E-07	0.97	5.77	-	1443.9
PM-3, 5677	K.LQVELDNVTGLLSQSDSK.S	1947.13342	2	1.32E-05	0.93	3.94	-	1192.5
PM-5, 5657 - 5721	K.LQVELDNVTGLLSQSDSK.S	1947.13342	2	7.51E-07	0.94	4.55	-	947.8
PM-5, 5520 - 5594	K.LQVELDNVTGLLSQSDSK.S	1947.13342	2	1.03E-08	0.97	6.06	-	1477.2
PM-3, 5547 - 5619	K.LQVELDNVTGLLSQSDSK.S	1947.13342	2	2.00E-04	0.96	4.70	-	1332.7
PM-1, 4043 - 4121	R.LTEM*ETLQSQLMAEK.L	1769.03151	2	7.40E-04	0.98	4.47	-	2678.4
PM-2, 4267	R.LTEMETLQSQLM*AEK.L	1769.03151	2	3.56E-05	0.81	3.06	-	817.3
PM-3, 4217 - 4255	R.LTEMETLQSQLM*AEK.L	1769.03151	2	7.48E-07	0.88	3.09	-	1166.7
PM-1, 2595	K.NKHEAMITDLEER.L	1586.75247	2	7.13E-05	0.87	3.31	-	789.1
PM-2, 2149 - 2162	K.NKHEAMITDLEER.L	1586.75247	2	1.31E-04	0.92	3.36	-	1090.9
PM-1, 5268 - 5277	K.NLPIYSEEIVEM*YK.G	1744.98660	3	3.98E-05	0.92	3.77	-	1455.9
PM-1, 5137 - 5215	K.NM*DPLNDNIATLLHQSSDK.F	2143.32109	2	7.31E-06	0.67	2.63	-	610.2
PM-4, 4641 - 4645	K.NM*DPLNDNIATLLHQSSDK.F	2143.32109	2	8.19E-05	0.63	2.61	-	604.9
PM-4, 6461	K.PAGPPGILALLDEECWFPK.A	2111.44835	2	5.90E-05	0.95	4.19	-	1459.0
PM-1, 3183	R.QAQQERDELADEIANSSGK.G	2090.15168	2	6.88E-04	0.76	3.27	-	458.2
PM-1, 3187	R.QAQQERDELADEIANSSGK.G	2090.15168	3	2.21E-04	0.87	3.44	-	1127.5
PM-3, 2357	K.QIATLHAQVADMK.K	1426.66697	1	7.88E-05	0.65	2.81	-	169.6
PM-5, 3221	R.RGDLPFVVPR.R	1156.36217	2	2.48E-06	0.95	3.90	-	1269.8
PM-2, 3089 - 3093	R.RGDLPFVVPR.R	1156.36217	2	8.41E-07	0.96	4.37	-	1298.0
PM-1, 3568	R.RGDLPFVVPR.R	1156.36217	2	1.50E-05	0.93	3.91	-	1055.2
PM-3, 2987 - 3056	R.RGDLPFVVPR.R	1156.36217	2	3.56E-06	0.96	4.18	-	1320.9
PM-3, 6367	R.RKLEGDSTDLSDQIAELQAQIAELK.M	2773.04667	2	8.83E-06	0.85	4.19	-	428.4
PM-3, 2489	K.RQAQQERDELADEIANSSGK.G	2246.33803	3	2.03E-04	0.93	4.03	-	1334.3
PM-1, 3361	R.SMMQDREDQSILCTGESGAGK.T	2301.52207	2	1.44E-04	0.93	4.67	-	1201.1
PM-3, 2955 - 2959	R.SMMQDREDQSILCTGESGAGK.T	2301.52207	2	1.12E-04	0.90	4.43	-	1141.8
PM-1, 3681	K.TDLLLEPYNK.Y	1206.36989	2	1.36E-05	0.85	3.38	-	887.1
PM-5, 4069 - 4080	K.TELEDTLDSTAAQQELR.S	1921.00969	2	2.47E-05	0.81	3.74	-	825.8
PM-1, 4371 - 4399	K.TELEDTLDSTAAQQELR.S	1921.00969	2	5.84E-05	0.94	3.91	-	1486.8
PM-3, 4080	K.TELEDTLDSTAAQQELR.S	1921.00969	2	5.08E-06	0.91	3.26	-	1289.7
PM-2, 4147	K.TELEDTLDSTAAQQELR.S	1921.00969	2	4.17E-06	0.87	3.30	-	1225.1
PM-1, 3292	K.VEAQLQELQVK.F	1285.47179	2	1.12E-04	0.95	3.65	-	1563.5
PM-1, 5164	K.VEDM*AELTCLNEASVLHNLK.E	2303.59917	2	7.13E-08	0.85	4.00	-	642.8
PM-2, 4845	K.VEDM*AELTCLNEASVLHNLK.E	2303.59917	2	5.87E-06	0.90	3.95	-	836.6
PM-2, 5633 - 5667	K.VEDMAELTCLNEASVLHNLK.E	2287.59977	2	1.04E-07	0.63	3.55	-	249.4
PM-1, 5908 - 5964	K.VEDMAELTCLNEASVLHNLK.E	2287.59977	2	1.11E-07	0.93	4.69	-	688.3
PM-4, 5541 - 5597	K.VEDMAELTCLNEASVLHNLK.E	2287.59977	2	1.88E-04	0.78	3.60	-	470.3
PM-3, 4716	K.VEDM*AELTCLNEASVLHNLK.E	2303.59917	2	5.83E-05	0.40	2.78	-	480.9
PM-3, 5463 - 5519	K.VEDMAELTCLNEASVLHNLK.E	2287.59977	2	1.56E-07	0.85	4.13	-	522.0
PM-1, 6019 - 6075	K.VEDMAELTCLNEASVLHNLK.E	2287.59977	2	8.80E-05	0.66	3.42	-	267.8
PM-5, 4836	K.VEDM*AELTCLNEASVLHNLK.E	2303.59917	2	2.50E-05	0.88	3.60	-	663.2
PM-4, 5422 - 5480	K.VEDMAELTCLNEASVLHNLK.E	2287.59977	2	7.03E-06	0.91	4.35	-	677.0
PM-5, 5609 - 5634	K.VEDMAELTCLNEASVLHNLK.E	2287.59977	2	6.98E-04	0.66	3.01	-	509.2
PM-3, 5507	K.VEDMAELTCLNEASVLHNLK.E	2287.59977	2	3.81E-06	0.88	3.78	-	645.9
PM-1, 6472	R.VISGVLQLGNIVFK.K	1487.81155	2	1.62E-06	0.98	5.07	-	2195.8
PM-1, 5013 - 5071	K.VSHLLGINVTDFTR.G	1572.79026	2	2.25E-05	0.85	2.97	-	987.9
PM-1, 4084	R.YEILTPNSIPK.G	1275.47536	2	3.61E-04	0.78	2.73	-	850.9
PM-2, 5931	K.YLYVDKNFINNPLAQADWAAK.K	2455.75199	3	3.28E-04	0.87	3.83	-	835.7
PM-1, 6253	K.YLYVDKNFINNPLAQADWAAK.K	2455.75199	3	4.69E-06	0.84	3.42	-	1010.1

gi|4507843|ref|NP_003357.1| ubiquinol-cytochrome c reductase core protein II [Homo sapiens]				2.21E-11	0.98	10.28	4.00	48469.7
PM-7, 6170 - 6171	K.LPNGLVIASLENYSPVSR.I	1930.19405	2	2.21E-11	0.98	5.54	-	1971.8
gi|4504517|ref|NP_001531.1| heat shock 27kDa protein 1; heat shock 27kD protein 1 [Homo sapiens] [M				3.36E-11	2.78	30.29	41.50	22782.3
PM-9, 5640 - 5699	R.KYTLPPGVDPTQVSSSLSPEGTLTVEAPMPK.L	3228.65914	3	1.26E-04	0.91	5.73	-	1110.2
PM-9, 6264	R.LPEEWSQWLGGSSWPGYVRPLPPAAIESPAVAAPAYSR.A	4095.56787	3	3.36E-11	0.96	5.64	-	1415.8
PM-9, 4693	R.VSLDVNHFAPDELTVK.T	1784.99058	2	9.30E-10	0.90	3.62	-	1327.6
PM-11, 4624 - 4681	R.VSLDVNHFAPDELTVK.T	1784.99058	2	3.37E-09	0.92	3.83	-	959.2
PM-11, 4739 - 4740	R.VSLDVNHFAPDELTVK.T	1784.99058	2	9.60E-04	0.27	2.55	-	327.8
PM-9, 4447 - 4512	R.VSLDVNHFAPDELTVK.T	1784.99058	2	4.09E-06	0.97	5.10	-	1399.8
gi|5453740|ref|NP_006462.1| myosin regulatory light chain MRCL3; myosin, light polypeptide, regulat				2.01E-10	1.94	20.27	10.50	19794.0
PM-10, 5419 - 5476	R.ATSNVFAM*FDQSQIQEFK.E	2108.31681	2	2.01E-10	0.96	5.15	-	1179.4
PM-10, 5531 - 5588	R.ATSNVFAM*FDQSQIQEFK.E	2108.31681	2	1.13E-04	0.84	3.45	-	678.5
PM-10, 6135 - 6195	R.ATSNVFAMFDQSQIQEFK.E	2092.31741	2	7.61E-08	0.98	5.40	-	2007.5
PM-11, 6096 - 6172	R.ATSNVFAMFDQSQIQEFK.E	2092.31741	2	2.02E-05	0.96	4.74	-	1525.6
PM-1, 6440	R.ATSNVFAMFDQSQIQEFK.E	2092.31741	2	4.84E-07	0.95	4.29	-	1433.0
gi|13124879|ref|NP_002465.1| smooth muscle myosin heavy chain 11 isoform SM1 [Homo sapiens]				3.77E-10	4.10	50.30	3.70	227336.7
PM-3, 2035	K.KEEELQAALAR.L	1258.40643	1	6.91E-04	0.45	2.52	-	588.7
PM-2, 2405 - 2406	R.KFDQLLAEEK.N	1221.38456	2	7.99E-04	0.81	3.40	-	814.5
PM-2, 5887	K.LQQLFNHTMFILEQEEYQR.E	2468.77258	2	3.42E-05	0.92	3.78	-	1242.8
PM-4, 5744 - 5745	K.LQQLFNHTMFILEQEEYQR.E	2468.77258	2	3.37E-09	0.96	4.88	-	1361.9
PM-3, 5756	K.LQQLFNHTMFILEQEEYQR.E	2468.77258	2	3.77E-10	0.94	4.07	-	1199.5
PM-1, 6341 - 6395	K.QLLQANPILEAFGNAK.T	1727.98540	2	6.37E-05	0.95	3.78	-	1287.9
PM-4, 5340 - 5420	K.TQLEELEDELQATEDAK.L	1963.04334	2	2.52E-04	0.85	3.31	-	1072.7
PM-4, 5476	K.TQLEELEDELQATEDAK.L	1963.04334	2	6.26E-07	0.97	5.20	-	1597.6
PM-2, 5510 - 5590	K.TQLEELEDELQATEDAK.L	1963.04334	2	1.29E-05	0.98	5.11	-	2175.5
PM-5, 5496 - 5552	K.TQLEELEDELQATEDAK.L	1963.04334	2	5.61E-09	0.96	5.30	-	1309.8
PM-8, 5458	K.TQLEELEDELQATEDAK.L	1963.04334	2	1.62E-04	0.91	3.92	-	966.6
PM-3, 5483 - 5541	K.TQLEELEDELQATEDAK.L	1963.04334	2	1.44E-05	0.98	6.03	-	1989.0
gi|5031635|ref|NP_005498.1| cofilin 1 (non-muscle) [Homo sapiens]				4.40E-10	3.26	40.30	36.70	18502.4
PM-11, 2967	K.AVLFCLSEDKK.N	1310.54450	2	3.47E-04	0.84	3.10	-	748.8
PM-10, 2832 - 2887	K.AVLFCLSEDKK.N	1310.54450	2	7.78E-04	0.85	3.16	-	915.6
PM-10, 5720 - 5747	K.EILVGDVGQTVDDPYATFVK.M	2167.40069	2	4.40E-10	0.98	5.92	-	1654.2
PM-1, 6044 - 6069	K.EILVGDVGQTVDDPYATFVK.M	2167.40069	2	2.71E-05	0.65	2.92	-	531.3
PM-10, 2643	K.LTGIKHELQANCYEEVKDR.C	2304.56861	2	2.08E-05	0.91	3.82	-	811.2
PM-10, 2995 - 3052	R.YALYDATYETK.E	1338.44364	1	6.05E-06	0.54	2.87	-	391.0
PM-5, 2986	R.YALYDATYETK.E	1338.44364	1	2.98E-05	0.56	2.75	-	417.0
gi|4503715|ref|NP_000500.1| fibrinogen, gamma chain isoform gamma-A precursor [Homo sapiens]				4.61E-10	5.78	70.23	24.90	49481.2
PM-2, 6427	K.ANQQFLVYCEIDGSGNGWTVFQK.R	2662.91741	2	4.61E-10	0.94	4.47	-	1117.5
PM-4, 6289	K.ANQQFLVYCEIDGSGNGWTVFQK.R	2662.91741	2	6.45E-07	0.91	4.02	-	831.9
PM-2, 5073	K.EGFGHLSPTGTTEFWLGNEK.I	2208.37146	2	1.71E-04	0.66	2.91	-	445.1
PM-6, 4811 - 4875	K.EGFGHLSPTGTTEFWLGNEK.I	2208.37146	2	1.01E-05	0.88	3.90	-	598.1
PM-1, 6443	R.FGSYCPTTCGIADFLSTYQTK.V	2418.68696	2	1.06E-08	0.87	4.27	-	369.5
PM-5, 6176	R.FGSYCPTTCGIADFLSTYQTK.V	2418.68696	2	3.57E-09	0.75	3.01	-	517.4
PM-6, 4010 - 4018	K.IHLISTQSAIPYALR.V	1683.97576	2	4.26E-04	0.70	2.57	-	613.5
PM-6, 3102 - 3158	K.VAQLEAQCQEPCKDTVQIHDITGK.D	2770.09251	2	1.30E-04	0.79	3.72	-	315.5
PM-4, 3133 - 3193	K.VAQLEAQCQEPCKDTVQIHDITGK.D	2770.09251	2	2.13E-05	0.64	3.03	-	383.2
PM-1, 3820	K.VAQLEAQCQEPCKDTVQIHDITGKDCQDIANK.G	3715.10553	3	5.28E-04	0.91	4.58	-	911.1
PM-4, 2520 - 2524	K.YEASILTHDSSIR.Y	1492.61564	2	4.48E-04	0.89	3.20	-	895.5
PM-2, 2685 - 2727	K.YEASILTHDSSIR.Y	1492.61564	2	2.84E-06	0.67	2.59	-	532.4
PM-1, 3033 - 3051	K.YEASILTHDSSIR.Y	1492.61564	2	2.86E-09	0.95	4.24	-	1018.1
gi|4507485|ref|NP_003237.1| thrombospondin 1 [Homo sapiens]				4.80E-10	19.44	270.37	38.10	129351.8
PM-2, 2077 - 2137	R.AQLYIDCEK.M	1140.29156	1	7.18E-04	0.33	2.53	-	413.0
PM-4, 2018	R.AQLYIDCEK.M	1140.29156	1	5.37E-04	0.42	2.51	-	443.8
PM-2, 6346	R.AQLYIDCEKMENAELDVPIQSVFTR.D	2971.35503	2	1.08E-04	0.80	3.68	-	422.0
PM-2, 6227	R.AQLYIDCEKMENAELDVPIQSVFTR.D	2971.35503	2	3.45E-06	0.89	4.14	-	707.6
PM-1, 4256 - 4319	R.CDNCPYNHNPDQADTDNNGEGDACAADIDGDGILNER.D	4095.11797	3	5.39E-07	0.98	7.42	-	2628.5
PM-3, 4017 - 4051	R.CDNCPYNHNPDQADTDNNGEGDACAADIDGDGILNER.D	4095.11797	3	6.81E-06	0.97	5.69	-	1783.8
PM-2, 2979 - 3049	R.CENTDPGYNCLPCPPR.F	1951.15360	2	7.27E-04	0.84	3.45	-	460.7
PM-2, 3650 - 3714	K.CGACPPGYSGNGIQCTDVDECKEVPDACFNHNGEHR.C	4110.39652	3	9.81E-04	0.63	3.17	-	483.2
PM-2, 3545	K.CGACPPGYSGNGIQCTDVDECKEVPDACFNHNGEHR.C	4110.39652	3	4.80E-10	0.75	3.70	-	518.9
PM-3, 5949 - 6088	K.DDFDHDSVPDIDDICPENVDISETDFR.R	3182.24511	3	5.74E-04	0.72	3.26	-	525.0
PM-2, 2411	K.DHSGQVFSVVSNGK.A	1461.56164	1	1.99E-04	0.53	2.79	-	451.7
PM-2, 2351 - 2410	K.DHSGQVFSVVSNGK.A	1461.56164	2	2.62E-04	0.66	2.63	-	451.6
PM-2, 2549 - 2602	R.DNCQYVYNVDQR.D	1574.65686	2	4.77E-06	0.74	2.71	-	828.3
PM-3, 5543 - 5560	K.DSDGDGRGDACKDDFDHDSVPDIDDICPENVDISETDFR.R	4416.46140	3	1.95E-05	0.87	4.60	-	596.6
PM-2, 5606 - 5665	K.DSDGDGRGDACKDDFDHDSVPDIDDICPENVDISETDFR.R	4416.46140	3	2.69E-05	0.88	4.42	-	791.8
PM-2, 3481 - 3559	R.DTDMDGVGDQCDNCPLEHNPDQLDSDSDR.I	3322.34922	3	9.45E-04	0.85	4.30	-	486.1
PM-4, 3513 - 3568	R.DTDMDGVGDQCDNCPLEHNPDQLDSDSDR.I	3322.34922	3	2.00E-04	0.90	4.60	-	604.2
PM-1, 3863	R.DTDMDGVGDQCDNCPLEHNPDQLDSDSDR.I	3322.34922	3	3.23E-07	0.94	5.27	-	785.1
PM-2, 3245	K.FQDLVDAVR.A	1063.18877	1	9.00E-04	0.21	1.81	-	359.9
PM-2, 5179 - 5298	R.FVFGTTPEDILR.N	1395.58470	1	1.08E-05	0.06	2.12	-	106.6
PM-5, 5337	R.FVFGTTPEDILR.N	1395.58470	1	5.07E-04	0.13	2.12	-	132.5
PM-2, 5183 - 5301	R.FVFGTTPEDILR.N	1395.58470	1	2.12E-05	0.28	2.97	-	116.6
PM-4, 5209	R.FVFGTTPEDILR.N	1395.58470	1	3.92E-05	0.08	2.29	-	109.3
PM-2, 5259 - 5337	R.GDACKDDFDHDSVPDIDDICPENVDISETDFRR.F	3870.01705	3	4.55E-07	0.83	4.12	-	613.1
PM-2, 4401	K.GGVNDNFQGVLQNVR.F	1617.74799	2	5.73E-04	0.47	2.83	-	446.2
PM-2, 4517	K.GGVNDNFQGVLQNVR.F	1617.74799	2	4.97E-05	0.88	3.91	-	705.0
PM-1, 4543 - 4621	K.GGVNDNFQGVLQNVR.F	1617.74799	2	1.26E-04	0.90	3.89	-	980.6
PM-2, 4254 - 4326	K.GGVNDNFQGVLQNVR.F	1617.74799	2	1.44E-07	0.75	3.19	-	565.9
PM-2, 4074 - 4153	K.GGVNDNFQGVLQNVR.F	1617.74799	2	7.49E-05	0.80	3.77	-	573.1
PM-3, 2243 - 2305	K.GTSQNDPNWVVR.H	1373.45608	2	5.74E-04	0.14	2.59	-	311.9
PM-9, 6191	R.IEDANLIPPVPDDKFQDLVDAVR.A	2580.87450	2	2.53E-07	0.44	2.78	-	212.2
PM-4, 6290	R.IEDANLIPPVPDDKFQDLVDAVR.A	2580.87450	2	3.03E-07	0.68	3.25	-	313.3
PM-5, 6204	R.IEDANLIPPVPDDKFQDLVDAVR.A	2580.87450	2	2.74E-05	0.67	3.13	-	338.4
PM-2, 6357 - 6417	R.IEDANLIPPVPDDKFQDLVDAVR.A	2580.87450	2	4.80E-07	0.76	3.47	-	310.7
PM-8, 6179	R.IEDANLIPPVPDDKFQDLVDAVR.A	2580.87450	2	8.87E-06	0.48	2.71	-	223.5
PM-3, 6292 - 6335	R.IEDANLIPPVPDDKFQDLVDAVR.A	2580.87450	2	7.27E-04	0.42	2.63	-	198.6
PM-2, 3322 - 3399	R.IGDTCDNNQDIDEDGHQNNLDNCPYVPNANQADHDK.D	4128.16954	3	9.18E-04	0.84	4.85	-	277.0
PM-2, 2999 - 3057	R.IGDTCDNNQDIDEDGHQNNLDNCPYVPNANQADHDKDGK.G	4428.48187	3	1.27E-06	0.82	4.07	-	549.7
PM-2, 2257 - 2326	K.IMADSGPIYDK.T	1210.38204	2	2.70E-05	0.70	2.79	-	556.7
PM-4, 6369	R.IPESGGDNSVFDIFELTGAAR.K	2196.35899	2	1.32E-09	0.91	3.69	-	917.3
PM-1, 6525	R.IPESGGDNSVFDIFELTGAAR.K	2196.35899	2	1.90E-09	0.97	5.27	-	1202.9
PM-9, 6259	R.IPESGGDNSVFDIFELTGAAR.K	2196.35899	2	1.99E-08	0.95	4.80	-	875.7
PM-5, 6273	R.IPESGGDNSVFDIFELTGAAR.K	2196.35899	2	7.84E-08	0.96	5.61	-	861.4
PM-2, 6442 - 6502	R.IPESGGDNSVFDIFELTGAAR.K	2196.35899	2	5.52E-09	0.98	6.29	-	1411.0
PM-2, 1905 - 1979	R.KDHSGQVFSVVSNGK.A	1589.73455	2	6.97E-05	0.54	2.54	-	440.8
PM-2, 2051	R.KDHSGQVFSVVSNGK.A	1589.73455	2	5.64E-05	0.89	3.22	-	935.7
PM-2, 3361	K.KDNCPNLPNSGQEDYDKDGIGDACDDDDDNDKIPDDR.D	4213.22315	3	1.51E-07	0.95	5.59	-	1075.8
PM-2, 2102	R.LCNNPAPQFGGK.D	1303.47214	2	1.98E-04	0.73	2.88	-	551.7
PM-2, 2053 - 2117	R.LCNSPSPQMNGKPCEGEAR.E	2133.37597	2	4.21E-04	0.71	3.52	-	384.8
PM-2, 1941 - 1997	R.LCNSPSPQMNGKPCEGEAR.E	2133.37597	2	3.88E-07	0.88	4.15	-	472.8
PM-2, 3482	R.TIVTTLQDSIRK.V	1375.59647	2	3.21E-05	0.87	3.14	-	987.8
PM-2, 6453 - 6455	K.TKDLQAICGISCDELSSMVLELR.G	2640.05170	3	1.58E-04	0.89	3.78	-	1080.8
PM-2, 5817 - 5818	K.TKDLQAICGISCDELSSM*VLELR.G	2656.05110	2	8.00E-04	0.79	3.18	-	633.6
PM-2, 6454 - 6519	K.TKDLQAICGISCDELSSMVLELR.G	2640.05170	2	1.05E-06	0.96	4.57	-	1274.3
PM-2, 6611	K.TKDLQAICGISCDELSSMVLELR.G	2640.05170	2	4.51E-05	0.86	2.97	-	821.4
gi|4502051|ref|NP_000688.1| arachidonate 12-lipoxygenase; 12(S)-lipoxygenase [Homo sapiens]				5.10E-10	3.01	40.31	11.80	75721.8

PM-5, 4304 - 4362	R.AAAQLTYCSLCPPDDLADR.G	2138.36748	2	2.30E-04	0.76	3.33	-	493.4
PM-5, 6296 - 6376	R.GEEEEFDHDVAEDLGLLQFVR.L	2448.58352	2	5.10E-10	0.98	6.25	-	2272.2
PM-5, 6258	R.NEQLDWPYEYLKPSCIENSVTI	2699.97273	2	2.07E-04	0.49	3.13	-	362.7
PM-5, 4904 - 4978	R.WVQGEDILSLPEGTAR.L	1771.95190	2	1.07E-04	0.78	3.21	-	650.3
gi|4826898|ref|NP_005013.1| profilin 1; profilin-1 [Homo sapiens]				5.41E-10	0.45	10.12	10.00	15054.2
PM-11, 4456	R.SSFYVNGLTLGGQK.C	1471.63957	1	4.24E-05	0.35	2.37	-	351.1
PM-11, 4231	R.SSFYVNGLTLGGQK.C	1471.63957	1	5.41E-10	0.45	2.27	-	338.8
gi|29741246|ref|XP_294070.1| similar to Glyceraldehyde 3-phosphate dehydrogenase, liver (GAPDH) [Ho				7.82E-10	1.65	20.21	11.80	35032.0
PM-8, 5486 - 5562	K.LVINGNPITIFQER.D	1614.87029	2	7.82E-10	0.89	3.58	-	778.0
PM-8, 6190	K.VLHDNFGIVKGLM*TTVHAITATQK.T	2612.04471	3	1.89E-06	0.75	3.24	-	1067.8
gi|4507729|ref|NP_001060.1| tubulin, beta polypeptide [Homo sapiens]				9.15E-10	3.05	40.34	18.20	49906.7
PM-6, 2495	K.EVDEQMLNVQNK.N	1447.59668	1	3.06E-05	0.55	3.15	-	314.9
PM-11, 2727	K.EVDEQMLNVQNK.N	1447.59668	1	2.39E-05	0.27	2.79	-	292.4
PM-4, 6254	K.GHYTEGAELVDSVLDVVR.K	1960.13388	2	4.58E-05	0.98	5.28	-	2077.0
PM-6, 6076 - 6134	K.GHYTEGAELVDSVLDVVR.K	1960.13388	2	2.40E-08	0.99	6.72	-	2683.4
PM-3, 6301	K.GHYTEGAELVDSVLDVVR.K	1960.13388	2	1.16E-07	0.99	6.63	-	3171.1
PM-6, 5866	K.LTTPTYGDLNHLVSATMSGVTTCLR.F	2710.08047	2	2.81E-04	0.67	3.00	-	576.7
PM-6, 6234	K.LTTPTYGDLNHLVSATMSGVTTCLR.F	2710.08047	2	1.84E-06	0.92	5.15	-	711.5
PM-1, 6519	K.LTTPTYGDLNHLVSATMSGVTTCLR.F	2710.08047	2	9.15E-10	0.96	5.78	-	1109.5
PM-6, 6095 - 6154	R.SGPFGQIFRPDNFVFGQSGAGNNWAK.G	2800.03898	3	1.50E-05	0.83	3.49	-	927.2
gi|29739414|ref|XP_294015.1| similar to Glyceraldehyde 3-phosphate dehydrogenase, liver (GAPDH) [Ho				1.28E-09	0.78	10.19	5.80	26916.7
PM-8, 4858	K.LISWYDNEFGYSNR.V	1764.87478	2	1.28E-09	0.78	2.84	-	703.2
gi|29742827|ref|XP_294099.1| similar to myosin:SUBUNIT=regulatory light chain [Homo sapiens]				1.53E-09	0.53	10.13	7.40	17094.0
PM-10, 3655 - 3719	R.FTDEEVDELYR.E	1416.47127	1	1.53E-09	0.53	2.68	-	377.0
PM-10, 3640 - 3695	R.FTDEEVDELYR.E	1416.47127	1	3.02E-06	0.29	1.89	-	468.9
gi|7669492|ref|NP_002037.2| glyceraldehyde-3-phosphate dehydrogenase [Homo sapiens]				2.56E-09	2.45	30.33	11.60	36053.0
PM-8, 3270 - 3342	R.GALQNIIPASTGAAK.A	1412.61680	1	5.98E-07	0.62	3.09	-	357.8
PM-8, 6278 - 6279	K.VIHDNFGIVEGLMTTVHAITATQK.T	2596.98694	3	1.43E-07	0.98	6.70	-	2070.4
PM-8, 4748 - 4806	R.VIISAPSADAPMFVMGVNHEK.Y	2214.59387	2	2.56E-09	0.86	4.00	-	446.9
gi|4759080|ref|NP_004159.1| succinate dehydrogenase complex, subunit A, flavoprotein precursor; suc				5.37E-09	1.72	20.27	8.70	72689.1
PM-5, 6216	R.AGLPCQDLEFVQFHPTGIYGAGCLITEGCR.G	3368.81096	3	5.04E-05	0.76	3.43	-	498.6
PM-5, 4338	R.HVNGQDQIVPGLYACGEAACASVHGANR.L	2953.22296	3	5.37E-09	0.96	5.48	-	1478.5
gi|4502565|ref|NP_001740.1| calpain, small subunit 1; calpain, small polypeptide; calcium-dependent				5.72E-09	4.34	50.22	26.10	28315.5
PM-10, 6239	R.LFAQLAGDDM*EVSATELMNILNK.V	2540.89556	2	5.72E-09	0.87	3.62	-	762.1
PM-10, 2584	R.SMVAVMDSDTTGK.L	1342.52228	1	4.91E-05	0.87	3.63	-	635.0
PM-10, 2139	R.THYSNIEANESEEVR.Q	1778.81502	2	5.39E-08	0.87	2.90	-	1061.4
PM-10, 6205	R.YSDESGNM*DFDNFISCLVR.L	2286.44088	2	4.11E-06	0.93	4.31	-	942.1
PM-9, 6275	R.YSDESGNMDFDNFISCLVR.L	2270.44148	2	1.91E-07	0.80	3.28	-	580.8
gi|7705767|ref|NP_057086.1| CGI-49 protein [Homo sapiens]				5.89E-09	0.85	10.19	6.80	46920.8
PM-7, 6523	K.GPEAGYVATPIAMVQAAMTLLSDASHLPK.A	2941.41545	3	5.89E-09	0.85	3.71	-	782.7
gi|4501885|ref|NP_001092.1| beta actin; beta cytoskeletal actin [Homo sapiens]				6.26E-09	6.23	70.32	34.90	41736.5
PM-7, 6244 - 6327	R.CPEALFQPSFLGMESCGIHETTFNSIMK.C	3233.70748	3	2.68E-04	0.93	5.08	-	726.2
PM-7, 2063 - 2136	R.GYSFTTTAER.E	1133.19273	2	1.34E-04	0.89	2.93	-	1329.7
PM-7, 4688 - 4696	R.KDLYANTVLSGGTTMYPGIADR.M	2344.62976	2	2.66E-04	0.88	3.31	-	938.7
PM-7, 4322 - 4380	R.KDLYANTVLSGGTTMYPGIADR.M	2344.62976	2	9.30E-09	0.88	3.70	-	608.5
PM-8, 4532	R.KDLYANTVLSGGTTMYPGIADR.M	2344.62976	2	4.43E-04	0.76	2.60	-	800.1
PM-7, 6490	K.LCYVALDFEQEMATAASSSSLEK.S	2551.83185	2	3.92E-05	0.76	2.77	-	634.5
PM-4, 5673 - 5700	R.TTGIVMDSGDGVTHTVPIYEGYALPHAILR.L	3185.59928	3	7.57E-07	0.97	5.89	-	1887.8
PM-3, 5711	R.TTGIVMDSGDGVTHTVPIYEGYALPHAILR.L	3185.59928	3	1.10E-05	0.88	3.91	-	848.6
PM-2, 5825 - 5829	R.TTGIVMDSGDGVTHTVPIYEGYALPHAILR.L	3185.59928	3	1.22E-08	0.98	5.77	-	2502.8
PM-8, 5739 - 5794	R.TTGIVMDSGDGVTHTVPIYEGYALPHAILR.L	3185.59928	3	7.45E-04	0.87	3.84	-	910.8
PM-9, 5389 - 5447	R.TTGIVM*DSGDGVTHTVPIYEGYALPHAILR.L	3201.59868	3	7.94E-05	0.69	3.04	-	786.0
PM-9, 5737 - 5799	R.TTGIVMDSGDGVTHTVPIYEGYALPHAILR.L	3185.59928	3	4.35E-08	0.97	5.89	-	1688.5
PM-7, 5878 - 5956	R.TTGIVMDSGDGVTHTVPIYEGYALPHAILR.L	3185.59928	3	1.19E-05	0.89	4.34	-	1012.3
PM-7, 5778 - 5820	R.TTGIVMDSGDGVTHTVPIYEGYALPHAILR.L	3185.59928	3	1.92E-05	0.95	5.52	-	1257.6
PM-7, 5666 - 5722	R.TTGIVMDSGDGVTHTVPIYEGYALPHAILR.L	3185.59928	3	5.62E-04	0.97	6.01	-	1513.5
PM-7, 5550 - 5610	R.TTGIVMDSGDGVTHTVPIYEGYALPHAILR.L	3185.59928	3	1.35E-06	0.96	5.54	-	1307.0
PM-7, 5438 - 5495	R.TTGIVMDSGDGVTHTVPIYEGYALPHAILR.L	3185.59928	3	2.67E-05	0.84	3.82	-	862.5
PM-5, 5786 - 5793	R.TTGIVMDSGDGVTHTVPIYEGYALPHAILR.L	3185.59928	3	1.47E-05	0.92	4.37	-	1196.3
PM-7, 5089 - 5147	R.TTGIVM*DSGDGVTHTVPIYEGYALPHAILR.L	3201.59868	3	4.76E-05	0.91	4.89	-	972.3
PM-7, 5322 - 5382	R.TTGIVMDSGDGVTHTVPIYEGYALPHAILR.L	3185.59928	3	1.30E-05	0.82	4.01	-	658.6
PM-7, 5211 - 5271	R.TTGIVMDSGDGVTHTVPIYEGYALPHAILR.L	3185.59928	3	6.26E-09	0.96	6.33	-	1054.9
PM-10, 5839	R.TTGIVMDSGDGVTHTVPIYEGYALPHAILR.L	3185.59928	3	1.14E-05	0.72	3.22	-	516.3
PM-7, 3442 - 3499	R.VAPEEHPVLLTEAPLNPK.A	1955.24351	2	2.38E-05	0.92	4.54	-	717.6
PM-7, 3306 - 3384	R.VAPEEHPVLLTEAPLNPK.A	1955.24351	2	2.34E-05	0.93	4.46	-	887.2
PM-7, 3223 - 3282	R.VAPEEHPVLLTEAPLNPK.A	1955.24351	3	5.18E-04	0.69	3.20	-	893.2
PM-7, 3180 - 3251	R.VAPEEHPVLLTEAPLNPK.A	1955.24351	2	5.05E-05	0.90	4.47	-	673.0
PM-7, 3103 - 3159	R.VAPEEHPVLLTEAPLNPK.A	1955.24351	2	3.83E-05	0.91	4.13	-	835.0
PM-6, 3434 - 3455	R.VAPEEHPVLLTEAPLNPK.A	1955.24351	2	8.55E-07	0.89	3.84	-	811.0
PM-5, 3632	R.VAPEEHPVLLTEAPLNPK.A	1955.24351	2	6.01E-04	0.93	4.07	-	988.0
PM-8, 3564 - 3627	R.VAPEEHPVLLTEAPLNPK.A	1955.24351	2	1.83E-05	0.92	4.64	-	758.4
PM-3, 3556	R.VAPEEHPVLLTEAPLNPK.A	1955.24351	2	1.83E-04	0.88	3.66	-	633.2
PM-2, 3618 - 3635	R.VAPEEHPVLLTEAPLNPK.A	1955.24351	3	6.69E-04	0.77	3.77	-	490.0
PM-9, 3629 - 3704	R.VAPEEHPVLLTEAPLNPK.A	1955.24351	2	1.50E-05	0.92	4.63	-	840.2
PM-2, 3609	R.VAPEEHPVLLTEAPLNPK.A	1955.24351	2	3.22E-04	0.89	3.78	-	862.6
PM-1, 4047 - 4104	R.VAPEEHPVLLTEAPLNPK.A	1955.24351	3	2.10E-05	0.66	3.69	-	660.2
PM-10, 3779	R.VAPEEHPVLLTEAPLNPK.A	1955.24351	2	7.20E-06	0.87	4.01	-	699.5
PM-1, 4035 - 4105	R.VAPEEHPVLLTEAPLNPK.A	1955.24351	2	6.98E-04	0.92	4.84	-	663.4
PM-11, 3780	R.VAPEEHPVLLTEAPLNPK.A	1955.24351	2	3.67E-05	0.89	4.46	-	535.6
gi|17986258|ref|NP_066299.2| smooth muscle and non-muscle myosin alkali light chain isoform 1 [Homo				7.32E-09	3.57	40.22	37.10	16930.0
PM-11, 2012 - 2075	R.HVLVTLGEK.M	996.18547	2	4.73E-06	0.84	2.93	-	577.1
PM-11, 3920 - 3923	K.NKDQGTYEDYVEGLR.V	1787.86505	2	1.48E-05	0.83	3.36	-	640.4
PM-11, 3435 - 3504	K.NKDQGTYEDYVEGLR.V	1787.86505	2	7.32E-09	0.95	4.31	-	1325.3
PM-10, 3520 - 3601	K.NKDQGTYEDYVEGLR.V	1787.86505	2	5.18E-05	0.91	3.71	-	1060.5
PM-11, 3071 - 3127	R.VFDKEGNGTVMGAEIR.H	1723.93228	2	5.61E-06	0.88	2.94	-	1192.8
PM-11, 6199	K.VLDFEHFLPM*LQTVAK.N	1905.24957	2	6.65E-04	0.90	3.84	-	745.2
gi|27498267|ref|XP_209737.1| similar to tyrosine 3/tryptophan 5 -monooxygenase activation protein,				7.67E-09	0.22	10.20	12.60	11063.6
PM-9, 6253	R.DICNDVLSLLEK.F	1419.62613	1	2.09E-05	0.71	3.29	-	604.8
PM-9, 5688	R.DICNDVLSLLEK.F	1419.62613	1	7.67E-09	0.22	1.81	-	696.7
gi|4557677|ref|NP_000203.1| integrin beta chain, beta 3 precursor; platelet glycoprotein IIIa precu				7.95E-09	6.47	90.25	22.30	87185.6
PM-4, 2414 - 2416	R.AKWDTANNPLYK.E	1421.58225	1	2.09E-04	0.20	2.41	-	246.4
PM-4, 4668	R.CDLKENLLKDNCAPESIEFPVSEAR.V	2936.26729	3	3.32E-04	0.78	4.24	-	448.7
PM-4, 4605	K.DDLWSIQNLGTK.L	1390.52332	1	7.95E-09	0.51	2.82	-	361.3
PM-4, 2348	R.GKCECGSCVCIQPGSYGDTCEK.C	2553.83574	2	2.77E-05	0.92	4.82	-	380.6
PM-4, 2186 - 2253	K.HVLTLTDQVTR.F	1283.45912	2	1.13E-05	0.90	3.43	-	760.9
PM-4, 5124 - 5185	R.LAGIVQPNDGQCHVGSDNHYSASTTMDYPSLGLMTEK.L	3996.37037	3	7.78E-06	0.57	3.10	-	441.7
PM-4, 4638 - 4696	R.NRDAPEGGFDAIMQATVCDEK.I	2325.52182	2	1.15E-05	0.96	4.94	-	1374.0
PM-4, 4738 - 4808	R.NRDAPEGGFDAIMQATVCDEK.I	2325.52182	2	9.72E-07	0.90	4.14	-	863.6
PM-4, 2372 - 2433	R.VLEDRPLSDKGSGDSSQVTQVSPQR.I	2686.87396	3	5.29E-04	0.84	3.70	-	1002.3
PM-4, 2412	R.VLEDRPLSDKGSGDSSQVTQVSPQR.I	2686.87396	2	5.90E-04	0.59	2.97	-	297.2
PM-4, 2440 - 2501	K.YCECDDFSCVR.Y	1511.64234	2	6.36E-06	0.85	3.05	-	596.0
gi|6681259|ref|NP_031377.1| multimerin [Homo sapiens]				9.59E-09	5.34	60.26	6.20	138070.8
PM-1, 4071	R.DQALQLQVLNSR.F	1385.55144	2	9.43E-06	0.97	4.29	-	1948.0
PM-1, 3869	K.HSLPDIQLLQK.G	1292.50915	2	5.38E-05	0.92	3.57	-	754.4
PM-2, 3475	K.LAFESENINSEIHCDR.V	1935.06453	2	9.59E-09	0.86	3.65	-	647.3
PM-9, 3463	K.LAFESENINSEIHCDR.V	1935.06453	2	2.02E-08	0.91	3.83	-	836.8
PM-3, 2452 - 2457	K.LKEVHEQLLSTEQVSDQK.N	2112.32666	2	5.99E-04	0.97	5.18	-	1572.4

PM-2, 6521	K.TM*TIINNAIDFIQDNYALK.E	2215.51196	2	1.09E-06	0.79	3.98	-	344.0
PM-3, 6475	K.TMTIINNAIDFIQDNYALK.E	2199.51256	2	6.07E-06	0.83	3.50	-	529.4
PM-1, 6551 - 6625	K.TM*TIINNAIDFIQDNYALK.E	2215.51196	2	6.36E-05	0.53	3.48	-	286.2
PM-1, 6581	K.TMTIINNAIDFIQDNYALK.E	2199.51256	2	1.02E-04	0.91	4.02	-	662.0
gi|4506677|ref|NP_002942.1| ribophorin II [Homo sapiens]				1.28E-08	1.53	20.19	6.70	69301.6
PM-5, 4548 - 4574	K.ISTEVGITNVDLSTVDKDQSIAPK.T	2531.79855	2	1.28E-08	0.81	3.88	-	717.7
PM-5, 6292	K.TSFTPVGDVFELNFMNVK.F	2046.33178	2	1.73E-06	0.72	2.56	-	492.7
gi|5729887|ref|NP_006624.1| IQ motif containing GTPase activating protein 2 [Homo sapiens]				1.38E-08	0.98	10.26	1.10	180611.0
PM-3, 4593	K.LPYDVTTEQALTYPEVK.N	1968.19269	2	2.25E-05	0.93	3.78	-	1397.1
PM-4, 4561 - 4568	K.LPYDVTTEQALTYPEVK.N	1968.19269	2	1.38E-08	0.98	5.12	-	2613.5
PM-2, 4671	K.LPYDVTTEQALTYPEVK.N	1968.19269	2	2.75E-06	0.86	3.22	-	971.2
gi|4506877|ref|NP_002996.1| selectin P precursor; Selectin P (granulocyte membrane protein, 140kD;				1.52E-08	1.73	20.28	5.70	90844.7
PM-3, 4031	K.AVQCQHLEAPSEGTM*DCVHPLTAFAYGSSCK.F	3469.85079	3	1.70E-04	0.81	4.10	-	515.3
PM-3, 2032 - 2065	K.HALCYTASCQDMSCSK.Q	1920.16132	2	1.52E-08	0.92	4.16	-	550.0
gi|4758086|ref|NP_004069.1| cysteine and glycine-rich protein 1; cysteine-rich protein; LIM-domain				1.59E-08	1.76	20.24	20.20	20567.3
PM-9, 3323 - 3383	K.GYGYGQGAGTLSTDKGESLGIK.H	2160.32665	2	5.82E-04	0.79	3.80	-	491.4
PM-11, 3259	K.NLDSTTVAVHGEEIYCK.S	1937.12034	2	1.59E-08	0.96	4.51	-	1285.1
PM-9, 3107 - 3111	K.NLDSTTVAVHGEEIYCK.S	1937.12034	2	7.16E-07	0.96	4.89	-	1259.1
gi|4501881|ref|NP_001091.1| alpha 1 actin precursor; alpha skeletal muscle actin [Homo sapiens] [MA				1.73E-08	2.60	30.25	8.50	42050.8
PM-7, 2307 - 2367	K.EITALAPSTMK.I	1162.38206	2	7.18E-04	0.80	3.01	-	478.0
PM-1, 3003	K.EITALAPSTMK.I	1162.38206	2	3.25E-04	0.73	2.50	-	695.1
PM-6, 4526 - 4595	K.SYELPDGQVITIGNER.F	1791.93998	2	5.69E-08	0.86	3.52	-	658.4
PM-2, 4793 - 4849	K.SYELPDGQVITIGNER.F	1791.93998	2	3.59E-05	0.91	3.94	-	870.0
PM-1, 5069 - 5127	K.SYELPDGQVITIGNER.F	1791.93998	2	5.81E-06	0.91	4.35	-	801.1
PM-7, 4204 - 4264	K.SYELPDGQVITIGNER.F	1791.93998	2	7.83E-07	0.95	4.34	-	968.8
PM-10, 4839 - 4871	K.SYELPDGQVITIGNER.F	1791.93998	2	6.42E-07	0.92	3.77	-	823.4
PM-7, 4631 - 4707	K.SYELPDGQVITIGNER.F	1791.93998	2	1.27E-05	0.96	4.69	-	979.9
PM-7, 4796 - 4879	K.SYELPDGQVITIGNER.F	1791.93998	2	1.73E-08	0.94	3.92	-	1045.7
PM-9, 4697 - 4780	K.SYELPDGQVITIGNER.F	1791.93998	2	5.95E-06	0.94	4.39	-	959.9
PM-12, 5112	K.SYELPDGQVITIGNER.F	1791.93998	2	1.15E-04	0.70	2.78	-	412.0
PM-7, 4244 - 4300	K.YPIEHGIITNWDDMEK.I	1962.17174	2	2.59E-05	0.94	5.08	-	730.9
PM-8, 4144 - 4219	K.YPIEHGIITNWDDMEK.I	1962.17174	2	9.54E-04	0.88	3.98	-	639.1
PM-9, 4149 - 4207	K.YPIEHGIITNWDDMEK.I	1962.17174	2	3.97E-04	0.88	4.40	-	543.5
gi|29737978|ref|XP_290747.1| similar to Myosin heavy chain, nonmuscle type B (Cellular myosin heavy				1.74E-08	2.32	30.22	2.00	191380.7
PM-4, 3056 - 3058	K.ADFCIIHYAGK.V	1295.49161	2	1.35E-04	0.95	4.00	-	1213.2
PM-1, 3619	K.ADFCIIHYAGK.V	1295.49161	2	3.00E-06	0.95	4.08	-	1433.4
PM-4, 3077	K.ADFCIIHYAGK.V	1295.49161	1	6.16E-06	0.59	2.46	-	575.5
PM-2, 3210	K.ADFCIIHYAGK.V	1295.49161	2	9.70E-07	0.66	2.68	-	547.8
PM-4, 2341	R.AGVLAHLEEER.D	1224.34854	1	1.56E-04	0.39	2.47	-	410.2
PM-4, 2344 - 2366	R.AGVLAHLEEER.D	1224.34854	2	4.43E-07	0.95	4.45	-	1369.4
PM-1, 2947	R.AGVLAHLEEER.D	1224.34854	2	2.05E-07	0.96	4.34	-	1533.6
PM-10, 2675 - 2736	R.AGVLAHLEEER.D	1224.34854	2	7.40E-08	0.78	2.85	-	852.2
PM-2, 2481	R.AGVLAHLEEER.D	1224.34854	2	8.59E-07	0.93	4.11	-	1269.3
PM-8, 2534 - 2550	R.AGVLAHLEEER.D	1224.34854	2	5.09E-04	0.81	3.23	-	740.1
PM-5, 2646 - 2661	R.AGVLAHLEEER.D	1224.34854	2	7.49E-05	0.89	3.68	-	980.0
PM-11, 2732	R.AGVLAHLEEER.D	1224.34854	2	1.78E-05	0.82	3.00	-	909.7
PM-2, 3518 - 3522	K.VDYKADEWLMK.N	1398.60886	2	1.74E-08	0.89	3.53	-	863.8
PM-4, 3378 - 3386	K.VDYKADEWLMK.N	1398.60886	2	3.83E-05	0.86	3.38	-	718.3
gi|4758012|ref|NP_004850.1| clathrin heavy chain; clathrin, heavy polypeptide-like 2 [Homo sapiens]				1.93E-08	4.13	50.23	5.20	191612.6
PM-2, 6589 - 6590	K.AFMTADLPNELIELLEK.I	1948.26956	2	4.71E-07	0.81	3.68	-	375.0
PM-4, 3025	R.KFNALFAQGNYSEAAK.V	1759.94281	2	5.50E-06	0.87	3.44	-	688.1
PM-4, 4066	R.LASTLVHLGEYQAAVDGAR.K	1972.19103	2	7.01E-06	0.86	3.67	-	754.2
PM-2, 4246	R.LASTLVHLGEYQAAVDGAR.K	1972.19103	2	3.39E-07	0.92	4.28	-	1006.8
PM-3, 4192	R.LASTLVHLGEYQAAVDGAR.K	1972.19103	2	1.02E-06	0.93	4.53	-	867.4
PM-5, 4230	R.LASTLVHLGEYQAAVDGAR.K	1972.19103	2	7.46E-06	0.91	4.16	-	974.9
PM-1, 4579	R.LASTLVHLGEYQAAVDGAR.K	1972.19103	2	1.80E-05	0.95	4.51	-	1257.3
PM-8, 4044	K.LHIIEVGTPPTGNQPFPK.K	1946.23831	2	7.35E-04	0.82	3.56	-	516.1
PM-4, 3890 - 3893	K.LHIIEVGTPPTGNQPFPK.K	1946.23831	2	1.71E-04	0.63	3.19	-	330.7
PM-1, 5039 - 5095	R.TSIDAYDNFDNISLAQR.L	1944.04818	2	1.93E-08	0.89	3.63	-	1029.6
PM-3, 4755	R.TSIDAYDNFDNISLAQR.L	1944.04818	2	8.79E-07	0.91	4.30	-	645.3
PM-2, 4846 - 4911	R.TSIDAYDNFDNISLAQR.L	1944.04818	2	3.20E-08	0.86	3.48	-	1182.2
gi|30147623|ref|XP_293669.2| similar to actinin, alpha 4 [Homo sapiens]				2.21E-08	0.78	10.16	3.50	57794.4
PM-4, 3650 - 3714	K.LSGSNPYTTVTPQIINSK.W	1921.14079	2	2.21E-08	0.78	3.16	-	723.0
gi|13518026|ref|NP_004978.2| LIM and senescent cell antigen-like domains 1 [Homo sapiens]				2.29E-08	2.37	30.27	12.60	37251.2
PM-8, 6258	R.CDLCQEVLADIGFVK.N	1768.04797	2	2.29E-08	0.98	5.41	-	2102.9
PM-8, 2562 - 2618	R.ELKGELYCLPCHDK.M	1763.03141	2	6.29E-04	0.50	3.02	-	373.2
PM-8, 3170 - 3215	R.VIEGDVVSALNK.A	1244.41966	1	7.58E-05	0.90	4.08	-	711.4
gi|28373103|ref|NP_005164.2| sarco/endoplasmic reticulum Ca2+ -ATPase isoform a; ATPase, Ca(2+)-tra				2.34E-08	2.24	30.25	6.00	109255.5
PM-2, 6258 - 6261	R.DIVPGDIVEVAVGDKVPADLR.L	2178.47138	2	1.96E-06	0.93	4.60	-	835.9
PM-4, 6036	R.IVENLQSFNEITAMTGDGVNDAPALK.K	2749.04707	2	2.34E-08	0.94	5.02	-	906.8
PM-2, 6169	R.IVENLQSFNEITAMTGDGVNDAPALK.K	2749.04707	2	2.49E-04	0.66	3.02	-	558.3
PM-4, 3272	K.NMLFSGTNITSGK.A	1370.55705	1	2.67E-06	0.36	2.87	-	212.0
gi|4504391|ref|NP_000179.1| hexokinase 1 isoform HKI; brain form hexokinase [Homo sapiens]				2.35E-08	1.55	20.20	4.40	102502.4
PM-4, 5508 - 5513	R.AILQQLGLNSTCDDSILVK.T	2089.39939	2	1.32E-04	0.69	2.76	-	703.1
PM-4, 5881 - 5941	R.TPDGTENGDFLALDLGGTNFR.V	2211.33051	2	2.35E-08	0.86	4.08	-	600.0
gi|21361755|ref|NP_004090.3| stomatin; erythrocyte membrane protein band 7.2 (stomatin) [Homo sapie				2.61E-08	3.51	40.32	17.00	31708.5
PM-9, 3149 - 3152	K.NLSQILSDR.E	1046.15975	2	3.81E-04	0.75	2.69	-	659.7
PM-8, 6250	K.NLSQILSDREEIAHNMQSTLDDATDAWGIK.V	3373.65339	3	8.86E-04	0.95	5.21	-	1496.9
PM-9, 6263	K.NLSQILSDREEIAHNMQSTLDDATDAWGIK.V	3373.65339	3	4.68E-06	0.97	6.47	-	1785.0
PM-9, 5923 - 5995	K.NLSQILSDREEIAHNM*QSTLDDATDAWGIK.V	3389.65279	3	2.88E-05	0.93	4.58	-	1330.5
PM-9, 3591 - 3647	R.VQNATLAVANITNADSATR.L	1931.09731	2	7.32E-05	0.96	4.81	-	1048.0
PM-9, 3703 - 3764	R.VQNATLAVANITNADSATR.L	1931.09731	2	5.74E-08	0.95	4.84	-	855.3
PM-9, 3435 - 3516	R.VQNATLAVANITNADSATR.L	1931.09731	2	7.17E-05	0.87	4.19	-	520.9
PM-9, 3931 - 4000	R.VQNATLAVANITNADSATR.L	1931.09731	2	4.11E-07	0.97	5.44	-	1108.2
PM-9, 4041 - 4108	R.VQNATLAVANITNADSATR.L	1931.09731	2	9.73E-05	0.88	3.70	-	869.8
PM-9, 4164 - 4235	R.VQNATLAVANITNADSATR.L	1931.09731	2	3.86E-07	0.95	5.22	-	783.5
PM-9, 4259	R.VQNATLAVANITNADSATR.L	1931.09731	2	3.02E-08	0.95	4.61	-	907.7
PM-9, 4465	R.VQNATLAVANITNADSATR.L	1931.09731	2	1.03E-06	0.67	3.07	-	433.4
PM-9, 3819 - 3876	R.VQNATLAVANITNADSATR.L	1931.09731	2	8.39E-08	0.95	4.87	-	978.4
PM-8, 3550	R.VQNATLAVANITNADSATR.L	1931.09731	2	7.49E-08	0.91	4.22	-	640.1
PM-10, 3635	R.VQNATLAVANITNADSATR.L	1931.09731	2	2.61E-08	0.86	3.66	-	655.6
PM-11, 3704	R.VQNATLAVANITNADSATR.L	1931.09731	2	2.43E-06	0.90	4.01	-	844.9
gi|5729997|ref|NP_004154.2| RAB27B, member RAS oncogene family [Homo sapiens]				2.80E-08	2.80	30.29	18.80	24607.7
PM-9, 5672	R.NWM*SQLQANAYCENPDIVLIGNK.A	2696.01232	2	1.52E-07	0.89	3.95	-	622.4
PM-9, 6100	R.NWMSQLQANAYCENPDIVLIGNK.A	2680.01292	2	9.42E-05	0.83	2.86	-	667.9
PM-9, 6187	R.NWMSQLQANAYCENPDIVLIGNK.A	2680.01292	2	2.80E-08	0.97	5.79	-	1071.6
PM-9, 4268 - 4323	K.YGIPYFETSAATGQNVEK.A	1976.13177	2	3.92E-06	0.94	4.18	-	1054.1
gi|28626504|ref|NP_113659.3| UNC-112 related protein 2 [Homo sapiens]				2.95E-08	12.87	160.32	37.40	75429.7
PM-8, 2868	K.AGDALWLR.F	902.03223	2	7.28E-05	0.92	3.82	-	1270.7
PM-5, 6246	R.ARGEELDEDLFLQLTGGHEAF	2348.51032	2	1.07E-06	0.73	2.60	-	621.5
PM-1, 6455 - 6456	R.ASFSQPLFQAVAAICR.L	1767.04487	2	2.95E-08	0.95	5.09	-	921.3
PM-8, 6152 - 6162	R.ASFSQPLFQAVAAICR.L	1767.04487	2	4.21E-06	0.94	4.26	-	969.0
PM-3, 6267	R.ASFSQPLFQAVAAICR.L	1767.04487	2	2.32E-04	0.84	3.38	-	591.2
PM-5, 6170 - 6180	R.ASFSQPLFQAVAAICR.L	1767.04487	2	4.60E-06	0.96	4.91	-	1050.9
PM-2, 6313	R.ASFSQPLFQAVAAICR.L	1767.04487	2	1.42E-05	0.86	3.35	-	762.9
PM-1, 3668 - 3727	K.EKEPEEELYDLSK.V	1609.71294	2	1.04E-04	0.63	2.97	-	280.3
PM-5, 6274 - 6346	R.FIQAWQSLPDFGISYVM*VR.F	2274.62593	2	2.51E-04	0.88	4.17	-	486.3

PM-8, 2678 - 2680	K.GCEVVPDVNVSGQK.F	1488.64855	1	6.24E-04	0.81	3.17	-	828.1
PM-10, 2793	K.GCEVVPDVNVSGQK.F	1488.64855	1	2.67E-05	0.90	4.25	-	755.5
PM-10, 2773	K.GCEVVPDVNVSGQK.F	1488.64855	1	2.05E-04	0.89	3.03	-	1136.3
PM-2, 2730	K.GCEVVPDVNVSGQK.F	1488.64855	1	2.03E-04	0.87	3.19	-	917.9
PM-5, 2742	K.GCEVVPDVNVSGQK.F	1488.64855	1	7.88E-06	0.85	3.84	-	651.5
PM-5, 6293	R.GEELDEDLFLQLTGGHEAF	2121.24574	2	1.77E-04	0.78	3.38	-	946.1
PM-5, 2485	R.HPEELSLLR.A	1094.24601	1	9.43E-05	0.12	1.85	-	143.2
PM-5, 4396 - 4456	R.ILEAHQNVAQLSLAEAQLR.F	2105.38372	2	4.05E-04	0.92	4.19	-	890.7
PM-5, 4284 - 4341	R.ILEAHQNVAQLSLAEAQLR.F	2105.38372	2	5.18E-07	0.98	6.32	-	2252.8
PM-2, 4310	R.ILEAHQNVAQLSLAEAQLR.F	2105.38372	2	1.28E-04	0.98	5.39	-	2818.9
PM-3, 4224 - 4283	R.ILEAHQNVAQLSLAEAQLR.F	2105.38372	2	5.01E-06	0.81	3.67	-	583.4
PM-11, 4416 - 4471	R.ILEAHQNVAQLSLAEAQLR.F	2105.38372	2	3.89E-04	0.72	2.98	-	710.3
PM-1, 6495	K.LEGSAPTDVLDSLTTIPELK.D	2100.35256	2	1.18E-04	0.86	3.96	-	445.2
PM-5, 3817 - 3884	K.SQDEAPGDPIQQLNLK.G	1753.89174	2	7.70E-04	0.78	3.13	-	435.3
PM-5, 3922	R.TGSGGPGNHPHGPDASAEGLNPYGLVAPR.F	2783.95312	2	1.04E-04	0.76	3.49	-	390.4
PM-5, 3622 - 3682	R.TGSGGPGNHPHGPDASAEGLNPYGLVAPR.F	2783.95312	2	2.87E-04	0.93	4.77	-	726.7
PM-5, 3782	R.TGSGGPGNHPHGPDASAEGLNPYGLVAPR.F	2783.95312	2	3.57E-04	0.84	3.58	-	671.6
PM-10, 3761	R.TGSGGPGNHPHGPDASAEGLNPYGLVAPR.F	2783.95312	2	1.69E-04	0.90	4.34	-	544.3
PM-1, 4057	R.TGSGGPGNHPHGPDASAEGLNPYGLVAPR.F	2783.95312	2	7.85E-04	0.90	4.26	-	643.5
PM-4, 3441 - 3466	R.TGSGGPGNHPHGPDASAEGLNPYGLVAPR.F	2783.95312	2	3.39E-04	0.85	4.13	-	487.0
PM-2, 3610	R.TGSGGPGNHPHGPDASAEGLNPYGLVAPR.F	2783.95312	2	3.62E-04	0.92	4.40	-	645.9
PM-3, 3559	R.TGSGGPGNHPHGPDASAEGLNPYGLVAPR.F	2783.95312	2	3.96E-05	0.92	4.94	-	661.4
PM-5, 6301 - 6377	R.TM*ADSSYTSEVQAILAFLSLQR.T	2448.73471	3	8.17E-04	0.86	4.02	-	1037.8
PM-1, 4133	R.VFVGEEDPEAESVTLR.V	1777.91007	2	6.95E-05	0.91	3.73	-	1166.0
PM-6, 3552 - 3622	R.VFVGEEDPEAESVTLR.V	1777.91007	2	2.00E-05	0.67	2.56	-	659.9
PM-10, 3871 - 3928	R.VFVGEEDPEAESVTLR.V	1777.91007	2	7.97E-05	0.88	3.49	-	1101.2
PM-7, 3658	R.VFVGEEDPEAESVTLR.V	1777.91007	2	1.35E-05	0.84	3.32	-	904.7
PM-3, 3756 - 3779	R.VFVGEEDPEAESVTLR.V	1777.91007	2	4.86E-05	0.89	3.38	-	1295.1
PM-2, 3797 - 3798	R.VFVGEEDPEAESVTLR.V	1777.91007	2	1.66E-06	0.95	4.62	-	1387.5
PM-5, 3780 - 3844	R.VFVGEEDPEAESVTLR.V	1777.91007	2	7.63E-07	0.92	4.43	-	1025.0
PM-8, 2423 - 2478	R.VTGESHIGGVLLK.I	1310.52448	2	1.06E-06	0.94	4.37	-	1360.2
PM-2, 2426	R.VTGESHIGGVLLK.I	1310.52448	2	4.20E-04	0.72	2.92	-	428.6
PM-7, 2209	R.VTGESHIGGVLLK.I	1310.52448	2	3.08E-04	0.90	3.18	-	1348.8
PM-6, 2278 - 2294	R.VTGESHIGGVLLK.I	1310.52448	2	8.33E-06	0.89	3.29	-	1070.1
PM-5, 2530	R.VTGESHIGGVLLK.I	1310.52448	2	9.46E-05	0.95	4.27	-	1682.7
PM-5, 2461 - 2534	R.VTGESHIGGVLLK.I	1310.52448	1	4.96E-04	0.51	2.42	-	614.9
PM-8, 2487	R.VTGESHIGGVLLK.I	1310.52448	1	3.72E-05	0.71	3.00	-	723.6
PM-5, 4598	K.VVLAGGVAPALFR.G	1270.54832	1	4.82E-04	0.74	2.98	-	258.7
gi|4507357|ref|NP_003555.1| transgelin 2; SM22-alpha homolog [Homo sapiens]				3.18E-08	1.49	20.24	20.10	22391.3
PM-10, 6277	K.DGTVLCELINALYPEGQAPVKK.I	2416.77877	2	2.37E-05	0.53	2.57	-	412.8
PM-10, 6251	R.YGINTTDIFQTVDLWEGK.N	2101.30080	2	3.18E-08	0.95	4.88	-	799.4
gi|4507879|ref|NP_003365.1| voltage-dependent anion channel 1 [Homo sapiens]				3.22E-08	1.83	20.22	13.10	30772.3
PM-8, 3928	K.KLETAVNLAWTAGNSNTR.F	1947.14157	2	1.74E-05	0.93	4.32	-	932.3
PM-8, 2628	K.SENGLEFTSSGSANTETTK.V	1960.98754	2	3.22E-08	0.90	3.93	-	694.5
gi|4502285|ref|NP_001672.1| ATPase, Ca++ transporting, cardiac muscle, slow twitch 2 isoform 2; ATP				3.41E-08	1.82	20.24	3.30	109690.3
PM-4, 4345 - 4422	R.SLPSVETLGCTSVICSDK.T	1954.21236	2	3.41E-08	0.89	3.99	-	592.5
PM-2, 4502	R.SLPSVETLGCTSVICSDK.T	1954.21236	2	3.30E-04	0.60	2.81	-	253.4
PM-3, 4423 - 4431	R.SLPSVETLGCTSVICSDK.T	1954.21236	2	2.78E-04	0.78	3.38	-	418.4
PM-1, 4771 - 4833	R.SLPSVETLGCTSVICSDK.T	1954.21236	2	1.95E-05	0.89	3.64	-	690.4
PM-4, 4053 - 4112	K.VGEATETALTCLVEK.M	1621.83478	2	2.41E-06	0.93	4.50	-	823.3
PM-2, 4199 - 4274	K.VGEATETALTCLVEK.M	1621.83478	2	5.53E-06	0.94	4.75	-	1064.7
gi|4504085|ref|NP_000399.1| glycerol-3-phosphate dehydrogenase 2 (mitochondrial) [Homo sapiens] [MA				3.55E-08	4.34	60.23	12.00	80814.1
PM-5, 2998	K.LVGAIVYYDGQHNDAR.M	1791.94490	2	6.57E-05	0.73	3.31	-	490.9
PM-5, 5574 - 5649	R.LVQDYGLESEVAQHLAATYGDK.A	2408.60567	2	3.55E-08	0.96	4.54	-	1516.9
PM-5, 3566	K.LYDLVAGSNCLK.S	1353.56937	1	9.51E-06	0.52	2.86	-	396.5
PM-5, 4158	R.SEISLLPSDIDR.Y	1345.48086	1	3.38E-07	0.51	3.00	-	348.5
PM-5, 6305	R.VLESINVQMDENTLHEILNEVDLNK.N	2911.23474	3	1.47E-06	0.91	4.39	-	722.5
PM-5, 6284	R.VLESINVQM*DENTLHEILNEVDLNK.N	2927.23414	3	2.39E-05	0.70	3.94	-	331.0
gi|11761631|ref|NP_005132.1| fibrinogen, beta chain preproprotein [Homo sapiens]				4.40E-08	3.35	40.20	11.40	55901.8
PM-6, 2334 - 2336	K.KREEAPSLRPAPPPISGGGYR.A	2236.51908	3	1.08E-04	0.90	3.90	-	1102.4
PM-6, 5996 - 6063	R.MGPTELLIEMEDWKGDK.V	1993.29069	2	3.90E-05	0.76	2.69	-	678.8
PM-6, 3872 - 3932	R.VYCDMNTENGGWTVIQNR.Q	2158.36073	2	4.40E-08	0.91	4.01	-	788.9
PM-6, 3408 - 3474	R.VYCDM*NTENGGWTVIQNR.Q	2174.36013	2	1.18E-04	0.78	3.44	-	662.5
gi|4507651|ref|NP_003281.1| tropomyosin 4 [Homo sapiens]				4.47E-08	0.95	10.22	6.00	28521.6
PM-8, 2139	R.KIQALQQQADEAEDR.M	1743.85695	3	4.47E-08	0.95	4.33	-	1684.7
gi|27485844|ref|XP_113743.3| similar to chromosome 11 open reading frame 25 [Homo sapiens]				5.14E-08	0.83	10.21	2.50	102449.4
PM-3, 6359 - 6360	K.VLSVDESIIKPEQEFFTAPFEK.N	2554.87541	2	5.14E-08	0.83	4.13	-	289.5
gi|4504745|ref|NP_000410.1| integrin alpha 2b precursor [Homo sapiens]				6.37E-08	8.39	100.28	17.20	113374.2
PM-3, 3887	R.DGYNDIAVAAPYGGPSGR.G	1780.87574	2	6.53E-05	0.73	2.86	-	582.5
PM-3, 3631 - 3675	R.DGYNDIAVAAPYGGPSGR.G	1780.87574	2	6.24E-05	0.54	2.97	-	537.7
PM-3, 3785 - 3843	R.DGYNDIAVAAPYGGPSGR.G	1780.87574	2	5.30E-05	0.76	3.85	-	396.7
PM-3, 4169	R.FGSAIAPLGDLDR.D	1332.48700	1	4.84E-06	0.59	2.82	-	394.1
PM-3, 6040 - 6108	R.GAVDIDDNGYPDLIVGAYGANQVAVYR.A	2827.05458	2	6.98E-07	0.92	4.82	-	466.6
PM-3, 6575 - 6632	R.GAVDIDDNGYPDLIVGAYGANQVAVYR.A	2827.05458	2	7.06E-06	0.94	5.39	-	582.7
PM-9, 4671 - 4727	R.GEAQVWTQLLR.A	1301.47614	2	3.23E-06	0.87	3.41	-	773.4
PM-10, 6139	R.LQDPVLVSCDSAPCTVVQCDLQEMAR.G	2993.40704	2	7.72E-05	0.91	4.46	-	557.1
PM-3, 5911 - 5979	R.SRPSQVLDSPFPTGSAFGFSLR.G	2354.60606	2	7.72E-07	0.86	3.87	-	461.7
PM-3, 5793 - 5855	R.SRPSQVLDSPFPTGSAFGFSLR.G	2354.60606	2	6.37E-08	0.92	4.57	-	576.1
PM-3, 3075 - 3152	K.TEEAEKTPVGSCFLAQPESGR.R	2294.48422	2	3.51E-05	0.80	3.87	-	442.6
PM-3, 2713 - 2715	K.TEEAEKTPVGSCFLAQPESGRR.A	2450.67057	3	1.62E-05	0.86	4.34	-	536.2
PM-3, 5048 - 5115	R.TLGPSQEETGGVFLCPWR.A	2035.26884	2	3.45E-05	0.80	3.15	-	799.0
PM-3, 4259 - 4319	K.TPVSCFNIQMCVGATGHNIPQK.L	2460.84162	3	3.52E-07	0.95	5.60	-	1074.6
gi|29568111|ref|NP_006088.2| myosin, light polypeptide 9, regulatory; myosin regulatory light chain				7.05E-08	0.65	10.13	6.40	19827.1
PM-10, 3296	R.FTDEEVDEMYR.E	1434.51009	1	7.05E-08	0.65	2.61	-	563.1
PM-10, 3293	R.FTDEEVDEMYR.E	1434.51009	1	7.22E-04	0.42	2.06	-	510.1
gi|14149734|ref|NP_065174.1| coronin, actin binding protein, 1B [Homo sapiens]				7.43E-08	1.80	20.20	13.50	54234.4
PM-5, 6252 - 6253	R.KSDLFQDDLYPDTAGPEAALEAEEWVSGR.D	3211.39431	3	1.40E-04	0.87	4.09	-	933.1
PM-5, 3737 - 3750	R.NVLSDSRPAMAPGSSHLGAPASTTTAADATPSGSLAR.A	3524.82150	3	7.43E-08	0.93	3.84	-	1777.2
gi|5453704|ref|NP_006398.1| cytoskeleton related vitamin A responsive protein; glutamate transporte				1.09E-07	1.85	20.35	16.00	21614.6
PM-10, 4784 - 4791	R.AWDDFFPGSDR.F	1313.35597	2	1.98E-06	0.87	3.12	-	864.3
PM-10, 6235 - 6236	R.TPMGIVLDALEQQEEGINR.L	2114.36606	2	1.09E-07	0.98	7.00	-	2312.5
gi|4507841|ref|NP_003356.1| ubiquinol-cytochrome c reductase core protein I [Homo sapiens]				1.37E-07	0.84	10.16	4.80	52618.5
PM-6, 5183	K.YIYDQCPAVAGYGPIEQLPDYNR.I	2703.96630	2	1.37E-07	0.84	3.12	-	665.2
gi|30148492|ref|XP_293610.2| similar to Heat shock cognate 71 kDa protein [Homo sapiens]				1.39E-07	0.88	10.19	2.60	72113.4
PM-5, 4038 - 4112	R.IINEPTAAAIAYGLDKK.V	1789.06550	2	1.39E-07	0.88	3.81	-	612.0
gi|11321601|ref|NP_002618.1| phosphofructokinase, platelet; Phosphofructokinase, platelet type [Hom				1.62E-07	0.83	10.20	2.20	85595.6
PM-4, 5169	R.IIEVVDAIMTTAQSHQR.T	1913.18853	2	1.62E-07	0.83	3.16	-	834.3
gi|5031571|ref|NP_005713.1| actin-related protein 2; ARP2 (actin-related protein 2, yeast) homolog				1.85E-07	0.95	10.19	4.30	44760.5
PM-7, 3476 - 3486	K.HIVLSGGSTMYPGLPSR.L	1773.04967	2	1.85E-07	0.95	3.72	-	1330.6
gi|4504165|ref|NP_000168.1| gelsolin (amyloidosis, Finnish type); Gelsolin [Homo sapiens]				1.87E-07	5.10	70.29	17.50	85696.9
PM-4, 3436 - 3541	K.AGALNSNDAFVLK.T	1320.47622	1	1.87E-07	0.52	2.32	-	487.3
PM-4, 5537 - 5596	R.AQPVQVAEGSEPDGFWEALGGK.A	2273.44345	2	4.08E-07	0.89	4.24	-	734.8
PM-4, 1897	K.HVVPNEVVVQR.L	1276.46981	1	2.95E-04	0.50	2.94	-	247.1
PM-4, 5349	K.NWRDPDQTDGLGLSYLSSHIANVER.V	2845.03323	2	1.51E-04	0.75	3.50	-	270.8
PM-4, 4584 - 4649	R.VHVSEEGTEPEAMLQVLGPK.P	2151.42630	2	3.62E-06	0.96	4.87	-	1054.4
PM-4, 4716	R.VHVSEEGTEPEAMLQVLGPKPALPAGTEDTAKEDAANR.K	3960.33442	3	4.96E-05	0.50	3.05	-	380.0
PM-4, 3554 - 3634	R.VPFDAATLHTSTAMAAQHGMDDDGTGQK.Q	2875.10141	3	7.93E-04	0.97	5.88	-	1776.9

gi|13562114|ref|NP_110400.1| beta tubulin 1, class VI [Homo sapiens]				1.91E-07	6.47	90.25	23.90	50326.6
PM-8, 2906	K.EVDQQLLSVQTR.N	1416.56231	1	1.23E-04	0.12	1.84	-	134.1
PM-6, 6230	K.GHYTEGAELIENVLEVVR.H	2029.23935	2	3.36E-06	0.97	4.96	-	1736.9
PM-8, 6210	R.GLSM*AATFIGNNTAIQEIFNR.V	2285.56551	2	2.69E-05	0.87	3.85	-	588.9
PM-6, 6279 - 6292	R.GLSMAATFIGNNTAIQEIFNR.V	2269.56611	2	2.72E-04	0.69	2.93	-	460.2
PM-6, 6203	R.GLSM*AATFIGNNTAIQEIFNR.V	2285.56551	2	1.91E-07	0.91	4.01	-	946.3
PM-6, 3359	R.IMNSFSVMPSPK.V	1338.62160	1	3.02E-05	0.40	2.47	-	426.4
PM-3, 4777	K.LGALFQPDSFVHGNSGAGNNWAK.G	2388.58260	2	5.88E-05	0.61	2.85	-	461.9
PM-2, 5014	R.LHFFM*PGFAPLTAQGSQQYR.A	2313.62224	2	3.28E-05	0.95	4.22	-	1167.9
PM-8, 5796 - 5810	R.LHFFMPGFAPLTAQGSQQYR.A	2297.62284	2	5.48E-06	0.93	4.49	-	613.3
PM-3, 4955	R.LHFFM*PGFAPLTAQGSQQYR.A	2313.62224	2	1.73E-05	0.91	3.73	-	849.8
PM-7, 5272	R.LHFFMPGFAPLTAQGSQQYR.A	2297.62284	2	6.74E-04	0.87	3.28	-	899.0
PM-5, 6337 - 6338	K.LTTPTYGDLNHLVSLTMSGITTSLR.F	2693.06979	2	7.63E-06	0.52	2.82	-	464.5
PM-3, 6468	K.LTTPTYGDLNHLVSLTMSGITTSLR.F	2693.06979	2	1.46E-05	0.94	5.02	-	696.6
PM-8, 6307	K.LTTPTYGDLNHLVSLTMSGITTSLR.F	2693.06979	2	2.07E-06	0.93	4.63	-	760.5
PM-6, 6306	K.LTTPTYGDLNHLVSLTMSGITTSLR.F	2693.06979	2	2.08E-05	0.93	4.73	-	830.7
gi|10346135|ref|NP_055083.1| microtubule-associated protein, RP/EB family, member 2; T-cell activat				2.13E-07	0.96	10.25	6.10	37031.1
PM-8, 4667	K.SDKDLETQVIQLNEQVHSLK.L	2325.56134	2	2.13E-07	0.96	4.94	-	1285.4
PM-2, 4839	K.SDKDLETQVIQLNEQVHSLK.L	2325.56134	2	3.60E-05	0.55	2.73	-	447.4
gi|30151108|ref|XP_301187.1| similar to actin beta, cytoskeletal - Kenyan clawed frog [Homo sapiens				2.46E-07	1.58	20.27	7.40	32164.8
PM-7, 5052	K.DLYANTVLSGGTTMYPGIADR.M	2216.45684	2	3.43E-05	0.89	3.40	-	1185.4
PM-7, 5386 - 5442	K.DLYANTVLSGGTTMYPGIADR.M	2216.45684	2	2.42E-04	0.96	5.34	-	993.6
PM-7, 5930 - 5990	K.DLYANTVLSGGTTMYPGIADR.M	2216.45684	2	5.84E-04	0.89	4.28	-	665.0
PM-9, 5155 - 5167	K.DLYANTVLSGGTTMYPGIADR.M	2216.45684	2	2.46E-07	0.87	4.06	-	682.0
PM-1, 5119	K.DLYANTVLSGGTTM*YPGIADR.M	2232.45624	2	4.26E-07	0.72	2.72	-	649.0
gi|17986273|ref|NP_524144.1| fast skeletal myosin alkali light chain 1 isoform 1f; A1 catalytic; A2				2.81E-07	0.84	10.18	8.20	21145.0
PM-11, 3183 - 3239	R.VFDKEGNGTVMGAELR.H	1723.93228	2	2.81E-07	0.84	3.56	-	652.1
gi|4506675|ref|NP_002941.1| ribophorin I [Homo sapiens]				3.25E-07	1.50	20.19	5.60	68569.0
PM-5, 6029	K.ISVIVETVYTHVLHPYPTQITQSEK.Q	2884.27545	3	3.25E-07	0.83	3.86	-	760.2
PM-5, 1930 - 1932	R.LAHLGVQVK.G	965.17461	1	1.73E-05	0.67	2.49	-	484.5
gi|29729616|ref|XP_292963.1| similar to peptidylprolyl isomerase A (cyclophilin A) [Homo sapiens] [				4.04E-07	1.70	20.25	16.40	18025.4
PM-10, 5389 - 5395	K.HTGPGILSMANAGPNTNGSQFFICTAK.S	2793.13125	2	4.04E-07	0.93	5.06	-	747.3
PM-11, 5483 - 5541	K.HTGPGILSMANAGPNTNGSQFFICTAK.S	2793.13125	2	4.74E-06	0.84	3.87	-	466.8
PM-10, 4725	K.HTGPGILSM*ANAGPNTNGSQFFICTAK.S	2809.13065	2	3.31E-05	0.77	3.77	-	450.3
gi|5031973|ref|NP_005733.1| protein disulfide isomerase-related protein [Homo sapiens]				4.24E-07	1.82	20.26	11.10	48121.0
PM-6, 6250	R.DGELPVEDDIDLSDVELDDLGKDEL	2759.86565	2	4.24E-07	0.96	5.27	-	1095.0
PM-6, 5214 - 5227	R.TCEEHQLCVVAVLPHILDTGAAGR.N	2648.01534	3	6.58E-07	0.86	4.02	-	867.1
gi|21361657|ref|NP_005304.3| glucose regulated protein, 58kDa; glucose regulated protein, 58kD [Hom				4.34E-07	0.96	10.23	5.10	56782.0
PM-6, 6256	K.FISDKDASIVGFFDDSFSEAHSEFLK.A	2940.16492	3	4.34E-07	0.96	4.65	-	1969.4
gi|4759154|ref|NP_004773.1| synaptosomal-associated protein 29; vesicle-membrane fusion protein SNA				4.87E-07	0.48	10.15	12.40	28970.1
PM-3, 2712	K.DIALGMQTEIEEQDDILDRLTTKVDKLDVNIK.S	3674.13012	3	4.87E-07	0.48	3.04	-	424.9
gi|4502295|ref|NP_001677.1| ATP synthase, H+ transporting, mitochondrial F1 complex, beta polypepti				6.44E-07	1.23	20.20	6.50	57955.2
PM-6, 6314 - 6378	R.DQEGQDVLLFIDNIFR.F	1923.11524	2	2.94E-05	0.91	3.93	-	921.4
PM-6, 4318 - 4330	K.VLDSGAPIKIPVGPETLGR.I	1920.24231	2	6.44E-07	0.32	2.71	-	274.8
gi|5902128|ref|NP_008880.1| syntaxin binding protein 2; Hunc18b [Homo sapiens]				6.66E-07	0.91	10.21	3.40	66438.1
PM-5, 6076	K.AAHIFFTDTCPEPLFSELGR.S	2309.58531	2	6.66E-07	0.91	4.11	-	990.6
gi|4503301|ref|NP_001350.1| 2,4-dienoyl CoA reductase 1 precursor; 4-enoyl-CoA reductase [Homo sapi				7.87E-07	0.94	10.23	6.60	36067.5
PM-8, 4039 - 4040	K.VAGHPNIVINNAAGNFISPTER.L	2292.53950	2	7.87E-07	0.94	4.63	-	1077.4
gi|4505981|ref|NP_002695.1| pro-platelet basic protein (includes platelet basic protein, bet; Pro-p				8.67E-07	0.98	10.26	11.70	13894.1
PM-11, 4012 - 4068	K.GKEESLDSDLYAELR.C	1725.83517	2	8.67E-07	0.98	5.23	-	2664.0
PM-11, 4123 - 4203	K.GKEESLDSDLYAELR.C	1725.83517	2	1.93E-06	0.96	4.46	-	1644.9
PM-1, 4389	K.GKEESLDSDLYAELR.C	1725.83517	2	6.55E-06	0.89	3.08	-	1082.7
gi|5031573|ref|NP_005712.1| ARP3 actin-related protein 3 homolog; ARP3 (actin-related protein 3, ye				8.87E-07	0.83	10.15	5.00	47370.9
PM-6, 4611 - 4626	R.AEPEDHYFLLTEPPLNTPENR.E	2483.67423	2	8.87E-07	0.83	3.09	-	618.7
gi|17450333|ref|XP_060887.1| similar to peptidyl-Pro cis trans isomerase [Homo sapiens]				9.45E-07	0.95	10.24	11.00	17503.9
PM-11, 6248	-.M*VNPTVFDIAVDGKPLGR.V	1946.26009	2	9.45E-07	0.95	4.71	-	959.3
gi|25188179|ref|NP_005653.3| voltage-dependent anion channel 3 [Homo sapiens]				9.46E-07	1.27	20.17	12.70	30658.4
PM-9, 4383 - 4439	K.VNNASLIGLGYTQTLRPGVK.L	2102.42298	2	6.58E-05	0.84	3.34	-	567.8
PM-9, 4631	K.VNNASLIGLGYTQTLRPGVK.L	2102.42298	2	9.46E-07	0.69	2.55	-	693.1
PM-9, 4208 - 4267	K.VNNASLIGLGYTQTLRPGVK.L	2102.42298	2	6.93E-06	0.72	3.16	-	458.5
PM-9, 4523 - 4591	K.WNTDNTLGTEISWENK.L	1909.00229	2	5.73E-06	0.59	2.64	-	434.9
gi|4501891|ref|NP_001093.1| actinin, alpha 1 [Homo sapiens]				9.99E-07	8.87	110.25	17.60	103056.9
PM-2, 5115	K.AGTQIENIEEDFRDGLK.L	1936.06918	2	1.55E-05	0.82	4.15	-	954.0
PM-3, 5080	K.AGTQIENIEEDFRDGLK.L	1936.06918	2	5.19E-05	0.48	3.96	-	384.9
PM-4, 2825	R.ASFNHFDRDHSGTLGPEEFK.A	2292.40855	2	9.13E-05	0.93	3.77	-	1319.9
PM-4, 3724 - 3786	K.ILAGDKNYITMDELR.R	1753.01353	2	5.59E-05	0.80	3.53	-	632.6
PM-4, 4521 - 4522	K.IVQTYHVNMAGTNPYTTITPQEINGK.W	2892.23635	2	2.32E-04	0.94	4.92	-	889.4
PM-4, 3829	K.IVQTYHVNM*AGTNPYTTITPQEINGK.W	2908.23575	2	2.92E-04	0.71	2.92	-	531.1
PM-4, 4362 - 4428	K.IVQTYHVNMAGTNPYTTITPQEINGK.W	2892.23635	2	9.99E-07	0.89	4.28	-	695.6
PM-4, 2820	R.LAILGIHNEVSK.I	1294.52505	2	4.03E-04	0.91	3.00	-	1126.7
PM-1, 6275	K.LASDLLEWIR.R	1216.41115	2	1.51E-05	0.95	3.44	-	2067.8
PM-4, 3125 - 3190	K.M*LDAEDIVGTARPDEK.A	1776.94709	2	3.90E-04	0.85	3.47	-	713.8
PM-1, 3652	K.M*LDAEDIVGTARPDEK.A	1776.94709	2	3.60E-04	0.64	2.95	-	435.7
PM-9, 4331 - 4333	K.NVNIQNFHISWK.D	1500.68583	2	5.45E-06	0.71	2.95	-	548.1
PM-1, 6508	R.VGWEQLLTTIAR.T	1387.60894	2	1.92E-06	0.91	3.57	-	976.7
PM-1, 4359 - 4416	K.VLAVNQENEQLMEDYEK.L	2053.23635	2	7.08E-04	0.60	2.70	-	592.1
gi|19557677|ref|NP_002108.3| major histocompatibility complex, class I, C precursor; HLA class I hi				1.30E-06	0.73	10.13	3.30	40994.6
PM-7, 4397	R.AYLEGTCVEWLR.R	1497.70027	2	1.30E-06	0.73	2.52	-	801.5
gi|4506005|ref|NP_002700.1| protein phosphatase 1, catalytic subunit, beta isoform [Homo sapiens] [				1.46E-06	0.97	10.25	5.50	37186.6
PM-8, 6228 - 6230	K.TFTDCFNCLPIAAIVDEK.I	2115.41542	2	1.46E-06	0.97	5.06	-	1576.5
gi|4557419|ref|NP_000063.1| CD36 antigen (collagen type I receptor, thrombospondin receptor); CD36				1.55E-06	0.97	10.26	3.60	53052.1
PM-4, 6388 - 6389	R.TYLDIQPITGFTLQFAK.R	1957.25787	2	1.55E-06	0.97	5.14	-	1350.3
gi|19913446|ref|NP_002134.2| hippocalcin; neuron specific calcium-binding protein hippocalcin [Homo				1.70E-06	0.31	10.11	6.20	22427.3
PM-10, 3404	R.LLQCDPSSASQF	1353.48310	1	1.70E-06	0.31	2.24	-	300.2
gi|4507953|ref|NP_003397.1| tyrosine 3/tryptophan 5 -monooxygenase activation protein, zeta polypep				1.93E-06	0.71	10.12	4.90	27744.9
PM-9, 3232	K.FLIPNASQAESK.V	1305.46156	1	1.93E-06	0.71	2.37	-	688.9
gi|4759082|ref|NP_004648.1| serum deprivation response protein; serum deprivation response; phospha				2.09E-06	2.65	30.23	11.10	47172.9
PM-11, 6200	R.DNSQVNAVTVLTLLDK.L	1730.94122	2	3.40E-05	0.87	3.30	-	900.0
PM-8, 3139 - 3150	K.LVNMLDAVQENQHK.M	1639.85851	2	2.09E-06	0.85	3.66	-	754.4
PM-9, 3259	K.LVNMLDAVQENQHK.M	1639.85851	2	6.21E-04	0.91	4.02	-	800.5
PM-4, 6117	K.VLIFQEENEIPASVFVK.Q	1963.26241	2	2.38E-04	0.91	4.57	-	568.4
PM-8, 6075	K.VLIFQEENEIPASVFVK.Q	1963.26241	2	1.84E-05	0.69	3.22	-	602.3
PM-3, 6191 - 6201	K.VLIFQEENEIPASVFVK.Q	1963.26241	2	3.70E-05	0.89	4.13	-	673.8
PM-2, 6237 - 6250	K.VLIFQEENEIPASVFVK.Q	1963.26241	2	4.69E-05	0.91	4.45	-	680.2
PM-9, 6111 - 6141	K.VLIFQEENEIPASVFVK.Q	1963.26241	2	2.45E-04	0.89	4.18	-	581.3
PM-11, 6155 - 6167	K.VLIFQEENEIPASVFVK.Q	1963.26241	2	4.34E-06	0.92	4.37	-	788.5
PM-1, 6424 - 6429	K.VLIFQEENEIPASVFVK.Q	1963.26241	2	2.53E-04	0.94	4.57	-	937.7
PM-12, 6235	K.VLIFQEENEIPASVFVK.Q	1963.26241	2	2.32E-04	0.93	4.45	-	925.7
gi|21361122|ref|NP_001440.2| four and a half LIM domains 1; Four-and-a-half LIM domains 1 [Homo sap				2.20E-06	3.48	40.27	22.10	31894.7
PM-8, 2186 - 2203	K.AIVAGDQNVEYK.G	1307.43431	2	5.81E-04	0.89	3.46	-	986.2
PM-8, 3938	R.FTAVEDQYYCVDCYK.N	1962.14837	2	1.90E-05	0.93	4.14	-	905.6
PM-8, 3051	R.FVFHQEQVYCPDCAK.K	1929.16798	2	6.48E-05	0.98	5.32	-	1776.4
PM-8, 3658	K.GSSVVAYEGQSWHDYCFHCK.K	2418.60884	2	2.20E-06	0.69	2.61	-	431.3
gi|27597085|ref|NP_000357.3| tropomyosin 1 (alpha) [Homo sapiens]				2.22E-06	0.95	10.25	5.60	32865.7
PM-8, 2863 - 2864	R.AQKDEEKMEIQEIQLK.E	1961.22673	3	1.24E-05	0.88	4.18	-	1059.7
PM-9, 2923	R.AQKDEEKMEIQEIQLK.E	1961.22673	2	1.27E-04	0.94	4.81	-	994.4
PM-8, 2858	R.AQKDEEKMEIQEIQLK.E	1961.22673	2	2.22E-06	0.95	4.94	-	1181.4

gi|22059949|ref|XP_114617.2| similar to tubulin, beta 5 [Homo sapiens]				2.29E-06	0.88	10.19	5.80	22810.8
PM-6, 2287 - 2351	R.ISVYYNEATGGK.Y	1302.41453	2	2.29E-06	0.88	3.70	-	696.2
gi|21361905|ref|NP_116190.2| junctional adhesion molecule 3 precursor [Homo sapiens]				2.34E-06	0.76	10.15	5.10	39602.0
PM-8, 3008	K.DDSGQYYCIASNDAGSAR.C	1950.97769	2	2.34E-06	0.76	3.10	-	611.9
gi|17921989|ref|NP_005991.1| tubulin, alpha 1; testis-specific alpha tubulin; tubulin alpha-1 chain				2.63E-06	4.41	60.24	17.90	49924.1
PM-6, 5882 - 5958	R.AVFVDLEPTVIDEIR.N	1716.95615	2	2.71E-05	0.81	2.57	-	1319.4
PM-10, 5249	K.AYHEQLSVAEITNACFEPANQMVK.C	2752.07590	2	3.37E-05	0.93	4.34	-	1129.6
PM-6, 5150	K.AYHEQLSVAEITNACFEPANQMVK.C	2752.07590	2	7.12E-04	0.71	2.84	-	769.0
PM-2, 5083	K.AYHEQLSVAEITNACFEPANQMVK.C	2752.07590	2	4.03E-04	0.87	3.49	-	1076.8
PM-7, 4952	K.AYHEQLSVAEITNACFEPANQMVK.C	2752.07590	2	6.55E-04	0.93	4.90	-	896.4
PM-10, 3159	K.DVNAAIAAIK.T	986.14745	1	9.17E-05	0.88	3.19	-	961.0
PM-6, 2670	K.DVNAAIAAIK.T	986.14745	1	4.14E-04	0.65	2.15	-	865.1
PM-6, 4516 - 4582	R.IHFPLATYAPVISAEK.A	1758.05310	2	8.85E-04	0.97	4.86	-	1185.5
PM-6, 4624 - 4700	R.IHFPLATYAPVISAEK.A	1758.05310	2	3.88E-04	0.94	3.58	-	1062.3
PM-6, 4638	R.IHFPLATYAPVISAEK.A	1758.05310	2	7.68E-05	0.86	3.59	-	434.4
PM-1, 5349 - 5408	R.IHFPLATYAPVISAEK.A	1758.05310	2	3.14E-04	0.89	3.92	-	489.1
PM-6, 2516	R.LDHKFDLMYAK.R	1381.62467	1	1.71E-04	0.34	1.94	-	456.1
PM-11, 5444 - 5500	K.TIGGGDDSFTTFFCETGAGK.H	2069.19409	2	2.63E-06	0.59	2.90	-	379.5
gi|4557435|ref|NP_001242.1| CD68 antigen; Macrophage antigen CD68 (microsialin); macrosialin; scave				2.85E-06	0.54	10.16	6.20	37408.0
PM-5, 4280	R.LQAAQLPHTGVFGQSFSCPSDR.S	2404.64673	2	8.00E-04	0.83	3.26	-	744.2
PM-4, 4137	R.LQAAQLPHTGVFGQSFSCPSDR.S	2404.64673	2	2.85E-06	0.54	2.55	-	577.5
gi|12025678|ref|NP_004915.2| actinin, alpha 4 [Homo sapiens]				3.31E-06	0.65	10.16	1.90	104853.3
PM-4, 4176	K.AGTQIENIDEDFRDGLK.L	1922.04251	2	3.31E-06	0.65	3.18	-	637.1
gi|4501893|ref|NP_001094.1| actinin, alpha 2 [Homo sapiens]				3.40E-06	2.21	30.21	4.40	103853.1
PM-10, 5332	K.AGTQIENIEEDFRNGLK.L	1935.08442	2	5.19E-05	0.81	4.28	-	764.3
PM-4, 4436	K.AGTQIENIEEDFRNGLK.L	1935.08442	2	5.71E-04	0.43	2.62	-	591.7
PM-4, 5024 - 5082	K.AGTQIENIEEDFRNGLK.L	1935.08442	2	3.40E-06	0.67	3.76	-	693.2
PM-1, 5579	K.AGTQIENIEEDFRNGLK.L	1935.08442	2	1.36E-04	0.68	3.76	-	638.2
PM-4, 5838	K.GYEEWLLNEIR.R	1422.56701	1	1.64E-05	0.75	3.22	-	429.9
PM-4, 2477	K.TFTAWCNSHLR.K	1393.55538	2	2.90E-05	0.79	2.59	-	671.8
gi|18104967|ref|NP_000953.2| prostaglandin-endoperoxide synthase 1 isoform 1 precursor; prostagland				3.66E-06	5.12	70.26	16.50	68686.0
PM-5, 3716 - 3777	K.AEHPTWGDEQLFQTTR.L	1917.02765	2	6.58E-05	0.94	4.09	-	863.6
PM-5, 3644 - 3700	K.ALGHGVDLGHIYGDNLER.Q	1937.10524	2	3.66E-06	0.97	5.23	-	1572.5
PM-4, 3473 - 3488	K.ALGHGVDLGHIYGDNLER.Q	1937.10524	2	1.68E-04	0.92	4.41	-	1172.3
PM-5, 6386	K.EM*AAELEELYGDIDALEFYPGLLLEK.C	2989.33908	2	1.23E-04	0.59	2.69	-	529.4
PM-5, 3570 - 3574	R.NMDHHILHVAVDVIR.E	1770.05224	2	5.35E-04	0.73	3.52	-	526.0
PM-5, 3038	R.NM*DHHILHVAVDVIR.E	1786.05164	2	1.14E-04	0.51	2.51	-	440.1
PM-5, 5812 - 5821	K.YQVLDGEMYPPSVEEAPVLMHYPR.G	2822.20799	2	4.00E-05	0.66	3.69	-	149.8
PM-5, 5334	K.YQVLDGEMYPPSVEEAPVLM*HYPR.G	2838.20739	2	7.03E-05	0.74	3.27	-	348.2
gi|4507877|ref|NP_003364.1| vinculin isoform VCL [Homo sapiens]				3.74E-06	3.33	40.20	5.00	116721.6
PM-3, 3136 - 3197	K.AQQVSQGLDVLTAK.V	1458.64233	1	3.74E-06	0.77	3.90	-	193.1
PM-4, 3045 - 3096	K.AQQVSQGLDVLTAK.V	1458.64233	1	1.63E-04	0.72	3.59	-	268.5
PM-3, 5355	K.CDRVDQLTAQLADLAAR.G	1917.13702	2	2.66E-05	0.77	2.90	-	876.9
PM-10, 4027 - 4077	R.MALLMAEMSR.L	1153.46531	2	4.66E-04	0.96	4.01	-	1639.7
PM-3, 2913	K.SLLDASEEAIKK.D	1304.47187	2	1.09E-05	0.83	2.99	-	910.0
gi|24308400|ref|NP_612366.1| hypothetical protein BC010682 [Homo sapiens]				3.80E-06	0.97	10.21	4.30	39866.6
PM-8, 3039 - 3064	R.DLLSHENAATLNDVK.T	1640.77663	2	3.80E-06	0.97	4.21	-	1994.4
gi|4502303|ref|NP_001688.1| mitochondrial ATP synthase, O subunit; ATP synthase, H+ transporting, m				3.87E-06	1.78	20.20	15.50	23277.2
PM-10, 5439 - 5495	R.GEVPCTVTSASPLEEATLSELK.T	2319.57189	2	3.87E-06	0.89	3.94	-	690.1
PM-9, 5376 - 5380	R.GEVPCTVTSASPLEEATLSELK.T	2319.57189	2	5.87E-04	0.89	4.03	-	749.2
PM-10, 3080	K.VAASVLNPYVK.R	1161.37549	2	2.86E-04	0.89	3.42	-	812.3
gi|24431935|ref|NP_065393.1| reticulon 4; neuroendocrine-specific protein C like (foocen) [Homo sap				3.90E-06	1.70	20.26	6.50	129930.3
PM-8, 4684	K.LPEDDEPPARPPPPPPASVSPQAEPVWTPPAPAPAAPPSTPAAPK.R	4495.00685	3	1.23E-05	0.92	5.18	-	582.7
PM-9, 4791	K.LPEDDEPPARPPPPPPASVSPQAEPVWTPPAPAPAAPPSTPAAPK.R	4495.00685	3	3.90E-06	0.87	4.80	-	423.7
PM-6, 6051	K.YQFVREPEDEEEEEEEEEDEDEDLEELEVLER.K	4189.14063	3	7.45E-05	0.83	3.99	-	564.3
gi|4504041|ref|NP_002061.1| guanine nucleotide binding protein (G protein), alpha inhibiting activi				4.36E-06	0.94	10.17	3.70	40450.7
PM-8, 1828	K.IIHEDGYSEEECR.Q	1637.70972	2	4.36E-06	0.94	3.42	-	1336.7
gi|8922609|ref|NP_060658.1| hypothetical protein FLJ10709 [Homo sapiens]				4.41E-06	0.86	10.19	3.40	66217.8
PM-5, 5398 - 5402	K.DALNLAQMQEQTLQLEQQSK.L	2317.56271	2	4.41E-06	0.86	3.73	-	892.1
gi|23510362|ref|NP_694592.1| protein-tyrosine kinase fyn isoform b; proto-oncogene tyrosine-protein				4.61E-06	0.88	10.14	3.00	60140.6
PM-6, 5056	R.AQFETLQQLVQHYSEK.A	1950.14069	2	4.61E-06	0.88	2.75	-	1142.7
gi|4504073|ref|NP_000398.1| glycoprotein Ib beta polypeptide precursor [Homo sapiens]				5.01E-06	0.91	10.22	5.30	21717.6
PM-9, 4363 - 4420	R.LLPYLAEDELR.A	1332.52691	1	5.01E-06	0.91	4.49	-	442.2
gi|5902016|ref|NP_008939.1| reticulon 4; neuroendocrine-specific protein C like (foocen) [Homo sapi				5.14E-06	0.88	10.16	7.00	22395.3
PM-10, 2764 - 2820	R.HQAQIDHYLGLANK.N	1608.78270	2	1.03E-04	0.86	3.23	-	758.7
PM-8, 2647 - 2656	R.HQAQIDHYLGLANK.N	1608.78270	2	5.14E-06	0.88	2.86	-	1051.2
gi|4507907|ref|NP_000543.1| von Willebrand factor precursor; Coagulation factor VIII VWF (von Wille				5.30E-06	2.13	30.20	1.60	309296.7
PM-2, 6130 - 6138	R.IEDLPTMVTLGNSFLHK.L	1916.23079	2	2.55E-04	0.88	4.07	-	641.9
PM-1, 3507	R.ILTSDVFQDCNK.L	1440.60389	1	6.90E-06	0.50	3.20	-	327.4
PM-2, 3167	R.SGFTYVLHEGECCGR.C	1772.94325	2	5.30E-06	0.75	2.87	-	693.8
gi|21361070|ref|NP_002617.2| phosphofructokinase, liver; Phosphofructokinase, liver type; human liv				5.78E-06	0.37	10.16	2.10	90202.5
PM-1, 5008	R.IMEVIDAITTTAQSHQR.T	1915.16129	2	5.78E-06	0.37	3.23	-	374.4
gi|5031987|ref|NP_005720.1| peptidylprolyl isomerase F (cyclophilin F) [Homo sapiens]				5.83E-06	0.31	10.25	14.00	22040.1
PM-10, 5139 - 5217	K.GSGDPSSSSSSGNPLVYLDVDANGKPLGR.V	2834.99028	2	5.83E-06	0.31	2.70	-	233.2
gi|13128974|ref|NP_076933.1| hypothetical protein MGC3265 [Homo sapiens]				6.36E-06	0.96	10.23	4.50	46867.3
PM-5, 2916	K.ANVIHATVTSVTLHSTEGK.A	1966.18479	2	6.36E-06	0.96	4.70	-	1217.4
gi|6912536|ref|NP_036475.1| nicotinamide nucleotide transhydrogenase [Homo sapiens]				6.46E-06	0.25	10.14	2.50	113946.9
PM-4, 6209	R.SLGVGYAAVDNPIFYKPNTAMLLGDAK.K	2827.24740	2	6.46E-06	0.25	2.66	-	251.2
gi|10835049|ref|NP_001655.1| ras homolog gene family, member A; Aplysia ras-related homolog 12; Rho				6.54E-06	0.84	10.25	12.40	21768.0
PM-9, 6328 - 6329	K.DQFPEVYVPTVFENYVADIEVDGK.Q	2775.01481	2	6.54E-06	0.84	4.99	-	417.7
gi|10716563|ref|NP_001737.1| calnexin [Homo sapiens]				6.90E-06	0.89	10.17	2.70	67567.8
PM-5, 4765 - 4838	K.APVPTGEVYFADSFDR.G	1771.90722	2	1.06E-05	0.89	3.38	-	931.9
PM-4, 4677 - 4708	K.APVPTGEVYFADSFDR.G	1771.90722	2	6.90E-06	0.89	3.20	-	966.0
gi|4503631|ref|NP_000120.1| coagulation factor XIII A1 subunit precursor; Coagulation factor XIII,				7.30E-06	0.96	10.25	2.30	83232.8
PM-4, 6376	R.GVNLQEFLNVTSVHLFK.E	1946.23831	2	7.30E-06	0.96	4.95	-	1162.0
gi|5174393|ref|NP_006001.1| arginine-rich, mutated in early stage tumors; arginine-rich protein [Ho				7.32E-06	1.33	20.22	11.50	26906.2
PM-10, 5652 - 5724	K.DRDVTFSPATIENELIK.F	1949.15100	2	7.32E-06	0.96	4.40	-	1628.0
PM-10, 5737	K.DRDVTFSPATIENELIK.F	1949.15100	2	3.38E-04	0.96	4.26	-	2055.2
PM-10, 2893	K.ILDDWGETCK.G	1237.36410	1	7.82E-06	0.37	2.10	-	355.2
gi|5730031|ref|NP_005688.2| secretory carrier membrane protein 2 [Homo sapiens]				7.74E-06	0.64	10.13	6.10	36648.6
PM-10, 5572 - 5627	R.TGASFQQAQEEFSQGIFSSR.T	2206.31396	2	7.74E-06	0.64	2.67	-	510.3
gi|4757826|ref|NP_004039.1| beta-2-microglobulin [Homo sapiens]				9.08E-06	0.88	10.21	18.50	13714.5
PM-11, 6223	K.SNFLNCYVSGFHPSDIEVDLLK.N	2555.84675	2	9.08E-06	0.88	4.10	-	536.7
gi|6005902|ref|NP_009043.1| thrombospondin 3 [Homo sapiens]				9.13E-06	0.46	10.14	0.70	104200.6
PM-5, 4294	R.FYVVMWK.Q	973.21547	1	2.98E-04	0.59	1.87	-	855.3
PM-2, 4167	R.FYVVMWK.Q	973.21547	1	9.13E-06	0.46	2.19	-	562.2
gi|5803225|ref|NP_006752.1| tyrosine 3/tryptophan 5 -monooxygenase activation protein, epsilon poly				1.09E-05	1.67	20.20	11.40	29173.7
PM-9, 6247	K.AAFDDAIAELDTLSEESYK.D	2089.19882	2	1.09E-05	0.92	3.92	-	965.5
PM-9, 6391 - 6393	K.AAFDDAIAELDTLSEESYKDSTLIM*QLLR.D	3276.61426	3	3.55E-04	0.75	3.14	-	607.6
gi|7657056|ref|NP_055415.1| EH-domain containing 3; EH domain containing 3 [Homo sapiens]				1.22E-05	0.95	10.25	5.00	60886.7
PM-3, 6479	R.FVCAQLPNPVLESISVIDTPGILSGEK.Q	2885.32491	2	7.30E-05	0.95	5.02	-	1130.6
PM-5, 6340	R.FVCAQLPNPVLESISVIDTPGILSGEK.Q	2885.32491	2	3.45E-04	0.85	3.85	-	767.4
PM-7, 6388	R.FVCAQLPNPVLESISVIDTPGILSGEK.Q	2885.32491	2	1.22E-05	0.95	4.71	-	1265.8
PM-1, 6585	R.FVCAQLPNPVLESISVIDTPGILSGEK.Q	2885.32491	2	3.35E-04	0.95	4.70	-	1286.8
gi|30150022|ref|XP_300862.1| similar to beta-tubulin 4Q [Homo sapiens]				1.27E-05	1.54	20.21	7.90	33545.2
PM-6, 3942 - 3998	K.LAVNMVPFPR.L	1144.41480	2	6.50E-04	0.82	3.05	-	567.1
PM-5, 5768	R.LHFFMPGFAPLTSR.G	1621.92962	2	1.27E-05	0.73	2.80	-	636.5

gi|4557305|ref|NP_000025.1| aldolase A; fructose-bisphosphate aldolase; Aldolase A, fructose-bispho				1.27E-05	0.86	10.17	7.70	39419.7
PM-7, 3698	K.ALSDHHIYLEGTLLKPNMVTPGHACTQK.F	3133.59399	3	1.27E-05	0.86	3.36	-	1008.9
gi|11321585|ref|NP_002065.1| guanine nucleotide-binding protein, beta-1 subunit; G protein, beta-1				1.27E-05	1.31	20.20	8.50	37376.8
PM-8, 4020	K.LIIWDSYTTNK.V	1354.53258	1	1.27E-05	0.37	2.39	-	260.4
PM-8, 6242 - 6319	R.LLLAGYDDFNCNVWDALK.A	2128.39264	2	1.29E-05	0.94	4.07	-	1315.4
gi|4502549|ref|NP_001734.1| calmodulin 2 (phosphorylase kinase, delta); phosphorylase kinase delta				1.35E-05	0.80	10.15	10.70	16837.5
PM-10, 3680 - 3757	R.VFDKDGNGYISAAELR.H	1755.90933	2	1.35E-05	0.80	3.01	-	826.6
PM-10, 3539 - 3556	R.VFDKDGNGYISAAELR.H	1755.90933	2	3.94E-04	0.47	2.57	-	447.8
gi|24307969|ref|NP_055423.1| cytoplasmic FMR1 interacting protein 1; selective hybridizing clone (m				1.40E-05	1.04	20.14	2.50	145181.2
PM-3, 3468	K.DIVEYAELK.T	1080.21287	1	8.47E-04	0.53	2.29	-	767.3
PM-3, 4935	R.WTCTSSGSSPQYNICEQMIQIR.E	2647.94934	2	1.40E-05	0.51	2.81	-	351.2
gi|4504011|ref|NP_002052.1| glutamate-cysteine ligase regulatory protein; gamma-glutamylcysteine sy				1.44E-05	0.83	10.17	5.50	30726.8
PM-9, 4547	K.TLNEWSSQINPDLVR.E	1772.93989	2	1.44E-05	0.83	3.34	-	697.2
gi|4504743|ref|NP_002194.1| integrin alpha 2 precursor; Integrin, alpha-2 (CD49B; alpha-2 subunit o				1.48E-05	2.55	30.19	4.70	129294.4
PM-3, 6309	K.DCGEDGLCISDLVLDVR.Q	1937.14189	2	1.48E-05	0.93	3.74	-	1487.8
PM-3, 3195	R.QIPAAQEQPFIVSNQNK.R	1913.12358	2	7.83E-05	0.73	3.01	-	396.6
PM-3, 4881	R.VDISLENPGTSPALEAYSETAK.V	2293.46974	2	4.67E-05	0.89	3.58	-	863.2
PM-2, 4962 - 5021	R.VDISLENPGTSPALEAYSETAK.V	2293.46974	2	2.38E-04	0.87	3.53	-	724.7
gi|4502097|ref|NP_001142.1| solute carrier family 25 (mitochondrial carrier; adenine nucleotide tra				1.61E-05	1.53	20.16	6.70	33148.4
PM-9, 2725	K.GAWSNVLR.G	903.02023	2	4.72E-04	0.77	2.76	-	766.7
PM-9, 5705	R.YFPTQALNFAFK.D	1447.66130	1	1.61E-05	0.76	3.22	-	499.2
gi|12408656|ref|NP_005177.2| calpain 1, large subunit; calpain, large polypeptide L1; calcium-activ				1.66E-05	0.72	10.17	3.50	81889.4
PM-4, 6329	R.DMETIGFAVYEVPPELVGQPAVHLK.R	2741.15451	2	1.66E-05	0.72	3.43	-	287.7
gi|4557251|ref|NP_001101.1| a disintegrin and metalloprotease domain 10 [Homo sapiens]				1.67E-05	1.89	20.24	6.30	84141.7
PM-5, 2382 - 2384	R.HTQVCINGQCAGSICEK.Y	1963.20906	2	1.67E-05	0.96	4.86	-	1230.8
PM-5, 2764	K.VSHITFAHEVGHNFGSPHDSGTECTPGESK.N	3223.39489	3	2.59E-05	0.93	4.39	-	1264.0
gi|10863927|ref|NP_066953.1| peptidylprolyl isomerase A (cyclophilin A) [Homo sapiens]				1.74E-05	3.39	40.21	20.60	18012.4
PM-11, 3811	K.FEDENFILK.H	1155.28144	2	6.90E-05	0.82	3.73	-	521.7
PM-11, 3427 - 3428	K.VKEGMNIVEAMER.F	1506.77390	2	1.74E-05	0.93	4.10	-	1145.0
PM-11, 4319	R.VSFELFADK.V	1056.19300	1	1.66E-04	0.83	2.55	-	1103.9
PM-10, 4465 - 4491	R.VSFELFADKVPK.T	1380.61317	2	1.33E-04	0.81	3.24	-	795.3
gi|5031599|ref|NP_005722.1| actin related protein 2/3 complex subunit 2; ARP2/3 protein complex sub				1.89E-05	0.54	20.13	8.30	34332.8
PM-8, 2371	R.AVIHYRDDETM*YVESK.K	1973.15290	2	1.89E-05	0.38	2.63	-	387.9
PM-8, 3102	R.NCFASVFEK.Y	1102.24502	1	5.10E-04	0.16	1.85	-	259.2
gi|4502905|ref|NP_001822.1| clusterin; complement-associated protein SP-40 [Homo sapiens]				2.12E-05	0.94	10.26	5.10	52494.3
PM-8, 3263	R.VTTVASHTSDSDVPSGVTEVVVK.L	2315.52008	2	2.12E-05	0.94	5.12	-	798.1
gi|20127528|ref|NP_037459.2| parvin, beta; CGI-56 protein; beta-parvin [Homo sapiens]				2.13E-05	1.64	20.17	7.10	41714.0
PM-8, 3056	K.ARPEDVVNLDLK.S	1369.54878	1	2.97E-05	0.51	2.87	-	283.9
PM-8, 3040 - 3042	K.ARPEDVVNLDLK.S	1369.54878	2	2.13E-05	0.77	3.18	-	714.9
PM-8, 6283 - 6284	K.VLLDWINDVLVEER.I	1713.95549	2	3.80E-04	0.87	2.84	-	1068.5
gi|5031569|ref|NP_005727.1| ARP1 actin-related protein 1 homolog A, centractin alpha; ARP1 (actin-r				2.23E-05	0.85	10.16	5.90	42613.4
PM-7, 5041	K.DQLQTFSEEHPVLLTEAPLNPR.K	2535.79382	2	2.23E-05	0.85	3.23	-	422.5
gi|19743813|ref|NP_002202.2| integrin beta 1 isoform 1A precursor; integrin VLA-4 beta subunit; fib				2.33E-05	1.85	20.28	4.90	88414.9
PM-3, 6431 - 6432	K.LSENNIQTIFAVTEEFQPVYK.E	2471.74650	2	2.33E-05	0.88	4.15	-	489.3
PM-3, 5047 - 5052	K.NVLSLTNKGEVFNELVGK.Q	1962.23605	2	3.28E-05	0.97	5.63	-	1079.5
gi|4758950|ref|NP_000933.1| peptidylprolyl isomerase B (cyclophilin B) [Homo sapiens]				2.46E-05	1.94	40.14	20.40	23742.4
PM-10, 4223	K.DTNGSQFFITTVK.T	1458.59766	1	4.47E-04	0.29	2.36	-	433.6
PM-10, 1956 - 2011	K.DVIIADCGK.I	991.14411	1	1.62E-04	0.62	2.09	-	635.8
PM-10, 2900 - 2979	K.HYGPGWVSMANAGK.D	1475.65664	2	2.46E-05	0.85	2.62	-	927.8
PM-10, 4689	R.VIFGLFGK.T	881.09607	1	2.33E-04	0.18	2.20	-	367.5
gi|24797067|ref|NP_002107.3| major histocompatibility complex, class I, A precursor; HLA-A1 class I				2.61E-05	1.12	20.20	10.10	40840.5
PM-8, 3095	R.DGEDQTQDTELVETRPAGDGTFQK.W	2638.69666	2	1.17E-04	0.45	2.97	-	478.1
PM-7, 2869	K.WAAVVVPSGEEQR.Y	1428.57478	1	2.61E-05	0.67	3.99	-	296.8
gi|5174727|ref|NP_005989.1| chaperonin containing TCP1, subunit 3 (gamma); TCP1 (t-complex-1) ring				2.70E-05	1.49	20.18	5.10	60330.4
PM-5, 4406 - 4465	K.GISDLAQHYLMR.A	1404.61986	2	2.70E-05	0.79	2.72	-	821.8
PM-5, 4772 - 4776	K.IGDEYFTFITDCKDPK.A	1950.15748	2	2.52E-04	0.70	3.58	-	299.9
gi|4502693|ref|NP_001760.1| CD9 antigen; motility related protein; leukocyte antigen MIC3; antigen				2.77E-05	0.98	10.31	11.00	25415.9
PM-10, 6252	K.AIHYALNCCGLAGGVEQFISDICPK.K	2795.21006	2	2.77E-05	0.98	6.17	-	1517.7
PM-9, 6276	K.AIHYALNCCGLAGGVEQFISDICPK.K	2795.21006	2	1.34E-04	0.98	5.88	-	1582.4
gi|7657581|ref|NP_055066.1| solute carrier family 25, member 13 (citrin) [Homo sapiens]				3.20E-05	0.71	10.18	2.70	74175.1
PM-5, 5669	R.IAPLEEGTLPFNLAEAQR.Q	1970.21504	2	3.20E-05	0.71	3.55	-	256.4
gi|21361361|ref|NP_006835.2| ilvB (bacterial acetolactate synthase)-like isoform 1; acetolactate sy				3.46E-05	0.88	10.18	3.30	67867.3
PM-5, 4424	R.EQVPSLGSNVACGLAYTDYHK.A	2310.52848	2	3.46E-05	0.88	3.68	-	613.6
gi|21464101|ref|NP_036611.2| tyrosine 3-monooxygenase/tryptophan 5-monooxygenase activation protein				3.46E-05	1.52	20.23	11.70	28302.4
PM-9, 6231 - 6232	K.TAFDDAIAELDTLNEDSYK.D	2132.22370	2	3.46E-05	0.96	4.65	-	1529.3
PM-9, 1959	R.YLAEVATGEK.R	1081.20087	1	8.74E-04	0.56	2.24	-	840.5
gi|5031593|ref|NP_005708.1| actin related protein 2/3 complex subunit 5; Arp2/3 protein complex sub				3.61E-05	1.63	20.32	12.60	16320.3
PM-10, 4105	K.GFESPSDNSSAM*LLQWHEK.A	2180.33989	2	3.61E-05	0.66	2.66	-	438.8
PM-10, 4215 - 4217	R.KVDVDEYDENKFVDEEDGGDGQAGPDEGEVDSCLR.Q	3889.93551	3	9.41E-04	0.97	6.43	-	1126.9
gi|7706224|ref|NP_056969.1| PCF11p homolog [Homo sapiens]				3.83E-05	0.45	10.09	1.20	82599.9
PM-2, 2478	R.RM*KKTEEER.P	1223.38556	1	3.83E-05	0.45	1.83	-	725.4
gi|6005786|ref|NP_009214.1| monoglyceride lipase; lysophospholipase-like [Homo sapiens]				3.85E-05	0.89	10.21	5.10	34292.3
PM-8, 3674	R.NKTEVDIYNSDPLICR.A	1938.15147	2	3.85E-05	0.89	3.66	-	779.8
gi|24119203|ref|NP_705935.1| tropomyosin 3 [Homo sapiens]				4.14E-05	0.95	10.21	4.40	29032.5
PM-9, 2959 - 3021	R.RIQLVEEELDR.A	1400.56288	2	4.41E-05	0.96	4.12	-	1575.8
PM-8, 2903 - 2926	R.RIQLVEEELDR.A	1400.56288	2	4.14E-05	0.95	3.91	-	1527.8
gi|4502201|ref|NP_001649.1| ADP-ribosylation factor 1 [Homo sapiens]				4.30E-05	0.75	10.19	11.60	20696.6
PM-10, 6191 - 6192	K.LGEIVTTIPTIGFNVETVEYK.N	2324.65485	2	4.30E-05	0.75	3.71	-	182.0
gi|20149570|ref|NP_005816.2| RAS guanyl releasing protein 2 isoform 1; calcium and diacylglycerol-r				5.55E-05	0.91	10.18	3.10	75547.0
PM-5, 6308 - 6369	R.YWISAFPAEFDLNPELAEQIK.E	2482.77098	2	5.55E-05	0.91	3.63	-	744.1
gi|4759086|ref|NP_004883.1| vesicle trafficking protein sec22b [Homo sapiens]				5.64E-05	0.88	10.18	8.80	24740.4
PM-9, 3803	R.VADGLPLAASMQEDEQSGR.D	1975.12722	2	5.64E-05	0.88	3.66	-	635.8
gi|12545410|ref|NP_031394.1| RAS p21 protein activator 3; GTPase activating protein III; Ins(1,3,4,				5.74E-05	1.75	20.25	6.10	95912.5
PM-4, 6258	R.IVECQGLPIVNGQCDPYATVTLAGPFR.S	2977.40738	2	8.19E-04	0.79	3.49	-	532.1
PM-4, 4966	K.SVYDGPEQEEYSTFVIDDPQETYK.T	2840.94215	2	5.74E-05	0.96	5.04	-	1219.2
gi|4506671|ref|NP_000995.1| ribosomal protein P2; 60S acidic ribosomal protein P2; acidic ribosomal				5.80E-05	0.80	10.20	28.70	11664.9
PM-11, 3677	K.LASVPAGGAVAVSAAPGSAAPAAGSAPAAAEEK.K	2776.05225	2	5.80E-05	0.80	3.96	-	373.5
gi|4503327|ref|NP_000389.1| cytochrome b5 reductase membrane-bound isoform; diaphorase (NADH) [Homo				7.32E-05	0.95	10.21	6.00	34234.6
PM-8, 5522 - 5578	R.APEAWDYGQGFVNEEMIR.D	2113.29517	2	7.32E-05	0.95	4.11	-	1224.4
gi|4505733|ref|NP_002610.1| platelet factor 4 (chemokine (C-X-C motif) ligand 4); platelet factor 4				7.34E-05	1.76	20.19	20.80	10844.9
PM-11, 2284 - 2359	R.HITSLEVIK.A	1040.23825	2	5.92E-04	0.87	3.21	-	583.5
PM-11, 4103 - 4181	R.KICLDLQAPLYK.K	1462.78211	2	9.31E-04	0.93	3.75	-	1327.0
PM-11, 4056 - 4117	R.KICLDLQAPLYK.K	1462.78211	1	7.34E-05	0.89	3.88	-	770.0
gi|20452464|ref|NP_620411.1| similar to endothelial cell-selective adhesion molecule; likely orthol				7.35E-05	0.68	10.14	4.40	41208.1
PM-6, 6328	R.QLPSFQTFFAPALDVIR.G	1951.25656	2	7.35E-05	0.68	2.81	-	400.4
gi|30159310|ref|XP_301899.1| similar to beta-actin [Homo sapiens]				7.69E-05	0.33	10.13	3.80	32376.4
PM-7, 1472 - 1764	K.DSYVGNEAQSK.R	1198.22158	1	7.69E-05	0.33	2.54	-	364.1
gi|5729877|ref|NP_006588.1| heat shock 70kDa protein 8 isoform 1; heat shock cognate protein, 71-kD				7.84E-05	1.72	20.24	7.30	70897.6
PM-5, 5426	K.QTQTFTTYSDNQPGVLIQVYEGER.A	2775.96443	2	9.60E-04	0.79	3.50	-	334.5
PM-5, 6250 - 6316	K.SINPDEAVAYGAAVQAAILSGDK.S	2261.47411	2	7.84E-05	0.94	4.79	-	900.6
gi|7705855|ref|NP_057226.1| steroid dehydrogenase homolog; likely ortholog of mouse Kik1 steroid de				8.19E-05	0.97	10.22	4.80	34323.9
PM-8, 6024	K.TFVDFFSQCLHEEYR.S	1979.16060	2	8.19E-05	0.97	4.32	-	1806.1
gi|19913373|ref|NP_079536.2| G6B protein isoform G6b-A precursor; G6B protein; immunoglobulin recep				8.47E-05	1.52	20.18	22.40	25002.6
PM-8, 3167 - 3168	R.GPGPGTEPALCGSGPSSPQQAPPAVHSGPC	2856.10082	2	8.47E-05	0.69	3.69	-	203.1
PM-10, 6089 - 6092	R.RPILWASSSGTPTVPPLQPFVGR.L	2464.84993	2	3.68E-04	0.82	3.48	-	537.7
gi|14765644|ref|XP_037574.1| similar to PTPL1-associated RhoGAP 1 [Homo sapiens]				9.04E-05	0.86	10.19	1.60	124613.7
PM-1, 5019	K.ELVELSQASPHDISNVLK.L	1980.20824	2	9.04E-05	0.86	3.80	-	663.8

PM-3, 6423	-.CPCSTKSNAEQPFELKQVK.G	2252.55686	2	9.13E-04	0.47	3.03	-	525.6
gi|5902090|ref|NP_008862.1| solute carrier family 2 (facilitated glucose transporter), member 3; GL				1.07E-04	0.82	10.17	0.00	53924.0
PM-7, 4973	R.LWGTQDVSQDIQEMKDESAR.M	2337.50934	2	1.07E-04	0.82	2.77	-	765.5
PM-1, 5569	R.LWGTQDVSQDIQEMKDESAR.M	2337.50934	2	2.74E-04	0.74	2.57	-	954.1
gi|24497612|ref|NP_116262.2| nm23-phosphorylated unknown substrate; SH3 domain-containing 70 kDa pr				1.10E-04	1.64	20.20	6.00	72695.6
PM-5, 4149 - 4164	R.EYVLYLRPTGPLAQK.L	1749.04621	2	1.10E-04	0.83	2.87	-	916.5
PM-5, 6306 - 6309	R.SVQAACDWLFSHVGDPFLDDPLPR.E	2744.03366	2	6.23E-04	0.81	3.93	-	332.2
gi|4507949|ref|NP_003395.1| tyrosine 3-monooxygenase/tryptophan 5-monooxygenase activation protein,				1.14E-04	0.84	10.16	4.90	28082.2
PM-9, 2611	K.YLIPNATQPESK.V	1361.52512	1	1.14E-04	0.84	3.23	-	818.3
gi|5032007|ref|NP_005850.1| purine-rich element binding protein A; purine-rich single-stranded DNA-				1.18E-04	0.82	10.18	0.00	34910.5
PM-8, 4546	K.LIDDYGVEEEPAELPEGTSLTVDNKR.F	2891.09052	3	1.18E-04	0.82	3.59	-	757.4
gi|6005846|ref|NP_009215.1| PTK9L protein tyrosine kinase 9-like (A6-related protein); protein tyro				1.20E-04	0.46	10.13	5.40	39547.7
PM-9, 3992	R.ETIELVHTEPTDVAQLPSR.V	2136.34822	2	1.20E-04	0.46	2.55	-	325.8
gi|5031611|ref|NP_005859.1| BET1 homolog; Golgi vesicular membrane trafficking protein p18; Bet1p h				1.20E-04	0.92	10.20	15.30	13289.4
PM-11, 4768	K.LLAEM*DSQFDSTTGFLGK.T	1977.18145	2	1.20E-04	0.92	4.01	-	919.1
gi|21735621|ref|NP_005909.2| mitochondrial malate dehydrogenase precursor [Homo sapiens]				1.22E-04	0.93	10.18	5.00	35503.0
PM-8, 5188	K.SQETECTYFSTPLLLGK.K	1975.20848	2	1.22E-04	0.93	3.70	-	1059.2
gi|4502107|ref|NP_001145.1| annexin V; endonexin II; anchorin CII; lipocortin V; placental anticoag				1.32E-04	0.65	10.15	7.80	35936.5
PM-8, 5192	K.QVYEEEYGSSLEDDVVGDTSGYYQR.M	2889.93166	2	1.32E-04	0.65	3.08	-	444.1
gi|10835220|ref|NP_004311.1| ATPase, Ca++ transporting, fast twitch 1 isoform b; sarcoplasmic/endop				1.36E-04	0.98	10.26	2.10	109282.7
PM-4, 6152 - 6156	R.DIVPGDIVEVAVGDKVPADIR.I	2178.47138	2	1.36E-04	0.98	5.20	-	1857.6
gi|17986260|ref|NP_524147.1| smooth muscle and non-muscle myosin alkali light chain isoform 2 [Homo				1.48E-04	1.47	20.20	23.30	12969.7
PM-11, 6371 - 6429	K.M*TEEEVEMLVAGHEDSNGCINYEELVR.M	3171.44016	3	9.10E-04	0.82	3.51	-	1015.4
PM-11, 5695 - 5739	K.M*TEEEVEM*LVAGHEDSNGCINYEELVR.M	3187.43956	3	1.48E-04	0.64	3.07	-	495.7
gi|7661746|ref|NP_054760.1| signal peptidase 12kDa [Homo sapiens]				1.53E-04	0.82	10.17	18.70	10567.3
PM-11, 2340	K.WLPVQESSTDDKKPGER.K	1973.13265	2	1.53E-04	0.82	3.41	-	568.5
gi|11386183|ref|NP_008921.1| WAS protein family, member 2; suppressor of cyclic-AMP receptor (WASP-				1.59E-04	0.80	10.18	3.80	54283.7
PM-9, 3753 - 3757	K.VTQLDPKEEEVSLQGINTR.K	2157.36743	2	1.59E-04	0.80	3.50	-	623.2
gi|4505879|ref|NP_002655.1| pleckstrin; p47 [Homo sapiens]				1.60E-04	0.94	10.29	6.90	40082.5
PM-7, 5220 - 5224	R.EDPAYLHYYDPAGAEDPLGAIHLR.G	2684.89982	3	1.60E-04	0.94	4.79	-	1297.4
gi|20472432|ref|XP_166699.1| similar to bicaudal-C [Homo sapiens]				1.67E-04	0.64	10.10	9.20	13337.0
PM-4, 4822	R.KMLLAISELNK.N	1260.57201	1	1.67E-04	0.64	1.99	-	1121.3
gi|4503545|ref|NP_001961.1| eukaryotic translation initiation factor 5A; eIF5AI; eIF5A [Homo sapien				1.68E-04	0.79	10.18	11.00	16832.2
PM-10, 3885	R.EDLRLPEGDLGKEIEQK.Y	1970.17021	2	1.68E-04	0.79	3.68	-	391.0
gi|28395033|ref|NP_786886.1| ras homolog gene family, member C; Aplysia RAS-related homolog 9 (onco				1.80E-04	0.40	10.15	12.40	22006.2
PM-9, 6343	K.DQFPEVYVPTVFENYIADIEVDGK.Q	2789.04149	2	1.80E-04	0.40	3.02	-	178.4
gi|21614520|ref|NP_000393.2| glucose-6-phosphate dehydrogenase [Homo sapiens]				1.91E-04	0.94	10.22	3.10	59257.4
PM-6, 3747	R.LQFHDVAGDIFHQQCK.R	1944.16262	2	1.91E-04	0.94	4.46	-	976.1
gi|5803201|ref|NP_006818.1| transmembrane trafficking protein [Homo sapiens]				1.95E-04	0.92	10.17	5.50	24975.8
PM-10, 2215 - 2277	K.ITDSAGHILYSK.E	1305.46156	2	1.95E-04	0.92	3.36	-	1263.2
gi|21361478|ref|NP_055944.2| septin 6 isoform B; septin 2 [Homo sapiens]				2.04E-04	0.83	10.17	4.60	49716.3
PM-6, 2696	K.TAAELLQSQGSQAGGSQTLK.R	1976.13485	2	2.04E-04	0.83	3.35	-	959.6
gi|8393638|ref|NP_058642.1| F11 receptor isoform a precursor; platelet F11 receptor; platelet adhes				2.31E-04	0.82	10.17	7.70	32582.9
PM-8, 4792 - 4859	K.LIVLVPPSKPTVNIPSSATIGNR.A	2374.80814	2	2.31E-04	0.82	3.48	-	482.3
gi|22042381|ref|XP_065237.3| similar to FKSG30 [Homo sapiens]				2.35E-04	0.26	10.18	4.80	42016.0
PM-7, 3352 - 3426	R.VAPEEHPILLTEAPLNPK.A	1969.27019	2	2.35E-04	0.26	2.57	-	173.5
gi|29729345|ref|XP_290995.1| similar to speckle-type POZ protein [Homo sapiens]				2.35E-04	0.72	10.19	10.90	18703.4
PM-3, 5776 - 5836	K.NRVEINDLDPEVFKEMM*R.F	2252.55733	2	2.35E-04	0.72	3.83	-	763.0
gi|8922673|ref|NP_060692.1| parvin, alpha; alpha-parvin; actopaxin [Homo sapiens]				2.48E-04	0.86	10.16	3.50	42243.4
PM-8, 4372	R.DAFDTLFDHAPDK.L	1492.57096	1	2.48E-04	0.86	3.21	-	762.8
gi|18640750|ref|NP_570138.1| regulator of G-protein signalling 18; regulator of G-protein signaling				2.50E-04	0.73	10.16	8.90	27582.0
PM-9, 4063	K.EVITNSITQPTLHSFDAAQSR.V	2316.51300	2	2.50E-04	0.73	2.93	-	425.8
gi|4757812|ref|NP_004880.1| ATP synthase, H+ transporting, mitochondrial F0 complex, subunit f, iso				2.54E-04	0.62	10.13	11.70	10917.8
PM-11, 6119	K.LGELPSWILMR.D	1315.60936	2	2.54E-04	0.62	2.52	-	547.0
gi|21361456|ref|NP_055425.2| alpha glucosidase II alpha subunit; likely ortholog of mouse G2an alph				2.58E-04	0.58	10.14	1.80	109437.2
PM-4, 3398	K.MMDYLQGSGETPQTDVR.W	1929.12196	2	2.58E-04	0.58	2.79	-	501.9
gi|4507231|ref|NP_003134.1| single-stranded DNA binding protein; single-stranded DNA-binding protei				2.66E-04	0.47	10.10	5.40	17259.5
PM-11, 2215	R.DVAYQYVK.K	986.10277	1	2.66E-04	0.47	2.07	-	646.1
gi|8923579|ref|NP_060377.1| hypothetical protein FLJ20625 [Homo sapiens]				2.70E-04	0.70	10.19	18.60	17744.7
PM-10, 6176 - 6177	K.LPPLPSLTSQPHQVLASEPIPFSDLQQVSR.I	3283.72259	3	2.70E-04	0.70	3.72	-	383.7
gi|30150532|ref|XP_291282.2| similar to proliferation related acidic leucine rich protein PAL31 [Ho				2.71E-04	0.24	10.14	3.80	48984.1
PM-4, 5372 - 5432	K.DAREEDGVDTIEEDLTR.A	1963.99143	2	2.71E-04	0.24	2.72	-	422.8
gi|29734623|ref|XP_299768.1| hypothetical protein XP_299768 [Homo sapiens]				2.72E-04	0.33	10.12	7.50	14905.7
PM-8, 3318 - 3319	K.RMVPVKEIDR.L	1243.50477	1	2.72E-04	0.33	2.46	-	446.2
gi|24797093|ref|NP_005383.2| PHD finger protein 2 isoform a [Homo sapiens]				2.84E-04	0.48	10.16	3.40	121231.7
PM-2, 6427	K.KDGLGLAVPAPTFYVSDVENYVGPERSVDVTDVTKQK.D	3996.42799	3	2.84E-04	0.48	3.18	-	314.5
gi|4885611|ref|NP_005461.1| spectrin SH3 domain binding protein 1; eps8 binding protein; interactor				3.20E-04	0.62	10.14	2.20	55156.6
PM-10, 3445 - 3463	R.ALIESYQNLTR.V	1308.46544	2	3.20E-04	0.62	2.74	-	407.3
gi|13637631|ref|XP_016625.1| similar to voltage-dependent anion channel isoform 2 [Homo sapiens] [M				3.38E-04	0.34	10.15	3.80	31479.2
PM-8, 4172	K.WCEYGLTFTEK.W	1434.59781	1	3.38E-04	0.34	2.65	-	337.6
gi|6912638|ref|NP_036557.1| ras suppressor protein 1 [Homo sapiens]				3.39E-04	0.27	10.13	4.30	31540.1
PM-9, 4516 - 4573	K.LTMVPPNIAELK.N	1326.63045	1	3.39E-04	0.27	2.66	-	268.4
gi|4758460|ref|NP_004479.1| glycoprotein V (platelet) [Homo sapiens]				3.50E-04	1.87	20.29	8.90	60958.9
PM-4, 5644	R.LMISDSHISAVAPGTFSDLIK.L	2203.54435	2	5.22E-04	0.92	4.39	-	774.3
PM-4, 6500 - 6502	K.LVNLQELALNQNQLDFLPASLFTNLENLK.L	3314.77659	3	3.50E-04	0.95	5.80	-	815.5
gi|4758128|ref|NP_004725.1| doublecortin and CaM kinase-like 1; doublecortin-like kinase [Homo sapi				3.51E-04	0.46	10.12	1.20	81099.6
PM-8, 2014 - 2070	R.EYALKIIKK.S	1106.38297	1	3.51E-04	0.46	2.42	-	668.0
PM-7, 1787	R.EYALKIIKK.S	1106.38297	1	4.89E-04	0.22	2.26	-	510.5
gi|4506413|ref|NP_002875.1| RAP1A, member of RAS oncogene family; RAS-related protein RAP1A [Homo s				3.70E-04	0.91	10.19	6.50	20987.1
PM-10, 6257	K.INVNEIFYDLVR.Q	1495.70427	2	3.70E-04	0.91	3.74	-	1053.2
gi|4757712|ref|NP_004026.1| acyl-Coenzyme A oxidase isoform a; acyl-coenzyme A oxidase 1 [Homo sapi				3.81E-04	0.75	10.18	3.90	74745.4
PM-5, 5673	R.GLETTATYDPETQEFILNSPTVTSIK.W	2857.11567	2	3.81E-04	0.75	3.63	-	277.4
gi|4502989|ref|NP_001856.1| cytochrome c oxidase subunit VIIa polypeptide 2 (liver) precursor; hepa				3.83E-04	0.92	10.17	15.70	9395.9
PM-12, 5563 - 5619	K.LFQEDDEIPLYLK.G	1623.82758	2	3.83E-04	0.92	3.48	-	1346.5
gi|8922712|ref|NP_060713.1| hypothetical protein FLJ10849 [Homo sapiens]				4.29E-04	0.79	10.16	4.40	49397.9
PM-6, 2210	K.AAAQLLQSQAQQSGAQQTK.K	1958.12276	2	4.29E-04	0.79	3.26	-	715.5
gi|5453597|ref|NP_006126.1| F-actin capping protein alpha-1 subunit; Cap Z [Homo sapiens]				4.36E-04	0.28	10.13	7.00	32922.5
PM-8, 5176	K.TIDGQQTIIACIESHQFQPK.N	2315.59140	2	4.36E-04	0.28	2.55	-	238.7
gi|4758984|ref|NP_004654.1| RAB11A, member RAS oncogene family; RAB 11A, member oncogene family [Ho				4.65E-04	0.80	10.15	5.10	24393.3
PM-9, 2548 - 2555	K.VVLIGDSGVGK.S	1044.22690	2	4.65E-04	0.80	2.97	-	963.5
gi|7657431|ref|NP_055205.1| EBNA-2 co-activator (100kD) [Homo sapiens]				4.83E-04	0.58	10.15	2.40	99689.7
PM-4, 6264	R.NLPGLVQEGEPFSEEATLFTK.E	2307.54125	2	4.83E-04	0.58	3.07	-	261.4
gi|11496277|ref|NP_068805.1| mitogen-activated protein kinase kinase 1 interacting protein 1; MEK p				4.87E-04	0.92	10.23	22.60	13622.6
PM-11, 5441 - 5508	K.VANDNAPEHALRPGFLSTFALATDQGSK.L	2929.19327	3	4.87E-04	0.92	4.51	-	1079.2
gi|6680033|ref|NP_031379.1| guanine nucleotide binding protein (G protein) alpha 12 [Homo sapiens]				5.35E-04	0.66	10.12	2.90	44250.9
PM-8, 2886	K.ILLLGAGESGK.S	1058.25357	1	5.35E-04	0.66	2.47	-	796.9
gi|4826854|ref|NP_004995.1| NADH dehydrogenase (ubiquinone) 1 beta subcomplex, 8, 19kDa; NADH dehyd				5.63E-04	0.36	10.13	10.20	21765.6
PM-10, 4052 - 4064	R.VEDYEPYPDDGMGYGDYPK.L	2211.30288	2	5.63E-04	0.36	2.54	-	386.6
gi|4502167|ref|NP_000475.1| amyloid beta (A4) precursor protein (protease nexin-II, Alzheimer disea				5.68E-04	0.84	10.17	2.20	86942.8
PM-3, 4477	K.STNLHDYGMLLPCGIDK.F	1935.21422	2	5.68E-04	0.84	3.35	-	823.9
gi|21314617|ref|NP_000433.2| platelet/endothelial cell adhesion molecule (CD31 antigen); platelet/e				5.77E-04	0.23	10.21	1.90	82449.6
PM-3, 2616	K.SDSGTYICTAGIDK.V	1488.60218	1	5.77E-04	0.23	2.51	-	242.4
gi|27479431|ref|XP_050793.2| similar to KIAA1393 protein [Homo sapiens]				5.92E-04	0.19	10.09	1.60	58245.9
PM-4, 4378	-.M*VLWILWR.P	1133.43358	1	5.92E-04	0.19	1.82	-	461.4
gi|16933542|ref|NP_002017.1| fibronectin 1 isoform 1 preproprotein; cold-insoluble globulin [Homo s				6.02E-04	0.64	10.15	0.70	259222.8
PM-1, 6265	R.FLATTPNSLLVSWQPPR.A	1928.22299	2	6.02E-04	0.64	2.88	-	398.8
gi|15553127|ref|NP_000180.2| hexokinase 2; hexokinase-2, muscle [Homo sapiens]				6.45E-04	0.23	10.12	0.90	102379.3

PM-4, 3820	R.NILIDFTK.R	964.14024	1	6.45E-04	0.23	1.93	-	367.3
gi|4505303|ref|NP_002466.1| myosin alkali light chain 1 slow a; myosin light chain 1, slow-twitch m				6.50E-04	0.12	10.14	5.30	22763.8
PM-11, 2756 - 2811	K.ILYSQCGDVMR.A	1342.57009	1	6.50E-04	0.12	2.27	-	146.0
gi|5453559|ref|NP_006347.1| ATP synthase, H+ transporting, mitochondrial F0 complex, subunit d; ATP				6.50E-04	0.86	10.18	10.60	18491.1
PM-10, 5640 - 5697	R.LAALPENPPAIDWAYYK.A	1933.19486	2	6.50E-04	0.86	3.68	-	498.7
gi|29738224|ref|XP_292615.1| similar to type I hair keratin 6 [Homo sapiens]				6.66E-04	0.04	10.09	2.00	51806.5
PM-8, 2175	K.ICTITKEIK.D	1106.36139	1	6.66E-04	0.04	1.85	-	216.7
gi|16904381|ref|NP_006717.1| LPS-responsive vesicle trafficking, beach and anchor containing; vesic				6.93E-04	0.49	10.14	0.70	319158.3
PM-4, 4246	K.DNPSIFVHSPHALMLQDVK.A	2149.45847	2	6.93E-04	0.49	2.67	-	429.4
gi|18104998|ref|NP_001119.2| adaptor-related protein complex 1, gamma 1 subunit; gamma adaptin; cla				7.76E-04	0.80	10.13	1.70	91679.3
PM-5, 5978 - 6045	K.ELLYFLDSCEPEFK.A	1791.01361	2	7.76E-04	0.80	2.56	-	886.2
gi|17105400|ref|NP_477352.1| phosphatidylinositol 4-kinase, catalytic, alpha polypeptide isoform 2;				9.73E-04	0.51	10.14	1.00	231289.6
PM-1, 6619	K.ASSSAGNLGVLIPVIAVLTR.R	1939.28876	2	9.73E-04	0.51	2.86	-	447.3
gi|4507857|ref|NP_003461.1| ubiquitin specific protease 7 (herpes virus-associated); Herpes virus-a				9.74E-04	0.07	10.10	1.00	128271.0
PM-10, 3128	R.LQEEKRIEAQK.R	1372.55267	1	9.74E-04	0.07	1.99	-	345.8
gi|20539300|ref|XP_088391.4| similar to tropomyosin 4 [Homo sapiens]				9.79E-04	0.93	10.17	6.90	18592.1
PM-9, 3312	R.KLVILEGELER.A	1299.54160	2	9.79E-04	0.93	3.34	-	1484.4
