# Supplementary material for: Proteomic and Phospho-Proteomic Profile of Human Platelets in Basal, Resting State: Insights into Integrin Signaling
Source: PLoS One. 2009 Oct 27;4(10):e7627. doi: 10.1371/journal.pone.0007627 (PMC2762604; doi:10.1371/journal.pone.0007627)
Supplement: Table S5 — A list of 262 unique phosphorylated proteins identified using four independent samples in this study. Protein uniprot name, Gene name, Uniprot accession number, Protein name, Gene ontology classification, Predicted sub-cellular localization and Protein family are shown based on its descrition in the Uniprot database (www.uniprot.org). (0.15 MB PDF) [file pone.0007627.s010.pdf]

	Entry name	Gene names	Uniprot Accession	Protein names	Gene Ontology	Protein family
**1**	AAKB1_HUMAN	PRKAB1 (AMPK)	Q9Y478	5'-AMP-activated protein kinase subunit beta-1 (AMPK beta-1 chain) (AMPKb)	cytoplasm; fatty acid biosynthetic process; nucleus; protein binding; signal transduction	5'-AMP-activated protein kinase beta subunit family
**2**	ABCA5_HUMAN	ABCA5 (KIAA1888)	Q8WWZ7	ATP-binding cassette sub-family A member 5	ATP binding; ATPase activity; Golgi membrane; integral to membrane; late endosome membrane; lysosomal membrane; transport	ABC transporter family, ABCA subfamily
**3**	ABCF1_HUMAN	ABCF1 (ABC50)	Q8NE71	ATP-binding cassette sub-family F member 1 (ATP-binding cassette 50) (TNF-alpha-stimulated ABC protein)	ATP binding; ATPase activity, coupled to transmembrane movement of substances; inflammatory response; translation; translation factor activity, nucleic acid binding	ABC transporter family, EF3 subfamily
**4**	ABLM3_HUMAN	ABLIM3 (KIAA0843) (HMFN1661)	O94929	Actin-binding LIM protein 3 (Actin-binding LIM protein family member 3) (abLIM-3)	actin binding; cytoplasm; cytoskeleton organization; zinc ion binding	
**5**	ACTG_HUMAN	ACTG1 (ACTB) (ACTG)	P63261	Actin, cytoplasmic 2 (Gamma-actin) [Cleaved into: Actin, cytoplasmic 2, N-terminally processed]	ATP binding; cell motion; cytoplasm; cytoskeleton; identical protein binding; sensory perception of sound; structural constituent of cytoskeleton	Actin family
**6**	ACTH_HUMAN	ACTG2 (ACTA3) (ACTL3) (ACTSG)	P63267	Actin, gamma-enteric smooth muscle (Smooth muscle gamma-actin) (Gamma-2-actin) (Alpha-actin-3)	ATP binding; cytoplasm; cytoskeleton; protein binding	Actin family
**7**	ACTN1_HUMAN	ACTN1	P12814	Alpha-actinin-1 (Alpha-actinin cytoskeletal isoform) (Non-muscle alpha-actinin-1) (F-actin cross-linking protein)	Z disc; actin binding; calcium ion binding; cytoskeleton; focal adhesion; focal adhesion formation; integrin binding; negative regulation of cell motion; nucleolus; pseudopodium; regulation of apoptosis; vinculin binding	Alpha-actinin family
**8**	ACTN3_HUMAN	ACTN3	Q08043	Alpha-actinin-3 (Alpha-actinin skeletal muscle isoform 3) (F-actin cross-linking protein)	actin binding; actin filament; calcium ion binding; focal adhesion; focal adhesion formation; integrin binding; protein homodimerization activity; pseudopodium; regulation of apoptosis; structural constituent of muscle	Alpha-actinin family
**9**	ADAM9_HUMAN	ADAM9 (KIAA0021) (MCMP) (MDC9) (MLTNG)	Q13443	Disintegrin and metalloproteinase domain-containing protein 9 (ADAM 9) (EC 3.4.24.-) (Metalloprotease/disintegrin/cysteine-rich protein 9) (Myeloma cell metalloproteinase) (Meltrin-gamma) (Cellular disintegrin-related protein)	PMA-inducible membrane protein ectodomain proteolysis; SH3 domain binding; activation of MAPKK activity; cell-cell adhesion mediated by integrin; cell-matrix adhesion; collagen binding; extracellular space; integral to membrane; integrin binding; integrin-mediated signaling pathway; intrinsic to external side of plasma membrane; keratinocyte differentiation; laminin binding; metalloendopeptidase activity; monocyte activation; phosphate binding; positive regulation of cell adhesion mediated by integrin; positive regulation of keratinocyte migration; positive regulation of macrophage fusion; positive regulation of membrane protein ectodomain proteolysis; positive regulation of protein secretion; protein kinase C binding; response to calcium ion; response to glucocorticoid stimulus; response to hydrogen peroxide; response to manganese ion; response to tumor necrosis factor; transforming growth factor beta receptor signaling	
**10**	ADCY6_HUMAN	ADCY6 (KIAA0422)	O43306	Adenylate cyclase type 6 (EC 4.6.1.1) (Adenylate cyclase type VI) (ATP pyrophosphate-lyase 6) (Adenylyl cyclase 6) (Ca(2+)-inhibitable adenylyl cyclase)	ATP binding; adenylate cyclase activity; cAMP biosynthetic process; integral to membrane; intracellular signaling cascade; magnesium ion binding	Adenylyl cyclase class-4/guanylyl cyclase family
**11**	ADDA_HUMAN	ADD1 (ADDA)	P35611	Alpha-adducin (Erythrocyte adducin subunit alpha)	F-actin capping protein complex; actin filament binding; actin filament bundle formation; barbed-end actin filament capping; calmodulin binding; cytosol; metal ion binding; nucleus; plasma membrane; positive regulation of protein binding; protein heterodimerization activity; protein homodimerization activity; spectrin binding; transcription factor binding	Aldolase class II family, Adducin subfamily

	Entry name	Gene names	Uniprot Accession	Protein names	Gene Ontology	Protein family
**12**	ADDG_HUMAN	ADD3 (ADDL)	Q9UEY8	Gamma-adducin (Adducin-like protein 70)	actin binding; calmodulin binding; cytoplasm; cytoskeleton; metal ion binding; plasma membrane; structural constituent of cytoskeleton	Aldolase class II family, Adducin subfamily
**13**	AFAP1_HUMAN	AFAP1 (AFAP)	Q8N556	Actin filament-associated protein 1 (110 kDa actin filament-associated protein) (AFAP-110)	actin binding; cytoplasm; cytoskeleton	
**14**	ALDOA_HUMAN	ALDOA (ALDA)	P04075	Fructose-bisphosphate aldolase A (EC 4.1.2.13) (Muscle-type aldolase) (Lung cancer antigen NY-LU-1)	ATP biosynthetic process; I band; actin binding; actin cytoskeleton; actin filament organization; extracellular vesicular exosome; fructose 1,6-bisphosphate metabolic process; fructose binding; fructose-bisphosphate aldolase activity; glycolysis; identical protein binding; muscle maintenance; nucleus; regulation of cell shape; striated muscle contraction; tubulin binding	Class I fructose-bisphosphate aldolase family
**15**	AMPD2_HUMAN	AMPD2	Q01433	AMP deaminase 2 (EC 3.5.4.6) (AMP deaminase isoform L)	AMP deaminase activity; purine base metabolic process; purine ribonucleoside monophosphate biosynthetic process	Adenosine and AMP deaminases family
**16**	ANLN_HUMAN	ANLN	Q9NQW6	Actin-binding protein anillin	actin binding; contractile ring; cytokinesis; mitosis; nucleus; regulation of exit from mitosis; septin ring assembly	
**17**	APOL1_HUMAN	APOL1 (APOL)	O14791	Apolipoprotein L1 (Apolipoprotein L-I) (ApoL-I) (Apolipoprotein L) (Apo-L) (ApoL)	chloride channel activity; chloride transport; cholesterol metabolic process; cytolysis; high-density lipoprotein particle; innate immune response; intrinsic to membrane; killing of cells of another organism; lipid binding; lipid transport; lipoprotein metabolic process; protein binding	Apolipoprotein L family
**18**	ARAP1_HUMAN	ARAP1 (CENTD2) (KIAA0782)	Q96P48	Arf-GAP, Rho-GAP domain, ANK repeat and PH domain-containing protein 1 (Centaurin-delta-2) (Cnt-d2)	ARF GTPase activator activity; Golgi cisterna membrane; Rho GTPase activator activity; actin filament reorganization during cell cycle; negative regulation of stress fiber formation; phosphatidylinositol-3,4,5-trisphosphate binding; plasma membrane; positive regulation of Cdc42 GTPase activity; positive regulation of filopodium assembly; protein binding; regulation of ARF GTPase activity; regulation of cell motion; regulation of cell shape; signal transduction; zinc ion binding	
**19**	ARHG6_HUMAN	ARHGEF6 (COOL2) (KIAA0006) (PIXA)	Q15052	Rho guanine nucleotide exchange factor 6 (Rac/Cdc42 guanine nucleotide exchange factor 6) (PAK-interacting exchange factor alpha) (Alpha-Pix) (COOL-2)	GTPase activator activity; JNK cascade; Rho guanyl-nucleotide exchange factor activity; apoptosis; cytoplasm; regulation of Rho protein signal transduction	
**20**	ARRB1_HUMAN	ARRB1 (ARR1)	P49407	Beta-arrestin-1 (Arrestin beta-1)	cytoplasm; enzyme inhibitor activity; membrane fraction; plasma membrane; protein binding; signal transduction; soluble fraction	Arrestin family
**21**	ASAP1_HUMAN	ASAP1 (DDEF1) (KIAA1249)	Q9ULH1	Arf-GAP with SH3 domain, ANK repeat and PH domain-containing protein 1 (130 kDa phosphatidylinositol 4,5-biphosphate-dependent ARF1 GTPase-activating protein) (PIP2-dependent ARF1 GAP) (ADP-ribosylation factor-directed GTPase-activating protein 1) (ARF GTPase-activating protein 1) (Development and differentiation-enhancing factor 1) (Differentiation-enhancing factor 1) (DEF-1)	ARF GTPase activator activity; cytoplasm; membrane; protein binding; regulation of ARF GTPase activity; zinc ion binding	
**22**	ASAP2_HUMAN	ASAP2 (DDEF2) (KIAA0400)	O43150	Arf-GAP with SH3 domain, ANK repeat and PH domain-containing protein 2 (Development and differentiation-enhancing factor 2) (Pyk2 C-terminus-associated protein) (PAP) (Paxillin-associated protein with ARFGAP activity 3) (PAG3)	ARF GTPase activator activity; Golgi cisterna membrane; plasma membrane; protein binding; regulation of ARF GTPase activity; zinc ion binding	
**23**	AT2A2_HUMAN	ATP2A2 (ATP2B)	P16615	Sarcoplasmic/endoplasmic reticulum calcium ATPase 2 (SERCA2) (EC 3.6.3.8) (Calcium pump 2) (Calcium-transporting ATPase sarcoplasmic reticulum type, slow twitch skeletal muscle isoform) (SR Ca(2+)-ATPase 2) (Endoplasmic reticulum class 1/2 Ca(2+) ATPase)	ATP binding; ATP biosynthetic process; S100 alpha binding; calcium ion binding; calcium-transporting ATPase activity; cell adhesion; epidermis development; integral to plasma membrane; magnesium ion binding; microsome; sarcoplasmic reticulum calcium ion transport; sarcoplasmic reticulum membrane	Cation transport ATPase (P-type) family, Type IIA subfamily

	Entry name	Gene names	Uniprot Accession	Protein names	Gene Ontology	Protein family
**24**	BCAT1_HUMAN	BCAT1 (BCT1) (ECA39)	P54687	Branched-chain-amino-acid aminotransferase, cytosolic (BCAT(c)) (EC 2.6.1.42) (Protein ECA39)	G1/S transition of mitotic cell cycle; branched chain family amino acid biosynthetic process; branched-chain-amino-acid transaminase activity; cell proliferation	Class-IV pyridoxal-phosphate-dependent aminotransferase family
**25**	BCL3_HUMAN	BCL3 (BCL4) (D19S37)	P20749	B-cell lymphoma 3-encoded protein (Protein Bcl-3)	Bcl3-Bcl10 complex; Bcl3/NF-kappaB2 complex; DNA damage response, signal transduction by p53 class mediator resulting in induction of apoptosis; I-kappaB kinase/NF-kappaB cascade; cytoplasm; maintenance of protein location in nucleus; negative regulation of apoptosis; negative regulation of interleukin-8 biosynthetic process; negative regulation of transcription; nucleus; positive regulation of translation; protein binding, bridging; protein import into nucleus, translocation; regulation of DNA binding; regulation of NF-kappaB import into nucleus; response to UV-C; response to virus; transcription; transcription factor binding	
**26**	BET1L_HUMAN	BET1L (GS15)	Q9NYM9	BET1-like protein (Vesicle transport protein GOS15) (Golgi SNARE with a size of 15 kDa) (GOS-15) (GS15)	Golgi membrane; SNAP receptor activity; SNARE complex; endosome; integral to membrane; protein transport; retrograde transport, endosome to Golgi	
**27**	BIN2_HUMAN	BIN2 (BRAP1)	Q9UBW5	Bridging integrator 2 (Breast cancer-associated protein 1)	cytoplasm; protein binding	
**28**	BNIP2_HUMAN	BNIP2 (NIP2)	Q12982	BCL2/adenovirus E1B 19 kDa protein-interacting protein 2	GTPase activator activity; anti-apoptosis; apoptosis; calcium ion binding; nuclear envelope; perinuclear region of cytoplasm; protein binding	
**29**	C2C2L_HUMAN	C2CD2L (KIAA0285) (TMEM24) (DLNB23)	O14523	C2 domain-containing protein 2-like (Transmembrane protein 24)	integral to membrane	
**30**	CA062_HUMAN	C1orf62	Q5T1N1	Uncharacterized protein C1orf62		
**31**	CALD1_HUMAN	CALD1 (CAD) (CDM)	Q05682	Caldesmon (CDM)	actin binding; calmodulin binding; cell motion; cytoskeleton; muscle contraction; myosin binding; tropomyosin binding	Caldesmon family
**32**	CALX_HUMAN	CANX	P27824	Calnexin (Major histocompatibility complex class I antigen-binding protein p88) (p90) (IP90)	calcium ion binding; endoplasmic reticulum membrane; integral to membrane; melanosome; protein folding; protein secretion; sugar binding; unfolded protein binding	Calreticulin family
**33**	CAP1_HUMAN	CAP1 (CAP)	Q01518	Adenylyl cyclase-associated protein 1 (CAP 1)	actin binding; activation of adenylate cyclase activity; establishment or maintenance of cell polarity; plasma membrane; signal transduction	CAP family
**34**	CASS4_HUMAN	CASS4 (C20orf32) (HEFL)	Q9NQ75	Cas scaffolding protein family member 4 (HEF1-EFS-p130Cas-like protein) (HEPL) (HEF-like protein)	cell adhesion; cytoplasm; cytoskeleton; focal adhesion; protein binding; signal transduction; two-component sensor activity	CAS family
**35**	CCM2_HUMAN	CCM2 (C7orf22) (PP10187)	Q9BSQ5	Malcavernin (Cerebral cavernous malformations 2 protein)	cytoplasm; integrin-mediated signaling pathway; protein binding; stress-activated MAPK cascade; vasculogenesis	
**36**	CCNY_HUMAN	CCNY (C10orf9) (CBCP1) (CFP1)	Q8ND76	Cyclin-Y (Cyclin fold protein 1) (Cyclin box protein 1)	nucleus	Cyclin family, Cyclin Y subfamily
**37**	CD2AP_HUMAN	CD2AP	Q9Y5K6	CD2-associated protein (Cas ligand with multiple SH3 domains) (Adapter protein CMS)	SH3 domain binding; cell division; filamentous actin; mitosis; nucleolus; plasma membrane; protein complex assembly; ruffle; signal transduction; structural constituent of cytoskeleton; substrate-bound cell migration, cell extension	
**38**	CE170_HUMAN	CEP170 (FAM68A) (KAB) (KIAA0470)	Q5SW79	Centrosomal protein of 170 kDa (Cep170) (KARP-1-binding protein) (KARP1-binding protein)	centriole; microtubule	CEP170 family
**39**	CI167_HUMAN	C9orf167	Q9NXH8	Torsin family protein C9orf167	ATP binding; chaperone mediated protein folding requiring cofactor; endoplasmic reticulum; integral to membrane; nucleoside-triphosphatase activity	ClpA/clpB family, Torsin subfamily
**40**	CIP4_HUMAN	TRIP10 (CIP4) (STOT) (STP)	Q15642	Cdc42-interacting protein 4 (Thyroid receptor-interacting protein 10) (TRIP-10) (Protein Felic) (Salt tolerant protein) (hSTP)	Golgi apparatus; actin cytoskeleton organization; cell cortex; cytoskeleton; endocytosis; identical protein binding; lipid binding; lysosome; perinuclear region of cytoplasm; plasma membrane; signal transduction	FNBP1 family

	Entry name	Gene names	Uniprot Accession	Protein names	Gene Ontology	Protein family
**41**	CKLF6_HUMAN	CMTM6 (CKLFSF6)	Q9NX76	CKLF-like MARVEL transmembrane domain-containing protein 6 (Chemokine-like factor superfamily member 6)	chemotaxis; cytokine activity; extracellular space; integral to membrane	Chemokine-like factor family
**42**	CLP1_HUMAN	CLP1 (HEAB)	Q92989	Polyribonucleotide 5'-hydroxyl-kinase Clp1 (EC 2.7.1.78) (Polynucleotide kinase Clp1) (Pre-mRNA cleavage complex II protein Clp1)	ATP binding; ATP-dependent polydeoxyribonucleotide 5'-hydroxyl-kinase activity; ATP-dependent polyribonucleotide 5'-hydroxyl-kinase activity; GTP binding; RNA interference, siRNA loading onto RISC; RNA interference, targeting of mRNA for destruction; mRNA 3'-end processing; nuclear mRNA splicing, via spliceosome; tRNA splicing, via endonucleolytic cleavage and ligation; tRNA-intron endonuclease complex	Clp1 family
**43**	CO4A3_HUMAN	COL4A3	Q01955	Collagen alpha-3(IV) chain (Goodpasture antigen) [Cleaved into: Tumstatin]	activation of caspase activity; blood circulation; cell adhesion; cell proliferation; cell surface receptor linked signal transduction; collagen type IV; extracellular matrix structural constituent; extracellular space; induction of apoptosis; integrin binding; metalloendopeptidase inhibitor activity; negative regulation of angiogenesis; negative regulation of cell proliferation; sensory perception of sound	Type IV collagen family
**44**	CSRP1_HUMAN	CSRP1 (CSRP) (CYRP)	P21291	Cysteine and glycine-rich protein 1 (Cysteine-rich protein 1) (CRP1) (CRP)	nucleus; zinc ion binding	
**45**	CXCL7_HUMAN	PPBP (CTAP3) (CXCL7) (SCYB7) (TGB1) (THBGB1)	P02775	Platelet basic protein (PBP) (C-X-C motif chemokine 7) (Small-inducible cytokine B7) (Leukocyte-derived growth factor) (LDGF) (Macrophage-derived growth factor) (MDGF) [Cleaved into: Connective tissue-activating peptide III (CTAP-III) (Low-affinity platelet factor IV) (LA-PF4); TC-2; Connective tissue-activating peptide III(1-81) (CTAP-III(1-81)); Beta-thromboglobulin (Beta-TG); Neutrophil-activating peptide 2(74) (NAP-2(74)); Neutrophil-activating peptide 2(73) (NAP-2(73)); Neutrophil-activating peptide 2 (NAP-2); TC-1; Neutrophil-activating peptide 2(1-66) (NAP-2(1-66)); Neutrophil-activating peptide 2(1-63) (NAP-2(1-63))]	cell proliferation; chemokine activity; chemotaxis; defense response to bacterium; extracellular space; glucose transmembrane transporter activity; glucose transport; growth factor activity; immune response; platelet alpha granule lumen	Intercrine alpha (chemokine CxC) family
**46**	DAP1_HUMAN	DAP (DAP1)	P51397	Death-associated protein 1 (DAP-1)	apoptosis; induction of apoptosis by extracellular signals	
**47**	DAPP1_HUMAN	DAPP1 (BAM32) (HSPC066)	Q9UN19	Dual adapter for phosphotyrosine and 3-phosphotyrosine and 3-phosphoinositide (hDAPP1) (B-cell adapter molecule of 32 kDa) (B lymphocyte adapter protein Bam32)	cytoplasm; membrane; phospholipid binding; protein amino acid dephosphorylation; protein binding; protein tyrosine phosphatase activity; signal transduction	
**48**	DBNL_HUMAN	DBNL (CMAP) (SH3P7) (PP5423)	Q9UJU6	Drebrin-like protein (Drebrin-F) (SH3 domain-containing protein 7) (Cervical SH3P7) (HPK1-interacting protein of 55 kDa) (HIP-55) (Cervical mucin-associated protein)	Rac protein signal transduction; activation of JUN kinase activity; cell cortex; cytoskeleton; endocytosis; enzyme activator activity; identical protein binding; immune response; lamellipodium	ABP1 family
**49**	DC1L1_HUMAN	DYNC1LI1 (DNCLI1)	Q9Y6G9	Cytoplasmic dynein 1 light intermediate chain 1 (Dynein light intermediate chain 1, cytosolic) (Dynein light chain A) (DLC-A)	ATP binding; dynein complex; microtubule; motor activity	Dynein light intermediate chain family
**50**	DEMA_HUMAN	EPB49 (DMT)	Q08495	Dematin (Erythrocyte membrane protein band 4.9)	actin binding; actin cytoskeleton; actin filament bundle formation; barbed-end actin filament capping	Villin/gelsolin family
**51**	DEN2C_HUMAN	DENND2C	Q68D51	DENN domain-containing protein 2C		
**52**	DNJC5_HUMAN	DNAJC5 (CSP)	Q9H3Z4	DnaJ homolog subfamily C member 5 (Cysteine string protein) (CSP)	heat shock protein binding; melanosome; membrane; protein folding; unfolded protein binding	
**53**	DOK3_HUMAN	DOK3	Q7L591	Docking protein 3 (Downstream of tyrosine kinase 3)	cytoplasm; insulin receptor binding; plasma membrane	DOK family, Type A subfamily
**54**	DREB_HUMAN	DBN1 (D0S117E)	Q16643	Drebrin (Developmentally-regulated brain protein)	actin binding; actin filament organization; actomyosin; cytoplasm; dendrite; profilin binding; regulation of dendrite development; regulation of neuronal synaptic plasticity	
**55**	DTD1_HUMAN	DTD1 (C20orf88) (HARS2)	Q8TEA8	D-tyrosyl-tRNA(Tyr) deacylase 1 (EC 3.1.-.-) (Histidyl-tRNA synthetase-related)	D-amino acid catabolic process; cytoplasm; hydrolase activity, acting on ester bonds	DTD family
**56**	DYR1A_HUMAN	DYRK1A (DYRK) (MNB) (MNBH)	Q13627	Dual specificity tyrosine-phosphorylation-regulated kinase 1A (EC 2.7.12.1) (Protein kinase minibrain homolog) (MNBH) (hMNB) (HP86) (Dual specificity YAK1-related kinase)	ATP binding; nervous system development; non-membrane spanning protein tyrosine kinase activity; nuclear speck; peptidyl-tyrosine phosphorylation; protein amino acid autophosphorylation; protein self-association; protein serine/threonine kinase activity	Protein kinase superfamily, CMGC Ser/Thr protein kinase family, MNB/DYRK subfamily

	Entry name	Gene names	Uniprot Accession	Protein names	Gene Ontology	Protein family
**57**	EF1B_HUMAN	EEF1B2 (EEF1B) (EF1B)	P24534	Elongation factor 1-beta (EF-1-beta)	cytosol; eukaryotic translation elongation factor 1 complex; protein binding; translation elongation factor activity; translational elongation	EF-1-beta/EF-1-delta family
**58**	EPHA6_HUMAN	EPHA6 (EHK2)	Q9UF33	Ephrin type-A receptor 6 (EC 2.7.10.1) (Tyrosine-protein kinase receptor EHK-2) (EPH homology kinase 2)	ATP binding; ephrin receptor activity; integral to membrane; protein amino acid phosphorylation; transmembrane receptor protein tyrosine kinase signaling pathway	Protein kinase superfamily, Tyr protein kinase family, Ephrin receptor subfamily
**59**	EPS15_HUMAN	EPS15 (AF1P)	P42566	Epidermal growth factor receptor substrate 15 (Protein Eps15) (Protein AF-1p)	SH3 domain binding; calcium ion binding; cell proliferation; coated pit; cytosol; early endosome membrane; epidermal growth factor receptor signaling pathway; protein transport; vesicle organization	
**60**	ESAM_HUMAN	ESAM (UNQ220/PRO246)	Q96AP7	Endothelial cell-selective adhesion molecule	adherens junction; integral to membrane; protein binding; tight junction	
**61**	ESYT2_HUMAN	FAM62B (ESYT2) (KIAA1228)	A0FGR8	Extended synaptotagmin-2 (E-Syt2) (Chr2Syt) (Protein FAM62B)	integral to membrane; plasma membrane	Extended synaptotagmin family
**62**	EXOC7_HUMAN	EXOC7 (EXO70) (KIAA1067)	Q9UPT5	Exocyst complex component 7 (Exocyst complex component Exo70)	centriolar satellite; cytosol; exocyst; exocytosis; plasma membrane; protein transport	EXO70 family
**63**	F10A1_HUMAN	ST13 (FAM10A1) (HIP) (SNC6)	P50502	Hsc70-interacting protein (Hip) (Suppression of tumorigenicity protein 13) (Putative tumor suppressor ST13) (Protein FAM10A1) (Progesterone receptor-associated p48 protein) (Renal carcinoma antigen NY-REN-33)	cytoplasm; protein binding, bridging; protein folding	FAM10 family
**64**	FA5_HUMAN	F5	P12259	Coagulation factor V (Activated protein C cofactor) (Proaccelerin, labile factor) [Cleaved into: Coagulation factor V heavy chain; Coagulation factor V light chain]	blood coagulation; calcium ion binding; cell adhesion; copper ion binding; oxidoreductase activity; plasma membrane; platelet alpha granule lumen	Multicopper oxidase family
**65**	FA63A_HUMAN	FAM63A (KIAA1390)	Q8N5J2	Protein FAM63A	protein binding	FAM63 family
**66**	FA65C_HUMAN	FAM65C (C20orf175)	Q96MK2	Protein FAM65C		FAM65 family
**67**	FARP2_HUMAN	FARP2 (KIAA0793)	O94887	FERM, RhoGEF and pleckstrin domain-containing protein 2 (FERM domain including RhoGEF) (FIR)	Rac protein signal transduction; Rho guanyl-nucleotide exchange factor activity; cytoplasm; cytoskeletal protein binding; cytoskeleton; extrinsic to membrane; neuron remodeling; regulation of Rho protein signal transduction	
**68**	FBP1L_HUMAN	FNBP1L (C1orf39) (TOCA1)	Q5T0N5	Formin-binding protein 1-like (Transducer of Cdc42-dependent actin assembly protein 1) (Toca-1)	cell cortex; cytoplasmic vesicle; cytoskeleton; endocytosis; lipid binding; plasma membrane	FNBP1 family
**69**	FCERG_HUMAN	FCER1G	P30273	High affinity immunoglobulin epsilon receptor subunit gamma (IgE Fc receptor subunit gamma) (FceRI gamma) (Fc-epsilon RI-gamma)	integral to plasma membrane	CD3Z/FCER1G family
**70**	FETUA_HUMAN	AHSG (FETUA) (PRO2743)	P02765	Alpha-2-HS-glycoprotein (Ba-alpha-2-glycoprotein) (Alpha-2-Z-globulin) (Fetuin-A) [Cleaved into: Alpha-2-HS-glycoprotein chain A; Alpha-2-HS-glycoprotein chain B]	acute-phase response; cysteine-type endopeptidase inhibitor activity; extracellular space; negative regulation of bone mineralization; negative regulation of insulin receptor signaling pathway; pinocytosis; positive regulation of phagocytosis; protein binding; regulation of inflammatory response	Fetuin family
**71**	FGD3_HUMAN	FGD3 (ZFYVE5)	Q5JSP0	FYVE, RhoGEF and PH domain-containing protein 3 (Zinc finger FYVE domain-containing protein 5)	Golgi apparatus; Rho guanyl-nucleotide exchange factor activity; actin cytoskeleton organization; cytoskeleton; filopodium assembly; lamellipodium; regulation of Cdc42 GTPase activity; regulation of cell shape; ruffle; small GTPase binding; zinc ion binding	
**72**	FHOD1_HUMAN	FHOD1 (FHOS) (FHOS1)	Q9Y613	FH1/FH2 domain-containing protein 1 (Formin homolog overexpressed in spleen 1) (FHOS) (Formin homology 2 domain-containing protein 1)	actin binding; actin cytoskeleton organization; cytoplasm; cytoskeleton; nucleus	Formin homology family
**73**	FIBA_HUMAN	FGA	P02671	Fibrinogen alpha chain [Cleaved into: Fibrinopeptide A]	eukaryotic cell surface binding; external side of plasma membrane; fibrinogen complex; platelet activation; platelet alpha granule lumen; protein binding, bridging; protein polymerization; receptor binding; response to calcium ion; signal transduction	

	Entry name	Gene names	Uniprot Accession	Protein names	Gene Ontology	Protein family
**74**	FINC_HUMAN	FN1 (FN)	P02751	Fibronectin (FN) (Cold-insoluble globulin) (CIG) [Cleaved into: Ugl-Y1; Ugl-Y2; Ugl-Y3]	ER-Golgi intermediate compartment; acute-phase response; cell migration; collagen binding; extracellular matrix structural constituent; fibrinogen complex; heparin binding; peptide cross-linking; platelet alpha granule lumen; proteinaceous extracellular matrix; regulation of cell shape; substrate adhesion-dependent cell spreading; transmembrane receptor protein tyrosine kinase signaling pathway	
**75**	FKB15_HUMAN	FKBP15 (KIAA0674)	Q5T1M5	FK506-binding protein 15	axon; cytoplasm; protein folding	FKBP-type PPIase family
**76**	FLNA_HUMAN	FLNA (FLN) (FLN1)	P21333	Filamin-A (Alpha-filamin) (Filamin-1) (Endothelial actin-binding protein) (Actin-binding protein 280) (ABP-280) (Non-muscle filamin)	Fc-gamma receptor I complex binding; GTP-Ral binding; Rac GTPase binding; actin crosslink formation; actin cytoskeleton; actin cytoskeleton reorganization; actin filament binding; cell cortex; cytoplasmic sequestering of protein; establishment of protein localization; glycoprotein binding; inhibition of adenylate cyclase activity by dopamine receptor signaling pathway; negative regulation of protein catabolic process; negative regulation of transcription factor activity; nucleus; plasma membrane; positive regulation of I-kappaB kinase/NF-kappaB cascade; positive regulation of transcription factor import into nucleus; protein homodimerization activity; protein localization at cell surface; protein stabilization; receptor clustering; signal transducer activity; transcription factor binding	Filamin family
**77**	FRIH_HUMAN	FTH1 (FTH) (FTHL6) (OK/SW-cl.84) (PIG15)	P02794	Ferritin heavy chain (Ferritin H subunit) (EC 1.16.3.1) (Cell proliferation-inducing gene 15 protein)	cell proliferation; ferric iron binding; ferroxidase activity; immune response; intracellular ferritin complex; intracellular sequestering of iron ion; iron ion transport; negative regulation of cell proliferation; oxidation reduction; protein binding	Ferritin family
**78**	FRM4B_HUMAN	FRMD4B (KIAA1013)	Q9Y2L6	FERM domain-containing protein 4B (GRP1-binding protein GRSP1)	binding; cytoplasm; cytoskeleton	
**79**	FYB_HUMAN	FYB (SLAP130)	O15117	FYN-binding protein (FYN-T-binding protein) (FYB-120/130) (p120/p130) (SLP-76-associated phosphoprotein) (SLAP-130) (Adhesion and degranulation promoting adaptor protein) (ADAP)	NLS-bearing substrate import into nucleus; cytoplasm; immune response; nucleus; protein amino acid phosphorylation; protein binding; protein kinase cascade	
**80**	G3P_HUMAN	GAPDH (GAPD) (CDABP0047) (OK/SW-cl.12)	P04406	Glyceraldehyde-3-phosphate dehydrogenase (GAPDH) (EC 1.2.1.12)	NAD or NADH binding; glyceraldehyde-3-phosphate dehydrogenase (phosphorylating) activity; glycolysis; membrane; oxidation reduction; perinuclear region of cytoplasm; protein binding	Glyceraldehyde-3-phosphate dehydrogenase family
**81**	G6B_HUMAN	G6B (C6orf25)	O95866	Protein G6b	Golgi apparatus; endoplasmic reticulum; heparin binding; integral to membrane; plasma membrane; receptor activity	
**82**	GA2L1_HUMAN	GAS2L1 (GAR22)	Q99501	GAS2-like protein 1 (Growth arrest-specific protein 2-like 1) (GAS2-related protein on chromosome 22)	cell cycle arrest; cytoplasm; cytoskeleton	GAS2 family
**83**	GI24_HUMAN	C10orf54 (PP2135) (UNQ730/PRO1412)	Q9H7M9	Platelet receptor Gi24	integral to membrane; receptor activity	
**84**	GP1BA_HUMAN	GP1BA	P07359	Platelet glycoprotein Ib alpha chain (Glycoprotein Ibalpha) (GP-Ib alpha) (GPIb-alpha) (GPIbA) (Antigen CD42b-alpha) (CD antigen CD42b) [Cleaved into: Glycocalicin]	cell adhesion; cell surface receptor linked signal transduction; fibrinolysis; integral to plasma membrane; membrane fraction; platelet activation; platelet alpha granule membrane; protein binding; thrombin receptor activity	
**85**	GP1BB_HUMAN	GP1BB	P13224	Platelet glycoprotein Ib beta chain (GP-Ib beta) (GPIb-beta) (GPIbB) (Antigen CD42b-beta) (CD antigen CD42c)	cell adhesion; cell surface receptor linked signal transduction; integral to plasma membrane; platelet activation; platelet alpha granule membrane; protein binding; transmembrane receptor activity	
**86**	GPSM3_HUMAN	GPSM3 (AGS4) (C6orf9) (G18)	Q9Y4H4	G-protein-signaling modulator 3 (Activator of G-protein signaling 4) (Protein G18) (G18.1b)	GTPase activator activity; cytoplasm; protein binding; signal transduction	

	Entry name	Gene names	Uniprot Accession	Protein names	Gene Ontology	Protein family
**87**	GRAP2_HUMAN	GRAP2 (GADS) (GRB2L) (GRID)	O75791	GRB2-related adapter protein 2 (Protein GADS) (Growth factor receptor-binding protein) (GRBLG) (Grf40 adapter protein) (Grf-40) (GRB-2-like protein) (GRB2L) (GRBX) (P38) (Hematopoietic cell-associated adapter protein GrpL) (Adapter protein GRID) (SH3-SH2-SH3 adapter Mona)	Ras protein signal transduction; SH3/SH2 adaptor activity; cell-cell signaling; cytosol; nucleus	GRB2/sem-5/DRK family
**88**	GRK6_HUMAN	GRK6 (GPRK6)	P43250	G protein-coupled receptor kinase 6 (EC 2.7.11.16) (G protein-coupled receptor kinase GRK6)	ATP binding; G-protein coupled receptor kinase activity; membrane; protein amino acid phosphorylation; regulation of G-protein coupled receptor protein signaling pathway; signal transducer activity; signal transduction	Protein kinase superfamily, AGC Ser/Thr protein kinase family, GPRK subfamily
**89**	GRP2_HUMAN	RASGRP2 (CDC25L) (MCG7)	Q7LDG7	RAS guanyl-releasing protein 2 (Calcium and DAG-regulated guanine nucleotide exchange factor I) (CalDAG-GEFI) (F25B3.3 kinase-like protein) (Cdc25-like protein) (HCDC25L)	Ras protein signal transduction; calcium ion binding; cell junction; cytosol; diacylglycerol binding; guanyl-nucleotide exchange factor activity; regulation of cell growth; regulation of small GTPase mediated signal transduction; ruffle membrane; synapse; synaptosome; zinc ion binding	RASGRP family
**90**	GSK3B_HUMAN	GSK3B	P49841	Glycogen synthase kinase-3 beta (GSK-3 beta) (EC 2.7.11.26)	ATP binding; Axin-APC-beta-catenin-GSK3B complex; ER overload response; NF-kappaB binding; Wnt receptor signaling pathway through beta-catenin; beta-catenin binding; beta-catenin destruction complex; cytosol; glycogen metabolic process; glycogen synthase kinase 3 activity; nucleus; p53 binding; peptidyl-serine phosphorylation; positive regulation of protein complex assembly; positive regulation of protein export from nucleus; protein kinase A catalytic subunit binding; tau-protein kinase [illegible]	Protein kinase superfamily, CMGC Ser/Thr protein kinase family, GSK-3 subfamily
**91**	GTR3_HUMAN	SLC2A3 (GLUT3)	P11169	Solute carrier family 2, facilitated glucose transporter member 3 (Glucose transporter type 3, brain) (GLUT-3)	carbohydrate metabolic process; cytoplasm; glucose transmembrane transporter activity; glucose transport; integral to membrane; plasma membrane; sugar:hydrogen symporter activity; transmembrane transport	Major facilitator superfamily, Sugar transporter (TC 2.A.1.1) family, Glucose transporter subfamily
**92**	H1BP3_HUMAN	HS1BP3	Q53T59	HCLS1-binding protein 3 (HS1-binding protein 3) (HSP1BP-3)	cell communication; phosphoinositide binding; protein binding	
**93**	HMHA1_HUMAN	HMHA1 (KIAA0223)	Q92619	Minor histocompatibility protein HA-1 [Cleaved into: Minor histocompatibility antigen HA-1 (mHag HA-1)]	GTPase activator activity; diacylglycerol binding; intracellular; intracellular signaling cascade; zinc ion binding	
**94**	HS90A_HUMAN	HSP90AA1 (HSP90A) (HSPC1) (HSPCA)	P07900	Heat shock protein HSP 90-alpha (HSP 86) (Renal carcinoma antigen NY-REN-38)	ATP binding; TPR domain binding; cellular chaperone-mediated protein complex assembly; cytosol; melanosome; mitochondrial outer membrane translocase complex assembly; mitochondrial transport; nitric-oxide synthase regulator activity; positive regulation of nitric oxide biosynthetic process; protein homodimerization activity; protein refolding; response to unfolded protein; signal transduction; unfolded protein binding	Heat shock protein 90 family
**95**	HS90B_HUMAN	HSP90AB1 (HSP90B) (HSPC2) (HSPCB)	P08238	Heat shock protein HSP 90-beta (HSP 90) (HSP 84)	ATP binding; TPR domain binding; melanosome; negative regulation of proteasomal ubiquitin-dependent protein catabolic process; nitric-oxide synthase regulator activity; positive regulation of nitric oxide biosynthetic process; protein folding; regulation of interferon-gamma-mediated signaling pathway; regulation of type I interferon-mediated signaling pathway; response to unfolded protein; unfolded protein binding	Heat shock protein 90 family
**96**	HSP71_HUMAN	HSPA1A (HSPA1); HSPA1B	P08107	Heat shock 70 kDa protein 1 (HSP70.1) (HSP70-1/HSP70-2)	ATP binding; anti-apoptosis; endoplasmic reticulum; mRNA catabolic process; mitochondrion; nucleus; response to unfolded protein; unfolded protein binding	Heat shock protein 70 family

	Entry name	Gene names	Uniprot Accession	Protein names	Gene Ontology	Protein family
**97**	HSPB1_HUMAN	HSPB1 (HSP27) (HSP28)	P04792	Heat shock protein beta-1 (HspB1) (Heat shock 27 kDa protein) (HSP 27) (Stress-responsive protein 27) (SRP27) (Estrogen-regulated 24 kDa protein) (28 kDa heat shock protein)	anti-apoptosis; cell death; cell motion; cell surface; cytoplasm; identical protein binding; nucleus; regulation of translational initiation; response to heat; response to unfolded protein; spindle	Small heat shock protein (HSP20) family
**98**	HUWE1_HUMAN	HUWE1 (KIAA0312) (KIAA1578) (UREB1) (HSPC272)	Q7Z6Z7	E3 ubiquitin-protein ligase HUWE1 (EC 6.3.2.-) (HECT, UBA and WWE domain-containing protein 1) (Upstream regulatory element-binding protein 1) (URE-binding protein 1) (URE-B1) (Mcl-1 ubiquitin ligase E3) (Mule) (ARF-binding protein 1) (ARF-BP1) (Large structure of UREB1) (LASU1) (Homologous to E6AP carboxyl terminus homologous protein 9) (HectH9)	DNA binding; cell differentiation; cytoplasm; histone ubiquitination; modification-dependent protein catabolic process; nucleus; protein binding; protein polyubiquitination; ubiquitin-protein ligase activity	TOM1/PTR1 family
**99**	IF2P_HUMAN	EIF5B (IF2) (KIAA0741)	O60841	Eukaryotic translation initiation factor 5B (eIF-5B) (Translation initiation factor IF-2)	GTP binding; GTPase activity; cytoplasm; protein binding; regulation of translational initiation; translation; translation initiation factor activity	IF-2 family
**100**	ILK_HUMAN	ILK (ILK1) (ILK2)	Q13418	Integrin-linked protein kinase (EC 2.7.11.1) (ILK-1) (ILK-2) (59 kDa serine/threonine-protein kinase) (p59ILK)	ATP binding; cell proliferation; cell-matrix adhesion; cytoplasm; focal adhesion; integrin-mediated signaling pathway; protein amino acid phosphorylation; protein binding; protein serine/threonine kinase activity; protein tyrosine kinase activity; regulation of signal transduction	Protein kinase superfamily, TKL Ser/Thr protein kinase family
**101**	INF2_HUMAN	INF2 (C14orf151) (C14orf173)	Q27J81	Inverted formin-2 (HBEBP2-binding protein C)	Rho GTPase binding; actin binding; actin cytoskeleton organization; endoplasmic reticulum; nucleus	Formin homology family
**102**	IPP2_HUMAN	PPP1R2 (IPP2)	P41236	Protein phosphatase inhibitor 2 (IPP-2)	glycogen metabolic process; protein binding; protein serine/threonine phosphatase inhibitor activity; regulation of phosphoprotein phosphatase activity; regulation of signal transduction	Protein phosphatase inhibitor 2 family
**103**	IQGA2_HUMAN	IQGAP2	Q13576	Ras GTPase-activating-like protein IQGAP2	GTPase inhibitor activity; Ras GTPase activator activity; actin binding; actin cytoskeleton; calmodulin binding; regulation of small GTPase mediated signal transduction; small GTPase mediated signal transduction	
**104**	ITB3_HUMAN	ITGB3 (GP3A)	P05106	Integrin beta-3 (Platelet membrane glycoprotein IIIa) (GPIIIa) (CD antigen CD61)	blood coagulation; cell-matrix adhesion; identical protein binding; integrin complex; integrin-mediated signaling pathway; interspecies interaction between organisms; negative regulation of foam cell differentiation; negative regulation of lipid storage; negative regulation of lipid transport; negative regulation of lipoprotein metabolic process; negative regulation of low-density lipoprotein receptor biosynthetic process; platelet alpha granule membrane; receptor activity	Integrin beta chain family
**105**	ITPR1_HUMAN	ITPR1 (INSP3R1)	Q14643	Inositol 1,4,5-trisphosphate receptor type 1 (Type 1 inositol 1,4,5-trisphosphate receptor) (Type 1 InsP3 receptor) (IP3 receptor isoform 1) (InsP3R1) (IP3R)	calcium ion binding; calcium ion transmembrane transporter activity; calcium ion transport; cell death; endoplasmic reticulum membrane; inositol 1,4,5-trisphosphate-sensitive calcium-release channel activity; inositol-1,4,5-trisphosphate receptor activity; integral to membrane; platelet dense granule membrane; platelet dense tubular network membrane; protein binding; response to hypoxia; signal transduction	InsP3 receptor family
**106**	JAM1_HUMAN	F11R (JAM1) (JCAM) (UNQ264/PRO301)	Q9Y624	Junctional adhesion molecule A (JAM-A) (Junctional adhesion molecule 1) (JAM-1) (Platelet adhesion molecule 1) (PAM-1) (Platelet F11 receptor) (CD antigen CD321)	inflammatory response; integral to membrane; interspecies interaction between organisms; tight junction	Immunoglobulin superfamily
**107**	KALRN_HUMAN	KALRN (DUET) (DUO) (HAPIP) (TRAD)	O60229	Kalirin (EC 2.7.11.1) (Huntingtin-associated protein-interacting protein) (Protein Duo) (Serine/threonine kinase with Dbl- and pleckstrin homology domain)	ATP binding; Rho guanyl-nucleotide exchange factor activity; actin cytoskeleton; cytoplasm; intracellular signaling cascade; magnesium ion binding; nervous system development; protein amino acid phosphorylation; protein serine/threonine kinase activity; regulation of Rho protein signal transduction; vesicle-mediated transport	Protein kinase superfamily, CAMK Ser/Thr protein kinase family

	Entry name	Gene names	Uniprot Accession	Protein names	Gene Ontology	Protein family
**108**	KAP2_HUMAN	PRKAR2A (PKR2) (PRKAR2)	P13861	cAMP-dependent protein kinase type II-alpha regulatory subunit	cAMP binding; cAMP-dependent protein kinase complex; cAMP-dependent protein kinase regulator activity; cytoplasm; hormone-mediated signaling; membrane fraction; plasma membrane; regulation of protein amino acid phosphorylation	CAMP-dependent kinase regulatory chain family
**109**	KAP3_HUMAN	PRKAR2B	P31323	cAMP-dependent protein kinase type II-beta regulatory subunit	Golgi apparatus; cAMP binding; cAMP-dependent protein kinase complex; cAMP-dependent protein kinase regulator activity; centrosome; hormone-mediated signaling; mitochondrion; regulation of protein amino acid phosphorylation	CAMP-dependent kinase regulatory chain family
**110**	KAPCG_HUMAN	PRKACG	P22612	cAMP-dependent protein kinase catalytic subunit gamma (PKA C-gamma) (EC 2.7.11.11)	ATP binding; cAMP-dependent protein kinase activity; hormone-mediated signaling; male gonad development; protein amino acid phosphorylation; protein kinase cascade; spermatogenesis	Protein kinase superfamily, AGC Ser/Thr protein kinase family, cAMP subfamily
**111**	KGP1B_HUMAN	PRKG1 (PRKG1B) (PRKGR1B)	P14619	cGMP-dependent protein kinase 1, beta isozyme (cGK 1 beta) (cGKI-beta) (EC 2.7.11.12)	ATP binding; actin cytoskeleton organization; cGMP binding; cGMP-dependent protein kinase activity; cytoplasm; nucleus; protein amino acid phosphorylation; signal transduction	Protein kinase superfamily, AGC Ser/Thr protein kinase family, cGMP subfamily
**112**	KIF11_HUMAN	KIF11 (EG5) (KNSL1) (TRIP5)	P52732	Kinesin-like protein KIF11 (Kinesin-related motor protein Eg5) (Kinesin-like spindle protein HKSP) (Thyroid receptor-interacting protein 5) (TRIP-5) (Kinesin-like protein 1)	ATP binding; cell division; chromatin remodeling complex; cytoplasm; kinesin complex; microtubule; microtubule motor activity; microtubule-based movement; mitosis; mitotic spindle organization; spindle; spindle pole body organization	Kinesin-like protein family, BimC subfamily
**113**	KNG1_HUMAN	KNG1 (BDK) (KNG)	P01042	Kininogen-1 (High molecular weight kininogen) (HMWK) (Williams-Fitzgerald-Flaujeac factor) (Fitzgerald factor) (Alpha-2-thiol proteinase inhibitor) [Cleaved into: Kininogen-1 heavy chain; T-kinin (Ile-Ser-Bradykinin); Bradykinin (Kallidin I); Lysyl-bradykinin (Kallidin II); Kininogen-1 light chain; Low molecular weight growth-promoting factor]	blood coagulation; centrosome; cysteine-type endopeptidase inhibitor activity; diuresis; elevation of cytosolic calcium ion concentration; extracellular space; heparin binding; inflammatory response; natriuresis; negative regulation of blood coagulation; negative regulation of cell adhesion; nucleus; positive regulation of apoptosis; receptor binding; smooth muscle contraction; vasodilation; zinc ion binding	
**114**	KPCA_HUMAN	PRKCA (PKCA) (PRKACA)	P17252	Protein kinase C alpha type (PKC-alpha) (PKC-A) (EC 2.7.11.13)	ATP binding; calcium ion binding; cytosol; diacylglycerol binding; induction of apoptosis by extracellular signals; membrane fraction; plasma membrane; protein amino acid phosphorylation; protein binding; zinc ion binding	Protein kinase superfamily, AGC Ser/Thr protein kinase family, PKC subfamily
**115**	KPCB_HUMAN	PRKCB (PKCB) (PRKCB1)	P05771	Protein kinase C beta type (PKC-beta) (PKC-B) (EC 2.7.11.13)	ATP binding; calcium ion binding; cytosol; diacylglycerol binding; intracellular signaling cascade; lipoprotein transport; plasma membrane; protein amino acid phosphorylation; protein binding; protein kinase C activity; zinc ion binding	Protein kinase superfamily, AGC Ser/Thr protein kinase family, PKC subfamily
**116**	KPCD_HUMAN	PRKCD	Q05655	Protein kinase C delta type (EC 2.7.11.13) (nPKC-delta)	ATP binding; calcium-independent protein kinase C activity; cytosol; diacylglycerol binding; enzyme activator activity; enzyme binding; insulin receptor substrate binding; intracellular signaling cascade; membrane; negative regulation of insulin receptor signaling pathway; negative regulation of peptidyl-tyrosine phosphorylation; negative regulation of protein binding; nucleus; protein C-terminus binding; protein amino acid phosphorylation; protein stabilization; regulation of receptor activity; senescence; zinc ion binding	Protein kinase superfamily, AGC Ser/Thr protein kinase family, PKC subfamily

	Entry name	Gene names	Uniprot Accession	Protein names	Gene Ontology	Protein family
**117**	KPCT_HUMAN	PRKCQ (PRKCT)	Q04759	Protein kinase C theta type (EC 2.7.11.13) (nPKC-theta)	ATP binding; diacylglycerol binding; intracellular; intracellular signaling cascade; magnesium ion binding; membrane protein ectodomain proteolysis; protein amino acid phosphorylation; protein binding; protein kinase C activity; regulation of cell growth; zinc ion binding	Protein kinase superfamily, AGC Ser/Thr protein kinase family, PKC subfamily
**118**	KPYM_HUMAN	PKM2 (PK2) (PK3) (PKM)	P14618	Pyruvate kinase isozymes M1/M2 (EC 2.7.1.40) (Pyruvate kinase muscle isozyme) (Pyruvate kinase 2/3) (Cytosolic thyroid hormone-binding protein) (CTHBP) (THBP1)	ATP binding; cytosol; glycolysis; magnesium ion binding; potassium ion binding; protein binding; pyruvate kinase activity	Pyruvate kinase family
**119**	KSR1_HUMAN	KSR1 (KSR)	Q8IVT5	Kinase suppressor of Ras 1	ATP binding; Ras protein signal transduction; cytoplasm; diacylglycerol binding; membrane; protein amino acid phosphorylation; protein binding; protein serine/threonine kinase activity; protein tyrosine kinase activity; zinc ion binding	Protein kinase superfamily, TKL Ser/Thr protein kinase family
**120**	LAT_HUMAN	LAT	O43561	Linker for activation of T-cells family member 1 (36 kDa phospho-tyrosine adapter protein) (pp36) (p36-38)	Ras protein signal transduction; SH3/SH2 adaptor activity; calcium-mediated signaling; immunological synapse; integral to membrane; integrin-mediated signaling pathway; mast cell degranulation; membrane raft; regulation of T cell activation	
**121**	LEGL_HUMAN	GRP (HSPC159)	Q3ZCW2	Galectin-related protein	intracellular; sugar binding	
**122**	LRC47_HUMAN	LRRC47 (KIAA1185)	Q8N1G4	Leucine-rich repeat-containing protein 47	RNA binding; phenylalanine-tRNA ligase activity; protein binding; translation	
**123**	LRMP_HUMAN	LRMP (JAW1)	Q12912	Lymphoid-restricted membrane protein (Protein Jaw1) [Cleaved into: Processed lymphoid-restricted membrane protein]	endoplasmic reticulum membrane; integral to plasma membrane; nucleus; vesicle fusion; vesicle targeting	
**124**	LRRF2_HUMAN	LRRFIP2	Q9Y608	Leucine-rich repeat flightless-interacting protein 2 (LRR FLII-interacting protein 2)	LRR domain binding; Wnt receptor signaling pathway	LRRFIP family
**125**	LY66F_HUMAN	LY6G6F (C6orf21) (G6F) (LY6G6D) (NG32)	Q5SQ64	Lymphocyte antigen 6 complex locus protein G6f	integral to membrane; plasma membrane	
**126**	LYN_HUMAN	LYN	P07948	Tyrosine-protein kinase Lyn (EC 2.7.10.2)	ATP binding; Golgi apparatus; erythrocyte differentiation; interspecies interaction between organisms; membrane raft; non-membrane spanning protein tyrosine kinase activity; nucleus; plasma membrane; positive regulation of cell proliferation; positive regulation of tyrosine phosphorylation of STAT protein; protein amino acid phosphorylation; protein binding; receptor signaling protein tyrosine kinase activity; response to hormone stimulus; signal transduction	Protein kinase superfamily, Tyr protein kinase family, SRC subfamily
**127**	M6PBP_HUMAN	M6PRBP1 (TIP47)	O60664	Mannose-6-phosphate receptor-binding protein 1 (Cargo selection protein TIP47) (47 kDa mannose 6-phosphate receptor-binding protein) (47 kDa MPR-binding protein) (Placental protein 17) (PP17)	Golgi apparatus; endosome membrane; monolayer-surrounded lipid storage body outer lipid monolayer; vesicle-mediated transport	Perilipin family
**128**	MAVS_HUMAN	MAVS (IPS1) (KIAA1271) (VISA)	Q7Z434	Mitochondrial antiviral-signaling protein (Interferon-beta promoter stimulator protein 1) (IPS-1) (Virus-induced-signaling adapter) (CARD adapter inducing interferon-beta) (Cardif) (Putative NF-kappa-B-activating protein 031N)	innate immune response; integral to membrane; interspecies interaction between organisms; mitochondrial outer membrane; positive regulation of I-kappaB kinase/NF-kappaB cascade; protein binding; response to virus; signal transducer activity	
**129**	MED7_HUMAN	MED7 (ARC34) (CRSP9)	O43513	Mediator of RNA polymerase II transcription subunit 7 (Mediator complex subunit 7) (Cofactor required for Sp1 transcriptional activation subunit 9) (CRSP complex subunit 9) (Transcriptional coactivator CRSP33) (RNA polymerase transcriptional regulation mediator subunit 7 homolog) (hMED7) (Activator-recruited cofactor 34 kDa component) (ARC34)	RNA polymerase II transcription mediator activity; mediator complex; regulation of transcription from RNA polymerase II promoter; transcription coactivator activity; transcription factor complex; transcription initiation from RNA polymerase II promoter	Mediator complex subunit 7 family
**130**	MFSD6_HUMAN	MFSD6 (MMR2)	Q6ZSS7	Major facilitator superfamily domain-containing protein 6 (Macrophage MHC class I receptor 2 homolog)	integral to membrane	Major facilitator superfamily, MFSD6 family
**131**	ML12B_HUMAN	MYL12B (MRLC2) (MYLC2B)	O14950	Myosin regulatory light chain 12B (Myosin regulatory light chain MRLC2) (Myosin regulatory light chain 2-B, smooth muscle isoform) (MLC-2A) (MLC-2) (SHUJUN-1)	calcium ion binding; motor activity; myosin complex	

	Entry name	Gene names	Uniprot Accession	Protein names	Gene Ontology	Protein family
**132**	MOES_HUMAN	MSN	P26038	Moesin (Membrane-organizing extension spike protein)	apical plasma membrane; cell adhesion molecule binding; cytoplasm; cytoskeletal protein binding; cytoskeleton; extrinsic to membrane; filopodium; leukocyte adhesion; leukocyte migration; membrane to membrane docking; microvillus membrane; receptor binding; structural constituent of cytoskeleton	
**133**	MOL1B_HUMAN	MOBKL1B (C2orf6) (MOB4B) (MOBK1B)	Q9H8S9	Mps one binder kinase activator-like 1B (Mob1 homolog 1B) (Mob1 alpha) (Mob1A) (Protein Mob4B)	protein binding; zinc ion binding	MOB1/phocein family
**134**	MRVI1_HUMAN	MRVI1 (IRAG) (JAW1L)	Q9Y6F6	Protein MRVI1 (Inositol 1,4,5-triphosphate receptor-associated cGMP kinase substrate) (JAW1-related protein MRVI1)	endoplasmic reticulum membrane; integral to membrane; nucleus; perinuclear region of cytoplasm; sarcoplasmic reticulum	
**135**	MTPN_HUMAN	MTPN	P58546	Myotrophin (Protein V-1)	cell growth; cytoplasm; protein binding; regulation of striated muscle development; regulation of translation	Myotrophin family
**136**	MY18A_HUMAN	MYO18A (KIAA0216) (MYSPDZ)	Q92614	Myosin-XVIIIa (Myosin containing a PDZ domain) (Molecule associated with JAK3 N-terminus) (MAJN)	ATP binding; ER-Golgi intermediate compartment; anti-apoptosis; identical protein binding; motor activity; myosin complex	
**137**	MYCPP_HUMAN	DENND4A (IRLB) (MYCPBP)	Q7Z401	C-myc promoter-binding protein (DENN domain-containing protein 4A)	DNA binding; nucleus; protein binding; regulation of transcription, DNA-dependent; transcription	
**138**	MYCT1_HUMAN	MYCT1 (MTLC) (MTMC1)	Q8N699	Myc target protein 1 (Myc target in myeloid cells protein 1)	nucleus	MYCT1 family
**139**	MYH9_HUMAN	MYH9	P35579	Myosin-9 (Myosin heavy chain 9) (Myosin heavy chain, non-muscle IIa) (Non-muscle myosin heavy chain IIa) (NMMHC II-a) (NMMHC-IIA) (Cellular myosin heavy chain, type A) (Non-muscle myosin heavy chain A) (NMMHC-A)	ADP binding; ATP binding; actin cytoskeleton reorganization; actin filament binding; actin filament-based movement; actin-dependent ATPase activity; angiogenesis; blood vessel endothelial cell migration; calmodulin binding; cleavage furrow; contractile ring; cytokinesis; cytosol; integrin-mediated signaling pathway; leukocyte migration; membrane protein ectodomain proteolysis; microfilament motor activity; monocyte differentiation; myosin complex; nucleus; platelet formation; protein anchor; protein homodimerization activity; protein transport; regulation of cell shape; ruffle; sensory perception of sound; stress fiber; uropod	
**140**	MYLK_HUMAN	MYLK (MLCK)	Q15746	Myosin light chain kinase, smooth muscle (MLCK) (EC 2.7.11.18) (Telokin) (Kinase-related protein) (KRP)	ATP binding; calcium ion binding; calmodulin binding; magnesium ion binding; myosin light chain kinase activity; protein amino acid phosphorylation	Protein kinase superfamily, CAMK Ser/Thr protein kinase family
**141**	MYPT1_HUMAN	PPP1R12A (MBS) (MYPT1)	O14974	Protein phosphatase 1 regulatory subunit 12A (Myosin phosphatase-targeting subunit 1) (Myosin phosphatase target subunit 1) (Protein phosphatase myosin-binding subunit)	cytoplasm; protein binding; signal transducer activity	
**142**	NAAA_HUMAN	NAAA (ASAHL) (PLT)	Q02083	N-acylethanolamine-hydrolyzing acid amidase (EC 3.5.1.-) (N-acylsphingosine amidohydrolase-like) (ASAH-like protein) (Acid ceramidase-like protein)	hydrolase activity; lipid metabolic process; lysosome	Acid ceramidase family
**143**	NBEL2_HUMAN	NBEAL2 (KIAA0540) (UNQ253/PRO290)	Q6ZNJ1	Neurobeachin-like protein 2	binding	WD repeat neurobeachin family
**144**	NEB2_HUMAN	PPP1R9B (PPP1R6)	Q96SB3	Neurabin-2 (Neurabin-II) (Spinophilin) (Protein phosphatase 1 regulatory subunit 9B)	RNA splicing; actin binding; cell cycle arrest; cell differentiation; cell junction; cytoskeleton; dendritic spine; negative regulation of cell growth; nervous system development; nucleoplasm; phosphoprotein phosphatase inhibitor activity; protein phosphatase 1 binding; protein phosphatase type 1 complex; regulation of cell growth by extracellular stimulus; regulation of cell proliferation; regulation of exit from mitosis; synapse	
**145**	NEXN_HUMAN	NEXN	Q0ZGT2	Nexilin (F-actin binding protein) (Nelin)	actin filament binding; cell-substrate adherens junction; cytoplasm; cytoskeleton; regulation of cell migration; regulation of cytoskeleton organization	

	Entry name	Gene names	Uniprot Accession	Protein names	Gene Ontology	Protein family
**146**	NFAC1_HUMAN	NFATC1 (NFAT2) (NFATC)	O95644	Nuclear factor of activated T-cells, cytoplasmic 1 (NF-ATc1) (NFAT transcription complex cytosolic component) (NF-ATc) (NFATc)	FK506 binding; cytoplasm; intracellular signaling cascade; nucleus; regulation of transcription, DNA-dependent; transcription activator activity; transcription factor activity; transcription from RNA polymerase II promoter	
**147**	NHRF1_HUMAN	SLC9A3R1 (NHERF) (NHERF1)	O14745	Na(+)/H(+) exchange regulatory cofactor NHE-RF1 (NHERF-1) (Ezrin-radixin-moesin-binding phosphoprotein 50) (EBP50) (Regulatory cofactor of Na(+)/H(+) exchanger) (Sodium-hydrogen exchanger regulatory factor 1) (Solute carrier family 9 isoform A3 regulatory factor 1)	PDZ domain binding; Wnt receptor signaling pathway; actin cytoskeleton; beta-2 adrenergic receptor binding; endomembrane system; filopodium; microvillus; protein complex assembly; regulation of sodium:hydrogen antiporter activity; ruffle	
**148**	NP1L1_HUMAN	NAP1L1 (NRP)	P55209	Nucleosome assembly protein 1-like 1 (NAP-1-related protein) (hNRP)	DNA replication; chromatin assembly complex; melanosome; nucleosome assembly; positive regulation of cell proliferation; protein binding	Nucleosome assembly protein (NAP) family
**149**	NP1L4_HUMAN	NAP1L4 (NAP2)	Q99733	Nucleosome assembly protein 1-like 4 (Nucleosome assembly protein 2) (NAP2)	chromatin assembly complex; cytoplasm; nucleosome assembly; unfolded protein binding	Nucleosome assembly protein (NAP) family
**150**	NSF1C_HUMAN	NSFL1C (UBXN2C)	Q9UNZ2	NSFL1 cofactor p47 (p97 cofactor p47) (UBX domain-containing protein 2C)	Golgi stack; lipid binding; nucleus; protein binding	NSFL1C family
**151**	ODPA_HUMAN	PDHA1 (PHE1A)	P08559	Pyruvate dehydrogenase E1 component subunit alpha, somatic form, mitochondrial (EC 1.2.4.1) (PDHE1-A type I)	glycolysis; mitochondrial matrix; oxidation reduction; protein binding; pyruvate dehydrogenase (acetyl-transferring) activity	
**152**	OSTF1_HUMAN	OSTF1	Q92882	Osteoclast-stimulating factor 1	cytoplasm; identical protein binding; ossification; signal transduction	
**153**	PA24A_HUMAN	PLA2G4A (CPLA2) (PLA2G4)	P47712	Cytosolic phospholipase A2 (cPLA2) (Phospholipase A2 group IVA) [Includes: Phospholipase A2 (EC 3.1.1.4) (Phosphatidylcholine 2-acylhydrolase); Lysophospholipase (EC 3.1.1.5)]	calcium ion binding; cytoplasmic vesicle; cytosol; icosanoid metabolic process; integral to membrane; lysophospholipase activity; phospholipase A2 activity; phospholipid catabolic process; platelet activating factor biosynthetic process	
**154**	PAK2_HUMAN	PAK2	Q13177	Serine/threonine-protein kinase PAK 2 (EC 2.7.11.1) (p21-activated kinase 2) (PAK-2) (Gamma-PAK) (PAK65) (S6/H4 kinase) (p58) [Cleaved into: PAK-2p27 (p27); PAK-2p34 (p34) (C-t-PAK2)]	ATP binding; apoptosis; cytosol; identical protein binding; interspecies interaction between organisms; membrane; negative regulation of protein kinase activity; nucleus; perinuclear region of cytoplasm; protein amino acid autophosphorylation; protein serine/threonine kinase activity; regulation of growth; signal transduction	Protein kinase superfamily, STE Ser/Thr protein kinase family, STE20 subfamily
**155**	PARD3_HUMAN	PARD3 (PAR3) (PAR3A)	Q8TEW0	Partitioning defective 3 homolog (PARD-3) (PAR-3) (Atypical PKC isotype-specific-interacting protein) (ASIP) (CTCL tumor antigen se2-5) (PAR3-alpha)	activation of protein kinase C activity by G-protein coupled receptor protein signaling pathway; asymmetric cell division; axonogenesis; cell cortex; cell cycle; endomembrane system; establishment or maintenance of cell polarity; protein binding; protein complex assembly; tight junction	PAR3 family
**156**	PARVB_HUMAN	PARVB (CGI-56)	Q9HBI1	Beta-parvin (Affixin)	actin binding; cell adhesion; cytoplasm; cytoskeleton; focal adhesion	Parvin family
**157**	PDLI1_HUMAN	PDLIM1 (CLIM1) (CLP36)	O00151	PDZ and LIM domain protein 1 (Elfin) (LIM domain protein CLP-36) (C-terminal LIM domain protein 1)	cytoplasm; cytoskeleton; response to oxidative stress; zinc ion binding	
**158**	PDLI5_HUMAN	PDLIM5 (ENH) (L9)	Q96HC4	PDZ and LIM domain protein 5 (Enigma homolog) (Enigma-like PDZ and LIM domains protein)	actin binding; actin cytoskeleton; actinin binding; cytosol; membrane fraction; protein kinase C binding; zinc ion binding	
**159**	PEAR1_HUMAN	PEAR1 (MEGF12)	Q5VY43	Platelet endothelial aggregation receptor 1 (hPEAR1) (Multiple epidermal growth factor-like domains 12) (Multiple EGF-like-domains 12)	integral to membrane; plasma membrane	MEGF family
**160**	PGK1_HUMAN	PGK1 (PGKA) (MIG10) (OK/SW-cl.110)	P00558	Phosphoglycerate kinase 1 (EC 2.7.2.3) (Primer recognition protein 2) (PRP 2) (Cell migration-inducing gene 10 protein)	ATP binding; cytoplasm; glycolysis; phosphoglycerate kinase activity; phosphorylation	Phosphoglycerate kinase family
**161**	PGM2L_HUMAN	PGM2L1 (BM32A)	Q6PCE3	Glucose 1,6-bisphosphate synthase (EC 2.7.1.106) (Phosphoglucomutase-2-like 1) (PMMLP)	glucose metabolic process; glucose-1,6-bisphosphate synthase activity; intramolecular transferase activity, phosphotransferases	Phosphohexose mutase family
**162**	PGRC1_HUMAN	PGRMC1 (HPR6.6) (PGRMC)	O00264	Membrane-associated progesterone receptor component 1 (mPR)	cell surface; endoplasmic reticulum membrane; heme binding; integral to plasma membrane; microsome; nucleolus; protein binding; receptor activity; steroid binding	Cytochrome b5 family, MAPR subfamily

	Entry name	Gene names	Uniprot Accession	Protein names	Gene Ontology	Protein family
**163**	PGRC2_HUMAN	PGRMC2 (DG6) (PMBP)	O15173	Membrane-associated progesterone receptor component 2 (Progesterone membrane-binding protein) (Steroid receptor protein DG6)	heme binding; integral to membrane; steroid binding; steroid hormone receptor activity	Cytochrome b5 family, MAPR subfamily
**164**	PIWL3_HUMAN	PIWIL3	Q7Z3Z3	Piwi-like protein 3	RNA binding; cell differentiation; cytoplasm; gene silencing by RNA; meiosis; multicellular organismal development; regulation of translation; spermatogenesis	Argonaute family, Piwi subfamily
**165**	PKHO2_HUMAN	PLEKHO2 (PLEKHQ1) (PP9099)	Q8TD55	Pleckstrin homology domain-containing family O member 2 (Pleckstrin homology domain-containing family Q member 1)		
**166**	PLEC1_HUMAN	PLEC1	Q15149	Plectin-1 (PLTN) (PCN) (Hemidesmosomal protein 1) (HD1)	actin binding; cytoplasm; cytoskeleton; plasma membrane; structural constituent of muscle	Plakin or cytolinker family
**167**	PLEK_HUMAN	PLEK (P47)	P08567	Pleckstrin (Platelet p47 protein)	actin cytoskeleton reorganization; cortical actin cytoskeleton organization; cytoplasm; hemopoietic progenitor cell differentiation; inhibition of phospholipase C activity involved in G-protein coupled receptor signaling pathway; integrin-mediated signaling pathway; membrane fraction; negative regulation of calcium-mediated signaling; negative regulation of inositol phosphate biosynthetic process; phosphate binding; phosphatidylinositol-3,4-bisphosphate binding; phosphoinositide metabolic process; platelet aggregation; positive regulation of actin filament bundle formation; positive regulation of actin filament depolymerization; positive regulation of inositol-polyphosphate 5-phosphatase activity; positive regulation of integrin activation; positive regulation of platelet activation; protein homodimerization activity; protein kinase C binding; protein kinase C signaling cascade; protein secretion by	
**168**	PP12C_HUMAN	PPP1R12C (LENG3) (MBS85)	Q9BZL4	Protein phosphatase 1 regulatory subunit 12C (Protein phosphatase 1 myosin-binding subunit of 85 kDa) (Protein phosphatase 1 myosin-binding subunit p85)	cytoplasm	
**169**	PP14A_HUMAN	PPP1R14A (CPI17) (PPP1INL)	Q96A00	Protein phosphatase 1 regulatory subunit 14A (17 kDa PKC-potentiated inhibitory protein of PP1) (CPI-17)	cytoplasm; phosphoprotein phosphatase inhibitor activity; protein binding; regulation of phosphorylation	PP1 inhibitor family
**170**	PPR3E_HUMAN	PPP1R3E	Q9H7J1	Protein phosphatase 1 regulatory subunit 3E	glycogen metabolic process	
**171**	PSA3_HUMAN	PSMA3 (PSC8)	P25788	Proteasome subunit alpha type-3 (EC 3.4.25.1) (Proteasome component C8) (Macropain subunit C8) (Multicatalytic endopeptidase complex subunit C8)	anaphase-promoting complex-dependent proteasomal ubiquitin-dependent protein catabolic process; cytoskeleton; cytosol; negative regulation of ubiquitin-protein ligase activity during mitotic cell cycle; nucleus; positive regulation of ubiquitin-protein ligase activity during mitotic cell cycle; proteasome core complex; protein binding; threonine-type endopeptidase activity	Peptidase T1A family
**172**	PTN12_HUMAN	PTPN12	Q05209	Tyrosine-protein phosphatase non-receptor type 12 (EC 3.1.3.48) (Protein-tyrosine phosphatase G1) (PTPG1) (PTP-PEST)	SH3 domain binding; non-membrane spanning protein tyrosine phosphatase activity; protein amino acid dephosphorylation; soluble fraction	Protein-tyrosine phosphatase family, Non-receptor class 4 subfamily
**173**	PTN6_HUMAN	PTPN6 (HCP) (PTP1C)	P29350	Tyrosine-protein phosphatase non-receptor type 6 (EC 3.1.3.48) (Protein-tyrosine phosphatase 1C) (PTP-1C) (Hematopoietic cell protein-tyrosine phosphatase) (SH-PTP1) (Protein-tyrosine phosphatase SHP-1)	G-protein coupled receptor protein signaling pathway; apoptosis; cytosol; membrane; nucleus; protein amino acid dephosphorylation; protein binding; protein tyrosine phosphatase activity	Protein-tyrosine phosphatase family, Non-receptor class 2 subfamily
**174**	PTPRA_HUMAN	PTPRA (PTPA) (PTPRL2)	P18433	Receptor-type tyrosine-protein phosphatase alpha (Protein-tyrosine phosphatase alpha) (R-PTP-alpha) (EC 3.1.3.48)	integral to plasma membrane; protein amino acid dephosphorylation; protein amino acid phosphorylation; transmembrane receptor protein tyrosine phosphatase activity	Protein-tyrosine phosphatase family, Receptor class 4 subfamily

	Entry name	Gene names	Uniprot Accession	Protein names	Gene Ontology	Protein family
**175**	PURA_HUMAN	PURA (PUR1)	Q00577	Transcriptional activator protein Pur-alpha (Purine-rich single-stranded DNA-binding protein alpha)	DNA replication factor A complex; DNA replication initiation; DNA unwinding during replication; RNA polymerase II transcription factor activity, enhancer binding; double-stranded telomeric DNA binding; nuclear chromosome, telomeric region; single-stranded DNA binding; transcription; transcription factor binding	PUR DNA-binding protein family
**176**	PURB_HUMAN	PURB	Q96QR8	Transcriptional activator protein Pur-beta (Purine-rich element-binding protein B)	DNA replication factor A complex; mRNA binding; regulation of myeloid cell differentiation; single-stranded DNA binding; transcription; transcription factor binding	PUR DNA-binding protein family
**177**	PXDC2_HUMAN	PLXDC2 (TEM7R) (UNQ2514/PRO6003)	Q6UX71	Plexin domain-containing protein 2 (Tumor endothelial marker 7-related protein)	integral to membrane	Plexin family
**178**	RAB7A_HUMAN	RAB7A (RAB7)	P51149	Ras-related protein Rab-7a	GTP binding; GTPase activity; Golgi apparatus; endocytosis; late endosome; lysosome; melanosome; phagocytic vesicle; protein binding; protein transport; small GTPase mediated signal transduction	Small GTPase superfamily, Rab family
**179**	RGC32_HUMAN	RGC32 (C13orf15)	Q9H4X1	Response gene to complement 32 protein (RGC-32)	cell cycle; centrosome; nucleus; regulation of cyclin-dependent protein kinase activity	
**180**	RGS18_HUMAN	RGS18 (RGS13)	Q9NS28	Regulator of G-protein signaling 18 (RGS18)	cytoplasm; negative regulation of signal transduction; signal transducer activity	
**181**	RHDF2_HUMAN	RHBDF2 (RHBDL5) (RHBDL6)	Q6PJF5	Rhomboid family member 2 (Rhomboid 5 homolog 2) (Rhomboid veinlet-like protein 5) (Rhomboid veinlet-like protein 6)	endoplasmic reticulum membrane; integral to membrane	Peptidase S54 family
**182**	RHG01_HUMAN	ARHGAP1 (CDC42GAP) (RHOGAP1)	Q07960	Rho GTPase-activating protein 1 (Rho-type GTPase-activating protein 1) (Rho-related small GTPase protein activator) (GTPase-activating protein rhoOGAP) (p50-RhoGAP) (CDC42 GTPase-activating protein)	Rho protein signal transduction; SH3 domain binding; SH3/SH2 adaptor activity; cytoplasm; intracellular membrane-bounded organelle	
**183**	RHG06_HUMAN	ARHGAP6 (RHOGAP6)	O43182	Rho GTPase-activating protein 6 (Rho-type GTPase-activating protein 6) (Rho-type GTPase-activating protein RhoGAPX-1)	Rho GTPase activator activity; Rho protein signal transduction; SH3 domain binding; SH3/SH2 adaptor activity; actin filament; actin filament polymerization; cytoplasm; negative regulation of focal adhesion formation; negative regulation of stress fiber formation; regulation of GTPase activity	
**184**	RHG17_HUMAN	ARHGAP17 (RICH1) (MSTP066) (MSTP110)	Q68EM7	Rho GTPase-activating protein 17 (Rho-type GTPase-activating protein 17) (RhoGAP interacting with CIP4 homologs protein 1) (RICH-1)	GTPase activator activity; SH3 domain binding; cytoplasm; signal transduction; tight junction	
**185**	RHG18_HUMAN	ARHGAP18	Q8N392	Rho GTPase-activating protein 18 (Rho-type GTPase-activating protein 18) (MacGAP)	GTPase activator activity; intracellular; protein binding; signal transduction	
**186**	RICS_HUMAN	RICS (GRIT) (KIAA0712)	A7KAX9	Rho/Cdc42/Rac GTPase-activating protein RICS (p200RhoGAP) (p250GAP) (RhoGAP involved in the beta-catenin-N-cadherin and NMDA receptor signaling) (Brain-specific Rho GTPase-activating protein) (GAB-associated Cdc42/Rac GTPase-activating protein) (GC-GAP) (GTPase regulator interacting with TrkA)	GTPase activator activity; Golgi membrane; cell cortex; cell junction; dendritic spine; endoplasmic reticulum membrane; endosome membrane; phosphoinositide binding; postsynaptic density; postsynaptic membrane; protein binding; signal transduction	PX domain-containing GAP family
**187**	RLA1_HUMAN	RPLP1 (RRP1)	P05386	60S acidic ribosomal protein P1	RNA binding; cytosolic large ribosomal subunit; protein binding; structural constituent of ribosome; translational elongation	Ribosomal protein L12P family
**188**	RPGP2_HUMAN	GARNL4 (KIAA1039) (RAP1GA2)	Q684P5	Rap1 GTPase-activating protein 2 (Rap1GAP2) (GTPase-activating Rap/Ran-GAP domain-like protein 4)	GTPase activator activity; perinuclear region of cytoplasm; regulation of small GTPase mediated signal transduction	
**189**	RTN1_HUMAN	RTN1 (NSP)	Q16799	Reticulon-1 (Neuroendocrine-specific protein)	integral to endoplasmic reticulum membrane; neuron differentiation; signal transducer activity; signal transduction	
**190**	RTN4_HUMAN	RTN4 (KIAA0886) (NOGO) (My043) (SP1507)	Q9NQC3	Reticulon-4 (Neurite outgrowth inhibitor) (Nogo protein) (Foocen) (Neuroendocrine-specific protein) (NSP) (Neuroendocrine-specific protein C homolog) (RTN-x) (Reticulon-5)	apoptosis; integral to endoplasmic reticulum membrane; negative regulation of anti-apoptosis; negative regulation of axon extension; nuclear envelope; protein binding; regulation of apoptosis; regulation of cell migration	
**191**	S39A9_HUMAN	SLC39A9 (ZIP9) (UNQ714/PRO1377)	Q9NUM3	Zinc transporter ZIP9 (Zrt- and Irt-like protein 9) (ZIP-9) (Solute carrier family 39 member 9)	Golgi apparatus; integral to membrane; metal ion transmembrane transporter activity; nucleolus; zinc ion binding; zinc ion transport	ZIP transporter (TC 2.A.5) family
**192**	SAM14_HUMAN	SAMD14	Q8IZD0	Sterile alpha motif domain-containing protein 14		

	Entry name	Gene names	Uniprot Accession	Protein names	Gene Ontology	Protein family
**193**	SCAM2_HUMAN	SCAMP2	O15127	Secretory carrier-associated membrane protein 2 (Secretory carrier membrane protein 2)	integral to membrane; post-Golgi vesicle-mediated transport; protein binding; protein transport; recycling endosome membrane; trans-Golgi network membrane	SCAMP family
**194**	SDPR_HUMAN	SDPR	O95810	Serum deprivation-response protein (Phosphatidylserine-binding protein) (PS-p68)	caveola; cytosol; phosphatidylserine binding; protein binding	PTRF/SDPR family
**195**	SEPT2_HUMAN	SEPT2 (DIFF6) (KIAA0158) (NEDD5)	Q15019	Septin-2 (Neural precursor cell expressed developmentally down-regulated protein 5) (NEDD-5)	GTP binding; cell division; mitosis; nucleus; protein binding; septin complex; spindle	Septin family
**196**	SEPT5_HUMAN	SEPT5 (PNUTL1)	Q99719	Septin-5 (Peanut-like protein 1) (Cell division control-related protein 1) (CDCrel-1)	GTP binding; GTPase activity; cell cycle; cytokinesis; plasma membrane; septin complex; structural molecule activity; synaptic vesicle; synaptic vesicle targeting	Septin family
**197**	SEPT6_HUMAN	SEPT6 (KIAA0128) (SEP2)	Q14141	Septin-6	GTP binding; cell cycle; cytokinesis; nucleus; protein binding; septin complex	Septin family
**198**	SEPT7_HUMAN	SEPT7 (CDC10)	Q16181	Septin-7 (CDC10 protein homolog)	GTP binding; condensed chromosome kinetochore; cytokinesis; mitosis; nucleus; protein binding; protein heterooligomerization; septin complex; spindle; stress fiber; structural molecule activity	Septin family
**199**	SFR19_HUMAN	SCAF1 (SFRS19) (SRA1)	Q9H7N4	Splicing factor, arginine/serine-rich 19 (Serine arginine-rich pre-mRNA splicing factor SR-A1) (SR-A1) (SR-related-CTD-associated factor) (SCAF)	RNA binding; RNA splicing; mRNA processing; nucleus	Splicing factor SR family
**200**	SKA2_HUMAN	FAM33A (SKA2)	Q8WVK7	Spindle and kinetochore-associated protein 2 (Protein FAM33A)	cell division; chromosome segregation; microtubule binding; mitotic anaphase; outer kinetochore of condensed chromosome; regulation of microtubule polymerization or depolymerization; spindle microtubule	SKA2 family
**201**	SKAP2_HUMAN	SKAP2 (PRAP) (RA70) (SAPS) (SCAP2) (SKAP55R)	O75563	Src kinase-associated phosphoprotein 2 (Src family-associated phosphoprotein 2) (Src kinase-associated phosphoprotein 55-related protein) (SKAP55 homolog) (SKAP-55HOM) (SKAP-HOM) (Src-associated adapter protein with PH and SH3 domains) (Pyk2/RAFTK-associated protein) (Retinoic acid-induced protein 70)	B cell activation; SH3/SH2 adaptor activity; cytoplasm; plasma membrane; protein complex assembly; signal transduction	SKAP family
**202**	SKIV2_HUMAN	SKIV2L (DDX13) (SKI2W) (SKIV2) (W)	Q15477	Helicase SKI2W (EC 3.6.1.-) (Helicase-like protein) (HLP)	ATP binding; ATP-dependent RNA helicase activity; RNA binding; nucleus; protein binding	Helicase family, SKI2 subfamily
**203**	SL9A1_HUMAN	SLC9A1 (APNH1) (NHE1)	P19634	Sodium/hydrogen exchanger 1 (Na(+)/H(+) exchanger 1) (NHE-1) (Solute carrier family 9 member 1) (Na(+)/H(+) antiporter, amiloride-sensitive) (APNH)	integral to membrane; regulation of pH; sodium ion binding; sodium ion transport	Monovalent cation:proton antiporter 1 (CPA1) transporter (TC 2.A.36) family
**204**	SL9A9_HUMAN	SLC9A9 (NHE9) (Nbla00118)	Q8IVB4	Sodium/hydrogen exchanger 9 (Na(+)/H(+) exchanger 9) (NHE-9) (Solute carrier family 9 member 9)	integral to membrane; late endosome membrane; recycling endosome; regulation of pH; sodium ion binding; sodium ion transport; sodium:hydrogen antiporter activity	Monovalent cation:proton antiporter 1 (CPA1) transporter (TC 2.A.36) family
**205**	SLAI2_HUMAN	SLAIN2 (KIAA1458)	Q9P270	SLAIN motif-containing protein 2		SLAIN motif-containing family
**206**	SLK_HUMAN	SLK (KIAA0204) (STK2)	Q9H2G2	STE20-like serine/threonine-protein kinase (STE20-like kinase) (hSLK) (EC 2.7.11.1) (STE20-related serine/threonine-protein kinase) (STE20-related kinase) (Serine/threonine-protein kinase 2) (CTCL tumor antigen se20-9)	ATP binding; DNA binding; apoptosis; cytoplasm; nuclease activity; nucleotide-excision repair; protein amino acid phosphorylation; protein serine/threonine kinase activity	Protein kinase superfamily, STE Ser/Thr protein kinase family, STE20 subfamily
**207**	SNTB1_HUMAN	SNTB1 (SNT2B1)	Q13884	Beta-1-syntrophin (59 kDa dystrophin-associated protein A1 basic component 1) (DAPA1B) (Tax interaction protein 43) (TIP-43) (Syntrophin-2) (BSYN2)	actin binding; calcium ion binding; calmodulin binding; cell junction; cytoplasm; cytoskeleton; dystrophin-associated glycoprotein complex; muscle contraction; sarcolemma	Syntrophin family
**208**	SPAG1_HUMAN	SPAG1	Q07617	Sperm-associated antigen 1 (Infertility-related sperm protein Spag-1) (HSD-3.8)	GTP binding; cytoplasm; hydrolase activity; single fertilization	
**209**	SPTB2_HUMAN	SPTBN1 (SPTB2)	Q01082	Spectrin beta chain, brain 1 (Spectrin, non-erythroid beta chain 1) (Beta-II spectrin) (Fodrin beta chain)	M band; actin binding; barbed-end actin filament capping; calmodulin binding; nucleolus; plasma membrane; spectrin; structural constituent of cytoskeleton	Spectrin family
**210**	SREC_HUMAN	SCARF1 (KIAA0149) (SREC)	Q14162	Endothelial cells scavenger receptor (Acetyl LDL receptor) (Scavenger receptor class F member 1)	cell adhesion; cholesterol catabolic process; integral to membrane; low-density lipoprotein binding; receptor-mediated endocytosis; scavenger receptor activity	

	Entry name	Gene names	Uniprot Accession	Protein names	Gene Ontology	Protein family
**211**	STIM1_HUMAN	STIM1 (GOK)	Q13586	Stromal interaction molecule 1	activation of store-operated calcium channel activity; calcium ion binding; calcium ion transport; detection of calcium ion; integral to endoplasmic reticulum membrane; integral to plasma membrane; protein binding	
**212**	SYTL3_HUMAN	SYTL3 (SLP3)	Q4VX76	Synaptotagmin-like protein 3 (Exophilin-6)	Rab GTPase binding; endomembrane system; intracellular protein transport; membrane; zinc ion binding	
**213**	SYTL4_HUMAN	SYTL4	Q96C24	Synaptotagmin-like protein 4 (Exophilin-2) (Granuphilin)	Rab GTPase binding; exocytosis; extrinsic to membrane; intracellular protein transport; neurexin binding; phospholipid binding; plasma membrane; secretory granule membrane; synaptic vesicle; transporter activity; zinc ion binding	
**214**	T22D4_HUMAN	TSC22D4 (TILZ2)	Q9Y3Q8	TSC22 domain family protein 4 (TSC22-related inducible leucine zipper protein 2) (Tsc-22-like protein THG-1)	cytoplasm; nucleus; protein binding; regulation of transcription, DNA-dependent; transcription; transcription factor activity; transcription repressor activity	TSC-22/Dip/Bun family
**215**	TA2R_HUMAN	TBXA2R	P21731	Thromboxane A2 receptor (TXA2-R) (Prostanoid TP receptor)	G-protein coupled receptor protein signaling pathway; integral to plasma membrane; thromboxane A2 receptor activity	G-protein coupled receptor 1 family
**216**	TAC2N_HUMAN	TC2N (C14orf47) (MTAC2D1)	Q8N9U0	Tandem C2 domains nuclear protein (Membrane targeting tandem C2 domain-containing protein 1) (Tandem C2 protein in nucleus) (Tac2-N)	nucleus	
**217**	TAGL2_HUMAN	TAGLN2 (KIAA0120) (CDABP0035)	P37802	Transgelin-2 (SM22-alpha homolog)	cytoskeleton; muscle organ development; nuclear membrane; plasma membrane; protein binding	Calponin family
**218**	TBA3C_HUMAN	TUBA3C (TUBA2); TUBA3D	Q13748	Tubulin alpha-3C/D chain (Alpha-tubulin 3C/D) (Tubulin alpha-2 chain) (Alpha-tubulin 2)	GTP binding; GTPase activity; microtubule; microtubule-based movement; protein binding; protein complex; protein polymerization; structural molecule activity	Tubulin family
**219**	TBA4A_HUMAN	TUBA4A (TUBA1)	P68366	Tubulin alpha-4A chain (Tubulin alpha-1 chain) (Alpha-tubulin 1) (Testis-specific alpha-tubulin) (Tubulin H2-alpha)	GTP binding; GTPase activity; microtubule; microtubule-based movement; protein binding; protein complex; protein polymerization; structural molecule activity	Tubulin family
**220**	TBB1_HUMAN	TUBB1	Q9H4B7	Tubulin beta-1 chain	GTP binding; GTPase activity; microtubule; microtubule-based movement; protein complex; protein polymerization	Tubulin family
**221**	TBB3_HUMAN	TUBB3 (TUBB4)	Q13509	Tubulin beta-3 chain (Tubulin beta-III) (Tubulin beta-4)	GTP binding; GTPase activity; microtubule; microtubule-based movement; protein complex; protein polymerization; structural molecule activity	Tubulin family
**222**	TBB4_HUMAN	TUBB4 (TUBB5)	P04350	Tubulin beta-4 chain (Tubulin 5 beta)	GTP binding; GTPase activity; microtubule; microtubule-based movement; protein complex; protein polymerization; structural molecule activity	Tubulin family
**223**	TBB5_HUMAN	TUBB (TUBB5) (OK/SW-cl.56)	P07437	Tubulin beta chain (Tubulin beta-5 chain)	GTP binding; GTPase activity; MHC class I protein binding; cell motion; microtubule; microtubule-based movement; natural killer cell mediated cytotoxicity; protein polymerization	Tubulin family
**224**	TBB6_HUMAN	TUBB6	Q9BUF5	Tubulin beta-6 chain	GTP binding; GTPase activity; microtubule; microtubule-based movement; protein complex; protein polymerization; structural molecule activity	Tubulin family
**225**	TBB8_HUMAN	TUBB8	Q3ZCM7	Tubulin beta-8 chain	GTP binding; GTPase activity; microtubule; microtubule-based movement; protein complex; protein polymerization; structural molecule activity	Tubulin family
**226**	TEBP_HUMAN	PTGES3 (P23) (TEBP)	Q15185	Prostaglandin E synthase 3 (EC 5.3.99.3) (Cytosolic prostaglandin E2 synthase) (cPGES) (Telomerase-binding protein p23) (Hsp90 co-chaperone) (Progesterone receptor complex p23)	chromosome, telomeric region; cytoplasm; prostaglandin biosynthetic process; prostaglandin-E synthase activity; signal transduction; telomerase activity; telomerase holoenzyme complex; telomere maintenance; unfolded protein binding	P23/wos2 family
**227**	TENS1_HUMAN	TNS1 (TNS)	Q9HBL0	Tensin-1	actin binding; cytoplasm; cytoskeleton; focal adhesion	

	Entry name	Gene names	Uniprot Accession	Protein names	Gene Ontology	Protein family
**228**	TGFI1_HUMAN	TGFB1I1 (ARA55)	O43294	Transforming growth factor beta-1-induced transcript 1 protein (Hydrogen peroxide-inducible clone 5 protein) (Hic-5) (Androgen receptor-associated protein of 55 kDa)	I-SMAD binding; Wnt receptor signaling pathway; androgen receptor binding; androgen receptor signaling pathway; cell adhesion; cell differentiation; cytoplasm; cytoskeleton; focal adhesion; negative regulation of cell proliferation; negative regulation of transforming growth factor beta receptor signaling pathway; nuclear matrix; positive regulation of epithelial to mesenchymal transition; positive regulation of transcription, DNA-dependent; positive regulation of transforming growth factor beta receptor signaling pathway; transcription coactivator activity; transcription from RNA polymerase II promoter; ubiquitin-dependent SMAD protein catabolic process; zinc ion binding	Paxillin family
**229**	TJAP1_HUMAN	TJAP1 (PILT) (TJP4)	Q5JTD0	Tight junction-associated protein 1 (Tight junction protein 4) (Protein incorporated later into tight junctions)	Golgi apparatus; protein binding; tight junction	
**230**	TLN1_HUMAN	TLN1 (KIAA1027) (TLN)	Q9Y490	Talin-1	LIM domain binding; actin binding; cell motion; cell-cell junction; cell-cell junction assembly; cytoplasm; cytoskeletal anchoring at plasma membrane; cytoskeleton; focal adhesion; intracellular membrane-bounded organelle; ruffle membrane; structural constituent of cytoskeleton; vinculin binding	
**231**	TMBI1_HUMAN	TMBIM1 (RECS1) (PP1201) (PSEC0158)	Q969X1	Transmembrane BAX inhibitor motif-containing protein 1 (Protein RECS1 homolog)	integral to membrane	BI1 family
**232**	TMM40_HUMAN	TMEM40	Q8WWA1	Transmembrane protein 40	integral to membrane	
**233**	TMOD3_HUMAN	TMOD3	Q9NYL9	Tropomodulin-3 (Ubiquitous tropomodulin) (U-Tmod)	actin binding; cytoplasm; cytoskeleton; tropomyosin binding	Tropomodulin family
**234**	TMX1_HUMAN	TMX1 (TMX) (TXNDC) (TXNDC1) (PSEC0085) (UNQ235/PRO268)	Q9H3N1	Thioredoxin-related transmembrane protein 1 (Thioredoxin domain-containing protein 1) (Transmembrane Trx-related protein)	DNA replication; ER to Golgi vesicle-mediated transport; anti-apoptosis; arsenate reductase (thioredoxin) activity; cell proliferation; cell redox homeostasis; disulfide oxidoreductase activity; electron transport chain; endoplasmic reticulum membrane; integral to membrane; leukocyte activation; membrane fraction; nucleus; positive regulation of growth; positive regulation of transcription, DNA-dependent; response to stress; signal transduction	
**235**	TNIK_HUMAN	TNIK (KIAA0551)	Q9UKE5	TRAF2 and NCK-interacting protein kinase (EC 2.7.11.1)	ATP binding; JNK cascade; protein amino acid phosphorylation; protein binding; protein serine/threonine kinase activity; small GTPase regulator activity	Protein kinase superfamily, STE Ser/Thr protein kinase family, STE20 subfamily
**236**	TPC1_HUMAN	TPCN1 (KIAA1169) (TPC1)	Q9ULQ1	Two pore calcium channel protein 1 (Voltage-dependent calcium channel protein TPC1)	calcium channel activity; calcium ion binding; calcium ion transport; integral to membrane; voltage-gated ion channel activity	Calcium channel alpha-1 subunit (TC 1.A.1.11) family
**237**	TPM1_HUMAN	TPM1 (C15orf13) (TMSA)	P09493	Tropomyosin alpha-1 chain (Tropomyosin-1) (Alpha-tropomyosin)	actin binding; bleb; cardiac muscle contraction; cell motion; cellular response to reactive oxygen species; muscle filament sliding; muscle thin filament tropomyosin; negative regulation of cell migration; positive regulation of ATPase activity; positive regulation of cell adhesion; positive regulation of heart rate by epinephrine; positive regulation of stress fiber formation; regulation of muscle contraction; ruffle membrane; ruffle organization; sarcomere organization; stress fiber; structural constituent of cytoskeleton; structural constituent of muscle; ventricular cardiac muscle morphogenesis; wound healing	Tropomyosin family
**238**	TRAD1_HUMAN	TRAFD1 (FLN29)	O14545	TRAF-type zinc finger domain-containing protein 1 (Protein FLN29)	intracellular; protein binding; zinc ion binding	

	Entry name	Gene names	Uniprot Accession	Protein names	Gene Ontology	Protein family
**239**	TRPC6_HUMAN	TRPC6 (TRP6)	Q9Y210	Short transient receptor potential channel 6 (TrpC6)	calcium ion binding; calcium ion transport; integral to membrane; plasma membrane; protein binding	Transient receptor family, STrpC subfamily
**240**	TSP1_HUMAN	THBS1 (TSP) (TSP1)	P07996	Thrombospondin-1	activation of MAPK activity; anti-apoptosis; calcium ion binding; cell adhesion; cell cycle arrest; cell migration; cellular response to heat; chronic inflammatory response; collagen V binding; engulfment of apoptotic cell; eukaryotic cell surface binding; external side of plasma membrane; extracellular matrix; fibrinogen binding; fibrinogen complex; fibroblast growth factor 2 binding; fibronectin binding; heparin binding; identical protein binding; immune response; integrin binding; laminin binding; low-density lipoprotein binding; negative regulation of angiogenesis; negative regulation of antigen processing and presentation of peptide or polysaccharide antigen via MHC class II; negative regulation of blood vessel endothelial cell migration; negative regulation of cGMP-mediated signaling; negative regulation of dendritic cell antigen processing and presentation; negative regulation of endothelial cell proliferation; negative regulation of fibrinolysis;	Thrombospondin family
**241**	TTC7B_HUMAN	TTC7B (TTC7L1)	Q86TV6	Tetratricopeptide repeat protein 7B (TPR repeat protein 7B) (Tetratricopeptide repeat protein 7-like-1)	binding	
**242**	TTL12_HUMAN	TTLL12 (KIAA0153)	Q14166	Tubulin--tyrosine ligase-like protein 12	protein modification process; tubulin-tyrosine ligase activity	
**243**	UBE2O_HUMAN	UBE2O (KIAA1734)	Q9C0C9	Ubiquitin-conjugating enzyme E2 O (EC 6.3.2.19) (Ubiquitin-protein ligase O) (Ubiquitin carrier protein O) (Ubiquitin-conjugating enzyme E2 of 230 kDa) (E2-230K)	ATP binding; modification-dependent protein catabolic process; post-translational protein modification; regulation of protein metabolic process; ubiquitin-protein ligase activity	Ubiquitin-conjugating enzyme family
**244**	UBP24_HUMAN	USP24 (KIAA1057)	Q9UPU5	Ubiquitin carboxyl-terminal hydrolase 24 (EC 3.1.2.15) (Ubiquitin thioesterase 24) (Ubiquitin-specific-processing protease 24) (Deubiquitinating enzyme 24)	cysteine-type peptidase activity; ubiquitin thiolesterase activity; ubiquitin-dependent protein catabolic process	Peptidase C19 family
**245**	UN13D_HUMAN	UNC13D	Q70J99	Protein unc-13 homolog D (Munc13-4)	cytoplasm; membrane	Unc-13 family
**246**	URP2_HUMAN	FERMT3 (KIND3) (MIG2B) (URP2)	Q86UX7	Fermitin family homolog 3 (Unc-112-related protein 2) (Kindlin-3) (MIG2-like protein)	cell adhesion; cytoplasm; plasma membrane; protein binding	Kindlin family
**247**	VASP_HUMAN	VASP	P50552	Vasodilator-stimulated phosphoprotein (VASP)	SH3 domain binding; actin binding; actin cytoskeleton; cytoplasm; filopodium membrane; focal adhesion; lamellipodium membrane	Ena/VASP family
**248**	VINC_HUMAN	VCL	P18206	Vinculin (Metavinculin)	actin binding; actin cytoskeleton; alpha-catenin binding; apical junction assembly; cell adhesion; cell motion; cell-cell junction; costamere; focal adhesion; lamellipodium assembly; negative regulation of cell migration; oxidoreductase activity; protein complex; structural molecule activity	Vinculin/alpha-catenin family
**249**	WDR1_HUMAN	WDR1	O75083	WD repeat-containing protein 1 (Actin-interacting protein 1) (AIP1) (NORI-1)	actin binding; cytoplasm; cytoskeleton; sensory perception of sound	WD repeat AIP1 family
**250**	WDR44_HUMAN	WDR44	Q5JSH3	WD repeat-containing protein 44 (Rabphilin-11)	Golgi apparatus; cytosol; endosome membrane; perinuclear region of cytoplasm	
**251**	XRN1_HUMAN	XRN1 (SEP1)	Q8IZH2	5'-3' exoribonuclease 1 (EC 3.1.11.-) (Strand-exchange protein 1 homolog)	5'-3' exonuclease activity; DNA binding; RNA binding; cell cycle; cytoplasm; negative regulation of cell cycle; nucleobase, nucleoside, nucleotide and nucleic acid metabolic process; protein binding	5'-3' exonuclease family
**252**	ZCCHL_HUMAN	ZC3HAV1L (C7orf39)	Q96H79	Zinc finger CCCH-type antiviral protein 1-like		
**253**	ZN185_HUMAN	ZNF185	O15231	Zinc finger protein 185 (LIM domain protein ZNF185) (P1-A)	cytoplasm; nucleus; plasma membrane; zinc ion binding	
**254**	ZO2_HUMAN	TJP2 (X104) (ZO2)	Q9UDY2	Tight junction protein ZO-2 (Zonula occludens protein 2) (Zona occludens protein 2) (Tight junction protein 2)	adherens junction; cytoplasm; guanylate kinase activity; nucleus; protein binding; tight junction	MAGUK family

	Entry name	Gene names	Uniprot Accession	Protein names	Gene Ontology	Protein family
**255**	ZYX_HUMAN	ZYX	Q15942	Zyxin (Zyxin-2)	cell adhesion; cell-cell adherens junction; cell-cell signaling; cytoplasm; focal adhesion; integral to plasma membrane; interspecies interaction between organisms; nucleus; protein binding; signal transduction; stress fiber; zinc ion binding	Zyxin/ajuba family
**256**	Q2NKW9_HUMAN	EIF4G2	Q2NKW9	Eukaryotic translation initiation factor 4 gamma, 2	RNA metabolic process; protein binding; translation initiation factor activity	
**257**	Q5JV32_HUMAN		Q5JV32	Deleted.		
**258**	Q6NXR8_HUMAN	RPS3A	Q6NXR8	Ribosomal protein S3A	ribosome; structural constituent of ribosome; translation	Ribosomal protein S3Ae family
**259**	Q8N1V6_HUMAN	hCG_2032358	Q8N1V6	cDNA FLJ37514 fis, clone BRCAN2000639 (HCG2032358)		
**260**	Q96H99_HUMAN	CTTN (hCG_23463)	Q96H99	Cortactin (cDNA FLJ77423, highly similar to Homo sapiens cortactin (CTTN), transcript variant 2, mRNA) (Cortactin, isoform CRA_c)		
**261**	Q9NWG2_HUMAN		Q9NWG2	cDNA FLJ10044 fis, clone HEMBA1001088, moderately similar to PINCH PROTEIN	zinc ion binding	
**262**	Q9UBY6_HUMAN	MYLK (hCG_2022707)	Q9UBY6	Kinase-related protein (Kinase-related protein isoform 2) (Myosin light chain kinase) (Myosin, light polypeptide kinase, isoform CRA_e) (cDNA, FLJ95241, Homo sapiens myosin, light polypeptide kinase (MYLK), transcriptvariant 8, mRNA)	kinase activity	
