# Supplementary material for: Proteomic and Phospho-Proteomic Profile of Human Platelets in Basal, Resting State: Insights into Integrin Signaling
Source: PLoS One. 2009 Oct 27;4(10):e7627. doi: 10.1371/journal.pone.0007627 (PMC2762604; doi:10.1371/journal.pone.0007627)
Supplement: Table S6 — A list of all non-phosphorylated proteins identified during phospho-proteomic profiling of platelets. Protein uniprot name, Gene name, Uniprot accession number, Protein name, Gene ontology classification, Predicted sub-cellular localization and Protein family are shown based on its descrition in the Uniprot database (www.uniprot.org). (0.09 MB PDF) [file pone.0007627.s011.pdf]

	Entry name	Gene names	Uniprot Accession	Protein names	Gene Ontology	Subcellular Localization	Protein family
**1**	1433E_HUMAN	YWHAE	P62258	14-3-3 protein epsilon (14-3-3E)	enzyme binding; interspecies interaction between organisms; intracellular signaling cascade; melanosome	Cytoplasm. Melanosome.	14-3-3 family
**2**	1433F_HUMAN	YWHAH (YWHA1)	Q04917	14-3-3 protein eta (Protein AS1)	cytoplasm; enzyme binding; glucocorticoid catabolic process; glucocorticoid receptor binding; glucocorticoid receptor signaling pathway; insulin-like growth factor receptor binding; intracellular protein transport; negative regulation of dendrite morphogenesis; positive regulation of transcription; protein domain specific binding; regulation of synaptic plasticity; transcription activator activity		14-3-3 family
**3**	1433G_HUMAN	YWHAG	P61981	14-3-3 protein gamma (Protein kinase C inhibitor protein 1) (KCIP-1) [Cleaved into: 14-3-3 protein gamma, N-terminally processed]	cytoplasm; insulin-like growth factor receptor binding; negative regulation of protein kinase activity; protein kinase C binding; protein kinase C inhibitor activity; regulation of neuron differentiation; regulation of signal transduction; regulation of synaptic plasticity	Cytoplasm.	14-3-3 family
**4**	1433T_HUMAN	YWHAQ	P27348	14-3-3 protein theta (14-3-3 protein tau) (14-3-3 protein T-cell) (Protein HS1)	centrosome; negative regulation of transcription, DNA-dependent; nucleus; protein N-terminus binding; protein domain specific binding	Cytoplasm.	14-3-3 family
**5**	1433Z_HUMAN	YWHAZ	P63104	14-3-3 protein zeta/delta (Protein kinase C inhibitor protein 1) (KCIP-1)	anti-apoptosis; melanosome; signal transduction; transcription factor binding	Cytoplasm. Melanosome.	14-3-3 family
**6**	1A03_HUMAN	HLA-A (HLAA)	P04439	HLA class I histocompatibility antigen, A-3 alpha chain (MHC class I antigen A*3)	MHC class I protein complex; MHC class I receptor activity; antigen processing and presentation of peptide antigen via MHC class I; immune response; integral to plasma membrane; interspecies interaction between organisms; protein binding	Membrane; Single-pass type I membrane protein.	MHC class I family
**7**	1B07_HUMAN	HLA-B (HLAB)	P01889	HLA class I histocompatibility antigen, B-7 alpha chain (MHC class I antigen B*7)	MHC class I protein complex; MHC class I receptor activity; antigen processing and presentation of peptide antigen via MHC class I; immune response; integral to plasma membrane; interspecies interaction between organisms	Membrane; Single-pass type I membrane protein.	MHC class I family
**8**	ACTB_HUMAN	ACTB	P60709	Actin, cytoplasmic 1 (Beta-actin) [Cleaved into: Actin, cytoplasmic 1, N-terminally processed]	ATP binding; NuA4 histone acetyltransferase complex; cell motion; histone methyltransferase complex; nitric-oxide synthase binding; sensory perception of sound; structural constituent of cytoskeleton	Cytoplasm ,cytoskeleton.	Actin family
**9**	ACTBM_HUMAN	ACTBL3 (FKSG30)	Q9BYX7	Beta-actin-like protein 3 (Kappa-actin)	ATP binding; cytoplasm; cytoskeleton; protein binding	Cytoplasm ,cytoskeleton.	Actin family
**10**	ACTN4_HUMAN	ACTN4	O43707	Alpha-actinin-4 (Non-muscle alpha-actinin 4) (F-actin cross-linking protein)	actin filament binding; calcium ion binding; integrin binding; nucleolus; nucleoside binding; perinuclear region of cytoplasm; positive regulation of cell motion; positive regulation of sodium:hydrogen antiporter activity; protein complex; protein homodimerization activity; pseudopodium; regulation of apoptosis	Nucleus. Cytoplasm.	Alpha-actinin family
**11**	ALBU_HUMAN	ALB (GIG20) (GIG42) (PRO0903) (PRO1708) (PRO2044) (PRO2619) (PRO2675) (UNQ696/PRO1341)	P02768	Serum albumin	DNA binding; antioxidant activity; cellular response to starvation; copper ion binding; drug binding; extracellular space; fatty acid binding; hemolysis by symbiont of host erythrocytes; maintenance of mitochondrion location; negative regulation of apoptosis; platelet alpha granule lumen; protein binding; protein complex; pyridoxal phosphate binding; toxin binding; transport	Secreted.	ALB/AFP/VDB family
**12**	ALDOC_HUMAN	ALDOC (ALDC)	P09972	Fructose-bisphosphate aldolase C (EC 4.1.2.13) (Brain-type aldolase)	cytoskeletal protein binding; cytoskeleton; fructose 1,6-bisphosphate metabolic process; fructose-bisphosphate aldolase activity; glycolysis		Class I fructose-bisphosphate aldolase family

	Entry name	Gene names	Uniprot Accession	Protein names	Gene Ontology	Subcellular Localization	Protein family
**13**	ANGT_HUMAN	AGT (SERPINA8)	P01019	Angiotensinogen (Serpin A8) [Cleaved into: Angiotensin-1 (Angiotensin I) (Ang I); Angiotensin-2 (Angiotensin II) (Ang II); Angiotensin-3 (Angiotensin III) (Ang III) (Des-Asp[1]-angiotensin II)]	G-protein signaling, coupled to cGMP nucleotide second messenger; acetyltransferase activator activity; activation of phospholipase C activity by G-protein coupled receptor protein signaling pathway coupled to IP3 second messenger; blood vessel remodeling; cell-cell signaling; cytoplasm; extracellular space; growth factor activity; hormone activity; low-density lipoprotein particle remodeling; negative regulation of nerve growth factor receptor signaling pathway; nitric oxide mediated signal transduction; oxygen and reactive oxygen species metabolic process; positive regulation of NAD(P)H oxidase activity; positive regulation of NF-kappaB transcription factor activity; positive regulation of apoptosis; positive regulation of cardiac muscle hypertrophy; positive regulation of cholesterol esterification; positive regulation of cytokine production; positive regulation of endothelial cell migration; positive regulation of epidermal growth factor receptor [illegible]	Secreted.	Serpin family
**14**	APOA1_HUMAN	APOA1	P02647	Apolipoprotein A-I (Apo-AI) (ApoA-I) [Cleaved into: Apolipoprotein A-I(1-242)]	Cdc42 protein signal transduction; G-protein coupled receptor protein signaling pathway; apolipoprotein A-I receptor binding; beta-amyloid binding; cholesterol binding; cholesterol efflux; cholesterol homeostasis; cholesterol import; cholesterol metabolic process; cholesterol transporter activity; endocytic vesicle; endoplasmic reticulum lumen; enzyme binding; high-density lipoprotein particle assembly; high-density lipoprotein particle clearance; identical protein binding; lipoprotein metabolic process; lipoprotein receptor binding; negative regulation of cytokine secretion during immune response; negative regulation of interleukin-1 beta secretion; negative regulation of very-low-density lipoprotein particle remodeling; phosphatidylcholine biosynthetic process; phosphatidylcholine-sterol O-acyltransferase activator activity; phospholipid binding; phospholipid efflux; positive regulation of cholesterol [illegible]	Secreted.	Apolipoprotein A1/A4/E family
**15**	APOC3_HUMAN	APOC3	P02656	Apolipoprotein C-III (Apo-CIII) (ApoC-III)	Cdc42 protein signal transduction; G-protein coupled receptor protein signaling pathway; cholesterol binding; cholesterol efflux; cholesterol homeostasis; chylomicron; chylomicron remnant clearance; high-density lipoprotein particle clearance; high-density lipoprotein particle remodeling; intermediate-density lipoprotein particle; lipase inhibitor activity; lipoprotein metabolic process; lipoprotein receptor binding; low-density lipoprotein particle clearance; negative regulation of cholesterol transport; negative regulation of fatty acid biosynthetic process; negative regulation of lipoprotein lipase activity; negative regulation of receptor-mediated endocytosis; negative regulation of triglyceride catabolic process; negative regulation of very-low-density lipoprotein particle clearance; negative regulation of very-low-density lipoprotein particle remodeling; phospholipid binding; phospholipid efflux; reverse cholesterol transport; spherical [illegible]	Secreted.	Apolipoprotein C3 family

	Entry name	Gene names	Uniprot Accession	Protein names	Gene Ontology	Subcellular Localization	Protein family
**16**	ARC1B_HUMAN	ARPC1B (ARC41)	O15143	Actin-related protein 2/3 complex subunit 1B (Arp2/3 complex 41 kDa subunit) (p41-ARC)	Arp2/3 protein complex; actin binding; cell motion; cytoplasm; regulation of actin filament polymerization; structural constituent of cytoskeleton	Cytoplasm ,cytoskeleton.	WD repeat ARPC1 family
**17**	ARPC2_HUMAN	ARPC2 (ARC34) (PRO2446)	O15144	Actin-related protein 2/3 complex subunit 2 (Arp2/3 complex 34 kDa subunit) (p34-ARC)	Arp2/3 protein complex; actin binding; cell motion; cell projection; cytoplasm; structural constituent of cytoskeleton	Cytoplasm ,cytoskeleton. Cell projection.	ARPC2 family
**18**	ATPB_HUMAN	ATP5B (ATPMB) (ATPSB)	P06576	ATP synthase subunit beta, mitochondrial (EC 3.6.3.14)	ATP binding; ATP synthesis coupled proton transport; MHC class I protein binding; angiogenesis; cell surface; eukaryotic cell surface binding; hydrogen ion transporting ATP synthase activity, rotational mechanism; hydrogen-exporting ATPase activity, phosphorylative mechanism; mitochondrial nucleoid; mitochondrial proton-transporting ATP synthase, catalytic core; plasma membrane; proton-transporting ATPase activity, rotational mechanism; regulation of intracellular pH	Mitochondrion. Mitochondrion inner membrane.	ATPase alpha/beta chains family
**19**	CALM_HUMAN	CALM1 (CALM) (CAM) (CAM1); CALM2 (CAM2) (CAMB); CALM3 (CALML2) (CAM3) (CAMC) (CAMIII)	P62158	Calmodulin (CaM)	G-protein coupled receptor protein signaling pathway; N-terminal myristoylation domain binding; calcium ion binding; centrosome; cytosol; negative regulation of ryanodine-sensitive calcium-release channel activity; plasma membrane; positive regulation of ryanodine-sensitive calcium-release channel activity; protein domain specific binding; regulation of cytokinesis; regulation of release of sequestered calcium ion into cytosol by sarcoplasmic reticulum; response to calcium ion; spindle microtubule; spindle pole; titin binding	Spindle.	Calmodulin family
**20**	CAPZB_HUMAN	CAPZB	P47756	F-actin-capping protein subunit beta (CapZ beta)	F-actin capping protein complex; actin binding; actin cytoskeleton organization; barbed-end actin filament capping; cell motion; cytoplasm	Cytoplasm ,cytoskeleton.	F-actin-capping protein beta subunit family
**21**	CAZA1_HUMAN	CAPZA1	P52907	F-actin-capping protein subunit alpha-1 (CapZ alpha-1)	F-actin capping protein complex; actin binding; actin cytoskeleton organization; barbed-end actin filament capping; cell motion; protein complex assembly		F-actin-capping protein alpha subunit family
**22**	CDN1B_HUMAN	CDKN1B (KIP1)	P46527	Cyclin-dependent kinase inhibitor 1B (Cyclin-dependent kinase inhibitor p27) (p27Kip1)	G1/S transition of mitotic cell cycle; autophagic cell death; cell cycle arrest; cyclin-dependent protein kinase inhibitor activity; cytosol; induction of apoptosis; negative regulation of cell growth; negative regulation of cell proliferation; negative regulation of phosphorylation; nucleus; protein binding; regulation of cyclin-dependent protein kinase activity; transforming growth factor beta receptor, cytoplasmic mediator activity	Nucleus. Cytoplasm.	CDI family

	Entry name	Gene names	Uniprot Accession	Protein names	Gene Ontology	Subcellular Localization	Protein family
**23**	CH60_HUMAN	HSPD1 (HSP60)	P10809	60 kDa heat shock protein, mitochondrial (Heat shock protein 60) (HSP-60) (Hsp60) (60 kDa chaperonin) (Chaperonin 60) (CPN60) (Mitochondrial matrix protein P1) (P60 lymphocyte protein) (HuCHA60)	'de novo' protein folding; ATP binding; ATPase activity; B cell cytokine production; B cell proliferation; DNA replication origin binding; MyD88-dependent toll-like receptor signaling pathway; T cell activation; activation of caspase activity; cell surface; cell surface binding; chaperone binding; chaperone-mediated protein complex assembly; coated pit; coated vesicle; cytosol; early endosome; extracellular space; interspecies interaction between organisms; isotype switching to IgG isotypes; lipopolysaccharide binding; lipopolysaccharide receptor complex; mitochondrial inner membrane; mitochondrial matrix; negative regulation of apoptosis; p53 binding; positive regulation of T cell activation; positive regulation of T cell mediated immune response to tumor cell; positive regulation of apoptosis; positive regulation of interferon-alpha production; positive regulation of interferon-gamma production; positive regulation of interleukin-10 production; [illegible]	Mitochondrion matrix.	Chaperonin (HSP60) family
**24**	CLH1_HUMAN	CLTC (CLH17) (CLTCL2) (KIAA0034)	Q00610	Clathrin heavy chain 1 (CLH-17)	clathrin coat of coated pit; clathrin coat of trans-Golgi network vesicle; intracellular protein transport; melanosome; protein binding; structural molecule activity; vesicle-mediated transport	Cytoplasmic vesicle membrane; Peripheral membrane protein; Cytoplasmic side. Membrane ,coated pit; Peripheral membrane protein; Cytoplasmic side. Melanosome.	Clathrin heavy chain family
**25**	CLIC1_HUMAN	CLIC1 (NCC27)	O00299	Chloride intracellular channel protein 1 (Nuclear chloride ion channel 27) (NCC27) (Chloride channel ABP) (Regulatory nuclear chloride ion channel protein) (hRNCC)	brush border; chloride ion binding; chloride transport; integral to membrane; membrane fraction; nuclear membrane; plasma membrane; signal transduction; soluble fraction; voltage-gated chloride channel activity	Nucleus. Nucleus membrane; Single-pass membrane protein. Cytoplasm. Cell membrane; Single-pass membrane protein.	Chloride channel CLIC family
**26**	CLIC4_HUMAN	CLIC4	Q9Y696	Chloride intracellular channel protein 4 (Intracellular chloride ion channel protein p64H1)	actin cytoskeleton; cell differentiation; chloride ion binding; chloride transport; cytoplasmic vesicle membrane; integral to membrane; microvillus; negative regulation of cell migration; nucleus; plasma membrane; protein binding; soluble fraction; voltage-gated chloride channel activity	Cytoplasm. Cytoplasmic vesicle membrane; Single-pass membrane protein. Nucleus. Cell membrane; Single-pass membrane protein.	Chloride channel CLIC family
**27**	CLUS_HUMAN	CLU (APOJ) (CLI) (KUB1) (AAG4)	P10909	Clusterin (Complement-associated protein SP-40,40) (Complement cytolysis inhibitor) (CLI) (NA1/NA2) (Apolipoprotein J) (Apo-J) (Testosterone-repressed prostate message 2) (TRPM-2) (Ku70-binding protein 1) (Aging-associated gene 4 protein) [Cleaved into: Clusterin beta chain (ApoJalpha) (Complement cytolysis inhibitor a chain); Clusterin alpha chain (ApoJbeta) (Complement cytolysis inhibitor b chain)]	apoptosis; complement activation, classical pathway; innate immune response; lipid metabolic process; protein binding; reverse cholesterol transport; spherical high-density lipoprotein particle	Secreted.	Clusterin family
**28**	CO3_HUMAN	C3 (CPAMD1)	P01024	Complement C3 (C3 and PZP-like alpha-2-macroglobulin domain-containing protein 1) [Cleaved into: Complement C3 beta chain; Complement C3 alpha chain; C3a anaphylatoxin; Complement C3b alpha' chain; Complement C3c alpha' chain fragment 1; Complement C3dg fragment; Complement C3g fragment; Complement C3d fragment; Complement C3f fragment; Complement C3c alpha' chain fragment 2]	G-protein coupled receptor protein signaling pathway; complement activation, alternative pathway; complement activation, classical pathway; endopeptidase inhibitor activity; extracellular space; receptor binding	Secreted.	
**29**	COF1_HUMAN	CFL1 (CFL)	P23528	Cofilin-1 (Cofilin, non-muscle isoform) (18 kDa phosphoprotein) (p18)	Rho protein signal transduction; actin binding; anti-apoptosis; nuclear matrix	Nucleus matrix. Cytoplasm ,cytoskeleton.	Actin-binding proteins ADF family
**30**	COTL1_HUMAN	COTL1 (CLP)	Q14019	Coactosin-like protein	actin binding; cytoplasm; cytoskeleton; enzyme binding	Cytoplasm ,cytoskeleton.	Actin-binding proteins ADF family, Coactosin subfamily
**31**	CPNS1_HUMAN	CAPNS1 (CAPN4) (CAPNS)	P04632	Calpain small subunit 1 (CSS1) (Calcium-dependent protease small subunit 1) (Calcium-dependent protease small subunit) (CDPS) (Calpain regulatory subunit) (Calcium-activated neutral proteinase small subunit) (CANP small subunit)	calcium ion binding; calcium-dependent cysteine-type endopeptidase activity; cytoplasm; nucleus; plasma membrane; positive regulation of cell proliferation; protein binding	Cytoplasm. Cell membrane.	

	Entry name	Gene names	Uniprot Accession	Protein names	Gene Ontology	Subcellular Localization	Protein family
**32**	DUS3_HUMAN	DUSP3 (VHR)	P51452	Dual specificity protein phosphatase 3 (EC 3.1.3.48) (EC 3.1.3.16) (Dual specificity protein phosphatase VHR)	nucleoplasm; protein amino acid dephosphorylation; protein tyrosine phosphatase activity		Protein-tyrosine phosphatase family, Non-receptor class dual specificity subfamily
**33**	EHD3_HUMAN	EHD3 (EHD2) (PAST3)	Q9NZN3	EH domain-containing protein 3	ATP binding; GTP binding; GTPase activity; calcium ion binding; nucleic acid binding; nucleus; plasma membrane; recycling endosome membrane	Cell membrane; Peripheral membrane protein; Cytoplasmic side. Endosome membrane; Peripheral membrane protein. Recycling endosome membrane; Peripheral membrane protein.	
**34**	EMIL1_HUMAN	EMILIN1 (EMI)	Q9Y6C2	EMILIN-1 (Elastin microfibril interface-located protein 1) (Elastin microfibril interfacer 1)	cell adhesion; centrosome; extracellular space; nucleus; proteinaceous extracellular matrix	Secreted ,extracellular space ,extracellular matrix.	
**35**	ENDD1_HUMAN	ENDOD1 (KIAA0830)	O94919	Endonuclease domain-containing 1 protein (EC 3.1.30.-)	endonuclease activity; extracellular region; metal ion binding; nucleic acid binding	Secreted.	DNA/RNA non-specific endonuclease family
**36**	ENOA_HUMAN	ENO1 (ENO1L1) (MBPB1) (MPB1)	P06733	Alpha-enolase (EC 4.2.1.11) (2-phospho-D-glycerate hydro-lyase) (Non-neural enolase) (NNE) (Enolase 1) (Phosphopyruvate hydratase) (C-myc promoter-binding protein) (MBP-1) (MPB-1) (Plasminogen-binding protein)	M band; glycolysis; magnesium ion binding; negative regulation of cell growth; negative regulation of transcription from RNA polymerase II promoter; nucleus; phosphopyruvate hydratase activity; phosphopyruvate hydratase complex; plasma membrane; serine-type endopeptidase activity; transcription; transcription corepressor activity; transcription factor activity	Cytoplasm. Cell membrane. Cytoplasm ,myofibril ,sarcomere ,M-band. Nucleus.	Enolase family
**37**	ENPL_HUMAN	HSP90B1 (TRA1)	P14625	Endoplasmin (Heat shock protein 90 kDa beta member 1) (94 kDa glucose-regulated protein) (GRP94) (gp96 homolog) (Tumor rejection antigen 1)	ATP binding; RNA binding; anti-apoptosis; calcium ion binding; cytosol; endoplasmic reticulum lumen; endoplasmic reticulum membrane; low-density lipoprotein receptor binding; melanosome; microsome; perinuclear region of cytoplasm; protein folding; protein transport; response to hypoxia; sequestering of calcium ion; unfolded protein binding; virion binding	Endoplasmic reticulum lumen. Melanosome.	Heat shock protein 90 family
**38**	F13A_HUMAN	F13A1 (F13A)	P00488	Coagulation factor XIII A chain (Coagulation factor XIIIa) (EC 2.3.2.13) (Protein-glutamine gamma-glutamyltransferase A chain) (Transglutaminase A chain)	acyltransferase activity; blood coagulation; calcium ion binding; cytoplasm; extracellular region; protein-glutamine gamma-glutamyltransferase activity	Cytoplasm. Secreted.	Transglutaminase superfamily, Transglutaminase family
**39**	FIBB_HUMAN	FGB	P02675	Fibrinogen beta chain [Cleaved into: Fibrinopeptide B]	eukaryotic cell surface binding; external side of plasma membrane; fibrinogen complex; nucleus; platelet activation; platelet alpha granule lumen; protein binding, bridging; protein polymerization; receptor binding; response to calcium ion; signal transduction; soluble fraction	Secreted.	
**40**	FIBG_HUMAN	FGG (PRO2061)	P02679	Fibrinogen gamma chain	calcium ion binding; eukaryotic cell surface binding; external side of plasma membrane; fibrinogen complex; platelet activation; platelet alpha granule lumen; protein binding, bridging; protein polymerization; receptor binding; response to calcium ion; signal transduction	Secreted.	
**41**	GANAB_HUMAN	GANAB (G2AN) (KIAA0088)	Q14697	Neutral alpha-glucosidase AB (EC 3.2.1.84) (Glucosidase II subunit alpha) (Alpha-glucosidase 2)	Golgi apparatus; carbohydrate metabolic process; endoplasmic reticulum; glucan 1,3-alpha-glucosidase activity; melanosome; protein binding	Endoplasmic reticulum. Golgi apparatus. Melanosome.	Glycosyl hydrolase 31 family
**42**	GBB1_HUMAN	GNB1	P62873	Guanine nucleotide-binding protein G(I)/G(S)/G(T) subunit beta-1 (Transducin beta chain 1)	GTPase activity; Ras protein signal transduction; hormone-mediated signaling; muscarinic acetylcholine receptor signaling pathway; signal transducer activity		WD repeat G protein beta family
**43**	GDIA_HUMAN	GDI1 (GDIL) (OPHN2) (RABGDIA) (XAP4)	P31150	Rab GDP dissociation inhibitor alpha (Rab GDI alpha) (Guanosine diphosphate dissociation inhibitor 1) (GDI-1) (Oligophrenin-2) (Protein XAP-4)	GTPase activator activity; Rab GDP-dissociation inhibitor activity; cytoplasm; protein binding; protein transport; regulation of GTPase activity	Cytoplasm.	Rab GDI family

	Entry name	Gene names	Uniprot Accession	Protein names	Gene Ontology	Subcellular Localization	Protein family
**44**	GDIR2_HUMAN	ARHGDIB (GDIA2) (GDID4) (RAP1GN1)	P52566	Rho GDP-dissociation inhibitor 2 (Rho GDI 2) (Rho-GDI beta) (Ly-GDI)	GTPase activator activity; Rho GDP-dissociation inhibitor activity; Rho protein signal transduction; actin cytoskeleton organization; cell motion; cytoplasmic membrane-bounded vesicle; cytoskeleton; immune response; multicellular organismal development; negative regulation of cell adhesion	Cytoplasm.	Rho GDI family
**45**	GELS_HUMAN	GSN	P06396	Gelsolin (Actin-depolymerizing factor) (ADF) (Brevin) (AGEL)	actin binding; actin cytoskeleton; actin filament polymerization; actin filament severing; barbed-end actin filament capping; calcium ion binding; cytosol; extracellular region	Cytoplasm ,cytoskeleton. Secreted.	Villin/gelsolin family
**46**	GMPR1_HUMAN	GMPR (GMPR1)	P36959	GMP reductase 1 (EC 1.7.1.7) (Guanosine 5'-monophosphate oxidoreductase 1) (Guanosine monophosphate reductase 1)	GMP reductase activity; nucleotide metabolic process; oxidation reduction; potassium ion binding; response to cold		IMPDH/GMPR family
**47**	GPSM1_HUMAN	GPSM1 (AGS3)	Q86YR5	G-protein-signaling modulator 1 (Activator of G-protein signaling 3)	GTPase activator activity; Golgi membrane; binding; cell differentiation; cytosol; endoplasmic reticulum membrane; nervous system development; plasma membrane; signal transduction	Cytoplasm ,cytosol. Endoplasmic reticulum membrane; Peripheral membrane protein; Cytoplasmic side. Golgi apparatus membrane; Peripheral membrane protein; Cytoplasmic side. Cell membrane; Peripheral membrane protein; Cytoplasmic side.	GPSM family
**48**	GRP78_HUMAN	HSPA5 (GRP78)	P11021	78 kDa glucose-regulated protein (GRP 78) (Heat shock 70 kDa protein 5) (Immunoglobulin heavy chain-binding protein) (BiP) (Endoplasmic reticulum lumenal Ca(2+)-binding protein grp78)	ATP binding; ER-Golgi intermediate compartment; anti-apoptosis; calcium ion binding; caspase inhibitor activity; cell surface; cellular response to glucose starvation; endoplasmic reticulum lumen; integral to endoplasmic reticulum membrane; melanosome; negative regulation of caspase activity; nucleus; perinuclear region of cytoplasm; protein binding, bridging; unfolded protein binding	Endoplasmic reticulum lumen. Melanosome.	Heat shock protein 70 family
**49**	GSTP1_HUMAN	GSTP1 (FAEES3) (GST3)	P09211	Glutathione S-transferase P (EC 2.5.1.18) (GST class-pi) (GSTP1-1)	anti-apoptosis; central nervous system development; cytoplasm; glutathione transferase activity; metabolic process; protein binding		GST superfamily, Pi family
**50**	HCDH_HUMAN	HADH (HAD) (HADHSC) (SCHAD)	Q16836	Hydroxyacyl-coenzyme A dehydrogenase, mitochondrial (HCDH) (EC 1.1.1.35) (Short chain 3-hydroxyacyl-CoA dehydrogenase) (Medium and short chain L-3-hydroxyacyl-coenzyme A dehydrogenase)	3-hydroxyacyl-CoA dehydrogenase activity; coenzyme binding; fatty acid metabolic process; mitochondrial matrix; oxidation reduction	Mitochondrion matrix.	3-hydroxyacyl-CoA dehydrogenase family
**51**	HPT_HUMAN	HP	P00738	Haptoglobin [Cleaved into: Haptoglobin alpha chain; Haptoglobin beta chain]	cellular iron ion homeostasis; defense response; extracellular region; hemoglobin binding; proteolysis; serine-type endopeptidase activity	Secreted.	Peptidase S1 family
**52**	HSP76_HUMAN	HSPA6 (HSP70B')	P17066	Heat shock 70 kDa protein 6 (Heat shock 70 kDa protein B')	ATP binding; response to unfolded protein		Heat shock protein 70 family
**53**	HSP7C_HUMAN	HSPA8 (HSC70) (HSP73) (HSPA10)	P11142	Heat shock cognate 71 kDa protein (Heat shock 70 kDa protein 8)	ATP binding; ATPase activity, coupled; cell surface; melanosome; protein binding; protein folding; response to unfolded protein	Cytoplasm. Melanosome.	Heat shock protein 70 family
**54**	IDHP_HUMAN	IDH2	P48735	Isocitrate dehydrogenase [NADP], mitochondrial (IDH) (EC 1.1.1.42) (Oxalosuccinate decarboxylase) (NADP(+)-specific ICDH) (IDP) (ICD-M)	NAD or NADH binding; glyoxylate cycle; isocitrate dehydrogenase (NADP+) activity; isocitrate metabolic process; magnesium ion binding; manganese ion binding; mitochondrion; oxidation reduction; tricarboxylic acid cycle	Mitochondrion.	Isocitrate and isopropylmalate dehydrogenase s family
**55**	ITA2B_HUMAN	ITGA2B (GP2B) (ITGAB)	P08514	Integrin alpha-IIb (Platelet membrane glycoprotein IIb) (GPalpha IIb) (GPIIb) (CD antigen CD41) [Cleaved into: Integrin alpha-IIb heavy chain; Integrin alpha-IIb light chain, form 1; Integrin alpha-IIb light chain, form 2]	calcium ion binding; cell adhesion; identical protein binding; integrin complex; integrin-mediated signaling pathway; platelet alpha granule membrane; receptor activity	Membrane; Single-pass type I membrane protein.	Integrin alpha chain family
**56**	ITA6_HUMAN	ITGA6	P23229	Integrin alpha-6 (VLA-6) (CD49 antigen-like family member F) (CD antigen CD49f) [Cleaved into: Integrin alpha-6 heavy chain; Integrin alpha-6 light chain]	calcium ion binding; cell adhesion; cell-substrate junction assembly; integrin complex; integrin-mediated signaling pathway; protein binding; receptor activity	Membrane; Single-pass type I membrane protein.	Integrin alpha chain family

	Entry name	Gene names	Uniprot Accession	Protein names	Gene Ontology	Subcellular Localization	Protein family
**57**	ITB1_HUMAN	ITGB1 (FNRB) (MDF2) (MSK12)	P05556	Integrin beta-1 (Fibronectin receptor subunit beta) (Integrin VLA-4 subunit beta) (CD antigen CD29)	B cell differentiation; cell surface; cell-cell adhesion mediated by integrin; cell-matrix adhesion; cellular defense response; homophilic cell adhesion; identical protein binding; integrin complex; integrin-mediated signaling pathway; interspecies interaction between organisms; leukocyte adhesion; melanosome; neuromuscular junction; protein heterodimerization activity; receptor activity; ruffle; sarcolemma	Cell membrane; Single-pass type I membrane protein. Melanosome.	Integrin beta chain family
**58**	K1C9_HUMAN	KRT9	P35527	Keratin, type I cytoskeletal 9 (Cytokeratin-9) (CK-9) (Keratin-9) (K9)	cytoplasm; intermediate filament; intermediate filament organization; protein binding; skin development; structural constituent of cytoskeleton		Intermediate filament family
**59**	K6PP_HUMAN	PFKP (PFKF)	Q01813	6-phosphofructokinase type C (EC 2.7.1.11) (Phosphofructokinase 1) (Phosphohexokinase) (Phosphofructo-1-kinase isozyme C) (PFK-C) (6-phosphofructokinase, platelet type)	6-phosphofructokinase activity; 6-phosphofructokinase complex; ATP binding; glycolysis; magnesium ion binding; protein binding		Phosphofructokinase family, Two domains subfamily
**60**	LIMS1_HUMAN	LIMS1 (PINCH) (PINCH1)	P48059	LIM and senescent cell antigen-like-containing domain protein 1 (Particularly interesting new Cys-His protein 1) (PINCH-1) (Renal carcinoma antigen NY-REN-48)	cell aging; focal adhesion; protein binding; zinc ion binding	Cell junction ,focal adhesion. Cell membrane; Peripheral membrane protein; Cytoplasmic side.	
**61**	LTBP1_HUMAN	LTBP1	Q14766	Latent-transforming growth factor beta-binding protein 1 (LTBP-1) (Transforming growth factor beta-1-binding protein 1) (TGF-beta1-BP-1)	calcium ion binding; cytoplasm; growth factor binding; nucleus; proteinaceous extracellular matrix; transforming growth factor beta receptor activity	Secreted.	LTBP family
**62**	LYAM3_HUMAN	SELP (GMRP) (GRMP)	P16109	P-selectin (Granule membrane protein 140) (GMP-140) (PADGEM) (Leukocyte-endothelial cell adhesion molecule 3) (LECAM3) (CD62 antigen-like family member P) (CD antigen CD62P)	defense response to Gram-negative bacterium; external side of plasma membrane; extracellular space; fucose binding; glycosphingolipid binding; heparin binding; integral to plasma membrane; leukocyte adhesion; lipopolysaccharide binding; membrane fraction; oligosaccharide binding; platelet alpha granule membrane; positive regulation of platelet activation; protein binding; response to lipopolysaccharide; sialic acid binding; soluble fraction	Membrane; Single-pass type I membrane protein.	Selectin/LECAM family
**63**	MARE1_HUMAN	MAPRE1	Q15691	Microtubule-associated protein RP/EB family member 1 (APC-binding protein EB1) (End-binding protein 1) (EB1)	cell division; cell proliferation; cortical microtubule cytoskeleton; microtubule plus-end binding; mitosis; negative regulation of microtubule polymerization; protein C-terminus binding	Cytoplasm.	MAPRE family
**64**	MARE2_HUMAN	MAPRE2 (RP1)	Q15555	Microtubule-associated protein RP/EB family member 2 (APC-binding protein EB2) (End-binding protein 2) (EB2)	cell division; cell proliferation; cytoplasm; microtubule; microtubule binding; mitosis; signal transduction	Cytoplasm.	MAPRE family
**65**	MLH1_HUMAN	MLH1 (COCA2)	P40692	DNA mismatch repair protein Mlh1 (MutL protein homolog 1)	ATP binding; cell cycle; mismatch repair; mismatched DNA binding; negative regulation of cell cycle; nucleus; protein binding	Nucleus.	DNA mismatch repair mutL/hexB family
**66**	MMRN1_HUMAN	MMRN1 (ECM) (EMILIN4) (GPIA*) (MMRN)	Q13201	Multimerin-1 (Endothelial cell multimerin) (EMILIN-4) (Elastin microfibril interface located protein 4) (Elastin microfibril interfacer 4) [Cleaved into: Platelet glycoprotein Ia*; 155 kDa platelet multimerin (p-155) (p155)]	blood coagulation; cell adhesion; extracellular region; platelet alpha granule lumen	Secreted.	
**67**	MPPH1_HUMAN	MPHOSPH1 (KRMP1)	Q96Q89	M-phase phosphoprotein 1 (MPP1) (Kinesin-related motor interacting with PIN1) (Cancer/testis antigen 90) (CT90)	ATP binding; ATPase activity; WW domain binding; cell cycle arrest; cell division; centrosome; microtubule; microtubule motor activity; microtubule-based movement; mitosis; nucleolus; nucleoplasm; regulation of mitosis	Cytoplasm. Nucleus.	Kinesin-like protein family
**68**	MYH10_HUMAN	MYH10	P35580	Myosin-10 (Myosin heavy chain 10) (Myosin heavy chain, non-muscle IIb) (Non-muscle myosin heavy chain IIb) (NMMHC II-b) (NMMHC-IIB) (Cellular myosin heavy chain, type B) (Non-muscle myosin heavy chain B) (NMMHC-B)	ADP binding; ATP binding; actin filament binding; actin filament-based movement; actin-dependent ATPase activity; calmodulin binding; cell cortex; cleavage furrow; cytokinesis after mitosis; microfilament motor activity; midbody; myosin complex; regulation of cell shape; stress fiber		
**69**	MYL6_HUMAN	MYL6	P60660	Myosin light polypeptide 6 (Smooth muscle and nonmuscle myosin light chain alkali 6) (Myosin light chain alkali 3) (Myosin light chain 3) (MLC-3) (LC17)	actin-dependent ATPase activity; calcium ion binding; motor activity; muscle filament sliding; skeletal muscle tissue development; structural constituent of muscle; unconventional myosin complex		

	Entry name	Gene names	Uniprot Accession	Protein names	Gene Ontology	Subcellular Localization	Protein family
**70**	PDIA1_HUMAN	P4HB (ERBA2L) (PDI) (PDIA1) (PO4DB)	P07237	Protein disulfide-isomerase (PDI) (EC 5.3.4.1) (Prolyl 4-hydroxylase subunit beta) (Cellular thyroid hormone-binding protein) (p55)	ER-Golgi intermediate compartment; cell redox homeostasis; cell surface; endoplasmic reticulum; endoplasmic reticulum lumen; extracellular region; melanosome; peptidyl-proline hydroxylation to 4-hydroxy-L-proline; plasma membrane; procollagen-proline 4-dioxygenase activity; protein binding; protein disulfide isomerase activity	Endoplasmic reticulum lumen. Melanosome. Cell membrane; Peripheral membrane protein.	Protein disulfide isomerase family
**71**	PDIA4_HUMAN	PDIA4 (ERP70) (ERP72)	P13667	Protein disulfide-isomerase A4 (EC 5.3.4.1) (Protein ERp-72) (ERp72)	cell redox homeostasis; endoplasmic reticulum; endoplasmic reticulum lumen; melanosome; protein binding; protein disulfide isomerase activity; protein secretion	Endoplasmic reticulum lumen. Melanosome.	Protein disulfide isomerase family
**72**	PECA1_HUMAN	PECAM1	P16284	Platelet endothelial cell adhesion molecule (PECAM-1) (EndoCAM) (GPIIA') (CD antigen CD31)	cell adhesion; cell recognition; extracellular space; integral to membrane; plasma membrane; platelet alpha granule membrane; protein binding; signal transduction	Membrane; Single-pass type I membrane protein.	
**73**	PLF4_HUMAN	PF4 (CXCL4) (SCYB4)	P02776	Platelet factor 4 (PF-4) (C-X-C motif chemokine 4) (Oncostatin-A) (Iroplact) [Cleaved into: Platelet factor 4, short form]	chemokine activity; cytokine-mediated signaling pathway; extracellular space; heparin binding; immune response; leukocyte chemotaxis; negative regulation of MHC class II biosynthetic process; negative regulation of angiogenesis; negative regulation of apoptosis; negative regulation of cytolysis; negative regulation of megakaryocyte differentiation; platelet activation; platelet alpha granule lumen; positive regulation of foam cell differentiation; positive regulation of gene expression; positive regulation of tumor necrosis factor production	Secreted.	Intercrine alpha (chemokine CxC) family
**74**	PNPH_HUMAN	NP (PNP)	P00491	Purine nucleoside phosphorylase (PNP) (EC 2.4.2.1) (Inosine phosphorylase)	NAD biosynthesis via nicotinamide riboside salvage pathway; cytosol; drug binding; immune response; inosine catabolic process; nicotinamide riboside catabolic process; nucleoside binding; phosphate binding; positive regulation of T cell proliferation; positive regulation of alpha-beta T cell differentiation; purine binding; purine-nucleoside phosphorylase activity; response to drug; urate biosynthetic process		PNP/MTAP phosphorylase family
**75**	PP1A_HUMAN	PPP1CA (PPP1A)	P62136	Serine/threonine-protein phosphatase PP1-alpha catalytic subunit (PP-1A) (EC 3.1.3.16)	cell cycle; cell division; cytoplasm; glycogen metabolic process; histone methyltransferase complex; iron ion binding; manganese ion binding; protein amino acid dephosphorylation; protein binding; protein serine/threonine phosphatase activity	Cytoplasm.	PPP phosphatase family, PP-1 subfamily
**76**	PPIA_HUMAN	PPIA (CYPA)	P62937	Peptidyl-prolyl cis-trans isomerase A (PPIase A) (Rotamase A) (EC 5.2.1.8) (Cyclophilin A) (Cyclosporin A-binding protein)	cytosol; extracellular region; initiation of viral infection; interspecies interaction between organisms; nucleus; peptide binding; peptidyl-prolyl cis-trans isomerase activity; protein folding; provirus integration; regulation of viral genome replication; unfolded protein binding; virion binding	Cytoplasm.	Cyclophilin-type PPIase family, PPIase A subfamily
**77**	PPIB_HUMAN	PPIB (CYPB)	P23284	Peptidyl-prolyl cis-trans isomerase B (PPIase) (Rotamase) (EC 5.2.1.8) (Cyclophilin B) (S-cyclophilin) (SCYLP) (CYP-S1)	endoplasmic reticulum; endoplasmic reticulum lumen; melanosome; peptide binding; peptidyl-prolyl cis-trans isomerase activity; protein folding; unfolded protein binding	Endoplasmic reticulum lumen. Melanosome.	Cyclophilin-type PPIase family, PPIase B subfamily
**78**	PRAF3_HUMAN	ARL6IP5 (DERP11) (JWA) (PRA2) (PRAF3) (HSPC127)	O75915	PRA1 family protein 3 (ADP-ribosylation factor-like protein 6-interacting protein 5) (ARL-6-interacting protein 5) (Aip-5) (Glutamate transporter EAAC1-interacting protein) (GTRAP3-18) (Prenylated Rab acceptor protein 2) (Protein JWa) (Dermal papilla-derived protein 11) (JM5) (Putative MAPK-activating protein PM27) (Cytoskeleton-related vitamin A-responsive protein)	L-glutamate transport; endoplasmic reticulum membrane; integral to membrane; protein binding	Endoplasmic reticulum membrane; Multi-pass membrane protein. Cytoplasm.	PRA1 family

	Entry name	Gene names	Uniprot Accession	Protein names	Gene Ontology	Subcellular Localization	Protein family
**79**	PRDX6_HUMAN	PRDX6 (AOP2) (KIAA0106)	P30041	Peroxiredoxin-6 (EC 1.11.1.15) (Antioxidant protein 2) (1-Cys peroxiredoxin) (1-Cys PRX) (Acidic calcium-independent phospholipase A2) (aiPLA2) (EC 3.1.1.-) (Non-selenium glutathione peroxidase) (NSGPx) (EC 1.11.1.7) (24 kDa protein) (Liver 2D page spot 40) (Red blood cells page spot 12)	cell redox homeostasis; cytoplasmic vesicle; cytosol; lysosome; nucleus; oxidation reduction; peroxiredoxin activity; phospholipase A2 activity; phospholipid catabolic process; response to oxidative stress	Cytoplasm. Lysosome. Cytoplasmic vesicle.	AhpC/TSA family, Rehydrin subfamily
**80**	PROF1_HUMAN	PFN1	P07737	Profilin-1 (Profilin I)	actin binding; actin cytoskeleton; actin cytoskeleton organization; cytoplasm	Cytoplasm ,cytoskeleton.	Profilin family
**81**	PTN3_HUMAN	PTPN3 (PTPH1)	P26045	Tyrosine-protein phosphatase non-receptor type 3 (EC 3.1.3.48) (Protein-tyrosine phosphatase H1) (PTP-H1)	cytoplasm; cytoskeletal protein binding; cytoskeleton; extrinsic to membrane; negative regulation of membrane protein ectodomain proteolysis; plasma membrane; protein amino acid dephosphorylation; protein tyrosine phosphatase activity	Cell membrane; Peripheral membrane protein; Cytoplasmic side. Cytoplasm ,cytoskeleton.	Protein-tyrosine phosphatase family, Non-receptor class subfamily
**82**	PTPRJ_HUMAN	PTPRJ (DEP1)	Q12913	Receptor-type tyrosine-protein phosphatase eta (Protein-tyrosine phosphatase eta) (R-PTP-eta) (EC 3.1.3.48) (HPTP eta) (Protein-tyrosine phosphatase receptor type J) (Density-enhanced phosphatase 1) (DEP-1) (CD antigen CD148)	cell junction; cell-cell signaling; integral to plasma membrane; nucleus; protein amino acid dephosphorylation; protein binding; transmembrane receptor protein tyrosine kinase signaling pathway; transmembrane receptor protein tyrosine phosphatase activity	Membrane; Single-pass type I membrane protein.	Protein-tyrosine phosphatase family, Receptor class 3 subfamily
**83**	QCR1_HUMAN	UQCRC1	P31930	Cytochrome b-c1 complex subunit 1, mitochondrial (Ubiquinol-cytochrome-c reductase complex core protein 1) (Core protein I) (Complex III subunit 1)	aerobic respiration; metalloendopeptidase activity; mitochondrial respiratory chain; protein binding; proteolysis; transport; ubiquinol-cytochrome-c reductase activity; zinc ion binding	Mitochondrion inner membrane.	Peptidase M16 family, UQCRC1/QCR1 subfamily
**84**	RAB32_HUMAN	RAB32	Q13637	Ras-related protein Rab-32	GTP binding; mitochondrion; protein transport; small GTPase mediated signal transduction	Mitochondrion.	Small GTPase superfamily, Rab family
**85**	RAP1A_HUMAN	RAP1A (KREV1)	P62834	Ras-related protein Rap-1A (GTP-binding protein smg-p21A) (Ras-related protein Krev-1) (C21KG) (G-22K)	GTP binding; GTPase activity; cell cycle; cytosol; negative regulation of cell cycle; plasma membrane	Cell membrane; Lipid-anchor.	Small GTPase superfamily, Ras family
**86**	RAP1B_HUMAN	RAP1B (OK/SW-cl.11)	P61224	Ras-related protein Rap-1b (GTP-binding protein smg p21B)	GTP binding; cytosol; plasma membrane; small GTPase mediated signal transduction	Cell membrane. Cytoplasm ,cytosol.	Small GTPase superfamily, Ras family
**87**	RHG08_HUMAN	ARHGAP8	P85298	Rho GTPase-activating protein 8 (Rho-type GTPase-activating protein 8)	GTPase activator activity; intracellular; signal transduction		
**88**	RSU1_HUMAN	RSU1 (RSP1)	Q15404	Ras suppressor protein 1 (Rsu-1) (RSP-1)	protein binding; signal transduction		
**89**	SEP11_HUMAN	11-Sep	Q9NVA2	Septin-11	GTP binding; cell cycle; cell division; nucleus; protein binding; protein heterooligomerization; septin complex; stress fiber		Septin family
**90**	SNP23_HUMAN	SNAP23	O00161	Synaptosomal-associated protein 23 (SNAP-23) (Vesicle-membrane fusion protein SNAP-23)	cell junction; cytoplasm; membrane fusion; nucleus; post-Golgi vesicle-mediated transport; protein binding; protein transport; synapse; synaptosome; vesicle targeting	Cell membrane; Peripheral membrane protein. Cell junction ,synapse ,synaptosome.	SNAP-25 family
**91**	SODC_HUMAN	SOD1	P00441	Superoxide dismutase [Cu-Zn] (EC 1.15.1.1)	DNA fragmentation involved in apoptosis; activation of MAPK activity; antioxidant activity; auditory receptor cell stereocilium organization; cell aging; cell soma; cellular iron ion homeostasis; chaperone binding; copper ion binding; cytoplasmic vesicle; cytosol; dendrite cytoplasm; double-strand break repair; embryo implantation; extracellular matrix; extracellular space; glutathione metabolic process; heart contraction; hydrogen peroxide biosynthetic process; locomotory behavior; mitochondrial matrix; muscle maintenance; myelin maintenance in the peripheral nervous system; myeloid cell homeostasis; negative regulation of cholesterol biosynthetic process; negative regulation of neuron apoptosis; neurofilament cytoskeleton organization; nucleus; ovarian follicle development; oxidation reduction; peroxisome; placenta development; positive regulation of apoptosis; positive regulation of cytokine production; protein [illegible]	Cytoplasm.	Cu-Zn superoxide dismutase family
**92**	SPB6_HUMAN	SERPINB6 (PI6) (PTI)	P35237	Serpin B6 (Placental thrombin inhibitor) (Cytoplasmic antiproteinase) (CAP) (Proteinase inhibitor 6) (PI-6)	centrosome; cytosol; protein binding; serine-type endopeptidase inhibitor activity	Cytoplasm.	Serpin family, Ov-serpin subfamily

	Entry name	Gene names	Uniprot Accession	Protein names	Gene Ontology	Subcellular Localization	Protein family
**93**	SPRC_HUMAN	SPARC (ON)	P09486	SPARC (Secreted protein acidic and rich in cysteine) (Osteonectin) (ON) (Basement-membrane protein 40) (BM-40)	basement membrane; calcium ion binding; collagen binding; copper ion binding; extracellular space; ossification; platelet alpha granule lumen; transmembrane receptor protein tyrosine kinase signaling pathway	Secreted ,extracellular space ,extracellular matrix ,basement membrane.	SPARC family
**94**	SRGN_HUMAN	SRGN (PRG) (PRG1)	P10124	Serglycin (Secretory granule proteoglycan core protein) (Platelet proteoglycan core protein) (P.PG) (Hematopoetic proteoglycan core protein)	Golgi apparatus; apoptosis; extracellular space; maintenance of granzyme B location in T cell secretory granule; maintenance of protease location in mast cell secretory granule; mast cell granule; negative regulation of bone mineralization; negative regulation of cytokine secretion; ossification; protein binding; protein maturation by peptide bond cleavage	Cytoplasmic granule. Secreted ,extracellular space. Golgi apparatus.	Serglycin family
**95**	SSF1_HUMAN	PPAN (SSF1)	Q9NQ55	Suppressor of SWI4 1 homolog (Ssf-1) (Peter Pan homolog)	RNA splicing; nucleolus; protein binding	Nucleus ,nucleolus.	
**96**	STOM_HUMAN	STOM (BND7) (EPB72)	P27105	Erythrocyte band 7 integral membrane protein (Stomatin) (Protein 7.2b)	cytoskeleton; integral to plasma membrane; melanosome; membrane raft; protein binding; protein homooligomerization	Cell membrane; Single-pass membrane protein; Cytoplasmic side. Cell membrane; Lipid-anchor; Cytoplasmic side. Melanosome.	Band 7/mec-2 family
**97**	SYUA_HUMAN	SNCA (NACP) (PARK1)	P37840	Alpha-synuclein (Non-A beta component of AD amyloid) (Non-A4 component of amyloid precursor) (NACP)	axon; cell cortex; cytosol; growth cone; histone binding; negative regulation of histone acetylation; negative regulation of monooxygenase activity; nucleus; plasma membrane; regulation of dopamine secretion; response to interferon-gamma	Cytoplasm. Membrane. Nucleus.	Synuclein family
**98**	TBAL3_HUMAN	TUBAL3	A6NHL2	Tubulin alpha chain-like 3	GTP binding; GTPase activity; microtubule; microtubule-based movement; protein complex; protein polymerization; structural molecule activity		Tubulin family
**99**	TPM2_HUMAN	TPM2 (TMSB)	P07951	Tropomyosin beta chain (Tropomyosin-2) (Beta tropomyosin)	actin binding; muscle thin filament tropomyosin; regulation of ATPase activity; structural constituent of muscle	Cytoplasm ,cytoskeleton.	Tropomyosin family
**100**	TPM3_HUMAN	TPM3	P06753	Tropomyosin alpha-3 chain (Tropomyosin-3) (Gamma-tropomyosin) (Tropomyosin-5) (hTM5)	actin binding; cell motion; muscle thin filament tropomyosin; regulation of muscle contraction	Cytoplasm ,cytoskeleton.	Tropomyosin family
**101**	TPM4_HUMAN	TPM4	P67936	Tropomyosin alpha-4 chain (Tropomyosin-4) (TM30p1)	actin binding; calcium ion binding; cell motion; muscle thin filament tropomyosin; structural constituent of muscle	Cytoplasm ,cytoskeleton.	Tropomyosin family
**102**	TYB4_HUMAN	TMSB4X (TB4X) (THYB4) (TMSB4)	P62328	Thymosin beta-4 (T beta-4) (Fx) [Cleaved into: Hematopoietic system regulatory peptide (Seraspenide)]	actin binding; actin cytoskeleton organization; cytoplasm; cytoskeleton; sequestering of actin monomers	Cytoplasm ,cytoskeleton.	Thymosin beta family
**103**	VDAC2_HUMAN	VDAC2	P45880	Voltage-dependent anion-selective channel protein 2 (VDAC-2) (hVDAC2) (Outer mitochondrial membrane protein porin 2)	anion transport; integral to membrane; mitochondrial nucleoid; mitochondrial outer membrane; nucleotide binding; protein binding; voltage-gated anion channel activity	Mitochondrion outer membrane.	Eukaryotic mitochondrial porin family
**104**	WASP_HUMAN	WAS (IMD2)	P42768	Wiskott-Aldrich syndrome protein (WASp)	actin cytoskeleton; blood coagulation; cytoplasm; defense response; epidermis development; identical protein binding; immune response; nucleus; small GTPase regulator activity	Cytoplasm ,cytoskeleton.	
