# Supplementary material for: Proteomic and Phospho-Proteomic Profile of Human Platelets in Basal, Resting State: Insights into Integrin Signaling
Source: PLoS One. 2009 Oct 27;4(10):e7627. doi: 10.1371/journal.pone.0007627 (PMC2762604; doi:10.1371/journal.pone.0007627)
Supplement: Table S2 — Ten lists of proteins and the identifying peptides from proteomic profiling of ten individual platelet samples. Protein identification is based on its refseq ID. Definition of additional terms is as follows: P (pro) is the protein probability (normalized to 1); P (pep) is the peptide probability (normalized to 1); Sf score is the quality of the match in a TurboSEQUEST search. The protein Sf score is the sum of peptide Sf scores for all the peptides associated with that protein; consensus Score is the quality of the match in a TurboSEQUEST search. (9.66 MB PDF) [file pone.0007627.s007.pdf]

Reference				P (pro)	Sf	Score	Coverage	MW
File, Scan(s)	Peptide	MH+	z	P (pep)	Sf	XC		Sp
gi|12667788|ref|NP_002464.1| myosin, heavy polypeptide 9, non-muscle [Homo sapiens]				1.00E-30	102.39	1150.38	48.20	226529.8
AHQ-1-2, 2081 - 2102	R.ALEEAMEQK.A	1049.18068	2	2.60E-04	0.90	3.40	-	1012.8
AHQ-1-3, 2030	R.ALEEAMEQK.A	1049.18068	1	8.60E-04	0.60	2.44	-	769.9
AHQ-1-2, 2058 - 2125	R.ALEEAMEQK.A	1049.18068	1	1.65E-04	0.33	2.22	-	626.6
AHQ-1-1, 2201	R.ALEEAMEQK.A	1049.18068	2	4.13E-05	0.92	3.58	-	1020.0
AHQ-1-4, 2020	R.ALEEAMEQK.A	1049.18068	2	5.24E-04	0.74	2.79	-	714.7
AHQ-1-1, 3891	K.ALELDSNLYR.I	1194.31920	2	9.12E-07	0.94	3.54	-	1216.7
AHQ-1-3, 3809	K.ALELDSNLYR.I	1194.31920	1	4.26E-06	0.10	2.26	-	212.2
AHQ-1-4, 3843	K.ALELDSNLYR.I	1194.31920	2	9.53E-07	0.95	4.14	-	1120.9
AHQ-1-3, 3806 - 3813	K.ALELDSNLYR.I	1194.31920	2	5.38E-08	0.94	4.04	-	1107.4
AHQ-1-5, 3721	K.ALELDSNLYR.I	1194.31920	1	7.75E-04	0.13	2.27	-	206.1
AHQ-1-2, 3821	K.ALELDSNLYR.I	1194.31920	2	9.25E-08	0.95	3.64	-	1496.1
AHQ-1-13- , 3863	K.ALELDSNLYR.I	1194.31920	2	8.93E-06	0.85	3.20	-	863.0
AHQ-1-5, 3707 - 3713	K.ALELDSNLYR.I	1194.31920	2	1.35E-07	0.95	4.03	-	1267.7
AHQ-1-2, 3943 - 4013	K.ALELDSNLYR.I	1194.31920	2	6.09E-07	0.95	4.07	-	1145.5
AHQ-1-3, 3905	K.ALELDSNLYR.I	1194.31920	2	9.05E-06	0.86	2.85	-	1006.6
AHQ-1-6, 2782	R.ALEQQVEEMK.T	1205.36380	2	4.06E-05	0.84	3.23	-	835.8
AHQ-1-3, 2873	R.ALEQQVEEMK.T	1205.36380	2	1.20E-04	0.83	3.32	-	974.6
AHQ-1-1, 3000	R.ALEQQVEEMK.T	1205.36380	2	3.10E-05	0.89	3.17	-	1083.9
AHQ-1-2, 2981 - 3059	R.ALEQQVEEMK.T	1205.36380	2	2.20E-05	0.90	3.66	-	1014.8
AHQ-1-2, 2215	R.ALEQQVEEM*K.T	1221.36320	2	5.09E-06	0.80	3.44	-	794.6
AHQ-1-7, 2813	R.ALEQQVEEMK.T	1205.36380	2	5.90E-04	0.77	2.94	-	919.0
AHQ-1-1, 2280	R.ALEQQVEEM*K.T	1221.36320	2	4.05E-06	0.82	2.97	-	928.1
AHQ-1-3, 2138	R.ALEQQVEEM*K.T	1221.36320	2	2.84E-05	0.82	3.40	-	874.5
AHQ-1-4, 2890 - 2891	R.ALEQQVEEMK.T	1205.36380	2	7.47E-05	0.90	3.65	-	1027.1
AHQ-1-5, 2006	R.ALEQQVEEM*K.T	1221.36320	2	4.34E-05	0.67	2.83	-	770.8
AHQ-1-3, 6527 - 6589	R.ALEQQVEEM*KTQLEELEDELQATEDAK.L	3165.38394	3	1.62E-12	0.97	6.15	-	1761.3
AHQ-1-2, 7349	R.ALEQQVEEMKTQLEELEDELQATEDAK.L	3149.38454	3	5.26E-07	0.96	6.21	-	1493.5
AHQ-1-2, 6951 - 7026	R.ALEQQVEEM*KTQLEELEDELQATEDAK.L	3165.38394	3	6.37E-09	0.97	5.76	-	1838.1
AHQ-1-1, 6456 - 6471	R.ALEQQVEEM*KTQLEELEDELQATEDAK.L	3165.38394	3	5.16E-14	0.97	5.91	-	1958.4
AHQ-1-2, 6713 - 6777	R.ALEQQVEEM*KTQLEELEDELQATEDAK.L	3165.38394	3	1.24E-11	0.97	6.07	-	1638.4
AHQ-1-3, 7209	R.ALEQQVEEMKTQLEELEDELQATEDAK.L	3149.38454	3	3.00E-08	0.96	5.58	-	1405.8
AHQ-1-6, 6470 - 6538	R.ALEQQVEEM*KTQLEELEDELQATEDAK.L	3165.38394	3	3.26E-07	0.96	5.99	-	1293.7
AHQ-1-4, 7386	R.ALEQQVEEMKTQLEELEDELQATEDAK.L	3149.38454	3	1.41E-05	0.96	6.15	-	1244.7
AHQ-1-5, 6634	R.ALEQQVEEM*KTQLEELEDELQATEDAK.L	3165.38394	3	1.56E-08	0.95	5.75	-	1044.2
AHQ-1-2, 5537	K.ANLQIDQINTDLNLER.S	1871.04187	2	7.19E-09	0.97	5.83	-	1302.7
AHQ-1-3, 5391	K.ANLQIDQINTDLNLER.S	1871.04187	2	1.03E-08	0.97	5.74	-	1435.3
AHQ-1-3, 5102 - 5175	K.ANLQIDQINTDLNLER.S	1871.04187	2	5.38E-11	0.98	6.06	-	1555.4
AHQ-1-10, 4577	K.ANLQIDQINTDLNLER.S	1871.04187	2	2.65E-06	0.96	5.08	-	1432.6
AHQ-1-8, 5091	K.ANLQIDQINTDLNLER.S	1871.04187	2	6.45E-05	0.97	5.10	-	1538.6
AHQ-1-2, 5093	K.ANLQIDQINTDLNLER.S	1871.04187	2	1.25E-06	0.96	5.22	-	874.6
AHQ-1-4, 4966 - 5034	K.ANLQIDQINTDLNLER.S	1871.04187	2	3.79E-05	0.83	4.17	-	408.8
AHQ-1-2, 5230 - 5301	K.ANLQIDQINTDLNLER.S	1871.04187	2	1.08E-10	0.98	6.18	-	1786.5
AHQ-1-4, 5242	K.ANLQIDQINTDLNLER.S	1871.04187	2	2.46E-06	0.87	3.74	-	643.4
AHQ-1-9, 5011	K.ANLQIDQINTDLNLER.S	1871.04187	2	4.98E-04	0.97	5.19	-	1550.6
AHQ-1-1, 5387	K.ANLQIDQINTDLNLER.S	1871.04187	2	3.74E-04	0.97	4.95	-	1762.1
AHQ-1-1, 5189 - 5251	K.ANLQIDQINTDLNLER.S	1871.04187	2	5.97E-04	0.97	5.35	-	1315.4
AHQ-1-2, 5369 - 5438	K.ANLQIDQINTDLNLER.S	1871.04187	2	3.56E-07	0.98	7.12	-	1738.0
AHQ-1-6, 5094 - 5096	K.ANLQIDQINTDLNLER.S	1871.04187	2	4.78E-07	0.96	4.82	-	1178.2
AHQ-1-1, 4980	K.ANLQIDQINTDLNLER.S	1871.04187	2	1.15E-08	0.94	4.83	-	700.2
AHQ-1-3, 5267	K.ANLQIDQINTDLNLER.S	1871.04187	2	3.73E-10	0.97	5.78	-	1276.1
AHQ-1-5, 2215	K.ASITALEAK.I	904.04321	2	5.19E-06	0.90	3.02	-	954.5
AHQ-1-4, 2367	K.ASITALEAK.I	904.04321	2	2.59E-06	0.89	2.91	-	913.0
AHQ-1-1, 2512	K.ASITALEAK.I	904.04321	2	5.98E-05	0.92	3.01	-	1125.4
AHQ-1-2, 2445	K.ASITALEAK.I	904.04321	2	2.30E-06	0.90	3.14	-	940.9
AHQ-1-3, 2350	K.ASITALEAK.I	904.04321	2	1.40E-04	0.92	2.94	-	1120.5
AHQ-1-1, 2237	R.ASREEILAQAK.E	1216.36955	2	1.84E-05	0.92	3.32	-	1417.8
AHQ-1-3, 2063	R.ASREEILAQAK.E	1216.36955	2	4.00E-06	0.93	3.54	-	1583.8
AHQ-1-2, 2115 - 2174	R.ASREEILAQAK.E	1216.36955	2	9.21E-05	0.91	3.24	-	1360.2
AHQ-1-4, 2044	R.ASREEILAQAK.E	1216.36955	2	1.41E-06	0.91	3.38	-	1467.0
AHQ-1-2, 3317	R.DELADEIANSSGK.G	1349.38323	2	2.19E-07	0.97	4.56	-	1896.4
AHQ-1-1, 3284	R.DELADEIANSSGK.G	1349.38323	2	1.64E-05	0.93	4.06	-	1012.7
AHQ-1-3, 3214	R.DELADEIANSSGK.G	1349.38323	2	3.87E-07	0.97	4.29	-	1792.8
AHQ-1-6, 6895	K.DFSALESQLQDTQELLQEENR.Q	2494.61059	3	3.22E-05	0.96	5.08	-	1529.2
AHQ-1-4, 7224	K.DFSALESQLQDTQELLQEENR.Q	2494.61059	3	1.48E-06	0.91	3.98	-	1459.1
AHQ-1-1, 6839 - 6911	K.DFSALESQLQDTQELLQEENR.Q	2494.61059	2	1.14E-07	0.94	4.79	-	879.4
AHQ-1-1, 6740	K.DFSALESQLQDTQELLQEENR.Q	2494.61059	3	3.25E-10	0.98	6.38	-	2039.0
AHQ-1-2, 7199 - 7227	K.DFSALESQLQDTQELLQEENR.Q	2494.61059	3	6.82E-08	0.93	4.17	-	1395.3
AHQ-1-5, 7038	K.DFSALESQLQDTQELLQEENR.Q	2494.61059	3	3.87E-05	0.95	4.68	-	1770.4
AHQ-1-3, 6898	K.DFSALESQLQDTQELLQEENR.Q	2494.61059	3	6.85E-05	0.93	4.32	-	1529.8
AHQ-1-3, 6646	K.DFSALESQLQDTQELLQEENRQK.L	2750.91328	3	1.09E-10	0.84	3.85	-	531.1
AHQ-1-1, 6413	K.DFSALESQLQDTQELLQEENRQK.L	2750.91328	3	3.50E-05	0.70	3.27	-	523.2
AHQ-1-2, 6607 - 6681	K.DFSALESQLQDTQELLQEENRQK.L	2750.91328	3	1.24E-10	0.93	5.54	-	590.2
AHQ-1-3, 6441 - 6511	K.DFSALESQLQDTQELLQEENRQK.L	2750.91328	3	1.19E-12	0.95	5.33	-	982.2
AHQ-1-2, 6749 - 6751	K.DFSALESQLQDTQELLQEENRQK.L	2750.91328	3	4.40E-06	0.62	3.11	-	587.6
AHQ-1-9, 7338	R.DLGEELEALKTELEDTLDSTAAQQELR.S	3019.21860	3	1.99E-06	0.96	5.30	-	1869.5
AHQ-1-2, 7437	R.DLGEELEALKTELEDTLDSTAAQQELR.S	3019.21860	3	1.18E-09	0.97	5.12	-	2058.3
AHQ-1-5, 7505	R.DLGEELEALKTELEDTLDSTAAQQELR.S	3019.21860	3	2.71E-07	0.97	5.53	-	2113.2
AHQ-1-7, 7528	R.DLGEELEALKTELEDTLDSTAAQQELR.S	3019.21860	3	4.46E-06	0.85	3.85	-	950.2
AHQ-1-3, 7154 - 7223	R.DLGEELEALKTELEDTLDSTAAQQELR.S	3019.21860	3	1.67E-15	0.99	7.55	-	3012.9
AHQ-1-6, 7314	R.DLGEELEALKTELEDTLDSTAAQQELR.S	3019.21860	3	1.46E-08	0.95	5.30	-	1573.8
AHQ-1-1, 7039	R.DLGEELEALKTELEDTLDSTAAQQELR.S	3019.21860	3	3.89E-09	0.93	4.51	-	1396.7
AHQ-1-3, 7361	R.DLGEELEALKTELEDTLDSTAAQQELR.S	3019.21860	3	1.29E-08	0.95	5.24	-	1638.8
AHQ-1-2, 3151	R.ELEDATETADAMNR.E	1566.63006	2	2.81E-05	0.75	2.61	-	786.1
AHQ-1-3, 2871	R.ELEDATETADAMNR.E	1566.63006	2	4.35E-08	0.87	3.82	-	845.6
AHQ-1-6, 2794 - 2806	R.ELEDATETADAMNR.E	1566.63006	2	1.78E-04	0.88	3.88	-	844.1
AHQ-1-5, 2806	R.ELEDATETADAMNR.E	1566.63006	2	5.54E-07	0.75	2.92	-	809.8
AHQ-1-2, 2333	R.ELEDATETADAM*NR.E	1582.62946	2	4.82E-05	0.68	2.85	-	643.9
AHQ-1-10, 2685	R.ELEDATETADAMNR.E	1566.63006	2	2.26E-04	0.87	3.51	-	863.0
AHQ-1-4, 2895 - 2900	R.ELEDATETADAMNR.E	1566.63006	2	4.49E-05	0.88	3.59	-	1012.1
AHQ-1-2, 2862	R.ELEDATETADAMNR.E	1566.63006	2	1.57E-05	0.52	2.61	-	306.6
AHQ-1-1, 2936 - 2937	R.ELEDATETADAMNR.E	1566.63006	2	3.31E-06	0.95	4.26	-	1321.7
AHQ-1-3, 2222	R.ELEDATETADAM*NR.E	1582.62946	2	1.51E-04	0.84	3.12	-	814.8
AHQ-1-9, 5834	R.ELESQISELQEDLESER.A	2035.10956	2	9.59E-04	0.92	4.32	-	772.7
AHQ-1-3, 5901	R.ELESQISELQEDLESER.A	2035.10956	2	2.23E-08	0.94	4.93	-	904.5
AHQ-1-3, 5835 - 5905	R.ELESQISELQEDLESER.A	2035.10956	2	2.71E-06	0.97	5.93	-	1426.7
AHQ-1-2, 6141	R.ELESQISELQEDLESER.A	2035.10956	2	2.68E-04	0.82	3.83	-	682.9
AHQ-1-3, 5470	R.ELESQISELQEDLESER.A	2035.10956	2	1.10E-06	0.96	4.74	-	1105.6
AHQ-1-4, 5982 - 6051	R.ELESQISELQEDLESER.A	2035.10956	2	2.43E-04	0.85	4.40	-	822.4
AHQ-1-4, 5484	R.ELESQISELQEDLESER.A	2035.10956	2	1.20E-04	0.83	3.39	-	897.4
AHQ-1-7, 5986	R.ELESQISELQEDLESER.A	2035.10956	2	1.30E-07	0.94	4.67	-	888.4
AHQ-1-2, 5999 - 6070	R.ELESQISELQEDLESER.A	2035.10956	2	1.06E-11	0.94	4.90	-	942.7
AHQ-1-2, 6387	R.ELESQISELQEDLESER.A	2035.10956	2	2.37E-07	0.93	3.99	-	1007.0
AHQ-1-2, 5659	R.ELESQISELQEDLESER.A	2035.10956	2	7.85E-06	0.89	3.98	-	669.2

AHQ-1-2, 6091	R.ELESQISELQEDLESER.A	2035.10956	3	2.47E-06	0.98	6.22	-	2334.7
AHQ-1-1, 5843 - 5907	R.ELESQISELQEDLESER.A	2035.10956	2	2.70E-08	0.94	4.74	-	918.8
AHQ-1-3, 2542	R.EMEAELEDER.K	1251.30306	2	6.75E-04	0.58	2.55	-	479.5
AHQ-1-2, 2285	R.EMEAELEDERK.Q	1379.47597	2	6.03E-05	0.93	3.50	-	1365.7
AHQ-1-1, 2347	R.EMEAELEDERK.Q	1379.47597	2	2.94E-04	0.84	2.80	-	1043.7
AHQ-1-4, 2203	R.EMEAELEDERK.Q	1379.47597	2	7.47E-07	0.90	3.21	-	1237.8
AHQ-1-3, 2205	R.EMEAELEDERK.Q	1379.47597	2	9.53E-05	0.86	2.82	-	1191.1
AHQ-1-2, 4747	K.EQADFAIEALAK.A	1306.44632	1	1.74E-05	0.36	2.64	-	272.6
AHQ-1-3, 4490	K.EQADFAIEALAK.A	1306.44632	2	4.53E-04	0.79	2.71	-	684.4
AHQ-1-2, 4939	K.EQADFAIEALAK.A	1306.44632	2	3.46E-05	0.71	2.75	-	500.1
AHQ-1-2, 4651	K.EQADFAIEALAK.A	1306.44632	1	6.54E-05	0.33	2.76	-	249.7
AHQ-1-4, 2406	R.EQLEEEEEAKHNLEK.Q	1855.93770	2	3.85E-07	0.89	4.23	-	669.4
AHQ-1-6, 2324	R.EQLEEEEEAKHNLEK.Q	1855.93770	2	6.33E-08	0.90	4.25	-	726.2
AHQ-1-1, 2552	R.EQLEEEEEAKHNLEK.Q	1855.93770	2	7.99E-07	0.91	4.41	-	790.9
AHQ-1-2, 2350	R.EQLEEEEEAKHNLEK.Q	1855.93770	2	6.78E-06	0.74	3.15	-	615.0
AHQ-1-5, 2301	R.EQLEEEEEAKHNLEK.Q	1855.93770	2	1.12E-04	0.83	3.81	-	546.4
AHQ-1-3, 2401 - 2409	R.EQLEEEEEAKHNLEK.Q	1855.93770	2	8.86E-05	0.90	4.13	-	833.0
AHQ-1-2, 3595	R.FLSNGHVTIPGQQDK.D	1641.80946	2	7.36E-06	0.69	3.04	-	464.9
AHQ-1-2, 5329 - 5397	R.FLSNGHVTIPGQQDKDMFQETMEAM*R.I	3028.39040	3	3.93E-06	0.86	5.08	-	946.9
AHQ-1-2, 6131 - 6210	R.FLSNGHVTIPGQQDKDMFQETMEAMR.I	3012.39100	3	1.76E-05	0.91	4.64	-	657.3
AHQ-1-6, 5971	R.FLSNGHVTIPGQQDKDMFQETMEAMR.I	3012.39100	3	3.07E-05	0.91	4.76	-	653.1
AHQ-1-2, 4438	R.GDLPFVVPR.R	1000.17581	2	2.38E-05	0.74	2.73	-	785.5
AHQ-1-1, 3044	K.HEAMITDLEER.L	1344.47646	2	1.71E-08	0.97	3.83	-	1614.0
AHQ-1-2, 2991	K.HEAMITDLEER.L	1344.47646	1	4.00E-05	0.68	2.93	-	652.2
AHQ-1-3, 2430	K.HEAM*ITDLEER.L	1360.47586	2	7.81E-04	0.96	3.79	-	1490.4
AHQ-1-2, 2978	K.HEAMITDLEER.L	1344.47646	2	1.28E-07	0.95	3.69	-	1120.6
AHQ-1-6, 3434	R.HEMPPHIYAITDTAYR.S	1916.14944	3	2.15E-09	0.97	4.37	-	2369.9
AHQ-1-6, 3151	R.HEM*PPHIYAITDTAYR.S	1932.14884	3	3.46E-08	0.96	4.61	-	1785.4
AHQ-1-5, 3163	R.HEM*PPHIYAITDTAYR.S	1932.14884	2	8.71E-04	0.78	2.74	-	650.9
AHQ-1-1, 3459	R.HEM*PPHIYAITDTAYR.S	1932.14884	2	1.80E-04	0.94	3.55	-	1152.4
AHQ-1-3, 3319	R.HEM*PPHIYAITDTAYR.S	1932.14884	3	8.89E-09	0.97	5.01	-	1973.7
AHQ-1-3, 3323	R.HEM*PPHIYAITDTAYR.S	1932.14884	2	7.93E-06	0.85	3.15	-	692.8
AHQ-1-2, 3423	R.HEM*PPHIYAITDTAYR.S	1932.14884	2	3.51E-04	0.82	2.88	-	627.2
AHQ-1-2, 3426 - 3437	R.HEM*PPHIYAITDTAYR.S	1932.14884	3	1.43E-07	0.97	4.70	-	2429.2
AHQ-1-2, 3843 - 3857	R.HEMPPHIYAITDTAYR.S	1916.14944	3	5.03E-07	0.97	4.21	-	2354.5
AHQ-1-2, 3758 - 3829	R.HEMPPHIYAITDTAYR.S	1916.14944	2	7.08E-05	0.95	4.30	-	1002.5
AHQ-1-7, 3178	R.HEM*PPHIYAITDTAYR.S	1932.14884	2	7.44E-04	0.58	2.70	-	464.5
AHQ-1-5, 3178	R.HEM*PPHIYAITDTAYR.S	1932.14884	3	5.42E-06	0.92	4.14	-	1204.9
AHQ-1-2, 3754	R.HEMPPHIYAITDTAYR.S	1916.14944	3	8.42E-10	0.97	4.25	-	2763.0
AHQ-1-3, 3627	R.HEMPPHIYAITDTAYR.S	1916.14944	3	1.97E-09	0.96	4.07	-	2083.3
AHQ-1-1, 3797	R.HEMPPHIYAITDTAYR.S	1916.14944	3	9.90E-07	0.94	3.72	-	1663.5
AHQ-1-10, 3317	R.HEMPPHIYAITDTAYR.S	1916.14944	2	1.65E-06	0.55	2.98	-	320.5
AHQ-1-2, 5023	K.HSQAVEELAEQLEQTK.R	1840.96940	2	1.22E-11	0.98	5.77	-	1925.6
AHQ-1-1, 4659	K.HSQAVEELAEQLEQTK.R	1840.96940	3	3.21E-05	0.94	4.38	-	1475.7
AHQ-1-3, 4597	K.HSQAVEELAEQLEQTK.R	1840.96940	2	4.16E-14	0.98	6.28	-	2331.1
AHQ-1-5, 4474 - 4481	K.HSQAVEELAEQLEQTK.R	1840.96940	2	2.02E-06	0.99	6.24	-	2646.6
AHQ-1-2, 4766	K.HSQAVEELAEQLEQTK.R	1840.96940	3	4.57E-04	0.97	4.97	-	1984.9
AHQ-1-2, 4758	K.HSQAVEELAEQLEQTK.R	1840.96940	2	6.22E-07	0.98	4.94	-	2202.1
AHQ-1-3, 4533 - 4609	K.HSQAVEELAEQLEQTK.R	1840.96940	2	5.54E-05	0.98	5.67	-	2429.0
AHQ-1-3, 4598 - 4611	K.HSQAVEELAEQLEQTK.R	1840.96940	3	6.86E-07	0.96	5.38	-	1500.0
AHQ-1-3, 4169 - 4237	K.HSQAVEELAEQLEQTKR.V	1997.15575	3	2.21E-12	0.98	7.60	-	2155.4
AHQ-1-2, 4321 - 4401	K.HSQAVEELAEQLEQTKR.V	1997.15575	3	1.88E-11	0.98	7.03	-	2331.5
AHQ-1-2, 4322 - 4327	K.HSQAVEELAEQLEQTKR.V	1997.15575	2	4.00E-09	0.98	6.05	-	2421.6
AHQ-1-3, 4183	K.HSQAVEELAEQLEQTKR.V	1997.15575	2	9.11E-11	0.98	6.09	-	2449.5
AHQ-1-3, 4485	K.HSQAVEELAEQLEQTKR.V	1997.15575	3	7.37E-09	0.97	5.92	-	1618.2
AHQ-1-5, 4071	K.HSQAVEELAEQLEQTKR.V	1997.15575	2	3.70E-06	0.98	6.16	-	1986.1
AHQ-1-6, 4023	K.HSQAVEELAEQLEQTKR.V	1997.15575	3	2.56E-05	0.95	4.98	-	1279.7
AHQ-1-2, 4415	K.HSQAVEELAEQLEQTKR.V	1997.15575	2	1.04E-11	0.98	5.36	-	2020.9
AHQ-1-1, 4241 - 4315	K.HSQAVEELAEQLEQTKR.V	1997.15575	2	4.79E-09	0.98	5.81	-	2456.8
AHQ-1-4, 4223	K.HSQAVEELAEQLEQTKR.V	1997.15575	3	8.22E-09	0.97	5.42	-	1621.8
AHQ-1-4, 4220 - 4228	K.HSQAVEELAEQLEQTKR.V	1997.15575	2	4.36E-05	0.96	5.10	-	1134.2
AHQ-1-5, 4061	K.HSQAVEELAEQLEQTKR.V	1997.15575	3	5.83E-07	0.97	5.73	-	1383.0
AHQ-1-1, 4311	K.HSQAVEELAEQLEQTKR.V	1997.15575	3	5.30E-09	0.97	5.48	-	1485.1
AHQ-1-2, 4069 - 4147	R.IAEFTTNLTEEEEK.S	1654.75371	2	2.01E-10	0.98	5.04	-	2370.2
AHQ-1-2, 4005 - 4074	R.IAEFTTNLTEEEEK.S	1654.75371	2	1.16E-10	0.95	4.53	-	1554.5
AHQ-1-3, 3783 - 3857	R.IAEFTTNLTEEEEK.S	1654.75371	2	1.67E-05	0.96	4.57	-	1715.3
AHQ-1-4, 4068	R.IAEFTTNLTEEEEK.S	1654.75371	2	2.23E-06	0.97	5.04	-	1940.1
AHQ-1-4, 3958	R.IAEFTTNLTEEEEK.S	1654.75371	2	6.47E-07	0.86	3.26	-	1202.9
AHQ-1-3, 3927 - 3999	R.IAEFTTNLTEEEEK.S	1654.75371	2	1.38E-09	0.96	4.30	-	1696.0
AHQ-1-6, 3608 - 3658	R.IAEFTTNLTEEEEK.S	1654.75371	2	3.02E-04	0.95	3.94	-	1527.3
AHQ-1-1, 4065	R.IAEFTTNLTEEEEK.S	1654.75371	2	9.21E-04	0.83	3.09	-	992.3
AHQ-1-2, 3918 - 3989	R.IAEFTTNLTEEEEK.S	1654.75371	2	5.70E-09	0.97	4.96	-	1684.4
AHQ-1-7, 3752	R.IAEFTTNLTEEEEK.S	1654.75371	2	4.15E-05	0.95	3.86	-	1423.5
AHQ-1-1, 3971 - 3993	R.IAEFTTNLTEEEEK.S	1654.75371	2	1.57E-07	0.89	3.34	-	1312.7
AHQ-1-4, 3806 - 3884	R.IAEFTTNLTEEEEK.S	1654.75371	2	1.24E-06	0.96	4.31	-	1745.8
AHQ-1-5, 3694	R.IAEFTTNLTEEEEK.S	1654.75371	2	5.20E-08	0.95	4.53	-	1224.5
AHQ-1-1, 3843 - 3907	R.IAEFTTNLTEEEEK.S	1654.75371	2	2.07E-10	0.97	4.69	-	1976.4
AHQ-1-2, 4222	R.IAEFTTNLTEEEEK.S	1654.75371	2	4.65E-06	0.97	4.30	-	2616.6
AHQ-1-3, 4013	R.IAEFTTNLTEEEEK.S	1654.75371	2	1.72E-06	0.95	3.81	-	1880.5
AHQ-1-1, 5321	R.IAQLEEELEEEQGNTELINDR.L	2473.58969	3	5.73E-06	0.78	3.45	-	850.4
AHQ-1-1, 5603	R.IAQLEEELEEEQGNTELINDRLK.K	2714.92085	3	8.77E-09	0.85	3.84	-	868.1
AHQ-1-1, 5608	R.IAQLEEELEEEQGNTELINDRLK.K	2714.92085	2	3.83E-06	0.92	4.53	-	932.9
AHQ-1-4, 3444 - 3460	K.IAQLEEQLDNETK.E	1531.64695	2	2.70E-05	0.97	4.82	-	1589.5
AHQ-1-10, 3128 - 3143	K.IAQLEEQLDNETK.E	1531.64695	2	4.53E-06	0.96	4.13	-	1501.4
AHQ-1-5, 3325 - 3395	K.IAQLEEQLDNETK.E	1531.64695	2	2.01E-04	0.91	3.14	-	1500.9
AHQ-1-1, 3888	K.IAQLEEQLDNETK.E	1531.64695	2	3.11E-06	0.86	3.39	-	880.7
AHQ-1-2, 3522 - 3594	K.IAQLEEQLDNETK.E	1531.64695	2	8.60E-06	0.96	4.24	-	1491.8
AHQ-1-6, 3294 - 3295	K.IAQLEEQLDNETK.E	1531.64695	2	1.13E-08	0.98	5.33	-	1854.4
AHQ-1-1, 3253	K.IAQLEEQLDNETK.E	1531.64695	2	4.88E-09	0.96	4.45	-	1752.8
AHQ-1-2, 3246	K.IAQLEEQLDNETK.E	1531.64695	2	3.29E-04	0.92	3.30	-	1250.9
AHQ-1-3, 3539 - 3587	K.IAQLEEQLDNETK.E	1531.64695	2	3.45E-04	0.91	3.23	-	1260.3
AHQ-1-3, 3623	K.IAQLEEQLDNETK.E	1531.64695	2	6.83E-10	0.96	4.61	-	1445.8
AHQ-1-8, 3198 - 3204	K.IAQLEEQLDNETK.E	1531.64695	2	2.63E-06	0.89	3.33	-	1175.3
AHQ-1-3, 3130 - 3207	K.IAQLEEQLDNETK.E	1531.64695	2	3.50E-08	0.96	3.99	-	1671.7
AHQ-1-11, 3198 - 3266	K.IAQLEEQLDNETK.E	1531.64695	2	5.91E-06	0.96	4.68	-	1470.3
AHQ-1-2, 3962 - 3997	K.IAQLEEQLDNETK.E	1531.64695	2	5.36E-08	0.84	3.22	-	559.7
AHQ-1-7, 3322	K.IAQLEEQLDNETK.E	1531.64695	2	6.23E-05	0.68	2.54	-	782.2
AHQ-1-1, 3463 - 3465	K.IAQLEEQLDNETK.E	1531.64695	2	3.90E-10	0.97	4.96	-	1899.9
AHQ-1-3, 3401 - 3437	K.IAQLEEQLDNETK.E	1531.64695	2	1.01E-06	0.96	4.47	-	1355.3
AHQ-1-9, 3214	K.IAQLEEQLDNETK.E	1531.64695	2	4.28E-06	0.97	5.12	-	1554.2
AHQ-1-4, 3224	K.IAQLEEQLDNETK.E	1531.64695	2	1.59E-04	0.84	3.10	-	913.3
AHQ-1-2, 3666 - 3746	K.IAQLEEQLDNETK.E	1531.64695	2	1.46E-07	0.97	5.42	-	1505.1
AHQ-1-2, 6781 - 6845	R.IIGLDQVAGMSETALPGAFK.T	2019.35101	2	1.09E-05	0.96	4.60	-	1462.8
AHQ-1-2, 6922	R.IIGLDQVAGMSETALPGAFK.T	2019.35101	2	4.86E-04	0.95	4.61	-	1281.0
AHQ-1-13- , 6453 - 6455	R.IIGLDQVAGMSETALPGAFK.T	2019.35101	2	2.81E-04	0.95	4.98	-	968.3
AHQ-1-11, 5930 - 5931	R.IIGLDQVAGMSETALPGAFK.T	2019.35101	2	8.77E-05	0.96	4.97	-	1159.9
AHQ-1-14- , 6220 - 6223	R.IIGLDQVAGMSETALPGAFK.T	2019.35101	2	1.06E-06	0.98	5.78	-	1513.9
AHQ-1-6, 6582 - 6610	R.IIGLDQVAGMSETALPGAFK.T	2019.35101	2	2.58E-07	0.97	5.91	-	1456.4

AHQ-1-3, 6031	R.IIGLDQVAGMSETALPGAFK.T	2019.35101	2	3.14E-05	0.89	4.04	-	822.4
AHQ-1-4, 6776	R.IIGLDQVAGMSETALPGAFK.T	2019.35101	2	5.58E-04	0.80	3.75	-	547.5
AHQ-1-10, 5824	R.IIGLDQVAGMSETALPGAFK.T	2019.35101	2	8.91E-04	0.86	3.57	-	750.8
AHQ-1-1, 5381	R.IIGLDQVAGM*SETALPGAFK.T	2035.35041	2	1.00E-04	0.72	3.12	-	487.4
AHQ-1-6, 5791 - 5870	R.IIGLDQVAGM*SETALPGAFK.T	2035.35041	2	4.99E-04	0.87	4.54	-	707.1
AHQ-1-4, 6791	R.IIGLDQVAGMSETALPGAFK.T	2019.35101	3	3.03E-04	0.96	5.02	-	1533.4
AHQ-1-2, 5917 - 5989	R.IIGLDQVAGM*SETALPGAFK.T	2035.35041	2	1.61E-05	0.96	5.40	-	945.3
AHQ-1-7, 6769	R.IIGLDQVAGMSETALPGAFK.T	2019.35101	2	7.36E-06	0.95	4.95	-	1054.4
AHQ-1-5, 6671 - 6737	R.IIGLDQVAGMSETALPGAFK.T	2019.35101	2	2.06E-06	0.98	6.47	-	1694.7
AHQ-1-1, 5968	R.IIGLDQVAGMSETALPGAFK.T	2019.35101	2	2.12E-04	0.94	4.23	-	1295.4
AHQ-1-2, 4182	R.IM*GIPEEEQM*GLLR.V	1648.92706	2	1.03E-07	0.95	4.36	-	1109.0
AHQ-1-2, 5475 - 5478	R.IMGIPEEEQMGLLR.V	1616.92826	2	2.10E-04	0.88	4.19	-	390.0
AHQ-1-3, 5330 - 5343	R.IMGIPEEEQMGLLR.V	1616.92826	2	2.61E-06	0.84	3.71	-	390.9
AHQ-1-1, 5411	R.IMGIPEEEQMGLLR.V	1616.92826	2	1.44E-05	0.71	2.99	-	347.5
AHQ-1-4, 5439 - 5444	R.IMGIPEEEQMGLLR.V	1616.92826	2	6.28E-06	0.89	4.21	-	440.2
AHQ-1-2, 4933	R.IM*GIPEEEQMGLLR.V	1632.92766	2	6.37E-06	0.94	3.77	-	884.5
AHQ-1-2, 4815 - 4865	R.IMGIPEEEQM*GLLR.V	1632.92766	2	2.42E-09	0.80	3.44	-	436.1
AHQ-1-7, 5313 - 5389	R.IMGIPEEEQMGLLR.V	1616.92826	2	1.06E-06	0.91	4.18	-	590.1
AHQ-1-5, 5353 - 5357	R.IMGIPEEEQMGLLR.V	1616.92826	2	5.08E-07	0.87	3.54	-	430.4
AHQ-1-1, 5928	K.IRELESQISELQEDLESER.A	2304.45416	2	5.84E-07	0.98	6.60	-	1007.9
AHQ-1-6, 5862	K.IRELESQISELQEDLESER.A	2304.45416	3	1.17E-08	0.98	5.52	-	2384.5
AHQ-1-6, 5858	K.IRELESQISELQEDLESER.A	2304.45416	2	9.40E-08	0.98	6.53	-	2199.6
AHQ-1-4, 6027	K.IRELESQISELQEDLESER.A	2304.45416	3	7.14E-08	0.98	6.79	-	2064.2
AHQ-1-4, 6016 - 6019	K.IRELESQISELQEDLESER.A	2304.45416	2	1.73E-12	0.98	6.84	-	1845.6
AHQ-1-3, 5894 - 5915	K.IRELESQISELQEDLESER.A	2304.45416	3	3.14E-11	0.98	7.03	-	1993.0
AHQ-1-5, 5994	K.IRELESQISELQEDLESER.A	2304.45416	2	1.55E-04	0.97	5.36	-	1002.5
AHQ-1-2, 6077 - 6145	K.IRELESQISELQEDLESER.A	2304.45416	2	4.08E-04	0.98	7.46	-	1532.8
AHQ-1-1, 5923	K.IRELESQISELQEDLESER.A	2304.45416	3	3.69E-09	0.97	5.57	-	1916.8
AHQ-1-2, 5867	K.ITDVIIGFQACCR.G	1555.80136	2	1.24E-05	0.91	3.33	-	1083.5
AHQ-1-3, 4807	K.KANLQIDQINTDLNLER.S	1999.21478	2	4.19E-09	0.97	5.48	-	1601.6
AHQ-1-2, 4966 - 4983	K.KANLQIDQINTDLNLER.S	1999.21478	3	1.02E-04	0.75	3.68	-	694.6
AHQ-1-2, 4607 - 4687	K.KANLQIDQINTDLNLER.S	1999.21478	2	9.14E-05	0.86	5.16	-	777.5
AHQ-1-1, 4667	K.KANLQIDQINTDLNLER.S	1999.21478	2	1.62E-06	0.97	5.96	-	1625.9
AHQ-1-5, 4550	K.KANLQIDQINTDLNLER.S	1999.21478	2	1.82E-06	0.97	5.48	-	1732.8
AHQ-1-7, 4500	K.KANLQIDQINTDLNLER.S	1999.21478	2	4.92E-04	0.95	5.25	-	1047.2
AHQ-1-2, 4773	K.KANLQIDQINTDLNLER.S	1999.21478	2	6.78E-07	0.98	6.07	-	1799.4
AHQ-1-3, 4634	K.KANLQIDQINTDLNLER.S	1999.21478	2	2.12E-06	0.97	5.85	-	1232.5
AHQ-1-2, 4774 - 4791	K.KANLQIDQINTDLNLER.S	1999.21478	3	1.62E-05	0.94	5.53	-	951.1
AHQ-1-2, 4627	K.KANLQIDQINTDLNLER.S	1999.21478	3	7.34E-04	0.49	3.48	-	482.2
AHQ-1-9, 4356 - 4360	K.KANLQIDQINTDLNLER.S	1999.21478	2	2.86E-04	0.96	4.96	-	1248.1
AHQ-1-2, 4978	K.KANLQIDQINTDLNLER.S	1999.21478	2	1.10E-10	0.96	5.49	-	1073.4
AHQ-1-3, 4457	K.KANLQIDQINTDLNLER.S	1999.21478	2	1.29E-05	0.95	5.04	-	884.6
AHQ-1-2, 2638	R.KKVEAQLQELQVK.F	1541.81761	2	3.07E-08	0.98	5.42	-	1947.6
AHQ-1-4, 2559 - 2560	R.KKVEAQLQELQVK.F	1541.81761	2	3.38E-09	0.98	5.45	-	1927.3
AHQ-1-3, 2553	R.KKVEAQLQELQVK.F	1541.81761	2	7.64E-06	0.97	4.71	-	1751.3
AHQ-1-6, 2446	R.KKVEAQLQELQVK.F	1541.81761	2	1.64E-08	0.96	4.54	-	1643.8
AHQ-1-5, 2411	R.KKVEAQLQELQVK.F	1541.81761	2	5.22E-08	0.98	5.43	-	2205.7
AHQ-1-1, 2740	R.KKVEAQLQELQVK.F	1541.81761	2	6.85E-09	0.95	4.43	-	1404.9
AHQ-1-2, 2954	R.KKVEAQLQELQVK.F	1541.81761	2	1.29E-07	0.97	5.43	-	1803.4
AHQ-1-2, 3714 - 3781	K.KLEEEQIILEDQNCK.L	1891.08992	2	1.41E-11	0.98	5.65	-	1971.6
AHQ-1-4, 3514	K.KLEEEQIILEDQNCK.L	1891.08992	2	9.32E-04	0.97	5.37	-	1932.4
AHQ-1-2, 3959	K.KLEEEQIILEDQNCK.L	1891.08992	2	2.01E-05	0.98	5.98	-	1639.5
AHQ-1-4, 3539 - 3608	K.KLEEEQIILEDQNCK.L	1891.08992	2	2.49E-10	0.98	6.02	-	1951.8
AHQ-1-3, 3489	K.KLEEEQIILEDQNCK.L	1891.08992	2	2.82E-06	0.98	5.72	-	1986.6
AHQ-1-2, 3725	K.KLEEEQIILEDQNCK.L	1891.08992	3	9.86E-05	0.91	4.41	-	1174.8
AHQ-1-3, 3753	K.KLEEEQIILEDQNCK.L	1891.08992	2	2.56E-04	0.91	3.60	-	694.5
AHQ-1-2, 3617 - 3630	K.KLEEEQIILEDQNCK.L	1891.08992	3	4.61E-04	0.92	4.47	-	1378.0
AHQ-1-3, 3505 - 3583	K.KLEEEQIILEDQNCK.L	1891.08992	2	8.48E-10	0.98	6.94	-	1812.5
AHQ-1-5, 3381	K.KLEEEQIILEDQNCK.L	1891.08992	2	1.06E-10	0.97	5.77	-	1386.7
AHQ-1-1, 3561 - 3587	K.KLEEEQIILEDQNCK.L	1891.08992	2	9.31E-05	0.98	6.04	-	1827.9
AHQ-1-2, 3878	K.KLEEEQIILEDQNCK.L	1891.08992	2	1.31E-05	0.96	4.60	-	1263.8
AHQ-1-1, 3680	K.KLEEEQIILEDQNCK.L	1891.08992	3	1.16E-05	0.87	4.10	-	1253.3
AHQ-1-1, 3675 - 3677	K.KLEEEQIILEDQNCK.L	1891.08992	2	5.27E-12	0.98	5.62	-	2144.5
AHQ-1-2, 3607	K.KLEEEQIILEDQNCK.L	1891.08992	2	4.34E-04	0.96	5.43	-	1164.7
AHQ-1-2, 4091	K.KLEEEQIILEDQNCKLAK.E	2203.49930	3	6.06E-05	0.94	4.77	-	1142.2
AHQ-1-3, 7102 - 7170	R.KLEGDSTDLSDQIAELQAQIAELK.M	2616.86032	2	2.47E-08	0.97	5.60	-	1481.9
AHQ-1-1, 6893	R.KLEGDSTDLSDQIAELQAQIAELK.M	2616.86032	2	4.72E-09	0.97	5.87	-	1379.9
AHQ-1-3, 6255	R.KLEGDSTDLSDQIAELQAQIAELK.M	2616.86032	3	2.79E-05	0.97	6.05	-	2157.9
AHQ-1-1, 6891	R.KLEGDSTDLSDQIAELQAQIAELK.M	2616.86032	3	2.97E-04	0.91	4.97	-	746.0
AHQ-1-5, 7277 - 7278	R.KLEGDSTDLSDQIAELQAQIAELK.M	2616.86032	3	8.40E-05	0.88	4.37	-	861.1
AHQ-1-2, 7223 - 7258	R.KLEGDSTDLSDQIAELQAQIAELK.M	2616.86032	2	6.96E-13	0.98	6.75	-	1473.4
AHQ-1-2, 7257 - 7266	R.KLEGDSTDLSDQIAELQAQIAELK.M	2616.86032	3	2.99E-06	0.94	5.66	-	891.0
AHQ-1-5, 7265 - 7281	R.KLEGDSTDLSDQIAELQAQIAELK.M	2616.86032	2	2.39E-07	0.94	4.54	-	1169.7
AHQ-1-4, 7278 - 7286	R.KLEGDSTDLSDQIAELQAQIAELK.M	2616.86032	3	4.25E-07	0.94	5.54	-	836.8
AHQ-1-3, 7101	R.KLEGDSTDLSDQIAELQAQIAELK.M	2616.86032	3	3.20E-05	0.88	4.28	-	791.1
AHQ-1-7, 7304	R.KLEGDSTDLSDQIAELQAQIAELK.M	2616.86032	2	2.16E-07	0.95	4.42	-	1178.0
AHQ-1-2, 6429 - 6499	R.KLEGDSTDLSDQIAELQAQIAELK.M	2616.86032	3	2.02E-09	0.96	5.83	-	1490.1
AHQ-1-4, 7280 - 7348	R.KLEGDSTDLSDQIAELQAQIAELK.M	2616.86032	2	4.96E-08	0.97	6.28	-	1193.8
AHQ-1-4, 2782	K.KLVWVPSDK.S	1072.28194	1	4.09E-04	0.50	2.40	-	476.4
AHQ-1-3, 2777	K.KLVWVPSDK.S	1072.28194	2	1.94E-04	0.86	3.48	-	772.5
AHQ-1-10, 2616	K.KLVWVPSDK.S	1072.28194	2	2.63E-04	0.89	3.77	-	750.1
AHQ-1-2, 2870	K.KLVWVPSDK.S	1072.28194	2	8.11E-06	0.74	3.02	-	597.5
AHQ-1-6, 2644	K.KLVWVPSDK.S	1072.28194	2	2.58E-05	0.88	3.25	-	1002.4
AHQ-1-1, 2973 - 2976	K.KLVWVPSDK.S	1072.28194	2	8.82E-04	0.87	3.38	-	833.3
AHQ-1-10, 2615 - 2635	K.KLVWVPSDK.S	1072.28194	1	9.51E-04	0.35	2.16	-	430.1
AHQ-1-2, 2871	K.KLVWVPSDK.S	1072.28194	1	1.50E-04	0.64	2.49	-	555.0
AHQ-1-4, 3682	K.KMEDSVGCLETAEEVK.R	1827.02450	2	1.45E-04	0.93	4.39	-	1199.3
AHQ-1-3, 3650	K.KMEDSVGCLETAEEVK.R	1827.02450	2	2.58E-07	0.92	3.85	-	1125.5
AHQ-1-1, 3317	K.KM*EDSVGCLETAEEVK.R	1843.02390	2	2.18E-04	0.78	3.01	-	648.6
AHQ-1-2, 3029	K.KM*EDSVGCLETAEEVKR.K	1999.21025	3	5.60E-06	0.96	5.02	-	1468.1
AHQ-1-1, 3397	K.KMEDSVGCLETAEEVKR.K	1983.21085	3	1.42E-06	0.91	4.05	-	1246.8
AHQ-1-2, 3414	K.KMEDSVGCLETAEEVKR.K	1983.21085	2	1.10E-10	0.97	4.87	-	1972.9
AHQ-1-5, 3182	K.KMEDSVGCLETAEEVKR.K	1983.21085	2	8.43E-09	0.95	4.54	-	1278.0
AHQ-1-4, 3340	K.KMEDSVGCLETAEEVKR.K	1983.21085	3	6.40E-05	0.91	4.42	-	1212.5
AHQ-1-1, 3051	K.KM*EDSVGCLETAEEVKR.K	1999.21025	3	1.19E-04	0.98	6.15	-	1958.8
AHQ-1-3, 3305	K.KMEDSVGCLETAEEVKR.K	1983.21085	2	1.32E-06	0.97	5.14	-	1307.7
AHQ-1-2, 3031 - 3034	K.KM*EDSVGCLETAEEVKR.K	1999.21025	2	2.70E-08	0.87	3.72	-	554.0
AHQ-1-4, 2919	K.KM*EDSVGCLETAEEVKR.K	1999.21025	3	1.46E-05	0.95	5.04	-	1376.1
AHQ-1-5, 3179	K.KMEDSVGCLETAEEVKR.K	1983.21085	3	7.23E-09	0.95	5.22	-	1019.9
AHQ-1-3, 3302	K.KMEDSVGCLETAEEVKR.K	1983.21085	3	6.24E-08	0.96	5.63	-	1212.4
AHQ-1-4, 4990	K.KM*QQNIQELEEQLEEEESAR.Q	2478.63294	2	1.52E-04	0.56	2.63	-	342.8
AHQ-1-1, 4992	K.KM*QQNIQELEEQLEEEESAR.Q	2478.63294	3	2.81E-08	0.95	5.04	-	1648.7
AHQ-1-3, 4905	K.KM*QQNIQELEEQLEEEESAR.Q	2478.63294	3	1.19E-06	0.90	4.47	-	1183.2
AHQ-1-3, 4907	K.KM*QQNIQELEEQLEEEESAR.Q	2478.63294	2	9.12E-08	0.85	3.88	-	514.8
AHQ-1-4, 4196	K.KQELEEICHDLEAR.V	1771.93036	3	5.66E-04	0.95	4.89	-	1535.7
AHQ-1-9, 3964	K.KQELEEICHDLEAR.V	1771.93036	2	4.36E-06	0.94	4.87	-	1165.6
AHQ-1-2, 4298 - 4369	K.KQELEEICHDLEAR.V	1771.93036	2	5.94E-06	0.96	5.07	-	1595.5

AHQ-1-6, 4008	K.KQELEEICHDLEAR.V	1771.93036	2	1.07E-05	0.97	5.33	-	1798.1
AHQ-1-5, 4054	K.KQELEEICHDLEAR.V	1771.93036	2	6.10E-05	0.95	4.95	-	1325.2
AHQ-1-4, 4188	K.KQELEEICHDLEAR.V	1771.93036	2	1.69E-06	0.95	4.65	-	1414.2
AHQ-1-1, 4252	K.KQELEEICHDLEAR.V	1771.93036	3	4.38E-06	0.95	5.10	-	1388.5
AHQ-1-5, 2757	K.KVEAQLQELQVK.F	1413.64470	2	1.42E-05	0.97	5.12	-	1806.0
AHQ-1-3, 2925 - 2998	K.KVEAQLQELQVK.F	1413.64470	2	2.65E-06	0.95	4.76	-	1161.0
AHQ-1-4, 2944	K.KVEAQLQELQVK.F	1413.64470	1	4.32E-04	0.36	2.52	-	394.3
AHQ-1-1, 3091	K.KVEAQLQELQVK.F	1413.64470	2	5.98E-06	0.95	4.15	-	1460.5
AHQ-1-4, 2930	K.KVEAQLQELQVK.F	1413.64470	2	1.67E-06	0.96	4.42	-	1828.8
AHQ-1-2, 3055	K.KVEAQLQELQVK.F	1413.64470	1	2.08E-07	0.53	2.96	-	396.5
AHQ-1-3, 2938	K.KVEAQLQELQVK.F	1413.64470	3	4.05E-07	0.93	4.88	-	1134.2
AHQ-1-2, 3051	K.KVEAQLQELQVK.F	1413.64470	3	1.04E-06	0.91	3.91	-	1390.0
AHQ-1-6, 2810	K.KVEAQLQELQVK.F	1413.64470	2	4.52E-07	0.95	4.19	-	1459.2
AHQ-1-2, 3045	K.KVEAQLQELQVK.F	1413.64470	1	2.31E-04	0.19	2.40	-	314.9
AHQ-1-2, 3977	K.KVIQYLAYVASSHK.S	1607.87775	2	1.48E-05	0.83	2.71	-	1008.2
AHQ-1-1, 3904 - 3984	K.LEEEQIILEDQNCK.L	1762.91700	2	3.46E-09	0.97	5.44	-	1164.2
AHQ-1-2, 4085 - 4109	K.LEEEQIILEDQNCK.L	1762.91700	2	1.88E-09	0.98	5.93	-	1595.9
AHQ-1-4, 4014	K.LEEEQIILEDQNCK.L	1762.91700	2	6.70E-09	0.96	5.02	-	1304.7
AHQ-1-2, 3987	K.LEEEQIILEDQNCK.L	1762.91700	2	1.57E-04	0.80	3.57	-	274.4
AHQ-1-4, 3922	K.LEEEQIILEDQNCK.L	1762.91700	2	1.58E-08	0.95	4.11	-	1279.2
AHQ-1-3, 3953 - 4030	K.LEEEQIILEDQNCK.L	1762.91700	2	5.18E-09	0.96	4.85	-	1247.0
AHQ-1-3, 7181 - 7182	K.LEGDSTDLSDQIAELQAQIAELK.M	2488.68740	2	3.36E-11	0.96	6.09	-	769.8
AHQ-1-2, 7325	K.LEGDSTDLSDQIAELQAQIAELK.M	2488.68740	3	2.79E-07	0.96	5.82	-	1372.2
AHQ-1-1, 6944	K.LEGDSTDLSDQIAELQAQIAELK.M	2488.68740	3	1.59E-05	0.91	4.63	-	941.3
AHQ-1-2, 3662	R.LEVNLQAMK.A	1046.26630	2	7.45E-05	0.91	3.87	-	788.0
AHQ-1-5, 3411	R.LEVNLQAMK.A	1046.26630	2	7.03E-07	0.77	2.72	-	500.0
AHQ-1-1, 3664 - 3715	R.LEVNLQAMK.A	1046.26630	2	3.51E-04	0.76	3.01	-	595.7
AHQ-1-3, 3555 - 3571	R.LEVNLQAMK.A	1046.26630	2	6.87E-05	0.85	3.54	-	581.6
AHQ-1-2, 2677	R.LEVNLQAM*K.A	1062.26570	2	1.55E-05	0.88	3.10	-	771.7
AHQ-1-6, 3402	R.LEVNLQAMK.A	1046.26630	2	1.47E-05	0.79	3.10	-	610.9
AHQ-1-2, 3634	K.LKDVLLQVDDER.R	1443.62767	2	8.21E-11	0.96	4.58	-	1573.2
AHQ-1-3, 3507 - 3579	K.LKDVLLQVDDER.R	1443.62767	2	8.89E-08	0.96	4.00	-	1883.6
AHQ-1-1, 3217	K.LKDVLLQVDDERR.N	1599.81402	2	3.29E-04	0.81	3.29	-	939.3
AHQ-1-2, 3169	K.LKDVLLQVDDERR.N	1599.81402	3	3.68E-04	0.87	3.02	-	1286.6
AHQ-1-5, 2878	K.LKDVLLQVDDERR.N	1599.81402	2	7.16E-05	0.88	3.43	-	1088.0
AHQ-1-3, 3061	K.LKDVLLQVDDERR.N	1599.81402	2	1.39E-05	0.87	3.88	-	909.1
AHQ-1-2, 2966	K.LKNKHEAMITDLEER.L	1828.08363	2	1.20E-07	0.93	3.59	-	1051.6
AHQ-1-2, 2065	K.LKQVEDEKNSFR.E	1493.64677	2	4.50E-05	0.63	2.77	-	532.3
AHQ-1-2, 7415 - 7481	K.LQLQEQLQAETELCAEAEELR.A	2503.72536	2	3.25E-06	0.86	3.13	-	886.9
AHQ-1-2, 7543	K.LQLQEQLQAETELCAEAEELR.A	2503.72536	2	2.82E-04	0.84	2.93	-	881.1
AHQ-1-2, 7469 - 7537	K.LQLQEQLQAETELCAEAEELR.A	2503.72536	3	1.19E-05	0.93	4.96	-	1240.8
AHQ-1-5, 6205	R.LQQELDDLLVDLDHQR.Q	1951.12699	3	3.52E-04	0.87	4.10	-	644.4
AHQ-1-1, 6160 - 6164	R.LQQELDDLLVDLDHQR.Q	1951.12699	3	1.70E-05	0.92	4.76	-	957.1
AHQ-1-2, 6503	R.LQQELDDLLVDLDHQR.Q	1951.12699	3	4.62E-06	0.74	3.23	-	735.6
AHQ-1-1, 6159	R.LQQELDDLLVDLDHQR.Q	1951.12699	2	2.37E-06	0.96	4.80	-	1443.5
AHQ-1-5, 6209 - 6210	R.LQQELDDLLVDLDHQR.Q	1951.12699	2	1.38E-09	0.98	5.89	-	1977.9
AHQ-1-2, 5875	R.LQQELDDLLVDLDHQR.Q	1951.12699	3	4.68E-06	0.92	4.57	-	895.3
AHQ-1-4, 6255	R.LQQELDDLLVDLDHQR.Q	1951.12699	2	5.90E-08	0.96	5.03	-	1458.8
AHQ-1-7, 6233	R.LQQELDDLLVDLDHQR.Q	1951.12699	2	5.68E-09	0.96	4.38	-	1608.4
AHQ-1-3, 5031 - 5035	R.LQQELDDLLVDLDHQR.Q	1951.12699	2	1.74E-12	0.96	4.62	-	1565.9
AHQ-1-6, 6106	R.LQQELDDLLVDLDHQR.Q	1951.12699	3	7.87E-05	0.80	3.51	-	752.8
AHQ-1-4, 6258	R.LQQELDDLLVDLDHQR.Q	1951.12699	3	3.82E-05	0.76	3.45	-	468.7
AHQ-1-3, 6141	R.LQQELDDLLVDLDHQR.Q	1951.12699	2	4.33E-11	0.96	4.81	-	1552.6
AHQ-1-2, 5859	R.LQQELDDLLVDLDHQR.Q	1951.12699	2	3.16E-06	0.90	4.02	-	1061.1
AHQ-1-2, 6495 - 6570	R.LQQELDDLLVDLDHQR.Q	1951.12699	2	1.92E-09	0.95	4.62	-	1260.3
AHQ-1-2, 6313 - 6385	R.LQQELDDLLVDLDHQR.Q	1951.12699	2	2.23E-11	0.97	5.61	-	1403.8
AHQ-1-6, 6110 - 6114	R.LQQELDDLLVDLDHQR.Q	1951.12699	2	5.91E-11	0.97	5.44	-	1616.9
AHQ-1-1, 5728	R.LQQELDDLLVDLDHQR.Q	1951.12699	3	1.41E-06	0.92	4.58	-	860.5
AHQ-1-3, 6123	K.LQVELDNVTGLLSQSDSK.S	1947.13342	2	5.37E-12	0.97	5.72	-	1297.2
AHQ-1-4, 5624	K.LQVELDNVTGLLSQSDSK.S	1947.13342	2	1.65E-07	0.96	5.22	-	1207.1
AHQ-1-3, 5459 - 5517	K.LQVELDNVTGLLSQSDSK.S	1947.13342	2	4.19E-08	0.94	4.57	-	1046.8
AHQ-1-2, 5671	K.LQVELDNVTGLLSQSDSK.S	1947.13342	2	1.65E-06	0.95	4.65	-	1009.0
AHQ-1-4, 5951 - 5983	K.LQVELDNVTGLLSQSDSK.S	1947.13342	2	3.37E-07	0.97	5.37	-	1394.9
AHQ-1-4, 6099	K.LQVELDNVTGLLSQSDSK.S	1947.13342	2	1.27E-07	0.88	4.00	-	686.1
AHQ-1-3, 5838 - 5909	K.LQVELDNVTGLLSQSDSK.S	1947.13342	2	1.00E-30	0.98	6.57	-	1394.4
AHQ-1-2, 6066 - 6133	K.LQVELDNVTGLLSQSDSK.S	1947.13342	2	2.93E-10	0.98	5.90	-	1541.2
AHQ-1-1, 5532	K.LQVELDNVTGLLSQSDSK.S	1947.13342	2	2.67E-07	0.95	4.35	-	1298.6
AHQ-1-1, 5824 - 5887	K.LQVELDNVTGLLSQSDSK.S	1947.13342	2	1.92E-06	0.98	5.69	-	1878.7
AHQ-1-3, 5945 - 6023	K.LQVELDNVTGLLSQSDSK.S	1947.13342	2	3.95E-08	0.98	6.50	-	1244.5
AHQ-1-2, 6206 - 6246	K.LQVELDNVTGLLSQSDSK.S	1947.13342	2	2.54E-10	0.97	5.92	-	1424.7
AHQ-1-5, 6039	K.LQVELDNVTGLLSQSDSK.S	1947.13342	2	5.14E-05	0.87	3.26	-	949.8
AHQ-1-2, 5983 - 6053	K.LQVELDNVTGLLSQSDSK.S	1947.13342	2	3.16E-07	0.98	6.20	-	1632.1
AHQ-1-5, 5914 - 5967	K.LQVELDNVTGLLSQSDSK.S	1947.13342	2	6.62E-12	0.98	6.29	-	1748.3
AHQ-1-6, 5834	K.LQVELDNVTGLLSQSDSK.S	1947.13342	2	1.58E-05	0.95	4.46	-	1273.6
AHQ-1-3, 6215	K.LQVELDNVTGLLSQSDSK.S	1947.13342	2	2.50E-12	0.97	5.77	-	1421.3
AHQ-1-1, 3651	R.LTEM*ETLQSQLM*AEK.L	1785.03091	2	1.38E-04	0.78	3.07	-	881.4
AHQ-1-3, 5201	R.LTEMETLQSQLMAEK.L	1753.03211	2	8.08E-04	0.61	2.85	-	669.5
AHQ-1-6, 5066	R.LTEMETLQSQLMAEK.L	1753.03211	2	2.78E-05	0.69	3.00	-	770.5
AHQ-1-3, 4074	R.LTEM*ETLQSQLMAEK.L	1769.03151	2	3.27E-06	0.97	4.31	-	2310.2
AHQ-1-2, 5322 - 5331	R.LTEMETLQSQLM*AEK.L	1769.03151	2	6.94E-04	0.57	3.43	-	684.7
AHQ-1-2, 4757	R.LTEMETLQSQLM*AEK.L	1769.03151	2	2.02E-06	0.95	4.73	-	1047.4
AHQ-1-3, 3566 - 3637	R.LTEM*ETLQSQLM*AEK.L	1785.03091	2	1.20E-06	0.95	4.09	-	1273.8
AHQ-1-2, 5381	R.LTEMETLQSQLMAEK.L	1753.03211	2	6.78E-05	0.97	4.73	-	1639.1
AHQ-1-2, 4225 - 4299	R.LTEM*ETLQSQLMAEK.L	1769.03151	2	2.93E-07	0.98	5.09	-	2313.5
AHQ-1-3, 4617 - 4694	R.LTEMETLQSQLM*AEK.L	1769.03151	2	1.84E-06	0.94	4.14	-	1241.5
AHQ-1-4, 4679	R.LTEMETLQSQLM*AEK.L	1769.03151	2	1.45E-05	0.87	3.72	-	883.8
AHQ-1-5, 5137 - 5143	R.LTEMETLQSQLMAEK.L	1753.03211	2	1.53E-06	0.95	4.51	-	1417.4
AHQ-1-2, 4850 - 4923	R.LTEMETLQSQLM*AEK.L	1769.03151	2	3.08E-06	0.76	3.52	-	651.4
AHQ-1-2, 3693 - 3774	R.LTEM*ETLQSQLM*AEK.L	1785.03091	2	7.57E-06	0.96	4.77	-	1179.7
AHQ-1-1, 6796	K.LTKDFSALESQLQDTQELLQEENR.Q	2837.04608	3	3.09E-06	0.82	3.73	-	950.8
AHQ-1-2, 6857	K.LTKDFSALESQLQDTQELLQEENR.Q	2837.04608	3	1.35E-07	0.94	5.12	-	1454.9
AHQ-1-2, 4110	K.MEDSVGCLETAEEVK.R	1698.85159	2	3.07E-06	0.93	3.82	-	919.0
AHQ-1-4, 4026	K.MEDSVGCLETAEEVK.R	1698.85159	2	3.17E-05	0.90	3.79	-	763.5
AHQ-1-3, 3981 - 3985	K.MEDSVGCLETAEEVK.R	1698.85159	2	5.03E-07	0.92	4.65	-	654.8
AHQ-1-2, 3713	K.MEDSVGCLETAEEVKR.K	1855.03794	3	2.05E-05	0.79	3.52	-	818.9
AHQ-1-3, 3595	K.MEDSVGCLETAEEVKR.K	1855.03794	3	6.84E-07	0.77	3.19	-	705.3
AHQ-1-1, 3345	K.M*EDSVGCLETAEEVKR.K	1871.03734	2	4.14E-05	0.81	3.16	-	494.4
AHQ-1-2, 3709	K.MEDSVGCLETAEEVKR.K	1855.03794	2	3.66E-04	0.76	3.43	-	412.5
AHQ-1-4, 3620	K.MEDSVGCLETAEEVKR.K	1855.03794	2	1.85E-06	0.77	3.25	-	511.7
AHQ-1-3, 3529 - 3603	K.MEDSVGCLETAEEVKR.K	1855.03794	2	6.47E-06	0.73	3.12	-	408.8
AHQ-1-2, 5761 - 5829	K.MQQNIQELEEQLEEEESAR.Q	2334.46063	3	9.56E-08	0.82	3.79	-	1022.8
AHQ-1-7, 5328 - 5341	K.NFINNPLAQADWAAK.K	1673.85315	2	2.25E-10	0.95	4.67	-	1142.1
AHQ-1-9, 5187	K.NFINNPLAQADWAAK.K	1673.85315	2	1.28E-06	0.93	4.00	-	1082.4
AHQ-1-2, 6125	K.NFINNPLAQADWAAK.K	1673.85315	2	3.43E-09	0.91	3.89	-	840.2
AHQ-1-14- , 5203 - 5214	K.NFINNPLAQADWAAK.K	1673.85315	2	2.18E-09	0.96	4.86	-	1137.7
AHQ-1-3, 5977	K.NFINNPLAQADWAAK.K	1673.85315	2	1.23E-08	0.92	4.15	-	756.0
AHQ-1-2, 5669	K.NFINNPLAQADWAAK.K	1673.85315	2	2.77E-08	0.88	3.74	-	722.2

AHQ-1-13- , 5279 - 5347	K.NFINNPLAQADWAAK.K	1673.85315	2	8.36E-07	0.97	5.77	-	1091.2
AHQ-1-3, 5362 - 5386	K.NFINNPLAQADWAAK.K	1673.85315	2	8.39E-04	0.45	2.66	-	406.7
AHQ-1-13, 5033	K.NFINNPLAQADWAAK.K	1673.85315	2	1.19E-09	0.96	5.04	-	1152.0
AHQ-1-2, 5463 - 5533	K.NFINNPLAQADWAAK.K	1673.85315	2	4.83E-11	0.96	5.32	-	1227.2
AHQ-1-2, 5899	K.NFINNPLAQADWAAK.K	1673.85315	2	1.50E-04	0.93	3.92	-	1007.9
AHQ-1-1, 5911	K.NFINNPLAQADWAAK.K	1673.85315	2	2.00E-07	0.89	3.25	-	883.2
AHQ-1-1, 5443 - 5452	K.NFINNPLAQADWAAK.K	1673.85315	2	2.24E-11	0.96	4.88	-	1081.3
AHQ-1-6, 5279 - 5283	K.NFINNPLAQADWAAK.K	1673.85315	2	5.99E-06	0.96	5.59	-	975.0
AHQ-1-10, 4725	K.NFINNPLAQADWAAK.K	1673.85315	2	2.49E-07	0.92	4.07	-	960.6
AHQ-1-4, 5470	K.NFINNPLAQADWAAK.K	1673.85315	2	2.65E-05	0.93	4.25	-	965.2
AHQ-1-5, 5373	K.NFINNPLAQADWAAK.K	1673.85315	2	2.69E-06	0.95	4.82	-	1053.1
AHQ-1-11, 4872 - 4882	K.NFINNPLAQADWAAK.K	1673.85315	2	3.54E-07	0.95	4.95	-	867.2
AHQ-1-2, 3155	K.NKHEAMITDLEER.L	1586.75247	2	1.19E-08	0.89	3.61	-	995.1
AHQ-1-3, 2199	K.NKHEAM*ITDLEER.L	1602.75187	3	5.97E-06	0.94	3.79	-	1813.9
AHQ-1-3, 2541	K.NKHEAMITDLEER.L	1586.75247	2	1.10E-08	0.94	3.63	-	1534.3
AHQ-1-2, 2637	K.NKHEAMITDLEER.L	1586.75247	2	9.74E-10	0.93	3.78	-	1211.1
AHQ-1-4, 2554	K.NKHEAMITDLEER.L	1586.75247	2	1.16E-07	0.92	3.50	-	1135.1
AHQ-1-11, 5258	K.NLPIYSEEIVEMYK.G	1728.98720	2	1.09E-04	0.81	3.47	-	402.1
AHQ-1-5, 5823 - 5841	K.NLPIYSEEIVEMYK.G	1728.98720	2	6.53E-04	0.91	3.09	-	867.9
AHQ-1-12, 5453	K.NLPIYSEEIVEMYK.G	1728.98720	2	2.31E-04	0.91	3.90	-	595.7
AHQ-1-3, 5847	K.NLPIYSEEIVEMYK.G	1728.98720	2	1.71E-05	0.84	3.48	-	553.6
AHQ-1-9, 5634 - 5643	K.NLPIYSEEIVEMYK.G	1728.98720	2	2.49E-04	0.76	3.15	-	513.1
AHQ-1-3, 5225	K.NLPIYSEEIVEM*YK.G	1744.98660	2	1.49E-04	0.92	3.66	-	882.2
AHQ-1-13, 5341 - 5400	K.NLPIYSEEIVEMYK.G	1728.98720	2	3.54E-04	0.86	3.41	-	623.5
AHQ-1-13- , 5739 - 5741	K.NLPIYSEEIVEMYK.G	1728.98720	2	8.92E-07	0.94	4.12	-	871.4
AHQ-1-6, 5143	K.NLPIYSEEIVEM*YK.G	1744.98660	2	2.22E-04	0.93	3.56	-	871.8
AHQ-1-4, 5302	K.NLPIYSEEIVEM*YK.G	1744.98660	2	1.62E-07	0.91	4.08	-	619.3
AHQ-1-2, 6009 - 6079	K.NLPIYSEEIVEMYK.G	1728.98720	2	4.68E-06	0.94	4.04	-	912.3
AHQ-1-2, 6211	K.NLPIYSEEIVEMYK.G	1728.98720	2	2.32E-04	0.83	3.02	-	656.1
AHQ-1-2, 5379	K.NLPIYSEEIVEM*YK.G	1744.98660	2	2.26E-09	0.94	4.08	-	873.1
AHQ-1-1, 5863	K.NLPIYSEEIVEMYK.G	1728.98720	2	4.87E-05	0.88	3.21	-	702.0
AHQ-1-1, 5299 - 5300	K.NLPIYSEEIVEM*YK.G	1744.98660	2	9.73E-06	0.95	4.34	-	1164.6
AHQ-1-5, 5538	K.NMDPLNDNIATLLHQSSDK.F	2127.32169	2	7.18E-08	0.93	4.42	-	751.4
AHQ-1-2, 5837	K.NMDPLNDNIATLLHQSSDK.F	2127.32169	2	6.97E-08	0.91	4.05	-	782.4
AHQ-1-2, 5945 - 6018	K.NMDPLNDNIATLLHQSSDK.F	2127.32169	2	1.24E-07	0.94	4.45	-	867.2
AHQ-1-3, 5117	K.NM*DPLNDNIATLLHQSSDK.F	2143.32109	3	3.09E-05	0.87	3.93	-	922.3
AHQ-1-2, 5254	K.NM*DPLNDNIATLLHQSSDK.F	2143.32109	3	2.62E-07	0.66	3.21	-	690.8
AHQ-1-6, 5460	K.NMDPLNDNIATLLHQSSDK.F	2127.32169	2	1.19E-04	0.89	4.55	-	617.9
AHQ-1-1, 5653	K.NMDPLNDNIATLLHQSSDK.F	2127.32169	2	5.79E-05	0.91	3.90	-	895.3
AHQ-1-2, 5746	K.NMDPLNDNIATLLHQSSDK.F	2127.32169	2	5.97E-05	0.84	3.93	-	534.5
AHQ-1-2, 5261 - 5339	K.NM*DPLNDNIATLLHQSSDK.F	2143.32109	2	1.22E-08	0.94	4.15	-	1207.4
AHQ-1-3, 7286 - 7287	K.PAGPPGILALLDEECWFPK.A	2112.43315	2	5.83E-08	0.98	5.58	-	1765.4
AHQ-1-2, 7425	K.PAGPPGILALLDEECWFPK.A	2112.43315	2	6.06E-07	0.98	6.43	-	2426.5
AHQ-1-2, 2442	K.PLLQVSR.Q	812.98013	1	5.40E-04	0.45	2.16	-	388.9
AHQ-1-1, 3320	R.QAQQERDELADEIANSSGK.G	2090.15168	2	1.29E-05	0.94	4.68	-	718.1
AHQ-1-3, 2826	R.QAQQERDELADEIANSSGK.G	2090.15168	2	9.49E-07	0.87	4.26	-	423.8
AHQ-1-1, 2939	R.QAQQERDELADEIANSSGK.G	2090.15168	2	2.58E-04	0.66	3.43	-	392.9
AHQ-1-3, 3299	R.QAQQERDELADEIANSSGK.G	2090.15168	2	1.06E-04	0.84	3.60	-	565.8
AHQ-1-3, 3213 - 3291	R.QAQQERDELADEIANSSGK.G	2090.15168	3	1.00E-03	0.75	3.56	-	604.4
AHQ-1-2, 3411	R.QAQQERDELADEIANSSGK.G	2090.15168	2	6.67E-06	0.86	4.14	-	396.4
AHQ-1-3, 2287	K.QIATLHAQVADM*K.K	1442.66637	2	4.82E-05	0.78	2.99	-	582.8
AHQ-1-2, 2378	K.QIATLHAQVADM*K.K	1442.66637	2	7.93E-04	0.68	3.09	-	342.4
AHQ-1-3, 2031	R.QLEEAEEEAQR.A	1332.35590	2	8.95E-06	0.81	3.56	-	585.1
AHQ-1-2, 2106	R.QLEEAEEEAQR.A	1332.35590	2	2.86E-04	0.82	3.31	-	692.8
AHQ-1-1, 2180	R.QLEEAEEEAQR.A	1332.35590	2	7.36E-06	0.86	3.36	-	811.6
AHQ-1-5, 1910	R.QLEEAEEEAQR.A	1332.35590	2	2.64E-06	0.76	3.29	-	681.7
AHQ-1-1, 3735	R.RGDLPFVVPR.R	1156.36217	2	6.64E-07	0.96	3.68	-	1524.6
AHQ-1-2, 3715 - 3791	R.RGDLPFVVPR.R	1156.36217	2	8.47E-06	0.95	3.76	-	1351.1
AHQ-1-3, 3602	R.RGDLPFVVPR.R	1156.36217	2	4.08E-05	0.93	3.27	-	1441.9
AHQ-1-4, 3616	R.RGDLPFVVPR.R	1156.36217	2	1.13E-05	0.94	3.43	-	1178.6
AHQ-1-10, 3263	R.RGDLPFVVPR.R	1156.36217	2	2.86E-06	0.93	3.32	-	1364.5
AHQ-1-6, 3415 - 3487	R.RGDLPFVVPR.R	1156.36217	2	1.58E-05	0.91	3.40	-	1056.6
AHQ-1-9, 3276	R.RGDLPFVVPR.R	1156.36217	2	1.42E-05	0.93	3.59	-	1024.8
AHQ-1-11, 3352	R.RGDLPFVVPR.R	1156.36217	2	2.53E-05	0.86	3.11	-	826.9
AHQ-1-3, 6803	R.RKLEGDSTDLSDQIAELQAQIAELK.M	2773.04667	3	2.26E-08	0.96	6.03	-	1275.3
AHQ-1-4, 6978	R.RKLEGDSTDLSDQIAELQAQIAELK.M	2773.04667	3	2.21E-07	0.95	5.77	-	1127.5
AHQ-1-2, 7126	R.RKLEGDSTDLSDQIAELQAQIAELK.M	2773.04667	3	1.72E-04	0.95	5.43	-	1297.9
AHQ-1-2, 7021	R.RKLEGDSTDLSDQIAELQAQIAELK.M	2773.04667	3	3.05E-09	0.97	6.11	-	1974.2
AHQ-1-1, 6744	R.RKLEGDSTDLSDQIAELQAQIAELK.M	2773.04667	3	1.18E-08	0.95	5.70	-	1392.1
AHQ-1-3, 2003	K.RQLEEAEEEAQR.A	1488.54225	2	1.46E-09	0.98	4.79	-	3347.0
AHQ-1-2, 2083	K.RQLEEAEEEAQR.A	1488.54225	2	2.04E-08	0.98	5.05	-	3426.6
AHQ-1-6, 6139	K.SGFEPASLKEEVGEEAIVELVENGKK.V	2790.07246	3	1.30E-08	0.93	4.97	-	1160.1
AHQ-1-2, 6373 - 6450	K.SGFEPASLKEEVGEEAIVELVENGKK.V	2790.07246	3	1.47E-08	0.97	6.26	-	1342.5
AHQ-1-2, 7523	K.SGFEPASLKEEVGEEAIVELVENGKK.V	2790.07246	3	3.35E-12	0.92	4.95	-	685.2
AHQ-1-2, 6606	K.SGFEPASLKEEVGEEAIVELVENGKK.V	2790.07246	3	7.38E-10	0.93	4.88	-	997.9
AHQ-1-2, 6518	K.SGFEPASLKEEVGEEAIVELVENGKK.V	2790.07246	3	4.62E-10	0.96	5.26	-	1458.5
AHQ-1-5, 6302	K.SGFEPASLKEEVGEEAIVELVENGKK.V	2790.07246	3	1.39E-09	0.71	3.28	-	576.8
AHQ-1-3, 6195	K.SGFEPASLKEEVGEEAIVELVENGKK.V	2790.07246	3	2.43E-05	0.88	4.74	-	471.7
AHQ-1-2, 7123	K.SGFEPASLKEEVGEEAIVELVENGKK.V	2790.07246	3	1.42E-05	0.88	4.27	-	697.3
AHQ-1-13, 5779 - 5835	K.SGFEPASLKEEVGEEAIVELVENGKK.V	2790.07246	3	6.80E-05	0.63	3.52	-	429.7
AHQ-1-1, 6176 - 6235	K.SGFEPASLKEEVGEEAIVELVENGKK.V	2790.07246	3	1.45E-11	0.94	5.23	-	1017.0
AHQ-1-1, 6328	K.SGFEPASLKEEVGEEAIVELVENGKK.V	2790.07246	3	3.06E-10	0.96	5.49	-	1370.6
AHQ-1-5, 6974 - 7045	K.SMEAEMIQLQEELAAAER.A	2050.30064	2	4.12E-06	0.95	3.98	-	1924.5
AHQ-1-3, 4802	K.SM*EAEM*IQLQEELAAAER.A	2082.29944	2	2.87E-05	0.93	4.16	-	1003.4
AHQ-1-2, 6002 - 6081	K.SM*EAEMIQLQEELAAAER.A	2066.30004	2	2.62E-07	0.95	5.40	-	1495.1
AHQ-1-3, 6877	K.SMEAEMIQLQEELAAAER.A	2050.30064	3	4.29E-08	0.97	5.10	-	2261.0
AHQ-1-2, 4974 - 5038	K.SM*EAEM*IQLQEELAAAER.A	2082.29944	2	2.87E-09	0.97	5.29	-	1747.9
AHQ-1-3, 5575 - 5646	K.SMEAEM*IQLQEELAAAER.A	2066.30004	2	9.06E-07	0.88	4.46	-	985.2
AHQ-1-1, 4812	K.SM*EAEM*IQLQEELAAAER.A	2082.29944	2	6.14E-10	0.97	4.97	-	1639.1
AHQ-1-1, 4941	K.SM*EAEM*IQLQEELAAAER.A	2082.29944	2	2.17E-06	0.87	3.69	-	846.6
AHQ-1-2, 7013 - 7078	K.SMEAEMIQLQEELAAAER.A	2050.30064	2	1.05E-06	0.98	6.33	-	1727.3
AHQ-1-2, 7058	K.SMEAEMIQLQEELAAAER.A	2050.30064	3	5.32E-08	0.95	5.02	-	1475.0
AHQ-1-3, 6825 - 6893	K.SMEAEMIQLQEELAAAER.A	2050.30064	2	4.07E-05	0.98	6.17	-	1676.0
AHQ-1-2, 7141 - 7209	K.SMEAEMIQLQEELAAAER.A	2050.30064	2	3.27E-07	0.95	4.79	-	1309.1
AHQ-1-5, 3435	R.SMMQDREDQSILCTGESGAGK.T	2302.50687	2	6.68E-07	0.97	5.68	-	1394.4
AHQ-1-7, 3486	R.SMMQDREDQSILCTGESGAGK.T	2302.50687	2	6.67E-04	0.78	3.45	-	795.4
AHQ-1-2, 3673	R.SMMQDREDQSILCTGESGAGK.T	2302.50687	2	4.95E-07	0.97	5.88	-	1588.2
AHQ-1-7, 3665 - 3674	K.TDLLLEPYNK.Y	1206.36989	2	1.36E-05	0.91	3.76	-	1028.1
AHQ-1-4, 3790 - 3791	K.TDLLLEPYNK.Y	1206.36989	2	4.54E-05	0.93	3.87	-	1133.2
AHQ-1-3, 3757	K.TDLLLEPYNK.Y	1206.36989	2	1.01E-06	0.83	3.32	-	920.1
AHQ-1-6, 3584 - 3595	K.TDLLLEPYNK.Y	1206.36989	2	1.36E-05	0.92	4.01	-	970.1
AHQ-1-5, 3618 - 3641	K.TDLLLEPYNK.Y	1206.36989	2	1.70E-04	0.81	3.10	-	929.4
AHQ-1-2, 3874 - 3897	K.TDLLLEPYNK.Y	1206.36989	2	1.41E-06	0.93	4.13	-	1043.2
AHQ-1-2, 3883	K.TDLLLEPYNK.Y	1206.36989	1	1.59E-04	0.29	2.21	-	350.3
AHQ-1-1, 3839	K.TDLLLEPYNK.Y	1206.36989	2	4.54E-05	0.91	3.89	-	1253.8
AHQ-1-2, 4082	K.TDLLLEPYNKYR.F	1525.73037	2	1.71E-06	0.62	2.84	-	524.1
AHQ-1-1, 4397 - 4419	K.TELEDTLDSTAAQQELR.S	1921.00969	2	3.21E-05	0.90	3.50	-	1154.6

AHQ-1-4, 4478	K.TELEDTLDSTAAQQELR.S	1921.00969	2	4.31E-08	0.97	5.43	-	1958.7
AHQ-1-2, 4583	K.TELEDTLDSTAAQQELR.S	1921.00969	3	1.42E-04	0.97	5.38	-	2131.7
AHQ-1-2, 4559	K.TELEDTLDSTAAQQELR.S	1921.00969	2	1.99E-07	0.94	4.30	-	1491.8
AHQ-1-5, 4414	K.TELEDTLDSTAAQQELR.S	1921.00969	2	9.81E-06	0.96	4.60	-	1615.5
AHQ-1-3, 4405 - 4411	K.TELEDTLDSTAAQQELR.S	1921.00969	2	2.23E-07	0.97	5.57	-	1769.5
AHQ-1-3, 3134 - 3135	R.TEMEDLMSSK.D	1171.32449	2	5.52E-04	0.85	2.78	-	742.9
AHQ-1-2, 3245 - 3249	R.TEMEDLMSSK.D	1171.32449	2	2.46E-05	0.88	3.09	-	799.1
AHQ-1-1, 3227	R.TEMEDLMSSK.D	1171.32449	2	2.80E-05	0.77	2.68	-	483.0
AHQ-1-6, 3279	R.TEMEDLMSSKDDVGK.S	1685.85626	2	5.76E-07	0.63	3.01	-	699.4
AHQ-1-2, 3533	R.TEMEDLMSSKDDVGK.S	1685.85626	2	5.63E-06	0.92	4.11	-	1048.5
AHQ-1-4, 3220	R.TEMEDLMSSKDDVGK.S	1685.85626	2	9.12E-05	0.67	3.10	-	597.4
AHQ-1-5, 3291	R.TEMEDLMSSKDDVGK.S	1685.85626	2	2.47E-05	0.85	3.13	-	973.8
AHQ-1-3, 6494	R.TFHIFYYLLSGAGEHLK.T	1997.28378	3	1.54E-06	0.93	4.19	-	1343.7
AHQ-1-4, 6676	R.TFHIFYYLLSGAGEHLK.T	1997.28378	3	7.29E-05	0.90	3.94	-	1007.5
AHQ-1-2, 6774	R.TFHIFYYLLSGAGEHLK.T	1997.28378	2	1.13E-07	0.97	5.27	-	1430.0
AHQ-1-2, 6675	R.TFHIFYYLLSGAGEHLK.T	1997.28378	3	1.48E-04	0.96	5.12	-	1550.6
AHQ-1-1, 6599	R.TFHIFYYLLSGAGEHLK.T	1997.28378	3	6.01E-08	0.94	4.20	-	1371.9
AHQ-1-2, 6677	R.TFHIFYYLLSGAGEHLK.T	1997.28378	2	5.06E-09	0.99	5.72	-	3195.9
AHQ-1-9, 6404	R.TFHIFYYLLSGAGEHLK.T	1997.28378	2	7.17E-05	0.97	4.97	-	1787.9
AHQ-1-1, 2121	K.VAAYDKLEK.T	1037.19123	2	1.29E-04	0.80	2.67	-	735.5
AHQ-1-3, 1894	K.VAAYDKLEK.T	1037.19123	2	8.48E-04	0.69	2.55	-	624.5
AHQ-1-2, 1978 - 2029	K.VAAYDKLEK.T	1037.19123	2	8.45E-04	0.83	3.02	-	603.2
AHQ-1-3, 3321	K.VEAQLQELQVK.F	1285.47179	2	6.11E-04	0.86	3.37	-	941.3
AHQ-1-4, 3350 - 3351	K.VEAQLQELQVK.F	1285.47179	1	4.65E-04	0.13	2.46	-	406.0
AHQ-1-5, 3197	K.VEAQLQELQVK.F	1285.47179	2	1.06E-04	0.90	3.64	-	1078.8
AHQ-1-6, 3204	K.VEAQLQELQVK.F	1285.47179	2	7.26E-06	0.80	2.82	-	915.1
AHQ-1-4, 3346	K.VEAQLQELQVK.F	1285.47179	2	2.44E-06	0.93	3.55	-	1393.6
AHQ-1-10, 5277	K.VEDMAELTCLNEASVLHNLK.E	2288.58457	3	1.48E-07	0.89	4.45	-	790.2
AHQ-1-5, 6094	K.VEDMAELTCLNEASVLHNLK.E	2288.58457	2	4.18E-07	0.91	4.05	-	728.7
AHQ-1-5, 5998 - 6075	K.VEDMAELTCLNEASVLHNLK.E	2288.58457	2	4.24E-04	0.89	4.01	-	711.6
AHQ-1-5, 5285 - 5359	K.VEDM*AELTCLNEASVLHNLK.E	2304.58397	2	1.29E-05	0.83	3.70	-	574.2
AHQ-1-2, 5498 - 5534	K.VEDM*AELTCLNEASVLHNLK.E	2304.58397	3	7.88E-07	0.93	4.33	-	1253.1
AHQ-1-2, 5507 - 5573	K.VEDM*AELTCLNEASVLHNLK.E	2304.58397	2	6.97E-10	0.88	4.09	-	571.7
AHQ-1-6, 5970 - 5982	K.VEDMAELTCLNEASVLHNLK.E	2288.58457	2	7.08E-11	0.96	4.99	-	1029.1
AHQ-1-9, 5874	K.VEDMAELTCLNEASVLHNLK.E	2288.58457	3	2.70E-06	0.72	3.38	-	590.8
AHQ-1-6, 5272	K.VEDM*AELTCLNEASVLHNLK.E	2304.58397	2	1.60E-08	0.85	3.65	-	663.6
AHQ-1-9, 5867 - 5936	K.VEDMAELTCLNEASVLHNLK.E	2288.58457	2	6.05E-05	0.66	3.38	-	309.0
AHQ-1-10, 5244 - 5276	K.VEDMAELTCLNEASVLHNLK.E	2288.58457	2	3.36E-12	0.92	4.65	-	598.9
AHQ-1-13- , 5316 - 5323	K.VEDM*AELTCLNEASVLHNLK.E	2304.58397	2	2.06E-04	0.63	3.07	-	509.1
AHQ-1-2, 6154 - 6221	K.VEDMAELTCLNEASVLHNLK.E	2288.58457	2	5.59E-06	0.84	3.82	-	596.9
AHQ-1-2, 6197	K.VEDMAELTCLNEASVLHNLK.E	2288.58457	3	1.01E-07	0.69	3.03	-	535.2
AHQ-1-4, 6152	K.VEDMAELTCLNEASVLHNLK.E	2288.58457	2	2.15E-08	0.96	4.92	-	931.6
AHQ-1-4, 6143	K.VEDMAELTCLNEASVLHNLK.E	2288.58457	3	3.08E-06	0.85	3.42	-	855.1
AHQ-1-4, 5455	K.VEDM*AELTCLNEASVLHNLK.E	2304.58397	2	5.33E-04	0.55	2.98	-	481.8
AHQ-1-2, 6399	K.VEDMAELTCLNEASVLHNLK.E	2288.58457	2	1.06E-12	0.96	5.16	-	1033.0
AHQ-1-1, 5400 - 5464	K.VEDM*AELTCLNEASVLHNLK.E	2304.58397	2	6.67E-08	0.90	3.98	-	649.0
AHQ-1-2, 6401	K.VEDMAELTCLNEASVLHNLK.E	2288.58457	3	5.32E-05	0.81	3.83	-	563.3
AHQ-1-10, 4708	K.VEDM*AELTCLNEASVLHNLK.E	2304.58397	2	1.76E-12	0.90	3.97	-	686.3
AHQ-1-6, 5986	K.VEDMAELTCLNEASVLHNLK.E	2288.58457	3	2.65E-08	0.85	3.65	-	748.6
AHQ-1-8, 5807	K.VEDMAELTCLNEASVLHNLK.E	2288.58457	2	5.16E-04	0.24	2.58	-	271.7
AHQ-1-1, 6184 - 6197	K.VEDMAELTCLNEASVLHNLK.E	2288.58457	2	8.32E-06	0.66	3.14	-	620.9
AHQ-1-3, 6038	K.VEDMAELTCLNEASVLHNLK.E	2288.58457	3	1.31E-04	0.82	3.73	-	491.3
AHQ-1-1, 6041	K.VEDMAELTCLNEASVLHNLK.E	2288.58457	3	2.18E-07	0.83	3.60	-	771.3
AHQ-1-3, 6035	K.VEDMAELTCLNEASVLHNLK.E	2288.58457	2	6.87E-11	0.91	4.09	-	703.3
AHQ-1-3, 5354	K.VEDM*AELTCLNEASVLHNLK.E	2304.58397	2	4.66E-06	0.91	4.43	-	665.8
AHQ-1-3, 5350 - 5367	K.VEDM*AELTCLNEASVLHNLK.E	2304.58397	3	1.29E-04	0.76	3.37	-	711.0
AHQ-1-7, 6061 - 6069	K.VEDMAELTCLNEASVLHNLK.E	2288.58457	2	2.46E-07	0.87	4.20	-	469.0
AHQ-1-7, 6074	K.VEDMAELTCLNEASVLHNLK.E	2288.58457	3	2.60E-10	0.93	4.55	-	948.7
AHQ-1-13, 4935 - 5015	K.VEDM*AELTCLNEASVLHNLK.E	2304.58397	2	1.03E-05	0.70	3.36	-	319.2
AHQ-1-1, 2164	R.VEEEEERCQHLQAEK.K	1916.01642	2	7.15E-04	0.85	3.64	-	883.9
AHQ-1-2, 2110	R.VEEEEERCQHLQAEK.K	1916.01642	3	6.55E-09	0.91	4.33	-	906.1
AHQ-1-10, 3585	K.VIQYLAYVASSHK.S	1479.70484	2	1.44E-06	0.95	3.84	-	1309.3
AHQ-1-3, 4015	K.VIQYLAYVASSHK.S	1479.70484	2	9.39E-04	0.93	3.78	-	1114.0
AHQ-1-6, 3796	K.VIQYLAYVASSHK.S	1479.70484	2	3.87E-05	0.95	3.99	-	1441.0
AHQ-1-6, 3795 - 3804	K.VIQYLAYVASSHK.S	1479.70484	3	8.76E-06	0.85	3.78	-	745.2
AHQ-1-7, 3884	K.VIQYLAYVASSHK.S	1479.70484	2	5.79E-08	0.94	4.17	-	1137.8
AHQ-1-1, 4125 - 4127	K.VIQYLAYVASSHK.S	1479.70484	2	3.42E-07	0.94	4.19	-	1084.9
AHQ-1-5, 3857	K.VIQYLAYVASSHK.S	1479.70484	3	1.08E-04	0.73	3.06	-	545.3
AHQ-1-9, 3648	K.VIQYLAYVASSHK.S	1479.70484	2	9.00E-04	0.94	3.35	-	1431.0
AHQ-1-4, 4043	K.VIQYLAYVASSHK.S	1479.70484	2	1.07E-06	0.94	3.83	-	1236.8
AHQ-1-5, 3851	K.VIQYLAYVASSHK.S	1479.70484	2	2.16E-08	0.95	3.95	-	1392.8
AHQ-1-2, 4154 - 4169	K.VIQYLAYVASSHK.S	1479.70484	2	1.57E-04	0.92	3.75	-	1101.6
AHQ-1-2, 4275	K.VIQYLAYVASSHK.S	1479.70484	2	9.57E-05	0.95	4.03	-	1358.6
AHQ-1-6, 6499	R.VISGVLQLGNIVFK.K	1487.81155	2	6.63E-06	0.96	4.01	-	1909.5
AHQ-1-4, 6690 - 6755	R.VISGVLQLGNIVFK.K	1487.81155	2	3.40E-10	0.97	4.79	-	1634.9
AHQ-1-13, 6016	R.VISGVLQLGNIVFK.K	1487.81155	2	4.14E-05	0.95	4.24	-	1155.2
AHQ-1-2, 6609 - 6682	R.VISGVLQLGNIVFK.K	1487.81155	2	6.30E-09	0.96	4.85	-	1324.9
AHQ-1-2, 6694	R.VISGVLQLGNIVFK.K	1487.81155	1	1.05E-04	0.30	2.89	-	330.6
AHQ-1-3, 6521 - 6585	R.VISGVLQLGNIVFK.K	1487.81155	2	4.23E-09	0.97	4.24	-	1979.3
AHQ-1-10, 5756	R.VISGVLQLGNIVFK.K	1487.81155	2	3.68E-04	0.96	4.47	-	1437.3
AHQ-1-5, 6629 - 6702	R.VISGVLQLGNIVFK.K	1487.81155	2	3.70E-05	0.96	4.23	-	1674.7
AHQ-1-2, 6722 - 6785	R.VISGVLQLGNIVFK.K	1487.81155	2	1.85E-07	0.97	4.46	-	2008.6
AHQ-1-2, 6939	R.VISGVLQLGNIVFK.K	1487.81155	2	6.60E-09	0.98	4.60	-	2370.5
AHQ-1-1, 6492 - 6496	R.VISGVLQLGNIVFK.K	1487.81155	2	1.20E-04	0.89	2.86	-	1294.6
AHQ-1-7, 6606	R.VISGVLQLGNIVFK.K	1487.81155	2	9.71E-04	0.97	4.58	-	1941.4
AHQ-1-1, 5971	R.VISGVLQLGNIVFKK.E	1615.98446	2	2.63E-07	0.94	4.41	-	1159.1
AHQ-1-5, 4859 - 4933	K.VSHLLGINVTDFTR.G	1572.79026	2	7.14E-11	0.96	4.53	-	1401.9
AHQ-1-1, 5091	K.VSHLLGINVTDFTR.G	1572.79026	2	1.34E-09	0.95	4.43	-	1340.2
AHQ-1-1, 5095	K.VSHLLGINVTDFTR.G	1572.79026	3	2.39E-05	0.91	3.89	-	1239.2
AHQ-1-2, 5095 - 5145	K.VSHLLGINVTDFTR.G	1572.79026	2	8.08E-11	0.96	4.33	-	1550.5
AHQ-1-2, 5139	K.VSHLLGINVTDFTR.G	1572.79026	3	5.16E-04	0.83	3.30	-	984.8
AHQ-1-4, 5066	K.VSHLLGINVTDFTR.G	1572.79026	2	2.83E-10	0.97	4.84	-	1470.5
AHQ-1-2, 5422	K.VSHLLGINVTDFTR.G	1572.79026	2	1.59E-06	0.95	3.68	-	1289.3
AHQ-1-3, 4985	K.VSHLLGINVTDFTR.G	1572.79026	2	7.94E-12	0.94	3.94	-	1240.0
AHQ-1-7, 4858	K.VSHLLGINVTDFTR.G	1572.79026	2	1.97E-11	0.96	4.15	-	1608.6
AHQ-1-3, 4993	K.VSHLLGINVTDFTR.G	1572.79026	3	1.32E-05	0.89	3.75	-	1080.2
AHQ-1-6, 4806 - 4854	K.VSHLLGINVTDFTR.G	1572.79026	2	3.41E-08	0.94	4.08	-	1293.7
AHQ-1-2, 3290	R.VVFQEFR.Q	925.06580	2	8.76E-04	0.79	2.74	-	467.6
AHQ-1-5, 3019	R.VVFQEFR.Q	925.06580	2	4.83E-05	0.74	2.52	-	493.0
AHQ-1-5, 3983 - 4001	R.YEILTPNSIPK.G	1275.47536	2	8.29E-05	0.91	3.86	-	1011.8
AHQ-1-2, 4265	R.YEILTPNSIPK.G	1275.47536	2	1.38E-05	0.85	3.44	-	901.1
AHQ-1-1, 4185 - 4188	R.YEILTPNSIPK.G	1275.47536	2	1.31E-04	0.92	3.66	-	1137.5
AHQ-1-2, 4178 - 4197	R.YEILTPNSIPK.G	1275.47536	2	7.77E-06	0.91	3.05	-	1145.7
AHQ-1-4, 4107 - 4115	R.YEILTPNSIPK.G	1275.47536	2	4.50E-05	0.93	3.03	-	1466.9
AHQ-1-2, 4429	R.YEILTPNSIPK.G	1275.47536	2	1.69E-04	0.86	2.83	-	1014.7
AHQ-1-2, 4262	R.YEILTPNSIPK.G	1275.47536	1	8.79E-04	0.53	2.54	-	585.9
AHQ-1-2, 4434	R.YEILTPNSIPK.G	1275.47536	1	5.31E-04	0.46	1.92	-	734.4

AHQ-1-3, 4042 - 4113	R.YEILTPNSIPK.G	1275.47536	2	1.16E-05	0.89	3.72	-	949.8
AHQ-1-1, 6147	K.YLYVDKNFINNPLAQADWAAK.K	2455.75199	3	1.67E-08	0.96	5.10	-	1370.2
AHQ-1-2, 6363	K.YLYVDKNFINNPLAQADWAAK.K	2455.75199	2	3.11E-10	0.93	4.21	-	952.4
AHQ-1-10, 5433	K.YLYVDKNFINNPLAQADWAAK.K	2455.75199	2	8.60E-04	0.55	2.58	-	291.9
AHQ-1-2, 6267	K.YLYVDKNFINNPLAQADWAAK.K	2455.75199	2	9.78E-04	0.65	2.67	-	560.3
gi|4507485|ref|NP_003237.1| thrombospondin 1 [Homo sapiens]				1.00E-30	29.55	350.31	30.50	129351.8
AHQ-1-2, 4279	K.AGTLDLSLTVQGK.Q	1303.48711	2	4.77E-04	0.94	3.70	-	1381.0
AHQ-1-2, 2842	R.AQLYIDCEK.M	1141.27636	2	6.54E-05	0.89	3.43	-	675.7
AHQ-1-2, 2838	R.AQLYIDCEK.M	1141.27636	1	4.57E-04	0.15	1.93	-	487.9
AHQ-1-2, 6301	R.AQLYIDCEKM*ENAELDVPIQSVFTR.D	2988.33923	3	1.91E-05	0.94	4.86	-	1181.3
AHQ-1-1, 4000	R.CENTDPGYNCLPCPPR.F	1954.10800	2	1.36E-06	0.70	3.00	-	425.3
AHQ-1-3, 3873	R.CENTDPGYNCLPCPPR.F	1954.10800	2	3.06E-11	0.89	3.78	-	591.2
AHQ-1-4, 4200	R.CENTDPGYNCLPCPPR.F	1954.10800	2	1.79E-05	0.88	3.80	-	458.7
AHQ-1-3, 4134	R.CENTDPGYNCLPCPPR.F	1954.10800	2	4.85E-06	0.88	4.00	-	475.9
AHQ-1-5, 4011 - 4014	R.CENTDPGYNCLPCPPR.F	1954.10800	2	9.65E-06	0.93	4.15	-	752.4
AHQ-1-4, 3930	R.CENTDPGYNCLPCPPR.F	1954.10800	2	3.44E-08	0.80	2.79	-	619.4
AHQ-1-2, 4257	R.CENTDPGYNCLPCPPR.F	1954.10800	2	4.42E-12	0.90	3.85	-	734.9
AHQ-1-4, 3971	K.CNYLGHYSDPMYR.C	1677.84222	2	1.13E-06	0.93	3.82	-	802.4
AHQ-1-5, 3311	K.CNYLGHYSDPM*YR.C	1693.84162	2	1.59E-04	0.89	3.76	-	737.2
AHQ-1-3, 3933	K.CNYLGHYSDPMYR.C	1677.84222	2	7.69E-09	0.93	3.60	-	938.4
AHQ-1-4, 3423	K.CNYLGHYSDPM*YR.C	1693.84162	2	8.28E-05	0.90	3.41	-	740.1
AHQ-1-4, 3450	K.DCVGDVTENQICNK.Q	1654.75869	2	1.18E-06	0.91	4.08	-	698.4
AHQ-1-2, 3510	K.DCVGDVTENQICNK.Q	1654.75869	2	3.99E-05	0.91	3.45	-	1011.2
AHQ-1-5, 2669	K.DHSGQVFSVVSNGK.A	1461.56164	2	3.31E-07	0.95	3.85	-	1451.4
AHQ-1-13- , 2833	K.DHSGQVFSVVSNGK.A	1461.56164	2	3.11E-06	0.92	3.38	-	1046.2
AHQ-1-2, 2859	K.DHSGQVFSVVSNGK.A	1461.56164	2	3.17E-08	0.95	3.70	-	1277.8
AHQ-1-5, 3087	R.DNCQYVYNVDQR.D	1575.64166	2	7.28E-05	0.91	3.50	-	1101.8
AHQ-1-2, 3418	R.DNCQYVYNVDQR.D	1575.64166	2	2.30E-05	0.84	3.27	-	891.3
AHQ-1-2, 3233 - 3309	R.DNCQYVYNVDQR.D	1575.64166	2	1.17E-04	0.86	3.53	-	694.3
AHQ-1-2, 3087	R.DNCQYVYNVDQR.D	1575.64166	2	4.09E-05	0.93	3.89	-	1222.2
AHQ-1-3, 3122 - 3137	R.DNCQYVYNVDQR.D	1575.64166	2	2.51E-05	0.93	4.30	-	922.3
AHQ-1-4, 3355	R.DNCQYVYNVDQR.D	1575.64166	2	6.60E-07	0.88	3.68	-	939.4
AHQ-1-7, 3102	R.DNCQYVYNVDQR.D	1575.64166	2	6.48E-05	0.95	4.34	-	1373.2
AHQ-1-3, 3297	R.DNCQYVYNVDQR.D	1575.64166	2	6.26E-07	0.91	3.96	-	847.6
AHQ-1-3, 4186 - 4257	R.DTDMDGVGDQCDNCPLEHNPDQLDSDSDR.I	3324.31882	3	7.83E-08	0.92	4.59	-	826.7
AHQ-1-6, 3824	R.DTDM*DGVGDQCDNCPLEHNPDQLDSDSDR.I	3340.31822	3	4.26E-13	0.88	4.00	-	829.7
AHQ-1-4, 4172	R.DTDMDGVGDQCDNCPLEHNPDQLDSDSDR.I	3324.31882	3	1.69E-04	0.90	4.57	-	674.2
AHQ-1-4, 4318	R.DTDMDGVGDQCDNCPLEHNPDQLDSDSDR.I	3324.31882	3	6.91E-09	0.93	5.03	-	673.6
AHQ-1-3, 3790 - 3859	R.DTDM*DGVGDQCDNCPLEHNPDQLDSDSDR.I	3340.31822	3	2.72E-06	0.90	4.50	-	913.5
AHQ-1-2, 4317 - 4385	R.DTDMDGVGDQCDNCPLEHNPDQLDSDSDR.I	3324.31882	3	4.00E-06	0.92	4.71	-	697.0
AHQ-1-1, 3880	R.DTDM*DGVGDQCDNCPLEHNPDQLDSDSDR.I	3340.31822	3	2.32E-04	0.87	4.81	-	558.2
AHQ-1-5, 3882	R.DTDM*DGVGDQCDNCPLEHNPDQLDSDSDR.I	3340.31822	3	3.64E-13	0.77	3.20	-	904.3
AHQ-1-4, 3908	R.DTDM*DGVGDQCDNCPLEHNPDQLDSDSDR.I	3340.31822	3	1.00E-30	0.84	3.97	-	744.3
AHQ-1-2, 3969 - 4042	R.DTDM*DGVGDQCDNCPLEHNPDQLDSDSDR.I	3340.31822	3	1.22E-14	0.94	5.17	-	914.4
AHQ-1-5, 3565	K.FQDLVDAVR.A	1063.18877	2	3.71E-04	0.90	3.06	-	1103.5
AHQ-1-10, 3315	K.FQDLVDAVR.A	1063.18877	1	2.48E-04	0.15	1.94	-	365.8
AHQ-1-11, 3423	K.FQDLVDAVR.A	1063.18877	2	4.98E-05	0.89	2.71	-	1042.4
AHQ-1-3, 3695	K.FQDLVDAVR.A	1063.18877	2	5.64E-05	0.89	2.75	-	1231.8
AHQ-1-10, 3320	K.FQDLVDAVR.A	1063.18877	2	2.24E-06	0.82	2.80	-	847.0
AHQ-1-3, 2929	R.FTGSQPFGQGVEHATANK.Q	1877.00666	2	7.04E-08	0.73	3.55	-	422.3
AHQ-1-2, 3114	R.FTGSQPFGQGVEHATANK.Q	1877.00666	2	3.59E-11	0.86	3.81	-	474.6
AHQ-1-1, 3025	R.FTGSQPFGQGVEHATANK.Q	1877.00666	2	7.29E-11	0.74	3.58	-	274.0
AHQ-1-4, 3084	R.FTGSQPFGQGVEHATANK.Q	1877.00666	2	1.63E-08	0.82	3.50	-	583.5
AHQ-1-2, 3198	R.FTGSQPFGQGVEHATANK.Q	1877.00666	2	3.79E-08	0.81	3.50	-	528.2
AHQ-1-6, 2750	R.FTGSQPFGQGVEHATANK.Q	1877.00666	2	1.20E-04	0.68	3.03	-	499.7
AHQ-1-2, 2969 - 3038	R.FTGSQPFGQGVEHATANK.Q	1877.00666	2	8.53E-07	0.92	4.47	-	691.0
AHQ-1-4, 2871 - 2898	R.FTGSQPFGQGVEHATANK.Q	1877.00666	2	5.86E-07	0.91	4.31	-	475.5
AHQ-1-4, 2836 - 2908	R.FTGSQPFGQGVEHATANK.Q	1877.00666	3	2.13E-05	0.63	3.13	-	489.9
AHQ-1-3, 2867	R.FTGSQPFGQGVEHATANK.Q	1877.00666	3	8.54E-06	0.89	4.39	-	627.4
AHQ-1-3, 3034	R.FTGSQPFGQGVEHATANK.Q	1877.00666	2	1.00E-10	0.95	4.71	-	739.3
AHQ-1-3, 2834 - 2905	R.FTGSQPFGQGVEHATANK.Q	1877.00666	2	3.61E-08	0.93	4.43	-	611.7
AHQ-1-1, 5673	R.FVFGTTPEDILR.N	1395.58470	2	5.22E-04	0.72	2.65	-	774.3
AHQ-1-6, 5520 - 5559	R.FVFGTTPEDILR.N	1395.58470	1	5.43E-07	0.03	1.95	-	89.3
AHQ-1-11, 5046 - 5066	R.FVFGTTPEDILR.N	1395.58470	2	8.22E-05	0.75	3.20	-	585.6
AHQ-1-6, 5540	R.FVFGTTPEDILR.N	1395.58470	1	3.24E-05	0.22	2.63	-	103.5
AHQ-1-10, 4889	R.FVFGTTPEDILR.N	1395.58470	2	2.35E-05	0.80	2.86	-	822.3
AHQ-1-4, 5722	R.FVFGTTPEDILR.N	1395.58470	2	1.05E-04	0.87	2.87	-	836.9
AHQ-1-4, 5732	R.FVFGTTPEDILR.N	1395.58470	1	3.27E-05	0.07	1.94	-	145.9
AHQ-1-2, 5789	R.FVFGTTPEDILR.N	1395.58470	2	1.98E-04	0.69	2.65	-	548.0
AHQ-1-3, 5710	K.GFLLLASLR.Q	990.22407	2	4.98E-05	0.97	3.63	-	2394.9
AHQ-1-6, 5608	K.GFLLLASLR.Q	990.22407	2	1.19E-04	0.97	4.34	-	2100.0
AHQ-1-2, 5895	K.GFLLLASLR.Q	990.22407	2	3.04E-04	0.97	3.61	-	2098.4
AHQ-1-7, 4448	K.GGVNDNFQGVLQNVR.F	1617.74799	2	1.28E-05	0.91	3.94	-	940.9
AHQ-1-5, 4469	K.GGVNDNFQGVLQNVR.F	1617.74799	2	8.75E-05	0.90	4.17	-	901.7
AHQ-1-1, 4595 - 4663	K.GGVNDNFQGVLQNVR.F	1617.74799	2	3.66E-05	0.92	4.33	-	1050.8
AHQ-1-10, 4115 - 4180	K.GGVNDNFQGVLQNVR.F	1617.74799	2	9.67E-06	0.71	3.35	-	615.4
AHQ-1-4, 4594 - 4607	K.GGVNDNFQGVLQNVR.F	1617.74799	2	3.73E-04	0.67	3.42	-	600.9
AHQ-1-2, 4897 - 4913	K.GGVNDNFQGVLQNVR.F	1617.74799	2	2.72E-04	0.87	4.18	-	667.5
AHQ-1-2, 4801	K.GGVNDNFQGVLQNVR.F	1617.74799	2	3.47E-10	0.91	4.23	-	809.1
AHQ-1-10, 4007 - 4017	K.GGVNDNFQGVLQNVR.F	1617.74799	2	4.34E-04	0.91	4.54	-	878.7
AHQ-1-5, 4567 - 4638	K.GGVNDNFQGVLQNVR.F	1617.74799	2	1.01E-09	0.93	4.22	-	994.1
AHQ-1-6, 4322 - 4390	K.GGVNDNFQGVLQNVR.F	1617.74799	2	4.65E-05	0.90	3.92	-	890.3
AHQ-1-5, 4803	K.GGVNDNFQGVLQNVR.F	1617.74799	2	1.28E-05	0.63	2.82	-	545.6
AHQ-1-13, 4273 - 4335	K.GGVNDNFQGVLQNVR.F	1617.74799	2	2.34E-07	0.83	4.18	-	482.8
AHQ-1-4, 4826 - 4894	K.GGVNDNFQGVLQNVR.F	1617.74799	2	2.92E-07	0.88	4.29	-	694.2
AHQ-1-3, 4378	K.GGVNDNFQGVLQNVR.F	1617.74799	2	3.12E-06	0.84	3.41	-	798.0
AHQ-1-6, 4539 - 4582	K.GGVNDNFQGVLQNVR.F	1617.74799	2	4.41E-10	0.90	4.07	-	736.6
AHQ-1-3, 4761 - 4826	K.GGVNDNFQGVLQNVR.F	1617.74799	2	2.23E-06	0.90	3.95	-	777.6
AHQ-1-2, 4715 - 4797	K.GGVNDNFQGVLQNVR.F	1617.74799	2	4.71E-08	0.94	4.67	-	1031.5
AHQ-1-10, 3911 - 3920	K.GGVNDNFQGVLQNVR.F	1617.74799	2	1.12E-06	0.92	4.45	-	941.8
AHQ-1-9, 4671	K.GGVNDNFQGVLQNVR.F	1617.74799	2	8.14E-04	0.83	3.70	-	665.5
AHQ-1-11, 4134 - 4151	K.GGVNDNFQGVLQNVR.F	1617.74799	2	9.13E-05	0.89	4.55	-	748.4
AHQ-1-13- , 4544	K.GGVNDNFQGVLQNVR.F	1617.74799	2	4.06E-04	0.75	3.53	-	566.0
AHQ-1-7, 4636	K.GGVNDNFQGVLQNVR.F	1617.74799	2	5.14E-07	0.92	4.29	-	839.9
AHQ-1-9, 4326 - 4403	K.GGVNDNFQGVLQNVR.F	1617.74799	2	1.52E-05	0.80	3.37	-	603.8
AHQ-1-9, 4531	K.GGVNDNFQGVLQNVR.F	1617.74799	2	1.01E-08	0.94	4.07	-	1267.1
AHQ-1-4, 2160 - 2175	K.GPDPSSPAFR.I	1031.10364	2	4.05E-06	0.70	3.02	-	481.3
AHQ-1-6, 2054	K.GPDPSSPAFR.I	1031.10364	2	2.57E-04	0.55	2.53	-	478.8
AHQ-1-5, 2039	K.GPDPSSPAFR.I	1031.10364	2	3.93E-06	0.65	2.63	-	419.2
AHQ-1-3, 2174	K.GPDPSSPAFR.I	1031.10364	2	5.28E-05	0.76	2.74	-	530.0
AHQ-1-2, 2250	K.GPDPSSPAFR.I	1031.10364	2	1.42E-04	0.60	2.78	-	409.2
AHQ-1-11, 2142	K.GPDPSSPAFR.I	1031.10364	2	1.21E-04	0.71	3.17	-	395.8
AHQ-1-10, 2089	K.GPDPSSPAFR.I	1031.10364	2	1.36E-04	0.82	3.18	-	585.3
AHQ-1-4, 3106	R.GTLLALER.K	873.03234	2	8.01E-04	0.91	3.49	-	1053.6
AHQ-1-6, 2950	R.GTLLALER.K	873.03234	2	7.78E-04	0.91	3.09	-	1047.7
AHQ-1-2, 3189	R.GTLLALER.K	873.03234	2	9.92E-04	0.92	3.32	-	1156.1
AHQ-1-12, 3026	R.GTLLALER.K	873.03234	2	1.05E-04	0.84	3.03	-	942.9

AHQ-1-3, 3079 - 3083	R.GTLLALER.K	873.03234	2	8.01E-04	0.90	3.62	-	869.7
AHQ-1-2, 2779	K.GTSQNDPNWVVR.H	1373.45608	2	1.37E-06	0.76	3.29	-	884.4
AHQ-1-7, 2593	K.GTSQNDPNWVVR.H	1373.45608	2	1.43E-04	0.49	2.61	-	709.0
AHQ-1-1, 6531 - 6596	R.IEDANLIPPVPDDKFQDLVDAVR.A	2580.87450	2	4.00E-05	0.51	2.67	-	253.3
AHQ-1-3, 6613 - 6631	R.IEDANLIPPVPDDKFQDLVDAVR.A	2580.87450	3	1.73E-06	0.60	3.27	-	274.0
AHQ-1-1, 6516 - 6587	R.IEDANLIPPVPDDKFQDLVDAVR.A	2580.87450	3	4.64E-10	0.67	3.44	-	344.9
AHQ-1-1, 6648	R.IEDANLIPPVPDDKFQDLVDAVR.A	2580.87450	3	1.32E-08	0.88	4.31	-	497.6
AHQ-1-3, 6765	R.IEDANLIPPVPDDKFQDLVDAVR.A	2580.87450	3	1.77E-08	0.60	3.29	-	312.9
AHQ-1-14- , 6344	R.IEDANLIPPVPDDKFQDLVDAVR.A	2580.87450	3	5.38E-05	0.82	3.64	-	535.6
AHQ-1-10, 5931 - 5939	R.IEDANLIPPVPDDKFQDLVDAVR.A	2580.87450	2	1.28E-06	0.73	3.42	-	390.7
AHQ-1-10, 5869 - 5932	R.IEDANLIPPVPDDKFQDLVDAVR.A	2580.87450	3	9.01E-07	0.74	3.93	-	365.9
AHQ-1-13, 6027 - 6105	R.IEDANLIPPVPDDKFQDLVDAVR.A	2580.87450	3	1.30E-05	0.73	3.73	-	365.6
AHQ-1-7, 6749 - 6761	R.IEDANLIPPVPDDKFQDLVDAVR.A	2580.87450	3	1.07E-06	0.60	3.15	-	354.0
AHQ-1-10, 5800 - 5865	R.IEDANLIPPVPDDKFQDLVDAVR.A	2580.87450	2	1.22E-05	0.84	3.76	-	347.4
AHQ-1-5, 6713 - 6787	R.IEDANLIPPVPDDKFQDLVDAVR.A	2580.87450	3	5.71E-08	0.67	3.25	-	400.7
AHQ-1-4, 6774 - 6854	R.IEDANLIPPVPDDKFQDLVDAVR.A	2580.87450	3	9.56E-12	0.64	3.30	-	330.7
AHQ-1-4, 6783 - 6855	R.IEDANLIPPVPDDKFQDLVDAVR.A	2580.87450	2	3.46E-08	0.65	3.13	-	198.3
AHQ-1-4, 6931 - 6932	R.IEDANLIPPVPDDKFQDLVDAVR.A	2580.87450	3	1.68E-08	0.72	3.51	-	347.6
AHQ-1-12, 6131 - 6134	R.IEDANLIPPVPDDKFQDLVDAVR.A	2580.87450	2	9.14E-09	0.72	3.36	-	211.7
AHQ-1-7, 6756 - 6770	R.IEDANLIPPVPDDKFQDLVDAVR.A	2580.87450	2	4.78E-06	0.76	3.34	-	279.9
AHQ-1-6, 6574 - 6640	R.IEDANLIPPVPDDKFQDLVDAVR.A	2580.87450	3	2.80E-09	0.76	3.95	-	369.6
AHQ-1-2, 6782 - 6849	R.IEDANLIPPVPDDKFQDLVDAVR.A	2580.87450	3	1.65E-08	0.78	3.48	-	535.1
AHQ-1-11, 5908 - 5974	R.IEDANLIPPVPDDKFQDLVDAVR.A	2580.87450	3	8.46E-09	0.73	3.21	-	355.9
AHQ-1-11, 5914	R.IEDANLIPPVPDDKFQDLVDAVR.A	2580.87450	2	3.94E-05	0.49	2.63	-	246.9
AHQ-1-2, 2715	K.IMADSGPIYDK.T	1210.38204	2	3.69E-05	0.88	3.30	-	762.3
AHQ-1-14- , 2332 - 2367	K.IM*ADSGPIYDK.T	1226.38144	2	9.80E-04	0.71	2.77	-	521.7
AHQ-1-11, 2550	K.IMADSGPIYDK.T	1210.38204	2	2.06E-06	0.91	3.54	-	688.8
AHQ-1-2, 2461	K.IM*ADSGPIYDK.T	1226.38144	2	4.35E-05	0.75	3.16	-	507.0
AHQ-1-13, 6343 - 6344	R.IPESGGDNSVFDIFELTGAAR.K	2196.35899	2	4.21E-10	0.97	6.11	-	922.2
AHQ-1-14- , 6543	R.IPESGGDNSVFDIFELTGAAR.K	2196.35899	2	9.45E-12	0.97	5.55	-	1088.1
AHQ-1-4, 7170	R.IPESGGDNSVFDIFELTGAAR.K	2196.35899	2	3.10E-10	0.95	5.03	-	1009.8
AHQ-1-10, 6117 - 6196	R.IPESGGDNSVFDIFELTGAAR.K	2196.35899	2	4.47E-08	0.96	5.30	-	1091.8
AHQ-1-2, 7177 - 7245	R.IPESGGDNSVFDIFELTGAAR.K	2196.35899	2	1.33E-08	0.97	5.33	-	1204.6
AHQ-1-9, 7083	R.IPESGGDNSVFDIFELTGAAR.K	2196.35899	2	6.33E-11	0.95	4.77	-	944.5
AHQ-1-9, 6930	R.IPESGGDNSVFDIFELTGAAR.K	2196.35899	2	3.54E-08	0.96	5.57	-	1030.5
AHQ-1-13- , 6795	R.IPESGGDNSVFDIFELTGAAR.K	2196.35899	2	5.75E-09	0.97	5.50	-	1252.1
AHQ-1-1, 6851	R.IPESGGDNSVFDIFELTGAAR.K	2196.35899	2	5.01E-06	0.97	5.49	-	1239.9
AHQ-1-3, 7006 - 7083	R.IPESGGDNSVFDIFELTGAAR.K	2196.35899	2	1.04E-09	0.97	5.25	-	1361.8
AHQ-1-7, 7145	R.IPESGGDNSVFDIFELTGAAR.K	2196.35899	2	4.01E-07	0.93	4.46	-	867.0
AHQ-1-6, 6970 - 6972	R.IPESGGDNSVFDIFELTGAAR.K	2196.35899	2	1.50E-10	0.96	5.83	-	914.5
AHQ-1-5, 7126 - 7133	R.IPESGGDNSVFDIFELTGAAR.K	2196.35899	2	3.39E-09	0.97	5.75	-	1338.6
AHQ-1-10, 2211 - 2256	R.KDHSGQVFSVVSNGK.A	1589.73455	2	1.90E-06	0.59	2.82	-	498.1
AHQ-1-6, 2198	R.KDHSGQVFSVVSNGK.A	1589.73455	3	3.72E-07	0.91	3.62	-	1405.3
AHQ-1-6, 2203	R.KDHSGQVFSVVSNGK.A	1589.73455	2	4.29E-05	0.90	3.78	-	982.7
AHQ-1-9, 2158 - 2227	R.KDHSGQVFSVVSNGK.A	1589.73455	2	1.13E-09	0.73	3.20	-	485.1
AHQ-1-5, 2305	R.KDHSGQVFSVVSNGK.A	1589.73455	2	1.27E-07	0.71	2.80	-	831.1
AHQ-1-3, 2357	R.KDHSGQVFSVVSNGK.A	1589.73455	2	2.32E-07	0.78	2.85	-	928.5
AHQ-1-4, 2346	R.KDHSGQVFSVVSNGK.A	1589.73455	2	3.58E-07	0.86	3.36	-	839.9
AHQ-1-3, 2462	R.KDHSGQVFSVVSNGK.A	1589.73455	2	2.01E-05	0.93	3.50	-	1195.6
AHQ-1-2, 2554	R.KDHSGQVFSVVSNGK.A	1589.73455	2	3.69E-08	0.94	3.71	-	1405.5
AHQ-1-2, 2469	R.KDHSGQVFSVVSNGK.A	1589.73455	2	9.08E-09	0.89	3.96	-	894.5
AHQ-1-4, 2444	R.KDHSGQVFSVVSNGK.A	1589.73455	2	5.28E-07	0.88	3.35	-	957.1
AHQ-1-2, 2507	K.KIMADSGPIYDK.T	1338.55496	1	2.61E-04	0.68	2.75	-	582.9
AHQ-1-4, 2395	K.KIMADSGPIYDK.T	1338.55496	2	4.90E-04	0.86	3.56	-	694.0
AHQ-1-2, 2509	K.KIMADSGPIYDK.T	1338.55496	2	1.24E-04	0.84	3.38	-	546.1
AHQ-1-3, 2373	R.KVTEENKELANELR.R	1673.84976	2	2.88E-10	0.93	4.18	-	1538.3
AHQ-1-1, 2531	R.KVTEENKELANELR.R	1673.84976	3	9.43E-04	0.68	3.13	-	625.2
AHQ-1-6, 2359	R.KVTEENKELANELR.R	1673.84976	2	4.85E-09	0.95	4.46	-	1332.2
AHQ-1-5, 2226	R.KVTEENKELANELR.R	1673.84976	3	4.07E-05	0.85	3.92	-	786.2
AHQ-1-3, 2378 - 2451	R.KVTEENKELANELR.R	1673.84976	3	3.82E-06	0.85	4.23	-	683.1
AHQ-1-4, 2360 - 2370	R.KVTEENKELANELR.R	1673.84976	2	3.07E-11	0.95	4.99	-	1204.8
AHQ-1-4, 2375	R.KVTEENKELANELR.R	1673.84976	3	7.23E-06	0.80	3.60	-	794.5
AHQ-1-1, 2476 - 2545	R.KVTEENKELANELR.R	1673.84976	2	7.98E-08	0.95	4.80	-	1386.4
AHQ-1-2, 2474	R.KVTEENKELANELR.R	1673.84976	2	1.38E-07	0.93	4.66	-	1119.1
AHQ-1-3, 2122	R.KVTEENKELANELR.R	1673.84976	2	5.49E-08	0.93	4.55	-	1146.0
AHQ-1-6, 2236	R.KVTEENKELANELR.R	1673.84976	2	6.98E-07	0.94	4.84	-	1215.9
AHQ-1-3, 2531	R.LCNSPSPQMNGKPCEGEAR.E	2135.34557	2	8.81E-07	0.80	3.55	-	357.6
AHQ-1-2, 2262	R.LCNSPSPQM*NGKPCEGEAR.E	2151.34497	2	2.64E-04	0.71	3.24	-	422.5
AHQ-1-5, 2478	R.LCNSPSPQMNGKPCEGEAR.E	2135.34557	2	4.67E-10	0.90	4.04	-	569.8
AHQ-1-2, 2609	R.LCNSPSPQMNGKPCEGEAR.E	2135.34557	3	5.98E-05	0.86	3.52	-	980.4
AHQ-1-2, 2605 - 2610	R.LCNSPSPQMNGKPCEGEAR.E	2135.34557	2	5.85E-09	0.82	3.83	-	502.6
AHQ-1-4, 2206	R.LCNSPSPQM*NGKPCEGEAR.E	2151.34497	3	1.93E-05	0.84	3.88	-	664.8
AHQ-1-2, 5905 - 5914	K.MENAELDVPIQSVFTR.D	1850.08607	2	2.60E-04	0.92	4.60	-	533.1
AHQ-1-1, 5764	K.MENAELDVPIQSVFTR.D	1850.08607	2	2.21E-04	0.64	3.08	-	267.1
AHQ-1-5, 5489 - 5494	K.M*ENAELDVPIQSVFTR.D	1866.08547	2	1.86E-04	0.80	3.49	-	635.7
AHQ-1-11, 5146 - 5159	K.MENAELDVPIQSVFTR.D	1850.08607	2	6.34E-07	0.93	4.57	-	595.2
AHQ-1-4, 5844	K.MENAELDVPIQSVFTR.D	1850.08607	2	6.38E-04	0.89	3.72	-	725.8
AHQ-1-3, 5747	K.MENAELDVPIQSVFTR.D	1850.08607	2	1.31E-08	0.89	4.10	-	486.0
AHQ-1-9, 5523 - 5531	K.MENAELDVPIQSVFTR.D	1850.08607	2	2.31E-09	0.93	4.61	-	550.9
AHQ-1-3, 5489	K.M*ENAELDVPIQSVFTR.D	1866.08547	2	1.33E-07	0.90	3.83	-	645.4
AHQ-1-5, 5710	K.MENAELDVPIQSVFTR.D	1850.08607	2	6.34E-04	0.88	3.53	-	613.0
AHQ-1-7, 5692	K.MENAELDVPIQSVFTR.D	1850.08607	2	3.32E-05	0.78	3.46	-	361.0
AHQ-1-2, 5665	K.M*ENAELDVPIQSVFTR.D	1866.08547	2	7.32E-10	0.93	4.31	-	728.7
AHQ-1-3, 3138	K.QVTQSYWDTNPTR.A	1596.68241	2	5.73E-07	0.71	2.67	-	695.9
AHQ-1-7, 3056	K.QVTQSYWDTNPTR.A	1596.68241	2	1.23E-06	0.87	3.11	-	762.4
AHQ-1-1, 3252	K.QVTQSYWDTNPTR.A	1596.68241	2	8.09E-06	0.76	3.17	-	528.3
AHQ-1-2, 3262	K.QVTQSYWDTNPTR.A	1596.68241	2	7.73E-06	0.92	3.64	-	903.9
AHQ-1-9, 2976	K.QVTQSYWDTNPTR.A	1596.68241	2	2.00E-05	0.71	2.58	-	504.1
AHQ-1-5, 3041	K.QVTQSYWDTNPTR.A	1596.68241	2	1.05E-06	0.90	3.66	-	657.4
AHQ-1-9, 2679	R.RPPLCYHNGVQYR.N	1661.86889	2	1.46E-04	0.89	3.32	-	946.7
AHQ-1-2, 2786	R.RPPLCYHNGVQYR.N	1661.86889	2	1.88E-04	0.86	3.27	-	628.9
AHQ-1-2, 2933	R.RPPLCYHNGVQYR.N	1661.86889	2	1.92E-06	0.88	3.82	-	593.9
AHQ-1-5, 2683 - 2753	R.RPPLCYHNGVQYR.N	1661.86889	2	9.98E-04	0.93	4.05	-	671.9
AHQ-1-4, 3946	K.SITLFVQEDR.A	1208.34588	2	2.21E-06	0.93	3.45	-	1287.3
AHQ-1-13, 3836	K.SITLFVQEDR.A	1208.34588	2	1.36E-05	0.93	3.33	-	1538.7
AHQ-1-2, 4067 - 4086	K.SITLFVQEDR.A	1208.34588	2	2.85E-06	0.95	3.89	-	1391.0
AHQ-1-6, 3704 - 3756	K.SITLFVQEDR.A	1208.34588	2	1.66E-07	0.93	2.93	-	1430.1
AHQ-1-11, 3616	K.SITLFVQEDR.A	1208.34588	2	9.80E-05	0.92	3.46	-	1270.8
AHQ-1-13- , 3975	K.SITLFVQEDR.A	1208.34588	2	2.79E-06	0.93	3.54	-	1242.9
AHQ-1-3, 3922	K.SITLFVQEDR.A	1208.34588	2	8.99E-07	0.91	3.01	-	1159.0
AHQ-1-10, 3516	K.SITLFVQEDR.A	1208.34588	2	7.48E-06	0.94	3.92	-	1195.6
AHQ-1-13, 3747 - 3827	K.SITLFVQEDR.A	1208.34588	2	2.67E-05	0.88	3.00	-	981.8
AHQ-1-12, 3738	K.SITLFVQEDR.A	1208.34588	2	2.17E-06	0.90	2.92	-	1191.1
AHQ-1-5, 3798	K.SITLFVQEDR.A	1208.34588	2	3.60E-06	0.87	2.92	-	1047.1
AHQ-1-1, 4583	R.TIVTTLQDSIR.K	1247.42355	2	5.18E-07	0.97	4.16	-	1850.6
AHQ-1-12, 4266	R.TIVTTLQDSIR.K	1247.42355	2	4.70E-07	0.95	3.45	-	1666.0
AHQ-1-6, 4335 - 4338	R.TIVTTLQDSIR.K	1247.42355	2	7.47E-07	0.97	3.95	-	1872.5

AHQ-1-4, 4620	R.TIVTTLQDSIR.K	1247.42355	2	6.97E-05	0.92	3.20	-	1213.6
AHQ-1-2, 4690	R.TIVTTLQDSIR.K	1247.42355	2	2.83E-07	0.96	4.41	-	1542.7
AHQ-1-4, 4536 - 4548	R.TIVTTLQDSIR.K	1247.42355	2	3.35E-07	0.97	4.06	-	1824.6
AHQ-1-7, 4416	R.TIVTTLQDSIR.K	1247.42355	2	5.35E-07	0.95	3.52	-	1470.3
AHQ-1-9, 4244 - 4314	R.TIVTTLQDSIR.K	1247.42355	2	3.66E-06	0.94	3.24	-	1439.5
AHQ-1-6, 3282	R.TIVTTLQDSIR.K	1247.42355	2	1.55E-05	0.95	3.30	-	1497.0
AHQ-1-3, 3386	R.TIVTTLQDSIR.K	1247.42355	2	3.34E-04	0.95	3.38	-	1748.3
AHQ-1-5, 4419	R.TIVTTLQDSIR.K	1247.42355	2	2.95E-06	0.96	4.09	-	1414.9
AHQ-1-3, 4513 - 4531	R.TIVTTLQDSIR.K	1247.42355	2	5.57E-06	0.97	4.21	-	1900.0
AHQ-1-6, 2475	K.VTEENKELANELR.R	1545.67685	2	4.09E-05	0.89	3.66	-	997.6
AHQ-1-9, 2452 - 2455	K.VTEENKELANELR.R	1545.67685	2	2.61E-06	0.88	3.98	-	791.8
AHQ-1-5, 2454	K.VTEENKELANELR.R	1545.67685	2	4.32E-08	0.79	3.23	-	791.1
gi|28626504|ref|NP_113659.3| UNC-112 related protein 2 [Homo sapiens]				1.00E-30	27.75	330.36	62.60	75429.7
AHQ-1-9, 3120	K.AGDALWLR.F	902.03223	2	4.22E-04	0.92	3.94	-	1148.3
AHQ-1-14- , 6440	R.ARGEELDEDLFLQLTGGHEAF	2348.51032	2	8.38E-04	0.92	4.13	-	1168.8
AHQ-1-6, 6740 - 6806	R.ASFSQPLFQAVAAICR.L	1768.02967	2	1.63E-04	0.96	4.85	-	896.2
AHQ-1-12, 6377 - 6378	R.ASFSQPLFQAVAAICR.L	1768.02967	2	7.51E-08	0.94	4.39	-	1044.6
AHQ-1-11, 6083 - 6151	R.ASFSQPLFQAVAAICR.L	1768.02967	2	4.16E-05	0.84	3.49	-	528.5
AHQ-1-10, 6045	R.ASFSQPLFQAVAAICR.L	1768.02967	2	1.95E-04	0.91	4.00	-	823.3
AHQ-1-1, 6785	R.ASFSQPLFQAVAAICR.L	1768.02967	2	1.03E-05	0.89	3.97	-	641.3
AHQ-1-9, 6732 - 6786	R.ASFSQPLFQAVAAICR.L	1768.02967	2	4.53E-06	0.96	5.13	-	936.7
AHQ-1-4, 7046	R.ASFSQPLFQAVAAICR.L	1768.02967	2	6.96E-07	0.96	4.90	-	1025.2
AHQ-1-6, 6787 - 6855	R.ASFSQPLFQAVAAICR.L	1768.02967	2	4.82E-08	0.94	4.41	-	898.5
AHQ-1-2, 7090	R.ASFSQPLFQAVAAICR.L	1768.02967	2	2.34E-04	0.90	3.90	-	711.3
AHQ-1-6, 6790	R.ASFSQPLFQAVAAICR.L	1768.02967	3	2.19E-06	0.92	4.46	-	1056.2
AHQ-1-6, 7074	R.ASFSQPLFQAVAAICR.L	1768.02967	2	1.13E-09	0.90	4.02	-	731.1
AHQ-1-7, 6986	R.ASFSQPLFQAVAAICR.L	1768.02967	2	1.41E-08	0.93	4.20	-	760.5
AHQ-1-5, 6985	R.ASFSQPLFQAVAAICR.L	1768.02967	2	1.43E-08	0.93	4.02	-	923.6
AHQ-1-3, 6882	R.ASFSQPLFQAVAAICR.L	1768.02967	2	1.03E-06	0.90	3.79	-	718.0
AHQ-1-9, 3443	K.EKEPEEELYDLSK.V	1609.71294	2	4.59E-10	0.82	3.55	-	608.2
AHQ-1-7, 3657	K.EKEPEEELYDLSK.V	1609.71294	2	2.31E-05	0.74	3.30	-	376.8
AHQ-1-3, 3750	K.EKEPEEELYDLSK.V	1609.71294	2	4.08E-04	0.66	2.72	-	374.0
AHQ-1-6, 3484 - 3530	K.EKEPEEELYDLSK.V	1609.71294	2	5.95E-04	0.55	2.93	-	305.4
AHQ-1-6, 7115 - 7194	R.FIQAWQSLPDFGISYVMVR.F	2258.62653	2	1.11E-09	0.96	5.33	-	757.7
AHQ-1-6, 7068	R.FIQAWQSLPDFGISYVM*VR.F	2274.62593	2	4.32E-05	0.92	4.46	-	578.4
AHQ-1-12, 6493	R.FIQAWQSLPDFGISYVM*VR.F	2274.62593	2	2.59E-04	0.94	4.57	-	653.3
AHQ-1-6, 3206	K.GCEVVPDVNVSGQK.F	1489.63335	1	5.75E-08	0.90	4.01	-	819.5
AHQ-1-9, 3155	K.GCEVVPDVNVSGQK.F	1489.63335	1	4.06E-06	0.86	3.39	-	774.5
AHQ-1-9, 3151	K.GCEVVPDVNVSGQK.F	1489.63335	1	6.25E-04	0.70	2.82	-	673.0
AHQ-1-7, 3257	K.GCEVVPDVNVSGQK.F	1489.63335	2	6.69E-04	0.82	3.09	-	694.7
AHQ-1-6, 7146 - 7147	R.GEELDEDLFLQLTGGHEAF	2121.24574	2	2.72E-08	0.93	4.10	-	1129.8
AHQ-1-14- , 6619	R.GEELDEDLFLQLTGGHEAF	2121.24574	2	1.90E-08	0.86	3.49	-	753.8
AHQ-1-6, 3067	R.GM*PAHFSDSAQTEACYHM*LSR.P	2430.63997	3	2.73E-05	0.91	4.32	-	1220.1
AHQ-1-6, 3990	R.GMPAHFSDSAQTEACYHMLSR.P	2398.64117	3	3.41E-08	0.97	5.97	-	1357.9
AHQ-1-9, 2611	R.HPEELSLLR.A	1094.24601	1	5.87E-05	0.22	1.95	-	282.1
AHQ-1-14- , 3888	R.IDLAVGDVVK.T	1029.21223	1	1.07E-04	0.58	2.35	-	575.3
AHQ-1-6, 3818 - 3886	R.IDLAVGDVVK.T	1029.21223	2	9.58E-05	0.88	3.32	-	899.6
AHQ-1-13- , 3987	R.IDLAVGDVVK.T	1029.21223	1	4.29E-04	0.82	3.13	-	643.6
AHQ-1-6, 3819 - 3887	R.IDLAVGDVVK.T	1029.21223	1	4.02E-05	0.24	2.00	-	414.2
AHQ-1-11, 3672	R.IDLAVGDVVK.T	1029.21223	1	4.24E-04	0.34	2.19	-	424.0
AHQ-1-1, 4061	R.IDLAVGDVVK.T	1029.21223	2	4.39E-04	0.93	3.54	-	1433.9
AHQ-1-6, 4471 - 4542	R.ILEAHQNVAQLSLAEAQLR.F	2105.38372	2	1.00E-30	0.98	6.56	-	2325.8
AHQ-1-6, 4706	R.ILEAHQNVAQLSLAEAQLR.F	2105.38372	2	1.80E-13	0.98	7.27	-	1553.9
AHQ-1-6, 4486 - 4562	R.ILEAHQNVAQLSLAEAQLR.F	2105.38372	3	5.48E-05	0.63	3.09	-	1132.1
AHQ-1-11, 4220	R.ILEAHQNVAQLSLAEAQLR.F	2105.38372	3	3.00E-05	0.95	5.92	-	1249.0
AHQ-1-5, 4599	R.ILEAHQNVAQLSLAEAQLR.F	2105.38372	2	2.07E-04	0.92	4.89	-	1128.4
AHQ-1-11, 4222	R.ILEAHQNVAQLSLAEAQLR.F	2105.38372	2	3.33E-14	0.98	6.82	-	2072.5
AHQ-1-12, 4401	R.ILEAHQNVAQLSLAEAQLR.F	2105.38372	2	1.00E-08	0.98	5.74	-	2006.8
AHQ-1-13- , 4567 - 4637	R.ILEAHQNVAQLSLAEAQLR.F	2105.38372	2	4.20E-05	0.98	5.54	-	2018.0
AHQ-1-9, 4639 - 4647	R.ILEAHQNVAQLSLAEAQLR.F	2105.38372	2	9.24E-06	0.95	4.67	-	1398.0
AHQ-1-9, 4418 - 4486	R.ILEAHQNVAQLSLAEAQLR.F	2105.38372	3	8.05E-07	0.91	4.84	-	1111.5
AHQ-1-1, 4709 - 4732	R.ILEAHQNVAQLSLAEAQLR.F	2105.38372	3	1.29E-05	0.85	3.82	-	898.8
AHQ-1-13, 4381	R.ILEAHQNVAQLSLAEAQLR.F	2105.38372	2	1.50E-11	0.98	5.72	-	1787.1
AHQ-1-9, 4383 - 4454	R.ILEAHQNVAQLSLAEAQLR.F	2105.38372	2	2.31E-06	0.98	5.85	-	2575.6
AHQ-1-6, 4887	R.ILEAHQNVAQLSLAEAQLR.F	2105.38372	2	5.00E-15	0.97	5.19	-	1430.3
AHQ-1-7, 4522	R.ILEAHQNVAQLSLAEAQLR.F	2105.38372	2	7.43E-08	0.98	6.05	-	2122.6
AHQ-1-3, 4666	R.ILEAHQNVAQLSLAEAQLR.F	2105.38372	3	8.54E-08	0.96	4.90	-	2134.2
AHQ-1-6, 4566	R.ILEAHQNVAQLSLAEAQLR.F	2105.38372	2	1.95E-13	0.98	7.06	-	2111.0
AHQ-1-9, 2486 - 2487	R.KDEILGIANNR.L	1243.39499	2	9.05E-04	0.88	3.06	-	894.5
AHQ-1-13, 2754 - 2811	R.KDEILGIANNR.L	1243.39499	2	2.11E-07	0.95	4.12	-	1387.2
AHQ-1-13- , 2767 - 2831	R.KDEILGIANNR.L	1243.39499	2	1.44E-07	0.94	3.96	-	1261.6
AHQ-1-6, 2674 - 2751	R.KDEILGIANNR.L	1243.39499	2	3.68E-06	0.79	3.35	-	870.0
AHQ-1-6, 2519 - 2590	R.KDEILGIANNR.L	1243.39499	1	2.83E-05	0.48	2.08	-	344.6
AHQ-1-6, 2515 - 2586	R.KDEILGIANNR.L	1243.39499	2	6.10E-07	0.95	4.28	-	1280.4
AHQ-1-12, 2619	R.KDEILGIANNR.L	1243.39499	2	5.21E-04	0.94	3.40	-	1370.2
AHQ-1-9, 2482 - 2555	R.KDEILGIANNR.L	1243.39499	1	1.52E-05	0.31	2.00	-	279.0
AHQ-1-11, 3863	R.KQDWSDHAIWWEQK.R	1858.00504	2	2.24E-04	0.69	2.96	-	634.7
AHQ-1-7, 3970 - 4048	R.KQDWSDHAIWWEQK.R	1858.00504	2	9.85E-04	0.79	3.44	-	631.0
AHQ-1-6, 3967	R.KQDWSDHAIWWEQK.R	1858.00504	3	1.26E-09	0.94	4.34	-	1363.9
AHQ-1-8, 6886 - 6895	K.LEGSAPTDVLDSLTTIPELK.D	2100.35256	2	4.54E-06	0.81	3.89	-	487.2
AHQ-1-9, 6766	K.LEGSAPTDVLDSLTTIPELK.D	2100.35256	2	2.95E-07	0.82	3.64	-	380.5
AHQ-1-6, 5770	K.LEGSAPTDVLDSLTTIPELK.D	2100.35256	2	1.17E-04	0.75	3.60	-	343.4
AHQ-1-7, 6952 - 6954	K.LEGSAPTDVLDSLTTIPELK.D	2100.35256	2	8.35E-06	0.96	5.31	-	1006.5
AHQ-1-6, 6359	K.LEGSAPTDVLDSLTTIPELK.D	2100.35256	2	2.32E-04	0.91	4.28	-	640.1
AHQ-1-7, 5826	K.LEGSAPTDVLDSLTTIPELK.D	2100.35256	2	1.88E-04	0.74	3.35	-	439.2
AHQ-1-10, 6008 - 6071	K.LEGSAPTDVLDSLTTIPELK.D	2100.35256	2	6.66E-04	0.74	3.35	-	455.9
AHQ-1-6, 6542 - 6554	K.LEGSAPTDVLDSLTTIPELK.D	2100.35256	2	4.19E-05	0.93	3.94	-	1056.1
AHQ-1-6, 6658 - 6735	K.LEGSAPTDVLDSLTTIPELK.D	2100.35256	2	8.15E-05	0.89	3.92	-	741.0
AHQ-1-5, 6961	K.LEGSAPTDVLDSLTTIPELK.D	2100.35256	2	2.00E-05	0.82	3.66	-	541.8
AHQ-1-6, 6775 - 6842	K.LEGSAPTDVLDSLTTIPELK.D	2100.35256	2	1.91E-06	0.95	4.66	-	942.2
AHQ-1-4, 7006	K.LEGSAPTDVLDSLTTIPELK.D	2100.35256	2	5.30E-05	0.48	3.10	-	355.8
AHQ-1-13- , 6669 - 6704	K.LEGSAPTDVLDSLTTIPELK.D	2100.35256	2	9.35E-04	0.63	2.87	-	459.5
AHQ-1-3, 6839 - 6915	K.LEGSAPTDVLDSLTTIPELK.D	2100.35256	2	1.67E-04	0.83	3.92	-	521.1
AHQ-1-6, 6478	K.LEGSAPTDVLDSLTTIPELKDHLR.I	2621.92493	3	3.71E-10	0.79	3.57	-	361.6
AHQ-1-6, 2395	R.LFFGPQHR.P	1002.15349	2	4.94E-05	0.84	2.57	-	892.9
AHQ-1-6, 5972	K.LLVPSPEGMSEIYLR.C	1705.01201	3	1.39E-09	0.98	5.36	-	2534.2
AHQ-1-12, 4687 - 4745	K.LLVPSPEGM*SEIYLR.C	1721.01141	2	1.16E-06	0.55	2.53	-	277.0
AHQ-1-5, 6098 - 6114	K.LLVPSPEGMSEIYLR.C	1705.01201	2	1.27E-05	0.57	3.05	-	182.4
AHQ-1-6, 1871 - 1930	R.LPRPSSLSDK.T	1100.25055	2	7.19E-04	0.88	2.65	-	1390.1
AHQ-1-4, 7207	K.LSQSGEVGEPAGTDPGLDDLDVALSNLEVK.L	3027.24073	3	4.61E-04	0.90	4.44	-	1106.2
AHQ-1-3, 7045	K.LSQSGEVGEPAGTDPGLDDLDVALSNLEVK.L	3027.24073	3	9.47E-07	0.86	3.39	-	1216.8
AHQ-1-7, 7176	K.LSQSGEVGEPAGTDPGLDDLDVALSNLEVK.L	3027.24073	3	1.04E-05	0.88	4.29	-	974.3
AHQ-1-6, 6980 - 7050	K.LSQSGEVGEPAGTDPGLDDLDVALSNLEVK.L	3027.24073	3	8.59E-10	0.94	5.32	-	1165.9
AHQ-1-2, 7217 - 7221	K.LSQSGEVGEPAGTDPGLDDLDVALSNLEVK.L	3027.24073	3	4.75E-11	0.88	3.98	-	1191.7
AHQ-1-1, 6859 - 6928	K.LSQSGEVGEPAGTDPGLDDLDVALSNLEVK.L	3027.24073	3	8.74E-06	0.96	5.19	-	1797.6
AHQ-1-7, 2309	R.LTQLYEQAR.W	1122.25622	2	3.85E-05	0.85	2.99	-	1155.6
AHQ-1-6, 2240 - 2306	R.LTQLYEQAR.W	1122.25622	2	5.55E-06	0.90	3.60	-	861.3

AHQ-1-6, 2294	R.LTQLYEQAR.W	1122.25622	1	1.21E-04	0.30	1.95	-	128.1
AHQ-1-9, 2234	R.LTQLYEQAR.W	1122.25622	2	4.55E-05	0.93	3.35	-	1503.6
AHQ-1-6, 5106	R.QWLLQTHWTLDK.Y	1569.78807	2	1.25E-08	0.44	2.96	-	200.7
AHQ-1-14- , 4080	K.SQDEAPGDPIQQLNLK.G	1753.89174	2	2.05E-06	0.85	3.38	-	486.5
AHQ-1-12, 4009 - 4081	K.SQDEAPGDPIQQLNLK.G	1753.89174	2	1.68E-08	0.89	4.00	-	502.6
AHQ-1-4, 4290	K.SQDEAPGDPIQQLNLK.G	1753.89174	2	2.41E-06	0.81	3.46	-	469.1
AHQ-1-7, 4152 - 4154	K.SQDEAPGDPIQQLNLK.G	1753.89174	2	1.44E-08	0.87	3.96	-	433.6
AHQ-1-3, 4249	K.SQDEAPGDPIQQLNLK.G	1753.89174	2	6.26E-04	0.83	3.66	-	424.5
AHQ-1-6, 4326	K.SQDEAPGDPIQQLNLK.G	1753.89174	2	1.12E-09	0.87	3.67	-	521.5
AHQ-1-6, 4064 - 4146	K.SQDEAPGDPIQQLNLK.G	1753.89174	2	4.35E-06	0.88	4.23	-	522.1
AHQ-1-6, 4214	K.SQDEAPGDPIQQLNLK.G	1753.89174	2	1.77E-05	0.83	3.63	-	443.2
AHQ-1-14- , 4424 - 4430	K.TASGDYIDSSWELR.V	1600.66783	2	7.17E-07	0.90	3.31	-	957.1
AHQ-1-9, 4342	K.TASGDYIDSSWELR.V	1600.66783	2	2.26E-04	0.92	3.55	-	934.2
AHQ-1-13, 4280	K.TASGDYIDSSWELR.V	1600.66783	2	1.83E-04	0.78	2.91	-	757.4
AHQ-1-6, 4382 - 4450	K.TASGDYIDSSWELR.V	1600.66783	2	7.71E-09	0.96	4.42	-	1145.6
AHQ-1-6, 4519 - 4586	K.TASGDYIDSSWELR.V	1600.66783	2	3.28E-08	0.90	3.23	-	1109.8
AHQ-1-7, 3893	R.TGSGGPGNHPHGPDASAEGLNPYGLVAPR.F	2783.95312	3	7.70E-05	0.79	3.96	-	584.2
AHQ-1-9, 3748	R.TGSGGPGNHPHGPDASAEGLNPYGLVAPR.F	2783.95312	2	4.95E-04	0.94	4.40	-	1003.2
AHQ-1-9, 3759	R.TGSGGPGNHPHGPDASAEGLNPYGLVAPR.F	2783.95312	2	2.75E-07	0.95	5.10	-	1001.8
AHQ-1-9, 3911	R.TGSGGPGNHPHGPDASAEGLNPYGLVAPR.F	2783.95312	2	3.13E-04	0.90	4.21	-	621.3
AHQ-1-1, 4135 - 4136	R.TGSGGPGNHPHGPDASAEGLNPYGLVAPR.F	2783.95312	3	4.07E-10	0.94	5.13	-	977.8
AHQ-1-3, 4021	R.TGSGGPGNHPHGPDASAEGLNPYGLVAPR.F	2783.95312	3	1.97E-05	0.94	5.46	-	1069.6
AHQ-1-6, 3854 - 3934	R.TGSGGPGNHPHGPDASAEGLNPYGLVAPR.F	2783.95312	3	7.66E-05	0.98	7.05	-	1653.6
AHQ-1-6, 3794 - 3866	R.TGSGGPGNHPHGPDASAEGLNPYGLVAPR.F	2783.95312	2	6.23E-06	0.95	4.79	-	854.7
AHQ-1-5, 3887	R.TGSGGPGNHPHGPDASAEGLNPYGLVAPR.F	2783.95312	2	2.68E-04	0.84	3.64	-	472.0
AHQ-1-6, 7310 - 7311	R.TM*ADSSYTSEVQAILAFLSLQR.T	2448.73471	3	5.35E-09	0.97	5.88	-	1795.6
AHQ-1-9, 3971 - 3974	R.VFVGEEDPEAESVTLR.V	1777.91007	2	9.09E-10	0.96	4.69	-	1449.6
AHQ-1-1, 4192 - 4257	R.VFVGEEDPEAESVTLR.V	1777.91007	2	2.22E-10	0.96	4.69	-	1509.7
AHQ-1-14- , 3983 - 4052	R.VFVGEEDPEAESVTLR.V	1777.91007	2	3.41E-10	0.96	4.67	-	1494.8
AHQ-1-4, 4192	R.VFVGEEDPEAESVTLR.V	1777.91007	2	1.61E-05	0.86	3.48	-	957.2
AHQ-1-10, 3732	R.VFVGEEDPEAESVTLR.V	1777.91007	2	2.79E-05	0.95	4.41	-	1231.9
AHQ-1-13- , 4108	R.VFVGEEDPEAESVTLR.V	1777.91007	2	8.97E-10	0.96	4.33	-	1774.3
AHQ-1-5, 4103	R.VFVGEEDPEAESVTLR.V	1777.91007	2	6.25E-09	0.94	4.08	-	1183.7
AHQ-1-6, 4032	R.VFVGEEDPEAESVTLR.V	1777.91007	3	4.11E-04	0.97	4.97	-	1993.3
AHQ-1-6, 3963 - 4034	R.VFVGEEDPEAESVTLR.V	1777.91007	2	3.49E-09	0.95	4.63	-	1313.7
AHQ-1-12, 3933	R.VFVGEEDPEAESVTLR.V	1777.91007	2	1.22E-07	0.92	4.20	-	1035.9
AHQ-1-8, 4047	R.VFVGEEDPEAESVTLR.V	1777.91007	2	1.19E-10	0.94	4.40	-	1052.0
AHQ-1-11, 3818	R.VFVGEEDPEAESVTLR.V	1777.91007	2	1.08E-05	0.95	4.24	-	1476.3
AHQ-1-6, 4102	R.VFVGEEDPEAESVTLR.V	1777.91007	2	1.37E-09	0.91	3.73	-	1108.1
AHQ-1-2, 4287 - 4326	R.VFVGEEDPEAESVTLR.V	1777.91007	2	2.95E-06	0.83	3.40	-	877.5
AHQ-1-7, 4100	R.VFVGEEDPEAESVTLR.V	1777.91007	2	4.23E-06	0.93	4.35	-	1075.5
AHQ-1-14- , 2788 - 2866	R.VTGESHIGGVLLK.I	1310.52448	2	9.01E-07	0.95	3.98	-	1450.3
AHQ-1-1, 3029	R.VTGESHIGGVLLK.I	1310.52448	2	1.31E-06	0.97	4.48	-	2088.1
AHQ-1-8, 2494	R.VTGESHIGGVLLK.I	1310.52448	2	2.39E-09	0.95	3.93	-	1636.0
AHQ-1-12, 2851	R.VTGESHIGGVLLK.I	1310.52448	2	2.78E-06	0.87	3.38	-	1115.6
AHQ-1-11, 2743	R.VTGESHIGGVLLK.I	1310.52448	2	9.60E-06	0.91	3.83	-	1377.0
AHQ-1-6, 2694	R.VTGESHIGGVLLK.I	1310.52448	1	2.11E-04	0.55	1.97	-	815.8
AHQ-1-10, 2689	R.VTGESHIGGVLLK.I	1310.52448	2	8.11E-05	0.56	2.51	-	808.4
AHQ-1-9, 2627	R.VTGESHIGGVLLK.I	1310.52448	2	8.87E-07	0.94	4.09	-	1433.3
AHQ-1-9, 2644	R.VTGESHIGGVLLK.I	1310.52448	1	1.06E-08	0.88	3.43	-	970.9
AHQ-1-6, 2710	R.VTGESHIGGVLLK.I	1310.52448	1	7.46E-08	0.88	3.21	-	931.6
AHQ-1-6, 2690 - 2760	R.VTGESHIGGVLLK.I	1310.52448	2	8.53E-10	0.97	4.80	-	2091.2
AHQ-1-11, 4550	K.VVLAGGVAPALFR.G	1270.54832	2	4.57E-09	0.94	3.51	-	1410.6
AHQ-1-1, 5096	K.VVLAGGVAPALFR.G	1270.54832	2	1.25E-06	0.97	4.64	-	1641.3
AHQ-1-10, 4412	K.VVLAGGVAPALFR.G	1270.54832	2	5.14E-08	0.97	4.34	-	1874.2
AHQ-1-6, 4818 - 4886	K.VVLAGGVAPALFR.G	1270.54832	2	1.14E-07	0.98	5.60	-	1815.1
AHQ-1-9, 4760 - 4762	K.VVLAGGVAPALFR.G	1270.54832	1	2.09E-04	0.75	3.41	-	244.2
AHQ-1-9, 4756	K.VVLAGGVAPALFR.G	1270.54832	2	9.49E-08	0.97	4.70	-	1832.5
AHQ-1-2, 5161	K.VVLAGGVAPALFR.G	1270.54832	2	5.81E-07	0.96	4.44	-	1535.2
AHQ-1-7, 4904	K.VVLAGGVAPALFR.G	1270.54832	2	6.07E-08	0.97	4.93	-	1703.7
AHQ-1-3, 5019	K.VVLAGGVAPALFR.G	1270.54832	2	6.81E-07	0.97	5.26	-	1678.0
AHQ-1-4, 5106	K.VVLAGGVAPALFR.G	1270.54832	2	4.66E-08	0.96	4.53	-	1443.0
AHQ-1-5, 4949	K.VVLAGGVAPALFR.G	1270.54832	2	5.12E-08	0.96	4.53	-	1409.2
AHQ-1-6, 2470 - 2471	K.YGILADAR.L	878.99544	2	6.11E-05	0.93	3.20	-	1269.8
AHQ-1-9, 5342	K.YYSFFDLDPK.T	1295.42046	2	4.89E-05	0.91	3.79	-	887.6
AHQ-1-6, 5411 - 5478	K.YYSFFDLDPK.T	1295.42046	2	6.79E-06	0.77	3.67	-	586.6
gi|4827056|ref|NP_005103.1| WD repeat-containing protein 1 isoform 2 [Homo sapiens]				1.00E-30	13.04	160.31	49.80	58001.9
AHQ-1-6, 5034	K.AHDGGIYAISWSPDSTHLLSASGDK.T	2586.75455	2	1.81E-05	0.91	3.98	-	908.2
AHQ-1-6, 5151	K.AHDGGIYAISWSPDSTHLLSASGDK.T	2586.75455	3	1.55E-05	0.93	5.21	-	965.3
AHQ-1-6, 5035	K.AHDGGIYAISWSPDSTHLLSASGDK.T	2586.75455	3	1.27E-08	0.97	5.94	-	1683.1
AHQ-1-9, 5702	K.CFSIDNPGYEPEVVAVHPGGDTVAIGGVDGNVR.L	3400.67708	3	1.75E-12	0.93	4.65	-	932.2
AHQ-1-6, 5708 - 5790	K.CFSIDNPGYEPEVVAVHPGGDTVAIGGVDGNVR.L	3400.67708	3	1.00E-30	0.96	5.62	-	1125.6
AHQ-1-13- , 5643	K.CFSIDNPGYEPEVVAVHPGGDTVAIGGVDGNVR.L	3400.67708	3	1.15E-06	0.82	3.64	-	687.6
AHQ-1-10, 5107	K.CFSIDNPGYEPEVVAVHPGGDTVAIGGVDGNVR.L	3400.67708	3	1.00E-30	0.96	5.44	-	1001.0
AHQ-1-6, 5855	K.CFSIDNPGYEPEVVAVHPGGDTVAIGGVDGNVR.L	3400.67708	3	1.82E-10	0.91	4.19	-	946.2
AHQ-1-7, 5893	K.CFSIDNPGYEPEVVAVHPGGDTVAIGGVDGNVR.L	3400.67708	3	6.88E-14	0.96	5.14	-	1427.4
AHQ-1-5, 5951	K.CFSIDNPGYEPEVVAVHPGGDTVAIGGVDGNVR.L	3400.67708	3	1.50E-08	0.80	3.72	-	592.8
AHQ-1-6, 3968 - 4010	R.FATASADGQIYIYDGK.T	1720.86021	2	1.92E-04	0.61	2.90	-	423.7
AHQ-1-6, 5831	K.FGAVFLWDSGSSVGEITGHNK.V	2209.40259	2	1.22E-04	0.93	4.23	-	970.1
AHQ-1-6, 5815 - 5832	K.FGAVFLWDSGSSVGEITGHNK.V	2209.40259	3	7.33E-08	0.79	3.51	-	838.2
AHQ-1-6, 4867	K.GPVTDVAYSHDGAFLAVCDASK.V	2282.47194	2	9.99E-05	0.71	3.55	-	603.1
AHQ-1-6, 4718 - 4795	K.GPVTDVAYSHDGAFLAVCDASK.V	2282.47194	2	5.59E-07	0.97	4.86	-	1633.0
AHQ-1-6, 1740	R.IAVVGEGR.E	800.92621	2	7.90E-04	0.73	2.55	-	676.2
AHQ-1-6, 2222	K.IKDIAWTEDSKR.I	1462.63267	2	1.16E-06	0.94	3.88	-	1329.1
AHQ-1-6, 3047	K.KVFASLPQVER.G	1274.49383	2	2.24E-05	0.83	2.91	-	903.3
AHQ-1-6, 5584 - 5654	R.LATGSDDNCAAFFEGPPFK.F	2046.20216	2	2.01E-09	0.94	4.65	-	883.2
AHQ-1-6, 5663 - 5726	R.LATGSDDNCAAFFEGPPFK.F	2046.20216	2	3.50E-06	0.89	4.10	-	873.6
AHQ-1-6, 5523 - 5590	R.LATGSDDNCAAFFEGPPFK.F	2046.20216	2	1.41E-07	0.84	3.78	-	630.7
AHQ-1-6, 5782 - 5812	R.LATGSDDNCAAFFEGPPFK.F	2046.20216	2	1.90E-09	0.91	4.75	-	708.4
AHQ-1-13, 3997 - 4021	R.LYSILGTTLKDEGK.L	1538.76736	2	4.64E-05	0.85	3.44	-	735.2
AHQ-1-6, 3930	R.LYSILGTTLKDEGK.L	1538.76736	3	2.00E-05	0.47	3.34	-	287.6
AHQ-1-7, 4012	R.LYSILGTTLKDEGK.L	1538.76736	2	1.67E-04	0.90	3.41	-	1073.1
AHQ-1-6, 3919 - 3950	R.LYSILGTTLKDEGK.L	1538.76736	2	8.78E-05	0.92	4.00	-	933.8
AHQ-1-6, 4982 - 5059	R.MTVDESGQLISCSMDDTVR.Y	2146.36365	2	6.97E-06	0.72	3.28	-	584.9
AHQ-1-6, 4299	R.NIDNPALADIYTEHAHQVVVAK.Y	2419.67805	2	1.68E-05	0.69	3.31	-	446.6
AHQ-1-7, 1896	K.SIQCLTVHKNGGK.S	1443.65418	2	8.84E-05	0.80	2.79	-	876.6
AHQ-1-6, 4198	K.SYIYSGSHDGHINYWDSETGENDSFAGK.G	3138.17570	3	7.57E-08	0.98	6.18	-	2306.3
AHQ-1-12, 3879	K.YAPSGFYIASGDVSGK.L	1619.75589	2	2.39E-08	0.87	3.52	-	588.9
AHQ-1-6, 3928 - 3999	K.YAPSGFYIASGDVSGK.L	1619.75589	2	9.64E-06	0.86	3.01	-	706.6
AHQ-1-11, 3775 - 3780	K.YAPSGFYIASGDVSGK.L	1619.75589	2	1.85E-08	0.89	3.85	-	608.2
AHQ-1-6, 2772	K.YEYQPFAGK.I	1103.20822	2	6.71E-07	0.78	2.80	-	615.1
gi|7669492|ref|NP_002037.2| glyceraldehyde-3-phosphate dehydrogenase [Homo sapiens]				1.00E-30	9.38	110.38	48.40	36053.0
AHQ-1-9, 3866 - 3872	R.GALQNIIPASTGAAK.A	1412.61680	1	1.49E-04	0.73	3.01	-	565.4
AHQ-1-11, 3540	R.GALQNIIPASTGAAK.A	1412.61680	1	2.93E-07	0.78	3.19	-	567.2
AHQ-1-9, 3460 - 3530	R.GALQNIIPASTGAAK.A	1412.61680	1	2.75E-04	0.75	3.05	-	573.0
AHQ-1-9, 3470 - 3539	R.GALQNIIPASTGAAK.A	1412.61680	1	3.42E-08	0.80	3.24	-	647.3
AHQ-1-9, 6095 - 6160	K.GILGYTEHQVVSSDFNSDTHSSTFDAGAGIALNDHFVK.L	4039.28524	3	1.38E-07	0.97	6.85	-	1387.6

AHQ-1-13, 4115	K.RVIISAPSADAPMFVM*GVNHEK.Y	2386.77962	3	5.36E-07	0.79	3.46	-	990.4
AHQ-1-9, 4042 - 4056	K.RVIISAPSADAPMFVM*GVNHEK.Y	2386.77962	3	2.48E-05	0.92	4.99	-	948.8
AHQ-1-9, 4543	K.RVIISAPSADAPMFVMGVNHEK.Y	2370.78022	3	3.66E-08	0.95	4.80	-	1262.8
AHQ-1-9, 4747 - 4755	K.RVIISAPSADAPMFVMGVNHEK.Y	2370.78022	3	1.73E-08	0.97	5.87	-	1613.1
AHQ-1-9, 7352 - 7354	K.VDIVAINDPFIDLNYMVYM*FQYDSTHGK.F	3326.74330	3	2.45E-05	0.79	4.83	-	485.6
AHQ-1-10, 6293	K.VIHDNFGIVEGLMTTVHAITATQK.T	2596.98694	3	1.00E-30	0.97	7.44	-	2083.7
AHQ-1-11, 6387	K.VIHDNFGIVEGLMTTVHAITATQK.T	2596.98694	2	7.99E-06	0.97	6.00	-	1984.4
AHQ-1-10, 6296	K.VIHDNFGIVEGLMTTVHAITATQK.T	2596.98694	2	2.63E-09	0.96	5.75	-	2225.0
AHQ-1-9, 7162 - 7166	K.VIHDNFGIVEGLMTTVHAITATQK.T	2596.98694	3	4.68E-12	0.99	7.53	-	2465.0
AHQ-1-9, 5159	R.VIISAPSADAPMFVMGVNHEK.Y	2214.59387	2	3.93E-07	0.95	4.41	-	914.2
AHQ-1-10, 4724 - 4779	R.VIISAPSADAPMFVMGVNHEK.Y	2214.59387	2	2.92E-04	0.56	3.24	-	627.4
AHQ-1-9, 4864 - 4910	R.VIISAPSADAPMFVMGVNHEK.Y	2214.59387	2	5.88E-06	0.90	3.78	-	678.9
AHQ-1-9, 4379 - 4380	R.VIISAPSADAPMFVM*GVNHEK.Y	2230.59327	2	7.90E-07	0.49	3.51	-	282.7
AHQ-1-10, 4593	R.VIISAPSADAPMFVMGVNHEK.Y	2214.59387	2	1.15E-04	0.55	3.04	-	255.7
AHQ-1-14, 4988	R.VPTANVSVVDLTCR.L	1532.74450	2	1.43E-06	0.90	3.52	-	894.8
AHQ-1-14- , 4098	R.VPTANVSVVDLTCR.L	1532.74450	2	1.27E-10	0.93	3.91	-	916.6
AHQ-1-13, 4029 - 4087	R.VPTANVSVVDLTCR.L	1532.74450	2	2.75E-04	0.95	4.23	-	1094.0
AHQ-1-12, 4003 - 4065	R.VPTANVSVVDLTCR.L	1532.74450	2	4.00E-09	0.93	3.98	-	1066.3
AHQ-1-9, 4340 - 4350	R.VPTANVSVVDLTCR.L	1532.74450	2	3.27E-06	0.94	4.14	-	984.2
AHQ-1-11, 3914	R.VPTANVSVVDLTCR.L	1532.74450	2	6.27E-08	0.95	4.26	-	1085.8
AHQ-1-14- , 4252	R.VPTANVSVVDLTCR.L	1532.74450	2	2.94E-04	0.86	3.08	-	845.0
AHQ-1-9, 4199 - 4270	R.VPTANVSVVDLTCR.L	1532.74450	2	4.58E-09	0.96	4.41	-	1285.9
AHQ-1-9, 4062 - 4122	R.VPTANVSVVDLTCR.L	1532.74450	2	1.42E-07	0.96	4.40	-	1105.3
AHQ-1-9, 3959 - 3976	R.VPTANVSVVDLTCR.L	1532.74450	2	1.92E-10	0.93	3.64	-	943.7
AHQ-1-9, 3874	R.VPTANVSVVDLTCR.L	1532.74450	2	9.76E-08	0.86	3.33	-	811.1
AHQ-1-6, 4184	R.VPTANVSVVDLTCR.L	1532.74450	2	5.33E-09	0.93	3.70	-	997.4
AHQ-1-10, 3805 - 3859	R.VPTANVSVVDLTCR.L	1532.74450	2	1.01E-08	0.96	4.20	-	1273.9
AHQ-1-13- , 4243 - 4307	R.VPTANVSVVDLTCR.L	1532.74450	2	1.59E-09	0.95	4.24	-	1033.1
AHQ-1-4, 4367	R.VPTANVSVVDLTCR.L	1532.74450	2	1.00E-07	0.88	3.41	-	757.4
AHQ-1-9, 7446 - 7462	K.WGDAGAEYVVESTGVFTTMEK.A	2278.48027	2	9.12E-07	0.93	4.41	-	804.2
AHQ-1-9, 7304 - 7375	K.WGDAGAEYVVESTGVFTTMEK.A	2278.48027	2	1.31E-08	0.96	5.04	-	1104.9
AHQ-1-9, 7187	K.WGDAGAEYVVESTGVFTTMEK.A	2278.48027	2	2.96E-08	0.95	4.77	-	916.4
AHQ-1-9, 6896 - 6962	K.WGDAGAEYVVESTGVFTTMEK.A	2278.48027	2	1.04E-06	0.92	3.70	-	995.2
AHQ-1-9, 6811	K.WGDAGAEYVVESTGVFTTMEK.A	2278.48027	2	2.69E-10	0.77	3.22	-	598.5
AHQ-1-9, 6736	K.WGDAGAEYVVESTGVFTTM*EK.A	2294.47967	2	4.40E-04	0.61	3.28	-	343.5
AHQ-1-9, 6712	K.WGDAGAEYVVESTGVFTTMEK.A	2278.48027	2	1.52E-06	0.89	4.03	-	718.7
AHQ-1-9, 6631 - 6648	K.WGDAGAEYVVESTGVFTTM*EK.A	2294.47967	2	3.16E-04	0.55	2.77	-	382.8
AHQ-1-9, 6628	K.WGDAGAEYVVESTGVFTTMEK.A	2278.48027	2	2.92E-07	0.89	3.88	-	735.9
AHQ-1-9, 6538 - 6615	K.WGDAGAEYVVESTGVFTTMEK.A	2278.48027	2	7.93E-10	0.93	4.56	-	730.4
AHQ-1-13- , 5703	K.WGDAGAEYVVESTGVFTTM*EK.A	2294.47967	2	1.46E-05	0.95	5.09	-	871.8
AHQ-1-13- , 5792	K.WGDAGAEYVVESTGVFTTM*EK.A	2294.47967	2	1.30E-07	0.94	4.39	-	887.5
AHQ-1-13- , 6285 - 6347	K.WGDAGAEYVVESTGVFTTMEK.A	2278.48027	2	4.50E-08	0.95	4.76	-	956.0
AHQ-1-13- , 6384	K.WGDAGAEYVVESTGVFTTMEK.A	2278.48027	2	1.96E-04	0.69	2.99	-	606.0
AHQ-1-9, 6387 - 6451	K.WGDAGAEYVVESTGVFTTMEK.A	2278.48027	2	3.47E-08	0.94	4.54	-	838.0
AHQ-1-9, 7031	K.WGDAGAEYVVESTGVFTTMEK.A	2278.48027	2	6.83E-04	0.41	3.35	-	162.3
AHQ-1-14- , 6055	K.WGDAGAEYVVESTGVFTTMEK.A	2278.48027	2	1.95E-06	0.94	4.16	-	961.7
gi|16753233|ref|NP_006280.2| talin 1 [Homo sapiens]				5.55E-16	116.92	1320.37	55.40	269665.3
AHQ-1-3, 3193 - 3201	K.AAAFEEQENETVVVK.E	1664.79496	2	3.56E-08	0.95	4.87	-	1305.2
AHQ-1-1, 4007	K.AAAFEEQENETVVVK.E	1664.79496	2	8.60E-04	0.80	3.19	-	817.9
AHQ-1-14- , 3147	K.AAAFEEQENETVVVK.E	1664.79496	2	3.79E-05	0.96	4.74	-	1418.2
AHQ-1-2, 3974	K.AAAFEEQENETVVVK.E	1664.79496	2	7.30E-04	0.84	3.68	-	936.6
AHQ-1-4, 3215 - 3290	K.AAAFEEQENETVVVK.E	1664.79496	2	2.84E-06	0.93	4.58	-	1120.6
AHQ-1-2, 3566	K.AAAFEEQENETVVVK.E	1664.79496	2	1.46E-04	0.94	4.25	-	1394.6
AHQ-1-2, 3322 - 3395	K.AAAFEEQENETVVVK.E	1664.79496	2	7.81E-05	0.93	4.01	-	1220.8
AHQ-1-1, 3288	K.AAAFEEQENETVVVK.E	1664.79496	2	3.39E-05	0.93	4.31	-	1244.8
AHQ-1-12, 3095	K.AAAFEEQENETVVVK.E	1664.79496	2	2.46E-05	0.85	3.96	-	738.8
AHQ-1-5, 2490	R.AAM*EPIVISAK.T	1146.38260	1	8.64E-04	0.43	2.48	-	262.2
AHQ-1-4, 3288	R.AAMEPIVISAK.T	1130.38320	1	3.07E-04	0.75	2.76	-	870.4
AHQ-1-4, 2596 - 2598	R.AAM*EPIVISAK.T	1146.38260	2	1.18E-05	0.79	2.51	-	785.3
AHQ-1-1, 2749	R.AAM*EPIVISAK.T	1146.38260	2	5.12E-07	0.84	3.08	-	773.9
AHQ-1-3, 2597	R.AAM*EPIVISAK.T	1146.38260	2	7.85E-05	0.74	2.84	-	643.4
AHQ-1-3, 2606	R.AAM*EPIVISAK.T	1146.38260	1	6.43E-04	0.38	2.50	-	173.9
AHQ-1-2, 2685	R.AAM*EPIVISAK.T	1146.38260	2	1.61E-06	0.84	2.93	-	777.4
AHQ-1-3, 3266	R.AAMEPIVISAK.T	1130.38320	2	2.68E-07	0.79	2.61	-	607.2
AHQ-1-2, 2683	R.AAM*EPIVISAK.T	1146.38260	1	3.28E-04	0.07	2.01	-	141.8
AHQ-1-6, 3142	R.AAMEPIVISAK.T	1130.38320	1	6.77E-05	0.24	2.17	-	560.3
AHQ-1-3, 1973	K.ADAEGESDLENSR.K	1393.35296	2	2.68E-07	0.88	3.45	-	906.4
AHQ-1-2, 2022	K.ADAEGESDLENSR.K	1393.35296	2	8.74E-08	0.93	3.42	-	1287.9
AHQ-1-1, 2123	K.ADAEGESDLENSR.K	1393.35296	2	4.27E-07	0.93	3.76	-	1066.5
AHQ-1-2, 2021 - 2089	K.ADAEGESDLENSR.K	1393.35296	2	8.40E-07	0.94	3.91	-	1249.2
AHQ-1-5, 1839	K.ADAEGESDLENSR.K	1393.35296	2	1.82E-07	0.83	3.00	-	782.1
AHQ-1-2, 1593 - 1666	K.ADAEGESDLENSRK.L	1521.52588	2	4.60E-06	0.83	2.80	-	1121.3
AHQ-1-3, 2517	K.AGALQCSPSDAYTK.K	1470.58689	2	2.11E-04	0.69	3.12	-	566.1
AHQ-1-1, 2745	K.AGALQCSPSDAYTK.K	1470.58689	2	8.91E-04	0.90	3.67	-	851.7
AHQ-1-6, 2562	K.AGALQCSPSDAYTK.K	1470.58689	2	2.19E-04	0.89	3.26	-	928.5
AHQ-1-4, 2646	K.AGALQCSPSDAYTK.K	1470.58689	2	1.75E-04	0.83	3.42	-	682.6
AHQ-1-2, 2742	K.AGALQCSPSDAYTK.K	1470.58689	1	2.34E-06	0.85	3.72	-	620.6
AHQ-1-8, 6286 - 6303	K.AGFLDLKDFLPK.E	1364.61374	2	4.85E-04	0.96	4.35	-	1938.4
AHQ-1-4, 4090 - 4170	K.AIAVTVQEMVTK.S	1290.55498	2	2.00E-04	0.92	3.73	-	983.6
AHQ-1-1, 3019	K.AIAVTVQEM*VTK.S	1306.55438	2	2.38E-04	0.88	3.05	-	1033.9
AHQ-1-3, 4050 - 4123	K.AIAVTVQEMVTK.S	1290.55498	2	9.21E-09	0.95	3.86	-	1502.0
AHQ-1-2, 4282	K.AIAVTVQEMVTK.S	1290.55498	2	1.92E-09	0.96	4.34	-	1516.1
AHQ-1-4, 2970	K.AIAVTVQEM*VTK.S	1306.55438	2	5.17E-04	0.95	3.94	-	1222.7
AHQ-1-3, 2945 - 2957	K.AIAVTVQEM*VTK.S	1306.55438	2	3.47E-08	0.96	4.18	-	1280.0
AHQ-1-5, 3946 - 3957	K.AIAVTVQEMVTK.S	1290.55498	2	2.49E-07	0.95	4.44	-	1185.9
AHQ-1-2, 3043	K.AIAVTVQEM*VTK.S	1306.55438	2	3.33E-04	0.92	3.77	-	800.9
AHQ-1-2, 4427	K.AIAVTVQEMVTK.S	1290.55498	2	3.11E-05	0.95	3.62	-	1425.0
AHQ-1-2, 2743	K.ALDGAFTEENR.A	1223.27427	2	1.02E-06	0.95	3.45	-	1894.8
AHQ-1-3, 2507	K.ALDGAFTEENR.A	1223.27427	2	3.99E-06	0.87	2.82	-	1006.8
AHQ-1-3, 2641 - 2715	K.ALDGAFTEENR.A	1223.27427	2	7.39E-06	0.94	3.59	-	1453.6
AHQ-1-13- , 2765	K.ALDGAFTEENR.A	1223.27427	2	3.01E-04	0.94	3.31	-	1456.8
AHQ-1-4, 2664	K.ALDGAFTEENR.A	1223.27427	2	1.41E-04	0.95	3.97	-	1499.3
AHQ-1-5, 2505 - 2578	K.ALDGAFTEENR.A	1223.27427	2	2.23E-06	0.92	3.52	-	1301.8
AHQ-1-4, 2503	K.ALDGAFTEENR.A	1223.27427	2	2.21E-07	0.95	3.45	-	1831.4
AHQ-1-2, 2593	K.ALDGAFTEENR.A	1223.27427	2	1.82E-04	0.89	2.88	-	1228.0
AHQ-1-13- , 3440	K.ALDYYM*LR.N	1061.23626	2	2.33E-05	0.91	2.73	-	1072.3
AHQ-1-13, 3923	K.ALDYYMLR.N	1045.23686	2	1.08E-04	0.94	3.48	-	847.1
AHQ-1-13- , 4083 - 4088	K.ALDYYMLR.N	1045.23686	2	1.27E-04	0.94	3.42	-	982.2
AHQ-1-8, 3739	K.ALDYYMLR.N	1045.23686	2	9.04E-06	0.93	3.35	-	796.9
AHQ-1-2, 2033 - 2046	R.ALEATTEHIR.Q	1141.25953	2	3.92E-08	0.82	3.11	-	962.4
AHQ-1-7, 1852	R.ALEATTEHIR.Q	1141.25953	2	2.28E-04	0.74	2.80	-	903.2
AHQ-1-2, 2161	R.ALEATTEHIR.Q	1141.25953	2	3.78E-06	0.86	3.06	-	1018.2
AHQ-1-5, 1786 - 1855	R.ALEATTEHIR.Q	1141.25953	2	3.83E-06	0.73	2.95	-	796.3
AHQ-1-3, 1985	R.ALEATTEHIR.Q	1141.25953	1	1.29E-05	0.11	1.89	-	346.2
AHQ-1-6, 1830 - 1883	R.ALEATTEHIR.Q	1141.25953	2	2.11E-05	0.64	2.61	-	765.6
AHQ-1-3, 1961 - 1993	R.ALEATTEHIR.Q	1141.25953	2	4.15E-04	0.89	2.95	-	1511.7
AHQ-1-4, 1934	R.ALEATTEHIR.Q	1141.25953	2	1.27E-05	0.93	3.61	-	1465.0

AHQ-1-7, 3410	K.ALGDLISATK.A	989.14811	1	8.85E-05	0.15	2.32	-	245.6
AHQ-1-4, 3516	K.ALGDLISATK.A	989.14811	2	2.21E-06	0.92	3.60	-	952.5
AHQ-1-3, 3483	K.ALGDLISATK.A	989.14811	2	2.97E-06	0.89	3.51	-	708.5
AHQ-1-2, 3581	K.ALGDLISATK.A	989.14811	2	2.14E-06	0.93	3.82	-	872.3
AHQ-1-6, 3366	K.ALGDLISATK.A	989.14811	2	5.14E-05	0.87	3.14	-	745.7
AHQ-1-1, 3553	K.ALGDLISATK.A	989.14811	2	5.64E-05	0.93	4.00	-	659.2
AHQ-1-5, 3389	K.ALGDLISATK.A	989.14811	2	3.08E-05	0.82	3.01	-	687.6
AHQ-1-5, 2499 - 2569	K.ALSTDPAAPNLK.S	1198.35099	2	2.38E-05	0.57	2.89	-	399.2
AHQ-1-3, 2635	K.ALSTDPAAPNLK.S	1198.35099	2	1.48E-07	0.78	2.90	-	668.8
AHQ-1-2, 2718 - 2745	K.ALSTDPAAPNLK.S	1198.35099	2	2.96E-06	0.78	3.23	-	621.0
AHQ-1-1, 2768 - 2775	K.ALSTDPAAPNLK.S	1198.35099	2	3.62E-05	0.83	3.33	-	606.8
AHQ-1-7, 2589	K.ALSTDPAAPNLK.S	1198.35099	2	2.79E-06	0.81	3.09	-	713.5
AHQ-1-4, 6647 - 6722	R.ANQAIQMACQSLGEPGCTQAQVLSAATIVAK.H	3220.64522	3	6.21E-04	0.90	4.63	-	686.5
AHQ-1-3, 6323	K.APGQLECETAIAALNSCLR.D	2077.32566	3	5.31E-05	0.86	4.27	-	685.9
AHQ-1-4, 6459 - 6464	K.APGQLECETAIAALNSCLR.D	2077.32566	2	7.67E-08	0.98	6.50	-	1646.0
AHQ-1-3, 5615	K.APGQLECETAIAALNSCLR.D	2077.32566	2	2.32E-08	0.97	5.04	-	1337.4
AHQ-1-4, 5727 - 5807	K.APGQLECETAIAALNSCLR.D	2077.32566	2	3.68E-06	0.93	4.10	-	1060.1
AHQ-1-2, 6493	K.APGQLECETAIAALNSCLR.D	2077.32566	2	6.99E-08	0.97	5.66	-	1335.6
AHQ-1-3, 6321	K.APGQLECETAIAALNSCLR.D	2077.32566	2	2.11E-08	0.97	5.57	-	1492.1
AHQ-1-1, 6264	K.APGQLECETAIAALNSCLR.D	2077.32566	2	3.22E-05	0.96	5.06	-	1245.1
AHQ-1-2, 6498	K.APGQLECETAIAALNSCLR.D	2077.32566	3	4.12E-05	0.84	3.92	-	778.3
AHQ-1-2, 6758	K.APGQLECETAIAALNSCLR.D	2077.32566	2	8.75E-11	0.97	4.84	-	1318.7
AHQ-1-3, 6905 - 6909	K.AQEACGPLEMDSALSVVQNLEK.D	2391.66165	3	7.46E-10	0.97	5.75	-	1809.9
AHQ-1-1, 6771 - 6784	K.AQEACGPLEMDSALSVVQNLEK.D	2391.66165	3	9.40E-06	0.93	4.37	-	1470.2
AHQ-1-6, 6875	K.AQEACGPLEMDSALSVVQNLEK.D	2391.66165	3	3.22E-04	0.91	4.51	-	1007.8
AHQ-1-5, 7019 - 7022	K.AQEACGPLEMDSALSVVQNLEK.D	2391.66165	3	1.34E-06	0.97	5.78	-	2159.5
AHQ-1-1, 6767	K.AQEACGPLEMDSALSVVQNLEK.D	2391.66165	2	6.08E-05	0.87	3.32	-	901.8
AHQ-1-2, 7165	K.AQEACGPLEMDSALSVVQNLEK.D	2391.66165	3	1.72E-05	0.95	4.69	-	1722.7
AHQ-1-2, 7082 - 7083	K.AQEACGPLEMDSALSVVQNLEK.D	2391.66165	3	2.15E-08	0.98	5.79	-	2428.1
AHQ-1-4, 3004	K.ASAGPQPLLVQSCK.A	1457.67763	1	6.83E-08	0.27	2.95	-	294.8
AHQ-1-1, 3117	K.ASAGPQPLLVQSCK.A	1457.67763	1	1.51E-04	0.26	2.27	-	346.6
AHQ-1-4, 3243	K.ASAGPQPLLVQSCK.A	1457.67763	2	2.40E-08	0.74	3.18	-	370.6
AHQ-1-4, 2947 - 3018	K.ASAGPQPLLVQSCK.A	1457.67763	2	1.51E-06	0.92	4.24	-	608.0
AHQ-1-5, 2846	K.ASAGPQPLLVQSCK.A	1457.67763	1	3.26E-11	0.68	3.11	-	474.2
AHQ-1-5, 2811	K.ASAGPQPLLVQSCK.A	1457.67763	2	2.18E-05	0.74	2.74	-	491.5
AHQ-1-2, 3030 - 3101	K.ASAGPQPLLVQSCK.A	1457.67763	2	1.05E-09	0.90	3.75	-	535.5
AHQ-1-4, 2994	K.ASAGPQPLLVQSCK.A	1457.67763	1	4.19E-06	0.18	2.05	-	415.7
AHQ-1-7, 2794 - 2873	K.ASAGPQPLLVQSCK.A	1457.67763	2	1.68E-07	0.89	3.86	-	583.8
AHQ-1-6, 2875	K.ASAGPQPLLVQSCK.A	1457.67763	1	2.28E-09	0.76	4.02	-	394.3
AHQ-1-6, 2856	K.ASAGPQPLLVQSCK.A	1457.67763	2	2.28E-06	0.69	3.00	-	328.4
AHQ-1-3, 2983 - 2989	K.ASAGPQPLLVQSCK.A	1457.67763	1	5.11E-07	0.16	2.07	-	333.0
AHQ-1-3, 2954 - 2973	K.ASAGPQPLLVQSCK.A	1457.67763	2	6.35E-06	0.86	3.61	-	468.7
AHQ-1-1, 3096	K.ASAGPQPLLVQSCK.A	1457.67763	2	2.16E-05	0.68	2.93	-	385.7
AHQ-1-1, 4387 - 4400	K.ASVPTIQDQASAMQLSQCAK.N	2136.39332	2	6.53E-05	0.90	4.02	-	638.0
AHQ-1-2, 4065	K.ASVPTIQDQASAM*QLSQCAK.N	2152.39272	2	1.47E-08	0.95	4.76	-	847.2
AHQ-1-2, 4485 - 4553	K.ASVPTIQDQASAMQLSQCAK.N	2136.39332	2	2.21E-10	0.94	4.66	-	998.7
AHQ-1-5, 3730 - 3731	K.ASVPTIQDQASAM*QLSQCAK.N	2152.39272	2	6.22E-07	0.94	4.98	-	667.2
AHQ-1-3, 3937	K.ASVPTIQDQASAM*QLSQCAK.N	2152.39272	2	2.43E-07	0.95	4.87	-	801.4
AHQ-1-5, 3879	K.ASVPTIQDQASAM*QLSQCAK.N	2152.39272	2	1.60E-09	0.87	3.78	-	581.5
AHQ-1-4, 3987 - 3994	K.ASVPTIQDQASAM*QLSQCAK.N	2152.39272	2	1.89E-07	0.91	4.38	-	617.0
AHQ-1-4, 3826	K.ASVPTIQDQASAM*QLSQCAK.N	2152.39272	2	6.31E-06	0.54	2.64	-	388.5
AHQ-1-7, 3758	K.ASVPTIQDQASAM*QLSQCAK.N	2152.39272	2	9.69E-05	0.86	3.71	-	533.5
AHQ-1-6, 3666 - 3674	K.ASVPTIQDQASAM*QLSQCAK.N	2152.39272	2	1.35E-06	0.90	4.03	-	725.1
AHQ-1-1, 3824 - 3827	K.ASVPTIQDQASAM*QLSQCAK.N	2152.39272	2	9.00E-08	0.92	4.28	-	668.3
AHQ-1-3, 4350 - 4426	K.ASVPTIQDQASAMQLSQCAK.N	2136.39332	2	6.79E-07	0.93	4.65	-	660.1
AHQ-1-4, 4414 - 4491	K.ASVPTIQDQASAMQLSQCAK.N	2136.39332	2	1.36E-09	0.95	5.39	-	660.6
AHQ-1-3, 3785	K.ASVPTIQDQASAM*QLSQCAK.N	2152.39272	2	2.80E-07	0.88	3.80	-	671.3
AHQ-1-1, 4481	K.ASVPTIQDQASAMQLSQCAK.N	2136.39332	2	4.07E-13	0.89	4.29	-	558.8
AHQ-1-1, 3967	K.ASVPTIQDQASAM*QLSQCAK.N	2152.39272	2	3.36E-08	0.87	3.89	-	723.6
AHQ-1-2, 4633 - 4674	K.ASVPTIQDQASAMQLSQCAK.N	2136.39332	2	4.26E-10	0.94	4.55	-	795.6
AHQ-1-4, 4583	K.ASVPTIQDQASAMQLSQCAK.N	2136.39332	2	9.44E-15	0.84	3.91	-	453.9
AHQ-1-2, 4975	K.ASVPTIQDQASAMQLSQCAK.N	2136.39332	2	1.09E-07	0.93	4.22	-	797.1
AHQ-1-6, 4238	K.ASVPTIQDQASAMQLSQCAK.N	2136.39332	2	2.97E-06	0.86	3.79	-	510.1
AHQ-1-5, 4325 - 4341	K.ASVPTIQDQASAMQLSQCAK.N	2136.39332	2	5.79E-08	0.92	4.22	-	789.6
AHQ-1-5, 4495	K.ASVPTIQDQASAMQLSQCAK.N	2136.39332	2	2.55E-10	0.92	4.60	-	601.0
AHQ-1-4, 4499	K.ASVPTIQDQASAMQLSQCAK.N	2136.39332	2	1.18E-05	0.67	2.91	-	399.7
AHQ-1-7, 4320 - 4332	K.ASVPTIQDQASAMQLSQCAK.N	2136.39332	2	5.85E-06	0.88	3.64	-	656.5
AHQ-1-6, 4408	K.ASVPTIQDQASAMQLSQCAK.N	2136.39332	2	3.91E-05	0.67	3.36	-	352.9
AHQ-1-4, 4402	K.ASVPTIQDQASAMQLSQCAK.N	2136.39332	2	5.94E-07	0.88	4.31	-	467.2
AHQ-1-3, 4519	K.ASVPTIQDQASAMQLSQCAK.N	2136.39332	2	6.78E-10	0.88	3.87	-	524.7
AHQ-1-5, 5042	K.AVAEQIPLLVQGVR.G	1493.77619	1	6.86E-07	0.27	2.94	-	118.0
AHQ-1-2, 5595	K.AVAEQIPLLVQGVR.G	1493.77619	2	1.28E-05	0.90	3.99	-	754.4
AHQ-1-5, 4989 - 5057	K.AVAEQIPLLVQGVR.G	1493.77619	2	1.03E-08	0.95	4.52	-	1074.7
AHQ-1-2, 5477	K.AVAEQIPLLVQGVR.G	1493.77619	2	2.25E-07	0.93	3.08	-	1195.8
AHQ-1-4, 5126 - 5134	K.AVAEQIPLLVQGVR.G	1493.77619	2	2.34E-07	0.89	3.41	-	747.6
AHQ-1-4, 5144	K.AVAEQIPLLVQGVR.G	1493.77619	1	6.50E-07	0.71	3.35	-	252.3
AHQ-1-2, 5225	K.AVAEQIPLLVQGVR.G	1493.77619	1	1.63E-05	0.74	3.61	-	358.0
AHQ-1-2, 5210	K.AVAEQIPLLVQGVR.G	1493.77619	3	4.13E-09	0.97	4.74	-	2583.7
AHQ-1-1, 5173	K.AVAEQIPLLVQGVR.G	1493.77619	3	2.49E-06	0.97	4.31	-	2592.4
AHQ-1-1, 5143	K.AVAEQIPLLVQGVR.G	1493.77619	2	1.73E-04	0.81	3.46	-	579.3
AHQ-1-7, 4985 - 4993	K.AVAEQIPLLVQGVR.G	1493.77619	2	8.97E-05	0.90	4.31	-	621.0
AHQ-1-2, 5187 - 5257	K.AVAEQIPLLVQGVR.G	1493.77619	2	1.78E-07	0.82	3.03	-	629.7
AHQ-1-6, 4943 - 4947	K.AVAEQIPLLVQGVR.G	1493.77619	2	7.80E-07	0.75	2.99	-	500.6
AHQ-1-3, 5057	K.AVAEQIPLLVQGVR.G	1493.77619	3	5.08E-06	0.95	4.45	-	1729.4
AHQ-1-6, 3747	K.AVASAAAALVLK.A	1085.32216	2	8.94E-07	0.96	3.06	-	2088.3
AHQ-1-2, 4161	K.AVASAAAALVLK.A	1085.32216	2	1.09E-04	0.95	3.79	-	1652.8
AHQ-1-2, 4077	K.AVASAAAALVLK.A	1085.32216	2	1.21E-05	0.92	3.69	-	1137.8
AHQ-1-4, 3976 - 4022	K.AVASAAAALVLK.A	1085.32216	2	5.46E-07	0.95	4.16	-	1341.8
AHQ-1-1, 3964 - 4031	K.AVASAAAALVLK.A	1085.32216	2	2.84E-06	0.97	4.05	-	2000.7
AHQ-1-3, 3993	K.AVASAAAALVLK.A	1085.32216	2	1.71E-04	0.96	3.89	-	1714.5
AHQ-1-5, 3793	K.AVASAAAALVLK.A	1085.32216	2	7.78E-08	0.97	4.23	-	2074.1
AHQ-1-1, 3872 - 3892	K.AVEGCVSASQAATEDGQLLR.G	2064.22056	2	6.07E-08	0.97	5.58	-	1618.7
AHQ-1-1, 3983	K.AVEGCVSASQAATEDGQLLR.G	2064.22056	2	8.14E-08	0.86	3.89	-	827.9
AHQ-1-4, 4294 - 4328	K.AVEGCVSASQAATEDGQLLR.G	2064.22056	2	2.66E-07	0.92	4.12	-	1127.8
AHQ-1-2, 5937 - 6011	K.AVEGCVSASQAATEDGQLLR.G	2064.22056	2	5.24E-04	0.41	2.80	-	507.2
AHQ-1-2, 4858	K.AVEGCVSASQAATEDGQLLR.G	2064.22056	2	1.10E-06	0.78	3.73	-	701.5
AHQ-1-2, 4554 - 4631	K.AVEGCVSASQAATEDGQLLR.G	2064.22056	2	3.42E-07	0.90	4.10	-	965.0
AHQ-1-1, 4165	K.AVEGCVSASQAATEDGQLLR.G	2064.22056	2	1.46E-06	0.97	5.21	-	1719.4
AHQ-1-6, 4048 - 4059	K.AVEGCVSASQAATEDGQLLR.G	2064.22056	2	2.69E-04	0.64	2.77	-	575.5
AHQ-1-4, 4151 - 4224	K.AVEGCVSASQAATEDGQLLR.G	2064.22056	2	4.50E-06	0.60	3.21	-	446.7
AHQ-1-2, 4405 - 4481	K.AVEGCVSASQAATEDGQLLR.G	2064.22056	2	2.92E-06	0.96	5.19	-	1310.5
AHQ-1-1, 4256	K.AVEGCVSASQAATEDGQLLR.G	2064.22056	2	3.77E-06	0.80	3.24	-	940.7
AHQ-1-3, 3845 - 3921	K.AVEGCVSASQAATEDGQLLR.G	2064.22056	2	3.49E-05	0.92	3.81	-	1238.6
AHQ-1-2, 5515	K.AVEGCVSASQAATEDGQLLR.G	2064.22056	2	6.30E-07	0.95	4.73	-	1153.4
AHQ-1-3, 4509	K.AVEGCVSASQAATEDGQLLR.G	2064.22056	2	1.08E-07	0.75	3.31	-	785.2
AHQ-1-5, 4261 - 4301	K.AVEGCVSASQAATEDGQLLR.G	2064.22056	2	2.43E-04	0.67	3.37	-	690.1
AHQ-1-4, 3907	K.AVEGCVSASQAATEDGQLLR.G	2064.22056	2	5.90E-05	0.87	3.65	-	1029.1

AHQ-1-3, 3995	K.AVEGCVSASQAATEDGQLLR.G	2064.22056	2	9.63E-04	0.65	3.24	-	719.3
AHQ-1-2, 4261 - 4330	K.AVEGCVSASQAATEDGQLLR.G	2064.22056	2	1.20E-06	0.87	4.22	-	680.9
AHQ-1-3, 4130 - 4201	K.AVEGCVSASQAATEDGQLLR.G	2064.22056	2	2.88E-07	0.96	4.89	-	1456.8
AHQ-1-2, 4153 - 4155	K.AVEGCVSASQAATEDGQLLR.G	2064.22056	2	5.66E-06	0.95	4.44	-	1582.1
AHQ-1-4, 3999	K.AVEGCVSASQAATEDGQLLR.G	2064.22056	2	4.14E-06	0.91	4.14	-	1235.7
AHQ-1-2, 3961	K.AVEGCVSASQAATEDGQLLR.G	2064.22056	2	1.34E-04	0.86	3.59	-	944.2
AHQ-1-5, 4430	K.AVEGCVSASQAATEDGQLLR.G	2064.22056	2	4.62E-05	0.71	2.98	-	441.7
AHQ-1-3, 4269 - 4338	K.AVEGCVSASQAATEDGQLLR.G	2064.22056	2	1.21E-05	0.83	3.19	-	888.4
AHQ-1-2, 5162	K.AVEGCVSASQAATEDGQLLR.G	2064.22056	2	7.86E-04	0.90	4.02	-	1111.9
AHQ-1-2, 4043	K.AVEGCVSASQAATEDGQLLR.G	2064.22056	2	7.96E-09	0.90	3.73	-	1046.0
AHQ-1-5, 7338 - 7346	K.AVSSAIAQLLGEVAQGNENYAGIAAR.D	2574.83158	3	2.75E-05	0.72	3.77	-	730.7
AHQ-1-3, 7085 - 7153	K.AVSSAIAQLLGEVAQGNENYAGIAAR.D	2574.83158	3	4.24E-06	0.97	6.02	-	2039.2
AHQ-1-6, 7170	K.AVSSAIAQLLGEVAQGNENYAGIAAR.D	2574.83158	3	6.03E-04	0.92	4.12	-	1419.9
AHQ-1-2, 7230 - 7294	K.AVSSAIAQLLGEVAQGNENYAGIAAR.D	2574.83158	3	7.74E-12	0.98	6.34	-	2599.8
AHQ-1-2, 5925	R.AVTDSINQLITM*CTQQAPGQK.E	2322.60252	3	1.20E-04	0.87	4.14	-	663.5
AHQ-1-6, 6090	R.AVTDSINQLITMCTQQAPGQK.E	2306.60312	2	3.50E-05	0.67	2.84	-	473.1
AHQ-1-5, 6233	R.AVTDSINQLITMCTQQAPGQK.E	2306.60312	2	5.43E-07	0.82	3.57	-	372.9
AHQ-1-1, 6059	R.AVTDSINQLITMCTQQAPGQK.E	2306.60312	2	7.91E-07	0.83	3.86	-	408.8
AHQ-1-3, 4833 - 4843	R.AVTDSINQLITMCTQQAPGQK.E	2306.60312	2	2.35E-06	0.86	4.11	-	418.4
AHQ-1-9, 6076	R.AVTDSINQLITMCTQQAPGQK.E	2306.60312	2	7.42E-04	0.59	3.05	-	340.0
AHQ-1-4, 5886	R.AVTDSINQLITM*CTQQAPGQK.E	2322.60252	2	7.82E-05	0.65	3.12	-	380.2
AHQ-1-2, 4891 - 4973	R.AVTDSINQLITMCTQQAPGQK.E	2306.60312	2	3.05E-06	0.50	3.21	-	226.8
AHQ-1-3, 6110	R.AVTDSINQLITMCTQQAPGQK.E	2306.60312	2	2.03E-04	0.86	3.77	-	623.4
AHQ-1-2, 6273 - 6281	R.AVTDSINQLITMCTQQAPGQK.E	2306.60312	2	1.48E-05	0.89	3.92	-	664.1
AHQ-1-1, 5720	R.AVTDSINQLITM*CTQQAPGQK.E	2322.60252	2	3.26E-05	0.77	3.60	-	443.9
AHQ-1-1, 5727	R.AVTDSINQLITM*CTQQAPGQK.E	2322.60252	3	1.14E-04	0.77	3.48	-	494.4
AHQ-1-4, 4912	R.AVTDSINQLITMCTQQAPGQK.E	2306.60312	2	2.17E-06	0.77	3.38	-	525.6
AHQ-1-3, 5875	R.AVTDSINQLITM*CTQQAPGQKECDNALR.E	3182.51068	3	1.85E-09	0.91	5.01	-	686.7
AHQ-1-6, 6150 - 6222	R.AVTDSINQLITMCTQQAPGQKECDNALR.E	3166.51128	3	8.45E-08	0.91	4.41	-	973.9
AHQ-1-1, 5864	R.AVTDSINQLITM*CTQQAPGQKECDNALR.E	3182.51068	3	3.82E-07	0.92	4.47	-	1099.9
AHQ-1-4, 6286 - 6354	R.AVTDSINQLITMCTQQAPGQKECDNALR.E	3166.51128	3	4.71E-11	0.92	4.70	-	888.3
AHQ-1-1, 6135	R.AVTDSINQLITMCTQQAPGQKECDNALR.E	3166.51128	3	1.01E-07	0.92	4.81	-	874.2
AHQ-1-2, 6357 - 6426	R.AVTDSINQLITMCTQQAPGQKECDNALR.E	3166.51128	3	9.44E-15	0.94	5.29	-	1190.7
AHQ-1-2, 5191	R.AVTDSINQLITMCTQQAPGQKECDNALR.E	3166.51128	3	1.22E-06	0.79	3.46	-	643.1
AHQ-1-3, 6261 - 6342	R.AVTDSINQLITMCTQQAPGQKECDNALR.E	3166.51128	3	4.05E-14	0.94	4.82	-	1398.9
AHQ-1-3, 6151 - 6197	R.AVTDSINQLITMCTQQAPGQKECDNALR.E	3166.51128	3	5.50E-13	0.95	5.48	-	1113.1
AHQ-1-4, 5988 - 6058	R.AVTDSINQLITM*CTQQAPGQKECDNALR.E	3182.51068	3	3.19E-08	0.90	4.93	-	752.1
AHQ-1-2, 6047 - 6117	R.AVTDSINQLITM*CTQQAPGQKECDNALR.E	3182.51068	3	9.73E-10	0.94	5.57	-	1077.9
AHQ-1-5, 6285 - 6359	R.AVTDSINQLITMCTQQAPGQKECDNALR.E	3166.51128	3	1.51E-13	0.95	5.36	-	1289.7
AHQ-1-4, 2431	R.CVSCLPGQR.D	1079.23309	2	1.44E-04	0.85	2.96	-	887.9
AHQ-1-5, 2353	R.CVSCLPGQR.D	1079.23309	2	9.06E-04	0.74	2.85	-	683.2
AHQ-1-4, 2436	R.CVSCLPGQR.D	1079.23309	1	1.25E-04	0.43	2.60	-	147.8
AHQ-1-3, 2427	R.CVSCLPGQR.D	1079.23309	2	1.89E-04	0.91	3.29	-	888.7
AHQ-1-3, 3609	R.DDILNGSHPVSFDK.A	1544.64741	2	3.13E-10	0.83	3.29	-	786.2
AHQ-1-10, 3209 - 3269	R.DDILNGSHPVSFDK.A	1544.64741	2	2.56E-05	0.30	2.52	-	455.0
AHQ-1-9, 3591	R.DDILNGSHPVSFDK.A	1544.64741	2	1.18E-07	0.95	4.11	-	1367.2
AHQ-1-13, 3462	R.DDILNGSHPVSFDK.A	1544.64741	2	1.61E-06	0.90	3.51	-	997.0
AHQ-1-1, 3660	R.DDILNGSHPVSFDK.A	1544.64741	2	5.78E-06	0.86	3.61	-	744.8
AHQ-1-7, 3765	R.DDILNGSHPVSFDK.A	1544.64741	2	6.47E-08	0.91	3.49	-	1080.3
AHQ-1-4, 3631	R.DDILNGSHPVSFDK.A	1544.64741	2	9.54E-08	0.93	3.92	-	1060.5
AHQ-1-7, 3552	R.DDILNGSHPVSFDK.A	1544.64741	2	5.48E-08	0.90	3.48	-	930.1
AHQ-1-8, 3562 - 3634	R.DDILNGSHPVSFDK.A	1544.64741	2	2.24E-09	0.96	4.21	-	1397.5
AHQ-1-10, 3433	R.DDILNGSHPVSFDK.A	1544.64741	2	1.22E-07	0.90	3.68	-	1002.8
AHQ-1-8, 4842	K.DHFGLEGDEESTMLEDSVSPK.K	2323.43307	2	2.81E-04	0.95	4.69	-	1072.2
AHQ-1-2, 6289	R.DKAPGQLECETAIAALNSCLR.D	2320.58644	3	7.54E-05	0.76	3.54	-	767.5
AHQ-1-2, 6290 - 6309	R.DKAPGQLECETAIAALNSCLR.D	2320.58644	2	1.93E-08	0.96	5.17	-	1222.7
AHQ-1-3, 5743	R.DKAPGQLECETAIAALNSCLR.D	2320.58644	2	1.94E-09	0.89	3.42	-	905.5
AHQ-1-2, 5531	R.DKAPGQLECETAIAALNSCLR.D	2320.58644	2	1.80E-11	0.87	3.50	-	802.1
AHQ-1-3, 6105 - 6107	R.DKAPGQLECETAIAALNSCLR.D	2320.58644	2	2.95E-08	0.97	5.59	-	1513.3
AHQ-1-2, 5919	R.DKAPGQLECETAIAALNSCLR.D	2320.58644	2	2.81E-07	0.94	4.65	-	893.4
AHQ-1-3, 6347	R.DKAPGQLECETAIAALNSCLR.D	2320.58644	2	6.71E-10	0.70	3.07	-	579.0
AHQ-1-5, 5405	R.DKAPGQLECETAIAALNSCLR.D	2320.58644	2	3.03E-10	0.95	4.71	-	900.3
AHQ-1-3, 6042 - 6109	R.DKAPGQLECETAIAALNSCLR.D	2320.58644	3	2.96E-05	0.86	4.71	-	809.1
AHQ-1-4, 6239	R.DKAPGQLECETAIAALNSCLR.D	2320.58644	2	6.02E-09	0.92	4.47	-	913.4
AHQ-1-6, 6078	R.DKAPGQLECETAIAALNSCLR.D	2320.58644	2	1.04E-09	0.95	4.73	-	1075.7
AHQ-1-9, 6060	R.DKAPGQLECETAIAALNSCLR.D	2320.58644	2	4.69E-10	0.86	3.56	-	730.2
AHQ-1-3, 6273 - 6346	R.DKAPGQLECETAIAALNSCLR.D	2320.58644	3	1.38E-10	0.91	3.88	-	1329.9
AHQ-1-4, 6499	R.DKAPGQLECETAIAALNSCLR.D	2320.58644	2	6.74E-12	0.94	4.25	-	1101.3
AHQ-1-4, 5452 - 5490	R.DKAPGQLECETAIAALNSCLR.D	2320.58644	2	5.01E-07	0.91	4.60	-	725.8
AHQ-1-1, 6091	R.DKAPGQLECETAIAALNSCLR.D	2320.58644	2	1.75E-10	0.96	5.04	-	1172.7
AHQ-1-1, 5417	R.DKAPGQLECETAIAALNSCLR.D	2320.58644	2	1.33E-13	0.96	4.91	-	1261.6
AHQ-1-3, 5327 - 5373	R.DKAPGQLECETAIAALNSCLR.D	2320.58644	2	2.70E-12	0.95	4.28	-	1258.9
AHQ-1-3, 5885	R.DKAPGQLECETAIAALNSCLR.D	2320.58644	2	6.99E-04	0.84	3.37	-	742.2
AHQ-1-8, 6154	R.DKAPGQLECETAIAALNSCLR.D	2320.58644	2	1.91E-11	0.94	4.56	-	1007.5
AHQ-1-4, 6403	R.DKAPGQLECETAIAALNSCLR.D	2320.58644	2	8.96E-05	0.89	3.72	-	820.7
AHQ-1-4, 5416 - 5482	R.DLDQASLAAVSQQLAPR.E	1783.96422	2	9.65E-11	0.96	5.61	-	814.0
AHQ-1-4, 5476 - 5492	R.DLDQASLAAVSQQLAPR.E	1783.96422	3	3.25E-05	0.94	4.45	-	1882.0
AHQ-1-1, 5371	R.DLDQASLAAVSQQLAPR.E	1783.96422	3	7.85E-06	0.96	4.37	-	2122.5
AHQ-1-2, 5505	R.DLDQASLAAVSQQLAPR.E	1783.96422	3	4.41E-05	0.95	4.48	-	2202.3
AHQ-1-5, 5421	R.DLDQASLAAVSQQLAPR.E	1783.96422	3	1.27E-08	0.93	4.46	-	1642.0
AHQ-1-14- , 5131	R.DLDQASLAAVSQQLAPR.E	1783.96422	2	1.73E-04	0.75	3.03	-	720.7
AHQ-1-2, 4875	R.DLDQASLAAVSQQLAPR.E	1783.96422	2	1.46E-05	0.65	2.57	-	789.0
AHQ-1-2, 5929	R.DLDQASLAAVSQQLAPR.E	1783.96422	2	1.20E-04	0.56	2.55	-	590.8
AHQ-1-3, 5355 - 5358	R.DLDQASLAAVSQQLAPR.E	1783.96422	3	4.78E-06	0.94	5.00	-	1763.7
AHQ-1-3, 5342 - 5409	R.DLDQASLAAVSQQLAPR.E	1783.96422	2	3.98E-06	0.86	3.77	-	709.9
AHQ-1-3, 4737	R.DLDQASLAAVSQQLAPR.E	1783.96422	2	7.95E-06	0.93	4.29	-	1052.4
AHQ-1-2, 3766	R.DPPSWSVLAGHSR.T	1409.53156	3	8.21E-05	0.87	3.91	-	757.7
AHQ-1-2, 3759	R.DPPSWSVLAGHSR.T	1409.53156	2	5.60E-07	0.81	2.84	-	378.1
AHQ-1-5, 3479 - 3495	R.DPPSWSVLAGHSR.T	1409.53156	2	1.20E-08	0.90	3.60	-	692.0
AHQ-1-3, 3621 - 3629	R.DPPSWSVLAGHSR.T	1409.53156	2	2.70E-08	0.94	3.98	-	692.9
AHQ-1-6, 3454	R.DPPSWSVLAGHSR.T	1409.53156	2	9.72E-09	0.80	3.26	-	413.8
AHQ-1-4, 3646 - 3648	R.DPPSWSVLAGHSR.T	1409.53156	2	4.04E-10	0.94	3.91	-	743.4
AHQ-1-7, 3520	R.DPPSWSVLAGHSR.T	1409.53156	2	1.13E-07	0.80	2.72	-	514.8
AHQ-1-4, 3656	R.DPPSWSVLAGHSR.T	1409.53156	3	3.02E-07	0.88	4.25	-	594.6
AHQ-1-3, 3625	R.DPPSWSVLAGHSR.T	1409.53156	3	9.40E-07	0.92	4.44	-	678.4
AHQ-1-1, 5360	R.DPVQLNLLYVQAR.D	1529.76539	2	3.86E-07	0.97	4.89	-	1549.1
AHQ-1-9, 5163	R.DPVQLNLLYVQAR.D	1529.76539	2	2.57E-08	0.94	4.24	-	1247.1
AHQ-1-8, 5230 - 5304	R.DPVQLNLLYVQAR.D	1529.76539	2	2.20E-05	0.96	4.73	-	1522.4
AHQ-1-13- , 5216	R.DPVQLNLLYVQAR.D	1529.76539	2	2.01E-06	0.81	3.34	-	737.6
AHQ-1-6, 2379	K.EAAYHPEVAPDVR.L	1454.56910	1	8.05E-04	0.22	1.90	-	370.5
AHQ-1-6, 2366	K.EAAYHPEVAPDVR.L	1454.56910	2	1.87E-06	0.82	3.06	-	651.0
AHQ-1-5, 2350	K.EAAYHPEVAPDVR.L	1454.56910	2	3.90E-04	0.84	2.67	-	1010.5
AHQ-1-2, 2551 - 2559	K.EAAYHPEVAPDVR.L	1454.56910	1	4.58E-05	0.58	2.48	-	430.9
AHQ-1-2, 2549 - 2550	K.EAAYHPEVAPDVR.L	1454.56910	2	1.63E-06	0.88	3.43	-	774.4
AHQ-1-3, 2465	K.EAAYHPEVAPDVR.L	1454.56910	2	2.15E-06	0.67	2.51	-	799.7
AHQ-1-3, 2467	K.EAAYHPEVAPDVR.L	1454.56910	1	5.85E-04	0.81	2.87	-	709.2
AHQ-1-7, 2392	K.EAAYHPEVAPDVR.L	1454.56910	2	6.69E-04	0.67	2.84	-	614.4

AHQ-1-2, 6169 - 6237	K.EADESLNFEEQILEAAK.S	1937.05072	2	1.34E-07	0.97	5.34	-	1564.4
AHQ-1-1, 6265	K.EADESLNFEEQILEAAK.S	1937.05072	2	5.66E-09	0.94	4.64	-	1275.2
AHQ-1-7, 6176	K.EADESLNFEEQILEAAK.S	1937.05072	2	2.27E-07	0.86	3.68	-	978.1
AHQ-1-5, 6246 - 6325	K.EADESLNFEEQILEAAK.S	1937.05072	2	2.25E-06	0.97	5.29	-	1584.9
AHQ-1-5, 6146	K.EADESLNFEEQILEAAK.S	1937.05072	2	1.50E-06	0.89	3.74	-	1078.8
AHQ-1-3, 6062 - 6101	K.EADESLNFEEQILEAAK.S	1937.05072	2	4.34E-08	0.94	4.53	-	1125.5
AHQ-1-6, 6030	K.EADESLNFEEQILEAAK.S	1937.05072	2	4.02E-05	0.94	4.53	-	1044.0
AHQ-1-3, 6159 - 6233	K.EADESLNFEEQILEAAK.S	1937.05072	2	3.23E-05	0.95	4.85	-	1246.2
AHQ-1-2, 6297 - 6371	K.EADESLNFEEQILEAAK.S	1937.05072	2	2.28E-08	0.97	5.49	-	1859.5
AHQ-1-4, 5979	K.EADESLNFEEQILEAAK.S	1937.05072	2	2.08E-10	0.89	4.07	-	711.1
AHQ-1-2, 6443 - 6519	K.EADESLNFEEQILEAAK.S	1937.05072	2	5.72E-09	0.97	5.25	-	1522.7
AHQ-1-4, 6140 - 6210	K.EADESLNFEEQILEAAK.S	1937.05072	2	5.08E-07	0.95	4.65	-	1308.2
AHQ-1-10, 5332	K.EADESLNFEEQILEAAK.S	1937.05072	2	6.97E-04	0.63	3.05	-	726.8
AHQ-1-2, 7551	K.EADESLNFEEQILEAAK.S	1937.05072	2	6.92E-04	0.61	2.70	-	676.1
AHQ-1-9, 5999	K.EADESLNFEEQILEAAK.S	1937.05072	2	2.63E-05	0.94	4.35	-	1366.1
AHQ-1-10, 5440 - 5499	K.EADESLNFEEQILEAAK.S	1937.05072	2	4.00E-04	0.62	3.32	-	788.4
AHQ-1-4, 6283 - 6352	K.EADESLNFEEQILEAAK.S	1937.05072	2	7.40E-08	0.96	4.97	-	1234.7
AHQ-1-4, 6444	K.EADESLNFEEQILEAAK.S	1937.05072	2	3.94E-07	0.91	3.99	-	1183.4
AHQ-1-1, 6027 - 6088	K.EADESLNFEEQILEAAK.S	1937.05072	2	2.67E-07	0.96	5.03	-	1632.7
AHQ-1-1, 7004	R.ECANGYLELLDHVLLTLQKPSPELK.Q	2883.30906	3	1.33E-06	0.90	3.93	-	1177.2
AHQ-1-5, 7459 - 7462	R.ECANGYLELLDHVLLTLQKPSPELK.Q	2883.30906	2	9.94E-05	0.89	4.38	-	630.1
AHQ-1-3, 7253	R.ECANGYLELLDHVLLTLQKPSPELK.Q	2883.30906	3	1.04E-09	0.93	4.56	-	1144.1
AHQ-1-9, 7286 - 7288	R.ECANGYLELLDHVLLTLQKPSPELK.Q	2883.30906	3	2.32E-06	0.84	4.01	-	900.3
AHQ-1-9, 7284	R.ECANGYLELLDHVLLTLQKPSPELK.Q	2883.30906	2	1.15E-04	0.83	3.77	-	627.4
AHQ-1-6, 7266	R.ECANGYLELLDHVLLTLQKPSPELK.Q	2883.30906	3	1.89E-14	0.94	4.86	-	1418.8
AHQ-1-4, 7430	R.ECANGYLELLDHVLLTLQKPSPELK.Q	2883.30906	3	3.12E-08	0.95	5.43	-	1210.8
AHQ-1-2, 7389 - 7395	R.ECANGYLELLDHVLLTLQKPSPELK.Q	2883.30906	3	2.33E-06	0.96	5.57	-	1516.8
AHQ-1-2, 7385 - 7450	R.EGISQEALHTQMLTAVQEISHLIEPLANAAR.A	3372.79804	3	7.60E-06	0.88	4.51	-	751.3
AHQ-1-8, 7542 - 7544	R.EGISQEALHTQMLTAVQEISHLIEPLANAAR.A	3372.79804	3	6.22E-07	0.98	7.11	-	1806.3
AHQ-1-10, 6444 - 6445	R.EGISQEALHTQMLTAVQEISHLIEPLANAAR.A	3372.79804	3	1.24E-08	0.97	6.31	-	1637.3
AHQ-1-4, 7400 - 7427	R.EGISQEALHTQMLTAVQEISHLIEPLANAAR.A	3372.79804	3	4.33E-07	0.78	3.49	-	674.7
AHQ-1-3, 7222 - 7289	R.EGISQEALHTQMLTAVQEISHLIEPLANAAR.A	3372.79804	3	2.42E-11	0.98	6.95	-	1901.4
AHQ-1-3, 7163	R.EGISQEALHTQM*LTAVQEISHLIEPLANAAR.A	3388.79744	3	5.58E-08	0.95	5.51	-	1009.0
AHQ-1-5, 7445 - 7515	R.EGISQEALHTQMLTAVQEISHLIEPLANAAR.A	3372.79804	3	1.35E-10	0.95	5.61	-	1177.4
AHQ-1-11, 6523	R.EGISQEALHTQMLTAVQEISHLIEPLANAAR.A	3372.79804	3	3.90E-08	0.90	4.40	-	937.1
AHQ-1-1, 6999	R.EGISQEALHTQMLTAVQEISHLIEPLANAAR.A	3372.79804	3	1.40E-05	0.69	3.01	-	1030.1
AHQ-1-7, 7532	R.EGISQEALHTQMLTAVQEISHLIEPLANAAR.A	3372.79804	3	2.43E-06	0.95	5.69	-	1608.0
AHQ-1-2, 7309	R.EGISQEALHTQM*LTAVQEISHLIEPLANAAR.A	3388.79744	3	3.07E-07	0.88	4.18	-	767.9
AHQ-1-6, 7264 - 7318	R.EGISQEALHTQMLTAVQEISHLIEPLANAAR.A	3372.79804	3	5.45E-09	0.95	5.74	-	1343.3
AHQ-1-2, 7314	R.ELLENPVQPINDMSYFGCLDSVMENSK.V	3132.48874	3	1.25E-07	0.94	4.62	-	1144.8
AHQ-1-4, 7251	R.ELLENPVQPINDM*SYFGCLDSVMENSK.V	3148.48814	3	4.58E-05	0.85	4.46	-	687.0
AHQ-1-2, 6919	R.ELLENPVQPINDMSYFGCLDSVM*ENSK.V	3148.48814	3	4.64E-06	0.62	3.43	-	326.3
AHQ-1-7, 3041 - 3042	R.EQGVEEHETLLLR.R	1553.69898	2	2.16E-04	0.85	3.44	-	515.5
AHQ-1-8, 2812 - 2885	R.EQGVEEHETLLLR.R	1553.69898	2	7.28E-04	0.84	3.14	-	686.3
AHQ-1-8, 5546 - 5548	R.ERIPEAPAGPPSDFGLFLSDDDPK.K	2571.77979	3	2.22E-15	0.97	6.52	-	1614.0
AHQ-1-13- , 5663	R.ERIPEAPAGPPSDFGLFLSDDDPK.K	2571.77979	3	2.33E-11	0.98	7.10	-	2149.9
AHQ-1-13, 5340	R.ERIPEAPAGPPSDFGLFLSDDDPK.K	2571.77979	3	5.55E-16	0.98	6.70	-	1764.0
AHQ-1-1, 5771 - 5833	R.ERIPEAPAGPPSDFGLFLSDDDPK.K	2571.77979	3	2.93E-08	0.92	5.07	-	1172.2
AHQ-1-14- , 5514	R.ERIPEAPAGPPSDFGLFLSDDDPK.K	2571.77979	3	5.07E-13	0.97	5.75	-	1777.5
AHQ-1-8, 5116 - 5195	R.ERIPEAPAGPPSDFGLFLSDDDPKK.G	2699.95270	3	2.36E-06	0.98	6.40	-	1880.9
AHQ-1-13- , 5275	R.ERIPEAPAGPPSDFGLFLSDDDPKK.G	2699.95270	3	2.92E-04	0.97	5.17	-	1803.2
AHQ-1-13, 4981	R.ERIPEAPAGPPSDFGLFLSDDDPKK.G	2699.95270	3	1.15E-08	0.97	5.47	-	1830.2
AHQ-1-14- , 5146	R.ERIPEAPAGPPSDFGLFLSDDDPKK.G	2699.95270	3	9.63E-08	0.97	5.59	-	1808.2
AHQ-1-4, 2364	K.EVANSTANLVK.T	1146.27608	2	4.36E-04	0.74	2.69	-	907.1
AHQ-1-13, 4541 - 4603	K.EVIQEWNLTNIK.R	1487.68214	2	4.32E-06	0.94	4.04	-	1331.7
AHQ-1-1, 4859 - 4935	K.EVIQEWNLTNIK.R	1487.68214	2	3.12E-04	0.81	2.90	-	734.9
AHQ-1-8, 4712	K.EVIQEWNLTNIK.R	1487.68214	1	5.46E-04	0.33	2.73	-	198.8
AHQ-1-8, 4706 - 4720	K.EVIQEWNLTNIK.R	1487.68214	2	4.07E-07	0.93	4.48	-	963.0
AHQ-1-11, 4384 - 4462	K.EVIQEWNLTNIK.R	1487.68214	2	9.62E-05	0.83	3.19	-	655.8
AHQ-1-8, 4075	K.EVIQEWNLTNIKR.W	1643.86849	2	4.05E-05	0.70	3.51	-	577.2
AHQ-1-8, 2936 - 3004	K.FFYSDQNVDSR.D	1378.42795	2	8.68E-06	0.93	3.58	-	1105.4
AHQ-1-9, 2928	K.FFYSDQNVDSR.D	1378.42795	2	1.65E-05	0.91	3.26	-	978.1
AHQ-1-10, 2855	K.FFYSDQNVDSR.D	1378.42795	2	3.26E-05	0.90	3.28	-	1008.5
AHQ-1-7, 3017	K.FFYSDQNVDSR.D	1378.42795	2	3.10E-06	0.83	3.08	-	664.5
AHQ-1-8, 6150 - 6175	K.FFYSDQNVDSRDPVQLNLLYVQAR.D	2889.17074	3	9.59E-09	0.98	5.59	-	2529.3
AHQ-1-1, 6137	K.FFYSDQNVDSRDPVQLNLLYVQAR.D	2889.17074	3	2.73E-05	0.82	3.31	-	851.3
AHQ-1-8, 6243	K.FFYSDQNVDSRDPVQLNLLYVQAR.D	2889.17074	3	5.64E-12	0.96	5.33	-	1756.1
AHQ-1-10, 5428	K.FFYSDQNVDSRDPVQLNLLYVQAR.D	2889.17074	3	8.15E-07	0.93	4.32	-	1537.5
AHQ-1-8, 6182	K.FFYSDQNVDSRDPVQLNLLYVQAR.D	2889.17074	2	7.63E-04	0.52	2.95	-	232.0
AHQ-1-3, 6394 - 6465	R.FGQDFSTFLEAGVEM*AGQAPSQEDR.A	2734.89265	3	3.02E-05	0.93	5.50	-	876.6
AHQ-1-2, 6550 - 6619	R.FGQDFSTFLEAGVEM*AGQAPSQEDR.A	2734.89265	3	6.17E-08	0.92	4.81	-	895.0
AHQ-1-4, 6563	R.FGQDFSTFLEAGVEM*AGQAPSQEDR.A	2734.89265	2	2.80E-05	0.88	3.36	-	1107.0
AHQ-1-4, 7291	R.FGQDFSTFLEAGVEMAGQAPSQEDR.A	2718.89325	3	1.16E-07	0.82	4.22	-	499.1
AHQ-1-4, 7288 - 7292	R.FGQDFSTFLEAGVEMAGQAPSQEDR.A	2718.89325	2	9.36E-04	0.90	4.13	-	931.1
AHQ-1-3, 7115	R.FGQDFSTFLEAGVEMAGQAPSQEDR.A	2718.89325	3	3.07E-11	0.85	3.95	-	658.2
AHQ-1-1, 6321	R.FGQDFSTFLEAGVEM*AGQAPSQEDR.A	2734.89265	2	2.51E-07	0.80	3.41	-	724.4
AHQ-1-1, 6312	R.FGQDFSTFLEAGVEM*AGQAPSQEDR.A	2734.89265	3	2.86E-06	0.85	4.24	-	650.7
AHQ-1-2, 6565 - 6637	R.FGQDFSTFLEAGVEM*AGQAPSQEDR.A	2734.89265	2	1.91E-07	0.93	4.26	-	1074.7
AHQ-1-2, 7470 - 7538	R.FGQDFSTFLEAGVEMAGQAPSQEDR.A	2718.89325	3	1.06E-05	0.79	4.00	-	718.2
AHQ-1-3, 7422	R.FGQDFSTFLEAGVEMAGQAPSQEDR.A	2718.89325	3	1.05E-07	0.86	4.25	-	596.3
AHQ-1-4, 6559 - 6627	R.FGQDFSTFLEAGVEM*AGQAPSQEDR.A	2734.89265	3	3.52E-06	0.88	4.40	-	683.3
AHQ-1-2, 7254	R.FGQDFSTFLEAGVEMAGQAPSQEDR.A	2718.89325	3	3.01E-06	0.87	4.62	-	689.3
AHQ-1-3, 6395 - 6475	R.FGQDFSTFLEAGVEM*AGQAPSQEDR.A	2734.89265	2	7.81E-06	0.83	3.12	-	1100.7
AHQ-1-3, 7341	R.FGQDFSTFLEAGVEMAGQAPSQEDR.A	2718.89325	3	1.29E-07	0.85	4.01	-	704.9
AHQ-1-4, 2791 - 2794	K.FLPSELRDEH	1243.35032	2	1.25E-04	0.69	2.79	-	345.2
AHQ-1-2, 5377	K.GLAGAVSELLR.S	1086.26701	2	3.47E-07	0.97	4.67	-	1994.3
AHQ-1-4, 5279	K.GLAGAVSELLR.S	1086.26701	2	1.01E-06	0.98	5.03	-	1956.4
AHQ-1-1, 5323	K.GLAGAVSELLR.S	1086.26701	2	9.24E-06	0.97	4.57	-	2011.7
AHQ-1-3, 5217	K.GLAGAVSELLR.S	1086.26701	2	4.74E-07	0.98	4.36	-	2406.3
AHQ-1-9, 4970	K.GLAGAVSELLR.S	1086.26701	2	1.86E-06	0.98	4.80	-	2185.5
AHQ-1-5, 5158	K.GLAGAVSELLR.S	1086.26701	2	7.71E-07	0.97	4.74	-	1973.3
AHQ-1-6, 5102	K.GLAGAVSELLR.S	1086.26701	2	7.92E-07	0.97	5.14	-	1650.4
AHQ-1-8, 4924	K.GLAGAVSELLR.S	1086.26701	2	7.19E-07	0.98	5.27	-	2207.6
AHQ-1-2, 6618	R.GSQAQPDSPSAQLALIAASQSFLQPGGK.M	2756.02103	3	5.90E-08	0.92	4.34	-	1371.5
AHQ-1-2, 6873 - 6938	R.GSQAQPDSPSAQLALIAASQSFLQPGGK.M	2756.02103	2	2.81E-06	0.93	5.14	-	762.5
AHQ-1-2, 6961 - 7025	R.GSQAQPDSPSAQLALIAASQSFLQPGGK.M	2756.02103	3	3.69E-06	0.91	4.70	-	1021.9
AHQ-1-5, 6821 - 6885	R.GSQAQPDSPSAQLALIAASQSFLQPGGK.M	2756.02103	2	4.65E-04	0.94	5.24	-	893.2
AHQ-1-2, 7009	R.GSQAQPDSPSAQLALIAASQSFLQPGGK.M	2756.02103	2	1.81E-09	0.86	4.11	-	524.9
AHQ-1-2, 7211	R.GSQAQPDSPSAQLALIAASQSFLQPGGK.M	2756.02103	2	5.85E-08	0.86	4.00	-	466.5
AHQ-1-6, 6662 - 6736	R.GSQAQPDSPSAQLALIAASQSFLQPGGK.M	2756.02103	3	2.59E-10	0.93	5.14	-	970.5
AHQ-1-6, 6590 - 6666	R.GSQAQPDSPSAQLALIAASQSFLQPGGK.M	2756.02103	2	2.45E-07	0.94	5.02	-	749.4
AHQ-1-5, 6749 - 6813	R.GSQAQPDSPSAQLALIAASQSFLQPGGK.M	2756.02103	3	5.90E-07	0.91	4.61	-	1054.0
AHQ-1-2, 6834 - 6897	R.GSQAQPDSPSAQLALIAASQSFLQPGGK.M	2756.02103	3	8.72E-06	0.89	4.29	-	881.4
AHQ-1-3, 6842	R.GSQAQPDSPSAQLALIAASQSFLQPGGK.M	2756.02103	2	5.46E-04	0.84	3.98	-	498.9
AHQ-1-4, 6799 - 6866	R.GSQAQPDSPSAQLALIAASQSFLQPGGK.M	2756.02103	2	2.92E-06	0.93	5.39	-	593.7
AHQ-1-1, 6543 - 6603	R.GSQAQPDSPSAQLALIAASQSFLQPGGK.M	2756.02103	3	8.64E-04	0.92	4.63	-	1293.4
AHQ-1-1, 6611 - 6680	R.GSQAQPDSPSAQLALIAASQSFLQPGGK.M	2756.02103	2	9.99E-05	0.96	5.75	-	1039.0

AHQ-1-1, 6659 - 6721	R.GSQAQPDSPSAQLALIAASQSFLQPGGK.M	2756.02103	3	7.04E-05	0.93	4.79	-	1115.5
AHQ-1-1, 6809 - 6881	R.GSQAQPDSPSAQLALIAASQSFLQPGGK.M	2756.02103	3	7.22E-09	0.88	3.62	-	1413.7
AHQ-1-5, 7537	K.GTEWVDPEDPTVIAENELLGAAAAIEAAAK.K	3053.32301	3	1.38E-11	0.98	6.14	-	2252.5
AHQ-1-6, 7342	K.GTEWVDPEDPTVIAENELLGAAAAIEAAAK.K	3053.32301	3	3.06E-11	0.97	6.21	-	1906.4
AHQ-1-7, 7556	K.GTEWVDPEDPTVIAENELLGAAAAIEAAAK.K	3053.32301	3	5.55E-14	0.97	6.39	-	1421.4
AHQ-1-13- , 7019	K.GTEWVDPEDPTVIAENELLGAAAAIEAAAK.K	3053.32301	3	1.16E-05	0.92	4.56	-	1530.5
AHQ-1-9, 7371	K.GTEWVDPEDPTVIAENELLGAAAAIEAAAK.K	3053.32301	3	3.82E-08	0.96	5.83	-	1526.6
AHQ-1-4, 7378	R.GVAALTSDPAVQAIVLDTASDVLDK.A	2470.75835	3	2.25E-05	0.98	7.30	-	2272.0
AHQ-1-4, 7375	R.GVAALTSDPAVQAIVLDTASDVLDK.A	2470.75835	2	9.51E-08	0.98	6.72	-	1178.3
AHQ-1-2, 7278 - 7345	R.GVAALTSDPAVQAIVLDTASDVLDK.A	2470.75835	3	1.11E-15	0.98	6.39	-	2786.6
AHQ-1-6, 7211	R.GVAALTSDPAVQAIVLDTASDVLDK.A	2470.75835	3	7.26E-10	0.98	6.32	-	1987.8
AHQ-1-8, 7483	R.GVGAAATAVTQALNELLQHVK.A	2092.38495	3	5.16E-07	0.94	4.64	-	1080.5
AHQ-1-2, 7529	R.GVGAAATAVTQALNELLQHVK.A	2092.38495	2	8.30E-04	0.47	2.94	-	452.9
AHQ-1-2, 7398	R.GVGAAATAVTQALNELLQHVK.A	2092.38495	2	1.97E-05	0.96	5.56	-	898.9
AHQ-1-2, 7397	R.GVGAAATAVTQALNELLQHVK.A	2092.38495	3	1.71E-06	0.95	4.70	-	1181.2
AHQ-1-4, 5980	R.GVGAAATAVTQALNELLQHVK.A	2092.38495	3	5.79E-08	0.93	4.56	-	1056.1
AHQ-1-2, 6031 - 6049	R.GVGAAATAVTQALNELLQHVK.A	2092.38495	3	6.77E-07	0.93	4.57	-	1209.3
AHQ-1-9, 7290	R.GVGAAATAVTQALNELLQHVK.A	2092.38495	2	2.60E-05	0.93	5.06	-	776.4
AHQ-1-7, 7486	R.GVGAAATAVTQALNELLQHVK.A	2092.38495	2	1.96E-05	0.85	4.13	-	547.0
AHQ-1-8, 7482	R.GVGAAATAVTQALNELLQHVK.A	2092.38495	2	1.45E-05	0.94	4.96	-	885.6
AHQ-1-7, 7484	R.GVGAAATAVTQALNELLQHVK.A	2092.38495	3	2.39E-08	0.92	4.00	-	1098.4
AHQ-1-12, 6658	R.GVGAAATAVTQALNELLQHVK.A	2092.38495	3	1.78E-09	0.94	4.44	-	1292.9
AHQ-1-13- , 6893 - 6968	R.GVGAAATAVTQALNELLQHVK.A	2092.38495	3	2.90E-06	0.93	5.22	-	1011.1
AHQ-1-3, 7261	R.GVGAAATAVTQALNELLQHVK.A	2092.38495	2	3.30E-07	0.94	5.08	-	912.2
AHQ-1-5, 7455	R.GVGAAATAVTQALNELLQHVK.A	2092.38495	2	3.58E-07	0.95	5.37	-	1051.8
AHQ-1-1, 5912	R.GVGAAATAVTQALNELLQHVK.A	2092.38495	2	1.87E-05	0.89	4.43	-	545.9
AHQ-1-14- , 6728	R.GVGAAATAVTQALNELLQHVK.A	2092.38495	2	1.89E-05	0.81	4.15	-	560.7
AHQ-1-3, 5862	R.GVGAAATAVTQALNELLQHVK.A	2092.38495	2	7.36E-06	0.92	4.75	-	557.6
AHQ-1-4, 7438	R.GVGAAATAVTQALNELLQHVK.A	2092.38495	3	2.90E-07	0.91	4.16	-	961.8
AHQ-1-6, 7274	R.GVGAAATAVTQALNELLQHVK.A	2092.38495	3	2.90E-08	0.91	4.32	-	961.6
AHQ-1-2, 6033	R.GVGAAATAVTQALNELLQHVK.A	2092.38495	2	1.93E-05	0.86	4.22	-	521.2
AHQ-1-6, 7275	R.GVGAAATAVTQALNELLQHVK.A	2092.38495	2	2.32E-04	0.92	4.71	-	821.4
AHQ-1-1, 7015	R.GVGAAATAVTQALNELLQHVK.A	2092.38495	2	1.03E-05	0.94	5.01	-	786.1
AHQ-1-1, 6996	R.GVGAAATAVTQALNELLQHVK.A	2092.38495	3	7.23E-07	0.88	4.08	-	978.4
AHQ-1-14- , 6726	R.GVGAAATAVTQALNELLQHVK.A	2092.38495	3	2.39E-04	0.84	3.16	-	1032.2
AHQ-1-5, 7453	R.GVGAAATAVTQALNELLQHVK.A	2092.38495	3	1.29E-09	0.94	4.94	-	1153.4
AHQ-1-7, 3265	R.IGITNHDEYSLVR.E	1517.66833	2	1.66E-10	0.90	3.71	-	1062.6
AHQ-1-10, 3115 - 3185	R.IGITNHDEYSLVR.E	1517.66833	2	1.31E-09	0.93	3.48	-	1374.2
AHQ-1-13, 3657	R.IGITNHDEYSLVR.E	1517.66833	2	6.25E-06	0.85	3.00	-	835.6
AHQ-1-13- , 3439 - 3445	R.IGITNHDEYSLVR.E	1517.66833	2	2.76E-08	0.94	3.57	-	1364.4
AHQ-1-11, 3183	R.IGITNHDEYSLVR.E	1517.66833	2	3.85E-09	0.92	3.79	-	1136.3
AHQ-1-13, 3366	R.IGITNHDEYSLVR.E	1517.66833	3	9.38E-05	0.82	3.55	-	672.3
AHQ-1-13, 3279 - 3357	R.IGITNHDEYSLVR.E	1517.66833	2	1.49E-04	0.94	3.76	-	1465.9
AHQ-1-13- , 3448 - 3461	R.IGITNHDEYSLVR.E	1517.66833	3	1.79E-09	0.93	4.36	-	908.0
AHQ-1-10, 3391	R.IGITNHDEYSLVR.E	1517.66833	2	8.49E-06	0.92	3.51	-	1079.0
AHQ-1-8, 3234 - 3308	R.IGITNHDEYSLVR.E	1517.66833	2	7.59E-09	0.95	4.02	-	1438.8
AHQ-1-12, 3593	R.IGITNHDEYSLVR.E	1517.66833	2	5.07E-04	0.73	2.76	-	600.5
AHQ-1-12, 3271	R.IGITNHDEYSLVR.E	1517.66833	2	1.62E-10	0.95	4.15	-	1353.4
AHQ-1-8, 4886	R.ILAQATSDLVNAIK.A	1457.69748	2	1.21E-07	0.98	5.03	-	1922.5
AHQ-1-2, 5274	R.ILAQATSDLVNAIK.A	1457.69748	2	6.97E-10	0.97	4.80	-	2015.1
AHQ-1-1, 4272	R.ILAQATSDLVNAIK.A	1457.69748	2	9.14E-07	0.98	4.82	-	2239.3
AHQ-1-7, 5064	R.ILAQATSDLVNAIK.A	1457.69748	2	3.72E-08	0.97	5.07	-	1781.2
AHQ-1-4, 5186	R.ILAQATSDLVNAIK.A	1457.69748	2	1.20E-08	0.98	5.66	-	2055.6
AHQ-1-2, 4609	R.ILAQATSDLVNAIK.A	1457.69748	2	1.92E-05	0.86	3.00	-	959.5
AHQ-1-2, 4373	R.ILAQATSDLVNAIK.A	1457.69748	2	5.43E-08	0.97	4.34	-	1994.1
AHQ-1-3, 5127 - 5147	R.ILAQATSDLVNAIK.A	1457.69748	2	8.54E-09	0.98	5.02	-	2106.1
AHQ-1-5, 4162	R.ILAQATSDLVNAIK.A	1457.69748	2	9.01E-08	0.96	3.45	-	1743.2
AHQ-1-2, 5449	R.ILAQATSDLVNAIK.A	1457.69748	2	2.60E-07	0.98	4.85	-	2148.6
AHQ-1-4, 4520	R.ILAQATSDLVNAIK.A	1457.69748	2	3.02E-08	0.89	3.11	-	926.0
AHQ-1-1, 5187 - 5191	R.ILAQATSDLVNAIK.A	1457.69748	2	1.16E-09	0.98	5.53	-	2485.0
AHQ-1-3, 4245	R.ILAQATSDLVNAIK.A	1457.69748	2	1.90E-09	0.97	4.63	-	1490.0
AHQ-1-4, 4282	R.ILAQATSDLVNAIK.A	1457.69748	2	1.11E-06	0.97	4.61	-	2274.2
AHQ-1-5, 5331	R.ILAQATSDLVNAIK.A	1457.69748	2	2.95E-04	0.82	2.95	-	1290.4
AHQ-1-1, 4713	R.ILAQATSDLVNAIK.A	1457.69748	2	1.59E-06	0.96	3.68	-	1957.6
AHQ-1-5, 5103 - 5135	R.ILAQATSDLVNAIK.A	1457.69748	2	4.71E-05	0.96	3.82	-	2063.3
AHQ-1-5, 5125	R.ILAQATSDLVNAIK.A	1457.69748	1	5.98E-04	0.88	3.80	-	821.8
AHQ-1-3, 4483	R.ILAQATSDLVNAIK.A	1457.69748	2	8.10E-07	0.96	3.94	-	1733.0
AHQ-1-6, 4099	R.ILAQATSDLVNAIK.A	1457.69748	2	1.16E-05	0.97	4.61	-	2144.1
AHQ-1-13, 5633	R.IPEAPAGPPSDFGLFLSDDDPK.K	2286.47890	3	2.43E-04	0.63	3.28	-	623.7
AHQ-1-9, 6056	R.IPEAPAGPPSDFGLFLSDDDPK.K	2286.47890	2	4.70E-04	0.93	4.57	-	692.9
AHQ-1-13- , 5972 - 5996	R.IPEAPAGPPSDFGLFLSDDDPK.K	2286.47890	2	1.84E-07	0.97	5.43	-	1107.9
AHQ-1-14- , 5794	R.IPEAPAGPPSDFGLFLSDDDPK.K	2286.47890	2	1.37E-05	0.93	4.60	-	746.5
AHQ-1-7, 6221	R.IPEAPAGPPSDFGLFLSDDDPK.K	2286.47890	2	7.50E-04	0.93	4.37	-	705.6
AHQ-1-13, 5589 - 5648	R.IPEAPAGPPSDFGLFLSDDDPK.K	2286.47890	2	3.53E-04	0.94	4.58	-	1118.0
AHQ-1-10, 5377 - 5381	R.IPEAPAGPPSDFGLFLSDDDPK.K	2286.47890	2	3.24E-04	0.91	4.37	-	675.0
AHQ-1-1, 6043 - 6099	R.IPEAPAGPPSDFGLFLSDDDPK.K	2286.47890	2	4.26E-05	0.85	4.04	-	721.3
AHQ-1-13, 5219 - 5283	R.IPEAPAGPPSDFGLFLSDDDPKK.G	2414.65181	3	1.76E-05	0.89	3.87	-	1200.0
AHQ-1-10, 4924 - 5001	R.IPEAPAGPPSDFGLFLSDDDPKK.G	2414.65181	3	2.48E-05	0.91	3.96	-	1373.4
AHQ-1-1, 5713	R.IPEAPAGPPSDFGLFLSDDDPKK.G	2414.65181	3	4.72E-08	0.95	5.23	-	1183.3
AHQ-1-9, 5399 - 5412	R.IPEAPAGPPSDFGLFLSDDDPKK.G	2414.65181	3	8.74E-05	0.90	4.10	-	1243.4
AHQ-1-14- , 5363 - 5446	R.IPEAPAGPPSDFGLFLSDDDPKK.G	2414.65181	3	1.20E-11	0.95	4.58	-	1664.5
AHQ-1-8, 5387 - 5394	R.IPEAPAGPPSDFGLFLSDDDPKK.G	2414.65181	2	5.58E-08	0.97	5.44	-	1309.8
AHQ-1-11, 5090	R.IPEAPAGPPSDFGLFLSDDDPKK.G	2414.65181	3	6.07E-10	0.93	4.25	-	1385.5
AHQ-1-14, 6274 - 6349	R.IPEAPAGPPSDFGLFLSDDDPKK.G	2414.65181	3	2.48E-06	0.94	4.84	-	1288.6
AHQ-1-8, 2461	R.KFFYSDQNVDSR.D	1506.60087	2	9.22E-05	0.83	3.48	-	706.3
AHQ-1-8, 2189	K.KGIWLEAGK.A	1002.19171	1	3.13E-04	0.21	2.22	-	395.8
AHQ-1-4, 2727	K.LAQAAQSSVATITR.L	1417.59344	2	5.56E-09	0.97	4.79	-	2006.5
AHQ-1-4, 2740	K.LAQAAQSSVATITR.L	1417.59344	1	8.20E-04	0.79	3.64	-	241.5
AHQ-1-2, 3050 - 3127	K.LAQAAQSSVATITR.L	1417.59344	2	5.26E-07	0.95	4.47	-	1466.2
AHQ-1-5, 2462 - 2465	K.LAQAAQSSVATITR.L	1417.59344	2	2.87E-08	0.97	4.62	-	1675.2
AHQ-1-2, 2645 - 2669	K.LAQAAQSSVATITR.L	1417.59344	2	4.08E-10	0.94	3.80	-	1419.2
AHQ-1-2, 2819	K.LAQAAQSSVATITR.L	1417.59344	1	1.34E-04	0.37	2.97	-	177.6
AHQ-1-9, 2590	K.LAQAAQSSVATITR.L	1417.59344	2	9.14E-07	0.98	4.44	-	2277.9
AHQ-1-5, 2617 - 2630	K.LAQAAQSSVATITR.L	1417.59344	2	1.78E-08	0.98	5.42	-	1971.7
AHQ-1-14- , 2744	K.LAQAAQSSVATITR.L	1417.59344	2	1.57E-07	0.96	4.03	-	1704.7
AHQ-1-4, 2567	K.LAQAAQSSVATITR.L	1417.59344	2	1.17E-09	0.97	4.92	-	1594.9
AHQ-1-7, 2648 - 2701	K.LAQAAQSSVATITR.L	1417.59344	2	7.85E-07	0.96	4.45	-	1629.7
AHQ-1-3, 2569	K.LAQAAQSSVATITR.L	1417.59344	2	3.35E-07	0.97	4.66	-	1525.6
AHQ-1-2, 2797 - 2833	K.LAQAAQSSVATITR.L	1417.59344	2	1.87E-09	0.97	4.91	-	1350.3
AHQ-1-1, 2843 - 2911	K.LAQAAQSSVATITR.L	1417.59344	2	3.65E-06	0.94	3.69	-	1539.8
AHQ-1-6, 2623 - 2632	K.LAQAAQSSVATITR.L	1417.59344	2	6.31E-08	0.95	4.03	-	1327.3
AHQ-1-3, 2725 - 2763	K.LAQAAQSSVATITR.L	1417.59344	2	1.15E-11	0.96	4.23	-	1614.3
AHQ-1-7, 2505	K.LAQAAQSSVATITR.L	1417.59344	2	2.04E-05	0.70	2.55	-	789.2
AHQ-1-6, 2479	K.LAQAAQSSVATITR.L	1417.59344	2	3.81E-11	0.95	4.08	-	1460.3
AHQ-1-2, 3139	R.LASEAKPAAVAAENEEIGSHIK.H	2236.46779	2	6.49E-12	0.95	4.68	-	941.0
AHQ-1-4, 3260 - 3263	R.LASEAKPAAVAAENEEIGSHIK.H	2236.46779	2	2.97E-07	0.96	5.67	-	825.7
AHQ-1-4, 3258 - 3294	R.LASEAKPAAVAAENEEIGSHIK.H	2236.46779	3	2.85E-08	0.96	5.40	-	1422.4

AHQ-1-2, 3366	R.LASEAKPAAVAAENEEIGSHIK.H	2236.46779	3	1.98E-09	0.97	5.73	-	1820.7
AHQ-1-2, 3371	R.LASEAKPAAVAAENEEIGSHIK.H	2236.46779	2	2.65E-09	0.94	4.90	-	920.5
AHQ-1-1, 3377 - 3385	R.LASEAKPAAVAAENEEIGSHIK.H	2236.46779	2	8.80E-09	0.95	5.30	-	772.3
AHQ-1-1, 3368	R.LASEAKPAAVAAENEEIGSHIK.H	2236.46779	3	2.49E-08	0.97	5.72	-	2369.2
AHQ-1-3, 3219 - 3262	R.LASEAKPAAVAAENEEIGSHIK.H	2236.46779	3	1.05E-11	0.97	5.96	-	1741.8
AHQ-1-3, 3261	R.LASEAKPAAVAAENEEIGSHIK.H	2236.46779	2	3.11E-06	0.91	3.92	-	710.3
AHQ-1-4, 3046	R.LASEAKPAAVAAENEEIGSHIK.H	2236.46779	3	4.19E-06	0.94	4.91	-	1319.8
AHQ-1-6, 3112	R.LASEAKPAAVAAENEEIGSHIK.H	2236.46779	2	5.68E-07	0.86	4.08	-	463.1
AHQ-1-3, 3565	R.LASEAKPAAVAAENEEIGSHIK.H	2236.46779	3	4.37E-12	0.95	5.24	-	1269.9
AHQ-1-6, 3100	R.LASEAKPAAVAAENEEIGSHIK.H	2236.46779	3	1.18E-06	0.97	5.19	-	2159.4
AHQ-1-2, 3137	R.LASEAKPAAVAAENEEIGSHIK.H	2236.46779	3	6.59E-07	0.95	5.10	-	1515.0
AHQ-1-1, 3667	R.LASEAKPAAVAAENEEIGSHIK.H	2236.46779	3	9.21E-07	0.96	4.86	-	1661.4
AHQ-1-5, 3113 - 3118	R.LASEAKPAAVAAENEEIGSHIK.H	2236.46779	3	1.99E-12	0.97	6.17	-	1848.0
AHQ-1-3, 5774	K.LGAASLGAEDPETQVVLINAVK.D	2196.48671	2	1.30E-04	0.95	4.76	-	932.5
AHQ-1-3, 5494 - 5569	K.LGAASLGAEDPETQVVLINAVK.D	2196.48671	2	3.79E-06	0.97	5.25	-	1194.5
AHQ-1-2, 5431 - 5511	K.LGAASLGAEDPETQVVLINAVK.D	2196.48671	2	4.72E-06	0.96	5.59	-	954.1
AHQ-1-13, 5155	K.LGAASLGAEDPETQVVLINAVK.D	2196.48671	2	1.26E-05	0.91	3.87	-	998.4
AHQ-1-3, 5538 - 5554	K.LGAASLGAEDPETQVVLINAVK.D	2196.48671	3	2.74E-08	0.92	4.46	-	1225.5
AHQ-1-3, 5279 - 5349	K.LGAASLGAEDPETQVVLINAVK.D	2196.48671	2	1.27E-07	0.97	5.37	-	1138.7
AHQ-1-6, 5262 - 5286	K.LGAASLGAEDPETQVVLINAVK.D	2196.48671	2	8.27E-10	0.96	5.13	-	1185.3
AHQ-1-6, 5450	K.LGAASLGAEDPETQVVLINAVK.D	2196.48671	2	5.01E-06	0.96	5.03	-	1102.8
AHQ-1-3, 5413	K.LGAASLGAEDPETQVVLINAVK.D	2196.48671	2	7.55E-09	0.92	4.50	-	708.3
AHQ-1-1, 5379	K.LGAASLGAEDPETQVVLINAVK.D	2196.48671	2	4.71E-07	0.95	4.50	-	1229.2
AHQ-1-5, 5330 - 5385	K.LGAASLGAEDPETQVVLINAVK.D	2196.48671	2	3.40E-09	0.96	5.20	-	1165.5
AHQ-1-7, 5524 - 5544	K.LGAASLGAEDPETQVVLINAVK.D	2196.48671	2	1.62E-04	0.97	5.28	-	1430.2
AHQ-1-2, 5673 - 5741	K.LGAASLGAEDPETQVVLINAVK.D	2196.48671	2	7.01E-06	0.97	5.60	-	1546.0
AHQ-1-7, 5337	K.LGAASLGAEDPETQVVLINAVK.D	2196.48671	2	1.16E-05	0.87	3.86	-	749.9
AHQ-1-4, 5604 - 5674	K.LGAASLGAEDPETQVVLINAVK.D	2196.48671	2	1.47E-04	0.96	5.22	-	1098.1
AHQ-1-1, 5581	K.LGAASLGAEDPETQVVLINAVK.D	2196.48671	3	6.83E-09	0.98	6.18	-	2827.9
AHQ-1-4, 5634 - 5648	K.LGAASLGAEDPETQVVLINAVK.D	2196.48671	3	7.24E-09	0.95	3.88	-	2204.4
AHQ-1-2, 5710 - 5718	K.LGAASLGAEDPETQVVLINAVK.D	2196.48671	3	1.64E-10	0.97	4.82	-	2433.6
AHQ-1-1, 5569 - 5575	K.LGAASLGAEDPETQVVLINAVK.D	2196.48671	2	1.11E-05	0.97	5.45	-	1071.6
AHQ-1-4, 5864 - 5883	K.LGAASLGAEDPETQVVLINAVK.D	2196.48671	2	6.65E-12	0.96	5.01	-	1248.6
AHQ-1-2, 5897 - 5941	K.LGAASLGAEDPETQVVLINAVK.D	2196.48671	2	4.64E-10	0.96	4.98	-	1095.8
AHQ-1-1, 5721	K.LGAASLGAEDPETQVVLINAVK.D	2196.48671	2	7.25E-05	0.95	4.42	-	989.4
AHQ-1-5, 5713	K.LGAASLGAEDPETQVVLINAVK.D	2196.48671	2	2.89E-11	0.97	5.52	-	1437.9
AHQ-1-5, 5518 - 5593	K.LGAASLGAEDPETQVVLINAVK.D	2196.48671	2	2.16E-05	0.97	5.26	-	1268.1
AHQ-1-11, 4831	K.LGAASLGAEDPETQVVLINAVK.D	2196.48671	2	1.23E-05	0.90	4.34	-	548.5
AHQ-1-14, 6162	K.LGAASLGAEDPETQVVLINAVK.D	2196.48671	2	5.90E-05	0.91	4.08	-	882.8
AHQ-1-8, 3429	K.LHTDDELNWLDHGR.T	1721.81153	2	3.17E-05	0.97	5.25	-	1865.0
AHQ-1-9, 3572 - 3584	K.LHTDDELNWLDHGR.T	1721.81153	2	1.72E-04	0.94	3.69	-	1332.9
AHQ-1-1, 4024	K.LHTDDELNWLDHGR.T	1721.81153	2	6.97E-06	0.96	3.96	-	1870.2
AHQ-1-9, 3588 - 3652	K.LHTDDELNWLDHGR.T	1721.81153	3	7.66E-04	0.78	3.38	-	748.9
AHQ-1-2, 4587	K.LLAALLEDEGGSGR.P	1401.54764	2	5.94E-05	0.96	3.99	-	1626.3
AHQ-1-3, 4425	K.LLAALLEDEGGSGR.P	1401.54764	2	5.20E-10	0.97	3.95	-	1941.2
AHQ-1-11, 4854 - 4912	K.LLAALLEDEGGSGRPLLQAAK.G	2123.43896	2	4.62E-09	0.97	5.55	-	1450.0
AHQ-1-2, 5373 - 5383	K.LLAALLEDEGGSGRPLLQAAK.G	2123.43896	2	1.44E-06	0.94	5.10	-	611.3
AHQ-1-4, 5410	K.LLAALLEDEGGSGRPLLQAAK.G	2123.43896	2	2.25E-04	0.98	6.23	-	1428.4
AHQ-1-7, 5284	K.LLAALLEDEGGSGRPLLQAAK.G	2123.43896	2	4.66E-12	0.98	6.70	-	1753.9
AHQ-1-11, 4852	K.LLAALLEDEGGSGRPLLQAAK.G	2123.43896	3	1.23E-09	0.95	5.21	-	1267.6
AHQ-1-9, 5119 - 5127	K.LLAALLEDEGGSGRPLLQAAK.G	2123.43896	2	2.90E-09	0.98	6.76	-	1480.2
AHQ-1-4, 5408 - 5420	K.LLAALLEDEGGSGRPLLQAAK.G	2123.43896	3	1.25E-05	0.97	5.91	-	1532.4
AHQ-1-7, 5290	K.LLAALLEDEGGSGRPLLQAAK.G	2123.43896	3	1.47E-10	0.96	5.17	-	1527.0
AHQ-1-9, 5122	K.LLAALLEDEGGSGRPLLQAAK.G	2123.43896	3	1.92E-10	0.97	5.47	-	1859.0
AHQ-1-2, 5371	K.LLAALLEDEGGSGRPLLQAAK.G	2123.43896	3	3.64E-05	0.94	4.66	-	1156.1
AHQ-1-3, 5317	K.LLAALLEDEGGSGRPLLQAAK.G	2123.43896	2	2.00E-06	0.96	5.38	-	1240.6
AHQ-1-3, 5321 - 5322	K.LLAALLEDEGGSGRPLLQAAK.G	2123.43896	3	5.59E-12	0.97	5.79	-	1886.3
AHQ-1-10, 4697	K.LLAALLEDEGGSGRPLLQAAK.G	2123.43896	2	7.31E-12	0.97	6.44	-	1095.7
AHQ-1-6, 5236	K.LLAALLEDEGGSGRPLLQAAK.G	2123.43896	2	6.78E-07	0.98	6.38	-	1415.2
AHQ-1-13- , 5331	K.LLAALLEDEGGSGRPLLQAAK.G	2123.43896	3	1.15E-08	0.93	4.28	-	1202.2
AHQ-1-5, 5309 - 5333	K.LLAALLEDEGGSGRPLLQAAK.G	2123.43896	3	2.64E-10	0.94	4.79	-	1308.5
AHQ-1-1, 5461	K.LLAALLEDEGGSGRPLLQAAK.G	2123.43896	3	9.27E-09	0.97	5.44	-	1825.4
AHQ-1-13, 5032	K.LLAALLEDEGGSGRPLLQAAK.G	2123.43896	2	1.28E-05	0.95	5.21	-	758.4
AHQ-1-8, 4987 - 5004	K.LLAALLEDEGGSGRPLLQAAK.G	2123.43896	3	3.19E-08	0.98	5.79	-	1995.0
AHQ-1-3, 5527	K.LLAALLEDEGGSGRPLLQAAK.G	2123.43896	2	5.60E-07	0.95	4.84	-	1055.5
AHQ-1-6, 5235	K.LLAALLEDEGGSGRPLLQAAK.G	2123.43896	3	1.85E-09	0.96	5.52	-	1557.0
AHQ-1-10, 4699	K.LLAALLEDEGGSGRPLLQAAK.G	2123.43896	3	2.97E-07	0.94	5.41	-	1146.0
AHQ-1-13, 5036	K.LLAALLEDEGGSGRPLLQAAK.G	2123.43896	3	9.94E-09	0.95	5.05	-	1331.7
AHQ-1-12, 5075	K.LLAALLEDEGGSGRPLLQAAK.G	2123.43896	2	3.16E-09	0.97	5.76	-	1213.8
AHQ-1-5, 5310	K.LLAALLEDEGGSGRPLLQAAK.G	2123.43896	2	8.66E-11	0.97	5.78	-	1249.0
AHQ-1-12, 5073	K.LLAALLEDEGGSGRPLLQAAK.G	2123.43896	3	2.27E-05	0.95	4.42	-	1374.3
AHQ-1-1, 5460	K.LLAALLEDEGGSGRPLLQAAK.G	2123.43896	2	4.76E-10	0.96	5.85	-	880.4
AHQ-1-2, 5462	K.LLAALLEDEGGSGRPLLQAAK.G	2123.43896	2	2.97E-04	0.98	6.17	-	1494.2
AHQ-1-2, 5465	K.LLAALLEDEGGSGRPLLQAAK.G	2123.43896	3	3.58E-09	0.96	5.54	-	1307.2
AHQ-1-8, 4988	K.LLAALLEDEGGSGRPLLQAAK.G	2123.43896	2	2.02E-08	0.97	6.45	-	1056.4
AHQ-1-3, 5329	K.LLAALLEDEGGSGRPLLQAAKGLAGAVSELLR.S	3190.68337	3	1.81E-05	0.84	4.38	-	828.5
AHQ-1-4, 5418	K.LLAALLEDEGGSGRPLLQAAKGLAGAVSELLR.S	3190.68337	3	1.21E-04	0.75	4.22	-	484.3
AHQ-1-5, 5318	K.LLAALLEDEGGSGRPLLQAAKGLAGAVSELLR.S	3190.68337	3	4.58E-06	0.82	5.07	-	405.9
AHQ-1-2, 4710 - 4723	R.LLSDSLPPSTGTFQEAQSR.L	2035.20076	2	2.23E-06	0.66	3.51	-	160.7
AHQ-1-4, 4442 - 4510	R.LLSDSLPPSTGTFQEAQSR.L	2035.20076	2	7.39E-08	0.78	3.78	-	263.4
AHQ-1-4, 4306 - 4374	R.LLSDSLPPSTGTFQEAQSR.L	2035.20076	2	1.62E-05	0.89	3.98	-	368.9
AHQ-1-7, 4225 - 4304	R.LLSDSLPPSTGTFQEAQSR.L	2035.20076	2	2.00E-05	0.77	3.83	-	221.0
AHQ-1-1, 4213 - 4279	R.LLSDSLPPSTGTFQEAQSR.L	2035.20076	2	5.54E-08	0.68	2.93	-	347.9
AHQ-1-2, 4557 - 4617	R.LLSDSLPPSTGTFQEAQSR.L	2035.20076	2	7.14E-07	0.94	4.44	-	551.4
AHQ-1-2, 4479 - 4549	R.LLSDSLPPSTGTFQEAQSR.L	2035.20076	2	2.71E-04	0.81	3.52	-	271.3
AHQ-1-3, 4461 - 4530	R.LLSDSLPPSTGTFQEAQSR.L	2035.20076	2	2.62E-04	0.78	3.55	-	280.2
AHQ-1-2, 4341 - 4413	R.LLSDSLPPSTGTFQEAQSR.L	2035.20076	2	1.66E-09	0.89	3.85	-	467.2
AHQ-1-3, 4311 - 4390	R.LLSDSLPPSTGTFQEAQSR.L	2035.20076	2	4.99E-06	0.91	4.56	-	427.4
AHQ-1-5, 4297 - 4366	R.LLSDSLPPSTGTFQEAQSR.L	2035.20076	2	3.24E-10	0.79	3.78	-	336.2
AHQ-1-1, 4348 - 4415	R.LLSDSLPPSTGTFQEAQSR.L	2035.20076	2	7.91E-12	0.62	2.94	-	247.7
AHQ-1-3, 4253 - 4321	R.LLSDSLPPSTGTFQEAQSR.L	2035.20076	2	1.24E-11	0.90	4.06	-	395.3
AHQ-1-5, 4153 - 4229	R.LLSDSLPPSTGTFQEAQSR.L	2035.20076	2	1.39E-14	0.93	4.66	-	418.1
AHQ-1-1, 4544	R.LLSDSLPPSTGTFQEAQSR.L	2035.20076	2	7.70E-04	0.29	2.64	-	233.7
AHQ-1-6, 4122 - 4191	R.LLSDSLPPSTGTFQEAQSR.L	2035.20076	2	2.29E-06	0.74	3.43	-	281.9
AHQ-1-3, 4233	R.LLSDSLPPSTGTFQEAQSR.L	2035.20076	3	5.26E-04	0.95	4.90	-	1328.2
AHQ-1-3, 5757 - 5829	R.LNEAAAGLNQAATELVQASR.G	2028.21299	2	2.26E-13	0.98	5.51	-	2261.6
AHQ-1-2, 6058 - 6129	R.LNEAAAGLNQAATELVQASR.G	2028.21299	2	3.96E-11	0.98	6.05	-	1914.8
AHQ-1-2, 5918 - 5987	R.LNEAAAGLNQAATELVQASR.G	2028.21299	2	2.18E-05	0.95	4.62	-	1050.4
AHQ-1-10, 4939	R.LNEAAAGLNQAATELVQASR.G	2028.21299	2	3.82E-04	0.90	3.69	-	900.2
AHQ-1-4, 5750	R.LNEAAAGLNQAATELVQASR.G	2028.21299	2	2.76E-12	0.98	6.01	-	1663.1
AHQ-1-4, 5758	R.LNEAAAGLNQAATELVQASR.G	2028.21299	3	1.17E-04	0.98	6.45	-	1849.4
AHQ-1-7, 5634	R.LNEAAAGLNQAATELVQASR.G	2028.21299	3	3.64E-10	0.98	5.30	-	2562.6
AHQ-1-2, 5375 - 5443	R.LNEAAAGLNQAATELVQASR.G	2028.21299	2	9.67E-05	0.95	4.36	-	1374.8
AHQ-1-3, 5641 - 5642	R.LNEAAAGLNQAATELVQASR.G	2028.21299	3	7.67E-11	0.98	6.72	-	2417.5
AHQ-1-6, 5550	R.LNEAAAGLNQAATELVQASR.G	2028.21299	2	1.41E-11	0.97	5.04	-	1688.9
AHQ-1-6, 5566	R.LNEAAAGLNQAATELVQASR.G	2028.21299	3	1.14E-04	0.98	6.30	-	3391.6
AHQ-1-3, 5637 - 5701	R.LNEAAAGLNQAATELVQASR.G	2028.21299	2	7.55E-14	0.98	6.20	-	1794.7

AHQ-1-5, 6021	R.LNEAAAGLNQAATELVQASR.G	2028.21299	2	2.23E-08	0.93	4.12	-	1084.8
AHQ-1-4, 5990 - 6007	R.LNEAAAGLNQAATELVQASR.G	2028.21299	2	1.81E-07	0.96	4.90	-	1406.0
AHQ-1-2, 6198	R.LNEAAAGLNQAATELVQASR.G	2028.21299	2	5.63E-13	0.98	5.92	-	1886.1
AHQ-1-1, 5687 - 5749	R.LNEAAAGLNQAATELVQASR.G	2028.21299	2	5.91E-13	0.98	5.77	-	2126.9
AHQ-1-4, 4566 - 4596	R.LNEAAAGLNQAATELVQASR.G	2028.21299	2	1.57E-05	0.85	3.94	-	601.0
AHQ-1-5, 5883	R.LNEAAAGLNQAATELVQASR.G	2028.21299	2	6.22E-09	0.95	4.97	-	968.6
AHQ-1-1, 5691	R.LNEAAAGLNQAATELVQASR.G	2028.21299	3	3.62E-04	0.96	5.16	-	1672.8
AHQ-1-5, 4483	R.LNEAAAGLNQAATELVQASR.G	2028.21299	2	1.06E-08	0.97	5.63	-	1169.4
AHQ-1-11, 5095	R.LNEAAAGLNQAATELVQASR.G	2028.21299	2	4.18E-08	0.97	4.64	-	1574.1
AHQ-1-3, 5543	R.LNEAAAGLNQAATELVQASR.G	2028.21299	2	5.17E-09	0.98	5.89	-	1454.8
AHQ-1-8, 5451	R.LNEAAAGLNQAATELVQASR.G	2028.21299	2	4.64E-07	0.95	4.44	-	1283.8
AHQ-1-2, 5591	R.LNEAAAGLNQAATELVQASR.G	2028.21299	2	5.90E-08	0.89	3.70	-	885.5
AHQ-1-2, 4650	R.LNEAAAGLNQAATELVQASR.G	2028.21299	2	3.67E-09	0.94	4.59	-	1019.2
AHQ-1-3, 5899 - 5978	R.LNEAAAGLNQAATELVQASR.G	2028.21299	2	1.37E-06	0.81	3.08	-	712.7
AHQ-1-2, 6113	R.LNEAAAGLNQAATELVQASR.G	2028.21299	3	6.39E-06	0.88	3.67	-	1054.4
AHQ-1-2, 5827 - 5835	R.LNEAAAGLNQAATELVQASR.G	2028.21299	3	2.11E-07	0.98	6.62	-	2391.8
AHQ-1-3, 5411	R.LNEAAAGLNQAATELVQASR.G	2028.21299	2	7.39E-12	0.97	5.34	-	1432.3
AHQ-1-9, 5468	R.LNEAAAGLNQAATELVQASR.G	2028.21299	2	1.37E-09	0.96	4.61	-	1580.6
AHQ-1-2, 5817 - 5847	R.LNEAAAGLNQAATELVQASR.G	2028.21299	2	3.58E-09	0.97	5.42	-	1525.7
AHQ-1-7, 5625	R.LNEAAAGLNQAATELVQASR.G	2028.21299	2	7.11E-11	0.97	5.59	-	1401.5
AHQ-1-3, 4499 - 4574	R.LNEAAAGLNQAATELVQASR.G	2028.21299	2	5.74E-08	0.94	4.80	-	853.7
AHQ-1-5, 5550 - 5625	R.LNEAAAGLNQAATELVQASR.G	2028.21299	2	8.56E-11	0.98	6.03	-	1636.8
AHQ-1-1, 5857 - 5883	R.LNEAAAGLNQAATELVQASR.G	2028.21299	2	2.33E-07	0.92	4.19	-	904.6
AHQ-1-2, 5583 - 5621	R.LNEAAAGLNQAATELVQASRGTPQDLAR.A	2867.12501	3	1.11E-04	0.54	3.07	-	541.6
AHQ-1-2, 2185	R.MATNAAAQNAIK.K	1204.38227	2	3.51E-05	0.87	3.12	-	1040.6
AHQ-1-3, 2046	R.MATNAAAQNAIK.K	1204.38227	2	8.19E-04	0.47	2.72	-	515.3
AHQ-1-6, 1920 - 1996	R.MATNAAAQNAIK.K	1204.38227	2	6.83E-04	0.61	2.82	-	638.5
AHQ-1-4, 1940 - 2006	R.MATNAAAQNAIK.K	1204.38227	2	7.15E-06	0.78	3.15	-	673.8
AHQ-1-2, 2079 - 2105	R.MATNAAAQNAIK.K	1204.38227	2	1.32E-06	0.83	3.63	-	574.4
AHQ-1-4, 4376	R.MVAAATNNLCEAANAAVQGHASQEK.L	2558.79098	3	5.14E-05	0.62	3.26	-	551.0
AHQ-1-1, 4223	R.M*VAAATNNLCEAANAAVQGHASQEK.L	2574.79038	3	5.06E-08	0.73	3.68	-	542.8
AHQ-1-3, 4162	R.M*VAAATNNLCEAANAAVQGHASQEK.L	2574.79038	3	6.89E-09	0.93	5.07	-	923.6
AHQ-1-3, 4538 - 4563	R.MVAAATNNLCEAANAAVQGHASQEK.L	2558.79098	3	2.65E-05	0.96	4.75	-	1736.3
AHQ-1-2, 4305 - 4306	R.M*VAAATNNLCEAANAAVQGHASQEK.L	2574.79038	3	2.42E-09	0.95	5.26	-	1068.1
AHQ-1-5, 3191	R.MVAAATNNLCEAANAAVQGHASQEK.L	2558.79098	3	1.75E-06	0.93	4.30	-	1307.1
AHQ-1-5, 4077 - 4079	R.M*VAAATNNLCEAANAAVQGHASQEK.L	2574.79038	3	2.07E-12	0.95	5.79	-	1126.3
AHQ-1-2, 4221	R.M*VAAATNNLCEAANAAVQGHASQEK.L	2574.79038	3	6.96E-09	0.90	4.70	-	845.6
AHQ-1-9, 4331	R.MVAAATNNLCEAANAAVQGHASQEK.L	2558.79098	3	9.26E-05	0.79	3.51	-	1013.6
AHQ-1-4, 4210	R.M*VAAATNNLCEAANAAVQGHASQEK.L	2574.79038	2	2.61E-06	0.68	3.01	-	475.5
AHQ-1-7, 4348 - 4361	R.MVAAATNNLCEAANAAVQGHASQEK.L	2558.79098	3	8.99E-07	0.82	3.43	-	888.9
AHQ-1-2, 4605	R.MVAAATNNLCEAANAAVQGHASQEK.L	2558.79098	2	1.09E-04	0.53	2.74	-	352.2
AHQ-1-6, 4196 - 4275	R.MVAAATNNLCEAANAAVQGHASQEK.L	2558.79098	3	3.57E-07	0.95	4.36	-	1691.1
AHQ-1-5, 4270 - 4345	R.MVAAATNNLCEAANAAVQGHASQEK.L	2558.79098	3	2.18E-05	0.77	4.00	-	752.9
AHQ-1-2, 4493 - 4562	R.MVAAATNNLCEAANAAVQGHASQEK.L	2558.79098	3	1.79E-04	0.90	4.39	-	1138.2
AHQ-1-1, 6608 - 6627	K.M*VGGIAQIIAAQEEMLR.K	1847.19347	2	4.18E-04	0.79	3.30	-	765.5
AHQ-1-13- , 6584	K.M*VGGIAQIIAAQEEMLR.K	1847.19347	2	3.77E-04	0.97	5.07	-	1595.7
AHQ-1-2, 6589	K.MVGGIAQIIAAQEEM*LR.K	1847.19347	2	2.20E-09	0.97	5.87	-	1483.9
AHQ-1-6, 6671 - 6672	K.M*VGGIAQIIAAQEEMLR.K	1847.19347	2	2.21E-07	0.98	6.48	-	2230.7
AHQ-1-5, 6778 - 6845	K.M*VGGIAQIIAAQEEMLR.K	1847.19347	2	5.47E-04	0.96	4.82	-	1531.8
AHQ-1-7, 6844	K.M*VGGIAQIIAAQEEMLR.K	1847.19347	2	1.23E-05	0.97	4.95	-	1771.3
AHQ-1-13- , 5853 - 5915	K.M*VGGIAQIIAAQEEM*LR.K	1863.19287	2	3.08E-08	0.96	4.55	-	1658.3
AHQ-1-4, 7156 - 7168	K.MVGGIAQIIAAQEEMLR.K	1831.19407	2	3.04E-10	0.98	5.80	-	1968.9
AHQ-1-2, 6911	K.M*VGGIAQIIAAQEEMLR.K	1847.19347	3	3.14E-06	0.98	6.11	-	2408.4
AHQ-1-2, 7191	K.MVGGIAQIIAAQEEMLR.K	1831.19407	2	7.61E-08	0.97	5.11	-	1434.4
AHQ-1-14- , 6334	K.M*VGGIAQIIAAQEEMLR.K	1847.19347	2	1.48E-04	0.97	5.24	-	1373.1
AHQ-1-4, 7026	K.MVGGIAQIIAAQEEMLR.K	1831.19407	2	5.96E-04	0.76	3.78	-	753.3
AHQ-1-3, 6133	K.M*VGGIAQIIAAQEEM*LR.K	1863.19287	2	3.01E-06	0.96	4.70	-	1318.9
AHQ-1-5, 7109	K.MVGGIAQIIAAQEEMLR.K	1831.19407	2	2.10E-11	0.97	5.55	-	1680.6
AHQ-1-4, 6256	K.M*VGGIAQIIAAQEEM*LR.K	1863.19287	2	3.49E-10	0.98	5.51	-	1872.9
AHQ-1-4, 6862 - 6863	K.M*VGGIAQIIAAQEEMLR.K	1847.19347	2	4.30E-04	0.95	4.92	-	1214.2
AHQ-1-12, 6214	K.M*VGGIAQIIAAQEEMLR.K	1847.19347	2	8.75E-07	0.96	4.52	-	1286.9
AHQ-1-9, 6640	K.M*VGGIAQIIAAQEEMLR.K	1847.19347	2	5.68E-10	0.98	5.51	-	1629.4
AHQ-1-6, 6966	K.MVGGIAQIIAAQEEMLR.K	1831.19407	2	7.62E-11	0.98	6.13	-	1614.5
AHQ-1-4, 6576	K.MVGGIAQIIAAQEEM*LR.K	1847.19347	2	4.37E-06	0.95	4.67	-	1289.0
AHQ-1-13, 6141	K.M*VGGIAQIIAAQEEMLR.K	1847.19347	2	9.94E-05	0.90	3.63	-	887.9
AHQ-1-1, 6131	K.M*VGGIAQIIAAQEEM*LR.K	1863.19287	2	3.07E-07	0.97	5.12	-	1633.3
AHQ-1-9, 6915	K.MVGGIAQIIAAQEEMLR.K	1831.19407	2	4.78E-12	0.97	4.92	-	1628.1
AHQ-1-4, 6486	K.MVGGIAQIIAAQEEM*LR.K	1847.19347	2	6.07E-07	0.93	4.27	-	889.7
AHQ-1-3, 6997 - 7063	K.MVGGIAQIIAAQEEMLR.K	1831.19407	2	1.12E-11	0.97	5.61	-	1413.8
AHQ-1-2, 4573 - 4642	K.NGNLPEFGDAISTASK.A	1621.73018	2	3.03E-05	0.93	3.92	-	791.4
AHQ-1-3, 4371 - 4429	K.NGNLPEFGDAISTASK.A	1621.73018	2	5.25E-06	0.86	3.49	-	527.0
AHQ-1-2, 4714	K.NGNLPEFGDAISTASK.A	1621.73018	2	1.34E-11	0.86	3.75	-	492.9
AHQ-1-3, 4497 - 4566	K.NGNLPEFGDAISTASK.A	1621.73018	2	2.69E-07	0.81	3.08	-	591.6
AHQ-1-1, 4656	K.NGNLPEFGDAISTASK.A	1621.73018	2	7.57E-06	0.78	3.02	-	537.8
AHQ-1-6, 4624	K.NGNLPEFGDAISTASK.A	1621.73018	2	5.58E-08	0.79	3.21	-	462.9
AHQ-1-1, 4559	K.NGNLPEFGDAISTASK.A	1621.73018	2	3.87E-04	0.56	3.07	-	255.0
AHQ-1-4, 4448 - 4518	K.NGNLPEFGDAISTASK.A	1621.73018	2	4.61E-06	0.89	3.99	-	522.3
AHQ-1-1, 4421 - 4495	K.NGNLPEFGDAISTASK.A	1621.73018	2	3.15E-07	0.92	3.85	-	784.1
AHQ-1-9, 4623	K.NGNLPEFGDAISTASK.A	1621.73018	2	2.20E-05	0.51	3.11	-	280.4
AHQ-1-3, 4231	K.NLGTALAELR.T	1058.21367	1	1.78E-04	0.21	2.21	-	113.1
AHQ-1-4, 4244	K.NLGTALAELR.T	1058.21367	2	5.40E-04	0.86	3.25	-	883.6
AHQ-1-2, 4375 - 4381	K.NLGTALAELR.T	1058.21367	2	3.35E-04	0.86	2.96	-	918.0
AHQ-1-1, 4343	K.NLGTALAELR.T	1058.21367	2	9.33E-04	0.86	3.02	-	887.3
AHQ-1-3, 2722	K.PAAVAAENEEIGSHIK.H	1636.78798	3	3.93E-09	0.97	5.33	-	1769.8
AHQ-1-5, 2601	K.PAAVAAENEEIGSHIK.H	1636.78798	3	9.25E-11	0.97	5.55	-	2012.3
AHQ-1-6, 2619	K.PAAVAAENEEIGSHIK.H	1636.78798	2	4.23E-10	0.98	5.72	-	2200.7
AHQ-1-1, 2891	K.PAAVAAENEEIGSHIK.H	1636.78798	2	8.69E-09	0.93	4.04	-	1131.8
AHQ-1-6, 2584	K.PAAVAAENEEIGSHIK.H	1636.78798	3	5.74E-07	0.96	4.97	-	1594.1
AHQ-1-3, 2731	K.PAAVAAENEEIGSHIK.H	1636.78798	2	2.13E-07	0.98	5.20	-	1940.6
AHQ-1-4, 2723	K.PAAVAAENEEIGSHIK.H	1636.78798	3	3.19E-12	0.97	5.51	-	2182.7
AHQ-1-5, 2603	K.PAAVAAENEEIGSHIK.H	1636.78798	2	1.74E-13	0.98	5.44	-	2106.7
AHQ-1-2, 2783 - 2837	K.PAAVAAENEEIGSHIK.H	1636.78798	2	9.94E-08	0.97	5.04	-	1707.3
AHQ-1-4, 2722	K.PAAVAAENEEIGSHIK.H	1636.78798	2	4.03E-07	0.98	5.59	-	2177.5
AHQ-1-7, 2637	K.PAAVAAENEEIGSHIK.H	1636.78798	2	2.06E-07	0.96	3.88	-	1683.8
AHQ-1-2, 2802 - 2814	K.PAAVAAENEEIGSHIK.H	1636.78798	3	1.51E-09	0.97	5.67	-	1888.0
AHQ-1-1, 2895	K.PAAVAAENEEIGSHIK.H	1636.78798	3	8.56E-07	0.96	4.97	-	1471.1
AHQ-1-6, 3154 - 3176	K.QAAASATQTIAAAQHAASTPK.A	1996.17099	3	4.96E-06	0.82	3.75	-	1094.5
AHQ-1-3, 3471 - 3478	K.QAAASATQTIAAAQHAASTPK.A	1996.17099	2	9.37E-10	0.87	4.45	-	466.3
AHQ-1-3, 3315	K.QAAASATQTIAAAQHAASTPK.A	1996.17099	2	9.90E-04	0.88	3.92	-	723.5
AHQ-1-1, 3577	K.QAAASATQTIAAAQHAASTPK.A	1996.17099	2	1.54E-07	0.72	3.51	-	527.5
AHQ-1-1, 3444 - 3460	K.QAAASATQTIAAAQHAASTPK.A	1996.17099	2	6.21E-06	0.78	3.69	-	425.5
AHQ-1-2, 3258	K.QAAASATQTIAAAQHAASTPK.A	1996.17099	2	2.91E-04	0.45	2.78	-	329.2
AHQ-1-1, 3413	K.QAAASATQTIAAAQHAASTPK.A	1996.17099	3	5.55E-06	0.76	3.22	-	857.1
AHQ-1-5, 3337	K.QAAASATQTIAAAQHAASTPK.A	1996.17099	2	2.30E-07	0.87	4.03	-	542.0
AHQ-1-5, 3170	K.QAAASATQTIAAAQHAASTPK.A	1996.17099	2	5.46E-04	0.94	5.24	-	750.0
AHQ-1-4, 3498	K.QAAASATQTIAAAQHAASTPK.A	1996.17099	3	2.26E-04	0.82	3.30	-	971.7
AHQ-1-5, 3167	K.QAAASATQTIAAAQHAASTPK.A	1996.17099	3	6.02E-04	0.71	3.32	-	570.4

AHQ-1-4, 3500 - 3508	K.QAAASATQTIAAAQHAASTPK.A	1996.17099	2	4.75E-07	0.93	4.98	-	653.1
AHQ-1-2, 4058	K.QAAASATQTIAAAQHAASTPK.A	1996.17099	2	2.27E-08	0.63	3.34	-	362.4
AHQ-1-2, 3605	K.QAAASATQTIAAAQHAASTPK.A	1996.17099	2	2.02E-09	0.88	4.45	-	617.8
AHQ-1-2, 3599	K.QAAASATQTIAAAQHAASTPK.A	1996.17099	3	8.20E-04	0.75	3.15	-	817.8
AHQ-1-6, 3163	K.QAAASATQTIAAAQHAASTPK.A	1996.17099	2	5.21E-04	0.85	3.54	-	646.8
AHQ-1-2, 3422	K.QAAASATQTIAAAQHAASTPK.A	1996.17099	2	5.23E-07	0.90	3.92	-	624.1
AHQ-1-1, 4075 - 4151	R.QELAVFCSPEPPAK.T	1574.77985	2	9.13E-08	0.83	2.97	-	738.8
AHQ-1-5, 4017 - 4030	R.QELAVFCSPEPPAK.T	1574.77985	2	5.75E-08	0.93	4.07	-	1017.5
AHQ-1-2, 4223 - 4294	R.QELAVFCSPEPPAK.T	1574.77985	2	2.20E-06	0.91	3.52	-	1037.8
AHQ-1-5, 7357 - 7437	R.QNLLQAAGNVGQASGELLQQIGESDTDPHFQDALMQLAK.A	4138.52644	3	3.02E-11	0.94	5.14	-	919.2
AHQ-1-6, 7250	R.QNLLQAAGNVGQASGELLQQIGESDTDPHFQDALMQLAK.A	4138.52644	3	3.52E-04	0.71	3.28	-	434.3
AHQ-1-2, 7354	R.QNLLQAAGNVGQASGELLQQIGESDTDPHFQDALM*QLAK.A	4154.52584	3	4.55E-08	0.94	4.94	-	1284.9
AHQ-1-4, 7394 - 7470	R.QNLLQAAGNVGQASGELLQQIGESDTDPHFQDALM*QLAK.A	4154.52584	3	4.65E-08	0.85	4.26	-	645.6
AHQ-1-3, 7237 - 7238	R.QNLLQAAGNVGQASGELLQQIGESDTDPHFQDALMQLAK.A	4138.52644	3	1.68E-09	0.94	5.44	-	968.3
AHQ-1-13- , 3879 - 3881	K.QVAASTAQLLVACK.V	1461.70942	2	3.08E-06	0.92	4.18	-	551.9
AHQ-1-2, 4017 - 4089	K.QVAASTAQLLVACK.V	1461.70942	2	2.76E-06	0.89	3.68	-	539.8
AHQ-1-10, 3464	K.QVAASTAQLLVACK.V	1461.70942	2	9.65E-05	0.76	2.73	-	515.3
AHQ-1-3, 4967	K.RVAGSVTELIQAAEAM*K.G	1791.06327	2	2.13E-04	0.91	4.32	-	497.9
AHQ-1-4, 6086	K.RVAGSVTELIQAAEAMK.G	1775.06387	2	9.48E-05	0.98	5.86	-	2350.5
AHQ-1-2, 5129	K.RVAGSVTELIQAAEAM*K.G	1791.06327	2	2.93E-05	0.95	4.42	-	817.4
AHQ-1-4, 5051	K.RVAGSVTELIQAAEAM*K.G	1791.06327	2	5.70E-05	0.92	3.97	-	644.0
AHQ-1-3, 5985	K.RVAGSVTELIQAAEAMK.G	1775.06387	2	4.15E-05	0.97	5.23	-	1556.6
AHQ-1-2, 6173	K.RVAGSVTELIQAAEAMK.G	1775.06387	2	3.73E-07	0.98	5.73	-	1965.8
AHQ-1-2, 6182	K.RVAGSVTELIQAAEAMK.G	1775.06387	3	3.52E-05	0.89	4.09	-	1025.2
AHQ-1-4, 3695	R.SGASGPENFQVGSM*PPAQQQITSGQMHR.G	2945.19807	3	1.12E-05	0.71	3.59	-	341.0
AHQ-1-1, 3389	R.SGASGPENFQVGSM*PPAQQQITSGQM*HR.G	2961.19747	3	6.97E-05	0.84	3.90	-	809.1
AHQ-1-4, 3927	R.SGASGPENFQVGSMPPAQQQITSGQM*HR.G	2945.19807	3	4.51E-05	0.59	3.52	-	333.8
AHQ-1-9, 2788	K.SIAAATSALVK.A	1032.21612	2	6.77E-04	0.91	3.08	-	1122.6
AHQ-1-4, 3048	K.SIAAATSALVK.A	1032.21612	2	5.04E-05	0.89	3.55	-	955.4
AHQ-1-10, 2797 - 2879	K.SIAAATSALVK.A	1032.21612	2	9.94E-04	0.82	2.99	-	852.0
AHQ-1-1, 3177	K.SIAAATSALVK.A	1032.21612	2	7.13E-06	0.85	2.97	-	828.1
AHQ-1-2, 3150	K.SIAAATSALVK.A	1032.21612	2	9.02E-06	0.91	3.14	-	1187.9
AHQ-1-13- , 3996	K.SKDHFGLEGDEESTM*LEDSVSPK.K	2554.68304	3	1.70E-04	0.95	4.96	-	1587.0
AHQ-1-8, 4208	K.SKDHFGLEGDEESTMLEDSVSPK.K	2538.68364	2	2.52E-11	0.96	5.08	-	1112.7
AHQ-1-8, 4209	K.SKDHFGLEGDEESTMLEDSVSPK.K	2538.68364	3	5.91E-06	0.97	5.77	-	1936.1
AHQ-1-9, 4276	K.SKDHFGLEGDEESTMLEDSVSPK.K	2538.68364	3	1.29E-05	0.95	4.17	-	1901.6
AHQ-1-12, 4230 - 4234	K.SKDHFGLEGDEESTMLEDSVSPK.K	2538.68364	3	1.44E-06	0.97	5.70	-	1916.8
AHQ-1-8, 3867	K.SKDHFGLEGDEESTM*LEDSVSPK.K	2554.68304	3	1.10E-08	0.96	5.63	-	1489.3
AHQ-1-1, 5493 - 5523	K.SNTSPEELGPLANQLTSDYGR.L	2250.36505	2	6.83E-07	0.80	3.58	-	389.1
AHQ-1-2, 5001 - 5022	K.SNTSPEELGPLANQLTSDYGR.L	2250.36505	2	5.72E-06	0.73	3.18	-	438.0
AHQ-1-4, 5695 - 5696	K.SNTSPEELGPLANQLTSDYGR.L	2250.36505	2	5.28E-05	0.91	4.13	-	708.4
AHQ-1-4, 5539 - 5611	K.SNTSPEELGPLANQLTSDYGR.L	2250.36505	2	5.29E-09	0.89	4.32	-	428.8
AHQ-1-2, 5251	K.SNTSPEELGPLANQLTSDYGR.L	2250.36505	2	9.19E-07	0.89	3.95	-	640.7
AHQ-1-4, 5450	K.SNTSPEELGPLANQLTSDYGR.L	2250.36505	2	3.97E-06	0.84	3.73	-	491.8
AHQ-1-4, 5362 - 5380	K.SNTSPEELGPLANQLTSDYGR.L	2250.36505	2	1.07E-06	0.74	3.40	-	404.7
AHQ-1-2, 5457 - 5525	K.SNTSPEELGPLANQLTSDYGR.L	2250.36505	2	1.03E-07	0.89	3.93	-	516.4
AHQ-1-7, 5301 - 5305	K.SNTSPEELGPLANQLTSDYGR.L	2250.36505	2	3.85E-05	0.85	3.88	-	441.7
AHQ-1-4, 4964	K.SNTSPEELGPLANQLTSDYGR.L	2250.36505	2	1.74E-08	0.74	3.19	-	456.6
AHQ-1-4, 4750 - 4759	K.SNTSPEELGPLANQLTSDYGR.L	2250.36505	2	1.84E-08	0.89	4.08	-	493.4
AHQ-1-3, 4695	K.SNTSPEELGPLANQLTSDYGR.L	2250.36505	2	1.27E-04	0.80	3.62	-	378.3
AHQ-1-8, 5219	K.SNTSPEELGPLANQLTSDYGR.L	2250.36505	2	3.76E-05	0.88	3.44	-	710.4
AHQ-1-3, 5337 - 5405	K.SNTSPEELGPLANQLTSDYGR.L	2250.36505	2	6.75E-07	0.78	3.34	-	439.8
AHQ-1-3, 4859	K.SNTSPEELGPLANQLTSDYGR.L	2250.36505	2	1.57E-04	0.55	3.00	-	322.5
AHQ-1-2, 5587 - 5657	K.SNTSPEELGPLANQLTSDYGR.L	2250.36505	2	2.32E-04	0.74	3.17	-	422.0
AHQ-1-1, 5355 - 5419	K.SNTSPEELGPLANQLTSDYGR.L	2250.36505	2	3.88E-05	0.85	3.94	-	433.9
AHQ-1-2, 5699 - 5773	K.SNTSPEELGPLANQLTSDYGR.L	2250.36505	2	5.37E-07	0.90	4.30	-	475.9
AHQ-1-2, 5869 - 5882	K.SNTSPEELGPLANQLTSDYGR.L	2250.36505	2	4.86E-05	0.39	3.08	-	405.1
AHQ-1-9, 5171	K.SNTSPEELGPLANQLTSDYGR.L	2250.36505	2	1.18E-04	0.52	2.83	-	247.7
AHQ-1-3, 5118	K.SNTSPEELGPLANQLTSDYGR.L	2250.36505	2	1.12E-07	0.83	3.39	-	518.6
AHQ-1-3, 5202 - 5277	K.SNTSPEELGPLANQLTSDYGR.L	2250.36505	2	2.18E-05	0.63	2.76	-	394.4
AHQ-1-5, 5570	K.SNTSPEELGPLANQLTSDYGR.L	2250.36505	2	1.78E-09	0.94	4.61	-	717.5
AHQ-1-3, 5593	K.SNTSPEELGPLANQLTSDYGR.L	2250.36505	2	2.05E-07	0.67	3.17	-	299.5
AHQ-1-5, 5473	K.SNTSPEELGPLANQLTSDYGR.L	2250.36505	2	2.44E-05	0.88	3.95	-	444.4
AHQ-1-3, 5426 - 5493	K.SNTSPEELGPLANQLTSDYGR.L	2250.36505	2	6.63E-10	0.87	3.95	-	391.7
AHQ-1-1, 4679	K.SNTSPEELGPLANQLTSDYGR.L	2250.36505	2	1.96E-06	0.73	3.55	-	264.4
AHQ-1-6, 5464	K.SNTSPEELGPLANQLTSDYGR.L	2250.36505	2	5.40E-06	0.78	3.35	-	468.0
AHQ-1-6, 4600	K.SNTSPEELGPLANQLTSDYGR.L	2250.36505	2	9.97E-07	0.69	3.17	-	335.3
AHQ-1-5, 4685	K.SNTSPEELGPLANQLTSDYGR.L	2250.36505	2	2.00E-08	0.82	3.74	-	392.1
AHQ-1-5, 5295 - 5377	K.SNTSPEELGPLANQLTSDYGR.L	2250.36505	2	4.95E-07	0.92	4.40	-	611.1
AHQ-1-2, 4831	K.SNTSPEELGPLANQLTSDYGR.L	2250.36505	2	5.11E-09	0.86	4.16	-	610.0
AHQ-1-6, 5223	K.SNTSPEELGPLANQLTSDYGR.L	2250.36505	2	2.95E-06	0.91	4.33	-	528.6
AHQ-1-2, 7113 - 7173	R.TEDSGLQTQVIAAATQCALSTSQLVACTK.V	3056.37009	3	8.89E-04	0.78	3.65	-	966.9
AHQ-1-2, 7351 - 7427	R.TEDSGLQTQVIAAATQCALSTSQLVACTK.V	3056.37009	3	6.50E-07	0.97	5.64	-	1904.4
AHQ-1-4, 7358 - 7367	R.TEDSGLQTQVIAAATQCALSTSQLVACTK.V	3056.37009	3	1.87E-10	0.97	5.50	-	2367.8
AHQ-1-5, 7374	R.TEDSGLQTQVIAAATQCALSTSQLVACTK.V	3056.37009	3	8.16E-14	0.97	6.31	-	1872.0
AHQ-1-2, 7446 - 7517	R.TEDSGLQTQVIAAATQCALSTSQLVACTK.V	3056.37009	3	1.07E-07	0.95	4.95	-	1345.0
AHQ-1-6, 7119 - 7186	R.TEDSGLQTQVIAAATQCALSTSQLVACTK.V	3056.37009	3	5.85E-13	0.96	5.69	-	1582.2
AHQ-1-7, 7397	R.TEDSGLQTQVIAAATQCALSTSQLVACTK.V	3056.37009	3	2.39E-11	0.98	6.54	-	2083.5
AHQ-1-3, 7429	R.TEDSGLQTQVIAAATQCALSTSQLVACTK.V	3056.37009	3	4.75E-08	0.88	4.17	-	871.4
AHQ-1-10, 3976	K.TKEVIQEWNLTNIK.R	1716.95938	2	2.78E-08	0.95	4.24	-	1439.5
AHQ-1-9, 4211	K.TKEVIQEWNLTNIK.R	1716.95938	2	1.17E-06	0.96	4.81	-	1422.8
AHQ-1-12, 4235	K.TKEVIQEWNLTNIK.R	1716.95938	2	2.10E-04	0.96	4.52	-	1582.4
AHQ-1-8, 4151 - 4155	K.TKEVIQEWNLTNIK.R	1716.95938	2	2.01E-04	0.96	4.77	-	1597.4
AHQ-1-8, 4159	K.TKEVIQEWNLTNIK.R	1716.95938	3	1.54E-06	0.93	4.91	-	990.9
AHQ-1-11, 4087	K.TKEVIQEWNLTNIK.R	1716.95938	2	1.22E-04	0.96	4.09	-	1863.2
AHQ-1-8, 3428	K.TKEVIQEWNLTNIKR.W	1873.14573	2	4.39E-06	0.96	4.60	-	1220.9
AHQ-1-9, 6460 - 6466	K.TLAESALQLLYTAK.E	1522.76793	2	5.63E-04	0.95	3.86	-	1386.8
AHQ-1-12, 6069	K.TLAESALQLLYTAK.E	1522.76793	2	1.51E-04	0.95	4.07	-	1070.2
AHQ-1-14- , 6198 - 6204	K.TLAESALQLLYTAK.E	1522.76793	2	7.29E-06	0.97	4.85	-	1448.1
AHQ-1-7, 6616 - 6617	K.TLAESALQLLYTAK.E	1522.76793	2	2.56E-06	0.94	4.00	-	953.6
AHQ-1-3, 6498 - 6561	K.TLAESALQLLYTAK.E	1522.76793	2	6.84E-09	0.97	4.80	-	1422.5
AHQ-1-6, 6487 - 6495	K.TLAESALQLLYTAK.E	1522.76793	2	3.32E-07	0.96	4.55	-	1252.2
AHQ-1-4, 6666 - 6730	K.TLAESALQLLYTAK.E	1522.76793	2	4.69E-08	0.97	5.25	-	1198.4
AHQ-1-2, 6669 - 6745	K.TLAESALQLLYTAK.E	1522.76793	2	1.10E-11	0.97	5.28	-	1326.2
AHQ-1-4, 6886	K.TLAESALQLLYTAK.E	1522.76793	2	1.35E-09	0.95	4.09	-	1070.0
AHQ-1-2, 6914 - 6917	K.TLAESALQLLYTAK.E	1522.76793	2	1.51E-04	0.94	4.01	-	1157.3
AHQ-1-1, 6643 - 6684	K.TLAESALQLLYTAK.E	1522.76793	2	3.16E-07	0.84	3.22	-	752.9
AHQ-1-5, 6605 - 6614	K.TLAESALQLLYTAK.E	1522.76793	2	1.92E-05	0.97	5.01	-	1197.4
AHQ-1-3, 6731 - 6801	K.TLAESALQLLYTAK.E	1522.76793	2	5.29E-06	0.93	3.68	-	1257.5
AHQ-1-1, 6469 - 6475	K.TLAESALQLLYTAK.E	1522.76793	2	1.45E-09	0.97	5.06	-	1163.9
AHQ-1-13, 6000	K.TLAESALQLLYTAK.E	1522.76793	2	1.27E-04	0.82	2.91	-	751.6
AHQ-1-1, 3391	K.TLSHPQQMALLDQTK.T	1711.96464	2	5.03E-05	0.95	4.73	-	841.0
AHQ-1-6, 3114	K.TLSHPQQMALLDQTK.T	1711.96464	2	6.49E-05	0.95	4.58	-	959.9
AHQ-1-2, 3409	K.TLSHPQQMALLDQTK.T	1711.96464	2	7.92E-06	0.92	4.07	-	758.8
AHQ-1-2, 3383 - 3387	K.TLSHPQQMALLDQTK.T	1711.96464	3	2.56E-06	0.93	4.91	-	937.0
AHQ-1-3, 3258	K.TLSHPQQMALLDQTK.T	1711.96464	3	3.25E-08	0.95	5.17	-	1311.9
AHQ-1-4, 3262	K.TLSHPQQMALLDQTK.T	1711.96464	3	3.45E-06	0.95	4.57	-	1442.6

AHQ-1-4, 3254	K.TLSHPQQMALLDQTK.T	1711.96464	2	5.29E-05	0.88	3.49	-	558.7
AHQ-1-5, 3122	K.TLSHPQQMALLDQTK.T	1711.96464	2	7.73E-07	0.95	4.59	-	870.7
AHQ-1-5, 3129	K.TLSHPQQMALLDQTK.T	1711.96464	3	3.87E-05	0.95	4.78	-	1642.5
AHQ-1-8, 3028	K.TLSHPQQMALLDQTK.T	1711.96464	2	2.49E-05	0.90	4.47	-	440.4
AHQ-1-3, 2509 - 2526	K.TLSHPQQM*ALLDQTK.T	1727.96404	2	2.02E-04	0.88	3.75	-	481.8
AHQ-1-5, 2398	K.TLSHPQQM*ALLDQTK.T	1727.96404	2	5.67E-04	0.81	3.16	-	466.0
AHQ-1-5, 4317 - 4322	K.TMLESAGGLIQTAR.A	1448.67095	2	2.63E-07	0.93	4.11	-	1315.5
AHQ-1-3, 4143	K.TMLESAGGLIQTAR.A	1448.67095	2	4.49E-09	0.91	4.02	-	1129.2
AHQ-1-1, 3611	K.TM*LESAGGLIQTAR.A	1464.67035	2	2.05E-06	0.97	5.16	-	2043.6
AHQ-1-3, 4330 - 4397	K.TMLESAGGLIQTAR.A	1448.67095	2	1.83E-07	0.96	4.71	-	1642.1
AHQ-1-5, 3374 - 3443	K.TM*LESAGGLIQTAR.A	1464.67035	2	1.07E-09	0.94	4.37	-	1185.9
AHQ-1-4, 4426 - 4440	K.TMLESAGGLIQTAR.A	1448.67095	2	9.81E-09	0.95	4.31	-	1646.8
AHQ-1-3, 4381	K.TM*LESAGGLIQTAR.A	1464.67035	2	3.62E-04	0.77	2.87	-	898.3
AHQ-1-4, 4447	K.TMLESAGGLIQTAR.A	1448.67095	1	5.23E-04	0.05	2.07	-	171.2
AHQ-1-1, 4371 - 4452	K.TMLESAGGLIQTAR.A	1448.67095	2	2.48E-07	0.93	4.22	-	1282.4
AHQ-1-2, 4482 - 4550	K.TMLESAGGLIQTAR.A	1448.67095	2	9.21E-12	0.95	4.76	-	1491.9
AHQ-1-2, 4302	K.TMLESAGGLIQTAR.A	1448.67095	2	3.86E-09	0.92	3.98	-	1215.4
AHQ-1-4, 3563 - 3592	K.TM*LESAGGLIQTAR.A	1464.67035	2	4.96E-04	0.93	3.84	-	1337.1
AHQ-1-6, 4250 - 4254	K.TMLESAGGLIQTAR.A	1448.67095	2	1.92E-07	0.94	4.51	-	1376.3
AHQ-1-8, 4201	K.TMLESAGGLIQTAR.A	1448.67095	2	1.25E-05	0.90	3.36	-	1520.4
AHQ-1-2, 3658 - 3730	K.TM*LESAGGLIQTAR.A	1464.67035	2	1.14E-05	0.95	4.36	-	1466.1
AHQ-1-10, 4405	K.TMQFEPSTMVYDACR.I	1838.07571	2	2.71E-04	0.88	3.33	-	585.1
AHQ-1-14- , 4826	K.TMQFEPSTMVYDACR.I	1838.07571	2	9.56E-04	0.65	2.96	-	446.4
AHQ-1-13- , 4063 - 4123	K.TMQFEPSTM*VYDACR.I	1854.07511	2	4.21E-05	0.88	3.61	-	437.1
AHQ-1-13- , 4936 - 4937	K.TMQFEPSTMVYDACR.I	1838.07571	2	1.81E-07	0.96	5.27	-	720.5
AHQ-1-8, 3398	K.TM*QFEPSTM*VYDACR.I	1870.07451	2	2.17E-05	0.45	2.65	-	347.7
AHQ-1-8, 4241	K.TM*QFEPSTMVYDACR.I	1854.07511	2	5.84E-05	0.80	3.35	-	558.9
AHQ-1-8, 4819	K.TMQFEPSTMVYDACR.I	1838.07571	2	1.76E-04	0.90	4.24	-	522.4
AHQ-1-13, 4180	K.TM*QFEPSTMVYDACR.I	1854.07511	2	1.53E-05	0.85	3.41	-	549.3
AHQ-1-8, 3683	K.TVTDM*LM*TICAR.I	1445.70887	2	1.29E-04	0.74	3.06	-	403.7
AHQ-1-8, 5114	K.TYGVSFFLVK.E	1161.37395	2	4.66E-06	0.90	3.36	-	693.5
AHQ-1-8, 5215	K.TYGVSFFLVK.E	1161.37395	2	3.42E-05	0.91	3.28	-	774.5
AHQ-1-12, 5129 - 5131	K.TYGVSFFLVK.E	1161.37395	2	5.03E-08	0.93	3.72	-	760.6
AHQ-1-11, 4920	K.TYGVSFFLVK.E	1161.37395	2	7.52E-07	0.91	3.84	-	813.8
AHQ-1-2, 6594	R.VAGSVTELIQAAEAMK.G	1618.87752	2	7.54E-11	0.97	4.54	-	1531.7
AHQ-1-7, 5365	R.VAGSVTELIQAAEAM*K.G	1634.87692	2	6.75E-05	0.92	3.95	-	1054.2
AHQ-1-4, 6586 - 6594	R.VAGSVTELIQAAEAMK.G	1618.87752	2	2.71E-06	0.97	4.86	-	1503.1
AHQ-1-5, 6457 - 6526	R.VAGSVTELIQAAEAMK.G	1618.87752	2	1.41E-09	0.96	5.02	-	1277.1
AHQ-1-4, 5498 - 5506	R.VAGSVTELIQAAEAM*K.G	1634.87692	2	4.23E-05	0.95	4.34	-	1381.4
AHQ-1-7, 6510	R.VAGSVTELIQAAEAMK.G	1618.87752	2	1.64E-06	0.97	5.23	-	1646.5
AHQ-1-3, 5390 - 5458	R.VAGSVTELIQAAEAM*K.G	1634.87692	2	5.06E-06	0.95	4.79	-	1231.6
AHQ-1-3, 6426	R.VAGSVTELIQAAEAMK.G	1618.87752	2	9.72E-06	0.91	3.96	-	1026.2
AHQ-1-13- , 6340	R.VAGSVTELIQAAEAMK.G	1618.87752	2	1.27E-05	0.96	4.95	-	1183.4
AHQ-1-6, 6398 - 6410	R.VAGSVTELIQAAEAMK.G	1618.87752	2	1.62E-07	0.98	5.79	-	2176.7
AHQ-1-1, 6415 - 6417	R.VAGSVTELIQAAEAMK.G	1618.87752	2	7.11E-07	0.98	5.11	-	2292.1
AHQ-1-1, 6553	R.VAGSVTELIQAAEAMK.G	1618.87752	2	1.06E-07	0.85	2.96	-	877.4
AHQ-1-9, 6346	R.VAGSVTELIQAAEAMK.G	1618.87752	2	9.22E-05	0.96	4.20	-	1692.4
AHQ-1-9, 5232	R.VAGSVTELIQAAEAM*K.G	1634.87692	2	1.14E-05	0.92	4.00	-	971.5
AHQ-1-4, 3708	K.VEHGSVALPAIMR.S	1380.64144	2	7.06E-07	0.89	3.07	-	1020.3
AHQ-1-2, 6285 - 6353	K.VGAIPANALDDGQWSQGLISAAR.M	2311.53959	2	7.89E-04	0.93	4.47	-	912.7
AHQ-1-3, 6025 - 6089	K.VGAIPANALDDGQWSQGLISAAR.M	2311.53959	2	1.14E-08	0.88	4.34	-	477.2
AHQ-1-2, 6109 - 6149	K.VGAIPANALDDGQWSQGLISAAR.M	2311.53959	2	8.24E-11	0.94	4.83	-	661.5
AHQ-1-5, 5894 - 5953	K.VGAIPANALDDGQWSQGLISAAR.M	2311.53959	2	4.36E-09	0.94	4.37	-	1014.4
AHQ-1-3, 6161	K.VGAIPANALDDGQWSQGLISAAR.M	2311.53959	2	2.90E-06	0.80	3.61	-	443.9
AHQ-1-1, 5996 - 6063	K.VGAIPANALDDGQWSQGLISAAR.M	2311.53959	2	2.44E-08	0.90	4.44	-	585.3
AHQ-1-13, 5453 - 5517	K.VGAIPANALDDGQWSQGLISAAR.M	2311.53959	2	1.73E-09	0.90	4.03	-	793.3
AHQ-1-5, 6033 - 6113	K.VGAIPANALDDGQWSQGLISAAR.M	2311.53959	2	1.26E-11	0.96	5.32	-	873.0
AHQ-1-2, 5979 - 6025	K.VGAIPANALDDGQWSQGLISAAR.M	2311.53959	2	4.32E-11	0.97	5.54	-	1051.0
AHQ-1-1, 5939 - 6003	K.VGAIPANALDDGQWSQGLISAAR.M	2311.53959	2	2.41E-10	0.95	4.97	-	816.4
AHQ-1-5, 6239 - 6250	K.VGAIPANALDDGQWSQGLISAAR.M	2311.53959	2	2.09E-06	0.75	3.04	-	420.4
AHQ-1-2, 5911 - 5955	K.VGAIPANALDDGQWSQGLISAAR.M	2311.53959	2	3.57E-07	0.93	4.62	-	755.7
AHQ-1-7, 6021 - 6100	K.VGAIPANALDDGQWSQGLISAAR.M	2311.53959	2	5.69E-08	0.96	5.82	-	822.5
AHQ-1-4, 6272	K.VGAIPANALDDGQWSQGLISAAR.M	2311.53959	2	7.83E-04	0.79	3.22	-	452.1
AHQ-1-4, 6138 - 6215	K.VGAIPANALDDGQWSQGLISAAR.M	2311.53959	2	1.08E-09	0.96	5.21	-	1145.3
AHQ-1-7, 5880	K.VGAIPANALDDGQWSQGLISAAR.M	2311.53959	2	8.63E-06	0.88	4.06	-	584.9
AHQ-1-1, 5837 - 5899	K.VGAIPANALDDGQWSQGLISAAR.M	2311.53959	2	4.24E-09	0.88	4.16	-	393.8
AHQ-1-4, 6043	K.VGAIPANALDDGQWSQGLISAAR.M	2311.53959	2	3.85E-05	0.83	3.51	-	612.6
AHQ-1-9, 5656 - 5683	K.VGAIPANALDDGQWSQGLISAAR.M	2311.53959	2	2.08E-07	0.93	4.95	-	623.4
AHQ-1-11, 5255	K.VGAIPANALDDGQWSQGLISAAR.M	2311.53959	2	1.79E-06	0.49	2.75	-	367.5
AHQ-1-4, 5924 - 5938	K.VGAIPANALDDGQWSQGLISAAR.M	2311.53959	2	7.50E-08	0.96	4.96	-	1186.2
AHQ-1-4, 5800	K.VGAIPANALDDGQWSQGLISAAR.M	2311.53959	2	2.47E-07	0.87	3.81	-	599.5
AHQ-1-9, 5898 - 5935	K.VGAIPANALDDGQWSQGLISAAR.M	2311.53959	2	9.16E-11	0.97	6.04	-	1089.7
AHQ-1-11, 5359 - 5426	K.VGAIPANALDDGQWSQGLISAAR.M	2311.53959	2	1.21E-06	0.96	5.24	-	934.2
AHQ-1-1, 5821	K.VGAIPANALDDGQWSQGLISAAR.M	2311.53959	3	4.16E-06	0.77	3.18	-	1005.2
AHQ-1-1, 5761 - 5825	K.VGAIPANALDDGQWSQGLISAAR.M	2311.53959	2	4.86E-04	0.69	3.43	-	352.5
AHQ-1-3, 5941 - 5949	K.VGAIPANALDDGQWSQGLISAAR.M	2311.53959	2	3.15E-11	0.91	4.53	-	440.0
AHQ-1-12, 5619	K.VGAIPANALDDGQWSQGLISAAR.M	2311.53959	2	1.41E-06	0.83	3.96	-	485.7
AHQ-1-9, 6051	K.VGAIPANALDDGQWSQGLISAAR.M	2311.53959	2	3.16E-05	0.51	2.97	-	208.7
AHQ-1-3, 5786 - 5857	K.VGAIPANALDDGQWSQGLISAAR.M	2311.53959	2	8.92E-08	0.96	5.63	-	977.5
AHQ-1-10, 5207 - 5287	K.VGAIPANALDDGQWSQGLISAAR.M	2311.53959	2	4.37E-08	0.95	5.79	-	649.7
AHQ-1-6, 5788 - 5814	K.VGAIPANALDDGQWSQGLISAAR.M	2311.53959	2	1.35E-06	0.95	4.33	-	1125.7
AHQ-1-6, 5894	K.VGAIPANALDDGQWSQGLISAAR.M	2311.53959	2	1.42E-09	0.95	4.75	-	761.5
AHQ-1-6, 5990	K.VGAIPANALDDGQWSQGLISAAR.M	2311.53959	2	7.17E-11	0.94	5.00	-	673.5
AHQ-1-6, 6100	K.VGAIPANALDDGQWSQGLISAAR.M	2311.53959	2	9.10E-11	0.95	5.01	-	767.9
AHQ-1-2, 6114 - 6189	K.VGAIPANALDDGQWSQGLISAAR.M	2311.53959	3	3.02E-05	0.95	4.73	-	1627.7
AHQ-1-8, 5962	K.VGAIPANALDDGQWSQGLISAAR.M	2311.53959	2	2.87E-12	0.96	5.36	-	991.2
AHQ-1-4, 3892	K.VGDDPAVWQLK.N	1228.37879	2	7.77E-09	0.92	3.71	-	903.0
AHQ-1-2, 4026	K.VGDDPAVWQLK.N	1228.37879	2	3.52E-08	0.93	3.82	-	884.8
AHQ-1-1, 3959	K.VGDDPAVWQLK.N	1228.37879	2	7.32E-08	0.94	4.16	-	879.7
AHQ-1-3, 3825 - 3893	K.VGDDPAVWQLK.N	1228.37879	2	7.25E-08	0.92	3.68	-	842.6
AHQ-1-7, 3792 - 3840	K.VGDDPAVWQLK.N	1228.37879	2	5.40E-04	0.90	3.27	-	989.9
AHQ-1-2, 3963 - 4037	K.VGDDPAVWQLK.N	1228.37879	2	5.34E-09	0.94	4.32	-	897.7
AHQ-1-6, 3710 - 3754	K.VGDDPAVWQLK.N	1228.37879	2	3.11E-08	0.89	3.30	-	771.0
AHQ-1-3, 3874	K.VGDDPAVWQLK.N	1228.37879	2	3.12E-08	0.93	4.37	-	770.6
AHQ-1-5, 3747	K.VGDDPAVWQLK.N	1228.37879	2	5.23E-09	0.92	3.99	-	825.3
AHQ-1-2, 3417	K.VGDDPAVWQLKNSAK.V	1628.81068	2	9.30E-07	0.81	3.31	-	571.9
AHQ-1-4, 2386 - 2403	K.VLGEAM*TGISQNAK.N	1435.62901	2	1.27E-04	0.94	4.27	-	1209.6
AHQ-1-4, 3362	K.VLGEAMTGISQNAK.N	1419.62961	2	6.13E-12	0.94	3.67	-	1418.1
AHQ-1-7, 3076	K.VLGEAMTGISQNAK.N	1419.62961	2	1.12E-08	0.96	3.81	-	1677.0
AHQ-1-4, 3196 - 3231	K.VLGEAMTGISQNAK.N	1419.62961	1	1.13E-04	0.77	3.13	-	478.2
AHQ-1-3, 3165 - 3166	K.VLGEAMTGISQNAK.N	1419.62961	1	8.90E-04	0.55	2.03	-	538.7
AHQ-1-14- , 2371 - 2451	K.VLGEAM*TGISQNAK.N	1435.62901	2	1.48E-04	0.95	3.83	-	1575.4
AHQ-1-2, 3286	K.VLGEAMTGISQNAK.N	1419.62961	1	6.52E-07	0.70	3.11	-	516.3
AHQ-1-2, 3259 - 3303	K.VLGEAMTGISQNAK.N	1419.62961	2	2.20E-08	0.97	4.99	-	1729.8
AHQ-1-2, 2589	K.VLGEAM*TGISQNAK.N	1435.62901	2	3.30E-06	0.95	3.57	-	1598.2
AHQ-1-3, 2495	K.VLGEAM*TGISQNAK.N	1435.62901	2	9.99E-05	0.93	3.54	-	1099.7
AHQ-1-2, 2478	K.VLGEAM*TGISQNAK.N	1435.62901	2	2.85E-07	0.90	4.07	-	753.2

AHQ-1-4, 3186	K.VLGEAMTGISQNAK.N	1419.62961	2	2.87E-09	0.97	3.62	-	2373.8
AHQ-1-2, 3430 - 3433	K.VLGEAMTGISQNAK.N	1419.62961	2	4.68E-07	0.97	3.94	-	1945.7
AHQ-1-1, 3275 - 3291	K.VLGEAMTGISQNAK.N	1419.62961	2	6.72E-05	0.98	5.41	-	1788.4
AHQ-1-1, 4901	K.VLGEAM*TGISQNAKNGNLPEFGDAISTASK.A	3038.33659	3	3.51E-04	0.85	4.31	-	362.9
AHQ-1-4, 5514 - 5582	K.VLGEAMTGISQNAKNGNLPEFGDAISTASK.A	3022.33719	3	6.56E-11	0.83	4.11	-	575.9
AHQ-1-3, 5401 - 5465	K.VLGEAMTGISQNAKNGNLPEFGDAISTASK.A	3022.33719	3	7.35E-10	0.93	5.25	-	935.3
AHQ-1-2, 5018 - 5089	K.VLGEAM*TGISQNAKNGNLPEFGDAISTASK.A	3038.33659	3	5.88E-05	0.87	4.33	-	604.5
AHQ-1-1, 4991	K.VLGEAM*TGISQNAKNGNLPEFGDAISTASK.A	3038.33659	3	1.59E-05	0.91	4.56	-	880.7
AHQ-1-1, 5472	K.VLGEAMTGISQNAKNGNLPEFGDAISTASK.A	3022.33719	3	8.22E-04	0.90	4.49	-	961.2
AHQ-1-2, 5585 - 5649	K.VLGEAMTGISQNAKNGNLPEFGDAISTASK.A	3022.33719	3	3.01E-08	0.93	5.28	-	780.3
AHQ-1-3, 1625 - 1657	K.VLVQNAAGSQEK.L	1244.37976	2	8.18E-07	0.93	3.63	-	1079.3
AHQ-1-6, 1791	K.VLVQNAAGSQEK.L	1244.37976	2	1.41E-07	0.92	3.59	-	1064.4
AHQ-1-6, 1596 - 1614	K.VLVQNAAGSQEK.L	1244.37976	2	1.54E-09	0.90	3.51	-	865.7
AHQ-1-1, 1932 - 2012	K.VLVQNAAGSQEK.L	1244.37976	2	6.08E-04	0.83	2.83	-	904.2
AHQ-1-2, 1758 - 1959	K.VLVQNAAGSQEK.L	1244.37976	1	3.67E-05	0.33	2.32	-	454.4
AHQ-1-3, 1717 - 1790	K.VLVQNAAGSQEK.L	1244.37976	2	6.92E-06	0.90	3.53	-	902.9
AHQ-1-2, 1665 - 1730	K.VLVQNAAGSQEK.L	1244.37976	2	1.71E-07	0.94	3.82	-	1074.6
AHQ-1-3, 1850 - 1929	K.VLVQNAAGSQEK.L	1244.37976	2	2.55E-08	0.90	3.30	-	969.5
AHQ-1-5, 1543 - 1606	K.VLVQNAAGSQEK.L	1244.37976	2	2.83E-06	0.92	3.66	-	1009.0
AHQ-1-2, 1989	K.VLVQNAAGSQEK.L	1244.37976	2	6.51E-08	0.94	3.43	-	1360.8
AHQ-1-4, 1611 - 1670	K.VLVQNAAGSQEK.L	1244.37976	2	4.03E-06	0.93	3.62	-	1170.8
AHQ-1-4, 1712 - 1835	K.VLVQNAAGSQEK.L	1244.37976	1	1.22E-05	0.33	2.35	-	293.1
AHQ-1-4, 1730 - 1790	K.VLVQNAAGSQEK.L	1244.37976	2	3.65E-04	0.76	3.00	-	649.9
AHQ-1-4, 1874 - 1952	K.VLVQNAAGSQEK.L	1244.37976	2	2.99E-10	0.87	3.38	-	703.3
AHQ-1-6, 1694	K.VLVQNAAGSQEK.L	1244.37976	2	2.13E-07	0.81	3.08	-	622.0
AHQ-1-2, 1793 - 1857	K.VLVQNAAGSQEK.L	1244.37976	2	1.72E-06	0.92	3.94	-	1002.5
AHQ-1-3, 4413	K.VM*VTNVTSLLK.T	1221.49262	2	4.91E-05	0.80	2.99	-	666.6
AHQ-1-2, 4569	K.VM*VTNVTSLLK.T	1221.49262	2	1.86E-04	0.76	3.04	-	730.3
AHQ-1-5, 4679 - 4689	K.VMVTNVTSLLK.T	1205.49322	2	1.16E-06	0.96	4.55	-	1155.4
AHQ-1-2, 4937	K.VMVTNVTSLLK.T	1205.49322	2	5.72E-06	0.91	3.77	-	839.7
AHQ-1-4, 4828 - 4838	K.VMVTNVTSLLK.T	1205.49322	2	5.29E-07	0.94	4.30	-	917.5
AHQ-1-2, 4958	K.VM*VTNVTSLLK.T	1221.49262	2	5.75E-04	0.63	2.82	-	736.1
AHQ-1-2, 5030	K.VMVTNVTSLLK.T	1205.49322	2	4.18E-05	0.88	3.59	-	728.5
AHQ-1-7, 4629 - 4646	K.VMVTNVTSLLK.T	1205.49322	2	7.24E-05	0.86	3.43	-	681.7
AHQ-1-3, 4785	K.VMVTNVTSLLK.T	1205.49322	2	2.02E-04	0.92	4.03	-	822.3
AHQ-1-4, 4455 - 4471	K.VM*VTNVTSLLK.T	1221.49262	2	5.87E-04	0.89	3.53	-	768.8
AHQ-1-6, 4255 - 4332	K.VM*VTNVTSLLK.T	1221.49262	2	8.27E-06	0.85	3.30	-	726.3
AHQ-1-1, 4489	K.VM*VTNVTSLLK.T	1221.49262	2	3.24E-04	0.62	2.65	-	514.7
AHQ-1-1, 4839 - 4840	K.VMVTNVTSLLK.T	1205.49322	2	1.60E-04	0.93	4.23	-	921.2
AHQ-1-6, 4610	K.VMVTNVTSLLK.T	1205.49322	2	1.37E-05	0.91	3.97	-	765.8
AHQ-1-5, 4314	K.VM*VTNVTSLLK.T	1221.49262	2	6.62E-07	0.81	3.38	-	625.4
AHQ-1-11, 2720	R.VQELGHGCAALVTK.A	1484.70307	2	5.09E-04	0.95	3.75	-	1450.9
AHQ-1-6, 2706	R.VQELGHGCAALVTK.A	1484.70307	2	1.24E-04	0.96	4.42	-	1465.1
AHQ-1-1, 2909 - 2980	R.VQELGHGCAALVTK.A	1484.70307	2	3.93E-04	0.94	4.34	-	1014.7
AHQ-1-4, 2806	R.VQELGHGCAALVTK.A	1484.70307	2	6.62E-05	0.98	5.05	-	1928.1
AHQ-1-3, 2823	R.VQELGHGCAALVTK.A	1484.70307	1	5.48E-06	0.83	3.80	-	497.4
AHQ-1-1, 2967	R.VQELGHGCAALVTK.A	1484.70307	2	2.71E-08	0.97	5.05	-	1626.2
AHQ-1-7, 2726	R.VQELGHGCAALVTK.A	1484.70307	2	9.66E-04	0.94	3.81	-	1298.1
AHQ-1-2, 2935	R.VQELGHGCAALVTK.A	1484.70307	1	3.13E-07	0.84	3.78	-	578.8
AHQ-1-2, 2914	R.VQELGHGCAALVTK.A	1484.70307	2	2.98E-04	0.95	4.38	-	1194.2
AHQ-1-2, 2739 - 2809	K.VSHVLAALQAGNR.G	1336.52525	2	9.66E-09	0.97	4.69	-	1756.2
AHQ-1-2, 2806	K.VSHVLAALQAGNR.G	1336.52525	1	5.54E-06	0.88	3.54	-	509.2
AHQ-1-5, 2554 - 2570	K.VSHVLAALQAGNR.G	1336.52525	2	6.68E-05	0.90	3.17	-	1006.3
AHQ-1-4, 2660 - 2730	K.VSHVLAALQAGNR.G	1336.52525	2	3.65E-11	0.96	4.12	-	1669.6
AHQ-1-4, 2707	K.VSHVLAALQAGNR.G	1336.52525	1	6.94E-06	0.88	3.37	-	553.7
AHQ-1-5, 2562	K.VSHVLAALQAGNR.G	1336.52525	1	1.25E-04	0.34	2.24	-	278.3
AHQ-1-7, 2600	K.VSHVLAALQAGNR.G	1336.52525	1	3.50E-06	0.67	2.62	-	482.3
AHQ-1-6, 2676	K.VSHVLAALQAGNR.G	1336.52525	2	1.57E-04	0.95	3.33	-	1694.5
AHQ-1-7, 2596	K.VSHVLAALQAGNR.G	1336.52525	2	9.46E-07	0.94	3.65	-	1311.9
AHQ-1-3, 2653 - 2727	K.VSHVLAALQAGNR.G	1336.52525	2	3.06E-05	0.96	4.19	-	1591.3
AHQ-1-4, 2714	K.VSHVLAALQAGNR.G	1336.52525	2	8.35E-09	0.98	5.20	-	1828.5
AHQ-1-3, 2706	K.VSHVLAALQAGNR.G	1336.52525	1	1.40E-07	0.80	3.35	-	423.6
AHQ-1-1, 2888	K.VSHVLAALQAGNR.G	1336.52525	1	2.18E-04	0.39	2.03	-	479.9
AHQ-1-1, 2887	K.VSHVLAALQAGNR.G	1336.52525	2	7.00E-07	0.97	4.36	-	2118.4
AHQ-1-6, 2570 - 2574	K.VSHVLAALQAGNR.G	1336.52525	2	2.31E-07	0.96	4.19	-	1713.7
AHQ-1-4, 2716	K.VSHVLAALQAGNR.G	1336.52525	1	1.63E-04	0.78	3.28	-	377.2
AHQ-1-7, 6934 - 6953	K.VSQMAQYFEPLTLAAVGAASK.T	2183.51313	2	5.31E-05	0.95	4.84	-	870.0
AHQ-1-6, 6734 - 6750	K.VSQMAQYFEPLTLAAVGAASK.T	2183.51313	2	2.87E-07	0.95	5.14	-	763.9
AHQ-1-6, 5927	K.VSQM*AQYFEPLTLAAVGAASK.T	2199.51253	2	2.02E-07	0.94	4.06	-	1244.8
AHQ-1-3, 5922 - 5989	K.VSQM*AQYFEPLTLAAVGAASK.T	2199.51253	2	1.75E-05	0.92	4.62	-	797.0
AHQ-1-12, 6307	K.VSQMAQYFEPLTLAAVGAASK.T	2183.51313	2	5.81E-04	0.76	3.71	-	335.1
AHQ-1-2, 7119	K.VSQMAQYFEPLTLAAVGAASK.T	2183.51313	2	4.52E-11	0.98	6.17	-	1260.1
AHQ-1-2, 6985 - 7055	K.VSQMAQYFEPLTLAAVGAASK.T	2183.51313	2	1.14E-05	0.96	5.51	-	954.8
AHQ-1-10, 5987 - 6032	K.VSQMAQYFEPLTLAAVGAASK.T	2183.51313	2	6.13E-04	0.87	3.82	-	777.2
AHQ-1-2, 6894 - 6973	K.VSQMAQYFEPLTLAAVGAASK.T	2183.51313	2	8.03E-07	0.96	5.74	-	853.3
AHQ-1-2, 6886	K.VSQMAQYFEPLTLAAVGAASK.T	2183.51313	2	3.91E-09	0.93	4.51	-	801.0
AHQ-1-11, 6102 - 6107	K.VSQMAQYFEPLTLAAVGAASK.T	2183.51313	2	7.42E-05	0.89	4.45	-	493.8
AHQ-1-4, 6100	K.VSQM*AQYFEPLTLAAVGAASK.T	2199.51253	2	1.18E-04	0.88	3.54	-	808.5
AHQ-1-4, 6867	K.VSQMAQYFEPLTLAAVGAASK.T	2183.51313	2	2.99E-04	0.87	4.25	-	549.7
AHQ-1-4, 6958	K.VSQMAQYFEPLTLAAVGAASK.T	2183.51313	3	2.16E-06	0.82	3.71	-	931.1
AHQ-1-11, 5395	K.VSQM*AQYFEPLTLAAVGAASK.T	2199.51253	2	2.24E-06	0.87	4.04	-	651.0
AHQ-1-3, 6854 - 6929	K.VSQMAQYFEPLTLAAVGAASK.T	2183.51313	2	6.82E-06	0.92	5.16	-	487.3
AHQ-1-3, 6797 - 6805	K.VSQMAQYFEPLTLAAVGAASK.T	2183.51313	3	2.38E-07	0.97	5.14	-	2147.5
AHQ-1-3, 5973	K.VSQM*AQYFEPLTLAAVGAASK.T	2199.51253	2	2.30E-05	0.92	4.36	-	775.3
AHQ-1-4, 7079 - 7142	K.VSQMAQYFEPLTLAAVGAASK.T	2183.51313	2	1.61E-08	0.97	5.34	-	1222.6
AHQ-1-3, 6707 - 6789	K.VSQMAQYFEPLTLAAVGAASK.T	2183.51313	2	2.15E-06	0.96	5.71	-	857.6
AHQ-1-3, 6119	K.VSQM*AQYFEPLTLAAVGAASK.T	2199.51253	2	1.51E-05	0.82	3.83	-	527.9
AHQ-1-3, 6010	K.VSQM*AQYFEPLTLAAVGAASK.T	2199.51253	2	3.24E-06	0.93	4.78	-	726.4
AHQ-1-5, 7098	K.VSQMAQYFEPLTLAAVGAASK.T	2183.51313	2	5.47E-06	0.76	2.93	-	591.0
AHQ-1-3, 5990 - 5997	K.VSQM*AQYFEPLTLAAVGAASK.T	2199.51253	3	4.01E-10	0.93	4.44	-	1353.0
AHQ-1-5, 6911 - 6913	K.VSQMAQYFEPLTLAAVGAASK.T	2183.51313	2	1.27E-06	0.97	6.35	-	1006.4
AHQ-1-7, 5950	K.VSQM*AQYFEPLTLAAVGAASK.T	2199.51253	2	3.14E-05	0.95	4.80	-	981.5
AHQ-1-1, 6807 - 6833	K.VSQMAQYFEPLTLAAVGAASK.T	2183.51313	2	2.52E-08	0.96	5.55	-	772.8
AHQ-1-1, 6677 - 6735	K.VSQMAQYFEPLTLAAVGAASK.T	2183.51313	2	2.18E-07	0.96	5.31	-	903.5
AHQ-1-5, 6037 - 6038	K.VSQM*AQYFEPLTLAAVGAASK.T	2199.51253	2	1.31E-04	0.96	5.09	-	1253.6
AHQ-1-14- , 6418	K.VSQMAQYFEPLTLAAVGAASK.T	2183.51313	2	1.10E-05	0.95	5.26	-	823.6
AHQ-1-1, 5989	K.VSQM*AQYFEPLTLAAVGAASK.T	2199.51253	2	1.22E-04	0.95	4.83	-	1028.9
AHQ-1-2, 4745	K.VVAPTISSPVCQEQLVEAGR.L	2142.41919	2	8.01E-06	0.90	4.23	-	415.6
AHQ-1-5, 4818 - 4897	K.VVAPTISSPVCQEQLVEAGR.L	2142.41919	2	2.95E-11	0.93	4.74	-	492.3
AHQ-1-5, 4710 - 4715	K.VVAPTISSPVCQEQLVEAGR.L	2142.41919	2	6.16E-07	0.96	5.05	-	1010.2
AHQ-1-14, 5353 - 5360	K.VVAPTISSPVCQEQLVEAGR.L	2142.41919	2	7.07E-07	0.92	4.22	-	765.9
AHQ-1-5, 4601	K.VVAPTISSPVCQEQLVEAGR.L	2142.41919	2	1.54E-07	0.93	4.70	-	537.3
AHQ-1-11, 4307	K.VVAPTISSPVCQEQLVEAGR.L	2142.41919	2	1.99E-04	0.81	3.86	-	381.3
AHQ-1-9, 4582	K.VVAPTISSPVCQEQLVEAGR.L	2142.41919	2	2.48E-07	0.95	4.91	-	871.9
AHQ-1-4, 5110 - 5124	K.VVAPTISSPVCQEQLVEAGR.L	2142.41919	2	3.94E-10	0.85	3.96	-	410.3
AHQ-1-1, 5065	K.VVAPTISSPVCQEQLVEAGR.L	2142.41919	2	6.11E-12	0.93	4.93	-	478.1
AHQ-1-4, 4979	K.VVAPTISSPVCQEQLVEAGR.L	2142.41919	2	7.16E-12	0.92	4.15	-	604.5

AHQ-1-2, 5175 - 5178	K.VVAPTISSPVCQEQLVEAGR.L	2142.41919	2	9.72E-12	0.94	4.82	-	689.3
AHQ-1-4, 4795 - 4870	K.VVAPTISSPVCQEQLVEAGR.L	2142.41919	2	4.69E-07	0.96	5.34	-	691.0
AHQ-1-1, 4896	K.VVAPTISSPVCQEQLVEAGR.L	2142.41919	2	2.95E-05	0.52	2.75	-	261.9
AHQ-1-4, 4670	K.VVAPTISSPVCQEQLVEAGR.L	2142.41919	2	1.49E-08	0.91	4.57	-	408.0
AHQ-1-1, 4723	K.VVAPTISSPVCQEQLVEAGR.L	2142.41919	2	7.78E-05	0.92	4.21	-	717.2
AHQ-1-6, 4622 - 4623	K.VVAPTISSPVCQEQLVEAGR.L	2142.41919	2	1.72E-07	0.97	5.87	-	1027.9
AHQ-1-1, 4620	K.VVAPTISSPVCQEQLVEAGR.L	2142.41919	2	5.70E-07	0.91	4.55	-	415.3
AHQ-1-2, 5035 - 5049	K.VVAPTISSPVCQEQLVEAGR.L	2142.41919	2	4.54E-13	0.96	5.06	-	904.8
AHQ-1-14- , 4544	K.VVAPTISSPVCQEQLVEAGR.L	2142.41919	2	2.27E-04	0.55	2.76	-	309.7
AHQ-1-6, 4959	K.VVAPTISSPVCQEQLVEAGR.L	2142.41919	2	1.10E-04	0.39	2.76	-	235.2
AHQ-1-12, 4467	K.VVAPTISSPVCQEQLVEAGR.L	2142.41919	2	2.48E-07	0.95	5.06	-	738.4
AHQ-1-2, 4861 - 4930	K.VVAPTISSPVCQEQLVEAGR.L	2142.41919	2	1.09E-04	0.93	4.47	-	627.1
AHQ-1-3, 5042 - 5110	K.VVAPTISSPVCQEQLVEAGR.L	2142.41919	2	4.97E-04	0.64	3.52	-	407.3
AHQ-1-3, 4902 - 4971	K.VVAPTISSPVCQEQLVEAGR.L	2142.41919	2	4.42E-13	0.89	4.02	-	523.0
AHQ-1-3, 4741 - 4750	K.VVAPTISSPVCQEQLVEAGR.L	2142.41919	3	4.98E-10	0.91	4.48	-	968.1
AHQ-1-3, 4733	K.VVAPTISSPVCQEQLVEAGR.L	2142.41919	2	1.47E-05	0.93	4.49	-	649.0
AHQ-1-10, 4183	K.VVAPTISSPVCQEQLVEAGR.L	2142.41919	2	1.04E-04	0.84	3.71	-	370.2
AHQ-1-5, 5049	K.VVAPTISSPVCQEQLVEAGR.L	2142.41919	2	1.38E-05	0.77	3.81	-	364.1
AHQ-1-3, 4607	K.VVAPTISSPVCQEQLVEAGR.L	2142.41919	2	2.98E-04	0.66	3.78	-	118.0
AHQ-1-8, 4667	K.VVAPTISSPVCQEQLVEAGR.L	2142.41919	2	2.43E-08	0.91	4.53	-	555.7
gi|4501885|ref|NP_001092.1| beta actin; beta cytoskeletal actin [Homo sapiens]				5.55E-16	13.29	150.37	36.00	41736.5
AHQ-1-14- , 6012	R.CPEALFQPSFLGM*ESCGIHETTFNSIM*K.C	3267.67588	3	3.56E-05	0.91	4.68	-	499.6
AHQ-1-12, 5881 - 5907	R.CPEALFQPSFLGM*ESCGIHETTFNSIM*K.C	3267.67588	3	2.56E-05	0.65	3.38	-	347.6
AHQ-1-13, 5776 - 5832	R.CPEALFQPSFLGM*ESCGIHETTFNSIM*K.C	3267.67588	3	1.36E-09	0.94	5.05	-	843.8
AHQ-1-13, 6217 - 6237	R.CPEALFQPSFLGM*ESCGIHETTFNSIMK.C	3251.67648	3	3.80E-08	0.82	4.64	-	601.1
AHQ-1-10, 6228	R.CPEALFQPSFLGMESCGIHETTFNSIMK.C	3235.67708	3	4.33E-04	0.83	4.04	-	568.3
AHQ-1-10, 6141 - 6207	R.CPEALFQPSFLGMESCGIHETTFNSIMK.C	3235.67708	3	2.03E-05	0.78	3.51	-	515.4
AHQ-1-10, 5991 - 6063	R.CPEALFQPSFLGM*ESCGIHETTFNSIMK.C	3251.67648	3	4.08E-06	0.79	4.25	-	576.9
AHQ-1-10, 5553 - 5607	R.CPEALFQPSFLGM*ESCGIHETTFNSIM*K.C	3267.67588	3	2.82E-04	0.44	3.07	-	327.6
AHQ-1-9, 7036 - 7118	R.CPEALFQPSFLGMESCGIHETTFNSIMK.C	3235.67708	3	5.09E-07	0.96	5.68	-	1041.7
AHQ-1-9, 6730 - 6795	R.CPEALFQPSFLGM*ESCGIHETTFNSIMK.C	3251.67648	3	4.70E-04	0.67	4.04	-	330.6
AHQ-1-9, 6636 - 6714	R.CPEALFQPSFLGM*ESCGIHETTFNSIMK.C	3251.67648	3	5.44E-07	0.64	3.93	-	263.8
AHQ-1-8, 7599	R.CPEALFQPSFLGMESCGIHETTFNSIMK.C	3235.67708	3	1.44E-09	0.91	4.24	-	781.7
AHQ-1-8, 7311 - 7382	R.CPEALFQPSFLGMESCGIHETTFNSIMK.C	3235.67708	3	4.83E-14	0.96	5.41	-	1181.6
AHQ-1-8, 7222 - 7290	R.CPEALFQPSFLGMESCGIHETTFNSIMK.C	3235.67708	3	3.20E-12	0.96	5.37	-	1130.1
AHQ-1-8, 7088 - 7159	R.CPEALFQPSFLGMESCGIHETTFNSIMK.C	3235.67708	3	2.09E-07	0.94	5.12	-	864.8
AHQ-1-8, 7079 - 7146	R.CPEALFQPSFLGM*ESCGIHETTFNSIMK.C	3251.67648	3	7.52E-05	0.80	4.38	-	529.9
AHQ-1-8, 6846 - 6915	R.CPEALFQPSFLGM*ESCGIHETTFNSIMK.C	3251.67648	3	9.56E-08	0.80	4.47	-	590.3
AHQ-1-8, 6755 - 6822	R.CPEALFQPSFLGM*ESCGIHETTFNSIMK.C	3251.67648	3	1.11E-04	0.72	3.92	-	386.6
AHQ-1-8, 6554	R.CPEALFQPSFLGM*ESCGIHETTFNSIM*K.C	3267.67588	3	5.20E-04	0.73	3.82	-	452.2
AHQ-1-5, 7303	R.CPEALFQPSFLGMESCGIHETTFNSIMK.C	3235.67708	3	2.28E-05	0.85	3.74	-	542.5
AHQ-1-8, 6402 - 6471	R.CPEALFQPSFLGM*ESCGIHETTFNSIM*K.C	3267.67588	3	1.46E-06	0.94	4.89	-	938.6
AHQ-1-13- , 6157 - 6239	R.CPEALFQPSFLGM*ESCGIHETTFNSIM*K.C	3267.67588	3	6.61E-04	0.86	4.10	-	524.0
AHQ-1-13- , 6495 - 6560	R.CPEALFQPSFLGM*ESCGIHETTFNSIMK.C	3251.67648	3	1.01E-04	0.92	5.21	-	731.5
AHQ-1-13- , 6623 - 6683	R.CPEALFQPSFLGMESCGIHETTFNSIM*K.C	3251.67648	3	3.54E-05	0.81	4.45	-	545.3
AHQ-1-13- , 6757	R.CPEALFQPSFLGM*ESCGIHETTFNSIMK.C	3251.67648	3	2.42E-04	0.66	3.54	-	365.4
AHQ-1-8, 7522 - 7591	K.EKLCYVALDFEQEMATAASSSSLEK.S	2810.10410	2	5.24E-09	0.93	4.46	-	854.5
AHQ-1-8, 7487 - 7559	K.EKLCYVALDFEQEMATAASSSSLEK.S	2810.10410	3	3.09E-05	0.93	5.00	-	1187.0
AHQ-1-8, 7628	K.EKLCYVALDFEQEMATAASSSSLEK.S	2810.10410	3	1.00E-06	0.89	4.40	-	845.3
AHQ-1-8, 7658 - 7659	K.EKLCYVALDFEQEMATAASSSSLEK.S	2810.10410	2	6.14E-08	0.88	3.86	-	739.3
AHQ-1-9, 7099 - 7171	K.EKLCYVALDFEQEMATAASSSSLEK.S	2810.10410	2	1.66E-05	0.91	4.03	-	771.0
AHQ-1-8, 7250 - 7318	K.EKLCYVALDFEQEMATAASSSSLEK.S	2810.10410	2	7.71E-08	0.89	4.31	-	552.9
AHQ-1-8, 5583 - 5658	K.EKLCYVALDFEQEM*ATAASSSSLEK.S	2826.10350	3	1.88E-05	0.82	4.03	-	599.7
AHQ-1-4, 7310	K.EKLCYVALDFEQEMATAASSSSLEK.S	2810.10410	3	4.32E-04	0.75	3.83	-	788.6
AHQ-1-8, 7528	K.EKLCYVALDFEQEMATAASSSSLEK.S	2810.10410	2	1.96E-08	0.91	4.71	-	854.7
AHQ-1-8, 7336 - 7414	K.EKLCYVALDFEQEMATAASSSSLEK.S	2810.10410	3	2.74E-05	0.93	4.91	-	1325.8
AHQ-1-10, 6259 - 6260	K.EKLCYVALDFEQEMATAASSSSLEK.S	2810.10410	3	1.55E-06	0.94	5.21	-	1159.7
AHQ-1-10, 6240	K.EKLCYVALDFEQEMATAASSSSLEK.S	2810.10410	2	3.14E-06	0.56	2.77	-	638.5
AHQ-1-8, 7386 - 7454	K.EKLCYVALDFEQEMATAASSSSLEK.S	2810.10410	2	2.24E-09	0.89	3.69	-	759.5
AHQ-1-8, 7466 - 7534	R.FRCPEALFQPSFLGMESCGIHETTFNSIMK.C	3539.03813	3	5.63E-06	0.93	4.86	-	988.4
AHQ-1-8, 6738 - 6851	R.FRCPEALFQPSFLGMESCGIHETTFNSIM*K.C	3555.03753	3	1.63E-08	0.94	5.61	-	1089.3
AHQ-1-8, 7090 - 7155	R.FRCPEALFQPSFLGMESCGIHETTFNSIMK.C	3539.03813	3	5.57E-11	0.98	7.44	-	1997.5
AHQ-1-13- , 6581 - 6645	R.FRCPEALFQPSFLGMESCGIHETTFNSIM*K.C	3555.03753	3	2.34E-06	0.88	4.42	-	704.2
AHQ-1-10, 5888 - 5959	R.FRCPEALFQPSFLGMESCGIHETTFNSIM*K.C	3555.03753	3	2.74E-05	0.81	4.65	-	746.2
AHQ-1-8, 2395 - 2467	R.GYSFTTTAER.E	1133.19273	2	4.57E-04	0.91	3.02	-	1158.8
AHQ-1-13- , 2624 - 2663	R.GYSFTTTAER.E	1133.19273	2	1.08E-04	0.91	3.45	-	971.2
AHQ-1-10, 2391 - 2463	R.GYSFTTTAER.E	1133.19273	2	4.47E-04	0.91	3.34	-	1015.5
AHQ-1-11, 2480 - 2490	R.GYSFTTTAER.E	1133.19273	2	6.89E-05	0.94	3.09	-	1278.2
AHQ-1-5, 2429	R.GYSFTTTAER.E	1133.19273	2	7.21E-05	0.89	3.13	-	980.5
AHQ-1-6, 2443 - 2494	R.GYSFTTTAER.E	1133.19273	2	3.37E-04	0.82	2.84	-	862.1
AHQ-1-14- , 2523 - 2527	R.GYSFTTTAER.E	1133.19273	2	5.34E-04	0.89	3.26	-	987.1
AHQ-1-3, 2533	R.GYSFTTTAER.E	1133.19273	2	1.10E-04	0.93	3.07	-	1254.2
AHQ-1-9, 2374 - 2446	R.GYSFTTTAER.E	1133.19273	2	7.58E-04	0.91	3.18	-	945.1
AHQ-1-2, 2627	R.GYSFTTTAER.E	1133.19273	2	5.22E-04	0.91	3.02	-	1184.4
AHQ-1-13, 2641 - 2661	R.GYSFTTTAER.E	1133.19273	2	2.52E-05	0.87	2.97	-	797.4
AHQ-1-8, 2281 - 2325	R.GYSFTTTAER.E	1133.19273	2	1.06E-04	0.91	3.15	-	1233.1
AHQ-1-7, 2473	R.GYSFTTTAER.E	1133.19273	2	9.61E-04	0.85	3.08	-	842.4
AHQ-1-13, 4607	R.KDLYANTVLSGGTTMYPGIADR.M	2344.62976	2	1.71E-05	0.91	3.96	-	766.7
AHQ-1-10, 3949 - 3959	R.KDLYANTVLSGGTTM*YPGIADR.M	2360.62916	2	3.94E-04	0.95	4.51	-	1159.2
AHQ-1-10, 4307	R.KDLYANTVLSGGTTMYPGIADR.M	2344.62976	2	1.68E-06	0.78	2.91	-	636.2
AHQ-1-8, 4730 - 4759	R.KDLYANTVLSGGTTMYPGIADR.M	2344.62976	2	9.17E-06	0.96	4.42	-	1395.6
AHQ-1-8, 4324	R.KDLYANTVLSGGTTM*YPGIADR.M	2360.62916	3	5.50E-05	0.96	4.80	-	1607.7
AHQ-1-14- , 4699 - 4752	R.KDLYANTVLSGGTTMYPGIADR.M	2344.62976	2	5.24E-04	0.72	2.93	-	526.1
AHQ-1-13, 4156	R.KDLYANTVLSGGTTM*YPGIADR.M	2360.62916	2	3.60E-05	0.63	2.65	-	571.3
AHQ-1-13- , 4369	R.KDLYANTVLSGGTTM*YPGIADR.M	2360.62916	2	4.92E-04	0.82	3.04	-	688.8
AHQ-1-10, 6495 - 6531	K.LCYVALDFEQEMATAASSSSLEK.S	2552.81665	2	5.91E-05	0.90	3.50	-	1003.7
AHQ-1-9, 7374 - 7444	K.LCYVALDFEQEMATAASSSSLEK.S	2552.81665	3	6.07E-06	0.96	5.55	-	1708.3
AHQ-1-10, 6219 - 6277	K.LCYVALDFEQEMATAASSSSLEK.S	2552.81665	2	1.65E-06	0.97	5.14	-	1852.8
AHQ-1-5, 5779	R.TTGIVM*DSGDGVTHTVPIYEGYALPHAILR.L	3201.59868	3	5.18E-06	0.93	4.80	-	1048.3
AHQ-1-5, 6050	R.TTGIVMDSGDGVTHTVPIYEGYALPHAILR.L	3185.59928	3	6.22E-08	0.97	5.95	-	1854.3
AHQ-1-5, 6147	R.TTGIVMDSGDGVTHTVPIYEGYALPHAILR.L	3185.59928	3	7.95E-10	0.97	5.23	-	2259.1
AHQ-1-8, 6204 - 6272	R.TTGIVMDSGDGVTHTVPIYEGYALPHAILR.L	3185.59928	3	3.98E-07	0.97	5.96	-	1838.7
AHQ-1-9, 5390 - 5410	R.TTGIVM*DSGDGVTHTVPIYEGYALPHAILR.L	3201.59868	3	6.05E-09	0.97	6.23	-	1392.1
AHQ-1-9, 5478 - 5550	R.TTGIVM*DSGDGVTHTVPIYEGYALPHAILR.L	3201.59868	3	4.62E-09	0.97	5.72	-	1976.5
AHQ-1-9, 5715 - 5795	R.TTGIVMDSGDGVTHTVPIYEGYALPHAILR.L	3185.59928	3	3.85E-12	0.96	6.17	-	1189.7
AHQ-1-9, 5851 - 5931	R.TTGIVMDSGDGVTHTVPIYEGYALPHAILR.L	3185.59928	3	2.00E-11	0.97	6.22	-	1465.4
AHQ-1-9, 5926 - 6004	R.TTGIVM*DSGDGVTHTVPIYEGYALPHAILR.L	3201.59868	3	9.24E-05	0.68	3.85	-	788.7
AHQ-1-9, 5998 - 6072	R.TTGIVMDSGDGVTHTVPIYEGYALPHAILR.L	3185.59928	3	9.01E-09	0.94	5.22	-	1182.4
AHQ-1-4, 5918	R.TTGIVM*DSGDGVTHTVPIYEGYALPHAILR.L	3201.59868	3	5.57E-07	0.93	4.78	-	1111.8
AHQ-1-4, 5819	R.TTGIVM*DSGDGVTHTVPIYEGYALPHAILR.L	3201.59868	3	7.37E-08	0.96	6.09	-	1089.4
AHQ-1-3, 6073	R.TTGIVMDSGDGVTHTVPIYEGYALPHAILR.L	3185.59928	3	8.10E-07	0.93	4.74	-	1066.2
AHQ-1-5, 5646	R.TTGIVM*DSGDGVTHTVPIYEGYALPHAILR.L	3201.59868	3	2.03E-06	0.92	5.16	-	723.6
AHQ-1-4, 6126	R.TTGIVMDSGDGVTHTVPIYEGYALPHAILR.L	3185.59928	3	1.93E-06	0.96	5.95	-	1329.5
AHQ-1-8, 5934 - 5998	R.TTGIVMDSGDGVTHTVPIYEGYALPHAILR.L	3185.59928	3	3.15E-11	0.98	6.82	-	1772.2
AHQ-1-8, 5796 - 5866	R.TTGIVMDSGDGVTHTVPIYEGYALPHAILR.L	3185.59928	3	5.55E-16	0.96	5.79	-	1562.0
AHQ-1-14, 6444	R.TTGIVM*DSGDGVTHTVPIYEGYALPHAILR.L	3201.59868	3	1.27E-06	0.78	4.13	-	649.3
AHQ-1-8, 5714 - 5782	R.TTGIVMDSGDGVTHTVPIYEGYALPHAILR.L	3185.59928	3	1.34E-13	0.98	6.78	-	2008.9

AHQ-1-8, 5466 - 5530	R.TTGIVM*DSGDGVTHTVPIYEGYALPHAILR.L	3201.59868	3	2.03E-09	0.96	5.76	-	1264.9
AHQ-1-10, 4857 - 4915	R.TTGIVM*DSGDGVTHTVPIYEGYALPHAILR.L	3201.59868	3	9.54E-08	0.95	5.17	-	1493.0
AHQ-1-10, 4975 - 5031	R.TTGIVM*DSGDGVTHTVPIYEGYALPHAILR.L	3201.59868	3	2.32E-10	0.98	6.81	-	1764.1
AHQ-1-10, 5191 - 5247	R.TTGIVMDSGDGVTHTVPIYEGYALPHAILR.L	3185.59928	3	6.00E-11	0.97	5.98	-	1747.2
AHQ-1-10, 5303 - 5359	R.TTGIVMDSGDGVTHTVPIYEGYALPHAILR.L	3185.59928	3	4.66E-10	0.97	6.37	-	1552.5
AHQ-1-3, 5733 - 5805	R.TTGIVM*DSGDGVTHTVPIYEGYALPHAILR.L	3201.59868	3	1.60E-06	0.96	5.53	-	1378.2
AHQ-1-8, 5370 - 5398	R.TTGIVM*DSGDGVTHTVPIYEGYALPHAILR.L	3201.59868	3	2.35E-08	0.96	5.93	-	1166.9
AHQ-1-8, 5287 - 5320	R.TTGIVM*DSGDGVTHTVPIYEGYALPHAILR.L	3201.59868	3	5.67E-06	0.61	3.16	-	605.2
AHQ-1-2, 6187 - 6253	R.TTGIVMDSGDGVTHTVPIYEGYALPHAILR.L	3185.59928	3	1.96E-06	0.94	5.46	-	914.2
AHQ-1-8, 6066 - 6131	R.TTGIVMDSGDGVTHTVPIYEGYALPHAILR.L	3185.59928	3	7.65E-11	0.96	5.87	-	1544.9
AHQ-1-2, 5913 - 5982	R.TTGIVM*DSGDGVTHTVPIYEGYALPHAILR.L	3201.59868	3	9.10E-05	0.96	5.84	-	1468.7
AHQ-1-13- , 5560 - 5636	R.TTGIVM*DSGDGVTHTVPIYEGYALPHAILR.L	3201.59868	3	6.25E-09	0.97	5.83	-	2136.4
AHQ-1-13- , 5823 - 5903	R.TTGIVMDSGDGVTHTVPIYEGYALPHAILR.L	3185.59928	3	2.78E-15	0.97	5.80	-	1954.5
AHQ-1-14- , 5419 - 5484	R.TTGIVM*DSGDGVTHTVPIYEGYALPHAILR.L	3201.59868	3	1.09E-05	0.95	5.35	-	1037.1
AHQ-1-6, 6034 - 6042	R.TTGIVMDSGDGVTHTVPIYEGYALPHAILR.L	3185.59928	3	5.51E-12	0.97	5.91	-	1376.6
AHQ-1-14, 6270 - 6324	R.TTGIVM*DSGDGVTHTVPIYEGYALPHAILR.L	3201.59868	3	8.39E-08	0.98	6.88	-	2224.4
AHQ-1-11, 5098	R.TTGIVM*DSGDGVTHTVPIYEGYALPHAILR.L	3201.59868	3	2.96E-13	0.97	6.55	-	1618.1
AHQ-1-11, 5186	R.TTGIVM*DSGDGVTHTVPIYEGYALPHAILR.L	3201.59868	3	4.36E-09	0.95	5.34	-	1283.5
AHQ-1-11, 5308 - 5368	R.TTGIVMDSGDGVTHTVPIYEGYALPHAILR.L	3185.59928	3	6.24E-07	0.93	4.79	-	1205.6
AHQ-1-11, 5446	R.TTGIVMDSGDGVTHTVPIYEGYALPHAILR.L	3185.59928	3	1.41E-08	0.96	5.41	-	1600.2
AHQ-1-7, 6130	R.TTGIVMDSGDGVTHTVPIYEGYALPHAILR.L	3185.59928	3	1.26E-09	0.95	5.35	-	1425.5
AHQ-1-1, 6001 - 6075	R.TTGIVMDSGDGVTHTVPIYEGYALPHAILR.L	3185.59928	3	1.28E-12	0.95	5.16	-	1499.9
AHQ-1-12, 5321 - 5383	R.TTGIVM*DSGDGVTHTVPIYEGYALPHAILR.L	3201.59868	3	4.76E-08	0.93	4.71	-	1094.3
AHQ-1-12, 5654 - 5714	R.TTGIVMDSGDGVTHTVPIYEGYALPHAILR.L	3185.59928	3	4.59E-10	0.91	4.98	-	653.5
AHQ-1-1, 5751 - 5820	R.TTGIVM*DSGDGVTHTVPIYEGYALPHAILR.L	3201.59868	3	3.45E-08	0.97	5.95	-	1514.8
AHQ-1-7, 5978 - 6052	R.TTGIVMDSGDGVTHTVPIYEGYALPHAILR.L	3185.59928	3	2.32E-07	0.74	3.39	-	810.1
AHQ-1-13, 5611	R.TTGIVMDSGDGVTHTVPIYEGYALPHAILR.L	3185.59928	3	8.09E-10	0.95	5.65	-	992.9
AHQ-1-13, 5472 - 5547	R.TTGIVMDSGDGVTHTVPIYEGYALPHAILR.L	3185.59928	3	2.18E-11	0.97	5.93	-	1787.5
AHQ-1-7, 5620 - 5689	R.TTGIVM*DSGDGVTHTVPIYEGYALPHAILR.L	3201.59868	3	1.30E-07	0.94	5.26	-	1053.9
AHQ-1-14- , 5678 - 5758	R.TTGIVMDSGDGVTHTVPIYEGYALPHAILR.L	3185.59928	3	9.75E-12	0.97	5.46	-	1924.4
AHQ-1-13, 5245 - 5308	R.TTGIVM*DSGDGVTHTVPIYEGYALPHAILR.L	3201.59868	3	3.44E-08	0.97	5.77	-	1664.3
AHQ-1-13, 3919	R.VAPEEHPVLLTEAPLNPK.A	1955.24351	3	1.43E-04	0.52	3.05	-	519.5
AHQ-1-13, 3883 - 3943	R.VAPEEHPVLLTEAPLNPK.A	1955.24351	2	1.32E-05	0.94	4.62	-	960.2
AHQ-1-12, 3801 - 3873	R.VAPEEHPVLLTEAPLNPK.A	1955.24351	3	3.82E-05	0.87	4.87	-	734.8
AHQ-1-12, 3793 - 3861	R.VAPEEHPVLLTEAPLNPK.A	1955.24351	2	1.30E-06	0.92	4.64	-	712.4
AHQ-1-14, 4824	R.VAPEEHPVLLTEAPLNPK.A	1955.24351	2	2.23E-07	0.90	4.04	-	721.9
AHQ-1-11, 3676	R.VAPEEHPVLLTEAPLNPK.A	1955.24351	2	7.14E-06	0.92	4.03	-	905.9
AHQ-1-10, 3587 - 3647	R.VAPEEHPVLLTEAPLNPK.A	1955.24351	3	8.43E-05	0.84	4.34	-	1046.6
AHQ-1-10, 3584 - 3643	R.VAPEEHPVLLTEAPLNPK.A	1955.24351	2	1.52E-09	0.91	4.55	-	757.8
AHQ-1-13- , 4043 - 4100	R.VAPEEHPVLLTEAPLNPK.A	1955.24351	3	9.63E-04	0.75	3.99	-	880.6
AHQ-1-13- , 4044 - 4113	R.VAPEEHPVLLTEAPLNPK.A	1955.24351	2	6.78E-10	0.94	4.55	-	1099.6
AHQ-1-9, 3606 - 3680	R.VAPEEHPVLLTEAPLNPK.A	1955.24351	2	7.21E-07	0.93	4.20	-	992.7
AHQ-1-8, 3872 - 3947	R.VAPEEHPVLLTEAPLNPK.A	1955.24351	2	3.51E-06	0.94	4.67	-	963.2
AHQ-1-8, 3847 - 3856	R.VAPEEHPVLLTEAPLNPK.A	1955.24351	2	2.25E-09	0.93	4.81	-	785.2
AHQ-1-8, 3717 - 3787	R.VAPEEHPVLLTEAPLNPK.A	1955.24351	2	5.07E-07	0.94	4.71	-	932.8
AHQ-1-8, 3572 - 3641	R.VAPEEHPVLLTEAPLNPK.A	1955.24351	2	1.18E-05	0.94	4.84	-	951.4
AHQ-1-8, 3556 - 3637	R.VAPEEHPVLLTEAPLNPK.A	1955.24351	3	9.23E-04	0.72	4.03	-	586.1
AHQ-1-8, 3476 - 3548	R.VAPEEHPVLLTEAPLNPK.A	1955.24351	2	2.18E-04	0.93	4.56	-	750.2
AHQ-1-7, 3900	R.VAPEEHPVLLTEAPLNPK.A	1955.24351	2	1.42E-04	0.88	3.92	-	830.5
AHQ-1-6, 3823	R.VAPEEHPVLLTEAPLNPK.A	1955.24351	3	1.87E-04	0.75	3.72	-	792.7
AHQ-1-14- , 3944 - 3963	R.VAPEEHPVLLTEAPLNPK.A	1955.24351	2	4.26E-05	0.91	4.39	-	928.1
AHQ-1-6, 3822	R.VAPEEHPVLLTEAPLNPK.A	1955.24351	2	3.14E-06	0.87	4.00	-	665.1
AHQ-1-5, 3893 - 3894	R.VAPEEHPVLLTEAPLNPK.A	1955.24351	2	1.50E-05	0.89	3.94	-	665.0
AHQ-1-9, 3750 - 3818	R.VAPEEHPVLLTEAPLNPK.A	1955.24351	2	7.75E-06	0.90	4.21	-	643.1
AHQ-1-1, 4133	R.VAPEEHPVLLTEAPLNPK.A	1955.24351	2	4.12E-06	0.89	3.82	-	815.1
gi|4507949|ref|NP_003395.1| tyrosine 3-monooxygenase/tryptophan 5-monooxygenase activation protein,				5.55E-16	4.54	50.28	30.10	28082.2
AHQ-1-13- , 1960	K.AVTEQGHELSNEER.N	1599.64147	2	1.16E-04	0.75	3.03	-	548.9
AHQ-1-10, 1796 - 1851	K.AVTEQGHELSNEER.N	1599.64147	2	8.66E-06	0.90	3.70	-	830.0
AHQ-1-13- , 4227 - 4237	K.QTTVSNSQQAYQEAFEISK.K	2160.28351	2	5.38E-07	0.76	4.00	-	385.1
AHQ-1-10, 3815 - 3816	K.QTTVSNSQQAYQEAFEISK.K	2160.28351	2	4.16E-08	0.92	4.80	-	607.5
AHQ-1-10, 4011 - 4016	K.QTTVSNSQQAYQEAFEISK.K	2160.28351	2	3.60E-09	0.96	5.58	-	1147.2
AHQ-1-13, 6319	K.TAFDEAIAELDTLNEESYK.D	2160.27705	2	1.44E-07	0.93	4.65	-	937.8
AHQ-1-13- , 6771 - 6828	K.TAFDEAIAELDTLNEESYK.D	2160.27705	2	5.94E-05	0.97	5.28	-	1334.4
AHQ-1-10, 6135 - 6208	K.TAFDEAIAELDTLNEESYK.D	2160.27705	2	8.00E-06	0.93	4.36	-	918.8
AHQ-1-10, 6144	K.TAFDEAIAELDTLNEESYK.D	2160.27705	3	5.64E-04	0.95	4.16	-	1840.7
AHQ-1-10, 6411	K.TAFDEAIAELDTLNEESYK.D	2160.27705	2	4.35E-04	0.57	3.16	-	356.8
AHQ-1-14- , 6530	K.TAFDEAIAELDTLNEESYK.D	2160.27705	2	3.89E-10	0.96	5.19	-	1145.9
AHQ-1-10, 6464	K.TAFDEAIAELDTLNEESYKDSTLIM*QLLR.D	3347.69249	3	5.55E-16	0.96	5.23	-	1922.7
AHQ-1-13- , 3004 - 3013	K.YLIPNATQPESK.V	1361.52512	2	3.70E-06	0.88	3.83	-	641.6
AHQ-1-10, 2739 - 2805	K.YLIPNATQPESK.V	1361.52512	2	1.71E-05	0.84	3.29	-	573.2
AHQ-1-10, 2737	K.YLIPNATQPESK.V	1361.52512	1	1.84E-05	0.83	3.56	-	642.4
AHQ-1-10, 2821	K.YLIPNATQPESK.V	1361.52512	1	9.71E-05	0.21	1.89	-	443.5
AHQ-1-10, 2913	K.YLIPNATQPESK.V	1361.52512	1	5.25E-08	0.75	2.43	-	835.8
gi|5729770|ref|NP_000382.3| ceroid-lipofuscinosis, neuronal 2, late infantile (Jansky-Bielschowsky				5.55E-16	0.96	10.25	4.80	61247.6
AHQ-1-8, 7468	R.VPIPWVSGTSASTPVFGGILSLINEHR.I	2836.23725	3	5.55E-16	0.96	4.91	-	1666.3
gi|9507215|ref|NP_061816.1| tubulin, alpha-like 2; tubulin, alpha 8 [Homo sapiens]				1.11E-15	4.32	50.22	13.10	50093.2
AHQ-1-7, 5473 - 5496	R.AVMIDLEPTVVDEVR.A	1686.95185	2	2.90E-04	0.76	3.45	-	643.9
AHQ-1-14- , 4920	R.AVM*IDLEPTVVDEVR.A	1702.95125	2	3.97E-07	0.92	4.33	-	792.7
AHQ-1-7, 3553	K.DVNVAIAAIK.T	1014.20080	2	1.68E-06	0.93	3.38	-	1233.9
AHQ-1-7, 5698 - 5733	R.IHFPLVTYAPIISAEK.A	1800.13312	2	2.77E-07	0.81	3.22	-	461.1
AHQ-1-9, 5554	R.IHFPLVTYAPIISAEK.A	1800.13312	2	9.36E-04	0.85	3.57	-	450.7
AHQ-1-14, 6189	K.INDDDSFTTFFSETGNGK.H	1996.03358	2	3.12E-10	0.94	4.34	-	994.9
AHQ-1-7, 5550 - 5626	K.INDDDSFTTFFSETGNGK.H	1996.03358	2	2.44E-11	0.87	3.86	-	669.1
AHQ-1-14- , 5286	K.INDDDSFTTFFSETGNGK.H	1996.03358	2	1.11E-15	0.91	3.98	-	799.7
AHQ-1-13, 5144	K.INDDDSFTTFFSETGNGK.H	1996.03358	2	1.49E-07	0.67	3.50	-	306.0
gi|4504517|ref|NP_001531.1| heat shock 27kDa protein 1; heat shock 27kD protein 1 [Homo sapiens] [M				1.11E-15	1.91	20.34	26.30	22782.3
AHQ-1-11, 6356	R.LPEEWSQWLGGSSWPGYVRPLPPAAIESPAVAAPAYSR.A	4095.56787	3	1.11E-15	0.97	6.87	-	1219.1
AHQ-1-13, 4520	R.VSLDVNHFAPDELTVK.T	1784.99058	2	3.18E-04	0.91	3.83	-	1046.6
AHQ-1-10, 4308	R.VSLDVNHFAPDELTVK.T	1784.99058	2	7.27E-08	0.93	4.18	-	985.9
gi|4503745|ref|NP_001447.1| filamin 1 (actin-binding protein-280); filamin 1; filamin A, alpha (act				1.67E-15	103.52	1210.36	56.00	280757.4
AHQ-1-4, 5438 - 5507	R.AEAGVPAEFSIWTR.E	1534.69735	2	2.98E-09	0.88	3.92	-	483.3
AHQ-1-5, 5341 - 5414	R.AEAGVPAEFSIWTR.E	1534.69735	2	4.65E-09	0.93	4.19	-	708.1
AHQ-1-1, 2424	R.AEFTVETR.S	953.03118	2	2.21E-04	0.87	2.78	-	1018.1
AHQ-1-5, 2147	R.AEFTVETR.S	953.03118	2	7.02E-04	0.87	2.72	-	983.7
AHQ-1-6, 2430	K.AEISFEDR.K	967.01472	2	3.74E-04	0.78	2.61	-	961.8
AHQ-1-1, 2664	K.AEISFEDR.K	967.01472	2	5.01E-04	0.88	3.02	-	1032.3
AHQ-1-4, 2642	K.AFGPGLQGGSAGSPAR.F	1430.55077	2	2.41E-06	0.92	3.30	-	1356.5
AHQ-1-9, 2531	K.AFGPGLQGGSAGSPAR.F	1430.55077	2	5.78E-05	0.88	2.68	-	1446.2
AHQ-1-7, 2584	K.AFGPGLQGGSAGSPAR.F	1430.55077	2	1.01E-05	0.84	2.81	-	931.4
AHQ-1-1, 2700 - 2771	K.AFGPGLQGGSAGSPAR.F	1430.55077	2	4.11E-10	0.93	3.37	-	1263.3
AHQ-1-1, 2915 - 2996	K.AFGPGLQGGSAGSPAR.F	1430.55077	2	4.16E-06	0.93	3.45	-	1302.9
AHQ-1-3, 2625 - 2695	K.AFGPGLQGGSAGSPAR.F	1430.55077	2	4.11E-06	0.92	3.02	-	1294.6
AHQ-1-5, 2538	K.AFGPGLQGGSAGSPAR.F	1430.55077	2	6.36E-05	0.93	2.73	-	2041.5
AHQ-1-2, 2711 - 2781	K.AFGPGLQGGSAGSPAR.F	1430.55077	2	7.59E-07	0.95	3.71	-	1579.8
AHQ-1-8, 2465	K.AFGPGLQGGSAGSPAR.F	1430.55077	2	1.93E-05	0.90	3.39	-	1105.6
AHQ-1-6, 2552 - 2580	K.AFGPGLQGGSAGSPAR.F	1430.55077	2	4.36E-08	0.96	3.38	-	1823.6
AHQ-1-14, 4396	K.AGNNMLLVGVHGPR.T	1435.68033	2	5.16E-07	0.74	2.93	-	572.3

AHQ-1-14, 4173	K.AGNNMLLVGVHGPR.T	1435.68033	3	1.27E-07	0.79	3.12	-	1108.1
AHQ-1-14- , 2908	K.AGNNM*LLVGVHGPR.T	1451.67973	2	1.55E-06	0.80	2.82	-	626.8
AHQ-1-14- , 2912 - 2934	K.AGNNM*LLVGVHGPR.T	1451.67973	3	1.29E-06	0.86	3.48	-	1098.5
AHQ-1-14, 4148	K.AGNNMLLVGVHGPR.T	1435.68033	2	2.92E-05	0.84	3.23	-	929.6
AHQ-1-14- , 3458 - 3482	K.AGNNMLLVGVHGPR.T	1435.68033	3	2.25E-08	0.92	4.47	-	1137.6
AHQ-1-14- , 3454 - 3504	K.AGNNMLLVGVHGPR.T	1435.68033	2	5.13E-09	0.85	3.35	-	739.4
AHQ-1-14, 3812	K.AGNNM*LLVGVHGPR.T	1451.67973	3	3.62E-07	0.94	3.58	-	1931.6
AHQ-1-14, 3638 - 3712	K.AGNNM*LLVGVHGPR.T	1451.67973	2	3.74E-05	0.78	3.03	-	710.4
AHQ-1-14- , 2782 - 2846	K.AGNNM*LLVGVHGPR.T	1451.67973	3	3.20E-06	0.90	3.90	-	1125.6
AHQ-1-5, 3123	K.AGNNMLLVGVHGPR.T	1435.68033	3	6.10E-06	0.90	3.29	-	1767.3
AHQ-1-14- , 3206 - 3211	K.AGNNMLLVGVHGPR.T	1435.68033	2	7.65E-06	0.76	3.27	-	552.0
AHQ-1-14- , 3227	K.AGNNMLLVGVHGPR.T	1435.68033	1	2.21E-07	0.45	2.29	-	126.1
AHQ-1-14- , 2778 - 2843	K.AGNNM*LLVGVHGPR.T	1451.67973	2	1.32E-04	0.88	3.60	-	881.9
AHQ-1-14- , 3207	K.AGNNMLLVGVHGPR.T	1435.68033	3	2.25E-08	0.92	3.79	-	1532.3
AHQ-1-2, 1761 - 1838	R.AGQSAAGAAPGGGVDTR.D	1443.50471	2	4.90E-04	0.80	3.53	-	607.1
AHQ-1-4, 1668	R.AGQSAAGAAPGGGVDTR.D	1443.50471	2	5.61E-05	0.53	2.92	-	653.5
AHQ-1-5, 2170	K.AGVAPLQVK.V	883.07036	2	1.35E-04	0.63	2.65	-	442.2
AHQ-1-4, 2304	K.AGVAPLQVK.V	883.07036	2	3.27E-04	0.63	2.54	-	529.1
AHQ-1-3, 2379	K.AGVAPLQVK.V	883.07036	1	7.75E-04	0.28	1.95	-	480.4
AHQ-1-1, 2451 - 2485	K.AGVAPLQVK.V	883.07036	2	1.18E-04	0.69	2.51	-	513.1
AHQ-1-3, 2310 - 2311	K.AGVAPLQVK.V	883.07036	2	2.98E-05	0.89	3.16	-	737.1
AHQ-1-2, 7474 - 7534	K.AHEPTYFTVDCAEAGQGDVSIGIK.C	2567.76960	3	4.69E-05	0.97	6.18	-	1447.3
AHQ-1-2, 6747	K.AHEPTYFTVDCAEAGQGDVSIGIK.C	2567.76960	3	1.70E-04	0.87	3.79	-	1098.0
AHQ-1-4, 2651 - 2663	K.AHVVPCFDASK.V	1232.39073	2	2.64E-04	0.70	2.61	-	503.7
AHQ-1-3, 2633	K.AHVVPCFDASK.V	1232.39073	2	5.38E-05	0.70	2.58	-	507.4
AHQ-1-1, 2787 - 2805	K.AHVVPCFDASK.V	1232.39073	2	6.17E-08	0.86	2.87	-	851.0
AHQ-1-2, 2726	K.AHVVPCFDASK.V	1232.39073	2	1.73E-04	0.66	2.63	-	549.2
AHQ-1-4, 6498 - 6570	R.ALGALVDSCAPGLCPDWDSWDASKPVTNAR.E	3233.53437	3	1.67E-15	0.95	5.66	-	958.5
AHQ-1-7, 6437 - 6440	R.ALGALVDSCAPGLCPDWDSWDASKPVTNAR.E	3233.53437	3	1.24E-06	0.94	5.42	-	811.5
AHQ-1-6, 6227 - 6306	R.ALGALVDSCAPGLCPDWDSWDASKPVTNAR.E	3233.53437	3	4.56E-12	0.95	5.70	-	885.5
AHQ-1-4, 6362 - 6427	R.ALGALVDSCAPGLCPDWDSWDASKPVTNAR.E	3233.53437	3	1.31E-05	0.66	3.53	-	408.7
AHQ-1-6, 6318 - 6382	R.ALGALVDSCAPGLCPDWDSWDASKPVTNAR.E	3233.53437	3	7.42E-11	0.94	5.41	-	784.8
AHQ-1-9, 6366	R.ALGALVDSCAPGLCPDWDSWDASKPVTNAR.E	3233.53437	3	1.47E-07	0.88	4.43	-	646.8
AHQ-1-1, 6116 - 6183	R.ALGALVDSCAPGLCPDWDSWDASKPVTNAR.E	3233.53437	3	7.26E-06	0.89	4.70	-	666.0
AHQ-1-3, 6330 - 6397	R.ALGALVDSCAPGLCPDWDSWDASKPVTNAR.E	3233.53437	3	2.98E-06	0.90	4.64	-	898.5
AHQ-1-1, 6247 - 6307	R.ALGALVDSCAPGLCPDWDSWDASKPVTNAR.E	3233.53437	3	1.63E-06	0.88	4.59	-	529.4
AHQ-1-1, 6367 - 6431	R.ALGALVDSCAPGLCPDWDSWDASKPVTNAR.E	3233.53437	3	1.97E-06	0.88	4.88	-	453.0
AHQ-1-2, 6286 - 6337	R.ALGALVDSCAPGLCPDWDSWDASKPVTNAR.E	3233.53437	3	3.44E-05	0.93	5.27	-	766.3
AHQ-1-3, 6213 - 6257	R.ALGALVDSCAPGLCPDWDSWDASKPVTNAR.E	3233.53437	3	6.16E-08	0.87	4.52	-	506.6
AHQ-1-5, 6518 - 6534	R.ALGALVDSCAPGLCPDWDSWDASKPVTNAR.E	3233.53437	3	1.04E-12	0.89	4.67	-	497.6
AHQ-1-10, 5663	R.ALGALVDSCAPGLCPDWDSWDASKPVTNAR.E	3233.53437	3	5.77E-09	0.60	3.16	-	325.7
AHQ-1-3, 6463 - 6542	R.ALGALVDSCAPGLCPDWDSWDASKPVTNAR.E	3233.53437	3	3.96E-09	0.87	4.21	-	748.0
AHQ-1-5, 6363 - 6442	R.ALGALVDSCAPGLCPDWDSWDASKPVTNAR.E	3233.53437	3	1.64E-08	0.95	5.84	-	951.2
AHQ-1-2, 6622 - 6699	R.ALGALVDSCAPGLCPDWDSWDASKPVTNAR.E	3233.53437	3	1.10E-05	0.92	5.12	-	512.8
AHQ-1-8, 6406 - 6482	R.ALGALVDSCAPGLCPDWDSWDASKPVTNAR.E	3233.53437	3	3.29E-07	0.87	4.43	-	486.4
AHQ-1-2, 6421 - 6489	R.ALGALVDSCAPGLCPDWDSWDASKPVTNAR.E	3233.53437	3	6.34E-10	0.95	5.32	-	993.4
AHQ-1-5, 6277	R.ALGALVDSCAPGLCPDWDSWDASKPVTNAR.E	3233.53437	3	4.53E-06	0.93	5.12	-	817.1
AHQ-1-2, 6529 - 6593	R.ALGALVDSCAPGLCPDWDSWDASKPVTNAR.E	3233.53437	3	3.44E-11	0.96	6.19	-	937.6
AHQ-1-9, 6282	R.ALGALVDSCAPGLCPDWDSWDASKPVTNAR.E	3233.53437	3	5.99E-11	0.94	5.89	-	625.5
AHQ-1-2, 1506	R.ALTQTGGPHVK.A	1109.26067	1	5.11E-05	0.05	1.89	-	169.8
AHQ-1-2, 1542 - 1602	R.ALTQTGGPHVK.A	1109.26067	1	6.28E-04	0.02	1.91	-	224.1
AHQ-1-3, 1547	R.ALTQTGGPHVK.A	1109.26067	2	9.04E-04	0.72	2.93	-	531.1
AHQ-1-4, 3375 - 3396	R.ANLPQSFQVDTSK.A	1435.56409	2	3.87E-07	0.70	2.77	-	367.5
AHQ-1-5, 3254 - 3329	R.ANLPQSFQVDTSK.A	1435.56409	2	3.24E-08	0.93	3.89	-	679.9
AHQ-1-2, 3374 - 3449	R.ANLPQSFQVDTSK.A	1435.56409	2	5.69E-09	0.91	3.55	-	658.6
AHQ-1-1, 3592 - 3659	R.ANLPQSFQVDTSK.A	1435.56409	2	9.58E-09	0.89	3.61	-	597.8
AHQ-1-3, 3339 - 3411	R.ANLPQSFQVDTSK.A	1435.56409	2	3.44E-05	0.88	3.43	-	648.2
AHQ-1-9, 3162	R.ANLPQSFQVDTSK.A	1435.56409	2	1.17E-05	0.79	2.93	-	533.2
AHQ-1-9, 3171	R.ANLPQSFQVDTSK.A	1435.56409	1	4.89E-04	0.52	2.64	-	390.5
AHQ-1-2, 3654	R.ANLPQSFQVDTSK.A	1435.56409	1	1.12E-07	0.33	2.62	-	334.0
AHQ-1-7, 3281	R.ANLPQSFQVDTSK.A	1435.56409	1	2.44E-06	0.53	2.66	-	450.5
AHQ-1-2, 3462	R.ANLPQSFQVDTSK.A	1435.56409	1	8.70E-06	0.78	3.30	-	489.6
AHQ-1-3, 3351 - 3353	R.ANLPQSFQVDTSK.A	1435.56409	1	1.51E-04	0.33	2.20	-	368.5
AHQ-1-1, 3552	R.APSVANVGSHCDLSLK.I	1656.84249	2	6.68E-05	0.83	3.69	-	485.7
AHQ-1-4, 3499	R.APSVANVGSHCDLSLK.I	1656.84249	2	1.84E-04	0.92	4.23	-	546.3
AHQ-1-5, 3345 - 3346	R.APSVANVGSHCDLSLK.I	1656.84249	2	1.59E-06	0.94	4.87	-	841.5
AHQ-1-5, 1707	K.ATCAPQHGAPGPGPADASK.V	1791.92336	2	1.67E-04	0.94	4.48	-	846.9
AHQ-1-1, 2029 - 2036	K.ATCAPQHGAPGPGPADASK.V	1791.92336	2	3.37E-08	0.95	4.16	-	1018.9
AHQ-1-13- , 1951 - 2031	K.ATCAPQHGAPGPGPADASK.V	1791.92336	2	2.51E-05	0.92	4.29	-	541.5
AHQ-1-4, 1799	K.ATCAPQHGAPGPGPADASK.V	1791.92336	2	1.13E-06	0.84	3.90	-	543.3
AHQ-1-11, 1894 - 1898	K.ATCAPQHGAPGPGPADASK.V	1791.92336	2	4.24E-04	0.90	4.27	-	590.6
AHQ-1-3, 1801 - 1862	K.ATCAPQHGAPGPGPADASK.V	1791.92336	2	6.10E-05	0.96	4.87	-	982.2
AHQ-1-7, 1725	K.ATCAPQHGAPGPGPADASK.V	1791.92336	2	1.26E-05	0.90	4.10	-	632.4
AHQ-1-1, 3783	R.AWGPGLEGGVVGK.S	1227.39403	2	4.95E-04	0.92	3.84	-	834.5
AHQ-1-5, 3570	R.AWGPGLEGGVVGK.S	1227.39403	2	6.90E-05	0.93	3.48	-	1059.2
AHQ-1-2, 3831 - 3901	R.AWGPGLEGGVVGK.S	1227.39403	2	1.92E-05	0.91	4.24	-	730.8
AHQ-1-4, 3743	R.AWGPGLEGGVVGK.S	1227.39403	2	2.80E-06	0.87	3.47	-	781.8
AHQ-1-6, 3546	R.AWGPGLEGGVVGK.S	1227.39403	2	7.29E-04	0.84	3.29	-	573.9
AHQ-1-7, 3636	R.AWGPGLEGGVVGK.S	1227.39403	2	4.51E-05	0.88	2.97	-	994.9
AHQ-1-3, 3703	R.AWGPGLEGGVVGK.S	1227.39403	2	5.26E-05	0.83	3.21	-	617.4
AHQ-1-2, 3283 - 3358	R.AYGPGIEPTGNMVK.K	1434.64274	2	3.59E-04	0.68	2.74	-	639.7
AHQ-1-5, 3075 - 3149	R.AYGPGIEPTGNMVK.K	1434.64274	2	2.72E-04	0.82	3.41	-	605.6
AHQ-1-1, 2872	R.AYGPGIEPTGNM*VK.K	1450.64214	2	6.38E-08	0.92	3.59	-	803.3
AHQ-1-4, 3503 - 3575	R.AYGPGIEPTGNMVK.K	1434.64274	2	6.45E-06	0.73	2.99	-	656.7
AHQ-1-7, 3152 - 3156	R.AYGPGIEPTGNMVK.K	1434.64274	2	2.99E-04	0.85	3.79	-	533.4
AHQ-1-4, 2635 - 2748	R.AYGPGIEPTGNM*VK.K	1450.64214	2	2.20E-04	0.42	2.91	-	261.2
AHQ-1-3, 2617 - 2669	R.AYGPGIEPTGNM*VK.K	1450.64214	2	2.69E-04	0.63	3.26	-	341.3
AHQ-1-2, 6459	K.CAPGVVGPAEADIDFDIIR.N	2017.24863	2	5.33E-06	0.74	3.12	-	505.8
AHQ-1-2, 6403	K.CAPGVVGPAEADIDFDIIR.N	2017.24863	3	4.19E-10	0.97	5.50	-	1769.9
AHQ-1-2, 6339 - 6386	K.CAPGVVGPAEADIDFDIIR.N	2017.24863	2	1.39E-04	0.96	5.15	-	874.2
AHQ-1-3, 6053	K.CAPGVVGPAEADIDFDIIR.N	2017.24863	2	7.46E-08	0.90	3.72	-	818.6
AHQ-1-3, 6221 - 6253	K.CAPGVVGPAEADIDFDIIR.N	2017.24863	2	5.56E-06	0.83	3.53	-	521.1
AHQ-1-3, 6235 - 6242	K.CAPGVVGPAEADIDFDIIR.N	2017.24863	3	1.60E-09	0.96	5.62	-	1524.8
AHQ-1-1, 6139 - 6148	K.CAPGVVGPAEADIDFDIIR.N	2017.24863	2	1.67E-05	0.82	3.88	-	442.4
AHQ-1-1, 6155	K.CAPGVVGPAEADIDFDIIR.N	2017.24863	3	2.23E-11	0.96	4.56	-	1663.9
AHQ-1-5, 6337 - 6403	K.CAPGVVGPAEADIDFDIIR.N	2017.24863	2	1.03E-06	0.79	3.73	-	394.4
AHQ-1-4, 6380	K.CAPGVVGPAEADIDFDIIR.N	2017.24863	2	2.08E-07	0.85	3.84	-	452.1
AHQ-1-2, 6837	K.CAPGVVGPAEADIDFDIIRNDNDTFTVK.Y	3052.31840	3	7.43E-06	0.77	3.59	-	544.0
AHQ-1-3, 6405 - 6469	K.CAPGVVGPAEADIDFDIIRNDNDTFTVK.Y	3052.31840	3	4.23E-04	0.69	3.47	-	329.8
AHQ-1-2, 6599 - 6665	K.CAPGVVGPAEADIDFDIIRNDNDTFTVK.Y	3052.31840	3	6.01E-05	0.54	3.34	-	472.6
AHQ-1-2, 6733 - 6753	K.CAPGVVGPAEADIDFDIIRNDNDTFTVK.Y	3052.31840	3	6.62E-05	0.88	4.36	-	577.1
AHQ-1-3, 2994 - 2995	K.CSGPGLSPGMVR.A	1219.41699	2	7.34E-05	0.80	3.02	-	627.1
AHQ-1-1, 3084	K.CSGPGLSPGMVR.A	1219.41699	2	1.69E-07	0.63	2.79	-	544.7
AHQ-1-5, 5450 - 5451	R.CSYQPTMEGVHTVHVTFAGVPIPR.S	2686.06233	2	7.81E-12	0.93	4.15	-	881.8
AHQ-1-2, 5149	R.CSYQPTM*EGVHTVHVTFAGVPIPR.S	2702.06173	2	9.21E-11	0.95	4.44	-	877.4
AHQ-1-5, 4953 - 4955	R.CSYQPTM*EGVHTVHVTFAGVPIPR.S	2702.06173	3	3.81E-05	0.77	3.22	-	542.6
AHQ-1-2, 5603 - 5670	R.CSYQPTMEGVHTVHVTFAGVPIPR.S	2686.06233	3	2.18E-11	0.94	5.30	-	762.5

AHQ-1-1, 5121	R.CSYQPTM*EGVHTVHVTFAGVPIPR.S	2702.06173	2	1.83E-06	0.83	3.30	-	634.0
AHQ-1-4, 5063 - 5067	R.CSYQPTM*EGVHTVHVTFAGVPIPR.S	2702.06173	3	8.01E-06	0.84	3.85	-	692.9
AHQ-1-4, 5554	R.CSYQPTMEGVHTVHVTFAGVPIPR.S	2686.06233	3	6.85E-08	0.87	3.79	-	804.6
AHQ-1-3, 5451	R.CSYQPTMEGVHTVHVTFAGVPIPR.S	2686.06233	3	2.48E-09	0.85	3.95	-	859.4
AHQ-1-3, 5466	R.CSYQPTMEGVHTVHVTFAGVPIPR.S	2686.06233	2	3.20E-07	0.92	3.56	-	1019.1
AHQ-1-6, 4902 - 4908	R.CSYQPTM*EGVHTVHVTFAGVPIPR.S	2702.06173	3	1.04E-04	0.82	4.04	-	239.6
AHQ-1-2, 5641 - 5645	R.CSYQPTMEGVHTVHVTFAGVPIPR.S	2686.06233	2	1.43E-08	0.96	5.02	-	982.9
AHQ-1-2, 4775	R.DAEMPATEKDLAEDAPWK.K	2018.19047	2	2.02E-04	0.94	4.43	-	1186.3
AHQ-1-1, 4097	R.DAEM*PATEKDLAEDAPWK.K	2034.18987	2	2.09E-04	0.88	3.68	-	698.1
AHQ-1-1, 3596 - 3672	R.DAEM*PATEKDLAEDAPWKK.I	2162.36278	3	1.14E-05	0.71	3.11	-	606.7
AHQ-1-2, 4295	R.DAEMPATEKDLAEDAPWKK.I	2146.36338	2	1.48E-05	0.90	3.45	-	1129.0
AHQ-1-1, 4067 - 4131	K.DAGEGGLSLAIEGPSK.A	1501.62084	2	5.48E-06	0.93	4.46	-	1331.1
AHQ-1-8, 4004	K.DAGEGGLSLAIEGPSK.A	1501.62084	2	7.74E-06	0.89	3.86	-	985.8
AHQ-1-13, 3880 - 3949	K.DAGEGGLSLAIEGPSK.A	1501.62084	2	3.87E-05	0.51	2.60	-	821.8
AHQ-1-5, 4025 - 4098	K.DAGEGGLSLAIEGPSK.A	1501.62084	2	1.13E-08	0.97	5.52	-	1448.1
AHQ-1-10, 3656	K.DAGEGGLSLAIEGPSK.A	1501.62084	2	7.43E-07	0.94	3.91	-	1440.8
AHQ-1-4, 4110 - 4183	K.DAGEGGLSLAIEGPSK.A	1501.62084	2	1.89E-08	0.96	4.80	-	1347.9
AHQ-1-13- , 4008	K.DAGEGGLSLAIEGPSK.A	1501.62084	2	2.46E-07	0.92	4.03	-	1048.9
AHQ-1-5, 4133	K.DAGEGGLSLAIEGPSK.A	1501.62084	2	4.58E-08	0.92	3.70	-	1177.4
AHQ-1-3, 3978 - 4058	K.DAGEGGLSLAIEGPSK.A	1501.62084	2	1.64E-05	0.94	4.32	-	1208.2
AHQ-1-1, 4208	K.DAGEGGLSLAIEGPSK.A	1501.62084	2	6.91E-06	0.92	4.27	-	1035.7
AHQ-1-6, 3975	K.DAGEGGLSLAIEGPSK.A	1501.62084	2	1.82E-06	0.84	3.56	-	756.7
AHQ-1-3, 4979	K.DAGEGLLAVQITDPEGK.P	1713.86752	2	8.07E-06	0.84	2.81	-	1258.8
AHQ-1-2, 5117	K.DAGEGLLAVQITDPEGK.P	1713.86752	2	2.97E-07	0.93	4.15	-	1264.7
AHQ-1-1, 4967	K.DAGEGLLAVQITDPEGK.P	1713.86752	2	4.75E-07	0.92	3.77	-	1387.1
AHQ-1-7, 4372 - 4385	K.DAGEGLLAVQITDPEGKPK.K	1939.15612	2	3.50E-08	0.93	4.11	-	1275.1
AHQ-1-2, 4618 - 4637	K.DAGEGLLAVQITDPEGKPK.K	1939.15612	3	4.50E-12	0.93	4.30	-	1328.5
AHQ-1-10, 3953	K.DAGEGLLAVQITDPEGKPK.K	1939.15612	2	2.95E-07	0.88	3.98	-	1006.0
AHQ-1-2, 4851	K.DAGEGLLAVQITDPEGKPK.K	1939.15612	2	1.32E-06	0.71	3.22	-	635.8
AHQ-1-1, 4492 - 4560	K.DAGEGLLAVQITDPEGKPK.K	1939.15612	3	3.70E-04	0.72	3.03	-	805.3
AHQ-1-1, 4491 - 4555	K.DAGEGLLAVQITDPEGKPK.K	1939.15612	2	1.31E-10	0.96	4.70	-	1529.9
AHQ-1-8, 4223	K.DAGEGLLAVQITDPEGKPK.K	1939.15612	2	9.43E-05	0.89	4.15	-	903.3
AHQ-1-9, 4250 - 4264	K.DAGEGLLAVQITDPEGKPK.K	1939.15612	2	8.78E-05	0.89	4.09	-	912.5
AHQ-1-5, 4395	K.DAGEGLLAVQITDPEGKPK.K	1939.15612	3	1.24E-08	0.79	3.03	-	938.1
AHQ-1-11, 4072	K.DAGEGLLAVQITDPEGKPK.K	1939.15612	2	3.62E-12	0.94	4.51	-	1105.8
AHQ-1-3, 4454 - 4522	K.DAGEGLLAVQITDPEGKPK.K	1939.15612	2	2.36E-06	0.94	4.40	-	1246.9
AHQ-1-2, 4751 - 4827	K.DAGEGLLAVQITDPEGKPK.K	1939.15612	2	8.51E-09	0.95	4.36	-	1179.6
AHQ-1-3, 4687	K.DAGEGLLAVQITDPEGKPK.K	1939.15612	2	7.16E-07	0.78	3.12	-	897.7
AHQ-1-6, 4308 - 4314	K.DAGEGLLAVQITDPEGKPK.K	1939.15612	2	6.55E-07	0.94	3.83	-	1515.9
AHQ-1-4, 4483 - 4562	K.DAGEGLLAVQITDPEGKPK.K	1939.15612	2	2.36E-05	0.74	2.96	-	859.4
AHQ-1-2, 4613 - 4685	K.DAGEGLLAVQITDPEGKPK.K	1939.15612	2	1.51E-06	0.96	4.36	-	1566.6
AHQ-1-1, 4636 - 4717	K.DAGEGLLAVQITDPEGKPK.K	1939.15612	2	8.50E-09	0.94	4.27	-	1109.4
AHQ-1-6, 4319	K.DAGEGLLAVQITDPEGKPK.K	1939.15612	3	2.48E-06	0.80	3.28	-	985.9
AHQ-1-2, 4706	K.DAGEGLLAVQITDPEGKPK.K	1939.15612	3	2.26E-06	0.90	3.73	-	1489.7
AHQ-1-7, 4620	R.DAGYGGLSLSIEGPSK.V	1551.67986	2	2.01E-04	0.63	3.04	-	420.6
AHQ-1-5, 4611 - 4687	R.DAGYGGLSLSIEGPSK.V	1551.67986	2	7.83E-11	0.95	4.16	-	1497.6
AHQ-1-1, 4704	R.DAGYGGLSLSIEGPSK.V	1551.67986	2	9.31E-06	0.58	3.17	-	544.0
AHQ-1-5, 4402	R.DAGYGGLSLSIEGPSK.V	1551.67986	2	8.54E-08	0.76	2.58	-	1158.2
AHQ-1-4, 4856	R.DAGYGGLSLSIEGPSK.V	1551.67986	2	6.43E-05	0.61	3.01	-	660.8
AHQ-1-3, 4627	R.DAGYGGLSLSIEGPSK.V	1551.67986	2	1.57E-13	0.96	4.34	-	1573.1
AHQ-1-1, 4603	R.DAGYGGLSLSIEGPSK.V	1551.67986	2	3.21E-10	0.94	4.34	-	1256.9
AHQ-1-5, 4882	R.DAGYGGLSLSIEGPSK.V	1551.67986	2	4.67E-07	0.81	3.40	-	757.0
AHQ-1-1, 4361 - 4435	R.DAGYGGLSLSIEGPSK.V	1551.67986	2	2.38E-09	0.96	4.56	-	1457.5
AHQ-1-2, 4759	R.DAGYGGLSLSIEGPSK.V	1551.67986	2	1.42E-06	0.95	4.38	-	1514.4
AHQ-1-5, 4753	R.DAGYGGLSLSIEGPSK.V	1551.67986	2	2.39E-04	0.75	2.94	-	1012.6
AHQ-1-3, 1883 - 1945	R.DAPQDFHPDR.V	1198.22650	2	6.17E-05	0.59	2.58	-	553.2
AHQ-1-1, 4881 - 4963	K.DGSCGVAYVVQEPGDYEVSVK.F	2260.41990	2	1.28E-04	0.78	4.03	-	511.6
AHQ-1-1, 5055	K.DGSCGVAYVVQEPGDYEVSVK.F	2260.41990	2	2.22E-05	0.38	3.01	-	305.3
AHQ-1-5, 4958 - 5029	K.DGSCGVAYVVQEPGDYEVSVK.F	2260.41990	2	1.52E-04	0.91	4.82	-	616.1
AHQ-1-5, 5255	K.DGSCGVAYVVQEPGDYEVSVK.F	2260.41990	2	2.15E-05	0.92	4.49	-	759.3
AHQ-1-4, 4996 - 5003	K.DGSCGVAYVVQEPGDYEVSVK.F	2260.41990	2	1.49E-05	0.90	4.55	-	608.2
AHQ-1-3, 4909 - 4910	K.DGSCGVAYVVQEPGDYEVSVK.F	2260.41990	2	9.49E-05	0.87	4.10	-	604.1
AHQ-1-2, 5046 - 5050	K.DGSCGVAYVVQEPGDYEVSVK.F	2260.41990	2	1.61E-05	0.89	4.55	-	587.2
AHQ-1-6, 4927 - 5002	K.DGSCGVAYVVQEPGDYEVSVK.F	2260.41990	2	6.26E-04	0.78	3.42	-	602.2
AHQ-1-6, 5088	K.DGSCGVAYVVQEPGDYEVSVK.F	2260.41990	2	1.22E-06	0.60	3.47	-	395.4
AHQ-1-8, 4942 - 4946	K.DGSCGVAYVVQEPGDYEVSVK.F	2260.41990	2	1.29E-04	0.87	4.56	-	650.7
AHQ-1-5, 5099 - 5178	K.DGSCGVAYVVQEPGDYEVSVK.F	2260.41990	2	9.63E-06	0.88	4.52	-	590.1
AHQ-1-14, 3353	K.DKGEYTLVVK.W	1152.32223	1	7.34E-04	0.37	2.39	-	613.4
AHQ-1-11, 2416	K.DKGEYTLVVK.W	1152.32223	1	2.31E-04	0.34	2.16	-	572.9
AHQ-1-12, 2469	K.DKGEYTLVVK.W	1152.32223	2	9.56E-04	0.74	2.52	-	615.9
AHQ-1-1, 2637 - 2641	K.DKGEYTLVVK.W	1152.32223	1	4.77E-04	0.48	2.76	-	509.1
AHQ-1-14- , 2466 - 2532	K.DKGEYTLVVK.W	1152.32223	2	2.11E-06	0.75	2.79	-	557.6
AHQ-1-1, 3315	K.DNGNGTYSCSYVPR.K	1591.64109	2	8.57E-05	0.81	2.81	-	1063.6
AHQ-1-2, 3427	K.DNGNGTYSCSYVPR.K	1591.64109	2	1.13E-04	0.84	2.72	-	1111.9
AHQ-1-2, 3223	R.DVDIIDHHDNTYTVK.Y	1785.89230	3	1.54E-05	0.71	3.15	-	526.3
AHQ-1-3, 3093 - 3095	R.DVDIIDHHDNTYTVK.Y	1785.89230	2	5.96E-07	0.92	3.94	-	1027.2
AHQ-1-2, 3265	R.DVDIIDHHDNTYTVK.Y	1785.89230	2	1.55E-07	0.96	4.56	-	1092.4
AHQ-1-3, 3342	R.DVDIIDHHDNTYTVK.Y	1785.89230	2	1.83E-06	0.79	3.00	-	941.7
AHQ-1-7, 3009	R.DVDIIDHHDNTYTVK.Y	1785.89230	2	2.28E-05	0.78	2.84	-	812.0
AHQ-1-1, 3123 - 3203	R.DVDIIDHHDNTYTVK.Y	1785.89230	2	1.76E-04	0.94	3.66	-	1340.2
AHQ-1-2, 3135 - 3205	R.DVDIIDHHDNTYTVK.Y	1785.89230	3	4.65E-05	0.70	3.19	-	413.6
AHQ-1-2, 3381 - 3447	R.DVDIIDHHDNTYTVK.Y	1785.89230	2	6.27E-07	0.84	3.33	-	617.1
AHQ-1-4, 3034 - 3110	R.DVDIIDHHDNTYTVK.Y	1785.89230	2	1.60E-06	0.90	3.73	-	961.4
AHQ-1-6, 2984	R.DVDIIDHHDNTYTVK.Y	1785.89230	2	4.28E-04	0.76	2.81	-	852.3
AHQ-1-3, 3185	R.DVDIIDHHDNTYTVK.Y	1785.89230	2	2.16E-06	0.86	3.58	-	907.2
AHQ-1-1, 3280	R.DVDIIDHHDNTYTVK.Y	1785.89230	2	4.05E-06	0.85	3.39	-	793.2
AHQ-1-9, 2899 - 2904	R.DVDIIDHHDNTYTVK.Y	1785.89230	2	6.60E-05	0.40	2.70	-	511.1
AHQ-1-6, 3334	R.EAGAGGLAIAVEGPSK.A	1427.58510	1	6.09E-09	0.65	3.60	-	269.7
AHQ-1-4, 3479	R.EAGAGGLAIAVEGPSK.A	1427.58510	2	2.81E-06	0.96	4.50	-	1331.4
AHQ-1-5, 3353	R.EAGAGGLAIAVEGPSK.A	1427.58510	1	2.15E-07	0.73	3.37	-	290.2
AHQ-1-8, 3201 - 3202	R.EAGAGGLAIAVEGPSK.A	1427.58510	1	1.40E-04	0.68	3.63	-	302.2
AHQ-1-5, 3350 - 3373	R.EAGAGGLAIAVEGPSK.A	1427.58510	2	6.13E-08	0.97	4.27	-	1785.9
AHQ-1-1, 3487 - 3556	R.EAGAGGLAIAVEGPSK.A	1427.58510	2	8.95E-08	0.96	4.34	-	1831.9
AHQ-1-3, 3461 - 3474	R.EAGAGGLAIAVEGPSK.A	1427.58510	2	4.31E-06	0.94	4.16	-	1047.8
AHQ-1-6, 3318 - 3319	R.EAGAGGLAIAVEGPSK.A	1427.58510	2	3.23E-10	0.95	4.17	-	1356.5
AHQ-1-6, 2530 - 2532	R.EATTEFSVDAR.A	1226.27493	2	1.67E-04	0.83	2.94	-	975.1
AHQ-1-2, 2701 - 2774	R.EATTEFSVDAR.A	1226.27493	2	1.09E-05	0.88	3.30	-	830.4
AHQ-1-1, 2731 - 2761	R.EATTEFSVDAR.A	1226.27493	2	7.18E-05	0.86	3.38	-	771.7
AHQ-1-3, 2611	R.EATTEFSVDAR.A	1226.27493	2	8.87E-06	0.86	3.04	-	991.7
AHQ-1-6, 6308 - 6383	R.EEGPYEVEVTYDGVPVPGSPFPLEAVAPTKPSK.V	3487.85286	3	1.71E-09	0.95	5.41	-	1068.2
AHQ-1-5, 6461 - 6538	R.EEGPYEVEVTYDGVPVPGSPFPLEAVAPTKPSK.V	3487.85286	3	4.83E-08	0.95	5.89	-	699.0
AHQ-1-3, 6473 - 6553	R.EEGPYEVEVTYDGVPVPGSPFPLEAVAPTKPSK.V	3487.85286	3	2.84E-07	0.87	4.41	-	508.3
AHQ-1-8, 6510	R.EEGPYEVEVTYDGVPVPGSPFPLEAVAPTKPSK.V	3487.85286	3	2.01E-08	0.89	4.73	-	647.7
AHQ-1-3, 6322 - 6401	R.EEGPYEVEVTYDGVPVPGSPFPLEAVAPTKPSK.V	3487.85286	3	1.03E-07	0.96	6.83	-	800.4
AHQ-1-9, 6371	R.EEGPYEVEVTYDGVPVPGSPFPLEAVAPTKPSK.V	3487.85286	3	4.87E-06	0.67	3.57	-	362.4
AHQ-1-1, 6259 - 6331	R.EEGPYEVEVTYDGVPVPGSPFPLEAVAPTKPSK.V	3487.85286	3	5.91E-07	0.95	6.00	-	735.8
AHQ-1-1, 6347 - 6405	R.EEGPYEVEVTYDGVPVPGSPFPLEAVAPTKPSK.V	3487.85286	3	5.13E-10	0.88	4.66	-	522.7

AHQ-1-7, 6461 - 6533	R.EEGPYEVEVTYDGVPVPGSPFPLEAVAPTKPSK.V	3487.85286	3	1.00E-04	0.91	4.94	-	527.6
AHQ-1-4, 6488 - 6558	R.EEGPYEVEVTYDGVPVPGSPFPLEAVAPTKPSK.V	3487.85286	3	4.00E-08	0.94	5.32	-	755.0
AHQ-1-2, 6470 - 6537	R.EEGPYEVEVTYDGVPVPGSPFPLEAVAPTKPSK.V	3487.85286	3	4.39E-09	0.93	5.36	-	705.9
AHQ-1-4, 6606 - 6623	R.EEGPYEVEVTYDGVPVPGSPFPLEAVAPTKPSK.V	3487.85286	3	7.20E-11	0.92	5.35	-	582.2
AHQ-1-2, 6601 - 6666	R.EEGPYEVEVTYDGVPVPGSPFPLEAVAPTKPSK.V	3487.85286	3	4.12E-05	0.91	5.05	-	524.2
AHQ-1-5, 5174 - 5243	R.EGPYSISVLYGDEEVPR.S	1911.05809	2	3.05E-05	0.74	3.42	-	409.3
AHQ-1-2, 5334	R.EGPYSISVLYGDEEVPR.S	1911.05809	3	3.97E-04	0.95	5.11	-	1463.9
AHQ-1-2, 5266 - 5345	R.EGPYSISVLYGDEEVPR.S	1911.05809	2	1.94E-09	0.90	3.81	-	1069.6
AHQ-1-3, 5125 - 5206	R.EGPYSISVLYGDEEVPR.S	1911.05809	2	4.08E-09	0.91	4.02	-	947.6
AHQ-1-10, 4569 - 4583	R.EGPYSISVLYGDEEVPR.S	1911.05809	2	1.04E-06	0.86	3.69	-	677.9
AHQ-1-2, 5026	R.EGPYSISVLYGDEEVPR.S	1911.05809	2	2.29E-06	0.86	3.89	-	620.7
AHQ-1-1, 5211	R.EGPYSISVLYGDEEVPR.S	1911.05809	3	1.16E-05	0.95	4.90	-	1523.1
AHQ-1-1, 5153 - 5219	R.EGPYSISVLYGDEEVPR.S	1911.05809	2	6.59E-08	0.92	3.98	-	1140.3
AHQ-1-1, 4872	R.EGPYSISVLYGDEEVPR.S	1911.05809	2	9.54E-07	0.39	3.18	-	378.6
AHQ-1-9, 5008 - 5027	R.EGPYSISVLYGDEEVPR.S	1911.05809	2	7.70E-06	0.93	4.18	-	983.4
AHQ-1-6, 5099	R.EGPYSISVLYGDEEVPR.S	1911.05809	2	2.62E-06	0.88	3.78	-	886.7
AHQ-1-7, 5078 - 5153	R.EGPYSISVLYGDEEVPR.S	1911.05809	2	7.59E-06	0.59	2.83	-	484.3
AHQ-1-7, 3913	R.ENGVYLIDVK.F	1150.30633	1	4.66E-04	0.11	1.93	-	252.4
AHQ-1-5, 3898 - 3969	R.ENGVYLIDVK.F	1150.30633	2	5.49E-04	0.67	2.79	-	471.3
AHQ-1-4, 4072	R.ENGVYLIDVK.F	1150.30633	2	2.75E-04	0.86	3.19	-	604.4
AHQ-1-5, 1490	K.ETGEHLVHVK.K	1149.28166	1	7.60E-05	0.30	2.38	-	259.6
AHQ-1-13- , 3539 - 3547	K.FADQHVPGSPFSVK.V	1516.68203	2	4.53E-06	0.80	3.28	-	586.4
AHQ-1-6, 3350	K.FADQHVPGSPFSVK.V	1516.68203	2	1.26E-06	0.87	3.65	-	504.4
AHQ-1-3, 3542	K.FADQHVPGSPFSVK.V	1516.68203	3	1.65E-07	0.91	3.74	-	1105.9
AHQ-1-5, 3378	K.FADQHVPGSPFSVK.V	1516.68203	3	1.60E-07	0.91	4.09	-	957.7
AHQ-1-1, 3617	K.FADQHVPGSPFSVK.V	1516.68203	2	1.86E-05	0.87	3.29	-	614.2
AHQ-1-1, 3619	K.FADQHVPGSPFSVK.V	1516.68203	3	1.60E-05	0.95	3.90	-	1535.5
AHQ-1-7, 3388 - 3390	K.FADQHVPGSPFSVK.V	1516.68203	2	6.99E-07	0.72	3.23	-	399.2
AHQ-1-3, 3525	K.FADQHVPGSPFSVK.V	1516.68203	2	7.03E-05	0.76	3.31	-	524.2
AHQ-1-4, 3551	K.FADQHVPGSPFSVK.V	1516.68203	3	2.04E-08	0.95	4.17	-	1600.1
AHQ-1-6, 3352	K.FADQHVPGSPFSVK.V	1516.68203	3	2.06E-06	0.87	3.84	-	835.4
AHQ-1-4, 3538	K.FADQHVPGSPFSVK.V	1516.68203	2	3.37E-04	0.81	3.53	-	400.1
AHQ-1-5, 5623 - 5627	R.FGGEHVPNSPFQVTALAGDQPSVQPPLR.S	2947.25342	2	4.03E-06	0.93	4.31	-	1033.5
AHQ-1-12, 5283	R.FGGEHVPNSPFQVTALAGDQPSVQPPLR.S	2947.25342	2	3.74E-06	0.95	5.14	-	1101.8
AHQ-1-13, 5197	R.FGGEHVPNSPFQVTALAGDQPSVQPPLR.S	2947.25342	3	4.21E-04	0.87	4.19	-	777.1
AHQ-1-4, 5811 - 5891	R.FGGEHVPNSPFQVTALAGDQPSVQPPLR.S	2947.25342	3	1.42E-10	0.94	5.09	-	838.7
AHQ-1-9, 5392 - 5459	R.FGGEHVPNSPFQVTALAGDQPSVQPPLR.S	2947.25342	3	1.86E-04	0.84	4.28	-	590.0
AHQ-1-1, 5499 - 5504	R.FGGEHVPNSPFQVTALAGDQPSVQPPLR.S	2947.25342	3	2.07E-04	0.89	4.49	-	738.3
AHQ-1-4, 5747 - 5770	R.FGGEHVPNSPFQVTALAGDQPSVQPPLR.S	2947.25342	2	2.50E-08	0.95	4.69	-	963.7
AHQ-1-12, 5255	R.FGGEHVPNSPFQVTALAGDQPSVQPPLR.S	2947.25342	3	2.52E-05	0.75	3.54	-	441.2
AHQ-1-2, 5926	R.FGGEHVPNSPFQVTALAGDQPSVQPPLR.S	2947.25342	2	2.56E-06	0.87	3.42	-	785.7
AHQ-1-10, 4900	R.FGGEHVPNSPFQVTALAGDQPSVQPPLR.S	2947.25342	3	1.66E-04	0.73	3.50	-	550.6
AHQ-1-2, 5893 - 5961	R.FGGEHVPNSPFQVTALAGDQPSVQPPLR.S	2947.25342	3	1.97E-06	0.91	4.72	-	801.3
AHQ-1-1, 5609 - 5671	R.FGGEHVPNSPFQVTALAGDQPSVQPPLR.S	2947.25342	3	1.50E-05	0.91	4.63	-	779.3
AHQ-1-3, 5709 - 5773	R.FGGEHVPNSPFQVTALAGDQPSVQPPLR.S	2947.25342	3	7.42E-11	0.93	5.72	-	779.5
AHQ-1-2, 5793 - 5861	R.FGGEHVPNSPFQVTALAGDQPSVQPPLR.S	2947.25342	2	7.60E-10	0.98	6.03	-	1506.3
AHQ-1-1, 5675 - 5680	R.FGGEHVPNSPFQVTALAGDQPSVQPPLR.S	2947.25342	2	2.94E-12	0.97	6.01	-	1276.8
AHQ-1-3, 5657 - 5685	R.FGGEHVPNSPFQVTALAGDQPSVQPPLR.S	2947.25342	2	4.72E-05	0.97	5.61	-	1228.2
AHQ-1-1, 5735 - 5799	R.FGGEHVPNSPFQVTALAGDQPSVQPPLR.S	2947.25342	3	7.84E-08	0.85	4.24	-	754.3
AHQ-1-2, 5757 - 5826	R.FGGEHVPNSPFQVTALAGDQPSVQPPLR.S	2947.25342	3	6.74E-10	0.95	5.75	-	718.9
AHQ-1-13- , 5615	R.FGGEHVPNSPFQVTALAGDQPSVQPPLR.S	2947.25342	3	2.95E-04	0.87	4.22	-	657.5
AHQ-1-8, 5415 - 5432	R.FGGEHVPNSPFQVTALAGDQPSVQPPLR.S	2947.25342	3	5.95E-07	0.94	5.23	-	1051.1
AHQ-1-3, 5578 - 5645	R.FGGEHVPNSPFQVTALAGDQPSVQPPLR.S	2947.25342	3	3.72E-07	0.94	4.94	-	872.2
AHQ-1-2, 5617 - 5691	R.FGGEHVPNSPFQVTALAGDQPSVQPPLR.S	2947.25342	3	2.70E-05	0.95	5.64	-	1062.9
AHQ-1-5, 5201 - 5269	K.FNEEHIPDSPFVVPVASPSGDAR.R	2468.66280	2	5.18E-08	0.94	5.26	-	663.2
AHQ-1-8, 5086	K.FNEEHIPDSPFVVPVASPSGDAR.R	2468.66280	2	5.53E-08	0.96	5.17	-	865.4
AHQ-1-5, 5401 - 5438	K.FNEEHIPDSPFVVPVASPSGDAR.R	2468.66280	2	2.87E-07	0.90	4.34	-	533.2
AHQ-1-9, 5034	K.FNEEHIPDSPFVVPVASPSGDAR.R	2468.66280	2	2.00E-04	0.83	3.13	-	777.6
AHQ-1-6, 5146	K.FNEEHIPDSPFVVPVASPSGDAR.R	2468.66280	2	5.79E-04	0.74	2.90	-	549.7
AHQ-1-1, 3937 - 4012	K.FNGTHIPGSPFK.I	1302.46259	2	9.80E-06	0.62	2.69	-	486.8
AHQ-1-3, 3879	K.FNGTHIPGSPFK.I	1302.46259	2	1.58E-05	0.74	3.02	-	580.8
AHQ-1-4, 3894	K.FNGTHIPGSPFK.I	1302.46259	2	1.54E-06	0.44	2.58	-	438.5
AHQ-1-2, 4030	K.FNGTHIPGSPFK.I	1302.46259	2	2.20E-06	0.84	2.77	-	797.0
AHQ-1-1, 3837	K.FNGTHIPGSPFK.I	1302.46259	2	1.27E-07	0.87	3.30	-	761.4
AHQ-1-5, 3729	K.FNGTHIPGSPFK.I	1302.46259	2	1.19E-05	0.86	3.58	-	558.6
AHQ-1-5, 2889	R.FVPAEMGTHTVSVK.Y	1503.74821	2	9.36E-06	0.92	3.75	-	800.1
AHQ-1-1, 3148	R.FVPAEMGTHTVSVK.Y	1503.74821	2	7.75E-08	0.74	2.79	-	622.3
AHQ-1-6, 2914	R.FVPAEMGTHTVSVK.Y	1503.74821	2	9.30E-05	0.75	2.63	-	562.1
AHQ-1-4, 6924 - 6938	R.GAGSYTIMVLFADQATPTSPIR.V	2297.61623	2	1.11E-05	0.89	4.14	-	524.5
AHQ-1-3, 6250	R.GAGSYTIM*VLFADQATPTSPIR.V	2313.61563	2	6.03E-05	0.39	2.64	-	280.2
AHQ-1-2, 6266	R.GAGSYTIM*VLFADQATPTSPIR.V	2313.61563	2	1.15E-05	0.93	4.59	-	743.4
AHQ-1-6, 3283 - 3298	R.GAGTGGLGLAVEGPSEAK.M	1571.71430	2	1.87E-07	0.86	3.10	-	969.9
AHQ-1-8, 3180	R.GAGTGGLGLAVEGPSEAK.M	1571.71430	2	4.56E-05	0.87	3.24	-	793.1
AHQ-1-1, 3621 - 3649	R.GAGTGGLGLAVEGPSEAK.M	1571.71430	2	2.06E-04	0.88	3.41	-	975.4
AHQ-1-3, 3631	R.GAGTGGLGLAVEGPSEAK.M	1571.71430	2	1.38E-05	0.21	2.66	-	255.5
AHQ-1-2, 3455 - 3529	R.GAGTGGLGLAVEGPSEAK.M	1571.71430	2	7.02E-07	0.93	4.57	-	828.5
AHQ-1-3, 3513	R.GAGTGGLGLAVEGPSEAK.M	1571.71430	2	1.73E-05	0.51	2.57	-	468.7
AHQ-1-3, 3361 - 3429	R.GAGTGGLGLAVEGPSEAK.M	1571.71430	2	6.27E-06	0.89	3.59	-	856.0
AHQ-1-1, 3464 - 3535	R.GAGTGGLGLAVEGPSEAK.M	1571.71430	2	6.68E-07	0.85	3.68	-	648.1
AHQ-1-5, 3306 - 3330	R.GAGTGGLGLAVEGPSEAK.M	1571.71430	2	1.07E-06	0.86	3.33	-	833.6
AHQ-1-4, 3442 - 3448	R.GAGTGGLGLAVEGPSEAK.M	1571.71430	2	1.82E-04	0.74	3.28	-	616.2
AHQ-1-2, 3674 - 3750	R.GAGTGGLGLAVEGPSEAK.M	1571.71430	2	7.34E-06	0.88	3.68	-	897.7
AHQ-1-3, 3446 - 3499	K.GKLDVQFSGLTK.G	1293.49392	2	2.81E-05	0.90	3.53	-	848.1
AHQ-1-1, 5067 - 5137	K.GLVEPVDVVDNADGTQTVNYVPSR.E	2545.74387	3	1.07E-05	0.72	3.27	-	567.0
AHQ-1-3, 5007 - 5081	K.GLVEPVDVVDNADGTQTVNYVPSR.E	2545.74387	2	5.52E-09	0.93	4.36	-	1112.6
AHQ-1-1, 5216 - 5279	K.GLVEPVDVVDNADGTQTVNYVPSR.E	2545.74387	2	1.79E-11	0.96	5.09	-	1182.3
AHQ-1-2, 5137 - 5205	K.GLVEPVDVVDNADGTQTVNYVPSR.E	2545.74387	2	1.84E-08	0.93	4.48	-	882.8
AHQ-1-1, 5064 - 5135	K.GLVEPVDVVDNADGTQTVNYVPSR.E	2545.74387	2	1.90E-10	0.96	5.17	-	944.5
AHQ-1-1, 4931 - 4995	K.GLVEPVDVVDNADGTQTVNYVPSR.E	2545.74387	2	4.39E-08	0.93	4.37	-	824.2
AHQ-1-4, 5187	K.GLVEPVDVVDNADGTQTVNYVPSR.E	2545.74387	2	1.81E-05	0.79	3.42	-	535.8
AHQ-1-1, 6437	K.GLVEPVDVVDNADGTQTVNYVPSREGPYSISVLYGDEEVPR.S	4437.77937	3	5.47E-08	0.82	3.70	-	541.5
AHQ-1-2, 6735 - 6738	K.GLVEPVDVVDNADGTQTVNYVPSREGPYSISVLYGDEEVPR.S	4437.77937	3	2.26E-13	0.95	5.92	-	882.7
AHQ-1-4, 2228 - 2235	K.GTVEPQLEAR.G	1100.20741	2	1.06E-04	0.80	2.92	-	555.5
AHQ-1-3, 2231	K.GTVEPQLEAR.G	1100.20741	2	3.40E-05	0.73	2.89	-	553.5
AHQ-1-6, 2115 - 2119	K.GTVEPQLEAR.G	1100.20741	2	3.35E-06	0.75	2.95	-	417.1
AHQ-1-2, 2315 - 2385	K.GTVEPQLEAR.G	1100.20741	2	1.73E-04	0.73	3.03	-	493.2
AHQ-1-1, 2367	K.GTVEPQLEAR.G	1100.20741	2	4.08E-04	0.78	2.76	-	566.6
AHQ-1-2, 5399 - 5421	K.HTAM*VSWGGVSIPNSPFR.V	1960.20541	2	1.06E-10	0.96	5.20	-	1149.1
AHQ-1-3, 5186 - 5255	K.HTAM*VSWGGVSIPNSPFR.V	1960.20541	2	3.60E-05	0.94	4.26	-	1043.4
AHQ-1-2, 5143	K.HTAM*VSWGGVSIPNSPFR.V	1960.20541	2	1.23E-05	0.94	4.31	-	1040.9
AHQ-1-1, 5681	K.HTAMVSWGGVSIPNSPFR.V	1944.20601	2	4.69E-04	0.96	5.20	-	1058.9
AHQ-1-4, 5272 - 5283	K.HTAM*VSWGGVSIPNSPFR.V	1960.20541	2	6.43E-06	0.96	4.69	-	1263.8
AHQ-1-4, 5688	K.HTAMVSWGGVSIPNSPFR.V	1944.20601	2	7.07E-05	0.97	4.93	-	1619.4
AHQ-1-2, 5393	K.HTAM*VSWGGVSIPNSPFR.V	1960.20541	3	4.03E-05	0.87	3.93	-	1148.9
AHQ-1-1, 5333 - 5336	K.HTAM*VSWGGVSIPNSPFR.V	1960.20541	3	8.59E-05	0.84	3.78	-	946.8
AHQ-1-1, 5327	K.HTAM*VSWGGVSIPNSPFR.V	1960.20541	2	3.79E-05	0.95	5.05	-	1009.2
AHQ-1-2, 5781 - 5782	K.HTAMVSWGGVSIPNSPFR.V	1944.20601	2	4.53E-04	0.96	4.53	-	1244.7

AHQ-1-5, 5185	K.HTAM*VSWGGVSIPNSPFR.V	1960.20541	2	1.15E-06	0.95	4.65	-	1187.8
AHQ-1-2, 5786 - 5795	K.HTAMVSWGGVSIPNSPFR.V	1944.20601	3	2.52E-05	0.72	3.99	-	432.5
AHQ-1-1, 4063	R.IANLQTDLSDGLR.L	1416.56231	2	2.21E-06	0.94	4.24	-	964.9
AHQ-1-9, 4111	R.IANLQTDLSDGLR.L	1416.56231	2	4.03E-07	0.92	4.14	-	792.1
AHQ-1-2, 4465 - 4541	R.IANLQTDLSDGLR.L	1416.56231	2	8.79E-07	0.92	4.15	-	827.2
AHQ-1-3, 3917	R.IANLQTDLSDGLR.L	1416.56231	2	2.21E-07	0.95	3.93	-	1246.3
AHQ-1-10, 3864	R.IANLQTDLSDGLR.L	1416.56231	2	1.17E-07	0.92	3.36	-	976.4
AHQ-1-2, 4355	R.IANLQTDLSDGLR.L	1416.56231	2	3.39E-06	0.95	4.34	-	1190.9
AHQ-1-1, 4247	R.IANLQTDLSDGLR.L	1416.56231	2	7.47E-06	0.95	4.30	-	1336.2
AHQ-1-5, 4233 - 4234	R.IANLQTDLSDGLR.L	1416.56231	2	5.34E-07	0.94	4.41	-	948.2
AHQ-1-2, 4139	R.IANLQTDLSDGLR.L	1416.56231	2	8.90E-07	0.95	3.86	-	1237.4
AHQ-1-1, 3963	R.IANLQTDLSDGLR.L	1416.56231	2	9.99E-07	0.92	3.66	-	926.2
AHQ-1-1, 4372	R.IANLQTDLSDGLR.L	1416.56231	1	5.66E-04	0.43	2.48	-	304.6
AHQ-1-5, 3843	R.IANLQTDLSDGLR.L	1416.56231	2	4.75E-05	0.92	3.83	-	915.1
AHQ-1-1, 4516	R.IANLQTDLSDGLR.L	1416.56231	2	6.56E-06	0.90	3.57	-	741.6
AHQ-1-13- , 4299 - 4308	R.IANLQTDLSDGLR.L	1416.56231	2	6.30E-07	0.94	4.01	-	994.7
AHQ-1-3, 4214	R.IANLQTDLSDGLR.L	1416.56231	2	2.94E-06	0.94	4.24	-	966.9
AHQ-1-2, 4061	R.IANLQTDLSDGLR.L	1416.56231	1	2.49E-05	0.39	2.63	-	244.5
AHQ-1-2, 4053 - 4066	R.IANLQTDLSDGLR.L	1416.56231	2	3.31E-06	0.94	4.02	-	1140.4
AHQ-1-2, 4614 - 4670	R.IANLQTDLSDGLR.L	1416.56231	2	1.26E-07	0.93	3.37	-	1227.0
AHQ-1-8, 4136	R.IANLQTDLSDGLR.L	1416.56231	2	1.51E-04	0.82	2.81	-	884.0
AHQ-1-13, 4107	R.IANLQTDLSDGLR.L	1416.56231	2	2.57E-08	0.95	4.02	-	1127.1
AHQ-1-6, 4166 - 4186	R.IANLQTDLSDGLR.L	1416.56231	2	8.24E-08	0.92	4.01	-	908.5
AHQ-1-11, 3939	R.IANLQTDLSDGLR.L	1416.56231	2	3.57E-04	0.92	3.82	-	1021.1
AHQ-1-7, 4242 - 4256	R.IANLQTDLSDGLR.L	1416.56231	2	6.48E-07	0.89	3.42	-	849.7
AHQ-1-12, 4073	R.IANLQTDLSDGLR.L	1416.56231	2	1.77E-04	0.92	3.99	-	881.6
AHQ-1-6, 3771	R.IANLQTDLSDGLR.L	1416.56231	2	1.75E-05	0.92	3.30	-	1384.8
AHQ-1-4, 3970	R.IANLQTDLSDGLR.L	1416.56231	2	1.04E-05	0.93	3.71	-	1064.4
AHQ-1-3, 4313 - 4383	R.IANLQTDLSDGLR.L	1416.56231	2	3.77E-06	0.92	4.12	-	823.6
AHQ-1-4, 4371	R.IANLQTDLSDGLR.L	1416.56231	2	2.31E-08	0.94	4.72	-	829.9
AHQ-1-3, 4514	R.IANLQTDLSDGLR.L	1416.56231	2	3.14E-05	0.78	2.73	-	727.0
AHQ-1-1, 5867 - 5875	K.IPEISIQDMTAQVTSPSGK.T	2003.26361	2	2.64E-05	0.93	4.66	-	639.4
AHQ-1-4, 4240 - 4270	K.IPEISIQDM*TAQVTSPSGK.T	2019.26301	2	2.23E-04	0.98	5.58	-	1809.2
AHQ-1-6, 4103 - 4108	K.IPEISIQDM*TAQVTSPSGK.T	2019.26301	2	2.04E-05	0.98	5.90	-	1880.7
AHQ-1-5, 5946 - 5955	K.IPEISIQDMTAQVTSPSGK.T	2003.26361	2	2.29E-13	0.97	5.08	-	1337.7
AHQ-1-1, 5621	K.IPEISIQDMTAQVTSPSGK.T	2003.26361	3	1.74E-04	0.89	4.26	-	703.6
AHQ-1-1, 5584 - 5643	K.IPEISIQDMTAQVTSPSGK.T	2003.26361	2	7.90E-08	0.98	5.47	-	1963.8
AHQ-1-5, 5611	K.IPEISIQDMTAQVTSPSGK.T	2003.26361	3	9.24E-07	0.93	4.57	-	1260.8
AHQ-1-5, 5589 - 5645	K.IPEISIQDMTAQVTSPSGK.T	2003.26361	2	1.04E-09	0.98	5.93	-	2116.8
AHQ-1-5, 4154 - 4225	K.IPEISIQDM*TAQVTSPSGK.T	2019.26301	2	2.17E-05	0.97	4.94	-	1829.4
AHQ-1-3, 5605	K.IPEISIQDMTAQVTSPSGK.T	2003.26361	2	3.41E-09	0.98	5.47	-	1859.6
AHQ-1-1, 4193 - 4261	K.IPEISIQDM*TAQVTSPSGK.T	2019.26301	2	3.24E-07	0.98	5.82	-	2145.4
AHQ-1-7, 5582	K.IPEISIQDMTAQVTSPSGK.T	2003.26361	2	1.50E-08	0.96	5.09	-	1115.8
AHQ-1-6, 5506	K.IPEISIQDMTAQVTSPSGK.T	2003.26361	2	3.95E-08	0.97	5.34	-	1695.0
AHQ-1-5, 5130	K.IPEISIQDMTAQVTSPSGK.T	2003.26361	2	5.07E-07	0.78	3.42	-	599.7
AHQ-1-5, 4951	K.IPEISIQDMTAQVTSPSGK.T	2003.26361	2	3.57E-07	0.91	3.80	-	1030.1
AHQ-1-8, 5471	K.IPEISIQDMTAQVTSPSGK.T	2003.26361	2	2.20E-11	0.97	5.21	-	1696.4
AHQ-1-12, 5249	K.IPEISIQDMTAQVTSPSGK.T	2003.26361	2	5.55E-12	0.98	5.75	-	1652.8
AHQ-1-3, 4225	K.IPEISIQDM*TAQVTSPSGK.T	2019.26301	2	2.31E-04	0.95	4.33	-	1407.1
AHQ-1-3, 2549	K.IVGPSGAAVPCK.V	1157.36530	2	1.34E-05	0.82	2.97	-	601.2
AHQ-1-5, 2466	K.IVGPSGAAVPCK.V	1157.36530	2	3.60E-07	0.91	3.51	-	886.1
AHQ-1-2, 2625	K.IVGPSGAAVPCK.V	1157.36530	2	1.95E-08	0.92	3.43	-	978.9
AHQ-1-4, 2571	K.IVGPSGAAVPCK.V	1157.36530	2	1.92E-06	0.90	3.94	-	716.6
AHQ-1-3, 4243 - 4319	K.IVGPSGAAVPCKVEPGLGADNSVVR.F	2451.78478	3	1.05E-07	0.93	4.71	-	1256.9
AHQ-1-3, 4259 - 4277	K.IVGPSGAAVPCKVEPGLGADNSVVR.F	2451.78478	2	1.69E-05	0.89	4.41	-	415.5
AHQ-1-4, 4320 - 4322	K.IVGPSGAAVPCKVEPGLGADNSVVR.F	2451.78478	2	3.97E-05	0.91	4.46	-	416.1
AHQ-1-4, 4314 - 4324	K.IVGPSGAAVPCKVEPGLGADNSVVR.F	2451.78478	3	4.74E-04	0.94	4.97	-	1463.6
AHQ-1-1, 4424	K.IVGPSGAAVPCKVEPGLGADNSVVR.F	2451.78478	3	1.25E-04	0.82	3.94	-	739.6
AHQ-1-5, 4210	K.IVGPSGAAVPCKVEPGLGADNSVVR.F	2451.78478	3	3.43E-07	0.81	3.95	-	785.2
AHQ-1-1, 4295 - 4296	K.IVGPSGAAVPCKVEPGLGADNSVVR.F	2451.78478	2	5.09E-07	0.92	4.84	-	335.3
AHQ-1-1, 4285 - 4360	K.IVGPSGAAVPCKVEPGLGADNSVVR.F	2451.78478	3	8.39E-08	0.94	5.19	-	1250.6
AHQ-1-2, 4391 - 4462	K.IVGPSGAAVPCKVEPGLGADNSVVR.F	2451.78478	3	2.02E-10	0.92	5.08	-	1015.9
AHQ-1-6, 4139	K.IVGPSGAAVPCKVEPGLGADNSVVR.F	2451.78478	3	1.23E-04	0.94	4.57	-	1251.2
AHQ-1-2, 4393 - 4463	K.IVGPSGAAVPCKVEPGLGADNSVVR.F	2451.78478	2	1.68E-05	0.90	4.30	-	579.7
AHQ-1-2, 4533	K.IVGPSGAAVPCKVEPGLGADNSVVR.F	2451.78478	3	1.99E-08	0.84	3.58	-	969.4
AHQ-1-2, 4538	K.IVGPSGAAVPCKVEPGLGADNSVVR.F	2451.78478	2	9.81E-04	0.88	3.81	-	467.8
AHQ-1-1, 4479 - 4549	R.KDGSCGVAYVVQEPGDYEVSVK.F	2388.59282	2	7.95E-06	0.91	4.38	-	816.5
AHQ-1-1, 4475 - 4547	R.KDGSCGVAYVVQEPGDYEVSVK.F	2388.59282	3	4.67E-06	0.91	4.83	-	991.6
AHQ-1-4, 4467 - 4472	K.KNGQHVASSPIPVVISQSEIGDASR.V	2577.83547	2	1.93E-06	0.94	5.01	-	727.0
AHQ-1-2, 4610 - 4686	K.KNGQHVASSPIPVVISQSEIGDASR.V	2577.83547	3	2.30E-05	0.66	3.28	-	822.7
AHQ-1-8, 4148	K.KNGQHVASSPIPVVISQSEIGDASR.V	2577.83547	2	5.95E-05	0.93	4.56	-	732.9
AHQ-1-1, 4523 - 4569	K.KNGQHVASSPIPVVISQSEIGDASR.V	2577.83547	3	8.83E-13	0.97	5.95	-	2059.0
AHQ-1-4, 4462	K.KNGQHVASSPIPVVISQSEIGDASR.V	2577.83547	3	5.09E-09	0.98	5.86	-	2417.4
AHQ-1-3, 4441 - 4445	K.KNGQHVASSPIPVVISQSEIGDASR.V	2577.83547	3	5.75E-09	0.97	5.63	-	1853.1
AHQ-1-1, 4428 - 4497	K.KNGQHVASSPIPVVISQSEIGDASR.V	2577.83547	3	2.06E-08	0.96	5.51	-	1723.3
AHQ-1-5, 4237	K.KNGQHVASSPIPVVISQSEIGDASR.V	2577.83547	2	3.32E-04	0.57	2.83	-	331.5
AHQ-1-5, 4315 - 4342	K.KNGQHVASSPIPVVISQSEIGDASR.V	2577.83547	3	1.65E-10	0.95	5.01	-	1601.0
AHQ-1-1, 4500	K.KNGQHVASSPIPVVISQSEIGDASR.V	2577.83547	2	1.59E-07	0.74	3.37	-	402.7
AHQ-1-3, 3447 - 3451	K.KTHIQDNHDGTYTVAYVPDVTGR.Y	2588.77367	2	1.03E-05	0.95	4.77	-	923.9
AHQ-1-1, 3543	K.KTHIQDNHDGTYTVAYVPDVTGR.Y	2588.77367	2	8.24E-05	0.92	4.67	-	638.7
AHQ-1-4, 3622	K.KTHIQDNHDGTYTVAYVPDVTGR.Y	2588.77367	3	4.65E-04	0.82	3.46	-	875.6
AHQ-1-1, 3643 - 3709	K.KTHIQDNHDGTYTVAYVPDVTGR.Y	2588.77367	3	1.19E-08	0.91	4.24	-	958.3
AHQ-1-2, 3722	K.KTHIQDNHDGTYTVAYVPDVTGR.Y	2588.77367	2	1.56E-05	0.80	3.41	-	485.7
AHQ-1-3, 3439 - 3457	K.KTHIQDNHDGTYTVAYVPDVTGR.Y	2588.77367	3	1.88E-08	0.94	4.62	-	1266.4
AHQ-1-6, 3280	K.KTHIQDNHDGTYTVAYVPDVTGR.Y	2588.77367	3	3.45E-04	0.86	3.79	-	872.8
AHQ-1-2, 3545 - 3613	K.KTHIQDNHDGTYTVAYVPDVTGR.Y	2588.77367	3	1.31E-08	0.95	5.50	-	1029.0
AHQ-1-2, 3690 - 3761	K.KTHIQDNHDGTYTVAYVPDVTGR.Y	2588.77367	3	1.15E-04	0.96	4.84	-	1467.5
AHQ-1-1, 3540	K.KTHIQDNHDGTYTVAYVPDVTGR.Y	2588.77367	3	1.60E-08	0.95	5.03	-	1110.2
AHQ-1-2, 4137 - 4165	K.LDVQFSGLTK.G	1108.26945	2	5.74E-05	0.92	3.70	-	912.7
AHQ-1-1, 4059	K.LDVQFSGLTK.G	1108.26945	2	8.39E-07	0.93	3.55	-	1088.4
AHQ-1-3, 4006	K.LDVQFSGLTK.G	1108.26945	2	8.22E-07	0.94	3.70	-	1173.4
AHQ-1-3, 4005 - 4081	K.LDVQFSGLTK.G	1108.26945	1	2.59E-04	0.08	2.03	-	222.8
AHQ-1-4, 4042	K.LDVQFSGLTK.G	1108.26945	1	3.12E-05	0.46	2.31	-	329.4
AHQ-1-2, 4151	K.LDVQFSGLTK.G	1108.26945	1	4.57E-05	0.16	2.17	-	213.9
AHQ-1-4, 4044 - 4060	K.LDVQFSGLTK.G	1108.26945	2	2.24E-04	0.83	3.11	-	787.0
AHQ-1-2, 4138	K.LDVQFSGLTK.G	1108.26945	1	3.96E-06	0.09	1.90	-	224.5
AHQ-1-1, 4068	K.LDVQFSGLTK.G	1108.26945	1	4.68E-05	0.16	2.09	-	233.7
AHQ-1-6, 4788 - 4798	K.LPQLPITNFSR.D	1286.50461	2	3.91E-05	0.88	3.45	-	622.1
AHQ-1-3, 4949 - 4959	K.LPQLPITNFSR.D	1286.50461	2	3.59E-05	0.75	2.64	-	604.6
AHQ-1-2, 5085	K.LPQLPITNFSR.D	1286.50461	2	5.57E-04	0.88	3.28	-	735.8
AHQ-1-11, 4502	K.LPQLPITNFSR.D	1286.50461	2	6.87E-05	0.89	2.98	-	851.4
AHQ-1-2, 5302 - 5347	K.LPQLPITNFSR.D	1286.50461	2	8.56E-06	0.85	3.14	-	590.0
AHQ-1-2, 5115	K.LPQLPITNFSR.D	1286.50461	1	6.02E-05	0.44	2.90	-	165.7
AHQ-1-3, 5194	K.LPQLPITNFSR.D	1286.50461	2	6.68E-06	0.83	3.24	-	550.9
AHQ-1-4, 5271	K.LPQLPITNFSR.D	1286.50461	2	1.18E-05	0.89	3.22	-	755.4
AHQ-1-2, 5105	K.LPQLPITNFSR.D	1286.50461	1	6.91E-04	0.40	2.15	-	274.3
AHQ-1-5, 4918	K.LPQLPITNFSR.D	1286.50461	1	3.83E-04	0.25	2.35	-	133.5

AHQ-1-1, 5152 - 5223	K.LPQLPITNFSR.D	1286.50461	2	2.56E-05	0.83	3.43	-	612.5
AHQ-1-5, 4847 - 4917	K.LPQLPITNFSR.D	1286.50461	2	3.95E-04	0.88	3.32	-	625.5
AHQ-1-1, 4989 - 5052	K.LPQLPITNFSR.D	1286.50461	2	3.24E-05	0.91	3.45	-	701.3
AHQ-1-2, 5213	K.LPQLPITNFSR.D	1286.50461	2	1.12E-06	0.84	2.86	-	886.9
AHQ-1-4, 5027 - 5036	K.LPQLPITNFSR.D	1286.50461	2	6.40E-05	0.87	2.98	-	679.3
AHQ-1-3, 5487 - 5553	K.LQVEPAVDTSGVQCYGPGIEGQGVFR.E	2766.03597	2	1.45E-06	0.95	5.42	-	840.3
AHQ-1-6, 5416 - 5494	K.LQVEPAVDTSGVQCYGPGIEGQGVFR.E	2766.03597	2	1.26E-10	0.97	5.61	-	1151.2
AHQ-1-1, 5477 - 5551	K.LQVEPAVDTSGVQCYGPGIEGQGVFR.E	2766.03597	3	3.89E-15	0.98	6.83	-	2337.5
AHQ-1-7, 5562 - 5572	K.LQVEPAVDTSGVQCYGPGIEGQGVFR.E	2766.03597	2	4.65E-08	0.93	4.28	-	858.2
AHQ-1-2, 5634 - 5701	K.LQVEPAVDTSGVQCYGPGIEGQGVFR.E	2766.03597	2	1.18E-07	0.96	5.73	-	692.1
AHQ-1-4, 5606 - 5687	K.LQVEPAVDTSGVQCYGPGIEGQGVFR.E	2766.03597	2	1.88E-08	0.96	5.50	-	1039.3
AHQ-1-2, 5823	K.LQVEPAVDTSGVQCYGPGIEGQGVFR.E	2766.03597	3	5.21E-05	0.93	4.85	-	1016.7
AHQ-1-1, 5648	K.LQVEPAVDTSGVQCYGPGIEGQGVFR.E	2766.03597	2	1.41E-10	0.96	5.09	-	1035.6
AHQ-1-5, 5549 - 5617	K.LQVEPAVDTSGVQCYGPGIEGQGVFR.E	2766.03597	2	2.36E-08	0.97	5.43	-	1063.6
AHQ-1-9, 5328 - 5408	K.LQVEPAVDTSGVQCYGPGIEGQGVFR.E	2766.03597	2	2.41E-06	0.90	3.81	-	957.9
AHQ-1-1, 5485 - 5559	K.LQVEPAVDTSGVQCYGPGIEGQGVFR.E	2766.03597	2	1.61E-05	0.85	3.51	-	845.4
AHQ-1-2, 5737 - 5806	K.LQVEPAVDTSGVQCYGPGIEGQGVFR.E	2766.03597	2	8.63E-05	0.94	3.95	-	1221.4
AHQ-1-4, 5595 - 5663	K.LQVEPAVDTSGVQCYGPGIEGQGVFR.E	2766.03597	3	3.95E-08	0.98	7.28	-	2288.2
AHQ-1-4, 5788 - 5808	K.LQVEPAVDTSGVQCYGPGIEGQGVFR.E	2766.03597	2	1.60E-08	0.91	4.30	-	718.2
AHQ-1-2, 5633 - 5653	K.LQVEPAVDTSGVQCYGPGIEGQGVFR.E	2766.03597	3	6.76E-05	0.95	4.96	-	1250.7
AHQ-1-3, 5654	K.LQVEPAVDTSGVQCYGPGIEGQGVFR.E	2766.03597	2	4.05E-09	0.93	4.72	-	758.4
AHQ-1-3, 5519	K.LQVEPAVDTSGVQCYGPGIEGQGVFR.E	2766.03597	2	9.42E-07	0.81	3.58	-	384.8
AHQ-1-6, 3019	R.LRNGHVGISFVPK.E	1424.67564	2	4.70E-04	0.62	2.75	-	496.5
AHQ-1-1, 3325	R.LRNGHVGISFVPK.E	1424.67564	3	5.62E-04	0.94	3.64	-	1778.6
AHQ-1-5, 2835	R.LRNGHVGISFVPK.E	1424.67564	3	1.69E-05	0.91	3.40	-	1498.7
AHQ-1-9, 2903	R.LRNGHVGISFVPK.E	1424.67564	3	1.61E-06	0.91	3.59	-	1734.0
AHQ-1-5, 2986	R.LRNGHVGISFVPK.E	1424.67564	2	5.95E-04	0.68	2.64	-	646.3
AHQ-1-1, 4191	R.LSPFMADIR.D	1050.25664	2	7.21E-04	0.60	2.55	-	344.4
AHQ-1-3, 4093 - 4097	R.LSPFMADIR.D	1050.25664	2	8.30E-06	0.60	2.63	-	450.1
AHQ-1-1, 3491 - 3571	R.LTVSSLQESGLK.V	1262.43499	2	1.06E-04	0.74	2.87	-	521.3
AHQ-1-1, 3588	R.LTVSSLQESGLK.V	1262.43499	2	2.88E-07	0.90	3.35	-	920.2
AHQ-1-5, 3938	R.LVSNHSLHETSSVFVDSLTK.A	2201.42190	3	1.43E-04	0.87	3.57	-	841.1
AHQ-1-13, 4056	R.LVSNHSLHETSSVFVDSLTK.A	2201.42190	2	2.70E-04	0.93	4.42	-	840.1
AHQ-1-11, 4118	R.LVSNHSLHETSSVFVDSLTK.A	2201.42190	2	1.90E-08	0.97	5.55	-	1067.3
AHQ-1-13, 4069	R.LVSNHSLHETSSVFVDSLTK.A	2201.42190	3	6.03E-04	0.81	3.30	-	879.9
AHQ-1-1, 4587	R.LVSNHSLHETSSVFVDSLTK.A	2201.42190	2	4.20E-11	0.97	5.25	-	1112.2
AHQ-1-1, 4591	R.LVSNHSLHETSSVFVDSLTK.A	2201.42190	3	3.54E-11	0.97	5.81	-	1377.3
AHQ-1-7, 4093	R.LVSNHSLHETSSVFVDSLTK.A	2201.42190	2	1.16E-05	0.96	5.51	-	1082.9
AHQ-1-1, 4308	R.LVSNHSLHETSSVFVDSLTK.A	2201.42190	2	5.40E-06	0.96	5.45	-	1143.9
AHQ-1-14- , 4091	R.LVSNHSLHETSSVFVDSLTK.A	2201.42190	3	3.25E-06	0.96	4.71	-	1501.2
AHQ-1-5, 4266	R.LVSNHSLHETSSVFVDSLTK.A	2201.42190	2	4.68E-07	0.96	5.14	-	1166.0
AHQ-1-6, 4022	R.LVSNHSLHETSSVFVDSLTK.A	2201.42190	2	6.18E-06	0.93	4.81	-	924.3
AHQ-1-1, 4307	R.LVSNHSLHETSSVFVDSLTK.A	2201.42190	3	7.51E-09	0.96	5.09	-	1550.3
AHQ-1-13- , 4241	R.LVSNHSLHETSSVFVDSLTK.A	2201.42190	3	1.14E-06	0.95	4.98	-	1087.2
AHQ-1-7, 4413	R.LVSNHSLHETSSVFVDSLTK.A	2201.42190	2	3.84E-05	0.90	4.28	-	545.0
AHQ-1-5, 4073	R.LVSNHSLHETSSVFVDSLTK.A	2201.42190	2	7.10E-08	0.98	5.80	-	1650.1
AHQ-1-5, 4409 - 4426	R.LVSNHSLHETSSVFVDSLTK.A	2201.42190	3	2.69E-10	0.96	4.77	-	1582.9
AHQ-1-8, 4027	R.LVSNHSLHETSSVFVDSLTK.A	2201.42190	2	2.31E-07	0.97	5.18	-	1419.6
AHQ-1-8, 4029	R.LVSNHSLHETSSVFVDSLTK.A	2201.42190	3	2.98E-07	0.96	4.88	-	1435.8
AHQ-1-5, 4415 - 4497	R.LVSNHSLHETSSVFVDSLTK.A	2201.42190	2	1.55E-08	0.97	5.23	-	1353.7
AHQ-1-6, 4354	R.LVSNHSLHETSSVFVDSLTK.A	2201.42190	3	2.60E-08	0.92	3.96	-	1136.2
AHQ-1-6, 4362	R.LVSNHSLHETSSVFVDSLTK.A	2201.42190	2	8.93E-08	0.94	4.74	-	878.7
AHQ-1-10, 3795	R.LVSNHSLHETSSVFVDSLTK.A	2201.42190	3	4.09E-06	0.92	4.32	-	874.6
AHQ-1-11, 3886	R.LVSNHSLHETSSVFVDSLTK.A	2201.42190	2	1.72E-06	0.95	5.05	-	811.6
AHQ-1-14- , 4088	R.LVSNHSLHETSSVFVDSLTK.A	2201.42190	2	1.48E-10	0.96	5.24	-	1092.8
AHQ-1-4, 4231	R.LVSNHSLHETSSVFVDSLTK.A	2201.42190	2	1.14E-08	0.97	5.66	-	1431.1
AHQ-1-4, 4236	R.LVSNHSLHETSSVFVDSLTK.A	2201.42190	3	7.54E-10	0.97	5.89	-	1743.3
AHQ-1-4, 4576 - 4604	R.LVSNHSLHETSSVFVDSLTK.A	2201.42190	2	7.38E-10	0.97	5.09	-	1342.2
AHQ-1-4, 2524	K.MDCQECPEGYR.V	1447.55375	2	8.30E-06	0.84	3.26	-	709.8
AHQ-1-1, 2341	K.M*DCQECPEGYR.V	1463.55315	2	1.65E-05	0.93	2.82	-	1789.2
AHQ-1-1, 2600	K.MDCQECPEGYR.V	1447.55375	2	5.45E-05	0.76	3.08	-	640.5
AHQ-1-5, 2433	K.MDCQECPEGYR.V	1447.55375	2	3.47E-06	0.85	3.37	-	696.3
AHQ-1-5, 2995	R.NGHVGISFVPK.E	1155.33104	1	8.06E-07	0.39	2.34	-	408.8
AHQ-1-3, 4837	K.NGQHVASSPIPVVISQSEIGDASR.V	2449.66256	3	1.69E-07	0.76	3.13	-	1161.0
AHQ-1-4, 4914	K.NGQHVASSPIPVVISQSEIGDASR.V	2449.66256	2	3.97E-07	0.93	4.44	-	694.2
AHQ-1-1, 4844	K.NGQHVASSPIPVVISQSEIGDASR.V	2449.66256	2	7.23E-04	0.98	5.48	-	1742.7
AHQ-1-5, 4957	K.NGQHVASSPIPVVISQSEIGDASR.V	2449.66256	3	3.96E-05	0.73	3.29	-	757.4
AHQ-1-5, 4789	K.NGQHVASSPIPVVISQSEIGDASR.V	2449.66256	2	3.58E-09	0.96	4.82	-	1240.4
AHQ-1-5, 1833	K.RAEFTVETR.S	1109.21754	2	1.19E-05	0.94	3.53	-	945.5
AHQ-1-1, 2212	K.RAEFTVETR.S	1109.21754	2	3.02E-05	0.90	3.40	-	774.8
AHQ-1-3, 2038 - 2045	K.RAEFTVETR.S	1109.21754	2	4.15E-06	0.94	3.43	-	1136.9
AHQ-1-2, 2101	K.RAEFTVETR.S	1109.21754	2	2.31E-06	0.92	3.53	-	972.4
AHQ-1-4, 3082	R.RAPSVANVGSHCDLSLK.I	1813.02884	3	2.43E-05	0.97	5.13	-	2325.6
AHQ-1-5, 2919	R.RAPSVANVGSHCDLSLK.I	1813.02884	3	1.12E-06	0.97	5.97	-	1931.0
AHQ-1-1, 3201 - 3204	R.RAPSVANVGSHCDLSLK.I	1813.02884	3	5.13E-05	0.98	5.45	-	2108.2
AHQ-1-3, 3049	R.RAPSVANVGSHCDLSLK.I	1813.02884	3	6.55E-06	0.98	5.04	-	2653.5
AHQ-1-1, 3143 - 3220	R.RLTVSSLQESGLK.V	1418.62134	2	3.99E-04	0.97	4.33	-	2196.5
AHQ-1-5, 2810 - 2883	R.RLTVSSLQESGLK.V	1418.62134	2	1.63E-05	0.98	4.87	-	2385.5
AHQ-1-4, 2940 - 3086	R.RLTVSSLQESGLK.V	1418.62134	2	6.88E-05	0.97	4.38	-	1877.4
AHQ-1-5, 3047 - 3095	R.RLTVSSLQESGLK.V	1418.62134	2	1.39E-05	0.96	3.87	-	2025.2
AHQ-1-3, 7345 - 7406	K.SADFVVEAIGDDVGTLGFSVEGPSQAK.I	2696.90399	3	4.95E-07	0.96	6.20	-	1546.8
AHQ-1-2, 7561 - 7562	K.SADFVVEAIGDDVGTLGFSVEGPSQAK.I	2696.90399	2	4.65E-04	0.88	3.37	-	1063.2
AHQ-1-7, 3886	R.SAGQGEVLVYVEDPAGHQEEAK.V	2314.45074	3	1.07E-09	0.92	4.05	-	1339.9
AHQ-1-2, 4370	R.SAGQGEVLVYVEDPAGHQEEAK.V	2314.45074	2	3.22E-07	0.88	4.20	-	469.4
AHQ-1-4, 3947 - 4010	R.SAGQGEVLVYVEDPAGHQEEAK.V	2314.45074	2	1.57E-06	0.90	4.37	-	568.9
AHQ-1-1, 4177	R.SAGQGEVLVYVEDPAGHQEEAK.V	2314.45074	2	1.62E-07	0.87	4.09	-	567.5
AHQ-1-3, 4105	R.SAGQGEVLVYVEDPAGHQEEAK.V	2314.45074	2	1.20E-07	0.87	4.18	-	470.3
AHQ-1-4, 4011	R.SAGQGEVLVYVEDPAGHQEEAK.V	2314.45074	3	1.63E-10	0.95	4.70	-	1187.9
AHQ-1-1, 4119 - 4199	R.SAGQGEVLVYVEDPAGHQEEAK.V	2314.45074	3	1.82E-07	0.90	4.19	-	1093.7
AHQ-1-1, 4316	R.SAGQGEVLVYVEDPAGHQEEAK.V	2314.45074	2	9.85E-06	0.60	3.33	-	227.7
AHQ-1-7, 3880 - 3881	R.SAGQGEVLVYVEDPAGHQEEAK.V	2314.45074	2	1.52E-05	0.90	4.45	-	530.9
AHQ-1-4, 4100	R.SAGQGEVLVYVEDPAGHQEEAK.V	2314.45074	2	4.77E-05	0.88	4.23	-	564.2
AHQ-1-5, 3869	R.SAGQGEVLVYVEDPAGHQEEAK.V	2314.45074	2	6.35E-06	0.93	5.18	-	605.2
AHQ-1-3, 3969	R.SAGQGEVLVYVEDPAGHQEEAK.V	2314.45074	2	8.31E-09	0.93	4.87	-	635.9
AHQ-1-2, 4253 - 4254	R.SAGQGEVLVYVEDPAGHQEEAK.V	2314.45074	2	1.02E-07	0.95	5.13	-	843.1
AHQ-1-3, 3973	R.SAGQGEVLVYVEDPAGHQEEAK.V	2314.45074	3	6.57E-06	0.91	3.87	-	999.3
AHQ-1-2, 4417 - 4437	R.SAGQGEVLVYVEDPAGHQEEAK.V	2314.45074	3	4.52E-04	0.92	4.10	-	1319.8
AHQ-1-2, 4105 - 4173	R.SAGQGEVLVYVEDPAGHQEEAK.V	2314.45074	3	3.65E-08	0.93	4.16	-	1330.3
AHQ-1-2, 4103 - 4174	R.SAGQGEVLVYVEDPAGHQEEAK.V	2314.45074	2	1.87E-07	0.94	4.97	-	741.8
AHQ-1-2, 4527	R.SAGQGEVLVYVEDPAGHQEEAK.V	2314.45074	2	2.16E-07	0.92	4.37	-	669.5
AHQ-1-5, 3853 - 3931	R.SAGQGEVLVYVEDPAGHQEEAK.V	2314.45074	3	7.18E-09	0.94	4.60	-	1112.8
AHQ-1-6, 3788	R.SAGQGEVLVYVEDPAGHQEEAK.V	2314.45074	3	1.63E-07	0.89	3.98	-	1012.9
AHQ-1-1, 3992 - 4055	R.SAGQGEVLVYVEDPAGHQEEAK.V	2314.45074	3	9.62E-10	0.94	4.38	-	1453.3
AHQ-1-1, 4039 - 4103	R.SAGQGEVLVYVEDPAGHQEEAK.V	2314.45074	2	1.62E-06	0.91	4.13	-	716.0
AHQ-1-8, 3839	R.SAGQGEVLVYVEDPAGHQEEAK.V	2314.45074	2	2.37E-05	0.92	4.42	-	655.8
AHQ-1-9, 3760 - 3806	R.SAGQGEVLVYVEDPAGHQEEAK.V	2314.45074	2	1.57E-04	0.78	3.49	-	382.6
AHQ-1-1, 3509	K.SPFEVYVDK.S	1084.20321	1	3.39E-04	0.77	3.18	-	836.4

AHQ-1-4, 3446	K.SPFEVYVDK.S	1084.20321	2	1.65E-05	0.90	3.02	-	1038.9
AHQ-1-1, 3501 - 3504	K.SPFEVYVDK.S	1084.20321	2	4.18E-06	0.94	3.56	-	1163.3
AHQ-1-1, 3500	K.SPFEVYVDK.S	1084.20321	1	2.32E-04	0.74	2.38	-	1005.2
AHQ-1-3, 3413	K.SPFEVYVDK.S	1084.20321	2	2.50E-05	0.93	3.60	-	1098.7
AHQ-1-3, 3431	K.SPFEVYVDK.S	1084.20321	1	6.79E-08	0.77	2.54	-	792.8
AHQ-1-7, 3341	K.SPFEVYVDK.S	1084.20321	2	1.98E-05	0.94	3.29	-	1450.1
AHQ-1-2, 3509 - 3521	K.SPFEVYVDK.S	1084.20321	2	9.69E-06	0.95	3.48	-	1405.3
AHQ-1-2, 3517	K.SPFEVYVDK.S	1084.20321	1	1.61E-05	0.86	3.29	-	869.2
AHQ-1-5, 3322	K.SPFEVYVDK.S	1084.20321	2	1.78E-06	0.90	2.78	-	1247.0
AHQ-1-5, 3341	K.SPFEVYVDK.S	1084.20321	1	2.59E-06	0.84	2.99	-	868.0
AHQ-1-6, 4958 - 4972	K.SPFSVAVSPSLDLSK.I	1534.73557	2	3.58E-05	0.77	3.05	-	765.1
AHQ-1-2, 5262	K.SPFSVAVSPSLDLSK.I	1534.73557	2	5.20E-04	0.84	3.42	-	640.9
AHQ-1-3, 5122	K.SPFSVAVSPSLDLSK.I	1534.73557	2	2.47E-04	0.88	3.15	-	1039.9
AHQ-1-2, 5146 - 5221	K.SPFSVAVSPSLDLSK.I	1534.73557	2	2.27E-04	0.94	4.54	-	1026.3
AHQ-1-3, 5013 - 5082	K.SPFSVAVSPSLDLSK.I	1534.73557	2	1.18E-05	0.85	4.24	-	549.7
AHQ-1-1, 5036 - 5093	K.SPFSVAVSPSLDLSK.I	1534.73557	2	3.78E-06	0.92	3.80	-	1111.8
AHQ-1-5, 5037 - 5038	K.SPFSVAVSPSLDLSK.I	1534.73557	2	3.30E-07	0.95	4.63	-	1048.4
AHQ-1-2, 3787	R.SPYTVTVGQACNPSACR.A	1871.04208	2	4.24E-05	0.86	3.90	-	362.1
AHQ-1-1, 3708	R.SPYTVTVGQACNPSACR.A	1871.04208	2	2.41E-08	0.90	3.88	-	653.7
AHQ-1-5, 3417	R.SPYTVTVGQACNPSACR.A	1871.04208	2	5.41E-05	0.91	4.22	-	534.3
AHQ-1-6, 3328 - 3399	R.SPYTVTVGQACNPSACR.A	1871.04208	2	7.91E-04	0.88	3.69	-	502.8
AHQ-1-1, 3531 - 3565	R.SPYTVTVGQACNPSACR.A	1871.04208	2	5.12E-04	0.41	2.74	-	318.5
AHQ-1-6, 3379	R.SPYTVTVGQACNPSACR.A	1871.04208	2	2.66E-05	0.89	4.07	-	463.5
AHQ-1-3, 3453 - 3521	R.SPYTVTVGQACNPSACR.A	1871.04208	2	1.16E-04	0.84	4.05	-	335.1
AHQ-1-2, 2841	K.SQGDASKVTAQGPGLEPSGNIANK.T	2327.49419	2	4.46E-04	0.77	4.10	-	329.1
AHQ-1-14- , 2188	K.SSFTVDCSK.A	1032.10676	1	1.85E-05	0.58	2.65	-	416.5
AHQ-1-14- , 2163 - 2234	K.SSFTVDCSK.A	1032.10676	1	3.01E-04	0.49	1.95	-	505.0
AHQ-1-3, 5066 - 5133	R.TFSVWYVPEVTGTHK.V	1751.96228	2	5.79E-04	0.78	3.20	-	435.4
AHQ-1-6, 4879 - 4955	R.TFSVWYVPEVTGTHK.V	1751.96228	2	9.85E-04	0.32	2.73	-	206.9
AHQ-1-7, 4988 - 5061	R.TFSVWYVPEVTGTHK.V	1751.96228	2	8.22E-04	0.46	2.76	-	298.2
AHQ-1-5, 5002 - 5011	R.TFSVWYVPEVTGTHK.V	1751.96228	3	3.20E-06	0.64	3.46	-	399.2
AHQ-1-3, 5083	R.TFSVWYVPEVTGTHK.V	1751.96228	3	4.18E-06	0.71	3.27	-	466.8
AHQ-1-1, 3337 - 3344	K.TGVAVNKPAEFTVDAK.H	1647.85391	2	4.18E-05	0.88	4.11	-	732.7
AHQ-1-5, 2955	K.TGVAVNKPAEFTVDAK.H	1647.85391	3	7.81E-09	0.93	4.42	-	900.9
AHQ-1-2, 3138 - 3211	K.TGVAVNKPAEFTVDAK.H	1647.85391	3	1.14E-06	0.87	3.56	-	854.0
AHQ-1-4, 3087 - 3090	K.TGVAVNKPAEFTVDAK.H	1647.85391	2	1.94E-07	0.91	4.13	-	685.0
AHQ-1-4, 3096	K.TGVAVNKPAEFTVDAK.H	1647.85391	3	6.11E-07	0.75	3.06	-	916.6
AHQ-1-6, 2962	K.TGVAVNKPAEFTVDAK.H	1647.85391	2	9.06E-06	0.87	3.70	-	549.1
AHQ-1-3, 3065	K.TGVAVNKPAEFTVDAK.H	1647.85391	3	3.42E-08	0.76	3.12	-	779.2
AHQ-1-1, 3137 - 3209	K.TGVAVNKPAEFTVDAK.H	1647.85391	3	1.59E-05	0.93	4.14	-	1185.2
AHQ-1-2, 3133 - 3202	K.TGVAVNKPAEFTVDAK.H	1647.85391	2	9.17E-06	0.94	4.42	-	873.1
AHQ-1-2, 3070 - 3142	R.TGVELGKPTHFTVNAK.A	1699.93205	2	1.55E-07	0.88	3.74	-	651.4
AHQ-1-3, 2950 - 3021	R.TGVELGKPTHFTVNAK.A	1699.93205	2	4.09E-04	0.82	3.53	-	568.1
AHQ-1-3, 2851 - 2866	R.TGVELGKPTHFTVNAK.A	1699.93205	3	5.53E-06	0.82	4.12	-	512.0
AHQ-1-1, 3016	R.TGVELGKPTHFTVNAK.A	1699.93205	3	1.43E-04	0.63	3.26	-	538.3
AHQ-1-1, 3147	R.TGVELGKPTHFTVNAK.A	1699.93205	2	7.44E-05	0.89	3.84	-	710.4
AHQ-1-2, 2953	R.TGVELGKPTHFTVNAK.A	1699.93205	2	3.11E-06	0.92	4.43	-	657.2
AHQ-1-5, 2702	R.TGVELGKPTHFTVNAK.A	1699.93205	2	2.59E-06	0.87	3.81	-	539.5
AHQ-1-1, 3200 - 3273	K.THEAEIVEGENHTYCIR.F	2060.19046	3	8.16E-11	0.94	4.73	-	1034.1
AHQ-1-8, 2996	K.THEAEIVEGENHTYCIR.F	2060.19046	2	5.99E-13	0.98	5.01	-	1883.4
AHQ-1-1, 3285	K.THEAEIVEGENHTYCIR.F	2060.19046	2	1.30E-05	0.92	4.84	-	553.5
AHQ-1-2, 3231	K.THEAEIVEGENHTYCIR.F	2060.19046	3	8.67E-08	0.92	4.40	-	896.7
AHQ-1-5, 3091	K.THEAEIVEGENHTYCIR.F	2060.19046	2	7.36E-13	0.98	5.55	-	2102.3
AHQ-1-5, 2890 - 2961	K.THEAEIVEGENHTYCIR.F	2060.19046	3	1.16E-06	0.93	4.17	-	1047.0
AHQ-1-5, 2958 - 3013	K.THEAEIVEGENHTYCIR.F	2060.19046	2	3.57E-10	0.99	6.48	-	2472.8
AHQ-1-4, 3239	K.THEAEIVEGENHTYCIR.F	2060.19046	2	2.20E-08	0.97	4.79	-	1798.9
AHQ-1-4, 3150	K.THEAEIVEGENHTYCIR.F	2060.19046	2	6.14E-12	0.98	5.27	-	1901.2
AHQ-1-3, 3090	K.THEAEIVEGENHTYCIR.F	2060.19046	3	5.77E-05	0.85	3.62	-	831.2
AHQ-1-7, 2990	K.THEAEIVEGENHTYCIR.F	2060.19046	2	6.34E-04	0.98	5.29	-	1754.1
AHQ-1-4, 3076 - 3146	K.THEAEIVEGENHTYCIR.F	2060.19046	3	1.60E-07	0.91	4.16	-	1165.2
AHQ-1-1, 3985	K.THIQDNHDGTYTVAYVPDVTGR.Y	2460.60076	2	4.27E-11	0.97	4.63	-	1635.5
AHQ-1-1, 3859 - 3932	K.THIQDNHDGTYTVAYVPDVTGR.Y	2460.60076	3	8.77E-04	0.73	3.25	-	381.2
AHQ-1-10, 2920 - 2928	R.TPCEEILVK.H	1090.27249	2	3.25E-04	0.80	2.96	-	783.6
AHQ-1-4, 3190	R.TPCEEILVK.H	1090.27249	2	5.77E-04	0.89	3.34	-	771.5
AHQ-1-4, 3203	R.TPCEEILVK.H	1090.27249	1	1.98E-06	0.52	2.49	-	496.0
AHQ-1-13, 3123 - 3138	R.TPCEEILVK.H	1090.27249	2	3.60E-05	0.87	3.03	-	866.9
AHQ-1-1, 3259	R.TPCEEILVK.H	1090.27249	2	2.08E-06	0.79	2.69	-	630.5
AHQ-1-14- , 3095 - 3158	R.TPCEEILVK.H	1090.27249	2	3.48E-05	0.87	3.30	-	666.1
AHQ-1-7, 3088 - 3097	R.TPCEEILVK.H	1090.27249	2	4.46E-06	0.91	3.17	-	928.3
AHQ-1-3, 3163	R.TPCEEILVK.H	1090.27249	2	2.43E-05	0.68	2.89	-	520.6
AHQ-1-13- , 3169	R.TPCEEILVK.H	1090.27249	2	3.79E-05	0.84	2.66	-	1018.8
AHQ-1-14, 4014 - 4017	R.TPCEEILVK.H	1090.27249	2	9.42E-07	0.75	2.73	-	600.4
AHQ-1-14- , 3107	R.TPCEEILVK.H	1090.27249	1	3.58E-05	0.59	2.82	-	501.6
AHQ-1-11, 3002	R.TPCEEILVK.H	1090.27249	2	8.91E-07	0.81	2.76	-	640.2
AHQ-1-6, 3054	R.TPCEEILVK.H	1090.27249	2	7.28E-05	0.80	3.06	-	622.1
AHQ-1-5, 3069	R.TPCEEILVK.H	1090.27249	2	6.40E-05	0.89	3.19	-	792.0
AHQ-1-2, 3582 - 3653	R.VANPSGNLTETYVQDR.G	1764.87462	2	6.34E-13	0.93	4.17	-	703.8
AHQ-1-1, 3235	R.VANPSGNLTETYVQDR.G	1764.87462	2	7.10E-09	0.94	4.27	-	876.9
AHQ-1-7, 3268	R.VANPSGNLTETYVQDR.G	1764.87462	2	1.70E-04	0.90	3.85	-	870.2
AHQ-1-1, 3615	R.VANPSGNLTETYVQDR.G	1764.87462	2	8.03E-13	0.59	2.50	-	555.2
AHQ-1-4, 3504	R.VANPSGNLTETYVQDR.G	1764.87462	2	7.45E-10	0.89	3.85	-	609.6
AHQ-1-3, 3325	R.VANPSGNLTETYVQDR.G	1764.87462	2	1.73E-13	0.89	4.03	-	484.7
AHQ-1-2, 3726	R.VANPSGNLTETYVQDR.G	1764.87462	2	2.36E-05	0.93	4.06	-	1006.3
AHQ-1-1, 3511 - 3591	R.VANPSGNLTETYVQDR.G	1764.87462	2	8.00E-12	0.96	4.64	-	1089.9
AHQ-1-3, 3462 - 3537	R.VANPSGNLTETYVQDR.G	1764.87462	2	7.95E-10	0.95	4.79	-	908.7
AHQ-1-2, 3415 - 3485	R.VANPSGNLTETYVQDR.G	1764.87462	2	3.62E-11	0.93	4.08	-	857.3
AHQ-1-13- , 3369	R.VANPSGNLTETYVQDR.G	1764.87462	2	3.11E-05	0.86	3.67	-	599.4
AHQ-1-5, 3410	R.VANPSGNLTETYVQDR.G	1764.87462	2	2.72E-08	0.88	3.99	-	756.7
AHQ-1-8, 3154	R.VANPSGNLTETYVQDR.G	1764.87462	2	1.58E-05	0.90	3.51	-	919.3
AHQ-1-5, 3218 - 3278	R.VANPSGNLTETYVQDR.G	1764.87462	2	3.91E-10	0.91	3.82	-	658.4
AHQ-1-3, 3306 - 3377	R.VANPSGNLTETYVQDR.G	1764.87462	2	5.89E-12	0.95	4.14	-	1267.6
AHQ-1-1, 3373 - 3443	R.VANPSGNLTETYVQDR.G	1764.87462	2	4.02E-13	0.94	4.05	-	972.4
AHQ-1-8, 2647	K.VAQPTITDNKDGTVTVR.Y	1816.00621	2	3.49E-06	0.82	3.25	-	613.5
AHQ-1-10, 2495	K.VAQPTITDNKDGTVTVR.Y	1816.00621	2	2.76E-04	0.34	2.67	-	264.2
AHQ-1-4, 2858	K.VAQPTITDNKDGTVTVR.Y	1816.00621	2	2.20E-05	0.89	4.27	-	490.6
AHQ-1-3, 2846	K.VAQPTITDNKDGTVTVR.Y	1816.00621	2	4.16E-04	0.88	3.87	-	613.8
AHQ-1-9, 2690	K.VAQPTITDNKDGTVTVR.Y	1816.00621	2	5.84E-04	0.57	2.90	-	408.2
AHQ-1-5, 2515	K.VAQPTITDNKDGTVTVR.Y	1816.00621	2	8.37E-06	0.67	3.14	-	456.7
AHQ-1-1, 2861 - 2927	K.VAQPTITDNKDGTVTVR.Y	1816.00621	2	6.11E-07	0.92	4.08	-	795.4
AHQ-1-5, 2739	K.VAQPTITDNKDGTVTVR.Y	1816.00621	2	1.98E-05	0.93	4.17	-	909.0
AHQ-1-1, 2979	K.VAQPTITDNKDGTVTVR.Y	1816.00621	2	8.51E-07	0.87	3.70	-	675.8
AHQ-1-4, 2776	K.VAQPTITDNKDGTVTVR.Y	1816.00621	2	5.55E-06	0.82	3.35	-	643.9
AHQ-1-2, 2949	K.VAQPTITDNKDGTVTVR.Y	1816.00621	2	3.21E-05	0.81	3.52	-	489.7
AHQ-1-13- , 2915	K.VAQPTITDNKDGTVTVR.Y	1816.00621	2	2.44E-04	0.81	3.19	-	587.7
AHQ-1-3, 3654	K.VDINTEDLEDGTCR.V	1638.69283	2	2.88E-07	0.81	3.21	-	877.3
AHQ-1-4, 3438	K.VDINTEDLEDGTCR.V	1638.69283	2	1.87E-04	0.89	3.45	-	883.6
AHQ-1-9, 3548	K.VDINTEDLEDGTCR.V	1638.69283	2	9.22E-07	0.89	3.49	-	958.0

AHQ-1-1, 3387	K.VDINTEDLEDGTCR.V	1638.69283	2	7.90E-06	0.88	3.50	-	830.1
AHQ-1-5, 3307 - 3377	K.VDINTEDLEDGTCR.V	1638.69283	2	4.98E-04	0.92	3.54	-	943.1
AHQ-1-6, 3567	K.VDINTEDLEDGTCR.V	1638.69283	2	3.03E-05	0.87	3.35	-	830.3
AHQ-1-1, 3609 - 3669	K.VDINTEDLEDGTCR.V	1638.69283	2	5.28E-06	0.87	3.38	-	852.7
AHQ-1-13, 3505	K.VDINTEDLEDGTCR.V	1638.69283	2	2.69E-04	0.91	3.35	-	1214.7
AHQ-1-8, 3609	K.VDINTEDLEDGTCR.V	1638.69283	2	1.26E-04	0.91	3.96	-	898.8
AHQ-1-5, 3463	K.VDINTEDLEDGTCR.V	1638.69283	2	3.29E-06	0.87	3.27	-	888.8
AHQ-1-5, 3613	K.VDINTEDLEDGTCR.V	1638.69283	2	1.74E-05	0.92	3.80	-	991.3
AHQ-1-1, 3492	K.VDINTEDLEDGTCR.V	1638.69283	2	6.43E-08	0.91	3.53	-	1132.5
AHQ-1-5, 3761	K.VDINTEDLEDGTCR.V	1638.69283	2	7.30E-09	0.89	3.22	-	995.7
AHQ-1-7, 3664 - 3737	K.VDINTEDLEDGTCR.V	1638.69283	2	7.56E-06	0.85	3.19	-	850.3
AHQ-1-2, 3785	K.VDINTEDLEDGTCR.V	1638.69283	2	1.27E-06	0.92	3.93	-	876.5
AHQ-1-3, 3385	K.VDINTEDLEDGTCR.V	1638.69283	2	4.40E-08	0.87	3.68	-	847.7
AHQ-1-6, 2482	K.VDVGKDQEFTVK.S	1365.51377	2	6.94E-09	0.78	3.07	-	609.2
AHQ-1-1, 2703	K.VDVGKDQEFTVK.S	1365.51377	2	4.03E-09	0.86	3.32	-	740.4
AHQ-1-5, 2461	K.VDVGKDQEFTVK.S	1365.51377	2	1.64E-07	0.75	3.41	-	400.2
AHQ-1-3, 2565	K.VDVGKDQEFTVK.S	1365.51377	2	2.02E-06	0.87	3.32	-	655.5
AHQ-1-4, 2566 - 2572	K.VDVGKDQEFTVK.S	1365.51377	2	3.43E-09	0.88	3.62	-	706.6
AHQ-1-3, 2575	K.VDVGKDQEFTVK.S	1365.51377	1	4.73E-06	0.26	2.55	-	281.2
AHQ-1-2, 2647 - 2659	K.VDVGKDQEFTVK.S	1365.51377	2	3.14E-05	0.85	3.18	-	738.1
AHQ-1-3, 2710 - 2789	K.VEPGLGADNSVVR.F	1313.44209	2	9.36E-08	0.74	2.98	-	259.8
AHQ-1-2, 2805 - 2878	K.VEPGLGADNSVVR.F	1313.44209	2	1.02E-07	0.89	3.74	-	438.7
AHQ-1-1, 2840 - 2917	K.VEPGLGADNSVVR.F	1313.44209	2	2.79E-04	0.70	2.70	-	271.3
AHQ-1-3, 6297 - 6369	K.VEYTPYEEGLHSVDVTYDGSPVPSSPFQVPVTEGCDPSR.V	4300.57494	3	2.61E-07	0.96	6.38	-	1170.2
AHQ-1-1, 6339	K.VEYTPYEEGLHSVDVTYDGSPVPSSPFQVPVTEGCDPSR.V	4300.57494	3	3.83E-07	0.82	4.06	-	496.1
AHQ-1-3, 6438 - 6449	K.VEYTPYEEGLHSVDVTYDGSPVPSSPFQVPVTEGCDPSR.V	4300.57494	3	2.69E-04	0.86	4.45	-	367.9
AHQ-1-4, 6534	K.VEYTPYEEGLHSVDVTYDGSPVPSSPFQVPVTEGCDPSR.V	4300.57494	3	2.19E-05	0.90	5.12	-	457.6
AHQ-1-2, 6465 - 6533	K.VEYTPYEEGLHSVDVTYDGSPVPSSPFQVPVTEGCDPSR.V	4300.57494	3	6.72E-06	0.94	5.15	-	994.0
AHQ-1-1, 6219 - 6279	K.VEYTPYEEGLHSVDVTYDGSPVPSSPFQVPVTEGCDPSR.V	4300.57494	3	4.05E-05	0.93	5.11	-	849.3
AHQ-1-5, 6422 - 6502	K.VEYTPYEEGLHSVDVTYDGSPVPSSPFQVPVTEGCDPSR.V	4300.57494	3	6.34E-05	0.95	5.64	-	713.6
AHQ-1-3, 7214 - 7221	K.VGSAADIPINISETDLSLLTATVVPPSGR.E	2895.25495	2	3.50E-04	0.89	4.02	-	716.6
AHQ-1-1, 6907 - 6971	K.VGSAADIPINISETDLSLLTATVVPPSGR.E	2895.25495	2	1.51E-09	0.94	4.86	-	786.9
AHQ-1-8, 7434 - 7472	K.VGSAADIPINISETDLSLLTATVVPPSGR.E	2895.25495	2	1.93E-05	0.93	4.96	-	582.8
AHQ-1-10, 6343 - 6344	K.VGSAADIPINISETDLSLLTATVVPPSGR.E	2895.25495	2	2.80E-07	0.95	5.17	-	801.1
AHQ-1-5, 7407	K.VGSAADIPINISETDLSLLTATVVPPSGR.E	2895.25495	2	7.46E-09	0.96	5.74	-	779.1
AHQ-1-8, 7435 - 7440	K.VGSAADIPINISETDLSLLTATVVPPSGR.E	2895.25495	3	8.29E-04	0.79	3.86	-	598.0
AHQ-1-2, 1507 - 1583	R.VHGPGIQSGTTNKPNK.F	1635.80645	2	2.63E-04	0.90	3.45	-	922.3
AHQ-1-1, 4532	K.VHSPSGALEECYVTEIDQDK.Y	2279.42322	2	4.01E-11	0.96	5.40	-	1146.3
AHQ-1-4, 4523	K.VHSPSGALEECYVTEIDQDK.Y	2279.42322	2	1.86E-06	0.89	3.75	-	1121.2
AHQ-1-4, 4608	K.VHSPSGALEECYVTEIDQDK.Y	2279.42322	2	4.83E-06	0.94	4.41	-	1162.0
AHQ-1-7, 4393	K.VHSPSGALEECYVTEIDQDK.Y	2279.42322	2	2.81E-04	0.96	4.75	-	1272.4
AHQ-1-3, 4489	K.VHSPSGALEECYVTEIDQDK.Y	2279.42322	3	1.18E-04	0.95	4.87	-	1451.1
AHQ-1-6, 4323	K.VHSPSGALEECYVTEIDQDK.Y	2279.42322	2	6.30E-08	0.97	5.51	-	1241.4
AHQ-1-5, 4389	K.VHSPSGALEECYVTEIDQDK.Y	2279.42322	2	1.52E-08	0.94	4.84	-	714.7
AHQ-1-9, 4615 - 4624	K.VHSPSGALEECYVTEIDQDKYAVR.F	2768.99349	3	4.80E-13	0.93	4.91	-	897.4
AHQ-1-5, 4801	K.VHSPSGALEECYVTEIDQDKYAVR.F	2768.99349	3	7.19E-11	0.90	4.57	-	659.3
AHQ-1-5, 4741	K.VHSPSGALEECYVTEIDQDKYAVR.F	2768.99349	2	1.67E-15	0.94	4.63	-	868.3
AHQ-1-5, 4703 - 4726	K.VHSPSGALEECYVTEIDQDKYAVR.F	2768.99349	3	6.95E-11	0.91	4.65	-	676.2
AHQ-1-3, 4817	K.VHSPSGALEECYVTEIDQDKYAVR.F	2768.99349	3	3.86E-06	0.69	3.27	-	355.1
AHQ-1-2, 4993	K.VHSPSGALEECYVTEIDQDKYAVR.F	2768.99349	3	1.11E-14	0.86	3.83	-	705.4
AHQ-1-4, 4882	K.VHSPSGALEECYVTEIDQDKYAVR.F	2768.99349	3	1.94E-09	0.92	4.83	-	631.3
AHQ-1-4, 4892	K.VHSPSGALEECYVTEIDQDKYAVR.F	2768.99349	2	2.77E-06	0.90	4.62	-	445.1
AHQ-1-6, 4662	K.VHSPSGALEECYVTEIDQDKYAVR.F	2768.99349	3	7.61E-05	0.92	4.69	-	673.6
AHQ-1-1, 4925	K.VHSPSGALEECYVTEIDQDKYAVR.F	2768.99349	2	1.04E-09	0.94	4.45	-	1235.0
AHQ-1-7, 4713	K.VHSPSGALEECYVTEIDQDKYAVR.F	2768.99349	3	1.67E-06	0.92	5.26	-	553.5
AHQ-1-8, 4596	K.VHSPSGALEECYVTEIDQDKYAVR.F	2768.99349	2	1.48E-08	0.92	4.28	-	794.8
AHQ-1-8, 4587 - 4659	K.VHSPSGALEECYVTEIDQDKYAVR.F	2768.99349	3	6.46E-07	0.84	3.61	-	748.3
AHQ-1-1, 4855 - 4920	K.VHSPSGALEECYVTEIDQDKYAVR.F	2768.99349	3	7.47E-09	0.95	5.26	-	861.9
AHQ-1-4, 3814	K.VNQPASFAVSLNGAK.G	1503.68480	2	3.86E-08	0.83	3.66	-	511.8
AHQ-1-4, 3926	K.VNQPASFAVSLNGAK.G	1503.68480	2	4.34E-06	0.91	3.69	-	673.9
AHQ-1-8, 3538	K.VNQPASFAVSLNGAK.G	1503.68480	2	4.39E-09	0.91	4.05	-	666.8
AHQ-1-5, 3693 - 3719	K.VNQPASFAVSLNGAK.G	1503.68480	2	1.02E-07	0.82	3.68	-	478.9
AHQ-1-5, 3787 - 3862	K.VNQPASFAVSLNGAK.G	1503.68480	2	5.18E-06	0.91	4.35	-	559.0
AHQ-1-2, 4014	K.VNQPASFAVSLNGAK.G	1503.68480	2	1.15E-05	0.92	4.12	-	620.2
AHQ-1-9, 3575	K.VNQPASFAVSLNGAK.G	1503.68480	2	7.27E-05	0.87	3.49	-	652.1
AHQ-1-1, 3855 - 3919	K.VNQPASFAVSLNGAK.G	1503.68480	2	3.71E-08	0.82	3.41	-	480.5
AHQ-1-3, 3789 - 3870	K.VNQPASFAVSLNGAK.G	1503.68480	2	9.58E-07	0.87	3.85	-	548.7
AHQ-1-6, 3650 - 3728	K.VNQPASFAVSLNGAK.G	1503.68480	2	2.36E-07	0.92	4.36	-	601.2
AHQ-1-2, 3930	K.VNQPASFAVSLNGAK.G	1503.68480	2	5.11E-07	0.94	4.09	-	708.5
AHQ-1-3, 3877	K.VNQPASFAVSLNGAK.G	1503.68480	2	3.16E-08	0.65	2.85	-	481.2
AHQ-1-7, 3809 - 3825	K.VNQPASFAVSLNGAK.G	1503.68480	2	1.64E-06	0.78	3.12	-	477.9
AHQ-1-2, 1589 - 1662	K.VPVHDVTDASK.V	1168.28175	1	5.73E-04	0.62	2.39	-	739.4
AHQ-1-1, 1804 - 1865	K.VPVHDVTDASK.V	1168.28175	2	1.36E-07	0.96	3.71	-	1548.2
AHQ-1-3, 1673	K.VPVHDVTDASK.V	1168.28175	2	6.01E-07	0.88	2.86	-	848.5
AHQ-1-3, 1579	K.VPVHDVTDASK.V	1168.28175	2	5.19E-08	0.92	3.27	-	1181.3
AHQ-1-4, 1596	K.VPVHDVTDASK.V	1168.28175	2	1.35E-08	0.93	3.41	-	1196.2
AHQ-1-2, 1559 - 1617	K.VPVHDVTDASK.V	1168.28175	2	6.95E-08	0.96	3.25	-	1750.4
AHQ-1-4, 4243	R.VQVQDNEGCPVEALVK.D	1786.98493	2	1.28E-06	0.94	3.99	-	1367.4
AHQ-1-3, 4310	R.VQVQDNEGCPVEALVK.D	1786.98493	2	4.11E-04	0.93	4.10	-	958.9
AHQ-1-3, 4198 - 4218	R.VQVQDNEGCPVEALVK.D	1786.98493	2	1.36E-09	0.97	5.41	-	1384.6
AHQ-1-2, 4466	R.VQVQDNEGCPVEALVK.D	1786.98493	2	9.75E-10	0.94	4.67	-	1124.5
AHQ-1-2, 4325 - 4397	R.VQVQDNEGCPVEALVK.D	1786.98493	2	4.67E-07	0.97	5.32	-	1409.7
AHQ-1-4, 4387	R.VQVQDNEGCPVEALVK.D	1786.98493	2	1.12E-10	0.88	3.98	-	693.3
AHQ-1-1, 4239	R.VQVQDNEGCPVEALVK.D	1786.98493	2	1.53E-06	0.95	4.45	-	1200.0
AHQ-1-1, 4412	R.VQVQDNEGCPVEALVK.D	1786.98493	2	1.18E-06	0.92	4.35	-	1151.9
AHQ-1-5, 4305	R.VQVQDNEGCPVEALVK.D	1786.98493	2	6.62E-07	0.94	4.08	-	1265.8
AHQ-1-1, 5481 - 5497	R.VQVQDNEGCPVEALVKDNGNGTYSCSYVPR.K	3359.60342	3	1.43E-06	0.86	3.92	-	900.9
AHQ-1-2, 5509 - 5582	R.VQVQDNEGCPVEALVKDNGNGTYSCSYVPR.K	3359.60342	3	2.22E-15	0.95	5.00	-	1491.6
AHQ-1-2, 5374 - 5441	R.VQVQDNEGCPVEALVKDNGNGTYSCSYVPR.K	3359.60342	3	6.24E-06	0.92	4.37	-	1153.5
AHQ-1-3, 5410	R.VQVQDNEGCPVEALVKDNGNGTYSCSYVPR.K	3359.60342	3	6.21E-08	0.91	4.12	-	1317.4
AHQ-1-3, 5138 - 5209	R.VQVQDNEGCPVEALVKDNGNGTYSCSYVPR.K	3359.60342	3	9.59E-09	0.95	5.30	-	1301.7
AHQ-1-5, 5261 - 5329	R.VQVQDNEGCPVEALVKDNGNGTYSCSYVPR.K	3359.60342	3	4.06E-07	0.77	3.59	-	912.6
AHQ-1-1, 5147	R.VQVQDNEGCPVEALVKDNGNGTYSCSYVPR.K	3359.60342	3	8.92E-07	0.94	4.69	-	1417.3
AHQ-1-1, 5351 - 5415	R.VQVQDNEGCPVEALVKDNGNGTYSCSYVPR.K	3359.60342	3	3.92E-08	0.93	4.84	-	1097.6
AHQ-1-7, 4701	R.VSGQGLHEGHTFEPAEFIIDTR.D	2441.64058	3	4.63E-06	0.95	4.84	-	1050.8
AHQ-1-1, 4959	R.VSGQGLHEGHTFEPAEFIIDTR.D	2441.64058	2	3.84E-04	0.96	5.16	-	1236.6
AHQ-1-1, 4943 - 5019	R.VSGQGLHEGHTFEPAEFIIDTR.D	2441.64058	3	3.49E-10	0.96	5.83	-	1399.8
AHQ-1-3, 4861	R.VSGQGLHEGHTFEPAEFIIDTR.D	2441.64058	3	2.79E-09	0.93	4.65	-	941.1
AHQ-1-5, 4862	R.VSGQGLHEGHTFEPAEFIIDTR.D	2441.64058	3	5.17E-11	0.95	5.23	-	1164.3
AHQ-1-5, 4694 - 4765	R.VSGQGLHEGHTFEPAEFIIDTR.D	2441.64058	3	1.87E-09	0.95	5.45	-	1086.2
AHQ-1-4, 4926 - 5004	R.VSGQGLHEGHTFEPAEFIIDTR.D	2441.64058	3	6.67E-10	0.96	5.52	-	1365.4
AHQ-1-9, 4600	R.VSGQGLHEGHTFEPAEFIIDTR.D	2441.64058	2	2.04E-04	0.89	4.06	-	567.4
AHQ-1-13, 4608	R.VSGQGLHEGHTFEPAEFIIDTR.D	2441.64058	2	5.82E-06	0.72	2.92	-	415.9
AHQ-1-9, 4616	R.VSGQGLHEGHTFEPAEFIIDTR.D	2441.64058	3	7.50E-06	0.94	4.57	-	1145.7
AHQ-1-8, 4432	R.VSGQGLHEGHTFEPAEFIIDTR.D	2441.64058	3	5.01E-08	0.93	4.71	-	1009.8
AHQ-1-13- , 4879	R.VSGQGLHEGHTFEPAEFIIDTR.D	2441.64058	3	2.67E-06	0.95	5.27	-	1410.0
AHQ-1-2, 5057	R.VSGQGLHEGHTFEPAEFIIDTR.D	2441.64058	3	1.95E-06	0.95	4.99	-	1122.6

AHQ-1-6, 4675	R.VSGQGLHEGHTFEPAEFIIDTR.D	2441.64058	2	6.51E-04	0.81	3.33	-	622.3
AHQ-1-2, 5065	R.VSGQGLHEGHTFEPAEFIIDTR.D	2441.64058	2	3.74E-05	0.87	3.73	-	647.1
AHQ-1-13, 4543 - 4611	R.VSGQGLHEGHTFEPAEFIIDTR.D	2441.64058	3	8.27E-04	0.89	4.30	-	696.1
AHQ-1-1, 2796 - 2868	K.VTAQGPGLEPSGNIANK.T	1653.81855	2	7.52E-07	0.91	4.52	-	657.0
AHQ-1-4, 2731	K.VTAQGPGLEPSGNIANK.T	1653.81855	2	1.22E-04	0.78	3.53	-	422.9
AHQ-1-6, 2724	K.VTAQGPGLEPSGNIANK.T	1653.81855	2	1.17E-06	0.92	4.20	-	861.3
AHQ-1-6, 2626	K.VTAQGPGLEPSGNIANK.T	1653.81855	2	2.26E-07	0.70	3.08	-	402.2
AHQ-1-2, 2775 - 2846	K.VTAQGPGLEPSGNIANK.T	1653.81855	2	1.77E-05	0.92	4.44	-	617.2
AHQ-1-3, 2721 - 2790	K.VTAQGPGLEPSGNIANK.T	1653.81855	2	3.38E-06	0.84	4.03	-	482.8
AHQ-1-3, 2829 - 2901	K.VTAQGPGLEPSGNIANK.T	1653.81855	2	4.29E-10	0.89	4.41	-	463.0
AHQ-1-5, 2703 - 2713	K.VTAQGPGLEPSGNIANK.T	1653.81855	2	5.76E-05	0.77	3.44	-	409.2
AHQ-1-2, 2917 - 2985	K.VTAQGPGLEPSGNIANK.T	1653.81855	2	1.23E-07	0.88	4.08	-	480.6
AHQ-1-8, 2489	K.VTAQGPGLEPSGNIANK.T	1653.81855	2	3.37E-04	0.77	3.06	-	639.3
AHQ-1-9, 2586	K.VTAQGPGLEPSGNIANK.T	1653.81855	2	6.27E-04	0.89	3.61	-	615.7
AHQ-1-1, 3369	K.VTVLFAGQHIAK.S	1284.53185	3	5.60E-05	0.80	3.60	-	690.6
AHQ-1-1, 3375	K.VTVLFAGQHIAK.S	1284.53185	1	8.53E-04	0.24	1.84	-	573.1
AHQ-1-3, 3226	K.VTVLFAGQHIAK.S	1284.53185	3	1.89E-05	0.78	3.69	-	585.9
AHQ-1-6, 3076	K.VTVLFAGQHIAK.S	1284.53185	3	7.57E-05	0.87	4.00	-	654.2
AHQ-1-3, 3149 - 3230	K.VTVLFAGQHIAK.S	1284.53185	2	5.06E-08	0.91	3.76	-	831.6
AHQ-1-2, 3334 - 3363	K.VTVLFAGQHIAK.S	1284.53185	2	6.90E-07	0.93	3.85	-	976.5
AHQ-1-5, 3058	K.VTVLFAGQHIAK.S	1284.53185	2	1.47E-07	0.91	3.43	-	1070.0
AHQ-1-5, 3067	K.VTVLFAGQHIAK.S	1284.53185	1	1.62E-04	0.22	1.86	-	493.8
AHQ-1-2, 3349	K.VTVLFAGQHIAK.S	1284.53185	1	5.63E-04	0.66	3.19	-	484.8
AHQ-1-1, 3367	K.VTVLFAGQHIAK.S	1284.53185	2	6.99E-07	0.92	4.14	-	825.6
AHQ-1-6, 3062	K.VTVLFAGQHIAK.S	1284.53185	2	6.30E-05	0.86	3.29	-	788.1
AHQ-1-7, 3080	K.VTVLFAGQHIAK.S	1284.53185	2	8.40E-05	0.82	3.09	-	634.1
AHQ-1-4, 3240	K.VTVLFAGQHIAK.S	1284.53185	2	2.88E-05	0.90	3.74	-	754.5
AHQ-1-5, 5290 - 5321	R.VTYCPTEPGNYIINIK.F	1884.14221	2	3.04E-08	0.85	3.67	-	377.6
AHQ-1-1, 5183	R.VTYCPTEPGNYIINIK.F	1884.14221	2	7.67E-08	0.90	3.84	-	463.9
AHQ-1-2, 5293 - 5319	R.VTYCPTEPGNYIINIK.F	1884.14221	2	1.26E-05	0.87	3.65	-	412.5
AHQ-1-1, 4828	R.VTYCPTEPGNYIINIK.F	1884.14221	2	7.01E-04	0.57	2.78	-	247.0
AHQ-1-4, 5075	R.VTYCPTEPGNYIINIK.F	1884.14221	2	1.99E-09	0.78	3.37	-	332.0
AHQ-1-1, 5199	R.VTYCPTEPGNYIINIK.F	1884.14221	3	1.03E-04	0.86	3.96	-	627.4
AHQ-1-1, 5309	R.VTYCPTEPGNYIINIK.F	1884.14221	2	6.88E-06	0.77	3.57	-	304.2
AHQ-1-6, 4923	R.VTYCPTEPGNYIINIK.F	1884.14221	2	1.61E-05	0.88	3.57	-	544.1
AHQ-1-5, 5123 - 5193	R.VTYCPTEPGNYIINIK.F	1884.14221	2	5.24E-07	0.92	4.47	-	520.0
AHQ-1-7, 4997	R.VTYCPTEPGNYIINIK.F	1884.14221	2	6.86E-04	0.42	2.56	-	267.8
AHQ-1-4, 5212	R.VTYCPTEPGNYIINIK.F	1884.14221	2	5.35E-05	0.68	3.46	-	301.4
AHQ-1-3, 5143	R.VTYCPTEPGNYIINIK.F	1884.14221	2	3.56E-05	0.88	3.60	-	451.2
AHQ-1-8, 5351	R.VTYTPMAPGSYLISIK.Y	1742.07225	2	1.19E-05	0.71	2.79	-	393.0
AHQ-1-6, 4987	R.VTYTPM*APGSYLISIK.Y	1758.07165	2	9.42E-04	0.62	2.69	-	326.9
AHQ-1-13, 5217	R.VTYTPMAPGSYLISIK.Y	1742.07225	2	7.87E-04	0.70	2.83	-	443.8
AHQ-1-13- , 5527	R.VTYTPMAPGSYLISIK.Y	1742.07225	2	2.45E-04	0.89	3.66	-	748.4
AHQ-1-6, 5490	R.VTYTPMAPGSYLISIK.Y	1742.07225	2	8.04E-04	0.72	2.62	-	614.7
AHQ-1-11, 5027	R.VTYTPMAPGSYLISIK.Y	1742.07225	2	4.51E-04	0.81	3.07	-	510.2
AHQ-1-4, 5698 - 5763	R.VTYTPMAPGSYLISIK.Y	1742.07225	2	8.16E-04	0.80	3.03	-	712.4
AHQ-1-5, 5515 - 5561	R.VTYTPMAPGSYLISIK.Y	1742.07225	2	1.45E-04	0.82	3.17	-	600.1
AHQ-1-14- , 4923 - 4943	R.VTYTPM*APGSYLISIK.Y	1758.07165	2	1.89E-04	0.52	2.94	-	311.2
AHQ-1-2, 5785 - 5803	R.VTYTPMAPGSYLISIK.Y	1742.07225	2	5.14E-05	0.46	2.71	-	328.9
AHQ-1-9, 5374 - 5376	R.VTYTPMAPGSYLISIK.Y	1742.07225	2	6.48E-04	0.77	3.42	-	432.8
AHQ-1-14- , 5382	R.VTYTPMAPGSYLISIK.Y	1742.07225	2	6.85E-04	0.84	3.24	-	636.1
AHQ-1-4, 5614 - 5694	R.VTYTPMAPGSYLISIK.Y	1742.07225	2	2.08E-05	0.80	2.76	-	745.5
AHQ-1-4, 2671	R.YAPSEAGLHEM*DIR.Y	1605.75422	2	3.43E-05	0.84	2.81	-	1005.8
AHQ-1-13- , 3472	R.YAPSEAGLHEMDIR.Y	1589.75482	2	2.54E-07	0.88	3.48	-	739.0
AHQ-1-1, 2784	R.YAPSEAGLHEM*DIR.Y	1605.75422	2	4.67E-05	0.82	2.89	-	880.7
AHQ-1-13- , 2748	R.YAPSEAGLHEM*DIR.Y	1605.75422	2	8.39E-04	0.68	2.53	-	692.1
AHQ-1-13, 3382	R.YAPSEAGLHEMDIR.Y	1589.75482	2	9.51E-04	0.74	2.80	-	776.3
AHQ-1-5, 3287	R.YAPSEAGLHEMDIR.Y	1589.75482	2	2.17E-04	0.91	3.79	-	993.5
AHQ-1-9, 3188	R.YAPSEAGLHEMDIR.Y	1589.75482	2	2.02E-04	0.70	2.76	-	645.5
AHQ-1-6, 3287	R.YAPSEAGLHEMDIR.Y	1589.75482	2	4.62E-06	0.67	2.64	-	621.0
AHQ-1-8, 3161	R.YAPSEAGLHEMDIR.Y	1589.75482	2	2.08E-05	0.75	3.01	-	761.0
AHQ-1-1, 4392	K.YGGDEIPFSPYR.V	1401.50466	1	2.22E-04	0.17	2.02	-	356.5
AHQ-1-2, 4497 - 4498	K.YGGDEIPFSPYR.V	1401.50466	1	7.78E-04	0.39	2.75	-	430.9
AHQ-1-12, 4113 - 4114	K.YGGDEIPFSPYR.V	1401.50466	2	6.13E-04	0.76	3.42	-	471.9
AHQ-1-1, 4409	K.YGGDEIPFSPYR.V	1401.50466	1	4.16E-07	0.25	2.26	-	426.8
AHQ-1-1, 4380 - 4443	K.YGGDEIPFSPYR.V	1401.50466	2	2.69E-05	0.72	3.10	-	438.8
AHQ-1-3, 4346	K.YGGDEIPFSPYR.V	1401.50466	1	1.00E-06	0.57	2.78	-	495.5
AHQ-1-4, 4403 - 4404	K.YGGDEIPFSPYR.V	1401.50466	1	5.16E-04	0.27	2.31	-	381.4
AHQ-1-3, 4345	K.YGGDEIPFSPYR.V	1401.50466	1	9.45E-04	0.22	1.85	-	457.6
AHQ-1-10, 3127	K.YGGPYHIGGSPFK.A	1380.53182	2	3.86E-06	0.96	4.42	-	1636.6
AHQ-1-9, 3123	K.YGGPYHIGGSPFK.A	1380.53182	1	6.36E-04	0.88	3.09	-	795.3
AHQ-1-11, 3191 - 3194	K.YGGPYHIGGSPFK.A	1380.53182	2	7.88E-07	0.98	5.10	-	1982.0
AHQ-1-1, 3493	K.YGGPYHIGGSPFK.A	1380.53182	2	7.08E-06	0.93	3.69	-	1179.1
AHQ-1-5, 3213	K.YGGPYHIGGSPFK.A	1380.53182	2	5.98E-07	0.96	4.39	-	1583.6
AHQ-1-6, 3214	K.YGGPYHIGGSPFK.A	1380.53182	2	4.29E-07	0.93	3.86	-	1335.0
AHQ-1-13- , 3488	K.YGGPYHIGGSPFK.A	1380.53182	2	3.31E-05	0.95	3.98	-	1329.5
AHQ-1-3, 3383	K.YGGPYHIGGSPFK.A	1380.53182	2	3.56E-07	0.97	4.13	-	1915.1
AHQ-1-5, 3219	K.YGGPYHIGGSPFK.A	1380.53182	1	3.09E-07	0.87	3.54	-	562.6
AHQ-1-4, 3384	K.YGGPYHIGGSPFK.A	1380.53182	2	3.75E-08	0.97	4.48	-	2163.4
AHQ-1-5, 3222	K.YGGPYHIGGSPFK.A	1380.53182	3	9.62E-05	0.88	4.24	-	687.3
AHQ-1-2, 3499	K.YGGPYHIGGSPFK.A	1380.53182	2	4.10E-07	0.94	3.71	-	1275.9
AHQ-1-8, 3092	K.YGGPYHIGGSPFK.A	1380.53182	2	6.00E-06	0.93	3.24	-	1606.9
AHQ-1-8, 3097	K.YGGPYHIGGSPFK.A	1380.53182	2	1.35E-10	0.95	3.99	-	1370.7
AHQ-1-3, 3195 - 3197	K.YGGQPVPNFPSK.L	1291.43658	2	9.66E-04	0.86	3.15	-	559.7
AHQ-1-1, 3164	K.YGGQPVPNFPSK.L	1291.43658	1	1.21E-04	0.33	2.64	-	289.5
AHQ-1-5, 2966 - 2997	K.YGGQPVPNFPSK.L	1291.43658	2	1.81E-05	0.86	2.96	-	513.0
AHQ-1-1, 3144	K.YGGQPVPNFPSK.L	1291.43658	2	1.30E-04	0.88	3.25	-	587.1
AHQ-1-3, 3062 - 3063	K.YGGQPVPNFPSK.L	1291.43658	1	6.53E-05	0.28	1.87	-	446.7
AHQ-1-3, 3053 - 3054	K.YGGQPVPNFPSK.L	1291.43658	2	2.28E-05	0.93	4.11	-	642.4
AHQ-1-6, 2975 - 2986	K.YGGQPVPNFPSK.L	1291.43658	2	8.19E-06	0.91	3.79	-	564.0
AHQ-1-2, 3147 - 3217	K.YGGQPVPNFPSK.L	1291.43658	2	7.05E-05	0.92	3.62	-	524.0
AHQ-1-1, 6207	K.YGGQPVPNFPSKLQVEPAVDTSGVQCYGPGIEGQGVFR.E	4038.44995	3	6.40E-09	0.93	4.76	-	1034.7
AHQ-1-4, 4411	K.YKGQHVPGSPFQFTVGPLGEGGAHK.V	2596.88377	3	1.55E-05	0.86	3.77	-	887.8
AHQ-1-5, 4197	K.YKGQHVPGSPFQFTVGPLGEGGAHK.V	2596.88377	3	5.06E-04	0.87	3.99	-	777.9
AHQ-1-1, 2989	K.YNEQHVPGSPFTAR.V	1603.71978	2	6.82E-07	0.90	3.62	-	630.9
AHQ-1-8, 2498	K.YNEQHVPGSPFTAR.V	1603.71978	2	1.03E-05	0.91	3.77	-	622.2
AHQ-1-4, 2852 - 2863	K.YNEQHVPGSPFTAR.V	1603.71978	2	7.26E-08	0.95	4.54	-	680.4
AHQ-1-4, 3130	K.YNEQHVPGSPFTAR.V	1603.71978	2	1.40E-08	0.84	3.33	-	425.3
AHQ-1-5, 2991	K.YNEQHVPGSPFTAR.V	1603.71978	2	1.62E-06	0.67	2.91	-	460.2
AHQ-1-1, 2999	K.YNEQHVPGSPFTAR.V	1603.71978	3	3.49E-06	0.91	4.52	-	1012.7
AHQ-1-3, 2847	K.YNEQHVPGSPFTAR.V	1603.71978	2	1.58E-04	0.65	2.78	-	404.5
AHQ-1-3, 3127	K.YNEQHVPGSPFTAR.V	1603.71978	2	1.30E-06	0.69	2.63	-	522.9
AHQ-1-2, 2965	K.YNEQHVPGSPFTAR.V	1603.71978	2	4.55E-04	0.77	2.63	-	505.7
AHQ-1-5, 2701	K.YNEQHVPGSPFTAR.V	1603.71978	2	1.02E-05	0.93	3.61	-	907.5
AHQ-1-10, 2661	K.YNEQHVPGSPFTAR.V	1603.71978	2	1.32E-04	0.50	2.55	-	344.7
AHQ-1-5, 2706	K.YNEQHVPGSPFTAR.V	1603.71978	3	2.65E-08	0.92	4.08	-	1138.1
AHQ-1-13- , 2943	K.YNEQHVPGSPFTAR.V	1603.71978	2	3.50E-08	0.80	2.95	-	442.9

AHQ-1-6, 2744	K.YNEQHVPGSPFTAR.V	1603.71978	2	5.77E-04	0.90	3.33	-	770.1
AHQ-1-11, 2731	K.YNEQHVPGSPFTAR.V	1603.71978	2	7.05E-04	0.65	2.73	-	399.8
AHQ-1-3, 4446 - 4455	K.YTPVQQGPVGVNVTYGGDPIPK.S	2287.55640	2	3.66E-04	0.59	2.64	-	581.3
AHQ-1-2, 4598	K.YTPVQQGPVGVNVTYGGDPIPK.S	2287.55640	2	1.66E-06	0.84	3.57	-	687.4
AHQ-1-10, 4111	K.YTPVQQGPVGVNVTYGGDPIPK.S	2287.55640	2	1.54E-04	0.85	4.13	-	390.5
AHQ-1-11, 4226	K.YTPVQQGPVGVNVTYGGDPIPK.S	2287.55640	2	4.38E-08	0.84	3.22	-	756.5
AHQ-1-3, 4589 - 4657	K.YTPVQQGPVGVNVTYGGDPIPK.S	2287.55640	2	1.78E-07	0.92	4.37	-	618.8
AHQ-1-1, 4729 - 4803	K.YTPVQQGPVGVNVTYGGDPIPK.S	2287.55640	2	1.13E-07	0.94	4.77	-	718.4
AHQ-1-2, 4725 - 4805	K.YTPVQQGPVGVNVTYGGDPIPK.S	2287.55640	2	1.29E-05	0.96	4.80	-	1283.9
AHQ-1-9, 4492	K.YTPVQQGPVGVNVTYGGDPIPK.S	2287.55640	2	3.27E-05	0.89	3.52	-	736.3
AHQ-1-1, 4588 - 4655	K.YTPVQQGPVGVNVTYGGDPIPK.S	2287.55640	2	8.52E-07	0.95	5.27	-	619.3
AHQ-1-1, 4468	K.YTPVQQGPVGVNVTYGGDPIPK.S	2287.55640	2	5.41E-05	0.86	3.96	-	454.5
AHQ-1-2, 4909 - 4977	K.YTPVQQGPVGVNVTYGGDPIPK.S	2287.55640	2	1.92E-08	0.91	4.40	-	537.7
AHQ-1-3, 4757 - 4825	K.YTPVQQGPVGVNVTYGGDPIPK.S	2287.55640	2	6.12E-07	0.95	5.24	-	697.8
AHQ-1-4, 4846	K.YTPVQQGPVGVNVTYGGDPIPK.S	2287.55640	2	4.42E-09	0.90	4.02	-	662.3
AHQ-1-4, 4662 - 4730	K.YTPVQQGPVGVNVTYGGDPIPK.S	2287.55640	2	3.32E-08	0.94	4.49	-	803.7
AHQ-1-5, 4553 - 4625	K.YTPVQQGPVGVNVTYGGDPIPK.S	2287.55640	2	5.09E-05	0.95	4.73	-	811.8
AHQ-1-6, 4546	K.YTPVQQGPVGVNVTYGGDPIPK.S	2287.55640	2	4.84E-05	0.93	4.41	-	775.9
AHQ-1-8, 4559	K.YTPVQQGPVGVNVTYGGDPIPK.S	2287.55640	2	1.33E-04	0.74	3.33	-	381.3
AHQ-1-3, 6733 - 6807	R.YWPQEAGEYAVHVLCNSEDIR.L	2538.73318	2	1.44E-05	0.82	3.08	-	700.6
AHQ-1-2, 6566	R.YWPQEAGEYAVHVLCNSEDIR.L	2538.73318	2	3.04E-11	0.94	3.90	-	1235.3
AHQ-1-1, 6667	R.YWPQEAGEYAVHVLCNSEDIR.L	2538.73318	2	2.69E-06	0.86	3.51	-	772.1
AHQ-1-1, 6805	R.YWPQEAGEYAVHVLCNSEDIR.L	2538.73318	2	1.23E-07	0.87	3.27	-	749.9
AHQ-1-3, 6726 - 6794	R.YWPQEAGEYAVHVLCNSEDIR.L	2538.73318	3	2.83E-06	0.67	3.03	-	545.6
AHQ-1-2, 6779	R.YWPQEAGEYAVHVLCNSEDIR.L	2538.73318	2	2.72E-08	0.92	3.79	-	1141.1
AHQ-1-2, 7033 - 7101	R.YWPQEAGEYAVHVLCNSEDIR.L	2538.73318	3	7.06E-12	0.82	3.47	-	653.6
AHQ-1-5, 6846	R.YWPQEAGEYAVHVLCNSEDIR.L	2538.73318	2	4.16E-05	0.75	2.53	-	771.0
AHQ-1-2, 6925 - 6989	R.YWPQEAGEYAVHVLCNSEDIR.L	2538.73318	2	1.86E-06	0.95	4.73	-	1017.1
AHQ-1-1, 6355 - 6379	R.YWPQEAGEYAVHVLCNSEDIR.L	2538.73318	2	2.65E-09	0.93	3.66	-	1000.6
gi|13124879|ref|NP_002465.1| smooth muscle myosin heavy chain 11 isoform SM1 [Homo sapiens]				3.89E-15	7.60	90.35	5.00	227336.7
AHQ-1-1, 4889	R.DLGEELEALK.T	1117.23151	2	1.55E-04	0.86	3.41	-	683.7
AHQ-1-2, 5039	R.DLGEELEALK.T	1117.23151	2	3.32E-06	0.89	3.44	-	855.8
AHQ-1-6, 2702	R.EDQSILCTGESGAGK.T	1553.63107	2	2.87E-04	0.63	2.95	-	386.1
AHQ-1-1, 3027	K.FDQLLAEEK.N	1093.21165	2	3.78E-05	0.90	2.86	-	1225.9
AHQ-1-4, 2883	K.FDQLLAEEK.N	1093.21165	1	4.41E-05	0.56	2.07	-	971.8
AHQ-1-2, 2993	K.FDQLLAEEK.N	1093.21165	2	1.43E-04	0.93	3.29	-	1627.7
AHQ-1-2, 3005	K.FDQLLAEEK.N	1093.21165	1	1.25E-04	0.44	2.41	-	694.5
AHQ-1-3, 2890	K.FDQLLAEEK.N	1093.21165	2	1.79E-05	0.92	3.25	-	1426.5
AHQ-1-1, 3032	K.FDQLLAEEK.N	1093.21165	1	7.50E-04	0.79	2.71	-	949.3
AHQ-1-3, 2481	K.KEEELQAALAR.L	1258.40643	3	8.40E-05	0.93	3.35	-	1812.9
AHQ-1-1, 2647	K.KEEELQAALAR.L	1258.40643	3	5.31E-04	0.91	3.61	-	1526.6
AHQ-1-3, 2469	K.KEEELQAALAR.L	1258.40643	2	4.14E-04	0.89	3.37	-	1049.8
AHQ-1-2, 2565 - 2566	K.KEEELQAALAR.L	1258.40643	2	1.51E-04	0.83	3.20	-	905.0
AHQ-1-2, 2594	K.KEEELQAALAR.L	1258.40643	3	2.36E-06	0.94	3.64	-	1848.0
AHQ-1-6, 2654	R.KFDQLLAEEK.N	1221.38456	2	8.85E-04	0.87	3.05	-	1089.7
AHQ-1-3, 2801	R.KFDQLLAEEK.N	1221.38456	1	1.44E-06	0.31	2.04	-	689.1
AHQ-1-3, 2797 - 2874	R.KFDQLLAEEK.N	1221.38456	2	1.71E-04	0.82	3.36	-	720.0
AHQ-1-1, 5440 - 5509	K.LQQLFNHTM*FILEQEEYQR.E	2484.77198	3	4.61E-14	0.98	6.85	-	2274.9
AHQ-1-2, 5563 - 5630	K.LQQLFNHTM*FILEQEEYQR.E	2484.77198	3	2.39E-14	0.98	6.74	-	1697.4
AHQ-1-3, 5289	K.LQQLFNHTM*FILEQEEYQR.E	2484.77198	3	7.66E-14	0.98	6.62	-	2429.9
AHQ-1-5, 6137 - 6174	K.LQQLFNHTMFILEQEEYQR.E	2468.77258	3	4.66E-11	0.98	6.00	-	2743.4
AHQ-1-2, 6265 - 6287	K.LQQLFNHTMFILEQEEYQR.E	2468.77258	3	3.89E-15	0.98	6.96	-	3070.0
AHQ-1-2, 6269	K.LQQLFNHTMFILEQEEYQR.E	2468.77258	2	4.42E-10	0.96	4.51	-	1574.3
AHQ-1-3, 5230 - 5297	K.LQQLFNHTM*FILEQEEYQR.E	2484.77198	2	2.02E-04	0.66	2.81	-	437.8
AHQ-1-4, 6218	K.LQQLFNHTMFILEQEEYQR.E	2468.77258	3	4.65E-05	0.95	5.05	-	1478.7
AHQ-1-2, 6481 - 6549	K.LQQLFNHTMFILEQEEYQR.E	2468.77258	3	1.44E-13	0.97	5.62	-	2271.4
AHQ-1-6, 6040	K.LQQLFNHTMFILEQEEYQR.E	2468.77258	3	2.72E-09	0.98	6.25	-	3443.9
AHQ-1-1, 6083 - 6108	K.LQQLFNHTMFILEQEEYQR.E	2468.77258	3	8.39E-08	0.96	5.46	-	1651.4
AHQ-1-4, 6226	K.LQQLFNHTMFILEQEEYQR.E	2468.77258	2	4.15E-06	0.97	4.37	-	1840.8
AHQ-1-2, 6610 - 6611	K.QLLQANPILEAFGNAK.T	1727.98540	2	3.45E-11	0.97	5.43	-	1043.5
AHQ-1-2, 6447 - 6513	K.QLLQANPILEAFGNAK.T	1727.98540	2	1.91E-10	0.97	4.54	-	1424.3
AHQ-1-5, 6519	K.QLLQANPILEAFGNAK.T	1727.98540	2	4.72E-04	0.61	2.83	-	561.6
AHQ-1-2, 6338 - 6346	K.QLLQANPILEAFGNAK.T	1727.98540	2	5.07E-06	0.95	4.73	-	1015.8
AHQ-1-6, 6230 - 6240	K.QLLQANPILEAFGNAK.T	1727.98540	2	4.26E-07	0.91	3.93	-	663.7
AHQ-1-5, 6353 - 6378	K.QLLQANPILEAFGNAK.T	1727.98540	2	1.82E-06	0.93	4.03	-	903.8
AHQ-1-9, 6174 - 6183	K.QLLQANPILEAFGNAK.T	1727.98540	2	6.96E-04	0.90	3.84	-	680.1
AHQ-1-3, 6282 - 6329	K.QLLQANPILEAFGNAK.T	1727.98540	2	1.07E-06	0.95	4.72	-	881.6
AHQ-1-3, 6461	K.QLLQANPILEAFGNAK.T	1727.98540	2	2.76E-06	0.93	3.91	-	824.8
AHQ-1-1, 6171 - 6243	K.QLLQANPILEAFGNAK.T	1727.98540	2	1.33E-10	0.96	4.57	-	1213.4
AHQ-1-7, 6356 - 6373	K.QLLQANPILEAFGNAK.T	1727.98540	2	1.84E-05	0.94	4.32	-	922.3
AHQ-1-4, 6612	K.QLLQANPILEAFGNAK.T	1727.98540	2	3.84E-10	0.96	4.62	-	1190.7
AHQ-1-1, 6303 - 6351	K.QLLQANPILEAFGNAK.T	1727.98540	2	2.29E-06	0.92	3.59	-	1042.6
AHQ-1-1, 6407	K.QLLQANPILEAFGNAK.T	1727.98540	2	3.00E-08	0.94	4.72	-	671.3
AHQ-1-4, 6458	K.QLLQANPILEAFGNAK.T	1727.98540	2	2.87E-05	0.71	3.07	-	483.6
AHQ-1-4, 5948	K.TQLEELEDELQATEDAK.L	1963.04334	3	1.11E-07	0.96	5.81	-	1302.9
AHQ-1-5, 5829 - 5830	K.TQLEELEDELQATEDAK.L	1963.04334	2	2.23E-07	0.98	6.40	-	2436.1
AHQ-1-4, 5895 - 5931	K.TQLEELEDELQATEDAK.L	1963.04334	2	4.51E-08	0.98	6.26	-	1863.0
AHQ-1-3, 5797 - 5803	K.TQLEELEDELQATEDAK.L	1963.04334	2	6.78E-07	0.96	4.73	-	1571.0
AHQ-1-1, 5788	K.TQLEELEDELQATEDAK.L	1963.04334	3	9.99E-05	0.92	5.04	-	989.4
AHQ-1-1, 5803	K.TQLEELEDELQATEDAK.L	1963.04334	2	5.33E-04	0.92	3.38	-	1200.2
AHQ-1-1, 4117	K.TQLEELEDELQATEDAK.L	1963.04334	2	1.49E-04	0.93	4.38	-	831.6
AHQ-1-6, 5755	K.TQLEELEDELQATEDAK.L	1963.04334	2	1.37E-07	0.98	6.39	-	2175.4
AHQ-1-2, 5974 - 5990	K.TQLEELEDELQATEDAK.L	1963.04334	3	3.07E-08	0.96	6.06	-	1221.4
AHQ-1-2, 5965 - 6037	K.TQLEELEDELQATEDAK.L	1963.04334	2	2.41E-08	0.98	5.94	-	2037.4
AHQ-1-7, 5832	K.TQLEELEDELQATEDAK.L	1963.04334	2	2.32E-08	0.97	5.39	-	1652.7
AHQ-1-2, 4214 - 4217	K.TQLEELEDELQATEDAK.L	1963.04334	2	6.57E-13	0.97	5.54	-	1181.0
AHQ-1-3, 5809 - 5823	K.TQLEELEDELQATEDAK.L	1963.04334	3	3.99E-06	0.96	5.72	-	1281.6
gi|21956645|ref|NP_665807.1| myotrophin; granule cell differentiation protein [Homo sapiens]				3.89E-15	3.86	40.32	49.20	12894.7
AHQ-1-14- , 4512	K.GADINAPDKHHITPLLSAVYEGHVSCVK.L	3031.39193	3	1.53E-06	0.95	5.74	-	930.5
AHQ-1-14, 5412	K.GADINAPDKHHITPLLSAVYEGHVSCVK.L	3031.39193	3	7.38E-07	0.97	6.16	-	1793.2
AHQ-1-14- , 4028 - 4090	K.GPDGLTAFEATDNQAIK.A	1748.87196	2	5.78E-08	0.96	4.94	-	1190.8
AHQ-1-13, 3908 - 3963	K.GPDGLTAFEATDNQAIK.A	1748.87196	2	5.99E-07	0.97	5.08	-	1638.7
AHQ-1-14, 4902 - 4960	K.GPDGLTAFEATDNQAIK.A	1748.87196	2	7.69E-07	0.94	4.40	-	971.2
AHQ-1-14, 4920	K.GPDGLTAFEATDNQAIK.A	1748.87196	2	7.57E-08	0.97	5.59	-	1093.7
AHQ-1-14, 5020 - 5086	K.GPDGLTAFEATDNQAIK.A	1748.87196	2	9.98E-04	0.74	3.56	-	547.5
AHQ-1-14, 4804 - 4864	K.GPDGLTAFEATDNQAIK.A	1748.87196	2	9.43E-04	0.96	4.67	-	1394.2
AHQ-1-13, 4019 - 4088	K.GPDGLTAFEATDNQAIK.A	1748.87196	2	3.41E-08	0.94	5.04	-	953.6
AHQ-1-14- , 4043	K.GPDGLTAFEATDNQAIK.A	1748.87196	3	1.75E-07	0.88	4.05	-	929.8
AHQ-1-13- , 4107 - 4171	K.GPDGLTAFEATDNQAIK.A	1748.87196	2	4.03E-06	0.97	4.93	-	1753.8
AHQ-1-13- , 4236	K.GPDGLTAFEATDNQAIK.A	1748.87196	2	2.61E-07	0.85	3.10	-	774.5
AHQ-1-14- , 3938 - 4007	K.GPDGLTAFEATDNQAIK.A	1748.87196	2	3.89E-15	0.98	5.91	-	1681.1
AHQ-1-14- , 4015	K.HHITPLLSAVYEGHVSCVK.L	2149.45825	3	4.35E-09	0.98	6.37	-	2520.8
AHQ-1-14- , 3598	K.NGDLDEVKDYVAK.G	1466.57496	2	8.10E-10	0.92	3.10	-	1395.2
AHQ-1-14, 4472	K.NGDLDEVKDYVAK.G	1466.57496	2	3.51E-08	0.95	3.82	-	1689.7
AHQ-1-13- , 3801	K.NGDLDEVKDYVAK.G	1466.57496	2	1.35E-08	0.88	3.70	-	829.1
AHQ-1-14- , 3707 - 3708	K.NGDLDEVKDYVAK.G	1466.57496	2	3.79E-08	0.95	4.02	-	1647.6
AHQ-1-13, 3687 - 3757	K.NGDLDEVKDYVAK.G	1466.57496	2	7.38E-08	0.87	3.02	-	1076.8

gi|4505839|ref|NP_002645.1| pyruvate kinase, muscle; Pyruvate kinase-3; Thyroid hormone-binding pro				6.11E-15	18.70	210.30	46.00	57913.6
AHQ-1-7, 5013	R.AGKPVICATQMLESM*IK.K	1895.29809	2	4.67E-04	0.91	4.33	-	1024.6
AHQ-1-7, 6546	R.AGKPVICATQMLESMIK.K	1879.29869	3	1.83E-05	0.93	4.76	-	1402.4
AHQ-1-7, 6556 - 6576	R.AGKPVICATQMLESMIK.K	1879.29869	2	1.93E-07	0.97	5.01	-	1096.5
AHQ-1-7, 5588	K.CDENILWLDYK.N	1470.62849	1	3.23E-07	0.75	3.35	-	493.7
AHQ-1-7, 6222 - 6292	R.EAEAAIYHLQLFEELR.R	1933.15326	2	4.38E-08	0.93	4.08	-	838.3
AHQ-1-7, 6110 - 6181	R.EAEAAIYHLQLFEELR.R	1933.15326	2	2.42E-07	0.95	4.30	-	1086.2
AHQ-1-7, 7440 - 7441	K.FGVEQDVDM*VFASFIR.K	1877.10995	2	1.53E-09	0.98	5.72	-	1657.2
AHQ-1-7, 7504 - 7505	K.FGVEQDVDMVFASFIR.K	1861.11055	2	1.43E-10	0.97	5.17	-	1402.5
AHQ-1-7, 5312	K.GADFLVTEVENGGSLGSK.K	1780.91396	2	2.98E-12	0.95	4.41	-	1428.8
AHQ-1-7, 4876 - 4946	K.GADFLVTEVENGGSLGSK.K	1780.91396	2	2.94E-07	0.94	4.61	-	1142.1
AHQ-1-7, 4708 - 4742	K.GADFLVTEVENGGSLGSK.K	1780.91396	2	2.43E-05	0.91	4.16	-	951.5
AHQ-1-7, 5022 - 5060	K.GADFLVTEVENGGSLGSK.K	1780.91396	2	1.84E-06	0.92	4.42	-	934.6
AHQ-1-7, 6916 - 6984	R.GIFPVLCKDPVQEAWAEDVDLR.V	2559.87857	3	6.11E-15	0.97	5.77	-	1836.9
AHQ-1-10, 6039	R.GIFPVLCKDPVQEAWAEDVDLR.V	2559.87857	2	9.49E-07	0.74	3.40	-	386.4
AHQ-1-14- , 6450	R.GIFPVLCKDPVQEAWAEDVDLR.V	2559.87857	3	2.91E-11	0.96	4.95	-	1835.9
AHQ-1-7, 6957 - 7020	R.GIFPVLCKDPVQEAWAEDVDLR.V	2559.87857	2	9.91E-05	0.96	4.85	-	1099.4
AHQ-1-7, 6969	R.GIFPVLCKDPVQEAWAEDVDLR.V	2559.87857	2	1.24E-10	0.91	3.93	-	623.6
AHQ-1-7, 7049	R.GIFPVLCKDPVQEAWAEDVDLR.V	2559.87857	3	1.17E-04	0.93	4.28	-	1276.9
AHQ-1-8, 6940	R.GIFPVLCKDPVQEAWAEDVDLR.V	2559.87857	3	1.33E-04	0.97	5.09	-	2090.6
AHQ-1-13- , 6705 - 6717	R.GIFPVLCKDPVQEAWAEDVDLR.V	2559.87857	3	1.81E-08	0.97	5.74	-	1635.8
AHQ-1-9, 6814	R.GIFPVLCKDPVQEAWAEDVDLR.V	2559.87857	3	2.66E-04	0.95	3.85	-	1977.7
AHQ-1-3, 4839	K.GVNLPGAAVDLPAVSEK.D	1637.85902	2	1.22E-06	0.89	3.53	-	728.6
AHQ-1-7, 4682 - 4753	K.GVNLPGAAVDLPAVSEK.D	1637.85902	2	6.66E-08	0.97	6.02	-	971.8
AHQ-1-1, 4845	K.GVNLPGAAVDLPAVSEK.D	1637.85902	2	5.48E-04	0.53	2.57	-	451.9
AHQ-1-7, 3126 - 3197	K.ITLDNAYMEK.C	1198.37126	2	4.89E-05	0.58	2.62	-	439.5
AHQ-1-7, 6401 - 6481	K.ITLDNAYMEKCDENILWLDYK.N	2649.97716	2	6.95E-06	0.88	3.89	-	785.5
AHQ-1-7, 6070 - 6121	K.ITLDNAYM*EKCDENILWLDYK.N	2665.97656	3	3.40E-05	0.88	4.30	-	798.4
AHQ-1-8, 4101 - 4107	K.KGVNLPGAAVDLPAVSEK.D	1766.03194	2	1.36E-05	0.97	5.05	-	1502.7
AHQ-1-7, 4184 - 4208	K.KGVNLPGAAVDLPAVSEK.D	1766.03194	2	4.09E-05	0.97	5.46	-	1525.9
AHQ-1-7, 4197	K.KGVNLPGAAVDLPAVSEK.D	1766.03194	3	6.36E-06	0.92	4.17	-	1251.4
AHQ-1-10, 3861	K.KGVNLPGAAVDLPAVSEK.D	1766.03194	2	2.22E-04	0.96	5.14	-	1187.4
AHQ-1-7, 5426	K.KGVNLPGAAVDLPAVSEKDIQDLK.F	2478.82685	3	9.26E-08	0.98	5.56	-	2544.1
AHQ-1-13, 5492	R.LAPITSDPTEATAVGAVEASFK.C	2176.40912	2	2.06E-07	0.93	4.50	-	721.6
AHQ-1-11, 5403	R.LAPITSDPTEATAVGAVEASFK.C	2176.40912	2	2.44E-06	0.94	4.61	-	885.2
AHQ-1-7, 6196 - 6264	R.LAPITSDPTEATAVGAVEASFK.C	2176.40912	2	7.96E-06	0.97	5.63	-	1358.2
AHQ-1-7, 6469 - 6549	R.LAPITSDPTEATAVGAVEASFK.C	2176.40912	2	1.01E-06	0.96	4.48	-	1422.6
AHQ-1-7, 5629	R.LAPITSDPTEATAVGAVEASFK.C	2176.40912	2	1.63E-06	0.95	4.29	-	1087.3
AHQ-1-7, 6060 - 6129	R.LAPITSDPTEATAVGAVEASFK.C	2176.40912	2	1.27E-06	0.98	5.93	-	1630.4
AHQ-1-7, 6625 - 6701	R.LAPITSDPTEATAVGAVEASFK.C	2176.40912	2	2.02E-04	0.74	3.34	-	424.5
AHQ-1-10, 5283	R.LAPITSDPTEATAVGAVEASFK.C	2176.40912	2	1.39E-08	0.94	4.48	-	870.3
AHQ-1-3, 6007 - 6087	R.LAPITSDPTEATAVGAVEASFK.C	2176.40912	2	9.94E-04	0.48	3.06	-	472.7
AHQ-1-9, 5890 - 5967	R.LAPITSDPTEATAVGAVEASFK.C	2176.40912	2	2.37E-06	0.94	4.49	-	846.5
AHQ-1-7, 6332 - 6400	R.LAPITSDPTEATAVGAVEASFK.C	2176.40912	2	2.37E-04	0.97	5.33	-	1288.8
AHQ-1-1, 5929 - 5969	R.LAPITSDPTEATAVGAVEASFK.C	2176.40912	2	1.08E-05	0.90	4.10	-	557.0
AHQ-1-14- , 3463	R.LDIDSPPITAR.N	1198.35099	2	7.00E-07	0.95	3.68	-	1475.7
AHQ-1-13, 3443	R.LDIDSPPITAR.N	1198.35099	2	8.17E-04	0.79	3.10	-	628.6
AHQ-1-7, 3438 - 3472	R.LDIDSPPITAR.N	1198.35099	2	2.17E-05	0.93	3.74	-	959.2
AHQ-1-7, 2617	R.LNFSHGTHEYHAETIK.N	1885.02879	2	3.53E-11	0.70	3.23	-	434.4
AHQ-1-9, 2230	R.LNFSHGTHEYHAETIK.N	1885.02879	2	1.05E-07	0.89	4.14	-	503.8
AHQ-1-7, 4080 - 4082	R.NTGIICTIGPASR.S	1361.54994	2	3.17E-06	0.86	3.40	-	897.5
AHQ-1-13, 3455 - 3467	R.NTGIICTIGPASR.S	1361.54994	2	4.18E-05	0.94	3.71	-	1236.7
AHQ-1-3, 3581	R.NTGIICTIGPASR.S	1361.54994	2	1.76E-05	0.87	2.97	-	1139.0
AHQ-1-11, 3304	R.NTGIICTIGPASR.S	1361.54994	2	2.34E-05	0.88	3.45	-	869.5
AHQ-1-7, 3801	R.NTGIICTIGPASR.S	1361.54994	2	9.20E-05	0.91	3.78	-	1072.6
AHQ-1-7, 3424 - 3452	R.NTGIICTIGPASR.S	1361.54994	2	1.86E-05	0.94	3.95	-	1182.1
AHQ-1-14- , 3484	R.NTGIICTIGPASR.S	1361.54994	2	6.45E-04	0.93	3.81	-	1135.9
AHQ-1-13- , 3548 - 3559	R.NTGIICTIGPASR.S	1361.54994	2	2.62E-06	0.93	3.97	-	1045.3
AHQ-1-7, 4922	R.RFDEILEASDGIMVAR.G	1823.06385	2	2.92E-09	0.95	4.48	-	926.6
AHQ-1-7, 5484	R.RFDEILEASDGIMVAR.G	1823.06385	2	1.05E-04	0.91	4.19	-	798.5
AHQ-1-7, 5009	R.RFDEILEASDGIMVAR.G	1823.06385	2	4.00E-04	0.90	4.53	-	482.2
AHQ-1-7, 5241 - 5280	R.RFDEILEASDGIMVAR.G	1823.06385	2	9.59E-13	0.98	5.70	-	1582.4
AHQ-1-7, 5834 - 5904	R.TATESFASDPILYRPVAVALDTK.G	2466.77140	2	2.87E-04	0.44	2.89	-	147.1
AHQ-1-11, 5372 - 5387	R.TATESFASDPILYRPVAVALDTK.G	2466.77140	2	9.37E-05	0.76	3.50	-	313.8
AHQ-1-11, 5366	R.TATESFASDPILYRPVAVALDTK.G	2466.77140	3	7.33E-07	0.66	3.07	-	695.1
AHQ-1-13, 5391 - 5469	R.TATESFASDPILYRPVAVALDTK.G	2466.77140	3	1.27E-05	0.80	3.32	-	841.4
AHQ-1-14- , 5706 - 5782	R.TATESFASDPILYRPVAVALDTK.G	2466.77140	3	5.90E-05	0.86	3.78	-	967.3
AHQ-1-7, 2869 - 2880	R.VNFAMNVGK.A	980.16641	2	2.30E-06	0.89	2.94	-	810.9
gi|13562114|ref|NP_110400.1| beta tubulin 1, class VI [Homo sapiens]				1.05E-14	19.74	220.38	61.40	50326.6
AHQ-1-7, 7384	K.AFVHWYTSEGM*DINEFGEAENNIHDLVSEYQQFQDAK.A	4380.62526	3	1.40E-04	0.91	4.76	-	876.4
AHQ-1-1, 6443 - 6445	R.ALSVAELTQQMFDAR.N	1680.90740	2	7.85E-06	0.88	3.82	-	1031.1
AHQ-1-10, 5703	R.ALSVAELTQQMFDAR.N	1680.90740	2	2.15E-04	0.97	5.15	-	1542.8
AHQ-1-9, 6394	R.ALSVAELTQQMFDAR.N	1680.90740	2	1.01E-06	0.94	4.39	-	1289.9
AHQ-1-3, 6477	R.ALSVAELTQQMFDAR.N	1680.90740	2	2.08E-05	0.96	4.76	-	1501.2
AHQ-1-9, 4675	R.ALSVAELTQQM*FDAR.N	1696.90680	2	3.38E-05	0.89	3.98	-	917.0
AHQ-1-13- , 6369 - 6379	R.ALSVAELTQQMFDAR.N	1680.90740	2	1.08E-04	0.92	3.54	-	1322.5
AHQ-1-6, 6452	R.ALSVAELTQQMFDAR.N	1680.90740	2	4.37E-06	0.91	3.78	-	1028.9
AHQ-1-8, 6474 - 6478	R.ALSVAELTQQMFDAR.N	1680.90740	2	3.75E-04	0.96	4.58	-	1767.3
AHQ-1-11, 5807 - 5824	R.ALSVAELTQQMFDAR.N	1680.90740	2	3.59E-04	0.80	3.00	-	846.1
AHQ-1-7, 6544 - 6548	R.ALSVAELTQQMFDAR.N	1680.90740	2	6.19E-04	0.97	5.34	-	1290.0
AHQ-1-7, 4785 - 4792	R.ALSVAELTQQM*FDAR.N	1696.90680	2	3.69E-08	0.97	5.40	-	1650.9
AHQ-1-14- , 4108	R.AVLVDLEPGTM*DSIR.S	1632.86102	2	6.79E-05	0.80	3.53	-	515.3
AHQ-1-7, 4954 - 5025	R.AVLVDLEPGTMDSIR.S	1616.86162	2	4.91E-08	0.93	4.75	-	662.5
AHQ-1-7, 3856	R.EIVHIQIGQCGNQIGAK.F	1867.11965	3	1.04E-04	0.94	4.66	-	1229.6
AHQ-1-7, 4021 - 4094	R.EIVHIQIGQCGNQIGAK.F	1867.11965	2	3.36E-05	0.75	3.43	-	436.3
AHQ-1-14- , 3838 - 3842	R.EIVHIQIGQCGNQIGAK.F	1867.11965	2	2.48E-04	0.93	4.38	-	865.9
AHQ-1-12, 3123 - 3195	K.EVDQQLLSVQTR.N	1416.56231	2	3.44E-04	0.58	2.65	-	464.1
AHQ-1-3, 3335	K.EVDQQLLSVQTR.N	1416.56231	2	5.53E-04	0.79	3.45	-	600.5
AHQ-1-10, 3059 - 3067	K.EVDQQLLSVQTR.N	1416.56231	2	8.05E-06	0.94	4.39	-	1255.9
AHQ-1-7, 4802 - 4806	K.FWEM*IGEEHGIDLAGSDR.G	2079.23556	2	6.70E-08	0.94	4.53	-	783.2
AHQ-1-11, 4886	K.FWEMIGEEHGIDLAGSDR.G	2063.23616	2	5.24E-06	0.97	5.63	-	1394.7
AHQ-1-14- , 4691	K.FWEM*IGEEHGIDLAGSDR.G	2079.23556	2	1.34E-07	0.97	4.72	-	1267.5
AHQ-1-7, 5393 - 5428	K.FWEMIGEEHGIDLAGSDR.G	2063.23616	2	8.40E-09	0.98	7.10	-	1776.3
AHQ-1-7, 5514 - 5585	K.FWEMIGEEHGIDLAGSDR.G	2063.23616	2	3.40E-05	0.96	4.27	-	1638.3
AHQ-1-7, 5600	K.FWEMIGEEHGIDLAGSDR.G	2063.23616	2	3.22E-06	0.96	4.85	-	1135.2
AHQ-1-13- , 5345	K.FWEMIGEEHGIDLAGSDR.G	2063.23616	2	9.79E-12	0.98	5.92	-	2429.0
AHQ-1-12, 5082	K.FWEMIGEEHGIDLAGSDR.G	2063.23616	2	2.22E-06	0.98	5.61	-	1921.7
AHQ-1-13- , 5340	K.FWEMIGEEHGIDLAGSDR.G	2063.23616	3	2.58E-08	0.93	3.95	-	1429.5
AHQ-1-14- , 5196 - 5218	K.FWEMIGEEHGIDLAGSDR.G	2063.23616	3	2.07E-10	0.95	5.13	-	1082.1
AHQ-1-13, 5052 - 5112	K.FWEMIGEEHGIDLAGSDR.G	2063.23616	2	1.46E-06	0.94	3.96	-	1062.8
AHQ-1-8, 5231	K.FWEMIGEEHGIDLAGSDR.G	2063.23616	2	2.53E-06	0.98	6.34	-	2062.6
AHQ-1-14- , 5198	K.FWEMIGEEHGIDLAGSDR.G	2063.23616	2	1.05E-14	0.98	5.67	-	2123.7
AHQ-1-7, 7153	K.GHYTEGAELIENVLEVVR.H	2029.23935	2	6.02E-04	0.95	4.75	-	1476.7
AHQ-1-14- , 6563	K.GHYTEGAELIENVLEVVR.H	2029.23935	3	9.25E-06	0.93	4.42	-	1730.6
AHQ-1-13- , 6817	K.GHYTEGAELIENVLEVVR.H	2029.23935	2	8.89E-07	0.98	6.61	-	2393.8
AHQ-1-13- , 6815	K.GHYTEGAELIENVLEVVR.H	2029.23935	3	5.04E-04	0.93	4.35	-	1624.1
AHQ-1-12, 6477 - 6482	K.GHYTEGAELIENVLEVVR.H	2029.23935	2	4.48E-08	0.99	7.53	-	3093.3

AHQ-1-7, 7069	K.GHYTEGAELIENVLEVVR.H	2029.23935	3	1.97E-04	0.92	4.41	-	1577.3
AHQ-1-12, 6478	K.GHYTEGAELIENVLEVVR.H	2029.23935	3	2.00E-06	0.86	4.38	-	1103.1
AHQ-1-14- , 6566 - 6567	K.GHYTEGAELIENVLEVVR.H	2029.23935	2	1.89E-12	0.99	7.21	-	2927.0
AHQ-1-8, 7020	K.GHYTEGAELIENVLEVVR.H	2029.23935	2	1.01E-09	0.98	6.08	-	2675.0
AHQ-1-8, 7031	K.GHYTEGAELIENVLEVVR.H	2029.23935	3	1.28E-04	0.92	4.42	-	1708.8
AHQ-1-10, 6165	K.GHYTEGAELIENVLEVVR.H	2029.23935	2	5.10E-07	0.98	5.99	-	1982.8
AHQ-1-11, 6280	K.GHYTEGAELIENVLEVVR.H	2029.23935	3	3.37E-04	0.90	3.98	-	1604.0
AHQ-1-4, 7196	K.GHYTEGAELIENVLEVVR.H	2029.23935	2	1.71E-06	0.98	5.68	-	2849.0
AHQ-1-11, 6275 - 6352	K.GHYTEGAELIENVLEVVR.H	2029.23935	2	2.81E-05	0.98	5.64	-	1982.9
AHQ-1-7, 7068 - 7070	K.GHYTEGAELIENVLEVVR.H	2029.23935	2	8.96E-09	0.99	7.50	-	2438.9
AHQ-1-9, 6922	K.GHYTEGAELIENVLEVVR.H	2029.23935	3	1.45E-06	0.77	4.14	-	698.1
AHQ-1-9, 6914 - 6992	K.GHYTEGAELIENVLEVVR.H	2029.23935	2	6.59E-11	0.98	6.24	-	2071.6
AHQ-1-6, 6987 - 7007	K.GHYTEGAELIENVLEVVR.H	2029.23935	2	3.31E-06	0.93	4.09	-	1435.3
AHQ-1-13, 6359	K.GHYTEGAELIENVLEVVR.H	2029.23935	2	6.80E-12	0.99	7.28	-	2839.4
AHQ-1-13, 6360	K.GHYTEGAELIENVLEVVR.H	2029.23935	3	2.20E-04	0.96	4.72	-	2500.4
AHQ-1-13- , 6596	R.GLSM*AATFIGNNTAIQEIFNR.V	2285.56551	2	1.71E-08	0.89	4.32	-	734.3
AHQ-1-14- , 6284 - 6366	R.GLSM*AATFIGNNTAIQEIFNR.V	2285.56551	2	9.81E-08	0.95	4.69	-	992.2
AHQ-1-13, 6169	R.GLSM*AATFIGNNTAIQEIFNR.V	2285.56551	2	3.22E-06	0.90	4.27	-	711.9
AHQ-1-7, 6882 - 6888	R.GLSM*AATFIGNNTAIQEIFNR.V	2285.56551	2	9.49E-08	0.93	4.51	-	845.5
AHQ-1-9, 6704	R.GLSM*AATFIGNNTAIQEIFNR.V	2285.56551	2	4.99E-11	0.93	4.36	-	779.5
AHQ-1-7, 3050	R.IMNSFSVM*PSPK.V	1354.62100	2	4.63E-04	0.83	2.83	-	807.5
AHQ-1-7, 4032	R.IMNSFSVMPSPK.V	1338.62160	2	5.04E-06	0.93	3.56	-	1095.8
AHQ-1-7, 3902 - 3917	R.IMNSFSVMPSPK.V	1338.62160	2	3.25E-05	0.95	3.93	-	1364.4
AHQ-1-13- , 3436	R.ISVYYNEAYGR.K	1335.44622	2	8.34E-06	0.75	2.77	-	571.6
AHQ-1-14, 4068 - 4072	R.ISVYYNEAYGR.K	1335.44622	2	6.32E-07	0.89	3.13	-	968.7
AHQ-1-7, 3172	R.ISVYYNEAYGR.K	1335.44622	2	4.65E-07	0.94	3.24	-	1323.5
AHQ-1-1, 3364 - 3372	R.ISVYYNEAYGR.K	1335.44622	2	5.53E-04	0.64	2.69	-	446.8
AHQ-1-8, 3088	R.ISVYYNEAYGR.K	1335.44622	2	2.99E-08	0.93	3.13	-	1296.9
AHQ-1-14- , 3174	R.ISVYYNEAYGR.K	1335.44622	2	1.25E-08	0.92	3.46	-	1023.4
AHQ-1-7, 3261	R.ISVYYNEAYGR.K	1335.44622	2	4.06E-08	0.92	3.11	-	1158.4
AHQ-1-7, 3190 - 3262	R.ISVYYNEAYGR.K	1335.44622	1	1.93E-04	0.25	2.37	-	190.9
AHQ-1-7, 5252	K.LGALFQPDSFVHGNSGAGNNWAK.G	2388.58260	2	3.55E-08	0.67	2.81	-	547.4
AHQ-1-13- , 5160	K.LGALFQPDSFVHGNSGAGNNWAK.G	2388.58260	3	8.36E-08	0.86	3.73	-	877.0
AHQ-1-7, 5249	R.LHFFM*PGFAPLTAQGSQQYR.A	2313.62224	2	7.89E-06	0.95	4.72	-	895.2
AHQ-1-10, 5304	R.LHFFMPGFAPLTAQGSQQYR.A	2297.62284	3	8.99E-04	0.96	4.85	-	1796.3
AHQ-1-10, 5297	R.LHFFMPGFAPLTAQGSQQYR.A	2297.62284	2	9.10E-05	0.92	3.99	-	727.6
AHQ-1-11, 4858	R.LHFFM*PGFAPLTAQGSQQYR.A	2313.62224	2	1.18E-06	0.98	5.86	-	1347.3
AHQ-1-14- , 5830	R.LHFFMPGFAPLTAQGSQQYR.A	2297.62284	3	2.29E-04	0.72	3.28	-	1102.6
AHQ-1-8, 5659	R.LHFFMPGFAPLTAQGSQQYR.A	2297.62284	2	5.51E-06	0.97	5.50	-	957.5
AHQ-1-9, 5790 - 5794	R.LHFFMPGFAPLTAQGSQQYR.A	2297.62284	2	1.95E-05	0.95	4.92	-	749.4
AHQ-1-9, 5100 - 5108	R.LHFFM*PGFAPLTAQGSQQYR.A	2313.62224	3	1.96E-04	0.98	5.95	-	2300.1
AHQ-1-11, 5476	R.LHFFMPGFAPLTAQGSQQYR.A	2297.62284	2	3.17E-08	0.96	5.42	-	797.3
AHQ-1-7, 7018 - 7082	K.LTTPTYGDLNHLVSLTMSGITTSLR.F	2693.06979	2	5.39E-11	0.94	4.43	-	958.4
AHQ-1-1, 6815	K.LTTPTYGDLNHLVSLTM*SGITTSLR.F	2709.06919	3	1.28E-06	0.83	3.74	-	832.2
AHQ-1-9, 6839 - 6908	K.LTTPTYGDLNHLVSLTM*SGITTSLR.F	2709.06919	3	4.16E-04	0.91	4.92	-	910.2
AHQ-1-8, 7447	K.LTTPTYGDLNHLVSLTMSGITTSLR.F	2693.06979	3	1.25E-08	0.94	5.02	-	1119.5
AHQ-1-9, 6888	K.LTTPTYGDLNHLVSLTMSGITTSLR.F	2693.06979	3	3.64E-09	0.87	3.87	-	875.6
AHQ-1-9, 7186 - 7255	K.LTTPTYGDLNHLVSLTMSGITTSLR.F	2693.06979	2	8.57E-08	0.95	5.18	-	786.3
AHQ-1-7, 7445	K.LTTPTYGDLNHLVSLTMSGITTSLR.F	2693.06979	2	1.17E-06	0.95	5.25	-	733.8
AHQ-1-7, 7444 - 7446	K.LTTPTYGDLNHLVSLTMSGITTSLR.F	2693.06979	3	1.49E-05	0.94	5.12	-	1125.7
AHQ-1-7, 7078	K.LTTPTYGDLNHLVSLTMSGITTSLR.F	2693.06979	3	7.84E-06	0.93	4.76	-	1168.2
AHQ-1-8, 7446	K.LTTPTYGDLNHLVSLTMSGITTSLR.F	2693.06979	2	2.14E-09	0.95	5.29	-	805.4
AHQ-1-7, 7013 - 7040	K.LTTPTYGDLNHLVSLTM*SGITTSLR.F	2709.06919	3	2.29E-05	0.91	5.17	-	667.7
AHQ-1-9, 7256	K.LTTPTYGDLNHLVSLTMSGITTSLR.F	2693.06979	3	3.91E-09	0.93	4.77	-	1219.5
AHQ-1-10, 6100	K.LTTPTYGDLNHLVSLTMSGITTSLR.F	2693.06979	2	3.93E-04	0.77	3.62	-	434.3
AHQ-1-14- , 6496 - 6534	K.LTTPTYGDLNHLVSLTM*SGITTSLR.F	2709.06919	3	6.96E-06	0.95	5.40	-	1109.9
AHQ-1-4, 7411	K.LTTPTYGDLNHLVSLTMSGITTSLR.F	2693.06979	3	4.73E-04	0.80	3.38	-	975.4
AHQ-1-9, 5424	R.NSSCFVEWIPNNVK.V	1695.87718	2	1.43E-06	0.89	3.52	-	863.5
AHQ-1-7, 3001	R.VSEHFSAMFK.R	1183.36152	2	9.03E-07	0.87	2.68	-	1041.2
AHQ-1-9, 5266	R.YLTVACIFR.G	1144.36822	2	1.02E-04	0.86	2.79	-	758.9
AHQ-1-11, 4956	R.YLTVACIFR.G	1144.36822	2	5.29E-04	0.88	2.66	-	896.6
gi|4758012|ref|NP_004850.1| clathrin heavy chain; clathrin, heavy polypeptide-like 2 [Homo sapiens]				2.55E-14	20.14	220.35	19.50	191612.6
AHQ-1-4, 7464	K.AFMTADLPNELIELLEK.I	1948.26956	2	4.34E-04	0.23	2.71	-	195.0
AHQ-1-3, 7285	K.AFMTADLPNELIELLEK.I	1948.26956	2	6.10E-05	0.92	4.31	-	610.9
AHQ-1-3, 4527	R.ALEHFTDLYDIK.R	1465.63179	2	5.73E-06	0.90	3.24	-	969.0
AHQ-1-3, 7281	R.FQSVPAQPGQTSPLLQYFGILLDQGQLNK.Y	3189.60926	3	2.92E-06	0.89	4.18	-	1016.2
AHQ-1-6, 7300	R.FQSVPAQPGQTSPLLQYFGILLDQGQLNK.Y	3189.60926	3	3.94E-04	0.70	3.40	-	543.5
AHQ-1-3, 4913	R.GQFSTDELVAEVEK.R	1552.66462	2	2.35E-07	0.95	4.51	-	1292.4
AHQ-1-1, 4945	R.GQFSTDELVAEVEK.R	1552.66462	2	3.98E-05	0.88	3.22	-	1044.4
AHQ-1-4, 4896	R.GQFSTDELVAEVEKR.N	1708.85097	2	6.14E-10	0.85	3.53	-	784.9
AHQ-1-5, 4738	R.GQFSTDELVAEVEKR.N	1708.85097	2	6.97E-07	0.83	3.40	-	727.3
AHQ-1-3, 4821	R.GQFSTDELVAEVEKR.N	1708.85097	2	3.10E-09	0.88	3.53	-	909.8
AHQ-1-3, 3378	K.HDVVFLITK.Y	1072.28194	2	1.35E-07	0.92	3.06	-	1134.8
AHQ-1-3, 3517	R.HSSLAGCQIINYR.T	1520.69551	2	2.16E-06	0.96	4.21	-	1247.4
AHQ-1-3, 2085	R.IHEGCEEPATHNALAK.I	1778.92458	2	2.06E-05	0.91	3.83	-	831.7
AHQ-1-3, 5357	R.ISGETIFVTAPHEATAGIIGVNR.K	2354.64751	3	2.55E-14	0.97	5.96	-	1352.0
AHQ-1-3, 7094	R.KFDVNTSAVQVLIEHIGNLDR.A	2369.66218	3	9.35E-13	0.98	6.49	-	2046.6
AHQ-1-6, 4423	R.LASTLVHLGEYQAAVDGAR.K	1972.19103	3	4.15E-05	0.83	3.09	-	1071.3
AHQ-1-3, 4578	R.LASTLVHLGEYQAAVDGAR.K	1972.19103	3	4.47E-04	0.91	4.18	-	1134.0
AHQ-1-3, 4583 - 4585	R.LASTLVHLGEYQAAVDGAR.K	1972.19103	2	1.90E-11	0.97	5.34	-	1196.9
AHQ-1-3, 4415	K.LHIIEVGTPPTGNQPFPK.K	1946.23831	2	7.60E-09	0.89	4.12	-	506.8
AHQ-1-3, 3795	K.LLYNNVSNFGR.L	1297.44435	2	6.98E-04	0.89	3.13	-	886.6
AHQ-1-3, 6865	R.LPVVIGGLLDVDCSEDVIK.N	2043.36744	2	2.69E-05	0.96	5.29	-	1184.0
AHQ-1-3, 6374	R.NLQNLLILTAIK.A	1354.66353	2	1.26E-06	0.97	5.05	-	1820.7
AHQ-1-3, 4398	R.NNLAGAEELFAR.K	1305.42165	2	4.68E-08	0.90	3.43	-	977.5
AHQ-1-4, 7092	R.RPLIDQVVQTALSETQDPEEVSVTVK.A	2883.20103	3	5.50E-07	0.93	4.74	-	941.3
AHQ-1-5, 7041	R.RPLIDQVVQTALSETQDPEEVSVTVK.A	2883.20103	3	3.38E-04	0.95	5.30	-	1354.5
AHQ-1-3, 6918 - 6922	R.RPLIDQVVQTALSETQDPEEVSVTVK.A	2883.20103	3	1.64E-06	0.98	6.93	-	1643.7
AHQ-1-5, 5105	R.TSIDAYDNFDNISLAQR.L	1944.04818	2	8.84E-08	0.96	4.52	-	1908.5
AHQ-1-3, 7062	K.VGYTPDWIFLLR.N	1480.73443	2	1.55E-08	0.89	3.69	-	572.6
AHQ-1-4, 7228	K.VGYTPDWIFLLR.N	1480.73443	2	1.20E-07	0.83	3.54	-	428.2
AHQ-1-3, 3153	K.VIQCFAETGQVQK.I	1509.70940	2	5.73E-06	0.96	3.88	-	1929.2
AHQ-1-3, 3830	R.VVGAMQLYSVDR.K	1338.55819	2	4.90E-06	0.93	3.50	-	1212.4
AHQ-1-3, 3218	R.VVGAM*QLYSVDR.K	1354.55759	2	3.95E-04	0.64	2.71	-	573.3
gi|4501881|ref|NP_001091.1| alpha 1 actin precursor; alpha skeletal muscle actin [Homo sapiens] [MA				3.05E-14	12.38	180.27	46.40	42050.8
AHQ-1-1, 2093	K.AGFAGDDAPR.A	977.01283	2	4.50E-05	0.85	2.75	-	1047.6
AHQ-1-8, 1551 - 1620	K.AGFAGDDAPR.A	977.01283	2	5.45E-06	0.88	3.66	-	799.2
AHQ-1-8, 1687 - 1768	K.AGFAGDDAPR.A	977.01283	2	7.78E-06	0.89	3.37	-	965.6
AHQ-1-13- , 2027 - 2096	K.AGFAGDDAPR.A	977.01283	2	6.29E-05	0.89	3.28	-	993.5
AHQ-1-13, 2034 - 2090	K.AGFAGDDAPR.A	977.01283	2	5.22E-04	0.88	3.38	-	949.8
AHQ-1-8, 1908 - 1975	K.AGFAGDDAPR.A	977.01283	2	1.61E-05	0.89	3.31	-	989.3
AHQ-1-12, 2013	K.AGFAGDDAPR.A	977.01283	2	2.13E-04	0.87	2.65	-	1203.0
AHQ-1-12, 1890 - 1953	K.AGFAGDDAPR.A	977.01283	2	3.29E-04	0.86	3.33	-	967.7
AHQ-1-10, 1829 - 1849	K.AGFAGDDAPR.A	977.01283	2	3.89E-04	0.84	2.78	-	1003.7
AHQ-1-8, 2040	K.AGFAGDDAPR.A	977.01283	2	3.69E-04	0.86	2.86	-	978.4
AHQ-1-9, 1676 - 1746	K.AGFAGDDAPR.A	977.01283	2	1.22E-06	0.87	3.03	-	937.0
AHQ-1-4, 3276	R.AVFPSIVGRPR.H	1199.43018	2	3.98E-05	0.21	2.50	-	397.8

AHQ-1-8, 4055	R.DLTDYLMK.I	999.16326	1	8.88E-05	0.50	2.49	-	488.1
AHQ-1-8, 3789 - 3860	R.DLTDYLM*K.I	1015.16266	2	2.90E-04	0.88	3.55	-	546.9
AHQ-1-8, 3309	R.DLTDYLMK.I	999.16326	1	5.97E-05	0.23	1.86	-	376.1
AHQ-1-5, 4593	R.DLTDYLMK.I	999.16326	2	5.60E-04	0.62	2.51	-	413.8
AHQ-1-12, 4287	R.DLTDYLMK.I	999.16326	2	3.83E-04	0.78	2.74	-	454.9
AHQ-1-8, 4497 - 4540	R.DLTDYLMK.I	999.16326	2	1.02E-04	0.46	2.55	-	322.2
AHQ-1-9, 3711	R.DLTDYLM*K.I	1015.16266	1	6.02E-05	0.15	2.11	-	109.2
AHQ-1-1, 4573	R.DLTDYLMK.I	999.16326	2	2.78E-04	0.68	2.88	-	399.7
AHQ-1-13- , 3803	R.DLTDYLM*K.I	1015.16266	1	5.64E-04	0.20	2.20	-	140.6
AHQ-1-14- , 4850 - 4911	K.DLYANNVMSGGTTM*YPGIADR.M	2263.49384	2	9.17E-05	0.65	3.74	-	373.7
AHQ-1-8, 1969	K.DSYVGDEAQSK.R	1199.20634	1	1.78E-04	0.23	2.06	-	250.9
AHQ-1-8, 1479 - 1547	K.DSYVGDEAQSK.R	1199.20634	2	2.50E-05	0.69	3.28	-	539.3
AHQ-1-8, 1943 - 2017	K.DSYVGDEAQSK.R	1199.20634	2	4.47E-04	0.67	3.24	-	567.8
AHQ-1-13, 2983 - 3006	K.EITALAPSTMK.I	1162.38206	2	4.75E-05	0.87	3.01	-	734.3
AHQ-1-2, 2529	K.EITALAPSTM*K.I	1178.38146	2	2.35E-06	0.80	3.09	-	617.1
AHQ-1-12, 2935 - 2937	K.EITALAPSTMK.I	1162.38206	1	8.76E-05	0.70	3.00	-	414.2
AHQ-1-12, 2933 - 2934	K.EITALAPSTMK.I	1162.38206	2	5.10E-04	0.84	3.02	-	722.5
AHQ-1-6, 2895 - 2910	K.EITALAPSTMK.I	1162.38206	2	2.33E-04	0.61	2.61	-	441.6
AHQ-1-13- , 2428 - 2503	K.EITALAPSTM*K.I	1178.38146	2	1.09E-04	0.76	3.13	-	512.2
AHQ-1-14- , 2882 - 2956	K.EITALAPSTMK.I	1162.38206	1	3.50E-05	0.57	2.92	-	280.4
AHQ-1-14, 3894	K.EITALAPSTMK.I	1162.38206	1	4.79E-07	0.34	2.68	-	196.8
AHQ-1-10, 2261 - 2275	K.EITALAPSTM*K.I	1178.38146	2	9.01E-04	0.58	2.60	-	451.5
AHQ-1-13- , 2975 - 3027	K.EITALAPSTMK.I	1162.38206	2	1.69E-04	0.78	2.80	-	613.3
AHQ-1-14, 3244 - 3276	K.EITALAPSTM*K.I	1178.38146	2	8.01E-05	0.50	2.79	-	385.1
AHQ-1-14, 3872 - 3928	K.EITALAPSTMK.I	1162.38206	1	5.69E-06	0.61	2.68	-	401.7
AHQ-1-9, 2255 - 2259	K.EITALAPSTM*K.I	1178.38146	2	5.83E-04	0.90	3.17	-	927.2
AHQ-1-3, 3027	K.EITALAPSTMK.I	1162.38206	1	3.89E-06	0.35	2.19	-	319.6
AHQ-1-14- , 2880 - 2954	K.EITALAPSTMK.I	1162.38206	2	1.00E-04	0.74	2.99	-	529.8
AHQ-1-4, 3030	K.EITALAPSTMK.I	1162.38206	1	2.84E-07	0.58	2.38	-	356.8
AHQ-1-10, 2708 - 2767	K.EITALAPSTMK.I	1162.38206	1	4.68E-04	0.65	2.32	-	439.1
AHQ-1-1, 3140	K.EITALAPSTMK.I	1162.38206	1	9.43E-04	0.27	2.41	-	307.0
AHQ-1-4, 2423	K.EITALAPSTM*K.I	1178.38146	2	6.37E-04	0.81	2.81	-	746.1
AHQ-1-14- , 2378 - 2402	K.EITALAPSTM*K.I	1178.38146	2	3.35E-06	0.90	3.68	-	713.1
AHQ-1-8, 2557 - 2626	K.EITALAPSTMK.I	1162.38206	2	3.41E-04	0.83	2.93	-	722.9
AHQ-1-8, 2553 - 2766	K.EITALAPSTMK.I	1162.38206	1	3.20E-04	0.72	3.28	-	341.4
AHQ-1-8, 7310 - 7390	K.EKLCYVALDFENEM*ATAASSSSLEK.S	2812.07683	2	7.05E-07	0.79	4.03	-	425.6
AHQ-1-8, 7239 - 7320	K.EKLCYVALDFENEM*ATAASSSSLEK.S	2812.07683	3	9.60E-04	0.57	3.19	-	686.3
AHQ-1-8, 7338 - 7407	K.EKLCYVALDFENEM*ATAASSSSLEK.S	2812.07683	3	1.44E-04	0.66	3.57	-	579.9
AHQ-1-12, 1878 - 1939	R.HQGVMVGMGQK.D	1172.40691	2	1.32E-06	0.81	3.02	-	620.9
AHQ-1-8, 1525 - 1591	R.HQGVMVGMGQK.D	1172.40691	2	2.71E-05	0.94	3.97	-	773.2
AHQ-1-8, 2485	R.HQGVMVGMGQKDSYVGDEAQSK.R	2352.59065	2	1.71E-04	0.90	3.76	-	652.1
AHQ-1-8, 2487	R.HQGVMVGMGQKDSYVGDEAQSK.R	2352.59065	3	5.90E-05	0.98	4.33	-	3199.5
AHQ-1-5, 2870	K.IWHHTFYNELR.V	1516.68695	3	1.61E-05	0.92	4.39	-	1485.0
AHQ-1-13, 3121	K.IWHHTFYNELR.V	1516.68695	3	1.38E-05	0.93	4.12	-	1258.5
AHQ-1-13, 3117 - 3183	K.IWHHTFYNELR.V	1516.68695	2	1.18E-06	0.80	3.44	-	671.7
AHQ-1-12, 3354	K.IWHHTFYNELR.V	1516.68695	2	1.40E-05	0.59	2.74	-	413.6
AHQ-1-8, 2985 - 3056	K.IWHHTFYNELR.V	1516.68695	2	5.07E-08	0.87	3.74	-	898.9
AHQ-1-8, 2901 - 2965	K.IWHHTFYNELR.V	1516.68695	3	9.34E-08	0.93	3.94	-	1557.1
AHQ-1-8, 2681	K.IWHHTFYNELR.V	1516.68695	3	1.68E-05	0.94	4.55	-	1663.2
AHQ-1-12, 3006 - 3013	K.IWHHTFYNELR.V	1516.68695	3	2.25E-05	0.87	3.80	-	1240.9
AHQ-1-12, 2995 - 3059	K.IWHHTFYNELR.V	1516.68695	2	8.09E-07	0.79	3.19	-	832.0
AHQ-1-8, 2589 - 2657	K.IWHHTFYNELR.V	1516.68695	2	1.02E-05	0.82	3.43	-	872.0
AHQ-1-11, 2954	K.IWHHTFYNELR.V	1516.68695	3	1.28E-04	0.95	4.65	-	1444.0
AHQ-1-11, 2946 - 2960	K.IWHHTFYNELR.V	1516.68695	2	3.76E-05	0.89	3.75	-	1104.4
AHQ-1-6, 2916	K.IWHHTFYNELR.V	1516.68695	3	1.80E-05	0.90	4.12	-	1455.4
AHQ-1-10, 2976	K.IWHHTFYNELR.V	1516.68695	2	3.13E-07	0.90	3.35	-	942.9
AHQ-1-10, 2820	K.IWHHTFYNELR.V	1516.68695	3	5.69E-05	0.92	4.14	-	1127.6
AHQ-1-10, 2757 - 2832	K.IWHHTFYNELR.V	1516.68695	2	1.30E-04	0.73	3.16	-	813.4
AHQ-1-8, 2840 - 2920	K.IWHHTFYNELR.V	1516.68695	2	1.46E-04	0.69	2.74	-	615.6
AHQ-1-8, 2493 - 2561	K.IWHHTFYNELR.V	1516.68695	2	4.46E-06	0.86	3.93	-	803.1
AHQ-1-12, 3135	K.IWHHTFYNELR.V	1516.68695	2	1.67E-09	0.93	3.37	-	1164.1
AHQ-1-5, 2861 - 2931	K.IWHHTFYNELR.V	1516.68695	2	1.64E-05	0.52	2.60	-	602.2
AHQ-1-8, 2517 - 2586	K.IWHHTFYNELR.V	1516.68695	3	1.11E-04	0.96	4.29	-	1747.1
AHQ-1-9, 2734 - 2803	K.IWHHTFYNELR.V	1516.68695	2	3.02E-06	0.79	3.67	-	651.1
AHQ-1-9, 2743	K.IWHHTFYNELR.V	1516.68695	3	9.54E-04	0.92	3.98	-	1694.8
AHQ-1-9, 2982 - 2994	K.IWHHTFYNELR.V	1516.68695	2	5.82E-10	0.92	3.57	-	1101.0
AHQ-1-3, 3002 - 3074	K.IWHHTFYNELR.V	1516.68695	2	4.77E-04	0.38	2.57	-	529.0
AHQ-1-13- , 3155 - 3159	K.IWHHTFYNELR.V	1516.68695	2	3.53E-08	0.72	3.23	-	735.4
AHQ-1-13- , 3156 - 3173	K.IWHHTFYNELR.V	1516.68695	3	2.42E-06	0.94	4.27	-	1374.1
AHQ-1-9, 5316 - 5334	R.LDLAGRDLTDYLMK.I	1624.88375	2	9.77E-04	0.57	2.76	-	560.3
AHQ-1-9, 4468 - 4551	K.SYELPDGQVITIGNER.F	1791.93998	2	9.55E-12	0.95	4.67	-	871.6
AHQ-1-2, 5430	K.SYELPDGQVITIGNER.F	1791.93998	2	2.36E-07	0.91	3.83	-	723.0
AHQ-1-9, 4562 - 4586	K.SYELPDGQVITIGNER.F	1791.93998	2	3.44E-13	0.92	4.02	-	783.9
AHQ-1-9, 4684	K.SYELPDGQVITIGNER.F	1791.93998	2	4.43E-12	0.96	4.30	-	1229.8
AHQ-1-13- , 4596	K.SYELPDGQVITIGNER.F	1791.93998	2	1.57E-11	0.95	4.63	-	992.4
AHQ-1-9, 4838 - 4906	K.SYELPDGQVITIGNER.F	1791.93998	2	9.21E-11	0.94	4.15	-	968.8
AHQ-1-9, 4974 - 5026	K.SYELPDGQVITIGNER.F	1791.93998	2	3.90E-09	0.96	4.92	-	1095.3
AHQ-1-7, 4677	K.SYELPDGQVITIGNER.F	1791.93998	2	1.67E-06	0.88	3.33	-	604.2
AHQ-1-9, 5095 - 5166	K.SYELPDGQVITIGNER.F	1791.93998	2	1.09E-11	0.95	4.00	-	1346.1
AHQ-1-4, 4776	K.SYELPDGQVITIGNER.F	1791.93998	2	4.27E-08	0.89	3.42	-	925.7
AHQ-1-7, 5044 - 5066	K.SYELPDGQVITIGNER.F	1791.93998	2	1.39E-04	0.59	2.68	-	623.9
AHQ-1-2, 5245	K.SYELPDGQVITIGNER.F	1791.93998	2	7.41E-08	0.89	3.82	-	659.3
AHQ-1-6, 5238	K.SYELPDGQVITIGNER.F	1791.93998	2	1.53E-07	0.96	4.79	-	1286.6
AHQ-1-6, 4990 - 5020	K.SYELPDGQVITIGNER.F	1791.93998	2	7.01E-06	0.95	4.12	-	1095.2
AHQ-1-13- , 4696 - 4720	K.SYELPDGQVITIGNER.F	1791.93998	2	5.50E-13	0.94	4.02	-	1056.5
AHQ-1-8, 5234 - 5302	K.SYELPDGQVITIGNER.F	1791.93998	2	1.86E-11	0.97	5.19	-	1489.3
AHQ-1-8, 5127 - 5194	K.SYELPDGQVITIGNER.F	1791.93998	2	4.96E-11	0.96	4.85	-	1030.1
AHQ-1-13- , 4989 - 5071	K.SYELPDGQVITIGNER.F	1791.93998	2	4.94E-10	0.96	4.73	-	1112.3
AHQ-1-10, 4067 - 4141	K.SYELPDGQVITIGNER.F	1791.93998	2	6.57E-07	0.95	4.46	-	942.0
AHQ-1-1, 5131 - 5177	K.SYELPDGQVITIGNER.F	1791.93998	2	9.60E-10	0.93	3.69	-	862.3
AHQ-1-10, 4237 - 4300	K.SYELPDGQVITIGNER.F	1791.93998	2	3.42E-07	0.94	4.62	-	782.2
AHQ-1-10, 4409 - 4471	K.SYELPDGQVITIGNER.F	1791.93998	2	5.27E-11	0.95	4.58	-	1047.8
AHQ-1-10, 4453 - 4527	K.SYELPDGQVITIGNER.F	1791.93998	2	8.87E-13	0.97	5.38	-	1236.4
AHQ-1-10, 4484	K.SYELPDGQVITIGNER.F	1791.93998	3	2.93E-08	0.95	4.66	-	1371.4
AHQ-1-10, 4624 - 4679	K.SYELPDGQVITIGNER.F	1791.93998	2	3.51E-11	0.96	4.77	-	1196.2
AHQ-1-8, 4994 - 5058	K.SYELPDGQVITIGNER.F	1791.93998	2	1.58E-10	0.96	4.66	-	1296.3
AHQ-1-8, 4894 - 4958	K.SYELPDGQVITIGNER.F	1791.93998	2	3.05E-14	0.97	5.20	-	1035.9
AHQ-1-11, 4280	K.SYELPDGQVITIGNER.F	1791.93998	2	1.32E-06	0.76	2.75	-	680.6
AHQ-1-11, 4379	K.SYELPDGQVITIGNER.F	1791.93998	2	9.66E-11	0.91	4.01	-	824.4
AHQ-1-11, 4760 - 4824	K.SYELPDGQVITIGNER.F	1791.93998	2	2.04E-12	0.96	4.62	-	1264.8
AHQ-1-8, 4750 - 4812	K.SYELPDGQVITIGNER.F	1791.93998	2	1.03E-12	0.96	4.63	-	1198.2
AHQ-1-2, 4969	K.SYELPDGQVITIGNER.F	1791.93998	2	9.93E-05	0.91	4.01	-	673.3
AHQ-1-2, 4863 - 4867	K.SYELPDGQVITIGNER.F	1791.93998	2	3.46E-06	0.89	3.90	-	714.9
AHQ-1-5, 5263 - 5337	K.SYELPDGQVITIGNER.F	1791.93998	2	2.78E-07	0.92	4.13	-	968.5
AHQ-1-5, 5089	K.SYELPDGQVITIGNER.F	1791.93998	2	1.05E-06	0.90	3.61	-	953.9
AHQ-1-8, 4623	K.SYELPDGQVITIGNER.F	1791.93998	3	4.54E-09	0.94	4.40	-	1556.9
AHQ-1-8, 4618 - 4682	K.SYELPDGQVITIGNER.F	1791.93998	2	1.97E-10	0.95	4.66	-	1124.1

AHQ-1-13- , 5145 - 5223	K.SYELPDGQVITIGNER.F	1791.93998	2	7.32E-12	0.96	4.76	-	1186.3
AHQ-1-8, 4485	K.SYELPDGQVITIGNER.F	1791.93998	2	1.09E-07	0.82	3.46	-	557.9
AHQ-1-14, 5756	K.SYELPDGQVITIGNER.F	1791.93998	2	2.80E-06	0.85	3.72	-	661.0
AHQ-1-5, 4681	K.SYELPDGQVITIGNER.F	1791.93998	2	3.09E-09	0.86	3.48	-	674.0
AHQ-1-12, 4318	K.SYELPDGQVITIGNER.F	1791.93998	2	3.99E-09	0.88	3.09	-	807.8
AHQ-1-12, 4441 - 4442	K.SYELPDGQVITIGNER.F	1791.93998	2	1.12E-11	0.95	4.47	-	909.1
AHQ-1-12, 4525	K.SYELPDGQVITIGNER.F	1791.93998	2	7.51E-10	0.95	4.27	-	975.6
AHQ-1-12, 4767 - 4830	K.SYELPDGQVITIGNER.F	1791.93998	2	1.73E-10	0.87	3.26	-	772.5
AHQ-1-12, 4963	K.SYELPDGQVITIGNER.F	1791.93998	2	3.61E-04	0.81	3.12	-	773.1
AHQ-1-1, 5297 - 5301	K.SYELPDGQVITIGNER.F	1791.93998	2	2.20E-07	0.96	4.89	-	1166.7
AHQ-1-14- , 4542	K.SYELPDGQVITIGNER.F	1791.93998	2	3.27E-09	0.87	3.42	-	848.1
AHQ-1-3, 4841	K.SYELPDGQVITIGNER.F	1791.93998	2	1.68E-06	0.80	3.06	-	564.8
AHQ-1-3, 5090 - 5091	K.SYELPDGQVITIGNER.F	1791.93998	2	1.67E-10	0.95	4.69	-	786.5
AHQ-1-13, 4292	K.SYELPDGQVITIGNER.F	1791.93998	2	1.42E-07	0.89	3.47	-	829.3
AHQ-1-3, 5251	K.SYELPDGQVITIGNER.F	1791.93998	2	1.19E-04	0.93	3.90	-	795.7
AHQ-1-13, 4391	K.SYELPDGQVITIGNER.F	1791.93998	2	3.95E-08	0.77	3.01	-	612.6
AHQ-1-9, 5214	K.SYELPDGQVITIGNER.F	1791.93998	2	1.10E-11	0.96	4.64	-	1078.2
AHQ-1-13, 4487 - 4488	K.SYELPDGQVITIGNER.F	1791.93998	2	9.86E-07	0.95	4.11	-	1217.8
AHQ-1-13, 4737 - 4795	K.SYELPDGQVITIGNER.F	1791.93998	2	1.18E-08	0.92	4.06	-	817.2
AHQ-1-13, 4876 - 4948	K.SYELPDGQVITIGNER.F	1791.93998	2	3.52E-11	0.96	4.73	-	1171.1
AHQ-1-4, 5148	K.SYELPDGQVITIGNER.F	1791.93998	2	9.78E-09	0.95	4.01	-	1237.3
AHQ-1-10, 3625	R.VAPEEHPTLLTEAPLNPK.A	1957.21627	2	7.22E-06	0.60	2.82	-	352.5
AHQ-1-12, 3749 - 3815	K.YPIEHGIITNWDDM*EK.I	1978.17114	2	2.44E-04	0.50	2.91	-	371.3
AHQ-1-10, 4088 - 4104	K.YPIEHGIITNWDDMEK.I	1962.17174	3	4.08E-07	0.83	4.12	-	835.8
AHQ-1-13, 4331	K.YPIEHGIITNWDDMEK.I	1962.17174	2	6.85E-04	0.84	3.97	-	676.8
AHQ-1-13, 4485	K.YPIEHGIITNWDDMEK.I	1962.17174	2	7.06E-06	0.83	3.80	-	455.9
AHQ-1-9, 5064 - 5143	K.YPIEHGIITNWDDMEK.I	1962.17174	2	1.83E-04	0.82	3.54	-	627.4
AHQ-1-9, 4702 - 4724	K.YPIEHGIITNWDDMEK.I	1962.17174	2	9.21E-06	0.86	3.56	-	727.2
AHQ-1-9, 4506	K.YPIEHGIITNWDDMEK.I	1962.17174	2	8.72E-05	0.38	2.58	-	518.4
AHQ-1-9, 4140	K.YPIEHGIITNWDDMEK.I	1962.17174	2	1.08E-04	0.90	4.12	-	781.3
AHQ-1-13- , 4069 - 4145	K.YPIEHGIITNWDDM*EK.I	1978.17114	2	5.13E-04	0.90	3.91	-	651.2
AHQ-1-13- , 4543 - 4611	K.YPIEHGIITNWDDMEK.I	1962.17174	2	4.73E-04	0.92	4.57	-	823.7
AHQ-1-9, 3962	K.YPIEHGIITNWDDM*EK.I	1978.17114	2	1.45E-04	0.83	3.47	-	729.9
AHQ-1-13- , 4644 - 4724	K.YPIEHGIITNWDDMEK.I	1962.17174	2	4.27E-05	0.80	3.81	-	531.1
AHQ-1-4, 4842	K.YPIEHGIITNWDDMEK.I	1962.17174	2	4.35E-05	0.78	3.54	-	549.9
AHQ-1-8, 5094 - 5096	K.YPIEHGIITNWDDMEK.I	1962.17174	2	5.28E-05	0.88	4.22	-	583.6
AHQ-1-8, 4523 - 4595	K.YPIEHGIITNWDDMEK.I	1962.17174	2	1.53E-04	0.87	4.04	-	562.8
AHQ-1-3, 4650	K.YPIEHGIITNWDDMEK.I	1962.17174	2	7.09E-05	0.68	3.20	-	554.1
AHQ-1-4, 4694	K.YPIEHGIITNWDDMEK.I	1962.17174	2	3.40E-04	0.52	2.82	-	474.4
AHQ-1-6, 4480	K.YPIEHGIITNWDDMEK.I	1962.17174	2	8.39E-04	0.92	4.10	-	1005.0
AHQ-1-9, 4068	K.YPIEHGIITNWDDM*EK.I	1978.17114	2	7.21E-04	0.58	3.00	-	439.9
AHQ-1-8, 5544	K.YPIEHGIITNWDDM*EKIWHHTFYNELR.V	3475.83549	3	1.27E-04	0.92	5.39	-	690.4
gi|29741246|ref|XP_294070.1| similar to Glyceraldehyde 3-phosphate dehydrogenase, liver (GAPDH) [Ho				3.28E-14	4.99	60.34	17.70	35032.0
AHQ-1-13- , 5417 - 5497	K.AENGKLVINGNPITIFQER.D	2114.39062	2	6.69E-09	0.65	2.74	-	682.2
AHQ-1-9, 5291 - 5359	K.AENGKLVINGNPITIFQER.D	2114.39062	2	7.76E-07	0.74	3.64	-	503.6
AHQ-1-9, 5435 - 5502	K.AENGKLVINGNPITIFQER.D	2114.39062	2	2.71E-06	0.65	3.60	-	316.8
AHQ-1-9, 5570 - 5572	K.AENGKLVINGNPITIFQER.D	2114.39062	2	2.87E-07	0.81	3.44	-	796.9
AHQ-1-13, 5185	K.AENGKLVINGNPITIFQER.D	2114.39062	2	1.53E-07	0.63	3.10	-	432.7
AHQ-1-12, 5238	K.AENGKLVINGNPITIFQER.D	2114.39062	2	7.61E-06	0.23	2.53	-	298.2
AHQ-1-14- , 5036	K.LISWYENEFGYSNR.V	1778.90145	2	1.16E-06	0.89	3.22	-	954.7
AHQ-1-9, 5648 - 5718	K.LVINGNPITIFQER.D	1614.87029	2	2.06E-12	0.94	3.87	-	1164.0
AHQ-1-10, 5260	K.LVINGNPITIFQER.D	1614.87029	2	6.67E-06	0.94	3.87	-	1321.9
AHQ-1-12, 5603 - 5678	K.LVINGNPITIFQER.D	1614.87029	2	9.00E-07	0.80	3.16	-	569.4
AHQ-1-11, 5288 - 5360	K.LVINGNPITIFQER.D	1614.87029	2	3.25E-11	0.95	3.86	-	1199.9
AHQ-1-12, 5473 - 5539	K.LVINGNPITIFQER.D	1614.87029	2	1.67E-10	0.89	3.69	-	852.9
AHQ-1-9, 5854 - 5856	K.LVINGNPITIFQER.D	1614.87029	2	1.33E-08	0.93	4.11	-	918.4
AHQ-1-13- , 5747 - 5811	K.LVINGNPITIFQER.D	1614.87029	2	4.32E-08	0.96	4.33	-	1479.4
AHQ-1-9, 5215 - 5258	K.LVINGNPITIFQERDPSK.I	2042.32440	2	1.41E-05	0.58	3.43	-	477.9
AHQ-1-11, 5934	K.VLHDNFGIVKGLM*TTVHAITATQK.T	2612.04471	3	1.48E-12	0.96	5.58	-	2454.8
AHQ-1-10, 5821	K.VLHDNFGIVKGLM*TTVHAITATQK.T	2612.04471	3	1.15E-08	0.97	5.61	-	1338.2
AHQ-1-9, 7111 - 7182	K.VLHDNFGIVKGLMTTVHAITATQK.T	2596.04531	2	1.78E-04	0.96	5.64	-	1862.9
AHQ-1-9, 6659	K.VLHDNFGIVKGLM*TTVHAITATQK.T	2612.04471	3	2.58E-05	0.91	4.72	-	1615.7
AHQ-1-9, 6512 - 6586	K.VLHDNFGIVKGLM*TTVHAITATQK.T	2612.04471	3	3.28E-14	0.97	6.86	-	2321.7
AHQ-1-11, 5944	K.VLHDNFGIVKGLM*TTVHAITATQK.T	2612.04471	2	9.04E-05	0.76	3.71	-	855.4
AHQ-1-11, 6386	K.VLHDNFGIVKGLMTTVHAITATQK.T	2596.04531	3	7.61E-08	0.96	6.90	-	1873.2
gi|4507729|ref|NP_001060.1| tubulin, beta polypeptide [Homo sapiens]				3.39E-14	10.24	120.36	36.60	49906.7
AHQ-1-14, 5760	R.AILVDLEPGTMDSVR.S	1616.86162	2	1.57E-06	0.64	3.46	-	296.3
AHQ-1-13- , 4964 - 4988	R.AILVDLEPGTMDSVR.S	1616.86162	2	4.14E-04	0.87	3.38	-	672.9
AHQ-1-11, 4583	R.AILVDLEPGTMDSVR.S	1616.86162	2	5.02E-04	0.81	3.52	-	449.7
AHQ-1-14- , 4875 - 4890	R.AILVDLEPGTMDSVR.S	1616.86162	2	2.39E-04	0.91	4.47	-	660.8
AHQ-1-12, 2925	R.EIVHIQAGQCGNQIGAK.F	1825.03962	2	8.15E-08	0.89	3.56	-	847.6
AHQ-1-7, 2914	K.EVDEQMLNVQNK.N	1447.59668	2	1.54E-04	0.91	4.28	-	1019.2
AHQ-1-9, 2852	K.EVDEQMLNVQNK.N	1447.59668	2	1.11E-04	0.78	3.27	-	705.8
AHQ-1-7, 2925	K.EVDEQMLNVQNK.N	1447.59668	1	8.96E-07	0.60	3.23	-	328.1
AHQ-1-13- , 6459	K.GHYTEGAELVDSVLDVVR.K	1960.13388	2	4.00E-08	0.99	7.25	-	2436.4
AHQ-1-9, 6459	K.GHYTEGAELVDSVLDVVR.K	1960.13388	3	7.45E-05	0.91	4.74	-	1038.4
AHQ-1-10, 5759	K.GHYTEGAELVDSVLDVVR.K	1960.13388	3	5.92E-04	0.83	3.75	-	1098.9
AHQ-1-14- , 6218	K.GHYTEGAELVDSVLDVVR.K	1960.13388	2	3.39E-14	0.98	6.73	-	2492.1
AHQ-1-11, 5871	K.GHYTEGAELVDSVLDVVR.K	1960.13388	2	1.93E-07	0.96	4.81	-	1220.0
AHQ-1-13, 6004 - 6015	K.GHYTEGAELVDSVLDVVR.K	1960.13388	3	7.18E-06	0.90	4.83	-	863.2
AHQ-1-13- , 6451 - 6452	K.GHYTEGAELVDSVLDVVR.K	1960.13388	3	8.28E-06	0.94	5.31	-	1247.0
AHQ-1-7, 4717	K.GHYTEGAELVDSVLDVVR.K	1960.13388	2	9.37E-11	0.98	6.52	-	1313.7
AHQ-1-9, 6447 - 6454	K.GHYTEGAELVDSVLDVVR.K	1960.13388	2	9.91E-07	0.98	6.45	-	2556.9
AHQ-1-7, 6584 - 6636	K.GHYTEGAELVDSVLDVVR.K	1960.13388	2	3.35E-07	0.98	6.48	-	2627.4
AHQ-1-7, 6585 - 6614	K.GHYTEGAELVDSVLDVVR.K	1960.13388	3	1.11E-04	0.88	4.40	-	815.4
AHQ-1-13, 6003	K.GHYTEGAELVDSVLDVVR.K	1960.13388	2	2.43E-08	0.99	6.55	-	3013.5
AHQ-1-12, 6073	K.GHYTEGAELVDSVLDVVR.K	1960.13388	2	3.85E-08	0.98	6.45	-	2253.1
AHQ-1-8, 6535 - 6542	K.GHYTEGAELVDSVLDVVR.K	1960.13388	2	1.49E-12	0.99	6.80	-	2859.3
AHQ-1-10, 5751	K.GHYTEGAELVDSVLDVVR.K	1960.13388	2	1.12E-09	0.98	5.49	-	2087.8
AHQ-1-7, 4653	R.ISEQFTAMFR.R	1230.41818	2	6.53E-06	0.93	3.51	-	1151.5
AHQ-1-7, 3633	R.ISEQFTAM*FR.R	1246.41758	2	5.09E-06	0.90	3.46	-	883.1
AHQ-1-7, 4550 - 4558	R.ISEQFTAMFR.R	1230.41818	2	7.30E-06	0.92	3.86	-	1088.6
AHQ-1-9, 7154 - 7156	K.LTTPTYGDLNHLVSATMSGVTTCLR.F	2711.06527	3	5.35E-07	0.91	4.41	-	1300.1
AHQ-1-7, 6386 - 6460	K.LTTPTYGDLNHLVSATM*SGVTTCLR.F	2727.06467	3	2.94E-04	0.67	3.10	-	681.3
AHQ-1-7, 7352	K.LTTPTYGDLNHLVSATMSGVTTCLR.F	2711.06527	2	2.99E-09	0.96	5.95	-	718.5
AHQ-1-9, 7155	K.LTTPTYGDLNHLVSATMSGVTTCLR.F	2711.06527	2	1.39E-08	0.92	4.60	-	764.7
AHQ-1-7, 7353	K.LTTPTYGDLNHLVSATMSGVTTCLR.F	2711.06527	3	8.45E-07	0.89	4.12	-	1180.6
AHQ-1-8, 7346	K.LTTPTYGDLNHLVSATMSGVTTCLR.F	2711.06527	3	1.28E-05	0.88	4.01	-	1062.6
AHQ-1-7, 6488	K.LTTPTYGDLNHLVSATMSGVTTCLR.F	2711.06527	2	9.88E-06	0.85	3.31	-	716.1
AHQ-1-12, 6150	K.M*SATFIGNSTAIQELFK.R	1875.13550	2	2.42E-06	0.79	3.38	-	981.2
AHQ-1-7, 6800	K.M*SATFIGNSTAIQELFK.R	1875.13550	2	1.84E-08	0.92	4.62	-	1125.2
AHQ-1-14- , 6003	K.M*SATFIGNSTAIQELFK.R	1875.13550	2	1.66E-05	0.66	3.60	-	645.2
AHQ-1-7, 6368 - 6369	K.M*SATFIGNSTAIQELFK.R	1875.13550	2	7.12E-05	0.86	4.29	-	996.1
AHQ-1-9, 6063	K.NSSYFVEWIPNNVK.T	1697.87148	2	1.30E-04	0.47	2.66	-	464.3
AHQ-1-7, 5645 - 5721	K.NSSYFVEWIPNNVK.T	1697.87148	2	1.02E-05	0.91	4.52	-	762.4
AHQ-1-7, 5800	K.NSSYFVEWIPNNVK.T	1697.87148	2	4.11E-09	0.88	3.43	-	873.4
AHQ-1-7, 6212	K.NSSYFVEWIPNNVK.T	1697.87148	2	2.24E-04	0.84	3.59	-	692.6

AHQ-1-7, 6297 - 6310	K.NSSYFVEWIPNNVK.T	1697.87148	2	8.28E-05	0.46	2.88	-	490.3
AHQ-1-7, 6382 - 6456	K.NSSYFVEWIPNNVK.T	1697.87148	2	8.34E-07	0.88	4.04	-	759.3
AHQ-1-7, 5548 - 5624	K.NSSYFVEWIPNNVK.T	1697.87148	2	6.06E-09	0.95	5.14	-	917.1
AHQ-1-12, 5378 - 5446	K.NSSYFVEWIPNNVK.T	1697.87148	2	9.87E-07	0.88	3.87	-	723.1
AHQ-1-8, 6572	R.SGPFGQIFRPDNFVFGQSGAGNNWAK.G	2800.03898	3	2.79E-05	0.96	5.87	-	1379.8
AHQ-1-7, 6732 - 6797	R.SGPFGQIFRPDNFVFGQSGAGNNWAK.G	2800.03898	3	1.10E-09	0.98	6.62	-	2545.6
AHQ-1-8, 6576 - 6646	R.SGPFGQIFRPDNFVFGQSGAGNNWAK.G	2800.03898	2	3.54E-06	0.95	4.94	-	973.3
AHQ-1-7, 6700 - 6768	R.SGPFGQIFRPDNFVFGQSGAGNNWAK.G	2800.03898	2	3.78E-07	0.97	6.12	-	1026.3
AHQ-1-3, 6614	R.SGPFGQIFRPDNFVFGQSGAGNNWAK.G	2800.03898	3	2.76E-10	0.97	5.44	-	1885.5
AHQ-1-13, 6045 - 6103	R.SGPFGQIFRPDNFVFGQSGAGNNWAK.G	2800.03898	3	2.87E-08	0.95	5.50	-	1490.0
AHQ-1-11, 5867 - 5927	R.SGPFGQIFRPDNFVFGQSGAGNNWAK.G	2800.03898	3	3.87E-11	0.97	6.58	-	1631.9
AHQ-1-9, 6452 - 6532	R.SGPFGQIFRPDNFVFGQSGAGNNWAK.G	2800.03898	2	3.76E-04	0.96	5.46	-	1103.7
AHQ-1-10, 5769	R.SGPFGQIFRPDNFVFGQSGAGNNWAK.G	2800.03898	2	2.68E-04	0.92	4.49	-	593.3
AHQ-1-9, 6458 - 6527	R.SGPFGQIFRPDNFVFGQSGAGNNWAK.G	2800.03898	3	4.39E-14	0.97	6.68	-	1706.0
AHQ-1-13- , 6500 - 6564	R.SGPFGQIFRPDNFVFGQSGAGNNWAK.G	2800.03898	3	2.06E-07	0.93	5.07	-	1112.2
AHQ-1-13, 5963 - 6019	R.SGPFGQIFRPDNFVFGQSGAGNNWAK.G	2800.03898	3	8.40E-05	0.94	5.33	-	1227.2
AHQ-1-10, 5757 - 5832	R.SGPFGQIFRPDNFVFGQSGAGNNWAK.G	2800.03898	3	1.80E-04	0.88	4.68	-	730.5
AHQ-1-14- , 6166 - 6227	R.SGPFGQIFRPDNFVFGQSGAGNNWAK.G	2800.03898	3	7.95E-10	0.98	6.53	-	2260.6
AHQ-1-14- , 6308	R.SGPFGQIFRPDNFVFGQSGAGNNWAK.G	2800.03898	3	5.19E-07	0.98	6.38	-	2408.7
AHQ-1-7, 2361	K.TAVCDIPPR.G	1030.18038	2	1.61E-05	0.90	2.86	-	1072.0
gi|21361657|ref|NP_005304.3| glucose regulated protein, 58kDa; glucose regulated protein, 58kD [Hom				3.66E-14	13.02	160.36	41.40	56782.0
AHQ-1-7, 2077 - 2110	R.EATNPPVIQEEKPK.K	1580.76433	2	6.38E-07	0.82	3.28	-	605.4
AHQ-1-7, 6022 - 6037	R.ELSDFISYLQR.E	1371.52000	2	2.07E-04	0.96	4.25	-	1246.2
AHQ-1-7, 6298	R.FAHTNVESLVNEYDDNGEGIILFR.P	2753.96046	3	1.30E-07	0.88	4.14	-	846.0
AHQ-1-7, 6666	R.FAHTNVESLVNEYDDNGEGIILFR.P	2753.96046	2	2.37E-10	0.96	4.95	-	1260.2
AHQ-1-7, 6830	R.FAHTNVESLVNEYDDNGEGIILFR.P	2753.96046	2	1.06E-06	0.46	2.60	-	360.8
AHQ-1-7, 6665 - 6676	R.FAHTNVESLVNEYDDNGEGIILFR.P	2753.96046	3	2.09E-06	0.94	4.60	-	1603.7
AHQ-1-7, 5702	K.FIQENIFGICPHMTEDNKDLIQGK.D	2850.21952	2	1.96E-04	0.73	3.17	-	573.7
AHQ-1-9, 7140	K.FISDKDASIVGFFDDSFSEAHSEFLK.A	2940.16492	3	9.59E-10	0.98	5.93	-	2834.3
AHQ-1-7, 7336 - 7344	K.FISDKDASIVGFFDDSFSEAHSEFLK.A	2940.16492	3	3.66E-14	0.98	7.27	-	2824.5
AHQ-1-7, 7305 - 7338	K.FISDKDASIVGFFDDSFSEAHSEFLK.A	2940.16492	2	2.92E-07	0.83	3.59	-	677.7
AHQ-1-7, 4169 - 4240	R.FLQDYFDGNLKR.Y	1516.68203	2	4.58E-04	0.83	3.41	-	766.3
AHQ-1-7, 2470	K.FVM*QEEFSR.D	1189.32290	2	3.17E-05	0.91	3.23	-	971.4
AHQ-1-7, 4700 - 4704	R.GFPTIYFSPANK.K	1342.52349	2	2.21E-07	0.78	2.84	-	571.8
AHQ-1-7, 6032	R.KTFSHELSDFGLESTAGEIPVVAIR.T	2705.01562	2	9.69E-06	0.95	4.78	-	1056.2
AHQ-1-7, 6029	R.KTFSHELSDFGLESTAGEIPVVAIR.T	2705.01562	3	2.79E-05	0.92	4.37	-	1180.4
AHQ-1-7, 2249 - 2260	K.LSKDPNIVIAK.M	1198.43727	2	2.79E-05	0.85	3.11	-	1048.0
AHQ-1-7, 3514 - 3524	K.MDATANDVPSPYEVR.G	1665.80646	2	1.68E-05	0.53	2.78	-	368.4
AHQ-1-7, 3122 - 3148	K.M*DATANDVPSPYEVR.G	1681.80586	2	4.23E-05	0.87	3.71	-	432.6
AHQ-1-7, 2482	K.SEPIPESNDGPVK.V	1369.45928	1	2.83E-04	0.29	2.22	-	368.0
AHQ-1-9, 6370	K.TFSHELSDFGLESTAGEIPVVAIR.T	2576.84271	3	3.22E-05	0.78	3.81	-	683.5
AHQ-1-7, 6518	K.TFSHELSDFGLESTAGEIPVVAIR.T	2576.84271	2	7.16E-05	0.96	4.62	-	1403.5
AHQ-1-7, 4506 - 4578	K.VVVAENFDEIVNNENK.D	1833.97686	2	1.34E-04	0.89	4.28	-	749.0
AHQ-1-7, 3124	K.YGVSGYPTLK.I	1085.23435	1	2.08E-04	0.57	2.60	-	376.0
AHQ-1-7, 3120 - 3136	K.YGVSGYPTLK.I	1085.23435	2	9.90E-05	0.89	3.37	-	627.0
gi|4501891|ref|NP_001093.1| actinin, alpha 1 [Homo sapiens]				4.22E-14	39.48	440.32	50.60	103056.9
AHQ-1-6, 7299 - 7379	R.AAPFNNWM*EGAM*EDLQDTFIVHTIEEIQGLTTAHEQFK.A	4396.81902	3	1.69E-05	0.94	5.56	-	687.9
AHQ-1-5, 7541 - 7543	R.AAPFNNWMEGAM*EDLQDTFIVHTIEEIQGLTTAHEQFK.A	4380.81962	3	8.42E-08	0.94	6.32	-	1396.3
AHQ-1-5, 7489 - 7490	R.AAPFNNWM*EGAM*EDLQDTFIVHTIEEIQGLTTAHEQFK.A	4396.81902	3	4.52E-07	0.92	5.15	-	945.2
AHQ-1-4, 5594	K.AGTQIENIEEDFRDGLK.L	1936.06918	2	2.90E-05	0.72	4.13	-	612.7
AHQ-1-10, 4825	K.AGTQIENIEEDFRDGLK.L	1936.06918	2	1.54E-04	0.47	3.36	-	501.7
AHQ-1-5, 5454	K.AGTQIENIEEDFRDGLK.L	1936.06918	2	3.48E-06	0.85	4.37	-	866.3
AHQ-1-12, 5174	K.AGTQIENIEEDFRDGLK.L	1936.06918	2	1.01E-04	0.85	4.04	-	579.0
AHQ-1-6, 5391 - 5459	K.AGTQIENIEEDFRDGLK.L	1936.06918	2	4.76E-06	0.77	4.16	-	741.8
AHQ-1-11, 4966	K.AGTQIENIEEDFRDGLK.L	1936.06918	2	5.15E-05	0.84	3.79	-	605.1
AHQ-1-6, 5400	K.AGTQIENIEEDFRDGLK.L	1936.06918	3	1.88E-04	0.60	3.37	-	373.6
AHQ-1-4, 6771	K.AIMTYVSSFYHAFSGAQK.A	2009.27323	3	1.59E-07	0.85	3.90	-	685.4
AHQ-1-5, 5733	K.AIM*TYVSSFYHAFSGAQK.A	2025.27263	3	8.48E-07	0.76	3.22	-	872.1
AHQ-1-5, 5667 - 5742	K.AIM*TYVSSFYHAFSGAQK.A	2025.27263	2	2.50E-09	0.87	3.92	-	578.0
AHQ-1-7, 6680	K.AIMTYVSSFYHAFSGAQK.A	2009.27323	2	9.13E-05	0.75	3.35	-	380.1
AHQ-1-5, 6601 - 6670	K.AIMTYVSSFYHAFSGAQK.A	2009.27323	2	9.79E-09	0.93	4.44	-	906.3
AHQ-1-5, 6645 - 6653	K.AIMTYVSSFYHAFSGAQK.A	2009.27323	3	5.02E-05	0.79	3.14	-	708.8
AHQ-1-4, 6766	K.AIMTYVSSFYHAFSGAQK.A	2009.27323	2	2.10E-07	0.96	5.14	-	1124.4
AHQ-1-6, 5636	K.AIM*TYVSSFYHAFSGAQK.A	2025.27263	2	2.76E-04	0.72	3.23	-	485.3
AHQ-1-5, 3241	R.ASFNHFDRDHSGTLGPEEFK.A	2292.40855	3	3.70E-06	0.95	4.52	-	1923.9
AHQ-1-5, 3251	R.ASFNHFDRDHSGTLGPEEFK.A	2292.40855	2	1.21E-05	0.94	4.03	-	1198.1
AHQ-1-5, 3467 - 3483	R.ASFNHFDRDHSGTLGPEEFK.A	2292.40855	2	3.19E-08	0.79	3.24	-	538.3
AHQ-1-5, 1514 - 1529	K.ATLPDADKER.L	1116.20684	2	7.80E-05	0.66	2.76	-	464.7
AHQ-1-1, 5791	K.DDPLTNLNTAFDVAEK.Y	1763.88340	2	4.95E-06	0.90	4.29	-	760.4
AHQ-1-3, 5831	K.DDPLTNLNTAFDVAEK.Y	1763.88340	2	3.70E-07	0.89	3.86	-	762.7
AHQ-1-9, 5724	K.DDPLTNLNTAFDVAEK.Y	1763.88340	2	4.05E-05	0.90	4.08	-	758.7
AHQ-1-5, 5969	K.DDPLTNLNTAFDVAEK.Y	1763.88340	2	7.01E-06	0.96	4.25	-	1448.4
AHQ-1-5, 5870 - 5937	K.DDPLTNLNTAFDVAEK.Y	1763.88340	2	1.07E-12	0.97	5.71	-	1343.7
AHQ-1-2, 6007	K.DDPLTNLNTAFDVAEK.Y	1763.88340	2	4.76E-06	0.88	3.44	-	944.7
AHQ-1-6, 5778 - 5779	K.DDPLTNLNTAFDVAEK.Y	1763.88340	2	2.21E-05	0.97	5.50	-	1293.2
AHQ-1-5, 5299	K.DDPLTNLNTAFDVAEK.Y	1763.88340	2	4.94E-09	0.92	3.89	-	973.3
AHQ-1-4, 5944 - 5966	K.DDPLTNLNTAFDVAEK.Y	1763.88340	2	3.28E-06	0.97	5.11	-	1704.8
AHQ-1-5, 4981 - 4982	K.DGLGFCALIHR.H	1260.44730	2	1.71E-05	0.92	3.01	-	1304.4
AHQ-1-4, 5112	K.DGLGFCALIHR.H	1260.44730	2	1.03E-04	0.96	3.00	-	2087.2
AHQ-1-5, 5214	K.DGLGFCALIHR.H	1260.44730	2	2.75E-06	0.94	3.17	-	1578.6
AHQ-1-5, 2406 - 2409	R.DHSGTLGPEEFK.A	1317.38606	2	2.11E-06	0.94	3.97	-	951.1
AHQ-1-4, 2540	R.DHSGTLGPEEFK.A	1317.38606	2	7.89E-05	0.91	3.56	-	960.2
AHQ-1-6, 2435	R.DHSGTLGPEEFK.A	1317.38606	2	9.99E-06	0.91	3.27	-	837.0
AHQ-1-6, 3738	K.DYETATLSEIK.A	1270.36761	2	9.23E-07	0.94	3.61	-	1234.9
AHQ-1-5, 3799 - 3803	K.DYETATLSEIK.A	1270.36761	2	2.06E-07	0.94	3.86	-	1302.4
AHQ-1-5, 3393 - 3445	R.ELPPDQAEYCIAR.M	1563.71392	2	2.19E-04	0.40	2.51	-	132.0
AHQ-1-4, 3652 - 3732	R.ELPPDQAEYCIAR.M	1563.71392	2	4.15E-05	0.41	2.56	-	132.6
AHQ-1-6, 3166	R.ETADTDTADQVM*ASFK.I	1746.83148	2	4.23E-05	0.72	2.89	-	709.7
AHQ-1-1, 4592 - 4593	R.ETADTDTADQVMASFK.I	1730.83208	2	2.35E-08	0.92	3.83	-	1028.9
AHQ-1-13, 4285	R.ETADTDTADQVMASFK.I	1730.83208	2	1.59E-07	0.94	3.88	-	1103.5
AHQ-1-10, 4055	R.ETADTDTADQVMASFK.I	1730.83208	2	3.14E-05	0.81	3.52	-	596.3
AHQ-1-7, 3230	R.ETADTDTADQVM*ASFK.I	1746.83148	2	1.15E-04	0.88	4.16	-	544.1
AHQ-1-13- , 3281	R.ETADTDTADQVM*ASFK.I	1746.83148	2	1.18E-05	0.89	3.81	-	663.8
AHQ-1-2, 4733	R.ETADTDTADQVMASFK.I	1730.83208	2	1.29E-07	0.91	3.70	-	1064.5
AHQ-1-13- , 4484	R.ETADTDTADQVMASFK.I	1730.83208	2	4.01E-09	0.95	4.20	-	1415.9
AHQ-1-5, 3205	R.ETADTDTADQVM*ASFK.I	1746.83148	2	1.26E-07	0.94	4.22	-	1053.8
AHQ-1-3, 4575	R.ETADTDTADQVMASFK.I	1730.83208	2	1.67E-05	0.93	4.12	-	968.4
AHQ-1-6, 4454	R.ETADTDTADQVMASFK.I	1730.83208	2	6.25E-06	0.95	3.68	-	1336.5
AHQ-1-5, 4530 - 4607	R.ETADTDTADQVMASFK.I	1730.83208	2	6.24E-05	0.97	5.52	-	1330.7
AHQ-1-4, 4639	R.ETADTDTADQVMASFK.I	1730.83208	2	2.34E-07	0.93	4.18	-	1007.9
AHQ-1-5, 2149	K.GISQEQM*NEFR.A	1355.45931	2	4.92E-04	0.82	2.96	-	831.0
AHQ-1-4, 3024	K.GISQEQMNEFR.A	1339.45991	2	4.77E-05	0.85	3.06	-	889.3
AHQ-1-3, 3026	K.GISQEQMNEFR.A	1339.45991	2	1.26E-07	0.74	2.59	-	773.7
AHQ-1-6, 2894	K.GISQEQMNEFR.A	1339.45991	2	3.40E-05	0.86	3.14	-	879.9
AHQ-1-5, 2851 - 2867	K.GISQEQMNEFR.A	1339.45991	2	1.22E-05	0.89	3.46	-	846.7
AHQ-1-6, 2171	K.GISQEQM*NEFR.A	1355.45931	2	8.25E-06	0.83	3.10	-	777.7
AHQ-1-5, 3094	K.GISQEQMNEFR.A	1339.45991	2	1.14E-06	0.81	2.83	-	721.2

AHQ-1-2, 3146	K.GISQEQMNEFR.A	1339.45991	2	9.63E-05	0.70	2.65	-	687.1
AHQ-1-4, 2280	K.GISQEQM*NEFR.A	1355.45931	2	7.19E-06	0.90	3.20	-	990.2
AHQ-1-5, 1973	R.HRPELIDYGK.L	1228.38202	3	1.23E-04	0.91	3.84	-	937.0
AHQ-1-6, 2027	R.HRPELIDYGK.L	1228.38202	3	2.16E-04	0.94	3.88	-	1369.4
AHQ-1-7, 2012	R.HRPELIDYGK.L	1228.38202	2	3.77E-04	0.88	3.15	-	679.4
AHQ-1-4, 2154	R.HRPELIDYGK.L	1228.38202	2	3.36E-04	0.92	3.75	-	609.7
AHQ-1-9, 1975	R.HRPELIDYGK.L	1228.38202	2	6.89E-05	0.88	3.45	-	606.7
AHQ-1-5, 1965 - 2029	R.HRPELIDYGK.L	1228.38202	2	4.43E-04	0.89	3.66	-	564.5
AHQ-1-7, 5050 - 5082	K.ICDQWDNLGALTQK.R	1663.83349	2	1.96E-05	0.92	3.78	-	1254.5
AHQ-1-4, 5060 - 5132	K.ICDQWDNLGALTQK.R	1663.83349	2	6.11E-08	0.97	4.35	-	2107.8
AHQ-1-3, 5074	K.ICDQWDNLGALTQK.R	1663.83349	2	2.64E-05	0.98	5.17	-	2418.7
AHQ-1-5, 5439	K.ICDQWDNLGALTQK.R	1663.83349	2	3.97E-07	0.94	3.78	-	1401.3
AHQ-1-6, 4974	K.ICDQWDNLGALTQK.R	1663.83349	2	8.77E-06	0.97	4.13	-	2150.6
AHQ-1-5, 4655 - 4730	K.ICDQWDNLGALTQK.R	1663.83349	2	5.39E-12	0.96	5.23	-	1284.3
AHQ-1-5, 5058 - 5066	K.ICDQWDNLGALTQK.R	1663.83349	2	1.58E-10	0.98	5.42	-	2732.9
AHQ-1-10, 4464	K.ICDQWDNLGALTQK.R	1663.83349	2	8.69E-05	0.88	3.54	-	1168.5
AHQ-1-6, 5983 - 6000	K.IDQLEGDHQLIQEALIFDNK.H	2340.57447	3	1.41E-07	0.93	4.90	-	1215.4
AHQ-1-10, 5284 - 5345	K.IDQLEGDHQLIQEALIFDNK.H	2340.57447	2	7.89E-04	0.92	4.04	-	1016.6
AHQ-1-5, 6185	K.IDQLEGDHQLIQEALIFDNK.H	2340.57447	3	8.38E-07	0.96	4.94	-	1828.8
AHQ-1-5, 5950	K.IDQLEGDHQLIQEALIFDNK.H	2340.57447	3	4.78E-08	0.97	5.37	-	2279.0
AHQ-1-5, 3474	K.ILAGDKNYITM*DELR.R	1769.01293	2	4.14E-04	0.87	3.78	-	555.5
AHQ-1-5, 4159 - 4195	K.ILAGDKNYITMDELR.R	1753.01353	2	1.56E-05	0.96	4.80	-	1131.6
AHQ-1-5, 4190	K.ILAGDKNYITMDELR.R	1753.01353	3	5.91E-06	0.90	4.17	-	828.4
AHQ-1-5, 5046 - 5118	R.ISIEMHGTLEDQLSHLR.Q	1980.23497	2	1.39E-04	0.92	4.08	-	956.7
AHQ-1-5, 4411	R.ISIEM*HGTLEDQLSHLR.Q	1996.23437	3	1.84E-07	0.95	4.44	-	1829.4
AHQ-1-5, 4413	R.ISIEM*HGTLEDQLSHLR.Q	1996.23437	2	1.16E-05	0.85	3.76	-	371.8
AHQ-1-5, 5043	R.ISIEMHGTLEDQLSHLR.Q	1980.23497	3	2.89E-12	0.97	5.23	-	2006.4
AHQ-1-5, 4370	K.IVQTYHVNM*AGTNPYTTITPQEINGK.W	2908.23575	2	9.27E-07	0.91	3.97	-	910.9
AHQ-1-5, 4739	K.IVQTYHVNMAGTNPYTTITPQEINGK.W	2892.23635	2	1.33E-05	0.66	2.89	-	423.2
AHQ-1-5, 4867	K.IVQTYHVNMAGTNPYTTITPQEINGK.W	2892.23635	2	4.18E-13	0.93	4.63	-	940.2
AHQ-1-5, 4222	K.IVQTYHVNM*AGTNPYTTITPQEINGK.W	2908.23575	2	1.00E-04	0.78	3.03	-	596.2
AHQ-1-5, 4869 - 4873	R.KAGTQIENIEEDFRDGLK.L	2064.24210	2	1.02E-04	0.88	4.50	-	959.2
AHQ-1-5, 4485 - 4507	R.KDDPLTNLNTAFDVAEK.Y	1892.05631	2	2.96E-09	0.95	4.70	-	1166.9
AHQ-1-7, 4980	R.KDDPLTNLNTAFDVAEK.Y	1892.05631	2	6.11E-04	0.97	4.90	-	1927.1
AHQ-1-9, 4840	R.KDDPLTNLNTAFDVAEK.Y	1892.05631	2	4.99E-07	0.96	5.29	-	1021.5
AHQ-1-6, 4931	R.KDDPLTNLNTAFDVAEK.Y	1892.05631	2	1.15E-05	0.95	4.64	-	1014.4
AHQ-1-5, 4975 - 5045	R.KDDPLTNLNTAFDVAEK.Y	1892.05631	2	1.54E-07	0.97	5.11	-	1333.8
AHQ-1-5, 5134	R.KDDPLTNLNTAFDVAEK.Y	1892.05631	2	3.57E-05	0.94	4.39	-	920.6
AHQ-1-4, 5100	R.KDDPLTNLNTAFDVAEK.Y	1892.05631	2	3.18E-07	0.97	5.44	-	1280.1
AHQ-1-6, 4315	R.KQFGAQANVIGPWIQTK.M	1887.17410	2	9.02E-04	0.93	4.59	-	912.1
AHQ-1-7, 3301 - 3305	R.LAILGIHNEVSK.I	1294.52505	2	1.22E-06	0.92	3.61	-	979.8
AHQ-1-5, 3249	R.LAILGIHNEVSK.I	1294.52505	2	7.40E-09	0.95	4.17	-	1034.9
AHQ-1-4, 6307	K.LASDLLEWIR.R	1216.41115	2	1.16E-07	0.97	4.40	-	2345.4
AHQ-1-7, 6236	K.LASDLLEWIR.R	1216.41115	2	9.05E-07	0.97	4.59	-	2086.7
AHQ-1-4, 6314	K.LASDLLEWIR.R	1216.41115	1	3.97E-04	0.22	2.21	-	397.6
AHQ-1-1, 6203 - 6204	K.LASDLLEWIR.R	1216.41115	2	3.93E-08	0.98	4.99	-	2537.2
AHQ-1-5, 6211 - 6278	K.LASDLLEWIR.R	1216.41115	2	4.55E-07	0.97	4.17	-	1954.2
AHQ-1-3, 6191	K.LASDLLEWIR.R	1216.41115	2	6.28E-08	0.97	4.20	-	1782.3
AHQ-1-6, 6119	K.LASDLLEWIR.R	1216.41115	2	9.41E-08	0.96	3.40	-	1984.6
AHQ-1-14- , 6291	K.LLETIDQLYLEYAK.R	1712.96427	2	1.48E-08	0.94	4.07	-	1159.3
AHQ-1-5, 6742 - 6805	K.LLETIDQLYLEYAK.R	1712.96427	2	2.30E-10	0.98	5.52	-	1480.3
AHQ-1-3, 6675	K.LLETIDQLYLEYAK.R	1712.96427	2	2.31E-09	0.97	5.18	-	1152.2
AHQ-1-10, 5889 - 5897	K.LLETIDQLYLEYAK.R	1712.96427	2	5.78E-05	0.82	3.38	-	601.6
AHQ-1-2, 6853 - 6865	K.LLETIDQLYLEYAK.R	1712.96427	2	2.60E-04	0.92	3.71	-	1053.2
AHQ-1-6, 6630	K.LLETIDQLYLEYAK.R	1712.96427	2	5.79E-08	0.96	4.87	-	1207.5
AHQ-1-7, 6824	K.LLETIDQLYLEYAK.R	1712.96427	2	1.71E-07	0.96	4.79	-	1147.8
AHQ-1-1, 6572	K.LLETIDQLYLEYAK.R	1712.96427	2	1.32E-07	0.95	4.16	-	1329.1
AHQ-1-5, 6874 - 6942	K.LLETIDQLYLEYAK.R	1712.96427	2	1.29E-08	0.98	5.27	-	1439.4
AHQ-1-5, 4678	K.LRKDDPLTNLNTAFDVAEK.Y	2161.40091	3	3.23E-04	0.86	3.81	-	831.3
AHQ-1-6, 2535 - 2551	R.LSNRPAFMPSEGR.M	1462.65941	2	8.33E-05	0.87	3.94	-	694.1
AHQ-1-5, 3427 - 3430	K.M*LDAEDIVGTARPDEK.A	1776.94709	2	5.97E-04	0.63	3.09	-	484.7
AHQ-1-5, 3674	K.MLDAEDIVGTARPDEK.A	1760.94769	2	8.45E-06	0.86	3.63	-	1008.3
AHQ-1-8, 3989	K.MLDAEDIVGTARPDEK.A	1760.94769	2	7.27E-06	0.62	3.20	-	485.4
AHQ-1-6, 3951	K.MLDAEDIVGTARPDEK.A	1760.94769	3	5.28E-07	0.86	4.02	-	596.3
AHQ-1-6, 3946	K.MLDAEDIVGTARPDEK.A	1760.94769	2	3.64E-06	0.89	4.27	-	874.0
AHQ-1-4, 3712	K.M*LDAEDIVGTARPDEK.A	1776.94709	2	5.02E-05	0.83	3.47	-	470.2
AHQ-1-5, 3981	K.MLDAEDIVGTARPDEK.A	1760.94769	2	8.40E-09	0.93	4.34	-	1091.9
AHQ-1-4, 4130 - 4139	K.MLDAEDIVGTARPDEK.A	1760.94769	2	1.74E-06	0.92	3.98	-	1079.6
AHQ-1-5, 3989 - 3990	K.MLDAEDIVGTARPDEK.A	1760.94769	3	1.39E-08	0.80	3.56	-	691.1
AHQ-1-9, 3887	K.MLDAEDIVGTARPDEK.A	1760.94769	2	7.25E-07	0.84	3.79	-	798.9
AHQ-1-9, 3888	K.MLDAEDIVGTARPDEK.A	1760.94769	3	7.48E-08	0.61	3.11	-	416.4
AHQ-1-5, 3547	K.M*LDAEDIVGTARPDEK.A	1776.94709	3	4.18E-04	0.87	3.51	-	955.7
AHQ-1-5, 3545	K.M*LDAEDIVGTARPDEK.A	1776.94709	2	6.20E-05	0.92	3.71	-	1084.0
AHQ-1-7, 5780	R.MVSDINNAWGCLEQVEK.G	1995.22333	2	4.37E-05	0.90	4.11	-	733.0
AHQ-1-5, 5411 - 5485	R.M*VSDINNAWGCLEQVEK.G	2011.22273	2	3.69E-10	0.98	5.33	-	1508.3
AHQ-1-4, 5922	R.MVSDINNAWGCLEQVEK.G	1995.22333	2	3.34E-06	0.64	3.02	-	373.6
AHQ-1-4, 5835	R.M*VSDINNAWGCLEQVEK.G	2011.22273	2	1.70E-07	0.92	4.17	-	668.3
AHQ-1-4, 5623	R.MVSDINNAWGCLEQVEK.G	1995.22333	2	5.28E-04	0.88	3.81	-	657.6
AHQ-1-5, 5281 - 5287	R.M*VSDINNAWGCLEQVEK.G	2011.22273	2	8.46E-09	0.95	4.47	-	1037.0
AHQ-1-6, 5442	R.MVSDINNAWGCLEQVEK.G	1995.22333	2	2.33E-07	0.95	4.55	-	1292.3
AHQ-1-3, 5798	R.MVSDINNAWGCLEQVEK.G	1995.22333	2	1.63E-06	0.73	2.90	-	635.3
AHQ-1-5, 6226	R.MVSDINNAWGCLEQVEK.G	1995.22333	2	1.03E-11	0.95	4.31	-	1167.0
AHQ-1-5, 5478	R.M*VSDINNAWGCLEQVEK.G	2011.22273	2	4.93E-08	0.85	4.03	-	479.8
AHQ-1-9, 5632	R.MVSDINNAWGCLEQVEK.G	1995.22333	2	2.33E-04	0.45	2.72	-	428.7
AHQ-1-5, 5530 - 5531	R.MVSDINNAWGCLEQVEK.G	1995.22333	2	2.05E-08	0.97	5.50	-	1167.7
AHQ-1-5, 6045 - 6115	R.MVSDINNAWGCLEQVEK.G	1995.22333	2	1.35E-07	0.96	4.88	-	1292.0
AHQ-1-5, 5654 - 5699	R.MVSDINNAWGCLEQVEK.G	1995.22333	2	1.57E-06	0.95	4.80	-	1227.8
AHQ-1-5, 5695 - 5703	R.M*VSDINNAWGCLEQVEK.G	2011.22273	2	1.01E-07	0.95	4.81	-	902.0
AHQ-1-5, 5891	R.M*VSDINNAWGCLEQVEK.G	2011.22273	2	3.51E-10	0.94	4.67	-	847.8
AHQ-1-6, 5911	R.MVSDINNAWGCLEQVEK.G	1995.22333	2	1.26E-07	0.93	4.43	-	940.6
AHQ-1-6, 5188	R.M*VSDINNAWGCLEQVEK.G	2011.22273	2	1.83E-10	0.96	4.66	-	1128.6
AHQ-1-6, 5728	R.MVSDINNAWGCLEQVEK.G	1995.22333	2	3.14E-05	0.95	4.74	-	1138.2
AHQ-1-5, 5785	R.MVSDINNAWGCLEQVEK.G	1995.22333	2	1.36E-07	0.97	5.31	-	1269.2
AHQ-1-6, 5630	R.M*VSDINNAWGCLEQVEK.G	2011.22273	2	8.86E-06	0.92	4.37	-	666.2
AHQ-1-6, 5594	R.MVSDINNAWGCLEQVEK.G	1995.22333	2	2.49E-09	0.95	4.45	-	1157.6
AHQ-1-9, 4642 - 4710	K.NVNIQNFHISWK.D	1500.68583	2	4.17E-09	0.96	4.63	-	1320.4
AHQ-1-4, 4946	K.NVNIQNFHISWK.D	1500.68583	2	1.86E-07	0.95	4.32	-	1127.6
AHQ-1-5, 4717 - 4734	K.NVNIQNFHISWK.D	1500.68583	2	3.02E-06	0.94	4.04	-	1182.0
AHQ-1-6, 4712	K.NVNIQNFHISWK.D	1500.68583	2	2.08E-05	0.93	3.33	-	1191.3
AHQ-1-4, 5287	K.QFGAQANVIGPWIQTK.M	1759.00118	2	1.34E-05	0.76	3.14	-	635.2
AHQ-1-6, 5130	K.QFGAQANVIGPWIQTK.M	1759.00118	2	5.07E-04	0.82	3.97	-	459.9
AHQ-1-5, 4145	R.TINEVENQILTR.D	1430.58898	1	1.15E-05	0.44	2.38	-	405.6
AHQ-1-3, 4061	R.TINEVENQILTR.D	1430.58898	2	2.40E-05	0.91	3.85	-	997.7
AHQ-1-7, 3992	R.TINEVENQILTR.D	1430.58898	2	7.71E-04	0.78	3.53	-	784.3
AHQ-1-5, 4078 - 4149	R.TINEVENQILTR.D	1430.58898	2	4.81E-08	0.86	3.64	-	670.5
AHQ-1-4, 3658	R.TINEVENQILTR.D	1430.58898	2	6.21E-07	0.77	2.90	-	554.9
AHQ-1-5, 3014 - 3085	R.TINEVENQILTR.D	1430.58898	2	3.04E-07	0.85	3.18	-	886.4

AHQ-1-5, 3954	R.TINEVENQILTR.D	1430.58898	1	7.96E-04	0.14	2.50	-	152.0
AHQ-1-6, 3914	R.TINEVENQILTR.D	1430.58898	2	5.10E-06	0.87	3.49	-	807.1
AHQ-1-8, 3945	R.TINEVENQILTR.D	1430.58898	2	2.78E-05	0.82	3.17	-	869.3
AHQ-1-4, 4082	R.TINEVENQILTR.D	1430.58898	2	7.32E-08	0.91	3.83	-	1125.3
AHQ-1-9, 3863 - 3867	R.TINEVENQILTR.D	1430.58898	2	1.91E-04	0.78	3.09	-	665.8
AHQ-1-2, 7215	R.VEQIAAIAQELNELDYYDSPSVNAR.C	2810.02233	3	1.86E-07	0.95	5.38	-	1190.4
AHQ-1-1, 6855	R.VEQIAAIAQELNELDYYDSPSVNAR.C	2810.02233	3	1.64E-06	0.98	6.46	-	1782.0
AHQ-1-5, 7269 - 7299	R.VEQIAAIAQELNELDYYDSPSVNAR.C	2810.02233	2	7.34E-05	0.91	4.04	-	799.2
AHQ-1-3, 7042	R.VEQIAAIAQELNELDYYDSPSVNAR.C	2810.02233	3	4.98E-05	0.94	5.04	-	945.8
AHQ-1-1, 6856 - 6860	R.VEQIAAIAQELNELDYYDSPSVNAR.C	2810.02233	2	7.28E-08	0.93	4.85	-	887.6
AHQ-1-5, 7137 - 7214	R.VEQIAAIAQELNELDYYDSPSVNAR.C	2810.02233	3	1.58E-05	0.89	4.16	-	1147.3
AHQ-1-11, 6078	R.VGWEQLLTTIAR.T	1387.60894	2	9.82E-08	0.94	3.66	-	1354.6
AHQ-1-9, 6683 - 6703	R.VGWEQLLTTIAR.T	1387.60894	2	2.18E-06	0.95	4.46	-	1173.2
AHQ-1-7, 6889	R.VGWEQLLTTIAR.T	1387.60894	2	1.84E-06	0.93	4.13	-	1234.1
AHQ-1-13- , 6671	R.VGWEQLLTTIAR.T	1387.60894	2	3.01E-07	0.95	3.61	-	1757.2
AHQ-1-6, 6722	R.VGWEQLLTTIAR.T	1387.60894	2	9.49E-06	0.96	4.28	-	1499.4
AHQ-1-14- , 6410	R.VGWEQLLTTIAR.T	1387.60894	2	1.49E-04	0.86	3.30	-	823.7
AHQ-1-8, 6791	R.VGWEQLLTTIAR.T	1387.60894	2	4.40E-07	0.94	3.83	-	1360.7
AHQ-1-2, 6986	R.VGWEQLLTTIAR.T	1387.60894	2	1.28E-06	0.95	3.90	-	1506.7
AHQ-1-13, 6207	R.VGWEQLLTTIAR.T	1387.60894	2	5.87E-06	0.89	3.60	-	1291.0
AHQ-1-3, 6782	R.VGWEQLLTTIAR.T	1387.60894	2	7.76E-08	0.97	4.33	-	1825.9
AHQ-1-10, 5955	R.VGWEQLLTTIAR.T	1387.60894	2	7.26E-07	0.95	3.95	-	1414.4
AHQ-1-4, 6922 - 6926	R.VGWEQLLTTIAR.T	1387.60894	2	2.70E-09	0.96	4.35	-	1436.8
AHQ-1-5, 6801 - 6879	R.VGWEQLLTTIAR.T	1387.60894	2	1.57E-05	0.96	4.19	-	1700.5
AHQ-1-1, 6645 - 6703	R.VGWEQLLTTIAR.T	1387.60894	2	5.50E-07	0.94	3.82	-	1233.5
AHQ-1-9, 4232	K.VLAVNQENEQLMEDYEK.L	2053.23635	2	1.11E-04	0.92	4.07	-	942.6
AHQ-1-8, 4207	K.VLAVNQENEQLMEDYEK.L	2053.23635	2	4.09E-06	0.89	4.39	-	605.2
AHQ-1-7, 4346	K.VLAVNQENEQLMEDYEK.L	2053.23635	2	7.40E-08	0.96	5.31	-	1131.6
AHQ-1-6, 4248	K.VLAVNQENEQLMEDYEK.L	2053.23635	2	6.74E-06	0.94	4.79	-	912.5
AHQ-1-5, 4786 - 4791	K.VLAVNQENEQLMEDYEK.L	2053.23635	2	4.22E-14	0.97	5.29	-	1605.9
AHQ-1-5, 4642	K.VLAVNQENEQLMEDYEK.L	2053.23635	2	2.71E-09	0.93	4.46	-	857.8
AHQ-1-5, 4527	K.VLAVNQENEQLMEDYEK.L	2053.23635	2	5.55E-12	0.98	5.99	-	1707.8
AHQ-1-5, 4438	K.VLAVNQENEQLMEDYEK.L	2053.23635	2	3.50E-10	0.96	5.03	-	1056.1
AHQ-1-5, 4350	K.VLAVNQENEQLMEDYEK.L	2053.23635	3	5.00E-10	0.91	4.39	-	1130.0
AHQ-1-5, 4327	K.VLAVNQENEQLMEDYEK.L	2053.23635	2	2.53E-07	0.96	5.00	-	1209.8
AHQ-1-5, 3859	K.VLAVNQENEQLM*EDYEK.L	2069.23575	2	2.15E-05	0.93	4.00	-	1093.1
AHQ-1-5, 3775 - 3783	K.VLAVNQENEQLM*EDYEK.L	2069.23575	2	1.20E-08	0.95	4.99	-	887.6
AHQ-1-5, 3591 - 3661	K.VLAVNQENEQLM*EDYEK.L	2069.23575	2	2.27E-07	0.96	4.68	-	1637.1
AHQ-1-5, 3502	K.VLAVNQENEQLM*EDYEK.L	2069.23575	2	3.12E-09	0.93	4.36	-	689.1
AHQ-1-4, 3730 - 3734	K.VLAVNQENEQLM*EDYEK.L	2069.23575	2	4.11E-06	0.96	5.07	-	1070.0
AHQ-1-3, 4406	K.VLAVNQENEQLMEDYEK.L	2053.23635	2	3.16E-06	0.96	5.12	-	1364.6
AHQ-1-2, 4575 - 4577	K.VLAVNQENEQLMEDYEK.L	2053.23635	2	6.79E-05	0.95	4.64	-	1273.6
AHQ-1-1, 4451	K.VLAVNQENEQLMEDYEK.L	2053.23635	2	4.10E-05	0.92	4.24	-	1007.7
AHQ-1-1, 3757	K.VLAVNQENEQLM*EDYEK.L	2069.23575	2	3.90E-07	0.82	4.14	-	528.5
gi|4557305|ref|NP_000025.1| aldolase A; fructose-bisphosphate aldolase; Aldolase A, fructose-bispho				6.05E-14	14.05	160.29	62.40	39419.7
AHQ-1-8, 2111 - 2115	R.ALANSLACQGK.Y	1134.28859	2	1.07E-04	0.80	3.31	-	629.2
AHQ-1-14- , 2308	R.ALANSLACQGK.Y	1134.28859	2	5.80E-05	0.71	2.53	-	805.5
AHQ-1-8, 4475	R.ALANSLACQGKYTPSGQAGAAASESLFVSNHAY	3344.61352	3	7.69E-04	0.60	3.16	-	311.1
AHQ-1-8, 4582 - 4651	R.ALANSLACQGKYTPSGQAGAAASESLFVSNHAY	3344.61352	3	2.32E-06	0.70	3.42	-	342.1
AHQ-1-8, 4156	K.ALSDHHIYLEGTLLKPNMVTPGHACTQK.F	3134.57879	3	7.07E-09	0.96	5.82	-	1061.4
AHQ-1-9, 4334	K.ALSDHHIYLEGTLLKPNMVTPGHACTQK.F	3134.57879	3	6.19E-05	0.86	3.54	-	954.1
AHQ-1-8, 6246 - 6259	K.CPLLKPWALTFSYGR.A	1811.13929	2	5.21E-09	0.94	4.58	-	659.3
AHQ-1-9, 6126 - 6190	K.CPLLKPWALTFSYGR.A	1811.13929	2	1.21E-05	0.74	3.36	-	452.4
AHQ-1-9, 4298 - 4310	K.FSHEEIAMATVTALR.R	1676.91875	2	1.50E-09	0.96	4.65	-	1323.8
AHQ-1-9, 3631	K.FSHEEIAM*ATVTALR.R	1692.91815	2	8.87E-04	0.90	4.11	-	830.9
AHQ-1-8, 4233	K.FSHEEIAMATVTALR.R	1676.91875	2	8.87E-09	0.95	4.16	-	1590.7
AHQ-1-11, 2848	K.GILAADESTGSIAK.R	1333.47008	2	1.06E-08	0.96	4.43	-	1266.2
AHQ-1-9, 2815 - 2826	K.GILAADESTGSIAK.R	1333.47008	2	2.39E-09	0.97	5.03	-	1613.9
AHQ-1-12, 2909	K.GILAADESTGSIAK.R	1333.47008	2	1.41E-05	0.94	3.90	-	1164.7
AHQ-1-8, 2770 - 2790	K.GILAADESTGSIAK.R	1333.47008	2	5.74E-08	0.97	4.90	-	1540.7
AHQ-1-8, 2772	K.GILAADESTGSIAK.R	1333.47008	1	6.63E-05	0.75	2.78	-	603.1
AHQ-1-9, 4614 - 4683	K.GVVPLAGTNGETTTQGLDGLSER.C	2273.44329	2	6.05E-14	0.91	5.64	-	764.3
AHQ-1-8, 4435	K.GVVPLAGTNGETTTQGLDGLSER.C	2273.44329	2	2.89E-07	0.76	4.62	-	519.5
AHQ-1-9, 4274 - 4302	K.GVVPLAGTNGETTTQGLDGLSER.C	2273.44329	2	1.45E-08	0.90	4.97	-	1123.7
AHQ-1-1, 4777	K.GVVPLAGTNGETTTQGLDGLSER.C	2273.44329	2	4.96E-08	0.47	3.41	-	355.9
AHQ-1-9, 4095	K.IGEHTPSALAIM*ENANVLAR.Y	2124.40728	2	5.03E-05	0.96	4.30	-	1206.7
AHQ-1-9, 5039	K.IGEHTPSALAIMENANVLAR.Y	2108.40788	2	2.99E-11	0.96	5.09	-	1024.4
AHQ-1-9, 5398 - 5467	K.IGEHTPSALAIMENANVLAR.Y	2108.40788	2	3.08E-06	0.89	3.81	-	736.9
AHQ-1-8, 5364 - 5367	K.IGEHTPSALAIMENANVLAR.Y	2108.40788	2	1.24E-04	0.96	5.42	-	838.3
AHQ-1-9, 7467	R.TVPPAVTGITFLSGGQSEEEASINLNAINK.C	3059.37393	3	6.95E-05	0.79	3.58	-	701.3
AHQ-1-9, 4316	K.VDKGVVPLAGTNGETTTQGLDGLSER.C	2615.83564	3	1.31E-07	0.77	3.92	-	1063.7
AHQ-1-9, 6040 - 6084	R.VNPCIGGVILFHETLYQK.A	2090.43072	2	5.08E-07	0.93	5.01	-	675.3
AHQ-1-9, 5888 - 5964	R.VNPCIGGVILFHETLYQK.A	2090.43072	2	3.13E-06	0.97	5.09	-	1602.5
AHQ-1-9, 6054 - 6124	R.YASICQQNGIVPIVEPEILPDGDHDLK.R	3023.36334	3	4.16E-04	0.83	4.42	-	514.9
AHQ-1-9, 5582 - 5652	R.YASICQQNGIVPIVEPEILPDGDHDLKR.C	3179.54969	3	6.91E-08	0.92	5.16	-	651.6
AHQ-1-8, 4475	K.YTPSGQAGAAASESLFVSNHAY	2229.34753	2	7.01E-05	0.89	3.98	-	534.9
AHQ-1-8, 4582 - 4651	K.YTPSGQAGAAASESLFVSNHAY	2229.34753	2	1.99E-07	0.84	3.92	-	411.7
AHQ-1-9, 4487 - 4554	K.YTPSGQAGAAASESLFVSNHAY	2229.34753	2	4.91E-04	0.82	3.24	-	652.0
gi|4504745|ref|NP_000410.1| integrin alpha 2b precursor [Homo sapiens]				1.12E-13	32.78	370.32	43.40	113374.2
AHQ-1-4, 2030	R.AEAQVELR.G	916.01408	2	6.80E-05	0.91	3.22	-	1289.2
AHQ-1-4, 1774 - 1838	R.AEAQVELR.G	916.01408	2	6.57E-04	0.84	2.86	-	1006.9
AHQ-1-4, 1764 - 2050	R.AEAQVELR.G	916.01408	1	7.87E-04	0.04	1.81	-	174.0
AHQ-1-11, 4892	R.AEGGQCPSLLFDLR.D	1564.74505	2	1.10E-05	0.93	4.33	-	858.6
AHQ-1-1, 5473 - 5480	R.AEGGQCPSLLFDLR.D	1564.74505	2	2.93E-09	0.95	4.39	-	949.3
AHQ-1-10, 4743	R.AEGGQCPSLLFDLR.D	1564.74505	2	5.85E-07	0.83	2.94	-	731.4
AHQ-1-13, 5071 - 5072	R.AEGGQCPSLLFDLR.D	1564.74505	2	5.36E-07	0.96	4.58	-	1073.2
AHQ-1-9, 5231	R.AEGGQCPSLLFDLR.D	1564.74505	2	3.65E-05	0.94	4.42	-	906.7
AHQ-1-4, 5136 - 5215	R.AEGGQCPSLLFDLR.D	1564.74505	2	1.62E-05	0.81	2.92	-	765.8
AHQ-1-7, 5357 - 5376	R.AEGGQCPSLLFDLR.D	1564.74505	2	7.34E-04	0.94	4.17	-	870.2
AHQ-1-13- , 5372	R.AEGGQCPSLLFDLR.D	1564.74505	2	3.20E-05	0.90	3.33	-	1000.8
AHQ-1-4, 5456 - 5522	R.AEGGQCPSLLFDLR.D	1564.74505	2	1.25E-06	0.93	4.15	-	1010.9
AHQ-1-4, 5655	R.AEGGQCPSLLFDLR.D	1564.74505	2	8.37E-10	0.95	4.39	-	1029.6
AHQ-1-5, 5354 - 5389	R.AEGGQCPSLLFDLR.D	1564.74505	2	1.88E-08	0.94	3.36	-	1250.8
AHQ-1-6, 5294 - 5314	R.AEGGQCPSLLFDLR.D	1564.74505	2	8.73E-09	0.93	3.56	-	1151.8
AHQ-1-3, 5313 - 5389	R.AEGGQCPSLLFDLR.D	1564.74505	2	7.22E-07	0.92	3.68	-	986.1
AHQ-1-1, 3008 - 3011	R.ALSNVEGFER.L	1122.21308	2	6.15E-06	0.91	3.58	-	811.9
AHQ-1-4, 2835 - 2884	R.ALSNVEGFER.L	1122.21308	2	4.94E-06	0.88	3.37	-	790.1
AHQ-1-6, 2754	R.ALSNVEGFER.L	1122.21308	2	2.84E-07	0.82	2.69	-	746.1
AHQ-1-5, 2722	R.ALSNVEGFER.L	1122.21308	2	5.94E-05	0.90	3.01	-	1199.6
AHQ-1-3, 2857 - 2859	R.ALSNVEGFER.L	1122.21308	2	4.49E-07	0.90	3.22	-	952.4
AHQ-1-7, 2781	R.ALSNVEGFER.L	1122.21308	2	1.12E-05	0.90	3.10	-	986.3
AHQ-1-6, 2752	R.ALSNVEGFER.L	1122.21308	1	8.74E-04	0.14	2.05	-	68.1
AHQ-1-4, 5210 - 5282	K.ASVQLLVQDSLNPAVK.S	1682.94293	2	3.67E-07	0.88	4.32	-	640.7
AHQ-1-3, 5165 - 5199	K.ASVQLLVQDSLNPAVK.S	1682.94293	2	8.10E-04	0.54	2.69	-	590.6
AHQ-1-5, 5129 - 5133	K.ASVQLLVQDSLNPAVK.S	1682.94293	2	1.28E-05	0.94	4.80	-	926.5
AHQ-1-4, 3108	R.DETRNVGSQTLQTFK.A	1724.85363	2	4.44E-06	0.90	3.84	-	1001.3
AHQ-1-3, 3987	R.DGYNDIAVAAPYGGPSGR.G	1780.87574	2	1.03E-04	0.65	3.14	-	408.4

AHQ-1-4, 4180 - 4258	R.DGYNDIAVAAPYGGPSGR.G	1780.87574	2	3.72E-08	0.94	4.73	-	887.3
AHQ-1-6, 3902	R.DGYNDIAVAAPYGGPSGR.G	1780.87574	2	7.85E-08	0.90	3.92	-	831.6
AHQ-1-5, 4203	R.DGYNDIAVAAPYGGPSGR.G	1780.87574	2	4.70E-10	0.89	4.16	-	812.7
AHQ-1-5, 3971	R.DGYNDIAVAAPYGGPSGR.G	1780.87574	2	8.36E-10	0.91	4.25	-	664.0
AHQ-1-4, 4642 - 4643	R.FGSAIAPLGDLDR.D	1332.48700	1	1.98E-04	0.60	2.60	-	447.7
AHQ-1-4, 5942 - 6023	R.FGSAIAPLGDLDRDGYNDIAVAAPYGGPSGR.G	3094.34015	3	1.66E-12	0.93	5.04	-	1021.2
AHQ-1-7, 6009 - 6050	R.FGSAIAPLGDLDRDGYNDIAVAAPYGGPSGR.G	3094.34015	3	2.75E-06	0.85	4.65	-	481.1
AHQ-1-4, 6090 - 6163	R.FGSAIAPLGDLDRDGYNDIAVAAPYGGPSGR.G	3094.34015	3	8.95E-07	0.93	5.61	-	669.2
AHQ-1-5, 6006 - 6077	R.FGSAIAPLGDLDRDGYNDIAVAAPYGGPSGR.G	3094.34015	3	1.65E-05	0.95	5.84	-	991.6
AHQ-1-6, 5839 - 5907	R.FGSAIAPLGDLDRDGYNDIAVAAPYGGPSGR.G	3094.34015	3	2.09E-06	0.81	3.66	-	890.2
AHQ-1-1, 6072	R.FGSAIAPLGDLDRDGYNDIAVAAPYGGPSGR.G	3094.34015	3	1.86E-05	0.82	3.44	-	993.3
AHQ-1-6, 5980	R.FGSAIAPLGDLDRDGYNDIAVAAPYGGPSGR.G	3094.34015	3	6.60E-04	0.85	4.45	-	793.2
AHQ-1-3, 5910 - 5958	R.FGSAIAPLGDLDRDGYNDIAVAAPYGGPSGR.G	3094.34015	3	1.90E-06	0.87	4.43	-	550.9
AHQ-1-4, 6618 - 6687	R.GAVDIDDNGYPDLIVGAYGANQVAVYR.A	2827.05458	3	1.13E-05	0.85	4.15	-	725.4
AHQ-1-4, 6751 - 6818	R.GAVDIDDNGYPDLIVGAYGANQVAVYR.A	2827.05458	2	4.21E-08	0.95	5.10	-	681.9
AHQ-1-4, 6491 - 6562	R.GAVDIDDNGYPDLIVGAYGANQVAVYR.A	2827.05458	2	8.16E-06	0.93	4.94	-	613.9
AHQ-1-4, 6890 - 6963	R.GAVDIDDNGYPDLIVGAYGANQVAVYR.A	2827.05458	2	2.64E-08	0.97	5.99	-	863.4
AHQ-1-4, 6484 - 6551	R.GAVDIDDNGYPDLIVGAYGANQVAVYR.A	2827.05458	3	6.82E-05	0.69	3.67	-	490.0
AHQ-1-4, 6400 - 6467	R.GAVDIDDNGYPDLIVGAYGANQVAVYR.A	2827.05458	2	4.71E-08	0.96	5.29	-	988.9
AHQ-1-4, 7018 - 7082	R.GAVDIDDNGYPDLIVGAYGANQVAVYR.A	2827.05458	3	1.41E-05	0.95	5.31	-	1470.6
AHQ-1-4, 7044 - 7115	R.GAVDIDDNGYPDLIVGAYGANQVAVYR.A	2827.05458	2	1.76E-05	0.95	5.18	-	686.5
AHQ-1-4, 7190 - 7238	R.GAVDIDDNGYPDLIVGAYGANQVAVYR.A	2827.05458	2	2.01E-05	0.94	4.72	-	739.0
AHQ-1-4, 7510 - 7584	R.GAVDIDDNGYPDLIVGAYGANQVAVYR.A	2827.05458	3	5.62E-05	0.82	3.91	-	765.4
AHQ-1-4, 6610 - 6684	R.GAVDIDDNGYPDLIVGAYGANQVAVYR.A	2827.05458	2	3.90E-13	0.97	5.77	-	845.6
AHQ-1-4, 6891 - 6954	R.GAVDIDDNGYPDLIVGAYGANQVAVYR.A	2827.05458	3	8.94E-04	0.86	4.43	-	725.9
AHQ-1-13, 4911 - 4921	R.GEAQVWTQLLR.A	1301.47614	2	5.51E-05	0.93	4.17	-	915.2
AHQ-1-10, 4536 - 4595	R.GEAQVWTQLLR.A	1301.47614	2	9.79E-06	0.93	3.82	-	1169.5
AHQ-1-1, 5312	R.GEAQVWTQLLR.A	1301.47614	2	5.94E-06	0.89	3.31	-	1198.0
AHQ-1-11, 4716	R.GEAQVWTQLLR.A	1301.47614	2	1.06E-06	0.93	3.92	-	1003.4
AHQ-1-10, 4795 - 4797	R.GEAQVWTQLLR.A	1301.47614	1	9.28E-04	0.23	2.25	-	249.0
AHQ-1-11, 4710	R.GEAQVWTQLLR.A	1301.47614	2	3.03E-06	0.94	4.42	-	931.4
AHQ-1-12, 4942	R.GEAQVWTQLLR.A	1301.47614	2	5.28E-06	0.91	3.67	-	935.7
AHQ-1-13- , 5196 - 5204	R.GEAQVWTQLLR.A	1301.47614	2	1.95E-06	0.95	4.20	-	1060.7
AHQ-1-4, 5612 - 5679	R.GNSFPASLVVAAEEGER.E	1733.86053	2	1.79E-08	0.95	4.75	-	889.4
AHQ-1-4, 5878 - 5947	R.GNSFPASLVVAAEEGER.E	1733.86053	2	6.34E-12	0.93	4.35	-	897.6
AHQ-1-4, 5746 - 5812	R.GNSFPASLVVAAEEGER.E	1733.86053	2	1.12E-13	0.93	4.43	-	937.2
AHQ-1-4, 5535 - 5610	R.GNSFPASLVVAAEEGER.E	1733.86053	2	1.94E-06	0.89	3.95	-	658.6
AHQ-1-4, 5407 - 5474	R.GNSFPASLVVAAEEGER.E	1733.86053	2	1.15E-07	0.78	3.51	-	483.0
AHQ-1-4, 6074	R.GNSFPASLVVAAEEGER.E	1733.86053	2	1.03E-09	0.88	3.81	-	679.0
AHQ-1-4, 6835 - 6902	R.GNSFPASLVVAAEEGEREQNSLDSWGPK.V	2976.16043	3	2.62E-04	0.72	3.58	-	504.5
AHQ-1-4, 6611 - 6642	R.GNSFPASLVVAAEEGEREQNSLDSWGPK.V	2976.16043	3	2.31E-07	0.73	3.69	-	415.2
AHQ-1-4, 6470 - 6544	R.GNSFPASLVVAAEEGEREQNSLDSWGPK.V	2976.16043	3	1.83E-04	0.76	4.27	-	435.4
AHQ-1-4, 6710 - 6767	R.GNSFPASLVVAAEEGEREQNSLDSWGPK.V	2976.16043	3	4.15E-09	0.82	4.14	-	486.7
AHQ-1-4, 5182	R.GPHALGAPSLLLTGTQLYGR.F	2023.32431	2	2.03E-07	0.96	5.05	-	849.2
AHQ-1-6, 5190	R.GPHALGAPSLLLTGTQLYGR.F	2023.32431	2	8.90E-08	0.96	5.49	-	996.0
AHQ-1-2, 5467	R.GPHALGAPSLLLTGTQLYGR.F	2023.32431	2	3.42E-09	0.95	4.98	-	732.5
AHQ-1-7, 5152	R.GPHALGAPSLLLTGTQLYGR.F	2023.32431	2	1.16E-05	0.95	5.03	-	892.1
AHQ-1-4, 5322 - 5384	R.GPHALGAPSLLLTGTQLYGR.F	2023.32431	2	1.38E-06	0.98	6.30	-	1244.5
AHQ-1-2, 5474	R.GPHALGAPSLLLTGTQLYGR.F	2023.32431	3	2.33E-04	0.89	3.44	-	1309.1
AHQ-1-5, 5238	R.GPHALGAPSLLLTGTQLYGR.F	2023.32431	2	2.22E-06	0.95	4.68	-	815.4
AHQ-1-1, 5435	R.GPHALGAPSLLLTGTQLYGR.F	2023.32431	3	3.29E-05	0.86	3.76	-	874.9
AHQ-1-6, 5186 - 5187	R.GPHALGAPSLLLTGTQLYGR.F	2023.32431	3	8.85E-06	0.85	3.44	-	1197.0
AHQ-1-5, 5241 - 5242	R.GPHALGAPSLLLTGTQLYGR.F	2023.32431	3	1.33E-05	0.84	3.98	-	739.9
AHQ-1-4, 5324 - 5400	R.GPHALGAPSLLLTGTQLYGR.F	2023.32431	3	4.51E-05	0.86	3.60	-	929.9
AHQ-1-4, 5435	R.GPHALGAPSLLLTGTQLYGR.F	2023.32431	2	4.87E-07	0.91	4.30	-	609.3
AHQ-1-4, 5436 - 5508	R.GQVLVFLGQSEGLR.S	1503.72793	2	9.90E-10	0.97	4.77	-	1617.3
AHQ-1-4, 5290 - 5359	R.GQVLVFLGQSEGLR.S	1503.72793	2	1.60E-07	0.96	4.28	-	1509.5
AHQ-1-4, 5188 - 5262	R.GQVLVFLGQSEGLR.S	1503.72793	2	1.01E-06	0.97	5.25	-	1471.4
AHQ-1-3, 5154	R.GQVLVFLGQSEGLR.S	1503.72793	2	3.17E-08	0.97	5.04	-	1432.1
AHQ-1-6, 5026	R.GQVLVFLGQSEGLR.S	1503.72793	2	2.22E-05	0.94	4.00	-	1037.9
AHQ-1-5, 5085	R.GQVLVFLGQSEGLR.S	1503.72793	2	4.15E-07	0.97	4.88	-	1681.9
AHQ-1-7, 4410 - 4485	R.HDLLVGAPLYMESR.A	1601.85188	2	3.28E-05	0.93	3.94	-	1102.3
AHQ-1-5, 4417	R.HDLLVGAPLYMESR.A	1601.85188	2	3.51E-07	0.97	4.56	-	1712.6
AHQ-1-4, 3990	R.HDLLVGAPLYM*ESR.A	1617.85128	3	3.55E-04	0.84	3.48	-	959.6
AHQ-1-4, 4532 - 4602	R.HDLLVGAPLYMESR.A	1601.85188	2	2.05E-05	0.96	4.75	-	1253.4
AHQ-1-4, 3819 - 3847	K.HSPICHTTMAFLR.D	1572.83705	2	1.00E-07	0.59	2.61	-	274.8
AHQ-1-11, 4142 - 4143	K.IVLLDVPVR.A	1024.28196	2	8.58E-04	0.88	3.33	-	763.7
AHQ-1-3, 4553	K.IVLLDVPVR.A	1024.28196	2	4.94E-04	0.90	3.35	-	931.2
AHQ-1-12, 4306	K.IVLLDVPVR.A	1024.28196	2	7.50E-04	0.90	3.60	-	758.5
AHQ-1-5, 4450	K.IVLLDVPVR.A	1024.28196	2	9.01E-04	0.94	3.89	-	1168.0
AHQ-1-6, 4363 - 4376	K.IVLLDVPVR.A	1024.28196	2	2.56E-04	0.85	3.37	-	736.4
AHQ-1-4, 4588	R.IYVENDFSWDK.R	1416.51610	1	1.28E-06	0.48	2.35	-	437.8
AHQ-1-6, 3855 - 3923	R.IYVENDFSWDKR.Y	1572.70245	2	2.84E-04	0.62	2.74	-	589.7
AHQ-1-7, 3985	R.IYVENDFSWDKR.Y	1572.70245	2	5.29E-07	0.92	3.47	-	1199.7
AHQ-1-4, 4066 - 4138	R.IYVENDFSWDKR.Y	1572.70245	2	9.38E-07	0.96	4.30	-	1492.5
AHQ-1-13- , 6756	R.LQDPVLVSCDSAPCTVVQCDLQEMAR.G	2996.36144	2	3.25E-05	0.69	2.99	-	298.8
AHQ-1-13- , 5875	R.LQDPVLVSCDSAPCTVVQCDLQEM*AR.G	3012.36084	3	2.99E-04	0.92	4.08	-	1290.5
AHQ-1-11, 6228	R.LQDPVLVSCDSAPCTVVQCDLQEMAR.G	2996.36144	2	1.17E-06	0.85	3.47	-	513.7
AHQ-1-10, 6052 - 6112	R.LQDPVLVSCDSAPCTVVQCDLQEMAR.G	2996.36144	2	7.49E-06	0.93	4.88	-	441.9
AHQ-1-10, 6033 - 6115	R.LQDPVLVSCDSAPCTVVQCDLQEMAR.G	2996.36144	3	3.40E-06	0.77	3.72	-	500.1
AHQ-1-10, 6247	R.LQDPVLVSCDSAPCTVVQCDLQEMAR.G	2996.36144	2	5.30E-06	0.31	2.52	-	180.5
AHQ-1-4, 4626 - 4666	K.LSLNAELQLDR.Q	1272.43310	2	3.13E-08	0.92	3.65	-	958.0
AHQ-1-5, 4547	K.LSLNAELQLDR.Q	1272.43310	2	3.50E-05	0.93	3.64	-	1320.4
AHQ-1-10, 2084	R.NRPPLEEDDEEGE	1529.50164	2	1.04E-10	0.93	3.68	-	1327.5
AHQ-1-11, 2138	R.NRPPLEEDDEEGE	1529.50164	2	1.88E-06	0.86	3.47	-	857.7
AHQ-1-13- , 2215	R.NRPPLEEDDEEGE	1529.50164	2	1.42E-08	0.91	3.22	-	1191.9
AHQ-1-7, 2778	R.NVGSQTLQTFK.A	1223.36055	2	1.46E-05	0.69	2.90	-	354.9
AHQ-1-9, 2691 - 2694	R.NVGSQTLQTFK.A	1223.36055	1	6.78E-04	0.12	1.88	-	194.9
AHQ-1-9, 2700	R.NVGSQTLQTFK.A	1223.36055	2	1.00E-04	0.84	3.13	-	543.3
AHQ-1-4, 3332	R.NVGSQTLQTFK.A	1223.36055	1	7.16E-06	0.50	2.43	-	346.0
AHQ-1-10, 2695	R.NVGSQTLQTFK.A	1223.36055	2	3.63E-07	0.89	3.77	-	568.0
AHQ-1-11, 2779	R.NVGSQTLQTFK.A	1223.36055	2	5.71E-04	0.82	3.10	-	509.9
AHQ-1-4, 3062 - 3127	R.NVGSQTLQTFK.A	1223.36055	2	7.08E-04	0.73	2.89	-	455.9
AHQ-1-4, 3119	R.NVGSQTLQTFK.A	1223.36055	1	5.03E-04	0.10	2.03	-	154.8
AHQ-1-4, 7426	R.QGLGASVVSWSDVIVACAPWQHWNVLEK.T	3139.53208	3	1.43E-07	0.91	5.30	-	531.8
AHQ-1-5, 6286 - 6362	R.SRPSQVLDSPFPTGSAFGFSLR.G	2354.60606	3	1.91E-08	0.97	6.19	-	1522.6
AHQ-1-4, 6240 - 6306	R.SRPSQVLDSPFPTGSAFGFSLR.G	2354.60606	3	2.05E-08	0.97	6.15	-	1564.9
AHQ-1-4, 6244 - 6311	R.SRPSQVLDSPFPTGSAFGFSLR.G	2354.60606	2	1.38E-07	0.95	5.53	-	539.0
AHQ-1-6, 6102 - 6147	R.SRPSQVLDSPFPTGSAFGFSLR.G	2354.60606	3	2.39E-11	0.97	6.00	-	1855.4
AHQ-1-4, 6358 - 6422	R.SRPSQVLDSPFPTGSAFGFSLR.G	2354.60606	3	8.09E-08	0.96	6.20	-	1276.7
AHQ-1-7, 6208 - 6224	R.SRPSQVLDSPFPTGSAFGFSLR.G	2354.60606	3	5.47E-09	0.97	5.71	-	1886.6
AHQ-1-1, 6173	R.SRPSQVLDSPFPTGSAFGFSLR.G	2354.60606	3	6.37E-09	0.97	5.87	-	1940.2
AHQ-1-3, 6174 - 6186	R.SRPSQVLDSPFPTGSAFGFSLR.G	2354.60606	3	2.38E-09	0.96	6.26	-	1265.9
AHQ-1-5, 6199 - 6221	R.SRPSQVLDSPFPTGSAFGFSLR.G	2354.60606	3	1.44E-11	0.96	5.86	-	1083.5
AHQ-1-14- , 5878	R.SRPSQVLDSPFPTGSAFGFSLR.G	2354.60606	3	1.91E-05	0.85	4.04	-	1001.8
AHQ-1-4, 3980	K.TEEAEKTPVGSCFLAQPESGR.R	2295.46902	2	1.82E-11	0.81	3.62	-	488.5
AHQ-1-6, 3659	K.TEEAEKTPVGSCFLAQPESGR.R	2295.46902	2	1.34E-06	0.90	3.75	-	671.2

AHQ-1-10, 3429	K.TEEAEKTPVGSCFLAQPESGR.R	2295.46902	2	1.35E-09	0.84	3.65	-	494.2
AHQ-1-4, 3759 - 3831	K.TEEAEKTPVGSCFLAQPESGR.R	2295.46902	2	2.24E-07	0.95	4.74	-	716.3
AHQ-1-7, 3734 - 3762	K.TEEAEKTPVGSCFLAQPESGR.R	2295.46902	2	5.95E-04	0.82	3.35	-	510.9
AHQ-1-5, 3689	K.TEEAEKTPVGSCFLAQPESGR.R	2295.46902	2	2.74E-08	0.95	4.85	-	699.7
AHQ-1-4, 5856	R.TLGPSQEETGGVFLCPWR.A	2036.25364	3	4.66E-06	0.92	4.47	-	1036.6
AHQ-1-5, 5723 - 5789	R.TLGPSQEETGGVFLCPWR.A	2036.25364	2	9.42E-07	0.96	5.34	-	1098.1
AHQ-1-4, 5914 - 5975	R.TLGPSQEETGGVFLCPWR.A	2036.25364	2	1.48E-06	0.97	5.20	-	1269.8
AHQ-1-13, 5319	R.TLGPSQEETGGVFLCPWR.A	2036.25364	3	4.52E-04	0.60	3.06	-	474.9
AHQ-1-11, 5174 - 5240	R.TLGPSQEETGGVFLCPWR.A	2036.25364	2	4.41E-05	0.84	3.78	-	922.1
AHQ-1-6, 5666	R.TLGPSQEETGGVFLCPWR.A	2036.25364	2	1.10E-07	0.92	4.30	-	716.9
AHQ-1-10, 5025 - 5092	R.TLGPSQEETGGVFLCPWR.A	2036.25364	2	4.45E-06	0.92	4.47	-	758.4
AHQ-1-1, 5715 - 5780	R.TLGPSQEETGGVFLCPWR.A	2036.25364	2	4.06E-05	0.88	4.00	-	727.8
AHQ-1-3, 5683 - 5703	R.TLGPSQEETGGVFLCPWR.A	2036.25364	2	1.82E-04	0.59	2.92	-	588.1
AHQ-1-4, 5771 - 5848	R.TLGPSQEETGGVFLCPWR.A	2036.25364	2	3.39E-09	0.95	4.47	-	1108.0
AHQ-1-13- , 5612 - 5644	R.TLGPSQEETGGVFLCPWR.A	2036.25364	2	1.44E-06	0.94	4.85	-	738.8
AHQ-1-7, 5716	R.TLGPSQEETGGVFLCPWR.A	2036.25364	2	3.13E-04	0.95	4.89	-	1110.5
AHQ-1-9, 5566	R.TLGPSQEETGGVFLCPWR.A	2036.25364	2	4.91E-06	0.92	4.13	-	788.3
AHQ-1-12, 5358 - 5431	R.TLGPSQEETGGVFLCPWR.A	2036.25364	2	3.30E-08	0.94	4.30	-	1114.9
AHQ-1-4, 3842 - 3911	K.TPVGSCFLAQPESGR.R	1607.76993	2	1.54E-07	0.94	4.74	-	848.2
AHQ-1-3, 3839	K.TPVGSCFLAQPESGR.R	1607.76993	2	9.50E-05	0.76	3.05	-	719.3
AHQ-1-9, 3622	K.TPVGSCFLAQPESGR.R	1607.76993	2	1.84E-06	0.89	3.48	-	871.4
AHQ-1-7, 3780	K.TPVGSCFLAQPESGR.R	1607.76993	2	6.91E-06	0.91	4.20	-	701.7
AHQ-1-5, 3750	K.TPVGSCFLAQPESGR.R	1607.76993	2	1.87E-07	0.90	3.97	-	597.0
AHQ-1-6, 3694	K.TPVGSCFLAQPESGR.R	1607.76993	2	1.11E-07	0.86	3.29	-	825.3
AHQ-1-4, 4030 - 4032	K.TPVGSCFLAQPESGR.R	1607.76993	2	8.48E-08	0.94	4.21	-	1084.5
AHQ-1-1, 3901	K.TPVGSCFLAQPESGR.R	1607.76993	2	7.41E-08	0.91	4.08	-	647.2
AHQ-1-10, 3448	K.TPVGSCFLAQPESGR.R	1607.76993	2	3.43E-06	0.94	4.30	-	825.4
AHQ-1-11, 3547	K.TPVGSCFLAQPESGR.R	1607.76993	2	1.30E-07	0.90	3.78	-	694.6
AHQ-1-4, 4978	K.TPVSCFNIQMCVGATGHNIPQK.L	2462.81122	3	1.94E-05	0.91	4.26	-	988.4
AHQ-1-4, 5214 - 5255	K.TPVSCFNIQMCVGATGHNIPQK.L	2462.81122	3	8.77E-05	0.95	5.06	-	1373.0
AHQ-1-4, 5440	K.TPVSCFNIQMCVGATGHNIPQK.L	2462.81122	3	9.42E-06	0.91	4.79	-	957.7
AHQ-1-4, 6383	K.TPVSCFNIQMCVGATGHNIPQK.L	2462.81122	3	1.51E-04	0.93	4.71	-	1029.5
AHQ-1-4, 4771	K.TPVSCFNIQM*CVGATGHNIPQK.L	2478.81062	3	1.41E-06	0.91	4.06	-	1230.0
AHQ-1-4, 4680 - 4702	K.TPVSCFNIQM*CVGATGHNIPQK.L	2478.81062	3	8.68E-07	0.96	5.02	-	1555.7
AHQ-1-4, 5776	K.TPVSCFNIQMCVGATGHNIPQK.L	2462.81122	3	6.52E-08	0.96	4.96	-	1402.9
AHQ-1-5, 4594	K.TPVSCFNIQM*CVGATGHNIPQK.L	2478.81062	3	3.02E-08	0.93	4.57	-	1292.6
AHQ-1-13, 2719	R.VAIVVGAPR.T	882.08560	2	2.75E-04	0.93	3.32	-	1168.1
AHQ-1-7, 2540	R.VAIVVGAPR.T	882.08560	2	5.81E-06	0.93	3.45	-	1427.2
AHQ-1-5, 2494 - 2495	R.VAIVVGAPR.T	882.08560	2	5.76E-06	0.94	3.69	-	1277.6
AHQ-1-13- , 2719	R.VAIVVGAPR.T	882.08560	2	3.54E-05	0.90	2.68	-	1101.2
AHQ-1-12, 2606	R.VAIVVGAPR.T	882.08560	2	2.89E-05	0.92	3.14	-	1179.6
AHQ-1-4, 2602 - 2606	R.VAIVVGAPR.T	882.08560	2	4.43E-04	0.95	3.82	-	1395.4
AHQ-1-6, 2514	R.VAIVVGAPR.T	882.08560	2	3.03E-06	0.92	3.53	-	1238.8
AHQ-1-4, 7526 - 7594	R.VLLLGSQQAGTTLNLDLGGK.H	1999.29783	2	4.75E-08	0.98	5.82	-	1942.3
AHQ-1-4, 6134 - 6207	R.VLLLGSQQAGTTLNLDLGGK.H	1999.29783	2	2.77E-07	0.97	5.67	-	1478.2
AHQ-1-4, 6947 - 6966	R.VLLLGSQQAGTTLNLDLGGK.H	1999.29783	2	3.43E-06	0.97	5.52	-	1696.9
AHQ-1-4, 6802 - 6875	R.VLLLGSQQAGTTLNLDLGGK.H	1999.29783	2	5.13E-08	0.96	5.91	-	1163.1
AHQ-1-4, 6707 - 6788	R.VLLLGSQQAGTTLNLDLGGK.H	1999.29783	2	3.16E-12	0.96	5.43	-	1437.3
AHQ-1-4, 6620 - 6624	R.VLLLGSQQAGTTLNLDLGGK.H	1999.29783	2	1.40E-04	0.97	5.65	-	1228.4
AHQ-1-5, 5987	R.VLLLGSQQAGTTLNLDLGGK.H	1999.29783	2	6.64E-07	0.92	4.26	-	1052.0
AHQ-1-4, 6278 - 6302	R.VLLLGSQQAGTTLNLDLGGK.H	1999.29783	2	3.17E-06	0.98	6.38	-	2184.0
AHQ-1-4, 7035 - 7098	R.VLLLGSQQAGTTLNLDLGGK.H	1999.29783	2	5.73E-06	0.95	5.47	-	924.8
AHQ-1-4, 4259 - 4266	R.VVLCELGNPMK.K	1261.53692	2	3.93E-07	0.96	4.46	-	1380.0
AHQ-1-4, 4267	R.VVLCELGNPMK.K	1261.53692	1	1.59E-05	0.78	3.00	-	754.3
AHQ-1-5, 4165 - 4174	R.VVLCELGNPMK.K	1261.53692	2	3.14E-08	0.95	4.00	-	1306.6
AHQ-1-4, 3419 - 3430	R.VVLCELGNPM*K.K	1277.53632	2	4.59E-04	0.94	4.18	-	1226.8
AHQ-1-4, 4303 - 4379	R.VYLFLQPR.G	1036.25129	2	8.11E-06	0.95	3.94	-	1284.0
AHQ-1-6, 4142	R.VYLFLQPR.G	1036.25129	2	2.40E-05	0.95	3.26	-	1329.2
AHQ-1-5, 4189 - 4193	R.VYLFLQPR.G	1036.25129	2	8.87E-06	0.95	3.67	-	1392.3
gi|10835002|ref|NP_001166.1| Rho GDP dissociation inhibitor (GDI) beta; Ly-GDI [Homo sapiens]				1.22E-13	5.51	60.30	47.30	22987.8
AHQ-1-10, 2384 - 2385	K.APEPHVEEDDDDELDSK.L	1940.90995	2	3.01E-11	0.98	6.06	-	1747.4
AHQ-1-10, 2404 - 2440	K.APEPHVEEDDDDELDSK.L	1940.90995	3	2.21E-05	0.89	4.08	-	788.4
AHQ-1-13- , 2561	K.APEPHVEEDDDDELDSK.L	1940.90995	2	1.22E-13	0.97	5.69	-	1484.5
AHQ-1-13, 2581	K.APEPHVEEDDDDELDSK.L	1940.90995	2	6.49E-10	0.97	5.50	-	1320.6
AHQ-1-13, 5372	K.ATFM*VGSYGPRPEEYEFLTPVEEAPK.G	2962.27864	3	5.51E-05	0.73	3.32	-	543.1
AHQ-1-14- , 5556 - 5579	K.ATFM*VGSYGPRPEEYEFLTPVEEAPK.G	2962.27864	3	4.95E-04	0.87	4.16	-	771.4
AHQ-1-12, 5433 - 5438	K.ATFM*VGSYGPRPEEYEFLTPVEEAPK.G	2962.27864	3	1.26E-09	0.93	4.96	-	833.5
AHQ-1-10, 5289 - 5347	K.ATFMVGSYGPRPEEYEFLTPVEEAPK.G	2946.27924	2	3.72E-05	0.92	4.56	-	704.3
AHQ-1-14- , 5771	K.ATFMVGSYGPRPEEYEFLTPVEEAPK.G	2946.27924	3	8.04E-04	0.71	3.18	-	597.9
AHQ-1-10, 2979	K.ELQEMDKDDESLIK.Y	1693.85489	2	1.32E-07	0.91	4.26	-	837.5
AHQ-1-13- , 6763 - 6764	R.LTLVCESAPGPITMDLTGDLEALKK.E	2675.11274	3	1.43E-08	0.85	3.68	-	758.1
AHQ-1-10, 6095 - 6153	R.LTLVCESAPGPITMDLTGDLEALKK.E	2675.11274	3	1.02E-07	0.83	3.63	-	574.4
AHQ-1-11, 3262 - 3291	K.TLLGDGPVVTDPK.A	1312.49400	2	6.26E-05	0.93	3.31	-	1432.9
AHQ-1-13- , 3423 - 3503	K.TLLGDGPVVTDPK.A	1312.49400	2	8.40E-09	0.94	3.79	-	1379.5
AHQ-1-13, 3418	K.TLLGDGPVVTDPK.A	1312.49400	2	6.11E-04	0.90	3.72	-	986.0
gi|4505879|ref|NP_002655.1| pleckstrin; p47 [Homo sapiens]				1.23E-13	9.34	110.41	41.40	40082.5
AHQ-1-11, 5315 - 5339	R.EDPAYLHYYDPAGAEDPLGAIHLR.G	2684.89982	3	2.46E-05	0.97	6.57	-	1032.9
AHQ-1-12, 5469	R.EDPAYLHYYDPAGAEDPLGAIHLR.G	2684.89982	3	7.76E-07	0.97	6.08	-	2086.8
AHQ-1-10, 5153	R.EDPAYLHYYDPAGAEDPLGAIHLR.G	2684.89982	2	2.10E-04	0.56	2.63	-	429.3
AHQ-1-8, 5695 - 5754	R.EDPAYLHYYDPAGAEDPLGAIHLR.G	2684.89982	3	4.07E-09	0.98	6.86	-	1850.0
AHQ-1-8, 5715 - 5790	R.EDPAYLHYYDPAGAEDPLGAIHLR.G	2684.89982	2	7.24E-05	0.80	3.40	-	481.9
AHQ-1-10, 5125 - 5155	R.EDPAYLHYYDPAGAEDPLGAIHLR.G	2684.89982	3	1.10E-05	0.93	5.37	-	800.7
AHQ-1-13- , 5764	R.EDPAYLHYYDPAGAEDPLGAIHLR.G	2684.89982	3	9.43E-05	0.96	6.22	-	1330.4
AHQ-1-13, 5423 - 5425	R.EDPAYLHYYDPAGAEDPLGAIHLR.G	2684.89982	3	8.65E-12	0.97	6.44	-	1521.2
AHQ-1-8, 2509 - 2539	K.GSTLTSPCQDFGKR.M	1555.69502	2	1.93E-09	0.32	2.65	-	238.7
AHQ-1-11, 2800	K.GSTLTSPCQDFGKR.M	1555.69502	2	3.32E-05	0.55	2.51	-	451.2
AHQ-1-8, 7298 - 7347	K.IFNHCFTGNCVIDWLVSNQSVR.N	2669.97641	2	9.49E-05	0.97	4.62	-	1632.8
AHQ-1-13, 6555 - 6557	R.KSEEENLFEIITADEVHYFLQAATPK.E	3024.32650	3	1.23E-13	0.99	7.74	-	3272.0
AHQ-1-10, 6412 - 6413	R.KSEEENLFEIITADEVHYFLQAATPK.E	3024.32650	3	3.89E-08	0.98	7.73	-	2394.2
AHQ-1-12, 6661 - 6734	R.KSEEENLFEIITADEVHYFLQAATPK.E	3024.32650	3	9.36E-09	0.98	7.24	-	2094.8
AHQ-1-8, 7502 - 7503	R.KSEEENLFEIITADEVHYFLQAATPK.E	3024.32650	3	3.30E-10	0.99	8.22	-	3039.0
AHQ-1-11, 6491	R.KSEEENLFEIITADEVHYFLQAATPK.E	3024.32650	3	1.56E-12	0.98	7.41	-	2707.5
AHQ-1-13- , 6975	R.KSEEENLFEIITADEVHYFLQAATPK.E	3024.32650	3	6.64E-12	0.98	7.07	-	2862.7
AHQ-1-12, 5545	R.LPETIDLGALYLSM*K.D	1680.98719	2	7.04E-05	0.95	4.49	-	1137.1
AHQ-1-8, 5795 - 5871	R.LPETIDLGALYLSM*K.D	1680.98719	2	5.51E-04	0.95	4.29	-	1189.6
AHQ-1-10, 5915	R.LPETIDLGALYLSMK.D	1664.98779	2	5.22E-05	0.95	3.92	-	1394.3
AHQ-1-9, 6670	R.LPETIDLGALYLSMK.D	1664.98779	2	2.05E-05	0.96	4.61	-	1467.8
AHQ-1-8, 6932	R.LPETIDLGALYLSMK.D	1664.98779	2	1.18E-05	0.96	3.55	-	1858.1
AHQ-1-11, 6026 - 6098	R.LPETIDLGALYLSMK.D	1664.98779	2	9.26E-05	0.97	3.86	-	2490.9
AHQ-1-8, 6778 - 6860	R.LPETIDLGALYLSMK.D	1664.98779	2	1.61E-06	0.98	5.02	-	1865.0
AHQ-1-12, 6213 - 6237	R.LPETIDLGALYLSMK.D	1664.98779	2	5.41E-09	0.98	4.86	-	2048.5
AHQ-1-1, 5980	R.LPETIDLGALYLSM*K.D	1680.98719	2	3.55E-05	0.90	3.85	-	759.6
AHQ-1-12, 5137 - 5197	R.LPETIDLGALYLSM*KDTEK.G	2154.46684	3	1.03E-05	0.84	3.90	-	842.9
AHQ-1-8, 6274 - 6298	R.NRQEGLMIASSLLNEGYLQPAGDMSK.S	2824.18382	2	2.14E-04	0.98	6.54	-	1756.6
AHQ-1-8, 6256 - 6278	R.NRQEGLMIASSLLNEGYLQPAGDMSK.S	2824.18382	3	8.14E-04	0.89	4.42	-	767.1
AHQ-1-10, 5579	R.NRQEGLMIASSLLNEGYLQPAGDMSK.S	2824.18382	2	7.82E-05	0.90	4.06	-	707.8
AHQ-1-10, 5563 - 5565	R.NRQEGLMIASSLLNEGYLQPAGDMSK.S	2824.18382	3	7.48E-06	0.76	3.59	-	630.7

AHQ-1-10, 5321	R.NRQEGLMIASSLLNEGYLQPAGDM*SK.S	2840.18322	2	2.12E-04	0.91	4.35	-	518.8
AHQ-1-11, 4352	K.QQDHFFQAAFLEER.D	1766.89390	2	3.92E-07	0.42	2.54	-	117.3
AHQ-1-8, 4488	K.QQDHFFQAAFLEER.D	1766.89390	2	1.49E-07	0.75	2.98	-	237.7
AHQ-1-8, 7568	K.SEEENLFEIITADEVHYFLQAATPK.E	2896.15359	3	1.61E-10	0.93	4.76	-	1080.5
AHQ-1-10, 6465	K.SEEENLFEIITADEVHYFLQAATPK.E	2896.15359	2	6.32E-06	0.85	4.44	-	400.9
AHQ-1-10, 6467	K.SEEENLFEIITADEVHYFLQAATPK.E	2896.15359	3	4.07E-09	0.91	4.48	-	969.9
AHQ-1-12, 6701	K.SEEENLFEIITADEVHYFLQAATPK.E	2896.15359	3	3.34E-08	0.89	4.40	-	705.1
AHQ-1-9, 7366	K.SEEENLFEIITADEVHYFLQAATPK.E	2896.15359	3	1.77E-12	0.95	5.18	-	1256.2
AHQ-1-13- , 7016	K.SEEENLFEIITADEVHYFLQAATPK.E	2896.15359	3	9.52E-09	0.89	4.55	-	773.1
gi|7656991|ref|NP_055140.1| coronin, actin binding protein, 1C; coronin, actin-binding protein, 1C;				1.91E-13	6.55	80.31	23.40	53248.7
AHQ-1-6, 5964 - 6031	R.AIFLADGNVFTTGFSR.M	1716.91793	2	1.13E-09	0.96	4.89	-	1349.2
AHQ-1-6, 6112 - 6171	R.AIFLADGNVFTTGFSR.M	1716.91793	2	2.09E-05	0.97	4.86	-	1761.8
AHQ-1-7, 6136	R.AIFLADGNVFTTGFSR.M	1716.91793	2	6.05E-05	0.96	5.00	-	1217.1
AHQ-1-6, 3262	R.KCEPIIMTVPR.K	1345.65706	2	2.16E-05	0.61	2.74	-	512.2
AHQ-1-6, 2614	R.KCEPIIM*TVPR.K	1361.65646	2	2.16E-05	0.71	2.57	-	547.8
AHQ-1-7, 7364 - 7438	R.KSDLFQDDLYPDTAGPEAALEAEEWFEGK.N	3273.46087	3	8.00E-10	0.97	6.15	-	1386.2
AHQ-1-6, 7142	R.KSDLFQDDLYPDTAGPEAALEAEEWFEGK.N	3273.46087	3	9.79E-08	0.90	4.74	-	699.8
AHQ-1-6, 3972	K.NADPILISLK.H	1084.29103	1	1.16E-04	0.06	1.94	-	301.8
AHQ-1-6, 3974	K.NADPILISLK.H	1084.29103	2	1.96E-05	0.87	2.99	-	730.4
AHQ-1-7, 2368	K.SIKDTICNQDER.I	1480.58332	2	3.67E-05	0.93	3.27	-	1496.0
AHQ-1-6, 2330	K.SIKDTICNQDER.I	1480.58332	2	2.57E-05	0.82	2.91	-	937.6
AHQ-1-6, 4410 - 4455	R.VTWDSSFCAVNPR.F	1540.68205	2	1.30E-04	0.67	2.69	-	626.5
AHQ-1-7, 6308	R.YFEITDESPYVHYLNTFSSK.E	2441.63258	2	9.44E-07	0.96	4.99	-	1011.5
AHQ-1-7, 6312	R.YFEITDESPYVHYLNTFSSK.E	2441.63258	3	7.81E-08	0.93	4.45	-	857.1
AHQ-1-6, 6190 - 6199	R.YFEITDESPYVHYLNTFSSK.E	2441.63258	3	1.11E-04	0.83	3.28	-	737.9
AHQ-1-6, 6194	R.YFEITDESPYVHYLNTFSSK.E	2441.63258	2	1.91E-13	0.93	4.76	-	728.2
AHQ-1-9, 6166	R.YFEITDESPYVHYLNTFSSK.E	2441.63258	2	6.48E-05	0.74	3.03	-	526.5
AHQ-1-10, 5504	R.YFEITDESPYVHYLNTFSSK.E	2441.63258	3	8.56E-04	0.77	3.02	-	749.2
AHQ-1-10, 5508	R.YFEITDESPYVHYLNTFSSK.E	2441.63258	2	7.36E-05	0.74	3.04	-	735.7
gi|30151108|ref|XP_301187.1| similar to actin beta, cytoskeletal - Kenyan clawed frog [Homo sapiens				2.14E-13	1.93	20.31	7.40	32164.8
AHQ-1-1, 4981	K.DLYANTVLSGGTTM*YPGIADR.M	2232.45624	2	4.08E-06	0.91	4.26	-	669.1
AHQ-1-1, 5123 - 5133	K.DLYANTVLSGGTTM*YPGIADR.M	2232.45624	2	2.39E-04	0.93	4.43	-	831.2
AHQ-1-2, 5125	K.DLYANTVLSGGTTM*YPGIADR.M	2232.45624	2	4.08E-09	0.86	3.81	-	585.2
AHQ-1-2, 5275	K.DLYANTVLSGGTTM*YPGIADR.M	2232.45624	2	3.22E-05	0.84	4.34	-	475.6
AHQ-1-2, 5622	K.DLYANTVLSGGTTMYPGIADR.M	2216.45684	2	8.79E-05	0.78	3.27	-	571.1
AHQ-1-3, 4997	K.DLYANTVLSGGTTM*YPGIADR.M	2232.45624	2	6.06E-13	0.94	4.61	-	820.5
AHQ-1-3, 5126 - 5131	K.DLYANTVLSGGTTM*YPGIADR.M	2232.45624	2	1.84E-06	0.93	4.64	-	838.4
AHQ-1-4, 5082	K.DLYANTVLSGGTTM*YPGIADR.M	2232.45624	2	8.47E-09	0.95	4.68	-	997.6
AHQ-1-4, 5236	K.DLYANTVLSGGTTM*YPGIADR.M	2232.45624	2	2.29E-04	0.92	4.96	-	736.2
AHQ-1-4, 5563	K.DLYANTVLSGGTTMYPGIADR.M	2216.45684	2	8.46E-08	0.94	4.42	-	1115.5
AHQ-1-5, 5062	K.DLYANTVLSGGTTM*YPGIADR.M	2232.45624	2	2.61E-06	0.97	4.87	-	1381.3
AHQ-1-5, 5182	K.DLYANTVLSGGTTM*YPGIADR.M	2232.45624	2	7.22E-05	0.87	3.97	-	684.5
AHQ-1-5, 5529	K.DLYANTVLSGGTTMYPGIADR.M	2216.45684	2	2.66E-07	0.93	4.50	-	795.9
AHQ-1-6, 4951	K.DLYANTVLSGGTTM*YPGIADR.M	2232.45624	2	5.77E-08	0.92	4.29	-	850.9
AHQ-1-6, 5086	K.DLYANTVLSGGTTM*YPGIADR.M	2232.45624	2	4.09E-06	0.94	5.01	-	789.4
AHQ-1-6, 5407	K.DLYANTVLSGGTTMYPGIADR.M	2216.45684	2	6.86E-06	0.97	5.97	-	1485.2
AHQ-1-7, 5674	K.DLYANTVLSGGTTMYPGIADR.M	2216.45684	2	2.09E-05	0.89	4.17	-	705.9
AHQ-1-8, 5002 - 5066	K.DLYANTVLSGGTTM*YPGIADR.M	2232.45624	2	1.54E-07	0.95	5.13	-	785.2
AHQ-1-8, 5132 - 5198	K.DLYANTVLSGGTTM*YPGIADR.M	2232.45624	2	1.43E-05	0.96	5.33	-	1006.4
AHQ-1-8, 5266 - 5342	K.DLYANTVLSGGTTM*YPGIADR.M	2232.45624	2	4.24E-05	0.95	5.10	-	954.2
AHQ-1-8, 5355 - 5427	K.DLYANTVLSGGTTMYPGIADR.M	2216.45684	2	1.18E-04	0.94	4.62	-	870.1
AHQ-1-8, 5362 - 5442	K.DLYANTVLSGGTTM*YPGIADR.M	2232.45624	2	4.49E-05	0.90	3.87	-	843.8
AHQ-1-8, 5702 - 5768	K.DLYANTVLSGGTTMYPGIADR.M	2216.45684	2	1.90E-04	0.95	5.11	-	869.4
AHQ-1-8, 5834 - 5902	K.DLYANTVLSGGTTMYPGIADR.M	2216.45684	2	1.68E-06	0.96	5.29	-	983.9
AHQ-1-8, 5966 - 6035	K.DLYANTVLSGGTTMYPGIADR.M	2216.45684	2	2.46E-05	0.96	5.37	-	804.1
AHQ-1-8, 6102 - 6167	K.DLYANTVLSGGTTMYPGIADR.M	2216.45684	2	1.80E-06	0.96	6.11	-	821.1
AHQ-1-8, 6234 - 6310	K.DLYANTVLSGGTTMYPGIADR.M	2216.45684	2	5.29E-04	0.95	5.11	-	767.1
AHQ-1-8, 6590 - 6667	K.DLYANTVLSGGTTMYPGIADR.M	2216.45684	2	2.68E-05	0.95	5.89	-	657.0
AHQ-1-8, 6979 - 7019	K.DLYANTVLSGGTTMYPGIADR.M	2216.45684	2	4.64E-04	0.94	5.04	-	697.8
AHQ-1-8, 7032 - 7106	K.DLYANTVLSGGTTMYPGIADR.M	2216.45684	2	7.47E-05	0.90	4.31	-	763.5
AHQ-1-8, 7411	K.DLYANTVLSGGTTMYPGIADR.M	2216.45684	2	1.45E-04	0.85	3.51	-	780.7
AHQ-1-8, 7642	K.DLYANTVLSGGTTMYPGIADR.M	2216.45684	2	1.07E-07	0.93	4.73	-	861.4
AHQ-1-9, 4903	K.DLYANTVLSGGTTM*YPGIADR.M	2232.45624	2	4.93E-09	0.92	4.26	-	713.6
AHQ-1-9, 5014 - 5090	K.DLYANTVLSGGTTM*YPGIADR.M	2232.45624	2	3.49E-04	0.92	4.25	-	700.0
AHQ-1-9, 5511 - 5586	K.DLYANTVLSGGTTMYPGIADR.M	2216.45684	2	8.44E-05	0.96	5.14	-	1245.7
AHQ-1-9, 5791 - 5858	K.DLYANTVLSGGTTMYPGIADR.M	2216.45684	2	5.16E-04	0.78	3.47	-	674.0
AHQ-1-10, 4408 - 4427	K.DLYANTVLSGGTTM*YPGIADR.M	2232.45624	2	2.14E-13	0.96	5.64	-	782.1
AHQ-1-10, 4507 - 4633	K.DLYANTVLSGGTTM*YPGIADR.M	2232.45624	2	7.78E-04	0.96	4.88	-	1281.0
AHQ-1-10, 4691 - 4701	K.DLYANTVLSGGTTM*YPGIADR.M	2232.45624	2	6.49E-08	0.93	4.85	-	849.0
AHQ-1-10, 4835	K.DLYANTVLSGGTTMYPGIADR.M	2216.45684	2	4.29E-05	0.93	4.43	-	865.4
AHQ-1-10, 4916 - 4991	K.DLYANTVLSGGTTMYPGIADR.M	2216.45684	2	4.99E-06	0.96	5.49	-	835.8
AHQ-1-10, 5163 - 5179	K.DLYANTVLSGGTTMYPGIADR.M	2216.45684	2	4.63E-06	0.96	5.06	-	964.3
AHQ-1-11, 4534	K.DLYANTVLSGGTTM*YPGIADR.M	2232.45624	2	4.50E-07	0.97	5.58	-	1267.6
AHQ-1-11, 4650	K.DLYANTVLSGGTTM*YPGIADR.M	2232.45624	2	2.41E-05	0.92	4.46	-	764.6
AHQ-1-11, 4918	K.DLYANTVLSGGTTMYPGIADR.M	2216.45684	2	2.56E-05	0.96	5.52	-	1152.6
AHQ-1-12, 4689	K.DLYANTVLSGGTTM*YPGIADR.M	2232.45624	2	6.37E-07	0.93	4.80	-	595.1
AHQ-1-12, 4998	K.DLYANTVLSGGTTM*YPGIADR.M	2232.45624	2	8.19E-04	0.94	4.90	-	962.8
AHQ-1-12, 5090	K.DLYANTVLSGGTTMYPGIADR.M	2216.45684	2	3.61E-04	0.93	3.90	-	1140.2
AHQ-1-12, 5222 - 5278	K.DLYANTVLSGGTTMYPGIADR.M	2216.45684	2	6.90E-04	0.91	4.51	-	609.8
AHQ-1-12, 5422	K.DLYANTVLSGGTTMYPGIADR.M	2216.45684	2	3.18E-06	0.86	3.97	-	676.1
AHQ-1-13, 4639 - 4703	K.DLYANTVLSGGTTM*YPGIADR.M	2232.45624	2	7.80E-09	0.95	5.21	-	970.5
AHQ-1-13, 4763 - 4784	K.DLYANTVLSGGTTM*YPGIADR.M	2232.45624	2	1.11E-04	0.94	4.86	-	858.2
AHQ-1-13, 5011 - 5067	K.DLYANTVLSGGTTMYPGIADR.M	2216.45684	2	2.70E-05	0.96	4.96	-	1375.0
AHQ-1-13, 5053	K.DLYANTVLSGGTTM*YPGIADR.M	2232.45624	2	3.36E-05	0.85	3.95	-	598.6
AHQ-1-14, 5529 - 5588	K.DLYANTVLSGGTTM*YPGIADR.M	2232.45624	2	4.50E-06	0.76	3.46	-	527.3
AHQ-1-14, 5648 - 5704	K.DLYANTVLSGGTTM*YPGIADR.M	2232.45624	2	4.92E-06	0.96	5.24	-	1140.2
AHQ-1-14, 5764 - 5820	K.DLYANTVLSGGTTM*YPGIADR.M	2232.45624	2	7.34E-07	0.97	5.45	-	1138.1
AHQ-1-14, 5876 - 5936	K.DLYANTVLSGGTTM*YPGIADR.M	2232.45624	2	1.77E-04	0.95	5.31	-	980.8
AHQ-1-14, 5960 - 6016	K.DLYANTVLSGGTTMYPGIADR.M	2216.45684	2	1.55E-06	0.97	5.70	-	1495.9
AHQ-1-14, 6357 - 6426	K.DLYANTVLSGGTTMYPGIADR.M	2216.45684	2	6.91E-04	0.76	3.61	-	389.1
AHQ-1-13- , 4811 - 4876	K.DLYANTVLSGGTTM*YPGIADR.M	2232.45624	2	2.75E-10	0.98	5.67	-	1644.5
AHQ-1-13- , 4943 - 5011	K.DLYANTVLSGGTTM*YPGIADR.M	2232.45624	2	1.73E-04	0.95	5.17	-	937.9
AHQ-1-13- , 5059	K.DLYANTVLSGGTTM*YPGIADR.M	2232.45624	2	1.69E-04	0.93	4.46	-	967.4
AHQ-1-13- , 5183 - 5203	K.DLYANTVLSGGTTM*YPGIADR.M	2232.45624	2	4.11E-08	0.96	5.15	-	1032.6
AHQ-1-13- , 5264 - 5327	K.DLYANTVLSGGTTMYPGIADR.M	2216.45684	2	1.77E-08	0.96	5.56	-	1079.5
AHQ-1-13- , 5305	K.DLYANTVLSGGTTM*YPGIADR.M	2232.45624	2	2.37E-04	0.82	3.62	-	659.3
AHQ-1-13- , 5468 - 5531	K.DLYANTVLSGGTTMYPGIADR.M	2216.45684	2	4.94E-06	0.97	6.20	-	924.1
AHQ-1-13- , 5595 - 5675	K.DLYANTVLSGGTTMYPGIADR.M	2216.45684	2	7.38E-04	0.96	5.40	-	1053.0
AHQ-1-13- , 5753	K.DLYANTVLSGGTTMYPGIADR.M	2216.45684	2	2.47E-08	0.94	4.71	-	920.0
AHQ-1-14- , 4711 - 4782	K.DLYANTVLSGGTTM*YPGIADR.M	2232.45624	2	8.61E-07	0.94	5.01	-	691.8
AHQ-1-14- , 4820 - 4902	K.DLYANTVLSGGTTM*YPGIADR.M	2232.45624	2	1.01E-04	0.97	5.35	-	1180.8
AHQ-1-14- , 4962 - 5035	K.DLYANTVLSGGTTM*YPGIADR.M	2232.45624	2	5.35E-06	0.95	4.98	-	987.3
AHQ-1-14- , 5098 - 5170	K.DLYANTVLSGGTTM*YPGIADR.M	2232.45624	2	1.60E-08	0.95	5.16	-	765.3
AHQ-1-14- , 5130 - 5191	K.DLYANTVLSGGTTMYPGIADR.M	2216.45684	2	2.85E-12	0.97	5.84	-	1157.8
AHQ-1-14- , 5280 - 5342	K.DLYANTVLSGGTTMYPGIADR.M	2216.45684	2	1.71E-06	0.95	4.99	-	999.6
AHQ-1-14- , 5402 - 5474	K.DLYANTVLSGGTTMYPGIADR.M	2216.45684	2	2.78E-05	0.95	5.27	-	863.1
gi|27498332|ref|XP_212565.1| similar to tubulin, beta 5 [Homo sapiens]				2.28E-13	5.03	60.32	11.90	49670.5

AHQ-1-11, 5312	R.ALTVPELTQQVFDAK.N	1660.89259	2	4.02E-05	0.73	2.89	-	348.8
AHQ-1-8, 5640	R.ALTVPELTQQVFDAK.N	1660.89259	2	8.47E-04	0.89	3.70	-	590.8
AHQ-1-7, 5805 - 5876	R.ALTVPELTQQVFDAK.N	1660.89259	2	2.23E-04	0.87	3.47	-	413.9
AHQ-1-11, 5266 - 5323	R.ALTVPELTQQVFDAK.N	1660.89259	2	1.82E-04	0.86	3.84	-	388.5
AHQ-1-7, 4041	R.IMNTFSVVPSPK.V	1320.58277	2	1.67E-06	0.84	3.25	-	690.3
AHQ-1-7, 3372	R.IM*NTFSVVPSPK.V	1336.58217	2	1.49E-04	0.87	3.01	-	931.7
AHQ-1-7, 3530 - 3600	R.IM*NTFSVVPSPK.V	1336.58217	2	1.44E-04	0.64	2.90	-	427.6
AHQ-1-7, 3896 - 3969	R.IMNTFSVVPSPK.V	1320.58277	2	1.71E-05	0.91	3.15	-	1363.0
AHQ-1-7, 7194	K.MAVTFIGNSTAIQELFK.R	1871.19002	2	8.71E-04	0.85	3.59	-	630.5
AHQ-1-7, 7288	K.MAVTFIGNSTAIQELFK.R	1871.19002	2	7.26E-07	0.96	5.06	-	1030.1
AHQ-1-14- , 6411 - 6432	K.M*AVTFIGNSTAIQELFK.R	1887.18942	2	5.15E-07	0.95	4.72	-	1052.0
AHQ-1-7, 7021 - 7084	K.M*AVTFIGNSTAIQELFK.R	1887.18942	2	2.28E-13	0.97	5.04	-	1204.7
AHQ-1-7, 6709 - 6714	K.M*AVTFIGNSTAIQELFK.R	1887.18942	2	3.36E-04	0.92	3.94	-	849.6
AHQ-1-1, 6564	K.M*AVTFIGNSTAIQELFK.R	1887.18942	2	1.49E-07	0.98	4.75	-	2063.9
AHQ-1-13, 6213	K.M*AVTFIGNSTAIQELFK.R	1887.18942	2	1.08E-04	0.67	2.99	-	573.5
AHQ-1-13, 6300	K.M*AVTFIGNSTAIQELFK.R	1887.18942	2	2.09E-08	0.95	4.50	-	973.1
AHQ-1-13- , 6448 - 6515	K.M*AVTFIGNSTAIQELFK.R	1887.18942	2	7.96E-04	0.91	3.96	-	949.9
AHQ-1-13- , 6700 - 6747	K.M*AVTFIGNSTAIQELFK.R	1887.18942	2	1.22E-11	0.96	4.76	-	1064.6
AHQ-1-14- , 6200 - 6259	K.M*AVTFIGNSTAIQELFK.R	1887.18942	2	3.05E-08	0.98	6.45	-	1784.5
AHQ-1-14- , 6499	K.M*AVTFIGNSTAIQELFK.R	1887.18942	2	6.52E-08	0.94	4.38	-	785.2
AHQ-1-7, 4481	R.YLTVAAVFR.G	1040.23994	2	2.66E-05	0.90	3.32	-	819.3
gi|11968039|ref|NP_071930.1| hypothetical protein FLJ22405 [Homo sapiens]				2.34E-13	0.95	10.21	4.60	46880.2
AHQ-1-14- , 6544	R.AVAPSPSGAIGGLLEQFAR.G	1842.08850	2	2.34E-13	0.95	4.27	-	837.7
gi|4826898|ref|NP_005013.1| profilin 1; profilin-1 [Homo sapiens]				2.64E-13	18.00	200.29	71.40	15054.2
AHQ-1-13, 4881 - 4892	K.CSVIRDSLLQDGEFSM*DLR.T	2259.50329	3	4.08E-05	0.94	4.84	-	1157.1
AHQ-1-13- , 5044	K.CSVIRDSLLQDGEFSM*DLR.T	2259.50329	3	1.37E-04	0.94	5.00	-	1243.8
AHQ-1-13- , 5155	K.CSVIRDSLLQDGEFSM*DLR.T	2259.50329	3	2.70E-05	0.97	5.57	-	1773.5
AHQ-1-13- , 1805 - 1863	K.CYEM*ASHLR.R	1184.32794	2	2.69E-05	0.87	2.73	-	1302.4
AHQ-1-13- , 1923 - 1983	K.CYEM*ASHLR.R	1184.32794	2	4.98E-05	0.95	3.36	-	1672.4
AHQ-1-13- , 2455 - 2477	K.CYEMASHLR.R	1168.32854	2	6.50E-04	0.95	3.51	-	1649.8
AHQ-1-14- , 2438 - 2450	K.CYEMASHLR.R	1168.32854	2	5.99E-04	0.95	2.96	-	2002.0
AHQ-1-13- , 1700 - 1769	K.CYEM*ASHLR.R	1184.32794	2	4.00E-05	0.77	2.59	-	834.0
AHQ-1-13, 1973 - 2029	K.CYEM*ASHLR.R	1184.32794	2	6.26E-04	0.83	2.72	-	1079.9
AHQ-1-13, 2094	K.CYEM*ASHLR.R	1184.32794	2	3.35E-04	0.88	2.65	-	1253.7
AHQ-1-13, 2557 - 2569	K.CYEMASHLR.R	1168.32854	2	3.60E-04	0.95	3.81	-	1661.9
AHQ-1-13- , 4203	K.DRSSFYVNGLTLGGQK.C	1742.91379	2	7.82E-10	0.97	5.58	-	1333.3
AHQ-1-13, 4023 - 4096	K.DRSSFYVNGLTLGGQK.C	1742.91379	2	1.66E-06	0.97	5.58	-	1098.8
AHQ-1-13- , 4344	K.DRSSFYVNGLTLGGQK.C	1742.91379	2	7.61E-12	0.97	5.34	-	1150.5
AHQ-1-13, 4153 - 4157	K.DRSSFYVNGLTLGGQK.C	1742.91379	2	2.64E-13	0.95	5.24	-	779.7
AHQ-1-12, 5398	R.DSLLQDGEFSMDLR.T	1626.77023	2	3.93E-07	0.87	2.96	-	1180.1
AHQ-1-13- , 4765	R.DSLLQDGEFSM*DLR.T	1642.76963	3	5.24E-08	0.94	4.42	-	1686.8
AHQ-1-13- , 4707 - 4785	R.DSLLQDGEFSM*DLR.T	1642.76963	2	3.39E-08	0.96	3.66	-	2032.6
AHQ-1-12, 5254 - 5317	R.DSLLQDGEFSMDLR.T	1626.77023	2	2.00E-10	0.97	4.55	-	1869.2
AHQ-1-13- , 4472 - 4483	R.DSLLQDGEFSM*DLR.T	1642.76963	2	8.05E-05	0.96	4.78	-	1538.8
AHQ-1-12, 5122 - 5193	R.DSLLQDGEFSMDLR.T	1626.77023	2	1.97E-09	0.97	4.97	-	1503.5
AHQ-1-12, 4547 - 4621	R.DSLLQDGEFSM*DLR.T	1642.76963	2	6.47E-09	0.95	3.78	-	1843.8
AHQ-1-13- , 4831 - 4912	R.DSLLQDGEFSM*DLR.T	1642.76963	2	5.50E-04	0.96	4.03	-	1741.1
AHQ-1-12, 4458 - 4459	R.DSLLQDGEFSM*DLR.T	1642.76963	2	1.96E-08	0.95	4.40	-	1133.5
AHQ-1-14- , 5426 - 5438	R.DSLLQDGEFSMDLR.T	1626.77023	2	9.80E-11	0.97	4.85	-	2035.6
AHQ-1-13- , 4979	R.DSLLQDGEFSM*DLR.T	1642.76963	2	2.99E-04	0.95	4.19	-	1496.9
AHQ-1-13- , 6977 - 7044	R.DSLLQDGEFSMDLR.T	1626.77023	2	1.29E-06	0.92	3.51	-	1240.3
AHQ-1-13- , 5336 - 5363	R.DSLLQDGEFSMDLR.T	1626.77023	2	1.83E-07	0.92	4.05	-	1138.4
AHQ-1-14- , 5508 - 5586	R.DSLLQDGEFSMDLR.T	1626.77023	2	7.71E-06	0.96	4.32	-	1801.2
AHQ-1-13- , 5371 - 5449	R.DSLLQDGEFSM*DLR.T	1642.76963	2	4.29E-05	0.85	3.27	-	1116.0
AHQ-1-13- , 5495 - 5559	R.DSLLQDGEFSMDLR.T	1626.77023	2	2.65E-07	0.97	4.54	-	2033.9
AHQ-1-14- , 5163 - 5242	R.DSLLQDGEFSMDLR.T	1626.77023	2	2.74E-06	0.95	3.94	-	1512.6
AHQ-1-13- , 5563	R.DSLLQDGEFSM*DLR.T	1642.76963	2	6.73E-04	0.94	3.62	-	1593.5
AHQ-1-14- , 4680 - 4698	R.DSLLQDGEFSM*DLR.T	1642.76963	2	4.39E-06	0.94	3.68	-	1512.5
AHQ-1-13- , 5623 - 5692	R.DSLLQDGEFSMDLR.T	1626.77023	2	2.61E-08	0.97	4.81	-	2074.3
AHQ-1-13- , 4576 - 4643	R.DSLLQDGEFSM*DLR.T	1642.76963	2	1.07E-09	0.97	5.65	-	1355.3
AHQ-1-14, 5338 - 5396	R.DSLLQDGEFSM*DLR.T	1642.76963	2	6.03E-05	0.78	2.96	-	856.6
AHQ-1-13- , 5759 - 5825	R.DSLLQDGEFSMDLR.T	1626.77023	2	1.56E-08	0.97	4.49	-	2106.2
AHQ-1-13, 5424 - 5487	R.DSLLQDGEFSMDLR.T	1626.77023	2	2.05E-08	0.95	3.44	-	1897.6
AHQ-1-13, 5307 - 5367	R.DSLLQDGEFSMDLR.T	1626.77023	2	1.47E-07	0.98	5.08	-	2163.8
AHQ-1-13, 4287 - 4368	R.DSLLQDGEFSM*DLR.T	1642.76963	2	5.14E-09	0.95	4.54	-	1095.1
AHQ-1-14- , 4550	R.DSLLQDGEFSM*DLR.T	1642.76963	2	1.46E-05	0.88	3.35	-	912.4
AHQ-1-13, 4373 - 4440	R.DSLLQDGEFSM*DLR.T	1642.76963	2	1.54E-06	0.95	4.11	-	1320.8
AHQ-1-13, 5183 - 5248	R.DSLLQDGEFSMDLR.T	1626.77023	2	4.64E-10	0.97	4.83	-	2123.8
AHQ-1-13, 4499 - 4565	R.DSLLQDGEFSM*DLR.T	1642.76963	2	7.44E-10	0.97	4.25	-	1979.8
AHQ-1-13, 4623	R.DSLLQDGEFSM*DLR.T	1642.76963	2	1.05E-06	0.94	3.98	-	1652.1
AHQ-1-13, 4721 - 4725	R.DSLLQDGEFSM*DLR.T	1642.76963	2	2.90E-06	0.96	3.74	-	1762.7
AHQ-1-14, 5544	R.DSLLQDGEFSM*DLR.T	1642.76963	2	7.50E-05	0.89	3.11	-	1337.6
AHQ-1-13, 5069 - 5127	R.DSLLQDGEFSMDLR.T	1626.77023	2	9.80E-06	0.96	4.01	-	1873.2
AHQ-1-13, 5100 - 5173	R.DSLLQDGEFSM*DLR.T	1642.76963	2	8.12E-06	0.90	3.83	-	956.3
AHQ-1-13- , 3617 - 3683	K.DSPSVWAAVPGK.T	1214.35211	2	2.74E-06	0.88	3.61	-	841.0
AHQ-1-13- , 3435 - 3444	K.DSPSVWAAVPGK.T	1214.35211	2	1.22E-06	0.89	3.12	-	1122.8
AHQ-1-14- , 3632 - 3644	K.DSPSVWAAVPGK.T	1214.35211	2	3.65E-08	0.89	3.09	-	1057.9
AHQ-1-13, 3373 - 3375	K.DSPSVWAAVPGK.T	1214.35211	2	2.41E-06	0.89	3.53	-	1090.9
AHQ-1-13, 3534 - 3599	K.DSPSVWAAVPGK.T	1214.35211	2	2.75E-05	0.93	3.51	-	1248.7
AHQ-1-13- , 3748	K.DSPSVWAAVPGK.T	1214.35211	2	3.56E-06	0.90	3.60	-	896.4
AHQ-1-13, 3648 - 3712	K.DSPSVWAAVPGK.T	1214.35211	2	4.49E-05	0.86	2.65	-	1048.7
AHQ-1-12, 3533 - 3553	K.DSPSVWAAVPGK.T	1214.35211	2	3.09E-06	0.87	3.18	-	881.0
AHQ-1-13, 6252 - 6256	K.DSPSVWAAVPGKTFVNITPAEVGVLVGK.D	2840.26581	3	1.92E-04	0.94	4.62	-	1532.4
AHQ-1-13- , 6675	K.DSPSVWAAVPGKTFVNITPAEVGVLVGK.D	2840.26581	3	5.47E-09	0.93	4.50	-	1620.1
AHQ-1-13, 2305	K.KCYEMASHLR.R	1296.50145	2	2.02E-07	0.79	2.77	-	782.3
AHQ-1-14- , 4400	R.SSFYVNGLTLGGQK.C	1471.63957	1	2.25E-09	0.51	2.77	-	391.6
AHQ-1-14, 5272	R.SSFYVNGLTLGGQK.C	1471.63957	2	1.54E-07	0.93	4.22	-	828.4
AHQ-1-14, 5281	R.SSFYVNGLTLGGQK.C	1471.63957	1	5.23E-06	0.44	2.18	-	511.7
AHQ-1-12, 4246	R.SSFYVNGLTLGGQK.C	1471.63957	1	2.78E-06	0.39	2.17	-	345.8
AHQ-1-12, 4383 - 4462	R.SSFYVNGLTLGGQK.C	1471.63957	2	6.37E-05	0.97	4.79	-	1296.1
AHQ-1-13, 4197 - 4237	R.SSFYVNGLTLGGQK.C	1471.63957	1	1.39E-06	0.75	3.06	-	476.7
AHQ-1-13, 4200 - 4561	R.SSFYVNGLTLGGQK.C	1471.63957	1	2.55E-05	0.58	3.22	-	393.4
AHQ-1-13, 4283	R.SSFYVNGLTLGGQK.C	1471.63957	2	1.52E-08	0.92	3.74	-	836.8
AHQ-1-13, 4195 - 4223	R.SSFYVNGLTLGGQK.C	1471.63957	2	1.46E-07	0.95	4.22	-	974.6
AHQ-1-14- , 4390	R.SSFYVNGLTLGGQK.C	1471.63957	2	2.55E-08	0.95	4.17	-	972.4
AHQ-1-14- , 4539 - 4616	R.SSFYVNGLTLGGQK.C	1471.63957	1	2.50E-09	0.53	2.34	-	408.6
AHQ-1-13- , 4940	R.SSFYVNGLTLGGQK.C	1471.63957	2	9.54E-05	0.77	2.58	-	660.9
AHQ-1-12, 4473 - 4543	R.SSFYVNGLTLGGQK.C	1471.63957	2	1.06E-10	0.95	3.86	-	1419.7
AHQ-1-13- , 4767 - 4833	R.SSFYVNGLTLGGQK.C	1471.63957	2	3.43E-08	0.89	3.62	-	795.5
AHQ-1-13- , 4619 - 4699	R.SSFYVNGLTLGGQK.C	1471.63957	2	1.12E-08	0.97	4.63	-	1669.4
AHQ-1-13- , 4471 - 4551	R.SSFYVNGLTLGGQK.C	1471.63957	2	9.58E-08	0.95	4.29	-	952.8
AHQ-1-13- , 4391 - 4792	R.SSFYVNGLTLGGQK.C	1471.63957	1	4.12E-05	0.44	3.02	-	314.1
AHQ-1-13- , 4387 - 4691	R.SSFYVNGLTLGGQK.C	1471.63957	1	2.30E-09	0.79	3.31	-	538.7
AHQ-1-13- , 4385 - 4451	R.SSFYVNGLTLGGQK.C	1471.63957	2	2.44E-07	0.92	4.14	-	733.8
AHQ-1-13, 4364 - 4443	R.SSFYVNGLTLGGQK.C	1471.63957	2	4.84E-09	0.97	4.40	-	1703.2
AHQ-1-13, 4495 - 4575	R.SSFYVNGLTLGGQK.C	1471.63957	2	8.81E-06	0.90	3.92	-	782.8
AHQ-1-12, 4241	R.SSFYVNGLTLGGQK.C	1471.63957	2	4.35E-04	0.94	4.03	-	936.6

AHQ-1-12, 4389 - 4463	R.SSFYVNGLTLGGQK.C	1471.63957	1	4.02E-07	0.73	2.68	-	569.8
AHQ-1-13- , 5125	R.SSFYVNGLTLGGQKCSVIR.D	2088.37322	2	1.53E-07	0.86	3.32	-	760.2
AHQ-1-12, 3014 - 3094	K.STGGAPTFNVTVTK.T	1380.52843	2	1.32E-08	0.89	4.03	-	442.5
AHQ-1-13- , 3323 - 3387	K.STGGAPTFNVTVTK.T	1380.52843	2	1.16E-06	0.90	3.55	-	551.7
AHQ-1-13- , 3239 - 3299	K.STGGAPTFNVTVTK.T	1380.52843	2	4.89E-07	0.81	3.32	-	423.6
AHQ-1-13- , 3001 - 3071	K.STGGAPTFNVTVTK.T	1380.52843	2	6.41E-06	0.80	3.33	-	381.7
AHQ-1-13- , 3100	K.STGGAPTFNVTVTK.T	1380.52843	1	3.63E-04	0.71	3.07	-	435.5
AHQ-1-13, 3015 - 3078	K.STGGAPTFNVTVTK.T	1380.52843	2	3.21E-06	0.94	4.31	-	651.6
AHQ-1-13, 3122 - 3165	K.STGGAPTFNVTVTK.T	1380.52843	2	2.52E-07	0.71	2.65	-	436.6
AHQ-1-13, 3230 - 3289	K.STGGAPTFNVTVTK.T	1380.52843	2	1.29E-05	0.83	3.31	-	511.3
AHQ-1-13- , 3140	K.STGGAPTFNVTVTKTDK.T	1724.89354	2	2.87E-05	0.84	3.55	-	530.9
AHQ-1-13- , 2964	K.STGGAPTFNVTVTKTDK.T	1724.89354	2	2.73E-04	0.73	3.25	-	403.8
AHQ-1-13- , 3621 - 3699	K.TDKTLVLLMGK.E	1219.51989	2	2.42E-07	0.96	4.15	-	1542.1
AHQ-1-12, 3573	K.TDKTLVLLMGK.E	1219.51989	2	1.20E-05	0.94	3.51	-	1171.6
AHQ-1-13- , 2987 - 2991	K.TDKTLVLLM*GK.E	1235.51929	2	5.84E-05	0.91	3.56	-	957.3
AHQ-1-13, 3537 - 3600	K.TDKTLVLLMGK.E	1219.51989	2	3.27E-04	0.77	2.61	-	891.5
AHQ-1-12, 5553 - 5609	K.TFVNITPAEVGVLVGK.D	1644.93630	2	6.03E-04	0.57	2.52	-	406.5
AHQ-1-12, 5670 - 5691	K.TFVNITPAEVGVLVGK.D	1644.93630	3	3.35E-07	0.95	4.73	-	1370.6
AHQ-1-13, 5659 - 5715	K.TFVNITPAEVGVLVGK.D	1644.93630	2	4.33E-06	0.89	3.96	-	712.5
AHQ-1-13, 5552	K.TFVNITPAEVGVLVGK.D	1644.93630	3	2.72E-06	0.96	4.79	-	1523.4
AHQ-1-14- , 5911	K.TFVNITPAEVGVLVGK.D	1644.93630	2	1.84E-05	0.83	3.54	-	580.0
AHQ-1-12, 5646 - 5705	K.TFVNITPAEVGVLVGK.D	1644.93630	2	2.42E-07	0.94	4.57	-	773.6
AHQ-1-13, 5527	K.TFVNITPAEVGVLVGK.D	1644.93630	2	3.68E-05	0.95	4.35	-	1065.7
AHQ-1-13, 5255 - 5335	K.TFVNITPAEVGVLVGK.D	1644.93630	2	4.40E-04	0.62	3.16	-	419.6
AHQ-1-12, 5401	K.TFVNITPAEVGVLVGK.D	1644.93630	2	8.32E-05	0.83	3.36	-	519.8
AHQ-1-14- , 5826	K.TFVNITPAEVGVLVGK.D	1644.93630	2	3.54E-06	0.82	3.26	-	799.0
AHQ-1-13- , 5637 - 5653	K.TFVNITPAEVGVLVGK.D	1644.93630	2	2.15E-04	0.85	3.38	-	818.9
AHQ-1-13- , 5744 - 5799	K.TFVNITPAEVGVLVGK.D	1644.93630	2	6.02E-04	0.90	4.23	-	667.2
AHQ-1-13, 5515 - 5576	K.TFVNITPAEVGVLVGK.D	1644.93630	2	7.20E-07	0.94	4.42	-	948.5
AHQ-1-13- , 5827 - 5907	K.TFVNITPAEVGVLVGK.D	1644.93630	2	4.65E-08	0.95	4.74	-	970.0
AHQ-1-13- , 5877 - 5939	K.TFVNITPAEVGVLVGK.D	1644.93630	3	2.75E-09	0.97	5.83	-	1436.4
AHQ-1-13- , 6092 - 6159	K.TFVNITPAEVGVLVGK.D	1644.93630	2	1.80E-06	0.95	4.79	-	1011.3
AHQ-1-13- , 5543 - 5551	K.TFVNITPAEVGVLVGKDR.S	1916.21052	2	6.44E-08	0.94	4.58	-	845.7
AHQ-1-13, 5233	K.TFVNITPAEVGVLVGKDR.S	1916.21052	3	7.47E-05	0.65	3.09	-	592.5
AHQ-1-13, 5236 - 5303	K.TFVNITPAEVGVLVGKDR.S	1916.21052	2	6.66E-06	0.91	4.11	-	790.5
AHQ-1-13, 5936 - 5980	K.TFVNITPAEVGVLVGKDR.S	1916.21052	2	1.84E-04	0.68	3.50	-	591.2
AHQ-1-13- , 2899 - 2919	R.TKSTGGAPTFNVTVTK.T	1609.80568	3	1.74E-05	0.82	3.50	-	897.5
AHQ-1-13, 2771 - 2849	R.TKSTGGAPTFNVTVTK.T	1609.80568	2	1.01E-04	0.89	3.87	-	676.8
AHQ-1-13- , 2821 - 2885	R.TKSTGGAPTFNVTVTK.T	1609.80568	2	7.15E-04	0.95	4.43	-	983.9
AHQ-1-12, 2803	R.TKSTGGAPTFNVTVTK.T	1609.80568	2	1.40E-05	0.92	3.99	-	894.9
AHQ-1-13- , 3776 - 3855	K.TLVLLMGK.E	875.15478	2	1.08E-05	0.71	2.54	-	462.6
AHQ-1-14- , 3023	K.TLVLLM*GK.E	891.15418	2	4.68E-04	0.81	2.71	-	559.1
AHQ-1-13, 3592 - 3780	K.TLVLLMGK.E	875.15478	1	8.58E-04	0.57	2.60	-	625.7
AHQ-1-14- , 3816	K.TLVLLMGK.E	875.15478	2	1.67E-05	0.81	2.96	-	536.1
AHQ-1-12, 3665 - 3666	K.TLVLLMGK.E	875.15478	2	1.05E-05	0.75	2.82	-	502.7
AHQ-1-12, 2955	K.TLVLLM*GK.E	891.15418	2	1.50E-04	0.73	2.70	-	578.9
AHQ-1-13, 3656 - 3731	K.TLVLLMGK.E	875.15478	2	1.46E-05	0.82	2.94	-	465.3
AHQ-1-13- , 3060 - 3127	K.TLVLLM*GK.E	891.15418	2	6.65E-04	0.82	2.65	-	703.9
gi|4502549|ref|NP_001734.1| calmodulin 2 (phosphorylase kinase, delta); phosphorylase kinase delta				2.93E-13	1.92	20.26	22.10	16837.5
AHQ-1-11, 3895 - 3950	K.EAFSLFDKDGDGTITTK.E	1845.98441	2	1.65E-04	0.93	3.74	-	1070.1
AHQ-1-12, 4077 - 4141	K.EAFSLFDKDGDGTITTK.E	1845.98441	2	8.35E-09	0.95	4.28	-	1496.5
AHQ-1-12, 3745 - 3809	R.VFDKDGNGYISAAELR.H	1755.90933	2	2.93E-13	0.97	5.10	-	1457.2
AHQ-1-12, 3874 - 3946	R.VFDKDGNGYISAAELR.H	1755.90933	2	1.51E-04	0.81	3.50	-	712.0
AHQ-1-11, 3730	R.VFDKDGNGYISAAELR.H	1755.90933	2	5.15E-09	0.97	4.79	-	1580.9
AHQ-1-13, 3828	R.VFDKDGNGYISAAELR.H	1755.90933	2	1.92E-10	0.95	4.31	-	1238.9
AHQ-1-14, 4744	R.VFDKDGNGYISAAELR.H	1755.90933	2	8.23E-07	0.60	2.53	-	610.9
AHQ-1-11, 3623 - 3668	R.VFDKDGNGYISAAELR.H	1755.90933	2	1.08E-04	0.84	3.43	-	945.5
gi|21735621|ref|NP_005909.2| mitochondrial malate dehydrogenase precursor [Homo sapiens]				3.05E-13	6.33	70.34	37.00	35503.0
AHQ-1-9, 3100	K.AGAGSATLSMAYAGAR.F	1455.62204	2	6.36E-05	0.84	3.62	-	675.7
AHQ-1-9, 6390 - 6470	K.GYLGPEQLPDCLKGCDVVVIPAGVPR.K	2813.24211	2	6.69E-08	0.87	4.16	-	412.4
AHQ-1-9, 5308	K.IFGVTTLDIVR.A	1234.46961	2	6.43E-07	0.94	3.92	-	1337.4
AHQ-1-9, 5714	R.LTLYDIAHTPGVAADLSHIETK.A	2366.65506	3	5.48E-09	0.98	6.71	-	2519.9
AHQ-1-9, 5711	R.LTLYDIAHTPGVAADLSHIETK.A	2366.65506	2	3.59E-04	0.80	3.01	-	617.6
AHQ-1-9, 5608 - 5616	K.SQETECTYFSTPLLLGK.K	1976.19328	2	1.14E-08	0.97	5.49	-	1748.2
AHQ-1-9, 6744	K.VAVLGASGGIGQPLSLLLK.N	1794.17156	2	3.05E-13	0.97	5.69	-	1343.9
AHQ-1-13, 6244 - 6272	K.VAVLGASGGIGQPLSLLLK.N	1794.17156	2	6.94E-04	0.94	4.28	-	1172.6
AHQ-1-13- , 6688	K.VAVLGASGGIGQPLSLLLK.N	1794.17156	2	1.39E-09	0.93	3.89	-	1156.7
AHQ-1-9, 4991 - 5062	K.VDFPQDQLTALTGR.I	1561.72111	2	1.08E-05	0.75	3.05	-	619.6
gi|4502295|ref|NP_001677.1| ATP synthase, H+ transporting, mitochondrial F1 complex, beta polypepti				3.50E-13	10.58	120.27	28.80	57955.2
AHQ-1-7, 7560	R.DQEGQDVLLFIDNIFR.F	1923.11524	2	3.90E-11	0.96	5.14	-	1147.2
AHQ-1-8, 7570	R.DQEGQDVLLFIDNIFR.F	1923.11524	2	3.50E-13	0.97	5.34	-	1264.0
AHQ-1-7, 4618 - 4669	R.FTQAGSEVSALLGR.I	1436.59522	2	3.43E-06	0.92	3.58	-	1229.7
AHQ-1-8, 4431	R.FTQAGSEVSALLGR.I	1436.59522	2	1.55E-09	0.94	4.24	-	1043.9
AHQ-1-8, 7455	K.GFQQILAGEYDHLPEQAFYMVGPIEEAVAK.A	3352.76051	3	4.52E-08	0.87	4.52	-	541.4
AHQ-1-7, 7456	K.GFQQILAGEYDHLPEQAFYMVGPIEEAVAK.A	3352.76051	3	1.28E-06	0.71	3.38	-	576.3
AHQ-1-7, 7341	K.GFQQILAGEYDHLPEQAFYM*VGPIEEAVAK.A	3368.75991	3	3.99E-05	0.92	5.26	-	897.1
AHQ-1-8, 3769 - 3799	R.IMNVIGEPIDER.G	1386.59962	2	5.19E-05	0.85	3.54	-	757.6
AHQ-1-7, 4854	R.IPSAVGYQPTLATDMGTMQER.I	2267.56880	2	2.18E-04	0.88	4.03	-	419.0
AHQ-1-7, 4106	R.IPSAVGYQPTLATDM*GTMQER.I	2283.56820	2	9.27E-05	0.74	4.46	-	392.3
AHQ-1-7, 4362	R.IPSAVGYQPTLATDMGTM*QER.I	2283.56820	2	6.09E-04	0.78	4.59	-	531.6
AHQ-1-8, 4772	R.IPSAVGYQPTLATDMGTMQER.I	2267.56880	2	3.10E-05	0.84	3.52	-	474.9
AHQ-1-7, 4280	R.LVLEVAQHLGESTVR.T	1651.88893	2	2.66E-07	0.97	4.38	-	1569.3
AHQ-1-8, 4141	R.LVLEVAQHLGESTVR.T	1651.88893	2	3.81E-07	0.97	4.38	-	1650.0
AHQ-1-7, 5934 - 5936	R.VALTGLTVAEYFR.D	1440.66861	2	1.58E-05	0.98	4.41	-	2119.6
AHQ-1-8, 5675	R.VALTGLTVAEYFR.D	1440.66861	2	2.83E-04	0.89	3.26	-	1106.8
AHQ-1-8, 3301 - 3306	K.VALVYGQMNQPPGAR.A	1601.85511	2	2.29E-05	0.82	3.35	-	719.0
AHQ-1-7, 4825	K.VLDSGAPIKIPVGPETLGR.I	1920.24231	2	1.81E-07	0.90	4.43	-	397.5
AHQ-1-8, 4740	K.VLDSGAPIKIPVGPETLGR.I	1920.24231	2	1.18E-04	0.63	3.08	-	320.7
gi|4505981|ref|NP_002695.1| pro-platelet basic protein (includes platelet basic protein, bet; Pro-p				3.68E-13	7.12	80.30	40.60	13894.1
AHQ-1-14, 5392 - 5457	K.EESLDSDLYAELR.C	1540.61070	2	8.36E-08	0.68	2.85	-	586.8
AHQ-1-14, 5709 - 5769	K.EESLDSDLYAELR.C	1540.61070	2	2.00E-04	0.89	3.30	-	1086.0
AHQ-1-14- , 4740 - 4806	K.EESLDSDLYAELR.C	1540.61070	2	4.97E-09	0.97	4.92	-	1381.6
AHQ-1-14, 5604 - 5664	K.EESLDSDLYAELR.C	1540.61070	2	6.29E-09	0.97	4.87	-	1505.5
AHQ-1-14- , 4450 - 4518	K.EESLDSDLYAELR.C	1540.61070	2	2.24E-10	0.88	3.62	-	581.9
AHQ-1-14- , 4683	K.GKEESLDSDLYAELR.C	1725.83517	2	1.89E-11	0.98	6.05	-	2537.1
AHQ-1-13, 4149	K.GKEESLDSDLYAELR.C	1725.83517	3	8.21E-04	0.74	3.71	-	628.6
AHQ-1-14- , 4483	K.GKEESLDSDLYAELR.C	1725.83517	2	3.43E-05	0.87	2.76	-	1155.9
AHQ-1-14- , 4684	K.GKEESLDSDLYAELR.C	1725.83517	3	1.80E-04	0.89	3.39	-	1241.7
AHQ-1-14- , 4398 - 4466	K.GKEESLDSDLYAELR.C	1725.83517	2	5.67E-11	0.98	5.48	-	2721.8
AHQ-1-14- , 4310 - 4370	K.GKEESLDSDLYAELR.C	1725.83517	2	2.06E-11	0.98	5.66	-	2279.2
AHQ-1-14- , 4262 - 4338	K.GKEESLDSDLYAELR.C	1725.83517	3	1.36E-06	0.90	4.34	-	966.8
AHQ-1-14- , 4171 - 4244	K.GKEESLDSDLYAELR.C	1725.83517	2	1.29E-11	0.98	5.12	-	2598.2
AHQ-1-12, 4134 - 4137	K.GKEESLDSDLYAELR.C	1725.83517	2	1.42E-11	0.98	5.41	-	2082.9
AHQ-1-14- , 4087 - 4154	K.GKEESLDSDLYAELR.C	1725.83517	2	1.59E-10	0.98	5.59	-	2617.2
AHQ-1-14- , 4056 - 4060	K.GKEESLDSDLYAELR.C	1725.83517	3	2.06E-05	0.89	4.23	-	823.2
AHQ-1-14- , 3967 - 3975	K.GKEESLDSDLYAELR.C	1725.83517	3	1.38E-05	0.89	4.38	-	1019.0
AHQ-1-14- , 3966 - 4031	K.GKEESLDSDLYAELR.C	1725.83517	2	1.66E-10	0.98	6.03	-	2280.6

AHQ-1-14- , 3827	K.GKEESLDSDLYAELR.C	1725.83517	2	2.08E-06	0.97	4.65	-	1790.6
AHQ-1-13- , 4352	K.GKEESLDSDLYAELR.C	1725.83517	3	3.65E-05	0.91	3.95	-	1192.3
AHQ-1-13- , 4311 - 4345	K.GKEESLDSDLYAELR.C	1725.83517	2	9.97E-10	0.98	5.56	-	2412.7
AHQ-1-14, 5961	K.GKEESLDSDLYAELR.C	1725.83517	2	4.79E-07	0.92	3.99	-	1177.5
AHQ-1-14, 5877 - 5900	K.GKEESLDSDLYAELR.C	1725.83517	2	7.15E-07	0.96	4.27	-	1438.4
AHQ-1-14, 5750 - 5818	K.GKEESLDSDLYAELR.C	1725.83517	2	3.75E-08	0.98	5.39	-	2082.6
AHQ-1-11, 4011	K.GKEESLDSDLYAELR.C	1725.83517	2	9.33E-08	0.97	4.84	-	2247.8
AHQ-1-14, 5606 - 5678	K.GKEESLDSDLYAELR.C	1725.83517	2	3.08E-10	0.98	4.72	-	2173.7
AHQ-1-14- , 4140 - 4219	K.GKEESLDSDLYAELR.C	1725.83517	3	1.42E-05	0.95	4.95	-	1598.1
AHQ-1-14, 4816 - 4872	K.GKEESLDSDLYAELR.C	1725.83517	2	3.24E-10	0.98	5.53	-	1939.8
AHQ-1-14, 5465 - 5536	K.GKEESLDSDLYAELR.C	1725.83517	2	5.99E-08	0.97	4.51	-	2256.3
AHQ-1-14, 4844	K.GKEESLDSDLYAELR.C	1725.83517	3	2.36E-04	0.94	4.52	-	1377.1
AHQ-1-14, 4928 - 5001	K.GKEESLDSDLYAELR.C	1725.83517	2	1.93E-05	0.97	4.76	-	1939.1
AHQ-1-10, 3908	K.GKEESLDSDLYAELR.C	1725.83517	2	7.91E-10	0.95	4.03	-	1560.5
AHQ-1-14, 5045 - 5121	K.GKEESLDSDLYAELR.C	1725.83517	3	2.85E-06	0.93	4.18	-	1314.4
AHQ-1-14, 5060 - 5140	K.GKEESLDSDLYAELR.C	1725.83517	2	5.23E-11	0.98	5.52	-	2351.7
AHQ-1-14, 5317 - 5349	K.GKEESLDSDLYAELR.C	1725.83517	2	3.68E-13	0.98	5.20	-	2604.5
AHQ-1-14, 5200 - 5257	K.GKEESLDSDLYAELR.C	1725.83517	2	2.48E-11	0.98	5.86	-	2787.8
AHQ-1-14, 5174 - 5245	K.GKEESLDSDLYAELR.C	1725.83517	3	4.20E-06	0.83	3.77	-	739.3
AHQ-1-14- , 4970 - 5050	K.GKEESLDSDLYAELR.C	1725.83517	2	6.19E-09	0.91	3.84	-	1085.2
AHQ-1-14, 5229 - 5289	K.GTHCNQVEVIATLK.D	1571.78073	2	1.73E-04	0.89	3.49	-	837.3
AHQ-1-14, 5097 - 5166	K.GTHCNQVEVIATLK.D	1571.78073	2	3.60E-07	0.96	4.50	-	1483.8
AHQ-1-14, 5361 - 5377	K.GTHCNQVEVIATLK.D	1571.78073	2	6.59E-09	0.91	3.69	-	975.7
AHQ-1-14, 4974 - 4998	K.GTHCNQVEVIATLK.D	1571.78073	2	8.70E-06	0.96	4.73	-	1435.0
AHQ-1-14, 4836 - 4917	K.GTHCNQVEVIATLK.D	1571.78073	2	9.91E-08	0.98	4.85	-	2094.3
AHQ-1-14, 5493	K.GTHCNQVEVIATLK.D	1571.78073	2	5.71E-05	0.94	4.03	-	1141.8
AHQ-1-14, 4709 - 4780	K.GTHCNQVEVIATLK.D	1571.78073	2	3.98E-10	0.98	5.42	-	2279.7
AHQ-1-13, 3401 - 3415	K.GTHCNQVEVIATLK.D	1571.78073	2	2.38E-05	0.92	3.68	-	1059.0
AHQ-1-14, 4580 - 4650	K.GTHCNQVEVIATLK.D	1571.78073	2	1.07E-08	0.97	4.73	-	1882.7
AHQ-1-14, 4464 - 4521	K.GTHCNQVEVIATLK.D	1571.78073	2	1.68E-10	0.98	5.23	-	1778.9
AHQ-1-14- , 4288 - 4350	K.GTHCNQVEVIATLK.D	1571.78073	2	3.00E-05	0.97	4.29	-	1866.7
AHQ-1-14, 4368	K.GTHCNQVEVIATLK.D	1571.78073	2	2.94E-05	0.96	4.35	-	1499.4
AHQ-1-14- , 4027 - 4055	K.GTHCNQVEVIATLK.D	1571.78073	2	5.93E-08	0.98	5.23	-	2101.9
AHQ-1-13- , 3484	K.GTHCNQVEVIATLK.D	1571.78073	2	1.33E-07	0.97	4.07	-	2252.3
AHQ-1-14, 4250 - 4312	K.GTHCNQVEVIATLK.D	1571.78073	2	3.25E-05	0.96	4.60	-	1423.3
AHQ-1-14, 4336 - 4408	K.GTHCNQVEVIATLK.D	1571.78073	2	8.13E-05	0.97	4.42	-	1719.4
AHQ-1-14- , 3886 - 3960	K.GTHCNQVEVIATLK.D	1571.78073	2	7.35E-06	0.97	5.11	-	1685.5
AHQ-1-14- , 3762 - 3826	K.GTHCNQVEVIATLK.D	1571.78073	2	2.39E-06	0.96	4.78	-	1291.6
AHQ-1-14- , 3674 - 3746	K.GTHCNQVEVIATLK.D	1571.78073	2	2.95E-06	0.97	4.43	-	1626.3
AHQ-1-14- , 3607 - 3682	K.GTHCNQVEVIATLK.D	1571.78073	2	5.07E-07	0.98	5.59	-	2085.7
AHQ-1-14- , 3478 - 3554	K.GTHCNQVEVIATLK.D	1571.78073	2	2.64E-07	0.98	5.08	-	2109.4
AHQ-1-14- , 3347 - 3426	K.GTHCNQVEVIATLK.D	1571.78073	2	7.81E-08	0.97	4.74	-	1588.5
AHQ-1-14- , 3078 - 3154	K.GTHCNQVEVIATLKDGR.K	1900.10650	2	3.73E-09	0.98	6.09	-	2318.2
AHQ-1-14- , 3223	K.GTHCNQVEVIATLKDGR.K	1900.10650	2	1.93E-07	0.97	4.97	-	1196.2
AHQ-1-14- , 3224 - 3290	K.GTHCNQVEVIATLKDGR.K	1900.10650	3	2.81E-10	0.97	5.55	-	1241.2
AHQ-1-14, 3977	K.GTHCNQVEVIATLKDGR.K	1900.10650	2	3.44E-07	0.97	4.76	-	1539.2
AHQ-1-14- , 3074 - 3150	K.GTHCNQVEVIATLKDGR.K	1900.10650	3	4.57E-07	0.95	4.89	-	1399.2
AHQ-1-14, 4044 - 4122	K.GTHCNQVEVIATLKDGR.K	1900.10650	3	6.07E-07	0.92	4.04	-	1148.0
AHQ-1-14, 4076 - 4132	K.GTHCNQVEVIATLKDGR.K	1900.10650	2	4.12E-04	0.97	5.19	-	1685.0
AHQ-1-14- , 3511 - 3575	K.GTHCNQVEVIATLKDGR.K	1900.10650	3	2.24E-09	0.91	4.58	-	732.1
AHQ-1-14, 4346	K.GTHCNQVEVIATLKDGR.K	1900.10650	3	2.47E-09	0.89	4.30	-	797.4
AHQ-1-14, 4257	K.GTHCNQVEVIATLKDGR.K	1900.10650	2	9.14E-05	0.92	4.19	-	919.5
AHQ-1-14- , 3655	K.GTHCNQVEVIATLKDGR.K	1900.10650	3	1.37E-04	0.92	4.20	-	1049.7
AHQ-1-14, 3676	K.GTHCNQVEVIATLKDGRK.I	2028.27941	3	4.02E-07	0.81	3.52	-	903.5
AHQ-1-14- , 2808	K.GTHCNQVEVIATLKDGRK.I	2028.27941	2	7.34E-05	0.96	5.85	-	977.3
AHQ-1-14, 3760 - 3820	K.ICLDPDAPR.I	1058.19059	2	1.45E-05	0.84	3.16	-	848.1
AHQ-1-14- , 2562 - 2660	R.KICLDPDAPR.I	1186.36350	1	2.55E-05	0.17	2.54	-	153.9
AHQ-1-14- , 2542 - 2564	R.KICLDPDAPR.I	1186.36350	2	5.52E-05	0.86	3.31	-	846.7
AHQ-1-14- , 2748	R.KICLDPDAPR.I	1186.36350	2	7.11E-05	0.83	3.16	-	733.6
AHQ-1-14, 3516 - 3596	R.KICLDPDAPR.I	1186.36350	2	2.39E-06	0.83	3.21	-	699.1
AHQ-1-14, 3404 - 3460	R.KICLDPDAPR.I	1186.36350	2	3.10E-05	0.89	3.25	-	901.7
AHQ-1-13- , 2699	R.KICLDPDAPR.I	1186.36350	2	8.44E-06	0.92	3.50	-	1064.5
AHQ-1-14, 3802	R.KICLDPDAPR.I	1186.36350	2	7.93E-05	0.72	2.80	-	597.1
AHQ-1-14- , 2626 - 2687	R.KICLDPDAPR.I	1186.36350	2	1.65E-05	0.85	3.02	-	875.8
AHQ-1-13, 2697	R.KICLDPDAPR.I	1186.36350	2	2.07E-05	0.84	3.16	-	682.2
AHQ-1-14- , 3239 - 3302	K.NIQSLEVIGK.G	1101.27845	2	1.35E-06	0.83	3.32	-	673.1
AHQ-1-14- , 4183	K.NIQSLEVIGK.G	1101.27845	2	2.25E-04	0.94	3.35	-	1637.3
AHQ-1-14- , 4186 - 4266	K.NIQSLEVIGK.G	1101.27845	1	4.58E-04	0.82	2.67	-	798.0
AHQ-1-14, 4197 - 4261	K.NIQSLEVIGK.G	1101.27845	2	4.80E-06	0.83	3.19	-	652.1
AHQ-1-14, 4377 - 4436	K.NIQSLEVIGK.G	1101.27845	2	3.40E-04	0.79	2.66	-	778.4
AHQ-1-14- , 3559 - 3623	K.NIQSLEVIGK.G	1101.27845	2	9.58E-05	0.67	2.79	-	600.4
AHQ-1-14, 4288 - 4348	K.NIQSLEVIGK.G	1101.27845	2	1.42E-06	0.87	3.44	-	745.3
AHQ-1-14- , 3752	K.NIQSLEVIGK.G	1101.27845	2	1.97E-04	0.81	2.66	-	1145.3
AHQ-1-14- , 3714 - 3778	K.NIQSLEVIGK.G	1101.27845	1	2.26E-04	0.89	3.16	-	1130.4
AHQ-1-14, 4680 - 4682	K.NIQSLEVIGK.G	1101.27845	2	3.76E-04	0.80	2.76	-	943.0
AHQ-1-14- , 3366 - 3446	K.NIQSLEVIGK.G	1101.27845	2	2.43E-06	0.81	3.18	-	587.0
AHQ-1-12, 3298	K.NIQSLEVIGK.G	1101.27845	2	5.80E-05	0.90	3.75	-	813.6
AHQ-1-14, 5120	K.NIQSLEVIGK.G	1101.27845	1	3.16E-05	0.82	2.88	-	763.2
gi|4759082|ref|NP_004648.1| serum deprivation response protein; serum deprivation response; phospha				3.94E-13	5.33	60.27	25.20	47172.9
AHQ-1-13, 6007	R.DNSQVNAVTVLTLLDK.L	1730.94122	2	4.96E-05	0.95	4.31	-	1111.8
AHQ-1-1, 6520	R.DNSQVNAVTVLTLLDK.L	1730.94122	2	1.48E-04	0.75	3.27	-	712.3
AHQ-1-12, 6209	R.DNSQVNAVTVLTLLDK.L	1730.94122	2	1.14E-04	0.70	3.03	-	559.7
AHQ-1-12, 6078 - 6098	R.DNSQVNAVTVLTLLDK.L	1730.94122	2	2.31E-06	0.97	5.17	-	1726.7
AHQ-1-11, 5910	R.DNSQVNAVTVLTLLDK.L	1730.94122	2	6.72E-04	0.95	4.15	-	1495.2
AHQ-1-13- , 6416	R.DNSQVNAVTVLTLLDK.L	1730.94122	2	1.53E-04	0.90	3.43	-	906.3
AHQ-1-6, 6575	R.DNSQVNAVTVLTLLDK.L	1730.94122	2	3.55E-05	0.92	3.94	-	934.2
AHQ-1-9, 6526 - 6603	R.DNSQVNAVTVLTLLDK.L	1730.94122	2	2.12E-05	0.96	4.88	-	1202.5
AHQ-1-7, 6717	R.DNSQVNAVTVLTLLDK.L	1730.94122	2	5.92E-04	0.90	3.21	-	1122.9
AHQ-1-13- , 3651 - 3669	K.LVNMLDAVQENQHK.M	1639.85851	2	1.19E-04	0.90	3.82	-	857.4
AHQ-1-11, 3386 - 3446	K.LVNMLDAVQENQHK.M	1639.85851	2	7.46E-07	0.90	4.07	-	816.6
AHQ-1-9, 3382	K.LVNMLDAVQENQHK.M	1639.85851	3	1.56E-06	0.62	3.26	-	268.7
AHQ-1-9, 3891	R.QEKPSSPSPM*PSSTPSPSLNLGNTEEAIR.D	3056.30877	3	1.47E-04	0.82	4.39	-	481.3
AHQ-1-6, 3354	R.SDGDPVQPAVLQVHQTS	1778.90130	2	4.50E-07	0.74	2.85	-	607.3
AHQ-1-9, 6354 - 6422	K.VLIFQEENEIPASVFVK.Q	1963.26241	2	3.99E-07	0.96	5.28	-	968.4
AHQ-1-6, 6316 - 6334	K.VLIFQEENEIPASVFVK.Q	1963.26241	2	7.01E-06	0.95	4.83	-	829.5
AHQ-1-10, 5593	K.VLIFQEENEIPASVFVK.Q	1963.26241	2	5.10E-05	0.91	3.82	-	843.3
AHQ-1-9, 6231 - 6286	K.VLIFQEENEIPASVFVK.Q	1963.26241	2	1.10E-04	0.96	5.02	-	847.0
AHQ-1-7, 6436 - 6468	K.VLIFQEENEIPASVFVK.Q	1963.26241	2	8.53E-06	0.94	4.70	-	980.3
AHQ-1-11, 5687 - 5702	K.VLIFQEENEIPASVFVK.Q	1963.26241	2	6.17E-05	0.94	4.15	-	959.6
AHQ-1-5, 6477	K.VLIFQEENEIPASVFVK.Q	1963.26241	2	1.72E-05	0.96	5.23	-	787.4
AHQ-1-13- , 6228	K.VLIFQEENEIPASVFVK.Q	1963.26241	2	3.91E-09	0.95	4.62	-	933.6
AHQ-1-12, 5905 - 5926	K.VLIFQEENEIPASVFVK.Q	1963.26241	2	6.92E-05	0.95	5.07	-	741.0
AHQ-1-4, 6547 - 6548	K.VLIFQEENEIPASVFVK.Q	1963.26241	2	4.27E-06	0.96	5.43	-	897.1
AHQ-1-3, 6382	K.VLIFQEENEIPASVFVK.Q	1963.26241	2	5.05E-05	0.95	4.53	-	1110.9
AHQ-1-13, 5836	K.VLIFQEENEIPASVFVK.Q	1963.26241	2	5.97E-06	0.96	4.91	-	1085.5
AHQ-1-8, 6366	K.VLIFQEENEIPASVFVK.Q	1963.26241	2	9.02E-05	0.94	4.59	-	780.8
AHQ-1-1, 6337 - 6348	K.VLIFQEENEIPASVFVK.Q	1963.26241	2	4.63E-04	0.90	4.15	-	716.1

AHQ-1-14- , 6030 - 6031	K.VLIFQEENEIPASVFVK.Q	1963.26241	2	4.03E-05	0.93	4.29	-	899.4
AHQ-1-6, 2823 - 2827	R.YEGSYALTSEEAER.S	1605.64124	2	3.94E-13	0.94	3.98	-	897.5
AHQ-1-12, 2845	R.YEGSYALTSEEAER.S	1605.64124	2	1.64E-10	0.89	3.19	-	832.7
gi|23510338|ref|NP_003325.2| ubiquitin-activating enzyme E1; A1S9T and BN75 temperature sensitivity				4.11E-13	5.22	60.24	8.30	117848.1
AHQ-1-4, 4835	R.ALPAVQQNNLDEDLIR.K	1810.00167	2	4.11E-13	0.82	3.20	-	455.8
AHQ-1-4, 4739	R.ALPAVQQNNLDEDLIR.K	1810.00167	2	5.93E-06	0.89	3.81	-	603.9
AHQ-1-4, 2443	R.KPLLESGTLGTK.G	1244.46280	2	2.40E-04	0.82	3.29	-	695.8
AHQ-1-5, 5458	R.LAGTQPLEVLEAVQR.S	1624.86348	2	2.58E-07	0.85	3.48	-	540.2
AHQ-1-4, 5543	R.LAGTQPLEVLEAVQR.S	1624.86348	2	3.10E-05	0.80	3.53	-	399.2
AHQ-1-4, 3810	R.LQTSSVLVSGLR.G	1260.46546	2	4.73E-04	0.88	3.01	-	1046.2
AHQ-1-4, 6108	R.NEEDAAELVALAQAVNAR.A	1885.02541	2	1.90E-05	0.96	4.32	-	1818.2
AHQ-1-4, 4168	R.YDGQVAVFGSDLQEK.L	1656.77453	2	3.18E-06	0.89	3.26	-	940.0
gi|4506025|ref|NP_000936.1| protein phosphatase 3, regulatory subunit B, alpha isoform 1; protein p				4.27E-13	1.83	20.25	16.50	19299.8
AHQ-1-12, 4307	K.EFIEGVSQFSVK.G	1370.53201	2	1.73E-04	0.86	3.26	-	755.3
AHQ-1-12, 5333 - 5343	R.VIDIFDTDGNGEVDFK.E	1784.90107	2	4.27E-13	0.97	5.05	-	1609.8
gi|10835187|ref|NP_000627.1| superoxide dismutase 2, mitochondrial [Homo sapiens]				4.34E-13	4.36	50.24	39.60	24733.0
AHQ-1-11, 5684 - 5758	K.AIWNVINWENVTER.Y	1744.93137	2	5.55E-07	0.86	3.71	-	677.0
AHQ-1-13, 5913	K.AIWNVINWENVTER.Y	1744.93137	2	7.13E-04	0.92	3.86	-	1117.7
AHQ-1-12, 5977	K.AIWNVINWENVTER.Y	1744.93137	2	1.40E-06	0.92	3.45	-	1263.2
AHQ-1-11, 5772	K.AIWNVINWENVTER.Y	1744.93137	3	1.57E-08	0.95	4.88	-	1408.3
AHQ-1-14- , 6104	K.AIWNVINWENVTER.Y	1744.93137	2	2.87E-05	0.84	3.42	-	776.5
AHQ-1-11, 4332	K.FNGGGHINHSIFWTNLSPNGGGEPK.G	2638.83761	3	2.27E-05	0.65	3.10	-	595.3
AHQ-1-11, 4334	K.FNGGGHINHSIFWTNLSPNGGGEPK.G	2638.83761	2	5.47E-08	0.85	3.94	-	466.5
AHQ-1-11, 3694 - 3755	K.GDVTAQIALQPALK.F	1425.65548	2	6.63E-06	0.80	2.57	-	922.8
AHQ-1-11, 2419 - 2486	K.HHAAYVNNLNVTEEK.Y	1739.86999	2	5.79E-06	0.51	2.56	-	162.5
AHQ-1-11, 2546	K.HHAAYVNNLNVTEEK.Y	1739.86999	2	4.31E-07	0.85	3.27	-	733.4
AHQ-1-11, 2282 - 2355	K.HHAAYVNNLNVTEEK.Y	1739.86999	2	4.34E-13	0.97	4.86	-	1414.6
AHQ-1-11, 6555	K.LTAASVGVQGSGWGWLGFNK.E	2036.27841	2	2.05E-07	0.79	3.56	-	501.5
AHQ-1-11, 5778	K.LTAASVGVQGSGWGWLGFNK.E	2036.27841	2	8.32E-04	0.91	4.18	-	777.5
gi|4502027|ref|NP_000468.1| albumin precursor; PRO0883 protein [Homo sapiens]				5.95E-13	26.99	310.33	54.50	69366.4
AHQ-1-6, 2466	K.AAFTECCQAADK.A	1374.47927	2	1.10E-04	0.90	3.00	-	1126.2
AHQ-1-5, 2455 - 2470	K.AAFTECCQAADK.A	1374.47927	2	1.89E-07	0.90	3.33	-	1072.2
AHQ-1-6, 2480	K.AAFTECCQAADK.A	1374.47927	1	4.11E-04	0.56	2.45	-	620.9
AHQ-1-6, 7231 - 7302	K.ALVLIAFAQYLQQCPFEDHVK.L	2492.87697	3	1.32E-09	0.96	5.14	-	1310.3
AHQ-1-5, 7415	K.ALVLIAFAQYLQQCPFEDHVK.L	2492.87697	3	2.24E-11	0.95	4.88	-	1236.4
AHQ-1-6, 4478 - 4548	K.AVM*DDFAAFVEK.C	1359.52946	2	3.05E-08	0.93	3.32	-	1216.7
AHQ-1-13- , 5579	K.AVMDDFAAFVEK.C	1343.53006	2	1.48E-06	0.94	3.70	-	1181.5
AHQ-1-3, 5674	K.AVMDDFAAFVEK.C	1343.53006	2	1.14E-04	0.87	3.56	-	757.3
AHQ-1-5, 4631	K.AVM*DDFAAFVEK.C	1359.52946	2	2.36E-05	0.96	3.97	-	1562.9
AHQ-1-6, 5578	K.AVMDDFAAFVEK.C	1343.53006	2	4.75E-10	0.96	4.04	-	1460.5
AHQ-1-6, 5583 - 5586	K.AVMDDFAAFVEK.C	1343.53006	1	3.61E-04	0.77	2.40	-	1137.4
AHQ-1-5, 5665 - 5666	K.AVMDDFAAFVEK.C	1343.53006	2	2.00E-09	0.95	4.09	-	1314.6
AHQ-1-14- , 5428	K.AVMDDFAAFVEK.C	1343.53006	2	1.90E-07	0.95	4.14	-	1267.4
AHQ-1-6, 2110	K.CCAAADPHECYAK.V	1556.67999	2	2.65E-07	0.90	3.79	-	734.6
AHQ-1-5, 2378	K.CCTESLVNR.R	1141.25805	2	2.62E-04	0.82	2.67	-	972.8
AHQ-1-6, 6932 - 6955	K.DVFLGM*FLYEYAR.R	1640.88331	2	1.74E-06	0.96	4.69	-	970.2
AHQ-1-5, 2573	R.FKDLGEENFK.A	1227.34766	2	2.37E-04	0.83	3.06	-	829.5
AHQ-1-14- , 2699 - 2759	R.FKDLGEENFK.A	1227.34766	2	5.96E-05	0.65	2.70	-	498.7
AHQ-1-5, 2877	K.FQNALLVR.Y	961.14282	2	8.39E-06	0.87	2.74	-	898.2
AHQ-1-6, 2899 - 2930	K.FQNALLVR.Y	961.14282	2	3.80E-04	0.91	2.98	-	1166.2
AHQ-1-6, 6670 - 6686	R.HPYFYAPELLFFAK.R	1744.02658	2	2.27E-05	0.50	2.52	-	266.8
AHQ-1-6, 6682	R.HPYFYAPELLFFAK.R	1744.02658	3	1.91E-07	0.95	4.59	-	1097.1
AHQ-1-5, 6786	R.HPYFYAPELLFFAK.R	1744.02658	3	9.38E-06	0.91	3.90	-	1022.4
AHQ-1-5, 2522	K.KQTALVELVK.H	1129.37494	1	1.17E-04	0.24	2.26	-	347.4
AHQ-1-5, 3211	K.KVPQVSTPTLVEVSR.N	1640.90605	2	2.46E-08	0.86	3.17	-	606.4
AHQ-1-5, 3490	K.KVPQVSTPTLVEVSR.N	1640.90605	2	4.36E-08	0.88	3.43	-	616.6
AHQ-1-9, 3339	K.KVPQVSTPTLVEVSR.N	1640.90605	2	2.90E-05	0.71	2.67	-	530.3
AHQ-1-10, 3280 - 3344	K.KVPQVSTPTLVEVSR.N	1640.90605	2	9.50E-08	0.62	2.88	-	438.9
AHQ-1-2, 3806	K.KVPQVSTPTLVEVSR.N	1640.90605	2	3.76E-07	0.93	3.91	-	832.7
AHQ-1-6, 3450	K.KVPQVSTPTLVEVSR.N	1640.90605	3	2.77E-06	0.95	4.73	-	1524.0
AHQ-1-6, 3448 - 3455	K.KVPQVSTPTLVEVSR.N	1640.90605	2	2.70E-08	0.95	4.08	-	1315.0
AHQ-1-6, 3186	K.KVPQVSTPTLVEVSR.N	1640.90605	2	3.27E-05	0.74	2.88	-	439.1
AHQ-1-5, 3494	K.KVPQVSTPTLVEVSR.N	1640.90605	3	1.65E-08	0.96	5.18	-	1655.2
AHQ-1-4, 3690	K.KVPQVSTPTLVEVSR.N	1640.90605	2	6.17E-06	0.85	3.23	-	652.8
AHQ-1-5, 2265 - 2293	K.LKECCEKPLLEK.S	1549.83503	2	1.36E-04	0.91	3.76	-	1062.7
AHQ-1-6, 2316 - 2336	K.LKECCEKPLLEK.S	1549.83503	2	2.37E-04	0.93	4.32	-	996.4
AHQ-1-5, 3262 - 3270	K.LVNEVTEFAK.T	1150.30633	2	2.54E-06	0.86	3.23	-	705.3
AHQ-1-3, 3374	K.LVNEVTEFAK.T	1150.30633	2	1.00E-05	0.86	3.28	-	739.4
AHQ-1-1, 3481	K.LVNEVTEFAK.T	1150.30633	2	9.73E-06	0.78	2.72	-	674.1
AHQ-1-6, 3140	K.LVNEVTEFAK.T	1150.30633	1	2.83E-06	0.50	2.27	-	408.6
AHQ-1-13, 3329 - 3335	K.LVNEVTEFAK.T	1150.30633	2	1.54E-05	0.73	3.03	-	412.3
AHQ-1-6, 5871	R.LVRPEVDVMCTAFHDNEETFLK.K	2652.98428	3	5.30E-04	0.97	5.49	-	1938.1
AHQ-1-6, 5439	R.LVRPEVDVMCTAFHDNEETFLKK.Y	2781.15719	3	9.42E-10	0.97	6.27	-	1467.0
AHQ-1-5, 5490 - 5519	R.LVRPEVDVMCTAFHDNEETFLKK.Y	2781.15719	3	1.15E-09	0.98	6.53	-	1908.8
AHQ-1-6, 5124	K.QNCELFEQLGEYK.F	1659.79847	2	3.66E-05	0.84	3.36	-	613.2
AHQ-1-6, 3982 - 4011	R.RHPDYSVVLLLR.L	1468.72842	2	3.78E-08	0.93	3.32	-	1315.3
AHQ-1-5, 4038 - 4105	R.RHPDYSVVLLLR.L	1468.72842	2	2.21E-04	0.75	2.88	-	576.4
AHQ-1-3, 4209	R.RHPDYSVVLLLR.L	1468.72842	2	1.47E-04	0.88	3.14	-	905.6
AHQ-1-6, 6098	R.RHPYFYAPELLFFAK.R	1900.21293	2	5.36E-06	0.98	5.14	-	2994.7
AHQ-1-7, 6125	R.RHPYFYAPELLFFAK.R	1900.21293	2	6.78E-05	0.95	4.40	-	1187.8
AHQ-1-5, 6187	R.RHPYFYAPELLFFAK.R	1900.21293	3	1.26E-11	0.97	5.53	-	1900.6
AHQ-1-6, 6095	R.RHPYFYAPELLFFAK.R	1900.21293	3	1.37E-09	0.98	5.54	-	2051.3
AHQ-1-6, 7222 - 7223	K.RMPCAEDYLSVVLNQLCVLHEK.T	2678.10170	3	1.48E-04	0.98	6.68	-	1660.2
AHQ-1-5, 7405 - 7406	K.RMPCAEDYLSVVLNQLCVLHEK.T	2678.10170	3	2.88E-06	0.97	6.08	-	1742.1
AHQ-1-6, 7187 - 7188	K.RM*PCAEDYLSVVLNQLCVLHEK.T	2694.10110	3	3.11E-05	0.96	5.65	-	1361.2
AHQ-1-6, 4754 - 4822	R.RPCFSALEVDETYVPK.E	1913.14041	3	1.15E-08	0.89	4.09	-	771.0
AHQ-1-4, 4911 - 4980	R.RPCFSALEVDETYVPK.E	1913.14041	2	1.18E-04	0.70	3.39	-	380.4
AHQ-1-10, 4320 - 4377	R.RPCFSALEVDETYVPK.E	1913.14041	2	2.53E-04	0.90	4.12	-	613.4
AHQ-1-7, 4817	R.RPCFSALEVDETYVPK.E	1913.14041	2	2.79E-05	0.69	3.22	-	380.1
AHQ-1-13- , 4844	R.RPCFSALEVDETYVPK.E	1913.14041	3	7.19E-04	0.87	3.87	-	791.5
AHQ-1-3, 4893	R.RPCFSALEVDETYVPK.E	1913.14041	2	9.69E-04	0.80	3.99	-	519.8
AHQ-1-5, 4827	R.RPCFSALEVDETYVPK.E	1913.14041	2	1.62E-04	0.89	4.32	-	482.8
AHQ-1-6, 4748	R.RPCFSALEVDETYVPK.E	1913.14041	2	7.72E-04	0.89	3.66	-	774.3
AHQ-1-11, 4471	R.RPCFSALEVDETYVPK.E	1913.14041	2	7.10E-06	0.74	3.42	-	359.4
AHQ-1-7, 7140	K.SHCIAEVENDEMPADLPSLAADFVESK.D	2977.22697	3	1.39E-04	0.92	4.87	-	774.0
AHQ-1-5, 7115 - 7121	K.SHCIAEVENDEMPADLPSLAADFVESK.D	2977.22697	2	1.42E-04	0.95	4.84	-	1172.7
AHQ-1-6, 6906 - 6974	K.SHCIAEVENDEMPADLPSLAADFVESK.D	2977.22697	3	1.00E-09	0.95	5.63	-	970.2
AHQ-1-3, 7018 - 7081	K.SHCIAEVENDEMPADLPSLAADFVESK.D	2977.22697	3	1.16E-04	0.81	4.47	-	300.8
AHQ-1-5, 7161 - 7177	K.SHCIAEVENDEMPADLPSLAADFVESK.D	2977.22697	3	2.43E-04	0.93	5.01	-	958.6
AHQ-1-5, 7075 - 7151	K.SHCIAEVENDEMPADLPSLAADFVESK.D	2977.22697	3	9.47E-10	0.95	5.84	-	705.0
AHQ-1-5, 6589	K.SHCIAEVENDEM*PADLPSLAADFVESK.D	2993.22637	2	9.48E-05	0.95	4.62	-	1107.0
AHQ-1-5, 2501	K.SLHTLFGDK.L	1018.14800	1	2.28E-05	0.56	2.08	-	606.7
AHQ-1-5, 5150	K.SLHTLFGDKLCTVATLR.E	1934.24913	2	7.51E-05	0.88	3.30	-	889.2
AHQ-1-5, 6051	K.VFDEFKPLVEEPQNLIK.Q	2046.35142	2	3.17E-08	0.92	4.40	-	546.0
AHQ-1-3, 5993	K.VFDEFKPLVEEPQNLIK.Q	2046.35142	2	2.97E-05	0.87	3.52	-	607.5
AHQ-1-5, 5857	K.VFDEFKPLVEEPQNLIK.Q	2046.35142	2	2.24E-04	0.91	4.51	-	532.3
AHQ-1-2, 6170	K.VFDEFKPLVEEPQNLIK.Q	2046.35142	2	5.99E-06	0.90	4.28	-	439.4

AHQ-1-5, 6053	K.VFDEFKPLVEEPQNLIK.Q	2046.35142	3	1.80E-06	0.97	5.52	-	1555.5
AHQ-1-6, 5914 - 5915	K.VFDEFKPLVEEPQNLIK.Q	2046.35142	3	5.38E-06	0.97	5.37	-	1440.5
AHQ-1-1, 5977	K.VFDEFKPLVEEPQNLIK.Q	2046.35142	2	1.91E-05	0.86	3.43	-	489.6
AHQ-1-9, 5812	K.VFDEFKPLVEEPQNLIK.Q	2046.35142	2	7.77E-05	0.80	3.79	-	355.6
AHQ-1-4, 6106	K.VFDEFKPLVEEPQNLIK.Q	2046.35142	2	1.66E-04	0.79	3.70	-	349.2
AHQ-1-7, 6004	K.VFDEFKPLVEEPQNLIK.Q	2046.35142	2	5.17E-05	0.70	3.44	-	240.3
AHQ-1-7, 6024	K.VFDEFKPLVEEPQNLIK.Q	2046.35142	3	1.50E-10	0.96	5.06	-	1568.4
AHQ-1-6, 5878 - 5946	K.VFDEFKPLVEEPQNLIK.Q	2046.35142	2	5.95E-13	0.92	4.70	-	467.3
AHQ-1-5, 3017 - 3051	K.VHTECCHGDLLECADDR.A	2091.20307	2	9.21E-07	0.93	4.14	-	878.3
AHQ-1-5, 3762	K.VHTECCHGDLLECADDRADLAK.Y	2589.77855	2	5.56E-06	0.66	2.97	-	474.7
AHQ-1-5, 3745	K.VHTECCHGDLLECADDRADLAK.Y	2589.77855	3	6.24E-05	0.89	4.43	-	602.7
AHQ-1-6, 3910	K.VPQVSTPTLVEVSR.N	1512.73313	2	3.00E-07	0.86	3.15	-	827.7
AHQ-1-5, 3961	K.VPQVSTPTLVEVSR.N	1512.73313	2	2.15E-04	0.88	3.22	-	814.2
AHQ-1-5, 2297 - 2298	K.YICENQDSISSK.L	1445.53420	2	3.81E-09	0.93	3.61	-	947.3
AHQ-1-7, 2341	K.YICENQDSISSK.L	1445.53420	2	8.05E-05	0.82	3.14	-	756.3
AHQ-1-6, 2420	K.YICENQDSISSK.L	1445.53420	2	4.42E-04	0.92	3.89	-	767.2
AHQ-1-6, 2314	K.YICENQDSISSK.L	1445.53420	1	1.42E-07	0.53	2.79	-	283.0
AHQ-1-6, 2307	K.YICENQDSISSK.L	1445.53420	2	4.28E-06	0.92	3.72	-	656.3
AHQ-1-1, 2493	K.YICENQDSISSK.L	1445.53420	2	1.01E-05	0.83	3.02	-	717.6
gi|6912638|ref|NP_036557.1| ras suppressor protein 1 [Homo sapiens]				5.99E-13	6.12	70.34	45.80	31540.1
AHQ-1-10, 6395 - 6396	K.AENNPWVTPIADQFQLGVSHVFEYIR.S	3032.35678	3	1.16E-06	0.86	3.93	-	610.7
AHQ-1-10, 5735 - 5791	R.ALYLSDNDFEILPPDIGK.L	2021.25559	2	1.63E-05	0.93	4.72	-	491.7
AHQ-1-10, 5685 - 5689	R.ALYLSDNDFEILPPDIGK.L	2021.25559	3	7.29E-06	0.88	4.41	-	713.6
AHQ-1-10, 5604 - 5679	R.ALYLSDNDFEILPPDIGK.L	2021.25559	2	2.15E-10	0.93	4.65	-	594.0
AHQ-1-13- , 6272 - 6335	R.ALYLSDNDFEILPPDIGK.L	2021.25559	2	8.03E-06	0.89	4.32	-	443.3
AHQ-1-10, 6375	R.GISNMLDVNGLFTLSHITQLVLSHNK.L	2853.28963	3	8.46E-09	0.60	3.61	-	361.9
AHQ-1-10, 6376	R.GISNMLDVNGLFTLSHITQLVLSHNK.L	2853.28963	2	1.93E-08	0.86	4.15	-	334.9
AHQ-1-10, 6313 - 6317	R.GISNM*LDVNGLFTLSHITQLVLSHNK.L	2869.28903	3	1.17E-11	0.90	4.80	-	674.5
AHQ-1-11, 5440 - 5511	K.LQILSLRDNDLISLPK.E	1839.16919	2	3.40E-04	0.54	3.18	-	663.9
AHQ-1-10, 5296	K.LQILSLRDNDLISLPK.E	1839.16919	2	7.06E-07	0.94	5.16	-	1362.3
AHQ-1-10, 5379 - 5453	R.LTVLPPELGNLDLTGQK.Q	1809.09672	2	9.88E-05	0.96	4.60	-	965.2
AHQ-1-10, 5389	R.LTVLPPELGNLDLTGQK.Q	1809.09672	3	3.72E-07	0.96	5.59	-	1247.1
AHQ-1-10, 5492 - 5561	R.LTVLPPELGNLDLTGQK.Q	1809.09672	2	3.10E-07	0.87	3.79	-	453.5
AHQ-1-11, 5514 - 5570	R.LTVLPPELGNLDLTGQK.Q	1809.09672	2	2.04E-04	0.84	3.67	-	384.3
AHQ-1-13- , 6012 - 6083	R.LTVLPPELGNLDLTGQK.Q	1809.09672	2	2.45E-05	0.93	4.02	-	777.9
AHQ-1-12, 5734 - 5793	R.LTVLPPELGNLDLTGQK.Q	1809.09672	2	4.16E-04	0.52	3.00	-	262.0
AHQ-1-12, 5737	R.LTVLPPELGNLDLTGQK.Q	1809.09672	2	5.60E-10	0.91	4.04	-	499.8
AHQ-1-10, 6291 - 6361	K.NLEVLNFFNNQIEELPTQISSLQK.L	2820.14663	2	9.56E-05	0.87	4.21	-	375.2
AHQ-1-11, 6447	K.NLEVLNFFNNQIEELPTQISSLQK.L	2820.14663	3	5.99E-13	0.97	6.85	-	1300.3
gi|4503571|ref|NP_001419.1| enolase 1; phosphopyruvate hydratase; MYC promoter-binding protein 1; n				6.19E-13	13.19	160.38	45.20	47168.7
AHQ-1-14- , 5299 - 5300	R.AAVPSGASTGIYEALELR.D	1806.00979	2	4.54E-10	0.87	4.18	-	327.2
AHQ-1-13- , 5441 - 5451	R.AAVPSGASTGIYEALELR.D	1806.00979	2	6.35E-06	0.96	4.99	-	946.0
AHQ-1-12, 5169	R.AAVPSGASTGIYEALELR.D	1806.00979	2	1.99E-05	0.88	4.18	-	477.5
AHQ-1-8, 5274 - 5344	R.AAVPSGASTGIYEALELR.D	1806.00979	2	2.62E-06	0.92	3.93	-	620.9
AHQ-1-13, 5139	R.AAVPSGASTGIYEALELR.D	1806.00979	2	3.59E-10	0.95	5.23	-	526.3
AHQ-1-14, 6216 - 6286	R.AAVPSGASTGIYEALELR.D	1806.00979	2	7.65E-08	0.94	4.21	-	918.0
AHQ-1-11, 4970	R.AAVPSGASTGIYEALELR.D	1806.00979	2	1.00E-04	0.83	3.69	-	375.5
AHQ-1-8, 4679 - 4754	K.DATNVGDEGGFAPNILENK.E	1962.06350	2	1.54E-08	0.88	4.18	-	898.7
AHQ-1-8, 6231	K.DATNVGDEGGFAPNILENKEGLELLK.T	2744.99178	2	6.19E-13	0.81	4.72	-	533.0
AHQ-1-11, 6284	K.DYPVVSIEDPFDQDDWGAWQK.F	2511.63977	2	9.94E-06	0.54	2.64	-	429.7
AHQ-1-8, 4782	K.FTASAGIQVVGDDLTVTNPK.R	2034.25590	2	1.52E-07	0.92	4.24	-	948.5
AHQ-1-13- , 4381 - 4384	R.GNPTVEVDLFTSK.G	1407.55065	1	8.59E-04	0.32	2.61	-	439.0
AHQ-1-8, 4196	R.GNPTVEVDLFTSK.G	1407.55065	1	4.86E-04	0.24	1.98	-	564.2
AHQ-1-8, 4203 - 4204	R.GNPTVEVDLFTSK.G	1407.55065	2	1.04E-07	0.82	3.36	-	749.9
AHQ-1-8, 4205	R.GNPTVEVDLFTSK.G	1407.55065	1	9.86E-09	0.52	3.26	-	455.0
AHQ-1-8, 6188	R.HIADLAGNSEVILPVPAFNVINGGSHAGNK.L	3013.35654	3	1.84E-08	0.96	5.55	-	1277.7
AHQ-1-8, 2140	R.IGAEVYHNLK.N	1144.30502	2	5.24E-05	0.94	3.92	-	1249.4
AHQ-1-8, 5570	K.LAM*QEFM*ILPVGAANFR.E	1941.30692	2	1.30E-06	0.96	4.99	-	961.9
AHQ-1-8, 6491	K.LAMQEFM*ILPVGAANFR.E	1925.30752	2	1.29E-04	0.91	4.63	-	741.1
AHQ-1-9, 6099 - 6167	K.LAM*QEFMILPVGAANFR.E	1925.30752	2	1.12E-04	0.49	3.18	-	412.2
AHQ-1-9, 6963	K.LAMQEFMILPVGAANFR.E	1909.30812	2	1.76E-04	0.82	3.98	-	395.4
AHQ-1-8, 7082	K.LAMQEFMILPVGAANFR.E	1909.30812	2	6.73E-04	0.93	4.76	-	632.0
AHQ-1-8, 3133 - 3220	K.LAQANGWGVMVSHR.S	1526.74832	2	1.70E-04	0.66	2.59	-	964.8
AHQ-1-8, 3038 - 3042	K.LMIEMDGTENK.S	1281.48208	2	4.23E-04	0.90	3.68	-	705.7
AHQ-1-8, 7111	K.SFIKDYPVVSIEDPFDQDDWGAWQK.F	2987.22328	3	3.73E-10	0.98	7.57	-	2481.9
AHQ-1-8, 7104	K.SFIKDYPVVSIEDPFDQDDWGAWQK.F	2987.22328	2	3.68E-05	0.63	3.30	-	468.3
AHQ-1-8, 4866 - 4882	R.YISPDQLADLYK.S	1426.59557	2	2.63E-09	0.96	4.94	-	871.5
AHQ-1-8, 4876 - 4955	R.YISPDQLADLYK.S	1426.59557	1	8.65E-06	0.67	3.13	-	501.0
AHQ-1-9, 4878	R.YISPDQLADLYK.S	1426.59557	2	3.51E-07	0.87	3.38	-	700.4
gi|4758086|ref|NP_004069.1| cysteine and glycine-rich protein 1; cysteine-rich protein; LIM-domain				6.68E-13	5.59	60.25	45.60	20567.3
AHQ-1-11, 4030 - 4095	K.GFGFGQGAGALVHSE	1434.53788	2	3.09E-07	0.93	3.41	-	1163.4
AHQ-1-13, 4185	K.GFGFGQGAGALVHSE	1434.53788	2	4.46E-04	0.74	2.98	-	583.3
AHQ-1-13- , 4377	K.GFGFGQGAGALVHSE	1434.53788	2	1.89E-05	0.86	3.28	-	811.3
AHQ-1-12, 3315	K.GLESTTLADKDGEIYCK.G	1902.06957	2	1.57E-06	0.97	4.98	-	1473.6
AHQ-1-11, 3230	K.GLESTTLADKDGEIYCK.G	1902.06957	3	8.27E-06	0.92	4.59	-	814.3
AHQ-1-11, 3162 - 3210	K.GLESTTLADKDGEIYCK.G	1902.06957	2	1.26E-07	0.96	5.09	-	1263.2
AHQ-1-13- , 2685	K.GYGYGQGAGTLSTDK.G	1475.54194	2	1.67E-04	0.86	3.12	-	839.0
AHQ-1-11, 2522	K.GYGYGQGAGTLSTDK.G	1475.54194	2	3.20E-07	0.95	3.97	-	1337.5
AHQ-1-13- , 3737	K.GYGYGQGAGTLSTDKGESLGIK.H	2160.32665	2	8.31E-09	0.90	4.40	-	608.2
AHQ-1-12, 3526	K.GYGYGQGAGTLSTDKGESLGIK.H	2160.32665	2	9.10E-11	0.94	4.59	-	1085.1
AHQ-1-13, 3605	K.GYGYGQGAGTLSTDKGESLGIK.H	2160.32665	2	5.88E-10	0.78	3.57	-	465.6
AHQ-1-11, 3358 - 3434	K.GYGYGQGAGTLSTDKGESLGIK.H	2160.32665	3	8.57E-07	0.72	3.04	-	765.3
AHQ-1-13- , 3739	K.GYGYGQGAGTLSTDKGESLGIK.H	2160.32665	3	4.34E-10	0.76	3.04	-	675.6
AHQ-1-11, 3430 - 3438	K.GYGYGQGAGTLSTDKGESLGIK.H	2160.32665	2	3.24E-11	0.89	3.83	-	970.7
AHQ-1-11, 3391 - 3392	K.NLDSTTVAVHGEEIYCK.S	1938.10514	2	6.68E-13	0.96	4.38	-	1119.3
AHQ-1-12, 3493	K.NLDSTTVAVHGEEIYCK.S	1938.10514	2	6.53E-04	0.91	3.91	-	907.8
AHQ-1-11, 3840	K.TVYFAEEVQCEGNSFHK.S	2047.19015	2	3.67E-09	0.89	4.08	-	647.3
gi|20535366|ref|XP_053177.4| similar to Tubulin alpha-3/alpha-7 chain (Alpha-tubulin 3/7) [Homo sap				7.43E-13	2.73	30.25	9.60	49858.2
AHQ-1-12, 3074	K.DVNAAIATIK.T	1016.17355	2	6.55E-05	0.94	3.41	-	1559.5
AHQ-1-8, 2882	K.DVNAAIATIK.T	1016.17355	1	1.41E-04	0.15	1.88	-	398.8
AHQ-1-7, 3280	K.DVNAAIATIK.T	1016.17355	1	3.24E-04	0.44	2.11	-	491.7
AHQ-1-7, 3114	K.DVNAAIATIK.T	1016.17355	2	8.46E-04	0.87	3.49	-	969.0
AHQ-1-4, 5472	K.TIGGGDDSFNTFFSETGAGK.H	2009.07549	2	7.57E-06	0.88	3.33	-	975.1
AHQ-1-3, 5363	K.TIGGGDDSFNTFFSETGAGK.H	2009.07549	2	8.97E-06	0.69	2.96	-	607.1
AHQ-1-7, 5130 - 5201	K.TIGGGDDSFNTFFSETGAGK.H	2009.07549	2	9.34E-05	0.76	3.57	-	399.8
AHQ-1-7, 5361 - 5433	K.TIGGGDDSFNTFFSETGAGK.H	2009.07549	2	1.95E-10	0.95	4.62	-	1108.8
AHQ-1-7, 5468	K.TIGGGDDSFNTFFSETGAGK.H	2009.07549	2	3.70E-09	0.94	4.65	-	904.4
AHQ-1-7, 5614	K.TIGGGDDSFNTFFSETGAGK.H	2009.07549	2	1.04E-09	0.93	4.90	-	625.2
AHQ-1-7, 4976	K.TIGGGDDSFNTFFSETGAGK.H	2009.07549	2	1.65E-06	0.80	3.55	-	374.5
AHQ-1-6, 5303	K.TIGGGDDSFNTFFSETGAGK.H	2009.07549	2	3.68E-07	0.88	3.69	-	843.0
AHQ-1-9, 5230	K.TIGGGDDSFNTFFSETGAGK.H	2009.07549	2	5.83E-07	0.86	3.74	-	625.1
AHQ-1-10, 4736 - 4811	K.TIGGGDDSFNTFFSETGAGK.H	2009.07549	2	1.29E-04	0.28	2.93	-	345.3
AHQ-1-11, 4538	K.TIGGGDDSFNTFFSETGAGK.H	2009.07549	2	1.31E-05	0.89	3.99	-	476.7
AHQ-1-1, 5425	K.TIGGGDDSFNTFFSETGAGK.H	2009.07549	2	1.41E-07	0.94	4.80	-	739.1
AHQ-1-12, 5053	K.TIGGGDDSFNTFFSETGAGK.H	2009.07549	2	1.64E-07	0.91	4.53	-	485.2
AHQ-1-14- , 5148 - 5211	K.TIGGGDDSFNTFFSETGAGK.H	2009.07549	2	7.43E-13	0.95	5.09	-	791.3
AHQ-1-13, 5020 - 5084	K.TIGGGDDSFNTFFSETGAGK.H	2009.07549	2	4.49E-05	0.82	3.44	-	681.5

AHQ-1-14, 5981 - 6040	K.TIGGGDDSFNTFFSETGAGK.H	2009.07549	2	7.51E-04	0.40	2.98	-	281.2
AHQ-1-14, 6100 - 6156	K.TIGGGDDSFNTFFSETGAGK.H	2009.07549	2	8.22E-05	0.64	2.94	-	458.6
AHQ-1-14, 6281	K.TIGGGDDSFNTFFSETGAGK.H	2009.07549	2	2.30E-08	0.74	3.56	-	457.8
AHQ-1-14- , 5371	K.TIGGGDDSFNTFFSETGAGK.H	2009.07549	2	9.22E-07	0.80	3.24	-	717.4
AHQ-1-12, 5514 - 5573	R.TIQFVDWCPTGFK.V	1600.81899	2	1.42E-05	0.84	3.72	-	647.5
gi|4503631|ref|NP_000120.1| coagulation factor XIII A1 subunit precursor; Coagulation factor XIII,				7.59E-13	24.00	280.29	41.90	83232.8
AHQ-1-5, 5423 - 5545	R.AVPPNNSNAAEDDLPTVELQGVVPR.G	2603.82656	2	5.45E-05	0.39	3.01	-	133.2
AHQ-1-5, 2235 - 2257	R.CGPASVQAIK.H	1032.19627	2	3.11E-05	0.91	2.96	-	1177.5
AHQ-1-5, 2513	R.CLGIPAR.I	787.95073	1	3.10E-04	0.49	2.17	-	333.7
AHQ-1-5, 2477	K.DGTHVVENVDATHIGK.L	1692.81163	3	4.46E-07	0.90	4.11	-	1012.1
AHQ-1-6, 2499	K.DGTHVVENVDATHIGK.L	1692.81163	3	3.76E-05	0.88	4.03	-	914.8
AHQ-1-6, 2500	K.DGTHVVENVDATHIGK.L	1692.81163	2	1.21E-05	0.83	3.59	-	620.0
AHQ-1-9, 2472	K.DGTHVVENVDATHIGK.L	1692.81163	2	8.64E-13	0.95	4.39	-	1220.3
AHQ-1-5, 2619	K.DGTHVVENVDATHIGK.L	1692.81163	2	4.60E-05	0.94	3.99	-	1272.8
AHQ-1-5, 2473	K.DGTHVVENVDATHIGK.L	1692.81163	2	3.53E-10	0.94	4.13	-	1073.4
AHQ-1-9, 4339	R.EIRPNSTVQWEEVCRPWVSGHR.K	2724.99524	3	2.51E-04	0.75	3.59	-	436.2
AHQ-1-5, 5927 - 6001	K.ETFDVTLEPLSFK.K	1526.71190	2	4.10E-09	0.93	4.05	-	860.9
AHQ-1-9, 5746 - 5748	K.ETFDVTLEPLSFK.K	1526.71190	2	2.75E-06	0.74	2.96	-	642.0
AHQ-1-10, 5187	K.ETFDVTLEPLSFK.K	1526.71190	2	2.42E-06	0.92	3.47	-	1047.0
AHQ-1-11, 5287 - 5334	K.ETFDVTLEPLSFK.K	1526.71190	2	1.32E-07	0.91	3.61	-	948.1
AHQ-1-5, 7194	R.GTQVVGSDMTVTVQFTNPLK.E	2123.41609	2	2.95E-08	0.90	3.78	-	1080.5
AHQ-1-5, 6986	R.GTQVVGSDMTVTVQFTNPLK.E	2123.41609	2	1.05E-05	0.91	4.00	-	720.6
AHQ-1-5, 6803	R.GTQVVGSDMTVTVQFTNPLK.E	2123.41609	2	2.90E-11	0.98	4.79	-	2254.5
AHQ-1-5, 6598 - 6667	R.GTQVVGSDMTVTVQFTNPLK.E	2123.41609	2	7.25E-05	0.92	4.03	-	938.7
AHQ-1-3, 5863	K.GTYIPVPIVSELQSGK.W	1688.94594	2	5.31E-06	0.64	2.60	-	403.6
AHQ-1-5, 5765 - 5833	K.GTYIPVPIVSELQSGK.W	1688.94594	2	3.81E-04	0.83	3.25	-	495.0
AHQ-1-12, 5439	K.GTYIPVPIVSELQSGK.W	1688.94594	2	2.73E-05	0.66	2.84	-	352.6
AHQ-1-13- , 5724 - 5729	K.GTYIPVPIVSELQSGK.W	1688.94594	2	2.28E-04	0.59	2.90	-	239.0
AHQ-1-6, 5736 - 5739	K.GTYIPVPIVSELQSGK.W	1688.94594	2	1.60E-04	0.73	3.19	-	319.3
AHQ-1-7, 7252 - 7257	R.GVNLQEFLNVTSVHLFK.E	1946.23831	2	4.28E-08	0.97	5.12	-	1431.7
AHQ-1-8, 7158	R.GVNLQEFLNVTSVHLFK.E	1946.23831	2	6.92E-06	0.95	4.74	-	959.2
AHQ-1-12, 6513	R.GVNLQEFLNVTSVHLFK.E	1946.23831	2	2.90E-07	0.95	4.65	-	1125.5
AHQ-1-14- , 6594 - 6595	R.GVNLQEFLNVTSVHLFK.E	1946.23831	2	4.07E-08	0.97	5.84	-	1500.8
AHQ-1-10, 6235	R.GVNLQEFLNVTSVHLFK.E	1946.23831	2	5.62E-08	0.95	5.30	-	919.5
AHQ-1-5, 7321	R.GVNLQEFLNVTSVHLFK.E	1946.23831	2	1.32E-08	0.96	4.95	-	1653.1
AHQ-1-11, 6307	R.GVNLQEFLNVTSVHLFK.E	1946.23831	2	1.12E-07	0.96	5.46	-	1103.9
AHQ-1-6, 7086 - 7094	R.GVNLQEFLNVTSVHLFK.E	1946.23831	2	4.70E-09	0.95	4.59	-	992.5
AHQ-1-5, 7209 - 7219	R.GVNLQEFLNVTSVHLFK.E	1946.23831	3	1.66E-07	0.86	3.81	-	869.6
AHQ-1-13- , 6843	R.GVNLQEFLNVTSVHLFK.E	1946.23831	3	4.60E-06	0.89	4.04	-	707.0
AHQ-1-9, 7038 - 7039	R.GVNLQEFLNVTSVHLFK.E	1946.23831	2	4.21E-08	0.96	4.90	-	1131.7
AHQ-1-2, 7265	R.GVNLQEFLNVTSVHLFK.E	1946.23831	2	1.63E-06	0.89	3.84	-	980.2
AHQ-1-1, 6908	R.GVNLQEFLNVTSVHLFK.E	1946.23831	3	4.08E-07	0.80	3.39	-	692.7
AHQ-1-5, 7186 - 7253	R.GVNLQEFLNVTSVHLFK.E	1946.23831	2	4.56E-06	0.96	4.92	-	1314.6
AHQ-1-5, 6863 - 6939	R.IVTNYFSAHDNDANLQMDIFLEEDGNVNSK.L	3415.64559	3	1.58E-05	0.94	5.77	-	902.7
AHQ-1-5, 7051 - 7118	R.IVTNYFSAHDNDANLQMDIFLEEDGNVNSK.L	3415.64559	3	1.64E-07	0.96	5.46	-	1568.9
AHQ-1-5, 2089 - 2101	K.KDGTHVVENVDATHIGK.L	1820.98455	3	5.38E-04	0.95	4.04	-	1815.0
AHQ-1-5, 2094 - 2095	K.KDGTHVVENVDATHIGK.L	1820.98455	2	2.22E-09	0.97	4.89	-	1576.8
AHQ-1-5, 2221 - 2289	K.KDGTHVVENVDATHIGK.L	1820.98455	2	7.59E-13	0.93	3.85	-	936.0
AHQ-1-5, 2223 - 2295	K.KDGTHVVENVDATHIGK.L	1820.98455	3	5.27E-05	0.97	5.88	-	1547.5
AHQ-1-6, 2132	K.KDGTHVVENVDATHIGK.L	1820.98455	2	5.04E-07	0.94	4.26	-	1012.9
AHQ-1-9, 2107	K.KDGTHVVENVDATHIGK.L	1820.98455	3	1.91E-04	0.79	3.28	-	1193.7
AHQ-1-7, 2124	K.KDGTHVVENVDATHIGK.L	1820.98455	2	1.17E-05	0.92	3.79	-	1048.6
AHQ-1-11, 4786	K.KETFDVTLEPLSFK.K	1654.88482	2	6.08E-04	0.91	3.54	-	1409.9
AHQ-1-5, 5355	K.KETFDVTLEPLSFK.K	1654.88482	2	1.00E-04	0.80	3.26	-	979.5
AHQ-1-5, 5226	K.KETFDVTLEPLSFK.K	1654.88482	2	2.59E-06	0.97	4.61	-	1798.5
AHQ-1-6, 5174	K.KETFDVTLEPLSFK.K	1654.88482	2	1.21E-05	0.94	3.92	-	1342.4
AHQ-1-5, 2599	R.KLIASMSSDSLR.H	1308.53039	2	9.30E-08	0.95	4.18	-	1299.3
AHQ-1-9, 4651	R.LALETALMYGAK.K	1281.54655	2	5.97E-08	0.97	4.05	-	1717.2
AHQ-1-9, 4176	R.LALETALM*YGAK.K	1297.54595	2	7.73E-05	0.71	2.65	-	879.9
AHQ-1-5, 4763 - 4779	R.LALETALMYGAK.K	1281.54655	2	1.43E-04	0.97	4.26	-	1908.6
AHQ-1-14- , 2283	K.LIASM*SSDSLR.H	1196.35688	2	3.10E-05	0.86	3.04	-	717.3
AHQ-1-5, 2929	K.LIASMSSDSLR.H	1180.35748	2	6.67E-06	0.94	3.57	-	1166.2
AHQ-1-5, 2141 - 2150	K.LIASM*SSDSLR.H	1196.35688	2	6.46E-04	0.91	3.30	-	830.6
AHQ-1-5, 5706 - 5709	R.M*YVAVWTPYGVLR.T	1571.86734	2	1.95E-05	0.96	4.37	-	1202.2
AHQ-1-7, 5704	R.M*YVAVWTPYGVLR.T	1571.86734	2	2.62E-08	0.95	3.83	-	1215.0
AHQ-1-11, 5634	R.MYVAVWTPYGVLR.T	1555.86794	2	6.88E-04	0.78	3.10	-	461.2
AHQ-1-6, 6180 - 6246	R.MYVAVWTPYGVLR.T	1555.86794	2	6.37E-05	0.93	3.85	-	1038.5
AHQ-1-6, 5670	R.M*YVAVWTPYGVLR.T	1571.86734	2	1.14E-04	0.92	3.83	-	874.0
AHQ-1-5, 6331	R.MYVAVWTPYGVLR.T	1555.86794	2	1.04E-05	0.91	4.38	-	648.4
AHQ-1-6, 6716	R.NPETDTYILFNPWCEDDAVYLDNEKER.E	3349.53903	3	2.12E-11	0.88	4.33	-	731.6
AHQ-1-9, 6718	R.NPETDTYILFNPWCEDDAVYLDNEKER.E	3349.53903	3	3.57E-06	0.94	4.99	-	1001.1
AHQ-1-5, 3586	R.NVWVHLDGPGVTR.P	1450.62682	2	4.46E-06	0.92	3.57	-	1031.6
AHQ-1-5, 3627 - 3647	R.NVWVHLDGPGVTRPMK.K	1807.11248	2	2.07E-06	0.90	3.69	-	668.6
AHQ-1-5, 3209	R.NVWVHLDGPGVTRPM*K.K	1823.11188	2	5.62E-06	0.92	4.24	-	789.6
AHQ-1-5, 4445	K.QIGGDGMMDITDTYK.F	1645.83696	2	1.65E-05	0.71	2.62	-	552.0
AHQ-1-5, 5885 - 5909	R.SNVDMDFEVENAVLGK.D	1767.93869	2	4.45E-08	0.90	4.14	-	1054.6
AHQ-1-5, 4757 - 4758	R.SNVDM*DFEVENAVLGK.D	1783.93809	2	1.42E-08	0.97	5.35	-	1421.6
AHQ-1-6, 5383 - 5430	R.SNVDMDFEVENAVLGK.D	1767.93869	2	4.75E-05	0.85	3.67	-	972.3
AHQ-1-5, 5479 - 5493	R.SNVDMDFEVENAVLGK.D	1767.93869	2	7.70E-09	0.97	5.27	-	1709.4
AHQ-1-6, 4680	R.SNVDM*DFEVENAVLGK.D	1783.93809	2	1.28E-06	0.97	4.70	-	2049.0
AHQ-1-11, 5358 - 5362	K.STVLTIPEIIIK.V	1327.63485	2	7.68E-05	0.92	3.96	-	967.8
AHQ-1-13, 5507	K.STVLTIPEIIIK.V	1327.63485	2	2.34E-04	0.91	3.08	-	1409.8
AHQ-1-9, 5694	K.STVLTIPEIIIK.V	1327.63485	2	1.62E-04	0.90	3.28	-	1167.8
AHQ-1-13- , 5851 - 5852	K.STVLTIPEIIIK.V	1327.63485	2	2.64E-05	0.94	3.66	-	1565.8
AHQ-1-3, 5991	K.STVLTIPEIIIK.V	1327.63485	2	4.57E-05	0.77	2.93	-	712.6
AHQ-1-7, 5877	K.STVLTIPEIIIK.V	1327.63485	2	3.73E-05	0.88	3.24	-	1322.4
AHQ-1-10, 5215	K.STVLTIPEIIIK.V	1327.63485	2	6.87E-04	0.88	3.28	-	1180.2
AHQ-1-5, 5578 - 5594	R.YPQENKGTYIPVPIVSELQSGK.W	2448.75607	2	3.27E-04	0.66	2.94	-	455.7
gi|5453595|ref|NP_006358.1| adenylyl cyclase-associated protein [Homo sapiens]				8.62E-13	11.48	140.34	43.40	51672.7
AHQ-1-7, 7508 - 7510	K.AGAAPYVQAFDSLLAGPVAEYLK.I	2352.66999	3	2.04E-07	0.94	5.43	-	956.9
AHQ-1-7, 2890 - 2961	R.ALLVTASQCQQPAENK.L	1759.96271	2	1.14E-06	0.81	3.64	-	566.2
AHQ-1-9, 2704 - 2715	R.ALLVTASQCQQPAENK.L	1759.96271	2	6.73E-07	0.96	4.92	-	1051.1
AHQ-1-7, 2780 - 2784	R.ALLVTASQCQQPAENK.L	1759.96271	2	5.07E-04	0.96	5.02	-	867.0
AHQ-1-7, 7029	R.ALLVTASQCQQPAENKLSDLLAPISEQIK.E	3168.60826	3	1.23E-08	0.93	5.16	-	1047.4
AHQ-1-7, 6776 - 6858	R.ALLVTASQCQQPAENKLSDLLAPISEQIK.E	3168.60826	3	5.94E-06	0.90	4.78	-	792.6
AHQ-1-7, 2566 - 2636	K.KEPAVLELEGK.K	1213.40557	2	4.64E-07	0.68	2.52	-	468.2
AHQ-1-11, 2642	K.KEPAVLELEGK.K	1213.40557	2	1.73E-05	0.86	3.43	-	525.7
AHQ-1-7, 1566	R.LEAVSHTSDMHR.G	1383.51591	1	9.77E-04	0.36	2.86	-	331.3
AHQ-1-7, 1549	R.LEAVSHTSDMHR.G	1383.51591	2	6.47E-04	0.95	4.21	-	1455.0
AHQ-1-7, 7116	K.LFNHLSAVSESIQALGWVAM*APK.P	2486.87415	3	5.57E-05	0.93	4.52	-	1224.4
AHQ-1-7, 7584 - 7588	K.LGLVFDDVVGIVEIINSK.D	1931.26186	2	2.09E-11	0.98	6.85	-	1656.1
AHQ-1-10, 4588	K.LSDLLAPISEQIK.E	1427.66814	1	1.79E-05	0.29	2.29	-	443.4
AHQ-1-13, 4893	K.LSDLLAPISEQIK.E	1427.66814	2	2.34E-06	0.86	3.30	-	837.4
AHQ-1-11, 4732	K.LSDLLAPISEQIK.E	1427.66814	2	1.23E-05	0.82	3.39	-	604.7
AHQ-1-9, 4990	K.LSDLLAPISEQIK.E	1427.66814	2	7.05E-05	0.66	2.89	-	495.2
AHQ-1-7, 5116 - 5134	K.LSDLLAPISEQIK.E	1427.66814	2	1.20E-06	0.94	4.30	-	1059.6
AHQ-1-7, 5121	K.LSDLLAPISEQIK.E	1427.66814	1	7.43E-06	0.72	3.16	-	485.0

AHQ-1-7, 5077 - 5148	K.LSDLLAPISEQIK.E	1427.66814	1	6.96E-05	0.26	2.21	-	333.6
AHQ-1-7, 3389 - 3393	K.NSLDCEIVSAK.S	1237.36245	1	6.38E-06	0.32	2.04	-	517.9
AHQ-1-7, 3085 - 3110	K.NSLDCEIVSAK.S	1237.36245	2	2.24E-04	0.88	3.17	-	848.8
AHQ-1-7, 4721	R.SALFAQINQGESITHALK.H	1929.16615	2	8.10E-09	0.85	3.72	-	501.5
AHQ-1-9, 4720	R.SALFAQINQGESITHALK.H	1929.16615	2	4.03E-08	0.95	4.77	-	947.0
AHQ-1-10, 4375	R.SALFAQINQGESITHALK.H	1929.16615	2	2.08E-07	0.82	4.05	-	486.8
AHQ-1-7, 4936	R.SALFAQINQGESITHALK.H	1929.16615	2	1.26E-07	0.86	4.21	-	455.5
AHQ-1-7, 4829 - 4864	R.SALFAQINQGESITHALK.H	1929.16615	3	1.91E-05	0.74	3.55	-	543.1
AHQ-1-7, 4830 - 4840	R.SALFAQINQGESITHALK.H	1929.16615	2	2.55E-07	0.92	4.33	-	769.1
AHQ-1-7, 5041	R.SALFAQINQGESITHALK.H	1929.16615	2	1.81E-08	0.74	3.64	-	451.2
AHQ-1-7, 2101	R.SGPKPFSAPKPQTSPSPK.R	1839.08461	3	3.80E-04	0.81	3.58	-	781.0
AHQ-1-14- , 6507	K.SSEMNVLIPTEGGDFNEFPVPEQFK.T	2813.08809	2	1.96E-05	0.44	2.79	-	228.6
AHQ-1-14- , 5994	K.SSEM*NVLIPTEGGDFNEFPVPEQFK.T	2829.08749	2	2.26E-04	0.82	4.00	-	271.4
AHQ-1-13- , 6664 - 6676	K.SSEMNVLIPTEGGDFNEFPVPEQFK.T	2813.08809	2	4.51E-05	0.66	2.88	-	348.1
AHQ-1-9, 4780	R.VENQENVSNLVIEDTELK.Q	2074.23206	2	4.62E-04	0.55	2.69	-	457.4
AHQ-1-10, 4364 - 4368	R.VENQENVSNLVIEDTELK.Q	2074.23206	2	1.40E-04	0.91	4.43	-	670.1
AHQ-1-8, 4799	R.VENQENVSNLVIEDTELK.Q	2074.23206	2	3.09E-08	0.93	4.98	-	661.4
AHQ-1-7, 5185	R.VENQENVSNLVIEDTELK.Q	2074.23206	2	1.80E-06	0.95	4.62	-	877.3
AHQ-1-7, 5032 - 5101	R.VENQENVSNLVIEDTELK.Q	2074.23206	2	1.51E-06	0.94	4.49	-	938.6
AHQ-1-11, 4499	R.VENQENVSNLVIEDTELK.Q	2074.23206	2	2.44E-04	0.90	4.02	-	835.5
AHQ-1-7, 4901	R.VENQENVSNLVIEDTELK.Q	2074.23206	3	1.83E-06	0.90	4.41	-	817.4
AHQ-1-12, 4665	R.VENQENVSNLVIEDTELK.Q	2074.23206	2	8.62E-13	0.93	4.50	-	793.7
AHQ-1-13, 4649	R.VENQENVSNLVIEDTELK.Q	2074.23206	2	5.36E-04	0.93	3.85	-	1012.8
AHQ-1-7, 4888 - 4956	R.VENQENVSNLVIEDTELK.Q	2074.23206	2	6.02E-08	0.95	5.02	-	926.2
AHQ-1-14, 5636	R.VENQENVSNLVIEDTELK.Q	2074.23206	2	5.65E-06	0.83	3.73	-	539.7
AHQ-1-7, 4689	R.VENQENVSNLVIEDTELK.Q	2074.23206	2	6.27E-05	0.59	3.17	-	352.5
AHQ-1-14- , 4786	R.VENQENVSNLVIEDTELK.Q	2074.23206	2	2.82E-04	0.92	4.18	-	832.8
AHQ-1-7, 4366	R.VENQENVSNLVIEDTELK.Q	2074.23206	2	2.38E-05	0.89	4.18	-	530.7
AHQ-1-5, 4923	R.VENQENVSNLVIEDTELK.Q	2074.23206	2	4.05E-06	0.68	3.40	-	699.5
gi|7705296|ref|NP_057271.1| bridging integrator 2; bridging integrator-2; breast cancer associated				9.16E-13	4.82	60.31	19.70	61746.8
AHQ-1-13- , 6121 - 6200	K.AIVWNNDLLWEDYEEK.L	2038.20157	2	3.02E-08	0.96	4.69	-	1295.0
AHQ-1-6, 6208	K.AIVWNNDLLWEDYEEK.L	2038.20157	2	7.75E-05	0.75	3.13	-	712.2
AHQ-1-10, 5503 - 5569	K.AIVWNNDLLWEDYEEK.L	2038.20157	2	1.56E-07	0.95	4.95	-	1002.5
AHQ-1-7, 6434	K.AIVWNNDLLWEDYEEK.L	2038.20157	2	9.16E-13	0.96	4.60	-	1414.2
AHQ-1-13, 5764	K.AIVWNNDLLWEDYEEK.L	2038.20157	2	2.73E-05	0.88	3.63	-	995.8
AHQ-1-7, 6337	K.AIVWNNDLLWEDYEEK.L	2038.20157	2	5.70E-06	0.65	3.13	-	388.6
AHQ-1-10, 6397 - 6439	K.AQTVFEDLNQELLEELPILYNSR.I	2736.02649	2	4.45E-05	0.85	3.56	-	642.1
AHQ-1-10, 6447 - 6448	K.AQTVFEDLNQELLEELPILYNSR.I	2736.02649	3	2.73E-04	0.95	6.20	-	716.0
AHQ-1-11, 2776	R.FEQSASNFYQQQAEGHK.L	2000.07352	2	6.69E-06	0.86	4.13	-	500.4
AHQ-1-7, 2789	R.FEQSASNFYQQQAEGHK.L	2000.07352	2	7.78E-05	0.90	4.20	-	680.1
AHQ-1-9, 2687	R.FEQSASNFYQQQAEGHK.L	2000.07352	2	8.76E-08	0.93	4.15	-	928.7
AHQ-1-13- , 2928	R.FEQSASNFYQQQAEGHK.L	2000.07352	2	2.49E-05	0.95	4.85	-	836.8
AHQ-1-10, 2545	R.FEQSASNFYQQQAEGHK.L	2000.07352	2	2.13E-06	0.66	3.15	-	392.7
AHQ-1-10, 2663	R.FEQSASNFYQQQAEGHK.L	2000.07352	2	4.70E-10	0.93	4.63	-	685.8
AHQ-1-13, 2897	R.FEQSASNFYQQQAEGHK.L	2000.07352	2	1.37E-04	0.84	3.53	-	670.8
AHQ-1-9, 6828 - 6895	R.IGCYVTIFQNISNLR.D	1800.07167	2	4.88E-08	0.92	4.87	-	679.0
AHQ-1-9, 6758 - 6830	R.IGCYVTIFQNISNLR.D	1800.07167	2	9.58E-06	0.91	3.70	-	963.3
AHQ-1-6, 6866	R.IGCYVTIFQNISNLR.D	1800.07167	2	6.83E-06	0.93	3.64	-	1109.0
AHQ-1-13, 6257 - 6275	R.IGCYVTIFQNISNLR.D	1800.07167	2	2.36E-04	0.73	3.40	-	723.5
AHQ-1-7, 7000	R.IGCYVTIFQNISNLR.D	1800.07167	2	2.51E-07	0.93	4.06	-	952.3
AHQ-1-7, 4344	R.TATVSSPLTSPTSPSTLSLK.S	1976.21467	2	2.06E-04	0.75	3.58	-	532.9
AHQ-1-6, 4282	R.TATVSSPLTSPTSPSTLSLK.S	1976.21467	2	1.53E-06	0.55	2.74	-	494.3
AHQ-1-9, 4215	R.TATVSSPLTSPTSPSTLSLK.S	1976.21467	2	6.90E-04	0.74	3.38	-	562.1
AHQ-1-10, 4609	R.VSETLQEIYSSEWDGHEELK.A	2380.50595	2	3.38E-04	0.60	2.85	-	341.5
gi|4504165|ref|NP_000168.1| gelsolin (amyloidosis, Finnish type); Gelsolin [Homo sapiens]				1.04E-12	23.39	260.33	40.90	85696.9
AHQ-1-5, 3757	K.AGALNSNDAFVLK.T	1320.47622	1	2.90E-08	0.41	2.39	-	412.4
AHQ-1-5, 3837 - 3910	K.AGALNSNDAFVLK.T	1320.47622	2	6.88E-09	0.93	3.83	-	870.7
AHQ-1-5, 3755	K.AGALNSNDAFVLK.T	1320.47622	2	3.23E-08	0.84	3.69	-	574.6
AHQ-1-5, 3655	K.AGALNSNDAFVLK.T	1320.47622	2	1.42E-04	0.63	3.23	-	354.7
AHQ-1-5, 3506 - 3585	K.AGALNSNDAFVLK.T	1320.47622	2	3.97E-06	0.90	3.67	-	968.3
AHQ-1-5, 3874	K.AGALNSNDAFVLK.T	1320.47622	1	1.28E-06	0.50	2.32	-	545.9
AHQ-1-6, 3492 - 3566	K.AGALNSNDAFVLK.T	1320.47622	2	8.74E-07	0.88	3.78	-	669.3
AHQ-1-5, 2951	K.AGKEPGLQIWR.V	1255.45060	3	5.99E-06	0.92	4.35	-	1153.5
AHQ-1-6, 5844	R.AQPVQVAEGSEPDGFWEALGGK.A	2273.44345	2	2.95E-05	0.75	3.41	-	375.3
AHQ-1-13, 5443	R.AQPVQVAEGSEPDGFWEALGGK.A	2273.44345	2	6.55E-05	0.91	4.10	-	791.9
AHQ-1-7, 5736	R.AQPVQVAEGSEPDGFWEALGGK.A	2273.44345	2	7.33E-08	0.93	4.88	-	601.8
AHQ-1-7, 5933	R.AQPVQVAEGSEPDGFWEALGGK.A	2273.44345	2	2.40E-07	0.93	4.47	-	704.3
AHQ-1-5, 5718 - 5729	R.AQPVQVAEGSEPDGFWEALGGK.A	2273.44345	2	6.15E-05	0.97	6.12	-	1079.2
AHQ-1-6, 5642	R.AQPVQVAEGSEPDGFWEALGGK.A	2273.44345	2	3.58E-06	0.80	3.76	-	437.2
AHQ-1-11, 5318	R.AQPVQVAEGSEPDGFWEALGGK.A	2273.44345	2	1.40E-07	0.93	4.98	-	566.2
AHQ-1-11, 5167	R.AQPVQVAEGSEPDGFWEALGGK.A	2273.44345	2	1.17E-05	0.90	4.37	-	539.3
AHQ-1-6, 5944	R.AQPVQVAEGSEPDGFWEALGGK.A	2273.44345	2	1.05E-04	0.84	4.09	-	286.5
AHQ-1-9, 5575	R.AQPVQVAEGSEPDGFWEALGGK.A	2273.44345	2	7.44E-05	0.91	4.57	-	431.1
AHQ-1-5, 5858	R.AQPVQVAEGSEPDGFWEALGGK.A	2273.44345	2	2.43E-06	0.96	5.25	-	718.4
AHQ-1-10, 5019 - 5024	R.AQPVQVAEGSEPDGFWEALGGK.A	2273.44345	2	3.30E-05	0.95	5.40	-	744.0
AHQ-1-5, 5949 - 6017	R.AQPVQVAEGSEPDGFWEALGGK.A	2273.44345	2	3.79E-07	0.97	6.02	-	1040.7
AHQ-1-2, 6106	R.AQPVQVAEGSEPDGFWEALGGK.A	2273.44345	2	5.66E-04	0.48	2.64	-	388.0
AHQ-1-5, 6167	R.AQPVQVAEGSEPDGFWEALGGK.A	2273.44345	2	6.37E-04	0.84	3.61	-	503.9
AHQ-1-5, 6067 - 6085	R.AQPVQVAEGSEPDGFWEALGGK.A	2273.44345	2	3.08E-06	0.97	6.65	-	840.8
AHQ-1-8, 5732 - 5800	R.AQPVQVAEGSEPDGFWEALGGK.A	2273.44345	2	2.24E-07	0.94	4.55	-	821.4
AHQ-1-9, 5731	R.AQPVQVAEGSEPDGFWEALGGK.A	2273.44345	2	6.81E-04	0.85	3.55	-	534.2
AHQ-1-5, 5825 - 5903	R.DPDQTDGLGLSYLSSHIANVER.V	2388.53285	3	3.48E-10	0.95	5.01	-	1208.2
AHQ-1-7, 5814	R.DPDQTDGLGLSYLSSHIANVER.V	2388.53285	3	1.16E-08	0.86	3.53	-	1246.8
AHQ-1-6, 5774	R.DPDQTDGLGLSYLSSHIANVER.V	2388.53285	3	4.86E-05	0.96	5.13	-	1374.0
AHQ-1-13, 2329 - 2387	K.DSQEEEKTEALTSAK.R	1666.72289	2	4.95E-07	0.93	3.69	-	1279.5
AHQ-1-8, 2087	K.DSQEEEKTEALTSAK.R	1666.72289	2	9.69E-08	0.90	3.50	-	1132.4
AHQ-1-13, 2333	K.DSQEEEKTEALTSAK.R	1666.72289	3	4.79E-09	0.95	4.30	-	1878.1
AHQ-1-11, 2190	K.DSQEEEKTEALTSAK.R	1666.72289	2	3.85E-05	0.89	3.62	-	983.9
AHQ-1-5, 2187 - 2259	K.DSQEEEKTEALTSAK.R	1666.72289	2	1.94E-07	0.95	4.21	-	1383.9
AHQ-1-7, 2149	K.DSQEEEKTEALTSAK.R	1666.72289	2	1.99E-05	0.85	3.10	-	1186.5
AHQ-1-5, 2106	K.DSQEEEKTEALTSAK.R	1666.72289	3	3.86E-06	0.95	3.74	-	2090.3
AHQ-1-5, 2099 - 2113	K.DSQEEEKTEALTSAK.R	1666.72289	2	2.50E-06	0.92	3.50	-	1346.5
AHQ-1-9, 2118 - 2120	K.DSQEEEKTEALTSAK.R	1666.72289	2	1.50E-07	0.96	4.79	-	1483.6
AHQ-1-13- , 2279	K.DSQEEEKTEALTSAK.R	1666.72289	2	9.98E-09	0.94	3.78	-	1460.1
AHQ-1-10, 2131	K.DSQEEEKTEALTSAK.R	1666.72289	2	1.21E-09	0.94	3.83	-	1457.7
AHQ-1-13- , 2287	K.DSQEEEKTEALTSAK.R	1666.72289	3	4.96E-06	0.97	4.11	-	2794.4
AHQ-1-6, 2135	K.DSQEEEKTEALTSAK.R	1666.72289	3	6.86E-06	0.91	3.36	-	1583.3
AHQ-1-2, 2338	K.DSQEEEKTEALTSAK.R	1666.72289	2	1.38E-08	0.91	3.65	-	1323.2
AHQ-1-1, 2364	K.DSQEEEKTEALTSAK.R	1666.72289	2	9.93E-06	0.93	3.71	-	1563.3
AHQ-1-6, 2120 - 2122	K.DSQEEEKTEALTSAK.R	1666.72289	2	2.89E-09	0.95	3.94	-	1539.3
AHQ-1-5, 6474 - 6549	R.EVQGFESATFLGYFK.S	1723.90570	2	2.12E-11	0.97	5.10	-	1455.1
AHQ-1-13- , 6225 - 6273	R.EVQGFESATFLGYFK.S	1723.90570	2	6.53E-11	0.96	4.59	-	1331.8
AHQ-1-5, 6597 - 6665	R.EVQGFESATFLGYFK.S	1723.90570	2	6.06E-05	0.84	3.53	-	718.8
AHQ-1-13, 5869 - 5891	R.EVQGFESATFLGYFK.S	1723.90570	2	2.23E-07	0.97	4.34	-	1871.9
AHQ-1-12, 5925 - 6006	R.EVQGFESATFLGYFK.S	1723.90570	2	8.21E-07	0.94	4.03	-	990.7
AHQ-1-5, 7606	R.EVQGFESATFLGYFK.S	1723.90570	2	1.13E-06	0.94	3.27	-	1439.1
AHQ-1-5, 6841 - 6870	R.EVQGFESATFLGYFK.S	1723.90570	2	2.43E-06	0.91	3.24	-	1152.8

AHQ-1-5, 6937 - 7017	R.EVQGFESATFLGYFK.S	1723.90570	2	2.19E-07	0.96	4.16	-	1382.9
AHQ-1-14- , 6051	R.EVQGFESATFLGYFK.S	1723.90570	2	4.99E-09	0.90	3.91	-	817.1
AHQ-1-5, 7483 - 7557	K.FDLVPVPTNLYGDFFTGDAYVILK.T	2706.08359	2	2.50E-04	0.79	3.19	-	448.3
AHQ-1-7, 2212 - 2216	K.HVVPNEVVVQR.L	1276.46981	2	1.08E-08	0.91	3.20	-	730.1
AHQ-1-9, 2174	K.HVVPNEVVVQR.L	1276.46981	2	9.54E-08	0.91	3.29	-	662.4
AHQ-1-5, 2395	K.HVVPNEVVVQR.L	1276.46981	1	1.41E-08	0.46	2.14	-	481.2
AHQ-1-5, 2389 - 2414	K.HVVPNEVVVQR.L	1276.46981	2	6.11E-05	0.87	3.03	-	610.8
AHQ-1-5, 2185 - 2186	K.HVVPNEVVVQR.L	1276.46981	1	3.26E-09	0.47	2.53	-	309.9
AHQ-1-5, 2181 - 2253	K.HVVPNEVVVQR.L	1276.46981	2	2.95E-09	0.90	3.17	-	726.8
AHQ-1-6, 2202	K.HVVPNEVVVQR.L	1276.46981	2	8.77E-10	0.88	3.45	-	567.0
AHQ-1-11, 2266	K.HVVPNEVVVQR.L	1276.46981	2	2.24E-04	0.87	3.02	-	547.8
AHQ-1-5, 6419 - 6493	R.IEGSNKVPVDPATYGQFYGGDSYIILYNYR.H	3401.72499	3	1.58E-11	0.89	4.00	-	1086.0
AHQ-1-5, 6907 - 6919	R.IEGSNKVPVDPATYGQFYGGDSYIILYNYR.H	3401.72499	3	6.28E-05	0.96	5.31	-	1353.3
AHQ-1-5, 6789	R.IEGSNKVPVDPATYGQFYGGDSYIILYNYR.H	3401.72499	3	1.25E-06	0.95	4.93	-	1461.4
AHQ-1-5, 6573 - 6641	R.IEGSNKVPVDPATYGQFYGGDSYIILYNYR.H	3401.72499	3	3.91E-05	0.91	4.24	-	1088.1
AHQ-1-6, 6362 - 6388	R.IEGSNKVPVDPATYGQFYGGDSYIILYNYR.H	3401.72499	3	6.68E-04	0.87	4.05	-	992.8
AHQ-1-5, 5714 - 5715	K.NWRDPDQTDGLGLSYLSSHIANVER.V	2845.03323	2	8.66E-04	0.75	3.53	-	337.3
AHQ-1-5, 5689 - 5761	K.NWRDPDQTDGLGLSYLSSHIANVER.V	2845.03323	3	5.49E-08	0.95	5.49	-	978.2
AHQ-1-5, 6329	K.NWRDPDQTDGLGLSYLSSHIANVER.V	2845.03323	3	1.36E-12	0.93	4.89	-	967.7
AHQ-1-5, 2017	K.PALPAGTEDTAK.E	1171.28241	2	1.86E-07	0.81	2.87	-	458.7
AHQ-1-5, 2415	K.PALPAGTEDTAKEDAANR.K	1827.93072	2	1.09E-06	0.93	4.04	-	778.4
AHQ-1-5, 4833 - 4903	K.QTQVSVLPEGGETPLFK.Q	1831.05925	2	1.02E-04	0.77	3.09	-	399.2
AHQ-1-5, 5030 - 5035	K.QTQVSVLPEGGETPLFK.Q	1831.05925	2	2.36E-07	0.88	3.80	-	486.4
AHQ-1-5, 2942	K.SEDCFILDHGK.D	1322.42590	3	1.08E-05	0.73	3.04	-	423.8
AHQ-1-7, 2980	K.SEDCFILDHGK.D	1322.42590	2	5.59E-05	0.96	3.71	-	1657.4
AHQ-1-5, 2921	K.SEDCFILDHGK.D	1322.42590	2	1.94E-04	0.95	3.90	-	1405.8
AHQ-1-6, 2967	K.SEDCFILDHGK.D	1322.42590	2	3.87E-06	0.91	3.00	-	1047.7
AHQ-1-8, 2075	K.TGAQELLR.V	888.00387	2	7.43E-05	0.81	2.66	-	907.2
AHQ-1-5, 2054	K.TGAQELLR.V	888.00387	2	1.02E-04	0.85	2.88	-	892.3
AHQ-1-6, 2087	K.TGAQELLR.V	888.00387	2	9.37E-06	0.88	2.93	-	1011.0
AHQ-1-5, 4603 - 4673	K.TPSAAYLWVGTGASEAEK.T	1838.99510	2	9.27E-06	0.95	4.59	-	1142.0
AHQ-1-6, 4298	K.TPSAAYLWVGTGASEAEK.T	1838.99510	2	1.28E-04	0.72	3.13	-	580.9
AHQ-1-5, 4491 - 4561	K.TPSAAYLWVGTGASEAEK.T	1838.99510	2	1.30E-05	0.98	5.16	-	2021.2
AHQ-1-10, 3964 - 4032	K.TPSAAYLWVGTGASEAEK.T	1838.99510	2	4.69E-04	0.89	3.55	-	1061.7
AHQ-1-5, 4663 - 4742	K.TPSAAYLWVGTGASEAEK.T	1838.99510	2	1.04E-12	0.97	5.62	-	1492.2
AHQ-1-6, 4935	R.VHVSEEGTEPEAMLQVLGPK.P	2151.42630	2	3.48E-07	0.97	5.69	-	1064.4
AHQ-1-8, 4815	R.VHVSEEGTEPEAMLQVLGPK.P	2151.42630	2	1.59E-04	0.95	4.70	-	754.0
AHQ-1-5, 4927 - 4997	R.VHVSEEGTEPEAMLQVLGPK.P	2151.42630	2	2.55E-07	0.97	5.83	-	1268.7
AHQ-1-5, 5314	R.VHVSEEGTEPEAMLQVLGPKPALPAGTEDTAK.E	3303.68611	3	1.64E-08	0.95	6.02	-	688.9
AHQ-1-5, 4385	R.VHVSEEGTEPEAM*LQVLGPKPALPAGTEDTAK.E	3319.68551	3	9.87E-05	0.92	4.76	-	765.3
AHQ-1-6, 5234	R.VHVSEEGTEPEAMLQVLGPKPALPAGTEDTAK.E	3303.68611	3	6.76E-09	0.92	4.76	-	674.6
AHQ-1-5, 5098	R.VHVSEEGTEPEAMLQVLGPKPALPAGTEDTAKEDAANR.K	3960.33442	3	4.99E-10	0.97	6.29	-	1184.5
AHQ-1-5, 3549 - 3626	R.VPFDAATLHTSTAMAAQHGM*DDDGTGQK.Q	2891.10081	3	1.90E-05	0.92	5.47	-	1120.2
AHQ-1-5, 3141	R.VPFDAATLHTSTAM*AAQHGM*DDDGTGQK.Q	2907.10021	3	6.55E-04	0.80	3.70	-	568.9
AHQ-1-5, 4026	R.VPFDAATLHTSTAMAAQHGMDDDGTGQK.Q	2875.10141	3	2.26E-08	0.97	6.22	-	1231.1
AHQ-1-5, 6978	K.VPVDPATYGQFYGGDSYIILYNYR.H	2773.04698	2	2.38E-06	0.74	3.05	-	632.9
AHQ-1-5, 6914	K.VPVDPATYGQFYGGDSYIILYNYR.H	2773.04698	3	9.67E-04	0.81	4.49	-	876.5
AHQ-1-5, 6681	K.VPVDPATYGQFYGGDSYIILYNYR.H	2773.04698	2	3.16E-07	0.93	4.16	-	901.0
AHQ-1-5, 5537 - 5607	K.VSNGAGTM*SVSLVADENPFAQGALK.S	2480.73680	2	2.89E-06	0.93	4.86	-	767.0
AHQ-1-6, 5458 - 5527	K.VSNGAGTM*SVSLVADENPFAQGALK.S	2480.73680	2	1.12E-07	0.83	3.15	-	747.8
AHQ-1-5, 1705	R.YIETDPANR.D	1079.14506	2	2.04E-05	0.64	2.67	-	502.5
gi|23308579|ref|NP_006592.3| unactive progesterone receptor, 23 kD; likely ortholog of mouse telome				1.17E-12	0.94	10.24	10.60	18697.3
AHQ-1-11, 4599	K.HLNEIDLFHCIDPNDSK.H	2069.24049	3	1.17E-12	0.94	4.78	-	1090.2
gi|14251209|ref|NP_001279.2| chloride intracellular channel 1; p64CLCP; chloride channel ABP [Homo				1.19E-12	9.19	110.26	51.00	26922.5
AHQ-1-13- , 5867	R.EEFASTCPDDEEIELAYEQVAK.A	2575.69730	2	1.13E-08	0.46	2.57	-	406.3
AHQ-1-12, 5602	R.EEFASTCPDDEEIELAYEQVAK.A	2575.69730	3	1.26E-10	0.96	5.02	-	1690.9
AHQ-1-13, 5520	R.EEFASTCPDDEEIELAYEQVAK.A	2575.69730	2	6.39E-07	0.41	2.62	-	370.5
AHQ-1-10, 5313	R.EEFASTCPDDEEIELAYEQVAK.A	2575.69730	3	1.86E-06	0.91	4.70	-	971.9
AHQ-1-9, 6456	K.FLDGNELTLADCNLLPK.L	1935.18752	2	1.64E-08	0.70	3.56	-	757.9
AHQ-1-10, 5595 - 5669	K.FLDGNELTLADCNLLPK.L	1935.18752	2	4.63E-07	0.92	5.04	-	986.5
AHQ-1-12, 5882	K.FLDGNELTLADCNLLPK.L	1935.18752	2	1.86E-04	0.89	4.29	-	1086.7
AHQ-1-10, 5704	K.FLDGNELTLADCNLLPK.L	1935.18752	2	3.76E-07	0.90	4.38	-	1020.0
AHQ-1-10, 3503	K.FSAYIKNSNPALNDNLEK.G	2039.23424	2	1.19E-12	0.88	4.68	-	795.6
AHQ-1-9, 3280	K.GVTFNVTTVDTK.R	1282.42476	2	5.48E-06	0.92	3.74	-	1038.6
AHQ-1-13- , 3477 - 3543	K.GVTFNVTTVDTK.R	1282.42476	2	2.40E-06	0.93	3.99	-	948.0
AHQ-1-13, 3434	K.GVTFNVTTVDTK.R	1282.42476	2	8.85E-07	0.93	3.72	-	934.6
AHQ-1-10, 3181 - 3245	K.GVTFNVTTVDTK.R	1282.42476	2	3.08E-06	0.91	3.52	-	1029.5
AHQ-1-10, 2337	K.IGNCPFSQR.L	1080.19948	2	8.83E-04	0.82	3.10	-	558.6
AHQ-1-9, 5835	R.KFLDGNELTLADCNLLPK.L	2063.36044	2	1.67E-04	0.75	3.41	-	733.6
AHQ-1-10, 5337	R.KFLDGNELTLADCNLLPK.L	2063.36044	2	4.18E-07	0.88	4.84	-	982.7
AHQ-1-10, 5164 - 5219	R.KFLDGNELTLADCNLLPK.L	2063.36044	2	3.78E-04	0.87	4.52	-	1016.9
AHQ-1-10, 4863	K.LAALNPESNTAGLDIFAK.F	1846.07392	2	3.90E-06	0.96	5.26	-	917.2
AHQ-1-10, 5135	K.LAALNPESNTAGLDIFAK.F	1846.07392	2	1.75E-06	0.94	4.68	-	790.4
AHQ-1-10, 5017	K.LAALNPESNTAGLDIFAK.F	1846.07392	2	6.77E-05	0.87	4.12	-	367.2
AHQ-1-9, 5378 - 5400	K.LAALNPESNTAGLDIFAK.F	1846.07392	2	1.94E-06	0.96	5.22	-	753.5
AHQ-1-13- , 5613	K.LAALNPESNTAGLDIFAK.F	1846.07392	2	2.64E-07	0.92	4.43	-	624.2
AHQ-1-14, 6266 - 6344	K.LAALNPESNTAGLDIFAK.F	1846.07392	2	3.54E-04	0.85	4.14	-	385.1
AHQ-1-12, 5262	K.LAALNPESNTAGLDIFAK.F	1846.07392	2	3.00E-04	0.96	4.47	-	1038.2
AHQ-1-9, 5590 - 5671	K.LAALNPESNTAGLDIFAK.F	1846.07392	2	3.49E-06	0.90	3.77	-	725.7
AHQ-1-14- , 5359	K.LAALNPESNTAGLDIFAK.F	1846.07392	2	2.86E-05	0.93	4.60	-	659.0
AHQ-1-13- , 5491	K.LAALNPESNTAGLDIFAK.F	1846.07392	2	1.92E-06	0.94	4.52	-	791.7
AHQ-1-13, 5300	K.LAALNPESNTAGLDIFAK.F	1846.07392	2	2.96E-11	0.94	4.92	-	607.2
AHQ-1-13, 5133 - 5191	K.LAALNPESNTAGLDIFAK.F	1846.07392	2	1.26E-05	0.89	3.73	-	567.1
AHQ-1-14- , 5736	R.LFM*VLWLK.G	1066.38391	2	4.11E-04	0.88	2.70	-	1039.3
AHQ-1-13- , 2923	K.LHIVQVVCK.K	1097.35623	1	3.28E-04	0.74	2.31	-	774.6
AHQ-1-10, 2177	K.NSNPALNDNLEK.G	1329.39838	2	1.79E-07	0.55	3.23	-	370.4
AHQ-1-10, 4683	K.VLDNYLTSPLPEEVDETSAEDEGVSQR.K	2994.12446	2	5.21E-04	0.38	2.85	-	215.6
AHQ-1-13- , 5351	K.VLDNYLTSPLPEEVDETSAEDEGVSQR.K	2994.12446	2	1.28E-05	0.68	3.60	-	248.9
AHQ-1-12, 5135	K.VLDNYLTSPLPEEVDETSAEDEGVSQR.K	2994.12446	2	1.30E-08	0.90	4.77	-	321.3
AHQ-1-12, 5118	K.VLDNYLTSPLPEEVDETSAEDEGVSQR.K	2994.12446	2	6.77E-06	0.75	3.48	-	326.4
AHQ-1-10, 4760 - 4824	K.VLDNYLTSPLPEEVDETSAEDEGVSQR.K	2994.12446	3	5.45E-07	0.94	5.19	-	937.5
AHQ-1-10, 4765 - 4823	K.VLDNYLTSPLPEEVDETSAEDEGVSQR.K	2994.12446	2	3.30E-07	0.91	4.59	-	404.7
AHQ-1-10, 4967	K.VLDNYLTSPLPEEVDETSAEDEGVSQR.K	2994.12446	2	2.93E-04	0.60	2.75	-	385.7
AHQ-1-13, 5076	K.VLDNYLTSPLPEEVDETSAEDEGVSQR.K	2994.12446	2	4.83E-06	0.50	2.81	-	290.7
gi|5453549|ref|NP_006397.1| thioredoxin peroxidase; thioredoxin peroxidase (antioxidant enzyme) [Ho				1.26E-12	2.65	30.23	10.00	30539.7
AHQ-1-13, 3768	K.DYGVYLEDSGHTLR.G	1625.72052	2	3.60E-07	0.96	4.53	-	1107.2
AHQ-1-10, 3504	K.DYGVYLEDSGHTLR.G	1625.72052	2	1.26E-12	0.97	4.27	-	1802.7
AHQ-1-10, 4629	R.IPLLSDLTHQISK.D	1465.71961	2	2.55E-08	0.96	3.94	-	1366.6
AHQ-1-13- , 3092	R.LVQAFQYTDK.H	1213.36412	2	1.51E-04	0.71	2.63	-	910.5
gi|13994151|ref|NP_066272.1| PDZ and LIM domain 1 (elfin); carboxy terminal LIM domain protein 1 [H				1.29E-12	16.63	200.29	60.50	36071.5
AHQ-1-13, 6307	K.AALANLCIGDVITAIDGENTSNM*THLEAQNR.I	3331.63807	3	1.99E-08	0.91	4.85	-	526.4
AHQ-1-14- , 6546	K.AALANLCIGDVITAIDGENTSNM*THLEAQNR.I	3331.63807	3	1.92E-08	0.86	4.14	-	577.0
AHQ-1-14- , 6462 - 6518	K.AALANLCIGDVITAIDGENTSNM*THLEAQNR.I	3331.63807	3	4.23E-08	0.91	4.80	-	617.2
AHQ-1-14- , 6047	K.AALANLCIGDVITAIDGENTSNM*THLEAQNR.I	3331.63807	3	4.28E-10	0.89	4.45	-	505.8
AHQ-1-14- , 5922 - 5987	K.AALANLCIGDVITAIDGENTSNM*THLEAQNR.I	3331.63807	3	6.14E-05	0.77	3.74	-	673.6
AHQ-1-13- , 6851 - 6853	K.AALANLCIGDVITAIDGENTSNMTHLEAQNR.I	3315.63867	3	1.29E-12	0.95	5.75	-	738.4
AHQ-1-13- , 6715 - 6789	K.AALANLCIGDVITAIDGENTSNM*THLEAQNR.I	3331.63807	3	2.39E-05	0.92	5.05	-	483.3

AHQ-1-14- , 6606	K.AALANLCIGDVITAIDGENTSNMTHLEAQNR.I	3315.63867	3	6.67E-08	0.91	4.97	-	643.6
AHQ-1-11, 6198 - 6272	K.AALANLCIGDVITAIDGENTSNM*THLEAQNR.I	3331.63807	3	7.87E-04	0.81	4.26	-	374.8
AHQ-1-13- , 4579	K.CGTGIVGVFVK.L	1138.36198	2	1.80E-06	0.91	3.30	-	1069.2
AHQ-1-12, 4399	K.CGTGIVGVFVK.L	1138.36198	2	8.70E-04	0.93	3.00	-	1196.4
AHQ-1-14- , 3638 - 3640	K.DFEQPLAISR.V	1176.30388	2	1.24E-06	0.94	4.21	-	981.9
AHQ-1-14, 4562	K.DFEQPLAISR.V	1176.30388	1	3.16E-04	0.37	1.87	-	395.9
AHQ-1-14, 4557 - 4560	K.DFEQPLAISR.V	1176.30388	2	5.97E-05	0.88	3.67	-	723.1
AHQ-1-13, 3613 - 3631	K.DFEQPLAISR.V	1176.30388	2	5.87E-05	0.87	3.15	-	799.8
AHQ-1-12, 3569	K.DFEQPLAISR.V	1176.30388	2	6.79E-07	0.92	3.67	-	973.3
AHQ-1-13- , 3459	K.GCTDNLTLTVAR.S	1322.47058	2	7.44E-04	0.84	3.39	-	724.1
AHQ-1-10, 3756	K.GHFFVEDQIYCEK.H	1673.82684	2	7.33E-06	0.91	3.57	-	862.9
AHQ-1-13- , 4156 - 4228	K.GHFFVEDQIYCEK.H	1673.82684	2	3.71E-05	0.96	4.44	-	1190.2
AHQ-1-12, 3922 - 3945	K.GHFFVEDQIYCEK.H	1673.82684	2	5.25E-05	0.94	4.02	-	1034.4
AHQ-1-12, 3995 - 4018	K.GHFFVEDQIYCEK.H	1673.82684	2	1.71E-06	0.96	4.31	-	1200.2
AHQ-1-13, 3980 - 4044	K.GHFFVEDQIYCEK.H	1673.82684	2	2.17E-05	0.96	4.09	-	1333.5
AHQ-1-14, 4916 - 4996	K.GHFFVEDQIYCEK.H	1673.82684	2	5.26E-04	0.93	3.74	-	1012.8
AHQ-1-14- , 4039	K.GHFFVEDQIYCEK.H	1673.82684	2	7.41E-05	0.93	3.70	-	1067.3
AHQ-1-9, 3970	K.GHFFVEDQIYCEK.H	1673.82684	2	1.66E-05	0.96	4.27	-	1380.2
AHQ-1-12, 2997	R.HPECYVCTDCGTNLK.Q	1858.02023	2	6.07E-04	0.83	3.35	-	500.4
AHQ-1-12, 2669	R.HRHPECYVCTDCGTNLK.Q	2151.34648	3	4.93E-10	0.97	4.95	-	1653.7
AHQ-1-14- , 2751	R.HRHPECYVCTDCGTNLK.Q	2151.34648	3	1.83E-06	0.97	5.01	-	1917.0
AHQ-1-13- , 2789	R.HRHPECYVCTDCGTNLK.Q	2151.34648	2	2.42E-04	0.89	4.24	-	449.9
AHQ-1-13, 3227	R.IKGCTDNLTLTVAR.S	1563.80174	2	5.59E-07	0.83	3.53	-	703.1
AHQ-1-9, 3052 - 3054	R.IKGCTDNLTLTVAR.S	1563.80174	2	3.47E-08	0.94	4.11	-	1051.2
AHQ-1-14- , 3178	R.IKGCTDNLTLTVAR.S	1563.80174	2	1.71E-09	0.94	4.04	-	1009.7
AHQ-1-11, 3056	R.IKGCTDNLTLTVAR.S	1563.80174	3	3.37E-05	0.82	3.66	-	1136.0
AHQ-1-12, 3122	R.IKGCTDNLTLTVAR.S	1563.80174	2	2.65E-08	0.93	4.06	-	810.3
AHQ-1-14, 4069	R.IKGCTDNLTLTVAR.S	1563.80174	2	8.12E-08	0.91	3.60	-	943.2
AHQ-1-11, 3048	R.IKGCTDNLTLTVAR.S	1563.80174	2	4.60E-07	0.93	4.23	-	1009.4
AHQ-1-13- , 3341	R.IKGCTDNLTLTVAR.S	1563.80174	2	2.39E-08	0.90	3.95	-	703.2
AHQ-1-13- , 3480 - 3531	R.IKGCTDNLTLTVAR.S	1563.80174	2	4.86E-04	0.87	3.58	-	606.5
AHQ-1-13- , 4932	K.LPM*CDKCGTGIVGVFVK.L	1900.27298	2	8.09E-04	0.89	3.50	-	686.5
AHQ-1-13, 4668	K.LPM*CDKCGTGIVGVFVK.L	1900.27298	2	7.87E-04	0.53	2.67	-	385.0
AHQ-1-13- , 5380	K.LPMCDKCGTGIVGVFVK.L	1884.27358	3	2.30E-05	0.78	3.29	-	860.5
AHQ-1-12, 5113	K.LPMCDKCGTGIVGVFVK.L	1884.27358	3	2.31E-07	0.79	3.63	-	699.4
AHQ-1-12, 4682	K.LPM*CDKCGTGIVGVFVK.L	1900.27298	3	1.04E-04	0.56	3.03	-	723.3
AHQ-1-14, 4606 - 4664	R.LVGGKDFEQPLAISR.V	1630.86972	2	2.88E-08	0.96	4.36	-	1501.9
AHQ-1-9, 3586	R.LVGGKDFEQPLAISR.V	1630.86972	2	1.03E-04	0.77	3.29	-	558.3
AHQ-1-14- , 3792	R.LVGGKDFEQPLAISR.V	1630.86972	2	1.23E-08	0.96	4.62	-	1372.3
AHQ-1-9, 3374	K.M*NLASEPQEVLHIGSAHNR.S	2120.33559	2	1.35E-07	0.91	3.96	-	820.6
AHQ-1-13, 3667 - 3699	K.M*NLASEPQEVLHIGSAHNR.S	2120.33559	3	8.74E-04	0.61	3.16	-	590.2
AHQ-1-13- , 3833	K.M*NLASEPQEVLHIGSAHNR.S	2120.33559	2	5.14E-06	0.92	3.95	-	761.1
AHQ-1-13, 3668 - 3683	K.M*NLASEPQEVLHIGSAHNR.S	2120.33559	2	2.76E-05	0.89	3.86	-	566.6
AHQ-1-13, 6308	K.QSTSFLVLQEILESEEK.G	1981.18977	2	4.91E-07	0.91	3.49	-	1203.9
AHQ-1-9, 6859	K.QSTSFLVLQEILESEEK.G	1981.18977	2	2.17E-04	0.94	3.95	-	1144.7
AHQ-1-12, 6410	K.QSTSFLVLQEILESEEK.G	1981.18977	2	1.24E-07	0.90	3.33	-	1261.3
AHQ-1-13- , 6752	K.QSTSFLVLQEILESEEK.G	1981.18977	2	2.96E-11	0.96	4.82	-	1583.3
AHQ-1-9, 2890	R.SAMPFTASPASSTTAR.V	1583.74859	2	9.79E-06	0.55	2.77	-	373.0
AHQ-1-12, 2417 - 2418	R.SAM*PFTASPASSTTAR.V	1599.74799	2	5.94E-07	0.91	3.58	-	1138.3
AHQ-1-11, 2390	R.SAM*PFTASPASSTTAR.V	1599.74799	2	6.09E-07	0.79	2.66	-	968.3
AHQ-1-9, 2308	R.SAM*PFTASPASSTTAR.V	1599.74799	2	3.40E-07	0.85	3.16	-	938.6
AHQ-1-13- , 2511 - 2512	R.SAM*PFTASPASSTTAR.V	1599.74799	2	6.61E-04	0.91	3.67	-	1132.6
AHQ-1-12, 5301	R.VITNQYNNPAGLYSSENISNFNNALESK.T	3103.30221	3	1.07E-04	0.90	4.62	-	687.5
AHQ-1-13- , 4913 - 4980	R.VTPPEGYEVVTVFPK	1662.90695	2	1.99E-04	0.59	2.63	-	198.6
AHQ-1-12, 3551	K.VWSPLVTEEGK.R	1245.40612	1	2.70E-05	0.59	2.01	-	837.6
AHQ-1-13- , 3215	K.VWSPLVTEEGKR.H	1401.59247	2	9.73E-07	0.80	2.85	-	837.6
AHQ-1-11, 3010	K.VWSPLVTEEGKR.H	1401.59247	2	1.83E-06	0.76	2.77	-	670.3
AHQ-1-14- , 3180 - 3188	K.VWSPLVTEEGKR.H	1401.59247	2	1.51E-08	0.77	2.55	-	879.7
gi|27500274|ref|XP_113950.3| similar to Munc13-4 protein [Homo sapiens]				1.41E-12	12.26	140.31	21.30	123281.1
AHQ-1-4, 7391 - 7536	K.AFGELCPNTAPLPQLVTEALQTGTTEWFHLK.Q	3472.91108	3	1.00E-06	0.71	3.67	-	377.4
AHQ-1-4, 7347	K.ALLGLVQDVIGDLHQCQR.T	2037.32944	3	4.50E-07	0.97	4.56	-	1982.8
AHQ-1-4, 6654	R.ALLYEDALYTVLHR.L	1677.92484	2	3.59E-10	0.97	4.48	-	1993.9
AHQ-1-4, 6047 - 6056	R.AVQMDELVPLGELTK.H	1643.92698	2	5.44E-09	0.80	3.44	-	506.8
AHQ-1-4, 2488	R.DLELQAASSR.E	1090.16939	2	1.03E-05	0.94	3.56	-	1596.2
AHQ-1-4, 4863	K.HSTSAVDLSTCFAQISHTAR.Q	2191.36726	3	1.05E-09	0.98	6.13	-	1940.7
AHQ-1-4, 4876	K.HSTSAVDLSTCFAQISHTAR.Q	2191.36726	2	1.12E-06	0.97	4.81	-	1760.3
AHQ-1-4, 3594 - 3614	R.IQQQAETTSEELGAVTVK.A	1933.10674	2	2.33E-08	0.98	5.72	-	1808.5
AHQ-1-4, 3468	R.LGHPEPNHVTEASELLR.Y	1900.08490	2	4.34E-04	0.57	2.81	-	339.4
AHQ-1-4, 5599 - 5660	K.LPAQLAWEALEQR.V	1525.73360	2	1.41E-12	0.94	4.20	-	1089.7
AHQ-1-4, 7387 - 7402	R.LPLTYPAPNGDPILQLLEGR.K	2178.51621	2	6.30E-06	0.86	3.76	-	635.9
AHQ-1-4, 4238 - 4248	R.SVFPLSVSDSPAR.L	1362.51311	2	1.77E-04	0.82	2.87	-	909.6
AHQ-1-4, 4384	R.TLAEQLEVGIAK.H	1272.47301	2	3.06E-05	0.95	3.44	-	1575.4
AHQ-1-4, 6446 - 6456	R.WFQPAIPSWLQK.T	1501.75533	2	6.30E-05	0.84	3.50	-	459.1
AHQ-1-4, 3531	R.YLQEAFHVEPEEHQQTLQR.V	2383.56113	2	8.34E-04	0.88	3.69	-	686.3
AHQ-1-4, 3451 - 3528	R.YLQEAFHVEPEEHQQTLQR.V	2383.56113	3	5.86E-04	0.93	4.27	-	1455.5
gi|4502303|ref|NP_001688.1| mitochondrial ATP synthase, O subunit; ATP synthase, H+ transporting, m				1.71E-12	2.68	30.23	23.50	23277.2
AHQ-1-11, 6330	R.FSPLTTNLINLLAENGR.L	1874.13050	2	1.71E-12	0.83	3.78	-	731.3
AHQ-1-14- , 5588	R.GEVPCTVTSASPLEEATLSELK.T	2320.55669	2	2.31E-05	0.93	4.69	-	729.9
AHQ-1-11, 3182	K.VAASVLNPYVK.R	1161.37549	2	2.45E-07	0.92	3.98	-	678.8
gi|4503689|ref|NP_000499.1| fibrinogen, alpha chain isoform alpha-E preproprotein [Homo sapiens] [M				1.84E-12	15.29	190.26	32.60	94972.5
AHQ-1-7, 6073 - 6092	K.DSDWPFCSDEDWNYK.C	1965.98609	2	2.40E-07	0.92	3.54	-	707.8
AHQ-1-6, 5903	K.DSDWPFCSDEDWNYK.C	1965.98609	2	7.69E-10	0.91	3.70	-	586.4
AHQ-1-8, 6068	K.DSDWPFCSDEDWNYK.C	1965.98609	2	9.00E-04	0.84	3.28	-	476.2
AHQ-1-9, 5922 - 5924	K.DSDWPFCSDEDWNYK.C	1965.98609	2	1.41E-06	0.89	3.96	-	571.0
AHQ-1-6, 5884	K.DSHSLTTNIMEILR.G	1630.84839	2	4.84E-06	0.97	4.41	-	2061.2
AHQ-1-10, 5213	K.DSHSLTTNIMEILR.G	1630.84839	2	2.20E-04	0.95	4.23	-	1146.1
AHQ-1-6, 2186	K.ESSSHHPGIAEFPSR.G	1638.72252	2	1.69E-05	0.81	2.88	-	717.1
AHQ-1-11, 2275	K.ESSSHHPGIAEFPSR.G	1638.72252	2	2.31E-04	0.67	2.66	-	536.3
AHQ-1-9, 2499	R.EVDLKDYEDQQK.Q	1510.58460	2	2.83E-05	0.74	3.15	-	434.9
AHQ-1-13- , 2717	R.EVDLKDYEDQQK.Q	1510.58460	2	4.33E-06	0.88	3.54	-	792.9
AHQ-1-2, 2707	R.EVDLKDYEDQQK.Q	1510.58460	2	3.55E-05	0.80	2.95	-	717.1
AHQ-1-6, 2526	R.EVDLKDYEDQQK.Q	1510.58460	2	4.75E-06	0.81	3.01	-	830.1
AHQ-1-14- , 2666	R.EVDLKDYEDQQK.Q	1510.58460	2	4.81E-08	0.68	2.70	-	597.2
AHQ-1-8, 2459	R.EVDLKDYEDQQK.Q	1510.58460	2	1.29E-06	0.86	3.09	-	769.6
AHQ-1-4, 2631	R.EVDLKDYEDQQK.Q	1510.58460	2	1.38E-05	0.75	3.06	-	550.7
AHQ-1-6, 6622	K.EVVTSEDGSDCPEAMDLGTLSGIGTLDGFR.H	3131.34937	3	2.18E-05	0.91	4.66	-	838.6
AHQ-1-7, 6804	K.EVVTSEDGSDCPEAMDLGTLSGIGTLDGFR.H	3131.34937	3	2.64E-05	0.91	4.30	-	1234.3
AHQ-1-6, 6880	K.EVVTSEDGSDCPEAMDLGTLSGIGTLDGFR.H	3131.34937	3	3.17E-08	0.93	4.73	-	1133.2
AHQ-1-13- , 6432	K.EVVTSEDGSDCPEAMDLGTLSGIGTLDGFR.H	3131.34937	3	1.52E-06	0.85	3.87	-	966.6
AHQ-1-13- , 6031	K.EVVTSEDGSDCPEAM*DLGTLSGIGTLDGFR.H	3147.34877	3	1.39E-05	0.90	4.56	-	915.6
AHQ-1-13- , 6304	K.EVVTSEDGSDCPEAM*DLGTLSGIGTLDGFR.H	3147.34877	3	1.96E-04	0.92	4.45	-	1154.2
AHQ-1-1, 6376 - 6380	K.EVVTSEDGSDCPEAM*DLGTLSGIGTLDGFR.H	3147.34877	3	6.47E-04	0.91	4.87	-	911.8
AHQ-1-6, 1659	R.GGSTSYGTGSETESPR.N	1573.55765	2	1.71E-05	0.70	2.96	-	525.0
AHQ-1-7, 1640 - 1692	R.GGSTSYGTGSETESPR.N	1573.55765	2	8.03E-05	0.42	2.51	-	253.1
AHQ-1-5, 4881	K.GLIDEVNQDFTNR.I	1521.61384	2	1.47E-06	0.95	4.31	-	1127.1
AHQ-1-10, 4281 - 4311	K.GLIDEVNQDFTNR.I	1521.61384	2	4.89E-07	0.91	3.47	-	933.3
AHQ-1-1, 4852 - 4853	K.GLIDEVNQDFTNR.I	1521.61384	2	1.90E-08	0.95	4.77	-	1134.4

AHQ-1-4, 4950	K.GLIDEVNQDFTNR.I	1521.61384	2	2.97E-06	0.82	2.93	-	776.5
AHQ-1-9, 4754 - 4759	K.GLIDEVNQDFTNR.I	1521.61384	2	8.46E-09	0.93	4.13	-	886.2
AHQ-1-9, 5192	K.GLIDEVNQDFTNR.I	1521.61384	2	1.56E-07	0.93	4.17	-	1047.2
AHQ-1-6, 4155	K.GLIDEVNQDFTNR.I	1521.61384	2	5.26E-07	0.87	3.78	-	646.5
AHQ-1-6, 4723 - 4791	K.GLIDEVNQDFTNR.I	1521.61384	2	1.97E-08	0.93	4.03	-	813.7
AHQ-1-12, 4561	K.GLIDEVNQDFTNR.I	1521.61384	2	9.76E-09	0.93	3.80	-	1027.2
AHQ-1-3, 4865	K.GLIDEVNQDFTNR.I	1521.61384	2	8.14E-06	0.84	3.07	-	796.6
AHQ-1-8, 4807	K.GLIDEVNQDFTNR.I	1521.61384	2	1.72E-09	0.85	3.73	-	691.8
AHQ-1-2, 5019	K.GLIDEVNQDFTNR.I	1521.61384	2	1.79E-06	0.93	4.21	-	979.3
AHQ-1-4, 2258	R.GSESGIFTNTK.E	1141.21317	1	5.55E-04	0.11	1.89	-	219.3
AHQ-1-13- , 2363	R.GSESGIFTNTK.E	1141.21317	1	2.53E-04	0.26	2.06	-	266.1
AHQ-1-3, 2273	R.GSESGIFTNTK.E	1141.21317	2	1.43E-04	0.71	2.56	-	697.7
AHQ-1-6, 2127	R.GSESGIFTNTK.E	1141.21317	1	3.90E-04	0.23	2.07	-	315.8
AHQ-1-6, 2276	R.GSESGIFTNTK.E	1141.21317	1	4.86E-04	0.14	1.85	-	192.5
AHQ-1-6, 2131	R.GSESGIFTNTK.E	1141.21317	2	1.15E-07	0.83	2.93	-	843.5
AHQ-1-12, 2582	R.HRHPDEAAFFDTASTGK.T	1887.98954	3	4.60E-04	0.96	4.57	-	1930.2
AHQ-1-13- , 2739	R.HRHPDEAAFFDTASTGK.T	1887.98954	2	5.16E-06	0.96	4.93	-	1042.5
AHQ-1-10, 2481	R.HRHPDEAAFFDTASTGK.T	1887.98954	2	1.61E-04	0.94	4.55	-	862.8
AHQ-1-6, 2444	R.HRHPDEAAFFDTASTGK.T	1887.98954	2	5.41E-06	0.97	5.27	-	1280.3
AHQ-1-6, 2372 - 2448	R.HRHPDEAAFFDTASTGK.T	1887.98954	3	8.89E-05	0.96	4.13	-	2161.1
AHQ-1-13- , 2735 - 2808	R.HRHPDEAAFFDTASTGK.T	1887.98954	3	4.43E-06	0.93	4.15	-	1212.3
AHQ-1-11, 2519	R.HRHPDEAAFFDTASTGK.T	1887.98954	2	6.28E-04	0.97	5.03	-	1344.0
AHQ-1-7, 2452	R.HRHPDEAAFFDTASTGK.T	1887.98954	2	6.81E-04	0.94	4.35	-	858.6
AHQ-1-13, 2759	R.HRHPDEAAFFDTASTGK.T	1887.98954	2	1.93E-05	0.91	3.74	-	822.7
AHQ-1-7, 2746	R.MELERPGGNEITR.G	1502.67870	2	7.60E-05	0.85	3.48	-	667.0
AHQ-1-1, 3457	R.NPGSSGTGGTATWKPGSSGPGSTGSWNSGSSGTGSTGNQNPGSPR.P	4123.15285	3	1.22E-04	0.62	3.58	-	335.8
AHQ-1-6, 2100 - 2170	R.NPSSAGSWNSGSSGPGSTGNR.N	1964.94443	2	2.01E-08	0.96	4.47	-	1129.6
AHQ-1-7, 2120 - 2196	R.NPSSAGSWNSGSSGPGSTGNR.N	1964.94443	2	1.84E-12	0.95	4.84	-	837.7
AHQ-1-9, 2094	R.NPSSAGSWNSGSSGPGSTGNR.N	1964.94443	2	5.46E-08	0.92	4.23	-	790.4
AHQ-1-1, 2329	R.NPSSAGSWNSGSSGPGSTGNR.N	1964.94443	2	3.89E-04	0.84	3.40	-	732.1
AHQ-1-6, 2683	K.NSLFEYQK.N	1029.12765	1	8.15E-06	0.14	2.08	-	165.9
AHQ-1-9, 2106	R.PGSTGTWNPGSSER.G	1433.46515	2	6.20E-04	0.91	3.88	-	693.6
AHQ-1-1, 2384	R.PGSTGTWNPGSSER.G	1433.46515	2	7.13E-07	0.82	3.35	-	523.3
AHQ-1-8, 2080	R.PGSTGTWNPGSSER.G	1433.46515	2	4.82E-06	0.85	3.67	-	505.0
AHQ-1-6, 4779 - 4850	R.PNNPDWGTFEEVSGNVSPGTR.R	2261.34962	2	2.72E-10	0.96	4.99	-	1133.5
AHQ-1-10, 4292 - 4356	R.PNNPDWGTFEEVSGNVSPGTR.R	2261.34962	2	1.32E-04	0.59	3.46	-	403.9
AHQ-1-12, 4585 - 4647	R.PNNPDWGTFEEVSGNVSPGTR.R	2261.34962	2	3.24E-07	0.90	4.10	-	730.7
AHQ-1-13, 4620	R.PNNPDWGTFEEVSGNVSPGTR.R	2261.34962	2	2.71E-04	0.82	3.48	-	338.4
AHQ-1-10, 6363 - 6364	K.TFPGFFSPMLGEFVSETESR.G	2266.51432	2	1.11E-09	0.88	4.15	-	453.2
AHQ-1-7, 7453	K.TFPGFFSPMLGEFVSETESR.G	2266.51432	2	1.47E-05	0.69	3.02	-	291.3
AHQ-1-3, 7233 - 7235	K.TFPGFFSPMLGEFVSETESR.G	2266.51432	2	8.27E-05	0.89	3.86	-	538.8
AHQ-1-11, 6243 - 6248	K.TFPGFFSPM*LGEFVSETESR.G	2282.51372	2	8.58E-04	0.56	3.33	-	188.6
AHQ-1-11, 6442	K.TFPGFFSPMLGEFVSETESR.G	2266.51432	2	1.84E-08	0.81	3.36	-	345.8
AHQ-1-2, 7303 - 7378	K.TFPGFFSPMLGEFVSETESR.G	2266.51432	2	6.66E-05	0.74	3.17	-	312.3
AHQ-1-6, 7242 - 7322	K.TFPGFFSPMLGEFVSETESR.G	2266.51432	2	1.35E-05	0.85	3.54	-	417.2
AHQ-1-5, 7107 - 7145	K.TFPGFFSPM*LGEFVSETESR.G	2282.51372	2	3.08E-04	0.59	2.91	-	177.1
AHQ-1-12, 6455	K.TFPGFFSPM*LGEFVSETESR.G	2282.51372	2	4.97E-04	0.73	3.49	-	200.6
AHQ-1-13- , 6793	K.TFPGFFSPM*LGEFVSETESR.G	2282.51372	2	1.21E-04	0.52	2.82	-	217.0
AHQ-1-13- , 6941 - 6999	K.TFPGFFSPMLGEFVSETESR.G	2266.51432	2	1.13E-09	0.77	3.51	-	310.8
AHQ-1-13, 6333	K.TFPGFFSPM*LGEFVSETESR.G	2282.51372	2	3.95E-04	0.68	3.14	-	187.1
AHQ-1-3, 7038	K.TFPGFFSPM*LGEFVSETESR.G	2282.51372	2	6.86E-07	0.75	3.00	-	340.0
AHQ-1-3, 2419	K.VQHIQLLQK.N	1107.33106	2	4.63E-04	0.81	2.75	-	721.2
AHQ-1-7, 2242	K.VQHIQLLQK.N	1107.33106	1	2.48E-04	0.64	2.68	-	552.3
AHQ-1-3, 2414	K.VQHIQLLQK.N	1107.33106	1	9.00E-04	0.22	2.11	-	482.5
AHQ-1-6, 2235	K.VQHIQLLQK.N	1107.33106	2	5.25E-05	0.83	2.97	-	777.4
AHQ-1-8, 2117 - 2119	K.VQHIQLLQK.N	1107.33106	1	4.51E-04	0.70	2.82	-	678.4
AHQ-1-10, 2236	K.VQHIQLLQK.N	1107.33106	1	4.89E-04	0.74	2.93	-	600.4
AHQ-1-10, 2231	K.VQHIQLLQK.N	1107.33106	1	4.89E-04	0.66	2.84	-	676.4
AHQ-1-9, 2166	K.VQHIQLLQK.N	1107.33106	1	5.72E-04	0.65	2.49	-	711.5
AHQ-1-9, 2172	K.VQHIQLLQK.N	1107.33106	1	3.51E-04	0.72	2.81	-	596.4
gi|29729616|ref|XP_292963.1| similar to peptidylprolyl isomerase A (cyclophilin A) [Homo sapiens] [				1.88E-12	1.87	20.32	16.40	18025.4
AHQ-1-12, 4743 - 4805	K.HTGPGILSM*ANAGPNTNGSQFFICTAK.S	2810.11545	3	6.16E-08	0.97	6.44	-	1368.2
AHQ-1-12, 4893	K.HTGPGILSM*ANAGPNTNGSQFFICTAK.S	2810.11545	2	3.36E-04	0.58	3.27	-	241.5
AHQ-1-12, 5365 - 5425	K.HTGPGILSMANAGPNTNGSQFFICTAK.S	2794.11605	2	1.22E-05	0.91	4.75	-	731.9
AHQ-1-12, 5379 - 5437	K.HTGPGILSMANAGPNTNGSQFFICTAK.S	2794.11605	3	1.88E-12	0.91	4.91	-	1126.3
AHQ-1-12, 5485 - 5486	K.HTGPGILSMANAGPNTNGSQFFICTAK.S	2794.11605	2	7.51E-05	0.91	4.36	-	686.3
AHQ-1-13, 4747	K.HTGPGILSM*ANAGPNTNGSQFFICTAK.S	2810.11545	3	8.85E-04	0.89	4.61	-	900.9
AHQ-1-13, 4755	K.HTGPGILSM*ANAGPNTNGSQFFICTAK.S	2810.11545	2	6.39E-04	0.83	3.82	-	466.2
AHQ-1-13, 4861	K.HTGPGILSM*ANAGPNTNGSQFFICTAK.S	2810.11545	3	3.33E-04	0.62	3.43	-	514.9
AHQ-1-13- , 5003	K.HTGPGILSM*ANAGPNTNGSQFFICTAK.S	2810.11545	3	1.54E-05	0.90	4.60	-	827.8
AHQ-1-14- , 4883	K.HTGPGILSM*ANAGPNTNGSQFFICTAK.S	2810.11545	3	1.07E-05	0.96	5.56	-	1393.5
gi|4505257|ref|NP_002435.1| moesin [Homo sapiens]				2.12E-12	13.91	150.29	31.90	67819.5
AHQ-1-6, 2114 - 2116	R.ALELEQER.K	988.07707	2	1.52E-04	0.89	2.96	-	1278.8
AHQ-1-6, 2474	K.ALTSELANAR.D	1046.15975	2	2.41E-06	0.90	3.38	-	805.6
AHQ-1-6, 7207	K.EGILNDDIYCPPETAVLLASYAVQSK.Y	2869.19288	2	3.27E-07	0.76	3.94	-	241.4
AHQ-1-6, 7208	K.EGILNDDIYCPPETAVLLASYAVQSK.Y	2869.19288	3	1.99E-09	0.93	5.25	-	902.6
AHQ-1-6, 3167	K.ERQEAEEAKEALLQASR.D	1959.10752	3	1.44E-08	0.84	3.60	-	866.9
AHQ-1-6, 6251 - 6319	R.EVWFFGLQYQDTK.G	1661.83760	2	4.95E-05	0.95	3.91	-	1216.4
AHQ-1-13, 6416	K.FYPEDVSEELIQDITQR.L	2083.24065	2	1.80E-06	0.94	4.32	-	1123.5
AHQ-1-6, 7148 - 7150	K.FYPEDVSEELIQDITQR.L	2083.24065	2	1.28E-09	0.96	5.81	-	915.8
AHQ-1-6, 7151 - 7154	K.FYPEDVSEELIQDITQR.L	2083.24065	3	5.43E-10	0.95	5.01	-	1339.7
AHQ-1-13- , 6875	K.FYPEDVSEELIQDITQR.L	2083.24065	2	7.81E-06	0.92	4.00	-	990.4
AHQ-1-7, 7361	K.FYPEDVSEELIQDITQR.L	2083.24065	2	9.40E-07	0.91	3.89	-	1137.1
AHQ-1-6, 5047	R.GMLREDAVLEYLK.I	1537.80610	2	2.35E-04	0.89	3.91	-	876.1
AHQ-1-6, 6256	K.IAQDLEMYGVNYFSIK.N	1892.16455	2	6.82E-07	0.93	4.51	-	921.9
AHQ-1-6, 6370	K.IAQDLEMYGVNYFSIK.N	1892.16455	2	8.14E-07	0.81	3.17	-	667.2
AHQ-1-6, 5574 - 5650	K.IAQDLEM*YGVNYFSIK.N	1908.16395	2	1.05E-07	0.97	4.78	-	1454.0
AHQ-1-6, 6144	K.IAQDLEMYGVNYFSIK.N	1892.16455	2	9.21E-07	0.91	3.95	-	885.9
AHQ-1-6, 1826	R.IQVWHEEHR.G	1234.34835	2	1.32E-04	0.94	3.69	-	1320.1
AHQ-1-6, 2194	R.ISQLEMAR.Q	948.12272	2	1.18E-04	0.86	2.95	-	904.2
AHQ-1-6, 4730 - 4731	K.KTQEQLALEMAELTAR.I	1833.10018	2	2.75E-10	0.97	4.97	-	1519.9
AHQ-1-6, 3070	K.TAM*STPHVAEPAENEQDEQDENGAEASADLR.A	3330.36796	3	2.99E-11	0.97	5.89	-	1696.9
AHQ-1-6, 3607	K.TAMSTPHVAEPAENEQDEQDENGAEASADLR.A	3314.36856	3	2.12E-12	0.96	5.35	-	1396.9
AHQ-1-6, 5378 - 5382	K.TQEQLALEMAELTAR.I	1704.92727	2	2.53E-09	0.98	5.53	-	1699.2
gi|4507877|ref|NP_003364.1| vinculin isoform VCL [Homo sapiens]				2.15E-12	49.20	600.29	50.20	116721.6
AHQ-1-14- , 6742	K.AIPDLTAPVAAVQAAVSNLVR.V	2077.41342	2	2.83E-07	0.97	5.12	-	1278.3
AHQ-1-4, 7462 - 7535	K.AIPDLTAPVAAVQAAVSNLVR.V	2077.41342	2	3.85E-08	0.97	5.44	-	980.3
AHQ-1-13- , 6988	K.AIPDLTAPVAAVQAAVSNLVR.V	2077.41342	2	1.64E-11	0.96	5.00	-	1007.2
AHQ-1-4, 7466	K.AIPDLTAPVAAVQAAVSNLVR.V	2077.41342	3	2.97E-05	0.95	4.40	-	1651.3
AHQ-1-12, 6675 - 6677	K.AIPDLTAPVAAVQAAVSNLVR.V	2077.41342	2	2.08E-04	0.96	4.97	-	969.0
AHQ-1-11, 6498 - 6566	K.AIPDLTAPVAAVQAAVSNLVR.V	2077.41342	2	4.14E-10	0.97	5.31	-	1343.4
AHQ-1-10, 6425 - 6501	K.AIPDLTAPVAAVQAAVSNLVR.V	2077.41342	2	2.79E-08	0.97	5.85	-	1185.2
AHQ-1-9, 7326	K.AIPDLTAPVAAVQAAVSNLVR.V	2077.41342	2	3.85E-04	0.96	4.91	-	981.2
AHQ-1-8, 7526	K.AIPDLTAPVAAVQAAVSNLVR.V	2077.41342	2	6.21E-07	0.97	5.32	-	982.4
AHQ-1-1, 7031 - 7104	K.AIPDLTAPVAAVQAAVSNLVR.V	2077.41342	2	4.33E-06	0.96	4.34	-	1111.0
AHQ-1-3, 7218 - 7290	K.AIPDLTAPVAAVQAAVSNLVR.V	2077.41342	2	2.72E-06	0.93	4.19	-	982.1
AHQ-1-5, 7349	K.AIPDLTAPVAAVQAAVSNLVR.V	2077.41342	2	8.42E-08	0.95	4.35	-	1032.4

AHQ-1-5, 7493 - 7494	K.AIPDLTAPVAAVQAAVSNLVR.V	2077.41342	2	5.65E-10	0.97	5.55	-	1062.8
AHQ-1-6, 7306 - 7370	K.AIPDLTAPVAAVQAAVSNLVR.V	2077.41342	2	9.29E-11	0.97	5.01	-	1068.9
AHQ-1-7, 7512	K.AIPDLTAPVAAVQAAVSNLVR.V	2077.41342	2	1.11E-06	0.97	5.34	-	1039.4
AHQ-1-2, 7429	K.AIPDLTAPVAAVQAAVSNLVR.V	2077.41342	2	1.79E-06	0.97	5.31	-	1075.5
AHQ-1-5, 3273 - 3299	R.ALASQLQDSLK.D	1174.32943	2	1.30E-05	0.91	3.69	-	1001.7
AHQ-1-4, 3399 - 3472	R.ALASQLQDSLK.D	1174.32943	2	2.39E-05	0.83	3.33	-	755.4
AHQ-1-3, 3389	R.ALASQLQDSLK.D	1174.32943	2	1.69E-05	0.84	3.23	-	782.7
AHQ-1-8, 3184	R.ALASQLQDSLK.D	1174.32943	2	1.22E-04	0.83	2.68	-	1018.3
AHQ-1-7, 3504 - 3572	K.AQQVSQGLDVLTAK.V	1458.64233	2	2.18E-04	0.89	3.24	-	1066.7
AHQ-1-4, 3607 - 3678	K.AQQVSQGLDVLTAK.V	1458.64233	2	8.46E-11	0.97	5.38	-	1676.5
AHQ-1-5, 3458 - 3530	K.AQQVSQGLDVLTAK.V	1458.64233	2	1.20E-09	0.96	4.20	-	1307.5
AHQ-1-4, 3671	K.AQQVSQGLDVLTAK.V	1458.64233	1	2.17E-04	0.34	2.51	-	406.8
AHQ-1-4, 3675	K.AQQVSQGLDVLTAK.V	1458.64233	1	1.63E-05	0.70	3.56	-	354.5
AHQ-1-5, 3522	K.AQQVSQGLDVLTAK.V	1458.64233	1	2.16E-04	0.75	3.56	-	397.7
AHQ-1-1, 3748	K.AQQVSQGLDVLTAK.V	1458.64233	2	3.75E-04	0.95	3.46	-	1410.5
AHQ-1-8, 3330	K.AQQVSQGLDVLTAK.V	1458.64233	2	1.97E-11	0.96	4.64	-	1363.4
AHQ-1-2, 3809	K.AQQVSQGLDVLTAK.V	1458.64233	2	6.37E-10	0.96	4.64	-	1282.3
AHQ-1-7, 3588	K.AQQVSQGLDVLTAK.V	1458.64233	2	3.74E-05	0.95	3.67	-	1446.1
AHQ-1-4, 2720	K.AVAGNISDPGLQK.S	1270.41721	2	8.58E-05	0.59	2.55	-	533.9
AHQ-1-7, 2440	K.AVAGNISDPGLQK.S	1270.41721	2	3.75E-04	0.70	2.97	-	515.2
AHQ-1-5, 2401 - 2417	K.AVAGNISDPGLQK.S	1270.41721	2	1.19E-04	0.86	3.65	-	640.7
AHQ-1-5, 2530	K.AVAGNISDPGLQK.S	1270.41721	2	2.21E-05	0.76	3.40	-	578.7
AHQ-1-2, 2581 - 2599	K.AVAGNISDPGLQK.S	1270.41721	2	1.02E-05	0.78	3.10	-	609.0
AHQ-1-4, 2490	K.AVAGNISDPGLQK.S	1270.41721	2	9.51E-06	0.69	2.78	-	690.3
AHQ-1-1, 2631	K.AVAGNISDPGLQK.S	1270.41721	2	3.20E-06	0.89	3.47	-	736.6
AHQ-1-3, 2515	K.AVAGNISDPGLQK.S	1270.41721	2	3.72E-05	0.85	3.15	-	819.2
AHQ-1-3, 6050	K.CDRVDQLTAQLADLAAR.G	1918.12182	2	2.90E-04	0.94	3.88	-	1359.3
AHQ-1-6, 6018	K.CDRVDQLTAQLADLAAR.G	1918.12182	3	1.38E-05	0.96	5.12	-	1565.0
AHQ-1-4, 6154	K.CDRVDQLTAQLADLAAR.G	1918.12182	2	6.35E-06	0.94	4.08	-	1252.6
AHQ-1-4, 6160	K.CDRVDQLTAQLADLAAR.G	1918.12182	3	9.76E-05	0.89	4.25	-	1126.9
AHQ-1-4, 6367	K.CDRVDQLTAQLADLAAR.G	1918.12182	2	7.81E-07	0.92	3.77	-	1010.6
AHQ-1-5, 6126 - 6141	K.CDRVDQLTAQLADLAAR.G	1918.12182	2	5.31E-05	0.94	3.50	-	1349.1
AHQ-1-5, 6130 - 6145	K.CDRVDQLTAQLADLAAR.G	1918.12182	3	7.64E-07	0.96	5.59	-	1480.7
AHQ-1-6, 6011 - 6068	K.CDRVDQLTAQLADLAAR.G	1918.12182	2	6.15E-05	0.78	2.99	-	789.3
AHQ-1-3, 6055	K.CDRVDQLTAQLADLAAR.G	1918.12182	3	1.94E-06	0.89	4.13	-	1233.5
AHQ-1-4, 6587	K.CDRVDQLTAQLADLAAR.G	1918.12182	2	2.04E-05	0.69	3.17	-	417.0
AHQ-1-4, 2359	R.DPSASPGDAGEQAIR.Q	1471.51169	1	2.02E-05	0.27	2.07	-	215.4
AHQ-1-4, 2343	R.DPSASPGDAGEQAIR.Q	1471.51169	2	2.15E-12	0.87	3.22	-	725.8
AHQ-1-5, 2290	R.DPSASPGDAGEQAIR.Q	1471.51169	1	6.71E-04	0.18	2.02	-	115.3
AHQ-1-4, 2244	R.DPSASPGDAGEQAIR.Q	1471.51169	2	7.52E-06	0.88	3.66	-	706.4
AHQ-1-3, 2293 - 2361	R.DPSASPGDAGEQAIR.Q	1471.51169	2	7.35E-07	0.92	3.93	-	720.7
AHQ-1-7, 2325	R.DPSASPGDAGEQAIR.Q	1471.51169	2	1.81E-04	0.91	3.38	-	821.8
AHQ-1-1, 2447	R.DPSASPGDAGEQAIR.Q	1471.51169	2	1.17E-07	0.86	3.24	-	692.0
AHQ-1-6, 2284	R.DPSASPGDAGEQAIR.Q	1471.51169	2	1.69E-08	0.91	3.78	-	676.4
AHQ-1-5, 2587	R.DYLIDGSR.G	939.00451	1	9.22E-05	0.21	1.95	-	199.0
AHQ-1-5, 2491	R.DYLIDGSR.G	939.00451	1	6.17E-04	0.59	2.59	-	389.6
AHQ-1-3, 4874	K.ELLPVLISAM*K.I	1230.54265	2	6.44E-04	0.64	2.75	-	660.5
AHQ-1-5, 6007	K.ELLPVLISAMK.I	1214.54325	2	6.31E-07	0.86	3.32	-	659.8
AHQ-1-5, 4762	K.ELLPVLISAM*K.I	1230.54265	2	3.02E-06	0.86	3.27	-	806.7
AHQ-1-4, 6148	K.ELLPVLISAMK.I	1214.54325	2	2.15E-06	0.68	2.83	-	541.5
AHQ-1-4, 4915 - 4948	K.ELLPVLISAM*K.I	1230.54265	2	3.75E-05	0.87	3.64	-	733.1
AHQ-1-6, 5966	K.ELLPVLISAMK.I	1214.54325	2	9.32E-05	0.68	3.02	-	414.6
AHQ-1-6, 2634 - 2642	R.ELTPQVVSAAR.I	1171.32878	2	8.65E-04	0.92	3.00	-	1320.1
AHQ-1-5, 2633 - 2646	R.ELTPQVVSAAR.I	1171.32878	2	6.59E-04	0.93	3.40	-	1281.9
AHQ-1-5, 3082	K.ETVQTTEDQILK.R	1405.53306	1	7.87E-06	0.08	2.26	-	113.7
AHQ-1-14- , 3134	K.ETVQTTEDQILK.R	1405.53306	2	4.97E-04	0.76	2.61	-	668.7
AHQ-1-4, 3202	K.ETVQTTEDQILK.R	1405.53306	1	9.92E-10	0.13	2.62	-	156.4
AHQ-1-14- , 3054	K.ETVQTTEDQILKR.D	1561.71942	2	2.85E-06	0.57	2.89	-	405.6
AHQ-1-4, 3043	K.ETVQTTEDQILKR.D	1561.71942	2	1.14E-05	0.90	3.54	-	976.3
AHQ-1-14, 3914	K.ETVQTTEDQILKR.D	1561.71942	2	2.26E-08	0.69	3.07	-	610.3
AHQ-1-5, 2894	K.ETVQTTEDQILKR.D	1561.71942	2	2.28E-07	0.71	2.90	-	707.0
AHQ-1-9, 2848	K.ETVQTTEDQILKR.D	1561.71942	2	6.75E-06	0.80	3.11	-	749.4
AHQ-1-6, 2923 - 2938	K.ETVQTTEDQILKR.D	1561.71942	2	1.78E-05	0.54	2.94	-	543.6
AHQ-1-12, 2943	K.ETVQTTEDQILKR.D	1561.71942	2	5.90E-05	0.74	2.89	-	912.4
AHQ-1-3, 3041	K.ETVQTTEDQILKR.D	1561.71942	2	6.39E-05	0.86	2.96	-	1161.5
AHQ-1-1, 6824 - 6904	R.GILSGTSDLLLTFDEAEVR.K	2037.25656	2	1.12E-05	0.79	3.54	-	592.3
AHQ-1-4, 7570	R.GILSGTSDLLLTFDEAEVR.K	2037.25656	2	2.59E-04	0.80	3.53	-	558.6
AHQ-1-4, 7227 - 7298	R.GILSGTSDLLLTFDEAEVR.K	2037.25656	2	4.26E-05	0.91	3.96	-	822.0
AHQ-1-4, 7100 - 7179	R.GILSGTSDLLLTFDEAEVR.K	2037.25656	2	4.11E-05	0.81	3.48	-	622.0
AHQ-1-5, 7273	R.GILSGTSDLLLTFDEAEVR.K	2037.25656	2	2.09E-04	0.94	4.61	-	887.8
AHQ-1-11, 6338	R.GILSGTSDLLLTFDEAEVR.K	2037.25656	2	8.34E-07	0.93	4.63	-	804.3
AHQ-1-4, 6830	R.GILSGTSDLLLTFDEAEVRK.I	2165.42947	3	2.43E-09	0.81	3.57	-	715.9
AHQ-1-4, 6822	R.GILSGTSDLLLTFDEAEVRK.I	2165.42947	2	2.78E-08	0.89	3.80	-	1012.5
AHQ-1-12, 2139	K.GNDIIAAAK.R	872.98920	2	1.18E-04	0.87	2.77	-	1150.8
AHQ-1-4, 1531 - 2227	K.GNDIIAAAK.R	872.98920	1	2.84E-04	0.27	2.06	-	315.5
AHQ-1-5, 3509	K.GWLRDPSASPGDAGEQAIR.Q	1984.11876	2	3.40E-04	0.92	4.65	-	666.1
AHQ-1-4, 3662	K.GWLRDPSASPGDAGEQAIR.Q	1984.11876	2	8.42E-07	0.87	4.01	-	609.9
AHQ-1-4, 3668	K.GWLRDPSASPGDAGEQAIR.Q	1984.11876	3	6.62E-08	0.88	4.23	-	829.2
AHQ-1-4, 2223	K.IAELCDDPKER.D	1347.47690	3	3.14E-06	0.83	3.48	-	710.0
AHQ-1-4, 2218	K.IAELCDDPKER.D	1347.47690	2	1.92E-06	0.81	3.08	-	571.5
AHQ-1-3, 3157	R.IPTISTQLK.I	1001.20202	2	1.87E-04	0.74	2.52	-	634.5
AHQ-1-12, 3050	R.IPTISTQLK.I	1001.20202	2	5.48E-04	0.77	2.55	-	679.3
AHQ-1-5, 3174	R.KIAELCDDPKERDDILR.S	2088.32839	3	5.30E-06	0.62	3.18	-	508.9
AHQ-1-4, 3323	R.KIAELCDDPKERDDILR.S	2088.32839	3	4.17E-04	0.81	3.29	-	929.7
AHQ-1-5, 3175	R.KIAELCDDPKERDDILR.S	2088.32839	2	1.59E-04	0.65	3.60	-	275.2
AHQ-1-4, 4460 - 4530	K.KIDAAQNWLADPNGGPEGEEQIR.G	2509.67332	2	1.59E-09	0.87	3.92	-	513.6
AHQ-1-5, 4365 - 4433	K.KIDAAQNWLADPNGGPEGEEQIR.G	2509.67332	2	1.03E-05	0.74	3.29	-	332.8
AHQ-1-4, 3458	R.LANVMMGPYR.Q	1152.41560	2	2.02E-04	0.90	3.73	-	783.9
AHQ-1-5, 3301 - 3334	R.LANVMMGPYR.Q	1152.41560	2	1.08E-04	0.93	3.79	-	845.9
AHQ-1-6, 2802 - 2816	R.LANVMM*GPYR.Q	1168.41500	2	1.39E-04	0.78	3.16	-	714.8
AHQ-1-4, 2924 - 2928	R.LANVMM*GPYR.Q	1168.41500	2	2.66E-04	0.83	3.69	-	610.8
AHQ-1-2, 4558	K.LLAVAATAPPDAPNREEVFDER.A	2382.61458	3	5.73E-04	0.84	3.74	-	705.6
AHQ-1-4, 4486 - 4487	K.LLAVAATAPPDAPNREEVFDER.A	2382.61458	3	5.46E-10	0.62	3.11	-	603.0
AHQ-1-3, 4401 - 4402	K.LLAVAATAPPDAPNREEVFDER.A	2382.61458	3	3.79E-07	0.66	3.78	-	585.2
AHQ-1-5, 4302 - 4374	K.LLAVAATAPPDAPNREEVFDER.A	2382.61458	3	2.57E-09	0.82	3.81	-	806.9
AHQ-1-4, 4582 - 4590	K.LLAVAATAPPDAPNREEVFDER.A	2382.61458	2	5.14E-05	0.89	3.82	-	761.3
AHQ-1-4, 4382 - 4451	K.LLAVAATAPPDAPNREEVFDER.A	2382.61458	2	3.89E-04	0.77	3.81	-	268.2
AHQ-1-4, 4458	R.LTDELAPPKPPLPEGEVPPPRPPPPEEK.D	3025.44391	3	6.07E-05	0.68	4.10	-	463.2
AHQ-1-4, 4648 - 4718	R.LTDELAPPKPPLPEGEVPPPRPPPPEEKDEEFPEQK.A	4028.46845	3	4.04E-05	0.86	4.08	-	796.5
AHQ-1-6, 3682	K.LVQAAQM*LQSDPYSVPAR.D	1991.25773	2	1.03E-05	0.94	4.24	-	929.5
AHQ-1-3, 4662 - 4677	K.LVQAAQMLQSDPYSVPAR.D	1975.25833	2	2.70E-04	0.74	3.02	-	596.5
AHQ-1-6, 4570 - 4571	K.LVQAAQMLQSDPYSVPAR.D	1975.25833	2	2.85E-05	0.94	5.16	-	675.2
AHQ-1-4, 4726 - 4748	K.LVQAAQMLQSDPYSVPAR.D	1975.25833	3	6.00E-07	0.95	4.80	-	1418.0
AHQ-1-3, 3835 - 3838	K.LVQAAQM*LQSDPYSVPAR.D	1991.25773	2	4.87E-05	0.89	3.98	-	584.2
AHQ-1-1, 3871	K.LVQAAQM*LQSDPYSVPAR.D	1991.25773	2	2.19E-06	0.73	2.91	-	569.1
AHQ-1-4, 3888	K.LVQAAQM*LQSDPYSVPAR.D	1991.25773	3	1.68E-06	0.95	4.81	-	1486.1
AHQ-1-4, 4480	R.MALLMAEMSR.L	1153.46531	2	7.12E-08	0.96	4.42	-	1715.6

AHQ-1-2, 4419	K.MLGQMTDQVADLR.A	1478.72055	2	1.90E-07	0.93	4.01	-	1227.2
AHQ-1-4, 2958 - 2960	K.M*LGQM*TDQVADLR.A	1510.71935	2	6.71E-05	0.95	4.06	-	1570.9
AHQ-1-2, 4009	K.M*LGQMTDQVADLR.A	1494.71995	2	1.20E-05	0.84	3.02	-	709.4
AHQ-1-8, 4116	K.MLGQMTDQVADLR.A	1478.72055	2	3.70E-06	0.93	4.61	-	987.7
AHQ-1-5, 3754 - 3766	K.M*LGQMTDQVADLR.A	1494.71995	2	7.73E-08	0.97	4.08	-	2467.9
AHQ-1-6, 3699	K.M*LGQMTDQVADLR.A	1494.71995	2	5.90E-05	0.95	3.40	-	1720.6
AHQ-1-5, 2849	K.M*LGQM*TDQVADLR.A	1510.71935	2	2.38E-05	0.96	4.07	-	1673.8
AHQ-1-6, 4135 - 4140	K.MLGQMTDQVADLR.A	1478.72055	2	4.72E-07	0.96	4.44	-	1608.7
AHQ-1-3, 4274	K.MLGQMTDQVADLR.A	1478.72055	2	2.44E-05	0.94	3.95	-	1329.1
AHQ-1-7, 4192	K.MLGQMTDQVADLR.A	1478.72055	2	4.41E-07	0.95	3.91	-	1659.9
AHQ-1-4, 3820 - 3878	K.M*LGQMTDQVADLR.A	1494.71995	2	8.24E-06	0.93	3.53	-	1393.3
AHQ-1-4, 4312	K.M*LGQM*TDQVADLR.A	1510.71935	2	8.31E-04	0.86	3.35	-	971.0
AHQ-1-5, 3242 - 3250	K.MLGQM*TDQVADLR.A	1494.71995	2	3.07E-06	0.90	3.62	-	1304.4
AHQ-1-4, 4292	K.MLGQMTDQVADLR.A	1478.72055	2	8.52E-04	0.80	2.92	-	1119.6
AHQ-1-5, 4181 - 4182	K.MLGQMTDQVADLR.A	1478.72055	2	5.18E-07	0.97	5.08	-	1533.3
AHQ-1-4, 3374	K.MLGQM*TDQVADLR.A	1494.71995	2	1.25E-04	0.94	3.59	-	1734.4
AHQ-1-5, 3105	K.M*SAEINEIIR.V	1192.36823	2	5.36E-06	0.91	2.82	-	1231.5
AHQ-1-4, 3235	K.M*SAEINEIIR.V	1192.36823	2	8.61E-04	0.86	3.01	-	863.8
AHQ-1-4, 3988	K.MSAEINEIIR.V	1176.36883	2	7.02E-06	0.96	4.27	-	1920.0
AHQ-1-5, 3753 - 3759	K.MSAEINEIIR.V	1176.36883	2	6.48E-06	0.94	3.80	-	1536.1
AHQ-1-4, 3667	K.MTGLVDEAIDTK.S	1293.46936	2	5.53E-07	0.96	3.72	-	1789.1
AHQ-1-6, 3490	K.MTGLVDEAIDTK.S	1293.46936	2	3.83E-08	0.97	4.13	-	1590.2
AHQ-1-5, 3057	K.M*TGLVDEAIDTK.S	1309.46876	2	6.89E-07	0.94	3.80	-	1260.2
AHQ-1-4, 3123 - 3198	K.M*TGLVDEAIDTK.S	1309.46876	2	2.28E-08	0.91	3.11	-	1190.9
AHQ-1-5, 3525	K.MTGLVDEAIDTK.S	1293.46936	2	1.08E-09	0.97	3.98	-	1703.5
AHQ-1-4, 3212	K.M*TGLVDEAIDTK.S	1309.46876	1	9.18E-04	0.23	2.27	-	205.7
AHQ-1-4, 3176	K.M*TGLVDEAIDTK.S	1309.46876	2	9.15E-07	0.94	3.58	-	1375.6
AHQ-1-5, 2857	R.NPGNQAAYEHFETMK.N	1737.87437	3	6.42E-04	0.78	3.27	-	551.5
AHQ-1-5, 2855	R.NPGNQAAYEHFETMK.N	1737.87437	2	2.03E-05	0.95	4.14	-	1189.4
AHQ-1-4, 3022	R.NPGNQAAYEHFETMK.N	1737.87437	2	1.74E-04	0.96	4.32	-	1268.5
AHQ-1-4, 2495	R.NPGNQAAYEHFETM*K.N	1753.87377	2	4.89E-05	0.90	4.02	-	673.2
AHQ-1-4, 2247	K.NQGIEEALKNR.N	1272.39320	2	9.21E-05	0.83	2.79	-	1199.0
AHQ-1-4, 2991	K.NQWIDNVEK.M	1146.23464	1	2.14E-04	0.38	2.36	-	240.7
AHQ-1-5, 2826	K.NQWIDNVEK.M	1146.23464	1	5.95E-04	0.18	1.83	-	274.3
AHQ-1-4, 3142	K.QVATALQNLQTK.T	1315.50112	2	1.79E-04	0.93	4.07	-	1041.5
AHQ-1-5, 2769 - 2777	K.QVATALQNLQTK.T	1315.50112	2	3.66E-07	0.91	3.83	-	660.8
AHQ-1-4, 2926 - 2927	K.QVATALQNLQTK.T	1315.50112	2	3.72E-09	0.90	3.47	-	778.5
AHQ-1-6, 3374	R.SLGEISALTSK.L	1106.25186	2	9.07E-06	0.92	3.44	-	908.6
AHQ-1-4, 3536	R.SLGEISALTSK.L	1106.25186	2	8.80E-07	0.94	3.96	-	966.0
AHQ-1-4, 3543	R.SLGEISALTSK.L	1106.25186	1	1.20E-06	0.20	2.44	-	188.7
AHQ-1-5, 3386	R.SLGEISALTSK.L	1106.25186	1	2.63E-07	0.20	2.10	-	187.9
AHQ-1-3, 3545	R.SLGEISALTSK.L	1106.25186	2	1.63E-05	0.94	3.74	-	1079.7
AHQ-1-7, 3416	R.SLGEISALTSK.L	1106.25186	1	2.46E-05	0.62	2.82	-	335.3
AHQ-1-1, 3633	R.SLGEISALTSK.L	1106.25186	2	3.18E-07	0.95	3.90	-	1144.7
AHQ-1-5, 3261	K.SLLDASEEAIKK.D	1304.47187	2	7.90E-05	0.80	2.98	-	782.6
AHQ-1-7, 3296 - 3300	K.SLLDASEEAIKK.D	1304.47187	2	7.40E-06	0.79	2.91	-	685.1
AHQ-1-4, 3394 - 3470	K.SLLDASEEAIKK.D	1304.47187	2	2.04E-06	0.89	3.34	-	827.1
AHQ-1-6, 3276	K.SLLDASEEAIKK.D	1304.47187	2	6.95E-06	0.73	2.65	-	568.4
AHQ-1-3, 3409	K.SLLDASEEAIKK.D	1304.47187	2	5.81E-05	0.75	2.73	-	768.9
AHQ-1-3, 2431	K.STVEGIQASVK.T	1119.25064	2	1.90E-06	0.86	3.04	-	745.5
AHQ-1-4, 2404	K.STVEGIQASVK.T	1119.25064	2	3.63E-07	0.92	3.70	-	779.9
AHQ-1-12, 2494 - 2495	R.TDAGFTLR.W	880.96819	2	9.47E-06	0.86	2.65	-	903.2
AHQ-1-4, 2506	R.TDAGFTLR.W	880.96819	2	9.13E-06	0.89	2.79	-	988.6
AHQ-1-9, 7276	R.TIESILEPVAQQISHLVIMHEEGEVDGK.A	3103.49334	3	7.49E-05	0.79	3.60	-	502.4
AHQ-1-11, 6436	R.TIESILEPVAQQISHLVIM*HEEGEVDGK.A	3119.49274	3	6.04E-04	0.79	3.60	-	411.4
AHQ-1-1, 4892	R.TNISDEESEQATEM*LVHNAQNLM*QSVK.E	3080.30897	3	6.87E-04	0.80	3.75	-	822.5
AHQ-1-12, 5330 - 5391	R.TNISDEESEQATEM*LVHNAQNLM*QSVK.E	3080.30897	3	2.57E-05	0.91	4.43	-	695.0
AHQ-1-12, 5689 - 5746	R.TNISDEESEQATEMLVHNAQNLM*QSVK.E	3064.30957	3	2.85E-04	0.90	4.97	-	736.8
AHQ-1-4, 5367	R.TNISDEESEQATEMLVHNAQNLM*QSVK.E	3064.30957	3	2.71E-04	0.82	4.14	-	496.1
AHQ-1-12, 5697 - 5729	R.TNISDEESEQATEMLVHNAQNLMQSVK.E	3048.31017	3	4.89E-06	0.86	4.12	-	580.1
AHQ-1-4, 4935	R.TNISDEESEQATEM*LVHNAQNLM*QSVK.E	3080.30897	3	4.80E-04	0.59	3.01	-	326.6
AHQ-1-10, 6251	R.TNISDEESEQATEMLVHNAQNLMQSVK.E	3048.31017	3	2.35E-06	0.92	4.94	-	602.6
AHQ-1-13, 6391 - 6468	R.TNISDEESEQATEMLVHNAQNLMQSVK.E	3048.31017	3	2.15E-08	0.46	3.11	-	415.0
AHQ-1-13- , 6740 - 6792	R.TNISDEESEQATEM*LVHNAQNLMQSVK.E	3064.30957	3	9.95E-09	0.57	3.28	-	363.9
AHQ-1-14- , 5460 - 5463	R.TNISDEESEQATEM*LVHNAQNLM*QSVK.E	3080.30897	3	1.37E-04	0.78	3.61	-	496.7
AHQ-1-4, 6338 - 6339	R.TNISDEESEQATEMLVHNAQNLM*QSVK.E	3064.30957	3	5.11E-05	0.82	4.27	-	561.1
AHQ-1-10, 5449	R.TNISDEESEQATEMLVHNAQNLMQSVK.E	3048.31017	3	7.03E-07	0.83	4.26	-	536.5
AHQ-1-5, 7297 - 7301	R.TNISDEESEQATEMLVHNAQNLMQSVK.E	3048.31017	3	3.67E-08	0.94	5.28	-	797.7
AHQ-1-9, 7119	R.TNISDEESEQATEMLVHNAQNLMQSVK.E	3048.31017	3	5.03E-05	0.90	4.57	-	659.9
AHQ-1-1, 6825	R.TNISDEESEQATEM*LVHNAQNLMQSVK.E	3064.30957	3	1.58E-04	0.81	4.02	-	488.6
AHQ-1-8, 7279	R.TNISDEESEQATEMLVHNAQNLMQSVK.E	3048.31017	3	6.60E-04	0.70	3.14	-	573.4
AHQ-1-4, 7024	R.TNISDEESEQATEM*LVHNAQNLMQSVK.E	3064.30957	3	2.27E-04	0.54	3.41	-	404.6
AHQ-1-12, 3170	R.TNLLQVCER.I	1134.28859	2	7.00E-04	0.87	3.29	-	1021.2
AHQ-1-4, 5626	K.VAM*ANIQPQMLVAGATSIAR.R	2059.44338	2	4.64E-05	0.88	4.44	-	567.2
AHQ-1-5, 5505	K.VAM*ANIQPQMLVAGATSIAR.R	2059.44338	3	2.60E-05	0.93	4.77	-	1818.0
AHQ-1-5, 5021 - 5095	K.VAM*ANIQPQM*LVAGATSIAR.R	2075.44278	2	1.08E-05	0.80	3.68	-	534.4
AHQ-1-7, 5385 - 5456	K.VAM*ANIQPQMLVAGATSIAR.R	2059.44338	2	1.73E-04	0.89	4.47	-	559.9
AHQ-1-4, 5531 - 5598	K.VAM*ANIQPQMLVAGATSIAR.R	2059.44338	3	1.32E-08	0.87	3.93	-	1715.1
AHQ-1-4, 6234	K.VAMANIQPQMLVAGATSIAR.R	2043.44398	3	8.51E-05	0.94	4.89	-	1301.9
AHQ-1-2, 5689	K.VAM*ANIQPQMLVAGATSIAR.R	2059.44338	2	2.76E-06	0.88	4.46	-	612.3
AHQ-1-4, 5802	K.VAMANIQPQM*LVAGATSIAR.R	2059.44338	3	9.01E-04	0.92	4.65	-	1419.6
AHQ-1-4, 5111	K.VAM*ANIQPQM*LVAGATSIAR.R	2075.44278	3	8.72E-05	0.94	4.26	-	1982.3
AHQ-1-5, 6197	K.VAMANIQPQMLVAGATSIAR.R	2043.44398	3	8.91E-10	0.96	5.34	-	1628.0
AHQ-1-3, 5643 - 5713	K.VAMANIQPQM*LVAGATSIAR.R	2059.44338	2	7.91E-04	0.55	3.45	-	364.4
AHQ-1-5, 6179 - 6193	K.VAMANIQPQMLVAGATSIAR.R	2043.44398	2	3.13E-06	0.95	5.03	-	799.2
AHQ-1-5, 5690	K.VAMANIQPQM*LVAGATSIAR.R	2059.44338	3	7.11E-06	0.90	4.58	-	1330.9
AHQ-1-4, 5335	R.VDQLTAQLADLAAR.G	1485.66778	2	5.36E-06	0.95	3.83	-	1652.9
AHQ-1-4, 5234 - 5235	R.VDQLTAQLADLAAR.G	1485.66778	1	5.57E-04	0.66	2.79	-	338.5
AHQ-1-5, 5149 - 5157	R.VDQLTAQLADLAAR.G	1485.66778	2	1.94E-07	0.98	4.99	-	2421.9
AHQ-1-2, 5726	R.VLQLTSWDEDAWASK.D	1749.90156	2	1.21E-05	0.86	2.57	-	1078.4
AHQ-1-4, 5518 - 5579	R.VLQLTSWDEDAWASK.D	1749.90156	2	2.40E-04	0.78	2.83	-	765.0
AHQ-1-7, 5532 - 5533	R.VLQLTSWDEDAWASK.D	1749.90156	2	3.20E-08	0.98	5.42	-	1625.9
AHQ-1-3, 5533	R.VLQLTSWDEDAWASK.D	1749.90156	2	7.69E-06	0.78	2.54	-	847.4
AHQ-1-1, 5595 - 5596	R.VLQLTSWDEDAWASK.D	1749.90156	2	2.56E-08	0.97	5.31	-	1590.1
AHQ-1-5, 5442 - 5517	R.VLQLTSWDEDAWASK.D	1749.90156	2	1.71E-07	0.95	4.02	-	1301.9
AHQ-1-4, 5836	R.VLQLTSWDEDAWASK.D	1749.90156	2	2.69E-08	0.94	4.22	-	925.0
AHQ-1-5, 5525 - 5587	R.VLQLTSWDEDAWASK.D	1749.90156	2	1.64E-05	0.97	5.20	-	1475.3
AHQ-1-6, 5438 - 5507	R.VLQLTSWDEDAWASK.D	1749.90156	2	4.11E-05	0.79	3.07	-	688.5
AHQ-1-4, 5622 - 5654	R.VLQLTSWDEDAWASK.D	1749.90156	2	2.94E-11	0.97	5.34	-	1427.5
AHQ-1-4, 3343	R.VMLVNSMNTVK.E	1236.53081	1	1.18E-04	0.29	1.97	-	392.0
AHQ-1-4, 3280	R.VMLVNSMNTVK.E	1236.53081	2	2.47E-04	0.89	3.16	-	987.0
AHQ-1-4, 2484	R.VMLVNSM*NTVK.E	1252.53021	2	4.14E-05	0.93	3.34	-	1114.5
AHQ-1-5, 3157	R.VMLVNSMNTVK.E	1236.53081	2	8.78E-04	0.87	3.23	-	899.8
AHQ-1-4, 3647	R.VMLVNSMNTVK.E	1236.53081	2	7.93E-07	0.75	3.06	-	615.2
gi|4504515|ref|NP_002146.1| heat shock 70kDa protein 6 (HSP70B'); heat shock 70kD protein 6 (HSP70B				2.29E-12	3.92	50.24	6.40	70853.4
AHQ-1-6, 5063 - 5075	R.ARFEELCSDLFR.S	1544.71384	2	5.38E-06	0.95	4.72	-	998.9
AHQ-1-6, 5138	R.FEELCSDLFR.S	1317.44926	2	7.58E-05	0.86	3.53	-	778.3

AHQ-1-6, 4942 - 4956	R.IINEPTAAAIAYGLDR.R	1688.90603	2	2.29E-12	0.95	4.43	-	1117.4
AHQ-1-6, 4504	K.LLQDFFNGK.E	1082.23369	1	4.16E-04	0.39	2.31	-	436.0
AHQ-1-6, 4403 - 4407	K.LLQDFFNGK.E	1082.23369	1	5.97E-06	0.25	2.22	-	340.9
AHQ-1-6, 4488	K.LLQDFFNGKELNK.S	1566.78249	2	1.04E-04	0.93	4.06	-	1174.6
AHQ-1-6, 4343 - 4400	K.LLQDFFNGKELNK.S	1566.78249	2	1.60E-06	0.91	3.84	-	1020.3
gi|5802974|ref|NP_006784.1| peroxiredoxin 3; antioxidant protein 1; thioredoxin-dependent peroxide				2.34E-12	4.09	60.29	28.90	27692.5
AHQ-1-11, 4359	K.AFQYVETHGEVCPANWTPDSPTIKPSPAASK.E	3388.70790	3	2.40E-06	0.93	4.65	-	1131.4
AHQ-1-11, 3883 - 3884	K.DLSLDDFK.G	953.02795	1	4.32E-05	0.30	2.16	-	451.0
AHQ-1-11, 5110 - 5112	R.DYGVLLEGSGLALR.G	1463.66058	2	2.34E-12	0.98	5.72	-	2746.7
AHQ-1-11, 5336	R.GLFIIDPNGVIK.H	1286.54452	2	1.25E-05	0.87	3.59	-	821.8
AHQ-1-11, 5326	R.GLFIIDPNGVIK.H	1286.54452	1	3.33E-06	0.24	2.01	-	286.4
AHQ-1-11, 5059	R.GLFIIDPNGVIK.H	1286.54452	2	6.53E-06	0.87	3.07	-	1120.1
AHQ-1-11, 5187	R.GLFIIDPNGVIK.H	1286.54452	2	7.45E-04	0.38	2.56	-	389.0
AHQ-1-11, 4894	K.GTAVVNGEFKDLSLDDFK.G	1956.14200	2	1.43E-04	0.86	3.85	-	730.8
AHQ-1-11, 2878 - 2926	K.HLSVNDLPVGR.S	1207.36435	2	1.12E-05	0.78	3.02	-	493.1
gi|29732955|ref|XP_291603.1| similar to glyceraldehyde 3-phosphate dehydrogenase [Homo sapiens] [MA				2.50E-12	0.83	10.23	16.80	11173.0
AHQ-1-9, 4471 - 4518	K.NISNASCTTNCIAPLAK.M	1838.05353	2	2.55E-08	0.75	3.36	-	689.0
AHQ-1-10, 3671 - 3739	K.NISNASCTTNCIAPLAK.M	1838.05353	2	4.40E-08	0.94	4.54	-	1092.0
AHQ-1-10, 3848 - 3872	K.NISNASCTTNCIAPLAK.M	1838.05353	2	2.50E-12	0.83	3.77	-	1229.8
AHQ-1-11, 4082	K.NISNASCTTNCIAPLAK.M	1838.05353	2	1.62E-05	0.49	3.14	-	615.6
AHQ-1-12, 3947	K.NISNASCTTNCIAPLAK.M	1838.05353	2	2.81E-07	0.66	3.29	-	861.9
AHQ-1-9, 4278 - 4354	K.NISNASCTTNCIAPLAK.M	1838.05353	2	6.13E-07	0.86	4.24	-	1130.3
gi|5453898|ref|NP_006212.1| protein (peptidyl-prolyl cis/trans isomerase) NIMA-interacting 1; dod;				2.75E-12	0.96	10.21	9.20	18243.2
AHQ-1-12, 4050	R.TKEEALELINGYIQK.I	1749.98614	2	2.75E-12	0.96	4.29	-	1689.8
gi|5031987|ref|NP_005720.1| peptidylprolyl isomerase F (cyclophilin F) [Homo sapiens]				2.82E-12	5.11	70.28	45.40	22040.1
AHQ-1-12, 3814 - 3890	R.FPDENFTLK.H	1111.22866	2	8.58E-04	0.79	2.90	-	608.0
AHQ-1-12, 3731 - 3778	R.FPDENFTLK.H	1111.22866	2	4.91E-05	0.76	2.75	-	607.4
AHQ-1-12, 3999 - 4030	R.FPDENFTLK.H	1111.22866	2	1.47E-04	0.77	2.67	-	609.1
AHQ-1-12, 5021	K.GSGDPSSSSSSGNPLVYLDVDANGKPLGR.V	2834.99028	2	7.68E-06	0.57	2.74	-	296.8
AHQ-1-11, 4819	K.GSGDPSSSSSSGNPLVYLDVDANGKPLGR.V	2834.99028	3	2.82E-12	0.85	3.70	-	804.2
AHQ-1-14- , 5318 - 5319	K.HVGPGVLSM*ANAGPNTNGSQFFICTIK.T	2836.19605	3	1.38E-04	0.94	4.91	-	1186.3
AHQ-1-12, 5721	K.HVGPGVLSMANAGPNTNGSQFFICTIK.T	2820.19665	2	1.02E-07	0.67	3.29	-	307.5
AHQ-1-12, 5810 - 5874	K.HVGPGVLSMANAGPNTNGSQFFICTIK.T	2820.19665	3	2.40E-05	0.91	4.12	-	1586.4
AHQ-1-12, 5175 - 5233	K.HVGPGVLSM*ANAGPNTNGSQFFICTIK.T	2836.19605	3	1.80E-05	0.96	5.67	-	1324.7
AHQ-1-12, 5677 - 5754	K.HVGPGVLSMANAGPNTNGSQFFICTIK.T	2820.19665	3	1.42E-05	0.89	3.87	-	1313.5
AHQ-1-12, 1550 - 1595	K.HVVFGHVK.E	923.09628	1	5.30E-04	0.25	2.15	-	341.9
AHQ-1-12, 4558	R.VIPSFMCQAGDFTNHNGTGGK.S	2240.46178	2	7.62E-06	0.81	3.47	-	718.7
AHQ-1-12, 3955	R.VIPSFM*CQAGDFTNHNGTGGK.S	2256.46118	2	1.72E-05	0.82	3.55	-	384.7
gi|5031635|ref|NP_005498.1| cofilin 1 (non-muscle) [Homo sapiens]				2.90E-12	10.80	120.35	59.60	18502.4
AHQ-1-12, 4115	K.AVLFCLSEDK.K	1183.35638	2	2.31E-04	0.88	3.20	-	764.0
AHQ-1-12, 3485 - 3549	K.AVLFCLSEDKK.N	1311.52930	2	1.79E-05	0.72	2.83	-	639.6
AHQ-1-12, 3362 - 3425	K.AVLFCLSEDKK.N	1311.52930	2	5.41E-09	0.91	3.60	-	910.2
AHQ-1-12, 3395	K.AVLFCLSEDKK.N	1311.52930	1	6.42E-04	0.68	3.00	-	460.9
AHQ-1-12, 5581 - 5598	K.EILVGDVGQTVDDPYATFVK.M	2167.40069	2	1.08E-07	0.96	5.13	-	1035.1
AHQ-1-13- , 5548 - 5611	K.EILVGDVGQTVDDPYATFVK.M	2167.40069	2	9.88E-05	0.53	2.68	-	504.7
AHQ-1-14- , 5634	K.EILVGDVGQTVDDPYATFVK.M	2167.40069	2	5.91E-07	0.97	5.20	-	1533.6
AHQ-1-12, 5261 - 5314	K.EILVGDVGQTVDDPYATFVK.M	2167.40069	2	3.85E-06	0.79	3.29	-	454.6
AHQ-1-13- , 5772	K.EILVGDVGQTVDDPYATFVK.M	2167.40069	2	2.88E-08	0.97	5.32	-	1314.1
AHQ-1-11, 5275 - 5346	K.EILVGDVGQTVDDPYATFVK.M	2167.40069	2	1.71E-08	0.96	5.06	-	1045.6
AHQ-1-12, 5459 - 5534	K.EILVGDVGQTVDDPYATFVK.M	2167.40069	2	5.85E-10	0.98	6.36	-	1374.0
AHQ-1-13, 5436	K.EILVGDVGQTVDDPYATFVK.M	2167.40069	2	1.11E-05	0.96	4.60	-	1339.4
AHQ-1-12, 5351 - 5409	K.EILVGDVGQTVDDPYATFVK.M	2167.40069	2	2.24E-06	0.97	4.97	-	1606.9
AHQ-1-12, 5887 - 5893	K.ESKKEDLVFIFWAPESAPLK.S	2335.68256	3	5.94E-05	0.78	3.82	-	576.5
AHQ-1-12, 2433 - 2501	K.HELQANCYEEVKDR.C	1792.90812	3	2.05E-09	0.98	6.32	-	2116.3
AHQ-1-12, 2491	K.HELQANCYEEVKDR.C	1792.90812	2	5.59E-04	0.89	3.87	-	481.8
AHQ-1-12, 2571	K.HELQANCYEEVKDR.C	1792.90812	3	1.77E-08	0.97	5.09	-	1973.7
AHQ-1-12, 2573 - 2578	K.HELQANCYEEVKDR.C	1792.90812	2	2.95E-07	0.96	4.21	-	1258.7
AHQ-1-12, 2362 - 2425	K.HELQANCYEEVKDR.C	1792.90812	2	1.44E-06	0.98	4.96	-	2177.4
AHQ-1-12, 2323 - 2385	K.HELQANCYEEVKDR.C	1792.90812	3	3.05E-07	0.98	5.88	-	3112.6
AHQ-1-14- , 2470	K.HELQANCYEEVKDR.C	1792.90812	3	7.47E-06	0.93	3.83	-	1537.0
AHQ-1-12, 2950	K.KAVLFCLSEDKK.N	1439.70221	2	2.99E-04	0.84	3.57	-	652.7
AHQ-1-12, 2957	K.KAVLFCLSEDKK.N	1439.70221	3	2.12E-06	0.95	5.03	-	1217.8
AHQ-1-12, 6221 - 6285	K.KEDLVFIFWAPESAPLK.S	1991.31745	3	2.84E-11	0.95	5.41	-	1111.9
AHQ-1-13- , 6633	K.KEDLVFIFWAPESAPLK.S	1991.31745	3	1.20E-06	0.95	5.32	-	1255.5
AHQ-1-13- , 6639	K.KEDLVFIFWAPESAPLK.S	1991.31745	2	2.56E-04	0.96	5.15	-	1019.7
AHQ-1-12, 6345	K.KEDLVFIFWAPESAPLK.S	1991.31745	2	1.28E-06	0.95	4.80	-	957.4
AHQ-1-11, 6042 - 6043	K.KEDLVFIFWAPESAPLK.S	1991.31745	2	5.80E-07	0.94	4.34	-	1013.3
AHQ-1-12, 6217 - 6274	K.KEDLVFIFWAPESAPLK.S	1991.31745	2	2.83E-09	0.97	5.56	-	1326.7
AHQ-1-12, 6254	K.KNIILEEGKEILVGDVGQTVDDPYATFVK.M	3192.60499	3	1.20E-07	0.98	6.67	-	2071.0
AHQ-1-12, 4002	K.LGGSAVISLEGKPL	1341.57848	2	9.14E-07	0.95	4.14	-	1323.1
AHQ-1-14- , 4492 - 4507	K.LGGSAVISLEGKPL	1341.57848	2	5.36E-04	0.89	3.54	-	1116.5
AHQ-1-12, 4374 - 4438	K.LGGSAVISLEGKPL	1341.57848	2	1.43E-07	0.95	4.11	-	1232.7
AHQ-1-13- , 4532	K.LGGSAVISLEGKPL	1341.57848	2	4.16E-04	0.95	4.64	-	985.0
AHQ-1-12, 4231 - 4310	K.LGGSAVISLEGKPL	1341.57848	2	2.18E-04	0.93	3.45	-	1416.1
AHQ-1-12, 4437 - 4497	K.LGGSAVISLEGKPL	1341.57848	2	4.18E-06	0.95	3.87	-	1220.0
AHQ-1-12, 6283 - 6354	K.NIILEEGKEILVGDVGQTVDDPYATFVK.M	3064.43208	3	2.08E-04	0.90	5.31	-	764.6
AHQ-1-12, 6635 - 6695	K.NIILEEGKEILVGDVGQTVDDPYATFVK.M	3064.43208	3	6.47E-04	0.95	5.30	-	1399.6
AHQ-1-13, 6259 - 6335	K.NIILEEGKEILVGDVGQTVDDPYATFVK.M	3064.43208	3	2.90E-12	0.97	6.37	-	1977.5
AHQ-1-12, 6409 - 6465	K.NIILEEGKEILVGDVGQTVDDPYATFVK.M	3064.43208	3	9.22E-12	0.97	6.98	-	1522.5
AHQ-1-12, 6690 - 6750	K.NIILEEGKEILVGDVGQTVDDPYATFVK.M	3064.43208	3	2.47E-09	0.97	6.66	-	1755.6
AHQ-1-12, 3130 - 3251	R.YALYDATYETK.E	1338.44364	1	3.33E-06	0.46	2.60	-	332.7
AHQ-1-12, 3119 - 3189	R.YALYDATYETK.E	1338.44364	2	2.04E-05	0.92	3.49	-	987.5
AHQ-1-12, 3213	R.YALYDATYETK.E	1338.44364	2	2.62E-07	0.95	3.70	-	1219.5
AHQ-1-13, 3294	R.YALYDATYETK.E	1338.44364	1	1.86E-08	0.54	2.61	-	298.8
AHQ-1-13- , 3373	R.YALYDATYETK.E	1338.44364	1	1.58E-06	0.47	2.70	-	375.0
AHQ-1-11, 3096 - 3155	R.YALYDATYETK.E	1338.44364	2	4.21E-06	0.90	3.33	-	938.0
AHQ-1-12, 2866	R.YALYDATYETKESK.K	1682.80875	2	1.05E-05	0.94	3.72	-	1344.6
gi|4757826|ref|NP_004039.1| beta-2-microglobulin [Homo sapiens]				3.36E-12	3.20	40.22	38.70	13714.5
AHQ-1-14- , 6408	K.SNFLNCYVSGFHPSDIEVDLLK.N	2556.83155	2	1.24E-05	0.78	3.21	-	476.3
AHQ-1-14- , 6415 - 6479	K.SNFLNCYVSGFHPSDIEVDLLK.N	2556.83155	3	1.49E-04	0.65	3.17	-	306.7
AHQ-1-14- , 6555 - 6559	K.SNFLNCYVSGFHPSDIEVDLLK.N	2556.83155	3	5.47E-07	0.83	4.32	-	422.5
AHQ-1-14- , 6459 - 6531	K.SNFLNCYVSGFHPSDIEVDLLKNGER.I	3013.28710	3	3.36E-12	0.90	4.11	-	945.7
AHQ-1-14- , 6107 - 6187	K.SNFLNCYVSGFHPSDIEVDLLKNGER.I	3013.28710	3	6.01E-12	0.72	3.56	-	516.5
AHQ-1-14- , 6326 - 6387	K.SNFLNCYVSGFHPSDIEVDLLKNGER.I	3013.28710	3	1.30E-08	0.84	3.82	-	760.6
AHQ-1-14- , 2142 - 2274	K.VEHSDLSFSK.D	1149.23530	1	2.40E-05	0.82	3.21	-	613.3
AHQ-1-14- , 1992 - 2058	R.VNHVTLSQPK.I	1123.28735	2	1.58E-04	0.82	2.70	-	716.5
AHQ-1-14- , 1844 - 1903	R.VNHVTLSQPK.I	1123.28735	1	4.20E-04	0.48	2.51	-	347.4
AHQ-1-14- , 2190 - 2216	R.VNHVTLSQPK.I	1123.28735	1	1.22E-08	0.66	2.93	-	337.7
gi|4557677|ref|NP_000203.1| integrin beta chain, beta 3 precursor; platelet glycoprotein IIIa precu				3.76E-12	18.94	230.30	37.40	87185.6
AHQ-1-3, 5505	R.CDLKENLLKDNCAPESIEFPVSEAR.V	2938.23689	3	3.43E-06	0.89	4.27	-	841.3
AHQ-1-6, 5344	R.CDLKENLLKDNCAPESIEFPVSEAR.V	2938.23689	3	6.67E-05	0.79	3.66	-	709.9
AHQ-1-4, 5524 - 5596	R.CDLKENLLKDNCAPESIEFPVSEAR.V	2938.23689	3	7.19E-04	0.91	4.79	-	820.5
AHQ-1-9, 5332	R.CDLKENLLKDNCAPESIEFPVSEAR.V	2938.23689	3	9.62E-04	0.93	4.37	-	1340.8
AHQ-1-3, 5377 - 5443	R.CDLKENLLKDNCAPESIEFPVSEAR.V	2938.23689	2	8.62E-06	0.86	3.69	-	682.2
AHQ-1-3, 5374 - 5442	R.CDLKENLLKDNCAPESIEFPVSEAR.V	2938.23689	3	1.21E-06	0.90	4.61	-	773.8
AHQ-1-1, 5516	R.CDLKENLLKDNCAPESIEFPVSEAR.V	2938.23689	3	7.17E-05	0.92	4.80	-	1114.0
AHQ-1-2, 4586	R.CGPGWLGSQCECSEEDYRPSQQDECSPR.E	3380.49342	3	1.05E-04	0.87	4.46	-	409.2

AHQ-1-5, 4441	R.CGPGWLGSQCECSEEDYRPSQQDECSPR.E	3380.49342	3	7.98E-07	0.52	3.00	-	481.9
AHQ-1-4, 4492 - 4511	R.CGPGWLGSQCECSEEDYRPSQQDECSPR.E	3380.49342	3	9.03E-06	0.72	3.38	-	427.3
AHQ-1-1, 4429	R.CGPGWLGSQCECSEEDYRPSQQDECSPR.E	3380.49342	3	3.30E-07	0.86	4.12	-	613.8
AHQ-1-3, 4430 - 4502	R.CGPGWLGSQCECSEEDYRPSQQDECSPR.E	3380.49342	3	4.09E-09	0.87	4.42	-	545.5
AHQ-1-6, 4358	R.CGPGWLGSQCECSEEDYRPSQQDECSPR.E	3380.49342	3	1.40E-07	0.82	4.13	-	335.1
AHQ-1-1, 4533 - 4551	R.DAPEGGFDAIM*QATVCDEK.I	2072.21656	2	8.50E-05	0.95	4.32	-	1147.8
AHQ-1-3, 5998 - 6017	R.DAPEGGFDAIMQATVCDEK.I	2056.21716	2	2.50E-05	0.96	5.29	-	830.3
AHQ-1-3, 5930 - 5950	R.DAPEGGFDAIMQATVCDEK.I	2056.21716	2	5.79E-07	0.90	3.69	-	715.7
AHQ-1-1, 5885 - 5925	R.DAPEGGFDAIMQATVCDEK.I	2056.21716	2	2.21E-09	0.92	4.16	-	855.0
AHQ-1-3, 4549 - 4619	R.DAPEGGFDAIM*QATVCDEK.I	2072.21656	2	4.82E-05	0.89	3.75	-	653.3
AHQ-1-3, 6098 - 6167	R.DAPEGGFDAIMQATVCDEK.I	2056.21716	2	2.09E-06	0.96	5.19	-	692.1
AHQ-1-3, 6030 - 6099	R.DAPEGGFDAIMQATVCDEK.I	2056.21716	2	1.04E-05	0.93	4.25	-	944.6
AHQ-1-3, 5009	K.DDLWSIQNLGTK.L	1390.52332	2	5.49E-07	0.93	4.18	-	906.6
AHQ-1-4, 5094	K.DDLWSIQNLGTK.L	1390.52332	2	3.50E-04	0.93	3.92	-	797.4
AHQ-1-1, 5043	K.DDLWSIQNLGTK.L	1390.52332	2	1.50E-04	0.90	3.98	-	702.0
AHQ-1-4, 5104	K.DDLWSIQNLGTK.L	1390.52332	1	2.05E-06	0.55	2.59	-	430.1
AHQ-1-2, 5174	K.DDLWSIQNLGTK.L	1390.52332	2	1.07E-05	0.79	2.94	-	856.7
AHQ-1-5, 4521 - 4523	K.DNCAPESIEFPVSEAR.V	1822.93099	2	7.89E-05	0.90	3.48	-	1048.1
AHQ-1-1, 4508	K.DNCAPESIEFPVSEAR.V	1822.93099	2	4.06E-06	0.54	2.73	-	572.3
AHQ-1-3, 4431 - 4506	K.DNCAPESIEFPVSEAR.V	1822.93099	2	3.22E-04	0.95	3.81	-	1482.0
AHQ-1-9, 4396	K.DNCAPESIEFPVSEAR.V	1822.93099	2	4.77E-04	0.92	3.09	-	1450.1
AHQ-1-6, 4427	K.DNCAPESIEFPVSEAR.V	1822.93099	2	3.76E-06	0.72	3.03	-	667.5
AHQ-1-7, 3232 - 3308	K.EATSTFTNITYR.G	1404.50685	2	1.96E-07	0.42	2.59	-	511.7
AHQ-1-6, 3286	K.EATSTFTNITYR.G	1404.50685	2	3.76E-06	0.51	2.68	-	343.0
AHQ-1-1, 3467	K.EATSTFTNITYR.G	1404.50685	2	3.68E-06	0.83	3.30	-	664.9
AHQ-1-4, 3426	K.EATSTFTNITYR.G	1404.50685	2	2.72E-05	0.84	2.92	-	915.8
AHQ-1-5, 3294	K.EATSTFTNITYR.G	1404.50685	2	2.42E-07	0.64	2.61	-	555.6
AHQ-1-2, 2466 - 2467	R.FQYYEDSSGK.S	1224.25749	1	7.76E-04	0.61	2.30	-	622.1
AHQ-1-4, 2338 - 2344	R.FQYYEDSSGK.S	1224.25749	1	8.36E-05	0.40	1.86	-	534.7
AHQ-1-3, 3662 - 3663	R.GECLCGQCVCHSSDFGK.I	2006.16137	3	1.74E-06	0.83	3.66	-	655.4
AHQ-1-2, 3805	R.GECLCGQCVCHSSDFGK.I	2006.16137	2	8.00E-09	0.87	3.57	-	727.7
AHQ-1-6, 3575	R.GECLCGQCVCHSSDFGK.I	2006.16137	2	1.31E-05	0.77	3.02	-	875.9
AHQ-1-3, 3655 - 3658	R.GECLCGQCVCHSSDFGK.I	2006.16137	2	2.03E-06	0.96	4.25	-	1491.4
AHQ-1-4, 3702 - 3735	R.GECLCGQCVCHSSDFGK.I	2006.16137	2	2.66E-07	0.94	4.14	-	1190.6
AHQ-1-1, 3707 - 3776	R.GECLCGQCVCHSSDFGK.I	2006.16137	2	2.89E-07	0.92	3.52	-	1260.0
AHQ-1-5, 3609	R.GECLCGQCVCHSSDFGK.I	2006.16137	2	4.37E-09	0.95	4.34	-	1297.5
AHQ-1-3, 4354	R.GKCECGSCVCIQPGSYGDTCEKCPTCPDACTFKK.E	4028.42584	3	2.12E-05	0.90	4.71	-	579.8
AHQ-1-4, 1876 - 1946	K.GSGDSSQVTQVSPQR.I	1533.58302	2	2.87E-04	0.40	2.76	-	456.3
AHQ-1-4, 2012 - 2078	K.GSGDSSQVTQVSPQR.I	1533.58302	2	8.38E-04	0.78	3.37	-	703.6
AHQ-1-3, 2002 - 2067	K.GSGDSSQVTQVSPQR.I	1533.58302	2	4.64E-05	0.58	3.20	-	664.2
AHQ-1-1, 2081	K.GSGDSSQVTQVSPQR.I	1533.58302	2	5.77E-04	0.70	2.69	-	883.6
AHQ-1-7, 1778 - 1840	K.GSGDSSQVTQVSPQR.I	1533.58302	2	2.77E-04	0.81	3.25	-	978.4
AHQ-1-2, 2811	K.HVLTLTDQVTR.F	1283.45912	2	9.83E-08	0.88	3.50	-	533.1
AHQ-1-6, 2592	K.HVLTLTDQVTR.F	1283.45912	2	3.64E-05	0.86	3.48	-	556.2
AHQ-1-4, 2712	K.HVLTLTDQVTR.F	1283.45912	2	1.67E-05	0.82	3.33	-	537.5
AHQ-1-3, 2534	K.HVLTLTDQVTR.F	1283.45912	2	1.10E-05	0.93	3.41	-	901.4
AHQ-1-3, 2703 - 2705	K.HVLTLTDQVTR.F	1283.45912	2	3.39E-07	0.92	3.45	-	768.4
AHQ-1-7, 2606	K.HVLTLTDQVTR.F	1283.45912	2	6.37E-04	0.78	3.14	-	427.6
AHQ-1-5, 2589	K.HVLTLTDQVTR.F	1283.45912	2	3.16E-07	0.94	3.79	-	925.9
AHQ-1-4, 2715	K.HVLTLTDQVTR.F	1283.45912	1	6.40E-04	0.39	2.11	-	400.5
AHQ-1-4, 2719	K.HVLTLTDQVTR.F	1283.45912	1	1.83E-04	0.56	2.75	-	312.5
AHQ-1-8, 2473	K.HVLTLTDQVTR.F	1283.45912	2	1.09E-06	0.90	3.55	-	636.9
AHQ-1-3, 4605	K.IGDTVSFSIEAK.V	1267.41009	2	4.82E-05	0.93	3.66	-	951.0
AHQ-1-3, 4414 - 4482	K.IGDTVSFSIEAK.V	1267.41009	2	1.52E-06	0.79	2.60	-	704.3
AHQ-1-1, 4085	K.IGDTVSFSIEAK.V	1267.41009	2	1.25E-08	0.92	3.34	-	1079.9
AHQ-1-3, 3667	K.IGDTVSFSIEAK.V	1267.41009	2	6.70E-05	0.93	3.03	-	1291.0
AHQ-1-3, 5161	R.LAGIVQPNDGQCHVGSDNHYSASTTM*DYPSLGLMTEK.L	4013.35457	3	7.28E-05	0.83	4.36	-	844.2
AHQ-1-3, 5523 - 5591	R.LAGIVQPNDGQCHVGSDNHYSASTTMDYPSLGLMTEK.L	3997.35517	3	5.44E-11	0.96	6.06	-	1014.2
AHQ-1-4, 5584	R.LAGIVQPNDGQCHVGSDNHYSASTTMDYPSLGLMTEK.L	3997.35517	3	6.74E-04	0.89	4.44	-	765.4
AHQ-1-3, 5393 - 5457	R.LAGIVQPNDGQCHVGSDNHYSASTTMDYPSLGLMTEK.L	3997.35517	3	5.77E-04	0.80	3.56	-	677.3
AHQ-1-2, 4126 - 4141	R.NDASHLLVFTTDAK.T	1532.67977	2	7.74E-06	0.91	3.85	-	883.0
AHQ-1-1, 4501	R.NDASHLLVFTTDAK.T	1532.67977	2	8.68E-06	0.93	3.90	-	986.4
AHQ-1-6, 3768 - 3774	R.NDASHLLVFTTDAK.T	1532.67977	2	6.93E-06	0.96	4.65	-	1404.7
AHQ-1-3, 3950 - 3959	R.NDASHLLVFTTDAK.T	1532.67977	3	4.80E-06	0.97	5.29	-	1823.9
AHQ-1-5, 3805 - 3821	R.NDASHLLVFTTDAK.T	1532.67977	2	5.93E-09	0.97	5.12	-	1626.8
AHQ-1-3, 4299	R.NDASHLLVFTTDAK.T	1532.67977	2	7.28E-06	0.88	3.53	-	822.3
AHQ-1-1, 4049 - 4073	R.NDASHLLVFTTDAK.T	1532.67977	2	9.72E-10	0.97	5.28	-	1366.6
AHQ-1-4, 4484	R.NDASHLLVFTTDAK.T	1532.67977	2	1.12E-04	0.85	3.46	-	753.1
AHQ-1-3, 3942 - 3947	R.NDASHLLVFTTDAK.T	1532.67977	2	8.78E-09	0.97	4.41	-	1728.8
AHQ-1-3, 4449 - 4459	R.NDASHLLVFTTDAK.T	1532.67977	2	3.76E-12	0.96	4.52	-	1223.3
AHQ-1-5, 4405	R.NDASHLLVFTTDAK.T	1532.67977	2	1.02E-08	0.91	3.86	-	870.4
AHQ-1-8, 3462	R.NDASHLLVFTTDAK.T	1532.67977	2	1.39E-09	0.97	5.19	-	1281.2
AHQ-1-4, 4522 - 4528	K.SILYVVEEPECPK.G	1564.78173	2	2.57E-06	0.85	2.90	-	996.3
AHQ-1-1, 4513 - 4515	K.SILYVVEEPECPK.G	1564.78173	2	1.89E-04	0.91	3.93	-	845.9
AHQ-1-3, 2922	R.TDTCM*SSNGLLCSGR.G	1677.81726	2	2.83E-08	0.93	3.70	-	1340.3
AHQ-1-5, 2815	R.TDTCM*SSNGLLCSGR.G	1677.81726	2	2.97E-06	0.85	3.08	-	1077.0
AHQ-1-5, 2910	R.TDTCM*SSNGLLCSGR.G	1677.81726	2	2.14E-06	0.88	3.34	-	969.8
AHQ-1-3, 3007	R.TDTCM*SSNGLLCSGR.G	1677.81726	2	6.96E-09	0.92	3.66	-	1042.6
AHQ-1-4, 3776	R.TDTCMSSNGLLCSGR.G	1661.81786	2	2.26E-07	0.92	3.56	-	1062.9
AHQ-1-7, 3585	R.TDTCMSSNGLLCSGR.G	1661.81786	2	5.73E-04	0.50	2.52	-	621.4
AHQ-1-4, 3602 - 3651	R.TDTCMSSNGLLCSGR.G	1661.81786	2	1.86E-09	0.81	3.19	-	731.8
AHQ-1-3, 3635 - 3702	R.TDTCMSSNGLLCSGR.G	1661.81786	2	6.00E-09	0.74	2.78	-	605.3
AHQ-1-4, 3070	R.TDTCM*SSNGLLCSGR.G	1677.81726	2	3.52E-07	0.66	2.65	-	464.3
AHQ-1-1, 3679	R.TDTCMSSNGLLCSGR.G	1661.81786	2	7.10E-07	0.88	3.50	-	899.7
AHQ-1-4, 2972	R.TDTCM*SSNGLLCSGR.G	1677.81726	2	1.64E-08	0.96	4.05	-	1661.4
AHQ-1-6, 3479 - 3495	R.TDTCMSSNGLLCSGR.G	1661.81786	2	1.01E-07	0.77	2.75	-	750.7
AHQ-1-3, 2830	R.TDTCM*SSNGLLCSGR.G	1677.81726	2	1.98E-06	0.87	3.29	-	935.3
AHQ-1-4, 2882	R.TDTCM*SSNGLLCSGR.G	1677.81726	2	5.20E-08	0.93	3.77	-	1172.2
AHQ-1-1, 2904	R.TDTCM*SSNGLLCSGR.G	1677.81726	2	2.97E-06	0.96	4.42	-	1327.7
AHQ-1-1, 2141	R.VLEDRPLSDK.G	1172.31354	2	1.18E-05	0.92	3.72	-	1054.3
AHQ-1-3, 1914 - 1989	R.VLEDRPLSDK.G	1172.31354	2	2.07E-05	0.93	3.73	-	967.5
AHQ-1-7, 1817	R.VLEDRPLSDK.G	1172.31354	2	3.84E-04	0.85	2.76	-	1119.8
AHQ-1-4, 1911	R.VLEDRPLSDK.G	1172.31354	2	8.16E-06	0.85	3.26	-	807.6
AHQ-1-3, 2791	R.VLEDRPLSDKGSGDSSQVTQVSPQR.I	2686.87396	3	6.17E-09	0.93	4.65	-	1472.9
AHQ-1-2, 2891	R.VLEDRPLSDKGSGDSSQVTQVSPQR.I	2686.87396	3	4.49E-09	0.93	4.30	-	1660.8
AHQ-1-3, 3275 - 3346	K.WDTANNPLYK.E	1222.33111	2	9.18E-04	0.81	3.20	-	512.4
AHQ-1-1, 3427	K.WDTANNPLYK.E	1222.33111	2	7.16E-05	0.66	2.86	-	567.6
AHQ-1-4, 3740 - 3766	K.YCECDDFSCVR.Y	1514.59674	2	1.10E-05	0.91	3.24	-	970.1
AHQ-1-3, 3691 - 3761	K.YCECDDFSCVR.Y	1514.59674	2	7.86E-04	0.93	3.63	-	1151.8
AHQ-1-1, 3733	K.YCECDDFSCVR.Y	1514.59674	2	3.68E-05	0.94	3.34	-	1281.4
gi|18104967|ref|NP_000953.2| prostaglandin-endoperoxide synthase 1 isoform 1 precursor; prostagland				4.20E-12	7.08	80.27	23.20	68686.0
AHQ-1-6, 3966	K.AEHPTWGDEQLFQTTR.L	1917.02765	2	3.50E-07	0.76	3.11	-	466.5
AHQ-1-6, 3883	K.ALGHGVDLGHIYGDNLER.Q	1937.10524	3	6.76E-05	0.80	3.42	-	774.8
AHQ-1-6, 3874	K.ALGHGVDLGHIYGDNLER.Q	1937.10524	2	7.05E-04	0.95	4.93	-	1140.0
AHQ-1-6, 5192	R.FGMKPYTSFQELVGEK.E	1862.13845	2	3.68E-08	0.91	3.98	-	579.9
AHQ-1-6, 7014 - 7079	K.GLLGNPICSPEYWKPSTFGGEVGFNIVK.T	3069.47853	3	4.20E-08	0.87	4.49	-	451.3
AHQ-1-6, 7324	K.IVIEEYVQQLSGYFLQLK.F	2171.52045	3	4.20E-12	0.96	5.44	-	1494.5

AHQ-1-6, 3326 - 3396	R.VPDASQDDGPAVERPSTEL	1984.06747	2	2.63E-09	0.95	4.85	-	1069.7
AHQ-1-6, 5454 - 5484	K.YQVLDGEMYPPSVEEAPVLM*HYPR.G	2838.20739	3	4.36E-04	0.89	4.79	-	677.3
AHQ-1-6, 5987 - 6006	K.YQVLDGEMYPPSVEEAPVLMHYPR.G	2822.20799	3	1.46E-10	0.94	5.41	-	799.6
gi|27498267|ref|XP_209737.1| similar to tyrosine 3/tryptophan 5 -monooxygenase activation protein,				4.60E-12	3.56	40.39	33.70	11063.6
AHQ-1-11, 5622	R.DICNDVLSLLEK.F	1420.61093	2	6.08E-08	0.91	3.60	-	975.7
AHQ-1-13, 5740	R.DICNDVLSLLEK.F	1420.61093	2	2.38E-05	0.96	3.89	-	1613.0
AHQ-1-13- , 6908	R.DICNDVLSLLEK.F	1420.61093	2	1.95E-06	0.98	5.69	-	2131.2
AHQ-1-13- , 6095 - 6100	R.DICNDVLSLLEK.F	1420.61093	2	1.12E-06	0.95	3.81	-	1551.1
AHQ-1-13, 5749	R.DICNDVLSLLEK.F	1420.61093	1	5.48E-06	0.20	2.16	-	531.4
AHQ-1-10, 5853	R.DICNDVLSLLEK.F	1420.61093	2	5.44E-06	0.86	2.98	-	1020.0
AHQ-1-10, 5515	R.DICNDVLSLLEK.F	1420.61093	1	3.75E-10	0.76	2.75	-	800.0
AHQ-1-10, 4763 - 4821	K.GIVDQSQQAYQEAFEISK.K	2042.19176	2	1.17E-08	0.98	5.57	-	1636.6
AHQ-1-10, 4881 - 4944	K.GIVDQSQQAYQEAFEISK.K	2042.19176	2	4.87E-11	0.98	5.90	-	1891.4
AHQ-1-10, 4980 - 5037	K.GIVDQSQQAYQEAFEISK.K	2042.19176	2	2.76E-07	0.94	4.30	-	1212.1
AHQ-1-10, 5096 - 5151	K.GIVDQSQQAYQEAFEISK.K	2042.19176	2	3.60E-09	0.97	5.13	-	1639.0
AHQ-1-10, 5217 - 5281	K.GIVDQSQQAYQEAFEISK.K	2042.19176	2	4.20E-06	0.97	5.54	-	1394.1
AHQ-1-10, 4720 - 4731	K.GIVDQSQQAYQEAFEISK.K	2042.19176	3	7.00E-10	0.99	7.15	-	2797.0
AHQ-1-10, 5617 - 5680	K.GIVDQSQQAYQEAFEISK.K	2042.19176	2	1.91E-05	0.84	3.84	-	657.0
AHQ-1-10, 4659 - 4707	K.GIVDQSQQAYQEAFEISK.K	2042.19176	2	2.86E-08	0.98	5.66	-	1985.6
AHQ-1-10, 5925 - 5985	K.GIVDQSQQAYQEAFEISK.K	2042.19176	2	1.83E-07	0.93	4.14	-	1034.9
AHQ-1-10, 6504	K.GIVDQSQQAYQEAFEISK.K	2042.19176	2	8.19E-09	0.96	5.27	-	1086.8
AHQ-1-10, 4451	K.GIVDQSQQAYQEAFEISK.K	2042.19176	2	1.92E-09	0.95	4.80	-	1078.2
AHQ-1-11, 4842	K.GIVDQSQQAYQEAFEISK.K	2042.19176	2	2.39E-07	0.96	5.50	-	990.3
AHQ-1-11, 4848	K.GIVDQSQQAYQEAFEISK.K	2042.19176	3	2.36E-05	0.96	4.93	-	1770.4
AHQ-1-11, 5012 - 5088	K.GIVDQSQQAYQEAFEISK.K	2042.19176	2	1.72E-04	0.69	3.06	-	488.0
AHQ-1-11, 5248	K.GIVDQSQQAYQEAFEISK.K	2042.19176	2	1.67E-07	0.97	5.59	-	1245.7
AHQ-1-1, 5384	K.GIVDQSQQAYQEAFEISK.K	2042.19176	2	1.61E-05	0.94	4.59	-	990.7
AHQ-1-12, 4954 - 5013	K.GIVDQSQQAYQEAFEISK.K	2042.19176	2	3.34E-07	0.97	5.50	-	1662.7
AHQ-1-12, 5010	K.GIVDQSQQAYQEAFEISK.K	2042.19176	2	7.55E-09	0.96	5.26	-	1264.2
AHQ-1-12, 5018	K.GIVDQSQQAYQEAFEISK.K	2042.19176	3	1.46E-08	0.97	5.15	-	2241.1
AHQ-1-13, 4632	K.GIVDQSQQAYQEAFEISK.K	2042.19176	2	1.30E-10	0.96	5.00	-	1348.2
AHQ-1-13, 4715	K.GIVDQSQQAYQEAFEISK.K	2042.19176	2	1.01E-09	0.96	4.65	-	1335.6
AHQ-1-13, 4959 - 5028	K.GIVDQSQQAYQEAFEISK.K	2042.19176	2	5.87E-10	0.97	5.35	-	1439.9
AHQ-1-13, 4968 - 4988	K.GIVDQSQQAYQEAFEISK.K	2042.19176	3	1.65E-09	0.98	6.13	-	2126.8
AHQ-1-13, 5088 - 5152	K.GIVDQSQQAYQEAFEISK.K	2042.19176	2	5.20E-05	0.91	4.12	-	799.8
AHQ-1-13, 5345	K.GIVDQSQQAYQEAFEISK.K	2042.19176	2	4.60E-12	0.97	5.27	-	1748.3
AHQ-1-10, 4303 - 4367	K.GIVDQSQQAYQEAFEISK.K	2042.19176	2	5.64E-09	0.96	4.47	-	1442.6
AHQ-1-13- , 5676	K.GIVDQSQQAYQEAFEISK.K	2042.19176	2	7.49E-10	0.97	5.48	-	1336.5
AHQ-1-14, 5940	K.GIVDQSQQAYQEAFEISK.K	2042.19176	2	1.02E-07	0.96	5.10	-	1158.2
AHQ-1-13- , 5433 - 5501	K.GIVDQSQQAYQEAFEISK.K	2042.19176	2	4.41E-09	0.97	5.90	-	1381.4
AHQ-1-13- , 4852	K.GIVDQSQQAYQEAFEISK.K	2042.19176	2	4.04E-11	0.95	4.68	-	1376.7
AHQ-1-13- , 4960	K.GIVDQSQQAYQEAFEISK.K	2042.19176	2	2.48E-10	0.96	4.65	-	1223.9
AHQ-1-13- , 5224 - 5267	K.GIVDQSQQAYQEAFEISK.K	2042.19176	2	8.48E-08	0.97	5.98	-	1283.8
AHQ-1-13- , 5235 - 5243	K.GIVDQSQQAYQEAFEISK.K	2042.19176	3	1.43E-08	0.99	7.84	-	3166.3
AHQ-1-14- , 5118	K.GIVDQSQQAYQEAFEISK.K	2042.19176	2	1.08E-07	0.97	5.11	-	1421.8
AHQ-1-10, 4341	K.GIVDQSQQAYQEAFEISKK.E	2170.36467	3	8.81E-04	0.77	3.25	-	785.2
AHQ-1-10, 4335	K.GIVDQSQQAYQEAFEISKK.E	2170.36467	2	9.67E-06	0.85	4.17	-	438.7
AHQ-1-11, 4363 - 4368	K.KGIVDQSQQAYQEAFEISK.K	2170.36467	2	9.71E-06	0.98	6.42	-	1784.3
AHQ-1-13- , 4727	K.KGIVDQSQQAYQEAFEISK.K	2170.36467	2	1.08E-06	0.98	5.58	-	1910.6
gi|4507357|ref|NP_003555.1| transgelin 2; SM22-alpha homolog [Homo sapiens]				4.74E-12	12.01	150.27	66.80	22391.3
AHQ-1-11, 5486 - 5559	R.DDGLFSGDPNWFPK.K	1595.69288	2	7.21E-09	0.97	4.91	-	1630.6
AHQ-1-12, 5669 - 5722	R.DDGLFSGDPNWFPK.K	1595.69288	2	1.90E-05	0.92	3.60	-	1003.1
AHQ-1-12, 5877	R.DDGLFSGDPNWFPK.K	1595.69288	2	2.24E-04	0.94	4.27	-	1153.9
AHQ-1-12, 5731 - 5779	R.DDGLFSGDPNWFPK.K	1595.69288	2	2.99E-06	0.96	4.42	-	1314.8
AHQ-1-11, 5707	R.DDGLFSGDPNWFPK.K	1595.69288	2	4.63E-04	0.94	4.23	-	923.2
AHQ-1-11, 5614 - 5647	R.DDGLFSGDPNWFPK.K	1595.69288	2	8.56E-09	0.96	4.40	-	1472.9
AHQ-1-11, 6046	K.DGTVLCELINALYPEGQAPVK.K	2289.59066	2	2.76E-06	0.89	3.87	-	801.0
AHQ-1-13, 5961 - 5972	K.DGTVLCELINALYPEGQAPVK.K	2289.59066	2	8.67E-06	0.74	3.30	-	493.9
AHQ-1-13- , 6359	K.DGTVLCELINALYPEGQAPVK.K	2289.59066	2	4.30E-09	0.95	4.85	-	878.5
AHQ-1-11, 5832	K.DGTVLCELINALYPEGQAPVK.K	2289.59066	2	1.25E-04	0.83	3.59	-	634.5
AHQ-1-14- , 6732	K.DGTVLCELINALYPEGQAPVK.K	2289.59066	2	5.51E-06	0.93	4.48	-	765.8
AHQ-1-12, 6029	K.DGTVLCELINALYPEGQAPVK.K	2289.59066	2	4.61E-04	0.92	4.32	-	809.3
AHQ-1-11, 5398 - 5399	K.DGTVLCELINALYPEGQAPVKK.I	2417.76357	2	1.04E-05	0.85	3.95	-	552.2
AHQ-1-11, 5406	K.DGTVLCELINALYPEGQAPVKK.I	2417.76357	3	3.06E-07	0.74	3.16	-	854.9
AHQ-1-12, 6605 - 6611	K.DGTVLCELINALYPEGQAPVKK.I	2417.76357	3	1.52E-10	0.95	5.43	-	1101.1
AHQ-1-11, 5602	K.DGTVLCELINALYPEGQAPVKK.I	2417.76357	3	4.18E-08	0.83	3.53	-	1042.6
AHQ-1-12, 6613 - 6614	K.DGTVLCELINALYPEGQAPVKK.I	2417.76357	2	1.22E-07	0.88	4.62	-	504.9
AHQ-1-13, 6488 - 6491	K.DGTVLCELINALYPEGQAPVKK.I	2417.76357	3	1.34E-06	0.91	4.40	-	1058.1
AHQ-1-11, 6435 - 6443	K.DGTVLCELINALYPEGQAPVKK.I	2417.76357	3	1.49E-07	0.89	4.20	-	912.5
AHQ-1-12, 5594	K.DGTVLCELINALYPEGQAPVKK.I	2417.76357	3	7.07E-07	0.84	3.70	-	762.8
AHQ-1-13- , 6932	K.DGTVLCELINALYPEGQAPVKK.I	2417.76357	3	3.56E-08	0.76	3.51	-	565.2
AHQ-1-14- , 6690	K.DGTVLCELINALYPEGQAPVKK.I	2417.76357	3	2.55E-08	0.92	4.54	-	912.7
AHQ-1-14- , 6694	K.DGTVLCELINALYPEGQAPVKK.I	2417.76357	2	3.55E-06	0.63	2.84	-	521.4
AHQ-1-11, 2663	R.GASQAGMTGYGMPR.Q	1384.56732	2	1.15E-04	0.81	3.25	-	816.4
AHQ-1-12, 1966 - 2025	R.GPAYGLSR.E	820.91599	2	2.89E-04	0.83	3.07	-	793.5
AHQ-1-12, 1991	R.GPAYGLSR.E	820.91599	1	2.25E-04	0.22	2.11	-	205.9
AHQ-1-11, 2502	K.IQASTMAFK.Q	997.19374	1	7.33E-04	0.63	2.76	-	468.1
AHQ-1-12, 2298	K.KIQASTMAFK.Q	1125.36665	2	4.57E-04	0.84	2.91	-	1042.5
AHQ-1-11, 2162 - 2218	R.NFSDNQLQEGK.N	1280.32582	2	9.96E-04	0.90	3.43	-	892.7
AHQ-1-12, 2101 - 2161	R.NFSDNQLQEGK.N	1280.32582	2	1.72E-04	0.88	3.49	-	842.4
AHQ-1-12, 2162 - 2245	R.NFSDNQLQEGK.N	1280.32582	1	3.25E-07	0.17	2.20	-	299.4
AHQ-1-11, 2334 - 2411	R.NFSDNQLQEGK.N	1280.32582	1	6.98E-05	0.41	2.28	-	316.7
AHQ-1-12, 2381	R.NFSDNQLQEGK.N	1280.32582	1	9.82E-06	0.11	2.14	-	181.4
AHQ-1-12, 2586	R.NFSDNQLQEGK.N	1280.32582	1	4.75E-06	0.14	1.93	-	376.9
AHQ-1-14- , 2298	R.NFSDNQLQEGK.N	1280.32582	2	8.38E-04	0.81	2.99	-	778.5
AHQ-1-11, 2410	R.NFSDNQLQEGK.N	1280.32582	2	1.05E-05	0.87	3.37	-	971.2
AHQ-1-11, 2558	R.NFSDNQLQEGK.N	1280.32582	1	1.14E-06	0.20	1.93	-	393.0
AHQ-1-13- , 2325 - 2328	R.NFSDNQLQEGK.N	1280.32582	2	5.37E-05	0.87	3.22	-	849.4
AHQ-1-11, 2959 - 3018	K.NVIGLQMGTNR.G	1203.39751	2	1.57E-07	0.93	3.41	-	1260.1
AHQ-1-11, 2322 - 2330	K.NVIGLQM*GTNR.G	1219.39691	2	1.62E-06	0.93	3.47	-	1146.6
AHQ-1-12, 5318	K.QMEQISQFLQAAER.Y	1679.87950	3	3.29E-10	0.95	4.86	-	1507.2
AHQ-1-12, 5821 - 5845	K.QMEQISQFLQAAER.Y	1679.87950	2	1.48E-05	0.97	4.57	-	2126.1
AHQ-1-11, 5654	K.QMEQISQFLQAAER.Y	1679.87950	2	5.26E-05	0.95	4.01	-	1505.0
AHQ-1-11, 5264 - 5320	K.QMEQISQFLQAAER.Y	1679.87950	2	2.83E-05	0.97	4.97	-	1339.9
AHQ-1-12, 4786	K.QM*EQISQFLQAAER.Y	1695.87890	2	6.76E-07	0.88	3.90	-	645.2
AHQ-1-14- , 5456	K.QMEQISQFLQAAER.Y	1679.87950	2	1.00E-06	0.93	3.59	-	1162.0
AHQ-1-11, 5102	K.QMEQISQFLQAAER.Y	1679.87950	2	5.97E-04	0.94	4.29	-	975.5
AHQ-1-11, 4246 - 4263	R.TLMNLGGLAVAR.D	1216.47932	2	4.85E-05	0.88	3.91	-	936.8
AHQ-1-11, 3466	R.TLM*NLGGLAVAR.D	1232.47872	2	2.03E-05	0.90	3.59	-	990.4
AHQ-1-12, 3953	R.TLM*NLGGLAVAR.D	1232.47872	2	2.32E-05	0.93	3.32	-	1301.1
AHQ-1-12, 4367 - 4447	R.TLMNLGGLAVAR.D	1216.47932	2	3.29E-06	0.93	4.11	-	1056.2
AHQ-1-13- , 4628 - 4631	R.TLMNLGGLAVAR.D	1216.47932	2	6.39E-07	0.91	4.26	-	906.0
AHQ-1-14- , 4600	R.TLMNLGGLAVAR.D	1216.47932	2	1.58E-04	0.92	3.61	-	1167.4
AHQ-1-13- , 6867 - 6868	R.YGINTTDIFQTVDLWEGK.N	2101.30080	2	1.40E-09	0.97	5.30	-	1180.6
AHQ-1-11, 6235	R.YGINTTDIFQTVDLWEGK.N	2101.30080	2	1.71E-06	0.94	4.82	-	728.9
AHQ-1-11, 6226 - 6304	R.YGINTTDIFQTVDLWEGK.N	2101.30080	2	3.23E-05	0.92	4.30	-	804.0
AHQ-1-12, 6371 - 6441	R.YGINTTDIFQTVDLWEGK.N	2101.30080	2	3.07E-11	0.97	4.84	-	1460.7

AHQ-1-11, 5996 - 6076	R.YGINTTDIFQTVDLWEGK.N	2101.30080	2	3.79E-05	0.81	3.58	-	673.0
AHQ-1-13, 6332 - 6411	R.YGINTTDIFQTVDLWEGK.N	2101.30080	2	2.24E-09	0.95	4.91	-	1018.6
AHQ-1-14- , 6620	R.YGINTTDIFQTVDLWEGK.N	2101.30080	2	4.74E-12	0.96	4.94	-	1276.1
AHQ-1-11, 6440 - 6514	R.YGINTTDIFQTVDLWEGK.N	2101.30080	2	9.17E-07	0.95	4.47	-	1045.8
AHQ-1-12, 6537	R.YGINTTDIFQTVDLWEGK.N	2101.30080	2	2.19E-08	0.96	5.20	-	1089.1
AHQ-1-11, 6596	R.YGINTTDIFQTVDLWEGK.N	2101.30080	2	1.52E-07	0.91	4.05	-	865.0
gi|5453740|ref|NP_006462.1| myosin regulatory light chain MRCL3; myosin, light polypeptide, regulat				4.79E-12	6.33	70.29	32.70	19794.0
AHQ-1-11, 5875 - 5952	R.ATSNVFAMFDQSQIQEFK.E	2092.31741	2	1.36E-05	0.80	3.53	-	561.9
AHQ-1-14- , 5664	R.ATSNVFAM*FDQSQIQEFK.E	2108.31681	2	5.43E-08	0.73	3.25	-	447.4
AHQ-1-14- , 5447 - 5448	R.ATSNVFAM*FDQSQIQEFK.E	2108.31681	2	1.20E-08	0.97	5.38	-	1260.8
AHQ-1-12, 5969 - 5973	R.ATSNVFAMFDQSQIQEFK.E	2092.31741	2	2.71E-04	0.97	5.00	-	1717.9
AHQ-1-12, 5322	R.ATSNVFAM*FDQSQIQEFK.E	2108.31681	2	3.26E-07	0.96	4.30	-	1495.9
AHQ-1-14- , 6108 - 6110	R.ATSNVFAMFDQSQIQEFK.E	2092.31741	2	6.44E-09	0.98	4.99	-	2072.9
AHQ-1-11, 5118 - 5188	R.ATSNVFAM*FDQSQIQEFK.E	2108.31681	2	1.60E-07	0.97	4.66	-	1563.8
AHQ-1-11, 5700 - 5770	R.ATSNVFAMFDQSQIQEFK.E	2092.31741	2	2.65E-06	0.96	4.55	-	1654.9
AHQ-1-11, 5523	R.DGFIDKEDLHDMLASLGK.N	2005.23806	3	9.12E-09	0.90	3.31	-	1732.9
AHQ-1-11, 5626 - 5635	R.DGFIDKEDLHDMLASLGK.N	2005.23806	3	4.79E-12	0.97	4.82	-	1885.6
AHQ-1-11, 5627	R.DGFIDKEDLHDMLASLGK.N	2005.23806	2	8.19E-10	0.97	5.29	-	1740.8
AHQ-1-11, 5518 - 5574	R.DGFIDKEDLHDMLASLGK.N	2005.23806	2	1.58E-06	0.98	5.82	-	2454.0
AHQ-1-11, 5708	R.DGFIDKEDLHDMLASLGK.N	2005.23806	2	6.54E-04	0.89	3.77	-	816.2
AHQ-1-14- , 5879	R.DGFIDKEDLHDMLASLGK.N	2005.23806	2	5.25E-12	0.97	4.80	-	1570.9
AHQ-1-11, 4318 - 4320	R.DGFIDKEDLHDM*LASLGK.N	2021.23746	2	3.86E-05	0.96	5.29	-	1026.5
AHQ-1-12, 4505	R.DGFIDKEDLHDM*LASLGK.N	2021.23746	2	2.75E-04	0.90	3.93	-	661.6
AHQ-1-12, 5745	R.DGFIDKEDLHDMLASLGK.N	2005.23806	2	8.99E-12	0.95	4.93	-	1201.3
AHQ-1-12, 5742	R.DGFIDKEDLHDMLASLGK.N	2005.23806	3	2.68E-05	0.80	3.43	-	857.4
AHQ-1-11, 2600	R.ELLTTMGDR.F	1036.18513	2	9.12E-05	0.81	2.73	-	678.8
AHQ-1-12, 4146	K.GNFNYIEFTR.I	1261.36733	2	4.20E-04	0.78	3.31	-	623.5
AHQ-1-13, 4176 - 4179	K.GNFNYIEFTR.I	1261.36733	2	7.57E-06	0.70	2.75	-	677.1
AHQ-1-14, 5122	K.GNFNYIEFTR.I	1261.36733	2	5.02E-05	0.76	2.81	-	686.9
AHQ-1-13- , 4363 - 4371	K.GNFNYIEFTR.I	1261.36733	2	8.67E-04	0.82	3.34	-	700.3
AHQ-1-11, 4139 - 4184	K.GNFNYIEFTR.I	1261.36733	2	8.91E-04	0.84	3.11	-	1035.2
AHQ-1-11, 4362	K.GNFNYIEFTR.I	1261.36733	1	3.79E-04	0.41	2.14	-	359.5
AHQ-1-11, 4355	K.GNFNYIEFTR.I	1261.36733	2	6.02E-04	0.78	2.95	-	882.2
AHQ-1-11, 3436	K.KGNFNYIEFTR.I	1389.54025	2	2.96E-09	0.95	4.19	-	980.1
gi|18860900|ref|NP_002834.2| protein tyrosine phosphatase, receptor type, J precursor; protein tyro				4.82E-12	7.95	90.26	10.60	145926.2
AHQ-1-1, 3468	R.AGSPTAPVHDESLVGPVDPSSGQQSR.D	2576.71806	3	1.08E-04	0.92	4.60	-	1201.2
AHQ-1-1, 4432 - 4444	K.AYAVILTTGEAGHPSADVLK.Y	2014.26782	3	2.76E-09	0.96	4.67	-	1601.0
AHQ-1-1, 4431	K.AYAVILTTGEAGHPSADVLK.Y	2014.26782	2	2.33E-07	0.98	5.27	-	2074.4
AHQ-1-1, 3079	K.GDPLGTEGGLDASNTER.S	1689.71978	2	1.17E-08	0.94	4.05	-	1051.1
AHQ-1-1, 5263 - 5267	K.GLIDGAESYVSFSR.Y	1501.62253	2	5.52E-09	0.97	4.33	-	2276.2
AHQ-1-1, 4968	K.SFCTDPASM*ASFDCEVVPK.E	2167.37954	2	5.17E-04	0.60	2.93	-	568.0
AHQ-1-1, 5739	K.SFCTDPASMASFDCEVVPK.E	2151.38014	2	6.38E-06	0.87	4.21	-	655.8
AHQ-1-1, 5404	K.SPDGASEYVYHLVIESK.H	1895.05866	2	4.82E-12	0.93	4.21	-	1059.4
AHQ-1-1, 4325	K.VITEPIPVSDLR.V	1339.56259	2	5.41E-04	0.83	3.02	-	565.5
AHQ-1-1, 3899	R.VLLESIGSHEELTQDSR.L	1914.06352	2	3.40E-07	0.86	3.54	-	896.9
AHQ-1-1, 3911	R.VLLESIGSHEELTQDSR.L	1914.06352	3	3.75E-10	0.93	4.63	-	748.4
gi|12408656|ref|NP_005177.2| calpain 1, large subunit; calpain, large polypeptide L1; calcium-activ				5.11E-12	9.84	110.30	15.80	81889.4
AHQ-1-6, 3634	R.DFFLANASR.A	1041.14166	2	7.89E-05	0.87	2.85	-	1163.7
AHQ-1-6, 6416 - 6448	R.DM*ETIGFAVYEVPPELVGQPAVHLK.R	2757.15391	3	5.11E-12	0.93	4.08	-	1191.0
AHQ-1-6, 6760	R.DMETIGFAVYEVPPELVGQPAVHLK.R	2741.15451	3	1.36E-07	0.83	3.73	-	782.8
AHQ-1-6, 6154	R.FRLPPGEYVVVPSTFEPNKEGDFVLR.F	2994.39160	3	1.93E-07	0.96	5.95	-	1363.0
AHQ-1-6, 4391 - 4411	R.KAPSDLYQIILK.A	1389.66474	2	3.01E-06	0.95	4.50	-	1148.9
AHQ-1-6, 3783	R.KWNTTLYEGTWR.R	1555.71826	2	3.51E-09	0.91	3.62	-	921.1
AHQ-1-6, 4875	R.LPPGEYVVVPSTFEPNK.E	1874.12573	2	6.80E-05	0.89	3.87	-	706.1
AHQ-1-6, 5898	R.LPPGEYVVVPSTFEPNKEGDFVLR.F	2691.03055	2	4.21E-04	0.70	3.20	-	258.0
AHQ-1-6, 5886	R.LPPGEYVVVPSTFEPNKEGDFVLR.F	2691.03055	3	2.65E-07	0.83	4.02	-	451.4
AHQ-1-6, 6584	K.LVFVHSAEGNEFWSALLEK.A	2177.44364	3	2.64E-08	0.90	4.18	-	893.8
AHQ-1-6, 3083	R.SEQFINLR.E	1007.12521	2	8.40E-04	0.85	2.68	-	1032.5
AHQ-1-6, 3302	K.YLGQDYEQLR.V	1285.38720	2	2.37E-07	0.91	3.41	-	979.3
gi|5453555|ref|NP_006316.1| ras-related nuclear protein [Homo sapiens]				5.33E-12	1.56	20.19	12.50	24422.9
AHQ-1-10, 5279	K.SNYNFEKPFLWLAR.K	1786.02509	2	5.33E-12	0.82	3.86	-	524.4
AHQ-1-10, 4085	R.VCENIPIVLCGNK.V	1518.78102	2	1.90E-04	0.73	3.01	-	554.7
gi|21361755|ref|NP_004090.3| stomatin; erythrocyte membrane protein band 7.2 (stomatin) [Homo sapie				5.41E-12	9.87	110.38	51.00	31708.5
AHQ-1-14- , 4687 - 4688	K.EASMVITESPAALQLR.Y	1716.98119	2	3.81E-08	0.93	4.13	-	1078.9
AHQ-1-1, 4863 - 4875	K.EASMVITESPAALQLR.Y	1716.98119	2	1.50E-05	0.79	3.55	-	774.4
AHQ-1-14- , 4100	K.EASM*VITESPAALQLR.Y	1732.98059	2	1.38E-07	0.93	4.12	-	1006.0
AHQ-1-13- , 4240	K.EASM*VITESPAALQLR.Y	1732.98059	2	5.10E-05	0.94	4.36	-	1261.5
AHQ-1-1, 4320	K.EASM*VITESPAALQLR.Y	1732.98059	2	2.66E-07	0.95	4.10	-	1518.1
AHQ-1-13- , 4751	K.EASMVITESPAALQLR.Y	1716.98119	2	7.89E-08	0.93	4.31	-	927.1
AHQ-1-9, 7250	K.GPGLFFILPCTDSFIK.V	1814.13672	2	4.19E-05	0.94	4.57	-	758.2
AHQ-1-10, 6348 - 6349	K.GPGLFFILPCTDSFIK.V	1814.13672	2	6.83E-05	0.95	5.20	-	770.5
AHQ-1-13- , 6935	K.GPGLFFILPCTDSFIK.V	1814.13672	2	1.71E-06	0.94	4.49	-	833.7
AHQ-1-1, 2547	R.LLAQTTLR.N	916.10035	2	3.09E-05	0.83	2.96	-	621.4
AHQ-1-10, 6271	K.NLSQILSDREEIAHNMQSTLDDATDAWGIK.V	3373.65339	3	1.20E-09	0.98	7.59	-	2373.6
AHQ-1-10, 6073	K.NLSQILSDREEIAHNMQSTLDDATDAWGIK.V	3373.65339	3	2.04E-06	0.91	4.28	-	1180.8
AHQ-1-13- , 7023	K.NSTIVFPLPIDMLQGIIGAK.H	2128.56375	2	3.68E-04	0.89	3.98	-	456.5
AHQ-1-10, 6383	K.NSTIVFPLPIDM*LQGIIGAK.H	2144.56315	2	4.63E-05	0.90	4.27	-	471.2
AHQ-1-1, 5604 - 5668	R.TISFDIPPQEILTK.D	1602.85305	2	3.39E-04	0.60	2.56	-	283.6
AHQ-1-1, 2400	K.VIAAEGEMNASR.A	1248.39191	2	1.57E-08	0.97	4.67	-	1438.6
AHQ-1-13- , 2319	K.VIAAEGEMNASR.A	1248.39191	2	8.13E-08	0.97	4.66	-	1411.7
AHQ-1-13, 2375	K.VIAAEGEMNASR.A	1248.39191	2	6.35E-06	0.95	4.06	-	1271.1
AHQ-1-10, 2156	K.VIAAEGEMNASR.A	1248.39191	2	6.01E-06	0.96	3.49	-	1692.1
AHQ-1-10, 3695	R.VQNATLAVANITNADSATR.L	1931.09731	2	5.91E-10	0.96	5.48	-	882.1
AHQ-1-10, 3563	R.VQNATLAVANITNADSATR.L	1931.09731	2	2.63E-06	0.93	4.80	-	654.4
AHQ-1-1, 3920	R.VQNATLAVANITNADSATR.L	1931.09731	2	5.41E-12	0.96	4.90	-	1087.8
AHQ-1-1, 4008 - 4032	R.VQNATLAVANITNADSATR.L	1931.09731	2	1.17E-08	0.97	4.96	-	1458.4
AHQ-1-1, 4277	R.VQNATLAVANITNADSATR.L	1931.09731	2	9.26E-10	0.96	4.75	-	1179.6
AHQ-1-2, 4113	R.VQNATLAVANITNADSATR.L	1931.09731	2	3.18E-08	0.92	3.76	-	1110.4
AHQ-1-13- , 4116	R.VQNATLAVANITNADSATR.L	1931.09731	2	7.37E-08	0.93	4.31	-	885.0
AHQ-1-1, 4629	R.VQNATLAVANITNADSATR.L	1931.09731	2	6.42E-08	0.93	4.54	-	757.8
AHQ-1-10, 3779 - 3855	R.VQNATLAVANITNADSATR.L	1931.09731	2	3.88E-11	0.96	5.26	-	1072.1
AHQ-1-13, 3811	R.YLQTLTTIAAEK.N	1352.55813	2	3.58E-04	0.81	2.65	-	1249.5
AHQ-1-10, 3539	R.YLQTLTTIAAEK.N	1352.55813	2	1.32E-04	0.90	3.26	-	1202.1
AHQ-1-1, 4019	R.YLQTLTTIAAEK.N	1352.55813	2	1.14E-07	0.95	3.77	-	1710.8
AHQ-1-14, 4756 - 4758	R.YLQTLTTIAAEK.N	1352.55813	2	5.07E-04	0.85	3.14	-	850.1
gi|4507953|ref|NP_003397.1| tyrosine 3/tryptophan 5 -monooxygenase activation protein, zeta polypep				6.33E-12	9.66	110.29	53.10	27744.9
AHQ-1-10, 3233 - 3376	K.FLIPNASQAESK.V	1305.46156	1	3.94E-07	0.35	2.03	-	382.5
AHQ-1-10, 3019 - 3080	K.FLIPNASQAESK.V	1305.46156	2	1.29E-09	0.88	2.78	-	700.5
AHQ-1-11, 3166	K.FLIPNASQAESK.V	1305.46156	2	4.96E-07	0.77	2.79	-	529.4
AHQ-1-13, 3501	K.FLIPNASQAESK.V	1305.46156	1	1.58E-04	0.30	1.93	-	475.7
AHQ-1-13- , 6963	K.IETELRDICNDVLSLLEK.F	2162.44718	3	7.50E-04	0.83	4.21	-	963.1
AHQ-1-10, 6393	K.IETELRDICNDVLSLLEK.F	2162.44718	3	1.21E-07	0.91	5.14	-	1090.6
AHQ-1-10, 5675 - 5731	K.IETELRDICNDVLSLLEK.F	2162.44718	2	4.59E-06	0.93	4.61	-	1077.7
AHQ-1-10, 6405 - 6415	R.LGLALNFSVFYYEILNSPEK.A	2318.65200	2	4.68E-05	0.95	5.15	-	807.6
AHQ-1-10, 6401 - 6404	R.LGLALNFSVFYYEILNSPEK.A	2318.65200	2	1.35E-06	0.81	4.00	-	292.0
AHQ-1-10, 2283 - 2320	K.SVTEQGAELSNEER.N	1549.57922	2	5.73E-09	0.96	5.05	-	958.5
AHQ-1-13- , 2376	K.SVTEQGAELSNEER.N	1549.57922	2	8.41E-10	0.90	3.92	-	1039.4

AHQ-1-10, 2472 - 2539	K.SVTEQGAELSNEER.N	1549.57922	2	1.25E-06	0.96	4.66	-	1571.0
AHQ-1-14- , 2288 - 2346	K.SVTEQGAELSNEER.N	1549.57922	2	9.14E-10	0.95	4.29	-	1554.0
AHQ-1-12, 2329 - 2330	K.SVTEQGAELSNEER.N	1549.57922	2	3.33E-09	0.92	4.32	-	1054.3
AHQ-1-11, 2296 - 2299	K.SVTEQGAELSNEER.N	1549.57922	2	9.98E-06	0.95	4.12	-	1261.7
AHQ-1-14- , 2186 - 2256	K.SVTEQGAELSNEER.N	1549.57922	2	6.52E-07	0.96	4.48	-	1542.9
AHQ-1-13, 2421	K.SVTEQGAELSNEER.N	1549.57922	2	1.46E-06	0.93	4.25	-	1194.4
AHQ-1-10, 2223 - 2264	K.SVTEQGAELSNEER.N	1549.57922	2	1.00E-06	0.93	3.36	-	1587.2
AHQ-1-9, 2239	K.SVTEQGAELSNEER.N	1549.57922	2	7.16E-06	0.92	3.92	-	1134.0
AHQ-1-10, 4571	K.SVTEQGAELSNEERNLLSVAYK.N	2438.63331	2	8.46E-04	0.81	3.34	-	713.9
AHQ-1-10, 4709 - 4712	K.SVTEQGAELSNEERNLLSVAYK.N	2438.63331	2	4.44E-05	0.95	5.17	-	794.5
AHQ-1-10, 5656 - 5729	K.TAFDEAIAELDTLSEESYK.D	2133.25160	2	1.23E-09	0.83	3.44	-	634.1
AHQ-1-13, 6431	K.TAFDEAIAELDTLSEESYK.D	2133.25160	2	1.20E-06	0.97	5.44	-	1400.2
AHQ-1-10, 6299	K.TAFDEAIAELDTLSEESYK.D	2133.25160	3	3.01E-07	0.96	5.78	-	1274.4
AHQ-1-13, 6432 - 6445	K.TAFDEAIAELDTLSEESYK.D	2133.25160	3	4.84E-10	0.96	5.19	-	1490.8
AHQ-1-14- , 6568 - 6640	K.TAFDEAIAELDTLSEESYK.D	2133.25160	2	7.62E-09	0.96	5.09	-	1048.3
AHQ-1-13- , 5797	K.TAFDEAIAELDTLSEESYK.D	2133.25160	2	3.02E-06	0.96	4.96	-	1094.8
AHQ-1-13- , 6811 - 6887	K.TAFDEAIAELDTLSEESYK.D	2133.25160	2	2.10E-08	0.97	5.36	-	1357.5
AHQ-1-13- , 6891	K.TAFDEAIAELDTLSEESYK.D	2133.25160	3	1.08E-05	0.95	5.39	-	1108.9
AHQ-1-11, 6388	K.TAFDEAIAELDTLSEESYK.D	2133.25160	2	6.33E-12	0.97	5.56	-	1188.3
AHQ-1-9, 7200	K.TAFDEAIAELDTLSEESYK.D	2133.25160	2	2.57E-05	0.96	5.31	-	976.8
AHQ-1-12, 6566	K.TAFDEAIAELDTLSEESYK.D	2133.25160	2	2.87E-09	0.89	3.48	-	1108.6
AHQ-1-10, 6252 - 6295	K.TAFDEAIAELDTLSEESYK.D	2133.25160	2	3.26E-11	0.96	5.17	-	1252.1
AHQ-1-10, 6369 - 6441	K.TAFDEAIAELDTLSEESYKDSTLIM*QLLR.D	3320.66704	3	4.53E-04	0.89	4.56	-	803.1
AHQ-1-10, 6511	K.TAFDEAIAELDTLSEESYKDSTLIM*QLLR.D	3320.66704	3	2.55E-11	0.96	5.73	-	1376.0
AHQ-1-10, 1487 - 1557	R.VVSSIEQK.T	890.01653	2	9.11E-07	0.66	3.14	-	400.2
AHQ-1-13- , 2628	R.YDDMAACMK.S	1106.27579	2	3.48E-06	0.92	3.09	-	1105.5
AHQ-1-10, 2445	R.YDDMAACMK.S	1106.27579	1	6.21E-04	0.26	1.81	-	314.6
AHQ-1-10, 2137 - 2216	R.YLAEVAAGDDK.K	1152.23595	2	4.46E-05	0.92	3.13	-	994.6
AHQ-1-13- , 2388	R.YLAEVAAGDDK.K	1152.23595	1	2.49E-05	0.79	2.53	-	1400.7
AHQ-1-13- , 2391	R.YLAEVAAGDDK.K	1152.23595	2	5.20E-04	0.90	2.95	-	1045.2
AHQ-1-10, 2133 - 2212	R.YLAEVAAGDDK.K	1152.23595	1	2.27E-05	0.77	2.86	-	1031.3
AHQ-1-13, 2435	R.YLAEVAAGDDK.K	1152.23595	1	5.81E-05	0.58	2.20	-	991.5
AHQ-1-10, 2027	R.YLAEVAAGDDKK.G	1280.40887	2	3.35E-05	0.84	3.18	-	873.6
AHQ-1-10, 1940	R.YLAEVAAGDDKK.G	1280.40887	3	1.16E-05	0.78	3.09	-	996.9
AHQ-1-10, 1929 - 1988	R.YLAEVAAGDDKK.G	1280.40887	1	2.45E-04	0.88	3.62	-	865.8
AHQ-1-10, 1912 - 1967	R.YLAEVAAGDDKK.G	1280.40887	2	2.86E-05	0.96	4.30	-	1614.0
AHQ-1-11, 2030 - 2099	R.YLAEVAAGDDKK.G	1280.40887	2	2.62E-04	0.90	3.09	-	1453.5
AHQ-1-13, 2157 - 2229	R.YLAEVAAGDDKK.G	1280.40887	2	5.70E-05	0.86	3.08	-	1024.5
AHQ-1-13- , 2069 - 2135	R.YLAEVAAGDDKK.G	1280.40887	2	5.34E-08	0.95	3.82	-	1553.7
gi|4885063|ref|NP_005156.1| aldolase C, fructose-bisphosphate; Aldolase C, fructose-bisphosphatase				6.50E-12	0.80	10.26	6.30	39455.6
AHQ-1-9, 4343 - 4382	K.GVVPLAGTDGETTTQGLDGLSER.C	2274.42806	2	2.67E-07	0.79	4.65	-	615.1
AHQ-1-9, 4786	K.GVVPLAGTDGETTTQGLDGLSER.C	2274.42806	2	1.21E-10	0.69	4.01	-	358.4
AHQ-1-10, 4236	K.GVVPLAGTDGETTTQGLDGLSER.C	2274.42806	2	8.61E-08	0.52	3.86	-	273.4
AHQ-1-12, 4506	K.GVVPLAGTDGETTTQGLDGLSER.C	2274.42806	2	6.50E-12	0.80	4.18	-	682.9
AHQ-1-8, 4671 - 4742	K.GVVPLAGTDGETTTQGLDGLSER.C	2274.42806	2	6.01E-10	0.88	5.21	-	740.4
gi|30159830|ref|XP_302246.1| similar to HSPC280 [Homo sapiens]				6.92E-12	1.92	20.24	3.90	45644.4
AHQ-1-14- , 6682 - 6752	K.CANLFEALVGTLK.A	1437.68632	2	4.35E-07	0.97	4.74	-	1522.5
AHQ-1-14- , 6639	R.DDKCANLFEALVGTLK.A	1796.03496	3	2.90E-04	0.93	4.20	-	1710.9
AHQ-1-14- , 6630 - 6707	R.DDKCANLFEALVGTLK.A	1796.03496	2	6.92E-12	0.96	4.30	-	1314.4
gi|17986273|ref|NP_524144.1| fast skeletal myosin alkali light chain 1 isoform 1f; A1 catalytic; A2				7.42E-12	1.85	20.20	8.20	21145.0
AHQ-1-12, 3429	R.VFDKEGNGTVMGAELR.H	1723.93228	2	1.61E-07	0.52	2.54	-	467.2
AHQ-1-13, 3327	R.VFDKEGNGTVMGAELR.H	1723.93228	2	4.19E-05	0.83	2.90	-	904.0
AHQ-1-14- , 2690 - 2692	R.VFDKEGNGTVM*GAELR.H	1739.93168	2	1.31E-09	0.93	3.92	-	980.0
AHQ-1-14- , 3340	R.VFDKEGNGTVMGAELR.H	1723.93228	2	7.42E-12	0.92	3.98	-	963.6
AHQ-1-12, 2630 - 2694	R.VFDKEGNGTVM*GAELR.H	1739.93168	2	6.28E-06	0.86	3.61	-	840.6
gi|4504523|ref|NP_002148.1| heat shock 10kDa protein 1 (chaperonin 10); heat shock 10kD protein 1 (				8.07E-12	1.89	20.28	25.50	10931.6
AHQ-1-14- , 3007	K.VLQATVVAVGSGSK.G	1316.52902	2	4.02E-04	0.89	3.19	-	810.0
AHQ-1-14- , 2834 - 2836	K.VLQATVVAVGSGSK.G	1316.52902	2	6.89E-11	0.98	5.53	-	1807.5
AHQ-1-14, 3728	K.VLQATVVAVGSGSK.G	1316.52902	2	8.07E-12	0.97	4.92	-	1336.6
AHQ-1-14- , 5028 - 5106	K.VVLDDKDYFLFR.D	1530.74861	2	8.30E-10	0.90	3.38	-	1022.7
AHQ-1-14- , 5030	K.VVLDDKDYFLFR.D	1530.74861	3	2.88E-07	0.86	3.41	-	584.0
AHQ-1-14, 5928 - 6001	K.VVLDDKDYFLFR.D	1530.74861	2	2.81E-10	0.92	3.60	-	1023.1
gi|4503327|ref|NP_000389.1| cytochrome b5 reductase membrane-bound isoform; diaphorase (NADH) [Homo				8.19E-12	9.01	110.33	49.50	34234.6
AHQ-1-9, 4532	R.AIMKDPDDHTVCHLLFANQTEK.D	2585.89793	3	8.19E-12	0.88	4.19	-	837.6
AHQ-1-9, 5114	R.APEAWDYGQGFVNEEM*IR.D	2129.29457	2	2.92E-05	0.91	3.79	-	834.8
AHQ-1-9, 4790 - 4791	K.DILLRPELEELR.N	1496.73370	2	2.97E-07	0.84	3.88	-	575.2
AHQ-1-9, 6238 - 6246	R.FALPSPQHILGLPVGQHIYLSAR.I	2515.94007	3	4.51E-10	0.95	5.58	-	1110.1
AHQ-1-9, 6250 - 6254	R.FALPSPQHILGLPVGQHIYLSAR.I	2515.94007	2	1.14E-07	0.95	5.29	-	679.2
AHQ-1-9, 2872	R.GPSGLLVYQGK.G	1119.29547	1	1.15E-05	0.79	3.39	-	464.9
AHQ-1-9, 2866	R.GPSGLLVYQGK.G	1119.29547	2	3.32E-05	0.96	4.27	-	1398.1
AHQ-1-9, 6086	R.IDGNLVVRPYTPISSDDDKGFVDLVIK.V	2977.35765	3	2.51E-05	0.97	6.57	-	1251.8
AHQ-1-9, 3627	R.STPAITLESPDIK.Y	1372.54621	1	2.88E-05	0.64	3.01	-	428.4
AHQ-1-11, 3603	R.STPAITLESPDIK.Y	1372.54621	2	3.15E-04	0.53	2.68	-	559.9
AHQ-1-12, 3719	R.STPAITLESPDIK.Y	1372.54621	2	2.58E-05	0.62	2.70	-	449.6
AHQ-1-9, 4638	R.STPAITLESPDIKYPLR.L	1902.18061	2	1.56E-05	0.88	3.94	-	651.2
AHQ-1-9, 6012	K.SVGMIAGGTGITPMLQVIR.A	1902.31543	2	3.35E-05	0.78	3.66	-	475.2
AHQ-1-9, 5528	K.SVGM*IAGGTGITPMLQVIR.A	1918.31483	2	1.06E-04	0.82	3.83	-	538.9
AHQ-1-9, 5080 - 5104	K.SVGMIAGGTGITPM*LQVIR.A	1918.31483	2	1.71E-04	0.56	3.15	-	371.1
gi|5729877|ref|NP_006588.1| heat shock 70kDa protein 8 isoform 1; heat shock cognate protein, 71-kD				8.52E-12	14.41	160.27	27.20	70897.6
AHQ-1-7, 4486	R.ARFEELNADLFR.G	1481.63768	2	1.05E-04	0.82	2.90	-	704.3
AHQ-1-6, 4387	R.ARFEELNADLFR.G	1481.63768	3	1.91E-04	0.97	5.37	-	1952.1
AHQ-1-6, 4380 - 4460	R.ARFEELNADLFR.G	1481.63768	2	2.16E-07	0.96	4.50	-	1104.2
AHQ-1-6, 4746	R.FEELNADLFR.G	1254.37310	2	1.10E-06	0.91	3.70	-	977.9
AHQ-1-6, 5362	K.GPAVGIDLGTTYSCVGVFQHGK.V	2265.53088	2	4.11E-10	0.95	5.22	-	764.6
AHQ-1-14- , 5166 - 5244	K.GPAVGIDLGTTYSCVGVFQHGK.V	2265.53088	3	5.17E-05	0.86	4.00	-	588.8
AHQ-1-6, 5754 - 5772	K.ILDKCNEIINWLDK.N	1776.04688	2	4.55E-05	0.93	4.46	-	1116.2
AHQ-1-6, 2834 - 2835	K.MKEIAEAYLGK.T	1253.49320	2	3.04E-07	0.92	3.55	-	1146.8
AHQ-1-6, 3378	R.MVNHFIAEFK.R	1236.46756	2	6.64E-08	0.94	3.88	-	1186.6
AHQ-1-6, 2778	K.NQVAM*NPTNTVFDAK.R	1666.83756	2	8.52E-12	0.90	3.45	-	853.1
AHQ-1-13- , 3756	K.NQVAMNPTNTVFDAK.R	1650.83816	2	2.36E-04	0.91	3.74	-	826.3
AHQ-1-6, 3446	K.NQVAMNPTNTVFDAK.R	1650.83816	2	2.54E-11	0.93	4.23	-	772.6
AHQ-1-6, 3427	K.NQVAM*NPTNTVFDAK.R	1666.83756	2	4.73E-06	0.89	3.53	-	817.5
AHQ-1-6, 4098	K.NSLESYAFNMK.A	1304.45393	2	3.83E-06	0.89	3.59	-	832.4
AHQ-1-6, 3304	K.NSLESYAFNM*K.A	1320.45333	2	1.07E-05	0.86	3.34	-	819.3
AHQ-1-6, 2598	R.RFDDAVVQSDMK.H	1411.56603	2	2.46E-06	0.96	4.33	-	1539.9
AHQ-1-12, 5093 - 5107	K.SFYPEEVSSMVLTK.M	1617.84485	2	1.72E-04	0.64	3.04	-	294.1
AHQ-1-6, 5256 - 5323	K.SFYPEEVSSMVLTK.M	1617.84485	2	3.35E-04	0.74	3.20	-	440.8
AHQ-1-6, 4484 - 4555	K.SFYPEEVSSM*VLTK.M	1633.84425	2	1.06E-04	0.87	3.11	-	756.1
AHQ-1-6, 6922 - 6986	K.SINPDEAVAYGAAVQAAILSGDK.S	2261.47411	2	3.82E-08	0.97	5.38	-	1438.3
AHQ-1-7, 7090	K.SINPDEAVAYGAAVQAAILSGDK.S	2261.47411	2	8.76E-06	0.94	4.42	-	981.9
AHQ-1-6, 3010	K.SQIHDIVLVGGSTR.I	1482.66712	1	1.05E-11	0.92	4.27	-	719.3
AHQ-1-9, 2916	K.SQIHDIVLVGGSTR.I	1482.66712	2	2.03E-08	0.97	4.02	-	1985.0
AHQ-1-6, 3000 - 3002	K.SQIHDIVLVGGSTR.I	1482.66712	2	2.07E-08	0.97	4.53	-	1844.5
AHQ-1-6, 3003	K.SQIHDIVLVGGSTR.I	1482.66712	1	3.95E-05	0.91	3.69	-	894.5
AHQ-1-7, 3034	K.SQIHDIVLVGGSTR.I	1482.66712	2	1.76E-10	0.98	4.42	-	2585.0
AHQ-1-8, 2802	K.SQIHDIVLVGGSTR.I	1482.66712	1	8.98E-04	0.86	3.45	-	730.1
AHQ-1-6, 4580 - 4650	K.TVTNAVVTVPAYFNDSQR.Q	1983.17068	2	1.14E-04	0.87	4.10	-	442.7

AHQ-1-6, 4722 - 4770	K.TVTNAVVTVPAYFNDSQR.Q	1983.17068	2	7.87E-04	0.77	3.59	-	308.9
AHQ-1-6, 4840 - 4872	K.TVTNAVVTVPAYFNDSQR.Q	1983.17068	2	2.38E-04	0.56	3.26	-	234.2
AHQ-1-6, 4666	K.TVTNAVVTVPAYFNDSQR.Q	1983.17068	3	2.00E-04	0.92	4.28	-	1266.3
gi|18201905|ref|NP_000166.2| glucose phosphate isomerase; neuroleukin; glucose-6-phosphate isomeras				8.67E-12	6.87	80.29	24.40	63146.7
AHQ-1-7, 6926	K.ILLANFLAQTEALM*R.G	1721.05778	2	1.38E-04	0.94	3.62	-	1419.9
AHQ-1-7, 7481	K.ILLANFLAQTEALMR.G	1705.05838	2	6.87E-09	0.97	5.74	-	1432.6
AHQ-1-7, 5140 - 5141	K.KIEPELDGSAQVTSHDASTNGLINFIK.Q	2886.16347	3	3.07E-04	0.90	4.73	-	803.6
AHQ-1-7, 3028	K.TFTTQETITNAETAK.E	1656.77283	2	1.08E-05	0.80	3.32	-	692.4
AHQ-1-7, 7301 - 7317	K.TITDVINIGIGGSDLGPLM*VTEALKPYSSGGPR.V	3346.79723	3	6.51E-07	0.84	3.77	-	814.3
AHQ-1-7, 6358 - 6372	K.TLAQLNPESSLFIIASK.T	1833.11828	2	7.10E-05	0.74	3.41	-	417.1
AHQ-1-7, 3289	K.VFEGNRPTNSIVFTK.L	1709.92693	2	3.90E-04	0.79	3.36	-	741.8
AHQ-1-7, 3938 - 3958	R.VWYVSNIDGTHIAK.T	1603.80282	2	8.67E-12	0.88	3.20	-	808.4
gi|10834976|ref|NP_000572.1| glutathione peroxidase 1 [Homo sapiens]				8.73E-12	5.04	60.29	42.80	21909.0
AHQ-1-11, 5126 - 5200	K.CEVNGAGAHPLFAFLR.E	1760.99723	2	8.73E-12	0.86	3.60	-	550.9
AHQ-1-11, 3934	K.FLVGPDGVPLR.R	1170.38561	2	1.29E-04	0.74	2.60	-	682.4
AHQ-1-11, 4758 - 4759	R.GLVVLGFPCNQFGHQENAK.N	2117.37312	2	1.16E-04	0.86	4.02	-	473.6
AHQ-1-11, 3750 - 3754	K.LITWSPVCR.N	1133.34543	2	2.23E-04	0.65	2.59	-	496.7
AHQ-1-14, 4846	R.PLAGGEPVSLGSLR.G	1353.54935	2	3.05E-06	0.96	4.74	-	1124.3
AHQ-1-14- , 3974	R.PLAGGEPVSLGSLR.G	1353.54935	2	1.24E-07	0.96	4.23	-	1063.8
AHQ-1-11, 3735 - 3742	R.PLAGGEPVSLGSLR.G	1353.54935	2	3.76E-09	0.96	5.03	-	1105.5
AHQ-1-11, 5023	K.YVRPGGGFEPNFMLFEK.C	1989.28515	2	3.91E-06	0.97	5.72	-	1624.1
AHQ-1-11, 5031	K.YVRPGGGFEPNFMLFEK.C	1989.28515	3	9.10E-04	0.95	4.82	-	1421.0
gi|16507237|ref|NP_005338.1| heat shock 70kDa protein 5 (glucose-regulated protein, 78kDa); BiP; he				9.14E-12	11.26	130.30	22.50	72332.5
AHQ-1-6, 5023	R.AKFEELNMDLFR.S	1513.74309	2	8.58E-08	0.92	3.89	-	984.1
AHQ-1-6, 4162	R.AKFEELNM*DLFR.S	1529.74249	2	1.37E-06	0.87	2.77	-	1143.0
AHQ-1-6, 5751	K.DNHLLGTFDLTGIPPAPR.G	1935.17239	2	8.98E-09	0.95	4.44	-	1060.6
AHQ-1-6, 5660	K.DNHLLGTFDLTGIPPAPR.G	1935.17239	2	6.20E-09	0.95	4.43	-	1071.3
AHQ-1-6, 6067 - 6142	R.IEIESFYEGEDFSETLTR.A	2166.28328	2	4.60E-08	0.95	4.08	-	1450.1
AHQ-1-6, 7415	R.IEIESFYEGEDFSETLTR.A	2166.28328	2	1.05E-06	0.95	4.34	-	1275.5
AHQ-1-6, 6211 - 6279	R.IEIESFYEGEDFSETLTR.A	2166.28328	2	2.23E-04	0.92	3.98	-	960.1
AHQ-1-6, 6350	R.IEIESFYEGEDFSETLTR.A	2166.28328	2	1.95E-06	0.95	4.61	-	1057.0
AHQ-1-6, 4768 - 4794	K.IEWLESHQDADIEDFK.A	1976.08864	2	9.14E-12	0.98	5.96	-	1904.9
AHQ-1-6, 4446 - 4459	R.IINEPTAAAIAYGLDKR.E	1817.07894	2	1.37E-10	0.76	3.77	-	804.5
AHQ-1-6, 3731	K.KSDIDEIVLVGGSTR.I	1589.77277	2	2.55E-08	0.96	4.30	-	1648.9
AHQ-1-6, 3256	K.KSQIFSTASDNQPTVTIK.V	1966.18156	2	5.63E-05	0.88	4.21	-	734.6
AHQ-1-6, 3442	K.KVTHAVVTVPAYFNDAQR.Q	2017.27663	2	8.99E-08	0.97	5.79	-	1280.7
AHQ-1-6, 4386	K.SDIDEIVLVGGSTR.I	1461.59985	1	2.36E-06	0.35	2.31	-	364.8
AHQ-1-6, 3742	K.SQIFSTASDNQPTVTIK.V	1838.00865	2	5.53E-05	0.81	3.27	-	784.7
AHQ-1-7, 3833 - 3836	K.SQIFSTASDNQPTVTIK.V	1838.00865	2	1.05E-06	0.92	4.23	-	863.4
AHQ-1-6, 2928 - 3008	K.TKPYIQVDIGGGQTK.T	1605.81703	2	1.54E-04	0.79	3.26	-	664.7
AHQ-1-6, 3898	K.VTHAVVTVPAYFNDAQR.Q	1889.10372	2	2.38E-11	0.95	4.83	-	751.4
AHQ-1-7, 3982	K.VTHAVVTVPAYFNDAQR.Q	1889.10372	2	4.66E-08	0.88	3.96	-	487.8
AHQ-1-6, 4031	K.VTHAVVTVPAYFNDAQR.Q	1889.10372	2	1.33E-05	0.80	3.20	-	432.3
gi|4758606|ref|NP_004508.1| integrin-linked kinase [Homo sapiens]				9.81E-12	11.75	140.32	34.30	51418.9
AHQ-1-13, 5019 - 5023	R.EVPFADLSNMEIGM*K.V	1697.95498	2	5.07E-04	0.74	3.10	-	598.7
AHQ-1-10, 5197	R.EVPFADLSNMEIGMK.V	1681.95558	2	9.60E-06	0.55	2.57	-	281.8
AHQ-1-7, 5350 - 5364	R.EVPFADLSNMEIGM*K.V	1697.95498	2	4.18E-05	0.89	3.88	-	561.7
AHQ-1-13- , 5299	R.EVPFADLSNMEIGM*K.V	1697.95498	2	3.51E-06	0.71	3.35	-	366.7
AHQ-1-7, 5797	K.FDMIVPILEK.M	1205.49168	2	4.35E-05	0.91	3.42	-	868.5
AHQ-1-7, 2138	R.GDDTPLHLAASHGHR.D	1584.67809	2	1.31E-10	0.89	3.20	-	1162.6
AHQ-1-9, 1983 - 1984	R.GDDTPLHLAASHGHR.D	1584.67809	2	1.85E-07	0.47	2.79	-	365.6
AHQ-1-10, 2063	R.GDDTPLHLAASHGHR.D	1584.67809	2	2.38E-07	0.78	2.93	-	802.6
AHQ-1-7, 1909 - 2028	R.GDDTPLHLAASHGHR.D	1584.67809	2	1.48E-10	0.94	3.82	-	1203.5
AHQ-1-7, 5205	R.GM*AFLHTLEPLIPR.H	1611.93301	2	2.27E-05	0.86	3.20	-	816.4
AHQ-1-7, 6114 - 6142	R.GMAFLHTLEPLIPR.H	1595.93361	2	8.90E-05	0.94	3.78	-	1298.7
AHQ-1-7, 2233	K.LNENHSGELWK.G	1327.42732	2	3.85E-04	0.76	2.97	-	957.8
AHQ-1-7, 2429	K.LNENHSGELWK.G	1327.42732	2	4.36E-07	0.84	3.68	-	849.9
AHQ-1-7, 2428 - 2433	K.LNENHSGELWK.G	1327.42732	1	2.23E-05	0.42	2.23	-	386.3
AHQ-1-7, 6449	R.LWLDNTENDLNQGDDHGFSPLHWACR.E	3113.28217	3	1.06E-08	0.93	5.03	-	1028.3
AHQ-1-7, 6301 - 6381	R.LWLDNTENDLNQGDDHGFSPLHWACR.E	3113.28217	3	6.37E-09	0.97	6.48	-	1343.5
AHQ-1-7, 4321 - 4392	R.M*YAPAWVAPEALQK.K	1591.85542	2	6.03E-05	0.93	3.98	-	864.4
AHQ-1-7, 4822	R.MYAPAWVAPEALQK.K	1575.85602	2	3.87E-08	0.93	3.38	-	1097.7
AHQ-1-13- , 4317 - 4389	R.M*YAPAWVAPEALQK.K	1591.85542	2	1.01E-05	0.87	3.05	-	710.5
AHQ-1-9, 4730	R.MYAPAWVAPEALQK.K	1575.85602	2	9.81E-12	0.94	3.84	-	1158.9
AHQ-1-13, 4196	R.M*YAPAWVAPEALQK.K	1591.85542	2	4.54E-05	0.89	3.31	-	820.5
AHQ-1-10, 4352	R.MYAPAWVAPEALQK.K	1575.85602	2	1.32E-04	0.89	3.29	-	864.3
AHQ-1-11, 4096	R.M*YAPAWVAPEALQK.K	1591.85542	2	8.28E-04	0.83	2.76	-	804.2
AHQ-1-11, 4491 - 4498	R.MYAPAWVAPEALQK.K	1575.85602	2	9.65E-06	0.94	3.66	-	1192.1
AHQ-1-7, 4838	R.SAVVEMLIMR.G	1149.45316	2	3.04E-07	0.96	4.15	-	1393.9
AHQ-1-7, 3026	R.SVMIDEDMTAR.I	1268.44340	2	7.63E-06	0.78	2.60	-	839.9
AHQ-1-7, 4656	K.VALEGLRPTIPPGISPHVCK.L	2143.53829	3	6.10E-10	0.79	3.54	-	912.0
AHQ-1-7, 4649 - 4661	K.VALEGLRPTIPPGISPHVCK.L	2143.53829	2	6.25E-04	0.94	4.98	-	613.0
AHQ-1-7, 2909	R.WQGNDIVVK.V	1059.20012	1	2.46E-04	0.75	2.34	-	955.3
gi|4506413|ref|NP_002875.1| RAP1A, member of RAS oncogene family; RAS-related protein RAP1A [Homo s				1.15E-11	5.49	60.28	33.20	20987.1
AHQ-1-11, 2442	K.CDLEDERVVGK.E	1321.43945	2	1.44E-05	0.92	3.54	-	1191.6
AHQ-1-14- , 5867 - 5871	K.INVNEIFYDLVR.Q	1495.70427	2	5.92E-04	0.92	3.88	-	1033.6
AHQ-1-13- , 6863	K.INVNEIFYDLVR.Q	1495.70427	2	4.41E-09	0.97	5.66	-	1374.5
AHQ-1-13, 6407	K.INVNEIFYDLVR.Q	1495.70427	2	1.12E-07	0.95	4.98	-	948.1
AHQ-1-12, 6534	K.INVNEIFYDLVR.Q	1495.70427	2	1.49E-05	0.93	4.03	-	1054.6
AHQ-1-11, 5527	K.INVNEIFYDLVR.Q	1495.70427	2	5.26E-07	0.89	3.71	-	876.0
AHQ-1-11, 6402	K.INVNEIFYDLVR.Q	1495.70427	2	1.01E-06	0.97	5.20	-	1524.9
AHQ-1-11, 5751 - 5790	K.INVNEIFYDLVR.Q	1495.70427	2	1.39E-08	0.95	4.52	-	965.3
AHQ-1-11, 6316	K.INVNEIFYDLVR.Q	1495.70427	1	1.59E-06	0.48	3.20	-	292.9
AHQ-1-11, 6314 - 6384	K.INVNEIFYDLVR.Q	1495.70427	1	4.34E-04	0.35	2.90	-	238.4
AHQ-1-11, 6311 - 6312	K.INVNEIFYDLVR.Q	1495.70427	2	4.63E-08	0.95	4.42	-	1105.1
AHQ-1-11, 6178 - 6180	K.INVNEIFYDLVR.Q	1495.70427	2	1.39E-06	0.95	4.14	-	1268.8
AHQ-1-14- , 6610	K.INVNEIFYDLVR.Q	1495.70427	2	3.89E-09	0.96	4.61	-	1373.1
AHQ-1-13- , 2879 - 2880	K.LVVLGSGGVGK.S	986.19059	2	4.31E-04	0.94	4.15	-	1083.8
AHQ-1-12, 2758 - 2763	K.LVVLGSGGVGK.S	986.19059	2	3.26E-06	0.88	3.38	-	948.1
AHQ-1-14- , 2823	K.LVVLGSGGVGK.S	986.19059	1	1.10E-04	0.54	2.85	-	199.5
AHQ-1-11, 5990 - 6048	K.SALTVQFVQGIFVEK.Y	1666.94196	2	2.78E-05	0.95	4.95	-	1215.0
AHQ-1-11, 5858 - 5928	K.SALTVQFVQGIFVEK.Y	1666.94196	2	8.36E-04	0.92	3.66	-	1375.2
AHQ-1-11, 5742 - 5803	K.SALTVQFVQGIFVEK.Y	1666.94196	2	1.79E-06	0.96	4.80	-	1387.9
AHQ-1-11, 5752 - 5754	K.SKINVNEIFYDLVR.Q	1710.95483	2	1.15E-11	0.96	4.82	-	1124.6
AHQ-1-14- , 6142	K.SKINVNEIFYDLVR.Q	1710.95483	3	6.39E-08	0.92	4.19	-	1330.0
AHQ-1-11, 5171 - 5195	K.SKINVNEIFYDLVR.Q	1710.95483	2	1.32E-07	0.95	4.41	-	1041.4
AHQ-1-11, 5756	K.SKINVNEIFYDLVR.Q	1710.95483	3	1.77E-06	0.93	4.04	-	1639.1
AHQ-1-11, 4979	K.SKINVNEIFYDLVR.Q	1710.95483	2	1.30E-09	0.90	3.97	-	747.7
AHQ-1-11, 5939 - 6004	K.SKINVNEIFYDLVR.Q	1710.95483	2	6.45E-11	0.95	4.59	-	1138.1
AHQ-1-14- , 6068 - 6139	K.SKINVNEIFYDLVR.Q	1710.95483	2	1.73E-10	0.95	4.26	-	1121.8
AHQ-1-11, 4747 - 4755	K.SKINVNEIFYDLVR.Q	1710.95483	2	5.38E-04	0.46	3.05	-	377.0
AHQ-1-11, 6111	K.SKINVNEIFYDLVR.Q	1710.95483	2	3.49E-08	0.95	4.13	-	1379.8
AHQ-1-11, 2730 - 2794	K.YDPTIEDSYR.K	1259.30338	2	2.24E-07	0.80	2.84	-	418.6
AHQ-1-13- , 2904	K.YDPTIEDSYR.K	1259.30338	2	4.01E-05	0.89	3.13	-	495.1
AHQ-1-12, 2795 - 2797	K.YDPTIEDSYR.K	1259.30338	2	3.02E-04	0.69	2.76	-	299.4
AHQ-1-13, 2885 - 2893	K.YDPTIEDSYR.K	1259.30338	2	3.95E-08	0.81	3.06	-	407.5
AHQ-1-14- , 2830 - 2851	K.YDPTIEDSYR.K	1259.30338	2	5.66E-05	0.75	2.61	-	400.9

gi|5031857|ref|NP_005557.1| lactate dehydrogenase A [Homo sapiens]				1.16E-11	5.10	60.29	19.00	36688.5
AHQ-1-14- , 6494 - 6554	K.DLADELALVDVIEDK.L	1658.82864	2	2.85E-05	0.89	4.02	-	774.8
AHQ-1-13, 6355 - 6356	K.DLADELALVDVIEDK.L	1658.82864	2	1.16E-11	0.98	5.71	-	1903.7
AHQ-1-11, 6287	K.DLADELALVDVIEDK.L	1658.82864	2	5.74E-04	0.45	2.74	-	368.7
AHQ-1-13- , 6803 - 6808	K.DLADELALVDVIEDK.L	1658.82864	2	1.86E-10	0.97	5.31	-	1505.5
AHQ-1-9, 7026 - 7048	K.DLADELALVDVIEDK.L	1658.82864	2	6.86E-06	0.96	3.78	-	1677.7
AHQ-1-9, 7147 - 7227	K.DLADELALVDVIEDKLK.G	1900.15980	2	1.29E-05	0.86	3.77	-	893.5
AHQ-1-9, 2602	K.LVIITAGAR.Q	914.12760	2	1.93E-06	0.89	2.90	-	1007.1
AHQ-1-13- , 2893	K.LVIITAGAR.Q	914.12760	2	3.01E-06	0.90	2.72	-	1105.0
AHQ-1-9, 3275 - 3344	K.QVVESAYEVIK.L	1265.43734	2	7.85E-04	0.66	3.08	-	303.9
AHQ-1-9, 2322	K.TLHPDLGTDKDKEQWK.E	1912.09245	2	3.39E-04	0.81	3.14	-	653.7
AHQ-1-11, 2118	K.VTLTSEEEAR.L	1135.20693	2	8.80E-05	0.88	2.96	-	1019.5
AHQ-1-9, 2003 - 2018	K.VTLTSEEEAR.L	1135.20693	2	1.49E-07	0.90	2.87	-	1143.2
gi|24431935|ref|NP_065393.1| reticulon 4; neuroendocrine-specific protein C like (foocen) [Homo sap				1.17E-11	1.82	20.40	3.90	129930.3
AHQ-1-1, 3335	R.GPLPAAPPVAPER.Q	1272.47793	2	2.26E-06	0.84	3.23	-	682.6
AHQ-1-13- , 6213 - 6220	K.YQFVREPEDEEEEEEEEEDEDEDLEELEVLER.K	4189.14063	3	1.17E-11	0.98	7.92	-	2303.3
gi|5031985|ref|NP_005787.1| nuclear transport factor 2; placental protein 15 [Homo sapiens]				1.48E-11	4.43	50.34	63.00	14478.4
AHQ-1-14- , 5770	K.ADEDPIM*GFHQMFLLK.N	1909.21859	2	1.42E-04	0.88	3.76	-	824.0
AHQ-1-14- , 5454 - 5519	K.IQHSITAQDHQPTPDSCIISM*VVGQLK.A	3022.40358	3	6.17E-07	0.98	6.71	-	2023.0
AHQ-1-14- , 6042 - 6086	K.IQHSITAQDHQPTPDSCIISMVVGQLK.A	3006.40418	3	1.48E-11	0.96	5.44	-	1496.5
AHQ-1-14- , 4536	K.NINDAWVCTNDM*FR.L	1773.92828	2	8.14E-05	0.91	3.73	-	962.4
AHQ-1-14- , 6331	R.TQLGAIYIDASCLTWEGQQFQGK.A	2616.88699	3	8.65E-06	0.71	3.14	-	763.9
gi|7657007|ref|NP_055210.1| dual adaptor of phosphotyrosine and 3-phosphoinositides [Homo sapiens]				1.57E-11	2.59	30.23	18.90	32044.2
AHQ-1-13- , 3989	R.DSNETTGLYSLSVR.A	1542.62982	2	6.77E-05	0.79	3.40	-	610.6
AHQ-1-13, 4964	R.HAAEALLLSNGCDGSYLLR.D	2062.29263	2	8.53E-04	0.85	3.39	-	814.5
AHQ-1-13- , 6140 - 6153	R.SDGEAELLQDLGWYHGNLTR.H	2275.41943	3	1.57E-11	0.95	4.70	-	1590.8
gi|4503715|ref|NP_000500.1| fibrinogen, gamma chain isoform gamma-A precursor [Homo sapiens]				1.82E-11	12.95	150.28	48.70	49481.2
AHQ-1-7, 4709 - 4744	K.AIQLTYNPDESSKPNMIDAATLK.S	2521.82886	3	6.39E-04	0.73	3.03	-	501.5
AHQ-1-7, 4176	K.AIQLTYNPDESSKPNM*IDAATLK.S	2537.82826	3	1.44E-04	0.66	3.23	-	593.7
AHQ-1-7, 4848 - 4849	K.AIQLTYNPDESSKPNMIDAATLK.S	2521.82886	2	8.91E-06	0.83	4.01	-	443.1
AHQ-1-11, 3900	K.AIQLTYNPDESSKPNM*IDAATLK.S	2537.82826	3	9.62E-05	0.68	3.45	-	589.3
AHQ-1-9, 4612 - 4618	K.AIQLTYNPDESSKPNMIDAATLK.S	2521.82886	3	1.25E-06	0.84	4.08	-	553.2
AHQ-1-5, 4769 - 4770	K.AIQLTYNPDESSKPNMIDAATLK.S	2521.82886	3	3.29E-04	0.54	3.06	-	298.6
AHQ-1-7, 5897	K.ASTPNGYDNGIIWATWK.T	1895.06358	2	1.08E-07	0.58	2.67	-	457.4
AHQ-1-14- , 5414 - 5479	K.ASTPNGYDNGIIWATWK.T	1895.06358	2	5.75E-11	0.81	3.21	-	671.4
AHQ-1-7, 5650 - 5720	K.ASTPNGYDNGIIWATWK.T	1895.06358	2	7.25E-06	0.82	3.44	-	572.8
AHQ-1-14- , 5550 - 5616	K.ASTPNGYDNGIIWATWK.T	1895.06358	2	9.25E-08	0.65	2.90	-	402.4
AHQ-1-13, 5261	K.ASTPNGYDNGIIWATWK.T	1895.06358	2	4.98E-08	0.53	2.55	-	411.6
AHQ-1-13- , 5555 - 5620	K.ASTPNGYDNGIIWATWK.T	1895.06358	2	9.43E-10	0.78	3.37	-	518.3
AHQ-1-14, 6428	K.ASTPNGYDNGIIWATWK.T	1895.06358	2	3.81E-06	0.54	2.64	-	333.5
AHQ-1-5, 5853 - 5862	K.ASTPNGYDNGIIWATWK.T	1895.06358	2	8.96E-07	0.80	3.41	-	572.6
AHQ-1-1, 5848 - 5861	K.ASTPNGYDNGIIWATWK.T	1895.06358	2	1.08E-05	0.63	2.52	-	517.3
AHQ-1-7, 2770	K.CHAGHLNGVYYQGGTYSK.A	2014.16662	3	6.26E-06	0.95	4.80	-	1359.7
AHQ-1-9, 2606 - 2680	K.CHAGHLNGVYYQGGTYSK.A	2014.16662	2	2.70E-04	0.89	4.03	-	747.5
AHQ-1-13- , 2820 - 2897	K.CHAGHLNGVYYQGGTYSK.A	2014.16662	3	1.80E-04	0.89	4.04	-	1138.9
AHQ-1-5, 2643	K.CHAGHLNGVYYQGGTYSK.A	2014.16662	2	2.38E-11	0.92	4.52	-	659.7
AHQ-1-7, 2764 - 2768	K.CHAGHLNGVYYQGGTYSK.A	2014.16662	2	1.82E-11	0.98	5.28	-	1528.7
AHQ-1-4, 2812	K.CHAGHLNGVYYQGGTYSK.A	2014.16662	2	8.66E-09	0.95	4.72	-	1016.2
AHQ-1-1, 3301	R.DNCCILDER.F	1197.27847	2	2.22E-05	0.86	3.16	-	936.2
AHQ-1-7, 5325 - 5380	K.EGFGHLSPTGTTEFWLGNEK.I	2208.37146	2	4.04E-05	0.92	4.46	-	653.3
AHQ-1-7, 5334 - 5405	K.EGFGHLSPTGTTEFWLGNEK.I	2208.37146	3	5.61E-04	0.94	4.49	-	1552.8
AHQ-1-5, 5513	K.EGFGHLSPTGTTEFWLGNEK.I	2208.37146	2	1.33E-05	0.81	3.29	-	612.8
AHQ-1-12, 5102	K.EGFGHLSPTGTTEFWLGNEK.I	2208.37146	2	2.59E-06	0.73	3.19	-	470.5
AHQ-1-3, 5421	K.EGFGHLSPTGTTEFWLGNEK.I	2208.37146	2	1.59E-04	0.77	3.37	-	502.3
AHQ-1-2, 5614	K.EGFGHLSPTGTTEFWLGNEK.I	2208.37146	2	3.75E-04	0.87	3.79	-	537.5
AHQ-1-9, 5323	K.EGFGHLSPTGTTEFWLGNEK.I	2208.37146	2	2.48E-07	0.84	3.21	-	867.7
AHQ-1-9, 5196 - 5204	K.EGFGHLSPTGTTEFWLGNEK.I	2208.37146	3	5.09E-05	0.96	4.38	-	2116.1
AHQ-1-9, 5188	K.EGFGHLSPTGTTEFWLGNEK.I	2208.37146	2	7.76E-06	0.95	4.54	-	969.1
AHQ-1-1, 5541	K.EGFGHLSPTGTTEFWLGNEK.I	2208.37146	2	3.75E-06	0.92	4.38	-	676.3
AHQ-1-8, 5200	K.EGFGHLSPTGTTEFWLGNEK.I	2208.37146	2	2.41E-06	0.91	4.13	-	599.1
AHQ-1-5, 5402 - 5426	K.EGFGHLSPTGTTEFWLGNEK.I	2208.37146	2	7.39E-07	0.95	4.96	-	840.7
AHQ-1-7, 5446 - 5516	K.EGFGHLSPTGTTEFWLGNEK.I	2208.37146	2	4.19E-07	0.93	4.84	-	580.9
AHQ-1-12, 6314	R.FGSYCPTTCGIADFLSTYQTK.V	2420.65656	3	2.57E-07	0.88	3.91	-	908.9
AHQ-1-13, 6233	R.FGSYCPTTCGIADFLSTYQTK.V	2420.65656	2	1.09E-05	0.84	3.74	-	501.1
AHQ-1-14- , 6423 - 6435	R.FGSYCPTTCGIADFLSTYQTK.V	2420.65656	2	1.08E-05	0.85	3.89	-	524.1
AHQ-1-13- , 6663	R.FGSYCPTTCGIADFLSTYQTK.V	2420.65656	2	5.38E-05	0.66	2.94	-	480.1
AHQ-1-1, 6711	R.FGSYCPTTCGIADFLSTYQTK.V	2420.65656	3	1.65E-04	0.60	3.05	-	530.5
AHQ-1-12, 6321	R.FGSYCPTTCGIADFLSTYQTK.V	2420.65656	2	8.47E-05	0.86	4.25	-	443.5
AHQ-1-11, 6115	R.FGSYCPTTCGIADFLSTYQTK.V	2420.65656	2	6.78E-05	0.78	3.50	-	457.4
AHQ-1-9, 4426 - 4434	K.IHLISTQSAIPYALR.V	1683.97576	2	1.15E-07	0.97	5.07	-	1269.2
AHQ-1-10, 4173	K.IHLISTQSAIPYALR.V	1683.97576	2	4.59E-08	0.91	3.70	-	813.5
AHQ-1-8, 4327 - 4332	K.IHLISTQSAIPYALR.V	1683.97576	2	1.86E-09	0.97	5.06	-	1182.5
AHQ-1-8, 4333	K.IHLISTQSAIPYALR.V	1683.97576	3	8.11E-05	0.63	3.00	-	644.3
AHQ-1-6, 4551 - 4619	K.IHLISTQSAIPYALR.V	1683.97576	2	2.09E-04	0.80	3.40	-	828.0
AHQ-1-7, 4538 - 4608	K.IHLISTQSAIPYALR.V	1683.97576	2	1.00E-08	0.97	5.31	-	1450.9
AHQ-1-4, 4768 - 4839	K.IHLISTQSAIPYALR.V	1683.97576	2	1.65E-05	0.93	4.31	-	960.9
AHQ-1-7, 1913 - 1918	R.LDGSVDFKK.N	1009.13788	2	1.56E-04	0.69	2.82	-	497.6
AHQ-1-7, 2040	R.LTIGEGQQHHLGGAK.Q	1546.71290	2	6.30E-06	0.90	3.63	-	908.4
AHQ-1-7, 1940	R.LTIGEGQQHHLGGAK.Q	1546.71290	2	6.15E-07	0.69	3.18	-	551.7
AHQ-1-11, 2092	R.LTIGEGQQHHLGGAK.Q	1546.71290	2	5.33E-06	0.82	2.98	-	843.9
AHQ-1-14- , 2147	R.LTIGEGQQHHLGGAK.Q	1546.71290	2	5.86E-07	0.84	3.28	-	903.9
AHQ-1-7, 4121	K.VAQLEAQCQEPCKDTVQIHDITGK.D	2772.06211	3	1.62E-04	0.83	4.02	-	565.3
AHQ-1-1, 4129	K.VAQLEAQCQEPCKDTVQIHDITGK.D	2772.06211	3	6.57E-06	0.92	4.62	-	925.0
AHQ-1-8, 3972	K.VAQLEAQCQEPCKDTVQIHDITGK.D	2772.06211	2	5.75E-05	0.36	2.67	-	288.4
AHQ-1-7, 3996	K.VAQLEAQCQEPCKDTVQIHDITGK.D	2772.06211	3	8.33E-04	0.89	3.91	-	1005.7
AHQ-1-11, 3752	K.VAQLEAQCQEPCKDTVQIHDITGK.D	2772.06211	3	7.95E-04	0.70	3.48	-	638.1
AHQ-1-5, 4226	K.VAQLEAQCQEPCKDTVQIHDITGKDCQDIANK.G	3718.05993	3	8.87E-04	0.88	4.06	-	948.8
AHQ-1-9, 4103	K.VAQLEAQCQEPCKDTVQIHDITGKDCQDIANK.G	3718.05993	3	5.76E-10	0.95	5.43	-	1324.2
AHQ-1-8, 4113	K.VAQLEAQCQEPCKDTVQIHDITGKDCQDIANK.G	3718.05993	3	6.10E-04	0.89	4.51	-	916.2
AHQ-1-1, 4324	K.VAQLEAQCQEPCKDTVQIHDITGKDCQDIANK.G	3718.05993	3	5.40E-06	0.96	5.67	-	1399.3
AHQ-1-7, 3208 - 3212	R.VELEDWNGR.T	1118.18129	2	3.40E-06	0.87	3.21	-	998.9
AHQ-1-7, 3324	R.VELEDWNGR.T	1118.18129	2	1.20E-04	0.81	2.97	-	800.0
AHQ-1-8, 3168 - 3170	R.VELEDWNGR.T	1118.18129	2	3.70E-04	0.84	2.76	-	1014.0
AHQ-1-12, 2985	K.YEASILTHDSSIR.Y	1492.61564	2	1.95E-08	0.83	3.09	-	663.0
AHQ-1-11, 2931	K.YEASILTHDSSIR.Y	1492.61564	2	2.91E-05	0.84	3.51	-	519.0
AHQ-1-7, 2937 - 3008	K.YEASILTHDSSIR.Y	1492.61564	2	1.70E-07	0.87	3.65	-	636.0
AHQ-1-7, 2946 - 2949	K.YEASILTHDSSIR.Y	1492.61564	1	3.68E-04	0.15	2.21	-	257.0
AHQ-1-9, 2854	K.YEASILTHDSSIR.Y	1492.61564	2	6.52E-09	0.89	3.27	-	820.8
AHQ-1-13, 3074	K.YEASILTHDSSIR.Y	1492.61564	2	5.30E-06	0.52	2.54	-	402.4
AHQ-1-10, 2851	K.YEASILTHDSSIR.Y	1492.61564	2	1.95E-06	0.79	2.75	-	709.3
AHQ-1-1, 3125 - 3195	K.YEASILTHDSSIR.Y	1492.61564	2	8.64E-04	0.70	2.80	-	487.8
AHQ-1-7, 2785 - 2802	R.YLQEIYNSNNQK.I	1514.62131	2	6.03E-06	0.93	4.21	-	1050.0
AHQ-1-7, 2646 - 2716	R.YLQEIYNSNNQK.I	1514.62131	2	8.87E-04	0.94	4.58	-	1072.6
AHQ-1-3, 2837	R.YLQEIYNSNNQK.I	1514.62131	2	3.94E-04	0.96	4.17	-	1931.0
gi|4557032|ref|NP_002291.1| lactate dehydrogenase B [Homo sapiens]				2.01E-11	9.19	110.27	36.50	36638.3
AHQ-1-9, 4423	K.FIIPQIVK.Y	958.22198	2	3.91E-04	0.80	2.66	-	755.6
AHQ-1-9, 7243 - 7314	K.GM*YGIENEVFLSLPCILNAR.G	2314.66682	2	2.57E-07	0.92	4.70	-	518.3

AHQ-1-9, 2008 - 2079	K.IVADKDYSVTANSK.I	1511.65887	2	2.01E-11	0.97	4.84	-	1868.3
AHQ-1-13- , 2600	K.IVVVTAGVR.Q	914.12760	2	1.27E-04	0.89	3.40	-	835.6
AHQ-1-13- , 3496	K.LIAPVAEEEATVPNNK.I	1695.89534	2	3.60E-04	0.87	3.39	-	767.0
AHQ-1-9, 3532	K.LIAPVAEEEATVPNNK.I	1695.89534	2	3.77E-06	0.65	2.85	-	490.2
AHQ-1-14- , 3548	K.LIAPVAEEEATVPNNK.I	1695.89534	2	1.61E-05	0.87	3.23	-	805.9
AHQ-1-9, 2039 - 2042	K.LKDDEVAQLK.K	1159.31477	1	4.06E-04	0.31	2.49	-	485.4
AHQ-1-14- , 2243	K.LKDDEVAQLK.K	1159.31477	2	1.52E-05	0.65	2.64	-	495.2
AHQ-1-9, 2038 - 2043	K.LKDDEVAQLK.K	1159.31477	2	7.92E-05	0.77	3.21	-	527.6
AHQ-1-9, 4026	K.LKGEMM*DLQHGSLFLQTPK.I	2190.57228	2	6.34E-05	0.53	3.83	-	423.9
AHQ-1-9, 4572	K.LKGEMMDLQHGSLFLQTPK.I	2174.57288	2	3.74E-04	0.94	4.08	-	1162.5
AHQ-1-9, 3814 - 3883	K.MVVESAYEVIK.L	1268.50463	2	7.66E-09	0.93	3.79	-	901.2
AHQ-1-14- , 3931	K.MVVESAYEVIK.L	1268.50463	2	7.68E-07	0.85	2.88	-	801.8
AHQ-1-13- , 4039	K.MVVESAYEVIK.L	1268.50463	2	4.47E-06	0.86	3.10	-	868.6
AHQ-1-14- , 6532	K.SLADELALVDVLEDK.L	1630.81843	2	3.58E-05	0.93	3.44	-	1455.5
AHQ-1-13- , 6780 - 6783	K.SLADELALVDVLEDK.L	1630.81843	2	5.20E-08	0.96	5.37	-	1374.0
AHQ-1-9, 6932 - 6938	K.SLADELALVDVLEDK.L	1630.81843	2	1.71E-04	0.96	4.48	-	1652.7
AHQ-1-9, 6506	K.SLADELALVDVLEDK.L	1630.81843	2	1.71E-04	0.80	2.87	-	872.3
AHQ-1-11, 6239 - 6246	K.SLADELALVDVLEDK.L	1630.81843	2	8.34E-06	0.86	3.96	-	716.9
AHQ-1-13, 6331	K.SLADELALVDVLEDK.L	1630.81843	2	4.39E-08	0.97	5.22	-	1483.1
AHQ-1-14- , 6603 - 6631	K.SLADELALVDVLEDKLK.G	1872.14959	2	3.52E-06	0.93	3.96	-	1196.0
AHQ-1-9, 7094 - 7172	K.SLADELALVDVLEDKLK.G	1872.14959	2	1.14E-05	0.84	3.42	-	745.7
gi|4759096|ref|NP_004252.1| 15 kDa selenoprotein [Homo sapiens]				2.17E-11	0.93	10.24	9.90	17640.3
AHQ-1-13, 5824	K.LLDDNGNIAEELSILK.W	1757.96344	2	8.87E-09	0.97	4.84	-	1416.0
AHQ-1-12, 5855 - 5886	K.LLDDNGNIAEELSILK.W	1757.96344	2	2.17E-11	0.93	4.30	-	872.4
gi|13775198|ref|NP_112576.1| SH3 domain binding glutamic acid-rich protein like 3; SH3BGRL3-like pr				2.22E-11	4.17	50.27	34.40	10437.7
AHQ-1-13- , 4920 - 4987	R.IQYQLVDISQDNALR.D	1776.97168	2	3.69E-11	0.98	5.19	-	2176.7
AHQ-1-14- , 4726 - 4804	R.IQYQLVDISQDNALR.D	1776.97168	2	2.48E-08	0.98	5.35	-	2015.8
AHQ-1-13- , 4823	R.IQYQLVDISQDNALR.D	1776.97168	2	2.22E-11	0.97	5.05	-	1824.6
AHQ-1-14, 5573 - 5584	R.IQYQLVDISQDNALR.D	1776.97168	2	7.08E-09	0.98	5.36	-	2097.5
AHQ-1-13, 4588	R.IQYQLVDISQDNALR.D	1776.97168	2	7.48E-04	0.94	4.08	-	1372.6
AHQ-1-13, 4675 - 4735	R.IQYQLVDISQDNALR.D	1776.97168	2	8.78E-06	0.97	5.13	-	1546.2
AHQ-1-13, 4685	R.IQYQLVDISQDNALRDEMR.A	2308.55751	3	9.97E-04	0.87	3.60	-	755.5
AHQ-1-13, 4447	R.IQYQLVDISQDNALRDEM*R.A	2324.55691	3	2.29E-07	0.56	3.03	-	419.9
AHQ-1-13, 4159 - 4239	R.IQYQLVDISQDNALRDEM*R.A	2324.55691	3	4.53E-08	0.87	4.26	-	609.3
AHQ-1-13- , 5255 - 5284	R.IQYQLVDISQDNALRDEMR.A	2308.55751	3	3.90E-06	0.79	3.38	-	836.3
AHQ-1-13- , 5241 - 5245	R.IQYQLVDISQDNALRDEMR.A	2308.55751	2	5.95E-04	0.89	4.08	-	617.1
AHQ-1-13- , 5119 - 5188	R.IQYQLVDISQDNALRDEMR.A	2308.55751	3	6.74E-06	0.88	3.45	-	965.7
AHQ-1-13- , 4981 - 5053	R.IQYQLVDISQDNALRDEMR.A	2308.55751	3	6.66E-06	0.71	3.66	-	459.3
AHQ-1-13- , 4351	R.IQYQLVDISQDNALRDEM*R.A	2324.55691	3	5.39E-04	0.78	3.48	-	380.9
AHQ-1-14- , 5000 - 5063	R.IQYQLVDISQDNALRDEMR.A	2308.55751	3	6.23E-08	0.87	3.92	-	790.8
AHQ-1-13- , 4656 - 4719	R.IQYQLVDISQDNALRDEM*R.A	2324.55691	3	7.78E-06	0.83	4.18	-	429.6
AHQ-1-14- , 4599	R.IQYQLVDISQDNALRDEM*R.A	2324.55691	3	2.72E-07	0.73	3.04	-	691.7
AHQ-1-13- , 4825	R.IQYQLVDISQDNALRDEM*R.A	2324.55691	3	1.89E-04	0.58	3.20	-	284.0
AHQ-1-13- , 4555 - 4557	R.IQYQLVDISQDNALRDEM*R.A	2324.55691	3	2.68E-06	0.75	3.45	-	518.1
AHQ-1-14- , 1816	R.VYSTSVTGSR.E	1057.13940	2	8.18E-06	0.86	2.85	-	933.6
AHQ-1-13- , 2008	R.VYSTSVTGSR.E	1057.13940	2	5.67E-05	0.91	2.60	-	1337.2
AHQ-1-13- , 1883 - 1948	R.VYSTSVTGSR.E	1057.13940	2	1.34E-05	0.95	3.95	-	1270.8
AHQ-1-13- , 1759 - 1825	R.VYSTSVTGSR.E	1057.13940	2	7.21E-06	0.89	2.94	-	936.2
AHQ-1-13, 1958 - 2038	R.VYSTSVTGSR.E	1057.13940	2	1.80E-06	0.86	3.03	-	848.9
AHQ-1-13- , 1623 - 1703	R.VYSTSVTGSR.E	1057.13940	2	1.61E-05	0.83	2.64	-	826.0
AHQ-1-13, 2363	R.VYSTSVTGSREIK.S	1427.58510	2	3.10E-05	0.82	3.11	-	797.4
AHQ-1-13- , 2316	R.VYSTSVTGSREIK.S	1427.58510	2	1.07E-05	0.59	3.00	-	565.6
AHQ-1-14- , 2291	R.VYSTSVTGSREIK.S	1427.58510	2	6.05E-04	0.64	2.80	-	551.4
gi|4506005|ref|NP_002700.1| protein phosphatase 1, catalytic subunit, beta isoform [Homo sapiens] [				2.35E-11	2.88	30.30	16.50	37186.6
AHQ-1-9, 7230 - 7232	R.EIFLSQPILLELEAPLK.I	1954.33857	2	2.15E-07	0.96	5.77	-	707.9
AHQ-1-14- , 6671	R.EIFLSQPILLELEAPLK.I	1954.33857	2	2.74E-05	0.94	4.55	-	781.4
AHQ-1-13- , 6919	R.EIFLSQPILLELEAPLK.I	1954.33857	2	5.49E-08	0.95	5.01	-	700.4
AHQ-1-12, 6601	R.EIFLSQPILLELEAPLK.I	1954.33857	2	2.87E-07	0.95	5.38	-	598.7
AHQ-1-9, 5263	K.IFCCHGGLSPDLQSMEQIR.R	2251.54930	3	2.08E-05	0.89	4.38	-	1236.4
AHQ-1-9, 5259	K.IFCCHGGLSPDLQSMEQIR.R	2251.54930	2	1.24E-05	0.96	4.74	-	1480.4
AHQ-1-9, 7163 - 7164	K.TFTDCFNCLPIAAIVDEK.I	2117.38502	2	2.44E-06	0.98	6.06	-	1212.3
AHQ-1-9, 7004	K.TFTDCFNCLPIAAIVDEK.I	2117.38502	2	2.35E-11	0.97	4.57	-	1423.8
gi|4504349|ref|NP_000509.1| beta globin [Homo sapiens]				2.36E-11	8.99	100.28	70.70	15998.3
AHQ-1-13- , 3105 - 3175	K.EFTPPVQAAYQK.V	1379.54213	2	5.05E-07	0.82	2.85	-	680.1
AHQ-1-14- , 5046 - 5047	R.FFESFGDLSTPDAVM*GNPK.V	2076.27158	2	3.36E-07	0.95	5.14	-	850.1
AHQ-1-13- , 5732	R.FFESFGDLSTPDAVMGNPK.V	2060.27218	2	1.15E-06	0.95	4.84	-	864.5
AHQ-1-13, 5499	R.FFESFGDLSTPDAVMGNPK.V	2060.27218	2	8.83E-05	0.86	3.29	-	844.0
AHQ-1-13, 5403	R.FFESFGDLSTPDAVMGNPK.V	2060.27218	2	1.07E-04	0.92	4.41	-	616.8
AHQ-1-13, 4903	R.FFESFGDLSTPDAVM*GNPK.V	2076.27158	3	5.89E-05	0.92	3.53	-	1545.4
AHQ-1-13- , 5140 - 5151	R.FFESFGDLSTPDAVM*GNPK.V	2076.27158	2	6.83E-05	0.90	4.29	-	598.5
AHQ-1-13, 3519 - 3525	K.GTFATLSELHCDK.L	1480.62492	2	3.80E-09	0.84	3.42	-	627.8
AHQ-1-13- , 3615	K.GTFATLSELHCDK.L	1480.62492	2	2.32E-08	0.77	3.40	-	496.6
AHQ-1-13- , 5173 - 5200	K.GTFATLSELHCDKLHVDPENFR.L	2588.83687	3	2.36E-11	0.96	5.38	-	1052.0
AHQ-1-13, 4885	K.GTFATLSELHCDKLHVDPENFR.L	2588.83687	3	3.06E-09	0.95	5.18	-	1133.8
AHQ-1-13, 4905	K.GTFATLSELHCDKLHVDPENFR.L	2588.83687	2	2.34E-06	0.76	3.04	-	561.7
AHQ-1-13- , 4963 - 5017	K.KVLGAFSDGLAHLDNLK.G	1799.06362	2	5.06E-06	0.94	4.46	-	1096.9
AHQ-1-13- , 4969	K.KVLGAFSDGLAHLDNLK.G	1799.06362	3	7.21E-06	0.93	4.99	-	908.2
AHQ-1-13- , 3584	K.SAVTALWGK.V	933.08624	2	6.18E-05	0.90	3.01	-	817.4
AHQ-1-13, 3474	K.SAVTALWGK.V	933.08624	2	1.58E-04	0.76	2.60	-	699.3
AHQ-1-13, 4999 - 5056	K.VLGAFSDGLAHLDNLK.G	1670.89071	2	7.13E-11	0.97	5.65	-	1688.3
AHQ-1-13, 4767 - 4841	K.VLGAFSDGLAHLDNLK.G	1670.89071	2	1.27E-10	0.97	4.91	-	1850.9
AHQ-1-13- , 5300	K.VLGAFSDGLAHLDNLK.G	1670.89071	3	1.60E-07	0.84	3.50	-	596.9
AHQ-1-13- , 5111	K.VLGAFSDGLAHLDNLK.G	1670.89071	2	1.97E-10	0.97	5.20	-	1548.1
AHQ-1-13- , 5293	K.VLGAFSDGLAHLDNLK.G	1670.89071	2	3.92E-07	0.95	4.50	-	1393.9
AHQ-1-13- , 3345	K.VNVDEVGGEALGR.L	1315.41484	1	2.28E-05	0.46	2.51	-	360.1
AHQ-1-13- , 3343	K.VNVDEVGGEALGR.L	1315.41484	2	1.85E-04	0.97	4.52	-	1919.3
AHQ-1-13- , 3349	K.VNVDEVGGEALGR.L	1315.41484	1	2.81E-07	0.73	3.40	-	337.2
AHQ-1-13, 3273 - 3275	K.VNVDEVGGEALGR.L	1315.41484	1	9.10E-05	0.61	2.81	-	347.2
AHQ-1-13- , 2535 - 2543	K.VVAGVANALAHK.Y	1150.35593	2	7.77E-08	0.93	3.15	-	1455.5
gi|24119203|ref|NP_705935.1| tropomyosin 3 [Homo sapiens]				2.46E-11	5.63	60.29	20.20	29032.5
AHQ-1-9, 2278	R.AREQAEAEVASLNR.R	1544.65218	2	9.03E-06	0.98	5.70	-	3481.3
AHQ-1-1, 3887 - 3903	R.IQLVEEELDR.A	1244.37653	2	8.34E-05	0.79	2.66	-	643.8
AHQ-1-13- , 3827 - 3840	R.IQLVEEELDR.A	1244.37653	2	1.02E-06	0.90	3.70	-	843.0
AHQ-1-11, 3515	R.IQLVEEELDR.A	1244.37653	2	2.79E-05	0.87	2.94	-	969.8
AHQ-1-10, 3408 - 3425	R.IQLVEEELDR.A	1244.37653	2	8.76E-08	0.92	3.75	-	1017.8
AHQ-1-9, 3538	R.IQLVEEELDR.A	1244.37653	1	9.35E-04	0.71	2.94	-	682.4
AHQ-1-9, 3528 - 3598	R.IQLVEEELDR.A	1244.37653	1	1.80E-05	0.60	2.74	-	578.7
AHQ-1-9, 3510 - 3590	R.IQLVEEELDR.A	1244.37653	2	2.59E-07	0.91	3.63	-	855.9
AHQ-1-13- , 3829	R.IQLVEEELDR.A	1244.37653	1	8.14E-06	0.43	2.41	-	384.6
AHQ-1-10, 2672	K.IQVLQQQADDAEER.A	1643.73738	2	6.38E-07	0.96	4.29	-	1460.5
AHQ-1-9, 2706	K.IQVLQQQADDAEER.A	1643.73738	2	1.33E-08	0.97	4.51	-	1878.9
AHQ-1-9, 2423	R.KIQVLQQQADDAEER.A	1771.91030	2	6.49E-08	0.97	4.86	-	1724.3
AHQ-1-9, 2523	R.KIQVLQQQADDAEER.A	1771.91030	2	3.32E-05	0.96	4.90	-	1332.3
AHQ-1-9, 2427 - 2440	R.KIQVLQQQADDAEER.A	1771.91030	3	2.46E-11	0.98	5.30	-	2262.3
AHQ-1-10, 2449	R.KIQVLQQQADDAEER.A	1771.91030	2	4.38E-04	0.96	4.98	-	1225.9
AHQ-1-10, 3131	R.RIQLVEEELDR.A	1400.56288	2	2.57E-07	0.98	5.23	-	2012.3
AHQ-1-9, 3019 - 3026	K.TIDDLEDKLK.C	1190.32563	2	2.08E-05	0.81	3.07	-	639.6

gi|4506877|ref|NP_002996.1| selectin P precursor; Selectin P (granulocyte membrane protein, 140kD;				2.52E-11	11.31	130.26	27.50	90844.7
AHQ-1-3, 4881 - 4895	K.AVQCQHLEAPSEGTM*DCVHPLTAFAYGSSCK.F	3472.80519	3	4.54E-10	0.94	5.24	-	900.3
AHQ-1-3, 6673	R.CDNLGQWTAPAPVCQALQCQDLPVPNEAR.V	3313.64431	3	4.75E-04	0.59	3.32	-	473.6
AHQ-1-4, 6852	R.CDNLGQWTAPAPVCQALQCQDLPVPNEAR.V	3313.64431	3	6.57E-06	0.83	4.23	-	604.8
AHQ-1-3, 5965	R.CIDSGHWSAPLPTCEAISCEPLESPVHGSM*DCSPSLR.A	4162.54346	3	1.28E-06	0.90	4.64	-	732.0
AHQ-1-3, 4591 - 4646	K.CPELFAPEQGSLDCSDTR.G	2085.21515	2	5.17E-04	0.91	4.15	-	536.2
AHQ-1-3, 2290 - 2294	K.FECQPGYR.V	1058.14914	2	5.24E-04	0.66	2.60	-	555.7
AHQ-1-3, 5953	K.LECLASGIWTNKPPQCLAAQCPPLK.I	2857.31818	3	2.52E-11	0.91	4.51	-	896.3
AHQ-1-3, 6153 - 6194	K.LECLASGIWTNKPPQCLAAQCPPLKIPER.G	3352.89300	3	1.09E-09	0.93	4.82	-	1211.0
AHQ-1-3, 3141	R.PSGQWTAVTPACR.A	1432.58672	2	1.65E-05	0.94	3.79	-	1080.3
AHQ-1-1, 4999	K.STCQFICDEGYSLSGPER.L	2109.23671	2	3.69E-06	0.87	3.21	-	982.6
AHQ-1-3, 4958 - 4961	K.STCQFICDEGYSLSGPER.L	2109.23671	2	2.91E-09	0.92	3.44	-	1110.4
AHQ-1-4, 5056	K.STCQFICDEGYSLSGPER.L	2109.23671	2	1.64E-06	0.89	3.96	-	703.9
AHQ-1-3, 6367 - 6379	K.VLPYYSSYYWIGIR.K	1781.04522	2	6.34E-09	0.88	4.08	-	450.6
AHQ-1-3, 2491	R.WSATPPTCK.G	1049.18216	2	6.18E-05	0.79	2.76	-	674.9
AHQ-1-3, 3949	R.WTDSPPMCEAIK.C	1436.63555	2	2.58E-06	0.80	2.79	-	558.2
AHQ-1-3, 2906 - 2913	R.YTDLVAIQNK.N	1165.32100	1	9.83E-04	0.62	2.86	-	697.4
AHQ-1-3, 2907	R.YTDLVAIQNK.N	1165.32100	2	1.11E-05	0.92	2.93	-	1276.9
gi|5031569|ref|NP_005727.1| ARP1 actin-related protein 1 homolog A, centractin alpha; ARP1 (actin-r				2.63E-11	1.90	20.28	10.60	42613.4
AHQ-1-8, 3976	R.ACYLSINPQKDETLETEK.A	2141.34169	2	7.82E-05	0.94	4.52	-	794.1
AHQ-1-8, 5371 - 5384	K.DQLQTFSEEHPVLLTEAPLNPR.K	2535.79382	2	2.63E-11	0.96	5.57	-	853.0
gi|22749499|ref|NP_689979.1| hypothetical protein MGC40107 [Homo sapiens]				2.68E-11	0.97	10.29	24.80	11741.6
AHQ-1-14- , 5646 - 5648	R.LGALSGAAALGFASYGAHGAQFPDAYGK.E	2669.93152	3	2.68E-11	0.97	5.90	-	1551.7
gi|17986283|ref|NP_006000.2| tubulin, alpha 3; tubulin, alpha, brain-specific; hum-a-tub1; hum-a-tu				2.95E-11	0.95	10.26	3.30	50135.3
AHQ-1-3, 5946	R.AVFVDLEPTVIDEVR.T	1702.92947	2	5.26E-09	0.95	4.87	-	877.4
AHQ-1-4, 6072	R.AVFVDLEPTVIDEVR.T	1702.92947	2	3.33E-07	0.96	4.37	-	1506.2
AHQ-1-7, 5906 - 5984	R.AVFVDLEPTVIDEVR.T	1702.92947	2	2.95E-11	0.95	5.24	-	685.9
AHQ-1-9, 5740	R.AVFVDLEPTVIDEVR.T	1702.92947	2	1.77E-06	0.83	3.69	-	612.4
AHQ-1-12, 5517	R.AVFVDLEPTVIDEVR.T	1702.92947	2	1.37E-05	0.93	4.19	-	1009.9
AHQ-1-14- , 5628 - 5659	R.AVFVDLEPTVIDEVR.T	1702.92947	2	1.78E-08	0.92	4.75	-	702.1
AHQ-1-1, 5943 - 5952	R.AVFVDLEPTVIDEVR.T	1702.92947	2	1.65E-06	0.88	4.34	-	605.0
gi|11761631|ref|NP_005132.1| fibrinogen, beta chain preproprotein [Homo sapiens]				3.29E-11	17.42	200.29	38.70	55901.8
AHQ-1-7, 2284	K.AHYGGFTVQNEANK.Y	1536.63020	2	2.28E-08	0.92	4.03	-	947.5
AHQ-1-11, 2344	K.AHYGGFTVQNEANK.Y	1536.63020	2	9.03E-07	0.92	4.16	-	676.2
AHQ-1-9, 2226	K.AHYGGFTVQNEANK.Y	1536.63020	2	2.39E-06	0.96	4.20	-	1396.7
AHQ-1-6, 2278	K.AHYGGFTVQNEANK.Y	1536.63020	2	2.89E-07	0.95	4.06	-	1277.3
AHQ-1-8, 2176	K.AHYGGFTVQNEANK.Y	1536.63020	2	1.01E-05	0.93	3.64	-	1080.3
AHQ-1-7, 3213	R.DNDGWLTSDPR.K	1276.29403	2	6.90E-05	0.88	3.32	-	850.8
AHQ-1-6, 3422	K.DNENVVNEYSSELEK.H	1769.80167	2	3.79E-09	0.79	3.31	-	486.6
AHQ-1-3, 4298	K.DNENVVNEYSSELEK.H	1769.80167	2	1.99E-06	0.90	4.28	-	699.6
AHQ-1-9, 4172 - 4184	K.DNENVVNEYSSELEK.H	1769.80167	2	6.81E-10	0.89	3.88	-	734.3
AHQ-1-13- , 4225	K.DNENVVNEYSSELEK.H	1769.80167	2	7.44E-04	0.84	3.58	-	647.9
AHQ-1-13, 4061	K.DNENVVNEYSSELEK.H	1769.80167	2	4.61E-05	0.84	3.75	-	625.7
AHQ-1-6, 4091 - 4174	K.DNENVVNEYSSELEK.H	1769.80167	2	1.89E-05	0.67	3.48	-	587.0
AHQ-1-9, 4432	K.DNENVVNEYSSELEK.H	1769.80167	2	4.73E-05	0.80	3.44	-	681.3
AHQ-1-7, 3766	K.DNENVVNEYSSELEK.H	1769.80167	2	6.24E-06	0.94	4.37	-	989.6
AHQ-1-12, 4054	K.DNENVVNEYSSELEK.H	1769.80167	2	1.35E-06	0.74	2.94	-	727.2
AHQ-1-7, 3461 - 3534	K.DNENVVNEYSSELEK.H	1769.80167	2	4.35E-06	0.60	3.04	-	475.3
AHQ-1-8, 4189	K.DNENVVNEYSSELEK.H	1769.80167	2	5.11E-04	0.82	3.36	-	765.2
AHQ-1-7, 4220 - 4288	K.DNENVVNEYSSELEK.H	1769.80167	2	3.67E-08	0.94	4.66	-	931.5
AHQ-1-1, 4321	K.DNENVVNEYSSELEK.H	1769.80167	2	2.24E-05	0.85	3.74	-	742.5
AHQ-1-8, 4072	K.GGETSEMYLIQPDSSVKPYR.V	2258.49373	3	4.62E-06	0.73	3.16	-	756.8
AHQ-1-8, 4081	K.GGETSEMYLIQPDSSVKPYR.V	2258.49373	2	4.00E-07	0.90	4.48	-	547.0
AHQ-1-9, 4043	K.GGETSEMYLIQPDSSVKPYR.V	2258.49373	2	1.58E-05	0.84	3.75	-	490.7
AHQ-1-7, 4164	K.GGETSEMYLIQPDSSVKPYR.V	2258.49373	2	8.45E-05	0.79	3.51	-	550.0
AHQ-1-7, 4156	K.GGETSEMYLIQPDSSVKPYR.V	2258.49373	3	3.53E-05	0.86	3.88	-	927.3
AHQ-1-7, 4150	K.GGETSEMYLIQPDSSVKPYR.V	2258.49373	2	2.53E-06	0.73	3.41	-	363.8
AHQ-1-6, 4110 - 4119	K.GGETSEMYLIQPDSSVKPYR.V	2258.49373	3	9.69E-08	0.85	3.90	-	842.6
AHQ-1-6, 3619 - 3626	K.GGETSEM*YLIQPDSSVKPYR.V	2274.49313	3	1.49E-07	0.87	3.90	-	881.7
AHQ-1-6, 4118	K.GGETSEMYLIQPDSSVKPYR.V	2258.49373	2	1.45E-06	0.86	4.11	-	492.6
AHQ-1-7, 3669	K.GGETSEM*YLIQPDSSVKPYR.V	2274.49313	2	9.60E-04	0.33	2.99	-	278.2
AHQ-1-7, 3670	K.GGETSEM*YLIQPDSSVKPYR.V	2274.49313	3	1.43E-05	0.77	3.30	-	899.6
AHQ-1-9, 4031	K.GGETSEMYLIQPDSSVKPYR.V	2258.49373	3	1.88E-07	0.85	3.13	-	1313.0
AHQ-1-11, 4068	K.HGTDDGVVWMNWK.G	1545.70374	2	7.81E-05	0.94	4.00	-	1044.4
AHQ-1-7, 4312	K.HGTDDGVVWMNWK.G	1545.70374	2	1.01E-05	0.93	3.67	-	1181.9
AHQ-1-6, 3183	R.KGGETSEM*YLIQPDSSVKPYR.V	2402.66604	3	4.95E-05	0.87	4.27	-	876.2
AHQ-1-8, 3086	R.KGGETSEM*YLIQPDSSVKPYR.V	2402.66604	3	5.17E-05	0.74	3.52	-	670.6
AHQ-1-6, 3651	R.KGGETSEMYLIQPDSSVKPYR.V	2386.66664	2	2.58E-05	0.94	4.60	-	1148.4
AHQ-1-7, 3217	R.KGGETSEM*YLIQPDSSVKPYR.V	2402.66604	3	3.73E-04	0.87	4.38	-	625.3
AHQ-1-9, 3490	R.KGGETSEMYLIQPDSSVKPYR.V	2386.66664	2	9.01E-04	0.71	3.26	-	443.9
AHQ-1-6, 3646 - 3727	R.KGGETSEMYLIQPDSSVKPYR.V	2386.66664	3	1.09E-09	0.93	5.45	-	756.1
AHQ-1-8, 3405	R.KGGETSEMYLIQPDSSVKPYR.V	2386.66664	3	3.91E-04	0.84	3.78	-	732.0
AHQ-1-7, 3666 - 3736	R.KGGETSEMYLIQPDSSVKPYR.V	2386.66664	3	2.70E-10	0.85	3.98	-	689.2
AHQ-1-7, 2744	K.KREEAPSLRPAPPPISGGGYR.A	2236.51908	3	2.27E-06	0.92	3.57	-	1378.6
AHQ-1-6, 2547	K.LESDVSAQM*EYCR.T	1605.72945	2	3.08E-07	0.72	2.90	-	497.5
AHQ-1-7, 2561	K.LESDVSAQM*EYCR.T	1605.72945	2	2.31E-04	0.79	2.96	-	674.6
AHQ-1-11, 5887 - 5924	R.M*GPTELLIEMEDWK.G	1708.97776	2	3.84E-06	0.93	3.99	-	1036.7
AHQ-1-7, 6660 - 6678	R.M*GPTELLIEMEDWK.G	1708.97776	2	3.46E-08	0.95	4.39	-	1365.3
AHQ-1-7, 7189 - 7196	R.MGPTELLIEMEDWK.G	1692.97836	2	2.47E-07	0.96	4.48	-	1273.2
AHQ-1-9, 6507	R.M*GPTELLIEMEDWK.G	1708.97776	2	1.38E-07	0.93	3.77	-	1205.7
AHQ-1-7, 4957 - 5037	R.M*GPTELLIEM*EDWK.G	1724.97716	2	1.22E-04	0.80	3.21	-	736.3
AHQ-1-8, 7150	R.MGPTELLIEMEDWK.G	1692.97836	2	6.61E-06	0.89	3.46	-	883.3
AHQ-1-10, 5772 - 5788	R.M*GPTELLIEMEDWK.G	1708.97776	2	5.96E-05	0.91	4.00	-	953.1
AHQ-1-6, 7035	R.MGPTELLIEMEDWK.G	1692.97836	2	3.73E-05	0.92	3.87	-	1128.6
AHQ-1-6, 6526 - 6534	R.M*GPTELLIEMEDWK.G	1708.97776	2	1.89E-05	0.95	4.11	-	1297.1
AHQ-1-7, 5996	K.NYCGLPGEYWLGNDK.I	1787.92994	2	1.13E-04	0.83	3.86	-	273.1
AHQ-1-6, 5276	K.NYCGLPGEYWLGNDK.I	1787.92994	2	3.29E-05	0.84	3.60	-	390.7
AHQ-1-11, 4875	K.NYCGLPGEYWLGNDK.I	1787.92994	2	1.19E-05	0.71	3.09	-	322.8
AHQ-1-7, 3892 - 3914	R.TPCTVSCNIPVVSGK.E	1621.85811	2	6.55E-07	0.66	3.09	-	403.0
AHQ-1-8, 3884	R.TPCTVSCNIPVVSGK.E	1621.85811	2	4.93E-05	0.61	2.80	-	692.2
AHQ-1-7, 5054 - 5068	R.TPCTVSCNIPVVSGKECEEIIR.K	2552.88440	2	1.58E-06	0.76	3.66	-	414.4
AHQ-1-7, 5052 - 5072	R.TPCTVSCNIPVVSGKECEEIIR.K	2552.88440	3	1.44E-05	0.93	4.36	-	1147.1
AHQ-1-6, 5008	R.TPCTVSCNIPVVSGKECEEIIR.K	2552.88440	3	7.04E-06	0.91	4.16	-	853.4
AHQ-1-1, 5156	R.TPCTVSCNIPVVSGKECEEIIR.K	2552.88440	3	6.11E-06	0.74	3.29	-	821.3
AHQ-1-6, 5006	R.TPCTVSCNIPVVSGKECEEIIR.K	2552.88440	2	1.24E-05	0.80	3.57	-	290.3
AHQ-1-8, 4991	R.TPCTVSCNIPVVSGKECEEIIR.K	2552.88440	3	7.99E-08	0.96	5.01	-	1537.7
AHQ-1-9, 4952	R.TPCTVSCNIPVVSGKECEEIIR.K	2552.88440	3	1.97E-06	0.90	3.78	-	1185.0
AHQ-1-9, 4924	R.TPCTVSCNIPVVSGKECEEIIR.K	2552.88440	2	1.08E-04	0.77	3.14	-	345.5
AHQ-1-9, 4596	R.VYCDMNTENGGWTVIQNR.Q	2159.34553	2	5.10E-09	0.92	4.62	-	713.5
AHQ-1-7, 4109 - 4182	R.VYCDM*NTENGGWTVIQNR.Q	2175.34493	2	5.90E-06	0.88	3.78	-	780.2
AHQ-1-8, 4668	R.VYCDMNTENGGWTVIQNR.Q	2159.34553	2	3.29E-11	0.93	4.56	-	759.7
AHQ-1-7, 4642 - 4668	R.VYCDMNTENGGWTVIQNR.Q	2159.34553	2	6.27E-10	0.93	4.61	-	980.9
AHQ-1-7, 4736 - 4810	R.VYCDMNTENGGWTVIQNR.Q	2159.34553	2	7.27E-10	0.96	4.84	-	1217.0
AHQ-1-6, 4043 - 4056	R.VYCDM*NTENGGWTVIQNR.Q	2175.34493	2	5.25E-07	0.95	4.77	-	973.0
AHQ-1-6, 4127	R.VYCDM*NTENGGWTVIQNR.Q	2175.34493	2	1.01E-05	0.94	3.98	-	1168.7
AHQ-1-6, 4594 - 4667	R.VYCDMNTENGGWTVIQNR.Q	2159.34553	2	9.01E-09	0.96	4.98	-	1120.9
AHQ-1-12, 4937 - 4945	R.YYWGGQYTWDMAK.H	1669.84009	2	1.30E-05	0.92	3.70	-	850.6

AHQ-1-7, 5200 - 5204	R.YYWGGQYTWDMAK.H	1669.84009	2	6.33E-04	0.89	3.20	-	826.9
AHQ-1-10, 4140	R.YYWGGQYTWDM*AK.H	1685.83949	2	3.69E-05	0.91	3.83	-	968.2
AHQ-1-14- , 4567 - 4598	R.YYWGGQYTWDM*AK.H	1685.83949	2	2.86E-06	0.86	3.11	-	733.8
AHQ-1-14- , 5086 - 5090	R.YYWGGQYTWDMAK.H	1669.84009	2	2.30E-04	0.96	4.18	-	1444.8
AHQ-1-7, 5285	R.YYWGGQYTWDMAK.H	1669.84009	2	7.44E-04	0.93	3.31	-	1286.8
gi|5031601|ref|NP_005711.1| actin related protein 2/3 complex subunit 1B; ARP2/3 protein complex su				3.35E-11	5.21	60.25	21.50	40949.5
AHQ-1-9, 2254 - 2256	K.ASSEGGTAAGAGLDSLHK.N	1629.71071	2	4.70E-05	0.51	2.98	-	500.9
AHQ-1-9, 4136	K.ASSEGGTAAGAGLDSLHKNSVSQISVLSGGK.A	2887.10986	3	3.35E-11	0.91	4.70	-	1073.1
AHQ-1-9, 2426	K.EVEERPAPTPWGSK.M	1583.72678	2	7.44E-08	0.94	4.27	-	962.9
AHQ-1-9, 3286	K.NSVSQISVLSGGK.A	1276.42175	1	2.86E-06	0.16	1.98	-	270.8
AHQ-1-9, 2926	K.NSVSQISVLSGGK.A	1276.42175	2	2.21E-06	0.95	3.71	-	1347.4
AHQ-1-9, 3452	R.TQIAICPNNHEVHIYEK.S	2068.29887	2	3.15E-05	0.93	4.46	-	1007.1
AHQ-1-9, 3338 - 3352	R.TQIAICPNNHEVHIYEK.S	2068.29887	2	1.87E-07	0.95	5.00	-	894.4
AHQ-1-9, 3192	R.VAWVSHDSTVCLADADKK.M	2004.20995	2	2.25E-06	0.95	4.82	-	1049.4
gi|22043610|ref|XP_171081.1| similar to ras-related C3 botulinum toxin substrate 1 isoform Rac1; rh				3.49E-11	2.73	30.28	24.00	21543.1
AHQ-1-11, 2755	R.HHCPNTPIILVGTK.L	1588.85612	3	7.29E-04	0.87	3.39	-	1257.5
AHQ-1-11, 3814 - 3815	K.KLTPITYPQGLAMAK.E	1632.99216	2	7.56E-05	0.88	3.89	-	645.7
AHQ-1-11, 4595	K.PVNLGLWDTAGQEDYDR.L	1950.05441	2	3.49E-11	0.98	5.64	-	2428.8
gi|21624607|ref|NP_066972.1| coactosin-like 1 [Homo sapiens]				3.53E-11	5.00	60.28	52.80	15944.9
AHQ-1-13, 2357	R.AAYNLVR.D	806.93245	2	4.34E-04	0.82	2.72	-	823.2
AHQ-1-12, 2215 - 2217	R.AAYNLVR.D	806.93245	2	1.06E-04	0.84	2.72	-	650.8
AHQ-1-13- , 5429 - 5445	R.DDGSAVIWVTFK.Y	1338.49001	2	2.40E-07	0.93	4.00	-	985.9
AHQ-1-13- , 5459	R.DDGSAVIWVTFK.Y	1338.49001	2	1.84E-06	0.95	3.74	-	1311.3
AHQ-1-13- , 4301	K.ELEEDFIKSELK.K	1480.64154	2	8.27E-04	0.60	3.05	-	448.3
AHQ-1-13, 6435	K.FALITWIGENVSGLQR.A	1805.06986	2	2.67E-08	0.97	5.55	-	1889.2
AHQ-1-13- , 2944	R.KELEEDFIK.S	1151.29110	2	9.47E-05	0.75	2.91	-	711.4
AHQ-1-13- , 2947	R.KELEEDFIK.S	1151.29110	1	6.14E-04	0.37	2.43	-	479.3
AHQ-1-13, 2905	R.KELEEDFIK.S	1151.29110	1	2.15E-04	0.59	2.73	-	523.2
AHQ-1-13, 2902 - 2906	R.KELEEDFIK.S	1151.29110	2	3.70E-04	0.80	3.02	-	767.5
AHQ-1-12, 2815	R.KELEEDFIK.S	1151.29110	2	4.23E-04	0.84	2.92	-	902.7
AHQ-1-13- , 5568	K.YDGSTIVPGEQGAEYQHFIQQCTDDVR.L	3116.27790	3	1.34E-08	0.60	3.13	-	306.6
AHQ-1-13, 5159 - 5221	K.YDGSTIVPGEQGAEYQHFIQQCTDDVR.L	3116.27790	3	3.53E-11	0.90	4.93	-	570.0
gi|4759300|ref|NP_004772.1| vesicle-associated membrane protein 3 (cellubrevin); vesicle-associated				3.55E-11	2.85	30.28	33.00	11309.0
AHQ-1-14- , 3604	R.ADALQAGASQFETSAAK.L	1666.77095	2	8.72E-09	0.93	4.07	-	968.7
AHQ-1-13- , 3679	R.ADALQAGASQFETSAAK.L	1666.77095	2	3.55E-11	0.94	4.74	-	931.3
AHQ-1-13, 3558	R.ADALQAGASQFETSAAK.L	1666.77095	2	3.12E-04	0.86	3.59	-	720.4
AHQ-1-13- , 3835	R.LQQTQNQVDEVVDIM*R.V	1933.13344	3	4.54E-08	0.93	4.20	-	1347.5
AHQ-1-13- , 4889	R.LQQTQNQVDEVVDIMR.V	1917.13404	2	7.78E-04	0.55	3.22	-	546.8
AHQ-1-13- , 5025	R.LQQTQNQVDEVVDIMR.V	1917.13404	2	3.63E-08	0.98	5.51	-	2464.4
AHQ-1-13- , 3823 - 3831	R.LQQTQNQVDEVVDIM*R.V	1933.13344	2	5.39E-06	0.96	5.06	-	1295.2
AHQ-1-13, 4768	R.LQQTQNQVDEVVDIMR.V	1917.13404	2	1.51E-07	0.98	5.56	-	1839.6
gi|4885413|ref|NP_005331.1| histidine triad nucleotide binding protein 1; Histidine triad nucleotid				3.83E-11	2.68	30.25	42.10	13801.8
AHQ-1-13, 5373 - 5387	R.CLAFHDISPQAPTHFLVIPK.K	2293.67220	3	4.93E-06	0.94	4.73	-	1354.6
AHQ-1-13- , 4005	R.MVVNEGSDGGQSVYHVHLHVLGGR.Q	2548.82255	3	3.83E-11	0.84	3.63	-	718.4
AHQ-1-13- , 2465	R.PGGDTIFGK.I	891.99098	2	1.76E-06	0.89	3.36	-	676.3
gi|7657056|ref|NP_055415.1| EH-domain containing 3; EH domain containing 3 [Homo sapiens]				4.16E-11	8.07	90.34	34.00	60886.7
AHQ-1-7, 2836 - 2866	K.ADQIETQQLMR.V	1333.49681	2	3.40E-05	0.89	3.23	-	1399.8
AHQ-1-7, 6365	R.DKPMYDEIFYTLSPVDGK.I	2119.37953	2	4.14E-05	0.96	5.21	-	1107.3
AHQ-1-7, 5941	R.FHEFHSPALEDADFDNKPMVLLVGQYSTGK.T	3394.75749	3	4.16E-11	0.97	6.62	-	1389.2
AHQ-1-10, 6385	R.FVCAQLPNPVLESISVIDTPGILSGEK.Q	2886.30971	2	3.99E-04	0.87	3.78	-	724.8
AHQ-1-8, 7478 - 7479	R.FVCAQLPNPVLESISVIDTPGILSGEK.Q	2886.30971	2	2.44E-04	0.95	4.78	-	1113.0
AHQ-1-7, 5741	K.GGAFEGTLHGPFGHGYGEGAGEGIDDAEWVVAR.D	3317.48513	3	2.68E-06	0.98	6.75	-	1966.3
AHQ-1-7, 6033	K.LADIDKDGMLDDDEFALANHLIK.V	2573.86063	3	5.07E-05	0.57	3.12	-	634.2
AHQ-1-7, 5045	K.LFEAEEQDLFR.D	1397.51432	2	8.08E-07	0.95	3.33	-	1825.7
AHQ-1-7, 2857 - 2865	R.MQDQLQAQDFSK.F	1439.57624	2	5.92E-04	0.95	4.63	-	1427.9
AHQ-1-8, 6158 - 6162	R.VYIGSFWSHPLLIPDNR.K	2015.30234	2	3.09E-04	0.91	3.58	-	1046.6
AHQ-1-7, 6333 - 6338	R.VYIGSFWSHPLLIPDNR.K	2015.30234	2	1.63E-05	0.85	3.71	-	547.1
gi|7661678|ref|NP_056461.1| RAP1B, member of RAS oncogene family; K-REV; RAS-related protein RAP1B				4.20E-11	8.75	100.30	28.80	20824.7
AHQ-1-11, 4171	K.DTDDVPMILVGNK.C	1417.61048	2	2.20E-07	0.85	3.48	-	796.8
AHQ-1-11, 6206 - 6279	R.KQVEVDAQQCMLEILDTAGTEQFTAM*R.D	3131.50548	3	2.02E-08	0.95	4.63	-	1518.2
AHQ-1-14- , 6447 - 6512	R.KQVEVDAQQCMLEILDTAGTEQFTAM*R.D	3131.50548	3	2.89E-06	0.93	4.61	-	1180.8
AHQ-1-11, 6560	R.KQVEVDAQQCMLEILDTAGTEQFTAM*R.D	3131.50548	3	4.18E-09	0.94	5.33	-	839.9
AHQ-1-13- , 6689 - 6759	R.KQVEVDAQQCMLEILDTAGTEQFTAM*R.D	3131.50548	3	4.78E-06	0.91	4.23	-	1386.8
AHQ-1-14- , 5268	R.KQVEVDAQQCM*LEILDTAGTEQFTAM*R.D	3147.50488	3	2.19E-08	0.95	5.04	-	1339.6
AHQ-1-11, 6516 - 6590	K.QVEVDAQQCM*LEILDTAGTEQFTAM*R.D	3019.33197	3	3.05E-04	0.73	3.26	-	644.2
AHQ-1-11, 3868 - 3927	R.QWNNCAFLESSAK.S	1556.68148	2	3.15E-05	0.51	2.86	-	394.2
AHQ-1-11, 3986 - 4058	R.QWNNCAFLESSAK.S	1556.68148	2	4.85E-05	0.55	2.89	-	204.5
AHQ-1-11, 3607 - 3620	R.VKDTDDVPMILVGNK.C	1644.91497	3	6.50E-06	0.94	4.34	-	1691.5
AHQ-1-11, 3598 - 3658	R.VKDTDDVPMILVGNK.C	1644.91497	2	4.41E-10	0.97	5.09	-	1904.1
AHQ-1-13- , 4003	R.VKDTDDVPMILVGNK.C	1644.91497	2	3.23E-07	0.97	5.13	-	1489.1
AHQ-1-13, 3839	R.VKDTDDVPMILVGNK.C	1644.91497	2	9.29E-06	0.98	4.89	-	1957.2
AHQ-1-12, 3759	R.VKDTDDVPMILVGNK.C	1644.91497	2	7.37E-07	0.97	4.71	-	1772.8
AHQ-1-11, 3202	R.VKDTDDVPM*ILVGNK.C	1660.91437	3	2.21E-04	0.89	3.77	-	1323.1
AHQ-1-11, 3218 - 3254	R.VKDTDDVPM*ILVGNK.C	1660.91437	2	1.37E-04	0.96	4.78	-	1229.9
AHQ-1-14- , 3900 - 3914	R.VKDTDDVPMILVGNK.C	1644.91497	2	3.45E-06	0.96	4.27	-	1534.0
AHQ-1-13- , 4012	R.VKDTDDVPMILVGNK.C	1644.91497	3	3.04E-06	0.95	4.56	-	1931.5
AHQ-1-11, 4351	R.VKDTDDVPMILVGNKCDLEDER.V	2563.84420	2	7.35E-06	0.80	3.38	-	481.4
AHQ-1-11, 4278 - 4358	R.VKDTDDVPMILVGNKCDLEDER.V	2563.84420	3	8.43E-09	0.93	4.79	-	1057.7
AHQ-1-13, 4073	R.VKDTDDVPM*ILVGNKCDLEDER.V	2579.84360	3	1.08E-04	0.87	4.46	-	680.9
AHQ-1-14, 5009	R.VKDTDDVPM*ILVGNKCDLEDER.V	2579.84360	3	2.29E-04	0.87	4.24	-	825.8
AHQ-1-11, 4200 - 4258	R.VKDTDDVPMILVGNKCDLEDER.V	2563.84420	2	2.72E-05	0.85	3.21	-	892.0
AHQ-1-11, 4148 - 4219	R.VKDTDDVPMILVGNKCDLEDER.V	2563.84420	3	4.20E-11	0.97	5.96	-	1809.7
AHQ-1-13- , 4612	R.VKDTDDVPMILVGNKCDLEDER.V	2563.84420	3	3.15E-05	0.93	4.51	-	1010.3
AHQ-1-11, 4020 - 4092	R.VKDTDDVPM*ILVGNKCDLEDER.V	2579.84360	3	7.39E-10	0.94	5.16	-	1557.4
AHQ-1-11, 3916	R.VKDTDDVPM*ILVGNKCDLEDER.V	2579.84360	2	7.31E-04	0.79	3.38	-	524.5
AHQ-1-11, 3906 - 3954	R.VKDTDDVPM*ILVGNKCDLEDER.V	2579.84360	3	9.85E-05	0.83	3.86	-	861.2
AHQ-1-11, 4378	R.VKDTDDVPMILVGNKCDLEDERVVGK.E	2947.33182	3	8.34E-08	0.93	5.30	-	1273.5
gi|20149570|ref|NP_005816.2| RAS guanyl releasing protein 2 isoform 1; calcium and diacylglycerol-r				4.53E-11	1.84	20.21	6.30	75547.0
AHQ-1-6, 5419	K.ILFQDYHSFVTHGCTVDNPVLER.F	2750.03824	3	4.53E-11	0.92	4.14	-	1030.0
AHQ-1-6, 6838	K.LWEGLTELVTATGNYGNYR.R	2158.35558	2	5.91E-04	0.92	3.40	-	1416.1
gi|5803225|ref|NP_006752.1| tyrosine 3/tryptophan 5 -monooxygenase activation protein, epsilon poly				4.64E-11	1.55	20.28	11.80	29173.7
AHQ-1-9, 6983 - 6986	K.AAFDDAIAELDTLSEESYK.D	2089.19882	2	4.64E-11	0.93	4.48	-	909.7
AHQ-1-10, 6183	K.AAFDDAIAELDTLSEESYK.D	2089.19882	3	1.06E-05	0.75	3.27	-	695.9
AHQ-1-10, 6179 - 6180	K.AAFDDAIAELDTLSEESYK.D	2089.19882	2	2.72E-07	0.97	5.05	-	1431.2
AHQ-1-13, 6263 - 6339	K.AAFDDAIAELDTLSEESYK.D	2089.19882	2	1.19E-08	0.98	5.64	-	1633.5
AHQ-1-10, 2464	K.EAAENSLVAYK.A	1195.30397	2	2.10E-05	0.62	3.02	-	698.2
AHQ-1-9, 2663	K.EAAENSLVAYK.A	1195.30397	1	7.05E-10	0.62	2.42	-	360.9
gi|5902128|ref|NP_008880.1| syntaxin binding protein 2; Hunc18b [Homo sapiens]				4.65E-11	9.20	100.27	25.10	66438.1
AHQ-1-6, 6418	K.AAHIFFTDTCPEPLFSELGR.S	2310.57011	2	4.65E-11	0.97	5.49	-	1143.1
AHQ-1-6, 6422 - 6438	K.AAHIFFTDTCPEPLFSELGR.S	2310.57011	3	1.73E-06	0.95	5.36	-	951.6
AHQ-1-6, 6694 - 6695	R.EPIPSLEAIYLLSPTEK.S	1901.18939	2	6.10E-07	0.92	4.32	-	838.8
AHQ-1-14- , 6351	R.EPIPSLEAIYLLSPTEK.S	1901.18939	2	7.45E-07	0.75	3.04	-	648.7
AHQ-1-6, 3175	R.KGPEDTAQLAHAVLAK.L	1649.87303	3	4.84E-06	0.96	5.10	-	1710.2
AHQ-1-6, 3180	R.KGPEDTAQLAHAVLAK.L	1649.87303	2	1.27E-10	0.85	3.52	-	718.9
AHQ-1-6, 3716	K.LCSVEQDLAM*GSDAEGEK.I	1957.08386	2	3.41E-10	0.95	4.15	-	1436.7
AHQ-1-6, 2332	K.LIQHANVQAHSSLIR.N	1687.92773	2	9.28E-08	0.95	4.34	-	1442.0
AHQ-1-6, 6488	K.LIVPVLLDAAVPAYDK.I	1698.03911	2	8.04E-11	0.97	4.59	-	2154.8

AHQ-1-6, 5542	R.NLWPFVSDPAPTASSQAAVSAR.F	2273.48981	2	1.13E-04	0.81	3.66	-	437.8
AHQ-1-6, 6044 - 6124	R.REPIPSLEAIYLLSPTEK.S	2057.37574	2	4.14E-04	0.96	5.06	-	1417.5
AHQ-1-6, 4858 - 4928	K.WEVLIGSSHILTPTR.F	1709.97007	2	2.25E-09	0.96	4.57	-	1333.2
AHQ-1-6, 2032	R.YETTGLSEAR.E	1127.18649	2	1.08E-04	0.85	2.54	-	815.5
gi|6005942|ref|NP_009057.1| valosin-containing protein; yeast Cdc48p homolog; transitional endoplas				4.74E-11	8.42	100.28	18.90	89321.3
AHQ-1-5, 4146 - 4219	K.AIANECQANFISIK.G	1580.78762	2	6.66E-05	0.81	3.87	-	827.5
AHQ-1-5, 3781	R.ELQELVQYPVEHPDK.F	1825.01157	2	1.12E-04	0.90	4.18	-	671.0
AHQ-1-5, 3789 - 3790	R.ELQELVQYPVEHPDK.F	1825.01157	3	1.72E-04	0.73	3.31	-	707.7
AHQ-1-5, 3631	R.GILLYGPPGTGK.T	1173.38627	2	8.86E-04	0.63	3.01	-	646.9
AHQ-1-5, 6945	R.IVSQLLTLMDGLK.Q	1431.76657	2	2.47E-08	0.97	4.72	-	1466.3
AHQ-1-5, 5895 - 5897	R.LDQLIYIPLPDEK.S	1557.81228	2	2.80E-06	0.85	3.71	-	651.4
AHQ-1-5, 3055	R.LEILQIHTK.N	1095.31705	1	2.66E-06	0.60	2.51	-	452.8
AHQ-1-5, 5155 - 5225	R.LIVDEAINEDNSVVSLSQPK.M	2171.39088	2	1.09E-04	0.97	5.67	-	879.5
AHQ-1-5, 7433 - 7434	K.NAPAIIFIDELDAIAPK.R	1812.09907	2	4.74E-11	0.96	4.97	-	1275.3
AHQ-1-5, 5079	K.VVETDPSPYCIVAPDTVIHCEGEPIKR.E	3085.45473	3	2.42E-09	0.84	4.03	-	385.8
AHQ-1-5, 3491	R.WALSQSNPSALR.E	1330.47434	2	7.77E-06	0.90	3.75	-	1069.6
gi|4501893|ref|NP_001094.1| actinin, alpha 2 [Homo sapiens]				5.16E-11	9.18	100.28	11.40	103853.1
AHQ-1-4, 4695	K.AGTQIENIEEDFR.N	1522.59861	2	5.27E-04	0.81	3.25	-	775.5
AHQ-1-5, 5121 - 5189	K.CQLEINFNTLQTK.L	1610.81373	2	1.75E-09	0.98	5.54	-	1400.5
AHQ-1-6, 5027 - 5100	K.CQLEINFNTLQTK.L	1610.81373	2	5.68E-04	0.85	2.92	-	1037.4
AHQ-1-4, 5195 - 5196	K.CQLEINFNTLQTK.L	1610.81373	2	1.87E-09	0.96	4.65	-	1447.1
AHQ-1-1, 5140	K.CQLEINFNTLQTK.L	1610.81373	2	6.82E-07	0.90	4.12	-	663.9
AHQ-1-5, 4965	K.EGLLLWCQR.K	1176.37031	2	5.43E-06	0.94	3.62	-	1030.8
AHQ-1-9, 4123 - 4202	R.FAIQDISVEETSAK.E	1538.68108	2	1.53E-09	0.96	4.70	-	1215.8
AHQ-1-3, 4347	R.FAIQDISVEETSAK.E	1538.68108	2	1.15E-04	0.91	3.44	-	1031.2
AHQ-1-7, 4281 - 4293	R.FAIQDISVEETSAK.E	1538.68108	2	4.39E-04	0.79	3.41	-	604.6
AHQ-1-2, 4514	R.FAIQDISVEETSAK.E	1538.68108	2	9.84E-04	0.78	2.75	-	883.9
AHQ-1-6, 4211 - 4215	R.FAIQDISVEETSAK.E	1538.68108	2	5.14E-06	0.95	3.95	-	1404.1
AHQ-1-10, 3900	R.FAIQDISVEETSAK.E	1538.68108	2	1.56E-04	0.68	2.75	-	730.7
AHQ-1-1, 4383 - 4399	R.FAIQDISVEETSAK.E	1538.68108	2	8.92E-04	0.89	2.99	-	1132.6
AHQ-1-4, 4391	R.FAIQDISVEETSAK.E	1538.68108	2	4.64E-09	0.92	3.57	-	1094.7
AHQ-1-13, 4124 - 4183	R.FAIQDISVEETSAK.E	1538.68108	2	9.20E-05	0.86	3.47	-	794.7
AHQ-1-5, 4265 - 4333	R.FAIQDISVEETSAK.E	1538.68108	2	5.90E-07	0.96	3.98	-	1601.7
AHQ-1-5, 4150	R.FAIQDISVEETSAK.E	1538.68108	2	5.52E-05	0.94	3.64	-	1488.3
AHQ-1-5, 6377	K.GYEEWLLNEIR.R	1422.56701	2	3.33E-07	0.94	3.33	-	1507.5
AHQ-1-1, 6132	K.GYEEWLLNEIR.R	1422.56701	2	1.37E-06	0.95	3.73	-	1410.7
AHQ-1-5, 6234	K.GYEEWLLNEIR.R	1422.56701	1	2.29E-05	0.39	2.34	-	270.3
AHQ-1-4, 5711	K.GYEEWLLNEIR.R	1422.56701	2	4.28E-07	0.93	3.39	-	1366.0
AHQ-1-6, 6108	K.GYEEWLLNEIR.R	1422.56701	2	1.90E-07	0.96	4.32	-	1679.4
AHQ-1-6, 5518	K.GYEEWLLNEIR.R	1422.56701	2	3.60E-07	0.92	2.92	-	1519.3
AHQ-1-5, 6225	K.GYEEWLLNEIR.R	1422.56701	1	6.45E-05	0.18	2.05	-	261.9
AHQ-1-4, 6276	K.GYEEWLLNEIR.R	1422.56701	2	5.23E-08	0.97	4.73	-	1711.8
AHQ-1-5, 5586	K.GYEEWLLNEIR.R	1422.56701	2	1.31E-05	0.90	3.27	-	1142.3
AHQ-1-5, 6222	K.GYEEWLLNEIR.R	1422.56701	2	2.22E-07	0.97	4.75	-	1809.5
AHQ-1-4, 2306	K.HEAFESDLAAHQDR.V	1626.66861	2	6.70E-08	0.97	4.31	-	2081.3
AHQ-1-5, 2153	K.HEAFESDLAAHQDR.V	1626.66861	2	5.16E-11	0.97	4.67	-	1493.3
AHQ-1-5, 2157 - 2158	K.HEAFESDLAAHQDR.V	1626.66861	3	1.66E-08	0.98	5.22	-	2148.4
AHQ-1-5, 3883	R.KAGTQIENIEEDFR.N	1650.77152	2	4.55E-07	0.91	4.08	-	698.8
AHQ-1-5, 1877 - 1955	R.KHEAFESDLAAHQDR.V	1754.84152	2	1.96E-07	0.95	3.83	-	1429.4
AHQ-1-6, 1867 - 1938	R.KHEAFESDLAAHQDR.V	1754.84152	2	9.57E-09	0.96	4.43	-	1447.7
AHQ-1-7, 1924	R.KHEAFESDLAAHQDR.V	1754.84152	2	4.37E-08	0.95	4.35	-	1160.9
AHQ-1-5, 4843 - 4845	K.LVSIGAEEIVDGNVK.M	1543.74400	2	7.31E-04	0.90	3.18	-	1104.5
AHQ-1-5, 4613 - 4683	K.LVSIGAEEIVDGNVK.M	1543.74400	2	2.14E-05	0.95	4.05	-	1099.1
AHQ-1-5, 4381 - 4457	K.LVSIGAEEIVDGNVK.M	1543.74400	2	8.08E-05	0.87	4.15	-	971.2
AHQ-1-5, 4249	K.LVSIGAEEIVDGNVK.M	1543.74400	2	3.10E-06	0.80	3.90	-	513.7
AHQ-1-5, 3469	K.TFTAWCNSHLR.K	1394.54018	2	5.93E-08	0.89	2.99	-	980.2
AHQ-1-6, 3456	K.TFTAWCNSHLR.K	1394.54018	2	3.66E-06	0.83	2.93	-	806.2
AHQ-1-5, 3590	K.TFTAWCNSHLR.K	1394.54018	2	2.01E-06	0.88	2.74	-	901.3
gi|4504391|ref|NP_000179.1| hexokinase 1 isoform HKI; brain form hexokinase [Homo sapiens]				5.26E-11	7.15	90.24	14.10	102502.4
AHQ-1-4, 4610	K.ATDCVGHDVVTLLR.D	1557.75406	2	5.26E-11	0.88	3.43	-	857.5
AHQ-1-4, 2800 - 2803	K.FLSQIESDR.L	1095.18763	1	3.02E-05	0.20	2.21	-	207.2
AHQ-1-4, 2799	K.FLSQIESDR.L	1095.18763	2	4.80E-05	0.77	2.93	-	687.3
AHQ-1-4, 3372	K.GAALITAVGVR.L	1028.23070	2	2.11E-04	0.97	4.06	-	2501.8
AHQ-1-4, 5830 - 5834	K.GDFIALDLGGSSFR.I	1455.59700	2	8.60E-09	0.97	4.63	-	1857.9
AHQ-1-4, 2200 - 2252	R.HIDLVEGDEGR.M	1240.30483	2	5.42E-06	0.94	3.84	-	1091.7
AHQ-1-4, 5940	R.LGVEPSDDDCVSVQHVCTIVSFR.S	2622.86999	3	8.13E-06	0.67	3.80	-	223.8
AHQ-1-4, 6170	K.LPVGFTFSFPCQQSK.I	1744.99134	2	5.70E-06	0.96	4.82	-	1023.5
AHQ-1-5, 6117 - 6175	K.LPVGFTFSFPCQQSK.I	1744.99134	2	6.75E-04	0.90	3.84	-	659.7
AHQ-1-4, 2822	R.LVNEYSLNAGK.Q	1208.34588	1	1.75E-07	0.61	2.20	-	576.3
AHQ-1-4, 6387 - 6391	R.TPDGTENGDFLALDLGGTNFR.V	2211.33051	2	2.48E-10	0.95	4.47	-	1107.3
AHQ-1-4, 6303 - 6322	R.TPDGTENGDFLALDLGGTNFR.V	2211.33051	2	2.26E-07	0.95	4.45	-	954.0
gi|4557597|ref|NP_001449.1| gamma filamin; filamin 2; filamin C, gamma (actin-binding protein-280);				5.60E-11	1.83	30.28	1.70	288897.4
AHQ-1-1, 4776 - 4800	R.GQHVPGSPFQFTVGPLGEGGAHK.V	2305.53673	3	2.98E-08	0.89	4.56	-	516.6
AHQ-1-8, 4255	R.GQHVPGSPFQFTVGPLGEGGAHK.V	2305.53673	3	1.43E-04	0.92	4.64	-	816.7
AHQ-1-7, 4508	R.GQHVPGSPFQFTVGPLGEGGAHK.V	2305.53673	3	7.56E-06	0.87	4.29	-	500.9
AHQ-1-6, 4499	R.GQHVPGSPFQFTVGPLGEGGAHK.V	2305.53673	2	2.04E-08	0.92	4.62	-	485.2
AHQ-1-6, 4487	R.GQHVPGSPFQFTVGPLGEGGAHK.V	2305.53673	3	5.11E-05	0.87	4.35	-	516.2
AHQ-1-4, 4734 - 4738	R.GQHVPGSPFQFTVGPLGEGGAHK.V	2305.53673	3	2.06E-07	0.93	5.16	-	701.4
AHQ-1-4, 4735	R.GQHVPGSPFQFTVGPLGEGGAHK.V	2305.53673	2	3.38E-07	0.94	5.02	-	474.5
AHQ-1-5, 4537	R.GQHVPGSPFQFTVGPLGEGGAHK.V	2305.53673	2	5.60E-11	0.96	5.57	-	689.3
AHQ-1-8, 4260	R.GQHVPGSPFQFTVGPLGEGGAHK.V	2305.53673	2	2.44E-07	0.95	5.19	-	571.4
AHQ-1-3, 1906 - 1978	K.IECDDKGDGSCDVR.Y	1628.67809	2	2.65E-06	0.78	3.17	-	623.2
AHQ-1-2, 2011	K.IECDDKGDGSCDVR.Y	1628.67809	3	6.17E-06	0.69	3.39	-	335.8
AHQ-1-4, 1907	K.IECDDKGDGSCDVR.Y	1628.67809	2	5.94E-04	0.67	2.79	-	517.5
AHQ-1-3, 2006 - 2115	K.NDNDTFTVK.Y	1054.09237	1	6.01E-05	0.09	1.87	-	174.2
gi|4885609|ref|NP_005408.1| v-src sarcoma (Schmidt-Ruppin A-2) viral oncogene homolog; Protooncogen				5.68E-11	3.03	40.23	15.10	59834.4
AHQ-1-7, 7496	R.KEPEERPTFEYLQAFLEDYFTSTEPQYQPGENL	3998.26826	3	2.11E-08	0.68	3.30	-	277.6
AHQ-1-7, 4153 - 4157	K.LFGGFNSSDTVTSPQR.A	1713.82931	2	5.68E-11	0.95	4.61	-	1067.1
AHQ-1-7, 3872	K.LFGGFNSSDTVTSPQR.A	1713.82931	2	4.27E-07	0.93	4.00	-	956.3
AHQ-1-7, 2538	R.LTTVCPTSKPQTQGLAK.D	1832.11198	2	8.36E-05	0.55	2.85	-	270.1
AHQ-1-7, 6574 - 6644	R.TQFNSLQQLVAYYSK.H	1790.99681	2	2.91E-06	0.95	4.14	-	1250.8
AHQ-1-7, 6426	R.TQFNSLQQLVAYYSK.H	1790.99681	2	3.06E-07	0.85	3.23	-	766.8
gi|4759212|ref|NP_004598.1| beta-tubulin cofactor A [Homo sapiens]				5.78E-11	3.81	40.32	26.90	12854.8
AHQ-1-13, 3526	R.ILENEKDLEEAEEYK.E	1852.97372	2	1.87E-10	0.97	5.20	-	1833.3
AHQ-1-13- , 3460	R.ILENEKDLEEAEEYKEAR.L	2209.35284	3	4.83E-10	0.97	6.30	-	1441.7
AHQ-1-13, 3371	R.ILENEKDLEEAEEYKEAR.L	2209.35284	2	2.49E-09	0.96	4.85	-	1259.1
AHQ-1-13, 3367	R.ILENEKDLEEAEEYKEAR.L	2209.35284	3	5.78E-11	0.96	5.02	-	1436.8
AHQ-1-13- , 3845 - 3885	R.LEAAYLDLQR.I	1192.34645	2	5.90E-06	0.97	3.88	-	2016.9
AHQ-1-13- , 3508 - 3533	R.RLEAAYLDLQR.I	1348.53280	2	1.17E-07	0.92	4.16	-	645.2
AHQ-1-13, 3403	R.RLEAAYLDLQR.I	1348.53280	2	1.48E-05	0.89	3.95	-	553.4
gi|17986260|ref|NP_524147.1| smooth muscle and non-muscle myosin alkali light chain isoform 2 [Homo				5.88E-11	3.29	40.26	23.30	12969.7
AHQ-1-12, 5441	K.M*TEEEVEM*LVAGHEDSNGCINYEELVR.M	3188.42436	3	5.88E-11	0.94	4.72	-	1332.3
AHQ-1-12, 5769 - 5827	K.M*TEEEVEMLVAGHEDSNGCINYEELVR.M	3172.42496	3	4.17E-05	0.76	4.69	-	609.7
AHQ-1-12, 5819 - 5899	K.MTEEEVEMLVAGHEDSNGCINYEELVR.M	3156.42556	3	3.06E-05	0.92	4.96	-	1123.7
AHQ-1-12, 5885 - 5957	K.M*TEEEVEMLVAGHEDSNGCINYEELVR.M	3172.42496	3	2.24E-07	0.89	4.98	-	815.2
AHQ-1-12, 5902	K.MTEEEVEMLVAGHEDSNGCINYEELVR.M	3156.42556	3	5.72E-04	0.94	4.77	-	1566.8
AHQ-1-12, 5994	K.MTEEEVEMLVAGHEDSNGCINYEELVR.M	3156.42556	3	4.32E-04	0.65	3.29	-	764.4

AHQ-1-12, 6055 - 6135	K.M*TEEEVEMLVAGHEDSNGCINYEELVR.M	3172.42496	3	8.64E-05	0.87	4.50	-	759.1
AHQ-1-12, 6631 - 6689	K.M*TEEEVEMLVAGHEDSNGCINYEELVR.M	3172.42496	3	3.30E-06	0.87	4.61	-	708.8
AHQ-1-13- , 6057 - 6131	K.MTEEEVEM*LVAGHEDSNGCINYEELVR.M	3172.42496	3	4.84E-04	0.53	3.30	-	683.7
AHQ-1-14- , 5504 - 5558	K.M*TEEEVEM*LVAGHEDSNGCINYEELVR.M	3188.42436	3	1.30E-09	0.96	5.25	-	1554.3
AHQ-1-14- , 5772 - 5854	K.M*TEEEVEMLVAGHEDSNGCINYEELVR.M	3172.42496	3	4.19E-04	0.64	4.02	-	547.5
gi|4506713|ref|NP_002945.1| ubiquitin and ribosomal protein S27a precursor; ubiquitin carboxyl exte				5.95E-11	0.80	10.21	10.30	17964.8
AHQ-1-14- , 4446 - 4464	K.TITLEVEPSDTIENVK.A	1788.97431	2	5.95E-11	0.80	4.14	-	673.0
AHQ-1-14- , 4352 - 4383	K.TITLEVEPSDTIENVK.A	1788.97431	2	3.87E-05	0.88	4.07	-	758.0
gi|6681259|ref|NP_031377.1| multimerin [Homo sapiens]				6.57E-11	20.77	230.28	21.90	138070.8
AHQ-1-3, 2358 - 2441	R.AQEQQSLIHTNQAESHTAVGR.G	2306.43830	3	1.01E-07	0.93	4.85	-	917.3
AHQ-1-3, 2365 - 2370	R.AQEQQSLIHTNQAESHTAVGR.G	2306.43830	2	3.63E-09	0.94	5.21	-	531.3
AHQ-1-2, 2459	R.AQEQQSLIHTNQAESHTAVGR.G	2306.43830	2	1.71E-06	0.94	4.84	-	742.4
AHQ-1-3, 7129	K.CTSDMETILTFIPQFHR.L	2098.38838	2	8.01E-05	0.84	4.10	-	473.5
AHQ-1-3, 6425	K.CTSDM*ETILTFIPQFHR.L	2114.38778	3	2.87E-09	0.86	3.85	-	686.3
AHQ-1-4, 4186	R.DQALQLQVLNSR.F	1385.55144	2	6.22E-04	0.63	2.71	-	748.3
AHQ-1-3, 4318	R.DQALQLQVLNSR.F	1385.55144	2	4.40E-06	0.94	3.34	-	1549.5
AHQ-1-3, 2821	K.EVHEQLLSTEQVSDQK.N	1870.99550	2	7.05E-05	0.96	4.99	-	892.4
AHQ-1-14- , 6118	K.FPPVTTFSGYLLYR.T	1661.92388	2	1.61E-08	0.93	3.59	-	937.8
AHQ-1-10, 5683	K.FPPVTTFSGYLLYR.T	1661.92388	2	2.14E-09	0.93	3.52	-	911.2
AHQ-1-13, 5940	K.FPPVTTFSGYLLYR.T	1661.92388	2	5.78E-04	0.92	3.27	-	929.6
AHQ-1-13- , 6348 - 6353	K.FPPVTTFSGYLLYR.T	1661.92388	2	5.68E-04	0.93	4.36	-	793.9
AHQ-1-2, 6630 - 6642	K.FPPVTTFSGYLLYR.T	1661.92388	2	4.46E-06	0.90	3.63	-	659.7
AHQ-1-3, 6467 - 6479	K.FPPVTTFSGYLLYR.T	1661.92388	2	7.14E-10	0.93	4.05	-	789.4
AHQ-1-3, 5925	K.FVLVQENRPTLTDIVELR.N	2143.47187	3	7.99E-09	0.91	4.41	-	950.3
AHQ-1-1, 5957	K.FVLVQENRPTLTDIVELR.N	2143.47187	3	3.68E-05	0.95	4.35	-	1429.5
AHQ-1-3, 6165 - 6190	K.GLTEFVEPIIQIK.T	1487.76518	2	1.08E-05	0.93	4.34	-	1011.8
AHQ-1-1, 6167	K.GLTEFVEPIIQIK.T	1487.76518	2	9.68E-05	0.91	3.66	-	946.3
AHQ-1-3, 2445	R.GVAEQQQQQGCGDPEVMQK.M	2119.27971	2	1.05E-04	0.94	4.84	-	759.4
AHQ-1-3, 2114	R.GVAEQQQQQGCGDPEVM*QK.M	2135.27911	2	8.93E-04	0.81	3.67	-	462.0
AHQ-1-2, 4075	K.HSLPDIQLLQK.G	1292.50915	2	7.15E-07	0.87	3.16	-	683.9
AHQ-1-3, 3881	K.HSLPDIQLLQK.G	1292.50915	2	9.92E-07	0.90	3.42	-	779.5
AHQ-1-3, 6061	K.IENLTSAVNSLNFIIK.E	1777.05472	2	5.65E-05	0.94	4.67	-	821.0
AHQ-1-3, 5174	K.IFQNDM*QETVAQLFK.T	1829.06683	2	1.56E-05	0.89	3.69	-	1014.1
AHQ-1-1, 5405	K.IFQNDM*QETVAQLFK.T	1829.06683	2	6.08E-05	0.69	2.89	-	533.1
AHQ-1-1, 6541	K.IFQNDMQETVAQLFK.T	1813.06743	2	3.32E-04	0.94	4.21	-	1125.4
AHQ-1-3, 6606 - 6609	K.IFQNDMQETVAQLFK.T	1813.06743	2	8.18E-07	0.98	5.64	-	2024.2
AHQ-1-3, 6710 - 6717	K.IFQNDMQETVAQLFK.T	1813.06743	2	2.58E-06	0.96	4.81	-	1607.7
AHQ-1-5, 6701 - 6709	K.IFQNDMQETVAQLFK.T	1813.06743	2	2.08E-08	0.98	5.22	-	2012.9
AHQ-1-3, 6806	K.IFQNDMQETVAQLFK.T	1813.06743	2	2.76E-06	0.88	3.83	-	803.9
AHQ-1-3, 5347	K.IFQNDM*QETVAQLFK.T	1829.06683	2	8.31E-07	0.88	4.05	-	834.3
AHQ-1-13, 5289	R.IPYLGVYVFK.Y	1199.46532	2	6.11E-05	0.71	2.62	-	891.3
AHQ-1-10, 4941	R.IPYLGVYVFK.Y	1199.46532	2	1.39E-05	0.94	3.67	-	1187.0
AHQ-1-14- , 5476	R.IPYLGVYVFK.Y	1199.46532	2	8.92E-04	0.93	2.83	-	1333.3
AHQ-1-13- , 4013	K.LAFESENINSEIHCDR.V	1936.04933	2	7.35E-06	0.94	4.77	-	946.7
AHQ-1-10, 3620	K.LAFESENINSEIHCDR.V	1936.04933	2	1.91E-05	0.91	3.52	-	1132.0
AHQ-1-3, 2870	K.LKEVHEQLLSTEQVSDQK.N	2112.32666	3	2.14E-04	0.95	5.19	-	1496.6
AHQ-1-3, 2865 - 2943	K.LKEVHEQLLSTEQVSDQK.N	2112.32666	2	1.05E-06	0.97	5.38	-	1773.1
AHQ-1-10, 3417 - 3491	K.LVEENALAPDFSK.G	1433.58810	2	4.31E-05	0.94	4.20	-	964.2
AHQ-1-13- , 3872	K.LVEENALAPDFSK.G	1433.58810	2	5.85E-05	0.72	2.88	-	584.6
AHQ-1-1, 3940 - 3996	K.LVEENALAPDFSK.G	1433.58810	2	6.38E-05	0.90	2.94	-	1146.2
AHQ-1-3, 7155	K.M*SEQLNDLTYDM*EILQPLLEQGASLR.Q	3041.40062	3	1.30E-04	0.92	4.25	-	1130.8
AHQ-1-3, 2134	K.QTHLEGALEQEHSR.S	1635.72018	2	1.48E-08	0.76	3.06	-	341.8
AHQ-1-3, 6781 - 6799	K.TMTIINNAIDFIQDNYALK.E	2199.51256	2	4.04E-05	0.70	3.45	-	445.5
AHQ-1-3, 7034	K.TMTIINNAIDFIQDNYALK.E	2199.51256	2	1.97E-04	0.73	3.41	-	555.4
AHQ-1-3, 7234	K.TMTIINNAIDFIQDNYALK.E	2199.51256	2	6.57E-11	0.95	5.10	-	815.4
AHQ-1-4, 3524	K.TVSSLSEDLESTR.Q	1424.49324	2	5.81E-05	0.69	3.21	-	552.6
AHQ-1-3, 4103	R.YNFVLQVAK.T	1082.27683	2	1.97E-05	0.93	3.10	-	1338.6
gi|4885183|ref|NP_005210.1| diaphanous 1; deafness, autosomal dominant 1; hDia1 [Homo sapiens] [MAS				6.73E-11	10.19	120.29	16.60	138977.7
AHQ-1-3, 6259 - 6271	R.DM*PLLSCLESLR.V	1451.69163	2	7.04E-05	0.90	3.59	-	721.8
AHQ-1-3, 3295	R.DVQNFPAATDEKDKFVEK.M	2082.25588	2	3.17E-06	0.57	3.22	-	285.5
AHQ-1-3, 4601	R.LGLHQVLQDLR.E	1292.51238	2	3.38E-07	0.78	2.90	-	710.2
AHQ-1-3, 2359	K.LQDLQGEKDALHSEK.Q	1711.85486	2	7.23E-05	0.91	3.78	-	916.2
AHQ-1-3, 6554	K.LQFSEQVENIKPEIVSVTAACEELR.K	2892.23121	3	2.24E-06	0.96	5.74	-	1514.2
AHQ-1-3, 5269	K.LVAEDLSQDCFWTK.V	1713.88927	2	1.45E-07	0.89	3.63	-	725.4
AHQ-1-4, 5376	K.LVAEDLSQDCFWTK.V	1713.88927	2	1.45E-07	0.86	3.56	-	618.0
AHQ-1-3, 7311	R.M*EM*DDFNEVFQILLNTVK.D	2219.52085	2	6.73E-11	0.97	4.86	-	1503.4
AHQ-1-3, 6283	K.MLSELKDEYDDLAESEQFGVVM*GTVPR.L	3076.40186	3	2.82E-04	0.65	3.48	-	412.3
AHQ-1-3, 5806 - 5874	K.QDLEAEVSQLTGEVAK.L	1717.85617	2	2.91E-05	0.87	4.19	-	958.5
AHQ-1-3, 6021	K.TAQNLSIFLGSFR.M	1454.65537	2	3.24E-06	0.88	3.56	-	653.3
AHQ-1-3, 7282	K.VGCLQLINALITPAEELDFR.V	2274.62236	2	9.40E-06	0.96	5.33	-	831.7
AHQ-1-3, 7135	K.VGCLQLINALITPAEELDFR.V	2274.62236	2	3.59E-07	0.89	4.23	-	628.5
AHQ-1-3, 5069	R.VQLNVFDEQGEEDSYDLK.G	2129.22304	2	1.04E-04	0.94	4.33	-	1124.2
gi|4557703|ref|NP_000414.1| keratin 2a [Homo sapiens]				6.80E-11	2.75	30.26	12.90	65864.9
AHQ-1-1, 6832	R.FGGFGGPGGVGGLGGPGGFGPGGYPGGIHEVSVNQSLLQPLNVK.V	4094.54151	3	2.30E-04	0.91	4.56	-	954.4
AHQ-1-14- , 6502	R.FGGFGGPGGVGGLGGPGGFGPGGYPGGIHEVSVNQSLLQPLNVK.V	4094.54151	3	6.80E-11	0.84	3.85	-	1006.2
AHQ-1-4, 3111 - 3112	R.FLEQQNQVLQTK.W	1476.65935	2	3.59E-07	0.97	4.82	-	2097.1
AHQ-1-5, 2998	R.FLEQQNQVLQTK.W	1476.65935	2	1.72E-06	0.97	5.22	-	2241.6
AHQ-1-6, 2988	R.FLEQQNQVLQTK.W	1476.65935	2	1.78E-06	0.98	4.82	-	2340.8
AHQ-1-11, 2928	R.FLEQQNQVLQTK.W	1476.65935	2	1.65E-04	0.92	3.46	-	1538.7
AHQ-1-12, 2962	R.FLEQQNQVLQTK.W	1476.65935	2	1.41E-08	0.96	4.00	-	2192.8
AHQ-1-13, 3046	R.FLEQQNQVLQTK.W	1476.65935	2	1.77E-04	0.95	4.22	-	1604.9
AHQ-1-13- , 3080 - 3088	R.FLEQQNQVLQTK.W	1476.65935	2	3.00E-07	0.97	5.03	-	2417.7
AHQ-1-14- , 3031	R.FLEQQNQVLQTK.W	1476.65935	2	7.75E-06	0.97	4.67	-	1825.4
AHQ-1-1, 3145	R.FLEQQNQVLQTK.W	1476.65935	2	2.99E-08	0.97	4.67	-	2005.0
AHQ-1-14- , 5647	K.VLYDAEISQIHQSVTDTNVILSM*DNSR.N	3066.34680	3	9.83E-08	0.95	5.13	-	1440.8
gi|11321601|ref|NP_002618.1| phosphofructokinase, platelet; Phosphofructokinase, platelet type [Hom				7.18E-11	3.80	40.28	9.30	85595.6
AHQ-1-5, 3667	K.AIGVLTSGGDAQGMNAAVR.A	1789.00750	2	7.18E-11	0.97	5.58	-	1468.9
AHQ-1-5, 3449	R.DLQSNVEHLTEK.M	1413.51529	2	8.03E-05	0.93	3.95	-	1259.3
AHQ-1-5, 6238 - 6241	K.EIGWTDVGGWTGQGGSILGTK.R	2120.30735	2	2.13E-04	0.95	4.69	-	954.4
AHQ-1-5, 6626	R.GITNLCVIGGDGSLTGANLFR.K	2137.40264	2	6.23E-10	0.95	5.36	-	691.7
gi|13518026|ref|NP_004978.2| LIM and senescent cell antigen-like domains 1 [Homo sapiens]				7.59E-11	4.11	50.24	20.00	37251.2
AHQ-1-9, 4810	K.AWCVNCFACSTCNTK.L	1884.08097	2	1.79E-04	0.58	2.75	-	315.5
AHQ-1-13- , 6956	R.CDLCQEVLADIGFVK.N	1770.01757	2	1.64E-07	0.85	2.56	-	1362.3
AHQ-1-11, 4314	K.CHAIIDEQPLIFK.N	1585.84900	2	9.49E-04	0.94	3.82	-	1406.7
AHQ-1-12, 4471	K.CHAIIDEQPLIFK.N	1585.84900	2	7.39E-04	0.90	3.57	-	971.6
AHQ-1-8, 4484	K.CHAIIDEQPLIFK.N	1585.84900	2	1.06E-05	0.96	4.32	-	1421.6
AHQ-1-10, 4164	K.CHAIIDEQPLIFK.N	1585.84900	2	4.98E-06	0.93	3.70	-	1109.3
AHQ-1-9, 3087	K.MGVPICGACR.R	1123.35250	2	4.76E-04	0.80	2.67	-	454.2
AHQ-1-12, 3497 - 3538	R.VIEGDVVSALNK.A	1244.41966	2	7.59E-11	0.96	3.93	-	1545.1
AHQ-1-8, 3374	R.VIEGDVVSALNK.A	1244.41966	2	1.38E-09	0.96	4.66	-	1228.8
AHQ-1-14, 4478	R.VIEGDVVSALNK.A	1244.41966	2	4.05E-07	0.96	4.36	-	1298.6
AHQ-1-13- , 3603	R.VIEGDVVSALNK.A	1244.41966	1	9.36E-05	0.31	1.97	-	493.5
AHQ-1-11, 3394	R.VIEGDVVSALNK.A	1244.41966	2	5.03E-09	0.93	4.26	-	913.6
AHQ-1-9, 3398 - 3411	R.VIEGDVVSALNK.A	1244.41966	2	5.29E-07	0.96	4.77	-	1274.5
AHQ-1-14- , 3574	R.VIEGDVVSALNK.A	1244.41966	2	3.46E-07	0.96	4.19	-	1367.7
gi|10863927|ref|NP_066953.1| peptidylprolyl isomerase A (cyclophilin A) [Homo sapiens]				7.61E-11	7.79	90.24	30.90	18012.4
AHQ-1-12, 4085 - 4098	K.EGMNIVEAMER.F	1279.46942	2	2.00E-04	0.85	3.18	-	713.6

AHQ-1-14, 4932	K.FEDENFILK.H	1155.28144	2	6.50E-05	0.74	3.01	-	590.2
AHQ-1-12, 3969 - 3970	K.FEDENFILK.H	1155.28144	2	1.29E-04	0.81	3.53	-	530.8
AHQ-1-13- , 4147	K.FEDENFILK.H	1155.28144	2	1.17E-04	0.75	2.86	-	500.0
AHQ-1-13, 3972	K.FEDENFILK.H	1155.28144	2	6.69E-04	0.77	3.19	-	538.8
AHQ-1-12, 3847 - 3909	K.FEDENFILK.H	1155.28144	2	5.18E-04	0.84	3.50	-	633.6
AHQ-1-12, 3767	K.ITIADCGQLE	1121.24345	2	1.87E-04	0.91	3.27	-	1525.7
AHQ-1-12, 3402 - 3478	K.KITIADCGQLE	1249.41637	2	8.04E-08	0.83	3.16	-	651.7
AHQ-1-14- , 3372	K.KITIADCGQLE	1249.41637	2	1.76E-05	0.90	3.84	-	1260.1
AHQ-1-12, 3277 - 3337	K.KITIADCGQLE	1249.41637	2	1.47E-06	0.92	3.81	-	1414.6
AHQ-1-13- , 4840	K.SIYGEKFEDENFILK.H	1833.03047	2	7.47E-08	0.94	4.19	-	1204.6
AHQ-1-14, 5580 - 5601	K.SIYGEKFEDENFILK.H	1833.03047	2	3.39E-06	0.94	4.28	-	1134.4
AHQ-1-12, 4551 - 4613	K.SIYGEKFEDENFILK.H	1833.03047	2	1.74E-08	0.96	4.57	-	1303.5
AHQ-1-12, 4762 - 4831	K.SIYGEKFEDENFILK.H	1833.03047	2	4.82E-06	0.88	3.68	-	998.2
AHQ-1-14- , 4735	K.SIYGEKFEDENFILK.H	1833.03047	2	7.61E-11	0.94	4.80	-	1014.7
AHQ-1-13, 4591	K.SIYGEKFEDENFILK.H	1833.03047	2	7.34E-08	0.93	4.09	-	1161.1
AHQ-1-12, 3791	K.VKEGMNIVEAMER.F	1506.77390	2	1.51E-05	0.87	3.68	-	1245.6
AHQ-1-12, 3613	K.VKEGMNIVEAMER.F	1506.77390	2	7.64E-07	0.94	3.85	-	1407.3
AHQ-1-14- , 2120	K.VKEGM*NIVEAM*ER.F	1538.77270	2	6.62E-04	0.83	3.01	-	689.9
AHQ-1-12, 2941	K.VKEGM*NIVEAMER.F	1522.77330	2	4.31E-04	0.87	3.36	-	941.6
AHQ-1-12, 4290	R.VSFELFADKVPK.T	1380.61317	2	1.28E-09	0.87	3.24	-	1017.6
AHQ-1-12, 4519	R.VSFELFADKVPK.T	1380.61317	3	5.31E-06	0.64	3.38	-	423.2
AHQ-1-12, 4513 - 4517	R.VSFELFADKVPK.T	1380.61317	2	2.07E-08	0.83	3.47	-	807.8
gi|5174393|ref|NP_006001.1|	arginine-rich, mutated in early stage tumors; arginine-rich protein [Ho			8.04E-11	3.44	40.30	25.20	26906.2
AHQ-1-12, 4545	K.DRDVTFSPATIENELIK.F	1949.15100	2	1.18E-08	0.97	4.98	-	1915.8
AHQ-1-12, 5435 - 5498	K.DRDVTFSPATIENELIK.F	1949.15100	2	1.98E-08	0.98	5.90	-	1921.3
AHQ-1-12, 2885	K.IINEVSKPLAHHIPVEK.I	1925.26378	2	9.81E-04	0.88	3.75	-	753.2
AHQ-1-12, 3186 - 3249	K.ILDDWGETCKGCAEK.S	1784.94596	2	8.04E-11	0.82	3.53	-	496.5
AHQ-1-12, 3765	K.QIDLSTVDLK.K	1132.28932	2	3.29E-04	0.77	2.55	-	604.9
gi|4507109|ref|NP_000336.1|	alpha-synuclein isoform NACP140; non A4 component of amyloid precursor			8.26E-11	4.98	60.31	34.30	14460.1
AHQ-1-12, 2197 - 2267	K.EGVVHGVATVAEK.T	1296.45466	2	7.52E-06	0.84	2.81	-	769.5
AHQ-1-13- , 2291 - 2295	K.EGVVHGVATVAEK.T	1296.45466	2	6.89E-07	0.78	3.04	-	555.5
AHQ-1-13- , 4801	K.EQVTNVGGAVVTGVTAVAQK.T	1929.16446	2	1.63E-06	0.91	3.77	-	842.6
AHQ-1-13, 4528	K.EQVTNVGGAVVTGVTAVAQK.T	1929.16446	2	4.72E-08	0.95	5.12	-	790.4
AHQ-1-14, 5498	K.EQVTNVGGAVVTGVTAVAQK.T	1929.16446	2	1.09E-06	0.96	5.13	-	1018.8
AHQ-1-12, 4557 - 4619	K.EQVTNVGGAVVTGVTAVAQK.T	1929.16446	2	8.26E-11	0.97	5.69	-	1152.6
AHQ-1-14- , 4672 - 4676	K.EQVTNVGGAVVTGVTAVAQK.T	1929.16446	2	1.89E-08	0.98	5.95	-	1532.9
AHQ-1-12, 2117	K.TKEGVLYVGSK.T	1181.36357	2	5.75E-06	0.83	3.03	-	703.6
AHQ-1-13- , 2177	K.TKEGVVHGVATVAEK.T	1525.73191	2	3.81E-06	0.95	3.94	-	1406.6
AHQ-1-12, 4181 - 4191	K.TKEQVTNVGGAVVTGVTAVAQK.T	2158.44170	3	4.58E-04	0.93	5.06	-	1141.7
AHQ-1-13, 4173	K.TKEQVTNVGGAVVTGVTAVAQK.T	2158.44170	3	8.12E-04	0.86	3.67	-	1112.6
AHQ-1-12, 4175	K.TKEQVTNVGGAVVTGVTAVAQK.T	2158.44170	2	1.49E-05	0.96	4.72	-	1302.9
AHQ-1-13- , 4368	K.TKEQVTNVGGAVVTGVTAVAQK.T	2158.44170	2	1.33E-07	0.98	6.28	-	1645.5
AHQ-1-14- , 4378	K.TKEQVTNVGGAVVTGVTAVAQK.T	2158.44170	2	2.16E-05	0.97	5.72	-	1337.4
AHQ-1-14- , 4372 - 4379	K.TKEQVTNVGGAVVTGVTAVAQK.T	2158.44170	3	1.83E-05	0.92	4.68	-	1107.3
AHQ-1-13, 4175	K.TKEQVTNVGGAVVTGVTAVAQK.T	2158.44170	2	1.50E-06	0.96	4.80	-	1350.8
AHQ-1-12, 4094 - 4101	K.TVEGAGSIAAATGFVK.K	1479.66001	1	8.61E-04	0.48	2.78	-	421.1
gi|4505733|ref|NP_002610.1|	platelet factor 4 (chemokine (C-X-C motif) ligand 4); platelet factor 4			8.34E-11	5.57	60.29	38.60	10844.9
AHQ-1-14- , 4475	K.AGPHCPTAQLIATLK.N	1579.84600	2	7.10E-06	0.55	2.61	-	578.2
AHQ-1-13, 4121 - 4188	K.AGPHCPTAQLIATLK.N	1579.84600	2	8.63E-07	0.93	3.93	-	1026.6
AHQ-1-14- , 4242 - 4308	K.AGPHCPTAQLIATLK.N	1579.84600	2	3.01E-08	0.91	3.78	-	764.3
AHQ-1-14, 5030 - 5048	K.AGPHCPTAQLIATLK.N	1579.84600	2	1.25E-06	0.94	4.06	-	828.4
AHQ-1-13- , 4316	K.AGPHCPTAQLIATLK.N	1579.84600	2	7.01E-04	0.91	3.41	-	924.2
AHQ-1-14- , 4111 - 4115	K.AGPHCPTAQLIATLK.N	1579.84600	2	1.11E-04	0.95	4.23	-	1084.7
AHQ-1-14, 4944 - 4965	K.AGPHCPTAQLIATLKNGR.K	1907.18700	3	8.34E-11	0.97	5.22	-	1698.1
AHQ-1-14, 4948 - 5006	K.AGPHCPTAQLIATLKNGR.K	1907.18700	2	1.50E-06	0.90	4.28	-	638.7
AHQ-1-14- , 3926 - 3935	K.AGPHCPTAQLIATLKNGR.K	1907.18700	3	9.65E-10	0.98	5.83	-	2686.5
AHQ-1-14- , 4059	K.AGPHCPTAQLIATLKNGR.K	1907.18700	2	2.03E-09	0.94	4.51	-	942.5
AHQ-1-14- , 4044	K.AGPHCPTAQLIATLKNGR.K	1907.18700	3	3.62E-08	0.95	4.99	-	1408.6
AHQ-1-14, 4813	K.AGPHCPTAQLIATLKNGR.K	1907.18700	3	4.43E-08	0.94	4.64	-	1303.5
AHQ-1-14, 4808 - 4821	K.AGPHCPTAQLIATLKNGR.K	1907.18700	2	3.30E-10	0.97	4.95	-	1736.2
AHQ-1-14, 4686	K.AGPHCPTAQLIATLKNGRK.I	2035.35992	3	5.86E-06	0.90	3.71	-	1333.5
AHQ-1-14- , 2534 - 2599	R.HITSLEVIK.A	1040.23825	2	1.11E-05	0.88	3.34	-	575.4
AHQ-1-14- , 2658 - 2735	R.HITSLEVIK.A	1040.23825	2	1.33E-06	0.88	3.48	-	469.8
AHQ-1-14, 3584 - 3640	R.HITSLEVIK.A	1040.23825	2	5.61E-05	0.88	3.35	-	568.7
AHQ-1-14, 3470 - 3528	R.HITSLEVIK.A	1040.23825	2	1.82E-04	0.85	2.95	-	554.1
AHQ-1-13, 2715	R.HITSLEVIK.A	1040.23825	2	7.03E-04	0.74	2.97	-	414.6
AHQ-1-13- , 2705	R.HITSLEVIK.A	1040.23825	2	4.97E-05	0.80	2.95	-	534.9
AHQ-1-14, 5812 - 5813	K.ICLDLQAPLYK.K	1335.59400	2	3.29E-05	0.94	4.20	-	1172.2
AHQ-1-14- , 4936	K.ICLDLQAPLYK.K	1335.59400	1	4.66E-04	0.60	2.87	-	532.9
AHQ-1-14- , 4926	K.ICLDLQAPLYK.K	1335.59400	2	1.20E-05	0.95	4.10	-	1414.8
AHQ-1-14- , 5139	K.ICLDLQAPLYK.K	1335.59400	2	4.03E-05	0.93	4.15	-	1185.6
AHQ-1-14- , 4546 - 4606	R.KICLDLQAPLYK.K	1463.76691	2	4.03E-07	0.97	5.19	-	1501.7
AHQ-1-14- , 4602	R.KICLDLQAPLYK.K	1463.76691	1	6.56E-06	0.87	3.75	-	741.0
AHQ-1-14, 5417 - 5482	R.KICLDLQAPLYK.K	1463.76691	2	6.98E-06	0.97	4.90	-	1575.7
AHQ-1-14, 5437 - 5444	R.KICLDLQAPLYK.K	1463.76691	1	4.79E-05	0.83	3.04	-	952.4
AHQ-1-14- , 4560 - 4624	R.KICLDLQAPLYK.K	1463.76691	1	8.48E-04	0.58	2.32	-	865.3
AHQ-1-14- , 4558 - 4559	R.KICLDLQAPLYK.K	1463.76691	3	1.13E-06	0.89	3.69	-	1171.7
gi|5729887|ref|NP_006624.1|	IQ motif containing GTPase activating protein 2 [Homo sapiens]			8.74E-11	10.73	120.27	14.30	180611.0
AHQ-1-3, 4035	K.AWVNQLETQTGEASK.L	1662.78230	2	1.47E-06	0.86	3.64	-	674.1
AHQ-1-5, 5786	K.GVLLDIDDLQTNQFK.N	1719.91690	2	7.23E-06	0.95	4.22	-	1281.7
AHQ-1-3, 7363	K.IGGILANELSVDEAALHAAVIAINEAVEK.G	2932.31842	3	7.11E-04	0.90	4.34	-	931.8
AHQ-1-3, 7130	K.KPEAQLPAVYPFAAAMYQNELFNLQK.Q	2983.43222	3	1.82E-04	0.76	3.65	-	594.4
AHQ-1-3, 5929	K.LGIAPQIQDLLGK.V	1366.63117	2	8.80E-08	0.88	3.53	-	572.1
AHQ-1-3, 4925 - 4937	K.LPYDVTTEQALTYPEVK.N	1968.19269	2	1.14E-09	0.98	4.92	-	2106.5
AHQ-1-5, 4931	K.LPYDVTTEQALTYPEVK.N	1968.19269	2	8.74E-11	0.96	4.52	-	1669.8
AHQ-1-3, 6046	R.NPNAVLTLVDDNLAPEYQK.E	2115.32901	2	5.58E-08	0.91	4.29	-	614.8
AHQ-1-3, 6957	R.NQPGNTLTEILETPATAQQEVDHATDMVSR.A	3268.51558	3	7.51E-08	0.72	3.05	-	510.0
AHQ-1-3, 4577	K.SLIINTNPVEVYK.A	1490.72593	2	1.62E-06	0.92	3.57	-	1115.3
AHQ-1-4, 4263	K.TLVGSENPPLTVIR.K	1496.73370	2	4.64E-06	0.92	3.61	-	1091.9
AHQ-1-3, 4238	K.TLVGSENPPLTVIR.K	1496.73370	2	1.35E-04	0.85	3.83	-	493.0
AHQ-1-5, 4257 - 4259	K.VDQVQDIVTGNPTVIK.M	1726.95257	2	1.99E-04	0.89	3.72	-	975.5
AHQ-1-4, 4355 - 4359	K.VDQVQDIVTGNPTVIK.M	1726.95257	2	1.33E-04	0.93	3.92	-	1219.1
AHQ-1-3, 4223 - 4294	K.VDQVQDIVTGNPTVIK.M	1726.95257	2	2.21E-05	0.96	4.88	-	1357.5
AHQ-1-3, 5974	R.VVAVGYINEAIDEGNPLR.T	1930.15092	2	1.62E-07	0.98	5.32	-	1867.1
gi|6005727|ref|NP_006576.1|	chaperonin containing TCP1, subunit 8 (theta); T-complex protein 1, the			9.31E-11	1.77	20.19	5.70	59662.4
AHQ-1-7, 2709 - 2712	K.AIADTGANVVVTGGK.V	1373.53743	2	9.31E-11	0.92	3.28	-	1198.5
AHQ-1-7, 5424	K.IAVYSCPFDGMITETK.G	1834.10347	2	3.37E-04	0.86	3.72	-	487.9
gi|7661728|ref|NP_054736.1|	HSPC003 protein [Homo sapiens]			1.23E-10	0.93	10.25	21.60	13507.4
AHQ-1-14- , 5571	R.VTAAIASNIWAAYDRNGNQAFNEDNLK.F	2968.18636	3	1.23E-10	0.93	5.07	-	735.1
gi|4504183|ref|NP_000843.1|	glutathione transferase; deafness, X-linked 7; fatty acid ethyl ester s			1.26E-10	9.12	110.29	64.30	23355.7
AHQ-1-11, 5056	K.AFLASPEYVNLPINGNGK.Q	1905.14305	2	8.56E-06	0.83	3.14	-	882.1
AHQ-1-14, 6170	K.AFLASPEYVNLPINGNGKQ	2033.27283	2	6.72E-04	0.64	2.74	-	730.5
AHQ-1-13- , 5549	K.ALPGQLKPFETLLSQNQGGK.T	2127.42930	2	3.71E-05	0.20	2.56	-	234.3
AHQ-1-11, 5026 - 5086	K.ALPGQLKPFETLLSQNQGGK.T	2127.42930	2	2.08E-07	0.91	4.21	-	546.4
AHQ-1-11, 5028 - 5042	K.ALPGQLKPFETLLSQNQGGK.T	2127.42930	3	3.69E-06	0.81	3.75	-	628.6
AHQ-1-11, 5127	K.ALPGQLKPFETLLSQNQGGK.T	2127.42930	2	3.33E-06	0.80	3.48	-	408.9
AHQ-1-11, 3139	K.ASCLYGQLPK.F	1138.31884	1	4.78E-04	0.44	2.45	-	242.1

AHQ-1-11, 3062 - 3135	K.ASCLYGQLPK.F	1138.31884	2	1.38E-06	0.88	2.79	-	969.6
AHQ-1-11, 4962	K.DQQEAALVDM*VNDGVEDLR.C	2134.26783	2	8.37E-06	0.96	4.93	-	1056.4
AHQ-1-11, 4958	K.DQQEAALVDMVNDGVEDLR.C	2118.26843	2	7.57E-09	0.93	4.49	-	730.0
AHQ-1-11, 6202 - 6271	K.DQQEAALVDMVNDGVEDLR.C	2118.26843	2	1.63E-08	0.96	5.26	-	935.9
AHQ-1-11, 5784 - 5852	K.DQQEAALVDMVNDGVEDLR.C	2118.26843	2	1.59E-05	0.92	4.11	-	948.1
AHQ-1-11, 5947 - 6022	K.DQQEAALVDMVNDGVEDLR.C	2118.26843	2	1.26E-10	0.98	5.87	-	1370.1
AHQ-1-11, 4578 - 4644	K.FQDGDLTLYQSNTILR.H	1885.06701	2	7.51E-10	0.92	4.18	-	777.0
AHQ-1-11, 4702 - 4783	K.FQDGDLTLYQSNTILR.H	1885.06701	2	5.15E-09	0.97	5.24	-	1462.6
AHQ-1-14- , 4928 - 4955	K.FQDGDLTLYQSNTILR.H	1885.06701	2	9.37E-09	0.94	4.64	-	895.4
AHQ-1-11, 5726 - 5796	R.M*LLADQGQSWKEEVVTVETWQEGSLK.A	3009.33685	3	2.77E-08	0.95	5.81	-	1100.7
AHQ-1-11, 5866	R.MLLADQGQSWKEEVVTVETWQEGSLK.A	2993.33745	3	4.16E-04	0.68	3.38	-	533.6
AHQ-1-11, 2276	R.TLGLYGK.D	751.89356	1	2.74E-04	0.44	1.97	-	664.0
AHQ-1-11, 4304 - 4331	K.YISLIYTNYEAGKDDYVK.A	2156.37637	2	1.63E-06	0.90	4.09	-	674.2
AHQ-1-13- , 4740	K.YISLIYTNYEAGKDDYVK.A	2156.37637	2	6.66E-09	0.94	4.58	-	805.4
AHQ-1-11, 4420	K.YISLIYTNYEAGKDDYVK.A	2156.37637	2	5.84E-07	0.77	3.25	-	535.2
gi|13899317|ref|NP_113657.1| SH3 domain binding glutamic acid-rich protein like 2 [Homo sapiens] [M				1.28E-10	6.37	70.30	72.00	12326.0
AHQ-1-14- , 5490 - 5492	K.ESNTVFSFLGLKPR.L	1595.82383	2	9.40E-08	0.81	3.42	-	574.6
AHQ-1-13- , 5665	K.ESNTVFSFLGLKPR.L	1595.82383	2	4.39E-08	0.73	3.43	-	394.8
AHQ-1-14- , 4982	R.FLEANKIEFEEVDITM*SEEQR.Q	2574.80223	2	2.06E-04	0.92	4.32	-	919.2
AHQ-1-14, 5865 - 5925	R.FLEANKIEFEEVDITM*SEEQR.Q	2574.80223	3	4.59E-04	0.96	5.08	-	1614.5
AHQ-1-14, 5874	R.FLEANKIEFEEVDITM*SEEQR.Q	2574.80223	2	3.51E-04	0.88	3.41	-	1097.6
AHQ-1-14- , 4974 - 5042	R.FLEANKIEFEEVDITM*SEEQR.Q	2574.80223	3	3.08E-08	0.98	5.95	-	2110.7
AHQ-1-14, 5141 - 5149	K.IEFEEVDITM*SEEQR.Q	1872.00051	2	1.14E-04	0.80	3.20	-	807.2
AHQ-1-14- , 4291	K.IEFEEVDITM*SEEQR.Q	1872.00051	2	1.28E-10	0.96	4.77	-	1161.1
AHQ-1-14, 4700	K.KPTQGNPLPPQIFNGDR.Y	1880.09682	2	2.73E-04	0.90	3.68	-	831.9
AHQ-1-14- , 3756 - 3771	K.KPTQGNPLPPQIFNGDR.Y	1880.09682	2	2.83E-04	0.85	3.32	-	741.0
AHQ-1-14- , 5722 - 5762	K.KPTQGNPLPPQIFNGDRYCGDYDSFFESK.E	3380.64432	3	5.24E-05	0.86	4.13	-	845.2
AHQ-1-14- , 4454 - 4532	R.VFIASSSGFVAIK.K	1326.56551	2	3.80E-07	0.98	4.19	-	2584.6
AHQ-1-14, 5352	R.YCGDYDSFFESK.E	1519.57010	2	1.28E-06	0.96	3.88	-	1111.2
AHQ-1-14- , 4463	R.YCGDYDSFFESK.E	1519.57010	2	7.24E-07	0.96	4.16	-	1185.2
gi|18104989|ref|NP_002822.2| protein tyrosine phosphatase, non-receptor type 6 isoform 1; protein-t				1.32E-10	2.59	30.24	9.40	67560.7
AHQ-1-14- , 6670	K.FATLTELVEYYTQQQGVLQDR.D	2503.74859	3	9.30E-07	0.94	4.71	-	1184.6
AHQ-1-6, 4443 - 4463	R.IQNSGDFYDLYGGEK.F	1706.79040	2	3.92E-05	0.73	3.54	-	333.3
AHQ-1-6, 4111 - 4188	R.IQNSGDFYDLYGGEK.F	1706.79040	2	1.45E-05	0.76	3.35	-	460.3
AHQ-1-6, 7219	R.YTVGGLETFDSLTDLVEHFK.K	2272.49536	2	1.32E-10	0.89	4.36	-	410.9
gi|4502051|ref|NP_000688.1| arachidonate 12-lipoxygenase; 12(S)-lipoxygenase [Homo sapiens]				1.44E-10	7.74	90.21	17.60	75721.8
AHQ-1-6, 4966	R.AAAQLTYCSLCPPDDLADR.G	2140.33708	2	1.41E-08	0.86	3.93	-	346.3
AHQ-1-6, 2258	K.AVSTGGGGHVQLLR.R	1352.52468	2	1.44E-10	0.86	3.34	-	1004.6
AHQ-1-6, 3428	K.GDPELQAWCR.E	1233.33558	2	3.37E-07	0.61	2.78	-	395.2
AHQ-1-6, 5515	R.GLLGLPGALYAHDALR.L	1637.90708	2	2.06E-10	0.93	3.82	-	845.9
AHQ-1-6, 5207	R.ITVQGPGACAEVAFPCYR.W	1999.25659	2	1.24E-09	0.87	3.43	-	893.9
AHQ-1-6, 4634	R.LPGDNALDMFQK.H	1349.53784	2	3.30E-06	0.95	3.82	-	1365.5
AHQ-1-6, 3760	R.LPGDNALDM*FQK.H	1365.53724	2	1.48E-04	0.88	3.20	-	896.6
AHQ-1-6, 5098	R.WVQGEDILSLPEGTAR.L	1771.95190	2	4.96E-05	0.86	3.98	-	595.7
AHQ-1-6, 4995	R.YVEGIVHLFYQR.D	1524.74730	2	7.03E-07	0.92	3.11	-	1154.9
gi|4506925|ref|NP_003013.1| SH3 domain binding glutamic acid-rich protein like; SH3-binding domain				1.46E-10	2.83	30.33	50.00	12774.2
AHQ-1-13- , 6061 - 6135	R.ENNAVYAFLGLTAPPGSK.E	1850.06426	2	3.09E-05	0.92	4.66	-	572.6
AHQ-1-13- , 6145 - 6207	R.ENNAVYAFLGLTAPPGSK.E	1850.06426	2	1.58E-04	0.88	4.05	-	608.4
AHQ-1-13, 4915 - 4980	R.ENVPENSRPATGYPLPPQIFNESQYR.G	3005.24660	3	8.54E-08	0.89	4.43	-	713.9
AHQ-1-13- , 5291	R.ENVPENSRPATGYPLPPQIFNESQYR.G	3005.24660	3	4.19E-10	0.93	4.77	-	1007.9
AHQ-1-13- , 5068 - 5147	R.ENVPENSRPATGYPLPPQIFNESQYR.G	3005.24660	3	1.46E-10	0.97	6.52	-	1275.5
AHQ-1-13- , 5159 - 5227	R.ENVPENSRPATGYPLPPQIFNESQYR.G	3005.24660	3	4.75E-08	0.96	5.68	-	1277.1
AHQ-1-13, 4799 - 4859	R.ENVPENSRPATGYPLPPQIFNESQYR.G	3005.24660	3	1.87E-05	0.94	4.79	-	1174.9
AHQ-1-13- , 2432 - 2435	R.VYIASSSGSTAIK.K	1284.44065	2	1.50E-05	0.94	4.20	-	1512.8
AHQ-1-13- , 2524	R.VYIASSSGSTAIK.K	1284.44065	2	2.82E-05	0.92	3.73	-	1559.7
gi|21618359|ref|NP_004095.3| fatty acid synthase [Homo sapiens]				1.68E-10	1.81	20.31	1.60	273262.0
AHQ-1-1, 6439	R.LHLSGIDANPNALFPPVEFPAPR.G	2473.81368	2	1.99E-07	0.82	3.30	-	462.3
AHQ-1-2, 7122	K.LPEDPLLSGLLDSPALK.A	1779.06739	2	1.68E-10	0.98	6.24	-	2232.4
AHQ-1-3, 6937 - 6959	K.LPEDPLLSGLLDSPALK.A	1779.06739	2	1.68E-05	0.97	5.08	-	1556.8
AHQ-1-1, 6811 - 6817	K.LPEDPLLSGLLDSPALK.A	1779.06739	2	4.00E-09	0.98	4.86	-	1931.8
gi|4506365|ref|NP_002856.1| RAB2, member RAS oncogene family [Homo sapiens]				1.88E-10	0.96	10.18	6.10	23545.4
AHQ-1-14, 5386	K.LQIWDTAGQESFR.S	1551.68478	2	2.30E-04	0.65	2.56	-	352.6
AHQ-1-11, 4218	K.LQIWDTAGQESFR.S	1551.68478	2	1.88E-10	0.96	3.56	-	1765.5
gi|4758304|ref|NP_004902.1| protein disulfide isomerase related protein (calcium-binding protein, i				1.93E-10	6.51	80.22	16.90	72931.9
AHQ-1-6, 4720	K.DLGLSESGEDVNAAILDESGKK.F	2248.38737	2	1.93E-10	0.95	4.42	-	1236.8
AHQ-1-6, 6166 - 6170	R.EVSQPDWTPPPEVTLVLTK.E	2137.41772	2	7.87E-04	0.39	3.19	-	220.1
AHQ-1-6, 4707	K.FAMEPEEFDSDTLR.E	1687.80890	2	1.44E-08	0.86	3.46	-	617.6
AHQ-1-6, 3686	R.FDVSGYPTIK.I	1127.27123	1	1.52E-04	0.26	2.10	-	437.8
AHQ-1-6, 3688	R.FDVSGYPTIK.I	1127.27123	2	6.40E-05	0.90	2.99	-	1162.7
AHQ-1-6, 3779	R.FDVSGYPTLK.I	1127.27123	2	2.54E-06	0.82	2.57	-	920.0
AHQ-1-6, 2882	K.IDATSASVLASR.F	1191.31686	2	1.76E-06	0.93	3.48	-	1278.9
AHQ-1-6, 1970 - 2034	K.RSPPIPLAK.V	979.20128	2	3.39E-04	0.81	2.52	-	654.7
AHQ-1-6, 2927	K.VSQGQLVVMQPEK.F	1443.69431	2	6.46E-05	0.85	2.68	-	1260.7
gi|12025678|ref|NP_004915.2| actinin, alpha 4 [Homo sapiens]				1.96E-10	10.60	130.27	22.30	104853.3
AHQ-1-5, 3462	R.ASFNHFDKDHGGALGPEEFK.A	2204.34290	2	1.88E-08	0.66	2.94	-	525.3
AHQ-1-5, 2077	R.EAILAIHK.E	895.08114	2	1.85E-04	0.89	2.94	-	952.0
AHQ-1-5, 2086	R.EAILAIHK.E	895.08114	1	4.54E-05	0.49	1.94	-	345.2
AHQ-1-6, 4724	R.ETTDTDTADQVIASFK.V	1742.81936	2	1.05E-05	0.90	3.18	-	1425.5
AHQ-1-4, 4908	R.ETTDTDTADQVIASFK.V	1742.81936	2	1.40E-07	0.95	4.53	-	1362.1
AHQ-1-14, 5558 - 5617	R.ETTDTDTADQVIASFK.V	1742.81936	2	7.34E-07	0.84	3.29	-	925.6
AHQ-1-5, 4811	R.ETTDTDTADQVIASFK.V	1742.81936	2	1.21E-07	0.91	4.20	-	1023.7
AHQ-1-5, 2159	K.GISQEQM*QEFR.A	1369.48598	2	5.66E-04	0.47	2.70	-	559.5
AHQ-1-5, 2445 - 2453	K.HRDYETATLSDIK.A	1549.66719	2	2.48E-04	0.92	3.65	-	891.3
AHQ-1-5, 4901	K.ICDQWDALGSLTHSR.R	1760.90926	2	5.57E-10	0.93	4.10	-	1009.3
AHQ-1-5, 3819	R.KDDPVTNLNNAFEVAEK.Y	1905.05509	2	3.69E-09	0.91	3.73	-	917.4
AHQ-1-5, 4443	R.KDDPVTNLNNAFEVAEK.Y	1905.05509	2	1.93E-05	0.97	5.36	-	1144.9
AHQ-1-4, 4104	R.M*APYQGPDAVPGALDYK.S	1810.02037	2	1.67E-05	0.87	3.30	-	752.2
AHQ-1-5, 4286	R.MAPYQGPDAVPGALDYK.S	1794.02097	2	4.78E-05	0.90	4.10	-	669.5
AHQ-1-5, 4217	K.MLDAEDIVNTARPDEK.A	1817.99924	2	3.95E-06	0.90	3.85	-	883.6
AHQ-1-5, 4095	K.MVSDINNGWQHLEQAEK.G	2000.18160	2	1.96E-10	0.90	4.27	-	593.5
AHQ-1-4, 7290	R.SIVDYKPNLDLLEQQHQLIQEALIFDNK.H	3326.74423	3	5.70E-04	0.83	4.30	-	664.0
AHQ-1-4, 6807	R.VEQIAAIAQELNELDYYDSHNVNTR.C	2907.09810	3	8.15E-05	0.83	4.08	-	518.0
AHQ-1-5, 6717 - 6729	R.VEQIAAIAQELNELDYYDSHNVNTR.C	2907.09810	3	2.73E-10	0.91	4.75	-	944.9
gi|21361370|ref|NP_002853.2| phosphorylase, glycogen; brain [Homo sapiens]				1.99E-10	5.67	60.24	10.60	96695.3
AHQ-1-5, 3813	R.GLAGLGDVAEVR.K	1157.30210	2	8.89E-06	0.96	4.39	-	1481.1
AHQ-1-5, 3635 - 3662	R.HLDHVAALFPGDVDR.L	1662.83036	2	7.76E-04	0.97	4.33	-	1936.3
AHQ-1-5, 6058 - 6059	K.LLPLVSDEVFIR.D	1401.67552	2	1.99E-10	0.90	3.47	-	647.1
AHQ-1-5, 7419	K.LQDFNVGDYIEAVLDR.N	1868.03645	2	2.59E-09	0.96	4.69	-	1199.0
AHQ-1-5, 4313	K.LVTSIGDVVNHDPVVGDR.L	1893.09067	2	1.25E-08	0.92	4.16	-	695.2
AHQ-1-5, 6769 - 6830	R.WLLLCNPGLADTIVEK.I	1844.16436	2	2.74E-05	0.96	4.83	-	1385.1
gi|28269681|ref|NP_783313.1| NADH-ubiquinone oxidoreductase subunit B14.7 [Homo sapiens]				2.07E-10	1.35	20.28	27.00	14852.0
AHQ-1-13- , 6708	R.EKPDDPLNYFLGGCAGGLTLGAR.T	2423.68676	3	2.07E-10	0.96	5.67	-	1524.3
AHQ-1-14- , 6463	R.EKPDDPLNYFLGGCAGGLTLGAR.T	2423.68676	3	3.87E-05	0.94	4.25	-	1747.5
AHQ-1-13, 4800 - 4867	R.VTLNPPGTFLEGVAK.V	1543.78883	2	1.72E-04	0.39	2.51	-	273.2
gi|9257257|ref|NP_059830.1| WD repeat-containing protein 1 isoform 1 [Homo sapiens]				2.12E-10	1.73	20.24	7.90	66193.1
AHQ-1-10, 3935	R.LHHVSSLAWLDEHTLVTTSHDASVK.E	2785.06406	3	2.11E-06	0.91	4.09	-	1105.6
AHQ-1-6, 4203	R.LHHVSSLAWLDEHTLVTTSHDASVK.E	2785.06406	3	2.12E-10	0.94	4.74	-	1119.4

AHQ-1-6, 5087	K.VVTVFSVADGYSENNVFYGHHAK.I	2541.75861	3	3.91E-08	0.78	3.36	-	695.9
gi|4507383|ref|NP_001052.1| thromboxane A synthase 1 (platelet, cytochrome P450, family 5, subfamil				2.12E-10	7.17	90.23	21.50	60675.3
AHQ-1-7, 2414	R.DVFSSTGCKPNPSR.Q	1553.67913	2	3.87E-08	0.56	2.82	-	501.0
AHQ-1-7, 2477	R.EAAQDCEVLGQR.I	1377.46310	2	1.98E-04	0.86	3.61	-	1050.8
AHQ-1-7, 4476 - 4556	R.FQACPETQVPLQLESK.S	1877.10807	2	2.12E-10	0.96	4.50	-	1541.9
AHQ-1-10, 4033	R.FQACPETQVPLQLESK.S	1877.10807	2	1.54E-05	0.96	4.45	-	1276.9
AHQ-1-7, 3466	R.GALMSAFSPEK.L	1138.31906	1	1.08E-04	0.37	2.32	-	601.6
AHQ-1-7, 4101	R.HSASPMGVQDFDIVR.D	1659.84828	2	4.75E-06	0.95	3.98	-	1257.6
AHQ-1-7, 5273	K.LYGPLCGYYLGR.R	1433.65622	2	1.09E-05	0.89	2.92	-	1061.1
AHQ-1-7, 3544	K.NRDELNGFFNK.L	1354.45276	2	5.49E-04	0.87	3.17	-	888.9
AHQ-1-14- , 5974	K.PSPFIGNLTFFR.Q	1396.61752	2	2.50E-06	0.76	2.62	-	566.3
AHQ-1-7, 3092	R.YAESGDAFDIQR.C	1372.42172	2	4.83E-10	0.95	4.06	-	1383.6
gi|7661818|ref|NP_054900.1| HSPC159 protein [Homo sapiens]				2.16E-10	4.29	50.27	43.00	19011.6
AHQ-1-13, 6379	R.GEEQSAIPYFPFIPDQPFR.V	2239.47029	2	1.89E-05	0.84	3.91	-	279.2
AHQ-1-13- , 6835 - 6836	R.GEEQSAIPYFPFIPDQPFR.V	2239.47029	2	1.62E-05	0.84	3.58	-	384.7
AHQ-1-14- , 6586	R.GEEQSAIPYFPFIPDQPFR.V	2239.47029	2	3.99E-05	0.92	4.61	-	355.7
AHQ-1-12, 6502	R.GEEQSAIPYFPFIPDQPFR.V	2239.47029	2	4.19E-06	0.82	3.60	-	308.1
AHQ-1-11, 6310	R.GEEQSAIPYFPFIPDQPFR.V	2239.47029	2	2.15E-04	0.92	4.42	-	418.9
AHQ-1-13- , 3873 - 3884	R.IQTLSAIDTIK.I	1203.41068	2	7.81E-04	0.80	3.10	-	578.5
AHQ-1-11, 4623	K.LDDGHLNNSLSSPVQADVYFPR.L	2445.62923	2	2.10E-06	0.79	3.34	-	721.2
AHQ-1-11, 4736 - 4796	K.LDDGHLNNSLSSPVQADVYFPR.L	2445.62923	2	1.24E-06	0.97	5.34	-	1595.8
AHQ-1-13- , 6711	R.NSCISGERGEEQSAIPYFPFIPDQPFR.V	3144.41922	3	3.18E-04	0.67	3.37	-	421.9
AHQ-1-13- , 6844	R.NSCISGERGEEQSAIPYFPFIPDQPFR.V	3144.41922	3	1.14E-05	0.79	3.55	-	671.2
AHQ-1-11, 4594	R.VFVDGHQLFDFYHR.I	1780.96540	2	6.45E-10	0.96	4.62	-	1653.9
AHQ-1-11, 4704	R.VFVDGHQLFDFYHR.I	1780.96540	2	2.16E-10	0.91	3.47	-	1112.2
gi|21464101|ref|NP_036611.2| tyrosine 3-monooxygenase/tryptophan 5-monooxygenase activation protein				2.18E-10	3.75	40.31	29.60	28302.4
AHQ-1-10, 6279	R.LGLALNYSVFYYEIQNAPEQACHLAK.T	3015.38773	3	2.18E-10	0.96	6.15	-	1136.4
AHQ-1-10, 3963 - 4036	R.NLLSVAYKNVVGAR.R	1504.75906	2	6.34E-06	0.95	4.18	-	1253.1
AHQ-1-10, 3768	R.NLLSVAYKNVVGAR.R	1504.75906	2	4.51E-07	0.92	3.31	-	1373.3
AHQ-1-13- , 3128	K.NVTELNEPLSNEER.N	1644.72215	2	9.25E-08	0.94	4.49	-	864.3
AHQ-1-14- , 3083	K.NVTELNEPLSNEER.N	1644.72215	2	7.71E-07	0.93	3.94	-	907.8
AHQ-1-10, 2876	K.NVTELNEPLSNEER.N	1644.72215	2	5.48E-04	0.91	3.53	-	875.1
AHQ-1-11, 2966 - 2968	K.NVTELNEPLSNEER.N	1644.72215	2	4.12E-04	0.81	3.03	-	682.5
AHQ-1-14, 3952	K.NVTELNEPLSNEER.N	1644.72215	2	5.85E-04	0.86	3.81	-	489.4
AHQ-1-10, 6012 - 6075	K.TAFDDAIAELDTLNEDSYK.D	2132.22370	2	4.15E-05	0.64	3.36	-	627.1
AHQ-1-13- , 6668 - 6681	K.TAFDDAIAELDTLNEDSYK.D	2132.22370	2	3.40E-05	0.87	3.99	-	808.6
AHQ-1-10, 5840	K.TAFDDAIAELDTLNEDSYK.D	2132.22370	2	2.14E-09	0.83	3.91	-	656.4
AHQ-1-14- , 6451 - 6452	K.TAFDDAIAELDTLNEDSYK.D	2132.22370	2	7.27E-06	0.93	4.23	-	1005.6
AHQ-1-13, 6229 - 6251	K.TAFDDAIAELDTLNEDSYK.D	2132.22370	2	3.87E-10	0.93	3.94	-	1073.9
gi|30159310|ref|XP_301899.1| similar to beta-actin [Homo sapiens]				2.32E-10	1.46	20.18	9.20	32376.4
AHQ-1-8, 1475 - 2015	K.DSYVGNEAQSK.R	1198.22158	1	2.32E-10	0.59	2.71	-	357.5
AHQ-1-8, 1705	K.DSYVGNEAQSK.R	1198.22158	2	6.24E-06	0.72	3.27	-	532.9
AHQ-1-8, 1801 - 1873	K.DSYVGNEAQSK.R	1198.22158	2	2.56E-05	0.72	3.08	-	664.4
AHQ-1-13- , 1661 - 1737	K.DSYVGNEAQSK.R	1198.22158	2	8.15E-04	0.49	2.88	-	428.4
AHQ-1-13, 1819 - 1877	K.DSYVGNEAQSK.R	1198.22158	1	9.33E-07	0.38	2.17	-	406.8
AHQ-1-9, 1542 - 1602	K.DSYVGNEAQSK.R	1198.22158	1	5.21E-08	0.71	2.80	-	512.5
AHQ-1-9, 1856	K.DSYVGNEAQSK.R	1198.22158	1	1.17E-08	0.56	2.58	-	405.7
AHQ-1-13- , 1705	K.DSYVGNEAQSK.R	1198.22158	1	4.05E-04	0.05	1.81	-	209.2
AHQ-1-12, 1638 - 1702	K.DSYVGNEAQSK.R	1198.22158	2	2.30E-06	0.64	3.00	-	597.1
AHQ-1-12, 1707 - 1846	K.DSYVGNEAQSK.R	1198.22158	1	1.26E-08	0.42	2.49	-	338.2
AHQ-1-12, 1757 - 1821	K.DSYVGNEAQSK.R	1198.22158	2	3.47E-06	0.56	3.10	-	500.5
AHQ-1-8, 4723 - 4795	K.SYKLLDGQVITIGNER.F	1807.04092	2	1.22E-05	0.80	3.41	-	679.7
AHQ-1-9, 4570 - 4575	K.SYKLLDGQVITIGNER.F	1807.04092	2	4.23E-04	0.72	2.96	-	541.6
AHQ-1-8, 4892 - 4971	K.SYKLLDGQVITIGNER.F	1807.04092	2	2.25E-06	0.87	3.49	-	724.1
gi|4507745|ref|NP_003320.1| thioredoxin [Homo sapiens]				2.33E-10	0.93	10.17	12.40	11693.3
AHQ-1-13- , 4000	K.TAFQEALDAAGDK.L	1337.41728	1	7.56E-06	0.53	2.10	-	916.3
AHQ-1-14- , 3891 - 3898	K.TAFQEALDAAGDK.L	1337.41728	2	2.33E-10	0.93	3.48	-	1470.7
AHQ-1-14- , 3899	K.TAFQEALDAAGDK.L	1337.41728	1	3.57E-05	0.79	2.88	-	1010.6
AHQ-1-14, 4788	K.TAFQEALDAAGDK.L	1337.41728	2	6.39E-06	0.92	3.39	-	1275.0
gi|20127528|ref|NP_037459.2| parvin, beta; CGI-56 protein; beta-parvin [Homo sapiens]				2.41E-10	5.13	60.26	21.70	41714.0
AHQ-1-14- , 3544	K.ARPEDVVNLDLK.S	1369.54878	2	3.72E-04	0.94	4.26	-	998.1
AHQ-1-9, 3287 - 3290	K.ARPEDVVNLDLK.S	1369.54878	2	1.34E-05	0.93	4.14	-	875.9
AHQ-1-9, 3294	K.ARPEDVVNLDLK.S	1369.54878	1	3.93E-05	0.41	2.35	-	298.4
AHQ-1-9, 3306	K.ARPEDVVNLDLK.S	1369.54878	1	1.08E-06	0.67	3.12	-	318.0
AHQ-1-13, 3482	K.ARPEDVVNLDLK.S	1369.54878	2	1.86E-04	0.95	4.23	-	1216.2
AHQ-1-10, 3244	K.ARPEDVVNLDLK.S	1369.54878	2	6.54E-05	0.93	3.94	-	937.5
AHQ-1-13- , 3587	K.ARPEDVVNLDLK.S	1369.54878	2	1.35E-05	0.88	3.72	-	762.1
AHQ-1-14, 4438	K.ARPEDVVNLDLK.S	1369.54878	2	1.93E-05	0.93	3.99	-	1068.9
AHQ-1-9, 5438	R.DAFDTLFDHAPDKLSVVK.K	2019.24293	2	3.17E-05	0.83	3.63	-	667.2
AHQ-1-9, 3703	K.LNVAEVTQSEIGQK.Q	1516.67865	2	4.65E-06	0.96	4.75	-	1262.2
AHQ-1-13, 3748	K.LNVAEVTQSEIGQK.Q	1516.67865	2	1.08E-05	0.83	3.20	-	998.5
AHQ-1-13- , 3888	K.LNVAEVTQSEIGQK.Q	1516.67865	2	1.98E-08	0.95	4.27	-	1351.4
AHQ-1-9, 3464	R.LPEHVTVQVVVVR.K	1475.76086	2	5.40E-05	0.90	3.75	-	895.7
AHQ-1-9, 3223	K.SLITFVNK.H	922.10336	2	2.92E-05	0.83	2.60	-	781.5
AHQ-1-9, 6375 - 6444	K.VLLDWINDVLVEER.I	1713.95549	2	3.26E-08	0.90	4.11	-	795.5
AHQ-1-9, 7202 - 7220	K.VLLDWINDVLVEER.I	1713.95549	2	2.41E-10	0.95	5.14	-	1136.3
AHQ-1-9, 7464	K.VLLDWINDVLVEER.I	1713.95549	2	7.22E-04	0.59	3.01	-	664.4
gi|4758442|ref|NP_004115.1| glia maturation factor, beta [Homo sapiens]				2.47E-10	2.33	30.25	35.20	16713.1
AHQ-1-12, 5954	R.LVVLDEELEGISPDELKDELPER.Q	2638.90589	3	2.47E-10	0.96	5.06	-	1717.9
AHQ-1-12, 6257 - 6318	R.VSYPLCFIFSSPVGCKPEQQM*MYAGSK.N	3131.61197	3	7.04E-08	0.62	3.45	-	688.2
AHQ-1-12, 6101	R.VSYPLCFIFSSPVGCKPEQQM*M*YAGSK.N	3147.61137	3	1.40E-05	0.75	3.41	-	626.1
gi|4557361|ref|NP_001187.1| BH3 interacting domain death agonist; BH3-interacting domain death agon				2.52E-10	2.76	30.23	28.70	21994.6
AHQ-1-11, 5836	R.DVFHTTVNFINQNLR.T	1819.01348	2	1.98E-05	0.90	3.99	-	794.8
AHQ-1-11, 5880	R.ELDALGHELPVLAPQWEGYDELQTDGNR.S	3167.38790	3	6.27E-05	0.89	4.24	-	824.9
AHQ-1-11, 2715	R.IEADSESQEDIIR.N	1505.56635	2	2.52E-10	0.97	4.54	-	2071.1
gi|12707570|ref|NP_004083.2| mitochondrial short-chain enoyl-coenzyme A hydratase 1 precursor [Homo				2.61E-10	1.50	20.21	13.10	31371.2
AHQ-1-10, 4925	K.AQFAQPEILIGTIPGAGGTQR.L	2126.40140	2	2.94E-04	0.55	2.58	-	571.6
AHQ-1-10, 6400	K.ICPVETLVEEAIQCAEK.I	1992.25744	2	2.61E-10	0.96	4.23	-	1372.5
gi|27480104|ref|XP_208215.1| similar to triosephosphate isomerase 1 [Homo sapiens]				2.67E-10	5.27	60.24	27.70	26942.6
AHQ-1-13, 3070	R.HVFGESDELIGQK.V	1459.58565	2	4.36E-05	0.94	4.03	-	818.3
AHQ-1-10, 2816 - 2843	R.HVFGESDELIGQK.V	1459.58565	2	2.67E-10	0.96	4.73	-	839.3
AHQ-1-13- , 3113	R.HVFGESDELIGQK.V	1459.58565	2	6.95E-09	0.96	4.71	-	791.0
AHQ-1-13- , 2835	R.IIYGGSVTGATCK.E	1328.51672	2	2.13E-08	0.90	3.77	-	585.1
AHQ-1-10, 2625	R.IIYGGSVTGATCK.E	1328.51672	1	5.64E-05	0.42	2.57	-	335.1
AHQ-1-10, 2611	R.IIYGGSVTGATCK.E	1328.51672	2	1.34E-04	0.90	3.33	-	798.1
AHQ-1-13, 2826 - 2829	R.IIYGGSVTGATCK.E	1328.51672	2	2.87E-07	0.88	3.77	-	589.3
AHQ-1-13- , 2168 - 2236	K.SNVSDAVAQSTR.I	1235.28659	2	8.87E-04	0.88	3.26	-	1072.8
AHQ-1-10, 2039	K.SNVSDAVAQSTR.I	1235.28659	2	9.42E-06	0.96	3.78	-	2283.7
AHQ-1-10, 3971 - 4015	K.VTNGAFTGEISPGMIK.D	1622.86786	2	5.00E-06	0.83	3.34	-	818.4
AHQ-1-10, 3512 - 3571	K.VTNGAFTGEISPGM*IK.D	1638.86726	2	2.60E-07	0.75	3.03	-	700.7
AHQ-1-10, 3997	K.VTNGAFTGEISPGM*IK.D	1638.86726	2	2.79E-05	0.60	2.58	-	620.8
AHQ-1-10, 4936 - 5016	K.VVLAYEPVWAIGTGK.T	1603.88587	2	1.13E-05	0.87	3.51	-	775.0
gi|20357552|ref|NP_005222.2| cortactin isoform a; oncogene EMS1 [Homo sapiens]				2.80E-10	1.90	20.30	8.00	61585.7
AHQ-1-14- , 4707	K.ASAGHAVSIAQDDAGADDWETDPDFVNDVSEK.E	3334.37643	3	2.64E-06	0.93	5.03	-	823.8
AHQ-1-14- , 4851 - 4922	K.ASAGHAVSIAQDDAGADDWETDPDFVNDVSEK.E	3334.37643	3	1.27E-04	0.90	4.78	-	582.4
AHQ-1-14- , 4992	K.ASAGHAVSIAQDDAGADDWETDPDFVNDVSEK.E	3334.37643	3	2.80E-10	0.95	5.98	-	789.3
AHQ-1-12, 5198 - 5257	R.YGLFPANYVELR.Q	1442.64306	2	9.98E-10	0.94	4.32	-	681.7

gi|30150448|ref|XP_293066.2| similar to Stress-70 protein, mitochondrial precursor (75 kDa glucose				2.81E-10	0.97	10.29	2.90	58788.2
AHQ-1-6, 4686 - 4696	R.VINEPTAAALAYGLDK.S	1646.86592	2	2.81E-10	0.97	5.73	-	834.3
gi|4503987|ref|NP_003869.1| gamma-glutamyl hydrolase precursor; conjugase; folylpolygammaglutamyl h				3.22E-10	0.93	10.22	4.10	35964.1
AHQ-1-14- , 5311	R.LDLTEKDYEILFK.S	1627.85937	2	3.22E-10	0.93	4.44	-	869.6
gi|4757900|ref|NP_004334.1| calreticulin precursor; Sicca syndrome antigen A (autoantigen Ro; calre				3.22E-10	9.15	110.29	41.20	48141.1
AHQ-1-7, 6117 - 6185	R.CKDDEFTHLYTLIVRPDNTYEVK.I	2859.15977	3	1.32E-04	0.97	5.81	-	1484.9
AHQ-1-7, 6126 - 6138	R.CKDDEFTHLYTLIVRPDNTYEVK.I	2859.15977	2	1.01E-06	0.86	3.17	-	980.5
AHQ-1-14- , 3958	K.EQFLDGDGWTSR.W	1411.45795	2	4.12E-05	0.85	2.91	-	1042.1
AHQ-1-14, 6413 - 6448	R.FYALSASFEPFSNK.G	1608.77470	2	7.44E-04	0.85	3.12	-	1036.9
AHQ-1-7, 5581 - 5652	R.FYALSASFEPFSNK.G	1608.77470	2	2.78E-08	0.97	5.25	-	1595.2
AHQ-1-7, 3690	K.IDDPTDSKPEDWDKPEHIPDPDAK.K	2761.89139	3	1.69E-04	0.79	3.98	-	750.6
AHQ-1-7, 3693	K.IDDPTDSKPEDWDKPEHIPDPDAK.K	2761.89139	2	6.61E-06	0.44	2.80	-	353.0
AHQ-1-7, 5308 - 5381	K.IDNSQVESGSLEDDWDFLPPKK.I	2520.68965	2	4.80E-09	0.80	3.35	-	594.1
AHQ-1-7, 2432	K.IKDPDASKPEDWDER.A	1801.89173	2	2.62E-06	0.93	3.79	-	980.7
AHQ-1-7, 5638	K.KPEDWDEEMDGEWEPPVIQNPEYK.G	2962.14926	3	2.09E-06	0.87	4.27	-	767.6
AHQ-1-7, 5636	K.KPEDWDEEMDGEWEPPVIQNPEYK.G	2962.14926	2	4.88E-09	0.92	4.40	-	587.4
AHQ-1-7, 5026	K.KPEDWDEEM*DGEWEPPVIQNPEYK.G	2978.14866	3	3.40E-06	0.83	4.01	-	660.8
AHQ-1-7, 2262	K.KVHVIFNYK.G	1148.38164	2	1.74E-05	0.84	2.90	-	720.6
AHQ-1-7, 2254	K.KVHVIFNYK.G	1148.38164	1	2.82E-06	0.80	2.92	-	564.0
AHQ-1-7, 7448	K.SGTIFDNFLITNDEAYAEEFGNETWGVTK.A	3270.46022	3	3.22E-10	0.97	5.89	-	1790.0
AHQ-1-7, 2670	K.VHVIFNYK.G	1020.20872	1	1.95E-06	0.76	2.78	-	746.9
gi|17921989|ref|NP_005991.1| tubulin, alpha 1; testis-specific alpha tubulin; tubulin alpha-1 chain				3.30E-10	13.63	170.29	46.90	49924.1
AHQ-1-13- , 4747 - 4812	R.AFVHWYVGEGM*EEGEFSEAR.E	2347.50435	3	8.13E-04	0.97	5.10	-	2323.8
AHQ-1-7, 4754	R.AFVHWYVGEGM*EEGEFSEAR.E	2347.50435	3	2.02E-05	0.93	3.67	-	1470.7
AHQ-1-14- , 4667 - 4739	R.AFVHWYVGEGM*EEGEFSEAR.E	2347.50435	3	5.12E-05	0.93	4.39	-	1610.2
AHQ-1-13, 5060 - 5125	R.AVCM*LSNTTAIAEAWAR.L	1883.13932	2	1.25E-04	0.95	3.97	-	1592.4
AHQ-1-12, 5049 - 5105	R.AVCM*LSNTTAIAEAWAR.L	1883.13932	2	6.30E-08	0.92	3.92	-	1289.5
AHQ-1-13- , 6167 - 6245	R.AVCMLSNTTAIAEAWAR.L	1867.13992	2	7.71E-07	0.96	4.66	-	1709.5
AHQ-1-14- , 5960 - 5980	R.AVCMLSNTTAIAEAWAR.L	1867.13992	2	2.66E-05	0.93	3.81	-	1445.7
AHQ-1-13- , 5583 - 5592	R.AVCM*LSNTTAIAEAWAR.L	1883.13932	2	5.39E-05	0.96	4.21	-	1936.7
AHQ-1-13- , 5348	R.AVCM*LSNTTAIAEAWAR.L	1883.13932	2	3.26E-06	0.94	3.97	-	1231.5
AHQ-1-7, 6084	R.AVCMLSNTTAIAEAWAR.L	1867.13992	2	6.23E-05	0.92	3.82	-	1182.6
AHQ-1-7, 6512	R.AVCMLSNTTAIAEAWAR.L	1867.13992	2	2.40E-05	0.77	2.86	-	1084.9
AHQ-1-7, 5772	R.AVCM*LSNTTAIAEAWAR.L	1883.13932	2	4.41E-10	0.95	4.40	-	1192.4
AHQ-1-13- , 5840	R.AVCMLSNTTAIAEAWAR.L	1867.13992	2	2.61E-05	0.85	3.17	-	934.5
AHQ-1-7, 6377 - 6444	R.AVCMLSNTTAIAEAWAR.L	1867.13992	2	4.00E-08	0.95	4.38	-	1404.1
AHQ-1-14, 6117	R.AVCM*LSNTTAIAEAWAR.L	1883.13932	2	1.50E-05	0.74	3.13	-	680.0
AHQ-1-13, 5849 - 5908	R.AVCMLSNTTAIAEAWAR.L	1867.13992	2	6.55E-05	0.62	2.79	-	529.6
AHQ-1-12, 5839	R.AVCMLSNTTAIAEAWAR.L	1867.13992	3	9.87E-06	0.96	4.25	-	2420.9
AHQ-1-3, 6355	R.AVFVDLEPTVIDEIR.N	1716.95615	2	1.52E-04	0.87	2.92	-	1272.4
AHQ-1-9, 6234 - 6235	R.AVFVDLEPTVIDEIR.N	1716.95615	2	4.13E-07	0.92	4.20	-	1242.3
AHQ-1-12, 5863	R.AVFVDLEPTVIDEIR.N	1716.95615	2	3.99E-05	0.78	3.24	-	826.0
AHQ-1-7, 6384 - 6409	R.AVFVDLEPTVIDEIR.N	1716.95615	2	1.96E-09	0.86	3.15	-	1201.8
AHQ-1-13, 5793	R.AVFVDLEPTVIDEIR.N	1716.95615	2	1.19E-07	0.65	2.65	-	936.4
AHQ-1-7, 5709 - 5770	K.AYHEQLSVAEITNACFEPANQMVK.C	2753.06070	2	2.13E-04	0.92	4.17	-	879.0
AHQ-1-7, 5564	K.AYHEQLSVAEITNACFEPANQMVK.C	2753.06070	2	2.35E-07	0.97	5.77	-	1269.3
AHQ-1-7, 5865	K.AYHEQLSVAEITNACFEPANQMVK.C	2753.06070	2	1.36E-07	0.91	3.90	-	887.2
AHQ-1-7, 3640	K.DVNAAIAAIK.T	986.14745	1	6.27E-05	0.93	3.27	-	1291.7
AHQ-1-12, 3353	K.DVNAAIAAIK.T	986.14745	2	1.91E-04	0.87	3.10	-	963.9
AHQ-1-7, 3421 - 3433	K.DVNAAIAAIK.T	986.14745	2	3.02E-05	0.93	3.98	-	1068.2
AHQ-1-12, 3029	K.DVNAAIAAIK.T	986.14745	1	1.51E-04	0.49	1.80	-	815.7
AHQ-1-7, 3096 - 3169	K.DVNAAIAAIK.T	986.14745	1	3.71E-04	0.71	2.36	-	890.7
AHQ-1-7, 1477	K.EDAANNYAR.G	1024.02636	1	1.50E-04	0.14	2.11	-	241.1
AHQ-1-12, 5047	R.IHFPLATYAPVISAEK.A	1758.05310	2	7.05E-05	0.79	3.31	-	450.7
AHQ-1-11, 4834	R.IHFPLATYAPVISAEK.A	1758.05310	2	4.68E-04	0.93	3.93	-	754.4
AHQ-1-7, 5128 - 5196	R.IHFPLATYAPVISAEK.A	1758.05310	2	3.04E-06	0.96	4.58	-	881.1
AHQ-1-10, 4643 - 4700	R.IHFPLATYAPVISAEK.A	1758.05310	2	9.05E-04	0.82	3.40	-	480.0
AHQ-1-9, 4947 - 5023	R.IHFPLATYAPVISAEK.A	1758.05310	2	1.55E-06	0.87	3.61	-	503.6
AHQ-1-13, 4976 - 5040	R.IHFPLATYAPVISAEK.A	1758.05310	2	4.61E-05	0.65	2.83	-	426.0
AHQ-1-7, 5224 - 5264	R.IHFPLATYAPVISAEK.A	1758.05310	2	7.26E-10	0.96	4.80	-	810.5
AHQ-1-7, 3052	R.LDHKFDLMYAK.R	1381.62467	2	5.72E-04	0.74	3.07	-	553.5
AHQ-1-7, 7088 - 7164	R.LISQIVSSITASLR.F	1488.75471	2	5.18E-09	0.97	4.98	-	1212.8
AHQ-1-7, 7225 - 7297	R.LISQIVSSITASLR.F	1488.75471	2	8.79E-08	0.95	4.70	-	1043.7
AHQ-1-14- , 6556	R.LISQIVSSITASLR.F	1488.75471	2	1.80E-08	0.95	4.64	-	1072.4
AHQ-1-9, 6934	R.LISQIVSSITASLR.F	1488.75471	2	2.90E-06	0.93	4.07	-	1087.5
AHQ-1-13- , 6812	R.LISQIVSSITASLR.F	1488.75471	2	1.35E-07	0.95	4.48	-	1031.2
AHQ-1-8, 7066	R.LISQIVSSITASLR.F	1488.75471	2	5.14E-09	0.92	4.05	-	862.8
AHQ-1-7, 6189	R.LISQIVSSITASLR.F	1488.75471	2	1.40E-04	0.94	4.37	-	962.9
AHQ-1-5, 3455	R.NLDIERPTYTNLNR.L	1719.88022	2	6.45E-05	0.43	2.82	-	284.6
AHQ-1-9, 3304	R.NLDIERPTYTNLNR.L	1719.88022	2	1.87E-04	0.60	3.06	-	439.2
AHQ-1-7, 3465 - 3488	R.NLDIERPTYTNLNR.L	1719.88022	3	7.80E-06	0.73	3.37	-	498.6
AHQ-1-14, 4358	R.NLDIERPTYTNLNR.L	1719.88022	2	1.01E-04	0.60	2.66	-	488.7
AHQ-1-7, 3540 - 3620	R.NLDIERPTYTNLNR.L	1719.88022	2	7.26E-05	0.36	2.77	-	244.1
AHQ-1-7, 3602 - 3676	R.NLDIERPTYTNLNR.L	1719.88022	2	3.88E-06	0.71	3.41	-	586.7
AHQ-1-13, 3463	R.NLDIERPTYTNLNR.L	1719.88022	2	4.48E-04	0.50	3.13	-	323.4
AHQ-1-6, 3424	R.NLDIERPTYTNLNR.L	1719.88022	2	1.95E-07	0.33	2.89	-	225.4
AHQ-1-7, 3441 - 3512	R.NLDIERPTYTNLNR.L	1719.88022	2	4.65E-05	0.65	3.20	-	383.6
AHQ-1-14- , 3523	R.NLDIERPTYTNLNR.L	1719.88022	2	2.08E-04	0.42	2.94	-	282.2
AHQ-1-7, 4276	R.QLFHPEQLITGK.E	1411.63050	2	6.81E-04	0.68	3.31	-	337.1
AHQ-1-3, 5970	R.SIQFVDWCPTGFK.V	1586.79232	2	4.75E-04	0.71	2.61	-	631.6
AHQ-1-11, 5371	R.SIQFVDWCPTGFK.V	1586.79232	2	2.99E-06	0.50	2.52	-	460.4
AHQ-1-4, 6094 - 6096	R.SIQFVDWCPTGFK.V	1586.79232	2	1.44E-05	0.96	4.86	-	1324.4
AHQ-1-6, 5910	R.SIQFVDWCPTGFK.V	1586.79232	2	2.11E-04	0.83	2.82	-	787.7
AHQ-1-1, 5967	R.SIQFVDWCPTGFK.V	1586.79232	2	1.50E-05	0.92	3.87	-	761.8
AHQ-1-7, 5928 - 6000	R.SIQFVDWCPTGFK.V	1586.79232	2	3.26E-09	0.96	4.70	-	1192.8
AHQ-1-7, 5878 - 5948	K.TIGGGDDSFTTFFCETGAGK.H	2070.17889	2	3.95E-08	0.91	3.98	-	831.7
AHQ-1-9, 5755 - 5828	K.TIGGGDDSFTTFFCETGAGK.H	2070.17889	2	1.70E-06	0.66	3.27	-	371.1
AHQ-1-12, 5462	K.TIGGGDDSFTTFFCETGAGK.H	2070.17889	2	1.38E-07	0.72	3.32	-	406.0
AHQ-1-7, 6016 - 6097	K.TIGGGDDSFTTFFCETGAGK.H	2070.17889	2	3.77E-08	0.89	3.97	-	593.3
AHQ-1-7, 6168 - 6240	K.TIGGGDDSFTTFFCETGAGK.H	2070.17889	2	7.34E-10	0.68	3.06	-	380.1
AHQ-1-14- , 5563 - 5623	K.TIGGGDDSFTTFFCETGAGK.H	2070.17889	2	3.30E-10	0.96	5.14	-	887.6
AHQ-1-4, 6031	K.TIGGGDDSFTTFFCETGAGK.H	2070.17889	2	2.48E-09	0.68	3.19	-	293.9
AHQ-1-13- , 5760	K.TIGGGDDSFTTFFCETGAGK.H	2070.17889	2	3.38E-10	0.86	3.74	-	595.2
AHQ-1-13- , 4595	K.VGINYQPPTVVPGGDLAK.V	1826.08584	2	3.33E-08	0.72	3.66	-	278.5
AHQ-1-13- , 4449 - 4521	K.VGINYQPPTVVPGGDLAK.V	1826.08584	2	1.70E-06	0.70	3.24	-	302.6
AHQ-1-13, 4344	K.VGINYQPPTVVPGGDLAK.V	1826.08584	2	1.20E-04	0.65	2.78	-	364.6
AHQ-1-6, 4514 - 4531	K.VGINYQPPTVVPGGDLAK.V	1826.08584	2	2.28E-05	0.57	3.22	-	259.5
AHQ-1-7, 4349 - 4352	K.VGINYQPPTVVPGGDLAK.V	1826.08584	2	4.19E-04	0.73	3.06	-	353.9
AHQ-1-7, 4444 - 4512	K.VGINYQPPTVVPGGDLAK.V	1826.08584	2	7.50E-07	0.78	3.41	-	397.6
AHQ-1-10, 4075	K.VGINYQPPTVVPGGDLAK.V	1826.08584	2	6.87E-04	0.62	3.31	-	304.2
AHQ-1-7, 4580 - 4648	K.VGINYQPPTVVPGGDLAK.V	1826.08584	2	2.50E-04	0.82	3.38	-	419.7
AHQ-1-12, 4406 - 4481	K.VGINYQPPTVVPGGDLAK.V	1826.08584	2	4.66E-06	0.83	3.36	-	481.1
AHQ-1-12, 4313 - 4377	K.VGINYQPPTVVPGGDLAK.V	1826.08584	2	7.52E-07	0.80	3.41	-	360.6
AHQ-1-7, 4560 - 4592	K.YMACCLLYR.G	1252.50864	2	4.14E-04	0.96	3.84	-	1136.7
AHQ-1-12, 4349	K.YMACCLLYR.G	1252.50864	2	1.56E-06	0.95	3.58	-	1012.3
AHQ-1-7, 4056 - 4058	K.YM*ACCLLYR.G	1268.50804	2	8.39E-04	0.74	2.79	-	494.9
gi|4557705|ref|NP_000217.1| keratin 9 [Homo sapiens]				3.32E-10	5.14	60.29	21.70	61986.9

AHQ-1-1, 6349 - 6427	K.DIENQYETQITQIEHEVSSSGQEVQSSAK.E	3266.38667	3	3.27E-07	0.95	5.45	-	1103.2
AHQ-1-3, 6502 - 6514	K.DIENQYETQITQIEHEVSSSGQEVQSSAK.E	3266.38667	3	6.98E-05	0.92	4.52	-	1063.6
AHQ-1-4, 6648	K.DIENQYETQITQIEHEVSSSGQEVQSSAK.E	3266.38667	3	4.03E-04	0.92	4.68	-	766.1
AHQ-1-6, 6467	K.DIENQYETQITQIEHEVSSSGQEVQSSAK.E	3266.38667	3	8.74E-08	0.93	4.76	-	1011.3
AHQ-1-14- , 6120 - 6127	K.DIENQYETQITQIEHEVSSSGQEVQSSAK.E	3266.38667	3	5.15E-07	0.96	5.75	-	1587.4
AHQ-1-12, 6001	K.DIENQYETQITQIEHEVSSSGQEVQSSAK.E	3266.38667	3	2.78E-04	0.94	4.79	-	1325.9
AHQ-1-14- , 5018	R.GGGGSFGYSYGGGSGGGFSASSLGGGFGGGSR.G	2706.73770	2	1.76E-09	0.92	4.49	-	742.8
AHQ-1-13- , 5292	R.HGVQELEIELQSQLSK.K	1839.03978	2	3.32E-10	0.96	5.10	-	1389.8
AHQ-1-14- , 6616	K.NYSPYYNTIDDLKDQIVDLTVGNNK.T	2904.13412	3	5.03E-06	0.88	4.00	-	636.0
AHQ-1-14- , 6484	K.SDLEM*QYETLQEELM*ALK.K	2204.46135	2	9.60E-08	0.92	4.10	-	938.9
AHQ-1-12, 6411	K.SDLEM*QYETLQEELM*ALK.K	2204.46135	2	3.19E-05	0.71	2.83	-	841.9
AHQ-1-8, 4847	K.TLNDMRQEYEQLIAK.N	1853.08996	2	6.43E-06	0.53	2.84	-	641.3
gi|5453832|ref|NP_006380.1|	oxygen regulated protein precursor; oxygen regulated protein (150kD) [H			3.36E-10	2.58	30.22	6.20	111334.8
AHQ-1-3, 6667	R.VEFEELCADLFER.V	1658.81048	2	3.36E-10	0.96	4.40	-	1491.0
AHQ-1-3, 6487 - 6562	K.VLQLINDNTATALSYGVFR.R	2096.37206	2	2.26E-08	0.96	4.36	-	1409.0
AHQ-1-3, 7430	R.VPGPVQQALQSAEM*SLDEIEQVILVGGATR.V	3153.55387	3	6.80E-04	0.66	3.65	-	337.3
gi|5174727|ref|NP_005989.1|	chaperonin containing TCP1, subunit 3 (gamma); TCP1 (t-complex-1) ring			3.65E-10	3.53	40.23	9.20	60330.4
AHQ-1-6, 3802	R.AVAQALEVIPR.T	1167.38326	2	2.48E-04	0.89	3.41	-	1108.6
AHQ-1-6, 4502	K.IPGGIIEDSCVLR.G	1430.65218	2	4.12E-07	0.95	4.64	-	1147.9
AHQ-1-6, 2822	R.TLIQNCGASTIR.L	1335.51249	2	2.61E-04	0.73	2.88	-	561.8
AHQ-1-6, 5551 - 5568	R.WSSLACNIALDAVK.M	1549.77353	2	3.65E-10	0.96	4.60	-	1377.7
gi|4502201|ref|NP_001649.1|	ADP-ribosylation factor 1 [Homo sapiens]			3.86E-10	8.06	90.30	46.40	20696.6
AHQ-1-12, 4523 - 4589	R.DAVLLVFANK.Q	1090.29726	1	4.84E-05	0.85	2.94	-	946.7
AHQ-1-12, 4515 - 4578	R.DAVLLVFANK.Q	1090.29726	2	5.86E-06	0.97	4.52	-	2175.5
AHQ-1-12, 4774	R.HYFQNTQGLIFVVDSNDR.E	2154.32702	2	5.20E-07	0.98	5.61	-	1960.4
AHQ-1-12, 4919	R.HYFQNTQGLIFVVDSNDR.E	2154.32702	2	3.97E-08	0.98	5.96	-	1855.0
AHQ-1-12, 5143 - 5211	R.HYFQNTQGLIFVVDSNDR.E	2154.32702	2	1.64E-09	0.98	5.53	-	1813.3
AHQ-1-12, 4959 - 5029	R.HYFQNTQGLIFVVDSNDRER.V	2439.62792	3	3.63E-04	0.44	3.31	-	274.5
AHQ-1-12, 4445 - 4509	R.HYFQNTQGLIFVVDSNDRER.V	2439.62792	3	6.18E-08	0.92	4.26	-	1186.3
AHQ-1-12, 4871 - 4885	R.HYFQNTQGLIFVVDSNDRER.V	2439.62792	3	3.86E-10	0.94	4.94	-	1054.7
AHQ-1-11, 4606 - 4684	R.HYFQNTQGLIFVVDSNDRER.V	2439.62792	3	1.29E-04	0.80	3.79	-	673.4
AHQ-1-12, 3673	R.ILMVGLDAAGK.T	1088.34632	1	6.08E-05	0.78	2.59	-	1254.3
AHQ-1-12, 3127	R.ILM*VGLDAAGK.T	1104.34572	2	3.48E-04	0.92	3.44	-	1349.7
AHQ-1-12, 3667 - 3669	R.ILMVGLDAAGK.T	1088.34632	2	6.33E-06	0.95	4.04	-	1222.6
AHQ-1-12, 3683	R.ILMVGLDAAGK.T	1088.34632	1	9.58E-04	0.80	3.50	-	634.5
AHQ-1-14- , 6268	K.LGEIVTTIPTIGFNVETVEYK.N	2324.65485	2	6.11E-04	0.48	2.79	-	188.7
AHQ-1-12, 6177 - 6234	K.LGEIVTTIPTIGFNVETVEYK.N	2324.65485	2	4.32E-04	0.72	3.86	-	162.9
AHQ-1-13- , 6512 - 6523	K.LGEIVTTIPTIGFNVETVEYK.N	2324.65485	2	3.69E-04	0.49	3.38	-	171.4
AHQ-1-12, 2609 - 2611	R.MLAEDELR.D	977.11769	2	7.61E-04	0.89	2.99	-	982.5
AHQ-1-12, 6567	R.M*LAEDELRDAVLLVFANK.Q	2064.39175	3	7.82E-10	0.96	4.30	-	2039.8
AHQ-1-12, 6565 - 6625	R.M*LAEDELRDAVLLVFANK.Q	2064.39175	2	1.01E-04	0.89	3.90	-	712.9
AHQ-1-13- , 5244 - 5252	K.NISFTVWDVGGQDK.I	1566.69621	2	3.48E-08	0.93	4.13	-	773.4
AHQ-1-11, 4780	K.NISFTVWDVGGQDK.I	1566.69621	2	1.53E-04	0.87	3.13	-	1002.0
AHQ-1-14- , 5110 - 5114	K.NISFTVWDVGGQDK.I	1566.69621	2	2.60E-06	0.87	3.77	-	624.3
AHQ-1-12, 4913 - 4969	K.NISFTVWDVGGQDK.I	1566.69621	2	6.80E-09	0.95	4.48	-	1006.4
AHQ-1-13, 4952 - 4963	K.NISFTVWDVGGQDK.I	1566.69621	2	4.47E-04	0.90	4.12	-	678.2
gi|11496277|ref|NP_068805.1|	mitogen-activated protein kinase kinase 1 interacting protein 1; MEK p			4.48E-10	1.85	20.27	35.50	13622.6
AHQ-1-14- , 5451	K.SIICYYNTYQVVQFNR.L	2070.31322	2	3.19E-06	0.92	3.81	-	1113.8
AHQ-1-14- , 5274	K.VANDNAPEHALRPGFLSTFALATDQGSK.L	2929.19327	3	4.48E-10	0.93	5.43	-	673.5
gi|14149680|ref|NP_056107.1|	KIAA0747 protein [Homo sapiens]			4.59E-10	5.01	60.26	7.90	122855.6
AHQ-1-4, 5752	K.AVYSTNCPVWEEAFR.F	1830.99795	2	1.28E-04	0.82	2.99	-	819.2
AHQ-1-12, 5382	R.FEWELPLDEAQR.R	1533.66622	2	1.67E-07	0.82	3.49	-	537.8
AHQ-1-4, 3320 - 3391	R.GSNPHLQTFTFTR.V	1506.64724	2	4.04E-06	0.87	3.02	-	940.0
AHQ-1-4, 4020	R.IHVLEAQDLIAK.D	1350.58860	2	4.59E-10	0.74	2.84	-	534.9
AHQ-1-7, 7246	R.LLVPLVPDLQDVAQLR.S	1790.13977	2	6.24E-06	0.79	3.51	-	416.4
AHQ-1-12, 3975	R.LTHVDSPLEAPAGPLGQVK.L	1930.19405	2	1.93E-06	0.97	5.13	-	1746.8
AHQ-1-4, 4219	R.LTHVDSPLEAPAGPLGQVK.L	1930.19405	2	2.07E-04	0.84	3.24	-	757.1
gi|4504521|ref|NP_002147.1|	heat shock 60kDa protein 1 (chaperonin); mitochondrial matrix protein P			4.75E-10	3.46	40.31	11.30	61024.2
AHQ-1-14- , 6627	R.ALM*LQGVDLLADAVAVTM*GPK.G	2146.55768	3	4.75E-10	0.98	6.27	-	2329.6
AHQ-1-7, 5750	K.CEFQDAYVLLSEK.K	1603.77482	2	9.54E-07	0.95	3.68	-	1739.8
AHQ-1-7, 5537	K.ISSIQSIVPALEIANAHR.K	1920.20240	2	3.09E-04	0.69	3.19	-	346.4
AHQ-1-7, 5889	K.TLNDELEIIEGMK.F	1505.71604	2	3.11E-06	0.83	2.68	-	1124.8
gi|4504345|ref|NP_000508.1|	alpha 2 globin [Homo sapiens]			5.00E-10	2.60	30.27	30.30	15257.4
AHQ-1-13- , 4388	K.FLASVSTVLTSK.Y	1253.46970	2	1.77E-06	0.91	3.45	-	1145.7
AHQ-1-13- , 4187	K.TYFPHFDLSHGSAQVK.G	1835.01138	3	2.66E-05	0.88	4.16	-	573.4
AHQ-1-13- , 4115 - 4183	K.TYFPHFDLSHGSAQVK.G	1835.01138	2	3.52E-04	0.75	3.17	-	641.8
AHQ-1-13, 4003	K.TYFPHFDLSHGSAQVK.G	1835.01138	3	1.08E-05	0.72	3.26	-	464.6
AHQ-1-13- , 2651	K.VGAHAGEYGAEALER.M	1530.62397	3	2.50E-05	0.97	5.44	-	2334.9
AHQ-1-13- , 2647	K.VGAHAGEYGAEALER.M	1530.62397	2	3.69E-08	0.96	4.09	-	1657.5
AHQ-1-13, 2654 - 2657	K.VGAHAGEYGAEALER.M	1530.62397	2	5.00E-10	0.97	4.94	-	1594.8
gi|4758950|ref|NP_000933.1|	peptidylprolyl isomerase B (cyclophilin B) [Homo sapiens]			5.24E-10	6.99	80.21	36.60	23742.4
AHQ-1-12, 4119	K.DFMIQGGDFTR.G	1287.42660	2	4.52E-04	0.87	2.90	-	934.1
AHQ-1-11, 4006	K.DFMIQGGDFTR.G	1287.42660	2	3.10E-04	0.89	3.19	-	1173.3
AHQ-1-11, 2882	R.DKPLKDVIIADCGK.I	1573.83653	2	2.71E-08	0.89	3.53	-	963.5
AHQ-1-12, 2946 - 2954	R.DKPLKDVIIADCGK.I	1573.83653	3	6.13E-07	0.94	4.23	-	1290.4
AHQ-1-12, 2951	R.DKPLKDVIIADCGK.I	1573.83653	2	4.30E-07	0.91	3.81	-	876.9
AHQ-1-11, 4122	K.DTNGSQFFITTVK.T	1458.59766	2	5.28E-07	0.92	3.59	-	1366.3
AHQ-1-11, 2396	K.DVIIADCGK.I	992.12891	1	1.18E-05	0.68	2.29	-	616.4
AHQ-1-12, 3103	K.HYGPGWVSMANAGK.D	1475.65664	2	5.24E-10	0.95	4.13	-	1232.4
AHQ-1-12, 3146 - 3158	K.IEVEKPFAIAK.E	1245.49240	2	1.83E-05	0.92	3.54	-	1274.3
AHQ-1-11, 4166	K.TVDNFVALATGEK.G	1365.51377	2	6.62E-05	0.92	3.67	-	1106.7
AHQ-1-11, 4172 - 4174	K.TVDNFVALATGEK.G	1365.51377	1	7.83E-04	0.25	2.09	-	308.0
AHQ-1-14, 5402	K.TVDNFVALATGEK.G	1365.51377	2	1.92E-04	0.88	3.89	-	648.7
AHQ-1-12, 4602	K.TVDNFVALATGEK.G	1365.51377	2	2.65E-06	0.90	3.23	-	880.6
AHQ-1-12, 4305	K.TVDNFVALATGEK.G	1365.51377	2	2.05E-05	0.87	3.52	-	826.3
AHQ-1-11, 3987	R.VIKDFMIQGGDFTR.G	1627.88933	2	1.27E-06	0.85	3.32	-	790.3
AHQ-1-12, 4133	R.VIKDFMIQGGDFTR.G	1627.88933	2	1.85E-06	0.88	3.54	-	901.7
gi|4758484|ref|NP_004823.1|	glutathione-S-transferase like; glutathione transferase omega [Homo sap			5.27E-10	4.23	60.18	19.50	27565.7
AHQ-1-10, 3720	K.EDPTVSALLTSEK.D	1390.51840	1	1.27E-04	0.48	2.78	-	252.4
AHQ-1-10, 2955	K.EDYAGLKEEFR.K	1357.45019	2	5.27E-10	0.87	2.91	-	1012.5
AHQ-1-10, 2461	K.GSAPPGPVPEGSIR.I	1321.46422	1	1.86E-04	0.27	2.59	-	205.0
AHQ-1-13- , 2989 - 2992	R.HEVININLK.N	1080.26247	2	2.72E-07	0.92	3.24	-	993.9
AHQ-1-10, 2677	R.HEVININLK.N	1080.26247	1	2.48E-04	0.75	3.00	-	543.6
AHQ-1-13- , 2924	R.SQNKEDYAGLKEEFR.K	1814.93364	3	2.87E-08	0.82	3.62	-	780.9
AHQ-1-12, 4582	K.VPSLVGSFIR.S	1075.28583	2	1.90E-06	0.86	2.78	-	856.2
gi|24497612|ref|NP_116262.2|	nm23-phosphorylated unknown substrate; SH3 domain-containing 70 kDa pr			5.27E-10	2.86	30.28	9.20	72695.6
AHQ-1-6, 6752	K.LGCDWVATIFSR.D	1426.62208	2	5.46E-07	0.97	3.56	-	1988.0
AHQ-1-6, 6598	R.LVGEALLESNTIIDHVYCSPSLR.C	2588.91822	2	5.27E-10	0.96	4.72	-	1252.1
AHQ-1-6, 6210	R.RPYEDQGLGETTPLTIICQPMQPLR.V	2916.32264	3	6.94E-05	0.94	5.50	-	868.4
gi|7706497|ref|NP_057392.1|	UMP-CMP kinase [Homo sapiens]			6.11E-10	4.48	50.23	27.60	25854.7
AHQ-1-11, 5740	R.IQTYLQSTKPIIDLYEEMGK.V	2371.73502	2	6.11E-10	0.90	4.67	-	362.2
AHQ-1-11, 2578	R.KNPDSQYGELIEK.Y	1521.65375	2	3.58E-07	0.93	4.36	-	755.6
AHQ-1-11, 6492	K.SVDEVFDEVVQIFDK.E	1769.92954	2	2.92E-04	0.79	3.07	-	681.2
AHQ-1-11, 6494	K.SVDEVFDEVVQIFDKEG	1956.09563	2	5.34E-04	0.93	4.31	-	916.4
AHQ-1-11, 3594 - 3667	K.YGYTHLSAGELLR.D	1480.64969	2	3.36E-07	0.93	3.75	-	1097.9
gi|4502011|ref|NP_000467.1|	adenylate kinase 1 [Homo sapiens]			6.27E-10	5.76	70.24	47.90	21634.7
AHQ-1-11, 3680 - 3684	K.ATEPVIAFYEK.R	1268.43969	1	7.51E-05	0.31	2.09	-	463.5

AHQ-1-11, 6483	K.GQLVPLETVLDMLR.D	1584.90590	2	6.27E-10	0.95	4.79	-	693.2
AHQ-1-11, 6222 - 6303	K.GQLVPLETVLDM*LR.D	1600.90530	2	4.38E-06	0.87	4.11	-	396.5
AHQ-1-11, 5016	R.IGQPTLLLYVDAGPETM*TQR.L	2220.53174	2	9.14E-05	0.85	3.61	-	703.8
AHQ-1-11, 3380 - 3460	K.IIFVVGGPGSGK.G	1131.34939	2	1.56E-04	0.91	3.59	-	1515.6
AHQ-1-11, 5900 - 5971	R.KVNAEGSVDSVFSQVCTHLDALK	2506.77407	3	9.80E-05	0.93	4.40	-	1359.8
AHQ-1-11, 3651	K.YGYTHLSTGDLLR.S	1496.64912	2	4.87E-04	0.93	3.76	-	1127.8
gi|19923437|ref|NP_057366.2| adenylate kinase 3 alpha like [Homo sapiens]				6.66E-10	2.24	30.21	19.40	25565.2
AHQ-1-10, 3119	K.AYEDQTKPVLEYYQK.K	1876.05535	2	3.05E-04	0.78	3.50	-	487.8
AHQ-1-10, 6024 - 6040	K.NLTQYSWLLDGFPR.T	1710.91339	2	6.66E-10	0.95	4.17	-	1440.8
AHQ-1-10, 4415 - 4417	K.TVGIDDLTGEPLIQR.E	1627.82100	2	6.16E-04	0.51	2.92	-	327.8
gi|4507907|ref|NP_000543.1| von Willebrand factor precursor; Coagulation factor VIII VWF (von Wille				6.96E-10	22.80	260.30	12.90	309296.7
AHQ-1-1, 5053	K.AFVLSSVDELEQQR.D	1621.77332	2	7.68E-05	0.93	3.54	-	1835.0
AHQ-1-1, 4025	K.AHLLSLVDVM*QR.E	1398.65673	2	2.15E-08	0.94	3.68	-	1185.2
AHQ-1-1, 4201 - 4267	R.CLPTACTIQLR.G	1335.57568	2	2.39E-06	0.57	2.52	-	496.3
AHQ-1-1, 5109 - 5175	R.EGGPSQIGDALGFAVR.Y	1574.71988	2	2.08E-06	0.63	3.18	-	504.7
AHQ-1-1, 5756	R.EQAPNLVYMVTGNPASDEIK.R	2177.42062	2	2.43E-05	0.93	4.12	-	685.8
AHQ-1-1, 4820	R.EQAPNLVYM*VTGNPASDEIK.R	2193.42002	2	4.35E-04	0.88	3.77	-	627.5
AHQ-1-1, 4187	K.EQDLEVILHNGACSPGAR.Q	1968.13770	2	2.02E-07	0.92	4.36	-	958.1
AHQ-1-1, 6273	R.IEDLPTMVTLGNSFLHK.L	1916.23079	2	4.24E-04	0.89	3.92	-	906.6
AHQ-1-1, 6867	R.IGWPNAPILIQDFETLPR.E	2081.40223	2	2.76E-08	0.92	4.31	-	728.7
AHQ-1-1, 6968	R.IGWPNAPILIQDFETLPR.E	2081.40223	2	1.28E-07	0.96	5.06	-	1029.3
AHQ-1-3, 7211	R.IGWPNAPILIQDFETLPR.E	2081.40223	2	6.07E-06	0.90	4.14	-	582.5
AHQ-1-3, 2615	R.ILAGPAGDSNVVK.L	1241.41901	2	2.26E-04	0.87	2.89	-	1019.6
AHQ-1-2, 2695	R.ILAGPAGDSNVVK.L	1241.41901	2	2.75E-06	0.93	3.57	-	1064.2
AHQ-1-1, 2728	R.ILAGPAGDSNVVK.L	1241.41901	2	2.10E-07	0.88	3.54	-	823.3
AHQ-1-1, 6313	K.ILDELLQTCVDPEDCPVCEVAGR.R	2692.97859	2	1.82E-04	0.89	4.35	-	461.7
AHQ-1-1, 3780 - 3852	R.ILTSDVFQDCNK.L	1441.58869	2	7.04E-05	0.89	3.39	-	962.5
AHQ-1-1, 5849	R.LPGDIQVVPIGVGPNANVQELER.I	2415.73085	3	4.32E-06	0.86	3.49	-	1130.3
AHQ-1-1, 5845	R.LPGDIQVVPIGVGPNANVQELER.I	2415.73085	2	7.06E-08	0.72	3.09	-	377.0
AHQ-1-1, 4263	K.LTGSCSYVLFQNK.E	1518.71629	2	3.62E-04	0.94	3.68	-	1269.8
AHQ-1-1, 5555	K.RLPGDIQVVPIGVGPNANVQELER.I	2571.91720	3	7.06E-06	0.98	5.98	-	2701.3
AHQ-1-1, 5664	K.RLPGDIQVVPIGVGPNANVQELER.I	2571.91720	3	4.48E-04	0.96	5.49	-	1719.1
AHQ-1-1, 4077	R.SGFTYVLHEGECCGR.C	1774.91285	2	6.96E-10	0.90	3.35	-	932.6
AHQ-1-1, 3499	R.SKEFMEEVIQR.M	1396.59450	2	1.13E-05	0.91	3.03	-	1168.2
AHQ-1-1, 2588	R.SKEFM*EEVIQR.M	1412.59390	2	1.25E-05	0.94	3.55	-	1081.9
AHQ-1-1, 5051 - 5060	K.SVGSQWASPENPCLINECVR.V	2306.51832	2	6.08E-06	0.95	4.80	-	749.4
AHQ-1-2, 5171 - 5201	K.SVGSQWASPENPCLINECVR.V	2306.51832	2	3.73E-06	0.85	3.81	-	484.2
AHQ-1-3, 5025 - 5045	K.SVGSQWASPENPCLINECVR.V	2306.51832	2	6.84E-07	0.82	3.81	-	330.2
AHQ-1-2, 6323	K.TYGLCGICDENGANDFMLR.D	2209.42291	2	5.91E-07	0.91	4.14	-	621.7
AHQ-1-1, 4584 - 4585	K.VIVIPVGIGPHANLK.Q	1527.87891	2	2.56E-07	0.89	4.00	-	435.6
AHQ-1-1, 4692	R.VTVFPIGIGDR.Y	1174.37426	2	4.49E-06	0.94	3.68	-	1097.1
AHQ-1-2, 3035	K.YAGSQVASTSEVLK.Y	1440.58064	2	1.75E-06	0.93	3.85	-	982.0
AHQ-1-1, 3017 - 3023	K.YAGSQVASTSEVLK.Y	1440.58064	2	1.15E-09	0.96	4.72	-	953.4
AHQ-1-1, 6641	K.YLFPGECQYVLVQDYCGSNPGTFR.I	2874.15305	2	1.90E-06	0.79	2.90	-	790.0
AHQ-1-1, 3472 - 3545	R.YLSDHSFLVSQGDR.E	1624.73576	2	7.84E-06	0.91	3.04	-	1444.4
AHQ-1-1, 6064	K.YTLFQIFSK.I	1147.34728	2	8.58E-08	0.93	3.21	-	1002.8
gi|4503643|ref|NP_000121.1| coagulation factor V precursor; labile factor; factor V Leiden [Homo sa				7.01E-10	4.33	50.22	3.00	251716.5
AHQ-1-3, 6026 - 6094	R.AADIEQQAVFAVFDENK.S	1896.04666	2	2.30E-06	0.95	4.36	-	1362.0
AHQ-1-1, 3309	R.AEVDDVIQVR.F	1144.26019	2	1.54E-04	0.97	4.00	-	2146.4
AHQ-1-1, 5268	R.DPDNIAAWYLR.S	1334.46145	2	7.71E-05	0.61	2.60	-	521.6
AHQ-1-1, 3277	K.EVIITGIQTQGAK.H	1358.56590	2	2.05E-05	0.88	3.00	-	1415.0
AHQ-1-5, 4986	R.GEYEEHLGILGPIIR.A	1696.92816	2	7.01E-10	0.93	3.58	-	1220.1
AHQ-1-2, 5207 - 5277	R.GEYEEHLGILGPIIR.A	1696.92816	2	3.30E-05	0.91	3.65	-	886.7
gi|4501993|ref|NP_003650.1| alkylglycerone phosphate synthase precursor [Homo sapiens]				7.10E-10	0.86	10.18	2.60	72911.4
AHQ-1-6, 6136	R.GISDPLTVFEQTEAAAR.E	1805.96665	2	7.10E-10	0.86	3.63	-	595.7
gi|14211923|ref|NP_115982.1| PKCI-1-related HIT protein [Homo sapiens]				7.27E-10	1.82	20.30	22.70	17161.6
AHQ-1-13, 5924	R.ISQAEEEDQQLLGHLLLVAK.Q	2235.52293	2	2.53E-09	0.95	4.65	-	1333.4
AHQ-1-13- , 6360	R.ISQAEEEDQQLLGHLLLVAK.Q	2235.52293	2	1.54E-06	0.97	5.93	-	1379.2
AHQ-1-13- , 6355 - 6364	R.ISQAEEEDQQLLGHLLLVAK.Q	2235.52293	3	2.98E-06	0.92	4.73	-	953.4
AHQ-1-13, 5920 - 5923	R.ISQAEEEDQQLLGHLLLVAK.Q	2235.52293	3	7.27E-10	0.88	4.21	-	826.1
AHQ-1-13, 5928	K.SLPADILYEDQQCLVFR.D	2069.32354	2	7.10E-06	0.94	3.89	-	1037.7
gi|30160191|ref|XP_294035.2| similar to Pyruvate kinase, M1 isozyme (Pyruvate kinase muscle isozyme				7.33E-10	0.94	10.21	2.80	50222.2
AHQ-1-7, 5188 - 5213	K.IYVDDGLISLQVK.Q	1463.70048	2	7.33E-10	0.94	3.94	-	1195.3
AHQ-1-7, 5292 - 5320	K.IYVDDGLISLQVK.Q	1463.70048	2	9.39E-09	0.96	4.26	-	1766.8
AHQ-1-7, 5048	K.IYVDDGLISLQVK.Q	1463.70048	2	1.97E-08	0.96	4.24	-	1758.2
gi|5803011|ref|NP_001966.1| enolase 2; enolase-2, gamma, neuronal; neurone-specific enolase; neuron				7.35E-10	0.97	10.25	3.70	47268.3
AHQ-1-8, 6795	R.YITGDQLGALYQDFVR.D	1860.05915	2	7.35E-10	0.97	4.98	-	1199.3
gi|10346135|ref|NP_055083.1| microtubule-associated protein, RP/EB family, member 2; T-cell activat				7.51E-10	1.86	20.28	9.80	37031.1
AHQ-1-13, 6393	R.FQDNLDFIQWFK.K	1601.78539	2	8.73E-05	0.91	3.53	-	995.1
AHQ-1-9, 7056	R.FQDNLDFIQWFK.K	1601.78539	2	3.16E-07	0.96	4.32	-	1429.2
AHQ-1-12, 6510	R.FQDNLDFIQWFK.K	1601.78539	2	7.51E-10	0.95	4.55	-	1156.7
AHQ-1-13- , 6848	R.FQDNLDFIQWFK.K	1601.78539	2	1.17E-09	0.96	4.71	-	1137.7
AHQ-1-12, 4853	K.SDKDLETQVIQLNEQVHSLK.L	2325.56134	3	9.80E-08	0.90	4.08	-	1017.9
AHQ-1-9, 4859	K.SDKDLETQVIQLNEQVHSLK.L	2325.56134	3	3.00E-07	0.96	5.50	-	1412.6
AHQ-1-9, 4858	K.SDKDLETQVIQLNEQVHSLK.L	2325.56134	2	1.33E-04	0.92	4.32	-	767.1
gi|4507651|ref|NP_003281.1| tropomyosin 4 [Homo sapiens]				7.95E-10	6.20	70.28	25.40	28521.6
AHQ-1-9, 1894	K.AEGDVAALNR.R	1016.09051	2	1.62E-04	0.91	2.98	-	1466.3
AHQ-1-9, 4096	R.AEVSELKCGDLEEELK.N	1851.02256	2	1.27E-05	0.92	4.27	-	796.1
AHQ-1-9, 6216	R.AEVSELKCGDLEEELKNVTNNLK.S	2634.89893	3	9.16E-07	0.86	4.46	-	923.0
AHQ-1-9, 6114 - 6127	R.AEVSELKCGDLEEELKNVTNNLK.S	2634.89893	3	1.02E-08	0.92	4.75	-	1134.2
AHQ-1-9, 1718 - 1791	R.EKAEGDVAALNR.R	1273.37796	2	8.79E-05	0.79	2.71	-	925.0
AHQ-1-10, 2524	K.IQALQQQADEAEDR.A	1615.68404	2	3.99E-05	0.93	3.51	-	1224.2
AHQ-1-9, 2528 - 2535	K.IQALQQQADEAEDR.A	1615.68404	2	7.95E-10	0.98	5.59	-	1737.7
AHQ-1-11, 2560	K.IQALQQQADEAEDR.A	1615.68404	2	2.51E-07	0.97	4.31	-	1673.4
AHQ-1-9, 2251 - 2288	R.KIQALQQQADEAEDR.A	1743.85695	3	2.77E-08	0.94	4.67	-	1307.7
AHQ-1-9, 2574	R.KIQALQQQADEAEDR.A	1743.85695	2	1.43E-09	0.96	4.83	-	1317.8
AHQ-1-10, 2321	R.KIQALQQQADEAEDR.A	1743.85695	2	7.10E-07	0.96	5.03	-	1738.7
AHQ-1-9, 2484	R.KIQALQQQADEAEDR.A	1743.85695	2	8.12E-05	0.95	4.68	-	1257.6
AHQ-1-9, 1967	K.YSEKEDKYEEEIK.L	1690.78605	3	2.00E-06	0.73	3.41	-	889.6
AHQ-1-9, 2059	K.YSEKEDKYEEEIK.L	1690.78605	3	2.12E-05	0.85	3.65	-	1219.1
gi|4507869|ref|NP_003361.1| vasodilator-stimulated phosphoprotein [Homo sapiens]				8.05E-10	2.94	40.24	13.40	39829.4
AHQ-1-8, 2121	R.ATVMLYDDGNKR.W	1383.55582	2	1.36E-04	0.63	2.69	-	490.9
AHQ-1-8, 6258	K.VKEEIIEAFVQELR.K	1703.96061	2	8.05E-10	0.97	4.79	-	1636.3
AHQ-1-14- , 3035	R.VQIYHNPTANSFR.V	1547.69936	2	1.01E-05	0.48	2.76	-	415.1
AHQ-1-13- , 3137	K.YNQATPNFHQWR.D	1562.67258	2	7.37E-09	0.86	3.61	-	564.2
gi|4507951|ref|NP_003396.1| tyrosine 3/tryptophan 5 -monooxygenase activation protein, eta polypept				8.07E-10	6.18	70.25	34.60	28218.5
AHQ-1-14- , 3019	K.AVTELNEPLSNEDR.N	1587.67060	2	2.78E-04	0.66	2.88	-	447.6
AHQ-1-10, 2795 - 2865	K.AVTELNEPLSNEDR.N	1587.67060	2	1.60E-06	0.95	4.93	-	915.1
AHQ-1-13- , 3053	K.AVTELNEPLSNEDR.N	1587.67060	2	1.38E-06	0.85	3.33	-	711.3
AHQ-1-10, 5495 - 5507	K.ELETVCNDVLSLLDK.F	1749.96137	2	8.07E-10	0.93	4.36	-	776.2
AHQ-1-13, 6403	K.ELETVCNDVLSLLDK.F	1749.96137	2	3.29E-07	0.79	3.50	-	495.7
AHQ-1-13- , 6864	K.ELETVCNDVLSLLDK.F	1749.96137	2	5.88E-07	0.93	4.31	-	855.1
AHQ-1-10, 6263	K.ELETVCNDVLSLLDK.F	1749.96137	2	4.37E-06	0.94	4.72	-	829.1
AHQ-1-10, 3889	K.FLIKNCNDFQYESK.V	1808.00430	2	1.09E-04	0.91	3.85	-	701.4
AHQ-1-13- , 2333	K.KNSVVEASEAAYK.E	1396.52786	2	2.82E-08	0.90	3.27	-	1061.8
AHQ-1-10, 2629	K.NSVVEASEAAYK.E	1268.35495	1	1.31E-05	0.63	2.21	-	578.2
AHQ-1-13- , 2672	K.NSVVEASEAAYK.E	1268.35495	2	1.20E-07	0.87	3.33	-	751.1

AHQ-1-10, 2468	K.NSVVEASEAAYK.E	1268.35495	2	1.36E-08	0.90	3.13	-	990.4
AHQ-1-13- , 6232 - 6235	K.QAFDDAIAELDTLNEDSYK.D	2159.24915	2	5.89E-07	0.94	4.61	-	761.3
AHQ-1-14- , 6040	K.QAFDDAIAELDTLNEDSYK.D	2159.24915	2	7.55E-05	0.84	3.77	-	565.3
AHQ-1-10, 5545 - 5619	K.QAFDDAIAELDTLNEDSYK.D	2159.24915	2	8.54E-06	0.29	2.63	-	153.9
AHQ-1-10, 2100	R.YLAEVASGEK.K	1067.17419	2	2.52E-05	0.87	2.99	-	1086.2
AHQ-1-10, 2099	R.YLAEVASGEK.K	1067.17419	1	1.72E-04	0.44	2.81	-	282.4
AHQ-1-10, 2093	R.YLAEVASGEK.K	1067.17419	1	1.16E-04	0.16	2.02	-	361.6
AHQ-1-13- , 2257 - 2267	R.YLAEVASGEK.K	1067.17419	2	6.49E-07	0.75	2.83	-	860.2
gi|4502565|ref|NP_001740.1| calpain, small subunit 1; calpain, small polypeptide; calcium-dependent				8.11E-10	3.95	50.24	36.20	28315.5
AHQ-1-12, 6597	R.LFAQLAGDDMEVSATELM*NILNK.V	2540.89556	3	5.53E-05	0.73	3.87	-	479.9
AHQ-1-12, 6413	R.SGTICSSELPGAFEAAGFHLNEHLYNM*IIR.R	3353.73016	3	8.41E-04	0.69	3.04	-	587.4
AHQ-1-12, 2819 - 2883	K.TDGFGIDTCR.S	1143.20921	2	9.15E-06	0.68	2.50	-	495.6
AHQ-1-11, 2307	R.THYSNIEANESEEVR.Q	1778.81502	2	2.42E-05	0.96	4.14	-	1668.1
AHQ-1-10, 2220 - 2281	R.THYSNIEANESEEVR.Q	1778.81502	2	5.89E-05	0.95	4.27	-	1290.5
AHQ-1-12, 6381 - 6418	R.YSDESGNM*DFDNFISCLVR.L	2287.42568	2	2.75E-08	0.95	4.62	-	1171.8
AHQ-1-11, 6154 - 6210	R.YSDESGNM*DFDNFISCLVR.L	2287.42568	2	4.97E-08	0.81	3.52	-	517.3
AHQ-1-10, 6120	R.YSDESGNM*DFDNFISCLVR.L	2287.42568	2	3.29E-07	0.95	4.75	-	1214.7
AHQ-1-13, 6320	R.YSDESGNM*DFDNFISCLVR.L	2287.42568	2	4.77E-06	0.83	3.52	-	577.4
AHQ-1-13- , 6773	R.YSDESGNM*DFDNFISCLVR.L	2287.42568	2	2.68E-07	0.90	3.98	-	713.7
AHQ-1-14- , 6523	R.YSDESGNM*DFDNFISCLVR.L	2287.42568	2	8.11E-10	0.90	3.77	-	887.1
gi|4505775|ref|NP_002626.1| phosphate carrier precursor isoform 1b; phosphate carrier, mitochondria				8.71E-10	2.43	30.21	15.20	39958.5
AHQ-1-9, 6559	K.GWAPTFLGYSMQGLCK.F	1818.10896	2	5.34E-06	0.94	4.15	-	979.3
AHQ-1-9, 2470	R.IQTQPGYANTLR.D	1362.51634	2	2.73E-04	0.67	3.02	-	393.8
AHQ-1-9, 7239	K.YYALCGFGGVLSCGLTHTAVVPLDLVK.C	2914.38804	2	8.71E-10	0.82	3.68	-	552.5
gi|17986258|ref|NP_066299.2| smooth muscle and non-muscle myosin alkali light chain isoform 1 [Homo				8.80E-10	7.08	80.27	42.40	16930.0
AHQ-1-14- , 4302	K.DQGTYEDYVEGLR.V	1545.58904	2	1.39E-05	0.85	3.46	-	1162.8
AHQ-1-13- , 4355	K.DQGTYEDYVEGLR.V	1545.58904	2	2.77E-06	0.95	3.76	-	1492.9
AHQ-1-12, 3913	K.EAFQLFDR.T	1026.12699	2	1.21E-04	0.77	2.93	-	701.5
AHQ-1-12, 2229 - 2273	R.HVLVTLGEK.M	996.18547	2	6.99E-07	0.88	3.30	-	638.9
AHQ-1-13- , 2359	R.HVLVTLGEK.M	996.18547	2	9.42E-07	0.82	2.88	-	608.8
AHQ-1-14, 3216	R.HVLVTLGEK.M	996.18547	1	7.00E-04	0.40	2.12	-	457.7
AHQ-1-13, 2414	R.HVLVTLGEK.M	996.18547	2	4.62E-06	0.86	3.27	-	682.5
AHQ-1-12, 2333	R.HVLVTLGEK.M	996.18547	2	4.72E-05	0.86	3.09	-	752.4
AHQ-1-12, 2239 - 2285	R.HVLVTLGEK.M	996.18547	1	2.00E-05	0.70	3.24	-	565.9
AHQ-1-14- , 2312 - 2366	R.HVLVTLGEK.M	996.18547	2	7.85E-05	0.85	2.74	-	736.0
AHQ-1-12, 2226 - 2374	R.HVLVTLGEK.M	996.18547	1	8.00E-04	0.74	2.48	-	803.9
AHQ-1-14- , 2320	R.HVLVTLGEK.M	996.18547	1	8.20E-04	0.79	2.84	-	726.5
AHQ-1-13- , 2356	R.HVLVTLGEK.M	996.18547	1	4.90E-04	0.61	2.52	-	661.2
AHQ-1-12, 3978 - 3990	K.NKDQGTYEDYVEGLR.V	1787.86505	3	2.53E-06	0.79	3.35	-	932.3
AHQ-1-14- , 3774	K.NKDQGTYEDYVEGLR.V	1787.86505	2	1.00E-07	0.94	3.84	-	1416.7
AHQ-1-12, 3967 - 3977	K.NKDQGTYEDYVEGLR.V	1787.86505	2	1.21E-09	0.89	3.82	-	910.7
AHQ-1-12, 3729	K.NKDQGTYEDYVEGLR.V	1787.86505	3	7.12E-06	0.83	3.69	-	1092.1
AHQ-1-12, 3723	K.NKDQGTYEDYVEGLR.V	1787.86505	2	6.10E-05	0.93	4.04	-	985.8
AHQ-1-12, 3601 - 3661	K.NKDQGTYEDYVEGLR.V	1787.86505	2	1.87E-09	0.96	4.38	-	1460.3
AHQ-1-13, 3729	K.NKDQGTYEDYVEGLR.V	1787.86505	2	1.56E-06	0.93	4.18	-	1106.9
AHQ-1-13, 4012	K.NKDQGTYEDYVEGLR.V	1787.86505	2	5.65E-09	0.86	3.40	-	806.2
AHQ-1-13- , 3876 - 3887	K.NKDQGTYEDYVEGLR.V	1787.86505	2	2.82E-09	0.95	4.25	-	1430.5
AHQ-1-12, 3594 - 3653	K.NKDQGTYEDYVEGLR.V	1787.86505	3	3.41E-04	0.93	4.38	-	1342.5
AHQ-1-14- , 3947	K.NKDQGTYEDYVEGLR.V	1787.86505	2	5.51E-06	0.89	3.69	-	1007.8
AHQ-1-14, 4652 - 4725	K.NKDQGTYEDYVEGLR.V	1787.86505	2	1.66E-07	0.91	4.11	-	1029.5
AHQ-1-12, 4057	K.NKDQGTYEDYVEGLR.V	1787.86505	2	3.81E-07	0.65	3.21	-	683.3
AHQ-1-14, 3504	R.VFDKEGNGTVM*GAEIR.H	1739.93168	2	1.98E-04	0.84	3.19	-	770.5
AHQ-1-13- , 3407 - 3429	R.VFDKEGNGTVMGAEIR.H	1723.93228	2	1.07E-08	0.94	4.38	-	1206.8
AHQ-1-12, 3257 - 3259	R.VFDKEGNGTVM*GAEIR.H	1739.93168	2	8.55E-04	0.94	3.92	-	1282.2
AHQ-1-14- , 2715	R.VFDKEGNGTVM*GAEIR.H	1739.93168	2	8.62E-05	0.81	2.63	-	838.2
AHQ-1-12, 3219 - 3281	R.VFDKEGNGTVMGAEIR.H	1723.93228	2	8.80E-10	0.95	4.33	-	1305.1
AHQ-1-12, 6186	K.VLDFEHFLPM*LQTVAK.N	1905.24957	2	5.82E-06	0.90	4.35	-	477.8
AHQ-1-12, 6525	K.VLDFEHFLPMLQTVAK.N	1889.25017	2	8.59E-08	0.94	4.69	-	864.0
AHQ-1-12, 6087 - 6157	K.VLDFEHFLPM*LQTVAK.N	1905.24957	3	1.22E-05	0.89	3.80	-	1143.4
AHQ-1-13, 6048	K.VLDFEHFLPM*LQTVAK.N	1905.24957	3	9.57E-05	0.73	3.16	-	600.9
AHQ-1-12, 6386 - 6445	K.VLDFEHFLPM*LQTVAK.N	1905.24957	2	2.08E-05	0.88	3.74	-	771.9
AHQ-1-13- , 6865	K.VLDFEHFLPMLQTVAK.N	1889.25017	3	1.15E-05	0.88	3.90	-	1166.0
AHQ-1-14- , 6234 - 6264	K.VLDFEHFLPM*LQTVAK.N	1905.24957	2	6.78E-04	0.93	3.97	-	898.9
gi|4758638|ref|NP_004896.1| peroxiredoxin 6; antioxidant protein 2; non-selenium glutathione peroxi				9.94E-10	8.82	110.33	53.10	25034.8
AHQ-1-10, 3669	R.DFTPVCTTELGR.A	1397.53591	1	1.04E-04	0.46	2.81	-	140.7
AHQ-1-10, 3660	R.DFTPVCTTELGR.A	1397.53591	1	9.40E-04	0.32	2.24	-	202.0
AHQ-1-13- , 3960 - 4007	R.DFTPVCTTELGR.A	1397.53591	2	5.53E-06	0.81	2.87	-	630.9
AHQ-1-10, 4481 - 4539	K.DGDSVMVLPTIPEEEAK.K	1831.03469	2	8.85E-05	0.74	2.99	-	784.0
AHQ-1-10, 3961 - 3984	K.DGDSVM*VLPTIPEEEAK.K	1847.03409	2	1.60E-05	0.48	2.52	-	375.2
AHQ-1-13- , 2683	K.DINAYNCEEPTEK.L	1584.64363	2	9.69E-05	0.66	2.77	-	645.8
AHQ-1-10, 5180 - 5249	K.DINAYNCEEPTEKLPFPIIDDR.N	2651.88650	2	5.85E-04	0.90	4.49	-	528.2
AHQ-1-10, 5651 - 5727	R.ELAILLGMLDPAEKDEK.G	1886.19976	2	6.20E-08	0.97	5.14	-	1227.8
AHQ-1-13- , 6323 - 6387	R.ELAILLGMLDPAEKDEK.G	1886.19976	2	1.80E-04	0.94	4.62	-	936.9
AHQ-1-10, 5860 - 5921	R.FHDFLGDSWGILFSHPR.D	2032.24831	2	1.91E-05	0.99	6.59	-	2624.4
AHQ-1-10, 5859 - 5861	R.FHDFLGDSWGILFSHPR.D	2032.24831	3	9.94E-10	0.97	5.80	-	1610.8
AHQ-1-14- , 5928 - 5995	K.LIALSIDSVEDHLAWSK.D	1898.14883	2	1.38E-06	0.96	4.68	-	1062.5
AHQ-1-13- , 6124	K.LIALSIDSVEDHLAWSK.D	1898.14883	3	3.80E-05	0.50	3.37	-	249.4
AHQ-1-10, 5524	K.LIALSIDSVEDHLAWSK.D	1898.14883	2	2.84E-09	0.94	4.22	-	1178.8
AHQ-1-10, 5476	K.LIALSIDSVEDHLAWSK.D	1898.14883	3	1.43E-05	0.53	3.48	-	346.5
AHQ-1-13- , 6123	K.LIALSIDSVEDHLAWSK.D	1898.14883	2	2.52E-07	0.86	2.96	-	888.7
AHQ-1-14- , 5915	K.LIALSIDSVEDHLAWSK.D	1898.14883	3	1.90E-04	0.54	3.17	-	356.4
AHQ-1-10, 5420 - 5475	K.LIALSIDSVEDHLAWSK.D	1898.14883	2	9.90E-08	0.96	4.85	-	1332.6
AHQ-1-13, 4483	K.LPFPIIDDR.N	1086.26548	2	5.25E-05	0.86	3.25	-	611.8
AHQ-1-10, 4196	K.LPFPIIDDR.N	1086.26548	2	1.98E-05	0.92	3.36	-	896.1
AHQ-1-13- , 4693	K.LPFPIIDDR.N	1086.26548	2	4.08E-05	0.95	3.17	-	1463.3
AHQ-1-10, 4696	R.VATPVDWKDGDSVM*VLPTIPEEEAK.K	2744.06717	3	8.72E-04	0.56	3.16	-	297.3
AHQ-1-10, 3591	R.VVFVFGPDK.K	1008.19471	1	1.86E-04	0.76	2.33	-	969.7
AHQ-1-10, 3599	R.VVFVFGPDK.K	1008.19471	2	4.80E-06	0.88	3.19	-	654.1
gi|4507677|ref|NP_003290.1| tumor rejection antigen (gp96) 1; Tumor rejection antigen-1 (gp96); glu				9.94E-10	9.17	110.28	18.40	92468.2
AHQ-1-5, 6593	R.EAVEKEFEPLLNWMK.D	1864.15434	2	3.70E-04	0.86	3.30	-	879.2
AHQ-1-5, 3929	R.FQSSHHPTDITSLDQYVER.M	2261.39276	3	2.43E-08	0.98	5.53	-	2393.7
AHQ-1-5, 3927 - 3949	R.FQSSHHPTDITSLDQYVER.M	2261.39276	2	4.99E-06	0.96	4.94	-	1225.0
AHQ-1-5, 4047 - 4117	K.GVVDSDDLPLNVSR.E	1486.60940	1	7.51E-04	0.63	2.53	-	474.5
AHQ-1-6, 4038	K.GVVDSDDLPLNVSR.E	1486.60940	2	1.06E-09	0.93	4.18	-	881.3
AHQ-1-5, 7317 - 7318	K.GYEVIYLTEPVDEYCIQALPEFDGK.R	2951.24921	2	4.27E-05	0.62	2.85	-	310.6
AHQ-1-5, 7011 - 7074	K.GYEVIYLTEPVDEYCIQALPEFDGKR.F	3107.43557	3	9.94E-10	0.87	4.43	-	612.7
AHQ-1-5, 3365 - 3437	K.IADDKYNDTFWK.E	1516.63566	2	1.20E-04	0.88	3.39	-	861.8
AHQ-1-5, 6454 - 6521	K.KYSQFINFPIYVWSSK.T	2008.30657	2	1.04E-08	0.97	5.04	-	1841.0
AHQ-1-5, 5339	R.LISLTDENALSGNEELTVK.I	2047.24975	2	3.20E-05	0.90	4.11	-	793.6
AHQ-1-5, 5229	R.LISLTDENALSGNEELTVK.I	2047.24975	2	7.99E-07	0.95	5.20	-	1115.8
AHQ-1-5, 2653	R.SGYLLPDTK.A	994.12321	1	1.35E-06	0.70	2.89	-	629.0
AHQ-1-5, 2642	R.SGYLLPDTK.A	994.12321	1	6.92E-05	0.79	2.66	-	768.4
AHQ-1-5, 2649 - 2650	R.SGYLLPDTK.A	994.12321	2	2.71E-05	0.88	2.86	-	944.2
AHQ-1-5, 2662	K.TETVEEPMEEEEAAKEEK.E	2109.20871	2	5.87E-05	0.45	2.73	-	312.3
AHQ-1-5, 7065 - 7086	K.YSQFINFPIYVWSSK.T	1880.13366	2	5.79E-06	0.96	4.91	-	1035.7
gi|29739414|ref|XP_294015.1| similar to Glyceraldehyde 3-phosphate dehydrogenase, liver (GAPDH) [Ho				1.03E-09	4.54	50.25	17.70	26916.7
AHQ-1-11, 3902	K.IISNASCTTNCLAPLAK.V	1837.10868	2	9.82E-08	0.80	3.73	-	806.7

AHQ-1-14- , 3995	K.IISNASCTTNCLAPLAK.V	1837.10868	2	3.71E-06	0.92	3.78	-	1519.4
AHQ-1-11, 3762 - 3822	K.IISNASCTTNCLAPLAK.V	1837.10868	2	1.46E-07	0.87	4.20	-	1282.1
AHQ-1-9, 3926 - 3947	K.IISNASCTTNCLAPLAK.V	1837.10868	2	3.54E-08	0.89	4.37	-	1202.3
AHQ-1-9, 4020 - 4087	K.IISNASCTTNCLAPLAK.V	1837.10868	2	3.26E-05	0.62	3.37	-	773.9
AHQ-1-9, 4142 - 4212	K.IISNASCTTNCLAPLAK.V	1837.10868	2	6.17E-09	0.81	4.06	-	961.5
AHQ-1-9, 5007 - 5038	K.LISWYDNEFGYSNR.V	1764.87478	2	2.13E-07	0.95	4.30	-	1243.8
AHQ-1-9, 5174 - 5242	K.LISWYDNEFGYSNR.V	1764.87478	2	9.49E-08	0.94	4.42	-	887.8
AHQ-1-9, 5363	K.LISWYDNEFGYSNR.V	1764.87478	2	2.62E-04	0.88	3.63	-	772.6
AHQ-1-9, 5495	K.LISWYDNEFGYSNR.V	1764.87478	2	1.56E-04	0.85	3.71	-	687.6
AHQ-1-14- , 5022 - 5102	K.LISWYDNEFGYSNR.V	1764.87478	2	1.10E-07	0.96	4.57	-	1510.2
AHQ-1-9, 4827	K.LISWYDNEFGYSNR.V	1764.87478	2	1.03E-09	0.94	4.35	-	716.4
AHQ-1-13- , 5137 - 5139	K.LISWYDNEFGYSNR.V	1764.87478	2	1.44E-09	0.97	4.55	-	1380.1
AHQ-1-10, 4591 - 4611	K.LISWYDNEFGYSNR.V	1764.87478	2	2.00E-08	0.95	4.70	-	992.7
AHQ-1-11, 4686 - 4751	K.LISWYDNEFGYSNR.V	1764.87478	2	1.29E-06	0.59	2.91	-	747.8
AHQ-1-11, 4827	K.LISWYDNEFGYSNR.V	1764.87478	2	3.97E-06	0.95	4.20	-	1075.7
AHQ-1-12, 4894	K.LISWYDNEFGYSNR.V	1764.87478	2	5.13E-04	0.75	2.53	-	998.3
AHQ-1-13, 4864	K.LISWYDNEFGYSNR.V	1764.87478	2	4.61E-09	0.93	4.25	-	829.3
AHQ-1-14, 5966 - 6028	K.LISWYDNEFGYSNR.V	1764.87478	2	2.20E-09	0.97	4.96	-	1345.2
AHQ-1-9, 2730	R.VVDLMAHMASK.E	1202.47292	2	2.35E-04	0.95	3.64	-	1160.3
AHQ-1-9, 2134	R.VVDLMAHM*ASKE	1347.58686	2	4.67E-04	0.87	3.97	-	1119.6
AHQ-1-9, 2942	R.VVDLMAHMASKE	1331.58746	2	1.55E-05	0.97	4.21	-	1881.0
gi|13489087|ref|NP_109591.1|	serine (or cysteine) proteinase inhibitor, clade B (ovalbumin), member			1.07E-09	4.58	50.23	22.20	42741.5
AHQ-1-9, 4363	K.FAYGYIEDLK.C	1219.36713	2	2.39E-08	0.95	3.31	-	1327.2
AHQ-1-9, 4567	R.LGVQDLFNSSK.A	1208.34588	2	2.60E-05	0.91	2.71	-	1250.5
AHQ-1-9, 4036	K.TYGADLASVDFQHASEDAR.K	2054.11949	3	5.36E-09	0.88	4.09	-	640.2
AHQ-1-9, 4010 - 4078	K.TYGADLASVDFQHASEDAR.K	2054.11949	2	1.07E-09	0.93	4.06	-	953.7
AHQ-1-8, 4065 - 4144	K.TYGADLASVDFQHASEDAR.K	2054.11949	3	6.90E-05	0.61	3.26	-	418.8
AHQ-1-8, 4059	K.TYGADLASVDFQHASEDAR.K	2054.11949	2	3.14E-09	0.90	3.69	-	901.9
AHQ-1-9, 6340	K.TYNFLPEFLVSTQK.T	1687.91650	2	2.99E-04	0.94	4.67	-	578.9
AHQ-1-9, 7234 - 7306	R.VLELPYQGEELSMVILLPDDIEDESTGLKK.I	3375.82891	3	6.31E-09	0.86	4.57	-	557.5
gi|5729997|ref|NP_004154.2|	RAB27B, member RAS oncogene family [Homo sapiens]			1.07E-09	5.93	70.24	35.30	24607.7
AHQ-1-11, 6286	K.AVETLLDLIMK.R	1246.54211	2	1.96E-08	0.90	3.73	-	843.3
AHQ-1-10, 5295	K.AVETLLDLIM*K.R	1262.54151	2	5.79E-06	0.88	3.43	-	938.6
AHQ-1-10, 6160 - 6163	K.AVETLLDLIMK.R	1246.54211	2	9.83E-08	0.91	3.90	-	761.9
AHQ-1-10, 4917	K.AVETLLDLIM*KR.M	1418.72786	2	5.27E-04	0.71	2.67	-	647.2
AHQ-1-13- , 3132	K.LLALGDSGVGK.T	1030.20023	2	1.06E-06	0.84	2.68	-	1030.5
AHQ-1-13- , 3133	K.LLALGDSGVGK.T	1030.20023	1	5.74E-04	0.48	2.03	-	548.7
AHQ-1-11, 2930	K.LLALGDSGVGK.T	1030.20023	1	5.54E-04	0.30	2.07	-	354.9
AHQ-1-10, 2836	K.LLALGDSGVGK.T	1030.20023	1	1.67E-04	0.58	2.16	-	723.2
AHQ-1-13, 3097	K.LLALGDSGVGK.T	1030.20023	1	5.60E-04	0.57	2.35	-	704.7
AHQ-1-10, 5720 - 5767	R.NWMSQLQANAYCENPDIVLIGNK.A	2680.99772	2	1.20E-07	0.71	3.16	-	508.9
AHQ-1-11, 5987	R.NWMSQLQANAYCENPDIVLIGNK.A	2680.99772	2	5.95E-05	0.88	4.03	-	443.4
AHQ-1-10, 5887	R.NWMSQLQANAYCENPDIVLIGNK.A	2680.99772	2	8.68E-04	0.80	3.32	-	578.2
AHQ-1-10, 3399 - 3401	K.VHLQLWDTAGQER.F	1553.70390	2	2.76E-08	0.95	4.09	-	1209.2
AHQ-1-12, 3637	K.VHLQLWDTAGQER.F	1553.70390	2	5.54E-08	0.94	3.62	-	1245.9
AHQ-1-13, 3743	K.VHLQLWDTAGQER.F	1553.70390	2	6.59E-06	0.92	3.73	-	1159.7
AHQ-1-11, 3444 - 3510	K.VHLQLWDTAGQER.F	1553.70390	2	7.43E-06	0.92	3.62	-	821.8
AHQ-1-13- , 3868	K.VHLQLWDTAGQER.F	1553.70390	2	4.31E-08	0.96	3.87	-	1769.6
AHQ-1-10, 4272 - 4312	K.YGIPYFETSAATGQNVEK.A	1976.13177	2	4.54E-06	0.74	3.45	-	710.3
AHQ-1-10, 4124 - 4179	K.YGIPYFETSAATGQNVEK.A	1976.13177	2	1.07E-09	0.95	4.87	-	752.2
AHQ-1-11, 4302 - 4339	K.YGIPYFETSAATGQNVEK.A	1976.13177	2	4.93E-08	0.95	4.26	-	1179.8
AHQ-1-12, 4470	K.YGIPYFETSAATGQNVEK.A	1976.13177	2	4.48E-04	0.78	3.54	-	408.8
AHQ-1-13- , 4647 - 4711	K.YGIPYFETSAATGQNVEK.A	1976.13177	2	3.07E-08	0.91	4.12	-	757.7
gi|4557395|ref|NP_000058.1|	carbonic anhydrase II; carbonate dehydratase II; carbonic dehydratase;			1.07E-09	4.68	60.28	32.30	29245.8
AHQ-1-10, 6292	K.AVQQPDGLAVLGIFLK.V	1669.98899	2	3.16E-09	0.95	4.79	-	664.3
AHQ-1-13- , 6895	K.AVQQPDGLAVLGIFLK.V	1669.98899	2	1.68E-08	0.94	4.55	-	636.3
AHQ-1-10, 3048	K.EPISVSSEQVLK.F	1316.48265	1	9.76E-04	0.41	3.00	-	161.8
AHQ-1-10, 3931 - 4004	R.ILNNGHAFNVEFDDSQDK.A	2064.15751	2	1.07E-09	0.97	5.61	-	1757.9
AHQ-1-10, 3763	R.ILNNGHAFNVEFDDSQDK.A	2064.15751	2	2.39E-06	0.87	3.90	-	947.2
AHQ-1-10, 3129	K.SADFTNFDPR.G	1170.21306	2	1.66E-06	0.85	3.02	-	818.2
AHQ-1-10, 3559	K.VVDVLDSIK.T	988.16011	2	4.58E-05	0.87	3.66	-	436.7
AHQ-1-13, 4472	K.YDPSLKPLSVSYDQATSLR.I	2141.36647	2	4.61E-07	0.64	3.00	-	418.8
gi|4505303|ref|NP_002466.1|	myosin alkali light chain 1 slow a; myosin light chain 1, slow-twitch m			1.09E-09	2.69	30.20	11.50	22763.8
AHQ-1-12, 2677 - 2757	R.ALGQNPTNAEVLK.V	1355.52211	2	1.09E-09	0.85	3.97	-	435.1
AHQ-1-12, 2779	R.ALGQNPTNAEVLK.V	1355.52211	2	5.10E-06	0.84	3.13	-	622.6
AHQ-1-12, 2877 - 2905	R.ALGQNPTNAEVLK.V	1355.52211	2	1.29E-07	0.84	3.71	-	425.2
AHQ-1-12, 2585	R.ALGQNPTNAEVLK.V	1355.52211	2	4.03E-07	0.70	3.32	-	350.9
AHQ-1-13- , 2743	K.ILYSQCGDVM*R.A	1359.55429	2	2.97E-06	0.92	3.11	-	1154.1
AHQ-1-13- , 3395	K.ILYSQCGDVMR.A	1343.55489	2	5.09E-07	0.90	3.09	-	975.6
AHQ-1-14- , 2694 - 2768	K.ILYSQCGDVM*R.A	1359.55429	2	1.17E-05	0.94	3.46	-	1192.3
AHQ-1-12, 3174 - 3237	K.ILYSQCGDVMR.A	1343.55489	2	1.74E-08	0.91	3.23	-	1020.1
gi|4507645|ref|NP_000356.1|	triosephosphate isomerase 1 [Homo sapiens]			1.18E-09	3.55	40.27	30.90	26669.3
AHQ-1-13- , 4023	K.DCGATWVVLGHSER.R	1588.72671	2	2.38E-07	0.89	3.78	-	701.4
AHQ-1-13, 3868	K.DCGATWVVLGHSER.R	1588.72671	2	8.03E-07	0.91	3.72	-	951.8
AHQ-1-10, 3581 - 3653	K.DCGATWVVLGHSER.R	1588.72671	2	1.18E-09	0.95	4.28	-	1256.3
AHQ-1-14- , 6574	K.ELASQPDVDGFLVGGASLKPEFVDIINAK.Q	3031.40532	3	1.78E-06	0.58	3.48	-	171.1
AHQ-1-10, 6007 - 6085	K.ELASQPDVDGFLVGGASLKPEFVDIINAK.Q	3031.40532	3	7.64E-07	0.80	3.84	-	238.4
AHQ-1-13- , 6684	K.ELASQPDVDGFLVGGASLKPEFVDIINAK.Q	3031.40532	3	7.58E-06	0.69	3.47	-	233.9
AHQ-1-10, 4815	K.QSLGELIGTLNAAK.V	1415.61746	2	8.42E-05	0.83	3.14	-	706.0
AHQ-1-13, 5349	K.VPADTEVVCAPPTAYIDFAR.Q	2194.44943	2	3.54E-07	0.96	5.31	-	871.4
AHQ-1-13- , 5656	K.VPADTEVVCAPPTAYIDFAR.Q	2194.44943	2	6.60E-06	0.93	4.28	-	763.8
gi|4503573|ref|NP_001967.1|	enolase 3; enolase-3, beta, muscle; muscle specific enolase; beta enola			1.25E-09	0.71	10.21	3.50	46958.5
AHQ-1-8, 3776	K.VNQIGSVTESIQACK.L	1635.82159	2	7.73E-06	0.90	4.19	-	582.5
AHQ-1-8, 3979	K.VNQIGSVTESIQACK.L	1635.82159	2	1.25E-09	0.71	3.24	-	632.4
AHQ-1-13, 3751	K.VNQIGSVTESIQACK.L	1635.82159	2	6.49E-04	0.89	3.59	-	745.3
AHQ-1-14, 4594 - 4648	K.VNQIGSVTESIQACK.L	1635.82159	2	2.88E-05	0.75	2.96	-	590.7
AHQ-1-13- , 3889	K.VNQIGSVTESIQACK.L	1635.82159	2	5.98E-07	0.83	3.02	-	796.8
AHQ-1-8, 3693	K.VNQIGSVTESIQACK.L	1635.82159	2	1.52E-06	0.94	3.99	-	1051.2
gi|29568101|ref|NP_006467.3|	ATP synthase, H+ transporting, mitochondrial F0 complex, subunit g; AT			1.33E-09	2.44	30.22	14.60	11400.3
AHQ-1-14- , 3660 - 3731	K.TPALVNAAVTYSK.P	1335.53080	2	1.33E-09	0.93	4.19	-	869.6
AHQ-1-14- , 3678 - 3715	K.TPALVNAAVTYSKPR.L	1588.83283	2	4.85E-05	0.96	4.47	-	1196.4
AHQ-1-14, 4512 - 4570	K.TPALVNAAVTYSKPR.L	1588.83283	2	2.84E-06	0.91	4.03	-	869.5
AHQ-1-14- , 4388	K.VELVPPTPAEIPR.A	1418.66294	2	9.83E-05	0.60	2.53	-	259.6
gi|16933567|ref|NP_005361.2|	mel transforming oncogene; ras-associated protein RAB8 [Homo sapiens]			1.33E-09	0.92	10.20	6.80	23668.1
AHQ-1-11, 5723	K.ANINVENAFFTLAR.D	1580.76926	2	1.33E-09	0.92	3.92	-	909.7
gi|29730023|ref|XP_291482.1|	similar to 78 kDa glucose-regulated protein precursor (GRP 78) (Immuno			1.39E-09	5.31	60.25	13.30	63563.0
AHQ-1-5, 4902	R.IINKPTAAAIAYGLDK.R	1659.95097	2	3.59E-07	0.70	3.26	-	701.9
AHQ-1-7, 4874	R.IINKPTAAAIAYGLDK.R	1659.95097	2	1.15E-05	0.87	3.30	-	989.4
AHQ-1-6, 4782 - 4863	R.IINKPTAAAIAYGLDK.R	1659.95097	2	1.96E-08	0.82	4.16	-	774.9
AHQ-1-9, 4758	R.IINKPTAAAIAYGLDK.R	1659.95097	2	1.88E-05	0.83	4.32	-	726.1
AHQ-1-6, 3468 - 3523	K.KKELEEIVQPIISK.L	1654.97263	2	3.20E-07	0.96	4.79	-	1452.4
AHQ-1-5, 3595	K.LYGSAGPPPTGEEDTAEKDEL	2177.26447	2	8.47E-09	0.93	4.32	-	847.4
AHQ-1-6, 3512 - 3594	K.LYGSAGPPPTGEEDTAEKDEL	2177.26447	2	1.18E-05	0.96	5.02	-	866.5
AHQ-1-6, 3430	K.LYGSAGPPPTGEEDTAEKDEL	2177.26447	2	1.00E-03	0.83	3.32	-	787.3
AHQ-1-6, 4030	R.NELESYAYSLK.N	1317.42597	2	3.74E-04	0.87	2.79	-	1089.5
AHQ-1-5, 5855	K.TFAPKEISAMVLTK.M	1536.86124	2	9.24E-06	0.90	2.96	-	1300.7
AHQ-1-6, 5764	K.TFAPKEISAMVLTK.M	1536.86124	2	1.39E-09	0.87	3.12	-	1269.1

AHQ-1-6, 4919 - 4992	K.TFAPKEISAM*VLTK.M	1552.86064	2	1.19E-05	0.85	3.61	-	1115.2
gi|29742827|ref|XP_294099.1| similar to myosin:SUBUNIT=regulatory light chain [Homo sapiens]				1.43E-09	1.63	20.23	14.20	17094.0
AHQ-1-11, 2267 - 2339	K.EAFNM*IDQNR.D	1254.35498	2	6.81E-04	0.66	2.70	-	540.9
AHQ-1-11, 3132	R.FTDEEVDELYR.E	1416.47127	2	1.80E-04	0.94	3.69	-	1267.6
AHQ-1-11, 3615 - 3678	R.FTDEEVDELYR.E	1416.47127	2	3.58E-08	0.97	4.63	-	1707.0
AHQ-1-11, 3679 - 3739	R.FTDEEVDELYR.E	1416.47127	1	9.65E-06	0.37	1.82	-	427.7
AHQ-1-11, 3692	R.FTDEEVDELYR.E	1416.47127	1	4.78E-05	0.61	2.54	-	443.4
AHQ-1-11, 3738 - 3751	R.FTDEEVDELYR.E	1416.47127	2	1.43E-09	0.97	4.55	-	1901.3
AHQ-1-12, 3706 - 3769	R.FTDEEVDELYR.E	1416.47127	2	1.31E-08	0.97	4.56	-	1686.9
AHQ-1-13, 3847	R.FTDEEVDELYR.E	1416.47127	2	8.77E-08	0.95	3.44	-	1575.9
AHQ-1-14, 4760 - 4761	R.FTDEEVDELYR.E	1416.47127	2	5.83E-08	0.97	4.40	-	1664.6
AHQ-1-13- , 3991	R.FTDEEVDELYR.E	1416.47127	2	2.47E-09	0.96	3.57	-	1753.8
AHQ-1-14- , 3894 - 3895	R.FTDEEVDELYR.E	1416.47127	2	1.05E-08	0.97	4.64	-	1698.5
gi|21361621|ref|NP_002624.2| phosphoglucomutase 1 [Homo sapiens]				1.44E-09	1.45	20.17	5.00	61448.7
AHQ-1-7, 3806	K.FNISNGGPAPEAITDK.I	1631.76820	2	1.44E-09	0.93	3.44	-	1638.9
AHQ-1-7, 4078 - 4086	K.TIEEYAVCPDLK.V	1439.61271	2	6.11E-04	0.52	2.52	-	767.2
gi|5803227|ref|NP_006817.1| tyrosine 3/tryptophan 5 -monooxygenase activation protein, theta polype				1.58E-09	4.46	50.26	30.20	27764.1
AHQ-1-14- , 2287	K.AVTEQGAELSNEER.N	1533.57979	2	5.43E-06	0.89	2.92	-	1374.4
AHQ-1-13- , 2311	K.AVTEQGAELSNEER.N	1533.57979	2	1.58E-09	0.93	3.91	-	1172.7
AHQ-1-10, 2141 - 2165	K.AVTEQGAELSNEER.N	1533.57979	2	5.92E-07	0.84	3.16	-	950.0
AHQ-1-13- , 4217	K.QTIDNSQGAYQEAFDISK.K	2016.11116	2	1.50E-05	0.86	3.53	-	793.5
AHQ-1-10, 3823	K.QTIDNSQGAYQEAFDISK.K	2016.11116	2	3.20E-04	0.45	2.77	-	133.3
AHQ-1-13- , 6767	K.TAFDEAIAELDTLNEDSYK.D	2146.25037	2	4.41E-09	0.95	4.72	-	1160.8
AHQ-1-10, 6127	K.TAFDEAIAELDTLNEDSYK.D	2146.25037	2	2.59E-05	0.96	4.83	-	1082.8
AHQ-1-13, 6315 - 6316	K.TAFDEAIAELDTLNEDSYK.D	2146.25037	2	4.10E-06	0.95	4.83	-	1004.4
AHQ-1-14- , 6522 - 6528	K.TAFDEAIAELDTLNEDSYK.D	2146.25037	2	8.64E-06	0.95	5.14	-	989.2
AHQ-1-10, 2551	R.YLAEVACGDDR.K	1270.35099	2	7.22E-08	0.91	2.92	-	1249.9
AHQ-1-10, 2553	K.YLIANATNPESK.V	1321.46099	2	2.39E-04	0.94	3.37	-	1726.3
AHQ-1-10, 2643 - 2707	K.YLIANATNPESK.V	1321.46099	1	1.45E-07	0.81	3.03	-	814.2
AHQ-1-10, 2648	K.YLIANATNPESK.V	1321.46099	2	1.30E-04	0.92	3.60	-	1183.3
gi|12545410|ref|NP_031394.1| RAS p21 protein activator 3; GTPase activating protein III; Ins(1,3,4,				1.60E-09	1.77	20.24	4.30	95912.5
AHQ-1-5, 5375	K.SADVEPVSASAAHILGEVCR.E	2070.26993	2	1.60E-09	0.95	4.88	-	1055.6
AHQ-1-5, 6513	K.SLCPFYGEDFYCEIPR.S	2056.26024	2	1.90E-05	0.82	3.60	-	429.9
gi|20127408|ref|NP_000173.2| hydroxyacyl dehydrogenase, subunit A; trifunctional protein, alpha sub				1.76E-09	4.81	60.25	12.70	82999.1
AHQ-1-6, 6556	R.DSIFSNLTGQLDYQGFEK.A	2063.20943	2	6.76E-08	0.96	4.92	-	1075.3
AHQ-1-6, 2616	K.DTSASAVAVGLK.Q	1119.25064	1	2.83E-04	0.20	2.22	-	392.8
AHQ-1-6, 4704	R.FGGGNPELLTQMVSK.G	1578.81508	2	4.98E-06	0.91	3.83	-	806.3
AHQ-1-6, 3094	K.TGIEQGSDAGYLCESQK.F	1844.93497	2	1.76E-09	0.92	3.83	-	875.2
AHQ-1-6, 3596	K.TLQEVTQLSQEAQR.I	1631.76974	2	1.18E-05	0.95	3.91	-	1896.1
AHQ-1-6, 6202	K.TVLGTPEVLLGALPGAGGTQR.L	2008.30795	3	1.65E-07	0.87	4.18	-	836.4
gi|8922673|ref|NP_060692.1| parvin, alpha; alpha-parvin; actopaxin [Homo sapiens]				1.79E-09	0.96	10.20	3.50	42243.4
AHQ-1-9, 4644 - 4656	R.DAFDTLFDHAPDK.L	1492.57096	2	8.48E-08	0.93	3.89	-	1137.1
AHQ-1-9, 4654	R.DAFDTLFDHAPDK.L	1492.57096	1	1.82E-04	0.47	2.34	-	520.3
AHQ-1-9, 4664	R.DAFDTLFDHAPDK.L	1492.57096	1	2.60E-06	0.87	3.91	-	605.0
AHQ-1-13- , 4779	R.DAFDTLFDHAPDK.L	1492.57096	2	4.31E-09	0.93	3.47	-	1288.4
AHQ-1-9, 4307	R.DAFDTLFDHAPDK.L	1492.57096	2	1.79E-09	0.96	3.68	-	1632.3
gi|4505759|ref|NP_002622.1| phosphogluconate dehydrogenase; 6-phosphogluconate dehydrogenase [Homo				1.79E-09	2.78	30.28	11.20	53151.7
AHQ-1-14, 5518	R.DYFGAHTYELLAK.P	1528.68958	2	1.67E-06	0.96	4.04	-	1603.4
AHQ-1-8, 6438	K.LVPLLDTGDIIIDGGNSEYR.D	2161.39768	2	5.75E-05	0.97	5.51	-	1555.0
AHQ-1-8, 6622	K.LVPLLDTGDIIIDGGNSEYR.D	2161.39768	2	1.79E-09	0.98	4.84	-	2241.5
AHQ-1-8, 4465	K.VGTGEPCCDWVGDEGAGHFVK.M	2280.43619	2	4.92E-05	0.81	3.31	-	587.9
AHQ-1-8, 4464	K.VGTGEPCCDWVGDEGAGHFVK.M	2280.43619	3	9.80E-09	0.84	4.12	-	352.1
gi|30150022|ref|XP_300862.1| similar to beta-tubulin 4Q [Homo sapiens]				1.85E-09	4.60	50.22	22.20	33545.2
AHQ-1-7, 6493	K.EAESCDCLQGFQLTHSLGGGTGSGMGTLLISK.I	3315.65550	3	5.74E-06	0.93	4.49	-	1401.9
AHQ-1-13- , 6252	K.EAESCDCLQGFQLTHSLGGGTGSGMGTLLISK.I	3315.65550	3	3.96E-04	0.85	3.86	-	944.0
AHQ-1-9, 3224 - 3236	R.FPGQLNADLR.K	1131.26634	2	1.26E-06	0.85	3.05	-	792.0
AHQ-1-8, 3197 - 3236	R.FPGQLNADLR.K	1131.26634	2	3.57E-05	0.90	3.13	-	852.8
AHQ-1-7, 3616	R.FPGQLNADLR.K	1131.26634	2	9.21E-07	0.85	3.06	-	714.8
AHQ-1-7, 3480 - 3496	R.FPGQLNADLR.K	1131.26634	2	6.19E-05	0.88	3.17	-	760.6
AHQ-1-7, 3340 - 3409	R.FPGQLNADLR.K	1131.26634	2	1.41E-04	0.91	3.54	-	888.3
AHQ-1-7, 3956	R.KLAVNMVPFPR.L	1272.58771	2	3.11E-07	0.95	4.07	-	1261.0
AHQ-1-8, 4329	K.LAVNMVPFPR.L	1144.41480	2	3.37E-05	0.94	3.83	-	899.1
AHQ-1-7, 4536	K.LAVNMVPFPR.L	1144.41480	2	3.23E-06	0.94	4.01	-	888.2
AHQ-1-10, 4116	K.LAVNMVPFPR.L	1144.41480	2	1.52E-06	0.90	3.41	-	731.2
AHQ-1-9, 4400 - 4406	K.LAVNMVPFPR.L	1144.41480	2	2.76E-08	0.94	4.29	-	773.3
AHQ-1-7, 5864 - 5892	R.LHFFMPGFAPLTSR.G	1621.92962	2	1.24E-08	0.94	3.67	-	895.7
AHQ-1-11, 5416	R.LHFFMPGFAPLTSR.G	1621.92962	2	6.91E-05	0.93	3.57	-	1037.1
AHQ-1-7, 5874	R.LHFFMPGFAPLTSR.G	1621.92962	3	7.20E-04	0.82	3.55	-	931.9
AHQ-1-9, 5695 - 5698	R.LHFFMPGFAPLTSR.G	1621.92962	2	1.85E-09	0.92	3.76	-	737.3
gi|19913424|ref|NP_001681.2| ATPase, H+ transporting, lysosomal 70kD, V1 subunit A, isoform 1; V-AT				1.91E-09	2.85	30.25	7.10	68303.7
AHQ-1-6, 4318	R.LAEMPADSGYPAYLGAR.L	1782.99819	2	1.12E-08	0.95	4.58	-	949.5
AHQ-1-6, 3078	R.TALVANTSNMPVAAR.E	1516.74852	2	1.91E-09	0.98	5.09	-	1974.1
AHQ-1-6, 3192	R.VGHSELVGEIIR.L	1309.49658	2	3.19E-08	0.92	3.38	-	1140.0
gi|4502097|ref|NP_001142.1| solute carrier family 25 (mitochondrial carrier; adenine nucleotide tra				1.92E-09	2.74	30.18	12.10	33148.4
AHQ-1-10, 3176 - 3247	R.AAYFGVYDTAK.G	1206.32845	2	1.19E-06	0.92	3.62	-	802.5
AHQ-1-10, 4248	K.DFLAGAVAAAVSK.T	1220.39980	2	5.22E-04	0.93	3.34	-	2189.0
AHQ-1-10, 5240	R.YFPTQALNFAFK.D	1447.66130	1	3.20E-06	0.58	2.92	-	409.8
AHQ-1-13, 5519	R.YFPTQALNFAFK.D	1447.66130	2	1.92E-09	0.89	2.91	-	831.3
AHQ-1-13- , 5789 - 5855	R.YFPTQALNFAFK.D	1447.66130	2	3.08E-07	0.86	3.23	-	695.7
AHQ-1-10, 5231 - 5252	R.YFPTQALNFAFK.D	1447.66130	2	1.06E-07	0.92	3.08	-	868.8
gi|4503117|ref|NP_000091.1| cystatin B; stefin B; liver thiol proteinase inhibitor; CPI-B [Homo sap				2.16E-09	2.45	30.21	45.90	11139.5
AHQ-1-13- , 3600 - 3655	K.SQVVAGTNYFIK.V	1327.51036	2	1.59E-06	0.92	3.76	-	988.7
AHQ-1-13- , 3804	R.VFQSLPHENKPLTLSNYQTNK.A	2459.74218	3	5.10E-06	0.70	3.39	-	542.5
AHQ-1-13- , 3813	R.VFQSLPHENKPLTLSNYQTNK.A	2459.74218	2	6.97E-04	0.91	4.21	-	890.6
AHQ-1-13, 3295	K.VHVGDEDFVHLR.V	1423.55823	2	2.16E-09	0.83	2.94	-	515.5
gi|7657431|ref|NP_055205.1| EBNA-2 co-activator (100kD) [Homo sapiens]				2.17E-09	3.59	40.25	10.50	99689.7
AHQ-1-4, 6378 - 6402	R.DIQNTQCLLNVEHLSAGCPHVTLQFADSK.G	3299.63749	3	2.17E-09	0.96	5.00	-	1638.6
AHQ-1-4, 6574	K.DTNGENIAESLVAEGLATR.R	1961.07704	2	1.10E-08	0.96	4.36	-	1767.3
AHQ-1-4, 6680	R.NLPGLVQEGEPFSEEATLFTK.E	2307.54125	2	3.24E-04	0.90	4.30	-	515.9
AHQ-1-4, 5043	K.VNVTVDYIRPASPATETVPAFSER.T	2620.89872	2	2.11E-04	0.61	2.83	-	376.9
AHQ-1-4, 5039	K.VNVTVDYIRPASPATETVPAFSER.T	2620.89872	3	3.90E-05	0.78	3.55	-	671.5
gi|4505773|ref|NP_002625.1| prohibitin [Homo sapiens]				2.21E-09	1.81	20.25	9.60	29803.9
AHQ-1-10, 3473	R.ILFRPVASQLPR.I	1397.69333	2	4.46E-04	0.84	3.09	-	778.5
AHQ-1-10, 3948	R.KLEAAEDIAYQLSR.S	1607.78978	2	2.21E-09	0.98	4.92	-	2169.0
gi|4507171|ref|NP_003109.1| secreted protein, acidic, cysteine-rich (osteonectin); Osteonectin (sec				2.22E-09	3.35	40.28	21.10	34632.0
AHQ-1-9, 3055	R.LEAGDHPVELLAR.D	1420.59579	2	1.32E-04	0.93	3.90	-	866.5
AHQ-1-8, 3057	R.LEAGDHPVELLAR.D	1420.59579	2	2.22E-09	0.94	4.37	-	1053.6
AHQ-1-9, 7126	K.NYNMYIFPVHWQFGQLDQHPIDGYLSHTELAPLR.A	4102.58560	3	2.69E-07	0.94	5.58	-	685.2
AHQ-1-9, 2723	K.RLEAGDHPVELLAR.D	1576.78214	2	2.29E-07	0.90	3.89	-	595.4
AHQ-1-9, 6550 - 6560	K.YIPPCLDSELTEFPLR.M	1952.21655	2	6.65E-08	0.58	2.94	-	199.1
gi|4504973|ref|NP_002292.1| lactate dehydrogenase C [Homo sapiens]				2.25E-09	0.91	10.18	3.60	36311.0
AHQ-1-9, 2475	R.VIGSGCNLDSAR.F	1250.36445	2	2.25E-09	0.91	3.63	-	721.7
AHQ-1-10, 2361	R.VIGSGCNLDSAR.F	1250.36445	2	3.67E-07	0.87	3.33	-	579.9
AHQ-1-11, 2348 - 2415	R.VIGSGCNLDSAR.F	1250.36445	2	7.77E-07	0.88	3.25	-	723.8
AHQ-1-9, 2379	R.VIGSGCNLDSAR.F	1250.36445	2	2.83E-06	0.85	3.48	-	788.2
gi|25188179|ref|NP_005653.3| voltage-dependent anion channel 3 [Homo sapiens]				2.34E-09	5.71	70.23	24.00	30658.4
AHQ-1-10, 3240	K.LSQNNFALGYK.A	1255.40424	1	1.09E-04	0.58	2.38	-	691.2

AHQ-1-13- , 3365 - 3431	K.LSQNNFALGYK.A	1255.40424	2	2.22E-04	0.82	3.05	-	749.8
AHQ-1-10, 3120	K.LSQNNFALGYK.A	1255.40424	2	3.44E-04	0.89	2.75	-	1228.6
AHQ-1-10, 4705	K.LTLDTIFVPNTGK.K	1419.64771	2	2.41E-07	0.93	3.72	-	1261.8
AHQ-1-10, 4596 - 4615	K.LTLDTIFVPNTGK.K	1419.64771	2	9.89E-05	0.92	3.89	-	816.8
AHQ-1-10, 4047	K.LTLDTIFVPNTGKK.S	1547.82062	2	4.90E-04	0.57	2.87	-	447.0
AHQ-1-10, 4288	K.LTLSALIDGK.N	1031.22813	2	8.34E-05	0.89	3.91	-	698.7
AHQ-1-13, 3696	K.VCNYGLTFTQK.W	1332.50706	2	5.10E-04	0.79	3.08	-	638.6
AHQ-1-10, 3423 - 3424	K.VCNYGLTFTQK.W	1332.50706	2	7.69E-05	0.94	4.33	-	958.5
AHQ-1-10, 4391	K.VNNASLIGLGYTQTLRPGVK.L	2102.42298	3	1.03E-06	0.88	4.36	-	755.3
AHQ-1-10, 4095	K.VNNASLIGLGYTQTLRPGVK.L	2102.42298	3	3.85E-07	0.89	3.99	-	1079.2
AHQ-1-10, 4093	K.VNNASLIGLGYTQTLRPGVK.L	2102.42298	2	1.77E-08	0.85	3.93	-	524.5
AHQ-1-10, 4223 - 4299	K.VNNASLIGLGYTQTLRPGVK.L	2102.42298	2	2.93E-09	0.85	3.76	-	637.0
AHQ-1-10, 4384	K.VNNASLIGLGYTQTLRPGVK.L	2102.42298	2	2.34E-09	0.85	3.88	-	517.3
AHQ-1-13, 4521	K.VNNASLIGLGYTQTLRPGVK.L	2102.42298	3	8.52E-07	0.79	4.10	-	587.5
AHQ-1-13, 4525	K.VNNASLIGLGYTQTLRPGVK.L	2102.42298	2	5.56E-07	0.86	3.63	-	642.1
AHQ-1-13- , 4744	K.VNNASLIGLGYTQTLRPGVK.L	2102.42298	2	8.64E-09	0.88	4.04	-	549.4
AHQ-1-10, 3619	K.YKVCNYGLTFTQK.W	1623.85410	2	3.02E-07	0.96	4.67	-	1350.3
gi|20539300|ref|XP_088391.4| similar to tropomyosin 4 [Homo sapiens]				2.40E-09	1.40	20.22	6.90	18592.1
AHQ-1-9, 3412 - 3488	R.KLVILEGELER.A	1299.54160	2	5.33E-06	0.95	4.10	-	1575.7
AHQ-1-9, 3576	R.KLVILEGELER.A	1299.54160	2	1.76E-06	0.90	3.75	-	934.4
AHQ-1-10, 3411	R.KLVILEGELER.A	1299.54160	2	1.45E-04	0.96	4.39	-	1529.8
AHQ-1-9, 3407 - 3484	R.KLVILEGELER.A	1299.54160	1	2.40E-09	0.45	2.48	-	388.4
AHQ-1-9, 4014	K.LVILEGELER.A	1171.36868	2	1.80E-08	0.95	3.70	-	1642.1
gi|4557419|ref|NP_000063.1| CD36 antigen (collagen type I receptor, thrombospondin receptor); CD36				2.40E-09	3.65	40.25	12.90	53052.1
AHQ-1-6, 4755	K.AFASPVENPDNYCFCTEK.I	2152.30319	2	9.03E-05	0.69	3.14	-	439.8
AHQ-1-5, 4857 - 4934	K.AFASPVENPDNYCFCTEK.I	2152.30319	2	2.62E-06	0.90	3.85	-	621.5
AHQ-1-5, 4781 - 4849	K.AFASPVENPDNYCFCTEK.I	2152.30319	2	1.76E-07	0.88	3.63	-	722.9
AHQ-1-1, 4831	K.AFASPVENPDNYCFCTEK.I	2152.30319	2	1.26E-04	0.41	2.54	-	354.1
AHQ-1-5, 2818 - 2895	K.RLQVNLLVKPSEK.I	1524.83342	3	9.89E-04	0.83	3.64	-	903.5
AHQ-1-6, 5616 - 5635	K.SQVLQFFSSDICR.S	1588.76661	2	3.24E-05	0.94	3.48	-	1541.2
AHQ-1-5, 5681 - 5705	K.SQVLQFFSSDICR.S	1588.76661	2	4.02E-07	0.97	4.91	-	1717.6
AHQ-1-1, 5755	K.SQVLQFFSSDICR.S	1588.76661	2	4.51E-06	0.97	4.96	-	1477.0
AHQ-1-7, 5693	K.SQVLQFFSSDICR.S	1588.76661	2	1.66E-04	0.76	2.58	-	1037.8
AHQ-1-5, 7309 - 7314	R.TYLDIQPITGFTLQFAK.R	1957.25787	2	2.40E-09	0.96	4.54	-	1328.8
gi|13129150|ref|NP_005339.1| heat shock 90kDa protein 1, alpha; heat shock 90kD protein 1, alpha [H				2.43E-09	4.32	50.25	8.70	84659.1
AHQ-1-5, 2446	K.DQVANSAFVER.L	1236.31618	2	3.28E-04	0.94	3.55	-	1369.4
AHQ-1-5, 2382	K.FYEQFSK.N	949.04099	1	7.12E-04	0.73	2.51	-	732.4
AHQ-1-5, 6970	K.HGLEVIYMIEPIDEYCVQQLK.E	2579.97320	3	1.72E-05	0.87	4.04	-	1167.7
AHQ-1-5, 4137	R.NPDDITNEEYGEFYK.S	1834.87381	2	2.43E-09	0.97	4.99	-	1505.5
AHQ-1-6, 4080	R.NPDDITNEEYGEFYK.S	1834.87381	2	3.88E-07	0.92	4.35	-	694.6
AHQ-1-5, 4063	R.RAPFDLFENR.K	1265.40235	2	7.52E-05	0.80	3.17	-	544.5
gi|4507521|ref|NP_001055.1| transketolase [Homo sapiens]				2.64E-09	2.23	30.20	8.20	67877.3
AHQ-1-6, 4732	K.ILATPPQEDAPSVDIANIR.M	2021.26036	2	2.08E-06	0.91	4.05	-	410.6
AHQ-1-6, 3848 - 3894	R.SVPTSTVFYPSDGVATEK.A	1886.04854	2	5.61E-04	0.55	2.84	-	269.7
AHQ-1-6, 7174	R.TVPFCSTFAAFFTR.A	1653.88190	2	2.64E-09	0.77	3.16	-	312.9
gi|4504077|ref|NP_000165.1| glycoprotein IX (platelet) [Homo sapiens]				2.70E-09	5.34	60.22	30.50	19046.0
AHQ-1-11, 4750	R.ALETMGLWVDCR.G	1452.68135	2	3.19E-09	0.93	3.08	-	1241.4
AHQ-1-12, 4157	R.ALETM*GLWVDCR.G	1468.68075	2	2.88E-04	0.90	2.60	-	1108.2
AHQ-1-12, 4927	R.ALETMGLWVDCR.G	1452.68135	2	2.12E-04	0.93	3.87	-	941.2
AHQ-1-11, 4024 - 4035	R.ALETM*GLWVDCR.G	1468.68075	2	1.52E-04	0.87	3.66	-	781.8
AHQ-1-12, 2635 - 2715	R.CASPSLAAHGPLGR.L	1395.56962	2	2.70E-09	0.83	3.43	-	909.7
AHQ-1-11, 2604 - 2624	R.CASPSLAAHGPLGR.L	1395.56962	2	1.20E-06	0.92	3.35	-	1221.9
AHQ-1-14- , 2758	R.CASPSLAAHGPLGR.L	1395.56962	2	6.07E-07	0.93	3.43	-	1316.3
AHQ-1-11, 3232 - 3298	R.GHGLTALPALPAR.T	1274.49706	2	4.88E-05	0.96	4.35	-	1335.4
AHQ-1-12, 3427	R.GHGLTALPALPAR.T	1274.49706	2	2.80E-04	0.93	3.33	-	967.3
AHQ-1-11, 3295 - 3296	R.GHGLTALPALPAR.T	1274.49706	3	2.38E-05	0.89	3.30	-	1092.9
AHQ-1-11, 3299	R.GHGLTALPALPAR.T	1274.49706	1	9.71E-05	0.63	2.47	-	408.8
AHQ-1-11, 5108	R.LWLEDRTPEALLQVR.C	1840.11574	2	3.95E-05	0.89	4.13	-	735.1
AHQ-1-11, 3075 - 3098	R.TPEALLQVR.C	1027.19957	2	4.75E-04	0.84	3.00	-	832.1
AHQ-1-14- , 3228	R.TPEALLQVR.C	1027.19957	2	3.60E-06	0.94	3.02	-	1586.6
AHQ-1-14, 4189	R.TPEALLQVR.C	1027.19957	2	2.87E-05	0.92	3.27	-	1145.1
gi|4758756|ref|NP_004528.1| nucleosome assembly protein 1-like 1; HSP22-like protein interacting pr				2.71E-09	4.18	50.24	15.10	45373.8
AHQ-1-7, 7370	K.GIPEFWLTVFK.N	1337.58999	2	2.71E-09	0.81	2.97	-	282.5
AHQ-1-7, 3786	R.KYAVLYQPLFDKR.F	1641.93733	2	5.64E-04	0.55	2.81	-	369.7
AHQ-1-7, 5757 - 5828	R.LDGLVETPTGYIESLPR.V	1861.08535	2	4.92E-05	0.93	3.98	-	952.6
AHQ-1-7, 5633 - 5676	K.NVDLLSDMVQEHDEPILK.H	2096.34751	2	3.07E-08	0.95	4.85	-	1000.5
AHQ-1-7, 4966	K.YAVLYQPLFDK.R	1357.57807	2	7.72E-06	0.94	3.35	-	1226.9
gi|5453990|ref|NP_006254.1| proteasome (prosome, macropain) activator subunit 1 (PA28 alpha); Prote				2.74E-09	1.72	20.19	13.70	28722.9
AHQ-1-10, 5637	K.ISELDAFLKEPALNEANLSNLK.A	2430.73906	2	2.74E-09	0.82	3.86	-	398.7
AHQ-1-10, 6105	R.NAYAVLYDIILK.N	1396.65574	2	3.71E-06	0.90	3.43	-	789.7
gi|4757766|ref|NP_004299.1| Rho GTPase activating protein 1; RhoGAP; p50rhoGAP; CDC42 GTPase-activa				2.89E-09	2.05	30.24	8.70	50435.4
AHQ-1-11, 5663	K.FLLDHQGELFPSPDPSGL	1970.17037	2	3.74E-05	0.45	2.83	-	483.5
AHQ-1-7, 6412	K.FLLDHQGELFPSPDPSGL	1970.17037	2	1.03E-04	0.63	2.84	-	467.1
AHQ-1-8, 4060 - 4132	K.NPEQEPIPIVLR.E	1405.62426	2	2.43E-04	0.67	2.54	-	717.1
AHQ-1-8, 6391 - 6392	R.VPATLQVLQTLPEENYQVLR.F	2312.65053	2	2.67E-06	0.93	4.68	-	712.1
AHQ-1-11, 5715	R.VPATLQVLQTLPEENYQVLR.F	2312.65053	2	2.66E-04	0.74	3.50	-	331.8
AHQ-1-7, 6457	R.VPATLQVLQTLPEENYQVLR.F	2312.65053	2	2.89E-09	0.93	4.80	-	619.0
gi|4502695|ref|NP_001779.1| cell division cycle 10; cell division cycle 10 (homolog to CDC10 of S.c				3.05E-09	1.81	20.25	9.60	48786.6
AHQ-1-8, 5531	K.NLEGYVGFANLPNQVYR.K	1955.16216	2	7.51E-06	0.95	4.91	-	1057.8
AHQ-1-13- , 6701	K.STLINSLFLTDLYSPEYPGPSHR.I	2608.88640	3	4.69E-05	0.68	3.14	-	430.1
AHQ-1-14- , 6215	K.STLINSLFLTDLYSPEYPGPSHR.I	2608.88640	3	2.58E-07	0.63	3.07	-	378.3
AHQ-1-14- , 6434	K.STLINSLFLTDLYSPEYPGPSHR.I	2608.88640	3	3.05E-09	0.86	4.03	-	587.5
AHQ-1-8, 6842	K.STLINSLFLTDLYSPEYPGPSHR.I	2608.88640	3	1.76E-05	0.75	3.32	-	698.1
gi|4505369|ref|NP_002486.1| NADH dehydrogenase (ubiquinone) Fe-S protein 4, 18kDa (NADH-coenzyme Q				3.38E-09	0.81	10.17	8.60	20107.8
AHQ-1-12, 4554	K.LDITTLTGVPEEHIK.T	1666.89713	2	3.38E-09	0.81	3.46	-	570.2
gi|22027538|ref|NP_037506.2| programmed cell death 6 interacting protein; ALG-2 interacting protein				3.45E-09	1.80	20.18	3.10	96022.6
AHQ-1-5, 4831	K.LANQAADYFGDAFK.Q	1531.65033	2	3.45E-09	0.89	3.57	-	810.6
AHQ-1-5, 3127 - 3133	R.LLDEEEATDNDLR.A	1533.57656	2	1.36E-06	0.91	3.56	-	1153.9
gi|10716563|ref|NP_001737.1| calnexin [Homo sapiens]				3.58E-09	1.74	20.22	4.20	67567.8
AHQ-1-5, 2329	K.AEEDEILNR.S	1089.13826	2	1.70E-04	0.85	2.79	-	1226.1
AHQ-1-4, 5167	K.APVPTGEVYFADSFDR.G	1771.90722	2	1.69E-04	0.91	3.63	-	1018.8
AHQ-1-3, 5115	K.APVPTGEVYFADSFDR.G	1771.90722	2	1.66E-06	0.87	3.28	-	820.9
AHQ-1-5, 5065	K.APVPTGEVYFADSFDR.G	1771.90722	2	3.58E-09	0.88	3.79	-	867.7
AHQ-1-6, 4998 - 5007	K.APVPTGEVYFADSFDR.G	1771.90722	2	1.57E-07	0.93	4.42	-	1066.0
AHQ-1-1, 5160	K.APVPTGEVYFADSFDR.G	1771.90722	2	1.96E-07	0.90	3.70	-	846.0
gi|8922835|ref|NP_060776.1| hypothetical protein FLJ11036 [Homo sapiens]				3.58E-09	0.97	10.22	6.90	22353.4
AHQ-1-13- , 6209	R.IHSVLQGFIPLFQK.F	1627.95380	2	3.58E-09	0.97	4.45	-	1552.8
gi|5032161|ref|NP_005639.1| elongin C; transcription elongation factor B (SIII), polypeptide 1 (15k				3.61E-09	1.75	20.24	28.60	12473.1
AHQ-1-13, 4651 - 4723	K.AMLSGPGQFAENETNEVNFR.E	2212.38515	2	2.43E-07	0.94	4.84	-	1206.5
AHQ-1-13- , 3180	K.LISSDGHEFIVK.R	1345.52569	2	3.61E-09	0.81	3.27	-	615.9
gi|27482573|ref|XP_208709.1| similar to Ubiquitin-conjugating enzyme E2-18 kDa UbcH7 (Ubiquitin-pro				3.62E-09	1.90	20.26	26.00	17875.4
AHQ-1-12, 4801	K.GQVCLPVISAENWKPATK.T	2000.30757	2	7.08E-07	0.94	4.48	-	754.5
AHQ-1-12, 6491 - 6494	K.TDQVIQSLIALVNDPQPEHPLR.A	2484.79318	2	1.70E-06	0.93	5.00	-	547.0
AHQ-1-12, 6479 - 6490	K.TDQVIQSLIALVNDPQPEHPLR.A	2484.79318	3	3.62E-09	0.96	5.25	-	1430.0
gi|14149734|ref|NP_065174.1| coronin, actin binding protein, 1B [Homo sapiens]				3.68E-09	0.96	10.26	5.90	54234.4
AHQ-1-7, 7214	R.KSDLFQDDLYPDTAGPEAALEAEEWVSGR.D	3211.39431	3	3.68E-09	0.96	5.25	-	1426.1
gi|29737978|ref|XP_290747.1| similar to Myosin heavy chain, nonmuscle type B (Cellular myosin heavy				4.22E-09	4.49	50.24	2.10	191380.7

AHQ-1-3, 4037 - 4073	K.ADFCIIHYAGK.V	1296.47641	2	1.38E-04	0.87	2.71	-	1123.4
AHQ-1-2, 4185 - 4186	K.ADFCIIHYAGK.V	1296.47641	1	4.06E-04	0.76	3.09	-	696.3
AHQ-1-4, 4078	K.ADFCIIHYAGK.V	1296.47641	2	4.22E-09	0.92	3.70	-	970.0
AHQ-1-1, 4123	K.ADFCIIHYAGK.V	1296.47641	2	1.84E-04	0.87	3.24	-	882.8
AHQ-1-6, 3871 - 3884	K.ADFCIIHYAGK.V	1296.47641	2	1.40E-07	0.95	3.62	-	1328.7
AHQ-1-5, 3926	K.ADFCIIHYAGK.V	1296.47641	2	3.33E-05	0.88	3.50	-	724.7
AHQ-1-2, 4198	K.ADFCIIHYAGK.V	1296.47641	2	7.39E-06	0.96	3.95	-	1315.8
AHQ-1-5, 2781 - 2797	R.AGVLAHLEEER.D	1224.34854	2	3.15E-07	0.95	4.14	-	1590.9
AHQ-1-12, 2930	R.AGVLAHLEEER.D	1224.34854	2	1.05E-07	0.82	2.93	-	927.6
AHQ-1-1, 3124	R.AGVLAHLEEER.D	1224.34854	1	8.00E-05	0.36	1.84	-	657.3
AHQ-1-2, 3023 - 3102	R.AGVLAHLEEER.D	1224.34854	2	1.06E-06	0.96	4.14	-	1778.2
AHQ-1-6, 2758 - 2826	R.AGVLAHLEEER.D	1224.34854	2	4.89E-05	0.92	3.30	-	1288.0
AHQ-1-4, 2950 - 2963	R.AGVLAHLEEER.D	1224.34854	2	5.93E-07	0.96	4.42	-	1492.7
AHQ-1-13- , 3087	R.AGVLAHLEEER.D	1224.34854	2	2.54E-08	0.95	4.01	-	1388.6
AHQ-1-3, 2953	R.AGVLAHLEEER.D	1224.34854	2	5.58E-07	0.94	3.84	-	1464.5
AHQ-1-1, 3121	R.AGVLAHLEEER.D	1224.34854	2	1.64E-06	0.94	3.82	-	1390.1
AHQ-1-3, 3794	K.DKADFCIIHYAGK.V	1539.73719	3	1.07E-06	0.94	3.99	-	1761.0
AHQ-1-3, 3787	K.DKADFCIIHYAGK.V	1539.73719	2	4.25E-09	0.97	4.62	-	1699.6
AHQ-1-1, 3884	K.DKADFCIIHYAGK.V	1539.73719	2	1.41E-08	0.93	3.71	-	1176.6
AHQ-1-2, 3926	K.DKADFCIIHYAGK.V	1539.73719	2	4.85E-08	0.97	4.71	-	1896.5
AHQ-1-2, 3929	K.DKADFCIIHYAGK.V	1539.73719	3	4.81E-07	0.86	3.34	-	1107.2
AHQ-1-2, 4949 - 5017	K.LDPHLVLDQLR.C	1319.53460	2	9.67E-06	0.74	2.61	-	625.0
AHQ-1-1, 4860	K.LDPHLVLDQLR.C	1319.53460	3	2.98E-06	0.78	3.06	-	781.4
AHQ-1-1, 4843 - 4847	K.LDPHLVLDQLR.C	1319.53460	2	5.30E-05	0.73	2.77	-	542.7
AHQ-1-3, 4813	K.LDPHLVLDQLR.C	1319.53460	3	4.10E-06	0.79	3.64	-	668.6
AHQ-1-2, 4961 - 4965	K.LDPHLVLDQLR.C	1319.53460	3	2.86E-06	0.88	3.09	-	951.4
AHQ-1-2, 4127	K.VDYKADEWLMK.N	1398.60886	2	9.17E-09	0.77	3.03	-	645.8
gi|5031597|ref|NP_005710.1| actin related protein 2/3 complex subunit 3; ARP2/3 protein complex sub				4.60E-09	1.92	20.27	9.00	20546.5
AHQ-1-11, 6127	K.DTDIVDEAIYYFK.A	1592.72721	2	1.49E-06	0.94	3.79	-	1092.2
AHQ-1-12, 6317 - 6319	K.DTDIVDEAIYYFK.A	1592.72721	2	4.60E-09	0.97	5.44	-	1611.1
AHQ-1-14- , 6446	K.DTDIVDEAIYYFK.A	1592.72721	2	6.31E-06	0.92	3.62	-	1332.2
AHQ-1-11, 5651	R.ETKDTDIVDEAIYYFK.A	1951.11899	2	1.37E-06	0.94	4.07	-	1148.0
gi|29740995|ref|XP_291768.1| similar to Proteasome activator complex subunit 2 (Proteasome activato				4.63E-09	0.96	10.25	9.60	19192.6
AHQ-1-10, 6231	R.AFYAELYHIISSNLEK.T	1899.13528	2	4.63E-09	0.96	5.06	-	1284.6
gi|6005846|ref|NP_009215.1| PTK9L protein tyrosine kinase 9-like (A6-related protein); protein tyro				4.64E-09	4.82	50.29	26.90	39547.7
AHQ-1-12, 6230	R.AVLPLLDAQQPCYLLYR.L	2035.39506	2	6.82E-07	0.79	3.25	-	457.9
AHQ-1-13- , 6679	R.AVLPLLDAQQPCYLLYR.L	2035.39506	2	3.65E-07	0.96	4.98	-	863.7
AHQ-1-9, 6767 - 6768	R.AVLPLLDAQQPCYLLYR.L	2035.39506	2	4.64E-09	0.96	5.36	-	914.7
AHQ-1-13- , 6568 - 6632	R.AVLPLLDAQQPCYLLYR.L	2035.39506	2	7.20E-05	0.53	2.82	-	287.7
AHQ-1-12, 6334	R.AVLPLLDAQQPCYLLYR.L	2035.39506	3	1.09E-07	0.91	3.75	-	1250.6
AHQ-1-12, 6323	R.AVLPLLDAQQPCYLLYR.L	2035.39506	2	1.23E-05	0.94	4.57	-	780.9
AHQ-1-14- , 6422	R.AVLPLLDAQQPCYLLYR.L	2035.39506	2	1.10E-06	0.96	5.28	-	938.2
AHQ-1-11, 3970	R.ETIELVHTEPTDVAQLPSR.V	2136.34822	2	7.28E-06	0.94	4.63	-	1009.1
AHQ-1-9, 4155	R.ETIELVHTEPTDVAQLPSR.V	2136.34822	2	2.48E-04	0.94	4.40	-	947.9
AHQ-1-11, 4163	K.HQTLQGLAFPLQPEAQR.A	1935.17562	2	6.77E-09	0.97	5.15	-	1459.3
AHQ-1-9, 4311	K.HQTLQGLAFPLQPEAQR.A	1935.17562	2	9.88E-08	0.95	4.96	-	765.7
AHQ-1-11, 5959	K.KIEIGDGAELTAEFLYDEVHPK.Q	2475.73515	3	1.13E-08	0.96	5.83	-	1424.4
AHQ-1-13- , 4824	K.VVIEDEQLVLGASQEPVGR.W	2039.27568	2	1.58E-05	0.97	4.50	-	2358.8
AHQ-1-13, 4600	K.VVIEDEQLVLGASQEPVGR.W	2039.27568	2	1.43E-06	0.96	3.79	-	2003.4
AHQ-1-12, 4629	K.VVIEDEQLVLGASQEPVGR.W	2039.27568	3	7.43E-04	0.97	4.92	-	2354.9
AHQ-1-12, 4642	K.VVIEDEQLVLGASQEPVGR.W	2039.27568	2	4.14E-07	0.98	5.75	-	2160.3
gi|24797067|ref|NP_002107.3| major histocompatibility complex, class I, A precursor; HLA-A1 class I				4.70E-09	5.33	60.28	23.30	40840.5
AHQ-1-6, 5119	R.APWIEQEGPEYWDQETR.N	2135.23420	2	4.75E-07	0.90	4.10	-	617.4
AHQ-1-9, 5056	R.APWIEQEGPEYWDQETR.N	2135.23420	2	1.21E-05	0.92	4.01	-	840.5
AHQ-1-8, 5100	R.APWIEQEGPEYWDQETR.N	2135.23420	2	4.70E-09	0.97	5.59	-	1369.1
AHQ-1-5, 5195	R.APWIEQEGPEYWDQETR.N	2135.23420	2	2.88E-08	0.95	4.44	-	925.1
AHQ-1-8, 3314 - 3318	R.DGEDQTQDTELVETRPAGDGTFQK.W	2638.69666	2	5.78E-08	0.84	4.06	-	411.4
AHQ-1-12, 4905	R.FIAVGYVDDTQFVR.F	1630.82504	2	1.65E-08	0.96	4.32	-	1616.5
AHQ-1-8, 3217	K.WAAVVVPSGEEQR.Y	1428.57478	1	2.80E-05	0.69	3.81	-	270.8
AHQ-1-8, 2044	R.YTCHVQHEGLPK.P	1470.63495	2	4.57E-04	0.92	3.68	-	812.7
AHQ-1-8, 3005 - 3076	R.YTCHVQHEGLPKPLTLR.W	2051.35781	2	8.49E-09	0.94	4.28	-	1495.1
AHQ-1-8, 3070	R.YTCHVQHEGLPKPLTLR.W	2051.35781	3	2.42E-06	0.93	4.99	-	1558.9
gi|9966867|ref|NP_065123.1| eIF-5A2 protein; eIF5AII [Homo sapiens]				4.79E-09	2.85	30.27	19.60	16793.1
AHQ-1-12, 3622	K.KYEDICPSTHNMDVPNIK.R	2163.41723	3	4.79E-09	0.95	4.97	-	1665.9
AHQ-1-12, 3626	K.KYEDICPSTHNMDVPNIK.R	2163.41723	2	2.39E-06	0.97	4.77	-	1445.3
AHQ-1-12, 3051	K.KYEDICPSTHNM*DVPNIK.R	2179.41663	3	1.07E-04	0.95	5.32	-	1212.8
AHQ-1-12, 4761	K.VHLVGIDIFTGK.K	1299.54329	2	1.47E-08	0.95	4.46	-	1079.3
gi|5803187|ref|NP_006746.1| transaldolase 1; dihydroxyacetone transferase; glycerone transferase [H				4.89E-09	2.49	30.20	10.70	37539.9
AHQ-1-9, 4860 - 4886	K.ALAGCDFLTISPK.L	1394.61830	2	4.89E-09	0.91	3.95	-	900.1
AHQ-1-9, 3015	K.LSSTWEGIQAGK.E	1277.40821	2	2.08E-04	0.94	3.69	-	1300.3
AHQ-1-9, 2698	K.SYEPLEDPGVK.S	1234.33696	2	6.85E-04	0.64	2.78	-	416.1
gi|17149842|ref|NP_004461.2| FK506-binding protein 2 precursor; FK506-binding protein 2 (13kD); pep				4.92E-09	3.48	40.21	19.00	15649.2
AHQ-1-13, 4119	K.GWDQGLLGM*CEGEK.R	1597.75215	2	8.50E-06	0.89	3.68	-	952.7
AHQ-1-13- , 5108	K.GWDQGLLGMCEGEK.R	1581.75275	2	1.34E-04	0.82	3.05	-	657.4
AHQ-1-13- , 4296	K.GWDQGLLGM*CEGEK.R	1597.75215	2	3.29E-04	0.75	2.63	-	800.6
AHQ-1-13- , 3993	R.KLVIPSELGYGER.G	1461.68782	2	2.67E-06	0.92	4.22	-	701.1
AHQ-1-13, 3755 - 3825	R.KLVIPSELGYGER.G	1461.68782	2	1.19E-04	0.75	3.12	-	425.5
AHQ-1-13- , 4325	K.LVIPSELGYGER.G	1333.51491	2	1.27E-07	0.79	3.00	-	412.7
AHQ-1-13, 4135 - 4140	K.LVIPSELGYGER.G	1333.51491	2	4.92E-09	0.84	2.99	-	540.6
gi|5031599|ref|NP_005722.1| actin related protein 2/3 complex subunit 2; ARP2/3 protein complex sub				5.08E-09	6.22	80.22	40.70	34332.8
AHQ-1-9, 2159 - 2167	R.ASHTAPQVLFSHR.E	1451.61481	2	3.47E-08	0.82	3.13	-	539.6
AHQ-1-9, 2558	K.DSIVHQAGMLK.R	1199.40563	1	5.08E-09	0.54	3.06	-	478.3
AHQ-1-9, 2582	K.ELQAHGADELLK.R	1324.46487	1	1.31E-05	0.34	2.01	-	439.3
AHQ-1-9, 2596	K.ELQAHGADELLK.R	1324.46487	2	3.49E-07	0.76	2.98	-	789.2
AHQ-1-14- , 2787	K.ELQAHGADELLK.R	1324.46487	2	1.57E-05	0.89	3.43	-	1128.8
AHQ-1-9, 2483 - 2559	K.ELQAHGADELLKR.V	1480.65123	2	7.91E-06	0.89	2.93	-	995.3
AHQ-1-14- , 6582	K.FENAAAGNKPEAVEVTFADFDGVLYHISNPNGDK.T	3638.89648	3	8.87E-06	0.86	3.84	-	699.9
AHQ-1-9, 3190	R.IIEETLALK.F	1030.24013	2	2.59E-05	0.73	2.67	-	534.6
AHQ-1-10, 6339 - 6340	R.VYGSFLVNPESGYNVSLLYDLENLPASK.D	3090.42808	3	5.44E-07	0.69	3.77	-	195.5
AHQ-1-9, 2607	K.YFQFQEEGKEGENR.A	1761.82929	2	1.91E-04	0.95	4.30	-	1329.1
AHQ-1-10, 2617	K.YFQFQEEGKEGENR.A	1761.82929	2	6.36E-05	0.93	3.82	-	1128.8
gi|4507725|ref|NP_000362.1| transthyretin (prealbumin, amyloidosis type I); Transthyretin (prealbum				5.17E-09	4.73	50.33	49.00	15886.9
AHQ-1-13- , 3943 - 4015	K.AADDTWEPFASGK.T	1395.45529	2	2.22E-06	0.87	3.11	-	959.9
AHQ-1-13- , 4179 - 4184	R.GSPAINVAVHVFR.K	1367.58095	2	7.28E-08	0.95	4.54	-	1134.6
AHQ-1-13, 3999 - 4007	R.GSPAINVAVHVFR.K	1367.58095	2	1.27E-06	0.90	3.78	-	861.8
AHQ-1-13, 3199	R.KAADDTWEPFASGK.T	1523.62820	2	3.50E-04	0.97	4.92	-	1366.4
AHQ-1-13- , 3301	R.KAADDTWEPFASGK.T	1523.62820	2	8.43E-04	0.96	4.66	-	1390.4
AHQ-1-13- , 6051	R.RYTIAALLSPYSYSTTAVVTNPK.E	2517.86154	2	2.61E-06	0.98	5.85	-	2034.1
AHQ-1-13- , 5683 - 5693	K.TSESGELHGLTTEEEFVEGIYK.V	2456.60073	3	9.95E-08	0.98	6.60	-	1653.9
AHQ-1-13- , 5687 - 5689	K.TSESGELHGLTTEEEFVEGIYK.V	2456.60073	2	1.15E-06	0.98	6.16	-	1446.9
AHQ-1-13, 5273 - 5336	K.TSESGELHGLTTEEEFVEGIYK.V	2456.60073	3	3.21E-06	0.97	5.97	-	1636.3
AHQ-1-13, 5331 - 5333	K.TSESGELHGLTTEEEFVEGIYK.V	2456.60073	2	5.17E-09	0.96	5.23	-	1092.9
gi|5803133|ref|NP_006825.1| RAB32, member RAS oncogene family [Homo sapiens]				5.37E-09	1.70	20.20	12.40	24997.1
AHQ-1-10, 4485	K.VHLPNGSPIPAVLLANK.C	1741.07044	2	5.37E-09	0.77	2.88	-	366.3
AHQ-1-10, 3945	K.VLVIGELGVGK.T	1084.33417	2	4.49E-08	0.94	3.92	-	1275.1
gi|6912238|ref|NP_036226.1| peroxiredoxin 5; antioxidant enzyme B166 [Homo sapiens]				5.46E-09	6.32	70.26	31.30	22026.2
AHQ-1-12, 6557	K.ETDLLLDDSLVSIFGNR.R	1908.09888	2	5.46E-09	0.97	5.08	-	1290.8

AHQ-1-12, 3839 - 3902	R.FSMVVQDGIVK.A	1223.46709	2	4.58E-05	0.86	3.46	-	626.9
AHQ-1-12, 2998	R.FSM*VVQDGIVK.A	1239.46649	2	6.80E-04	0.89	2.99	-	1222.4
AHQ-1-12, 5186 - 5191	K.GVLFGVPGAFTPGCSK.T	1595.84389	2	6.25E-04	0.88	4.10	-	542.9
AHQ-1-12, 4626 - 4633	K.KGVLFGVPGAFTPGCSK.T	1724.01680	2	1.88E-08	0.98	5.25	-	1850.7
AHQ-1-12, 4197	K.THLPGFVEQAEALK.A	1540.74504	3	7.24E-07	0.95	4.09	-	1862.6
AHQ-1-12, 4710	K.VNLAELFK.G	934.11414	2	2.75E-04	0.80	2.86	-	711.3
gi|21361322|ref|NP_006078.2| tubulin, beta, 5 [Homo sapiens]				5.69E-09	2.46	30.28	13.70	49585.5
AHQ-1-7, 2940	R.EIVHLQAGQCGNQIGAK.F	1825.03962	3	3.94E-05	0.90	3.62	-	1544.2
AHQ-1-7, 3036	R.EIVHLQAGQCGNQIGAK.F	1825.03962	2	5.69E-09	0.90	4.27	-	770.2
AHQ-1-13, 3009	R.EIVHLQAGQCGNQIGAK.F	1825.03962	2	9.38E-06	0.88	3.86	-	868.4
AHQ-1-14- , 3011	R.EIVHLQAGQCGNQIGAK.F	1825.03962	2	2.98E-06	0.92	3.95	-	1138.3
AHQ-1-7, 2924 - 2932	R.EIVHLQAGQCGNQIGAK.F	1825.03962	2	1.55E-04	0.95	4.69	-	1256.2
AHQ-1-7, 5501 - 5577	K.FWEVISDEHGIDPTGTYHGDSDLQLER.I	3118.27220	3	5.54E-06	0.95	5.58	-	993.8
AHQ-1-7, 6884 - 6948	K.M*AATFIGNSTAIQELFK.R	1859.13607	2	1.52E-04	0.61	3.29	-	320.6
gi|20149594|ref|NP_031381.2| heat shock 90kDa protein 1, beta; heat shock 90kD protein 1, beta; Hea				5.76E-09	5.85	70.26	11.50	83263.6
AHQ-1-5, 4075	R.ALLFIPR.R	830.05229	2	3.79E-04	0.80	2.54	-	949.6
AHQ-1-5, 2435	K.EDQTEYLEER.R	1312.32145	2	4.56E-05	0.51	2.57	-	495.3
AHQ-1-5, 5711	K.HSQFIGYPITLYLEK.E	1810.08487	2	7.90E-05	0.97	5.18	-	1623.9
AHQ-1-5, 3418 - 3431	K.IDIIPNPQER.T	1195.35034	2	4.39E-04	0.76	2.61	-	488.5
AHQ-1-6, 4151	R.NPDDITQEEYGEFYK.S	1848.90048	2	1.11E-08	0.96	4.82	-	1308.6
AHQ-1-5, 3979 - 3982	K.SLTNDWEDHLAVK.H	1528.64798	2	5.76E-09	0.88	3.71	-	834.2
AHQ-1-5, 4335 - 4343	R.TLTLVDTGIGMTK.A	1350.60718	2	1.04E-04	0.96	4.01	-	1283.9
gi|5803201|ref|NP_006818.1| transmembrane trafficking protein [Homo sapiens]				6.41E-09	2.70	40.22	17.40	24975.8
AHQ-1-11, 2386	K.ITDSAGHILYSK.E	1305.46156	2	6.41E-09	0.97	4.31	-	1569.7
AHQ-1-11, 5951	R.LEDLSESIVNDFAYMK.K	1875.08916	2	9.81E-05	0.95	4.37	-	1377.4
AHQ-1-11, 2980	K.LKPLEVELR.R	1097.33294	1	2.62E-04	0.13	1.96	-	225.8
AHQ-1-11, 5243	R.RLEDLSESIVNDFAYM*K.K	2047.27492	2	6.24E-04	0.65	2.69	-	458.8
gi|27764873|ref|NP_057070.2| cytokine receptor-like factor 3; cytokine receptor-like molecule 9; cy				7.06E-09	0.95	10.22	3.20	49737.6
AHQ-1-7, 3366	K.LIEHGVNTAEDLVR.E	1566.74089	2	7.06E-09	0.95	4.42	-	1140.2
gi|4757810|ref|NP_004037.1| ATP synthase, H+ transporting, mitochondrial F1 complex, alpha subunit,				7.08E-09	8.13	90.26	21.50	59750.3
AHQ-1-7, 5809	K.FENAFLSHVVSQHQALLGTIR.A	2368.67911	3	7.08E-09	0.97	5.13	-	1769.3
AHQ-1-7, 3597	K.GIRPAINVGLSVSR.V	1439.68861	2	9.05E-06	0.94	3.98	-	962.6
AHQ-1-7, 6326	K.GMSLNLEPDNVGVVVFGNDK.L	2105.35763	2	3.85E-07	0.90	4.42	-	471.3
AHQ-1-7, 5677	K.GM*SLNLEPDNVGVVVFGNDK.L	2121.35703	2	3.80E-06	0.79	3.75	-	328.4
AHQ-1-7, 2842	R.GYLDKLEPSK.I	1150.30633	2	1.67E-05	0.80	2.70	-	859.8
AHQ-1-7, 3489	K.HALIIYDDLSK.Q	1288.47413	2	1.19E-06	0.95	3.57	-	1341.2
AHQ-1-7, 6846	K.LKEIVTNFLAGFEA	1552.79572	2	1.42E-05	0.89	3.28	-	1021.0
AHQ-1-14- , 5298 - 5376	R.TGAIVDVPVGEELLGR.V	1625.84824	2	5.99E-04	0.65	3.36	-	382.7
AHQ-1-7, 5476 - 5477	R.TGAIVDVPVGEELLGR.V	1625.84824	2	3.06E-06	0.95	4.81	-	839.4
AHQ-1-7, 3865	K.TGTAEMSSILEER.I	1424.55988	2	4.26E-06	0.96	4.07	-	1669.6
gi|4758714|ref|NP_004519.1| microsomal glutathione S-transferase 3; microsomal glutathione S-transf				7.11E-09	0.95	10.22	9.20	16516.2
AHQ-1-12, 3702	R.VLYAYGYYTGEPSK.R	1611.77536	2	7.11E-09	0.95	4.33	-	1124.9
gi|29150259|ref|NP_803875.1| fibrocystin L; polycystic kidney and hepatic disease-like 1 [Homo sapi				7.35E-09	0.73	10.15	0.40	465745.4
AHQ-1-1, 4696	K.IVGEDYPGWSEDSFGAR.V	1885.96719	2	7.35E-09	0.73	3.06	-	441.5
gi|4757768|ref|NP_004300.1| Rho GDP dissociation inhibitor (GDI) alpha [Homo sapiens]				7.42E-09	2.78	30.25	15.70	23206.9
AHQ-1-13, 4527	R.AEEYEFLTPVEEAPK.G	1752.89898	2	7.42E-09	0.91	3.45	-	980.1
AHQ-1-10, 4243	R.AEEYEFLTPVEEAPK.G	1752.89898	2	5.56E-05	0.63	2.92	-	479.6
AHQ-1-12, 4565	R.AEEYEFLTPVEEAPK.G	1752.89898	2	5.88E-05	0.91	3.40	-	1116.7
AHQ-1-13- , 4753	R.AEEYEFLTPVEEAPK.G	1752.89898	2	8.61E-07	0.91	4.14	-	786.1
AHQ-1-13, 3848	K.SIQEIQELDKDDESLR.K	1919.03693	2	1.92E-05	0.94	4.46	-	1014.2
AHQ-1-13- , 4016	K.SIQEIQELDKDDESLR.K	1919.03693	2	9.78E-06	0.90	4.08	-	872.5
AHQ-1-10, 3588	K.SIQEIQELDKDDESLR.K	1919.03693	2	2.11E-07	0.92	4.10	-	963.6
AHQ-1-10, 3212	K.SIQEIQELDKDDESLRK.Y	2047.20984	2	1.06E-04	0.94	5.01	-	998.9
gi|4505763|ref|NP_000282.1| phosphoglycerate kinase 1 [Homo sapiens]				7.65E-09	6.11	70.27	29.70	44614.4
AHQ-1-8, 5920	K.ACANPAAGSVILLENLR.F	1771.03187	2	3.92E-04	0.95	4.50	-	1283.0
AHQ-1-9, 5927	K.ACANPAAGSVILLENLR.F	1771.03187	2	7.65E-09	0.90	3.91	-	953.6
AHQ-1-8, 2285	R.AHSSMVGVNLPQK.A	1368.58752	1	3.70E-07	0.89	3.66	-	756.5
AHQ-1-8, 2283	R.AHSSMVGVNLPQK.A	1368.58752	2	4.38E-05	0.97	4.34	-	1876.0
AHQ-1-9, 5439	K.ALESPERPFLAILGGAK.V	1770.06542	2	9.53E-05	0.97	4.88	-	1641.9
AHQ-1-8, 5360	K.ALESPERPFLAILGGAK.V	1770.06542	2	1.64E-07	0.97	5.47	-	1736.1
AHQ-1-8, 4771 - 4843	K.DVLFLKDCVGPEVEK.A	1750.00620	2	1.28E-06	0.96	4.55	-	1452.4
AHQ-1-9, 5383	K.ITLPVDFVTADKFDENAK.T	2024.25948	2	5.31E-07	0.83	3.76	-	514.3
AHQ-1-8, 5374	K.ITLPVDFVTADKFDENAK.T	2024.25948	2	1.80E-08	0.85	3.94	-	498.1
AHQ-1-8, 4858 - 4860	K.TGQATVASGIPAGWMGLDCGPESSKK.Y	2607.90190	2	4.76E-05	0.64	3.34	-	181.6
AHQ-1-8, 5267	K.VLNNMEIGTSLFDEEGAK.I	1968.17459	2	9.99E-08	0.89	4.49	-	474.3
gi|4504041|ref|NP_002061.1| guanine nucleotide binding protein (G protein), alpha inhibiting activi				7.74E-09	5.14	60.23	20.60	40450.7
AHQ-1-9, 4574	K.AMGNLQIDFADPSR.A	1535.70716	2	1.44E-04	0.72	2.85	-	764.9
AHQ-1-9, 5668	R.EYQLNDSAAYYLNDLER.I	2078.18096	2	7.74E-09	0.96	4.58	-	1294.7
AHQ-1-9, 2151	K.IIHEDGYSEEECR.Q	1638.69452	2	1.01E-05	0.86	2.99	-	919.7
AHQ-1-9, 4763	K.ITHSPLTICFPEYTGANK.Y	2051.30821	2	2.37E-04	0.80	3.56	-	487.9
AHQ-1-9, 5606	K.ITHSPLTICFPEYTGANKYDEAASYIQSK.F	3307.63156	3	3.54E-05	0.89	4.66	-	459.6
AHQ-1-9, 2520	K.YDEAASYIQSK.F	1275.34595	2	1.35E-04	0.91	3.19	-	1222.5
gi|4557731|ref|NP_000618.1| latent transforming growth factor beta binding protein 1 precursor [Hom				7.98E-09	9.90	110.28	10.80	152790.9
AHQ-1-1, 3760	R.ASGLGDHCEDINECLEDK.S	2065.13910	2	2.48E-06	0.96	5.01	-	1400.2
AHQ-1-3, 3721	R.ASGLGDHCEDINECLEDK.S	2065.13910	2	3.01E-06	0.94	4.57	-	1311.5
AHQ-1-1, 4004	R.ASGLGDHCEDINECLEDKSVCQR.G	2696.84428	3	3.57E-06	0.85	3.69	-	925.5
AHQ-1-1, 2689 - 2709	R.CTCGQGYQLSAAK.D	1446.58862	2	1.14E-04	0.95	4.30	-	1015.1
AHQ-1-1, 4204	K.CVDIDECTQVQHLCSQGR.C	2209.38110	2	7.98E-09	0.97	4.62	-	1599.3
AHQ-1-3, 2135	K.DQCEDIDECQHR.H	1607.61898	2	1.35E-07	0.86	3.39	-	651.8
AHQ-1-1, 3447 - 3449	K.EAQPGQSQVSYQGLPVQK.T	1945.12244	2	1.36E-05	0.94	4.17	-	940.7
AHQ-1-4, 6178	K.IGFGPDPTFSSCVPDPPVISEEKGPCYR.L	3112.43550	3	1.01E-04	0.74	4.06	-	292.4
AHQ-1-1, 5983 - 5987	K.IGFGPDPTFSSCVPDPPVISEEKGPCYR.L	3112.43550	3	3.24E-07	0.95	5.58	-	1150.4
AHQ-1-1, 3136	K.LCQIPVHGASVPK.L	1407.66344	2	7.52E-08	0.88	3.54	-	558.5
AHQ-1-3, 2927	R.TSTDLDVDVDQPK.E	1433.50014	2	5.22E-06	0.92	3.97	-	1034.6
AHQ-1-1, 2929 - 2987	R.TSTDLDVDVDQPK.E	1433.50014	2	4.65E-07	0.86	3.26	-	812.9
AHQ-1-3, 2686 - 2694	R.TSTDLDVDVDQPKEEK.K	1819.90213	2	3.04E-07	0.91	3.99	-	1206.5
AHQ-1-4, 2691	R.TSTDLDVDVDQPKEEK.K	1819.90213	2	2.98E-07	0.94	4.18	-	1484.6
AHQ-1-5, 2586	R.TSTDLDVDVDQPKEEK.K	1819.90213	2	9.39E-08	0.78	2.97	-	1049.3
AHQ-1-2, 2770	R.TSTDLDVDVDQPKEEK.K	1819.90213	2	1.87E-04	0.87	3.57	-	1183.9
AHQ-1-1, 3671	R.YTCICYEGYR.F	1387.51964	2	1.57E-06	0.90	3.08	-	719.5
gi|21361670|ref|NP_054782.2| src homology 3 domain-containing protein HIP-55; HIP-55 protein [Homo				7.99E-09	3.49	40.23	16.00	48294.0
AHQ-1-11, 3302	R.FQDVGPQAPVGSVYQK.T	1720.90658	2	5.35E-08	0.89	3.29	-	920.7
AHQ-1-12, 3413	R.FQDVGPQAPVGSVYQK.T	1720.90658	2	2.81E-08	0.95	4.51	-	1027.9
AHQ-1-10, 3215 - 3217	R.FQDVGPQAPVGSVYQK.T	1720.90658	2	7.99E-09	0.96	4.41	-	1263.4
AHQ-1-12, 5463	K.FVLINWTGEGVNDVR.K	1719.92182	2	4.28E-06	0.95	4.13	-	1263.0
AHQ-1-12, 5410 - 5479	K.SPTDWALFTYEGNSNDIR.V	2087.19108	2	2.20E-04	0.68	3.07	-	613.6
AHQ-1-13, 5500 - 5508	R.VAGTGEGGLEEMVEELNSGK.V	2007.16599	2	4.71E-04	0.82	3.56	-	557.1
AHQ-1-12, 5549	R.VAGTGEGGLEEMVEELNSGK.V	2007.16599	2	1.32E-05	0.91	4.33	-	801.3
gi|4557801|ref|NP_000261.1| purine nucleoside phosphorylase [Homo sapiens]				8.52E-09	13.14	150.33	61.90	32147.7
AHQ-1-9, 1520	K.ANHEEVLAAGK.Q	1139.24364	1	3.55E-05	0.74	2.77	-	740.4
AHQ-1-9, 4402 - 4410	R.DHINLPGFSGQNPLR.G	1665.83425	2	2.84E-08	0.91	4.65	-	514.1
AHQ-1-9, 5662	K.FEVGDIMLIR.D	1193.44099	2	1.07E-05	0.92	3.57	-	1188.8
AHQ-1-9, 4556	R.FHMYEGYPLWK.V	1471.70636	2	2.33E-05	0.87	3.17	-	923.1
AHQ-1-9, 4558	R.FHMYEGYPLWK.V	1471.70636	1	3.58E-06	0.81	2.79	-	918.3
AHQ-1-9, 3068	R.FPAMSDAYDR.T	1173.28036	2	2.67E-04	0.94	3.35	-	1343.0
AHQ-1-13- , 4167	K.HRPQVAIICGSGLGGLTDK.L	1981.26589	3	3.68E-06	0.98	5.31	-	4012.6
AHQ-1-9, 7312	K.LEQFVSILMASIPLPDK.A	1902.28716	2	4.34E-07	0.95	5.28	-	764.9

AHQ-1-9, 4871 - 4943	K.LGADAVGMSTVPEVIVAR.H	1786.08666	2	4.00E-04	0.92	3.87	-	953.8
AHQ-1-13, 4139	K.LGADAVGM*STVPEVIVAR.H	1802.08606	2	4.89E-05	0.94	4.67	-	851.8
AHQ-1-10, 3916	K.LGADAVGM*STVPEVIVAR.H	1802.08606	2	5.36E-04	0.89	3.62	-	869.9
AHQ-1-13- , 4321	K.LGADAVGM*STVPEVIVAR.H	1802.08606	2	7.64E-05	0.94	4.57	-	770.8
AHQ-1-9, 4222 - 4223	K.LGADAVGM*STVPEVIVAR.H	1802.08606	2	1.84E-05	0.95	4.15	-	1223.9
AHQ-1-11, 4656	K.LGADAVGMSTVPEVIVAR.H	1786.08666	2	9.80E-06	0.52	2.71	-	593.1
AHQ-1-11, 4027	K.LGADAVGM*STVPEVIVAR.H	1802.08606	2	4.21E-04	0.84	3.55	-	709.3
AHQ-1-13- , 6003 - 6015	K.LTQAQIFDYSEIPNFPR.S	2040.26383	2	1.18E-05	0.91	4.54	-	516.8
AHQ-1-9, 5996 - 6070	K.LTQAQIFDYSEIPNFPR.S	2040.26383	2	3.21E-05	0.79	3.52	-	427.8
AHQ-1-13- , 6025	K.LTQAQIFDYSEIPNFPR.S	2040.26383	3	8.46E-05	0.86	4.08	-	894.6
AHQ-1-9, 5115	R.LVFGFLNGR.A	1023.21261	2	3.15E-06	0.91	2.89	-	1386.7
AHQ-1-9, 4931	R.VFGFSLITNK.V	1126.32960	2	3.85E-05	0.92	3.84	-	777.1
AHQ-1-13- , 5180 - 5184	R.VFGFSLITNK.V	1126.32960	2	3.45E-04	0.92	3.60	-	731.1
AHQ-1-9, 6058 - 6096	R.VFHLLGVDTLVVTNAAGGLNPK.F	2236.59890	2	8.52E-09	0.99	6.63	-	2850.1
AHQ-1-9, 2775	K.VIM*DYESLEK.A	1243.40878	2	3.83E-05	0.88	3.15	-	673.4
AHQ-1-9, 3506 - 3526	K.VIMDYESLEK.A	1227.40938	2	6.81E-05	0.81	2.80	-	458.6
gi|4507649|ref|NP_003280.1| tropomyosin 2 (beta) [Homo sapiens]				9.40E-09	1.91	20.23	4.90	32989.6
AHQ-1-9, 2650	K.ATDAEADVASLNR.R	1333.38703	2	4.40E-07	0.93	3.34	-	1654.1
AHQ-1-9, 2406	K.KATDAEADVASLNR.R	1461.55994	2	9.40E-09	0.97	4.58	-	2225.9
gi|5031593|ref|NP_005708.1| actin related protein 2/3 complex subunit 5; Arp2/3 protein complex sub				1.00E-08	6.39	70.34	53.60	16320.3
AHQ-1-12, 2978 - 3047	K.ALAAGGVGSIVR.V	1071.25558	2	1.73E-07	0.98	4.35	-	2384.6
AHQ-1-12, 3561	K.AVQSLDKNGVDLLM*K.Y	1647.91883	2	5.95E-06	0.89	3.84	-	632.5
AHQ-1-12, 4019 - 4082	K.AVQSLDKNGVDLLMK.Y	1631.91943	2	1.74E-06	0.97	5.29	-	1471.5
AHQ-1-12, 4026	K.AVQSLDKNGVDLLMK.Y	1631.91943	3	5.46E-04	0.89	3.43	-	1160.4
AHQ-1-12, 3469 - 3498	K.AVQSLDKNGVDLLM*K.Y	1647.91883	2	1.00E-08	0.92	4.12	-	628.4
AHQ-1-12, 4125	K.GFESPSDNSSAM*LLQWHEK.A	2180.33989	2	1.59E-06	0.95	4.35	-	1295.5
AHQ-1-12, 4210 - 4215	K.GFESPSDNSSAM*LLQWHEK.A	2180.33989	2	1.51E-07	0.66	2.98	-	472.4
AHQ-1-12, 4527	K.GFESPSDNSSAMLLQWHEK.A	2164.34049	2	6.03E-07	0.93	4.68	-	865.5
AHQ-1-12, 4649 - 4663	K.GFESPSDNSSAMLLQWHEK.A	2164.34049	2	1.12E-06	0.92	4.25	-	622.0
AHQ-1-12, 4271 - 4334	R.KVDVDEYDENKFVDEEDGGDGQAGPDEGEVDSCLR.Q	3890.92031	3	1.46E-05	0.98	6.85	-	1849.6
AHQ-1-12, 4535 - 4598	K.VDVDEYDENKFVDEEDGGDGQAGPDEGEVDSCLR.Q	3762.74739	3	8.02E-07	0.96	5.69	-	1379.1
gi|4758208|ref|NP_004081.1| dual specificity phosphatase 3; vaccinia virus phosphatase VH1-related;				1.02E-08	3.46	40.23	31.90	20478.2
AHQ-1-11, 4106	R.AADFIDQALAQK.N	1291.43488	2	5.78E-05	0.94	4.12	-	1236.4
AHQ-1-11, 5211 - 5283	K.ANDTQEFNLSAYFER.A	1805.88207	2	1.67E-05	0.76	3.06	-	753.5
AHQ-1-14- , 5539	K.ANDTQEFNLSAYFER.A	1805.88207	2	1.02E-08	0.63	3.25	-	366.5
AHQ-1-11, 5704 - 5724	R.EIGPNDGFLAQLCQLNDR.L	2062.24949	2	4.49E-06	0.93	4.53	-	889.2
AHQ-1-11, 3962 - 3968	R.IYVGNASVAQDIPK.L	1475.67136	2	4.06E-05	0.95	4.56	-	1182.8
AHQ-1-11, 3619	R.IYVGNASVAQDIPK.L	1475.67136	2	3.54E-05	0.96	4.05	-	1439.6
gi|28077085|ref|NP_004559.3| serine (or cysteine) proteinase inhibitor, clade B (ovalbumin), member				1.16E-08	1.82	20.22	10.10	42586.7
AHQ-1-9, 4783	K.SGGGGDIHQGFQSLLTEVNK.T	2045.19887	3	1.16E-08	0.94	4.37	-	1422.4
AHQ-1-9, 4772	K.SGGGGDIHQGFQSLLTEVNK.T	2045.19887	2	3.55E-05	0.84	3.76	-	692.0
AHQ-1-9, 7372	K.TYIGEIFTQILVLPYVGK.E	2055.44459	2	5.95E-07	0.89	4.01	-	918.3
gi|6598323|ref|NP_001485.2| GDP dissociation inhibitor 2; rab GDP-dissociation inhibitor, beta [Hom				1.19E-08	3.63	40.31	16.20	50662.9
AHQ-1-14, 6210	R.NPYYGGESASITPLEDLYK.R	2118.28499	2	1.29E-06	0.78	3.37	-	714.7
AHQ-1-9, 6907	K.SPYLYPLYGLGELPQGFAR.L	2142.44089	2	1.96E-06	0.95	4.63	-	822.1
AHQ-1-8, 7022 - 7023	K.SPYLYPLYGLGELPQGFAR.L	2142.44089	2	3.19E-05	0.97	5.68	-	1048.5
AHQ-1-7, 7089	K.SPYLYPLYGLGELPQGFAR.L	2142.44089	2	4.16E-06	0.96	5.03	-	950.4
AHQ-1-6, 6954	K.SPYLYPLYGLGELPQGFAR.L	2142.44089	2	1.44E-05	0.97	5.52	-	946.0
AHQ-1-8, 4157	R.TDDYLDQPCYETINR.I	1904.98887	2	2.28E-04	0.93	4.53	-	507.0
AHQ-1-8, 7511	K.VPSTEAEALASSLMGLFEK.R	1981.25641	2	1.19E-08	0.97	6.22	-	1414.6
gi|4502687|ref|NP_003865.1| CD84 antigen (leukocyte antigen); leukocyte antigen CD84 [Homo sapiens]				1.24E-08	1.56	20.26	11.60	36870.6
AHQ-1-7, 5030 - 5080	R.IHALGPNYNLVISDLR.M	1796.06296	2	1.24E-08	0.97	5.23	-	1577.4
AHQ-1-7, 3778	K.TSVAYVTPGDSETAPVVTVTHR.N	2288.49956	2	4.80E-04	0.58	3.10	-	327.2
gi|17318569|ref|NP_006112.2| keratin 1; Keratin-1; cytokeratin 1; hair alpha protein [Homo sapiens]				1.27E-08	4.03	50.25	9.90	66066.3
AHQ-1-14- , 3951	K.LNDLEDALQQAK.E	1358.47963	2	1.54E-05	0.94	4.07	-	1579.8
AHQ-1-3, 4034	K.LNDLEDALQQAK.E	1358.47963	2	4.83E-04	0.92	3.48	-	1387.8
AHQ-1-4, 4080	K.LNDLEDALQQAK.E	1358.47963	2	6.58E-04	0.89	3.23	-	1429.0
AHQ-1-13- , 4048	K.LNDLEDALQQAK.E	1358.47963	2	5.73E-06	0.96	4.53	-	1647.6
AHQ-1-13, 5813	R.SLDLDSIIAEVK.A	1303.48388	2	4.00E-07	0.89	3.19	-	1062.6
AHQ-1-12, 5861	R.SLDLDSIIAEVK.A	1303.48388	2	4.71E-06	0.95	4.38	-	1291.6
AHQ-1-1, 6272	R.SLDLDSIIAEVK.A	1303.48388	2	8.35E-08	0.94	4.08	-	1394.5
AHQ-1-13- , 6197	R.SLDLDSIIAEVK.A	1303.48388	2	1.27E-08	0.97	5.08	-	1604.0
AHQ-1-14- , 5982 - 5992	R.SLDLDSIIAEVK.A	1303.48388	2	7.82E-05	0.93	3.37	-	1315.1
AHQ-1-14- , 4678 - 4706	K.SLNNQFASFIDK.V	1384.51877	2	3.82E-04	0.59	2.53	-	449.8
AHQ-1-14- , 6362	R.THNLEPYFESFINNLR.R	1995.18315	2	3.43E-05	0.68	3.10	-	334.9
AHQ-1-1, 4705 - 4748	K.WELLQQVDTSTR.T	1476.61621	2	3.75E-06	0.83	2.85	-	996.4
gi|4758970|ref|NP_004150.1| proteasome beta 8 subunit isoform E1 proprotein; proteasome subunit, be				1.28E-08	1.59	20.17	9.60	29769.4
AHQ-1-11, 2496	K.FQHGVIAAVDSR.A	1300.44824	2	1.27E-04	0.76	2.60	-	885.3
AHQ-1-11, 3980 - 3983	K.VESTDVSDLLHQYR.E	1662.78230	2	1.28E-08	0.83	3.40	-	815.6
gi|21361399|ref|NP_055040.2| protein phosphatase 2 (formerly 2A), regulatory subunit A (PR 65), alp				1.34E-08	3.66	40.29	11.70	65308.3
AHQ-1-6, 3911	R.AISHEHSPSDLEAHFVPLVK.R	2214.46551	3	1.65E-08	0.97	5.78	-	1596.3
AHQ-1-6, 5466 - 5538	K.IGPILDNSTLQSEVKPILEK.L	2195.54185	3	1.34E-08	0.90	3.68	-	1189.4
AHQ-1-6, 3708	R.LAGGDWFTSR.T	1110.20399	2	1.10E-06	0.87	2.93	-	698.8
AHQ-1-6, 5934	K.SEIIPMFSNLASDEQDSVR.L	2139.32924	2	7.18E-08	0.92	4.20	-	823.7
AHQ-1-6, 5868 - 5938	K.SEIIPMFSNLASDEQDSVR.L	2139.32924	2	1.88E-04	0.93	4.44	-	832.2
gi|29742410|ref|XP_291183.1| similar to TREM-like transcript 1 [Homo sapiens]				1.41E-08	2.33	30.22	16.70	32678.3
AHQ-1-13- , 4392 - 4456	R.FLPEGCQPLVSSAVDR.R	1776.99174	2	8.09E-08	0.91	3.76	-	592.4
AHQ-1-11, 4131	R.FLPEGCQPLVSSAVDR.R	1776.99174	2	1.41E-08	0.82	3.58	-	385.6
AHQ-1-13- , 4305	R.FLPEGCQPLVSSAVDR.R	1776.99174	2	4.42E-06	0.87	3.78	-	429.6
AHQ-1-9, 5099	K.VLVCSKPVTYATVIFPGGNK.G	2152.54196	2	5.25E-05	0.88	3.97	-	517.4
AHQ-1-11, 3662	R.VSLNILPPEEEEETHK.I	1865.03087	3	5.79E-04	0.63	3.38	-	548.7
gi|4557014|ref|NP_001743.1| catalase [Homo sapiens]				1.51E-08	4.30	60.23	15.40	59755.8
AHQ-1-7, 2581	K.DAQIFIQK.K	963.11234	1	1.97E-05	0.48	2.29	-	673.1
AHQ-1-7, 5058	R.DPILFPSFIHSQK.R	1529.76385	2	1.75E-06	0.85	3.02	-	703.7
AHQ-1-7, 2576	R.FNTANDDNVTQVR.A	1494.54849	2	5.05E-04	0.16	2.53	-	303.6
AHQ-1-7, 2462	R.FNTANDDNVTQVR.A	1494.54849	2	4.17E-05	0.85	3.12	-	963.5
AHQ-1-7, 4692	K.GAGAFGYFEVTHDITK.Y	1713.87091	2	1.20E-05	0.93	4.41	-	819.6
AHQ-1-7, 4670 - 4730	R.LGPNYLHIPVNCPYR.A	1815.08803	2	4.16E-04	0.26	2.70	-	306.7
AHQ-1-7, 3974	K.NAIHTFVQSGSHLAAR.E	1709.89026	2	1.51E-08	0.95	4.65	-	1230.1
gi|5123454|ref|NP_005336.2| heat shock 70kDa protein 1A; heat shock 70kD protein 1A; heat shock-ind				1.51E-08	2.65	40.19	10.50	70037.7
AHQ-1-6, 5354 - 5358	K.AAAIGIDLGTTYSCVGVFQHGK.V	2267.54677	3	3.57E-06	0.73	3.53	-	461.1
AHQ-1-6, 4163	K.NALESYAFNMK.S	1288.45450	2	4.55E-05	0.83	2.74	-	928.5
AHQ-1-6, 6828 - 6899	K.SINPDEAVAYGAAVQAAILM*GDK.S	2321.59293	2	1.51E-08	0.88	3.82	-	700.2
AHQ-1-6, 2168	K.VEIIANDQGNR.T	1229.32518	1	5.09E-04	0.21	1.86	-	307.8
gi|4506183|ref|NP_002779.1| proteasome alpha 3 subunit isoform 1; proteasome subunit C8; macropain				1.58E-08	0.95	10.21	5.50	28433.1
AHQ-1-10, 3881	R.HVGMAVAGLLADAR.S	1381.62943	2	1.58E-08	0.95	4.10	-	1022.0
gi|30154095|ref|XP_293967.2| similar to gamma actin-like protein [Homo sapiens]				1.61E-08	1.53	20.18	12.20	19259.0
AHQ-1-8, 5022 - 5098	K.DLYTSTVLSGGTTM*YPGIADR.M	2235.45690	2	1.61E-08	0.76	3.34	-	495.7
AHQ-1-8, 6374 - 6452	K.DLYTSTVLSGGTTMYPGIADR.M	2219.45750	2	2.89E-06	0.34	2.86	-	371.9
AHQ-1-8, 6531 - 6532	K.DLYTSTVLSGGTTMYPGIADR.M	2219.45750	2	1.12E-04	0.44	2.70	-	376.2
AHQ-1-10, 4423	K.DLYTSTVLSGGTTM*YPGIADR.M	2235.45690	2	5.41E-06	0.70	3.50	-	366.4
AHQ-1-13, 4724 - 4787	K.DLYTSTVLSGGTTM*YPGIADR.M	2235.45690	2	1.14E-05	0.46	3.09	-	345.5
AHQ-1-13, 4949	K.DLYTSTVLSGGTTM*YPGIADR.M	2235.45690	2	8.04E-05	0.64	3.38	-	486.4
AHQ-1-14, 5490 - 5560	K.DLYTSTVLSGGTTM*YPGIADR.M	2235.45690	2	3.76E-05	0.66	3.45	-	489.6
AHQ-1-14, 5652 - 5724	K.DLYTSTVLSGGTTM*YPGIADR.M	2235.45690	2	1.16E-05	0.57	3.08	-	376.6
AHQ-1-14, 5912	K.DLYTSTVLSGGTTM*YPGIADR.M	2235.45690	2	5.85E-05	0.55	3.10	-	449.0
AHQ-1-14, 6017	K.DLYTSTVLSGGTTMYPGIADR.M	2219.45750	2	1.48E-07	0.77	3.34	-	397.4

AHQ-1-13- , 4992	K.DLYTSTVLSGGTTM*YPGIADR.M	2235.45690	2	1.55E-04	0.56	3.06	-	377.4
AHQ-1-13- , 5301	K.DLYTSTVLSGGTTMYPGIADR.M	2219.45750	2	3.67E-06	0.42	3.46	-	413.8
AHQ-1-14- , 4758	K.DLYTSTVLSGGTTM*YPGIADR.M	2235.45690	2	3.52E-07	0.76	3.37	-	449.4
AHQ-1-14- , 4843	K.DLYTSTVLSGGTTM*YPGIADR.M	2235.45690	2	4.08E-06	0.55	3.13	-	452.3
AHQ-1-14- , 4968	K.DLYTSTVLSGGTTM*YPGIADR.M	2235.45690	2	2.37E-04	0.28	2.71	-	287.1
AHQ-1-14- , 5074 - 5091	K.DLYTSTVLSGGTTM*YPGIADR.M	2235.45690	2	5.61E-05	0.62	3.60	-	527.1
gi|4503747|ref|NP_001448.1| filamin B, beta (actin binding protein 278); beta filamin; filamin 1 (a				1.62E-08	4.10	60.24	2.00	278191.4
AHQ-1-1, 3701 - 3703	K.DLAEDAPWK.K	1045.12708	1	7.03E-06	0.48	2.42	-	616.2
AHQ-1-7, 3628	K.DLAEDAPWK.K	1045.12708	1	7.23E-04	0.27	2.10	-	445.1
AHQ-1-4, 3696	K.DLAEDAPWK.K	1045.12708	1	1.47E-05	0.26	2.15	-	465.1
AHQ-1-2, 3790	K.DLAEDAPWK.K	1045.12708	2	2.23E-04	0.80	3.14	-	768.2
AHQ-1-2, 3786	K.DLAEDAPWK.K	1045.12708	1	2.31E-06	0.52	1.84	-	819.0
AHQ-1-3, 3015	K.DLAEDAPWKK.I	1173.29999	2	6.50E-04	0.71	2.60	-	875.5
AHQ-1-13, 6001	R.LIALLEVLSQK.R	1227.51852	2	8.75E-05	0.94	3.71	-	1252.5
AHQ-1-2, 6678	R.LIALLEVLSQK.R	1227.51852	2	1.03E-07	0.97	4.12	-	1704.3
AHQ-1-6, 6475	R.LIALLEVLSQK.R	1227.51852	2	9.42E-08	0.96	4.44	-	1134.3
AHQ-1-3, 6509	R.LIALLEVLSQK.R	1227.51852	2	8.38E-08	0.97	4.28	-	1637.4
AHQ-1-1, 6491	R.LIALLEVLSQK.R	1227.51852	2	4.82E-07	0.97	4.25	-	1461.7
AHQ-1-4, 6671 - 6672	R.LIALLEVLSQK.R	1227.51852	2	1.79E-06	0.97	4.20	-	1458.1
AHQ-1-5, 6577	R.LIALLEVLSQK.R	1227.51852	2	1.62E-08	0.98	4.87	-	1680.1
AHQ-1-14- , 6219 - 6222	R.LIALLEVLSQK.R	1227.51852	2	4.66E-06	0.97	4.59	-	1270.2
AHQ-1-11, 5834	R.LIALLEVLSQK.R	1227.51852	2	8.35E-06	0.97	4.58	-	1229.6
AHQ-1-10, 5724	R.LIALLEVLSQK.R	1227.51852	2	2.86E-07	0.96	4.25	-	1292.7
AHQ-1-9, 6396	R.LIALLEVLSQK.R	1227.51852	2	9.63E-07	0.96	4.40	-	1256.8
AHQ-1-7, 3593	R.LLGWIQNK.I	972.16560	2	8.47E-04	0.78	2.74	-	633.2
AHQ-1-2, 1930	K.LVSIDSK.A	761.88676	1	4.19E-04	0.20	2.02	-	279.4
AHQ-1-2, 6719 - 6794	R.QM*QLENVSVALEFLDR.E	1909.15348	2	4.34E-05	0.89	3.92	-	832.3
AHQ-1-1, 6463	R.QM*QLENVSVALEFLDR.E	1909.15348	2	4.69E-06	0.91	4.03	-	1061.4
gi|4504743|ref|NP_002194.1| integrin alpha 2 precursor; Integrin, alpha-2 (CD49B; alpha-2 subunit o				1.63E-08	3.40	40.25	5.80	129294.4
AHQ-1-3, 3765	K.AGDISCNADINPLK.I	1489.63335	2	2.07E-05	0.66	3.15	-	575.2
AHQ-1-3, 4847	K.KGILGQHQFLEGPEGIENTR.F	2224.46193	2	1.23E-05	0.95	4.94	-	699.7
AHQ-1-3, 3418	K.TQVGLIQYANNPR.V	1474.64668	2	1.63E-08	0.89	3.03	-	1061.0
AHQ-1-4, 5294	R.VDISLENPGTSPALEAYSETAK.V	2293.46974	2	4.27E-06	0.91	4.16	-	688.8
AHQ-1-3, 5195	R.VDISLENPGTSPALEAYSETAK.V	2293.46974	2	4.90E-08	0.91	4.35	-	706.6
gi|14745898|ref|XP_016144.2| similar to tyrosine 3-monooxygenase/tryptophan 5-monooxygenase activat				1.81E-08	0.97	10.25	7.50	18201.0
AHQ-1-10, 6164 - 6176	K.LLCCDILDVLDK.N	1479.74157	2	1.81E-08	0.97	4.99	-	979.7
AHQ-1-9, 6979 - 6998	K.LLCCDILDVLDK.N	1479.74157	2	2.79E-05	0.96	4.41	-	1139.2
gi|5453597|ref|NP_006126.1| F-actin capping protein alpha-1 subunit; Cap Z [Homo sapiens]				1.82E-08	4.14	50.25	26.20	32922.5
AHQ-1-9, 3180	K.DHYSNGFCTVYAK.T	1563.67248	2	5.84E-04	0.75	2.79	-	748.6
AHQ-1-13- , 5488	K.FTITPPTAQVVGVLK.I	1571.88532	2	3.27E-06	0.87	3.53	-	588.9
AHQ-1-9, 3170	K.IQVHYYEDGNVQLVSHK.D	2030.22904	2	1.67E-06	0.97	5.10	-	1638.1
AHQ-1-9, 4044	R.LLLNNDNLLR.E	1198.39736	2	1.62E-04	0.79	3.07	-	839.0
AHQ-1-9, 5514	K.TIDGQQTIIACIESHQFQPK.N	2316.57620	3	3.19E-05	0.55	3.17	-	209.9
AHQ-1-9, 5510 - 5515	K.TIDGQQTIIACIESHQFQPK.N	2316.57620	2	1.82E-08	0.76	3.89	-	295.7
gi|4502985|ref|NP_001854.1| cytochrome c oxidase subunit VIb; human cytochrome oxidase subunit VIb				1.84E-08	0.97	10.23	20.90	10192.3
AHQ-1-14- , 6138	R.VYQSLCPTSWVTDWDEQR.A	2272.43560	2	1.84E-08	0.97	4.68	-	1479.2
gi|14165439|ref|NP_002131.2| heterogeneous nuclear ribonucleoprotein K isoform a; dC-stretch bindin				1.85E-08	0.82	10.18	4.10	51027.9
AHQ-1-7, 5297 - 5368	R.GSYGDLGGPIITTQVTIPK.D	1918.18004	2	1.85E-08	0.82	3.38	-	634.5
AHQ-1-6, 5247 - 5312	R.GSYGDLGGPIITTQVTIPK.D	1918.18004	2	5.39E-05	0.82	3.69	-	582.5
gi|4759140|ref|NP_004243.1| solute carrier family 9 (sodium/hydrogen exchanger), isoform 3 regulato				1.87E-08	0.93	10.19	3.90	38868.2
AHQ-1-13- , 3931	R.LLVVDPETDEQLQK.L	1627.81777	2	1.87E-08	0.93	3.83	-	745.1
AHQ-1-9, 3779	R.LLVVDPETDEQLQK.L	1627.81777	2	3.75E-05	0.78	3.01	-	596.4
gi|4507855|ref|NP_003472.1| Ubiquitin isopeptidase T; Ubiquitin-specific protease-5 (ubiquitin isop				1.89E-08	1.68	20.25	5.40	93393.2
AHQ-1-5, 4262	K.IFQNAPTDPTQDFSTQVAK.L	2109.28133	2	1.11E-04	0.72	2.85	-	678.4
AHQ-1-5, 7274	R.IGEWELIQESGVPLKPLFGPGYTGIR.N	2858.28283	3	1.89E-08	0.96	5.00	-	1737.2
gi|4757812|ref|NP_004880.1| ATP synthase, H+ transporting, mitochondrial F0 complex, subunit f, iso				1.90E-08	2.73	30.22	25.50	10917.8
AHQ-1-13- , 6144	R.DFSPSGIFGAFQR.G	1429.56124	2	6.05E-08	0.94	3.90	-	1122.9
AHQ-1-14- , 5934	R.DFSPSGIFGAFQR.G	1429.56124	2	2.42E-06	0.94	3.94	-	1030.8
AHQ-1-13, 5752	R.DFSPSGIFGAFQR.G	1429.56124	2	1.90E-08	0.94	3.65	-	1301.7
AHQ-1-14- , 5970 - 5978	K.LGELPSWILMR.D	1315.60936	2	9.56E-07	0.93	4.36	-	656.5
AHQ-1-14- , 5534	K.LGELPSWILM*R.D	1331.60876	2	8.25E-06	0.86	3.32	-	581.9
gi|4505753|ref|NP_002620.1| phosphoglycerate mutase 1 (brain); Phosphoglycerate mutase A, nonmuscle				1.98E-08	1.69	20.22	16.50	28803.7
AHQ-1-10, 3809 - 3824	R.SYDVPPPPM*EPDHPFYSNISK.D	2434.66506	2	9.03E-05	0.78	3.32	-	374.4
AHQ-1-10, 4604	R.YADLTEDQLPSCESLKDTIAR.A	2427.62735	2	1.98E-08	0.91	4.49	-	513.2
AHQ-1-10, 4597	R.YADLTEDQLPSCESLKDTIAR.A	2427.62735	3	4.53E-05	0.76	3.59	-	502.9
gi|5031571|ref|NP_005713.1| actin-related protein 2; ARP2 (actin-related protein 2, yeast) homolog				2.02E-08	4.51	60.25	19.00	44760.5
AHQ-1-9, 5804 - 5819	K.CGYAGSNFPEHIFPALVGR.P	2094.33801	2	7.63E-04	0.35	2.82	-	379.5
AHQ-1-9, 6115	K.CGYAGSNFPEHIFPALVGRPIIR.S	2573.95654	3	5.82E-06	0.48	3.25	-	277.1
AHQ-1-8, 3853	K.DLMVGDEASELR.S	1335.46633	2	6.21E-08	0.95	3.89	-	1176.0
AHQ-1-9, 3768	K.DLMVGDEASELR.S	1335.46633	2	2.02E-08	0.90	2.97	-	1107.2
AHQ-1-9, 3355 - 3356	K.HIVLSGGSTM*YPGLPSR.L	1789.04907	2	6.42E-06	0.97	4.84	-	1802.2
AHQ-1-9, 4119	K.HLWDYTFGPEK.L	1393.52736	2	1.47E-06	0.92	3.47	-	697.8
AHQ-1-13- , 4357	K.HLWDYTFGPEK.L	1393.52736	2	4.79E-05	0.86	2.99	-	537.9
AHQ-1-8, 4140	K.HLWDYTFGPEK.L	1393.52736	2	3.18E-06	0.86	3.07	-	534.9
AHQ-1-8, 3441	K.ILLTEPPMNPTK.N	1354.64066	2	9.02E-04	0.70	3.14	-	439.9
AHQ-1-9, 3518 - 3556	K.ILLTEPPMNPTK.N	1354.64066	2	2.59E-04	0.89	3.56	-	577.1
gi|4557675|ref|NP_000201.1| integrin alpha chain, alpha 6 [Homo sapiens]				2.08E-08	2.65	30.22	4.90	119509.5
AHQ-1-4, 3515	K.FGSCQQGVAATFTK.D	1503.66172	2	6.25E-08	0.87	3.19	-	911.7
AHQ-1-4, 6227	R.NSYPDVAVGSLSDSVTIFR.S	2028.20822	2	2.08E-08	0.92	4.31	-	632.4
AHQ-1-4, 3960	K.VFIYHGSANGINTKPTQVLK.G	2188.51433	3	3.75E-05	0.85	3.55	-	1204.4
gi|5453603|ref|NP_006422.1| chaperonin containing TCP1, subunit 2 (beta); chaperonin containing t-c				2.09E-08	1.91	30.18	7.90	57487.9
AHQ-1-7, 3145	R.EALLSSAVDHGSDEVK.F	1657.76083	2	2.09E-08	0.92	3.58	-	1156.9
AHQ-1-7, 3201 - 3210	R.GATQQILDEAER.S	1331.41427	1	7.20E-04	0.08	2.41	-	191.1
AHQ-1-7, 5820	R.SLHDALCVLAQTVK.D	1556.80920	2	3.73E-08	0.91	2.75	-	1568.3
gi|18570595|ref|XP_095590.1| similar to myosin regulatory light chain [Homo sapiens]				2.20E-08	0.92	10.18	8.90	13951.5
AHQ-1-11, 3086 - 3107	K.LNGTDPEDVIR.H	1229.32195	2	2.20E-08	0.92	3.59	-	998.9
AHQ-1-12, 3099	K.LNGTDPEDVIR.H	1229.32195	2	7.97E-08	0.90	3.30	-	954.7
AHQ-1-14, 4030	K.LNGTDPEDVIR.H	1229.32195	2	1.19E-04	0.91	3.53	-	974.6
AHQ-1-11, 2955 - 3026	K.LNGTDPEDVIR.H	1229.32195	2	8.39E-07	0.94	3.62	-	1360.1
gi|28376654|ref|NP_778250.1| hypothetical protein INM01 [Homo sapiens]				2.29E-08	0.92	10.25	10.90	28153.2
AHQ-1-13- , 5704 - 5708	K.LALEDISADPEDTVGGHPSWSGWEDDAK.G	2999.10449	3	2.29E-08	0.92	4.99	-	841.0
gi|25306267|ref|NP_001700.2| brain-derived neurotrophic factor isoform a preproprotein [Homo sapien				2.37E-08	0.95	10.22	6.10	27817.8
AHQ-1-13- , 3785	K.TAVDMSGGTVTVLEK.V	1508.71993	2	2.37E-08	0.95	4.45	-	1154.2
gi|4758076|ref|NP_004068.1| citrate synthase precursor; Citrate synthase, mitochondrial [Homo sapie				2.39E-08	0.88	10.15	2.40	51706.2
AHQ-1-8, 3392	K.IVPNVLLEQGK.A	1210.44805	2	2.39E-08	0.88	3.01	-	509.4
gi|5729842|ref|NP_006699.1| glyoxalase I; lactoyl glutathione lyase; lactoylglutathione lyase [Homo				2.44E-08	1.88	20.32	20.10	20719.6
AHQ-1-11, 4610 - 4614	R.GFGHIGIAVPDVYSACK.R	1793.03600	2	2.27E-05	0.91	3.62	-	1156.5
AHQ-1-11, 6382 - 6454	K.GLAFIQDPDGYWIEILNPNK.M	2304.58542	2	2.44E-08	0.98	6.30	-	1142.1
gi|20070125|ref|NP_000909.2| prolyl 4-hydroxylase, beta subunit; v-erb-a avian erythroblastic leuke				2.45E-08	8.47	100.29	27.00	57115.9
AHQ-1-7, 5217	K.HNQLPLVIEFTEQTAPK.I	1966.22639	2	5.90E-04	0.97	5.80	-	1258.0
AHQ-1-7, 3750 - 3822	K.IKPHLMSQELPEDWDKQPVK.V	2419.78460	3	1.35E-04	0.91	4.09	-	806.0
AHQ-1-7, 5404	R.ILEFFGLK.K	967.18573	2	5.41E-04	0.84	3.13	-	520.6
AHQ-1-7, 5317	K.ILFIFIDSDHTDNQR.I	1835.00968	2	6.83E-05	0.89	3.62	-	1023.7
AHQ-1-7, 2632	K.LGETYKDHENIVIAK.M	1730.94291	2	3.91E-05	0.93	3.76	-	1340.0
AHQ-1-7, 2682 - 2690	R.LITLEEEM*TK.Y	1223.41900	2	5.25E-04	0.77	2.55	-	767.6
AHQ-1-7, 2264	K.MDSTANEVEAVK.V	1294.41421	2	1.77E-06	0.81	3.37	-	831.4
AHQ-1-7, 3926 - 4006	K.VDATEESDLAQQYGVR.G	1781.85882	2	4.06E-05	0.88	3.29	-	1297.9

AHQ-1-7, 2142	K.YKPESEELTAER.I	1452.54828	1	3.45E-05	0.11	1.85	-	260.2
AHQ-1-7, 2140	K.YKPESEELTAER.I	1452.54828	2	2.45E-08	0.61	2.74	-	311.2
AHQ-1-7, 4057 - 4128	K.YQLDKDGVVLFK.K	1425.65394	2	7.25E-06	0.85	3.63	-	1014.1
gi|7657313|ref|NP_055277.1| Lsm1 protein [Homo sapiens]				2.59E-08	0.88	10.21	12.00	15179.3
AHQ-1-12, 5755	R.SIDQFANLVLHQTVER.I	1871.08670	2	2.59E-08	0.88	4.13	-	662.6
gi|4504483|ref|NP_000185.1| hypoxanthine phosphoribosyltransferase 1 [Homo sapiens]				2.60E-08	1.86	20.19	10.60	24579.2
AHQ-1-10, 6068	K.FFADLLDYIK.A	1245.44772	2	2.60E-08	0.94	3.86	-	871.4
AHQ-1-10, 5052	K.NVLIVEDIIDTGK.T	1429.64090	2	3.62E-04	0.92	3.37	-	1234.3
gi|28603830|ref|NP_776155.1| hypothetical protein MGC51029 [Homo sapiens]				2.82E-08	0.89	10.21	11.00	17177.5
AHQ-1-12, 6634	K.AAPEINNLIEEATEFIK.Q	1903.12224	2	2.82E-08	0.89	4.16	-	540.0
gi|22042381|ref|XP_065237.3| similar to FKSG30 [Homo sapiens]				2.86E-08	1.43	20.19	3.50	42016.0
AHQ-1-8, 2219	K.QEYDESGPSIVHR.K	1517.58206	3	3.15E-05	0.70	3.08	-	689.8
AHQ-1-13- , 2980	K.QEYDESGPSIVHR.K	1517.58206	2	1.56E-04	0.57	2.85	-	495.6
AHQ-1-13- , 2453 - 2492	K.QEYDESGPSIVHR.K	1517.58206	2	2.86E-08	0.63	2.75	-	311.1
AHQ-1-10, 2296 - 2299	K.QEYDESGPSIVHR.K	1517.58206	2	6.20E-08	0.83	3.46	-	451.1
AHQ-1-14, 3810	K.QEYDESGPSIVHR.K	1517.58206	2	4.84E-04	0.40	2.65	-	461.4
AHQ-1-13, 2457 - 2518	K.QEYDESGPSIVHR.K	1517.58206	2	6.20E-04	0.65	2.76	-	345.8
AHQ-1-14- , 2395 - 2408	K.QEYDESGPSIVHR.K	1517.58206	2	5.39E-05	0.40	2.56	-	244.2
AHQ-1-8, 3449 - 3524	R.VAPEEHPILLTEAPLNPK.A	1969.27019	2	2.40E-04	0.80	3.85	-	451.9
AHQ-1-8, 3660 - 3731	R.VAPEEHPILLTEAPLNPK.A	1969.27019	2	4.58E-04	0.73	3.43	-	415.8
AHQ-1-10, 3611	R.VAPEEHPILLTEAPLNPK.A	1969.27019	2	8.98E-04	0.21	2.52	-	179.6
gi|20070158|ref|NP_003567.2| serine/threonine kinase 24 (STE20 homolog, yeast); STE20-like kinase 3				2.95E-08	0.72	10.16	3.40	49307.6
AHQ-1-7, 3949	K.SQACGGNLGSIEELR.G	1592.71366	2	2.95E-08	0.72	3.18	-	854.3
gi|5032041|ref|NP_005605.1| Ras homolog enriched in brain 2 [Homo sapiens]				3.02E-08	0.92	10.18	8.70	20497.4
AHQ-1-12, 5070	K.ALAESWNAAFLESSAK.E	1695.85389	2	6.16E-05	0.81	3.54	-	459.9
AHQ-1-12, 4882	K.ALAESWNAAFLESSAK.E	1695.85389	2	3.02E-08	0.92	3.59	-	995.6
gi|5803217|ref|NP_006821.1| ubiquinol-cytochrome c reductase (6.4kD) subunit [Homo sapiens]				3.05E-08	0.77	10.14	21.40	6569.6
AHQ-1-14- , 6286	R.LILDWVPYINGK.F	1431.70332	2	3.05E-08	0.77	2.73	-	766.5
AHQ-1-14- , 6140 - 6206	R.LILDWVPYINGK.F	1431.70332	2	2.18E-04	0.46	2.58	-	397.1
gi|18426915|ref|NP_004386.2| drebrin 1 isoform a; drebrin E; drebrin-1; drebrin E2 [Homo sapiens] [				3.38E-08	1.92	20.26	5.40	71438.9
AHQ-1-13- , 4199	K.LAASGEGGLQELSGHFENQK.V	2073.20908	2	2.61E-05	0.93	4.75	-	792.8
AHQ-1-8, 4031	K.LAASGEGGLQELSGHFENQK.V	2073.20908	2	3.38E-08	0.96	5.25	-	1098.5
AHQ-1-13- , 5715	K.YVLINWVGEDVPDAR.K	1746.94404	2	4.45E-04	0.95	3.98	-	1547.3
AHQ-1-13, 5376	K.YVLINWVGEDVPDAR.K	1746.94404	2	1.11E-05	0.96	4.61	-	1414.2
gi|9910542|ref|NP_064535.1| SAR1a gene homolog 1; SAR1 protein [Homo sapiens]				3.53E-08	2.19	30.21	33.30	22366.6
AHQ-1-14- , 3863	R.EIFGLYGQTTGK.G	1314.46845	1	2.00E-04	0.40	2.48	-	227.8
AHQ-1-11, 3606 - 3611	R.EIFGLYGQTTGK.G	1314.46845	2	7.38E-04	0.93	3.50	-	1173.9
AHQ-1-11, 5895	R.LGQHVPTLHPTSEELTIAGMTFTTFDLGGHEQAR.R	3694.08659	3	3.53E-08	0.88	4.24	-	592.8
AHQ-1-11, 6451 - 6452	K.NYLPAINGIVFLVDCADHSR.L	2276.55690	2	3.70E-06	0.91	4.05	-	728.2
AHQ-1-11, 6187	K.NYLPAINGIVFLVDCADHSR.L	2276.55690	2	2.10E-04	0.34	2.50	-	314.8
gi|11968045|ref|NP_071934.1| hypothetical protein FLJ22056 [Homo sapiens]				3.54E-08	0.94	10.24	8.00	36010.3
AHQ-1-11, 6431	K.DPTSLLGVLQAEADSTSEGLEDAVHSR.G	2798.95471	3	3.54E-08	0.94	4.87	-	1208.4
gi|5901944|ref|NP_008977.1| elastin microfibril interface located protein; TNF? elastin microfibril				3.65E-08	4.31	50.27	9.60	106694.7
AHQ-1-3, 3610	R.APAPASAPPGPSEELLR.Q	1660.85268	2	3.65E-08	0.65	2.94	-	274.2
AHQ-1-3, 3029	R.GLEEGQAQAGQCPSLEGR.L	1888.99421	2	2.00E-05	0.90	4.03	-	781.0
AHQ-1-3, 3131	R.GTELGGAAGQGGHPPGYTSLASR.L	2142.27464	2	6.36E-05	0.84	3.84	-	353.6
AHQ-1-3, 3687 - 3694	R.LDTVAGGLQGLR.E	1200.37012	2	2.65E-07	0.96	4.26	-	1571.0
AHQ-1-3, 3793	R.PARPNLSGSSAGSPLSGLGGEGPGESEK.V	2596.74928	3	3.60E-07	0.96	5.40	-	1493.1
gi|10863977|ref|NP_067000.1| LSM2 homolog, U6 small nuclear RNA associated; U6 snRNA-associated Sm-				3.76E-08	0.97	10.26	20.00	10834.5
AHQ-1-14- , 5398 - 5400	R.YVQLPADEVDTQLLQDAAR.K	2146.34311	2	3.76E-08	0.97	5.28	-	1366.2
AHQ-1-14, 6262 - 6325	R.YVQLPADEVDTQLLQDAAR.K	2146.34311	2	1.61E-06	0.94	4.49	-	890.5
gi|15147250|ref|NP_150281.1| protein phosphatase 1, regulatory (inhibitor) subunit 14A; 17-kDa PKC-				3.79E-08	0.86	10.20	10.90	16692.9
AHQ-1-12, 6497	K.SCGKPVEDFIQELLAK.L	1836.09909	2	3.79E-08	0.86	4.02	-	491.7
gi|21361198|ref|NP_000286.2| serine (or cysteine) proteinase inhibitor, clade A (alpha-1 antiprotei				3.87E-08	1.91	20.24	7.90	46722.2
AHQ-1-7, 3738	K.LQHLENELTHDIITK.F	1805.02503	2	6.87E-04	0.95	4.20	-	1338.0
AHQ-1-7, 4681 - 4761	K.VFSNGADLSGVTEEAPLK.L	1835.00476	2	3.87E-08	0.96	4.75	-	1441.8
gi|23111030|ref|NP_037478.2| sorting nexin 12 [Homo sapiens]				3.88E-08	0.95	10.25	8.60	18884.4
AHQ-1-12, 4923 - 4926	R.GDEGIFEESFIEER.R	1657.71615	2	3.88E-08	0.95	5.03	-	697.7
gi|8923114|ref|NP_060140.1| ubiquitin-specific protease otubain 1 [Homo sapiens]				3.93E-08	0.52	10.15	6.60	31311.8
AHQ-1-9, 2603	R.GEGGTTNPHIFPEGSEPK.V	1854.95463	2	3.93E-08	0.52	2.96	-	367.8
gi|5031595|ref|NP_005709.1| actin related protein 2/3 complex subunit 4; Arp2/3 protein complex sub				4.09E-08	3.75	40.26	31.00	19666.9
AHQ-1-11, 4179	R.AENFFILR.R	1010.17070	2	7.31E-04	0.97	4.25	-	1764.8
AHQ-1-12, 4219 - 4229	K.ELLLQPVTISR.N	1269.51549	2	2.01E-04	0.89	3.45	-	732.2
AHQ-1-12, 5583	R.KPVEGYDISFLITNFHTEQM*YK.H	2678.01211	3	2.83E-05	0.94	5.23	-	1210.3
AHQ-1-12, 3142	K.VLIEGSINSVR.V	1187.37134	2	2.01E-06	0.90	3.21	-	1118.7
AHQ-1-11, 3120	K.VLIEGSINSVR.V	1187.37134	2	4.09E-08	0.95	3.57	-	1610.5
gi|21361884|ref|NP_116235.2| RAB2B [Homo sapiens]				4.23E-08	0.80	10.16	8.30	24214.2
AHQ-1-11, 2830	K.IGPQQSISTSVGPSASQR.N	1800.95164	2	4.23E-08	0.80	3.28	-	672.6
gi|5031973|ref|NP_005733.1| protein disulfide isomerase-related protein [Homo sapiens]				4.54E-08	4.34	50.20	18.20	48121.0
AHQ-1-7, 7337	R.DGELPVEDDIDLSDVELDDLGKDEL	2759.86565	2	1.35E-05	0.84	3.14	-	997.3
AHQ-1-7, 2054	K.GESPVDYDGGR.T	1152.15291	2	3.13E-04	0.83	2.77	-	902.6
AHQ-1-7, 4940	K.GSFSEQGINEFLR.E	1484.59520	2	8.84E-08	0.86	3.13	-	884.5
AHQ-1-13- , 4192 - 4197	R.GSTAPVGGGAFPTIVER.E	1616.79990	2	1.30E-06	0.86	3.90	-	736.6
AHQ-1-7, 4108 - 4126	R.GSTAPVGGGAFPTIVER.E	1616.79990	2	2.66E-05	0.91	3.98	-	845.8
AHQ-1-8, 4052	R.GSTAPVGGGAFPTIVER.E	1616.79990	2	1.11E-04	0.90	3.79	-	836.1
AHQ-1-11, 3866	R.GSTAPVGGGAFPTIVER.E	1616.79990	2	5.58E-04	0.30	2.52	-	446.8
AHQ-1-12, 3997	R.GSTAPVGGGAFPTIVER.E	1616.79990	2	5.05E-05	0.81	3.24	-	789.3
AHQ-1-13, 4015	R.GSTAPVGGGAFPTIVER.E	1616.79990	2	1.53E-04	0.50	2.69	-	508.4
AHQ-1-13- , 6275	R.TGEAIVDAALSALR.Q	1387.56411	2	4.54E-08	0.94	3.45	-	1864.3
AHQ-1-7, 6442	R.TGEAIVDAALSALR.Q	1387.56411	2	4.83E-07	0.90	3.38	-	1329.1
gi|13128974|ref|NP_076933.1| hypothetical protein MGC3265 [Homo sapiens]				4.64E-08	0.93	10.21	4.50	46867.3
AHQ-1-7, 4397	R.SYYSVQTAEWQAHPLYGSR.P	2244.40703	2	4.64E-08	0.93	4.22	-	1012.4
gi|27480842|ref|XP_208238.1| similar to bA92K2.2 (similar to ubiquitin) [Homo sapiens]				4.76E-08	1.89	30.18	19.90	17966.9
AHQ-1-14, 4000 - 4001	K.ESTLHLVLR.L	1068.25169	2	1.62E-07	0.85	3.50	-	723.3
AHQ-1-14- , 3036	K.ESTLHLVLR.L	1068.25169	1	3.14E-04	0.37	2.25	-	211.2
AHQ-1-14- , 2039 - 2099	K.IQDKEGIPPDQQR.L	1524.66087	2	4.76E-08	0.79	3.00	-	653.8
AHQ-1-14, 3176 - 3232	R.TLSDYNIQK.E	1082.18886	1	2.42E-04	0.25	2.02	-	480.7
gi|30158844|ref|XP_292513.2| similar to KIAA1879 protein [Homo sapiens]				4.81E-08	2.06	30.20	1.90	203894.8
AHQ-1-7, 3337	K.AVDSLVPIGR.G	1027.19957	1	1.21E-04	0.18	2.02	-	167.1
AHQ-1-7, 4253	K.TSIAIDTIINQK.H	1317.51378	2	1.07E-04	0.93	3.37	-	1565.3
AHQ-1-7, 3089	R.VVDALGNAIDGK.G	1172.31354	2	4.81E-08	0.95	3.91	-	1308.1
gi|4826848|ref|NP_004991.1| NADH dehydrogenase (ubiquinone) 1 alpha subcomplex, 5; NADH dehydrogena				4.82E-08	0.98	10.35	22.40	13458.6
AHQ-1-14- , 6650	K.KLEDQLQGGQLEEVILQAEHELNLAR.K	2975.30339	3	4.82E-08	0.98	6.98	-	2166.7
gi|4507767|ref|NP_003326.1| ubiquitin-activating enzyme E1-like; Ubiquitin-activating enzyme-2; ubi				4.84E-08	3.44	40.25	7.70	111719.5
AHQ-1-5, 3510	R.APASAAASEDAPYPVCTVR.Y	1935.10448	2	1.02E-05	0.76	3.22	-	502.4
AHQ-1-5, 7333	R.GLNPDLQVIPLTYPLDPTTEHIYGDNFFSR.V	3434.79811	3	1.22E-04	0.73	3.70	-	279.7
AHQ-1-4, 4971	R.VDGVAAALDSFQAR.R	1420.55265	2	3.69E-05	0.97	3.88	-	2519.7
AHQ-1-4, 4814	R.VLVSGLQGLGAEVAK.N	1441.69805	2	4.84E-08	0.97	4.98	-	2049.8
gi|4502021|ref|NP_003680.1| aldo-keto reductase family 7, member A2 (aflatoxin aldehyde reductase);				4.88E-08	1.86	20.22	10.30	36648.3
AHQ-1-9, 3811	K.ALQAAYGASAPSVTSAALR.W	1806.01302	2	4.88E-08	0.91	4.46	-	637.9
AHQ-1-9, 5610 - 5612	R.FYAYNPLAGGLLTGK.Y	1585.82741	2	8.13E-04	0.95	4.44	-	1002.0
gi|11128019|ref|NP_061820.1| cytochrome c [Homo sapiens]				4.95E-08	0.91	10.17	13.30	11748.7
AHQ-1-13- , 2213 - 2216	K.TGQAPGYSYTAANK.N	1429.51641	2	4.95E-08	0.91	3.49	-	784.2
gi|4504557|ref|NP_000864.1| intercellular adhesion molecule 2 precursor [Homo sapiens]				5.01E-08	0.94	10.24	5.80	30653.1
AHQ-1-7, 7536 - 7540	R.VPTVEPLDSLTLFLFR.G	1848.17455	2	5.01E-08	0.94	4.73	-	590.7
gi|4826657|ref|NP_004333.1| caldesmon 1 isoform 3; H-CAD; L-CAD [Homo sapiens]				5.07E-08	2.60	30.21	10.00	62662.9
AHQ-1-13- , 2687	K.GNVFSSPTAAGTPNK.E	1448.56286	2	1.16E-05	0.85	3.10	-	870.5

AHQ-1-6, 2667 - 2675	K.SAKPTKPAASDLPVPAEGVR.N	1992.26539	3	2.58E-04	0.89	3.96	-	982.9
AHQ-1-13, 3760	K.TTTTNTQVEGDDEAAFLER.L	2099.15534	2	2.18E-04	0.86	3.76	-	784.5
AHQ-1-10, 3513	K.TTTTNTQVEGDDEAAFLER.L	2099.15534	2	5.21E-06	0.95	4.28	-	1253.3
AHQ-1-6, 3751 - 3759	K.TTTTNTQVEGDDEAAFLER.L	2099.15534	2	5.07E-08	0.86	3.91	-	657.5
gi|20270259|ref|NP_620061.1| phosphoglycerate kinase 2; phosphoglycerate kinase 1, pseudogene 2 [Ho				5.23E-08	0.93	10.21	3.60	44895.0
AHQ-1-8, 3384	K.LGDVYVNDAFGTAHR.A	1635.76178	2	5.23E-08	0.93	4.10	-	1024.8
gi|4504071|ref|NP_000164.1| platelet glycoprotein Ib alpha polypeptide precursor [Homo sapiens] [MA				5.26E-08	5.20	60.24	10.50	68955.0
AHQ-1-12, 5681 - 5690	R.AWLLFLR.G	919.14753	2	7.94E-05	0.92	3.22	-	997.4
AHQ-1-4, 5146	R.GLGELQELYLK.G	1263.46458	2	2.26E-05	0.92	3.72	-	1186.9
AHQ-1-1, 5157	R.GLGELQELYLK.G	1263.46458	2	1.33E-04	0.94	3.91	-	1380.1
AHQ-1-3, 5046	R.GLGELQELYLK.G	1263.46458	2	6.40E-06	0.95	4.03	-	1434.5
AHQ-1-9, 4887	R.GLGELQELYLK.G	1263.46458	2	4.71E-05	0.94	3.89	-	1374.7
AHQ-1-13, 4036 - 4053	R.GQDLLSTVSIR.Y	1189.34410	2	2.88E-07	0.94	3.62	-	1261.7
AHQ-1-14, 5014	R.GQDLLSTVSIR.Y	1189.34410	2	8.73E-05	0.95	3.40	-	1534.2
AHQ-1-12, 4001 - 4023	R.GQDLLSTVSIR.Y	1189.34410	2	5.26E-08	0.95	4.46	-	1113.8
AHQ-1-14- , 4082 - 4102	R.GQDLLSTVSIR.Y	1189.34410	2	9.40E-04	0.93	3.29	-	1393.9
AHQ-1-4, 4255	R.GQDLLSTVSIR.Y	1189.34410	2	2.31E-07	0.95	4.32	-	1170.9
AHQ-1-13- , 4215 - 4245	R.GQDLLSTVSIR.Y	1189.34410	2	1.40E-05	0.95	3.93	-	1406.1
AHQ-1-1, 4292	R.GQDLLSTVSIR.Y	1189.34410	2	2.70E-07	0.96	4.30	-	1300.6
AHQ-1-3, 4189	R.GQDLLSTVSIR.Y	1189.34410	2	2.27E-06	0.95	3.82	-	1397.6
AHQ-1-3, 2105	R.GVLQGHLESSR.N	1183.29965	2	2.87E-04	0.72	2.85	-	585.8
AHQ-1-4, 2103 - 2110	R.GVLQGHLESSR.N	1183.29965	2	6.74E-04	0.90	3.17	-	996.6
AHQ-1-3, 3697	R.LTQLNLDRCELTK.L	1605.83862	2	3.56E-04	0.69	3.86	-	399.1
AHQ-1-1, 5056	R.WLQDNAENVYVWK.Q	1665.82948	2	9.39E-07	0.97	4.58	-	1636.9
AHQ-1-3, 4981	R.WLQDNAENVYVWK.Q	1665.82948	2	1.04E-06	0.95	4.12	-	1423.1
AHQ-1-3, 5178 - 5253	R.WLQDNAENVYVWK.Q	1665.82948	2	1.58E-07	0.97	4.90	-	1697.7
gi|4507487|ref|NP_003238.1| thrombospondin 2 [Homo sapiens]				5.40E-08	1.34	20.18	2.60	129954.5
AHQ-1-1, 5317	R.DDDYAGFVFGYQSSSR.F	1814.84582	2	5.40E-08	0.91	3.36	-	1337.6
AHQ-1-2, 5514	R.DDDYAGFVFGYQSSSR.F	1814.84582	2	1.84E-07	0.78	3.37	-	640.3
AHQ-1-6, 2559	R.NALWHTGNTPGQVR.T	1551.69124	2	1.76E-04	0.90	3.51	-	1097.3
AHQ-1-7, 2570	R.NALWHTGNTPGQVR.T	1551.69124	2	1.19E-04	0.76	3.26	-	669.0
AHQ-1-8, 2446	R.NALWHTGNTPGQVR.T	1551.69124	2	6.38E-05	0.78	2.77	-	759.1
AHQ-1-9, 2507 - 2576	R.NALWHTGNTPGQVR.T	1551.69124	2	6.87E-06	0.43	2.66	-	565.2
AHQ-1-11, 2584 - 2587	R.NALWHTGNTPGQVR.T	1551.69124	2	1.45E-05	0.88	3.40	-	973.5
AHQ-1-1, 2844 - 2849	R.NALWHTGNTPGQVR.T	1551.69124	2	9.40E-04	0.87	3.30	-	909.9
gi|4505667|ref|NP_001074.1| phosphodiesterase 5A isoform 1; cGMP-binding cGMP-specific 3',5'-cyclic				5.70E-08	1.83	30.19	4.50	100012.4
AHQ-1-5, 3822 - 3830	R.FNAEVDQITGYK.T	1385.50354	2	3.64E-07	0.94	3.70	-	1165.4
AHQ-1-5, 4318	R.FPWTTENTGNVNQQCIR.S	2067.22783	2	1.45E-04	0.12	2.57	-	155.3
AHQ-1-5, 3863	K.VIGVCQLVNK.M	1131.37098	2	5.70E-08	0.77	3.05	-	527.7
gi|20532885|ref|XP_086931.2| similar to epsilon isoform of 14-3-3 protein [Homo sapiens]				5.75E-08	2.58	30.28	13.70	29380.0
AHQ-1-13- , 2680	K.EAAKNSIVAYK.A	1194.36234	2	7.62E-04	0.67	2.81	-	652.1
AHQ-1-11, 6264	K.LICCDILDVLDK.H	1479.74157	2	1.12E-04	0.92	3.88	-	873.9
AHQ-1-13, 6347	K.LICCDILDVLDK.H	1479.74157	2	2.84E-06	0.96	4.33	-	1067.1
AHQ-1-13- , 6797	K.LICCDILDVLDK.H	1479.74157	2	5.75E-08	0.97	5.57	-	1113.8
AHQ-1-10, 2513 - 2584	R.YLAEFATGNDRK.E	1385.50677	2	8.18E-07	0.94	3.71	-	1413.3
AHQ-1-10, 2379	R.YLAEFATGNDRK.E	1385.50677	2	1.78E-04	0.84	3.12	-	849.1
gi|30151001|ref|XP_170195.2| similar to ADP,ATP carrier protein, fibroblast isoform (ADP/ATP transl				6.00E-08	0.89	10.18	2.60	46759.8
AHQ-1-10, 3500	R.AAYFGIYDTAK.G	1220.35512	2	2.66E-04	0.89	2.90	-	841.9
AHQ-1-13, 3804	R.AAYFGIYDTAK.G	1220.35512	2	6.00E-08	0.89	3.07	-	1041.9
AHQ-1-13- , 3952	R.AAYFGIYDTAK.G	1220.35512	2	4.41E-04	0.92	3.60	-	932.6
AHQ-1-10, 3496 - 3499	R.AAYFGIYDTAK.G	1220.35512	1	4.67E-04	0.72	2.39	-	890.0
gi|5454170|ref|NP_006393.1| hepatitis B virus x-interacting protein; hepatitis B virus x-interactin				6.16E-08	0.98	10.30	22.00	9613.9
AHQ-1-14- , 5874	K.NPSIVGVLCTDSQGLNLGCR.G	2163.41855	2	6.16E-08	0.98	5.94	-	2008.6
gi|20127517|ref|NP_009148.2| programmed cell death 10; apoptosis-related protein 15 [Homo sapiens]				6.35E-08	0.87	10.16	6.10	24701.4
AHQ-1-10, 3952	R.LIHQTNLILQTFK.T	1569.87265	2	6.35E-08	0.87	3.28	-	759.6
gi|29568111|ref|NP_006088.2| myosin, light polypeptide 9, regulatory; myosin regulatory light chain				6.37E-08	3.33	40.19	12.20	19827.1
AHQ-1-11, 2611 - 2686	R.FTDEEVDEM*YR.E	1450.50949	2	6.04E-06	0.94	3.59	-	1128.4
AHQ-1-11, 3350 - 3362	R.FTDEEVDEMYR.E	1434.51009	2	2.59E-07	0.86	3.12	-	907.6
AHQ-1-11, 3354	R.FTDEEVDEMYR.E	1434.51009	1	1.50E-05	0.04	1.81	-	265.5
AHQ-1-11, 3367	R.FTDEEVDEMYR.E	1434.51009	1	6.37E-08	0.68	2.66	-	476.1
AHQ-1-12, 2737	R.FTDEEVDEM*YR.E	1450.50949	2	1.26E-05	0.88	3.30	-	734.2
AHQ-1-12, 3446 - 3462	R.FTDEEVDEMYR.E	1434.51009	2	8.65E-06	0.90	3.07	-	1322.2
AHQ-1-12, 3798	K.GNFNYVEFTR.I	1247.34066	2	3.11E-05	0.82	2.93	-	765.3
AHQ-1-11, 3682 - 3710	K.GNFNYVEFTR.I	1247.34066	2	1.98E-06	0.93	3.74	-	893.3
AHQ-1-11, 3783 - 3796	K.GNFNYVEFTR.I	1247.34066	2	7.93E-05	0.76	2.79	-	520.1
AHQ-1-11, 3903	K.GNFNYVEFTR.I	1247.34066	2	8.89E-05	0.87	3.06	-	891.9
AHQ-1-12, 3734 - 3799	K.GNFNYVEFTR.I	1247.34066	2	1.48E-04	0.68	2.63	-	668.5
AHQ-1-14- , 3962	K.GNFNYVEFTR.I	1247.34066	2	5.55E-07	0.87	3.14	-	890.0
AHQ-1-11, 5598	R.NAFACFDEEASGFIHEDHLR.E	2367.49548	2	3.97E-05	0.84	3.58	-	615.0
gi|4503545|ref|NP_001961.1| eukaryotic translation initiation factor 5A; eIF5AI; eIF5A [Homo sapien				6.40E-08	0.93	10.20	11.00	16832.2
AHQ-1-12, 3937	R.EDLRLPEGDLGKEIEQK.Y	1970.17021	3	6.40E-08	0.93	4.01	-	1395.6
gi|4758984|ref|NP_004654.1| RAB11A, member RAS oncogene family; RAB 11A, member oncogene family [Ho				6.88E-08	6.14	70.30	30.60	24393.3
AHQ-1-12, 2802	K.AQIWDTAGQER.Y	1275.35241	2	3.69E-04	0.90	2.94	-	1204.3
AHQ-1-11, 2726 - 2744	K.AQIWDTAGQER.Y	1275.35241	2	2.48E-07	0.95	3.80	-	1370.4
AHQ-1-11, 4543 - 4622	R.DHADSNIVIMLVGNK.S	1626.85974	2	4.60E-07	0.97	4.73	-	1832.3
AHQ-1-11, 4054 - 4132	R.DHADSNIVIM*LVGNK.S	1642.85914	2	7.90E-06	0.96	4.72	-	1609.6
AHQ-1-14- , 5172	R.GAVGALLVYDIAK.H	1290.53317	2	1.58E-06	0.93	3.84	-	1291.5
AHQ-1-11, 4794	R.GAVGALLVYDIAK.H	1290.53317	2	6.88E-08	0.98	5.99	-	1990.7
AHQ-1-11, 2054	K.HLTYENVER.W	1161.24931	2	1.61E-06	0.78	2.58	-	563.2
AHQ-1-11, 2320	R.NEFNLESK.S	981.04139	1	1.17E-04	0.57	2.83	-	326.9
AHQ-1-11, 3016	K.STIGVEFATR.S	1081.20410	2	1.24E-06	0.92	3.21	-	991.2
gi|19913412|ref|NP_005106.2| major vault protein [Homo sapiens]				6.97E-08	0.80	10.16	1.80	99326.3
AHQ-1-4, 6012	R.LAQDPFPLYPGEVLEK.D	1817.07418	2	6.97E-08	0.80	3.24	-	730.9
gi|4507793|ref|NP_003339.1| ubiquitin-conjugating enzyme E2N (UBC13 homolog, yeast); ubiquitin-conj				7.12E-08	4.94	60.26	42.10	17137.7
AHQ-1-12, 5182 - 5203	K.LELFLPEEYPM*AAPK.V	1765.06265	2	1.72E-04	0.76	3.71	-	391.1
AHQ-1-13- , 5864 - 5865	K.LELFLPEEYPMAAPK.V	1749.06325	2	7.12E-08	0.85	4.11	-	593.0
AHQ-1-13, 5153	K.LELFLPEEYPM*AAPK.V	1765.06265	2	5.03E-04	0.47	3.03	-	382.8
AHQ-1-13- , 3501	R.LLAEPVPGIK.A	1037.27750	2	1.35E-04	0.66	2.73	-	493.6
AHQ-1-12, 2202 - 2203	K.TNEAQAIETAR.A	1204.27249	2	1.39E-06	0.92	3.96	-	1377.0
AHQ-1-12, 3517	K.WSPALQIR.T	971.13770	2	3.78E-05	0.78	2.94	-	538.8
AHQ-1-13- , 5415	R.YFHVVIAGPQDSPFEGGTFK.L	2197.43341	3	7.98E-07	0.96	5.23	-	1699.1
AHQ-1-12, 5133	R.YFHVVIAGPQDSPFEGGTFK.L	2197.43341	2	3.92E-05	0.95	4.54	-	1053.1
gi|4503823|ref|NP_002028.1| protein-tyrosine kinase fyn isoform a; proto-oncogene tyrosine-protein				7.16E-08	0.87	10.18	2.00	60761.5
AHQ-1-7, 3800 - 3805	K.WTAPEAALYGR.F	1235.37302	2	7.16E-08	0.87	3.55	-	635.7
gi|22748619|ref|NP_689476.1| tropomyosin 3 [Homo sapiens]				7.24E-08	4.30	50.23	21.80	28222.6
AHQ-1-9, 3771 - 3843	R.ALKDEEKMELQEIQLK.E	1946.25520	2	4.33E-04	0.94	4.16	-	1392.0
AHQ-1-10, 3159	R.KLVIIEGDLER.T	1285.51492	2	5.58E-05	0.90	3.52	-	980.3
AHQ-1-9, 3446 - 3503	R.LATALQKLEEAEK.A	1444.65557	2	1.56E-06	0.95	4.53	-	1435.0
AHQ-1-9, 3786	K.LVIIEGDLER.T	1157.34201	2	7.24E-08	0.81	2.72	-	904.5
AHQ-1-10, 2041 - 2117	K.YSQKEDKYEEEIK.I	1689.80129	2	6.33E-04	0.58	2.99	-	762.9
AHQ-1-9, 1979 - 2050	K.YSQKEDKYEEEIK.I	1689.80129	2	2.05E-05	0.69	3.20	-	618.0
gi|27436929|ref|NP_005518.2| heat shock 70kDa protein 1-like; heat shock 70kD protein-like 1 [Homo				7.57E-08	0.97	10.22	2.20	70404.7
AHQ-1-6, 3174	K.AKIHDIVLVGGSTR.I	1466.71083	2	7.57E-08	0.97	4.43	-	1985.9
gi|4507231|ref|NP_003134.1| single-stranded DNA binding protein; single-stranded DNA-binding protei				7.70E-08	3.24	40.28	25.70	17259.5
AHQ-1-13- , 2473	R.DVAYQYVK.K	986.10277	1	8.47E-04	0.49	2.16	-	717.8
AHQ-1-13, 2530	R.DVAYQYVK.K	986.10277	1	5.84E-05	0.47	1.96	-	728.6
AHQ-1-13, 5737 - 5801	K.NPVTIFSLATNEM*WR.S	1796.04007	2	1.00E-04	0.92	3.95	-	927.7

AHQ-1-13- , 6824	K.NPVTIFSLATNEMWR.S	1780.04067	2	7.70E-08	0.98	5.60	-	1896.7
AHQ-1-13- , 3304	R.SGDSEVYQLGDVSQK.T	1612.67692	2	8.91E-07	0.93	3.86	-	1003.4
AHQ-1-13, 3231	R.SGDSEVYQLGDVSQK.T	1612.67692	2	4.90E-07	0.87	3.74	-	808.6
gi|4503477|ref|NP_001950.1| eukaryotic translation elongation factor 1 beta 2; eukaryotic translati				7.85E-08	1.82	20.22	14.70	24763.6
AHQ-1-9, 5762 - 5768	K.SPAGLQVLNDYLADK.S	1604.78589	2	2.70E-05	0.89	3.72	-	920.6
AHQ-1-9, 5456 - 5522	K.SPAGLQVLNDYLADK.S	1604.78589	2	5.97E-07	0.93	4.06	-	924.0
AHQ-1-9, 4602	K.SPAGLQVLNDYLADK.S	1604.78589	2	7.85E-08	0.96	4.48	-	1296.2
AHQ-1-9, 5110	K.SSILLDVKPWDDETDMAK.L	2064.30228	2	1.21E-04	0.85	3.88	-	490.5
gi|30153515|ref|XP_293925.2| similar to phosphoglycerate mutase (EC 5.4.2.1) B chain - rat [Homo sa				8.25E-08	0.90	10.19	2.70	57163.7
AHQ-1-13- , 6316	R.ALPFWNEEIVPQIK.E	1684.95898	2	8.25E-08	0.90	3.84	-	565.4
gi|5031677|ref|NP_005681.1| dynamin 1-like protein isoform 3; dynamin-like protein [Homo sapiens] [				8.40E-08	9.85	110.34	20.90	78060.6
AHQ-1-6, 4800	K.ALQGASQIIAEIR.E	1370.57991	2	2.83E-05	0.91	3.80	-	1223.1
AHQ-1-6, 2811	R.CVELVHEEMQR.I	1431.62054	2	5.61E-05	0.82	3.17	-	733.0
AHQ-1-13, 5031	K.DTLQSELVGQLYK.S	1494.67144	2	8.40E-08	0.90	3.19	-	1438.6
AHQ-1-14, 6074	K.DTLQSELVGQLYK.S	1494.67144	2	5.65E-05	0.71	2.63	-	893.5
AHQ-1-6, 4448	K.GHAVNLLDVPVPVAR.K	1557.82197	2	6.39E-06	0.92	3.61	-	1181.5
AHQ-1-6, 6494	K.IFSPNVVNLTLVDLPGM*TK.V	2075.45768	2	7.11E-04	0.95	4.54	-	1262.8
AHQ-1-6, 7028 - 7034	K.IFSPNVVNLTLVDLPGMTK.V	2059.45828	2	1.62E-05	0.93	4.40	-	886.2
AHQ-1-7, 7208	K.IFSPNVVNLTLVDLPGMTK.V	2059.45828	2	8.42E-06	0.87	3.44	-	804.9
AHQ-1-7, 6646	K.IFSPNVVNLTLVDLPGM*TK.V	2075.45768	2	6.90E-06	0.69	2.83	-	685.2
AHQ-1-5, 7193	K.IFSPNVVNLTLVDLPGMTK.V	2059.45828	2	9.28E-05	0.92	4.09	-	981.3
AHQ-1-6, 3383	K.LGIIGVVNR.S	941.15305	2	1.56E-05	0.92	3.07	-	1138.0
AHQ-1-6, 7254 - 7255	K.LQDVFNTVGADIIQLPQIVVVGTQSSGK.S	2928.32977	3	2.42E-06	0.97	6.78	-	1746.6
AHQ-1-14- , 6155	K.SSLLDDLLTESEDM*AQR.R	1940.07638	2	9.24E-08	0.96	5.04	-	1321.2
AHQ-1-13- , 6829 - 6901	K.SSLLDDLLTESEDMAQR.R	1924.07698	2	1.81E-05	0.92	3.94	-	990.0
AHQ-1-6, 7230	R.TLESVDPLGGLNTIDILTAIR.N	2212.52928	2	5.50E-06	0.96	5.15	-	1068.0
gi|4758018|ref|NP_004359.1| calponin 2; Calonin 2 [Homo sapiens]				8.59E-08	1.88	20.22	9.10	33696.9
AHQ-1-12, 2863 - 2865	K.GLQSGVDIGVK.Y	1073.22510	2	1.09E-05	0.92	3.65	-	1156.4
AHQ-1-11, 5950 - 6024	R.TWIEGLTGLSIGPDFQK.G	1863.10294	2	2.41E-05	0.94	4.15	-	1118.2
AHQ-1-9, 6604	R.TWIEGLTGLSIGPDFQK.G	1863.10294	2	5.65E-06	0.95	3.55	-	1637.1
AHQ-1-12, 6146 - 6173	R.TWIEGLTGLSIGPDFQK.G	1863.10294	2	8.59E-08	0.96	4.03	-	1637.3
AHQ-1-12, 6270	R.TWIEGLTGLSIGPDFQK.G	1863.10294	2	6.90E-04	0.56	2.80	-	424.6
AHQ-1-13- , 6449 - 6524	R.TWIEGLTGLSIGPDFQK.G	1863.10294	2	1.22E-05	0.95	4.26	-	1149.6
AHQ-1-14- , 6195 - 6267	R.TWIEGLTGLSIGPDFQK.G	1863.10294	2	1.37E-06	0.96	4.39	-	1293.6
AHQ-1-1, 6573	R.TWIEGLTGLSIGPDFQK.G	1863.10294	2	2.97E-05	0.94	3.72	-	1362.8
gi|5453599|ref|NP_006127.1| capping protein (actin filament) muscle Z-line, alpha 2; F-actin cappin				8.74E-08	2.54	30.35	12.20	32948.9
AHQ-1-9, 5279 - 5286	K.FTITPSTTQVVGILK.I	1605.90007	2	1.33E-04	0.92	4.27	-	527.1
AHQ-1-13- , 5436 - 5504	K.FTITPSTTQVVGILK.I	1605.90007	2	1.19E-06	0.81	3.29	-	428.9
AHQ-1-9, 5379	K.IDGQQTIIACIESHQFQAK.N	2189.43441	2	8.74E-08	0.75	3.32	-	504.1
AHQ-1-9, 4855	K.KIDGQQTIIACIESHQFQAK.N	2317.60733	3	9.28E-08	0.98	6.96	-	2682.1
gi|10835242|ref|NP_006249.1| protein kinase, cGMP-dependent, type I; Protein kinase, cGMP-dependent				9.35E-08	0.93	10.19	2.50	77803.3
AHQ-1-6, 6884	K.LSDFNIIDTLGVGGFGR.V	1781.98992	2	9.35E-08	0.93	3.90	-	1044.9
gi|7661922|ref|NP_055814.1| RAB21, member RAS oncogene family [Homo sapiens]				1.02E-07	0.97	10.28	7.60	24347.4
AHQ-1-10, 3999 - 4008	R.HVSIQEAESYAESVGAK.H	1805.92351	2	1.02E-07	0.97	5.58	-	1225.3
gi|4504073|ref|NP_000398.1| glycoprotein Ib beta polypeptide precursor [Homo sapiens]				1.05E-07	2.41	30.21	15.50	21717.6
AHQ-1-1, 4907	R.LLPYLAEDELR.A	1332.52691	2	1.05E-07	0.85	3.14	-	545.3
AHQ-1-3, 4869 - 4870	R.LLPYLAEDELR.A	1332.52691	2	4.13E-06	0.77	3.06	-	418.6
AHQ-1-4, 4942	R.LLPYLAEDELR.A	1332.52691	2	8.80E-07	0.81	3.03	-	632.4
AHQ-1-6, 4727 - 4738	R.LLPYLAEDELR.A	1332.52691	2	8.46E-05	0.78	2.78	-	590.2
AHQ-1-8, 4699	R.LLPYLAEDELR.A	1332.52691	2	2.74E-07	0.78	3.05	-	608.4
AHQ-1-9, 4666	R.LLPYLAEDELR.A	1332.52691	2	2.32E-06	0.69	2.89	-	330.2
AHQ-1-13, 4557	R.LLPYLAEDELR.A	1332.52691	2	5.41E-05	0.90	3.06	-	783.7
AHQ-1-10, 4259 - 4264	R.LLPYLAEDELR.A	1332.52691	2	2.62E-05	0.74	2.62	-	467.8
AHQ-1-12, 4590	R.LLPYLAEDELR.A	1332.52691	2	4.05E-05	0.69	2.62	-	356.0
AHQ-1-11, 4390 - 4422	R.LLPYLAEDELR.A	1332.52691	2	2.78E-07	0.88	3.51	-	467.8
AHQ-1-10, 3980	R.LSLTDPLVAER.A	1214.39356	2	2.75E-04	0.90	3.30	-	1287.4
AHQ-1-11, 4083 - 4094	R.LSLTDPLVAER.A	1214.39356	2	2.99E-06	0.96	4.13	-	1547.8
AHQ-1-11, 2319	R.TAHLGANPWR.C	1123.24913	2	2.68E-06	0.60	2.78	-	458.4
gi|29731325|ref|XP_293023.1| similar to peptidyl-Pro cis trans isomerase [Homo sapiens]				1.11E-07	0.88	10.19	6.00	28867.8
AHQ-1-13- , 4231 - 4235	K.AGPNTNGSQFFICTAK.T	1714.88050	2	8.02E-06	0.74	3.30	-	511.5
AHQ-1-12, 4045 - 4046	K.AGPNTNGSQFFICTAK.T	1714.88050	2	1.11E-07	0.88	3.72	-	644.5
gi|30148346|ref|XP_302059.1| similar to MLRQ subunit of the NADH: ubiquinone oxidoreductase complex				1.14E-07	0.88	10.15	6.60	17253.8
AHQ-1-14, 4233	K.FYSVNVDYSK.L	1222.32788	1	2.30E-06	0.49	2.46	-	547.4
AHQ-1-14- , 3403	K.FYSVNVDYSK.L	1222.32788	2	1.14E-07	0.88	3.10	-	646.5
AHQ-1-14, 4232	K.FYSVNVDYSK.L	1222.32788	2	2.14E-04	0.68	2.82	-	405.1
gi|28395033|ref|NP_786886.1| ras homolog gene family, member C; Aplysia RAS-related homolog 9 (onco				1.16E-07	5.31	60.22	44.00	22006.2
AHQ-1-13- , 4877 - 4887	K.HFCPNVPIILVGNK.K	1609.91693	2	6.35E-05	0.95	3.96	-	1565.8
AHQ-1-11, 4482	K.HFCPNVPIILVGNK.K	1609.91693	2	1.23E-04	0.95	3.63	-	1439.4
AHQ-1-10, 4316 - 4319	K.HFCPNVPIILVGNK.K	1609.91693	2	5.91E-04	0.97	4.31	-	1627.4
AHQ-1-10, 4000 - 4005	K.HFCPNVPIILVGNKK.D	1738.08985	2	5.01E-06	0.85	3.44	-	916.6
AHQ-1-10, 4096	R.ISAFGYLECSAK.T	1347.51850	2	4.93E-04	0.93	3.37	-	1063.0
AHQ-1-13- , 6833	R.LRPLSYPDTDVILM*CFSIDSPDSLENIPEK.W	3483.90759	3	3.87E-04	0.87	4.23	-	659.5
AHQ-1-10, 2604	K.LVIVGDGACGK.T	1090.27572	1	1.16E-07	0.75	3.23	-	533.1
AHQ-1-10, 2603 - 2605	K.LVIVGDGACGK.T	1090.27572	2	1.05E-04	0.85	3.33	-	816.3
AHQ-1-10, 2599	K.LVIVGDGACGK.T	1090.27572	1	7.78E-05	0.53	2.04	-	621.5
AHQ-1-13- , 5185 - 5212	K.QVELALWDTAGQEDYDR.L	2010.10662	2	4.35E-06	0.95	4.38	-	1148.8
AHQ-1-10, 4625	K.QVELALWDTAGQEDYDR.L	2010.10662	2	5.47E-04	0.94	3.88	-	1261.6
gi|28827809|ref|NP_789797.1| nucleoside diphosphate kinase B-like [Homo sapiens]				1.17E-07	3.03	40.21	29.20	15529.0
AHQ-1-12, 3317 - 3387	R.GDFCIQVGR.N	1053.17404	2	1.46E-05	0.89	3.25	-	993.5
AHQ-1-12, 3321	R.GDFCIQVGR.N	1053.17404	1	2.43E-04	0.34	1.82	-	253.5
AHQ-1-12, 2151	R.NIIHGSDSVKSAEK.E	1485.62464	1	4.06E-06	0.56	3.09	-	327.9
AHQ-1-12, 2137	R.NIIHGSDSVKSAEK.E	1485.62464	2	6.93E-06	0.63	3.43	-	553.9
AHQ-1-12, 2829 - 2906	R.VMLGETNPADSKPGTIR.G	1787.03151	2	1.61E-06	0.78	3.71	-	535.8
AHQ-1-12, 2830	R.VMLGETNPADSKPGTIR.G	1787.03151	3	7.97E-07	0.78	3.60	-	562.1
AHQ-1-12, 2649	R.VM*LGETNPADSKPGTIR.G	1803.03091	2	1.32E-06	0.79	3.49	-	666.3
AHQ-1-12, 2722	R.VMLGETNPADSKPGTIR.G	1787.03151	2	3.77E-05	0.76	3.34	-	565.2
AHQ-1-12, 2650	R.VM*LGETNPADSKPGTIR.G	1803.03091	3	1.17E-07	0.80	3.01	-	739.1
gi|4505735|ref|NP_002611.1| platelet factor 4 variant 1; Platelet factor 4, variant 1 (PF4-like) [H				1.20E-07	2.81	30.24	26.00	11552.6
AHQ-1-14- , 3884	R.AEAEEDGDLQCLCVK.T	1739.86036	2	1.20E-07	0.97	4.81	-	1816.4
AHQ-1-14- , 3767 - 3784	R.AEAEEDGDLQCLCVK.T	1739.86036	2	2.27E-04	0.94	4.60	-	752.9
AHQ-1-14- , 4942	K.ICLDLQALLYK.K	1351.63656	2	9.51E-04	0.89	3.54	-	1145.9
AHQ-1-14- , 5834	R.KICLDLQALLYK.K	1479.80948	2	3.33E-04	0.94	4.21	-	956.6
gi|4507149|ref|NP_000445.1| superoxide dismutase 1, soluble (amyotrophic lateral sclerosis 1 (adult				1.20E-07	3.56	40.30	57.80	15935.7
AHQ-1-12, 5231	K.AVCVLKGDGPVQGIINFEQK.E	2174.50602	2	2.85E-05	0.93	4.46	-	901.4
AHQ-1-11, 3911	K.GDGPVQGIINFEQK.E	1502.65366	2	2.38E-05	0.73	2.95	-	697.8
AHQ-1-12, 4810	K.GLTEGLHGFHVHEFGDNTAGCTSAGPHFNPLSR.K	3522.76733	3	1.20E-07	0.95	4.82	-	1479.5
AHQ-1-12, 5778	R.HVGDLGNVTADKDGVADVSIEDSVISLSGDHCIIGR.T	3724.02282	3	1.55E-05	0.90	4.33	-	827.9
AHQ-1-11, 5587	R.HVGDLGNVTADKDGVADVSIEDSVISLSGDHCIIGR.T	3724.02282	3	3.95E-07	0.95	5.95	-	991.1
gi|4503971|ref|NP_001484.1| GDP dissociation inhibitor 1; mental retardation, X-linked 41; mental r				1.21E-07	0.86	10.28	4.30	50582.4
AHQ-1-7, 5450	R.NPYYGGESSSITPLEELYK.R	2148.31110	2	1.05E-06	0.97	5.51	-	1138.6
AHQ-1-12, 5151	R.NPYYGGESSSITPLEELYK.R	2148.31110	2	1.21E-07	0.86	3.83	-	709.1
AHQ-1-6, 5388	R.NPYYGGESSSITPLEELYK.R	2148.31110	2	4.06E-05	0.93	4.49	-	917.4
gi|4503291|ref|NP_001346.1| D-dopachrome tautomerase [Homo sapiens]				1.37E-07	1.83	20.18	22.00	12711.7
AHQ-1-14- , 5624 - 5643	R.FFPLESWQIGK.I	1352.56151	2	1.37E-07	0.90	2.99	-	803.0
AHQ-1-14, 4525	R.LCAAAASILGKPADR.V	1515.76031	2	3.37E-05	0.92	3.68	-	1217.3
gi|29729950|ref|XP_293759.1| similar to Heat shock protein HSP 90-alpha (HSP 86) [Homo sapiens] [MA				1.40E-07	0.86	10.17	5.00	39776.4
AHQ-1-5, 5113	K.CLELFTELAEDKENYKK.F	2132.37640	2	1.40E-07	0.86	3.45	-	963.9

gi|4758988|ref|NP_004152.1| RAB1A, member RAS oncogene family; RAB1, member RAS oncogene family [Ho				1.40E-07	3.21	40.23	25.40	22677.6
AHQ-1-14- , 6152	K.EFADSLGIPFLETSAK.N	1725.91990	2	2.21E-05	0.34	2.78	-	269.8
AHQ-1-13- , 6332 - 6409	K.EFADSLGIPFLETSAK.N	1725.91990	2	2.93E-06	0.80	3.48	-	399.6
AHQ-1-14- , 5890	R.FADDTYTESYISTIGVDFK.I	2173.31742	2	3.37E-05	0.94	4.21	-	970.7
AHQ-1-11, 5564 - 5623	R.FADDTYTESYISTIGVDFK.I	2173.31742	2	1.40E-07	0.96	4.59	-	1405.0
AHQ-1-11, 5943	R.FADDTYTESYISTIGVDFK.I	2173.31742	2	6.48E-06	0.79	3.33	-	554.1
AHQ-1-11, 6603	R.FADDTYTESYISTIGVDFK.I	2173.31742	2	6.13E-06	0.54	2.98	-	396.4
AHQ-1-13- , 6099	R.FADDTYTESYISTIGVDFK.I	2173.31742	2	3.65E-06	0.96	4.69	-	1275.4
AHQ-1-11, 5810 - 5879	R.FADDTYTESYISTIGVDFK.I	2173.31742	2	3.14E-07	0.93	4.43	-	809.8
AHQ-1-11, 5670 - 5743	R.FADDTYTESYISTIGVDFK.I	2173.31742	2	1.07E-06	0.97	4.64	-	1821.6
AHQ-1-11, 5220	K.NATNVEQSFM*TMAAEIK.K	1902.13962	2	8.46E-04	0.78	3.74	-	801.3
AHQ-1-11, 5776 - 5835	K.NATNVEQSFMTMAAEIK.K	1886.14022	2	9.30E-04	0.66	3.44	-	741.2
gi|20070156|ref|NP_003565.2| vessicle-associated membrane protein (VAMP)-associated protein of 33 k				1.42E-07	0.94	10.19	5.00	27317.4
AHQ-1-12, 4047	K.GPFTDVVTTNLK.L	1292.46278	2	1.42E-07	0.94	3.85	-	1421.3
gi|5902016|ref|NP_008939.1| reticulon 4; neuroendocrine-specific protein C like (foocen) [Homo sapi				1.44E-07	1.71	20.23	14.10	22395.3
AHQ-1-1, 3263 - 3340	R.HQAQIDHYLGLANK.N	1608.78270	2	3.07E-04	0.90	3.52	-	769.3
AHQ-1-12, 3041	R.HQAQIDHYLGLANK.N	1608.78270	2	2.84E-06	0.96	4.58	-	1421.0
AHQ-1-1, 3072 - 3149	K.YSNSALGHVNCTIK.E	1565.73305	2	1.44E-07	0.75	3.23	-	557.5
gi|20536967|ref|XP_115812.1| similar to Latent transforming growth factor beta binding protein 1L p				1.47E-07	0.35	10.13	12.00	13416.2
AHQ-1-1, 3631	K.GISGEQSTEGSFPLR.Y	1565.66662	2	1.47E-07	0.35	2.55	-	269.1
gi|4506181|ref|NP_002778.1| proteasome alpha 2 subunit; proteasome subunit HC3; proteasome componen				1.56E-07	4.78	60.27	34.20	25898.4
AHQ-1-11, 2171	K.AANGVVLATEK.K	1073.22510	1	7.95E-04	0.40	2.26	-	407.8
AHQ-1-11, 4618	R.GYSFSLTTFSPSGK.L	1479.61533	2	7.33E-06	0.75	2.95	-	528.2
AHQ-1-11, 3495	K.HIGLVYSGMGPDYR.V	1565.77809	2	2.29E-04	0.96	4.24	-	1530.3
AHQ-1-11, 4934	R.KLAQQYYLVYQEPIPTAQLVQR.V	2651.05593	2	3.84E-05	0.95	4.94	-	910.8
AHQ-1-11, 5298	K.LAQQYYLVYQEPIPTAQLVQR.V	2522.88301	2	3.93E-04	0.75	2.93	-	695.7
AHQ-1-11, 6394	R.YNEDLELEDAIHTAILTLK.E	2202.44658	2	1.56E-07	0.96	5.18	-	1289.5
gi|7657486|ref|NP_055217.1| low molecular mass ubiquinone-binding protein [Homo sapiens]				1.60E-07	0.97	10.27	14.00	11316.3
AHQ-1-14- , 3976	R.HVISYSLSPFEQR.A	1563.73870	2	1.60E-07	0.97	5.36	-	1353.8
gi|7305503|ref|NP_038470.1| stomatin (EPB72)-like 2; stomatin-like protein 2; stomatin-like 2 [Homo				1.62E-07	0.91	10.18	4.20	38533.8
AHQ-1-9, 7106 - 7116	R.ILEPGLNILIPVLDR.I	1676.03667	2	1.62E-07	0.91	3.52	-	756.6
gi|6912494|ref|NP_036457.1| microtubule-associated protein, RP/EB family, member 1; ADENOMATOUS POL				1.64E-07	4.32	50.27	25.40	29998.9
AHQ-1-12, 3918	K.FFDANYDGKDYDPVAAR.Q	1965.06754	2	2.25E-05	0.53	2.92	-	412.7
AHQ-1-12, 3915	K.FFDANYDGKDYDPVAAR.Q	1965.06754	3	6.08E-06	0.78	3.91	-	371.5
AHQ-1-12, 6341	K.FQDNFEFVQWFK.K	1635.80184	2	1.64E-07	0.95	3.87	-	1373.7
AHQ-1-12, 2673	K.LEHEYIQNFK.I	1321.46268	1	2.16E-04	0.50	2.13	-	758.4
AHQ-1-12, 2666	K.LEHEYIQNFK.I	1321.46268	2	3.70E-06	0.81	2.82	-	846.9
AHQ-1-13, 3234 - 3293	K.LTVEDLEKER.D	1232.36575	2	9.87E-05	0.81	2.70	-	953.8
AHQ-1-13, 4345	R.NIELICQENEGENDPVLQR.I	2272.43545	2	1.69E-07	0.97	5.33	-	1749.5
gi|29747201|ref|XP_290702.1| similar to My016 protein [Homo sapiens]				1.68E-07	0.90	10.19	10.50	16932.3
AHQ-1-14- , 3535	R.DLEAEHVEVEDTTLNR.C	1870.95236	2	6.47E-06	0.90	3.65	-	813.6
AHQ-1-14, 4362	R.DLEAEHVEVEDTTLNR.C	1870.95236	2	1.68E-07	0.90	3.72	-	922.5
gi|4503531|ref|NP_001958.1| eukaryotic translation initiation factor 4A, isoform 2 [Homo sapiens] [				1.68E-07	0.95	10.21	3.90	46393.9
AHQ-1-8, 3417	R.GIYAYGFEKPSAIQQR.A	1829.04828	2	1.68E-07	0.95	4.17	-	1198.9
gi|10864055|ref|NP_067069.1| megakaryocyte-enhanced gene transcript 1 protein; lymphocyte antigen-6				1.79E-07	0.95	10.23	4.20	40891.9
AHQ-1-9, 4260	R.YWCAVLGQHHNYQNWR.V	2134.32402	2	1.79E-07	0.95	4.61	-	1289.7
gi|4502389|ref|NP_003851.1| barrier to autointegration factor 1; breakpoint cluster region protein,				1.81E-07	0.96	10.23	13.50	10058.5
AHQ-1-14- , 6164	K.AYVVLGQFLVLK.K	1350.67334	2	1.81E-07	0.96	4.62	-	1598.2
gi|4508047|ref|NP_003452.1| zyxin [Homo sapiens]				1.81E-07	1.84	30.21	9.80	61277.0
AHQ-1-8, 3936 - 3940	K.LGHPEALSAGTGSPQPPSFTYAQQR.E	2598.81170	3	9.00E-06	0.84	4.30	-	626.7
AHQ-1-6, 3922	K.LGHPEALSAGTGSPQPPSFTYAQQR.E	2598.81170	3	1.61E-05	0.76	3.16	-	717.3
AHQ-1-9, 2619 - 2626	R.SPGAPGPLTLK.E	1038.22236	1	7.66E-04	0.07	1.81	-	213.7
AHQ-1-9, 7362	K.VSSIDLEIDSLSSLLDDMTK.N	2182.43225	2	1.81E-07	0.93	4.22	-	1018.6
gi|21614499|ref|NP_003370.2| villin 2; Villin-2; cytovillin [Homo sapiens]				1.83E-07	3.40	50.22	8.00	69412.3
AHQ-1-6, 4366	K.APDFVFYAPR.L	1183.33971	2	1.83E-07	0.97	4.34	-	1353.4
AHQ-1-6, 2440	K.FVIKPIDK.K	960.19473	1	2.53E-04	0.13	1.97	-	376.8
AHQ-1-6, 5198	K.IGFPWSEIR.N	1105.27049	2	1.28E-05	0.89	2.56	-	1265.3
AHQ-1-6, 3743	K.KAPDFVFYAPR.L	1311.51262	2	1.26E-06	0.68	2.65	-	558.7
AHQ-1-6, 4418	R.VTTM*DAELEFAIQPNTTGK.Q	2083.30556	2	8.20E-05	0.72	3.09	-	463.7
gi|10835189|ref|NP_000628.1| glutathione reductase [Homo sapiens]				1.86E-07	2.66	30.23	10.60	51700.3
AHQ-1-7, 4910	K.ADFDNTVAIHPTSSEELVTLR	2316.50977	2	7.64E-06	0.81	4.07	-	373.7
AHQ-1-7, 4116	K.ALLTPVAIAAGR.K	1153.39973	2	2.04E-04	0.93	3.24	-	1418.4
AHQ-1-7, 2529	R.GHAAFTSDPKPTIEVSGK.K	1843.03012	2	1.86E-07	0.92	4.60	-	381.0
gi|4507287|ref|NP_003755.1| syntaxin 11 [Homo sapiens]				1.89E-07	2.73	30.20	15.70	32955.3
AHQ-1-9, 4210 - 4214	R.DIQDENQLLVADVK.R	1600.75241	2	8.09E-05	0.93	3.54	-	1893.4
AHQ-1-9, 1971	K.ELSEAAEAQHGPHSAVAR.I	1860.96563	2	1.89E-07	0.84	3.44	-	748.6
AHQ-1-9, 6486 - 6492	K.WDVFSENLLADVK.G	1536.71002	2	4.69E-05	0.96	4.01	-	1411.3
gi|4507789|ref|NP_003338.1| ubiquitin-conjugating enzyme E2L 3 [Homo sapiens]				2.03E-07	1.69	20.17	15.60	17861.5
AHQ-1-12, 2163	R.ADLAEEYSK.D	1026.07893	2	6.90E-05	0.87	2.77	-	1065.0
AHQ-1-12, 5138	R.IEINFPAEYPFKPPK.I	1791.08155	3	2.03E-07	0.82	3.29	-	873.1
AHQ-1-12, 5081 - 5142	R.IEINFPAEYPFKPPK.I	1791.08155	2	2.63E-07	0.84	3.43	-	800.1
gi|30149327|ref|XP_293672.2| similar to ebiP7687 [Homo sapiens]				2.06E-07	1.48	20.19	4.80	59404.3
AHQ-1-5, 3677	K.HLEINPNHPIVETLR.Q	1783.02428	2	2.06E-07	0.91	3.89	-	779.3
AHQ-1-5, 2761	K.SIYYITGESK.E	1161.28598	1	6.47E-04	0.57	1.88	-	1038.0
gi|4503725|ref|NP_000792.1| FK506-binding protein 1A; FK506-binding protein 1; FK506-binding protei				2.19E-07	1.80	20.26	13.00	11950.6
AHQ-1-13- , 3091	R.GWEEGVAQM*SVGQR.A	1550.67866	2	2.19E-07	0.97	5.24	-	1159.8
AHQ-1-14- , 3039	R.GWEEGVAQM*SVGQR.A	1550.67866	2	2.85E-07	0.96	4.62	-	1119.7
AHQ-1-14- , 3922 - 3930	R.GWEEGVAQMSVGQR.A	1534.67926	2	1.42E-06	0.83	3.20	-	1076.3
AHQ-1-13, 3876	R.GWEEGVAQMSVGQR.A	1534.67926	2	1.70E-05	0.88	4.02	-	811.5
gi|4506363|ref|NP_002861.1| RAB13, member RAS oncogene family; RAS-associated protein RAB13 [Homo s				2.24E-07	1.77	20.18	10.80	22774.0
AHQ-1-11, 3564 - 3590	K.LLLIGDSGVGK.T	1072.28025	2	2.24E-07	0.92	3.35	-	1013.1
AHQ-1-11, 3578	K.LLLIGDSGVGK.T	1072.28025	1	2.97E-04	0.76	2.70	-	628.1
AHQ-1-13, 3769 - 3776	K.LLLIGDSGVGK.T	1072.28025	2	1.23E-04	0.89	3.59	-	806.8
AHQ-1-11, 2948	K.LQVWDTAGQER.F	1303.40576	2	1.09E-06	0.86	3.54	-	992.9
gi|5902090|ref|NP_008862.1| solute carrier family 2 (facilitated glucose transporter), member 3; GL				2.38E-07	1.33	20.25	4.00	53924.0
AHQ-1-1, 5271	R.LWGTQDVSQDIQEMK.D	1778.96471	2	2.38E-07	0.93	4.01	-	822.4
AHQ-1-1, 4183	R.LWGTQDVSQDIQEM*KDESAR.M	2353.50874	3	2.12E-04	0.40	3.24	-	164.9
gi|5031573|ref|NP_005712.1| ARP3 actin-related protein 3 homolog; ARP3 (actin-related protein 3, ye				2.39E-07	6.35	70.25	28.70	47370.9
AHQ-1-8, 7516	R.DITYFIQQLLR.D	1410.64250	2	3.77E-06	0.97	4.76	-	1574.7
AHQ-1-8, 3614	K.DYEEIGPSICR.H	1340.44122	2	1.08E-05	0.85	2.59	-	1269.9
AHQ-1-8, 5936	K.GVDDLDFFIGDEAIEKPTYATK.W	2445.66268	2	2.39E-07	0.90	4.26	-	700.3
AHQ-1-8, 4289	R.HGIVEDWDLMER.F	1500.66127	2	2.19E-05	0.95	3.85	-	1290.5
AHQ-1-9, 6339	K.LGYAGNTEPQFIIPSCIAIK.E	2194.53570	2	2.43E-05	0.80	3.82	-	367.4
AHQ-1-8, 6550 - 6615	K.LGYAGNTEPQFIIPSCIAIK.E	2194.53570	2	4.06E-06	0.86	4.15	-	553.9
AHQ-1-8, 3719 - 3720	R.LPACVVDCGTGYTK.L	1543.74421	2	1.02E-04	0.91	3.99	-	866.6
AHQ-1-8, 6724	R.TLTGTVIDSGDGVTHVIPVAEGYVIGSCIK.H	3061.45302	3	3.33E-04	0.91	4.73	-	719.3
gi|5802966|ref|NP_006861.1| destrin (actin depolymerizing factor); destrin [Homo sapiens]				2.41E-07	1.76	20.17	14.50	18505.6
AHQ-1-12, 1985 - 2065	K.HECQANGPEDLNR.A	1541.58530	2	1.50E-05	0.87	3.12	-	878.7
AHQ-1-12, 3733	R.YALYDASFETK.E	1308.41754	1	2.41E-07	0.89	3.42	-	802.3
gi|10880989|ref|NP_067075.1| RAB18, member RAS oncogene family; RAB18 small GTPase [Homo sapiens] [				2.55E-07	2.73	30.20	19.40	22976.9
AHQ-1-11, 3431	K.LAIWDTAGQER.F	1260.38088	2	3.38E-07	0.93	3.66	-	1279.1
AHQ-1-11, 5932 - 5938	K.LDNWLNELETYCTR.N	1828.98037	2	2.55E-07	0.92	4.09	-	802.4
AHQ-1-11, 5554	K.TCDGVQCAFEELVEK.I	1787.94661	2	1.68E-04	0.87	3.89	-	776.0
gi|5803181|ref|NP_006810.1| stress-induced-phosphoprotein 1 (Hsp70/Hsp90-organizing protein) [Homo				2.59E-07	0.85	10.19	3.50	62638.9
AHQ-1-14- , 6248	K.ALSVGNIDDALQCYSEAIK.L	2069.27871	2	2.59E-07	0.85	3.58	-	491.6
gi|9910280|ref|NP_064505.1| UDP-glucose ceramide glucosyltransferase-like 1; UDP-glucose:glycoprote				2.62E-07	0.97	10.22	0.90	177187.7
AHQ-1-3, 5687	K.VWQLQDLSFQTAAR.I	1663.85826	2	2.62E-07	0.97	4.39	-	2103.8

gi|4505813|ref|NP_003737.1| cytoplasmic dynein light polypeptide; dynein, cytoplasmic, light chain				2.65E-07	2.35	30.17	24.70	10365.8
AHQ-1-14- , 3866	K.NADM*SEEM*QQDSVECATQALEK.Y	2548.67874	2	4.45E-04	0.85	3.49	-	603.8
AHQ-1-14- , 3162	R.NFGSYVTHETK.H	1283.37131	2	2.65E-07	0.66	2.67	-	677.4
AHQ-1-14- , 2387	R.NFGSYVTHETK.H	1283.37131	2	3.88E-07	0.91	3.44	-	935.8
AHQ-1-14- , 3636 - 3671	K.YNPTWHCIVGR.N	1404.57820	2	3.43E-07	0.83	3.28	-	548.4
gi|18765729|ref|NP_003816.2| synaptosomal-associated protein 23 isoform SNAP23A; synaptosomal-assoc				2.67E-07	2.37	30.20	19.40	23353.9
AHQ-1-10, 2497	R.IEEGLDQINK.D	1159.27163	2	5.42E-05	0.52	2.55	-	609.9
AHQ-1-12, 4386 - 4466	R.ILGLAIESQDAGIK.T	1428.65614	2	2.16E-06	0.93	3.70	-	1170.9
AHQ-1-11, 4239	R.ILGLAIESQDAGIK.T	1428.65614	2	5.64E-05	0.96	3.95	-	1639.9
AHQ-1-12, 3242	K.TTWGDGGENSPCNVVSK.Q	1809.89231	2	2.67E-07	0.92	3.91	-	1250.1
gi|4757714|ref|NP_004291.1| acid phosphatase 1 isoform c; acid phosphatase of erythrocyte; red cell				2.72E-07	2.66	30.22	24.10	18042.4
AHQ-1-11, 3646 - 3647	K.IELLGSYDPQK.Q	1263.42144	2	2.16E-05	0.95	4.48	-	1027.6
AHQ-1-11, 3179	R.SPIAEAVFR.K	990.13779	2	2.72E-07	0.91	3.24	-	884.9
AHQ-1-11, 3382	R.VDSAATSGYEIGNPPDYR.G	1912.99094	2	1.91E-05	0.80	3.38	-	705.8
gi|6005854|ref|NP_009204.1| repressor of estrogen receptor activity; B-cell associated protein [Hom				2.74E-07	2.72	30.24	16.40	33296.1
AHQ-1-9, 4900	R.IGGVQQDTILAEGLHFR.I	1855.08727	2	2.74E-07	0.90	3.72	-	911.8
AHQ-1-9, 7107	R.IPWFQYPIIYDIR.A	1725.02480	2	2.71E-04	0.97	4.73	-	1467.9
AHQ-1-9, 6658	R.IYLTADNLVLNLQDESFTR.G	2226.47137	2	3.02E-04	0.86	3.55	-	735.2
gi|17463729|ref|XP_069253.1| similar to prohibitin [Homo sapiens]				2.74E-07	0.87	10.15	2.50	43846.2
AHQ-1-10, 3524	R.FDAGELITQR.E	1150.26643	2	2.74E-07	0.87	2.94	-	1215.7
gi|4557235|ref|NP_000009.1| acyl-Coenzyme A dehydrogenase, very long chain precursor [Homo sapiens]				2.84E-07	0.93	10.20	2.30	70389.7
AHQ-1-6, 4523 - 4524	R.VPSENVLGEVGSGFK.V	1519.68100	2	2.84E-07	0.93	3.99	-	1008.0
gi|7705849|ref|NP_057215.1| ras-related GTP-binding protein RAB10; ras-related GTP-binding protein				2.89E-07	1.90	20.23	11.50	22540.8
AHQ-1-11, 6008 - 6023	K.AFLTLAEDILR.K	1262.47982	2	2.89E-07	0.96	4.20	-	1791.3
AHQ-1-13- , 6631	K.AFLTLAEDILR.K	1262.47982	2	4.24E-07	0.95	4.13	-	1381.4
AHQ-1-14- , 6370 - 6372	K.AFLTLAEDILR.K	1262.47982	2	1.03E-06	0.95	4.54	-	1419.8
AHQ-1-11, 1787 - 1859	R.NIDEHANEDVER.M	1441.44245	2	4.51E-04	0.94	3.53	-	1322.7
gi|21361122|ref|NP_001440.2| four and a half LIM domains 1; Four-and-a-half LIM domains 1 [Homo sap				2.89E-07	3.64	40.26	18.90	31894.7
AHQ-1-9, 2388	K.AIVAGDQNVEYK.G	1307.43431	2	6.47E-06	0.91	3.67	-	862.9
AHQ-1-13- , 2552	K.AIVAGDQNVEYK.G	1307.43431	2	2.71E-05	0.90	3.78	-	703.1
AHQ-1-10, 2373	K.AIVAGDQNVEYK.G	1307.43431	2	6.61E-05	0.75	2.88	-	609.2
AHQ-1-9, 3210	K.CLHPLANETFVAK.D	1501.73210	2	2.73E-04	0.88	3.21	-	620.7
AHQ-1-13, 3707 - 3787	R.FVFHQEQVYCPDCAK.K	1931.13758	2	4.84E-05	0.80	3.41	-	729.6
AHQ-1-13- , 3933	R.FVFHQEQVYCPDCAK.K	1931.13758	2	1.06E-05	0.97	5.22	-	1391.7
AHQ-1-13- , 3927	R.FVFHQEQVYCPDCAK.K	1931.13758	3	8.19E-06	0.78	3.59	-	1032.9
AHQ-1-9, 3718 - 3754	R.FVFHQEQVYCPDCAK.K	1931.13758	2	2.89E-07	0.93	3.63	-	1138.8
AHQ-1-9, 3039	K.GEDFYCVTCHETK.F	1648.75260	2	4.20E-06	0.91	3.36	-	867.7
gi|4826659|ref|NP_004921.1| F-actin capping protein beta subunit; Cap Z [Homo sapiens]				2.94E-07	3.48	40.24	19.10	30628.5
AHQ-1-10, 3832	K.GCWDSIHVVEVQEK.S	1687.85505	2	2.94E-07	0.95	4.80	-	1408.7
AHQ-1-10, 3296	R.KLEVEANNAFDQYR.D	1697.82988	2	1.61E-04	0.93	4.49	-	914.3
AHQ-1-10, 3213	R.KLEVEANNAFDQYR.D	1697.82988	2	5.20E-07	0.95	4.11	-	1463.7
AHQ-1-9, 3278 - 3279	R.KLEVEANNAFDQYR.D	1697.82988	2	3.36E-05	0.95	3.95	-	1655.2
AHQ-1-13- , 2999	K.SGSGTMNLGGSLTR.Q	1338.47345	2	1.53E-04	0.57	2.53	-	549.9
AHQ-1-9, 2716	K.SGSGTMNLGGSLTR.Q	1338.47345	2	6.93E-06	0.81	3.06	-	705.7
AHQ-1-10, 3449	R.STLNEIYFGK.T	1172.31200	2	1.21E-05	0.84	2.93	-	851.6
AHQ-1-9, 3552	R.STLNEIYFGK.T	1172.31200	2	3.34E-06	0.77	2.75	-	652.8
gi|30158852|ref|XP_301583.1| similar to actin CA15 - sea squirt (Styela clava) (fragments) [Homo sa				2.96E-07	0.76	10.15	6.50	19197.0
AHQ-1-6, 2908	K.IWHHTFYNKLR.V	1515.74533	2	7.64E-05	0.46	2.85	-	512.8
AHQ-1-8, 2686	K.IWHHTFYNKLR.V	1515.74533	2	2.96E-07	0.76	2.83	-	848.5
AHQ-1-1, 3279 - 3356	K.IWHHTFYNKLR.V	1515.74533	2	1.34E-05	0.52	2.95	-	460.7
gi|4507509|ref|NP_003245.1| tissue inhibitor of metalloproteinase 1 precursor; Erythroid-potentiati				2.98E-07	0.96	10.20	5.80	23170.8
AHQ-1-10, 3517	K.GFQALGDAADIR.F	1234.34342	2	2.98E-07	0.96	3.90	-	1774.1
gi|4507643|ref|NP_003279.1| tumor protein D52-like 2; hD54 [Homo sapiens]				2.99E-07	1.70	20.23	14.60	22209.6
AHQ-1-10, 5073	R.TPAVEGLTEAEEEELRAELTK.V	2316.50485	3	2.99E-07	0.86	4.31	-	588.4
AHQ-1-10, 5072	R.TPAVEGLTEAEEEELRAELTK.V	2316.50485	2	7.86E-04	0.91	4.57	-	650.3
AHQ-1-10, 6372 - 6388	R.TPAVEGLTEAEEEELRAELTKVEEEIVTLR.Q	3385.71879	3	1.48E-05	0.84	4.14	-	613.1
gi|4503481|ref|NP_001395.1| eukaryotic translation elongation factor 1 gamma; elongation factor 1-g				3.55E-07	1.25	20.20	6.40	50118.5
AHQ-1-12, 4909 - 4911	R.EYFSWEGAFQHVGK.A	1685.81925	2	3.55E-07	0.65	3.23	-	422.5
AHQ-1-14- , 2728	R.KLDPGSEETQTLVR.E	1573.73020	2	9.03E-07	0.60	3.25	-	321.8
AHQ-1-8, 2469 - 2471	R.KLDPGSEETQTLVR.E	1573.73020	2	1.13E-06	0.87	3.92	-	536.8
gi|12408675|ref|NP_036526.2| prefoldin 2 [Homo sapiens]				3.59E-07	0.96	10.25	7.80	16647.7
AHQ-1-12, 3713 - 3715	K.IIETLTQQLQAK.G	1386.61925	2	3.59E-07	0.96	5.06	-	1509.6
gi|30153852|ref|XP_171113.3| similar to Moesin (Membrane-organizing extension spike protein) [Homo				3.61E-07	0.97	10.24	9.30	15601.7
AHQ-1-6, 1866	R.RKPDTIEVQQMK.A	1473.72364	2	3.61E-07	0.97	4.71	-	1658.5
gi|22059949|ref|XP_114617.2| similar to tubulin, beta 5 [Homo sapiens]				3.63E-07	1.87	20.34	18.80	22810.8
AHQ-1-10, 4839	K.FWEVISDEHGIDPTGTYHGDSDLQLDR.I	3104.24553	3	1.70E-05	0.94	5.75	-	908.8
AHQ-1-13- , 5400	K.FWEVISDEHGIDPTGTYHGDSDLQLDR.I	3104.24553	3	4.58E-04	0.82	4.27	-	602.3
AHQ-1-7, 5509 - 5536	K.FWEVISDEHGIDPTGTYHGDSDLQLDR.I	3104.24553	3	3.63E-07	0.97	6.84	-	1587.6
AHQ-1-7, 5621	K.FWEVISDEHGIDPTGTYHGDSDLQLDR.I	3104.24553	3	1.08E-04	0.93	5.26	-	1006.0
AHQ-1-14, 6116	K.FWEVISDEHGIDPTGTYHGDSDLQLDR.I	3104.24553	3	7.47E-04	0.87	4.31	-	878.2
AHQ-1-9, 5371	K.FWEVISDEHGIDPTGTYHGDSDLQLDR.I	3104.24553	3	7.34E-04	0.57	3.21	-	595.6
AHQ-1-14- , 5271	K.FWEVISDEHGIDPTGTYHGDSDLQLDR.I	3104.24553	3	1.23E-05	0.97	6.33	-	1532.0
AHQ-1-12, 5178	K.FWEVISDEHGIDPTGTYHGDSDLQLDR.I	3104.24553	3	6.50E-04	0.95	5.91	-	1006.4
AHQ-1-9, 2640 - 2642	R.ISVYYNEATGGK.Y	1302.41453	1	3.46E-05	0.05	1.86	-	221.9
AHQ-1-7, 2688 - 2696	R.ISVYYNEATGGK.Y	1302.41453	2	5.62E-07	0.90	3.66	-	752.9
AHQ-1-14- , 2742 - 2786	R.ISVYYNEATGGK.Y	1302.41453	2	1.31E-05	0.91	3.48	-	817.5
AHQ-1-7, 2685 - 2686	R.ISVYYNEATGGK.Y	1302.41453	1	1.90E-05	0.56	2.57	-	431.5
AHQ-1-13, 2825 - 2838	R.ISVYYNEATGGK.Y	1302.41453	2	1.08E-06	0.87	2.93	-	886.4
gi|16933542|ref|NP_002017.1| fibronectin 1 isoform 1 preproprotein; cold-insoluble globulin [Homo s				3.67E-07	4.95	60.24	4.50	259222.8
AHQ-1-1, 4461 - 4472	R.EESPLLIGQQSTVSDVPR.D	1956.14354	2	2.65E-06	0.93	4.26	-	776.8
AHQ-1-1, 4525	R.ITYGETGGNSPVQEFTVPGSK.S	2169.33354	2	3.38E-05	0.73	3.19	-	394.6
AHQ-1-1, 6153 - 6165	R.NTFAEVTGLSPGVTYYFK.V	1995.21983	2	1.66E-05	0.95	4.71	-	910.1
AHQ-1-1, 4416	R.PAQGVVTTLENVSPPR.R	1665.87246	2	6.65E-04	0.79	2.94	-	871.7
AHQ-1-1, 4819	R.SSPVVIDASTAIDAPSNLR.F	1914.10666	2	3.91E-05	0.89	4.02	-	727.3
AHQ-1-1, 4105	R.VDVIPVNLPGEHGQR.L	1630.82981	2	3.67E-07	0.66	2.60	-	475.4
gi|28178832|ref|NP_002159.2| isocitrate dehydrogenase 2 (NADP+), mitochondrial precursor; isocitrat				3.68E-07	3.19	40.23	11.90	50909.0
AHQ-1-8, 4108 - 4180	K.DLAGCIHGLSNVK.L	1385.57150	1	8.35E-05	0.54	2.75	-	391.0
AHQ-1-8, 4024 - 4100	K.DLAGCIHGLSNVK.L	1385.57150	2	1.76E-04	0.95	3.76	-	1428.4
AHQ-1-8, 4832 - 4835	R.NILGGTVFREPIICK.N	1719.04170	2	3.68E-07	0.96	4.60	-	1431.4
AHQ-1-13- , 2167	K.TIEAEAAHGTVTR.H	1356.46696	2	8.40E-07	0.91	3.32	-	1179.6
AHQ-1-9, 1940	K.TIEAEAAHGTVTR.H	1356.46696	2	1.18E-06	0.77	2.81	-	669.2
AHQ-1-11, 2078	K.TIEAEAAHGTVTR.H	1356.46696	2	1.75E-06	0.75	3.00	-	917.3
AHQ-1-8, 1909	K.TIEAEAAHGTVTR.H	1356.46696	2	9.85E-06	0.92	3.53	-	1121.4
AHQ-1-8, 3106	K.VCVETVESGAMTK.D	1412.61230	2	6.33E-04	0.79	2.61	-	859.3
gi|7705614|ref|NP_057132.1| CGI-111 protein [Homo sapiens]				3.73E-07	1.79	20.23	17.10	22087.6
AHQ-1-9, 7303	K.FSMVLPEVEAALAEIPGVR.S	2029.38904	2	3.73E-07	0.93	4.53	-	800.3
AHQ-1-9, 4291 - 4358	K.GLGSTVQEIDLTGVK.L	1517.70655	2	5.88E-04	0.85	3.21	-	805.8
gi|5453559|ref|NP_006347.1| ATP synthase, H+ transporting, mitochondrial F0 complex, subunit d; ATP				3.74E-07	0.78	10.17	10.60	18491.1
AHQ-1-11, 5239	R.LAALPENPPAIDWAYYK.A	1933.19486	2	3.74E-07	0.78	3.36	-	349.7
gi|14043026|ref|NP_003752.2| vesicle-associated membrane protein 8; endobrevin [Homo sapiens]				3.91E-07	0.91	10.21	14.00	11438.2
AHQ-1-13- , 2575	R.NKTEDLEATSEHFK.T	1649.74039	2	2.00E-05	0.95	3.87	-	1432.6
AHQ-1-13- , 2571	R.NKTEDLEATSEHFK.T	1649.74039	3	3.91E-07	0.91	4.22	-	1317.4
gi|4503821|ref|NP_001456.1| FYN binding protein (FYB-120/130); FYN-binding protein (FYB-120/130) [H				4.06E-07	0.88	10.27	2.60	85450.5
AHQ-1-10, 3876 - 3877	R.DLQVKPGESLEVIQTTDDTK.V	2217.41641	2	6.59E-07	0.96	5.05	-	806.6
AHQ-1-12, 4111	R.DLQVKPGESLEVIQTTDDTK.V	2217.41641	2	4.06E-07	0.88	4.35	-	294.8
AHQ-1-13, 4091	R.DLQVKPGESLEVIQTTDDTK.V	2217.41641	2	1.34E-06	0.97	5.37	-	924.5
AHQ-1-13- , 4272	R.DLQVKPGESLEVIQTTDDTK.V	2217.41641	2	3.63E-04	0.93	4.54	-	721.1

AHQ-1-6, 4227	R.DLQVKPGESLEVIQTTDDTK.V	2217.41641	2	1.55E-05	0.88	4.09	-	484.3
gi|15553127|ref|NP_000180.2|	hexokinase 2; hexokinase-2, muscle [Homo sapiens]			4.16E-07	0.93	10.16	1.20	102379.3
AHQ-1-4, 2379	K.GAAM*VTAVAYR.L	1126.31148	2	4.16E-07	0.93	3.19	-	1532.9
gi|14249382|ref|NP_116139.1|	hypothetical protein MGC15429 [Homo sapiens]			4.17E-07	1.87	20.19	11.00	22345.5
AHQ-1-11, 3552	R.AVAIDLPGLGHSK.E	1278.48248	2	1.06E-05	0.94	3.80	-	1213.6
AHQ-1-11, 4243	R.FSVLLLHGIR.F	1155.41731	2	4.17E-07	0.93	3.31	-	1143.1
gi|10047092|ref|NP_037391.1|	neuronal protein [Homo sapiens]			4.33E-07	0.94	10.19	5.00	31454.0
AHQ-1-14- , 6022	R.TTDIFQTVDLWEGK.D	1653.81378	2	6.52E-05	0.89	3.44	-	1261.0
AHQ-1-12, 5878	R.TTDIFQTVDLWEGK.D	1653.81378	2	4.33E-07	0.94	3.89	-	1180.6
gi|29727565|ref|XP_291384.1|	similar to Cytochrome c, somatic [Homo sapiens]			4.36E-07	1.96	20.31	16.20	11621.4
AHQ-1-13- , 6556 - 6569	K.GIIWGEDTLM*EYLENPK.K	2025.26771	2	4.36E-07	0.98	5.62	-	1762.9
AHQ-1-13- , 6876	K.GIIWGEDTLMEYLENPK.K	2009.26831	2	5.73E-07	0.98	6.22	-	1834.2
AHQ-1-13, 6107 - 6112	K.GIIWGEDTLM*EYLENPK.K	2025.26771	2	1.42E-06	0.97	5.00	-	1542.4
gi|4506505|ref|NP_002916.1|	regulator of G-protein signaling 10 [Homo sapiens]			4.41E-07	1.35	20.18	16.20	19607.9
AHQ-1-11, 4558	K.LQDQIFNLMK.Y	1250.49254	2	3.55E-04	0.78	3.66	-	538.9
AHQ-1-11, 5175	K.WAASLENLLEDPEGVKR.F	1928.13502	2	4.41E-07	0.57	3.36	-	326.6
gi|21264318|ref|NP_004028.3|	adenosine monophosphate deaminase 2 (isoform L) [Homo sapiens]			4.50E-07	1.62	20.18	3.50	100687.2
AHQ-1-5, 6189	K.CGVPFTDLLDAAK.S	1408.60184	2	4.50E-07	0.80	3.06	-	522.6
AHQ-1-5, 5675	R.SAPYEFPEESPIEQLEER.R	2151.27185	2	1.37E-04	0.81	3.35	-	466.8
gi|17986001|ref|NP_005505.2|	major histocompatibility complex, class I, B; HLA class I histocompati			4.56E-07	2.78	30.25	13.30	40459.8
AHQ-1-8, 4867	R.APWIEQEGPEYWDR.N	1776.88556	2	4.56E-07	0.91	3.97	-	866.4
AHQ-1-12, 4770	R.APWIEQEGPEYWDR.N	1776.88556	2	5.11E-04	0.64	3.00	-	597.1
AHQ-1-9, 4875	R.APWIEQEGPEYWDR.N	1776.88556	2	6.52E-06	0.94	3.87	-	1342.0
AHQ-1-14, 5774	R.APWIEQEGPEYWDR.N	1776.88556	2	1.49E-05	0.72	3.11	-	674.5
AHQ-1-8, 5055 - 5088	R.FISVGYVDDTQFVR.F	1646.82447	2	5.10E-04	0.91	3.78	-	1054.2
AHQ-1-8, 4064	R.GHDQYAYDGKDYIALNEDLR.S	2357.47731	3	2.03E-06	0.95	5.03	-	1389.6
gi|7019375|ref|NP_037373.1|	formin homology 2 domain containing 1; FH1/FH2 domain-containing protei			4.65E-07	2.54	30.20	5.40	126496.5
AHQ-1-6, 6988	R.FGPCATLWASLDPVSVDTAR.L	2165.41131	2	4.65E-07	0.85	3.57	-	764.0
AHQ-1-6, 3927	K.FSGVAGEAPSNPSVPVAVSSGPGR.G	2227.41945	2	3.16E-04	0.79	3.85	-	310.4
AHQ-1-8, 5972	R.VQYLEDTDPFACANFPEPR.R	2271.44761	2	1.98E-05	0.90	3.92	-	861.7
gi|4502209|ref|NP_001653.1|	ADP-ribosylation factor 5 [Homo sapiens]			4.79E-07	0.79	10.13	7.80	20529.5
AHQ-1-11, 5079	K.NICFTVWDVGGQDK.I	1640.79838	2	4.79E-07	0.79	2.55	-	1078.8
gi|7019545|ref|NP_037481.1|	secreted protein of unknown function [Homo sapiens]			4.82E-07	0.74	10.17	8.70	18856.2
AHQ-1-12, 3954	R.YGGEEEDQPIYLAVK.G	1711.85009	2	4.82E-07	0.74	3.39	-	513.9
gi|23110942|ref|NP_002781.2|	proteasome alpha 5 subunit; proteasome component 5; macropain subunit			4.93E-07	1.79	20.20	12.90	26410.9
AHQ-1-10, 3041	R.AIGSASEGAQSSLQEVYHK.S	1963.09463	2	4.93E-07	0.83	3.98	-	531.7
AHQ-1-10, 5188	R.LFQVEYAIEAIK.L	1424.66595	2	6.50E-07	0.96	3.72	-	2086.0
gi|13376181|ref|NP_079079.1|	hypothetical protein FLJ21665 [Homo sapiens]			4.98E-07	0.63	10.15	3.80	49908.5
AHQ-1-7, 5096	R.SFGGGTGSGFTSLLM*ER.L	1720.88522	2	4.98E-07	0.63	3.07	-	577.9
gi|4557297|ref|NP_000022.1|	delta-aminolevulinic acid dehydratase; porphobilinogen synthase; aminol			5.01E-07	0.93	10.22	5.80	36294.7
AHQ-1-9, 5016 - 5047	R.GSAADSEESPAIEAIHLLR.K	1967.12642	2	5.01E-07	0.93	4.34	-	683.4
gi|7705819|ref|NP_057181.1|	HSPC039 protein [Homo sapiens]			5.05E-07	0.87	10.22	24.40	8968.7
AHQ-1-14- , 5116	K.NIGWGTDQGIGGFGEEPGIK.S	2033.18655	2	5.05E-07	0.87	3.84	-	619.4
AHQ-1-14, 5996	K.NIGWGTDQGIGGFGEEPGIK.S	2033.18655	2	3.44E-06	0.89	4.32	-	630.8
gi|4504505|ref|NP_000405.1|	hydroxysteroid (17-beta) dehydrogenase 4 [Homo sapiens]			5.42E-07	1.78	20.20	3.90	79685.9
AHQ-1-6, 2783	R.ATSTATSGFAGAIGQK.L	1468.59408	2	1.18E-05	0.78	3.10	-	703.8
AHQ-1-5, 2749	R.ATSTATSGFAGAIGQK.L	1468.59408	2	5.42E-07	0.82	3.24	-	877.6
AHQ-1-6, 3156	R.VVLVTGAGAGLGR.A	1170.38715	2	9.30E-06	0.96	4.01	-	1590.6
gi|4557697|ref|NP_000412.1|	keratin 10; Keratin-10 [Homo sapiens]			5.64E-07	2.63	30.20	10.30	57247.1
AHQ-1-5, 2654	R.ALEESNYELEGK.I	1382.45482	2	1.36E-06	0.92	3.82	-	1057.5
AHQ-1-13- , 2824	R.ALEESNYELEGK.I	1382.45482	2	1.72E-04	0.69	2.94	-	652.9
AHQ-1-14- , 2762	R.ALEESNYELEGK.I	1382.45482	2	5.64E-07	0.93	3.67	-	1278.6
AHQ-1-1, 4349	K.GSLGGGFSSGGFSGGSFSR.G	1708.76962	2	7.57E-06	0.84	3.38	-	646.7
AHQ-1-14- , 5591	R.NVSTGDVNVEM*NAAPGVDLTQLLNNM*R.N	2906.20003	3	9.38E-05	0.86	4.04	-	975.5
gi|5453605|ref|NP_006421.1|	chaperonin containing TCP1, subunit 4 (delta); chaperonin containing t-			5.69E-07	0.92	10.16	2.40	57838.8
AHQ-1-7, 3648	K.VIDPATATSVDLR.D	1358.52277	2	5.69E-07	0.92	3.07	-	1123.7
gi|8923812|ref|NP_060943.1|	uncharacterized hypothalamus protein HT012 [Homo sapiens]			6.29E-07	0.91	10.18	8.60	14960.4
AHQ-1-13- , 4248	K.TLAFTSVDLTNK.A	1310.47811	2	3.96E-06	0.93	3.62	-	1030.8
AHQ-1-13, 4068	K.TLAFTSVDLTNK.A	1310.47811	2	6.29E-07	0.91	2.79	-	1398.3
gi|4502219|ref|NP_001656.1|	ras homolog gene family, member G (rho G); RhoG [Homo sapiens]			6.46E-07	1.24	20.17	18.80	21338.4
AHQ-1-11, 4712	K.EYIPTVFDNYSAQSAVDGR.T	2133.25975	2	6.46E-07	0.80	3.35	-	478.0
AHQ-1-11, 4706	R.TVNLNLWDTAGQEEYDR.L	2025.12129	2	9.42E-04	0.44	2.63	-	339.4
gi|30157434|ref|XP_300550.1|	similar to cDNA sequence, BC023835 [Homo sapiens]			6.85E-07	1.17	20.18	20.10	23169.2
AHQ-1-11, 4512	K.DSGACFSASEPDAVHVVLDR.H	2134.26934	2	4.65E-04	0.27	2.74	-	198.5
AHQ-1-11, 5595	R.YHALLIPSCPGALTDLASSGSLAR.I	2472.80246	3	6.85E-07	0.90	3.62	-	1783.4
gi|30158489|ref|XP_301421.1|	similar to Chloride intracellular channel protein 1 (Nuclear chloride			7.31E-07	1.26	30.23	11.60	28267.5
AHQ-1-10, 5485 - 5539	K.FLNGNELTLADCNLLPK.L	1934.20276	2	1.89E-05	0.54	3.25	-	576.1
AHQ-1-13- , 6192	K.FLNGNELTLADCNLLPK.L	1934.20276	2	3.42E-05	0.90	4.63	-	1204.9
AHQ-1-12, 5550	R.KFLNGNELTLADCNLLPK.L	2062.37568	2	9.98E-04	0.58	3.57	-	612.5
AHQ-1-10, 2483	K.NSNPALNDNLQK.G	1328.41362	1	1.28E-04	0.16	2.21	-	260.1
AHQ-1-10, 2333	K.NSNPALNDNLQK.G	1328.41362	1	1.34E-04	0.09	2.36	-	199.2
AHQ-1-10, 2175	K.NSNPALNDNLQK.G	1328.41362	1	7.31E-07	0.13	2.06	-	222.6
gi|13489091|ref|NP_066949.1|	3-mercaptopyruvate sulfurtransferase [Homo sapiens]			7.40E-07	3.37	40.31	24.90	33178.2
AHQ-1-9, 3244	R.AFGHHAVSLLDGGLR.H	1550.74638	2	6.87E-05	0.85	3.41	-	926.8
AHQ-1-9, 6710	R.AGQPLQLLDASWYLPK.L	1801.07798	2	7.40E-07	0.97	4.87	-	1870.5
AHQ-1-9, 7071 - 7139	R.DGIEPGHIPGTVNIPFTDFLSQEGLEK.S	2912.19939	3	9.01E-04	0.96	6.27	-	1230.2
AHQ-1-9, 4779 - 4792	R.HIPGAAFFDIDQCSDR.T	1850.98926	2	4.29E-05	0.59	2.58	-	365.1
gi|4507207|ref|NP_003121.1|	sorcin; Sorcin (class 4 gene) [Homo sapiens]			7.57E-07	0.94	10.24	10.10	21676.3
AHQ-1-11, 4468 - 4470	R.CLTQSGIAGGYKPFNLETCR.L	2275.54736	2	7.57E-07	0.94	4.83	-	537.9
gi|24307939|ref|NP_036205.1|	chaperonin containing TCP1, subunit 5 (epsilon) [Homo sapiens]			7.81E-07	1.45	20.16	4.40	59670.7
AHQ-1-7, 4537	K.IAILTCPFEPPKPK.T	1612.95750	2	9.77E-04	0.70	3.15	-	401.1
AHQ-1-7, 3318	R.SLHDALCVIR.N	1185.37874	2	7.81E-07	0.74	2.72	-	650.4
gi|21314617|ref|NP_000433.2|	platelet/endothelial cell adhesion molecule (CD31 antigen); platelet/e			7.91E-07	3.97	50.30	9.90	82449.6
AHQ-1-3, 5995	R.CESISGTLPISYQLLK.T	1811.08954	2	4.94E-05	0.81	3.06	-	432.0
AHQ-1-4, 6103	R.CESISGTLPISYQLLK.T	1811.08954	2	1.37E-04	0.90	4.23	-	489.2
AHQ-1-1, 5948 - 5956	R.CESISGTLPISYQLLK.T	1811.08954	2	3.61E-04	0.73	3.20	-	296.4
AHQ-1-4, 6686	K.CTIQVTHLAQEFPEIIIQK.D	2270.63371	2	2.74E-04	0.97	5.97	-	1258.1
AHQ-1-5, 3442	K.EDTIVSQTQDFTK.I	1512.60049	2	5.38E-04	0.57	2.98	-	371.6
AHQ-1-4, 2491	R.IISGIHMQTSESTK.S	1532.74472	2	7.91E-07	0.95	4.13	-	977.3
AHQ-1-5, 4127	K.STESYFIPEVR.I	1328.45199	2	4.71E-04	0.68	2.58	-	498.4
gi|30147105|ref|XP_210550.2|	similar to Chain , Aldose Reductase (E.C.1.1.1.21) Mutant With Tyr 48			7.92E-07	0.91	10.19	2.20	61339.9
AHQ-1-5, 4271 - 4281	K.ADLINNLGTIAK.S	1243.43490	2	7.92E-07	0.91	3.80	-	832.5
AHQ-1-5, 4055	K.ADLINNLGTIAK.S	1243.43490	2	9.86E-06	0.86	3.54	-	548.4
gi|4502693|ref|NP_001760.1|	CD9 antigen; motility related protein; leukocyte antigen MIC3; antigen			8.22E-07	2.17	30.20	9.60	25415.9
AHQ-1-11, 2228	K.EVQEFYK.D	943.03476	1	7.65E-04	0.57	2.93	-	286.4
AHQ-1-1, 2824	K.EVQEFYKDTYNK.L	1564.67709	2	2.84E-04	0.80	3.01	-	816.7
AHQ-1-10, 3163 - 3173	K.KDVLETFTVK.S	1180.37558	2	6.86E-05	0.79	3.16	-	691.0
AHQ-1-9, 3176	K.KDVLETFTVK.S	1180.37558	2	8.22E-07	0.79	2.85	-	882.5
AHQ-1-12, 3330	K.KDVLETFTVK.S	1180.37558	2	5.12E-05	0.87	3.31	-	882.1
AHQ-1-1, 3600	K.KDVLETFTVK.S	1180.37558	2	1.97E-05	0.92	3.43	-	1159.6
gi|4507143|ref|NP_003786.1|	sorting nexin 3 isoform a; sorting nexin 3A [Homo sapiens]			8.25E-07	1.78	20.19	14.80	18762.2
AHQ-1-12, 4886 - 4887	R.GDDGIFDDNFIEER.K	1642.66158	2	8.25E-07	0.91	3.74	-	963.2
AHQ-1-12, 2759	R.KQGLEQFINK.V	1205.38836	2	8.40E-05	0.87	3.41	-	895.9
gi|4503743|ref|NP_002009.1|	flightless I homolog [Homo sapiens]			8.28E-07	1.80	20.22	2.00	144750.1
AHQ-1-3, 4066	R.LLQSLLDTR.C	1059.24157	2	8.30E-05	0.84	2.92	-	980.6
AHQ-1-3, 6690	K.VGLGLGYLELPQINYK.L	1778.08432	2	8.28E-07	0.95	4.46	-	1180.6
gi|10835035|ref|NP_003157.1|	sulfotransferase family, cytosolic, 1A, phenol-preferring, member 3; t			8.32E-07	1.63	20.19	14.20	34196.0

AHQ-1-9, 5847	K.SHLPLALLPQTLLDQK.V	1788.12388	2	2.41E-06	0.85	3.11	-	773.2
AHQ-1-9, 6752	K.YFAEALGPLQSFQARPDDLLINTYPK.S	2969.33891	3	8.32E-07	0.78	3.49	-	472.3
gi|20330805|ref|NP_005263.1| guanine nucleotide binding protein, alpha transducing activity polypep				8.46E-07	0.94	10.19	3.10	40175.6
AHQ-1-9, 3143 - 3152	K.LLLLGAGESGK.S	1058.25357	2	8.46E-07	0.94	3.79	-	1329.2
gi|4506193|ref|NP_002784.1| proteasome beta 1 subunit; proteasome subunit HC5; proteasome component				8.50E-07	3.51	40.25	27.40	26489.2
AHQ-1-11, 5408 - 5418	K.AGGSASAMLQPLLDNQVGFK.N	2005.28443	2	7.07E-04	0.88	4.20	-	488.7
AHQ-1-11, 6419	R.FFPYYVYNIIGGLDEEGK.G	2125.36396	2	1.40E-04	0.97	5.05	-	1412.8
AHQ-1-11, 3892	K.GAVYSFDPVGSYQR.D	1546.66500	2	2.70E-04	0.85	3.38	-	735.9
AHQ-1-11, 3435	K.NMQNVEHVPLSLDR.A	1652.85728	2	8.50E-07	0.80	3.64	-	579.1
gi|5902726|ref|NP_005800.1| peroxiredoxin 2; thioredoxin-dependent peroxide reductase 1 (thiol-spec				8.55E-07	1.73	20.26	9.10	21856.8
AHQ-1-11, 6299	K.EGGLGPLNIPLLADVTR.R	1736.00583	2	5.71E-04	0.77	2.95	-	464.9
AHQ-1-11, 5820	R.KEGGLGPLNIPLLADVTR.R	1864.17875	2	8.55E-07	0.97	5.26	-	1109.8
gi|9966825|ref|NP_065089.1| HEF like Protein [Homo sapiens]				8.67E-07	1.53	20.29	4.30	86696.2
AHQ-1-13- , 4333	K.AISAFHGSLSSSQPAEIITQSK.L	2260.48935	2	8.67E-07	0.97	5.86	-	1580.6
AHQ-1-13, 3457	K.LVDTLCMETQER.D	1496.68930	2	7.66E-04	0.56	2.66	-	404.6
gi|4759086|ref|NP_004883.1| vesicle trafficking protein sec22b [Homo sapiens]				9.05E-07	2.73	30.22	20.00	24740.4
AHQ-1-12, 2069	R.DLQQYQSQAK.Q	1209.29073	2	9.52E-04	0.93	3.39	-	1256.5
AHQ-1-12, 3921	R.NLGSINTELQDVQR.I	1587.71696	2	9.05E-07	0.92	4.46	-	823.3
AHQ-1-12, 3973	R.VADGLPLAASMQEDEQSGR.D	1975.12722	2	1.88E-04	0.88	3.44	-	747.5
gi|14042953|ref|NP_114420.1| FKSG17 [Homo sapiens]				9.67E-07	0.66	10.15	6.10	23305.9
AHQ-1-11, 4507	K.NILFVITKPDVYK.S	1550.86611	2	9.67E-07	0.66	2.90	-	651.6
gi|13637631|ref|XP_016625.1| similar to voltage-dependent anion channel isoform 2 [Homo sapiens] [M				9.72E-07	1.29	20.21	11.30	31479.2
AHQ-1-9, 4115 - 4183	K.VNNSSLIGVGYTQTLRPGVK.L	2104.39573	2	8.19E-04	0.33	2.72	-	297.3
AHQ-1-9, 4839	K.YKWCEYGLTFTEK.W	1726.92965	2	9.72E-07	0.97	4.29	-	1479.9
gi|4759044|ref|NP_004841.1| Rho-associated, coiled-coil containing protein kinase 2 [Homo sapiens]				9.80E-07	2.27	30.20	4.00	160883.4
AHQ-1-3, 5218	K.EFINLQSALESER.R	1536.66842	2	9.80E-07	0.69	2.80	-	758.8
AHQ-1-3, 3725	R.GAFGEVQLVR.H	1076.23068	2	1.39E-04	0.82	2.96	-	1150.3
AHQ-1-3, 5427 - 5503	R.SQLQALHIGLDSSSIGSGPGDAEADDGFPESR.L	3215.34459	3	2.70E-04	0.76	3.91	-	808.3
gi|5453714|ref|NP_006448.1| LIM protein (similar to rat protein kinase C-binding enigma) [Homo sapi				1.01E-06	1.33	20.16	4.90	64027.9
AHQ-1-13- , 2049	R.ASAAPKPEPVPVQK.G	1419.65094	2	5.74E-05	0.69	3.12	-	481.8
AHQ-1-13- , 2951	K.EVVKPVPITSPAVSK.V	1551.85241	2	1.01E-06	0.63	2.73	-	322.2
AHQ-1-13, 2933	K.EVVKPVPITSPAVSK.V	1551.85241	2	3.48E-05	0.69	2.94	-	310.7
gi|19923457|ref|NP_057447.2| glycoprotein VI (platelet); platelet glycoprotein VI [Homo sapiens] [M				1.01E-06	0.92	10.20	3.20	67308.6
AHQ-1-7, 5773	K.PSLQALPSSLVPLEKPVTLR.C	2146.55880	2	4.39E-06	0.84	3.45	-	555.9
AHQ-1-7, 5756	K.PSLQALPSSLVPLEKPVTLR.C	2146.55880	3	1.01E-06	0.92	4.05	-	1177.1
gi|20547107|ref|XP_114482.1| similar to HEAT SHOCK 70 KDA PROTEIN 4 (HEAT SHOCK 70-RELATED PROTEIN				1.01E-06	2.42	30.23	5.60	94330.3
AHQ-1-4, 7038	R.EFSITDVVPYPISLR.W	1736.98906	2	7.10E-06	0.67	3.07	-	317.4
AHQ-1-4, 3318	K.ELSTTLNADEAVTR.G	1520.62416	2	6.29E-04	0.82	3.14	-	670.0
AHQ-1-4, 6814 - 6831	K.SNLAYDIVQLPTGLTGIK.V	1904.19651	2	1.01E-06	0.93	4.62	-	781.8
gi|4506675|ref|NP_002941.1| ribophorin I [Homo sapiens]				1.12E-06	0.93	10.21	2.80	68569.0
AHQ-1-1, 5880	R.FVDHVFDEQVIDSLTVK.I	1992.21748	2	1.12E-06	0.93	4.17	-	829.5
gi|11496891|ref|NP_000662.2| class III alcohol dehydrogenase 5 chi subunit; Alcohol dehydrogenase (				1.20E-06	0.95	10.26	5.90	39738.1
AHQ-1-11, 4932 - 4963	K.AAVAWEAGKPLSIEEIEVAPPK.A	2306.64276	2	2.72E-04	0.91	4.56	-	546.3
AHQ-1-9, 5200 - 5270	K.AAVAWEAGKPLSIEEIEVAPPK.A	2306.64276	2	1.20E-06	0.95	5.13	-	885.7
gi|30147623|ref|XP_293669.2| similar to actinin, alpha 4 [Homo sapiens]				1.22E-06	0.87	10.18	3.50	57794.4
AHQ-1-5, 4250	K.LSGSNPYTTVTPQIINSK.W	1921.14079	2	1.22E-06	0.87	3.58	-	724.0
gi|5901922|ref|NP_008996.1| CDC37 homolog [Homo sapiens]				1.25E-06	0.91	10.21	5.60	44468.0
AHQ-1-8, 6899	R.LGPGGLDPVEVYESLPEELQK.C	2270.52091	2	1.25E-06	0.91	4.25	-	514.3
gi|5454090|ref|NP_006271.1| signal sequence receptor, delta; translocon-associated protein delta [H				1.26E-06	1.70	20.18	17.30	18998.5
AHQ-1-12, 4725	R.FFDEESYSLLR.K	1406.52121	2	1.26E-06	0.91	3.62	-	917.4
AHQ-1-12, 5991	R.NNEDISIIPPLFTVSVDHR.G	2167.40715	3	1.58E-06	0.78	3.50	-	689.3
gi|4502171|ref|NP_000476.1| adenine phosphoribosyltransferase; AMP pyrophosphorylase; AMP diphospho				1.26E-06	4.31	50.28	41.10	19607.6
AHQ-1-11, 3666	R.DISPVLKDPASFR.A	1445.64525	2	5.60E-06	0.80	3.18	-	569.6
AHQ-1-11, 6506	R.GFLFGPSLAQELGLGCVLIR.K	2149.54130	2	2.98E-06	0.98	5.70	-	1753.0
AHQ-1-11, 4907	R.GKLPGPTLWASYSLEYGK.A	1968.24075	2	1.92E-05	0.81	3.26	-	543.0
AHQ-1-11, 3479	R.IDYIAGLDSR.G	1123.24098	2	1.37E-05	0.94	3.56	-	1235.7
AHQ-1-11, 5396 - 5402	R.SFPDFPTPGVVFR.D	1466.66462	2	1.26E-06	0.78	2.76	-	478.0
gi|29737976|ref|XP_290742.1| similar to Phosphoribosylformylglycinamidine synthase (FGAM synthase)				1.29E-06	0.68	10.18	2.10	144733.3
AHQ-1-3, 7087	R.LGGTALAQCFSQLGEHPPDLDLPENLVR.A	3050.39201	3	1.29E-06	0.68	3.51	-	403.5
gi|30149460|ref|XP_210540.2| similar to Heat shock protein HSP 90-alpha (HSP 86) [Homo sapiens] [MA				1.34E-06	0.93	10.20	4.00	29287.8
AHQ-1-5, 2202 - 2210	K.HIYYITGETK.D	1225.37490	1	2.54E-04	0.81	2.63	-	879.9
AHQ-1-5, 2201	K.HIYYITGETK.D	1225.37490	2	1.34E-06	0.93	3.19	-	907.0
gi|5453722|ref|NP_006321.1| lysophospholipase I; lysophospholipase 1; lysophospholipid-specific lys				1.34E-06	1.71	20.21	12.60	24669.5
AHQ-1-11, 2962	K.ALIDQEVKNGIPSNR.I	1654.84968	2	1.34E-06	0.74	2.71	-	752.5
AHQ-1-11, 6196	K.LAGVTALSCWLPLR.A	1558.86992	2	1.19E-05	0.97	4.26	-	1779.6
gi|29788766|ref|NP_064424.2| tubulin, beta polypeptide 4, member Q [Homo sapiens]				1.40E-06	2.28	30.31	11.10	48377.2
AHQ-1-13- , 4045	R.AVLVDLEPGTM*DSVR.S	1618.83435	2	2.69E-04	0.86	3.46	-	533.0
AHQ-1-14- , 3939 - 4016	R.AVLVDLEPGTM*DSVR.S	1618.83435	2	2.56E-05	0.54	2.85	-	317.4
AHQ-1-9, 6335 - 6336	K.EAESCDCLQGFQLTHSLGGGTGSGMGTLLLSK.I	3315.65550	3	1.40E-06	0.76	3.90	-	649.0
AHQ-1-7, 5504 - 5513	R.KEAESCDCLQGFQLTHSLGGGTGSGM*GTLLLSK.I	3459.82781	3	4.04E-04	0.97	6.06	-	1816.5
gi|4505145|ref|NP_002387.1| malic enzyme 2, NAD(+)-dependent, mitochondrial; Malic enzyme, mitochon				1.41E-06	2.69	30.20	6.30	65443.3
AHQ-1-7, 3644	K.ALTSQLTDEELAQGR.L	1632.75450	2	3.06E-04	0.89	3.91	-	932.8
AHQ-1-6, 3220	R.HISDSVFLEAAK.A	1317.47234	2	5.94E-06	0.94	3.74	-	843.7
AHQ-1-6, 2972	K.IETQDIQALR.F	1187.32821	2	1.41E-06	0.87	3.23	-	1049.5
gi|5729875|ref|NP_006658.1| progesterone receptor membrane component 1; progesterone binding protei				1.45E-06	2.41	30.17	17.90	21671.0
AHQ-1-11, 4343 - 4371	K.FYGPEGPYGVFAGR.D	1517.66849	2	4.41E-04	0.84	3.18	-	749.0
AHQ-1-11, 2818 - 2838	R.GDQPAASGDSDDDEPPPLPR.L	2037.04410	2	3.47E-05	0.73	3.22	-	295.7
AHQ-1-11, 3854 - 3915	R.KFYGPEGPYGVFAGR.D	1645.84140	2	1.45E-06	0.83	3.48	-	606.3
gi|4506045|ref|NP_002718.1| proteoglycan 1, secretory granule; Proteoglycan 1, secretory granule (p				1.45E-06	2.79	30.20	17.10	17623.6
AHQ-1-1, 2449	R.CNPDSNSANCLEEK.G	1640.68887	2	9.56E-06	0.91	3.48	-	907.5
AHQ-1-1, 4816 - 4821	K.GPMFELLPGESNK.I	1419.62807	2	1.45E-06	0.94	3.87	-	1195.3
AHQ-1-1, 4209 - 4212	K.GPM*FELLPGESNK.I	1435.62747	2	4.18E-06	0.94	4.07	-	883.9
gi|4506381|ref|NP_002863.1| ras-related C3 botulinum toxin substrate 2; Ras-related C3 botulinum to				1.47E-06	1.71	20.20	7.80	21428.7
AHQ-1-11, 3946	K.KLAPITYPQGLALAK.E	1584.92723	2	2.21E-05	0.90	3.93	-	617.3
AHQ-1-11, 4347 - 4348	K.LAPITYPQGLALAK.E	1456.75431	2	1.47E-06	0.81	2.71	-	476.1
gi|29731000|ref|XP_291064.1| similar to KIAA0540 protein [Homo sapiens]				1.53E-06	1.71	20.23	2.10	224374.8
AHQ-1-1, 3721	K.LIVVVAGQPSEVR.S	1367.61917	2	1.53E-06	0.96	4.51	-	1433.3
AHQ-1-1, 6193	R.VLNTSSLESATDEAGSPLAAAAAAAAER.C	2716.89684	3	1.35E-04	0.75	3.31	-	817.6
gi|17402865|ref|NP_003303.2| thiosulfate sulfurtransferase; rhodanese [Homo sapiens]				1.58E-06	2.56	30.20	14.80	33428.7
AHQ-1-9, 4038	R.FLGTEPEPDAVGLDSGHIR.G	2011.18089	2	4.05E-05	0.84	3.72	-	517.5
AHQ-1-9, 3335	K.KVDLSQPLIATCR.K	1502.76154	2	4.79E-04	0.80	2.96	-	694.8
AHQ-1-9, 4716	K.TYEQVLENLESK.R	1453.57618	2	1.58E-06	0.92	4.06	-	833.5
gi|9845502|ref|NP_002286.2| laminin receptor 1; Laminin receptor-1 (67kD); laminin receptor 1 (67kD				1.59E-06	0.83	10.16	5.10	32853.8
AHQ-1-10, 5175	R.FTPGTFTNQIQAAFR.E	1699.89060	2	1.59E-06	0.83	3.19	-	444.4
gi|29729857|ref|XP_293567.1| similar to 60 kDa heat shock protein, mitochondrial precursor (Hsp60)				1.62E-06	0.93	10.16	3.40	38427.8
AHQ-1-14- , 3583	R.TVIIEQSWGSPK.V	1345.52569	2	5.62E-04	0.93	3.28	-	1383.2
AHQ-1-7, 3516	R.TVIIEQSWGSPK.V	1345.52569	2	1.62E-06	0.93	3.00	-	1546.3
gi|5729991|ref|NP_006494.1| proteasome 26S ATPase subunit 4 isoform 1; protease 26S subunit 6; Tat-				1.69E-06	0.88	10.17	2.90	47365.9
AHQ-1-5, 3827	R.GVLM*YGPPGCGK.T	1253.47332	2	1.69E-06	0.88	3.35	-	950.7
gi|4504085|ref|NP_000399.1| glycerol-3-phosphate dehydrogenase 2 (mitochondrial) [Homo sapiens] [MA				1.71E-06	4.44	50.21	10.30	80814.1
AHQ-1-6, 3648	R.CKDVLTGQEFDVR.A	1568.73370	2	5.66E-06	0.89	3.34	-	952.7
AHQ-1-6, 6072 - 6082	R.LAFLNVQAAEEALPR.I	1642.88050	2	1.22E-04	0.89	3.82	-	851.1
AHQ-1-6, 5780	R.LVQDYGLESEVAQHLAATYGDK.A	2408.60567	3	1.11E-05	0.84	3.47	-	762.2
AHQ-1-6, 6291	R.VIFFLPWQK.M	1178.44934	2	1.71E-06	0.91	3.34	-	812.6
AHQ-1-6, 6342 - 6414	R.YGAATANYMEVVSLLK.K	1731.00632	2	1.00E-05	0.91	4.27	-	683.6
gi|10863873|ref|NP_000651.1| transforming growth factor, beta 1 (Camurati-Engelmann disease); trans				1.74E-06	2.74	30.21	11.00	44422.0
AHQ-1-13, 3349	R.ALDTNYCFSSTEK.N	1537.63010	2	2.37E-04	0.90	3.34	-	896.8

AHQ-1-13- , 3424	R.ALDTNYCFSSTEK.N	1537.63010	2	1.74E-06	0.93	3.85	-	1037.6
AHQ-1-9, 6827	R.LLAPSDSPEWLSFDVTGVVR.Q	2189.45272	2	2.18E-05	0.90	4.06	-	470.4
AHQ-1-13- , 3611	K.VEQLSNMIVR.S	1189.41074	2	3.29E-04	0.95	4.30	-	1325.9
AHQ-1-13, 3509	K.VEQLSNMIVR.S	1189.41074	2	5.10E-06	0.91	3.50	-	1206.7
gi|4557797|ref|NP_000260.1| non-metastatic cells 1 protein [Homo sapiens]				1.79E-06	2.59	30.16	20.40	17148.6
AHQ-1-12, 4061	K.DRPFFAGLVK.Y	1150.35439	2	1.61E-04	0.91	3.28	-	1093.6
AHQ-1-12, 2917	R.GLVGEIIKR.F	985.20582	2	1.05E-04	0.87	2.56	-	1062.4
AHQ-1-14- , 3064	R.TFIAIKPDGVQR.G	1345.57205	2	1.79E-06	0.80	3.02	-	781.1
AHQ-1-12, 2949	R.TFIAIKPDGVQR.G	1345.57205	2	2.59E-05	0.85	3.14	-	808.8
gi|17450333|ref|XP_060887.1| similar to peptidyl-Pro cis trans isomerase [Homo sapiens]				1.89E-06	0.91	10.23	11.00	17503.9
AHQ-1-12, 6417	-.M*VNPTVFDIAVDGKPLGR.V	1946.26009	2	2.55E-04	0.93	4.47	-	1033.5
AHQ-1-13- , 6768	-.M*VNPTVFDIAVDGKPLGR.V	1946.26009	2	1.42E-05	0.95	4.56	-	1201.4
AHQ-1-12, 6198 - 6261	-.M*VNPTVFDIAVDGKPLGR.V	1946.26009	2	1.89E-06	0.91	3.65	-	1028.7
gi|19913414|ref|NP_055018.2| adaptor-related protein complex 2, alpha 1 subunit isoform 1; adaptin,				1.92E-06	0.59	20.17	3.90	107545.3
AHQ-1-5, 5547 - 5551	K.TSVQFQNFSPTVVHPGDLQTQLAVQTK.R	2972.30119	3	1.01E-04	0.55	3.33	-	525.7
AHQ-1-12, 3757 - 3826	R.YGGAPQALTLK.L	1119.29547	1	1.92E-06	0.04	1.81	-	297.6
gi|4502297|ref|NP_001678.1| ATP synthase, H+ transporting, mitochondrial F1 complex, delta subunit				1.96E-06	1.82	20.16	13.70	17489.8
AHQ-1-13, 2625	K.AQAELVGTADEATR.A	1432.51860	1	7.36E-04	0.22	2.29	-	208.4
AHQ-1-13- , 2611	K.AQAELVGTADEATR.A	1432.51860	1	2.72E-05	0.71	2.74	-	417.1
AHQ-1-13, 2621	K.AQAELVGTADEATR.A	1432.51860	2	1.96E-06	0.92	3.29	-	1150.6
AHQ-1-13- , 2368	R.IEANEALVK.A	987.13221	2	2.94E-04	0.90	2.94	-	891.4
gi|13569962|ref|NP_112243.1| RAB1B, member RAS oncogene family; small GTP-binding protein [Homo sap				1.98E-06	2.43	30.29	18.90	22171.0
AHQ-1-11, 4336	R.GAHGIIVVYDVTDQESYANVK.Q	2279.49113	2	1.98E-06	0.98	5.83	-	1912.9
AHQ-1-11, 4435	R.GAHGIIVVYDVTDQESYANVK.Q	2279.49113	2	2.58E-05	0.96	4.92	-	1249.3
AHQ-1-11, 2059 - 2096	R.MGPGAASGGERPNLK.I	1442.62650	2	8.69E-04	0.48	2.70	-	451.2
AHQ-1-11, 6298 - 6302	K.NATNVEQAFMTMAAEIK.K	1870.14079	2	3.67E-06	0.97	5.29	-	1729.8
gi|15306356|ref|XP_035638.2| similar to interleukin 25; lymphocyte antigen 6 complex, locus E ligan				2.05E-06	2.65	30.19	20.80	18795.1
AHQ-1-13, 3473	R.ESDVPLKTEEFEVTK.T	1751.91253	2	6.39E-05	0.76	3.32	-	462.2
AHQ-1-12, 3465	R.ESDVPLKTEEFEVTK.T	1751.91253	2	2.05E-06	0.78	3.48	-	384.5
AHQ-1-13- , 3596	R.GAEIEYAMAYSK.A	1333.49204	2	2.34E-04	0.94	3.85	-	1361.3
AHQ-1-13- , 4796	K.SYLYFTQFK.A	1197.36315	2	2.31E-05	0.93	3.55	-	1121.3
gi|8923579|ref|NP_060377.1| hypothetical protein FLJ20625 [Homo sapiens]				2.05E-06	1.90	20.27	26.70	17744.7
AHQ-1-12, 6174 - 6175	K.LPPLPSLTSQPHQVLASEPIPFSDLQQVSR.I	3283.72259	3	1.88E-04	0.95	5.47	-	1157.5
AHQ-1-14- , 6250	K.LPPLPSLTSQPHQVLASEPIPFSDLQQVSR.I	3283.72259	3	5.99E-04	0.94	5.38	-	627.2
AHQ-1-12, 5265	R.TDEQALLSSILAK.T	1389.57677	2	2.05E-06	0.95	4.33	-	1219.9
gi|6041669|ref|NP_004538.2| NADH dehydrogenase (ubiquinone) 1 beta subcomplex, 4, 15kDa; NADH dehyd				2.10E-06	0.87	10.18	14.00	15208.5
AHQ-1-13, 4691	R.TLPETLDPAEYNISPETR.R	2047.20831	2	2.10E-06	0.87	3.54	-	545.3
gi|4504045|ref|NP_002063.1| guanine nucleotide binding protein (G protein), q polypeptide [Homo sap				2.13E-06	3.37	40.27	15.90	42142.8
AHQ-1-9, 3183	K.LLLLGTGESGK.S	1088.27968	2	8.19E-04	0.82	3.37	-	794.8
AHQ-1-9, 4544 - 4592	K.SLWNDPGIQECYDR.R	1754.85835	2	2.13E-06	0.85	3.04	-	903.7
AHQ-1-9, 7228	R.VPTTGIIEYPFDLQSVIFR.M	2196.53000	2	3.50E-04	0.96	5.37	-	943.0
AHQ-1-9, 4539	K.VSAFENPYVDAIK.S	1453.62101	2	4.75E-04	0.74	2.64	-	818.7
gi|4506765|ref|NP_002952.1| S100 calcium-binding protein A4; 18A2; 42A; S100 calcium-binding protei				2.26E-06	0.96	20.16	18.80	11728.5
AHQ-1-14- , 3514 - 3579	K.ALDVMVSTFHK.Y	1248.47665	2	2.26E-06	0.85	3.23	-	870.0
AHQ-1-14, 4718	R.ELPSFLGK.R	891.04613	1	7.06E-05	0.10	1.83	-	132.9
gi|4757952|ref|NP_001782.1| cell division cycle 42 isoform 1; cell division cycle 42 (GTP-binding p				2.28E-06	3.54	40.26	25.70	21258.5
AHQ-1-11, 2304	K.CVVVGDGAVGK.T	1062.22237	1	5.02E-04	0.77	2.61	-	745.1
AHQ-1-11, 2295 - 2298	K.CVVVGDGAVGK.T	1062.22237	2	6.76E-05	0.79	2.83	-	853.5
AHQ-1-11, 6300	K.NVFDEAILAALEPPEPK.K	1854.09282	3	2.28E-06	0.93	5.16	-	912.8
AHQ-1-11, 3790	K.TCLLISYTTNK.F	1315.51795	2	1.03E-05	0.92	3.19	-	1323.6
AHQ-1-11, 2530	K.YVECSALTQK.G	1200.34381	2	8.38E-06	0.90	3.68	-	602.1
gi|10835049|ref|NP_001655.1| ras homolog gene family, member A; Aplysia ras-related homolog 12; Rho				2.36E-06	0.90	10.18	6.20	21768.0
AHQ-1-13- , 3964	R.IGAFGYMECSAK.T	1335.53122	2	2.36E-06	0.90	3.54	-	746.6
AHQ-1-10, 3544	R.IGAFGYMECSAK.T	1335.53122	2	2.34E-05	0.57	2.80	-	505.2
gi|5803013|ref|NP_006808.1| endoplasmic reticulum protein 29 precursor; endoplasmic reticulum lumen				2.39E-06	1.80	20.23	14.90	28993.2
AHQ-1-10, 3787	R.DGDFENPVPYTGAVK.V	1609.71786	2	2.39E-06	0.86	3.73	-	605.1
AHQ-1-10, 3655 - 3663	R.DGDFENPVPYTGAVK.V	1609.71786	2	5.44E-06	0.83	3.61	-	475.1
AHQ-1-10, 6380	K.GQGVYLGMPGCLPVYDALAGEFIR.A	2585.98267	2	3.16E-06	0.93	4.63	-	615.6
gi|13699868|ref|NP_005947.2| methylenetetrahydrofolate dehydrogenase (NADP+ dependent), methenyltet				2.40E-06	0.83	10.18	1.70	101530.5
AHQ-1-5, 4407 - 4462	K.YVVVTGITPTPLGEGK.S	1631.89439	2	2.40E-06	0.83	3.61	-	541.3
gi|30148456|ref|XP_293602.2| similar to glutathione-S-transferase like; glutathione transferase ome				2.45E-06	0.91	10.18	3.90	29139.6
AHQ-1-13- , 4800	K.VPSLVGSFLR.T	1075.28583	2	4.87E-05	0.91	3.62	-	925.3
AHQ-1-10, 4239	K.VPSLVGSFLR.T	1075.28583	2	2.45E-06	0.91	3.38	-	1167.4
gi|4758790|ref|NP_004543.1| NADH dehydrogenase (ubiquinone) Fe-S protein 5, 15kDa (NADH-coenzyme Q				2.48E-06	0.97	10.26	11.30	12517.4
AHQ-1-13- , 6743	K.IEYDDFVECLLR.Q	1573.74872	2	2.48E-06	0.97	5.15	-	1092.7
AHQ-1-13, 6284	K.IEYDDFVECLLR.Q	1573.74872	2	3.12E-05	0.90	3.21	-	1163.6
gi|29729345|ref|XP_290995.1| similar to speckle-type POZ protein [Homo sapiens]				2.54E-06	1.62	20.20	10.90	18703.4
AHQ-1-3, 6146	K.NRVEINDLDPEVFKEM*MR.F	2252.55733	2	2.16E-04	0.67	3.08	-	859.5
AHQ-1-4, 6260 - 6274	K.NRVEINDLDPEVFKEM*MR.F	2252.55733	2	2.54E-06	0.74	3.63	-	770.5
AHQ-1-5, 6214	K.NRVEINDLDPEVFKEMM*R.F	2252.55733	2	6.13E-06	0.88	3.74	-	811.0
AHQ-1-6, 6107 - 6116	K.NRVEINDLDPEVFKEM*MR.F	2252.55733	2	2.64E-04	0.87	3.76	-	887.1
AHQ-1-1, 6163	K.NRVEINDLDPEVFKEM*MR.F	2252.55733	2	3.03E-04	0.79	4.02	-	867.3
gi|29727317|ref|XP_298144.1| hypothetical protein XP_298144 [Homo sapiens]				2.64E-06	0.80	10.15	8.00	22108.6
AHQ-1-4, 6688 - 6704	K.GEYNLTLDTALTSNLK.T	1753.93165	2	2.64E-06	0.80	3.03	-	933.3
gi|4503529|ref|NP_001407.1| eukaryotic translation initiation factor 4A, isoform 1 [Homo sapiens] [				2.68E-06	0.84	10.19	3.70	46153.7
AHQ-1-8, 3879	K.LQMEAPHIIVGTPGR.V	1619.91357	2	2.68E-06	0.84	3.78	-	677.0
gi|4759342|ref|NP_004808.1| tight junction protein 2 (zona occludens 2); Friedreich ataxia region g				2.80E-06	0.97	10.25	1.60	124661.6
AHQ-1-7, 3769	R.TFLRPSPEDEAIYGPNTK.M	2036.23035	2	2.80E-06	0.97	4.98	-	1543.2
gi|4502861|ref|NP_001275.1| adaptor-related protein complex 3, sigma 1 subunit; clathrin-associated				2.93E-06	0.94	10.20	7.80	21732.0
AHQ-1-11, 3748	K.FYQPYSEDTQQIIR.E	1917.06756	2	2.93E-06	0.94	4.08	-	979.2
gi|11496885|ref|NP_005442.2| enigma protein; LIM domain protein [Homo sapiens]				2.96E-06	0.94	10.19	2.60	49844.4
AHQ-1-11, 4576	K.VVLEGPAPWGFR.L	1328.54318	2	2.96E-06	0.94	3.83	-	1233.3
gi|30157216|ref|XP_292133.2| similar to genethonin 1 [Homo sapiens]				3.01E-06	1.08	20.19	4.50	39693.8
AHQ-1-14, 5280	K.PSDTIENVK.A	1003.08850	1	4.49E-04	0.37	2.25	-	455.7
AHQ-1-5, 4582	K.TITLEVKPSDTIENVK.A	1788.03268	2	2.57E-05	0.57	3.00	-	579.3
AHQ-1-6, 4483	K.TITLEVKPSDTIENVK.A	1788.03268	2	9.20E-05	0.50	3.48	-	396.2
AHQ-1-9, 4414	K.TITLEVKPSDTIENVK.A	1788.03268	2	1.55E-04	0.25	2.53	-	236.3
AHQ-1-14, 5201 - 5277	K.TITLEVKPSDTIENVK.A	1788.03268	2	3.01E-06	0.71	3.74	-	565.2
AHQ-1-2, 4761	K.TITLEVKPSDTIENVK.A	1788.03268	2	5.12E-05	0.80	3.55	-	789.8
AHQ-1-1, 4637	K.TITLEVKPSDTIENVK.A	1788.03268	2	8.75E-05	0.37	2.58	-	315.8
gi|7656952|ref|NP_055227.1| calcyclin binding protein [Homo sapiens]				3.03E-06	0.86	10.19	10.10	26209.8
AHQ-1-10, 4647	K.IYITLTGVHQVPTENVQVHFTER.S	2683.01488	3	3.03E-06	0.86	3.76	-	986.0
gi|7705632|ref|NP_057152.1| CGI-135 protein [Homo sapiens]				3.27E-06	0.93	10.21	7.20	16979.6
AHQ-1-13- , 6777	K.GIVLLEELLPK.G	1224.51463	2	5.10E-06	0.94	4.11	-	1676.0
AHQ-1-12, 6383	K.GIVLLEELLPK.G	1224.51463	2	3.27E-06	0.93	3.24	-	1745.3
gi|5453998|ref|NP_006382.1| importin 7; RAN-binding protein 7 [Homo sapiens]				3.31E-06	0.82	10.16	1.40	119515.7
AHQ-1-4, 6927	R.ENIVEAIIHSPELIR.V	1733.98994	2	3.31E-06	0.82	3.28	-	725.4
gi|19913373|ref|NP_079536.2| G6B protein isoform G6b-A precursor; G6B protein; immunoglobulin recep				3.36E-06	2.52	30.16	13.90	25002.6
AHQ-1-12, 2510	R.TVLHVLGDR.T	1010.17224	2	3.36E-06	0.88	2.78	-	1080.5
AHQ-1-11, 2444	R.TVLHVLGDR.T	1010.17224	2	4.78E-05	0.94	3.13	-	1478.9
AHQ-1-12, 2643 - 2721	R.VNLSCGGVSHPIR.W	1397.58551	2	2.41E-05	0.89	2.93	-	1271.6
AHQ-1-11, 5018	R.WVWAPSFPACK.G	1350.56891	2	2.39E-04	0.75	2.87	-	531.2
gi|6005786|ref|NP_009214.1| monoglyceride lipase; lysophospholipase-like [Homo sapiens]				3.38E-06	1.55	20.22	12.10	34292.3
AHQ-1-13- , 6260	K.LTVPFLLLQGSADR.L	1530.79329	2	3.38E-06	0.65	2.73	-	410.3
AHQ-1-14- , 6044 - 6046	K.LTVPFLLLQGSADR.L	1530.79329	2	5.87E-05	0.77	3.20	-	547.7
AHQ-1-9, 6428	R.TPQSIPYQDLPHLVNADGQYLFCR.Y	2835.14314	2	6.45E-04	0.79	3.41	-	504.4
AHQ-1-9, 6407 - 6438	R.TPQSIPYQDLPHLVNADGQYLFCR.Y	2835.14314	3	1.11E-05	0.89	4.50	-	621.3

gi|27500115|ref|XP_208901.1|	similar to ubiquitin-conjugating enzyme E2M; UBC12 homolog, yeast [Hom			3.44E-06	0.92	10.19	8.70	20964.0
AHQ-1-11, 5711	K.TCDISFSDPDDLLNFK.L	1889.02935	2	3.44E-06	0.92	3.81	-	940.8
gi|24307969|ref|NP_055423.1|	cytoplasmic FMR1 interacting protein 1; selective hybridizing clone (m			3.44E-06	0.79	10.17	1.00	145181.2
AHQ-1-3, 4581 - 4586	R.SSLEGPTILDIEK.F	1402.57231	2	3.44E-06	0.79	3.34	-	727.6
gi|6715607|ref|NP_000175.1|	G-gamma globin [Homo sapiens]			3.66E-06	0.89	10.19	6.80	16126.3
AHQ-1-13- , 5019 - 5032	R.LLVVYPWTQR.F	1275.52342	2	3.66E-06	0.89	3.20	-	972.0
gi|6005749|ref|NP_009193.1|	RNA-binding protein regulatory subunit; oncogene DJ1 [Homo sapiens] [MA			3.67E-06	2.51	30.22	30.20	19846.9
AHQ-1-11, 3330	R.DVVICPDASLEDAKK.E	1661.85581	2	3.67E-06	0.86	3.28	-	806.1
AHQ-1-11, 5082 - 5138	K.EGPYDVVVLPGGNLGAQNLSESAAVK.E	2585.85114	2	2.45E-04	0.86	4.31	-	315.8
AHQ-1-11, 3095 - 3158	K.VTVAGLAGKDPVQCSR.D	1659.88952	2	3.69E-05	0.79	3.24	-	569.4
gi|7661948|ref|NP_055545.1|	KIAA0152 gene product [Homo sapiens]			3.71E-06	0.89	10.18	4.50	32233.6
AHQ-1-10, 3260 - 3303	R.SNPEDQILYQTER.Y	1593.67683	2	7.36E-06	0.81	3.57	-	505.0
AHQ-1-9, 3422	R.SNPEDQILYQTER.Y	1593.67683	2	3.71E-06	0.89	3.53	-	862.1
gi|19923483|ref|NP_057406.2|	GTPase Rab14 [Homo sapiens]			3.72E-06	2.97	40.23	17.20	23896.8
AHQ-1-10, 4039	R.GAAGALMVYDITR.R	1338.55819	2	1.78E-04	0.95	4.24	-	1498.9
AHQ-1-11, 4168	R.GAAGALMVYDITR.R	1338.55819	2	4.13E-06	0.95	3.65	-	1442.1
AHQ-1-11, 3650	R.GAAGALM*VYDITR.R	1354.55759	2	3.72E-06	0.94	3.72	-	1414.0
AHQ-1-11, 3180	K.SCLLHQFTEK.K	1264.43272	2	6.62E-05	0.91	3.25	-	838.5
AHQ-1-10, 3092	K.SCLLHQFTEK.K	1264.43272	2	1.93E-05	0.88	3.44	-	777.3
AHQ-1-10, 4215 - 4216	K.TGENVEDAFLEAAK.K	1494.58517	1	5.99E-04	0.22	2.41	-	460.1
AHQ-1-11, 4338	K.TGENVEDAFLEAAK.K	1494.58517	1	2.99E-04	0.20	2.23	-	624.9
AHQ-1-10, 4211 - 4256	K.TGENVEDAFLEAAK.K	1494.58517	2	8.00E-04	0.90	3.73	-	886.7
gi|19743813|ref|NP_002202.2|	integrin beta 1 isoform 1A precursor; integrin VLA-4 beta subunit; fib			3.86E-06	0.78	10.24	2.10	88414.9
AHQ-1-5, 5315	K.LKPEDITQIQPQQLVLR.L	2020.36187	2	1.59E-04	0.78	3.65	-	411.0
AHQ-1-3, 5293 - 5295	K.LKPEDITQIQPQQLVLR.L	2020.36187	3	3.86E-06	0.78	3.87	-	731.1
gi|24111250|ref|NP_006563.2|	guanine nucleotide binding protein (G protein), alpha 13 [Homo sapiens			3.88E-06	0.34	10.13	4.50	44049.3
AHQ-1-9, 5172	K.LGEPDYIPSQQDILLAR.R	1929.16292	2	3.88E-06	0.34	2.63	-	254.1
gi|4885153|ref|NP_005198.1|	v-crk sarcoma virus CT10 oncogene homolog (avian)-like; v-crk avian sar			3.92E-06	1.90	20.22	9.60	33776.7
AHQ-1-9, 6632	K.IGDQEFDHLPALLEFYK.I	2036.27195	2	3.92E-06	0.93	4.43	-	931.1
AHQ-1-14- , 4799	K.TALALEVGDIVK.V	1229.44814	2	2.34E-05	0.96	4.14	-	1888.9
gi|9625002|ref|NP_000879.2|	integrin, beta 6 [Homo sapiens]			3.93E-06	0.91	10.13	1.10	85935.7
AHQ-1-3, 4002	R.LGFGSFVEK.P	984.13002	2	3.93E-06	0.91	2.59	-	1101.8
gi|4885405|ref|NP_005326.1|	hematopoietic cell-specific Lyn substrate 1 [Homo sapiens]			4.04E-06	0.96	10.20	2.70	53997.7
AHQ-1-12, 2375 - 2438	K.SAVGHEYVAEVEK.H	1418.53353	2	4.04E-06	0.96	4.04	-	1928.4
gi|22267436|ref|NP_056284.1|	DKFZp564D177 protein [Homo sapiens]			4.05E-06	2.62	30.21	18.20	28466.4
AHQ-1-11, 6114	K.DKEWQEQFLIPNLALIDK.Q	2201.50664	2	1.20E-04	0.92	4.28	-	753.6
AHQ-1-11, 3582 - 3644	K.LVGVFHTEYGALNR.V	1576.78061	2	1.65E-05	0.80	2.69	-	817.2
AHQ-1-11, 4080	R.VHVLWWNESADSR.A	1599.73113	2	4.05E-06	0.90	3.45	-	465.6
gi|4503609|ref|NP_001976.1|	electron-transfer-flavoprotein, beta polypeptide; electron-transferring			4.06E-06	1.71	20.20	11.40	27843.4
AHQ-1-10, 3540	K.EVIAVSCGPAQCQETIR.T	1921.14254	2	4.06E-06	0.90	4.04	-	823.1
AHQ-1-13, 3759	K.EVIAVSCGPAQCQETIR.T	1921.14254	2	4.52E-04	0.62	2.74	-	489.5
AHQ-1-10, 3139 - 3152	K.LSVISVEDPPQR.T	1340.50744	2	1.37E-04	0.81	3.05	-	777.5
gi|5803121|ref|NP_006801.1|	for protein disulfide isomerase-related [Homo sapiens]			4.09E-06	2.39	30.21	10.80	59594.0
AHQ-1-7, 4160	R.FHISEFPTLK.Y	1219.41350	2	4.09E-06	0.73	2.53	-	783.1
AHQ-1-7, 3174	K.GPPLWEEDPGAK.D	1296.40999	2	2.22E-04	0.77	2.59	-	652.4
AHQ-1-7, 6860 - 6930	K.NPQPPQPQVPETPWADEGGSVYHLTDEDFDQFVK.E	3870.10181	3	9.89E-06	0.90	4.27	-	876.4
gi|4504111|ref|NP_002077.1|	growth factor receptor-bound protein 2 [Homo sapiens]			4.23E-06	0.67	10.16	5.50	25206.2
AHQ-1-10, 3277	K.VLNEECDQNWYK.A	1599.70313	2	4.23E-06	0.67	3.06	-	502.8
gi|20127450|ref|NP_002729.2|	protein kinase C, beta 1; Protein kinase C, beta 1 polypeptide [Homo s			4.23E-06	0.51	10.13	1.80	77011.7
AHQ-1-9, 4411	K.CSLNPEWNETFR.F	1554.66558	2	4.23E-06	0.51	2.60	-	373.3
gi|7706244|ref|NP_057005.1|	divalent cation tolerant protein CUTA [Homo sapiens]			4.26E-06	0.91	10.21	17.30	16832.5
AHQ-1-13, 6464	R.SVHPYEVAEVIALPVEQGNFPYLQWVR.Q	3142.55428	3	4.26E-06	0.91	4.10	-	1084.6
gi|29736026|ref|XP_085123.3|	similar to Ras-related protein Rab-15 [Homo sapiens]			4.36E-06	0.47	10.14	5.30	23517.8
AHQ-1-11, 3324	R.IQIWDTAGQER.Y	1317.43243	1	4.36E-06	0.47	2.79	-	315.4
gi|20070272|ref|NP_057110.2|	androgen-regulated short-chain dehydrogenase/reductase 1; prostate sho			4.41E-06	1.79	20.21	10.40	35414.1
AHQ-1-9, 3463	K.GSGVTTYSVHPGTVQSELVR.H	2075.26811	2	1.45E-05	0.83	3.43	-	537.5
AHQ-1-9, 2494 - 2500	K.VVVVTGANTGIGK.E	1215.42469	2	4.41E-06	0.96	4.29	-	1583.8
gi|19913441|ref|NP_002140.2|	hippocalcin-like 1; visinin-like protein 3; calcium-binding protein BD			4.44E-06	1.81	20.21	14.00	22313.1
AHQ-1-11, 4138	R.ENTEFTDHELQEWYK.G	1970.04095	2	6.63E-05	0.93	4.14	-	943.3
AHQ-1-11, 5615	R.SEMLEIVQAIYK.M	1424.68776	2	4.44E-06	0.89	2.93	-	1056.4
gi|4557251|ref|NP_001101.1|	a disintegrin and metalloprotease domain 10 [Homo sapiens]			4.60E-06	0.52	10.15	1.90	84141.7
AHQ-1-5, 4139	R.TITLQPGSPCNDFR.G	1607.76993	2	4.60E-06	0.52	2.99	-	256.3
gi|21281669|ref|NP_060899.1|	transcription factor-like nuclear regulator; putative transcription re			4.66E-06	0.31	10.16	0.80	252842.5
AHQ-1-2, 4895	K.NNSLEIDQTENVKPMLR.G	2002.23894	2	4.66E-06	0.31	3.15	-	386.1
AHQ-1-1, 4683	K.NNSLEIDQTENVKPMLR.G	2002.23894	2	5.78E-05	0.42	3.03	-	468.8
gi|4506265|ref|NP_000954.1|	prostaglandin-endoperoxide synthase 2 precursor; prostaglandin G/H synt			4.88E-06	0.81	10.17	1.50	68995.7
AHQ-1-6, 4106	R.LILIGETIK.I	1000.25717	2	4.88E-06	0.81	3.35	-	562.3
gi|4505989|ref|NP_000299.1|	protective protein for beta-galactosidase; Protective protein for beta-			4.91E-06	1.78	20.20	5.00	54495.8
AHQ-1-12, 4594	K.EFSHIAFLTIK.G	1306.53429	2	6.76E-04	0.81	3.14	-	595.4
AHQ-1-12, 3494	K.YGDSGEQIAGFVK.E	1371.47686	2	4.91E-06	0.97	4.00	-	1828.9
AHQ-1-14- , 3599	K.YGDSGEQIAGFVK.E	1371.47686	1	1.04E-04	0.37	2.56	-	342.5
AHQ-1-11, 3406	K.YGDSGEQIAGFVK.E	1371.47686	2	3.59E-04	0.97	3.88	-	2517.7
gi|4557871|ref|NP_001054.1|	transferrin [Homo sapiens]			5.27E-06	1.57	20.16	4.20	77049.5
AHQ-1-5, 4218	K.ADRDQYELLCLDNTR.K	1884.01756	2	5.27E-06	0.68	2.72	-	702.5
AHQ-1-5, 4235	R.FDEFFSEGCAPGSK.K	1579.66868	2	3.27E-05	0.89	3.20	-	1199.5
gi|5453607|ref|NP_006420.1|	chaperonin containing TCP1, subunit 7 (eta); chaperonin containing t-co			5.29E-06	1.85	20.24	6.60	59366.3
AHQ-1-7, 5253	K.LPIGDVATQYFADR.D	1566.73935	2	1.63E-05	0.90	3.90	-	882.5
AHQ-1-7, 7276 - 7281	K.SQDAEVGDGTTSVTLLAAEFLK.Q	2253.44875	2	5.29E-06	0.95	4.87	-	838.9
gi|21361358|ref|NP_006365.2|	serine/threonine kinase 25 (STE20 homolog, yeast); sterile 20 (oxidant			5.35E-06	0.96	10.23	3.80	48111.3
AHQ-1-7, 4809	K.LADFGVAGQLTDTQIK.R	1677.88001	2	5.35E-06	0.96	4.55	-	1652.7
gi|4506189|ref|NP_002783.1|	proteasome alpha 7 subunit isoform 1; proteasome subunit RC6-1; proteas			5.45E-06	1.79	20.18	10.50	27886.7
AHQ-1-10, 2880 - 2943	K.ALLEVVQSGGK.N	1101.27845	2	5.45E-06	0.91	3.07	-	1234.3
AHQ-1-10, 3863	K.NYTDEAIETDDLTIK.L	1741.83137	2	5.97E-05	0.89	3.55	-	799.6
gi|22060072|ref|XP_088293.2|	similar to cytochrome c [Homo sapiens]			5.63E-06	1.58	20.15	18.10	11887.8
AHQ-1-13- , 4375	R.ADLIAYLK.K	907.08869	2	1.11E-05	0.89	2.95	-	948.7
AHQ-1-13, 4184	R.ADLIAYLK.K	907.08869	2	5.63E-06	0.83	2.76	-	865.6
AHQ-1-13- , 3567 - 3645	K.TGPNLHGLFGR.K	1169.31780	2	5.88E-05	0.76	2.69	-	590.3
gi|21361619|ref|NP_061882.2|	TOLLIP protein; Toll-interacting protein [Homo sapiens]			5.65E-06	1.70	20.22	9.90	30281.6
AHQ-1-11, 5463	R.GPVYIGELPQDFLR.I	1604.83072	2	5.65E-06	0.95	4.37	-	1231.1
AHQ-1-11, 4679 - 4742	R.IAWTHITIPESLR.Q	1537.78752	2	2.46E-04	0.75	2.83	-	762.0
gi|4557321|ref|NP_000030.1|	apolipoprotein A-I precursor [Homo sapiens]			5.69E-06	0.91	10.20	6.00	30777.6
AHQ-1-11, 5632 - 5643	R.EQLGPVTQEFWDNLEK.E	1934.09489	2	5.69E-06	0.91	3.91	-	711.4
gi|4505273|ref|NP_002442.1|	5'-methylthioadenosine phosphorylase [Homo sapiens]			5.79E-06	0.92	10.22	6.40	31249.9
AHQ-1-9, 5647	K.IGIIGGTGLDDPEILEGR.T	1826.04101	2	5.79E-06	0.92	4.35	-	707.8
gi|4501841|ref|NP_001596.1|	alanyl-tRNA synthetase [Homo sapiens]			5.83E-06	1.39	20.17	3.40	106800.7
AHQ-1-4, 7318	R.EIADLGEALATAVIPQWQK.D	2054.33195	2	5.83E-06	0.80	3.36	-	609.9
AHQ-1-4, 3492	K.ITCLCQVPQNAANR.G	1647.85889	2	4.44E-04	0.60	3.23	-	294.8
gi|8922804|ref|NP_060760.1|	hypothetical protein FLJ10983 [Homo sapiens]			6.01E-06	0.71	10.15	1.80	68268.9
AHQ-1-6, 2311	R.IVLANDPADR.L	1199.29585	2	6.01E-06	0.71	2.92	-	775.0
gi|4502553|ref|NP_003647.1|	calcium/calmodulin-dependent protein kinase I [Homo sapiens]			6.24E-06	0.55	10.14	4.60	41336.9
AHQ-1-9, 5487 - 5507	R.DLKPENLLYYSLDEDSK.I	2043.21643	2	6.24E-06	0.55	2.75	-	389.2
gi|21389549|ref|NP_653263.1|	hypothetical protein MGC26605 [Homo sapiens]			6.24E-06	1.31	20.17	14.90	26927.8
AHQ-1-10, 5156	R.AITVFSPDGHLFQVEYAQEAVK.K	2450.73052	2	4.40E-04	0.58	2.93	-	544.7
AHQ-1-10, 5157	R.AITVFSPDGHLFQVEYAQEAVK.K	2450.73052	3	6.24E-06	0.66	3.27	-	590.5
AHQ-1-10, 4245 - 4315	K.LTVEDPVTVEYITR.F	1635.83990	2	8.06E-05	0.65	3.17	-	640.8
gi|13027638|ref|NP_006750.2|	UDP-glucose pyrophosphorylase 2; UTP-glucose-1-phosphate uridyltransfe			6.37E-06	0.90	10.23	3.50	56964.9
AHQ-1-7, 6728	R.NENTFLDLTVQQIEHLNK.T	2157.36913	2	6.37E-06	0.90	4.52	-	856.5

gi|6005745|ref|NP_009165.1| dynactin 3 isoform 1; dynactin light chain [Homo sapiens]				6.51E-06	0.82	10.15	5.90	21119.3
AHQ-1-11, 2816	K.VQVALGNISSK.R	1116.29312	2	6.51E-06	0.82	2.93	-	671.4
gi|29734141|ref|XP_291117.1| similar to succinate dehydrogenase flavoprotein subunit [Homo sapiens]				6.60E-06	0.05	10.09	8.90	16590.8
AHQ-1-6, 2091	R.KHTLSYVDVGTGK.V	1405.58112	1	6.60E-06	0.05	1.81	-	327.2
gi|4506407|ref|NP_002873.1| RAN binding protein 1 [Homo sapiens]				6.65E-06	0.88	10.18	5.50	23310.0
AHQ-1-10, 4121	K.TLEEDEEELFK.M	1382.45159	2	6.65E-06	0.88	3.56	-	646.8
gi|13786129|ref|NP_112586.1| RAB33B, member RAS oncogene family; likely ortholog of mouse RAB33b [H				6.65E-06	0.35	10.16	4.80	25717.4
AHQ-1-13, 3485	K.IQLWDTAGQER.F	1317.43243	2	9.63E-04	0.88	3.26	-	792.9
AHQ-1-14- , 3667	K.IQLWDTAGQER.F	1317.43243	2	2.41E-05	0.90	3.14	-	1023.9
AHQ-1-11, 3439 - 3440	K.IQLWDTAGQER.F	1317.43243	1	6.65E-06	0.35	2.45	-	261.6
gi|29729329|ref|XP_291459.1| similar to Cofilin, non-muscle isoform (18 kDa phosphoprotein) (P18) [				6.88E-06	1.44	20.20	18.30	17172.9
AHQ-1-12, 5509 - 5571	K.EILVGDVGQTVNNLYATFVK.M	2181.47373	2	6.88E-06	0.92	4.09	-	965.2
AHQ-1-12, 2351	K.NIILEEGK.E	916.05399	1	3.15E-04	0.52	2.67	-	577.1
AHQ-1-12, 2345 - 2409	K.NIILEEGK.E	916.05399	1	2.37E-04	0.52	2.32	-	435.6
gi|7330335|ref|NP_039234.1| chloride intracellular channel 4; chloride intracellular channel 4 like				7.05E-06	3.00	40.18	24.50	28771.9
AHQ-1-10, 4424 - 4441	R.DEFTNTCPSDKEVEIAYSDVAK.R	2520.66487	2	1.16E-04	0.74	3.53	-	416.0
AHQ-1-10, 5393	K.FLDGNEMTLADCNLLPK.L	1953.22635	2	7.05E-06	0.69	3.18	-	505.0
AHQ-1-10, 5007	R.KFLDGNEMTLADCNLLPK.L	2081.39926	2	1.37E-05	0.83	3.65	-	711.8
AHQ-1-10, 5120	K.LDEYLNSPLPDEIDENSMEDIK.F	2580.76043	2	2.02E-04	0.74	2.83	-	530.4
gi|19923233|ref|NP_002970.2| sterol carrier protein 2 [Homo sapiens]				7.25E-06	0.85	10.18	2.20	58993.3
AHQ-1-13- , 2771 - 2836	K.LQNLQLQPGNAK.L	1324.51124	2	7.25E-06	0.85	3.55	-	811.6
gi|4501867|ref|NP_001089.1| aconitase 2 [Homo sapiens]				7.40E-06	0.85	10.19	2.10	85424.8
AHQ-1-5, 3434	K.IVYGHLDDPASQEIER.G	1842.98698	2	7.40E-06	0.85	3.77	-	644.6
gi|21614520|ref|NP_000393.2| glucose-6-phosphate dehydrogenase [Homo sapiens]				7.88E-06	2.49	30.23	9.10	59257.4
AHQ-1-14- , 5592	R.DGLLPENTFIVGYAR.S	1665.87093	2	1.18E-05	0.88	3.39	-	717.6
AHQ-1-7, 7096	R.LFYLALPPTVYEAVTK.N	1826.16735	2	7.88E-06	0.69	3.58	-	256.5
AHQ-1-7, 4473	R.LQFHDVAGDIFHQQCK.R	1945.14742	3	1.36E-05	0.92	4.53	-	855.0
AHQ-1-7, 4472	R.LQFHDVAGDIFHQQCK.R	1945.14742	2	1.62E-04	0.95	3.95	-	1207.5
gi|4505185|ref|NP_002406.1| macrophage migration inhibitory factor (glycosylation-inhibiting factor				8.17E-06	0.96	10.21	7.80	12476.2
AHQ-1-14- , 4618 - 4630	K.LLCGLLAER.L	1046.26608	2	8.17E-06	0.96	4.29	-	1328.8
gi|5902134|ref|NP_009005.1| coronin, actin binding protein, 1A; coronin, actin-binding, 1A; coronin				8.35E-06	5.26	60.24	14.30	51026.0
AHQ-1-7, 1861 - 1866	R.AAPEASGTPSSDAVSR.L	1503.55369	2	1.25E-04	0.92	4.03	-	717.9
AHQ-1-7, 2045	K.ADQCYEDVR.V	1157.19275	2	1.90E-04	0.84	2.86	-	893.8
AHQ-1-7, 4460	R.DAGPLLISLK.D	1027.23948	2	1.46E-05	0.88	3.01	-	903.5
AHQ-1-7, 4456	R.DAGPLLISLK.D	1027.23948	1	1.60E-04	0.32	2.27	-	576.0
AHQ-1-7, 2197	R.KLQATVQELQK.R	1286.50292	2	3.89E-04	0.92	3.81	-	1325.7
AHQ-1-7, 2441	K.LQATVQELQK.R	1158.33000	2	1.09E-05	0.77	3.06	-	566.3
AHQ-1-7, 7437	R.YFEITSEAPFLHYLSMFSSK.E	2398.71748	3	2.48E-05	0.92	4.44	-	838.2
AHQ-1-7, 7436	R.YFEITSEAPFLHYLSMFSSK.E	2398.71748	2	8.35E-06	0.91	4.78	-	465.3
gi|11321585|ref|NP_002065.1| guanine nucleotide-binding protein, beta-1 subunit; G protein, beta-1				8.37E-06	2.38	30.19	12.10	37376.8
AHQ-1-9, 4963	R.KACADATLSQITNNIDPVGR.I	2146.36794	2	8.37E-06	0.68	3.07	-	444.4
AHQ-1-9, 4247	K.LIIWDSYTTNK.V	1354.53258	2	1.22E-04	0.84	3.14	-	807.5
AHQ-1-9, 1859	R.LLVSASQDGK.L	1018.14631	2	2.24E-05	0.87	2.88	-	944.3
gi|4506353|ref|NP_002854.1| phosphorylase, glycogen; liver (Hers disease, glycogen storage disease				8.61E-06	0.85	10.16	1.70	97147.8
AHQ-1-5, 5673 - 5691	K.LVIDQIDNGFFSPK.Q	1593.80471	2	8.61E-06	0.85	3.15	-	936.7
gi|21450721|ref|NP_659447.1| hypothetical protein MGC26778 [Homo sapiens]				8.81E-06	0.02	10.12	3.10	29454.0
AHQ-1-14- , 4064	K.KAQEDYDR.Y	1025.05426	1	8.81E-06	0.02	1.81	-	70.1
gi|27597085|ref|NP_000357.3| tropomyosin 1 (alpha) [Homo sapiens]				8.82E-06	2.44	30.17	8.50	32865.7
AHQ-1-9, 3164	R.KLVIIESDLER.A	1315.54103	1	2.89E-04	0.30	1.84	-	379.0
AHQ-1-9, 3150	R.KLVIIESDLER.A	1315.54103	2	8.82E-06	0.88	3.24	-	1004.1
AHQ-1-10, 3541	K.LVIIESDLER.A	1187.36811	2	2.05E-04	0.85	2.88	-	936.6
AHQ-1-10, 2049	K.YSQKEDRYEEEIK.V	1717.81473	2	2.10E-04	0.71	3.21	-	351.4
gi|30148492|ref|XP_293610.2| similar to Heat shock cognate 71 kDa protein [Homo sapiens]				8.94E-06	2.65	30.22	4.40	72113.4
AHQ-1-6, 4342	K.DAGTIAGLNVLR.I	1200.37012	2	3.49E-04	0.93	3.63	-	1249.6
AHQ-1-10, 4285 - 4361	R.IINEPTAAAIAYGLDK.K	1660.89259	2	8.94E-06	0.80	3.49	-	895.1
AHQ-1-6, 4291 - 4302	R.IINEPTAAAIAYGLDKK.V	1789.06550	3	1.69E-05	0.75	3.33	-	530.2
AHQ-1-7, 4377	R.IINEPTAAAIAYGLDKK.V	1789.06550	2	3.11E-04	0.64	2.94	-	422.3
AHQ-1-7, 4386	R.IINEPTAAAIAYGLDKK.V	1789.06550	3	3.63E-04	0.68	3.28	-	384.1
AHQ-1-6, 4287	R.IINEPTAAAIAYGLDKK.V	1789.06550	2	4.03E-05	0.83	3.74	-	693.4
AHQ-1-6, 4232 - 4303	R.IINEPTAAAIAYGLDKK.V	1789.06550	2	9.15E-06	0.92	4.44	-	612.5
gi|7705704|ref|NP_057001.1| glutathione S-transferase subunit 13 homolog [Homo sapiens]				8.95E-06	1.84	20.24	13.70	25496.7
AHQ-1-11, 4392	R.FLTAVNLEHPEMLEK.A	1772.05845	2	2.84E-05	0.96	4.70	-	1548.1
AHQ-1-11, 4382	R.NEDITEPQSILAAAEK.A	1729.86695	2	8.95E-06	0.88	3.71	-	613.3
gi|4503141|ref|NP_001805.1| cathepsin C isoform a preproprotein; dipeptidyl-peptidase I; dipeptidyl				9.04E-06	1.81	20.22	4.30	51841.5
AHQ-1-14- , 5427 - 5450	R.GTDECAIESIAVAATPIPK.L	1945.18072	2	9.04E-06	0.95	4.45	-	1210.4
AHQ-1-14, 5953	R.RGTDECAIESIAVAATPIPK.L	2101.36707	2	7.26E-05	0.86	4.00	-	606.1
gi|14149777|ref|NP_115500.1| hypothetical protein DKFZp564D1378 [Homo sapiens]				9.28E-06	0.96	10.26	8.10	28535.7
AHQ-1-10, 6301 - 6373	K.DGLALGPGPFVTALEYATDTK.A	2137.37459	2	9.28E-06	0.96	5.30	-	968.1
gi|21536449|ref|NP_002613.2| prefoldin 1; prefoldin subunit 1 [Homo sapiens]				9.62E-06	0.97	10.22	9.00	14210.4
AHQ-1-13- , 3917	K.LADIQIEQLNR.T	1313.48523	2	9.62E-06	0.97	4.35	-	1992.5
AHQ-1-13, 3783	K.LADIQIEQLNR.T	1313.48523	2	8.40E-05	0.95	4.50	-	1455.7
gi|4506191|ref|NP_002792.1| proteasome beta 10 subunit proprotein; proteasome subunit MECL1; protea				9.73E-06	0.90	10.22	7.30	28936.1
AHQ-1-13, 6089 - 6095	R.LPFTALGSGQDAALAVLEDR.F	2045.28192	2	4.14E-04	0.96	4.43	-	1443.1
AHQ-1-10, 5868	R.LPFTALGSGQDAALAVLEDR.F	2045.28192	2	9.73E-06	0.90	3.72	-	837.2
gi|4505591|ref|NP_002565.1| peroxiredoxin 1; natural killer-enhancing factor A; proliferation-assoc				9.73E-06	2.40	30.16	14.60	22110.2
AHQ-1-11, 3782	R.GLFIIDDK.G	921.07223	1	6.70E-04	0.80	3.11	-	1103.6
AHQ-1-11, 3518 - 3543	R.LVQAFQFTDK.H	1197.36469	2	9.73E-06	0.89	3.09	-	1066.8
AHQ-1-11, 3363	R.QITVNDLPVGR.S	1212.38090	2	5.80E-04	0.71	3.00	-	404.9
gi|20270343|ref|NP_620150.1| hypothetical protein BC015408 [Homo sapiens]				9.86E-06	0.88	10.22	12.40	21415.8
AHQ-1-11, 5658 - 5662	K.NELHNLLDKPQLQGIPVLVLGNK.R	2553.98492	3	9.86E-06	0.88	4.37	-	343.3
gi|20543399|ref|XP_116396.1| similar to peptidylprolyl isomerase A (cyclophilin A) [Homo sapiens] [				9.89E-06	0.86	10.17	5.00	19852.7
AHQ-1-12, 4335 - 4398	R.VSFKLFADK.F	1055.25138	2	9.89E-06	0.86	3.48	-	957.3
gi|18558709|ref|XP_087572.1| similar to bA291L22.2 (similar to CDC10 (cell division cycle 10, S. ce				9.94E-06	0.96	10.18	10.90	9988.5
AHQ-1-8, 3090	K.FEDYLNAESR.V	1244.29194	2	9.94E-06	0.96	3.58	-	1711.8
gi|4502211|ref|NP_001654.1| ADP-ribosylation factor 6 [Homo sapiens]				1.00E-05	3.26	40.20	24.60	20082.0
AHQ-1-12, 3998	K.FNVWDVGGQDK.I	1265.35598	2	1.00E-05	0.78	2.87	-	862.2
AHQ-1-12, 4193	R.ILMLGLDAAGK.T	1102.37299	2	1.59E-05	0.96	3.94	-	1508.0
AHQ-1-12, 3678	R.ILM*LGLDAAGK.T	1118.37239	2	1.90E-04	0.96	3.36	-	1998.1
AHQ-1-12, 5366	K.LGQSVTTIPTVGFNVETVTYK.N	2255.55261	2	1.07E-05	0.57	3.14	-	170.5
gi|4758038|ref|NP_004246.1| cytochrome c oxidase subunit Va precursor; cytochrome c oxidase polypep				1.02E-05	1.25	20.21	20.70	16774.1
AHQ-1-14, 5745	K.GINTLVTYDMVPEPK.I	1677.94342	2	4.87E-05	0.59	2.51	-	764.4
AHQ-1-13, 4677 - 4736	K.GINTLVTYDMVPEPK.I	1677.94342	2	7.85E-04	0.93	4.02	-	1131.9
AHQ-1-13, 5592	R.WVTYFNKPDIDAWELR.K	2054.29220	2	1.02E-05	0.66	3.20	-	306.9
gi|21361456|ref|NP_055425.2| alpha glucosidase II alpha subunit; likely ortholog of mouse G2an alph				1.02E-05	1.78	20.20	4.60	109437.2
AHQ-1-4, 6655	R.FSFSGNTLVSSSADPEGHFETPIWIER.V	3012.23437	3	9.23E-05	0.85	3.58	-	964.9
AHQ-1-5, 3871	R.VVIIGAGKPAAVVLQTK.G	1665.05702	2	1.02E-05	0.93	3.83	-	1010.5
AHQ-1-4, 4047	R.VVIIGAGKPAAVVLQTK.G	1665.05702	2	1.13E-05	0.90	3.97	-	879.1
gi|20551618|ref|XP_167021.1| similar to calponin 2; Calonin 2 [Homo sapiens]				1.03E-05	0.78	10.16	3.70	47327.9
AHQ-1-12, 5218	R.SM*QNWHQLENLSNFIK.A	2006.23095	3	1.03E-05	0.78	3.25	-	819.4
gi|23510451|ref|NP_001631.3| N-acylaminoacyl-peptide hydrolase; oxidized protein hydrolase; acylami				1.04E-05	0.90	10.15	1.80	81224.1
AHQ-1-6, 2960	K.ALDVSASDDEIAR.L	1362.42514	2	1.04E-05	0.90	2.91	-	1229.1
gi|21361478|ref|NP_055944.2| septin 6 isoform B; septin 2 [Homo sapiens]				1.08E-05	1.91	20.28	9.40	49716.3
AHQ-1-7, 3005	R.KTAAELLQSQGSQAGGSQTLK.R	2104.30776	2	1.08E-05	0.97	5.65	-	1322.8
AHQ-1-7, 5216	R.TVPLAGHVGFDSLPDQLVNK.S	2108.38284	2	5.48E-05	0.94	4.59	-	811.8
gi|29727204|ref|XP_290872.1| similar to Pyruvate kinase, M2 isozyme [Homo sapiens]				1.10E-05	1.80	20.21	14.80	16297.7
AHQ-1-7, 2297	R.APIIAVTR.N	841.03348	2	2.19E-04	0.90	3.04	-	973.2
AHQ-1-7, 5454	K.KGDVVIVLTGWRPGSGFTNTMR.V	2392.76561	3	1.10E-05	0.90	4.28	-	1004.0

gi|4758440|ref|NP_004868.1|	glia maturation factor, gamma [Homo sapiens]			1.10E-05	0.93	10.23	9.20	16801.2
AHQ-1-12, 4297	R.TTDDLTEAWLQEK.L	1550.64872	2	1.10E-05	0.93	4.04	-	1349.2
gi|10800412|ref|NP_036458.2|	microtubule-associated protein, RP/EB family, member 3; APC binding pr			1.13E-05	0.69	10.14	3.60	31982.0
AHQ-1-9, 2108	K.LEHEYIHNFK.V	1330.47280	2	1.13E-05	0.69	2.71	-	824.4
gi|5174539|ref|NP_005908.1|	cytosolic malate dehydrogenase; soluble malate dehydrogenase [Homo sapi			1.19E-05	1.88	20.22	9.00	36425.9
AHQ-1-13, 3065	K.DVIATDKEDVAFK.D	1451.60343	2	1.19E-05	0.94	3.54	-	1367.6
AHQ-1-9, 3594	K.VIVVGNPANTNCLTASK.S	1760.00585	2	9.80E-05	0.95	4.41	-	912.6
gi|11993943|ref|NP_055419.1|	Tax interaction protein 1 [Homo sapiens]			1.21E-05	0.96	10.23	13.70	13734.6
AHQ-1-13, 4072 - 4079	R.VSEGGPAEIAGLQIGDK.I	1641.80454	2	1.21E-05	0.96	4.68	-	1555.4
gi|4502981|ref|NP_001852.1|	cytochrome c oxidase subunit IV isoform 1 precursor; cytochrome c oxida			1.29E-05	2.48	30.18	21.30	19576.6
AHQ-1-13- , 2320	R.DHPLPEVAHVK.H	1242.40869	3	9.47E-05	0.91	3.53	-	1240.9
AHQ-1-12, 3534	K.HYVYGPLPQSFDK.E	1551.72638	2	1.29E-05	0.92	3.57	-	911.5
AHQ-1-13- , 3963	K.SEDFSLPAYM*DR.R	1447.55197	2	2.20E-04	0.66	2.73	-	358.5
gi|5729873|ref|NP_006656.1|	heparanase; heparanase-1 [Homo sapiens]			1.33E-05	0.95	10.19	1.70	61176.4
AHQ-1-14- , 5010	K.TDFLIFDPK.K	1096.25713	2	1.33E-05	0.95	3.89	-	1438.3
AHQ-1-14- , 5008	K.TDFLIFDPK.K	1096.25713	1	1.86E-04	0.44	2.27	-	536.6
gi|4557469|ref|NP_001273.1|	adaptor-related protein complex 2, beta 1 subunit; adaptin, beta 2 (bet			1.34E-05	0.93	10.21	1.30	104551.9
AHQ-1-6, 6235	R.CVSTLLDLIQTK.V	1392.64385	2	1.34E-05	0.93	4.20	-	945.5
gi|19923262|ref|NP_004153.2|	RAB5A, member RAS oncogene family; RAS-associated protein RAB5A [Homo			1.34E-05	1.79	20.17	10.20	23658.5
AHQ-1-10, 3676 - 3735	K.FEIWDTAGQER.Y	1352.43364	2	2.72E-04	0.90	2.52	-	1523.2
AHQ-1-10, 3319 - 3323	K.LVLLGESAVGK.S	1086.30692	2	1.34E-05	0.89	3.40	-	1037.6
gi|30149697|ref|XP_070805.2|	similar to prohibitin [Homo sapiens]			1.36E-05	0.93	10.18	3.30	33918.9
AHQ-1-10, 3456	K.DLQNVNLTLR.I	1186.34344	2	1.36E-05	0.93	3.67	-	1142.5
gi|21070997|ref|NP_003147.2|	stromal interaction molecule 1 precursor [Homo sapiens]			1.43E-05	0.94	10.21	2.30	77422.9
AHQ-1-7, 3650	K.ELESHSSWYAPEALQK.W	1876.01544	2	1.43E-05	0.94	4.28	-	974.8
gi|4505357|ref|NP_002480.1|	NADH dehydrogenase (ubiquinone) 1 alpha subcomplex, 4, 9kDa; NADH dehyd			1.44E-05	0.89	10.20	14.80	9369.8
AHQ-1-14- , 5555 - 5615	R.LALFNPDVCWDR.N	1507.69520	2	1.44E-05	0.89	3.99	-	577.5
gi|4557385|ref|NP_000055.1|	complement component 3 precursor; acylation-stimulating protein cleavag			1.48E-05	0.95	10.22	1.30	187162.3
AHQ-1-6, 4652	R.VPVAVQGEDTVQSLTQGDGVAK.L	2199.40432	2	1.48E-05	0.95	4.37	-	1062.2
gi|4502107|ref|NP_001145.1|	annexin V; endonexin II; anchorin CII; lipocortin V; placental anticoag			1.49E-05	1.48	20.19	8.80	35936.5
AHQ-1-9, 6964	K.GLGTDEESILTLLTSR.S	1705.88853	2	1.49E-05	0.89	3.77	-	808.4
AHQ-1-9, 3878	R.GTVTDFPGFDER.A	1341.40762	2	8.18E-04	0.60	2.55	-	460.6
gi|18079216|ref|NP_065815.1|	CASK-interacting protein 1 [Homo sapiens]			1.50E-05	0.26	10.19	1.30	149812.6
AHQ-1-1, 6939 - 7007	R.PGSPQALGGPHGPAPATAK.V	1711.90292	2	1.50E-05	0.26	2.58	-	427.1
gi|22538465|ref|NP_002786.2|	proteasome beta 3 subunit; proteasome theta chain; proteasome chain 13			1.52E-05	1.87	20.22	16.60	22948.8
AHQ-1-10, 4784 - 4789	R.FGPYYTEPVIAGLDPK.T	1768.00162	2	1.52E-05	0.91	3.68	-	1132.7
AHQ-1-10, 5793	R.LYIGLAGLATDVQTVAQR.L	1890.17306	2	5.28E-05	0.96	4.45	-	1892.3
gi|4507747|ref|NP_003321.1|	thioredoxin reductase 1 [Homo sapiens]			1.54E-05	0.82	10.21	7.20	54419.1
AHQ-1-7, 7457	K.IPVTDEEQTNVPYIYAIGDILEDKVELTPVAIQAGR.L	3972.44634	3	1.54E-05	0.82	4.18	-	373.5
gi|4759270|ref|NP_004613.1|	translin; recombination hotspot associated factor; recombination hotspo			1.58E-05	0.83	10.18	6.60	26182.8
AHQ-1-10, 3919	R.EAVTEILGIEPDREK.G	1699.88399	2	1.58E-05	0.83	3.62	-	781.5
gi|6857820|ref|NP_005019.2|	phosphatidylinositol-4-phosphate 5-kinase type II alpha; 1-phosphatidyl			1.73E-05	0.79	10.15	3.40	46224.3
AHQ-1-7, 4764 - 4766	R.FGIDDQDFQNSLTR.S	1656.73462	2	1.73E-05	0.79	3.06	-	904.3
gi|7706675|ref|NP_057661.1|	RAB6B, member RAS oncogene family; small GTPase RAB6B [Homo sapiens] [M			1.75E-05	2.28	30.23	16.30	23461.5
AHQ-1-11, 4515	R.GSDVIIMLVGNK.T	1246.50220	2	2.19E-04	0.98	4.60	-	2401.4
AHQ-1-11, 3400 - 3458	R.LQLWDTAGQER.F	1317.43243	2	1.75E-05	0.88	3.05	-	1003.3
AHQ-1-11, 3247 - 3319	R.LQLWDTAGQER.F	1317.43243	2	4.84E-05	0.88	3.40	-	763.2
AHQ-1-10, 3200 - 3255	R.LQLWDTAGQER.F	1317.43243	2	4.23E-05	0.83	3.07	-	786.3
AHQ-1-11, 3514	K.LVFLGEQSVGK.T	1177.37492	1	5.17E-04	0.43	2.38	-	428.5
gi|21361409|ref|NP_036515.2|	osteoclast stimulating factor 1 [Homo sapiens]			1.76E-05	0.31	10.13	11.50	24063.9
AHQ-1-10, 5008	R.TGLIPSNYVAEQAESIDNPLHEAAK.R	2668.89701	2	1.76E-05	0.31	2.69	-	275.6
gi|22538814|ref|NP_002976.2|	small inducible cytokine A5 precursor; T-cell specific protein p288; T			1.79E-05	0.87	10.15	12.10	9989.6
AHQ-1-14- , 3832	K.CSNPAVVFVTR.K	1251.43718	2	1.79E-05	0.87	2.97	-	793.3
gi|23110944|ref|NP_002782.1|	proteasome alpha 6 subunit; prosomal P27K protein; proteasome subunit			1.80E-05	0.69	10.14	3.70	27399.3
AHQ-1-10, 3580	R.LYQVEYAFK.A	1161.33081	2	1.80E-05	0.69	2.83	-	593.6
gi|29736622|ref|XP_293924.1|	similar to cytoplasmic beta-actin [Homo sapiens]			1.83E-05	1.52	20.25	9.80	42002.9
AHQ-1-8, 1477 - 1512	K.CDVDIRK.D	907.02724	2	4.46E-04	0.76	2.60	-	604.3
AHQ-1-8, 6956 - 7030	R.FRCPEAIFQPSFLGIESSGIHETTFNSIMK.C	3446.89713	3	1.83E-05	0.76	3.53	-	414.1
gi|7705501|ref|NP_057546.1|	hypothetical protein HSPC194 [Homo sapiens]			1.89E-05	1.90	20.21	15.20	11564.5
AHQ-1-14- , 6320 - 6394	K.FMPAGLIAGASLLM*VAK.V	1707.13765	2	8.37E-04	0.94	4.12	-	946.6
AHQ-1-14- , 5910 - 5979	K.FM*PAGLIAGASLLM*VAK.V	1723.13705	2	1.89E-05	0.97	3.83	-	2129.8
gi|29743626|ref|XP_297123.1|	hypothetical protein XP_297123 [Homo sapiens]			1.89E-05	0.63	10.15	13.80	13908.9
AHQ-1-13, 6477	R.M*EQKNVRSNNVWELAK.I	1963.20761	2	1.89E-05	0.63	2.94	-	741.1
gi|4505237|ref|NP_002427.1|	palmitoylated membrane protein 1; membrane protein, palmitoylated 1 (55			1.91E-05	0.91	10.18	2.60	52296.2
AHQ-1-9, 4958	K.IAILDIEPQTLK.I	1354.61716	2	1.91E-05	0.91	3.57	-	1002.3
gi|5803009|ref|NP_006786.1|	EH-domain containing 1; homolog of Drosophila past; EH domain containin			2.11E-05	0.95	10.22	2.80	60844.7
AHQ-1-7, 5840 - 5913	K.LLDTVDDMLANDIAR.L	1675.88593	2	2.11E-05	0.95	3.94	-	1763.9
gi|4503377|ref|NP_001377.1|	dihydropyrimidinase-like 2; collapsin response mediator protein hCRMP-2			2.22E-05	1.45	20.16	7.30	62293.3
AHQ-1-7, 4245	R.GLYDGPVCEVSVTPK.T	1622.82128	2	2.22E-05	0.76	2.87	-	632.7
AHQ-1-7, 5314	R.ILDLGITGPEGHVLSRPEEVEAEAVNR.A	2902.20927	3	8.18E-05	0.69	3.28	-	506.2
gi|4759274|ref|NP_004777.1|	thioredoxin-like, 32kDa; thioredoxin-related 32 kDa protein; thioredoxi			2.27E-05	0.90	10.18	5.20	32251.1
AHQ-1-9, 3083	R.IDQYQGADAVGLEEK.I	1636.74162	2	2.27E-05	0.90	3.63	-	1107.6
gi|8922712|ref|NP_060713.1|	hypothetical protein FLJ10849 [Homo sapiens]			2.28E-05	1.57	20.18	8.60	49397.9
AHQ-1-7, 2546	K.AAAQLLQSQAQQSGAQQTK.K	1958.12276	2	2.53E-04	0.84	3.64	-	479.6
AHQ-1-7, 3649	K.DTDPDSKPFSLQETYEAK.R	2072.17149	2	2.28E-05	0.74	3.03	-	347.7
gi|4506417|ref|NP_002877.1|	RAP2B, member of RAS oncogene family [Homo sapiens]			2.30E-05	4.03	50.23	35.00	20494.2
AHQ-1-11, 4416	K.ALAEEWSCPFM*ETSAK.N	1875.06928	2	4.12E-04	0.49	2.92	-	531.3
AHQ-1-11, 4914 - 4916	K.ALAEEWSCPFMETSAK.N	1859.06988	2	4.82E-05	0.92	4.32	-	766.2
AHQ-1-11, 5838	K.ASVDELFAEIVR.Q	1349.51434	2	2.30E-05	0.89	3.71	-	1059.5
AHQ-1-11, 6294	K.EIEVDSSPSVLEILDTAGTEQFASM*R.D	2842.08287	3	9.85E-05	0.89	4.52	-	850.1
AHQ-1-11, 4078	K.YDPTIEDFYR.K	1319.40042	2	1.48E-04	0.84	2.88	-	414.4
gi|10190678|ref|NP_065691.1|	RP42 homolog; squamous cell carcinoma-related oncogene [Homo sapiens]			2.30E-05	0.87	10.21	6.60	30124.1
AHQ-1-10, 5708	K.LDVATDNFFQNPELYIR.E	2056.26326	2	2.30E-05	0.87	4.28	-	601.0
gi|21361565|ref|NP_001679.2|	ATP synthase, H+ transporting, mitochondrial F0 complex, subunit b, is			2.34E-05	1.81	20.19	9.00	28908.4
AHQ-1-11, 2903	R.HYLFDVQR.N	1078.20513	2	2.34E-05	0.95	3.43	-	1422.3
AHQ-1-11, 6510	R.YGLIPEEFFQFLYPK.T	1892.18434	2	3.43E-05	0.86	3.76	-	387.8
gi|4757818|ref|NP_004879.1|	ATPase, H+ transporting, lysosomal, V1 subunit G isoform 1; vacuolar H(			2.54E-05	0.92	10.18	9.30	13757.4
AHQ-1-13- , 2856 - 2863	K.EEAQAEIEQYR.L	1366.41548	2	2.54E-05	0.92	3.67	-	1083.6
gi|18543899|ref|XP_086916.1|	similar to Phosphatidylethanolamine-binding protein (PEBP) (Prostatic			2.64E-05	0.85	10.21	11.80	21265.8
AHQ-1-11, 5002	R.YVWLVYEQDRPLKCDEPILSNR.S	2796.15005	3	2.64E-05	0.85	4.12	-	590.6
gi|4507657|ref|NP_003282.1|	tripeptidyl peptidase II [Homo sapiens]			2.66E-05	1.57	20.22	2.70	138447.8
AHQ-1-3, 4615	K.IVDIIDTTGSGDVNTATEVEPK.D	2275.45273	2	8.10E-04	0.95	4.33	-	1626.8
AHQ-1-3, 3379	R.LSTMETGTGLIR.A	1279.48905	2	2.66E-05	0.62	2.63	-	719.3
gi|13376282|ref|NP_079131.1|	hypothetical protein FLJ12785 [Homo sapiens]			2.72E-05	0.77	10.14	0.00	68298.1
AHQ-1-6, 3996	K.YPGHLAAITLSR.M	1299.50338	2	2.80E-04	0.54	2.52	-	776.0
AHQ-1-4, 4118 - 4135	K.YPGHLAAITLSR.M	1299.50338	2	2.72E-05	0.77	2.73	-	932.6
gi|5031691|ref|NP_004640.1|	chromosome 21 open reading frame 33; human HES1 protein, homolog to E.c			2.75E-05	1.83	20.24	19.00	28142.2
AHQ-1-11, 3791	R.GVEVTVGHEQEEGGKWPYAGTAEAIK.A	2743.96557	3	1.19E-04	0.92	4.88	-	857.8
AHQ-1-11, 5068	K.ITDLANLSAANHDAAIFPGGFGAAK.N	2443.69961	2	2.75E-05	0.91	4.35	-	635.0
AHQ-1-11, 5063	K.ITDLANLSAANHDAAIFPGGFGAAK.N	2443.69961	3	2.81E-04	0.85	3.95	-	896.5
gi|19913446|ref|NP_002134.2|	hippocalcin; neuron specific calcium-binding protein hippocalcin [Homo			2.79E-05	1.21	20.15	7.80	22427.3
AHQ-1-11, 2260	K.FAEHVFR.T	906.02258	1	2.79E-05	0.28	1.84	-	438.6
AHQ-1-11, 4807	K.LSLEEFIR.G	1007.16512	2	5.71E-04	0.93	3.04	-	1598.5
gi|21359955|ref|NP_076982.2|	SECIS binding protein 2 [Homo sapiens]			2.91E-05	0.17	10.15	2.10	95477.3
AHQ-1-12, 6026 - 6041	K.NVSIPSSEALSSDPSYNK.E	1896.00183	2	2.91E-05	0.17	2.61	-	295.6
gi|24308263|ref|NP_067045.1|	TcD37 homolog; prune [Homo sapiens]			2.93E-05	0.96	10.25	3.80	50199.3
AHQ-1-7, 7605	K.LLPLLQEALSAYFDSMK.I	1940.29226	2	2.93E-05	0.96	4.94	-	1415.5

gi|4504051|ref|NP_002064.1|	guanine nucleotide binding protein, alpha z polypeptide; transducin alp			3.00E-05	1.60	20.19	9.00	40923.5
AHQ-1-9, 5494 - 5500	R.AYDAVQLFALTGPAESK.G	1781.98669	2	3.00E-05	0.76	3.28	-	689.3
AHQ-1-9, 6627 - 6646	R.IAAADYIPTVEDILR.S	1660.89259	2	1.17E-04	0.83	3.84	-	354.4
gi|16950601|ref|NP_005956.2|	myosin light chain kinase isoform 6; myosin light chain kinase [Homo s			3.30E-05	1.62	20.17	2.90	110074.8
AHQ-1-12, 5402	K.IEGYPDPEVVWFK.D	1579.77650	2	1.32E-04	0.90	3.35	-	532.2
AHQ-1-6, 5080	R.STSFNVQDLLPDHEYK.F	1894.03076	2	3.30E-05	0.72	3.34	-	378.2
gi|4758158|ref|NP_004395.1|	neural precursor cell expressed, developmentally down-regulated 5 [Homo			3.30E-05	0.88	10.19	4.40	41487.2
AHQ-1-9, 6943	K.STLINSLFLTDLYPER.V	1883.13416	2	3.30E-05	0.88	3.85	-	784.4
gi|4506405|ref|NP_002872.1|	v-ral simian leukemia viral oncogene homolog B; RAS-like protein B; GTP			3.32E-05	2.79	30.25	15.50	23408.4
AHQ-1-10, 3761	K.AEEWGVQYVETSAK.T	1597.70708	2	5.05E-04	0.87	3.25	-	776.2
AHQ-1-10, 3345 - 3420	R.SKAEEWGVQYVETSAK.T	1812.95765	2	3.32E-05	0.97	5.01	-	1430.4
AHQ-1-10, 3332	R.VKAEEDKIPLLVVGNK.S	1753.07630	2	2.29E-04	0.96	4.90	-	1181.3
gi|4506285|ref|NP_003470.1|	protein tyrosine phosphatase type IVA, member 2 isoform 1; protein tyro			3.36E-05	0.93	10.17	8.40	19127.1
AHQ-1-11, 2895	R.FLITHNPTNATLNK.F	1584.80104	2	3.36E-05	0.93	3.46	-	1565.0
gi|5454152|ref|NP_006285.1|	ubiquinol-cytochrome c reductase binding protein [Homo sapiens]			3.37E-05	0.97	10.24	11.70	13530.4
AHQ-1-13- , 5352 - 5359	K.YEEENFYLEPYLK.E	1737.88601	2	3.37E-05	0.97	4.84	-	1798.2
gi|19923231|ref|NP_002860.2|	RAB6A, member RAS oncogene family; Oncogene RAB6; RAB6, member RAS onc			3.47E-05	0.73	10.17	6.70	23548.6
AHQ-1-11, 2870	R.VAAALPGMESTQDR.S	1446.61192	2	3.47E-05	0.73	3.00	-	456.5
gi|18105037|ref|NP_004709.2|	cytochrome c oxidase subunit VIIa polypeptide 2 like; estrogen recepto			3.55E-05	0.89	10.21	28.90	12614.6
AHQ-1-14- , 6048	K.LAGAWASEAYSPQGLKPVVSTEAPPIIFATPTK.L	3399.87995	3	3.55E-05	0.89	4.20	-	851.6
gi|4758032|ref|NP_004757.1|	coatomer protein complex, subunit beta 2 (beta prime); coatomer binding			3.61E-05	0.80	10.17	2.10	102486.5
AHQ-1-5, 6542	K.AAESLADPTEYENLFPGLK.E	2066.25322	2	3.61E-05	0.80	3.47	-	532.7
gi|4758504|ref|NP_004484.1|	hydroxyacyl-Coenzyme A dehydrogenase, type II [Homo sapiens]			3.64E-05	1.53	20.21	16.50	26922.9
AHQ-1-10, 4663	R.LVGQGASAVLLDLPNSGGEAQAK.K	2196.44680	2	3.64E-05	0.66	3.16	-	338.4
AHQ-1-10, 4463	R.LVGQGASAVLLDLPNSGGEAQAK.K	2196.44680	2	2.63E-04	0.89	4.17	-	669.8
AHQ-1-13- , 6827	R.VMTIAPGLFGTPLLTSLPEK.V	2086.52363	2	5.29E-05	0.87	3.71	-	695.4
gi|6005902|ref|NP_009043.1|	thrombospondin 3 [Homo sapiens]			3.70E-05	0.77	10.14	0.70	104200.6
AHQ-1-2, 4925	R.FYVVMWK.Q	973.21547	2	1.82E-04	0.92	2.80	-	961.8
AHQ-1-3, 4747	R.FYVVMWK.Q	973.21547	2	5.48E-04	0.81	2.67	-	664.9
AHQ-1-6, 4536	R.FYVVMWK.Q	973.21547	1	2.83E-04	0.80	2.10	-	1059.8
AHQ-1-7, 4566	R.FYVVMWK.Q	973.21547	1	3.70E-05	0.77	2.19	-	1001.3
AHQ-1-11, 4287	R.FYVVMWK.Q	973.21547	1	7.32E-04	0.75	2.00	-	1098.1
AHQ-1-2, 4906 - 4917	R.FYVVMWK.Q	973.21547	1	1.43E-04	0.74	2.03	-	991.5
gi|4826643|ref|NP_005130.1|	annexin A3; Annexin III (lipocortin III); annexin III (lipocortin III,			3.87E-05	0.87	10.20	5.00	36375.0
AHQ-1-9, 6155	K.GAGTNEDALIEILTTR.T	1674.83453	2	3.87E-05	0.87	3.90	-	709.3
gi|30149555|ref|XP_301154.1|	similar to heat shock protein 86 [Homo sapiens]			3.91E-05	0.96	10.23	10.60	15781.6
AHQ-1-5, 5958	K.HSQFIGYPITLFVEK.E	1780.05877	2	3.91E-05	0.96	4.66	-	1193.2
gi|5453710|ref|NP_006139.1|	LIM and SH3 protein 1; Lasp-1 [Homo sapiens]			3.92E-05	0.71	10.16	5.00	29717.0
AHQ-1-13- , 4084	K.GFSVVADTPELQR.I	1419.56466	2	3.92E-05	0.71	2.65	-	855.9
AHQ-1-9, 3903 - 3912	K.GFSVVADTPELQR.I	1419.56466	1	9.70E-05	0.22	2.19	-	231.6
gi|4502923|ref|NP_001830.1|	calponin 3; calponin, acidic [Homo sapiens]			4.08E-05	0.90	10.16	3.30	36413.4
AHQ-1-12, 2641 - 2647	K.GPSYGLSAEVK.N	1108.22631	2	4.08E-05	0.90	3.16	-	888.6
gi|29728979|ref|XP_060316.2|	similar to Olfactory receptor 2T1 (Olfactory receptor 1-25) (OR1-25) [			4.39E-05	0.23	10.13	2.10	75271.9
AHQ-1-13- , 4620	R.FKGPQRVSGELWSR.A	1647.86206	2	4.39E-05	0.23	2.57	-	249.8
gi|4502989|ref|NP_001856.1|	cytochrome c oxidase subunit VIIa polypeptide 2 (liver) precursor; hepa			4.43E-05	0.95	10.19	15.70	9395.9
AHQ-1-14- , 5314 - 5370	K.LFQEDDEIPLYLK.G	1623.82758	2	4.43E-05	0.95	3.81	-	1547.4
gi|5729779|ref|NP_006701.1|	COP9 homolog [Homo sapiens]			4.61E-05	0.97	10.20	6.20	23225.5
AHQ-1-11, 2987	R.KPVAGALDVSFNK.F	1346.55682	2	4.61E-05	0.97	4.07	-	1703.8
gi|23200010|ref|NP_112585.2|	Williams Beuren syndrome chromosome region 21 isoform 4 [Homo sapiens]			4.69E-05	0.84	10.21	20.80	10639.1
AHQ-1-10, 6432	R.LLDGEAALPAVVFLHGLFGSK.T	2155.52425	2	4.69E-05	0.84	4.11	-	271.0
gi|4503785|ref|NP_000135.1|	frataxin; Friedreich ataxia (frataxin) [Homo sapiens]			4.78E-05	0.96	10.21	5.70	23218.1
AHQ-1-13- , 3869	K.LGGDLGTYVINK.Q	1250.42590	2	4.78E-05	0.96	4.13	-	1346.1
AHQ-1-13, 3741	K.LGGDLGTYVINK.Q	1250.42590	2	9.91E-05	0.93	3.81	-	985.6
gi|6996014|ref|NP_002100.2|	histidyl-tRNA synthetase; histidine-tRNA ligase; HisRS; histidine trans			4.80E-05	0.73	10.13	2.60	57410.2
AHQ-1-7, 2618	R.TTETQVLVASAQK.K	1376.53809	2	4.80E-05	0.73	2.68	-	1077.3
gi|17482508|ref|XP_058957.1|	similar to QIL1 [Homo sapiens]			4.87E-05	0.97	10.26	18.60	13087.2
AHQ-1-14- , 5683 - 5688	K.GSVAGGAVYLVYDQELLGPSDK.S	2239.46691	2	4.87E-05	0.97	5.27	-	1439.5
gi|4506237|ref|NP_002809.1|	proteasome (prosome, macropain) activator subunit 2 (PA28 beta); Protea			5.03E-05	0.89	10.22	6.70	27348.4
AHQ-1-10, 6275	K.VLSLLALVKPEVWTLK.E	1810.25573	2	5.03E-05	0.89	4.35	-	659.5
gi|4505621|ref|NP_002558.1|	prostatic binding protein; phosphatidylethanolamine binding protein [Ho			5.03E-05	2.85	30.25	37.40	21056.6
AHQ-1-11, 4762	R.APVAGTCYQAEWDDYVPK.L	2072.23961	2	5.03E-05	0.95	4.22	-	1440.3
AHQ-1-11, 4183	K.GNDISSGTVLSDYVGSGPPK.G	1951.08062	2	5.98E-05	0.95	4.29	-	1206.3
AHQ-1-11, 5111 - 5178	K.WSGPLSLQEVDEQPQHPLHVTYAGAAVDELGK.V	3473.79274	3	6.42E-04	0.95	5.03	-	1455.9
gi|4758784|ref|NP_004540.1|	NADH dehydrogenase (ubiquinone) 1, subcomplex unknown, 2, 14.5kDa; NADH			5.10E-05	0.72	10.13	7.60	14187.4
AHQ-1-13, 2818	K.REDYLYAVR.D	1185.31400	2	5.10E-05	0.72	2.69	-	698.2
gi|27477800|ref|XP_208313.1|	similar to tropomyosin 4 [Homo sapiens]			5.25E-05	1.43	20.23	15.20	18426.7
AHQ-1-9, 5227	K.CGDLEEELKNVTNNLK.S	1878.05124	2	1.53E-04	0.95	4.53	-	1121.9
AHQ-1-9, 5674 - 5682	K.CGDLEEELKNVTNNLK.S	1878.05124	2	5.25E-05	0.95	4.39	-	1515.4
AHQ-1-9, 1852 - 1916	K.TIDDLEEK.L	963.02115	2	2.75E-04	0.48	2.66	-	247.3
gi|4507195|ref|NP_003119.1|	spectrin, beta, non-erythrocytic 1; Spectrin, beta, nonerythrocytic-1 (			5.45E-05	0.89	10.21	0.80	274628.5
AHQ-1-1, 6420	R.DASVAEAWLLGQEPYLSSR.E	2093.28190	2	5.45E-05	0.89	4.13	-	590.1
gi|4758460|ref|NP_004479.1|	glycoprotein V (platelet) [Homo sapiens]			5.51E-05	1.65	20.21	6.30	60958.9
AHQ-1-5, 5671	R.CAGPGAHAGLPLWALPGGDAECPGPR.G	2587.87539	3	9.27E-05	0.81	4.16	-	668.7
AHQ-1-5, 3371	R.YLGVTLSPR.L	1006.18036	2	5.51E-05	0.84	3.32	-	912.7
gi|5032137|ref|NP_005629.1|	synaptobrevin-like 1 [Homo sapiens]			5.53E-05	1.88	20.23	13.20	24934.9
AHQ-1-13- , 4755 - 4756	K.LTYSHGNYLFHYICQDR.I	2189.39466	2	5.53E-05	0.94	4.53	-	959.9
AHQ-1-13- , 2853	K.VMETQAQVDELK.G	1391.57303	2	5.21E-04	0.94	3.74	-	1426.8
gi|30148978|ref|XP_291520.2|	similar to Keratin, type I cytoskeletal 18 (Cytokeratin 18) (K18) (CK			5.53E-05	0.78	10.15	2.20	68413.1
AHQ-1-11, 4590 - 4620	R.DWSHYFKTIKDLR.A	1709.92863	2	5.53E-05	0.78	2.92	-	888.9
gi|4503447|ref|NP_001389.1|	peroxisomal enoyl-coenzyme A hydratase-like protein; delta3,5-delta2,4-			5.66E-05	0.90	10.16	3.40	35994.1
AHQ-1-9, 4262	R.YCAQDAFFQVK.E	1378.53429	2	5.66E-05	0.90	3.21	-	1066.1
gi|19923142|ref|NP_002256.2|	karyopherin beta 1; nuclear factor p97; importin 90; importin beta-1 s			5.77E-05	1.76	20.21	4.00	97169.7
AHQ-1-5, 7410	K.GALQYLVPILTQTLTK.Q	1760.11044	2	5.89E-05	0.83	3.83	-	523.6
AHQ-1-5, 6407	K.YLEVVLNTLQQASQAQVDK.S	2148.40214	2	5.77E-05	0.92	4.15	-	1380.2
gi|4757712|ref|NP_004026.1|	acyl-Coenzyme A oxidase isoform a; acyl-coenzyme A oxidase 1 [Homo sapi			6.02E-05	0.82	10.18	3.60	74745.4
AHQ-1-6, 6492	R.INEGIGQGDLSELPELHALTAGLK.A	2476.76782	3	6.02E-05	0.82	3.55	-	784.0
gi|4507295|ref|NP_003560.1|	syntaxin 7 [Homo sapiens]			6.40E-05	0.77	10.15	5.70	29845.3
AHQ-1-13- , 3453	R.TLNQLGTPQDSPELR.Q	1669.81798	2	6.40E-05	0.77	2.99	-	561.5
gi|6912536|ref|NP_036475.1|	nicotinamide nucleotide transhydrogenase [Homo sapiens]			6.43E-05	0.71	10.18	1.80	113946.9
AHQ-1-4, 6579	K.TSGTLISFIYPAQNPELLNK.L	2207.51119	2	6.43E-05	0.71	3.58	-	301.3
gi|22065448|ref|XP_084610.2|	similar to tubulin alpha-1 chain - Chinese hamster [Homo sapiens] [MAS			6.49E-05	0.79	10.16	2.40	41009.4
AHQ-1-11, 4694	K.EIIDLVLDR.I	1086.26378	2	6.49E-05	0.79	3.17	-	811.6
gi|7705827|ref|NP_057187.1|	GTP-binding protein Sara [Homo sapiens]			6.52E-05	0.85	10.15	5.60	22409.7
AHQ-1-11, 4102	K.LVFLGLDNAGK.T	1147.34882	2	6.52E-05	0.85	2.84	-	978.2
gi|4504215|ref|NP_000848.1|	guanylate cyclase 1, soluble, beta 3 [Homo sapiens]			7.68E-05	0.83	10.14	1.80	70514.1
AHQ-1-6, 4300	K.LTQELEILTDR.L	1331.49732	2	7.68E-05	0.83	2.53	-	1458.9
gi|18201911|ref|NP_000629.2|	vitronectin precursor; serum spreading factor; somatomedin B; compleme			8.03E-05	0.88	10.16	3.10	54335.4
AHQ-1-13, 5463	R.SIAQYWLGCPAPGHL	1671.90045	2	8.03E-05	0.88	3.24	-	765.4
gi|30158159|ref|XP_301350.1|	similar to 60S RIBOSOMAL PROTEIN L34 [Homo sapiens]			8.37E-05	0.63	10.16	21.00	14102.3
AHQ-1-9, 6866	R.SIVQFATNFMTINKWFKFSEKQQQK.I	3080.55113	3	8.37E-05	0.63	3.25	-	593.7
gi|4557377|ref|NP_000052.1|	Bruton agammaglobulinemia tyrosine kinase [Homo sapiens]			8.39E-05	1.25	20.24	4.70	76280.8
AHQ-1-6, 2994	K.ITCVETVVPEKNPPPER.Q	1967.23275	2	3.71E-04	0.50	2.70	-	319.0
AHQ-1-6, 3368 - 3414	R.YVLDDEYTSSVGSK.F	1563.64427	2	8.39E-05	0.75	3.55	-	302.2
gi|14249348|ref|NP_116120.1|	hypothetical protein MGC14353 [Homo sapiens]			8.58E-05	0.78	10.14	11.40	13940.7
AHQ-1-13, 4039	K.SWCPDCVQAEPVVR.E	1705.89366	2	8.58E-05	0.78	2.57	-	969.3
gi|4502285|ref|NP_001672.1|	ATPase, Ca++ transporting, cardiac muscle, slow twitch 2 isoform 2; ATP			8.67E-05	0.92	10.20	1.50	109690.3
AHQ-1-1, 4789 - 4799	K.VGEATETALTCLVEK.M	1622.81958	2	8.67E-05	0.92	3.98	-	935.7

<table>
<tr><td>gi|21389337|ref|NP_653164.1| ribose 5-phosphate isomerase A (ribose 5-phosphate epimerase); RIBOSE</td><td></td><td></td><td></td><td>8.75E-05</td><td>0.90</td><td>10.21</td><td>7.60</td><td>26090.9</td></tr>
<tr><td>AHQ-1-10, 4496</td><td>R.VKQENLNLVCIPTSFQAR.Q</td><td>2119.43046</td><td>2</td><td>8.75E-05</td><td>0.90</td><td>4.11</td><td>-</td><td>639.8</td></tr>
<tr><td>gi|5730118|ref|NP_006514.1| X-prolyl aminopeptidase (aminopeptidase P) 1, soluble; X-prolyl aminope</td><td></td><td></td><td></td><td>8.91E-05</td><td>1.80</td><td>20.19</td><td>4.80</td><td>69858.4</td></tr>
<tr><td>AHQ-1-6, 6327 - 6403</td><td>R.GSLTFEPLTLVPIQTK.M</td><td>1745.05263</td><td>2</td><td>8.91E-05</td><td>0.87</td><td>3.83</td><td>-</td><td>648.9</td></tr>
<tr><td>AHQ-1-6, 6266 - 6268</td><td>R.VGVDPLIIPTDYWK.K</td><td>1616.88141</td><td>2</td><td>2.39E-04</td><td>0.94</td><td>3.89</td><td>-</td><td>962.6</td></tr>
<tr><td>gi|27481323|ref|XP_208234.1| similar to unactive progesterone receptor, 23 kD; likely ortholog of m</td><td></td><td></td><td></td><td>9.31E-05</td><td>0.75</td><td>10.14</td><td>7.50</td><td>18694.3</td></tr>
<tr><td>AHQ-1-11, 5907</td><td>K.LNWLSVDFNNWK.D</td><td>1536.71494</td><td>2</td><td>9.31E-05</td><td>0.75</td><td>2.75</td><td>-</td><td>631.3</td></tr>
<tr><td>gi|19923264|ref|NP_004571.2| RAB27A, member RAS oncogene family [Homo sapiens]</td><td></td><td></td><td></td><td>9.36E-05</td><td>0.85</td><td>10.16</td><td>4.50</td><td>24867.9</td></tr>
<tr><td>AHQ-1-10, 4372</td><td>K.FITTVGIDFR.E</td><td>1169.35448</td><td>2</td><td>9.36E-05</td><td>0.85</td><td>3.21</td><td>-</td><td>661.2</td></tr>
<tr><td>gi|7662651|ref|NP_054778.1| RGC32 protein [Homo sapiens]</td><td></td><td></td><td></td><td>9.60E-05</td><td>1.86</td><td>20.21</td><td>26.50</td><td>12924.1</td></tr>
<tr><td>AHQ-1-12, 5162 - 5167</td><td>K.ELEAFIADLDK.T</td><td>1264.40621</td><td>2</td><td>1.88E-04</td><td>0.95</td><td>3.66</td><td>-</td><td>1475.9</td></tr>
<tr><td>AHQ-1-12, 4186 - 4249</td><td>K.LNSPTDSTPALLSATVTPQK.A</td><td>2042.27634</td><td>2</td><td>9.60E-05</td><td>0.91</td><td>4.23</td><td>-</td><td>559.5</td></tr>
<tr><td>gi|4502205|ref|NP_001651.1| ADP-ribosylation factor 4 [Homo sapiens]</td><td></td><td></td><td></td><td>9.72E-05</td><td>0.77</td><td>10.15</td><td>5.60</td><td>20510.6</td></tr>
<tr><td>AHQ-1-11, 2476</td><td>R.IQEVADELQK.M</td><td>1173.29830</td><td>2</td><td>9.72E-05</td><td>0.77</td><td>3.05</td><td>-</td><td>666.4</td></tr>
<tr><td>gi|21245108|ref|NP_631960.1| TGFB-induced factor 2-like, Y-linked [Homo sapiens]</td><td></td><td></td><td></td><td>9.87E-05</td><td>0.65</td><td>10.14</td><td>4.90</td><td>20813.6</td></tr>
<tr><td>AHQ-1-13, 5664</td><td>R.CQERSNQIR.S</td><td>1192.28823</td><td>2</td><td>9.87E-05</td><td>0.65</td><td>2.87</td><td>-</td><td>712.9</td></tr>
<tr><td>gi|10880136|ref|NP_004032.2| arrestin beta 1 isoform A [Homo sapiens]</td><td></td><td></td><td></td><td>1.05E-04</td><td>0.59</td><td>10.16</td><td>4.30</td><td>47065.3</td></tr>
<tr><td>AHQ-1-7, 2374</td><td>K.VQYAPERPGPQPTAETTR.Q</td><td>1999.17334</td><td>2</td><td>1.05E-04</td><td>0.59</td><td>3.15</td><td>-</td><td>275.7</td></tr>
<tr><td>gi|28461290|ref|NP_443728.2| TRALPUSH; no opposite paired repeat protein [Homo sapiens]</td><td></td><td></td><td></td><td>1.05E-04</td><td>0.55</td><td>10.14</td><td>0.40</td><td>240073.5</td></tr>
<tr><td>AHQ-1-8, 3526</td><td>K.KKPQVNAK.D</td><td>913.09970</td><td>1</td><td>1.16E-04</td><td>0.39</td><td>2.30</td><td>-</td><td>419.0</td></tr>
<tr><td>AHQ-1-9, 3550</td><td>K.KKPQVNAK.D</td><td>913.09970</td><td>1</td><td>3.73E-04</td><td>0.61</td><td>2.63</td><td>-</td><td>591.8</td></tr>
<tr><td>AHQ-1-10, 3377</td><td>K.KKPQVNAK.D</td><td>913.09970</td><td>1</td><td>1.05E-04</td><td>0.55</td><td>2.60</td><td>-</td><td>447.6</td></tr>
<tr><td>AHQ-1-11, 3463</td><td>K.KKPQVNAK.D</td><td>913.09970</td><td>1</td><td>4.42E-04</td><td>0.50</td><td>2.55</td><td>-</td><td>450.5</td></tr>
<tr><td>AHQ-1-7, 3692</td><td>K.KKPQVNAK.D</td><td>913.09970</td><td>1</td><td>3.25E-04</td><td>0.41</td><td>2.31</td><td>-</td><td>429.7</td></tr>
<tr><td>gi|7657548|ref|NP_055378.1| spondyloepiphyseal dysplasia, late; sedlin [Homo sapiens]</td><td></td><td></td><td></td><td>1.07E-04</td><td>0.68</td><td>10.13</td><td>10.00</td><td>16444.6</td></tr>
<tr><td>AHQ-1-13, 4165</td><td>K.FSM*NPFYEPNSPIR.S</td><td>1715.91027</td><td>2</td><td>1.07E-04</td><td>0.68</td><td>2.61</td><td>-</td><td>695.8</td></tr>
<tr><td>gi|17444699|ref|XP_017966.4| similar to Reticulon protein 3 (Neuroendocrine-specific protein-like 2</td><td></td><td></td><td></td><td>1.07E-04</td><td>0.88</td><td>10.15</td><td>4.70</td><td>25522.7</td></tr>
<tr><td>AHQ-1-1, 2960 - 2972</td><td>K.TQIDHYVGIAR.H</td><td>1273.42279</td><td>2</td><td>1.07E-04</td><td>0.88</td><td>2.97</td><td>-</td><td>1117.2</td></tr>
<tr><td>gi|6005848|ref|NP_009126.1| polymerase (DNA directed) iota; RAD30 (S. cerevisiae) homolog B; polyme</td><td></td><td></td><td></td><td>1.08E-04</td><td>0.40</td><td>10.14</td><td>2.40</td><td>80345.8</td></tr>
<tr><td>AHQ-1-8, 4068 - 4123</td><td>K.QMQDIPINPRDHLSSSK.Q</td><td>1967.19629</td><td>2</td><td>1.08E-04</td><td>0.40</td><td>2.84</td><td>-</td><td>451.4</td></tr>
<tr><td>gi|4557773|ref|NP_000248.1| myosin, heavy polypeptide 7, cardiac muscle, beta [Homo sapiens]</td><td></td><td></td><td></td><td>1.08E-04</td><td>0.76</td><td>10.16</td><td>0.50</td><td>223110.8</td></tr>
<tr><td>AHQ-1-2, 3350</td><td>R.CNGVLEGIR.I</td><td>1019.15759</td><td>1</td><td>7.31E-04</td><td>0.22</td><td>2.31</td><td>-</td><td>127.3</td></tr>
<tr><td>AHQ-1-1, 3328</td><td>R.CNGVLEGIR.I</td><td>1019.15759</td><td>2</td><td>1.08E-04</td><td>0.76</td><td>3.00</td><td>-</td><td>626.3</td></tr>
<tr><td>gi|19923750|ref|NP_002858.2| RAB3B, member RAS oncogene family; Brain antigen RAB3B [Homo sapiens]</td><td></td><td></td><td></td><td>1.08E-04</td><td>0.75</td><td>10.13</td><td>5.00</td><td>24757.7</td></tr>
<tr><td>AHQ-1-12, 3406</td><td>K.LQIWDTAGQER.Y</td><td>1317.43243</td><td>2</td><td>1.08E-04</td><td>0.75</td><td>2.70</td><td>-</td><td>724.0</td></tr>
<tr><td>gi|8922699|ref|NP_060705.1| hypothetical protein FLJ10830 [Homo sapiens]</td><td></td><td></td><td></td><td>1.10E-04</td><td>0.86</td><td>10.15</td><td>2.30</td><td>52779.0</td></tr>
<tr><td>AHQ-1-7, 3972</td><td>K.TVFGVEPDLTR.E</td><td>1234.38333</td><td>2</td><td>1.10E-04</td><td>0.86</td><td>2.82</td><td>-</td><td>1050.5</td></tr>
<tr><td>gi|20428652|ref|NP_005347.2| lymphocyte-specific protein tyrosine kinase; oncogene LCK; membrane as</td><td></td><td></td><td></td><td>1.16E-04</td><td>0.74</td><td>10.17</td><td>2.00</td><td>57969.3</td></tr>
<tr><td>AHQ-1-7, 2317</td><td>R.LIEDNEYTAR.E</td><td>1224.30217</td><td>2</td><td>1.16E-04</td><td>0.74</td><td>3.43</td><td>-</td><td>477.3</td></tr>
<tr><td>gi|17448269|ref|XP_069750.1| similar to GDP dissociation inhibitor isoform 2; GDI-2 [Homo sapiens]</td><td></td><td></td><td></td><td>1.19E-04</td><td>0.93</td><td>10.17</td><td>4.20</td><td>32024.9</td></tr>
<tr><td>AHQ-1-13, 4155</td><td>K.DLGTESQIFISR.A</td><td>1366.50176</td><td>2</td><td>1.19E-04</td><td>0.93</td><td>3.31</td><td>-</td><td>1485.1</td></tr>
<tr><td>gi|4507303|ref|NP_001045.1| sulfotransferase family, cytosolic, 1A, phenol-preferring, member 2; th</td><td></td><td></td><td></td><td>1.20E-04</td><td>0.96</td><td>10.24</td><td>5.40</td><td>34310.4</td></tr>
<tr><td>AHQ-1-9, 5959 - 5971</td><td>K.THLPLALLPQTLLDQK.V</td><td>1802.15055</td><td>2</td><td>1.20E-04</td><td>0.96</td><td>4.88</td><td>-</td><td>1005.7</td></tr>
<tr><td>gi|29728414|ref|XP_290989.1| similar to myosin-I, Myr 1c (alternatively spliced) - rat [Homo sapien</td><td></td><td></td><td></td><td>1.23E-04</td><td>0.85</td><td>10.21</td><td>1.90</td><td>124949.7</td></tr>
<tr><td>AHQ-1-2, 7273</td><td>K.CRTHFLLMKKSQIVIAAWYR.R</td><td>2524.05044</td><td>3</td><td>1.23E-04</td><td>0.85</td><td>4.11</td><td>-</td><td>992.7</td></tr>
<tr><td>gi|4557525|ref|NP_000099.1| dihydrolipoamide dehydrogenase precursor; E3 component of pyruvate dehy</td><td></td><td></td><td></td><td>1.23E-04</td><td>0.94</td><td>10.18</td><td>2.60</td><td>54149.9</td></tr>
<tr><td>AHQ-1-7, 2485</td><td>K.SEEQLKEEGIEYK.V</td><td>1582.69072</td><td>2</td><td>1.23E-04</td><td>0.94</td><td>3.52</td><td>-</td><td>1352.5</td></tr>
<tr><td>gi|4885539|ref|NP_005380.1| protein-L-isoaspartate (D-aspartate) O-methyltransferase [Homo sapiens]</td><td></td><td></td><td></td><td>1.25E-04</td><td>0.91</td><td>10.21</td><td>15.00</td><td>24636.2</td></tr>
<tr><td>AHQ-1-11, 5823</td><td>R.M*GYAEEAPYDAIHVGAAAPVVPQALIDQLKPGGR.L</td><td>3522.97505</td><td>3</td><td>1.25E-04</td><td>0.91</td><td>4.29</td><td>-</td><td>904.3</td></tr>
<tr><td>gi|4557241|ref|NP_001095.1| skeletal muscle specific actinin, alpha 3 [Homo sapiens]</td><td></td><td></td><td></td><td>1.26E-04</td><td>0.92</td><td>10.17</td><td>0.90</td><td>103293.7</td></tr>
<tr><td>AHQ-1-6, 2884</td><td>K.ALDFIASK.G</td><td>865.00867</td><td>2</td><td>2.09E-04</td><td>0.83</td><td>2.78</td><td>-</td><td>622.3</td></tr>
<tr><td>AHQ-1-12, 2942</td><td>K.ALDFIASK.G</td><td>865.00867</td><td>1</td><td>2.40E-04</td><td>0.44</td><td>1.83</td><td>-</td><td>872.7</td></tr>
<tr><td>AHQ-1-5, 2830 - 2834</td><td>K.ALDFIASK.G</td><td>865.00867</td><td>2</td><td>1.26E-04</td><td>0.92</td><td>3.35</td><td>-</td><td>780.0</td></tr>
<tr><td>gi|13376187|ref|NP_079082.1| hypothetical protein FLJ23554 [Homo sapiens]</td><td></td><td></td><td></td><td>1.27E-04</td><td>0.72</td><td>10.15</td><td>2.20</td><td>82410.2</td></tr>
<tr><td>AHQ-1-13- , 4837 - 4839</td><td>R.GNGM*EPDSLDEEESPR.W</td><td>1778.79043</td><td>3</td><td>1.27E-04</td><td>0.72</td><td>3.07</td><td>-</td><td>1139.6</td></tr>
<tr><td>gi|29743324|ref|XP_293276.1| similar to Heat shock 27 kDa protein (HSP 27) (Stress-responsive prote</td><td></td><td></td><td></td><td>1.34E-04</td><td>0.86</td><td>10.27</td><td>16.00</td><td>21244.7</td></tr>
<tr><td>AHQ-1-13, 5151</td><td>R.KYTLPPGVDPTKVSSSLSPEGTLTVEAPM*PK.L</td><td>3244.70168</td><td>3</td><td>1.34E-04</td><td>0.86</td><td>5.36</td><td>-</td><td>723.0</td></tr>
<tr><td>gi|13899297|ref|NP_113646.1| B aggressive lymphoma gene [Homo sapiens]</td><td></td><td></td><td></td><td>1.35E-04</td><td>0.13</td><td>10.13</td><td>1.30</td><td>96282.7</td></tr>
<tr><td>AHQ-1-1, 3004</td><td>K.SILQQAGVEMK.S</td><td>1204.42218</td><td>1</td><td>1.35E-04</td><td>0.13</td><td>2.12</td><td>-</td><td>483.0</td></tr>
<tr><td>gi|15718761|ref|NP_004976.2| c-K-ras2 protein isoform b; Kirsten rat sarcoma-2 viral (v-Ki-ras2) on</td><td></td><td></td><td></td><td>1.39E-04</td><td>0.95</td><td>10.20</td><td>8.00</td><td>21424.4</td></tr>
<tr><td>AHQ-1-11, 3612</td><td>R.VKDSEDVPM*VLVGNK.C</td><td>1646.88770</td><td>2</td><td>1.39E-04</td><td>0.95</td><td>4.08</td><td>-</td><td>1484.2</td></tr>
<tr><td>gi|6005886|ref|NP_009200.1| syntaxin binding protein 3; syntaxin 4 binding protein [Homo sapiens] [</td><td></td><td></td><td></td><td>1.45E-04</td><td>0.91</td><td>10.20</td><td>2.50</td><td>67573.7</td></tr>
<tr><td>AHQ-1-6, 3767</td><td>K.SCEVIIGSTHVLTPK.K</td><td>1642.89886</td><td>2</td><td>1.45E-04</td><td>0.91</td><td>4.02</td><td>-</td><td>834.3</td></tr>
<tr><td>gi|5453629|ref|NP_006391.1| dynactin 2; dynactin complex 50 kD subunit; dynamitin; 50 kD dynein-ass</td><td></td><td></td><td></td><td>1.46E-04</td><td>0.91</td><td>10.23</td><td>4.70</td><td>44819.3</td></tr>
<tr><td>AHQ-1-9, 5255</td><td>K.LLGPDAAINLTDPDGALAK.R</td><td>1866.10514</td><td>2</td><td>1.46E-04</td><td>0.91</td><td>4.52</td><td>-</td><td>766.2</td></tr>
<tr><td>gi|5803135|ref|NP_006852.1| RAB35, member RAS oncogene family; ras-related protein rab-1c (GTP-bind</td><td></td><td></td><td></td><td>1.48E-04</td><td>0.91</td><td>10.20</td><td>5.50</td><td>23025.1</td></tr>
<tr><td>AHQ-1-14- , 3818 - 3819</td><td>K.LLIIGDSGVGK.S</td><td>1072.28025</td><td>2</td><td>1.48E-04</td><td>0.91</td><td>4.00</td><td>-</td><td>891.2</td></tr>
<tr><td>AHQ-1-11, 3566 - 3627</td><td>K.LLIIGDSGVGK.S</td><td>1072.28025</td><td>1</td><td>2.51E-04</td><td>0.21</td><td>1.89</td><td>-</td><td>500.1</td></tr>
<tr><td>gi|29725611|ref|NP_066954.2| regulatory subunit PR 53 of protein phosphatase 2A isoform b; phosphot</td><td></td><td></td><td></td><td>1.51E-04</td><td>0.93</td><td>10.25</td><td>9.00</td><td>36775.0</td></tr>
<tr><td>AHQ-1-9, 7222</td><td>K.LDEEAENLVATVVPTHLAAAVPEVAVYLK.E</td><td>3063.49045</td><td>3</td><td>1.51E-04</td><td>0.93</td><td>4.97</td><td>-</td><td>1162.2</td></tr>
<tr><td>gi|14589893|ref|NP_001785.2| cadherin 4, type 1 preproprotein; cadherin 4, R-cadherin (retinal); R-</td><td></td><td></td><td></td><td>1.55E-04</td><td>0.42</td><td>10.15</td><td>2.00</td><td>100279.9</td></tr>
<tr><td>AHQ-1-5, 4751 - 4823</td><td>K.VENPIDLYIYVIDM*NDNR.P</td><td>2213.45293</td><td>2</td><td>1.96E-04</td><td>0.33</td><td>3.07</td><td>-</td><td>315.7</td></tr>
<tr><td>AHQ-1-11, 4291</td><td>K.VENPIDLYIYVIDM*NDNR.P</td><td>2213.45293</td><td>2</td><td>3.37E-04</td><td>0.44</td><td>2.90</td><td>-</td><td>493.8</td></tr>
<tr><td>AHQ-1-1, 4911</td><td>K.VENPIDLYIYVIDM*NDNR.P</td><td>2213.45293</td><td>2</td><td>1.55E-04</td><td>0.42</td><td>2.65</td><td>-</td><td>495.0</td></tr>
<tr><td>gi|6005731|ref|NP_009167.1| calcium binding protein P22; SLC9A1 binding protein; calcineurin homolo</td><td></td><td></td><td></td><td>1.57E-04</td><td>0.70</td><td>10.16</td><td>6.20</td><td>22456.0</td></tr>
<tr><td>AHQ-1-11, 4479</td><td>R.IPELAINPLGDR.I</td><td>1308.50858</td><td>2</td><td>1.57E-04</td><td>0.70</td><td>3.13</td><td>-</td><td>755.5</td></tr>
<tr><td>gi|4758788|ref|NP_004542.1| NADH dehydrogenase (ubiquinone) Fe-S protein 3, 30kDa (NADH-coenzyme Q</td><td></td><td></td><td></td><td>1.58E-04</td><td>0.70</td><td>10.17</td><td>6.40</td><td>30241.3</td></tr>
<tr><td>AHQ-1-10, 5852</td><td>K.TYTDELTPIESAVSVFK.A</td><td>1901.10311</td><td>2</td><td>1.58E-04</td><td>0.70</td><td>3.41</td><td>-</td><td>291.5</td></tr>
<tr><td>gi|20540302|ref|XP_166540.1| similar to Heat shock cognate 71 kDa protein [Homo sapiens]</td><td></td><td></td><td></td><td>1.59E-04</td><td>0.43</td><td>10.10</td><td>6.10</td><td>12714.2</td></tr>
<tr><td>AHQ-1-6, 1623 - 1626</td><td>K.DNNLLGK.F</td><td>773.85763</td><td>1</td><td>1.59E-04</td><td>0.43</td><td>1.99</td><td>-</td><td>412.1</td></tr>
<tr><td>gi|29740132|ref|XP_299177.1| hypothetical protein XP_299177 [Homo sapiens]</td><td></td><td></td><td></td><td>1.61E-04</td><td>0.56</td><td>10.16</td><td>2.70</td><td>114488.9</td></tr>
<tr><td>AHQ-1-3, 5285</td><td>R.RCHEEKLPLSAGTASHKGQASAQPITPR.Q</td><td>3030.36879</td><td>3</td><td>1.61E-04</td><td>0.56</td><td>3.23</td><td>-</td><td>460.7</td></tr>
<tr><td>gi|4503253|ref|NP_001335.1| defender against cell death 1 [Homo sapiens]</td><td></td><td></td><td></td><td>1.62E-04</td><td>0.92</td><td>10.17</td><td>10.60</td><td>12496.6</td></tr>
<tr><td>AHQ-1-14, 4921</td><td>R.FLEEYLSSTPQR.L</td><td>1470.60844</td><td>2</td><td>1.62E-04</td><td>0.92</td><td>3.38</td><td>-</td><td>1399.3</td></tr>
<tr><td>gi|29732170|ref|XP_291550.1| similar to Heat shock protein HSP 90-alpha (HSP 86) [Homo sapiens] [MA</td><td></td><td></td><td></td><td>1.64E-04</td><td>0.85</td><td>10.18</td><td>13.60</td><td>13262.9</td></tr>
<tr><td>AHQ-1-5, 3519</td><td>K.KHLEINPDHSIIETLR.Q</td><td>1916.17061</td><td>2</td><td>1.64E-04</td><td>0.85</td><td>3.50</td><td>-</td><td>1078.9</td></tr>
<tr><td>gi|30156394|ref|XP_062437.6| similar to Keratin, type I cytoskeletal 18 (Cytokeratin 18) (K18) (CK</td><td></td><td></td><td></td><td>1.70E-04</td><td>0.18</td><td>10.13</td><td>3.30</td><td>44396.0</td></tr>
<tr><td>AHQ-1-13- , 5015</td><td>R.DWSHYFKIIDDLR.A</td><td>1708.89749</td><td>2</td><td>1.70E-04</td><td>0.18</td><td>2.51</td><td>-</td><td>299.5</td></tr>
<tr><td>gi|4503445|ref|NP_001944.1| endothelial cell growth factor 1 (platelet-derived); thymidine phosphor</td><td></td><td></td><td></td><td>1.72E-04</td><td>0.96</td><td>10.25</td><td>3.90</td><td>49955.2</td></tr>
<tr><td>AHQ-1-7, 4404</td><td>R.AREQEELLAPADGTVELVR.A</td><td>2097.31523</td><td>2</td><td>1.72E-04</td><td>0.96</td><td>5.08</td><td>-</td><td>934.9</td></tr>
<tr><td>gi|12408677|ref|NP_002614.2| prefoldin 4 [Homo sapiens]</td><td></td><td></td><td></td><td>1.76E-04</td><td>0.94</td><td>10.19</td><td>9.00</td><td>15314.1</td></tr>
<tr><td>AHQ-1-12, 4878</td><td>K.NLQEEIDALESR.V</td><td>1417.50394</td><td>2</td><td>1.76E-04</td><td>0.94</td><td>3.72</td><td>-</td><td>1386.3</td></tr>
<tr><td>gi|10140853|ref|NP_065438.1| diazepam binding inhibitor; GABA receptor modulator; endozepine; acyl</td><td></td><td></td><td></td><td>1.77E-04</td><td>0.80</td><td>10.19</td><td>15.40</td><td>11793.3</td></tr>
<tr><td>AHQ-1-14- , 3815</td><td>K.TKPSDEEM*LFIYGHYK.Q</td><td>1975.21039</td><td>3</td><td>1.77E-04</td><td>0.80</td><td>3.77</td><td>-</td><td>406.3</td></tr>
<tr><td>gi|18491024|ref|NP_005691.2| dipeptidylpeptidase III isoform 1; dipeptidylpeptidase III [Homo sapie</td><td></td><td></td><td></td><td>1.77E-04</td><td>0.73</td><td>10.14</td><td>2.30</td><td>82588.6</td></tr>
<tr><td>AHQ-1-6, 4672</td><td>K.LAQDFLDSQNLSAYNTR.L</td><td>1957.09009</td><td>2</td><td>1.77E-04</td><td>0.73</td><td>2.79</td><td>-</td><td>519.7</td></tr>
<tr><td>gi|29739581|ref|XP_084467.3| similar to eukaryotic initiation factor 5A [Homo sapiens]</td><td></td><td></td><td></td><td>1.87E-04</td><td>0.90</td><td>10.19</td><td>8.40</td><td>16773.1</td></tr>
<tr><td>AHQ-1-12, 2967</td><td>R.LPEGDLGKEIEQK.Y</td><td>1456.62321</td><td>2</td><td>1.87E-04</td><td>0.90</td><td>3.85</td><td>-</td><td>1001.0</td></tr>
<tr><td>gi|4885281|ref|NP_005262.1| glutamate dehydrogenase 1 [Homo sapiens]</td><td></td><td></td><td></td><td>1.95E-04</td><td>0.73</td><td>10.15</td><td>2.90</td><td>61397.5</td></tr>
<tr><td>AHQ-1-7, 3994</td><td>K.HGGTIPIVPTAEFQDR.I</td><td>1738.92514</td><td>2</td><td>1.95E-04</td><td>0.73</td><td>2.94</td><td>-</td><td>363.4</td></tr>
<tr><td>gi|4501889|ref|NP_001606.1| actin, gamma 2 propeptide; actin, alpha-3 [Homo sapiens]</td><td></td><td></td><td></td><td>1.96E-04</td><td>0.21</td><td>10.17</td><td>5.60</td><td>41876.7</td></tr>
<tr><td>AHQ-1-14- , 4866 - 4882</td><td>K.DLYANNVLSGGTTM*YPGIADR.M</td><td>2245.45502</td><td>2</td><td>2.62E-04</td><td>0.74</td><td>3.36</td><td>-</td><td>532.6</td></tr>
<tr><td>AHQ-1-10, 4952</td><td>K.DLYANNVLSGGTTM*YPGIADR.M</td><td>2245.45502</td><td>2</td><td>1.96E-04</td><td>0.21</td><td>2.76</td><td>-</td><td>225.5</td></tr>
<tr><td>gi|4505329|ref|NP_003818.1| N-ethylmaleimide-sensitive factor attachment protein, alpha; alpha-SNAP</td><td></td><td></td><td></td><td>1.98E-04</td><td>0.96</td><td>10.24</td><td>6.10</td><td>33246.6</td></tr>
<tr><td>AHQ-1-9, 3232</td><td>K.LLEAHEEQNVDSYTESVK.E</td><td>2092.20594</td><td>2</td><td>1.98E-04</td><td>0.96</td><td>4.85</td><td>-</td><td>1413.4</td></tr>
<tr><td>gi|4505755|ref|NP_000281.1| phosphoglycerate mutase 2 (muscle); Phosphoglycerate mutase, muscle for</td><td></td><td></td><td></td><td>2.01E-04</td><td>0.37</td><td>10.15</td><td>5.10</td><td>28850.0</td></tr>
<tr><td>AHQ-1-6, 7244</td><td>K.M*EFDICYTSVLKR.A</td><td>1679.93944</td><td>2</td><td>2.01E-04</td><td>0.37</td><td>2.56</td><td>-</td><td>519.6</td></tr>
</table>

gi|8393638|ref|NP_058642.1|	F11 receptor isoform a precursor; platelet F11 receptor; platelet adhes			2.09E-04	0.80	10.15	3.70	32582.9
AHQ-1-9, 5247	R.VTFLPTGITFK.S	1224.47318	2	2.09E-04	0.80	3.08	-	477.3
gi|14765644|ref|XP_037574.1|	similar to PTPL1-associated RhoGAP 1 [Homo sapiens]			2.15E-04	0.67	10.14	1.70	124613.7
AHQ-1-13- , 6885	K.YPLLNTVETLTAAGTLIAK.V	1990.32931	2	2.15E-04	0.67	2.75	-	873.0
gi|4505409|ref|NP_002503.1|	non-metastatic cells 2, protein (NM23B) expressed in; Non-metastatic ce			2.17E-04	0.88	10.19	0.00	17297.9
AHQ-1-12, 5865 - 5941	K.EISLWFKPEELVDYK.S	1897.15929	2	2.17E-04	0.88	3.90	-	600.3
gi|7705300|ref|NP_057701.1|	hypothetical protein BM-002 [Homo sapiens]			2.18E-04	0.69	10.13	17.60	9117.5
AHQ-1-14- , 5394	K.VLSVPESTPFTAVLK.F	1588.86951	2	2.18E-04	0.69	2.64	-	330.1
gi|4506195|ref|NP_002785.1|	proteasome beta 2 subunit; proteasome subunit, beta type, 2; macropain			2.34E-04	0.78	10.17	5.50	22836.1
AHQ-1-11, 5782	R.FILNLPTFSVR.I	1307.56542	2	2.34E-04	0.78	3.41	-	501.7
gi|21361827|ref|NP_062826.2|	putative methyltransferase M6A; putative methyltransferase [Homo sapie			2.35E-04	0.64	10.17	3.10	64473.6
AHQ-1-2, 4489	K.YVHYEIDACMDSEAPGSK.D	2074.23418	2	2.35E-04	0.64	3.34	-	429.7
gi|4502013|ref|NP_001616.1|	adenylate kinase 2 isoform a; adenylate kinase, mitochondrial; adenylat			2.35E-04	0.80	10.17	10.50	26477.6
AHQ-1-13- , 6989	R.GIHSAIDASQTPDVVFASILAAFSK.A	2546.85974	3	2.35E-04	0.80	3.36	-	867.7
gi|4557609|ref|NP_000806.1|	gamma-aminobutyric acid (GABA) A receptor, delta [Homo sapiens]			2.41E-04	0.39	10.15	4.90	50689.0
AHQ-1-9, 2452 - 2455	R.NAIVLFSLSAAGVTQELAISRR.Q	2317.67354	3	2.41E-04	0.39	3.07	-	487.7
gi|4885079|ref|NP_005165.1|	ATP synthase, H+ transporting, mitochondrial F1 complex, gamma polypept			2.43E-04	0.77	10.15	4.00	32880.7
AHQ-1-12, 4967	R.IYGLGSLALYEK.A	1327.55027	2	2.43E-04	0.77	2.72	-	669.0
gi|21361822|ref|NP_057049.3|	cell death-regulatory protein GRIM19; CGI-39 protein [Homo sapiens] [M			2.48E-04	0.66	10.13	5.70	25864.7
AHQ-1-13- , 6904	R.WVPPLIGELYGLR.T	1513.80756	2	2.48E-04	0.66	2.61	-	354.7
gi|21624643|ref|NP_660202.1|	similar to CG3714 gene product [Homo sapiens]			2.56E-04	0.64	10.13	9.00	14620.8
AHQ-1-7, 3220 - 3294	R.SPAQYQVVLSER.L	1377.52778	2	2.56E-04	0.64	2.58	-	485.0
gi|27485813|ref|XP_208062.1|	similar to ARP2/3 complex 21 kDa subunit (P21-ARC) (Actin-related prot			2.61E-04	0.78	10.13	6.30	12870.0
AHQ-1-11, 2432	R.AYLQQLR.Q	892.03735	2	2.61E-04	0.78	2.63	-	731.5
gi|5454148|ref|NP_006368.1|	UNC13 (C. elegans)-like; homolog of rat Munc13 (diacylglycerol-binding)			2.61E-04	0.92	10.19	0.80	180682.0
AHQ-1-5, 4041	R.EDRVLGLAVMPLR.D	1469.77813	3	2.61E-04	0.92	3.87	-	1741.7
gi|7706485|ref|NP_057376.1|	tumor necrosis factor type 1 receptor associated protein [Homo sapiens]			2.70E-04	0.93	10.21	2.00	80010.4
AHQ-1-5, 4509 - 4510	R.GVVDSEDIPLNLSR.E	1514.66275	2	2.70E-04	0.93	4.14	-	877.8
gi|4501865|ref|NP_001087.1|	ATP citrate lyase [Homo sapiens]			2.78E-04	0.64	10.15	1.50	121419.0
AHQ-1-4, 5316	R.DEPSVAAMVYPFTGDHK.Q	1865.05606	2	2.78E-04	0.64	2.97	-	533.4
gi|30156116|ref|XP_301571.1|	similar to Dual specificity protein phosphatase 3 (Dual specificity pr			2.90E-04	0.74	10.13	1.70	64164.4
AHQ-1-11, 3604	K.DSGITYLGIK.A	1067.21733	2	2.90E-04	0.74	2.64	-	656.4
gi|7657649|ref|NP_055362.1|	tropomodulin 3 (ubiquitous) [Homo sapiens]			2.92E-04	0.53	10.14	5.70	39594.5
AHQ-1-9, 5314	K.ILPVFDEPPNPTNVEESLKR.T	2295.57683	2	2.92E-04	0.53	2.88	-	322.8
gi|11140817|ref|NP_068557.1|	retinoic acid induced 2 [Homo sapiens]			3.04E-04	0.37	10.17	3.40	57147.9
AHQ-1-12, 6335	R.HTVIKM*GSENEALDLSM*K.S	2036.31705	2	3.04E-04	0.37	3.05	-	261.5
gi|7705369|ref|NP_057535.1|	coatomer protein complex, subunit beta; beta coat protein [Homo sapiens			3.06E-04	0.88	10.18	1.90	107141.5
AHQ-1-5, 3439 - 3446	K.EAGELKPEEEITVGPVQK.L	1954.16755	2	3.06E-04	0.88	3.52	-	872.2
gi|18087855|ref|NP_542408.1|	dynein light chain 2 [Homo sapiens]			3.09E-04	0.43	10.09	0.00	10349.8
AHQ-1-14- , 3114	K.DIAAYIK.K	793.93045	1	3.09E-04	0.43	1.88	-	717.6
gi|8923582|ref|NP_060378.1|	hypothetical protein FLJ20626 [Homo sapiens]			3.12E-04	0.77	10.14	2.70	48956.7
AHQ-1-9, 2851 - 2918	R.AFDWKSQLVIHR.K	1500.72897	2	3.12E-04	0.77	2.87	-	600.3
gi|27500963|ref|XP_028810.2|	similar to KIAA1755 protein [Homo sapiens]			3.16E-04	0.33	10.11	1.90	53033.5
AHQ-1-7, 4357	K.DGSLETVEK.A	978.03581	1	3.16E-04	0.33	2.20	-	492.7
gi|7019485|ref|NP_037364.1|	programmed cell death 6; apoptosis-linked gene 2 [Homo sapiens]			3.22E-04	0.74	10.14	5.20	21868.3
AHQ-1-11, 4922	K.YITDWQNVFR.T	1342.48358	2	3.22E-04	0.74	2.86	-	638.8
gi|16357474|ref|NP_006026.2|	CDC42-binding protein kinase beta; CDC42-binding protein kinase beta (			3.24E-04	0.70	10.15	0.90	194327.5
AHQ-1-4, 4002	R.FQFPSHVTDVSEEAK.D	1721.84821	2	3.24E-04	0.70	2.91	-	543.1
gi|5174389|ref|NP_005882.1|	acetyl-Coenzyme A acetyltransferase 2; acetoacetyl Coenzyme A thiolase			3.38E-04	0.90	10.17	4.00	41295.5
AHQ-1-9, 3607	K.AGHFDKEIVPVLVSTR.K	1769.03752	2	3.38E-04	0.90	3.39	-	1334.0
gi|11386183|ref|NP_008921.1|	WAS protein family, member 2; suppressor of cyclic-AMP receptor (WASP-			3.40E-04	0.90	10.19	3.20	54283.7
AHQ-1-14- , 6095	R.SELECVTNITLANVIR.Q	1834.08473	2	3.40E-04	0.90	3.88	-	1011.3
gi|6912586|ref|NP_036220.1|	6-phosphogluconolactonase [Homo sapiens]			3.49E-04	0.63	10.21	8.10	27546.7
AHQ-1-10, 3955	R.ILEDQEENPLPAALVQPHTGK.L	2300.55348	2	3.49E-04	0.63	3.00	-	346.2
AHQ-1-10, 3939	R.ILEDQEENPLPAALVQPHTGK.L	2300.55348	3	4.08E-04	0.87	4.17	-	887.8
gi|5453854|ref|NP_006187.1|	poly(rC) binding protein 1; heterogenous nuclear ribonucleoprotein X; p			3.50E-04	0.71	10.26	3.90	37525.7
AHQ-1-13- , 4079 - 4151	R.LVVPATQCGSLIGK.G	1444.72199	2	3.50E-04	0.71	2.85	-	389.5
gi|4505695|ref|NP_002604.1|	3-phosphoinositide dependent protein kinase-1; PkB kinase [Homo sapiens			3.51E-04	0.52	10.11	1.60	63151.5
AHQ-1-4, 2396	R.EYAIKILEK.R	1107.32460	1	3.51E-04	0.52	2.28	-	691.7
gi|7305053|ref|NP_038479.1|	myoferlin isoform a; fer-1-like 3 [Homo sapiens]			3.55E-04	0.37	10.14	0.80	234705.6
AHQ-1-2, 5006	R.ILVELATFLEKTPPDK.K	1815.14287	2	3.55E-04	0.37	2.80	-	377.8
gi|30156971|ref|XP_301321.1|	similar to Tubulin alpha-4 chain (Alpha-tubulin 4) [Homo sapiens] [MAS			3.63E-04	0.40	10.12	3.80	20833.0
AHQ-1-8, 3229	K.FNLMYAK.R	887.08098	1	3.63E-04	0.40	2.01	-	459.5
gi|7524354|ref|NP_039268.1|	dimethylarginine dimethylaminohydrolase 2; dimethylarginine dimethylami			3.66E-04	0.33	10.13	5.60	29643.6
AHQ-1-10, 3495	R.DFAVSTVPVSGPSHLR.G	1669.86281	2	3.66E-04	0.33	2.60	-	379.7
gi|23097264|ref|NP_690870.1|	hypothetical protein DKFZp761P1121 [Homo sapiens]			3.67E-04	0.81	10.21	7.90	24194.3
AHQ-1-10, 5384	K.LAALTNYLQPQLDWQAR.G	2002.26196	2	3.67E-04	0.81	4.14	-	272.2
gi|4557896|ref|NP_000243.1|	myotubularin [Homo sapiens]			3.74E-04	0.30	10.13	2.30	69931.3
AHQ-1-12, 3727 - 3790	K.DDEKYLDVIRETNK.Q	1738.87708	2	3.74E-04	0.30	2.54	-	338.1
gi|30149013|ref|XP_302138.1|	similar to alpha tubulin [Homo sapiens]			3.81E-04	0.79	10.16	4.60	22444.5
AHQ-1-7, 5112 - 5124	R.EIINLVLDR.I	1085.27902	2	3.81E-04	0.79	3.29	-	846.6
AHQ-1-13- , 5131	R.EIINLVLDR.I	1085.27902	2	5.04E-04	0.80	2.97	-	792.9
AHQ-1-14- , 4996 - 5006	R.EIINLVLDR.I	1085.27902	2	7.17E-04	0.80	2.93	-	732.2
AHQ-1-7, 5108	R.EIINLVLDR.I	1085.27902	1	6.61E-04	0.32	2.05	-	245.2
gi|9845522|ref|NP_000365.2|	uroporphyrinogen decarboxylase; uroporphyrinogen III decarboxylase [Hom			3.82E-04	0.87	10.19	6.80	40802.7
AHQ-1-9, 7142	K.TVTLQGNLDPCALYASEEEIGQLVK.Q	2751.05952	2	3.82E-04	0.87	3.87	-	563.3
gi|4502905|ref|NP_001822.1|	clusterin; complement-associated protein SP-40 [Homo sapiens]			3.83E-04	0.89	10.23	3.80	52494.3
AHQ-1-11, 5087	K.LFDSDPITVTVPVEVSR.K	1875.11203	2	3.83E-04	0.89	3.78	-	816.9
gi|7705963|ref|NP_057454.1|	RAB9-like protein [Homo sapiens]			3.84E-04	0.87	10.17	7.00	22719.3
AHQ-1-11, 3784	R.FVTLQIWDTAGQER.F	1664.84302	2	3.84E-04	0.87	3.38	-	1197.0
gi|27478732|ref|XP_210750.1|	similar to ATP synthase, H+ transporting, mitochondrial F0 complex, su			3.84E-04	0.81	10.15	7.10	16272.9
AHQ-1-11, 3427	K.SCAEWVSLSK.A	1168.30181	2	3.84E-04	0.81	2.85	-	785.1
gi|14161692|ref|NP_115706.1|	calpain small subunit 2 [Homo sapiens]			3.87E-04	0.80	10.14	2.80	27660.1
AHQ-1-12, 3379	K.LGFEEFK.Y	869.98378	2	3.87E-04	0.80	2.87	-	691.0
gi|4758986|ref|NP_004209.1|	RAB11B, member RAS oncogene family; RAB11B, member of RAS oncogene fami			3.95E-04	0.86	10.17	9.60	24573.4
AHQ-1-11, 5892	K.NNLSFIETSALDSTNVEEAFK.N	2330.49008	2	3.95E-04	0.86	3.36	-	739.0
gi|20149637|ref|NP_057526.2|	type 1 tumor necrosis factor receptor shedding aminopeptidase regulato			3.98E-04	0.26	10.18	1.90	107761.4
AHQ-1-4, 5775	R.SELLLLACVHNYQPCVQR.A	2203.52751	2	3.98E-04	0.26	2.70	-	305.8
gi|21361553|ref|NP_003168.2|	spleen tyrosine kinase [Homo sapiens]			4.03E-04	0.78	10.18	3.10	72065.7
AHQ-1-13- , 6481	R.EEAEDYLVQGGMSDGLYLLR.Q	2259.47849	2	4.03E-04	0.78	3.61	-	705.8
gi|4503101|ref|NP_001312.1|	cysteine and glycine-rich protein 2; LIM domain only 5, smooth muscle;			4.24E-04	0.39	10.16	9.30	20953.8
AHQ-1-14, 4172	K.GYGYGQGAGTLNM*DRGER.L	1919.02542	2	4.24E-04	0.39	3.16	-	265.7
gi|4503069|ref|NP_001306.1|	mitogen-activated protein kinase 14 isoform 1; cytokine suppressive ant			4.35E-04	0.85	10.18	4.20	41493.2
AHQ-1-9, 6894	K.LTDDHVQFLIYQILR.G	1875.16009	2	4.35E-04	0.85	3.56	-	724.5
gi|5729808|ref|NP_006557.1|	adhesion glycoprotein; platelet and T cell activation antigen 1; DNAX a			4.39E-04	0.67	10.16	5.40	38583.7
AHQ-1-6, 6718	R.WSVIVIPDVTVSDSGLYR.C	2007.27528	2	4.39E-04	0.67	3.12	-	406.7
gi|27479431|ref|XP_050793.2|	similar to KIAA1393 protein [Homo sapiens]			4.40E-04	0.30	10.10	1.60	58245.9
AHQ-1-5, 4846	-.M*VLWILWR.P	1133.43358	1	4.40E-04	0.30	1.93	-	553.7
gi|29734623|ref|XP_299768.1|	hypothetical protein XP_299768 [Homo sapiens]			4.42E-04	0.24	10.10	7.50	14905.7
AHQ-1-10, 3409	K.RMVPVKEIDR.L	1243.50477	1	4.42E-04	0.24	2.03	-	469.2
gi|14780596|ref|XP_033004.1|	hypothetical protein BC005107 [Homo sapiens]			4.42E-04	0.42	10.13	5.00	18127.9
AHQ-1-2, 1958 - 2025	R.EQIIAKAK.A	901.08569	1	4.42E-04	0.42	2.53	-	288.5
gi|6680033|ref|NP_031379.1|	guanine nucleotide binding protein (G protein) alpha 12 [Homo sapiens]			4.43E-04	0.57	10.13	2.90	44250.9
AHQ-1-9, 3140	K.ILLLGAGESGK.S	1058.25357	1	4.43E-04	0.57	2.22	-	738.5
gi|21618331|ref|NP_000746.2|	carnitine acetyltransferase isoform 1 precursor [Homo sapiens]			4.46E-04	0.43	10.15	4.00	70875.3
AHQ-1-9, 4536	R.FKAHQDALPRLPVPPLQQSLDHYLK.A	2913.36808	3	4.46E-04	0.43	3.03	-	517.3
gi|5174441|ref|NP_005886.1|	Golgi autoantigen, golgin subfamily a, 3; Golgin-160; male enhanced ant			4.51E-04	0.10	10.13	0.60	170266.8

AHQ-1-2, 2579	K.EEQVRLQAR.K	1129.25198	1	4.51E-04	0.10	1.96	-	127.9
gi|29729703|ref|XP_293563.1|	similar to huntingtin-associated protein interacting protein (duo) [Ho			4.59E-04	0.20	10.13	6.40	26758.5
AHQ-1-14- , 3936	K.ETDEERVLMDWEKVK.D	1908.12238	2	4.59E-04	0.20	2.63	-	259.6
gi|17439599|ref|XP_067423.1|	similar to destrin - pig [Homo sapiens]			4.71E-04	0.58	10.12	4.40	19819.2
AHQ-1-12, 2338 - 2341	K.CIIVEEGK.E	949.10404	1	4.71E-04	0.58	2.46	-	572.7
gi|30155280|ref|XP_294126.2|	similar to Keratin, type I cytoskeletal 18 (Cytokeratin 18) (K18) (CK			4.72E-04	0.22	10.15	7.90	36155.8
AHQ-1-13, 4020 - 4024	R.RTVQSLEIDLDLMRNPKASLENSLR.E	2900.30469	3	4.72E-04	0.22	3.01	-	221.4
gi|30153966|ref|XP_293932.2|	similar to hypothetical protein BC013073 [Homo sapiens]			4.73E-04	0.66	10.18	9.00	21711.3
AHQ-1-3, 4018	R.MIFTLFTINVSKDIRHH	2073.44997	2	4.73E-04	0.66	3.48	-	321.9
AHQ-1-6, 4096	R.MIFTLFTINVSKDIRHH	2073.44997	2	8.43E-04	0.39	3.05	-	373.4
AHQ-1-2, 4350	R.MIFTLFTINVSKDIRHH	2073.44997	3	5.20E-04	0.80	3.54	-	1173.3
gi|28373103|ref|NP_005164.2|	sarco/endoplasmic reticulum Ca2+ -ATPase isoform a; ATPase, Ca(2+)-tra			4.76E-04	0.90	10.19	1.50	109255.5
AHQ-1-1, 3792	R.VDQSILTGESVSVTK.H	1563.73208	2	4.76E-04	0.90	3.73	-	773.5
gi|25777600|ref|NP_002798.2|	proteasome 26S non-ATPase subunit 1; 26S proteasome non-ATPase regulat			4.79E-04	0.93	10.20	2.20	105835.6
AHQ-1-4, 5664	R.TPEQCPSVVSLLSESYNPHVR.Y	2401.63796	3	4.79E-04	0.93	4.03	-	1964.5
gi|21361741|ref|NP_057569.2|	hypothetical protein HSPC228; My012 protein [Homo sapiens]			4.88E-04	0.63	10.15	6.50	33879.0
AHQ-1-9, 5082	K.YAGSALQYEDVSTAVQNLQK.A	2186.36410	2	4.88E-04	0.63	2.96	-	363.0
gi|24475861|ref|NP_054891.2|	phosphohistidine phosphatase; sex-regulated protein janus-a [Homo sapi			4.90E-04	0.87	10.20	16.80	13832.4
AHQ-1-13- , 4141	K.IHVYGYSMAYGPAQHAISTEK.I	2324.60023	3	4.90E-04	0.87	4.04	-	659.0
gi|10835121|ref|NP_000289.1|	pyruvate kinase, liver and RBC; Pyruvate kinase, liver and RBC type [H			4.90E-04	0.94	10.20	1.90	61829.8
AHQ-1-7, 3708 - 3754	R.GDLGIEIPAEK.V	1142.28420	2	4.90E-04	0.94	3.98	-	1116.8
gi|13027380|ref|NP_055864.1|	amyotrophic lateral sclerosis 2 (juvenile) chromosome region, candidat			5.12E-04	0.70	10.17	2.30	101432.5
AHQ-1-13, 4999 - 5056	R.VEQMTKTYNDIDMVTHLLAER.D	2508.85682	3	5.12E-04	0.70	3.39	-	754.7
gi|4759080|ref|NP_004159.1|	succinate dehydrogenase complex, subunit A, flavoprotein precursor; suc			5.57E-04	0.50	10.16	4.50	72689.1
AHQ-1-6, 7082	R.AGLPCQDLEFVQFHPTGIYGAGCLITEGCR.G	3371.76536	3	5.57E-04	0.50	3.12	-	427.0
gi|7661624|ref|NP_056202.1|	preimplantation protein 3; likely ortholog of preimplantation protein 3			5.77E-04	0.50	10.14	7.20	22256.6
AHQ-1-10, 3555	K.ILEPPEGQDEGVWK.Y	1597.75022	2	5.77E-04	0.50	2.71	-	216.5
gi|6996010|ref|NP_002038.1|	glycyl-tRNA synthetase; GlyRS; glycine tRNA ligase [Homo sapiens]			5.96E-04	1.18	20.18	5.70	77530.3
AHQ-1-6, 6566	K.SPITGNDLSPPVSFNLMFK.T	2065.37824	2	5.96E-04	0.39	3.15	-	245.3
AHQ-1-6, 6883	K.TLYVEEVVPNVIEPSFGLGR.I	2219.52197	2	7.46E-04	0.79	3.60	-	517.9
gi|5174737|ref|NP_006077.1|	tubulin, beta, 4 [Homo sapiens]			6.26E-04	0.70	10.16	3.30	50517.5
AHQ-1-7, 4652 - 4654	R.ALTVPELTQQM*FDAK.N	1708.95749	2	6.26E-04	0.70	3.12	-	425.6
gi|30156349|ref|XP_301652.1|	similar to Eukaryotic initiation factor 4A-II (eIF-4A-II) (eIF4A-II) [			6.33E-04	0.23	10.14	9.30	21450.7
AHQ-1-8, 4379 - 4461	K.M*HAADLTVSALRGDM*DEK.E	1993.20912	2	6.33E-04	0.23	2.74	-	340.6
gi|4502417|ref|NP_000703.1|	biliverdin reductase A [Homo sapiens]			6.43E-04	0.46	10.14	4.70	33488.3
AHQ-1-9, 2911	K.SGSLENVPNVGVNK.N	1414.54642	2	6.43E-04	0.46	2.75	-	234.0
gi|6912396|ref|NP_036335.1|	glyoxylate reductase/hydroxypyruvate reductase [Homo sapiens]			6.74E-04	0.67	10.18	5.50	35668.0
AHQ-1-9, 4180	R.AADCEVEQWDSDEPIPAK.E	2062.15676	2	6.74E-04	0.67	2.82	-	509.3
gi|5902154|ref|NP_008994.1|	vascular Rab-GAP/TBC-containing [Homo sapiens]			6.74E-04	0.31	10.10	1.10	102676.0
AHQ-1-4, 2795	K.QM*IKDLAKEK.D	1220.46471	1	6.74E-04	0.31	1.93	-	628.4
gi|4506077|ref|NP_002734.1|	protein kinase C substrate 80K-H; glucosidase II, beta subunit; AGE-bin			6.80E-04	0.92	10.15	1.90	59296.0
AHQ-1-5, 4167	K.SLEDQVEMLR.T	1220.37847	2	6.80E-04	0.92	3.02	-	1500.4
gi|14249478|ref|NP_116189.1|	hypothetical protein FLJ14525 [Homo sapiens]			6.93E-04	0.08	10.14	2.40	36346.2
AHQ-1-2, 1962 - 2030	R.KIM*QDKEK.I	1036.22824	1	6.93E-04	0.08	2.01	-	289.7
gi|30157774|ref|XP_301845.1|	similar to Kunitz-type protease inhibitor 3 (HKIB9) [Homo sapiens] [MA			7.02E-04	0.41	10.15	12.00	24113.1
AHQ-1-12, 5435 - 5498	-.MQLQASLSFLLILTLCLELRSELAR.D	2921.51174	3	7.02E-04	0.41	3.04	-	435.0
gi|13385594|ref|NP_085056.1|	cytochrome b5 outer mitochondrial membrane precursor; type 2 cyt-b5 [H			7.20E-04	0.83	10.17	23.30	16332.0
AHQ-1-11, 5722	R.FLNEHPGGEEVLLEQAGVDASESFEDVGHSSDAR.E	3629.75848	3	7.20E-04	0.83	3.45	-	779.6
gi|28872728|ref|NP_002808.2|	proteasome 26S non-ATPase subunit 13 isoform 1; 26S proteasome subunit			7.50E-04	0.42	10.16	4.80	42918.2
AHQ-1-9, 4996	K.TAWGQQPDLAANEAQLLR.K	1983.17391	2	7.50E-04	0.42	3.11	-	230.9
gi|13376539|ref|NP_079295.1|	hypothetical protein FLJ13940 [Homo sapiens]			7.61E-04	0.49	10.14	5.00	27546.1
AHQ-1-11, 3862	R.QIFHPEQLITGK.E	1411.63050	2	7.61E-04	0.49	2.82	-	161.2
gi|11321611|ref|NP_006868.1|	guanosine monophosphate reductase; guanine monophosphate reductase [Ho			7.68E-04	0.90	10.23	7.50	37414.6
AHQ-1-9, 4174	K.LFATNHPECLQNVAVSSGSGQNDLEK.M	2818.02614	3	7.68E-04	0.90	4.57	-	1225.5
gi|22059372|ref|XP_113714.2|	similar to Density-regulated protein (DRP) (DRP1 protein) (Smooth musc			7.85E-04	0.49	10.15	8.60	22091.9
AHQ-1-10, 4371	K.WPEVDDDSIEDLGEVKK	1975.09895	2	7.85E-04	0.49	2.93	-	543.6
gi|24432013|ref|NP_078982.2|	hypothetical protein FLJ13479 [Homo sapiens]			7.96E-04	0.01	10.10	1.50	67976.0
AHQ-1-9, 2763	K.AYAELKDLR.N	1079.23134	1	7.96E-04	0.01	2.03	-	90.3
gi|6912662|ref|NP_036372.1|	sirtuin 4; sir2-like 4; sirtuin type 4; sirtuin silent mating type info			8.02E-04	0.47	10.16	8.60	35187.3
AHQ-1-9, 5234	K.AGSRRLTELHGCMDRVLCLDCGEQTPR.G	3192.57848	3	8.02E-04	0.47	3.13	-	476.4
gi|29745994|ref|XP_290546.1|	similar to KIAA0830 protein [Homo sapiens]			8.03E-04	0.95	10.21	2.60	55016.4
AHQ-1-9, 4200	K.ILEVVNQIQDEER.M	1585.74098	2	8.03E-04	0.95	4.26	-	1288.2
gi|20546215|ref|XP_115639.1|	similar to leukocyte immunoglobulin-like receptor, subfamily B (with T			8.20E-04	0.31	10.14	4.70	42748.2
AHQ-1-11, 4463 - 4476	R.CSREVNSREDNVLYRVVK.P	2225.47152	2	8.20E-04	0.31	2.74	-	501.9
gi|4504061|ref|NP_002067.1|	glucosamine (N-acetyl)-6-sulfatase precursor; N-acetylglucosamine-6-sul			8.21E-04	0.30	10.14	3.30	62081.8
AHQ-1-3, 5689 - 5761	K.RWQTLLSVDDLVEKLVKR.L	2199.58179	2	8.21E-04	0.30	2.66	-	321.3
gi|18588342|ref|XP_085748.1|	similar to KIAA1039 protein [Homo sapiens]			8.36E-04	0.65	10.14	14.40	10510.0
AHQ-1-7, 3578	K.LPFTDGDAQQLQR.K	1489.61498	2	8.36E-04	0.65	2.79	-	806.8
gi|7662502|ref|NP_054779.1|	MCT-1 protein [Homo sapiens]			8.37E-04	0.92	10.22	10.50	20555.2
AHQ-1-11, 5014	K.FVLSGANIMCPGLTSPGAK.L	1922.25862	2	8.37E-04	0.92	4.45	-	747.3
gi|5803003|ref|NP_006720.1|	diaphanous 2 isoform 156 [Homo sapiens]			8.71E-04	0.63	10.17	1.60	125568.3
AHQ-1-1, 5015	K.TAREQYEKLSTMHNNMMK.L	2213.54787	3	8.71E-04	0.63	3.38	-	764.9
gi|6912324|ref|NP_036266.1|	leiomodin 1 (smooth muscle); thyroid and eye muscle autoantigen D1 (64k			8.77E-04	0.38	10.13	3.00	63737.1
AHQ-1-14, 6114 - 6186	K.GEKKDLLEVPKAGAVAK.G	1754.06430	2	8.77E-04	0.38	2.69	-	557.0
gi|21361547|ref|NP_002930.2|	ribonuclease/angiogenin inhibitor; Placental ribonuclease inhibitor [H			8.83E-04	0.82	10.17	3.30	49973.3
AHQ-1-7, 6921	R.WAELLPLLQQCQVVR.L	1855.19360	2	8.83E-04	0.82	3.41	-	432.4
gi|4502167|ref|NP_000475.1|	amyloid beta (A4) precursor protein (protease nexin-II, Alzheimer disea			8.87E-04	0.42	10.13	2.30	86942.8
AHQ-1-4, 7012	R.CLVGEFVSDALLVPDKCK.F	2053.38561	2	8.87E-04	0.42	2.52	-	557.7
gi|4507947|ref|NP_003671.1|	tyrosyl-tRNA synthetase; tyrosyl-tRNA synthetase; tyrosyl tRNA ligase [			8.94E-04	0.90	10.19	3.40	59143.1
AHQ-1-7, 6225	K.AFCEPGNVENNGVLSFIK.H	1997.21741	2	8.94E-04	0.90	3.87	-	987.5
gi|4506185|ref|NP_002780.1|	proteasome alpha 4 subunit; proteasome component C9; proteasome subunit			9.35E-04	0.71	10.16	9.20	29483.6
AHQ-1-10, 6091	K.LNEDMACSVAGITSDANVLTNELR.L	2595.84629	2	9.35E-04	0.71	3.21	-	467.2
gi|11072091|ref|NP_057736.2|	Crn, crooked neck-like 1; CGI-201 protein; crooked neck protein (crn);			9.61E-04	0.47	10.10	1.10	99200.7
AHQ-1-4, 3214	R.FVLVHPDVK.N	1054.26662	1	9.61E-04	0.47	1.82	-	804.7

Reference				P (pro)	Sf	Score	Coverage	MW
File, Scan(s)	Peptide	MH+	z	P (pep)	Sf	XC		Sp
gi|4507485|ref|NP_003237.1| thrombospondin 1 [Homo sapiens]				1.00E-30	31.15	370.29	32.80	129351.8
AHQ-2-2, 4316 - 4386	K.AGTLDLSLTVQGK.Q	1303.48711	2	8.12E-06	0.93	3.68	-	1377.6
AHQ-2-6, 2503	R.AQLYIDCEK.M	1141.27636	1	2.19E-04	0.15	2.57	-	218.1
AHQ-2-3, 6700	R.AQLYIDCEKMENAELDVPIQSVFTR.D	2972.33983	3	6.44E-06	0.95	5.68	-	899.4
AHQ-2-10, 5959	R.AQLYIDCEKMENAELDVPIQSVFTR.D	2972.33983	2	5.61E-08	0.78	3.23	-	496.8
AHQ-2-2, 6745	R.AQLYIDCEKMENAELDVPIQSVFTR.D	2972.33983	3	2.18E-05	0.93	4.94	-	1081.7
AHQ-2-2, 7069	R.AQLYIDCEKMENAELDVPIQSVFTR.D	2972.33983	3	1.17E-05	0.94	5.06	-	1038.1
AHQ-2-4, 4244	R.CENTDPGYNCLPCPPR.F	1954.10800	2	4.82E-12	0.91	3.94	-	551.4
AHQ-2-3, 4060 - 4072	R.CENTDPGYNCLPCPPR.F	1954.10800	2	1.61E-04	0.80	3.09	-	592.5
AHQ-2-1, 4209	R.CENTDPGYNCLPCPPR.F	1954.10800	2	9.99E-06	0.71	3.26	-	296.6
AHQ-2-1, 3929	R.CENTDPGYNCLPCPPR.F	1954.10800	2	8.00E-06	0.83	3.20	-	498.0
AHQ-2-2, 3945 - 4017	R.CENTDPGYNCLPCPPR.F	1954.10800	2	8.39E-09	0.94	4.43	-	735.7
AHQ-2-2, 4096	R.CENTDPGYNCLPCPPR.F	1954.10800	2	2.55E-05	0.61	3.13	-	276.5
AHQ-2-4, 3354	K.CNYLGHYSDPM*YR.C	1693.84162	2	4.87E-05	0.93	3.43	-	971.5
AHQ-2-2, 3389 - 3392	K.CNYLGHYSDPM*YR.C	1693.84162	2	1.47E-04	0.92	3.78	-	779.0
AHQ-2-2, 3917	K.CNYLGHYSDPMYR.C	1677.84222	2	3.51E-05	0.91	3.90	-	635.4
AHQ-2-4, 3872 - 3948	K.CNYLGHYSDPMYR.C	1677.84222	2	5.43E-04	0.72	2.91	-	742.7
AHQ-2-4, 3477 - 3554	K.DCVGDVTENQICNK.Q	1654.75869	2	5.76E-05	0.87	3.22	-	867.3
AHQ-2-3, 3464 - 3467	K.DCVGDVTENQICNK.Q	1654.75869	2	9.38E-07	0.96	4.44	-	1340.8
AHQ-2-2, 3525	K.DCVGDVTENQICNK.Q	1654.75869	2	5.37E-06	0.94	3.91	-	1192.7
AHQ-2-4, 6610	K.DDFDHDSVPDIDDICPENVDISETDFR.R	3183.22991	3	2.56E-04	0.73	3.58	-	785.8
AHQ-2-5, 2584	K.DHSGQVFSVVSNGK.A	1461.56164	2	5.86E-04	0.92	3.86	-	925.3
AHQ-2-6, 2521	K.DHSGQVFSVVSNGK.A	1461.56164	2	2.05E-04	0.77	2.69	-	700.8
AHQ-2-2, 2693	K.DHSGQVFSVVSNGK.A	1461.56164	2	8.23E-07	0.94	3.24	-	1501.7
AHQ-2-10, 2496	K.DHSGQVFSVVSNGK.A	1461.56164	2	2.93E-04	0.94	3.91	-	1152.5
AHQ-2-2, 2706	K.DHSGQVFSVVSNGK.A	1461.56164	1	2.18E-08	0.37	2.24	-	344.3
AHQ-2-4, 2653	K.DHSGQVFSVVSNGK.A	1461.56164	2	1.17E-08	0.95	4.12	-	1147.7
AHQ-2-1, 3189	R.DNCQYVYNVDQR.D	1575.64166	2	7.58E-07	0.92	4.39	-	781.8
AHQ-2-1, 3353	R.DNCQYVYNVDQR.D	1575.64166	2	5.51E-05	0.91	3.45	-	1363.2
AHQ-2-4, 3144	R.DNCQYVYNVDQR.D	1575.64166	2	2.03E-05	0.93	3.86	-	1047.1
AHQ-2-3, 3140	R.DNCQYVYNVDQR.D	1575.64166	2	1.47E-04	0.90	3.65	-	948.7
AHQ-2-2, 6294	K.DSDGDGRGDACKDDFDHDSVPDIDDICPENVDISETDFR.R	4418.43100	3	7.42E-09	0.74	4.05	-	331.8
AHQ-2-3, 4309 - 4363	R.DTDMDGVGDQCDNCPLEHNPDQLDSDSDR.I	3324.31882	3	7.78E-11	0.90	4.37	-	665.2
AHQ-2-2, 3972 - 4040	R.DTDM*DGVGDQCDNCPLEHNPDQLDSDSDR.I	3340.31822	3	5.96E-13	0.91	4.57	-	855.6
AHQ-2-5, 4288	R.DTDMDGVGDQCDNCPLEHNPDQLDSDSDR.I	3324.31882	3	1.46E-05	0.88	4.49	-	555.1
AHQ-2-7, 4275	R.DTDMDGVGDQCDNCPLEHNPDQLDSDSDR.I	3324.31882	3	2.68E-05	0.86	4.36	-	512.1
AHQ-2-2, 4346 - 4417	R.DTDMDGVGDQCDNCPLEHNPDQLDSDSDR.I	3324.31882	3	1.16E-13	0.92	5.05	-	570.8
AHQ-2-4, 4234	R.DTDMDGVGDQCDNCPLEHNPDQLDSDSDR.I	3324.31882	3	3.39E-09	0.92	4.49	-	836.3
AHQ-2-4, 3932 - 4008	R.DTDM*DGVGDQCDNCPLEHNPDQLDSDSDR.I	3340.31822	3	4.01E-08	0.81	3.58	-	678.1
AHQ-2-3, 3917 - 3973	R.DTDM*DGVGDQCDNCPLEHNPDQLDSDSDR.I	3340.31822	3	2.62E-10	0.91	5.34	-	523.3
AHQ-2-2, 4108	R.DTDM*DGVGDQCDNCPLEHNPDQLDSDSDR.I	3340.31822	3	3.07E-04	0.76	3.45	-	608.5
AHQ-2-4, 4320	R.DTDMDGVGDQCDNCPLEHNPDQLDSDSDR.I	3324.31882	3	1.47E-07	0.80	3.65	-	449.2
AHQ-2-1, 3889 - 3890	R.DTDM*DGVGDQCDNCPLEHNPDQLDSDSDR.I	3340.31822	3	6.04E-09	0.91	4.54	-	822.9
AHQ-2-2, 4545	R.DTDMDGVGDQCDNCPLEHNPDQLDSDSDR.I	3324.31882	3	1.90E-07	0.75	3.40	-	458.7
AHQ-2-1, 4310	R.DTDMDGVGDQCDNCPLEHNPDQLDSDSDR.I	3324.31882	3	1.87E-05	0.73	3.48	-	463.4
AHQ-2-2, 3644	K.FQDLVDAVR.A	1063.18877	1	2.79E-04	0.24	2.03	-	346.3
AHQ-2-2, 3645	K.FQDLVDAVR.A	1063.18877	2	2.87E-06	0.94	3.39	-	1279.7
AHQ-2-1, 3782	K.FQDLVDAVR.A	1063.18877	2	1.23E-04	0.86	2.57	-	998.3
AHQ-2-6, 3346	K.FQDLVDAVR.A	1063.18877	2	1.61E-05	0.88	2.84	-	1050.2
AHQ-2-9, 3299	K.FQDLVDAVR.A	1063.18877	1	2.72E-05	0.24	2.07	-	348.1
AHQ-2-7, 2455	R.FTGSQPFGQGVEHATANK.Q	1877.00666	2	1.20E-05	0.86	3.98	-	431.7
AHQ-2-1, 2962	R.FTGSQPFGQGVEHATANK.Q	1877.00666	2	5.11E-09	0.81	3.79	-	373.7
AHQ-2-3, 2925	R.FTGSQPFGQGVEHATANK.Q	1877.00666	2	3.31E-12	0.72	3.34	-	409.8
AHQ-2-2, 2833 - 2913	R.FTGSQPFGQGVEHATANK.Q	1877.00666	2	2.22E-06	0.90	4.17	-	513.9
AHQ-2-2, 2734	R.FTGSQPFGQGVEHATANK.Q	1877.00666	3	5.63E-04	0.60	3.25	-	481.2
AHQ-2-5, 2479	R.FTGSQPFGQGVEHATANK.Q	1877.00666	2	4.36E-04	0.87	3.90	-	542.3
AHQ-2-3, 2831	R.FTGSQPFGQGVEHATANK.Q	1877.00666	2	1.27E-07	0.78	3.67	-	337.0
AHQ-2-4, 2926	R.FTGSQPFGQGVEHATANK.Q	1877.00666	2	2.94E-06	0.88	4.14	-	452.8
AHQ-2-4, 5689	R.FVFGTTPEDILR.N	1395.58470	2	3.47E-04	0.84	3.11	-	708.8
AHQ-2-4, 5697	R.FVFGTTPEDILR.N	1395.58470	1	4.07E-07	0.08	2.12	-	88.6
AHQ-2-9, 5330 - 5331	R.FVFGTTPEDILR.N	1395.58470	1	4.86E-06	0.03	1.95	-	98.3
AHQ-2-9, 5326 - 5334	R.FVFGTTPEDILR.N	1395.58470	2	6.40E-04	0.81	3.31	-	697.9
AHQ-2-3, 5660 - 5661	R.FVFGTTPEDILR.N	1395.58470	1	1.23E-04	0.12	2.06	-	170.6
AHQ-2-12, 5465	R.FVFGTTPEDILR.N	1395.58470	2	3.05E-04	0.89	2.80	-	1036.5
AHQ-2-5, 5417	R.FVFGTTPEDILR.N	1395.58470	1	2.77E-05	0.15	2.00	-	132.9
AHQ-2-5, 5420	R.FVFGTTPEDILR.N	1395.58470	1	2.45E-06	0.12	2.25	-	69.2
AHQ-2-6, 5337 - 5418	R.FVFGTTPEDILR.N	1395.58470	1	2.53E-04	0.08	2.04	-	139.4
AHQ-2-6, 4425 - 4433	K.GGVNDNFQGVLQNVR.F	1617.74799	2	3.27E-10	0.92	4.37	-	868.7
AHQ-2-5, 4245	K.GGVNDNFQGVLQNVR.F	1617.74799	2	4.16E-04	0.78	3.07	-	642.9
AHQ-2-2, 4829 - 4908	K.GGVNDNFQGVLQNVR.F	1617.74799	2	1.09E-05	0.95	4.79	-	1083.8
AHQ-2-2, 4725 - 4752	K.GGVNDNFQGVLQNVR.F	1617.74799	2	1.74E-05	0.89	4.11	-	798.0
AHQ-2-2, 4581	K.GGVNDNFQGVLQNVR.F	1617.74799	2	1.71E-04	0.91	3.91	-	813.6
AHQ-2-6, 4190 - 4191	K.GGVNDNFQGVLQNVR.F	1617.74799	2	1.44E-05	0.94	4.24	-	1140.7
AHQ-2-1, 4653	K.GGVNDNFQGVLQNVR.F	1617.74799	2	7.06E-05	0.83	3.75	-	707.4
AHQ-2-4, 4890	K.GGVNDNFQGVLQNVR.F	1617.74799	2	2.57E-08	0.91	4.21	-	840.8
AHQ-2-4, 4716 - 4746	K.GGVNDNFQGVLQNVR.F	1617.74799	2	5.10E-08	0.86	3.53	-	776.9
AHQ-2-4, 4625 - 4701	K.GGVNDNFQGVLQNVR.F	1617.74799	2	7.36E-07	0.81	3.37	-	590.9
AHQ-2-4, 4530	K.GGVNDNFQGVLQNVR.F	1617.74799	2	3.40E-05	0.81	3.25	-	897.2
AHQ-2-7, 4039 - 4111	K.GGVNDNFQGVLQNVR.F	1617.74799	2	2.46E-04	0.73	3.58	-	408.4
AHQ-2-10, 4327 - 4330	K.GGVNDNFQGVLQNVR.F	1617.74799	2	2.85E-07	0.91	4.18	-	878.3
AHQ-2-2, 4422	K.GGVNDNFQGVLQNVR.F	1617.74799	2	8.26E-05	0.72	3.29	-	494.5
AHQ-2-7, 4401 - 4413	K.GGVNDNFQGVLQNVR.F	1617.74799	2	2.01E-08	0.88	4.17	-	770.3
AHQ-2-10, 3898	K.GGVNDNFQGVLQNVR.F	1617.74799	2	1.85E-07	0.74	3.09	-	686.3
AHQ-2-1, 4749	K.GGVNDNFQGVLQNVR.F	1617.74799	2	8.96E-06	0.90	4.16	-	717.7
AHQ-2-3, 4887	K.GGVNDNFQGVLQNVR.F	1617.74799	2	1.59E-05	0.88	4.02	-	728.1
AHQ-2-8, 3943	K.GGVNDNFQGVLQNVR.F	1617.74799	2	2.90E-05	0.63	3.12	-	497.8
AHQ-2-5, 4493 - 4544	K.GGVNDNFQGVLQNVR.F	1617.74799	2	9.51E-05	0.89	4.04	-	836.3
AHQ-2-9, 4166 - 4174	K.GGVNDNFQGVLQNVR.F	1617.74799	2	2.12E-04	0.76	3.76	-	470.6
AHQ-2-9, 4408 - 4442	K.GGVNDNFQGVLQNVR.F	1617.74799	2	3.44E-05	0.92	4.02	-	1089.1
AHQ-2-3, 4707 - 4783	K.GGVNDNFQGVLQNVR.F	1617.74799	2	9.78E-10	0.93	4.36	-	1019.6
AHQ-2-10, 3990	K.GGVNDNFQGVLQNVR.F	1617.74799	2	8.60E-04	0.67	3.50	-	529.9
AHQ-2-3, 4391	K.GGVNDNFQGVLQNVR.F	1617.74799	2	1.55E-04	0.84	3.55	-	904.9
AHQ-2-5, 4664	K.GGVNDNFQGVLQNVR.F	1617.74799	2	1.85E-06	0.82	3.57	-	652.4
AHQ-2-3, 4543	K.GGVNDNFQGVLQNVR.F	1617.74799	2	1.28E-06	0.86	3.67	-	719.2
AHQ-2-10, 4162 - 4163	K.GGVNDNFQGVLQNVR.F	1617.74799	2	6.77E-11	0.90	4.28	-	807.4
AHQ-2-1, 2234 - 2253	K.GPDPSSPAFR.I	1031.10364	2	6.05E-05	0.70	2.91	-	516.5
AHQ-2-2, 2074	K.GPDPSSPAFR.I	1031.10364	2	1.20E-05	0.87	3.06	-	686.1
AHQ-2-5, 1857 - 1868	K.GPDPSSPAFR.I	1031.10364	2	8.55E-04	0.77	3.19	-	409.9
AHQ-2-6, 1855 - 1881	K.GPDPSSPAFR.I	1031.10364	2	8.98E-05	0.75	3.00	-	455.9
AHQ-2-3, 2004 - 2083	K.GPDPSSPAFR.I	1031.10364	2	1.79E-05	0.84	3.11	-	570.4
AHQ-2-11, 2104	K.GPDPSSPAFR.I	1031.10364	2	4.15E-04	0.79	3.04	-	623.2
AHQ-2-4, 2073	K.GPDPSSPAFR.I	1031.10364	2	2.09E-05	0.87	3.13	-	683.1
AHQ-2-10, 2648 - 2656	R.GTLLALER.K	873.03234	2	1.89E-04	0.88	3.05	-	939.2
AHQ-2-2, 2910	R.GTLLALER.K	873.03234	2	3.77E-04	0.90	3.32	-	1022.9
AHQ-2-11, 2877 - 2878	R.GTLLALER.K	873.03234	2	5.53E-04	0.83	2.92	-	886.9

AHQ-2-4, 2910	R.GTLLALER.K	873.03234	2	6.52E-04	0.93	3.49	-	1149.8
AHQ-2-3, 2544 - 2608	K.GTSQNDPNWVVR.H	1373.45608	2	6.47E-04	0.47	2.73	-	588.2
AHQ-2-2, 2557 - 2586	K.GTSQNDPNWVVR.H	1373.45608	2	1.69E-04	0.72	3.13	-	810.7
AHQ-2-4, 2557 - 2566	K.GTSQNDPNWVVR.H	1373.45608	2	4.16E-05	0.72	3.40	-	601.4
AHQ-2-1, 2772	K.GTSQNDPNWVVR.H	1373.45608	2	2.11E-04	0.48	2.53	-	608.8
AHQ-2-13, 6260	R.IEDANLIPPVPDDKFQDLVDAVR.A	2580.87450	3	6.37E-06	0.71	3.24	-	317.7
AHQ-2-5, 6659 - 6660	R.IEDANLIPPVPDDKFQDLVDAVR.A	2580.87450	2	2.55E-07	0.76	3.32	-	316.2
AHQ-2-12, 6465 - 6539	R.IEDANLIPPVPDDKFQDLVDAVR.A	2580.87450	2	1.23E-07	0.81	3.63	-	274.2
AHQ-2-12, 6399 - 6462	R.IEDANLIPPVPDDKFQDLVDAVR.A	2580.87450	3	2.21E-05	0.85	4.17	-	447.4
AHQ-2-5, 6869	R.IEDANLIPPVPDDKFQDLVDAVR.A	2580.87450	3	4.77E-04	0.84	4.02	-	416.7
AHQ-2-2, 6960 - 7038	R.IEDANLIPPVPDDKFQDLVDAVR.A	2580.87450	2	1.59E-05	0.54	2.77	-	235.2
AHQ-2-13, 6261	R.IEDANLIPPVPDDKFQDLVDAVR.A	2580.87450	2	1.46E-06	0.69	3.39	-	189.1
AHQ-2-11, 6261 - 6304	R.IEDANLIPPVPDDKFQDLVDAVR.A	2580.87450	2	8.26E-06	0.69	3.17	-	310.2
AHQ-2-11, 6164 - 6184	R.IEDANLIPPVPDDKFQDLVDAVR.A	2580.87450	3	1.14E-08	0.82	4.05	-	431.1
AHQ-2-11, 6162 - 6170	R.IEDANLIPPVPDDKFQDLVDAVR.A	2580.87450	2	3.80E-04	0.48	2.97	-	215.9
AHQ-2-14-, 6304 - 6385	R.IEDANLIPPVPDDKFQDLVDAVR.A	2580.87450	3	8.25E-07	0.76	3.12	-	630.2
AHQ-2-3, 6983	R.IEDANLIPPVPDDKFQDLVDAVR.A	2580.87450	3	4.57E-05	0.80	4.01	-	360.5
AHQ-2-13-, 6445 - 6449	R.IEDANLIPPVPDDKFQDLVDAVR.A	2580.87450	3	3.62E-07	0.67	3.04	-	448.4
AHQ-2-10, 6124 - 6199	R.IEDANLIPPVPDDKFQDLVDAVR.A	2580.87450	3	2.33E-07	0.82	4.13	-	334.7
AHQ-2-10, 6042 - 6114	R.IEDANLIPPVPDDKFQDLVDAVR.A	2580.87450	2	3.76E-05	0.63	3.29	-	186.2
AHQ-2-10, 6038 - 6050	R.IEDANLIPPVPDDKFQDLVDAVR.A	2580.87450	3	2.15E-10	0.88	4.28	-	444.6
AHQ-2-3, 6791 - 6823	R.IEDANLIPPVPDDKFQDLVDAVR.A	2580.87450	3	1.61E-06	0.68	3.45	-	418.4
AHQ-2-3, 6932 - 6984	R.IEDANLIPPVPDDKFQDLVDAVR.A	2580.87450	2	5.60E-06	0.58	2.57	-	316.2
AHQ-2-1, 6273	R.IEDANLIPPVPDDKFQDLVDAVR.A	2580.87450	3	3.56E-04	0.88	4.35	-	413.1
AHQ-2-3, 6892	R.IEDANLIPPVPDDKFQDLVDAVR.A	2580.87450	3	4.99E-11	0.80	3.66	-	423.5
AHQ-2-6, 6393 - 6469	R.IEDANLIPPVPDDKFQDLVDAVR.A	2580.87450	2	9.49E-06	0.34	2.55	-	164.4
AHQ-2-9, 6736	R.IEDANLIPPVPDDKFQDLVDAVR.A	2580.87450	3	7.11E-08	0.79	3.93	-	341.8
AHQ-2-9, 6610 - 6612	R.IEDANLIPPVPDDKFQDLVDAVR.A	2580.87450	2	3.81E-04	0.36	2.64	-	274.7
AHQ-2-1, 6612	R.IEDANLIPPVPDDKFQDLVDAVR.A	2580.87450	3	9.04E-05	0.71	3.40	-	333.2
AHQ-2-1, 6722 - 6800	R.IEDANLIPPVPDDKFQDLVDAVR.A	2580.87450	3	5.85E-08	0.87	4.14	-	462.9
AHQ-2-1, 6736 - 6801	R.IEDANLIPPVPDDKFQDLVDAVR.A	2580.87450	2	2.31E-05	0.55	2.87	-	245.3
AHQ-2-1, 6865	R.IEDANLIPPVPDDKFQDLVDAVR.A	2580.87450	3	5.94E-06	0.82	3.92	-	358.9
AHQ-2-4, 6844 - 6848	R.IEDANLIPPVPDDKFQDLVDAVR.A	2580.87450	3	3.27E-09	0.68	3.03	-	472.8
AHQ-2-7, 6551	R.IEDANLIPPVPDDKFQDLVDAVR.A	2580.87450	3	2.17E-08	0.72	3.47	-	356.4
AHQ-2-2, 7041	R.IEDANLIPPVPDDKFQDLVDAVR.A	2580.87450	3	1.93E-06	0.79	3.81	-	436.7
AHQ-2-6, 6657	R.IEDANLIPPVPDDKFQDLVDAVR.A	2580.87450	3	1.86E-04	0.69	3.22	-	353.2
AHQ-2-6, 6497 - 6570	R.IEDANLIPPVPDDKFQDLVDAVR.A	2580.87450	3	2.46E-08	0.86	4.46	-	338.3
AHQ-2-2, 2309 - 2380	K.IM*ADSGPIYDK.T	1226.38144	2	1.53E-04	0.76	3.37	-	415.5
AHQ-2-8, 2250	K.IMADSGPIYDK.T	1210.38204	2	7.99E-06	0.91	3.18	-	945.0
AHQ-2-3, 2528	K.IMADSGPIYDK.T	1210.38204	2	5.10E-06	0.92	3.59	-	720.0
AHQ-2-2, 2542	K.IMADSGPIYDK.T	1210.38204	2	2.21E-04	0.91	3.55	-	716.3
AHQ-2-11, 2496	K.IMADSGPIYDK.T	1210.38204	2	7.49E-04	0.85	3.11	-	668.4
AHQ-2-4, 2536 - 2549	K.IMADSGPIYDK.T	1210.38204	2	1.24E-04	0.88	3.27	-	623.0
AHQ-2-6, 2379	K.IMADSGPIYDK.T	1210.38204	1	8.26E-04	0.73	2.64	-	720.9
AHQ-2-6, 2374	K.IMADSGPIYDK.T	1210.38204	2	7.40E-05	0.93	3.68	-	879.4
AHQ-2-12, 2542	K.IMADSGPIYDK.T	1210.38204	2	3.45E-06	0.87	3.50	-	771.0
AHQ-2-13, 2704 - 2715	K.IMADSGPIYDK.T	1210.38204	2	6.07E-05	0.83	3.04	-	660.3
AHQ-2-14-, 2263	K.IM*ADSGPIYDK.T	1226.38144	2	9.36E-06	0.83	3.55	-	471.0
AHQ-2-14-, 6687 - 6757	R.IPESGGDNSVFDIFELTGAAR.K	2196.35899	2	1.62E-08	0.97	5.74	-	1034.2
AHQ-2-1, 7092	R.IPESGGDNSVFDIFELTGAAR.K	2196.35899	2	9.03E-13	0.97	5.68	-	1367.0
AHQ-2-10, 6410 - 6471	R.IPESGGDNSVFDIFELTGAAR.K	2196.35899	2	2.03E-08	0.97	5.29	-	1243.5
AHQ-2-10, 6550 - 6615	R.IPESGGDNSVFDIFELTGAAR.K	2196.35899	2	1.00E-30	0.97	5.38	-	1313.0
AHQ-2-2, 7286 - 7352	R.IPESGGDNSVFDIFELTGAAR.K	2196.35899	2	3.43E-11	0.97	5.81	-	1285.4
AHQ-2-3, 7217 - 7285	R.IPESGGDNSVFDIFELTGAAR.K	2196.35899	2	1.18E-06	0.96	5.28	-	988.8
AHQ-2-3, 7353	R.IPESGGDNSVFDIFELTGAAR.K	2196.35899	2	5.82E-11	0.97	5.66	-	1425.2
AHQ-2-3, 7437	R.IPESGGDNSVFDIFELTGAAR.K	2196.35899	2	1.38E-05	0.91	4.24	-	817.6
AHQ-2-9, 7119 - 7186	R.IPESGGDNSVFDIFELTGAAR.K	2196.35899	2	8.74E-07	0.92	4.46	-	638.8
AHQ-2-13, 6616	R.IPESGGDNSVFDIFELTGAAR.K	2196.35899	2	3.23E-10	0.96	5.38	-	1071.4
AHQ-2-6, 7042	R.IPESGGDNSVFDIFELTGAAR.K	2196.35899	2	2.88E-05	0.92	4.20	-	829.5
AHQ-2-6, 6901 - 6911	R.IPESGGDNSVFDIFELTGAAR.K	2196.35899	2	1.20E-12	0.95	5.11	-	947.8
AHQ-2-9, 6996 - 6998	R.IPESGGDNSVFDIFELTGAAR.K	2196.35899	2	5.15E-05	0.94	4.77	-	903.4
AHQ-2-4, 7273 - 7316	R.IPESGGDNSVFDIFELTGAAR.K	2196.35899	2	4.88E-14	0.96	5.62	-	862.8
AHQ-2-11, 6509	R.IPESGGDNSVFDIFELTGAAR.K	2196.35899	2	1.05E-04	0.93	4.47	-	745.6
AHQ-2-12, 6863	R.IPESGGDNSVFDIFELTGAAR.K	2196.35899	2	9.07E-08	0.93	4.97	-	856.9
AHQ-2-4, 7378	R.IPESGGDNSVFDIFELTGAAR.K	2196.35899	2	6.93E-08	0.90	4.20	-	657.9
AHQ-2-5, 7071 - 7135	R.IPESGGDNSVFDIFELTGAAR.K	2196.35899	2	1.51E-06	0.96	5.63	-	958.2
AHQ-2-4, 7466	R.IPESGGDNSVFDIFELTGAAR.K	2196.35899	2	4.21E-08	0.85	3.58	-	711.0
AHQ-2-2, 7425	R.IPESGGDNSVFDIFELTGAAR.K	2196.35899	2	6.99E-12	0.97	5.63	-	1199.5
AHQ-2-4, 2214 - 2282	R.KDHSGQVFSVVSNGK.A	1589.73455	2	1.48E-11	0.90	3.63	-	997.4
AHQ-2-3, 2215 - 2289	R.KDHSGQVFSVVSNGK.A	1589.73455	2	2.28E-08	0.90	3.70	-	966.2
AHQ-2-2, 2230 - 2301	R.KDHSGQVFSVVSNGK.A	1589.73455	2	6.86E-11	0.94	3.80	-	1347.8
AHQ-2-1, 2438 - 2516	R.KDHSGQVFSVVSNGK.A	1589.73455	2	1.50E-05	0.26	2.52	-	303.4
AHQ-2-5, 2091	R.KDHSGQVFSVVSNGK.A	1589.73455	2	1.05E-07	0.89	3.44	-	937.6
AHQ-2-9, 2050 - 2127	R.KDHSGQVFSVVSNGK.A	1589.73455	2	1.40E-06	0.77	2.94	-	796.0
AHQ-2-2, 2322	K.KIMADSGPIYDK.T	1338.55496	2	5.23E-08	0.93	4.03	-	849.7
AHQ-2-4, 2341	R.KVTEENKELANELR.R	1673.84976	2	6.71E-09	0.95	4.73	-	1416.0
AHQ-2-5, 1956 - 2027	R.KVTEENKELANELR.R	1673.84976	3	5.03E-09	0.78	3.19	-	818.8
AHQ-2-3, 2233	R.KVTEENKELANELR.R	1673.84976	2	4.76E-06	0.81	3.62	-	769.9
AHQ-2-4, 2236 - 2237	R.KVTEENKELANELR.R	1673.84976	2	3.80E-09	0.94	4.97	-	1112.4
AHQ-2-2, 2368	R.KVTEENKELANELR.R	1673.84976	2	4.86E-10	0.97	5.23	-	1684.3
AHQ-2-6, 2031	R.KVTEENKELANELR.R	1673.84976	2	4.07E-08	0.95	5.39	-	1105.5
AHQ-2-5, 1964 - 2035	R.KVTEENKELANELR.R	1673.84976	2	2.24E-06	0.93	4.71	-	1062.1
AHQ-2-2, 1969	R.KVTEENKELANELR.R	1673.84976	2	1.05E-08	0.88	3.68	-	843.5
AHQ-2-6, 2171	R.KVTEENKELANELR.R	1673.84976	2	2.92E-04	0.93	4.68	-	671.7
AHQ-2-2, 2272	R.KVTEENKELANELR.R	1673.84976	2	3.28E-08	0.94	5.04	-	1309.6
AHQ-2-9, 2082	R.KVTEENKELANELR.R	1673.84976	2	1.11E-08	0.94	4.84	-	1282.4
AHQ-2-1, 2454	R.KVTEENKELANELR.R	1673.84976	2	4.06E-10	0.95	5.19	-	1369.8
AHQ-2-4, 1993	R.KVTEENKELANELR.R	1673.84976	2	1.57E-09	0.94	4.33	-	1435.6
AHQ-2-7, 1873 - 1951	R.KVTEENKELANELR.R	1673.84976	2	7.50E-06	0.93	4.54	-	1023.0
AHQ-2-3, 2345	R.KVTEENKELANELR.R	1673.84976	2	1.24E-07	0.94	4.61	-	1228.6
AHQ-2-1, 2637	R.LCNSPSPQMNGKPCEGEAR.E	2135.34557	2	1.98E-07	0.91	4.35	-	616.4
AHQ-2-5, 2388	R.LCNSPSPQMNGKPCEGEAR.E	2135.34557	2	1.93E-06	0.93	4.87	-	636.9
AHQ-2-3, 2513	R.LCNSPSPQMNGKPCEGEAR.E	2135.34557	2	2.72E-07	0.83	3.64	-	518.9
AHQ-2-2, 2532	R.LCNSPSPQMNGKPCEGEAR.E	2135.34557	2	8.94E-05	0.82	3.73	-	557.0
AHQ-2-3, 2148 - 2149	R.LCNSPSPQM*NGKPCEGEAR.E	2151.34497	2	5.47E-06	0.87	3.71	-	533.6
AHQ-2-4, 2149	R.LCNSPSPQM*NGKPCEGEAR.E	2151.34497	2	9.06E-04	0.72	2.65	-	538.6
AHQ-2-7, 5420 - 5421	K.MENAELDVPIQSVFTR.D	1850.08607	2	9.21E-07	0.93	4.66	-	571.8
AHQ-2-4, 5586	K.M*ENAELDVPIQSVFTR.D	1866.08547	2	5.26E-05	0.40	2.58	-	274.2
AHQ-2-2, 5964	K.MENAELDVPIQSVFTR.D	1850.08607	2	1.94E-05	0.94	4.91	-	604.2
AHQ-2-2, 5840	K.MENAELDVPIQSVFTR.D	1850.08607	2	9.57E-10	0.95	4.87	-	654.4
AHQ-2-3, 5811	K.MENAELDVPIQSVFTR.D	1850.08607	2	2.36E-09	0.93	4.57	-	654.0
AHQ-2-4, 5812 - 5813	K.MENAELDVPIQSVFTR.D	1850.08607	2	3.57E-09	0.80	3.73	-	416.6
AHQ-2-2, 5572	K.M*ENAELDVPIQSVFTR.D	1866.08547	2	3.17E-09	0.94	4.86	-	734.4
AHQ-2-6, 5219	K.M*ENAELDVPIQSVFTR.D	1866.08547	2	9.12E-04	0.96	5.14	-	807.5
AHQ-2-11, 5309	K.MENAELDVPIQSVFTR.D	1850.08607	2	2.98E-05	0.87	3.87	-	456.5
AHQ-2-5, 5519 - 5552	K.MENAELDVPIQSVFTR.D	1850.08607	2	8.03E-10	0.92	4.50	-	521.0
AHQ-2-3, 5544	K.M*ENAELDVPIQSVFTR.D	1866.08547	2	6.73E-10	0.94	4.91	-	637.0

AHQ-2-4, 5946	K.MENAELDVPIQSVFTR.D	1850.08607	2	8.62E-07	0.87	3.61	-	566.3
AHQ-2-3, 3060 - 3067	K.QVTQSYWDTNPTR.A	1596.68241	2	5.34E-08	0.91	3.74	-	953.6
AHQ-2-5, 2831 - 2832	K.QVTQSYWDTNPTR.A	1596.68241	2	1.71E-06	0.84	3.48	-	501.6
AHQ-2-4, 3054 - 3056	K.QVTQSYWDTNPTR.A	1596.68241	2	9.16E-07	0.90	3.77	-	670.8
AHQ-2-2, 3076 - 3088	K.QVTQSYWDTNPTR.A	1596.68241	2	1.84E-06	0.93	3.85	-	1019.7
AHQ-2-11, 2941	K.QVTQSYWDTNPTR.A	1596.68241	2	5.04E-08	0.89	3.76	-	677.5
AHQ-2-4, 2786	R.RPPLCYHNGVQYR.N	1661.86889	2	1.50E-06	0.88	3.40	-	642.5
AHQ-2-2, 2526 - 2604	R.RPPLCYHNGVQYR.N	1661.86889	2	4.00E-04	0.64	3.05	-	434.3
AHQ-2-6, 2525	R.RPPLCYHNGVQYR.N	1661.86889	2	1.75E-08	0.94	4.51	-	822.5
AHQ-2-4, 2684 - 2752	R.RPPLCYHNGVQYR.N	1661.86889	2	1.56E-06	0.91	3.81	-	764.5
AHQ-2-5, 2340	R.RPPLCYHNGVQYR.N	1661.86889	2	4.96E-04	0.85	3.46	-	555.4
AHQ-2-2, 2717 - 2794	R.RPPLCYHNGVQYR.N	1661.86889	2	9.12E-07	0.91	3.96	-	710.5
AHQ-2-3, 2711 - 2785	R.RPPLCYHNGVQYR.N	1661.86889	2	1.03E-08	0.94	4.14	-	737.1
AHQ-2-2, 3906	K.SITLFVQEDR.A	1208.34588	2	4.25E-06	0.94	3.70	-	1384.1
AHQ-2-3, 3853	K.SITLFVQEDR.A	1208.34588	2	6.45E-06	0.92	3.58	-	1107.7
AHQ-2-6, 3563	K.SITLFVQEDR.A	1208.34588	2	1.87E-06	0.86	2.71	-	1017.1
AHQ-2-12, 3747 - 3750	K.SITLFVQEDR.A	1208.34588	2	3.95E-05	0.90	3.25	-	1129.2
AHQ-2-4, 3840	K.SITLFVQEDR.A	1208.34588	2	1.93E-05	0.93	3.38	-	1283.5
AHQ-2-1, 4020	K.SITLFVQEDR.A	1208.34588	2	4.38E-04	0.90	3.46	-	1158.6
AHQ-2-5, 3647	K.SITLFVQEDR.A	1208.34588	2	8.63E-07	0.90	3.02	-	1145.3
AHQ-2-10, 3460	K.SITLFVQEDR.A	1208.34588	2	1.27E-06	0.88	3.08	-	1089.2
AHQ-2-2, 4013	K.SITLFVQEDR.A	1208.34588	2	4.88E-07	0.86	2.58	-	1063.0
AHQ-2-5, 3100	R.TIVTTLQDSIR.K	1247.42355	2	9.57E-06	0.97	3.15	-	2436.3
AHQ-2-4, 4461 - 4533	R.TIVTTLQDSIR.K	1247.42355	2	3.27E-06	0.96	3.70	-	1619.6
AHQ-2-1, 4628 - 4636	R.TIVTTLQDSIR.K	1247.42355	2	5.86E-06	0.97	4.32	-	1536.2
AHQ-2-7, 4047	R.TIVTTLQDSIR.K	1247.42355	2	1.58E-06	0.96	3.57	-	1650.1
AHQ-2-5, 4135 - 4179	R.TIVTTLQDSIR.K	1247.42355	2	3.27E-07	0.97	4.48	-	1511.3
AHQ-2-8, 3908	R.TIVTTLQDSIR.K	1247.42355	2	9.23E-07	0.97	3.76	-	1907.7
AHQ-2-2, 4524 - 4605	R.TIVTTLQDSIR.K	1247.42355	2	7.81E-07	0.97	4.17	-	1807.8
AHQ-2-3, 4603	R.TIVTTLQDSIR.K	1247.42355	2	3.19E-07	0.95	3.62	-	1551.7
AHQ-2-6, 4110 - 4141	R.TIVTTLQDSIR.K	1247.42355	2	1.35E-07	0.97	3.63	-	2298.3
AHQ-2-3, 4479 - 4485	R.TIVTTLQDSIR.K	1247.42355	2	2.83E-07	0.97	4.39	-	1552.9
AHQ-2-9, 4108 - 4128	R.TIVTTLQDSIR.K	1247.42355	2	7.23E-07	0.96	3.81	-	1326.4
AHQ-2-6, 4114	R.TIVTTLQDSIR.K	1247.42355	1	7.90E-04	0.22	2.02	-	139.5
AHQ-2-1, 6597	K.TKDLQAICGISCDELSSM*VLELR.G	2658.02070	3	5.26E-10	0.81	3.77	-	898.2
AHQ-2-6, 2267 - 2274	K.VTEENKELANELR.R	1545.67685	2	3.84E-05	0.90	4.04	-	956.6
AHQ-2-4, 2444	K.VTEENKELANELR.R	1545.67685	2	8.93E-07	0.85	3.58	-	951.5
AHQ-2-7, 2215 - 2227	K.VTEENKELANELR.R	1545.67685	2	5.88E-04	0.68	3.33	-	525.4
AHQ-2-9, 2339	K.VTEENKELANELR.R	1545.67685	2	1.47E-04	0.78	3.24	-	786.7
AHQ-2-5, 2267	K.VTEENKELANELR.R	1545.67685	2	4.06E-08	0.93	3.93	-	1308.9
AHQ-2-1, 2673	K.VTEENKELANELR.R	1545.67685	2	2.93E-06	0.87	3.48	-	942.6
AHQ-2-5, 2273	K.VTEENKELANELR.R	1545.67685	3	4.87E-08	0.80	3.48	-	807.2
gi|4507729|ref|NP_001060.1| tubulin, beta polypeptide [Homo sapiens]				1.00E-30	7.27	90.36	30.80	49906.7
AHQ-2-11, 4684	R.AILVDLEPGTMDSVR.S	1616.86162	2	2.43E-04	0.62	2.71	-	494.0
AHQ-2-13, 3000	R.EIVHIQAGQCGNQIGAK.F	1825.03962	2	6.60E-04	0.66	2.58	-	556.2
AHQ-2-12, 2870	K.EVDEQMLNVQNK.N	1447.59668	2	8.20E-04	0.87	3.35	-	1150.8
AHQ-2-7, 2691	K.EVDEQMLNVQNK.N	1447.59668	1	5.86E-06	0.61	3.54	-	281.8
AHQ-2-7, 2687 - 2703	K.EVDEQMLNVQNK.N	1447.59668	2	6.05E-04	0.88	3.69	-	1217.8
AHQ-2-13-, 6419	K.GHYTEGAELVDSVLDVVR.K	1960.13388	2	1.78E-14	0.99	6.74	-	3027.6
AHQ-2-13, 6204	K.GHYTEGAELVDSVLDVVR.K	1960.13388	3	7.37E-04	0.92	4.58	-	1162.3
AHQ-2-7, 6236	K.GHYTEGAELVDSVLDVVR.K	1960.13388	2	1.36E-11	0.98	5.93	-	2544.0
AHQ-2-13, 6144 - 6203	K.GHYTEGAELVDSVLDVVR.K	1960.13388	2	3.66E-11	0.97	5.56	-	1656.4
AHQ-2-7, 6180	K.GHYTEGAELVDSVLDVVR.K	1960.13388	3	5.31E-05	0.92	4.76	-	1103.8
AHQ-2-12, 6331	K.GHYTEGAELVDSVLDVVR.K	1960.13388	2	1.68E-07	0.98	6.29	-	2617.9
AHQ-2-14-, 6283	K.GHYTEGAELVDSVLDVVR.K	1960.13388	3	1.26E-04	0.87	3.78	-	1057.1
AHQ-2-11, 6049 - 6056	K.GHYTEGAELVDSVLDVVR.K	1960.13388	2	1.37E-04	0.98	6.46	-	2699.9
AHQ-2-13, 6201	K.GHYTEGAELVDSVLDVVR.K	1960.13388	2	1.34E-09	0.99	6.52	-	2791.4
AHQ-2-10, 5886	K.GHYTEGAELVDSVLDVVR.K	1960.13388	2	1.75E-05	0.98	6.25	-	2496.6
AHQ-2-8, 6202	K.GHYTEGAELVDSVLDVVR.K	1960.13388	2	1.23E-05	0.97	4.52	-	1787.2
AHQ-2-14-, 6291	K.GHYTEGAELVDSVLDVVR.K	1960.13388	2	2.41E-06	0.98	6.31	-	2951.5
AHQ-2-9, 6262 - 6340	K.GHYTEGAELVDSVLDVVR.K	1960.13388	2	2.77E-07	0.98	6.01	-	1909.7
AHQ-2-9, 6406	K.GHYTEGAELVDSVLDVVR.K	1960.13388	2	2.20E-07	0.98	5.92	-	2358.1
AHQ-2-12, 4509	R.ISEQFTAMFR.R	1230.41818	2	1.44E-08	0.90	3.20	-	1191.4
AHQ-2-12, 4421 - 4435	R.ISEQFTAMFR.R	1230.41818	2	4.06E-05	0.68	3.07	-	744.3
AHQ-2-9, 7299	K.LTTPTYGDLNHLVSATMSGVTTCLR.F	2711.06527	3	6.67E-06	0.84	3.63	-	930.0
AHQ-2-7, 6327	K.LTTPTYGDLNHLVSATMSGVTTCLR.F	2711.06527	3	1.51E-05	0.84	4.00	-	617.1
AHQ-2-9, 7298 - 7300	K.LTTPTYGDLNHLVSATMSGVTTCLR.F	2711.06527	2	8.29E-05	0.94	4.64	-	904.1
AHQ-2-8, 7263	K.LTTPTYGDLNHLVSATMSGVTTCLR.F	2711.06527	2	1.69E-05	0.92	4.53	-	809.9
AHQ-2-7, 7355 - 7425	K.LTTPTYGDLNHLVSATMSGVTTCLR.F	2711.06527	2	7.89E-05	0.76	3.35	-	569.4
AHQ-2-10, 6651	K.LTTPTYGDLNHLVSATMSGVTTCLR.F	2711.06527	2	2.20E-04	0.73	3.55	-	559.6
AHQ-2-7, 7347 - 7419	K.LTTPTYGDLNHLVSATMSGVTTCLR.F	2711.06527	3	4.35E-07	0.91	4.37	-	1186.9
AHQ-2-9, 6303 - 6384	K.LTTPTYGDLNHLVSATM*SGVTTCLR.F	2727.06467	2	1.98E-05	0.68	3.20	-	520.1
AHQ-2-12, 5473	K.NSSYFVEWIPNNVK.T	1697.87148	2	5.10E-05	0.84	3.56	-	623.0
AHQ-2-7, 6245 - 6316	K.NSSYFVEWIPNNVK.T	1697.87148	2	1.71E-04	0.71	3.02	-	773.8
AHQ-2-13, 5549	K.NSSYFVEWIPNNVK.T	1697.87148	2	6.95E-04	0.55	2.54	-	574.0
AHQ-2-7, 5271 - 5348	K.NSSYFVEWIPNNVK.T	1697.87148	2	1.64E-06	0.93	4.14	-	913.1
AHQ-2-12, 5583	K.NSSYFVEWIPNNVK.T	1697.87148	2	3.40E-05	0.49	2.60	-	449.3
AHQ-2-9, 6570	R.SGPFGQIFRPDNFVFGQSGAGNNWAK.G	2800.03898	3	1.03E-05	0.89	4.16	-	911.6
AHQ-2-9, 6572 - 6582	R.SGPFGQIFRPDNFVFGQSGAGNNWAK.G	2800.03898	2	7.81E-09	0.97	5.31	-	1185.3
AHQ-2-7, 6447 - 6517	R.SGPFGQIFRPDNFVFGQSGAGNNWAK.G	2800.03898	2	2.23E-07	0.97	5.78	-	1098.4
AHQ-2-12, 6342	R.SGPFGQIFRPDNFVFGQSGAGNNWAK.G	2800.03898	2	4.18E-08	0.96	5.34	-	1023.8
AHQ-2-7, 6681 - 6749	R.SGPFGQIFRPDNFVFGQSGAGNNWAK.G	2800.03898	3	4.21E-07	0.94	5.28	-	1098.4
AHQ-2-7, 6384 - 6451	R.SGPFGQIFRPDNFVFGQSGAGNNWAK.G	2800.03898	3	3.56E-09	0.96	5.71	-	1371.1
AHQ-2-7, 6599 - 6667	R.SGPFGQIFRPDNFVFGQSGAGNNWAK.G	2800.03898	2	7.61E-08	0.95	5.28	-	990.3
AHQ-2-9, 6418	R.SGPFGQIFRPDNFVFGQSGAGNNWAK.G	2800.03898	3	5.05E-05	0.87	4.01	-	1028.7
AHQ-2-7, 6307 - 6380	R.SGPFGQIFRPDNFVFGQSGAGNNWAK.G	2800.03898	2	9.48E-06	0.95	4.71	-	1283.5
AHQ-2-7, 6281 - 6352	R.SGPFGQIFRPDNFVFGQSGAGNNWAK.G	2800.03898	3	2.43E-05	0.97	7.10	-	1325.6
AHQ-2-13, 6239 - 6299	R.SGPFGQIFRPDNFVFGQSGAGNNWAK.G	2800.03898	3	1.74E-13	0.97	5.82	-	1885.0
AHQ-2-11, 6160	R.SGPFGQIFRPDNFVFGQSGAGNNWAK.G	2800.03898	2	6.98E-08	0.89	4.35	-	601.6
AHQ-2-14-, 6249 - 6315	R.SGPFGQIFRPDNFVFGQSGAGNNWAK.G	2800.03898	3	1.00E-30	0.98	7.06	-	2425.8
AHQ-2-12, 6359 - 6438	R.SGPFGQIFRPDNFVFGQSGAGNNWAK.G	2800.03898	2	1.90E-04	0.90	4.21	-	753.6
AHQ-2-11, 6096 - 6165	R.SGPFGQIFRPDNFVFGQSGAGNNWAK.G	2800.03898	3	1.18E-13	0.98	7.13	-	2018.8
gi|21361399|ref|NP_055040.2| protein phosphatase 2 (formerly 2A), regulatory subunit A (PR 65), alp				1.00E-30	0.99	10.38	3.90	65308.3
AHQ-2-6, 7233	K.DNTIEHLLPLFLAQLKDECPEVR.L	2753.12348	3	1.00E-30	0.99	7.60	-	3759.0
gi|4501885|ref|NP_001092.1| beta actin; beta cytoskeletal actin [Homo sapiens]				1.11E-16	13.78	150.46	36.80	41736.5
AHQ-2-8, 6187	R.CPEALFQPSFLGM*ESCGIHETTFNSIM*K.C	3267.67588	3	6.87E-07	0.89	4.52	-	607.8
AHQ-2-14-, 6484 - 6544	R.CPEALFQPSFLGM*ESCGIHETTFNSIMK.C	3251.67648	3	1.92E-04	0.76	4.61	-	521.2
AHQ-2-13-, 6865 - 6907	R.CPEALFQPSFLGMESCGIHETTFNSIMK.C	3235.67708	3	6.61E-08	0.92	4.41	-	736.7
AHQ-2-13-, 6609 - 6669	R.CPEALFQPSFLGMESCGIHETTFNSIM*K.C	3251.67648	3	6.23E-04	0.71	4.33	-	448.1
AHQ-2-1, 6962	R.CPEALFQPSFLGM*ESCGIHETTFNSIMK.C	3251.67648	3	2.02E-04	0.80	3.73	-	563.8
AHQ-2-13-, 6310	R.CPEALFQPSFLGM*ESCGIHETTFNSIM*K.C	3267.67588	3	4.50E-07	0.88	4.08	-	733.2
AHQ-2-13, 6647	R.CPEALFQPSFLGMESCGIHETTFNSIMK.C	3235.67708	3	6.35E-08	0.90	4.44	-	619.4
AHQ-2-13, 6439 - 6508	R.CPEALFQPSFLGMESCGIHETTFNSIM*K.C	3251.67648	3	1.53E-05	0.81	4.19	-	570.7
AHQ-2-13, 6313 - 6381	R.CPEALFQPSFLGM*ESCGIHETTFNSIMK.C	3251.67648	3	6.18E-07	0.68	3.78	-	510.5
AHQ-2-13, 6159	R.CPEALFQPSFLGM*ESCGIHETTFNSIM*K.C	3267.67588	3	2.77E-05	0.78	3.47	-	503.3
AHQ-2-12, 6947 - 7013	R.CPEALFQPSFLGMESCGIHETTFNSIMK.C	3235.67708	3	1.79E-06	0.92	4.27	-	1025.6
AHQ-2-8, 6950 - 6984	R.CPEALFQPSFLGMESCGIHETTFNSIMK.C	3235.67708	3	1.20E-08	0.94	5.21	-	1016.7

AHQ-2-3, 7333 - 7337	R.CPEALFQPSFLGMESCGIHETTFNSIMK.C	3235.67708	3	5.20E-06	0.88	4.39	-	642.6
AHQ-2-11, 6620 - 6669	R.CPEALFQPSFLGMESCGIHETTFNSIMK.C	3235.67708	3	2.21E-09	0.93	4.85	-	690.0
AHQ-2-11, 6249 - 6317	R.CPEALFQPSFLGMESCGIHETTFNSIM*K.C	3251.67648	3	7.60E-04	0.49	3.51	-	267.7
AHQ-2-7, 7191	R.CPEALFQPSFLGMESCGIHETTFNSIMK.C	3235.67708	3	1.39E-04	0.81	3.80	-	683.3
AHQ-2-10, 6610	R.CPEALFQPSFLGMESCGIHETTFNSIMK.C	3235.67708	3	3.41E-07	0.94	5.08	-	855.1
AHQ-2-8, 6998	R.CPEALFQPSFLGM*ESCGIHETTFNSIMK.C	3251.67648	3	4.05E-04	0.47	3.17	-	437.1
AHQ-2-8, 7047 - 7082	R.CPEALFQPSFLGMESCGIHETTFNSIMK.C	3235.67708	3	6.27E-06	0.94	5.02	-	880.4
AHQ-2-10, 6458 - 6520	R.CPEALFQPSFLGMESCGIHETTFNSIMK.C	3235.67708	3	2.41E-04	0.91	4.63	-	916.4
AHQ-2-8, 7154 - 7220	R.CPEALFQPSFLGMESCGIHETTFNSIMK.C	3235.67708	3	1.07E-07	0.94	5.49	-	855.5
AHQ-2-9, 7242 - 7311	R.CPEALFQPSFLGMESCGIHETTFNSIMK.C	3235.67708	3	3.27E-07	0.92	5.01	-	580.6
AHQ-2-9, 7139 - 7202	R.CPEALFQPSFLGMESCGIHETTFNSIMK.C	3235.67708	3	5.86E-08	0.89	4.57	-	641.7
AHQ-2-8, 7290 - 7359	R.CPEALFQPSFLGMESCGIHETTFNSIMK.C	3235.67708	3	3.34E-10	0.96	5.66	-	1210.6
AHQ-2-8, 7566 - 7586	R.CPEALFQPSFLGMESCGIHETTFNSIMK.C	3235.67708	3	1.01E-06	0.96	5.99	-	928.6
AHQ-2-8, 7426 - 7499	R.CPEALFQPSFLGMESCGIHETTFNSIMK.C	3235.67708	3	5.28E-11	0.96	5.69	-	1091.8
AHQ-2-8, 6730 - 6803	R.CPEALFQPSFLGM*ESCGIHETTFNSIMK.C	3251.67648	3	1.10E-06	0.83	4.49	-	653.4
AHQ-2-8, 7419 - 7490	K.EKLCYVALDFEQEMATAASSSSLEK.S	2810.10410	2	1.63E-05	0.85	3.43	-	754.7
AHQ-2-8, 7574	K.EKLCYVALDFEQEMATAASSSSLEK.S	2810.10410	3	7.76E-08	0.93	5.33	-	1028.0
AHQ-2-8, 7283 - 7362	K.EKLCYVALDFEQEMATAASSSSLEK.S	2810.10410	3	3.59E-07	0.95	5.07	-	1438.9
AHQ-2-8, 7282 - 7350	K.EKLCYVALDFEQEMATAASSSSLEK.S	2810.10410	2	1.43E-04	0.90	3.72	-	832.0
AHQ-2-8, 7179 - 7251	K.EKLCYVALDFEQEMATAASSSSLEK.S	2810.10410	3	3.25E-05	0.93	5.29	-	1322.2
AHQ-2-9, 7326 - 7334	K.EKLCYVALDFEQEMATAASSSSLEK.S	2810.10410	2	5.78E-08	0.91	4.42	-	779.3
AHQ-2-8, 7135 - 7212	K.EKLCYVALDFEQEMATAASSSSLEK.S	2810.10410	2	2.30E-05	0.89	3.91	-	688.2
AHQ-2-4, 7482	K.EKLCYVALDFEQEMATAASSSSLEK.S	2810.10410	2	7.46E-06	0.89	3.66	-	904.1
AHQ-2-10, 6591 - 6659	K.EKLCYVALDFEQEMATAASSSSLEK.S	2810.10410	2	1.30E-04	0.90	4.29	-	636.3
AHQ-2-10, 6699 - 6712	K.EKLCYVALDFEQEMATAASSSSLEK.S	2810.10410	2	5.09E-05	0.63	2.68	-	643.7
AHQ-2-11, 6684 - 6689	K.EKLCYVALDFEQEMATAASSSSLEK.S	2810.10410	2	2.35E-07	0.95	4.73	-	1067.7
AHQ-2-14-, 6820	K.EKLCYVALDFEQEMATAASSSSLEK.S	2810.10410	2	1.82E-04	0.51	2.53	-	287.3
AHQ-2-8, 7432 - 7502	K.EKLCYVALDFEQEMATAASSSSLEK.S	2810.10410	3	4.35E-10	0.97	6.73	-	1343.0
AHQ-2-8, 5784 - 5850	K.EKLCYVALDFEQEM*ATAASSSSLEK.S	2826.10350	3	1.32E-07	0.90	4.38	-	954.3
AHQ-2-8, 5634 - 5703	K.EKLCYVALDFEQEM*ATAASSSSLEK.S	2826.10350	3	3.06E-04	0.88	4.17	-	1120.6
AHQ-2-8, 5435 - 5504	K.EKLCYVALDFEQEM*ATAASSSSLEK.S	2826.10350	3	2.35E-05	0.70	3.12	-	892.2
AHQ-2-8, 7150 - 7219	R.FRCPEALFQPSFLGMESCGIHETTFNSIMK.C	3539.03813	3	1.89E-11	0.98	8.13	-	2448.3
AHQ-2-10, 6378 - 6455	R.FRCPEALFQPSFLGMESCGIHETTFNSIMK.C	3539.03813	3	2.02E-07	0.98	7.55	-	2016.5
AHQ-2-10, 6560 - 6619	R.FRCPEALFQPSFLGMESCGIHETTFNSIMK.C	3539.03813	3	4.72E-05	0.94	4.73	-	1157.9
AHQ-2-8, 7286 - 7366	R.FRCPEALFQPSFLGMESCGIHETTFNSIMK.C	3539.03813	3	4.01E-12	0.98	7.38	-	2161.4
AHQ-2-8, 7046 - 7115	R.FRCPEALFQPSFLGMESCGIHETTFNSIMK.C	3539.03813	3	2.12E-13	0.98	9.18	-	2466.9
AHQ-2-8, 7435 - 7508	R.FRCPEALFQPSFLGMESCGIHETTFNSIMK.C	3539.03813	3	3.79E-05	0.97	6.12	-	1464.4
AHQ-2-8, 6850 - 6868	R.FRCPEALFQPSFLGMESCGIHETTFNSIMK.C	3539.03813	3	6.81E-07	0.98	7.35	-	1924.3
AHQ-2-8, 6934 - 7006	R.FRCPEALFQPSFLGMESCGIHETTFNSIMK.C	3539.03813	3	2.26E-10	0.97	6.58	-	1407.4
AHQ-2-8, 7559	R.FRCPEALFQPSFLGMESCGIHETTFNSIMK.C	3539.03813	3	2.59E-04	0.92	5.01	-	854.7
AHQ-2-5, 2247 - 2255	R.GYSFTTTAER.E	1133.19273	2	6.00E-05	0.92	3.39	-	1168.9
AHQ-2-4, 2436	R.GYSFTTTAER.E	1133.19273	2	1.29E-04	0.91	3.13	-	1093.5
AHQ-2-8, 2242 - 2258	R.GYSFTTTAER.E	1133.19273	2	1.49E-04	0.90	3.01	-	1110.4
AHQ-2-7, 2201 - 2209	R.GYSFTTTAER.E	1133.19273	2	1.29E-04	0.93	3.52	-	1092.8
AHQ-2-8, 2104 - 2188	R.GYSFTTTAER.E	1133.19273	2	3.75E-04	0.90	3.49	-	853.1
AHQ-2-3, 2443 - 2444	R.GYSFTTTAER.E	1133.19273	2	1.31E-04	0.91	3.25	-	1089.4
AHQ-2-14-, 2415 - 2416	R.GYSFTTTAER.E	1133.19273	2	4.87E-06	0.93	3.26	-	1027.1
AHQ-2-10, 2266 - 2326	R.GYSFTTTAER.E	1133.19273	2	1.64E-04	0.92	3.50	-	1083.4
AHQ-2-13-, 2529 - 2530	R.GYSFTTTAER.E	1133.19273	2	7.86E-06	0.92	3.19	-	1025.4
AHQ-2-10, 4347 - 4358	R.KDLYANTVLSGGTTMYPGIADR.M	2344.62976	2	1.53E-04	0.96	4.62	-	1389.1
AHQ-2-10, 4631	R.KDLYANTVLSGGTTMYPGIADR.M	2344.62976	2	3.94E-04	0.73	3.01	-	502.4
AHQ-2-14-, 4255 - 4259	R.KDLYANTVLSGGTTM*YPGIADR.M	2360.62916	2	4.02E-06	0.90	3.59	-	1036.3
AHQ-2-14, 5725	R.KDLYANTVLSGGTTMYPGIADR.M	2344.62976	2	5.49E-04	0.84	3.11	-	958.9
AHQ-2-11, 4130	R.KDLYANTVLSGGTTM*YPGIADR.M	2360.62916	2	2.73E-09	0.93	3.63	-	1277.9
AHQ-2-8, 4298	R.KDLYANTVLSGGTTMYPGIADR.M	2344.62976	3	4.40E-04	0.90	4.51	-	1002.9
AHQ-2-8, 4295	R.KDLYANTVLSGGTTMYPGIADR.M	2344.62976	2	2.87E-06	0.95	4.43	-	1302.8
AHQ-2-14-, 4752 - 4760	R.KDLYANTVLSGGTTMYPGIADR.M	2344.62976	2	6.41E-04	0.92	3.87	-	970.3
AHQ-2-11, 4558	R.KDLYANTVLSGGTTMYPGIADR.M	2344.62976	2	1.91E-05	0.89	3.48	-	915.3
AHQ-2-14, 5266 - 5329	R.KDLYANTVLSGGTTM*YPGIADR.M	2360.62916	2	4.92E-05	0.94	4.59	-	867.9
AHQ-2-14, 5830	R.KDLYANTVLSGGTTMYPGIADR.M	2344.62976	2	3.12E-05	0.96	4.53	-	1487.1
AHQ-2-8, 7610 - 7611	K.LCYVALDFEQEMATAASSSSLEK.S	2552.81665	3	6.25E-08	0.97	6.11	-	1902.5
AHQ-2-8, 7472 - 7544	K.LCYVALDFEQEMATAASSSSLEK.S	2552.81665	3	2.51E-08	0.97	6.18	-	1678.9
AHQ-2-7, 5571 - 5583	R.TTGIVMDSGDGVTHTVPIYEGYALPHAILR.L	3185.59928	3	4.99E-11	0.97	6.04	-	1719.6
AHQ-2-6, 5839 - 5915	R.TTGIVMDSGDGVTHTVPIYEGYALPHAILR.L	3185.59928	3	5.55E-16	0.96	5.63	-	1501.7
AHQ-2-7, 5693 - 5767	R.TTGIVMDSGDGVTHTVPIYEGYALPHAILR.L	3185.59928	3	4.03E-09	0.94	5.08	-	1390.9
AHQ-2-1, 6209	R.TTGIVMDSGDGVTHTVPIYEGYALPHAILR.L	3185.59928	3	6.94E-07	0.94	4.94	-	1276.5
AHQ-2-6, 5477	R.TTGIVM*DSGDGVTHTVPIYEGYALPHAILR.L	3201.59868	3	6.67E-04	0.82	3.56	-	1111.3
AHQ-2-6, 5379	R.TTGIVM*DSGDGVTHTVPIYEGYALPHAILR.L	3201.59868	3	2.74E-05	0.90	4.60	-	872.4
AHQ-2-13-, 5815 - 5873	R.TTGIVMDSGDGVTHTVPIYEGYALPHAILR.L	3185.59928	3	1.33E-09	0.97	5.84	-	1770.7
AHQ-2-7, 5836	R.TTGIVMDSGDGVTHTVPIYEGYALPHAILR.L	3185.59928	3	1.25E-09	0.97	5.90	-	1432.7
AHQ-2-6, 5671 - 5685	R.TTGIVMDSGDGVTHTVPIYEGYALPHAILR.L	3185.59928	3	1.08E-07	0.97	6.69	-	1383.7
AHQ-2-1, 5874	R.TTGIVM*DSGDGVTHTVPIYEGYALPHAILR.L	3201.59868	3	1.67E-07	0.95	5.58	-	1105.1
AHQ-2-5, 5893 - 5907	R.TTGIVMDSGDGVTHTVPIYEGYALPHAILR.L	3185.59928	3	1.10E-13	0.97	5.99	-	1580.7
AHQ-2-8, 5944	R.TTGIVMDSGDGVTHTVPIYEGYALPHAILR.L	3185.59928	3	5.03E-10	0.97	6.15	-	1810.2
AHQ-2-7, 5244 - 5255	R.TTGIVM*DSGDGVTHTVPIYEGYALPHAILR.L	3201.59868	3	2.78E-06	0.94	5.21	-	1231.5
AHQ-2-13-, 5722 - 5749	R.TTGIVMDSGDGVTHTVPIYEGYALPHAILR.L	3185.59928	3	1.46E-06	0.97	6.80	-	1756.9
AHQ-2-13-, 5459 - 5538	R.TTGIVM*DSGDGVTHTVPIYEGYALPHAILR.L	3201.59868	3	2.53E-05	0.95	5.43	-	1168.6
AHQ-2-9, 5295 - 5366	R.TTGIVM*DSGDGVTHTVPIYEGYALPHAILR.L	3201.59868	3	7.72E-06	0.96	6.32	-	1243.9
AHQ-2-9, 5438 - 5507	R.TTGIVM*DSGDGVTHTVPIYEGYALPHAILR.L	3201.59868	3	3.86E-05	0.94	4.63	-	1284.4
AHQ-2-9, 5576 - 5648	R.TTGIVMDSGDGVTHTVPIYEGYALPHAILR.L	3185.59928	3	2.90E-13	0.97	6.20	-	1741.0
AHQ-2-9, 5667 - 5699	R.TTGIVM*DSGDGVTHTVPIYEGYALPHAILR.L	3201.59868	3	8.76E-05	0.87	4.46	-	715.0
AHQ-2-9, 5718 - 5786	R.TTGIVMDSGDGVTHTVPIYEGYALPHAILR.L	3185.59928	3	1.48E-11	0.96	6.25	-	1187.2
AHQ-2-9, 5807 - 5874	R.TTGIVMDSGDGVTHTVPIYEGYALPHAILR.L	3185.59928	3	6.04E-09	0.97	6.18	-	1551.1
AHQ-2-9, 5943	R.TTGIVMDSGDGVTHTVPIYEGYALPHAILR.L	3185.59928	3	2.46E-09	0.97	6.00	-	1574.3
AHQ-2-5, 5396	R.TTGIVM*DSGDGVTHTVPIYEGYALPHAILR.L	3201.59868	3	7.14E-07	0.94	5.08	-	1051.7
AHQ-2-1, 6128	R.TTGIVMDSGDGVTHTVPIYEGYALPHAILR.L	3185.59928	3	3.01E-06	0.97	6.06	-	1784.0
AHQ-2-14, 6589	R.TTGIVM*DSGDGVTHTVPIYEGYALPHAILR.L	3201.59868	3	9.51E-08	0.95	5.18	-	1373.9
AHQ-2-8, 5674 - 5738	R.TTGIVMDSGDGVTHTVPIYEGYALPHAILR.L	3185.59928	3	9.99E-14	0.96	6.29	-	1198.7
AHQ-2-14-, 5739 - 5803	R.TTGIVMDSGDGVTHTVPIYEGYALPHAILR.L	3185.59928	3	1.18E-11	0.98	7.19	-	1671.7
AHQ-2-8, 5583 - 5650	R.TTGIVM*DSGDGVTHTVPIYEGYALPHAILR.L	3201.59868	3	1.55E-04	0.75	4.17	-	692.5
AHQ-2-8, 5530 - 5602	R.TTGIVMDSGDGVTHTVPIYEGYALPHAILR.L	3185.59928	3	5.22E-14	0.97	6.15	-	1984.9
AHQ-2-10, 5019 - 5084	R.TTGIVM*DSGDGVTHTVPIYEGYALPHAILR.L	3201.59868	3	1.78E-07	0.94	5.35	-	999.3
AHQ-2-10, 5274 - 5338	R.TTGIVMDSGDGVTHTVPIYEGYALPHAILR.L	3185.59928	3	6.91E-09	0.96	6.19	-	1251.5
AHQ-2-10, 5362 - 5434	R.TTGIVMDSGDGVTHTVPIYEGYALPHAILR.L	3185.59928	3	1.11E-16	0.98	6.77	-	1704.8
AHQ-2-8, 5398 - 5470	R.TTGIVMDSGDGVTHTVPIYEGYALPHAILR.L	3185.59928	3	5.67E-12	0.97	6.26	-	1660.9
AHQ-2-8, 5367 - 5426	R.TTGIVM*DSGDGVTHTVPIYEGYALPHAILR.L	3201.59868	3	8.11E-05	0.91	4.46	-	915.2
AHQ-2-8, 5231 - 5310	R.TTGIVMDSGDGVTHTVPIYEGYALPHAILR.L	3185.59928	3	6.74E-12	0.97	6.46	-	1226.4
AHQ-2-4, 6249 - 6252	R.TTGIVMDSGDGVTHTVPIYEGYALPHAILR.L	3185.59928	3	8.19E-09	0.97	6.64	-	1356.3
AHQ-2-4, 6088 - 6157	R.TTGIVMDSGDGVTHTVPIYEGYALPHAILR.L	3185.59928	3	1.73E-08	0.96	5.49	-	1657.1
AHQ-2-4, 5721	R.TTGIVM*DSGDGVTHTVPIYEGYALPHAILR.L	3201.59868	3	8.05E-05	0.95	5.48	-	1223.8
AHQ-2-8, 5087 - 5170	R.TTGIVM*DSGDGVTHTVPIYEGYALPHAILR.L	3201.59868	3	1.80E-10	0.96	5.56	-	1546.1
AHQ-2-8, 4943 - 5015	R.TTGIVM*DSGDGVTHTVPIYEGYALPHAILR.L	3201.59868	3	4.55E-08	0.97	6.19	-	1494.4
AHQ-2-14-, 5416 - 5492	R.TTGIVM*DSGDGVTHTVPIYEGYALPHAILR.L	3201.59868	3	8.11E-10	0.96	5.85	-	1515.3
AHQ-2-11, 5278 - 5329	R.TTGIVM*DSGDGVTHTVPIYEGYALPHAILR.L	3201.59868	3	2.45E-04	0.93	4.68	-	1331.8
AHQ-2-11, 5536 - 5596	R.TTGIVMDSGDGVTHTVPIYEGYALPHAILR.L	3185.59928	3	5.35E-13	0.98	6.04	-	2168.4
AHQ-2-14-, 5556 - 5559	R.TTGIVM*DSGDGVTHTVPIYEGYALPHAILR.L	3201.59868	3	2.46E-10	0.96	5.51	-	1722.6
AHQ-2-14-, 5717 - 5792	R.TTGIVM*DSGDGVTHTVPIYEGYALPHAILR.L	3201.59868	3	2.03E-04	0.78	3.95	-	548.6

AHQ-2-3, 6213	R.TTGIVMDSGDGVTHTVPIYEGYALPHAILR.L	3185.59928	3	3.36E-11	0.97	6.38	-	1316.5
AHQ-2-2, 6049 - 6128	R.TTGIVMDSGDGVTHTVPIYEGYALPHAILR.L	3185.59928	3	8.12E-04	0.91	4.38	-	1231.7
AHQ-2-2, 6241	R.TTGIVMDSGDGVTHTVPIYEGYALPHAILR.L	3185.59928	3	8.02E-07	0.96	5.91	-	1171.9
AHQ-2-12, 5486	R.TTGIVM*DSGDGVTHTVPIYEGYALPHAILR.L	3201.59868	3	1.73E-04	0.93	5.08	-	767.7
AHQ-2-12, 5574	R.TTGIVM*DSGDGVTHTVPIYEGYALPHAILR.L	3201.59868	3	3.07E-05	0.93	5.14	-	917.1
AHQ-2-12, 5763	R.TTGIVMDSGDGVTHTVPIYEGYALPHAILR.L	3185.59928	3	4.13E-05	0.83	3.91	-	728.9
AHQ-2-12, 5878 - 5959	R.TTGIVMDSGDGVTHTVPIYEGYALPHAILR.L	3185.59928	3	8.15E-10	0.97	6.12	-	1380.1
AHQ-2-3, 6092	R.TTGIVMDSGDGVTHTVPIYEGYALPHAILR.L	3185.59928	3	1.06E-09	0.97	6.13	-	1624.9
AHQ-2-5, 5713	R.TTGIVMDSGDGVTHTVPIYEGYALPHAILR.L	3185.59928	3	2.51E-09	0.98	7.09	-	1800.2
AHQ-2-13, 5347	R.TTGIVM*DSGDGVTHTVPIYEGYALPHAILR.L	3201.59868	3	2.62E-08	0.92	4.43	-	1078.7
AHQ-2-13, 5428	R.TTGIVM*DSGDGVTHTVPIYEGYALPHAILR.L	3201.59868	3	2.52E-07	0.94	5.17	-	1142.8
AHQ-2-13, 5601	R.TTGIVMDSGDGVTHTVPIYEGYALPHAILR.L	3185.59928	3	1.59E-09	0.96	5.30	-	1644.3
AHQ-2-13, 5725	R.TTGIVMDSGDGVTHTVPIYEGYALPHAILR.L	3185.59928	3	3.23E-08	0.96	5.42	-	1892.6
AHQ-2-13, 5808	R.TTGIVMDSGDGVTHTVPIYEGYALPHAILR.L	3185.59928	3	3.23E-06	0.96	5.02	-	1539.0
AHQ-2-3, 3935 - 3937	R.VAPEEHPVLLTEAPLNPK.A	1955.24351	2	7.18E-04	0.88	4.12	-	559.4
AHQ-2-13, 3920	R.VAPEEHPVLLTEAPLNPK.A	1955.24351	2	2.86E-06	0.93	4.34	-	966.4
AHQ-2-12, 3853	R.VAPEEHPVLLTEAPLNPK.A	1955.24351	2	8.25E-04	0.92	4.34	-	750.1
AHQ-2-12, 3718 - 3785	R.VAPEEHPVLLTEAPLNPK.A	1955.24351	2	4.09E-06	0.90	4.26	-	691.0
AHQ-2-14, 4794	R.VAPEEHPVLLTEAPLNPK.A	1955.24351	2	1.12E-04	0.94	4.52	-	961.3
AHQ-2-4, 3916 - 3984	R.VAPEEHPVLLTEAPLNPK.A	1955.24351	2	1.66E-04	0.85	3.94	-	597.3
AHQ-2-11, 3653	R.VAPEEHPVLLTEAPLNPK.A	1955.24351	2	2.51E-05	0.84	4.01	-	492.0
AHQ-2-8, 3296 - 3366	R.VAPEEHPVLLTEAPLNPK.A	1955.24351	2	9.07E-07	0.93	4.63	-	801.4
AHQ-2-5, 3591 - 3660	R.VAPEEHPVLLTEAPLNPK.A	1955.24351	2	6.26E-06	0.85	3.82	-	575.1
AHQ-2-5, 3597 - 3639	R.VAPEEHPVLLTEAPLNPK.A	1955.24351	3	7.90E-04	0.72	4.22	-	498.7
AHQ-2-13-, 3955 - 3969	R.VAPEEHPVLLTEAPLNPK.A	1955.24351	3	7.30E-05	0.85	4.75	-	862.4
AHQ-2-13-, 3958	R.VAPEEHPVLLTEAPLNPK.A	1955.24351	2	2.65E-04	0.87	4.07	-	683.1
AHQ-2-9, 3606	R.VAPEEHPVLLTEAPLNPK.A	1955.24351	2	1.91E-05	0.94	4.07	-	1143.2
AHQ-2-9, 3522 - 3590	R.VAPEEHPVLLTEAPLNPK.A	1955.24351	2	1.69E-04	0.91	4.26	-	815.6
AHQ-2-6, 3573	R.VAPEEHPVLLTEAPLNPK.A	1955.24351	2	3.49E-06	0.94	4.86	-	768.9
AHQ-2-2, 3964 - 4033	R.VAPEEHPVLLTEAPLNPK.A	1955.24351	3	9.99E-05	0.60	3.28	-	577.7
AHQ-2-7, 3456 - 3463	R.VAPEEHPVLLTEAPLNPK.A	1955.24351	2	6.34E-04	0.92	4.28	-	794.0
AHQ-2-8, 3067 - 3138	R.VAPEEHPVLLTEAPLNPK.A	1955.24351	3	8.06E-05	0.79	4.15	-	794.0
AHQ-2-8, 3170 - 3238	R.VAPEEHPVLLTEAPLNPK.A	1955.24351	3	1.31E-04	0.85	4.10	-	875.8
AHQ-2-8, 3194 - 3266	R.VAPEEHPVLLTEAPLNPK.A	1955.24351	2	1.73E-04	0.93	4.87	-	813.6
AHQ-2-8, 3768	R.VAPEEHPVLLTEAPLNPK.A	1955.24351	2	4.41E-06	0.92	4.51	-	880.5
AHQ-2-8, 3546	R.VAPEEHPVLLTEAPLNPK.A	1955.24351	2	1.25E-04	0.94	4.42	-	1098.3
AHQ-2-8, 3536 - 3616	R.VAPEEHPVLLTEAPLNPK.A	1955.24351	2	1.32E-04	0.95	4.98	-	922.6
AHQ-2-8, 3407 - 3478	R.VAPEEHPVLLTEAPLNPK.A	1955.24351	2	3.11E-06	0.94	5.05	-	879.7
AHQ-2-10, 3475 - 3542	R.VAPEEHPVLLTEAPLNPK.A	1955.24351	2	1.07E-10	0.94	4.54	-	981.5
AHQ-2-1, 4053 - 4132	R.VAPEEHPVLLTEAPLNPK.A	1955.24351	2	1.52E-04	0.93	4.57	-	920.7
AHQ-2-8, 2907	R.VAPEEHPVLLTEAPLNPKANR.E	2296.61120	2	1.48E-05	0.95	4.87	-	876.2
gi|4503571|ref|NP_001419.1| enolase 1; phosphopyruvate hydratase; MYC promoter-binding protein 1; n				1.11E-16	11.77	150.34	44.90	47168.7
AHQ-2-14-, 5300	R.AAVPSGASTGIYEALELR.D	1806.00979	2	4.21E-11	0.94	4.58	-	698.0
AHQ-2-13-, 5343 - 5418	R.AAVPSGASTGIYEALELR.D	1806.00979	2	2.28E-08	0.96	5.00	-	811.0
AHQ-2-11, 5098 - 5114	R.AAVPSGASTGIYEALELR.D	1806.00979	2	3.59E-06	0.96	5.05	-	831.3
AHQ-2-12, 5345 - 5354	R.AAVPSGASTGIYEALELR.D	1806.00979	2	4.55E-13	0.96	5.18	-	911.7
AHQ-2-8, 4868 - 4935	R.AAVPSGASTGIYEALELR.D	1806.00979	2	1.11E-16	0.96	5.15	-	947.8
AHQ-2-14, 6454 - 6509	R.AAVPSGASTGIYEALELR.D	1806.00979	2	1.90E-11	0.96	5.58	-	567.8
AHQ-2-13, 5247 - 5309	R.AAVPSGASTGIYEALELR.D	1806.00979	2	2.71E-10	0.92	4.25	-	535.7
AHQ-2-9, 5175 - 5198	R.AAVPSGASTGIYEALELR.D	1806.00979	2	7.29E-04	0.82	4.00	-	370.3
AHQ-2-7, 4540	K.DATNVGDEGGFAPNILENK.E	1962.06350	2	8.75E-06	0.94	4.69	-	801.8
AHQ-2-8, 6062	K.DATNVGDEGGFAPNILENKEGLELLK.T	2744.99178	3	3.45E-06	0.81	3.86	-	1041.8
AHQ-2-11, 6602	K.DYPVVSIEDPFDQDDWGAWQK.F	2511.63977	2	1.17E-07	0.79	2.99	-	677.4
AHQ-2-8, 5898 - 5930	K.FTASAGIQVVGDDLTVTNPK.R	2034.25590	2	7.80E-04	0.44	2.87	-	327.8
AHQ-2-13, 4239 - 4241	R.GNPTVEVDLFTSK.G	1407.55065	2	8.07E-04	0.68	3.18	-	544.4
AHQ-2-13-, 4286	R.GNPTVEVDLFTSK.G	1407.55065	1	2.31E-06	0.34	1.97	-	641.7
AHQ-2-13, 4240	R.GNPTVEVDLFTSK.G	1407.55065	1	1.29E-04	0.24	2.40	-	481.2
AHQ-2-8, 5940	R.HIADLAGNSEVILPVPAFNVINGGSHAGNK.L	3013.35654	3	1.06E-13	0.95	5.41	-	1400.7
AHQ-2-9, 2091 - 2092	R.IGAEVYHNLK.N	1144.30502	2	4.48E-04	0.88	3.36	-	1074.8
AHQ-2-9, 7015	K.LAMQEFMILPVGAANFR.E	1909.30812	2	1.89E-05	0.93	4.09	-	824.7
AHQ-2-10, 6443	K.LAMQEFMILPVGAANFR.E	1909.30812	2	3.41E-05	0.94	4.41	-	915.0
AHQ-2-8, 6200	K.LAMQEFM*ILPVGAANFR.E	1925.30752	2	1.18E-05	0.86	4.83	-	445.7
AHQ-2-7, 6999	K.LAMQEFMILPVGAANFR.E	1909.30812	2	5.14E-06	0.93	3.84	-	1006.0
AHQ-2-8, 6822 - 6895	K.LAMQEFMILPVGAANFR.E	1909.30812	2	4.77E-10	0.97	6.32	-	976.6
AHQ-2-8, 5830	K.LAM*QEFMILPVGAANFR.E	1925.30752	2	7.17E-06	0.94	5.06	-	1195.4
AHQ-2-8, 6939 - 6951	K.SFIKDYPVVSIEDPFDQDDWGAWQK.F	2987.22328	3	2.98E-06	0.98	6.86	-	2364.9
AHQ-2-8, 6930 - 6938	K.SFIKDYPVVSIEDPFDQDDWGAWQK.F	2987.22328	2	9.56E-04	0.82	3.62	-	586.7
AHQ-2-7, 2017	K.TIAPALVSK.K	900.09769	1	9.31E-04	0.10	1.93	-	322.9
AHQ-2-8, 1940	K.TIAPALVSK.K	900.09769	1	1.29E-04	0.28	2.06	-	387.9
AHQ-2-7, 3504	K.VNQIGSVTESLQACK.L	1635.82159	2	4.54E-07	0.76	2.89	-	723.7
AHQ-2-13, 3808	K.VNQIGSVTESLQACK.L	1635.82159	2	3.20E-06	0.94	4.28	-	882.1
AHQ-2-7, 4755	R.YISPDQLADLYK.S	1426.59557	2	1.31E-08	0.85	3.33	-	603.2
AHQ-2-8, 4581 - 4589	R.YISPDQLADLYK.S	1426.59557	2	1.09E-06	0.93	3.80	-	826.2
gi|4503745|ref|NP_001447.1| filamin 1 (actin-binding protein-280); filamin 1; filamin A, alpha (act				2.22E-16	103.55	1210.34	56.40	280757.4
AHQ-2-5, 5131 - 5203	R.AEAGVPAEFSIWTR.E	1534.69735	2	5.58E-07	0.76	3.68	-	234.5
AHQ-2-5, 2252	K.AEISFEDR.K	967.01472	2	9.16E-04	0.91	2.80	-	1188.0
AHQ-2-5, 2355 - 2357	K.AFGPGLQGGSAGSPAR.F	1430.55077	2	2.16E-11	0.96	4.65	-	1701.9
AHQ-2-7, 2319	K.AFGPGLQGGSAGSPAR.F	1430.55077	2	3.87E-04	0.90	3.07	-	1419.9
AHQ-2-4, 2532 - 2533	K.AFGPGLQGGSAGSPAR.F	1430.55077	2	4.49E-07	0.95	4.40	-	1340.0
AHQ-2-2, 2528 - 2552	K.AFGPGLQGGSAGSPAR.F	1430.55077	2	1.71E-10	0.95	3.40	-	1472.1
AHQ-2-4, 2548	K.AFGPGLQGGSAGSPAR.F	1430.55077	1	2.75E-07	0.53	2.94	-	178.1
AHQ-2-6, 2377	K.AFGPGLQGGSAGSPAR.F	1430.55077	2	3.53E-08	0.95	3.79	-	1664.5
AHQ-2-1, 2872	K.AFGPGLQGGSAGSPAR.F	1430.55077	2	2.53E-06	0.94	3.77	-	1182.4
AHQ-2-3, 2536	K.AFGPGLQGGSAGSPAR.F	1430.55077	1	3.28E-06	0.28	2.69	-	218.7
AHQ-2-1, 2706 - 2777	K.AFGPGLQGGSAGSPAR.F	1430.55077	2	3.15E-08	0.95	3.36	-	1626.9
AHQ-2-1, 2713 - 2717	K.AFGPGLQGGSAGSPAR.F	1430.55077	1	6.94E-05	0.04	1.94	-	92.0
AHQ-2-3, 2523 - 2552	K.AFGPGLQGGSAGSPAR.F	1430.55077	2	3.67E-06	0.92	3.65	-	1194.5
AHQ-2-14-, 3323 - 3363	K.AGNNMLLVGVHGPR.T	1435.68033	2	2.67E-06	0.87	3.70	-	717.4
AHQ-2-14, 3544 - 3614	K.AGNNM*LLVGVHGPR.T	1451.67973	2	1.47E-04	0.75	3.07	-	669.9
AHQ-2-14, 3761 - 3829	K.AGNNM*LLVGVHGPR.T	1451.67973	2	4.85E-06	0.70	2.70	-	624.2
AHQ-2-14-, 3032 - 3039	K.AGNNMLLVGVHGPR.T	1435.68033	2	7.32E-06	0.81	3.14	-	743.5
AHQ-2-13-, 2729	K.AGNNM*LLVGVHGPR.T	1451.67973	2	6.73E-05	0.80	3.08	-	887.0
AHQ-2-14, 4353	K.AGNNMLLVGVHGPR.T	1435.68033	2	1.34E-04	0.71	2.60	-	721.4
AHQ-2-14, 4314	K.AGNNMLLVGVHGPR.T	1435.68033	3	5.67E-07	0.89	3.58	-	1212.2
AHQ-2-14-, 2764	K.AGNNM*LLVGVHGPR.T	1451.67973	3	2.65E-07	0.81	3.22	-	1097.2
AHQ-2-14, 4233 - 4300	K.AGNNMLLVGVHGPR.T	1435.68033	2	2.16E-08	0.77	3.37	-	519.7
AHQ-2-14-, 2655 - 2733	K.AGNNM*LLVGVHGPR.T	1451.67973	2	9.38E-06	0.87	3.50	-	871.2
AHQ-2-14-, 3428 - 3499	K.AGNNMLLVGVHGPR.T	1435.68033	2	9.45E-06	0.75	2.77	-	714.0
AHQ-2-14-, 2652 - 2729	K.AGNNM*LLVGVHGPR.T	1451.67973	3	4.42E-05	0.83	3.60	-	1297.6
AHQ-2-14, 4046	K.AGNNMLLVGVHGPR.T	1435.68033	3	2.65E-07	0.93	4.03	-	1548.2
AHQ-2-4, 2188	K.AGVAPLQVK.V	883.07036	2	2.28E-05	0.78	2.73	-	615.6
AHQ-2-5, 1955	K.AGVAPLQVK.V	883.07036	2	8.19E-04	0.80	2.94	-	650.9
AHQ-2-2, 2157	K.AGVAPLQVK.V	883.07036	2	1.04E-05	0.79	2.76	-	596.0
AHQ-2-3, 2169 - 2172	K.AGVAPLQVK.V	883.07036	2	2.44E-04	0.68	2.82	-	405.0
AHQ-2-1, 2381	K.AGVAPLQVK.V	883.07036	2	1.89E-04	0.86	2.80	-	716.7
AHQ-2-2, 5194	K.AHEPTYFTVDCAEAGQGDVSIGIK.C	2567.76960	2	4.81E-04	0.50	2.90	-	284.0
AHQ-2-2, 7677 - 7757	K.AHEPTYFTVDCAEAGQGDVSIGIK.C	2567.76960	2	9.54E-04	0.92	3.60	-	1246.2

AHQ-2-1, 7428	K.AHEPTYFTVDCAEAGQGDVSIGIK.C	2567.76960	3	1.62E-04	0.72	3.31	-	836.9
AHQ-2-1, 7310 - 7372	K.AHEPTYFTVDCAEAGQGDVSIGIK.C	2567.76960	3	1.21E-05	0.96	5.09	-	1658.1
AHQ-2-4, 2514 - 2516	K.AHVVPCFDASK.V	1232.39073	2	1.10E-05	0.63	2.55	-	434.6
AHQ-2-3, 2500	K.AHVVPCFDASK.V	1232.39073	2	1.50E-04	0.63	2.54	-	690.8
AHQ-2-2, 2509 - 2510	K.AHVVPCFDASK.V	1232.39073	2	3.86E-06	0.89	3.10	-	922.3
AHQ-2-5, 2324	K.AHVVPCFDASK.V	1232.39073	2	8.49E-06	0.82	2.84	-	648.6
AHQ-2-1, 2728 - 2785	K.AHVVPCFDASK.V	1232.39073	2	4.24E-08	0.76	2.63	-	647.1
AHQ-2-4, 6490 - 6572	R.ALGALVDSCAPGLCPDWDSWDASKPVTNAR.E	3233.53437	3	5.29E-09	0.91	4.82	-	631.9
AHQ-2-9, 6432	R.ALGALVDSCAPGLCPDWDSWDASKPVTNAR.E	3233.53437	3	4.29E-05	0.90	4.68	-	643.4
AHQ-2-7, 6243 - 6321	R.ALGALVDSCAPGLCPDWDSWDASKPVTNAR.E	3233.53437	3	2.41E-07	0.94	5.65	-	604.5
AHQ-2-2, 6512 - 6580	R.ALGALVDSCAPGLCPDWDSWDASKPVTNAR.E	3233.53437	3	3.15E-12	0.93	5.42	-	624.9
AHQ-2-3, 6587 - 6661	R.ALGALVDSCAPGLCPDWDSWDASKPVTNAR.E	3233.53437	3	1.30E-11	0.94	5.30	-	815.1
AHQ-2-3, 6429 - 6499	R.ALGALVDSCAPGLCPDWDSWDASKPVTNAR.E	3233.53437	3	4.69E-11	0.89	4.61	-	682.8
AHQ-2-3, 6312 - 6356	R.ALGALVDSCAPGLCPDWDSWDASKPVTNAR.E	3233.53437	3	2.73E-04	0.70	3.31	-	665.7
AHQ-2-10, 5778	R.ALGALVDSCAPGLCPDWDSWDASKPVTNAR.E	3233.53437	3	7.16E-08	0.88	4.20	-	698.6
AHQ-2-2, 6648 - 6720	R.ALGALVDSCAPGLCPDWDSWDASKPVTNAR.E	3233.53437	3	2.22E-16	0.95	5.78	-	774.1
AHQ-2-1, 6448 - 6516	R.ALGALVDSCAPGLCPDWDSWDASKPVTNAR.E	3233.53437	3	1.38E-13	0.95	5.85	-	920.3
AHQ-2-1, 6357	R.ALGALVDSCAPGLCPDWDSWDASKPVTNAR.E	3233.53437	3	7.49E-08	0.84	3.92	-	773.0
AHQ-2-1, 6222 - 6285	R.ALGALVDSCAPGLCPDWDSWDASKPVTNAR.E	3233.53437	3	9.24E-06	0.85	4.24	-	623.3
AHQ-2-2, 6362 - 6444	R.ALGALVDSCAPGLCPDWDSWDASKPVTNAR.E	3233.53437	3	2.02E-04	0.93	5.29	-	756.6
AHQ-2-8, 6096 - 6159	R.ALGALVDSCAPGLCPDWDSWDASKPVTNAR.E	3233.53437	3	2.52E-08	0.84	3.99	-	541.6
AHQ-2-5, 6205 - 6276	R.ALGALVDSCAPGLCPDWDSWDASKPVTNAR.E	3233.53437	3	1.19E-08	0.85	4.34	-	544.5
AHQ-2-5, 6473 - 6539	R.ALGALVDSCAPGLCPDWDSWDASKPVTNAR.E	3233.53437	3	3.57E-09	0.90	4.60	-	572.6
AHQ-2-5, 6575	R.ALGALVDSCAPGLCPDWDSWDASKPVTNAR.E	3233.53437	3	2.30E-07	0.75	4.07	-	407.0
AHQ-2-9, 6288	R.ALGALVDSCAPGLCPDWDSWDASKPVTNAR.E	3233.53437	3	1.35E-09	0.92	5.28	-	648.2
AHQ-2-2, 1474	R.ALTQTGGPHVK.A	1109.26067	2	3.36E-04	0.85	3.29	-	750.9
AHQ-2-9, 3036	R.ANLPQSFQVDTSK.A	1435.56409	2	3.28E-04	0.44	2.57	-	299.5
AHQ-2-3, 3253 - 3265	R.ANLPQSFQVDTSK.A	1435.56409	2	5.58E-05	0.70	2.54	-	544.4
AHQ-2-3, 3507	R.ANLPQSFQVDTSK.A	1435.56409	1	2.45E-06	0.45	2.36	-	520.4
AHQ-2-1, 3608 - 3609	R.ANLPQSFQVDTSK.A	1435.56409	2	9.36E-06	0.70	3.39	-	364.9
AHQ-2-7, 2969	R.ANLPQSFQVDTSK.A	1435.56409	1	9.71E-06	0.28	2.68	-	272.2
AHQ-2-5, 3068	R.ANLPQSFQVDTSK.A	1435.56409	1	9.98E-08	0.65	3.02	-	393.1
AHQ-2-6, 3245	R.ANLPQSFQVDTSK.A	1435.56409	1	3.96E-04	0.33	2.27	-	376.8
AHQ-2-4, 3306	R.ANLPQSFQVDTSK.A	1435.56409	1	4.29E-08	0.61	2.97	-	349.3
AHQ-2-8, 2863 - 2878	R.ANLPQSFQVDTSK.A	1435.56409	2	1.09E-08	0.89	3.62	-	592.8
AHQ-2-5, 3051	R.ANLPQSFQVDTSK.A	1435.56409	2	4.59E-09	0.91	3.89	-	540.0
AHQ-2-1, 3330 - 3412	R.ANLPQSFQVDTSK.A	1435.56409	2	1.09E-04	0.88	3.27	-	754.4
AHQ-2-7, 2961 - 2984	R.ANLPQSFQVDTSK.A	1435.56409	2	4.74E-08	0.84	3.42	-	514.8
AHQ-2-4, 3284	R.ANLPQSFQVDTSK.A	1435.56409	2	3.29E-10	0.82	3.11	-	549.2
AHQ-2-6, 3042	R.ANLPQSFQVDTSK.A	1435.56409	2	3.09E-08	0.90	3.55	-	675.7
AHQ-2-2, 3264	R.ANLPQSFQVDTSK.A	1435.56409	1	2.49E-07	0.65	3.21	-	380.1
AHQ-2-1, 3450	R.ANLPQSFQVDTSK.A	1435.56409	1	5.65E-04	0.26	2.49	-	358.9
AHQ-2-2, 4690 - 4760	R.ANLPQSFQVDTSKAGVAPLQVK.V	2299.61185	2	3.36E-05	0.91	4.02	-	1055.9
AHQ-2-1, 3568 - 3626	R.APSVANVGSHCDLSLK.I	1656.84249	2	9.59E-05	0.83	3.44	-	507.2
AHQ-2-6, 3082	R.APSVANVGSHCDLSLK.I	1656.84249	2	1.65E-05	0.91	4.25	-	704.6
AHQ-2-5, 3087 - 3101	R.APSVANVGSHCDLSLK.I	1656.84249	2	2.22E-04	0.95	5.01	-	846.6
AHQ-2-1, 6116 - 6185	K.ASGPGLNTTGVPASLPVEFTIDAK.D	2343.61826	3	6.89E-05	0.83	3.98	-	622.6
AHQ-2-5, 1505	K.ATCAPQHGAPGPGPADASK.V	1791.92336	2	7.44E-05	0.88	3.71	-	668.2
AHQ-2-1, 1946	K.ATCAPQHGAPGPGPADASK.V	1791.92336	2	5.65E-07	0.95	4.74	-	945.6
AHQ-2-2, 3648 - 3718	R.AWGPGLEGGVVGK.S	1227.39403	2	3.21E-07	0.92	3.74	-	862.7
AHQ-2-1, 3792 - 3862	R.AWGPGLEGGVVGK.S	1227.39403	2	4.54E-04	0.94	4.10	-	990.8
AHQ-2-6, 3266 - 3341	R.AWGPGLEGGVVGK.S	1227.39403	2	1.19E-04	0.81	3.18	-	637.2
AHQ-2-6, 3351	R.AWGPGLEGGVVGK.S	1227.39403	2	1.52E-06	0.81	3.13	-	774.7
AHQ-2-3, 3623	R.AWGPGLEGGVVGK.S	1227.39403	2	9.20E-05	0.87	3.12	-	763.3
AHQ-2-7, 3257	R.AWGPGLEGGVVGK.S	1227.39403	2	1.42E-06	0.82	2.91	-	770.1
AHQ-2-2, 3653 - 3720	R.AWGPGLEGGVVGK.S	1227.39403	1	7.89E-06	0.73	2.86	-	676.9
AHQ-2-4, 3644	R.AWGPGLEGGVVGK.S	1227.39403	2	4.18E-05	0.95	4.11	-	1086.8
AHQ-2-5, 3352	R.AWGPGLEGGVVGK.S	1227.39403	2	2.17E-05	0.86	3.29	-	846.1
AHQ-2-3, 3171 - 3188	R.AYGPGIEPTGNMVK.K	1434.64274	2	5.52E-06	0.63	2.90	-	500.9
AHQ-2-1, 2692 - 2725	R.AYGPGIEPTGNM*VK.K	1450.64214	2	1.62E-04	0.52	2.51	-	425.2
AHQ-2-7, 2323 - 2372	R.AYGPGIEPTGNM*VK.K	1450.64214	2	3.38E-04	0.33	2.76	-	299.7
AHQ-2-7, 2837	R.AYGPGIEPTGNMVK.K	1434.64274	2	8.73E-04	0.57	2.54	-	435.9
AHQ-2-2, 3470 - 3541	R.AYGPGIEPTGNMVK.K	1434.64274	1	5.37E-05	0.31	2.81	-	380.2
AHQ-2-5, 3143 - 3221	R.AYGPGIEPTGNMVK.K	1434.64274	2	3.47E-05	0.81	2.95	-	544.9
AHQ-2-5, 2916	R.AYGPGIEPTGNMVK.K	1434.64274	2	6.17E-06	0.83	3.51	-	473.7
AHQ-2-4, 3105 - 3178	R.AYGPGIEPTGNMVK.K	1434.64274	1	2.92E-05	0.33	2.55	-	333.3
AHQ-2-6, 2883 - 2931	R.AYGPGIEPTGNMVK.K	1434.64274	2	7.77E-05	0.87	3.27	-	654.4
AHQ-2-4, 3372 - 3444	R.AYGPGIEPTGNMVK.K	1434.64274	2	9.57E-04	0.59	3.03	-	375.5
AHQ-2-1, 3429 - 3508	R.AYGPGIEPTGNMVK.K	1434.64274	2	4.76E-04	0.64	2.69	-	479.3
AHQ-2-2, 2529 - 2538	R.AYGPGIEPTGNM*VK.K	1450.64214	2	9.11E-04	0.62	2.97	-	334.3
AHQ-2-2, 2697 - 2742	R.AYGPGIEPTGNM*VK.K	1450.64214	2	8.04E-07	0.91	3.94	-	614.5
AHQ-2-3, 3368 - 3443	R.AYGPGIEPTGNMVK.K	1434.64274	2	3.20E-05	0.80	3.36	-	548.3
AHQ-2-4, 6474 - 6545	K.CAPGVVGPAEADIDFDIIR.N	2017.24863	2	2.94E-05	0.86	3.65	-	622.8
AHQ-2-3, 6399 - 6467	K.CAPGVVGPAEADIDFDIIR.N	2017.24863	2	3.44E-06	0.94	4.81	-	665.4
AHQ-2-2, 6433 - 6445	K.CAPGVVGPAEADIDFDIIR.N	2017.24863	2	2.89E-06	0.93	4.23	-	806.6
AHQ-2-5, 6304 - 6323	K.CAPGVVGPAEADIDFDIIR.N	2017.24863	2	1.15E-04	0.82	4.07	-	819.6
AHQ-2-2, 6516	K.CAPGVVGPAEADIDFDIIR.N	2017.24863	2	8.39E-08	0.90	3.66	-	673.9
AHQ-2-6, 6154	K.CAPGVVGPAEADIDFDIIR.N	2017.24863	2	2.21E-07	0.77	3.32	-	452.4
AHQ-2-2, 6281 - 6292	K.CAPGVVGPAEADIDFDIIR.N	2017.24863	2	2.01E-07	0.96	4.71	-	947.8
AHQ-2-2, 6150	K.CAPGVVGPAEADIDFDIIR.N	2017.24863	2	6.27E-05	0.82	3.27	-	576.3
AHQ-2-3, 6412	K.CAPGVVGPAEADIDFDIIR.N	2017.24863	3	1.10E-09	0.97	5.08	-	1684.2
AHQ-2-1, 6328 - 6361	K.CAPGVVGPAEADIDFDIIR.N	2017.24863	2	1.23E-05	0.93	4.35	-	684.0
AHQ-2-3, 6424 - 6500	K.CAPGVVGPAEADIDFDIIR.N	2017.24863	2	1.08E-07	0.85	3.74	-	458.6
AHQ-2-3, 6489	K.CAPGVVGPAEADIDFDIIR.N	2017.24863	2	2.72E-07	0.92	4.31	-	685.1
AHQ-2-1, 6149	K.CAPGVVGPAEADIDFDIIR.N	2017.24863	2	9.16E-06	0.80	3.06	-	621.9
AHQ-2-1, 6338	K.CAPGVVGPAEADIDFDIIR.N	2017.24863	3	3.14E-09	0.95	4.59	-	1372.4
AHQ-2-2, 6452	K.CAPGVVGPAEADIDFDIIR.N	2017.24863	3	1.10E-07	0.95	4.63	-	1573.3
AHQ-2-3, 6241	K.CAPGVVGPAEADIDFDIIR.N	2017.24863	2	1.68E-06	0.73	3.00	-	528.5
AHQ-2-2, 7320 - 7393	K.CAPGVVGPAEADIDFDIIRNDNDTFTVK.Y	3052.31840	3	1.02E-05	0.73	3.62	-	422.3
AHQ-2-2, 6725 - 6800	K.CAPGVVGPAEADIDFDIIRNDNDTFTVK.Y	3052.31840	3	2.39E-05	0.77	3.87	-	421.9
AHQ-2-3, 6629 - 6711	K.CAPGVVGPAEADIDFDIIRNDNDTFTVK.Y	3052.31840	3	5.55E-06	0.85	4.44	-	419.8
AHQ-2-3, 7272 - 7345	K.CAPGVVGPAEADIDFDIIRNDNDTFTVK.Y	3052.31840	3	7.21E-05	0.65	3.20	-	374.6
AHQ-2-1, 6501 - 6576	K.CAPGVVGPAEADIDFDIIRNDNDTFTVK.Y	3052.31840	3	1.05E-04	0.74	3.80	-	540.9
AHQ-2-2, 2928 - 2944	K.CSGPGLSPGMVR.A	1219.41699	2	9.55E-07	0.77	3.09	-	556.5
AHQ-2-5, 2755	K.CSGPGLSPGMVR.A	1219.41699	2	8.47E-06	0.81	2.88	-	805.1
AHQ-2-3, 2932 - 2935	K.CSGPGLSPGMVR.A	1219.41699	2	1.18E-05	0.57	2.59	-	448.5
AHQ-2-4, 5537 - 5544	R.CSYQPTMEGVHTVHVTFAGVPIPR.S	2686.06233	2	1.27E-11	0.97	5.17	-	1228.8
AHQ-2-4, 5000 - 5036	R.CSYQPTM*EGVHTVHVTFAGVPIPR.S	2702.06173	3	1.21E-06	0.88	4.37	-	544.2
AHQ-2-1, 5097	R.CSYQPTM*EGVHTVHVTFAGVPIPR.S	2702.06173	2	3.92E-05	0.68	2.59	-	405.2
AHQ-2-1, 5017 - 5088	R.CSYQPTM*EGVHTVHVTFAGVPIPR.S	2702.06173	3	4.19E-07	0.94	5.30	-	546.6
AHQ-2-5, 5227 - 5235	R.CSYQPTMEGVHTVHVTFAGVPIPR.S	2686.06233	2	2.44E-10	0.97	5.21	-	1265.8
AHQ-2-3, 5009 - 5031	R.CSYQPTM*EGVHTVHVTFAGVPIPR.S	2702.06173	3	2.92E-05	0.87	4.61	-	402.5
AHQ-2-5, 5224 - 5244	R.CSYQPTMEGVHTVHVTFAGVPIPR.S	2686.06233	3	8.83E-06	0.89	4.06	-	707.6
AHQ-2-2, 5517 - 5589	R.CSYQPTMEGVHTVHVTFAGVPIPR.S	2686.06233	2	3.84E-13	0.97	5.33	-	1344.0
AHQ-2-2, 5034	R.CSYQPTM*EGVHTVHVTFAGVPIPR.S	2702.06173	2	8.03E-05	0.86	3.29	-	825.0
AHQ-2-1, 5590 - 5661	R.CSYQPTMEGVHTVHVTFAGVPIPR.S	2686.06233	3	8.19E-10	0.82	3.59	-	709.9
AHQ-2-5, 4732 - 4743	R.CSYQPTM*EGVHTVHVTFAGVPIPR.S	2702.06173	2	1.25E-05	0.84	3.99	-	391.0

AHQ-2-3, 5015 - 5027	R.CSYQPTM*EGVHTVHVTFAGVPIPR.S	2702.06173	2	9.01E-07	0.87	3.85	-	524.4
AHQ-2-3, 4681 - 4721	R.DAEMPATEKDLAEDAPWK.K	2018.19047	2	1.94E-05	0.93	3.87	-	1128.1
AHQ-2-1, 4721	R.DAEMPATEKDLAEDAPWK.K	2018.19047	2	4.81E-07	0.93	4.01	-	1042.7
AHQ-2-5, 4439	R.DAEMPATEKDLAEDAPWK.K	2018.19047	2	1.16E-04	0.89	3.64	-	981.3
AHQ-2-1, 4117	R.DAEM*PATEKDLAEDAPWK.K	2034.18987	2	7.35E-06	0.90	3.84	-	696.0
AHQ-2-2, 4186	R.DAEMPATEKDLAEDAPWKK.I	2146.36338	3	3.92E-09	0.78	3.71	-	535.7
AHQ-2-13, 3959	K.DAGEGGLSLAIEGPSK.A	1501.62084	2	2.37E-04	0.44	2.55	-	535.1
AHQ-2-8, 3779	K.DAGEGGLSLAIEGPSK.A	1501.62084	2	1.34E-05	0.71	3.03	-	841.9
AHQ-2-1, 4118 - 4189	K.DAGEGGLSLAIEGPSK.A	1501.62084	2	5.63E-06	0.96	4.83	-	1384.7
AHQ-2-3, 4113	K.DAGEGGLSLAIEGPSK.A	1501.62084	2	3.53E-05	0.94	4.97	-	1181.7
AHQ-2-2, 4170	K.DAGEGGLSLAIEGPSK.A	1501.62084	2	6.32E-04	0.85	3.44	-	962.8
AHQ-2-12, 3971	K.DAGEGGLSLAIEGPSK.A	1501.62084	2	6.02E-05	0.87	3.55	-	964.0
AHQ-2-4, 4076 - 4146	K.DAGEGGLSLAIEGPSK.A	1501.62084	2	3.60E-05	0.81	3.32	-	849.9
AHQ-2-1, 5048	K.DAGEGLLAVQITDPEGK.P	1713.86752	2	4.99E-07	0.88	3.46	-	1050.1
AHQ-2-6, 4102 - 4117	K.DAGEGLLAVQITDPEGKPK.K	1939.15612	2	3.43E-05	0.93	4.08	-	1224.0
AHQ-2-3, 4456 - 4527	K.DAGEGLLAVQITDPEGKPK.K	1939.15612	2	3.89E-06	0.95	4.46	-	1280.1
AHQ-2-3, 4461 - 4469	K.DAGEGLLAVQITDPEGKPK.K	1939.15612	3	7.81E-10	0.93	4.16	-	1280.4
AHQ-2-3, 4625 - 4699	K.DAGEGLLAVQITDPEGKPK.K	1939.15612	2	8.81E-06	0.95	4.79	-	1195.6
AHQ-2-4, 4426 - 4508	K.DAGEGLLAVQITDPEGKPK.K	1939.15612	2	1.34E-08	0.96	4.95	-	1259.6
AHQ-2-1, 4777 - 4796	K.DAGEGLLAVQITDPEGKPK.K	1939.15612	2	6.00E-06	0.94	4.48	-	1190.5
AHQ-2-2, 4612 - 4680	K.DAGEGLLAVQITDPEGKPK.K	1939.15612	2	6.48E-10	0.94	4.44	-	1099.8
AHQ-2-2, 4500 - 4569	K.DAGEGLLAVQITDPEGKPK.K	1939.15612	3	7.12E-09	0.94	4.78	-	1264.5
AHQ-2-2, 4882	K.DAGEGLLAVQITDPEGKPK.K	1939.15612	2	4.02E-05	0.87	3.78	-	972.3
AHQ-2-11, 4108	K.DAGEGLLAVQITDPEGKPK.K	1939.15612	2	5.70E-07	0.96	4.85	-	1412.9
AHQ-2-2, 4470 - 4541	K.DAGEGLLAVQITDPEGKPK.K	1939.15612	2	2.37E-07	0.94	4.09	-	1330.8
AHQ-2-1, 4513 - 4548	K.DAGEGLLAVQITDPEGKPK.K	1939.15612	2	1.67E-07	0.90	3.72	-	1021.9
AHQ-2-5, 4185 - 4188	K.DAGEGLLAVQITDPEGKPK.K	1939.15612	2	2.90E-09	0.94	4.32	-	1257.7
AHQ-2-7, 4065	K.DAGEGLLAVQITDPEGKPK.K	1939.15612	2	2.65E-05	0.90	4.09	-	980.6
AHQ-2-8, 3912	K.DAGEGLLAVQITDPEGKPK.K	1939.15612	2	9.16E-06	0.89	3.73	-	1125.5
AHQ-2-1, 4553 - 4626	K.DAGEGLLAVQITDPEGKPK.K	1939.15612	3	2.94E-08	0.93	3.92	-	1298.3
AHQ-2-2, 4748 - 4754	K.DAGEGLLAVQITDPEGKPK.K	1939.15612	2	4.48E-06	0.93	4.35	-	1175.9
AHQ-2-5, 4305	K.DAGEGLLAVQITDPEGKPK.K	1939.15612	2	9.04E-06	0.73	2.84	-	847.7
AHQ-2-4, 4448	K.DAGEGLLAVQITDPEGKPK.K	1939.15612	3	4.79E-08	0.90	3.84	-	954.6
AHQ-2-10, 3935	K.DAGEGLLAVQITDPEGKPK.K	1939.15612	2	5.58E-05	0.86	3.89	-	822.5
AHQ-2-1, 4616	K.DAGEGLLAVQITDPEGKPK.K	1939.15612	2	4.59E-06	0.88	3.80	-	968.6
AHQ-2-5, 4235	R.DAGYGGLSLSIEGPSK.V	1551.67986	2	4.60E-04	0.77	2.99	-	955.0
AHQ-2-5, 4879	R.DAGYGGLSLSIEGPSK.V	1551.67986	2	4.79E-06	0.58	2.83	-	958.6
AHQ-2-5, 4739 - 4809	R.DAGYGGLSLSIEGPSK.V	1551.67986	2	2.52E-05	0.70	3.36	-	708.8
AHQ-2-1, 4688	R.DAGYGGLSLSIEGPSK.V	1551.67986	2	7.16E-08	0.91	3.51	-	1265.6
AHQ-2-2, 1825	R.DAPQDFHPDR.V	1198.22650	2	4.10E-07	0.54	2.64	-	446.9
AHQ-2-1, 1973 - 2034	R.DAPQDFHPDR.V	1198.22650	2	2.20E-05	0.56	2.68	-	455.6
AHQ-2-3, 1695 - 1760	R.DAPQDFHPDR.V	1198.22650	2	9.67E-08	0.59	2.51	-	680.5
AHQ-2-2, 1692 - 1756	R.DAPQDFHPDR.V	1198.22650	2	8.83E-06	0.46	2.67	-	537.5
AHQ-2-5, 5060 - 5129	K.DGSCGVAYVVQEPGDYEVSVK.F	2260.41990	2	1.84E-04	0.88	4.23	-	657.7
AHQ-2-5, 4947 - 4989	K.DGSCGVAYVVQEPGDYEVSVK.F	2260.41990	2	1.50E-08	0.79	4.11	-	410.4
AHQ-2-4, 4992 - 5060	K.DGSCGVAYVVQEPGDYEVSVK.F	2260.41990	2	5.35E-04	0.89	4.31	-	567.1
AHQ-2-7, 5053	K.DGSCGVAYVVQEPGDYEVSVK.F	2260.41990	2	1.89E-05	0.79	3.83	-	405.8
AHQ-2-6, 4814 - 4891	K.DGSCGVAYVVQEPGDYEVSVK.F	2260.41990	2	4.97E-06	0.93	4.94	-	756.0
AHQ-2-10, 4536	K.DGSCGVAYVVQEPGDYEVSVK.F	2260.41990	2	5.36E-04	0.86	3.88	-	492.0
AHQ-2-14-, 2331	K.DKGEYTLVVK.W	1152.32223	1	1.54E-04	0.20	2.35	-	405.1
AHQ-2-14-, 2345	K.DKGEYTLVVK.W	1152.32223	1	3.40E-04	0.43	2.46	-	503.5
AHQ-2-14, 3368	K.DKGEYTLVVK.W	1152.32223	2	1.13E-04	0.72	2.81	-	502.2
AHQ-2-5, 2135 - 2139	K.DKGEYTLVVK.W	1152.32223	2	7.09E-06	0.65	2.62	-	457.8
AHQ-2-14, 3138 - 3424	K.DKGEYTLVVK.W	1152.32223	1	8.23E-04	0.17	2.19	-	402.7
AHQ-2-14-, 2328 - 2397	K.DKGEYTLVVK.W	1152.32223	2	3.59E-06	0.79	2.86	-	700.8
AHQ-2-14, 3248 - 3312	K.DKGEYTLVVK.W	1152.32223	2	1.97E-06	0.62	2.66	-	439.4
AHQ-2-13-, 2415	K.DKGEYTLVVK.W	1152.32223	1	5.96E-04	0.12	1.81	-	386.1
AHQ-2-4, 2348	K.DKGEYTLVVK.W	1152.32223	1	7.57E-07	0.54	2.80	-	511.9
AHQ-2-14, 3588 - 3636	K.DKGEYTLVVK.W	1152.32223	2	4.58E-05	0.61	2.52	-	437.9
AHQ-2-14, 3630	K.DKGEYTLVVK.W	1152.32223	1	8.66E-06	0.50	2.52	-	627.0
AHQ-2-1, 3384	K.DNGNGTYSCSYVPR.K	1591.64109	2	4.69E-06	0.83	3.04	-	909.4
AHQ-2-4, 3080	R.DVDIIDHHDNTYTVK.Y	1785.89230	2	2.43E-06	0.94	4.43	-	947.6
AHQ-2-4, 3000 - 3008	R.DVDIIDHHDNTYTVK.Y	1785.89230	3	2.91E-04	0.66	3.03	-	403.7
AHQ-2-6, 2786	R.DVDIIDHHDNTYTVK.Y	1785.89230	2	9.98E-05	0.81	3.06	-	732.7
AHQ-2-2, 3246	R.DVDIIDHHDNTYTVK.Y	1785.89230	2	7.81E-05	0.93	4.15	-	1072.5
AHQ-2-4, 2941 - 3012	R.DVDIIDHHDNTYTVK.Y	1785.89230	2	1.47E-06	0.92	4.05	-	904.4
AHQ-2-2, 3154	R.DVDIIDHHDNTYTVK.Y	1785.89230	2	1.02E-06	0.89	3.55	-	886.0
AHQ-2-2, 3028 - 3084	R.DVDIIDHHDNTYTVK.Y	1785.89230	2	6.93E-10	0.97	4.93	-	1351.3
AHQ-2-1, 3292 - 3365	R.DVDIIDHHDNTYTVK.Y	1785.89230	2	4.20E-06	0.72	3.02	-	411.4
AHQ-2-2, 2942 - 3016	R.DVDIIDHHDNTYTVK.Y	1785.89230	2	1.19E-04	0.89	3.53	-	784.8
AHQ-2-4, 3272	R.DVDIIDHHDNTYTVK.Y	1785.89230	2	9.85E-05	0.89	3.51	-	1067.7
AHQ-2-3, 3267 - 3344	R.DVDIIDHHDNTYTVK.Y	1785.89230	2	4.49E-05	0.91	3.93	-	605.8
AHQ-2-3, 3083	R.DVDIIDHHDNTYTVK.Y	1785.89230	2	6.47E-08	0.96	4.87	-	1134.3
AHQ-2-1, 3157 - 3224	R.DVDIIDHHDNTYTVK.Y	1785.89230	2	4.96E-07	0.95	4.42	-	1247.8
AHQ-2-3, 2940 - 3012	R.DVDIIDHHDNTYTVK.Y	1785.89230	2	1.06E-05	0.93	3.86	-	986.5
AHQ-2-9, 3110	R.EAGAGGLAIAVEGPSK.A	1427.58510	1	2.72E-05	0.59	3.04	-	394.7
AHQ-2-7, 3109	R.EAGAGGLAIAVEGPSK.A	1427.58510	2	3.02E-05	0.92	2.94	-	1743.7
AHQ-2-1, 3513 - 3517	R.EAGAGGLAIAVEGPSK.A	1427.58510	2	1.15E-06	0.94	4.38	-	1098.0
AHQ-2-6, 3127	R.EAGAGGLAIAVEGPSK.A	1427.58510	1	1.54E-08	0.65	3.36	-	315.9
AHQ-2-5, 3172	R.EAGAGGLAIAVEGPSK.A	1427.58510	1	3.55E-08	0.79	3.42	-	412.3
AHQ-2-5, 3164 - 3305	R.EAGAGGLAIAVEGPSK.A	1427.58510	1	6.87E-04	0.08	1.97	-	245.7
AHQ-2-4, 3401 - 3408	R.EAGAGGLAIAVEGPSK.A	1427.58510	2	3.97E-05	0.96	4.50	-	1637.2
AHQ-2-2, 3030	R.EATTEFSVDAR.A	1226.27493	2	5.40E-04	0.75	2.71	-	648.6
AHQ-2-2, 2540 - 2609	R.EATTEFSVDAR.A	1226.27493	2	5.16E-06	0.87	3.42	-	807.3
AHQ-2-5, 6391 - 6456	R.EEGPYEVEVTYDGVPVPGSPFPLEAVAPTKPSK.V	3487.85286	3	1.79E-06	0.88	4.05	-	733.2
AHQ-2-3, 6483 - 6552	R.EEGPYEVEVTYDGVPVPGSPFPLEAVAPTKPSK.V	3487.85286	3	2.90E-08	0.96	6.30	-	690.4
AHQ-2-2, 6493 - 6560	R.EEGPYEVEVTYDGVPVPGSPFPLEAVAPTKPSK.V	3487.85286	3	2.08E-10	0.96	6.06	-	948.7
AHQ-2-2, 7241	R.EEGPYEVEVTYDGVPVPGSPFPLEAVAPTKPSK.V	3487.85286	3	9.94E-04	0.59	3.19	-	416.7
AHQ-2-3, 6619 - 6688	R.EEGPYEVEVTYDGVPVPGSPFPLEAVAPTKPSK.V	3487.85286	3	2.70E-10	0.96	6.19	-	939.1
AHQ-2-1, 6432 - 6512	R.EEGPYEVEVTYDGVPVPGSPFPLEAVAPTKPSK.V	3487.85286	3	2.68E-05	0.95	6.15	-	695.3
AHQ-2-5, 6523	R.EEGPYEVEVTYDGVPVPGSPFPLEAVAPTKPSK.V	3487.85286	3	1.96E-09	0.94	5.61	-	687.7
AHQ-2-6, 6301 - 6303	R.EEGPYEVEVTYDGVPVPGSPFPLEAVAPTKPSK.V	3487.85286	3	5.73E-07	0.93	5.23	-	763.8
AHQ-2-2, 6696 - 6765	R.EEGPYEVEVTYDGVPVPGSPFPLEAVAPTKPSK.V	3487.85286	3	2.11E-08	0.94	5.18	-	826.6
AHQ-2-1, 6572 - 6646	R.EEGPYEVEVTYDGVPVPGSPFPLEAVAPTKPSK.V	3487.85286	3	3.62E-07	0.90	4.73	-	586.2
AHQ-2-2, 6593 - 6660	R.EEGPYEVEVTYDGVPVPGSPFPLEAVAPTKPSK.V	3487.85286	3	1.01E-07	0.94	5.56	-	824.0
AHQ-2-4, 6696 - 6768	R.EEGPYEVEVTYDGVPVPGSPFPLEAVAPTKPSK.V	3487.85286	3	1.27E-08	0.92	5.34	-	520.5
AHQ-2-4, 6552 - 6628	R.EEGPYEVEVTYDGVPVPGSPFPLEAVAPTKPSK.V	3487.85286	3	1.29E-09	0.94	5.96	-	665.1
AHQ-2-1, 5301 - 5302	R.EGPYSISVLYGDEEVPR.S	1911.05809	3	5.53E-07	0.96	4.81	-	1682.3
AHQ-2-2, 4976	R.EGPYSISVLYGDEEVPR.S	1911.05809	2	2.88E-07	0.86	3.76	-	739.5
AHQ-2-1, 5230 - 5304	R.EGPYSISVLYGDEEVPR.S	1911.05809	2	6.07E-08	0.93	4.08	-	1145.4
AHQ-2-2, 5252 - 5280	R.EGPYSISVLYGDEEVPR.S	1911.05809	2	8.43E-05	0.92	4.02	-	1190.9
AHQ-2-8, 4773 - 4845	R.EGPYSISVLYGDEEVPR.S	1911.05809	2	4.90E-06	0.93	4.23	-	778.4
AHQ-2-1, 5332	R.EGPYSISVLYGDEEVPR.S	1911.05809	2	5.78E-05	0.84	3.68	-	846.9
AHQ-2-4, 5244 - 5313	R.EGPYSISVLYGDEEVPR.S	1911.05809	2	1.42E-07	0.90	3.70	-	1145.3
AHQ-2-5, 5068	R.EGPYSISVLYGDEEVPR.S	1911.05809	2	9.15E-07	0.93	4.14	-	1047.5
AHQ-2-6, 4962	R.EGPYSISVLYGDEEVPR.S	1911.05809	2	1.08E-08	0.78	3.75	-	734.2
AHQ-2-7, 4956	R.EGPYSISVLYGDEEVPR.S	1911.05809	2	7.79E-09	0.93	4.45	-	1073.8

AHQ-2-9, 4978	R.EGPYSISVLYGDEEVPR.S	1911.05809	2	7.90E-05	0.82	3.24	-	719.2
AHQ-2-2, 5348 - 5352	R.EGPYSISVLYGDEEVPR.S	1911.05809	2	3.88E-10	0.94	4.08	-	1321.0
AHQ-2-1, 5368	R.EGPYSISVLYGDEEVPR.S	1911.05809	2	3.27E-05	0.94	4.37	-	1038.3
AHQ-2-3, 5220 - 5303	R.EGPYSISVLYGDEEVPR.S	1911.05809	2	4.62E-08	0.96	4.93	-	1300.6
AHQ-2-3, 4032 - 4033	R.ENGVYLIDVK.F	1150.30633	1	2.63E-06	0.18	2.34	-	167.0
AHQ-2-6, 3781	R.ENGVYLIDVK.F	1150.30633	1	4.46E-04	0.14	1.90	-	264.5
AHQ-2-5, 3847	R.ENGVYLIDVK.F	1150.30633	1	1.33E-04	0.21	2.12	-	286.1
AHQ-2-5, 3701	R.ENGVYLIDVK.F	1150.30633	1	1.92E-04	0.16	2.28	-	290.0
AHQ-2-5, 3241	K.FADQHVPGSPFSVK.V	1516.68203	2	4.52E-04	0.48	2.57	-	314.7
AHQ-2-1, 3616 - 3689	K.FADQHVPGSPFSVK.V	1516.68203	2	3.00E-05	0.41	2.65	-	301.3
AHQ-2-13-, 3417	K.FADQHVPGSPFSVK.V	1516.68203	2	1.01E-05	0.73	2.88	-	470.5
AHQ-2-7, 2991	K.FADQHVPGSPFSVK.V	1516.68203	2	2.19E-06	0.53	2.86	-	323.6
AHQ-2-2, 3466	K.FADQHVPGSPFSVK.V	1516.68203	2	1.98E-06	0.87	3.41	-	642.4
AHQ-2-8, 2872	K.FADQHVPGSPFSVK.V	1516.68203	3	9.48E-06	0.90	3.72	-	1045.0
AHQ-2-1, 3628	K.FADQHVPGSPFSVK.V	1516.68203	3	1.25E-05	0.92	3.64	-	1356.9
AHQ-2-6, 3107	K.FADQHVPGSPFSVK.V	1516.68203	3	6.08E-05	0.92	4.03	-	1305.6
AHQ-2-6, 3106	K.FADQHVPGSPFSVK.V	1516.68203	2	2.35E-07	0.86	3.70	-	556.7
AHQ-2-5, 3121 - 3131	K.FADQHVPGSPFSVK.V	1516.68203	3	7.67E-08	0.95	4.41	-	1396.2
AHQ-2-3, 3441	K.FADQHVPGSPFSVK.V	1516.68203	2	2.63E-07	0.88	3.75	-	519.6
AHQ-2-5, 3103 - 3171	K.FADQHVPGSPFSVK.V	1516.68203	2	1.64E-05	0.79	3.39	-	500.4
AHQ-2-1, 5800	R.FGGEHVPNSPFQVTALAGDQPSVQPPLR.S	2947.25342	2	1.14E-08	0.96	5.38	-	1040.9
AHQ-2-1, 5724 - 5788	R.FGGEHVPNSPFQVTALAGDQPSVQPPLR.S	2947.25342	3	1.94E-08	0.93	5.09	-	782.9
AHQ-2-1, 5856 - 5921	R.FGGEHVPNSPFQVTALAGDQPSVQPPLR.S	2947.25342	3	2.15E-08	0.94	5.41	-	1053.1
AHQ-2-1, 5910	R.FGGEHVPNSPFQVTALAGDQPSVQPPLR.S	2947.25342	2	2.02E-05	0.95	5.22	-	720.7
AHQ-2-8, 5103	R.FGGEHVPNSPFQVTALAGDQPSVQPPLR.S	2947.25342	2	3.15E-06	0.97	5.55	-	1164.1
AHQ-2-2, 5542	R.FGGEHVPNSPFQVTALAGDQPSVQPPLR.S	2947.25342	3	2.29E-04	0.86	3.97	-	682.1
AHQ-2-11, 5181	R.FGGEHVPNSPFQVTALAGDQPSVQPPLR.S	2947.25342	3	1.04E-04	0.88	4.18	-	627.3
AHQ-2-5, 5392	R.FGGEHVPNSPFQVTALAGDQPSVQPPLR.S	2947.25342	3	3.93E-04	0.87	4.02	-	620.8
AHQ-2-11, 5222	R.FGGEHVPNSPFQVTALAGDQPSVQPPLR.S	2947.25342	2	5.42E-06	0.94	4.33	-	957.7
AHQ-2-5, 5407	R.FGGEHVPNSPFQVTALAGDQPSVQPPLR.S	2947.25342	2	5.02E-07	0.94	4.80	-	916.4
AHQ-2-2, 5652 - 5720	R.FGGEHVPNSPFQVTALAGDQPSVQPPLR.S	2947.25342	3	5.50E-09	0.93	5.16	-	751.4
AHQ-2-2, 5654 - 5669	R.FGGEHVPNSPFQVTALAGDQPSVQPPLR.S	2947.25342	2	4.50E-08	0.96	5.63	-	901.3
AHQ-2-8, 5088 - 5107	R.FGGEHVPNSPFQVTALAGDQPSVQPPLR.S	2947.25342	3	1.39E-05	0.93	4.65	-	997.4
AHQ-2-9, 5335 - 5360	R.FGGEHVPNSPFQVTALAGDQPSVQPPLR.S	2947.25342	3	7.53E-06	0.91	4.61	-	697.5
AHQ-2-2, 5740 - 5808	R.FGGEHVPNSPFQVTALAGDQPSVQPPLR.S	2947.25342	2	9.03E-07	0.95	5.52	-	846.9
AHQ-2-6, 5346	R.FGGEHVPNSPFQVTALAGDQPSVQPPLR.S	2947.25342	3	4.36E-08	0.92	4.42	-	924.0
AHQ-2-13-, 5407 - 5421	R.FGGEHVPNSPFQVTALAGDQPSVQPPLR.S	2947.25342	3	4.66E-10	0.93	4.94	-	788.9
AHQ-2-2, 5789 - 5856	R.FGGEHVPNSPFQVTALAGDQPSVQPPLR.S	2947.25342	3	3.38E-09	0.91	4.42	-	956.6
AHQ-2-4, 5672 - 5746	R.FGGEHVPNSPFQVTALAGDQPSVQPPLR.S	2947.25342	2	1.02E-05	0.96	5.57	-	1019.1
AHQ-2-4, 5665 - 5693	R.FGGEHVPNSPFQVTALAGDQPSVQPPLR.S	2947.25342	3	2.91E-06	0.92	5.13	-	658.6
AHQ-2-2, 5870 - 5876	R.FGGEHVPNSPFQVTALAGDQPSVQPPLR.S	2947.25342	2	7.58E-10	0.98	6.88	-	1290.0
AHQ-2-3, 5640 - 5711	R.FGGEHVPNSPFQVTALAGDQPSVQPPLR.S	2947.25342	2	6.04E-05	0.96	5.18	-	1354.5
AHQ-2-3, 5656 - 5723	R.FGGEHVPNSPFQVTALAGDQPSVQPPLR.S	2947.25342	3	4.01E-09	0.93	4.77	-	974.7
AHQ-2-12, 5453	R.FGGEHVPNSPFQVTALAGDQPSVQPPLR.S	2947.25342	2	1.14E-05	0.95	4.89	-	937.7
AHQ-2-3, 5729	R.FGGEHVPNSPFQVTALAGDQPSVQPPLR.S	2947.25342	2	1.10E-06	0.97	6.01	-	1008.8
AHQ-2-3, 5779 - 5847	R.FGGEHVPNSPFQVTALAGDQPSVQPPLR.S	2947.25342	2	6.70E-11	0.95	5.00	-	899.9
AHQ-2-12, 5443	R.FGGEHVPNSPFQVTALAGDQPSVQPPLR.S	2947.25342	3	3.59E-06	0.87	3.80	-	916.1
AHQ-2-13, 5328	R.FGGEHVPNSPFQVTALAGDQPSVQPPLR.S	2947.25342	2	8.21E-05	0.66	2.98	-	575.9
AHQ-2-7, 5277 - 5283	R.FGGEHVPNSPFQVTALAGDQPSVQPPLR.S	2947.25342	3	1.77E-06	0.93	4.86	-	941.1
AHQ-2-7, 5288	R.FGGEHVPNSPFQVTALAGDQPSVQPPLR.S	2947.25342	2	2.64E-06	0.95	5.13	-	919.1
AHQ-2-5, 5075 - 5077	K.FNEEHIPDSPFVVPVASPSGDAR.R	2468.66280	2	2.29E-05	0.93	4.69	-	655.3
AHQ-2-5, 3219	K.FNGTHIPGSPFK.I	1302.46259	2	2.08E-05	0.84	3.21	-	665.6
AHQ-2-5, 3491 - 3495	K.FNGTHIPGSPFK.I	1302.46259	2	4.58E-06	0.85	3.29	-	708.1
AHQ-2-3, 3829	K.FNGTHIPGSPFK.I	1302.46259	2	1.47E-04	0.78	3.26	-	449.1
AHQ-2-6, 3362	K.FNGTHIPGSPFK.I	1302.46259	2	5.08E-04	0.80	2.85	-	724.4
AHQ-2-1, 3953	K.FNGTHIPGSPFK.I	1302.46259	2	2.79E-04	0.61	2.55	-	536.1
AHQ-2-5, 3672 - 3677	K.FNGTHIPGSPFK.I	1302.46259	1	1.33E-04	0.37	2.04	-	392.1
AHQ-2-3, 2927 - 2933	R.FVPAEMGTHTVSVK.Y	1503.74821	2	1.66E-11	0.92	3.84	-	777.6
AHQ-2-7, 2500 - 2572	R.FVPAEMGTHTVSVK.Y	1503.74821	2	1.39E-08	0.89	3.84	-	692.7
AHQ-2-1, 2470	R.FVPAEM*GTHTVSVK.Y	1519.74761	2	2.92E-04	0.73	2.64	-	430.2
AHQ-2-2, 2912	R.FVPAEMGTHTVSVK.Y	1503.74821	2	2.71E-04	0.77	2.51	-	692.7
AHQ-2-5, 2617	R.FVPAEMGTHTVSVK.Y	1503.74821	2	4.49E-11	0.89	3.03	-	866.0
AHQ-2-1, 3113	R.FVPAEMGTHTVSVK.Y	1503.74821	2	9.05E-06	0.76	2.90	-	503.3
AHQ-2-2, 6430	R.GAGSYTIM*VLFADQATPTSPIR.V	2313.61563	2	6.27E-04	0.60	3.11	-	266.2
AHQ-2-3, 6952 - 7019	R.GAGSYTIMVLFADQATPTSPIR.V	2297.61623	2	3.94E-04	0.76	3.98	-	571.5
AHQ-2-2, 6278 - 6358	R.GAGSYTIM*VLFADQATPTSPIR.V	2313.61563	2	2.12E-05	0.83	4.27	-	723.2
AHQ-2-2, 7057 - 7125	R.GAGSYTIMVLFADQATPTSPIR.V	2297.61623	2	9.87E-05	0.93	4.32	-	845.3
AHQ-2-2, 7197 - 7273	R.GAGSYTIMVLFADQATPTSPIR.V	2297.61623	2	1.11E-04	0.72	3.42	-	437.7
AHQ-2-2, 3341 - 3412	R.GAGTGGLGLAVEGPSEAK.M	1571.71430	2	1.32E-06	0.87	3.48	-	806.2
AHQ-2-3, 3529	R.GAGTGGLGLAVEGPSEAK.M	1571.71430	2	7.34E-05	0.69	2.96	-	550.6
AHQ-2-2, 3254 - 3325	R.GAGTGGLGLAVEGPSEAK.M	1571.71430	2	3.31E-06	0.88	3.80	-	731.5
AHQ-2-2, 3768 - 3784	R.GAGTGGLGLAVEGPSEAK.M	1571.71430	2	5.19E-05	0.80	2.84	-	929.0
AHQ-2-5, 3085	R.GAGTGGLGLAVEGPSEAK.M	1571.71430	2	6.92E-06	0.93	3.91	-	1057.5
AHQ-2-1, 3552 - 3553	R.GAGTGGLGLAVEGPSEAK.M	1571.71430	2	1.04E-04	0.85	3.46	-	783.2
AHQ-2-2, 3480 - 3553	R.GAGTGGLGLAVEGPSEAK.M	1571.71430	2	3.28E-05	0.85	3.85	-	828.6
AHQ-2-4, 3344 - 3386	R.GAGTGGLGLAVEGPSEAK.M	1571.71430	2	2.21E-04	0.65	2.89	-	544.7
AHQ-2-5, 3176	R.GAGTGGLGLAVEGPSEAK.M	1571.71430	2	2.97E-05	0.70	3.14	-	692.2
AHQ-2-8, 2875	R.GAGTGGLGLAVEGPSEAK.M	1571.71430	2	4.31E-05	0.73	3.05	-	696.8
AHQ-2-1, 3410 - 3481	R.GAGTGGLGLAVEGPSEAK.M	1571.71430	2	1.40E-04	0.89	3.58	-	803.6
AHQ-2-3, 3327 - 3401	R.GAGTGGLGLAVEGPSEAK.M	1571.71430	2	7.25E-08	0.94	4.87	-	746.2
AHQ-2-6, 3078 - 3085	R.GAGTGGLGLAVEGPSEAK.M	1571.71430	2	2.90E-05	0.83	3.47	-	693.9
AHQ-2-2, 3597	K.GKLDVQFSGLTK.G	1293.49392	2	1.67E-04	0.91	3.59	-	860.0
AHQ-2-2, 3286	K.GKLDVQFSGLTK.G	1293.49392	2	6.02E-05	0.95	3.82	-	1381.9
AHQ-2-2, 3232 - 3374	K.GKLDVQFSGLTK.G	1293.49392	1	3.13E-06	0.59	3.04	-	432.3
AHQ-2-2, 3284	K.GKLDVQFSGLTK.G	1293.49392	2	5.54E-05	0.96	4.08	-	1520.9
AHQ-2-4, 3298 - 3314	K.GKLDVQFSGLTK.G	1293.49392	2	5.44E-04	0.91	3.50	-	930.5
AHQ-2-3, 5084 - 5152	K.GLVEPVDVVDNADGTQTVNYVPSR.E	2545.74387	2	2.14E-08	0.98	6.64	-	1294.7
AHQ-2-1, 5034 - 5105	K.GLVEPVDVVDNADGTQTVNYVPSR.E	2545.74387	2	3.50E-06	0.73	3.47	-	408.8
AHQ-2-1, 5168 - 5244	K.GLVEPVDVVDNADGTQTVNYVPSR.E	2545.74387	2	1.85E-10	0.96	5.42	-	1120.9
AHQ-2-2, 5220 - 5293	K.GLVEPVDVVDNADGTQTVNYVPSR.E	2545.74387	2	2.94E-06	0.97	5.81	-	1390.9
AHQ-2-1, 6676 - 6678	K.GLVEPVDVVDNADGTQTVNYVPSREGPYSISVLYGDEEVPR.S	4437.77937	3	1.24E-10	0.95	5.53	-	900.3
AHQ-2-1, 2292 - 2368	K.GTVEPQLEAR.G	1100.20741	2	1.36E-05	0.72	2.60	-	504.5
AHQ-2-3, 2124	K.GTVEPQLEAR.G	1100.20741	2	3.22E-04	0.82	3.08	-	525.7
AHQ-2-2, 2116 - 2132	K.GTVEPQLEAR.G	1100.20741	2	8.78E-05	0.84	3.05	-	585.1
AHQ-2-6, 1925 - 1929	K.GTVEPQLEAR.G	1100.20741	2	4.16E-05	0.81	3.26	-	528.6
AHQ-2-5, 1891 - 1907	K.GTVEPQLEAR.G	1100.20741	2	9.54E-04	0.76	2.58	-	711.8
AHQ-2-2, 5628 - 5629	K.HTAMVSWGGVSIPNSPFR.V	1944.20601	2	1.19E-06	0.98	6.08	-	1290.8
AHQ-2-1, 5388	K.HTAM*VSWGGVSIPNSPFR.V	1960.20541	3	1.58E-04	0.71	3.83	-	455.4
AHQ-2-3, 5327	K.HTAM*VSWGGVSIPNSPFR.V	1960.20541	2	9.24E-05	0.93	4.12	-	1004.9
AHQ-2-2, 5248	K.HTAM*VSWGGVSIPNSPFR.V	1960.20541	3	1.40E-05	0.79	3.74	-	716.5
AHQ-2-5, 4905	K.HTAM*VSWGGVSIPNSPFR.V	1960.20541	2	6.82E-05	0.96	4.70	-	1356.6
AHQ-2-2, 5849	K.HTAMVSWGGVSIPNSPFR.V	1944.20601	2	3.14E-09	0.94	4.56	-	915.7
AHQ-2-3, 5599 - 5600	K.HTAMVSWGGVSIPNSPFR.V	1944.20601	2	7.04E-08	0.98	5.84	-	1651.9
AHQ-2-4, 5184 - 5185	K.HTAM*VSWGGVSIPNSPFR.V	1960.20541	2	1.82E-04	0.93	4.23	-	1020.9
AHQ-2-1, 5778 - 5784	K.HTAMVSWGGVSIPNSPFR.V	1944.20601	2	2.67E-04	0.98	6.37	-	1163.6
AHQ-2-2, 5333	K.HTAM*VSWGGVSIPNSPFR.V	1960.20541	2	2.96E-05	0.89	3.35	-	953.5
AHQ-2-3, 5215 - 5255	K.HTAM*VSWGGVSIPNSPFR.V	1960.20541	2	8.70E-04	0.96	4.70	-	1282.0

AHQ-2-5, 5308	K.HTAMVSWGGVSIPNSPFR.V	1944.20601	2	6.34E-05	0.98	6.16	-	1344.8
AHQ-2-3, 5236	K.HTAM*VSWGGVSIPNSPFR.V	1960.20541	3	2.48E-04	0.67	3.29	-	655.8
AHQ-2-2, 5237 - 5264	K.HTAM*VSWGGVSIPNSPFR.V	1960.20541	2	3.22E-07	0.97	5.67	-	1275.1
AHQ-2-4, 5622	K.HTAMVSWGGVSIPNSPFR.V	1944.20601	2	1.03E-07	0.95	4.49	-	994.6
AHQ-2-11, 3970	R.IANLQTDLSDGLR.L	1416.56231	2	5.20E-07	0.87	3.31	-	798.9
AHQ-2-3, 3897 - 3907	R.IANLQTDLSDGLR.L	1416.56231	2	5.18E-06	0.93	4.04	-	981.2
AHQ-2-1, 4405 - 4472	R.IANLQTDLSDGLR.L	1416.56231	2	7.47E-06	0.94	4.38	-	978.6
AHQ-2-5, 3989 - 4015	R.IANLQTDLSDGLR.L	1416.56231	2	9.29E-06	0.94	4.04	-	1172.5
AHQ-2-2, 3920	R.IANLQTDLSDGLR.L	1416.56231	2	7.16E-07	0.97	4.23	-	1657.7
AHQ-2-2, 4008	R.IANLQTDLSDGLR.L	1416.56231	2	3.06E-06	0.95	4.83	-	990.2
AHQ-2-3, 3983	R.IANLQTDLSDGLR.L	1416.56231	2	1.01E-05	0.94	4.69	-	838.1
AHQ-2-12, 4119	R.IANLQTDLSDGLR.L	1416.56231	2	1.18E-07	0.94	4.77	-	790.4
AHQ-2-2, 4089 - 4162	R.IANLQTDLSDGLR.L	1416.56231	2	4.43E-06	0.74	2.59	-	835.6
AHQ-2-7, 3919 - 3935	R.IANLQTDLSDGLR.L	1416.56231	2	6.32E-06	0.85	3.40	-	738.0
AHQ-2-1, 4294	R.IANLQTDLSDGLR.L	1416.56231	2	9.43E-07	0.90	3.90	-	785.7
AHQ-2-2, 4281 - 4358	R.IANLQTDLSDGLR.L	1416.56231	2	2.04E-06	0.95	4.31	-	1191.0
AHQ-2-5, 3691	R.IANLQTDLSDGLR.L	1416.56231	2	1.79E-05	0.92	3.33	-	1271.9
AHQ-2-13, 4156	R.IANLQTDLSDGLR.L	1416.56231	2	3.46E-06	0.93	3.56	-	1231.2
AHQ-2-1, 4094	R.IANLQTDLSDGLR.L	1416.56231	2	2.18E-06	0.95	4.13	-	1339.1
AHQ-2-9, 3967	R.IANLQTDLSDGLR.L	1416.56231	2	8.84E-05	0.94	4.07	-	1081.3
AHQ-2-4, 3869 - 3905	R.IANLQTDLSDGLR.L	1416.56231	2	2.52E-08	0.93	4.00	-	1064.4
AHQ-2-9, 3646	R.IANLQTDLSDGLR.L	1416.56231	2	3.66E-09	0.85	3.32	-	706.2
AHQ-2-3, 4305 - 4307	R.IANLQTDLSDGLR.L	1416.56231	1	9.12E-05	0.53	2.97	-	345.3
AHQ-2-4, 4284	R.IANLQTDLSDGLR.L	1416.56231	2	1.11E-04	0.87	3.16	-	879.8
AHQ-2-1, 4000	R.IANLQTDLSDGLR.L	1416.56231	2	1.15E-07	0.94	4.15	-	1103.7
AHQ-2-1, 4546 - 4612	R.IANLQTDLSDGLR.L	1416.56231	2	3.19E-07	0.92	3.43	-	1120.1
AHQ-2-2, 4722	R.IANLQTDLSDGLR.L	1416.56231	2	3.98E-06	0.96	4.47	-	1353.7
AHQ-2-4, 4289 - 4290	R.IANLQTDLSDGLR.L	1416.56231	1	4.34E-05	0.74	3.26	-	309.6
AHQ-2-2, 4618	R.IANLQTDLSDGLR.L	1416.56231	1	6.78E-05	0.37	2.09	-	326.3
AHQ-2-2, 4598	R.IANLQTDLSDGLR.L	1416.56231	2	1.61E-06	0.94	3.91	-	1340.9
AHQ-2-4, 5685	K.IPEISIQDMTAQVTSPSGK.T	2003.26361	2	1.91E-08	0.97	4.80	-	1861.3
AHQ-2-6, 5353	K.IPEISIQDMTAQVTSPSGK.T	2003.26361	2	1.60E-10	0.97	4.74	-	1914.8
AHQ-2-6, 5369 - 5442	K.IPEISIQDMTAQVTSPSGK.T	2003.26361	2	2.78E-11	0.99	6.79	-	2256.6
AHQ-2-13, 5325	K.IPEISIQDMTAQVTSPSGK.T	2003.26361	2	3.71E-08	0.97	5.01	-	1659.2
AHQ-2-11, 5189	K.IPEISIQDMTAQVTSPSGK.T	2003.26361	2	4.97E-10	0.97	4.69	-	1665.8
AHQ-2-5, 4815	K.IPEISIQDMTAQVTSPSGK.T	2003.26361	2	2.62E-09	0.97	4.86	-	1874.0
AHQ-2-12, 5442 - 5449	K.IPEISIQDMTAQVTSPSGK.T	2003.26361	2	2.91E-12	0.98	5.88	-	1925.1
AHQ-2-6, 3945	K.IPEISIQDM*TAQVTSPSGK.T	2019.26301	2	1.22E-08	0.97	5.55	-	1395.1
AHQ-2-1, 4285 - 4356	K.IPEISIQDM*TAQVTSPSGK.T	2019.26301	2	8.23E-10	0.98	5.56	-	1952.6
AHQ-2-13-, 5430	K.IPEISIQDMTAQVTSPSGK.T	2003.26361	2	7.51E-05	0.96	5.45	-	1014.4
AHQ-2-3, 5659 - 5728	K.IPEISIQDMTAQVTSPSGK.T	2003.26361	2	1.83E-06	0.94	4.28	-	1215.2
AHQ-2-8, 5190	K.IPEISIQDMTAQVTSPSGK.T	2003.26361	2	8.63E-09	0.98	5.50	-	1705.4
AHQ-2-7, 3923	K.IPEISIQDM*TAQVTSPSGK.T	2019.26301	2	8.74E-07	0.95	4.55	-	1097.0
AHQ-2-4, 4224	K.IPEISIQDM*TAQVTSPSGK.T	2019.26301	2	2.57E-05	0.94	4.54	-	1040.1
AHQ-2-14, 6444	K.IPEISIQDMTAQVTSPSGK.T	2003.26361	2	3.44E-04	0.97	4.98	-	1532.8
AHQ-2-7, 5347 - 5356	K.IPEISIQDMTAQVTSPSGK.T	2003.26361	2	4.12E-09	0.98	5.63	-	1973.5
AHQ-2-1, 5740	K.IPEISIQDMTAQVTSPSGK.T	2003.26361	2	8.01E-12	0.98	5.43	-	2033.0
AHQ-2-1, 5760	K.IPEISIQDMTAQVTSPSGK.T	2003.26361	3	1.83E-05	0.88	4.09	-	924.3
AHQ-2-5, 5589 - 5623	K.IPEISIQDMTAQVTSPSGK.T	2003.26361	2	9.93E-05	0.74	3.15	-	627.5
AHQ-2-4, 2468	K.IVGPSGAAVPCK.V	1157.36530	2	1.50E-08	0.70	2.60	-	574.7
AHQ-2-3, 2464	K.IVGPSGAAVPCK.V	1157.36530	2	2.17E-05	0.86	3.16	-	708.8
AHQ-2-2, 2480	K.IVGPSGAAVPCK.V	1157.36530	2	1.08E-08	0.92	3.45	-	940.3
AHQ-2-1, 2621 - 2622	K.IVGPSGAAVPCK.V	1157.36530	2	3.85E-07	0.88	3.38	-	689.6
AHQ-2-5, 2327	K.IVGPSGAAVPCK.V	1157.36530	2	2.92E-08	0.92	3.65	-	869.5
AHQ-2-2, 2617	K.IVGPSGAAVPCK.V	1157.36530	2	4.18E-09	0.92	3.67	-	985.3
AHQ-2-2, 4268 - 4336	K.IVGPSGAAVPCKVEPGLGADNSVVR.F	2451.78478	3	5.14E-07	0.96	4.97	-	1703.6
AHQ-2-3, 4264 - 4336	K.IVGPSGAAVPCKVEPGLGADNSVVR.F	2451.78478	2	2.08E-05	0.94	5.23	-	603.9
AHQ-2-5, 3999	K.IVGPSGAAVPCKVEPGLGADNSVVR.F	2451.78478	3	3.22E-04	0.93	5.11	-	1175.3
AHQ-2-5, 4119	K.IVGPSGAAVPCKVEPGLGADNSVVR.F	2451.78478	3	6.30E-07	0.94	4.56	-	1373.2
AHQ-2-3, 4369 - 4443	K.IVGPSGAAVPCKVEPGLGADNSVVR.F	2451.78478	3	3.59E-04	0.72	3.35	-	640.2
AHQ-2-2, 4404 - 4482	K.IVGPSGAAVPCKVEPGLGADNSVVR.F	2451.78478	3	1.53E-08	0.91	4.55	-	1017.5
AHQ-2-1, 4348 - 4416	K.IVGPSGAAVPCKVEPGLGADNSVVR.F	2451.78478	3	3.79E-09	0.92	5.13	-	986.3
AHQ-2-3, 4260 - 4331	K.IVGPSGAAVPCKVEPGLGADNSVVR.F	2451.78478	3	1.32E-07	0.91	4.08	-	1261.7
AHQ-2-2, 4277 - 4348	K.IVGPSGAAVPCKVEPGLGADNSVVR.F	2451.78478	2	3.22E-07	0.90	4.41	-	492.4
AHQ-2-2, 4421 - 4449	K.IVGPSGAAVPCKVEPGLGADNSVVR.F	2451.78478	2	1.23E-08	0.95	5.60	-	458.7
AHQ-2-6, 3954 - 3957	K.IVGPSGAAVPCKVEPGLGADNSVVR.F	2451.78478	3	1.33E-06	0.97	5.38	-	1860.1
AHQ-2-1, 4353 - 4421	K.IVGPSGAAVPCKVEPGLGADNSVVR.F	2451.78478	2	4.16E-06	0.95	5.06	-	704.9
AHQ-2-5, 4263	R.KDGSCGVAYVVQEPGDYEVSVK.F	2388.59282	2	1.48E-07	0.73	3.40	-	519.6
AHQ-2-1, 4545	K.KNGQHVASSPIPVVISQSEIGDASR.V	2577.83547	2	1.47E-04	0.81	3.01	-	651.4
AHQ-2-5, 3840 - 3911	K.KNGQHVASSPIPVVISQSEIGDASR.V	2577.83547	3	1.94E-08	0.97	5.90	-	1860.8
AHQ-2-1, 4544	K.KNGQHVASSPIPVVISQSEIGDASR.V	2577.83547	3	1.79E-09	0.96	5.15	-	1638.0
AHQ-2-6, 3995 - 4006	K.KNGQHVASSPIPVVISQSEIGDASR.V	2577.83547	2	1.11E-04	0.80	3.30	-	531.6
AHQ-2-1, 3708	K.KTHIQDNHDGTYTVAYVPDVTGR.Y	2588.77367	2	1.41E-06	0.88	3.21	-	1080.4
AHQ-2-3, 3263	K.KTHIQDNHDGTYTVAYVPDVTGR.Y	2588.77367	3	2.43E-04	0.93	4.36	-	1167.2
AHQ-2-5, 2987	K.KTHIQDNHDGTYTVAYVPDVTGR.Y	2588.77367	3	7.64E-05	0.84	3.59	-	1004.1
AHQ-2-4, 3402	K.KTHIQDNHDGTYTVAYVPDVTGR.Y	2588.77367	3	1.23E-07	0.94	4.49	-	1318.9
AHQ-2-2, 3390 - 3462	K.KTHIQDNHDGTYTVAYVPDVTGR.Y	2588.77367	3	1.96E-07	0.93	4.63	-	1229.1
AHQ-2-8, 2820	K.KTHIQDNHDGTYTVAYVPDVTGR.Y	2588.77367	3	1.61E-04	0.86	3.72	-	819.4
AHQ-2-1, 3661	K.KTHIQDNHDGTYTVAYVPDVTGR.Y	2588.77367	3	3.24E-05	0.93	4.06	-	1133.4
AHQ-2-2, 3233	K.KTHIQDNHDGTYTVAYVPDVTGR.Y	2588.77367	3	8.25E-09	0.93	4.51	-	1167.3
AHQ-2-1, 3540 - 3565	K.KTHIQDNHDGTYTVAYVPDVTGR.Y	2588.77367	2	2.36E-05	0.94	4.58	-	888.9
AHQ-2-1, 3506 - 3585	K.KTHIQDNHDGTYTVAYVPDVTGR.Y	2588.77367	3	4.41E-09	0.93	4.40	-	1031.6
AHQ-2-1, 4086	K.LDVQFSGLTK.G	1108.26945	2	4.69E-05	0.88	3.28	-	826.2
AHQ-2-1, 4092	K.LDVQFSGLTK.G	1108.26945	1	7.73E-07	0.62	2.92	-	338.5
AHQ-2-3, 3955	K.LDVQFSGLTK.G	1108.26945	1	3.35E-05	0.31	2.43	-	266.3
AHQ-2-4, 3934	K.LDVQFSGLTK.G	1108.26945	2	7.61E-05	0.91	3.67	-	840.1
AHQ-2-3, 3951	K.LDVQFSGLTK.G	1108.26945	2	1.42E-05	0.93	4.01	-	825.2
AHQ-2-2, 3985	K.LDVQFSGLTK.G	1108.26945	1	3.86E-05	0.37	2.42	-	273.8
AHQ-2-2, 3984 - 3994	K.LDVQFSGLTK.G	1108.26945	2	8.39E-06	0.95	3.53	-	1569.9
AHQ-2-2, 7149	R.LLGWIQNKLPQLPITNFSR.D	2239.64761	2	1.60E-07	0.84	4.05	-	442.0
AHQ-2-1, 5030 - 5100	K.LPQLPITNFSR.D	1286.50461	2	1.17E-04	0.90	3.04	-	900.5
AHQ-2-6, 4573 - 4581	K.LPQLPITNFSR.D	1286.50461	2	9.06E-04	0.85	2.92	-	617.7
AHQ-2-5, 4695	K.LPQLPITNFSR.D	1286.50461	2	5.30E-05	0.89	3.15	-	692.4
AHQ-2-2, 4961 - 5033	K.LPQLPITNFSR.D	1286.50461	2	2.13E-08	0.92	3.48	-	783.1
AHQ-2-2, 5285	K.LPQLPITNFSR.D	1286.50461	2	1.41E-07	0.89	3.20	-	786.5
AHQ-2-2, 4940 - 5008	K.LPQLPITNFSR.D	1286.50461	2	1.13E-07	0.90	3.13	-	778.1
AHQ-2-3, 5279	K.LPQLPITNFSR.D	1286.50461	2	7.42E-05	0.89	3.14	-	795.6
AHQ-2-11, 4573	K.LPQLPITNFSR.D	1286.50461	2	9.93E-04	0.82	3.08	-	595.4
AHQ-2-3, 5185	K.LPQLPITNFSR.D	1286.50461	2	5.72E-05	0.79	2.95	-	582.1
AHQ-2-3, 4935 - 5007	K.LPQLPITNFSR.D	1286.50461	2	1.10E-05	0.92	3.23	-	902.2
AHQ-2-2, 5618 - 5688	K.LQVEPAVDTSGVQCYGPGIEGQGVFR.E	2766.03597	2	3.69E-07	0.94	4.98	-	777.3
AHQ-2-3, 5699 - 5767	K.LQVEPAVDTSGVQCYGPGIEGQGVFR.E	2766.03597	2	4.01E-06	0.95	5.11	-	760.6
AHQ-2-2, 5637 - 5710	K.LQVEPAVDTSGVQCYGPGIEGQGVFR.E	2766.03597	3	6.11E-15	0.98	6.33	-	2152.5
AHQ-2-2, 5724 - 5792	K.LQVEPAVDTSGVQCYGPGIEGQGVFR.E	2766.03597	2	6.24E-05	0.81	3.42	-	598.7
AHQ-2-2, 5768 - 5837	K.LQVEPAVDTSGVQCYGPGIEGQGVFR.E	2766.03597	2	7.57E-07	0.96	5.85	-	688.2
AHQ-2-3, 5557 - 5628	K.LQVEPAVDTSGVQCYGPGIEGQGVFR.E	2766.03597	2	5.34E-06	0.93	4.77	-	740.6
AHQ-2-4, 5577 - 5652	K.LQVEPAVDTSGVQCYGPGIEGQGVFR.E	2766.03597	2	6.41E-05	0.85	3.54	-	626.3

AHQ-2-1, 5736 - 5808	K.LQVEPAVDTSGVQCYGPGIEGQGVFR.E	2766.03597	2	5.14E-08	0.94	4.66	-	752.8
AHQ-2-1, 5605 - 5673	K.LQVEPAVDTSGVQCYGPGIEGQGVFR.E	2766.03597	2	4.31E-06	0.89	3.75	-	754.8
AHQ-2-1, 5604 - 5676	K.LQVEPAVDTSGVQCYGPGIEGQGVFR.E	2766.03597	3	4.05E-12	0.98	6.58	-	2509.8
AHQ-2-6, 5318 - 5385	K.LQVEPAVDTSGVQCYGPGIEGQGVFR.E	2766.03597	2	3.80E-07	0.97	5.68	-	990.6
AHQ-2-2, 5776 - 5852	K.LQVEPAVDTSGVQCYGPGIEGQGVFR.E	2766.03597	3	1.30E-09	0.96	5.26	-	1518.0
AHQ-2-4, 5645 - 5720	K.LQVEPAVDTSGVQCYGPGIEGQGVFR.E	2766.03597	2	1.34E-11	0.97	6.24	-	1165.1
AHQ-2-2, 5860	K.LQVEPAVDTSGVQCYGPGIEGQGVFR.E	2766.03597	2	2.66E-08	0.93	4.94	-	633.5
AHQ-2-4, 5801	K.LQVEPAVDTSGVQCYGPGIEGQGVFR.E	2766.03597	2	4.87E-05	0.75	3.11	-	504.7
AHQ-2-8, 2279	R.LRNGHVGISFVPK.E	1424.67564	2	1.72E-05	0.74	2.87	-	434.6
AHQ-2-5, 2643	R.LRNGHVGISFVPK.E	1424.67564	2	1.77E-04	0.78	2.69	-	651.5
AHQ-2-5, 2547	R.LRNGHVGISFVPK.E	1424.67564	2	2.42E-04	0.78	2.96	-	740.1
AHQ-2-4, 2993	R.LRNGHVGISFVPK.E	1424.67564	2	1.77E-04	0.75	2.74	-	809.8
AHQ-2-1, 3125	R.LRNGHVGISFVPK.E	1424.67564	3	7.56E-05	0.87	3.21	-	1410.8
AHQ-2-5, 2645	R.LRNGHVGISFVPK.E	1424.67564	3	3.03E-05	0.91	3.24	-	1694.8
AHQ-2-1, 4204	R.LSPFMADIR.D	1050.25664	2	1.82E-04	0.67	2.70	-	378.2
AHQ-2-6, 3110 - 3111	R.LTVSSLQESGLK.V	1262.43499	2	9.52E-05	0.91	3.50	-	961.3
AHQ-2-4, 3422 - 3428	R.LTVSSLQESGLK.V	1262.43499	2	6.87E-05	0.84	3.30	-	681.3
AHQ-2-8, 2887	R.LTVSSLQESGLK.V	1262.43499	2	6.92E-05	0.84	3.02	-	794.3
AHQ-2-7, 3012	R.LTVSSLQESGLK.V	1262.43499	2	1.09E-04	0.90	3.30	-	969.0
AHQ-2-11, 3873	R.LVSNHSLHETSSVFVDSLTK.A	2201.42190	3	6.92E-10	0.97	5.05	-	1754.4
AHQ-2-7, 3655	R.LVSNHSLHETSSVFVDSLTK.A	2201.42190	3	1.77E-08	0.97	5.96	-	1482.9
AHQ-2-7, 3651	R.LVSNHSLHETSSVFVDSLTK.A	2201.42190	2	5.80E-06	0.96	5.02	-	1130.5
AHQ-2-14-, 3980	R.LVSNHSLHETSSVFVDSLTK.A	2201.42190	3	1.77E-07	0.97	5.49	-	1679.1
AHQ-2-4, 4098 - 4101	R.LVSNHSLHETSSVFVDSLTK.A	2201.42190	2	1.32E-07	0.97	5.59	-	1365.4
AHQ-2-4, 4104 - 4105	R.LVSNHSLHETSSVFVDSLTK.A	2201.42190	3	1.08E-06	0.93	4.19	-	1147.5
AHQ-2-5, 3867 - 3944	R.LVSNHSLHETSSVFVDSLTK.A	2201.42190	3	4.07E-05	0.96	4.77	-	1734.8
AHQ-2-3, 4172	R.LVSNHSLHETSSVFVDSLTK.A	2201.42190	2	3.06E-04	0.95	4.70	-	1242.8
AHQ-2-3, 4167	R.LVSNHSLHETSSVFVDSLTK.A	2201.42190	3	2.52E-08	0.97	4.52	-	2422.6
AHQ-2-14, 5585	R.LVSNHSLHETSSVFVDSLTK.A	2201.42190	2	2.12E-05	0.89	3.98	-	552.5
AHQ-2-5, 4117 - 4183	R.LVSNHSLHETSSVFVDSLTK.A	2201.42190	3	4.05E-09	0.98	5.49	-	2260.2
AHQ-2-13-, 4139	R.LVSNHSLHETSSVFVDSLTK.A	2201.42190	3	1.43E-05	0.95	4.89	-	1395.2
AHQ-2-10, 3931	R.LVSNHSLHETSSVFVDSLTK.A	2201.42190	2	1.40E-05	0.91	4.53	-	594.0
AHQ-2-4, 4472 - 4474	R.LVSNHSLHETSSVFVDSLTK.A	2201.42190	2	2.67E-08	0.97	5.39	-	1317.5
AHQ-2-13, 4071	R.LVSNHSLHETSSVFVDSLTK.A	2201.42190	2	2.97E-07	0.93	4.83	-	713.8
AHQ-2-8, 3678	R.LVSNHSLHETSSVFVDSLTK.A	2201.42190	2	3.78E-04	0.96	5.08	-	1108.0
AHQ-2-14, 5322	R.LVSNHSLHETSSVFVDSLTK.A	2201.42190	2	1.35E-06	0.93	4.21	-	956.5
AHQ-2-7, 4024	R.LVSNHSLHETSSVFVDSLTK.A	2201.42190	2	8.28E-08	0.94	4.77	-	714.3
AHQ-2-5, 4120 - 4196	R.LVSNHSLHETSSVFVDSLTK.A	2201.42190	2	3.05E-09	0.96	5.55	-	913.4
AHQ-2-5, 3753 - 3835	R.LVSNHSLHETSSVFVDSLTK.A	2201.42190	2	3.19E-08	0.97	6.15	-	1029.8
AHQ-2-5, 3712 - 3784	R.LVSNHSLHETSSVFVDSLTK.A	2201.42190	3	1.21E-11	0.97	5.82	-	1614.3
AHQ-2-13, 4252	R.LVSNHSLHETSSVFVDSLTK.A	2201.42190	2	2.62E-04	0.84	4.09	-	411.0
AHQ-2-6, 4134	R.LVSNHSLHETSSVFVDSLTK.A	2201.42190	2	6.43E-09	0.95	4.64	-	990.6
AHQ-2-5, 4276	R.LVSNHSLHETSSVFVDSLTK.A	2201.42190	2	2.19E-07	0.95	4.77	-	940.4
AHQ-2-5, 3885	R.LVSNHSLHETSSVFVDSLTK.A	2201.42190	2	1.85E-05	0.94	4.65	-	955.6
AHQ-2-5, 4660 - 4735	R.LVSNHSLHETSSVFVDSLTK.A	2201.42190	2	2.92E-04	0.87	4.05	-	516.3
AHQ-2-1, 4652	R.LVSNHSLHETSSVFVDSLTK.A	2201.42190	3	2.10E-07	0.92	4.27	-	1009.2
AHQ-2-1, 4641	R.LVSNHSLHETSSVFVDSLTK.A	2201.42190	2	9.16E-09	0.98	5.47	-	1818.7
AHQ-2-6, 3763 - 3773	R.LVSNHSLHETSSVFVDSLTK.A	2201.42190	3	2.36E-04	0.96	5.07	-	1517.3
AHQ-2-1, 4350	R.LVSNHSLHETSSVFVDSLTK.A	2201.42190	2	1.30E-04	0.98	5.61	-	1721.7
AHQ-2-13-, 4137	R.LVSNHSLHETSSVFVDSLTK.A	2201.42190	2	5.83E-05	0.97	5.61	-	1363.5
AHQ-2-11, 3864	R.LVSNHSLHETSSVFVDSLTK.A	2201.42190	2	4.16E-05	0.94	4.49	-	856.9
AHQ-2-1, 4352 - 4368	R.LVSNHSLHETSSVFVDSLTK.A	2201.42190	3	2.49E-06	0.97	5.66	-	1627.8
AHQ-2-6, 3771	R.LVSNHSLHETSSVFVDSLTK.A	2201.42190	2	7.29E-07	0.96	5.08	-	1205.1
AHQ-2-5, 2383	K.MDCQECPEGYR.V	1447.55375	1	1.84E-05	0.17	2.07	-	151.8
AHQ-2-5, 2369	K.MDCQECPEGYR.V	1447.55375	2	5.53E-04	0.85	2.95	-	802.7
AHQ-2-4, 2482 - 2558	K.MDCQECPEGYR.V	1447.55375	2	2.98E-04	0.86	2.96	-	827.9
AHQ-2-6, 2358	K.MDCQECPEGYR.V	1447.55375	2	7.95E-04	0.72	3.01	-	618.5
AHQ-2-5, 2704	R.NGHVGISFVPK.E	1155.33104	1	6.28E-07	0.59	2.29	-	460.6
AHQ-2-4, 4882	K.NGQHVASSPIPVVISQSEIGDASR.V	2449.66256	2	5.81E-07	0.80	2.99	-	727.5
AHQ-2-5, 4595 - 4603	K.NGQHVASSPIPVVISQSEIGDASR.V	2449.66256	2	2.00E-04	0.91	3.84	-	590.7
AHQ-2-1, 4922 - 4924	K.NGQHVASSPIPVVISQSEIGDASR.V	2449.66256	2	4.98E-06	0.97	5.41	-	1290.9
AHQ-2-5, 4792 - 4793	K.NGQHVASSPIPVVISQSEIGDASR.V	2449.66256	2	3.60E-06	0.69	3.08	-	345.9
AHQ-2-8, 4324	K.NGQHVASSPIPVVISQSEIGDASR.V	2449.66256	2	1.32E-06	0.94	3.73	-	1540.5
AHQ-2-2, 1774 - 1797	K.RAEFTVETR.S	1109.21754	2	2.86E-06	0.94	3.60	-	1052.7
AHQ-2-1, 2106	K.RAEFTVETR.S	1109.21754	2	8.53E-06	0.94	3.23	-	1141.2
AHQ-2-2, 1870	K.RAEFTVETR.S	1109.21754	2	9.48E-05	0.90	3.40	-	783.0
AHQ-2-5, 2612 - 2615	R.RAPSVANVGSHCDLSLK.I	1813.02884	2	7.00E-04	0.95	4.47	-	1272.7
AHQ-2-1, 3984	K.RIANLQTDLSDGLR.L	1572.74866	2	1.36E-06	0.63	3.02	-	461.4
AHQ-2-9, 2655	R.RLTVSSLQESGLK.V	1418.62134	2	2.03E-04	0.97	4.69	-	1728.8
AHQ-2-5, 2540 - 2616	R.RLTVSSLQESGLK.V	1418.62134	2	1.78E-04	0.97	4.70	-	2080.6
AHQ-2-7, 2536 - 2539	R.RLTVSSLQESGLK.V	1418.62134	2	2.42E-04	0.95	4.40	-	1335.1
AHQ-2-2, 2924	R.RLTVSSLQESGLK.V	1418.62134	2	4.09E-05	0.95	4.40	-	1201.7
AHQ-2-3, 2948	R.RLTVSSLQESGLK.V	1418.62134	2	1.13E-05	0.97	4.57	-	2263.6
AHQ-2-2, 7712 - 7780	K.SADFVVEAIGDDVGTLGFSVEGPSQAK.I	2696.90399	3	2.55E-05	0.96	5.76	-	1358.0
AHQ-2-4, 3885 - 3956	R.SAGQGEVLVYVEDPAGHQEEAK.V	2314.45074	2	3.17E-06	0.87	3.98	-	557.4
AHQ-2-8, 3503	R.SAGQGEVLVYVEDPAGHQEEAK.V	2314.45074	2	9.80E-11	0.86	4.14	-	477.8
AHQ-2-7, 3537	R.SAGQGEVLVYVEDPAGHQEEAK.V	2314.45074	2	2.13E-07	0.91	4.48	-	489.7
AHQ-2-2, 3921 - 3992	R.SAGQGEVLVYVEDPAGHQEEAK.V	2314.45074	3	5.93E-07	0.94	4.77	-	1212.2
AHQ-2-2, 4202	R.SAGQGEVLVYVEDPAGHQEEAK.V	2314.45074	3	9.82E-10	0.93	4.15	-	1329.1
AHQ-2-2, 4181 - 4258	R.SAGQGEVLVYVEDPAGHQEEAK.V	2314.45074	2	1.01E-06	0.94	4.99	-	637.1
AHQ-2-2, 3910 - 3981	R.SAGQGEVLVYVEDPAGHQEEAK.V	2314.45074	2	6.92E-06	0.93	4.75	-	744.6
AHQ-2-4, 3890	R.SAGQGEVLVYVEDPAGHQEEAK.V	2314.45074	2	1.69E-06	0.94	5.14	-	589.6
AHQ-2-1, 4056 - 4136	R.SAGQGEVLVYVEDPAGHQEEAK.V	2314.45074	2	6.31E-06	0.94	5.05	-	865.6
AHQ-2-2, 4062 - 4132	R.SAGQGEVLVYVEDPAGHQEEAK.V	2314.45074	3	1.17E-06	0.93	4.03	-	1419.4
AHQ-2-1, 4054 - 4129	R.SAGQGEVLVYVEDPAGHQEEAK.V	2314.45074	3	4.00E-07	0.95	4.86	-	1406.5
AHQ-2-2, 4052 - 4109	R.SAGQGEVLVYVEDPAGHQEEAK.V	2314.45074	2	7.65E-07	0.94	4.81	-	805.6
AHQ-2-4, 3902 - 3909	R.SAGQGEVLVYVEDPAGHQEEAK.V	2314.45074	3	6.74E-09	0.91	4.14	-	1389.4
AHQ-2-2, 4410	R.SAGQGEVLVYVEDPAGHQEEAK.V	2314.45074	2	8.81E-06	0.91	4.54	-	595.1
AHQ-2-1, 4205 - 4222	R.SAGQGEVLVYVEDPAGHQEEAK.V	2314.45074	2	3.42E-07	0.93	4.96	-	673.2
AHQ-2-3, 3901 - 3972	R.SAGQGEVLVYVEDPAGHQEEAK.V	2314.45074	3	4.27E-09	0.93	4.10	-	1343.2
AHQ-2-3, 3903 - 3971	R.SAGQGEVLVYVEDPAGHQEEAK.V	2314.45074	2	2.41E-10	0.96	5.55	-	729.2
AHQ-2-6, 3606 - 3637	R.SAGQGEVLVYVEDPAGHQEEAK.V	2314.45074	3	3.51E-07	0.90	4.29	-	1128.8
AHQ-2-3, 4045	R.SAGQGEVLVYVEDPAGHQEEAK.V	2314.45074	2	2.77E-07	0.89	4.43	-	403.5
AHQ-2-3, 4173	R.SAGQGEVLVYVEDPAGHQEEAK.V	2314.45074	2	1.46E-07	0.90	4.15	-	637.7
AHQ-2-3, 4256	R.SAGQGEVLVYVEDPAGHQEEAK.V	2314.45074	2	1.54E-07	0.87	3.84	-	596.5
AHQ-2-2, 3320 - 3388	K.SPFEVYVDK.S	1084.20321	1	9.91E-05	0.79	3.06	-	786.7
AHQ-2-2, 3321 - 3400	K.SPFEVYVDK.S	1084.20321	2	8.23E-06	0.93	3.49	-	996.9
AHQ-2-7, 3029	K.SPFEVYVDK.S	1084.20321	1	3.12E-06	0.81	3.03	-	717.7
AHQ-2-3, 3337	K.SPFEVYVDK.S	1084.20321	1	1.25E-04	0.64	2.35	-	776.2
AHQ-2-1, 3466 - 3492	K.SPFEVYVDK.S	1084.20321	2	7.36E-06	0.87	2.84	-	1017.6
AHQ-2-1, 3485	K.SPFEVYVDK.S	1084.20321	1	9.71E-07	0.82	2.96	-	911.0
AHQ-2-4, 3352	K.SPFEVYVDK.S	1084.20321	1	8.40E-05	0.35	2.20	-	728.6
AHQ-2-3, 3343	K.SPFEVYVDK.S	1084.20321	2	3.10E-05	0.92	3.39	-	1080.1
AHQ-2-4, 3362	K.SPFEVYVDK.S	1084.20321	1	5.72E-05	0.71	2.93	-	656.7
AHQ-2-3, 3348	K.SPFEVYVDK.S	1084.20321	1	7.83E-06	0.78	3.04	-	745.5
AHQ-2-4, 3364	K.SPFEVYVDK.S	1084.20321	2	2.66E-06	0.91	3.39	-	916.0
AHQ-2-7, 3033	K.SPFEVYVDK.S	1084.20321	2	6.81E-06	0.92	3.12	-	1172.8

AHQ-2-2, 2968	K.SPFEVYVDKSQGDASK.V	1757.87886	2	1.42E-04	0.95	4.15	-	1349.9
AHQ-2-1, 5096 - 5173	K.SPFSVAVSPSLDLSK.I	1534.73557	2	1.26E-07	0.87	4.09	-	687.7
AHQ-2-2, 5308	K.SPFSVAVSPSLDLSK.I	1534.73557	2	1.52E-06	0.40	2.65	-	405.4
AHQ-2-3, 5076 - 5125	K.SPFSVAVSPSLDLSK.I	1534.73557	2	7.84E-06	0.79	3.49	-	522.0
AHQ-2-4, 5068 - 5080	K.SPFSVAVSPSLDLSK.I	1534.73557	2	9.52E-08	0.94	5.20	-	790.3
AHQ-2-2, 4944 - 5014	K.SPFSVAVSPSLDLSK.I	1534.73557	2	3.62E-04	0.83	3.49	-	563.2
AHQ-2-6, 4791	K.SPFSVAVSPSLDLSK.I	1534.73557	2	2.83E-06	0.93	4.23	-	1049.1
AHQ-2-4, 4932	K.SPFSVAVSPSLDLSK.I	1534.73557	2	1.13E-04	0.87	3.61	-	784.9
AHQ-2-2, 5184 - 5200	K.SPFSVAVSPSLDLSK.I	1534.73557	2	9.57E-07	0.94	4.65	-	942.9
AHQ-2-2, 5097 - 5132	K.SPFSVAVSPSLDLSK.I	1534.73557	2	3.03E-06	0.95	4.85	-	1080.6
AHQ-2-5, 4871	K.SPFSVAVSPSLDLSK.I	1534.73557	2	8.23E-06	0.90	3.55	-	948.9
AHQ-2-2, 3552	R.SPYTVTVGQACNPSACR.A	1871.04208	2	7.22E-07	0.91	4.43	-	538.1
AHQ-2-7, 3217	R.SPYTVTVGQACNPSACR.A	1871.04208	2	4.40E-05	0.81	3.76	-	472.1
AHQ-2-5, 3285	R.SPYTVTVGQACNPSACR.A	1871.04208	3	3.33E-04	0.88	3.86	-	875.2
AHQ-2-4, 3430 - 3488	R.SPYTVTVGQACNPSACR.A	1871.04208	2	1.92E-04	0.92	4.57	-	495.1
AHQ-2-5, 3272	R.SPYTVTVGQACNPSACR.A	1871.04208	2	6.12E-05	0.92	4.65	-	440.0
AHQ-2-2, 3468 - 3536	R.SPYTVTVGQACNPSACR.A	1871.04208	2	9.91E-07	0.91	4.57	-	427.0
AHQ-2-1, 3525 - 3596	R.SPYTVTVGQACNPSACR.A	1871.04208	2	4.93E-05	0.90	3.92	-	559.8
AHQ-2-1, 3698	R.SPYTVTVGQACNPSACR.A	1871.04208	2	3.57E-04	0.56	2.76	-	449.0
AHQ-2-3, 3435 - 3515	R.SPYTVTVGQACNPSACR.A	1871.04208	2	2.87E-06	0.88	4.21	-	415.6
AHQ-2-6, 3235	R.SPYTVTVGQACNPSACR.A	1871.04208	2	1.00E-05	0.91	4.20	-	470.5
AHQ-2-3, 3480	R.SPYTVTVGQACNPSACR.A	1871.04208	3	3.26E-04	0.92	4.21	-	1361.3
AHQ-2-5, 3233 - 3303	R.SPYTVTVGQACNPSACR.A	1871.04208	2	6.77E-04	0.58	2.94	-	319.8
AHQ-2-2, 2674	K.SQGDASKVTAQGPGLEPSGNIANK.T	2327.49419	2	1.50E-06	0.90	4.53	-	666.8
AHQ-2-14-, 2092 - 2152	K.SSFTVDCSK.A	1032.10676	1	7.17E-04	0.70	2.81	-	295.1
AHQ-2-14-, 4569 - 4648	K.SSFTVDCSKAGNNMLLVGVHGPR.T	2448.76449	3	6.09E-05	0.70	3.16	-	790.7
AHQ-2-2, 5065 - 5138	R.TFSVWYVPEVTGTHK.V	1751.96228	3	1.25E-05	0.72	3.22	-	480.5
AHQ-2-2, 5045 - 5124	R.TFSVWYVPEVTGTHK.V	1751.96228	2	3.72E-07	0.85	3.74	-	462.0
AHQ-2-5, 4704 - 4777	R.TFSVWYVPEVTGTHK.V	1751.96228	2	9.36E-04	0.54	2.69	-	314.8
AHQ-2-4, 2958	K.TGVAVNKPAEFTVDAK.H	1647.85391	3	4.87E-06	0.85	3.54	-	713.5
AHQ-2-3, 2955	K.TGVAVNKPAEFTVDAK.H	1647.85391	3	6.27E-06	0.92	3.96	-	1015.1
AHQ-2-2, 2861 - 2932	K.TGVAVNKPAEFTVDAK.H	1647.85391	2	6.43E-06	0.95	4.51	-	1069.3
AHQ-2-1, 3142 - 3216	K.TGVAVNKPAEFTVDAK.H	1647.85391	3	2.11E-06	0.85	3.55	-	806.0
AHQ-2-2, 2933	K.TGVAVNKPAEFTVDAK.H	1647.85391	3	2.21E-06	0.74	3.12	-	623.9
AHQ-2-4, 2949	K.TGVAVNKPAEFTVDAK.H	1647.85391	2	6.93E-05	0.92	3.95	-	747.4
AHQ-2-3, 2920 - 2991	K.TGVAVNKPAEFTVDAK.H	1647.85391	2	6.95E-06	0.90	4.32	-	541.2
AHQ-2-2, 2673	R.TGVELGKPTHFTVNAK.A	1699.93205	3	6.77E-04	0.79	3.82	-	688.6
AHQ-2-4, 2817	R.TGVELGKPTHFTVNAK.A	1699.93205	2	3.22E-04	0.90	3.73	-	799.1
AHQ-2-3, 2679 - 2697	R.TGVELGKPTHFTVNAK.A	1699.93205	3	9.45E-06	0.52	3.30	-	484.6
AHQ-2-2, 2704 - 2773	R.TGVELGKPTHFTVNAK.A	1699.93205	2	5.86E-04	0.92	4.20	-	650.6
AHQ-2-1, 2940	R.TGVELGKPTHFTVNAK.A	1699.93205	2	7.22E-04	0.85	3.86	-	437.5
AHQ-2-1, 3044 - 3112	R.TGVELGKPTHFTVNAK.A	1699.93205	2	3.57E-06	0.82	3.50	-	455.0
AHQ-2-2, 2558 - 2636	R.TGVELGKPTHFTVNAK.A	1699.93205	2	3.04E-04	0.84	4.01	-	427.1
AHQ-2-7, 2760 - 2831	K.THEAEIVEGENHTYCIR.F	2060.19046	3	5.12E-05	0.88	3.54	-	1214.8
AHQ-2-7, 2755	K.THEAEIVEGENHTYCIR.F	2060.19046	2	2.74E-11	0.98	5.81	-	2375.2
AHQ-2-4, 2948 - 3018	K.THEAEIVEGENHTYCIR.F	2060.19046	3	4.50E-07	0.90	3.79	-	1105.1
AHQ-2-4, 2953 - 3028	K.THEAEIVEGENHTYCIR.F	2060.19046	2	2.00E-04	0.98	6.38	-	2488.8
AHQ-2-5, 2811 - 2812	K.THEAEIVEGENHTYCIR.F	2060.19046	2	2.28E-12	0.98	5.85	-	2174.1
AHQ-2-7, 2648	K.THEAEIVEGENHTYCIR.F	2060.19046	2	2.04E-05	0.98	5.34	-	1810.7
AHQ-2-3, 3045	K.THEAEIVEGENHTYCIR.F	2060.19046	2	6.77E-14	0.98	5.69	-	2083.4
AHQ-2-5, 2712	K.THEAEIVEGENHTYCIR.F	2060.19046	3	1.80E-05	0.93	4.12	-	1506.7
AHQ-2-5, 2711	K.THEAEIVEGENHTYCIR.F	2060.19046	2	1.39E-06	0.98	5.61	-	1695.0
AHQ-2-1, 3172 - 3246	K.THEAEIVEGENHTYCIR.F	2060.19046	3	4.20E-06	0.80	3.26	-	974.8
AHQ-2-6, 2817 - 2821	K.THEAEIVEGENHTYCIR.F	2060.19046	2	3.57E-13	0.99	6.61	-	2548.9
AHQ-2-1, 3301	K.THEAEIVEGENHTYCIR.F	2060.19046	2	9.27E-12	0.97	4.90	-	1691.2
AHQ-2-1, 3873	K.THIQDNHDGTYTVAYVPDVTGR.Y	2460.60076	2	4.85E-08	0.94	4.07	-	1053.6
AHQ-2-14, 3840 - 3913	R.TPCEEILVK.H	1090.27249	2	1.48E-04	0.80	2.77	-	722.1
AHQ-2-13, 3088	R.TPCEEILVK.H	1090.27249	2	9.29E-07	0.85	3.30	-	770.6
AHQ-2-13-, 3045 - 3054	R.TPCEEILVK.H	1090.27249	2	9.10E-06	0.77	2.85	-	742.4
AHQ-2-4, 3092	R.TPCEEILVK.H	1090.27249	2	2.08E-05	0.86	3.06	-	680.3
AHQ-2-14-, 2967	R.TPCEEILVK.H	1090.27249	2	7.79E-05	0.82	3.00	-	683.0
AHQ-2-7, 2812	R.TPCEEILVK.H	1090.27249	2	3.38E-05	0.85	2.99	-	758.4
AHQ-2-14, 3937	R.TPCEEILVK.H	1090.27249	1	3.43E-05	0.38	2.64	-	344.2
AHQ-2-3, 3109	R.TPCEEILVK.H	1090.27249	2	2.77E-05	0.90	3.35	-	872.6
AHQ-2-10, 2811	R.TPCEEILVK.H	1090.27249	2	2.47E-04	0.66	2.80	-	467.2
AHQ-2-4, 3098	R.TPCEEILVK.H	1090.27249	1	4.95E-04	0.56	3.05	-	393.8
AHQ-2-14, 3976 - 4044	R.TPCEEILVK.H	1090.27249	2	1.52E-05	0.70	2.90	-	505.0
AHQ-2-11, 2972	R.TPCEEILVK.H	1090.27249	2	1.75E-06	0.74	2.85	-	625.2
AHQ-2-5, 2867	R.TPCEEILVK.H	1090.27249	2	8.91E-07	0.83	2.98	-	617.3
AHQ-2-6, 2871	R.TPCEEILVK.H	1090.27249	2	5.23E-06	0.74	2.52	-	636.6
AHQ-2-1, 3238	R.TPCEEILVK.H	1090.27249	2	1.77E-05	0.78	2.64	-	652.9
AHQ-2-6, 3033	R.VANPSGNLTETYVQDR.G	1764.87462	2	2.35E-11	0.95	4.46	-	1160.3
AHQ-2-1, 3221 - 3244	R.VANPSGNLTETYVQDR.G	1764.87462	2	5.41E-11	0.93	3.99	-	911.0
AHQ-2-2, 3593	R.VANPSGNLTETYVQDR.G	1764.87462	2	1.85E-05	0.87	3.13	-	850.7
AHQ-2-4, 3438 - 3509	R.VANPSGNLTETYVQDR.G	1764.87462	2	4.24E-12	0.95	4.25	-	1128.2
AHQ-2-4, 3242 - 3296	R.VANPSGNLTETYVQDR.G	1764.87462	2	4.52E-08	0.92	4.17	-	718.3
AHQ-2-3, 3152	R.VANPSGNLTETYVQDR.G	1764.87462	2	6.41E-10	0.96	4.54	-	1195.2
AHQ-2-2, 3242 - 3324	R.VANPSGNLTETYVQDR.G	1764.87462	2	4.76E-11	0.95	4.73	-	987.6
AHQ-2-2, 3090 - 3160	R.VANPSGNLTETYVQDR.G	1764.87462	2	1.83E-10	0.92	3.82	-	881.7
AHQ-2-6, 3211 - 3289	R.VANPSGNLTETYVQDR.G	1764.87462	2	9.64E-05	0.88	3.90	-	650.7
AHQ-2-3, 3240 - 3319	R.VANPSGNLTETYVQDR.G	1764.87462	2	8.69E-11	0.96	4.82	-	997.5
AHQ-2-5, 3063	R.VANPSGNLTETYVQDR.G	1764.87462	2	1.96E-13	0.93	4.36	-	795.2
AHQ-2-3, 3396 - 3469	R.VANPSGNLTETYVQDR.G	1764.87462	2	7.65E-11	0.91	4.20	-	586.0
AHQ-2-2, 3376 - 3448	R.VANPSGNLTETYVQDR.G	1764.87462	2	4.17E-12	0.92	3.90	-	805.4
AHQ-2-9, 3040	R.VANPSGNLTETYVQDR.G	1764.87462	2	7.73E-07	0.84	3.71	-	495.2
AHQ-2-1, 3380 - 3448	R.VANPSGNLTETYVQDR.G	1764.87462	2	1.50E-11	0.91	4.05	-	664.9
AHQ-2-7, 2971 - 2972	R.VANPSGNLTETYVQDR.G	1764.87462	2	3.14E-07	0.94	4.50	-	905.1
AHQ-2-1, 3537 - 3612	R.VANPSGNLTETYVQDR.G	1764.87462	2	2.03E-11	0.98	5.86	-	1248.8
AHQ-2-5, 2364	K.VAQPTITDNKDGTVTVR.Y	1816.00621	2	3.91E-04	0.85	3.63	-	606.3
AHQ-2-6, 2595	K.VAQPTITDNKDGTVTVR.Y	1816.00621	2	7.80E-08	0.92	3.98	-	876.5
AHQ-2-4, 2769	K.VAQPTITDNKDGTVTVR.Y	1816.00621	2	6.92E-05	0.83	3.42	-	589.1
AHQ-2-10, 2534	K.VAQPTITDNKDGTVTVR.Y	1816.00621	2	2.06E-06	0.76	2.97	-	505.9
AHQ-2-10, 2382 - 2451	K.VAQPTITDNKDGTVTVR.Y	1816.00621	2	5.33E-05	0.70	3.02	-	498.0
AHQ-2-13-, 2626	K.VAQPTITDNKDGTVTVR.Y	1816.00621	2	2.03E-05	0.72	3.12	-	506.6
AHQ-2-1, 2698	K.VAQPTITDNKDGTVTVR.Y	1816.00621	2	7.40E-05	0.79	3.27	-	616.6
AHQ-2-13, 2861	K.VAQPTITDNKDGTVTVR.Y	1816.00621	2	1.93E-04	0.79	3.24	-	569.5
AHQ-2-1, 2894	K.VAQPTITDNKDGTVTVR.Y	1816.00621	2	3.04E-06	0.89	3.63	-	860.0
AHQ-2-9, 2603	K.VAQPTITDNKDGTVTVR.Y	1816.00621	2	9.36E-04	0.79	3.68	-	389.0
AHQ-2-2, 2808 - 2809	K.VAQPTITDNKDGTVTVR.Y	1816.00621	2	6.52E-07	0.87	4.23	-	517.8
AHQ-2-5, 2524 - 2539	K.VAQPTITDNKDGTVTVR.Y	1816.00621	2	9.94E-10	0.90	4.21	-	599.1
AHQ-2-8, 2374	K.VAQPTITDNKDGTVTVR.Y	1816.00621	2	1.46E-05	0.57	2.79	-	330.1
AHQ-2-4, 2534	K.VAQPTITDNKDGTVTVR.Y	1816.00621	2	3.40E-04	0.81	3.84	-	392.6
AHQ-2-8, 2263	K.VAQPTITDNKDGTVTVR.Y	1816.00621	2	4.80E-05	0.84	3.46	-	615.1
AHQ-2-7, 3536	K.VDINTEDLEDGTCR.V	1638.69283	2	5.62E-06	0.93	3.75	-	1138.3
AHQ-2-1, 3542	K.VDINTEDLEDGTCR.V	1638.69283	2	5.80E-07	0.95	4.42	-	1091.8
AHQ-2-1, 3436	K.VDINTEDLEDGTCR.V	1638.69283	2	3.11E-11	0.95	4.23	-	1284.3
AHQ-2-5, 3559 - 3567	K.VDINTEDLEDGTCR.V	1638.69283	2	5.26E-06	0.94	4.58	-	925.1

AHQ-2-6, 3271	K.VDINTEDLEDGTCR.V	1638.69283	2	1.11E-05	0.85	3.33	-	882.3
AHQ-2-13, 3505 - 3569	K.VDINTEDLEDGTCR.V	1638.69283	2	7.37E-07	0.82	3.56	-	904.9
AHQ-2-4, 3442	K.VDINTEDLEDGTCR.V	1638.69283	2	6.73E-05	0.63	2.90	-	514.0
AHQ-2-6, 3363	K.VDINTEDLEDGTCR.V	1638.69283	2	1.83E-04	0.81	2.85	-	955.9
AHQ-2-3, 3679 - 3703	K.VDINTEDLEDGTCR.V	1638.69283	2	1.80E-05	0.85	3.10	-	757.6
AHQ-2-6, 3490	K.VDINTEDLEDGTCR.V	1638.69283	2	5.97E-08	0.89	3.74	-	855.6
AHQ-2-5, 3464 - 3473	K.VDINTEDLEDGTCR.V	1638.69283	2	9.48E-09	0.94	4.27	-	1285.5
AHQ-2-8, 3287	K.VDINTEDLEDGTCR.V	1638.69283	2	7.05E-04	0.92	3.45	-	1374.0
AHQ-2-1, 3721 - 3725	K.VDINTEDLEDGTCR.V	1638.69283	2	3.60E-05	0.91	3.62	-	1155.1
AHQ-2-12, 3554	K.VDINTEDLEDGTCR.V	1638.69283	2	6.14E-08	0.91	3.54	-	876.2
AHQ-2-1, 3838	K.VDINTEDLEDGTCR.V	1638.69283	2	5.90E-08	0.93	3.52	-	1228.3
AHQ-2-4, 3681	K.VDINTEDLEDGTCR.V	1638.69283	2	4.30E-09	0.93	3.57	-	1313.3
AHQ-2-5, 3349	K.VDINTEDLEDGTCR.V	1638.69283	2	3.30E-07	0.80	2.99	-	779.6
AHQ-2-3, 2451	K.VDVGKDQEFTVK.S	1365.51377	1	9.16E-06	0.39	2.72	-	282.8
AHQ-2-2, 2460 - 2533	K.VDVGKDQEFTVK.S	1365.51377	2	1.80E-07	0.91	3.69	-	778.8
AHQ-2-6, 2297	K.VDVGKDQEFTVK.S	1365.51377	2	2.02E-09	0.89	3.47	-	765.3
AHQ-2-1, 2632 - 2633	K.VDVGKDQEFTVK.S	1365.51377	2	6.20E-08	0.88	3.36	-	674.8
AHQ-2-2, 2466	K.VDVGKDQEFTVK.S	1365.51377	1	1.58E-04	0.59	3.34	-	334.2
AHQ-2-5, 2279	K.VDVGKDQEFTVK.S	1365.51377	2	6.63E-08	0.92	3.58	-	824.5
AHQ-2-1, 2800 - 2868	K.VEPGLGADNSVVR.F	1313.44209	2	2.26E-06	0.84	3.40	-	286.2
AHQ-2-2, 2864 - 2866	K.VEPGLGADNSVVR.F	1313.44209	2	3.99E-08	0.86	3.23	-	477.6
AHQ-2-4, 6604 - 6616	K.VEYTPYEEGLHSVDVTYDGSPVPSSPFQVPVTEGCDPSR.V	4300.57494	3	5.89E-06	0.89	4.62	-	594.2
AHQ-2-4, 6482 - 6549	K.VEYTPYEEGLHSVDVTYDGSPVPSSPFQVPVTEGCDPSR.V	4300.57494	3	9.77E-04	0.51	3.29	-	389.8
AHQ-2-1, 6488 - 6554	K.VEYTPYEEGLHSVDVTYDGSPVPSSPFQVPVTEGCDPSR.V	4300.57494	3	1.15E-04	0.88	4.65	-	593.8
AHQ-2-5, 6463 - 6545	K.VEYTPYEEGLHSVDVTYDGSPVPSSPFQVPVTEGCDPSR.V	4300.57494	3	3.00E-05	0.92	5.13	-	694.3
AHQ-2-3, 7528	K.VGSAADIPINISETDLSLLTATVVPPSGR.E	2895.25495	2	4.89E-08	0.93	4.71	-	733.2
AHQ-2-3, 7531 - 7532	K.VGSAADIPINISETDLSLLTATVVPPSGR.E	2895.25495	3	5.15E-07	0.87	4.35	-	599.0
AHQ-2-5, 7415	K.VGSAADIPINISETDLSLLTATVVPPSGR.E	2895.25495	2	4.82E-12	0.95	4.87	-	809.4
AHQ-2-7, 7475	K.VGSAADIPINISETDLSLLTATVVPPSGR.E	2895.25495	2	1.17E-06	0.85	3.81	-	516.2
AHQ-2-1, 7172 - 7237	K.VGSAADIPINISETDLSLLTATVVPPSGR.E	2895.25495	3	9.77E-04	0.66	3.10	-	562.9
AHQ-2-6, 7219 - 7274	K.VGSAADIPINISETDLSLLTATVVPPSGR.E	2895.25495	2	2.65E-06	0.86	3.52	-	920.8
AHQ-2-9, 7332 - 7402	K.VGSAADIPINISETDLSLLTATVVPPSGR.E	2895.25495	2	8.12E-07	0.90	4.37	-	488.9
AHQ-2-1, 7168 - 7242	K.VGSAADIPINISETDLSLLTATVVPPSGR.E	2895.25495	2	2.00E-07	0.92	4.69	-	592.8
AHQ-2-6, 4123	K.VHSPSGALEECYVTEIDQDK.Y	2279.42322	2	5.15E-05	0.95	4.98	-	963.4
AHQ-2-4, 4478	K.VHSPSGALEECYVTEIDQDK.Y	2279.42322	2	3.25E-04	0.95	5.10	-	1076.5
AHQ-2-1, 4580	K.VHSPSGALEECYVTEIDQDK.Y	2279.42322	2	1.21E-08	0.96	4.91	-	1305.3
AHQ-2-5, 4189	K.VHSPSGALEECYVTEIDQDK.Y	2279.42322	2	8.88E-10	0.96	5.18	-	1428.8
AHQ-2-7, 4075	K.VHSPSGALEECYVTEIDQDK.Y	2279.42322	2	4.03E-11	0.98	4.65	-	2160.6
AHQ-2-8, 4255	K.VHSPSGALEECYVTEIDQDKYAVR.F	2768.99349	3	8.09E-10	0.84	4.10	-	559.2
AHQ-2-7, 4285 - 4356	K.VHSPSGALEECYVTEIDQDKYAVR.F	2768.99349	3	4.39E-10	0.88	4.51	-	638.9
AHQ-2-6, 4445	K.VHSPSGALEECYVTEIDQDKYAVR.F	2768.99349	3	7.85E-07	0.93	4.88	-	938.6
AHQ-2-5, 4471 - 4545	K.VHSPSGALEECYVTEIDQDKYAVR.F	2768.99349	3	1.70E-08	0.93	5.33	-	627.9
AHQ-2-1, 4918	K.VHSPSGALEECYVTEIDQDKYAVR.F	2768.99349	2	2.56E-09	0.95	4.46	-	1551.4
AHQ-2-1, 4912	K.VHSPSGALEECYVTEIDQDKYAVR.F	2768.99349	3	4.21E-04	0.88	4.30	-	640.2
AHQ-2-3, 4837	K.VHSPSGALEECYVTEIDQDKYAVR.F	2768.99349	3	7.65E-09	0.85	4.42	-	437.5
AHQ-2-11, 4444	K.VHSPSGALEECYVTEIDQDKYAVR.F	2768.99349	3	7.12E-10	0.86	4.44	-	517.9
AHQ-2-8, 4263	K.VHSPSGALEECYVTEIDQDKYAVR.F	2768.99349	2	2.73E-08	0.92	3.89	-	1162.0
AHQ-2-8, 3484	K.VNQPASFAVSLNGAK.G	1503.68480	2	1.43E-06	0.79	3.13	-	552.5
AHQ-2-5, 3599 - 3668	K.VNQPASFAVSLNGAK.G	1503.68480	2	5.31E-07	0.83	3.24	-	491.8
AHQ-2-4, 3844 - 3912	K.VNQPASFAVSLNGAK.G	1503.68480	2	1.02E-08	0.76	3.19	-	464.1
AHQ-2-2, 3817 - 3889	K.VNQPASFAVSLNGAK.G	1503.68480	2	6.42E-05	0.86	3.40	-	692.2
AHQ-2-7, 3531	K.VNQPASFAVSLNGAK.G	1503.68480	2	3.54E-07	0.89	3.82	-	633.9
AHQ-2-7, 3436	K.VNQPASFAVSLNGAK.G	1503.68480	2	2.70E-08	0.79	3.53	-	434.8
AHQ-2-5, 3456 - 3527	K.VNQPASFAVSLNGAK.G	1503.68480	2	2.96E-07	0.67	2.79	-	409.1
AHQ-2-6, 3462 - 3542	K.VNQPASFAVSLNGAK.G	1503.68480	2	1.02E-07	0.93	4.03	-	678.9
AHQ-2-8, 3356	K.VNQPASFAVSLNGAK.G	1503.68480	2	2.40E-04	0.93	4.13	-	694.9
AHQ-2-1, 3952 - 4016	K.VNQPASFAVSLNGAK.G	1503.68480	2	5.13E-06	0.88	3.49	-	664.9
AHQ-2-2, 1477 - 1490	K.VPVHDVTDASK.V	1168.28175	2	3.33E-07	0.95	3.61	-	1495.0
AHQ-2-3, 1497	K.VPVHDVTDASK.V	1168.28175	2	9.94E-07	0.94	3.24	-	1249.3
AHQ-2-1, 4288 - 4358	R.VQVQDNEGCPVEALVK.D	1786.98493	2	8.89E-07	0.97	5.28	-	1598.4
AHQ-2-4, 4170 - 4248	R.VQVQDNEGCPVEALVK.D	1786.98493	2	4.01E-10	0.95	4.70	-	1068.6
AHQ-2-3, 4195 - 4263	R.VQVQDNEGCPVEALVK.D	1786.98493	2	6.07E-06	0.96	4.89	-	1363.4
AHQ-2-5, 3948 - 3964	R.VQVQDNEGCPVEALVK.D	1786.98493	2	3.89E-06	0.97	5.09	-	1468.5
AHQ-2-2, 4444 - 4448	R.VQVQDNEGCPVEALVK.D	1786.98493	2	9.23E-09	0.96	4.66	-	1807.8
AHQ-2-2, 4304 - 4373	R.VQVQDNEGCPVEALVK.D	1786.98493	2	8.03E-06	0.96	4.46	-	1399.1
AHQ-2-6, 3893 - 3898	R.VQVQDNEGCPVEALVK.D	1786.98493	2	2.77E-05	0.77	3.77	-	539.9
AHQ-2-1, 4404 - 4428	R.VQVQDNEGCPVEALVK.D	1786.98493	2	1.47E-10	0.94	4.97	-	956.1
AHQ-2-2, 4221 - 4232	R.VQVQDNEGCPVEALVK.D	1786.98493	2	5.48E-09	0.95	4.78	-	954.2
AHQ-2-3, 4332	R.VQVQDNEGCPVEALVK.D	1786.98493	2	7.22E-07	0.96	4.49	-	1741.0
AHQ-2-2, 5584	R.VQVQDNEGCPVEALVKDNGNGTYSCSYVPR.K	3359.60342	3	1.44E-05	0.87	3.73	-	1011.6
AHQ-2-4, 5524	R.VQVQDNEGCPVEALVKDNGNGTYSCSYVPR.K	3359.60342	3	1.33E-05	0.79	3.57	-	854.8
AHQ-2-1, 5276 - 5346	R.VQVQDNEGCPVEALVKDNGNGTYSCSYVPR.K	3359.60342	3	5.67E-07	0.93	4.77	-	1418.0
AHQ-2-2, 5312 - 5380	R.VQVQDNEGCPVEALVKDNGNGTYSCSYVPR.K	3359.60342	3	2.75E-12	0.90	4.42	-	1336.7
AHQ-2-1, 5552	R.VQVQDNEGCPVEALVKDNGNGTYSCSYVPR.K	3359.60342	3	4.78E-04	0.92	4.09	-	1562.9
AHQ-2-3, 5272	R.VQVQDNEGCPVEALVKDNGNGTYSCSYVPR.K	3359.60342	3	4.55E-14	0.94	4.56	-	1710.2
AHQ-2-3, 5483 - 5551	R.VQVQDNEGCPVEALVKDNGNGTYSCSYVPR.K	3359.60342	3	1.70E-06	0.94	4.78	-	1743.6
AHQ-2-5, 5319	R.VQVQDNEGCPVEALVKDNGNGTYSCSYVPR.K	3359.60342	3	3.60E-09	0.80	3.22	-	1111.4
AHQ-2-7, 4217	R.VSGQGLHEGHTFEPAEFIIDTR.D	2441.64058	3	2.24E-09	0.97	5.65	-	1514.9
AHQ-2-13, 4639 - 4643	R.VSGQGLHEGHTFEPAEFIIDTR.D	2441.64058	3	2.96E-10	0.95	5.55	-	1120.4
AHQ-2-8, 3947	R.VSGQGLHEGHTFEPAEFIIDTR.D	2441.64058	2	4.14E-10	0.95	5.17	-	811.4
AHQ-2-13-, 4737	R.VSGQGLHEGHTFEPAEFIIDTR.D	2441.64058	3	4.35E-04	0.86	3.43	-	1003.2
AHQ-2-8, 4235	R.VSGQGLHEGHTFEPAEFIIDTR.D	2441.64058	3	1.39E-04	0.94	4.64	-	1199.4
AHQ-2-5, 4361 - 4443	R.VSGQGLHEGHTFEPAEFIIDTR.D	2441.64058	3	4.89E-11	0.96	5.40	-	1283.5
AHQ-2-4, 4792 - 4804	R.VSGQGLHEGHTFEPAEFIIDTR.D	2441.64058	3	3.05E-08	0.95	5.32	-	1318.7
AHQ-2-1, 4960 - 5033	R.VSGQGLHEGHTFEPAEFIIDTR.D	2441.64058	3	1.43E-04	0.90	4.31	-	745.3
AHQ-2-6, 4373 - 4446	R.VSGQGLHEGHTFEPAEFIIDTR.D	2441.64058	3	1.71E-09	0.97	6.15	-	1388.3
AHQ-2-10, 4120	R.VSGQGLHEGHTFEPAEFIIDTR.D	2441.64058	2	3.42E-04	0.84	3.40	-	345.4
AHQ-2-8, 3946	R.VSGQGLHEGHTFEPAEFIIDTR.D	2441.64058	3	1.49E-07	0.91	4.70	-	833.9
AHQ-2-1, 4964	R.VSGQGLHEGHTFEPAEFIIDTR.D	2441.64058	2	4.21E-05	0.94	4.04	-	1161.9
AHQ-2-1, 2893 - 2934	K.VTAQGPGLEPSGNIANK.T	1653.81855	2	7.83E-05	0.77	3.89	-	341.1
AHQ-2-4, 2740	K.VTAQGPGLEPSGNIANK.T	1653.81855	2	9.48E-07	0.91	3.74	-	882.6
AHQ-2-3, 2753	K.VTAQGPGLEPSGNIANK.T	1653.81855	2	2.69E-06	0.74	3.23	-	432.2
AHQ-2-1, 2748 - 2816	K.VTAQGPGLEPSGNIANK.T	1653.81855	2	8.70E-04	0.82	3.28	-	727.2
AHQ-2-3, 2607 - 2675	K.VTAQGPGLEPSGNIANK.T	1653.81855	2	2.39E-05	0.74	3.08	-	698.8
AHQ-2-5, 2435 - 2481	K.VTAQGPGLEPSGNIANK.T	1653.81855	2	3.57E-05	0.93	5.00	-	530.7
AHQ-2-6, 2586	K.VTAQGPGLEPSGNIANK.T	1653.81855	2	2.77E-05	0.85	3.86	-	503.8
AHQ-2-6, 2471 - 2483	K.VTAQGPGLEPSGNIANK.T	1653.81855	2	1.43E-04	0.78	3.43	-	423.8
AHQ-2-8, 2290	K.VTAQGPGLEPSGNIANK.T	1653.81855	2	1.66E-05	0.45	2.65	-	300.7
AHQ-2-2, 2601 - 2613	K.VTAQGPGLEPSGNIANK.T	1653.81855	2	7.83E-05	0.82	3.90	-	424.2
AHQ-2-2, 2860	K.VTAQGPGLEPSGNIANK.T	1653.81855	2	2.75E-11	0.91	3.92	-	624.0
AHQ-2-5, 2579	K.VTAQGPGLEPSGNIANK.T	1653.81855	2	6.45E-09	0.82	3.79	-	444.7
AHQ-2-2, 2684 - 2776	K.VTAQGPGLEPSGNIANK.T	1653.81855	2	6.12E-08	0.93	4.52	-	665.2
AHQ-2-6, 2777	K.VTVLFAGQHIAK.S	1284.53185	2	8.91E-05	0.81	3.67	-	518.6
AHQ-2-4, 3082	K.VTVLFAGQHIAK.S	1284.53185	1	8.38E-06	0.67	2.92	-	508.5
AHQ-2-2, 2978 - 3049	K.VTVLFAGQHIAK.S	1284.53185	2	5.96E-07	0.92	3.87	-	904.9
AHQ-2-7, 2627	K.VTVLFAGQHIAK.S	1284.53185	2	5.07E-05	0.83	3.29	-	689.8
AHQ-2-5, 2695	K.VTVLFAGQHIAK.S	1284.53185	2	4.14E-06	0.92	3.95	-	871.8

AHQ-2-4, 3033	K.VTVLFAGQHIAK.S	1284.53185	1	8.91E-05	0.29	1.81	-	443.4
AHQ-2-4, 3037	K.VTVLFAGQHIAK.S	1284.53185	2	2.78E-07	0.76	3.27	-	544.8
AHQ-2-3, 3043	K.VTVLFAGQHIAK.S	1284.53185	2	3.04E-04	0.69	3.02	-	501.5
AHQ-2-1, 3316 - 3317	K.VTVLFAGQHIAK.S	1284.53185	2	6.00E-05	0.97	4.47	-	1489.0
AHQ-2-4, 5194	R.VTYCPTEPGNYIINIK.F	1884.14221	2	8.10E-04	0.84	3.29	-	594.9
AHQ-2-9, 4930	R.VTYCPTEPGNYIINIK.F	1884.14221	2	2.17E-04	0.65	3.19	-	295.6
AHQ-2-6, 5066	R.VTYCPTEPGNYIINIK.F	1884.14221	2	5.29E-05	0.60	2.80	-	290.3
AHQ-2-8, 4739 - 4791	R.VTYCPTEPGNYIINIK.F	1884.14221	2	1.16E-08	0.92	3.70	-	694.6
AHQ-2-7, 4819 - 4887	R.VTYCPTEPGNYIINIK.F	1884.14221	2	9.57E-10	0.92	4.46	-	499.9
AHQ-2-5, 4835 - 4911	R.VTYCPTEPGNYIINIK.F	1884.14221	2	1.18E-09	0.93	4.28	-	645.2
AHQ-2-5, 5047	R.VTYCPTEPGNYIINIK.F	1884.14221	2	2.52E-06	0.93	4.33	-	560.3
AHQ-2-13-, 4899	R.VTYCPTEPGNYIINIK.F	1884.14221	2	2.63E-05	0.67	3.39	-	251.6
AHQ-2-2, 5240	R.VTYCPTEPGNYIINIK.F	1884.14221	2	6.77E-04	0.79	2.82	-	509.8
AHQ-2-5, 5169	R.VTYCPTEPGNYIINIK.F	1884.14221	2	3.41E-05	0.73	3.16	-	217.2
AHQ-2-1, 5073	R.VTYCPTEPGNYIINIK.F	1884.14221	2	4.41E-06	0.86	3.59	-	602.8
AHQ-2-5, 4933 - 5003	R.VTYCPTEPGNYIINIK.F	1884.14221	2	6.25E-09	0.92	4.59	-	396.7
AHQ-2-3, 5208	R.VTYCPTEPGNYIINIK.F	1884.14221	2	1.27E-05	0.88	3.83	-	480.4
AHQ-2-1, 5257	R.VTYCPTEPGNYIINIK.F	1884.14221	2	2.83E-04	0.67	2.89	-	359.5
AHQ-2-8, 4996	R.VTYTPMAPGSYLISIK.Y	1742.07225	2	1.40E-05	0.82	3.11	-	730.5
AHQ-2-5, 4831	R.VTYTPM*APGSYLISIK.Y	1758.07165	2	2.35E-05	0.88	3.68	-	654.2
AHQ-2-2, 5668	R.VTYTPMAPGSYLISIK.Y	1742.07225	2	1.94E-04	0.67	2.86	-	555.8
AHQ-2-14-, 5379 - 5403	R.VTYTPMAPGSYLISIK.Y	1742.07225	2	1.22E-04	0.72	3.03	-	703.0
AHQ-2-13, 5327	R.VTYTPMAPGSYLISIK.Y	1742.07225	2	1.84E-04	0.72	2.96	-	522.3
AHQ-2-7, 5191 - 5227	R.VTYTPMAPGSYLISIK.Y	1742.07225	2	5.89E-05	0.73	3.25	-	486.8
AHQ-2-3, 5639	R.VTYTPMAPGSYLISIK.Y	1742.07225	2	4.06E-04	0.63	2.57	-	511.7
AHQ-2-13-, 5426	R.VTYTPMAPGSYLISIK.Y	1742.07225	2	2.99E-05	0.88	3.28	-	731.4
AHQ-2-6, 4785	R.VTYTPM*APGSYLISIK.Y	1758.07165	2	3.83E-05	0.71	3.12	-	387.7
AHQ-2-11, 5156 - 5162	R.VTYTPMAPGSYLISIK.Y	1742.07225	2	7.06E-04	0.81	3.43	-	481.9
AHQ-2-10, 4915 - 4931	R.VTYTPMAPGSYLISIK.Y	1742.07225	2	3.61E-04	0.79	3.45	-	588.3
AHQ-2-10, 3022	R.YAPSEAGLHEMDIR.Y	1589.75482	2	1.03E-04	0.81	3.29	-	657.6
AHQ-2-1, 3526 - 3536	R.YAPSEAGLHEMDIR.Y	1589.75482	2	2.07E-05	0.88	3.14	-	869.6
AHQ-2-2, 3333 - 3361	R.YAPSEAGLHEMDIR.Y	1589.75482	2	7.72E-05	0.41	2.50	-	385.1
AHQ-2-13-, 3375 - 3381	R.YAPSEAGLHEMDIR.Y	1589.75482	2	3.80E-05	0.91	3.59	-	946.0
AHQ-2-3, 3336	R.YAPSEAGLHEMDIR.Y	1589.75482	2	1.83E-05	0.85	3.01	-	748.8
AHQ-2-4, 3336 - 3368	R.YAPSEAGLHEMDIR.Y	1589.75482	2	2.71E-04	0.46	2.66	-	444.3
AHQ-2-5, 2363	R.YAPSEAGLHEM*DIR.Y	1605.75422	2	7.03E-04	0.79	3.04	-	688.9
AHQ-2-5, 3015 - 3083	R.YAPSEAGLHEMDIR.Y	1589.75482	2	2.10E-06	0.89	3.23	-	906.4
AHQ-2-7, 2947	R.YAPSEAGLHEMDIR.Y	1589.75482	2	9.96E-05	0.89	3.16	-	1096.4
AHQ-2-6, 3974 - 4045	K.YGGDEIPFSPYR.V	1401.50466	2	1.38E-04	0.75	2.85	-	603.6
AHQ-2-12, 4185	K.YGGDEIPFSPYR.V	1401.50466	2	1.93E-05	0.80	3.27	-	552.8
AHQ-2-2, 4380	K.YGGDEIPFSPYR.V	1401.50466	1	9.93E-04	0.27	2.34	-	385.2
AHQ-2-10, 3887	K.YGGDEIPFSPYR.V	1401.50466	1	3.07E-05	0.22	2.19	-	398.7
AHQ-2-10, 3886	K.YGGDEIPFSPYR.V	1401.50466	2	4.12E-04	0.70	3.48	-	443.3
AHQ-2-9, 4054	K.YGGDEIPFSPYR.V	1401.50466	2	2.32E-04	0.52	2.59	-	419.7
AHQ-2-4, 4324 - 4393	K.YGGDEIPFSPYR.V	1401.50466	2	5.67E-04	0.65	3.00	-	360.0
AHQ-2-12, 4190 - 4193	K.YGGDEIPFSPYR.V	1401.50466	1	1.28E-04	0.36	2.43	-	433.1
AHQ-2-9, 2948	K.YGGPYHIGGSPFK.A	1380.53182	2	9.93E-04	0.89	3.25	-	1138.5
AHQ-2-7, 2763 - 2835	K.YGGPYHIGGSPFK.A	1380.53182	2	5.55E-04	0.82	3.17	-	867.3
AHQ-2-4, 3268	K.YGGPYHIGGSPFK.A	1380.53182	1	4.04E-09	0.90	3.83	-	521.5
AHQ-2-5, 2911 - 2984	K.YGGPYHIGGSPFK.A	1380.53182	2	2.89E-07	0.96	4.60	-	1623.9
AHQ-2-7, 2829	K.YGGPYHIGGSPFK.A	1380.53182	1	2.00E-05	0.82	3.11	-	429.5
AHQ-2-10, 2934	K.YGGPYHIGGSPFK.A	1380.53182	2	7.92E-07	0.95	3.91	-	1568.8
AHQ-2-5, 2919	K.YGGPYHIGGSPFK.A	1380.53182	3	1.15E-05	0.87	3.98	-	617.3
AHQ-2-1, 3461	K.YGGPYHIGGSPFK.A	1380.53182	2	2.19E-05	0.96	3.99	-	1486.6
AHQ-2-14-, 3277	K.YGGPYHIGGSPFK.A	1380.53182	2	1.65E-07	0.97	4.75	-	1927.9
AHQ-2-5, 2928	K.YGGPYHIGGSPFK.A	1380.53182	1	1.33E-06	0.86	3.53	-	578.7
AHQ-2-4, 3249	K.YGGPYHIGGSPFK.A	1380.53182	2	7.24E-07	0.89	3.16	-	1213.9
AHQ-2-13-, 3337 - 3347	K.YGGPYHIGGSPFK.A	1380.53182	2	1.15E-05	0.95	4.07	-	1151.8
AHQ-2-2, 2989 - 3062	K.YGGQPVPNFPSK.L	1291.43658	2	1.04E-05	0.84	3.60	-	374.2
AHQ-2-1, 3121	K.YGGQPVPNFPSK.L	1291.43658	2	8.52E-05	0.93	3.51	-	782.3
AHQ-2-4, 2989	K.YGGQPVPNFPSK.L	1291.43658	1	6.37E-04	0.34	2.52	-	219.5
AHQ-2-4, 2984 - 3016	K.YGGQPVPNFPSK.L	1291.43658	2	8.06E-07	0.92	3.69	-	564.7
AHQ-2-2, 3002	K.YGGQPVPNFPSK.L	1291.43658	1	3.14E-04	0.43	2.63	-	478.2
AHQ-2-3, 2993	K.YGGQPVPNFPSK.L	1291.43658	1	1.25E-04	0.17	2.28	-	239.6
AHQ-2-3, 2987 - 2992	K.YGGQPVPNFPSK.L	1291.43658	2	1.73E-05	0.90	3.28	-	560.3
AHQ-2-3, 4297	K.YKGQHVPGSPFQFTVGPLGEGGAHK.V	2596.88377	3	6.86E-08	0.90	4.79	-	798.2
AHQ-2-7, 3667	K.YKGQHVPGSPFQFTVGPLGEGGAHK.V	2596.88377	3	1.37E-06	0.87	4.06	-	762.2
AHQ-2-6, 3829	K.YKGQHVPGSPFQFTVGPLGEGGAHK.V	2596.88377	2	1.08E-06	0.90	4.44	-	372.9
AHQ-2-6, 3826	K.YKGQHVPGSPFQFTVGPLGEGGAHK.V	2596.88377	3	6.02E-09	0.95	5.57	-	1050.3
AHQ-2-5, 3791 - 3865	K.YKGQHVPGSPFQFTVGPLGEGGAHK.V	2596.88377	3	7.04E-07	0.91	4.50	-	819.0
AHQ-2-5, 2475	K.YNEQHVPGSPFTAR.V	1603.71978	2	5.57E-07	0.86	3.56	-	448.3
AHQ-2-5, 2485 - 2489	K.YNEQHVPGSPFTAR.V	1603.71978	3	4.68E-06	0.94	4.75	-	1151.2
AHQ-2-5, 2792 - 2795	K.YNEQHVPGSPFTAR.V	1603.71978	2	3.47E-09	0.91	3.46	-	798.7
AHQ-2-9, 2531	K.YNEQHVPGSPFTAR.V	1603.71978	2	1.42E-07	0.83	3.65	-	451.4
AHQ-2-6, 2534	K.YNEQHVPGSPFTAR.V	1603.71978	2	7.37E-06	0.75	2.99	-	471.6
AHQ-2-6, 2545 - 2579	K.YNEQHVPGSPFTAR.V	1603.71978	3	1.29E-04	0.89	3.91	-	912.8
AHQ-2-4, 2701	K.YNEQHVPGSPFTAR.V	1603.71978	2	1.16E-05	0.66	3.37	-	382.6
AHQ-2-7, 2461	K.YNEQHVPGSPFTAR.V	1603.71978	2	9.00E-04	0.35	2.58	-	232.2
AHQ-2-1, 2932 - 2949	K.YNEQHVPGSPFTAR.V	1603.71978	2	2.31E-05	0.88	3.35	-	608.5
AHQ-2-10, 2502	K.YNEQHVPGSPFTAR.V	1603.71978	2	2.41E-04	0.89	3.25	-	661.5
AHQ-2-8, 2243 - 2291	K.YNEQHVPGSPFTAR.V	1603.71978	2	1.09E-04	0.91	3.49	-	715.4
AHQ-2-4, 3030	K.YNEQHVPGSPFTAR.V	1603.71978	2	1.22E-05	0.81	3.17	-	444.1
AHQ-2-3, 4632 - 4703	K.YTPVQQGPVGVNVTYGGDPIPK.S	2287.55640	2	1.78E-07	0.89	4.10	-	527.1
AHQ-2-3, 4771 - 4839	K.YTPVQQGPVGVNVTYGGDPIPK.S	2287.55640	2	4.30E-08	0.93	4.64	-	700.2
AHQ-2-7, 4340	K.YTPVQQGPVGVNVTYGGDPIPK.S	2287.55640	2	6.96E-04	0.93	4.34	-	783.8
AHQ-2-8, 4258	K.YTPVQQGPVGVNVTYGGDPIPK.S	2287.55640	2	3.90E-07	0.80	3.59	-	368.6
AHQ-2-6, 4406	K.YTPVQQGPVGVNVTYGGDPIPK.S	2287.55640	2	2.71E-04	0.88	4.02	-	493.2
AHQ-2-5, 4643	K.YTPVQQGPVGVNVTYGGDPIPK.S	2287.55640	2	5.25E-05	0.92	3.92	-	773.9
AHQ-2-1, 4558	K.YTPVQQGPVGVNVTYGGDPIPK.S	2287.55640	2	1.51E-05	0.80	3.33	-	530.9
AHQ-2-4, 4660 - 4677	K.YTPVQQGPVGVNVTYGGDPIPK.S	2287.55640	2	1.07E-04	0.92	4.61	-	657.1
AHQ-2-4, 4748 - 4749	K.YTPVQQGPVGVNVTYGGDPIPK.S	2287.55640	2	2.49E-07	0.89	4.44	-	506.0
AHQ-2-1, 4808 - 4881	K.YTPVQQGPVGVNVTYGGDPIPK.S	2287.55640	2	5.01E-06	0.93	4.87	-	575.4
AHQ-2-5, 4483 - 4499	K.YTPVQQGPVGVNVTYGGDPIPK.S	2287.55640	2	3.76E-06	0.93	4.37	-	743.3
AHQ-2-2, 4884 - 4901	K.YTPVQQGPVGVNVTYGGDPIPK.S	2287.55640	2	5.57E-07	0.92	4.13	-	642.5
AHQ-2-2, 4746 - 4816	K.YTPVQQGPVGVNVTYGGDPIPK.S	2287.55640	2	2.11E-06	0.92	4.34	-	654.6
AHQ-2-2, 4658 - 4728	K.YTPVQQGPVGVNVTYGGDPIPK.S	2287.55640	2	1.91E-06	0.92	4.21	-	894.1
AHQ-2-2, 4526 - 4532	K.YTPVQQGPVGVNVTYGGDPIPK.S	2287.55640	2	1.58E-06	0.92	4.34	-	768.9
AHQ-2-4, 4836	K.YTPVQQGPVGVNVTYGGDPIPK.S	2287.55640	2	2.68E-07	0.89	3.98	-	586.3
AHQ-2-3, 4492	K.YTPVQQGPVGVNVTYGGDPIPK.S	2287.55640	2	1.13E-04	0.82	3.36	-	464.2
AHQ-2-4, 6988	R.YWPQEAGEYAVHVLCNSEDIR.L	2538.73318	2	3.75E-07	0.81	3.49	-	527.2
AHQ-2-2, 6570 - 6640	R.YWPQEAGEYAVHVLCNSEDIR.L	2538.73318	2	9.09E-09	0.95	3.92	-	1452.5
AHQ-2-1, 7022	R.YWPQEAGEYAVHVLCNSEDIR.L	2538.73318	2	7.80E-08	0.91	3.53	-	1115.4
AHQ-2-1, 7013	R.YWPQEAGEYAVHVLCNSEDIR.L	2538.73318	3	5.81E-10	0.72	3.05	-	448.4
AHQ-2-1, 6854 - 6925	R.YWPQEAGEYAVHVLCNSEDIR.L	2538.73318	2	5.88E-07	0.91	3.69	-	873.4
AHQ-2-1, 6810 - 6876	R.YWPQEAGEYAVHVLCNSEDIR.L	2538.73318	3	4.42E-09	0.85	3.33	-	559.7
AHQ-2-1, 6506 - 6526	R.YWPQEAGEYAVHVLCNSEDIR.L	2538.73318	3	6.42E-07	0.71	3.23	-	447.3
AHQ-2-2, 6572 - 6645	R.YWPQEAGEYAVHVLCNSEDIR.L	2538.73318	3	2.16E-12	0.81	3.53	-	582.8

AHQ-2-2, 6945 - 6990	R.YWPQEAGEYAVHVLCNSEDIR.L	2538.73318	2	3.44E-08	0.94	4.21	-	889.8
AHQ-2-2, 6957 - 7028	R.YWPQEAGEYAVHVLCNSEDIR.L	2538.73318	3	9.20E-12	0.84	3.48	-	490.7
AHQ-2-5, 6767 - 6784	R.YWPQEAGEYAVHVLCNSEDIR.L	2538.73318	2	1.53E-05	0.92	4.27	-	726.9
AHQ-2-2, 7004 - 7072	R.YWPQEAGEYAVHVLCNSEDIR.L	2538.73318	3	1.45E-09	0.88	3.99	-	514.9
AHQ-2-3, 6899 - 6967	R.YWPQEAGEYAVHVLCNSEDIR.L	2538.73318	2	3.00E-04	0.89	3.80	-	704.0
AHQ-2-3, 6889 - 6972	R.YWPQEAGEYAVHVLCNSEDIR.L	2538.73318	3	1.69E-10	0.78	3.22	-	567.0
AHQ-2-4, 6626 - 6649	R.YWPQEAGEYAVHVLCNSEDIR.L	2538.73318	2	4.69E-07	0.75	2.82	-	662.8
gi|16753233|ref|NP_006280.2| talin 1 [Homo sapiens]				1.11E-15	118.32	1330.37	57.10	269665.3
AHQ-2-1, 3198 - 3272	K.AAAFEEQENETVVVK.E	1664.79496	2	2.35E-04	0.89	3.79	-	928.7
AHQ-2-5, 3151	K.AAAFEEQENETVVVK.E	1664.79496	2	4.99E-09	0.94	4.30	-	1065.8
AHQ-2-2, 3854	K.AAAFEEQENETVVVK.E	1664.79496	2	5.44E-04	0.44	2.95	-	390.1
AHQ-2-2, 4010	K.AAAFEEQENETVVVK.E	1664.79496	2	6.08E-05	0.84	3.84	-	761.0
AHQ-2-3, 3205	K.AAAFEEQENETVVVK.E	1664.79496	2	8.46E-04	0.22	2.52	-	361.7
AHQ-2-1, 3473	K.AAAFEEQENETVVVK.E	1664.79496	2	7.66E-08	0.90	3.73	-	859.3
AHQ-2-4, 3213 - 3220	K.AAAFEEQENETVVVK.E	1664.79496	2	2.70E-09	0.92	4.52	-	927.1
AHQ-2-4, 3365	K.AAAFEEQENETVVVK.E	1664.79496	2	1.47E-08	0.87	3.83	-	877.9
AHQ-2-2, 3113 - 3193	K.AAAFEEQENETVVVK.E	1664.79496	2	2.71E-06	0.93	4.20	-	1554.8
AHQ-2-3, 2469	R.AAM*EPIVISAK.T	1146.38260	2	1.11E-05	0.80	2.69	-	679.2
AHQ-2-4, 3140	R.AAMEPIVISAK.T	1130.38320	2	2.16E-05	0.85	2.53	-	754.4
AHQ-2-4, 2469	R.AAM*EPIVISAK.T	1146.38260	1	1.99E-04	0.50	2.35	-	221.3
AHQ-2-5, 2291	R.AAM*EPIVISAK.T	1146.38260	2	2.58E-06	0.84	2.99	-	742.6
AHQ-2-4, 2465	R.AAM*EPIVISAK.T	1146.38260	2	3.31E-05	0.89	2.75	-	1156.6
AHQ-2-3, 2467 - 2471	R.AAM*EPIVISAK.T	1146.38260	1	4.00E-04	0.26	2.20	-	190.1
AHQ-2-1, 2678	R.AAM*EPIVISAK.T	1146.38260	2	1.82E-05	0.84	2.64	-	832.6
AHQ-2-1, 2693	R.AAM*EPIVISAK.T	1146.38260	1	1.65E-05	0.59	2.92	-	265.0
AHQ-2-10, 2824	R.AAMEPIVISAK.T	1130.38320	1	1.50E-04	0.33	1.97	-	658.5
AHQ-2-2, 2496	R.AAM*EPIVISAK.T	1146.38260	1	4.87E-05	0.46	2.81	-	190.3
AHQ-2-2, 2489	R.AAM*EPIVISAK.T	1146.38260	2	1.92E-07	0.92	3.07	-	1126.8
AHQ-2-2, 1833	K.ADAEGESDLENSR.K	1393.35296	2	5.98E-08	0.74	2.83	-	613.0
AHQ-2-1, 2037	K.ADAEGESDLENSR.K	1393.35296	2	5.30E-08	0.92	3.36	-	1096.7
AHQ-2-5, 2417 - 2419	K.AGALQCSPSDAYTK.K	1470.58689	2	2.33E-07	0.92	3.96	-	825.6
AHQ-2-2, 2594	K.AGALQCSPSDAYTK.K	1470.58689	1	1.44E-05	0.82	3.90	-	476.0
AHQ-2-5, 2428	K.AGALQCSPSDAYTK.K	1470.58689	1	2.76E-07	0.81	3.57	-	472.8
AHQ-2-4, 2565	K.AGALQCSPSDAYTK.K	1470.58689	2	5.20E-07	0.93	3.72	-	1010.1
AHQ-2-6, 2426	K.AGALQCSPSDAYTK.K	1470.58689	2	3.77E-06	0.90	3.59	-	799.6
AHQ-2-4, 2585	K.AGALQCSPSDAYTK.K	1470.58689	1	3.34E-05	0.73	3.17	-	492.3
AHQ-2-1, 2710 - 2712	K.AGALQCSPSDAYTK.K	1470.58689	2	1.83E-06	0.93	4.19	-	877.3
AHQ-2-3, 2587	K.AGALQCSPSDAYTK.K	1470.58689	1	9.53E-07	0.68	3.23	-	378.0
AHQ-2-1, 2526	K.AGALQCSPSDAYTK.K	1470.58689	2	9.53E-05	0.83	3.18	-	721.6
AHQ-2-2, 2312	K.AGALQCSPSDAYTKK.E	1598.75981	2	3.16E-04	0.89	3.72	-	736.6
AHQ-2-4, 2264 - 2281	K.AGALQCSPSDAYTKK.E	1598.75981	2	1.59E-04	0.79	3.25	-	583.9
AHQ-2-9, 6210	K.AGFLDLKDFLPK.E	1364.61374	2	4.53E-04	0.90	3.18	-	1389.6
AHQ-2-1, 4248	K.AIAVTVQEMVTK.S	1290.55498	2	2.23E-06	0.96	4.27	-	1329.8
AHQ-2-2, 4041 - 4053	K.AIAVTVQEMVTK.S	1290.55498	2	1.19E-04	0.96	5.29	-	1228.5
AHQ-2-4, 3980 - 4000	K.AIAVTVQEMVTK.S	1290.55498	2	2.10E-09	0.96	5.04	-	1121.8
AHQ-2-1, 3028	K.AIAVTVQEM*VTK.S	1306.55438	2	3.45E-05	0.91	3.60	-	812.9
AHQ-2-2, 4126 - 4196	K.AIAVTVQEMVTK.S	1290.55498	2	3.38E-09	0.94	4.12	-	1221.2
AHQ-2-3, 4005 - 4085	K.AIAVTVQEMVTK.S	1290.55498	2	5.38E-07	0.95	4.08	-	1265.5
AHQ-2-4, 2576 - 2582	K.ALDGAFTEENR.A	1223.27427	2	3.52E-07	0.94	3.92	-	1228.7
AHQ-2-3, 2441	K.ALDGAFTEENR.A	1223.27427	2	5.66E-06	0.93	3.57	-	1269.8
AHQ-2-2, 2458 - 2465	K.ALDGAFTEENR.A	1223.27427	2	5.68E-06	0.95	3.98	-	1758.7
AHQ-2-5, 2401 - 2404	K.ALDGAFTEENR.A	1223.27427	1	6.87E-04	0.22	1.96	-	233.6
AHQ-2-4, 2440 - 2510	K.ALDGAFTEENR.A	1223.27427	2	4.84E-07	0.94	3.49	-	1485.7
AHQ-2-12, 3799	K.ALDYYMLR.N	1045.23686	2	1.55E-04	0.90	2.68	-	1067.1
AHQ-2-13-, 3306	K.ALDYYM*LR.N	1061.23626	2	5.58E-06	0.82	2.55	-	732.4
AHQ-2-13, 3320	K.ALDYYM*LR.N	1061.23626	2	2.16E-06	0.92	3.03	-	1056.2
AHQ-2-13, 3952	K.ALDYYMLR.N	1045.23686	2	1.46E-06	0.95	4.13	-	942.1
AHQ-2-13-, 3994 - 3999	K.ALDYYMLR.N	1045.23686	2	3.56E-07	0.93	3.70	-	818.5
AHQ-2-7, 1573	R.ALEATTEHIR.Q	1141.25953	2	4.18E-05	0.93	3.51	-	1428.0
AHQ-2-5, 1559	R.ALEATTEHIR.Q	1141.25953	2	5.35E-04	0.73	3.01	-	742.6
AHQ-2-2, 1724 - 1792	R.ALEATTEHIR.Q	1141.25953	2	1.38E-05	0.92	3.44	-	1340.5
AHQ-2-4, 1741 - 1806	R.ALEATTEHIR.Q	1141.25953	2	2.10E-06	0.88	2.73	-	1309.1
AHQ-2-1, 2077	R.ALEATTEHIR.Q	1141.25953	1	3.85E-05	0.02	1.86	-	193.4
AHQ-2-1, 2062	R.ALEATTEHIR.Q	1141.25953	2	5.63E-07	0.85	3.00	-	1106.8
AHQ-2-4, 3440	K.ALGDLISATK.A	989.14811	2	6.11E-05	0.93	3.64	-	912.2
AHQ-2-2, 3433	K.ALGDLISATK.A	989.14811	2	5.58E-05	0.90	4.01	-	680.5
AHQ-2-7, 3113	K.ALGDLISATK.A	989.14811	2	3.40E-08	0.92	3.39	-	902.8
AHQ-2-5, 3199	K.ALGDLISATK.A	989.14811	2	1.08E-06	0.91	3.58	-	832.1
AHQ-2-1, 3564	K.ALGDLISATK.A	989.14811	2	5.31E-05	0.91	3.54	-	798.4
AHQ-2-6, 3178 - 3187	K.ALGDLISATK.A	989.14811	2	1.59E-07	0.95	4.41	-	954.9
AHQ-2-3, 3431 - 3432	K.ALGDLISATK.A	989.14811	2	3.08E-06	0.91	3.62	-	763.8
AHQ-2-1, 2729 - 2733	K.ALSTDPAAPNLK.S	1198.35099	2	4.82E-05	0.66	2.67	-	480.4
AHQ-2-4, 2485 - 2556	K.ALSTDPAAPNLK.S	1198.35099	2	1.48E-04	0.80	3.47	-	577.2
AHQ-2-2, 2553 - 2554	K.ALSTDPAAPNLK.S	1198.35099	2	1.24E-06	0.78	3.11	-	610.8
AHQ-2-5, 2377 - 2457	K.ALSTDPAAPNLK.S	1198.35099	2	4.91E-08	0.73	2.98	-	613.4
AHQ-2-6, 2389	K.ALSTDPAAPNLK.S	1198.35099	2	7.72E-07	0.59	2.63	-	581.8
AHQ-2-3, 2548 - 2565	K.ALSTDPAAPNLK.S	1198.35099	2	3.93E-07	0.84	3.27	-	775.7
AHQ-2-13-, 2653	K.ALSTDPAAPNLK.S	1198.35099	2	8.78E-05	0.69	2.73	-	638.0
AHQ-2-2, 3580	K.ALSTDPAAPNLKSQLAAAAR.A	1967.21592	2	1.09E-08	0.83	3.43	-	506.0
AHQ-2-3, 3555 - 3560	K.ALSTDPAAPNLKSQLAAAAR.A	1967.21592	2	9.62E-06	0.91	3.93	-	693.6
AHQ-2-3, 6676 - 6744	R.ANQAIQMACQSLGEPGCTQAQVLSAATIVAK.H	3220.64522	3	4.16E-04	0.90	4.69	-	833.0
AHQ-2-2, 7384 - 7456	R.ANQAIQMACQSLGEPGCTQAQVLSAATIVAK.H	3220.64522	3	3.49E-06	0.91	4.89	-	711.4
AHQ-2-1, 6600 - 6673	R.ANQAIQMACQSLGEPGCTQAQVLSAATIVAK.H	3220.64522	3	6.55E-05	0.94	4.88	-	1066.4
AHQ-2-2, 6498	K.APGQLECETAIAALNSCLR.D	2077.32566	2	2.35E-09	0.97	4.82	-	1445.7
AHQ-2-4, 6534	K.APGQLECETAIAALNSCLR.D	2077.32566	2	1.70E-07	0.97	5.26	-	1283.2
AHQ-2-5, 6295	K.APGQLECETAIAALNSCLR.D	2077.32566	2	1.94E-07	0.98	5.98	-	1546.8
AHQ-2-4, 5782	K.APGQLECETAIAALNSCLR.D	2077.32566	2	3.78E-05	0.68	3.46	-	434.7
AHQ-2-3, 5763 - 5768	K.APGQLECETAIAALNSCLR.D	2077.32566	2	1.65E-07	0.97	5.32	-	1187.6
AHQ-2-2, 5822	K.APGQLECETAIAALNSCLR.D	2077.32566	2	8.79E-09	0.96	4.61	-	1446.7
AHQ-2-1, 6424	K.APGQLECETAIAALNSCLR.D	2077.32566	2	1.48E-06	0.91	3.98	-	1172.5
AHQ-2-3, 6464	K.APGQLECETAIAALNSCLR.D	2077.32566	2	9.29E-05	0.96	4.85	-	1196.7
AHQ-2-1, 7002	K.AQEACGPLEMDSALSVVQNLEK.D	2391.66165	3	5.64E-06	0.96	5.38	-	1257.6
AHQ-2-2, 7177 - 7178	K.AQEACGPLEMDSALSVVQNLEK.D	2391.66165	3	2.93E-11	0.96	5.19	-	1853.4
AHQ-2-5, 2624 - 2625	K.ASAGPQPLLVQSCK.A	1457.67763	1	4.86E-04	0.25	2.07	-	326.8
AHQ-2-3, 2855 - 2895	K.ASAGPQPLLVQSCK.A	1457.67763	2	1.03E-05	0.87	3.84	-	406.7
AHQ-2-2, 3118	K.ASAGPQPLLVQSCK.A	1457.67763	2	5.43E-09	0.77	2.88	-	455.8
AHQ-2-6, 2626 - 2677	K.ASAGPQPLLVQSCK.A	1457.67763	2	2.40E-06	0.87	3.94	-	481.7
AHQ-2-7, 2568	K.ASAGPQPLLVQSCK.A	1457.67763	2	1.02E-09	0.82	3.70	-	467.3
AHQ-2-2, 2878	K.ASAGPQPLLVQSCK.A	1457.67763	1	1.45E-05	0.46	2.86	-	403.2
AHQ-2-2, 2846 - 2916	K.ASAGPQPLLVQSCK.A	1457.67763	2	2.28E-09	0.90	4.32	-	475.4
AHQ-2-4, 2825 - 2896	K.ASAGPQPLLVQSCK.A	1457.67763	2	1.24E-09	0.93	4.36	-	690.3
AHQ-2-4, 2872	K.ASAGPQPLLVQSCK.A	1457.67763	1	1.04E-05	0.59	3.04	-	397.1
AHQ-2-4, 3186	K.ASAGPQPLLVQSCK.A	1457.67763	2	1.95E-05	0.80	2.94	-	575.5
AHQ-2-7, 2577	K.ASAGPQPLLVQSCK.A	1457.67763	1	1.10E-13	0.75	3.48	-	471.5
AHQ-2-1, 3001 - 3076	K.ASAGPQPLLVQSCK.A	1457.67763	2	3.17E-10	0.93	4.52	-	583.6
AHQ-2-6, 2673 - 2743	K.ASAGPQPLLVQSCK.A	1457.67763	1	2.71E-05	0.11	2.07	-	287.3
AHQ-2-5, 2608 - 2623	K.ASAGPQPLLVQSCK.A	1457.67763	2	6.52E-07	0.80	3.41	-	361.1

AHQ-2-3, 4079	K.ASVPTIQDQASAM*QLSQCAK.N	2152.39272	2	2.49E-04	0.65	3.20	-	338.3
AHQ-2-4, 4352 - 4420	K.ASVPTIQDQASAMQLSQCAK.N	2136.39332	2	3.11E-14	0.95	5.30	-	665.7
AHQ-2-7, 4083	K.ASVPTIQDQASAMQLSQCAK.N	2136.39332	2	5.43E-07	0.93	4.25	-	1018.3
AHQ-2-2, 4412 - 4484	K.ASVPTIQDQASAMQLSQCAK.N	2136.39332	2	1.68E-07	0.94	4.80	-	737.1
AHQ-2-4, 4045 - 4049	K.ASVPTIQDQASAM*QLSQCAK.N	2152.39272	2	1.40E-05	0.87	3.83	-	532.7
AHQ-2-3, 4515 - 4592	K.ASVPTIQDQASAMQLSQCAK.N	2136.39332	2	3.84E-08	0.96	5.10	-	857.8
AHQ-2-5, 4187	K.ASVPTIQDQASAMQLSQCAK.N	2136.39332	2	5.32E-06	0.92	3.97	-	934.7
AHQ-2-4, 3764	K.ASVPTIQDQASAM*QLSQCAK.N	2152.39272	2	3.13E-05	0.57	2.74	-	393.0
AHQ-2-3, 4376 - 4447	K.ASVPTIQDQASAMQLSQCAK.N	2136.39332	2	7.20E-09	0.92	4.48	-	657.4
AHQ-2-6, 4258	K.ASVPTIQDQASAMQLSQCAK.N	2136.39332	2	4.25E-04	0.81	3.71	-	447.2
AHQ-2-2, 4548 - 4616	K.ASVPTIQDQASAMQLSQCAK.N	2136.39332	2	1.36E-08	0.89	4.24	-	418.5
AHQ-2-6, 4093	K.ASVPTIQDQASAMQLSQCAK.N	2136.39332	2	1.13E-10	0.90	4.29	-	590.3
AHQ-2-1, 4446 - 4460	K.ASVPTIQDQASAMQLSQCAK.N	2136.39332	2	9.18E-05	0.89	4.01	-	575.1
AHQ-2-5, 4343	K.ASVPTIQDQASAMQLSQCAK.N	2136.39332	2	1.22E-09	0.82	3.75	-	385.9
AHQ-2-6, 3530	K.ASVPTIQDQASAM*QLSQCAK.N	2152.39272	2	1.63E-06	0.93	4.54	-	664.0
AHQ-2-2, 3966 - 3973	K.ASVPTIQDQASAM*QLSQCAK.N	2152.39272	2	8.29E-07	0.95	5.00	-	872.9
AHQ-2-4, 4452 - 4520	K.ASVPTIQDQASAMQLSQCAK.N	2136.39332	2	6.78E-07	0.87	4.10	-	521.2
AHQ-2-1, 5150 - 5161	K.AVAEQIPLLVQGVR.G	1493.77619	2	2.54E-06	0.75	3.08	-	499.8
AHQ-2-6, 4767	K.AVAEQIPLLVQGVR.G	1493.77619	1	5.94E-06	0.60	2.87	-	163.0
AHQ-2-6, 4725	K.AVAEQIPLLVQGVR.G	1493.77619	2	2.46E-06	0.88	3.57	-	643.7
AHQ-2-3, 5071	K.AVAEQIPLLVQGVR.G	1493.77619	1	8.65E-07	0.77	3.56	-	385.8
AHQ-2-2, 5146	K.AVAEQIPLLVQGVR.G	1493.77619	3	6.34E-06	0.94	4.59	-	1492.9
AHQ-2-4, 5078	K.AVAEQIPLLVQGVR.G	1493.77619	1	1.31E-06	0.43	3.23	-	145.5
AHQ-2-4, 5044 - 5057	K.AVAEQIPLLVQGVR.G	1493.77619	2	8.21E-04	0.86	3.80	-	523.4
AHQ-2-3, 5044 - 5119	K.AVAEQIPLLVQGVR.G	1493.77619	2	5.33E-07	0.92	4.27	-	675.8
AHQ-2-5, 4773	K.AVAEQIPLLVQGVR.G	1493.77619	1	3.94E-07	0.41	2.70	-	189.5
AHQ-2-2, 5077 - 5098	K.AVAEQIPLLVQGVR.G	1493.77619	2	1.19E-07	0.79	3.22	-	694.9
AHQ-2-1, 4042	K.AVASAAAALVLK.A	1085.32216	2	7.22E-06	0.96	4.36	-	1352.0
AHQ-2-2, 3902	K.AVASAAAALVLK.A	1085.32216	2	6.36E-08	0.97	4.24	-	1777.7
AHQ-2-2, 4012	K.AVASAAAALVLK.A	1085.32216	2	1.39E-05	0.96	4.19	-	1720.7
AHQ-2-1, 3914	K.AVEGCVSASQAATEDGQLLR.G	2064.22056	2	6.66E-05	0.93	4.41	-	1054.9
AHQ-2-2, 5006	K.AVEGCVSASQAATEDGQLLR.G	2064.22056	2	3.75E-06	0.88	3.81	-	960.3
AHQ-2-5, 4964	K.AVEGCVSASQAATEDGQLLR.G	2064.22056	2	6.89E-04	0.46	2.77	-	591.9
AHQ-2-2, 3957 - 4034	K.AVEGCVSASQAATEDGQLLR.G	2064.22056	2	1.42E-08	0.95	4.13	-	1532.3
AHQ-2-1, 4021	K.AVEGCVSASQAATEDGQLLR.G	2064.22056	2	4.52E-07	0.79	3.36	-	940.2
AHQ-2-2, 4265 - 4338	K.AVEGCVSASQAATEDGQLLR.G	2064.22056	2	6.91E-06	0.92	4.49	-	903.8
AHQ-2-2, 4838 - 4898	K.AVEGCVSASQAATEDGQLLR.G	2064.22056	2	3.08E-06	0.94	4.33	-	1303.3
AHQ-2-1, 4388	K.AVEGCVSASQAATEDGQLLR.G	2064.22056	2	3.58E-04	0.95	4.59	-	1455.6
AHQ-2-4, 4298	K.AVEGCVSASQAATEDGQLLR.G	2064.22056	2	2.29E-04	0.77	3.37	-	732.1
AHQ-2-3, 4163 - 4231	K.AVEGCVSASQAATEDGQLLR.G	2064.22056	2	4.37E-06	0.81	3.63	-	739.9
AHQ-2-2, 4409	K.AVEGCVSASQAATEDGQLLR.G	2064.22056	2	1.13E-07	0.96	4.99	-	1477.7
AHQ-2-1, 4213 - 4245	K.AVEGCVSASQAATEDGQLLR.G	2064.22056	2	2.26E-07	0.92	3.94	-	1051.5
AHQ-2-1, 4233	K.AVEGCVSASQAATEDGQLLR.G	2064.22056	3	2.17E-05	0.87	3.80	-	1250.3
AHQ-2-3, 7449 - 7459	K.AVSSAIAQLLGEVAQGNENYAGIAAR.D	2574.83158	3	1.80E-08	0.97	5.78	-	2051.6
AHQ-2-6, 6010 - 6021	R.AVTDSINQLITMCTQQAPGQK.E	2306.60312	2	1.49E-06	0.86	4.14	-	589.3
AHQ-2-3, 4911	R.AVTDSINQLITMCTQQAPGQK.E	2306.60312	2	7.78E-06	0.72	3.66	-	384.9
AHQ-2-1, 6216 - 6225	R.AVTDSINQLITMCTQQAPGQK.E	2306.60312	2	1.87E-04	0.55	3.08	-	472.8
AHQ-2-4, 4929	R.AVTDSINQLITMCTQQAPGQK.E	2306.60312	2	1.52E-07	0.84	3.48	-	538.5
AHQ-2-2, 5904	R.AVTDSINQLITM*CTQQAPGQK.E	2322.60252	2	7.05E-04	0.34	2.93	-	203.0
AHQ-2-2, 4962	R.AVTDSINQLITMCTQQAPGQK.E	2306.60312	2	1.03E-07	0.89	4.40	-	461.4
AHQ-2-4, 5897 - 5904	R.AVTDSINQLITM*CTQQAPGQK.E	2322.60252	2	5.32E-04	0.63	3.23	-	286.3
AHQ-2-1, 6304 - 6372	R.AVTDSINQLITMCTQQAPGQKECDNALR.E	3166.51128	3	8.48E-09	0.92	4.77	-	982.6
AHQ-2-7, 6077	R.AVTDSINQLITMCTQQAPGQKECDNALR.E	3166.51128	3	5.16E-12	0.91	5.03	-	774.0
AHQ-2-3, 6347 - 6415	R.AVTDSINQLITMCTQQAPGQKECDNALR.E	3166.51128	3	2.27E-11	0.96	6.11	-	1183.0
AHQ-2-1, 6004	R.AVTDSINQLITM*CTQQAPGQKECDNALR.E	3182.51068	3	2.84E-09	0.93	5.18	-	870.9
AHQ-2-5, 6268	R.AVTDSINQLITMCTQQAPGQKECDNALR.E	3166.51128	3	4.10E-10	0.95	5.24	-	1329.8
AHQ-2-4, 6364 - 6432	R.AVTDSINQLITMCTQQAPGQKECDNALR.E	3166.51128	3	2.81E-11	0.94	5.40	-	1085.9
AHQ-2-2, 5173	R.AVTDSINQLITMCTQQAPGQKECDNALR.E	3166.51128	3	8.90E-08	0.84	4.41	-	590.9
AHQ-2-5, 6179	R.AVTDSINQLITMCTQQAPGQKECDNALR.E	3166.51128	3	1.11E-15	0.93	4.77	-	1240.2
AHQ-2-4, 6017 - 6032	R.AVTDSINQLITM*CTQQAPGQKECDNALR.E	3182.51068	3	6.38E-10	0.92	5.09	-	890.5
AHQ-2-6, 6058	R.AVTDSINQLITMCTQQAPGQKECDNALR.E	3166.51128	3	8.05E-10	0.92	4.54	-	1050.5
AHQ-2-2, 7389 - 7402	R.AVTDSINQLITMCTQQAPGQKECDNALRELETVR.E	3894.32086	3	8.58E-04	0.51	3.54	-	227.4
AHQ-2-2, 2416 - 2417	R.CVSCLPGQR.D	1079.23309	2	1.75E-05	0.89	3.14	-	927.0
AHQ-2-5, 2261 - 2263	R.CVSCLPGQR.D	1079.23309	1	1.82E-05	0.29	2.19	-	117.8
AHQ-2-2, 2406	R.CVSCLPGQR.D	1079.23309	1	6.86E-04	0.08	1.88	-	146.7
AHQ-2-3, 2380 - 2391	R.CVSCLPGQR.D	1079.23309	2	9.92E-05	0.83	2.95	-	724.6
AHQ-2-10, 3395	R.DDILNGSHPVSFDK.A	1544.64741	2	6.04E-08	0.96	3.95	-	1762.0
AHQ-2-11, 3358	R.DDILNGSHPVSFDK.A	1544.64741	2	2.59E-04	0.59	2.55	-	604.3
AHQ-2-10, 3227 - 3291	R.DDILNGSHPVSFDK.A	1544.64741	2	1.57E-07	0.87	3.66	-	890.3
AHQ-2-7, 3455 - 3508	R.DDILNGSHPVSFDK.A	1544.64741	2	4.14E-11	0.92	3.83	-	1030.4
AHQ-2-8, 3424	R.DDILNGSHPVSFDK.A	1544.64741	2	1.31E-07	0.90	3.59	-	977.8
AHQ-2-7, 3323 - 3335	R.DDILNGSHPVSFDK.A	1544.64741	2	9.79E-10	0.94	3.97	-	1036.0
AHQ-2-8, 3243	R.DDILNGSHPVSFDK.A	1544.64741	2	1.58E-07	0.91	3.77	-	910.4
AHQ-2-1, 3694	R.DDILNGSHPVSFDK.A	1544.64741	2	7.21E-04	0.66	2.72	-	489.5
AHQ-2-9, 3259 - 3330	R.DDILNGSHPVSFDK.A	1544.64741	2	3.51E-10	0.95	4.02	-	1318.7
AHQ-2-7, 4783	K.DHFGLEGDEESTMLEDSVSPK.K	2323.43307	2	4.93E-04	0.84	3.60	-	752.8
AHQ-2-3, 5808 - 5875	R.DKAPGQLECETAIAALNSCLR.D	2320.58644	2	2.19E-09	0.79	3.34	-	833.6
AHQ-2-3, 5491 - 5493	R.DKAPGQLECETAIAALNSCLR.D	2320.58644	2	1.14E-10	0.95	4.66	-	1161.1
AHQ-2-7, 5960	R.DKAPGQLECETAIAALNSCLR.D	2320.58644	2	1.26E-11	0.87	4.05	-	608.1
AHQ-2-4, 6013	R.DKAPGQLECETAIAALNSCLR.D	2320.58644	2	7.60E-04	0.82	3.34	-	622.1
AHQ-2-1, 5512	R.DKAPGQLECETAIAALNSCLR.D	2320.58644	2	2.03E-05	0.91	4.40	-	809.2
AHQ-2-6, 5953	R.DKAPGQLECETAIAALNSCLR.D	2320.58644	2	4.13E-09	0.96	4.96	-	1207.8
AHQ-2-3, 6009	R.DKAPGQLECETAIAALNSCLR.D	2320.58644	2	1.17E-05	0.91	4.34	-	761.4
AHQ-2-2, 5477 - 5549	R.DKAPGQLECETAIAALNSCLR.D	2320.58644	2	3.99E-11	0.93	4.11	-	950.7
AHQ-2-1, 6232	R.DKAPGQLECETAIAALNSCLR.D	2320.58644	2	4.73E-10	0.92	4.29	-	609.4
AHQ-2-9, 6024	R.DKAPGQLECETAIAALNSCLR.D	2320.58644	2	8.03E-08	0.91	4.11	-	872.2
AHQ-2-4, 6310	R.DKAPGQLECETAIAALNSCLR.D	2320.58644	2	2.26E-08	0.96	5.30	-	855.6
AHQ-2-2, 6042	R.DKAPGQLECETAIAALNSCLR.D	2320.58644	2	3.06E-06	0.91	4.02	-	894.0
AHQ-2-3, 6255 - 6264	R.DKAPGQLECETAIAALNSCLR.D	2320.58644	2	2.09E-06	0.97	5.20	-	1383.3
AHQ-2-4, 6565	R.DKAPGQLECETAIAALNSCLR.D	2320.58644	2	2.22E-15	0.93	4.37	-	920.6
AHQ-2-1, 5853	R.DKAPGQLECETAIAALNSCLR.D	2320.58644	2	8.08E-07	0.88	3.63	-	890.1
AHQ-2-2, 6304 - 6376	R.DKAPGQLECETAIAALNSCLR.D	2320.58644	2	1.57E-10	0.96	4.77	-	1175.3
AHQ-2-5, 6068	R.DKAPGQLECETAIAALNSCLR.D	2320.58644	2	1.38E-07	0.94	4.67	-	978.3
AHQ-2-5, 6325	R.DKAPGQLECETAIAALNSCLR.D	2320.58644	2	6.59E-10	0.94	4.74	-	842.9
AHQ-2-3, 6417 - 6492	R.DKAPGQLECETAIAALNSCLR.D	2320.58644	2	4.05E-14	0.95	5.09	-	927.1
AHQ-2-5, 5297	R.DKAPGQLECETAIAALNSCLR.D	2320.58644	2	2.78E-15	0.93	4.53	-	946.9
AHQ-2-2, 6520	R.DKAPGQLECETAIAALNSCLR.D	2320.58644	2	6.02E-11	0.92	3.96	-	786.6
AHQ-2-4, 6484	R.DKAPGQLECETAIAALNSCLR.D	2320.58644	2	4.25E-08	0.95	4.44	-	1004.8
AHQ-2-4, 4848 - 4857	R.DLDQASLAAVSQQLAPR.E	1783.96422	2	2.33E-06	0.85	3.58	-	757.8
AHQ-2-3, 5463 - 5535	R.DLDQASLAAVSQQLAPR.E	1783.96422	2	2.79E-04	0.93	4.74	-	647.5
AHQ-2-2, 5506 - 5580	R.DLDQASLAAVSQQLAPR.E	1783.96422	2	2.31E-04	0.93	4.48	-	745.9
AHQ-2-3, 5476	R.DLDQASLAAVSQQLAPR.E	1783.96422	3	7.10E-06	0.90	3.94	-	1596.8
AHQ-2-2, 5528	R.DLDQASLAAVSQQLAPR.E	1783.96422	3	3.62E-07	0.96	4.88	-	1940.0
AHQ-2-4, 5488 - 5568	R.DLDQASLAAVSQQLAPR.E	1783.96422	2	3.74E-09	0.96	4.63	-	1550.3
AHQ-2-4, 5509	R.DLDQASLAAVSQQLAPR.E	1783.96422	3	1.35E-05	0.95	4.47	-	1984.8
AHQ-2-11, 5032 - 5033	R.DLDQASLAAVSQQLAPR.E	1783.96422	2	3.48E-04	0.43	2.88	-	395.1
AHQ-2-1, 5488 - 5493	R.DLDQASLAAVSQQLAPR.E	1783.96422	3	1.83E-06	0.93	4.58	-	1587.1

AHQ-2-5, 3227	R.DPPSWSVLAGHSR.T	1409.53156	3	9.41E-07	0.90	4.30	-	753.1
AHQ-2-1, 3750	R.DPPSWSVLAGHSR.T	1409.53156	3	3.69E-07	0.84	3.66	-	577.9
AHQ-2-2, 3526 - 3606	R.DPPSWSVLAGHSR.T	1409.53156	2	6.56E-08	0.90	3.13	-	633.2
AHQ-2-4, 3524 - 3526	R.DPPSWSVLAGHSR.T	1409.53156	2	3.67E-10	0.95	3.97	-	881.3
AHQ-2-2, 3545	R.DPPSWSVLAGHSR.T	1409.53156	3	3.61E-08	0.92	4.10	-	980.6
AHQ-2-3, 3516 - 3539	R.DPPSWSVLAGHSR.T	1409.53156	2	4.08E-08	0.94	3.99	-	732.8
AHQ-2-10, 4790	R.DPVQLNLLYVQAR.D	1529.76539	2	1.02E-06	0.96	4.04	-	1725.9
AHQ-2-7, 5219	R.DPVQLNLLYVQAR.D	1529.76539	2	1.56E-05	0.96	3.63	-	2076.7
AHQ-2-9, 5152	R.DPVQLNLLYVQAR.D	1529.76539	2	3.57E-06	0.91	3.76	-	1141.7
AHQ-2-3, 2357	K.EAAYHPEVAPDVR.L	1454.56910	2	2.00E-04	0.89	2.77	-	1127.8
AHQ-2-7, 2152	K.EAAYHPEVAPDVR.L	1454.56910	1	3.11E-05	0.50	2.19	-	292.4
AHQ-2-4, 2352 - 2353	K.EAAYHPEVAPDVR.L	1454.56910	2	4.38E-06	0.78	3.02	-	622.9
AHQ-2-4, 2362	K.EAAYHPEVAPDVR.L	1454.56910	1	3.63E-10	0.83	3.27	-	526.1
AHQ-2-5, 2185	K.EAAYHPEVAPDVR.L	1454.56910	2	3.88E-05	0.73	2.66	-	734.3
AHQ-2-8, 2088 - 2096	K.EAAYHPEVAPDVR.L	1454.56910	2	1.12E-08	0.85	3.08	-	868.8
AHQ-2-1, 6245 - 6313	K.EADESLNFEEQILEAAK.S	1937.05072	2	1.50E-05	0.91	3.85	-	1316.4
AHQ-2-10, 5518 - 5579	K.EADESLNFEEQILEAAK.S	1937.05072	2	8.77E-09	0.94	4.50	-	1302.6
AHQ-2-14-, 5820	K.EADESLNFEEQILEAAK.S	1937.05072	2	5.94E-05	0.62	2.96	-	739.7
AHQ-2-3, 6397 - 6471	K.EADESLNFEEQILEAAK.S	1937.05072	2	4.43E-07	0.83	3.65	-	925.7
AHQ-2-5, 6052 - 6132	K.EADESLNFEEQILEAAK.S	1937.05072	2	4.24E-09	0.96	4.98	-	1282.8
AHQ-2-4, 6254 - 6328	K.EADESLNFEEQILEAAK.S	1937.05072	2	6.38E-06	0.96	5.46	-	1309.1
AHQ-2-9, 6151	K.EADESLNFEEQILEAAK.S	1937.05072	2	5.71E-06	0.72	3.01	-	618.1
AHQ-2-6, 5914	K.EADESLNFEEQILEAAK.S	1937.05072	2	1.64E-06	0.82	3.50	-	853.2
AHQ-2-9, 5999 - 6075	K.EADESLNFEEQILEAAK.S	1937.05072	2	3.28E-05	0.87	3.84	-	818.1
AHQ-2-5, 6201	K.EADESLNFEEQILEAAK.S	1937.05072	2	9.68E-04	0.89	4.15	-	761.7
AHQ-2-5, 6340	K.EADESLNFEEQILEAAK.S	1937.05072	2	3.55E-08	0.95	5.23	-	1056.5
AHQ-2-6, 6029	K.EADESLNFEEQILEAAK.S	1937.05072	2	8.69E-07	0.97	4.75	-	1540.7
AHQ-2-2, 6714 - 6786	K.EADESLNFEEQILEAAK.S	1937.05072	2	2.99E-04	0.28	2.58	-	424.5
AHQ-2-3, 6715	K.EADESLNFEEQILEAAK.S	1937.05072	2	3.29E-04	0.62	3.74	-	361.4
AHQ-2-4, 6788	K.EADESLNFEEQILEAAK.S	1937.05072	2	1.02E-04	0.79	3.55	-	559.6
AHQ-2-11, 5636	K.EADESLNFEEQILEAAK.S	1937.05072	2	8.08E-05	0.91	3.92	-	1258.3
AHQ-2-7, 6009	K.EADESLNFEEQILEAAK.S	1937.05072	2	2.67E-04	0.82	3.91	-	800.3
AHQ-2-11, 5770 - 5789	K.EADESLNFEEQILEAAK.S	1937.05072	2	3.37E-04	0.69	3.03	-	795.1
AHQ-2-2, 6252 - 6325	K.EADESLNFEEQILEAAK.S	1937.05072	2	1.35E-08	0.96	4.90	-	1465.1
AHQ-2-1, 6122 - 6192	K.EADESLNFEEQILEAAK.S	1937.05072	2	7.74E-09	0.96	5.39	-	1393.6
AHQ-2-2, 6344 - 6412	K.EADESLNFEEQILEAAK.S	1937.05072	2	7.91E-09	0.98	5.76	-	1782.2
AHQ-2-2, 6484 - 6486	K.EADESLNFEEQILEAAK.S	1937.05072	2	3.45E-06	0.95	4.99	-	1350.2
AHQ-2-4, 6396 - 6464	K.EADESLNFEEQILEAAK.S	1937.05072	2	5.42E-06	0.97	5.43	-	1710.8
AHQ-2-3, 6208 - 6276	K.EADESLNFEEQILEAAK.S	1937.05072	2	7.28E-07	0.96	5.02	-	1615.1
AHQ-2-4, 6018	K.EADESLNFEEQILEAAK.S	1937.05072	2	4.76E-05	0.91	4.50	-	771.7
AHQ-2-3, 7561 - 7563	R.ECANGYLELLDHVLLTLQKPSPELK.Q	2883.30906	3	7.55E-10	0.96	5.10	-	1654.0
AHQ-2-8, 7427	R.ECANGYLELLDHVLLTLQKPSPELK.Q	2883.30906	3	7.39E-06	0.81	3.24	-	991.5
AHQ-2-2, 7606 - 7608	R.ECANGYLELLDHVLLTLQKPSPELK.Q	2883.30906	3	5.19E-12	0.96	5.43	-	1434.1
AHQ-2-9, 7450	R.ECANGYLELLDHVLLTLQKPSPELK.Q	2883.30906	2	6.90E-09	0.88	3.66	-	790.7
AHQ-2-4, 7620 - 7638	R.ECANGYLELLDHVLLTLQKPSPELK.Q	2883.30906	3	7.25E-11	0.97	5.87	-	1295.2
AHQ-2-6, 7262 - 7263	R.ECANGYLELLDHVLLTLQKPSPELK.Q	2883.30906	3	6.67E-10	0.91	4.66	-	934.4
AHQ-2-5, 7429 - 7509	R.ECANGYLELLDHVLLTLQKPSPELK.Q	2883.30906	2	2.94E-05	0.90	4.23	-	537.0
AHQ-2-5, 7507	R.ECANGYLELLDHVLLTLQKPSPELK.Q	2883.30906	3	2.81E-12	0.96	5.91	-	1252.5
AHQ-2-6, 7298 - 7318	R.EGISQEALHTQMLTAVQEISHLIEPLANAAR.A	3372.79804	3	3.33E-15	0.97	6.23	-	1822.7
AHQ-2-3, 7533 - 7607	R.EGISQEALHTQMLTAVQEISHLIEPLANAAR.A	3372.79804	3	1.11E-09	0.95	5.84	-	860.3
AHQ-2-3, 7627	R.EGISQEALHTQMLTAVQEISHLIEPLANAAR.A	3372.79804	3	6.89E-07	0.96	5.86	-	1193.2
AHQ-2-2, 7604 - 7674	R.EGISQEALHTQMLTAVQEISHLIEPLANAAR.A	3372.79804	3	1.87E-10	0.93	5.05	-	979.4
AHQ-2-1, 7274 - 7341	R.EGISQEALHTQMLTAVQEISHLIEPLANAAR.A	3372.79804	3	7.54E-04	0.73	3.77	-	436.7
AHQ-2-5, 7540 - 7600	R.EGISQEALHTQMLTAVQEISHLIEPLANAAR.A	3372.79804	3	6.45E-07	0.97	5.06	-	2291.9
AHQ-2-7, 7575	R.EGISQEALHTQMLTAVQEISHLIEPLANAAR.A	3372.79804	3	1.92E-08	0.93	5.04	-	1199.3
AHQ-2-9, 7515 - 7518	R.EGISQEALHTQMLTAVQEISHLIEPLANAAR.A	3372.79804	3	4.44E-06	0.95	5.57	-	1295.1
AHQ-2-4, 7618 - 7689	R.EGISQEALHTQMLTAVQEISHLIEPLANAAR.A	3372.79804	3	1.39E-12	0.96	6.21	-	1258.4
AHQ-2-5, 7427	R.EGISQEALHTQM*LTAVQEISHLIEPLANAAR.A	3388.79744	3	8.49E-06	0.91	4.47	-	863.2
AHQ-2-4, 7516	R.EGISQEALHTQM*LTAVQEISHLIEPLANAAR.A	3388.79744	3	3.36E-04	0.88	4.44	-	742.7
AHQ-2-2, 7469 - 7544	R.EGISQEALHTQM*LTAVQEISHLIEPLANAAR.A	3388.79744	3	1.85E-06	0.95	5.38	-	1131.9
AHQ-2-8, 7486	R.EGISQEALHTQMLTAVQEISHLIEPLANAAR.A	3372.79804	3	7.23E-08	0.95	5.19	-	1449.1
AHQ-2-2, 7270 - 7349	R.ELLENPVQPINDM*SYFGCLDSVMENSK.V	3148.48814	3	2.53E-05	0.60	3.51	-	335.6
AHQ-2-4, 7342 - 7409	R.ELLENPVQPINDM*SYFGCLDSVMENSK.V	3148.48814	3	2.52E-05	0.65	4.25	-	330.8
AHQ-2-7, 2704	R.ELMEEKKEEITGTLR.K	1807.05950	2	2.53E-09	0.80	3.71	-	467.0
AHQ-2-7, 2147	R.ELM*EEKKEEITGTLR.K	1823.05890	2	5.73E-04	0.36	2.52	-	266.9
AHQ-2-7, 2613 - 2695	R.EQGVEEHETLLLR.R	1553.69898	2	7.17E-04	0.85	3.75	-	455.8
AHQ-2-7, 2860	R.EQGVEEHETLLLR.R	1553.69898	2	8.57E-09	0.58	3.00	-	257.9
AHQ-2-13-, 5557	R.ERIPEAPAGPPSDFGLFLSDDDPK.K	2571.77979	3	4.03E-12	0.98	6.95	-	1421.2
AHQ-2-7, 5447	R.ERIPEAPAGPPSDFGLFLSDDDPK.K	2571.77979	3	3.81E-09	0.97	6.45	-	1515.4
AHQ-2-13, 5436 - 5461	R.ERIPEAPAGPPSDFGLFLSDDDPK.K	2571.77979	3	9.29E-08	0.97	6.03	-	1684.0
AHQ-2-13-, 5203 - 5205	R.ERIPEAPAGPPSDFGLFLSDDDPKK.G	2699.95270	3	2.33E-11	0.98	6.46	-	1843.3
AHQ-2-13, 5113	R.ERIPEAPAGPPSDFGLFLSDDDPKK.G	2699.95270	3	6.96E-11	0.97	6.23	-	1832.3
AHQ-2-7, 4903 - 4912	R.ERIPEAPAGPPSDFGLFLSDDDPKK.G	2699.95270	3	5.63E-12	0.98	6.71	-	1951.4
AHQ-2-4, 2278	K.EVANSTANLVK.T	1146.27608	2	2.75E-04	0.73	2.50	-	797.5
AHQ-2-2, 2046	K.EVANSTANLVK.T	1146.27608	2	4.47E-04	0.85	2.73	-	1015.9
AHQ-2-4, 2276	K.EVANSTANLVK.T	1146.27608	1	1.95E-04	0.25	1.89	-	279.4
AHQ-2-3, 2048 - 2071	K.EVANSTANLVK.T	1146.27608	2	5.35E-04	0.74	3.09	-	677.4
AHQ-2-8, 4340	K.EVIQEWNLTNIK.R	1487.68214	2	5.54E-05	0.93	3.75	-	1522.4
AHQ-2-7, 4457	K.EVIQEWNLTNIK.R	1487.68214	1	1.79E-05	0.72	3.64	-	260.5
AHQ-2-10, 4292	K.EVIQEWNLTNIK.R	1487.68214	2	1.68E-05	0.76	3.11	-	725.9
AHQ-2-12, 4709	K.EVIQEWNLTNIK.R	1487.68214	2	2.44E-04	0.86	3.48	-	982.4
AHQ-2-11, 4549	K.EVIQEWNLTNIK.R	1487.68214	2	3.70E-07	0.84	3.24	-	873.0
AHQ-2-7, 3748	K.EVIQEWNLTNIKR.W	1643.86849	2	5.29E-06	0.81	3.46	-	696.9
AHQ-2-8, 2735	K.FFYSDQNVDSR.D	1378.42795	2	6.39E-06	0.86	2.94	-	838.7
AHQ-2-7, 2872	K.FFYSDQNVDSR.D	1378.42795	2	3.37E-04	0.71	2.60	-	430.5
AHQ-2-8, 5936	K.FFYSDQNVDSRDPVQLNLLYVQAR.D	2889.17074	3	1.10E-10	0.97	5.91	-	1672.6
AHQ-2-7, 5955 - 6025	K.FFYSDQNVDSRDPVQLNLLYVQAR.D	2889.17074	3	3.82E-11	0.98	6.78	-	2150.9
AHQ-2-2, 7792	R.FGQDFSTFLEAGVEMAGQAPSQEDR.A	2718.89325	3	1.37E-08	0.83	4.40	-	660.3
AHQ-2-3, 6596 - 6665	R.FGQDFSTFLEAGVEM*AGQAPSQEDR.A	2734.89265	2	1.09E-06	0.90	4.14	-	1046.1
AHQ-2-3, 7651 - 7727	R.FGQDFSTFLEAGVEMAGQAPSQEDR.A	2718.89325	2	5.24E-09	0.88	3.16	-	1184.8
AHQ-2-3, 7733	R.FGQDFSTFLEAGVEMAGQAPSQEDR.A	2718.89325	3	2.19E-05	0.78	3.77	-	609.4
AHQ-2-4, 6664 - 6738	R.FGQDFSTFLEAGVEM*AGQAPSQEDR.A	2734.89265	2	1.33E-07	0.86	3.74	-	888.4
AHQ-2-1, 7145	R.FGQDFSTFLEAGVEMAGQAPSQEDR.A	2718.89325	2	2.11E-06	0.75	2.96	-	782.3
AHQ-2-1, 6505	R.FGQDFSTFLEAGVEM*AGQAPSQEDR.A	2734.89265	2	9.09E-07	0.92	4.38	-	952.2
AHQ-2-1, 6497 - 6566	R.FGQDFSTFLEAGVEM*AGQAPSQEDR.A	2734.89265	3	4.97E-08	0.92	5.09	-	684.0
AHQ-2-2, 7482	R.FGQDFSTFLEAGVEMAGQAPSQEDR.A	2718.89325	2	6.51E-04	0.68	2.58	-	1002.1
AHQ-2-2, 7497	R.FGQDFSTFLEAGVEMAGQAPSQEDR.A	2718.89325	3	8.42E-05	0.81	3.40	-	813.3
AHQ-2-1, 2890	K.FLPSELRDEH	1243.35032	2	1.89E-07	0.62	2.50	-	310.2
AHQ-2-5, 4903	K.GLAGAVSELLR.S	1086.26701	2	1.15E-06	0.98	5.11	-	2177.4
AHQ-2-3, 5231	K.GLAGAVSELLR.S	1086.26701	2	3.76E-05	0.97	4.28	-	1864.9
AHQ-2-2, 5265 - 5272	K.GLAGAVSELLR.S	1086.26701	2	1.72E-07	0.97	5.10	-	1793.0
AHQ-2-8, 4599	K.GLAGAVSELLR.S	1086.26701	2	1.77E-06	0.98	5.21	-	2090.9
AHQ-2-4, 5200	K.GLAGAVSELLR.S	1086.26701	2	2.08E-06	0.98	5.05	-	2326.4
AHQ-2-6, 4878	K.GLAGAVSELLR.S	1086.26701	2	8.09E-07	0.98	5.09	-	2219.0
AHQ-2-1, 5353	K.GLAGAVSELLR.S	1086.26701	2	7.23E-05	0.97	4.47	-	1728.0
AHQ-2-9, 4831	K.GLAGAVSELLR.S	1086.26701	2	3.73E-06	0.98	5.04	-	2189.9
AHQ-2-5, 6727 - 6799	R.GSQAQPDSPSAQLALIAASQSFLQPGGK.M	2756.02103	2	1.44E-04	0.96	5.62	-	825.9

AHQ-2-2, 6972 - 7052	R.GSQAQPDSPSAQLALIAASQSFLQPGGK.M	2756.02103	2	1.30E-05	0.92	4.97	-	717.5
AHQ-2-2, 7414	R.GSQAQPDSPSAQLALIAASQSFLQPGGK.M	2756.02103	2	3.55E-04	0.92	4.37	-	730.1
AHQ-2-2, 7412 - 7420	R.GSQAQPDSPSAQLALIAASQSFLQPGGK.M	2756.02103	3	3.12E-07	0.93	4.85	-	1040.4
AHQ-2-6, 6566 - 6633	R.GSQAQPDSPSAQLALIAASQSFLQPGGK.M	2756.02103	2	1.51E-06	0.95	5.50	-	782.8
AHQ-2-4, 6948	R.GSQAQPDSPSAQLALIAASQSFLQPGGK.M	2756.02103	2	1.55E-07	0.94	5.69	-	707.9
AHQ-2-3, 6869 - 6951	R.GSQAQPDSPSAQLALIAASQSFLQPGGK.M	2756.02103	2	3.11E-05	0.94	5.03	-	725.7
AHQ-2-3, 7312 - 7335	R.GSQAQPDSPSAQLALIAASQSFLQPGGK.M	2756.02103	2	7.17E-05	0.88	4.18	-	581.3
AHQ-2-2, 7256 - 7324	R.GSQAQPDSPSAQLALIAASQSFLQPGGK.M	2756.02103	2	1.28E-04	0.72	3.10	-	503.8
AHQ-2-2, 6852 - 6928	R.GSQAQPDSPSAQLALIAASQSFLQPGGK.M	2756.02103	3	1.23E-05	0.92	4.90	-	868.0
AHQ-2-1, 6817 - 6888	R.GSQAQPDSPSAQLALIAASQSFLQPGGK.M	2756.02103	3	5.06E-07	0.95	5.39	-	1052.9
AHQ-2-6, 6506 - 6569	R.GSQAQPDSPSAQLALIAASQSFLQPGGK.M	2756.02103	2	4.86E-08	0.94	5.11	-	866.4
AHQ-2-4, 6941 - 7012	R.GSQAQPDSPSAQLALIAASQSFLQPGGK.M	2756.02103	2	1.77E-07	0.96	5.52	-	1096.5
AHQ-2-8, 6470 - 6479	R.GSQAQPDSPSAQLALIAASQSFLQPGGK.M	2756.02103	2	5.03E-05	0.92	4.47	-	729.6
AHQ-2-2, 6988 - 6996	R.GSQAQPDSPSAQLALIAASQSFLQPGGK.M	2756.02103	3	3.00E-05	0.94	5.39	-	1041.9
AHQ-2-7, 7611	K.GTEWVDPEDPTVIAENELLGAAAAIEAAAK.K	3053.32301	3	3.33E-11	0.97	6.31	-	1852.5
AHQ-2-13-, 7101	K.GTEWVDPEDPTVIAENELLGAAAAIEAAAK.K	3053.32301	3	1.73E-05	0.96	5.25	-	1979.9
AHQ-2-6, 7345 - 7346	K.GTEWVDPEDPTVIAENELLGAAAAIEAAAK.K	3053.32301	3	8.07E-11	0.96	5.96	-	1231.5
AHQ-2-1, 7344	K.GTEWVDPEDPTVIAENELLGAAAAIEAAAK.K	3053.32301	3	1.33E-05	0.92	4.82	-	1061.0
AHQ-2-5, 7603 - 7676	K.GTEWVDPEDPTVIAENELLGAAAAIEAAAK.K	3053.32301	3	1.15E-13	0.97	6.45	-	1851.6
AHQ-2-4, 7560 - 7561	R.GVAALTSDPAVQAIVLDTASDVLDK.A	2470.75835	2	3.12E-08	0.97	6.46	-	969.8
AHQ-2-1, 7228	R.GVAALTSDPAVQAIVLDTASDVLDK.A	2470.75835	3	1.07E-11	0.98	7.22	-	2782.6
AHQ-2-2, 7556 - 7560	R.GVAALTSDPAVQAIVLDTASDVLDK.A	2470.75835	3	7.26E-04	0.98	6.40	-	3438.5
AHQ-2-5, 5515 - 5517	R.GVGAAATAVTQALNELLQHVK.A	2092.38495	2	2.74E-07	0.95	5.16	-	972.1
AHQ-2-9, 7454 - 7456	R.GVGAAATAVTQALNELLQHVK.A	2092.38495	2	6.15E-05	0.95	5.43	-	848.7
AHQ-2-8, 7420	R.GVGAAATAVTQALNELLQHVK.A	2092.38495	3	2.13E-05	0.94	4.97	-	941.9
AHQ-2-8, 7418	R.GVGAAATAVTQALNELLQHVK.A	2092.38495	2	2.62E-06	0.96	5.64	-	913.4
AHQ-2-10, 6779	R.GVGAAATAVTQALNELLQHVK.A	2092.38495	2	8.23E-06	0.92	4.94	-	673.7
AHQ-2-7, 7515	R.GVGAAATAVTQALNELLQHVK.A	2092.38495	3	8.92E-08	0.89	4.62	-	636.8
AHQ-2-14-, 6905	R.GVGAAATAVTQALNELLQHVK.A	2092.38495	2	2.36E-06	0.94	5.41	-	843.6
AHQ-2-5, 7504 - 7505	R.GVGAAATAVTQALNELLQHVK.A	2092.38495	2	3.07E-04	0.96	5.41	-	1184.6
AHQ-2-3, 7568	R.GVGAAATAVTQALNELLQHVK.A	2092.38495	3	1.47E-05	0.89	3.36	-	1267.8
AHQ-2-2, 7613 - 7614	R.GVGAAATAVTQALNELLQHVK.A	2092.38495	3	8.65E-10	0.96	5.58	-	1257.0
AHQ-2-2, 7612	R.GVGAAATAVTQALNELLQHVK.A	2092.38495	2	1.13E-06	0.92	4.82	-	643.6
AHQ-2-7, 7516 - 7517	R.GVGAAATAVTQALNELLQHVK.A	2092.38495	2	2.56E-05	0.93	5.42	-	746.8
AHQ-2-1, 7288	R.GVGAAATAVTQALNELLQHVK.A	2092.38495	3	4.14E-07	0.86	3.92	-	930.5
AHQ-2-1, 7289	R.GVGAAATAVTQALNELLQHVK.A	2092.38495	2	2.67E-08	0.95	5.48	-	1038.3
AHQ-2-6, 7265 - 7266	R.GVGAAATAVTQALNELLQHVK.A	2092.38495	2	5.42E-09	0.96	5.57	-	1053.0
AHQ-2-6, 7261	R.GVGAAATAVTQALNELLQHVK.A	2092.38495	3	3.64E-09	0.89	3.93	-	869.7
AHQ-2-3, 5892	R.GVGAAATAVTQALNELLQHVK.A	2092.38495	2	7.03E-06	0.93	4.82	-	797.7
AHQ-2-3, 7567 - 7569	R.GVGAAATAVTQALNELLQHVK.A	2092.38495	2	8.29E-06	0.93	4.99	-	759.1
AHQ-2-4, 7616 - 7625	R.GVGAAATAVTQALNELLQHVK.A	2092.38495	2	4.44E-07	0.90	4.31	-	676.7
AHQ-2-4, 7624 - 7629	R.GVGAAATAVTQALNELLQHVK.A	2092.38495	3	5.37E-07	0.93	4.40	-	1115.7
AHQ-2-14-, 6904	R.GVGAAATAVTQALNELLQHVK.A	2092.38495	3	2.29E-06	0.95	4.94	-	1225.0
AHQ-2-5, 7503	R.GVGAAATAVTQALNELLQHVK.A	2092.38495	3	1.15E-07	0.92	4.96	-	1147.7
AHQ-2-3, 2292	K.HRVQELGHGCAALVTK.A	1778.02933	2	9.17E-07	0.94	3.99	-	1248.0
AHQ-2-13, 3333	R.IGITNHDEYSLVR.E	1517.66833	2	3.37E-11	0.91	3.64	-	966.1
AHQ-2-13-, 3327	R.IGITNHDEYSLVR.E	1517.66833	3	6.58E-09	0.91	4.33	-	655.8
AHQ-2-13-, 3761 - 3834	R.IGITNHDEYSLVR.E	1517.66833	2	1.29E-06	0.81	3.57	-	527.8
AHQ-2-7, 3252 - 3324	R.IGITNHDEYSLVR.E	1517.66833	2	2.47E-04	0.87	3.21	-	1191.9
AHQ-2-13-, 3310 - 3329	R.IGITNHDEYSLVR.E	1517.66833	2	6.90E-06	0.95	4.06	-	1450.5
AHQ-2-8, 3302	R.IGITNHDEYSLVR.E	1517.66833	2	3.89E-06	0.85	3.07	-	857.1
AHQ-2-7, 2852 - 2923	R.IGITNHDEYSLVR.E	1517.66833	2	2.86E-05	0.93	3.86	-	1190.2
AHQ-2-10, 2999	R.IGITNHDEYSLVR.E	1517.66833	2	9.59E-05	0.91	3.74	-	1064.7
AHQ-2-2, 5216 - 5226	R.ILAQATSDLVNAIK.A	1457.69748	2	1.90E-08	0.98	5.25	-	1990.5
AHQ-2-5, 3965	R.ILAQATSDLVNAIK.A	1457.69748	2	1.38E-09	0.97	4.01	-	2108.9
AHQ-2-2, 5229	R.ILAQATSDLVNAIK.A	1457.69748	1	1.57E-05	0.73	3.33	-	511.6
AHQ-2-5, 4912 - 4983	R.ILAQATSDLVNAIK.A	1457.69748	2	2.11E-09	0.98	5.70	-	2170.3
AHQ-2-5, 4929	R.ILAQATSDLVNAIK.A	1457.69748	1	6.27E-04	0.78	3.56	-	726.3
AHQ-2-2, 4726	R.ILAQATSDLVNAIK.A	1457.69748	2	1.58E-08	0.98	5.59	-	2228.5
AHQ-2-1, 4334 - 4354	R.ILAQATSDLVNAIK.A	1457.69748	2	3.80E-04	0.96	4.17	-	1787.8
AHQ-2-2, 4542 - 4546	R.ILAQATSDLVNAIK.A	1457.69748	2	5.20E-08	0.95	4.04	-	1351.1
AHQ-2-3, 4259	R.ILAQATSDLVNAIK.A	1457.69748	1	6.55E-05	0.15	1.84	-	486.4
AHQ-2-9, 4830	R.ILAQATSDLVNAIK.A	1457.69748	2	4.98E-06	0.90	3.52	-	959.1
AHQ-2-2, 4249 - 4256	R.ILAQATSDLVNAIK.A	1457.69748	2	7.92E-06	0.97	4.37	-	2258.0
AHQ-2-8, 4607	R.ILAQATSDLVNAIK.A	1457.69748	2	4.53E-08	0.97	5.19	-	1651.4
AHQ-2-4, 4206	R.ILAQATSDLVNAIK.A	1457.69748	2	2.82E-08	0.97	4.16	-	1864.6
AHQ-2-14, 6148	R.ILAQATSDLVNAIK.A	1457.69748	2	7.38E-08	0.96	4.49	-	1522.9
AHQ-2-6, 4849	R.ILAQATSDLVNAIK.A	1457.69748	2	4.74E-05	0.96	3.74	-	1821.9
AHQ-2-7, 4795 - 4809	R.ILAQATSDLVNAIK.A	1457.69748	2	5.57E-08	0.98	4.77	-	1986.2
AHQ-2-1, 5250	R.ILAQATSDLVNAIK.A	1457.69748	2	1.71E-07	0.95	4.07	-	1569.0
AHQ-2-6, 3933	R.ILAQATSDLVNAIK.A	1457.69748	2	5.41E-09	0.96	4.00	-	1780.9
AHQ-2-3, 4236 - 4243	R.ILAQATSDLVNAIK.A	1457.69748	2	2.56E-06	0.98	4.61	-	2288.6
AHQ-2-4, 4498	R.ILAQATSDLVNAIK.A	1457.69748	2	1.70E-04	0.92	3.30	-	1365.1
AHQ-2-3, 5175 - 5179	R.ILAQATSDLVNAIK.A	1457.69748	2	7.50E-07	0.97	5.39	-	1661.5
AHQ-2-3, 5197	R.ILAQATSDLVNAIK.A	1457.69748	1	6.65E-06	0.78	3.97	-	440.9
AHQ-2-1, 6264	R.IPEAPAGPPSDFGLFLSDDDPK.K	2286.47890	2	3.54E-04	0.87	3.73	-	705.7
AHQ-2-13, 5812 - 5871	R.IPEAPAGPPSDFGLFLSDDDPK.K	2286.47890	2	1.93E-06	0.96	5.01	-	1353.2
AHQ-2-8, 5900 - 5924	R.IPEAPAGPPSDFGLFLSDDDPK.K	2286.47890	2	7.96E-04	0.89	4.09	-	667.8
AHQ-2-7, 5951 - 5964	R.IPEAPAGPPSDFGLFLSDDDPK.K	2286.47890	2	6.22E-05	0.92	4.43	-	694.3
AHQ-2-14-, 5795 - 5865	R.IPEAPAGPPSDFGLFLSDDDPK.K	2286.47890	2	5.66E-07	0.96	5.52	-	864.1
AHQ-2-13-, 5403 - 5439	R.IPEAPAGPPSDFGLFLSDDDPKK.G	2414.65181	3	4.28E-13	0.94	4.36	-	1580.3
AHQ-2-8, 4997 - 5069	R.IPEAPAGPPSDFGLFLSDDDPKK.G	2414.65181	3	2.16E-04	0.94	4.06	-	1655.3
AHQ-2-13, 5379 - 5457	R.IPEAPAGPPSDFGLFLSDDDPKK.G	2414.65181	3	6.43E-04	0.77	3.35	-	1092.3
AHQ-2-7, 5264 - 5343	R.IPEAPAGPPSDFGLFLSDDDPKK.G	2414.65181	3	1.77E-12	0.96	5.36	-	1177.7
AHQ-2-14-, 5397 - 5417	R.IPEAPAGPPSDFGLFLSDDDPKK.G	2414.65181	3	3.94E-14	0.96	5.11	-	1529.4
AHQ-2-13, 2881	K.ISIGNVVK.T	830.00746	2	7.06E-05	0.74	2.69	-	425.4
AHQ-2-7, 1991	K.KGIWLEAGK.A	1002.19171	2	8.08E-05	0.90	3.70	-	912.1
AHQ-2-1, 2650 - 2653	K.LAQAAQSSVATITR.L	1417.59344	2	1.67E-05	0.94	3.70	-	1338.9
AHQ-2-9, 2503	K.LAQAAQSSVATITR.L	1417.59344	2	2.87E-08	0.93	3.62	-	1394.4
AHQ-2-4, 2614	K.LAQAAQSSVATITR.L	1417.59344	1	5.45E-04	0.14	2.69	-	97.2
AHQ-2-6, 2313	K.LAQAAQSSVATITR.L	1417.59344	2	2.68E-11	0.96	4.71	-	1278.3
AHQ-2-11, 2601	K.LAQAAQSSVATITR.L	1417.59344	2	5.01E-08	0.94	3.59	-	1565.5
AHQ-2-4, 2466	K.LAQAAQSSVATITR.L	1417.59344	2	1.46E-10	0.97	4.72	-	1761.5
AHQ-2-2, 2620 - 2688	K.LAQAAQSSVATITR.L	1417.59344	2	1.23E-09	0.97	5.32	-	1615.3
AHQ-2-2, 2485	K.LAQAAQSSVATITR.L	1417.59344	2	1.89E-07	0.96	4.60	-	1405.8
AHQ-2-7, 2403 - 2408	K.LAQAAQSSVATITR.L	1417.59344	2	7.96E-08	0.97	4.99	-	1829.0
AHQ-2-6, 2462 - 2465	K.LAQAAQSSVATITR.L	1417.59344	2	2.29E-07	0.97	5.18	-	1784.0
AHQ-2-14-, 2651	K.LAQAAQSSVATITR.L	1417.59344	2	2.21E-09	0.91	3.27	-	1411.2
AHQ-2-6, 2489	K.LAQAAQSSVATITR.L	1417.59344	1	2.39E-05	0.26	2.47	-	119.7
AHQ-2-1, 2796	K.LAQAAQSSVATITR.L	1417.59344	2	7.79E-09	0.97	4.54	-	1710.6
AHQ-2-3, 2456 - 2457	K.LAQAAQSSVATITR.L	1417.59344	2	1.26E-07	0.93	3.77	-	1304.0
AHQ-2-10, 2454	K.LAQAAQSSVATITR.L	1417.59344	2	1.59E-06	0.94	3.51	-	1455.3
AHQ-2-5, 2433 - 2443	K.LAQAAQSSVATITR.L	1417.59344	2	2.54E-09	0.97	4.70	-	1717.5
AHQ-2-4, 2604 - 2610	K.LAQAAQSSVATITR.L	1417.59344	2	9.98E-10	0.97	4.65	-	1656.1
AHQ-2-4, 3128 - 3164	R.LASEAKPAAVAAENEEIGSHIK.H	2236.46779	3	1.33E-09	0.95	4.99	-	1538.1
AHQ-2-1, 3310 - 3357	R.LASEAKPAAVAAENEEIGSHIK.H	2236.46779	3	2.10E-05	0.97	5.78	-	1916.9
AHQ-2-3, 3124	R.LASEAKPAAVAAENEEIGSHIK.H	2236.46779	3	1.87E-07	0.96	5.88	-	1468.3

AHQ-2-3, 3120	R.LASEAKPAAVAAENEEIGSHIK.H	2236.46779	2	2.06E-05	0.89	4.20	-	661.1
AHQ-2-6, 2855 - 2869	R.LASEAKPAAVAAENEEIGSHIK.H	2236.46779	3	3.96E-05	0.96	5.31	-	1615.9
AHQ-2-3, 2937	R.LASEAKPAAVAAENEEIGSHIK.H	2236.46779	2	1.98E-08	0.96	5.37	-	1111.2
AHQ-2-2, 2934	R.LASEAKPAAVAAENEEIGSHIK.H	2236.46779	2	1.35E-05	0.96	5.01	-	1131.9
AHQ-2-9, 2851	R.LASEAKPAAVAAENEEIGSHIK.H	2236.46779	2	1.27E-04	0.86	4.04	-	426.3
AHQ-2-9, 2846	R.LASEAKPAAVAAENEEIGSHIK.H	2236.46779	3	5.68E-06	0.85	3.89	-	911.5
AHQ-2-2, 3128	R.LASEAKPAAVAAENEEIGSHIK.H	2236.46779	2	6.84E-07	0.95	5.19	-	711.2
AHQ-2-6, 2879	R.LASEAKPAAVAAENEEIGSHIK.H	2236.46779	2	4.44E-06	0.92	4.76	-	553.9
AHQ-2-5, 2839	R.LASEAKPAAVAAENEEIGSHIK.H	2236.46779	2	1.14E-07	0.91	4.82	-	508.5
AHQ-2-4, 3133	R.LASEAKPAAVAAENEEIGSHIK.H	2236.46779	2	2.18E-06	0.95	4.98	-	729.6
AHQ-2-13, 5124	K.LGAASLGAEDPETQVVLINAVK.D	2196.48671	2	7.80E-05	0.85	3.71	-	793.9
AHQ-2-7, 5117 - 5125	K.LGAASLGAEDPETQVVLINAVK.D	2196.48671	2	1.62E-10	0.94	4.58	-	758.6
AHQ-2-7, 5268 - 5272	K.LGAASLGAEDPETQVVLINAVK.D	2196.48671	2	4.65E-06	0.97	5.58	-	1251.4
AHQ-2-2, 5790 - 5865	K.LGAASLGAEDPETQVVLINAVK.D	2196.48671	2	4.85E-07	0.96	5.06	-	1054.8
AHQ-2-2, 5638 - 5641	K.LGAASLGAEDPETQVVLINAVK.D	2196.48671	3	1.09E-11	0.98	6.84	-	2644.4
AHQ-2-9, 5302	K.LGAASLGAEDPETQVVLINAVK.D	2196.48671	2	2.88E-05	0.64	3.21	-	464.5
AHQ-2-3, 5564 - 5643	K.LGAASLGAEDPETQVVLINAVK.D	2196.48671	2	2.53E-07	0.97	5.22	-	1343.3
AHQ-2-5, 5383 - 5453	K.LGAASLGAEDPETQVVLINAVK.D	2196.48671	2	2.37E-04	0.96	5.23	-	1161.8
AHQ-2-2, 5536 - 5610	K.LGAASLGAEDPETQVVLINAVK.D	2196.48671	2	2.20E-08	0.97	5.23	-	1277.3
AHQ-2-4, 5598 - 5676	K.LGAASLGAEDPETQVVLINAVK.D	2196.48671	2	1.31E-06	0.97	5.56	-	1402.1
AHQ-2-4, 5640 - 5662	K.LGAASLGAEDPETQVVLINAVK.D	2196.48671	3	1.14E-04	0.96	4.45	-	2318.4
AHQ-2-2, 5437 - 5465	K.LGAASLGAEDPETQVVLINAVK.D	2196.48671	2	4.69E-08	0.91	4.13	-	736.4
AHQ-2-1, 5857	K.LGAASLGAEDPETQVVLINAVK.D	2196.48671	2	7.47E-04	0.90	4.32	-	628.9
AHQ-2-5, 5225 - 5251	K.LGAASLGAEDPETQVVLINAVK.D	2196.48671	2	5.65E-06	0.97	5.17	-	1443.8
AHQ-2-4, 5748 - 5817	K.LGAASLGAEDPETQVVLINAVK.D	2196.48671	2	9.23E-08	0.97	5.61	-	1443.6
AHQ-2-3, 5615 - 5620	K.LGAASLGAEDPETQVVLINAVK.D	2196.48671	3	1.22E-05	0.89	3.60	-	1376.2
AHQ-2-1, 5700	K.LGAASLGAEDPETQVVLINAVK.D	2196.48671	3	8.75E-06	0.95	4.63	-	1743.3
AHQ-2-3, 5417 - 5487	K.LGAASLGAEDPETQVVLINAVK.D	2196.48671	2	1.12E-06	0.92	4.51	-	742.1
AHQ-2-1, 5541	K.LGAASLGAEDPETQVVLINAVK.D	2196.48671	2	1.03E-06	0.94	4.33	-	1015.3
AHQ-2-14-, 5116	K.LGAASLGAEDPETQVVLINAVK.D	2196.48671	2	8.45E-08	0.93	4.49	-	930.8
AHQ-2-3, 5841 - 5912	K.LGAASLGAEDPETQVVLINAVK.D	2196.48671	2	1.77E-08	0.92	4.27	-	805.6
AHQ-2-14-, 5296	K.LGAASLGAEDPETQVVLINAVK.D	2196.48671	2	2.47E-04	0.95	4.72	-	999.6
AHQ-2-1, 5688 - 5693	K.LGAASLGAEDPETQVVLINAVK.D	2196.48671	2	5.22E-06	0.97	5.32	-	1350.3
AHQ-2-6, 5303 - 5305	K.LGAASLGAEDPETQVVLINAVK.D	2196.48671	2	3.74E-08	0.95	5.24	-	913.1
AHQ-2-6, 5313 - 5325	K.LGAASLGAEDPETQVVLINAVK.D	2196.48671	3	6.26E-06	0.90	4.42	-	1411.4
AHQ-2-6, 5482	K.LGAASLGAEDPETQVVLINAVK.D	2196.48671	2	8.38E-06	0.91	4.44	-	763.2
AHQ-2-4, 5405 - 5457	K.LGAASLGAEDPETQVVLINAVK.D	2196.48671	2	4.88E-10	0.97	5.22	-	1607.8
AHQ-2-1, 4030	K.LHTDDELNWLDHGR.T	1721.81153	3	9.26E-06	0.85	3.72	-	688.7
AHQ-2-8, 3063	K.LHTDDELNWLDHGR.T	1721.81153	3	2.24E-06	0.92	4.23	-	1171.8
AHQ-2-9, 3354	K.LHTDDELNWLDHGR.T	1721.81153	2	8.09E-06	0.95	4.08	-	1428.8
AHQ-2-7, 3275 - 3343	K.LHTDDELNWLDHGR.T	1721.81153	2	3.19E-06	0.97	4.72	-	1649.3
AHQ-2-8, 3062	K.LHTDDELNWLDHGR.T	1721.81153	2	5.36E-06	0.96	4.65	-	1428.0
AHQ-2-7, 3327	K.LHTDDELNWLDHGR.T	1721.81153	3	3.65E-04	0.86	3.25	-	1196.4
AHQ-2-3, 4371 - 4439	K.LLAALLEDEGGSGR.P	1401.54764	2	1.32E-04	0.74	3.49	-	418.6
AHQ-2-1, 4530	K.LLAALLEDEGGSGR.P	1401.54764	2	2.06E-11	0.97	4.56	-	1584.3
AHQ-2-1, 5522	K.LLAALLEDEGGSGRPLLQAAK.G	2123.43896	2	6.96E-09	0.98	6.31	-	1430.4
AHQ-2-1, 5521 - 5528	K.LLAALLEDEGGSGRPLLQAAK.G	2123.43896	3	5.88E-09	0.93	4.53	-	1302.0
AHQ-2-3, 5256	K.LLAALLEDEGGSGRPLLQAAK.G	2123.43896	2	3.17E-08	0.92	4.52	-	803.6
AHQ-2-2, 5388 - 5413	K.LLAALLEDEGGSGRPLLQAAK.G	2123.43896	3	4.22E-12	0.97	6.02	-	1660.5
AHQ-2-8, 4609	K.LLAALLEDEGGSGRPLLQAAK.G	2123.43896	3	1.40E-09	0.96	5.27	-	1671.1
AHQ-2-12, 5209 - 5214	K.LLAALLEDEGGSGRPLLQAAK.G	2123.43896	3	4.48E-05	0.95	4.73	-	1509.2
AHQ-2-3, 5361	K.LLAALLEDEGGSGRPLLQAAK.G	2123.43896	2	1.10E-09	0.98	6.46	-	1785.2
AHQ-2-3, 5363	K.LLAALLEDEGGSGRPLLQAAK.G	2123.43896	3	1.34E-07	0.96	4.91	-	1666.8
AHQ-2-2, 5389	K.LLAALLEDEGGSGRPLLQAAK.G	2123.43896	2	9.23E-11	0.97	6.04	-	1553.7
AHQ-2-10, 4710	K.LLAALLEDEGGSGRPLLQAAK.G	2123.43896	3	3.72E-05	0.92	4.86	-	868.2
AHQ-2-8, 4608 - 4623	K.LLAALLEDEGGSGRPLLQAAK.G	2123.43896	2	2.88E-10	0.98	6.85	-	1368.3
AHQ-2-6, 5001	K.LLAALLEDEGGSGRPLLQAAK.G	2123.43896	2	6.70E-05	0.90	4.21	-	754.0
AHQ-2-9, 4982 - 4983	K.LLAALLEDEGGSGRPLLQAAK.G	2123.43896	2	7.98E-09	0.98	6.65	-	1545.2
AHQ-2-9, 4990	K.LLAALLEDEGGSGRPLLQAAK.G	2123.43896	3	6.44E-08	0.95	5.24	-	1163.3
AHQ-2-5, 5033 - 5103	K.LLAALLEDEGGSGRPLLQAAK.G	2123.43896	2	7.72E-09	0.98	6.55	-	1411.4
AHQ-2-5, 5027 - 5039	K.LLAALLEDEGGSGRPLLQAAK.G	2123.43896	3	8.08E-09	0.95	5.06	-	1319.7
AHQ-2-10, 4715	K.LLAALLEDEGGSGRPLLQAAK.G	2123.43896	2	3.11E-07	0.96	5.53	-	1129.4
AHQ-2-7, 4875 - 4885	K.LLAALLEDEGGSGRPLLQAAK.G	2123.43896	2	9.57E-10	0.98	6.21	-	1563.0
AHQ-2-4, 5236	K.LLAALLEDEGGSGRPLLQAAK.G	2123.43896	2	3.93E-05	0.87	4.29	-	410.9
AHQ-2-4, 5372 - 5376	K.LLAALLEDEGGSGRPLLQAAK.G	2123.43896	2	4.78E-09	0.98	7.40	-	1348.3
AHQ-2-4, 5360 - 5362	K.LLAALLEDEGGSGRPLLQAAK.G	2123.43896	3	1.00E-10	0.96	5.23	-	1500.2
AHQ-2-3, 5375	K.LLAALLEDEGGSGRPLLQAAKGLAGAVSELLR.S	3190.68337	3	1.59E-04	0.60	3.57	-	615.0
AHQ-2-2, 5402	K.LLAALLEDEGGSGRPLLQAAKGLAGAVSELLR.S	3190.68337	3	5.30E-04	0.82	4.54	-	476.0
AHQ-2-5, 5411	R.LNEAAAGLNQAATELVQASR.G	2028.21299	2	5.69E-11	0.98	5.78	-	1864.5
AHQ-2-2, 4558 - 4589	R.LNEAAAGLNQAATELVQASR.G	2028.21299	2	2.20E-10	0.92	4.99	-	649.3
AHQ-2-3, 6079 - 6084	R.LNEAAAGLNQAATELVQASR.G	2028.21299	2	7.16E-12	0.98	5.69	-	2180.9
AHQ-2-4, 5633 - 5704	R.LNEAAAGLNQAATELVQASR.G	2028.21299	2	2.22E-09	0.98	5.92	-	2035.9
AHQ-2-5, 5419 - 5424	R.LNEAAAGLNQAATELVQASR.G	2028.21299	3	6.15E-05	0.98	5.88	-	2540.8
AHQ-2-5, 5888	R.LNEAAAGLNQAATELVQASR.G	2028.21299	2	3.78E-06	0.93	4.04	-	984.0
AHQ-2-11, 5242	R.LNEAAAGLNQAATELVQASR.G	2028.21299	2	4.13E-06	0.92	4.33	-	790.6
AHQ-2-4, 5710 - 5733	R.LNEAAAGLNQAATELVQASR.G	2028.21299	3	1.32E-06	0.98	5.74	-	3004.1
AHQ-2-4, 4529 - 4570	R.LNEAAAGLNQAATELVQASR.G	2028.21299	2	2.86E-13	0.97	5.41	-	1249.8
AHQ-2-3, 5460	R.LNEAAAGLNQAATELVQASR.G	2028.21299	2	7.21E-06	0.78	3.07	-	743.5
AHQ-2-1, 5369	R.LNEAAAGLNQAATELVQASR.G	2028.21299	2	4.87E-09	0.96	4.39	-	1678.7
AHQ-2-3, 6401	R.LNEAAAGLNQAATELVQASR.G	2028.21299	2	2.50E-09	0.91	4.13	-	740.6
AHQ-2-8, 5111	R.LNEAAAGLNQAATELVQASR.G	2028.21299	2	2.46E-07	0.96	4.59	-	1374.9
AHQ-2-3, 5827	R.LNEAAAGLNQAATELVQASR.G	2028.21299	2	8.17E-08	0.98	6.04	-	1795.6
AHQ-2-9, 5374	R.LNEAAAGLNQAATELVQASR.G	2028.21299	2	8.95E-13	0.98	6.15	-	1765.1
AHQ-2-6, 5358 - 5373	R.LNEAAAGLNQAATELVQASR.G	2028.21299	2	2.37E-11	0.98	5.75	-	1801.1
AHQ-2-1, 5592	R.LNEAAAGLNQAATELVQASR.G	2028.21299	2	2.66E-06	0.67	2.79	-	668.8
AHQ-2-5, 4347	R.LNEAAAGLNQAATELVQASR.G	2028.21299	2	5.64E-08	0.96	5.26	-	1081.1
AHQ-2-3, 5739 - 5740	R.LNEAAAGLNQAATELVQASR.G	2028.21299	2	1.42E-11	0.98	5.59	-	1816.1
AHQ-2-4, 6093 - 6098	R.LNEAAAGLNQAATELVQASR.G	2028.21299	2	3.26E-10	0.97	5.00	-	1632.2
AHQ-2-6, 5775	R.LNEAAAGLNQAATELVQASR.G	2028.21299	2	8.42E-09	0.95	4.96	-	994.2
AHQ-2-3, 5676 - 5696	R.LNEAAAGLNQAATELVQASR.G	2028.21299	3	1.14E-04	0.98	5.59	-	2692.2
AHQ-2-1, 5694	R.LNEAAAGLNQAATELVQASR.G	2028.21299	2	1.90E-04	0.94	4.31	-	900.0
AHQ-2-1, 5804 - 5872	R.LNEAAAGLNQAATELVQASR.G	2028.21299	2	8.91E-09	0.96	5.02	-	1352.1
AHQ-2-1, 5810 - 5824	R.LNEAAAGLNQAATELVQASR.G	2028.21299	3	6.21E-09	0.98	6.05	-	2302.0
AHQ-2-5, 5785	R.LNEAAAGLNQAATELVQASR.G	2028.21299	2	5.05E-13	0.98	5.50	-	1892.6
AHQ-2-3, 5604 - 5675	R.LNEAAAGLNQAATELVQASR.G	2028.21299	2	1.67E-11	0.98	5.97	-	1634.0
AHQ-2-1, 6009	R.LNEAAAGLNQAATELVQASR.G	2028.21299	2	7.21E-06	0.96	4.70	-	1331.7
AHQ-2-2, 5514	R.LNEAAAGLNQAATELVQASR.G	2028.21299	2	9.85E-08	0.94	4.37	-	1106.4
AHQ-2-1, 6129 - 6137	R.LNEAAAGLNQAATELVQASR.G	2028.21299	2	4.61E-14	0.98	6.08	-	2000.8
AHQ-2-10, 5011 - 5012	R.LNEAAAGLNQAATELVQASR.G	2028.21299	2	9.65E-09	0.96	4.16	-	1556.8
AHQ-2-7, 5700	R.LNEAAAGLNQAATELVQASR.G	2028.21299	2	4.86E-04	0.88	4.23	-	551.7
AHQ-2-2, 5657 - 5701	R.LNEAAAGLNQAATELVQASR.G	2028.21299	2	1.62E-09	0.97	5.82	-	1374.8
AHQ-2-2, 6129	R.LNEAAAGLNQAATELVQASR.G	2028.21299	3	4.87E-09	0.95	4.66	-	1820.0
AHQ-2-2, 5713 - 5729	R.LNEAAAGLNQAATELVQASR.G	2028.21299	3	1.36E-09	0.98	6.53	-	2105.4
AHQ-2-2, 6120	R.LNEAAAGLNQAATELVQASR.G	2028.21299	2	6.60E-09	0.97	5.10	-	1777.1
AHQ-2-2, 5769 - 5845	R.LNEAAAGLNQAATELVQASR.G	2028.21299	2	2.50E-08	0.97	5.73	-	1338.0
AHQ-2-7, 5313	R.LNEAAAGLNQAATELVQASR.G	2028.21299	2	5.97E-11	0.97	5.36	-	1474.9
AHQ-2-2, 5916 - 5986	R.LNEAAAGLNQAATELVQASR.G	2028.21299	2	2.77E-08	0.97	5.32	-	1409.9

AHQ-2-1, 4590 - 4592	R.LNEAAAGLNQAATELVQASR.G	2028.21299	2	8.95E-10	0.95	5.03	-	846.9
AHQ-2-3, 5928 - 5940	R.LNEAAAGLNQAATELVQASR.G	2028.21299	2	4.42E-09	0.98	5.99	-	1402.3
AHQ-2-3, 5456	R.LNEAAAGLNQAATELVQASRGTPQDLAR.A	2867.12501	3	3.07E-06	0.69	3.47	-	747.8
AHQ-2-4, 2040	R.MATNAAAQNAIK.K	1204.38227	2	4.40E-06	0.79	3.11	-	889.4
AHQ-2-2, 2017	R.MATNAAAQNAIK.K	1204.38227	2	4.36E-06	0.87	3.57	-	837.4
AHQ-2-2, 2102	R.MATNAAAQNAIK.K	1204.38227	2	2.27E-10	0.90	3.77	-	969.3
AHQ-2-2, 4297	R.M*VAAATNNLCEAANAAVQGHASQEK.L	2574.79038	3	2.01E-05	0.93	5.11	-	762.7
AHQ-2-2, 4182	R.M*VAAATNNLCEAANAAVQGHASQEK.L	2574.79038	3	7.20E-09	0.91	4.63	-	926.4
AHQ-2-4, 4100	R.M*VAAATNNLCEAANAAVQGHASQEK.L	2574.79038	3	2.20E-09	0.84	3.89	-	713.6
AHQ-2-2, 4477	R.MVAAATNNLCEAANAAVQGHASQEK.L	2558.79098	2	1.34E-07	0.86	3.41	-	811.1
AHQ-2-2, 4488 - 4562	R.MVAAATNNLCEAANAAVQGHASQEK.L	2558.79098	3	7.03E-10	0.96	5.60	-	1528.3
AHQ-2-1, 4257	R.M*VAAATNNLCEAANAAVQGHASQEK.L	2574.79038	3	1.21E-12	0.91	4.17	-	1045.7
AHQ-2-4, 4305 - 4376	R.MVAAATNNLCEAANAAVQGHASQEK.L	2558.79098	3	4.16E-08	0.97	6.00	-	1641.4
AHQ-2-6, 4050 - 4051	R.MVAAATNNLCEAANAAVQGHASQEK.L	2558.79098	3	8.61E-09	0.90	4.38	-	871.0
AHQ-2-7, 3967 - 3987	R.MVAAATNNLCEAANAAVQGHASQEK.L	2558.79098	3	5.90E-06	0.91	4.48	-	908.5
AHQ-2-7, 4133	R.MVAAATNNLCEAANAAVQGHASQEK.L	2558.79098	3	4.32E-06	0.77	3.47	-	520.2
AHQ-2-5, 4064 - 4075	R.MVAAATNNLCEAANAAVQGHASQEK.L	2558.79098	3	6.06E-05	0.78	3.64	-	622.5
AHQ-2-3, 4152 - 4153	R.M*VAAATNNLCEAANAAVQGHASQEK.L	2574.79038	3	7.24E-09	0.96	5.56	-	1308.2
AHQ-2-3, 4339	R.MVAAATNNLCEAANAAVQGHASQEK.L	2558.79098	3	5.64E-08	0.85	3.72	-	884.3
AHQ-2-10, 3695	R.M*VAAATNNLCEAANAAVQGHASQEK.L	2574.79038	3	1.09E-09	0.93	4.36	-	1106.9
AHQ-2-1, 4385 - 4400	R.M*VAAATNNLCEAANAAVQGHASQEK.L	2574.79038	3	2.00E-05	0.63	3.17	-	566.5
AHQ-2-3, 4368 - 4441	R.MVAAATNNLCEAANAAVQGHASQEK.L	2558.79098	2	6.52E-06	0.58	2.77	-	579.8
AHQ-2-1, 4537 - 4540	R.MVAAATNNLCEAANAAVQGHASQEK.L	2558.79098	3	3.70E-06	0.94	4.69	-	1085.9
AHQ-2-10, 3854 - 3914	R.MVAAATNNLCEAANAAVQGHASQEK.L	2558.79098	3	9.44E-07	0.94	4.66	-	1276.9
AHQ-2-3, 4424 - 4425	R.MVAAATNNLCEAANAAVQGHASQEK.L	2558.79098	3	1.88E-05	0.72	3.38	-	667.1
AHQ-2-10, 3991	R.MVAAATNNLCEAANAAVQGHASQEK.L	2558.79098	3	1.32E-09	0.71	3.58	-	607.3
AHQ-2-8, 3848	R.MVAAATNNLCEAANAAVQGHASQEK.L	2558.79098	2	7.23E-08	0.90	4.05	-	646.7
AHQ-2-8, 3840	R.MVAAATNNLCEAANAAVQGHASQEK.L	2558.79098	3	1.35E-07	0.82	3.61	-	905.4
AHQ-2-4, 7236 - 7274	K.MVGGIAQIIAAQEEMLR.K	1831.19407	2	1.40E-06	0.96	4.43	-	1525.5
AHQ-2-5, 7019	K.MVGGIAQIIAAQEEMLR.K	1831.19407	2	2.15E-08	0.98	5.09	-	2197.6
AHQ-2-14-, 6567 - 6579	K.MVGGIAQIIAAQEEMLR.K	1831.19407	2	1.57E-04	0.71	3.43	-	533.3
AHQ-2-2, 6501	K.MVGGIAQIIAAQEEM*LR.K	1847.19347	2	6.77E-04	0.91	3.53	-	1115.8
AHQ-2-4, 6529	K.MVGGIAQIIAAQEEM*LR.K	1847.19347	2	1.19E-06	0.95	4.50	-	1365.9
AHQ-2-6, 6849 - 6854	K.MVGGIAQIIAAQEEMLR.K	1831.19407	2	1.02E-13	0.98	6.09	-	1893.6
AHQ-2-10, 6370	K.MVGGIAQIIAAQEEMLR.K	1831.19407	2	6.68E-09	0.97	5.46	-	1631.1
AHQ-2-10, 5783	K.MVGGIAQIIAAQEEM*LR.K	1847.19347	2	2.07E-04	0.83	3.22	-	941.6
AHQ-2-9, 6914 - 6978	K.MVGGIAQIIAAQEEMLR.K	1831.19407	2	2.31E-06	0.96	4.73	-	1317.7
AHQ-2-7, 6893	K.MVGGIAQIIAAQEEMLR.K	1831.19407	2	2.38E-06	0.95	4.65	-	1415.9
AHQ-2-4, 6229	K.M*VGGIAQIIAAQEEM*LR.K	1863.19287	2	1.58E-04	0.67	2.59	-	772.8
AHQ-2-2, 6237 - 6238	K.M*VGGIAQIIAAQEEM*LR.K	1863.19287	2	8.36E-11	0.98	6.06	-	1643.6
AHQ-2-3, 6228	K.M*VGGIAQIIAAQEEM*LR.K	1863.19287	2	1.38E-07	0.97	5.23	-	1400.7
AHQ-2-13, 6359	K.M*VGGIAQIIAAQEEMLR.K	1847.19347	2	3.59E-05	0.96	4.78	-	1392.9
AHQ-2-3, 7181 - 7192	K.MVGGIAQIIAAQEEMLR.K	1831.19407	2	2.44E-09	0.97	5.71	-	1492.0
AHQ-2-1, 6816 - 6837	K.M*VGGIAQIIAAQEEMLR.K	1847.19347	2	4.17E-11	0.94	4.60	-	1022.9
AHQ-2-14-, 6383 - 6428	K.M*VGGIAQIIAAQEEMLR.K	1847.19347	2	1.79E-05	0.97	5.09	-	1878.3
AHQ-2-3, 6547	K.MVGGIAQIIAAQEEM*LR.K	1847.19347	2	1.98E-09	0.97	4.99	-	1598.9
AHQ-2-2, 7248 - 7312	K.MVGGIAQIIAAQEEMLR.K	1831.19407	2	2.27E-06	0.97	5.64	-	1814.8
AHQ-2-4, 6940 - 6950	K.M*VGGIAQIIAAQEEMLR.K	1847.19347	2	2.50E-04	0.98	6.05	-	2100.4
AHQ-2-4, 6638	K.MVGGIAQIIAAQEEMLRK.E	1959.36698	2	1.23E-04	0.93	4.61	-	753.0
AHQ-2-7, 2095	K.NCGQMSEIEAK.V	1268.40005	2	8.94E-05	0.84	3.15	-	1028.7
AHQ-2-3, 4521 - 4591	K.NGNLPEFGDAISTASK.A	1621.73018	2	1.85E-04	0.70	2.88	-	527.9
AHQ-2-3, 4793	K.NGNLPEFGDAISTASK.A	1621.73018	2	1.40E-08	0.91	3.91	-	636.5
AHQ-2-2, 4593	K.NGNLPEFGDAISTASK.A	1621.73018	2	2.06E-04	0.74	2.93	-	407.1
AHQ-2-5, 4669 - 4745	K.NGNLPEFGDAISTASK.A	1621.73018	2	5.31E-10	0.85	3.66	-	472.3
AHQ-2-4, 4788 - 4856	K.NGNLPEFGDAISTASK.A	1621.73018	2	5.78E-06	0.70	3.57	-	290.6
AHQ-2-1, 4576	K.NGNLPEFGDAISTASK.A	1621.73018	2	6.78E-05	0.50	2.85	-	286.3
AHQ-2-6, 4558 - 4578	K.NGNLPEFGDAISTASK.A	1621.73018	2	2.98E-07	0.82	3.39	-	489.2
AHQ-2-1, 4344	K.NLGTALAELR.T	1058.21367	2	3.18E-04	0.86	3.09	-	948.4
AHQ-2-5, 3764	K.NLGTALAELR.T	1058.21367	2	2.87E-04	0.82	2.81	-	828.7
AHQ-2-4, 4093 - 4096	K.NLGTALAELR.T	1058.21367	2	3.01E-06	0.83	3.41	-	828.8
AHQ-2-1, 2833	K.PAAVAAENEEIGSHIK.H	1636.78798	3	3.06E-08	0.95	4.79	-	1481.6
AHQ-2-1, 2829	K.PAAVAAENEEIGSHIK.H	1636.78798	2	9.55E-06	0.97	4.41	-	1848.3
AHQ-2-4, 2584	K.PAAVAAENEEIGSHIK.H	1636.78798	2	1.48E-06	0.98	5.88	-	2076.7
AHQ-2-2, 2592	K.PAAVAAENEEIGSHIK.H	1636.78798	2	1.73E-10	0.98	5.75	-	2273.0
AHQ-2-4, 2588	K.PAAVAAENEEIGSHIK.H	1636.78798	3	2.12E-10	0.97	5.48	-	1829.0
AHQ-2-3, 2599	K.PAAVAAENEEIGSHIK.H	1636.78798	3	7.35E-11	0.98	5.42	-	2300.9
AHQ-2-6, 2407	K.PAAVAAENEEIGSHIK.H	1636.78798	2	4.16E-09	0.98	5.44	-	2304.0
AHQ-2-2, 2593	K.PAAVAAENEEIGSHIK.H	1636.78798	3	1.43E-11	0.97	5.27	-	1703.6
AHQ-2-5, 2385 - 2391	K.PAAVAAENEEIGSHIK.H	1636.78798	2	8.17E-05	0.98	5.55	-	1675.8
AHQ-2-6, 2405	K.PAAVAAENEEIGSHIK.H	1636.78798	3	1.60E-07	0.95	4.50	-	1812.9
AHQ-2-3, 2595	K.PAAVAAENEEIGSHIK.H	1636.78798	2	2.39E-07	0.98	5.69	-	1920.6
AHQ-2-6, 2911	K.QAAASATQTIAAAQHAASTPK.A	1996.17099	2	1.56E-05	0.92	4.54	-	719.8
AHQ-2-2, 3413	K.QAAASATQTIAAAQHAASTPK.A	1996.17099	2	1.10E-07	0.85	4.07	-	538.6
AHQ-2-2, 3189	K.QAAASATQTIAAAQHAASTPK.A	1996.17099	2	6.99E-05	0.90	4.44	-	540.6
AHQ-2-5, 2887	K.QAAASATQTIAAAQHAASTPK.A	1996.17099	2	1.93E-06	0.94	5.05	-	734.5
AHQ-2-5, 2872 - 2883	K.QAAASATQTIAAAQHAASTPK.A	1996.17099	3	9.21E-04	0.61	3.03	-	654.8
AHQ-2-1, 3566	K.QAAASATQTIAAAQHAASTPK.A	1996.17099	2	7.01E-06	0.90	4.21	-	700.8
AHQ-2-7, 2803	K.QAAASATQTIAAAQHAASTPK.A	1996.17099	2	2.72E-04	0.77	3.66	-	418.1
AHQ-2-1, 3394	K.QAAASATQTIAAAQHAASTPK.A	1996.17099	3	4.18E-06	0.83	3.66	-	820.1
AHQ-2-1, 3392	K.QAAASATQTIAAAQHAASTPK.A	1996.17099	2	4.17E-06	0.86	4.04	-	603.6
AHQ-2-3, 3187	K.QAAASATQTIAAAQHAASTPK.A	1996.17099	2	3.35E-06	0.85	4.11	-	559.3
AHQ-2-4, 3388	K.QAAASATQTIAAAQHAASTPK.A	1996.17099	2	3.41E-10	0.93	4.73	-	744.5
AHQ-2-3, 3191	K.QAAASATQTIAAAQHAASTPK.A	1996.17099	3	5.06E-04	0.84	3.86	-	782.1
AHQ-2-3, 3384	K.QAAASATQTIAAAQHAASTPK.A	1996.17099	2	5.32E-06	0.81	4.29	-	586.7
AHQ-2-4, 3188 - 3190	K.QAAASATQTIAAAQHAASTPK.A	1996.17099	2	5.19E-06	0.94	5.10	-	802.5
AHQ-2-1, 3069	R.QEDVIATANLSR.R	1317.43074	2	5.69E-04	0.75	2.91	-	513.5
AHQ-2-3, 4055 - 4133	R.QELAVFCSPEPPAK.T	1574.77985	2	4.12E-06	0.88	3.71	-	788.3
AHQ-2-4, 4064 - 4072	R.QELAVFCSPEPPAK.T	1574.77985	2	1.44E-08	0.92	3.64	-	1076.6
AHQ-2-1, 4106 - 4185	R.QELAVFCSPEPPAK.T	1574.77985	2	3.10E-05	0.74	2.97	-	701.4
AHQ-2-5, 3763 - 3843	R.QELAVFCSPEPPAK.T	1574.77985	2	1.94E-06	0.88	3.34	-	781.2
AHQ-2-2, 4088 - 4158	R.QELAVFCSPEPPAK.T	1574.77985	2	1.71E-05	0.92	4.05	-	805.8
AHQ-2-8, 7406	R.QNLLQAAGNVGQASGELLQQIGESDTDPHFQDALMQLAK.A	4138.52644	3	1.35E-13	0.92	5.61	-	556.9
AHQ-2-5, 7471 - 7473	R.QNLLQAAGNVGQASGELLQQIGESDTDPHFQDALM*QLAK.A	4154.52584	3	1.13E-07	0.86	4.52	-	619.5
AHQ-2-5, 7495	R.QNLLQAAGNVGQASGELLQQIGESDTDPHFQDALMQLAK.A	4138.52644	3	1.14E-10	0.97	6.85	-	1156.2
AHQ-2-1, 7240	R.QNLLQAAGNVGQASGELLQQIGESDTDPHFQDALM*QLAK.A	4154.52584	3	2.37E-06	0.83	4.16	-	594.8
AHQ-2-2, 7576	R.QNLLQAAGNVGQASGELLQQIGESDTDPHFQDALM*QLAK.A	4154.52584	3	1.27E-05	0.92	5.36	-	807.1
AHQ-2-7, 7503	R.QNLLQAAGNVGQASGELLQQIGESDTDPHFQDALMQLAK.A	4138.52644	3	8.55E-07	0.92	5.34	-	518.4
AHQ-2-2, 3856 - 3857	K.QVAASTAQLLVACK.V	1461.70942	2	1.77E-05	0.94	3.93	-	879.8
AHQ-2-3, 3816 - 3817	K.QVAASTAQLLVACK.V	1461.70942	2	9.74E-08	0.94	4.21	-	738.2
AHQ-2-2, 2453	R.RAIADMLR.A	946.15319	2	1.83E-05	0.84	2.89	-	743.3
AHQ-2-3, 4951	K.RVAGSVTELIQAAEAM*K.G	1791.06327	2	2.64E-07	0.84	3.68	-	338.9
AHQ-2-4, 6002 - 6005	K.RVAGSVTELIQAAEAMK.G	1775.06387	2	1.20E-05	0.98	5.56	-	1950.3
AHQ-2-1, 6142	K.RVAGSVTELIQAAEAMK.G	1775.06387	2	6.35E-06	0.98	5.09	-	2215.8
AHQ-2-1, 5085	K.RVAGSVTELIQAAEAM*K.G	1791.06327	2	4.78E-06	0.92	4.38	-	689.1
AHQ-2-3, 5995	K.RVAGSVTELIQAAEAMK.G	1775.06387	2	2.27E-06	0.97	5.20	-	1447.1
AHQ-2-5, 5581	K.RVAGSVTELIQAAEAMK.G	1775.06387	2	3.32E-09	0.98	5.70	-	1723.6
AHQ-2-2, 6025	K.RVAGSVTELIQAAEAMK.G	1775.06387	2	7.09E-07	0.98	6.13	-	2491.8

AHQ-2-2, 2888	K.SIAAATSALVK.A	1032.21612	2	3.38E-04	0.91	3.51	-	995.7
AHQ-2-3, 2896 - 2899	K.SIAAATSALVK.A	1032.21612	2	8.54E-04	0.87	3.16	-	802.5
AHQ-2-4, 2870 - 2888	K.SIAAATSALVK.A	1032.21612	2	2.67E-04	0.83	3.01	-	702.3
AHQ-2-6, 2667	K.SIAAATSALVK.A	1032.21612	2	5.57E-05	0.84	2.91	-	972.8
AHQ-2-5, 2620	K.SIAAATSALVK.A	1032.21612	2	5.02E-05	0.87	3.01	-	987.6
AHQ-2-7, 3603	K.SKDHFGLEGDEESTM*LEDSVSPK.K	2554.68304	3	2.02E-06	0.90	4.19	-	879.5
AHQ-2-10, 3572	K.SKDHFGLEGDEESTM*LEDSVSPK.K	2554.68304	3	5.95E-04	0.68	3.02	-	874.2
AHQ-2-7, 3985 - 4059	K.SKDHFGLEGDEESTMLEDSVSPK.K	2538.68364	2	1.03E-09	0.89	3.89	-	664.2
AHQ-2-7, 3988 - 4055	K.SKDHFGLEGDEESTMLEDSVSPK.K	2538.68364	3	5.73E-07	0.97	5.57	-	1849.9
AHQ-2-7, 4071	K.SKDHFGLEGDEESTMLEDSVSPK.K	2538.68364	2	2.85E-06	0.81	2.99	-	882.5
AHQ-2-5, 5352	K.SNTSPEELGPLANQLTSDYGR.L	2250.36505	2	1.42E-07	0.94	4.74	-	638.9
AHQ-2-3, 5647	K.SNTSPEELGPLANQLTSDYGR.L	2250.36505	2	5.31E-07	0.90	4.15	-	489.3
AHQ-2-2, 5370 - 5441	K.SNTSPEELGPLANQLTSDYGR.L	2250.36505	2	6.89E-07	0.88	4.05	-	421.7
AHQ-2-3, 4767	K.SNTSPEELGPLANQLTSDYGR.L	2250.36505	2	5.44E-09	0.73	3.77	-	489.6
AHQ-2-4, 5558 - 5594	K.SNTSPEELGPLANQLTSDYGR.L	2250.36505	2	7.16E-05	0.51	2.58	-	246.5
AHQ-2-5, 5491 - 5568	K.SNTSPEELGPLANQLTSDYGR.L	2250.36505	2	1.03E-05	0.91	4.06	-	626.7
AHQ-2-1, 4769 - 4842	K.SNTSPEELGPLANQLTSDYGR.L	2250.36505	2	1.64E-06	0.92	4.22	-	719.4
AHQ-2-2, 5050	K.SNTSPEELGPLANQLTSDYGR.L	2250.36505	2	1.80E-08	0.89	4.32	-	573.7
AHQ-2-3, 5431 - 5511	K.SNTSPEELGPLANQLTSDYGR.L	2250.36505	2	2.84E-09	0.83	3.72	-	325.8
AHQ-2-9, 5111	K.SNTSPEELGPLANQLTSDYGR.L	2250.36505	2	4.22E-05	0.88	3.92	-	685.3
AHQ-2-6, 4489 - 4498	K.SNTSPEELGPLANQLTSDYGR.L	2250.36505	2	5.06E-09	0.84	3.79	-	484.6
AHQ-2-4, 5354 - 5425	K.SNTSPEELGPLANQLTSDYGR.L	2250.36505	2	2.07E-06	0.89	3.99	-	504.2
AHQ-2-4, 4780	K.SNTSPEELGPLANQLTSDYGR.L	2250.36505	2	1.87E-13	0.95	4.75	-	814.4
AHQ-2-4, 5204	K.SNTSPEELGPLANQLTSDYGR.L	2250.36505	2	1.32E-06	0.91	4.58	-	463.1
AHQ-2-4, 5664 - 5666	K.SNTSPEELGPLANQLTSDYGR.L	2250.36505	2	5.58E-07	0.88	4.09	-	493.2
AHQ-2-1, 5598 - 5670	K.SNTSPEELGPLANQLTSDYGR.L	2250.36505	2	5.20E-07	0.93	4.31	-	667.8
AHQ-2-5, 4583 - 4584	K.SNTSPEELGPLANQLTSDYGR.L	2250.36505	2	4.35E-11	0.94	4.47	-	843.1
AHQ-2-3, 5187 - 5199	K.SNTSPEELGPLANQLTSDYGR.L	2250.36505	2	7.84E-06	0.89	4.13	-	557.5
AHQ-2-6, 5059 - 5130	K.SNTSPEELGPLANQLTSDYGR.L	2250.36505	2	2.62E-07	0.87	3.67	-	643.0
AHQ-2-2, 4808	K.SNTSPEELGPLANQLTSDYGR.L	2250.36505	2	1.11E-07	0.85	3.75	-	379.7
AHQ-2-3, 5335 - 5403	K.SNTSPEELGPLANQLTSDYGR.L	2250.36505	2	1.26E-07	0.71	3.04	-	380.4
AHQ-2-2, 5217 - 5234	K.SNTSPEELGPLANQLTSDYGR.L	2250.36505	2	5.68E-06	0.81	3.71	-	389.5
AHQ-2-6, 5407	K.SNTSPEELGPLANQLTSDYGR.L	2250.36505	2	6.90E-06	0.82	3.20	-	625.6
AHQ-2-1, 5452 - 5516	K.SNTSPEELGPLANQLTSDYGR.L	2250.36505	2	1.09E-05	0.76	3.36	-	471.0
AHQ-2-2, 5617 - 5693	K.SNTSPEELGPLANQLTSDYGR.L	2250.36505	2	3.33E-07	0.92	4.22	-	644.1
AHQ-2-5, 5163	K.SNTSPEELGPLANQLTSDYGR.L	2250.36505	2	2.24E-07	0.89	3.86	-	619.4
AHQ-2-6, 7183 - 7185	R.TEDSGLQTQVIAAATQCALSTSQLVACTK.V	3056.37009	3	1.11E-14	0.97	5.69	-	1833.9
AHQ-2-2, 7472 - 7542	R.TEDSGLQTQVIAAATQCALSTSQLVACTK.V	3056.37009	3	8.79E-13	0.98	6.23	-	2128.7
AHQ-2-1, 7250 - 7286	R.TEDSGLQTQVIAAATQCALSTSQLVACTK.V	3056.37009	3	5.43E-05	0.90	4.26	-	1192.1
AHQ-2-7, 3843 - 3912	K.TKEVIQEWNLTNIK.R	1716.95938	2	3.29E-06	0.96	4.85	-	1418.0
AHQ-2-8, 3786	K.TKEVIQEWNLTNIK.R	1716.95938	2	1.02E-05	0.93	4.58	-	1044.2
AHQ-2-9, 3955	K.TKEVIQEWNLTNIK.R	1716.95938	2	3.58E-06	0.96	4.69	-	1631.7
AHQ-2-13, 4253 - 4257	K.TKEVIQEWNLTNIK.R	1716.95938	2	1.37E-08	0.96	4.81	-	1476.4
AHQ-2-7, 3857	K.TKEVIQEWNLTNIK.R	1716.95938	3	4.75E-05	0.93	4.68	-	1175.8
AHQ-2-9, 6422	K.TLAESALQLLYTAK.E	1522.76793	2	3.78E-05	0.95	4.07	-	1242.4
AHQ-2-2, 6668 - 6748	K.TLAESALQLLYTAK.E	1522.76793	2	4.08E-08	0.97	5.25	-	1436.5
AHQ-2-3, 6639 - 6707	K.TLAESALQLLYTAK.E	1522.76793	2	6.76E-10	0.97	5.41	-	1388.8
AHQ-2-2, 6838	K.TLAESALQLLYTAK.E	1522.76793	2	4.84E-06	0.93	3.57	-	1047.3
AHQ-2-4, 6712 - 6780	K.TLAESALQLLYTAK.E	1522.76793	2	4.08E-11	0.97	4.91	-	1410.5
AHQ-2-1, 6650 - 6654	K.TLAESALQLLYTAK.E	1522.76793	2	7.81E-10	0.96	4.78	-	1039.5
AHQ-2-10, 5915	K.TLAESALQLLYTAK.E	1522.76793	2	8.83E-04	0.82	3.44	-	860.3
AHQ-2-3, 6904	K.TLAESALQLLYTAK.E	1522.76793	2	3.21E-07	0.94	4.16	-	1034.9
AHQ-2-11, 6064	K.TLAESALQLLYTAK.E	1522.76793	2	4.46E-04	0.92	3.87	-	861.4
AHQ-2-5, 6411 - 6491	K.TLAESALQLLYTAK.E	1522.76793	2	1.04E-05	0.97	4.82	-	1430.4
AHQ-2-2, 2420	K.TLSHPQQM*ALLDQTK.T	1727.96404	2	1.63E-05	0.89	3.71	-	582.7
AHQ-2-2, 3134	K.TLSHPQQMALLDQTK.T	1711.96464	3	4.26E-08	0.96	5.18	-	1801.4
AHQ-2-3, 3125	K.TLSHPQQMALLDQTK.T	1711.96464	3	2.30E-07	0.91	5.00	-	879.1
AHQ-2-10, 2820	K.TLSHPQQMALLDQTK.T	1711.96464	2	7.76E-04	0.78	3.28	-	394.1
AHQ-2-4, 3124	K.TLSHPQQMALLDQTK.T	1711.96464	2	1.34E-05	0.94	5.11	-	647.7
AHQ-2-5, 2845	K.TLSHPQQMALLDQTK.T	1711.96464	3	1.49E-05	0.91	4.50	-	990.4
AHQ-2-2, 3125 - 3126	K.TLSHPQQMALLDQTK.T	1711.96464	2	2.40E-04	0.95	5.08	-	735.3
AHQ-2-1, 3369	K.TLSHPQQMALLDQTK.T	1711.96464	3	4.75E-05	0.83	3.90	-	674.8
AHQ-2-4, 3129	K.TLSHPQQMALLDQTK.T	1711.96464	3	1.64E-07	0.93	4.93	-	997.6
AHQ-2-9, 2850	K.TLSHPQQMALLDQTK.T	1711.96464	2	6.67E-05	0.93	4.32	-	714.8
AHQ-2-3, 3087 - 3155	K.TLSHPQQMALLDQTK.T	1711.96464	2	2.30E-04	0.95	4.64	-	821.2
AHQ-2-7, 2753 - 2792	K.TLSHPQQMALLDQTK.T	1711.96464	2	2.55E-04	0.88	4.14	-	661.8
AHQ-2-6, 2861	K.TLSHPQQMALLDQTK.T	1711.96464	2	1.25E-04	0.95	4.46	-	871.1
AHQ-2-5, 2835 - 2843	K.TLSHPQQMALLDQTK.T	1711.96464	2	7.55E-04	0.96	4.92	-	829.3
AHQ-2-5, 4085 - 4091	K.TMLESAGGLIQTAR.A	1448.67095	2	9.01E-07	0.95	3.82	-	1833.3
AHQ-2-4, 4349 - 4360	K.TMLESAGGLIQTAR.A	1448.67095	2	3.77E-05	0.86	3.56	-	850.8
AHQ-2-5, 3203	K.TM*LESAGGLIQTAR.A	1464.67035	2	2.32E-05	0.89	3.46	-	1174.0
AHQ-2-2, 3493 - 3494	K.TM*LESAGGLIQTAR.A	1464.67035	2	1.17E-07	0.97	4.91	-	1655.7
AHQ-2-3, 3468 - 3549	K.TM*LESAGGLIQTAR.A	1464.67035	2	5.67E-04	0.95	4.04	-	1468.5
AHQ-2-6, 3173	K.TM*LESAGGLIQTAR.A	1464.67035	2	1.15E-05	0.90	3.27	-	1091.7
AHQ-2-1, 3620	K.TM*LESAGGLIQTAR.A	1464.67035	2	1.71E-05	0.95	4.17	-	1463.2
AHQ-2-2, 4514	K.TMLESAGGLIQTAR.A	1448.67095	2	2.20E-05	0.93	3.65	-	1599.4
AHQ-2-6, 4046	K.TMLESAGGLIQTAR.A	1448.67095	2	7.51E-07	0.89	2.57	-	1765.1
AHQ-2-1, 4418 - 4498	K.TMLESAGGLIQTAR.A	1448.67095	2	1.92E-06	0.95	4.02	-	1843.7
AHQ-2-2, 4428	K.TMLESAGGLIQTAR.A	1448.67095	2	8.93E-07	0.77	3.10	-	1005.7
AHQ-2-4, 4357	K.TMLESAGGLIQTAR.A	1448.67095	1	6.37E-04	0.09	1.82	-	296.4
AHQ-2-13-, 4883	K.TMQFEPSTMVYDACR.I	1838.07571	2	1.28E-05	0.92	4.52	-	608.1
AHQ-2-7, 4679	K.TMQFEPSTMVYDACR.I	1838.07571	2	4.70E-06	0.94	5.26	-	553.3
AHQ-2-7, 3859	K.TMQFEPSTM*VYDACR.I	1854.07511	2	3.66E-04	0.82	3.30	-	471.3
AHQ-2-12, 4838 - 4855	K.TMQFEPSTMVYDACR.I	1838.07571	2	2.90E-04	0.89	4.41	-	499.4
AHQ-2-13, 4235 - 4236	K.TM*QFEPSTMVYDACR.I	1854.07511	2	3.63E-06	0.93	4.64	-	738.2
AHQ-2-7, 4116 - 4123	K.TM*QFEPSTMVYDACR.I	1854.07511	2	3.89E-06	0.95	4.92	-	678.7
AHQ-2-14-, 4837	K.TMQFEPSTMVYDACR.I	1838.07571	2	1.24E-06	0.94	4.64	-	567.0
AHQ-2-13, 4789 - 4795	K.TMQFEPSTMVYDACR.I	1838.07571	2	6.65E-04	0.94	4.33	-	635.9
AHQ-2-13-, 4289	K.TM*QFEPSTMVYDACR.I	1854.07511	2	5.68E-06	0.91	4.64	-	658.6
AHQ-2-13-, 5394 - 5462	K.TVTDMLMTICAR.I	1413.71007	2	9.21E-04	0.86	3.24	-	804.3
AHQ-2-7, 3533 - 3608	K.TVTDM*LM*TICAR.I	1445.70887	2	3.56E-06	0.91	3.16	-	1014.8
AHQ-2-13-, 3737 - 3801	K.TVTDM*LM*TICAR.I	1445.70887	2	1.53E-06	0.91	3.34	-	805.4
AHQ-2-13, 5352	K.TVTDMLMTICAR.I	1413.71007	2	5.96E-05	0.86	2.62	-	1176.7
AHQ-2-13, 3719 - 3784	K.TVTDM*LM*TICAR.I	1445.70887	2	7.71E-08	0.90	3.77	-	764.2
AHQ-2-12, 5258	K.TYGVSFFLVK.E	1161.37395	2	1.66E-06	0.75	2.71	-	627.3
AHQ-2-7, 4923	K.TYGVSFFLVK.E	1161.37395	2	2.83E-06	0.89	3.62	-	581.3
AHQ-2-11, 5017	K.TYGVSFFLVK.E	1161.37395	2	1.83E-04	0.78	2.62	-	681.8
AHQ-2-9, 5132	R.VAGSVTELIQAAEAM*K.G	1634.87692	2	1.86E-04	0.81	3.01	-	968.2
AHQ-2-9, 6278 - 6306	R.VAGSVTELIQAAEAMK.G	1618.87752	2	7.78E-06	0.94	3.87	-	1428.5
AHQ-2-3, 6553	R.VAGSVTELIQAAEAMK.G	1618.87752	2	6.03E-09	0.94	4.23	-	957.1
AHQ-2-5, 6337 - 6355	R.VAGSVTELIQAAEAMK.G	1618.87752	2	3.89E-11	0.98	5.85	-	1625.3
AHQ-2-13, 6132	R.VAGSVTELIQAAEAMK.G	1618.87752	2	8.18E-05	0.94	3.79	-	1535.7
AHQ-2-1, 6573 - 6593	R.VAGSVTELIQAAEAMK.G	1618.87752	2	5.50E-11	0.98	6.07	-	2033.3
AHQ-2-4, 6589 - 6666	R.VAGSVTELIQAAEAMK.G	1618.87752	2	3.58E-09	0.97	5.21	-	1452.4
AHQ-2-3, 5408 - 5479	R.VAGSVTELIQAAEAM*K.G	1634.87692	2	8.37E-06	0.97	4.66	-	1763.9
AHQ-2-2, 5460 - 5496	R.VAGSVTELIQAAEAM*K.G	1634.87692	2	9.45E-04	0.79	3.35	-	797.6
AHQ-2-6, 6234 - 6238	R.VAGSVTELIQAAEAMK.G	1618.87752	2	7.93E-07	0.97	4.73	-	1730.5

AHQ-2-2, 6526 - 6592	R.VAGSVTELIQAAEAMK.G	1618.87752	2	1.10E-10	0.98	6.26	-	1684.1
AHQ-2-2, 6781	R.VAGSVTELIQAAEAMK.G	1618.87752	2	1.39E-08	0.95	4.13	-	1463.6
AHQ-2-7, 6155	R.VAGSVTELIQAAEAMK.G	1618.87752	2	1.39E-06	0.76	2.80	-	903.2
AHQ-2-1, 6152 - 6217	K.VGAIPANALDDGQWSQGLISAAR.M	2311.53959	2	1.05E-11	0.94	5.12	-	679.0
AHQ-2-12, 5865 - 5875	K.VGAIPANALDDGQWSQGLISAAR.M	2311.53959	2	8.52E-08	0.95	5.58	-	745.8
AHQ-2-1, 6053 - 6082	K.VGAIPANALDDGQWSQGLISAAR.M	2311.53959	2	1.74E-12	0.93	4.63	-	751.6
AHQ-2-2, 5836	K.VGAIPANALDDGQWSQGLISAAR.M	2311.53959	2	4.19E-07	0.90	4.32	-	594.2
AHQ-2-1, 6254	K.VGAIPANALDDGQWSQGLISAAR.M	2311.53959	2	4.99E-06	0.68	3.04	-	465.4
AHQ-2-4, 5917 - 5988	K.VGAIPANALDDGQWSQGLISAAR.M	2311.53959	2	3.79E-09	0.96	5.34	-	961.1
AHQ-2-2, 5930 - 6000	K.VGAIPANALDDGQWSQGLISAAR.M	2311.53959	2	5.64E-08	0.96	5.41	-	977.3
AHQ-2-11, 5446	K.VGAIPANALDDGQWSQGLISAAR.M	2311.53959	2	1.55E-06	0.92	4.29	-	789.0
AHQ-2-3, 6075 - 6101	K.VGAIPANALDDGQWSQGLISAAR.M	2311.53959	2	1.25E-10	0.95	4.79	-	1100.5
AHQ-2-1, 5901 - 5965	K.VGAIPANALDDGQWSQGLISAAR.M	2311.53959	2	2.11E-05	0.86	3.99	-	517.4
AHQ-2-3, 6171 - 6243	K.VGAIPANALDDGQWSQGLISAAR.M	2311.53959	2	1.42E-07	0.93	4.70	-	703.9
AHQ-2-3, 5899 - 5967	K.VGAIPANALDDGQWSQGLISAAR.M	2311.53959	2	1.66E-04	0.88	3.99	-	579.2
AHQ-2-10, 5188 - 5262	K.VGAIPANALDDGQWSQGLISAAR.M	2311.53959	2	3.75E-09	0.92	4.42	-	612.2
AHQ-2-10, 5582 - 5654	K.VGAIPANALDDGQWSQGLISAAR.M	2311.53959	2	2.22E-08	0.90	4.23	-	621.7
AHQ-2-7, 5831	K.VGAIPANALDDGQWSQGLISAAR.M	2311.53959	2	5.86E-09	0.91	4.34	-	585.2
AHQ-2-11, 5540 - 5601	K.VGAIPANALDDGQWSQGLISAAR.M	2311.53959	2	4.62E-12	0.98	6.23	-	1460.8
AHQ-2-6, 5586 - 5633	K.VGAIPANALDDGQWSQGLISAAR.M	2311.53959	2	1.42E-07	0.95	4.72	-	830.0
AHQ-2-5, 5884 - 5951	K.VGAIPANALDDGQWSQGLISAAR.M	2311.53959	2	2.08E-11	0.96	5.47	-	922.9
AHQ-2-3, 5709 - 5787	K.VGAIPANALDDGQWSQGLISAAR.M	2311.53959	2	3.63E-09	0.91	4.25	-	651.7
AHQ-2-2, 6068 - 6136	K.VGAIPANALDDGQWSQGLISAAR.M	2311.53959	2	1.57E-09	0.95	4.93	-	790.3
AHQ-2-4, 6350 - 6429	K.VGAIPANALDDGQWSQGLISAAR.M	2311.53959	2	3.37E-06	0.83	3.43	-	608.1
AHQ-2-5, 6125 - 6133	K.VGAIPANALDDGQWSQGLISAAR.M	2311.53959	2	3.76E-10	0.96	5.44	-	980.3
AHQ-2-6, 5801 - 5853	K.VGAIPANALDDGQWSQGLISAAR.M	2311.53959	2	8.39E-12	0.96	5.58	-	845.8
AHQ-2-7, 5672 - 5680	K.VGAIPANALDDGQWSQGLISAAR.M	2311.53959	2	2.12E-06	0.92	4.37	-	834.9
AHQ-2-4, 6078 - 6149	K.VGAIPANALDDGQWSQGLISAAR.M	2311.53959	2	1.41E-08	0.92	4.38	-	519.4
AHQ-2-12, 5998	K.VGAIPANALDDGQWSQGLISAAR.M	2311.53959	2	1.14E-10	0.93	3.82	-	953.3
AHQ-2-2, 6172	K.VGAIPANALDDGQWSQGLISAAR.M	2311.53959	2	1.29E-09	0.89	3.86	-	652.5
AHQ-2-14-, 5769	K.VGAIPANALDDGQWSQGLISAAR.M	2311.53959	2	2.58E-09	0.94	4.98	-	646.0
AHQ-2-10, 5375 - 5439	K.VGAIPANALDDGQWSQGLISAAR.M	2311.53959	2	1.60E-13	0.95	5.25	-	773.7
AHQ-2-10, 5279	K.VGAIPANALDDGQWSQGLISAAR.M	2311.53959	2	2.18E-06	0.94	4.65	-	842.3
AHQ-2-9, 5887 - 5904	K.VGAIPANALDDGQWSQGLISAAR.M	2311.53959	2	3.30E-08	0.97	5.21	-	1519.1
AHQ-2-9, 5690	K.VGAIPANALDDGQWSQGLISAAR.M	2311.53959	2	1.55E-08	0.95	4.78	-	1006.0
AHQ-2-8, 5536	K.VGAIPANALDDGQWSQGLISAAR.M	2311.53959	2	4.36E-07	0.82	3.61	-	559.8
AHQ-2-8, 5680	K.VGAIPANALDDGQWSQGLISAAR.M	2311.53959	2	7.22E-09	0.95	5.05	-	696.5
AHQ-2-3, 5983	K.VGAIPANALDDGQWSQGLISAAR.M	2311.53959	2	1.03E-06	0.96	5.23	-	940.8
AHQ-2-1, 3976	K.VGDDPAVWQLK.N	1228.37879	2	6.73E-09	0.94	4.09	-	965.4
AHQ-2-5, 3503	K.VGDDPAVWQLK.N	1228.37879	2	2.57E-08	0.91	3.71	-	885.0
AHQ-2-7, 3424	K.VGDDPAVWQLK.N	1228.37879	2	5.71E-06	0.90	3.49	-	854.5
AHQ-2-10, 3400	K.VGDDPAVWQLK.N	1228.37879	2	4.74E-07	0.85	3.25	-	664.2
AHQ-2-2, 3844	K.VGDDPAVWQLK.N	1228.37879	2	7.73E-09	0.94	4.28	-	929.4
AHQ-2-4, 3784	K.VGDDPAVWQLK.N	1228.37879	2	1.95E-09	0.90	3.29	-	832.8
AHQ-2-2, 3804 - 3881	K.VGDDPAVWQLK.N	1228.37879	2	2.43E-08	0.93	4.17	-	799.7
AHQ-2-6, 3491	K.VGDDPAVWQLK.N	1228.37879	2	6.32E-09	0.95	3.99	-	1057.0
AHQ-2-2, 3192	K.VGDDPAVWQLKNSAK.V	1628.81068	2	2.74E-08	0.79	3.39	-	682.9
AHQ-2-4, 3182	K.VGDDPAVWQLKNSAK.V	1628.81068	2	2.95E-11	0.80	3.34	-	583.2
AHQ-2-3, 3091 - 3160	K.VLGEAMTGISQNAK.N	1419.62961	1	2.28E-09	0.88	3.77	-	527.3
AHQ-2-4, 3084 - 3165	K.VLGEAMTGISQNAK.N	1419.62961	1	7.84E-05	0.49	2.38	-	370.1
AHQ-2-2, 3092	K.VLGEAMTGISQNAK.N	1419.62961	2	2.46E-10	0.98	4.92	-	2259.1
AHQ-2-4, 3270	K.VLGEAMTGISQNAK.N	1419.62961	2	3.30E-08	0.96	4.04	-	2056.1
AHQ-2-1, 2460	K.VLGEAM*TGISQNAK.N	1435.62901	2	5.81E-05	0.92	3.92	-	934.8
AHQ-2-2, 3108 - 3178	K.VLGEAMTGISQNAK.N	1419.62961	1	9.19E-08	0.63	3.12	-	342.8
AHQ-2-2, 2324 - 2396	K.VLGEAM*TGISQNAK.N	1435.62901	2	5.13E-06	0.97	5.06	-	1498.8
AHQ-2-3, 3156	K.VLGEAMTGISQNAK.N	1419.62961	2	3.03E-08	0.98	4.59	-	2529.5
AHQ-2-6, 2859 - 2862	K.VLGEAMTGISQNAK.N	1419.62961	2	1.62E-04	0.88	3.15	-	1078.9
AHQ-2-5, 2859 - 2860	K.VLGEAMTGISQNAK.N	1419.62961	2	9.35E-05	0.97	4.42	-	1702.0
AHQ-2-3, 4920 - 4932	K.VLGEAM*TGISQNAKNGNLPEFGDAISTASK.A	3038.33659	3	5.89E-07	0.87	4.52	-	382.5
AHQ-2-5, 5408 - 5445	K.VLGEAMTGISQNAKNGNLPEFGDAISTASK.A	3022.33719	3	1.01E-09	0.84	3.92	-	613.7
AHQ-2-1, 4954	K.VLGEAM*TGISQNAKNGNLPEFGDAISTASK.A	3038.33659	3	3.55E-05	0.89	4.39	-	669.1
AHQ-2-3, 5497 - 5567	K.VLGEAMTGISQNAKNGNLPEFGDAISTASK.A	3022.33719	3	8.12E-12	0.88	4.62	-	612.8
AHQ-2-2, 5088	K.VLGEAM*TGISQNAKNGNLPEFGDAISTASK.A	3038.33659	3	2.03E-07	0.85	4.19	-	564.8
AHQ-2-2, 5538 - 5608	K.VLGEAMTGISQNAKNGNLPEFGDAISTASK.A	3022.33719	3	3.31E-04	0.89	4.78	-	686.0
AHQ-2-3, 1848	K.VLVQNAAGSQEK.L	1244.37976	2	2.18E-04	0.79	2.99	-	682.8
AHQ-2-3, 1661 - 1737	K.VLVQNAAGSQEK.L	1244.37976	2	5.68E-07	0.88	3.23	-	710.0
AHQ-2-5, 1459	K.VLVQNAAGSQEK.L	1244.37976	2	1.10E-04	0.74	3.18	-	527.4
AHQ-2-3, 1520 - 1579	K.VLVQNAAGSQEK.L	1244.37976	2	3.19E-04	0.91	3.49	-	964.9
AHQ-2-1, 1786 - 1845	K.VLVQNAAGSQEK.L	1244.37976	2	4.43E-05	0.88	3.53	-	670.0
AHQ-2-4, 1505 - 1577	K.VLVQNAAGSQEK.L	1244.37976	2	2.35E-08	0.93	3.51	-	1027.1
AHQ-2-4, 1754	K.VLVQNAAGSQEK.L	1244.37976	2	1.87E-04	0.95	4.13	-	1203.2
AHQ-2-6, 1486	K.VLVQNAAGSQEK.L	1244.37976	2	8.54E-07	0.83	3.41	-	577.2
AHQ-2-2, 1661 - 1730	K.VLVQNAAGSQEK.L	1244.37976	2	1.75E-08	0.93	4.04	-	927.6
AHQ-2-1, 1904	K.VLVQNAAGSQEK.L	1244.37976	2	5.71E-04	0.67	2.78	-	407.2
AHQ-2-2, 1614	K.VLVQNAAGSQEK.L	1244.37976	1	2.87E-04	0.24	1.99	-	423.0
AHQ-2-2, 1522 - 1592	K.VLVQNAAGSQEK.L	1244.37976	2	9.26E-05	0.88	3.49	-	892.3
AHQ-2-4, 4737 - 4738	K.VMVTNVTSLLK.T	1205.49322	2	1.24E-05	0.92	4.36	-	741.3
AHQ-2-3, 4759	K.VMVTNVTSLLK.T	1205.49322	2	7.13E-06	0.92	4.30	-	811.7
AHQ-2-3, 4375	K.VM*VTNVTSLLK.T	1221.49262	2	8.23E-05	0.79	3.13	-	679.9
AHQ-2-5, 4367 - 4371	K.VMVTNVTSLLK.T	1205.49322	2	2.10E-06	0.93	4.28	-	928.5
AHQ-2-1, 4896	K.VMVTNVTSLLK.T	1205.49322	2	2.80E-05	0.91	3.52	-	887.6
AHQ-2-1, 4528	K.VM*VTNVTSLLK.T	1221.49262	2	7.32E-04	0.86	3.41	-	718.0
AHQ-2-5, 4019	K.VM*VTNVTSLLK.T	1221.49262	2	2.22E-04	0.79	3.25	-	582.9
AHQ-2-2, 4388 - 4389	K.VM*VTNVTSLLK.T	1221.49262	2	7.40E-05	0.82	3.54	-	670.2
AHQ-2-6, 4333	K.VMVTNVTSLLK.T	1205.49322	2	2.56E-04	0.88	3.79	-	605.1
AHQ-2-2, 4772	K.VMVTNVTSLLK.T	1205.49322	2	2.24E-05	0.90	3.90	-	869.6
AHQ-2-7, 4208	K.VMVTNVTSLLK.T	1205.49322	2	4.87E-05	0.87	3.55	-	784.5
AHQ-2-4, 4341	K.VM*VTNVTSLLK.T	1221.49262	2	1.61E-05	0.80	3.39	-	611.1
AHQ-2-3, 2691 - 2693	R.VQELGHGCAALVTK.A	1484.70307	2	9.01E-06	0.97	4.54	-	1764.3
AHQ-2-2, 2705	R.VQELGHGCAALVTK.A	1484.70307	1	1.72E-05	0.91	4.06	-	854.2
AHQ-2-2, 2696 - 2708	R.VQELGHGCAALVTK.A	1484.70307	2	1.90E-04	0.97	4.47	-	1592.1
AHQ-2-3, 2703	R.VQELGHGCAALVTK.A	1484.70307	1	4.40E-05	0.86	3.72	-	585.9
AHQ-2-6, 2505	R.VQELGHGCAALVTK.A	1484.70307	2	2.46E-04	0.95	4.01	-	1346.0
AHQ-2-1, 2904	R.VQELGHGCAALVTK.A	1484.70307	2	1.68E-09	0.97	5.65	-	1386.2
AHQ-2-4, 2750	R.VQELGHGCAALVTK.A	1484.70307	2	4.03E-04	0.95	4.11	-	1200.1
AHQ-2-4, 2661 - 2672	R.VQELGHGCAALVTK.A	1484.70307	2	1.20E-05	0.97	4.21	-	1701.8
AHQ-2-3, 2511	K.VSHVLAALQAGNR.G	1336.52525	1	7.95E-05	0.85	3.47	-	513.7
AHQ-2-3, 2512 - 2592	K.VSHVLAALQAGNR.G	1336.52525	2	3.20E-11	0.97	4.68	-	1792.2
AHQ-2-7, 2276	K.VSHVLAALQAGNR.G	1336.52525	1	1.59E-05	0.72	3.11	-	378.8
AHQ-2-2, 2520 - 2598	K.VSHVLAALQAGNR.G	1336.52525	1	4.92E-04	0.30	2.66	-	227.8
AHQ-2-2, 2521 - 2522	K.VSHVLAALQAGNR.G	1336.52525	2	8.35E-11	0.97	5.15	-	1781.6
AHQ-2-3, 2525	K.VSHVLAALQAGNR.G	1336.52525	1	4.19E-06	0.91	4.13	-	520.4
AHQ-2-2, 2524	K.VSHVLAALQAGNR.G	1336.52525	1	5.20E-05	0.85	3.46	-	415.2
AHQ-2-5, 2308	K.VSHVLAALQAGNR.G	1336.52525	1	8.32E-05	0.77	3.18	-	356.8
AHQ-2-3, 2661	K.VSHVLAALQAGNR.G	1336.52525	1	1.73E-08	0.34	1.95	-	545.3
AHQ-2-1, 2810	K.VSHVLAALQAGNR.G	1336.52525	1	3.86E-04	0.65	2.45	-	462.6
AHQ-2-10, 2392	K.VSHVLAALQAGNR.G	1336.52525	1	8.38E-06	0.67	2.73	-	355.5

AHQ-2-5, 2325	K.VSHVLAALQAGNR.G	1336.52525	1	1.06E-05	0.67	2.97	-	214.9
AHQ-2-4, 2638	K.VSHVLAALQAGNR.G	1336.52525	2	1.26E-09	0.96	3.71	-	1663.0
AHQ-2-6, 2335 - 2339	K.VSHVLAALQAGNR.G	1336.52525	2	1.24E-06	0.97	4.39	-	1822.0
AHQ-2-9, 2395	K.VSHVLAALQAGNR.G	1336.52525	1	7.92E-04	0.51	2.51	-	328.7
AHQ-2-1, 2730 - 2809	K.VSHVLAALQAGNR.G	1336.52525	2	8.00E-11	0.97	4.59	-	1717.1
AHQ-2-4, 2525 - 2526	K.VSHVLAALQAGNR.G	1336.52525	1	1.03E-07	0.89	3.74	-	570.2
AHQ-2-5, 2307 - 2319	K.VSHVLAALQAGNR.G	1336.52525	2	3.38E-09	0.98	5.17	-	2143.9
AHQ-2-5, 5747	K.VSQM*AQYFEPLTLAAVGAASK.T	2199.51253	2	1.86E-04	0.87	3.79	-	683.5
AHQ-2-11, 6352 - 6354	K.VSQMAQYFEPLTLAAVGAASK.T	2183.51313	2	4.31E-04	0.96	5.21	-	965.0
AHQ-2-3, 6037 - 6107	K.VSQM*AQYFEPLTLAAVGAASK.T	2199.51253	2	6.37E-06	0.94	4.56	-	887.0
AHQ-2-3, 6068	K.VSQM*AQYFEPLTLAAVGAASK.T	2199.51253	3	3.31E-05	0.93	4.58	-	1175.7
AHQ-2-3, 6876	K.VSQMAQYFEPLTLAAVGAASK.T	2183.51313	2	9.94E-06	0.93	4.64	-	759.0
AHQ-2-3, 6959 - 7027	K.VSQMAQYFEPLTLAAVGAASK.T	2183.51313	2	2.92E-06	0.97	5.78	-	1138.2
AHQ-2-3, 6969 - 6989	K.VSQMAQYFEPLTLAAVGAASK.T	2183.51313	3	4.08E-09	0.97	6.15	-	1542.8
AHQ-2-3, 7116	K.VSQMAQYFEPLTLAAVGAASK.T	2183.51313	2	4.23E-10	0.96	5.42	-	854.9
AHQ-2-5, 6825 - 6835	K.VSQMAQYFEPLTLAAVGAASK.T	2183.51313	2	6.59E-06	0.98	6.63	-	1264.6
AHQ-2-9, 6786	K.VSQMAQYFEPLTLAAVGAASK.T	2183.51313	2	6.13E-05	0.85	3.60	-	574.4
AHQ-2-6, 5705	K.VSQM*AQYFEPLTLAAVGAASK.T	2199.51253	2	1.80E-08	0.95	4.51	-	1257.4
AHQ-2-6, 6645 - 6653	K.VSQMAQYFEPLTLAAVGAASK.T	2183.51313	2	8.73E-07	0.96	5.43	-	1061.9
AHQ-2-10, 6214 - 6226	K.VSQMAQYFEPLTLAAVGAASK.T	2183.51313	2	1.08E-07	0.97	5.47	-	1157.5
AHQ-2-4, 7177	K.VSQMAQYFEPLTLAAVGAASK.T	2183.51313	2	1.30E-06	0.93	4.42	-	875.0
AHQ-2-4, 7040 - 7042	K.VSQMAQYFEPLTLAAVGAASK.T	2183.51313	3	2.69E-09	0.96	5.03	-	1581.8
AHQ-2-4, 7036 - 7100	K.VSQMAQYFEPLTLAAVGAASK.T	2183.51313	2	1.06E-07	0.96	5.56	-	1106.0
AHQ-2-4, 6953 - 7032	K.VSQMAQYFEPLTLAAVGAASK.T	2183.51313	2	5.11E-07	0.97	5.63	-	1278.3
AHQ-2-2, 6096	K.VSQM*AQYFEPLTLAAVGAASK.T	2199.51253	2	8.45E-08	0.95	4.89	-	1049.1
AHQ-2-4, 6222	K.VSQM*AQYFEPLTLAAVGAASK.T	2199.51253	2	6.20E-07	0.95	4.58	-	1232.6
AHQ-2-4, 6072	K.VSQM*AQYFEPLTLAAVGAASK.T	2199.51253	2	1.53E-06	0.94	4.18	-	1023.7
AHQ-2-2, 6921	K.VSQMAQYFEPLTLAAVGAASK.T	2183.51313	2	1.40E-07	0.93	4.82	-	628.2
AHQ-2-2, 7020 - 7089	K.VSQMAQYFEPLTLAAVGAASK.T	2183.51313	2	1.90E-06	0.96	5.64	-	882.1
AHQ-2-2, 7188	K.VSQMAQYFEPLTLAAVGAASK.T	2183.51313	2	3.75E-09	0.96	5.50	-	931.2
AHQ-2-1, 7034	K.VSQMAQYFEPLTLAAVGAASK.T	2183.51313	2	1.94E-07	0.96	5.28	-	805.8
AHQ-2-4, 6006 - 6076	K.VSQM*AQYFEPLTLAAVGAASK.T	2199.51253	2	3.51E-07	0.94	4.66	-	1004.8
AHQ-2-13-, 6662	K.VSQMAQYFEPLTLAAVGAASK.T	2183.51313	2	8.68E-06	0.90	4.39	-	555.0
AHQ-2-1, 6924 - 6938	K.VSQMAQYFEPLTLAAVGAASK.T	2183.51313	3	4.14E-07	0.97	5.72	-	1785.2
AHQ-2-8, 6528	K.VSQMAQYFEPLTLAAVGAASK.T	2183.51313	2	8.70E-06	0.95	4.90	-	825.0
AHQ-2-7, 6675	K.VSQMAQYFEPLTLAAVGAASK.T	2183.51313	2	3.98E-07	0.96	5.55	-	818.9
AHQ-2-1, 6860 - 6921	K.VSQMAQYFEPLTLAAVGAASK.T	2183.51313	2	7.80E-07	0.95	4.67	-	1040.2
AHQ-2-1, 6048 - 6124	K.VSQM*AQYFEPLTLAAVGAASK.T	2199.51253	2	7.21E-05	0.85	3.94	-	618.4
AHQ-2-1, 6133	K.VSQM*AQYFEPLTLAAVGAASK.T	2199.51253	3	2.10E-10	0.96	4.91	-	1635.6
AHQ-2-7, 5639	K.VSQM*AQYFEPLTLAAVGAASK.T	2199.51253	2	1.39E-06	0.94	4.81	-	729.6
AHQ-2-12, 6667	K.VSQMAQYFEPLTLAAVGAASK.T	2183.51313	2	9.69E-05	0.74	3.35	-	425.1
AHQ-2-4, 4664	K.VVAPTISSPVCQEQLVEAGR.L	2142.41919	2	1.89E-05	0.93	4.76	-	482.3
AHQ-2-4, 4800 - 4825	K.VVAPTISSPVCQEQLVEAGR.L	2142.41919	2	1.99E-06	0.96	5.26	-	992.4
AHQ-2-10, 4178 - 4246	K.VVAPTISSPVCQEQLVEAGR.L	2142.41919	2	1.06E-05	0.90	4.04	-	699.8
AHQ-2-13-, 4606	K.VVAPTISSPVCQEQLVEAGR.L	2142.41919	2	4.00E-07	0.94	4.42	-	749.7
AHQ-2-8, 4262	K.VVAPTISSPVCQEQLVEAGR.L	2142.41919	2	1.00E-07	0.92	4.71	-	456.3
AHQ-2-8, 4358 - 4427	K.VVAPTISSPVCQEQLVEAGR.L	2142.41919	2	9.06E-07	0.94	5.10	-	543.3
AHQ-2-8, 4448	K.VVAPTISSPVCQEQLVEAGR.L	2142.41919	3	8.70E-12	0.95	5.14	-	1275.6
AHQ-2-14, 5497	K.VVAPTISSPVCQEQLVEAGR.L	2142.41919	2	7.62E-05	0.45	2.65	-	400.4
AHQ-2-1, 4980	K.VVAPTISSPVCQEQLVEAGR.L	2142.41919	2	5.58E-05	0.82	3.66	-	280.4
AHQ-2-4, 4957	K.VVAPTISSPVCQEQLVEAGR.L	2142.41919	2	1.21E-09	0.91	4.70	-	400.2
AHQ-2-1, 4812	K.VVAPTISSPVCQEQLVEAGR.L	2142.41919	2	2.95E-06	0.96	5.17	-	972.4
AHQ-2-1, 4693	K.VVAPTISSPVCQEQLVEAGR.L	2142.41919	2	4.47E-05	0.83	3.96	-	278.4
AHQ-2-14-, 4572 - 4576	K.VVAPTISSPVCQEQLVEAGR.L	2142.41919	2	2.22E-13	0.95	4.89	-	789.4
AHQ-2-3, 4668 - 4671	K.VVAPTISSPVCQEQLVEAGR.L	2142.41919	2	1.02E-07	0.93	4.57	-	513.4
AHQ-2-6, 4501 - 4513	K.VVAPTISSPVCQEQLVEAGR.L	2142.41919	2	1.41E-11	0.98	6.30	-	1043.9
AHQ-2-3, 4791 - 4860	K.VVAPTISSPVCQEQLVEAGR.L	2142.41919	2	1.40E-05	0.95	5.07	-	769.8
AHQ-2-2, 4689	K.VVAPTISSPVCQEQLVEAGR.L	2142.41919	2	2.78E-04	0.77	4.13	-	166.7
AHQ-2-5, 4485	K.VVAPTISSPVCQEQLVEAGR.L	2142.41919	2	2.21E-05	0.88	4.46	-	423.7
AHQ-2-5, 4587 - 4600	K.VVAPTISSPVCQEQLVEAGR.L	2142.41919	2	1.33E-08	0.97	5.85	-	1079.4
AHQ-2-2, 5001 - 5002	K.VVAPTISSPVCQEQLVEAGR.L	2142.41919	2	9.66E-11	0.94	4.69	-	775.1
AHQ-2-3, 4953	K.VVAPTISSPVCQEQLVEAGR.L	2142.41919	2	3.40E-06	0.85	3.54	-	453.1
AHQ-2-12, 4605	K.VVAPTISSPVCQEQLVEAGR.L	2142.41919	2	6.56E-05	0.63	3.15	-	318.3
AHQ-2-2, 4824 - 4897	K.VVAPTISSPVCQEQLVEAGR.L	2142.41919	2	2.17E-04	0.97	5.77	-	901.4
AHQ-2-7, 4475 - 4487	K.VVAPTISSPVCQEQLVEAGR.L	2142.41919	2	2.02E-07	0.94	5.06	-	732.2
gi|21956645|ref|NP_665807.1| myotrophin; granule cell differentiation protein [Homo sapiens]				1.11E-15	4.72	50.34	66.90	12894.7
AHQ-2-14-, 4531	K.GADINAPDKHHITPLLSAVYEGHVSCVK.L	3031.39193	3	7.13E-09	0.98	6.61	-	1640.5
AHQ-2-13, 4357	K.GADINAPDKHHITPLLSAVYEGHVSCVK.L	3031.39193	3	7.81E-07	0.97	6.06	-	1765.6
AHQ-2-14, 5752	K.GADINAPDKHHITPLLSAVYEGHVSCVK.L	3031.39193	3	4.36E-08	0.97	6.10	-	1358.3
AHQ-2-13-, 3995 - 4069	K.GPDGLTAFEATDNQAIK.A	1748.87196	2	2.80E-10	0.97	5.32	-	1781.4
AHQ-2-13, 4031 - 4111	K.GPDGLTAFEATDNQAIK.A	1748.87196	2	1.11E-15	0.98	5.97	-	1427.5
AHQ-2-13, 3907 - 3967	K.GPDGLTAFEATDNQAIK.A	1748.87196	2	4.18E-04	0.88	3.69	-	918.9
AHQ-2-14-, 4084	K.GPDGLTAFEATDNQAIK.A	1748.87196	2	3.86E-05	0.95	4.94	-	1079.8
AHQ-2-14, 4860 - 4934	K.GPDGLTAFEATDNQAIK.A	1748.87196	2	2.78E-06	0.97	5.17	-	1369.3
AHQ-2-14, 4992 - 5060	K.GPDGLTAFEATDNQAIK.A	1748.87196	2	6.72E-04	0.94	4.21	-	1123.6
AHQ-2-13-, 4162	K.GPDGLTAFEATDNQAIK.A	1748.87196	2	8.52E-09	0.97	5.41	-	1373.1
AHQ-2-13-, 4023 - 4093	K.GPDGLTAFEATDNQAIK.A	1748.87196	2	6.10E-07	0.97	5.27	-	1512.6
AHQ-2-14-, 3876 - 3951	K.GPDGLTAFEATDNQAIK.A	1748.87196	2	1.00E-09	0.98	5.91	-	1845.8
AHQ-2-13-, 4090	K.GPDGLTAFEATDNQAIK.A	1748.87196	3	1.99E-07	0.74	3.32	-	795.2
AHQ-2-13, 4003 - 4005	K.HHITPLLSAVYEGHVSCVK.L	2149.45825	2	4.42E-05	0.97	5.13	-	1168.4
AHQ-2-13, 3999	K.HHITPLLSAVYEGHVSCVK.L	2149.45825	3	4.56E-06	0.99	6.89	-	3169.8
AHQ-2-14, 5293 - 5365	K.HHITPLLSAVYEGHVSCVK.L	2149.45825	3	7.76E-04	0.97	5.48	-	1758.3
AHQ-2-14-, 6836 - 6843	R.KPLHYAADCGQLEILEFLLLK.G	2473.91510	3	3.38E-05	0.86	3.53	-	1032.7
AHQ-2-14-, 3587	K.NGDLDEVKDYVAK.G	1466.57496	2	5.42E-08	0.95	3.83	-	1399.1
AHQ-2-14-, 3673	K.NGDLDEVKDYVAK.G	1466.57496	2	1.15E-08	0.94	3.71	-	1315.9
AHQ-2-13-, 3770	K.NGDLDEVKDYVAK.G	1466.57496	2	3.00E-08	0.93	3.66	-	1177.1
AHQ-2-14, 4537	K.NGDLDEVKDYVAK.G	1466.57496	2	5.53E-08	0.95	4.17	-	1456.6
AHQ-2-13-, 3658	K.NGDLDEVKDYVAK.G	1466.57496	2	1.58E-09	0.92	3.58	-	1103.8
AHQ-2-13-, 3777	K.NGDLDEVKDYVAK.G	1466.57496	1	1.30E-06	0.41	2.60	-	269.5
AHQ-2-13, 3751 - 3759	K.NGDLDEVKDYVAK.G	1466.57496	2	4.93E-09	0.93	3.98	-	1116.5
gi|4504165|ref|NP_000168.1| gelsolin (amyloidosis, Finnish type); Gelsolin [Homo sapiens]				2.22E-15	21.02	230.37	35.30	85696.9
AHQ-2-5, 3572	K.AGALNSNDAFVLK.T	1320.47622	1	1.62E-07	0.40	2.42	-	406.7
AHQ-2-5, 3657 - 3727	K.AGALNSNDAFVLK.T	1320.47622	1	2.61E-06	0.40	2.28	-	414.7
AHQ-2-5, 3509 - 3587	K.AGALNSNDAFVLK.T	1320.47622	2	1.54E-06	0.87	3.74	-	711.4
AHQ-2-5, 3311 - 3347	K.AGALNSNDAFVLK.T	1320.47622	2	2.11E-05	0.75	3.12	-	619.0
AHQ-2-5, 3663 - 3716	K.AGALNSNDAFVLK.T	1320.47622	2	1.11E-07	0.88	3.49	-	722.7
AHQ-2-6, 3543	K.AGALNSNDAFVLK.T	1320.47622	2	3.99E-04	0.91	3.67	-	952.1
AHQ-2-6, 3627	K.AGALNSNDAFVLK.T	1320.47622	2	9.53E-05	0.40	3.00	-	375.1
AHQ-2-7, 3253	K.AGALNSNDAFVLK.T	1320.47622	2	3.45E-06	0.78	3.26	-	555.1
AHQ-2-6, 5497 - 5501	R.AQPVQVAEGSEPDGFWEALGGK.A	2273.44345	2	8.86E-08	0.96	5.69	-	715.4
AHQ-2-6, 5593 - 5673	R.AQPVQVAEGSEPDGFWEALGGK.A	2273.44345	2	4.89E-07	0.95	5.20	-	715.0
AHQ-2-11, 5494	R.AQPVQVAEGSEPDGFWEALGGK.A	2273.44345	2	1.02E-04	0.91	4.07	-	662.8
AHQ-2-5, 5575	R.AQPVQVAEGSEPDGFWEALGGK.A	2273.44345	2	1.39E-04	0.93	4.78	-	719.5
AHQ-2-10, 5124 - 5126	R.AQPVQVAEGSEPDGFWEALGGK.A	2273.44345	2	3.12E-05	0.94	5.06	-	623.5
AHQ-2-7, 5540	R.AQPVQVAEGSEPDGFWEALGGK.A	2273.44345	2	4.62E-05	0.94	4.97	-	635.4
AHQ-2-9, 5680 - 5711	R.AQPVQVAEGSEPDGFWEALGGK.A	2273.44345	2	1.04E-05	0.94	4.68	-	695.9
AHQ-2-5, 5715 - 5783	R.AQPVQVAEGSEPDGFWEALGGK.A	2273.44345	2	2.09E-06	0.96	5.60	-	850.4

AHQ-2-9, 5522	R.AQPVQVAEGSEPDGFWEALGGK.A	2273.44345	2	3.73E-10	0.91	4.73	-	464.4
AHQ-2-5, 5900 - 5979	R.AQPVQVAEGSEPDGFWEALGGK.A	2273.44345	2	2.66E-06	0.97	6.26	-	764.4
AHQ-2-7, 5657 - 5723	R.AQPVQVAEGSEPDGFWEALGGK.A	2273.44345	2	3.68E-08	0.95	5.50	-	563.2
AHQ-2-1, 6058	R.AQPVQVAEGSEPDGFWEALGGK.A	2273.44345	2	2.80E-04	0.75	3.67	-	284.0
AHQ-2-3, 6032	R.AQPVQVAEGSEPDGFWEALGGK.A	2273.44345	2	3.40E-05	0.89	4.12	-	619.5
AHQ-2-10, 5260 - 5322	R.AQPVQVAEGSEPDGFWEALGGK.A	2273.44345	2	8.37E-07	0.93	4.86	-	689.6
AHQ-2-8, 5511 - 5590	R.AQPVQVAEGSEPDGFWEALGGK.A	2273.44345	2	9.55E-06	0.92	4.17	-	765.3
AHQ-2-5, 6047	R.AQPVQVAEGSEPDGFWEALGGK.A	2273.44345	2	3.39E-08	0.96	5.40	-	786.4
AHQ-2-6, 5558	R.DPDQTDGLGLSYLSSHIANVER.V	2388.53285	3	1.02E-05	0.94	4.41	-	1305.9
AHQ-2-5, 5641 - 5723	R.DPDQTDGLGLSYLSSHIANVER.V	2388.53285	3	1.50E-04	0.56	3.05	-	337.4
AHQ-2-5, 2136	K.DSQEEEKTEALTSAK.R	1666.72289	2	1.39E-07	0.84	3.30	-	858.6
AHQ-2-7, 1903	K.DSQEEEKTEALTSAK.R	1666.72289	2	2.50E-10	0.94	3.50	-	1654.1
AHQ-2-11, 2149	K.DSQEEEKTEALTSAK.R	1666.72289	2	1.35E-04	0.82	3.29	-	884.7
AHQ-2-9, 2034 - 2043	K.DSQEEEKTEALTSAK.R	1666.72289	2	3.09E-05	0.88	3.39	-	1344.9
AHQ-2-5, 2043	K.DSQEEEKTEALTSAK.R	1666.72289	2	8.81E-04	0.91	3.74	-	1201.0
AHQ-2-13, 2311	K.DSQEEEKTEALTSAK.R	1666.72289	2	6.43E-05	0.87	3.59	-	894.4
AHQ-2-5, 1945	K.DSQEEEKTEALTSAK.R	1666.72289	3	4.70E-05	0.97	4.62	-	2225.1
AHQ-2-5, 1941 - 1968	K.DSQEEEKTEALTSAK.R	1666.72289	2	1.24E-08	0.95	4.27	-	1434.7
AHQ-2-10, 2026	K.DSQEEEKTEALTSAK.R	1666.72289	2	1.25E-05	0.88	3.50	-	1071.0
AHQ-2-13-, 2205	K.DSQEEEKTEALTSAK.R	1666.72289	2	1.51E-07	0.96	4.24	-	1709.7
AHQ-2-13-, 2219	K.DSQEEEKTEALTSAK.R	1666.72289	3	3.58E-08	0.97	4.45	-	2372.4
AHQ-2-8, 1854	K.DSQEEEKTEALTSAK.R	1666.72289	2	9.00E-08	0.88	3.63	-	1050.9
AHQ-2-6, 1950	K.DSQEEEKTEALTSAK.R	1666.72289	3	1.72E-05	0.88	3.27	-	1253.1
AHQ-2-6, 1941	K.DSQEEEKTEALTSAK.R	1666.72289	2	3.89E-08	0.91	3.59	-	1126.4
AHQ-2-5, 6363 - 6369	R.EVQGFESATFLGYFK.S	1723.90570	2	7.65E-10	0.96	4.13	-	1576.4
AHQ-2-13, 6087 - 6156	R.EVQGFESATFLGYFK.S	1723.90570	2	4.96E-05	0.80	2.74	-	1051.6
AHQ-2-12, 6238	R.EVQGFESATFLGYFK.S	1723.90570	2	8.87E-04	0.88	3.19	-	1027.5
AHQ-2-5, 6927	R.EVQGFESATFLGYFK.S	1723.90570	2	2.02E-04	0.86	2.95	-	912.6
AHQ-2-5, 7065	R.EVQGFESATFLGYFK.S	1723.90570	2	6.00E-07	0.88	3.28	-	1036.0
AHQ-2-13-, 6258 - 6331	R.EVQGFESATFLGYFK.S	1723.90570	2	4.91E-09	0.97	4.86	-	1760.0
AHQ-2-5, 2233	K.HVVPNEVVVQR.L	1276.46981	1	2.20E-06	0.75	2.59	-	640.6
AHQ-2-6, 2022	K.HVVPNEVVVQR.L	1276.46981	2	1.78E-06	0.84	3.12	-	546.8
AHQ-2-5, 2228	K.HVVPNEVVVQR.L	1276.46981	2	1.35E-04	0.83	2.90	-	572.7
AHQ-2-7, 1952	K.HVVPNEVVVQR.L	1276.46981	2	2.46E-07	0.86	3.02	-	560.6
AHQ-2-9, 2067	K.HVVPNEVVVQR.L	1276.46981	2	1.15E-07	0.84	2.95	-	470.7
AHQ-2-11, 2204 - 2212	K.HVVPNEVVVQR.L	1276.46981	2	3.76E-06	0.94	3.51	-	842.2
AHQ-2-11, 2208 - 2210	K.HVVPNEVVVQR.L	1276.46981	1	5.46E-06	0.60	2.47	-	427.3
AHQ-2-5, 2019	K.HVVPNEVVVQR.L	1276.46981	1	1.83E-06	0.27	2.53	-	204.7
AHQ-2-12, 2242 - 2243	K.HVVPNEVVVQR.L	1276.46981	1	9.70E-07	0.25	2.33	-	261.4
AHQ-2-12, 2237	K.HVVPNEVVVQR.L	1276.46981	2	4.67E-06	0.92	3.50	-	653.2
AHQ-2-5, 2007 - 2076	K.HVVPNEVVVQR.L	1276.46981	2	1.69E-08	0.90	3.44	-	545.2
AHQ-2-5, 6864	R.IEGSNKVPVDPATYGQFYGGDSYIILYNYR.H	3401.72499	3	7.43E-04	0.78	3.66	-	543.2
AHQ-2-5, 6415 - 6431	R.IEGSNKVPVDPATYGQFYGGDSYIILYNYR.H	3401.72499	3	3.62E-07	0.97	5.77	-	1617.7
AHQ-2-5, 5467 - 5535	K.NWRDPDQTDGLGLSYLSSHIANVER.V	2845.03323	3	4.24E-09	0.97	7.01	-	1209.9
AHQ-2-6, 5445	K.NWRDPDQTDGLGLSYLSSHIANVER.V	2845.03323	3	7.20E-06	0.98	7.40	-	2024.2
AHQ-2-5, 6172 - 6183	K.NWRDPDQTDGLGLSYLSSHIANVER.V	2845.03323	3	2.22E-15	0.95	5.56	-	1046.1
AHQ-2-5, 1760 - 1811	K.PALPAGTEDTAK.E	1171.28241	2	1.92E-08	0.83	2.90	-	512.6
AHQ-2-5, 2269	K.PALPAGTEDTAKEDAANR.K	1827.93072	2	2.24E-08	0.81	3.58	-	412.9
AHQ-2-7, 2257	K.PALPAGTEDTAKEDAANR.K	1827.93072	2	3.78E-05	0.38	2.77	-	123.6
AHQ-2-5, 2080	K.PALPAGTEDTAKEDAANRK.L	1956.10363	2	2.69E-04	0.86	3.61	-	524.2
AHQ-2-7, 4555	K.QTQVSVLPEGGETPLFK.Q	1831.05925	2	3.80E-05	0.76	3.38	-	308.4
AHQ-2-6, 4655	K.QTQVSVLPEGGETPLFK.Q	1831.05925	2	3.85E-07	0.93	4.35	-	576.0
AHQ-2-5, 4856	K.QTQVSVLPEGGETPLFK.Q	1831.05925	2	6.73E-06	0.84	3.45	-	423.3
AHQ-2-8, 4370	K.QTQVSVLPEGGETPLFK.Q	1831.05925	2	9.44E-04	0.82	3.24	-	424.6
AHQ-2-5, 4688 - 4763	K.QTQVSVLPEGGETPLFK.Q	1831.05925	2	4.52E-05	0.87	3.85	-	525.5
AHQ-2-5, 2697	K.SEDCFILDHGK.D	1322.42590	2	2.89E-06	0.95	3.71	-	1519.9
AHQ-2-7, 2661	K.SEDCFILDHGK.D	1322.42590	2	1.54E-06	0.92	3.21	-	1220.8
AHQ-2-6, 2749	K.SEDCFILDHGK.D	1322.42590	2	2.76E-06	0.96	3.58	-	1707.3
AHQ-2-5, 1881	K.TGAQELLR.V	888.00387	2	2.33E-04	0.89	3.02	-	1067.4
AHQ-2-8, 3938	K.TPSAAYLWVGTGASEAEK.T	1838.99510	2	6.63E-04	0.92	3.92	-	986.7
AHQ-2-5, 4252 - 4323	K.TPSAAYLWVGTGASEAEK.T	1838.99510	2	1.81E-10	0.98	5.17	-	1734.4
AHQ-2-5, 4693	K.TPSAAYLWVGTGASEAEK.T	1838.99510	2	6.91E-05	0.78	3.02	-	604.0
AHQ-2-7, 4135	K.TPSAAYLWVGTGASEAEK.T	1838.99510	2	2.04E-06	0.85	3.48	-	837.3
AHQ-2-6, 4371	K.TPSAAYLWVGTGASEAEK.T	1838.99510	2	8.02E-05	0.59	2.79	-	561.6
AHQ-2-5, 4391 - 4469	K.TPSAAYLWVGTGASEAEK.T	1838.99510	2	1.66E-10	0.96	4.51	-	1465.9
AHQ-2-9, 4646	R.VHVSEEGTEPEAMLQVLGPK.P	2151.42630	2	2.55E-04	0.96	4.78	-	1103.5
AHQ-2-5, 3541 - 3563	R.VHVSEEGTEPEAM*LQVLGPK.P	2167.42570	2	1.14E-04	0.95	4.85	-	1119.5
AHQ-2-7, 4571	R.VHVSEEGTEPEAMLQVLGPK.P	2151.42630	2	3.74E-06	0.94	4.43	-	772.8
AHQ-2-5, 4697 - 4767	R.VHVSEEGTEPEAMLQVLGPK.P	2151.42630	2	6.50E-06	0.97	5.40	-	1211.2
AHQ-2-5, 5071	R.VHVSEEGTEPEAMLQVLGPKPALPAGTEDTAK.E	3303.68611	3	7.23E-06	0.86	4.10	-	653.0
AHQ-2-6, 5013	R.VHVSEEGTEPEAMLQVLGPKPALPAGTEDTAK.E	3303.68611	3	2.34E-09	0.92	4.89	-	718.5
AHQ-2-8, 4813	R.VHVSEEGTEPEAMLQVLGPKPALPAGTEDTAK.E	3303.68611	3	1.00E-05	0.87	4.04	-	607.9
AHQ-2-5, 4147	R.VHVSEEGTEPEAM*LQVLGPKPALPAGTEDTAK.E	3319.68551	3	1.24E-04	0.89	4.66	-	694.3
AHQ-2-5, 4821	R.VHVSEEGTEPEAMLQVLGPKPALPAGTEDTAKEDAANR.K	3960.33442	3	9.41E-06	0.94	5.32	-	1043.1
AHQ-2-5, 3435	R.VPFDAATLHTSTAM*AAQHGMDDDGTGQK.Q	2891.10081	3	9.38E-04	0.94	5.46	-	1128.0
AHQ-2-5, 3804	R.VPFDAATLHTSTAMAAQHGMDDDGTGQK.Q	2875.10141	3	1.20E-06	0.97	5.98	-	1573.3
AHQ-2-5, 6199 - 6267	K.VSNGAGTMSVSLVADENPFAQGALK.S	2464.73740	2	2.01E-09	0.80	3.23	-	691.6
gi|4503327|ref|NP_000389.1| cytochrome b5 reductase membrane-bound isoform; diaphorase (NADH) [Homo				2.22E-15	7.72	90.29	42.20	34234.6
AHQ-2-9, 5659 - 5732	R.APEAWDYGQGFVNEEMIR.D	2113.29517	2	1.02E-06	0.96	4.94	-	1139.9
AHQ-2-9, 5056 - 5058	R.APEAWDYGQGFVNEEM*IR.D	2129.29457	2	6.94E-04	0.97	4.77	-	1878.3
AHQ-2-9, 4650	K.DILLRPELEELR.N	1496.73370	2	4.01E-09	0.86	3.29	-	884.6
AHQ-2-9, 6116 - 6134	R.FALPSPQHILGLPVGQHIYLSAR.I	2515.94007	2	6.01E-05	0.89	4.37	-	539.1
AHQ-2-11, 5888 - 5913	R.FALPSPQHILGLPVGQHIYLSAR.I	2515.94007	3	8.25E-04	0.77	3.70	-	756.6
AHQ-2-12, 6146 - 6166	R.FALPSPQHILGLPVGQHIYLSAR.I	2515.94007	3	7.04E-09	0.95	5.33	-	1151.8
AHQ-2-9, 6115 - 6182	R.FALPSPQHILGLPVGQHIYLSAR.I	2515.94007	3	2.22E-15	0.97	5.88	-	1844.5
AHQ-2-9, 2707 - 2712	R.GPSGLLVYQGK.G	1119.29547	1	1.35E-05	0.81	3.61	-	381.4
AHQ-2-9, 2732	R.GPSGLLVYQGK.G	1119.29547	2	3.02E-04	0.93	3.41	-	1310.7
AHQ-2-12, 6003	R.IDGNLVVRPYTPISSDDDKGFVDLVIK.V	2977.35765	3	6.37E-05	0.49	3.12	-	398.3
AHQ-2-9, 6160 - 6239	R.IDGNLVVRPYTPISSDDDKGFVDLVIK.V	2977.35765	3	1.56E-05	0.93	5.49	-	878.4
AHQ-2-9, 6006 - 6076	R.IDGNLVVRPYTPISSDDDKGFVDLVIK.V	2977.35765	3	5.89E-05	0.95	5.38	-	1508.5
AHQ-2-9, 4479	R.STPAITLESPDIKYPLR.L	1902.18061	2	1.30E-08	0.88	4.13	-	554.1
AHQ-2-9, 5940 - 6008	K.SVGMIAGGTGITPMLQVIR.A	1902.31543	2	1.23E-04	0.63	3.49	-	538.6
AHQ-2-9, 5010	K.SVGMIAGGTGITPM*LQVIR.A	1918.31483	2	6.29E-06	0.71	3.24	-	442.8
gi|21361657|ref|NP_005304.3| glucose regulated protein, 58kDa; glucose regulated protein, 58kD [Hom				3.33E-15	9.86	120.33	27.90	56782.0
AHQ-2-7, 5839	R.ELSDFISYLQR.E	1371.52000	2	2.03E-06	0.97	4.39	-	1588.1
AHQ-2-7, 7283 - 7292	K.FISDKDASIVGFFDDSFSEAHSEFLK.A	2940.16492	3	3.33E-15	0.98	6.59	-	3103.6
AHQ-2-10, 6632	K.FISDKDASIVGFFDDSFSEAHSEFLK.A	2940.16492	3	7.23E-06	0.89	4.84	-	691.9
AHQ-2-7, 4297	R.GFPTIYFSPANK.K	1342.52349	1	6.18E-04	0.46	2.75	-	504.8
AHQ-2-7, 5607 - 5611	R.KTFSHELSDFGLESTAGEIPVVAIR.T	2705.01562	3	3.13E-06	0.92	4.65	-	1080.2
AHQ-2-7, 2343	K.LNFAVASR.K	878.01067	2	2.19E-04	0.80	2.93	-	776.0
AHQ-2-7, 1955	K.LSKDPNIVIAK.M	1198.43727	2	1.02E-05	0.86	3.23	-	935.5
AHQ-2-7, 3269 - 3347	K.MDATANDVPSPYEVR.G	1665.80646	2	2.07E-04	0.84	3.86	-	476.1
AHQ-2-7, 2916	K.M*DATANDVPSPYEVR.G	1681.80586	2	4.52E-05	0.84	3.71	-	397.0
AHQ-2-7, 2131	K.SEPIPESNDGPVK.V	1369.45928	1	2.11E-04	0.47	2.59	-	467.8
AHQ-2-7, 1867	R.TADGIVSHLK.K	1041.18311	2	8.95E-05	0.84	2.77	-	919.1
AHQ-2-9, 6354	K.TFSHELSDFGLESTAGEIPVVAIR.T	2576.84271	2	9.78E-08	0.97	4.93	-	1659.8
AHQ-2-7, 6263 - 6273	K.TFSHELSDFGLESTAGEIPVVAIR.T	2576.84271	3	5.86E-05	0.89	4.33	-	740.4

AHQ-2-7, 2785 - 2799	K.YGVSGYPTLK.I	1085.23435	2	6.17E-04	0.92	3.39	-	666.1
gi|22043610|ref|XP_171081.1| similar to ras-related C3 botulinum toxin substrate 1 isoform Rac1; rh				3.89E-15	2.86	40.26	21.40	21543.1
AHQ-2-11, 2538	R.AVLCPPPVK.K	982.22199	1	1.97E-04	0.15	1.97	-	289.5
AHQ-2-11, 3732 - 3800	K.KLTPITYPQGLAMAK.E	1632.99216	2	9.79E-05	0.87	3.72	-	591.4
AHQ-2-11, 3714 - 3717	K.LTPITYPQGLAM*AK.E	1520.81864	2	7.99E-05	0.86	3.03	-	553.3
AHQ-2-11, 4702 - 4705	K.PVNLGLWDTAGQEDYDR.L	1950.05441	2	3.89E-15	0.98	5.26	-	2272.8
gi|4502295|ref|NP_001677.1| ATP synthase, H+ transporting, mitochondrial F1 complex, beta polypepti				4.44E-15	10.95	120.33	38.40	57955.2
AHQ-2-7, 2513	K.AHGGYSVFAGVGER.T	1407.51566	2	2.72E-08	0.80	2.97	-	519.0
AHQ-2-7, 5581 - 5596	R.AIAELGIYPAVDPLDSTSR.I	1989.21513	2	1.88E-07	0.90	4.03	-	539.5
AHQ-2-7, 7615	R.DQEGQDVLLFIDNIFR.F	1923.11524	2	2.12E-10	0.96	5.51	-	975.9
AHQ-2-7, 6035	R.FLSQPFQVAEVFTGHMGK.L	2024.33104	3	5.50E-08	0.76	3.86	-	506.2
AHQ-2-7, 6029 - 6107	R.FLSQPFQVAEVFTGHMGK.L	2024.33104	2	3.06E-07	0.85	3.69	-	605.0
AHQ-2-7, 4252 - 4256	R.FTQAGSEVSALLGR.I	1436.59522	2	1.72E-09	0.97	4.98	-	1359.8
AHQ-2-7, 7351	K.GFQQILAGEYDHLPEQAFYM*VGPIEEAVAK.A	3368.75991	3	4.44E-15	0.97	6.65	-	1217.1
AHQ-2-7, 3369	R.IMDPNIVGSEHYDVAR.G	1817.01617	2	1.75E-10	0.95	4.37	-	1016.3
AHQ-2-7, 3563 - 3573	R.IMNVIGEPIDER.G	1386.59962	2	3.43E-04	0.93	4.28	-	979.8
AHQ-2-7, 4563	R.IPSAVGYQPTLATDMGTMQER.I	2267.56880	2	1.56E-05	0.88	4.20	-	415.1
AHQ-2-7, 3928 - 3943	R.LVLEVAQHLGESTVR.T	1651.88893	2	6.42E-09	0.97	5.19	-	1674.3
AHQ-2-7, 5588 - 5589	R.VALTGLTVAEYFR.D	1440.66861	2	5.53E-05	0.91	3.19	-	1301.6
AHQ-2-7, 4495 - 4507	K.VLDSGAPIKIPVGPETLGR.I	1920.24231	2	8.79E-08	0.88	4.23	-	362.2
AHQ-2-7, 4496	K.VLDSGAPIKIPVGPETLGR.I	1920.24231	3	5.51E-09	0.94	4.45	-	1519.2
gi|4827056|ref|NP_005103.1| WD repeat-containing protein 1 isoform 2 [Homo sapiens]				8.88E-15	11.37	130.38	45.90	58001.9
AHQ-2-9, 4774	K.AHDGGIYAISWSPDSTHLLSASGDK.T	2586.75455	2	4.13E-04	0.90	3.92	-	808.6
AHQ-2-6, 4813	K.AHDGGIYAISWSPDSTHLLSASGDK.T	2586.75455	2	6.60E-09	0.96	5.39	-	1170.0
AHQ-2-6, 4997	K.AHDGGIYAISWSPDSTHLLSASGDK.T	2586.75455	3	2.70E-04	0.95	5.41	-	1016.3
AHQ-2-10, 5288	K.CFSIDNPGYEPEVVAVHPGGDTVAIGGVDGNVR.L	3400.67708	3	5.72E-05	0.92	4.50	-	731.9
AHQ-2-6, 5769 - 5813	K.CFSIDNPGYEPEVVAVHPGGDTVAIGGVDGNVR.L	3400.67708	3	3.81E-11	0.94	5.37	-	870.0
AHQ-2-6, 5630 - 5701	K.CFSIDNPGYEPEVVAVHPGGDTVAIGGVDGNVR.L	3400.67708	3	8.88E-15	0.97	6.07	-	1235.5
AHQ-2-7, 5701	K.CFSIDNPGYEPEVVAVHPGGDTVAIGGVDGNVR.L	3400.67708	3	2.62E-09	0.71	3.48	-	516.9
AHQ-2-9, 5730	K.CFSIDNPGYEPEVVAVHPGGDTVAIGGVDGNVR.L	3400.67708	3	3.72E-09	0.95	5.06	-	940.2
AHQ-2-6, 7415 - 7437	K.CVAVGPGGYAVVVCIGQIVLLK.D	2275.75982	2	1.91E-05	0.48	2.88	-	370.7
AHQ-2-10, 4319	K.GPVTDVAYSHDGAFLAVCDASK.V	2282.47194	2	9.73E-10	0.93	4.19	-	1078.9
AHQ-2-6, 4533 - 4601	K.GPVTDVAYSHDGAFLAVCDASK.V	2282.47194	2	1.46E-09	0.98	5.55	-	1848.6
AHQ-2-13, 2427	K.IKDIAWTEDSKR.I	1462.63267	2	9.44E-06	0.90	3.61	-	830.0
AHQ-2-11, 5322	R.LATGSDDNCAAFFEGPPFK.F	2046.20216	2	1.11E-07	0.89	3.51	-	932.1
AHQ-2-6, 5457 - 5521	R.LATGSDDNCAAFFEGPPFK.F	2046.20216	2	3.78E-06	0.95	4.94	-	1053.5
AHQ-2-7, 5443	R.LATGSDDNCAAFFEGPPFK.F	2046.20216	2	8.25E-07	0.81	3.55	-	846.5
AHQ-2-6, 5829	R.LATGSDDNCAAFFEGPPFK.F	2046.20216	2	1.42E-08	0.79	3.31	-	698.1
AHQ-2-7, 3549	R.LYSILGTTLKDEGK.L	1538.76736	2	3.27E-08	0.85	3.44	-	740.3
AHQ-2-6, 3631	R.LYSILGTTLKDEGK.L	1538.76736	2	4.52E-06	0.86	3.29	-	794.3
AHQ-2-9, 3656	R.LYSILGTTLKDEGK.L	1538.76736	2	4.09E-04	0.80	2.92	-	820.2
AHQ-2-6, 4947 - 5010	R.MTVDESGQLISCSMDDTVR.Y	2146.36365	2	2.69E-04	0.87	3.60	-	791.2
AHQ-2-6, 4375	R.M*TVDESGQLISCSMDDTVR.Y	2162.36305	2	5.68E-04	0.90	4.06	-	605.4
AHQ-2-6, 4033	R.NIDNPALADIYTEHAHQVVVAK.Y	2419.67805	3	9.64E-04	0.73	3.16	-	409.5
AHQ-2-13, 4227	R.NIDNPALADIYTEHAHQVVVAK.Y	2419.67805	2	2.69E-09	0.96	5.20	-	1130.7
AHQ-2-6, 1635	K.SIQCLTVHKNGGK.S	1443.65418	2	1.31E-04	0.81	3.61	-	816.9
AHQ-2-6, 3998	K.SYIYSGSHDGHINYWDSETGENDSFAGK.G	3138.17570	3	1.41E-11	0.98	7.58	-	1743.0
AHQ-2-6, 4125	K.SYIYSGSHDGHINYWDSETGENDSFAGK.G	3138.17570	3	6.34E-08	0.96	6.09	-	1189.4
AHQ-2-12, 3939	K.YAPSGFYIASGDVSGK.L	1619.75589	2	5.10E-05	0.87	3.32	-	738.0
AHQ-2-1, 4173	K.YAPSGFYIASGDVSGK.L	1619.75589	2	3.27E-06	0.76	3.10	-	542.9
AHQ-2-6, 3699 - 3769	K.YAPSGFYIASGDVSGK.L	1619.75589	2	6.66E-09	0.96	4.73	-	920.4
gi|4757810|ref|NP_004037.1| ATP synthase, H+ transporting, mitochondrial F1 complex, alpha subunit,				8.88E-15	3.49	40.30	11.60	59750.3
AHQ-2-7, 5361 - 5365	K.FENAFLSHVVSQHQALLGTIR.A	2368.67911	2	1.31E-08	0.93	4.49	-	948.3
AHQ-2-7, 5349 - 5352	K.FENAFLSHVVSQHQALLGTIR.A	2368.67911	3	8.88E-15	0.97	5.91	-	1882.7
AHQ-2-7, 6184	K.GMSLNLEPDNVGVVVFGNDK.L	2105.35763	2	5.31E-04	0.87	4.49	-	383.7
AHQ-2-7, 3496 - 3499	K.TGTAEMSSILEER.I	1424.55988	2	3.09E-04	0.91	3.20	-	1546.3
AHQ-2-7, 2816	R.VLSIGDGIAR.V	1001.16212	2	9.86E-05	0.73	2.72	-	593.3
gi|12667788|ref|NP_002464.1| myosin, heavy polypeptide 9, non-muscle [Homo sapiens]				9.99E-15	109.21	1260.38	49.00	226529.8
AHQ-2-3, 2488	R.AKQTLENERGELANEVK.V	1930.10932	2	5.99E-05	0.95	4.87	-	950.8
AHQ-2-4, 1934	R.ALEEAMEQK.A	1049.18068	2	8.75E-06	0.90	3.44	-	1008.0
AHQ-2-3, 1911	R.ALEEAMEQK.A	1049.18068	2	3.03E-05	0.89	3.55	-	728.9
AHQ-2-2, 1777 - 1912	R.ALEEAMEQK.A	1049.18068	1	1.72E-04	0.27	2.17	-	548.1
AHQ-2-1, 3910 - 3912	K.ALELDSNLYR.I	1194.31920	1	2.43E-04	0.18	2.49	-	283.9
AHQ-2-4, 3745	K.ALELDSNLYR.I	1194.31920	2	1.46E-05	0.95	4.16	-	1246.4
AHQ-2-2, 3797	K.ALELDSNLYR.I	1194.31920	2	4.79E-06	0.94	3.71	-	1337.6
AHQ-2-6, 3469 - 3470	K.ALELDSNLYR.I	1194.31920	2	7.24E-07	0.90	3.44	-	973.9
AHQ-2-5, 3488	K.ALELDSNLYR.I	1194.31920	2	6.73E-07	0.96	4.09	-	1458.6
AHQ-2-3, 3747 - 3764	K.ALELDSNLYR.I	1194.31920	2	2.48E-06	0.95	4.02	-	1174.6
AHQ-2-1, 3908	K.ALELDSNLYR.I	1194.31920	2	6.39E-06	0.94	3.49	-	1290.1
AHQ-2-4, 2788	R.ALEQQVEEMK.T	1205.36380	2	4.95E-05	0.89	3.71	-	1015.1
AHQ-2-2, 2034	R.ALEQQVEEM*K.T	1221.36320	2	4.61E-05	0.81	3.26	-	898.3
AHQ-2-4, 2054	R.ALEQQVEEM*K.T	1221.36320	2	7.18E-04	0.74	2.81	-	631.8
AHQ-2-1, 2954	R.ALEQQVEEMK.T	1205.36380	2	4.54E-06	0.92	3.81	-	1296.9
AHQ-2-2, 2786 - 2796	R.ALEQQVEEMK.T	1205.36380	2	1.35E-07	0.93	3.99	-	1056.1
AHQ-2-1, 2196	R.ALEQQVEEM*K.T	1221.36320	2	4.31E-06	0.83	3.55	-	798.8
AHQ-2-5, 2541	R.ALEQQVEEMK.T	1205.36380	2	2.86E-05	0.90	3.63	-	1015.2
AHQ-2-3, 2789	R.ALEQQVEEMK.T	1205.36380	2	4.35E-05	0.91	3.82	-	1126.5
AHQ-2-5, 7456	R.ALEQQVEEMKTQLEELEDELQATEDAK.L	3149.38454	3	2.12E-07	0.96	5.75	-	1239.8
AHQ-2-6, 6401	R.ALEQQVEEM*KTQLEELEDELQATEDAK.L	3165.38394	3	1.11E-10	0.98	6.25	-	2185.4
AHQ-2-3, 6708 - 6712	R.ALEQQVEEM*KTQLEELEDELQATEDAK.L	3165.38394	3	2.07E-10	0.97	6.38	-	1503.0
AHQ-2-1, 6712 - 6713	R.ALEQQVEEM*KTQLEELEDELQATEDAK.L	3165.38394	3	1.45E-09	0.96	5.92	-	1486.1
AHQ-2-1, 6585 - 6649	R.ALEQQVEEM*KTQLEELEDELQATEDAK.L	3165.38394	3	4.07E-05	0.97	6.95	-	1674.2
AHQ-2-5, 6585	R.ALEQQVEEM*KTQLEELEDELQATEDAK.L	3165.38394	3	2.06E-07	0.96	5.81	-	1518.2
AHQ-2-2, 6764	R.ALEQQVEEM*KTQLEELEDELQATEDAK.L	3165.38394	3	2.62E-12	0.98	7.60	-	1959.7
AHQ-2-2, 7568 - 7570	R.ALEQQVEEMKTQLEELEDELQATEDAK.L	3149.38454	3	7.41E-05	0.97	5.89	-	1664.6
AHQ-2-3, 7519	R.ALEQQVEEMKTQLEELEDELQATEDAK.L	3149.38454	3	2.61E-05	0.96	6.27	-	1270.0
AHQ-2-6, 7214	R.ALEQQVEEMKTQLEELEDELQATEDAK.L	3149.38454	3	1.89E-06	0.96	6.02	-	1252.5
AHQ-2-4, 7572	R.ALEQQVEEMKTQLEELEDELQATEDAK.L	3149.38454	3	3.54E-08	0.97	5.62	-	1939.6
AHQ-2-2, 5320 - 5390	K.ANLQIDQINTDLNLER.S	1871.04187	2	5.81E-04	0.97	5.76	-	1519.3
AHQ-2-2, 5474 - 5544	K.ANLQIDQINTDLNLER.S	1871.04187	2	2.55E-11	0.97	5.49	-	1442.9
AHQ-2-1, 5037	K.ANLQIDQINTDLNLER.S	1871.04187	2	4.37E-06	0.97	5.53	-	1233.2
AHQ-2-9, 4968	K.ANLQIDQINTDLNLER.S	1871.04187	2	2.29E-07	0.96	4.98	-	1401.2
AHQ-2-2, 5210 - 5284	K.ANLQIDQINTDLNLER.S	1871.04187	2	3.26E-08	0.98	5.70	-	1578.6
AHQ-2-5, 5052 - 5061	K.ANLQIDQINTDLNLER.S	1871.04187	2	1.47E-06	0.97	5.66	-	1544.5
AHQ-2-2, 5084 - 5094	K.ANLQIDQINTDLNLER.S	1871.04187	2	1.20E-05	0.95	4.74	-	835.5
AHQ-2-2, 4992 - 5004	K.ANLQIDQINTDLNLER.S	1871.04187	2	2.58E-06	0.93	4.19	-	1012.4
AHQ-2-4, 5338	K.ANLQIDQINTDLNLER.S	1871.04187	2	1.25E-04	0.94	4.24	-	1021.6
AHQ-2-6, 4941	K.ANLQIDQINTDLNLER.S	1871.04187	2	1.30E-07	0.93	4.44	-	968.1
AHQ-2-3, 5039 - 5072	K.ANLQIDQINTDLNLER.S	1871.04187	2	1.69E-05	0.93	4.72	-	765.6
AHQ-2-1, 5268 - 5344	K.ANLQIDQINTDLNLER.S	1871.04187	2	1.13E-05	0.96	5.04	-	1066.4
AHQ-2-1, 5432	K.ANLQIDQINTDLNLER.S	1871.04187	2	2.03E-04	0.95	4.83	-	1183.9
AHQ-2-3, 5184 - 5259	K.ANLQIDQINTDLNLER.S	1871.04187	2	2.81E-06	0.97	5.28	-	1527.5
AHQ-2-3, 5324	K.ANLQIDQINTDLNLER.S	1871.04187	2	3.81E-08	0.94	4.61	-	996.7
AHQ-2-4, 5230 - 5245	K.ANLQIDQINTDLNLER.S	1871.04187	2	1.38E-04	0.97	4.81	-	1879.3
AHQ-2-7, 4931	K.ANLQIDQINTDLNLER.S	1871.04187	2	5.84E-04	0.94	4.63	-	922.0
AHQ-2-3, 5477	K.ANLQIDQINTDLNLER.S	1871.04187	2	1.44E-08	0.97	4.78	-	1377.6
AHQ-2-10, 4654	K.ANLQIDQINTDLNLER.S	1871.04187	2	2.64E-08	0.97	5.28	-	1707.6

AHQ-2-4, 2240	K.ASITALEAK.I	904.04321	2	1.26E-06	0.94	3.21	-	1269.1
AHQ-2-5, 2009 - 2012	K.ASITALEAK.I	904.04321	2	5.72E-04	0.88	3.10	-	809.4
AHQ-2-2, 2238	K.ASITALEAK.I	904.04321	2	3.11E-05	0.92	2.85	-	1118.4
AHQ-2-1, 2440 - 2466	K.ASITALEAK.I	904.04321	2	9.65E-05	0.92	3.37	-	971.2
AHQ-2-3, 2231	K.ASITALEAK.I	904.04321	2	2.76E-06	0.91	2.91	-	970.8
AHQ-2-6, 2023	K.ASITALEAK.I	904.04321	2	9.37E-06	0.78	2.54	-	764.6
AHQ-2-3, 1893 - 1901	R.ASREEILAQAK.E	1216.36955	2	1.78E-04	0.95	3.59	-	1888.8
AHQ-2-2, 1861 - 1930	R.ASREEILAQAK.E	1216.36955	2	1.71E-06	0.94	3.79	-	1624.5
AHQ-2-4, 1902	R.ASREEILAQAK.E	1216.36955	2	3.22E-06	0.89	3.03	-	1292.9
AHQ-2-2, 1906	R.ASREEILAQAK.E	1216.36955	1	5.56E-04	0.58	1.97	-	385.7
AHQ-2-1, 2129	R.ASREEILAQAK.E	1216.36955	2	1.27E-05	0.92	3.75	-	1355.1
AHQ-2-2, 3429	R.DELADEIANSSGK.G	1349.38323	1	6.52E-08	0.51	2.30	-	437.7
AHQ-2-3, 3391	R.DELADEIANSSGK.G	1349.38323	1	8.76E-06	0.40	2.45	-	352.6
AHQ-2-4, 3234	R.DELADEIANSSGK.G	1349.38323	1	2.10E-05	0.07	1.83	-	275.3
AHQ-2-4, 3241	R.DELADEIANSSGK.G	1349.38323	2	2.25E-05	0.96	4.35	-	1633.2
AHQ-2-2, 3208 - 3245	R.DELADEIANSSGK.G	1349.38323	2	3.03E-07	0.93	3.17	-	1310.1
AHQ-2-2, 2920	R.DELADEIANSSGK.G	1349.38323	1	1.40E-07	0.64	2.43	-	487.6
AHQ-2-3, 3229	R.DELADEIANSSGK.G	1349.38323	2	3.20E-05	0.94	3.92	-	1138.6
AHQ-2-1, 3281	R.DELADEIANSSGK.G	1349.38323	2	5.29E-07	0.96	4.42	-	1528.3
AHQ-2-1, 6972	K.DFSALESQLQDTQELLQEENR.Q	2494.61059	3	5.10E-07	0.98	6.56	-	1691.1
AHQ-2-2, 7356 - 7422	K.DFSALESQLQDTQELLQEENR.Q	2494.61059	3	8.18E-05	0.85	3.85	-	1085.6
AHQ-2-2, 7201 - 7268	K.DFSALESQLQDTQELLQEENR.Q	2494.61059	3	1.11E-10	0.98	6.29	-	2119.2
AHQ-2-3, 7295	K.DFSALESQLQDTQELLQEENR.Q	2494.61059	3	9.16E-07	0.95	4.75	-	1635.6
AHQ-2-3, 7131 - 7143	K.DFSALESQLQDTQELLQEENR.Q	2494.61059	3	3.65E-04	0.96	4.72	-	2147.4
AHQ-2-3, 6872	K.DFSALESQLQDTQELLQEENR.Q	2494.61059	3	4.59E-04	0.89	4.24	-	736.6
AHQ-2-4, 7192 - 7200	K.DFSALESQLQDTQELLQEENR.Q	2494.61059	3	4.40E-12	0.97	6.21	-	2214.1
AHQ-2-1, 7096 - 7176	K.DFSALESQLQDTQELLQEENR.Q	2494.61059	3	7.32E-13	0.97	5.47	-	1962.2
AHQ-2-5, 7043	K.DFSALESQLQDTQELLQEENR.Q	2494.61059	3	5.05E-14	0.98	6.26	-	2141.0
AHQ-2-4, 7354	K.DFSALESQLQDTQELLQEENR.Q	2494.61059	3	5.96E-06	0.94	4.79	-	1575.9
AHQ-2-2, 6762	K.DFSALESQLQDTQELLQEENRQK.L	2750.91328	2	3.66E-10	0.69	3.39	-	386.0
AHQ-2-3, 6697 - 6716	K.DFSALESQLQDTQELLQEENRQK.L	2750.91328	3	2.32E-11	0.94	5.13	-	869.9
AHQ-2-3, 6705	K.DFSALESQLQDTQELLQEENRQK.L	2750.91328	2	7.45E-05	0.86	3.73	-	568.2
AHQ-2-2, 6756 - 6780	K.DFSALESQLQDTQELLQEENRQK.L	2750.91328	3	4.52E-11	0.87	4.25	-	682.8
AHQ-2-3, 2021 - 2051	K.DLEAHIDSANK.N	1213.27938	2	7.46E-07	0.97	4.21	-	1837.6
AHQ-2-2, 2026 - 2101	K.DLEAHIDSANK.N	1213.27938	2	6.52E-07	0.98	4.11	-	2515.5
AHQ-2-2, 2024 - 2093	K.DLEAHIDSANK.N	1213.27938	1	4.45E-04	0.53	2.00	-	709.5
AHQ-2-4, 2032	K.DLEAHIDSANK.N	1213.27938	2	7.82E-08	0.98	3.93	-	2675.0
AHQ-2-1, 2216	K.DLEAHIDSANK.N	1213.27938	2	2.50E-06	0.98	4.39	-	2492.6
AHQ-2-3, 2431	K.DLEAHIDSANKNRDEAIK.Q	2040.18063	2	7.77E-05	0.90	4.39	-	699.9
AHQ-2-2, 2581	K.DLEAHIDSANKNRDEAIK.Q	2040.18063	2	4.94E-07	0.95	4.97	-	1011.3
AHQ-2-2, 2442 - 2449	K.DLEAHIDSANKNRDEAIK.Q	2040.18063	2	1.87E-04	0.95	4.94	-	934.1
AHQ-2-4, 2230	K.DLEGLSQR.H	917.98683	2	2.79E-04	0.79	2.68	-	905.3
AHQ-2-1, 7185	R.DLGEELEALKTELEDTLDSTAAQQELR.S	3019.21860	3	4.06E-12	0.98	6.47	-	2559.3
AHQ-2-5, 7571	R.DLGEELEALKTELEDTLDSTAAQQELR.S	3019.21860	3	7.49E-10	0.97	6.30	-	1859.9
AHQ-2-1, 7290	R.DLGEELEALKTELEDTLDSTAAQQELR.S	3019.21860	3	1.96E-05	0.85	3.87	-	1136.4
AHQ-2-7, 7579	R.DLGEELEALKTELEDTLDSTAAQQELR.S	3019.21860	3	1.54E-08	0.96	4.86	-	1887.5
AHQ-2-2, 7508	R.DLGEELEALKTELEDTLDSTAAQQELR.S	3019.21860	3	1.17E-14	0.98	7.05	-	2181.7
AHQ-2-6, 7317	R.DLGEELEALKTELEDTLDSTAAQQELR.S	3019.21860	3	1.77E-11	0.97	5.48	-	1974.4
AHQ-2-3, 7584 - 7621	R.DLGEELEALKTELEDTLDSTAAQQELR.S	3019.21860	3	3.97E-11	0.96	5.97	-	1141.4
AHQ-2-2, 7740	R.DLGEELEALKTELEDTLDSTAAQQELR.S	3019.21860	3	3.07E-07	0.95	5.43	-	1500.2
AHQ-2-2, 7632 - 7666	R.DLGEELEALKTELEDTLDSTAAQQELR.S	3019.21860	3	1.20E-13	0.97	6.19	-	1809.2
AHQ-2-3, 5359	K.DMFQETMEAMR.I	1389.60414	1	7.53E-05	0.48	2.63	-	258.7
AHQ-2-2, 3434 - 3454	K.DVLLQVDDER.R	1202.29650	2	8.87E-07	0.91	3.27	-	1289.5
AHQ-2-1, 3432	K.DVLLQVDDER.R	1202.29650	2	1.82E-04	0.88	3.00	-	1142.9
AHQ-2-4, 2813	K.DVLLQVDDERR.N	1358.48286	2	6.14E-04	0.49	2.90	-	546.6
AHQ-2-6, 2627	K.DVLLQVDDERR.N	1358.48286	2	4.91E-04	0.61	2.71	-	584.1
AHQ-2-1, 2054	R.EEILAQAK.E	902.02731	1	2.45E-05	0.11	1.94	-	234.8
AHQ-2-5, 2665 - 2740	R.ELEDATETADAMNR.E	1566.63006	2	4.26E-06	0.88	3.32	-	1055.7
AHQ-2-11, 2770	R.ELEDATETADAMNR.E	1566.63006	2	5.19E-05	0.48	2.76	-	333.3
AHQ-2-4, 2860 - 2934	R.ELEDATETADAMNR.E	1566.63006	2	2.74E-10	0.94	4.42	-	1008.5
AHQ-2-6, 2643 - 2717	R.ELEDATETADAMNR.E	1566.63006	2	1.24E-05	0.76	3.05	-	884.4
AHQ-2-2, 2877	R.ELEDATETADAMNR.E	1566.63006	2	3.47E-06	0.94	3.69	-	1385.1
AHQ-2-3, 2861 - 2867	R.ELEDATETADAMNR.E	1566.63006	2	1.97E-08	0.96	5.18	-	1275.7
AHQ-2-2, 3834	R.ELEDATETADAMNREVSSLK.N	2210.36264	2	1.47E-04	0.81	3.31	-	596.9
AHQ-2-3, 5571	R.ELESQISELQEDLESER.A	2035.10956	2	3.37E-06	0.84	3.27	-	625.4
AHQ-2-4, 6037 - 6112	R.ELESQISELQEDLESER.A	2035.10956	2	2.31E-04	0.90	3.92	-	974.1
AHQ-2-4, 6366 - 6436	R.ELESQISELQEDLESER.A	2035.10956	2	4.83E-05	0.83	3.64	-	651.7
AHQ-2-2, 5614 - 5616	R.ELESQISELQEDLESER.A	2035.10956	2	6.43E-10	0.95	4.72	-	1043.6
AHQ-2-6, 5710	R.ELESQISELQEDLESER.A	2035.10956	2	9.47E-06	0.82	3.22	-	920.2
AHQ-2-4, 5982 - 6048	R.ELESQISELQEDLESER.A	2035.10956	3	1.12E-05	0.97	5.96	-	2013.2
AHQ-2-3, 6033	R.ELESQISELQEDLESER.A	2035.10956	3	2.38E-08	0.98	6.03	-	2019.5
AHQ-2-3, 6083	R.ELESQISELQEDLESER.A	2035.10956	2	7.23E-06	0.87	3.92	-	780.1
AHQ-2-3, 6375	R.ELESQISELQEDLESER.A	2035.10956	2	7.50E-10	0.91	4.17	-	833.6
AHQ-2-3, 6475	R.ELESQISELQEDLESER.A	2035.10956	2	2.00E-04	0.96	4.72	-	1586.9
AHQ-2-2, 6070 - 6085	R.ELESQISELQEDLESER.A	2035.10956	2	1.72E-05	0.96	5.29	-	1123.0
AHQ-2-1, 5628	R.ELESQISELQEDLESER.A	2035.10956	2	3.01E-07	0.90	4.04	-	653.3
AHQ-2-1, 5945 - 6018	R.ELESQISELQEDLESER.A	2035.10956	2	4.89E-07	0.94	4.67	-	1101.6
AHQ-2-2, 6080 - 6094	R.ELESQISELQEDLESER.A	2035.10956	3	2.92E-11	0.98	6.16	-	2178.6
AHQ-2-2, 6428 - 6429	R.ELESQISELQEDLESER.A	2035.10956	2	2.18E-06	0.97	5.12	-	1448.5
AHQ-2-3, 6031 - 6059	R.ELESQISELQEDLESER.A	2035.10956	2	3.12E-07	0.97	5.59	-	1121.1
AHQ-2-3, 2485	R.EMEAELEDER.K	1251.30306	2	4.01E-04	0.76	2.73	-	755.4
AHQ-2-5, 1883	R.EMEAELEDERK.Q	1379.47597	2	5.83E-05	0.85	3.05	-	1025.2
AHQ-2-2, 2096	R.EMEAELEDERK.Q	1379.47597	2	1.15E-04	0.92	3.37	-	1371.3
AHQ-2-1, 2272	R.EMEAELEDERK.Q	1379.47597	2	1.82E-04	0.92	3.61	-	1170.4
AHQ-2-3, 2092	R.EMEAELEDERK.Q	1379.47597	2	2.66E-04	0.84	2.87	-	1022.3
AHQ-2-1, 4685	K.EQADFAIEALAK.A	1306.44632	2	7.26E-04	0.78	2.94	-	615.6
AHQ-2-2, 4570 - 4640	K.EQADFAIEALAK.A	1306.44632	1	1.22E-07	0.36	2.91	-	278.6
AHQ-2-2, 2325	R.EQLEEEEEAKHNLEK.Q	1855.93770	2	1.98E-04	0.91	4.37	-	666.6
AHQ-2-4, 2292	R.EQLEEEEEAKHNLEK.Q	1855.93770	2	5.04E-05	0.88	3.93	-	722.2
AHQ-2-1, 2484	R.EQLEEEEEAKHNLEK.Q	1855.93770	2	2.24E-09	0.92	3.99	-	1017.3
AHQ-2-6, 2157	R.EQLEEEEEAKHNLEK.Q	1855.93770	2	7.57E-07	0.82	3.66	-	440.1
AHQ-2-3, 2299	R.EQLEEEEEAKHNLEK.Q	1855.93770	2	1.94E-04	0.83	3.74	-	525.3
AHQ-2-5, 2143	R.EQLEEEEEAKHNLEK.Q	1855.93770	2	6.72E-05	0.88	4.39	-	436.2
AHQ-2-2, 3405 - 3478	R.FLSNGHVTIPGQQDK.D	1641.80946	2	1.72E-10	0.91	3.84	-	696.8
AHQ-2-5, 5848	R.FLSNGHVTIPGQQDKDMFQETMEAMR.I	3012.39100	3	1.32E-06	0.91	4.60	-	712.1
AHQ-2-2, 6148	R.FLSNGHVTIPGQQDKDMFQETMEAMR.I	3012.39100	3	7.31E-07	0.93	5.62	-	611.0
AHQ-2-2, 5289 - 5357	R.FLSNGHVTIPGQQDKDMFQETM*EAMR.I	3028.39040	3	1.57E-04	0.69	4.40	-	471.4
AHQ-2-6, 5781	R.FLSNGHVTIPGQQDKDMFQETMEAMR.I	3012.39100	3	7.33E-05	0.87	4.29	-	806.7
AHQ-2-7, 5752	R.FLSNGHVTIPGQQDKDMFQETMEAMR.I	3012.39100	3	1.32E-04	0.66	3.63	-	418.3
AHQ-2-3, 6135	R.FLSNGHVTIPGQQDKDMFQETMEAMR.I	3012.39100	3	1.66E-05	0.93	4.83	-	814.4
AHQ-2-6, 3298	K.FVSELWK.D	909.06314	1	2.02E-04	0.37	1.97	-	739.3
AHQ-2-2, 4270	R.GDLPFVVPR.R	1000.17581	2	2.09E-04	0.78	2.58	-	822.4
AHQ-2-3, 4252	R.GDLPFVVPR.R	1000.17581	2	1.91E-04	0.65	2.82	-	597.3
AHQ-2-1, 2512	K.HEAM*ITDLEER.L	1360.47586	2	9.93E-04	0.94	3.58	-	1194.1
AHQ-2-2, 2721	K.HEAMITDLEER.L	1344.47646	2	2.27E-05	0.90	3.22	-	790.9
AHQ-2-1, 2960	K.HEAMITDLEER.L	1344.47646	2	2.30E-08	0.97	4.46	-	1577.7
AHQ-2-3, 2724 - 2725	K.HEAMITDLEER.L	1344.47646	2	6.34E-04	0.94	3.53	-	1090.5

AHQ-2-5, 3157 - 3205	R.HEMPPHIYAITDTAYR.S	1916.14944	2	8.31E-05	0.92	3.99	-	833.8
AHQ-2-10, 3115	R.HEMPPHIYAITDTAYR.S	1916.14944	3	1.60E-05	0.97	3.89	-	2517.2
AHQ-2-2, 3170 - 3244	R.HEM*PPHIYAITDTAYR.S	1932.14884	3	2.84E-04	0.95	4.05	-	1931.7
AHQ-2-10, 2870	R.HEM*PPHIYAITDTAYR.S	1932.14884	2	3.67E-04	0.56	2.55	-	415.1
AHQ-2-5, 2861	R.HEM*PPHIYAITDTAYR.S	1932.14884	2	6.17E-04	0.86	3.10	-	706.2
AHQ-2-10, 3112	R.HEMPPHIYAITDTAYR.S	1916.14944	2	2.46E-05	0.91	3.95	-	789.2
AHQ-2-2, 3492 - 3565	R.HEMPPHIYAITDTAYR.S	1916.14944	2	2.15E-06	0.93	3.84	-	814.6
AHQ-2-2, 3497	R.HEMPPHIYAITDTAYR.S	1916.14944	3	2.21E-09	0.98	4.62	-	3259.0
AHQ-2-3, 3652	R.HEMPPHIYAITDTAYR.S	1916.14944	3	7.28E-04	0.85	3.32	-	998.5
AHQ-2-1, 3790	R.HEMPPHIYAITDTAYR.S	1916.14944	2	1.36E-04	0.71	3.35	-	548.8
AHQ-2-1, 3406	R.HEM*PPHIYAITDTAYR.S	1932.14884	3	5.29E-07	0.95	4.46	-	1761.6
AHQ-2-4, 3174	R.HEM*PPHIYAITDTAYR.S	1932.14884	3	9.46E-07	0.97	5.03	-	1793.0
AHQ-2-6, 2897	R.HEM*PPHIYAITDTAYR.S	1932.14884	3	6.66E-06	0.97	5.14	-	1942.9
AHQ-2-3, 3177	R.HEM*PPHIYAITDTAYR.S	1932.14884	3	2.55E-06	0.97	5.05	-	2197.7
AHQ-2-2, 3582	R.HEMPPHIYAITDTAYR.S	1916.14944	3	1.57E-08	0.98	5.24	-	3224.8
AHQ-2-2, 3636 - 3637	R.HEMPPHIYAITDTAYR.S	1916.14944	2	1.27E-04	0.93	3.91	-	846.9
AHQ-2-2, 3664	R.HEMPPHIYAITDTAYR.S	1916.14944	3	2.88E-08	0.97	5.22	-	1830.0
AHQ-2-4, 3177	R.HEM*PPHIYAITDTAYR.S	1932.14884	2	4.21E-04	0.83	3.20	-	555.8
AHQ-2-3, 3476 - 3556	R.HEMPPHIYAITDTAYR.S	1916.14944	2	3.32E-08	0.87	3.56	-	743.1
AHQ-2-6, 3143	R.HEMPPHIYAITDTAYR.S	1916.14944	3	2.62E-10	0.98	5.42	-	2630.5
AHQ-2-2, 4588	K.HSQAVEELAEQLEQTK.R	1840.96940	3	2.28E-04	0.96	5.22	-	1546.1
AHQ-2-4, 4532	K.HSQAVEELAEQLEQTK.R	1840.96940	2	5.58E-11	0.98	6.16	-	1842.4
AHQ-2-3, 4540 - 4615	K.HSQAVEELAEQLEQTK.R	1840.96940	2	1.87E-09	0.98	5.94	-	2390.4
AHQ-2-1, 4702	K.HSQAVEELAEQLEQTK.R	1840.96940	2	2.72E-09	0.98	5.94	-	1743.1
AHQ-2-2, 4580 - 4653	K.HSQAVEELAEQLEQTK.R	1840.96940	2	6.99E-12	0.98	6.65	-	2276.1
AHQ-2-1, 4706	K.HSQAVEELAEQLEQTK.R	1840.96940	3	1.31E-05	0.97	5.08	-	2147.2
AHQ-2-5, 4153 - 4223	K.HSQAVEELAEQLEQTK.R	1840.96940	2	2.12E-09	0.94	4.25	-	1244.1
AHQ-2-3, 4545 - 4561	K.HSQAVEELAEQLEQTK.R	1840.96940	3	4.68E-08	0.97	5.52	-	2163.4
AHQ-2-5, 3708	K.HSQAVEELAEQLEQTKR.V	1997.15575	2	3.18E-09	0.99	7.27	-	2804.4
AHQ-2-5, 3692	K.HSQAVEELAEQLEQTKR.V	1997.15575	3	1.90E-08	0.98	6.40	-	2377.6
AHQ-2-4, 4040 - 4057	K.HSQAVEELAEQLEQTKR.V	1997.15575	3	7.27E-07	0.98	6.83	-	1951.0
AHQ-2-1, 4318	K.HSQAVEELAEQLEQTKR.V	1997.15575	2	4.49E-09	0.98	6.29	-	2620.5
AHQ-2-4, 4041	K.HSQAVEELAEQLEQTKR.V	1997.15575	2	8.86E-08	0.98	6.75	-	2218.7
AHQ-2-1, 4309	K.HSQAVEELAEQLEQTKR.V	1997.15575	3	1.55E-05	0.97	5.84	-	1703.7
AHQ-2-3, 4053	K.HSQAVEELAEQLEQTKR.V	1997.15575	2	2.30E-08	0.99	6.93	-	2772.8
AHQ-2-6, 3729	K.HSQAVEELAEQLEQTKR.V	1997.15575	3	8.91E-06	0.93	4.73	-	1069.4
AHQ-2-2, 4073 - 4146	K.HSQAVEELAEQLEQTKR.V	1997.15575	2	9.67E-11	0.98	5.77	-	1945.4
AHQ-2-2, 4076 - 4154	K.HSQAVEELAEQLEQTKR.V	1997.15575	3	1.03E-08	0.98	7.09	-	2020.3
AHQ-2-1, 3869 - 3940	R.IAEFTTNLTEEEEK.S	1654.75371	2	8.41E-09	0.97	5.01	-	1850.7
AHQ-2-1, 4010 - 4082	R.IAEFTTNLTEEEEK.S	1654.75371	2	3.01E-08	0.97	5.00	-	1760.2
AHQ-2-2, 4184	R.IAEFTTNLTEEEEK.S	1654.75371	2	4.84E-07	0.95	4.12	-	1304.0
AHQ-2-2, 4036 - 4105	R.IAEFTTNLTEEEEK.S	1654.75371	2	4.12E-10	0.97	4.75	-	2172.6
AHQ-2-2, 4284 - 4354	R.IAEFTTNLTEEEEK.S	1654.75371	2	5.81E-05	0.87	3.56	-	729.0
AHQ-2-5, 3436 - 3516	R.IAEFTTNLTEEEEK.S	1654.75371	2	1.42E-10	0.97	4.58	-	2148.8
AHQ-2-3, 4364	R.IAEFTTNLTEEEEK.S	1654.75371	2	1.24E-05	0.60	2.75	-	854.7
AHQ-2-4, 4106	R.IAEFTTNLTEEEEK.S	1654.75371	2	9.77E-06	0.77	2.95	-	611.3
AHQ-2-2, 3908 - 3976	R.IAEFTTNLTEEEEK.S	1654.75371	2	1.79E-10	0.97	4.93	-	1794.1
AHQ-2-7, 3447	R.IAEFTTNLTEEEEK.S	1654.75371	2	2.70E-05	0.91	3.01	-	1269.0
AHQ-2-4, 3828 - 3906	R.IAEFTTNLTEEEEK.S	1654.75371	2	1.82E-07	0.90	2.86	-	1484.0
AHQ-2-3, 4221 - 4291	R.IAEFTTNLTEEEEK.S	1654.75371	2	5.36E-04	0.84	3.26	-	904.5
AHQ-2-4, 3728 - 3796	R.IAEFTTNLTEEEEK.S	1654.75371	2	2.61E-10	0.96	4.29	-	1717.7
AHQ-2-3, 4035 - 4103	R.IAEFTTNLTEEEEK.S	1654.75371	2	2.65E-09	0.97	4.69	-	1780.3
AHQ-2-3, 3919 - 3967	R.IAEFTTNLTEEEEK.S	1654.75371	2	1.83E-08	0.98	5.06	-	2000.8
AHQ-2-2, 3794 - 3864	R.IAEFTTNLTEEEEK.S	1654.75371	2	3.71E-10	0.97	5.24	-	1871.6
AHQ-2-3, 3851 - 3892	R.IAEFTTNLTEEEEK.S	1654.75371	2	2.96E-09	0.96	4.97	-	1570.9
AHQ-2-3, 3712 - 3792	R.IAEFTTNLTEEEEK.S	1654.75371	2	1.24E-09	0.96	4.62	-	1650.9
AHQ-2-4, 3900 - 3977	R.IAEFTTNLTEEEEK.S	1654.75371	2	1.26E-09	0.97	4.54	-	1819.5
AHQ-2-2, 3298	R.IAEFTTNLTEEEEKSK.S	1870.00428	2	9.73E-10	0.82	3.57	-	686.7
AHQ-2-3, 3315	R.IAEFTTNLTEEEEKSK.S	1870.00428	2	5.59E-04	0.81	3.59	-	645.9
AHQ-2-4, 3332	R.IAEFTTNLTEEEEKSK.S	1870.00428	2	6.00E-05	0.67	3.16	-	468.3
AHQ-2-2, 5418 - 5501	R.IAQLEEELEEEQGNTELINDR.L	2473.58969	3	4.88E-08	0.65	3.24	-	408.4
AHQ-2-3, 5721	R.IAQLEEELEEEQGNTELINDRLK.K	2714.92085	3	9.11E-06	0.91	4.79	-	871.8
AHQ-2-1, 5718 - 5754	R.IAQLEEELEEEQGNTELINDRLK.K	2714.92085	3	3.16E-06	0.83	3.35	-	910.0
AHQ-2-1, 3625	K.IAQLEEQLDNETK.E	1531.64695	2	7.34E-07	0.91	3.65	-	1165.4
AHQ-2-3, 3364	K.IAQLEEQLDNETK.E	1531.64695	2	1.22E-05	0.92	3.45	-	1333.0
AHQ-2-2, 3825 - 3848	K.IAQLEEQLDNETK.E	1531.64695	2	1.53E-07	0.94	4.50	-	855.2
AHQ-2-5, 3045	K.IAQLEEQLDNETK.E	1531.64695	2	1.42E-07	0.83	3.26	-	721.7
AHQ-2-7, 3067	K.IAQLEEQLDNETK.E	1531.64695	2	3.28E-06	0.94	3.89	-	1359.1
AHQ-2-3, 3449	K.IAQLEEQLDNETK.E	1531.64695	2	9.34E-05	0.84	3.33	-	999.1
AHQ-2-6, 2990	K.IAQLEEQLDNETK.E	1531.64695	2	3.77E-07	0.44	2.61	-	406.6
AHQ-2-2, 2926	K.IAQLEEQLDNETK.E	1531.64695	2	7.90E-09	0.95	4.14	-	1300.3
AHQ-2-11, 3194	K.IAQLEEQLDNETK.E	1531.64695	2	2.38E-08	0.95	3.98	-	1306.9
AHQ-2-2, 3110 - 3184	K.IAQLEEQLDNETK.E	1531.64695	2	1.20E-09	0.97	4.76	-	2038.2
AHQ-2-1, 3465 - 3494	K.IAQLEEQLDNETK.E	1531.64695	2	1.47E-09	0.97	4.55	-	1925.4
AHQ-2-4, 3173	K.IAQLEEQLDNETK.E	1531.64695	2	3.11E-08	0.96	4.60	-	1583.3
AHQ-2-10, 3062	K.IAQLEEQLDNETK.E	1531.64695	2	5.51E-09	0.96	4.90	-	1365.3
AHQ-2-2, 3529	K.IAQLEEQLDNETK.E	1531.64695	2	7.87E-08	0.95	4.60	-	1360.6
AHQ-2-6, 3113	K.IAQLEEQLDNETK.E	1531.64695	2	9.57E-05	0.97	4.75	-	1905.7
AHQ-2-9, 3048 - 3106	K.IAQLEEQLDNETK.E	1531.64695	2	2.93E-05	0.96	3.84	-	1784.0
AHQ-2-4, 3373	K.IAQLEEQLDNETK.E	1531.64695	2	1.65E-07	0.95	3.82	-	1667.8
AHQ-2-2, 3386 - 3457	K.IAQLEEQLDNETK.E	1531.64695	2	5.74E-08	0.94	4.06	-	1381.5
AHQ-2-3, 3097 - 3167	K.IAQLEEQLDNETK.E	1531.64695	2	1.87E-07	0.97	5.01	-	1586.5
AHQ-2-2, 3033 - 3065	K.IAQLEEQLDNETKER.Q	1816.94784	2	7.36E-09	0.95	4.26	-	1110.2
AHQ-2-2, 3489 - 3558	K.IAQLEEQLDNETKER.Q	1816.94784	2	5.66E-09	0.96	4.77	-	1338.7
AHQ-2-2, 3486	K.IAQLEEQLDNETKER.Q	1816.94784	3	5.42E-06	0.69	3.41	-	438.1
AHQ-2-2, 5976	R.IIGLDQVAGM*SETALPGAFK.T	2035.35041	2	2.01E-04	0.95	5.16	-	838.9
AHQ-2-5, 6695 - 6700	R.IIGLDQVAGMSETALPGAFK.T	2019.35101	2	1.56E-05	0.96	5.01	-	1131.5
AHQ-2-8, 6458	R.IIGLDQVAGMSETALPGAFK.T	2019.35101	2	4.67E-09	0.96	5.03	-	1100.2
AHQ-2-4, 5473 - 5513	R.IIGLDQVAGM*SETALPGAFK.T	2035.35041	2	1.09E-05	0.84	3.86	-	654.6
AHQ-2-1, 5500	R.IIGLDQVAGM*SETALPGAFK.T	2035.35041	2	8.51E-07	0.95	4.33	-	1156.8
AHQ-2-3, 6804 - 6815	R.IIGLDQVAGMSETALPGAFK.T	2019.35101	2	5.42E-06	0.97	5.66	-	1478.9
AHQ-2-1, 6728 - 6746	R.IIGLDQVAGMSETALPGAFK.T	2019.35101	3	8.40E-04	0.96	5.75	-	1056.5
AHQ-2-3, 5453	R.IIGLDQVAGM*SETALPGAFK.T	2035.35041	2	1.88E-07	0.96	4.94	-	1036.8
AHQ-2-2, 5492	R.IIGLDQVAGM*SETALPGAFK.T	2035.35041	2	1.02E-05	0.94	4.23	-	1051.3
AHQ-2-1, 6720 - 6789	R.IIGLDQVAGMSETALPGAFK.T	2019.35101	2	1.38E-05	0.95	5.23	-	881.7
AHQ-2-6, 6495 - 6527	R.IIGLDQVAGMSETALPGAFK.T	2019.35101	2	5.77E-07	0.97	5.62	-	1223.1
AHQ-2-1, 6121	R.IIGLDQVAGMSETALPGAFK.T	2019.35101	2	1.64E-04	0.94	4.69	-	870.0
AHQ-2-2, 6848 - 6870	R.IIGLDQVAGMSETALPGAFK.T	2019.35101	3	4.82E-04	0.94	5.43	-	895.6
AHQ-2-4, 6837 - 6904	R.IIGLDQVAGMSETALPGAFK.T	2019.35101	2	3.70E-09	0.96	5.20	-	1215.2
AHQ-2-14-, 6285 - 6324	R.IIGLDQVAGMSETALPGAFK.T	2019.35101	2	1.80E-04	0.97	5.46	-	1250.2
AHQ-2-13, 6277	R.IIGLDQVAGMSETALPGAFK.T	2019.35101	2	5.85E-04	0.94	4.85	-	740.1
AHQ-2-7, 6607	R.IIGLDQVAGMSETALPGAFK.T	2019.35101	2	1.50E-05	0.97	5.40	-	1289.3
AHQ-2-3, 4804	R.IM*GIPEEEQMGLLR.V	1632.92766	2	5.35E-04	0.66	2.70	-	619.0
AHQ-2-6, 4411 - 4454	R.IM*GIPEEEQMGLLR.V	1632.92766	2	6.39E-04	0.85	3.20	-	611.7
AHQ-2-1, 5497	R.IMGIPEEEQMGLLR.V	1616.92826	2	1.09E-04	0.54	2.54	-	297.7
AHQ-2-4, 5417	R.IMGIPEEEQMGLLR.V	1616.92826	2	6.52E-06	0.89	4.01	-	387.3
AHQ-2-8, 4893 - 4965	R.IMGIPEEEQMGLLR.V	1616.92826	2	3.44E-06	0.92	3.90	-	607.0

AHQ-2-2, 4724 - 4800	R.IMGIPEEEQM*GLLR.V	1632.92766	2	4.66E-04	0.86	3.84	-	515.9
AHQ-2-2, 6021	K.IRELESQISELQEDLESER.A	2304.45416	2	1.76E-04	0.98	6.55	-	1169.6
AHQ-2-3, 5889 - 5968	K.IRELESQISELQEDLESER.A	2304.45416	2	7.27E-08	0.98	6.43	-	1315.9
AHQ-2-3, 5976 - 6016	K.IRELESQISELQEDLESER.A	2304.45416	3	2.00E-10	0.98	6.64	-	2072.4
AHQ-2-1, 6050	K.IRELESQISELQEDLESER.A	2304.45416	2	2.08E-05	0.98	6.87	-	1408.7
AHQ-2-6, 5619	K.IRELESQISELQEDLESER.A	2304.45416	3	1.45E-07	0.97	5.83	-	2189.8
AHQ-2-6, 5618	K.IRELESQISELQEDLESER.A	2304.45416	2	4.89E-06	0.98	6.18	-	1753.2
AHQ-2-4, 5993 - 6004	K.IRELESQISELQEDLESER.A	2304.45416	3	8.42E-08	0.98	6.82	-	1774.7
AHQ-2-5, 5653 - 5671	K.IRELESQISELQEDLESER.A	2304.45416	2	1.47E-05	0.98	6.93	-	1762.2
AHQ-2-3, 6039	K.IRELESQISELQEDLESER.A	2304.45416	2	7.53E-07	0.98	6.21	-	1086.3
AHQ-2-4, 5985	K.IRELESQISELQEDLESER.A	2304.45416	2	2.56E-05	0.98	6.85	-	1818.7
AHQ-2-2, 6024 - 6030	K.IRELESQISELQEDLESER.A	2304.45416	3	1.92E-08	0.97	6.01	-	1569.2
AHQ-2-1, 6052	K.IRELESQISELQEDLESER.A	2304.45416	3	6.46E-09	0.98	6.41	-	2003.4
AHQ-2-3, 4624 - 4636	K.KANLQIDQINTDLNLER.S	1999.21478	3	1.61E-06	0.91	4.50	-	1175.6
AHQ-2-3, 4616	K.KANLQIDQINTDLNLER.S	1999.21478	2	5.46E-06	0.97	6.12	-	1394.5
AHQ-2-6, 4230 - 4237	K.KANLQIDQINTDLNLER.S	1999.21478	2	8.08E-05	0.98	5.97	-	2118.8
AHQ-2-5, 4283	K.KANLQIDQINTDLNLER.S	1999.21478	2	1.94E-05	0.98	6.77	-	1456.7
AHQ-2-2, 4845 - 4921	K.KANLQIDQINTDLNLER.S	1999.21478	2	9.99E-15	0.98	6.08	-	2021.2
AHQ-2-2, 4494	K.KANLQIDQINTDLNLER.S	1999.21478	2	2.83E-06	0.95	5.19	-	945.7
AHQ-2-3, 4380 - 4453	K.KANLQIDQINTDLNLER.S	1999.21478	2	5.77E-06	0.97	6.07	-	1279.2
AHQ-2-3, 4811	K.KANLQIDQINTDLNLER.S	1999.21478	2	1.42E-04	0.97	5.42	-	1463.2
AHQ-2-3, 4909	K.KANLQIDQINTDLNLER.S	1999.21478	2	1.45E-06	0.93	4.62	-	794.9
AHQ-2-2, 4721	K.KANLQIDQINTDLNLER.S	1999.21478	2	1.23E-07	0.97	5.76	-	1371.0
AHQ-2-9, 4179	K.KANLQIDQINTDLNLER.S	1999.21478	2	2.27E-06	0.96	5.05	-	1326.5
AHQ-2-2, 4654	K.KANLQIDQINTDLNLER.S	1999.21478	3	1.60E-04	0.91	4.20	-	1322.9
AHQ-2-1, 4720	K.KANLQIDQINTDLNLER.S	1999.21478	3	9.84E-04	0.89	4.16	-	1084.9
AHQ-2-2, 4638 - 4652	K.KANLQIDQINTDLNLER.S	1999.21478	2	1.98E-10	0.98	5.96	-	1555.8
AHQ-2-1, 2642	R.KKVEAQLQELQVK.F	1541.81761	2	5.75E-08	0.97	5.10	-	1443.0
AHQ-2-4, 2389	R.KKVEAQLQELQVK.F	1541.81761	2	3.94E-04	0.97	5.01	-	1914.5
AHQ-2-2, 2408 - 2410	R.KKVEAQLQELQVK.F	1541.81761	2	3.12E-08	0.97	5.71	-	1509.0
AHQ-2-3, 3643	K.KLEEEQIILEDQNCK.L	1891.08992	2	5.53E-04	0.92	4.51	-	700.5
AHQ-2-4, 3534	K.KLEEEQIILEDQNCK.L	1891.08992	2	3.19E-11	0.98	6.11	-	1933.9
AHQ-2-2, 3766	K.KLEEEQIILEDQNCK.L	1891.08992	2	3.87E-06	0.98	5.99	-	1861.4
AHQ-2-1, 3696 - 3697	K.KLEEEQIILEDQNCK.L	1891.08992	3	1.06E-06	0.90	4.39	-	1074.8
AHQ-2-1, 3684	K.KLEEEQIILEDQNCK.L	1891.08992	2	3.84E-09	0.98	5.74	-	2208.5
AHQ-2-1, 3518 - 3588	K.KLEEEQIILEDQNCK.L	1891.08992	2	4.39E-04	0.96	5.04	-	1494.0
AHQ-2-3, 3565 - 3633	K.KLEEEQIILEDQNCK.L	1891.08992	3	1.38E-04	0.92	4.09	-	1345.8
AHQ-2-3, 3776	K.KLEEEQIILEDQNCK.L	1891.08992	2	2.12E-04	0.97	5.54	-	1673.1
AHQ-2-2, 3678	K.KLEEEQIILEDQNCK.L	1891.08992	2	6.14E-05	0.85	3.39	-	683.6
AHQ-2-3, 3531 - 3608	K.KLEEEQIILEDQNCK.L	1891.08992	2	2.09E-11	0.97	5.63	-	1604.2
AHQ-2-3, 3416 - 3436	K.KLEEEQIILEDQNCK.L	1891.08992	3	6.21E-06	0.90	4.54	-	1240.7
AHQ-2-2, 3581 - 3652	K.KLEEEQIILEDQNCK.L	1891.08992	2	4.92E-11	0.98	6.33	-	1550.7
AHQ-2-3, 7364 - 7391	R.KLEGDSTDLSDQIAELQAQIAELK.M	2616.86032	3	7.48E-10	0.88	4.59	-	791.0
AHQ-2-3, 7367 - 7431	R.KLEGDSTDLSDQIAELQAQIAELK.M	2616.86032	2	3.01E-07	0.97	5.53	-	1192.2
AHQ-2-6, 7067	R.KLEGDSTDLSDQIAELQAQIAELK.M	2616.86032	3	1.73E-09	0.91	5.00	-	814.9
AHQ-2-1, 7149 - 7225	R.KLEGDSTDLSDQIAELQAQIAELK.M	2616.86032	2	6.49E-08	0.97	6.47	-	1151.3
AHQ-2-1, 7073 - 7148	R.KLEGDSTDLSDQIAELQAQIAELK.M	2616.86032	3	4.28E-06	0.92	4.86	-	926.0
AHQ-2-4, 7382 - 7440	R.KLEGDSTDLSDQIAELQAQIAELK.M	2616.86032	2	4.74E-06	0.97	5.68	-	1486.4
AHQ-2-5, 7264 - 7265	R.KLEGDSTDLSDQIAELQAQIAELK.M	2616.86032	3	4.64E-06	0.90	5.06	-	1016.7
AHQ-2-4, 7424 - 7494	R.KLEGDSTDLSDQIAELQAQIAELK.M	2616.86032	3	3.98E-06	0.95	5.51	-	1047.0
AHQ-2-4, 7492 - 7556	R.KLEGDSTDLSDQIAELQAQIAELK.M	2616.86032	2	1.92E-05	0.92	4.78	-	621.1
AHQ-2-5, 7263	R.KLEGDSTDLSDQIAELQAQIAELK.M	2616.86032	2	6.87E-08	0.96	5.63	-	1304.6
AHQ-2-2, 7436	R.KLEGDSTDLSDQIAELQAQIAELK.M	2616.86032	2	3.75E-08	0.97	6.53	-	1234.0
AHQ-2-6, 2383	K.KLVWVPSDK.S	1072.28194	2	1.06E-05	0.77	2.69	-	736.5
AHQ-2-2, 2568	K.KLVWVPSDK.S	1072.28194	2	2.64E-04	0.83	3.03	-	896.5
AHQ-2-3, 2564	K.KLVWVPSDK.S	1072.28194	1	8.42E-04	0.52	2.58	-	484.4
AHQ-2-5, 2341	K.KLVWVPSDK.S	1072.28194	2	7.57E-05	0.69	2.71	-	711.4
AHQ-2-4, 3588	K.KMEDSVGCLETAEEVK.R	1827.02450	2	1.75E-06	0.82	3.69	-	793.1
AHQ-2-2, 3204	K.KMEDSVGCLETAEEVKR.K	1983.21085	3	2.11E-06	0.93	4.77	-	919.1
AHQ-2-2, 3197	K.KMEDSVGCLETAEEVKR.K	1983.21085	2	4.51E-07	0.92	3.65	-	1117.5
AHQ-2-2, 2817	K.KM*EDSVGCLETAEEVKR.K	1999.21025	2	2.20E-06	0.89	3.87	-	843.1
AHQ-2-2, 2805	K.KM*EDSVGCLETAEEVKR.K	1999.21025	3	3.91E-06	0.92	4.55	-	891.6
AHQ-2-4, 3208	K.KMEDSVGCLETAEEVKR.K	1983.21085	2	1.35E-05	0.96	4.52	-	1601.6
AHQ-2-3, 3200	K.KMEDSVGCLETAEEVKR.K	1983.21085	2	2.19E-09	0.97	5.16	-	1752.2
AHQ-2-5, 2529	K.KM*EDSVGCLETAEEVKR.K	1999.21025	3	2.62E-04	0.94	5.01	-	831.4
AHQ-2-6, 2947	K.KMEDSVGCLETAEEVKR.K	1983.21085	3	1.41E-08	0.88	4.04	-	778.1
AHQ-2-1, 3388	K.KMEDSVGCLETAEEVKR.K	1983.21085	3	1.07E-09	0.91	4.37	-	1019.7
AHQ-2-1, 3000	K.KM*EDSVGCLETAEEVKR.K	1999.21025	3	5.62E-04	0.93	4.54	-	1114.6
AHQ-2-3, 2816	K.KM*EDSVGCLETAEEVKR.K	1999.21025	2	3.22E-04	0.72	3.60	-	268.0
AHQ-2-2, 4964	K.KM*QQNIQELEEQLEEEESAR.Q	2478.63294	2	2.53E-04	0.52	2.80	-	292.5
AHQ-2-3, 5379 - 5404	K.KMQQNIQELEEQLEEEESAR.Q	2462.63354	2	1.33E-05	0.91	4.14	-	1059.2
AHQ-2-1, 5578	K.KMQQNIQELEEQLEEEESAR.Q	2462.63354	2	5.04E-05	0.87	3.87	-	704.9
AHQ-2-2, 5428	K.KMQQNIQELEEQLEEEESAR.Q	2462.63354	2	2.94E-05	0.96	5.52	-	1162.5
AHQ-2-3, 4919	K.KM*QQNIQELEEQLEEEESAR.Q	2478.63294	2	7.74E-04	0.63	2.99	-	348.3
AHQ-2-1, 5013	K.KM*QQNIQELEEQLEEEESAR.Q	2478.63294	2	6.47E-06	0.58	2.83	-	380.1
AHQ-2-3, 4923	K.KM*QQNIQELEEQLEEEESAR.Q	2478.63294	3	2.24E-04	0.85	3.92	-	1004.9
AHQ-2-4, 4922	K.KM*QQNIQELEEQLEEEESAR.Q	2478.63294	2	1.67E-09	0.51	3.00	-	381.7
AHQ-2-4, 4065 - 4080	K.KQELEEICHDLEAR.V	1771.93036	3	9.81E-06	0.97	5.26	-	2036.8
AHQ-2-1, 4264 - 4273	K.KQELEEICHDLEAR.V	1771.93036	3	5.72E-04	0.97	5.77	-	1930.4
AHQ-2-6, 3759	K.KQELEEICHDLEAR.V	1771.93036	2	6.24E-04	0.98	5.09	-	2702.2
AHQ-2-4, 4048 - 4053	K.KQELEEICHDLEAR.V	1771.93036	2	3.86E-04	0.97	4.64	-	2430.9
AHQ-2-7, 3661	K.KQELEEICHDLEAR.V	1771.93036	2	2.53E-04	0.97	5.04	-	2065.9
AHQ-2-8, 3690	K.KQELEEICHDLEAR.V	1771.93036	2	6.93E-04	0.96	4.84	-	1703.7
AHQ-2-11, 3857 - 3920	K.KQELEEICHDLEAR.V	1771.93036	2	3.10E-04	0.70	3.13	-	595.5
AHQ-2-1, 3037	K.KVEAQLQELQVK.F	1413.64470	3	4.68E-06	0.81	3.66	-	1038.4
AHQ-2-4, 2705	K.KVEAQLQELQVK.F	1413.64470	2	1.07E-05	0.97	5.05	-	1757.4
AHQ-2-1, 3032	K.KVEAQLQELQVK.F	1413.64470	2	1.12E-07	0.95	4.39	-	1316.6
AHQ-2-5, 2492	K.KVEAQLQELQVK.F	1413.64470	2	1.04E-07	0.97	4.88	-	1857.5
AHQ-2-6, 2565	K.KVEAQLQELQVK.F	1413.64470	2	3.89E-07	0.97	4.97	-	1430.6
AHQ-2-3, 2743 - 2745	K.KVEAQLQELQVK.F	1413.64470	2	1.43E-04	0.98	5.52	-	2061.0
AHQ-2-3, 2761	K.KVEAQLQELQVK.F	1413.64470	1	1.52E-04	0.60	3.18	-	388.9
AHQ-2-2, 2753	K.KVEAQLQELQVK.F	1413.64470	2	1.64E-06	0.96	5.03	-	1319.4
AHQ-2-2, 2761	K.KVEAQLQELQVK.F	1413.64470	1	3.93E-06	0.56	2.98	-	384.6
AHQ-2-2, 3570	K.KVIQYLAYVASSHK.S	1607.87775	2	1.06E-08	0.97	4.73	-	1494.9
AHQ-2-6, 3182	K.KVIQYLAYVASSHK.S	1607.87775	2	9.80E-04	0.92	3.60	-	1012.4
AHQ-2-2, 3970 - 3996	K.LEEEQIILEDQNCK.L	1762.91700	2	4.04E-10	0.97	5.83	-	1233.5
AHQ-2-1, 3948 - 4022	K.LEEEQIILEDQNCK.L	1762.91700	2	1.23E-08	0.96	5.24	-	1237.0
AHQ-2-1, 4038	K.LEEEQIILEDQNCK.L	1762.91700	2	1.03E-07	0.94	3.81	-	1181.4
AHQ-2-2, 3888	K.LEEEQIILEDQNCK.L	1762.91700	2	1.45E-07	0.95	4.56	-	1355.3
AHQ-2-3, 3961 - 4008	K.LEEEQIILEDQNCK.L	1762.91700	2	3.67E-08	0.97	4.58	-	1473.3
AHQ-2-4, 3850	K.LEEEQIILEDQNCK.L	1762.91700	2	4.09E-07	0.93	4.16	-	936.0
AHQ-2-2, 4065 - 4136	K.LEEEQIILEDQNCK.L	1762.91700	2	1.22E-09	0.97	5.46	-	1438.5
AHQ-2-1, 7208	K.LEGDSTDLSDQIAELQAQIAELK.M	2488.68740	2	1.66E-12	0.97	5.49	-	1298.9
AHQ-2-4, 7548 - 7549	K.LEGDSTDLSDQIAELQAQIAELK.M	2488.68740	2	2.33E-07	0.91	3.81	-	846.8
AHQ-2-3, 7487 - 7489	K.LEGDSTDLSDQIAELQAQIAELK.M	2488.68740	2	5.07E-10	0.97	6.14	-	1277.0
AHQ-2-1, 7212	K.LEGDSTDLSDQIAELQAQIAELK.M	2488.68740	3	5.22E-06	0.77	4.16	-	628.8
AHQ-2-1, 3653	R.LEVNLQAMK.A	1046.26630	2	7.45E-05	0.90	3.52	-	780.2

AHQ-2-3, 2483	R.LEVNLQAM*K.A	1062.26570	2	9.18E-04	0.81	2.88	-	644.8
AHQ-2-4, 2472	R.LEVNLQAM*K.A	1062.26570	1	1.58E-04	0.14	1.80	-	80.2
AHQ-2-3, 3448	R.LEVNLQAMK.A	1046.26630	2	1.19E-04	0.90	3.59	-	666.5
AHQ-2-1, 2660 - 2677	R.LEVNLQAM*K.A	1062.26570	2	1.59E-05	0.76	2.77	-	538.9
AHQ-2-4, 3453	R.LEVNLQAMK.A	1046.26630	2	5.62E-06	0.85	3.06	-	770.8
AHQ-2-5, 3148	R.LEVNLQAMK.A	1046.26630	2	1.56E-04	0.69	2.72	-	586.1
AHQ-2-1, 3660	R.LEVNLQAMK.A	1046.26630	1	2.69E-04	0.65	2.72	-	646.7
AHQ-2-2, 3441 - 3444	R.LEVNLQAMK.A	1046.26630	2	9.64E-06	0.88	3.60	-	621.7
AHQ-2-4, 3382	K.LKDVLLQVDDER.R	1443.62767	2	8.14E-09	0.94	3.44	-	1544.0
AHQ-2-4, 3398	K.LKDVLLQVDDER.R	1443.62767	1	1.02E-05	0.66	3.20	-	405.4
AHQ-2-2, 3377	K.LKDVLLQVDDER.R	1443.62767	2	2.65E-09	0.95	4.00	-	1567.6
AHQ-2-1, 3621 - 3673	K.LKDVLLQVDDER.R	1443.62767	2	1.93E-09	0.92	3.75	-	1184.3
AHQ-2-3, 3373	K.LKDVLLQVDDER.R	1443.62767	2	1.05E-07	0.95	3.72	-	1558.3
AHQ-2-2, 3272	K.LKDVLLQVDDER.R	1443.62767	2	5.59E-07	0.95	3.63	-	1823.9
AHQ-2-6, 2654	K.LKDVLLQVDDERR.N	1599.81402	2	2.73E-04	0.88	3.35	-	1066.4
AHQ-2-2, 2900	K.LKDVLLQVDDERR.N	1599.81402	2	5.21E-05	0.88	3.60	-	951.6
AHQ-2-3, 2915 - 2916	K.LKDVLLQVDDERR.N	1599.81402	2	3.05E-06	0.93	4.02	-	1273.5
AHQ-2-6, 2655	K.LKDVLLQVDDERR.N	1599.81402	3	5.37E-07	0.93	3.99	-	1179.0
AHQ-2-4, 2836 - 2904	K.LKDVLLQVDDERR.N	1599.81402	2	3.03E-04	0.90	3.18	-	1266.3
AHQ-2-3, 2683	K.LKNKHEAMITDLEER.L	1828.08363	2	1.83E-05	0.91	3.65	-	1030.6
AHQ-2-3, 5616 - 5689	R.LQQELDDLLVDLDHQR.Q	1951.12699	2	1.76E-05	0.90	4.12	-	733.9
AHQ-2-5, 5939 - 6011	R.LQQELDDLLVDLDHQR.Q	1951.12699	3	1.13E-05	0.86	4.52	-	631.4
AHQ-2-1, 5845	R.LQQELDDLLVDLDHQR.Q	1951.12699	2	7.07E-05	0.85	3.57	-	795.0
AHQ-2-2, 6236 - 6316	R.LQQELDDLLVDLDHQR.Q	1951.12699	3	4.33E-05	0.93	5.02	-	802.6
AHQ-2-2, 6345	R.LQQELDDLLVDLDHQR.Q	1951.12699	2	3.73E-10	0.97	5.48	-	1240.2
AHQ-2-6, 5885 - 5951	R.LQQELDDLLVDLDHQR.Q	1951.12699	2	3.22E-10	0.97	5.43	-	1338.9
AHQ-2-1, 6284 - 6325	R.LQQELDDLLVDLDHQR.Q	1951.12699	3	1.50E-06	0.82	3.75	-	660.9
AHQ-2-1, 6286 - 6289	R.LQQELDDLLVDLDHQR.Q	1951.12699	2	8.80E-12	0.98	5.37	-	1998.0
AHQ-2-4, 6245 - 6256	R.LQQELDDLLVDLDHQR.Q	1951.12699	3	3.84E-05	0.89	4.20	-	705.8
AHQ-2-4, 6238 - 6312	R.LQQELDDLLVDLDHQR.Q	1951.12699	2	6.34E-11	0.97	5.01	-	1901.9
AHQ-2-3, 6319	R.LQQELDDLLVDLDHQR.Q	1951.12699	2	3.04E-06	0.96	5.15	-	1189.0
AHQ-2-5, 5927 - 6008	R.LQQELDDLLVDLDHQR.Q	1951.12699	2	5.00E-10	0.98	5.63	-	1688.4
AHQ-2-2, 5732	R.LQQELDDLLVDLDHQR.Q	1951.12699	2	2.93E-07	0.95	4.85	-	1183.8
AHQ-2-2, 6197 - 6276	R.LQQELDDLLVDLDHQR.Q	1951.12699	2	3.32E-09	0.96	5.13	-	1406.2
AHQ-2-3, 6400 - 6419	R.LQQELDDLLVDLDHQR.Q	1951.12699	2	8.46E-07	0.98	5.12	-	2053.9
AHQ-2-3, 6176 - 6247	R.LQQELDDLLVDLDHQR.Q	1951.12699	2	4.00E-11	0.97	4.99	-	1456.6
AHQ-2-2, 5966 - 6032	K.LQVELDNVTGLLSQSDSK.S	1947.13342	2	9.84E-07	0.96	5.34	-	1155.9
AHQ-2-4, 5970 - 6040	K.LQVELDNVTGLLSQSDSK.S	1947.13342	2	8.38E-07	0.97	5.45	-	1467.8
AHQ-2-2, 6982	K.LQVELDNVTGLLSQSDSK.S	1947.13342	2	4.62E-06	0.92	4.02	-	980.6
AHQ-2-3, 6167 - 6239	K.LQVELDNVTGLLSQSDSK.S	1947.13342	2	2.34E-08	0.97	6.11	-	1182.7
AHQ-2-2, 6209 - 6280	K.LQVELDNVTGLLSQSDSK.S	1947.13342	2	1.19E-13	0.98	6.84	-	1341.7
AHQ-2-5, 5781 - 5851	K.LQVELDNVTGLLSQSDSK.S	1947.13342	2	1.12E-07	0.97	5.46	-	1614.2
AHQ-2-3, 6309 - 6329	K.LQVELDNVTGLLSQSDSK.S	1947.13342	2	9.25E-07	0.97	5.06	-	1367.9
AHQ-2-3, 6019 - 6096	K.LQVELDNVTGLLSQSDSK.S	1947.13342	2	9.91E-08	0.98	6.01	-	1584.5
AHQ-2-4, 6244	K.LQVELDNVTGLLSQSDSK.S	1947.13342	2	5.96E-05	0.96	4.84	-	1227.5
AHQ-2-1, 5649	K.LQVELDNVTGLLSQSDSK.S	1947.13342	2	1.46E-04	0.97	5.69	-	1349.5
AHQ-2-4, 6325 - 6394	K.LQVELDNVTGLLSQSDSK.S	1947.13342	2	2.24E-05	0.87	3.98	-	544.9
AHQ-2-3, 6403	K.LQVELDNVTGLLSQSDSK.S	1947.13342	2	1.53E-12	0.97	5.41	-	1190.9
AHQ-2-1, 5952 - 6032	K.LQVELDNVTGLLSQSDSK.S	1947.13342	2	3.35E-07	0.98	5.80	-	1692.0
AHQ-2-3, 6532	K.LQVELDNVTGLLSQSDSK.S	1947.13342	2	1.75E-08	0.96	5.21	-	992.5
AHQ-2-5, 6031	K.LQVELDNVTGLLSQSDSK.S	1947.13342	2	7.18E-05	0.96	4.89	-	1131.2
AHQ-2-2, 6100	K.LQVELDNVTGLLSQSDSK.S	1947.13342	2	4.57E-11	0.97	6.47	-	1230.3
AHQ-2-2, 6072 - 6140	K.LQVELDNVTGLLSQSDSK.S	1947.13342	2	5.56E-07	0.98	5.92	-	1546.7
AHQ-2-2, 7346	K.LQVELDNVTGLLSQSDSK.S	1947.13342	2	7.77E-05	0.93	4.10	-	1006.8
AHQ-2-2, 6348 - 6418	K.LQVELDNVTGLLSQSDSK.S	1947.13342	2	5.59E-10	0.98	6.16	-	1976.0
AHQ-2-2, 7092	K.LQVELDNVTGLLSQSDSK.S	1947.13342	2	1.74E-08	0.96	5.61	-	1099.2
AHQ-2-3, 7383	K.LQVELDNVTGLLSQSDSK.S	1947.13342	2	1.67E-05	0.65	3.05	-	555.2
AHQ-2-2, 6448	K.LQVELDNVTGLLSQSDSK.S	1947.13342	2	1.45E-09	0.96	5.21	-	1125.7
AHQ-2-4, 6110 - 6158	K.LQVELDNVTGLLSQSDSK.S	1947.13342	2	5.18E-05	0.95	5.31	-	848.1
AHQ-2-4, 6478	K.LQVELDNVTGLLSQSDSK.S	1947.13342	2	1.44E-07	0.97	4.90	-	1385.1
AHQ-2-1, 6100	K.LQVELDNVTGLLSQSDSK.S	1947.13342	2	1.44E-07	0.97	5.84	-	1106.8
AHQ-2-3, 6995	K.LQVELDNVTGLLSQSDSK.S	1947.13342	2	8.86E-06	0.88	4.21	-	566.0
AHQ-2-1, 6204	K.LQVELDNVTGLLSQSDSK.S	1947.13342	2	1.88E-06	0.94	4.52	-	937.5
AHQ-2-2, 6542	K.LQVELDNVTGLLSQSDSK.S	1947.13342	2	6.65E-11	0.97	5.29	-	1208.6
AHQ-2-3, 5901 - 5975	K.LQVELDNVTGLLSQSDSK.S	1947.13342	2	4.24E-11	0.98	6.58	-	1454.3
AHQ-2-3, 5192 - 5195	R.LTEMETLQSQLMAEK.L	1753.03211	2	8.84E-09	0.98	5.44	-	1860.7
AHQ-2-2, 5232	R.LTEMETLQSQLMAEK.L	1753.03211	2	3.40E-08	0.97	5.59	-	1709.2
AHQ-2-3, 5205	R.LTEM*ETLQSQLM*AEK.L	1785.03091	2	2.24E-05	0.90	3.50	-	1099.3
AHQ-2-2, 5349 - 5366	R.LTEMETLQSQLMAEK.L	1753.03211	2	1.34E-04	0.94	3.76	-	1508.6
AHQ-2-4, 4630 - 4662	R.LTEMETLQSQLM*AEK.L	1769.03151	2	1.66E-06	0.93	3.81	-	1117.6
AHQ-2-3, 4039 - 4081	R.LTEM*ETLQSQLMAEK.L	1769.03151	2	6.18E-05	0.96	4.07	-	2004.9
AHQ-2-2, 5149 - 5228	R.LTEMETLQSQLMAEK.L	1753.03211	2	2.37E-06	0.94	4.31	-	1211.2
AHQ-2-2, 4085 - 4156	R.LTEM*ETLQSQLMAEK.L	1769.03151	2	4.58E-06	0.96	3.66	-	2294.6
AHQ-2-1, 5288	R.LTEMETLQSQLMAEK.L	1753.03211	2	6.74E-06	0.96	4.53	-	1510.2
AHQ-2-1, 3666	R.LTEM*ETLQSQLM*AEK.L	1785.03091	2	2.15E-04	0.89	3.39	-	1089.1
AHQ-2-4, 5180	R.LTEMETLQSQLMAEK.L	1753.03211	2	1.30E-05	0.96	4.86	-	1522.7
AHQ-2-1, 7028	K.LTKDFSALESQLQDTQELLQEENR.Q	2837.04608	3	3.03E-08	0.95	4.90	-	1592.4
AHQ-2-5, 2785	K.LVWVPSDK.S	944.10903	1	7.66E-04	0.33	2.07	-	394.3
AHQ-2-3, 3985	K.MEDSVGCLETAEEVK.R	1698.85159	2	3.25E-06	0.82	3.29	-	716.8
AHQ-2-2, 4025	K.MEDSVGCLETAEEVK.R	1698.85159	2	1.49E-04	0.74	3.03	-	580.6
AHQ-2-2, 3198	K.M*EDSVGCLETAEEVKR.K	1871.03734	2	1.60E-07	0.92	3.99	-	772.8
AHQ-2-3, 3528	K.MEDSVGCLETAEEVKR.K	1855.03794	2	7.52E-05	0.76	3.28	-	458.9
AHQ-2-4, 3530	K.MEDSVGCLETAEEVKR.K	1855.03794	2	6.61E-08	0.91	3.96	-	768.6
AHQ-2-2, 3562 - 3568	K.MEDSVGCLETAEEVKR.K	1855.03794	3	7.09E-08	0.73	3.09	-	735.5
AHQ-2-6, 3250	K.MEDSVGCLETAEEVKR.K	1855.03794	2	7.76E-05	0.44	2.63	-	289.8
AHQ-2-2, 3554 - 3572	K.MEDSVGCLETAEEVKR.K	1855.03794	2	2.14E-07	0.72	3.29	-	461.7
AHQ-2-2, 5733 - 5801	K.MQQNIQELEEQLEEEESAR.Q	2334.46063	3	7.08E-08	0.82	4.09	-	514.0
AHQ-2-14-, 5192	K.NFINNPLAQADWAAK.K	1673.85315	2	1.83E-09	0.93	4.49	-	851.7
AHQ-2-5, 5167 - 5193	K.NFINNPLAQADWAAK.K	1673.85315	2	4.57E-06	0.96	4.82	-	1209.7
AHQ-2-1, 5525	K.NFINNPLAQADWAAK.K	1673.85315	2	1.93E-04	0.93	3.98	-	942.2
AHQ-2-13, 5116 - 5191	K.NFINNPLAQADWAAK.K	1673.85315	2	7.59E-06	0.95	5.05	-	881.8
AHQ-2-14, 6344	K.NFINNPLAQADWAAK.K	1673.85315	2	1.50E-06	0.93	4.60	-	819.3
AHQ-2-10, 4711 - 4779	K.NFINNPLAQADWAAK.K	1673.85315	2	7.52E-04	0.91	4.05	-	988.6
AHQ-2-3, 6144	K.NFINNPLAQADWAAK.K	1673.85315	2	7.74E-08	0.92	4.40	-	747.7
AHQ-2-11, 4997	K.NFINNPLAQADWAAK.K	1673.85315	2	1.76E-08	0.94	4.61	-	851.6
AHQ-2-7, 5027	K.NFINNPLAQADWAAK.K	1673.85315	2	1.14E-10	0.94	4.36	-	1003.0
AHQ-2-3, 5415 - 5416	K.NFINNPLAQADWAAK.K	1673.85315	2	2.06E-08	0.96	4.97	-	1270.6
AHQ-2-10, 4798	K.NFINNPLAQADWAAK.K	1673.85315	2	4.73E-06	0.93	3.91	-	1062.2
AHQ-2-9, 5098	K.NFINNPLAQADWAAK.K	1673.85315	2	2.19E-10	0.94	4.61	-	990.4
AHQ-2-12, 5238	K.NFINNPLAQADWAAK.K	1673.85315	2	3.48E-07	0.87	3.49	-	827.5
AHQ-2-2, 6160	K.NFINNPLAQADWAAK.K	1673.85315	2	1.67E-06	0.92	4.07	-	856.6
AHQ-2-4, 5430	K.NFINNPLAQADWAAK.K	1673.85315	2	6.66E-05	0.91	3.72	-	1015.6
AHQ-2-1, 6068	K.NFINNPLAQADWAAK.K	1673.85315	2	6.98E-07	0.94	4.15	-	1161.7
AHQ-2-4, 6164	K.NFINNPLAQADWAAK.K	1673.85315	2	2.55E-07	0.95	4.34	-	1357.7
AHQ-2-6, 5081 - 5089	K.NFINNPLAQADWAAK.K	1673.85315	2	2.42E-05	0.94	4.50	-	1013.1
AHQ-2-2, 5433	K.NFINNPLAQADWAAK.K	1673.85315	2	3.73E-06	0.96	5.18	-	1102.1
AHQ-2-3, 2908	K.NKHEAMITDLEER.L	1586.75247	2	1.81E-10	0.93	3.61	-	1252.1

AHQ-2-4, 2382 - 2384	K.NKHEAMITDLEER.L	1586.75247	2	4.69E-06	0.94	3.70	-	1204.5
AHQ-2-3, 2053	K.NKHEAM*ITDLEER.L	1602.75187	2	4.07E-04	0.90	3.86	-	820.7
AHQ-2-5, 2191	K.NKHEAMITDLEER.L	1586.75247	2	9.01E-10	0.94	3.79	-	1346.3
AHQ-2-3, 2383	K.NKHEAMITDLEER.L	1586.75247	2	3.52E-11	0.94	3.77	-	1284.4
AHQ-2-2, 2404 - 2405	K.NKHEAMITDLEER.L	1586.75247	2	7.84E-09	0.95	3.80	-	1358.2
AHQ-2-1, 2644	K.NKHEAMITDLEER.L	1586.75247	2	1.56E-05	0.96	3.70	-	1473.3
AHQ-2-2, 5294 - 5365	K.NLPIYSEEIVEM*YK.G	1744.98660	2	5.56E-06	0.94	4.58	-	924.3
AHQ-2-1, 5986	K.NLPIYSEEIVEMYK.G	1728.98720	2	3.83E-06	0.88	3.46	-	607.6
AHQ-2-4, 5278 - 5288	K.NLPIYSEEIVEM*YK.G	1744.98660	2	6.80E-06	0.95	4.41	-	1033.0
AHQ-2-3, 5273 - 5299	K.NLPIYSEEIVEM*YK.G	1744.98660	2	1.35E-06	0.93	3.97	-	929.6
AHQ-2-13, 5544	K.NLPIYSEEIVEMYK.G	1728.98720	2	1.65E-05	0.90	3.83	-	596.8
AHQ-2-12, 5670	K.NLPIYSEEIVEMYK.G	1728.98720	2	3.30E-07	0.90	3.42	-	796.4
AHQ-2-2, 5933 - 6004	K.NLPIYSEEIVEMYK.G	1728.98720	2	1.15E-09	0.94	4.64	-	635.7
AHQ-2-11, 5421 - 5422	K.NLPIYSEEIVEMYK.G	1728.98720	2	7.26E-07	0.92	4.22	-	719.1
AHQ-2-3, 5908 - 5939	K.NLPIYSEEIVEMYK.G	1728.98720	2	2.71E-07	0.93	4.63	-	634.4
AHQ-2-7, 5484	K.NLPIYSEEIVEMYK.G	1728.98720	2	2.14E-04	0.88	3.60	-	625.1
AHQ-2-10, 5162	K.NLPIYSEEIVEMYK.G	1728.98720	2	1.80E-05	0.92	4.05	-	617.4
AHQ-2-3, 5896 - 5964	K.NMDPLNDNIATLLHQSSDK.F	2127.32169	2	1.68E-04	0.92	4.55	-	720.4
AHQ-2-7, 5169	K.NMDPLNDNIATLLHQSSDK.F	2127.32169	2	7.02E-04	0.89	3.68	-	1034.9
AHQ-2-2, 5141	K.NM*DPLNDNIATLLHQSSDK.F	2143.32109	2	5.11E-06	0.91	4.03	-	862.5
AHQ-2-6, 5253	K.NMDPLNDNIATLLHQSSDK.F	2127.32169	2	4.97E-07	0.86	3.93	-	638.8
AHQ-2-5, 5311 - 5312	K.NMDPLNDNIATLLHQSSDK.F	2127.32169	2	2.95E-08	0.94	4.88	-	748.3
AHQ-2-2, 5604	K.NMDPLNDNIATLLHQSSDK.F	2127.32169	2	1.43E-04	0.77	3.45	-	560.4
AHQ-2-1, 5752	K.NMDPLNDNIATLLHQSSDK.F	2127.32169	2	1.02E-06	0.91	4.25	-	711.6
AHQ-2-4, 5098	K.NM*DPLNDNIATLLHQSSDK.F	2143.32109	2	2.25E-05	0.63	2.79	-	660.1
AHQ-2-2, 5620 - 5621	K.NMDPLNDNIATLLHQSSDK.F	2127.32169	3	4.08E-09	0.82	3.65	-	511.8
AHQ-2-3, 5585	K.NMDPLNDNIATLLHQSSDK.F	2127.32169	2	8.07E-07	0.90	4.31	-	710.0
AHQ-2-3, 5237	K.NM*DPLNDNIATLLHQSSDK.F	2143.32109	2	2.89E-09	0.60	2.89	-	495.4
AHQ-2-3, 6211	K.NMDPLNDNIATLLHQSSDK.F	2127.32169	2	5.31E-04	0.66	2.92	-	450.7
AHQ-2-3, 5743	K.NMDPLNDNIATLLHQSSDK.F	2127.32169	2	3.49E-07	0.91	4.03	-	773.8
AHQ-2-1, 5209	K.NM*DPLNDNIATLLHQSSDK.F	2143.32109	3	2.62E-06	0.81	3.01	-	982.5
AHQ-2-2, 5144	K.NM*DPLNDNIATLLHQSSDK.F	2143.32109	3	8.52E-09	0.86	3.73	-	659.4
AHQ-2-4, 5605 - 5624	K.NMDPLNDNIATLLHQSSDK.F	2127.32169	2	2.95E-06	0.93	4.92	-	729.1
AHQ-2-4, 5617	K.NMDPLNDNIATLLHQSSDK.F	2127.32169	3	3.92E-08	0.77	3.34	-	393.2
AHQ-2-1, 5136 - 5217	K.NM*DPLNDNIATLLHQSSDK.F	2143.32109	2	8.40E-05	0.73	3.14	-	934.7
AHQ-2-2, 5929	K.NMDPLNDNIATLLHQSSDK.F	2127.32169	2	1.27E-07	0.93	4.58	-	778.1
AHQ-2-2, 5760 - 5781	K.NMDPLNDNIATLLHQSSDK.F	2127.32169	2	2.60E-07	0.94	4.71	-	904.9
AHQ-2-3, 5100	K.NM*DPLNDNIATLLHQSSDK.F	2143.32109	2	8.95E-04	0.74	2.73	-	854.3
AHQ-2-2, 5261	K.NM*DPLNDNIATLLHQSSDK.F	2143.32109	2	1.20E-08	0.92	3.63	-	1103.6
AHQ-2-3, 7603	K.PAGPPGILALLDDEECWFPK.A	2112.43315	2	4.15E-06	0.98	5.69	-	2107.6
AHQ-2-2, 7648	K.PAGPPGILALLDDEECWFPK.A	2112.43315	2	5.31E-06	0.98	6.05	-	1543.0
AHQ-2-2, 3068 - 3136	R.QAQQERDELADEIANSSGK.G	2090.15168	2	3.42E-06	0.94	4.16	-	1029.8
AHQ-2-4, 2770 - 2810	R.QAQQERDELADEIANSSGK.G	2090.15168	2	2.02E-04	0.21	2.77	-	160.6
AHQ-2-1, 3294 - 3302	R.QAQQERDELADEIANSSGK.G	2090.15168	3	9.73E-04	0.93	4.44	-	1264.6
AHQ-2-3, 3211	R.QAQQERDELADEIANSSGK.G	2090.15168	2	4.45E-05	0.87	4.35	-	316.4
AHQ-2-5, 2885	R.QAQQERDELADEIANSSGK.G	2090.15168	2	1.46E-04	0.79	3.90	-	360.4
AHQ-2-3, 2771	R.QAQQERDELADEIANSSGK.G	2090.15168	2	8.50E-04	0.29	2.98	-	160.0
AHQ-2-2, 3220	R.QAQQERDELADEIANSSGK.G	2090.15168	3	1.35E-04	0.73	3.32	-	646.4
AHQ-2-6, 2885	R.QAQQERDELADEIANSSGK.G	2090.15168	2	3.06E-04	0.78	3.99	-	351.7
AHQ-2-2, 3213	R.QAQQERDELADEIANSSGK.G	2090.15168	2	1.82E-05	0.86	3.78	-	499.4
AHQ-2-2, 4646	K.QELEEICHDLEAR.V	1643.75744	2	2.43E-06	0.84	3.76	-	466.3
AHQ-2-2, 2186	K.QIATLHAQVADM*K.K	1442.66637	2	4.22E-04	0.49	2.81	-	216.9
AHQ-2-4, 2628	K.QIATLHAQVADMK.K	1426.66697	2	1.20E-04	0.78	2.80	-	567.8
AHQ-2-2, 1946	R.QLEEAEEEAQR.A	1332.35590	2	8.16E-07	0.73	2.97	-	550.3
AHQ-2-3, 1933	R.QLEEAEEEAQR.A	1332.35590	2	1.14E-04	0.66	2.89	-	575.9
AHQ-2-1, 2104	R.QLEEAEEEAQR.A	1332.35590	2	4.04E-06	0.64	3.10	-	441.3
AHQ-2-2, 2432	K.RALEQQVEEMK.T	1361.55016	2	9.79E-04	0.90	3.41	-	1009.2
AHQ-2-3, 3407	R.RGDLPFVVPR.R	1156.36217	2	9.84E-07	0.92	3.56	-	1028.3
AHQ-2-12, 3295	R.RGDLPFVVPR.R	1156.36217	2	2.05E-05	0.90	3.39	-	956.9
AHQ-2-10, 3080	R.RGDLPFVVPR.R	1156.36217	2	7.18E-04	0.89	3.09	-	1106.2
AHQ-2-11, 3286	R.RGDLPFVVPR.R	1156.36217	2	9.38E-06	0.94	3.67	-	1319.5
AHQ-2-5, 3057 - 3067	R.RGDLPFVVPR.R	1156.36217	2	6.25E-05	0.93	3.52	-	1154.7
AHQ-2-4, 3346 - 3424	R.RGDLPFVVPR.R	1156.36217	2	3.71E-07	0.96	4.29	-	1272.9
AHQ-2-1, 3712	R.RGDLPFVVPR.R	1156.36217	2	2.46E-06	0.94	3.27	-	1564.1
AHQ-2-2, 3340 - 3409	R.RGDLPFVVPR.R	1156.36217	2	2.94E-05	0.95	3.74	-	1433.9
AHQ-2-6, 3109	R.RGDLPFVVPR.R	1156.36217	2	1.18E-05	0.92	3.65	-	924.1
AHQ-2-1, 6944	R.RKLEGDSTDLSDQIAELQAQIAELK.M	2773.04667	3	1.54E-05	0.96	6.31	-	1392.5
AHQ-2-2, 6997 - 7064	R.RKLEGDSTDLSDQIAELQAQIAELK.M	2773.04667	3	1.39E-11	0.96	5.67	-	1487.2
AHQ-2-3, 6968	R.RKLEGDSTDLSDQIAELQAQIAELK.M	2773.04667	3	5.85E-08	0.96	6.18	-	1295.8
AHQ-2-3, 7049	R.RKLEGDSTDLSDQIAELQAQIAELK.M	2773.04667	3	8.13E-05	0.93	5.01	-	1161.6
AHQ-2-4, 7048	R.RKLEGDSTDLSDQIAELQAQIAELK.M	2773.04667	3	8.33E-10	0.98	7.42	-	1597.8
AHQ-2-2, 2816	K.RQAQQERDELADEIANSSGK.G	2246.33803	3	2.29E-05	0.92	4.05	-	1122.2
AHQ-2-11, 1970	K.RQLEEAEEEAQR.A	1488.54225	2	2.09E-04	0.92	4.89	-	939.0
AHQ-2-2, 1829	K.RQLEEAEEEAQR.A	1488.54225	2	3.37E-04	0.97	4.95	-	2527.2
AHQ-2-3, 1852	K.RQLEEAEEEAQR.A	1488.54225	2	1.27E-06	0.96	4.87	-	1910.2
AHQ-2-1, 6490	K.SGFEPASLKEEVGEEAIVELVENGKK.V	2790.07246	3	4.68E-11	0.92	4.60	-	909.4
AHQ-2-14-, 5909	K.SGFEPASLKEEVGEEAIVELVENGKK.V	2790.07246	3	2.55E-11	0.96	5.42	-	1207.9
AHQ-2-1, 6394	K.SGFEPASLKEEVGEEAIVELVENGKK.V	2790.07246	3	1.46E-09	0.91	4.58	-	930.7
AHQ-2-3, 6371	K.SGFEPASLKEEVGEEAIVELVENGKK.V	2790.07246	3	1.25E-06	0.91	4.52	-	926.0
AHQ-2-2, 6825	K.SGFEPASLKEEVGEEAIVELVENGKK.V	2790.07246	3	7.87E-08	0.96	5.61	-	1425.4
AHQ-2-2, 7081	K.SGFEPASLKEEVGEEAIVELVENGKK.V	2790.07246	3	3.22E-10	0.95	5.11	-	1275.0
AHQ-2-2, 6372 - 6405	K.SGFEPASLKEEVGEEAIVELVENGKK.V	2790.07246	3	1.03E-09	0.95	5.43	-	1131.1
AHQ-2-2, 6917	K.SGFEPASLKEEVGEEAIVELVENGKK.V	2790.07246	3	8.36E-08	0.90	4.30	-	931.3
AHQ-2-1, 4889 - 4969	K.SM*EAEM*IQLQEELAAAER.A	2082.29944	2	3.32E-11	0.98	5.26	-	1855.1
AHQ-2-2, 5081	K.SM*EAEM*IQLQEELAAAER.A	2082.29944	2	1.99E-04	0.94	4.57	-	1125.4
AHQ-2-1, 6973	K.SMEAEMIQLQEELAAAER.A	2050.30064	3	8.67E-07	0.96	5.31	-	1622.8
AHQ-2-2, 7496	K.SMEAEMIQLQEELAAAER.A	2050.30064	2	1.71E-08	0.97	5.11	-	1512.7
AHQ-2-3, 7069	K.SMEAEMIQLQEELAAAER.A	2050.30064	3	2.11E-04	0.92	4.74	-	1067.4
AHQ-2-2, 7269 - 7336	K.SMEAEMIQLQEELAAAER.A	2050.30064	2	2.49E-05	0.89	3.84	-	1040.1
AHQ-2-4, 7142	K.SMEAEMIQLQEELAAAER.A	2050.30064	3	1.62E-07	0.94	4.87	-	1677.2
AHQ-2-4, 4868 - 4874	K.SM*EAEM*IQLQEELAAAER.A	2082.29944	2	2.70E-07	0.97	4.89	-	1722.4
AHQ-2-6, 6735 - 6758	K.SMEAEMIQLQEELAAAER.A	2050.30064	2	9.84E-05	0.96	5.18	-	1648.1
AHQ-2-2, 7161	K.SM*EAEM*IQLQEELAAAER.A	2082.29944	2	1.25E-06	0.97	5.40	-	1844.9
AHQ-2-3, 5880 - 5955	K.SM*EAEMIQLQEELAAAER.A	2066.30004	2	7.33E-08	0.94	4.97	-	1660.7
AHQ-2-3, 5948 - 6028	K.SM*EAEMIQLQEELAAAER.A	2066.30004	2	9.08E-07	0.95	4.43	-	1839.6
AHQ-2-2, 6076 - 6081	K.SM*EAEMIQLQEELAAAER.A	2066.30004	2	3.40E-04	0.88	4.41	-	916.1
AHQ-2-3, 6993 - 7065	K.SMEAEMIQLQEELAAAER.A	2050.30064	2	6.98E-06	0.98	6.23	-	1778.9
AHQ-2-4, 7124 - 7128	K.SMEAEMIQLQEELAAAER.A	2050.30064	2	2.32E-06	0.97	5.51	-	1897.3
AHQ-2-5, 6923 - 6931	K.SMEAEMIQLQEELAAAER.A	2050.30064	2	6.78E-06	0.97	5.44	-	1759.2
AHQ-2-2, 7132 - 7200	K.SMEAEMIQLQEELAAAER.A	2050.30064	2	2.08E-09	0.97	6.08	-	1421.8
AHQ-2-2, 7144 - 7145	K.SMEAEMIQLQEELAAAER.A	2050.30064	3	1.57E-05	0.97	5.56	-	2108.5
AHQ-2-5, 3264	R.SMMQDREDQSILCTGESGAGK.T	2302.50687	2	5.23E-05	0.96	5.64	-	1057.0
AHQ-2-3, 2952 - 2953	R.SMM*QDREDQSILCTGESGAGK.T	2318.50627	2	9.24E-04	0.88	3.86	-	729.1
AHQ-2-3, 3488	R.SMMQDREDQSILCTGESGAGK.T	2302.50687	2	1.39E-04	0.94	4.80	-	1164.5
AHQ-2-9, 3210	R.SMMQDREDQSILCTGESGAGK.T	2302.50687	2	3.48E-05	0.93	4.78	-	862.9
AHQ-2-4, 3497	R.SMMQDREDQSILCTGESGAGK.T	2302.50687	2	7.31E-05	0.98	5.73	-	1652.6
AHQ-2-2, 3504	R.SMMQDREDQSILCTGESGAGK.T	2302.50687	2	6.34E-05	0.96	5.90	-	1082.5

AHQ-2-6, 3241	R.SMMQDREDQSILCTGESGAGK.T	2302.50687	2	2.63E-04	0.96	5.43	-	949.7
AHQ-2-2, 3685 - 3761	K.TDLLLEPYNK.Y	1206.36989	2	4.98E-05	0.92	4.09	-	1066.6
AHQ-2-2, 3694 - 3753	K.TDLLLEPYNK.Y	1206.36989	1	8.96E-04	0.06	2.13	-	215.8
AHQ-2-1, 3844	K.TDLLLEPYNK.Y	1206.36989	2	4.79E-06	0.93	3.87	-	1248.5
AHQ-2-5, 3408 - 3424	K.TDLLLEPYNK.Y	1206.36989	2	2.30E-05	0.86	3.35	-	946.5
AHQ-2-4, 3696	K.TDLLLEPYNK.Y	1206.36989	2	1.95E-06	0.92	4.03	-	968.4
AHQ-2-6, 3381 - 3382	K.TDLLLEPYNK.Y	1206.36989	2	9.73E-06	0.84	3.42	-	852.1
AHQ-2-3, 3676	K.TDLLLEPYNK.Y	1206.36989	2	2.78E-04	0.87	3.22	-	1229.6
AHQ-2-2, 3884	K.TDLLLEPYNKYR.F	1525.73037	2	6.76E-05	0.94	4.18	-	1104.6
AHQ-2-4, 4476	K.TELEDTLDSTAAQQELR.S	1921.00969	2	5.43E-06	0.94	3.75	-	1873.5
AHQ-2-5, 4301	K.TELEDTLDSTAAQQELR.S	1921.00969	2	2.45E-05	0.84	3.22	-	1354.8
AHQ-2-1, 4488	K.TELEDTLDSTAAQQELR.S	1921.00969	2	6.27E-10	0.97	4.84	-	1590.7
AHQ-2-2, 4517 - 4518	K.TELEDTLDSTAAQQELR.S	1921.00969	2	1.80E-07	0.93	4.06	-	1049.5
AHQ-2-2, 3104	R.TEMEDLMSSK.D	1171.32449	2	3.41E-05	0.89	3.58	-	821.5
AHQ-2-3, 3076 - 3085	R.TEMEDLMSSK.D	1171.32449	2	2.46E-04	0.91	3.54	-	833.1
AHQ-2-1, 3209	R.TEMEDLMSSK.D	1171.32449	2	2.62E-06	0.82	2.63	-	780.7
AHQ-2-1, 2584	R.TEMEDLM*SSKDDVGK.S	1701.85566	2	8.33E-05	0.70	3.03	-	574.0
AHQ-2-2, 3358	R.TEMEDLMSSKDDVGK.S	1685.85626	2	4.19E-06	0.90	3.69	-	1036.0
AHQ-2-5, 3137	R.TEMEDLMSSKDDVGK.S	1685.85626	2	2.19E-08	0.74	3.11	-	748.5
AHQ-2-1, 3457	R.TEMEDLMSSKDDVGK.S	1685.85626	2	5.50E-06	0.85	3.23	-	985.3
AHQ-2-4, 3350	R.TEMEDLMSSKDDVGK.S	1685.85626	2	9.72E-06	0.75	3.19	-	691.3
AHQ-2-3, 3340	R.TEMEDLMSSKDDVGK.S	1685.85626	2	3.16E-05	0.91	3.92	-	873.1
AHQ-2-2, 7257	R.TFHIFYYLLSGAGEHLK.T	1997.28378	2	4.01E-04	0.90	4.04	-	738.1
AHQ-2-3, 6693 - 6761	R.TFHIFYYLLSGAGEHLK.T	1997.28378	2	2.65E-04	0.95	4.58	-	1163.4
AHQ-2-5, 6243 - 6308	R.TFHIFYYLLSGAGEHLK.T	1997.28378	2	3.84E-08	0.97	5.19	-	1661.7
AHQ-2-7, 6019	R.TFHIFYYLLSGAGEHLK.T	1997.28378	2	8.33E-05	0.97	5.06	-	1421.0
AHQ-2-3, 6601	R.TFHIFYYLLSGAGEHLK.T	1997.28378	2	9.83E-06	0.97	5.15	-	1599.6
AHQ-2-2, 6558 - 6638	R.TFHIFYYLLSGAGEHLK.T	1997.28378	2	1.73E-05	0.98	6.33	-	1954.6
AHQ-2-5, 6252	R.TFHIFYYLLSGAGEHLK.T	1997.28378	3	1.10E-06	0.95	4.80	-	1343.6
AHQ-2-2, 6569	R.TFHIFYYLLSGAGEHLK.T	1997.28378	3	3.75E-08	0.92	4.25	-	1145.5
AHQ-2-6, 6241	R.TFHIFYYLLSGAGEHLK.T	1997.28378	3	1.57E-08	0.96	5.07	-	1484.3
AHQ-2-7, 6023	R.TFHIFYYLLSGAGEHLK.T	1997.28378	3	2.15E-05	0.96	5.48	-	1454.1
AHQ-2-2, 6817	R.TFHIFYYLLSGAGEHLK.T	1997.28378	2	6.36E-06	0.97	4.96	-	1690.5
AHQ-2-4, 3626	K.TLEEEAKTHEAQIQEMR.Q	2044.23269	2	3.80E-04	0.94	4.36	-	907.1
AHQ-2-2, 3733	K.TLEEEAKTHEAQIQEMR.Q	2044.23269	2	1.24E-04	0.96	4.54	-	1347.5
AHQ-2-4, 1730	K.VAAYDKLEK.T	1037.19123	2	7.40E-06	0.65	2.74	-	470.9
AHQ-2-5, 2979	K.VEAQLQELQVK.F	1285.47179	2	1.05E-04	0.94	3.74	-	1498.7
AHQ-2-2, 3225	K.VEAQLQELQVK.F	1285.47179	2	6.37E-05	0.96	4.32	-	1500.1
AHQ-2-4, 3229	K.VEAQLQELQVK.F	1285.47179	2	1.62E-05	0.94	3.82	-	1214.6
AHQ-2-1, 3404	K.VEAQLQELQVK.F	1285.47179	2	4.47E-06	0.95	4.00	-	1476.3
AHQ-2-4, 3240	K.VEAQLQELQVK.F	1285.47179	1	2.07E-04	0.53	2.92	-	474.4
AHQ-2-3, 3227	K.VEAQLQELQVK.F	1285.47179	2	2.47E-05	0.94	4.15	-	1240.3
AHQ-2-6, 5773	K.VEDMAELTCLNEASVLHNLK.E	2288.58457	2	2.58E-06	0.94	4.77	-	648.7
AHQ-2-11, 5584 - 5585	K.VEDMAELTCLNEASVLHNLK.E	2288.58457	2	4.57E-09	0.95	4.89	-	821.9
AHQ-2-3, 6137	K.VEDMAELTCLNEASVLHNLK.E	2288.58457	3	1.01E-07	0.86	3.91	-	695.1
AHQ-2-3, 6139	K.VEDMAELTCLNEASVLHNLK.E	2288.58457	2	7.63E-12	0.88	3.98	-	604.2
AHQ-2-10, 4766	K.VEDM*AELTCLNEASVLHNLK.E	2304.58397	3	5.19E-06	0.94	4.56	-	1222.9
AHQ-2-5, 5768 - 5784	K.VEDMAELTCLNEASVLHNLK.E	2288.58457	2	3.41E-06	0.89	3.98	-	724.7
AHQ-2-12, 5838	K.VEDMAELTCLNEASVLHNLK.E	2288.58457	2	1.21E-08	0.65	3.58	-	306.0
AHQ-2-2, 6394	K.VEDMAELTCLNEASVLHNLK.E	2288.58457	3	8.80E-10	0.83	3.82	-	699.7
AHQ-2-5, 5152	K.VEDM*AELTCLNEASVLHNLK.E	2304.58397	2	1.02E-09	0.79	3.73	-	493.5
AHQ-2-9, 5766	K.VEDMAELTCLNEASVLHNLK.E	2288.58457	2	1.07E-07	0.74	3.58	-	335.4
AHQ-2-10, 5364	K.VEDMAELTCLNEASVLHNLK.E	2288.58457	2	8.82E-09	0.91	4.29	-	740.2
AHQ-2-13, 5639	K.VEDMAELTCLNEASVLHNLK.E	2288.58457	2	4.25E-09	0.87	3.84	-	573.7
AHQ-2-1, 6157 - 6161	K.VEDMAELTCLNEASVLHNLK.E	2288.58457	2	8.73E-11	0.96	5.18	-	940.1
AHQ-2-2, 6141	K.VEDMAELTCLNEASVLHNLK.E	2288.58457	3	2.39E-08	0.88	4.44	-	617.5
AHQ-2-1, 5548	K.VEDM*AELTCLNEASVLHNLK.E	2304.58397	2	7.41E-13	0.94	4.79	-	770.5
AHQ-2-10, 4756	K.VEDM*AELTCLNEASVLHNLK.E	2304.58397	2	6.79E-13	0.92	3.97	-	982.4
AHQ-2-13-, 5250	K.VEDM*AELTCLNEASVLHNLK.E	2304.58397	2	5.65E-04	0.68	3.29	-	523.1
AHQ-2-10, 5368	K.VEDMAELTCLNEASVLHNLK.E	2288.58457	3	2.21E-07	0.85	3.82	-	716.1
AHQ-2-2, 6410	K.VEDMAELTCLNEASVLHNLK.E	2288.58457	2	2.20E-12	0.87	4.12	-	566.2
AHQ-2-2, 5417 - 5452	K.VEDM*AELTCLNEASVLHNLK.E	2304.58397	3	3.27E-10	0.96	5.86	-	1255.7
AHQ-2-7, 5691	K.VEDMAELTCLNEASVLHNLK.E	2288.58457	2	7.74E-12	0.93	4.25	-	730.6
AHQ-2-6, 5061	K.VEDM*AELTCLNEASVLHNLK.E	2304.58397	2	6.07E-08	0.88	4.16	-	567.0
AHQ-2-9, 5062	K.VEDM*AELTCLNEASVLHNLK.E	2304.58397	2	4.39E-05	0.57	3.05	-	294.4
AHQ-2-6, 5751	K.VEDMAELTCLNEASVLHNLK.E	2288.58457	3	8.01E-07	0.83	3.71	-	763.4
AHQ-2-2, 5382 - 5416	K.VEDM*AELTCLNEASVLHNLK.E	2304.58397	2	1.06E-09	0.91	4.25	-	732.2
AHQ-2-4, 6144 - 6150	K.VEDMAELTCLNEASVLHNLK.E	2288.58457	3	6.74E-09	0.94	4.71	-	998.6
AHQ-2-4, 6140 - 6156	K.VEDMAELTCLNEASVLHNLK.E	2288.58457	2	7.19E-10	0.94	4.95	-	687.3
AHQ-2-8, 5515 - 5582	K.VEDMAELTCLNEASVLHNLK.E	2288.58457	2	1.76E-07	0.95	4.40	-	1023.2
AHQ-2-2, 6142 - 6217	K.VEDMAELTCLNEASVLHNLK.E	2288.58457	2	1.94E-10	0.94	4.95	-	725.5
AHQ-2-1, 6102 - 6168	K.VEDMAELTCLNEASVLHNLK.E	2288.58457	3	7.34E-07	0.87	3.99	-	618.4
AHQ-2-1, 5658	K.VEDM*AELTCLNEASVLHNLK.E	2304.58397	3	3.59E-09	0.87	3.69	-	1007.2
AHQ-2-1, 2089	R.VEEEEERCQHLQAEK.K	1916.01642	3	3.77E-04	0.84	4.05	-	759.6
AHQ-2-4, 1908	R.VEEEEERCQHLQAEK.K	1916.01642	2	8.64E-05	0.84	3.39	-	996.7
AHQ-2-3, 1907	R.VEEEEERCQHLQAEK.K	1916.01642	3	8.11E-05	0.80	3.66	-	592.5
AHQ-2-2, 3924	K.VIQYLAYVASSHK.S	1479.70484	3	7.03E-05	0.87	3.78	-	803.6
AHQ-2-1, 4121	K.VIQYLAYVASSHK.S	1479.70484	3	2.06E-06	0.81	3.75	-	474.8
AHQ-2-4, 3886	K.VIQYLAYVASSHK.S	1479.70484	3	1.97E-07	0.86	3.82	-	674.4
AHQ-2-3, 3879	K.VIQYLAYVASSHK.S	1479.70484	2	1.02E-05	0.75	2.92	-	736.4
AHQ-2-7, 3387	K.VIQYLAYVASSHK.S	1479.70484	2	7.44E-05	0.91	3.17	-	1061.9
AHQ-2-1, 4112 - 4128	K.VIQYLAYVASSHK.S	1479.70484	2	6.19E-07	0.94	3.88	-	1250.7
AHQ-2-2, 3909	K.VIQYLAYVASSHK.S	1479.70484	2	4.99E-08	0.94	4.05	-	1097.1
AHQ-2-5, 3499	K.VIQYLAYVASSHK.S	1479.70484	3	2.49E-05	0.83	3.71	-	552.2
AHQ-2-5, 3496	K.VIQYLAYVASSHK.S	1479.70484	2	5.10E-04	0.94	4.08	-	1288.2
AHQ-2-8, 3068	K.VIQYLAYVASSHK.S	1479.70484	2	9.87E-06	0.95	3.95	-	1131.7
AHQ-2-10, 3431	K.VIQYLAYVASSHK.S	1479.70484	2	7.49E-09	0.93	4.03	-	1094.3
AHQ-2-4, 6732 - 6804	R.VISGVLQLGNIVFK.K	1487.81155	2	5.28E-09	0.97	5.16	-	1701.4
AHQ-2-7, 6239 - 6319	R.VISGVLQLGNIVFK.K	1487.81155	2	1.12E-08	0.96	4.53	-	1453.3
AHQ-2-3, 6664 - 6731	R.VISGVLQLGNIVFK.K	1487.81155	2	6.90E-08	0.97	4.50	-	1981.1
AHQ-2-6, 6346 - 6413	R.VISGVLQLGNIVFK.K	1487.81155	2	2.11E-07	0.97	5.44	-	1775.3
AHQ-2-5, 6420 - 6488	R.VISGVLQLGNIVFK.K	1487.81155	2	2.95E-05	0.95	3.65	-	1502.5
AHQ-2-2, 6673 - 6744	R.VISGVLQLGNIVFK.K	1487.81155	2	3.52E-09	0.98	5.64	-	1820.7
AHQ-2-2, 6772 - 6842	R.VISGVLQLGNIVFK.K	1487.81155	2	8.63E-09	0.97	5.08	-	1635.7
AHQ-2-5, 6435 - 6444	R.VISGVLQLGNIVFK.K	1487.81155	1	6.49E-04	0.54	3.07	-	510.7
AHQ-2-2, 6857	R.VISGVLQLGNIVFK.K	1487.81155	2	4.56E-04	0.86	2.52	-	1187.5
AHQ-2-10, 5919	R.VISGVLQLGNIVFK.K	1487.81155	2	9.20E-07	0.92	3.67	-	1155.9
AHQ-2-3, 6681	R.VISGVLQLGNIVFK.K	1487.81155	1	2.69E-04	0.67	3.22	-	673.1
AHQ-2-6, 4561 - 4569	K.VSHLLGINVTDFTR.G	1572.79026	2	3.60E-11	0.97	5.33	-	1326.3
AHQ-2-5, 4608	K.VSHLLGINVTDFTR.G	1572.79026	2	1.35E-08	0.95	4.16	-	1424.7
AHQ-2-7, 4429 - 4500	K.VSHLLGINVTDFTR.G	1572.79026	2	2.41E-04	0.86	3.42	-	792.3
AHQ-2-1, 5054 - 5094	K.VSHLLGINVTDFTR.G	1572.79026	2	3.16E-10	0.97	4.47	-	1600.3
AHQ-2-1, 5101	K.VSHLLGINVTDFTR.G	1572.79026	3	7.28E-07	0.89	3.66	-	1084.6
AHQ-2-4, 4958 - 4960	K.VSHLLGINVTDFTR.G	1572.79026	2	2.68E-10	0.96	4.60	-	1449.5
AHQ-2-2, 4942 - 5012	K.VSHLLGINVTDFTR.G	1572.79026	2	5.73E-12	0.95	4.17	-	1242.2
AHQ-2-3, 4964	K.VSHLLGINVTDFTR.G	1572.79026	2	5.34E-11	0.95	4.21	-	1437.6
AHQ-2-5, 2753	R.VVFQEFR.Q	925.06580	2	8.12E-05	0.80	2.54	-	536.1

AHQ-2-1, 3270	R.VVFQEFR.Q	925.06580	2	2.57E-04	0.68	2.56	-	546.4
AHQ-2-2, 4044 - 4056	R.YEILTPNSIPK.G	1275.47536	2	3.16E-07	0.93	3.99	-	977.6
AHQ-2-2, 4172	R.YEILTPNSIPK.G	1275.47536	2	3.22E-05	0.80	2.80	-	939.3
AHQ-2-3, 4283 - 4285	R.YEILTPNSIPK.G	1275.47536	1	8.40E-04	0.36	2.17	-	644.1
AHQ-2-3, 4268	R.YEILTPNSIPK.G	1275.47536	2	1.64E-04	0.85	3.00	-	743.8
AHQ-2-3, 4019	R.YEILTPNSIPK.G	1275.47536	2	4.23E-06	0.90	3.44	-	1143.6
AHQ-2-4, 4009	R.YEILTPNSIPK.G	1275.47536	2	1.39E-05	0.78	3.13	-	659.3
AHQ-2-1, 4077 - 4158	R.YEILTPNSIPK.G	1275.47536	2	3.99E-05	0.84	2.97	-	825.2
AHQ-2-1, 4225	R.YEILTPNSIPK.G	1275.47536	2	1.64E-04	0.92	3.57	-	1001.7
AHQ-2-2, 6357 - 6369	K.YLYVDKNFINNPLAQADWAAK.K	2455.75199	3	2.97E-07	0.96	5.02	-	1701.8
AHQ-2-10, 5590	K.YLYVDKNFINNPLAQADWAAK.K	2455.75199	2	7.36E-08	0.77	3.22	-	529.3
gi|30159830|ref|XP_302246.1| similar to HSPC280 [Homo sapiens]				1.17E-14	1.95	20.30	3.90	45644.4
AHQ-2-13, 6799	K.CANLFEALVGTLK.A	1437.68632	2	8.46E-09	0.98	6.05	-	1854.5
AHQ-2-14-, 6861	K.CANLFEALVGTLK.A	1437.68632	2	1.25E-06	0.97	5.39	-	1363.7
AHQ-2-14-, 6808 - 6817	R.DDKCANLFEALVGTLK.A	1796.03496	2	2.83E-10	0.96	4.79	-	1277.6
AHQ-2-14-, 6811 - 6812	R.DDKCANLFEALVGTLK.A	1796.03496	3	1.21E-04	0.95	4.28	-	2137.2
AHQ-2-13-, 6955	R.DDKCANLFEALVGTLK.A	1796.03496	2	9.88E-14	0.96	4.50	-	1404.3
AHQ-2-13, 6735 - 6736	R.DDKCANLFEALVGTLK.A	1796.03496	2	1.17E-14	0.96	4.94	-	1285.9
gi|4507521|ref|NP_001055.1| transketolase [Homo sapiens]				1.28E-14	1.42	20.20	5.90	67877.3
AHQ-2-6, 4597	K.ILATPPQEDAPSVDIANIR.M	2021.26036	2	1.28E-14	0.89	3.97	-	429.0
AHQ-2-6, 3689 - 3702	R.SVPTSTVFYPSDGVATEK.A	1886.04854	2	1.74E-06	0.53	2.65	-	220.2
gi|4885609|ref|NP_005408.1| v-src sarcoma (Schmidt-Ruppin A-2) viral oncogene homolog; Protooncogen				1.78E-14	4.56	50.37	20.10	59834.4
AHQ-2-7, 7007	R.AGPLAGGVTTFVALYDYESR.T	2088.30526	2	4.65E-05	0.84	3.65	-	530.0
AHQ-2-7, 7543	R.KEPEERPTFEYLQAFLEDYFTSTEPQYQPGENL	3998.26826	3	4.53E-09	0.90	5.16	-	352.2
AHQ-2-7, 7523	R.LPQLVDMAAQIASGMAYVER.M	2164.53485	2	1.78E-14	0.99	7.30	-	4979.6
AHQ-2-7, 6323	R.MPCPPECPESLHDLMCQCWR.K	2608.97680	3	6.47E-12	0.90	4.23	-	588.1
AHQ-2-7, 6303	R.TQFNSLQQLVAYYSK.H	1790.99681	2	4.95E-08	0.97	4.95	-	1359.8
AHQ-2-7, 6204 - 6217	R.TQFNSLQQLVAYYSK.H	1790.99681	2	2.27E-10	0.93	3.81	-	1066.3
gi|4501881|ref|NP_001091.1| alpha 1 actin precursor; alpha skeletal muscle actin [Homo sapiens] [MA				1.83E-14	15.20	200.27	46.70	42050.8
AHQ-2-13, 2043 - 2100	K.AGFAGDDAPR.A	977.01283	2	5.43E-04	0.90	3.16	-	1265.0
AHQ-2-8, 1594 - 1619	K.AGFAGDDAPR.A	977.01283	2	2.51E-04	0.92	3.61	-	1099.4
AHQ-2-12, 1901 - 1974	K.AGFAGDDAPR.A	977.01283	2	1.02E-04	0.87	3.09	-	969.3
AHQ-2-12, 1755 - 1834	K.AGFAGDDAPR.A	977.01283	2	1.08E-06	0.92	3.53	-	1158.5
AHQ-2-8, 1687 - 1762	K.AGFAGDDAPR.A	977.01283	2	3.89E-04	0.88	3.19	-	1053.0
AHQ-2-13-, 1925	K.AGFAGDDAPR.A	977.01283	2	4.77E-04	0.88	3.31	-	986.7
AHQ-2-8, 1449 - 1524	K.AGFAGDDAPR.A	977.01283	2	3.83E-07	0.88	3.48	-	882.4
AHQ-2-13-, 3541 - 3557	R.AVFPSIVGR.P	946.12815	2	2.38E-04	0.83	2.66	-	727.3
AHQ-2-8, 2742 - 2747	R.AVFPSIVGRPR.H	1199.43018	2	2.01E-04	0.70	2.69	-	626.3
AHQ-2-8, 2559 - 2610	R.AVFPSIVGRPR.H	1199.43018	2	1.94E-06	0.74	3.00	-	533.7
AHQ-2-8, 2344 - 2414	R.AVFPSIVGRPR.H	1199.43018	2	6.67E-04	0.56	2.57	-	380.9
AHQ-2-8, 2463 - 2534	R.AVFPSIVGRPR.H	1199.43018	2	5.75E-04	0.57	2.84	-	365.3
AHQ-2-9, 2822 - 2823	R.AVFPSIVGRPR.H	1199.43018	2	2.96E-05	0.60	2.65	-	524.8
AHQ-2-9, 3675	R.DLTDYLM*K.I	1015.16266	1	2.58E-04	0.25	2.52	-	97.8
AHQ-2-9, 4418	R.DLTDYLMK.I	999.16326	1	4.72E-04	0.43	2.71	-	294.0
AHQ-2-7, 4424	R.DLTDYLMK.I	999.16326	1	4.32E-05	0.39	2.47	-	383.9
AHQ-2-13, 4308 - 4384	R.DLTDYLMK.I	999.16326	1	5.67E-05	0.46	2.13	-	681.4
AHQ-2-9, 4430	R.DLTDYLMK.I	999.16326	2	9.76E-04	0.69	2.79	-	392.9
AHQ-2-11, 4285	R.DLTDYLMK.I	999.16326	2	7.02E-04	0.63	2.62	-	406.6
AHQ-2-8, 4043	R.DLTDYLMK.I	999.16326	1	4.54E-04	0.40	2.77	-	322.7
AHQ-2-8, 4346 - 4416	R.DLTDYLMK.I	999.16326	2	3.09E-04	0.86	3.10	-	483.4
AHQ-2-8, 3606	R.DLTDYLM*K.I	1015.16266	1	1.90E-05	0.49	2.35	-	173.4
AHQ-2-5, 4495	R.DLTDYLMK.I	999.16326	1	8.81E-06	0.63	2.19	-	721.9
AHQ-2-10, 3518	R.DLTDYLM*K.I	1015.16266	1	6.23E-04	0.18	1.92	-	122.1
AHQ-2-10, 3522 - 3534	R.DLTDYLM*K.I	1015.16266	2	8.39E-04	0.83	3.20	-	471.5
AHQ-2-10, 4122 - 4172	R.DLTDYLMK.I	999.16326	2	9.28E-04	0.69	2.90	-	375.2
AHQ-2-8, 4331 - 4461	R.DLTDYLMK.I	999.16326	1	1.26E-05	0.69	2.97	-	645.9
AHQ-2-8, 3822	R.DLTDYLMK.I	999.16326	1	8.47E-04	0.45	1.89	-	483.5
AHQ-2-13, 3781	R.DLTDYLM*K.I	1015.16266	1	3.90E-04	0.21	2.11	-	99.9
AHQ-2-10, 4999	K.DLYANNVMSGGTTMYPGIADR.M	2247.49444	2	3.47E-07	0.50	2.87	-	517.4
AHQ-2-8, 1480	K.DSYVGDEAQSK.R	1199.20634	1	7.37E-04	0.76	2.82	-	539.2
AHQ-2-13-, 1718	K.DSYVGDEAQSK.R	1199.20634	1	4.81E-06	0.30	1.94	-	431.9
AHQ-2-12, 1587 - 1649	K.DSYVGDEAQSK.R	1199.20634	2	1.04E-05	0.82	3.21	-	585.8
AHQ-2-8, 1453	K.DSYVGDEAQSKR.G	1355.39270	2	1.07E-06	0.53	2.52	-	551.8
AHQ-2-13, 2919 - 2992	K.EITALAPSTMK.I	1162.38206	2	3.77E-04	0.72	2.73	-	532.0
AHQ-2-9, 2586 - 2730	K.EITALAPSTMK.I	1162.38206	1	2.22E-04	0.40	2.34	-	294.6
AHQ-2-4, 2901	K.EITALAPSTMK.I	1162.38206	1	2.09E-05	0.19	1.98	-	277.6
AHQ-2-7, 2599	K.EITALAPSTMK.I	1162.38206	1	1.27E-05	0.25	2.24	-	277.4
AHQ-2-8, 1956 - 2026	K.EITALAPSTM*K.I	1178.38146	2	1.55E-06	0.89	3.24	-	840.2
AHQ-2-14, 3740 - 3864	K.EITALAPSTMK.I	1162.38206	1	4.72E-06	0.43	2.38	-	309.7
AHQ-2-8, 2330 - 2399	K.EITALAPSTMK.I	1162.38206	2	1.69E-04	0.74	2.78	-	526.2
AHQ-2-14, 3176 - 3217	K.EITALAPSTM*K.I	1178.38146	2	7.43E-04	0.71	2.66	-	659.4
AHQ-2-10, 2171 - 2244	K.EITALAPSTM*K.I	1178.38146	2	3.56E-04	0.80	2.89	-	688.5
AHQ-2-10, 2566 - 2643	K.EITALAPSTMK.I	1162.38206	2	5.02E-04	0.77	2.63	-	589.9
AHQ-2-10, 2607 - 2674	K.EITALAPSTMK.I	1162.38206	1	1.05E-04	0.31	2.66	-	221.9
AHQ-2-12, 2858 - 2859	K.EITALAPSTMK.I	1162.38206	2	8.24E-05	0.83	3.05	-	588.1
AHQ-2-8, 2732 - 2734	K.EITALAPSTMK.I	1162.38206	1	8.37E-05	0.24	2.35	-	166.8
AHQ-2-14-, 2275 - 2343	K.EITALAPSTM*K.I	1178.38146	2	1.04E-06	0.90	3.14	-	1045.3
AHQ-2-11, 2809	K.EITALAPSTMK.I	1162.38206	1	7.06E-06	0.33	2.21	-	310.0
AHQ-2-8, 2814	K.EITALAPSTMK.I	1162.38206	2	2.72E-04	0.72	2.55	-	593.7
AHQ-2-14-, 2763 - 2833	K.EITALAPSTMK.I	1162.38206	2	1.88E-04	0.81	3.21	-	525.1
AHQ-2-5, 2647	K.EITALAPSTMK.I	1162.38206	1	4.34E-04	0.20	2.12	-	258.1
AHQ-2-12, 2783 - 2857	K.EITALAPSTMK.I	1162.38206	1	5.38E-05	0.58	2.33	-	421.7
AHQ-2-8, 7271 - 7342	K.EKLCYVALDFENEM*ATAASSSSLEK.S	2812.07683	2	1.06E-04	0.59	3.45	-	361.1
AHQ-2-8, 7535 - 7572	K.EKLCYVALDFENEM*ATAASSSSLEK.S	2812.07683	3	4.66E-05	0.87	3.88	-	760.2
AHQ-2-8, 1532 - 1602	R.HQGVMVGMGQK.D	1172.40691	2	3.34E-04	0.89	3.57	-	618.0
AHQ-2-8, 2778 - 2852	K.IWHHTFYNELR.V	1516.68695	2	3.56E-08	0.90	3.49	-	886.9
AHQ-2-3, 2900	K.IWHHTFYNELR.V	1516.68695	2	6.08E-04	0.69	2.51	-	582.2
AHQ-2-6, 2629	K.IWHHTFYNELR.V	1516.68695	2	3.84E-06	0.77	3.16	-	899.4
AHQ-2-8, 2542 - 2614	K.IWHHTFYNELR.V	1516.68695	2	8.47E-10	0.94	4.17	-	1001.1
AHQ-2-12, 3017	K.IWHHTFYNELR.V	1516.68695	3	9.70E-06	0.92	3.53	-	1706.9
AHQ-2-10, 2539 - 2582	K.IWHHTFYNELR.V	1516.68695	2	2.96E-05	0.89	3.16	-	934.1
AHQ-2-2, 2881	K.IWHHTFYNELR.V	1516.68695	2	1.03E-05	0.58	3.02	-	529.7
AHQ-2-9, 2946	K.IWHHTFYNELR.V	1516.68695	2	1.88E-06	0.78	2.86	-	752.5
AHQ-2-12, 3142	K.IWHHTFYNELR.V	1516.68695	2	4.86E-09	0.94	3.36	-	1299.2
AHQ-2-8, 2208 - 2278	K.IWHHTFYNELR.V	1516.68695	2	1.87E-04	0.73	2.90	-	601.5
AHQ-2-13, 3044	K.IWHHTFYNELR.V	1516.68695	2	2.13E-05	0.76	3.22	-	802.6
AHQ-2-4, 2866	K.IWHHTFYNELR.V	1516.68695	2	3.08E-04	0.70	2.78	-	564.7
AHQ-2-12, 2829 - 2897	K.IWHHTFYNELR.V	1516.68695	3	1.98E-05	0.92	4.33	-	1530.8
AHQ-2-9, 2574 - 2646	K.IWHHTFYNELR.V	1516.68695	2	5.38E-07	0.89	4.22	-	963.3
AHQ-2-8, 2346 - 2415	K.IWHHTFYNELR.V	1516.68695	2	2.17E-06	0.87	3.55	-	919.2
AHQ-2-13-, 3006	K.IWHHTFYNELR.V	1516.68695	2	5.32E-06	0.89	3.11	-	837.6
AHQ-2-8, 4991 - 5059	R.LDLAGRDLTDYLMK.I	1624.88375	2	5.55E-05	0.89	3.73	-	866.3
AHQ-2-9, 5196	R.LDLAGRDLTDYLMK.I	1624.88375	2	1.91E-04	0.82	3.17	-	628.4
AHQ-2-8, 5004 - 5081	K.SYELPDGQVITIGNER.F	1791.93998	2	3.24E-06	0.96	4.99	-	1219.3
AHQ-2-8, 5150 - 5151	K.SYELPDGQVITIGNER.F	1791.93998	2	1.18E-08	0.96	4.51	-	1338.6
AHQ-2-14, 6138	K.SYELPDGQVITIGNER.F	1791.93998	2	3.06E-05	0.91	3.29	-	911.0
AHQ-2-8, 4851 - 4923	K.SYELPDGQVITIGNER.F	1791.93998	2	4.39E-11	0.97	5.02	-	1247.9

AHQ-2-1, 4697	K.SYELPDGQVITIGNER.F	1791.93998	2	3.76E-05	0.90	3.98	-	663.9
AHQ-2-8, 4735 - 4803	K.SYELPDGQVITIGNER.F	1791.93998	2	8.18E-13	0.95	4.54	-	1105.0
AHQ-2-3, 5344	K.SYELPDGQVITIGNER.F	1791.93998	2	3.91E-07	0.85	3.63	-	556.0
AHQ-2-8, 4729	K.SYELPDGQVITIGNER.F	1791.93998	3	1.15E-09	0.96	4.81	-	1877.4
AHQ-2-8, 4705 - 4783	K.SYELPDGQVITIGNER.F	1791.93998	2	1.62E-11	0.96	4.72	-	1060.2
AHQ-2-9, 4331 - 4410	K.SYELPDGQVITIGNER.F	1791.93998	2	5.40E-05	0.68	2.81	-	510.9
AHQ-2-3, 5153 - 5160	K.SYELPDGQVITIGNER.F	1791.93998	2	1.18E-07	0.96	4.80	-	1115.9
AHQ-2-3, 4769 - 4772	K.SYELPDGQVITIGNER.F	1791.93998	2	1.81E-08	0.93	4.23	-	880.0
AHQ-2-13, 5036 - 5095	K.SYELPDGQVITIGNER.F	1791.93998	2	6.50E-06	0.95	4.11	-	1168.4
AHQ-2-9, 4478 - 4528	K.SYELPDGQVITIGNER.F	1791.93998	2	8.05E-10	0.93	4.19	-	992.0
AHQ-2-13, 4869 - 4895	K.SYELPDGQVITIGNER.F	1791.93998	2	8.91E-07	0.96	4.84	-	956.2
AHQ-2-9, 4607	K.SYELPDGQVITIGNER.F	1791.93998	2	7.64E-10	0.91	3.90	-	797.7
AHQ-2-9, 4698 - 4708	K.SYELPDGQVITIGNER.F	1791.93998	2	7.57E-09	0.95	3.88	-	1446.9
AHQ-2-9, 4794 - 4862	K.SYELPDGQVITIGNER.F	1791.93998	2	1.83E-14	0.97	5.22	-	1397.2
AHQ-2-9, 5036 - 5106	K.SYELPDGQVITIGNER.F	1791.93998	2	3.57E-04	0.86	3.68	-	661.2
AHQ-2-9, 5144 - 5218	K.SYELPDGQVITIGNER.F	1791.93998	2	4.09E-12	0.95	4.28	-	1268.1
AHQ-2-14, 5956 - 6033	K.SYELPDGQVITIGNER.F	1791.93998	2	2.02E-04	0.92	4.22	-	869.4
AHQ-2-8, 4641	K.SYELPDGQVITIGNER.F	1791.93998	3	1.81E-06	0.95	4.61	-	1606.6
AHQ-2-8, 4603 - 4671	K.SYELPDGQVITIGNER.F	1791.93998	2	1.05E-11	0.97	5.33	-	1224.4
AHQ-2-8, 4516 - 4587	K.SYELPDGQVITIGNER.F	1791.93998	2	4.55E-11	0.96	5.18	-	1099.5
AHQ-2-8, 4366 - 4447	K.SYELPDGQVITIGNER.F	1791.93998	2	6.27E-13	0.96	4.46	-	1223.5
AHQ-2-3, 4680	K.SYELPDGQVITIGNER.F	1791.93998	2	8.11E-06	0.85	3.35	-	627.4
AHQ-2-4, 4666	K.SYELPDGQVITIGNER.F	1791.93998	2	2.35E-07	0.95	4.36	-	955.0
AHQ-2-13, 4504	K.SYELPDGQVITIGNER.F	1791.93998	2	1.53E-11	0.94	4.30	-	957.8
AHQ-2-10, 4104 - 4114	K.SYELPDGQVITIGNER.F	1791.93998	2	1.61E-04	0.86	3.50	-	577.1
AHQ-2-4, 4778 - 4784	K.SYELPDGQVITIGNER.F	1791.93998	2	1.66E-08	0.95	4.09	-	1149.4
AHQ-2-10, 4191 - 4202	K.SYELPDGQVITIGNER.F	1791.93998	2	1.78E-07	0.94	4.58	-	900.2
AHQ-2-10, 4302 - 4374	K.SYELPDGQVITIGNER.F	1791.93998	2	2.49E-10	0.94	4.10	-	1146.6
AHQ-2-10, 4515 - 4579	K.SYELPDGQVITIGNER.F	1791.93998	2	1.38E-08	0.95	4.71	-	1037.6
AHQ-2-10, 4750 - 4830	K.SYELPDGQVITIGNER.F	1791.93998	2	4.92E-04	0.76	3.09	-	531.1
AHQ-2-2, 5198	K.SYELPDGQVITIGNER.F	1791.93998	2	8.21E-06	0.90	3.54	-	914.5
AHQ-2-13-, 5129 - 5193	K.SYELPDGQVITIGNER.F	1791.93998	2	1.88E-13	0.97	5.10	-	1370.7
AHQ-2-2, 4818	K.SYELPDGQVITIGNER.F	1791.93998	2	2.72E-10	0.91	3.83	-	829.4
AHQ-2-8, 4226 - 4306	K.SYELPDGQVITIGNER.F	1791.93998	2	7.03E-11	0.96	4.82	-	1122.7
AHQ-2-11, 4306 - 4380	K.SYELPDGQVITIGNER.F	1791.93998	2	8.65E-10	0.90	3.93	-	742.8
AHQ-2-11, 4525	K.SYELPDGQVITIGNER.F	1791.93998	2	5.69E-11	0.95	4.44	-	1072.9
AHQ-2-11, 4697 - 4768	K.SYELPDGQVITIGNER.F	1791.93998	2	2.76E-12	0.92	4.22	-	752.6
AHQ-2-11, 4912 - 4990	K.SYELPDGQVITIGNER.F	1791.93998	2	1.68E-11	0.94	3.94	-	1041.9
AHQ-2-4, 5429	K.SYELPDGQVITIGNER.F	1791.93998	2	9.41E-09	0.93	4.16	-	930.0
AHQ-2-1, 5373 - 5441	K.SYELPDGQVITIGNER.F	1791.93998	2	3.80E-09	0.95	4.59	-	1042.3
AHQ-2-1, 5181 - 5197	K.SYELPDGQVITIGNER.F	1791.93998	2	2.54E-09	0.94	4.32	-	953.4
AHQ-2-13-, 4959 - 4981	K.SYELPDGQVITIGNER.F	1791.93998	2	4.30E-05	0.83	3.19	-	863.1
AHQ-2-5, 4563	K.SYELPDGQVITIGNER.F	1791.93998	2	5.55E-10	0.78	3.46	-	533.2
AHQ-2-6, 4471	K.SYELPDGQVITIGNER.F	1791.93998	2	3.71E-04	0.69	2.66	-	460.2
AHQ-2-6, 4838 - 4850	K.SYELPDGQVITIGNER.F	1791.93998	2	8.86E-07	0.83	3.15	-	674.2
AHQ-2-6, 5134 - 5138	K.SYELPDGQVITIGNER.F	1791.93998	2	2.45E-05	0.76	3.11	-	569.7
AHQ-2-1, 4790	K.SYELPDGQVITIGNER.F	1791.93998	2	1.55E-08	0.90	3.59	-	786.2
AHQ-2-13-, 4565	K.SYELPDGQVITIGNER.F	1791.93998	2	1.41E-09	0.94	4.02	-	957.0
AHQ-2-12, 4570	K.SYELPDGQVITIGNER.F	1791.93998	2	3.19E-07	0.87	3.40	-	770.5
AHQ-2-12, 4718	K.SYELPDGQVITIGNER.F	1791.93998	2	4.36E-12	0.93	4.06	-	924.9
AHQ-2-7, 5059 - 5075	K.SYELPDGQVITIGNER.F	1791.93998	2	2.05E-04	0.84	3.12	-	857.6
AHQ-2-12, 4930 - 4993	K.SYELPDGQVITIGNER.F	1791.93998	2	2.61E-13	0.96	4.77	-	1082.6
AHQ-2-12, 5111 - 5181	K.SYELPDGQVITIGNER.F	1791.93998	2	6.22E-08	0.93	4.53	-	746.1
AHQ-2-9, 4315 - 4394	K.SYELPDGQVITIGNER.F	1791.93998	2	8.52E-07	0.89	3.64	-	755.3
AHQ-2-14-, 4916	K.SYELPDGQVITIGNER.F	1791.93998	2	4.80E-06	0.91	3.88	-	972.6
AHQ-2-9, 3434 - 3502	R.VAPEEHPTLLTEAPLNPK.A	1957.21627	2	8.33E-04	0.89	4.22	-	596.8
AHQ-2-8, 3142 - 3215	R.VAPEEHPTLLTEAPLNPK.A	1957.21627	2	1.20E-04	0.85	3.67	-	642.5
AHQ-2-11, 4242	K.YPIEHGIITNWDDMEK.I	1962.17174	2	2.00E-04	0.85	3.88	-	655.7
AHQ-2-10, 4006 - 4039	K.YPIEHGIITNWDDMEK.I	1962.17174	2	1.11E-04	0.87	3.75	-	792.2
AHQ-2-9, 4548 - 4566	K.YPIEHGIITNWDDMEK.I	1962.17174	2	1.34E-04	0.90	4.52	-	725.7
AHQ-2-9, 4463 - 4474	K.YPIEHGIITNWDDMEK.I	1962.17174	2	1.44E-06	0.91	4.47	-	575.8
AHQ-2-9, 4139 - 4210	K.YPIEHGIITNWDDMEK.I	1962.17174	2	5.72E-04	0.89	4.34	-	668.3
AHQ-2-12, 4151	K.YPIEHGIITNWDDMEK.I	1962.17174	2	8.86E-04	0.78	3.88	-	607.0
AHQ-2-14, 5698	K.YPIEHGIITNWDDMEK.I	1962.17174	2	7.04E-04	0.43	2.58	-	535.7
AHQ-2-2, 4684	K.YPIEHGIITNWDDMEK.I	1962.17174	2	9.41E-04	0.81	3.64	-	692.9
AHQ-2-3, 4648	K.YPIEHGIITNWDDMEK.I	1962.17174	2	3.09E-04	0.73	3.44	-	666.2
AHQ-2-8, 4777	K.YPIEHGIITNWDDMEK.I	1962.17174	2	2.64E-08	0.91	4.46	-	683.2
AHQ-2-8, 4322	K.YPIEHGIITNWDDMEK.I	1962.17174	2	5.27E-04	0.91	4.38	-	698.4
AHQ-2-8, 3519 - 3563	K.YPIEHGIITNWDDM*EK.I	1978.17114	3	7.28E-04	0.78	3.29	-	918.4
AHQ-2-12, 4085	K.YPIEHGIITNWDDM*EK.I	1978.17114	2	7.05E-05	0.78	3.30	-	562.9
AHQ-2-13-, 4454 - 4526	K.YPIEHGIITNWDDMEK.I	1962.17174	2	2.70E-06	0.88	4.10	-	713.9
AHQ-2-13-, 4457	K.YPIEHGIITNWDDMEK.I	1962.17174	2	3.70E-04	0.83	3.97	-	576.2
AHQ-2-7, 4172	K.YPIEHGIITNWDDMEK.I	1962.17174	2	1.44E-04	0.84	3.70	-	709.6
AHQ-2-13-, 4671	K.YPIEHGIITNWDDMEK.I	1962.17174	2	5.30E-08	0.83	3.63	-	632.7
AHQ-2-5, 4299	K.YPIEHGIITNWDDMEK.I	1962.17174	2	7.85E-05	0.79	3.27	-	662.1
AHQ-2-4, 4814	K.YPIEHGIITNWDDMEK.I	1962.17174	2	7.29E-04	0.37	2.65	-	525.6
AHQ-2-12, 4735	K.YPIEHGIITNWDDMEK.I	1962.17174	2	1.31E-04	0.87	3.96	-	648.1
AHQ-2-8, 6512 - 6578	K.YPIEHGIITNWDDMEKIWHHTFYNELR.V	3459.83609	3	1.14E-07	0.92	5.03	-	721.3
AHQ-2-8, 5200 - 5204	K.YPIEHGIITNWDDM*EKIWHHTFYNELR.V	3475.83549	3	2.89E-06	0.93	5.11	-	855.7
gi|27500274|ref|XP_113950.3| similar to Munc13-4 protein [Homo sapiens]				1.89E-14	9.17	120.33	17.60	123281.1
AHQ-2-5, 7631	K.AFGELCPNTAPLPQLVTEALQTGTTEWFHLK.Q	3472.91108	3	1.35E-11	0.74	3.49	-	442.7
AHQ-2-6, 7373	K.AFGELCPNTAPLPQLVTEALQTGTTEWFHLK.Q	3472.91108	3	9.41E-09	0.80	3.93	-	339.9
AHQ-2-4, 6670	R.ALLYEDALYTVLHR.L	1677.92484	2	1.04E-11	0.95	4.37	-	1236.4
AHQ-2-4, 4988 - 4993	R.AVQM*DELVPLGELTK.H	1659.92638	2	1.52E-05	0.78	2.96	-	719.4
AHQ-2-4, 2424	R.DLELQAASSR.E	1090.16939	1	6.59E-04	0.10	1.84	-	257.8
AHQ-2-4, 3417 - 3434	R.EVPGLSGSEEPGEVPQTR.L	1868.97961	2	8.41E-04	0.55	2.78	-	321.1
AHQ-2-4, 4754	K.HSTSAVDLSTCFAQISHTAR.Q	2191.36726	3	1.89E-14	0.98	6.19	-	2344.4
AHQ-2-5, 4344	K.HSTSAVDLSTCFAQISHTAR.Q	2191.36726	3	1.99E-07	0.96	5.35	-	1262.8
AHQ-2-4, 3517 - 3532	R.IQQQAETTSEELGAVTVK.A	1933.10674	2	7.70E-11	0.98	6.58	-	1572.0
AHQ-2-4, 3520	K.LGELTDLHGLR.R	1224.39168	2	4.54E-04	0.87	3.20	-	1088.4
AHQ-2-4, 3349	R.LGHPEPNHVTEASELLR.Y	1900.08490	2	5.78E-04	0.72	3.10	-	489.6
AHQ-2-4, 5610	K.LPAQLAWEALEQR.V	1525.73360	2	1.70E-09	0.96	4.15	-	1431.6
AHQ-2-4, 4169	R.SVFPLSVSDSPAR.L	1362.51311	2	5.69E-06	0.76	2.63	-	858.0
AHQ-2-4, 6480	R.WFQPAIPSWLQK.T	1501.75533	2	1.05E-05	0.79	3.14	-	516.7
gi|4505879|ref|NP_002655.1| pleckstrin; p47 [Homo sapiens]				2.94E-14	9.70	120.41	44.30	40082.5
AHQ-2-1, 6069	R.EDPAYLHYYDPAGAEDPLGAIHLR.G	2684.89982	3	6.82E-06	0.96	5.95	-	1241.5
AHQ-2-13, 5573	R.EDPAYLHYYDPAGAEDPLGAIHLR.G	2684.89982	3	2.72E-06	0.78	3.20	-	600.8
AHQ-2-12, 5718 - 5721	R.EDPAYLHYYDPAGAEDPLGAIHLR.G	2684.89982	3	1.17E-09	0.96	5.88	-	1451.9
AHQ-2-11, 5468 - 5470	R.EDPAYLHYYDPAGAEDPLGAIHLR.G	2684.89982	3	9.46E-09	0.95	5.66	-	1516.2
AHQ-2-13-, 5662 - 5673	R.EDPAYLHYYDPAGAEDPLGAIHLR.G	2684.89982	3	1.28E-09	0.97	6.64	-	1779.8
AHQ-2-13, 5563 - 5577	R.EDPAYLHYYDPAGAEDPLGAIHLR.G	2684.89982	3	3.39E-08	0.98	6.89	-	1834.9
AHQ-2-8, 5442	R.EDPAYLHYYDPAGAEDPLGAIHLR.G	2684.89982	3	7.11E-12	0.97	6.48	-	1687.3
AHQ-2-8, 5448	R.EDPAYLHYYDPAGAEDPLGAIHLR.G	2684.89982	2	1.47E-05	0.84	3.54	-	569.3
AHQ-2-14-, 5652	R.EDPAYLHYYDPAGAEDPLGAIHLR.G	2684.89982	3	6.80E-09	0.95	5.52	-	1090.9
AHQ-2-12, 2451	R.GCVVTSVESNSNGR.K	1467.54464	2	5.50E-06	0.88	3.33	-	1081.4
AHQ-2-8, 2339	R.GCVVTSVESNSNGR.K	1467.54464	2	2.94E-06	0.92	3.42	-	1296.0
AHQ-2-11, 2442	R.GCVVTSVESNSNGR.K	1467.54464	2	7.57E-04	0.90	2.92	-	1605.0

AHQ-2-13-, 2463 - 2513	R.GCVVTSVESNSNGR.K	1467.54464	2	1.04E-05	0.92	3.87	-	1184.9
AHQ-2-8, 2156 - 2232	R.GCVVTSVESNSNGRK.S	1595.71755	2	3.32E-05	0.85	3.24	-	1149.0
AHQ-2-8, 2844	K.GSTLTSPCQDFGK.R	1399.50867	1	9.36E-06	0.07	2.07	-	204.7
AHQ-2-8, 2288 - 2302	K.GSTLTSPCQDFGKR.M	1555.69502	2	3.82E-07	0.40	2.66	-	322.6
AHQ-2-13-, 2865 - 2875	K.GSTLTSPCQDFGKR.M	1555.69502	2	7.79E-04	0.66	3.11	-	370.3
AHQ-2-8, 7367 - 7444	K.IFNHCFTGNCVIDWLVSNQSVR.N	2669.97641	2	6.90E-07	0.98	5.72	-	1805.1
AHQ-2-10, 6535	K.IFNHCFTGNCVIDWLVSNQSVR.N	2669.97641	2	9.65E-10	0.97	5.00	-	1425.2
AHQ-2-9, 7272 - 7351	K.IFNHCFTGNCVIDWLVSNQSVR.N	2669.97641	2	2.21E-04	0.95	4.77	-	1193.6
AHQ-2-8, 7050 - 7055	K.IFNHCFTGNCVIDWLVSNQSVR.N	2669.97641	2	3.23E-12	0.98	6.39	-	1803.4
AHQ-2-8, 7224 - 7295	K.IFNHCFTGNCVIDWLVSNQSVR.N	2669.97641	2	5.70E-08	0.98	6.44	-	1793.2
AHQ-2-13-, 7061	R.KSEEENLFEIITADEVHYFLQAATPK.E	3024.32650	3	6.35E-08	0.98	7.68	-	2039.8
AHQ-2-10, 6795	R.KSEEENLFEIITADEVHYFLQAATPK.E	3024.32650	3	6.31E-08	0.98	7.80	-	2794.7
AHQ-2-8, 7452 - 7526	R.KSEEENLFEIITADEVHYFLQAATPK.E	3024.32650	3	3.30E-06	0.99	8.20	-	3635.1
AHQ-2-9, 7478 - 7479	R.KSEEENLFEIITADEVHYFLQAATPK.E	3024.32650	3	2.60E-06	0.98	7.82	-	2629.7
AHQ-2-14-, 6915	R.KSEEENLFEIITADEVHYFLQAATPK.E	3024.32650	3	2.94E-14	0.99	7.89	-	3063.0
AHQ-2-12, 7194 - 7263	R.KSEEENLFEIITADEVHYFLQAATPK.E	3024.32650	3	9.18E-12	0.98	6.64	-	2062.7
AHQ-2-10, 6162	R.LPETIDLGALYLSMK.D	1664.98779	2	2.89E-05	0.95	4.48	-	1321.3
AHQ-2-12, 6567 - 6585	R.LPETIDLGALYLSMK.D	1664.98779	2	1.29E-06	0.97	4.37	-	2018.7
AHQ-2-9, 6734 - 6760	R.LPETIDLGALYLSMK.D	1664.98779	2	1.58E-05	0.97	4.09	-	1936.0
AHQ-2-8, 6618 - 6686	R.LPETIDLGALYLSMK.D	1664.98779	2	7.65E-05	0.97	3.90	-	1863.5
AHQ-2-8, 6531 - 6598	R.LPETIDLGALYLSMK.D	1664.98779	2	7.47E-06	0.98	4.40	-	2673.0
AHQ-2-12, 5799 - 5801	R.LPETIDLGALYLSM*K.D	1680.98719	2	1.67E-06	0.95	4.73	-	997.5
AHQ-2-8, 6018 - 6019	R.NRQEGLMIASSLLNEGYLQPAGDMSK.S	2824.18382	3	5.51E-05	0.94	4.95	-	1049.1
AHQ-2-8, 5946 - 5986	R.NRQEGLMIASSLLNEGYLQPAGDMSK.S	2824.18382	2	2.41E-04	0.97	5.60	-	1654.7
AHQ-2-8, 6132	R.NRQEGLMIASSLLNEGYLQPAGDMSK.S	2824.18382	2	8.84E-05	0.91	4.29	-	668.0
AHQ-2-8, 5856 - 5882	R.NRQEGLMIASSLLNEGYLQPAGDMSK.S	2824.18382	2	1.59E-07	0.89	4.09	-	672.0
AHQ-2-8, 5886 - 5951	R.NRQEGLMIASSLLNEGYLQPAGDMSK.S	2824.18382	3	9.57E-04	0.86	4.28	-	514.0
AHQ-2-9, 6166	R.NRQEGLMIASSLLNEGYLQPAGDMSK.S	2824.18382	2	2.63E-05	0.92	4.46	-	536.5
AHQ-2-10, 4107	K.QQDHFFQAAFLEER.D	1766.89390	2	1.07E-10	0.80	3.36	-	254.4
AHQ-2-8, 3970 - 3972	K.QQDHFFQAAFLEER.D	1766.89390	2	4.77E-07	0.86	3.49	-	295.0
AHQ-2-11, 4381	K.QQDHFFQAAFLEER.D	1766.89390	2	4.69E-05	0.72	3.26	-	169.8
AHQ-2-12, 4515	K.QQDHFFQAAFLEER.D	1766.89390	2	4.95E-10	0.83	3.24	-	244.0
AHQ-2-12, 7255	K.SEEENLFEIITADEVHYFLQAATPK.E	2896.15359	2	5.60E-07	0.88	4.39	-	554.9
AHQ-2-12, 7253 - 7254	K.SEEENLFEIITADEVHYFLQAATPK.E	2896.15359	3	1.72E-09	0.91	5.18	-	779.7
AHQ-2-10, 6871	K.SEEENLFEIITADEVHYFLQAATPK.E	2896.15359	3	9.75E-09	0.93	5.16	-	818.1
AHQ-2-10, 6870	K.SEEENLFEIITADEVHYFLQAATPK.E	2896.15359	2	1.49E-05	0.88	4.68	-	490.2
AHQ-2-9, 7544 - 7610	K.SEEENLFEIITADEVHYFLQAATPK.E	2896.15359	2	3.43E-05	0.73	3.96	-	312.2
AHQ-2-8, 7615	K.SEEENLFEIITADEVHYFLQAATPK.E	2896.15359	2	1.48E-09	0.56	2.87	-	459.4
AHQ-2-13-, 7102 - 7106	K.SEEENLFEIITADEVHYFLQAATPK.E	2896.15359	3	7.46E-07	0.93	4.99	-	1158.4
AHQ-2-8, 7560	K.SEEENLFEIITADEVHYFLQAATPK.E	2896.15359	3	2.98E-11	0.91	4.06	-	1230.0
AHQ-2-8, 7474 - 7543	K.SEEENLFEIITADEVHYFLQAATPK.E	2896.15359	2	3.48E-04	0.27	2.53	-	389.2
AHQ-2-14-, 6960	K.SEEENLFEIITADEVHYFLQAATPK.E	2896.15359	3	5.91E-10	0.85	3.99	-	769.6
gi|4505839|ref|NP_002645.1| pyruvate kinase, muscle; Pyruvate kinase-3; Thyroid hormone-binding pro				3.00E-14	14.83	160.36	40.90	57913.6
AHQ-2-7, 6127 - 6137	R.AGKPVICATQMLESMIK.K	1879.29869	3	2.03E-06	0.95	4.47	-	1732.7
AHQ-2-9, 6290	R.AGKPVICATQMLESMIK.K	1879.29869	2	6.44E-04	0.91	3.56	-	875.8
AHQ-2-7, 6119 - 6128	R.AGKPVICATQMLESMIK.K	1879.29869	2	7.60E-06	0.95	4.73	-	950.7
AHQ-2-7, 7567	K.FGVEQDVDMVFASFIR.K	1861.11055	2	6.58E-10	0.96	4.82	-	1342.1
AHQ-2-7, 7477	K.FGVEQDVDM*VFASFIR.K	1877.10995	2	3.82E-08	0.96	4.59	-	1193.7
AHQ-2-7, 4877 - 4880	K.GADFLVTEVENGGSLGSK.K	1780.91396	2	1.53E-10	0.95	4.72	-	1310.7
AHQ-2-7, 5123	K.GADFLVTEVENGGSLGSK.K	1780.91396	2	5.88E-06	0.97	4.96	-	1402.4
AHQ-2-7, 2512	K.GDYPLEAVR.M	1020.12076	2	1.59E-05	0.78	2.73	-	705.7
AHQ-2-7, 6908 - 6972	R.GIFPVLCKDPVQEAWAEDVDLR.V	2559.87857	3	3.69E-11	0.97	5.81	-	2010.1
AHQ-2-11, 6386 - 6393	R.GIFPVLCKDPVQEAWAEDVDLR.V	2559.87857	3	2.30E-06	0.96	3.97	-	2564.0
AHQ-2-9, 6880	R.GIFPVLCKDPVQEAWAEDVDLR.V	2559.87857	3	2.01E-06	0.93	4.45	-	1215.8
AHQ-2-7, 6815 - 6887	R.GIFPVLCKDPVQEAWAEDVDLR.V	2559.87857	3	7.84E-11	0.98	6.08	-	2153.5
AHQ-2-7, 6811 - 6879	R.GIFPVLCKDPVQEAWAEDVDLR.V	2559.87857	2	1.73E-05	0.95	4.48	-	1153.7
AHQ-2-9, 6870	R.GIFPVLCKDPVQEAWAEDVDLR.V	2559.87857	2	8.16E-09	0.92	4.15	-	666.2
AHQ-2-11, 6389 - 6394	R.GIFPVLCKDPVQEAWAEDVDLR.V	2559.87857	2	6.62E-06	0.93	4.47	-	810.4
AHQ-2-13-, 6717	R.GIFPVLCKDPVQEAWAEDVDLR.V	2559.87857	3	3.00E-14	0.98	6.14	-	2377.6
AHQ-2-1, 4926	K.GVNLPGAAVDLPAVSEK.D	1637.85902	2	1.55E-04	0.87	4.08	-	409.6
AHQ-2-7, 2891 - 2893	K.ITLDNAYMEK.C	1198.37126	2	2.18E-06	0.86	3.47	-	617.4
AHQ-2-7, 3816	K.KGVNLPGAAVDLPAVSEK.D	1766.03194	3	1.04E-06	0.90	4.22	-	1008.9
AHQ-2-9, 3879 - 3927	K.KGVNLPGAAVDLPAVSEK.D	1766.03194	2	2.10E-04	0.96	5.05	-	1100.1
AHQ-2-7, 3805	K.KGVNLPGAAVDLPAVSEK.D	1766.03194	2	2.84E-08	0.97	5.84	-	983.5
AHQ-2-7, 5036	K.KGVNLPGAAVDLPAVSEKDIQDLK.F	2478.82685	3	2.36E-09	0.98	7.22	-	2147.9
AHQ-2-7, 5413	R.LAPITSDPTEATAVGAVEASFK.C	2176.40912	2	1.95E-08	0.96	4.83	-	1346.7
AHQ-2-7, 6215	R.LAPITSDPTEATAVGAVEASFK.C	2176.40912	2	3.20E-07	0.96	4.73	-	1291.5
AHQ-2-7, 6300 - 6368	R.LAPITSDPTEATAVGAVEASFK.C	2176.40912	2	1.72E-10	0.97	5.31	-	1235.5
AHQ-2-11, 5580	R.LAPITSDPTEATAVGAVEASFK.C	2176.40912	2	8.94E-05	0.92	4.26	-	781.4
AHQ-2-7, 6071 - 6141	R.LAPITSDPTEATAVGAVEASFK.C	2176.40912	2	6.87E-09	0.97	6.06	-	1138.6
AHQ-2-7, 6637 - 6712	R.LAPITSDPTEATAVGAVEASFK.C	2176.40912	2	5.72E-06	0.94	4.75	-	742.7
AHQ-2-1, 6118	R.LAPITSDPTEATAVGAVEASFK.C	2176.40912	2	1.11E-08	0.94	4.53	-	870.3
AHQ-2-7, 5935 - 6004	R.LAPITSDPTEATAVGAVEASFK.C	2176.40912	2	1.20E-04	0.98	6.38	-	1262.7
AHQ-2-7, 6881	R.LAPITSDPTEATAVGAVEASFK.C	2176.40912	2	1.18E-05	0.92	3.69	-	1019.9
AHQ-2-9, 5855 - 5923	R.LAPITSDPTEATAVGAVEASFK.C	2176.40912	2	9.13E-04	0.67	2.97	-	527.4
AHQ-2-7, 7228 - 7300	R.LAPITSDPTEATAVGAVEASFK.C	2176.40912	2	4.71E-06	0.96	4.95	-	960.1
AHQ-2-7, 6441	R.LAPITSDPTEATAVGAVEASFK.C	2176.40912	2	9.54E-07	0.91	3.63	-	997.4
AHQ-2-7, 5828 - 5895	R.LAPITSDPTEATAVGAVEASFK.C	2176.40912	2	1.81E-11	0.97	5.87	-	1153.7
AHQ-2-7, 6552	R.LAPITSDPTEATAVGAVEASFK.C	2176.40912	2	4.14E-04	0.92	4.00	-	880.6
AHQ-2-7, 7321	R.LAPITSDPTEATAVGAVEASFK.C	2176.40912	2	2.53E-04	0.82	3.69	-	618.2
AHQ-2-7, 3172 - 3185	R.LDIDSPPITAR.N	1198.35099	2	3.12E-05	0.89	3.38	-	879.5
AHQ-2-7, 2003 - 2075	R.LNFSHGTHEYHAETIK.N	1885.02879	2	2.96E-06	0.86	3.58	-	655.8
AHQ-2-7, 3143 - 3160	R.NTGIICTIGPASR.S	1361.54994	2	1.47E-06	0.96	4.80	-	1287.4
AHQ-2-13-, 3446 - 3450	R.NTGIICTIGPASR.S	1361.54994	2	1.27E-04	0.93	3.67	-	1018.3
AHQ-2-11, 3292	R.NTGIICTIGPASR.S	1361.54994	2	3.91E-05	0.93	4.03	-	1082.0
AHQ-2-13, 3465	R.NTGIICTIGPASR.S	1361.54994	2	2.64E-05	0.89	3.53	-	953.4
AHQ-2-7, 3963 - 3983	R.NTGIICTIGPASR.S	1361.54994	2	4.47E-04	0.92	3.37	-	1362.4
AHQ-2-7, 5017 - 5029	R.RFDEILEASDGIMVAR.G	1823.06385	2	6.42E-12	0.97	5.22	-	1498.6
AHQ-2-7, 5392 - 5467	R.TATESFASDPILYRPVAVALDTK.G	2466.77140	3	1.48E-04	0.78	3.17	-	800.4
AHQ-2-12, 5759 - 5829	R.TATESFASDPILYRPVAVALDTK.G	2466.77140	2	2.70E-04	0.42	3.17	-	175.1
AHQ-2-13-, 5649 - 5717	R.TATESFASDPILYRPVAVALDTK.G	2466.77140	3	6.20E-05	0.88	3.78	-	1161.2
AHQ-2-7, 5535 - 5537	R.TATESFASDPILYRPVAVALDTK.G	2466.77140	3	2.38E-07	0.91	4.08	-	1020.9
AHQ-2-7, 5483 - 5551	R.TATESFASDPILYRPVAVALDTK.G	2466.77140	2	9.30E-05	0.90	4.24	-	427.4
gi|21361670|ref|NP_054782.2| src homology 3 domain-containing protein HIP-55; HIP-55 protein [Homo				3.55E-14	1.50	20.25	8.80	48294.0
AHQ-2-13-, 6509	R.GYGPDGHFGM*FPANYVELIE	2230.44050	2	6.86E-04	0.53	3.06	-	178.0
AHQ-2-12, 5518	K.SPTDWALFTYEGNSNDIR.V	2087.19108	2	3.55E-14	0.97	4.76	-	1362.3
gi|4885387|ref|NP_005318.1| L-3-hydroxyacyl-Coenzyme A dehydrogenase, short chain; L-3-hydroxyacyl-				4.05E-14	0.95	10.28	9.60	34286.3
AHQ-2-9, 7558	K.TLSTIATSTDAASVVHSTDLVVEAIVENLK.V	3086.43759	3	4.05E-14	0.95	5.67	-	1083.7
gi|4507953|ref|NP_003397.1| tyrosine 3/tryptophan 5 -monooxygenase activation protein, zeta polypep				5.55E-14	9.23	110.28	46.90	27744.9
AHQ-2-10, 6818	R.EKIETELRDICNDVLSLLEK.F	2419.73463	2	3.13E-04	0.59	3.89	-	321.9
AHQ-2-10, 3134 - 3247	K.FLIPNASQAESK.V	1305.46156	1	4.03E-06	0.50	2.48	-	451.7
AHQ-2-10, 2912 - 2974	K.FLIPNASQAESK.V	1305.46156	1	8.24E-05	0.37	2.16	-	454.9
AHQ-2-9, 2983 - 3008	K.FLIPNASQAESK.V	1305.46156	2	1.83E-04	0.62	2.59	-	397.4
AHQ-2-9, 2986	K.FLIPNASQAESK.V	1305.46156	1	1.20E-06	0.64	2.77	-	455.6
AHQ-2-10, 2911 - 2975	K.FLIPNASQAESK.V	1305.46156	2	2.37E-06	0.78	2.81	-	435.7
AHQ-2-10, 3620	K.FLIPNASQAESK.V	1305.46156	1	4.30E-06	0.27	1.90	-	491.2
AHQ-2-12, 3135 - 3143	K.FLIPNASQAESK.V	1305.46156	2	7.54E-07	0.59	2.64	-	430.2

AHQ-2-10, 3356	K.FLIPNASQAESK.V	1305.46156	1	1.21E-06	0.27	1.80	-	491.6
AHQ-2-10, 5892	K.IETELRDICNDVLSLLEK.F	2162.44718	3	4.06E-08	0.91	4.19	-	1120.5
AHQ-2-9, 7458	K.IETELRDICNDVLSLLEK.F	2162.44718	2	4.51E-05	0.95	4.70	-	1179.6
AHQ-2-10, 6791 - 6799	R.LGLALNFSVFYYEILNSPEK.A	2318.65200	2	2.21E-05	0.85	4.35	-	361.9
AHQ-2-13-, 2307	K.SVTEQGAELSNEER.N	1549.57922	2	2.41E-07	0.91	3.60	-	1063.8
AHQ-2-9, 2151 - 2199	K.SVTEQGAELSNEER.N	1549.57922	2	1.65E-08	0.95	4.10	-	1528.5
AHQ-2-14-, 2259 - 2320	K.SVTEQGAELSNEER.N	1549.57922	2	5.35E-09	0.95	4.67	-	1272.8
AHQ-2-10, 2108 - 2178	K.SVTEQGAELSNEER.N	1549.57922	2	1.09E-07	0.93	3.68	-	1265.8
AHQ-2-1, 2425	K.SVTEQGAELSNEER.N	1549.57922	2	2.51E-05	0.85	2.93	-	1141.3
AHQ-2-10, 4166	K.SVTEQGAELSNEERNLLSVAYK.N	2438.63331	2	3.44E-05	0.71	3.26	-	333.5
AHQ-2-11, 6736	K.TAFDEAIAELDTLSEESYK.D	2133.25160	2	3.98E-10	0.96	5.31	-	931.0
AHQ-2-10, 5474 - 5476	K.TAFDEAIAELDTLSEESYK.D	2133.25160	2	5.40E-07	0.96	4.47	-	1490.7
AHQ-2-13-, 6957	K.TAFDEAIAELDTLSEESYK.D	2133.25160	2	1.52E-06	0.96	5.22	-	1409.8
AHQ-2-10, 5899	K.TAFDEAIAELDTLSEESYK.D	2133.25160	2	9.79E-08	0.57	3.34	-	404.1
AHQ-2-10, 6682	K.TAFDEAIAELDTLSEESYK.D	2133.25160	2	7.83E-09	0.96	4.79	-	1244.7
AHQ-2-10, 6688 - 6690	K.TAFDEAIAELDTLSEESYK.D	2133.25160	3	2.12E-06	0.94	4.41	-	1270.2
AHQ-2-13, 6739	K.TAFDEAIAELDTLSEESYK.D	2133.25160	2	1.94E-10	0.97	5.51	-	1252.8
AHQ-2-12, 7077 - 7081	K.TAFDEAIAELDTLSEESYK.D	2133.25160	2	3.86E-08	0.97	5.57	-	1515.6
AHQ-2-14-, 6815 - 6816	K.TAFDEAIAELDTLSEESYK.D	2133.25160	2	5.28E-07	0.97	5.49	-	1575.4
AHQ-2-9, 7526 - 7598	K.TAFDEAIAELDTLSEESYKDSTLIM*QLLR.D	3320.66704	3	2.25E-08	0.91	4.81	-	914.1
AHQ-2-10, 6820 - 6854	K.TAFDEAIAELDTLSEESYKDSTLIM*QLLR.D	3320.66704	3	4.77E-11	0.89	4.36	-	832.0
AHQ-2-10, 6878 - 6879	K.TAFDEAIAELDTLSEESYKDSTLIMQLLR.D	3304.66764	3	5.55E-14	0.94	5.08	-	1047.8
AHQ-2-13-, 2299	R.YLAEVAAGDDK.K	1152.23595	1	1.02E-05	0.33	1.92	-	769.6
AHQ-2-10, 2116	R.YLAEVAAGDDK.K	1152.23595	1	1.54E-06	0.88	2.44	-	1713.8
AHQ-2-10, 1743 - 1810	R.YLAEVAAGDDKK.G	1280.40887	1	8.90E-04	0.86	3.84	-	680.5
AHQ-2-13, 2171	R.YLAEVAAGDDKK.G	1280.40887	2	3.21E-04	0.87	3.02	-	1117.3
AHQ-2-10, 1732 - 1806	R.YLAEVAAGDDKK.G	1280.40887	2	4.06E-05	0.96	3.95	-	1674.1
AHQ-2-13-, 2037	R.YLAEVAAGDDKK.G	1280.40887	2	2.15E-04	0.96	3.63	-	1833.4
AHQ-2-12, 1957	R.YLAEVAAGDDKK.G	1280.40887	2	3.24E-07	0.96	3.53	-	2027.3
AHQ-2-11, 1972 - 1980	R.YLAEVAAGDDKK.G	1280.40887	2	2.94E-04	0.96	3.68	-	1878.2
AHQ-2-10, 1851	R.YLAEVAAGDDKK.G	1280.40887	2	7.70E-05	0.96	3.96	-	2041.4
AHQ-2-9, 1682 - 1706	R.YLAEVAAGDDKK.G	1280.40887	2	7.50E-07	0.91	3.11	-	1365.8
AHQ-2-10, 1812	R.YLAEVAAGDDKK.G	1280.40887	3	1.55E-06	0.91	3.33	-	1197.9
gi|17986260|ref|NP_524147.1|	smooth muscle and non-muscle myosin alkali light chain isoform 2 [Homo			7.61E-14	3.64	40.36	23.30	12969.7
AHQ-2-12, 5726 - 5802	K.M*TEEEVEM*LVAGHEDSNGCINYEELVR.M	3188.42436	3	2.83E-06	0.96	5.40	-	1749.3
AHQ-2-12, 5874 - 5953	K.MTEEEVEM*LVAGHEDSNGCINYEELVR.M	3172.42496	3	8.29E-05	0.45	3.60	-	324.4
AHQ-2-12, 6018 - 6067	K.M*TEEEVEMLVAGHEDSNGCINYEELVR.M	3172.42496	3	2.58E-06	0.86	4.21	-	912.9
AHQ-2-12, 6133 - 6199	K.MTEEEVEM*LVAGHEDSNGCINYEELVR.M	3172.42496	3	5.99E-10	0.95	7.08	-	1404.4
AHQ-2-12, 6262 - 6326	K.MTEEEVEM*LVAGHEDSNGCINYEELVR.M	3172.42496	3	2.20E-04	0.84	4.70	-	1088.2
AHQ-2-12, 6329 - 6405	K.MTEEEVEMLVAGHEDSNGCINYEELVR.M	3156.42556	3	4.39E-10	0.98	7.21	-	2947.0
AHQ-2-12, 6346 - 6426	K.M*TEEEVEMLVAGHEDSNGCINYEELVR.M	3172.42496	3	7.61E-14	0.74	3.43	-	636.5
AHQ-2-12, 6414 - 6481	K.MTEEEVEMLVAGHEDSNGCINYEELVR.M	3156.42556	3	2.77E-04	0.98	6.17	-	2657.7
AHQ-2-12, 6489 - 6551	K.M*TEEEVEMLVAGHEDSNGCINYEELVR.M	3172.42496	3	2.43E-06	0.89	5.21	-	772.9
AHQ-2-12, 7178 - 7242	K.M*TEEEVEMLVAGHEDSNGCINYEELVR.M	3172.42496	3	4.74E-08	0.71	3.50	-	570.8
AHQ-2-12, 7297	K.MTEEEVEMLVAGHEDSNGCINYEELVR.M	3156.42556	3	2.85E-05	0.96	5.01	-	1716.8
AHQ-2-12, 7311	K.M*TEEEVEMLVAGHEDSNGCINYEELVR.M	3172.42496	3	3.05E-07	0.77	4.23	-	638.9
AHQ-2-14, 6552 - 6610	K.M*TEEEVEM*LVAGHEDSNGCINYEELVR.M	3188.42436	3	1.47E-07	0.94	4.42	-	1572.8
AHQ-2-14-, 5595	K.M*TEEEVEM*LVAGHEDSNGCINYEELVR.M	3188.42436	3	1.64E-12	0.96	6.10	-	1292.2
AHQ-2-14-, 5741 - 5817	K.M*TEEEVEMLVAGHEDSNGCINYEELVR.M	3172.42496	3	9.57E-08	0.84	5.08	-	693.1
AHQ-2-14-, 5895 - 5928	K.MTEEEVEM*LVAGHEDSNGCINYEELVR.M	3172.42496	3	1.64E-05	0.72	4.43	-	568.4
AHQ-2-14-, 5985 - 5992	K.MTEEEVEM*LVAGHEDSNGCINYEELVR.M	3172.42496	3	2.28E-04	0.80	4.33	-	862.9
gi|27498267|ref|XP_209737.1|	similar to tyrosine 3/tryptophan 5 -monooxygenase activation protein,			8.16E-14	2.38	40.32	41.10	11063.6
AHQ-2-10, 5720 - 5782	R.DICNDVLSLLEK.F	1420.61093	1	2.04E-06	0.79	2.79	-	823.8
AHQ-2-14-, 5933 - 6003	R.DICNDVLSLLEK.F	1420.61093	2	6.79E-05	0.83	2.78	-	1043.1
AHQ-2-11, 5869	R.DICNDVLSLLEK.F	1420.61093	1	1.71E-07	0.36	2.03	-	643.0
AHQ-2-10, 6686 - 6758	R.DICNDVLSLLEK.F	1420.61093	1	4.42E-04	0.88	3.56	-	765.0
AHQ-2-9, 6304	R.DICNDVLSLLEK.F	1420.61093	1	6.94E-12	0.39	1.88	-	700.5
AHQ-2-9, 6312	R.DICNDVLSLLEK.F	1420.61093	2	1.22E-04	0.96	3.97	-	1577.0
AHQ-2-9, 6316	R.DICNDVLSLLEK.F	1420.61093	1	2.33E-05	0.55	2.21	-	737.8
AHQ-2-9, 7371	R.DICNDVLSLLEK.F	1420.61093	1	1.34E-07	0.85	3.48	-	701.2
AHQ-2-9, 7374	R.DICNDVLSLLEK.F	1420.61093	2	1.72E-06	0.97	5.01	-	1714.5
AHQ-2-10, 6147	R.DICNDVLSLLEK.F	1420.61093	2	1.31E-06	0.94	3.76	-	1973.1
AHQ-2-10, 5766	R.DICNDVLSLLEK.F	1420.61093	2	1.26E-05	0.87	3.43	-	972.8
AHQ-2-10, 5764	R.DICNDVLSLLEK.F	1420.61093	1	5.23E-09	0.82	3.28	-	801.0
AHQ-2-14-, 6819 - 6832	R.DICNDVLSLLEK.F	1420.61093	2	1.39E-06	0.96	4.29	-	1703.4
AHQ-2-10, 1607 - 1666	R.EKIQTELR.D	1017.16155	1	1.70E-04	0.08	2.03	-	98.3
AHQ-2-10, 4795 - 4858	K.GIVDQSQQAYQEAFEISK.K	2042.19176	2	6.75E-10	0.98	5.60	-	1751.4
AHQ-2-10, 4870	K.GIVDQSQQAYQEAFEISK.K	2042.19176	3	3.14E-06	0.98	6.39	-	2301.0
AHQ-2-10, 4918 - 4998	K.GIVDQSQQAYQEAFEISK.K	2042.19176	2	1.12E-09	0.98	5.79	-	1672.0
AHQ-2-10, 5058 - 5122	K.GIVDQSQQAYQEAFEISK.K	2042.19176	2	1.27E-12	0.98	6.07	-	1588.2
AHQ-2-10, 5186 - 5264	K.GIVDQSQQAYQEAFEISK.K	2042.19176	2	8.95E-06	0.97	5.13	-	1208.1
AHQ-2-10, 5326 - 5386	K.GIVDQSQQAYQEAFEISK.K	2042.19176	2	8.16E-14	0.98	5.63	-	2265.2
AHQ-2-10, 5451 - 5530	K.GIVDQSQQAYQEAFEISK.K	2042.19176	2	4.04E-10	0.96	5.21	-	1190.7
AHQ-2-10, 5592 - 5660	K.GIVDQSQQAYQEAFEISK.K	2042.19176	2	1.10E-07	0.97	5.36	-	1259.0
AHQ-2-10, 4404 - 4466	K.GIVDQSQQAYQEAFEISK.K	2042.19176	2	1.54E-10	0.97	5.49	-	1625.0
AHQ-2-5, 5348	K.GIVDQSQQAYQEAFEISK.K	2042.19176	2	8.48E-06	0.93	4.35	-	1094.2
AHQ-2-14-, 5131 - 5139	K.GIVDQSQQAYQEAFEISK.K	2042.19176	2	1.05E-07	0.98	6.45	-	1938.6
AHQ-2-10, 4550	K.GIVDQSQQAYQEAFEISK.K	2042.19176	2	1.19E-10	0.98	5.15	-	1754.3
AHQ-2-10, 6179 - 6248	K.GIVDQSQQAYQEAFEISK.K	2042.19176	2	4.15E-05	0.94	4.17	-	1062.3
AHQ-2-10, 6238 - 6316	K.GIVDQSQQAYQEAFEISK.K	2042.19176	2	3.91E-08	0.97	5.51	-	1465.2
AHQ-2-10, 6380	K.GIVDQSQQAYQEAFEISK.K	2042.19176	2	1.28E-07	0.92	3.60	-	1152.9
AHQ-2-10, 6475	K.GIVDQSQQAYQEAFEISK.K	2042.19176	2	1.49E-08	0.95	4.53	-	1017.1
AHQ-2-10, 6595 - 6599	K.GIVDQSQQAYQEAFEISK.K	2042.19176	2	2.30E-06	0.88	4.09	-	555.7
AHQ-2-9, 5250 - 5251	K.GIVDQSQQAYQEAFEISK.K	2042.19176	2	4.35E-09	0.97	5.47	-	1320.5
AHQ-2-11, 4644	K.GIVDQSQQAYQEAFEISK.K	2042.19176	2	1.13E-07	0.96	5.09	-	1165.1
AHQ-2-11, 5012	K.GIVDQSQQAYQEAFEISK.K	2042.19176	2	2.86E-06	0.97	5.33	-	1491.7
AHQ-2-11, 5221 - 5273	K.GIVDQSQQAYQEAFEISK.K	2042.19176	2	3.22E-06	0.91	4.39	-	690.7
AHQ-2-9, 4888	K.GIVDQSQQAYQEAFEISK.K	2042.19176	2	2.40E-06	0.91	4.40	-	725.6
AHQ-2-12, 5245	K.GIVDQSQQAYQEAFEISK.K	2042.19176	2	7.89E-09	0.98	5.73	-	1719.4
AHQ-2-13, 5079 - 5128	K.GIVDQSQQAYQEAFEISK.K	2042.19176	2	2.29E-11	0.97	5.35	-	1540.2
AHQ-2-13, 5145	K.GIVDQSQQAYQEAFEISK.K	2042.19176	3	7.50E-08	0.98	6.09	-	2512.7
AHQ-2-14, 6120 - 6128	K.GIVDQSQQAYQEAFEISK.K	2042.19176	2	2.81E-08	0.97	5.25	-	1613.4
AHQ-2-13-, 5201	K.GIVDQSQQAYQEAFEISK.K	2042.19176	2	5.70E-07	0.95	4.62	-	1159.1
AHQ-2-13-, 5219 - 5299	K.GIVDQSQQAYQEAFEISK.K	2042.19176	3	1.25E-05	0.93	3.83	-	1290.1
AHQ-2-1, 5501	K.GIVDQSQQAYQEAFEISK.K	2042.19176	2	1.83E-07	0.94	4.52	-	990.5
AHQ-2-10, 4259	K.KGIVDQSQQAYQEAFEISK.K	2170.36467	3	3.20E-08	0.92	4.81	-	1380.5
AHQ-2-10, 4250	K.KGIVDQSQQAYQEAFEISK.K	2170.36467	2	1.26E-04	0.98	5.97	-	2223.3
gi|4505733|ref|NP_002610.1|	platelet factor 4 (chemokine (C-X-C motif) ligand 4); platelet factor 4			8.22E-14	4.73	50.27	38.60	10844.9
AHQ-2-14, 5400 - 5460	K.AGPHCPTAQLIATLK.N	1579.84600	2	3.21E-04	0.84	3.64	-	620.5
AHQ-2-13-, 4143 - 4209	K.AGPHCPTAQLIATLK.N	1579.84600	3	3.80E-04	0.84	3.80	-	998.4
AHQ-2-13-, 4302 - 4366	K.AGPHCPTAQLIATLK.N	1579.84600	2	3.75E-06	0.90	4.22	-	761.3
AHQ-2-14-, 3997 - 4080	K.AGPHCPTAQLIATLK.N	1579.84600	2	6.40E-10	0.91	3.71	-	813.7
AHQ-2-13, 4127	K.AGPHCPTAQLIATLK.N	1579.84600	2	3.75E-05	0.89	3.88	-	719.5
AHQ-2-14-, 3999 - 4077	K.AGPHCPTAQLIATLK.N	1579.84600	3	9.05E-05	0.83	3.85	-	847.2
AHQ-2-14-, 4152 - 4215	K.AGPHCPTAQLIATLK.N	1579.84600	2	4.72E-08	0.92	3.90	-	777.6
AHQ-2-14-, 4243	K.AGPHCPTAQLIATLK.N	1579.84600	3	4.17E-04	0.91	4.32	-	1328.7
AHQ-2-14, 5280 - 5361	K.AGPHCPTAQLIATLK.N	1579.84600	3	4.47E-04	0.72	3.51	-	772.4

AHQ-2-14, 5272	K.AGPHCPTAQLIATLK.N	1579.84600	2	2.47E-06	0.83	3.26	-	632.0
AHQ-2-14, 5265 - 5338	K.AGPHCPTAQLIATLK.N	1579.84600	2	1.42E-07	0.93	4.59	-	681.2
AHQ-2-13-, 4174 - 4210	K.AGPHCPTAQLIATLK.N	1579.84600	2	4.16E-07	0.89	3.82	-	736.7
AHQ-2-14, 5024 - 5028	K.AGPHCPTAQLIATLK.N	1579.84600	2	2.50E-09	0.92	4.13	-	694.7
AHQ-2-14, 4989	K.AGPHCPTAQLIATLKNGR.K	1907.18700	2	5.14E-09	0.96	4.85	-	1300.1
AHQ-2-14, 4941 - 5000	K.AGPHCPTAQLIATLKNGR.K	1907.18700	3	3.18E-10	0.98	5.41	-	3098.8
AHQ-2-14, 5153 - 5226	K.AGPHCPTAQLIATLKNGR.K	1907.18700	2	3.69E-06	0.94	4.32	-	923.9
AHQ-2-14, 4866 - 4938	K.AGPHCPTAQLIATLKNGR.K	1907.18700	2	5.70E-05	0.92	4.11	-	751.4
AHQ-2-14, 4781	K.AGPHCPTAQLIATLKNGR.K	1907.18700	2	6.80E-04	0.93	4.02	-	1046.9
AHQ-2-13-, 4113	K.AGPHCPTAQLIATLKNGR.K	1907.18700	2	2.11E-07	0.95	3.80	-	1165.9
AHQ-2-13-, 4081 - 4109	K.AGPHCPTAQLIATLKNGR.K	1907.18700	3	5.86E-08	0.98	5.32	-	2619.1
AHQ-2-14-, 3936	K.AGPHCPTAQLIATLKNGR.K	1907.18700	2	3.94E-12	0.95	4.72	-	1002.5
AHQ-2-14-, 3915	K.AGPHCPTAQLIATLKNGR.K	1907.18700	3	1.01E-09	0.95	4.50	-	1827.2
AHQ-2-13, 4045	K.AGPHCPTAQLIATLKNGR.K	1907.18700	2	8.22E-14	0.94	4.49	-	972.0
AHQ-2-14, 5213 - 5217	K.AGPHCPTAQLIATLKNGR.K	1907.18700	3	5.33E-09	0.98	5.32	-	2457.9
AHQ-2-14-, 2545 - 2615	R.HITSLEVIK.A	1040.23825	2	1.63E-04	0.90	3.65	-	523.1
AHQ-2-14, 3380 - 3690	R.HITSLEVIK.A	1040.23825	1	7.67E-04	0.26	2.03	-	367.1
AHQ-2-12, 2447	R.HITSLEVIK.A	1040.23825	2	8.27E-06	0.81	2.66	-	625.7
AHQ-2-13, 2639 - 2667	R.HITSLEVIK.A	1040.23825	2	2.48E-06	0.89	3.24	-	693.3
AHQ-2-13-, 2537 - 2607	R.HITSLEVIK.A	1040.23825	2	5.13E-06	0.91	3.26	-	646.8
AHQ-2-14, 3381 - 3441	R.HITSLEVIK.A	1040.23825	2	7.40E-04	0.81	2.92	-	580.8
AHQ-2-14, 3500 - 3562	R.HITSLEVIK.A	1040.23825	2	1.51E-04	0.76	2.91	-	414.5
AHQ-2-14-, 2391	R.HITSLEVIK.A	1040.23825	1	2.36E-05	0.40	2.22	-	431.8
AHQ-2-14-, 2387 - 2411	R.HITSLEVIK.A	1040.23825	2	4.16E-06	0.89	3.33	-	617.8
AHQ-2-14-, 2457 - 2535	R.HITSLEVIK.A	1040.23825	2	1.64E-06	0.93	4.01	-	663.7
AHQ-2-14, 6080 - 6140	K.ICLDLQAPLYK.K	1335.59400	2	5.80E-05	0.96	4.25	-	1753.6
AHQ-2-13, 4939 - 4951	K.ICLDLQAPLYK.K	1335.59400	2	1.70E-05	0.97	4.07	-	1916.1
AHQ-2-13-, 5013 - 5015	K.ICLDLQAPLYK.K	1335.59400	2	1.98E-06	0.97	4.64	-	2104.2
AHQ-2-14-, 4923 - 4936	K.ICLDLQAPLYK.K	1335.59400	2	3.23E-06	0.97	4.40	-	2089.6
AHQ-2-14-, 4929	K.ICLDLQAPLYK.K	1335.59400	1	1.37E-04	0.66	2.64	-	649.4
AHQ-2-14, 6093 - 6154	K.ICLDLQAPLYK.K	1335.59400	1	1.57E-05	0.70	2.97	-	596.4
AHQ-2-14, 6281	K.ICLDLQAPLYK.K	1335.59400	2	5.70E-04	0.75	3.21	-	681.5
AHQ-2-14-, 5148	K.ICLDLQAPLYK.K	1335.59400	2	2.66E-05	0.94	4.02	-	1348.9
AHQ-2-13-, 4477	R.KICLDLQAPLYK.K	1463.76691	3	4.98E-05	0.90	3.48	-	1572.4
AHQ-2-13-, 4473 - 4481	R.KICLDLQAPLYK.K	1463.76691	2	5.43E-09	0.98	5.07	-	1930.6
AHQ-2-14, 5965	R.KICLDLQAPLYK.K	1463.76691	2	1.15E-06	0.96	4.40	-	1417.9
AHQ-2-14, 5772 - 5773	R.KICLDLQAPLYK.K	1463.76691	2	5.43E-06	0.97	4.61	-	1605.8
AHQ-2-14, 5648 - 5708	R.KICLDLQAPLYK.K	1463.76691	2	2.34E-06	0.97	4.41	-	1870.1
AHQ-2-14, 5482 - 5560	R.KICLDLQAPLYK.K	1463.76691	2	3.11E-06	0.95	4.16	-	1305.7
AHQ-2-14-, 4295	R.KICLDLQAPLYK.K	1463.76691	2	1.06E-05	0.93	3.83	-	1137.0
AHQ-2-14-, 4435 - 4500	R.KICLDLQAPLYK.K	1463.76691	2	2.08E-05	0.97	4.73	-	1582.7
AHQ-2-14-, 4441 - 4507	R.KICLDLQAPLYK.K	1463.76691	3	8.81E-04	0.87	3.98	-	1282.9
AHQ-2-14-, 4563 - 4627	R.KICLDLQAPLYK.K	1463.76691	2	1.50E-04	0.95	4.72	-	1117.7
AHQ-2-13, 4415	R.KICLDLQAPLYK.K	1463.76691	2	1.55E-06	0.95	4.19	-	1261.4
AHQ-2-12, 4454 - 4477	R.KICLDLQAPLYK.K	1463.76691	2	2.21E-07	0.93	3.77	-	1107.2
AHQ-2-13-, 4478	R.KICLDLQAPLYK.K	1463.76691	1	8.98E-06	0.82	3.47	-	672.7
gi|4507949|ref|NP_003395.1| tyrosine 3-monooxygenase/tryptophan 5-monooxygenase activation protein,				1.17E-13	4.56	50.28	31.70	28082.2
AHQ-2-10, 6875	K.IEAELQDICNDVLELLDK.Y	2132.37471	2	8.95E-08	0.96	5.61	-	1133.0
AHQ-2-10, 4074	K.QTTVSNSQQAYQEAFEISK.K	2160.28351	2	1.09E-05	0.81	3.61	-	447.7
AHQ-2-13-, 6813	K.TAFDEAIAELDTLNEESYK.D	2160.27705	2	5.23E-07	0.93	4.42	-	946.0
AHQ-2-10, 6200	K.TAFDEAIAELDTLNEESYK.D	2160.27705	2	1.00E-05	0.77	3.58	-	515.7
AHQ-2-14-, 6844 - 6903	K.TAFDEAIAELDTLNEESYK.D	2160.27705	2	1.44E-04	0.86	4.05	-	593.9
AHQ-2-14-, 6675 - 6744	K.TAFDEAIAELDTLNEESYK.D	2160.27705	2	1.19E-05	0.95	4.96	-	783.9
AHQ-2-10, 6419	K.TAFDEAIAELDTLNEESYK.D	2160.27705	2	3.50E-04	0.96	4.74	-	1451.5
AHQ-2-10, 6874 - 6876	K.TAFDEAIAELDTLNEESYKDSTLIM*QLLR.D	3347.69249	3	1.17E-13	0.94	5.09	-	1044.2
AHQ-2-10, 2732	K.YLIPNATQPESK.V	1361.52512	1	7.50E-05	0.67	2.29	-	749.8
AHQ-2-10, 2618 - 2628	K.YLIPNATQPESK.V	1361.52512	2	3.13E-06	0.92	3.62	-	827.0
AHQ-2-10, 2823	K.YLIPNATQPESK.V	1361.52512	1	2.92E-05	0.77	2.49	-	915.7
gi|5031987|ref|NP_005720.1| peptidylprolyl isomerase F (cyclophilin F) [Homo sapiens]				1.23E-13	4.87	60.31	41.50	22040.1
AHQ-2-12, 3727	R.FPDENFTLK.H	1111.22866	2	1.55E-05	0.63	2.66	-	606.5
AHQ-2-12, 3817	R.FPDENFTLK.H	1111.22866	2	4.89E-04	0.81	2.61	-	874.6
AHQ-2-12, 4081	R.FPDENFTLK.H	1111.22866	1	2.11E-04	0.17	2.07	-	226.8
AHQ-2-12, 5178 - 5241	K.GSGDPSSSSSSGNPLVYLDVDANGKPLGR.V	2834.99028	3	1.23E-13	0.91	4.54	-	945.2
AHQ-2-12, 5195 - 5265	K.GSGDPSSSSSSGNPLVYLDVDANGKPLGR.V	2834.99028	2	1.01E-06	0.52	2.58	-	292.3
AHQ-2-12, 6046 - 6111	K.HVGPGVLSMANAGPNTNGSQFFICTIK.T	2820.19665	2	1.44E-06	0.90	4.35	-	649.1
AHQ-2-14-, 5321	K.HVGPGVLSM*ANAGPNTNGSQFFICTIK.T	2836.19605	3	7.52E-06	0.92	4.95	-	775.7
AHQ-2-11, 5684	K.HVGPGVLSMANAGPNTNGSQFFICTIK.T	2820.19665	2	2.90E-05	0.90	4.73	-	450.3
AHQ-2-12, 5373 - 5375	K.HVGPGVLSM*ANAGPNTNGSQFFICTIK.T	2836.19605	2	2.74E-05	0.91	4.85	-	384.9
AHQ-2-12, 5374 - 5398	K.HVGPGVLSM*ANAGPNTNGSQFFICTIK.T	2836.19605	3	1.65E-05	0.94	4.66	-	1483.4
AHQ-2-12, 5525	K.HVGPGVLSM*ANAGPNTNGSQFFICTIK.T	2836.19605	2	1.07E-04	0.67	3.02	-	326.8
AHQ-2-12, 5909 - 5973	K.HVGPGVLSMANAGPNTNGSQFFICTIK.T	2820.19665	2	1.50E-07	0.93	4.78	-	577.5
AHQ-2-12, 5918 - 5983	K.HVGPGVLSMANAGPNTNGSQFFICTIK.T	2820.19665	3	1.26E-06	0.97	6.14	-	1742.4
AHQ-2-14-, 5857	K.HVGPGVLSMANAGPNTNGSQFFICTIK.T	2820.19665	3	2.56E-08	0.94	4.98	-	1351.8
AHQ-2-12, 4023	R.VIPSFM*CQAGDFTNHNGTGGK.S	2256.46118	2	2.58E-05	0.65	2.87	-	141.1
AHQ-2-12, 4651 - 4723	R.VIPSFMCQAGDFTNHNGTGGK.S	2240.46178	2	1.71E-05	0.83	3.73	-	463.7
gi|4505257|ref|NP_002435.1| moesin [Homo sapiens]				1.42E-13	7.53	90.29	23.10	67819.5
AHQ-2-6, 2283	K.ALTSELANAR.D	1046.15975	2	2.09E-05	0.93	3.24	-	1192.8
AHQ-2-6, 3921	R.EDAVLEYLK.I	1080.21287	1	8.46E-05	0.41	2.71	-	253.0
AHQ-2-7, 7456	K.EGILNDDIYCPPETAVLLASYAVQSK.Y	2869.19288	2	9.73E-05	0.49	2.89	-	255.5
AHQ-2-10, 6658	K.FYPEDVSEELIQDITQR.L	2083.24065	2	3.31E-08	0.97	5.00	-	1980.1
AHQ-2-9, 7306	K.FYPEDVSEELIQDITQR.L	2083.24065	2	1.09E-09	0.95	4.57	-	1181.7
AHQ-2-11, 6710	K.FYPEDVSEELIQDITQR.L	2083.24065	2	1.77E-04	0.85	3.58	-	710.9
AHQ-2-6, 7133 - 7151	K.FYPEDVSEELIQDITQR.L	2083.24065	2	2.72E-11	0.96	5.20	-	1181.4
AHQ-2-7, 7361	K.FYPEDVSEELIQDITQR.L	2083.24065	2	7.28E-11	0.97	5.71	-	1101.1
AHQ-2-13, 6720	K.FYPEDVSEELIQDITQR.L	2083.24065	2	9.16E-11	0.97	5.22	-	1243.6
AHQ-2-6, 5486	K.IAQDLEM*YGVNYFSIK.N	1908.16395	2	4.36E-09	0.97	4.81	-	1801.8
AHQ-2-6, 1995	R.ISQLEMAR.Q	948.12272	2	1.42E-04	0.87	2.78	-	1168.8
AHQ-2-6, 4477	K.KTQEQLALEMAELTAR.I	1833.10018	2	3.98E-07	0.97	4.77	-	1510.0
AHQ-2-6, 3457	K.TAMSTPHVAEPAENEQDEQDENGAEASADLR.A	3314.36856	3	1.42E-13	0.94	5.21	-	1314.3
AHQ-2-6, 3310 - 3385	K.TAMSTPHVAEPAENEQDEQDENGAEASADLR.A	3314.36856	3	4.99E-09	0.93	4.89	-	1148.8
AHQ-2-6, 5185 - 5194	K.TQEQLALEMAELTAR.I	1704.92727	2	1.02E-10	0.98	5.59	-	2047.5
AHQ-2-7, 5148	K.TQEQLALEMAELTAR.I	1704.92727	2	3.74E-04	0.72	2.71	-	714.7
gi|13994151|ref|NP_066272.1| PDZ and LIM domain 1 (elfin); carboxy terminal LIM domain protein 1 [H				1.55E-13	15.15	200.26	55.00	36071.5
AHQ-2-14-, 6625	K.AALANLCIGDVITAIDGENTSNM*THLEAQNR.I	3331.63807	3	3.51E-07	0.80	4.25	-	424.7
AHQ-2-14-, 6375 - 6451	K.AALANLCIGDVITAIDGENTSNMTHLEAQNR.I	3315.63867	3	3.27E-09	0.93	4.98	-	976.0
AHQ-2-13-, 6917	K.AALANLCIGDVITAIDGENTSNMTHLEAQNR.I	3315.63867	3	1.55E-13	0.92	4.91	-	621.3
AHQ-2-13, 6697	K.AALANLCIGDVITAIDGENTSNMTHLEAQNR.I	3315.63867	3	2.04E-08	0.89	4.42	-	667.1
AHQ-2-13-, 6775 - 6810	K.AALANLCIGDVITAIDGENTSNM*THLEAQNR.I	3331.63807	3	4.71E-06	0.79	3.86	-	519.8
AHQ-2-12, 7022	K.AALANLCIGDVITAIDGENTSNMTHLEAQNR.I	3315.63867	3	1.07E-11	0.88	4.40	-	622.3
AHQ-2-14-, 6165	K.AALANLCIGDVITAIDGENTSNM*THLEAQNR.I	3331.63807	3	6.52E-05	0.92	4.69	-	791.3
AHQ-2-14-, 6024 - 6100	K.AALANLCIGDVITAIDGENTSNM*THLEAQNR.I	3331.63807	3	6.75E-07	0.87	4.28	-	472.1
AHQ-2-12, 6765	K.AALANLCIGDVITAIDGENTSNM*THLEAQNR.I	3331.63807	3	1.84E-08	0.74	3.68	-	305.8
AHQ-2-13, 6557	K.AALANLCIGDVITAIDGENTSNM*THLEAQNR.I	3331.63807	3	2.42E-04	0.80	3.90	-	418.4
AHQ-2-13-, 4490	K.CGTGIVGVFVK.L	1138.36198	2	6.06E-04	0.95	3.73	-	1354.1
AHQ-2-13, 4456	K.CGTGIVGVFVK.L	1138.36198	2	1.61E-04	0.92	3.24	-	1123.0
AHQ-2-14-, 4455	K.CGTGIVGVFVK.L	1138.36198	2	1.31E-04	0.94	3.12	-	1456.3
AHQ-2-14-, 3611 - 3619	K.DFEQPLAISR.V	1176.30388	1	6.36E-07	0.56	2.29	-	345.2

AHQ-2-12, 3610	K.DFEQPLAISR.V	1176.30388	2	3.08E-04	0.85	3.05	-	844.6
AHQ-2-13, 3700	K.DFEQPLAISR.V	1176.30388	2	2.05E-05	0.84	3.08	-	799.9
AHQ-2-14-, 3616 - 3617	K.DFEQPLAISR.V	1176.30388	2	7.50E-04	0.90	3.39	-	1183.0
AHQ-2-13, 3413	K.GCTDNLTLTVAR.S	1322.47058	2	4.31E-04	0.78	3.25	-	593.9
AHQ-2-13-, 3387	K.GCTDNLTLTVAR.S	1322.47058	2	3.70E-06	0.84	3.64	-	701.6
AHQ-2-14-, 3360	K.GCTDNLTLTVAR.S	1322.47058	2	1.19E-04	0.84	3.53	-	629.8
AHQ-2-12, 3997 - 4066	K.GHFFVEDQIYCEK.H	1673.82684	2	2.25E-04	0.95	4.50	-	1000.0
AHQ-2-13, 4025 - 4029	K.GHFFVEDQIYCEK.H	1673.82684	2	3.60E-05	0.96	4.40	-	1454.8
AHQ-2-14, 4910 - 4985	K.GHFFVEDQIYCEK.H	1673.82684	2	8.58E-06	0.93	3.55	-	912.1
AHQ-2-13-, 4077	K.GHFFVEDQIYCEK.H	1673.82684	2	4.51E-05	0.97	4.24	-	1669.3
AHQ-2-13-, 2999 - 3003	R.HPECYVCTDCGTNLK.Q	1858.02023	2	9.24E-04	0.93	3.77	-	789.3
AHQ-2-12, 2970	R.HPECYVCTDCGTNLK.Q	1858.02023	2	7.21E-05	0.81	2.90	-	601.5
AHQ-2-13, 2728	R.HRHPECYVCTDCGTNLK.Q	2151.34648	2	7.10E-06	0.82	3.49	-	419.7
AHQ-2-13-, 2671	R.HRHPECYVCTDCGTNLK.Q	2151.34648	3	1.17E-05	0.93	4.67	-	911.9
AHQ-2-11, 3022	R.IKGCTDNLTLTVAR.S	1563.80174	2	1.44E-08	0.93	3.33	-	1175.4
AHQ-2-14, 4033 - 4085	R.IKGCTDNLTLTVAR.S	1563.80174	2	5.11E-04	0.90	3.22	-	1074.2
AHQ-2-14, 3905 - 3956	R.IKGCTDNLTLTVAR.S	1563.80174	2	1.04E-07	0.91	3.31	-	1075.5
AHQ-2-13-, 3073	R.IKGCTDNLTLTVAR.S	1563.80174	2	1.23E-10	0.94	4.39	-	897.8
AHQ-2-12, 3057	R.IKGCTDNLTLTVAR.S	1563.80174	2	1.10E-11	0.86	3.06	-	829.6
AHQ-2-14-, 3033	R.IKGCTDNLTLTVAR.S	1563.80174	3	6.73E-04	0.92	4.25	-	1549.3
AHQ-2-14-, 3031 - 3043	R.IKGCTDNLTLTVAR.S	1563.80174	2	1.20E-10	0.96	4.98	-	1046.1
AHQ-2-13, 3111 - 3112	R.IKGCTDNLTLTVAR.S	1563.80174	2	1.76E-07	0.86	3.42	-	752.6
AHQ-2-13-, 5302	K.LPMCDKCGTGIVGVFVK.L	1884.27358	3	8.88E-07	0.60	3.32	-	530.2
AHQ-2-12, 4781 - 4807	K.LPM*CDKCGTGIVGVFVK.L	1900.27298	2	7.67E-05	0.80	2.94	-	604.0
AHQ-2-14-, 5248	K.LPMCDKCGTGIVGVFVK.L	1884.27358	3	7.17E-06	0.80	3.83	-	525.9
AHQ-2-13, 3803	R.LVGGKDFEQPLAISR.V	1630.86972	2	1.00E-04	0.94	4.60	-	913.9
AHQ-2-14-, 3724	R.LVGGKDFEQPLAISR.V	1630.86972	2	4.24E-08	0.97	5.19	-	1746.7
AHQ-2-14, 4589 - 4658	R.LVGGKDFEQPLAISR.V	1630.86972	2	1.60E-06	0.89	3.85	-	904.1
AHQ-2-13, 4079	K.MNLASEPQEVLHIGSAHNR.S	2104.33619	2	4.46E-05	0.89	4.08	-	623.8
AHQ-2-13-, 3523	K.M*NLASEPQEVLHIGSAHNR.S	2120.33559	3	1.97E-10	0.64	3.22	-	532.8
AHQ-2-12, 6771 - 6790	K.QSTSFLVLQEILESEEK.G	1981.18977	2	5.50E-09	0.96	4.98	-	1215.5
AHQ-2-13, 6573 - 6576	K.QSTSFLVLQEILESEEK.G	1981.18977	2	7.66E-10	0.97	4.73	-	1818.8
AHQ-2-13-, 6778	K.QSTSFLVLQEILESEEK.G	1981.18977	2	4.30E-07	0.96	4.62	-	1401.4
AHQ-2-13, 6095	K.QSTSFLVLQEILESEEKGDPNKPSGFR.S	3037.32681	3	5.91E-05	0.88	4.28	-	647.1
AHQ-2-13-, 6273 - 6275	K.QSTSFLVLQEILESEEKGDPNKPSGFR.S	3037.32681	3	2.22E-07	0.66	3.35	-	488.2
AHQ-2-12, 2365 - 2371	R.SAM*PFTASPASSTTAR.V	1599.74799	2	5.44E-04	0.77	3.08	-	637.4
AHQ-2-13, 3035	R.SAMPFTASPASSTTAR.V	1583.74859	2	3.59E-07	0.23	2.51	-	234.0
AHQ-2-12, 4813 - 4883	R.VTPPEGYEVVTVFPK	1662.90695	2	4.65E-04	0.30	2.62	-	111.4
AHQ-2-13-, 3061 - 3071	K.VWSPLVTEEGKR.H	1401.59247	2	5.39E-07	0.81	2.67	-	766.7
AHQ-2-14-, 2979	K.VWSPLVTEEGKR.H	1401.59247	2	6.24E-08	0.90	2.68	-	1197.6
AHQ-2-13, 3095	K.VWSPLVTEEGKR.H	1401.59247	2	5.10E-07	0.55	2.53	-	551.3
gi|5729770|ref|NP_000382.3| ceroid-lipofuscinosis, neuronal 2, late infantile (Jansky-Bielschowsky				1.73E-13	1.91	20.32	7.30	61247.6
AHQ-2-8, 2615	R.LFGGNFAHQASVAR.V	1475.63637	2	2.98E-06	0.93	4.24	-	1116.0
AHQ-2-8, 7410 - 7412	R.VPIPWVSGTSASTPVFGGILSLINEHR.I	2836.23725	3	1.73E-13	0.98	6.41	-	1688.0
gi|7669492|ref|NP_002037.2| glyceraldehyde-3-phosphate dehydrogenase [Homo sapiens]				2.12E-13	9.27	120.36	48.40	36053.0
AHQ-2-11, 3485	R.GALQNIIPASTGAAK.A	1412.61680	1	5.89E-08	0.76	3.39	-	522.7
AHQ-2-9, 3290 - 3358	R.GALQNIIPASTGAAK.A	1412.61680	1	1.37E-06	0.64	3.38	-	284.2
AHQ-2-9, 7218 - 7291	K.GILGYTEHQVVSSDFNSDTHSSTFDAGAGIALNDHFVK.L	4039.28524	3	6.17E-09	0.96	6.37	-	1207.8
AHQ-2-9, 6254 - 6327	K.GILGYTEHQVVSSDFNSDTHSSTFDAGAGIALNDHFVK.L	4039.28524	3	3.46E-05	0.90	4.82	-	814.5
AHQ-2-9, 4260	K.RVIISAPSADAPMFVMGVNHEK.Y	2370.78022	3	5.97E-06	0.91	4.36	-	994.6
AHQ-2-9, 7530 - 7531	K.VDIVAINDPFIDLNYMVYM*FQYDSTHGK.F	3326.74330	3	2.87E-05	0.61	4.07	-	262.5
AHQ-2-10, 6662	K.VIHDNFGIVEGLMTTVHAITATQK.T	2596.98694	3	5.01E-12	0.95	6.50	-	1441.2
AHQ-2-11, 6720 - 6724	K.VIHDNFGIVEGLMTTVHAITATQK.T	2596.98694	3	2.12E-13	0.97	7.11	-	2408.2
AHQ-2-9, 7294 - 7372	K.VIHDNFGIVEGLMTTVHAITATQK.T	2596.98694	2	3.99E-04	0.93	4.44	-	940.9
AHQ-2-9, 7286	K.VIHDNFGIVEGLMTTVHAITATQK.T	2596.98694	2	3.22E-07	0.97	6.49	-	1653.7
AHQ-2-11, 6721 - 6722	K.VIHDNFGIVEGLMTTVHAITATQK.T	2596.98694	2	3.93E-07	0.97	7.16	-	1781.4
AHQ-2-9, 6416 - 6474	K.VIHDNFGIVEGLM*TTVHAITATQK.T	2612.98634	2	6.84E-04	0.92	4.11	-	777.0
AHQ-2-9, 6466 - 6534	K.VIHDNFGIVEGLM*TTVHAITATQK.T	2612.98634	3	6.69E-13	0.98	6.56	-	1849.5
AHQ-2-9, 7268 - 7338	K.VIHDNFGIVEGLMTTVHAITATQK.T	2596.98694	3	1.12E-11	0.92	5.38	-	1396.3
AHQ-2-10, 6666	K.VIHDNFGIVEGLMTTVHAITATQK.T	2596.98694	2	1.63E-05	0.97	5.64	-	2279.0
AHQ-2-9, 6607 - 6660	K.VIHDNFGIVEGLM*TTVHAITATQK.T	2612.98634	3	1.15E-07	0.97	6.20	-	1494.0
AHQ-2-10, 4799 - 4800	R.VIISAPSADAPMFVMGVNHEK.Y	2214.59387	2	4.98E-08	0.61	2.97	-	346.9
AHQ-2-10, 4524	R.VIISAPSADAPMFVMGVNHEK.Y	2214.59387	2	1.60E-05	0.74	3.32	-	293.5
AHQ-2-10, 4328	R.VIISAPSADAPM*FVMGVNHEK.Y	2230.59327	2	9.96E-05	0.37	2.94	-	237.4
AHQ-2-12, 5261	R.VIISAPSADAPMFVMGVNHEK.Y	2214.59387	2	5.44E-08	0.35	2.63	-	309.8
AHQ-2-12, 5067	R.VIISAPSADAPMFVMGVNHEK.Y	2214.59387	2	6.67E-09	0.69	3.14	-	400.3
AHQ-2-9, 4454 - 4462	R.VIISAPSADAPMFVM*GVNHEK.Y	2230.59327	2	9.21E-05	0.48	3.23	-	472.1
AHQ-2-9, 5050	R.VIISAPSADAPMFVMGVNHEK.Y	2214.59387	2	3.99E-04	0.81	3.55	-	550.3
AHQ-2-9, 4787 - 4790	R.VIISAPSADAPMFVMGVNHEK.Y	2214.59387	2	2.08E-05	0.88	3.76	-	611.5
AHQ-2-9, 3802 - 3810	R.VPTANVSVVDLTCR.L	1532.74450	2	1.01E-07	0.90	3.52	-	961.3
AHQ-2-12, 4097 - 4166	R.VPTANVSVVDLTCR.L	1532.74450	2	2.71E-09	0.94	4.02	-	1145.0
AHQ-2-9, 4736	R.VPTANVSVVDLTCR.L	1532.74450	2	3.52E-04	0.87	2.86	-	849.9
AHQ-2-11, 3934 - 3960	R.VPTANVSVVDLTCR.L	1532.74450	2	3.65E-09	0.92	3.62	-	911.8
AHQ-2-9, 4070 - 4144	R.VPTANVSVVDLTCR.L	1532.74450	2	9.09E-09	0.94	4.16	-	1014.6
AHQ-2-10, 3770 - 3834	R.VPTANVSVVDLTCR.L	1532.74450	2	3.40E-07	0.93	3.78	-	883.5
AHQ-2-13, 4125 - 4141	R.VPTANVSVVDLTCR.L	1532.74450	2	1.10E-07	0.95	4.05	-	1020.7
AHQ-2-14, 4998	R.VPTANVSVVDLTCR.L	1532.74450	2	1.29E-10	0.94	4.08	-	979.6
AHQ-2-13-, 4163	R.VPTANVSVVDLTCR.L	1532.74450	2	7.06E-09	0.96	4.49	-	1290.7
AHQ-2-9, 3956 - 4002	R.VPTANVSVVDLTCR.L	1532.74450	2	4.52E-07	0.87	3.24	-	819.2
AHQ-2-9, 4162 - 4232	R.VPTANVSVVDLTCR.L	1532.74450	2	2.53E-10	0.89	3.51	-	706.6
AHQ-2-9, 7578	K.WGDAGAEYVVESTGVFTTMEK.A	2278.48027	2	6.28E-07	0.92	4.51	-	707.1
AHQ-2-9, 7551	K.WGDAGAEYVVESTGVFTTM*EK.A	2294.47967	2	1.62E-06	0.64	3.01	-	441.2
AHQ-2-9, 7436 - 7508	K.WGDAGAEYVVESTGVFTTMEK.A	2278.48027	2	2.13E-08	0.96	4.40	-	1308.0
AHQ-2-9, 7287 - 7363	K.WGDAGAEYVVESTGVFTTMEK.A	2278.48027	2	9.18E-06	0.89	4.03	-	629.6
AHQ-2-9, 6519 - 6591	K.WGDAGAEYVVESTGVFTTMEK.A	2278.48027	2	2.34E-06	0.90	4.29	-	695.2
AHQ-2-9, 6603 - 6672	K.WGDAGAEYVVESTGVFTTMEK.A	2278.48027	2	8.01E-06	0.95	5.07	-	851.0
AHQ-2-9, 7171 - 7219	K.WGDAGAEYVVESTGVFTTMEK.A	2278.48027	2	7.37E-05	0.91	4.45	-	686.7
AHQ-2-12, 6313	K.WGDAGAEYVVESTGVFTTMEK.A	2278.48027	2	1.01E-05	0.87	4.22	-	543.7
AHQ-2-9, 7036 - 7103	K.WGDAGAEYVVESTGVFTTMEK.A	2278.48027	2	5.18E-08	0.95	4.70	-	999.4
AHQ-2-13, 6080 - 6104	K.WGDAGAEYVVESTGVFTTMEK.A	2278.48027	2	1.66E-05	0.89	3.68	-	832.1
AHQ-2-13, 6175 - 6237	K.WGDAGAEYVVESTGVFTTMEK.A	2278.48027	2	4.21E-07	0.86	3.89	-	557.8
AHQ-2-9, 6954	K.WGDAGAEYVVESTGVFTTMEK.A	2278.48027	2	1.06E-07	0.90	3.86	-	775.0
AHQ-2-9, 6747 - 6815	K.WGDAGAEYVVESTGVFTTMEK.A	2278.48027	2	4.82E-05	0.96	4.30	-	1370.6
AHQ-2-13-, 5679	K.WGDAGAEYVVESTGVFTTM*EK.A	2294.47967	2	3.67E-04	0.92	4.05	-	921.9
AHQ-2-13-, 6245 - 6311	K.WGDAGAEYVVESTGVFTTMEK.A	2278.48027	2	2.79E-05	0.95	4.62	-	1077.5
AHQ-2-13-, 6382 - 6434	K.WGDAGAEYVVESTGVFTTMEK.A	2278.48027	2	4.27E-08	0.90	4.03	-	728.1
gi|4506353|ref|NP_002854.1| phosphorylase, glycogen; liver (Hers disease, glycogen storage disease				2.13E-13	2.22	30.27	7.30	97147.8
AHQ-2-5, 4808	K.EYAQNIWNVEPSDLK.I	1806.95311	2	1.04E-04	0.46	2.74	-	456.9
AHQ-2-5, 7431 - 7432	R.GAGTVFDAFPDQVAIQLNDTHPALAIPELMR.I	3309.74040	3	2.13E-13	0.84	4.17	-	473.8
AHQ-2-5, 6508	R.WLLLCNPGLAELIAEK.I	1842.19160	2	8.19E-10	0.92	4.55	-	1016.4
gi|27498332|ref|XP_212565.1| similar to tubulin, beta 5 [Homo sapiens]				2.14E-13	4.34	50.29	9.90	49670.5
AHQ-2-9, 5556 - 5558	R.ALTVPELTQQVFDAK.N	1660.89259	2	9.03E-04	0.83	3.57	-	412.8
AHQ-2-11, 5449	R.ALTVPELTQQVFDAK.N	1660.89259	2	1.55E-04	0.64	2.66	-	322.1
AHQ-2-7, 3256	R.IM*NTFSVVPSPK.V	1336.58217	2	3.48E-04	0.88	3.41	-	708.1
AHQ-2-7, 3503 - 3519	R.IMNTFSVVPSPK.V	1320.58277	2	3.74E-04	0.97	3.79	-	2236.7
AHQ-2-7, 6983	K.M*AVTFIGNSTAIQELFK.R	1887.18942	2	2.14E-13	0.96	4.87	-	866.0
AHQ-2-12, 6805	K.MAVTFIGNSTAIQELFK.R	1871.19002	2	4.19E-05	0.95	4.29	-	1191.0

AHQ-2-13, 6575	K.M*AVTFIGNSTAIQELFK.R	1887.18942	2	5.33E-13	0.95	4.49	-	955.8
AHQ-2-14-, 6289 - 6351	K.M*AVTFIGNSTAIQELFK.R	1887.18942	2	2.09E-11	0.97	5.76	-	1363.2
AHQ-2-14-, 6517 - 6533	K.M*AVTFIGNSTAIQELFK.R	1887.18942	2	2.11E-07	0.95	4.43	-	1190.4
AHQ-2-14-, 6631 - 6639	K.M*AVTFIGNSTAIQELFK.R	1887.18942	2	1.70E-07	0.98	5.40	-	1551.4
AHQ-2-7, 7348	K.MAVTFIGNSTAIQELFK.R	1871.19002	2	9.45E-07	0.90	3.98	-	674.9
gi|5453595|ref|NP_006358.1| adenylyl cyclase-associated protein [Homo sapiens]				2.59E-13	10.05	130.31	35.60	51672.7
AHQ-2-10, 2567 - 2570	R.ALLVTASQCQQPAENK.L	1759.96271	2	8.93E-04	0.95	4.69	-	1030.4
AHQ-2-9, 2630	R.ALLVTASQCQQPAENK.L	1759.96271	2	1.08E-07	0.90	3.92	-	790.3
AHQ-2-7, 2535 - 2547	R.ALLVTASQCQQPAENK.L	1759.96271	2	5.76E-05	0.97	5.38	-	1206.5
AHQ-2-13, 2867	R.ALLVTASQCQQPAENK.L	1759.96271	2	4.33E-05	0.93	3.98	-	1021.9
AHQ-2-7, 6641	R.ALLVTASQCQQPAENKLSDLLAPISEQIK.E	3168.60826	3	2.40E-10	0.94	4.98	-	1149.9
AHQ-2-7, 3295 - 3308	K.EMNDAAMFYTNR.V	1463.62130	2	2.25E-04	0.34	2.58	-	334.2
AHQ-2-7, 1891	K.KEPAVLELEGKK.W	1341.57848	1	4.87E-04	0.42	2.36	-	318.1
AHQ-2-7, 7673	K.LGLVFDDVVGIVEIINSK.D	1931.26186	2	2.59E-13	0.98	6.26	-	1385.7
AHQ-2-13-, 5107	K.LSDLLAPISEQIK.E	1427.66814	2	5.53E-04	0.92	3.15	-	1135.7
AHQ-2-7, 4843 - 4921	K.LSDLLAPISEQIK.E	1427.66814	2	4.22E-05	0.86	3.52	-	821.0
AHQ-2-7, 4851	K.LSDLLAPISEQIK.E	1427.66814	1	1.11E-05	0.57	2.72	-	546.6
AHQ-2-12, 5090	K.LSDLLAPISEQIK.E	1427.66814	2	2.41E-05	0.69	2.85	-	676.9
AHQ-2-11, 4849	K.LSDLLAPISEQIK.E	1427.66814	2	2.61E-04	0.84	3.14	-	796.2
AHQ-2-9, 4907	K.LSDLLAPISEQIK.E	1427.66814	1	1.62E-07	0.55	2.93	-	434.9
AHQ-2-9, 4900 - 4904	K.LSDLLAPISEQIK.E	1427.66814	2	5.80E-07	0.90	4.07	-	720.2
AHQ-2-14-, 3007 - 3013	K.NSLDCEIVSAK.S	1237.36245	2	1.21E-06	0.88	3.17	-	902.1
AHQ-2-7, 3528	K.NSLDCEIVSAK.S	1237.36245	1	7.60E-05	0.29	2.05	-	304.1
AHQ-2-11, 3428	K.NSLDCEIVSAK.S	1237.36245	1	3.46E-05	0.29	2.05	-	412.4
AHQ-2-8, 4308	R.SALFAQINQGESITHALK.H	1929.16615	3	5.48E-06	0.73	3.47	-	552.3
AHQ-2-7, 4369 - 4440	R.SALFAQINQGESITHALK.H	1929.16615	2	1.92E-09	0.94	5.07	-	729.8
AHQ-2-9, 4515 - 4535	R.SALFAQINQGESITHALK.H	1929.16615	2	2.20E-07	0.93	4.93	-	882.7
AHQ-2-8, 4299	R.SALFAQINQGESITHALK.H	1929.16615	2	4.62E-05	0.90	4.46	-	556.0
AHQ-2-7, 1761 - 1767	R.SGPKPFSAPKPQTSPSPK.R	1839.08461	2	5.91E-04	0.75	3.37	-	596.8
AHQ-2-14-, 6640	K.SSEMNVLIPTEGGDFNEFPVPEQFK.T	2813.08809	2	1.19E-06	0.85	4.00	-	279.3
AHQ-2-14-, 6292	K.SSEM*NVLIPTEGGDFNEFPVPEQFK.T	2829.08749	2	7.82E-04	0.64	3.29	-	189.9
AHQ-2-7, 6856 - 6936	K.SSEMNVLIPTEGGDFNEFPVPEQFK.T	2813.08809	2	2.33E-04	0.92	4.37	-	364.9
AHQ-2-14-, 6176	K.SSEM*NVLIPTEGGDFNEFPVPEQFK.T	2829.08749	2	1.03E-06	0.82	3.79	-	274.9
AHQ-2-14-, 6071 - 6075	K.SSEM*NVLIPTEGGDFNEFPVPEQFK.T	2829.08749	2	8.98E-05	0.82	3.91	-	246.4
AHQ-2-7, 6751 - 6767	K.SSEMNVLIPTEGGDFNEFPVPEQFK.T	2813.08809	2	4.95E-07	0.54	3.02	-	221.2
AHQ-2-14-, 6512 - 6515	K.SSEMNVLIPTEGGDFNEFPVPEQFK.T	2813.08809	2	1.33E-08	0.95	5.28	-	529.6
AHQ-2-7, 6421 - 6493	K.SSEM*NVLIPTEGGDFNEFPVPEQFK.T	2829.08749	2	1.14E-04	0.32	2.78	-	123.1
AHQ-2-13, 6476 - 6479	K.SSEMNVLIPTEGGDFNEFPVPEQFK.T	2813.08809	2	1.64E-05	0.60	3.22	-	184.9
AHQ-2-11, 6045	K.SSEM*NVLIPTEGGDFNEFPVPEQFK.T	2829.08749	2	9.68E-05	0.40	2.67	-	182.3
AHQ-2-11, 6297 - 6373	K.SSEMNVLIPTEGGDFNEFPVPEQFK.T	2813.08809	2	8.29E-06	0.71	3.08	-	397.9
AHQ-2-14-, 6424	K.SSEMNVLIPTEGGDFNEFPVPEQFK.T	2813.08809	2	5.31E-08	0.74	3.38	-	263.1
AHQ-2-12, 4814	R.VENQENVSNLVIEDTELK.Q	2074.23206	2	5.16E-06	0.92	4.35	-	684.2
AHQ-2-7, 4827	R.VENQENVSNLVIEDTELK.Q	2074.23206	2	3.73E-07	0.91	4.33	-	653.2
AHQ-2-13, 4767 - 4773	R.VENQENVSNLVIEDTELK.Q	2074.23206	2	7.78E-07	0.90	3.99	-	817.2
AHQ-2-11, 4864	R.VENQENVSNLVIEDTELK.Q	2074.23206	2	2.38E-09	0.94	4.77	-	892.2
AHQ-2-13-, 4861	R.VENQENVSNLVIEDTELK.Q	2074.23206	2	1.42E-07	0.92	4.71	-	624.6
AHQ-2-7, 4580 - 4652	R.VENQENVSNLVIEDTELK.Q	2074.23206	2	3.65E-08	0.95	5.00	-	774.4
AHQ-2-11, 4608	R.VENQENVSNLVIEDTELK.Q	2074.23206	2	5.44E-09	0.93	4.90	-	624.8
AHQ-2-14-, 4803	R.VENQENVSNLVIEDTELK.Q	2074.23206	2	1.07E-07	0.97	5.87	-	1159.5
AHQ-2-10, 4412	R.VENQENVSNLVIEDTELK.Q	2074.23206	2	1.37E-07	0.94	4.69	-	999.4
AHQ-2-10, 4398	R.VENQENVSNLVIEDTELK.Q	2074.23206	2	2.68E-04	0.92	4.05	-	860.3
AHQ-2-8, 4507	R.VENQENVSNLVIEDTELK.Q	2074.23206	2	2.60E-08	0.94	4.70	-	768.3
AHQ-2-7, 4984 - 5057	R.VENQENVSNLVIEDTELK.Q	2074.23206	2	4.20E-08	0.91	4.66	-	593.2
AHQ-2-9, 4676	R.VENQENVSNLVIEDTELK.Q	2074.23206	2	2.93E-04	0.92	4.16	-	719.9
AHQ-2-7, 2163 - 2243	K.VPTISINK.T	872.04435	1	7.67E-06	0.64	3.12	-	360.9
gi|4501891|ref|NP_001093.1| actinin, alpha 1 [Homo sapiens]				2.93E-13	36.65	410.33	46.30	103056.9
AHQ-2-4, 5534	K.AGTQIENIEEDFRDGLK.L	1936.06918	2	4.30E-05	0.70	3.46	-	296.8
AHQ-2-10, 4836	K.AGTQIENIEEDFRDGLK.L	1936.06918	2	5.20E-06	0.82	4.02	-	907.4
AHQ-2-5, 4629	K.AGTQIENIEEDFRDGLK.L	1936.06918	2	2.61E-05	0.89	4.69	-	1039.8
AHQ-2-11, 5088 - 5090	K.AGTQIENIEEDFRDGLK.L	1936.06918	2	5.99E-05	0.89	4.91	-	877.5
AHQ-2-1, 5697	K.AGTQIENIEEDFRDGLK.L	1936.06918	2	3.88E-05	0.43	3.24	-	462.5
AHQ-2-9, 5159	K.AGTQIENIEEDFRDGLK.L	1936.06918	2	7.14E-05	0.57	3.77	-	345.6
AHQ-2-5, 5223	K.AGTQIENIEEDFRDGLK.L	1936.06918	2	3.15E-04	0.79	3.98	-	820.1
AHQ-2-9, 4594	K.AGTQIENIEEDFRDGLK.L	1936.06918	2	9.40E-04	0.50	2.70	-	316.0
AHQ-2-6, 5181	K.AGTQIENIEEDFRDGLK.L	1936.06918	2	4.22E-06	0.80	4.39	-	630.1
AHQ-2-5, 5403	K.AIM*TYVSSFYHAFSGAQK.A	2025.27263	2	1.07E-06	0.82	3.71	-	502.3
AHQ-2-9, 6438 - 6510	K.AIMTYVSSFYHAFSGAQK.A	2009.27323	2	7.75E-04	0.66	3.23	-	327.8
AHQ-2-5, 6407 - 6480	K.AIMTYVSSFYHAFSGAQK.A	2009.27323	2	5.89E-09	0.93	4.66	-	772.7
AHQ-2-6, 6406	K.AIMTYVSSFYHAFSGAQK.A	2009.27323	2	2.20E-04	0.77	3.70	-	412.9
AHQ-2-5, 2963	R.ASFNHFDRDHSGTLGPEEFK.A	2292.40855	2	4.68E-04	0.92	3.75	-	1194.9
AHQ-2-5, 3240 - 3317	R.ASFNHFDRDHSGTLGPEEFK.A	2292.40855	2	4.60E-04	0.70	2.67	-	584.2
AHQ-2-5, 2964 - 2972	R.ASFNHFDRDHSGTLGPEEFK.A	2292.40855	3	4.88E-05	0.97	4.73	-	2593.0
AHQ-2-5, 1895	K.ASIHEAWTDGK.E	1215.29697	2	3.94E-05	0.79	3.04	-	882.2
AHQ-2-5, 1803 - 1880	K.ASIHEAWTDGK.E	1215.29697	2	5.19E-05	0.87	3.08	-	1025.9
AHQ-2-1, 5936	K.DDPLTNLNTAFDVAEK.Y	1763.88340	2	8.44E-07	0.81	3.46	-	656.7
AHQ-2-5, 5775 - 5855	K.DDPLTNLNTAFDVAEK.Y	1763.88340	2	1.82E-12	0.96	4.94	-	1363.8
AHQ-2-5, 6145	K.DDPLTNLNTAFDVAEK.Y	1763.88340	2	9.69E-13	0.96	4.73	-	1124.8
AHQ-2-6, 5646	K.DDPLTNLNTAFDVAEK.Y	1763.88340	2	1.64E-10	0.96	5.03	-	997.5
AHQ-2-4, 5978 - 5984	K.DDPLTNLNTAFDVAEK.Y	1763.88340	2	3.89E-10	0.95	4.84	-	1241.7
AHQ-2-5, 4781 - 4855	K.DGLGFCALIHR.H	1260.44730	2	4.97E-05	0.89	2.91	-	1292.0
AHQ-2-5, 5113	K.DGLGFCALIHR.H	1260.44730	2	1.83E-06	0.92	2.79	-	1428.4
AHQ-2-5, 2225 - 2227	R.DHSGTLGPEEFK.A	1317.38606	1	2.75E-06	0.63	2.80	-	385.6
AHQ-2-6, 2239	R.DHSGTLGPEEFK.A	1317.38606	2	3.22E-05	0.87	3.31	-	753.3
AHQ-2-5, 2223	R.DHSGTLGPEEFK.A	1317.38606	2	3.91E-04	0.91	3.49	-	893.3
AHQ-2-6, 3607	K.DYETATLSEIK.A	1270.36761	2	1.53E-04	0.77	2.53	-	843.2
AHQ-2-4, 4621	R.ETADTDTADQVMASFK.I	1730.83208	2	1.53E-07	0.86	3.40	-	737.5
AHQ-2-3, 4628 - 4635	R.ETADTDTADQVMASFK.I	1730.83208	2	8.67E-09	0.95	4.23	-	1185.3
AHQ-2-9, 4362 - 4376	R.ETADTDTADQVMASFK.I	1730.83208	2	2.30E-07	0.77	3.71	-	476.9
AHQ-2-11, 4237 - 4309	R.ETADTDTADQVMASFK.I	1730.83208	2	5.66E-09	0.88	3.51	-	860.8
AHQ-2-5, 3116 - 3195	R.ETADTDTADQVM*ASFK.I	1746.83148	2	8.42E-06	0.94	4.13	-	1114.2
AHQ-2-2, 4674	R.ETADTDTADQVMASFK.I	1730.83208	2	3.89E-04	0.79	2.86	-	678.1
AHQ-2-13, 4361	R.ETADTDTADQVMASFK.I	1730.83208	2	2.07E-04	0.65	2.92	-	643.1
AHQ-2-13-, 4430	R.ETADTDTADQVMASFK.I	1730.83208	2	2.58E-06	0.95	3.95	-	1396.0
AHQ-2-6, 4337	R.ETADTDTADQVMASFK.I	1730.83208	2	1.60E-05	0.96	4.26	-	1575.6
AHQ-2-1, 4650	R.ETADTDTADQVMASFK.I	1730.83208	2	1.26E-05	0.83	3.31	-	812.7
AHQ-2-5, 4399 - 4473	R.ETADTDTADQVMASFK.I	1730.83208	2	1.42E-05	0.94	3.65	-	1635.3
AHQ-2-5, 2685	K.GISQEQMNEFR.A	1339.45991	1	6.28E-04	0.40	2.41	-	176.5
AHQ-2-6, 2721	K.GISQEQMNEFR.A	1339.45991	2	2.34E-04	0.76	2.89	-	738.3
AHQ-2-5, 2675	K.GISQEQMNEFR.A	1339.45991	1	7.37E-04	0.11	2.12	-	146.8
AHQ-2-9, 2735	K.GISQEQMNEFR.A	1339.45991	2	2.66E-06	0.75	2.70	-	730.8
AHQ-2-5, 2673 - 2681	K.GISQEQMNEFR.A	1339.45991	2	4.37E-08	0.89	3.77	-	801.0
AHQ-2-11, 2845	K.GISQEQMNEFR.A	1339.45991	2	2.92E-05	0.85	3.31	-	816.7
AHQ-2-4, 2016	R.HRPELIDYGK.L	1228.38202	2	1.77E-04	0.89	3.46	-	609.5
AHQ-2-5, 1716	R.HRPELIDYGK.L	1228.38202	2	2.74E-04	0.88	3.26	-	564.5
AHQ-2-5, 1723	R.HRPELIDYGK.L	1228.38202	3	1.13E-04	0.88	3.46	-	801.7
AHQ-2-6, 4853 - 4854	K.ICDQWDNLGALTQK.R	1663.83349	2	2.00E-08	0.97	4.81	-	2339.9
AHQ-2-5, 4877 - 4955	K.ICDQWDNLGALTQK.R	1663.83349	2	8.16E-06	0.97	5.24	-	1948.6

AHQ-2-7, 4872 - 4881	K.ICDQWDNLGALTQK.R	1663.83349	2	3.35E-05	0.93	3.42	-	1742.6
AHQ-2-3, 5149 - 5155	K.ICDQWDNLGALTQK.R	1663.83349	2	3.53E-06	0.97	4.95	-	1758.9
AHQ-2-4, 5141 - 5145	K.ICDQWDNLGALTQK.R	1663.83349	2	9.41E-07	0.97	4.91	-	1914.0
AHQ-2-1, 5144	K.ICDQWDNLGALTQK.R	1663.83349	2	3.25E-07	0.93	3.89	-	1503.0
AHQ-2-5, 5388	K.ICDQWDNLGALTQK.R	1663.83349	2	9.58E-08	0.97	4.11	-	2084.3
AHQ-2-5, 4644	K.ICDQWDNLGALTQK.R	1663.83349	2	9.11E-08	0.93	4.58	-	994.2
AHQ-2-5, 5800 - 5872	K.IDQLEGDHQLIQEALIFDNK.H	2340.57447	3	7.63E-12	0.97	6.02	-	1873.4
AHQ-2-4, 6160	K.IDQLEGDHQLIQEALIFDNK.H	2340.57447	3	8.05E-05	0.90	3.95	-	1113.8
AHQ-2-6, 5793 - 5794	K.IDQLEGDHQLIQEALIFDNK.H	2340.57447	3	4.51E-07	0.96	5.48	-	1376.1
AHQ-2-5, 6032 - 6103	K.IDQLEGDHQLIQEALIFDNK.H	2340.57447	3	1.30E-04	0.93	4.54	-	1361.2
AHQ-2-5, 3941	K.ILAGDKNYITMDELR.R	1753.01353	2	2.89E-07	0.95	4.36	-	1286.4
AHQ-2-5, 3947 - 3955	K.ILAGDKNYITMDELR.R	1753.01353	3	4.21E-05	0.92	4.62	-	820.9
AHQ-2-5, 3248	K.ILAGDKNYITM*DELR.R	1769.01293	2	4.29E-05	0.91	4.38	-	522.7
AHQ-2-5, 2561	R.IMSIVDPNR.L	1045.23840	2	1.48E-04	0.80	3.12	-	623.3
AHQ-2-5, 4807 - 4833	R.ISIEMHGTLEDQLSHLR.Q	1980.23497	2	3.00E-04	0.94	4.07	-	1477.8
AHQ-2-5, 4799 - 4868	R.ISIEMHGTLEDQLSHLR.Q	1980.23497	3	3.11E-09	0.96	5.05	-	1738.7
AHQ-2-5, 4089	R.ISIEM*HGTLEDQLSHLR.Q	1996.23437	2	3.70E-08	0.79	3.45	-	432.0
AHQ-2-5, 4784	K.IVQTYHVNMAGTNPYTTITPQEINGK.W	2892.23635	3	1.31E-07	0.77	3.86	-	352.0
AHQ-2-5, 4548 - 4549	K.IVQTYHVNMAGTNPYTTITPQEINGK.W	2892.23635	2	1.68E-07	0.95	4.86	-	957.7
AHQ-2-5, 4789 - 4797	K.IVQTYHVNMAGTNPYTTITPQEINGK.W	2892.23635	2	1.03E-05	0.85	3.94	-	524.5
AHQ-2-5, 4640 - 4712	K.IVQTYHVNMAGTNPYTTITPQEINGK.W	2892.23635	3	2.84E-04	0.75	3.69	-	550.7
AHQ-2-5, 4659	K.IVQTYHVNMAGTNPYTTITPQEINGK.W	2892.23635	2	1.04E-08	0.95	4.84	-	940.6
AHQ-2-5, 4547 - 4568	R.KAGTQIENIEEDFRDGLK.L	2064.24210	3	5.48E-05	0.95	6.56	-	1037.7
AHQ-2-11, 4624	R.KAGTQIENIEEDFRDGLK.L	2064.24210	3	9.37E-05	0.76	4.11	-	732.1
AHQ-2-5, 4520 - 4575	R.KAGTQIENIEEDFRDGLK.L	2064.24210	2	3.33E-04	0.90	4.82	-	1038.2
AHQ-2-4, 5041	R.KDDPLTNLNTAFDVAEK.Y	1892.05631	2	1.37E-06	0.95	4.83	-	1050.1
AHQ-2-5, 4761 - 4771	R.KDDPLTNLNTAFDVAEK.Y	1892.05631	2	5.93E-06	0.97	5.32	-	1335.0
AHQ-2-5, 5001	R.KDDPLTNLNTAFDVAEK.Y	1892.05631	2	7.16E-05	0.97	5.53	-	1387.9
AHQ-2-4, 4336 - 4398	R.KQFGAQANVIGPWIQTK.M	1887.17410	2	1.88E-04	0.85	3.46	-	755.3
AHQ-2-6, 4025	R.KQFGAQANVIGPWIQTK.M	1887.17410	2	3.24E-04	0.94	4.37	-	1144.9
AHQ-2-5, 4044 - 4051	R.KQFGAQANVIGPWIQTK.M	1887.17410	2	5.83E-06	0.93	4.36	-	979.9
AHQ-2-6, 3001	R.LAILGIHNEVSK.I	1294.52505	2	2.09E-05	0.93	3.74	-	985.5
AHQ-2-5, 3315	R.LAILGIHNEVSK.I	1294.52505	2	6.07E-07	0.92	3.32	-	1118.3
AHQ-2-5, 2933 - 3003	R.LAILGIHNEVSK.I	1294.52505	2	2.33E-07	0.92	3.40	-	1005.7
AHQ-2-5, 2943	R.LAILGIHNEVSK.I	1294.52505	3	2.15E-04	0.64	3.09	-	570.0
AHQ-2-5, 2957	R.LAILGIHNEVSK.I	1294.52505	1	7.34E-06	0.71	3.15	-	423.8
AHQ-2-5, 3521	R.LAILGIHNEVSK.I	1294.52505	1	2.32E-05	0.48	2.10	-	499.9
AHQ-2-5, 5948 - 6019	K.LASDLLEWIR.R	1216.41115	2	7.57E-08	0.98	4.93	-	2339.4
AHQ-2-4, 6313	K.LASDLLEWIR.R	1216.41115	2	5.05E-08	0.96	3.87	-	1490.0
AHQ-2-5, 6056	K.LASDLLEWIR.R	1216.41115	2	2.16E-08	0.97	4.38	-	2093.9
AHQ-2-7, 5921 - 5928	K.LASDLLEWIR.R	1216.41115	2	3.01E-06	0.96	4.20	-	1530.4
AHQ-2-1, 6340 - 6344	K.LASDLLEWIR.R	1216.41115	2	2.11E-07	0.97	4.48	-	1804.7
AHQ-2-6, 5905 - 5909	K.LASDLLEWIR.R	1216.41115	2	9.24E-08	0.97	4.27	-	2212.6
AHQ-2-4, 6924	K.LLETIDQLYLEYAK.R	1712.96427	2	3.25E-08	0.97	4.58	-	1691.9
AHQ-2-1, 6776	K.LLETIDQLYLEYAK.R	1712.96427	2	1.55E-07	0.96	4.53	-	1335.3
AHQ-2-2, 6909 - 6976	K.LLETIDQLYLEYAK.R	1712.96427	2	1.36E-04	0.95	4.03	-	1191.2
AHQ-2-5, 6717 - 6791	K.LLETIDQLYLEYAK.R	1712.96427	2	2.17E-08	0.98	5.96	-	1412.2
AHQ-2-5, 7105	K.LLETIDQLYLEYAK.R	1712.96427	2	2.50E-06	0.92	3.69	-	1081.4
AHQ-2-3, 6860	K.LLETIDQLYLEYAK.R	1712.96427	2	7.19E-04	0.94	3.68	-	1212.8
AHQ-2-5, 5405	K.LLETIDQLYLEYAK.R	1712.96427	2	1.54E-06	0.94	4.18	-	760.2
AHQ-2-7, 6644 - 6653	K.LLETIDQLYLEYAK.R	1712.96427	2	1.13E-05	0.95	3.95	-	1370.7
AHQ-2-5, 6955 - 7023	K.LLETIDQLYLEYAK.R	1712.96427	2	1.21E-06	0.97	4.85	-	1447.9
AHQ-2-14-, 6407 - 6419	K.LLETIDQLYLEYAK.R	1712.96427	2	1.36E-05	0.95	4.12	-	1302.5
AHQ-2-5, 6811 - 6875	K.LLETIDQLYLEYAK.R	1712.96427	2	1.43E-04	0.93	3.46	-	1186.8
AHQ-2-6, 6529 - 6597	K.LLETIDQLYLEYAK.R	1712.96427	2	4.22E-09	0.97	5.01	-	1415.5
AHQ-2-6, 2330 - 2354	R.LSNRPAFMPSEGR.M	1462.65941	2	3.54E-04	0.87	3.52	-	735.9
AHQ-2-1, 3784	K.M*LDAEDIVGTARPDEK.A	1776.94709	2	7.83E-05	0.88	3.74	-	725.2
AHQ-2-5, 3779 - 3807	K.MLDAEDIVGTARPDEK.A	1760.94769	3	8.63E-05	0.79	3.70	-	678.8
AHQ-2-5, 3775 - 3776	K.MLDAEDIVGTARPDEK.A	1760.94769	2	1.99E-08	0.94	4.58	-	995.2
AHQ-2-7, 3692	K.MLDAEDIVGTARPDEK.A	1760.94769	2	9.87E-05	0.83	3.60	-	678.1
AHQ-2-8, 3686	K.MLDAEDIVGTARPDEK.A	1760.94769	3	5.49E-08	0.77	3.59	-	485.5
AHQ-2-6, 3753	K.MLDAEDIVGTARPDEK.A	1760.94769	3	1.27E-04	0.85	3.56	-	766.6
AHQ-2-6, 3719 - 3766	K.MLDAEDIVGTARPDEK.A	1760.94769	2	1.20E-06	0.94	4.62	-	1071.0
AHQ-2-5, 3355	K.M*LDAEDIVGTARPDEK.A	1776.94709	2	7.79E-05	0.86	3.83	-	586.1
AHQ-2-9, 3758	K.MLDAEDIVGTARPDEK.A	1760.94769	2	4.89E-04	0.87	3.68	-	742.3
AHQ-2-4, 4025	K.MLDAEDIVGTARPDEK.A	1760.94769	2	5.95E-06	0.89	3.71	-	900.3
AHQ-2-4, 3620	K.M*LDAEDIVGTARPDEK.A	1776.94709	2	2.77E-04	0.93	4.02	-	998.0
AHQ-2-8, 3683	K.MLDAEDIVGTARPDEK.A	1760.94769	2	2.22E-05	0.88	4.26	-	806.1
AHQ-2-6, 5166 - 5245	R.M*VSDINNAWGCLEQVEK.G	2011.22273	2	6.36E-05	0.69	2.87	-	656.5
AHQ-2-7, 5555 - 5557	R.MVSDINNAWGCLEQVEK.G	1995.22333	2	1.80E-06	0.93	4.27	-	901.4
AHQ-2-4, 5912	R.MVSDINNAWGCLEQVEK.G	1995.22333	2	4.27E-05	0.80	3.25	-	760.2
AHQ-2-5, 6159	R.MVSDINNAWGCLEQVEK.G	1995.22333	2	2.12E-10	0.96	4.83	-	1258.3
AHQ-2-5, 5988	R.MVSDINNAWGCLEQVEK.G	1995.22333	2	2.36E-07	0.94	4.30	-	1055.5
AHQ-2-6, 5795	R.MVSDINNAWGCLEQVEK.G	1995.22333	2	1.36E-05	0.96	4.57	-	1217.2
AHQ-2-5, 5876	R.MVSDINNAWGCLEQVEK.G	1995.22333	2	2.17E-08	0.98	5.25	-	2166.7
AHQ-2-5, 5617	R.MVSDINNAWGCLEQVEK.G	1995.22333	2	7.62E-11	0.98	6.10	-	1273.4
AHQ-2-6, 5071	R.M*VSDINNAWGCLEQVEK.G	2011.22273	2	8.16E-08	0.95	4.42	-	906.9
AHQ-2-5, 5560 - 5580	R.M*VSDINNAWGCLEQVEK.G	2011.22273	2	7.19E-08	0.94	4.59	-	978.4
AHQ-2-5, 5300 - 5327	R.M*VSDINNAWGCLEQVEK.G	2011.22273	2	1.09E-08	0.97	5.15	-	1608.7
AHQ-2-6, 5533	R.MVSDINNAWGCLEQVEK.G	1995.22333	2	3.66E-08	0.96	4.74	-	1446.7
AHQ-2-5, 5176 - 5180	R.M*VSDINNAWGCLEQVEK.G	2011.22273	2	1.08E-06	0.93	4.66	-	674.6
AHQ-2-6, 5405 - 5471	R.MVSDINNAWGCLEQVEK.G	1995.22333	2	1.23E-07	0.97	4.93	-	1771.8
AHQ-2-6, 5323	R.MVSDINNAWGCLEQVEK.G	1995.22333	2	9.18E-11	0.95	4.55	-	1140.8
AHQ-2-3, 5887 - 5893	R.MVSDINNAWGCLEQVEK.G	1995.22333	2	2.61E-04	0.95	4.71	-	856.6
AHQ-2-9, 4388	K.NVNIQNFHISWK.D	1500.68583	2	6.69E-04	0.91	3.57	-	987.4
AHQ-2-5, 3460	K.NYITMDELR.R	1155.30648	2	8.59E-04	0.75	3.02	-	570.3
AHQ-2-5, 4941 - 5011	K.QFGAQANVIGPWIQTK.M	1759.00118	2	4.28E-05	0.83	3.43	-	652.8
AHQ-2-5, 2629 - 2631	R.QKDYETATLSEIK.A	1526.67030	2	1.46E-05	0.66	3.42	-	298.4
AHQ-2-3, 4011 - 4020	R.TINEVENQILTR.D	1430.58898	2	7.90E-06	0.87	3.71	-	876.6
AHQ-2-4, 3997	R.TINEVENQILTR.D	1430.58898	2	2.56E-05	0.93	4.16	-	1181.1
AHQ-2-6, 3726 - 3731	R.TINEVENQILTR.D	1430.58898	2	9.70E-08	0.86	3.72	-	889.5
AHQ-2-2, 4050	R.TINEVENQILTR.D	1430.58898	2	2.43E-06	0.81	3.41	-	704.6
AHQ-2-5, 3728 - 3743	R.TINEVENQILTR.D	1430.58898	2	1.59E-06	0.92	4.02	-	995.4
AHQ-2-10, 3606 - 3631	R.TINEVENQILTR.D	1430.58898	2	2.65E-05	0.88	3.50	-	1024.6
AHQ-2-5, 3936 - 4007	R.TINEVENQILTR.D	1430.58898	1	4.46E-06	0.29	2.07	-	262.0
AHQ-2-5, 3400 - 3428	R.TINEVENQILTR.D	1430.58898	2	2.65E-04	0.73	3.29	-	465.9
AHQ-2-7, 3672	R.TINEVENQILTR.D	1430.58898	2	4.48E-04	0.81	3.13	-	751.5
AHQ-2-5, 3737	R.TINEVENQILTR.D	1430.58898	1	4.83E-04	0.14	2.30	-	148.4
AHQ-2-5, 7091 - 7156	R.VEQIAAIAQELNELDYYDSPSVNAR.C	2810.02233	3	8.62E-05	0.97	6.14	-	1856.0
AHQ-2-1, 7108	R.VEQIAAIAQELNELDYYDSPSVNAR.C	2810.02233	3	8.72E-08	0.96	5.74	-	1309.1
AHQ-2-5, 7219 - 7223	R.VEQIAAIAQELNELDYYDSPSVNAR.C	2810.02233	3	2.64E-05	0.94	4.91	-	1397.2
AHQ-2-7, 6548	R.VGWEQLLTTIAR.T	1387.60894	2	6.37E-06	0.93	4.15	-	1121.3
AHQ-2-1, 6904	R.VGWEQLLTTIAR.T	1387.60894	2	8.08E-07	0.90	3.77	-	894.6
AHQ-2-5, 6647 - 6725	R.VGWEQLLTTIAR.T	1387.60894	2	4.59E-07	0.97	4.97	-	1704.7
AHQ-2-6, 6571 - 6591	R.VGWEQLLTTIAR.T	1387.60894	2	1.66E-06	0.95	3.81	-	1618.7
AHQ-2-11, 6306 - 6322	R.VGWEQLLTTIAR.T	1387.60894	2	1.85E-06	0.95	3.98	-	1299.1
AHQ-2-2, 7005 - 7006	R.VGWEQLLTTIAR.T	1387.60894	2	3.42E-08	0.96	4.78	-	1509.8

AHQ-2-4, 7000	R.VGWEQLLTTIAR.T	1387.60894	2	9.39E-08	0.95	3.88	-	1419.4
AHQ-2-10, 6139	R.VGWEQLLTTIAR.T	1387.60894	2	3.18E-06	0.88	3.28	-	1112.8
AHQ-2-9, 6678 - 6690	R.VGWEQLLTTIAR.T	1387.60894	2	2.90E-07	0.96	4.69	-	1438.2
AHQ-2-3, 6949	R.VGWEQLLTTIAR.T	1387.60894	2	3.52E-07	0.94	3.76	-	1393.9
AHQ-2-14-, 6507	R.VGWEQLLTTIAR.T	1387.60894	2	1.66E-06	0.93	3.55	-	1376.7
AHQ-2-5, 4231 - 4300	K.VLAVNQENEQLMEDYEK.L	2053.23635	2	1.08E-06	0.94	5.04	-	801.7
AHQ-2-5, 4541 - 4612	K.VLAVNQENEQLMEDYEK.L	2053.23635	2	2.93E-13	0.97	5.71	-	1283.8
AHQ-2-5, 4176	K.VLAVNQENEQLMEDYEK.L	2053.23635	3	5.90E-11	0.93	4.51	-	1038.2
AHQ-2-6, 4465	K.VLAVNQENEQLMEDYEK.L	2053.23635	2	4.38E-09	0.95	4.56	-	1082.6
AHQ-2-3, 4431	K.VLAVNQENEQLMEDYEK.L	2053.23635	2	5.30E-06	0.88	3.78	-	889.4
AHQ-2-9, 4098 - 4176	K.VLAVNQENEQLMEDYEK.L	2053.23635	2	5.31E-06	0.85	3.78	-	626.4
AHQ-2-6, 4087	K.VLAVNQENEQLMEDYEK.L	2053.23635	2	2.98E-08	0.96	5.45	-	928.0
AHQ-2-5, 4093 - 4163	K.VLAVNQENEQLMEDYEK.L	2053.23635	2	1.15E-06	0.95	5.09	-	877.8
AHQ-2-2, 4485	K.VLAVNQENEQLMEDYEK.L	2053.23635	2	1.30E-07	0.93	4.39	-	1133.9
AHQ-2-6, 3419 - 3426	K.VLAVNQENEQLM*EDYEK.L	2069.23575	2	1.76E-06	0.96	4.98	-	1169.6
AHQ-2-8, 3892	K.VLAVNQENEQLMEDYEK.L	2053.23635	2	9.04E-09	0.95	5.05	-	1068.2
AHQ-2-5, 3417 - 3431	K.VLAVNQENEQLM*EDYEK.L	2069.23575	2	1.12E-07	0.97	5.56	-	1337.4
AHQ-2-5, 3665	K.VLAVNQENEQLM*EDYEK.L	2069.23575	2	2.45E-08	0.95	4.41	-	1049.2
AHQ-2-1, 4500	K.VLAVNQENEQLMEDYEK.L	2053.23635	2	7.51E-06	0.97	5.10	-	1500.7
AHQ-2-5, 4377 - 4447	K.VLAVNQENEQLMEDYEK.L	2053.23635	2	2.83E-09	0.97	5.49	-	1673.4
gi|4505981|ref|NP_002695.1| pro-platelet basic protein (includes platelet basic protein, bet; Pro-p				3.11E-13	7.76	90.33	40.60	13894.1
AHQ-2-14-, 5097 - 5173	K.EESLDSDLYAELR.C	1540.61070	2	6.17E-04	0.58	2.96	-	504.4
AHQ-2-14-, 4771 - 4839	K.EESLDSDLYAELR.C	1540.61070	2	1.02E-07	0.94	4.14	-	986.4
AHQ-2-14, 5833 - 5896	K.EESLDSDLYAELR.C	1540.61070	2	2.59E-07	0.95	4.01	-	1181.5
AHQ-2-14-, 4428	K.EESLDSDLYAELR.C	1540.61070	2	1.29E-06	0.75	2.98	-	389.2
AHQ-2-14-, 4431 - 4495	K.GKEESLDSDLYAELR.C	1725.83517	2	1.75E-10	0.98	5.62	-	2889.5
AHQ-2-14-, 4560 - 4623	K.GKEESLDSDLYAELR.C	1725.83517	2	4.94E-12	0.98	4.82	-	2295.6
AHQ-2-13, 4173 - 4175	K.GKEESLDSDLYAELR.C	1725.83517	2	6.76E-11	0.98	5.87	-	2484.8
AHQ-2-13, 4184	K.GKEESLDSDLYAELR.C	1725.83517	3	1.43E-04	0.91	4.28	-	1013.3
AHQ-2-14-, 4373 - 4436	K.GKEESLDSDLYAELR.C	1725.83517	3	1.35E-04	0.88	3.91	-	955.3
AHQ-2-14-, 4304	K.GKEESLDSDLYAELR.C	1725.83517	2	2.07E-11	0.98	5.60	-	2745.5
AHQ-2-14-, 4289 - 4368	K.GKEESLDSDLYAELR.C	1725.83517	2	2.19E-11	0.99	6.47	-	2861.7
AHQ-2-14-, 4219 - 4279	K.GKEESLDSDLYAELR.C	1725.83517	3	1.32E-04	0.92	4.50	-	1138.6
AHQ-2-14-, 4176 - 4240	K.GKEESLDSDLYAELR.C	1725.83517	2	3.11E-13	0.98	5.13	-	2478.0
AHQ-2-14-, 4104 - 4163	K.GKEESLDSDLYAELR.C	1725.83517	3	1.55E-07	0.94	4.77	-	1426.9
AHQ-2-14-, 4688	K.GKEESLDSDLYAELR.C	1725.83517	2	9.89E-08	0.97	4.88	-	2183.8
AHQ-2-14-, 3971 - 4041	K.GKEESLDSDLYAELR.C	1725.83517	3	5.06E-08	0.93	4.83	-	1159.3
AHQ-2-14-, 3895 - 3975	K.GKEESLDSDLYAELR.C	1725.83517	2	3.31E-12	0.98	6.54	-	2565.3
AHQ-2-14-, 3879 - 3955	K.GKEESLDSDLYAELR.C	1725.83517	3	2.54E-05	0.92	5.17	-	757.3
AHQ-2-14-, 3864 - 3903	K.GKEESLDSDLYAELR.C	1725.83517	2	5.28E-11	0.97	5.05	-	1751.5
AHQ-2-13-, 4242 - 4247	K.GKEESLDSDLYAELR.C	1725.83517	3	9.23E-10	0.91	4.58	-	955.1
AHQ-2-13-, 4239	K.GKEESLDSDLYAELR.C	1725.83517	2	1.40E-11	0.98	5.60	-	2349.5
AHQ-2-13-, 4029	K.GKEESLDSDLYAELR.C	1725.83517	2	3.85E-07	0.95	4.42	-	1261.7
AHQ-2-14, 6458	K.GKEESLDSDLYAELR.C	1725.83517	2	3.16E-07	0.92	3.90	-	1185.3
AHQ-2-14, 6188 - 6244	K.GKEESLDSDLYAELR.C	1725.83517	2	3.43E-05	0.97	4.95	-	1865.1
AHQ-2-14, 6089 - 6166	K.GKEESLDSDLYAELR.C	1725.83517	2	3.08E-04	0.97	5.16	-	1764.0
AHQ-2-14, 6040 - 6101	K.GKEESLDSDLYAELR.C	1725.83517	2	1.25E-05	0.97	4.16	-	2571.2
AHQ-2-14, 5937 - 6009	K.GKEESLDSDLYAELR.C	1725.83517	2	1.02E-06	0.97	4.71	-	2062.1
AHQ-2-14-, 4044 - 4115	K.GKEESLDSDLYAELR.C	1725.83517	2	2.16E-11	0.98	5.53	-	2304.4
AHQ-2-14, 5805 - 5854	K.GKEESLDSDLYAELR.C	1725.83517	2	3.12E-09	0.98	5.19	-	2453.6
AHQ-2-14, 5672 - 5732	K.GKEESLDSDLYAELR.C	1725.83517	2	2.59E-07	0.98	5.11	-	2403.4
AHQ-2-14, 5556 - 5612	K.GKEESLDSDLYAELR.C	1725.83517	2	5.45E-12	0.98	5.16	-	2737.1
AHQ-2-14, 5469	K.GKEESLDSDLYAELR.C	1725.83517	3	5.18E-04	0.79	3.71	-	827.1
AHQ-2-14, 4816 - 4876	K.GKEESLDSDLYAELR.C	1725.83517	2	5.12E-09	0.98	5.45	-	1880.9
AHQ-2-14, 4828 - 4896	K.GKEESLDSDLYAELR.C	1725.83517	3	3.14E-06	0.91	4.08	-	1064.2
AHQ-2-14, 5412 - 5492	K.GKEESLDSDLYAELR.C	1725.83517	2	4.23E-09	0.98	6.04	-	2277.3
AHQ-2-14, 4936 - 5001	K.GKEESLDSDLYAELR.C	1725.83517	2	2.51E-10	0.98	5.43	-	2225.4
AHQ-2-14, 4958 - 5036	K.GKEESLDSDLYAELR.C	1725.83517	3	6.83E-04	0.85	3.85	-	892.4
AHQ-2-14, 5333 - 5401	K.GKEESLDSDLYAELR.C	1725.83517	3	2.08E-05	0.87	4.12	-	829.1
AHQ-2-14, 5324 - 5388	K.GKEESLDSDLYAELR.C	1725.83517	2	6.15E-10	0.98	5.10	-	2716.9
AHQ-2-14, 5062 - 5129	K.GKEESLDSDLYAELR.C	1725.83517	3	1.89E-05	0.87	4.01	-	1135.7
AHQ-2-14, 5064 - 5137	K.GKEESLDSDLYAELR.C	1725.83517	2	8.69E-09	0.98	5.88	-	2398.0
AHQ-2-14, 5277 - 5340	K.GKEESLDSDLYAELR.C	1725.83517	3	4.30E-05	0.95	4.45	-	1631.8
AHQ-2-14, 5200 - 5281	K.GKEESLDSDLYAELR.C	1725.83517	2	1.98E-08	0.98	5.44	-	2463.3
AHQ-2-14, 5192 - 5268	K.GKEESLDSDLYAELR.C	1725.83517	3	2.54E-04	0.94	4.86	-	1352.2
AHQ-2-14, 5177 - 5249	K.GTHCNQVEVIATLK.D	1571.78073	2	3.08E-08	0.95	3.46	-	1819.4
AHQ-2-14-, 4799 - 4871	K.GTHCNQVEVIATLK.D	1571.78073	2	9.22E-05	0.92	4.04	-	852.0
AHQ-2-13, 3300 - 3376	K.GTHCNQVEVIATLK.D	1571.78073	2	5.30E-05	0.83	3.37	-	600.4
AHQ-2-14, 5312 - 5378	K.GTHCNQVEVIATLK.D	1571.78073	2	7.65E-06	0.92	3.88	-	1047.8
AHQ-2-14, 5050 - 5117	K.GTHCNQVEVIATLK.D	1571.78073	2	1.68E-05	0.97	4.83	-	1666.5
AHQ-2-14, 4970	K.GTHCNQVEVIATLK.D	1571.78073	3	8.53E-05	0.71	3.55	-	947.9
AHQ-2-14, 4916 - 4961	K.GTHCNQVEVIATLK.D	1571.78073	2	2.32E-06	0.96	4.56	-	1458.8
AHQ-2-14, 5440 - 5510	K.GTHCNQVEVIATLK.D	1571.78073	2	1.53E-05	0.96	4.62	-	1317.7
AHQ-2-14, 4796 - 4857	K.GTHCNQVEVIATLK.D	1571.78073	2	1.13E-09	0.96	4.65	-	1342.7
AHQ-2-14, 5552 - 5617	K.GTHCNQVEVIATLK.D	1571.78073	2	3.02E-06	0.93	3.91	-	1237.4
AHQ-2-14, 4660 - 4740	K.GTHCNQVEVIATLK.D	1571.78073	2	2.10E-11	0.97	4.86	-	1501.8
AHQ-2-14-, 4601	K.GTHCNQVEVIATLK.D	1571.78073	2	3.93E-05	0.96	3.92	-	1720.0
AHQ-2-14, 5681	K.GTHCNQVEVIATLK.D	1571.78073	2	1.47E-04	0.94	4.39	-	899.2
AHQ-2-14, 4536 - 4616	K.GTHCNQVEVIATLK.D	1571.78073	2	2.57E-08	0.98	5.39	-	1700.5
AHQ-2-14, 5822 - 5846	K.GTHCNQVEVIATLK.D	1571.78073	2	9.67E-06	0.95	4.24	-	1364.0
AHQ-2-14-, 3403 - 3476	K.GTHCNQVEVIATLK.D	1571.78073	2	7.15E-07	0.97	4.96	-	1611.7
AHQ-2-14, 4469 - 4540	K.GTHCNQVEVIATLK.D	1571.78073	2	5.01E-09	0.97	4.83	-	1956.1
AHQ-2-14, 5982 - 6045	K.GTHCNQVEVIATLK.D	1571.78073	2	4.72E-09	0.96	4.58	-	1504.6
AHQ-2-14, 4356 - 4420	K.GTHCNQVEVIATLK.D	1571.78073	2	2.10E-08	0.97	4.49	-	2149.0
AHQ-2-13, 3420	K.GTHCNQVEVIATLK.D	1571.78073	2	2.24E-04	0.94	3.83	-	1365.0
AHQ-2-14, 4224 - 4292	K.GTHCNQVEVIATLK.D	1571.78073	2	1.05E-07	0.97	4.79	-	1420.7
AHQ-2-14, 6304 - 6320	K.GTHCNQVEVIATLK.D	1571.78073	2	7.96E-04	0.87	3.24	-	1089.0
AHQ-2-14-, 3531 - 3552	K.GTHCNQVEVIATLK.D	1571.78073	2	3.57E-09	0.98	5.22	-	1808.7
AHQ-2-14, 6480	K.GTHCNQVEVIATLK.D	1571.78073	2	5.60E-04	0.89	3.68	-	850.1
AHQ-2-13, 3504	K.GTHCNQVEVIATLK.D	1571.78073	2	3.10E-06	0.92	4.08	-	1026.5
AHQ-2-14-, 4393 - 4460	K.GTHCNQVEVIATLK.D	1571.78073	2	8.36E-12	0.96	4.82	-	1422.4
AHQ-2-14-, 4247 - 4311	K.GTHCNQVEVIATLK.D	1571.78073	2	2.05E-06	0.98	5.33	-	1706.0
AHQ-2-14-, 4119 - 4184	K.GTHCNQVEVIATLK.D	1571.78073	2	2.53E-09	0.97	4.91	-	1480.6
AHQ-2-13-, 3358	K.GTHCNQVEVIATLK.D	1571.78073	2	1.35E-07	0.94	3.97	-	1187.0
AHQ-2-13-, 3491	K.GTHCNQVEVIATLK.D	1571.78073	2	1.01E-06	0.95	4.30	-	1211.2
AHQ-2-14-, 3269 - 3340	K.GTHCNQVEVIATLK.D	1571.78073	2	5.33E-08	0.98	4.90	-	1918.2
AHQ-2-14-, 3987 - 4059	K.GTHCNQVEVIATLK.D	1571.78073	2	1.23E-07	0.97	5.31	-	1575.3
AHQ-2-14-, 3615 - 3695	K.GTHCNQVEVIATLK.D	1571.78073	2	4.72E-09	0.98	5.05	-	2059.1
AHQ-2-14-, 3623 - 3701	K.GTHCNQVEVIATLK.D	1571.78073	3	1.12E-04	0.94	4.30	-	1572.4
AHQ-2-14-, 3763 - 3840	K.GTHCNQVEVIATLK.D	1571.78073	2	5.62E-10	0.98	5.54	-	2048.8
AHQ-2-14, 4084 - 4156	K.GTHCNQVEVIATLK.D	1571.78073	2	1.50E-05	0.97	4.64	-	1865.2
AHQ-2-14-, 3855 - 3921	K.GTHCNQVEVIATLK.D	1571.78073	2	5.63E-07	0.97	4.62	-	1735.9
AHQ-2-14-, 3387 - 3464	K.GTHCNQVEVIATLK.D	1571.78073	3	9.93E-04	0.90	4.39	-	1100.6
AHQ-2-14-, 3628	K.GTHCNQVEVIATLKDGR.K	1900.10650	3	6.33E-07	0.94	4.35	-	1282.0
AHQ-2-14-, 2935 - 3012	K.GTHCNQVEVIATLKDGR.K	1900.10650	3	1.85E-09	0.95	4.76	-	1206.8
AHQ-2-14-, 2940 - 3021	K.GTHCNQVEVIATLKDGR.K	1900.10650	2	2.10E-08	0.98	5.20	-	1871.3
AHQ-2-14, 4010 - 4076	K.GTHCNQVEVIATLKDGR.K	1900.10650	3	1.79E-07	0.92	4.01	-	905.6

AHQ-2-14, 3802 - 3869	K.GTHCNQVEVIATLKDGR.K	1900.10650	3	3.19E-04	0.86	3.60	-	705.1
AHQ-2-14, 4161	K.GTHCNQVEVIATLKDGR.K	1900.10650	3	1.17E-06	0.96	5.31	-	1321.5
AHQ-2-14, 4168	K.GTHCNQVEVIATLKDGR.K	1900.10650	2	8.08E-10	0.97	4.90	-	1653.0
AHQ-2-14, 4533	K.GTHCNQVEVIATLKDGR.K	1900.10650	3	9.52E-07	0.76	3.19	-	795.1
AHQ-2-14-, 3335	K.GTHCNQVEVIATLKDGR.K	1900.10650	3	1.00E-05	0.87	3.90	-	750.4
AHQ-2-14, 4069	K.GTHCNQVEVIATLKDGR.K	1900.10650	2	6.52E-07	0.96	5.04	-	1113.0
AHQ-2-14-, 2676	K.GTHCNQVEVIATLKDGRK.I	2028.27941	2	7.61E-05	0.94	4.82	-	710.4
AHQ-2-14-, 2668 - 2677	K.GTHCNQVEVIATLKDGRK.I	2028.27941	3	6.29E-04	0.89	3.91	-	1100.8
AHQ-2-13, 2932	K.ICLDPDAPR.I	1058.19059	2	1.12E-05	0.77	2.83	-	811.7
AHQ-2-13-, 2873 - 2877	K.ICLDPDAPR.I	1058.19059	2	2.38E-04	0.79	2.98	-	830.5
AHQ-2-14, 3669 - 3734	K.ICLDPDAPR.I	1058.19059	2	2.54E-05	0.79	2.90	-	908.5
AHQ-2-14-, 2916 - 2917	K.ICLDPDAPR.I	1058.19059	2	1.41E-04	0.87	3.02	-	1078.0
AHQ-2-14-, 2771 - 2820	K.ICLDPDAPR.I	1058.19059	2	6.68E-04	0.88	3.03	-	993.1
AHQ-2-14, 3013 - 3068	R.KICLDPDAPR.I	1186.36350	2	8.83E-06	0.63	2.89	-	522.4
AHQ-2-13-, 2567 - 2614	R.KICLDPDAPR.I	1186.36350	2	1.95E-06	0.92	3.39	-	1010.5
AHQ-2-14-, 2573	R.KICLDPDAPR.I	1186.36350	2	5.97E-04	0.86	3.18	-	858.6
AHQ-2-14-, 2445 - 2511	R.KICLDPDAPR.I	1186.36350	2	3.06E-05	0.85	3.15	-	809.6
AHQ-2-14, 3220 - 3282	R.KICLDPDAPR.I	1186.36350	2	2.60E-05	0.82	3.01	-	895.9
AHQ-2-14, 3340 - 3400	R.KICLDPDAPR.I	1186.36350	2	2.19E-05	0.87	3.66	-	707.0
AHQ-2-14, 3762 - 3825	R.KICLDPDAPR.I	1186.36350	2	1.62E-04	0.76	3.11	-	754.5
AHQ-2-14, 3674 - 3676	R.KICLDPDAPR.I	1186.36350	2	5.56E-05	0.67	2.87	-	527.7
AHQ-2-13, 2663	R.KICLDPDAPR.I	1186.36350	2	2.94E-06	0.84	3.33	-	785.8
AHQ-2-14, 1789	K.KLAGDESAD	905.92969	1	8.57E-04	0.39	1.98	-	441.5
AHQ-2-14, 4118 - 4188	K.NIQSLEVIGK.G	1101.27845	2	2.85E-06	0.94	3.86	-	1110.6
AHQ-2-13-, 3313	K.NIQSLEVIGK.G	1101.27845	2	2.23E-06	0.86	3.56	-	704.3
AHQ-2-14, 4090 - 4164	K.NIQSLEVIGK.G	1101.27845	2	1.52E-05	0.85	3.50	-	653.4
AHQ-2-14-, 3081 - 3143	K.NIQSLEVIGK.G	1101.27845	2	6.21E-06	0.87	3.51	-	728.7
AHQ-2-14-, 4180	K.NIQSLEVIGK.G	1101.27845	2	1.50E-04	0.93	3.10	-	1538.2
AHQ-2-13-, 3321	K.NIQSLEVIGK.G	1101.27845	1	7.56E-04	0.36	2.58	-	207.0
AHQ-2-14-, 3111 - 3171	K.NIQSLEVIGK.G	1101.27845	2	9.60E-06	0.86	3.63	-	653.9
AHQ-2-14-, 3289 - 3351	K.NIQSLEVIGK.G	1101.27845	2	2.70E-06	0.91	3.87	-	821.1
AHQ-2-14, 4252 - 4320	K.NIQSLEVIGK.G	1101.27845	2	1.78E-06	0.87	3.71	-	629.4
AHQ-2-13, 3340	K.NIQSLEVIGK.G	1101.27845	2	8.14E-06	0.85	3.33	-	762.9
AHQ-2-14, 4618 - 4682	K.NIQSLEVIGK.G	1101.27845	2	3.30E-04	0.83	3.26	-	757.6
AHQ-2-14, 5140 - 5205	K.NIQSLEVIGK.G	1101.27845	1	8.95E-04	0.71	2.59	-	653.4
AHQ-2-14-, 3416 - 3483	K.NIQSLEVIGK.G	1101.27845	2	3.82E-04	0.86	3.29	-	758.1
AHQ-2-14, 5141 - 5214	K.NIQSLEVIGK.G	1101.27845	2	9.03E-05	0.84	3.08	-	992.0
AHQ-2-14-, 3331	K.NIQSLEVIGK.G	1101.27845	2	1.06E-06	0.89	3.68	-	777.4
gi|4506877|ref|NP_002996.1| selectin P precursor; Selectin P (granulocyte membrane protein, 140kD;				3.18E-13	5.28	60.32	11.10	90844.7
AHQ-2-3, 4760 - 4761	K.CPELFAPEQGSLDCSDTR.G	2085.21515	2	3.18E-13	0.94	4.44	-	787.4
AHQ-2-2, 4810 - 4868	K.CPELFAPEQGSLDCSDTR.G	2085.21515	2	3.17E-06	0.94	4.36	-	802.2
AHQ-2-3, 6089	K.LECLASGIWTNKPPQCLAAQCPPLK.I	2857.31818	3	3.55E-04	0.73	3.23	-	966.7
AHQ-2-3, 6308	K.LECLASGIWTNKPPQCLAAQCPPLKIPER.G	3352.89300	3	1.13E-05	0.97	6.47	-	1317.1
AHQ-2-2, 3081	R.PSGQWTAVTPACR.A	1432.58672	2	1.15E-07	0.95	4.06	-	1131.9
AHQ-2-3, 5077	K.STCQFICDEGYSLSGPER.L	2109.23671	2	6.89E-08	0.91	3.73	-	973.0
AHQ-2-2, 5126	K.STCQFICDEGYSLSGPER.L	2109.23671	2	3.43E-08	0.86	3.63	-	680.3
AHQ-2-1, 5052	K.STCQFICDEGYSLSGPER.L	2109.23671	2	1.15E-06	0.93	4.53	-	853.8
AHQ-2-4, 5092	K.STCQFICDEGYSLSGPER.L	2109.23671	2	4.06E-08	0.91	4.02	-	798.4
AHQ-2-3, 6508	K.VLPYYSSYYWIGIR.K	1781.04522	2	7.75E-08	0.83	3.64	-	463.2
gi|5031635|ref|NP_005498.1| cofilin 1 (non-muscle) [Homo sapiens]				3.53E-13	10.16	110.39	59.60	18502.4
AHQ-2-13, 3495 - 3499	K.AVLFCLSEDKK.N	1311.52930	2	5.41E-05	0.54	2.60	-	499.5
AHQ-2-13-, 3493 - 3494	K.AVLFCLSEDKK.N	1311.52930	2	8.21E-07	0.88	3.13	-	832.6
AHQ-2-12, 3297 - 3366	K.AVLFCLSEDKK.N	1311.52930	2	8.75E-05	0.88	2.97	-	799.2
AHQ-2-12, 7090	K.EDLVFIFWAPESAPLK.S	1863.14454	2	3.78E-06	0.96	4.35	-	1482.8
AHQ-2-13, 5607	K.EILVGDVGQTVDDPYATFVK.M	2167.40069	2	8.88E-09	0.97	5.10	-	1345.4
AHQ-2-11, 5533	K.EILVGDVGQTVDDPYATFVK.M	2167.40069	2	1.01E-05	0.96	4.82	-	1219.7
AHQ-2-12, 5867 - 5933	K.EILVGDVGQTVDDPYATFVK.M	2167.40069	2	3.53E-13	0.97	5.48	-	1251.6
AHQ-2-12, 5730 - 5798	K.EILVGDVGQTVDDPYATFVK.M	2167.40069	2	1.76E-12	0.98	5.78	-	1630.3
AHQ-2-13-, 5706 - 5737	K.EILVGDVGQTVDDPYATFVK.M	2167.40069	2	9.53E-07	0.97	5.60	-	1449.3
AHQ-2-12, 5506	K.EILVGDVGQTVDDPYATFVK.M	2167.40069	2	1.14E-06	0.88	3.63	-	911.6
AHQ-2-14-, 5683 - 5684	K.EILVGDVGQTVDDPYATFVK.M	2167.40069	2	8.41E-08	0.97	5.99	-	1266.5
AHQ-2-12, 5622 - 5695	K.EILVGDVGQTVDDPYATFVK.M	2167.40069	2	7.69E-08	0.97	5.14	-	1327.6
AHQ-2-12, 6055 - 6117	K.ESKKEDLVFIFWAPESAPLK.S	2335.68256	3	6.45E-04	0.85	4.14	-	672.1
AHQ-2-12, 2317 - 2382	K.HELQANCYEEVKDR.C	1792.90812	3	2.70E-12	0.98	5.83	-	2182.8
AHQ-2-12, 2415 - 2494	K.HELQANCYEEVKDR.C	1792.90812	2	9.87E-04	0.63	3.20	-	169.5
AHQ-2-13-, 2449	K.HELQANCYEEVKDR.C	1792.90812	2	5.13E-04	0.95	4.07	-	1328.6
AHQ-2-13-, 2446	K.HELQANCYEEVKDR.C	1792.90812	3	6.11E-07	0.95	4.84	-	1397.2
AHQ-2-12, 2274 - 2342	K.HELQANCYEEVKDR.C	1792.90812	2	1.78E-10	0.98	5.45	-	1959.9
AHQ-2-12, 2805	K.KAVLFCLSEDKK.N	1439.70221	1	3.69E-07	0.66	3.08	-	391.4
AHQ-2-12, 2797	K.KAVLFCLSEDKK.N	1439.70221	1	1.18E-05	0.48	2.45	-	609.1
AHQ-2-12, 6726	K.KEDLVFIFWAPESAPLK.S	1991.31745	2	8.87E-06	0.91	4.10	-	856.1
AHQ-2-14-, 6467	K.KEDLVFIFWAPESAPLK.S	1991.31745	2	2.11E-08	0.96	4.69	-	1212.0
AHQ-2-13-, 6618	K.KEDLVFIFWAPESAPLK.S	1991.31745	3	2.80E-06	0.97	5.86	-	1459.8
AHQ-2-11, 6278 - 6356	K.KEDLVFIFWAPESAPLK.S	1991.31745	2	2.21E-04	0.93	4.31	-	789.3
AHQ-2-13-, 6605	K.KEDLVFIFWAPESAPLK.S	1991.31745	2	5.38E-07	0.97	5.91	-	1491.9
AHQ-2-13, 6379	K.KEDLVFIFWAPESAPLK.S	1991.31745	2	5.79E-04	0.96	4.71	-	1125.3
AHQ-2-12, 6666	K.KEDLVFIFWAPESAPLK.S	1991.31745	3	8.36E-05	0.95	5.21	-	1132.7
AHQ-2-12, 6493 - 6553	K.KEDLVFIFWAPESAPLK.S	1991.31745	2	1.35E-10	0.98	5.50	-	1719.4
AHQ-2-12, 6494 - 6570	K.KEDLVFIFWAPESAPLK.S	1991.31745	3	1.37E-09	0.97	5.70	-	1603.9
AHQ-2-12, 6581 - 6657	K.KEDLVFIFWAPESAPLK.S	1991.31745	2	4.76E-09	0.98	5.69	-	1601.4
AHQ-2-12, 6626	K.KEDLVFIFWAPESAPLK.S	1991.31745	2	2.88E-09	0.97	5.65	-	1366.0
AHQ-2-12, 6586 - 6593	K.KNIILEEGKEILVGDVGQTVDDPYATFVK.M	3192.60499	3	6.49E-09	0.98	7.79	-	2450.0
AHQ-2-12, 4029	K.LGGSAVISLEGKPL	1341.57848	2	5.49E-08	0.95	3.95	-	1589.3
AHQ-2-12, 5058 - 5121	K.LGGSAVISLEGKPL	1341.57848	2	2.60E-04	0.90	3.61	-	833.8
AHQ-2-12, 4331 - 4405	K.LGGSAVISLEGKPL	1341.57848	2	2.10E-06	0.96	4.29	-	1401.0
AHQ-2-12, 4961	K.LGGSAVISLEGKPL	1341.57848	2	9.08E-06	0.93	3.84	-	1153.6
AHQ-2-12, 4469 - 4545	K.LGGSAVISLEGKPL	1341.57848	2	2.54E-10	0.97	5.35	-	1264.2
AHQ-2-12, 4822 - 4894	K.LGGSAVISLEGKPL	1341.57848	2	8.51E-07	0.95	4.72	-	1073.3
AHQ-2-12, 4705 - 4773	K.LGGSAVISLEGKPL	1341.57848	2	7.20E-07	0.97	5.35	-	1292.6
AHQ-2-12, 4566 - 4637	K.LGGSAVISLEGKPL	1341.57848	2	4.66E-06	0.96	5.01	-	1289.8
AHQ-2-13, 6619	K.NIILEEGKEILVGDVGQTVDDPYATFVK.M	3064.43208	3	6.92E-08	0.97	7.08	-	1598.1
AHQ-2-12, 7217 - 7281	K.NIILEEGKEILVGDVGQTVDDPYATFVK.M	3064.43208	3	3.42E-09	0.98	7.15	-	1564.4
AHQ-2-12, 6949 - 7005	K.NIILEEGKEILVGDVGQTVDDPYATFVK.M	3064.43208	3	4.62E-11	0.98	7.07	-	2027.6
AHQ-2-1, 7113 - 7126	K.NIILEEGKEILVGDVGQTVDDPYATFVK.M	3064.43208	3	9.99E-04	0.88	3.72	-	1120.0
AHQ-2-13-, 6837 - 6838	K.NIILEEGKEILVGDVGQTVDDPYATFVK.M	3064.43208	3	2.49E-11	0.98	7.85	-	1614.7
AHQ-2-11, 6545 - 6612	K.NIILEEGKEILVGDVGQTVDDPYATFVK.M	3064.43208	3	2.90E-07	0.94	4.99	-	1238.9
AHQ-2-14-, 6691 - 6763	K.NIILEEGKEILVGDVGQTVDDPYATFVK.M	3064.43208	3	1.24E-09	0.96	5.45	-	1367.2
AHQ-2-12, 3118 - 3181	R.YALYDATYETK.E	1338.44364	1	7.39E-07	0.63	2.95	-	342.5
AHQ-2-12, 3117 - 3153	R.YALYDATYETK.E	1338.44364	2	4.77E-08	0.95	3.54	-	1189.7
AHQ-2-12, 3085 - 3158	R.YALYDATYETK.E	1338.44364	2	1.51E-07	0.92	3.40	-	973.6
AHQ-2-12, 3346	R.YALYDATYETK.E	1338.44364	2	2.27E-06	0.90	2.80	-	1062.9
AHQ-2-13, 3295	R.YALYDATYETK.E	1338.44364	1	2.03E-07	0.53	2.65	-	328.4
gi|4758638|ref|NP_004896.1| peroxiredoxin 6; antioxidant protein 2; non-selenium glutathione peroxi				3.73E-13	8.23	110.29	48.20	25034.8
AHQ-2-10, 4035	K.DGDSVM*VLPTIPEEEAK.K	1847.03409	2	6.55E-06	0.56	2.91	-	440.2
AHQ-2-10, 2588 - 2590	K.DINAYNCEEPTEK.L	1584.64363	2	9.73E-05	0.88	3.39	-	878.9
AHQ-2-10, 2483 - 2487	K.DINAYNCEEPTEK.L	1584.64363	2	1.17E-06	0.89	3.69	-	721.7
AHQ-2-10, 5354	K.DINAYNCEEPTEKLPFPIIDDR.N	2651.88650	3	6.66E-05	0.87	3.74	-	881.5

AHQ-2-10, 5454	K.DINAYNCEEPTEKLPFPIIDDR.N	2651.88650	2	6.31E-05	0.31	2.58	-	213.0
AHQ-2-10, 5819 - 5895	R.ELAILLGMLDPAEKDEK.G	1886.19976	2	2.71E-04	0.93	4.14	-	977.9
AHQ-2-12, 6257	R.ELAILLGMLDPAEKDEK.G	1886.19976	2	7.20E-05	0.79	3.38	-	596.0
AHQ-2-10, 5699 - 5758	R.ELAILLGMLDPAEKDEK.G	1886.19976	2	3.43E-06	0.96	4.78	-	1197.6
AHQ-2-10, 5998	R.FHDFLGDSWGILFSHPR.D	2032.24831	3	4.49E-09	0.96	4.91	-	1462.5
AHQ-2-10, 5995 - 6059	R.FHDFLGDSWGILFSHPR.D	2032.24831	2	1.13E-05	0.98	5.86	-	2124.4
AHQ-2-10, 6122	R.FHDFLGDSWGILFSHPR.D	2032.24831	2	4.26E-06	0.97	5.22	-	1462.8
AHQ-2-14-, 5968	K.LIALSIDSVEDHLAWSK.D	1898.14883	2	3.73E-13	0.98	5.34	-	2020.7
AHQ-2-10, 5572 - 5647	K.LIALSIDSVEDHLAWSK.D	1898.14883	2	1.30E-08	0.97	4.83	-	1607.3
AHQ-2-14-, 5979	K.LIALSIDSVEDHLAWSK.D	1898.14883	3	9.04E-05	0.36	3.09	-	213.3
AHQ-2-10, 5747	K.LIALSIDSVEDHLAWSK.D	1898.14883	2	4.63E-04	0.85	3.26	-	805.2
AHQ-2-10, 4143 - 4154	K.LPFPIIDDR.N	1086.26548	2	3.46E-05	0.91	3.26	-	825.4
AHQ-2-10, 5311	R.VATPVDWKDGDSVMVLPTIPEEEAK.K	2728.06777	2	4.48E-04	0.82	4.05	-	399.3
AHQ-2-10, 5214 - 5287	R.VATPVDWKDGDSVMVLPTIPEEEAK.K	2728.06777	2	1.22E-04	0.65	2.97	-	429.7
AHQ-2-10, 4735 - 4803	R.VATPVDWKDGDSVM*VLPTIPEEEAK.K	2744.06717	3	2.63E-04	0.35	3.02	-	205.3
AHQ-2-10, 3482 - 3484	R.VVFVFGPDK.K	1008.19471	1	1.36E-04	0.58	2.10	-	776.6
AHQ-2-10, 3502	R.VVFVFGPDK.K	1008.19471	2	3.25E-07	0.80	2.71	-	602.8
AHQ-2-10, 2682	R.VVFVFGPDKK.L	1136.36763	2	2.34E-05	0.88	3.18	-	890.4
gi|10863977|ref|NP_067000.1| LSM2 homolog, U6 small nuclear RNA associated; U6 snRNA-associated Sm-				3.96E-13	1.68	20.26	40.00	10834.5
AHQ-2-14-, 6707	K.NDLSICGTLHSVDQYLNIK.L	2192.43507	2	2.27E-07	0.94	4.38	-	999.4
AHQ-2-14-, 6709	K.NDLSICGTLHSVDQYLNIK.L	2192.43507	3	1.24E-07	0.76	3.47	-	478.4
AHQ-2-14-, 5420 - 5423	R.YVQLPADEVDTQLLQDAAR.K	2146.34311	2	4.36E-09	0.97	5.26	-	1459.9
AHQ-2-14, 6508	R.YVQLPADEVDTQLLQDAAR.K	2146.34311	2	3.96E-13	0.93	3.91	-	1268.2
AHQ-2-14, 6249	R.YVQLPADEVDTQLLQDAAR.K	2146.34311	2	8.62E-05	0.57	2.61	-	525.3
gi|24431935|ref|NP_065393.1| reticulon 4; neuroendocrine-specific protein C like (foocen) [Homo sap				4.87E-13	0.97	10.30	2.80	129930.3
AHQ-2-14-, 6089	K.YQFVREPEDEEEEEEEEEDEDEDLEELEVLER.K	4189.14063	3	4.87E-13	0.97	6.06	-	1640.6
gi|13562114|ref|NP_110400.1| beta tubulin 1, class VI [Homo sapiens]				5.25E-13	20.69	230.36	57.40	50326.6
AHQ-2-1, 6625	R.ALSVAELTQQMFDAR.N	1680.90740	2	1.80E-04	0.93	4.00	-	1221.2
AHQ-2-10, 5863	R.ALSVAELTQQMFDAR.N	1680.90740	2	6.94E-05	0.93	4.18	-	1450.3
AHQ-2-9, 6334	R.ALSVAELTQQMFDAR.N	1680.90740	2	7.00E-08	0.94	4.34	-	1194.1
AHQ-2-14-, 6225 - 6295	R.ALSVAELTQQMFDAR.N	1680.90740	2	1.16E-04	0.82	2.99	-	1045.2
AHQ-2-9, 4567 - 4568	R.ALSVAELTQQM*FDAR.N	1696.90680	2	1.86E-05	0.90	3.81	-	994.0
AHQ-2-8, 6170 - 6188	R.ALSVAELTQQMFDAR.N	1680.90740	2	3.04E-06	0.96	4.86	-	1450.7
AHQ-2-13, 6169 - 6179	R.ALSVAELTQQMFDAR.N	1680.90740	2	4.48E-05	0.97	5.25	-	1849.3
AHQ-2-11, 6008 - 6028	R.ALSVAELTQQMFDAR.N	1680.90740	2	1.77E-06	0.94	4.65	-	1094.5
AHQ-2-14, 4969 - 5029	R.AVLVDLEPGTM*DSIR.S	1632.86102	2	6.36E-04	0.53	2.74	-	444.2
AHQ-2-7, 4687 - 4767	R.AVLVDLEPGTMDSIR.S	1616.86162	2	8.52E-08	0.90	4.59	-	590.7
AHQ-2-14-, 4692	R.AVLVDLEPGTMDSIR.S	1616.86162	2	7.03E-06	0.86	3.44	-	621.6
AHQ-2-12, 4965	R.AVLVDLEPGTMDSIR.S	1616.86162	2	2.14E-05	0.81	3.45	-	656.1
AHQ-2-14, 4869 - 4933	R.EIVHIQIGQCGNQIGAK.F	1867.11965	2	7.79E-05	0.71	3.59	-	431.5
AHQ-2-7, 3539 - 3541	R.EIVHIQIGQCGNQIGAK.F	1867.11965	2	7.97E-04	0.94	4.31	-	1100.4
AHQ-2-13, 3981	R.EIVHIQIGQCGNQIGAK.F	1867.11965	2	4.16E-07	0.91	4.14	-	726.0
AHQ-2-14-, 3781 - 3784	R.EIVHIQIGQCGNQIGAK.F	1867.11965	2	2.20E-08	0.95	5.44	-	809.7
AHQ-2-12, 3734	R.EIVHIQIGQCGNQIGAK.F	1867.11965	2	3.11E-04	0.80	3.39	-	419.2
AHQ-2-9, 2984 - 3054	K.EVDQQLLSVQTR.N	1416.56231	2	8.75E-04	0.74	3.22	-	540.5
AHQ-2-8, 2908 - 2979	K.EVDQQLLSVQTR.N	1416.56231	1	7.17E-04	0.12	1.82	-	119.4
AHQ-2-10, 3012	K.EVDQQLLSVQTR.N	1416.56231	2	2.45E-04	0.92	3.83	-	1059.9
AHQ-2-13-, 3206 - 3270	K.EVDQQLLSVQTR.N	1416.56231	2	6.87E-04	0.85	3.42	-	806.2
AHQ-2-7, 5287 - 5301	K.FWEMIGEEHGIDLAGSDR.G	2063.23616	2	9.83E-05	0.71	3.35	-	326.8
AHQ-2-7, 5136 - 5216	K.FWEMIGEEHGIDLAGSDR.G	2063.23616	2	1.56E-04	0.83	3.61	-	470.2
AHQ-2-7, 5453	K.FWEMIGEEHGIDLAGSDR.G	2063.23616	2	1.50E-05	0.48	2.62	-	311.5
AHQ-2-14, 5821	K.FWEM*IGEEHGIDLAGSDR.G	2079.23556	2	2.93E-06	0.93	3.69	-	896.3
AHQ-2-12, 5179 - 5246	K.FWEMIGEEHGIDLAGSDR.G	2063.23616	2	4.06E-08	0.97	5.99	-	1514.3
AHQ-2-14, 6337 - 6406	K.FWEMIGEEHGIDLAGSDR.G	2063.23616	2	2.26E-11	0.97	5.00	-	1748.9
AHQ-2-13-, 5279	K.FWEMIGEEHGIDLAGSDR.G	2063.23616	3	1.82E-09	0.95	4.18	-	1477.6
AHQ-2-14-, 5191	K.FWEMIGEEHGIDLAGSDR.G	2063.23616	2	1.27E-09	0.98	6.57	-	2014.3
AHQ-2-14-, 5195 - 5276	K.FWEMIGEEHGIDLAGSDR.G	2063.23616	3	1.25E-09	0.96	5.13	-	1408.4
AHQ-2-13, 5176	K.FWEMIGEEHGIDLAGSDR.G	2063.23616	2	2.12E-07	0.98	5.72	-	1697.3
AHQ-2-13-, 5269	K.FWEMIGEEHGIDLAGSDR.G	2063.23616	2	1.67E-05	0.91	3.87	-	849.4
AHQ-2-7, 5552	K.FWEMIGEEHGIDLAGSDR.G	2063.23616	2	3.43E-07	0.95	4.07	-	1384.9
AHQ-2-8, 6650 - 6728	K.GHYTEGAELIENVLEVVR.H	2029.23935	2	3.39E-06	0.98	6.02	-	2442.6
AHQ-2-12, 6833	K.GHYTEGAELIENVLEVVR.H	2029.23935	2	3.98E-06	0.98	6.37	-	2489.4
AHQ-2-12, 6814 - 6815	K.GHYTEGAELIENVLEVVR.H	2029.23935	3	2.96E-05	0.94	5.20	-	1457.0
AHQ-2-12, 6778 - 6813	K.GHYTEGAELIENVLEVVR.H	2029.23935	2	1.62E-06	0.98	6.36	-	2400.9
AHQ-2-4, 7280	K.GHYTEGAELIENVLEVVR.H	2029.23935	2	1.26E-04	0.94	4.02	-	1623.5
AHQ-2-13, 6621	K.GHYTEGAELIENVLEVVR.H	2029.23935	2	5.22E-05	0.97	5.57	-	1643.2
AHQ-2-9, 6950	K.GHYTEGAELIENVLEVVR.H	2029.23935	2	9.65E-07	0.99	7.03	-	2474.5
AHQ-2-7, 6775 - 6851	K.GHYTEGAELIENVLEVVR.H	2029.23935	3	7.72E-04	0.85	4.40	-	1044.8
AHQ-2-7, 6772 - 6855	K.GHYTEGAELIENVLEVVR.H	2029.23935	2	5.88E-12	0.98	6.61	-	2573.9
AHQ-2-11, 6506	K.GHYTEGAELIENVLEVVR.H	2029.23935	3	1.85E-07	0.93	4.35	-	1960.1
AHQ-2-13-, 6850	K.GHYTEGAELIENVLEVVR.H	2029.23935	2	5.25E-13	0.98	7.25	-	2187.3
AHQ-2-11, 6504	K.GHYTEGAELIENVLEVVR.H	2029.23935	2	5.38E-10	0.98	6.58	-	2630.7
AHQ-2-1, 7134	K.GHYTEGAELIENVLEVVR.H	2029.23935	2	3.73E-06	0.97	5.46	-	1673.6
AHQ-2-10, 6350	K.GHYTEGAELIENVLEVVR.H	2029.23935	2	6.01E-08	0.98	5.56	-	2349.6
AHQ-2-13, 6635	K.GHYTEGAELIENVLEVVR.H	2029.23935	3	4.10E-04	0.88	4.16	-	1423.2
AHQ-2-14-, 6703	K.GHYTEGAELIENVLEVVR.H	2029.23935	3	1.18E-04	0.89	4.80	-	1219.3
AHQ-2-14-, 6443 - 6459	R.GLSM*AATFIGNNTAIQEIFNR.V	2285.56551	2	6.75E-09	0.92	4.49	-	811.0
AHQ-2-7, 7420	R.GLSMAATFIGNNTAIQEIFNR.V	2269.56611	2	2.84E-06	0.93	4.76	-	839.9
AHQ-2-7, 6659 - 6697	R.GLSM*AATFIGNNTAIQEIFNR.V	2285.56551	2	5.26E-09	0.92	4.26	-	810.0
AHQ-2-13-, 6594	R.GLSM*AATFIGNNTAIQEIFNR.V	2285.56551	2	9.45E-06	0.93	4.61	-	740.2
AHQ-2-13, 6391	R.GLSM*AATFIGNNTAIQEIFNR.V	2285.56551	2	1.33E-05	0.91	4.54	-	681.6
AHQ-2-11, 6036	R.HESESCDCLQGFQIVHSLGGGTGSGMGTLLMNK.I	3511.88634	3	2.80E-10	0.96	5.30	-	1656.1
AHQ-2-7, 3495 - 3524	R.IMNSFSVMPSPK.V	1338.62160	2	1.38E-06	0.91	3.59	-	1430.3
AHQ-2-7, 3760	R.IMNSFSVMPSPK.V	1338.62160	2	2.52E-06	0.92	3.42	-	816.4
AHQ-2-7, 3892	R.IMNSFSVMPSPK.V	1338.62160	2	6.15E-07	0.95	3.80	-	1448.4
AHQ-2-7, 2736	R.IMNSFSVM*PSPK.V	1354.62100	2	5.27E-04	0.81	3.29	-	783.8
AHQ-2-14, 3914 - 3988	R.ISVYYNEAYGR.K	1335.44622	2	3.34E-09	0.91	3.14	-	1002.7
AHQ-2-14-, 3219	R.ISVYYNEAYGR.K	1335.44622	2	3.74E-07	0.95	3.23	-	1484.6
AHQ-2-14-, 3055 - 3056	R.ISVYYNEAYGR.K	1335.44622	2	3.45E-06	0.92	3.22	-	1185.5
AHQ-2-13-, 3253	R.ISVYYNEAYGR.K	1335.44622	2	2.99E-06	0.95	3.41	-	1419.9
AHQ-2-14, 4020	R.ISVYYNEAYGR.K	1335.44622	2	8.27E-06	0.90	2.89	-	907.8
AHQ-2-7, 2895 - 2897	R.ISVYYNEAYGR.K	1335.44622	2	1.09E-07	0.95	3.40	-	1344.7
AHQ-2-13, 3264 - 3273	R.ISVYYNEAYGR.K	1335.44622	2	2.26E-05	0.71	2.52	-	767.9
AHQ-2-12, 5174	K.LGALFQPDSFVHGNSGAGNNWAK.G	2388.58260	2	2.11E-06	0.83	3.36	-	813.5
AHQ-2-13, 5117 - 5184	K.LGALFQPDSFVHGNSGAGNNWAK.G	2388.58260	2	4.08E-07	0.87	3.66	-	785.5
AHQ-2-7, 4688	K.LGALFQPDSFVHGNSGAGNNWAK.G	2388.58260	3	3.32E-10	0.83	3.45	-	803.7
AHQ-2-13-, 5185 - 5197	K.LGALFQPDSFVHGNSGAGNNWAK.G	2388.58260	3	9.80E-10	0.74	3.62	-	545.6
AHQ-2-7, 4811	R.LHFFM*PGFAPLTAQGSQQYR.A	2313.62224	2	1.14E-07	0.97	5.17	-	1075.7
AHQ-2-11, 5588	R.LHFFMPGFAPLTAQGSQQYR.A	2297.62284	2	5.61E-04	0.97	4.99	-	1108.2
AHQ-2-9, 4903 - 4918	R.LHFFM*PGFAPLTAQGSQQYR.A	2313.62224	2	4.16E-06	0.97	5.78	-	937.4
AHQ-2-8, 5263	R.LHFFMPGFAPLTAQGSQQYR.A	2297.62284	2	3.41E-06	0.96	5.38	-	807.1
AHQ-2-12, 5861	R.LHFFMPGFAPLTAQGSQQYR.A	2297.62284	3	1.34E-06	0.93	4.55	-	1238.4
AHQ-2-10, 5267	R.LHFFMPGFAPLTAQGSQQYR.A	2297.62284	2	3.70E-08	0.97	5.53	-	850.5
AHQ-2-11, 4950	R.LHFFM*PGFAPLTAQGSQQYR.A	2313.62224	2	2.79E-06	0.97	5.03	-	1221.6
AHQ-2-9, 5598 - 5603	R.LHFFMPGFAPLTAQGSQQYR.A	2297.62284	2	3.98E-05	0.96	5.02	-	804.3
AHQ-2-12, 5862	R.LHFFMPGFAPLTAQGSQQYR.A	2297.62284	2	1.95E-07	0.96	5.40	-	727.1
AHQ-2-9, 7414	K.LTTPTYGDLNHLVSLTMSGITTSLR.F	2693.06979	3	7.90E-08	0.96	5.82	-	1259.6

AHQ-2-7, 7433 - 7509	K.LTTPTYGDLNHLVSLTMSGITTSLR.F	2693.06979	2	6.86E-08	0.93	4.08	-	948.2
AHQ-2-7, 7479 - 7480	K.LTTPTYGDLNHLVSLTMSGITTSLR.F	2693.06979	3	4.55E-08	0.96	5.53	-	1573.2
AHQ-2-4, 7597	K.LTTPTYGDLNHLVSLTMSGITTSLR.F	2693.06979	3	8.42E-06	0.93	4.78	-	1147.8
AHQ-2-14-, 5879	K.LTTPTYGDLNHLVSLTM*SGITTSLR.F	2709.06919	3	1.71E-06	0.89	4.06	-	846.4
AHQ-2-14-, 6647	K.LTTPTYGDLNHLVSLTMSGITTSLR.F	2693.06979	3	3.45E-07	0.84	4.03	-	643.0
AHQ-2-9, 7335 - 7410	K.LTTPTYGDLNHLVSLTMSGITTSLR.F	2693.06979	2	1.37E-06	0.83	3.87	-	463.5
AHQ-2-8, 6806	K.LTTPTYGDLNHLVSLTMSGITTSLR.F	2693.06979	2	2.36E-05	0.89	3.83	-	893.9
AHQ-2-8, 7387 - 7388	K.LTTPTYGDLNHLVSLTMSGITTSLR.F	2693.06979	2	3.84E-08	0.95	5.45	-	795.3
AHQ-2-10, 6356 - 6360	K.LTTPTYGDLNHLVSLTMSGITTSLR.F	2693.06979	2	2.16E-07	0.76	3.47	-	476.1
AHQ-2-14-, 6612 - 6681	K.LTTPTYGDLNHLVSLTM*SGITTSLR.F	2709.06919	3	5.56E-06	0.94	5.33	-	1181.6
AHQ-2-9, 6895 - 6960	K.LTTPTYGDLNHLVSLTM*SGITTSLR.F	2709.06919	3	5.27E-04	0.92	4.62	-	986.2
AHQ-2-12, 7143	K.LTTPTYGDLNHLVSLTMSGITTSLR.F	2693.06979	2	1.42E-06	0.93	4.46	-	813.3
AHQ-2-10, 6680 - 6744	K.LTTPTYGDLNHLVSLTMSGITTSLR.F	2693.06979	3	1.31E-06	0.92	4.65	-	1008.4
AHQ-2-7, 6981	K.LTTPTYGDLNHLVSLTMSGITTSLR.F	2693.06979	3	2.79E-05	0.95	5.31	-	1216.3
AHQ-2-9, 6956	K.LTTPTYGDLNHLVSLTMSGITTSLR.F	2693.06979	3	7.22E-09	0.85	4.14	-	564.1
AHQ-2-10, 6742	K.LTTPTYGDLNHLVSLTMSGITTSLR.F	2693.06979	2	1.06E-06	0.95	4.86	-	1065.9
AHQ-2-7, 6099 - 6103	R.NSSCFVEWIPNNVK.V	1695.87718	2	3.59E-04	0.90	4.14	-	840.3
AHQ-2-11, 5108	R.NSSCFVEWIPNNVK.V	1695.87718	2	4.08E-04	0.89	3.92	-	672.2
AHQ-2-12, 2955	R.VSEHFSAMFK.R	1183.36152	2	5.09E-07	0.89	3.07	-	1010.6
AHQ-2-9, 5170	R.YLTVACIFR.G	1144.36822	2	5.48E-05	0.87	2.74	-	841.9
AHQ-2-13, 5259	R.YLTVACIFR.G	1144.36822	2	8.56E-04	0.87	3.02	-	784.0
AHQ-2-7, 5097	R.YLTVACIFR.G	1144.36822	2	8.10E-05	0.76	2.70	-	609.2
gi|28626504|ref|NP_113659.3| UNC-112 related protein 2 [Homo sapiens]				5.57E-13	24.84	290.35	56.60	75429.7
AHQ-2-6, 2933 - 2969	K.AGDALWLR.F	902.03223	2	3.61E-04	0.92	3.82	-	1132.9
AHQ-2-13, 6455	R.ARGEELDEDLFLQLTGGHEAF	2348.51032	2	7.68E-06	0.90	3.77	-	850.8
AHQ-2-11, 6345 - 6346	R.ARGEELDEDLFLQLTGGHEAF	2348.51032	2	5.75E-09	0.81	3.83	-	570.5
AHQ-2-12, 6643	R.ARGEELDEDLFLQLTGGHEAF	2348.51032	2	6.82E-10	0.88	3.62	-	791.5
AHQ-2-10, 6232	R.ARGEELDEDLFLQLTGGHEAF	2348.51032	2	1.44E-07	0.79	3.44	-	594.3
AHQ-2-14-, 6529	R.ARGEELDEDLFLQLTGGHEAF	2348.51032	2	3.21E-08	0.96	4.79	-	1516.2
AHQ-2-9, 6744 - 6826	R.ASFSQPLFQAVAAICR.L	1768.02967	2	3.19E-07	0.96	4.96	-	864.5
AHQ-2-1, 6996	R.ASFSQPLFQAVAAICR.L	1768.02967	2	4.56E-05	0.88	3.61	-	681.2
AHQ-2-9, 6890 - 6891	R.ASFSQPLFQAVAAICR.L	1768.02967	2	1.33E-06	0.95	4.66	-	958.2
AHQ-2-6, 6698	R.ASFSQPLFQAVAAICR.L	1768.02967	3	6.84E-07	0.93	4.41	-	1044.0
AHQ-2-6, 6627 - 6649	R.ASFSQPLFQAVAAICR.L	1768.02967	2	5.95E-04	0.95	4.68	-	814.2
AHQ-2-12, 6722	R.ASFSQPLFQAVAAICR.L	1768.02967	2	2.69E-07	0.94	4.28	-	914.1
AHQ-2-6, 6973	R.ASFSQPLFQAVAAICR.L	1768.02967	2	3.74E-05	0.91	3.94	-	628.8
AHQ-2-3, 7073	R.ASFSQPLFQAVAAICR.L	1768.02967	2	8.14E-07	0.96	4.91	-	1001.9
AHQ-2-8, 6587	R.ASFSQPLFQAVAAICR.L	1768.02967	2	5.33E-05	0.92	3.33	-	1091.6
AHQ-2-5, 6903	R.ASFSQPLFQAVAAICR.L	1768.02967	2	8.42E-05	0.95	4.42	-	1154.1
AHQ-2-7, 6724 - 6735	R.ASFSQPLFQAVAAICR.L	1768.02967	2	8.08E-07	0.96	5.13	-	1016.7
AHQ-2-4, 7132	R.ASFSQPLFQAVAAICR.L	1768.02967	2	1.63E-05	0.92	3.83	-	782.6
AHQ-2-13, 6528	R.ASFSQPLFQAVAAICR.L	1768.02967	2	2.13E-08	0.80	2.94	-	489.7
AHQ-2-11, 6396 - 6476	R.ASFSQPLFQAVAAICR.L	1768.02967	2	7.24E-04	0.89	3.50	-	828.5
AHQ-2-6, 6713 - 6774	R.ASFSQPLFQAVAAICR.L	1768.02967	2	1.02E-05	0.96	5.57	-	904.0
AHQ-2-11, 3452	K.EKEPEEELYDLSK.V	1609.71294	2	8.81E-06	0.84	3.68	-	489.8
AHQ-2-6, 3282 - 3354	K.EKEPEEELYDLSK.V	1609.71294	2	9.89E-09	0.89	3.83	-	528.9
AHQ-2-1, 3840 - 3841	K.EKEPEEELYDLSK.V	1609.71294	2	4.48E-05	0.74	3.28	-	391.4
AHQ-2-9, 3303 - 3336	K.EKEPEEELYDLSK.V	1609.71294	2	1.58E-04	0.85	3.25	-	647.8
AHQ-2-10, 3294	K.EKEPEEELYDLSK.V	1609.71294	2	1.21E-06	0.80	3.32	-	445.8
AHQ-2-6, 3425	K.EKEPEEELYDLSK.V	1609.71294	2	1.69E-05	0.74	3.19	-	429.7
AHQ-2-9, 7128	R.FIQAWQSLPDFGISYVM*VR.F	2274.62593	2	9.60E-08	0.92	4.46	-	542.1
AHQ-2-9, 7362	R.FIQAWQSLPDFGISYVMVR.F	2258.62653	2	2.28E-05	0.94	5.08	-	667.7
AHQ-2-12, 6921 - 6922	R.FIQAWQSLPDFGISYVM*VR.F	2274.62593	2	3.10E-06	0.93	4.77	-	606.1
AHQ-2-13, 6659	R.FIQAWQSLPDFGISYVM*VR.F	2274.62593	2	1.14E-04	0.88	4.15	-	514.1
AHQ-2-12, 3195 - 3198	K.GCEVVPDVNVSGQK.F	1489.63335	1	2.02E-05	0.70	2.36	-	752.2
AHQ-2-9, 3071	K.GCEVVPDVNVSGQK.F	1489.63335	1	3.46E-10	0.89	3.91	-	856.9
AHQ-2-12, 3189 - 3194	K.GCEVVPDVNVSGQK.F	1489.63335	2	2.50E-05	0.68	2.59	-	630.5
AHQ-2-13, 6711	R.GEELDEDLFLQLTGGHEAF	2121.24574	2	1.52E-06	0.89	4.19	-	759.1
AHQ-2-14-, 6713 - 6787	R.GEELDEDLFLQLTGGHEAF	2121.24574	2	6.33E-06	0.92	4.13	-	1074.4
AHQ-2-6, 7150	R.GEELDEDLFLQLTGGHEAF	2121.24574	2	3.94E-06	0.80	3.55	-	565.3
AHQ-2-12, 7047 - 7115	R.GEELDEDLFLQLTGGHEAF	2121.24574	2	1.33E-05	0.73	3.23	-	658.3
AHQ-2-6, 3765 - 3786	R.GMPAHFSDSAQTEACYHMLSR.P	2398.64117	3	6.15E-06	0.95	5.42	-	875.3
AHQ-2-10, 3562	R.IDLAVGDVVK.T	1029.21223	1	3.40E-04	0.57	2.15	-	636.3
AHQ-2-13, 3885	R.IDLAVGDVVK.T	1029.21223	2	1.08E-04	0.93	3.65	-	1275.2
AHQ-2-13-, 3910	R.IDLAVGDVVK.T	1029.21223	1	4.44E-05	0.64	2.61	-	582.4
AHQ-2-7, 3629	R.IDLAVGDVVK.T	1029.21223	1	7.50E-04	0.20	2.38	-	204.6
AHQ-2-6, 3635 - 3705	R.IDLAVGDVVK.T	1029.21223	1	4.38E-04	0.22	2.27	-	193.8
AHQ-2-12, 3773	R.IDLAVGDVVK.T	1029.21223	1	7.74E-04	0.28	2.12	-	516.5
AHQ-2-6, 3621 - 3697	R.IDLAVGDVVK.T	1029.21223	2	3.45E-04	0.90	3.81	-	918.2
AHQ-2-6, 3625 - 3693	R.IDLAVGDVVK.T	1029.21223	1	2.85E-04	0.22	1.91	-	488.9
AHQ-2-12, 3781	R.IDLAVGDVVK.T	1029.21223	1	2.62E-04	0.63	2.83	-	511.7
AHQ-2-12, 4437 - 4447	R.ILEAHQNVAQLSLAEAQLR.F	2105.38372	2	5.49E-08	0.99	6.99	-	2278.1
AHQ-2-12, 4434	R.ILEAHQNVAQLSLAEAQLR.F	2105.38372	3	1.18E-06	0.96	5.31	-	1846.4
AHQ-2-6, 4253 - 4321	R.ILEAHQNVAQLSLAEAQLR.F	2105.38372	2	7.49E-09	0.98	6.82	-	2138.3
AHQ-2-6, 4247 - 4319	R.ILEAHQNVAQLSLAEAQLR.F	2105.38372	3	5.09E-07	0.94	5.58	-	1158.9
AHQ-2-1, 4766	R.ILEAHQNVAQLSLAEAQLR.F	2105.38372	2	1.36E-11	0.98	5.59	-	1992.0
AHQ-2-9, 4375	R.ILEAHQNVAQLSLAEAQLR.F	2105.38372	2	1.00E-04	0.96	4.92	-	1503.5
AHQ-2-2, 4694	R.ILEAHQNVAQLSLAEAQLR.F	2105.38372	2	5.57E-13	0.98	6.59	-	1781.4
AHQ-2-9, 4223 - 4302	R.ILEAHQNVAQLSLAEAQLR.F	2105.38372	2	2.79E-12	0.98	7.01	-	2046.0
AHQ-2-11, 4249	R.ILEAHQNVAQLSLAEAQLR.F	2105.38372	2	1.83E-07	0.98	6.47	-	2525.1
AHQ-2-13-, 4463	R.ILEAHQNVAQLSLAEAQLR.F	2105.38372	2	8.01E-05	0.98	6.03	-	1485.5
AHQ-2-3, 4663	R.ILEAHQNVAQLSLAEAQLR.F	2105.38372	2	1.45E-05	0.97	4.79	-	1670.2
AHQ-2-13, 4401	R.ILEAHQNVAQLSLAEAQLR.F	2105.38372	2	6.07E-08	0.97	5.10	-	1913.5
AHQ-2-4, 4657	R.ILEAHQNVAQLSLAEAQLR.F	2105.38372	2	8.35E-11	0.98	5.61	-	1549.0
AHQ-2-7, 4179	R.ILEAHQNVAQLSLAEAQLR.F	2105.38372	2	1.73E-06	0.98	6.48	-	1909.8
AHQ-2-6, 4397	R.ILEAHQNVAQLSLAEAQLR.F	2105.38372	2	9.64E-12	0.98	5.93	-	1911.5
AHQ-2-13, 2724 - 2783	R.KDEILGIANNR.L	1243.39499	2	1.54E-05	0.96	3.96	-	1452.3
AHQ-2-6, 2305	R.KDEILGIANNR.L	1243.39499	1	1.32E-05	0.47	2.23	-	292.0
AHQ-2-9, 2355	R.KDEILGIANNR.L	1243.39499	2	2.09E-04	0.89	3.52	-	893.1
AHQ-2-6, 2301	R.KDEILGIANNR.L	1243.39499	2	5.33E-04	0.95	3.87	-	1342.8
AHQ-2-9, 2352	R.KDEILGIANNR.L	1243.39499	1	1.84E-04	0.29	2.05	-	231.6
AHQ-2-13-, 2659 - 2669	R.KDEILGIANNR.L	1243.39499	1	3.83E-05	0.58	2.24	-	381.8
AHQ-2-13-, 2658 - 2665	R.KDEILGIANNR.L	1243.39499	2	6.27E-04	0.92	3.65	-	1046.2
AHQ-2-12, 2525 - 2593	R.KDEILGIANNR.L	1243.39499	1	6.52E-09	0.62	2.38	-	372.4
AHQ-2-6, 2393 - 2395	R.KDEILGIANNR.L	1243.39499	2	1.47E-07	0.90	3.58	-	1161.1
AHQ-2-6, 3645 - 3646	R.KQDWSDHAIWWEQK.R	1858.00504	3	9.39E-04	0.70	3.43	-	721.4
AHQ-2-6, 3669 - 3677	R.KQDWSDHAIWWEQK.R	1858.00504	2	2.51E-04	0.90	4.15	-	909.6
AHQ-2-6, 6666 - 6737	K.LEGSAPTDVLDSLTTIPELK.D	2100.35256	2	1.02E-07	0.95	4.65	-	1010.3
AHQ-2-7, 6780	K.LEGSAPTDVLDSLTTIPELK.D	2100.35256	2	2.25E-06	0.86	3.84	-	620.8
AHQ-2-1, 6857 - 6937	K.LEGSAPTDVLDSLTTIPELK.D	2100.35256	2	6.32E-07	0.85	3.52	-	742.9
AHQ-2-14-, 6536	K.LEGSAPTDVLDSLTTIPELK.D	2100.35256	2	1.73E-04	0.87	3.94	-	555.7
AHQ-2-10, 6224 - 6264	K.LEGSAPTDVLDSLTTIPELK.D	2100.35256	2	9.02E-05	0.90	4.33	-	593.5
AHQ-2-8, 6648	K.LEGSAPTDVLDSLTTIPELK.D	2100.35256	2	8.68E-05	0.90	4.15	-	620.7
AHQ-2-6, 5599 - 5601	K.LEGSAPTDVLDSLTTIPELK.D	2100.35256	2	1.28E-06	0.95	4.59	-	1095.7
AHQ-2-6, 6253 - 6255	K.LEGSAPTDVLDSLTTIPELK.D	2100.35256	2	1.65E-07	0.94	4.84	-	753.0
AHQ-2-6, 6387 - 6454	K.LEGSAPTDVLDSLTTIPELK.D	2100.35256	2	3.43E-05	0.72	3.27	-	603.6
AHQ-2-9, 6840	K.LEGSAPTDVLDSLTTIPELK.D	2100.35256	2	4.32E-06	0.88	4.07	-	528.6

AHQ-2-6, 6554	K.LEGSAPTDVLDSLTTIPELK.D	2100.35256	2	2.49E-06	0.92	3.94	-	968.3
AHQ-2-6, 6141 - 6153	K.LEGSAPTDVLDSLTTIPELKDHLR.I	2621.92493	3	9.15E-11	0.93	5.24	-	693.7
AHQ-2-11, 4648	K.LLVPSPEGM*SEIYLR.C	1721.01141	2	7.81E-07	0.51	2.64	-	174.9
AHQ-2-1, 5062	K.LLVPSPEGM*SEIYLR.C	1721.01141	2	4.42E-04	0.37	2.61	-	182.1
AHQ-2-6, 5421	K.LLVPSPEGMSEIYLR.C	1705.01201	2	9.57E-07	0.63	2.61	-	443.2
AHQ-2-11, 5574 - 5576	K.LLVPSPEGMSEIYLR.C	1705.01201	2	5.01E-08	0.78	3.19	-	361.4
AHQ-2-6, 5721 - 5785	K.LLVPSPEGMSEIYLR.C	1705.01201	2	2.21E-10	0.88	3.36	-	599.2
AHQ-2-13, 5653	K.LLVPSPEGMSEIYLR.C	1705.01201	2	1.98E-05	0.66	2.75	-	352.0
AHQ-2-14-, 5776	K.LLVPSPEGMSEIYLR.C	1705.01201	2	2.77E-08	0.72	2.76	-	320.4
AHQ-2-6, 6913 - 6982	K.LSQSGEVGEPAGTDPGLDDLDVALSNLEVK.L	3027.24073	3	9.78E-06	0.95	5.21	-	1482.5
AHQ-2-7, 7068	K.LSQSGEVGEPAGTDPGLDDLDVALSNLEVK.L	3027.24073	3	4.19E-05	0.94	4.50	-	1501.2
AHQ-2-6, 7005	K.LSQSGEVGEPAGTDPGLDDLDVALSNLEVK.L	3027.24073	3	7.70E-07	0.91	4.30	-	1102.2
AHQ-2-7, 2056 - 2077	R.LTQLYEQAR.W	1122.25622	2	1.24E-06	0.91	3.35	-	1307.9
AHQ-2-12, 2298	R.LTQLYEQAR.W	1122.25622	2	8.15E-07	0.81	3.28	-	938.0
AHQ-2-13-, 2338	R.LTQLYEQAR.W	1122.25622	2	4.94E-05	0.84	3.51	-	892.2
AHQ-2-6, 2089 - 2165	R.LTQLYEQAR.W	1122.25622	2	5.01E-06	0.89	3.79	-	884.1
AHQ-2-9, 2140	R.LTQLYEQAR.W	1122.25622	2	8.72E-04	0.89	3.21	-	1134.4
AHQ-2-6, 4837	R.QWLLQTHWTLDK.Y	1569.78807	2	6.50E-05	0.31	2.70	-	200.4
AHQ-2-14-, 3979 - 3992	K.SQDEAPGDPIQQLNLK.G	1753.89174	2	6.66E-08	0.90	4.16	-	575.5
AHQ-2-6, 3882 - 3949	K.SQDEAPGDPIQQLNLK.G	1753.89174	2	6.29E-07	0.88	4.37	-	447.3
AHQ-2-11, 3853 - 3912	K.SQDEAPGDPIQQLNLK.G	1753.89174	2	9.39E-05	0.86	3.72	-	577.3
AHQ-2-13, 4083 - 4087	K.SQDEAPGDPIQQLNLK.G	1753.89174	2	1.01E-04	0.85	3.44	-	620.7
AHQ-2-6, 5343	K.SQDEAPGDPIQQLNLKGCEVVPDVNVSGQK.F	3224.50249	3	2.17E-07	0.86	4.45	-	611.4
AHQ-2-11, 4202	K.TASGDYIDSSWELR.V	1600.66783	2	9.20E-07	0.89	3.50	-	750.7
AHQ-2-9, 4256 - 4295	K.TASGDYIDSSWELR.V	1600.66783	2	2.12E-06	0.91	3.52	-	953.9
AHQ-2-6, 4229 - 4297	K.TASGDYIDSSWELR.V	1600.66783	2	4.21E-11	0.96	4.72	-	1077.2
AHQ-2-11, 3645	R.TGSGGPGNHPHGPDASAEGLNPYGLVAPR.F	2783.95312	3	5.63E-04	0.96	5.86	-	1382.1
AHQ-2-6, 3678 - 3754	R.TGSGGPGNHPHGPDASAEGLNPYGLVAPR.F	2783.95312	2	3.10E-04	0.84	3.97	-	488.3
AHQ-2-6, 3755 - 3831	R.TGSGGPGNHPHGPDASAEGLNPYGLVAPR.F	2783.95312	3	3.74E-04	0.96	5.54	-	1565.0
AHQ-2-6, 3541 - 3562	R.TGSGGPGNHPHGPDASAEGLNPYGLVAPR.F	2783.95312	2	8.19E-04	0.92	4.56	-	796.6
AHQ-2-6, 3534 - 3602	R.TGSGGPGNHPHGPDASAEGLNPYGLVAPR.F	2783.95312	3	7.92E-12	0.95	5.79	-	1055.3
AHQ-2-10, 3532	R.TGSGGPGNHPHGPDASAEGLNPYGLVAPR.F	2783.95312	2	3.42E-04	0.90	4.07	-	651.4
AHQ-2-9, 3559 - 3636	R.TGSGGPGNHPHGPDASAEGLNPYGLVAPR.F	2783.95312	2	2.47E-05	0.91	4.79	-	472.2
AHQ-2-4, 3920	R.TGSGGPGNHPHGPDASAEGLNPYGLVAPR.F	2783.95312	3	9.74E-04	0.96	5.73	-	1569.8
AHQ-2-11, 3652	R.TGSGGPGNHPHGPDASAEGLNPYGLVAPR.F	2783.95312	2	2.64E-06	0.94	5.09	-	739.9
AHQ-2-8, 3774 - 3851	R.VFVGEEDPEAESVTLR.V	1777.91007	2	1.63E-04	0.91	4.18	-	942.4
AHQ-2-1, 4236 - 4244	R.VFVGEEDPEAESVTLR.V	1777.91007	2	1.67E-08	0.95	4.28	-	1286.6
AHQ-2-2, 4144 - 4212	R.VFVGEEDPEAESVTLR.V	1777.91007	2	4.21E-10	0.95	5.03	-	1221.8
AHQ-2-14, 4830 - 4909	R.VFVGEEDPEAESVTLR.V	1777.91007	2	1.35E-05	0.94	4.20	-	1313.1
AHQ-2-7, 3856	R.VFVGEEDPEAESVTLR.V	1777.91007	2	1.61E-07	0.91	3.60	-	1160.2
AHQ-2-4, 4133 - 4152	R.VFVGEEDPEAESVTLR.V	1777.91007	2	6.75E-10	0.96	4.21	-	1579.2
AHQ-2-10, 3734	R.VFVGEEDPEAESVTLR.V	1777.91007	2	1.04E-06	0.89	3.99	-	851.8
AHQ-2-14-, 3920 - 3923	R.VFVGEEDPEAESVTLR.V	1777.91007	2	1.87E-09	0.96	4.18	-	1727.2
AHQ-2-12, 4019 - 4021	R.VFVGEEDPEAESVTLR.V	1777.91007	2	1.94E-07	0.96	4.74	-	1181.5
AHQ-2-13, 4004	R.VFVGEEDPEAESVTLR.V	1777.91007	2	1.06E-08	0.92	4.07	-	1049.2
AHQ-2-11, 3860	R.VFVGEEDPEAESVTLR.V	1777.91007	2	1.53E-08	0.95	4.31	-	1417.3
AHQ-2-3, 4171	R.VFVGEEDPEAESVTLR.V	1777.91007	2	4.00E-11	0.97	5.03	-	1406.3
AHQ-2-13-, 4034	R.VFVGEEDPEAESVTLR.V	1777.91007	2	1.80E-10	0.94	4.35	-	1243.6
AHQ-2-9, 3863 - 3943	R.VFVGEEDPEAESVTLR.V	1777.91007	2	2.81E-06	0.94	4.13	-	1334.9
AHQ-2-6, 3861 - 3930	R.VFVGEEDPEAESVTLR.V	1777.91007	2	9.65E-11	0.97	5.07	-	1400.3
AHQ-2-5, 3937	R.VFVGEEDPEAESVTLR.V	1777.91007	2	1.68E-06	0.96	4.54	-	1466.2
AHQ-2-13, 2889	R.VTGESHIGGVLLK.I	1310.52448	2	3.31E-07	0.96	3.97	-	1935.8
AHQ-2-11, 2669 - 2737	R.VTGESHIGGVLLK.I	1310.52448	2	3.77E-04	0.77	3.04	-	923.9
AHQ-2-9, 2487	R.VTGESHIGGVLLK.I	1310.52448	1	2.59E-08	0.84	3.12	-	852.6
AHQ-2-13-, 2835	R.VTGESHIGGVLLK.I	1310.52448	1	2.01E-04	0.35	2.69	-	338.2
AHQ-2-13-, 2833	R.VTGESHIGGVLLK.I	1310.52448	2	3.76E-07	0.96	4.52	-	1634.8
AHQ-2-1, 2953	R.VTGESHIGGVLLK.I	1310.52448	2	1.19E-06	0.96	3.83	-	1896.5
AHQ-2-6, 2461	R.VTGESHIGGVLLK.I	1310.52448	1	4.67E-08	0.84	3.44	-	703.3
AHQ-2-6, 2453	R.VTGESHIGGVLLK.I	1310.52448	2	1.51E-07	0.95	4.17	-	1524.2
AHQ-2-9, 2478 - 2486	R.VTGESHIGGVLLK.I	1310.52448	2	4.58E-07	0.96	4.16	-	1744.9
AHQ-2-7, 2379	R.VTGESHIGGVLLK.I	1310.52448	2	4.34E-08	0.93	4.04	-	1322.2
AHQ-2-12, 2714	R.VTGESHIGGVLLK.I	1310.52448	2	1.31E-07	0.87	3.45	-	1144.7
AHQ-2-14-, 2743 - 2744	R.VTGESHIGGVLLK.I	1310.52448	2	2.28E-09	0.98	4.64	-	2316.6
AHQ-2-5, 4696	K.VVLAGGVAPALFR.G	1270.54832	2	7.96E-08	0.97	4.84	-	1811.2
AHQ-2-1, 5121	K.VVLAGGVAPALFR.G	1270.54832	2	1.01E-07	0.98	5.26	-	2067.4
AHQ-2-9, 4648	K.VVLAGGVAPALFR.G	1270.54832	2	4.26E-06	0.89	2.99	-	1433.8
AHQ-2-9, 4588	K.VVLAGGVAPALFR.G	1270.54832	1	6.40E-04	0.63	3.13	-	154.7
AHQ-2-3, 5036	K.VVLAGGVAPALFR.G	1270.54832	2	8.93E-06	0.94	3.73	-	1406.0
AHQ-2-11, 4630	K.VVLAGGVAPALFR.G	1270.54832	2	5.01E-07	0.96	4.60	-	1423.7
AHQ-2-6, 4593 - 4673	K.VVLAGGVAPALFR.G	1270.54832	2	1.82E-08	0.97	5.04	-	1849.4
AHQ-2-6, 4611	K.VVLAGGVAPALFR.G	1270.54832	1	7.67E-05	0.21	1.95	-	116.3
AHQ-2-7, 4493	K.VVLAGGVAPALFR.G	1270.54832	2	1.88E-08	0.97	4.86	-	1674.1
AHQ-2-10, 4366	K.VVLAGGVAPALFR.G	1270.54832	2	2.99E-07	0.95	4.12	-	1452.3
AHQ-2-6, 2282	K.YGILADAR.L	878.99544	2	2.74E-05	0.94	3.74	-	1146.1
AHQ-2-7, 5217	K.YYSFFDLDPK.T	1295.42046	2	5.47E-05	0.93	3.21	-	942.3
AHQ-2-6, 5226	K.YYSFFDLDPK.T	1295.42046	2	1.68E-04	0.87	3.92	-	798.5
AHQ-2-9, 5227	K.YYSFFDLDPK.T	1295.42046	2	1.84E-06	0.81	3.50	-	648.3
gi|5031857|ref|NP_005557.1| lactate dehydrogenase A [Homo sapiens]				5.59E-13	4.49	60.28	24.70	36688.5
AHQ-2-13, 6651	K.DLADELALVDVIEDK.L	1658.82864	2	1.62E-10	0.97	5.29	-	1370.3
AHQ-2-9, 7156 - 7236	K.DLADELALVDVIEDK.L	1658.82864	2	5.59E-13	0.97	5.12	-	1751.1
AHQ-2-13-, 6861	K.DLADELALVDVIEDK.L	1658.82864	2	5.90E-11	0.97	5.69	-	1327.0
AHQ-2-14-, 6717 - 6727	K.DLADELALVDVIEDK.L	1658.82864	2	5.41E-05	0.90	3.16	-	1569.1
AHQ-2-9, 7383	K.DLADELALVDVIEDKLK.G	1900.15980	2	2.72E-07	0.96	4.93	-	1662.9
AHQ-2-9, 4770	K.DQLIYNLLKEEQTPQNK.I	2075.30802	2	3.59E-06	0.93	4.49	-	1413.1
AHQ-2-9, 7419	K.GLYGIKDDVFLSVPCILGQNGISDLVK.V	2923.37318	2	6.73E-07	0.88	4.65	-	339.1
AHQ-2-9, 3128	K.QVVESAYEVIK.L	1265.43734	2	7.66E-04	0.59	2.71	-	337.5
AHQ-2-11, 2069 - 2072	K.VTLTSEEEAR.L	1135.20693	2	1.52E-04	0.85	2.79	-	979.2
AHQ-2-11, 2073 - 2074	K.VTLTSEEEAR.L	1135.20693	1	4.05E-06	0.15	2.20	-	161.8
gi|21361478|ref|NP_055944.2| septin 6 isoform B; septin 2 [Homo sapiens]				6.53E-13	3.80	40.33	15.90	49716.3
AHQ-2-7, 3781	K.FEGEPATHTQPGVQLQSNTYDLQESNVR.L	3147.31508	3	6.53E-13	0.98	6.64	-	1909.6
AHQ-2-7, 2673	R.KTAAELLQSQGSQAGGSQTLK.R	2104.30776	2	5.46E-04	0.91	4.21	-	631.0
AHQ-2-7, 2879 - 2880	K.TAAELLQSQGSQAGGSQTLK.R	1976.13485	2	1.25E-08	0.96	4.81	-	1320.2
AHQ-2-7, 4876	R.TVPLAGHVGFDSLPDQLVNK.S	2108.38284	2	6.46E-07	0.96	5.21	-	1011.5
gi|13124879|ref|NP_002465.1| smooth muscle myosin heavy chain 11 isoform SM1 [Homo sapiens]				8.93E-13	9.68	110.39	6.10	227336.7
AHQ-2-3, 4949	R.DLGEELEALK.T	1117.23151	2	1.78E-04	0.94	3.95	-	1089.3
AHQ-2-2, 4986	R.DLGEELEALK.T	1117.23151	2	4.61E-05	0.90	3.42	-	921.8
AHQ-2-3, 4948	R.DLGEELEALK.T	1117.23151	1	3.00E-04	0.32	2.72	-	354.2
AHQ-2-6, 2610	R.EDQSILCTGESGAGK.T	1553.63107	2	5.41E-04	0.66	3.09	-	413.1
AHQ-2-3, 2764	R.EDQSILCTGESGAGK.T	1553.63107	2	2.17E-04	0.90	3.80	-	839.3
AHQ-2-3, 2801	K.FDQLLAEEK.N	1093.21165	1	1.48E-06	0.64	2.47	-	660.4
AHQ-2-2, 2810	K.FDQLLAEEK.N	1093.21165	1	4.37E-05	0.69	2.67	-	755.9
AHQ-2-2, 2804	K.FDQLLAEEK.N	1093.21165	2	1.69E-05	0.92	3.24	-	1487.8
AHQ-2-3, 2796	K.FDQLLAEEK.N	1093.21165	2	1.61E-05	0.93	3.09	-	1569.4
AHQ-2-1, 2977	K.FDQLLAEEK.N	1093.21165	2	1.24E-04	0.93	3.18	-	1522.2
AHQ-2-4, 2332	K.KEEELQAALAR.L	1258.40643	2	4.40E-04	0.93	3.40	-	1591.0
AHQ-2-2, 2349	K.KEEELQAALAR.L	1258.40643	2	7.10E-05	0.86	3.53	-	971.6
AHQ-2-3, 2324 - 2335	K.KEEELQAALAR.L	1258.40643	2	3.28E-04	0.76	3.08	-	855.6

AHQ-2-1, 2564	K.KEEELQAALAR.L	1258.40643	3	1.49E-05	0.90	3.64	-	1465.1
AHQ-2-6, 2454	R.KFDQLLAEEK.N	1221.38456	2	6.46E-04	0.87	3.12	-	1131.2
AHQ-2-5, 2423	R.KFDQLLAEEK.N	1221.38456	1	2.22E-05	0.31	1.99	-	542.2
AHQ-2-6, 2414 - 2447	R.KFDQLLAEEK.N	1221.38456	1	4.50E-04	0.19	1.81	-	218.5
AHQ-2-2, 2576 - 2628	R.KFDQLLAEEK.N	1221.38456	2	1.97E-05	0.92	3.26	-	1274.8
AHQ-2-3, 2627	R.KFDQLLAEEK.N	1221.38456	2	5.51E-04	0.81	3.26	-	820.5
AHQ-2-2, 5386 - 5392	K.LQQLFNHTM*FILEQEEYQR.E	2484.77198	3	5.35E-12	0.99	7.79	-	3672.9
AHQ-2-2, 5384 - 5385	K.LQQLFNHTM*FILEQEEYQR.E	2484.77198	2	4.22E-07	0.93	4.62	-	642.5
AHQ-2-5, 5788 - 5868	K.LQQLFNHTMFILEQEEYQR.E	2468.77258	2	4.12E-08	0.89	3.26	-	1133.5
AHQ-2-4, 6186	K.LQQLFNHTMFILEQEEYQR.E	2468.77258	2	1.39E-07	0.96	4.30	-	1672.3
AHQ-2-4, 6185	K.LQQLFNHTMFILEQEEYQR.E	2468.77258	3	5.24E-10	0.98	6.08	-	2523.6
AHQ-2-2, 6180	K.LQQLFNHTMFILEQEEYQR.E	2468.77258	2	1.96E-08	0.94	4.03	-	1406.0
AHQ-2-2, 6184 - 6188	K.LQQLFNHTMFILEQEEYQR.E	2468.77258	3	2.44E-11	0.98	6.45	-	2786.2
AHQ-2-2, 6270	K.LQQLFNHTMFILEQEEYQR.E	2468.77258	3	2.29E-12	0.98	6.47	-	2733.8
AHQ-2-2, 6290 - 6370	K.LQQLFNHTMFILEQEEYQR.E	2468.77258	2	2.93E-09	0.98	5.89	-	1842.8
AHQ-2-5, 5792	K.LQQLFNHTMFILEQEEYQR.E	2468.77258	3	5.96E-06	0.97	4.54	-	2616.9
AHQ-2-2, 6450 - 6521	K.LQQLFNHTMFILEQEEYQR.E	2468.77258	2	7.15E-09	0.98	5.70	-	2034.3
AHQ-2-2, 6506	K.LQQLFNHTMFILEQEEYQR.E	2468.77258	3	2.32E-09	0.98	5.48	-	2685.3
AHQ-2-3, 5348	K.LQQLFNHTM*FILEQEEYQR.E	2484.77198	3	3.68E-07	0.98	6.43	-	2249.0
AHQ-2-5, 5983 - 6027	K.LQQLFNHTMFILEQEEYQR.E	2468.77258	3	9.87E-11	0.97	5.50	-	1755.7
AHQ-2-1, 5430 - 5498	K.LQQLFNHTM*FILEQEEYQR.E	2484.77198	3	3.80E-10	0.98	6.48	-	3212.2
AHQ-2-3, 5343	K.LQQLFNHTM*FILEQEEYQR.E	2484.77198	2	8.41E-04	0.85	3.44	-	834.2
AHQ-2-5, 6219	K.LQQLFNHTMFILEQEEYQR.E	2468.77258	2	5.23E-04	0.83	3.47	-	809.4
AHQ-2-1, 6229 - 6250	K.LQQLFNHTMFILEQEEYQR.E	2468.77258	3	1.02E-11	0.98	5.90	-	2586.4
AHQ-2-1, 5745	K.LQQLFNHTM*FILEQEEYQR.E	2484.77198	3	3.94E-09	0.93	4.62	-	1374.9
AHQ-2-2, 5502	K.LQQLFNHTM*FILEQEEYQR.E	2484.77198	3	3.14E-10	0.98	6.21	-	2073.1
AHQ-2-3, 2572 - 2659	R.NCAAYLK.L	840.96726	1	7.70E-04	0.60	2.19	-	616.2
AHQ-2-1, 2421	R.NCAAYLK.L	840.96726	1	4.94E-04	0.72	1.87	-	996.7
AHQ-2-2, 1618	R.NTDQASMPDNTAAQK.V	1592.67074	2	2.28E-04	0.81	3.75	-	429.9
AHQ-2-2, 6573	K.QLLQANPILEAFGNAK.T	1727.98540	2	5.82E-06	0.92	4.02	-	836.8
AHQ-2-9, 6080 - 6146	K.QLLQANPILEAFGNAK.T	1727.98540	2	1.95E-05	0.88	3.71	-	665.8
AHQ-2-3, 6416 - 6431	K.QLLQANPILEAFGNAK.T	1727.98540	2	1.52E-06	0.94	4.27	-	808.4
AHQ-2-3, 6575	K.QLLQANPILEAFGNAK.T	1727.98540	2	5.16E-10	0.96	4.43	-	1120.0
AHQ-2-2, 6354 - 6432	K.QLLQANPILEAFGNAK.T	1727.98540	2	1.95E-08	0.96	4.70	-	1033.3
AHQ-2-7, 5992 - 5997	K.QLLQANPILEAFGNAK.T	1727.98540	2	5.39E-07	0.94	4.14	-	927.6
AHQ-2-6, 6077	K.QLLQANPILEAFGNAK.T	1727.98540	2	2.12E-08	0.96	4.77	-	1078.5
AHQ-2-5, 6352	K.QLLQANPILEAFGNAK.T	1727.98540	2	4.04E-10	0.95	4.45	-	1035.0
AHQ-2-1, 6396 - 6476	K.QLLQANPILEAFGNAK.T	1727.98540	2	9.52E-07	0.94	4.42	-	877.8
AHQ-2-4, 6450 - 6465	K.QLLQANPILEAFGNAK.T	1727.98540	2	1.02E-07	0.95	4.72	-	925.6
AHQ-2-5, 6109 - 6119	K.QLLQANPILEAFGNAK.T	1727.98540	2	1.94E-07	0.96	4.87	-	1103.5
AHQ-2-1, 6582	K.QLLQANPILEAFGNAK.T	1727.98540	2	2.15E-08	0.89	4.07	-	583.6
AHQ-2-9, 6223 - 6296	K.QLLQANPILEAFGNAK.T	1727.98540	2	1.46E-05	0.55	2.69	-	460.5
AHQ-2-2, 4166	K.TQLEELEDELQATEDAK.L	1963.04334	2	1.54E-06	0.93	4.34	-	985.2
AHQ-2-5, 5651 - 5668	K.TQLEELEDELQATEDAK.L	1963.04334	2	6.78E-06	0.98	6.13	-	1926.0
AHQ-2-2, 5924 - 5996	K.TQLEELEDELQATEDAK.L	1963.04334	2	1.30E-09	0.98	6.91	-	2060.3
AHQ-2-2, 5948	K.TQLEELEDELQATEDAK.L	1963.04334	3	1.53E-05	0.96	5.74	-	1213.1
AHQ-2-2, 6145 - 6213	K.TQLEELEDELQATEDAK.L	1963.04334	2	8.91E-05	0.91	4.05	-	849.7
AHQ-2-1, 6112	K.TQLEELEDELQATEDAK.L	1963.04334	2	8.93E-13	0.98	5.90	-	1809.3
AHQ-2-1, 5928	K.TQLEELEDELQATEDAK.L	1963.04334	3	1.44E-09	0.96	5.62	-	1448.5
AHQ-2-1, 5924	K.TQLEELEDELQATEDAK.L	1963.04334	2	5.15E-06	0.98	4.88	-	2195.2
AHQ-2-6, 5565 - 5566	K.TQLEELEDELQATEDAK.L	1963.04334	2	1.08E-06	0.98	5.98	-	2223.6
AHQ-2-4, 5885 - 5956	K.TQLEELEDELQATEDAK.L	1963.04334	2	1.60E-09	0.99	7.39	-	2442.5
AHQ-2-7, 5625	K.TQLEELEDELQATEDAK.L	1963.04334	2	4.01E-11	0.98	6.37	-	2566.8
AHQ-2-4, 4117	K.TQLEELEDELQATEDAK.L	1963.04334	2	1.27E-04	0.85	3.42	-	769.8
AHQ-2-3, 5904	K.TQLEELEDELQATEDAK.L	1963.04334	3	1.68E-06	0.97	6.00	-	1283.3
AHQ-2-3, 4132	K.TQLEELEDELQATEDAK.L	1963.04334	2	3.75E-05	0.97	5.53	-	1712.2
gi|4557305|ref|NP_000025.1|	aldolase A; fructose-bisphosphate aldolase; Aldolase A, fructose-bispho			9.87E-13	8.68	100.28	47.30	39419.7
AHQ-2-9, 2056	R.ALANSLACQGK.Y	1134.28859	2	1.64E-04	0.81	2.80	-	849.4
AHQ-2-8, 3880	K.ALSDHHIYLEGTLLKPNMVTPGHACTQK.F	3134.57879	3	1.10E-06	0.96	5.59	-	1283.8
AHQ-2-8, 3790	K.ALSDHHIYLEGTLLKPNMVTPGHACTQK.F	3134.57879	3	2.38E-09	0.94	5.59	-	709.0
AHQ-2-9, 6098 - 6103	K.CPLLKPWALTFSYGR.A	1811.13929	2	2.69E-09	0.93	4.42	-	641.1
AHQ-2-8, 5974 - 6044	K.CPLLKPWALTFSYGR.A	1811.13929	2	7.10E-06	0.94	4.55	-	712.9
AHQ-2-9, 4106	K.FSHEEIAMATVTALR.R	1676.91875	2	9.87E-13	0.97	5.14	-	1455.0
AHQ-2-8, 3883	K.FSHEEIAMATVTALR.R	1676.91875	2	5.56E-11	0.90	3.82	-	1073.6
AHQ-2-11, 2821	K.GILAADESTGSIAK.R	1333.47008	2	4.08E-06	0.95	3.54	-	1612.3
AHQ-2-8, 2543 - 2586	K.GILAADESTGSIAK.R	1333.47008	2	7.30E-10	0.97	4.99	-	1435.9
AHQ-2-9, 2720	K.GILAADESTGSIAK.R	1333.47008	1	2.62E-04	0.40	2.72	-	292.8
AHQ-2-12, 2861	K.GILAADESTGSIAK.R	1333.47008	2	2.12E-07	0.94	3.86	-	1219.9
AHQ-2-9, 4250	K.GVVPLAGTNGETTTQGLDGLSER.C	2273.44329	2	3.02E-06	0.65	3.77	-	469.9
AHQ-2-9, 5264	K.IGEHTPSALAIMENANVLAR.Y	2108.40788	2	9.13E-05	0.96	4.99	-	951.8
AHQ-2-10, 4944	K.IGEHTPSALAIMENANVLAR.Y	2108.40788	2	2.21E-04	0.80	3.56	-	551.2
AHQ-2-8, 4975 - 5003	K.IGEHTPSALAIMENANVLAR.Y	2108.40788	2	1.08E-04	0.95	4.86	-	868.6
AHQ-2-9, 4966	K.IGEHTPSALAIMENANVLAR.Y	2108.40788	2	2.16E-08	0.93	4.32	-	880.3
AHQ-2-8, 6206 - 6275	R.VNPCIGGVILFHETLYQK.A	2090.43072	2	9.27E-08	0.93	4.29	-	805.8
AHQ-2-9, 5720	R.YASICQQNGIVPIVEPEILPDGDHDLKR.C	3179.54969	3	4.11E-04	0.69	3.66	-	250.8
AHQ-2-8, 4218 - 4288	K.YTPSGQAGAAASESLFVSNHAY	2229.34753	2	3.96E-06	0.84	3.97	-	408.6
gi|20535366|ref|XP_053177.4|	similar to Tubulin alpha-3/alpha-7 chain (Alpha-tubulin 3/7) [Homo sap			1.44E-12	2.78	30.23	9.60	49858.2
AHQ-2-7, 2847	K.DVNAAIATIK.T	1016.17355	2	1.20E-05	0.94	4.01	-	1199.5
AHQ-2-7, 3060	K.DVNAAIATIK.T	1016.17355	1	1.20E-05	0.25	1.98	-	378.0
AHQ-2-7, 3163	K.DVNAAIATIK.T	1016.17355	1	4.22E-04	0.46	2.07	-	629.7
AHQ-2-1, 5526	K.TIGGGDDSFNTFFSETGAGK.H	2009.07549	2	5.18E-06	0.52	2.68	-	409.4
AHQ-2-7, 4864	K.TIGGGDDSFNTFFSETGAGK.H	2009.07549	2	1.44E-12	0.89	4.27	-	413.5
AHQ-2-7, 5000	K.TIGGGDDSFNTFFSETGAGK.H	2009.07549	2	1.15E-07	0.89	4.11	-	578.8
AHQ-2-7, 5151 - 5225	K.TIGGGDDSFNTFFSETGAGK.H	2009.07549	2	1.52E-07	0.94	4.55	-	1007.6
AHQ-2-7, 5307 - 5372	K.TIGGGDDSFNTFFSETGAGK.H	2009.07549	2	1.03E-05	0.82	3.79	-	490.3
AHQ-2-7, 5375 - 5445	K.TIGGGDDSFNTFFSETGAGK.H	2009.07549	2	1.09E-04	0.72	3.18	-	428.4
AHQ-2-13-, 5270 - 5274	K.TIGGGDDSFNTFFSETGAGK.H	2009.07549	2	4.96E-08	0.91	4.20	-	694.4
AHQ-2-9, 5162	K.TIGGGDDSFNTFFSETGAGK.H	2009.07549	2	1.15E-06	0.83	3.72	-	492.5
AHQ-2-11, 5009 - 5080	K.TIGGGDDSFNTFFSETGAGK.H	2009.07549	2	6.54E-10	0.93	4.49	-	777.2
AHQ-2-12, 5249 - 5257	K.TIGGGDDSFNTFFSETGAGK.H	2009.07549	2	3.96E-08	0.90	3.63	-	861.2
AHQ-2-13-, 4877	K.TIGGGDDSFNTFFSETGAGK.H	2009.07549	2	9.76E-08	0.67	3.43	-	312.5
AHQ-2-13, 5175	K.TIGGGDDSFNTFFSETGAGK.H	2009.07549	2	6.80E-08	0.58	3.10	-	294.6
AHQ-2-14, 5856	K.TIGGGDDSFNTFFSETGAGK.H	2009.07549	2	1.73E-08	0.82	3.86	-	367.4
AHQ-2-14, 6205 - 6265	K.TIGGGDDSFNTFFSETGAGK.H	2009.07549	2	1.17E-07	0.92	4.12	-	803.2
AHQ-2-14, 6324	K.TIGGGDDSFNTFFSETGAGK.H	2009.07549	2	1.42E-08	0.93	4.49	-	849.4
AHQ-2-14, 6516	K.TIGGGDDSFNTFFSETGAGK.H	2009.07549	2	4.71E-09	0.96	4.60	-	1433.0
AHQ-2-14-, 5167 - 5215	K.TIGGGDDSFNTFFSETGAGK.H	2009.07549	2	3.62E-06	0.93	4.52	-	761.9
AHQ-2-7, 5739 - 5740	R.TIQFVDWCPTGFK.V	1600.81899	2	2.75E-06	0.94	4.56	-	784.5
AHQ-2-12, 5742 - 5809	R.TIQFVDWCPTGFK.V	1600.81899	2	9.38E-06	0.67	3.00	-	478.9
gi|4758606|ref|NP_004508.1|	integrin-linked kinase [Homo sapiens]			1.57E-12	12.11	140.27	31.90	51418.9
AHQ-2-13-, 5710	R.EVPFADLSNMEIGMK.V	1681.95558	2	3.83E-04	0.91	3.64	-	545.8
AHQ-2-13, 5612 - 5689	R.EVPFADLSNMEIGMK.V	1681.95558	2	4.16E-06	0.91	4.13	-	555.9
AHQ-2-7, 4125 - 4195	R.EVPFADLSNM*EIGM*K.V	1713.95438	2	3.96E-05	0.70	2.89	-	489.0
AHQ-2-11, 5464 - 5532	R.EVPFADLSNMEIGMK.V	1681.95558	2	1.38E-05	0.88	4.39	-	579.0
AHQ-2-7, 4244 - 4279	K.FDM*IVPILEK.M	1221.49108	2	4.76E-05	0.85	2.76	-	749.1
AHQ-2-10, 5164	K.FDMIVPILEK.M	1205.49168	2	8.38E-04	0.65	2.51	-	619.2
AHQ-2-11, 2068 - 2077	R.GDDTPLHLAASHGHR.D	1584.67809	2	1.23E-07	0.93	3.95	-	1069.2

AHQ-2-7, 1651 - 1672	R.GDDTPLHLAASHGHR.D	1584.67809	2	3.42E-09	0.93	3.93	-	1099.2
AHQ-2-10, 1899 - 1903	R.GDDTPLHLAASHGHR.D	1584.67809	2	4.12E-07	0.92	3.86	-	1047.8
AHQ-2-9, 5722	R.GMAFLHTLEPLIPR.H	1595.93361	2	1.61E-05	0.91	3.66	-	915.1
AHQ-2-7, 4744	R.GM*AFLHTLEPLIPR.H	1611.93301	2	2.12E-05	0.90	3.54	-	869.8
AHQ-2-7, 5649	R.GMAFLHTLEPLIPR.H	1595.93361	3	9.44E-08	0.88	3.35	-	1247.5
AHQ-2-10, 4660	R.GM*AFLHTLEPLIPR.H	1611.93301	2	8.30E-05	0.87	3.48	-	619.0
AHQ-2-11, 4969	R.GM*AFLHTLEPLIPR.H	1611.93301	2	2.94E-05	0.89	3.48	-	773.6
AHQ-2-7, 1928 - 1936	K.LNENHSGELWK.G	1327.42732	2	2.28E-07	0.92	3.57	-	1292.8
AHQ-2-9, 6123	R.LWLDNTENDLNQGDDHGFSPLHWACR.E	3113.28217	3	1.19E-04	0.80	3.44	-	1133.5
AHQ-2-7, 6048 - 6117	R.LWLDNTENDLNQGDDHGFSPLHWACR.E	3113.28217	3	1.05E-05	0.95	5.35	-	1173.0
AHQ-2-11, 5796	R.LWLDNTENDLNQGDDHGFSPLHWACR.E	3113.28217	3	1.57E-12	0.93	4.54	-	1259.7
AHQ-2-14-, 5963 - 5973	R.LWLDNTENDLNQGDDHGFSPLHWACR.E	3113.28217	3	1.20E-09	0.95	5.48	-	1174.6
AHQ-2-11, 4146	R.M*YAPAWVAPEALQK.K	1591.85542	2	1.67E-04	0.91	3.38	-	830.2
AHQ-2-10, 3891 - 3956	R.M*YAPAWVAPEALQK.K	1591.85542	2	6.69E-05	0.89	3.37	-	637.0
AHQ-2-11, 4568 - 4576	R.MYAPAWVAPEALQK.K	1575.85602	2	8.29E-08	0.96	4.39	-	1070.3
AHQ-2-13-, 4805 - 4806	R.MYAPAWVAPEALQK.K	1575.85602	2	1.78E-06	0.94	3.72	-	1197.3
AHQ-2-13-, 4301	R.M*YAPAWVAPEALQK.K	1591.85542	2	2.56E-05	0.87	3.45	-	730.0
AHQ-2-10, 4338	R.MYAPAWVAPEALQK.K	1575.85602	2	2.48E-05	0.91	3.48	-	781.2
AHQ-2-12, 4294	R.M*YAPAWVAPEALQK.K	1591.85542	2	4.75E-04	0.61	2.55	-	441.8
AHQ-2-13, 4255	R.M*YAPAWVAPEALQK.K	1591.85542	2	6.33E-05	0.87	3.21	-	852.2
AHQ-2-11, 4014	R.SAVVEMLIM*R.G	1165.45256	2	1.81E-04	0.90	3.06	-	1388.4
AHQ-2-7, 4232 - 4307	K.VALEGLRPTIPPGISPHVCK.L	2143.53829	2	5.05E-09	0.96	5.02	-	920.4
AHQ-2-7, 4227	K.VALEGLRPTIPPGISPHVCK.L	2143.53829	3	8.90E-10	0.90	4.11	-	845.0
AHQ-2-11, 4385 - 4414	K.VALEGLRPTIPPGISPHVCK.L	2143.53829	2	5.86E-04	0.74	3.67	-	512.2
AHQ-2-7, 2688	R.WQGNDIVVK.V	1059.20012	1	9.76E-05	0.79	2.46	-	1005.6
gi|4504745|ref|NP_000410.1| integrin alpha 2b precursor [Homo sapiens]				1.85E-12	34.27	380.35	38.30	113374.2
AHQ-2-4, 1737	R.AEAQVELR.G	916.01408	2	1.06E-04	0.91	2.97	-	1311.3
AHQ-2-13-, 5307	R.AEGGQCPSLLFDLR.D	1564.74505	2	9.94E-05	0.83	3.12	-	810.7
AHQ-2-13, 5207	R.AEGGQCPSLLFDLR.D	1564.74505	2	8.54E-05	0.77	3.00	-	572.2
AHQ-2-1, 5485 - 5556	R.AEGGQCPSLLFDLR.D	1564.74505	2	1.04E-07	0.94	4.50	-	838.2
AHQ-2-11, 5020 - 5028	R.AEGGQCPSLLFDLR.D	1564.74505	2	1.00E-07	0.94	3.60	-	1189.0
AHQ-2-1, 5561	R.AEGGQCPSLLFDLR.D	1564.74505	2	7.23E-04	0.83	2.71	-	821.0
AHQ-2-10, 4814	R.AEGGQCPSLLFDLR.D	1564.74505	2	1.00E-09	0.96	4.85	-	1009.6
AHQ-2-4, 5653	R.AEGGQCPSLLFDLR.D	1564.74505	2	1.04E-11	0.95	4.00	-	1344.6
AHQ-2-9, 5156	R.AEGGQCPSLLFDLR.D	1564.74505	2	3.90E-04	0.85	3.61	-	533.6
AHQ-2-4, 5512	R.AEGGQCPSLLFDLR.D	1564.74505	2	6.33E-07	0.94	4.39	-	971.1
AHQ-2-4, 5130	R.AEGGQCPSLLFDLR.D	1564.74505	2	3.94E-04	0.86	3.36	-	932.3
AHQ-2-7, 5121	R.AEGGQCPSLLFDLR.D	1564.74505	2	3.17E-07	0.95	4.39	-	1113.6
AHQ-2-4, 5426 - 5496	R.AEGGQCPSLLFDLR.D	1564.74505	2	1.48E-08	0.96	5.15	-	1051.9
AHQ-2-6, 5129 - 5137	R.AEGGQCPSLLFDLR.D	1564.74505	2	3.45E-05	0.93	3.93	-	860.6
AHQ-2-5, 5140 - 5220	R.AEGGQCPSLLFDLR.D	1564.74505	2	1.24E-07	0.95	4.36	-	996.6
AHQ-2-4, 5065	R.AEGGQCPSLLFDLRDETR.N	2066.23814	3	1.67E-05	0.73	3.16	-	719.4
AHQ-2-4, 4946	R.AEGGQCPSLLFDLRDETR.N	2066.23814	3	5.33E-06	0.75	3.42	-	824.7
AHQ-2-4, 4934	R.AEGGQCPSLLFDLRDETR.N	2066.23814	2	5.10E-04	0.83	3.53	-	807.7
AHQ-2-6, 2562	R.ALSNVEGFER.L	1122.21308	2	1.42E-06	0.86	3.17	-	874.4
AHQ-2-4, 2709 - 2780	R.ALSNVEGFER.L	1122.21308	1	1.96E-04	0.14	2.38	-	137.9
AHQ-2-4, 2712 - 2781	R.ALSNVEGFER.L	1122.21308	2	2.69E-06	0.90	3.65	-	935.3
AHQ-2-4, 2716 - 2785	R.ALSNVEGFER.L	1122.21308	1	1.88E-05	0.30	2.63	-	98.5
AHQ-2-4, 2808	R.ALSNVEGFER.L	1122.21308	2	4.00E-06	0.69	2.89	-	639.0
AHQ-2-6, 2561	R.ALSNVEGFER.L	1122.21308	1	2.88E-06	0.19	2.06	-	192.2
AHQ-2-5, 2519	R.ALSNVEGFER.L	1122.21308	1	2.96E-04	0.14	2.17	-	132.0
AHQ-2-5, 2520	R.ALSNVEGFER.L	1122.21308	2	4.81E-06	0.77	2.70	-	893.8
AHQ-2-4, 3132	R.ALSNVEGFER.L	1122.21308	1	4.43E-04	0.30	2.59	-	279.6
AHQ-2-5, 2523	R.ALSNVEGFER.L	1122.21308	1	9.36E-04	0.08	1.81	-	54.0
AHQ-2-7, 2492	R.ALSNVEGFER.L	1122.21308	2	7.33E-07	0.85	3.21	-	790.1
AHQ-2-1, 2948	R.ALSNVEGFER.L	1122.21308	2	2.85E-05	0.83	3.10	-	686.7
AHQ-2-4, 5440	K.ASVQLLVQDSLNPAVK.S	1682.94293	2	4.33E-05	0.87	4.02	-	627.7
AHQ-2-4, 5280 - 5348	K.ASVQLLVQDSLNPAVK.S	1682.94293	2	2.49E-06	0.92	4.81	-	660.8
AHQ-2-4, 5144 - 5212	K.ASVQLLVQDSLNPAVK.S	1682.94293	2	2.41E-04	0.86	3.87	-	898.4
AHQ-2-4, 3224	R.DETRNVGSQTLQTFK.A	1724.85363	2	3.67E-06	0.73	2.90	-	586.0
AHQ-2-4, 3004	R.DETRNVGSQTLQTFK.A	1724.85363	2	4.16E-07	0.84	3.43	-	968.8
AHQ-2-3, 4047 - 4048	R.DGYNDIAVAAPYGGPSGR.G	1780.87574	2	1.41E-05	0.71	3.41	-	520.3
AHQ-2-5, 3891 - 3892	R.DGYNDIAVAAPYGGPSGR.G	1780.87574	2	1.46E-06	0.94	5.17	-	753.7
AHQ-2-6, 3813 - 3881	R.DGYNDIAVAAPYGGPSGR.G	1780.87574	2	2.30E-04	0.71	3.54	-	468.5
AHQ-2-4, 4013 - 4033	R.DGYNDIAVAAPYGGPSGR.G	1780.87574	2	1.38E-06	0.86	3.85	-	739.4
AHQ-2-4, 4225	R.DGYNDIAVAAPYGGPSGR.G	1780.87574	2	2.28E-07	0.91	4.18	-	765.3
AHQ-2-4, 4578 - 4581	R.FGSAIAPLGDLDR.D	1332.48700	2	3.20E-06	0.91	3.59	-	1102.2
AHQ-2-7, 4191	R.FGSAIAPLGDLDR.D	1332.48700	2	2.06E-04	0.84	2.69	-	883.9
AHQ-2-4, 4586	R.FGSAIAPLGDLDR.D	1332.48700	1	3.98E-06	0.80	3.18	-	499.4
AHQ-2-4, 6014 - 6084	R.FGSAIAPLGDLDRDGYNDIAVAAPYGGPSGR.G	3094.34015	3	2.41E-07	0.94	5.38	-	864.0
AHQ-2-4, 6153 - 6224	R.FGSAIAPLGDLDRDGYNDIAVAAPYGGPSGR.G	3094.34015	3	2.50E-06	0.92	5.34	-	865.8
AHQ-2-4, 6292 - 6360	R.FGSAIAPLGDLDRDGYNDIAVAAPYGGPSGR.G	3094.34015	3	1.62E-04	0.87	4.39	-	798.1
AHQ-2-5, 6055 - 6085	R.FGSAIAPLGDLDRDGYNDIAVAAPYGGPSGR.G	3094.34015	3	2.83E-07	0.90	4.50	-	797.4
AHQ-2-5, 5887 - 5957	R.FGSAIAPLGDLDRDGYNDIAVAAPYGGPSGR.G	3094.34015	3	3.78E-11	0.93	4.12	-	1416.1
AHQ-2-4, 5889 - 5957	R.FGSAIAPLGDLDRDGYNDIAVAAPYGGPSGR.G	3094.34015	3	2.50E-05	0.90	4.87	-	575.6
AHQ-2-6, 5731 - 5797	R.FGSAIAPLGDLDRDGYNDIAVAAPYGGPSGR.G	3094.34015	3	2.16E-06	0.91	4.87	-	878.1
AHQ-2-7, 5732 - 5811	R.FGSAIAPLGDLDRDGYNDIAVAAPYGGPSGR.G	3094.34015	3	6.46E-05	0.93	4.94	-	942.6
AHQ-2-1, 6025 - 6080	R.FGSAIAPLGDLDRDGYNDIAVAAPYGGPSGR.G	3094.34015	3	3.75E-07	0.87	4.54	-	685.2
AHQ-2-5, 5804 - 5873	R.FGSAIAPLGDLDRDGYNDIAVAAPYGGPSGR.G	3094.34015	3	8.35E-11	0.91	4.59	-	907.3
AHQ-2-4, 6826 - 6870	R.GAVDIDDNGYPDLIVGAYGANQVAVYR.A	2827.05458	2	1.02E-05	0.92	4.75	-	522.2
AHQ-2-4, 7037 - 7114	R.GAVDIDDNGYPDLIVGAYGANQVAVYR.A	2827.05458	2	3.42E-08	0.94	5.54	-	479.8
AHQ-2-4, 7256 - 7324	R.GAVDIDDNGYPDLIVGAYGANQVAVYR.A	2827.05458	2	1.13E-06	0.86	3.72	-	476.1
AHQ-2-4, 7532 - 7601	R.GAVDIDDNGYPDLIVGAYGANQVAVYR.A	2827.05458	2	7.28E-04	0.85	3.43	-	714.1
AHQ-2-4, 7392 - 7461	R.GAVDIDDNGYPDLIVGAYGANQVAVYR.A	2827.05458	2	6.31E-09	0.95	5.06	-	705.0
AHQ-2-4, 6585 - 6653	R.GAVDIDDNGYPDLIVGAYGANQVAVYR.A	2827.05458	2	2.32E-07	0.90	4.78	-	549.6
AHQ-2-4, 6937 - 6969	R.GAVDIDDNGYPDLIVGAYGANQVAVYR.A	2827.05458	2	2.00E-07	0.94	5.07	-	495.4
AHQ-2-10, 4500 - 4571	R.GEAQVWTQLLR.A	1301.47614	2	3.59E-05	0.93	3.81	-	1147.1
AHQ-2-10, 4822	R.GEAQVWTQLLR.A	1301.47614	1	8.19E-04	0.09	2.01	-	210.1
AHQ-2-1, 5341 - 5348	R.GEAQVWTQLLR.A	1301.47614	2	7.36E-05	0.94	3.80	-	1359.4
AHQ-2-10, 4498 - 4595	R.GEAQVWTQLLR.A	1301.47614	1	2.57E-05	0.17	2.35	-	168.2
AHQ-2-11, 4777	R.GEAQVWTQLLR.A	1301.47614	2	3.76E-07	0.88	3.68	-	771.7
AHQ-2-4, 5470 - 5549	R.GNSFPASLVVAAEEGER.E	1733.86053	2	5.87E-07	0.94	4.74	-	948.3
AHQ-2-4, 5722 - 5792	R.GNSFPASLVVAAEEGER.E	1733.86053	2	1.17E-09	0.96	5.09	-	1200.7
AHQ-2-4, 5878 - 5949	R.GNSFPASLVVAAEEGER.E	1733.86053	2	1.58E-04	0.82	3.10	-	880.0
AHQ-2-4, 6016	R.GNSFPASLVVAAEEGER.E	1733.86053	2	2.97E-12	0.90	4.22	-	747.4
AHQ-2-4, 5621 - 5696	R.GNSFPASLVVAAEEGER.E	1733.86053	2	6.12E-09	0.92	4.67	-	742.3
AHQ-2-4, 5385 - 5456	R.GNSFPASLVVAAEEGER.E	1733.86053	2	1.56E-04	0.89	3.97	-	691.2
AHQ-2-4, 6146	R.GNSFPASLVVAAEEGER.E	1733.86053	2	1.82E-06	0.73	2.94	-	676.1
AHQ-2-4, 6554 - 6625	R.GNSFPASLVVAAEEGEREQNSLDSWGPK.V	2976.16043	3	6.73E-08	0.80	3.94	-	384.9
AHQ-2-4, 6662 - 6702	R.GNSFPASLVVAAEEGEREQNSLDSWGPK.V	2976.16043	2	8.26E-05	0.72	3.26	-	554.8
AHQ-2-4, 6785 - 6856	R.GNSFPASLVVAAEEGEREQNSLDSWGPK.V	2976.16043	3	6.75E-08	0.75	3.97	-	391.8
AHQ-2-4, 7092 - 7169	R.GNSFPASLVVAAEEGEREQNSLDSWGPK.V	2976.16043	3	1.31E-10	0.87	4.60	-	481.5
AHQ-2-4, 6888 - 6913	R.GNSFPASLVVAAEEGEREQNSLDSWGPK.V	2976.16043	2	3.04E-05	0.52	3.15	-	260.6
AHQ-2-4, 5201 - 5272	R.GPHALGAPSLLLTGTQLYGR.F	2023.32431	2	1.22E-05	0.97	6.00	-	1202.5
AHQ-2-4, 5205 - 5285	R.GPHALGAPSLLLTGTQLYGR.F	2023.32431	3	7.91E-04	0.91	3.86	-	1320.9
AHQ-2-1, 5517	R.GPHALGAPSLLLTGTQLYGR.F	2023.32431	2	2.72E-04	0.95	5.06	-	891.2
AHQ-2-6, 4913 - 4938	R.GPHALGAPSLLLTGTQLYGR.F	2023.32431	3	2.77E-04	0.85	3.63	-	1017.7

AHQ-2-6, 4918 - 4919	R.GPHALGAPSLLLTGTQLYGR.F	2023.32431	2	9.46E-06	0.98	6.42	-	1111.8
AHQ-2-7, 4709	R.GPHALGAPSLLLTGTQLYGR.F	2023.32431	2	9.09E-06	0.97	5.37	-	1098.6
AHQ-2-4, 5320 - 5388	R.GPHALGAPSLLLTGTQLYGR.F	2023.32431	2	1.97E-05	0.96	5.17	-	911.8
AHQ-2-7, 4711	R.GPHALGAPSLLLTGTQLYGR.F	2023.32431	3	1.16E-05	0.87	4.11	-	860.6
AHQ-2-4, 5358	R.GPHALGAPSLLLTGTQLYGR.F	2023.32431	3	5.62E-05	0.90	3.81	-	1287.9
AHQ-2-5, 4860 - 4932	R.GPHALGAPSLLLTGTQLYGR.F	2023.32431	2	4.68E-06	0.96	5.10	-	1068.3
AHQ-2-3, 5317 - 5325	R.GPHALGAPSLLLTGTQLYGR.F	2023.32431	2	2.52E-05	0.95	5.18	-	724.5
AHQ-2-4, 5406 - 5477	R.GQVLVFLGQSEGLR.S	1503.72793	2	2.34E-05	0.88	2.95	-	1251.1
AHQ-2-4, 5264 - 5334	R.GQVLVFLGQSEGLR.S	1503.72793	2	1.20E-08	0.97	4.51	-	1622.5
AHQ-2-3, 5172	R.GQVLVFLGQSEGLR.S	1503.72793	2	3.51E-07	0.95	3.79	-	1509.4
AHQ-2-4, 5120 - 5196	R.GQVLVFLGQSEGLR.S	1503.72793	2	5.78E-08	0.97	5.31	-	1685.8
AHQ-2-5, 4108	R.HDLLVGAPLYMESR.A	1601.85188	2	1.46E-06	0.92	3.41	-	1306.4
AHQ-2-6, 4099 - 4153	R.HDLLVGAPLYMESR.A	1601.85188	2	2.18E-04	0.83	3.44	-	691.0
AHQ-2-4, 3829 - 3861	R.HDLLVGAPLYM*ESR.A	1617.85128	2	2.29E-06	0.97	4.60	-	1828.2
AHQ-2-4, 4416 - 4484	R.HDLLVGAPLYMESR.A	1601.85188	2	6.25E-06	0.96	4.80	-	1312.9
AHQ-2-5, 3267 - 3276	K.HSPICHTTMAFLR.D	1572.83705	2	6.31E-08	0.67	2.71	-	343.2
AHQ-2-4, 2966	K.HSPICHTTM*AFLR.D	1588.83645	2	4.61E-04	0.77	2.71	-	612.5
AHQ-2-5, 4159 - 4160	K.IVLLDVPVR.A	1024.28196	2	1.64E-04	0.95	3.97	-	1360.4
AHQ-2-4, 4424 - 4497	K.IVLLDVPVR.A	1024.28196	2	3.23E-04	0.90	3.96	-	830.9
AHQ-2-12, 4327	K.IVLLDVPVR.A	1024.28196	2	1.50E-04	0.88	3.33	-	835.4
AHQ-2-6, 4107	K.IVLLDVPVR.A	1024.28196	2	2.90E-04	0.88	3.22	-	902.4
AHQ-2-3, 4505	K.IVLLDVPVR.A	1024.28196	2	9.94E-05	0.93	3.68	-	1024.4
AHQ-2-11, 4162	K.IVLLDVPVR.A	1024.28196	2	2.68E-04	0.88	3.28	-	801.5
AHQ-2-4, 4566 - 4585	R.IYVENDFSWDK.R	1416.51610	2	4.40E-07	0.92	3.91	-	737.4
AHQ-2-4, 4569 - 4577	R.IYVENDFSWDK.R	1416.51610	1	7.52E-06	0.27	2.07	-	386.3
AHQ-2-4, 3925 - 3998	R.IYVENDFSWDKR.Y	1572.70245	2	7.03E-06	0.95	4.17	-	1467.2
AHQ-2-4, 4308	R.IYVENDFSWDKR.Y	1572.70245	2	7.50E-05	0.69	3.04	-	862.2
AHQ-2-4, 4213	R.IYVENDFSWDKR.Y	1572.70245	2	4.57E-07	0.85	3.69	-	699.7
AHQ-2-10, 6499	R.LQDPVLVSCDSAPCTVVQCDLQEMAR.G	2996.36144	2	8.61E-04	0.72	3.26	-	242.5
AHQ-2-10, 6363 - 6434	R.LQDPVLVSCDSAPCTVVQCDLQEMAR.G	2996.36144	2	1.85E-05	0.88	3.84	-	521.9
AHQ-2-4, 4652	K.LSLNAELQLDR.Q	1272.43310	2	3.25E-08	0.92	3.88	-	912.6
AHQ-2-4, 5177	K.LSLNAELQLDR.Q	1272.43310	2	2.25E-06	0.96	3.75	-	1815.0
AHQ-2-4, 4777 - 4854	K.LSLNAELQLDR.Q	1272.43310	2	1.10E-06	0.90	3.30	-	1002.8
AHQ-2-11, 2112	R.NRPPLEEDDEEGE	1529.50164	2	2.14E-09	0.91	3.28	-	1021.2
AHQ-2-11, 2706	R.NVGSQTLQTFK.A	1223.36055	2	3.41E-04	0.92	3.60	-	733.4
AHQ-2-4, 3262	R.NVGSQTLQTFK.A	1223.36055	1	7.68E-06	0.13	2.07	-	163.2
AHQ-2-6, 2547	R.NVGSQTLQTFK.A	1223.36055	2	2.34E-07	0.88	3.22	-	612.7
AHQ-2-4, 2950 - 2954	R.NVGSQTLQTFK.A	1223.36055	1	5.93E-05	0.17	2.33	-	170.1
AHQ-2-12, 2742	R.NVGSQTLQTFK.A	1223.36055	2	7.07E-04	0.83	3.03	-	468.7
AHQ-2-4, 2692 - 2766	R.NVGSQTLQTFK.A	1223.36055	2	5.10E-04	0.90	3.80	-	516.8
AHQ-2-7, 5812	R.SRPSQVLDSPFPTGSAFGFSLR.G	2354.60606	3	3.98E-05	0.97	5.22	-	2055.9
AHQ-2-3, 6283	R.SRPSQVLDSPFPTGSAFGFSLR.G	2354.60606	3	2.88E-06	0.89	4.30	-	1134.6
AHQ-2-4, 5925	R.SRPSQVLDSPFPTGSAFGFSLR.G	2354.60606	3	1.58E-10	0.97	6.24	-	1805.8
AHQ-2-4, 6062 - 6136	R.SRPSQVLDSPFPTGSAFGFSLR.G	2354.60606	3	5.50E-09	0.92	4.60	-	1281.4
AHQ-2-1, 6326	R.SRPSQVLDSPFPTGSAFGFSLR.G	2354.60606	3	9.18E-12	0.96	5.55	-	1240.1
AHQ-2-4, 6284 - 6352	R.SRPSQVLDSPFPTGSAFGFSLR.G	2354.60606	2	1.06E-07	0.95	4.99	-	708.9
AHQ-2-14-, 5917	R.SRPSQVLDSPFPTGSAFGFSLR.G	2354.60606	3	9.22E-07	0.94	4.90	-	1111.5
AHQ-2-5, 5899 - 5967	R.SRPSQVLDSPFPTGSAFGFSLR.G	2354.60606	3	1.67E-10	0.95	5.56	-	1494.4
AHQ-2-6, 5867 - 5933	R.SRPSQVLDSPFPTGSAFGFSLR.G	2354.60606	3	2.44E-04	0.97	6.32	-	1415.2
AHQ-2-4, 6137 - 6213	R.SRPSQVLDSPFPTGSAFGFSLR.G	2354.60606	2	7.30E-05	0.85	3.74	-	528.5
AHQ-2-4, 6348 - 6417	R.SRPSQVLDSPFPTGSAFGFSLR.G	2354.60606	3	4.02E-08	0.98	7.03	-	1607.2
AHQ-2-4, 6209 - 6281	R.SRPSQVLDSPFPTGSAFGFSLR.G	2354.60606	3	1.19E-09	0.97	6.46	-	1508.6
AHQ-2-6, 5877 - 5945	R.SRPSQVLDSPFPTGSAFGFSLR.G	2354.60606	2	7.51E-07	0.84	3.59	-	614.6
AHQ-2-3, 3759	K.TEEAEKTPVGSCFLAQPESGR.R	2295.46902	2	2.90E-05	0.90	4.37	-	615.3
AHQ-2-4, 3652 - 3721	K.TEEAEKTPVGSCFLAQPESGR.R	2295.46902	2	2.28E-06	0.92	4.18	-	632.0
AHQ-2-11, 3510	K.TEEAEKTPVGSCFLAQPESGR.R	2295.46902	2	2.61E-04	0.84	4.05	-	414.9
AHQ-2-7, 3407	K.TEEAEKTPVGSCFLAQPESGR.R	2295.46902	2	2.72E-07	0.93	4.60	-	614.9
AHQ-2-5, 3472 - 3481	K.TEEAEKTPVGSCFLAQPESGR.R	2295.46902	2	7.94E-06	0.94	4.89	-	568.0
AHQ-2-6, 3433	K.TEEAEKTPVGSCFLAQPESGR.R	2295.46902	2	4.51E-05	0.88	4.13	-	527.8
AHQ-2-4, 3877	K.TEEAEKTPVGSCFLAQPESGR.R	2295.46902	2	6.07E-11	0.90	3.95	-	581.1
AHQ-2-11, 5266 - 5333	R.TLGPSQEETGGVFLCPWR.A	2036.25364	2	2.82E-04	0.92	4.44	-	999.2
AHQ-2-5, 5555	R.TLGPSQEETGGVFLCPWR.A	2036.25364	2	8.54E-06	0.88	3.82	-	714.2
AHQ-2-7, 5385 - 5465	R.TLGPSQEETGGVFLCPWR.A	2036.25364	2	7.31E-10	0.93	4.28	-	787.9
AHQ-2-7, 5475	R.TLGPSQEETGGVFLCPWR.A	2036.25364	2	2.07E-07	0.96	5.30	-	1021.4
AHQ-2-4, 5972 - 6000	R.TLGPSQEETGGVFLCPWR.A	2036.25364	2	2.09E-05	0.43	2.91	-	301.0
AHQ-2-1, 5841 - 5908	R.TLGPSQEETGGVFLCPWR.A	2036.25364	2	2.12E-06	0.93	4.37	-	839.9
AHQ-2-3, 5828 - 5836	R.TLGPSQEETGGVFLCPWR.A	2036.25364	2	1.66E-07	0.66	3.29	-	672.8
AHQ-2-6, 5479 - 5511	R.TLGPSQEETGGVFLCPWR.A	2036.25364	2	6.97E-05	0.91	4.17	-	768.1
AHQ-2-10, 5106	R.TLGPSQEETGGVFLCPWR.A	2036.25364	2	4.40E-05	0.95	4.77	-	1019.5
AHQ-2-13-, 5562 - 5565	R.TLGPSQEETGGVFLCPWR.A	2036.25364	2	2.24E-10	0.97	6.03	-	1215.4
AHQ-2-9, 5495	R.TLGPSQEETGGVFLCPWR.A	2036.25364	2	1.46E-05	0.83	3.67	-	611.4
AHQ-2-4, 5900 - 5966	R.TLGPSQEETGGVFLCPWR.A	2036.25364	2	3.87E-07	0.94	4.65	-	948.6
AHQ-2-4, 5757 - 5833	R.TLGPSQEETGGVFLCPWR.A	2036.25364	2	5.32E-07	0.96	5.25	-	1155.0
AHQ-2-4, 5838	R.TLGPSQEETGGVFLCPWR.A	2036.25364	3	4.13E-04	0.94	4.80	-	1329.1
AHQ-2-12, 5571 - 5581	R.TLGPSQEETGGVFLCPWR.A	2036.25364	2	3.56E-07	0.94	4.74	-	836.7
AHQ-2-6, 3527	K.TPVGSCFLAQPESGR.R	1607.76993	2	3.03E-07	0.92	3.86	-	831.4
AHQ-2-11, 3560 - 3564	K.TPVGSCFLAQPESGR.R	1607.76993	2	1.58E-05	0.91	4.12	-	604.1
AHQ-2-5, 3575	K.TPVGSCFLAQPESGR.R	1607.76993	2	4.63E-06	0.86	3.05	-	928.1
AHQ-2-4, 3964	K.TPVGSCFLAQPESGR.R	1607.76993	2	4.05E-07	0.92	3.89	-	802.1
AHQ-2-1, 3933 - 3937	K.TPVGSCFLAQPESGR.R	1607.76993	2	1.47E-06	0.83	3.76	-	643.2
AHQ-2-7, 3509	K.TPVGSCFLAQPESGR.R	1607.76993	2	4.18E-06	0.87	4.14	-	592.4
AHQ-2-4, 3780 - 3814	K.TPVGSCFLAQPESGR.R	1607.76993	2	2.28E-04	0.85	3.53	-	637.7
AHQ-2-10, 3426	K.TPVGSCFLAQPESGR.R	1607.76993	2	1.68E-05	0.89	3.70	-	723.8
AHQ-2-4, 3665 - 3690	K.TPVGSCFLAQPESGR.R	1607.76993	2	7.39E-05	0.72	3.02	-	730.7
AHQ-2-4, 6384	K.TPVSCFNIQMCVGATGHNIPQK.L	2462.81122	3	8.01E-08	0.96	5.27	-	1347.6
AHQ-2-4, 5273 - 5346	K.TPVSCFNIQMCVGATGHNIPQK.L	2462.81122	3	6.68E-07	0.98	6.54	-	1882.8
AHQ-2-4, 4916	K.TPVSCFNIQMCVGATGHNIPQK.L	2462.81122	3	1.35E-04	0.97	4.79	-	2019.0
AHQ-2-4, 5413 - 5494	K.TPVSCFNIQMCVGATGHNIPQK.L	2462.81122	3	5.71E-10	0.96	5.29	-	1499.2
AHQ-2-4, 4646	K.TPVSCFNIQM*CVGATGHNIPQK.L	2478.81062	2	1.62E-06	0.86	3.34	-	947.5
AHQ-2-4, 6125	K.TPVSCFNIQMCVGATGHNIPQK.L	2462.81122	3	2.01E-06	0.92	4.57	-	1022.9
AHQ-2-5, 4420	K.TPVSCFNIQM*CVGATGHNIPQK.L	2478.81062	3	7.86E-04	0.91	3.89	-	1242.0
AHQ-2-4, 5932	K.TPVSCFNIQMCVGATGHNIPQK.L	2462.81122	3	4.70E-04	0.82	3.34	-	886.3
AHQ-2-4, 4741	K.TPVSCFNIQM*CVGATGHNIPQK.L	2478.81062	3	1.62E-10	0.96	4.55	-	1873.3
AHQ-2-4, 4632 - 4640	K.TPVSCFNIQM*CVGATGHNIPQK.L	2478.81062	3	4.14E-06	0.96	5.27	-	1319.4
AHQ-2-5, 2293	R.VAIVVGAPR.T	882.08560	2	5.09E-06	0.86	2.83	-	979.0
AHQ-2-4, 2478 - 2496	R.VAIVVGAPR.T	882.08560	2	8.33E-06	0.94	3.69	-	1400.5
AHQ-2-7, 2253	R.VAIVVGAPR.T	882.08560	2	2.27E-05	0.90	3.43	-	949.1
AHQ-2-13-, 2627	R.VAIVVGAPR.T	882.08560	2	3.10E-06	0.91	3.09	-	1163.7
AHQ-2-4, 6476 - 6514	R.VLLLGSQQAGTTLNLDLGGK.H	1999.29783	2	4.61E-07	0.96	5.55	-	1234.1
AHQ-2-4, 6724 - 6793	R.VLLLGSQQAGTTLNLDLGGK.H	1999.29783	2	1.05E-07	0.98	6.02	-	1851.4
AHQ-2-4, 6876 - 6944	R.VLLLGSQQAGTTLNLDLGGK.H	1999.29783	2	3.40E-08	0.98	6.49	-	2093.7
AHQ-2-4, 7013 - 7069	R.VLLLGSQQAGTTLNLDLGGK.H	1999.29783	2	9.97E-08	0.98	5.95	-	1800.6
AHQ-2-4, 6009	R.VLLLGSQQAGTTLNLDLGGK.H	1999.29783	2	5.11E-09	0.98	6.57	-	1910.0
AHQ-2-4, 7238 - 7266	R.VLLLGSQQAGTTLNLDLGGK.H	1999.29783	2	1.35E-08	0.98	6.48	-	2054.9
AHQ-2-4, 7298 - 7372	R.VLLLGSQQAGTTLNLDLGGK.H	1999.29783	2	6.65E-10	0.97	5.85	-	1620.9
AHQ-2-4, 6332 - 6408	R.VLLLGSQQAGTTLNLDLGGK.H	1999.29783	2	5.65E-08	0.98	6.18	-	1549.1
AHQ-2-4, 7446 - 7456	R.VLLLGSQQAGTTLNLDLGGK.H	1999.29783	2	1.08E-11	0.94	4.72	-	1058.0

AHQ-2-4, 7533 - 7606	R.VLLLGSQQAGTTLNLDLGGK.H	1999.29783	2	1.28E-07	0.98	5.91	-	1615.6
AHQ-2-4, 7685 - 7757	R.VLLLGSQQAGTTLNLDLGGK.H	1999.29783	2	1.85E-12	0.98	6.38	-	1717.4
AHQ-2-5, 6188	R.VLLLGSQQAGTTLNLDLGGK.H	1999.29783	2	5.50E-04	0.66	3.40	-	381.3
AHQ-2-4, 6241 - 6261	R.VLLLGSQQAGTTLNLDLGGK.H	1999.29783	2	4.59E-08	0.98	6.11	-	1932.0
AHQ-2-4, 7136 - 7201	R.VLLLGSQQAGTTLNLDLGGK.H	1999.29783	2	1.43E-09	0.98	6.10	-	1401.5
AHQ-2-4, 6096 - 6166	R.VLLLGSQQAGTTLNLDLGGK.H	1999.29783	2	1.16E-05	0.97	5.23	-	1729.2
AHQ-2-5, 3940 - 3977	R.VVLCELGNPMK.K	1261.53692	2	4.47E-06	0.93	3.99	-	925.4
AHQ-2-4, 3341 - 3370	R.VVLCELGNPM*K.K	1277.53632	2	7.85E-07	0.95	3.86	-	1364.9
AHQ-2-4, 4188 - 4189	R.VVLCELGNPMK.K	1261.53692	2	1.71E-06	0.96	4.50	-	1268.7
AHQ-2-5, 3283	R.VVLCELGNPMKK.N	1389.70984	2	5.68E-05	0.88	2.97	-	1097.1
AHQ-2-4, 3568	R.VVLCELGNPMKK.N	1389.70984	2	2.71E-05	0.74	2.81	-	628.2
AHQ-2-5, 3863 - 3873	R.VYLFLQPR.G	1036.25129	2	2.11E-05	0.94	3.90	-	1192.2
AHQ-2-4, 4145 - 4216	R.VYLFLQPR.G	1036.25129	2	1.70E-05	0.94	3.64	-	1096.8
gi|4503715|ref|NP_000500.1| fibrinogen, gamma chain isoform gamma-A precursor [Homo sapiens]				1.97E-12	10.87	130.29	44.60	49481.2
AHQ-2-7, 4385 - 4467	K.AIQLTYNPDESSKPNMIDAATLK.S	2521.82886	2	3.09E-06	0.87	4.38	-	350.4
AHQ-2-5, 4551 - 4580	K.AIQLTYNPDESSKPNMIDAATLK.S	2521.82886	3	1.64E-04	0.71	3.42	-	357.3
AHQ-2-9, 4490	K.AIQLTYNPDESSKPNMIDAATLK.S	2521.82886	2	1.68E-05	0.75	3.80	-	294.6
AHQ-2-7, 4535 - 4604	K.AIQLTYNPDESSKPNMIDAATLK.S	2521.82886	2	2.69E-06	0.91	4.72	-	635.0
AHQ-2-7, 4393 - 4463	K.AIQLTYNPDESSKPNMIDAATLK.S	2521.82886	3	1.71E-05	0.90	4.31	-	746.7
AHQ-2-5, 5779	K.ASTPNGYDNGIIWATWK.T	1895.06358	2	2.98E-05	0.67	2.96	-	354.8
AHQ-2-1, 5825 - 5888	K.ASTPNGYDNGIIWATWK.T	1895.06358	2	4.49E-04	0.75	3.35	-	463.9
AHQ-2-1, 5982	K.ASTPNGYDNGIIWATWK.T	1895.06358	2	1.10E-10	0.65	2.73	-	385.2
AHQ-2-14-, 5445 - 5512	K.ASTPNGYDNGIIWATWK.T	1895.06358	2	1.72E-06	0.89	3.44	-	726.5
AHQ-2-13-, 5641	K.ASTPNGYDNGIIWATWK.T	1895.06358	2	1.74E-07	0.52	2.98	-	378.9
AHQ-2-7, 5655 - 5724	K.ASTPNGYDNGIIWATWK.T	1895.06358	2	3.38E-11	0.65	3.05	-	479.8
AHQ-2-5, 5588	K.ASTPNGYDNGIIWATWK.T	1895.06358	2	2.01E-05	0.39	2.60	-	350.2
AHQ-2-7, 2413 - 2491	K.CHAGHLNGVYYQGGTYSK.A	2014.16662	2	2.13E-11	0.97	5.58	-	1307.3
AHQ-2-2, 2678	K.CHAGHLNGVYYQGGTYSK.A	2014.16662	2	2.11E-07	0.96	4.70	-	1084.3
AHQ-2-7, 2437	K.CHAGHLNGVYYQGGTYSK.A	2014.16662	3	9.68E-08	0.97	5.53	-	1805.9
AHQ-2-13-, 2717	K.CHAGHLNGVYYQGGTYSK.A	2014.16662	2	2.08E-11	0.97	5.30	-	1165.1
AHQ-2-4, 2646	K.CHAGHLNGVYYQGGTYSK.A	2014.16662	2	3.34E-08	0.92	3.94	-	988.2
AHQ-2-9, 2506	K.CHAGHLNGVYYQGGTYSK.A	2014.16662	2	1.97E-04	0.81	3.72	-	433.1
AHQ-2-13, 2769 - 2773	K.CHAGHLNGVYYQGGTYSK.A	2014.16662	2	4.47E-05	0.94	4.54	-	756.0
AHQ-2-1, 2838	K.CHAGHLNGVYYQGGTYSK.A	2014.16662	2	7.73E-07	0.95	4.70	-	776.4
AHQ-2-9, 5254	K.EGFGHLSPTGTTEFWLGNEK.I	2208.37146	2	2.68E-05	0.81	3.97	-	338.8
AHQ-2-8, 4856	K.EGFGHLSPTGTTEFWLGNEK.I	2208.37146	2	5.33E-06	0.95	4.62	-	1018.8
AHQ-2-1, 5609 - 5629	K.EGFGHLSPTGTTEFWLGNEK.I	2208.37146	3	2.22E-04	0.95	4.08	-	2123.0
AHQ-2-11, 5006	K.EGFGHLSPTGTTEFWLGNEK.I	2208.37146	2	3.84E-04	0.85	3.73	-	418.1
AHQ-2-3, 5439	K.EGFGHLSPTGTTEFWLGNEK.I	2208.37146	2	4.44E-04	0.86	3.76	-	555.7
AHQ-2-5, 5155	K.EGFGHLSPTGTTEFWLGNEK.I	2208.37146	2	9.93E-06	0.93	4.39	-	650.9
AHQ-2-5, 5156	K.EGFGHLSPTGTTEFWLGNEK.I	2208.37146	3	3.09E-05	0.93	4.55	-	1392.3
AHQ-2-5, 5279	K.EGFGHLSPTGTTEFWLGNEK.I	2208.37146	2	1.61E-06	0.84	3.93	-	444.3
AHQ-2-1, 5704 - 5725	K.EGFGHLSPTGTTEFWLGNEK.I	2208.37146	2	8.64E-06	0.73	3.34	-	438.0
AHQ-2-4, 5613	K.EGFGHLSPTGTTEFWLGNEK.I	2208.37146	2	1.14E-08	0.93	3.77	-	1155.2
AHQ-2-7, 5223 - 5297	K.EGFGHLSPTGTTEFWLGNEK.I	2208.37146	2	2.31E-06	0.94	4.78	-	854.1
AHQ-2-6, 5078	K.EGFGHLSPTGTTEFWLGNEK.I	2208.37146	2	4.60E-04	0.94	4.57	-	847.7
AHQ-2-7, 5080 - 5152	K.EGFGHLSPTGTTEFWLGNEK.I	2208.37146	2	2.31E-08	0.93	4.58	-	738.8
AHQ-2-7, 4968 - 5035	K.EGFGHLSPTGTTEFWLGNEK.I	2208.37146	2	3.12E-05	0.96	5.48	-	689.7
AHQ-2-2, 5493	K.EGFGHLSPTGTTEFWLGNEK.I	2208.37146	2	1.01E-07	0.94	4.69	-	847.7
AHQ-2-11, 6381 - 6461	R.FGSYCPTTCGIADFLSTYQTK.V	2420.65656	2	7.08E-04	0.87	3.77	-	594.8
AHQ-2-7, 6901 - 6971	R.FGSYCPTTCGIADFLSTYQTK.V	2420.65656	2	2.41E-04	0.77	3.27	-	556.8
AHQ-2-12, 6701 - 6769	R.FGSYCPTTCGIADFLSTYQTK.V	2420.65656	2	5.80E-10	0.92	4.49	-	799.3
AHQ-2-12, 6706	R.FGSYCPTTCGIADFLSTYQTK.V	2420.65656	3	2.49E-06	0.78	3.60	-	844.4
AHQ-2-14-, 6543	R.FGSYCPTTCGIADFLSTYQTK.V	2420.65656	2	4.67E-05	0.54	2.91	-	346.8
AHQ-2-4, 4650 - 4720	K.IHLISTQSAIPYALR.V	1683.97576	2	4.52E-09	0.96	5.07	-	1124.4
AHQ-2-9, 4222 - 4230	K.IHLISTQSAIPYALR.V	1683.97576	2	6.44E-06	0.97	4.91	-	1435.0
AHQ-2-7, 4103 - 4171	K.IHLISTQSAIPYALR.V	1683.97576	2	1.97E-12	0.97	5.79	-	1406.1
AHQ-2-3, 4743	K.IHLISTQSAIPYALR.V	1683.97576	2	4.71E-06	0.96	4.64	-	1106.0
AHQ-2-8, 3936	K.IHLISTQSAIPYALR.V	1683.97576	2	2.24E-07	0.97	4.71	-	1394.4
AHQ-2-10, 4096	K.IHLISTQSAIPYALR.V	1683.97576	2	5.13E-07	0.93	3.60	-	980.8
AHQ-2-2, 4673 - 4753	K.IHLISTQSAIPYALR.V	1683.97576	2	1.16E-07	0.97	4.91	-	1251.9
AHQ-2-5, 4332	K.IHLISTQSAIPYALR.V	1683.97576	2	1.74E-08	0.97	4.72	-	1474.1
AHQ-2-5, 4345	K.IHLISTQSAIPYALR.V	1683.97576	3	1.54E-04	0.57	3.09	-	555.0
AHQ-2-13-, 2101 - 2105	R.LTIGEGQQHHLGGAK.Q	1546.71290	2	1.11E-08	0.95	4.17	-	1357.6
AHQ-2-7, 1597 - 1603	R.LTIGEGQQHHLGGAK.Q	1546.71290	2	1.09E-07	0.92	3.54	-	1415.9
AHQ-2-7, 1691 - 1756	R.LTIGEGQQHHLGGAK.Q	1546.71290	2	3.44E-07	0.94	3.88	-	1493.7
AHQ-2-10, 1859	R.LTIGEGQQHHLGGAK.Q	1546.71290	2	3.79E-07	0.55	2.61	-	416.5
AHQ-2-11, 2030	R.LTIGEGQQHHLGGAK.Q	1546.71290	2	7.14E-06	0.86	3.26	-	854.9
AHQ-2-12, 2007	R.LTIGEGQQHHLGGAK.Q	1546.71290	2	1.66E-07	0.88	3.71	-	776.7
AHQ-2-4, 1966	R.LTIGEGQQHHLGGAK.Q	1546.71290	2	1.26E-04	0.85	3.05	-	882.3
AHQ-2-14-, 2085	R.LTIGEGQQHHLGGAK.Q	1546.71290	2	2.34E-09	0.96	4.46	-	1074.6
AHQ-2-7, 2637	R.TSTADYAMFK.V	1135.27203	1	4.84E-05	0.12	2.08	-	235.1
AHQ-2-9, 3763 - 3770	K.VAQLEAQCQEPCKDTVQIHDITGK.D	2772.06211	3	4.34E-05	0.94	4.97	-	1189.9
AHQ-2-7, 3713 - 3752	K.VAQLEAQCQEPCKDTVQIHDITGK.D	2772.06211	3	3.86E-04	0.91	4.36	-	782.4
AHQ-2-7, 3715 - 3716	K.VAQLEAQCQEPCKDTVQIHDITGK.D	2772.06211	2	6.47E-04	0.60	2.99	-	205.3
AHQ-2-4, 4044	K.VAQLEAQCQEPCKDTVQIHDITGK.D	2772.06211	3	5.88E-04	0.91	4.64	-	802.7
AHQ-2-1, 4162	K.VAQLEAQCQEPCKDTVQIHDITGK.D	2772.06211	3	4.44E-05	0.89	4.32	-	850.9
AHQ-2-8, 3691	K.VAQLEAQCQEPCKDTVQIHDITGK.D	2772.06211	3	4.96E-06	0.73	3.50	-	590.6
AHQ-2-8, 3695	K.VAQLEAQCQEPCKDTVQIHDITGK.D	2772.06211	2	2.62E-04	0.27	2.54	-	192.2
AHQ-2-10, 3815	K.VAQLEAQCQEPCKDTVQIHDITGKDCQDIANK.G	3718.05993	3	1.51E-05	0.94	5.05	-	1310.0
AHQ-2-1, 4380	K.VAQLEAQCQEPCKDTVQIHDITGKDCQDIANK.G	3718.05993	3	9.41E-10	0.94	5.59	-	1012.9
AHQ-2-7, 3899 - 3925	K.VAQLEAQCQEPCKDTVQIHDITGKDCQDIANK.G	3718.05993	3	2.90E-04	0.95	5.24	-	1536.8
AHQ-2-8, 3826	K.VAQLEAQCQEPCKDTVQIHDITGKDCQDIANK.G	3718.05993	3	4.83E-04	0.86	4.32	-	730.4
AHQ-2-5, 3111 - 3180	R.VELEDWNGR.T	1118.18129	2	6.72E-05	0.86	2.66	-	1022.9
AHQ-2-8, 3000	R.VELEDWNGR.T	1118.18129	2	1.81E-04	0.88	2.91	-	1193.6
AHQ-2-7, 3079	R.VELEDWNGR.T	1118.18129	2	6.26E-07	0.90	3.07	-	1158.2
AHQ-2-12, 2919	K.YEASILTHDSSIR.Y	1492.61564	2	5.54E-06	0.86	2.93	-	846.0
AHQ-2-12, 2922	K.YEASILTHDSSIR.Y	1492.61564	1	3.64E-04	0.61	2.81	-	336.1
AHQ-2-11, 2888	K.YEASILTHDSSIR.Y	1492.61564	2	1.64E-05	0.78	2.99	-	537.9
AHQ-2-5, 2663 - 2671	K.YEASILTHDSSIR.Y	1492.61564	2	2.69E-05	0.91	3.83	-	819.7
AHQ-2-2, 2937	K.YEASILTHDSSIR.Y	1492.61564	2	3.60E-05	0.50	2.62	-	399.2
AHQ-2-6, 2727	K.YEASILTHDSSIR.Y	1492.61564	2	1.08E-04	0.73	2.62	-	585.6
AHQ-2-9, 2692 - 2710	K.YEASILTHDSSIR.Y	1492.61564	2	1.03E-06	0.93	3.82	-	986.1
AHQ-2-7, 2564 - 2635	K.YEASILTHDSSIR.Y	1492.61564	2	5.54E-06	0.89	3.65	-	705.2
AHQ-2-12, 2911	K.YEASILTHDSSIR.Y	1492.61564	2	1.19E-05	0.83	3.49	-	490.8
AHQ-2-3, 2889 - 2949	K.YEASILTHDSSIR.Y	1492.61564	2	2.16E-05	0.82	3.01	-	650.4
AHQ-2-1, 2896 - 2908	R.YLQEIYNSNNQK.I	1514.62131	2	1.66E-06	0.94	4.45	-	1227.3
gi|11386183|ref|NP_008921.1| WAS protein family, member 2; suppressor of cyclic-AMP receptor (WASP-				1.99E-12	1.82	20.31	7.60	54283.7
AHQ-2-10, 3660	K.VTQLDPKEEEVSLQGINTR.K	2157.36743	2	1.23E-06	0.84	3.73	-	483.1
AHQ-2-14-, 6841	K.YAEDIFGELFTQANTFASR.V	2181.34601	2	1.99E-12	0.98	6.20	-	2134.1
gi|4506925|ref|NP_003013.1| SH3 domain binding glutamic acid-rich protein like; SH3-binding domain				2.00E-12	3.83	40.33	64.00	12774.2
AHQ-2-13, 5897 - 5956	R.ENNAVYAFLGLTAPPGSK.E	1850.06426	2	4.89E-04	0.84	4.25	-	407.1
AHQ-2-13-, 6425 - 6489	R.ENNAVYAFLGLTAPPGSK.E	1850.06426	2	6.40E-08	0.93	4.13	-	920.6
AHQ-2-13, 4873 - 4941	R.ENVPENSRPATGYPLPPQIFNESQYR.G	3005.24660	3	3.47E-06	0.89	3.99	-	1010.9
AHQ-2-13, 5004 - 5067	R.ENVPENSRPATGYPLPPQIFNESQYR.G	3005.24660	3	5.44E-09	0.96	5.92	-	1159.5
AHQ-2-13-, 5265	R.ENVPENSRPATGYPLPPQIFNESQYR.G	3005.24660	3	7.32E-11	0.92	4.71	-	868.9
AHQ-2-13-, 5173 - 5241	R.ENVPENSRPATGYPLPPQIFNESQYR.G	3005.24660	3	6.91E-09	0.97	6.36	-	1380.2

AHQ-2-13-, 5081 - 5149	R.ENVPENSRPATGYPLPPQIFNESQYR.G	3005.24660	3	2.00E-12	0.97	6.60	-	1195.5
AHQ-2-13-, 4983 - 5014	R.ENVPENSRPATGYPLPPQIFNESQYR.G	3005.24660	3	2.31E-12	0.96	6.18	-	1074.3
AHQ-2-13, 2768 - 2831	K.IGFEEKDIAANEENRK.W	1864.00620	2	6.14E-05	0.97	4.70	-	2029.1
AHQ-2-13, 2433 - 2443	R.VYIASSSGSTAIK.K	1284.44065	2	7.77E-05	0.96	4.43	-	1985.5
AHQ-2-13, 2435	R.VYIASSSGSTAIK.K	1284.44065	1	8.97E-04	0.10	2.04	-	226.4
gi|4759212|ref|NP_004598.1| beta-tubulin cofactor A [Homo sapiens]				2.10E-12	0.97	10.31	16.70	12854.8
AHQ-2-13, 3365	R.ILENEKDLEEAEEYKEAR.L	2209.35284	2	8.61E-06	0.97	5.10	-	1468.2
AHQ-2-13-, 3359	R.ILENEKDLEEAEEYKEAR.L	2209.35284	2	3.12E-05	0.89	3.77	-	684.5
AHQ-2-13, 3359	R.ILENEKDLEEAEEYKEAR.L	2209.35284	3	2.10E-12	0.97	6.28	-	1427.2
gi|4557677|ref|NP_000203.1| integrin beta chain, beta 3 precursor; platelet glycoprotein IIIa precu				2.24E-12	19.39	230.29	32.50	87185.6
AHQ-2-3, 2864 - 2873	R.AKWDTANNPLYK.E	1421.58225	2	7.24E-07	0.89	3.83	-	671.5
AHQ-2-3, 2868	R.AKWDTANNPLYK.E	1421.58225	1	6.15E-05	0.74	3.31	-	443.2
AHQ-2-6, 2834	R.AKWDTANNPLYK.E	1421.58225	1	5.44E-04	0.30	2.00	-	322.4
AHQ-2-1, 3097	R.AKWDTANNPLYK.E	1421.58225	2	1.49E-09	0.91	3.25	-	862.5
AHQ-2-4, 5600	R.CDLKENLLKDNCAPESIEFPVSEAR.V	2938.23689	2	4.53E-06	0.89	4.49	-	421.4
AHQ-2-3, 5461 - 5531	R.CDLKENLLKDNCAPESIEFPVSEAR.V	2938.23689	3	3.30E-04	0.92	4.57	-	962.5
AHQ-2-3, 5459 - 5516	R.CDLKENLLKDNCAPESIEFPVSEAR.V	2938.23689	2	1.06E-06	0.71	3.29	-	591.3
AHQ-2-5, 5303	R.CDLKENLLKDNCAPESIEFPVSEAR.V	2938.23689	3	3.86E-04	0.92	4.43	-	829.6
AHQ-2-4, 5542 - 5552	R.CDLKENLLKDNCAPESIEFPVSEAR.V	2938.23689	3	6.66E-05	0.94	5.21	-	1067.9
AHQ-2-3, 5608	R.CDLKENLLKDNCAPESIEFPVSEAR.V	2938.23689	3	3.56E-04	0.87	4.04	-	1008.3
AHQ-2-2, 4582	R.CGPGWLGSQCECSEEDYRPSQQDECSPR.E	3380.49342	3	4.35E-07	0.87	4.09	-	716.1
AHQ-2-1, 4508 - 4512	R.CGPGWLGSQCECSEEDYRPSQQDECSPR.E	3380.49342	3	4.40E-08	0.76	3.51	-	398.7
AHQ-2-3, 4468 - 4539	R.CGPGWLGSQCECSEEDYRPSQQDECSPR.E	3380.49342	3	1.86E-07	0.73	3.47	-	579.1
AHQ-2-4, 4522 - 4602	R.CGPGWLGSQCECSEEDYRPSQQDECSPR.E	3380.49342	3	2.22E-06	0.89	4.55	-	503.9
AHQ-2-6, 4298	R.CGPGWLGSQCECSEEDYRPSQQDECSPR.E	3380.49342	3	2.63E-04	0.73	3.56	-	410.9
AHQ-2-5, 4411	R.CGPGWLGSQCECSEEDYRPSQQDECSPR.E	3380.49342	3	2.24E-12	0.67	3.23	-	405.6
AHQ-2-1, 6045 - 6078	R.DAPEGGFDAIMQATVCDEK.I	2056.21716	2	1.04E-06	0.93	4.51	-	770.9
AHQ-2-1, 4620 - 4634	R.DAPEGGFDAIM*QATVCDEK.I	2072.21656	2	5.34E-08	0.95	4.84	-	790.6
AHQ-2-4, 4698 - 4761	R.DAPEGGFDAIM*QATVCDEK.I	2072.21656	2	5.26E-05	0.85	3.44	-	709.2
AHQ-2-3, 7303	R.DAPEGGFDAIMQATVCDEK.I	2056.21716	2	8.25E-05	0.84	3.44	-	470.8
AHQ-2-3, 4643 - 4716	R.DAPEGGFDAIM*QATVCDEK.I	2072.21656	2	6.76E-06	0.93	4.64	-	724.5
AHQ-2-3, 7087	R.DAPEGGFDAIMQATVCDEK.I	2056.21716	2	4.71E-07	0.68	2.94	-	475.2
AHQ-2-3, 6863	R.DAPEGGFDAIMQATVCDEK.I	2056.21716	2	3.27E-09	0.90	4.01	-	558.8
AHQ-2-3, 6579 - 6651	R.DAPEGGFDAIMQATVCDEK.I	2056.21716	2	3.89E-06	0.96	5.14	-	1110.3
AHQ-2-3, 6439 - 6511	R.DAPEGGFDAIMQATVCDEK.I	2056.21716	2	2.36E-04	0.95	4.80	-	1148.1
AHQ-2-3, 6296 - 6368	R.DAPEGGFDAIMQATVCDEK.I	2056.21716	2	2.76E-07	0.96	5.66	-	851.6
AHQ-2-3, 6156 - 6224	R.DAPEGGFDAIMQATVCDEK.I	2056.21716	2	9.14E-08	0.94	4.60	-	898.9
AHQ-2-4, 5104	K.DDLWSIQNLGTK.L	1390.52332	1	4.96E-04	0.53	2.76	-	366.7
AHQ-2-5, 4920 - 4921	K.DDLWSIQNLGTK.L	1390.52332	1	1.28E-06	0.15	1.93	-	272.8
AHQ-2-3, 5080 - 5099	K.DDLWSIQNLGTK.L	1390.52332	2	1.30E-05	0.94	4.57	-	871.1
AHQ-2-6, 4835	K.DDLWSIQNLGTK.L	1390.52332	1	5.51E-08	0.54	2.47	-	445.0
AHQ-2-3, 5088	K.DDLWSIQNLGTK.L	1390.52332	1	7.12E-07	0.74	2.90	-	560.0
AHQ-2-9, 4378	K.DNCAPESIEFPVSEAR.V	1822.93099	2	1.50E-04	0.76	3.05	-	696.2
AHQ-2-6, 4342 - 4354	K.DNCAPESIEFPVSEAR.V	1822.93099	2	2.48E-05	0.88	3.52	-	853.6
AHQ-2-3, 4785	K.DNCAPESIEFPVSEAR.V	1822.93099	2	1.22E-08	0.93	3.58	-	1470.0
AHQ-2-7, 4372	K.DNCAPESIEFPVSEAR.V	1822.93099	2	1.58E-05	0.36	2.56	-	374.6
AHQ-2-3, 4519 - 4587	K.DNCAPESIEFPVSEAR.V	1822.93099	2	4.40E-06	0.75	2.85	-	798.9
AHQ-2-4, 4596 - 4616	K.DNCAPESIEFPVSEAR.V	1822.93099	2	6.92E-07	0.93	3.71	-	1215.1
AHQ-2-1, 3464	K.EATSTFTNITYR.G	1404.50685	2	5.83E-07	0.83	2.87	-	755.2
AHQ-2-4, 3342	K.EATSTFTNITYR.G	1404.50685	2	7.41E-07	0.68	2.77	-	572.6
AHQ-2-3, 3325	K.EATSTFTNITYR.G	1404.50685	2	7.74E-07	0.70	2.86	-	480.6
AHQ-2-3, 2260 - 2283	R.FQYYEDSSGK.S	1224.25749	2	4.19E-06	0.72	2.72	-	500.5
AHQ-2-3, 3819	R.GECLCGQCVCHSSDFGK.I	2006.16137	2	1.29E-05	0.63	2.79	-	528.5
AHQ-2-4, 3669 - 3749	R.GECLCGQCVCHSSDFGK.I	2006.16137	2	1.76E-07	0.96	4.20	-	1719.1
AHQ-2-6, 3467	R.GECLCGQCVCHSSDFGK.I	2006.16137	2	3.50E-05	0.97	4.46	-	1607.8
AHQ-2-1, 3736	R.GECLCGQCVCHSSDFGK.I	2006.16137	2	9.91E-06	0.87	3.50	-	884.2
AHQ-2-5, 3524 - 3531	R.GECLCGQCVCHSSDFGK.I	2006.16137	2	5.20E-07	0.94	3.81	-	1557.6
AHQ-2-3, 3735	R.GECLCGQCVCHSSDFGK.I	2006.16137	2	2.36E-07	0.89	3.72	-	1066.3
AHQ-2-3, 3672 - 3687	R.GECLCGQCVCHSSDFGK.I	2006.16137	3	3.48E-09	0.83	3.35	-	776.0
AHQ-2-3, 3587 - 3665	R.GECLCGQCVCHSSDFGK.I	2006.16137	2	3.07E-05	0.91	4.20	-	907.6
AHQ-2-2, 3742 - 3790	R.GECLCGQCVCHSSDFGK.I	2006.16137	2	1.68E-07	0.89	3.78	-	864.3
AHQ-2-5, 1577	K.GSGDSSQVTQVSPQR.I	1533.58302	2	7.80E-04	0.72	3.20	-	822.8
AHQ-2-4, 1716 - 1780	K.GSGDSSQVTQVSPQR.I	1533.58302	2	5.69E-04	0.79	3.13	-	801.6
AHQ-2-7, 1581	K.GSGDSSQVTQVSPQR.I	1533.58302	2	1.35E-04	0.53	2.84	-	664.7
AHQ-2-7, 2336 - 2349	K.HVLTLTDQVTR.F	1283.45912	2	2.21E-07	0.92	3.38	-	692.6
AHQ-2-4, 2561	K.HVLTLTDQVTR.F	1283.45912	2	5.26E-07	0.90	3.60	-	605.4
AHQ-2-6, 2385	K.HVLTLTDQVTR.F	1283.45912	2	2.67E-07	0.92	3.88	-	680.6
AHQ-2-3, 2624	K.HVLTLTDQVTR.F	1283.45912	2	8.62E-04	0.77	2.58	-	573.9
AHQ-2-3, 2540 - 2551	K.HVLTLTDQVTR.F	1283.45912	2	6.93E-06	0.90	3.67	-	511.8
AHQ-2-4, 2573	K.HVLTLTDQVTR.F	1283.45912	1	4.03E-06	0.63	3.02	-	289.7
AHQ-2-5, 2371	K.HVLTLTDQVTR.F	1283.45912	2	2.40E-09	0.94	3.64	-	900.1
AHQ-2-1, 2738 - 2808	K.HVLTLTDQVTR.F	1283.45912	2	1.40E-08	0.89	2.94	-	756.9
AHQ-2-3, 4997	K.IGDTVSFSIEAK.V	1267.41009	2	1.10E-06	0.93	3.20	-	1096.0
AHQ-2-3, 4287 - 4355	R.NDASHLLVFTTDAK.T	1532.67977	2	1.05E-04	0.75	2.70	-	536.9
AHQ-2-5, 3501	R.NDASHLLVFTTDAK.T	1532.67977	2	2.13E-08	0.97	4.35	-	1660.3
AHQ-2-3, 4459 - 4532	R.NDASHLLVFTTDAK.T	1532.67977	2	9.17E-10	0.97	4.69	-	1412.4
AHQ-2-6, 3454 - 3525	R.NDASHLLVFTTDAK.T	1532.67977	2	1.13E-09	0.96	4.46	-	1496.1
AHQ-2-7, 3403	R.NDASHLLVFTTDAK.T	1532.67977	2	4.74E-07	0.89	3.74	-	830.3
AHQ-2-3, 3540 - 3557	R.NDASHLLVFTTDAK.T	1532.67977	2	7.01E-08	0.87	3.75	-	858.6
AHQ-2-1, 4060 - 4125	R.NDASHLLVFTTDAK.T	1532.67977	2	6.84E-05	0.87	3.34	-	887.7
AHQ-2-3, 3788 - 3864	R.NDASHLLVFTTDAK.T	1532.67977	2	5.44E-09	0.98	5.49	-	1697.8
AHQ-2-3, 3771 - 3847	R.NDASHLLVFTTDAK.T	1532.67977	2	2.34E-10	0.97	5.19	-	1551.3
AHQ-2-1, 4554	R.NDASHLLVFTTDAK.T	1532.67977	2	2.00E-10	0.95	4.28	-	1249.6
AHQ-2-3, 3861	R.NDASHLLVFTTDAK.T	1532.67977	3	1.01E-09	0.95	4.58	-	1402.1
AHQ-2-4, 3845	R.NDASHLLVFTTDAK.T	1532.67977	2	9.22E-09	0.97	4.78	-	1545.6
AHQ-2-4, 4454 - 4492	R.NDASHLLVFTTDAK.T	1532.67977	2	6.82E-09	0.94	4.28	-	1083.6
AHQ-2-4, 3230 - 3257	K.SFTIKPVGFK.D	1124.35685	2	1.97E-05	0.88	2.87	-	1221.0
AHQ-2-3, 3215	K.SFTIKPVGFK.D	1124.35685	1	4.11E-04	0.37	2.47	-	573.9
AHQ-2-1, 4574	K.SILYVVEEPECPK.G	1564.78173	2	1.04E-04	0.83	2.85	-	864.1
AHQ-2-3, 4499 - 4508	K.SILYVVEEPECPK.G	1564.78173	2	1.28E-05	0.95	3.48	-	1572.7
AHQ-2-4, 3714	R.TDTCMSSNGLLCSGR.G	1661.81786	2	6.53E-07	0.69	3.08	-	891.0
AHQ-2-3, 3827	R.TDTCMSSNGLLCSGR.G	1661.81786	2	2.82E-07	0.72	2.52	-	834.6
AHQ-2-4, 2978 - 2981	R.TDTCM*SSNGLLCSGR.G	1677.81726	2	4.04E-07	0.75	3.07	-	399.4
AHQ-2-4, 2876	R.TDTCM*SSNGLLCSGR.G	1677.81726	2	6.69E-08	0.94	3.79	-	1311.9
AHQ-2-1, 2885	R.TDTCM*SSNGLLCSGR.G	1677.81726	2	7.34E-08	0.96	3.95	-	1897.0
AHQ-2-1, 2984	R.TDTCM*SSNGLLCSGR.G	1677.81726	2	2.09E-06	0.88	3.38	-	789.7
AHQ-2-5, 3545	R.TDTCMSSNGLLCSGR.G	1661.81786	2	4.69E-07	0.92	3.59	-	1026.7
AHQ-2-5, 3651	R.TDTCMSSNGLLCSGR.G	1661.81786	2	2.35E-08	0.86	3.32	-	1048.4
AHQ-2-2, 3778	R.TDTCMSSNGLLCSGR.G	1661.81786	2	1.94E-07	0.77	2.71	-	893.5
AHQ-2-1, 3722 - 3768	R.TDTCMSSNGLLCSGR.G	1661.81786	2	5.90E-06	0.83	2.85	-	942.5
AHQ-2-1, 3622	R.TDTCMSSNGLLCSGR.G	1661.81786	2	7.64E-10	0.91	3.53	-	947.5
AHQ-2-3, 2957 - 3037	R.TDTCM*SSNGLLCSGR.G	1677.81726	2	2.33E-07	0.87	3.46	-	1103.2
AHQ-2-3, 2871 - 2881	R.TDTCM*SSNGLLCSGR.G	1677.81726	2	1.12E-10	0.96	3.59	-	1714.3
AHQ-2-3, 1763 - 1837	R.VLEDRPLSDK.G	1172.31354	2	7.00E-04	0.88	3.29	-	930.2
AHQ-2-7, 2495 - 2551	R.VLEDRPLSDKGSGDSSQVTQVSPQR.I	2686.87396	3	1.28E-07	0.90	3.80	-	1309.9
AHQ-2-1, 2869	R.VLEDRPLSDKGSGDSSQVTQVSPQR.I	2686.87396	3	1.07E-04	0.90	4.14	-	1106.6
AHQ-2-3, 2680 - 2755	R.VLEDRPLSDKGSGDSSQVTQVSPQR.I	2686.87396	3	2.09E-09	0.96	5.44	-	1599.0

AHQ-2-3, 3264 - 3268	K.WDTANNPLYK.E	1222.33111	2	3.03E-06	0.76	3.36	-	463.2
AHQ-2-1, 3776	K.YCECDDFSCVR.Y	1514.59674	2	9.60E-07	0.95	3.61	-	1190.7
AHQ-2-2, 3842	K.YCECDDFSCVR.Y	1514.59674	2	1.49E-09	0.91	3.52	-	975.4
AHQ-2-3, 3763	K.YCECDDFSCVR.Y	1514.59674	2	4.98E-07	0.91	3.44	-	990.2
AHQ-2-4, 3768 - 3774	K.YCECDDFSCVR.Y	1514.59674	2	7.30E-05	0.85	2.59	-	1044.0
AHQ-2-1, 2382	R.YCRDEIESVK.E	1300.42023	2	3.30E-05	0.87	3.29	-	1002.6
AHQ-2-4, 2229 - 2232	R.YCRDEIESVK.E	1300.42023	2	8.40E-05	0.90	3.05	-	1173.3
gi|10346135|ref|NP_055083.1| microtubule-associated protein, RP/EB family, member 2; T-cell activat				2.35E-12	1.94	20.27	9.80	37031.1
AHQ-2-13, 6680	R.FQDNLDFIQWFK.K	1601.78539	2	1.15E-07	0.96	4.46	-	1284.0
AHQ-2-9, 7118 - 7122	R.FQDNLDFIQWFK.K	1601.78539	2	3.52E-08	0.97	4.59	-	1356.8
AHQ-2-12, 6946 - 6950	R.FQDNLDFIQWFK.K	1601.78539	2	5.20E-07	0.96	4.28	-	1266.6
AHQ-2-13-, 6903	R.FQDNLDFIQWFK.K	1601.78539	2	2.51E-10	0.97	5.16	-	1426.5
AHQ-2-12, 4953	K.SDKDLETQVIQLNEQVHSLK.L	2325.56134	3	3.30E-08	0.94	4.92	-	1171.6
AHQ-2-9, 4686 - 4694	K.SDKDLETQVIQLNEQVHSLK.L	2325.56134	2	2.35E-12	0.97	5.30	-	1277.3
AHQ-2-9, 4684 - 4688	K.SDKDLETQVIQLNEQVHSLK.L	2325.56134	3	2.14E-09	0.94	4.96	-	1141.1
gi|4507767|ref|NP_003326.1| ubiquitin-activating enzyme E1-like; Ubiquitin-activating enzyme-2; ubi				3.29E-12	4.18	50.24	10.10	111719.5
AHQ-2-4, 5030 - 5101	K.FQHLHGRPPQPWDPVDAETVVGLAR.D	2824.14835	3	6.84E-05	0.83	3.59	-	670.4
AHQ-2-4, 7496	R.GLNPDLQVIPLTYPLDPTTEHIYGDNFFSR.V	3434.79811	3	1.45E-07	0.67	3.43	-	291.8
AHQ-2-5, 7376	R.GLNPDLQVIPLTYPLDPTTEHIYGDNFFSR.V	3434.79811	3	3.10E-05	0.75	3.62	-	308.6
AHQ-2-4, 4757	R.VLVSGLQGLGAEVAK.N	1441.69805	2	3.29E-12	0.98	4.70	-	2313.3
AHQ-2-4, 4316	R.VTELVQQLTGQAPAPGQR.V	1894.12181	2	8.12E-04	0.82	3.75	-	539.9
AHQ-2-4, 4124 - 4136	R.YFPSTAEHTLQWAR.H	1707.86959	2	5.66E-10	0.88	3.86	-	671.9
gi|23510338|ref|NP_003325.2| ubiquitin-activating enzyme E1; A1S9T and BN75 temperature sensitivity				3.43E-12	6.96	80.28	11.80	117848.1
AHQ-2-4, 6693 - 6700	R.AAVATFLQSVQVPEFTPK.S	1934.22430	2	2.71E-04	0.67	2.93	-	522.3
AHQ-2-4, 4682 - 4693	R.ALPAVQQNNLDEDLIR.K	1810.00167	2	3.43E-12	0.96	5.63	-	750.6
AHQ-2-4, 3682 - 3689	K.DNPGVVTCLDEAR.H	1447.55333	2	8.18E-04	0.90	3.49	-	855.3
AHQ-2-4, 2308	R.KPLLESGTLGTK.G	1244.46280	2	1.83E-06	0.92	3.31	-	1205.8
AHQ-2-4, 5474	R.LAGTQPLEVLEAVQR.S	1624.86348	2	4.33E-04	0.84	3.32	-	616.6
AHQ-2-4, 6124	R.NEEDAAELVALAQAVNAR.A	1885.02541	2	1.35E-06	0.95	4.18	-	1651.6
AHQ-2-5, 6667	K.SLVASLAEPDFVVTDFAK.F	1910.15638	2	3.29E-05	0.92	4.25	-	620.8
AHQ-2-4, 4201	R.YDGQVAVFGSDLQEK.L	1656.77453	2	1.09E-04	0.80	3.11	-	736.9
gi|29739414|ref|XP_294015.1| similar to Glyceraldehyde 3-phosphate dehydrogenase, liver (GAPDH) [Ho				3.62E-12	3.72	40.24	17.70	26916.7
AHQ-2-11, 3868	K.IISNASCTTNCLAPLAK.V	1837.10868	2	3.57E-05	0.84	3.72	-	1019.3
AHQ-2-11, 4190	K.IISNASCTTNCLAPLAK.V	1837.10868	2	1.01E-04	0.46	2.68	-	676.6
AHQ-2-10, 3699 - 3759	K.IISNASCTTNCLAPLAK.V	1837.10868	2	1.44E-05	0.73	3.09	-	1025.7
AHQ-2-12, 4035	K.IISNASCTTNCLAPLAK.V	1837.10868	2	3.09E-06	0.90	3.96	-	1188.8
AHQ-2-9, 5090 - 5160	K.LISWYDNEFGYSNR.V	1764.87478	2	3.62E-12	0.96	3.92	-	1343.8
AHQ-2-9, 5236	K.LISWYDNEFGYSNR.V	1764.87478	2	1.05E-06	0.92	4.12	-	850.4
AHQ-2-9, 5323	K.LISWYDNEFGYSNR.V	1764.87478	2	3.53E-05	0.67	2.90	-	501.3
AHQ-2-9, 4735	K.LISWYDNEFGYSNR.V	1764.87478	2	1.33E-06	0.79	2.98	-	610.6
AHQ-2-10, 4658	K.LISWYDNEFGYSNR.V	1764.87478	2	3.38E-09	0.92	3.94	-	852.1
AHQ-2-10, 4748	K.LISWYDNEFGYSNR.V	1764.87478	2	1.06E-06	0.94	4.20	-	1085.6
AHQ-2-14-, 5031 - 5035	K.LISWYDNEFGYSNR.V	1764.87478	2	3.34E-09	0.96	4.77	-	1286.2
AHQ-2-13-, 5085	K.LISWYDNEFGYSNR.V	1764.87478	2	1.24E-11	0.95	4.24	-	1053.0
AHQ-2-11, 4806 - 4868	K.LISWYDNEFGYSNR.V	1764.87478	2	1.08E-08	0.95	4.07	-	1230.9
AHQ-2-11, 4933 - 4998	K.LISWYDNEFGYSNR.V	1764.87478	2	1.11E-05	0.84	3.07	-	662.1
AHQ-2-9, 4950 - 5018	K.LISWYDNEFGYSNR.V	1764.87478	2	3.61E-09	0.96	4.53	-	1368.2
AHQ-2-12, 5070	K.LISWYDNEFGYSNR.V	1764.87478	2	4.54E-07	0.94	3.71	-	1217.6
AHQ-2-13, 4987 - 4988	K.LISWYDNEFGYSNR.V	1764.87478	2	1.27E-06	0.95	4.20	-	1297.1
AHQ-2-14, 6212 - 6278	K.LISWYDNEFGYSNR.V	1764.87478	2	4.97E-08	0.96	4.22	-	1600.0
AHQ-2-9, 2579	R.VVDLMAHMASK.E	1202.47292	2	1.85E-06	0.97	4.05	-	1604.6
AHQ-2-9, 2719 - 2790	R.VVDLMAHMASKE	1331.58746	2	8.16E-05	0.90	3.42	-	1196.1
gi|5729997|ref|NP_004154.2| RAB27B, member RAS oncogene family [Homo sapiens]				4.10E-12	4.59	50.23	29.80	24607.7
AHQ-2-10, 6303 - 6367	K.AVETLLDLIMK.R	1246.54211	2	7.02E-04	0.72	3.03	-	623.2
AHQ-2-11, 5598	K.AVETLLDLIM*K.R	1262.54151	2	1.10E-05	0.90	3.68	-	796.2
AHQ-2-11, 6540	K.AVETLLDLIMK.R	1246.54211	2	7.10E-07	0.94	3.96	-	1206.3
AHQ-2-10, 5358	K.AVETLLDLIM*K.R	1262.54151	2	4.25E-05	0.93	3.74	-	1221.0
AHQ-2-11, 6254 - 6257	R.NWMSQLQANAYCENPDIVLIGNK.A	2680.99772	2	3.64E-05	0.92	3.99	-	845.3
AHQ-2-10, 5970	R.NWMSQLQANAYCENPDIVLIGNK.A	2680.99772	2	3.05E-06	0.85	3.51	-	602.4
AHQ-2-11, 3469	K.VHLQLWDTAGQER.F	1553.70390	2	4.10E-12	0.96	4.21	-	1307.6
AHQ-2-10, 3255	K.VHLQLWDTAGQER.F	1553.70390	2	8.92E-11	0.95	4.24	-	1194.8
AHQ-2-10, 4186 - 4263	K.YGIPYFETSAATGQNVEK.A	1976.13177	2	4.62E-09	0.94	4.21	-	895.6
AHQ-2-11, 4329 - 4396	K.YGIPYFETSAATGQNVEK.A	1976.13177	2	4.23E-08	0.97	4.65	-	1506.0
AHQ-2-10, 4326 - 4391	K.YGIPYFETSAATGQNVEK.A	1976.13177	2	3.53E-07	0.69	3.60	-	540.3
gi|4758086|ref|NP_004069.1| cysteine and glycine-rich protein 1; cysteine-rich protein; LIM-domain				4.34E-12	6.01	70.26	46.10	20567.3
AHQ-2-11, 4173	K.GFGFGQGAGALVHSE	1434.53788	2	2.21E-06	0.91	3.12	-	1118.0
AHQ-2-13, 4228 - 4237	K.GFGFGQGAGALVHSE	1434.53788	2	4.82E-07	0.95	4.17	-	1187.8
AHQ-2-13-, 4279	K.GFGFGQGAGALVHSE	1434.53788	2	2.33E-05	0.94	3.67	-	1235.8
AHQ-2-11, 4060 - 4086	K.GFGFGQGAGALVHSE	1434.53788	2	6.83E-05	0.93	3.87	-	934.3
AHQ-2-11, 3209 - 3217	K.GLESTTLADKDGEIYCK.G	1902.06957	3	5.53E-06	0.85	3.92	-	773.0
AHQ-2-12, 3279	K.GLESTTLADKDGEIYCK.G	1902.06957	2	9.15E-06	0.96	5.23	-	877.7
AHQ-2-13, 3371	K.GLESTTLADKDGEIYCK.G	1902.06957	2	4.36E-10	0.95	4.66	-	1151.8
AHQ-2-13-, 3369	K.GLESTTLADKDGEIYCK.G	1902.06957	2	2.13E-08	0.95	4.75	-	1042.0
AHQ-2-11, 3160 - 3220	K.GLESTTLADKDGEIYCK.G	1902.06957	2	7.15E-09	0.94	4.96	-	882.5
AHQ-2-11, 2477	K.GYGYGQGAGTLSTDK.G	1475.54194	2	1.96E-04	0.87	3.40	-	766.1
AHQ-2-12, 3519	K.GYGYGQGAGTLSTDKGESLGIK.H	2160.32665	3	1.40E-08	0.88	4.07	-	596.4
AHQ-2-12, 3521	K.GYGYGQGAGTLSTDKGESLGIK.H	2160.32665	2	4.68E-09	0.94	4.78	-	958.7
AHQ-2-13-, 3698	K.GYGYGQGAGTLSTDKGESLGIK.H	2160.32665	2	4.34E-12	0.87	4.63	-	462.8
AHQ-2-11, 3409 - 3474	K.GYGYGQGAGTLSTDKGESLGIK.H	2160.32665	2	2.55E-06	0.85	4.02	-	564.3
AHQ-2-13, 3640	K.GYGYGQGAGTLSTDKGESLGIK.H	2160.32665	2	4.93E-06	0.90	3.84	-	806.4
AHQ-2-10, 3276	K.GYGYGQGAGTLSTDKGESLGIK.H	2160.32665	2	1.53E-08	0.66	3.30	-	324.6
AHQ-2-11, 2922	K.KNLDSTTVAVHGEEIYCK.S	2066.27805	2	5.70E-04	0.56	2.51	-	313.8
AHQ-2-11, 3380 - 3457	K.NLDSTTVAVHGEEIYCK.S	1938.10514	2	5.17E-10	0.96	5.13	-	1106.1
AHQ-2-11, 3882	K.TVYFAEEVQCEGNSFHK.S	2047.19015	2	1.24E-04	0.84	3.63	-	551.0
gi|4506413|ref|NP_002875.1| RAP1A, member of RAS oncogene family; RAS-related protein RAP1A [Homo s				4.87E-12	7.01	80.29	33.20	20987.1
AHQ-2-11, 1854	K.CDLEDER.V	937.95183	2	3.97E-04	0.76	2.67	-	729.5
AHQ-2-11, 2408 - 2412	K.CDLEDERVVGK.E	1321.43945	2	8.40E-05	0.89	3.45	-	1124.7
AHQ-2-10, 6578	K.INVNEIFYDLVR.Q	1495.70427	2	1.04E-08	0.94	4.28	-	1018.8
AHQ-2-14-, 6767 - 6839	K.INVNEIFYDLVR.Q	1495.70427	2	1.07E-09	0.95	5.21	-	1019.5
AHQ-2-11, 6566 - 6648	K.INVNEIFYDLVR.Q	1495.70427	2	1.17E-09	0.96	5.04	-	1278.6
AHQ-2-14-, 6771	K.INVNEIFYDLVR.Q	1495.70427	1	4.21E-06	0.23	2.69	-	209.9
AHQ-2-14-, 6637	K.INVNEIFYDLVR.Q	1495.70427	2	2.73E-08	0.95	4.50	-	1338.8
AHQ-2-11, 6576 - 6640	K.INVNEIFYDLVR.Q	1495.70427	1	2.65E-06	0.30	3.14	-	218.2
AHQ-2-13-, 6919	K.INVNEIFYDLVR.Q	1495.70427	1	8.60E-07	0.08	1.85	-	78.7
AHQ-2-13-, 6918	K.INVNEIFYDLVR.Q	1495.70427	2	4.93E-08	0.94	4.83	-	894.9
AHQ-2-13, 6700 - 6701	K.INVNEIFYDLVR.Q	1495.70427	2	2.12E-07	0.95	4.92	-	1061.7
AHQ-2-11, 6010	K.INVNEIFYDLVR.Q	1495.70427	1	2.46E-04	0.41	2.69	-	270.0
AHQ-2-11, 5686 - 5748	K.INVNEIFYDLVR.Q	1495.70427	2	2.40E-06	0.92	3.99	-	1095.0
AHQ-2-11, 5692	K.INVNEIFYDLVR.Q	1495.70427	1	1.10E-04	0.07	1.96	-	127.6
AHQ-2-11, 6685 - 6749	K.INVNEIFYDLVR.Q	1495.70427	2	3.52E-08	0.94	4.65	-	1065.7
AHQ-2-11, 6584 - 6636	K.INVNEIFYDLVR.Q	1495.70427	1	3.15E-04	0.18	3.10	-	141.7
AHQ-2-11, 6004	K.INVNEIFYDLVR.Q	1495.70427	2	1.78E-07	0.95	5.13	-	829.1
AHQ-2-11, 6425	K.INVNEIFYDLVR.Q	1495.70427	2	1.43E-08	0.94	4.20	-	1346.9
AHQ-2-10, 2494	K.LVVLGSGGVGK.S	986.19059	2	1.34E-05	0.80	3.18	-	752.1
AHQ-2-11, 2573 - 2624	K.LVVLGSGGVGK.S	986.19059	2	2.12E-08	0.92	3.68	-	1009.7
AHQ-2-13, 2827	K.LVVLGSGGVGK.S	986.19059	2	2.73E-04	0.92	3.27	-	1230.5
AHQ-2-10, 2479	K.LVVLGSGGVGK.S	986.19059	1	5.15E-05	0.81	3.01	-	541.1

AHQ-2-11, 6202 - 6268	K.SALTVQFVQGIFVEK.Y	1666.94196	2	8.00E-05	0.97	5.31	-	1630.4
AHQ-2-11, 6464 - 6525	K.SALTVQFVQGIFVEK.Y	1666.94196	2	5.04E-05	0.94	3.85	-	1450.9
AHQ-2-11, 6529 - 6594	K.SALTVQFVQGIFVEK.Y	1666.94196	2	1.42E-04	0.97	5.45	-	1690.4
AHQ-2-11, 6148 - 6208	K.SALTVQFVQGIFVEK.Y	1666.94196	2	6.30E-06	0.97	4.86	-	2070.1
AHQ-2-11, 6328 - 6404	K.SALTVQFVQGIFVEK.Y	1666.94196	2	6.16E-06	0.97	5.71	-	1851.3
AHQ-2-11, 5664	K.SALTVQFVQGIFVEK.Y	1666.94196	2	1.85E-06	0.85	3.43	-	730.7
AHQ-2-11, 6654 - 6712	K.SALTVQFVQGIFVEK.Y	1666.94196	2	6.11E-04	0.95	4.81	-	1303.6
AHQ-2-11, 6874 - 6949	K.SALTVQFVQGIFVEK.Y	1666.94196	2	1.47E-04	0.96	4.68	-	1737.4
AHQ-2-11, 5698	K.SKINVNEIFYDLVR.Q	1710.95483	2	5.58E-08	0.93	4.05	-	1128.2
AHQ-2-11, 5917 - 5918	K.SKINVNEIFYDLVR.Q	1710.95483	2	7.91E-12	0.96	4.66	-	1281.0
AHQ-2-11, 6132 - 6206	K.SKINVNEIFYDLVR.Q	1710.95483	2	5.18E-09	0.91	4.03	-	831.2
AHQ-2-11, 5240 - 5265	K.SKINVNEIFYDLVR.Q	1710.95483	2	1.25E-07	0.86	3.47	-	697.1
AHQ-2-14-, 6193 - 6212	K.SKINVNEIFYDLVR.Q	1710.95483	3	4.64E-04	0.83	3.88	-	958.0
AHQ-2-11, 5082 - 5084	K.SKINVNEIFYDLVR.Q	1710.95483	2	1.40E-09	0.89	4.17	-	636.6
AHQ-2-14-, 6191	K.SKINVNEIFYDLVR.Q	1710.95483	2	4.87E-12	0.96	4.86	-	1175.2
AHQ-2-13-, 2818 - 2821	K.YDPTIEDSYR.K	1259.30338	2	6.30E-07	0.85	3.19	-	350.8
AHQ-2-11, 2677 - 2745	K.YDPTIEDSYR.K	1259.30338	2	1.41E-05	0.85	2.73	-	459.0
AHQ-2-11, 2532	K.YDPTIEDSYR.K	1259.30338	2	9.88E-07	0.87	2.89	-	542.4
AHQ-2-13, 2875	K.YDPTIEDSYR.K	1259.30338	2	1.52E-04	0.77	2.66	-	417.3
AHQ-2-11, 2301 - 2330	K.YDPTIEDSYRK.Q	1387.47629	2	1.88E-06	0.82	3.04	-	444.6
gi|17986273|ref|NP_524144.1| fast skeletal myosin alkali light chain 1 isoform 1f; A1 catalytic; A2				5.26E-12	0.96	10.23	8.20	21145.0
AHQ-2-13, 3356	R.VFDKEGNGTVMGAELR.H	1723.93228	2	6.35E-09	0.93	3.78	-	1063.0
AHQ-2-13-, 3345 - 3353	R.VFDKEGNGTVMGAELR.H	1723.93228	2	5.26E-12	0.96	4.59	-	1274.8
AHQ-2-12, 3305 - 3374	R.VFDKEGNGTVMGAELR.H	1723.93228	2	5.24E-06	0.93	3.84	-	1127.6
gi|5031855|ref|NP_005556.1| lymphocyte cytosolic protein 2; SH2 domain-containing leukocyte protein				5.41E-12	2.84	30.28	8.30	60187.8
AHQ-2-12, 4154	K.ESQVYLLGTGLR.G	1336.51879	2	2.64E-05	0.90	3.69	-	1293.3
AHQ-2-14-, 6823	R.GKEDFLSVSDIIDYFR.K	1905.09669	2	5.41E-12	0.98	5.64	-	2364.7
AHQ-2-10, 6580	R.SEVLGWDPDSLADYFK.K	1842.98222	2	3.16E-07	0.95	4.44	-	1011.8
gi|4504349|ref|NP_000509.1| beta globin [Homo sapiens]				6.03E-12	9.86	110.28	67.30	15998.3
AHQ-2-14-, 5640	R.FFESFGDLSTPDAVMGNPK.V	2060.27218	2	1.94E-06	0.94	4.51	-	880.3
AHQ-2-13-, 5823	R.FFESFGDLSTPDAVMGNPK.V	2060.27218	2	2.92E-05	0.77	2.93	-	824.1
AHQ-2-13-, 5639 - 5665	R.FFESFGDLSTPDAVMGNPK.V	2060.27218	2	3.85E-06	0.95	4.81	-	953.3
AHQ-2-13-, 5125	R.FFESFGDLSTPDAVM*GNPK.V	2076.27158	2	1.97E-07	0.85	4.06	-	404.7
AHQ-2-14-, 5055	R.FFESFGDLSTPDAVM*GNPK.V	2076.27158	2	7.15E-09	0.89	4.00	-	706.0
AHQ-2-14, 6153 - 6232	R.FFESFGDLSTPDAVM*GNPK.V	2076.27158	2	8.14E-04	0.70	3.47	-	451.3
AHQ-2-13, 4960 - 5035	R.FFESFGDLSTPDAVM*GNPK.V	2076.27158	2	5.75E-06	0.90	4.27	-	539.0
AHQ-2-13, 5536 - 5599	R.FFESFGDLSTPDAVMGNPK.V	2060.27218	2	1.83E-05	0.89	4.07	-	711.7
AHQ-2-13, 5380	R.FFESFGDLSTPDAVMGNPK.V	2060.27218	2	1.47E-05	0.88	3.87	-	728.2
AHQ-2-13, 5043	R.FFESFGDLSTPDAVM*GNPK.V	2076.27158	3	3.38E-07	0.95	4.75	-	1501.2
AHQ-2-13, 3509	K.GTFATLSELHCDK.L	1480.62492	2	7.47E-08	0.72	3.25	-	338.4
AHQ-2-13-, 3505	K.GTFATLSELHCDK.L	1480.62492	2	1.12E-05	0.82	3.73	-	515.3
AHQ-2-13, 5001	K.GTFATLSELHCDKLHVDPENFR.L	2588.83687	3	2.58E-09	0.93	4.69	-	942.3
AHQ-2-13, 5011	K.GTFATLSELHCDKLHVDPENFR.L	2588.83687	2	1.98E-05	0.90	4.13	-	414.9
AHQ-2-13-, 5055 - 5121	K.GTFATLSELHCDKLHVDPENFR.L	2588.83687	3	5.44E-09	0.89	3.86	-	1099.2
AHQ-2-13-, 5119	K.GTFATLSELHCDKLHVDPENFR.L	2588.83687	2	8.58E-09	0.89	3.82	-	636.6
AHQ-2-13-, 4867	K.KVLGAFSDGLAHLDNLK.G	1799.06362	3	6.15E-09	0.97	5.62	-	1570.5
AHQ-2-13-, 2758	K.LHVDPENFR.L	1127.23455	2	3.44E-04	0.78	2.50	-	931.8
AHQ-2-13, 6459	R.LLGNVLVCVLAHHFGK.E	1779.14197	3	6.12E-08	0.84	3.80	-	529.4
AHQ-2-13, 3441	K.SAVTALWGK.V	933.08624	2	5.41E-04	0.92	2.65	-	1251.6
AHQ-2-14, 6349	K.VLGAFSDGLAHLDNLK.G	1670.89071	2	9.82E-05	0.92	3.88	-	1228.5
AHQ-2-13, 5069 - 5131	K.VLGAFSDGLAHLDNLK.G	1670.89071	2	2.58E-10	0.96	5.17	-	1269.0
AHQ-2-13-, 4973 - 5041	K.VLGAFSDGLAHLDNLK.G	1670.89071	2	1.60E-08	0.95	4.66	-	1186.9
AHQ-2-13-, 5042	K.VLGAFSDGLAHLDNLK.G	1670.89071	3	1.74E-09	0.87	3.53	-	768.0
AHQ-2-13, 4907 - 4931	K.VLGAFSDGLAHLDNLK.G	1670.89071	2	7.73E-07	0.96	5.30	-	1227.4
AHQ-2-13, 5105 - 5107	K.VLGAFSDGLAHLDNLK.G	1670.89071	3	8.37E-08	0.87	3.79	-	702.2
AHQ-2-13-, 5217	K.VLGAFSDGLAHLDNLK.G	1670.89071	3	4.45E-07	0.84	3.13	-	603.2
AHQ-2-13-, 5209 - 5277	K.VLGAFSDGLAHLDNLK.G	1670.89071	2	6.03E-12	0.96	5.53	-	1422.4
AHQ-2-13-, 3238 - 3269	K.VNVDEVGGEALGR.L	1315.41484	1	4.98E-06	0.65	2.95	-	333.9
AHQ-2-13, 3287	K.VNVDEVGGEALGR.L	1315.41484	1	9.63E-05	0.78	3.33	-	439.7
AHQ-2-13, 3281 - 3284	K.VNVDEVGGEALGR.L	1315.41484	2	4.38E-06	0.96	4.57	-	1601.6
AHQ-2-14-, 3243 - 3245	K.VNVDEVGGEALGR.L	1315.41484	2	8.18E-07	0.97	4.58	-	2112.5
AHQ-2-13-, 3265 - 3266	K.VNVDEVGGEALGR.L	1315.41484	2	1.87E-04	0.96	4.38	-	1459.3
AHQ-2-13-, 2662	K.VVAGVANALAHK.Y	1150.35593	2	2.17E-09	0.96	3.95	-	1649.5
AHQ-2-13, 2271	K.VVAGVANALAHK.Y	1150.35593	2	1.33E-04	0.86	2.96	-	961.7
gi|4507877|ref|NP_003364.1| vinculin isoform VCL [Homo sapiens]				6.61E-12	53.03	630.31	49.80	116721.6
AHQ-2-10, 3296 - 3306	K.AGEVINQPMMMAAR.Q	1519.83932	2	5.22E-04	0.91	4.02	-	819.1
AHQ-2-4, 3693	K.AGEVINQPMMMAAR.Q	1519.83932	2	1.25E-06	0.95	4.66	-	1089.0
AHQ-2-12, 7213	K.AIPDLTAPVAAVQAAVSNLVR.V	2077.41342	2	1.11E-08	0.95	4.37	-	988.2
AHQ-2-11, 6777 - 6856	K.AIPDLTAPVAAVQAAVSNLVR.V	2077.41342	2	1.52E-11	0.97	4.96	-	1260.6
AHQ-2-10, 6819 - 6822	K.AIPDLTAPVAAVQAAVSNLVR.V	2077.41342	2	1.34E-06	0.97	5.74	-	1308.0
AHQ-2-13, 6877	K.AIPDLTAPVAAVQAAVSNLVR.V	2077.41342	2	3.84E-08	0.97	5.52	-	1266.8
AHQ-2-13-, 7073	K.AIPDLTAPVAAVQAAVSNLVR.V	2077.41342	2	4.52E-06	0.96	5.22	-	864.8
AHQ-2-1, 7314	K.AIPDLTAPVAAVQAAVSNLVR.V	2077.41342	2	2.90E-08	0.96	4.51	-	1101.5
AHQ-2-9, 7498 - 7503	K.AIPDLTAPVAAVQAAVSNLVR.V	2077.41342	2	2.46E-07	0.97	5.11	-	1216.8
AHQ-2-8, 7467 - 7468	K.AIPDLTAPVAAVQAAVSNLVR.V	2077.41342	2	4.48E-05	0.97	5.68	-	1126.0
AHQ-2-7, 7544 - 7559	K.AIPDLTAPVAAVQAAVSNLVR.V	2077.41342	2	1.06E-07	0.93	4.35	-	741.2
AHQ-2-3, 7609 - 7689	K.AIPDLTAPVAAVQAAVSNLVR.V	2077.41342	2	3.54E-09	0.96	5.23	-	1006.8
AHQ-2-6, 7227 - 7306	K.AIPDLTAPVAAVQAAVSNLVR.V	2077.41342	2	3.54E-11	0.97	4.99	-	1446.5
AHQ-2-4, 7512 - 7589	K.AIPDLTAPVAAVQAAVSNLVR.V	2077.41342	2	6.16E-06	0.87	3.77	-	679.2
AHQ-2-5, 7639	K.AIPDLTAPVAAVQAAVSNLVR.V	2077.41342	2	2.53E-09	0.88	3.64	-	744.8
AHQ-2-5, 7548	K.AIPDLTAPVAAVQAAVSNLVR.V	2077.41342	2	6.61E-12	0.97	5.44	-	1261.9
AHQ-2-4, 7662	K.AIPDLTAPVAAVQAAVSNLVR.V	2077.41342	2	2.97E-06	0.97	5.05	-	1183.5
AHQ-2-14-, 6925 - 6927	K.AIPDLTAPVAAVQAAVSNLVR.V	2077.41342	2	8.35E-10	0.98	5.88	-	1275.4
AHQ-2-7, 2965 - 2973	R.ALASQLQDSLK.D	1174.32943	2	7.45E-04	0.83	2.91	-	839.3
AHQ-2-6, 3037	R.ALASQLQDSLK.D	1174.32943	2	2.05E-04	0.84	3.09	-	844.2
AHQ-2-5, 3035 - 3047	R.ALASQLQDSLK.D	1174.32943	2	8.83E-05	0.95	3.78	-	1436.9
AHQ-2-4, 3289 - 3361	R.ALASQLQDSLK.D	1174.32943	2	5.12E-04	0.88	3.22	-	952.3
AHQ-2-4, 3801	K.AQQVSQGLDVLTAK.V	1458.64233	1	7.34E-06	0.71	2.94	-	591.6
AHQ-2-6, 3281	K.AQQVSQGLDVLTAK.V	1458.64233	1	1.83E-05	0.64	3.76	-	294.8
AHQ-2-6, 3203 - 3269	K.AQQVSQGLDVLTAK.V	1458.64233	2	2.22E-08	0.96	4.26	-	1570.9
AHQ-2-1, 3760	K.AQQVSQGLDVLTAK.V	1458.64233	2	4.59E-05	0.94	3.91	-	1064.8
AHQ-2-3, 3513 - 3593	K.AQQVSQGLDVLTAK.V	1458.64233	2	5.15E-04	0.95	3.77	-	1533.1
AHQ-2-4, 3561	K.AQQVSQGLDVLTAK.V	1458.64233	1	5.49E-04	0.63	3.25	-	397.6
AHQ-2-4, 3513 - 3585	K.AQQVSQGLDVLTAK.V	1458.64233	2	2.25E-09	0.96	4.71	-	1431.4
AHQ-2-7, 3183 - 3195	K.AQQVSQGLDVLTAK.V	1458.64233	2	2.14E-10	0.97	4.71	-	1478.7
AHQ-2-8, 3008	K.AQQVSQGLDVLTAK.V	1458.64233	2	7.37E-11	0.96	4.41	-	1307.5
AHQ-2-9, 3224	K.AQQVSQGLDVLTAK.V	1458.64233	2	7.05E-05	0.84	3.24	-	790.2
AHQ-2-2, 3556 - 3624	K.AQQVSQGLDVLTAK.V	1458.64233	2	6.39E-04	0.95	4.45	-	1205.4
AHQ-2-5, 3280	K.AQQVSQGLDVLTAK.V	1458.64233	1	4.29E-04	0.28	2.67	-	256.0
AHQ-2-7, 2211	K.AVAGNISDPGLQK.S	1270.41721	2	2.94E-05	0.87	3.45	-	682.1
AHQ-2-5, 2251	K.AVAGNISDPGLQK.S	1270.41721	2	6.60E-07	0.90	3.55	-	774.0
AHQ-2-6, 2255	K.AVAGNISDPGLQK.S	1270.41721	2	8.62E-07	0.87	3.66	-	713.3
AHQ-2-3, 2424	K.AVAGNISDPGLQK.S	1270.41721	2	3.57E-05	0.82	3.37	-	643.8
AHQ-2-5, 2259	K.AVAGNISDPGLQK.S	1270.41721	1	4.19E-05	0.53	3.20	-	305.8
AHQ-2-4, 2529	K.AVAGNISDPGLQK.S	1270.41721	2	8.83E-04	0.82	3.47	-	589.0
AHQ-2-4, 2416	K.AVAGNISDPGLQK.S	1270.41721	2	3.74E-05	0.90	3.61	-	812.8
AHQ-2-8, 2135	K.AVAGNISDPGLQK.S	1270.41721	2	2.28E-04	0.86	3.30	-	716.4

AHQ-2-6, 2261	K.AVAGNISDPGLQK.S	1270.41721	1	5.34E-06	0.68	2.81	-	435.1
AHQ-2-3, 6197	K.CDRVDQLTAQLADLAAR.G	1918.12182	2	6.85E-05	0.92	3.69	-	1129.7
AHQ-2-4, 6201 - 6272	K.CDRVDQLTAQLADLAAR.G	1918.12182	2	6.64E-06	0.92	4.04	-	937.5
AHQ-2-6, 5861 - 5862	K.CDRVDQLTAQLADLAAR.G	1918.12182	2	3.33E-04	0.79	3.29	-	694.0
AHQ-2-4, 6228	K.CDRVDQLTAQLADLAAR.G	1918.12182	3	4.77E-07	0.93	4.81	-	1315.5
AHQ-2-1, 6166	K.CDRVDQLTAQLADLAAR.G	1918.12182	2	2.12E-06	0.84	3.50	-	758.2
AHQ-2-4, 6448	K.CDRVDQLTAQLADLAAR.G	1918.12182	2	6.06E-05	0.76	3.05	-	719.9
AHQ-2-6, 5874 - 5882	K.CDRVDQLTAQLADLAAR.G	1918.12182	3	2.05E-05	0.88	4.50	-	988.8
AHQ-2-5, 5991	K.CDRVDQLTAQLADLAAR.G	1918.12182	3	1.82E-07	0.92	4.31	-	1425.1
AHQ-2-1, 6169	K.CDRVDQLTAQLADLAAR.G	1918.12182	3	5.89E-05	0.91	4.64	-	1016.1
AHQ-2-4, 2233 - 2309	R.DPSASPGDAGEQAIR.Q	1471.51169	2	7.68E-08	0.94	4.48	-	756.7
AHQ-2-7, 2187	R.DPSASPGDAGEQAIR.Q	1471.51169	2	3.08E-06	0.90	3.76	-	682.2
AHQ-2-5, 2195 - 2209	R.DPSASPGDAGEQAIR.Q	1471.51169	2	4.41E-09	0.94	4.22	-	942.2
AHQ-2-1, 2416 - 2426	R.DPSASPGDAGEQAIR.Q	1471.51169	2	4.28E-07	0.88	3.55	-	720.7
AHQ-2-3, 2328	R.DPSASPGDAGEQAIR.Q	1471.51169	2	1.10E-08	0.92	3.65	-	861.9
AHQ-2-6, 2185	R.DPSASPGDAGEQAIR.Q	1471.51169	2	6.89E-07	0.93	4.55	-	650.3
AHQ-2-4, 2316 - 2320	R.DPSASPGDAGEQAIR.Q	1471.51169	2	2.66E-08	0.91	3.24	-	895.1
AHQ-2-5, 4504	K.ELLPVLISAM*K.I	1230.54265	2	3.32E-04	0.89	3.31	-	916.4
AHQ-2-4, 6068 - 6085	K.ELLPVLISAMK.I	1214.54325	2	1.73E-04	0.81	3.09	-	601.4
AHQ-2-4, 4833	K.ELLPVLISAM*K.I	1230.54265	2	1.59E-06	0.86	3.40	-	847.3
AHQ-2-5, 5695	K.ELLPVLISAMK.I	1214.54325	2	5.53E-05	0.82	3.27	-	601.5
AHQ-2-10, 2466	R.ELTPQVVSAAR.I	1171.32878	2	4.77E-04	0.87	2.57	-	1099.1
AHQ-2-11, 2960	K.ETVQTTEDQILK.R	1405.53306	2	3.47E-05	0.71	3.43	-	403.5
AHQ-2-5, 2875	K.ETVQTTEDQILK.R	1405.53306	1	1.80E-04	0.13	2.44	-	145.4
AHQ-2-4, 2916	K.ETVQTTEDQILKR.D	1561.71942	2	2.83E-05	0.67	2.74	-	601.3
AHQ-2-11, 2797 - 2874	K.ETVQTTEDQILKR.D	1561.71942	2	2.25E-04	0.54	3.03	-	373.3
AHQ-2-7, 2624	K.ETVQTTEDQILKR.D	1561.71942	2	2.95E-04	0.62	2.84	-	544.1
AHQ-2-5, 2660	K.ETVQTTEDQILKR.D	1561.71942	2	3.81E-04	0.71	2.64	-	673.4
AHQ-2-3, 2936	K.ETVQTTEDQILKR.D	1561.71942	2	9.10E-08	0.66	2.74	-	632.4
AHQ-2-4, 7749 - 7769	R.GILSGTSDLLLTFDEAEVR.K	2037.25656	2	5.67E-05	0.80	3.96	-	460.1
AHQ-2-5, 7297 - 7365	R.GILSGTSDLLLTFDEAEVR.K	2037.25656	2	7.88E-07	0.98	6.26	-	1266.1
AHQ-2-1, 7164 - 7166	R.GILSGTSDLLLTFDEAEVR.K	2037.25656	2	1.16E-06	0.93	4.21	-	1033.0
AHQ-2-11, 6676 - 6745	R.GILSGTSDLLLTFDEAEVR.K	2037.25656	2	4.86E-04	0.89	3.99	-	881.7
AHQ-2-4, 7368 - 7444	R.GILSGTSDLLLTFDEAEVR.K	2037.25656	2	1.41E-09	0.97	5.64	-	1032.0
AHQ-2-5, 6627 - 6644	R.GILSGTSDLLLTFDEAEVRK.I	2165.42947	2	6.58E-05	0.82	3.38	-	632.7
AHQ-2-3, 6825	R.GILSGTSDLLLTFDEAEVRK.I	2165.42947	2	2.88E-05	0.72	3.44	-	474.9
AHQ-2-4, 6874 - 6880	R.GILSGTSDLLLTFDEAEVRK.I	2165.42947	2	1.07E-07	0.93	4.11	-	1093.8
AHQ-2-4, 1929 - 2065	K.GNDIIAAAK.R	872.98920	1	1.50E-05	0.39	2.01	-	472.5
AHQ-2-4, 1998	K.GNDIIAAAK.R	872.98920	1	6.91E-05	0.23	1.95	-	486.6
AHQ-2-5, 3313	K.GWLRDPSASPGDAGEQAIR.Q	1984.11876	2	1.27E-04	0.88	4.15	-	675.5
AHQ-2-4, 3572	K.GWLRDPSASPGDAGEQAIR.Q	1984.11876	3	2.06E-09	0.93	4.46	-	1492.2
AHQ-2-4, 3564 - 3580	K.GWLRDPSASPGDAGEQAIR.Q	1984.11876	2	4.10E-04	0.86	4.08	-	652.0
AHQ-2-4, 2142	K.IAELCDDPKER.D	1347.47690	2	8.77E-05	0.75	3.05	-	575.0
AHQ-2-5, 1932	K.IAELCDDPKER.D	1347.47690	2	1.24E-04	0.89	3.26	-	1011.8
AHQ-2-5, 3212	K.IAELCDDPKERDDILR.S	1960.15548	3	2.87E-07	0.90	3.79	-	1338.5
AHQ-2-4, 3468	K.IAELCDDPKERDDILR.S	1960.15548	3	2.45E-04	0.86	3.10	-	1290.1
AHQ-2-5, 3211	K.IAELCDDPKERDDILR.S	1960.15548	2	1.55E-04	0.65	3.01	-	543.1
AHQ-2-13, 3084	R.IPTISTQLK.I	1001.20202	2	2.61E-04	0.68	2.59	-	636.0
AHQ-2-4, 2014	R.KIAELCDDPKER.D	1475.64981	2	6.04E-06	0.91	3.06	-	1272.7
AHQ-2-4, 3136 - 3210	R.KIAELCDDPKERDDILR.S	2088.32839	3	4.23E-04	0.74	3.24	-	555.4
AHQ-2-4, 4512	K.KIDAAQNWLADPNGGPEGEEQIR.G	2509.67332	2	1.46E-09	0.90	4.17	-	607.0
AHQ-2-5, 4191 - 4244	K.KIDAAQNWLADPNGGPEGEEQIR.G	2509.67332	3	4.03E-05	0.94	5.45	-	994.4
AHQ-2-4, 4429 - 4444	K.KIDAAQNWLADPNGGPEGEEQIR.G	2509.67332	2	5.64E-04	0.67	3.01	-	392.5
AHQ-2-6, 4071 - 4145	K.KIDAAQNWLADPNGGPEGEEQIR.G	2509.67332	3	2.14E-05	0.92	4.23	-	1398.5
AHQ-2-5, 2544	R.LANVMM*GPYR.Q	1168.41500	2	1.76E-04	0.71	2.70	-	700.1
AHQ-2-4, 2824	R.LANVM*MGPYR.Q	1168.41500	2	8.54E-04	0.86	3.65	-	910.5
AHQ-2-6, 3053	R.LANVMMGPYR.Q	1152.41560	2	3.06E-04	0.90	3.42	-	950.4
AHQ-2-6, 2625	R.LANVM*MGPYR.Q	1168.41500	2	8.48E-05	0.87	3.18	-	1004.5
AHQ-2-5, 3027	R.LANVMMGPYR.Q	1152.41560	2	2.15E-04	0.90	3.89	-	695.1
AHQ-2-5, 2980 - 2983	K.LLAVAATAPPDAPNR.E	1477.69048	2	8.61E-04	0.92	4.46	-	573.4
AHQ-2-4, 4541	K.LLAVAATAPPDAPNREEVFDER.A	2382.61458	2	8.76E-05	0.83	3.58	-	419.8
AHQ-2-4, 4597 - 4668	R.LTDELAPPKPPLPEGEVPPPRPPPPEEKDEEFPEQK.A	4028.46845	3	4.96E-07	0.82	3.94	-	651.0
AHQ-2-4, 4736	R.LTDELAPPKPPLPEGEVPPPRPPPPEEKDEEFPEQK.A	4028.46845	3	5.65E-05	0.84	4.05	-	494.8
AHQ-2-5, 4372 - 4385	R.LTDELAPPKPPLPEGEVPPPRPPPPEEKDEEFPEQK.A	4028.46845	3	1.68E-04	0.79	4.11	-	542.7
AHQ-2-4, 3750 - 3820	K.LVQAAQM*LQSDPYSVPAR.D	1991.25773	2	1.11E-09	0.94	4.76	-	633.4
AHQ-2-6, 3535	K.LVQAAQM*LQSDPYSVPAR.D	1991.25773	2	1.91E-07	0.94	4.89	-	706.6
AHQ-2-5, 3555	K.LVQAAQM*LQSDPYSVPAR.D	1991.25773	2	8.47E-05	0.67	3.15	-	451.7
AHQ-2-4, 4684 - 4753	K.LVQAAQMLQSDPYSVPAR.D	1975.25833	2	8.96E-07	0.91	4.37	-	673.4
AHQ-2-4, 4706	K.LVQAAQMLQSDPYSVPAR.D	1975.25833	3	2.12E-04	0.80	3.07	-	1031.9
AHQ-2-7, 4293 - 4303	K.LVQAAQMLQSDPYSVPAR.D	1975.25833	2	3.99E-06	0.84	4.06	-	601.2
AHQ-2-7, 3487	K.LVQAAQM*LQSDPYSVPAR.D	1991.25773	2	4.28E-04	0.87	3.61	-	783.3
AHQ-2-12, 4202	R.MALLMAEMSR.L	1153.46531	2	1.52E-07	0.96	4.52	-	1772.0
AHQ-2-12, 2167	R.M*ALLM*AEM*SR.L	1201.46351	2	5.19E-04	0.87	2.90	-	878.4
AHQ-2-4, 4353	R.MALLMAEMSR.L	1153.46531	2	9.43E-08	0.96	4.65	-	1548.0
AHQ-2-6, 3947	K.MLGQMTDQVADLR.A	1478.72055	2	2.22E-07	0.96	4.95	-	1512.4
AHQ-2-4, 3781	K.M*LGQMTDQVADLR.A	1494.71995	2	2.02E-06	0.70	2.52	-	652.7
AHQ-2-5, 3987	K.M*LGQM*TDQVADLR.A	1510.71935	2	9.38E-05	0.93	3.52	-	1519.1
AHQ-2-4, 3297	K.MLGQM*TDQVADLR.A	1494.71995	2	3.45E-06	0.92	3.85	-	1453.4
AHQ-2-4, 4233 - 4236	K.MLGQMTDQVADLR.A	1478.72055	2	4.85E-10	0.97	4.95	-	1638.2
AHQ-2-7, 3907	K.MLGQMTDQVADLR.A	1478.72055	2	1.26E-07	0.90	2.91	-	1693.0
AHQ-2-5, 3084	K.MLGQM*TDQVADLR.A	1494.71995	2	8.54E-07	0.93	3.86	-	1522.2
AHQ-2-8, 3811 - 3814	K.MLGQMTDQVADLR.A	1478.72055	2	1.84E-08	0.97	4.93	-	1876.3
AHQ-2-9, 3951	K.MLGQMTDQVADLR.A	1478.72055	2	2.67E-06	0.91	3.29	-	1267.0
AHQ-2-4, 2865	K.M*LGQM*TDQVADLR.A	1510.71935	2	8.97E-04	0.96	3.81	-	1966.1
AHQ-2-6, 3062	K.MLGQM*TDQVADLR.A	1494.71995	2	3.65E-04	0.73	2.87	-	827.7
AHQ-2-4, 4253	K.M*LGQM*TDQVADLR.A	1510.71935	2	2.41E-05	0.94	3.68	-	1347.3
AHQ-2-3, 4272	K.MLGQMTDQVADLR.A	1478.72055	2	4.15E-08	0.96	4.61	-	1725.6
AHQ-2-5, 3979 - 3983	K.MLGQMTDQVADLR.A	1478.72055	2	1.47E-06	0.97	4.70	-	1911.4
AHQ-2-4, 3101 - 3104	K.M*SAEINEIIR.V	1192.36823	2	9.40E-06	0.93	3.59	-	1116.5
AHQ-2-4, 3441	K.MSAEINEIIR.V	1176.36883	2	1.88E-04	0.86	2.67	-	949.4
AHQ-2-5, 3504	K.MSAEINEIIR.V	1176.36883	2	1.52E-04	0.81	3.02	-	800.8
AHQ-2-5, 2848	K.M*TGLVDEAIDTK.S	1309.46876	2	1.13E-07	0.96	3.84	-	1726.7
AHQ-2-4, 3593 - 3594	K.MTGLVDEAIDTK.S	1293.46936	1	1.40E-04	0.34	2.19	-	356.0
AHQ-2-4, 3086	K.M*TGLVDEAIDTK.S	1309.46876	2	3.20E-07	0.96	3.99	-	1535.7
AHQ-2-6, 3314	K.MTGLVDEAIDTK.S	1293.46936	2	2.20E-07	0.97	4.03	-	1663.6
AHQ-2-6, 2863	K.M*TGLVDEAIDTK.S	1309.46876	2	6.83E-06	0.89	3.05	-	1119.2
AHQ-2-5, 4041 - 4113	K.MTGLVDEAIDTK.S	1293.46936	2	4.91E-04	0.80	2.63	-	942.9
AHQ-2-6, 2659	R.NPGNQAAYEHFETMK.N	1737.87437	3	6.16E-07	0.68	3.05	-	475.9
AHQ-2-6, 2646 - 2651	R.NPGNQAAYEHFETMK.N	1737.87437	2	7.19E-05	0.89	3.90	-	520.7
AHQ-2-5, 2576	R.NPGNQAAYEHFETMK.N	1737.87437	2	1.20E-06	0.90	3.70	-	812.1
AHQ-2-4, 2381	R.NPGNQAAYEHFETM*K.N	1753.87377	2	4.21E-04	0.88	3.71	-	582.9
AHQ-2-4, 2886	R.NPGNQAAYEHFETMK.N	1737.87437	2	2.41E-05	0.93	3.82	-	1043.5
AHQ-2-5, 2577 - 2589	R.NPGNQAAYEHFETMK.N	1737.87437	3	1.53E-05	0.77	3.30	-	731.0
AHQ-2-5, 2648	K.NQWIDNVEK.M	1146.23464	1	2.96E-04	0.62	2.84	-	273.5
AHQ-2-6, 2671 - 2686	K.NQWIDNVEK.M	1146.23464	1	1.93E-06	0.26	2.22	-	225.9
AHQ-2-4, 2720 - 2800	K.QVATALQNLQTK.T	1315.50112	2	6.63E-05	0.89	3.76	-	515.1
AHQ-2-7, 2489 - 2505	K.QVATALQNLQTK.T	1315.50112	2	4.80E-06	0.85	3.03	-	657.9

AHQ-2-4, 3416	R.SLGEISALTSK.L	1106.25186	1	3.17E-09	0.68	3.35	-	297.3
AHQ-2-7, 3048	R.SLGEISALTSK.L	1106.25186	1	3.96E-06	0.33	2.49	-	170.7
AHQ-2-1, 3646	R.SLGEISALTSK.L	1106.25186	2	1.78E-04	0.81	2.68	-	693.1
AHQ-2-7, 3039	R.SLGEISALTSK.L	1106.25186	2	2.37E-04	0.90	3.41	-	728.0
AHQ-2-6, 3150	R.SLGEISALTSK.L	1106.25186	1	1.92E-06	0.63	3.02	-	183.5
AHQ-2-6, 3130 - 3149	R.SLGEISALTSK.L	1106.25186	2	3.39E-06	0.93	3.60	-	929.3
AHQ-2-8, 2842	K.SLLDASEEAIKK.D	1304.47187	2	3.34E-05	0.80	2.83	-	745.4
AHQ-2-4, 3276 - 3281	K.SLLDASEEAIKK.D	1304.47187	2	4.02E-05	0.89	3.13	-	1017.7
AHQ-2-5, 3007 - 3008	K.SLLDASEEAIKK.D	1304.47187	2	3.33E-06	0.79	2.89	-	751.0
AHQ-2-4, 2244 - 2312	K.STVEGIQASVK.T	1119.25064	2	3.21E-06	0.88	2.86	-	1065.0
AHQ-2-5, 2120	K.STVEGIQASVK.T	1119.25064	1	2.48E-05	0.15	2.11	-	103.5
AHQ-2-5, 2112 - 2119	K.STVEGIQASVK.T	1119.25064	2	1.26E-06	0.91	3.41	-	928.6
AHQ-2-4, 2392 - 2397	R.TDAGFTLR.W	880.96819	2	8.53E-05	0.84	2.52	-	873.3
AHQ-2-7, 7501	R.TIESILEPVAQQISHLVIMHEEGEVDGK.A	3103.49334	3	1.05E-06	0.74	3.59	-	495.7
AHQ-2-11, 6809 - 6814	R.TIESILEPVAQQISHLVIMHEEGEVDGK.A	3103.49334	3	6.71E-09	0.85	4.10	-	620.8
AHQ-2-14-, 6789 - 6860	R.TIESILEPVAQQISHLVIM*HEEGEVDGK.A	3119.49274	3	1.60E-05	0.68	3.52	-	261.7
AHQ-2-5, 7487	R.TIESILEPVAQQISHLVIMHEEGEVDGK.A	3103.49334	3	2.59E-06	0.82	3.79	-	457.7
AHQ-2-3, 7411	R.TNISDEESEQATEMLVHNAQNLMQSVK.E	3048.31017	3	8.52E-04	0.66	3.12	-	641.9
AHQ-2-4, 7460 - 7477	R.TNISDEESEQATEMLVHNAQNLMQSVK.E	3048.31017	3	4.65E-06	0.88	4.64	-	551.5
AHQ-2-12, 5930 - 6009	R.TNISDEESEQATEMLVHNAQNLM*QSVK.E	3064.30957	3	9.84E-08	0.94	5.31	-	940.7
AHQ-2-12, 5533 - 5597	R.TNISDEESEQATEM*LVHNAQNLM*QSVK.E	3080.30897	3	4.78E-06	0.59	3.02	-	607.7
AHQ-2-4, 7105 - 7172	R.TNISDEESEQATEM*LVHNAQNLMQSVK.E	3064.30957	3	5.99E-04	0.63	3.39	-	639.2
AHQ-2-12, 5149 - 5182	R.TNISDEESEQATEMLVHNAQNLM*QSVK.E	3064.30957	3	1.22E-06	0.88	4.78	-	655.4
AHQ-2-4, 5380	R.TNISDEESEQATEMLVHNAQNLM*QSVK.E	3064.30957	3	6.25E-04	0.72	3.25	-	713.4
AHQ-2-12, 6001	R.TNISDEESEQATEMLVHNAQNLMQSVK.E	3048.31017	3	5.70E-05	0.84	3.91	-	544.2
AHQ-2-6, 7093 - 7094	R.TNISDEESEQATEMLVHNAQNLMQSVK.E	3048.31017	3	2.91E-10	0.90	4.80	-	574.4
AHQ-2-12, 6993	R.TNISDEESEQATEMLVHNAQNLMQSVK.E	3048.31017	3	2.52E-09	0.88	4.75	-	439.4
AHQ-2-12, 5945	R.TNISDEESEQATEMLVHNAQNLM*QSVK.E	3064.30957	3	6.28E-06	0.77	3.94	-	488.9
AHQ-2-12, 6663 - 6725	R.TNISDEESEQATEM*LVHNAQNLMQSVK.E	3064.30957	3	1.00E-05	0.88	5.15	-	652.3
AHQ-2-4, 7374 - 7393	R.TNISDEESEQATEMLVHNAQNLMQSVK.E	3048.31017	3	6.60E-04	0.69	3.78	-	391.4
AHQ-2-10, 5635	R.TNISDEESEQATEMLVHNAQNLMQSVK.E	3048.31017	3	1.01E-07	0.64	3.29	-	464.0
AHQ-2-10, 6590	R.TNISDEESEQATEMLVHNAQNLMQSVK.E	3048.31017	3	8.04E-05	0.92	4.49	-	1001.5
AHQ-2-10, 2926	R.TNLLQVCER.I	1134.28859	1	1.52E-05	0.34	2.34	-	66.0
AHQ-2-4, 3148 - 3217	R.TNLLQVCER.I	1134.28859	2	2.02E-05	0.92	3.38	-	1231.4
AHQ-2-12, 3101	R.TNLLQVCER.I	1134.28859	2	7.02E-06	0.87	3.29	-	925.3
AHQ-2-12, 4931	K.VAM*ANIQPQM*LVAGATSIAR.R	2075.44278	2	3.15E-05	0.47	2.94	-	365.8
AHQ-2-5, 5965 - 6023	K.VAMANIQPQMLVAGATSIAR.R	2043.44398	3	1.89E-04	0.94	4.71	-	1439.3
AHQ-2-4, 5786	K.VAMANIQPQM*LVAGATSIAR.R	2059.44338	3	2.18E-05	0.93	4.94	-	1470.7
AHQ-2-6, 5881 - 5950	K.VAMANIQPQMLVAGATSIAR.R	2043.44398	2	3.99E-05	0.89	4.30	-	599.2
AHQ-2-5, 5931 - 5999	K.VAMANIQPQMLVAGATSIAR.R	2043.44398	2	7.59E-05	0.96	5.27	-	885.7
AHQ-2-5, 4987	R.VDQLTAQLADLAAR.G	1485.66778	2	6.14E-06	0.94	2.81	-	2229.1
AHQ-2-5, 4940 - 4993	R.VDQLTAQLADLAAR.G	1485.66778	1	5.66E-07	0.81	3.18	-	400.4
AHQ-2-5, 5296 - 5367	R.VLQLTSWDEDAWASK.D	1749.90156	2	8.27E-09	0.96	4.63	-	1384.0
AHQ-2-1, 5692	R.VLQLTSWDEDAWASK.D	1749.90156	2	1.41E-05	0.92	3.65	-	889.3
AHQ-2-6, 5270 - 5281	R.VLQLTSWDEDAWASK.D	1749.90156	2	2.11E-09	0.97	4.70	-	1396.4
AHQ-2-5, 5429	R.VLQLTSWDEDAWASK.D	1749.90156	2	2.94E-04	0.75	2.84	-	718.5
AHQ-2-2, 5649	R.VLQLTSWDEDAWASK.D	1749.90156	2	1.03E-08	0.94	4.29	-	1045.8
AHQ-2-4, 5608 - 5690	R.VLQLTSWDEDAWASK.D	1749.90156	2	1.10E-08	0.96	5.09	-	1168.5
AHQ-2-3, 5595 - 5597	R.VLQLTSWDEDAWASK.D	1749.90156	2	5.11E-07	0.95	4.44	-	1058.4
AHQ-2-4, 2372	R.VMLVNSM*NTVK.E	1252.53021	2	4.35E-04	0.95	3.79	-	1159.0
AHQ-2-5, 2863	R.VMLVNSMNTVK.E	1236.53081	2	4.18E-05	0.95	3.68	-	1344.5
AHQ-2-5, 2995	R.VMLVNSMNTVK.E	1236.53081	2	3.68E-05	0.92	3.41	-	1120.1
AHQ-2-4, 3145	R.VMLVNSMNTVK.E	1236.53081	2	5.10E-04	0.93	3.47	-	1237.8
AHQ-2-5, 2689 - 2703	R.WIDNPTVDDR.G	1231.29640	2	3.17E-05	0.93	3.59	-	1190.6
AHQ-2-6, 2701	R.WIDNPTVDDR.G	1231.29640	2	2.34E-04	0.96	4.18	-	1409.3
gi|4505735|ref|NP_002611.1| platelet factor 4 variant 1; Platelet factor 4, variant 1 (PF4-like) [H				6.69E-12	1.92	20.25	26.00	11552.6
AHQ-2-14-, 3843 - 3911	R.AEAEEDGDLQCLCVK.T	1739.86036	2	6.69E-12	0.96	4.91	-	1356.0
AHQ-2-14-, 3752	R.AEAEEDGDLQCLCVK.T	1739.86036	2	6.37E-06	0.92	3.44	-	1024.4
AHQ-2-14-, 5861	R.KICLDLQALLYK.K	1479.80948	2	2.29E-04	0.96	4.68	-	1124.4
gi|4758012|ref|NP_004850.1| clathrin heavy chain; clathrin, heavy polypeptide-like 2 [Homo sapiens]				6.73E-12	23.66	260.32	25.60	191612.6
AHQ-2-3, 6853	K.ADDPSSYMEVVQAANTSGNWEELVK.Y	2741.92512	2	6.55E-07	0.92	4.52	-	692.2
AHQ-2-3, 7599 - 7600	K.AFMTADLPNELIELLEK.I	1948.26956	2	7.93E-04	0.93	4.81	-	551.1
AHQ-2-3, 4488	R.ALEHFTDLYDIK.R	1465.63179	2	6.83E-06	0.93	3.66	-	1097.9
AHQ-2-1, 7308	R.FQSVPAQPGQTSPLLQYFGILLDQGQLNK.Y	3189.60926	3	1.54E-07	0.87	4.48	-	638.7
AHQ-2-3, 7596 - 7601	R.FQSVPAQPGQTSPLLQYFGILLDQGQLNK.Y	3189.60926	3	5.48E-05	0.91	5.03	-	792.1
AHQ-2-3, 4961	R.GQFSTDELVAEVEK.R	1552.66462	2	2.99E-04	0.87	3.57	-	915.7
AHQ-2-4, 4830 - 4844	R.GQFSTDELVAEVEKR.N	1708.85097	2	9.83E-09	0.93	3.99	-	1081.5
AHQ-2-3, 4827	R.GQFSTDELVAEVEKR.N	1708.85097	2	8.45E-09	0.95	3.83	-	1585.8
AHQ-2-3, 3207	K.HDVVFLITK.Y	1072.28194	2	4.10E-07	0.94	3.20	-	1181.5
AHQ-2-3, 3208	K.HDVVFLITK.Y	1072.28194	1	1.62E-04	0.59	2.19	-	680.2
AHQ-2-3, 5396 - 5413	R.ISGETIFVTAPHEATAGIIGVNR.K	2354.64751	3	2.42E-08	0.94	4.62	-	1101.8
AHQ-2-3, 3568	K.IVLDNSVFSEHR.N	1416.56400	2	5.01E-07	0.88	3.25	-	1055.1
AHQ-2-3, 7309	R.KFDVNTSAVQVLIEHIGNLDR.A	2369.66218	3	6.73E-12	0.98	6.12	-	2796.5
AHQ-2-3, 3500 - 3503	R.KFNALFAQGNYSEAAK.V	1759.94281	2	3.87E-07	0.98	5.79	-	1885.1
AHQ-2-3, 4551	R.LASTLVHLGEYQAAVDGAR.K	1972.19103	2	1.91E-06	0.93	3.99	-	1180.1
AHQ-2-4, 4560	R.LASTLVHLGEYQAAVDGAR.K	1972.19103	2	9.72E-05	0.94	4.67	-	1004.9
AHQ-2-1, 4692	R.LASTLVHLGEYQAAVDGAR.K	1972.19103	2	2.06E-06	0.93	4.43	-	949.5
AHQ-2-3, 4381	K.LHIIEVGTPPTGNQPFPK.K	1946.23831	2	1.52E-04	0.91	4.09	-	633.7
AHQ-2-3, 7420	R.LLEMNLMHAPQVADAILGNQMFTHYDR.A	3130.61385	3	9.90E-10	0.98	6.48	-	2002.2
AHQ-2-3, 3737	K.LLYNNVSNFGR.L	1297.44435	2	7.15E-08	0.91	3.34	-	970.7
AHQ-2-1, 6953	R.LPVVIGGLLDVDCSEDVIK.N	2043.36744	2	4.61E-05	0.92	4.29	-	723.6
AHQ-2-3, 7205 - 7267	R.LPVVIGGLLDVDCSEDVIK.N	2043.36744	2	7.31E-07	0.98	5.76	-	1693.9
AHQ-2-3, 6456	R.NLQNLLILTAIK.A	1354.66353	2	4.83E-04	0.97	5.15	-	1791.4
AHQ-2-3, 4361	R.NNLAGAEELFAR.K	1305.42165	2	1.96E-06	0.89	3.36	-	894.5
AHQ-2-3, 2356	K.RDPHLACVAYER.G	1488.65351	2	2.70E-04	0.78	2.92	-	521.1
AHQ-2-3, 7128 - 7129	R.RPLIDQVVQTALSETQDPEEVSVTVK.A	2883.20103	2	1.35E-05	0.93	5.24	-	477.9
AHQ-2-5, 6999	R.RPLIDQVVQTALSETQDPEEVSVTVK.A	2883.20103	3	5.57E-04	0.81	3.87	-	810.5
AHQ-2-3, 7105 - 7112	R.RPLIDQVVQTALSETQDPEEVSVTVK.A	2883.20103	3	1.90E-05	0.94	4.93	-	1097.0
AHQ-2-4, 5164	R.TSIDAYDNFDNISLAQR.L	1944.04818	2	2.09E-08	0.91	4.17	-	530.5
AHQ-2-1, 5180	R.TSIDAYDNFDNISLAQR.L	1944.04818	2	1.70E-07	0.97	5.44	-	1773.5
AHQ-2-3, 7253	K.VGYTPDWIFLLR.N	1480.73443	2	7.41E-08	0.69	2.79	-	448.4
AHQ-2-3, 3095	K.VIQCFAETGQVQK.I	1509.70940	2	1.09E-06	0.96	4.05	-	1744.4
AHQ-2-3, 3423 - 3493	K.VSQPIEGHAASFAQFK.M	1717.90593	2	4.53E-04	0.67	2.99	-	710.8
AHQ-2-3, 5016	K.WLKEDKLECSEELGDLVK.S	2193.45974	2	2.32E-09	0.98	5.61	-	2202.0
AHQ-2-3, 7523	K.YHEQLSTQSLIELFESFK.S	2200.43237	2	9.81E-07	0.98	5.60	-	1873.2
gi|21464101|ref|NP_036611.2| tyrosine 3-monooxygenase/tryptophan 5-monooxygenase activation protein				8.16E-12	5.03	70.26	44.90	28302.4
AHQ-2-10, 6930	K.ELEAVCQDVLSLLDNYLIK.N	2237.55566	2	6.46E-09	0.96	5.21	-	1096.9
AHQ-2-10, 6668	R.LGLALNYSVFYYEIQNAPEQACHLAK.T	3015.38773	3	8.16E-12	0.92	4.97	-	682.6
AHQ-2-10, 3870	R.NLLSVAYKNVVGAR.R	1504.75906	2	3.43E-06	0.95	3.52	-	1720.8
AHQ-2-9, 3796	R.NLLSVAYKNVVGAR.R	1504.75906	2	4.21E-04	0.92	3.72	-	1097.1
AHQ-2-10, 2790	K.NVTELNEPLSNEER.N	1644.72215	2	1.22E-05	0.91	3.77	-	771.9
AHQ-2-10, 2915	K.NVTELNEPLSNEER.N	1644.72215	2	4.10E-06	0.76	3.28	-	524.1
AHQ-2-10, 6567	K.TAFDDAIAELDTLNEDSYK.D	2132.22370	2	3.29E-04	0.79	3.62	-	574.4
AHQ-2-14-, 6521 - 6585	K.TAFDDAIAELDTLNEDSYK.D	2132.22370	2	1.21E-07	0.68	2.84	-	765.0
AHQ-2-10, 6126	K.TAFDDAIAELDTLNEDSYK.D	2132.22370	2	1.20E-05	0.78	3.66	-	595.7
AHQ-2-10, 6258 - 6330	K.TAFDDAIAELDTLNEDSYK.D	2132.22370	2	4.22E-08	0.95	4.44	-	1173.3
AHQ-2-10, 6334	K.TAFDDAIAELDTLNEDSYK.D	2132.22370	3	1.91E-06	0.76	3.52	-	711.7

AHQ-2-14-, 6548 - 6583	K.TAFDDAIAELDTLNEDSYK.D	2132.22370	2	3.89E-07	0.97	5.00	-	1303.6
AHQ-2-10, 2138	R.YDDMAAAMK.N	1016.17419	1	1.11E-05	0.27	2.21	-	340.2
AHQ-2-10, 2082	R.YLAEVATGEK.R	1081.20087	1	1.98E-05	0.21	2.11	-	459.3
AHQ-2-10, 2084	R.YLAEVATGEK.R	1081.20087	2	8.09E-05	0.90	3.38	-	891.2
AHQ-2-10, 2090	R.YLAEVATGEK.R	1081.20087	1	1.77E-04	0.41	2.41	-	278.3
gi|4504391|ref|NP_000179.1| hexokinase 1 isoform HKI; brain form hexokinase [Homo sapiens]				8.74E-12	11.01	120.25	15.50	102502.4
AHQ-2-4, 6378 - 6457	R.AILQQLGLNSTCDDSILVK.T	2090.38419	2	4.13E-11	0.93	4.46	-	725.1
AHQ-2-4, 4525 - 4538	K.ATDCVGHDVVTLLR.D	1557.75406	2	8.74E-12	0.92	3.53	-	949.5
AHQ-2-4, 2669 - 2749	K.FLSQIESDR.L	1095.18763	2	8.72E-05	0.87	3.47	-	724.5
AHQ-2-4, 3221	K.GAALITAVGVR.L	1028.23070	2	1.24E-06	0.96	4.25	-	1796.7
AHQ-2-4, 5809	K.GDFIALDLGGSSFR.I	1455.59700	2	8.78E-12	0.98	4.96	-	2145.6
AHQ-2-4, 2162	R.HIDLVEGDEGR.M	1240.30483	2	2.57E-07	0.97	4.75	-	1437.2
AHQ-2-4, 5561	K.KLPVGFTFSFPCQQSK.I	1873.16425	2	3.18E-07	0.91	3.77	-	949.2
AHQ-2-4, 6181 - 6182	K.LPVGFTFSFPCQQSK.I	1744.99134	2	6.57E-07	0.94	4.10	-	1042.4
AHQ-2-4, 6021	R.LSDETLIDIMTR.F	1407.61560	2	3.18E-08	0.95	4.05	-	1199.0
AHQ-2-4, 2729 - 2801	R.LVNEYSLNAGK.Q	1208.34588	2	3.26E-07	0.72	3.10	-	705.8
AHQ-2-4, 7521	R.SANLVAATLGAILNR.L	1484.72615	2	1.24E-07	0.95	4.93	-	1090.6
AHQ-2-4, 6416	R.TPDGTENGDFLALDLGGTNFR.V	2211.33051	2	1.04E-08	0.92	4.58	-	663.1
gi|9910542|ref|NP_064535.1| SAR1a gene homolog 1; SAR1 protein [Homo sapiens]				8.98E-12	4.46	50.31	46.00	22366.6
AHQ-2-11, 3620	R.EIFGLYGQTTGK.G	1314.46845	1	4.12E-05	0.66	3.05	-	283.2
AHQ-2-11, 6081	R.LGQHVPTLHPTSEELTIAGMTFTTFDLGGHEQAR.R	3694.08659	3	8.98E-12	0.96	6.10	-	1057.5
AHQ-2-11, 3654	K.LREIFGLYGQTTGK.G	1583.81305	2	1.06E-06	0.96	4.52	-	1230.8
AHQ-2-11, 6424	K.NYLPAINGIVFLVDCADHSR.L	2276.55690	2	3.33E-09	0.95	4.39	-	1151.0
AHQ-2-11, 6560 - 6621	K.VELNALM*TDETISNVPILILGNK.I	2514.92088	2	5.88E-04	0.93	4.37	-	888.9
gi|4507907|ref|NP_000543.1| von Willebrand factor precursor; Coagulation factor VIII VWF (von Wille				9.27E-12	30.36	340.33	14.60	309296.7
AHQ-2-1, 5082	K.AFVLSSVDELEQQR.D	1621.77332	2	1.85E-06	0.88	3.39	-	1085.0
AHQ-2-1, 4036	K.AHLLSLVDVM*QR.E	1398.65673	2	1.32E-09	0.95	3.62	-	1709.2
AHQ-2-1, 5038	K.AHLLSLVDVMQR.E	1382.65733	2	6.13E-09	0.95	4.00	-	1443.6
AHQ-2-1, 3081 - 3161	K.APTCGLCEVAR.L	1236.40097	2	8.42E-05	0.88	2.92	-	895.9
AHQ-2-1, 4324	R.CLPTACTIQLR.G	1335.57568	2	1.43E-04	0.54	2.63	-	494.7
AHQ-2-1, 5220	R.EGGPSQIGDALGFAVR.Y	1574.71988	2	1.41E-08	0.72	2.92	-	649.0
AHQ-2-1, 4696	R.EGGPSQIGDALGFAVR.Y	1574.71988	2	1.38E-06	0.69	3.05	-	639.5
AHQ-2-1, 4888	R.EQAPNLVYM*VTGNPASDEIK.R	2193.42002	2	6.98E-09	0.89	4.08	-	486.7
AHQ-2-1, 5813 - 5880	R.EQAPNLVYMVTGNPASDEIK.R	2177.42062	2	4.53E-04	0.82	3.54	-	445.8
AHQ-2-1, 4226	K.EQDLEVILHNGACSPGAR.Q	1968.13770	2	6.80E-10	0.87	3.81	-	562.7
AHQ-2-1, 4525 - 4598	K.EQDLEVILHNGACSPGAR.Q	1968.13770	2	8.11E-05	0.81	3.77	-	566.6
AHQ-2-1, 2420	R.GLRPSCPNSQSPVK.V	1528.71594	2	3.61E-05	0.73	3.47	-	499.7
AHQ-2-1, 6433	R.IEDLPTMVTLGNSFLHK.L	1916.23079	2	6.47E-06	0.96	5.20	-	1021.2
AHQ-2-1, 6426	R.IEDLPTMVTLGNSFLHK.L	1916.23079	3	2.19E-04	0.63	3.26	-	451.3
AHQ-2-1, 5260 - 5290	R.IEDLPTM*VTLGNSFLHK.L	1932.23019	2	5.48E-07	0.83	3.66	-	668.6
AHQ-2-2, 7564	R.IGWPNAPILIQDFETLPR.E	2081.40223	2	2.19E-06	0.91	4.07	-	599.1
AHQ-2-1, 7120	R.IGWPNAPILIQDFETLPR.E	2081.40223	2	1.11E-08	0.92	4.57	-	732.5
AHQ-2-1, 7234 - 7312	R.IGWPNAPILIQDFETLPR.E	2081.40223	2	2.49E-08	0.95	4.67	-	888.3
AHQ-2-1, 2684	R.ILAGPAGDSNVVK.L	1241.41901	2	2.46E-07	0.95	3.56	-	1390.5
AHQ-2-1, 2773	R.ILAGPAGDSNVVK.L	1241.41901	2	9.55E-05	0.70	2.78	-	680.0
AHQ-2-3, 2504	R.ILAGPAGDSNVVK.L	1241.41901	2	2.40E-06	0.80	2.89	-	705.4
AHQ-2-1, 3876 - 3880	R.ILTSDVFQDCNK.L	1441.58869	2	4.21E-07	0.96	4.64	-	1155.6
AHQ-2-2, 3818	R.ILTSDVFQDCNK.L	1441.58869	2	6.04E-08	0.86	3.22	-	872.3
AHQ-2-1, 5988 - 6005	R.LPGDIQVVPIGVGPNANVQELER.I	2415.73085	3	2.38E-05	0.95	4.87	-	1575.5
AHQ-2-1, 4329 - 4330	R.LSEAEFEVLK.A	1165.31777	2	7.18E-08	0.95	3.99	-	1257.0
AHQ-2-1, 4272 - 4293	K.LTGSCSYVLFQNK.E	1518.71629	2	1.40E-06	0.95	4.34	-	1276.0
AHQ-2-1, 5798 - 5869	K.RLPGDIQVVPIGVGPNANVQELER.I	2571.91720	3	4.37E-04	0.97	5.57	-	2164.3
AHQ-2-2, 5600	K.RLPGDIQVVPIGVGPNANVQELER.I	2571.91720	3	1.95E-06	0.97	5.50	-	1914.5
AHQ-2-1, 5654 - 5730	K.RLPGDIQVVPIGVGPNANVQELER.I	2571.91720	3	5.59E-09	0.98	6.54	-	1902.6
AHQ-2-1, 4072	R.SGFTYVLHEGECCGR.C	1774.91285	2	3.23E-11	0.94	3.62	-	1416.7
AHQ-2-1, 2524 - 2534	R.SKEFM*EEVIQR.M	1412.59390	2	1.94E-05	0.92	3.28	-	1144.2
AHQ-2-1, 3456 - 3470	R.SKEFMEEVIQR.M	1396.59450	2	7.21E-07	0.92	3.53	-	1087.9
AHQ-2-3, 5032 - 5112	K.SVGSQWASPENPCLINECVR.V	2306.51832	2	3.25E-05	0.82	3.63	-	396.8
AHQ-2-2, 5158	K.SVGSQWASPENPCLINECVR.V	2306.51832	2	1.99E-05	0.87	4.02	-	425.6
AHQ-2-1, 5093	K.SVGSQWASPENPCLINECVR.V	2306.51832	2	6.58E-05	0.91	4.16	-	588.2
AHQ-2-1, 4998 - 5021	K.SVGSQWASPENPCLINECVR.V	2306.51832	2	1.18E-06	0.89	3.86	-	707.0
AHQ-2-1, 5790 - 5837	K.TYGLCGICDENGANDFM*LR.D	2225.42231	2	3.77E-10	0.95	4.52	-	1128.6
AHQ-2-1, 6241 - 6242	K.TYGLCGICDENGANDFMLR.D	2209.42291	2	1.09E-06	0.94	4.37	-	927.4
AHQ-2-1, 4613	K.VIVIPVGIGPHANLK.Q	1527.87891	3	1.41E-04	0.68	3.23	-	486.4
AHQ-2-1, 4609 - 4621	K.VIVIPVGIGPHANLK.Q	1527.87891	2	5.66E-07	0.91	4.09	-	652.9
AHQ-2-1, 2408 - 2476	R.VKEEVFIQQR.N	1276.46658	2	1.93E-07	0.85	3.00	-	799.8
AHQ-2-1, 4744	R.VTVFPIGIGDR.Y	1174.37426	2	3.49E-04	0.93	3.61	-	1129.4
AHQ-2-1, 3633	R.WTCPCVCTGSSTR.H	1575.72645	2	2.38E-07	0.87	2.93	-	814.1
AHQ-2-3, 2841	K.YAGSQVASTSEVLK.Y	1440.58064	2	9.40E-06	0.96	4.36	-	1332.6
AHQ-2-1, 3010	K.YAGSQVASTSEVLK.Y	1440.58064	1	6.65E-06	0.81	3.11	-	792.4
AHQ-2-2, 2852	K.YAGSQVASTSEVLK.Y	1440.58064	2	1.31E-04	0.96	4.23	-	1181.1
AHQ-2-1, 2993 - 2994	K.YAGSQVASTSEVLK.Y	1440.58064	2	1.27E-07	0.94	4.38	-	866.5
AHQ-2-3, 7500	R.YFTFSGICQYLLAR.D	1741.00269	2	1.09E-06	0.94	4.41	-	851.3
AHQ-2-1, 6848	K.YLFPGECQYVLVQDYCGSNPGTFR.I	2874.15305	2	9.27E-12	0.88	3.78	-	753.4
AHQ-2-1, 6782 - 6836	K.YLFPGECQYVLVQDYCGSNPGTFR.I	2874.15305	3	2.13E-08	0.86	3.71	-	1336.9
AHQ-2-2, 3257	R.YLSDHSFLVSQGDR.E	1624.73576	2	1.78E-09	0.97	4.86	-	1836.0
AHQ-2-1, 3452	R.YLSDHSFLVSQGDR.E	1624.73576	2	1.85E-09	0.94	3.98	-	1453.4
AHQ-2-1, 6172	K.YTLFQIFSK.I	1147.34728	2	1.10E-08	0.93	3.18	-	1091.9
gi|9507215|ref|NP_061816.1| tubulin, alpha-like 2; tubulin, alpha 8 [Homo sapiens]				9.36E-12	3.71	40.23	10.90	50093.2
AHQ-2-14-, 5289	R.AVMIDLEPTVVDEVR.A	1686.95185	2	8.48E-10	0.95	4.27	-	1436.8
AHQ-2-14, 6029	R.AVM*IDLEPTVVDEVR.A	1702.95125	2	2.70E-05	0.90	3.69	-	1060.6
AHQ-2-7, 5291	R.IHFPLVTYAPIISAEK.A	1800.13312	2	6.73E-07	0.93	4.08	-	715.4
AHQ-2-14, 6325 - 6396	K.INDDDSFTTFFSETGNGK.H	1996.03358	2	1.20E-10	0.47	2.55	-	170.6
AHQ-2-14-, 5308	K.INDDDSFTTFFSETGNGK.H	1996.03358	2	9.36E-12	0.93	4.67	-	697.8
AHQ-2-13, 5293	K.INDDDSFTTFFSETGNGK.H	1996.03358	2	3.06E-07	0.75	3.75	-	397.3
gi|29741246|ref|XP_294070.1| similar to Glyceraldehyde 3-phosphate dehydrogenase, liver (GAPDH) [Ho				9.77E-12	4.26	50.25	14.60	35032.0
AHQ-2-9, 5291	K.AENGKLVINGNPITIFQER.D	2114.39062	2	1.01E-07	0.70	3.07	-	466.5
AHQ-2-9, 5471	K.AENGKLVINGNPITIFQER.D	2114.39062	2	6.09E-06	0.67	3.29	-	483.6
AHQ-2-13, 5295	K.AENGKLVINGNPITIFQER.D	2114.39062	2	5.97E-07	0.40	3.16	-	363.6
AHQ-2-9, 5706 - 5779	K.LVINGNPITIFQER.D	1614.87029	2	6.84E-09	0.96	4.64	-	1258.9
AHQ-2-14-, 5632 - 5705	K.LVINGNPITIFQER.D	1614.87029	2	1.28E-09	0.95	4.23	-	1112.7
AHQ-2-10, 5230	K.LVINGNPITIFQER.D	1614.87029	2	1.46E-05	0.82	2.77	-	1063.2
AHQ-2-9, 5550 - 5623	K.LVINGNPITIFQER.D	1614.87029	2	2.91E-10	0.92	3.92	-	947.8
AHQ-2-10, 5328	K.LVINGNPITIFQER.D	1614.87029	2	3.14E-11	0.94	3.52	-	1516.3
AHQ-2-13, 5652	K.LVINGNPITIFQER.D	1614.87029	2	1.25E-07	0.86	2.99	-	812.8
AHQ-2-12, 5711 - 5786	K.LVINGNPITIFQER.D	1614.87029	2	1.20E-09	0.92	3.42	-	1120.1
AHQ-2-13, 5525 - 5587	K.LVINGNPITIFQER.D	1614.87029	2	9.77E-12	0.92	3.63	-	1028.7
AHQ-2-9, 5112 - 5182	K.LVINGNPITIFQERDPSK.I	2042.32440	2	2.15E-08	0.86	3.46	-	616.3
AHQ-2-9, 6398	K.VLHDNFGIVKGLMTTVHAITATQK.T	2596.04531	3	1.99E-04	0.94	4.95	-	1868.1
AHQ-2-14-, 6825	K.VLHDNFGIVKGLMTTVHAITATQK.T	2596.04531	3	7.04E-09	0.90	4.77	-	1403.6
AHQ-2-10, 5962 - 6040	K.VLHDNFGIVKGLM*TTVHAITATQK.T	2612.04471	3	1.30E-05	0.88	5.01	-	1100.4
gi|18104967|ref|NP_000953.2| prostaglandin-endoperoxide synthase 1 isoform 1 precursor; prostagland				1.00E-11	6.22	70.28	20.20	68686.0
AHQ-2-6, 3730 - 3738	K.AEHPTWGDEQLFQTTR.L	1917.02765	2	6.00E-05	0.95	4.46	-	859.0
AHQ-2-7, 3637	K.AEHPTWGDEQLFQTTR.L	1917.02765	2	4.77E-07	0.91	3.80	-	807.3
AHQ-2-6, 3651 - 3653	K.ALGHGVDLGHIYGDNLER.Q	1937.10524	3	2.87E-07	0.92	3.89	-	1187.6
AHQ-2-6, 3642 - 3663	K.ALGHGVDLGHIYGDNLER.Q	1937.10524	2	1.00E-11	0.97	5.46	-	1576.7
AHQ-2-6, 4945	R.FGMKPYTSFQELVGEK.E	1862.13845	2	1.24E-06	0.91	3.84	-	513.0

AHQ-2-6, 7025 - 7090	K.GLLGNPICSPEYWKPSTFGGEVGFNIVK.T	3069.47853	3	1.15E-08	0.85	4.33	-	419.1
AHQ-2-6, 6935 - 7013	K.GLLGNPICSPEYWKPSTFGGEVGFNIVK.T	3069.47853	3	1.39E-07	0.81	4.27	-	495.6
AHQ-2-6, 3090	R.VPDASQDDGPAVERPSTEL	1984.06747	2	4.34E-05	0.76	2.61	-	768.6
AHQ-2-6, 3193 - 3267	R.VPDASQDDGPAVERPSTEL	1984.06747	2	9.93E-05	0.92	4.29	-	888.7
AHQ-2-6, 5802	K.YQVLDGEMYPPSVEEAPVLMHYPR.G	2822.20799	3	1.35E-09	0.95	5.62	-	818.9
AHQ-2-6, 5298	K.YQVLDGEMYPPSVEEAPVLM*HYPR.G	2838.20739	3	2.80E-11	0.87	4.16	-	643.9
gi|10835002|ref|NP_001166.1| Rho GDP dissociation inhibitor (GDI) beta; Ly-GDI [Homo sapiens]				1.22E-11	5.38	60.24	47.30	22987.8
AHQ-2-10, 2303	K.APEPHVEEDDDDELDSK.L	1940.90995	2	2.14E-07	0.94	4.14	-	1126.4
AHQ-2-10, 2263 - 2324	K.APEPHVEEDDDDELDSK.L	1940.90995	3	1.22E-11	0.95	4.29	-	1745.3
AHQ-2-13-, 5638	K.ATFM*VGSYGPRPEEYEFLTPVEEAPK.G	2962.27864	3	1.63E-04	0.88	3.98	-	826.1
AHQ-2-12, 5891	K.ATFMVGSYGPRPEEYEFLTPVEEAPK.G	2946.27924	3	1.71E-04	0.81	3.96	-	435.8
AHQ-2-10, 5154 - 5172	K.ATFM*VGSYGPRPEEYEFLTPVEEAPK.G	2962.27864	3	5.64E-08	0.92	4.77	-	847.7
AHQ-2-10, 5394 - 5458	K.ATFMVGSYGPRPEEYEFLTPVEEAPK.G	2946.27924	3	7.41E-04	0.67	3.35	-	520.4
AHQ-2-10, 5435 - 5502	K.ATFMVGSYGPRPEEYEFLTPVEEAPK.G	2946.27924	2	4.02E-04	0.65	3.00	-	422.7
AHQ-2-14-, 5607	K.ATFM*VGSYGPRPEEYEFLTPVEEAPK.G	2962.27864	3	6.08E-05	0.83	4.03	-	825.4
AHQ-2-10, 2904	K.ELQEMDKDDESLIK.Y	1693.85489	2	3.86E-05	0.89	4.21	-	804.3
AHQ-2-11, 6465	R.LTLVCESAPGPITMDLTGDLEALKK.E	2675.11274	3	3.82E-05	0.52	3.22	-	295.5
AHQ-2-10, 6343 - 6423	R.LTLVCESAPGPITMDLTGDLEALKK.E	2675.11274	3	4.16E-08	0.84	4.15	-	627.4
AHQ-2-10, 3068 - 3119	K.TLLGDGPVVTDPK.A	1312.49400	2	5.53E-10	0.97	4.33	-	1753.2
gi|13775198|ref|NP_112576.1| SH3 domain binding glutamic acid-rich protein like 3; SH3BGRL3-like pr				1.26E-11	5.04	60.29	35.50	10437.7
AHQ-2-14-, 4748	R.IQYQLVDISQDNALR.D	1776.97168	2	1.26E-11	0.98	4.83	-	2260.5
AHQ-2-13-, 4893	R.IQYQLVDISQDNALR.D	1776.97168	2	1.33E-10	0.98	5.18	-	2065.1
AHQ-2-14, 5800	R.IQYQLVDISQDNALR.D	1776.97168	2	1.68E-10	0.98	5.26	-	2056.3
AHQ-2-13-, 4826 - 4898	R.IQYQLVDISQDNALR.D	1776.97168	2	7.83E-08	0.95	4.53	-	1527.7
AHQ-2-13, 4843 - 4868	R.IQYQLVDISQDNALR.D	1776.97168	2	2.78E-09	0.95	5.17	-	1333.2
AHQ-2-13-, 4802	R.IQYQLVDISQDNALR.D	1776.97168	3	2.19E-07	0.96	5.77	-	1986.2
AHQ-2-13, 4707 - 4779	R.IQYQLVDISQDNALR.D	1776.97168	2	1.24E-10	0.98	5.83	-	1721.2
AHQ-2-13-, 4713 - 4779	R.IQYQLVDISQDNALR.D	1776.97168	2	2.55E-10	0.98	5.83	-	2052.8
AHQ-2-13, 5063 - 5144	R.IQYQLVDISQDNALRDEMR.A	2308.55751	3	2.12E-07	0.80	3.65	-	550.7
AHQ-2-13-, 4845 - 4851	R.IQYQLVDISQDNALRDEMR.A	2308.55751	3	5.55E-04	0.78	3.26	-	772.8
AHQ-2-13, 4937 - 4999	R.IQYQLVDISQDNALRDEMR.A	2308.55751	3	4.38E-09	0.83	3.44	-	623.4
AHQ-2-14-, 4456 - 4529	R.IQYQLVDISQDNALRDEM*R.A	2324.55691	3	6.60E-04	0.81	3.62	-	441.0
AHQ-2-13-, 4513 - 4577	R.IQYQLVDISQDNALRDEM*R.A	2324.55691	3	1.08E-04	0.79	3.54	-	470.0
AHQ-2-13-, 5043 - 5109	R.IQYQLVDISQDNALRDEMR.A	2308.55751	3	6.93E-06	0.80	3.17	-	654.8
AHQ-2-13-, 4274	R.IQYQLVDISQDNALRDEM*R.A	2324.55691	3	3.65E-04	0.82	3.32	-	535.9
AHQ-2-13-, 5045 - 5111	R.IQYQLVDISQDNALRDEMR.A	2308.55751	2	3.36E-04	0.92	4.29	-	665.8
AHQ-2-13-, 4298	K.RIQYQLVDISQDNALR.D	1933.15803	2	2.02E-08	0.97	5.45	-	1070.8
AHQ-2-13, 4244	K.RIQYQLVDISQDNALR.D	1933.15803	2	4.42E-07	0.96	4.71	-	1085.6
AHQ-2-13-, 1841 - 1855	R.VYSTSVTGSR.E	1057.13940	2	2.56E-07	0.91	3.23	-	1144.6
AHQ-2-13-, 1589 - 1649	R.VYSTSVTGSR.E	1057.13940	2	8.29E-06	0.88	2.98	-	1034.1
AHQ-2-13-, 1543 - 1969	R.VYSTSVTGSR.E	1057.13940	1	3.66E-04	0.09	1.85	-	72.1
AHQ-2-13-, 1930 - 1974	R.VYSTSVTGSR.E	1057.13940	2	4.79E-05	0.90	3.25	-	1013.1
AHQ-2-13, 2083 - 2145	R.VYSTSVTGSR.E	1057.13940	2	6.75E-07	0.75	2.80	-	743.4
AHQ-2-13, 1939 - 2005	R.VYSTSVTGSR.E	1057.13940	2	9.95E-06	0.89	3.30	-	914.5
AHQ-2-13, 1887 - 1987	R.VYSTSVTGSR.E	1057.13940	1	4.26E-05	0.24	2.38	-	71.1
AHQ-2-13-, 1709 - 1770	R.VYSTSVTGSR.E	1057.13940	2	1.38E-05	0.93	3.26	-	1261.5
AHQ-2-13, 1827 - 2279	R.VYSTSVTGSR.E	1057.13940	1	8.25E-04	0.02	1.91	-	41.7
AHQ-2-13, 2335	R.VYSTSVTGSREIK.S	1427.58510	2	2.90E-05	0.57	2.53	-	641.6
AHQ-2-13-, 2225	R.VYSTSVTGSREIK.S	1427.58510	2	8.76E-07	0.56	2.95	-	507.5
gi|4503689|ref|NP_000499.1| fibrinogen, alpha chain isoform alpha-E preproprotein [Homo sapiens] [M				1.38E-11	18.48	220.27	29.10	94972.5
AHQ-2-6, 5849 - 5854	K.DSDWPFCSDEDWNYK.C	1965.98609	2	4.10E-06	0.94	4.21	-	737.5
AHQ-2-7, 6017	K.DSDWPFCSDEDWNYK.C	1965.98609	2	2.37E-05	0.91	3.27	-	713.9
AHQ-2-9, 5555	K.DSHSLTTNIMEILR.G	1630.84839	2	2.82E-05	0.95	3.99	-	1300.5
AHQ-2-10, 4455	K.DSHSLTTNIM*EILR.G	1646.84779	2	4.02E-06	0.86	3.35	-	801.1
AHQ-2-1, 5193	K.DSHSLTTNIM*EILR.G	1646.84779	2	8.98E-04	0.90	3.18	-	1261.6
AHQ-2-7, 5495	K.DSHSLTTNIMEILR.G	1630.84839	2	1.14E-04	0.85	2.70	-	1057.4
AHQ-2-12, 5771	K.DSHSLTTNIMEILR.G	1630.84839	2	2.62E-05	0.96	4.00	-	1573.3
AHQ-2-10, 5216 - 5252	K.DSHSLTTNIMEILR.G	1630.84839	2	6.74E-07	0.95	4.07	-	1178.2
AHQ-2-11, 5501 - 5506	K.DSHSLTTNIMEILR.G	1630.84839	2	5.29E-04	0.82	3.51	-	533.6
AHQ-2-13, 2403	K.ESSSHHPGIAEFPSR.G	1638.72252	2	9.57E-05	0.78	3.27	-	404.9
AHQ-2-13, 2696	R.EVDLKDYEDQQK.Q	1510.58460	2	2.69E-06	0.74	2.73	-	693.2
AHQ-2-1, 2694	R.EVDLKDYEDQQK.Q	1510.58460	2	3.19E-07	0.66	2.85	-	585.7
AHQ-2-6, 2365	R.EVDLKDYEDQQK.Q	1510.58460	2	6.22E-08	0.93	3.89	-	1023.3
AHQ-2-5, 2360	R.EVDLKDYEDQQK.Q	1510.58460	2	4.61E-05	0.81	2.82	-	681.1
AHQ-2-9, 2420	R.EVDLKDYEDQQK.Q	1510.58460	2	1.08E-06	0.78	2.90	-	666.8
AHQ-2-7, 2331	R.EVDLKDYEDQQK.Q	1510.58460	2	2.78E-05	0.77	3.09	-	574.2
AHQ-2-8, 2247 - 2255	R.EVDLKDYEDQQK.Q	1510.58460	2	2.66E-07	0.88	3.47	-	863.5
AHQ-2-13-, 2630	R.EVDLKDYEDQQK.Q	1510.58460	2	5.65E-09	0.90	3.32	-	906.9
AHQ-2-13, 6527	K.EVVTSEDGSDCPEAMDLGTLSGIGTLDGFR.H	3131.34937	3	7.43E-06	0.72	3.24	-	760.4
AHQ-2-13, 6279 - 6285	K.EVVTSEDGSDCPEAMDLGTLSGIGTLDGFR.H	3131.34937	3	2.66E-08	0.94	5.39	-	958.3
AHQ-2-1, 6721	K.EVVTSEDGSDCPEAMDLGTLSGIGTLDGFR.H	3131.34937	3	3.50E-09	0.88	4.23	-	956.9
AHQ-2-11, 6212	K.EVVTSEDGSDCPEAMDLGTLSGIGTLDGFR.H	3131.34937	3	2.07E-05	0.91	4.63	-	1015.4
AHQ-2-1, 6581 - 6606	K.EVVTSEDGSDCPEAM*DLGTLSGIGTLDGFR.H	3147.34877	3	1.10E-04	0.91	4.83	-	640.6
AHQ-2-7, 6752	K.EVVTSEDGSDCPEAMDLGTLSGIGTLDGFR.H	3131.34937	3	3.17E-05	0.68	3.21	-	742.2
AHQ-2-7, 1495 - 1501	R.GGSTSYGTGSETESPR.N	1573.55765	2	3.64E-05	0.76	3.61	-	338.4
AHQ-2-1, 1886	R.GGSTSYGTGSETESPR.N	1573.55765	2	7.37E-07	0.76	2.67	-	664.0
AHQ-2-4, 4961 - 4974	K.GLIDEVNQDFTNR.I	1521.61384	2	6.18E-07	0.95	4.35	-	1089.9
AHQ-2-10, 4548	K.GLIDEVNQDFTNR.I	1521.61384	2	2.00E-07	0.72	2.51	-	830.9
AHQ-2-10, 4414 - 4426	K.GLIDEVNQDFTNR.I	1521.61384	2	5.54E-08	0.93	4.22	-	925.5
AHQ-2-12, 4758	K.GLIDEVNQDFTNR.I	1521.61384	2	2.54E-08	0.93	4.33	-	807.8
AHQ-2-9, 4627 - 4704	K.GLIDEVNQDFTNR.I	1521.61384	2	1.11E-06	0.74	3.19	-	713.9
AHQ-2-9, 5167	K.GLIDEVNQDFTNR.I	1521.61384	2	2.03E-08	0.86	3.45	-	876.0
AHQ-2-5, 4785 - 4788	K.GLIDEVNQDFTNR.I	1521.61384	2	6.35E-05	0.92	3.64	-	1100.9
AHQ-2-1, 4937 - 4940	K.GLIDEVNQDFTNR.I	1521.61384	2	1.70E-09	0.94	4.11	-	960.4
AHQ-2-6, 4671 - 4687	K.GLIDEVNQDFTNR.I	1521.61384	2	1.34E-09	0.92	3.84	-	860.9
AHQ-2-2, 5016	K.GLIDEVNQDFTNR.I	1521.61384	2	9.20E-11	0.91	3.78	-	883.2
AHQ-2-11, 4524 - 4585	K.GLIDEVNQDFTNR.I	1521.61384	2	1.46E-08	0.85	2.83	-	997.1
AHQ-2-3, 4971	K.GLIDEVNQDFTNR.I	1521.61384	2	1.39E-09	0.90	3.18	-	901.0
AHQ-2-8, 4571 - 4579	K.GLIDEVNQDFTNR.I	1521.61384	2	4.56E-08	0.92	4.12	-	863.0
AHQ-2-8, 4124	K.GLIDEVNQDFTNR.I	1521.61384	2	2.37E-05	0.87	3.02	-	712.2
AHQ-2-7, 4703	K.GLIDEVNQDFTNR.I	1521.61384	2	2.47E-07	0.82	3.26	-	753.1
AHQ-2-4, 2145 - 2150	R.GSESGIFTNTK.E	1141.21317	1	7.56E-04	0.51	2.22	-	426.0
AHQ-2-13, 2383	R.GSESGIFTNTK.E	1141.21317	2	4.29E-04	0.75	2.97	-	577.2
AHQ-2-6, 1910	R.GSESGIFTNTK.E	1141.21317	1	1.56E-04	0.20	2.09	-	255.8
AHQ-2-6, 1918	R.GSESGIFTNTK.E	1141.21317	1	8.48E-06	0.57	2.61	-	388.8
AHQ-2-13-, 2262	R.GSESGIFTNTK.E	1141.21317	1	1.77E-05	0.58	2.88	-	313.1
AHQ-2-13-, 3053	R.HPDEAAFFDTASTGK.T	1594.66329	2	3.38E-06	0.85	3.81	-	461.3
AHQ-2-13, 2669	R.HRHPDEAAFFDTASTGK.T	1887.98954	2	2.45E-04	0.91	3.79	-	819.3
AHQ-2-4, 2404	R.HRHPDEAAFFDTASTGK.T	1887.98954	2	8.91E-05	0.85	3.61	-	313.7
AHQ-2-7, 2168	R.HRHPDEAAFFDTASTGK.T	1887.98954	3	7.11E-07	0.97	4.99	-	2083.1
AHQ-2-6, 2158 - 2230	R.HRHPDEAAFFDTASTGK.T	1887.98954	3	1.55E-04	0.95	4.71	-	1518.9
AHQ-2-5, 2221	R.HRHPDEAAFFDTASTGK.T	1887.98954	2	7.63E-06	0.94	4.49	-	844.3
AHQ-2-13-, 2594	R.HRHPDEAAFFDTASTGK.T	1887.98954	2	2.24E-05	0.95	5.01	-	864.0
AHQ-2-9, 2258	R.HRHPDEAAFFDTASTGK.T	1887.98954	2	1.90E-04	0.96	4.42	-	1420.0
AHQ-2-13-, 2613	R.HRHPDEAAFFDTASTGK.T	1887.98954	3	2.65E-06	0.97	4.99	-	2382.7
AHQ-2-13, 2691	R.HRHPDEAAFFDTASTGK.T	1887.98954	3	8.78E-07	0.94	4.19	-	1518.9
AHQ-2-11, 2414	R.HRHPDEAAFFDTASTGK.T	1887.98954	2	1.41E-05	0.96	4.86	-	1139.8

AHQ-2-10, 2256	R.HRHPDEAAFFDTASTGK.T	1887.98954	2	4.39E-05	0.95	4.28	-	1163.2
AHQ-2-9, 3123	K.M*KPVPDLVPGNFK.S	1458.75054	2	4.91E-07	0.84	3.39	-	571.9
AHQ-2-10, 3144 - 3159	K.M*KPVPDLVPGNFK.S	1458.75054	2	1.54E-04	0.63	3.07	-	358.0
AHQ-2-10, 3594	K.MKPVPDLVPGNFK.S	1442.75114	2	1.94E-04	0.80	3.15	-	469.0
AHQ-2-9, 5127	K.NNKDSHSLTTNIMEILR.G	1987.22751	2	1.31E-05	0.86	3.97	-	688.2
AHQ-2-9, 5020	K.NNKDSHSLTTNIMEILR.G	1987.22751	2	4.81E-06	0.95	4.36	-	1109.3
AHQ-2-4, 2133	R.NPSSAGSWNSGSSGPGSTGNR.N	1964.94443	2	6.75E-06	0.88	3.73	-	551.9
AHQ-2-6, 1938	R.NPSSAGSWNSGSSGPGSTGNR.N	1964.94443	2	6.05E-06	0.84	3.08	-	692.9
AHQ-2-7, 2031	R.NPSSAGSWNSGSSGPGSTGNR.N	1964.94443	2	1.01E-09	0.92	4.58	-	558.4
AHQ-2-9, 2123	R.NPSSAGSWNSGSSGPGSTGNR.N	1964.94443	2	1.20E-06	0.90	3.70	-	759.9
AHQ-2-7, 1876	R.NPSSAGSWNSGSSGPGSTGNR.N	1964.94443	2	6.70E-11	0.96	4.76	-	1106.5
AHQ-2-1, 2282	R.NPSSAGSWNSGSSGPGSTGNR.N	1964.94443	2	3.13E-05	0.43	3.02	-	289.8
AHQ-2-9, 2518	K.NSLFEYQK.N	1029.12765	1	3.83E-04	0.28	2.65	-	199.4
AHQ-2-8, 1816 - 1820	R.PGSTGTWNPGSSER.G	1433.46515	2	1.83E-06	0.86	3.86	-	502.2
AHQ-2-7, 1857	R.PGSTGTWNPGSSER.G	1433.46515	2	9.74E-05	0.67	3.21	-	490.4
AHQ-2-11, 2150	R.PGSTGTWNPGSSER.G	1433.46515	2	3.26E-04	0.66	2.84	-	623.6
AHQ-2-6, 1930 - 1934	R.PGSTGTWNPGSSER.G	1433.46515	2	3.98E-09	0.78	3.20	-	396.7
AHQ-2-7, 4683	R.PNNPDWGTFEEVSGNVSPGTR.R	2261.34962	2	1.73E-07	0.95	4.91	-	979.6
AHQ-2-7, 4665 - 4668	R.PNNPDWGTFEEVSGNVSPGTR.R	2261.34962	2	1.73E-06	0.89	3.59	-	906.5
AHQ-2-11, 4610 - 4649	R.PNNPDWGTFEEVSGNVSPGTR.R	2261.34962	2	1.46E-07	0.90	4.25	-	666.7
AHQ-2-10, 4435	R.PNNPDWGTFEEVSGNVSPGTR.R	2261.34962	2	9.22E-07	0.82	3.64	-	576.7
AHQ-2-6, 4637 - 4703	R.PNNPDWGTFEEVSGNVSPGTR.R	2261.34962	2	1.03E-04	0.79	3.47	-	357.2
AHQ-2-13, 6813 - 6875	K.TFPGFFSPMLGEFVSETESR.G	2266.51432	2	3.26E-05	0.78	3.51	-	340.1
AHQ-2-13-, 7015 - 7085	K.TFPGFFSPMLGEFVSETESR.G	2266.51432	2	2.80E-08	0.90	4.45	-	382.2
AHQ-2-2, 7313	K.TFPGFFSPM*LGEFVSETESR.G	2282.51372	2	9.62E-04	0.47	2.92	-	202.2
AHQ-2-10, 6747	K.TFPGFFSPMLGEFVSETESR.G	2266.51432	2	3.45E-07	0.82	3.67	-	358.9
AHQ-2-3, 7543 - 7545	K.TFPGFFSPMLGEFVSETESR.G	2266.51432	2	2.10E-06	0.87	4.06	-	351.6
AHQ-2-4, 7604	K.TFPGFFSPMLGEFVSETESR.G	2266.51432	2	1.13E-06	0.83	3.33	-	505.4
AHQ-2-2, 7598	K.TFPGFFSPMLGEFVSETESR.G	2266.51432	2	2.33E-08	0.88	3.89	-	434.1
AHQ-2-6, 7203 - 7237	K.TFPGFFSPMLGEFVSETESR.G	2266.51432	2	6.97E-07	0.82	3.37	-	390.5
AHQ-2-9, 7380 - 7420	K.TFPGFFSPMLGEFVSETESR.G	2266.51432	2	3.88E-04	0.62	2.82	-	292.2
AHQ-2-1, 7086	K.TFPGFFSPM*LGEFVSETESR.G	2282.51372	2	2.99E-04	0.52	2.74	-	163.9
AHQ-2-7, 7483	K.TFPGFFSPMLGEFVSETESR.G	2266.51432	2	2.39E-06	0.84	3.76	-	287.9
AHQ-2-11, 6426 - 6493	K.TFPGFFSPM*LGEFVSETESR.G	2282.51372	2	8.01E-05	0.48	3.00	-	107.9
AHQ-2-11, 6792 - 6793	K.TFPGFFSPMLGEFVSETESR.G	2266.51432	2	1.38E-11	0.93	4.88	-	531.1
AHQ-2-13-, 6827 - 6829	K.TFPGFFSPM*LGEFVSETESR.G	2282.51372	2	5.89E-07	0.87	4.37	-	219.7
AHQ-2-9, 6992 - 6994	K.TFPGFFSPM*LGEFVSETESR.G	2282.51372	2	7.40E-04	0.75	3.33	-	303.7
AHQ-2-1, 7260 - 7261	K.TFPGFFSPMLGEFVSETESR.G	2266.51432	2	1.34E-05	0.86	3.63	-	472.6
AHQ-2-12, 6837	K.TFPGFFSPM*LGEFVSETESR.G	2282.51372	2	1.38E-05	0.77	3.51	-	184.5
AHQ-2-12, 7147 - 7153	K.TFPGFFSPMLGEFVSETESR.G	2266.51432	2	2.82E-04	0.86	3.86	-	471.7
AHQ-2-5, 7079	K.TFPGFFSPM*LGEFVSETESR.G	2282.51372	2	5.10E-04	0.67	2.90	-	221.9
AHQ-2-12, 2254 - 2334	K.VQHIQLLQK.N	1107.33106	1	4.15E-04	0.48	2.61	-	499.5
AHQ-2-13, 2451	K.VQHIQLLQK.N	1107.33106	1	5.72E-04	0.61	2.47	-	647.3
AHQ-2-11, 2242	K.VQHIQLLQK.N	1107.33106	1	1.16E-04	0.36	2.33	-	584.5
AHQ-2-10, 2059	K.VQHIQLLQK.N	1107.33106	1	3.51E-04	0.65	2.57	-	600.3
AHQ-2-10, 2056	K.VQHIQLLQK.N	1107.33106	2	1.89E-04	0.83	3.00	-	820.9
AHQ-2-9, 2040 - 2046	K.VQHIQLLQK.N	1107.33106	2	4.51E-06	0.90	3.25	-	813.2
AHQ-2-9, 2038	K.VQHIQLLQK.N	1107.33106	1	8.36E-04	0.48	2.84	-	426.2
AHQ-2-8, 1842	K.VQHIQLLQK.N	1107.33106	1	5.72E-04	0.67	2.77	-	540.7
AHQ-2-8, 1834	K.VQHIQLLQK.N	1107.33106	1	3.26E-04	0.66	2.63	-	641.1
AHQ-2-13-, 2343	K.VQHIQLLQK.N	1107.33106	1	4.88E-04	0.29	2.48	-	441.2
AHQ-2-7, 1904	K.VQHIQLLQK.N	1107.33106	2	5.70E-04	0.76	2.79	-	618.1
AHQ-2-5, 1987	K.VQHIQLLQK.N	1107.33106	1	4.89E-04	0.32	2.37	-	489.8
AHQ-2-4, 2248 - 2253	K.VQHIQLLQK.N	1107.33106	1	8.47E-04	0.72	2.65	-	667.2
AHQ-2-2, 2261	K.VQHIQLLQK.N	1107.33106	1	1.89E-04	0.06	2.05	-	407.2
AHQ-2-1, 2492	K.VQHIQLLQK.N	1107.33106	1	3.40E-04	0.62	2.51	-	662.2
AHQ-2-9, 2030 - 2099	K.VQHIQLLQK.N	1107.33106	1	1.42E-04	0.27	2.19	-	540.3
gi|24119203|ref|NP_705935.1| tropomyosin 3 [Homo sapiens]				1.53E-11	5.55	60.27	20.20	29032.5
AHQ-2-9, 2202	R.AREQAEAEVASLNR.R	1544.65218	2	1.95E-04	0.98	5.46	-	3011.3
AHQ-2-9, 3391	R.IQLVEEELDR.A	1244.37653	2	5.15E-07	0.90	3.44	-	974.0
AHQ-2-10, 3354 - 3355	R.IQLVEEELDR.A	1244.37653	2	4.36E-06	0.84	3.33	-	811.1
AHQ-2-10, 2571	K.IQVLQQQADDAEER.A	1643.73738	2	1.53E-11	0.97	4.70	-	1659.5
AHQ-2-9, 2638	K.IQVLQQQADDAEER.A	1643.73738	2	3.62E-06	0.97	4.70	-	1842.6
AHQ-2-10, 2312	R.KIQVLQQQADDAEER.A	1771.91030	2	2.07E-06	0.98	5.05	-	2107.0
AHQ-2-9, 2332	R.KIQVLQQQADDAEER.A	1771.91030	2	7.98E-07	0.97	5.16	-	1416.7
AHQ-2-10, 2983	R.RIQLVEEELDR.A	1400.56288	2	1.45E-07	0.96	4.21	-	1295.8
AHQ-2-9, 2966	R.RIQLVEEELDR.A	1400.56288	2	4.50E-08	0.98	4.72	-	2021.1
AHQ-2-9, 2894	K.TIDDLEDKLK.C	1190.32563	2	4.63E-06	0.77	3.06	-	536.8
gi|11761631|ref|NP_005132.1| fibrinogen, beta chain preproprotein [Homo sapiens]				1.65E-11	21.63	260.29	49.30	55901.8
AHQ-2-7, 2193 - 2195	K.AHYGGFTVQNEANK.Y	1536.63020	2	5.03E-08	0.94	4.17	-	1148.3
AHQ-2-6, 2077 - 2078	K.AHYGGFTVQNEANK.Y	1536.63020	2	1.91E-07	0.95	4.52	-	1296.9
AHQ-2-7, 1983 - 1985	K.AHYGGFTVQNEANK.Y	1536.63020	2	6.16E-09	0.90	3.79	-	905.7
AHQ-2-11, 4001 - 4017	K.DNENVVNEYSSELEK.H	1769.80167	2	2.17E-06	0.93	4.54	-	1009.2
AHQ-2-4, 4332	K.DNENVVNEYSSELEK.H	1769.80167	2	2.94E-05	0.84	3.46	-	741.5
AHQ-2-1, 4381	K.DNENVVNEYSSELEK.H	1769.80167	2	1.71E-10	0.93	3.90	-	1094.2
AHQ-2-7, 4072 - 4079	K.DNENVVNEYSSELEK.H	1769.80167	2	2.66E-08	0.92	4.11	-	808.2
AHQ-2-7, 4365	K.DNENVVNEYSSELEK.H	1769.80167	2	4.88E-05	0.61	3.00	-	621.9
AHQ-2-7, 4452	K.DNENVVNEYSSELEK.H	1769.80167	2	1.41E-08	0.88	3.72	-	802.4
AHQ-2-12, 4165	K.DNENVVNEYSSELEK.H	1769.80167	2	2.88E-04	0.70	3.24	-	540.1
AHQ-2-6, 4363	K.DNENVVNEYSSELEK.H	1769.80167	2	1.80E-05	0.90	3.89	-	703.2
AHQ-2-6, 4063 - 4077	K.DNENVVNEYSSELEK.H	1769.80167	2	5.42E-08	0.92	4.29	-	841.8
AHQ-2-9, 4096	K.DNENVVNEYSSELEK.H	1769.80167	2	1.21E-05	0.86	3.42	-	829.3
AHQ-2-7, 3359 - 3439	K.DNENVVNEYSSELEK.H	1769.80167	2	4.95E-07	0.76	3.73	-	475.9
AHQ-2-7, 3381	K.EDGGGWWYNR.C	1240.26508	2	1.78E-04	0.57	2.76	-	402.7
AHQ-2-6, 3413	K.EDGGGWWYNR.C	1240.26508	2	3.62E-07	0.80	2.94	-	470.8
AHQ-2-6, 3137 - 3142	R.EEAPSLRPAPPPISGGGYR.A	1952.15981	3	2.28E-05	0.76	3.07	-	695.5
AHQ-2-10, 3099	R.EEAPSLRPAPPPISGGGYR.A	1952.15981	2	7.61E-05	0.27	2.73	-	143.1
AHQ-2-6, 3141 - 3153	R.EEAPSLRPAPPPISGGGYR.A	1952.15981	2	1.34E-05	0.15	2.53	-	180.5
AHQ-2-6, 3867 - 3869	K.GGETSEMYLIQPDSSVKPYR.V	2258.49373	2	1.61E-04	0.88	4.30	-	561.6
AHQ-2-7, 3320	K.GGETSEM*YLIQPDSSVKPYR.V	2274.49313	2	7.44E-07	0.67	3.37	-	389.1
AHQ-2-7, 3815	K.GGETSEMYLIQPDSSVKPYR.V	2258.49373	2	9.72E-06	0.82	3.66	-	454.6
AHQ-2-8, 3755	K.GGETSEMYLIQPDSSVKPYR.V	2258.49373	2	1.53E-04	0.82	3.87	-	483.5
AHQ-2-8, 3759	K.GGETSEMYLIQPDSSVKPYR.V	2258.49373	3	4.66E-09	0.86	3.81	-	950.9
AHQ-2-7, 3819	K.GGETSEMYLIQPDSSVKPYR.V	2258.49373	3	4.18E-05	0.71	3.16	-	624.0
AHQ-2-9, 3859 - 3862	K.GGETSEMYLIQPDSSVKPYR.V	2258.49373	2	1.02E-06	0.93	4.51	-	754.0
AHQ-2-7, 4044 - 4107	K.HGTDDGVVWMNWK.G	1545.70374	2	6.44E-06	0.94	4.34	-	964.0
AHQ-2-10, 3872 - 3920	K.HGTDDGVVWMNWK.G	1545.70374	2	8.35E-09	0.95	3.92	-	1359.3
AHQ-2-6, 3219 - 3290	K.HGTDDGVVWM*NWK.G	1561.70314	2	7.96E-05	0.95	3.75	-	1404.5
AHQ-2-7, 3880 - 3956	K.HGTDDGVVWMNWK.G	1545.70374	2	1.68E-08	0.98	5.63	-	1329.2
AHQ-2-6, 3939 - 3975	K.HGTDDGVVWMNWK.G	1545.70374	2	2.15E-08	0.98	5.03	-	1798.0
AHQ-2-12, 3251	K.IRPFFPQQ	1033.20750	2	2.08E-04	0.62	2.53	-	504.6
AHQ-2-7, 6237 - 6309	R.KAPDAGGCLHADPDLGVLCPTGCQLQEALLQQERPIR.N	4089.58482	3	1.46E-04	0.81	4.10	-	350.3
AHQ-2-8, 3031	R.KGGETSEMYLIQPDSSVKPYR.V	2386.66664	2	7.48E-06	0.96	5.80	-	1032.1
AHQ-2-9, 3279	R.KGGETSEMYLIQPDSSVKPYR.V	2386.66664	3	4.17E-04	0.84	4.13	-	641.9
AHQ-2-8, 3027 - 3028	R.KGGETSEMYLIQPDSSVKPYR.V	2386.66664	3	5.89E-10	0.93	5.16	-	730.4
AHQ-2-6, 2953	R.KGGETSEM*YLIQPDSSVKPYR.V	2402.66604	3	5.60E-04	0.83	3.68	-	881.6
AHQ-2-6, 3373 - 3386	R.KGGETSEMYLIQPDSSVKPYR.V	2386.66664	3	1.65E-11	0.91	4.88	-	683.1

AHQ-2-7, 3241 - 3311	R.KGGETSEMYLIQPDSSVKPYR.V	2386.66664	3	4.36E-05	0.91	4.69	-	674.2
AHQ-2-11, 6118	R.M*GPTELLIEMEDWK.G	1708.97776	2	1.08E-05	0.87	3.34	-	946.6
AHQ-2-9, 7052	R.MGPTELLIEMEDWK.G	1692.97836	2	5.41E-05	0.83	3.33	-	672.8
AHQ-2-10, 5947	R.M*GPTELLIEMEDWK.G	1708.97776	2	8.60E-04	0.83	3.36	-	627.6
AHQ-2-10, 6479	R.MGPTELLIEMEDWK.G	1692.97836	2	2.09E-06	0.95	4.57	-	1178.0
AHQ-2-6, 6955 - 6965	R.MGPTELLIEMEDWK.G	1692.97836	2	1.35E-07	0.96	4.85	-	1353.1
AHQ-2-11, 6549 - 6558	R.MGPTELLIEMEDWK.G	1692.97836	2	1.84E-04	0.96	4.79	-	1271.3
AHQ-2-11, 5109	R.MGPTELLIEM*EDWK.G	1708.97776	2	2.06E-04	0.87	3.74	-	743.1
AHQ-2-7, 7131	R.MGPTELLIEMEDWK.G	1692.97836	2	4.00E-06	0.90	3.36	-	1094.1
AHQ-2-7, 7037 - 7099	R.MGPTELLIEMEDWK.G	1692.97836	2	4.67E-08	0.96	5.16	-	1181.7
AHQ-2-7, 6388 - 6403	R.M*GPTELLIEMEDWK.G	1708.97776	2	1.30E-06	0.93	3.47	-	1244.3
AHQ-2-6, 5243	R.MGPTELLIEM*EDWK.G	1708.97776	2	6.38E-05	0.93	4.36	-	708.8
AHQ-2-7, 6165	R.MGPTELLIEMEDWKGDK.V	1993.29069	3	1.21E-05	0.67	3.16	-	592.3
AHQ-2-7, 6171	R.MGPTELLIEMEDWKGDK.V	1993.29069	2	1.20E-08	0.93	4.08	-	1079.0
AHQ-2-7, 5163	K.NYCGLPGEYWLGNDK.I	1787.92994	2	6.45E-10	0.93	4.49	-	448.3
AHQ-2-11, 5013	K.NYCGLPGEYWLGNDK.I	1787.92994	2	5.34E-04	0.87	3.82	-	435.9
AHQ-2-6, 5985	K.NYCGLPGEYWLGNDKISQLTR.M	2486.74455	2	9.84E-09	0.80	3.43	-	387.2
AHQ-2-6, 5990	K.NYCGLPGEYWLGNDKISQLTR.M	2486.74455	3	4.70E-08	0.81	3.59	-	392.6
AHQ-2-7, 2591	R.SILENLR.S	844.97899	2	9.01E-04	0.81	2.61	-	893.4
AHQ-2-8, 3672 - 3694	R.TPCTVSCNIPVVSGK.E	1621.85811	2	3.00E-05	0.86	3.54	-	735.6
AHQ-2-6, 3701	R.TPCTVSCNIPVVSGK.E	1621.85811	2	3.72E-04	0.90	4.10	-	626.7
AHQ-2-6, 4867 - 4869	R.TPCTVSCNIPVVSGKECEEIIR.K	2552.88440	2	1.53E-08	0.93	4.74	-	550.0
AHQ-2-7, 4805 - 4883	R.TPCTVSCNIPVVSGKECEEIIR.K	2552.88440	3	1.01E-09	0.93	4.49	-	1327.8
AHQ-2-7, 5047	R.TPCTVSCNIPVVSGKECEEIIR.K	2552.88440	3	2.39E-05	0.86	3.95	-	833.9
AHQ-2-9, 4912	R.TPCTVSCNIPVVSGKECEEIIR.K	2552.88440	3	1.20E-04	0.67	3.25	-	767.2
AHQ-2-7, 4964	R.TPCTVSCNIPVVSGKECEEIIR.K	2552.88440	3	4.13E-04	0.84	3.63	-	1130.4
AHQ-2-6, 4486 - 4525	R.VYCDMNTENGGWTVIQNR.Q	2159.34553	2	3.60E-08	0.96	4.64	-	1204.8
AHQ-2-7, 4081	R.VYCDM*NTENGGWTVIQNR.Q	2175.34493	2	2.59E-05	0.93	4.20	-	958.5
AHQ-2-8, 4488 - 4500	R.VYCDMNTENGGWTVIQNR.Q	2159.34553	2	1.26E-10	0.97	4.78	-	1590.2
AHQ-2-6, 4594 - 4595	R.VYCDMNTENGGWTVIQNR.Q	2159.34553	2	1.48E-08	0.92	3.92	-	942.8
AHQ-2-7, 4693	R.VYCDMNTENGGWTVIQNR.Q	2159.34553	2	1.13E-04	0.87	3.70	-	819.1
AHQ-2-7, 3981	R.VYCDM*NTENGGWTVIQNR.Q	2175.34493	2	1.82E-04	0.96	4.68	-	1318.3
AHQ-2-6, 4001	R.VYCDM*NTENGGWTVIQNR.Q	2175.34493	2	7.57E-06	0.95	4.40	-	1219.6
AHQ-2-9, 4563	R.VYCDMNTENGGWTVIQNR.Q	2159.34553	2	5.95E-07	0.94	4.77	-	866.2
AHQ-2-7, 4548 - 4623	R.VYCDMNTENGGWTVIQNR.Q	2159.34553	2	1.21E-07	0.94	4.74	-	1042.0
AHQ-2-7, 1695	K.YQISVNK.Y	851.96999	1	1.85E-04	0.36	2.70	-	339.3
AHQ-2-7, 4960 - 5041	R.YYWGGQYTWDMAK.H	1669.84009	2	1.71E-08	0.95	4.10	-	1077.5
AHQ-2-7, 4288	R.YYWGGQYTWDM*AK.H	1685.83949	2	2.44E-06	0.94	3.66	-	967.2
AHQ-2-11, 4354 - 4370	R.YYWGGQYTWDM*AK.H	1685.83949	2	2.10E-05	0.87	2.90	-	972.6
AHQ-2-11, 4897	R.YYWGGQYTWDMAK.H	1669.84009	2	8.09E-07	0.95	3.77	-	1331.5
AHQ-2-6, 4994 - 4995	R.YYWGGQYTWDMAK.H	1669.84009	2	2.90E-06	0.95	3.89	-	1337.0
AHQ-2-6, 4382	R.YYWGGQYTWDM*AK.H	1685.83949	2	1.96E-05	0.96	4.08	-	1337.1
AHQ-2-12, 5113	R.YYWGGQYTWDMAK.H	1669.84009	2	1.51E-06	0.95	3.76	-	1318.1
AHQ-2-14, 5588 - 5660	R.YYWGGQYTWDM*AK.H	1685.83949	2	2.36E-05	0.91	3.37	-	819.8
AHQ-2-14, 6257	R.YYWGGQYTWDMAK.H	1669.84009	2	5.51E-08	0.95	3.43	-	1395.6
AHQ-2-14-, 4544 - 4600	R.YYWGGQYTWDM*AK.H	1685.83949	2	4.05E-05	0.96	4.34	-	1073.0
gi|4501893|ref|NP_001094.1| actinin, alpha 2 [Homo sapiens]				1.69E-11	9.72	120.24	12.40	103853.1
AHQ-2-5, 4319	K.AGTQIENIEEDFR.N	1522.59861	2	1.70E-05	0.93	4.78	-	1007.1
AHQ-2-5, 4264 - 4335	K.AGTQIENIEEDFR.N	1522.59861	2	1.37E-05	0.87	3.76	-	827.7
AHQ-2-5, 5057 - 5127	K.CQLEINFNTLQTK.L	1610.81373	2	1.51E-06	0.96	4.57	-	1320.0
AHQ-2-6, 4939 - 5006	K.CQLEINFNTLQTK.L	1610.81373	2	4.23E-07	0.92	4.34	-	914.9
AHQ-2-5, 5299 - 5371	K.CQLEINFNTLQTK.L	1610.81373	2	8.87E-06	0.94	4.03	-	1244.3
AHQ-2-4, 4329	R.FAIQDISVEETSAK.E	1538.68108	2	1.50E-10	0.93	3.37	-	1178.7
AHQ-2-13-, 4217 - 4222	R.FAIQDISVEETSAK.E	1538.68108	2	4.90E-04	0.85	3.34	-	858.5
AHQ-2-11, 4016 - 4040	R.FAIQDISVEETSAK.E	1538.68108	2	3.75E-06	0.95	3.75	-	1514.2
AHQ-2-1, 4434	R.FAIQDISVEETSAK.E	1538.68108	2	4.03E-05	0.69	2.71	-	594.2
AHQ-2-6, 3950 - 4026	R.FAIQDISVEETSAK.E	1538.68108	2	3.77E-10	0.84	3.06	-	946.6
AHQ-2-5, 6065 - 6148	K.GYEEWLLNEIR.R	1422.56701	2	5.92E-06	0.95	4.24	-	1438.0
AHQ-2-1, 6277	K.GYEEWLLNEIR.R	1422.56701	2	1.09E-06	0.93	4.25	-	1162.5
AHQ-2-6, 5977 - 6054	K.GYEEWLLNEIR.R	1422.56701	1	3.86E-05	0.41	2.84	-	109.4
AHQ-2-6, 5347 - 5362	K.GYEEWLLNEIR.R	1422.56701	1	1.64E-04	0.42	2.49	-	410.9
AHQ-2-4, 6365	K.GYEEWLLNEIR.R	1422.56701	2	8.29E-05	0.95	3.96	-	1483.2
AHQ-2-5, 6261	K.GYEEWLLNEIR.R	1422.56701	1	5.69E-05	0.27	2.32	-	311.8
AHQ-2-5, 6071 - 6140	K.GYEEWLLNEIR.R	1422.56701	1	2.25E-10	0.37	2.61	-	134.3
AHQ-2-5, 6064 - 6131	K.GYEEWLLNEIR.R	1422.56701	1	6.94E-05	0.34	2.56	-	246.9
AHQ-2-5, 1976	K.HEAFESDLAAHQDR.V	1626.66861	2	2.20E-09	0.97	4.79	-	1823.6
AHQ-2-5, 2265	R.ISNRPAFMPSEGK.M	1434.64597	2	5.18E-07	0.81	3.25	-	693.5
AHQ-2-5, 3569	R.KAGTQIENIEEDFR.N	1650.77152	2	1.36E-05	0.96	4.85	-	1178.1
AHQ-2-5, 4257	R.KAGTQIENIEEDFRNGLK.L	2063.25733	2	7.21E-04	0.77	3.60	-	707.4
AHQ-2-5, 1611 - 1643	R.KHEAFESDLAAHQDR.V	1754.84152	2	1.81E-09	0.95	4.32	-	1099.8
AHQ-2-6, 1670	R.KHEAFESDLAAHQDR.V	1754.84152	2	5.59E-09	0.97	4.86	-	1797.2
AHQ-2-5, 1620	R.KHEAFESDLAAHQDR.V	1754.84152	3	1.69E-11	0.90	4.40	-	892.8
AHQ-2-11, 2950	R.KTFTAWCNSHLR.K	1522.71309	2	2.95E-06	0.43	2.70	-	344.8
AHQ-2-5, 2493	R.KTFTAWCNSHLR.K	1522.71309	2	3.08E-06	0.70	3.27	-	363.7
AHQ-2-5, 4476 - 4507	K.LVSIGAEEIVDGNVK.M	1543.74400	2	3.85E-07	0.89	3.35	-	944.2
AHQ-2-5, 4731 - 4812	K.LVSIGAEEIVDGNVK.M	1543.74400	2	4.85E-08	0.97	4.49	-	1636.3
AHQ-2-5, 4116	K.LVSIGAEEIVDGNVK.M	1543.74400	2	1.16E-04	0.94	3.87	-	1224.7
AHQ-2-5, 4588 - 4663	K.LVSIGAEEIVDGNVK.M	1543.74400	2	1.91E-06	0.93	3.69	-	1106.7
AHQ-2-5, 3388	K.TFTAWCNSHLR.K	1394.54018	2	2.22E-07	0.81	2.94	-	727.2
gi|4507357|ref|NP_003555.1| transgelin 2; SM22-alpha homolog [Homo sapiens]				1.73E-11	15.40	190.28	88.40	22391.3
AHQ-2-11, 5745 - 5805	R.DDGLFSGDPNWFPK.K	1595.69288	2	2.16E-05	0.90	3.14	-	1117.9
AHQ-2-12, 6017 - 6097	R.DDGLFSGDPNWFPK.K	1595.69288	2	1.06E-06	0.97	4.86	-	1430.8
AHQ-2-12, 6162	R.DDGLFSGDPNWFPK.K	1595.69288	2	8.27E-08	0.96	4.40	-	1429.1
AHQ-2-11, 5992	R.DDGLFSGDPNWFPK.K	1595.69288	2	1.20E-08	0.95	4.23	-	1232.0
AHQ-2-11, 5865 - 5880	R.DDGLFSGDPNWFPK.K	1595.69288	2	1.91E-07	0.97	4.72	-	1566.8
AHQ-2-11, 5652 - 5721	R.DDGLFSGDPNWFPK.K	1595.69288	2	2.81E-05	0.97	4.50	-	1589.5
AHQ-2-12, 5887 - 5967	R.DDGLFSGDPNWFPK.K	1595.69288	2	8.53E-07	0.94	4.00	-	1146.8
AHQ-2-13, 5732 - 5792	R.DDGLFSGDPNWFPK.K	1595.69288	2	3.13E-05	0.87	3.28	-	949.3
AHQ-2-13, 5869	R.DDGLFSGDPNWFPK.K	1595.69288	2	3.33E-04	0.89	3.05	-	972.0
AHQ-2-13, 6207	K.DGTVLCELINALYPEGQAPVK.K	2289.59066	2	2.29E-04	0.83	3.78	-	579.1
AHQ-2-11, 6862	K.DGTVLCELINALYPEGQAPVK.K	2289.59066	3	6.97E-05	0.91	4.12	-	1231.9
AHQ-2-11, 6106 - 6128	K.DGTVLCELINALYPEGQAPVK.K	2289.59066	2	6.53E-06	0.93	4.64	-	676.1
AHQ-2-12, 7197 - 7199	K.DGTVLCELINALYPEGQAPVK.K	2289.59066	2	1.91E-05	0.91	4.42	-	658.3
AHQ-2-13-, 6366	K.DGTVLCELINALYPEGQAPVK.K	2289.59066	2	2.27E-09	0.94	4.98	-	698.0
AHQ-2-12, 6401 - 6463	K.DGTVLCELINALYPEGQAPVK.K	2289.59066	2	8.98E-05	0.90	4.20	-	608.2
AHQ-2-13, 6863	K.DGTVLCELINALYPEGQAPVK.K	2289.59066	2	8.62E-04	0.88	4.27	-	571.2
AHQ-2-11, 6324 - 6348	K.DGTVLCELINALYPEGQAPVK.K	2289.59066	2	1.43E-04	0.63	3.18	-	485.2
AHQ-2-11, 5530 - 5592	K.DGTVLCELINALYPEGQAPVKK.I	2417.76357	3	5.32E-09	0.87	3.94	-	1072.6
AHQ-2-14-, 6867	K.DGTVLCELINALYPEGQAPVKK.I	2417.76357	3	3.26E-08	0.83	3.91	-	651.4
AHQ-2-11, 5801	K.DGTVLCELINALYPEGQAPVKK.I	2417.76357	2	6.04E-06	0.45	2.60	-	463.4
AHQ-2-12, 5841 - 5843	K.DGTVLCELINALYPEGQAPVKK.I	2417.76357	2	8.67E-07	0.84	3.60	-	630.9
AHQ-2-13, 6812	K.DGTVLCELINALYPEGQAPVKK.I	2417.76357	2	9.73E-07	0.84	4.11	-	522.4
AHQ-2-13, 6811	K.DGTVLCELINALYPEGQAPVKK.I	2417.76357	3	2.09E-09	0.90	4.56	-	795.0
AHQ-2-13-, 7014	K.DGTVLCELINALYPEGQAPVKK.I	2417.76357	3	3.80E-08	0.85	3.72	-	1083.7
AHQ-2-11, 5570	K.DGTVLCELINALYPEGQAPVKK.I	2417.76357	2	3.24E-06	0.68	2.87	-	513.2
AHQ-2-13-, 7018	K.DGTVLCELINALYPEGQAPVKK.I	2417.76357	2	1.10E-08	0.78	3.86	-	559.7
AHQ-2-12, 7129 - 7145	K.DGTVLCELINALYPEGQAPVKK.I	2417.76357	3	3.22E-09	0.91	4.34	-	833.7

AHQ-2-12, 5845	K.DGTVLCELINALYPEGQAPVKK.I	2417.76357	3	9.60E-05	0.78	3.22	-	937.9
AHQ-2-11, 6784	K.DGTVLCELINALYPEGQAPVKK.I	2417.76357	2	7.32E-06	0.83	4.23	-	568.2
AHQ-2-11, 6790	K.DGTVLCELINALYPEGQAPVKK.I	2417.76357	3	2.37E-08	0.93	4.54	-	1071.4
AHQ-2-12, 4542	R.ENFQNWLK.D	1079.18989	1	4.57E-04	0.07	2.00	-	142.8
AHQ-2-12, 2406	K.ENPRNFSDNQLQEGK.N	1776.84549	2	4.53E-05	0.87	3.38	-	662.4
AHQ-2-11, 2382	K.ENPRNFSDNQLQEGK.N	1776.84549	2	3.32E-05	0.61	2.69	-	663.7
AHQ-2-11, 2610	R.GASQAGMTGYGMPR.Q	1384.56732	2	2.48E-06	0.92	3.47	-	1211.0
AHQ-2-11, 1905	R.GPAYGLSR.E	820.91599	2	1.77E-04	0.91	2.99	-	1170.3
AHQ-2-12, 1833 - 1893	R.GPAYGLSR.E	820.91599	2	2.76E-05	0.90	3.23	-	855.9
AHQ-2-12, 2078 - 2081	R.GPAYGLSREVQQK.I	1433.59456	2	5.64E-05	0.93	4.17	-	1302.5
AHQ-2-11, 2426	K.IQASTMAFK.Q	997.19374	1	8.91E-04	0.68	2.48	-	678.0
AHQ-2-12, 2170 - 2174	K.KIQASTMAFK.Q	1125.36665	2	1.14E-04	0.81	3.10	-	815.4
AHQ-2-11, 2157	K.KIQASTMAFK.Q	1125.36665	2	6.15E-05	0.88	3.24	-	981.1
AHQ-2-11, 2564	R.NFSDNQLQEGK.N	1280.32582	1	6.61E-05	0.15	1.82	-	212.1
AHQ-2-12, 2586	R.NFSDNQLQEGK.N	1280.32582	2	4.29E-04	0.77	2.76	-	967.2
AHQ-2-12, 2583	R.NFSDNQLQEGK.N	1280.32582	1	3.68E-07	0.14	2.14	-	307.8
AHQ-2-12, 2177 - 2202	R.NFSDNQLQEGK.N	1280.32582	2	2.40E-05	0.83	3.54	-	625.8
AHQ-2-11, 2396	R.NFSDNQLQEGK.N	1280.32582	1	1.35E-05	0.43	2.09	-	459.6
AHQ-2-11, 2297	R.NFSDNQLQEGK.N	1280.32582	1	1.64E-05	0.20	2.24	-	228.8
AHQ-2-12, 2293	R.NFSDNQLQEGK.N	1280.32582	1	3.92E-08	0.28	2.39	-	214.9
AHQ-2-13, 2356	R.NFSDNQLQEGK.N	1280.32582	2	1.87E-05	0.72	3.25	-	518.8
AHQ-2-11, 2262 - 2264	K.NVIGLQM*GTNR.G	1219.39691	2	1.71E-05	0.94	4.03	-	1052.7
AHQ-2-12, 4906	K.QM*EQISQFLQAAER.Y	1695.87890	2	4.91E-04	0.88	3.68	-	751.8
AHQ-2-13-, 5509	K.QMEQISQFLQAAER.Y	1679.87950	2	3.25E-04	0.96	4.93	-	1223.5
AHQ-2-11, 5236 - 5269	K.QMEQISQFLQAAER.Y	1679.87950	2	4.32E-08	0.96	5.02	-	1197.2
AHQ-2-11, 5249	K.QMEQISQFLQAAER.Y	1679.87950	3	2.19E-04	0.97	4.69	-	2374.6
AHQ-2-14-, 4816 - 4817	K.QM*EQISQFLQAAER.Y	1695.87890	2	5.92E-04	0.89	4.05	-	669.4
AHQ-2-14-, 5461	K.QMEQISQFLQAAER.Y	1679.87950	2	1.06E-04	0.94	4.14	-	1057.5
AHQ-2-11, 4577 - 4613	K.QM*EQISQFLQAAER.Y	1695.87890	2	9.67E-04	0.55	2.64	-	426.5
AHQ-2-13, 5395	K.QMEQISQFLQAAER.Y	1679.87950	2	1.96E-05	0.94	4.07	-	1051.1
AHQ-2-12, 5509	K.QMEQISQFLQAAER.Y	1679.87950	3	3.22E-07	0.98	4.89	-	3133.3
AHQ-2-13-, 7081	K.QYDADLEQILIQWITTQCR.K	2396.66131	3	1.72E-07	0.96	5.29	-	2109.4
AHQ-2-12, 7234	K.QYDADLEQILIQWITTQCR.K	2396.66131	3	5.84E-04	0.90	3.80	-	1169.6
AHQ-2-11, 4638	R.TLMNLGGLAVAR.D	1216.47932	2	1.88E-04	0.93	3.70	-	1526.8
AHQ-2-12, 3545 - 3574	R.TLM*NLGGLAVAR.D	1232.47872	2	5.03E-04	0.92	4.23	-	897.7
AHQ-2-12, 4417 - 4450	R.TLMNLGGLAVAR.D	1216.47932	2	5.66E-07	0.94	4.30	-	1160.7
AHQ-2-13, 3553	R.TLM*NLGGLAVAR.D	1232.47872	2	8.50E-04	0.94	3.75	-	1295.6
AHQ-2-13, 4440 - 4443	R.TLMNLGGLAVAR.D	1216.47932	2	3.81E-06	0.91	4.25	-	822.1
AHQ-2-14-, 4509	R.TLMNLGGLAVAR.D	1216.47932	2	1.38E-06	0.93	3.71	-	1281.0
AHQ-2-12, 4841	R.TLMNLGGLAVAR.D	1216.47932	2	7.86E-05	0.94	3.73	-	1553.9
AHQ-2-11, 4277 - 4340	R.TLMNLGGLAVAR.D	1216.47932	2	9.47E-04	0.93	4.02	-	1206.6
AHQ-2-11, 3880	R.TLM*NLGGLAVAR.D	1232.47872	2	1.47E-05	0.88	3.50	-	856.9
AHQ-2-13-, 4495 - 4497	R.TLMNLGGLAVAR.D	1216.47932	2	1.42E-06	0.93	4.22	-	1089.0
AHQ-2-12, 4521	R.TLMNLGGLAVAR.D	1216.47932	2	4.09E-06	0.74	2.94	-	765.9
AHQ-2-12, 6810 - 6825	R.YGINTTDIFQTVDLWEGK.N	2101.30080	2	2.24E-10	0.95	4.47	-	1046.2
AHQ-2-14-, 6721 - 6791	R.YGINTTDIFQTVDLWEGK.N	2101.30080	2	1.02E-09	0.97	5.58	-	1503.5
AHQ-2-13, 6669 - 6744	R.YGINTTDIFQTVDLWEGK.N	2101.30080	2	8.01E-11	0.97	5.39	-	1302.0
AHQ-2-11, 6456 - 6490	R.YGINTTDIFQTVDLWEGK.N	2101.30080	2	1.86E-09	0.97	4.46	-	1990.2
AHQ-2-11, 6638 - 6696	R.YGINTTDIFQTVDLWEGK.N	2101.30080	2	1.03E-08	0.97	5.62	-	1386.3
AHQ-2-13-, 6933	R.YGINTTDIFQTVDLWEGK.N	2101.30080	2	2.65E-07	0.97	5.30	-	1630.7
AHQ-2-11, 6740	R.YGINTTDIFQTVDLWEGK.N	2101.30080	2	1.73E-11	0.97	5.51	-	1073.4
AHQ-2-11, 6916 - 6938	R.YGINTTDIFQTVDLWEGK.N	2101.30080	2	2.80E-07	0.91	3.98	-	1023.0
AHQ-2-12, 7305 - 7313	R.YGINTTDIFQTVDLWEGK.N	2101.30080	2	3.06E-11	0.88	3.77	-	764.0
AHQ-2-12, 7029 - 7103	R.YGINTTDIFQTVDLWEGK.N	2101.30080	2	6.50E-10	0.96	5.46	-	972.1
AHQ-2-12, 7170 - 7245	R.YGINTTDIFQTVDLWEGK.N	2101.30080	2	1.28E-09	0.93	4.08	-	900.2
AHQ-2-11, 6264 - 6338	R.YGINTTDIFQTVDLWEGK.N	2101.30080	2	9.32E-07	0.97	4.85	-	1527.4
AHQ-2-12, 6967	R.YGINTTDIFQTVDLWEGKNMACVQR.T	2962.30673	3	8.86E-08	0.90	4.17	-	975.9
AHQ-2-12, 7021	R.YGINTTDIFQTVDLWEGKNMACVQR.T	2962.30673	2	1.91E-09	0.82	3.72	-	469.5
gi|5453740|ref|NP_006462.1| myosin regulatory light chain MRCL3; myosin, light polypeptide, regulat				1.78E-11	6.08	70.28	33.30	19794.0
AHQ-2-11, 6125 - 6192	R.ATSNVFAMFDQSQIQEFK.E	2092.31741	2	3.94E-07	0.96	4.94	-	1307.5
AHQ-2-14-, 5459	R.ATSNVFAM*FDQSQIQEFK.E	2108.31681	2	2.20E-11	0.96	4.66	-	1427.2
AHQ-2-14, 6572 - 6597	R.ATSNVFAM*FDQSQIQEFK.E	2108.31681	2	5.63E-06	0.80	3.47	-	759.2
AHQ-2-13, 6129	R.ATSNVFAMFDQSQIQEFK.E	2092.31741	2	3.46E-07	0.92	3.92	-	1005.6
AHQ-2-12, 5522 - 5538	R.ATSNVFAM*FDQSQIQEFK.E	2108.31681	2	1.94E-09	0.96	4.97	-	1190.2
AHQ-2-11, 6370	R.ATSNVFAMFDQSQIQEFK.E	2092.31741	2	4.80E-05	0.94	4.45	-	905.2
AHQ-2-14-, 6179 - 6180	R.ATSNVFAMFDQSQIQEFK.E	2092.31741	2	2.14E-10	0.98	5.44	-	1644.8
AHQ-2-11, 5224 - 5284	R.ATSNVFAM*FDQSQIQEFK.E	2108.31681	2	1.32E-05	0.97	5.08	-	1320.4
AHQ-2-11, 6001 - 6002	R.ATSNVFAMFDQSQIQEFK.E	2092.31741	3	6.93E-05	0.87	3.79	-	981.2
AHQ-2-11, 5996 - 6065	R.ATSNVFAMFDQSQIQEFK.E	2092.31741	2	4.19E-05	0.96	4.66	-	1556.0
AHQ-2-11, 5976 - 5982	R.ATSNVFAMFDQSQIQEFK.E	2092.31741	2	1.07E-05	0.94	4.45	-	686.8
AHQ-2-11, 5932	R.DGFIDKEDLHDMLASLGK.N	2005.23806	2	3.41E-05	0.84	3.36	-	750.6
AHQ-2-11, 5813 - 5820	R.DGFIDKEDLHDMLASLGK.N	2005.23806	3	4.61E-09	0.95	4.51	-	1554.4
AHQ-2-11, 5809	R.DGFIDKEDLHDMLASLGK.N	2005.23806	2	1.08E-06	0.97	5.01	-	1615.9
AHQ-2-11, 5660 - 5685	R.DGFIDKEDLHDMLASLGK.N	2005.23806	2	1.78E-11	0.97	5.60	-	1527.1
AHQ-2-11, 5196	R.DGFIDKEDLHDMLASLGK.N	2005.23806	2	3.51E-07	0.75	3.42	-	776.9
AHQ-2-14-, 5860 - 5924	R.DGFIDKEDLHDMLASLGK.N	2005.23806	2	3.46E-11	0.97	5.01	-	1257.1
AHQ-2-11, 4405	R.DGFIDKEDLHDM*LASLGK.N	2021.23746	2	3.58E-05	0.97	5.30	-	1045.6
AHQ-2-13-, 6038	R.DGFIDKEDLHDMLASLGK.N	2005.23806	2	3.17E-05	0.93	3.95	-	1129.2
AHQ-2-13, 5883	R.DGFIDKEDLHDMLASLGK.N	2005.23806	2	1.03E-07	0.95	4.48	-	1242.7
AHQ-2-12, 5993 - 6071	R.DGFIDKEDLHDMLASLGK.N	2005.23806	2	3.73E-07	0.98	5.15	-	1943.2
AHQ-2-12, 5994 - 5999	R.DGFIDKEDLHDMLASLGK.N	2005.23806	3	1.22E-09	0.92	3.76	-	1514.9
AHQ-2-11, 6141 - 6221	K.EAFNMIDQNRDGFIDKEDLHDMLASLGK.N	3224.57104	3	6.21E-05	0.92	5.09	-	884.7
AHQ-2-11, 4198 - 4256	K.GNFNYIEFTR.I	1261.36733	2	8.05E-05	0.77	3.16	-	797.8
AHQ-2-11, 3988 - 4048	K.GNFNYIEFTR.I	1261.36733	2	1.61E-05	0.84	3.18	-	840.6
AHQ-2-13-, 4273	K.GNFNYIEFTR.I	1261.36733	2	1.47E-04	0.76	2.94	-	682.5
AHQ-2-12, 4178	K.GNFNYIEFTR.I	1261.36733	2	3.96E-05	0.87	3.59	-	829.3
AHQ-2-11, 4246 - 4248	K.GNFNYIEFTR.I	1261.36733	1	3.23E-06	0.37	2.74	-	396.1
AHQ-2-13, 4217	K.GNFNYIEFTR.I	1261.36733	2	3.88E-05	0.78	3.22	-	709.3
AHQ-2-11, 3364	K.KGNFNYIEFTR.I	1389.54025	2	6.03E-07	0.92	3.65	-	992.3
AHQ-2-11, 3846	K.KGNFNYIEFTR.I	1389.54025	2	1.21E-04	0.83	3.16	-	703.8
gi|21361755|ref|NP_004090.3| stomatin; erythrocyte membrane protein band 7.2 (stomatin) [Homo sapie				1.84E-11	9.01	100.34	46.20	31708.5
AHQ-2-10, 3803 - 3824	K.EASM*VITESPAALQLR.Y	1732.98059	2	4.49E-06	0.57	2.51	-	750.8
AHQ-2-1, 4936	K.EASMVITESPAALQLR.Y	1716.98119	2	2.64E-05	0.92	4.06	-	971.3
AHQ-2-14-, 4699	K.EASMVITESPAALQLR.Y	1716.98119	2	2.18E-09	0.80	3.52	-	867.6
AHQ-2-12, 4686	K.EASMVITESPAALQLR.Y	1716.98119	2	5.28E-07	0.36	2.84	-	464.9
AHQ-2-1, 4369	K.EASM*VITESPAALQLR.Y	1732.98059	2	6.06E-07	0.86	3.33	-	794.2
AHQ-2-14, 5777	K.EASMVITESPAALQLR.Y	1716.98119	2	4.73E-06	0.71	3.06	-	539.0
AHQ-2-13, 4648	K.EASMVITESPAALQLR.Y	1716.98119	2	1.46E-05	0.72	3.13	-	810.3
AHQ-2-9, 4555 - 4570	K.EASMVITESPAALQLR.Y	1716.98119	2	1.39E-06	0.65	2.69	-	769.8
AHQ-2-9, 7406	K.GPGLFFILPCTDSFIK.V	1814.13672	2	6.04E-04	0.95	4.69	-	882.1
AHQ-2-2, 7585	K.GPGLFFILPCTDSFIK.V	1814.13672	2	6.28E-06	0.93	4.54	-	660.4
AHQ-2-10, 6730	K.GPGLFFILPCTDSFIK.V	1814.13672	2	1.71E-06	0.96	4.94	-	946.3
AHQ-2-1, 2480	R.LLAQTTLR.N	916.10035	2	3.46E-05	0.84	3.33	-	527.7
AHQ-2-1, 7190	K.NLSQILSDREEIAHNMQSTLDDATDAWGIK.V	3373.65339	3	3.93E-09	0.85	4.22	-	769.2
AHQ-2-1, 6212 - 6290	K.NLSQILSDREEIAHNM*QSTLDDATDAWGIK.V	3389.65279	3	5.14E-05	0.97	5.44	-	2523.3
AHQ-2-10, 6646	K.NLSQILSDREEIAHNMQSTLDDATDAWGIK.V	3373.65339	3	6.22E-07	0.97	5.77	-	1970.2
AHQ-2-9, 7302	K.NLSQILSDREEIAHNMQSTLDDATDAWGIK.V	3373.65339	3	6.19E-07	0.98	6.80	-	1661.3

AHQ-2-9, 7554	K.NSTIVFPLPIDMLQGIIGAK.H	2128.56375	2	7.16E-04	0.75	3.45	-	400.7
AHQ-2-14-, 6968	K.NSTIVFPLPIDMLQGIIGAK.H	2128.56375	2	2.51E-04	0.71	3.13	-	395.2
AHQ-2-13-, 7109	K.NSTIVFPLPIDMLQGIIGAK.H	2128.56375	2	1.14E-04	0.85	4.04	-	493.4
AHQ-2-12, 7262	K.NSTIVFPLPIDMLQGIIGAK.H	2128.56375	2	6.06E-04	0.81	3.52	-	565.9
AHQ-2-13-, 2235	K.VIAAEGEMNASR.A	1248.39191	2	2.63E-09	0.96	4.05	-	1537.6
AHQ-2-1, 2338	K.VIAAEGEMNASR.A	1248.39191	2	7.83E-10	0.97	4.23	-	1734.0
AHQ-2-10, 3712 - 3716	R.VQNATLAVANITNADSATR.L	1931.09731	2	8.24E-07	0.96	5.66	-	716.2
AHQ-2-10, 4127 - 4199	R.VQNATLAVANITNADSATR.L	1931.09731	2	5.01E-06	0.94	4.75	-	723.9
AHQ-2-10, 3624	R.VQNATLAVANITNADSATR.L	1931.09731	2	1.11E-07	0.90	4.77	-	419.0
AHQ-2-10, 3519 - 3554	R.VQNATLAVANITNADSATR.L	1931.09731	2	3.31E-05	0.82	3.84	-	375.4
AHQ-2-1, 4709 - 4712	R.VQNATLAVANITNADSATR.L	1931.09731	2	2.07E-10	0.92	4.41	-	664.9
AHQ-2-1, 4414	R.VQNATLAVANITNADSATR.L	1931.09731	2	4.91E-07	0.85	3.96	-	462.0
AHQ-2-1, 3960	R.VQNATLAVANITNADSATR.L	1931.09731	2	8.53E-10	0.93	4.48	-	761.2
AHQ-2-1, 4306	R.VQNATLAVANITNADSATR.L	1931.09731	2	2.74E-05	0.74	3.14	-	505.5
AHQ-2-1, 4201	R.VQNATLAVANITNADSATR.L	1931.09731	2	1.84E-11	0.96	5.18	-	1169.5
AHQ-2-1, 4045 - 4064	R.VQNATLAVANITNADSATR.L	1931.09731	2	1.56E-09	0.97	5.54	-	1463.9
AHQ-2-1, 4037 - 4040	R.YLQTLTTIAAEK.N	1352.55813	2	4.59E-05	0.94	4.27	-	1232.5
AHQ-2-13, 3851	R.YLQTLTTIAAEK.N	1352.55813	2	8.01E-05	0.93	3.38	-	1300.1
AHQ-2-10, 3454	R.YLQTLTTIAAEK.N	1352.55813	2	6.82E-04	0.92	3.63	-	1162.2
AHQ-2-13-, 3885	R.YLQTLTTIAAEK.N	1352.55813	2	6.98E-08	0.94	3.32	-	1477.0
gi|4826898|ref|NP_005013.1| profilin 1; profilin-1 [Homo sapiens]				1.88E-11	16.99	200.30	75.00	15054.2
AHQ-2-12, 1735	K.CYEM*ASHLR.R	1184.32794	2	2.96E-04	0.92	2.60	-	1695.0
AHQ-2-12, 2305	K.CYEMASHLR.R	1168.32854	2	2.38E-04	0.77	2.53	-	1019.9
AHQ-2-13-, 1607 - 1666	K.CYEM*ASHLR.R	1184.32794	2	1.85E-05	0.90	2.91	-	1351.8
AHQ-2-13-, 1754 - 1813	K.CYEM*ASHLR.R	1184.32794	2	1.37E-04	0.89	2.99	-	1153.6
AHQ-2-13-, 1873 - 1895	K.CYEM*ASHLR.R	1184.32794	2	1.39E-04	0.89	3.02	-	1177.3
AHQ-2-13-, 1961 - 2023	K.CYEM*ASHLR.R	1184.32794	2	2.89E-05	0.80	2.79	-	850.4
AHQ-2-13-, 2281 - 2354	K.CYEMASHLR.R	1168.32854	2	1.04E-04	0.95	3.28	-	1781.1
AHQ-2-13-, 2413 - 2485	K.CYEMASHLR.R	1168.32854	2	2.70E-05	0.97	3.74	-	2150.0
AHQ-2-13-, 2425	K.CYEMASHLR.R	1168.32854	2	4.42E-04	0.94	3.29	-	1392.9
AHQ-2-13-, 2701	K.CYEMASHLR.R	1168.32854	2	5.98E-04	0.94	3.52	-	1469.8
AHQ-2-13, 2389 - 2459	K.CYEMASHLR.R	1168.32854	2	1.54E-04	0.95	3.21	-	2003.0
AHQ-2-13, 2523 - 2587	K.CYEMASHLR.R	1168.32854	2	1.80E-04	0.92	2.80	-	1661.1
AHQ-2-13, 1951 - 2028	K.CYEM*ASHLR.R	1184.32794	2	1.89E-04	0.94	3.07	-	1593.3
AHQ-2-13-, 4263	K.DRSSFYVNGLTLGGQK.C	1742.91379	3	6.21E-07	0.95	4.54	-	1688.7
AHQ-2-13-, 4265	K.DRSSFYVNGLTLGGQK.C	1742.91379	2	1.88E-11	0.97	5.42	-	1146.1
AHQ-2-13, 4075 - 4139	K.DRSSFYVNGLTLGGQK.C	1742.91379	2	5.29E-09	0.96	5.16	-	1062.7
AHQ-2-13, 4201	K.DRSSFYVNGLTLGGQK.C	1742.91379	2	6.33E-05	0.92	3.76	-	906.1
AHQ-2-13-, 4123 - 4126	K.DRSSFYVNGLTLGGQK.C	1742.91379	3	1.45E-05	0.83	3.54	-	954.1
AHQ-2-13-, 4121 - 4197	K.DRSSFYVNGLTLGGQK.C	1742.91379	2	1.95E-10	0.97	5.73	-	1047.7
AHQ-2-12, 5633 - 5699	R.DSLLQDGEFSMDLR.T	1626.77023	2	1.38E-09	0.97	4.54	-	1932.2
AHQ-2-12, 5810 - 5818	R.DSLLQDGEFSMDLR.T	1626.77023	2	2.48E-09	0.97	5.13	-	1794.5
AHQ-2-13-, 5633 - 5701	R.DSLLQDGEFSMDLR.T	1626.77023	2	3.36E-10	0.98	5.31	-	2089.7
AHQ-2-13-, 5585 - 5653	R.DSLLQDGEFSMDLR.T	1626.77023	2	4.32E-10	0.98	5.39	-	2309.3
AHQ-2-13-, 5502 - 5578	R.DSLLQDGEFSM*DLR.T	1642.76963	2	7.45E-04	0.92	3.42	-	1517.5
AHQ-2-12, 5905 - 5971	R.DSLLQDGEFSMDLR.T	1626.77023	2	1.72E-06	0.95	3.99	-	1730.1
AHQ-2-12, 6039	R.DSLLQDGEFSMDLR.T	1626.77023	2	8.69E-05	0.95	4.37	-	1333.4
AHQ-2-13-, 5449 - 5517	R.DSLLQDGEFSMDLR.T	1626.77023	2	1.44E-10	0.97	4.81	-	2045.3
AHQ-2-12, 6237 - 6310	R.DSLLQDGEFSMDLR.T	1626.77023	2	5.68E-05	0.90	3.83	-	912.7
AHQ-2-12, 5498 - 5561	R.DSLLQDGEFSMDLR.T	1626.77023	2	1.11E-08	0.93	3.72	-	1121.8
AHQ-2-13-, 5893 - 5953	R.DSLLQDGEFSMDLR.T	1626.77023	2	2.38E-06	0.90	2.85	-	1271.9
AHQ-2-12, 5365 - 5433	R.DSLLQDGEFSMDLR.T	1626.77023	2	1.13E-09	0.95	4.42	-	1340.3
AHQ-2-13-, 5409	R.DSLLQDGEFSM*DLR.T	1642.76963	2	5.16E-04	0.92	3.48	-	1143.5
AHQ-2-13-, 5310 - 5381	R.DSLLQDGEFSMDLR.T	1626.77023	2	1.00E-09	0.97	4.93	-	1548.2
AHQ-2-13-, 5065 - 5083	R.DSLLQDGEFSM*DLR.T	1642.76963	2	1.12E-05	0.92	3.48	-	1484.5
AHQ-2-13-, 4922 - 4947	R.DSLLQDGEFSM*DLR.T	1642.76963	2	1.54E-07	0.98	4.53	-	2548.1
AHQ-2-13-, 4821 - 4874	R.DSLLQDGEFSM*DLR.T	1642.76963	2	1.91E-07	0.97	5.10	-	1710.0
AHQ-2-13-, 4689 - 4757	R.DSLLQDGEFSM*DLR.T	1642.76963	2	5.53E-11	0.96	4.49	-	1674.6
AHQ-2-13-, 4566 - 4626	R.DSLLQDGEFSM*DLR.T	1642.76963	2	1.95E-10	0.95	4.52	-	1236.3
AHQ-2-13-, 4467	R.DSLLQDGEFSM*DLR.T	1642.76963	2	1.14E-04	0.93	3.96	-	1030.4
AHQ-2-12, 5275 - 5282	R.DSLLQDGEFSMDLR.T	1626.77023	2	3.32E-06	0.90	3.73	-	831.2
AHQ-2-12, 4743 - 4769	R.DSLLQDGEFSM*DLR.T	1642.76963	2	1.81E-04	0.77	2.64	-	907.8
AHQ-2-12, 4617 - 4625	R.DSLLQDGEFSM*DLR.T	1642.76963	2	9.90E-06	0.93	4.14	-	1197.7
AHQ-2-13, 6183 - 6243	R.DSLLQDGEFSMDLR.T	1626.77023	2	5.56E-09	0.97	4.49	-	1960.1
AHQ-2-13-, 5979 - 6050	R.DSLLQDGEFSMDLR.T	1626.77023	2	3.65E-06	0.95	3.65	-	1805.2
AHQ-2-13, 6049 - 6107	R.DSLLQDGEFSMDLR.T	1626.77023	2	1.19E-08	0.96	4.19	-	2194.0
AHQ-2-13, 5933 - 5997	R.DSLLQDGEFSMDLR.T	1626.77023	2	3.67E-08	0.96	4.03	-	1744.7
AHQ-2-13, 5799 - 5859	R.DSLLQDGEFSMDLR.T	1626.77023	2	1.79E-08	0.96	4.14	-	1778.2
AHQ-2-13, 6900	R.DSLLQDGEFSMDLR.T	1626.77023	2	1.21E-07	0.95	3.53	-	1995.8
AHQ-2-14, 5662 - 5712	R.DSLLQDGEFSM*DLR.T	1642.76963	2	8.34E-05	0.96	3.87	-	1957.8
AHQ-2-13, 6429	R.DSLLQDGEFSMDLR.T	1626.77023	2	1.29E-04	0.93	3.80	-	1436.2
AHQ-2-13-, 6113 - 6177	R.DSLLQDGEFSMDLR.T	1626.77023	2	2.97E-09	0.98	4.85	-	2374.1
AHQ-2-13-, 6209 - 6274	R.DSLLQDGEFSMDLR.T	1626.77023	2	3.87E-07	0.96	3.27	-	2485.5
AHQ-2-14, 6505 - 6564	R.DSLLQDGEFSM*DLR.T	1642.76963	2	8.74E-05	0.92	3.33	-	1361.4
AHQ-2-13-, 6341 - 6406	R.DSLLQDGEFSMDLR.T	1626.77023	2	2.80E-04	0.97	4.96	-	2037.2
AHQ-2-13, 5580 - 5643	R.DSLLQDGEFSMDLR.T	1626.77023	2	2.21E-08	0.97	4.74	-	1873.9
AHQ-2-13, 5451 - 5516	R.DSLLQDGEFSMDLR.T	1626.77023	2	1.81E-09	0.97	5.00	-	1898.5
AHQ-2-13-, 6682	R.DSLLQDGEFSMDLR.T	1626.77023	2	2.27E-09	0.94	3.71	-	1430.4
AHQ-2-13, 4508 - 4627	R.DSLLQDGEFSM*DLR.T	1642.76963	2	2.85E-08	0.93	4.02	-	1234.4
AHQ-2-14-, 4588	R.DSLLQDGEFSM*DLR.T	1642.76963	2	9.70E-09	0.91	3.60	-	1107.0
AHQ-2-14-, 4705	R.DSLLQDGEFSM*DLR.T	1642.76963	2	6.10E-08	0.96	4.33	-	1470.7
AHQ-2-13, 4691 - 4759	R.DSLLQDGEFSM*DLR.T	1642.76963	2	4.71E-08	0.95	3.68	-	1796.1
AHQ-2-13, 4824 - 4887	R.DSLLQDGEFSM*DLR.T	1642.76963	2	1.85E-07	0.96	4.16	-	1635.1
AHQ-2-14, 6512 - 6584	R.DSLLQDGEFSMDLR.T	1626.77023	2	3.02E-08	0.92	3.28	-	1544.8
AHQ-2-13, 5245 - 5255	R.DSLLQDGEFSM*DLR.T	1642.76963	2	9.33E-07	0.92	3.79	-	1202.5
AHQ-2-13, 5323 - 5387	R.DSLLQDGEFSMDLR.T	1626.77023	2	8.50E-09	0.96	4.20	-	1447.2
AHQ-2-14-, 5264 - 5341	R.DSLLQDGEFSMDLR.T	1626.77023	2	7.25E-09	0.97	4.96	-	1479.8
AHQ-2-14-, 5444 - 5513	R.DSLLQDGEFSMDLR.T	1626.77023	2	1.17E-06	0.97	4.35	-	1809.1
AHQ-2-13, 4896	R.DSLLQDGEFSMDLRTK.S	1856.04748	2	2.71E-05	0.52	2.84	-	332.6
AHQ-2-12, 3573 - 3654	K.DSPSVWAAVPGK.T	1214.35211	2	2.83E-06	0.88	3.29	-	885.2
AHQ-2-12, 3854	K.DSPSVWAAVPGK.T	1214.35211	2	2.82E-07	0.80	2.50	-	1099.2
AHQ-2-13-, 3349 - 3374	K.DSPSVWAAVPGK.T	1214.35211	2	9.07E-06	0.85	2.73	-	1177.3
AHQ-2-13-, 3529 - 3598	K.DSPSVWAAVPGK.T	1214.35211	2	4.95E-05	0.89	3.54	-	944.5
AHQ-2-13, 3677 - 3753	K.DSPSVWAAVPGK.T	1214.35211	2	1.76E-05	0.84	2.99	-	935.3
AHQ-2-13-, 3625 - 3706	K.DSPSVWAAVPGK.T	1214.35211	2	1.63E-07	0.91	3.75	-	985.8
AHQ-2-13, 3392	K.DSPSVWAAVPGK.T	1214.35211	2	3.17E-05	0.86	3.13	-	1039.2
AHQ-2-13, 3548 - 3731	K.DSPSVWAAVPGK.T	1214.35211	1	4.46E-04	0.13	2.01	-	446.2
AHQ-2-13, 3547 - 3616	K.DSPSVWAAVPGK.T	1214.35211	2	1.29E-05	0.87	3.26	-	1066.8
AHQ-2-13, 3860 - 3921	K.DSPSVWAAVPGK.T	1214.35211	2	8.19E-04	0.87	2.81	-	1079.7
AHQ-2-13, 6652	K.DSPSVWAAVPGKTFVNITPAEVGVLVGK.D	2840.26581	3	3.78E-06	0.95	5.40	-	1399.2
AHQ-2-13, 6504	K.DSPSVWAAVPGKTFVNITPAEVGVLVGK.D	2840.26581	2	1.88E-04	0.56	3.27	-	211.7
AHQ-2-13, 6489 - 6492	K.DSPSVWAAVPGKTFVNITPAEVGVLVGK.D	2840.26581	3	1.21E-09	0.89	4.34	-	1298.1
AHQ-2-13-, 1538 - 1713	K.EGVHGGLINK.K	1024.15577	1	3.42E-04	0.43	2.03	-	200.3
AHQ-2-13-, 2153	K.KCYEMASHLR.R	1296.50145	3	1.18E-06	0.95	4.05	-	2020.1
AHQ-2-13-, 2155 - 2158	K.KCYEMASHLR.R	1296.50145	1	4.23E-04	0.24	1.95	-	272.3
AHQ-2-13-, 2154	K.KCYEMASHLR.R	1296.50145	2	2.09E-06	0.96	3.22	-	2103.2
AHQ-2-14, 5313	R.SSFYVNGLTLGGQK.C	1471.63957	2	5.86E-05	0.88	3.51	-	670.3

AHQ-2-14, 5328	R.SSFYVNGLTLGGQK.C	1471.63957	1	1.43E-10	0.47	2.46	-	388.6
AHQ-2-14, 5630 - 5689	R.SSFYVNGLTLGGQK.C	1471.63957	2	2.38E-09	0.96	4.29	-	1309.2
AHQ-2-13-, 4597 - 4657	R.SSFYVNGLTLGGQK.C	1471.63957	2	1.48E-07	0.97	4.54	-	1586.5
AHQ-2-12, 4305	R.SSFYVNGLTLGGQK.C	1471.63957	2	4.60E-05	0.92	3.87	-	816.6
AHQ-2-13-, 4471 - 4537	R.SSFYVNGLTLGGQK.C	1471.63957	2	3.33E-08	0.97	4.67	-	1270.2
AHQ-2-12, 4306 - 4309	R.SSFYVNGLTLGGQK.C	1471.63957	1	1.81E-09	0.43	2.95	-	394.7
AHQ-2-13, 4249 - 4311	R.SSFYVNGLTLGGQK.C	1471.63957	2	1.96E-07	0.93	3.86	-	889.5
AHQ-2-14-, 4213 - 4293	R.SSFYVNGLTLGGQK.C	1471.63957	1	2.64E-04	0.67	2.60	-	551.1
AHQ-2-13, 4564	R.SSFYVNGLTLGGQK.C	1471.63957	1	1.71E-04	0.62	2.47	-	534.9
AHQ-2-13, 4563 - 4629	R.SSFYVNGLTLGGQK.C	1471.63957	2	3.15E-07	0.86	3.11	-	748.4
AHQ-2-12, 4466 - 4601	R.SSFYVNGLTLGGQK.C	1471.63957	1	4.97E-09	0.37	2.61	-	324.7
AHQ-2-13-, 4305 - 4373	R.SSFYVNGLTLGGQK.C	1471.63957	2	5.14E-08	0.93	4.13	-	728.2
AHQ-2-14-, 4505 - 4571	R.SSFYVNGLTLGGQK.C	1471.63957	1	1.89E-04	0.56	2.35	-	508.4
AHQ-2-13, 4471 - 4539	R.SSFYVNGLTLGGQK.C	1471.63957	2	3.56E-10	0.96	4.55	-	1069.3
AHQ-2-13, 4332 - 4404	R.SSFYVNGLTLGGQK.C	1471.63957	2	1.02E-05	0.84	3.05	-	682.0
AHQ-2-13, 4261 - 4628	R.SSFYVNGLTLGGQK.C	1471.63957	1	8.45E-09	0.48	2.72	-	368.0
AHQ-2-13, 4259 - 4315	R.SSFYVNGLTLGGQK.C	1471.63957	1	1.65E-06	0.60	2.84	-	478.6
AHQ-2-13-, 4307 - 4694	R.SSFYVNGLTLGGQK.C	1471.63957	1	3.08E-07	0.44	2.85	-	354.3
AHQ-2-14-, 4508 - 4575	R.SSFYVNGLTLGGQK.C	1471.63957	2	5.07E-09	0.96	4.48	-	1210.3
AHQ-2-12, 4463 - 4533	R.SSFYVNGLTLGGQK.C	1471.63957	2	3.03E-09	0.96	4.79	-	1193.9
AHQ-2-13, 3317 - 3379	K.STGGAPTFNVTVTK.T	1380.52843	2	1.91E-05	0.84	3.27	-	630.1
AHQ-2-13-, 2934 - 3001	K.STGGAPTFNVTVTK.T	1380.52843	2	2.16E-06	0.88	3.79	-	510.4
AHQ-2-13, 3087 - 3168	K.STGGAPTFNVTVTK.T	1380.52843	2	1.47E-06	0.72	2.99	-	424.1
AHQ-2-13-, 3010 - 3293	K.STGGAPTFNVTVTK.T	1380.52843	1	8.64E-04	0.35	2.66	-	288.5
AHQ-2-13-, 3069 - 3137	K.STGGAPTFNVTVTK.T	1380.52843	2	3.33E-08	0.85	3.03	-	518.6
AHQ-2-13-, 3201 - 3267	K.STGGAPTFNVTVTK.T	1380.52843	2	6.12E-07	0.86	3.45	-	473.0
AHQ-2-12, 2913 - 3033	K.STGGAPTFNVTVTK.T	1380.52843	1	4.43E-05	0.17	1.92	-	412.7
AHQ-2-14-, 3023 - 3025	K.STGGAPTFNVTVTK.T	1380.52843	2	1.89E-05	0.73	2.68	-	424.3
AHQ-2-13, 3051 - 3315	K.STGGAPTFNVTVTK.T	1380.52843	1	4.21E-04	0.41	2.72	-	297.2
AHQ-2-13, 3216 - 3297	K.STGGAPTFNVTVTK.T	1380.52843	2	9.80E-05	0.89	3.37	-	549.1
AHQ-2-14, 3933 - 3957	K.STGGAPTFNVTVTK.T	1380.52843	2	1.70E-04	0.66	2.81	-	374.7
AHQ-2-13, 2997 - 3059	K.STGGAPTFNVTVTK.T	1380.52843	2	1.92E-08	0.89	3.78	-	539.7
AHQ-2-12, 3035	K.STGGAPTFNVTVTK.T	1380.52843	2	1.09E-04	0.84	3.18	-	480.7
AHQ-2-12, 2942	K.STGGAPTFNVTVTKTDK.T	1724.89354	2	4.32E-05	0.88	3.22	-	843.1
AHQ-2-13, 2895	K.STGGAPTFNVTVTKTDK.T	1724.89354	2	1.47E-06	0.89	3.62	-	653.3
AHQ-2-13-, 2847 - 2854	K.TDKTLVLLM*GK.E	1235.51929	2	1.66E-06	0.94	4.13	-	1056.0
AHQ-2-13-, 3533 - 3613	K.TDKTLVLLMGK.E	1219.51989	2	1.07E-06	0.95	4.13	-	1314.6
AHQ-2-13, 3541 - 3620	K.TDKTLVLLMGK.E	1219.51989	2	1.81E-06	0.95	4.02	-	1310.8
AHQ-2-13-, 6681 - 6693	K.TFVNITPAEVGVLVGK.D	1644.93630	2	4.96E-04	0.92	3.98	-	909.7
AHQ-2-13-, 5997 - 6069	K.TFVNITPAEVGVLVGK.D	1644.93630	2	7.68E-07	0.95	4.88	-	1062.4
AHQ-2-13, 5424 - 5503	K.TFVNITPAEVGVLVGK.D	1644.93630	2	2.00E-04	0.76	3.62	-	572.9
AHQ-2-13, 5644 - 5723	K.TFVNITPAEVGVLVGK.D	1644.93630	2	2.94E-06	0.95	4.75	-	944.6
AHQ-2-13, 5685 - 5749	K.TFVNITPAEVGVLVGK.D	1644.93630	3	1.46E-07	0.95	4.81	-	1448.8
AHQ-2-13, 5783 - 5843	K.TFVNITPAEVGVLVGK.D	1644.93630	2	3.90E-07	0.94	4.72	-	884.8
AHQ-2-13, 5895 - 5957	K.TFVNITPAEVGVLVGK.D	1644.93630	2	1.56E-04	0.82	3.33	-	547.9
AHQ-2-13, 5971 - 6023	K.TFVNITPAEVGVLVGK.D	1644.93630	2	5.78E-05	0.85	3.78	-	799.5
AHQ-2-13-, 5923	K.TFVNITPAEVGVLVGK.D	1644.93630	3	2.73E-07	0.98	5.93	-	1981.4
AHQ-2-13-, 5870 - 5935	K.TFVNITPAEVGVLVGK.D	1644.93630	2	2.19E-05	0.92	4.11	-	928.3
AHQ-2-13-, 5793 - 5818	K.TFVNITPAEVGVLVGK.D	1644.93630	3	1.32E-08	0.97	6.09	-	1503.6
AHQ-2-13-, 5781 - 5849	K.TFVNITPAEVGVLVGK.D	1644.93630	2	5.03E-07	0.95	4.32	-	1042.3
AHQ-2-12, 6073	K.TFVNITPAEVGVLVGK.D	1644.93630	2	3.63E-04	0.87	3.58	-	612.7
AHQ-2-12, 5897 - 5899	K.TFVNITPAEVGVLVGK.D	1644.93630	3	2.37E-08	0.96	5.23	-	1340.6
AHQ-2-12, 5885 - 5962	K.TFVNITPAEVGVLVGK.D	1644.93630	2	1.66E-05	0.89	3.92	-	734.5
AHQ-2-12, 5850 - 5921	K.TFVNITPAEVGVLVGK.D	1644.93630	2	4.48E-05	0.88	3.76	-	674.1
AHQ-2-14-, 5859	K.TFVNITPAEVGVLVGK.D	1644.93630	2	1.02E-04	0.91	4.11	-	734.8
AHQ-2-13, 5345 - 5353	K.TFVNITPAEVGVLVGKDR.S	1916.21052	3	1.72E-04	0.73	3.82	-	505.2
AHQ-2-13, 5336 - 5399	K.TFVNITPAEVGVLVGKDR.S	1916.21052	2	4.90E-07	0.92	4.50	-	576.2
AHQ-2-12, 5469	K.TFVNITPAEVGVLVGKDR.S	1916.21052	2	3.01E-06	0.85	3.90	-	476.5
AHQ-2-13-, 5438	K.TFVNITPAEVGVLVGKDR.S	1916.21052	2	4.88E-08	0.90	4.38	-	627.3
AHQ-2-13, 2731 - 2800	R.TKSTGGAPTFNVTVTK.T	1609.80568	2	3.94E-04	0.95	4.34	-	1092.0
AHQ-2-13, 3349	R.TKSTGGAPTFNVTVTK.T	1609.80568	2	4.97E-05	0.50	2.63	-	271.6
AHQ-2-13-, 2746 - 2795	R.TKSTGGAPTFNVTVTK.T	1609.80568	2	2.07E-04	0.95	4.25	-	946.8
AHQ-2-13-, 2753 - 2785	R.TKSTGGAPTFNVTVTK.T	1609.80568	3	4.60E-04	0.83	3.72	-	789.6
AHQ-2-13, 2839	R.TKSTGGAPTFNVTVTK.T	1609.80568	3	1.57E-05	0.84	3.62	-	932.3
AHQ-2-13, 2859 - 2860	R.TKSTGGAPTFNVTVTK.T	1609.80568	2	7.72E-06	0.92	3.72	-	757.6
AHQ-2-13, 3716	K.TLVLLMGK.E	875.15478	2	1.26E-05	0.85	2.66	-	787.5
AHQ-2-13-, 3005	K.TLVLLM*GK.E	891.15418	2	1.17E-05	0.75	2.75	-	728.8
AHQ-2-13-, 2907 - 2977	K.TLVLLM*GK.E	891.15418	2	4.39E-04	0.78	2.60	-	600.2
AHQ-2-13, 3587 - 3655	K.TLVLLMGK.E	875.15478	2	3.64E-05	0.88	3.19	-	601.3
AHQ-2-13-, 3701 - 3771	K.TLVLLMGK.E	875.15478	2	3.35E-05	0.89	3.37	-	626.9
AHQ-2-12, 3557	K.TLVLLMGK.E	875.15478	1	7.70E-05	0.40	2.14	-	556.7
AHQ-2-13-, 3578	K.TLVLLMGK.E	875.15478	1	8.91E-05	0.20	2.32	-	224.2
AHQ-2-13, 2960 - 3023	K.TLVLLM*GK.E	891.15418	2	2.82E-04	0.81	2.63	-	882.8
AHQ-2-12, 2831	K.TLVLLM*GK.E	891.15418	2	5.93E-04	0.86	2.61	-	762.5
AHQ-2-13-, 3574 - 3637	K.TLVLLMGK.E	875.15478	2	8.45E-06	0.85	2.79	-	619.8
gi|4759082|ref|NP_004648.1| serum deprivation response protein; serum deprivation response; phospha				1.93E-11	4.42	50.27	13.60	47172.9
AHQ-2-11, 6282	R.DNSQVNAVTVLTLLDK.L	1730.94122	2	7.37E-04	0.95	4.25	-	1643.6
AHQ-2-14-, 6268 - 6331	R.DNSQVNAVTVLTLLDK.L	1730.94122	2	2.57E-11	0.96	3.90	-	1803.5
AHQ-2-13-, 6426 - 6461	R.DNSQVNAVTVLTLLDK.L	1730.94122	2	1.75E-09	0.97	5.15	-	1808.2
AHQ-2-13, 6360	R.DNSQVNAVTVLTLLDK.L	1730.94122	2	1.06E-05	0.85	3.46	-	877.9
AHQ-2-13, 6264 - 6283	R.DNSQVNAVTVLTLLDK.L	1730.94122	2	5.28E-08	0.98	5.41	-	1728.1
AHQ-2-9, 6575 - 6646	R.DNSQVNAVTVLTLLDK.L	1730.94122	2	1.93E-05	0.97	4.75	-	1771.9
AHQ-2-12, 6369 - 6437	R.DNSQVNAVTVLTLLDK.L	1730.94122	2	5.04E-07	0.96	4.58	-	1354.2
AHQ-2-14-, 6384 - 6436	R.DNSQVNAVTVLTLLDK.L	1730.94122	2	3.09E-04	0.89	3.53	-	1309.5
AHQ-2-11, 6168	R.DNSQVNAVTVLTLLDK.L	1730.94122	2	1.75E-07	0.97	5.07	-	1897.8
AHQ-2-11, 6077 - 6156	R.DNSQVNAVTVLTLLDK.L	1730.94122	2	1.14E-09	0.96	5.10	-	1159.9
AHQ-2-11, 3356 - 3404	K.LVNMLDAVQENQHK.M	1639.85851	2	8.49E-08	0.92	3.91	-	1187.8
AHQ-2-12, 3443	K.LVNMLDAVQENQHK.M	1639.85851	2	9.10E-05	0.85	3.56	-	813.8
AHQ-2-11, 2089	K.RLENNHAQLLR.R	1364.53869	2	4.01E-06	0.83	2.86	-	874.5
AHQ-2-13-, 3386	R.SDGDPVQPAVLQVHQTS	1778.90130	2	3.95E-04	0.75	2.83	-	612.6
AHQ-2-12, 6169 - 6234	K.VLIFQEENEIPASVFVK.Q	1963.26241	2	1.19E-07	0.96	5.21	-	829.1
AHQ-2-10, 5750	K.VLIFQEENEIPASVFVK.Q	1963.26241	2	8.16E-06	0.89	3.78	-	612.4
AHQ-2-13, 6047	K.VLIFQEENEIPASVFVK.Q	1963.26241	2	1.92E-10	0.96	5.44	-	912.4
AHQ-2-9, 6546	K.VLIFQEENEIPASVFVK.Q	1963.26241	2	1.72E-08	0.96	4.92	-	1190.1
AHQ-2-9, 6394 - 6475	K.VLIFQEENEIPASVFVK.Q	1963.26241	2	2.79E-08	0.93	4.59	-	826.0
AHQ-2-11, 5886 - 5928	K.VLIFQEENEIPASVFVK.Q	1963.26241	2	6.97E-07	0.96	4.90	-	1186.2
AHQ-2-13-, 6213	K.VLIFQEENEIPASVFVK.Q	1963.26241	2	5.32E-04	0.96	5.40	-	1036.0
AHQ-2-9, 6159 - 6236	K.VLIFQEENEIPASVFVK.Q	1963.26241	2	2.80E-08	0.96	5.43	-	827.9
AHQ-2-14-, 6017 - 6091	K.VLIFQEENEIPASVFVK.Q	1963.26241	2	1.93E-11	0.95	5.18	-	892.3
AHQ-2-8, 6114 - 6131	K.VLIFQEENEIPASVFVK.Q	1963.26241	2	2.43E-06	0.95	4.80	-	1056.1
AHQ-2-6, 6205	K.VLIFQEENEIPASVFVK.Q	1963.26241	2	8.67E-08	0.94	4.78	-	819.6
gi|5901944|ref|NP_008977.1| elastin microfibril interface located protein; TNF? elastin microfibril				1.94E-11	4.45	50.27	9.40	106694.7
AHQ-2-3, 3553	R.APAPASAPPGPSEELLR.Q	1660.85268	2	9.57E-10	0.71	3.27	-	328.6
AHQ-2-4, 3565 - 3638	R.APAPASAPPGPSEELLR.Q	1660.85268	2	2.91E-07	0.76	3.22	-	351.7
AHQ-2-3, 3033	R.GTELGGAAGQGGHPPGYTSLASR.L	2142.27464	2	1.35E-08	0.88	4.61	-	320.2
AHQ-2-4, 3013	R.GTELGGAAGQGGHPPGYTSLASR.L	2142.27464	2	6.08E-06	0.58	3.06	-	304.1
AHQ-2-3, 3611	R.LDTVAGGLQGLR.E	1200.37012	2	4.29E-06	0.97	4.53	-	1590.4

AHQ-2-4, 2762	R.LVGSGLHTVEAAGEAR.Q	1567.72888	2	1.66E-06	0.89	3.52	-	995.5
AHQ-2-3, 2765	R.LVGSGLHTVEAAGEAR.Q	1567.72888	2	1.94E-11	0.93	3.66	-	1297.4
AHQ-2-3, 3711	R.PARPNLSGSSAGSPLSGLGGEGPGESEK.V	2596.74928	3	3.02E-07	0.96	5.42	-	1274.3
gi|5729877|ref|NP_006588.1| heat shock 70kDa protein 8 isoform 1; heat shock cognate protein, 71-kD				2.04E-11	11.87	130.27	21.10	70897.6
AHQ-2-6, 4227	R.ARFEELNADLFR.G	1481.63768	2	3.77E-08	0.94	3.90	-	1020.2
AHQ-2-6, 4146	R.ARFEELNADLFR.G	1481.63768	3	1.54E-04	0.93	4.29	-	1201.1
AHQ-2-6, 4559	R.FEELNADLFR.G	1254.37310	2	3.91E-07	0.93	3.38	-	1236.4
AHQ-2-6, 5186	K.GPAVGIDLGTTYSCVGVFQHGK.V	2265.53088	3	2.04E-11	0.90	4.33	-	1023.5
AHQ-2-6, 5182 - 5205	K.GPAVGIDLGTTYSCVGVFQHGK.V	2265.53088	2	1.80E-07	0.97	5.32	-	1449.8
AHQ-2-6, 5535	K.ILDKCNEIINWLDK.N	1776.04688	2	1.25E-07	0.96	5.04	-	1499.3
AHQ-2-6, 3259	K.LDKSQIHDIVLVGGSTR.I	1839.08615	2	1.09E-04	0.85	3.63	-	633.6
AHQ-2-6, 2719	R.M*VNHFIAEFK.R	1252.46696	2	5.55E-05	0.91	2.86	-	1117.3
AHQ-2-6, 3722	K.NQVAMNPTNTVFDAK.R	1650.83816	2	2.06E-04	0.87	3.50	-	738.4
AHQ-2-6, 2765	K.NQVAM*NPTNTVFDAK.R	1666.83756	2	1.07E-05	0.95	4.51	-	1084.5
AHQ-2-6, 3993	K.NQVAMNPTNTVFDAK.R	1650.83816	2	3.59E-07	0.90	4.39	-	558.8
AHQ-2-6, 3821 - 3865	K.NSLESYAFNMK.A	1304.45393	2	1.87E-05	0.86	3.36	-	772.7
AHQ-2-6, 3074	K.NSLESYAFNM*K.A	1320.45333	2	1.21E-05	0.78	2.75	-	664.7
AHQ-2-6, 2415	R.RFDDAVVQSDMK.H	1411.56603	2	4.76E-06	0.95	4.42	-	1320.9
AHQ-2-13, 2765	R.RFDDAVVQSDMK.H	1411.56603	2	9.18E-05	0.92	3.61	-	1179.8
AHQ-2-6, 6834 - 6905	K.SINPDEAVAYGAAVQAAILSGDK.S	2261.47411	2	6.78E-10	0.96	5.39	-	1043.7
AHQ-2-6, 6951 - 6987	K.SINPDEAVAYGAAVQAAILSGDK.S	2261.47411	2	4.65E-04	0.84	3.40	-	581.5
AHQ-2-7, 7003	K.SINPDEAVAYGAAVQAAILSGDK.S	2261.47411	2	1.09E-08	0.96	4.97	-	1021.4
AHQ-2-7, 2660	K.SQIHDIVLVGGSTR.I	1482.66712	2	1.92E-08	0.97	4.25	-	1812.0
AHQ-2-6, 2751	K.SQIHDIVLVGGSTR.I	1482.66712	2	3.72E-10	0.97	4.50	-	2004.5
AHQ-2-6, 2761	K.SQIHDIVLVGGSTR.I	1482.66712	1	9.36E-09	0.85	3.63	-	392.4
gi|4502027|ref|NP_000468.1| albumin precursor; PRO0883 protein [Homo sapiens]				2.13E-11	20.89	250.36	48.30	69366.4
AHQ-2-5, 2347 - 2348	K.AAFTECCQAADK.A	1374.47927	2	4.75E-05	0.87	3.31	-	794.5
AHQ-2-5, 5527 - 5528	K.AVMDDFAAFVEK.C	1343.53006	2	2.60E-07	0.97	4.54	-	1509.6
AHQ-2-6, 5429 - 5430	K.AVMDDFAAFVEK.C	1343.53006	2	1.01E-08	0.97	4.25	-	1836.4
AHQ-2-12, 5531	K.AVMDDFAAFVEK.C	1343.53006	2	7.50E-05	0.83	3.02	-	784.0
AHQ-2-5, 5541	K.AVMDDFAAFVEK.C	1343.53006	1	1.74E-06	0.87	3.63	-	900.0
AHQ-2-14-, 5448	K.AVMDDFAAFVEK.C	1343.53006	2	1.13E-09	0.96	4.00	-	1783.9
AHQ-2-6, 2298 - 2314	K.CCTESLVNR.R	1141.25805	2	4.33E-05	0.85	2.79	-	1035.9
AHQ-2-5, 2335	K.CCTESLVNR.R	1141.25805	1	7.95E-06	0.25	2.00	-	126.0
AHQ-2-5, 2328	K.CCTESLVNR.R	1141.25805	2	3.83E-05	0.84	2.68	-	1028.4
AHQ-2-6, 2537	R.ETYGEMADCCAK.Q	1437.55563	2	2.34E-05	0.51	2.94	-	355.0
AHQ-2-5, 2353	R.FKDLGEENFK.A	1227.34766	1	5.92E-04	0.27	1.85	-	492.8
AHQ-2-6, 2357 - 2362	R.FKDLGEENFK.A	1227.34766	2	9.91E-06	0.79	3.15	-	675.8
AHQ-2-13-, 2698 - 2707	R.FKDLGEENFK.A	1227.34766	2	1.50E-06	0.78	2.94	-	646.4
AHQ-2-5, 2275 - 2351	R.FKDLGEENFK.A	1227.34766	2	4.15E-06	0.83	3.17	-	714.3
AHQ-2-5, 2596	K.FQNALLVR.Y	961.14282	2	3.09E-05	0.84	2.71	-	947.1
AHQ-2-5, 2260	K.KQTALVELVK.H	1129.37494	2	2.56E-05	0.81	3.01	-	766.7
AHQ-2-5, 3209 - 3215	K.KVPQVSTPTLVEVSR.N	1640.90605	2	3.41E-07	0.89	3.83	-	648.6
AHQ-2-10, 3156 - 3162	K.KVPQVSTPTLVEVSR.N	1640.90605	2	1.54E-06	0.89	3.53	-	625.9
AHQ-2-6, 3151 - 3233	K.KVPQVSTPTLVEVSR.N	1640.90605	2	6.16E-09	0.93	3.71	-	1066.7
AHQ-2-7, 3072	K.KVPQVSTPTLVEVSR.N	1640.90605	2	3.62E-07	0.86	3.28	-	630.8
AHQ-2-2, 3610	K.KVPQVSTPTLVEVSR.N	1640.90605	2	6.11E-09	0.80	3.13	-	552.5
AHQ-2-4, 3470 - 3545	K.KVPQVSTPTLVEVSR.N	1640.90605	2	3.64E-05	0.55	2.95	-	491.8
AHQ-2-8, 2915	K.KVPQVSTPTLVEVSR.N	1640.90605	2	4.70E-06	0.52	2.73	-	443.5
AHQ-2-3, 3559	K.KVPQVSTPTLVEVSR.N	1640.90605	2	1.39E-09	0.82	2.99	-	721.6
AHQ-2-6, 2985 - 3051	K.LVNEVTEFAK.T	1150.30633	2	2.63E-07	0.77	3.06	-	520.6
AHQ-2-1, 3449	K.LVNEVTEFAK.T	1150.30633	2	2.16E-06	0.84	3.64	-	507.7
AHQ-2-6, 5605	R.LVRPEVDVMCTAFHDNEETFLK.K	2652.98428	3	7.27E-06	0.97	5.74	-	1719.8
AHQ-2-5, 5640	R.LVRPEVDVMCTAFHDNEETFLK.K	2652.98428	3	1.50E-06	0.98	7.13	-	2273.8
AHQ-2-5, 5253	R.LVRPEVDVMCTAFHDNEETFLKK.Y	2781.15719	3	1.77E-08	0.97	6.57	-	1148.2
AHQ-2-5, 5019	K.QNCELFEQLGEYK.F	1659.79847	2	5.15E-05	0.48	2.50	-	374.3
AHQ-2-6, 3630	R.RHPDYSVVLLLR.L	1468.72842	2	4.28E-07	0.93	3.51	-	1021.6
AHQ-2-5, 3609 - 3621	R.RHPDYSVVLLLR.L	1468.72842	2	8.71E-05	0.94	3.60	-	1189.6
AHQ-2-6, 5742	R.RHPYFYAPELLFFAK.R	1900.21293	2	4.51E-11	0.98	4.85	-	2456.0
AHQ-2-5, 5633	R.RHPYFYAPELLFFAK.R	1900.21293	3	4.24E-10	0.97	6.04	-	1954.3
AHQ-2-5, 5631	R.RHPYFYAPELLFFAK.R	1900.21293	2	2.54E-06	0.98	5.78	-	2733.8
AHQ-2-6, 7174	K.RM*PCAEDYLSVVLNQLCVLHEK.T	2694.10110	3	8.96E-04	0.95	5.63	-	1113.2
AHQ-2-6, 7213	K.RMPCAEDYLSVVLNQLCVLHEK.T	2678.10170	3	3.82E-08	0.97	6.21	-	1828.1
AHQ-2-5, 7420	K.RM*PCAEDYLSVVLNQLCVLHEK.T	2694.10110	3	3.76E-05	0.96	6.09	-	1284.8
AHQ-2-5, 7455 - 7457	K.RMPCAEDYLSVVLNQLCVLHEK.T	2678.10170	3	1.20E-05	0.98	6.47	-	1924.1
AHQ-2-7, 4523	R.RPCFSALEVDETYVPK.E	1913.14041	2	2.01E-04	0.89	3.81	-	611.4
AHQ-2-1, 4994	R.RPCFSALEVDETYVPK.E	1913.14041	2	2.98E-08	0.84	3.85	-	542.1
AHQ-2-5, 4636	R.RPCFSALEVDETYVPK.E	1913.14041	2	5.15E-06	0.91	4.37	-	645.3
AHQ-2-6, 6349 - 6357	K.SHCIAEVENDEM*PADLPSLAADFVESK.D	2993.22637	3	1.06E-05	0.92	5.30	-	598.5
AHQ-2-7, 7025 - 7043	K.SHCIAEVENDEMPADLPSLAADFVESK.D	2977.22697	3	1.52E-04	0.95	5.77	-	756.2
AHQ-2-6, 6893 - 6963	K.SHCIAEVENDEMPADLPSLAADFVESK.D	2977.22697	2	5.02E-08	0.96	5.29	-	966.2
AHQ-2-5, 7059 - 7124	K.SHCIAEVENDEMPADLPSLAADFVESK.D	2977.22697	3	2.13E-11	0.96	6.10	-	975.7
AHQ-2-5, 7061	K.SHCIAEVENDEMPADLPSLAADFVESK.D	2977.22697	2	3.57E-05	0.94	4.82	-	847.8
AHQ-2-6, 6859 - 6922	K.SHCIAEVENDEMPADLPSLAADFVESK.D	2977.22697	3	2.08E-07	0.95	5.43	-	939.5
AHQ-2-5, 2248	K.SLHTLFGDK.L	1018.14800	1	3.46E-05	0.63	2.53	-	489.3
AHQ-2-7, 2196	K.SLHTLFGDK.L	1018.14800	1	2.11E-04	0.55	2.34	-	505.6
AHQ-2-6, 4846	K.SLHTLFGDKLCTVATLR.E	1934.24913	3	9.95E-08	0.86	3.65	-	822.9
AHQ-2-6, 4855	K.SLHTLFGDKLCTVATLR.E	1934.24913	2	7.39E-06	0.93	4.28	-	870.8
AHQ-2-6, 5683 - 5765	K.VFDEFKPLVEEPQNLIK.Q	2046.35142	2	2.36E-04	0.87	4.44	-	449.0
AHQ-2-6, 5711 - 5778	K.VFDEFKPLVEEPQNLIK.Q	2046.35142	3	2.20E-06	0.95	4.38	-	1467.1
AHQ-2-1, 6126	K.VFDEFKPLVEEPQNLIK.Q	2046.35142	3	2.30E-07	0.97	5.06	-	1530.8
AHQ-2-5, 5971	K.VFDEFKPLVEEPQNLIK.Q	2046.35142	2	5.83E-05	0.73	3.60	-	413.3
AHQ-2-5, 5795 - 5805	K.VFDEFKPLVEEPQNLIK.Q	2046.35142	3	5.33E-06	0.95	4.41	-	1581.9
AHQ-2-5, 5787 - 5864	K.VFDEFKPLVEEPQNLIK.Q	2046.35142	2	3.23E-05	0.94	4.54	-	626.2
AHQ-2-9, 5776 - 5854	K.VFDEFKPLVEEPQNLIK.Q	2046.35142	2	6.92E-05	0.90	4.40	-	443.2
AHQ-2-3, 6108	K.VFDEFKPLVEEPQNLIK.Q	2046.35142	2	3.42E-04	0.89	4.00	-	555.2
AHQ-2-4, 6109	K.VFDEFKPLVEEPQNLIK.Q	2046.35142	2	7.24E-04	0.66	3.20	-	297.6
AHQ-2-6, 5562	K.VFDEFKPLVEEPQNLIK.Q	2046.35142	3	2.59E-06	0.90	4.13	-	1055.6
AHQ-2-7, 5711 - 5712	K.VFDEFKPLVEEPQNLIK.Q	2046.35142	2	3.22E-05	0.85	4.44	-	371.7
AHQ-2-1, 6120	K.VFDEFKPLVEEPQNLIK.Q	2046.35142	2	3.58E-06	0.91	4.30	-	466.9
AHQ-2-5, 3528	K.VHTECCHGDLLECADDRADLAK.Y	2589.77855	3	1.32E-04	0.94	5.40	-	826.7
AHQ-2-5, 3759 - 3760	K.VPQVSTPTLVEVSR.N	1512.73313	2	2.92E-04	0.88	3.21	-	894.7
AHQ-2-10, 3610	K.VPQVSTPTLVEVSR.N	1512.73313	2	1.56E-04	0.56	2.54	-	595.5
AHQ-2-6, 3725 - 3751	K.VPQVSTPTLVEVSR.N	1512.73313	2	1.61E-07	0.91	3.93	-	810.2
AHQ-2-6, 2177 - 2181	K.YICENQDSISSK.L	1445.53420	2	9.41E-07	0.95	4.81	-	735.5
AHQ-2-5, 2201	K.YICENQDSISSK.L	1445.53420	1	9.02E-07	0.44	2.74	-	323.9
AHQ-2-1, 2452	K.YICENQDSISSK.L	1445.53420	1	8.51E-04	0.05	1.96	-	183.0
AHQ-2-1, 2449	K.YICENQDSISSK.L	1445.53420	2	4.98E-07	0.93	3.99	-	759.1
gi|30151108|ref|XP_301187.1| similar to actin beta, cytoskeletal - Kenyan clawed frog [Homo sapiens				2.28E-11	2.61	30.34	13.10	32164.8
AHQ-2-1, 5056	K.DLYANTVLSGGTTM*YPGIADR.M	2232.45624	2	1.24E-05	0.95	4.15	-	1166.9
AHQ-2-1, 5584	K.DLYANTVLSGGTTMYPGIADR.M	2216.45684	2	5.16E-07	0.91	4.46	-	670.4
AHQ-2-2, 5286 - 5317	K.DLYANTVLSGGTTM*YPGIADR.M	2232.45624	2	3.00E-04	0.91	4.40	-	727.1
AHQ-2-2, 5846	K.DLYANTVLSGGTTMYPGIADR.M	2216.45684	2	1.42E-04	0.67	3.08	-	495.9
AHQ-2-3, 5135	K.DLYANTVLSGGTTM*YPGIADR.M	2232.45624	2	2.90E-06	0.85	3.58	-	541.9
AHQ-2-3, 5788	K.DLYANTVLSGGTTMYPGIADR.M	2216.45684	2	4.41E-04	0.92	4.84	-	723.7
AHQ-2-4, 5140	K.DLYANTVLSGGTTM*YPGIADR.M	2232.45624	2	1.46E-07	0.93	4.48	-	757.5
AHQ-2-4, 5632 - 5644	K.DLYANTVLSGGTTMYPGIADR.M	2216.45684	2	5.30E-07	0.74	3.47	-	543.1

AHQ-2-5, 5497	K.DLYANTVLSGGTTMYPGIADR.M	2216.45684	2	1.84E-07	0.92	4.64	-	796.8
AHQ-2-6, 5002 - 5003	K.DLYANTVLSGGTTM*YPGIADR.M	2232.45624	2	5.23E-06	0.92	4.21	-	812.3
AHQ-2-6, 5342	K.DLYANTVLSGGTTMYPGIADR.M	2216.45684	2	6.06E-05	0.91	4.28	-	913.0
AHQ-2-14-, 5523 - 5587	K.DLYANTVLSGGTTMYPGIADR.M	2216.45684	2	1.25E-05	0.92	4.21	-	893.2
AHQ-2-8, 4724	K.DLYANTVLSGGTTM*YPGIADR.M	2232.45624	2	3.42E-08	0.89	4.53	-	515.4
AHQ-2-8, 4824 - 4903	K.DLYANTVLSGGTTM*YPGIADR.M	2232.45624	2	2.88E-05	0.97	5.57	-	1114.7
AHQ-2-8, 4915 - 4987	K.DLYANTVLSGGTTM*YPGIADR.M	2232.45624	2	1.52E-05	0.96	5.14	-	932.1
AHQ-2-8, 5055 - 5126	K.DLYANTVLSGGTTM*YPGIADR.M	2232.45624	2	2.15E-09	0.96	4.98	-	1082.7
AHQ-2-8, 5159 - 5232	K.DLYANTVLSGGTTM*YPGIADR.M	2232.45624	2	2.13E-04	0.79	3.51	-	599.2
AHQ-2-8, 5234 - 5302	K.DLYANTVLSGGTTMYPGIADR.M	2216.45684	2	1.24E-04	0.98	6.86	-	1120.3
AHQ-2-8, 5331 - 5399	K.DLYANTVLSGGTTMYPGIADR.M	2216.45684	2	9.70E-06	0.87	3.67	-	718.0
AHQ-2-8, 5427 - 5498	K.DLYANTVLSGGTTMYPGIADR.M	2216.45684	2	7.23E-07	0.85	3.69	-	634.8
AHQ-2-8, 5447 - 5514	K.DLYANTVLSGGTTM*YPGIADR.M	2232.45624	2	4.11E-04	0.93	4.46	-	858.6
AHQ-2-8, 5656 - 5722	K.DLYANTVLSGGTTMYPGIADR.M	2216.45684	2	4.50E-05	0.91	4.44	-	623.7
AHQ-2-8, 5747 - 5826	K.DLYANTVLSGGTTMYPGIADR.M	2216.45684	2	2.21E-05	0.93	4.51	-	773.8
AHQ-2-8, 5878 - 5958	K.DLYANTVLSGGTTMYPGIADR.M	2216.45684	2	5.28E-06	0.97	5.83	-	1110.6
AHQ-2-8, 6026 - 6107	K.DLYANTVLSGGTTMYPGIADR.M	2216.45684	2	2.28E-04	0.97	5.86	-	1125.3
AHQ-2-8, 6174 - 6244	K.DLYANTVLSGGTTMYPGIADR.M	2216.45684	2	2.99E-06	0.95	5.02	-	834.2
AHQ-2-8, 6307 - 6371	K.DLYANTVLSGGTTMYPGIADR.M	2216.45684	2	3.03E-04	0.97	5.81	-	918.2
AHQ-2-8, 6438 - 6506	K.DLYANTVLSGGTTMYPGIADR.M	2216.45684	2	2.22E-05	0.95	5.23	-	828.9
AHQ-2-8, 6575 - 6642	K.DLYANTVLSGGTTMYPGIADR.M	2216.45684	2	8.17E-05	0.95	4.75	-	1034.5
AHQ-2-8, 6710 - 6790	K.DLYANTVLSGGTTMYPGIADR.M	2216.45684	2	9.23E-05	0.94	4.83	-	832.1
AHQ-2-8, 6848 - 6920	K.DLYANTVLSGGTTMYPGIADR.M	2216.45684	2	3.58E-06	0.96	5.73	-	855.2
AHQ-2-8, 7252 - 7323	K.DLYANTVLSGGTTMYPGIADR.M	2216.45684	2	9.57E-04	0.92	4.90	-	553.2
AHQ-2-9, 4951 - 4952	K.DLYANTVLSGGTTM*YPGIADR.M	2232.45624	2	6.79E-05	0.95	5.02	-	1056.4
AHQ-2-9, 5034 - 5108	K.DLYANTVLSGGTTM*YPGIADR.M	2232.45624	2	1.61E-05	0.94	4.50	-	934.2
AHQ-2-9, 5394	K.DLYANTVLSGGTTMYPGIADR.M	2216.45684	2	3.17E-05	0.89	4.29	-	695.7
AHQ-2-9, 6294 - 6366	K.DLYANTVLSGGTTMYPGIADR.M	2216.45684	2	1.80E-07	0.83	3.77	-	515.9
AHQ-2-10, 4583 - 4664	K.DLYANTVLSGGTTM*YPGIADR.M	2232.45624	2	3.71E-08	0.94	4.99	-	780.4
AHQ-2-10, 4722 - 4786	K.DLYANTVLSGGTTM*YPGIADR.M	2232.45624	2	2.24E-04	0.96	4.94	-	1008.7
AHQ-2-10, 4979 - 4994	K.DLYANTVLSGGTTMYPGIADR.M	2216.45684	2	9.99E-06	0.97	5.63	-	1328.6
AHQ-2-10, 5098 - 5166	K.DLYANTVLSGGTTMYPGIADR.M	2216.45684	2	5.56E-07	0.96	5.30	-	884.2
AHQ-2-10, 5351 - 5414	K.DLYANTVLSGGTTMYPGIADR.M	2216.45684	2	3.44E-04	0.96	4.92	-	1237.4
AHQ-2-10, 5478 - 5547	K.DLYANTVLSGGTTMYPGIADR.M	2216.45684	2	2.12E-04	0.77	3.68	-	500.8
AHQ-2-10, 6131	K.DLYANTVLSGGTTMYPGIADR.M	2216.45684	2	7.76E-06	0.82	3.74	-	635.8
AHQ-2-11, 4704	K.DLYANTVLSGGTTM*YPGIADR.M	2232.45624	2	4.15E-11	0.97	5.75	-	1014.5
AHQ-2-11, 4810 - 4829	K.DLYANTVLSGGTTM*YPGIADR.M	2232.45624	2	2.35E-04	0.79	3.38	-	546.0
AHQ-2-11, 5005	K.DLYANTVLSGGTTM*YPGIADR.M	2232.45624	2	1.01E-05	0.90	4.37	-	602.7
AHQ-2-11, 5116	K.DLYANTVLSGGTTMYPGIADR.M	2216.45684	2	5.65E-08	0.95	4.53	-	1111.9
AHQ-2-11, 5262 - 5330	K.DLYANTVLSGGTTMYPGIADR.M	2216.45684	2	2.25E-06	0.93	4.57	-	780.5
AHQ-2-12, 4925 - 4926	K.DLYANTVLSGGTTM*YPGIADR.M	2232.45624	2	2.36E-09	0.97	5.64	-	1413.6
AHQ-2-12, 5351	K.DLYANTVLSGGTTMYPGIADR.M	2216.45684	2	2.28E-11	0.96	5.18	-	1305.8
AHQ-2-12, 5501 - 5566	K.DLYANTVLSGGTTMYPGIADR.M	2216.45684	2	6.07E-04	0.92	4.35	-	812.4
AHQ-2-13, 4787 - 4849	K.DLYANTVLSGGTTM*YPGIADR.M	2232.45624	2	1.74E-08	0.96	5.38	-	1065.2
AHQ-2-13, 4871 - 4935	K.DLYANTVLSGGTTM*YPGIADR.M	2232.45624	2	1.09E-05	0.96	5.18	-	1166.1
AHQ-2-13, 5000 - 5073	K.DLYANTVLSGGTTM*YPGIADR.M	2232.45624	2	1.89E-05	0.93	4.18	-	961.1
AHQ-2-13, 5139 - 5200	K.DLYANTVLSGGTTM*YPGIADR.M	2232.45624	2	3.64E-07	0.90	4.33	-	769.5
AHQ-2-13, 5161 - 5231	K.DLYANTVLSGGTTMYPGIADR.M	2216.45684	2	1.14E-06	0.95	4.78	-	1092.4
AHQ-2-13, 5559 - 5624	K.DLYANTVLSGGTTMYPGIADR.M	2216.45684	2	4.96E-04	0.94	5.01	-	701.6
AHQ-2-14, 5668 - 5728	K.DLYANTVLSGGTTM*YPGIADR.M	2232.45624	2	2.62E-09	0.96	4.79	-	1252.0
AHQ-2-14, 5944 - 6006	K.DLYANTVLSGGTTM*YPGIADR.M	2232.45624	2	3.26E-05	0.97	5.01	-	1486.5
AHQ-2-14, 6068 - 6141	K.DLYANTVLSGGTTM*YPGIADR.M	2232.45624	2	4.22E-05	0.95	5.17	-	897.6
AHQ-2-14, 6144 - 6184	K.DLYANTVLSGGTTMYPGIADR.M	2216.45684	2	3.17E-07	0.97	5.91	-	1248.3
AHQ-2-14, 6200 - 6268	K.DLYANTVLSGGTTM*YPGIADR.M	2232.45624	2	3.03E-06	0.94	4.52	-	969.1
AHQ-2-14, 6248 - 6312	K.DLYANTVLSGGTTMYPGIADR.M	2216.45684	2	3.69E-05	0.94	4.76	-	923.1
AHQ-2-14, 6373 - 6436	K.DLYANTVLSGGTTMYPGIADR.M	2216.45684	2	2.43E-04	0.96	5.93	-	1011.3
AHQ-2-14, 6590 - 6609	K.DLYANTVLSGGTTM*YPGIADR.M	2232.45624	2	2.36E-08	0.97	5.17	-	1379.6
AHQ-2-13-, 4942 - 5009	K.DLYANTVLSGGTTM*YPGIADR.M	2232.45624	2	1.11E-05	0.96	5.44	-	1081.9
AHQ-2-13-, 5049	K.DLYANTVLSGGTTM*YPGIADR.M	2232.45624	2	8.29E-05	0.84	3.52	-	753.3
AHQ-2-13-, 5135 - 5181	K.DLYANTVLSGGTTM*YPGIADR.M	2232.45624	2	2.50E-04	0.92	4.45	-	822.9
AHQ-2-13-, 5243 - 5313	K.DLYANTVLSGGTTMYPGIADR.M	2216.45684	2	5.35E-05	0.95	5.12	-	963.0
AHQ-2-13-, 5386 - 5453	K.DLYANTVLSGGTTMYPGIADR.M	2216.45684	2	8.38E-05	0.96	5.80	-	928.3
AHQ-2-13-, 5518 - 5594	K.DLYANTVLSGGTTMYPGIADR.M	2216.45684	2	9.96E-04	0.95	5.41	-	835.3
AHQ-2-13-, 5658 - 5667	K.DLYANTVLSGGTTMYPGIADR.M	2216.45684	2	1.71E-05	0.96	5.25	-	884.8
AHQ-2-13-, 5811 - 5874	K.DLYANTVLSGGTTMYPGIADR.M	2216.45684	2	9.92E-06	0.94	4.82	-	780.5
AHQ-2-13-, 5951 - 6025	K.DLYANTVLSGGTTMYPGIADR.M	2216.45684	2	6.53E-04	0.91	4.07	-	790.5
AHQ-2-14-, 4677 - 4744	K.DLYANTVLSGGTTM*YPGIADR.M	2232.45624	2	3.81E-06	0.93	4.51	-	836.3
AHQ-2-14-, 4851 - 4915	K.DLYANTVLSGGTTM*YPGIADR.M	2232.45624	2	4.95E-05	0.96	5.06	-	1086.9
AHQ-2-14-, 4980 - 5044	K.DLYANTVLSGGTTM*YPGIADR.M	2232.45624	2	5.09E-05	0.95	5.26	-	795.6
AHQ-2-14-, 5083 - 5145	K.DLYANTVLSGGTTM*YPGIADR.M	2232.45624	2	4.61E-08	0.96	5.30	-	896.2
AHQ-2-14-, 5143 - 5211	K.DLYANTVLSGGTTMYPGIADR.M	2216.45684	2	2.35E-11	0.97	6.08	-	1439.6
AHQ-2-14-, 5212 - 5213	K.DLYANTVLSGGTTM*YPGIADR.M	2232.45624	2	5.30E-04	0.88	3.78	-	819.6
AHQ-2-14-, 5255 - 5333	K.DLYANTVLSGGTTMYPGIADR.M	2216.45684	2	2.72E-05	0.97	5.27	-	1272.5
AHQ-2-14-, 5395 - 5467	K.DLYANTVLSGGTTMYPGIADR.M	2216.45684	2	2.75E-05	0.96	5.58	-	889.5
AHQ-2-14-, 5656	K.DLYANTVLSGGTTMYPGIADR.M	2216.45684	2	7.53E-07	0.93	4.44	-	970.7
AHQ-2-8, 3534 - 3602	K.YPIEHSIITNWDDM*EK.I	2008.19724	2	6.24E-04	0.67	2.88	-	425.1
gi|5729887|ref|NP_006624.1| IQ motif containing GTPase activating protein 2 [Homo sapiens]				2.31E-11	5.41	60.29	7.00	180611.0
AHQ-2-6, 5530	K.GVLLDIDDLQTNQFK.N	1719.91690	2	3.98E-06	0.85	3.14	-	846.6
AHQ-2-3, 5883	K.GVLLDIDDLQTNQFK.N	1719.91690	2	2.31E-11	0.98	5.83	-	1673.2
AHQ-2-3, 5992	K.LGIAPQIQDLLGK.V	1366.63117	2	4.19E-05	0.81	3.20	-	436.7
AHQ-2-6, 5609	K.LGIAPQIQDLLGK.V	1366.63117	1	2.22E-04	0.14	1.99	-	291.3
AHQ-2-4, 4989	K.LPYDVTTEQALTYPEVK.N	1968.19269	2	2.95E-08	0.95	4.41	-	1530.2
AHQ-2-3, 4989	K.LPYDVTTEQALTYPEVK.N	1968.19269	2	3.75E-10	0.97	4.68	-	1684.4
AHQ-2-3, 6159	R.NPNAVLTLVDDNLAPEYQK.E	2115.32901	2	3.67E-04	0.87	3.67	-	548.3
AHQ-2-3, 7135	R.NQPGNTLTEILETPATAQQEVDHATDMVSR.A	3268.51558	3	9.82E-06	0.83	3.85	-	533.8
AHQ-2-5, 6992	R.NQPGNTLTEILETPATAQQEVDHATDMVSR.A	3268.51558	3	2.77E-04	0.71	3.23	-	503.7
AHQ-2-6, 6827	R.NQPGNTLTEILETPATAQQEVDHATDMVSR.A	3268.51558	3	1.53E-07	0.82	3.87	-	506.1
AHQ-2-4, 4304	K.VDQVQDIVTGNPTVIK.M	1726.95257	2	5.76E-06	0.91	3.86	-	1169.8
AHQ-2-3, 4313	K.VDQVQDIVTGNPTVIK.M	1726.95257	2	3.06E-08	0.96	4.91	-	1385.5
gi|4502985|ref|NP_001854.1| cytochrome c oxidase subunit VIb; human cytochrome oxidase subunit VIb				2.36E-11	0.93	10.22	20.90	10192.3
AHQ-2-14-, 6223	R.VYQSLCPTSWVTDWDEQR.A	2272.43560	2	2.36E-11	0.93	4.43	-	868.5
gi|4504523|ref|NP_002148.1| heat shock 10kDa protein 1 (chaperonin 10); heat shock 10kD protein 1 (				2.40E-11	1.84	20.20	25.50	10931.6
AHQ-2-14-, 2720 - 2732	K.VLQATVVAVGSGSK.G	1316.52902	2	1.44E-09	0.96	3.50	-	1879.8
AHQ-2-14, 3660	K.VLQATVVAVGSGSK.G	1316.52902	2	1.66E-05	0.91	3.73	-	849.5
AHQ-2-14-, 5020 - 5027	K.VVLDDKDYFLFR.D	1530.74861	2	1.66E-10	0.94	4.00	-	964.5
AHQ-2-14, 6260	K.VVLDDKDYFLFR.D	1530.74861	2	2.40E-11	0.88	3.16	-	972.7
gi|7661818|ref|NP_054900.1| HSPC159 protein [Homo sapiens]				2.61E-11	3.80	50.24	43.60	19011.6
AHQ-2-13-, 6889 - 6890	R.GEEQSAIPYFPFIPDQPFR.V	2239.47029	2	4.82E-07	0.89	4.53	-	340.8
AHQ-2-13, 6671	R.GEEQSAIPYFPFIPDQPFR.V	2239.47029	2	9.91E-05	0.90	4.06	-	454.4
AHQ-2-11, 6617 - 6628	R.GEEQSAIPYFPFIPDQPFR.V	2239.47029	2	9.10E-06	0.91	4.35	-	463.5
AHQ-2-14-, 6747	R.GEEQSAIPYFPFIPDQPFR.V	2239.47029	2	5.71E-06	0.90	4.36	-	394.9
AHQ-2-11, 2492 - 2502	K.INGDLQITK.L	1002.14688	1	1.85E-04	0.32	2.05	-	393.4
AHQ-2-13, 3711	R.IQTLSAIDTIK.I	1203.41068	2	5.01E-04	0.70	2.88	-	469.0
AHQ-2-13-, 3722 - 3795	R.IQTLSAIDTIK.I	1203.41068	2	7.84E-04	0.71	2.71	-	599.6
AHQ-2-11, 4886 - 4952	K.LDDGHLNNSLSSPVQADVYFPR.L	2445.62923	2	2.61E-11	0.95	4.86	-	1366.3
AHQ-2-11, 4892	K.LDDGHLNNSLSSPVQADVYFPR.L	2445.62923	3	6.09E-07	0.92	4.60	-	1102.2
AHQ-2-11, 4737	K.LDDGHLNNSLSSPVQADVYFPR.L	2445.62923	2	1.34E-07	0.92	4.73	-	829.0

AHQ-2-11, 4993 - 5073	K.LDDGHLNNSLSSPVQADVYFPR.L	2445.62923	3	7.46E-05	0.69	3.22	-	825.7
AHQ-2-13, 4881	R.VFVDGHQLFDFYHR.I	1780.96540	2	1.52E-05	0.97	4.65	-	1682.6
AHQ-2-11, 4678 - 4742	R.VFVDGHQLFDFYHR.I	1780.96540	2	3.81E-11	0.94	4.40	-	1089.2
AHQ-2-12, 4885	R.VFVDGHQLFDFYHR.I	1780.96540	2	1.46E-10	0.96	4.74	-	1318.4
gi|6005846|ref|NP_009215.1| PTK9L protein tyrosine kinase 9-like (A6-related protein); protein tyro				2.73E-11	5.51	60.28	29.80	39547.7
AHQ-2-12, 6690	R.AVLPLLDAQQPCYLLYR.L	2035.39506	3	5.14E-10	0.94	4.42	-	1254.7
AHQ-2-12, 6681	R.AVLPLLDAQQPCYLLYR.L	2035.39506	2	8.15E-06	0.95	4.64	-	897.0
AHQ-2-12, 6594	R.AVLPLLDAQQPCYLLYR.L	2035.39506	2	5.16E-06	0.85	3.59	-	516.5
AHQ-2-13, 6472 - 6484	R.AVLPLLDAQQPCYLLYR.L	2035.39506	2	2.73E-11	0.96	5.35	-	954.2
AHQ-2-12, 3183	K.DDLSFAGYQK.H	1144.21551	1	6.52E-06	0.68	2.54	-	523.4
AHQ-2-10, 3862 - 3863	R.ETIELVHTEPTDVAQLPSR.V	2136.34822	2	3.33E-05	0.91	4.66	-	700.4
AHQ-2-11, 4020 - 4022	R.ETIELVHTEPTDVAQLPSR.V	2136.34822	2	5.23E-06	0.95	4.83	-	940.7
AHQ-2-11, 4188 - 4189	K.HQTLQGLAFPLQPEAQR.A	1935.17562	2	9.76E-11	0.98	5.35	-	1418.2
AHQ-2-10, 3976	K.HQTLQGLAFPLQPEAQR.A	1935.17562	2	3.17E-10	0.97	4.60	-	1665.3
AHQ-2-11, 6178	K.KIEIGDGAELTAEFLYDEVHPK.Q	2475.73515	3	1.22E-10	0.97	5.59	-	1735.5
AHQ-2-12, 4789 - 4826	K.VVIEDEQLVLGASQEPVGR.W	2039.27568	2	2.52E-07	0.97	4.74	-	2028.3
gi|5174727|ref|NP_005989.1| chaperonin containing TCP1, subunit 3 (gamma); TCP1 (t-complex-1) ring				3.08E-11	3.70	40.22	9.20	60330.4
AHQ-2-6, 3553	R.AVAQALEVIPR.T	1167.38326	2	7.78E-05	0.95	4.37	-	1370.3
AHQ-2-6, 4353	K.IPGGIIEDSCVLR.G	1430.65218	2	3.08E-11	0.94	4.21	-	1191.6
AHQ-2-6, 2638	R.TLIQNCGASTIR.L	1335.51249	2	2.77E-04	0.88	3.45	-	821.0
AHQ-2-6, 5389	R.WSSLACNIALDAVK.M	1549.77353	2	1.71E-06	0.93	3.71	-	1188.0
gi|7661678|ref|NP_056461.1| RAP1B, member of RAS oncogene family; K-REV; RAS-related protein RAP1B				3.39E-11	11.06	120.27	35.90	20824.7
AHQ-2-11, 4258 - 4260	K.DTDDVPMILVGNK.C	1417.61048	1	6.81E-05	0.31	2.63	-	489.6
AHQ-2-11, 4257	K.DTDDVPMILVGNK.C	1417.61048	2	7.01E-08	0.94	3.85	-	948.5
AHQ-2-11, 6686	R.KQVEVDAQQCMLEILDTAGTEQFTAMR.D	3115.50608	3	2.16E-05	0.67	3.25	-	661.1
AHQ-2-14, 6313	R.KQVEVDAQQCM*LEILDTAGTEQFTAM*R.D	3147.50488	3	7.22E-04	0.90	4.16	-	1142.5
AHQ-2-11, 6437 - 6452	R.KQVEVDAQQCMLEILDTAGTEQFTAM*R.D	3131.50548	3	6.69E-09	0.94	4.72	-	1629.4
AHQ-2-11, 5117	R.KQVEVDAQQCM*LEILDTAGTEQFTAM*R.D	3147.50488	3	3.51E-09	0.93	4.65	-	961.0
AHQ-2-14-, 5280	R.KQVEVDAQQCM*LEILDTAGTEQFTAM*R.D	3147.50488	3	1.51E-08	0.83	3.39	-	974.3
AHQ-2-13-, 6925	R.KQVEVDAQQCMLEILDTAGTEQFTAMR.D	3115.50608	3	8.49E-08	0.92	4.99	-	818.2
AHQ-2-11, 5553	R.KQVEVDAQQCM*LEILDTAGTEQFTAMR.D	3131.50548	3	1.76E-04	0.93	4.54	-	1295.3
AHQ-2-11, 6770 - 6776	K.QVEVDAQQCMLEILDTAGTEQFTAMR.D	2987.33317	2	1.50E-07	0.89	4.49	-	453.2
AHQ-2-11, 4036 - 4102	R.QWNNCAFLESSAK.S	1556.68148	2	1.04E-06	0.61	2.73	-	246.3
AHQ-2-11, 3893 - 3973	R.QWNNCAFLESSAK.S	1556.68148	2	7.21E-07	0.80	3.81	-	220.4
AHQ-2-11, 3801	R.QWNNCAFLESSAK.S	1556.68148	2	1.38E-05	0.74	3.23	-	275.4
AHQ-2-14-, 3811 - 3823	R.VKDTDDVPMILVGNK.C	1644.91497	2	2.53E-06	0.93	3.27	-	1484.9
AHQ-2-13-, 3905	R.VKDTDDVPMILVGNK.C	1644.91497	3	9.13E-05	0.95	4.28	-	1849.5
AHQ-2-11, 3729 - 3758	R.VKDTDDVPMILVGNK.C	1644.91497	2	7.77E-09	0.98	5.36	-	2089.7
AHQ-2-11, 3576 - 3578	R.VKDTDDVPMILVGNK.C	1644.91497	3	8.89E-04	0.97	5.23	-	2422.7
AHQ-2-11, 3141 - 3200	R.VKDTDDVPM*ILVGNK.C	1660.91437	2	6.76E-04	0.95	4.54	-	1121.5
AHQ-2-11, 3533 - 3596	R.VKDTDDVPMILVGNK.C	1644.91497	2	2.11E-04	0.97	4.36	-	1911.7
AHQ-2-13, 3864	R.VKDTDDVPMILVGNK.C	1644.91497	2	6.61E-08	0.97	5.03	-	1836.0
AHQ-2-13-, 3902 - 3907	R.VKDTDDVPMILVGNK.C	1644.91497	2	4.41E-07	0.97	4.62	-	1976.2
AHQ-2-11, 4505 - 4554	R.VKDTDDVPMILVGNKCDLEDER.V	2563.84420	3	5.80E-10	0.97	5.38	-	2296.6
AHQ-2-14-, 4580 - 4587	R.VKDTDDVPMILVGNKCDLEDER.V	2563.84420	3	3.02E-08	0.93	4.17	-	1428.1
AHQ-2-11, 4422 - 4490	R.VKDTDDVPMILVGNKCDLEDER.V	2563.84420	2	1.03E-08	0.92	3.98	-	918.6
AHQ-2-12, 4478	R.VKDTDDVPMILVGNKCDLEDER.V	2563.84420	3	5.80E-05	0.84	3.62	-	812.3
AHQ-2-12, 4631	R.VKDTDDVPMILVGNKCDLEDER.V	2563.84420	3	3.39E-11	0.92	4.85	-	847.1
AHQ-2-11, 4373 - 4436	R.VKDTDDVPMILVGNKCDLEDER.V	2563.84420	3	1.19E-04	0.91	4.79	-	990.9
AHQ-2-13, 4481	R.VKDTDDVPMILVGNKCDLEDER.V	2563.84420	3	4.13E-04	0.86	3.85	-	957.1
AHQ-2-13, 4601	R.VKDTDDVPMILVGNKCDLEDER.V	2563.84420	3	1.37E-05	0.82	3.30	-	1173.1
AHQ-2-11, 4302	R.VKDTDDVPM*ILVGNKCDLEDER.V	2579.84360	3	6.95E-04	0.70	3.47	-	637.8
AHQ-2-11, 4268 - 4333	R.VKDTDDVPMILVGNKCDLEDER.V	2563.84420	2	2.02E-04	0.93	4.53	-	711.8
AHQ-2-11, 4229 - 4308	R.VKDTDDVPMILVGNKCDLEDER.V	2563.84420	3	4.62E-08	0.92	5.12	-	753.1
AHQ-2-13-, 4559	R.VKDTDDVPMILVGNKCDLEDER.V	2563.84420	3	4.06E-06	0.92	4.93	-	1026.2
AHQ-2-11, 4070 - 4145	R.VKDTDDVPM*ILVGNKCDLEDER.V	2579.84360	3	1.27E-08	0.95	5.07	-	1242.4
AHQ-2-11, 3948	R.VKDTDDVPM*ILVGNKCDLEDER.V	2579.84360	2	2.56E-04	0.44	2.76	-	340.6
AHQ-2-11, 4574	R.VKDTDDVPMILVGNKCDLEDERVVGK.E	2947.33182	3	1.68E-08	0.92	4.33	-	1508.6
AHQ-2-11, 4429	R.VKDTDDVPMILVGNKCDLEDERVVGK.E	2947.33182	3	7.01E-05	0.93	5.24	-	1180.7
gi|4502219|ref|NP_001656.1| ras homolog gene family, member G (rho G); RhoG [Homo sapiens]				3.51E-11	1.87	20.20	18.80	21338.4
AHQ-2-11, 4869	K.EYIPTVFDNYSAQSAVDGR.T	2133.25975	2	3.51E-11	0.93	3.89	-	904.9
AHQ-2-11, 4820	R.TVNLNLWDTAGQEEYDR.L	2025.12129	2	6.85E-08	0.94	4.06	-	1463.9
gi|10835187|ref|NP_000627.1| superoxide dismutase 2, mitochondrial [Homo sapiens]				3.70E-11	4.36	50.23	39.60	24733.0
AHQ-2-13-, 6309 - 6315	K.AIWNVINWENVTER.Y	1744.93137	2	3.92E-11	0.96	4.69	-	1426.6
AHQ-2-13, 6127	K.AIWNVINWENVTER.Y	1744.93137	2	9.69E-08	0.90	4.22	-	720.4
AHQ-2-11, 6289	K.AIWNVINWENVTER.Y	1744.93137	2	3.70E-11	0.93	3.63	-	1181.3
AHQ-2-11, 6012	K.AIWNVINWENVTER.Y	1744.93137	2	2.04E-06	0.89	3.12	-	1072.3
AHQ-2-11, 5885 - 5954	K.AIWNVINWENVTER.Y	1744.93137	2	2.42E-07	0.90	3.87	-	726.0
AHQ-2-14-, 6171	K.AIWNVINWENVTER.Y	1744.93137	2	1.28E-06	0.94	3.88	-	1146.8
AHQ-2-11, 4493 - 4516	K.FNGGGHINHSIFWTNLSPNGGGEPK.G	2638.83761	2	3.09E-07	0.87	3.94	-	552.5
AHQ-2-11, 4389	K.FNGGGHINHSIFWTNLSPNGGGEPK.G	2638.83761	2	4.45E-09	0.91	4.62	-	647.2
AHQ-2-11, 3748 - 3808	K.GDVTAQIALQPALK.F	1425.65548	2	1.04E-04	0.86	3.50	-	899.1
AHQ-2-11, 3720	K.GDVTAQIALQPALK.F	1425.65548	1	3.36E-06	0.80	3.33	-	858.6
AHQ-2-11, 3657 - 3724	K.GDVTAQIALQPALK.F	1425.65548	2	4.84E-06	0.77	3.50	-	585.4
AHQ-2-11, 3804	K.GDVTAQIALQPALK.F	1425.65548	1	2.41E-06	0.80	3.42	-	852.4
AHQ-2-11, 2293	K.HHAAYVNNLNVTEEK.Y	1739.86999	3	7.92E-06	0.95	4.44	-	2010.9
AHQ-2-11, 2205	K.HHAAYVNNLNVTEEK.Y	1739.86999	3	1.29E-04	0.94	4.39	-	1578.4
AHQ-2-11, 2292 - 2369	K.HHAAYVNNLNVTEEK.Y	1739.86999	2	8.01E-07	0.93	3.99	-	1031.5
AHQ-2-11, 2198	K.HHAAYVNNLNVTEEK.Y	1739.86999	2	2.00E-07	0.93	4.22	-	815.2
AHQ-2-11, 6893 - 6973	K.LTAASVGVQGSGWGWLGFNK.E	2036.27841	2	6.80E-06	0.78	3.47	-	619.1
gi|4507651|ref|NP_003281.1| tropomyosin 4 [Homo sapiens]				3.92E-11	4.49	50.27	20.20	28521.6
AHQ-2-9, 3896 - 3968	R.AEVSELKCGDLEEELK.N	1851.02256	2	3.12E-07	0.91	4.12	-	837.9
AHQ-2-9, 3895 - 3966	R.AEVSELKCGDLEEELK.N	1851.02256	2	3.39E-06	0.94	4.46	-	982.8
AHQ-2-9, 6031	R.AEVSELKCGDLEEELKNVTNNLK.S	2634.89893	3	3.92E-11	0.95	5.43	-	1344.0
AHQ-2-9, 1620	R.EKAEGDVAALNR.R	1273.37796	2	1.17E-05	0.73	2.76	-	681.8
AHQ-2-9, 2454 - 2483	K.IQALQQQADEAEDR.A	1615.68404	2	6.04E-11	0.97	4.73	-	1638.3
AHQ-2-11, 2508	K.IQALQQQADEAEDR.A	1615.68404	2	1.40E-04	0.97	3.61	-	2466.9
AHQ-2-9, 2592	K.IQALQQQADEAEDR.A	1615.68404	2	3.79E-06	0.80	2.84	-	488.7
AHQ-2-10, 2214	R.KIQALQQQADEAEDR.A	1743.85695	2	4.73E-07	0.93	4.29	-	1185.2
AHQ-2-9, 2230	R.KIQALQQQADEAEDR.A	1743.85695	3	3.86E-04	0.58	3.14	-	589.1
AHQ-2-9, 2351	R.KIQALQQQADEAEDR.A	1743.85695	2	5.45E-04	0.91	3.33	-	1320.5
gi|17986258|ref|NP_066299.2| smooth muscle and non-muscle myosin alkali light chain isoform 1 [Homo				4.01E-11	7.16	80.28	37.10	16930.0
AHQ-2-12, 4293 - 4310	K.DQGTYEDYVEGLR.V	1545.58904	2	1.28E-05	0.93	3.55	-	1101.4
AHQ-2-12, 3858 - 3927	K.EAFQLFDR.T	1026.12699	2	6.25E-04	0.73	2.87	-	640.6
AHQ-2-13, 2371	R.HVLVTLGEK.M	996.18547	2	3.73E-05	0.86	2.78	-	779.9
AHQ-2-13-, 2237	R.HVLVTLGEK.M	996.18547	2	4.46E-06	0.90	3.39	-	718.2
AHQ-2-14-, 2207	R.HVLVTLGEK.M	996.18547	2	7.56E-07	0.90	3.31	-	683.0
AHQ-2-14-, 2208 - 2209	R.HVLVTLGEK.M	996.18547	1	3.81E-04	0.71	3.01	-	430.1
AHQ-2-12, 2193	R.HVLVTLGEK.M	996.18547	2	2.60E-06	0.85	2.84	-	695.3
AHQ-2-12, 2105 - 2169	R.HVLVTLGEK.M	996.18547	1	3.47E-05	0.62	2.92	-	538.7
AHQ-2-13, 2376	R.HVLVTLGEK.M	996.18547	1	1.05E-05	0.66	2.85	-	582.1
AHQ-2-12, 2102 - 2166	R.HVLVTLGEK.M	996.18547	2	3.28E-06	0.88	3.50	-	588.0
AHQ-2-12, 3763 - 3770	K.NKDQGTYEDYVEGLR.V	1787.86505	3	2.53E-04	0.53	3.08	-	624.1
AHQ-2-12, 4171	K.NKDQGTYEDYVEGLR.V	1787.86505	2	7.01E-06	0.75	3.07	-	717.2
AHQ-2-12, 3743 - 3823	K.NKDQGTYEDYVEGLR.V	1787.86505	2	2.24E-08	0.86	3.68	-	708.1
AHQ-2-14-, 3709	K.NKDQGTYEDYVEGLR.V	1787.86505	2	5.23E-09	0.92	4.13	-	982.4
AHQ-2-12, 3601 - 3669	K.NKDQGTYEDYVEGLR.V	1787.86505	3	5.21E-06	0.95	4.90	-	1296.1

AHQ-2-12, 3598 - 3673	K.NKDQGTYEDYVEGLR.V	1787.86505	2	4.16E-09	0.95	4.03	-	1490.9
AHQ-2-13-, 3813 - 3819	K.NKDQGTYEDYVEGLR.V	1787.86505	2	1.36E-10	0.89	3.87	-	779.0
AHQ-2-14-, 3711 - 3716	K.NKDQGTYEDYVEGLR.V	1787.86505	3	1.42E-05	0.93	5.01	-	991.2
AHQ-2-14-, 3988	K.NKDQGTYEDYVEGLR.V	1787.86505	2	2.98E-08	0.80	3.17	-	782.0
AHQ-2-12, 3995 - 4065	K.NKDQGTYEDYVEGLR.V	1787.86505	2	1.43E-08	0.94	4.22	-	1175.0
AHQ-2-13, 3752 - 3775	K.NKDQGTYEDYVEGLR.V	1787.86505	2	6.77E-08	0.93	4.23	-	928.1
AHQ-2-14, 4805	K.NKDQGTYEDYVEGLR.V	1787.86505	2	3.58E-05	0.81	3.54	-	900.2
AHQ-2-14, 4594	K.NKDQGTYEDYVEGLR.V	1787.86505	3	1.05E-06	0.87	4.19	-	881.1
AHQ-2-14, 4592	K.NKDQGTYEDYVEGLR.V	1787.86505	2	1.56E-10	0.91	3.71	-	1067.7
AHQ-2-12, 3165 - 3234	R.VFDKEGNGTVMGAEIR.H	1723.93228	2	2.26E-10	0.94	4.21	-	1036.7
AHQ-2-14-, 3305	R.VFDKEGNGTVMGAEIR.H	1723.93228	2	4.01E-11	0.96	4.46	-	1553.3
AHQ-2-12, 3246 - 3254	R.VFDKEGNGTVM*GAEIR.H	1739.93168	2	1.65E-06	0.89	4.02	-	870.6
AHQ-2-12, 6973 - 7042	K.VLDFEHFLPMLQTVAK.N	1889.25017	2	6.76E-07	0.95	4.97	-	918.9
AHQ-2-13-, 6482	K.VLDFEHFLPM*LQTVAK.N	1905.24957	3	9.10E-06	0.88	3.77	-	861.6
AHQ-2-13-, 6922 - 6923	K.VLDFEHFLPMLQTVAK.N	1889.25017	2	3.16E-04	0.96	5.20	-	960.2
AHQ-2-12, 6975 - 6987	K.VLDFEHFLPMLQTVAK.N	1889.25017	3	1.24E-05	0.96	5.07	-	1385.9
AHQ-2-12, 6651	K.VLDFEHFLPM*LQTVAK.N	1905.24957	2	6.12E-06	0.88	3.71	-	655.8
AHQ-2-12, 6378 - 6449	K.VLDFEHFLPM*LQTVAK.N	1905.24957	3	5.56E-06	0.86	3.61	-	833.4
AHQ-2-13, 6256	K.VLDFEHFLPM*LQTVAK.N	1905.24957	2	3.12E-06	0.90	4.06	-	655.2
AHQ-2-12, 6366 - 6429	K.VLDFEHFLPM*LQTVAK.N	1905.24957	2	4.52E-06	0.91	4.28	-	694.1
AHQ-2-13-, 6927	K.VLDFEHFLPMLQTVAK.N	1889.25017	3	2.07E-05	0.92	4.06	-	1606.0
AHQ-2-13, 6699	K.VLDFEHFLPMLQTVAK.N	1889.25017	2	1.17E-06	0.91	4.20	-	658.8
AHQ-2-14-, 6312 - 6328	K.VLDFEHFLPM*LQTVAK.N	1905.24957	3	1.20E-04	0.78	3.38	-	914.2
AHQ-2-14-, 6316	K.VLDFEHFLPM*LQTVAK.N	1905.24957	2	4.82E-04	0.92	4.19	-	731.5
AHQ-2-14-, 6776 - 6777	K.VLDFEHFLPMLQTVAK.N	1889.25017	2	4.71E-06	0.97	5.52	-	1488.6
AHQ-2-14-, 6779 - 6780	K.VLDFEHFLPMLQTVAK.N	1889.25017	3	1.14E-05	0.96	4.78	-	1708.3
gi|6857820|ref|NP_005019.2| phosphatidylinositol-4-phosphate 5-kinase type II alpha; 1-phosphatidyl				4.25E-11	2.74	30.24	11.30	46224.3
AHQ-2-7, 4559	R.FGIDDQDFQNSLTR.S	1656.73462	2	3.71E-06	0.88	3.88	-	982.4
AHQ-2-7, 2449	K.HGAGAEISTVNPEQYSK.R	1788.89618	2	4.68E-10	0.97	4.89	-	2082.4
AHQ-2-7, 3947	K.TITSEDVAEMHNILK.K	1701.92338	2	4.25E-11	0.89	4.04	-	741.1
gi|4757826|ref|NP_004039.1| beta-2-microglobulin [Homo sapiens]				4.87E-11	3.13	40.24	38.70	13714.5
AHQ-2-14-, 6653 - 6720	K.SNFLNCYVSGFHPSDIEVDLLK.N	2556.83155	3	3.25E-06	0.78	3.66	-	642.3
AHQ-2-14-, 6519 - 6581	K.SNFLNCYVSGFHPSDIEVDLLK.N	2556.83155	2	2.49E-09	0.80	3.98	-	356.9
AHQ-2-14-, 6716	K.SNFLNCYVSGFHPSDIEVDLLK.N	2556.83155	2	5.02E-06	0.93	4.73	-	750.1
AHQ-2-14-, 6491	K.SNFLNCYVSGFHPSDIEVDLLKNGER.I	3013.28710	3	4.87E-11	0.89	4.41	-	962.8
AHQ-2-14-, 6255 - 6257	K.SNFLNCYVSGFHPSDIEVDLLKNGER.I	3013.28710	3	4.70E-08	0.85	4.05	-	429.6
AHQ-2-14, 3076	K.VEHSDLSFSK.D	1149.23530	1	4.29E-04	0.35	2.38	-	415.3
AHQ-2-14-, 2059 - 2061	K.VEHSDLSFSK.D	1149.23530	1	3.06E-04	0.86	3.33	-	650.5
AHQ-2-14-, 1723	R.VNHVTLSQPK.I	1123.28735	1	6.53E-04	0.23	2.26	-	173.1
AHQ-2-14-, 1848	R.VNHVTLSQPK.I	1123.28735	1	4.10E-04	0.27	2.09	-	243.5
AHQ-2-14-, 1961 - 2037	R.VNHVTLSQPK.I	1123.28735	1	1.75E-06	0.58	2.73	-	338.7
gi|27480104|ref|XP_208215.1| similar to triosephosphate isomerase 1 [Homo sapiens]				5.13E-11	3.55	40.27	17.30	26942.6
AHQ-2-10, 2686	R.HVFGESDELIGQK.V	1459.58565	1	3.52E-06	0.91	3.87	-	798.5
AHQ-2-10, 2676 - 2678	R.HVFGESDELIGQK.V	1459.58565	2	5.13E-11	0.97	5.19	-	1038.2
AHQ-2-10, 2679	R.HVFGESDELIGQK.V	1459.58565	1	9.63E-07	0.87	3.44	-	882.7
AHQ-2-11, 2900	R.HVFGESDELIGQK.V	1459.58565	2	2.27E-08	0.92	4.17	-	535.9
AHQ-2-10, 2506 - 2518	R.IIYGGSVTGATCK.E	1328.51672	2	4.78E-05	0.80	2.98	-	538.5
AHQ-2-10, 2378	R.RHVFGESDELIGQK.V	1615.77200	2	2.00E-09	0.98	5.40	-	2427.4
AHQ-2-10, 3440 - 3514	K.VTNGAFTGEISPGM*IK.D	1638.86726	2	4.93E-05	0.80	3.08	-	902.7
gi|5031677|ref|NP_005681.1| dynamin 1-like protein isoform 3; dynamin-like protein [Homo sapiens] [				5.49E-11	7.36	90.28	18.50	78060.6
AHQ-2-14-, 4833	K.ALQGASQIIAEIR.E	1370.57991	2	3.62E-08	0.96	4.50	-	1740.5
AHQ-2-14, 6032	K.ALQGASQIIAEIR.E	1370.57991	2	5.78E-08	0.95	4.05	-	1828.3
AHQ-2-14, 6293 - 6296	K.DTLQSELVGQLYK.S	1494.67144	2	6.64E-05	0.89	3.12	-	1041.1
AHQ-2-13, 5187	K.DTLQSELVGQLYK.S	1494.67144	2	1.78E-06	0.94	4.02	-	1400.2
AHQ-2-14-, 5199 - 5200	K.DTLQSELVGQLYK.S	1494.67144	1	1.59E-04	0.58	2.82	-	371.3
AHQ-2-6, 6941	K.IFSPNVVNLTLVDLPGMTK.V	2059.45828	2	9.48E-04	0.61	2.51	-	646.6
AHQ-2-6, 6399	K.IFSPNVVNLTLVDLPGM*TK.V	2075.45768	2	9.21E-06	0.87	3.69	-	833.2
AHQ-2-5, 7161	K.IFSPNVVNLTLVDLPGMTK.V	2059.45828	2	1.61E-06	0.76	3.00	-	744.0
AHQ-2-6, 7250 - 7251	K.LQDVFNTVGADIIQLPQIVVVGTQSSGK.S	2928.32977	2	1.94E-06	0.63	3.44	-	234.4
AHQ-2-6, 6155	K.LYTDFDEIRQEIENETER.I	2301.40883	2	1.66E-04	0.32	2.53	-	365.1
AHQ-2-13, 6755 - 6756	K.SSLLDDLLTESEDMAQR.R	1924.07698	2	1.55E-04	0.88	4.31	-	645.6
AHQ-2-14-, 6229 - 6235	K.SSLLDDLLTESEDM*AQR.R	1940.07638	2	5.49E-11	0.97	5.68	-	1343.2
AHQ-2-14-, 6827 - 6887	K.SSLLDDLLTESEDMAQR.R	1924.07698	2	1.76E-06	0.95	5.21	-	966.6
AHQ-2-7, 7471	R.TLESVDPLGGLNTIDILTAIR.N	2212.52928	2	9.10E-05	0.95	4.52	-	1146.0
gi|6598323|ref|NP_001485.2| GDP dissociation inhibitor 2; rab GDP-dissociation inhibitor, beta [Hom				5.58E-11	1.95	20.32	8.50	50662.9
AHQ-2-8, 6763	K.SPYLYPLYGLGELPQGFAR.L	2142.44089	2	5.82E-08	0.96	4.98	-	987.1
AHQ-2-10, 6379	K.SPYLYPLYGLGELPQGFAR.L	2142.44089	2	8.54E-07	0.95	5.06	-	754.2
AHQ-2-9, 6966	K.SPYLYPLYGLGELPQGFAR.L	2142.44089	2	5.92E-05	0.97	5.43	-	1163.9
AHQ-2-7, 6925 - 6947	K.SPYLYPLYGLGELPQGFAR.L	2142.44089	2	8.65E-05	0.97	5.55	-	1111.4
AHQ-2-8, 7454	K.VPSTEAEALASSLMGLFEK.R	1981.25641	2	5.58E-11	0.98	6.38	-	1685.5
gi|7656991|ref|NP_055140.1| coronin, actin binding protein, 1C; coronin, actin-binding protein, 1C;				6.27E-11	6.23	70.28	22.40	53248.7
AHQ-2-6, 5859 - 5897	R.AIFLADGNVFTTGFSR.M	1716.91793	2	1.87E-06	0.98	5.02	-	2162.8
AHQ-2-6, 3706	K.CDLISIPK.K	947.13128	2	1.86E-04	0.83	3.02	-	814.5
AHQ-2-7, 2888 - 2892	R.KCEPIIMTVPR.K	1345.65706	2	2.83E-04	0.86	3.34	-	704.8
AHQ-2-6, 3017 - 3021	R.KCEPIIMTVPR.K	1345.65706	2	1.68E-06	0.91	3.66	-	891.9
AHQ-2-7, 7391	R.KSDLFQDDLYPDTAGPEAALEAEEWFEGK.N	3273.46087	3	2.20E-08	0.96	5.60	-	1575.4
AHQ-2-9, 7323	R.KSDLFQDDLYPDTAGPEAALEAEEWFEGK.N	3273.46087	3	4.43E-08	0.96	5.52	-	1323.2
AHQ-2-7, 3632	K.NADPILISLK.H	1084.29103	1	4.45E-04	0.12	1.91	-	304.6
AHQ-2-6, 3707	K.NADPILISLK.H	1084.29103	1	4.38E-04	0.10	1.90	-	272.5
AHQ-2-6, 3714	K.NADPILISLK.H	1084.29103	2	8.39E-06	0.72	2.60	-	522.9
AHQ-2-6, 2201	K.SIKDTICNQDER.I	1480.58332	2	2.50E-06	0.90	2.81	-	1486.4
AHQ-2-7, 6028	R.YFEITDESPYVHYLNTFSSK.E	2441.63258	3	6.27E-11	0.92	4.46	-	874.7
AHQ-2-6, 6073	R.YFEITDESPYVHYLNTFSSK.E	2441.63258	3	1.19E-10	0.93	3.92	-	1095.3
AHQ-2-6, 6074 - 6085	R.YFEITDESPYVHYLNTFSSK.E	2441.63258	2	3.81E-08	0.95	4.49	-	1160.0
AHQ-2-7, 6024 - 6027	R.YFEITDESPYVHYLNTFSSK.E	2441.63258	2	5.59E-06	0.92	4.05	-	856.2
AHQ-2-10, 5659	R.YFEITDESPYVHYLNTFSSK.E	2441.63258	2	3.38E-04	0.94	4.13	-	1120.1
gi|4557731|ref|NP_000618.1| latent transforming growth factor beta binding protein 1 precursor [Hom				6.98E-11	14.22	170.32	19.20	152790.9
AHQ-2-2, 3764	R.ASGLGDHCEDINECLEDK.S	2065.13910	2	3.37E-05	0.96	4.94	-	1579.7
AHQ-2-1, 3797	R.ASGLGDHCEDINECLEDK.S	2065.13910	2	6.85E-05	0.97	5.69	-	1538.7
AHQ-2-3, 3699 - 3708	R.ASGLGDHCEDINECLEDK.S	2065.13910	2	6.98E-11	0.98	6.32	-	2172.3
AHQ-2-1, 3793	R.ASGLGDHCEDINECLEDK.S	2065.13910	3	4.07E-07	0.90	4.12	-	1112.3
AHQ-2-1, 2892	R.CFQETIGSQCGK.A	1417.54728	2	5.09E-04	0.83	2.87	-	899.9
AHQ-2-1, 6716	K.CLCLPGYVPSDKPNYCTPLNTALNLEK.D	3142.56947	3	9.46E-04	0.84	3.98	-	507.6
AHQ-2-1, 3710	R.CTCGQGYQLSAAKDQCEDIDECQHR.H	3035.18500	3	2.42E-09	0.88	4.41	-	714.9
AHQ-2-3, 4253	K.CVDIDECTQVQHLCSQGR.C	2209.38110	2	7.11E-04	0.89	3.90	-	700.0
AHQ-2-1, 4256	K.CVDIDECTQVQHLCSQGR.C	2209.38110	2	3.02E-06	0.97	5.08	-	1501.6
AHQ-2-3, 2059	K.DQCEDIDECQHR.H	1607.61898	2	7.00E-04	0.69	2.74	-	488.8
AHQ-2-1, 2173 - 2174	K.DQCEDIDECQHR.H	1607.61898	2	7.09E-06	0.84	3.45	-	651.8
AHQ-2-1, 3298	K.EAQPGQSQVSYQGLPVQK.T	1945.12244	2	8.41E-04	0.93	3.87	-	907.4
AHQ-2-1, 3638	K.EAQPGQSQVSYQGLPVQK.T	1945.12244	2	7.89E-06	0.78	3.20	-	672.7
AHQ-2-1, 3460	K.EAQPGQSQVSYQGLPVQK.T	1945.12244	2	1.35E-08	0.91	4.09	-	662.6
AHQ-2-1, 4052	K.EEPVEALTFSR.E	1278.39297	2	6.02E-05	0.70	2.77	-	492.8
AHQ-2-1, 3601	K.EICPGGMGYTVSGVHR.R	1721.93967	2	3.95E-04	0.62	2.72	-	661.8
AHQ-2-1, 2876	K.GFVPAGESSSEAGGENYK.D	1786.83392	2	2.00E-05	0.93	4.27	-	789.7
AHQ-2-4, 5744	R.GRCEDIDECLNPSTCPDEQCVNSPGSYQCVPCTEGFR.G	4402.64921	3	1.35E-04	0.75	3.72	-	462.1
AHQ-2-3, 5715 - 5720	R.GRCEDIDECLNPSTCPDEQCVNSPGSYQCVPCTEGFR.G	4402.64921	3	1.76E-06	0.91	4.72	-	867.9
AHQ-2-1, 2224	K.HPPEASVQIHQVSR.I	1585.74913	3	8.93E-09	0.66	3.13	-	533.7

AHQ-2-1, 3102	K.LCQIPVHGASVPK.L	1407.66344	2	7.52E-04	0.83	3.51	-	421.6
AHQ-2-1, 3702	R.NTEGSFQCVCDQGYR.A	1823.89914	2	2.32E-06	0.93	4.35	-	679.0
AHQ-2-1, 2980	R.TSTDLDVDVDQPK.E	1433.50014	2	1.51E-06	0.88	3.27	-	943.8
AHQ-2-4, 2590	R.TSTDLDVDVDQPKEEK.K	1819.90213	2	2.67E-04	0.76	3.31	-	805.6
AHQ-2-1, 3700	R.YTCICYEGYR.F	1387.51964	2	5.31E-04	0.74	3.01	-	405.2
gi|4502549|ref|NP_001734.1| calmodulin 2 (phosphorylase kinase, delta); phosphorylase kinase delta				7.54E-11	2.59	30.24	29.50	16837.5
AHQ-2-11, 3949 - 4012	K.EAFSLFDKDGDGTITTK.E	1845.98441	2	4.17E-05	0.89	4.07	-	729.0
AHQ-2-12, 4113 - 4181	K.EAFSLFDKDGDGTITTK.E	1845.98441	2	7.20E-10	0.95	3.81	-	1384.4
AHQ-2-12, 4249	K.EAFSLFDKDGDGTITTK.E	1845.98441	2	2.85E-07	0.93	4.20	-	1005.8
AHQ-2-13, 4085 - 4165	K.EAFSLFDKDGDGTITTK.E	1845.98441	2	1.64E-05	0.74	3.22	-	476.4
AHQ-2-12, 1630	K.MKDTDSEEEIR.E	1353.43852	2	2.05E-05	0.68	2.71	-	546.1
AHQ-2-12, 3933 - 4007	R.VFDKDGNGYISAAELR.H	1755.90933	2	9.57E-06	0.92	4.00	-	1160.1
AHQ-2-12, 3802 - 3835	R.VFDKDGNGYISAAELR.H	1755.90933	3	1.77E-06	0.88	3.76	-	1023.7
AHQ-2-12, 3798 - 3866	R.VFDKDGNGYISAAELR.H	1755.90933	2	7.54E-11	0.96	4.85	-	1380.7
AHQ-2-13, 3867	R.VFDKDGNGYISAAELR.H	1755.90933	2	9.57E-08	0.91	3.67	-	810.6
AHQ-2-11, 3621 - 3689	R.VFDKDGNGYISAAELR.H	1755.90933	2	4.80E-06	0.87	3.43	-	827.2
gi|17921989|ref|NP_005991.1| tubulin, alpha 1; testis-specific alpha tubulin; tubulin alpha-1 chain				8.92E-11	14.03	170.28	45.10	49924.1
AHQ-2-7, 6369 - 6377	R.AFVHWYVGEGMEEGEFSEAR.E	2331.50495	2	1.01E-04	0.93	3.69	-	1066.6
AHQ-2-12, 4623	R.AFVHWYVGEGM*EEGEFSEAR.E	2347.50435	3	3.43E-06	0.97	5.38	-	1787.4
AHQ-2-12, 5202	R.AFVHWYVGEGMEEGEFSEAR.E	2331.50495	2	2.19E-08	0.90	3.55	-	805.9
AHQ-2-13, 5181	R.AFVHWYVGEGMEEGEFSEAR.E	2331.50495	2	4.97E-07	0.94	4.10	-	1130.8
AHQ-2-12, 6121 - 6187	R.AVCMLSNTTAIAEAWAR.L	1867.13992	2	1.84E-07	0.92	3.96	-	1333.5
AHQ-2-12, 5595 - 5599	R.AVCM*LSNTTAIAEAWAR.L	1883.13932	2	9.27E-07	0.88	3.33	-	999.0
AHQ-2-14-, 6044	R.AVCMLSNTTAIAEAWAR.L	1867.13992	2	1.07E-06	0.95	4.37	-	1361.5
AHQ-2-13-, 5225 - 5293	R.AVCM*LSNTTAIAEAWAR.L	1883.13932	2	6.55E-06	0.94	4.58	-	1125.8
AHQ-2-7, 6281 - 6352	R.AVCMLSNTTAIAEAWAR.L	1867.13992	2	2.22E-06	0.95	5.09	-	1305.9
AHQ-2-13-, 6297 - 6365	R.AVCMLSNTTAIAEAWAR.L	1867.13992	2	1.69E-04	0.95	4.06	-	1502.0
AHQ-2-13-, 5309 - 5378	R.AVCM*LSNTTAIAEAWAR.L	1883.13932	2	4.57E-05	0.83	3.17	-	1201.2
AHQ-2-7, 6681 - 6749	R.AVCMLSNTTAIAEAWAR.L	1867.13992	2	5.94E-06	0.87	4.16	-	923.9
AHQ-2-7, 5181 - 5253	R.AVCM*LSNTTAIAEAWAR.L	1883.13932	2	4.95E-05	0.86	3.58	-	1065.6
AHQ-2-7, 6833	R.AVCMLSNTTAIAEAWAR.L	1867.13992	2	3.35E-04	0.85	3.47	-	928.1
AHQ-2-13-, 6149 - 6157	R.AVCMLSNTTAIAEAWAR.L	1867.13992	2	4.90E-06	0.94	4.77	-	988.8
AHQ-2-13, 5987 - 6063	R.AVCMLSNTTAIAEAWAR.L	1867.13992	2	7.64E-10	0.91	4.08	-	930.0
AHQ-2-14, 6332	R.AVCM*LSNTTAIAEAWAR.L	1883.13932	2	5.24E-04	0.94	3.89	-	1208.0
AHQ-2-11, 5941	R.AVCMLSNTTAIAEAWAR.L	1867.13992	2	2.88E-06	0.82	3.64	-	758.6
AHQ-2-13, 5167 - 5227	R.AVCM*LSNTTAIAEAWAR.L	1883.13932	2	2.98E-04	0.88	3.49	-	833.8
AHQ-2-7, 6181	R.AVFVDLEPTVIDEIR.N	1716.95615	2	4.15E-07	0.93	3.92	-	1465.6
AHQ-2-7, 6041 - 6111	R.AVFVDLEPTVIDEIR.N	1716.95615	2	1.90E-07	0.74	2.99	-	981.0
AHQ-2-13-, 6173 - 6175	R.AVFVDLEPTVIDEIR.N	1716.95615	2	1.04E-05	0.90	3.64	-	1336.6
AHQ-2-12, 6150 - 6157	R.AVFVDLEPTVIDEIR.N	1716.95615	2	5.31E-08	0.90	3.53	-	1203.9
AHQ-2-7, 6199 - 6203	R.AVFVDLEPTVIDEIRNGPYR.Q	2304.58696	2	1.46E-04	0.36	2.70	-	167.7
AHQ-2-7, 5263	K.AYHEQLSVAEITNACFEPANQMVK.C	2753.06070	2	3.31E-05	0.68	2.79	-	614.4
AHQ-2-7, 3377	K.DVNAAIAAIK.T	986.14745	1	1.44E-04	0.90	3.18	-	1131.8
AHQ-2-11, 3288	K.DVNAAIAAIK.T	986.14745	1	1.47E-04	0.43	1.88	-	714.4
AHQ-2-12, 3037	K.DVNAAIAAIK.T	986.14745	1	1.13E-07	0.87	2.43	-	1368.1
AHQ-2-12, 3359	K.DVNAAIAAIK.T	986.14745	2	3.86E-06	0.91	3.60	-	1076.2
AHQ-2-7, 3023	K.FDLMYAK.R	888.06574	1	6.33E-04	0.51	2.45	-	415.7
AHQ-2-13, 3428	K.FDLMYAK.R	888.06574	2	2.69E-04	0.83	2.57	-	864.1
AHQ-2-12, 3258	K.FDLMYAK.R	888.06574	2	6.24E-04	0.82	2.82	-	686.7
AHQ-2-12, 5093 - 5173	R.IHFPLATYAPVISAEK.A	1758.05310	2	3.91E-04	0.60	3.19	-	340.2
AHQ-2-7, 4636 - 4708	R.IHFPLATYAPVISAEK.A	1758.05310	2	2.21E-04	0.94	4.01	-	793.6
AHQ-2-11, 4896	R.IHFPLATYAPVISAEK.A	1758.05310	2	2.43E-07	0.89	3.85	-	493.1
AHQ-2-9, 4767 - 4839	R.IHFPLATYAPVISAEK.A	1758.05310	2	4.67E-06	0.96	4.62	-	896.9
AHQ-2-7, 4779 - 4780	R.IHFPLATYAPVISAEK.A	1758.05310	2	1.78E-06	0.96	4.56	-	976.5
AHQ-2-10, 4627 - 4702	R.IHFPLATYAPVISAEK.A	1758.05310	2	3.10E-05	0.90	3.81	-	637.8
AHQ-2-7, 6935	R.LISQIVSSITASLR.F	1488.75471	2	8.92E-11	0.97	4.79	-	1248.9
AHQ-2-9, 6995	R.LISQIVSSITASLR.F	1488.75471	2	1.22E-08	0.94	4.11	-	1117.5
AHQ-2-7, 7031 - 7095	R.LISQIVSSITASLR.F	1488.75471	2	9.20E-08	0.94	4.67	-	860.0
AHQ-2-7, 7159 - 7239	R.LISQIVSSITASLR.F	1488.75471	2	8.73E-08	0.94	3.48	-	1245.3
AHQ-2-14-, 6697 - 6699	R.LISQIVSSITASLR.F	1488.75471	2	3.13E-08	0.97	5.51	-	1313.3
AHQ-2-13, 3443 - 3455	R.NLDIERPTYTNLNR.L	1719.88022	2	7.87E-08	0.67	3.15	-	435.7
AHQ-2-4, 3502 - 3512	R.NLDIERPTYTNLNR.L	1719.88022	2	3.65E-05	0.37	3.26	-	249.1
AHQ-2-13-, 3447 - 3453	R.NLDIERPTYTNLNR.L	1719.88022	2	6.98E-06	0.46	2.96	-	290.3
AHQ-2-9, 3148	R.NLDIERPTYTNLNR.L	1719.88022	2	5.83E-04	0.47	2.65	-	437.2
AHQ-2-8, 2922	R.NLDIERPTYTNLNR.L	1719.88022	2	9.58E-04	0.74	3.18	-	481.1
AHQ-2-5, 3232	R.NLDIERPTYTNLNR.L	1719.88022	2	7.75E-10	0.54	2.90	-	314.3
AHQ-2-6, 3214	R.NLDIERPTYTNLNR.L	1719.88022	2	5.67E-04	0.32	2.97	-	298.2
AHQ-2-7, 3083 - 3151	R.NLDIERPTYTNLNR.L	1719.88022	2	4.95E-04	0.65	3.59	-	427.4
AHQ-2-12, 3361	R.NLDIERPTYTNLNR.L	1719.88022	2	4.06E-06	0.55	3.33	-	285.6
AHQ-2-10, 3131	R.NLDIERPTYTNLNR.L	1719.88022	2	7.38E-04	0.61	3.21	-	405.7
AHQ-2-13-, 5725 - 5726	R.SIQFVDWCPTGFK.V	1586.79232	2	9.81E-07	0.89	3.00	-	854.8
AHQ-2-3, 6067	R.SIQFVDWCPTGFK.V	1586.79232	2	4.33E-04	0.86	3.25	-	808.8
AHQ-2-12, 5769 - 5791	R.SIQFVDWCPTGFK.V	1586.79232	2	4.43E-04	0.90	3.15	-	1029.6
AHQ-2-1, 6110	R.SIQFVDWCPTGFK.V	1586.79232	2	9.01E-06	0.93	3.47	-	1073.3
AHQ-2-7, 5868 - 5943	K.TIGGGDDSFTTFFCETGAGK.H	2070.17889	2	2.00E-10	0.94	4.62	-	809.7
AHQ-2-9, 5794	K.TIGGGDDSFTTFFCETGAGK.H	2070.17889	2	4.43E-09	0.79	3.28	-	584.2
AHQ-2-7, 5760 - 5799	K.TIGGGDDSFTTFFCETGAGK.H	2070.17889	2	8.09E-10	0.89	4.02	-	618.6
AHQ-2-13-, 5650 - 5689	K.TIGGGDDSFTTFFCETGAGK.H	2070.17889	2	1.26E-07	0.89	3.94	-	585.2
AHQ-2-14-, 5655	K.TIGGGDDSFTTFFCETGAGK.H	2070.17889	2	3.00E-10	0.93	4.61	-	680.6
AHQ-2-4, 6038	K.TIGGGDDSFTTFFCETGAGK.H	2070.17889	2	2.17E-06	0.35	2.65	-	199.0
AHQ-2-7, 5963 - 6037	K.TIGGGDDSFTTFFCETGAGK.H	2070.17889	2	2.39E-08	0.94	4.17	-	1093.6
AHQ-2-10, 5344 - 5350	K.TIGGGDDSFTTFFCETGAGK.H	2070.17889	2	2.83E-07	0.92	4.32	-	519.3
AHQ-2-13, 5551 - 5591	K.TIGGGDDSFTTFFCETGAGK.H	2070.17889	2	6.90E-07	0.66	3.15	-	424.2
AHQ-2-1, 6034	K.TIGGGDDSFTTFFCETGAGK.H	2070.17889	2	9.14E-06	0.50	2.68	-	426.6
AHQ-2-13, 4355 - 4408	K.VGINYQPPTVVPGGDLAK.V	1826.08584	2	7.81E-08	0.86	3.76	-	459.5
AHQ-2-12, 4569	K.VGINYQPPTVVPGGDLAK.V	1826.08584	2	2.22E-08	0.78	3.32	-	370.9
AHQ-2-2, 4692	K.VGINYQPPTVVPGGDLAK.V	1826.08584	2	9.55E-06	0.35	2.68	-	185.7
AHQ-2-12, 4430 - 4502	K.VGINYQPPTVVPGGDLAK.V	1826.08584	2	5.01E-05	0.83	3.33	-	507.1
AHQ-2-14, 5377 - 5452	K.VGINYQPPTVVPGGDLAK.V	1826.08584	2	3.93E-04	0.45	3.09	-	180.4
AHQ-2-3, 4669	K.VGINYQPPTVVPGGDLAK.V	1826.08584	2	5.36E-05	0.66	3.47	-	293.0
AHQ-2-11, 4266 - 4334	K.VGINYQPPTVVPGGDLAK.V	1826.08584	2	4.90E-06	0.74	3.04	-	367.1
AHQ-2-13-, 4417 - 4423	K.VGINYQPPTVVPGGDLAK.V	1826.08584	2	2.47E-04	0.92	4.42	-	527.3
AHQ-2-13-, 4499	K.VGINYQPPTVVPGGDLAK.V	1826.08584	2	4.82E-04	0.78	3.39	-	396.0
AHQ-2-9, 4488 - 4498	K.VGINYQPPTVVPGGDLAK.V	1826.08584	2	8.21E-08	0.55	2.95	-	256.8
AHQ-2-6, 4362	K.VGINYQPPTVVPGGDLAK.V	1826.08584	2	3.50E-06	0.60	3.97	-	235.0
AHQ-2-7, 4513	K.VGINYQPPTVVPGGDLAK.V	1826.08584	2	9.06E-04	0.54	2.75	-	300.2
AHQ-2-7, 4212 - 4291	K.VGINYQPPTVVPGGDLAK.V	1826.08584	2	3.03E-07	0.90	4.13	-	455.6
AHQ-2-7, 4360 - 4431	K.VGINYQPPTVVPGGDLAK.V	1826.08584	2	2.56E-06	0.88	4.17	-	361.9
AHQ-2-13, 4480	K.VGINYQPPTVVPGGDLAK.V	1826.08584	2	1.19E-06	0.66	2.77	-	446.3
AHQ-2-12, 3978	K.YM*ACCLLYR.G	1268.50804	2	9.69E-04	0.78	3.23	-	475.5
AHQ-2-12, 4462	K.YMACCLLYR.G	1252.50864	2	1.34E-05	0.96	3.64	-	1194.0
AHQ-2-9, 4406	K.YMACCLLYR.G	1252.50864	1	8.00E-04	0.50	2.49	-	357.8
AHQ-2-7, 4364 - 4371	K.YMACCLLYR.G	1252.50864	2	1.07E-05	0.96	3.84	-	1237.4
gi|4502201|ref|NP_001649.1| ADP-ribosylation factor 1 [Homo sapiens]				9.06E-11	9.93	110.30	54.70	20696.6
AHQ-2-12, 4681	R.DAVLLVFANK.Q	1090.29726	2	1.76E-06	0.96	4.28	-	1678.9
AHQ-2-12, 4803 - 4830	R.DAVLLVFANK.Q	1090.29726	1	4.92E-04	0.85	3.31	-	833.8
AHQ-2-12, 4663 - 4738	R.DAVLLVFANK.Q	1090.29726	1	1.67E-04	0.72	2.73	-	677.6

AHQ-2-12, 6285	R.DAVLLVFANKQDLPNAMNAAEITDK.L	2703.06468	2	8.63E-07	0.91	4.54	-	661.9
AHQ-2-12, 4911	R.HYFQNTQGLIFVVDSNDR.E	2154.32702	2	8.16E-08	0.98	5.95	-	2171.7
AHQ-2-12, 5318	R.HYFQNTQGLIFVVDSNDR.E	2154.32702	2	9.06E-11	0.97	5.42	-	1572.6
AHQ-2-12, 5402 - 5451	R.HYFQNTQGLIFVVDSNDR.E	2154.32702	2	9.43E-06	0.98	5.88	-	2093.5
AHQ-2-12, 5602 - 5673	R.HYFQNTQGLIFVVDSNDR.E	2154.32702	2	1.28E-08	0.95	5.03	-	769.9
AHQ-2-12, 4999	R.HYFQNTQGLIFVVDSNDR.E	2154.32702	2	5.41E-06	0.98	5.90	-	1877.9
AHQ-2-12, 5094 - 5166	R.HYFQNTQGLIFVVDSNDR.E	2154.32702	2	8.56E-07	0.98	6.01	-	1996.0
AHQ-2-12, 4899 - 4971	R.HYFQNTQGLIFVVDSNDRER.V	2439.62792	3	3.42E-05	0.93	5.26	-	846.0
AHQ-2-12, 4362	R.HYFQNTQGLIFVVDSNDRER.V	2439.62792	3	2.77E-06	0.84	3.82	-	469.3
AHQ-2-12, 4507 - 4573	R.HYFQNTQGLIFVVDSNDRER.V	2439.62792	3	1.37E-06	0.80	3.92	-	449.4
AHQ-2-12, 5057	R.HYFQNTQGLIFVVDSNDRER.V	2439.62792	3	4.19E-06	0.91	4.16	-	1023.1
AHQ-2-12, 3090	R.ILM*VGLDAAGK.T	1104.34572	2	8.68E-07	0.95	3.46	-	1705.1
AHQ-2-11, 3541	R.ILMVGLDAAGK.T	1088.34632	2	3.24E-05	0.94	3.11	-	1368.7
AHQ-2-12, 3659 - 3725	R.ILMVGLDAAGK.T	1088.34632	2	4.16E-04	0.95	3.50	-	1565.8
AHQ-2-12, 3683	R.ILMVGLDAAGK.T	1088.34632	1	4.16E-04	0.74	2.86	-	711.0
AHQ-2-12, 6537 - 6601	K.LGEIVTTIPTIGFNVETVEYK.N	2324.65485	3	1.84E-04	0.91	4.28	-	1004.7
AHQ-2-13-, 6517 - 6519	K.LGEIVTTIPTIGFNVETVEYK.N	2324.65485	2	6.49E-04	0.92	4.73	-	311.1
AHQ-2-12, 6727 - 6803	K.LGEIVTTIPTIGFNVETVEYK.N	2324.65485	2	7.05E-06	0.45	2.99	-	164.8
AHQ-2-14-, 6379	K.LGEIVTTIPTIGFNVETVEYK.N	2324.65485	2	2.89E-06	0.76	3.65	-	264.9
AHQ-2-11, 6353	K.LGEIVTTIPTIGFNVETVEYK.N	2324.65485	2	2.21E-05	0.44	3.02	-	159.8
AHQ-2-11, 6232 - 6293	K.LGEIVTTIPTIGFNVETVEYK.N	2324.65485	2	9.03E-08	0.64	3.35	-	172.5
AHQ-2-12, 6475 - 6545	K.LGEIVTTIPTIGFNVETVEYK.N	2324.65485	2	4.61E-04	0.82	3.75	-	279.7
AHQ-2-12, 2545	R.MLAEDELR.D	977.11769	2	1.70E-04	0.92	3.43	-	1040.3
AHQ-2-12, 7067	R.M*LAEDELRDAVLLVFANK.Q	2064.39175	3	1.36E-08	0.95	4.65	-	1813.7
AHQ-2-13-, 5183	K.NISFTVWDVGGQDK.I	1566.69621	2	2.92E-05	0.92	3.55	-	1044.7
AHQ-2-12, 5137 - 5206	K.NISFTVWDVGGQDK.I	1566.69621	2	7.64E-09	0.96	4.89	-	903.7
AHQ-2-14-, 5109 - 5112	K.NISFTVWDVGGQDK.I	1566.69621	2	1.53E-04	0.87	3.55	-	792.9
AHQ-2-12, 4794 - 4809	K.NISFTVWDVGGQDK.I	1566.69621	2	2.10E-04	0.39	2.67	-	354.9
AHQ-2-12, 3675 - 3749	K.QDLPNAMNAAEITDK.L	1631.79001	2	3.33E-07	0.92	3.87	-	964.3
gi|4885063|ref|NP_005156.1| aldolase C, fructose-bisphosphate; Aldolase C, fructose-bisphosphatase				9.34E-11	1.76	20.29	7.10	39455.6
AHQ-2-8, 4271 - 4348	K.GVVPLAGTDGETTTQGLDGLSER.C	2274.42806	2	9.34E-11	0.80	5.00	-	520.0
AHQ-2-8, 4449 - 4515	K.GVVPLAGTDGETTTQGLDGLSER.C	2274.42806	2	1.93E-10	0.95	4.95	-	720.1
AHQ-2-9, 4504 - 4546	K.GVVPLAGTDGETTTQGLDGLSER.C	2274.42806	2	1.05E-06	0.78	4.61	-	513.1
AHQ-2-9, 4634	K.GVVPLAGTDGETTTQGLDGLSER.C	2274.42806	2	8.55E-09	0.85	4.73	-	884.0
AHQ-2-10, 4340	K.GVVPLAGTDGETTTQGLDGLSER.C	2274.42806	2	9.96E-07	0.85	4.94	-	724.2
AHQ-2-12, 4349	K.GVVPLAGTDGETTTQGLDGLSER.C	2274.42806	2	1.79E-06	0.69	3.95	-	619.6
AHQ-2-12, 4693	K.GVVPLAGTDGETTTQGLDGLSER.C	2274.42806	2	3.62E-10	0.69	4.21	-	445.0
AHQ-2-8, 4127 - 4199	K.VDKGVVPLAGTDGETTTQGLDGLSER.C	2616.82041	3	7.32E-10	0.96	5.77	-	1083.4
gi|7705704|ref|NP_057001.1| glutathione S-transferase subunit 13 homolog [Homo sapiens]				1.03E-10	5.53	60.25	35.80	25496.7
AHQ-2-11, 3270 - 3334	K.AGMSAEQAQGLLEK.I	1433.61314	2	1.64E-05	0.92	4.17	-	1334.2
AHQ-2-11, 2557	K.AGM*SAEQAQGLLEK.I	1449.61254	2	1.28E-04	0.92	3.68	-	1241.5
AHQ-2-11, 4440	R.FLTAVNLEHPEMLEK.A	1772.05845	2	4.78E-06	0.94	4.84	-	942.5
AHQ-2-11, 4793	R.MELLAHLLGEK.W	1254.52433	2	2.12E-05	0.93	3.58	-	1235.5
AHQ-2-11, 4445 - 4494	R.NEDITEPQSILAAAEK.A	1729.86695	2	1.03E-10	0.88	3.60	-	645.2
AHQ-2-11, 5741	R.YGAFGLPITVAHVDGQTHMLFGSDR.M	2691.01737	3	7.48E-08	0.93	5.01	-	757.3
gi|5453607|ref|NP_006420.1| chaperonin containing TCP1, subunit 7 (eta); chaperonin containing t-co				1.10E-10	2.73	30.24	11.20	59366.3
AHQ-2-14-, 6909	R.INALTAASEAACLIVSVDETIKNPR.S	2659.00999	3	1.10E-10	0.92	4.57	-	984.5
AHQ-2-7, 5008	K.LPIGDVATQYFADR.D	1566.73935	2	5.66E-05	0.85	2.73	-	1070.2
AHQ-2-7, 7175	K.SQDAEVGDGTTSVTLLAAEFLK.Q	2253.44875	2	3.62E-05	0.95	4.76	-	971.8
gi|4557705|ref|NP_000217.1| keratin 9 [Homo sapiens]				1.12E-10	5.50	60.32	20.30	61986.9
AHQ-2-5, 6531 - 6547	K.DIENQYETQITQIEHEVSSSGQEVQSSAK.E	3266.38667	3	7.51E-07	0.97	5.37	-	2390.0
AHQ-2-14-, 6219	K.DIENQYETQITQIEHEVSSSGQEVQSSAK.E	3266.38667	3	1.12E-10	0.95	4.89	-	1877.8
AHQ-2-5, 4472	K.EIETYHNLLEGGQEDFESSGAGK.I	2511.59648	3	4.63E-09	0.96	5.47	-	1803.5
AHQ-2-5, 5093	R.HGVQELEIELQSQLSK.K	1839.03978	2	2.25E-07	0.98	6.43	-	2179.0
AHQ-2-5, 7367 - 7369	K.NYSPYYNTIDDLKDQIVDLTVGNNK.T	2904.13412	3	2.02E-09	0.87	4.50	-	473.6
AHQ-2-5, 7368	K.NYSPYYNTIDDLKDQIVDLTVGNNK.T	2904.13412	2	1.53E-07	0.87	4.05	-	577.9
AHQ-2-5, 6587	K.SDLEMQYETLQEELMALKK.N	2300.63547	3	4.64E-08	0.75	3.31	-	556.3
AHQ-2-5, 6569 - 6584	K.SDLEMQYETLQEELMALKK.N	2300.63547	2	2.43E-05	0.85	3.97	-	536.2
AHQ-2-5, 2804 - 2805	K.VQALEEANNDLENK.I	1587.67060	2	3.07E-09	0.98	5.74	-	1789.2
gi|7705296|ref|NP_057271.1| bridging integrator 2; bridging integrator-2; breast cancer associated				1.12E-10	5.96	70.25	21.60	61746.8
AHQ-2-13, 5947	K.AIVWNNDLLWEDYEEK.L	2038.20157	2	8.20E-09	0.88	3.88	-	676.2
AHQ-2-10, 5808 - 5880	K.AIVWNNDLLWEDYEEK.L	2038.20157	2	5.80E-07	0.90	3.78	-	954.7
AHQ-2-10, 5674 - 5746	K.AIVWNNDLLWEDYEEK.L	2038.20157	2	1.12E-10	0.96	4.82	-	1398.5
AHQ-2-10, 6778 - 6850	K.AQTVFEDLNQELLEELPILYNSR.I	2736.02649	2	4.20E-06	0.93	4.99	-	759.2
AHQ-2-10, 6843 - 6851	K.AQTVFEDLNQELLEELPILYNSR.I	2736.02649	3	5.81E-05	0.65	3.54	-	374.4
AHQ-2-10, 2522	R.FEQSASNFYQQQAEGHK.L	2000.07352	2	2.09E-06	0.96	4.72	-	1340.2
AHQ-2-14-, 6587 - 6589	R.IGCYVTIFQNISNLR.D	1800.07167	2	4.63E-05	0.96	5.09	-	1037.3
AHQ-2-10, 6342	R.IGCYVTIFQNISNLR.D	1800.07167	2	2.21E-05	0.86	3.66	-	735.7
AHQ-2-9, 6902 - 6919	R.IGCYVTIFQNISNLR.D	1800.07167	2	1.33E-05	0.90	3.89	-	761.5
AHQ-2-10, 6102	R.IGCYVTIFQNISNLR.D	1800.07167	2	7.37E-04	0.50	2.65	-	190.3
AHQ-2-12, 6751	R.IGCYVTIFQNISNLR.D	1800.07167	2	5.13E-05	0.76	3.60	-	509.4
AHQ-2-10, 2559	K.LNHNLYEVMSK.L	1348.55307	2	3.93E-08	0.79	2.94	-	859.1
AHQ-2-7, 4008	R.TATVSSPLTSPTSPSTLSLK.S	1976.21467	2	1.75E-05	0.72	3.37	-	559.4
AHQ-2-10, 4630	R.VSETLQEIYSSEWDGHEELK.A	2380.50595	2	2.04E-05	0.69	3.45	-	374.2
gi|5902134|ref|NP_009005.1| coronin, actin binding protein, 1A; coronin, actin-binding, 1A; coronin				1.14E-10	3.75	40.28	16.30	51026.0
AHQ-2-7, 4145	R.DAGPLLISLK.D	1027.23948	2	3.18E-05	0.92	3.07	-	1159.1
AHQ-2-7, 7080 - 7148	R.KSDLFQEDLYPPTAGPDPALTAEEWLGGR.D	3175.44994	3	1.07E-09	0.95	5.54	-	532.4
AHQ-2-1, 7133	R.KSDLFQEDLYPPTAGPDPALTAEEWLGGR.D	3175.44994	3	3.23E-04	0.77	3.54	-	353.9
AHQ-2-7, 3999 - 4012	R.VSQTTWDSGFCAVNPK.F	1798.95426	2	2.01E-06	0.93	4.42	-	976.9
AHQ-2-7, 7467	R.YFEITSEAPFLHYLSMFSSK.E	2398.71748	3	1.14E-10	0.96	5.32	-	1175.3
gi|20070125|ref|NP_000909.2| prolyl 4-hydroxylase, beta subunit; v-erb-a avian erythroblastic leuke				1.20E-10	3.95	50.28	16.30	57115.9
AHQ-2-7, 4715	K.HNQLPLVIEFTEQTAPK.I	1966.22639	2	1.19E-06	0.91	4.35	-	660.1
AHQ-2-7, 2359	K.LGETYKDHENIVIAK.M	1730.94291	2	5.70E-04	0.86	3.38	-	761.2
AHQ-2-13, 5656 - 5903	K.QPVKVLVGK.N	968.21840	1	2.86E-04	0.37	2.16	-	472.6
AHQ-2-7, 7709	R.TGPAATTLPDGAAAESLVESSEVAVIGFFK.D	2937.24716	3	1.20E-10	0.93	4.87	-	1084.6
AHQ-2-7, 7601	R.TGPAATTLPDGAAAESLVESSEVAVIGFFK.D	2937.24716	3	3.61E-06	0.97	5.53	-	2512.2
AHQ-2-7, 3757	K.YQLDKDGVVLFK.K	1425.65394	2	2.90E-05	0.88	3.98	-	774.1
gi|4758970|ref|NP_004150.1| proteasome beta 8 subunit isoform E1 proprotein; proteasome subunit, be				1.43E-10	2.57	30.21	14.70	29769.4
AHQ-2-11, 2400	K.FQHGVIAAVDSR.A	1300.44824	1	9.02E-08	0.67	2.45	-	395.8
AHQ-2-11, 2300	K.KGPGLYYVDEHGTR.L	1592.73690	2	1.44E-07	0.96	4.23	-	1406.4
AHQ-2-11, 4008	K.VESTDVSDLLHQYR.E	1662.78230	2	1.43E-10	0.94	3.99	-	1299.4
gi|4503821|ref|NP_001456.1| FYN binding protein (FYB-120/130); FYN-binding protein (FYB-120/130) [H				1.44E-10	0.95	10.25	2.60	85450.5
AHQ-2-12, 4177	R.DLQVKPGESLEVIQTTDDTK.V	2217.41641	2	1.44E-10	0.95	4.97	-	576.7
AHQ-2-13, 4152	R.DLQVKPGESLEVIQTTDDTK.V	2217.41641	2	1.00E-07	0.95	4.95	-	788.2
AHQ-2-10, 3874	R.DLQVKPGESLEVIQTTDDTK.V	2217.41641	2	2.80E-09	0.92	4.32	-	566.7
gi|20357552|ref|NP_005222.2| cortactin isoform a; oncogene EMS1 [Homo sapiens]				1.45E-10	2.63	30.21	7.10	61585.7
AHQ-2-12, 3999	R.LPSSPVYEDAASFK.A	1511.65733	2	1.25E-04	0.60	2.72	-	455.8
AHQ-2-11, 3852 - 3905	R.LPSSPVYEDAASFK.A	1511.65733	2	9.22E-05	0.92	3.20	-	982.7
AHQ-2-13, 3099 - 3164	R.VDQSAVGFEYQGK.T	1428.52841	2	5.06E-05	0.77	2.84	-	799.3
AHQ-2-12, 5377 - 5381	R.YGLFPANYVELR.Q	1442.64306	2	1.45E-10	0.94	4.11	-	776.2
gi|4507231|ref|NP_003134.1| single-stranded DNA binding protein; single-stranded DNA-binding protei				1.77E-10	3.54	40.23	32.40	17259.5
AHQ-2-13, 5943	K.NPVTIFSLATNEM*WR.S	1796.04007	2	1.77E-10	0.93	4.34	-	855.8
AHQ-2-13, 6631	K.NPVTIFSLATNEMWR.S	1780.04067	2	1.50E-05	0.97	4.60	-	1963.7
AHQ-2-13, 6029 - 6100	R.QATTIIADNIIFLSDQTK.E	1993.24692	2	8.01E-04	0.68	3.19	-	490.8
AHQ-2-13-, 3213	R.SGDSEVYQLGDVSQK.T	1612.67692	2	8.86E-07	0.95	4.61	-	1049.9
AHQ-2-13, 3228	R.SGDSEVYQLGDVSQK.T	1612.67692	2	9.11E-07	0.90	4.01	-	855.9
gi|4502051|ref|NP_000688.1| arachidonate 12-lipoxygenase; 12(S)-lipoxygenase [Homo sapiens]				1.82E-10	5.71	70.23	16.10	75721.8

AHQ-2-6, 4877	R.AAAQLTYCSLCPPDDLADR.G	2140.33708	2	8.87E-10	0.92	4.53	-	443.2
AHQ-2-6, 2045	K.AVSTGGGGHVQLLR.R	1352.52468	2	6.69E-09	0.87	3.53	-	904.7
AHQ-2-6, 5289	R.GLLGLPGALYAHDALR.L	1637.90708	2	1.82E-10	0.92	3.64	-	774.7
AHQ-2-6, 5162	R.ITVQGPGACAEVAFPCYR.W	1999.25659	2	1.46E-07	0.88	3.81	-	951.0
AHQ-2-6, 4467	R.LPGDNALDMFQK.H	1349.53784	2	3.62E-08	0.92	3.84	-	828.0
AHQ-2-6, 3901 - 3902	R.TQLISDGGIFDK.A	1294.43554	1	2.43E-04	0.58	2.18	-	920.3
AHQ-2-6, 4933	R.WVQGEDILSLPEGTAR.L	1771.95190	2	6.92E-05	0.63	2.88	-	491.7
gi|4508047|ref|NP_003452.1| zyxin [Homo sapiens]				1.82E-10	2.46	30.27	8.90	61277.0
AHQ-2-8, 7340	K.EVEELEQLTQQLMQDMEHPQR.Q	2612.87876	3	1.82E-10	0.87	3.86	-	1230.1
AHQ-2-9, 7367	K.EVEELEQLTQQLMQDMEHPQR.Q	2612.87876	2	7.44E-07	0.92	4.51	-	593.2
AHQ-2-8, 7338 - 7402	K.EVEELEQLTQQLMQDMEHPQR.Q	2612.87876	2	1.71E-07	0.91	4.45	-	608.1
AHQ-2-9, 1959	K.FGPVVAPKPK.V	1040.28308	1	6.34E-04	0.62	2.27	-	676.6
AHQ-2-9, 7539	K.VSSIDLEIDSLSSLLDDMTK.N	2182.43225	2	1.78E-06	0.97	5.50	-	1095.5
gi|5803201|ref|NP_006818.1| transmembrane trafficking protein [Homo sapiens]				1.87E-10	3.83	50.28	17.40	24975.8
AHQ-2-11, 2314	K.ITDSAGHILYSK.E	1305.46156	1	4.49E-04	0.65	1.85	-	1002.8
AHQ-2-11, 2313	K.ITDSAGHILYSK.E	1305.46156	2	8.92E-09	0.96	4.55	-	1424.3
AHQ-2-11, 6209 - 6220	R.LEDLSESIVNDFAYMK.K	1875.08916	2	7.26E-07	0.95	4.82	-	1005.0
AHQ-2-11, 2902 - 2904	K.LKPLEVELR.R	1097.33294	1	1.05E-05	0.06	2.22	-	126.8
AHQ-2-11, 6090	R.RLEDLSESIVNDFAYMK.K	2031.27552	2	8.05E-08	0.98	5.59	-	2091.4
AHQ-2-11, 5414	R.RLEDLSESIVNDFAYM*K.K	2047.27492	2	4.04E-04	0.88	3.54	-	765.7
AHQ-2-11, 4989	R.RLEDLSESIVNDFAYMK.K	2031.27552	2	1.87E-10	0.97	4.90	-	1799.8
gi|21618359|ref|NP_004095.3| fatty acid synthase [Homo sapiens]				1.88E-10	5.69	70.24	4.60	273262.0
AHQ-2-1, 4250	K.CTVFHGAQVEDAFR.Y	1638.78572	2	2.64E-07	0.73	2.68	-	864.7
AHQ-2-1, 4246	R.FPQLDSTSFANSR.D	1470.56853	2	5.28E-08	0.90	3.65	-	882.8
AHQ-2-1, 6224	R.LFDHPESPTPNPTEPLFLAQAEVYK.E	2842.15075	3	1.88E-10	0.93	4.71	-	1090.0
AHQ-2-1, 6628	R.LHLSGIDANPNALFPPVEFPAPR.G	2473.81368	3	4.77E-04	0.79	3.58	-	618.2
AHQ-2-3, 7121 - 7193	K.LPEDPLLSGLLDSPALK.A	1779.06739	2	3.41E-04	0.94	3.69	-	1495.8
AHQ-2-1, 7033	K.LPEDPLLSGLLDSPALK.A	1779.06739	2	7.88E-06	0.97	4.34	-	1646.4
AHQ-2-1, 3832	R.LQVVDQPLPVR.G	1264.49894	2	2.68E-05	0.84	3.23	-	1083.5
AHQ-2-1, 3950	K.VLQGDLVM*NVYR.D	1423.66306	2	3.75E-04	0.52	2.77	-	579.1
gi|4507207|ref|NP_003121.1| sorcin; Sorcin (class 4 gene) [Homo sapiens]				1.89E-10	0.93	10.23	10.10	21676.3
AHQ-2-11, 4501 - 4578	R.CLTQSGIAGGYKPFNLETCR.L	2275.54736	2	1.89E-10	0.93	4.54	-	555.6
gi|4504041|ref|NP_002061.1| guanine nucleotide binding protein (G protein), alpha inhibiting activi				1.94E-10	1.79	20.25	9.90	40450.7
AHQ-2-9, 5811	R.EYQLNDSAAYYLNDLER.I	2078.18096	2	1.94E-10	0.89	3.94	-	837.9
AHQ-2-14, 6136	K.ITHSPLTICFPEYTGANK.Y	2051.30821	2	1.59E-05	0.90	4.18	-	489.9
gi|4759300|ref|NP_004772.1| vesicle-associated membrane protein 3 (cellubrevin); vesicle-associated				1.98E-10	3.82	40.31	40.00	11309.0
AHQ-2-13-, 3646	R.ADALQAGASQFETSAAK.L	1666.77095	2	3.19E-08	0.94	4.15	-	1048.0
AHQ-2-14-, 3556	R.ADALQAGASQFETSAAK.L	1666.77095	2	8.55E-10	0.95	4.37	-	1128.6
AHQ-2-13-, 3786	R.LQQTQNQVDEVVDIM*R.V	1933.13344	2	3.59E-07	0.98	6.26	-	1604.3
AHQ-2-13-, 4971	R.LQQTQNQVDEVVDIMR.V	1917.13404	2	7.27E-08	0.97	5.40	-	1720.9
AHQ-2-13, 4879	R.LQQTQNQVDEVVDIMR.V	1917.13404	2	1.98E-10	0.97	5.23	-	1371.2
AHQ-2-13-, 4673 - 4681	K.LSELDDRADALQAGASQFETSAAK.L	2495.64172	3	2.86E-05	0.92	4.45	-	1368.7
gi|29730023|ref|XP_291482.1| similar to 78 kDa glucose-regulated protein precursor (GRP 78) (Immuno				1.98E-10	4.51	50.23	13.30	63563.0
AHQ-2-6, 4605 - 4669	R.IINKPTAAAIAYGLDK.R	1659.95097	2	6.18E-09	0.87	4.03	-	875.3
AHQ-2-6, 3205	K.KKELEEIVQPIISK.L	1654.97263	2	3.51E-08	0.96	4.60	-	1466.0
AHQ-2-6, 3323 - 3405	K.LYGSAGPPPTGEEDTAEKDEL	2177.26447	2	8.52E-06	0.92	4.27	-	729.4
AHQ-2-6, 3833	R.NELESYAYSLK.N	1317.42597	2	5.12E-04	0.85	3.20	-	733.5
AHQ-2-6, 5525 - 5529	K.TFAPKEISAMVLTK.M	1536.86124	2	1.98E-10	0.92	4.37	-	1281.7
gi|10716563|ref|NP_001737.1| calnexin [Homo sapiens]				2.06E-10	4.53	50.21	12.30	67567.8
AHQ-2-5, 2204	K.AEEDEILNR.S	1089.13826	2	2.37E-04	0.93	3.06	-	1799.2
AHQ-2-1, 5200	K.APVPTGEVYFADSFDR.G	1771.90722	2	2.61E-07	0.78	3.05	-	562.1
AHQ-2-6, 4833	K.APVPTGEVYFADSFDR.G	1771.90722	2	1.25E-04	0.88	3.02	-	1109.5
AHQ-2-5, 4895 - 4969	K.APVPTGEVYFADSFDR.G	1771.90722	2	2.06E-10	0.93	4.15	-	1119.9
AHQ-2-5, 4193	R.CESAPGCGVWQRPVIDNPNYK.G	2450.69236	2	3.48E-05	0.79	3.00	-	634.8
AHQ-2-9, 6211	R.KIPNPDFFEDLEPFR.M	1865.07739	2	1.89E-04	0.88	3.88	-	661.3
AHQ-2-6, 6183	R.KIPNPDFFEDLEPFR.M	1865.07739	2	3.49E-06	0.87	3.20	-	770.3
AHQ-2-5, 6235 - 6307	R.KIPNPDFFEDLEPFR.M	1865.07739	2	1.28E-06	0.93	3.61	-	1091.2
AHQ-2-5, 3271 - 3340	K.TPELNLDQFHDK.T	1457.56976	2	1.05E-05	0.94	3.95	-	1512.6
gi|4507677|ref|NP_003290.1| tumor rejection antigen (gp96) 1; Tumor rejection antigen-1 (gp96); glu				2.25E-10	6.14	80.30	14.40	92468.2
AHQ-2-5, 2297 - 2301	K.DISTNYYASQK.K	1290.36061	1	2.25E-10	0.37	2.26	-	345.0
AHQ-2-5, 6396	R.EAVEKEFEPLLNWMK.D	1864.15434	2	2.70E-04	0.94	4.48	-	991.6
AHQ-2-6, 3685	R.FQSSHHPTDITSLDQYVER.M	2261.39276	3	1.37E-04	0.97	5.45	-	2403.4
AHQ-2-6, 3691	R.FQSSHHPTDITSLDQYVER.M	2261.39276	2	1.12E-06	0.79	3.39	-	609.3
AHQ-2-5, 3655 - 3659	R.FQSSHHPTDITSLDQYVER.M	2261.39276	2	1.21E-04	0.93	4.43	-	962.4
AHQ-2-5, 6980 - 7041	K.GYEVIYLTEPVDEYCIQALPEFDGKR.F	3107.43557	3	1.11E-05	0.87	4.40	-	603.7
AHQ-2-5, 6260	K.KYSQFINFPIYVWSSK.T	2008.30657	2	2.15E-09	0.98	5.21	-	1865.4
AHQ-2-5, 5329	R.LISLTDENALSGNEELTVK.I	2047.24975	2	6.73E-06	0.84	3.33	-	486.5
AHQ-2-5, 3273	R.LSLNIDPDAK.V	1086.22065	1	4.56E-05	0.38	2.24	-	561.9
AHQ-2-5, 6940 - 7007	K.YSQFINFPIYVWSSK.T	1880.13366	2	1.56E-04	0.98	5.95	-	1445.3
gi|4504345|ref|NP_000508.1| alpha 2 globin [Homo sapiens]				2.31E-10	2.83	30.30	30.30	15257.4
AHQ-2-13, 4247	K.FLASVSTVLTSK.Y	1253.46970	2	2.42E-06	0.90	3.38	-	1124.7
AHQ-2-13-, 4293	K.FLASVSTVLTSK.Y	1253.46970	2	3.92E-06	0.92	3.46	-	1286.8
AHQ-2-13-, 4063 - 4073	K.TYFPHFDLSHGSAQVK.G	1835.01138	2	5.03E-10	0.96	5.09	-	1160.3
AHQ-2-13, 3992 - 4007	K.TYFPHFDLSHGSAQVK.G	1835.01138	2	2.75E-06	0.95	4.14	-	1205.9
AHQ-2-13-, 2539 - 2558	K.VGAHAGEYGAEALER.M	1530.62397	2	5.46E-09	0.96	3.93	-	1671.0
AHQ-2-13, 2604 - 2609	K.VGAHAGEYGAEALER.M	1530.62397	2	2.31E-10	0.97	4.15	-	1815.0
gi|12408656|ref|NP_005177.2| calpain 1, large subunit; calpain, large polypeptide L1; calcium-activ				2.59E-10	8.22	100.26	19.90	81889.4
AHQ-2-6, 6702 - 6717	R.DMETIGFAVYEVPPELVGQPAVHLK.R	2741.15451	3	2.59E-10	0.87	4.20	-	775.0
AHQ-2-6, 6001	R.FRLPPGEYVVVPSTFEPNKEGDFVLR.F	2994.39160	3	1.99E-04	0.94	5.26	-	1024.4
AHQ-2-6, 4097 - 4109	R.KAPSDLYQIILK.A	1389.66474	2	4.01E-08	0.93	4.16	-	834.0
AHQ-2-10, 3992	R.KAPSDLYQIILK.A	1389.66474	2	8.31E-05	0.80	2.97	-	639.5
AHQ-2-6, 5645	R.LPPGEYVVVPSTFEPNKEGDFVLR.F	2691.03055	3	3.19E-07	0.77	3.68	-	527.7
AHQ-2-6, 6433	K.LVFVHSAEGNEFWSALLEK.A	2177.44364	2	9.69E-06	0.96	4.82	-	1355.4
AHQ-2-6, 5073	R.NYPATFWVNPQFK.I	1612.81141	2	3.96E-05	0.92	3.82	-	926.4
AHQ-2-6, 4335 - 4345	K.RPTELLSNPQFIVDGATR.T	2015.25904	3	6.52E-07	0.94	4.98	-	1254.4
AHQ-2-6, 2837 - 2842	R.SEQFINLR.E	1007.12521	1	4.65E-04	0.26	2.20	-	128.5
AHQ-2-6, 4161	K.WNTTLYEGTWR.R	1427.54534	2	5.81E-04	0.75	2.64	-	652.6
AHQ-2-6, 3123 - 3129	K.YLGQDYEQLR.V	1285.38720	2	3.60E-06	0.88	3.11	-	690.2
gi|27477800|ref|XP_208313.1| similar to tropomyosin 4 [Homo sapiens]				2.76E-10	0.95	10.25	10.10	18426.7
AHQ-2-9, 5234 - 5312	K.CGDLEEELKNVTNNLK.S	1878.05124	2	8.17E-07	0.92	4.32	-	1053.4
AHQ-2-9, 5506 - 5578	K.CGDLEEELKNVTNNLK.S	1878.05124	2	1.17E-07	0.80	3.72	-	648.4
AHQ-2-9, 5590 - 5652	K.CGDLEEELKNVTNNLK.S	1878.05124	2	2.76E-10	0.95	4.52	-	1406.7
AHQ-2-9, 4606 - 4614	K.CGDLEEELKNVTNNLK.S	1878.05124	2	1.11E-08	0.97	5.06	-	1673.5
gi|4757812|ref|NP_004880.1| ATP synthase, H+ transporting, mitochondrial F0 complex, subunit f, iso				3.07E-10	2.63	30.20	25.50	10917.8
AHQ-2-13-, 6125 - 6139	R.DFSPSGIFGAFQR.G	1429.56124	2	2.96E-08	0.94	4.10	-	1153.2
AHQ-2-13, 5969 - 5975	R.DFSPSGIFGAFQR.G	1429.56124	2	5.35E-09	0.93	3.61	-	1182.9
AHQ-2-14-, 6007	R.DFSPSGIFGAFQR.G	1429.56124	2	3.07E-10	0.93	3.57	-	1048.0
AHQ-2-13-, 6118	K.LGELPSWILMR.D	1315.60936	2	4.91E-08	0.92	3.85	-	743.1
AHQ-2-13-, 5606 - 5611	K.LGELPSWILM*R.D	1331.60876	2	5.97E-06	0.89	3.63	-	529.7
AHQ-2-14-, 5551	K.LGELPSWILM*R.D	1331.60876	2	3.63E-06	0.89	3.29	-	661.8
AHQ-2-13, 5948	K.LGELPSWILMR.D	1315.60936	2	1.71E-04	0.90	3.58	-	686.0
AHQ-2-13, 5496	K.LGELPSWILM*R.D	1331.60876	2	5.74E-07	0.78	3.13	-	464.9
AHQ-2-14-, 6023	K.LGELPSWILMR.D	1315.60936	2	2.84E-05	0.92	3.69	-	783.0
gi|7657124|ref|NP_055165.1| TNF-induced protein [Homo sapiens]				3.13E-10	0.91	10.21	10.50	22047.4
AHQ-2-12, 7174 - 7241	K.SIATTLIDDTSSEVLDELYR.V	2242.42274	2	3.13E-10	0.91	4.14	-	861.4
gi|24234688|ref|NP_004125.3| heat shock 70kDa protein 9B precursor; heat shock 70kD protein 9; stre				3.57E-10	0.88	10.19	2.80	73680.1
AHQ-2-6, 6011	K.STNGDTFLGGEDFDQALLR.H	2057.16328	2	3.57E-10	0.88	3.70	-	588.6

gi|30159310|ref|XP_301899.1| similar to beta-actin [Homo sapiens]				3.59E-10	1.41	20.18	9.20	32376.4
AHQ-2-8, 1448 - 1791	K.DSYVGNEAQSK.R	1198.22158	1	3.59E-10	0.57	2.73	-	338.9
AHQ-2-8, 1454	K.DSYVGNEAQSK.R	1198.22158	2	1.01E-05	0.81	3.08	-	848.8
AHQ-2-8, 1545 - 1603	K.DSYVGNEAQSK.R	1198.22158	2	5.69E-05	0.79	3.19	-	693.1
AHQ-2-13, 1820	K.DSYVGNEAQSK.R	1198.22158	1	2.09E-06	0.63	2.63	-	453.1
AHQ-2-12, 1611 - 1671	K.DSYVGNEAQSK.R	1198.22158	1	1.64E-07	0.20	2.16	-	283.9
AHQ-2-9, 1490 - 1566	K.DSYVGNEAQSK.R	1198.22158	1	9.56E-09	0.36	2.32	-	286.7
AHQ-2-13, 1912	K.DSYVGNEAQSK.R	1198.22158	1	1.38E-08	0.19	1.86	-	321.5
AHQ-2-8, 4535	K.SYKLLDGQVITIGNER.F	1807.04092	2	3.67E-05	0.80	3.05	-	752.5
AHQ-2-8, 4617 - 4695	K.SYKLLDGQVITIGNER.F	1807.04092	2	2.54E-05	0.84	3.18	-	588.1
AHQ-2-9, 4846 - 4874	K.SYKLLDGQVITIGNER.F	1807.04092	2	6.55E-04	0.73	2.90	-	639.2
gi|18426915|ref|NP_004386.2| drebrin 1 isoform a; drebrin E; drebrin-1; drebrin E2 [Homo sapiens] [				3.62E-10	3.54	40.26	11.70	71438.9
AHQ-2-14-, 5735	R.EESAADWALYTYEDGSDDLK.L	2279.31212	2	5.40E-04	0.84	3.61	-	668.8
AHQ-2-8, 3706	K.KSESEVEEAAAIIAQRPDNPR.E	2311.49476	3	1.87E-08	0.85	3.96	-	668.0
AHQ-2-12, 4006	K.LAASGEGGLQELSGHFENQK.V	2073.20908	2	2.59E-05	0.94	4.60	-	922.4
AHQ-2-12, 4010	K.LAASGEGGLQELSGHFENQK.V	2073.20908	3	3.53E-05	0.66	3.52	-	357.7
AHQ-2-13-, 4105	K.LAASGEGGLQELSGHFENQK.V	2073.20908	2	3.62E-10	0.96	5.22	-	1172.3
AHQ-2-13, 4024	K.LAASGEGGLQELSGHFENQK.V	2073.20908	2	4.10E-07	0.86	4.01	-	676.9
AHQ-2-8, 3702	K.LAASGEGGLQELSGHFENQK.V	2073.20908	2	8.14E-05	0.81	3.86	-	616.9
AHQ-2-13-, 5637	K.YVLINWVGEDVPDAR.K	1746.94404	2	1.90E-05	0.97	4.78	-	1760.6
AHQ-2-14-, 5589 - 5608	K.YVLINWVGEDVPDAR.K	1746.94404	2	2.73E-08	0.89	3.51	-	1062.6
AHQ-2-12, 5654	K.YVLINWVGEDVPDAR.K	1746.94404	2	2.34E-07	0.92	3.60	-	1290.9
AHQ-2-13, 5520	K.YVLINWVGEDVPDAR.K	1746.94404	2	5.20E-07	0.94	3.97	-	1197.3
gi|16507237|ref|NP_005338.1| heat shock 70kDa protein 5 (glucose-regulated protein, 78kDa); BiP; he				3.90E-10	11.06	130.29	28.00	72332.5
AHQ-2-6, 4777	R.AKFEELNMDLFR.S	1513.74309	2	4.67E-08	0.92	3.67	-	918.4
AHQ-2-6, 5499	K.DNHLLGTFDLTGIPPAPR.G	1935.17239	2	2.42E-05	0.86	3.40	-	699.8
AHQ-2-6, 6017 - 6094	R.IEIESFYEGEDFSETLTR.A	2166.28328	2	6.37E-06	0.77	3.22	-	687.9
AHQ-2-6, 6162 - 6231	R.IEIESFYEGEDFSETLTR.A	2166.28328	2	3.98E-08	0.94	4.52	-	962.4
AHQ-2-6, 4626	K.IEWLESHQDADIEDFK.A	1976.08864	3	3.90E-10	0.85	3.60	-	820.7
AHQ-2-6, 4617	K.IEWLESHQDADIEDFK.A	1976.08864	2	1.23E-06	0.98	5.74	-	1602.6
AHQ-2-6, 4094 - 4173	R.IINEPTAAAIAYGLDKR.E	1817.07894	2	1.09E-05	0.93	3.90	-	1121.8
AHQ-2-6, 3641 - 3709	R.ITPSYVAFTPEGER.L	1567.72412	2	2.08E-06	0.92	3.79	-	841.5
AHQ-2-7, 3605	R.ITPSYVAFTPEGER.L	1567.72412	2	8.04E-05	0.46	2.52	-	344.8
AHQ-2-6, 3475	K.KSDIDEIVLVGGSTR.I	1589.77277	2	1.11E-07	0.97	4.71	-	2052.9
AHQ-2-6, 3002	K.KSQIFSTASDNQPTVTIK.V	1966.18156	2	1.50E-09	0.98	5.65	-	2027.3
AHQ-2-6, 2061	K.MKETAEAYLGK.K	1241.43928	2	7.42E-05	0.93	3.91	-	893.3
AHQ-2-6, 3069	K.NQLTSNPENTVFDAK.R	1678.78173	2	4.92E-09	0.91	3.88	-	756.6
AHQ-2-6, 3814	K.SQIFSTASDNQPTVTIK.V	1838.00865	2	1.59E-07	0.92	3.93	-	1001.6
AHQ-2-7, 3544	K.SQIFSTASDNQPTVTIK.V	1838.00865	2	2.39E-04	0.78	4.19	-	489.4
AHQ-2-6, 3605 - 3675	K.SQIFSTASDNQPTVTIK.V	1838.00865	2	4.96E-08	0.96	5.63	-	909.3
AHQ-2-6, 2854	R.TWNDPSVQQDIK.F	1431.53230	1	3.57E-05	0.18	2.37	-	248.2
AHQ-2-6, 3661 - 3698	K.VTHAVVTVPAYFNDAQR.Q	1889.10372	2	4.07E-07	0.70	3.24	-	511.5
gi|29732955|ref|XP_291603.1| similar to glyceraldehyde 3-phosphate dehydrogenase [Homo sapiens] [MA				4.08E-10	0.90	10.24	16.80	11173.0
AHQ-2-9, 3984 - 4034	K.NISNASCTTNCIAPLAK.M	1838.05353	2	6.65E-06	0.71	3.85	-	795.1
AHQ-2-9, 4074 - 4114	K.NISNASCTTNCIAPLAK.M	1838.05353	2	4.08E-10	0.90	4.53	-	1261.8
AHQ-2-9, 4186 - 4259	K.NISNASCTTNCIAPLAK.M	1838.05353	2	3.35E-09	0.92	4.06	-	1764.2
AHQ-2-9, 4332	K.NISNASCTTNCIAPLAK.M	1838.05353	2	6.52E-10	0.73	3.16	-	1040.0
AHQ-2-9, 4440 - 4516	K.NISNASCTTNCIAPLAK.M	1838.05353	2	1.22E-07	0.94	4.25	-	1285.0
AHQ-2-10, 3710	K.NISNASCTTNCIAPLAK.M	1838.05353	2	1.67E-08	0.96	4.73	-	1401.7
AHQ-2-10, 3979 - 4058	K.NISNASCTTNCIAPLAK.M	1838.05353	2	1.04E-06	0.71	2.98	-	1148.8
AHQ-2-9, 3820 - 3902	K.NISNASCTTNCIAPLAK.M	1838.05353	2	1.55E-08	0.93	4.31	-	804.4
gi|5453549|ref|NP_006397.1| thioredoxin peroxidase; thioredoxin peroxidase (antioxidant enzyme) [Ho				4.25E-10	5.62	60.24	33.60	30539.7
AHQ-2-10, 3471	K.DYGVYLEDSGHTLR.G	1625.72052	2	8.81E-09	0.95	4.33	-	1169.4
AHQ-2-10, 3831	K.HGEVCPAGWKPGSETIIPDPAGK.L	2405.67144	2	2.69E-05	0.90	3.77	-	850.7
AHQ-2-10, 4546	R.IPLLSDLTHQISK.D	1465.71961	2	4.25E-10	0.96	4.16	-	1419.0
AHQ-2-10, 4454	K.ISKPAPYWEGTAVIDGEFK.E	2109.36607	2	2.31E-07	0.95	4.82	-	1169.3
AHQ-2-10, 2670	R.LVQAFQYTDK.H	1213.36412	2	8.78E-06	0.90	3.15	-	860.5
AHQ-2-10, 2104	R.VSVADHSLHLSK.A	1293.45401	2	1.73E-06	0.96	3.93	-	1493.2
gi|4504517|ref|NP_001531.1| heat shock 27kDa protein 1; heat shock 27kD protein 1 [Homo sapiens] [M				4.36E-10	6.55	80.35	59.00	22782.3
AHQ-2-13-, 2229	K.DGVVEITGK.H	918.02674	1	1.60E-04	0.20	2.15	-	423.9
AHQ-2-13-, 2206	K.DGVVEITGK.H	918.02674	1	5.03E-04	0.49	2.55	-	403.1
AHQ-2-13-, 5334 - 5397	R.KYTLPPGVDPTQVSSSLSPEGTLTVEAPM*PK.L	3244.65854	3	1.39E-04	0.96	6.57	-	1087.1
AHQ-2-13-, 5630 - 5703	R.KYTLPPGVDPTQVSSSLSPEGTLTVEAPMPK.L	3228.65914	3	1.59E-04	0.95	7.03	-	1394.9
AHQ-2-14, 5930	K.LATQSNEITIPVTFESR.A	1907.11412	2	1.97E-04	0.28	2.68	-	214.7
AHQ-2-11, 4636 - 4645	K.LATQSNEITIPVTFESR.A	1907.11412	2	3.66E-05	0.66	3.34	-	352.8
AHQ-2-11, 4762	K.LATQSNEITIPVTFESR.A	1907.11412	2	2.70E-05	0.61	3.29	-	253.0
AHQ-2-11, 4497	R.LFDQAFGLPR.L	1164.33793	2	3.04E-07	0.97	4.28	-	1616.7
AHQ-2-10, 4287	R.LFDQAFGLPR.L	1164.33793	2	2.83E-06	0.97	4.23	-	1645.0
AHQ-2-10, 6415 - 6432	R.LPEEWSQWLGGSSWPGYVR.P	2235.44173	2	4.15E-05	0.94	4.29	-	999.7
AHQ-2-10, 6638	R.LPEEWSQWLGGSSWPGYVRPLPPAAIESPAVAAPAYSR.A	4095.56787	3	1.09E-07	0.97	6.20	-	1359.2
AHQ-2-11, 6700	R.LPEEWSQWLGGSSWPGYVRPLPPAAIESPAVAAPAYSR.A	4095.56787	3	7.14E-04	0.87	3.78	-	957.1
AHQ-2-13-, 4701 - 4722	R.VSLDVNHFAPDELTVK.T	1784.99058	2	4.36E-10	0.96	4.80	-	1276.3
AHQ-2-13-, 4955	R.VSLDVNHFAPDELTVK.T	1784.99058	2	1.21E-08	0.95	4.63	-	962.4
AHQ-2-10, 4294 - 4295	R.VSLDVNHFAPDELTVK.T	1784.99058	2	3.35E-08	0.95	4.93	-	1047.8
AHQ-2-11, 4513 - 4522	R.VSLDVNHFAPDELTVK.T	1784.99058	2	2.04E-06	0.95	4.61	-	1228.3
AHQ-2-13, 4623	R.VSLDVNHFAPDELTVK.T	1784.99058	2	1.77E-07	0.93	4.43	-	1158.5
gi|22059949|ref|XP_114617.2| similar to tubulin, beta 5 [Homo sapiens]				4.70E-10	1.81	20.38	18.80	22810.8
AHQ-2-13, 5288	K.FWEVISDEHGIDPTGTYHGDSDLQLDR.I	3104.24553	3	3.47E-06	0.90	4.85	-	847.8
AHQ-2-12, 5409	K.FWEVISDEHGIDPTGTYHGDSDLQLDR.I	3104.24553	3	2.21E-04	0.93	5.49	-	870.6
AHQ-2-14-, 5283	K.FWEVISDEHGIDPTGTYHGDSDLQLDR.I	3104.24553	3	4.70E-10	0.98	7.62	-	1742.7
AHQ-2-13, 2809	R.ISVYYNEATGGK.Y	1302.41453	2	7.36E-05	0.78	2.82	-	723.4
AHQ-2-13-, 2747	R.ISVYYNEATGGK.Y	1302.41453	2	2.07E-06	0.83	3.07	-	688.0
AHQ-2-7, 2435	R.ISVYYNEATGGK.Y	1302.41453	1	1.33E-05	0.50	2.96	-	293.7
AHQ-2-7, 2432	R.ISVYYNEATGGK.Y	1302.41453	2	9.63E-06	0.90	3.55	-	839.8
gi|7657007|ref|NP_055210.1| dual adaptor of phosphotyrosine and 3-phosphoinositides [Homo sapiens]				4.75E-10	0.89	10.21	7.10	32044.2
AHQ-2-12, 6047	R.SDGEAELLQDLGWYHGNLTR.H	2275.41943	2	4.75E-10	0.89	4.17	-	719.3
gi|6005749|ref|NP_009193.1| RNA-binding protein regulatory subunit; oncogene DJ1 [Homo sapiens] [MA				4.83E-10	2.72	30.24	26.50	19846.9
AHQ-2-10, 3184 - 3198	R.DVVICPDASLEDAKK.E	1661.85581	2	4.40E-04	0.88	3.37	-	993.7
AHQ-2-10, 6883	R.GPGTSFEFALAIVEALNGK.E	1922.17039	2	1.05E-07	0.94	4.73	-	977.3
AHQ-2-11, 3037	K.VTVAGLAGKDPVQCSR.D	1659.88952	2	4.32E-05	0.86	3.53	-	623.7
AHQ-2-10, 2896	K.VTVAGLAGKDPVQCSR.D	1659.88952	2	4.83E-10	0.90	3.64	-	703.6
gi|4507869|ref|NP_003361.1| vasodilator-stimulated phosphoprotein [Homo sapiens]				5.02E-10	3.78	40.27	14.20	39829.4
AHQ-2-7, 3444	R.KMQPDQQVVINCAIVR.G	1901.24418	2	7.42E-06	0.93	4.02	-	976.1
AHQ-2-7, 6000 - 6013	K.VKEEIIEAFVQELR.K	1703.96061	2	5.02E-10	0.97	5.34	-	1795.6
AHQ-2-7, 1789	R.VKQELLEEVKK.E	1343.59438	2	9.47E-05	0.94	4.35	-	1261.3
AHQ-2-7, 2508	R.VQIYHNPTANSFR.V	1547.69936	2	4.39E-08	0.93	3.72	-	923.8
AHQ-2-13-, 2807	R.VQIYHNPTANSFR.V	1547.69936	2	3.70E-06	0.89	3.11	-	1127.7
AHQ-2-7, 2409	R.VQIYHNPTANSFR.V	1547.69936	2	2.20E-09	0.94	3.46	-	1209.9
gi|21361370|ref|NP_002853.2| phosphorylase, glycogen; brain [Homo sapiens]				5.65E-10	6.16	70.25	12.00	96695.3
AHQ-2-5, 3565	R.GLAGLGDVAEVR.K	1157.30210	2	3.90E-05	0.96	4.39	-	1347.2
AHQ-2-5, 3344	R.HLDHVAALFPGDVDR.L	1662.83036	2	2.77E-08	0.97	4.91	-	1675.2
AHQ-2-5, 6227	K.IGEEFLTDLSQLK.K	1493.68345	2	1.39E-06	0.97	4.99	-	1669.7
AHQ-2-5, 5829	K.IVNGWQVEEADDWLR.Y	1830.97790	2	5.65E-10	0.96	4.78	-	1100.3
AHQ-2-5, 5771 - 5776	K.LLPLVSDEVFIR.D	1401.67552	2	7.38E-07	0.85	3.61	-	493.2
AHQ-2-5, 4099	K.LVTSIGDVVNHDPVVGDR.L	1893.09067	2	2.40E-05	0.88	3.88	-	649.9
AHQ-2-5, 6747	R.WLLLCNPGLADTIVEK.I	1844.16436	2	1.77E-05	0.57	3.07	-	477.3
gi|10863927|ref|NP_066953.1| peptidylprolyl isomerase A (cyclophilin A) [Homo sapiens]				5.75E-10	6.33	80.24	30.90	18012.4

AHQ-2-13-, 4250	K.EGMNIVEAMER.F	1279.46942	2	9.43E-04	0.59	2.81	-	486.9
AHQ-2-12, 4045 - 4046	K.FEDENFILK.H	1155.28144	2	2.88E-05	0.84	3.37	-	610.9
AHQ-2-12, 4162	K.FEDENFILK.H	1155.28144	1	5.72E-04	0.71	2.52	-	1024.0
AHQ-2-12, 3946	K.ITIADCGQLE	1121.24345	1	2.76E-04	0.46	2.31	-	393.4
AHQ-2-12, 3454	K.KITIADCGQLE	1249.41637	2	1.22E-06	0.88	3.91	-	980.0
AHQ-2-13-, 3378	K.KITIADCGQLE	1249.41637	2	4.37E-06	0.89	3.45	-	787.3
AHQ-2-12, 3277 - 3345	K.KITIADCGQLE	1249.41637	2	1.34E-07	0.69	3.33	-	662.9
AHQ-2-14, 5936 - 5997	K.SIYGEKFEDENFILK.H	1833.03047	2	5.31E-04	0.86	3.86	-	733.7
AHQ-2-13, 4649	K.SIYGEKFEDENFILK.H	1833.03047	2	8.99E-04	0.95	4.73	-	1106.7
AHQ-2-14-, 4757	K.SIYGEKFEDENFILK.H	1833.03047	2	3.27E-09	0.94	4.63	-	1106.8
AHQ-2-12, 4741 - 4811	K.SIYGEKFEDENFILK.H	1833.03047	2	3.69E-09	0.95	4.35	-	1347.2
AHQ-2-12, 4649 - 4670	K.SIYGEKFEDENFILK.H	1833.03047	2	5.75E-10	0.96	4.78	-	1268.7
AHQ-2-12, 3567	K.VKEGMNIVEAMER.F	1506.77390	2	1.69E-05	0.95	4.37	-	1608.2
AHQ-2-12, 4382	R.VSFELFADK.V	1056.19300	2	2.05E-05	0.83	3.17	-	854.1
AHQ-2-12, 4511	R.VSFELFADK.V	1056.19300	1	1.39E-04	0.46	2.60	-	581.1
AHQ-2-12, 4517	R.VSFELFADK.V	1056.19300	2	9.94E-06	0.95	3.96	-	1087.1
AHQ-2-12, 4514	R.VSFELFADKVPK.T	1380.61317	2	5.25E-07	0.89	3.98	-	893.6
AHQ-2-12, 4302	R.VSFELFADKVPK.T	1380.61317	2	9.31E-07	0.88	3.77	-	885.5
gi|7705501|ref|NP_057546.1| hypothetical protein HSPC194 [Homo sapiens]				5.93E-10	1.84	20.22	41.10	11564.5
AHQ-2-14-, 6947	K.AGSVPSLAAGLLFGSLAGLGAYQLSQDPR.N	2818.17710	2	1.07E-04	0.88	4.33	-	622.3
AHQ-2-14-, 5971	K.FM*PAGLIAGASLLM*VAK.V	1723.13705	2	5.93E-10	0.97	4.43	-	2003.2
gi|4557419|ref|NP_000063.1| CD36 antigen (collagen type I receptor, thrombospondin receptor); CD36				5.96E-10	2.66	30.25	10.20	53052.1
AHQ-2-1, 4929	K.AFASPVENPDNYCFCTEK.I	2152.30319	2	2.28E-06	0.74	3.43	-	382.0
AHQ-2-5, 5488	K.SQVLQFFSSDICR.S	1588.76661	2	5.96E-10	0.95	3.98	-	1410.5
AHQ-2-5, 7341 - 7343	R.TYLDIQPITGFTLQFAK.R	1957.25787	2	1.88E-08	0.97	5.05	-	1461.9
gi|4557297|ref|NP_000022.1| delta-aminolevulinic acid dehydratase; porphobilinogen synthase; aminol				6.20E-10	1.73	20.21	10.00	36294.7
AHQ-2-9, 4843	R.GSAADSEESPAIEAIHLLR.K	1967.12642	2	2.97E-08	0.93	4.11	-	906.5
AHQ-2-9, 4866	R.GSAADSEESPAIEAIHLLR.K	1967.12642	3	6.20E-10	0.86	3.68	-	884.1
AHQ-2-9, 4683	R.LEEMLRPLVEEGLR.C	1684.98233	2	1.70E-04	0.87	3.36	-	1046.8
gi|4757786|ref|NP_004306.1| N-acylsphingosine amidohydrolase (acid ceramidase) 1; N-acylsphingosine				6.21E-10	0.98	10.28	4.30	44649.3
AHQ-2-13-, 6718 - 6785	K.LPGLLGNFPGPFEEEMK.G	1876.16512	2	3.90E-04	0.92	3.32	-	1211.3
AHQ-2-14-, 6513 - 6576	K.LPGLLGNFPGPFEEEMK.G	1876.16512	2	6.21E-10	0.98	5.58	-	1613.2
AHQ-2-13, 6437 - 6499	K.LPGLLGNFPGPFEEEMK.G	1876.16512	2	6.65E-07	0.97	4.76	-	1608.2
gi|4504515|ref|NP_002146.1| heat shock 70kDa protein 6 (HSP70B'); heat shock 70kD protein 6 (HSP70B				6.28E-10	4.62	60.23	10.70	70853.4
AHQ-2-6, 2229	K.ATAGDTHLGGEDFDNR.L	1676.68279	3	6.24E-07	0.64	3.02	-	338.8
AHQ-2-6, 2233	K.ATAGDTHLGGEDFDNR.L	1676.68279	2	6.32E-10	0.95	4.63	-	1197.3
AHQ-2-7, 4655	R.IINEPTAAAIAYGLDR.R	1688.90603	2	3.51E-06	0.92	3.24	-	1271.2
AHQ-2-6, 4753 - 4771	R.IINEPTAAAIAYGLDR.R	1688.90603	2	6.28E-10	0.95	4.67	-	1000.3
AHQ-2-6, 4341	K.LLQDFFNGK.E	1082.23369	2	8.39E-04	0.87	2.59	-	886.6
AHQ-2-6, 4206 - 4277	K.LLQDFFNGKELNK.S	1566.78249	2	5.13E-04	0.95	3.84	-	1750.7
AHQ-2-13-, 3457 - 3463	R.TTPSYVAFTDTER.L	1488.58062	2	3.23E-07	0.78	3.11	-	584.3
AHQ-2-13, 3467	R.TTPSYVAFTDTER.L	1488.58062	2	1.78E-04	0.87	2.79	-	922.1
AHQ-2-12, 3370	R.TTPSYVAFTDTER.L	1488.58062	2	6.40E-07	0.81	2.64	-	676.3
AHQ-2-6, 1985 - 2053	R.VEILANDQGNR.T	1229.32518	2	2.84E-04	0.94	3.94	-	1330.0
AHQ-2-6, 1989 - 2058	R.VEILANDQGNR.T	1229.32518	1	2.50E-04	0.12	1.90	-	237.7
gi|4758018|ref|NP_004359.1| calponin 2; Calonin 2 [Homo sapiens]				6.48E-10	2.77	30.27	12.60	33696.9
AHQ-2-12, 5155	K.DGTILCTLMNK.L	1267.49833	2	7.78E-04	0.88	3.35	-	947.4
AHQ-2-12, 2786	K.GLQSGVDIGVK.Y	1073.22510	2	1.85E-06	0.92	3.35	-	1178.1
AHQ-2-13-, 2861	K.GLQSGVDIGVK.Y	1073.22510	2	4.14E-05	0.91	2.87	-	1471.6
AHQ-2-10, 6028 - 6096	R.TWIEGLTGLSIGPDFQK.G	1863.10294	2	2.11E-06	0.74	3.18	-	337.5
AHQ-2-12, 6466 - 6525	R.TWIEGLTGLSIGPDFQK.G	1863.10294	2	6.48E-10	0.97	4.62	-	1720.9
AHQ-2-12, 6591	R.TWIEGLTGLSIGPDFQK.G	1863.10294	2	9.67E-05	0.79	2.87	-	953.9
AHQ-2-13, 6311 - 6315	R.TWIEGLTGLSIGPDFQK.G	1863.10294	2	3.30E-09	0.97	5.30	-	1334.3
AHQ-2-13, 6415 - 6480	R.TWIEGLTGLSIGPDFQK.G	1863.10294	2	1.40E-04	0.45	2.71	-	588.5
AHQ-2-1, 6770 - 6838	R.TWIEGLTGLSIGPDFQK.G	1863.10294	2	9.86E-06	0.94	3.99	-	1170.3
AHQ-2-13-, 6422 - 6494	R.TWIEGLTGLSIGPDFQK.G	1863.10294	2	2.05E-09	0.97	5.44	-	1418.0
AHQ-2-14-, 6360	R.TWIEGLTGLSIGPDFQK.G	1863.10294	2	2.30E-05	0.96	4.31	-	1447.9
gi|27478749|ref|XP_208411.1| similar to L-lactate dehydrogenase A chain (LDH-A) (LDH muscle subunit				6.81E-10	1.79	20.29	13.40	25628.5
AHQ-2-9, 4446	K.GEMMDLQHGSLFLR.T	1634.90537	2	1.30E-09	0.83	3.80	-	688.8
AHQ-2-9, 7474	K.LLIVSNPVDILTYVAWK.I	1945.33337	2	6.81E-10	0.96	5.90	-	913.9
gi|7657056|ref|NP_055415.1| EH-domain containing 3; EH domain containing 3 [Homo sapiens]				7.28E-10	5.66	60.30	20.20	60886.7
AHQ-2-7, 5517	R.FHEFHSPALEDADFDNKPMVLLVGQYSTGK.T	3394.75749	3	3.08E-08	0.94	5.11	-	843.2
AHQ-2-10, 6774 - 6775	R.FVCAQLPNPVLESISVIDTPGILSGEK.Q	2886.30971	2	3.12E-04	0.92	4.50	-	927.8
AHQ-2-7, 7519	R.FVCAQLPNPVLESISVIDTPGILSGEK.Q	2886.30971	2	1.13E-05	0.95	4.86	-	1034.5
AHQ-2-8, 7423	R.FVCAQLPNPVLESISVIDTPGILSGEK.Q	2886.30971	2	7.28E-10	0.97	6.04	-	1454.3
AHQ-2-13, 5303	K.LADIDKDGM*LDDDEFALANHLIK.V	2589.86003	3	6.75E-06	0.94	4.60	-	1434.0
AHQ-2-13, 5625 - 5693	K.LADIDKDGMLDDDEFALANHLIK.V	2573.86063	3	1.25E-06	0.96	4.60	-	2116.1
AHQ-2-7, 5936	R.VYIGSFWSHPLLIPDNR.K	2015.30234	2	1.64E-04	0.94	4.13	-	1106.0
AHQ-2-8, 4501	R.YLLEQDFPGMR.I	1369.57075	2	4.97E-05	0.92	3.43	-	1430.2
AHQ-2-13-, 4855	R.YLLEQDFPGMR.I	1369.57075	2	2.42E-04	0.84	3.47	-	1011.1
AHQ-2-8, 4508	R.YLLEQDFPGMR.I	1369.57075	1	2.67E-04	0.29	2.11	-	271.6
gi|16975484|ref|NP_056057.1| centaurin delta 2 isoform b; ARF-GAP, RHO-GAP, ankyrin repeat, and ple				8.11E-10	0.82	10.17	1.20	136262.7
AHQ-2-3, 5169	R.AVFPEGPCEEPLQLR.K	1743.96175	2	8.11E-10	0.82	3.35	-	461.7
gi|4758038|ref|NP_004246.1| cytochrome c oxidase subunit Va precursor; cytochrome c oxidase polypep				8.83E-10	1.81	20.28	30.00	16774.1
AHQ-2-13, 6731 - 6807	K.EIYPYVIQELRPTLNELGISTPEELGLDKV	3430.88913	3	8.83E-10	0.94	5.53	-	708.8
AHQ-2-13-, 6950	K.EIYPYVIQELRPTLNELGISTPEELGLDKV	3430.88913	3	1.59E-08	0.91	4.24	-	1127.4
AHQ-2-14-, 6868 - 6936	K.EIYPYVIQELRPTLNELGISTPEELGLDKV	3430.88913	3	1.77E-04	0.87	4.47	-	544.8
AHQ-2-13, 4207	K.GINTLVTYDM*VPEPK.I	1693.94282	2	1.36E-04	0.87	3.07	-	1246.8
gi|4504077|ref|NP_000165.1| glycoprotein IX (platelet) [Homo sapiens]				8.95E-10	5.03	60.22	30.50	19046.0
AHQ-2-11, 4076	R.ALETM*GLWVDCR.G	1468.68075	2	5.86E-04	0.89	3.10	-	1099.0
AHQ-2-11, 4902	R.ALETMGLWVDCR.G	1452.68135	2	1.47E-06	0.93	3.55	-	1034.8
AHQ-2-14, 3538 - 3596	R.CASPSLAAHGPLGR.L	1395.56962	2	7.56E-06	0.77	2.87	-	791.8
AHQ-2-12, 2610	R.CASPSLAAHGPLGR.L	1395.56962	2	2.56E-09	0.93	3.71	-	1249.8
AHQ-2-14-, 2660	R.CASPSLAAHGPLGR.L	1395.56962	2	4.08E-08	0.85	2.79	-	940.4
AHQ-2-11, 2614	R.CASPSLAAHGPLGR.L	1395.56962	1	6.67E-08	0.67	3.39	-	215.3
AHQ-2-11, 2572	R.CASPSLAAHGPLGR.L	1395.56962	2	8.95E-10	0.93	3.49	-	1082.6
AHQ-2-11, 3201 - 3265	R.GHGLTALPALPAR.T	1274.49706	2	5.59E-05	0.94	4.06	-	780.0
AHQ-2-11, 5229	R.LWLEDRTPEALLQVR.C	1840.11574	2	9.45E-04	0.90	4.35	-	641.0
AHQ-2-11, 3060	R.TPEALLQVR.C	1027.19957	1	3.37E-06	0.44	2.35	-	167.5
AHQ-2-11, 3057	R.TPEALLQVR.C	1027.19957	2	4.58E-05	0.94	3.73	-	1261.4
AHQ-2-14-, 3071	R.TPEALLQVR.C	1027.19957	2	4.46E-05	0.96	3.24	-	1787.2
AHQ-2-12, 3082	R.TPEALLQVR.C	1027.19957	2	2.99E-05	0.90	2.95	-	993.1
gi|6681259|ref|NP_031377.1| multimerin [Homo sapiens]				9.17E-10	17.27	190.33	21.60	138070.8
AHQ-2-1, 2522	R.AQEQQSLIHTNQAESHTAVGR.G	2306.43830	2	1.46E-04	0.77	3.36	-	520.9
AHQ-2-3, 2259	R.AQEQQSLIHTNQAESHTAVGR.G	2306.43830	2	6.09E-06	0.94	5.05	-	742.5
AHQ-2-3, 2351	R.AQEQQSLIHTNQAESHTAVGR.G	2306.43830	2	2.67E-05	0.89	3.96	-	892.4
AHQ-2-2, 2293	R.AQEQQSLIHTNQAESHTAVGR.G	2306.43830	2	3.44E-05	0.91	4.93	-	495.0
AHQ-2-3, 2261	R.AQEQQSLIHTNQAESHTAVGR.G	2306.43830	3	5.16E-04	0.95	4.78	-	1693.7
AHQ-2-3, 7405 - 7412	K.CTSDMETILTFIPQFHR.L	2098.38838	3	2.11E-05	0.91	4.57	-	801.9
AHQ-2-3, 7397 - 7407	K.CTSDMETILTFIPQFHR.L	2098.38838	2	3.57E-04	0.90	3.88	-	834.7
AHQ-2-3, 4140	R.DQALQLQVLNSR.F	1385.55144	2	2.32E-06	0.96	3.88	-	2203.7
AHQ-2-10, 5850 - 5851	K.FPPVTTFSGYLLYR.T	1661.92388	2	3.11E-05	0.93	4.09	-	725.5
AHQ-2-14-, 6196	K.FPPVTTFSGYLLYR.T	1661.92388	2	7.61E-07	0.93	3.92	-	670.0
AHQ-2-3, 5971 - 6004	K.FVLVQENRPTLTDIVELR.N	2143.47187	2	4.64E-04	0.78	3.88	-	574.3
AHQ-2-4, 6008	K.FVLVQENRPTLTDIVELR.N	2143.47187	3	4.01E-07	0.92	4.23	-	896.7
AHQ-2-3, 5979	K.FVLVQENRPTLTDIVELR.N	2143.47187	3	1.92E-06	0.94	4.16	-	1254.2
AHQ-2-6, 5989	K.GLTEFVEPIIQIK.T	1487.76518	2	8.16E-05	0.82	3.01	-	841.1

AHQ-2-1, 6312	K.GLTEFVEPIIQIK.T	1487.76518	2	1.75E-06	0.92	4.34	-	825.6
AHQ-2-3, 6288 - 6355	K.GLTEFVEPIIQIK.T	1487.76518	2	2.47E-04	0.93	3.48	-	1275.4
AHQ-2-3, 6311	K.GLTEFVEPIIQIK.T	1487.76518	1	1.02E-04	0.58	3.18	-	524.2
AHQ-2-3, 3748	K.HSLPDIQLLQK.G	1292.50915	2	2.05E-09	0.93	3.49	-	1041.9
AHQ-2-1, 4017 - 4028	K.HSLPDIQLLQK.G	1292.50915	2	2.59E-06	0.94	3.53	-	1066.2
AHQ-2-4, 3769	K.HSLPDIQLLQK.G	1292.50915	2	4.42E-06	0.50	2.68	-	516.0
AHQ-2-2, 3816	K.HSLPDIQLLQK.G	1292.50915	2	9.49E-04	0.93	3.37	-	1010.2
AHQ-2-3, 6195	K.IENLTSAVNSLNFIIK.E	1777.05472	2	4.00E-05	0.96	5.42	-	972.5
AHQ-2-3, 5209 - 5224	K.IFQNDM*QETVAQLFK.T	1829.06683	2	3.20E-07	0.95	4.19	-	1372.6
AHQ-2-5, 6571	K.IFQNDMQETVAQLFK.T	1813.06743	2	2.17E-05	0.96	4.16	-	1741.6
AHQ-2-3, 6771 - 6844	K.IFQNDMQETVAQLFK.T	1813.06743	2	1.42E-08	0.97	5.05	-	1885.4
AHQ-2-3, 6909	K.IFQNDMQETVAQLFK.T	1813.06743	2	3.28E-08	0.96	4.53	-	1586.2
AHQ-2-3, 5457	K.IFQNDM*QETVAQLFK.T	1829.06683	2	2.42E-08	0.92	4.09	-	910.8
AHQ-2-10, 3544 - 3546	K.LAFESENINSEIHCDR.V	1936.04933	2	8.80E-06	0.92	4.42	-	539.9
AHQ-2-3, 2735	K.LKEVHEQLLSTEQVSDQK.N	2112.32666	2	5.29E-06	0.97	5.49	-	1335.3
AHQ-2-1, 2970	K.LKEVHEQLLSTEQVSDQK.N	2112.32666	2	1.69E-06	0.96	4.96	-	1010.2
AHQ-2-5, 2507	K.LKEVHEQLLSTEQVSDQK.N	2112.32666	2	2.47E-08	0.97	5.94	-	1259.9
AHQ-2-4, 2741	K.LKEVHEQLLSTEQVSDQK.N	2112.32666	2	1.15E-06	0.97	5.91	-	1019.6
AHQ-2-3, 2740	K.LKEVHEQLLSTEQVSDQK.N	2112.32666	3	2.43E-07	0.96	5.14	-	1865.4
AHQ-2-11, 3593	K.LVEENALAPDFSK.G	1433.58810	2	9.37E-04	0.86	3.44	-	770.6
AHQ-2-10, 3439 - 3442	K.LVEENALAPDFSK.G	1433.58810	1	1.40E-04	0.13	2.16	-	353.2
AHQ-2-10, 3423 - 3430	K.LVEENALAPDFSK.G	1433.58810	2	7.57E-06	0.94	3.74	-	1065.3
AHQ-2-3, 7571	K.M*SEQLNDLTYDMEILQPLLEQGASLR.Q	3025.40122	3	1.12E-06	0.96	6.50	-	1587.4
AHQ-2-3, 1971 - 1984	K.QTHLEGALEQEHSR.S	1635.72018	2	5.14E-05	0.50	2.62	-	279.6
AHQ-2-3, 7483	K.TM*TIINNAIDFIQDNYALK.E	2215.51196	2	9.17E-10	0.94	4.95	-	963.2
AHQ-2-4, 7605	K.TMTIINNAIDFIQDNYALK.E	2199.51256	2	3.97E-08	0.89	4.05	-	837.5
AHQ-2-3, 3403	K.TVSSLSEDLESTR.Q	1424.49324	2	1.89E-05	0.86	2.88	-	1105.0
AHQ-2-3, 3417	K.TVSSLSEDLESTR.Q	1424.49324	1	1.17E-04	0.22	2.36	-	157.8
AHQ-2-1, 3576	K.TVSSLSEDLESTR.Q	1424.49324	2	8.42E-05	0.45	2.93	-	381.7
AHQ-2-3, 4017	R.YNFVLQVAK.T	1082.27683	2	4.68E-05	0.92	3.05	-	1019.4
gi|5902128|ref|NP_008880.1| syntaxin binding protein 2; Hunc18b [Homo sapiens]				9.25E-10	7.13	80.32	21.40	66438.1
AHQ-2-6, 6293	K.AAHIFFTDTCPEPLFSELGR.S	2310.57011	2	1.91E-07	0.98	6.44	-	1964.5
AHQ-2-6, 6295 - 6309	K.AAHIFFTDTCPEPLFSELGR.S	2310.57011	3	6.34E-07	0.94	4.57	-	1207.5
AHQ-2-6, 6590 - 6601	R.EPIPSLEAIYLLSPTEK.S	1901.18939	2	7.00E-06	0.95	4.71	-	910.7
AHQ-2-6, 2906 - 2907	R.KGPEDTAQLAHAVLAK.L	1649.87303	3	1.76E-08	0.96	4.71	-	1672.9
AHQ-2-6, 2903	R.KGPEDTAQLAHAVLAK.L	1649.87303	2	9.25E-10	0.95	4.04	-	1477.8
AHQ-2-6, 2129	K.LIQHANVQAHSSLIR.N	1687.92773	2	4.60E-06	0.92	3.74	-	1099.1
AHQ-2-6, 6345 - 6358	K.LIVPVLLDAAVPAYDK.I	1698.03911	2	1.11E-05	0.98	5.08	-	1929.2
AHQ-2-6, 3055	R.NLEQLGGTVTNPGGSGTSSR.L	1933.02693	2	5.38E-05	0.92	4.12	-	837.1
AHQ-2-6, 5410	R.NLWPFVSDPAPTASSQAAVSAR.F	2273.48981	2	1.03E-04	0.71	3.27	-	405.2
AHQ-2-6, 5865 - 5923	R.REPIPSLEAIYLLSPTEK.S	2057.37574	2	2.21E-05	0.98	6.03	-	1608.6
AHQ-2-6, 5866	R.REPIPSLEAIYLLSPTEK.S	2057.37574	3	1.24E-05	0.72	3.25	-	710.6
gi|12707570|ref|NP_004083.2| mitochondrial short-chain enoyl-coenzyme A hydratase 1 precursor [Homo				9.41E-10	0.97	10.26	5.90	31371.2
AHQ-2-10, 6787	K.ICPVETLVEEAIQCAEK.I	1992.25744	2	9.41E-10	0.97	5.24	-	1309.0
gi|29740995|ref|XP_291768.1| similar to Proteasome activator complex subunit 2 (Proteasome activato				9.54E-10	0.98	10.29	9.60	19192.6
AHQ-2-10, 6482 - 6483	R.AFYAELYHIISSNLEK.T	1899.13528	2	9.54E-10	0.98	5.75	-	1787.0
gi|4885413|ref|NP_005331.1| histidine triad nucleotide binding protein 1; Histidine triad nucleotid				9.74E-10	4.60	50.30	71.40	13801.8
AHQ-2-13-, 2705	K.AQVARPGGDTIFGK.I	1417.59513	2	1.57E-04	0.92	3.73	-	1234.0
AHQ-2-13, 2763	K.AQVARPGGDTIFGK.I	1417.59513	2	1.76E-04	0.81	3.30	-	895.9
AHQ-2-13-, 5621	R.CLAFHDISPQAPTHFLVIPK.K	2293.67220	2	8.02E-04	0.81	3.57	-	731.5
AHQ-2-13-, 5618	R.CLAFHDISPQAPTHFLVIPK.K	2293.67220	3	1.44E-06	0.94	4.49	-	1148.5
AHQ-2-13, 6041 - 6109	K.IIFEDDRCLAFHDISPQAPTHFLVIPK.K	3182.64001	3	3.03E-06	0.92	4.92	-	1172.5
AHQ-2-13-, 5543	K.KHISQISVAEDDDESLLGHLMIVGK.K	2736.09467	3	2.06E-06	0.98	6.05	-	2255.4
AHQ-2-13-, 3898	R.MVVNEGSDGGQSVYHVHLHVLGGR.Q	2548.82255	3	9.74E-10	0.85	3.84	-	793.0
gi|6912494|ref|NP_036457.1| microtubule-associated protein, RP/EB family, member 1; ADENOMATOUS POL				1.02E-09	3.12	40.24	15.30	29998.9
AHQ-2-12, 3990	K.FFDANYDGKDYDPVAAR.Q	1965.06754	2	5.69E-05	0.85	3.57	-	557.6
AHQ-2-12, 6686 - 6689	K.FQDNFEFVQWFK.K	1635.80184	2	1.02E-09	0.97	4.82	-	1419.8
AHQ-2-12, 6819	K.FQDNFEFVQWFK.K	1635.80184	2	6.86E-07	0.96	4.02	-	1320.0
AHQ-2-12, 6175	K.GKFQDNFEFVQWFK.K	1821.02630	2	1.96E-04	0.96	4.59	-	1270.2
AHQ-2-13-, 2709	K.LEHEYIQNFK.I	1321.46268	2	9.03E-04	0.67	2.58	-	951.4
AHQ-2-12, 2563	K.LEHEYIQNFK.I	1321.46268	1	1.01E-05	0.34	2.14	-	553.1
gi|29568111|ref|NP_006088.2| myosin, light polypeptide 9, regulatory; myosin regulatory light chain				1.07E-09	3.13	40.22	18.00	19827.1
AHQ-2-11, 3352	R.FTDEEVDEMYR.E	1434.51009	1	2.09E-07	0.47	2.18	-	470.5
AHQ-2-11, 3366	R.FTDEEVDEMYR.E	1434.51009	1	8.48E-05	0.33	2.31	-	347.2
AHQ-2-11, 2658	R.FTDEEVDEM*YR.E	1450.50949	2	7.24E-06	0.94	3.45	-	1257.9
AHQ-2-11, 3640 - 3704	K.GNFNYVEFTR.I	1247.34066	2	1.24E-05	0.84	3.34	-	792.5
AHQ-2-11, 3674	K.GNFNYVEFTR.I	1247.34066	2	2.14E-05	0.86	3.19	-	708.0
AHQ-2-11, 3820	K.GNFNYVEFTR.I	1247.34066	2	7.66E-04	0.73	2.75	-	515.4
AHQ-2-11, 3925	K.GNFNYVEFTR.I	1247.34066	2	1.71E-04	0.92	3.77	-	907.8
AHQ-2-11, 4129	K.GNFNYVEFTR.I	1247.34066	1	5.78E-06	0.21	1.85	-	221.1
AHQ-2-13-, 3935 - 3978	K.GNFNYVEFTR.I	1247.34066	2	1.83E-05	0.88	3.18	-	914.8
AHQ-2-12, 3766	K.GNFNYVEFTR.I	1247.34066	2	2.83E-07	0.78	2.95	-	614.3
AHQ-2-14-, 3867	K.GNFNYVEFTR.I	1247.34066	2	4.18E-05	0.84	2.97	-	909.8
AHQ-2-11, 5726 - 5765	R.NAFACFDEEASGFIHEDHLR.E	2367.49548	2	1.07E-09	0.94	4.38	-	1036.3
gi|17986283|ref|NP_006000.2| tubulin, alpha 3; tubulin, alpha, brain-specific; hum-a-tub1; hum-a-tu				1.07E-09	0.96	10.29	3.30	50135.3
AHQ-2-1, 6081 - 6089	R.AVFVDLEPTVIDEVR.T	1702.92947	2	4.26E-06	0.88	4.05	-	737.9
AHQ-2-3, 6047	R.AVFVDLEPTVIDEVR.T	1702.92947	2	6.39E-07	0.72	3.70	-	502.2
AHQ-2-4, 6056	R.AVFVDLEPTVIDEVR.T	1702.92947	2	6.69E-09	0.94	4.53	-	1002.4
AHQ-2-6, 5686 - 5743	R.AVFVDLEPTVIDEVR.T	1702.92947	2	1.07E-05	0.89	4.16	-	726.1
AHQ-2-7, 5315	R.AVFVDLEPTVIDEVR.T	1702.92947	2	7.35E-05	0.89	3.36	-	888.3
AHQ-2-7, 5605 - 5675	R.AVFVDLEPTVIDEVR.T	1702.92947	2	1.07E-09	0.96	5.73	-	1039.3
AHQ-2-10, 5270 - 5291	R.AVFVDLEPTVIDEVR.T	1702.92947	2	4.25E-08	0.96	5.17	-	1265.0
AHQ-2-11, 5492	R.AVFVDLEPTVIDEVR.T	1702.92947	2	1.28E-06	0.91	4.36	-	868.5
AHQ-2-12, 5767 - 5846	R.AVFVDLEPTVIDEVR.T	1702.92947	2	3.80E-04	0.51	2.84	-	299.1
AHQ-2-13, 5605	R.AVFVDLEPTVIDEVR.T	1702.92947	2	5.86E-06	0.84	4.11	-	498.3
AHQ-2-13-, 5705	R.AVFVDLEPTVIDEVR.T	1702.92947	2	3.51E-07	0.94	4.95	-	887.9
AHQ-2-14-, 5671 - 5749	R.AVFVDLEPTVIDEVR.T	1702.92947	2	6.78E-09	0.96	5.21	-	987.3
gi|23110944|ref|NP_002782.1| proteasome alpha 6 subunit; prosomal P27K protein; proteasome subunit				1.09E-09	0.87	10.17	4.90	27399.3
AHQ-2-10, 4034 - 4036	K.LLDSSTVTHLFK.I	1361.56825	2	1.09E-09	0.87	3.38	-	704.4
gi|5032161|ref|NP_005639.1| elongin C; transcription elongation factor B (SIII), polypeptide 1 (15k				1.13E-09	2.50	30.24	28.60	12473.1
AHQ-2-13-, 4634	K.AM*LSGPGQFAENETNEVNFR.E	2228.38455	2	2.24E-06	0.74	3.20	-	787.2
AHQ-2-13-, 4935	K.AMLSGPGQFAENETNEVNFR.E	2212.38515	2	1.13E-09	0.95	4.74	-	1282.4
AHQ-2-13-, 3041	K.LISSDGHEFIVK.R	1345.52569	2	4.63E-06	0.81	3.17	-	616.8
AHQ-2-13, 3077	K.LISSDGHEFIVK.R	1345.52569	2	1.62E-04	0.75	2.90	-	646.5
gi|4507109|ref|NP_000336.1| alpha-synuclein isoform NACP140; non A4 component of amyloid precursor				1.14E-09	5.18	60.29	45.70	14460.1
AHQ-2-12, 2118 - 2119	K.EGVVHGVATVAEK.T	1296.45466	2	7.81E-07	0.91	3.47	-	957.2
AHQ-2-13-, 4715 - 4718	K.EQVTNVGGAVVTGVTAVAQK.T	1929.16446	2	6.72E-09	0.96	5.36	-	873.0
AHQ-2-14, 5685	K.EQVTNVGGAVVTGVTAVAQK.T	1929.16446	2	7.45E-08	0.95	4.86	-	1200.3
AHQ-2-14-, 4695	K.EQVTNVGGAVVTGVTAVAQK.T	1929.16446	2	1.35E-07	0.94	4.87	-	773.4
AHQ-2-14-, 4640 - 4708	K.EQVTNVGGAVVTGVTAVAQK.T	1929.16446	3	1.31E-05	0.91	4.25	-	1195.7
AHQ-2-13-, 4719	K.EQVTNVGGAVVTGVTAVAQK.T	1929.16446	3	7.58E-04	0.97	5.50	-	1724.2
AHQ-2-12, 4699	K.EQVTNVGGAVVTGVTAVAQK.T	1929.16446	2	3.39E-07	0.95	5.23	-	907.4
AHQ-2-12, 4939	K.EQVTNVGGAVVTGVTAVAQK.T	1929.16446	2	2.88E-07	0.90	4.07	-	563.6
AHQ-2-13, 4633	K.EQVTNVGGAVVTGVTAVAQK.T	1929.16446	2	6.93E-06	0.95	4.91	-	1042.7
AHQ-2-12, 1993	K.TKEGVLYVGSK.T	1181.36357	2	1.22E-05	0.77	2.71	-	618.9
AHQ-2-12, 1989	K.TKEGVVHGVATVAEK.T	1525.73191	3	5.21E-04	0.92	3.97	-	1177.7
AHQ-2-14-, 2052	K.TKEGVVHGVATVAEK.T	1525.73191	2	1.08E-06	0.96	4.44	-	1462.6

AHQ-2-12, 1985	K.TKEGVVHGVATVAEK.T	1525.73191	2	2.83E-06	0.94	3.95	-	1298.1
AHQ-2-14, 5386 - 5409	K.TKEQVTNVGGAVVTGVTAVAQK.T	2158.44170	3	7.41E-04	0.85	3.77	-	1288.5
AHQ-2-14, 5393	K.TKEQVTNVGGAVVTGVTAVAQK.T	2158.44170	2	1.88E-06	0.97	5.80	-	1296.0
AHQ-2-13, 4209	K.TKEQVTNVGGAVVTGVTAVAQK.T	2158.44170	2	1.77E-05	0.95	4.63	-	1189.1
AHQ-2-14-, 4207 - 4223	K.TKEQVTNVGGAVVTGVTAVAQK.T	2158.44170	3	4.35E-04	0.77	3.50	-	931.6
AHQ-2-14-, 4231	K.TKEQVTNVGGAVVTGVTAVAQK.T	2158.44170	2	1.14E-09	0.97	5.17	-	1380.7
AHQ-2-12, 4209 - 4214	K.TKEQVTNVGGAVVTGVTAVAQK.T	2158.44170	3	7.51E-05	0.91	4.57	-	1391.5
AHQ-2-13-, 4211	K.TVEGAGSIAAATGFVK.K	1479.66001	1	3.68E-04	0.37	2.66	-	257.5
AHQ-2-13, 4171	K.TVEGAGSIAAATGFVK.K	1479.66001	1	9.02E-06	0.61	3.08	-	444.8
AHQ-2-13, 4159	K.TVEGAGSIAAATGFVK.K	1479.66001	2	8.07E-05	0.49	2.65	-	543.0
AHQ-2-12, 4158	K.TVEGAGSIAAATGFVK.K	1479.66001	1	7.28E-05	0.34	2.73	-	333.4
AHQ-2-14, 5025	K.TVEGAGSIAAATGFVK.K	1479.66001	2	7.07E-04	0.67	2.50	-	876.0
gi|21361741|ref|NP_057569.2| hypothetical protein HSPC228; My012 protein [Homo sapiens]				1.14E-09	0.37	10.14	6.50	33879.0
AHQ-2-9, 5040	K.YAGSALQYEDVSTAVQNLQK.A	2186.36410	2	1.14E-09	0.37	2.86	-	419.3
gi|12025678|ref|NP_004915.2| actinin, alpha 4 [Homo sapiens]				1.15E-09	8.27	100.26	15.80	104853.3
AHQ-2-5, 4049	K.DGLAFNALIHR.H	1227.39726	2	3.10E-08	0.86	2.99	-	939.7
AHQ-2-5, 4685	R.ETTDTDTADQVIASFK.V	1742.81936	2	2.10E-05	0.94	3.96	-	1467.7
AHQ-2-14, 5658 - 5733	R.ETTDTDTADQVIASFK.V	1742.81936	2	2.45E-05	0.79	3.35	-	441.0
AHQ-2-5, 2719 - 2723	K.GISQEQMQEFR.A	1353.48658	2	8.61E-07	0.79	3.02	-	1094.7
AHQ-2-5, 3816 - 3819	R.M*APYQGPDAVPGALDYK.S	1810.02037	2	1.29E-07	0.94	3.82	-	1222.1
AHQ-2-5, 4088	R.MAPYQGPDAVPGALDYK.S	1794.02097	2	3.94E-07	0.93	3.80	-	1192.2
AHQ-2-5, 4009	K.MLDAEDIVNTARPDEK.A	1817.99924	2	1.98E-06	0.76	3.50	-	579.8
AHQ-2-5, 4509 - 4523	R.QFASQANVVGPWIQTK.M	1775.00061	2	1.15E-09	0.89	4.12	-	490.6
AHQ-2-1, 6788	R.VEQIAAIAQELNELDYYDSHNVNTR.C	2907.09810	3	6.77E-04	0.64	3.31	-	479.9
AHQ-2-5, 3627	K.VLAGDKNFITAEELR.R	1676.89525	2	7.94E-06	0.85	4.15	-	593.9
AHQ-2-5, 3455 - 3463	K.VLAVNQENEHLMEDYEK.L	2062.24648	2	4.50E-05	0.94	5.10	-	577.7
AHQ-2-5, 3459	K.VLAVNQENEHLMEDYEK.L	2062.24648	3	5.20E-08	0.66	3.05	-	413.2
gi|4506505|ref|NP_002916.1| regulator of G-protein signaling 10 [Homo sapiens]				1.18E-09	2.70	30.22	23.40	19607.9
AHQ-2-11, 3518 - 3524	K.ILEEPHPLMFQK.L	1482.77213	2	4.46E-06	0.94	4.30	-	886.3
AHQ-2-11, 4628 - 4629	K.LQDQIFNLMK.Y	1250.49254	2	6.52E-05	0.89	3.65	-	732.7
AHQ-2-11, 5292 - 5308	K.WAASLENLLEDPEGVKR.F	1928.13502	2	1.18E-09	0.87	4.45	-	484.2
gi|10834976|ref|NP_000572.1| glutathione peroxidase 1 [Homo sapiens]				1.18E-09	8.06	90.30	58.70	21909.0
AHQ-2-11, 5345	K.CEVNGAGAHPLFAFLR.E	1760.99723	2	1.18E-09	0.93	4.08	-	1026.9
AHQ-2-11, 2738 - 2818	R.DYTQMNELQR.R	1298.40778	2	1.52E-05	0.83	3.10	-	816.1
AHQ-2-11, 3968	K.FLVGPDGVPLR.R	1170.38561	2	8.48E-06	0.94	3.61	-	1302.8
AHQ-2-11, 4846 - 4917	R.GLVVLGFPCNQFGHQENAK.N	2117.37312	2	6.26E-05	0.81	3.73	-	386.8
AHQ-2-11, 5938 - 6018	R.GLVVLGFPCNQFGHQENAKNEEILNSLK.Y	3158.53371	3	9.80E-07	0.88	4.58	-	604.9
AHQ-2-14, 4825	R.PLAGGEPVSLGSLR.G	1353.54935	2	6.49E-06	0.93	3.92	-	837.1
AHQ-2-11, 3646 - 3725	R.PLAGGEPVSLGSLR.G	1353.54935	2	8.24E-07	0.95	4.13	-	1332.3
AHQ-2-10, 3608 - 3619	R.PLAGGEPVSLGSLR.G	1353.54935	2	1.87E-06	0.94	4.10	-	980.5
AHQ-2-11, 6737	R.RFQTIDIEPDIEALLSQGPSCA	2462.71807	2	3.10E-07	0.95	4.77	-	1102.5
AHQ-2-11, 4865	K.YVRPGGGFEPNFM*LFEK.C	2005.28455	3	7.76E-04	0.81	3.71	-	763.2
AHQ-2-11, 5126	K.YVRPGGGFEPNFMLFEK.C	1989.28515	3	9.28E-08	0.95	4.47	-	1561.5
AHQ-2-11, 5124 - 5125	K.YVRPGGGFEPNFMLFEK.C	1989.28515	2	9.65E-05	0.98	6.01	-	2762.1
AHQ-2-11, 5234	K.YVRPGGGFEPNFMLFEK.C	1989.28515	2	2.05E-05	0.94	4.29	-	832.5
gi|4557395|ref|NP_000058.1| carbonic anhydrase II; carbonate dehydratase II; carbonic dehydratase;				1.20E-09	4.66	60.25	28.80	29245.8
AHQ-2-10, 6650	K.AVQQPDGLAVLGIFLK.V	1669.98899	2	1.19E-05	0.95	4.55	-	724.1
AHQ-2-10, 2048 - 2052	K.GGPLDGTYR.L	936.00385	1	1.69E-04	0.08	1.97	-	144.7
AHQ-2-10, 3963	R.ILNNGHAFNVEFDDSQDK.A	2064.15751	2	3.07E-07	0.96	4.96	-	1197.7
AHQ-2-10, 3852	R.ILNNGHAFNVEFDDSQDK.A	2064.15751	2	3.50E-06	0.76	3.61	-	388.6
AHQ-2-10, 3071	K.SADFTNFDPR.G	1170.21306	2	1.24E-07	0.86	3.36	-	775.5
AHQ-2-10, 3483	K.VVDVLDSIK.T	988.16011	2	3.08E-04	0.84	3.49	-	467.7
AHQ-2-10, 3146	K.YAAELHLVHWNTK.Y	1582.78684	2	1.20E-09	0.98	4.21	-	2631.7
gi|6005942|ref|NP_009057.1| valosin-containing protein; yeast Cdc48p homolog; transitional endoplas				1.34E-09	9.63	110.28	21.50	89321.3
AHQ-2-5, 3525 - 3539	R.ELQELVQYPVEHPDK.F	1825.01157	2	6.03E-04	0.90	3.86	-	905.6
AHQ-2-5, 3379	R.GILLYGPPGTGK.T	1173.38627	2	1.23E-04	0.84	3.42	-	891.3
AHQ-2-5, 6857	R.IVSQLLTLMDGLK.Q	1431.76657	2	5.91E-05	0.81	3.00	-	780.3
AHQ-2-5, 2988	R.KYEMFAQTLQQSR.G	1630.85008	2	8.24E-08	0.97	5.02	-	1808.7
AHQ-2-5, 2705	R.LEILQIHTK.N	1095.31705	1	1.43E-05	0.58	2.44	-	372.4
AHQ-2-5, 5181	R.LIVDEAINEDNSVVSLSQPK.M	2171.39088	2	3.05E-07	0.97	5.53	-	1350.1
AHQ-2-5, 4795	K.MTNGFSGADLTEICQR.A	1801.97996	2	2.78E-09	0.90	3.57	-	1049.0
AHQ-2-5, 7483 - 7484	K.NAPAIIFIDELDAIAPK.R	1812.09907	2	2.04E-08	0.96	5.43	-	1269.0
AHQ-2-5, 4324	R.QTNPSAMEVEEDDPVPEIR.R	2157.30143	2	3.36E-07	0.95	4.58	-	1362.0
AHQ-2-5, 4885	K.VVETDPSPYCIVAPDTVIHCEGEPIKR.E	3085.45473	3	1.34E-09	0.83	3.86	-	615.2
AHQ-2-5, 3244 - 3252	R.WALSQSNPSALR.E	1330.47434	2	5.21E-05	0.92	3.67	-	1161.4
gi|21624607|ref|NP_066972.1| coactosin-like 1 [Homo sapiens]				1.35E-09	4.06	60.30	31.70	15944.9
AHQ-2-13-, 2218	R.AAYNLVR.D	806.93245	2	5.88E-04	0.91	2.74	-	1110.2
AHQ-2-12, 5966	R.DDGSAVIWVTFK.Y	1338.49001	1	1.11E-05	0.43	2.44	-	488.6
AHQ-2-13, 2244	K.EVVQNFAK.E	935.05900	1	7.76E-05	0.02	2.15	-	87.3
AHQ-2-13-, 2082 - 2151	K.EVVQNFAK.E	935.05900	1	5.43E-05	0.22	2.52	-	138.7
AHQ-2-13, 6737	K.FALITWIGENVSGLQR.A	1805.06986	2	1.35E-09	0.98	6.05	-	2532.9
AHQ-2-12, 7053 - 7058	K.FALITWIGENVSGLQR.A	1805.06986	2	7.15E-05	0.98	5.71	-	2586.2
AHQ-2-12, 2670	R.KELEEDFIK.S	1151.29110	2	4.78E-04	0.80	2.90	-	791.0
AHQ-2-12, 5421 - 5487	K.YDGSTIVPGEQGAEYQHFIQQCTDDVR.L	3116.27790	3	7.61E-08	0.71	3.43	-	385.2
gi|21361322|ref|NP_006078.2| tubulin, beta, 5 [Homo sapiens]				1.48E-09	2.44	30.25	13.70	49585.5
AHQ-2-7, 2807	R.EIVHLQAGQCGNQIGAK.F	1825.03962	2	1.48E-09	0.94	4.15	-	1128.0
AHQ-2-9, 2740	R.EIVHLQAGQCGNQIGAK.F	1825.03962	2	7.15E-04	0.72	3.07	-	594.6
AHQ-2-7, 2647	R.EIVHLQAGQCGNQIGAK.F	1825.03962	2	1.92E-06	0.95	4.60	-	1160.4
AHQ-2-7, 5359	K.FWEVISDEHGIDPTGTYHGDSDLQLER.I	3118.27220	3	1.88E-04	0.92	5.09	-	918.3
AHQ-2-12, 6459 - 6461	K.M*AATFIGNSTAIQELFK.R	1859.13607	2	2.88E-04	0.53	3.10	-	584.1
AHQ-2-7, 6973	K.M*AATFIGNSTAIQELFK.R	1859.13607	2	5.28E-05	0.59	3.53	-	516.7
gi|13569962|ref|NP_112243.1| RAB1B, member RAS oncogene family; small GTP-binding protein [Homo sap				1.48E-09	3.63	40.33	18.90	22171.0
AHQ-2-11, 4541	R.GAHGIIVVYDVTDQESYANVK.Q	2279.49113	2	1.47E-08	0.98	5.49	-	1941.9
AHQ-2-11, 4410 - 4478	R.GAHGIIVVYDVTDQESYANVK.Q	2279.49113	2	2.32E-09	0.98	6.51	-	1917.9
AHQ-2-11, 6494 - 6553	K.NATNVEQAFMTMAAEIK.K	1870.14079	2	1.48E-09	0.97	5.24	-	1276.6
AHQ-2-11, 6658	K.NATNVEQAFM*TMAAEIK.K	1886.14019	2	5.74E-06	0.93	5.00	-	1296.8
AHQ-2-11, 6785 - 6786	K.NATNVEQAFMTMAAEIK.K	1870.14079	2	4.04E-08	0.96	4.96	-	1276.9
AHQ-2-11, 5061	K.NATNVEQAFM*TM*AAEIK.K	1902.13959	2	5.46E-06	0.75	3.27	-	845.0
gi|5031593|ref|NP_005708.1| actin related protein 2/3 complex subunit 5; Arp2/3 protein complex sub				1.48E-09	4.55	50.27	30.50	16320.3
AHQ-2-12, 2909	K.ALAAGGVGSIVR.V	1071.25558	2	3.00E-07	0.97	4.75	-	1929.7
AHQ-2-14-, 2939	K.ALAAGGVGSIVR.V	1071.25558	2	1.37E-06	0.95	3.83	-	1610.3
AHQ-2-12, 3542 - 3614	K.AVQSLDKNGVDLLM*K.Y	1647.91883	2	2.34E-05	0.85	3.90	-	505.2
AHQ-2-12, 4069 - 4137	K.AVQSLDKNGVDLLMK.Y	1631.91943	2	1.48E-09	0.98	5.45	-	1871.9
AHQ-2-12, 4591	K.GFESPSDNSSAMLLQWHEK.A	2164.34049	2	1.56E-07	0.93	4.43	-	886.9
AHQ-2-12, 4783 - 4806	K.GFESPSDNSSAMLLQWHEK.A	2164.34049	2	1.56E-07	0.83	4.11	-	633.3
AHQ-2-12, 4791	K.GFESPSDNSSAMLLQWHEK.A	2164.34049	3	2.10E-04	0.61	3.06	-	368.1
AHQ-2-12, 4271	K.GFESPSDNSSAM*LLQWHEK.A	2180.33989	2	8.56E-05	0.92	4.29	-	758.3
AHQ-2-12, 4174	K.GFESPSDNSSAM*LLQWHEK.A	2180.33989	2	6.80E-06	0.92	3.57	-	1069.9
gi|4504183|ref|NP_000843.1| glutathione transferase; deafness, X-linked 7; fatty acid ethyl ester s				1.61E-09	5.26	60.25	51.90	23355.7
AHQ-2-11, 5268	K.ALPGQLKPFETLLSQNQGGK.T	2127.42930	2	7.61E-09	0.79	3.35	-	514.9
AHQ-2-13, 5331 - 5340	K.ALPGQLKPFETLLSQNQGGK.T	2127.42930	2	4.64E-06	0.62	3.10	-	281.7
AHQ-2-10, 4874	K.ALPGQLKPFETLLSQNQGGK.T	2127.42930	2	2.67E-07	0.81	3.34	-	454.2
AHQ-2-11, 5142 - 5206	K.ALPGQLKPFETLLSQNQGGK.T	2127.42930	2	2.86E-05	0.84	3.70	-	337.0
AHQ-2-11, 3020 - 3094	K.ASCLYGQLPK.F	1138.31884	2	9.35E-05	0.91	3.22	-	842.2
AHQ-2-13-, 6558	K.DQQEAALVDMVNDGVEDLR.C	2118.26843	2	6.54E-06	0.91	3.92	-	1067.8
AHQ-2-11, 6470 - 6478	K.DQQEAALVDMVNDGVEDLR.C	2118.26843	2	4.91E-08	0.90	4.26	-	706.9
AHQ-2-11, 6272 - 6337	K.DQQEAALVDMVNDGVEDLR.C	2118.26843	2	6.68E-06	0.95	4.57	-	1067.4

AHQ-2-13, 6363 - 6367	K.DQQEAALVDMVNDGVEDLR.C	2118.26843	2	3.24E-08	0.94	4.95	-	814.8
AHQ-2-10, 6187	K.DQQEAALVDMVNDGVEDLR.C	2118.26843	2	7.47E-04	0.90	4.05	-	745.7
AHQ-2-14-, 6441	K.DQQEAALVDMVNDGVEDLR.C	2118.26843	2	4.43E-06	0.95	4.79	-	894.8
AHQ-2-11, 6041	K.DQQEAALVDMVNDGVEDLR.C	2118.26843	2	7.20E-04	0.95	4.67	-	1234.1
AHQ-2-11, 4944	K.FQDGDLTLYQSNTILR.H	1885.06701	2	1.61E-09	0.91	4.17	-	826.9
AHQ-2-11, 4840	K.FQDGDLTLYQSNTILR.H	1885.06701	2	1.21E-08	0.95	5.02	-	753.1
AHQ-2-11, 4720 - 4780	K.FQDGDLTLYQSNTILR.H	1885.06701	2	1.08E-04	0.92	4.23	-	800.3
AHQ-2-14-, 4981	K.FQDGDLTLYQSNTILR.H	1885.06701	2	7.81E-04	0.83	3.71	-	547.7
AHQ-2-14, 6062	K.FQDGDLTLYQSNTILR.H	1885.06701	2	1.37E-06	0.83	3.51	-	714.6
AHQ-2-10, 4622 - 4688	K.FQDGDLTLYQSNTILR.H	1885.06701	2	3.40E-07	0.94	4.93	-	838.7
AHQ-2-11, 6196 - 6269	R.MLLADQGQSWKEEVVTVETWQEGSLK.A	2993.33745	3	1.26E-05	0.85	4.61	-	665.3
AHQ-2-11, 4546	K.YISLIYTNYEAGKDDYVK.A	2156.37637	2	4.12E-04	0.85	3.73	-	618.1
AHQ-2-10, 4183	K.YISLIYTNYEAGKDDYVK.A	2156.37637	2	3.32E-07	0.85	3.83	-	621.6
gi|4507645|ref|NP_000356.1| triosephosphate isomerase 1 [Homo sapiens]				1.69E-09	4.58	50.33	32.90	26669.3
AHQ-2-10, 6612	K.ELASQPDVDGFLVGGASLKPEFVDIINAK.Q	3031.40532	3	1.69E-09	0.76	3.79	-	218.0
AHQ-2-10, 3998	R.KQSLGELIGTLNAAK.V	1543.79037	2	1.02E-05	0.96	4.18	-	1809.5
AHQ-2-10, 3760	R.KQSLGELIGTLNAAK.V	1543.79037	2	1.49E-04	0.94	4.03	-	1169.9
AHQ-2-10, 4852	K.QSLGELIGTLNAAK.V	1415.61746	2	3.27E-04	0.90	3.54	-	1013.1
AHQ-2-10, 4942 - 5022	K.VAHALAEGLGVIACIGEK.L	1810.10800	2	6.70E-06	0.99	6.67	-	3106.5
AHQ-2-10, 5108 - 5175	K.VPADTEVVCAPPTAYIDFAR.Q	2194.44943	2	1.79E-04	0.97	5.42	-	1030.2
gi|4502491|ref|NP_001203.1| complement component 1, q subcomponent binding protein precursor; hyalu				1.72E-09	0.87	10.20	5.00	31362.0
AHQ-2-9, 5352	K.AFVDFLSDEIKEER.K	1698.85455	2	1.72E-09	0.87	3.46	-	951.1
gi|4503423|ref|NP_001939.1| dUTP pyrophosphatase [Homo sapiens]				1.77E-09	0.95	10.22	10.40	17748.0
AHQ-2-11, 5458	R.IFYPEIEEVQALDDTER.G	2068.22598	2	1.77E-09	0.95	4.40	-	1135.5
gi|25188179|ref|NP_005653.3| voltage-dependent anion channel 3 [Homo sapiens]				1.79E-09	2.33	30.21	16.30	30658.4
AHQ-2-10, 4624 - 4648	K.LTLDTIFVPNTGK.K	1419.64771	2	1.92E-05	0.91	3.62	-	848.5
AHQ-2-10, 4619	K.LTLDTIFVPNTGK.K	1419.64771	1	1.04E-05	0.75	2.87	-	583.2
AHQ-2-11, 4856	K.LTLDTIFVPNTGK.K	1419.64771	2	1.32E-05	0.85	3.09	-	820.5
AHQ-2-9, 4154 - 4234	K.VNNASLIGLGYTQTLRPGVK.L	2102.42298	2	1.44E-06	0.88	4.30	-	801.7
AHQ-2-10, 4026 - 4028	K.VNNASLIGLGYTQTLRPGVK.L	2102.42298	3	6.38E-06	0.76	3.48	-	664.8
AHQ-2-14, 5690	K.VNNASLIGLGYTQTLRPGVK.L	2102.42298	2	1.52E-08	0.70	3.61	-	447.0
AHQ-2-10, 4410	K.VNNASLIGLGYTQTLRPGVK.L	2102.42298	2	2.72E-09	0.65	3.38	-	365.4
AHQ-2-10, 4022	K.VNNASLIGLGYTQTLRPGVK.L	2102.42298	2	2.13E-08	0.87	3.90	-	637.5
AHQ-2-9, 4674 - 4678	K.VNNASLIGLGYTQTLRPGVK.L	2102.42298	2	8.74E-07	0.67	3.14	-	379.9
AHQ-2-10, 4196	K.VNNASLIGLGYTQTLRPGVK.L	2102.42298	2	1.17E-05	0.71	2.93	-	655.1
AHQ-2-9, 3640	K.YKVCNYGLTFTQK.W	1623.85410	2	1.39E-05	0.95	4.14	-	1142.4
AHQ-2-10, 3564	K.YKVCNYGLTFTQK.W	1623.85410	2	1.79E-09	0.93	4.12	-	1006.3
gi|5803181|ref|NP_006810.1| stress-induced-phosphoprotein 1 (Hsp70/Hsp90-organizing protein) [Homo				1.81E-09	0.93	10.23	3.50	62638.9
AHQ-2-6, 6485	K.ALSVGNIDDALQCYSEAIK.L	2069.27871	2	1.81E-09	0.93	4.38	-	785.9
AHQ-2-14-, 6348	K.ALSVGNIDDALQCYSEAIK.L	2069.27871	2	8.50E-07	0.92	4.40	-	690.6
AHQ-2-6, 6402 - 6478	K.ALSVGNIDDALQCYSEAIK.L	2069.27871	2	1.16E-08	0.94	4.64	-	876.8
gi|4503609|ref|NP_001976.1| electron-transfer-flavoprotein, beta polypeptide; electron-transferring				1.86E-09	1.91	20.23	12.50	27843.4
AHQ-2-10, 4776	K.HSMNPFCEIAVEEAVR.L	1891.11834	2	1.86E-09	0.96	4.59	-	1246.2
AHQ-2-10, 4188	K.IEVIKPGDLGVDLTSK.L	1684.95560	2	4.51E-07	0.94	4.12	-	853.0
gi|5031595|ref|NP_005709.1| actin related protein 2/3 complex subunit 4; Arp2/3 protein complex sub				1.95E-09	5.17	60.35	42.90	19666.9
AHQ-2-12, 4333	R.AENFFILR.R	1010.17070	2	5.60E-04	0.97	4.18	-	1810.6
AHQ-2-11, 6357	R.ATLQAALCLENFSSQVVER.H	2138.38741	2	1.92E-04	0.41	2.66	-	420.9
AHQ-2-11, 4105	K.ELLLQPVTISR.N	1269.51549	1	7.13E-04	0.44	2.47	-	171.7
AHQ-2-12, 4273 - 4281	K.ELLLQPVTISR.N	1269.51549	2	8.42E-05	0.92	3.90	-	846.6
AHQ-2-11, 5849	R.KPVEGYDISFLITNFHTEQMYK.H	2662.01271	3	5.29E-08	0.98	7.04	-	2055.4
AHQ-2-12, 5741	R.RKPVEGYDISFLITNFHTEQMYK.H	2818.19906	3	1.13E-04	0.97	6.68	-	1510.5
AHQ-2-12, 3191	K.VLIEGSINSVR.V	1187.37134	2	1.95E-09	0.92	3.34	-	1144.0
AHQ-2-12, 3109	K.VLIEGSINSVR.V	1187.37134	2	1.12E-08	0.90	3.07	-	1083.9
AHQ-2-11, 3062	K.VLIEGSINSVR.V	1187.37134	2	2.17E-07	0.93	3.30	-	1153.3
gi|21361198|ref|NP_000286.2| serine (or cysteine) proteinase inhibitor, clade A (alpha-1 antiprotei				1.95E-09	1.93	20.27	7.90	46722.2
AHQ-2-7, 3189 - 3263	K.LQHLENELTHDIITK.F	1805.02503	2	1.07E-08	0.97	5.37	-	1650.3
AHQ-2-7, 4543 - 4611	K.VFSNGADLSGVTEEAPLK.L	1835.00476	2	1.95E-09	0.95	4.60	-	1084.0
gi|5031571|ref|NP_005713.1| actin-related protein 2; ARP2 (actin-related protein 2, yeast) homolog				2.13E-09	6.88	80.23	24.10	44760.5
AHQ-2-8, 3583 - 3655	K.DLMVGDEASELR.S	1335.46633	2	8.36E-08	0.83	3.13	-	779.1
AHQ-2-8, 2838	R.GYAFNHSADFETVR.M	1614.69943	2	2.13E-09	0.93	3.95	-	1016.1
AHQ-2-8, 3475	K.HIVLSGGSTMYPGLPSR.L	1773.04967	2	2.83E-04	0.97	4.58	-	1584.6
AHQ-2-8, 3794	K.HLWDYTFGPEK.L	1393.52736	2	1.99E-06	0.90	3.68	-	626.8
AHQ-2-8, 3796 - 3798	K.HLWDYTFGPEK.L	1393.52736	1	4.64E-04	0.68	2.80	-	672.5
AHQ-2-8, 2544	R.KVVVCDNGTGFVK.C	1424.64764	2	1.39E-04	0.89	3.64	-	960.7
AHQ-2-8, 3642	K.LCYVGYNIEQEQK.L	1645.81494	2	3.38E-07	0.96	3.77	-	1514.5
AHQ-2-8, 4809	R.SMLEVNYPMENGIVR.N	1753.03703	2	4.61E-05	0.79	3.21	-	553.8
AHQ-2-8, 3004	K.VVVCDNGTGFVK.C	1296.47472	2	1.79E-06	0.61	2.61	-	384.5
gi|13518026|ref|NP_004978.2| LIM and senescent cell antigen-like domains 1 [Homo sapiens]				2.17E-09	1.92	20.24	7.70	37251.2
AHQ-2-8, 4164 - 4166	K.CHAIIDEQPLIFK.N	1585.84900	2	7.63E-09	0.94	4.78	-	1060.0
AHQ-2-11, 4361	K.CHAIIDEQPLIFK.N	1585.84900	2	1.07E-04	0.94	4.07	-	1381.5
AHQ-2-9, 4358 - 4370	K.CHAIIDEQPLIFK.N	1585.84900	2	5.58E-05	0.94	4.20	-	1282.1
AHQ-2-10, 4112	K.CHAIIDEQPLIFK.N	1585.84900	2	6.97E-05	0.96	4.67	-	1390.6
AHQ-2-8, 3111 - 3127	R.VIEGDVVSALNK.A	1244.41966	2	1.81E-08	0.96	4.15	-	1286.9
AHQ-2-11, 3384	R.VIEGDVVSALNK.A	1244.41966	2	2.91E-07	0.95	4.12	-	1206.5
AHQ-2-9, 3295	R.VIEGDVVSALNK.A	1244.41966	1	2.45E-05	0.41	2.82	-	328.1
AHQ-2-9, 3286	R.VIEGDVVSALNK.A	1244.41966	2	6.40E-09	0.96	4.49	-	1343.3
AHQ-2-13-, 3498 - 3575	R.VIEGDVVSALNK.A	1244.41966	2	1.39E-08	0.96	4.48	-	1193.7
AHQ-2-14-, 3521	R.VIEGDVVSALNK.A	1244.41966	2	2.17E-09	0.97	4.85	-	1680.5
gi|4503747|ref|NP_001448.1| filamin B, beta (actin binding protein 278); beta filamin; filamin 1 (a				2.33E-09	4.75	60.26	1.70	278191.4
AHQ-2-2, 3712	K.DLAEDAPWK.K	1045.12708	2	5.07E-04	0.89	3.43	-	909.8
AHQ-2-1, 3737 - 3738	K.DLAEDAPWK.K	1045.12708	2	3.03E-04	0.87	3.38	-	753.3
AHQ-2-3, 3648	K.DLAEDAPWK.K	1045.12708	1	4.43E-05	0.49	2.01	-	662.8
AHQ-2-5, 3445	K.DLAEDAPWK.K	1045.12708	1	3.24E-06	0.31	2.07	-	502.8
AHQ-2-4, 3650	K.DLAEDAPWK.K	1045.12708	1	1.15E-04	0.29	2.00	-	483.0
AHQ-2-2, 3706	K.DLAEDAPWK.K	1045.12708	1	1.17E-05	0.48	2.44	-	501.1
AHQ-2-2, 2896	K.DLAEDAPWKK.I	1173.29999	2	1.89E-04	0.76	2.88	-	882.0
AHQ-2-1, 3084 - 3085	K.DLAEDAPWKK.I	1173.29999	2	6.35E-04	0.57	2.78	-	669.4
AHQ-2-11, 6024	R.LIALLEVLSQK.R	1227.51852	2	5.68E-08	0.96	4.87	-	1046.3
AHQ-2-1, 6657 - 6658	R.LIALLEVLSQK.R	1227.51852	2	9.63E-07	0.97	4.54	-	1545.7
AHQ-2-6, 6281	R.LIALLEVLSQK.R	1227.51852	2	2.33E-09	0.98	4.98	-	1723.2
AHQ-2-3, 6616	R.LIALLEVLSQK.R	1227.51852	2	3.98E-06	0.97	4.68	-	1499.8
AHQ-2-2, 6604 - 6672	R.LIALLEVLSQK.R	1227.51852	2	2.03E-06	0.97	4.36	-	1375.1
AHQ-2-5, 6311 - 6381	R.LIALLEVLSQK.R	1227.51852	2	8.01E-07	0.97	5.15	-	1280.1
AHQ-2-4, 6672 - 6680	R.LIALLEVLSQK.R	1227.51852	2	1.02E-08	0.97	4.82	-	1341.0
AHQ-2-2, 3596 - 3604	R.LLGWIQNK.I	972.16560	2	4.22E-04	0.85	3.30	-	655.5
AHQ-2-1, 6653	R.QM*QLENVSVALEFLDR.E	1909.15348	2	1.81E-08	0.94	4.34	-	1311.5
AHQ-2-2, 6757 - 6829	R.QM*QLENVSVALEFLDR.E	1909.15348	2	5.37E-04	0.92	3.97	-	848.4
AHQ-2-2, 7380	R.QMQLENVSVALEFLDR.E	1893.15408	2	1.53E-04	0.91	3.88	-	899.6
gi|4507725|ref|NP_000362.1| transthyretin (prealbumin, amyloidosis type I); Transthyretin (prealbum				2.35E-09	3.75	40.33	36.70	15886.9
AHQ-2-13-, 3870	K.AADDTWEPFASGK.T	1395.45529	2	6.74E-09	0.92	4.12	-	782.3
AHQ-2-13, 3839	K.AADDTWEPFASGK.T	1395.45529	2	4.75E-08	0.92	3.51	-	1055.7
AHQ-2-12, 3729	K.AADDTWEPFASGK.T	1395.45529	2	5.74E-06	0.67	2.85	-	498.2
AHQ-2-13-, 4067 - 4075	R.GSPAINVAVHVFR.K	1367.58095	2	1.58E-08	0.96	4.54	-	1439.9
AHQ-2-13, 4013 - 4016	R.GSPAINVAVHVFR.K	1367.58095	2	8.33E-09	0.96	4.52	-	1246.1
AHQ-2-13, 5460	K.TSESGELHGLTTEEEFVEGIYK.V	2456.60073	2	1.31E-07	0.92	4.43	-	808.0
AHQ-2-13-, 5601 - 5603	K.TSESGELHGLTTEEEFVEGIYK.V	2456.60073	3	3.47E-09	0.98	6.56	-	2523.4
AHQ-2-12, 5577	K.TSESGELHGLTTEEEFVEGIYK.V	2456.60073	2	2.96E-07	0.93	4.33	-	889.4

AHQ-2-13, 5453	K.TSESGELHGLTTEEEFVEGIYK.V	2456.60073	3	6.79E-04	0.85	3.15	-	1568.8
AHQ-2-12, 5582	K.TSESGELHGLTTEEEFVEGIYK.V	2456.60073	3	2.35E-09	0.97	5.79	-	1797.4
AHQ-2-13-, 5609 - 5677	K.TSESGELHGLTTEEEFVEGIYK.V	2456.60073	2	1.73E-06	0.97	5.35	-	1514.5
AHQ-2-13, 6384	K.TSESGELHGLTTEEEFVEGIYKVEIDTK.S	3142.37019	3	9.96E-06	0.90	4.91	-	536.6
gi|4758442|ref|NP_004115.1| glia maturation factor, beta [Homo sapiens]				2.36E-09	1.76	20.25	35.20	16713.1
AHQ-2-12, 6218 - 6291	R.LVVLDEELEGISPDELKDELPER.Q	2638.90589	3	1.05E-05	0.93	4.90	-	1095.0
AHQ-2-12, 6865 - 6931	R.VSYPLCFIFSSPVGCKPEQQMMYAGSK.N	3115.61257	3	2.36E-09	0.84	4.16	-	414.2
gi|5453832|ref|NP_006380.1| oxygen regulated protein precursor; oxygen regulated protein (150kD) [H				2.38E-09	2.85	30.31	5.30	111334.8
AHQ-2-3, 6405	K.NINADEAAAMGAVYQAAALSK.A	2080.30817	2	6.59E-07	0.97	6.28	-	958.8
AHQ-2-3, 6861	R.VEFEELCADLFER.V	1658.81048	2	2.38E-09	0.95	4.19	-	1139.5
AHQ-2-3, 6768	K.VLQLINDNTATALSYGVFR.R	2096.37206	2	1.85E-07	0.93	3.69	-	1051.9
gi|4503631|ref|NP_000120.1| coagulation factor XIII A1 subunit precursor; Coagulation factor XIII,				2.60E-09	17.27	210.27	32.50	83232.8
AHQ-2-5, 5539 - 5616	R.AVPPNNSNAAEDDLPTVELQGVVPR.G	2603.82656	2	1.80E-08	0.70	3.38	-	224.7
AHQ-2-5, 5399 - 5471	R.AVPPNNSNAAEDDLPTVELQGVVPR.G	2603.82656	2	1.31E-07	0.59	2.89	-	228.8
AHQ-2-5, 2095 - 2096	R.CGPASVQAIK.H	1032.19627	2	2.81E-04	0.87	3.08	-	853.9
AHQ-2-6, 2386	R.CLGIPAR.I	787.95073	1	2.80E-04	0.43	2.19	-	209.2
AHQ-2-5, 2305	K.DGTHVVENVDATHIGK.L	1692.81163	2	2.60E-09	0.94	4.08	-	1144.1
AHQ-2-6, 2321	K.DGTHVVENVDATHIGK.L	1692.81163	2	9.02E-09	0.95	3.86	-	1388.1
AHQ-2-9, 2372	K.DGTHVVENVDATHIGK.L	1692.81163	2	4.89E-05	0.93	4.12	-	1021.3
AHQ-2-11, 4216	R.EIRPNSTVQWEEVCRPWVSGHR.K	2724.99524	3	1.36E-04	0.59	3.27	-	338.1
AHQ-2-5, 4445	R.EIRPNSTVQWEEVCRPWVSGHR.K	2724.99524	3	2.81E-04	0.78	3.29	-	652.6
AHQ-2-5, 4175	R.EIRPNSTVQWEEVCRPWVSGHR.K	2724.99524	3	9.59E-04	0.89	4.36	-	681.5
AHQ-2-11, 5493 - 5509	K.ETFDVTLEPLSFK.K	1526.71190	2	4.88E-06	0.90	3.67	-	853.1
AHQ-2-6, 5669 - 5677	K.ETFDVTLEPLSFK.K	1526.71190	2	2.11E-06	0.82	3.05	-	762.1
AHQ-2-5, 5751 - 5823	K.ETFDVTLEPLSFK.K	1526.71190	2	2.24E-08	0.89	3.78	-	836.9
AHQ-2-5, 4817	K.ETFDVTLEPLSFKK.E	1654.88482	2	4.26E-04	0.37	2.69	-	349.5
AHQ-2-7, 5391 - 5441	K.GTYIPVPIVSELQSGK.W	1688.94594	2	6.42E-06	0.65	3.19	-	263.4
AHQ-2-9, 5463 - 5478	K.GTYIPVPIVSELQSGK.W	1688.94594	2	3.93E-05	0.81	3.54	-	391.5
AHQ-2-5, 5523 - 5543	K.GTYIPVPIVSELQSGK.W	1688.94594	2	5.94E-04	0.86	3.64	-	493.7
AHQ-2-9, 7063	R.GVNLQEFLNVTSVHLFK.E	1946.23831	2	6.26E-08	0.95	4.41	-	1357.5
AHQ-2-9, 7076	R.GVNLQEFLNVTSVHLFK.E	1946.23831	3	1.56E-07	0.85	4.03	-	703.9
AHQ-2-10, 6495 - 6575	R.GVNLQEFLNVTSVHLFK.E	1946.23831	2	3.82E-05	0.91	4.32	-	861.5
AHQ-2-8, 6844	R.GVNLQEFLNVTSVHLFK.E	1946.23831	2	2.09E-06	0.94	4.34	-	1150.5
AHQ-2-7, 7036	R.GVNLQEFLNVTSVHLFK.E	1946.23831	2	7.74E-08	0.97	5.02	-	1522.6
AHQ-2-5, 7108 - 7125	R.GVNLQEFLNVTSVHLFK.E	1946.23831	2	8.75E-08	0.95	5.00	-	1085.3
AHQ-2-5, 7127 - 7164	R.GVNLQEFLNVTSVHLFK.E	1946.23831	3	8.27E-08	0.88	3.90	-	900.5
AHQ-2-5, 7191 - 7256	R.GVNLQEFLNVTSVHLFK.E	1946.23831	2	2.23E-08	0.96	4.60	-	1379.8
AHQ-2-5, 7324	R.GVNLQEFLNVTSVHLFK.E	1946.23831	2	2.91E-07	0.94	4.88	-	838.0
AHQ-2-14-, 6755	R.GVNLQEFLNVTSVHLFK.E	1946.23831	2	9.58E-07	0.96	5.00	-	1300.6
AHQ-2-11, 6565	R.GVNLQEFLNVTSVHLFK.E	1946.23831	2	1.35E-07	0.97	5.04	-	1431.3
AHQ-2-13, 6673	R.GVNLQEFLNVTSVHLFK.E	1946.23831	2	1.79E-08	0.97	5.24	-	1310.8
AHQ-2-13-, 6901	R.GVNLQEFLNVTSVHLFK.E	1946.23831	2	1.41E-07	0.97	4.48	-	1590.0
AHQ-2-6, 6989	R.GVNLQEFLNVTSVHLFK.E	1946.23831	3	3.16E-09	0.89	4.24	-	731.3
AHQ-2-2, 7437	R.GVNLQEFLNVTSVHLFK.E	1946.23831	2	1.18E-06	0.90	3.86	-	882.3
AHQ-2-6, 6978	R.GVNLQEFLNVTSVHLFK.E	1946.23831	2	8.78E-07	0.95	4.70	-	1107.0
AHQ-2-5, 3833	R.HVYGELDVQIQR.R	1457.61612	2	5.54E-05	0.80	2.93	-	573.2
AHQ-2-6, 1917	K.KDGTHVVENVDATHIGK.L	1820.98455	2	9.20E-08	0.95	4.50	-	1362.3
AHQ-2-5, 2100	K.KDGTHVVENVDATHIGK.L	1820.98455	3	6.44E-06	0.95	4.86	-	1605.4
AHQ-2-5, 1903	K.KDGTHVVENVDATHIGK.L	1820.98455	2	2.89E-07	0.96	4.18	-	1656.0
AHQ-2-5, 2367	R.KLIASMSSDSLR.H	1308.53039	2	3.36E-09	0.96	4.23	-	1420.7
AHQ-2-6, 4510 - 4527	R.LALETALMYGAK.K	1281.54655	2	9.69E-07	0.97	4.04	-	1762.9
AHQ-2-5, 2749 - 2829	K.LIASMSSDSLR.H	1180.35748	2	2.45E-05	0.95	3.80	-	1196.8
AHQ-2-5, 5473	R.M*YVAVWTPYGVLR.T	1571.86734	2	1.03E-05	0.97	5.12	-	1150.7
AHQ-2-5, 6093 - 6160	R.MYVAVWTPYGVLR.T	1555.86794	2	1.07E-06	0.95	4.34	-	998.6
AHQ-2-6, 5441 - 5446	R.M*YVAVWTPYGVLR.T	1571.86734	2	2.13E-04	0.93	3.58	-	944.9
AHQ-2-6, 6046 - 6053	R.MYVAVWTPYGVLR.T	1555.86794	2	3.04E-08	0.95	4.10	-	1100.7
AHQ-2-7, 6725	R.NPETDTYILFNPWCEDDAVYLDNEKER.E	3349.53903	3	4.37E-04	0.70	3.07	-	868.5
AHQ-2-5, 6979	R.NPETDTYILFNPWCEDDAVYLDNEKER.E	3349.53903	3	4.62E-07	0.88	4.22	-	564.7
AHQ-2-5, 6819	R.NPETDTYILFNPWCEDDAVYLDNEKER.E	3349.53903	3	9.09E-09	0.94	5.48	-	841.5
AHQ-2-5, 3329 - 3332	R.NVWVHLDGPGVTR.P	1450.62682	2	3.15E-04	0.68	2.63	-	817.2
AHQ-2-5, 3297	R.NVWVHLDGPGVTRPMK.K	1807.11248	2	8.39E-06	0.94	4.56	-	883.5
AHQ-2-5, 4637	R.SNVDM*DFEVENAVLGK.D	1783.93809	2	1.27E-08	0.96	4.62	-	1715.2
AHQ-2-11, 5486	K.STVLTIPEIIIK.V	1327.63485	2	1.93E-07	0.89	3.38	-	1142.7
AHQ-2-9, 5580	K.STVLTIPEIIIK.V	1327.63485	2	5.16E-04	0.83	2.81	-	1157.5
AHQ-2-8, 5275	K.STVLTIPEIIIK.V	1327.63485	2	8.06E-04	0.92	3.52	-	1290.3
gi|15147250|ref|NP_150281.1| protein phosphatase 1, regulatory (inhibitor) subunit 14A; 17-kDa PKC-				2.66E-09	0.85	10.23	10.90	16692.9
AHQ-2-12, 6919	K.SCGKPVEDFIQELLAK.L	1836.09909	2	2.66E-09	0.85	4.55	-	503.4
gi|4505989|ref|NP_000299.1| protective protein for beta-galactosidase; Protective protein for beta-				2.68E-09	1.73	20.21	6.90	54495.8
AHQ-2-10, 5068	K.CNFYDNKDLECVTNLQEVAR.I	2491.69642	2	1.54E-05	0.75	2.77	-	873.4
AHQ-2-12, 3494 - 3507	K.YGDSGEQIAGFVK.E	1371.47686	2	2.68E-09	0.98	4.14	-	2739.3
AHQ-2-11, 3400	K.YGDSGEQIAGFVK.E	1371.47686	2	5.32E-06	0.97	3.97	-	2410.8
gi|5803227|ref|NP_006817.1| tyrosine 3/tryptophan 5 -monooxygenase activation protein, theta polype				2.68E-09	3.63	40.26	23.30	27764.1
AHQ-2-12, 2210	K.AVTEQGAELSNEER.N	1533.57979	2	2.18E-06	0.80	3.48	-	721.0
AHQ-2-10, 2086	K.AVTEQGAELSNEER.N	1533.57979	2	2.68E-09	0.96	5.02	-	1292.1
AHQ-2-10, 6663	R.SICTTVLELLDK.Y	1393.62862	2	9.15E-09	0.94	4.46	-	878.8
AHQ-2-10, 6411	K.TAFDEAIAELDTLNEDSYK.D	2146.25037	2	1.47E-08	0.96	5.15	-	1365.0
AHQ-2-14-, 6664	K.TAFDEAIAELDTLNEDSYK.D	2146.25037	2	5.89E-07	0.94	4.81	-	834.4
AHQ-2-10, 2574	K.YLIANATNPESK.V	1321.46099	1	3.53E-09	0.77	2.61	-	808.5
AHQ-2-10, 2435	K.YLIANATNPESK.V	1321.46099	2	5.57E-06	0.96	3.79	-	1723.0
gi|13489087|ref|NP_109591.1| serine (or cysteine) proteinase inhibitor, clade B (ovalbumin), member				2.69E-09	1.78	20.20	8.70	42741.5
AHQ-2-9, 3892	K.TYGADLASVDFQHASEDAR.K	2054.11949	2	1.36E-07	0.89	3.27	-	942.9
AHQ-2-11, 3896	K.TYGADLASVDFQHASEDAR.K	2054.11949	2	2.69E-09	0.92	4.01	-	858.7
AHQ-2-13, 4051	K.TYGADLASVDFQHASEDAR.K	2054.11949	2	1.12E-06	0.77	3.15	-	275.1
AHQ-2-13-, 4107	K.TYGADLASVDFQHASEDAR.K	2054.11949	2	3.87E-06	0.72	3.41	-	374.8
AHQ-2-8, 3758	K.TYGADLASVDFQHASEDAR.K	2054.11949	2	2.57E-05	0.79	3.18	-	648.5
AHQ-2-13, 6133	K.TYNFLPEFLVSTQK.T	1687.91650	2	7.49E-06	0.86	3.74	-	453.3
gi|7706497|ref|NP_057392.1| UMP-CMP kinase [Homo sapiens]				2.77E-09	2.82	30.27	20.20	25854.7
AHQ-2-11, 5953	R.IQTYLQSTKPIIDLYEEMGK.V	2371.73502	3	4.54E-04	0.74	3.32	-	695.6
AHQ-2-11, 5948 - 5949	R.IQTYLQSTKPIIDLYEEMGK.V	2371.73502	2	4.30E-07	0.96	5.46	-	802.4
AHQ-2-11, 2501	R.KNPDSQYGELIEK.Y	1521.65375	2	8.97E-05	0.89	3.62	-	914.1
AHQ-2-11, 3649	K.YGYTHLSAGELLR.D	1480.64969	2	2.77E-09	0.96	4.02	-	1673.1
gi|4507951|ref|NP_003396.1| tyrosine 3/tryptophan 5 -monooxygenase activation protein, eta polypept				2.81E-09	5.31	60.22	34.10	28218.5
AHQ-2-13-, 2958	K.AVTELNEPLSNEDR.N	1587.67060	2	1.01E-08	0.89	3.89	-	643.0
AHQ-2-13, 3007	K.AVTELNEPLSNEDR.N	1587.67060	2	6.02E-09	0.93	4.12	-	781.8
AHQ-2-10, 2694	K.AVTELNEPLSNEDR.N	1587.67060	2	6.80E-06	0.94	4.38	-	895.1
AHQ-2-10, 6622	K.ELETVCNDVLSLLDK.F	1749.96137	2	4.48E-06	0.75	3.54	-	513.8
AHQ-2-13, 6708	K.ELETVCNDVLSLLDK.F	1749.96137	2	9.89E-09	0.89	3.97	-	670.6
AHQ-2-14-, 6784	K.ELETVCNDVLSLLDK.F	1749.96137	2	4.26E-07	0.91	4.05	-	706.6
AHQ-2-14-, 5981	K.ELETVCNDVLSLLDK.F	1749.96137	2	5.70E-07	0.86	3.50	-	564.0
AHQ-2-10, 3888	K.FLIKNCNDFQYESK.V	1808.00430	2	5.40E-04	0.89	3.60	-	787.8
AHQ-2-10, 2556	K.NSVVEASEAAYK.E	1268.35495	1	4.48E-04	0.51	2.02	-	528.8
AHQ-2-10, 2350	K.NSVVEASEAAYK.E	1268.35495	2	2.81E-09	0.95	4.26	-	1021.8
AHQ-2-10, 2346 - 2354	K.NSVVEASEAAYK.E	1268.35495	1	1.37E-04	0.20	2.01	-	276.5
AHQ-2-10, 2772	K.NSVVEASEAAYK.E	1268.35495	1	3.29E-04	0.21	2.17	-	253.0
AHQ-2-14-, 6113	K.QAFDDAIAELDTLNEDSYK.D	2159.24915	2	3.04E-05	0.75	3.26	-	511.2
AHQ-2-10, 5846	K.QAFDDAIAELDTLNEDSYK.D	2159.24915	2	7.58E-07	0.80	3.88	-	421.2
AHQ-2-10, 1979	R.YLAEVASGEK.K	1067.17419	1	8.14E-06	0.74	3.00	-	512.6

AHQ-2-10, 1986	R.YLAEVASGEK.K	1067.17419	2	4.22E-06	0.86	3.15	-	945.5
gi|4502695|ref|NP_001779.1| cell division cycle 10; cell division cycle 10 (homolog to CDC10 of S.c				2.94E-09	2.45	30.25	13.60	48786.6
AHQ-2-8, 3708	K.IYEFPETDDEEENKLVK.K	2099.23685	2	2.69E-04	0.71	3.33	-	348.7
AHQ-2-13, 5393	K.NLEGYVGFANLPNQVYR.K	1955.16216	2	8.38E-05	0.91	4.21	-	729.8
AHQ-2-14-, 5393 - 5465	K.NLEGYVGFANLPNQVYR.K	1955.16216	2	4.93E-07	0.91	3.92	-	882.7
AHQ-2-13-, 5494	K.NLEGYVGFANLPNQVYR.K	1955.16216	2	4.25E-07	0.95	4.87	-	977.2
AHQ-2-8, 5220	K.NLEGYVGFANLPNQVYR.K	1955.16216	2	1.82E-08	0.95	5.08	-	1086.5
AHQ-2-14-, 6299	K.STLINSLFLTDLYSPEYPGPSHR.I	2608.88640	3	3.33E-09	0.63	3.08	-	315.8
AHQ-2-14-, 6531	K.STLINSLFLTDLYSPEYPGPSHR.I	2608.88640	3	2.94E-09	0.78	3.35	-	629.2
AHQ-2-13, 6468	K.STLINSLFLTDLYSPEYPGPSHR.I	2608.88640	3	7.05E-04	0.53	3.13	-	453.2
gi|6912638|ref|NP_036557.1| ras suppressor protein 1 [Homo sapiens]				2.97E-09	6.21	80.29	48.70	31540.1
AHQ-2-12, 7181 - 7185	K.AENNPWVTPIADQFQLGVSHVFEYIR.S	3032.35678	3	7.70E-08	0.81	3.69	-	503.4
AHQ-2-10, 5900 - 5958	R.ALYLSDNDFEILPPDIGK.L	2021.25559	2	2.06E-07	0.95	4.89	-	587.5
AHQ-2-10, 6010 - 6071	R.ALYLSDNDFEILPPDIGK.L	2021.25559	2	2.97E-09	0.87	4.03	-	464.7
AHQ-2-10, 1474 - 1536	K.ELHIQGNR.L	967.06432	1	2.08E-04	0.17	2.48	-	136.0
AHQ-2-14-, 6877	R.GISNM*LDVNGLFTLSHITQLVLSHNK.L	2869.28903	3	5.83E-04	0.80	3.73	-	708.7
AHQ-2-10, 6754	R.GISNMLDVNGLFTLSHITQLVLSHNK.L	2853.28963	2	4.42E-08	0.82	3.89	-	314.6
AHQ-2-10, 5507 - 5576	K.LQILSLRDNDLISLPK.E	1839.16919	2	6.16E-08	0.95	5.06	-	1562.1
AHQ-2-10, 5408 - 5431	K.LQILSLRDNDLISLPK.E	1839.16919	2	3.78E-09	0.95	4.93	-	1538.4
AHQ-2-10, 5536 - 5598	R.LTVLPPELGNLDLTGQK.Q	1809.09672	2	5.70E-06	0.94	4.46	-	768.4
AHQ-2-11, 5669 - 5728	R.LTVLPPELGNLDLTGQK.Q	1809.09672	2	8.69E-07	0.91	4.17	-	602.1
AHQ-2-10, 5594	R.LTVLPPELGNLDLTGQK.Q	1809.09672	3	8.03E-07	0.96	5.83	-	1156.0
AHQ-2-10, 5658 - 5727	R.LTVLPPELGNLDLTGQK.Q	1809.09672	2	3.96E-05	0.92	4.06	-	595.6
AHQ-2-12, 6013 - 6078	R.LTVLPPELGNLDLTGQK.Q	1809.09672	2	2.83E-07	0.88	3.68	-	551.5
AHQ-2-14-, 5896 - 5969	R.LTVLPPELGNLDLTGQK.Q	1809.09672	2	2.29E-04	0.87	4.01	-	456.6
AHQ-2-11, 6764 - 6766	K.NLEVLNFFNNQIEELPTQISSLQK.L	2820.14663	2	7.80E-09	0.90	4.60	-	606.0
AHQ-2-14-, 6873 - 6943	K.NLEVLNFFNNQIEELPTQISSLQK.L	2820.14663	3	3.33E-05	0.90	5.00	-	550.1
AHQ-2-10, 6648 - 6719	K.NLEVLNFFNNQIEELPTQISSLQK.L	2820.14663	2	4.99E-08	0.87	4.48	-	390.3
gi|21735621|ref|NP_005909.2| mitochondrial malate dehydrogenase precursor [Homo sapiens]				3.20E-09	5.00	60.27	32.50	35503.0
AHQ-2-9, 6462 - 6482	K.GYLGPEQLPDCLKGCDVVVIPAGVPR.K	2813.24211	3	6.91E-04	0.78	4.06	-	512.2
AHQ-2-9, 6467	K.GYLGPEQLPDCLKGCDVVVIPAGVPR.K	2813.24211	2	2.25E-07	0.90	4.69	-	376.8
AHQ-2-13, 6123	K.GYLGPEQLPDCLKGCDVVVIPAGVPR.K	2813.24211	3	2.36E-04	0.63	3.35	-	493.8
AHQ-2-9, 5560	R.LTLYDIAHTPGVAADLSHIETK.A	2366.65506	3	7.56E-09	0.96	5.49	-	1424.9
AHQ-2-9, 5554	R.LTLYDIAHTPGVAADLSHIETK.A	2366.65506	2	2.96E-06	0.56	2.76	-	324.9
AHQ-2-11, 5389	K.SQETECTYFSTPLLLGK.K	1976.19328	2	1.18E-07	0.96	4.84	-	1112.4
AHQ-2-9, 5527	K.SQETECTYFSTPLLLGK.K	1976.19328	2	2.54E-04	0.92	3.81	-	1117.7
AHQ-2-9, 6779 - 6782	K.VAVLGASGGIGQPLSLLLK.N	1794.17156	2	2.05E-04	0.96	5.35	-	1036.2
AHQ-2-9, 4986	K.VDFPQDQLTALTGR.I	1561.72111	2	3.20E-09	0.81	2.75	-	963.7
AHQ-2-9, 2284	R.VNVPVIGGHAGK.T	1148.34004	1	1.80E-05	0.42	2.29	-	429.5
gi|4557675|ref|NP_000201.1| integrin alpha chain, alpha 6 [Homo sapiens]				3.26E-09	3.35	40.26	5.00	119509.5
AHQ-2-4, 6025	K.DFHYIVFGAPGTYNWK.G	1916.12609	2	1.46E-07	0.97	4.84	-	1538.9
AHQ-2-4, 3456 - 3464	K.FGSCQQGVAATFTK.D	1503.66172	2	3.26E-09	0.95	4.01	-	1212.9
AHQ-2-4, 3574	K.FSYLPIQK.G	996.18393	1	4.58E-04	0.50	2.07	-	645.5
AHQ-2-4, 4912	R.IEDDMDGGDWSFCDGR.L	1876.91588	2	2.01E-04	0.94	3.56	-	1452.9
gi|18860900|ref|NP_002834.2| protein tyrosine phosphatase, receptor type, J precursor; protein tyro				3.30E-09	13.37	150.25	16.60	145926.2
AHQ-2-1, 3505	R.AGSPTAPVHDESLVGPVDPSSGQQSR.D	2576.71806	2	2.67E-07	0.84	3.53	-	557.1
AHQ-2-1, 5976 - 5993	K.AVSISPTNVILTWK.S	1529.80530	2	3.04E-04	0.88	3.33	-	674.0
AHQ-2-1, 4476	K.AYAVILTTGEAGHPSADVLK.Y	2014.26782	3	3.30E-09	0.97	5.01	-	1831.7
AHQ-2-1, 5108	R.DTEVLLVGLEPGTR.Y	1499.69122	2	8.77E-04	0.65	2.86	-	632.5
AHQ-2-1, 2813 - 2820	K.FSGFEASHGPIK.A	1277.40990	2	4.32E-07	0.90	3.51	-	851.6
AHQ-2-1, 5366	K.GLIDGAESYVSFSR.Y	1501.62253	2	6.77E-09	0.88	3.18	-	1159.7
AHQ-2-1, 4885	K.GLIDGAESYVSFSR.Y	1501.62253	2	3.07E-07	0.89	3.31	-	973.2
AHQ-2-1, 3977	K.KDFIATQGPLPNTLK.D	1643.90840	2	6.03E-06	0.95	4.23	-	1403.7
AHQ-2-1, 5041	K.SFCTDPASM*ASFDCEVVPK.E	2167.37954	2	5.68E-05	0.94	4.27	-	942.1
AHQ-2-1, 5890	K.SFCTDPASMASFDCEVVPK.E	2151.38014	2	7.96E-04	0.87	3.72	-	631.6
AHQ-2-1, 5468	K.SPDGASEYVYHLVIESK.H	1895.05866	2	1.21E-08	0.95	4.79	-	1153.7
AHQ-2-1, 2754	K.TKGDPLGTEGGLDASNTER.S	1918.99702	2	7.65E-07	0.93	4.48	-	847.6
AHQ-2-1, 3741	K.TPSSTGPSPVFDIK.A	1433.58810	2	7.44E-05	0.87	3.15	-	1000.3
AHQ-2-1, 4560	R.VENFEAYFK.K	1147.26100	2	1.33E-05	0.93	3.93	-	748.8
AHQ-2-1, 4384	K.VITEPIPVSDLR.V	1339.56259	2	2.17E-05	0.87	3.50	-	623.1
AHQ-2-1, 3920	R.VLLESIGSHEELTQDSR.L	1914.06352	2	5.68E-09	0.93	4.35	-	883.5
AHQ-2-1, 3926	R.VLLESIGSHEELTQDSR.L	1914.06352	3	2.66E-06	0.92	4.18	-	971.2
AHQ-2-1, 4004	R.VLLESIGSHEELTQDSR.L	1914.06352	2	7.15E-04	0.77	3.03	-	670.9
gi|8922673|ref|NP_060692.1| parvin, alpha; alpha-parvin; actopaxin [Homo sapiens]				3.31E-09	1.74	20.21	5.60	42243.4
AHQ-2-13, 4647 - 4725	R.DAFDTLFDHAPDK.L	1492.57096	2	1.92E-07	0.94	4.02	-	1067.2
AHQ-2-9, 4519	R.DAFDTLFDHAPDK.L	1492.57096	2	3.31E-09	0.96	4.27	-	1422.8
AHQ-2-9, 4526	R.DAFDTLFDHAPDK.L	1492.57096	1	3.11E-08	0.87	3.79	-	592.3
AHQ-2-13-, 4726	R.DAFDTLFDHAPDK.L	1492.57096	2	6.42E-09	0.95	4.06	-	1520.2
AHQ-2-13-, 4190	R.VLYNLFTK.Y	998.19983	2	2.95E-04	0.81	2.80	-	463.5
AHQ-2-9, 3854	R.VLYNLFTK.Y	998.19983	1	3.65E-04	0.34	2.32	-	397.2
AHQ-2-9, 3718	R.VLYNLFTK.Y	998.19983	2	3.22E-05	0.78	2.69	-	435.9
gi|4504743|ref|NP_002194.1| integrin alpha 2 precursor; Integrin, alpha-2 (CD49B; alpha-2 subunit o				3.44E-09	3.96	50.24	7.40	129294.4
AHQ-2-3, 4661	K.EKNPLMYLTGVQTDK.A	1737.99886	2	1.03E-04	0.36	2.66	-	317.5
AHQ-2-3, 6851	K.GNWLLVGSPWSGFPENR.M	1917.11562	2	1.27E-04	0.91	4.22	-	662.3
AHQ-2-4, 4876	K.KGILGQHQFLEGPEGIENTR.F	2224.46193	2	1.73E-06	0.95	4.81	-	813.3
AHQ-2-3, 3312	K.TQVGLIQYANNPR.V	1474.64668	2	9.58E-06	0.90	3.03	-	1303.8
AHQ-2-4, 5304	R.VDISLENPGTSPALEAYSETAK.V	2293.46974	2	3.44E-09	0.84	3.41	-	698.3
AHQ-2-3, 5288	R.VDISLENPGTSPALEAYSETAK.V	2293.46974	2	1.91E-04	0.92	4.17	-	803.3
gi|4507143|ref|NP_003786.1| sorting nexin 3 isoform a; sorting nexin 3A [Homo sapiens]				3.70E-09	1.86	20.22	16.70	18762.2
AHQ-2-12, 4594	R.CLHM*FLQDEIIDK.S	1679.93944	2	3.39E-04	0.91	3.73	-	1259.3
AHQ-2-12, 5117 - 5119	R.GDDGIFDDNFIEER.K	1642.66158	2	3.70E-09	0.95	4.49	-	1081.3
gi|20070156|ref|NP_003565.2| vessicle-associated membrane protein (VAMP)-associated protein of 33 k				3.76E-09	1.86	20.19	5.80	27317.4
AHQ-2-12, 4267	K.FKGPFTDVVTTNLK.L	1567.81039	2	3.75E-04	0.91	3.42	-	1070.1
AHQ-2-12, 4098 - 4105	K.GPFTDVVTTNLK.L	1292.46278	2	3.76E-09	0.95	3.82	-	1561.4
gi|14149734|ref|NP_065174.1| coronin, actin binding protein, 1B [Homo sapiens]				4.17E-09	0.95	10.27	5.90	54234.4
AHQ-2-7, 7101	R.KSDLFQDDLYPDTAGPEAALEAEEWVSGR.D	3211.39431	3	2.70E-07	0.93	4.82	-	1011.5
AHQ-2-6, 6967 - 6986	R.KSDLFQDDLYPDTAGPEAALEAEEWVSGR.D	3211.39431	3	4.17E-09	0.95	5.49	-	1226.1
gi|29727565|ref|XP_291384.1| similar to Cytochrome c, somatic [Homo sapiens]				4.20E-09	3.62	40.29	17.10	11621.4
AHQ-2-13, 6344 - 6423	K.GIIWGEDTLM*EYLENPK.K	2025.26771	2	1.53E-04	0.96	4.41	-	1645.1
AHQ-2-13-, 6946	K.GIIWGEDTLMEYLENPK.K	2009.26831	2	7.59E-07	0.97	5.77	-	1792.2
AHQ-2-13-, 6746	K.GIIWGEDTLMEYLENPKK.Y	2137.44123	2	4.20E-09	0.97	4.82	-	1492.7
AHQ-2-13-, 6751	K.GIIWGEDTLMEYLENPKK.Y	2137.44123	3	2.39E-07	0.72	3.20	-	439.7
AHQ-2-13, 6523	K.GIIWGEDTLMEYLENPKK.Y	2137.44123	2	6.39E-05	0.92	4.22	-	754.6
AHQ-2-13, 5981	K.GIIWGEDTLM*EYLENPKK.Y	2153.44063	2	3.23E-06	0.72	3.34	-	501.0
gi|7657486|ref|NP_055217.1| low molecular mass ubiquinone-binding protein [Homo sapiens]				4.55E-09	0.95	10.21	14.00	11316.3
AHQ-2-14-, 3871	R.HVISYSLSPFEQR.A	1563.73870	2	4.55E-09	0.95	4.25	-	1067.1
gi|4502013|ref|NP_001616.1| adenylate kinase 2 isoform a; adenylate kinase, mitochondrial; adenylat				4.76E-09	3.65	40.29	34.70	26477.6
AHQ-2-10, 6814 - 6815	R.GIHSAIDASQTPDVVFASILAAFSK.A	2546.85974	2	3.09E-04	0.97	5.81	-	1435.4
AHQ-2-10, 3632	R.LQAYHTQTTPLIEYYR.K	1998.22695	3	2.22E-06	0.61	3.42	-	666.3
AHQ-2-10, 3618	R.LQAYHTQTTPLIEYYR.K	1998.22695	2	7.51E-08	0.97	5.25	-	1724.3
AHQ-2-10, 5670	K.NLETPLCKNGFLLDGFPR.T	2093.39146	2	4.76E-09	0.82	3.29	-	653.0
AHQ-2-10, 3807	R.SYHEEFNPPKEPMKDDITGEPLIR.R	2844.14855	2	5.43E-04	0.89	3.66	-	736.3
gi|29742827|ref|XP_294099.1| similar to myosin:SUBUNIT=regulatory light chain [Homo sapiens]				5.08E-09	2.63	30.22	14.20	17094.0
AHQ-2-11, 2890	K.EAFNM*IDQNR.D	1254.35498	2	3.93E-04	0.82	3.20	-	710.8
AHQ-2-11, 2828 - 2882	K.EAFNMIDQNR.D	1238.35558	2	1.34E-04	0.84	3.44	-	700.7
AHQ-2-12, 2927 - 2935	K.EAFNMIDQNR.D	1238.35558	2	8.03E-04	0.68	2.73	-	663.2
AHQ-2-10, 3639	R.FTDEEVDELYR.E	1416.47127	1	3.43E-06	0.39	2.00	-	441.8

AHQ-2-11, 3636 - 3712	R.FTDEEVDELYR.E	1416.47127	2	2.18E-08	0.97	4.37	-	1989.9
AHQ-2-11, 3716 - 3780	R.FTDEEVDELYR.E	1416.47127	1	4.48E-07	0.32	1.89	-	491.8
AHQ-2-11, 3722 - 3784	R.FTDEEVDELYR.E	1416.47127	1	1.50E-08	0.42	2.32	-	369.8
AHQ-2-11, 3792	R.FTDEEVDELYR.E	1416.47127	2	5.08E-09	0.97	4.44	-	1794.9
AHQ-2-10, 3630	R.FTDEEVDELYR.E	1416.47127	2	3.12E-04	0.92	3.51	-	802.0
AHQ-2-12, 3849 - 3850	R.FTDEEVDELYR.E	1416.47127	2	4.78E-07	0.96	4.19	-	1563.1
AHQ-2-13, 3828 - 3899	R.FTDEEVDELYR.E	1416.47127	2	5.51E-06	0.93	2.92	-	1580.2
AHQ-2-14, 4754	R.FTDEEVDELYR.E	1416.47127	2	7.07E-09	0.96	3.99	-	1625.0
AHQ-2-13-, 3937 - 3942	R.FTDEEVDELYR.E	1416.47127	2	1.23E-07	0.97	3.99	-	1896.2
AHQ-2-14-, 3839	R.FTDEEVDELYR.E	1416.47127	2	4.85E-07	0.96	4.02	-	1469.3
AHQ-2-14-, 3845	R.FTDEEVDELYR.E	1416.47127	1	7.34E-07	0.29	1.94	-	283.7
gi|9966825|ref|NP_065089.1| HEF like Protein [Homo sapiens]				5.08E-09	1.89	20.28	4.30	86696.2
AHQ-2-13-, 4233	K.AISAFHGSLSSSQPAEIITQSK.L	2260.48935	2	1.75E-07	0.97	5.50	-	1530.1
AHQ-2-13-, 3454	K.LVDTLCMETQER.D	1496.68930	2	5.08E-09	0.92	3.60	-	877.3
gi|14149680|ref|NP_056107.1| KIAA0747 protein [Homo sapiens]				5.28E-09	2.71	30.19	4.30	122855.6
AHQ-2-12, 5591	R.FEWELPLDEAQR.R	1533.66622	2	5.28E-09	0.88	3.56	-	596.9
AHQ-2-7, 7067	R.LLVPLVPDLQDVAQLR.S	1790.13977	2	1.99E-07	0.90	3.66	-	617.0
AHQ-2-6, 6998	R.LLVPLVPDLQDVAQLR.S	1790.13977	2	5.21E-07	0.86	3.38	-	532.1
AHQ-2-4, 7384	R.LLVPLVPDLQDVAQLR.S	1790.13977	2	1.01E-06	0.70	2.65	-	451.8
AHQ-2-12, 4022	R.LTHVDSPLEAPAGPLGQVK.L	1930.19405	2	2.39E-04	0.93	3.89	-	1141.9
gi|4506405|ref|NP_002872.1| v-ral simian leukemia viral oncogene homolog B; RAS-like protein B; GTP				5.40E-09	2.82	30.28	15.50	23408.4
AHQ-2-10, 3560	K.AEEDKIPLLVVGNK.S	1525.77182	2	2.21E-07	0.90	3.40	-	864.5
AHQ-2-10, 3322	R.SKAEEWGVQYVETSAK.T	1812.95765	3	3.08E-07	0.94	4.04	-	1147.8
AHQ-2-10, 3303	R.SKAEEWGVQYVETSAK.T	1812.95765	2	5.40E-09	0.98	5.61	-	1698.4
AHQ-2-10, 3152	R.VKAEEDKIPLLVVGNK.S	1753.07630	2	6.40E-05	0.95	4.87	-	927.7
gi|5031931|ref|NP_005585.1| nascent-polypeptide-associated complex alpha polypeptide [Homo sapiens]				5.64E-09	0.94	10.20	6.50	23383.7
AHQ-2-13, 4035	K.SPASDTYIVFGEAK.I	1485.61987	2	5.64E-09	0.94	4.07	-	1076.5
gi|4507789|ref|NP_003338.1| ubiquitin-conjugating enzyme E2L 3 [Homo sapiens]				5.69E-09	0.93	10.19	9.70	17861.5
AHQ-2-12, 5279 - 5299	R.IEINFPAEYPFKPPK.I	1791.08155	3	5.69E-09	0.93	3.87	-	1345.4
AHQ-2-12, 5217 - 5294	R.IEINFPAEYPFKPPK.I	1791.08155	2	2.29E-06	0.80	3.65	-	581.7
gi|21361785|ref|NP_543010.2| histidyl-tRNA synthetase 2; histidyl-tRNA synthetase; bA379J5.3; chrom				5.94E-09	0.98	10.30	12.70	24013.4
AHQ-2-10, 6635	K.GNKPDFHLAMPTEQAEGFYNSFLEQLR.K	3141.46192	3	5.94E-09	0.98	6.09	-	2102.2
gi|4758460|ref|NP_004479.1| glycoprotein V (platelet) [Homo sapiens]				6.02E-09	2.95	40.27	14.80	60958.9
AHQ-2-5, 5487	R.CAGPGAHAGLPLWALPGGDAECPGPR.G	2587.87539	3	3.63E-05	0.78	4.10	-	594.2
AHQ-2-1, 5861	R.CAGPGAHAGLPLWALPGGDAECPGPR.G	2587.87539	3	5.84E-05	0.75	3.87	-	532.1
AHQ-2-5, 4611	R.GVLQSQSFSGMTVLQR.L	1738.99008	2	2.19E-06	0.96	4.25	-	1895.0
AHQ-2-5, 3732	R.LTEVLLGHNSWR.C	1425.61726	1	4.06E-05	0.25	2.16	-	201.3
AHQ-2-5, 7575 - 7616	K.LVNLQELALNQNQLDFLPASLFTNLENLK.L	3314.77659	3	6.02E-09	0.95	5.33	-	1353.9
gi|17149842|ref|NP_004461.2| FK506-binding protein 2 precursor; FK506-binding protein 2 (13kD); pep				6.04E-09	2.25	30.18	19.00	15649.2
AHQ-2-13-, 5058	K.GWDQGLLGMCEGEK.R	1581.75275	2	1.89E-07	0.86	3.24	-	869.8
AHQ-2-13-, 4469	K.GWDQGLLGMCEGEKR.K	1737.93910	2	6.13E-04	0.60	2.80	-	384.9
AHQ-2-13-, 4229 - 4241	K.LVIPSELGYGER.G	1333.51491	2	6.04E-09	0.78	2.76	-	519.2
AHQ-2-13, 4183	K.LVIPSELGYGER.G	1333.51491	2	2.32E-07	0.73	2.90	-	399.9
gi|4502565|ref|NP_001740.1| calpain, small subunit 1; calpain, small polypeptide; calcium-dependent				6.05E-09	2.59	30.23	10.80	28315.5
AHQ-2-12, 2874	K.TDGFGIDTCR.S	1143.20921	2	4.77E-04	0.73	3.04	-	412.2
AHQ-2-12, 6785	R.YSDESGNM*DFDNFISCLVR.L	2287.42568	2	6.05E-09	0.93	4.53	-	1133.3
AHQ-2-12, 7061	R.YSDESGNMDFDNFISCLVR.L	2271.42628	2	3.60E-08	0.93	4.52	-	933.3
AHQ-2-13, 6588	R.YSDESGNM*DFDNFISCLVR.L	2287.42568	2	1.38E-04	0.74	3.16	-	588.0
AHQ-2-14-, 6596 - 6663	R.YSDESGNM*DFDNFISCLVR.L	2287.42568	2	2.68E-08	0.87	3.82	-	808.1
AHQ-2-11, 6473	R.YSDESGNM*DFDNFISCLVR.L	2287.42568	2	2.93E-05	0.81	3.56	-	610.3
gi|4504521|ref|NP_002147.1| heat shock 60kDa protein 1 (chaperonin); mitochondrial matrix protein P				6.11E-09	2.73	40.31	12.90	61024.2
AHQ-2-14-, 6907	R.ALM*LQGVDLLADAVAVTMGPK.G	2130.55828	2	6.11E-09	0.98	6.22	-	2455.4
AHQ-2-7, 3871 - 3876	R.GYISPYFINTSK.G	1390.56492	1	2.60E-05	0.22	2.22	-	290.4
AHQ-2-14-, 6979 - 6980	R.TALLDAAGVASLLTTAEVVVTEIPKEEK.D	2870.28530	3	3.25E-04	0.68	3.32	-	546.0
AHQ-2-7, 5643	K.TLNDELEIIEGMK.F	1505.71604	2	4.60E-04	0.84	3.34	-	712.7
gi|29568101|ref|NP_006467.3| ATP synthase, H+ transporting, mitochondrial F0 complex, subunit g; AT				6.15E-09	3.28	40.21	23.30	11400.3
AHQ-2-14-, 4525	R.LATFWYYAK.V	1163.34840	2	2.42E-06	0.74	2.57	-	450.5
AHQ-2-14, 4566	K.TPALVNAAVTYSK.P	1335.53080	2	1.52E-05	0.84	3.33	-	726.3
AHQ-2-14-, 3659	K.TPALVNAAVTYSK.P	1335.53080	2	6.15E-09	0.97	4.28	-	1717.6
AHQ-2-14, 4504	K.TPALVNAAVTYSKPR.L	1588.83283	3	4.56E-04	0.88	3.43	-	1318.6
AHQ-2-14, 5292 - 5364	K.VELVPPTPAEIPR.A	1418.66294	2	8.10E-04	0.70	2.59	-	449.7
gi|4503725|ref|NP_000792.1| FK506-binding protein 1A; FK506-binding protein 1; FK506-binding protei				6.15E-09	3.75	40.29	29.60	11950.6
AHQ-2-13, 3380	R.GQTCVVHYTGMLEDGKK.F	1925.17623	2	1.28E-04	0.90	3.96	-	808.1
AHQ-2-13, 3924 - 3993	R.GWEEGVAQMSVGQR.A	1534.67926	2	1.20E-05	0.93	4.18	-	1142.5
AHQ-2-13-, 3954 - 4027	R.GWEEGVAQMSVGQR.A	1534.67926	2	1.40E-04	0.60	2.72	-	842.9
AHQ-2-13, 3403 - 3408	K.RGQTCVVHYTGMLEDGK.K	1953.18967	2	3.62E-08	0.98	5.85	-	1856.1
AHQ-2-13-, 3407 - 3411	K.RGQTCVVHYTGMLEDGK.K	1953.18967	2	3.47E-08	0.98	5.81	-	1710.1
AHQ-2-13-, 2987	K.RGQTCVVHYTGMLEDGKK.F	2081.36259	3	6.15E-09	0.94	4.44	-	1275.3
AHQ-2-13, 3021	K.RGQTCVVHYTGMLEDGKK.F	2081.36259	2	8.24E-05	0.96	4.47	-	1463.7
AHQ-2-13, 2923	K.RGQTCVVHYTGMLEDGKK.F	2081.36259	2	9.73E-04	0.94	4.62	-	814.0
gi|21314617|ref|NP_000433.2| platelet/endothelial cell adhesion molecule (CD31 antigen); platelet/e				6.27E-09	7.60	90.31	17.60	82449.6
AHQ-2-5, 5919	R.CESISGTLPISYQLLK.T	1811.08954	2	4.15E-06	0.93	4.24	-	685.8
AHQ-2-4, 6752	K.CTIQVTHLAQEFPEIIIQK.D	2270.63371	2	2.18E-07	0.98	6.14	-	1604.8
AHQ-2-3, 6701	K.CTIQVTHLAQEFPEIIIQK.D	2270.63371	2	5.82E-06	0.95	4.17	-	1227.3
AHQ-2-4, 6533 - 6548	R.DQNFVILEFPVEEQDR.V	1979.13566	2	9.25E-04	0.38	2.71	-	381.8
AHQ-2-5, 6351	R.DQNFVILEFPVEEQDR.V	1979.13566	2	3.14E-04	0.93	4.78	-	865.2
AHQ-2-6, 6191	R.DQNFVILEFPVEEQDR.V	1979.13566	2	2.00E-04	0.30	2.54	-	241.0
AHQ-2-4, 3489	K.EDTIVSQTQDFTK.I	1512.60049	2	2.43E-04	0.90	3.82	-	560.1
AHQ-2-4, 2361 - 2365	R.IISGIHMQTSESTK.S	1532.74472	2	1.86E-08	0.96	4.40	-	1190.3
AHQ-2-4, 4269	R.ISYDAQFEVIK.G	1313.48046	2	7.10E-06	0.87	3.33	-	709.8
AHQ-2-4, 3266	K.SDSGTYICTAGIDK.V	1489.58698	2	8.73E-05	0.91	3.91	-	904.9
AHQ-2-4, 4085 - 4162	K.STESYFIPEVR.I	1328.45199	2	3.68E-04	0.84	3.23	-	743.5
AHQ-2-3, 5667	K.VIAPVDEVQISILSSK.V	1698.98227	2	8.19E-07	0.84	3.34	-	443.4
AHQ-2-4, 5677 - 5684	K.VIAPVDEVQISILSSK.V	1698.98227	2	6.27E-09	0.91	3.82	-	682.1
gi|4503823|ref|NP_002028.1| protein-tyrosine kinase fyn isoform a; proto-oncogene tyrosine-protein				6.29E-09	0.90	10.18	2.00	60761.5
AHQ-2-7, 3409 - 3413	K.WTAPEAALYGR.F	1235.37302	2	6.29E-09	0.90	3.64	-	633.0
gi|18543899|ref|XP_086916.1| similar to Phosphatidylethanolamine-binding protein (PEBP) (Prostatic				6.51E-09	0.95	10.22	8.00	21265.8
AHQ-2-11, 3229 - 3230	K.NRPTSISWDGLDSGK.L	1633.74419	2	6.51E-09	0.95	4.30	-	1080.2
gi|30150271|ref|XP_292964.2| similar to Heat shock protein HSP 90-beta (HSP 84) (Tumor specific tra				6.67E-09	0.92	10.21	4.10	47348.8
AHQ-2-5, 4667	K.CLELFSELAEDKKNYKK.F	2117.40810	2	6.67E-09	0.92	4.23	-	1518.2
gi|5031597|ref|NP_005710.1| actin related protein 2/3 complex subunit 3; ARP2/3 protein complex sub				6.77E-09	2.37	30.27	23.60	20546.5
AHQ-2-11, 6398 - 6408	K.DTDIVDEAIYYFK.A	1592.72721	2	1.39E-08	0.97	5.11	-	1444.5
AHQ-2-12, 6729 - 6730	K.DTDIVDEAIYYFK.A	1592.72721	2	6.77E-09	0.98	5.30	-	1674.0
AHQ-2-13-, 6709	K.DTDIVDEAIYYFK.A	1592.72721	2	2.02E-06	0.94	3.40	-	1561.6
AHQ-2-13, 6517	K.DTDIVDEAIYYFK.A	1592.72721	2	4.17E-05	0.95	4.93	-	867.8
AHQ-2-14-, 6568 - 6571	K.DTDIVDEAIYYFK.A	1592.72721	2	1.84E-05	0.88	2.55	-	1367.4
AHQ-2-12, 7123	K.EM*YTLGITNFPIPGEPGFPLNAIYAK.P	2871.29861	2	1.99E-04	0.47	2.76	-	176.5
AHQ-2-11, 5824 - 5828	R.ETKDTDIVDEAIYYFK.A	1951.11899	2	4.11E-08	0.93	4.27	-	900.9
gi|4758504|ref|NP_004484.1| hydroxyacyl-Coenzyme A dehydrogenase, type II [Homo sapiens]				7.01E-09	3.44	40.23	26.40	26922.9
AHQ-2-10, 6806	R.LGDPAEYAHLVQAIIENPFLNGEVIR.L	2880.24689	2	7.01E-09	0.82	3.48	-	616.2
AHQ-2-10, 4747	R.LVGQGASAVLLDLPNSGGEAQAK.K	2196.44680	2	4.59E-05	0.87	4.07	-	693.3
AHQ-2-10, 6463 - 6470	R.VMTIAPGLFGTPLLTSLPEK.V	2086.52363	2	3.52E-05	0.87	3.72	-	673.6
AHQ-2-10, 6098	R.VM*TIAPGLFGTPLLTSLPEK.V	2102.52303	2	3.72E-06	0.88	4.35	-	401.6
gi|4758756|ref|NP_004528.1| nucleosome assembly protein 1-like 1; HSP22-like protein interacting pr				7.68E-09	3.59	40.27	14.60	45373.8
AHQ-2-7, 7275	K.GIPEFWLTVFK.N	1337.58999	2	7.68E-09	0.75	2.99	-	239.4
AHQ-2-7, 5523	R.LDGLVETPTGYIESLPR.V	1861.08535	2	6.70E-07	0.95	4.52	-	1022.2
AHQ-2-7, 5609	R.LDGLVETPTGYIESLPR.V	1861.08535	2	2.85E-04	0.87	3.67	-	652.9

AHQ-2-7, 5337 - 5351	K.NVDLLSDMVQEHDEPILK.H	2096.34751	2	8.65E-08	0.97	5.40	-	1394.4
AHQ-2-7, 4589	K.YAVLYQPLFDK.R	1357.57807	2	3.53E-07	0.92	3.16	-	1143.0
AHQ-2-7, 4508 - 4585	K.YAVLYQPLFDK.R	1357.57807	1	8.70E-04	0.58	2.62	-	601.2
gi|4505763|ref|NP_000282.1| phosphoglycerate kinase 1 [Homo sapiens]				8.22E-09	7.67	100.28	42.20	44614.4
AHQ-2-8, 5586	K.ACANPAAGSVILLENLR.F	1771.03187	2	2.65E-06	0.95	4.77	-	1092.5
AHQ-2-11, 5562 - 5641	K.ACANPAAGSVILLENLR.F	1771.03187	2	2.02E-06	0.96	4.62	-	1508.3
AHQ-2-10, 4938	K.ALESPERPFLAILGGAK.V	1770.06542	2	5.19E-07	0.95	4.63	-	1125.7
AHQ-2-11, 5253	K.ALESPERPFLAILGGAK.V	1770.06542	2	1.32E-04	0.88	3.83	-	1135.4
AHQ-2-8, 4924	K.ALESPERPFLAILGGAK.V	1770.06542	2	4.98E-06	0.96	4.94	-	1527.1
AHQ-2-8, 4932	K.ALESPERPFLAILGGAK.V	1770.06542	2	8.22E-09	0.97	5.19	-	1762.0
AHQ-2-9, 5255	K.ALESPERPFLAILGGAK.V	1770.06542	2	6.52E-06	0.95	4.58	-	1395.1
AHQ-2-8, 4496	K.DVLFLKDCVGPEVEK.A	1750.00620	2	7.56E-08	0.92	3.75	-	1035.4
AHQ-2-8, 4495	K.DVLFLKDCVGPEVEK.A	1750.00620	2	1.95E-06	0.97	5.16	-	1373.6
AHQ-2-11, 5198	K.ITLPVDFVTADKFDENAK.T	2024.25948	2	2.55E-05	0.51	2.77	-	400.0
AHQ-2-8, 5216	K.SVVLMSHLGRPDGVPMPDKYSLEPVAVELK.S	3265.83562	3	2.33E-06	0.96	5.54	-	1692.0
AHQ-2-8, 4547	K.TGQATVASGIPAGWMGLDCGPESSKK.Y	2607.90190	3	4.09E-05	0.83	3.85	-	487.2
AHQ-2-8, 4993	K.VLNNMEIGTSLFDEEGAK.I	1968.17459	2	1.61E-04	0.74	3.67	-	296.3
AHQ-2-8, 5315 - 5316	K.VLPGVDALSNI	1098.27456	1	8.89E-04	0.27	1.89	-	340.8
AHQ-2-8, 3834	K.WNTEDKVSHVSTGGGASLELLEGK.V	2515.71777	2	8.31E-04	0.73	3.01	-	697.5
AHQ-2-13-, 4291 - 4294	K.YSLEPVAVELK.S	1248.44991	2	1.28E-06	0.78	2.66	-	716.6
gi|20127528|ref|NP_037459.2| parvin, beta; CGI-56 protein; beta-parvin [Homo sapiens]				8.59E-09	4.74	60.28	27.20	41714.0
AHQ-2-9, 3130 - 3134	K.ARPEDVVNLDLK.S	1369.54878	1	5.13E-07	0.27	2.65	-	192.8
AHQ-2-9, 3131	K.ARPEDVVNLDLK.S	1369.54878	2	2.75E-05	0.91	4.32	-	806.9
AHQ-2-13, 3468	K.ARPEDVVNLDLK.S	1369.54878	2	8.94E-05	0.93	4.60	-	777.1
AHQ-2-11, 3297	K.ARPEDVVNLDLK.S	1369.54878	2	4.73E-05	0.91	3.47	-	961.7
AHQ-2-13-, 3413 - 3482	K.ARPEDVVNLDLK.S	1369.54878	2	5.24E-06	0.88	3.80	-	622.8
AHQ-2-9, 5311	R.DAFDTLFDHAPDKLSVVK.K	2019.24293	2	9.57E-05	0.84	4.00	-	500.2
AHQ-2-1, 3989	K.LNVAEVTQSEIGQK.Q	1516.67865	2	3.41E-06	0.94	4.60	-	802.2
AHQ-2-13, 3807	K.LNVAEVTQSEIGQK.Q	1516.67865	2	3.91E-06	0.90	4.00	-	824.4
AHQ-2-9, 4559	K.NAINSPMSPALVDVHPEDTQLEENEER.T	3036.23445	3	4.06E-08	0.78	3.64	-	422.4
AHQ-2-9, 3731	K.QLEEDLYDGQVLQK.L	1678.82164	2	4.06E-04	0.73	3.14	-	780.9
AHQ-2-9, 3938 - 3939	K.QLEEDLYDGQVLQK.L	1678.82164	2	1.61E-06	0.95	4.45	-	1086.6
AHQ-2-9, 7354 - 7364	K.VLLDWINDVLVEER.I	1713.95549	2	8.59E-09	0.97	5.65	-	1370.2
AHQ-2-9, 6579	K.VLLDWINDVLVEER.I	1713.95549	2	3.95E-06	0.79	3.40	-	607.0
gi|28827809|ref|NP_789797.1| nucleoside diphosphate kinase B-like [Homo sapiens]				8.67E-09	5.25	60.26	43.10	15529.0
AHQ-2-12, 3357 - 3367	R.GDFCIQVGR.N	1053.17404	2	2.37E-05	0.88	3.35	-	1016.5
AHQ-2-12, 2057	R.NIIHGSDSVKSAEK.E	1485.62464	1	7.62E-08	0.81	3.53	-	659.0
AHQ-2-12, 2049	R.NIIHGSDSVKSAEK.E	1485.62464	2	1.88E-07	0.88	4.14	-	986.7
AHQ-2-12, 2751	R.VMLGETNPADSKPGTIR.G	1787.03151	2	4.32E-08	0.81	3.86	-	478.4
AHQ-2-12, 2566	R.VM*LGETNPADSKPGTIR.G	1803.03091	2	7.52E-05	0.84	3.34	-	716.1
AHQ-2-12, 7109	K.YM*NSGPVVAMVWEGLNVVK.T	2110.48562	2	8.67E-09	0.96	5.27	-	1220.3
AHQ-2-12, 7106 - 7134	K.YMNSGPVVAMVWEGLNVVK.T	2094.48622	2	1.28E-05	0.94	4.88	-	576.6
gi|5902016|ref|NP_008939.1| reticulon 4; neuroendocrine-specific protein C like (foocen) [Homo sapi				8.76E-09	1.72	20.18	14.10	22395.3
AHQ-2-1, 3174	R.HQAQIDHYLGLANK.N	1608.78270	2	3.54E-04	0.91	3.65	-	837.4
AHQ-2-1, 3038	K.YSNSALGHVNCTIK.E	1565.73305	2	8.76E-09	0.82	3.15	-	715.1
gi|5803225|ref|NP_006752.1| tyrosine 3/tryptophan 5 -monooxygenase activation protein, epsilon poly				1.01E-08	3.32	40.25	20.40	29173.7
AHQ-2-13-, 6842 - 6849	K.AAFDDAIAELDTLSEESYK.D	2089.19882	2	5.73E-07	0.96	4.97	-	1215.8
AHQ-2-9, 7070 - 7078	K.AAFDDAIAELDTLSEESYK.D	2089.19882	2	4.07E-04	0.93	4.42	-	849.0
AHQ-2-13, 6627	K.AAFDDAIAELDTLSEESYK.D	2089.19882	2	3.17E-07	0.93	3.52	-	1297.6
AHQ-2-14-, 6629 - 6708	K.AAFDDAIAELDTLSEESYK.D	2089.19882	2	1.01E-08	0.95	4.53	-	1249.5
AHQ-2-10, 6898 - 6962	K.AAFDDAIAELDTLSEESYKDSTLIMQLLR.D	3260.61486	3	5.62E-08	0.91	4.72	-	801.2
AHQ-2-9, 2370	K.EAAENSLVAYK.A	1195.30397	2	4.91E-06	0.76	3.15	-	546.8
AHQ-2-9, 1552	K.HLIPAANTGESK.V	1238.37521	2	6.99E-04	0.71	2.61	-	576.8
gi|13899317|ref|NP_113657.1| SH3 domain binding glutamic acid-rich protein like 2 [Homo sapiens] [M				1.02E-08	4.31	50.22	72.00	12326.0
AHQ-2-14-, 5479 - 5484	K.ESNTVFSFLGLKPR.L	1595.82383	2	5.55E-08	0.67	3.10	-	359.4
AHQ-2-13, 5392 - 5443	K.ESNTVFSFLGLKPR.L	1595.82383	2	4.32E-06	0.90	3.78	-	745.9
AHQ-2-14, 6081	R.FLEANKIEFEEVDITM*SEEQR.Q	2574.80223	2	8.02E-04	0.78	3.26	-	685.7
AHQ-2-14-, 5071 - 5075	R.FLEANKIEFEEVDITM*SEEQR.Q	2574.80223	3	1.02E-08	0.90	4.03	-	1260.6
AHQ-2-13-, 3830	K.KPTQGNPLPPQIFNGDR.Y	1880.09682	2	2.69E-06	0.90	3.78	-	786.5
AHQ-2-13-, 6034	K.KPTQGNPLPPQIFNGDRYCGDYDSFFESK.E	3380.64432	3	3.96E-04	0.74	3.35	-	692.7
AHQ-2-13-, 5903 - 5966	K.KPTQGNPLPPQIFNGDRYCGDYDSFFESK.E	3380.64432	3	1.84E-06	0.88	4.36	-	729.8
AHQ-2-13, 5739 - 5801	K.KPTQGNPLPPQIFNGDRYCGDYDSFFESK.E	3380.64432	3	1.67E-05	0.86	4.40	-	923.0
AHQ-2-14-, 5871 - 5937	K.KPTQGNPLPPQIFNGDRYCGDYDSFFESK.E	3380.64432	3	1.59E-04	0.85	4.43	-	686.0
AHQ-2-14, 5601 - 5604	R.VFIASSSGFVAIK.K	1326.56551	2	1.11E-06	0.97	4.35	-	2021.9
AHQ-2-14-, 4555 - 4629	R.VFIASSSGFVAIK.K	1326.56551	2	2.99E-05	0.95	3.17	-	2063.4
gi|30150022|ref|XP_300862.1| similar to beta-tubulin 4Q [Homo sapiens]				1.10E-08	6.54	70.29	22.50	33545.2
AHQ-2-11, 5970	K.EAESCDCLQGFQLTHSLGGGTGSGMGTLLISK.I	3315.65550	3	9.68E-07	0.94	4.66	-	1666.0
AHQ-2-7, 6267 - 6337	K.EAESCDCLQGFQLTHSLGGGTGSGMGTLLISK.I	3315.65550	3	1.29E-08	0.95	5.38	-	998.0
AHQ-2-13-, 3403	R.FPGQLNADLR.K	1131.26634	2	3.35E-05	0.68	2.51	-	694.2
AHQ-2-8, 2882 - 2959	R.FPGQLNADLR.K	1131.26634	2	4.72E-04	0.88	2.91	-	978.2
AHQ-2-7, 3133	R.FPGQLNADLR.K	1131.26634	2	1.27E-05	0.89	2.82	-	1048.7
AHQ-2-7, 2995 - 3063	R.FPGQLNADLR.K	1131.26634	2	8.36E-04	0.85	3.06	-	686.4
AHQ-2-7, 5800 - 5804	R.KEAESCDCLQGFQLTHSLGGGTGSGMGTLLISK.I	3443.82841	3	1.10E-08	0.97	5.75	-	2083.2
AHQ-2-7, 3419 - 3432	R.KLAVNMVPFPR.L	1272.58771	2	2.26E-05	0.95	4.32	-	1167.9
AHQ-2-7, 4140 - 4216	K.LAVNMVPFPR.L	1144.41480	2	2.06E-06	0.92	3.57	-	823.2
AHQ-2-8, 3975	K.LAVNMVPFPR.L	1144.41480	2	4.75E-06	0.95	3.77	-	948.4
AHQ-2-9, 4212	K.LAVNMVPFPR.L	1144.41480	2	7.49E-04	0.89	3.06	-	732.6
AHQ-2-13-, 4486	K.LAVNMVPFPR.L	1144.41480	2	2.76E-06	0.69	2.57	-	648.7
AHQ-2-10, 4072	K.LAVNMVPFPR.L	1144.41480	2	1.63E-06	0.93	4.10	-	890.1
AHQ-2-11, 4272	K.LAVNMVPFPR.L	1144.41480	2	2.15E-06	0.88	3.41	-	663.9
AHQ-2-7, 3460	K.LAVNM*VPFPR.L	1160.41420	2	3.80E-04	0.93	3.88	-	965.9
AHQ-2-9, 5523	R.LHFFMPGFAPLTSR.G	1621.92962	2	2.59E-04	0.88	3.31	-	635.6
AHQ-2-7, 5405 - 5407	R.LHFFMPGFAPLTSR.G	1621.92962	2	1.86E-06	0.92	3.47	-	881.2
gi|21361553|ref|NP_003168.2| spleen tyrosine kinase [Homo sapiens]				1.10E-08	0.79	10.26	2.00	72065.7
AHQ-2-13, 4476	K.TGPFEDLKENLIR.E	1532.72291	2	1.10E-08	0.79	3.29	-	693.1
gi|4506005|ref|NP_002700.1| protein phosphatase 1, catalytic subunit, beta isoform [Homo sapiens] [				1.10E-08	4.11	50.27	24.20	37186.6
AHQ-2-9, 3703	R.AHQVVEDGYEFFAK.R	1640.77679	2	1.37E-04	0.92	3.54	-	1021.7
AHQ-2-13, 6787	R.EIFLSQPILLELEAPLK.I	1954.33857	2	1.71E-06	0.95	5.04	-	686.6
AHQ-2-9, 7387 - 7388	R.EIFLSQPILLELEAPLK.I	1954.33857	2	1.10E-08	0.95	5.32	-	749.5
AHQ-2-9, 5212 - 5222	K.IFCCHGGLSPDLQSMEQIR.R	2251.54930	2	3.93E-07	0.97	4.83	-	1653.8
AHQ-2-9, 4759 - 4760	K.IKYPENFFLLR.G	1440.71344	2	5.50E-04	0.30	2.72	-	246.0
AHQ-2-9, 7310	K.TFTDCFNCLPIAAIVDEK.I	2117.38502	2	2.41E-07	0.97	5.25	-	1137.4
gi|11496885|ref|NP_005442.2| enigma protein; LIM domain protein [Homo sapiens]				1.13E-08	3.61	40.25	16.40	49844.4
AHQ-2-13, 6205	K.AAQAGVAVGDWVLSIDGENAGSLTHIEAQNK.I	3123.37980	3	1.64E-04	0.81	4.20	-	549.5
AHQ-2-14-, 6263	K.AAQAGVAVGDWVLSIDGENAGSLTHIEAQNK.I	3123.37980	3	1.13E-08	0.91	4.82	-	619.6
AHQ-2-10, 3396	K.VLEEGGFFEEK.G	1284.39598	2	2.38E-08	0.77	3.06	-	642.6
AHQ-2-14-, 4887	K.VVLEGPAPWGFR.L	1328.54318	2	6.47E-08	0.96	3.77	-	1752.0
AHQ-2-14, 6185	K.VVLEGPAPWGFR.L	1328.54318	2	3.19E-07	0.93	3.50	-	1099.0
AHQ-2-13-, 4917	K.VVLEGPAPWGFR.L	1328.54318	2	9.45E-07	0.92	3.86	-	886.6
AHQ-2-11, 4657	K.VVLEGPAPWGFR.L	1328.54318	2	2.34E-05	0.92	3.51	-	1104.0
AHQ-2-9, 4464 - 4466	R.YLVALGHAYHPEEFVCSQCGK.V	2468.74928	2	1.15E-07	0.97	4.97	-	1384.3
AHQ-2-9, 4468	R.YLVALGHAYHPEEFVCSQCGK.V	2468.74928	3	1.86E-04	0.96	4.97	-	1479.5
gi|28178832|ref|NP_002159.2| isocitrate dehydrogenase 2 (NADP+), mitochondrial precursor; isocitrat				1.18E-08	4.54	50.24	15.00	50909.0
AHQ-2-8, 3767	K.DLAGCIHGLSNVK.L	1385.57150	2	2.47E-06	0.97	4.10	-	1914.2
AHQ-2-8, 1862 - 1932	R.GKLDGNQDLIR.F	1229.36832	2	1.89E-04	0.90	3.00	-	1189.2
AHQ-2-8, 5235	K.LNEHFLNTTDFLDTIK.S	1922.12725	2	1.18E-08	0.97	4.82	-	1465.2
AHQ-2-8, 4491	R.NILGGTVFREPIICK.N	1719.04170	2	1.97E-04	0.93	4.29	-	927.7

AHQ-2-8, 1620	K.TIEAEAAHGTVTR.H	1356.46696	2	4.11E-04	0.78	3.03	-	685.0
gi|4758988|ref|NP_004152.1| RAB1A, member RAS oncogene family; RAB1, member RAS oncogene family [Ho				1.20E-08	4.05	50.24	35.60	22677.6
AHQ-2-14-, 6236 - 6261	K.EFADSLGIPFLETSAK.N	1725.91990	2	1.46E-05	0.61	3.27	-	354.6
AHQ-2-11, 6076 - 6085	K.EFADSLGIPFLETSAK.N	1725.91990	2	1.16E-04	0.80	3.59	-	592.4
AHQ-2-12, 6362	K.EFADSLGIPFLETSAK.N	1725.91990	2	7.01E-05	0.72	3.58	-	431.0
AHQ-2-11, 5989 - 6048	K.EFADSLGIPFLETSAK.N	1725.91990	2	6.48E-05	0.72	3.73	-	403.3
AHQ-2-11, 5908	K.EFADSLGIPFLETSAK.N	1725.91990	2	7.24E-05	0.62	2.74	-	385.4
AHQ-2-11, 5973 - 6052	R.FADDTYTESYISTIGVDFK.I	2173.31742	2	2.09E-07	0.97	4.85	-	1619.9
AHQ-2-11, 5866 - 5930	R.FADDTYTESYISTIGVDFK.I	2173.31742	2	1.46E-06	0.88	3.24	-	1005.2
AHQ-2-11, 5561 - 5634	R.FADDTYTESYISTIGVDFK.I	2173.31742	2	2.07E-08	0.97	4.37	-	1666.4
AHQ-2-14-, 6040 - 6105	R.FADDTYTESYISTIGVDFK.I	2173.31742	2	1.20E-08	0.91	3.55	-	986.9
AHQ-2-11, 6112 - 6172	R.FADDTYTESYISTIGVDFK.I	2173.31742	2	4.29E-08	0.95	4.35	-	1007.5
AHQ-2-11, 6241 - 6316	R.FADDTYTESYISTIGVDFK.I	2173.31742	2	5.83E-06	0.94	4.15	-	1040.2
AHQ-2-11, 6842 - 6913	R.FADDTYTESYISTIGVDFK.I	2173.31742	2	2.08E-06	0.93	3.76	-	1075.2
AHQ-2-11, 6976	R.FADDTYTESYISTIGVDFK.I	2173.31742	2	2.86E-07	0.94	4.61	-	938.6
AHQ-2-14-, 5904 - 5977	R.FADDTYTESYISTIGVDFK.I	2173.31742	2	3.59E-08	0.94	4.02	-	1291.4
AHQ-2-13, 6025	R.FADDTYTESYISTIGVDFK.I	2173.31742	2	4.54E-05	0.69	3.21	-	636.1
AHQ-2-11, 5037	R.GAHGIIVVYDVTDQESFNNVK.Q	2306.51658	2	1.18E-04	0.91	4.08	-	744.6
AHQ-2-11, 4794	K.NATNVEQSFMTM*AAEIK.K	1902.13962	2	2.86E-04	0.79	3.67	-	814.1
AHQ-2-11, 6032	K.NATNVEQSFMTMAAEIK.K	1886.14022	2	7.20E-06	0.83	3.92	-	944.5
gi|4503971|ref|NP_001484.1| GDP dissociation inhibitor 1; mental retardation, X-linked 41; mental r				1.26E-08	3.56	40.27	14.80	50582.4
AHQ-2-7, 4151	R.FQLLEGPPESMGR.G	1461.66818	2	9.71E-05	0.89	3.96	-	908.5
AHQ-2-7, 5160	R.NPYYGGESSSITPLEELYK.R	2148.31110	2	8.10E-06	0.79	3.44	-	585.8
AHQ-2-6, 5217 - 5218	R.NPYYGGESSSITPLEELYK.R	2148.31110	2	1.35E-04	0.96	5.44	-	965.4
AHQ-2-6, 4274	R.TDDYLDQPCLETVNR.I	1840.94632	2	1.26E-08	0.90	4.32	-	626.8
AHQ-2-7, 7235	K.VPSTETEALASNLMGMFEK.R	2056.34679	2	4.81E-08	0.97	5.15	-	1191.0
AHQ-2-6, 7114	K.VPSTETEALASNLMGMFEK.R	2056.34679	2	3.51E-08	0.97	4.90	-	1764.8
gi|4505585|ref|NP_002563.1| platelet-activating factor acetylhydrolase, isoform Ib, beta subunit 30				1.33E-08	1.80	20.22	17.00	25569.1
AHQ-2-10, 5584 - 5587	R.ELFSPLHALNFGIGGDTTR.H	2046.27160	2	1.49E-05	0.83	3.50	-	611.4
AHQ-2-10, 6598	K.ICKPLHELIMQLLEETPEEK.Q	2452.87287	2	1.33E-08	0.96	4.46	-	1883.6
gi|5453760|ref|NP_006147.1| neural precursor cell expressed, developmentally down-regulated 8 [Homo				1.36E-08	0.86	10.17	17.30	9071.5
AHQ-2-14-, 3675	K.EIEIDIEPTDKVER.I	1686.84208	2	1.36E-08	0.86	3.39	-	821.6
gi|6679056|ref|NP_031387.1| nidogen 2 (osteonidogen); nidogen 2 [Homo sapiens]				1.39E-08	0.77	10.18	2.10	151153.0
AHQ-2-1, 6780 - 6809	R.AGLELGAEPETIVNSGLISPEGLAIDHIR.R	2973.32740	3	1.39E-08	0.77	3.70	-	682.6
gi|21361547|ref|NP_002930.2| ribonuclease/angiogenin inhibitor; Placental ribonuclease inhibitor [H				1.42E-08	0.90	10.20	3.30	49973.3
AHQ-2-14-, 6503	R.WAELLPLLQQCQVVR.L	1855.19360	2	1.42E-08	0.90	4.04	-	544.5
gi|22042381|ref|XP_065237.3| similar to FKSG30 [Homo sapiens]				1.42E-08	1.59	20.19	8.30	42016.0
AHQ-2-14-, 2301 - 2324	K.QEYDESGPSIVHR.K	1517.58206	2	2.12E-04	0.59	2.66	-	342.2
AHQ-2-13-, 2893	K.QEYDESGPSIVHR.K	1517.58206	2	1.96E-04	0.35	2.74	-	476.1
AHQ-2-13-, 2377 - 2443	K.QEYDESGPSIVHR.K	1517.58206	2	4.88E-08	0.87	3.42	-	527.6
AHQ-2-13, 2477	K.QEYDESGPSIVHR.K	1517.58206	2	1.42E-08	0.79	3.20	-	399.9
AHQ-2-8, 2022 - 2090	K.QEYDESGPSIVHR.K	1517.58206	2	1.44E-07	0.56	2.63	-	290.6
AHQ-2-9, 3435 - 3514	R.VAPEEHPILLTEAPLNPK.A	1969.27019	2	1.07E-04	0.65	3.14	-	428.4
AHQ-2-8, 3219 - 3286	R.VAPEEHPILLTEAPLNPK.A	1969.27019	2	1.35E-04	0.61	3.17	-	385.3
AHQ-2-8, 3098 - 3140	R.VAPEEHPILLTEAPLNPK.A	1969.27019	2	1.22E-05	0.79	3.65	-	485.9
gi|4505705|ref|NP_003759.1| phosphoprotein enriched in astrocytes 15; Phosphoprotein enriched in as				1.50E-08	0.88	10.21	13.10	15040.0
AHQ-2-12, 6109 - 6177	K.LDKDNLSYIEHIFEISR.R	2093.32504	2	1.50E-08	0.88	4.25	-	591.2
gi|4504483|ref|NP_000185.1| hypoxanthine phosphoribosyltransferase 1 [Homo sapiens]				1.61E-08	1.90	20.33	21.10	24579.2
AHQ-2-10, 6623	R.SPGVVISDDEPGYDLDLFCIPNHYAEDLER.V	3438.67572	3	1.61E-08	0.96	6.57	-	793.2
AHQ-2-10, 4422	R.SVGYKPDFVGFEIPDK.F	1799.01571	2	1.30E-07	0.93	4.50	-	738.4
gi|28077085|ref|NP_004559.3| serine (or cysteine) proteinase inhibitor, clade B (ovalbumin), member				1.62E-08	0.92	10.22	5.30	42586.7
AHQ-2-9, 4624	K.SGGGGDIHQGFQSLLTEVNK.T	2045.19887	2	1.62E-08	0.92	4.48	-	849.8
AHQ-2-8, 4371	K.SGGGGDIHQGFQSLLTEVNK.T	2045.19887	2	1.57E-04	0.82	3.50	-	873.5
gi|6005854|ref|NP_009204.1| repressor of estrogen receptor activity; B-cell associated protein [Hom				1.65E-08	1.86	20.24	10.70	33296.1
AHQ-2-9, 7182 - 7235	R.IPWFQYPIIYDIR.A	1725.02480	2	1.10E-04	0.95	3.82	-	1459.0
AHQ-2-9, 6743	R.IYLTADNLVLNLQDESFTR.G	2226.47137	2	1.65E-08	0.91	4.75	-	579.0
gi|4507295|ref|NP_003560.1| syntaxin 7 [Homo sapiens]				1.69E-08	0.91	10.20	5.70	29845.3
AHQ-2-13-, 3366	R.TLNQLGTPQDSPELR.Q	1669.81798	2	1.76E-07	0.85	3.41	-	538.0
AHQ-2-13, 3385 - 3391	R.TLNQLGTPQDSPELR.Q	1669.81798	2	1.69E-08	0.91	4.02	-	724.8
gi|4758790|ref|NP_004543.1| NADH dehydrogenase (ubiquinone) Fe-S protein 5, 15kDa (NADH-coenzyme Q				1.69E-08	2.57	30.23	34.90	12517.4
AHQ-2-13-, 3827	K.EWIECAHGIGYTR.A	1593.74495	2	7.43E-07	0.84	2.97	-	684.8
AHQ-2-13-, 6761	K.IEYDDFVECLLR.Q	1573.74872	2	2.69E-07	0.96	4.66	-	910.4
AHQ-2-13, 6552	K.IEYDDFVECLLR.Q	1573.74872	2	1.69E-08	0.88	3.31	-	809.8
AHQ-2-13-, 3897	R.WLTIQSGEQPYK.M	1450.62036	2	1.82E-04	0.85	3.56	-	728.4
gi|27436929|ref|NP_005518.2| heat shock 70kDa protein 1-like; heat shock 70kD protein-like 1 [Homo				1.73E-08	0.97	10.21	2.20	70404.7
AHQ-2-6, 2927	K.AKIHDIVLVGGSTR.I	1466.71083	2	1.73E-08	0.97	4.12	-	2243.4
gi|4557032|ref|NP_002291.1| lactate dehydrogenase B [Homo sapiens]				1.74E-08	11.71	160.29	43.70	36638.3
AHQ-2-13-, 4554	K.FIIPQIVK.Y	958.22198	1	4.10E-04	0.28	2.02	-	430.5
AHQ-2-9, 4084	K.GEM*MDLQHGSLFLQTPK.I	1949.24111	2	4.22E-04	0.67	3.61	-	687.0
AHQ-2-9, 4654	K.GEMMDLQHGSLFLQTPK.I	1933.24171	2	9.28E-06	0.95	4.64	-	941.7
AHQ-2-9, 2212	R.GLTSVINQK.L	960.10999	2	2.28E-04	0.60	2.57	-	536.1
AHQ-2-14-, 2380	R.GLTSVINQK.L	960.10999	2	9.43E-05	0.51	2.59	-	325.3
AHQ-2-9, 7407 - 7464	K.GMYGIENEVFLSLPCILNAR.G	2298.66742	2	6.28E-06	0.77	3.74	-	412.1
AHQ-2-9, 1600	R.IHPVSTMVK.G	1012.25154	1	1.57E-05	0.13	2.29	-	212.9
AHQ-2-9, 7611	K.ITVVGVGQVGMACAISILGK.S	1975.40611	2	6.63E-04	0.92	4.25	-	687.0
AHQ-2-13, 2248	K.IVADKDYSVTANSK.I	1511.65887	2	6.80E-06	0.93	3.67	-	1225.1
AHQ-2-9, 1982	K.IVADKDYSVTANSK.I	1511.65887	2	8.10E-05	0.87	3.62	-	749.7
AHQ-2-13-, 2127	K.IVADKDYSVTANSK.I	1511.65887	2	9.09E-07	0.90	3.09	-	948.8
AHQ-2-13-, 2611	K.IVVVTAGVR.Q	914.12760	2	2.38E-07	0.89	3.43	-	818.5
AHQ-2-14-, 3511	K.LIAPVAEEEATVPNNK.I	1695.89534	2	4.68E-05	0.91	3.61	-	1031.1
AHQ-2-14-, 3597	K.LIAPVAEEEATVPNNK.I	1695.89534	2	2.50E-06	0.70	2.69	-	626.7
AHQ-2-14-, 3313 - 3385	K.LIAPVAEEEATVPNNK.I	1695.89534	2	8.01E-04	0.93	3.86	-	1009.6
AHQ-2-9, 1922	K.LKDDEVAQLK.K	1159.31477	2	4.01E-05	0.69	2.61	-	489.7
AHQ-2-9, 3826	K.LKGEM*MDLQHGSLFLQTPK.I	2190.57228	3	8.92E-04	0.69	3.93	-	495.7
AHQ-2-11, 3713	K.MVVESAYEVIK.L	1268.50463	2	8.48E-05	0.94	3.95	-	1086.0
AHQ-2-9, 3664 - 3668	K.MVVESAYEVIK.L	1268.50463	2	1.46E-07	0.93	4.01	-	985.5
AHQ-2-10, 3592	K.MVVESAYEVIK.L	1268.50463	2	1.48E-07	0.92	3.48	-	1160.4
AHQ-2-14-, 3853	K.MVVESAYEVIK.L	1268.50463	2	4.73E-06	0.92	3.39	-	923.3
AHQ-2-9, 3378	K.SADTLWDIQK.D	1177.28864	2	7.51E-07	0.77	3.04	-	683.2
AHQ-2-13-, 6821 - 6830	K.SLADELALVDVLEDK.L	1630.81843	2	1.74E-08	0.97	5.75	-	1370.0
AHQ-2-14-, 6600 - 6671	K.SLADELALVDVLEDK.L	1630.81843	2	1.69E-04	0.91	4.04	-	864.6
AHQ-2-9, 7104 - 7176	K.SLADELALVDVLEDK.L	1630.81843	2	2.04E-05	0.94	3.92	-	1370.7
AHQ-2-9, 7014 - 7026	K.SLADELALVDVLEDK.L	1630.81843	2	3.05E-08	0.97	5.18	-	1520.7
AHQ-2-10, 6436	K.SLADELALVDVLEDK.L	1630.81843	2	1.26E-04	0.94	4.79	-	1070.8
AHQ-2-11, 6508	K.SLADELALVDVLEDK.L	1630.81843	2	8.25E-04	0.89	3.52	-	832.9
AHQ-2-13, 6612	K.SLADELALVDVLEDK.L	1630.81843	2	3.21E-07	0.96	4.37	-	1578.3
AHQ-2-14-, 6805	K.SLADELALVDVLEDKLK.G	1872.14959	2	2.97E-05	0.96	4.57	-	1665.5
AHQ-2-9, 7266 - 7315	K.SLADELALVDVLEDKLK.G	1872.14959	2	1.22E-06	0.94	3.92	-	1415.8
gi|19923142|ref|NP_002256.2| karyopherin beta 1; nuclear factor p97; importin 90; importin beta-1 s				1.78E-08	3.39	40.21	7.90	97169.7
AHQ-2-5, 6877	R.AAVENLPTFLVELSR.V	1659.90783	2	3.56E-04	0.75	2.98	-	354.3
AHQ-2-5, 7452 - 7453	K.GALQYLVPILTQTLTK.Q	1760.11044	2	1.84E-06	0.80	3.77	-	453.0
AHQ-2-5, 7672	R.IQFNDLQSLLCATLQNVLR.K	2248.58813	2	3.27E-07	0.89	4.14	-	880.1
AHQ-2-5, 6315	K.YLEVVLNTLQQASQAQVDK.S	2148.40214	2	1.78E-08	0.95	3.85	-	2159.4
gi|5031573|ref|NP_005712.1| ARP3 actin-related protein 3 homolog; ARP3 (actin-related protein 3, ye				1.88E-08	5.80	80.29	31.10	47370.9
AHQ-2-9, 7490 - 7491	R.DITYFIQQLLR.D	1410.64250	1	3.58E-04	0.39	2.49	-	261.3
AHQ-2-7, 7555	R.DITYFIQQLLR.D	1410.64250	1	1.33E-05	0.12	2.12	-	221.2
AHQ-2-7, 7557	R.DITYFIQQLLR.D	1410.64250	1	3.01E-05	0.18	2.00	-	109.2

AHQ-2-7, 5803 - 5877	K.GVDDLDFFIGDEAIEKPTYATK.W	2445.66268	2	1.04E-05	0.76	2.94	-	868.2
AHQ-2-7, 4189 - 4260	R.HGIVEDWDLMER.F	1500.66127	2	4.43E-06	0.90	3.27	-	1090.0
AHQ-2-7, 2112	R.HNPVFGVM*S	1004.14480	1	9.69E-05	0.31	1.83	-	293.1
AHQ-2-7, 6516	K.LGYAGNTEPQFIIPSCIAIK.E	2194.53570	2	2.35E-06	0.88	3.87	-	765.3
AHQ-2-7, 6395 - 6407	K.LGYAGNTEPQFIIPSCIAIK.E	2194.53570	2	1.88E-08	0.93	4.79	-	575.2
AHQ-2-12, 6245 - 6259	K.LGYAGNTEPQFIIPSCIAIK.E	2194.53570	2	2.02E-04	0.73	3.55	-	423.1
AHQ-2-10, 5818	K.LGYAGNTEPQFIIPSCIAIK.E	2194.53570	2	5.09E-04	0.68	3.57	-	341.1
AHQ-2-13, 6088	K.LGYAGNTEPQFIIPSCIAIK.E	2194.53570	2	4.42E-04	0.44	3.25	-	271.9
AHQ-2-9, 3576	R.LPACVVDCGTGYTK.L	1543.74421	2	3.28E-04	0.91	3.59	-	975.7
AHQ-2-7, 3669	K.NIVLSGGSTMFR.D	1282.49463	2	1.25E-06	0.94	3.91	-	1387.5
AHQ-2-13, 4055	K.NIVLSGGSTMFR.D	1282.49463	2	6.45E-04	0.87	2.99	-	1371.6
AHQ-2-9, 6692	R.TLTGTVIDSGDGVTHVIPVAEGYVIGSCIK.H	3061.45302	3	7.38E-04	0.90	4.30	-	1006.2
AHQ-2-8, 6492	R.TLTGTVIDSGDGVTHVIPVAEGYVIGSCIK.H	3061.45302	3	2.52E-04	0.83	3.80	-	801.9
AHQ-2-7, 6768	R.TLTGTVIDSGDGVTHVIPVAEGYVIGSCIK.H	3061.45302	3	1.18E-04	0.94	4.83	-	1228.1
gi|21389549|ref|NP_653263.1| hypothetical protein MGC26605 [Homo sapiens]				2.00E-08	1.59	20.22	14.90	26927.8
AHQ-2-10, 5203	R.AITVFSPDGHLFQVEYAQEAVK.K	2450.73052	3	6.56E-05	0.66	3.06	-	556.0
AHQ-2-10, 4370 - 4371	K.LTVEDPVTVEYITR.F	1635.83990	2	2.00E-08	0.93	4.25	-	749.4
gi|4557361|ref|NP_001187.1| BH3 interacting domain death agonist; BH3-interacting domain death agon				2.02E-08	2.87	30.30	29.70	21994.6
AHQ-2-11, 6861	R.DLATALEQLLQAYPR.D	1702.93270	2	3.67E-08	0.96	4.81	-	1138.5
AHQ-2-11, 6037	R.DVFHTTVNFINQNLR.T	1819.01348	2	7.01E-04	0.95	4.45	-	1406.4
AHQ-2-11, 6124 - 6129	R.ELDALGHELPVLAPQWEGYDELQTDGNR.S	3167.38790	3	2.02E-08	0.96	5.92	-	1328.1
gi|4758208|ref|NP_004081.1| dual specificity phosphatase 3; vaccinia virus phosphatase VH1-related;				2.17E-08	4.11	50.25	38.90	20478.2
AHQ-2-11, 4168	R.AADFIDQALAQK.N	1291.43488	1	7.25E-05	0.41	3.09	-	511.3
AHQ-2-11, 4152	R.AADFIDQALAQK.N	1291.43488	2	5.66E-04	0.90	3.82	-	922.0
AHQ-2-11, 5369	K.ANDTQEFNLSAYFER.A	1805.88207	2	2.65E-07	0.89	3.63	-	933.9
AHQ-2-11, 5912 - 5952	R.EIGPNDGFLAQLCQLNDR.L	2062.24949	2	2.17E-08	0.94	4.97	-	860.7
AHQ-2-11, 6097	R.EIGPNDGFLAQLCQLNDR.L	2062.24949	2	4.36E-05	0.61	3.11	-	422.5
AHQ-2-11, 3616	R.IYVGNASVAQDIPK.L	1475.67136	2	1.02E-05	0.96	4.77	-	1434.7
AHQ-2-11, 4046 - 4049	R.IYVGNASVAQDIPK.L	1475.67136	2	4.20E-08	0.96	4.18	-	1594.5
AHQ-2-11, 3266	K.LGITHVLNAAEGR.S	1351.53669	2	1.04E-06	0.91	3.24	-	1256.6
AHQ-2-11, 3277	K.LGITHVLNAAEGR.S	1351.53669	1	9.57E-05	0.50	2.48	-	467.3
gi|5802974|ref|NP_006784.1| peroxiredoxin 3; antioxidant protein 1; thioredoxin-dependent peroxide				2.21E-08	4.92	60.29	36.70	27692.5
AHQ-2-10, 4223	K.AFQYVETHGEVCPANWTPDSPTIKPSPAASK.E	3388.70790	3	2.21E-08	0.96	5.73	-	1246.3
AHQ-2-11, 3937	K.DLSLDDFK.G	953.02795	1	1.17E-05	0.46	2.15	-	519.1
AHQ-2-10, 5116	R.DYGVLLEGSGLALR.G	1463.66058	1	2.81E-04	0.06	2.10	-	152.8
AHQ-2-11, 5312	R.DYGVLLEGSGLALR.G	1463.66058	2	6.21E-07	0.96	3.90	-	1895.8
AHQ-2-11, 5485	R.GLFIIDPNGVIK.H	1286.54452	1	2.64E-05	0.40	2.16	-	521.8
AHQ-2-11, 5342	R.GLFIIDPNGVIK.H	1286.54452	2	9.99E-06	0.72	2.61	-	858.0
AHQ-2-10, 2662	K.HLSVNDLPVGR.S	1207.36435	2	7.58E-07	0.90	3.30	-	780.0
AHQ-2-10, 4882	R.KNGGLGHMNIALLSDLTK.Q	1883.20557	2	1.74E-04	0.93	4.25	-	827.3
gi|5453599|ref|NP_006127.1| capping protein (actin filament) muscle Z-line, alpha 2; F-actin cappin				2.23E-08	2.70	30.26	18.90	32948.9
AHQ-2-9, 4630	R.EGAAHAFAQYNLDQFTPVK.I	2108.29826	2	3.98E-05	0.93	4.26	-	924.7
AHQ-2-9, 5171 - 5186	K.FTITPSTTQVVGILK.I	1605.90007	2	2.23E-08	0.81	3.28	-	472.1
AHQ-2-9, 4700	K.KIDGQQTIIACIESHQFQAK.N	2317.60733	2	1.74E-05	0.95	5.22	-	743.7
gi|5453555|ref|NP_006316.1| ras-related nuclear protein [Homo sapiens]				2.23E-08	1.71	20.20	14.80	24422.9
AHQ-2-10, 5250 - 5258	K.SNYNFEKPFLWLAR.K	1786.02509	2	2.23E-08	0.90	3.98	-	620.5
AHQ-2-10, 3970 - 3994	K.YVATLGVEVHPLVFHTNR.G	2053.35211	2	2.05E-06	0.81	3.70	-	419.5
gi|16933567|ref|NP_005361.2| mel transforming oncogene; ras-associated protein RAB8 [Homo sapiens]				2.43E-08	0.88	10.19	6.80	23668.1
AHQ-2-11, 5680	K.ANINVENAFFTLAR.D	1580.76926	2	6.88E-05	0.89	3.52	-	925.3
AHQ-2-11, 5049	K.ANINVENAFFTLAR.D	1580.76926	2	2.43E-08	0.88	3.72	-	805.6
gi|27597085|ref|NP_000357.3| tropomyosin 1 (alpha) [Homo sapiens]				2.50E-08	2.71	30.24	16.90	32865.7
AHQ-2-9, 2835	K.ALMAAEDKYSQKEDRYEEEIK.V	2547.78004	2	2.50E-08	0.85	3.61	-	835.5
AHQ-2-9, 2864	R.AQKDEEKMEIQEIQLK.E	1961.22673	2	2.67E-05	0.95	4.76	-	1173.3
AHQ-2-10, 2955	R.KLVIIESDLER.A	1315.54103	1	2.14E-04	0.14	2.00	-	202.7
AHQ-2-9, 2958 - 2979	R.KLVIIESDLER.A	1315.54103	2	5.51E-05	0.91	3.84	-	1047.8
gi|4507649|ref|NP_003280.1| tropomyosin 2 (beta) [Homo sapiens]				2.66E-08	0.93	10.19	4.60	32989.6
AHQ-2-9, 2588	K.ATDAEADVASLNR.R	1333.38703	2	2.66E-08	0.93	3.77	-	1371.4
gi|4826643|ref|NP_005130.1| annexin A3; Annexin III (lipocortin III); annexin III (lipocortin III,				2.67E-08	0.91	10.21	5.00	36375.0
AHQ-2-9, 6074	K.GAGTNEDALIEILTTR.T	1674.83453	2	2.67E-08	0.91	4.26	-	908.3
gi|13385594|ref|NP_085056.1| cytochrome b5 outer mitochondrial membrane precursor; type 2 cyt-b5 [H				2.69E-08	0.86	10.22	23.30	16332.0
AHQ-2-11, 5934	R.FLNEHPGGEEVLLEQAGVDASESFEDVGHSSDAR.E	3629.75848	3	2.69E-08	0.86	4.32	-	604.6
gi|4505813|ref|NP_003737.1| cytoplasmic dynein light polypeptide; dynein, cytoplasmic, light chain				2.74E-08	2.74	40.17	49.40	10365.8
AHQ-2-14, 5373	K.NADM*SEEMQQDSVECATQALEK.Y	2532.67934	2	5.60E-04	0.58	3.10	-	556.4
AHQ-2-14, 5010	K.NADMSEEM*QQDSVECATQALEK.Y	2532.67934	2	7.57E-06	0.46	3.23	-	481.2
AHQ-2-14-, 2273	R.NFGSYVTHETK.H	1283.37131	2	1.09E-06	0.86	3.29	-	708.8
AHQ-2-14-, 3567 - 3568	K.YNPTWHCIVGR.N	1404.57820	2	2.74E-08	0.84	3.46	-	399.4
AHQ-2-13-, 3514	K.YNPTWHCIVGR.N	1404.57820	2	1.97E-05	0.83	3.40	-	527.2
gi|4504071|ref|NP_000164.1| platelet glycoprotein Ib alpha polypeptide precursor [Homo sapiens] [MA				2.96E-08	4.53	50.23	9.40	68955.0
AHQ-2-1, 5176	R.GLGELQELYLK.G	1263.46458	2	2.43E-05	0.94	4.10	-	1346.2
AHQ-2-4, 5090	R.GLGELQELYLK.G	1263.46458	2	3.67E-04	0.95	4.52	-	1252.8
AHQ-2-3, 5091 - 5095	R.GLGELQELYLK.G	1263.46458	2	2.79E-05	0.94	4.24	-	1144.5
AHQ-2-3, 4192	R.GQDLLSTVSIR.Y	1189.34410	2	6.02E-07	0.96	4.46	-	1308.0
AHQ-2-4, 4173	R.GQDLLSTVSIR.Y	1189.34410	2	2.96E-08	0.96	3.97	-	1635.6
AHQ-2-12, 4047	R.GQDLLSTVSIR.Y	1189.34410	2	6.74E-08	0.96	4.42	-	1377.6
AHQ-2-1, 4312 - 4316	R.GQDLLSTVSIR.Y	1189.34410	2	1.30E-07	0.96	4.54	-	1397.6
AHQ-2-14, 5009	R.GQDLLSTVSIR.Y	1189.34410	2	2.85E-04	0.86	2.71	-	1057.3
AHQ-2-4, 1973 - 1976	R.GVLQGHLESSR.N	1183.29965	1	2.32E-04	0.25	2.43	-	358.6
AHQ-2-3, 1955 - 1987	R.GVLQGHLESSR.N	1183.29965	2	3.85E-05	0.89	3.46	-	843.9
AHQ-2-4, 1964 - 1970	R.GVLQGHLESSR.N	1183.29965	2	2.38E-05	0.88	3.03	-	998.7
AHQ-2-3, 3607 - 3609	R.LTQLNLDRCELTK.L	1605.83862	2	2.16E-05	0.79	3.84	-	606.1
AHQ-2-4, 3616 - 3628	R.LTQLNLDRCELTK.L	1605.83862	2	2.92E-04	0.86	4.08	-	703.3
AHQ-2-4, 5364	R.WLQDNAENVYVWK.Q	1665.82948	2	3.73E-04	0.68	2.69	-	463.6
AHQ-2-3, 5019	R.WLQDNAENVYVWK.Q	1665.82948	2	6.00E-08	0.94	3.93	-	1217.1
AHQ-2-3, 5347	R.WLQDNAENVYVWK.Q	1665.82948	2	4.36E-08	0.96	3.88	-	1603.6
gi|4758076|ref|NP_004068.1| citrate synthase precursor; Citrate synthase, mitochondrial [Homo sapie				2.99E-08	1.77	20.19	5.80	51706.2
AHQ-2-8, 4499 - 4504	K.GLVYETSVLDPDEGIR.F	1763.92654	2	2.99E-08	0.87	3.70	-	667.4
AHQ-2-8, 3048 - 3056	K.IVPNVLLEQGK.A	1210.44805	2	5.36E-06	0.90	3.17	-	593.8
gi|4885183|ref|NP_005210.1| diaphanous 1; deafness, autosomal dominant 1; hDia1 [Homo sapiens] [MAS				2.99E-08	1.80	20.25	3.10	138977.7
AHQ-2-3, 6748	K.LQFSEQVENIKPEIVSVTAACEELR.K	2892.23121	3	3.88E-05	0.87	3.75	-	1300.3
AHQ-2-4, 5394	K.LVAEDLSQDCFWTK.V	1713.88927	2	6.00E-07	0.95	4.09	-	1127.6
AHQ-2-3, 5364	K.LVAEDLSQDCFWTK.V	1713.88927	2	2.99E-08	0.92	3.88	-	944.6
gi|6912240|ref|NP_036227.1| adaptor-related protein complex 3, mu 1 subunit; mu-adaptin 3A; AP-3 ad				3.24E-08	0.90	10.20	5.50	46938.9
AHQ-2-10, 4434	K.GLVNLQSGAPKPEENPSLNIQFK.I	2481.78929	2	3.24E-08	0.90	4.10	-	659.6
gi|4557597|ref|NP_001449.1| gamma filamin; filamin 2; filamin C, gamma (actin-binding protein-280);				3.29E-08	1.76	30.28	1.70	288897.4
AHQ-2-1, 4826 - 4836	R.GQHVPGSPFQFTVGPLGEGGAHK.V	2305.53673	2	1.24E-04	0.95	5.70	-	546.8
AHQ-2-4, 4608 - 4613	R.GQHVPGSPFQFTVGPLGEGGAHK.V	2305.53673	3	1.26E-07	0.83	4.07	-	391.1
AHQ-2-6, 4165	R.GQHVPGSPFQFTVGPLGEGGAHK.V	2305.53673	2	6.09E-05	0.88	4.36	-	363.0
AHQ-2-3, 4660	R.GQHVPGSPFQFTVGPLGEGGAHK.V	2305.53673	3	1.39E-05	0.89	4.47	-	608.9
AHQ-2-5, 4136 - 4213	R.GQHVPGSPFQFTVGPLGEGGAHK.V	2305.53673	2	4.35E-05	0.94	5.49	-	453.7
AHQ-2-2, 1705 - 1760	K.IECDDKGDGSCDVR.Y	1628.67809	2	3.29E-08	0.87	3.00	-	942.6
AHQ-2-1, 1993	K.IECDDKGDGSCDVR.Y	1628.67809	2	3.38E-04	0.86	3.35	-	829.5
AHQ-2-2, 1793 - 2009	K.NDNDTFTVK.Y	1054.09237	1	2.79E-04	0.06	1.96	-	165.8
gi|11321601|ref|NP_002618.1| phosphofructokinase, platelet; Phosphofructokinase, platelet type [Hom				3.43E-08	3.66	40.29	8.80	85595.6
AHQ-2-5, 3200 - 3207	R.DLQSNVEHLTEK.M	1413.51529	2	3.60E-06	0.87	3.62	-	856.4
AHQ-2-5, 6129	K.EIGWTDVGGWTGQGGSILGTK.R	2120.30735	2	1.40E-05	0.83	3.72	-	585.7
AHQ-2-5, 6595	R.GITNLCVIGGDGSLTGANLFR.K	2137.40264	2	3.43E-08	0.97	5.84	-	1016.3
AHQ-2-5, 4907	R.LPLMECVQMTQDVQK.A	1822.16087	2	1.60E-05	0.98	4.93	-	2422.1

gi|4507509|ref|NP_003245.1| tissue inhibitor of metalloproteinase 1 precursor; Erythroid-potentiati				3.48E-08	1.66	20.23	16.90	23170.8
AHQ-2-9, 5534	R.FVYTPAMESVCGYFHR.S	1966.22851	2	3.48E-08	0.73	2.93	-	682.0
AHQ-2-10, 4216	K.LQSGTHCLWTDQLLQGSEK.G	2203.41795	2	3.84E-04	0.93	4.54	-	927.0
gi|5174393|ref|NP_006001.1| arginine-rich, mutated in early stage tumors; arginine-rich protein [Ho				3.52E-08	2.47	30.29	13.70	26906.2
AHQ-2-12, 4678	K.DRDVTFSPATIENELIK.F	1949.15100	2	1.02E-05	0.94	4.53	-	1126.2
AHQ-2-12, 5651 - 5723	K.DRDVTFSPATIENELIK.F	1949.15100	2	8.12E-06	0.97	5.49	-	1648.8
AHQ-2-12, 5745	K.DRDVTFSPATIENELIK.F	1949.15100	2	3.52E-08	0.98	5.76	-	2252.9
AHQ-2-12, 6273 - 6305	R.DVTFSPATIENELIK.F	1677.87678	2	2.70E-04	0.65	2.81	-	476.7
AHQ-2-12, 3223	K.ILDDWGETCKGCAEK.S	1784.94596	2	3.70E-04	0.84	3.14	-	680.1
gi|5901922|ref|NP_008996.1| CDC37 homolog [Homo sapiens]				3.61E-08	0.94	10.24	5.60	44468.0
AHQ-2-7, 6733 - 6807	R.LGPGGLDPVEVYESLPEELQK.C	2270.52091	2	3.61E-08	0.94	4.79	-	572.1
gi|19913441|ref|NP_002140.2| hippocalcin-like 1; visinin-like protein 3; calcium-binding protein BD				3.67E-08	2.80	30.22	22.30	22313.1
AHQ-2-11, 4174 - 4181	R.ENTEFTDHELQEWYK.G	1970.04095	2	3.67E-08	0.91	4.14	-	903.4
AHQ-2-11, 5752	R.SEMLEIVQAIYK.M	1424.68776	2	8.51E-04	0.93	2.75	-	1468.6
AHQ-2-11, 5920	K.WAFSMYDLDGNGYISR.S	1896.07185	2	8.42E-08	0.96	4.24	-	1714.4
gi|20070158|ref|NP_003567.2| serine/threonine kinase 24 (STE20 homolog, yeast); STE20-like kinase 3				3.70E-08	0.92	10.18	3.40	49307.6
AHQ-2-7, 3641	K.SQACGGNLGSIEELR.G	1592.71366	2	3.70E-08	0.92	3.53	-	1541.3
gi|29737978|ref|XP_290747.1| similar to Myosin heavy chain, nonmuscle type B (Cellular myosin heavy				3.70E-08	4.37	50.25	3.80	191380.7
AHQ-2-3, 3975 - 3991	K.ADFCIIHYAGK.V	1296.47641	2	7.08E-07	0.94	3.22	-	1369.4
AHQ-2-4, 3965	K.ADFCIIHYAGK.V	1296.47641	2	6.94E-06	0.95	3.68	-	1628.8
AHQ-2-4, 3978	K.ADFCIIHYAGK.V	1296.47641	1	7.60E-06	0.71	2.75	-	622.3
AHQ-2-7, 3609	K.ADFCIIHYAGK.V	1296.47641	2	1.75E-07	0.89	2.78	-	1360.8
AHQ-2-6, 3650	K.ADFCIIHYAGK.V	1296.47641	2	1.71E-06	0.96	4.04	-	1404.8
AHQ-2-5, 3687	K.ADFCIIHYAGK.V	1296.47641	2	2.94E-06	0.95	3.47	-	1406.8
AHQ-2-2, 4000 - 4002	K.ADFCIIHYAGK.V	1296.47641	2	3.70E-08	0.97	4.16	-	1651.8
AHQ-2-1, 3066	R.AGVLAHLEEER.D	1224.34854	1	6.10E-07	0.51	2.65	-	546.3
AHQ-2-1, 3056 - 3061	R.AGVLAHLEEER.D	1224.34854	2	6.74E-07	0.94	4.35	-	1383.8
AHQ-2-2, 2806	R.AGVLAHLEEER.D	1224.34854	2	4.18E-07	0.95	4.04	-	1451.4
AHQ-2-2, 2762 - 2832	R.AGVLAHLEEER.D	1224.34854	2	7.13E-04	0.87	3.08	-	1104.0
AHQ-2-1, 3017 - 3089	R.AGVLAHLEEER.D	1224.34854	1	3.77E-04	0.20	1.84	-	544.9
AHQ-2-9, 2558	R.AGVLAHLEEER.D	1224.34854	1	7.87E-04	0.27	2.40	-	340.1
AHQ-2-3, 2744 - 2815	R.AGVLAHLEEER.D	1224.34854	2	1.16E-07	0.94	3.85	-	1297.1
AHQ-2-3, 2772 - 2775	R.AGVLAHLEEER.D	1224.34854	1	1.09E-04	0.63	2.85	-	573.6
AHQ-2-1, 6757 - 6794	R.ALEQQVEEM*RTQLEELEDELQATEDAK.L	3193.39738	3	6.73E-04	0.62	3.43	-	528.6
AHQ-2-2, 3762	K.DKADFCIIHYAGK.V	1539.73719	3	2.54E-06	0.95	4.58	-	1414.8
AHQ-2-2, 3750 - 3757	K.DKADFCIIHYAGK.V	1539.73719	2	3.86E-06	0.96	4.91	-	1395.1
AHQ-2-5, 3389	K.DKADFCIIHYAGK.V	1539.73719	3	9.67E-08	0.91	3.72	-	1491.8
AHQ-2-1, 3886	K.DKADFCIIHYAGK.V	1539.73719	2	1.08E-06	0.96	4.41	-	1623.5
AHQ-2-3, 3689	K.DKADFCIIHYAGK.V	1539.73719	2	3.71E-08	0.94	3.85	-	1455.6
AHQ-2-2, 4798 - 4837	K.LDPHLVLDQLR.C	1319.53460	2	1.18E-06	0.87	2.97	-	722.4
AHQ-2-2, 4822	K.LDPHLVLDQLR.C	1319.53460	3	1.06E-06	0.89	3.38	-	963.0
AHQ-2-1, 4916	K.LDPHLVLDQLR.C	1319.53460	3	8.66E-06	0.84	3.74	-	727.8
gi|14251209|ref|NP_001279.2| chloride intracellular channel 1; p64CLCP; chloride channel ABP [Homo				3.92E-08	6.48	80.30	46.10	26922.5
AHQ-2-9, 6487 - 6550	K.FLDGNELTLADCNLLPK.L	1935.18752	2	3.20E-06	0.85	4.78	-	679.7
AHQ-2-9, 6402	K.FLDGNELTLADCNLLPK.L	1935.18752	2	9.85E-05	0.23	2.59	-	350.2
AHQ-2-9, 3591	K.FSAYIKNSNPALNDNLEK.G	2039.23424	2	4.43E-04	0.87	4.19	-	771.4
AHQ-2-13-, 3418	K.GVTFNVTTVDTK.R	1282.42476	2	3.11E-06	0.89	3.61	-	916.3
AHQ-2-13, 3431 - 3436	K.GVTFNVTTVDTK.R	1282.42476	2	6.36E-06	0.95	3.96	-	1098.0
AHQ-2-10, 3087	K.GVTFNVTTVDTK.R	1282.42476	2	1.92E-05	0.92	3.75	-	1005.4
AHQ-2-14-, 3365	K.GVTFNVTTVDTK.R	1282.42476	2	4.40E-05	0.77	3.03	-	527.9
AHQ-2-9, 3111	K.GVTFNVTTVDTK.R	1282.42476	2	4.72E-06	0.90	3.26	-	959.2
AHQ-2-9, 2260	K.IGNCPFSQR.L	1080.19948	2	1.03E-04	0.76	3.06	-	484.5
AHQ-2-9, 5792 - 5822	R.KFLDGNELTLADCNLLPK.L	2063.36044	2	4.20E-06	0.95	5.34	-	1211.6
AHQ-2-9, 5535 - 5611	K.LAALNPESNTAGLDIFAK.F	1846.07392	2	3.92E-08	0.97	6.02	-	999.2
AHQ-2-13, 5311	K.LAALNPESNTAGLDIFAK.F	1846.07392	2	3.04E-05	0.91	4.20	-	705.9
AHQ-2-9, 5282 - 5350	K.LAALNPESNTAGLDIFAK.F	1846.07392	2	4.80E-05	0.93	4.74	-	637.1
AHQ-2-10, 4930	K.LAALNPESNTAGLDIFAK.F	1846.07392	2	7.93E-06	0.96	5.18	-	958.6
AHQ-2-10, 5167	K.LAALNPESNTAGLDIFAK.F	1846.07392	2	8.54E-04	0.90	3.94	-	629.1
AHQ-2-11, 5169 - 5192	K.LAALNPESNTAGLDIFAK.F	1846.07392	2	3.02E-04	0.92	4.26	-	737.1
AHQ-2-14-, 5369	K.LAALNPESNTAGLDIFAK.F	1846.07392	2	1.73E-05	0.95	5.26	-	758.6
AHQ-2-12, 5430	K.LAALNPESNTAGLDIFAK.F	1846.07392	2	2.75E-06	0.92	3.95	-	792.3
AHQ-2-9, 2442 - 2462	K.LHIVQVVCK.K	1097.35623	1	3.16E-04	0.59	2.39	-	449.1
AHQ-2-10, 2439	K.LHIVQVVCK.K	1097.35623	1	1.42E-05	0.76	2.62	-	780.3
AHQ-2-9, 2459	K.LHIVQVVCK.K	1097.35623	1	1.05E-05	0.78	2.79	-	574.5
AHQ-2-12, 2653	K.LHIVQVVCK.K	1097.35623	1	1.82E-05	0.73	2.41	-	830.8
AHQ-2-9, 5414 - 5482	K.VLDNYLTSPLPEEVDETSAEDEGVSQR.K	2994.12446	2	5.80E-04	0.65	3.01	-	381.5
AHQ-2-12, 5358	K.VLDNYLTSPLPEEVDETSAEDEGVSQR.K	2994.12446	2	4.98E-06	0.41	2.60	-	319.1
gi|4758484|ref|NP_004823.1| glutathione-S-transferase like; glutathione transferase omega [Homo sap				3.97E-08	2.67	50.19	24.10	27565.7
AHQ-2-10, 3682	K.EDPTVSALLTSEK.D	1390.51840	1	2.62E-06	0.55	2.70	-	263.9
AHQ-2-10, 3774	K.EFTKLEEVLTNK.K	1451.64657	2	3.97E-08	0.77	3.88	-	430.3
AHQ-2-10, 2328 - 2331	K.GSAPPGPVPEGSIR.I	1321.46422	1	8.31E-05	0.23	2.65	-	159.0
AHQ-2-10, 2475	R.HEVININLK.N	1080.26247	1	4.94E-05	0.85	3.57	-	490.9
AHQ-2-10, 2459 - 2463	R.HEVININLK.N	1080.26247	2	1.07E-06	0.89	3.25	-	727.1
AHQ-2-10, 2458	R.HEVININLK.N	1080.26247	1	4.94E-05	0.64	2.72	-	499.7
AHQ-2-9, 4344	K.VPSLVGSFIR.S	1075.28583	1	4.18E-04	0.24	1.88	-	237.7
gi|14249348|ref|NP_116120.1| hypothetical protein MGC14353 [Homo sapiens]				4.23E-08	0.99	20.18	19.50	13940.7
AHQ-2-13-, 4145	K.SWCPDCVQAEPVVR.E	1705.89366	2	4.23E-08	0.95	3.56	-	1525.1
AHQ-2-13, 4715 - 4781	K.TIFAYFTGSK.D	1135.29336	1	3.44E-05	0.04	1.96	-	77.5
gi|4505303|ref|NP_002466.1| myosin alkali light chain 1 slow a; myosin light chain 1, slow-twitch m				4.26E-08	2.66	30.20	11.50	22763.8
AHQ-2-12, 2971	R.ALGQNPTNAEVLK.V	1355.52211	1	6.29E-06	0.21	2.37	-	290.9
AHQ-2-12, 2598 - 2665	R.ALGQNPTNAEVLK.V	1355.52211	2	3.44E-07	0.86	3.94	-	567.9
AHQ-2-12, 2609 - 2673	R.ALGQNPTNAEVLK.V	1355.52211	1	9.97E-04	0.35	2.86	-	319.8
AHQ-2-12, 2963 - 2967	R.ALGQNPTNAEVLK.V	1355.52211	2	2.28E-04	0.81	3.28	-	619.8
AHQ-2-12, 2515	R.ALGQNPTNAEVLK.V	1355.52211	2	3.55E-05	0.88	3.76	-	588.4
AHQ-2-12, 3269	K.ILYSQCGDVMR.A	1343.55489	2	4.26E-08	0.91	2.92	-	1063.0
AHQ-2-14-, 3260	K.ILYSQCGDVMR.A	1343.55489	2	6.48E-05	0.90	2.80	-	1076.7
AHQ-2-12, 2581 - 2651	K.ILYSQCGDVM*R.A	1359.55429	2	1.49E-04	0.89	3.33	-	885.1
gi|30158489|ref|XP_301421.1| similar to Chloride intracellular channel protein 1 (Nuclear chloride				4.39E-08	1.64	20.22	6.90	28267.5
AHQ-2-9, 6250 - 6319	K.FLNGNELTLADCNLLPK.L	1934.20276	2	2.05E-06	0.86	4.27	-	977.7
AHQ-2-10, 5795 - 5859	K.FLNGNELTLADCNLLPK.L	1934.20276	2	2.80E-05	0.50	3.03	-	555.1
AHQ-2-12, 6203	K.FLNGNELTLADCNLLPK.L	1934.20276	2	4.39E-08	0.81	3.90	-	896.7
AHQ-2-9, 5708 - 5754	R.KFLNGNELTLADCNLLPK.L	2062.37568	2	2.76E-07	0.83	4.34	-	795.3
gi|18571222|ref|XP_088393.1| similar to destrin - pig [Homo sapiens]				4.48E-08	1.85	20.20	11.70	11560.5
AHQ-2-12, 2509	K.HFVGMLPEKDCR.C	1490.73281	3	4.48E-08	0.90	3.70	-	1150.1
AHQ-2-12, 2199	K.HFVGM*LPEKDCR.C	1506.73221	2	3.83E-04	0.95	3.98	-	1167.6
gi|4758304|ref|NP_004902.1| protein disulfide isomerase related protein (calcium-binding protein, i				4.49E-08	3.82	50.20	12.40	72931.9
AHQ-2-6, 4603	K.DLGLSESGEDVNAAILDESGKK.F	2248.38737	2	5.60E-08	0.92	4.05	-	1110.0
AHQ-2-6, 6047 - 6057	R.EVSQPDWTPPPEVTLVLTK.E	2137.41772	2	8.92E-04	0.35	2.94	-	143.4
AHQ-2-6, 4574	K.FAMEPEEFDSDTLR.E	1687.80890	2	4.49E-08	0.78	3.12	-	497.9
AHQ-2-6, 2753	K.IDATSASVLASR.F	1191.31686	2	2.05E-05	0.92	3.42	-	1129.0
AHQ-2-6, 2725 - 2734	K.VSQGQLVVMQPEK.F	1443.69431	2	3.74E-06	0.84	3.18	-	1324.3
gi|4506063|ref|NP_002725.1| protein kinase, cAMP-dependent, regulatory, type I, alpha; tissue-speci				4.53E-08	1.41	20.16	7.60	42981.4
AHQ-2-9, 5120	R.LTVADALEPVQFEDGQK.I	1861.04222	2	9.47E-05	0.49	2.66	-	281.1
AHQ-2-7, 2823	K.NVLFSHLDDNER.S	1459.54574	2	4.53E-08	0.92	3.15	-	1352.6
gi|27478053|ref|XP_015334.4| similar to heat shock 70kD protein binding protein; progesterone recep				4.71E-08	0.98	10.26	11.60	16603.8
AHQ-2-7, 4677	R.LLGHWEEAAHDLALACK.L	1936.18044	2	4.71E-08	0.98	5.24	-	2060.5
gi|24797067|ref|NP_002107.3| major histocompatibility complex, class I, A precursor; HLA-A1 class I				4.80E-08	4.09	50.23	21.60	40840.5

AHQ-2-8, 3178	R.DGEDQTQDTELVETRPAGDGTFQK.W	2638.69666	3	5.38E-06	0.62	3.18	-	477.3
AHQ-2-8, 3171	R.DGEDQTQDTELVETRPAGDGTFQK.W	2638.69666	2	9.92E-06	0.68	3.84	-	376.5
AHQ-2-14-, 5008	R.FIAVGYVDDTQFVR.F	1630.82504	2	1.18E-06	0.92	3.57	-	1249.1
AHQ-2-14, 6130 - 6208	R.FIAVGYVDDTQFVR.F	1630.82504	2	2.78E-04	0.93	3.08	-	1534.2
AHQ-2-8, 2902 - 2916	K.WAAVVVPSGEEQR.Y	1428.57478	2	2.39E-04	0.95	3.98	-	1182.1
AHQ-2-8, 1858	R.YLENGKETLQR.T	1351.49032	2	6.15E-05	0.66	2.57	-	721.8
AHQ-2-8, 2754	R.YTCHVQHEGLPKPLTLR.W	2051.35781	3	4.80E-08	0.94	4.68	-	1908.2
gi|4758950|ref|NP_000933.1| peptidylprolyl isomerase B (cyclophilin B) [Homo sapiens]				4.92E-08	7.72	90.24	39.80	23742.4
AHQ-2-12, 4231 - 4287	K.DFMIQGGDFTR.G	1287.42660	2	3.17E-04	0.81	2.95	-	891.9
AHQ-2-11, 2852	R.DKPLKDVIIADCGK.I	1573.83653	3	1.01E-05	0.95	4.71	-	1740.5
AHQ-2-11, 2830	R.DKPLKDVIIADCGK.I	1573.83653	2	4.92E-08	0.93	3.57	-	1147.6
AHQ-2-12, 4453	K.DTNGSQFFITTVK.T	1458.59766	2	1.95E-04	0.86	2.99	-	1039.2
AHQ-2-13-, 4438	K.DTNGSQFFITTVK.T	1458.59766	2	1.04E-06	0.84	3.09	-	826.3
AHQ-2-11, 4201 - 4270	K.DTNGSQFFITTVK.T	1458.59766	1	6.84E-05	0.17	2.28	-	349.7
AHQ-2-12, 4449	K.DTNGSQFFITTVK.T	1458.59766	1	5.18E-06	0.07	1.86	-	207.9
AHQ-2-11, 2388	K.DVIIADCGK.I	992.12891	1	4.17E-07	0.45	2.11	-	544.8
AHQ-2-12, 2426	K.DVIIADCGK.I	992.12891	1	3.38E-05	0.71	2.14	-	814.7
AHQ-2-12, 3003	K.HYGPGWVSMANAGK.D	1475.65664	2	9.79E-08	0.96	4.70	-	1063.5
AHQ-2-12, 3042 - 3049	K.IEVEKPFAIAK.E	1245.49240	2	6.50E-05	0.86	3.32	-	860.1
AHQ-2-11, 3004 - 3074	K.IEVEKPFAIAK.E	1245.49240	2	1.28E-05	0.93	3.61	-	1484.6
AHQ-2-11, 3193	K.IEVEKPFAIAKE	1374.60693	2	6.67E-04	0.97	4.39	-	2010.5
AHQ-2-12, 4366	K.TVDNFVALATGEK.G	1365.51377	2	5.15E-06	0.93	4.04	-	995.7
AHQ-2-12, 4371	K.TVDNFVALATGEK.G	1365.51377	1	2.72E-06	0.06	2.13	-	273.0
AHQ-2-12, 4350 - 4353	K.TVDNFVALATGEK.G	1365.51377	2	3.19E-04	0.91	3.78	-	786.6
AHQ-2-11, 4240	K.TVDNFVALATGEK.G	1365.51377	1	1.31E-05	0.46	3.00	-	329.7
AHQ-2-11, 4594 - 4666	K.TVDNFVALATGEK.G	1365.51377	2	5.51E-07	0.90	3.36	-	790.2
AHQ-2-14-, 4361	K.TVDNFVALATGEK.G	1365.51377	2	1.25E-04	0.76	3.00	-	603.8
AHQ-2-11, 2838 - 2913	K.VLEGMEVVR.K	1032.23962	2	3.11E-05	0.93	3.45	-	1211.9
gi|5174539|ref|NP_005908.1| cytosolic malate dehydrogenase; soluble malate dehydrogenase [Homo sapi				5.03E-08	2.29	30.20	12.00	36425.9
AHQ-2-13-, 2994	K.DVIATDKEDVAFK.D	1451.60343	2	5.03E-08	0.95	3.90	-	1619.9
AHQ-2-9, 5142	K.EVGVYEALKDDSWLK.G	1752.94535	2	7.14E-05	0.83	3.28	-	857.4
AHQ-2-9, 4632	K.FVEGLPINDFSR.E	1394.55680	1	8.95E-05	0.50	2.82	-	279.3
gi|22538814|ref|NP_002976.2| small inducible cytokine A5 precursor; T-cell specific protein p288; T				5.32E-08	1.41	20.20	20.90	9989.6
AHQ-2-14-, 3772 - 3780	K.CSNPAVVFVTR.K	1251.43718	2	5.32E-08	0.93	3.99	-	779.1
AHQ-2-14-, 2271	K.EYFYTSGK.C	995.06653	1	2.16E-04	0.48	2.25	-	253.9
gi|6912238|ref|NP_036226.1| peroxiredoxin 5; antioxidant enzyme B166 [Homo sapiens]				6.64E-08	6.43	70.27	44.90	22026.2
AHQ-2-12, 7069	K.ETDLLLDDSLVSIFGNR.R	1908.09888	2	8.49E-08	0.97	5.39	-	1416.7
AHQ-2-12, 3906 - 3982	R.FSMVVQDGIVK.A	1223.46709	2	4.13E-06	0.95	4.10	-	1302.6
AHQ-2-12, 4683	K.KGVLFGVPGAFTPGCSK.T	1724.01680	2	6.64E-08	0.94	4.28	-	908.6
AHQ-2-13-, 3490 - 3525	R.LLADPTGAFGK.E	1090.25413	2	1.22E-04	0.79	2.59	-	797.7
AHQ-2-12, 4206	K.THLPGFVEQAEALK.A	1540.74504	3	5.02E-05	0.94	3.97	-	2161.2
AHQ-2-12, 4886 - 4967	K.VGDAIPAVEVFEGEPGNK.V	1829.00022	2	5.30E-07	0.94	4.67	-	565.3
AHQ-2-12, 4765 - 4777	K.VNLAELFK.G	934.11414	2	8.05E-05	0.90	3.38	-	764.4
gi|5031985|ref|NP_005787.1| nuclear transport factor 2; placental protein 15 [Homo sapiens]				6.72E-08	3.95	50.30	63.00	14478.4
AHQ-2-14-, 6128	K.ADEDPIMGFHQMFLLK.N	1893.21919	3	1.92E-05	0.60	3.21	-	450.1
AHQ-2-14-, 6107 - 6119	K.IQHSITAQDHQPTPDSCIISMVVGQLK.A	3006.40418	3	1.39E-07	0.97	5.90	-	1857.0
AHQ-2-14-, 5387 - 5391	K.NINDAWVCTNDMFR.L	1757.92888	2	6.72E-08	0.97	4.98	-	1178.4
AHQ-2-14, 5550	K.NINDAWVCTNDM*FR.L	1773.92828	2	1.77E-05	0.46	2.89	-	381.1
AHQ-2-14-, 6445 - 6468	R.TQLGAIYIDASCLTWEGQQFQGK.A	2616.88699	2	1.54E-07	0.95	4.76	-	999.1
gi|14211923|ref|NP_115982.1| PKCI-1-related HIT protein [Homo sapiens]				7.12E-08	3.50	40.25	41.70	17161.6
AHQ-2-13-, 3277	K.AQQATPGGAAPTIFSR.I	1573.73512	2	6.26E-07	0.93	4.33	-	565.4
AHQ-2-13, 4753	R.DVAPQAPVHFLVIPK.K	1631.94245	2	9.11E-04	0.79	3.30	-	575.6
AHQ-2-13-, 6318	R.ISQAEEEDQQLLGHLLLVAK.Q	2235.52293	2	1.12E-05	0.95	4.92	-	1278.1
AHQ-2-13-, 6247 - 6314	R.ISQAEEEDQQLLGHLLLVAK.Q	2235.52293	3	7.12E-08	0.88	3.99	-	975.6
AHQ-2-13, 6119	R.ISQAEEEDQQLLGHLLLVAK.Q	2235.52293	2	8.12E-07	0.93	4.42	-	1150.9
AHQ-2-13-, 6333 - 6398	K.SLPADILYEDQQCLVFR.D	2069.32354	2	4.50E-04	0.92	3.70	-	1054.7
AHQ-2-13, 6172	K.SLPADILYEDQQCLVFR.D	2069.32354	2	2.96E-05	0.90	3.90	-	825.1
gi|4507745|ref|NP_003320.1| thioredoxin [Homo sapiens]				7.73E-08	0.94	10.19	12.40	11693.3
AHQ-2-14, 4769 - 4784	K.TAFQEALDAAGDK.L	1337.41728	2	4.20E-07	0.77	3.00	-	918.0
AHQ-2-13-, 2809	K.TAFQEALDAAGDK.L	1337.41728	2	2.02E-05	0.76	2.62	-	1161.1
AHQ-2-13-, 3837 - 3909	K.TAFQEALDAAGDK.L	1337.41728	2	2.30E-06	0.93	3.49	-	1682.1
AHQ-2-13-, 3914 - 3930	K.TAFQEALDAAGDK.L	1337.41728	1	7.54E-05	0.49	2.22	-	888.7
AHQ-2-14-, 3815 - 3816	K.TAFQEALDAAGDK.L	1337.41728	2	7.73E-08	0.94	3.72	-	1429.5
AHQ-2-14-, 3820 - 3896	K.TAFQEALDAAGDK.L	1337.41728	1	6.68E-07	0.75	2.84	-	829.2
AHQ-2-13, 3863	K.TAFQEALDAAGDK.L	1337.41728	2	1.14E-06	0.93	3.29	-	1477.0
gi|4505759|ref|NP_002622.1| phosphogluconate dehydrogenase; 6-phosphogluconate dehydrogenase [Homo				8.12E-08	3.73	40.33	15.50	53151.7
AHQ-2-8, 2923	R.HEMLPASLIQAQR.D	1494.74454	2	2.05E-04	0.88	3.10	-	753.2
AHQ-2-8, 6563	K.IKDAFDRNPELQNLLLDDFFK.S	2552.86598	3	1.36E-05	0.94	4.88	-	1151.4
AHQ-2-8, 6208	K.LVPLLDTGDIIIDGGNSEYR.D	2161.39768	2	3.54E-07	0.91	4.28	-	958.2
AHQ-2-7, 6304	K.LVPLLDTGDIIIDGGNSEYR.D	2161.39768	2	8.12E-08	0.98	6.56	-	1979.8
AHQ-2-8, 4223 - 4224	K.VGTGEPCCDWVGDEGAGHFVK.M	2280.43619	3	1.48E-06	0.92	4.72	-	697.7
AHQ-2-8, 4222	K.VGTGEPCCDWVGDEGAGHFVK.M	2280.43619	2	3.43E-05	0.46	2.57	-	349.3
gi|4557801|ref|NP_000261.1| purine nucleoside phosphorylase [Homo sapiens]				8.16E-08	8.01	100.33	46.70	32147.7
AHQ-2-9, 1478	K.ANHEEVLAAGK.Q	1139.24364	1	8.29E-05	0.39	2.52	-	381.9
AHQ-2-9, 4298 - 4303	R.FHMYEGYPLWK.V	1471.70636	1	7.16E-05	0.62	2.55	-	716.1
AHQ-2-9, 4299	R.FHMYEGYPLWK.V	1471.70636	2	1.82E-04	0.92	3.14	-	1175.1
AHQ-2-13-, 3993 - 4066	K.HRPQVAIICGSGLGGLTDK.L	1981.26589	3	8.16E-08	0.98	5.51	-	2889.7
AHQ-2-13, 4023 - 4039	K.HRPQVAIICGSGLGGLTDK.L	1981.26589	2	1.41E-06	0.93	4.19	-	992.9
AHQ-2-13-, 4082	K.HRPQVAIICGSGLGGLTDK.L	1981.26589	2	1.04E-06	0.91	4.07	-	712.8
AHQ-2-13, 6867	K.LEQFVSILMASIPLPDK.A	1902.28716	2	2.36E-06	0.94	4.60	-	846.9
AHQ-2-13-, 7058 - 7135	K.LEQFVSILMASIPLPDK.A	1902.28716	2	9.01E-04	0.93	4.35	-	725.1
AHQ-2-9, 7483 - 7484	K.LEQFVSILMASIPLPDK.A	1902.28716	2	7.35E-06	0.96	5.05	-	1082.0
AHQ-2-9, 4126	K.LGADAVGM*STVPEVIVAR.H	1802.08606	2	2.09E-07	0.91	3.71	-	817.2
AHQ-2-13-, 4243 - 4246	K.LGADAVGM*STVPEVIVAR.H	1802.08606	2	1.31E-06	0.94	4.62	-	832.8
AHQ-2-13, 4855 - 4920	K.LGADAVGMSTVPEVIVAR.H	1786.08666	2	4.24E-04	0.55	2.91	-	774.3
AHQ-2-11, 4096	K.LGADAVGM*STVPEVIVAR.H	1802.08606	2	1.60E-04	0.89	3.79	-	859.8
AHQ-2-13, 5851 - 5853	K.LTQAQIFDYSEIPNFPR.S	2040.26383	2	9.01E-07	0.90	4.46	-	555.3
AHQ-2-13-, 5987	K.LTQAQIFDYSEIPNFPR.S	2040.26383	2	1.03E-05	0.94	4.54	-	807.1
AHQ-2-9, 6034 - 6060	K.LTQAQIFDYSEIPNFPR.S	2040.26383	2	1.09E-04	0.91	4.54	-	477.2
AHQ-2-9, 4792	R.VFGFSLITNK.V	1126.32960	2	3.94E-05	0.93	3.81	-	768.5
AHQ-2-9, 5986 - 5995	R.VFHLLGVDTLVVTNAAGGLNPK.F	2236.59890	2	6.10E-06	0.99	6.67	-	3631.5
AHQ-2-9, 6095	R.VFHLLGVDTLVVTNAAGGLNPK.F	2236.59890	2	4.31E-07	0.92	4.04	-	1058.0
AHQ-2-9, 3371 - 3380	K.VIMDYESLEK.A	1227.40938	2	3.57E-05	0.87	3.37	-	501.2
gi|24497612|ref|NP_116262.2| nm23-phosphorylated unknown substrate; SH3 domain-containing 70 kDa pr				8.26E-08	2.56	30.22	5.70	72695.6
AHQ-2-6, 6658	K.LGCDWVATIFSR.D	1426.62208	2	8.26E-08	0.93	3.42	-	1304.0
AHQ-2-6, 6066	R.RPYEDQGLGETTPLTIICQPMQPLR.V	2916.32264	3	1.45E-04	0.90	4.41	-	768.2
AHQ-2-6, 5295	R.RPYEDQGLGETTPLTIICQPM*QPLR.V	2932.32204	3	1.25E-04	0.73	4.05	-	557.1
gi|30147105|ref|XP_210550.2| similar to Chain , Aldose Reductase (E.C.1.1.1.21) Mutant With Tyr 48				8.40E-08	0.92	10.21	2.20	61339.9
AHQ-2-5, 4033	K.ADLINNLGTIAK.S	1243.43490	2	8.40E-08	0.92	4.14	-	997.8
AHQ-2-6, 4003	K.ADLINNLGTIAK.S	1243.43490	2	9.11E-05	0.87	3.08	-	885.0
AHQ-2-5, 3896	K.ADLINNLGTIAK.S	1243.43490	2	7.68E-04	0.81	3.38	-	408.8
gi|4503743|ref|NP_002009.1| flightless I homolog [Homo sapiens]				8.75E-08	2.65	30.23	4.50	144750.1
AHQ-2-4, 3185	K.AVQGAQQPSLYQIR.T	1559.75158	2	4.46E-07	0.92	3.76	-	817.5
AHQ-2-3, 7323	R.TQSNLPTSLEGLSNLADVDLSCNDLTR.V	2936.15611	2	1.62E-05	0.77	3.13	-	674.1
AHQ-2-3, 6871	K.VGLGLGYLELPQINYK.L	1778.08432	2	8.75E-08	0.96	4.66	-	1318.6
gi|4502097|ref|NP_001142.1| solute carrier family 25 (mitochondrial carrier; adenine nucleotide tra				9.02E-08	0.80	10.17	4.00	33148.4
AHQ-2-10, 5271	R.YFPTQALNFAFK.D	1447.66130	1	9.02E-08	0.80	3.29	-	594.0

AHQ-2-10, 5266 - 5275	R.YFPTQALNFAFK.D	1447.66130	2	4.42E-06	0.86	3.40	-	546.9
gi|5802966|ref|NP_006861.1| destrin (actin depolymerizing factor); destrin [Homo sapiens]				9.11E-08	2.72	30.22	24.80	18505.6
AHQ-2-12, 1875 - 1907	K.HECQANGPEDLNR.A	1541.58530	2	1.47E-05	0.92	4.05	-	858.5
AHQ-2-12, 7065 - 7125	R.KEELMFFLWAPELAPLK.S	2063.49022	2	1.05E-04	0.91	4.39	-	837.5
AHQ-2-13-, 3857 - 3881	R.YALYDASFETK.E	1308.41754	2	9.11E-08	0.88	2.99	-	979.6
AHQ-2-12, 3714 - 3733	R.YALYDASFETK.E	1308.41754	2	1.79E-06	0.95	3.83	-	1201.6
gi|30160191|ref|XP_294035.2| similar to Pyruvate kinase, M1 isozyme (Pyruvate kinase muscle isozyme				9.24E-08	0.70	10.16	2.80	50222.2
AHQ-2-7, 5184	K.IYVDDGLISLQVK.Q	1463.70048	1	9.24E-08	0.70	3.17	-	618.2
AHQ-2-7, 5112 - 5188	K.IYVDDGLISLQVK.Q	1463.70048	2	3.72E-06	0.91	2.84	-	1607.1
gi|16950601|ref|NP_005956.2| myosin light chain kinase isoform 6; myosin light chain kinase [Homo s				9.35E-08	2.78	30.20	3.20	110074.8
AHQ-2-12, 2523	R.DLEVVEGSAAR.F	1146.23295	2	4.86E-06	0.92	4.02	-	1168.9
AHQ-2-6, 3429 - 3430	K.LVQCVDAFEEK.A	1339.49637	2	8.70E-04	0.92	2.91	-	1221.2
AHQ-2-6, 3329	K.VSDFYDIEER.L	1273.33005	2	9.35E-08	0.94	3.30	-	1215.4
gi|19913373|ref|NP_079536.2| G6B protein isoform G6b-A precursor; G6B protein; immunoglobulin recep				9.44E-08	1.82	20.21	9.30	25002.6
AHQ-2-11, 2365	R.TVLHVLGDR.T	1010.17224	2	6.07E-05	0.88	2.86	-	1301.2
AHQ-2-12, 2391	R.TVLHVLGDR.T	1010.17224	2	5.01E-04	0.92	3.31	-	1399.1
AHQ-2-12, 2394	R.TVLHVLGDR.T	1010.17224	1	8.47E-04	0.64	2.19	-	606.3
AHQ-2-10, 2187	R.TVLHVLGDR.T	1010.17224	1	6.03E-04	0.57	2.28	-	486.0
AHQ-2-12, 2574	R.VNLSCGGVSHPIR.W	1397.58551	2	9.44E-08	0.94	4.22	-	1399.5
gi|18104989|ref|NP_002822.2| protein tyrosine phosphatase, non-receptor type 6 isoform 1; protein-t				9.44E-08	2.67	30.19	7.10	67560.7
AHQ-2-6, 5893	K.AGFWEEFESLQK.Q	1471.59489	2	2.17E-06	0.94	3.72	-	1543.0
AHQ-2-14, 6066	R.DLSGLDAETLLK.G	1275.43053	2	9.44E-08	0.94	3.61	-	1539.4
AHQ-2-6, 3030	R.WYHGHMSGGQAETLLQAK.G	2015.24110	2	7.87E-07	0.79	3.87	-	557.3
gi|9966867|ref|NP_065123.1| eIF-5A2 protein; eIF5AII [Homo sapiens]				9.60E-08	2.66	30.30	19.60	16793.1
AHQ-2-12, 3631 - 3638	K.KYEDICPSTHNMDVPNIK.R	2163.41723	3	3.63E-06	0.97	6.00	-	1676.2
AHQ-2-12, 4823 - 4825	K.VHLVGIDIFTGK.K	1299.54329	1	9.60E-08	0.93	3.67	-	1142.2
AHQ-2-12, 4821	K.VHLVGIDIFTGK.K	1299.54329	2	4.04E-07	0.93	3.40	-	1254.4
AHQ-2-11, 4656	K.VHLVGIDIFTGK.K	1299.54329	2	9.32E-05	0.91	3.32	-	1185.8
AHQ-2-12, 3973	K.YEDICPSTHNMDVPNIK.R	2035.24432	2	1.36E-04	0.75	3.45	-	560.1
gi|4503377|ref|NP_001377.1| dihydropyrimidinase-like 2; collapsin response mediator protein hCRMP-2				9.93E-08	2.32	30.19	8.00	62293.3
AHQ-2-7, 5521	R.FQMPDQGMTSADDFFQGTK.A	2152.34998	2	8.20E-04	0.82	2.77	-	1006.3
AHQ-2-7, 2540	R.GSPLVVISQGK.I	1085.27902	1	9.93E-08	0.59	2.46	-	621.3
AHQ-2-7, 3031	K.IVLEDGTLHVTEGSGR.Y	1683.84465	2	9.66E-06	0.91	3.87	-	860.5
gi|4506417|ref|NP_002877.1| RAP2B, member of RAS oncogene family [Homo sapiens]				1.01E-07	3.98	50.21	37.20	20494.2
AHQ-2-11, 6062 - 6073	K.ASVDELFAEIVR.Q	1349.51434	2	1.01E-07	0.95	4.08	-	1338.8
AHQ-2-11, 6752	K.EIEVDSSPSVLEILDTAGTEQFASMR.D	2826.08347	2	1.76E-05	0.79	3.76	-	425.4
AHQ-2-11, 3033	R.VPMILVGNK.V	971.24256	2	4.23E-06	0.92	2.95	-	982.1
AHQ-2-11, 2304	K.VVVLGSGGVGK.S	972.16391	1	2.82E-04	0.50	2.28	-	552.0
AHQ-2-11, 4138	K.YDPTIEDFYR.K	1319.40042	2	3.16E-05	0.82	2.59	-	470.2
gi|5453605|ref|NP_006421.1| chaperonin containing TCP1, subunit 4 (delta); chaperonin containing t-				1.02E-07	1.89	20.23	4.80	57838.8
AHQ-2-7, 6536	R.DALSDLALHFLNK.M	1457.65603	2	1.02E-07	0.97	4.51	-	2145.6
AHQ-2-7, 3356	K.VIDPATATSVDLR.D	1358.52277	2	1.40E-05	0.92	3.38	-	1066.7
gi|4757768|ref|NP_004300.1| Rho GDP dissociation inhibitor (GDI) alpha [Homo sapiens]				1.07E-07	3.67	40.24	22.50	23206.9
AHQ-2-12, 4658 - 4674	R.AEEYEFLTPVEEAPK.G	1752.89898	2	3.28E-06	0.91	4.26	-	743.3
AHQ-2-10, 4262 - 4267	R.AEEYEFLTPVEEAPK.G	1752.89898	2	1.39E-05	0.92	3.84	-	984.5
AHQ-2-10, 2810	K.IDKTDYMVGSYGPR.A	1602.79350	2	5.53E-06	0.91	3.32	-	1296.0
AHQ-2-10, 3556	K.SIQEIQELDKDDESLR.K	1919.03693	2	1.07E-07	0.95	4.81	-	943.0
AHQ-2-10, 3098 - 3104	K.SIQEIQELDKDDESLRK.Y	2047.20984	3	1.02E-06	0.86	4.09	-	786.2
AHQ-2-10, 3084 - 3102	K.SIQEIQELDKDDESLRK.Y	2047.20984	2	4.60E-07	0.90	4.45	-	646.8
gi|7657431|ref|NP_055205.1| EBNA-2 co-activator (100kD) [Homo sapiens]				1.08E-07	0.88	10.22	2.40	99689.7
AHQ-2-5, 6572 - 6580	R.NLPGLVQEGEPFSEEATLFTK.E	2307.54125	2	2.24E-06	0.92	4.35	-	550.5
AHQ-2-6, 6405	R.NLPGLVQEGEPFSEEATLFTK.E	2307.54125	2	1.08E-07	0.88	3.74	-	600.3
AHQ-2-4, 6772	R.NLPGLVQEGEPFSEEATLFTK.E	2307.54125	2	8.17E-06	0.89	4.33	-	425.9
gi|19913426|ref|NP_001683.2| ATPase, H+ transporting, lysosomal 56/58kD, V1 subunit B, isoform 1; A				1.09E-07	0.91	10.21	3.90	56832.7
AHQ-2-7, 5409	K.IPIFSAAGLPHNEIAAQICR.Q	2180.51549	3	1.09E-07	0.91	4.17	-	907.2
gi|13129150|ref|NP_005339.1| heat shock 90kDa protein 1, alpha; heat shock 90kD protein 1, alpha [H				1.18E-07	3.51	40.25	6.10	84659.1
AHQ-2-5, 2684	K.ELHINLIPNKQDR.T	1590.80882	2	7.17E-04	0.92	3.81	-	787.1
AHQ-2-6, 2218	K.FYEQFSK.N	949.04099	1	4.69E-04	0.45	2.30	-	279.3
AHQ-2-5, 2203	K.FYEQFSK.N	949.04099	1	4.40E-04	0.77	2.53	-	676.8
AHQ-2-5, 4012	R.NPDDITNEEYGEFYK.S	1834.87381	2	3.26E-06	0.95	4.94	-	843.7
AHQ-2-6, 3951	R.NPDDITNEEYGEFYK.S	1834.87381	2	1.18E-07	0.97	5.00	-	1213.4
AHQ-2-5, 3749	R.RAPFDLFENR.K	1265.40235	2	4.25E-06	0.86	3.48	-	634.3
gi|4505753|ref|NP_002620.1| phosphoglycerate mutase 1 (brain); Phosphoglycerate mutase A, nonmuscle				1.19E-07	4.51	60.24	28.30	28803.7
AHQ-2-10, 3696	R.FSGWYDADLSPAGHEEAK.R	1981.06697	2	2.35E-07	0.93	4.18	-	1020.1
AHQ-2-10, 3304	R.FSGWYDADLSPAGHEEAKR.G	2137.25332	2	1.19E-07	0.96	4.87	-	1088.1
AHQ-2-10, 1852	R.HYGGLTGLNK.A	1060.18811	1	8.33E-04	0.68	2.80	-	395.6
AHQ-2-10, 3795 - 3860	R.RSYDVPPPPMEPDHPFYSNISK.D	2574.85201	3	2.62E-05	0.71	3.08	-	656.0
AHQ-2-10, 4130	R.SYDVPPPPMEPDHPFYSNISK.D	2418.66566	2	2.78E-05	0.36	2.79	-	253.4
AHQ-2-10, 4695	R.YADLTEDQLPSCESLKDTIAR.A	2427.62735	3	9.19E-06	0.86	3.44	-	892.3
gi|8923812|ref|NP_060943.1| uncharacterized hypothalamus protein HT012 [Homo sapiens]				1.21E-07	0.93	10.18	8.60	14960.4
AHQ-2-13-, 4166	K.TLAFTSVDLTNK.A	1310.47811	2	1.21E-07	0.93	3.63	-	1211.6
gi|4507171|ref|NP_003109.1| secreted protein, acidic, cysteine-rich (osteonectin); Osteonectin (sec				1.22E-07	2.18	30.20	9.90	34632.0
AHQ-2-9, 2882	R.LEAGDHPVELLAR.D	1420.59579	2	1.04E-06	0.96	4.06	-	1498.2
AHQ-2-8, 2244 - 2307	K.RLEAGDHPVELLAR.D	1576.78214	2	2.10E-07	0.78	3.46	-	427.9
AHQ-2-11, 6186	K.YIPPCLDSELTEFPLR.M	1952.21655	2	1.22E-07	0.44	2.94	-	123.8
AHQ-2-9, 6616 - 6622	K.YIPPCLDSELTEFPLR.M	1952.21655	2	2.55E-06	0.64	2.67	-	280.6
gi|11993943|ref|NP_055419.1| Tax interaction protein 1 [Homo sapiens]				1.24E-07	0.95	10.21	13.70	13734.6
AHQ-2-13-, 4167	R.VSEGGPAEIAGLQIGDK.I	1641.80454	2	1.24E-07	0.95	4.12	-	1504.0
AHQ-2-13, 4121 - 4195	R.VSEGGPAEIAGLQIGDK.I	1641.80454	2	2.01E-06	0.94	3.66	-	1415.9
gi|7657313|ref|NP_055277.1| Lsm1 protein [Homo sapiens]				1.24E-07	0.86	10.21	12.00	15179.3
AHQ-2-12, 5955 - 6034	R.SIDQFANLVLHQTVER.I	1871.08670	2	1.24E-07	0.86	4.17	-	555.2
gi|4757766|ref|NP_004299.1| Rho GTPase activating protein 1; RhoGAP; p50rhoGAP; CDC42 GTPase-activa				1.25E-07	3.33	40.23	11.40	50435.4
AHQ-2-7, 6247	K.FLLDHQGELFPSPDPSGL	1970.17037	2	4.49E-07	0.85	3.60	-	704.2
AHQ-2-11, 3965	K.NPEQEPIPIVLR.E	1405.62426	2	4.60E-07	0.89	3.36	-	974.1
AHQ-2-8, 6120	R.VPATLQVLQTLPEENYQVLR.F	2312.65053	2	4.23E-04	0.64	2.85	-	484.4
AHQ-2-11, 5926	R.VPATLQVLQTLPEENYQVLR.F	2312.65053	2	1.25E-07	0.93	4.63	-	806.7
AHQ-2-7, 6172	R.VPATLQVLQTLPEENYQVLR.F	2312.65053	2	6.34E-04	0.68	3.06	-	513.2
AHQ-2-11, 6678	K.YNMGLPVDFDQYNELHLPAVILK.T	2691.09719	3	5.13E-05	0.66	3.08	-	258.5
gi|30148346|ref|XP_302059.1| similar to MLRQ subunit of the NADH: ubiquinone oxidoreductase complex				1.30E-07	0.32	10.11	6.60	17253.8
AHQ-2-14-, 3349 - 3376	K.FYSVNVDYSK.L	1222.32788	1	1.30E-07	0.32	2.19	-	246.1
gi|5902090|ref|NP_008862.1| solute carrier family 2 (facilitated glucose transporter), member 3; GL				1.40E-07	0.83	10.18	3.00	53924.0
AHQ-2-1, 5361	R.LWGTQDVSQDIQEMK.D	1778.96471	2	1.40E-07	0.83	3.51	-	481.6
gi|7661922|ref|NP_055814.1| RAB21, member RAS oncogene family [Homo sapiens]				1.45E-07	0.96	10.25	7.60	24347.4
AHQ-2-10, 3946	R.HVSIQEAESYAESVGAK.H	1805.92351	2	1.45E-07	0.96	5.06	-	1244.7
gi|8923541|ref|NP_060357.1| hypothetical protein FLJ20580 [Homo sapiens]				1.52E-07	0.92	10.21	10.00	18048.3
AHQ-2-10, 4110	K.AQESVGIYEVTHQFVK.C	1836.03759	2	1.52E-07	0.92	4.24	-	823.7
gi|4502021|ref|NP_003680.1| aldo-keto reductase family 7, member A2 (aflatoxin aldehyde reductase);				1.55E-07	0.81	10.17	5.80	36648.3
AHQ-2-9, 3694	K.ALQAAYGASAPSVTSAALR.W	1806.01302	2	1.55E-07	0.81	3.34	-	685.8
gi|10337595|ref|NP_066283.1| protein phosphatase 1A isoform 1; protein phosphatase 2C alpha isoform				1.64E-07	0.89	10.19	4.70	42447.5
AHQ-2-7, 3401	K.GPTEQLVSPEPEVHDIER.S	2033.18486	3	1.64E-07	0.89	3.85	-	1097.9
gi|4505621|ref|NP_002558.1| prostatic binding protein; phosphatidylethanolamine binding protein [Ho				1.67E-07	1.91	20.24	20.30	21056.6
AHQ-2-11, 4916	R.APVAGTCYQAEWDDYVPK.L	2072.23961	2	6.31E-05	0.96	4.46	-	1273.5
AHQ-2-11, 4262	K.GNDISSGTVLSDYVGSGPPK.G	1951.08062	2	1.67E-07	0.95	4.44	-	1110.2
gi|4507487|ref|NP_003238.1| thrombospondin 2 [Homo sapiens]				1.71E-07	1.74	20.19	2.60	129954.5
AHQ-2-1, 5418	R.DDDYAGFVFGYQSSSR.F	1814.84582	2	1.61E-04	0.87	3.49	-	920.8
AHQ-2-1, 5502	R.DDDYAGFVFGYQSSSR.F	1814.84582	2	1.88E-04	0.86	3.05	-	1023.0
AHQ-2-1, 3041 - 3045	R.NALWHTGNTPGQVR.T	1551.69124	2	1.71E-07	0.86	3.43	-	844.9

AHQ-2-2, 2516	R.NALWHTGNTPGQVR.T	1551.69124	2	5.73E-05	0.70	2.55	-	737.4
AHQ-2-3, 2509	R.NALWHTGNTPGQVR.T	1551.69124	3	7.06E-04	0.87	3.76	-	785.8
AHQ-2-3, 3063	R.NALWHTGNTPGQVR.T	1551.69124	2	1.37E-04	0.73	2.78	-	778.8
AHQ-2-4, 2449 - 2520	R.NALWHTGNTPGQVR.T	1551.69124	2	2.91E-04	0.76	3.44	-	872.1
AHQ-2-5, 2296	R.NALWHTGNTPGQVR.T	1551.69124	2	6.45E-07	0.88	3.04	-	1003.7
AHQ-2-11, 2493 - 2494	R.NALWHTGNTPGQVR.T	1551.69124	2	1.19E-05	0.92	3.17	-	1303.5
AHQ-2-13-, 2690	R.NALWHTGNTPGQVR.T	1551.69124	2	1.46E-05	0.86	3.24	-	982.4
AHQ-2-14-, 2644	R.NALWHTGNTPGQVR.T	1551.69124	2	3.13E-06	0.87	3.26	-	978.4
gi|10863873|ref|NP_000651.1| transforming growth factor, beta 1 (Camurati-Engelmann disease); trans				1.72E-07	3.00	40.21	16.90	44422.0
AHQ-2-13, 3368	R.ALDTNYCFSSTEK.N	1537.63010	2	2.74E-06	0.85	3.30	-	730.8
AHQ-2-13-, 3350	R.ALDTNYCFSSTEK.N	1537.63010	2	1.72E-07	0.92	3.90	-	788.9
AHQ-2-13-, 6602	K.GYHANFCLGPCPYIWSLDTQYSK.V	2781.07085	2	3.47E-04	0.59	2.63	-	395.3
AHQ-2-9, 6959	R.LLAPSDSPEWLSFDVTGVVR.Q	2189.45272	2	7.65E-06	0.57	3.19	-	317.8
AHQ-2-13, 3481	K.VEQLSNMIVR.S	1189.41074	2	1.90E-05	0.92	3.40	-	1328.5
gi|23308579|ref|NP_006592.3| unactive progesterone receptor, 23 kD; likely ortholog of mouse telome				1.72E-07	0.93	10.22	10.60	18697.3
AHQ-2-11, 4689	K.HLNEIDLFHCIDPNDSK.H	2069.24049	3	1.72E-07	0.93	4.34	-	1117.2
gi|4759140|ref|NP_004243.1| solute carrier family 9 (sodium/hydrogen exchanger), isoform 3 regulato				1.77E-07	0.93	10.21	3.90	38868.2
AHQ-2-13-, 3861 - 3862	R.LLVVDPETDEQLQK.L	1627.81777	2	1.77E-07	0.93	4.17	-	740.1
AHQ-2-13, 3843	R.LLVVDPETDEQLQK.L	1627.81777	2	1.98E-07	0.91	3.76	-	694.9
gi|10047092|ref|NP_037391.1| neuronal protein [Homo sapiens]				1.79E-07	0.96	10.23	5.00	31454.0
AHQ-2-14-, 6087	R.TTDIFQTVDLWEGK.D	1653.81378	2	1.79E-07	0.96	4.69	-	1554.1
gi|27480253|ref|XP_208225.1| similar to hypothetical protein FLJ20420 [Homo sapiens]				1.81E-07	0.96	10.19	10.90	11294.5
AHQ-2-10, 2524	R.VAEELALEQAK.K	1201.35165	1	6.08E-04	0.63	2.70	-	892.6
AHQ-2-10, 2523	R.VAEELALEQAK.K	1201.35165	2	1.81E-07	0.96	3.71	-	2068.8
gi|4502303|ref|NP_001688.1| mitochondrial ATP synthase, O subunit; ATP synthase, H+ transporting, m				1.82E-07	5.06	60.25	40.80	23277.2
AHQ-2-11, 6652 - 6714	R.FSPLTTNLINLLAENGR.L	1874.13050	2	6.48E-07	0.66	2.89	-	758.8
AHQ-2-11, 5398 - 5472	R.GEVPCTVTSASPLEEATLSELK.T	2320.55669	2	1.30E-04	0.92	5.05	-	647.5
AHQ-2-13, 5564	R.GEVPCTVTSASPLEEATLSELK.T	2320.55669	2	5.08E-06	0.82	3.26	-	754.1
AHQ-2-14-, 5619	R.GEVPCTVTSASPLEEATLSELK.T	2320.55669	2	9.40E-04	0.62	3.14	-	409.6
AHQ-2-11, 3481	K.LVRPPVQVYGIEGR.Y	1583.85942	3	1.82E-07	0.97	4.55	-	2007.8
AHQ-2-11, 4369	K.TDPSILGGMIVR.I	1259.50097	2	3.35E-04	0.89	3.58	-	542.0
AHQ-2-11, 3130	K.VAASVLNPYVK.R	1161.37549	2	7.09E-07	0.90	3.79	-	704.1
AHQ-2-11, 2404	R.YATALYSAASK.Q	1146.27455	2	4.91E-04	0.82	2.54	-	1042.1
gi|18572267|ref|XP_088476.1| similar to thioredoxin domain containing 4; endoplasmic reticulum resi				1.89E-07	0.91	10.18	3.40	46970.9
AHQ-2-7, 4844	K.TPADCPVIAIDSFR.H	1563.75706	2	1.89E-07	0.91	3.52	-	916.4
gi|4503573|ref|NP_001967.1| enolase 3; enolase-3, beta, muscle; muscle specific enolase; beta enola				2.05E-07	0.94	10.20	3.50	46958.5
AHQ-2-8, 3447	K.VNQIGSVTESIQACK.L	1635.82159	2	3.71E-05	0.86	3.29	-	774.6
AHQ-2-14, 4590	K.VNQIGSVTESIQACK.L	1635.82159	2	9.83E-07	0.90	3.61	-	845.7
AHQ-2-8, 2991	K.VNQIGSVTESIQACK.L	1635.82159	2	2.05E-07	0.94	4.00	-	1127.3
gi|7657647|ref|NP_055363.1| tropomodulin 2 (neuronal) [Homo sapiens]				2.15E-07	0.87	10.15	3.10	39594.8
AHQ-2-11, 3168	K.FGYQFTKQGPR.T	1329.48804	2	2.15E-07	0.87	3.10	-	938.7
gi|4757900|ref|NP_004334.1| calreticulin precursor; Sicca syndrome antigen A (autoantigen Ro; calre				2.16E-07	8.10	90.27	28.30	48141.1
AHQ-2-7, 5721 - 5736	R.CKDDEFTHLYTLIVRPDNTYEVK.I	2859.15977	2	2.08E-05	0.83	3.46	-	766.0
AHQ-2-9, 5842	R.CKDDEFTHLYTLIVRPDNTYEVK.I	2859.15977	3	3.46E-07	0.95	5.37	-	1174.9
AHQ-2-7, 5719	R.CKDDEFTHLYTLIVRPDNTYEVK.I	2859.15977	3	7.34E-05	0.94	4.90	-	873.4
AHQ-2-12, 5435 - 5511	R.FYALSASFEPFSNK.G	1608.77470	2	3.13E-06	0.93	3.80	-	1349.8
AHQ-2-7, 5364 - 5435	R.FYALSASFEPFSNK.G	1608.77470	2	1.87E-06	0.96	3.25	-	1874.4
AHQ-2-7, 2252	K.FYGDEEKDKGLQTSQDAR.F	2088.17738	2	2.16E-07	0.90	3.69	-	970.0
AHQ-2-7, 1817	K.HEQNIDCGGGYVK.L	1478.56912	1	1.33E-04	0.86	4.19	-	552.6
AHQ-2-13, 2309	K.HEQNIDCGGGYVK.L	1478.56912	2	1.13E-04	0.95	4.17	-	1290.2
AHQ-2-7, 5060	K.IDNSQVESGSLEDDWDFLPPKK.I	2520.68965	3	3.81E-05	0.92	4.64	-	716.7
AHQ-2-7, 5341 - 5417	K.KPEDWDEEMDGEWEPPVIQNPEYK.G	2962.14926	3	9.51E-07	0.93	5.00	-	964.5
AHQ-2-7, 1907	K.KVHVIFNYK.G	1148.38164	1	1.20E-05	0.87	3.04	-	697.0
AHQ-2-7, 1905	K.KVHVIFNYK.G	1148.38164	2	1.46E-04	0.80	3.00	-	646.2
AHQ-2-7, 2129	K.NVLINKDIR.C	1085.28225	2	2.06E-05	0.86	2.87	-	807.8
AHQ-2-7, 2313	K.VHVIFNYK.G	1020.20872	1	1.35E-06	0.76	2.42	-	727.3
gi|15451907|ref|NP_006301.2| aminopeptidase puromycin sensitive; puromycin-sensitive aminopeptidase				2.19E-07	1.61	20.19	3.90	98502.8
AHQ-2-5, 7073	R.TIQQCCENILLNAAWLK.R	2078.39839	2	2.19E-07	0.90	3.82	-	1266.1
AHQ-2-5, 4897	K.VTLSFPSTLQTGTGTLK.I	1752.00203	2	5.64E-04	0.71	2.88	-	474.9
gi|4759044|ref|NP_004841.1| Rho-associated, coiled-coil containing protein kinase 2 [Homo sapiens]				2.22E-07	3.24	40.21	4.60	160883.4
AHQ-2-3, 6337	K.ELQDQLEAEQYFSTLYK.T	2106.27421	2	1.86E-05	0.94	4.09	-	1103.9
AHQ-2-3, 3099	K.LFHVRPVTQTDVYR.A	1731.97897	2	8.45E-05	0.58	2.75	-	443.9
AHQ-2-4, 2889	R.SIAEEQYSDLEKEK.I	1669.76838	2	3.52E-05	0.82	2.99	-	959.9
AHQ-2-3, 4312	R.TLTSDVANLANEKEELNNK.L	2104.26140	2	2.22E-07	0.90	4.17	-	1020.5
gi|28269681|ref|NP_783313.1| NADH-ubiquinone oxidoreductase subunit B14.7 [Homo sapiens]				2.24E-07	2.62	30.25	40.40	14852.0
AHQ-2-13, 5148 - 5188	K.AYSTTSIASVAGLTAAAYR.V	1875.07212	2	3.17E-05	0.89	4.01	-	515.3
AHQ-2-13, 6493 - 6496	R.EKPDDPLNYFLGGCAGGLTLGAR.T	2423.68676	3	2.24E-07	0.92	4.50	-	1261.7
AHQ-2-13-, 6710	R.EKPDDPLNYFLGGCAGGLTLGAR.T	2423.68676	3	9.07E-07	0.97	5.00	-	2314.3
AHQ-2-13-, 4966 - 5031	R.VTLNPPGTFLEGVAK.V	1543.78883	2	6.51E-06	0.81	3.14	-	480.7
AHQ-2-13, 4912 - 4952	R.VTLNPPGTFLEGVAK.V	1543.78883	2	3.79E-04	0.38	2.63	-	200.2
gi|5729808|ref|NP_006557.1| adhesion glycoprotein; platelet and T cell activation antigen 1; DNAX a				2.45E-07	0.98	10.25	6.00	38583.7
AHQ-2-6, 4797 - 4873	K.IGTQQDSIAIFSPTHGMVIR.K	2172.49357	2	2.05E-05	0.78	3.28	-	409.0
AHQ-2-6, 4789	K.IGTQQDSIAIFSPTHGMVIR.K	2172.49357	3	2.45E-07	0.98	5.08	-	2888.2
gi|19923483|ref|NP_057406.2| GTPase Rab14 [Homo sapiens]				2.51E-07	4.33	50.23	31.20	23896.8
AHQ-2-10, 3975	K.FMADCPHTIGVEFGTR.I	1840.07303	2	9.05E-04	0.70	3.37	-	482.7
AHQ-2-10, 4015	R.GAAGALMVYDITR.R	1338.55819	2	7.34E-07	0.95	4.59	-	1068.4
AHQ-2-11, 3117	K.SCLLHQFTEK.K	1264.43272	1	8.12E-04	0.36	2.21	-	468.5
AHQ-2-10, 2940	K.SCLLHQFTEK.K	1264.43272	2	4.08E-06	0.94	4.04	-	861.3
AHQ-2-11, 3982	R.STYNHLSSWLTDAR.N	1651.76121	2	1.22E-04	0.80	2.86	-	778.8
AHQ-2-10, 3798	R.STYNHLSSWLTDAR.N	1651.76121	2	8.53E-05	0.89	3.06	-	1000.6
AHQ-2-10, 4212	K.TGENVEDAFLEAAK.K	1494.58517	1	1.41E-06	0.77	3.59	-	726.5
AHQ-2-10, 4210	K.TGENVEDAFLEAAK.K	1494.58517	2	2.51E-07	0.86	3.69	-	620.1
AHQ-2-11, 4416 - 4425	K.TGENVEDAFLEAAK.K	1494.58517	2	3.43E-05	0.91	4.09	-	718.7
gi|19923457|ref|NP_057447.2| glycoprotein VI (platelet); platelet glycoprotein VI [Homo sapiens] [M				2.63E-07	2.32	30.25	6.60	67308.6
AHQ-2-7, 3188	R.CQGPPGVDLYR.L	1263.40482	2	8.96E-05	0.51	2.50	-	534.7
AHQ-2-7, 5355	K.PSLQALPSSLVPLEKPVTLR.C	2146.55880	3	4.80E-06	0.95	5.03	-	1317.1
AHQ-2-7, 5368	K.PSLQALPSSLVPLEKPVTLR.C	2146.55880	2	2.21E-06	0.85	3.88	-	425.0
AHQ-2-7, 4653	R.YGFDQFALYK.E	1252.39881	2	2.63E-07	0.95	3.77	-	1206.0
gi|4758984|ref|NP_004654.1| RAB11A, member RAS oncogene family; RAB 11A, member oncogene family [Ho				2.79E-07	5.25	60.28	31.90	24393.3
AHQ-2-10, 2551	K.AQIWDTAGQER.Y	1275.35241	2	2.76E-05	0.95	3.23	-	1428.2
AHQ-2-12, 2731	K.AQIWDTAGQER.Y	1275.35241	2	3.78E-06	0.90	2.76	-	1143.7
AHQ-2-13-, 2825	K.AQIWDTAGQER.Y	1275.35241	2	2.41E-06	0.92	3.07	-	1370.1
AHQ-2-11, 2693 - 2766	K.AQIWDTAGQER.Y	1275.35241	2	2.79E-07	0.97	3.98	-	1783.6
AHQ-2-11, 4696 - 4756	R.DHADSNIVIMLVGNK.S	1626.85974	2	4.70E-06	0.97	4.64	-	1823.3
AHQ-2-11, 4900 - 4974	R.GAVGALLVYDIAK.H	1290.53317	2	3.12E-07	0.98	5.70	-	1833.6
AHQ-2-11, 1998	K.HLTYENVER.W	1161.24931	2	1.00E-05	0.80	2.87	-	506.0
AHQ-2-11, 2989	K.STIGVEFATR.S	1081.20410	2	5.06E-07	0.93	3.08	-	1069.6
AHQ-2-11, 2720	K.VVLIGDSGVGK.S	1044.22690	1	1.51E-04	0.53	2.14	-	877.3
AHQ-2-10, 2560	K.VVLIGDSGVGK.S	1044.22690	2	5.08E-04	0.79	2.97	-	1027.2
AHQ-2-11, 2725	K.VVLIGDSGVGK.S	1044.22690	1	7.67E-05	0.60	2.87	-	747.7
AHQ-2-11, 2717	K.VVLIGDSGVGK.S	1044.22690	2	1.34E-04	0.66	3.25	-	705.1
gi|23110942|ref|NP_002781.2| proteasome alpha 5 subunit; proteasome component 5; macropain subunit				2.84E-07	2.66	30.22	21.20	26410.9
AHQ-2-10, 2950	R.AIGSASEGAQSSLQEVYHK.S	1963.09463	2	2.84E-07	0.92	4.13	-	818.0
AHQ-2-10, 4387	K.GPQLFHMDPSGTFVQCDAR.A	2165.39491	2	3.17E-04	0.94	4.42	-	938.7
AHQ-2-10, 3339 - 3346	R.ITSPLMEPSSIEK.I	1432.66506	2	2.61E-05	0.80	2.70	-	1000.4
gi|4885153|ref|NP_005198.1| v-crk sarcoma virus CT10 oncogene homolog (avian)-like; v-crk avian sar				2.92E-07	2.66	30.23	18.50	33776.7
AHQ-2-11, 3945	K.IHYLDTTTLIEPAPR.Y	1740.98094	2	3.71E-07	0.90	3.57	-	930.1

AHQ-2-10, 5130 - 5131	R.TLYDFPGNDAEDLPFK.K	1842.98222	2	2.92E-07	0.85	3.63	-	529.1
AHQ-2-10, 5020 - 5023	R.YPSPPMGSVSAPNLPTAEDNLEYVR.T	2705.98075	2	3.30E-07	0.91	4.65	-	451.5
gi|4507149|ref|NP_000445.1| superoxide dismutase 1, soluble (amyotrophic lateral sclerosis 1 (adult				3.01E-07	3.27	40.29	57.80	15935.7
AHQ-2-12, 5378 - 5386	K.AVCVLKGDGPVQGIINFEQK.E	2174.50602	2	4.46E-05	0.97	5.55	-	1289.5
AHQ-2-12, 4561	K.GDGPVQGIINFEQK.E	1502.65366	2	3.01E-07	0.69	3.33	-	522.4
AHQ-2-12, 4338 - 4357	K.GDGPVQGIINFEQK.E	1502.65366	2	1.20E-05	0.36	2.67	-	499.0
AHQ-2-12, 4887	K.GLTEGLHGFHVHEFGDNTAGCTSAGPHFNPLSR.K	3522.76733	3	3.14E-04	0.96	5.39	-	1514.2
AHQ-2-11, 4664	K.GLTEGLHGFHVHEFGDNTAGCTSAGPHFNPLSR.K	3522.76733	3	1.29E-05	0.96	5.71	-	1550.3
AHQ-2-13, 5913	R.HVGDLGNVTADKDGVADVSIEDSVISLSGDHCIIGR.T	3724.02282	3	9.38E-06	0.65	3.18	-	547.0
gi|10880989|ref|NP_067075.1| RAB18, member RAS oncogene family; RAB18 small GTPase [Homo sapiens] [				3.04E-07	1.81	20.21	14.10	22976.9
AHQ-2-11, 6180	K.LDNWLNELETYCTR.N	1828.98037	2	6.51E-06	0.88	3.61	-	729.5
AHQ-2-11, 5768 - 5769	K.TCDGVQCAFEELVEK.I	1787.94661	2	3.04E-07	0.92	4.29	-	920.4
gi|4502107|ref|NP_001145.1| annexin V; endonexin II; anchorin CII; lipocortin V; placental anticoag				3.15E-07	1.87	20.21	9.10	35936.5
AHQ-2-9, 6714	R.DLLDDLKSELTGK.F	1447.61309	2	3.15E-07	0.94	3.93	-	1153.3
AHQ-2-9, 7030 - 7048	K.GLGTDEESILTLLTSR.S	1705.88853	2	3.65E-06	0.93	4.19	-	945.7
gi|4504073|ref|NP_000398.1| glycoprotein Ib beta polypeptide precursor [Homo sapiens]				3.16E-07	2.54	30.21	15.50	21717.6
AHQ-2-1, 4957	R.LLPYLAEDELR.A	1332.52691	2	4.83E-07	0.78	2.95	-	478.2
AHQ-2-5, 4597	R.LLPYLAEDELR.A	1332.52691	2	2.79E-05	0.80	2.68	-	611.7
AHQ-2-6, 4537	R.LLPYLAEDELR.A	1332.52691	2	6.44E-07	0.88	3.51	-	613.6
AHQ-2-13, 4668	R.LLPYLAEDELR.A	1332.52691	2	2.37E-05	0.89	2.83	-	883.2
AHQ-2-10, 4234 - 4266	R.LLPYLAEDELR.A	1332.52691	2	1.78E-06	0.88	3.16	-	609.1
AHQ-2-10, 4252	R.LLPYLAEDELR.A	1332.52691	1	8.21E-06	0.48	3.28	-	176.2
AHQ-2-12, 4682	R.LLPYLAEDELR.A	1332.52691	2	1.07E-05	0.73	2.71	-	351.9
AHQ-2-11, 4484	R.LLPYLAEDELR.A	1332.52691	1	8.79E-04	0.70	3.01	-	438.9
AHQ-2-13-, 4741	R.LLPYLAEDELR.A	1332.52691	2	2.94E-06	0.81	2.85	-	557.2
AHQ-2-11, 4121 - 4124	R.LSLTDPLVAER.A	1214.39356	2	3.16E-07	0.94	4.25	-	1080.5
AHQ-2-10, 2074	R.TAHLGANPWR.C	1123.24913	2	5.09E-05	0.82	2.91	-	707.4
gi|4557703|ref|NP_000414.1| keratin 2a [Homo sapiens]				3.28E-07	2.30	30.26	10.50	65864.9
AHQ-2-14-, 6635	R.FGGFGGPGGVGGLGGPGGFGPGGYPGGIHEVSVNQSLLQPLNVK.V	4094.54151	3	3.32E-05	0.93	5.05	-	1188.4
AHQ-2-14-, 2931 - 2933	R.FLEQQNQVLQTK.W	1476.65935	2	3.28E-07	0.97	5.15	-	2319.0
AHQ-2-4, 3014	R.FLEQQNQVLQTK.W	1476.65935	2	2.41E-04	0.59	2.74	-	477.1
AHQ-2-1, 3154	R.FLEQQNQVLQTK.W	1476.65935	2	1.26E-05	0.78	2.96	-	1041.6
AHQ-2-14-, 5905	K.VDLLNQEIEFLK.V	1461.68459	2	6.01E-04	0.40	2.65	-	412.0
gi|9910280|ref|NP_064505.1| UDP-glucose ceramide glucosyltransferase-like 1; UDP-glucose:glycoprote				3.29E-07	2.14	30.16	4.10	177187.7
AHQ-2-5, 6924	R.AVYLGELPHDQDVVEYIMNQPNVVPR.I	2997.37416	2	4.13E-05	0.80	3.23	-	486.7
AHQ-2-3, 7605	R.IIGPLEDSELFNQDDFHLLENIILK.T	2927.29710	3	1.92E-04	0.70	3.08	-	715.0
AHQ-2-3, 4687	R.IVPEWQDYDQEIK.Q	1663.80866	2	3.29E-07	0.63	2.94	-	289.0
gi|30148456|ref|XP_293602.2| similar to glutathione-S-transferase like; glutathione transferase ome				3.31E-07	0.91	10.17	3.90	29139.6
AHQ-2-11, 4433	K.VPSLVGSFLR.T	1075.28583	2	3.31E-07	0.91	3.09	-	1144.2
AHQ-2-10, 4136 - 4138	K.VPSLVGSFLR.T	1075.28583	2	1.46E-05	0.94	3.33	-	1318.7
gi|29729616|ref|XP_292963.1| similar to peptidylprolyl isomerase A (cyclophilin A) [Homo sapiens] [				3.36E-07	1.86	20.28	16.40	18025.4
AHQ-2-12, 4919 - 4945	K.HTGPGILSM*ANAGPNTNGSQFFICTAK.S	2810.11545	3	6.05E-06	0.93	5.05	-	1167.5
AHQ-2-12, 5009 - 5089	K.HTGPGILSM*ANAGPNTNGSQFFICTAK.S	2810.11545	3	7.54E-06	0.91	4.96	-	973.0
AHQ-2-12, 5066 - 5129	K.HTGPGILSM*ANAGPNTNGSQFFICTAK.S	2810.11545	2	4.04E-04	0.85	4.16	-	592.5
AHQ-2-12, 5541 - 5621	K.HTGPGILSMANAGPNTNGSQFFICTAK.S	2794.11605	2	2.29E-05	0.93	4.53	-	734.3
AHQ-2-12, 5629 - 5701	K.HTGPGILSMANAGPNTNGSQFFICTAK.S	2794.11605	3	9.74E-05	0.93	5.04	-	1225.8
AHQ-2-12, 5853 - 5931	K.HTGPGILSMANAGPNTNGSQFFICTAK.S	2794.11605	2	2.66E-06	0.82	3.11	-	943.5
AHQ-2-13, 4880	K.HTGPGILSM*ANAGPNTNGSQFFICTAK.S	2810.11545	3	4.24E-06	0.91	4.50	-	1088.6
AHQ-2-13, 4888	K.HTGPGILSM*ANAGPNTNGSQFFICTAK.S	2810.11545	2	1.78E-04	0.62	3.25	-	313.9
AHQ-2-13, 5005	K.HTGPGILSM*ANAGPNTNGSQFFICTAK.S	2810.11545	3	7.10E-04	0.73	3.49	-	673.2
AHQ-2-13-, 4953	K.HTGPGILSM*ANAGPNTNGSQFFICTAK.S	2810.11545	3	3.78E-06	0.92	4.62	-	1086.9
AHQ-2-13-, 5554 - 5587	K.HTGPGILSMANAGPNTNGSQFFICTAK.S	2794.11605	3	3.36E-07	0.94	5.66	-	1300.7
gi|20149594|ref|NP_031381.2| heat shock 90kDa protein 1, beta; heat shock 90kD protein 1, beta; Hea				3.42E-07	3.61	40.25	7.30	83263.6
AHQ-2-14-, 3019	R.ELISNASDALDK.I	1276.37539	2	9.02E-04	0.66	3.06	-	432.7
AHQ-2-5, 2959 - 2971	R.ELISNASDALDK.I	1276.37539	2	7.72E-07	0.87	3.31	-	761.8
AHQ-2-5, 4107 - 4121	R.NPDDITQEEYGEFYK.S	1848.90048	2	3.42E-07	0.97	4.95	-	1358.8
AHQ-2-5, 3731 - 3733	K.SLTNDWEDHLAVK.H	1528.64798	2	3.83E-06	0.84	3.29	-	1017.0
AHQ-2-5, 4105	R.TLTLVDTGIGMTK.A	1350.60718	2	4.97E-06	0.94	3.70	-	1103.2
gi|16933542|ref|NP_002017.1| fibronectin 1 isoform 1 preproprotein; cold-insoluble globulin [Homo s				3.75E-07	6.26	90.20	6.70	259222.8
AHQ-2-1, 6274	K.FTQVTPTSLSAQWTPPNVQLTGYR.V	2693.99453	2	6.18E-06	0.69	3.47	-	297.6
AHQ-2-1, 2780	R.GDSPASSKPISINYR.T	1592.73521	2	1.57E-04	0.41	2.55	-	244.2
AHQ-2-1, 4601	R.ITYGETGGNSPVQEFTVPGSK.S	2169.33354	2	1.03E-06	0.83	3.94	-	422.6
AHQ-2-1, 5126	R.NLQPASEYTVSLVAIK.G	1733.98671	2	1.05E-05	0.81	2.88	-	484.2
AHQ-2-2, 6400	R.NTFAEVTGLSPGVTYYFK.V	1995.21983	2	6.12E-05	0.64	2.86	-	423.1
AHQ-2-1, 6796	R.NTFAEVTGLSPGVTYYFK.V	1995.21983	2	1.99E-04	0.71	3.29	-	448.7
AHQ-2-1, 4892	R.SSPVVIDASTAIDAPSNLR.F	1914.10666	2	5.09E-04	0.91	4.03	-	961.4
AHQ-2-1, 3482	R.VPGTSTSATLTGLTR.G	1462.63098	2	2.07E-06	0.77	2.92	-	664.5
AHQ-2-1, 7160	R.VTWAPPPSIDLTNFLVR.Y	1927.23500	2	3.75E-07	0.89	3.32	-	647.9
AHQ-2-2, 7448	R.VTWAPPPSIDLTNFLVR.Y	1927.23500	2	9.37E-07	0.86	3.34	-	518.2
AHQ-2-1, 3946	K.WLPSSSPVTGYR.V	1350.50402	2	8.97E-04	0.32	2.65	-	233.7
gi|4506185|ref|NP_002780.1| proteasome alpha 4 subunit; proteasome component C9; proteasome subunit				3.75E-07	1.87	20.25	12.30	29483.6
AHQ-2-10, 4444	K.LLDEVFFSEK.I	1227.38757	2	3.75E-07	0.94	3.52	-	918.9
AHQ-2-10, 6738 - 6739	R.YLLQYQEPIPCEQLVTALCDIK.Q	2698.10465	2	2.26E-06	0.93	4.93	-	590.2
gi|7705849|ref|NP_057215.1| ras-related GTP-binding protein RAB10; ras-related GTP-binding protein				3.85E-07	1.85	30.24	15.50	22540.8
AHQ-2-14-, 6463	K.AFLTLAEDILR.K	1262.47982	2	1.01E-06	0.95	4.22	-	1557.7
AHQ-2-11, 6236 - 6244	K.AFLTLAEDILR.K	1262.47982	2	3.85E-07	0.96	4.88	-	1393.2
AHQ-2-10, 6092	K.AFLTLAEDILR.K	1262.47982	2	4.89E-07	0.94	3.92	-	1397.0
AHQ-2-11, 2996 - 3012	K.KTYDLLFK.L	1028.22593	2	3.00E-04	0.72	2.68	-	544.5
AHQ-2-11, 1960	R.NIDEHANEDVER.M	1441.44245	1	2.05E-06	0.18	2.32	-	168.4
AHQ-2-11, 1826	R.NIDEHANEDVER.M	1441.44245	1	3.35E-04	0.63	2.77	-	307.9
AHQ-2-11, 1792	R.NIDEHANEDVER.M	1441.44245	2	5.16E-04	0.66	2.86	-	550.1
gi|22060072|ref|XP_088293.2| similar to cytochrome c [Homo sapiens]				3.86E-07	1.98	30.18	24.80	11887.8
AHQ-2-13-, 4267	R.ADLIAYLK.K	907.08869	2	4.09E-04	0.91	3.43	-	961.7
AHQ-2-13, 4220	R.ADLIAYLK.K	907.08869	2	1.09E-04	0.83	2.74	-	802.5
AHQ-2-13, 2956	K.M*IFVGIK.K	824.06630	1	5.94E-04	0.23	1.91	-	174.8
AHQ-2-13-, 3519	K.TGPNLHGLFGR.K	1169.31780	1	2.69E-05	0.37	2.24	-	162.6
AHQ-2-13-, 3513 - 3518	K.TGPNLHGLFGR.K	1169.31780	2	9.04E-07	0.92	3.64	-	820.4
AHQ-2-13, 3513 - 3515	K.TGPNLHGLFGR.K	1169.31780	2	3.86E-07	0.92	3.49	-	813.9
gi|29727317|ref|XP_298144.1| hypothetical protein XP_298144 [Homo sapiens]				3.87E-07	1.04	20.16	15.90	22108.6
AHQ-2-7, 5201	K.CAAM*SKVLTAMPNM*GK.A	1744.11508	2	4.14E-04	0.26	2.67	-	252.5
AHQ-2-5, 6477	K.GEYNLTLDTALTSNLK.T	1753.93165	2	1.21E-06	0.78	3.00	-	1074.4
AHQ-2-4, 6753	K.GEYNLTLDTALTSNLK.T	1753.93165	2	3.87E-07	0.78	3.24	-	779.4
gi|20149637|ref|NP_057526.2| type 1 tumor necrosis factor receptor shedding aminopeptidase regulato				4.07E-07	0.79	10.13	1.40	107761.4
AHQ-2-4, 3194	R.ILASTQFEPTAAR.M	1405.58112	2	4.07E-07	0.79	2.66	-	929.7
gi|5453722|ref|NP_006321.1| lysophospholipase I; lysophospholipase 1; lysophospholipid-specific lys				4.11E-07	2.66	30.22	16.50	24669.5
AHQ-2-11, 2716 - 2750	R.ASFPQGPIGGANR.D	1272.39489	2	4.11E-07	0.82	3.22	-	520.6
AHQ-2-11, 6438 - 6442	K.LAGVTALSCWLPLR.A	1558.86992	2	6.50E-04	0.96	4.48	-	1255.7
AHQ-2-11, 3493	K.TLVNPANVTFK.T	1204.40037	2	5.95E-05	0.88	3.56	-	599.9
gi|4758906|ref|NP_004146.1| serine (or cysteine) proteinase inhibitor, clade B (ovalbumin), member				4.21E-07	0.88	10.17	3.20	42403.4
AHQ-2-8, 2894	R.HLGIVDAFQQGK.A	1313.48692	2	4.21E-07	0.88	3.27	-	865.9
gi|5803187|ref|NP_006746.1| transaldolase 1; dihydroxyacetone transferase; glycerone transferase [H				4.24E-07	0.95	10.20	3.30	37539.9
AHQ-2-9, 3667	K.LLGELLQDNAK.L	1214.39356	2	4.24E-07	0.95	3.98	-	1229.6
AHQ-2-9, 3658 - 3659	K.LLGELLQDNAK.L	1214.39356	1	9.67E-05	0.34	2.22	-	462.6
gi|5031973|ref|NP_005733.1| protein disulfide isomerase-related protein [Homo sapiens]				4.39E-07	4.42	50.22	21.80	48121.0
AHQ-2-7, 7367 - 7369	R.DGELPVEDDIDLSDVELDDLGKDEL	2759.86565	2	6.88E-05	0.90	3.88	-	881.5
AHQ-2-7, 3695 - 3764	R.GSTAPVGGGAFPTIVER.E	1616.79990	2	4.39E-07	0.84	3.23	-	888.0
AHQ-2-7, 4641 - 4644	K.LAAVDATVNQVLASR.Y	1528.73579	2	4.51E-06	0.94	3.68	-	1532.4

AHQ-2-7, 6151 - 6152	R.TGEAIVDAALSALR.Q	1387.56411	2	2.78E-06	0.97	4.40	-	1958.5
AHQ-2-7, 3367 - 3397	K.VGAVDADKHHSLGGQYGVQGFPTIK.I	2582.85541	2	1.49E-06	0.76	3.39	-	389.5
gi|4502171|ref|NP_000476.1| adenine phosphoribosyltransferase; AMP pyrophosphorylase; AMP diphospho				4.90E-07	3.45	40.23	30.00	19607.6
AHQ-2-11, 3672	R.DISPVLKDPASFR.A	1445.64525	2	1.38E-05	0.78	3.19	-	541.6
AHQ-2-11, 4985	R.GKLPGPTLWASYSLEYGK.A	1968.24075	2	3.34E-05	0.94	4.65	-	771.2
AHQ-2-11, 3497 - 3500	R.IDYIAGLDSR.G	1123.24098	2	4.90E-07	0.96	4.04	-	1689.1
AHQ-2-11, 5548 - 5614	R.SFPDFPTPGVVFR.D	1466.66462	2	1.43E-04	0.76	3.03	-	506.8
gi|7330335|ref|NP_039234.1| chloride intracellular channel 4; chloride intracellular channel 4 like				4.91E-07	3.34	40.26	28.90	28771.9
AHQ-2-10, 2862	K.AGSDGESIGNCPFSQR.L	1683.73852	2	4.91E-07	0.91	3.92	-	782.3
AHQ-2-10, 5580 - 5588	K.FLDGNEMTLADCNLLPK.L	1953.22635	2	7.86E-06	0.90	3.84	-	975.9
AHQ-2-10, 5318	K.LDEYLNSPLPDEIDENSMEDIK.F	2580.76043	2	7.47E-04	0.56	2.78	-	566.6
AHQ-2-10, 6726 - 6798	K.TDVNKIEEFLEEVLCPPK.Y	2162.44564	2	6.15E-07	0.96	5.18	-	1084.0
gi|4504505|ref|NP_000405.1| hydroxysteroid (17-beta) dehydrogenase 4 [Homo sapiens]				4.94E-07	0.90	10.19	2.20	79685.9
AHQ-2-6, 2590	R.ATSTATSGFAGAIGQK.L	1468.59408	2	4.94E-07	0.90	3.71	-	1165.7
gi|27500115|ref|XP_208901.1| similar to ubiquitin-conjugating enzyme E2M; UBC12 homolog, yeast [Hom				4.95E-07	0.82	10.16	8.70	20964.0
AHQ-2-11, 5968 - 6042	K.TCDISFSDPDDLLNFK.L	1889.02935	2	4.95E-07	0.82	3.18	-	632.4
gi|5729875|ref|NP_006658.1| progesterone receptor membrane component 1; progesterone binding protei				4.99E-07	2.27	30.23	11.80	21671.0
AHQ-2-10, 4236 - 4239	K.FYGPEGPYGVFAGR.D	1517.66849	2	1.67E-06	0.96	4.57	-	1392.6
AHQ-2-10, 2536	R.ILMAINGK.V	860.10021	1	6.99E-05	0.35	1.88	-	599.8
AHQ-2-11, 3921	R.KFYGPEGPYGVFAGR.D	1645.84140	2	5.26E-05	0.92	3.39	-	1182.0
AHQ-2-10, 3746	R.KFYGPEGPYGVFAGR.D	1645.84140	2	4.99E-07	0.96	4.35	-	1316.8
gi|5454152|ref|NP_006285.1| ubiquinol-cytochrome c reductase binding protein [Homo sapiens]				5.03E-07	0.98	10.26	11.70	13530.4
AHQ-2-13-, 5289 - 5290	K.YEEENFYLEPYLK.E	1737.88601	2	5.03E-07	0.98	5.28	-	2166.6
AHQ-2-13, 5183	K.YEEENFYLEPYLK.E	1737.88601	2	5.52E-04	0.96	4.51	-	1353.0
gi|27481323|ref|XP_208234.1| similar to unactive progesterone receptor, 23 kD; likely ortholog of m				5.41E-07	0.97	20.15	15.60	18694.3
AHQ-2-11, 6122	K.LNWLSVDFNNWK.D	1536.71494	2	6.33E-06	0.80	3.03	-	604.1
AHQ-2-11, 4413	K.LTFSCLGGSDNFK.H	1447.59493	1	5.41E-07	0.17	1.88	-	276.7
AHQ-2-11, 4400 - 4448	K.LTFSCLGGSDNFK.H	1447.59493	2	2.22E-04	0.79	2.56	-	873.1
gi|4503785|ref|NP_000135.1| frataxin; Friedreich ataxia (frataxin) [Homo sapiens]				5.46E-07	0.96	10.22	5.70	23218.1
AHQ-2-13-, 3818	K.LGGDLGTYVINK.Q	1250.42590	2	5.46E-07	0.96	4.37	-	1218.1
gi|5729804|ref|NP_006694.1| nudix (nucleoside diphosphate linked moiety X)-type motif 3; diphosphoi				5.66E-07	0.57	10.16	11.60	19470.8
AHQ-2-11, 4356	R.WIVPGGGM*EPEEEPSVAAVR.E	2127.36326	2	5.66E-07	0.57	3.07	-	164.1
gi|13489091|ref|NP_066949.1| 3-mercaptopyruvate sulfurtransferase [Homo sapiens]				5.78E-07	0.97	10.26	5.40	33178.2
AHQ-2-9, 6772	R.AGQPLQLLDASWYLPK.L	1801.07798	2	1.31E-04	0.93	3.70	-	1337.7
AHQ-2-9, 6680 - 6754	R.AGQPLQLLDASWYLPK.L	1801.07798	2	5.78E-07	0.97	4.54	-	2133.6
gi|4826848|ref|NP_004991.1| NADH dehydrogenase (ubiquinone) 1 alpha subcomplex, 5; NADH dehydrogena				5.90E-07	0.83	10.29	13.80	13458.6
AHQ-2-13, 3748	K.TTGLVGLAVCNTPHER.L	1726.93595	2	5.90E-07	0.83	3.46	-	717.4
gi|5032041|ref|NP_005605.1| Ras homolog enriched in brain 2 [Homo sapiens]				5.97E-07	0.95	10.24	8.70	20497.4
AHQ-2-12, 5030 - 5107	K.ALAESWNAAFLESSAK.E	1695.85389	2	5.97E-07	0.95	4.84	-	973.4
gi|7705819|ref|NP_057181.1| HSPC039 protein [Homo sapiens]				6.00E-07	0.95	10.25	24.40	8968.7
AHQ-2-14-, 5117	K.NIGWGTDQGIGGFGEEPGIK.S	2033.18655	2	6.00E-07	0.95	5.00	-	1110.2
AHQ-2-14, 6221	K.NIGWGTDQGIGGFGEEPGIK.S	2033.18655	2	7.63E-05	0.76	3.35	-	546.2
gi|22267436|ref|NP_056284.1| DKFZp564D177 protein [Homo sapiens]				6.01E-07	0.95	10.18	5.30	28466.4
AHQ-2-10, 3896	R.VHVLWWNESADSR.A	1599.73113	2	6.01E-07	0.95	3.52	-	1610.3
gi|17318569|ref|NP_006112.2| keratin 1; Keratin-1; cytokeratin 1; hair alpha protein [Homo sapiens]				6.17E-07	3.89	50.22	9.50	66066.3
AHQ-2-5, 2775	R.DYQELMNTK.L	1142.26457	1	2.58E-04	0.66	2.38	-	498.0
AHQ-2-5, 6253 - 6272	R.SLDLDSIIAEVK.A	1303.48388	2	6.17E-07	0.96	4.31	-	1553.9
AHQ-2-14-, 6060 - 6061	R.SLDLDSIIAEVK.A	1303.48388	2	2.31E-06	0.95	4.36	-	1305.8
AHQ-2-5, 6619	R.THNLEPYFESFINNLR.R	1995.18315	2	1.27E-05	0.91	4.12	-	541.2
AHQ-2-5, 2224	R.TNAENEFVTIKK.D	1394.55511	2	2.42E-05	0.53	2.59	-	407.6
AHQ-2-14, 5670	K.WELLQQVDTSTR.T	1476.61621	2	5.47E-06	0.83	2.66	-	1131.8
gi|21618338|ref|NP_003141.2| signal transducer and activator of transcription 3 isoform 2; acute-ph				6.19E-07	1.51	20.16	5.20	87980.2
AHQ-2-6, 3427	R.FLQESNVLYQHNLR.R	1761.96193	2	6.19E-07	0.76	3.13	-	741.0
AHQ-2-6, 2841	R.LLQTAATAAQQGGQANHPTAAVVTEK.Q	2577.83547	3	9.66E-04	0.75	3.25	-	543.8
gi|21614499|ref|NP_003370.2| villin 2; Villin-2; cytovillin [Homo sapiens]				6.26E-07	1.91	20.22	3.20	69412.3
AHQ-2-7, 4084	K.APDFVFYAPR.L	1183.33971	2	3.82E-06	0.97	3.22	-	2000.6
AHQ-2-6, 4155 - 4158	K.APDFVFYAPR.L	1183.33971	2	6.26E-07	0.97	4.50	-	1577.0
AHQ-2-6, 4950 - 4963	K.IGFPWSEIR.N	1105.27049	2	1.01E-04	0.93	3.26	-	1111.1
gi|27544939|ref|NP_060932.2| uncharacterized hematopoietic stem/progenitor cells protein MDS027 [Ho				6.26E-07	0.95	10.23	17.30	8744.9
AHQ-2-14-, 5667	K.IADFLNSFDMSCR.S	1577.76410	2	6.26E-07	0.95	4.52	-	1220.9
AHQ-2-14-, 5303	K.IADFLNSFDMSCR.S	1577.76410	2	4.06E-04	0.92	3.19	-	1038.0
gi|4757952|ref|NP_001782.1| cell division cycle 42 isoform 1; cell division cycle 42 (GTP-binding p				6.30E-07	4.24	50.28	31.40	21258.5
AHQ-2-11, 2273	K.CVVVGDGAVGK.T	1062.22237	1	1.44E-04	0.86	2.87	-	1004.1
AHQ-2-11, 2272	K.CVVVGDGAVGK.T	1062.22237	2	3.90E-05	0.86	2.91	-	1186.4
AHQ-2-11, 6573 - 6577	K.NVFDEAILAALEPPEPK.K	1854.09282	3	6.30E-07	0.96	5.69	-	1271.8
AHQ-2-11, 3821 - 3840	K.TCLLISYTTNK.F	1315.51795	2	2.64E-05	0.83	2.73	-	1254.4
AHQ-2-11, 2457	K.WVPEITHHCPK.T	1405.60610	1	7.69E-07	0.71	3.40	-	440.2
AHQ-2-11, 2478 - 2486	K.YVECSALTQK.G	1200.34381	2	1.47E-05	0.88	3.81	-	477.2
gi|4507793|ref|NP_003339.1| ubiquitin-conjugating enzyme E2N (UBC13 homolog, yeast); ubiquitin-conj				6.35E-07	3.34	40.24	30.30	17137.7
AHQ-2-12, 5873	K.LELFLPEEYPMAAPK.V	1749.06325	2	6.35E-07	0.87	4.17	-	607.3
AHQ-2-12, 5355 - 5379	K.LELFLPEEYPM*AAPK.V	1765.06265	2	1.94E-04	0.76	3.85	-	326.0
AHQ-2-12, 2126	K.TNEAQAIETAR.A	1204.27249	2	3.73E-04	0.75	2.87	-	998.8
AHQ-2-12, 5273	R.YFHVVIAGPQDSPFEGGTFK.L	2197.43341	2	8.78E-05	0.97	4.73	-	1492.8
gi|4502285|ref|NP_001672.1| ATPase, Ca++ transporting, cardiac muscle, slow twitch 2 isoform 2; ATP				6.48E-07	0.92	10.21	1.50	109690.3
AHQ-2-1, 4868	K.VGEATETALTCLVEK.M	1622.81958	2	6.48E-07	0.92	4.12	-	847.5
gi|18570595|ref|XP_095590.1| similar to myosin regulatory light chain [Homo sapiens]				6.68E-07	0.91	10.18	0.00	13951.5
AHQ-2-11, 3041 - 3108	K.LNGTDPEDVIR.H	1229.32195	2	3.67E-05	0.93	3.00	-	1461.9
AHQ-2-12, 3159	K.LNGTDPEDVIR.H	1229.32195	2	3.32E-06	0.91	3.33	-	1030.8
AHQ-2-13-, 3221	K.LNGTDPEDVIR.H	1229.32195	2	5.58E-04	0.85	3.38	-	714.8
AHQ-2-11, 2952	K.LNGTDPEDVIR.H	1229.32195	2	6.68E-07	0.91	3.51	-	1075.7
gi|7657548|ref|NP_055378.1| spondyloepiphyseal dysplasia, late; sedlin [Homo sapiens]				6.70E-07	0.72	10.16	10.00	16444.6
AHQ-2-13, 5072 - 5080	K.FSMNPFYEPNSPIR.S	1699.91087	2	6.70E-07	0.72	3.25	-	519.2
gi|14745898|ref|XP_016144.2| similar to tyrosine 3-monooxygenase/tryptophan 5-monooxygenase activat				6.77E-07	0.96	10.23	7.50	18201.0
AHQ-2-11, 6541 - 6542	K.LLCCDILDVLDK.N	1479.74157	2	6.77E-07	0.96	4.61	-	1074.9
AHQ-2-10, 6478	K.LLCCDILDVLDK.N	1479.74157	2	7.45E-07	0.96	4.48	-	1055.7
gi|20551618|ref|XP_167021.1| similar to calponin 2; Calonin 2 [Homo sapiens]				6.89E-07	1.64	20.22	3.70	47327.9
AHQ-2-12, 5274 - 5341	R.SM*QNWHQLENLSNFIK.A	2006.23095	2	1.67E-06	0.82	3.75	-	497.4
AHQ-2-12, 5682	R.SMQNWHQLENLSNFIK.A	1990.23155	2	1.19E-04	0.93	4.26	-	885.9
AHQ-2-13, 5572	R.SMQNWHQLENLSNFIK.A	1990.23155	2	6.89E-07	0.82	3.60	-	635.1
AHQ-2-13-, 5702	R.SMQNWHQLENLSNFIK.A	1990.23155	2	8.43E-05	0.94	4.38	-	1187.9
AHQ-2-11, 5453	R.SMQNWHQLENLSNFIK.A	1990.23155	2	4.21E-04	0.95	4.35	-	1206.5
gi|5453597|ref|NP_006126.1| F-actin capping protein alpha-1 subunit; Cap Z [Homo sapiens]				7.00E-07	2.59	40.20	22.00	32922.5
AHQ-2-9, 4723 - 4731	K.FITHAPPGEFNEVFNDVR.L	2090.28294	2	1.62E-05	0.79	3.48	-	342.9
AHQ-2-10, 4896	K.FTITPPTAQVVGVLK.I	1571.88532	2	5.08E-06	0.51	2.73	-	184.7
AHQ-2-9, 4480	R.LLLNNDNLLR.E	1198.39736	2	2.28E-04	0.48	2.58	-	386.8
AHQ-2-9, 3876	R.LLLNNDNLLR.E	1198.39736	2	7.51E-04	0.84	3.80	-	702.4
AHQ-2-9, 5403	K.TIDGQQTIIACIESHQFQPK.N	2316.57620	3	7.00E-07	0.81	4.04	-	320.2
AHQ-2-10, 5040	K.TIDGQQTIIACIESHQFQPK.N	2316.57620	2	1.50E-06	0.63	2.96	-	303.1
AHQ-2-9, 5398 - 5466	K.TIDGQQTIIACIESHQFQPK.N	2316.57620	2	3.31E-06	0.81	3.85	-	347.2
gi|11496277|ref|NP_068805.1| mitogen-activated protein kinase kinase 1 interacting protein 1; MEK p				7.01E-07	1.89	20.29	35.50	13622.6
AHQ-2-14, 6556 - 6569	K.SIICYYNTYQVVQFNR.L	2070.31322	2	7.01E-07	0.94	4.52	-	1074.5
AHQ-2-14-, 5243	K.VANDNAPEHALRPGFLSTFALATDQGSK.L	2929.19327	3	2.02E-05	0.96	5.77	-	1095.8
gi|4502693|ref|NP_001760.1| CD9 antigen; motility related protein; leukocyte antigen MIC3; antigen				7.09E-07	3.03	40.31	24.10	25415.9
AHQ-2-11, 6805	K.AIHYALNCCGLAGGVEQFISDICPK.K	2798.16446	3	1.34E-06	0.91	4.36	-	810.5
AHQ-2-11, 6806 - 6810	K.AIHYALNCCGLAGGVEQFISDICPK.K	2798.16446	2	7.09E-07	0.98	6.17	-	1810.1
AHQ-2-11, 2168	K.EVQEFYK.D	943.03476	1	4.80E-04	0.30	2.37	-	234.4
AHQ-2-11, 3137 - 3174	K.KDVLETFTVK.S	1180.37558	2	2.27E-04	0.90	3.61	-	922.4

AHQ-2-13-, 3383	K.KDVLETFTVK.S	1180.37558	2	2.29E-05	0.84	2.89	-	783.9
AHQ-2-11, 3132 - 3192	K.KDVLETFTVK.S	1180.37558	1	3.21E-05	0.38	1.90	-	500.6
AHQ-2-10, 2962	K.KDVLETFTVK.S	1180.37558	2	3.10E-05	0.91	3.42	-	1035.6
AHQ-2-1, 3541 - 3549	K.KDVLETFTVK.S	1180.37558	2	5.24E-06	0.89	3.31	-	922.9
AHQ-2-13, 3389	K.KDVLETFTVK.S	1180.37558	2	1.27E-06	0.81	3.15	-	735.6
AHQ-2-11, 3685	K.SCPDAIKEVFDNK.F	1524.67770	2	1.23E-04	0.95	3.86	-	1319.9
gi|10835035|ref|NP_003157.1| sulfotransferase family, cytosolic, 1A, phenol-preferring, member 3; t				7.60E-07	1.84	30.19	20.70	34196.0
AHQ-2-9, 5640	K.SHLPLALLPQTLLDQK.V	1788.12388	2	1.67E-04	0.91	3.85	-	862.6
AHQ-2-9, 5382 - 5450	R.VPFLEVNDPGEPSGLETLK.D	2042.27480	2	6.75E-04	0.25	3.10	-	243.6
AHQ-2-9, 6814	K.YFAEALGPLQSFQARPDDLLINTYPK.S	2969.33891	3	7.60E-07	0.68	3.38	-	327.7
gi|4506025|ref|NP_000936.1| protein phosphatase 3, regulatory subunit B, alpha isoform 1; protein p				7.69E-07	0.79	10.14	11.80	19299.8
AHQ-2-12, 5963	R.IYDMDKDGYISNGELFQVLK.M	2349.64477	2	7.69E-07	0.79	2.81	-	737.4
gi|4505591|ref|NP_002565.1| peroxiredoxin 1; natural killer-enhancing factor A; proliferation-assoc				8.04E-07	4.17	60.23	45.70	22110.2
AHQ-2-10, 3622	R.GLFIIDDK.G	921.07223	1	2.19E-04	0.63	2.57	-	947.9
AHQ-2-11, 2966	K.HGEVCPAGWKPGSDTIKPDVQK.S	2408.67532	2	6.32E-06	0.25	3.19	-	115.2
AHQ-2-11, 5457	K.LNCQVIGASVDSHFCHLAWVNTPK.K	2757.09720	3	3.27E-04	0.62	3.46	-	447.7
AHQ-2-11, 3492 - 3536	R.LVQAFQFTDK.H	1197.36469	2	8.04E-07	0.92	3.43	-	1049.3
AHQ-2-11, 4758	K.QGGLGPMNIPLVSDPK.R	1623.89899	2	1.08E-05	0.92	3.87	-	958.9
AHQ-2-11, 3365 - 3368	R.QITVNDLPVGR.S	1212.38090	2	5.18E-06	0.83	3.33	-	481.5
gi|5453970|ref|NP_006245.1| protein kinase C, delta [Homo sapiens]				8.18E-07	0.86	10.16	1.80	77476.6
AHQ-2-6, 3905	K.LLAEALNQVTQR.A	1356.55324	2	8.18E-07	0.86	3.28	-	941.1
gi|4506077|ref|NP_002734.1| protein kinase C substrate 80K-H; glucosidase II, beta subunit; AGE-bin				8.22E-07	1.82	20.17	4.40	59296.0
AHQ-2-5, 2976	K.AQQEQELAADAFK.E	1449.54763	2	8.22E-07	0.92	3.41	-	1245.6
AHQ-2-5, 3177	K.LWEEQLAAAK.A	1159.31646	2	3.94E-05	0.91	3.50	-	780.5
gi|20452464|ref|NP_620411.1| similar to endothelial cell-selective adhesion molecule; likely orthol				8.28E-07	0.94	10.23	6.70	41208.1
AHQ-2-7, 3813	R.LPTTDGAHPQPISPIPGGVSSSGLSR.M	2529.79081	3	8.28E-07	0.94	4.61	-	1504.2
gi|5453998|ref|NP_006382.1| importin 7; RAN-binding protein 7 [Homo sapiens]				8.30E-07	0.90	10.20	1.40	119515.7
AHQ-2-4, 6997	R.ENIVEAIIHSPELIR.V	1733.98994	2	8.30E-07	0.90	4.07	-	789.2
gi|7661624|ref|NP_056202.1| preimplantation protein 3; likely ortholog of preimplantation protein 3				8.49E-07	0.64	10.14	7.20	22256.6
AHQ-2-10, 3550 - 3555	K.ILEPPEGQDEGVWK.Y	1597.75022	2	8.49E-07	0.64	2.76	-	555.9
gi|6715607|ref|NP_000175.1| G-gamma globin [Homo sapiens]				8.81E-07	0.92	10.18	6.80	16126.3
AHQ-2-13-, 4921 - 4925	R.LLVVYPWTQR.F	1275.52342	2	6.84E-06	0.90	3.38	-	966.1
AHQ-2-14, 6261	R.LLVVYPWTQR.F	1275.52342	2	8.81E-07	0.92	3.59	-	1029.0
gi|20539300|ref|XP_088391.4| similar to tropomyosin 4 [Homo sapiens]				8.87E-07	1.87	20.20	6.90	18592.1
AHQ-2-9, 3215 - 3250	R.KLVILEGELER.A	1299.54160	1	3.98E-04	0.34	2.09	-	441.3
AHQ-2-9, 3344	R.KLVILEGELER.A	1299.54160	2	1.39E-04	0.92	3.92	-	1108.8
AHQ-2-9, 3214 - 3218	R.KLVILEGELER.A	1299.54160	2	7.14E-04	0.88	3.16	-	1229.2
AHQ-2-9, 3944	K.LVILEGELER.A	1171.36868	2	8.87E-07	0.95	3.77	-	1693.8
gi|4504973|ref|NP_002292.1| lactate dehydrogenase C [Homo sapiens]				9.00E-07	0.82	10.18	3.60	36311.0
AHQ-2-11, 2372	R.VIGSGCNLDSAR.F	1250.36445	2	1.07E-06	0.84	3.54	-	506.8
AHQ-2-9, 2310	R.VIGSGCNLDSAR.F	1250.36445	2	9.00E-07	0.82	3.35	-	465.8
gi|5902726|ref|NP_005800.1| peroxiredoxin 2; thioredoxin-dependent peroxide reductase 1 (thiol-spec				9.08E-07	1.90	20.30	9.10	21856.8
AHQ-2-11, 6568	K.EGGLGPLNIPLLADVTR.R	1736.00583	2	1.48E-06	0.92	4.34	-	667.5
AHQ-2-11, 5990 - 5998	R.KEGGLGPLNIPLLADVTR.R	1864.17875	2	9.08E-07	0.97	5.94	-	1279.9
gi|29732788|ref|XP_297707.1| hypothetical protein XP_297707 [Homo sapiens]				9.30E-07	0.58	10.16	5.70	55232.0
AHQ-2-9, 5056 - 5058	R.KYEPSSLTAMLKHSGEPVQHKLSTTAVVR.T	3196.66997	3	9.30E-07	0.58	3.22	-	477.2
gi|5123454|ref|NP_005336.2| heat shock 70kDa protein 1A; heat shock 70kD protein 1A; heat shock-ind				9.31E-07	4.35	50.32	16.10	70037.7
AHQ-2-6, 5435	K.AAAIGIDLGTTYSCVGVFQHGK.V	2267.54677	2	1.88E-04	0.85	3.86	-	607.4
AHQ-2-6, 4733	K.DAGVIAGLNVLR.I	1198.39736	2	4.68E-06	0.95	3.00	-	1981.2
AHQ-2-6, 6233	K.ELEQVCNPIISGLYQGAGGPGPGGFGAQGPK.G	3058.37100	3	9.31E-07	0.98	6.35	-	1993.9
AHQ-2-13, 3809	K.NQVALNPQNTVFDAK.R	1659.82478	2	3.30E-04	0.37	2.54	-	503.3
AHQ-2-6, 3506 - 3511	K.NQVALNPQNTVFDAK.R	1659.82478	2	6.83E-05	0.94	4.52	-	739.8
AHQ-2-6, 7167	K.SINPDEAVAYGAAVQAAILMGDK.S	2305.59353	2	4.17E-04	0.63	2.69	-	547.5
gi|30147623|ref|XP_293669.2| similar to actinin, alpha 4 [Homo sapiens]				9.66E-07	0.83	10.22	3.50	57794.4
AHQ-2-5, 4052	K.LSGSNPYTTVTPQIINSK.W	1921.14079	2	9.66E-07	0.83	3.45	-	663.8
AHQ-2-6, 3934	K.LSGSNPYTTVTPQIINSK.W	1921.14079	2	2.93E-05	0.50	2.69	-	466.8
AHQ-2-5, 3908 - 3980	K.LSGSNPYTTVTPQIINSK.W	1921.14079	2	4.39E-05	0.88	4.34	-	739.5
gi|8922712|ref|NP_060713.1| hypothetical protein FLJ10849 [Homo sapiens]				9.66E-07	2.69	30.19	9.10	49397.9
AHQ-2-7, 2327	K.AAAQLLQSQAQQSGAQQTK.K	1958.12276	2	2.02E-06	0.91	3.75	-	964.3
AHQ-2-7, 2453	K.RNEFLGELQK.K	1234.38656	2	1.23E-04	0.94	3.65	-	1361.5
AHQ-2-7, 2084	R.SYELQESNVR.L	1225.29016	2	9.66E-07	0.84	2.93	-	972.6
gi|18201905|ref|NP_000166.2| glucose phosphate isomerase; neuroleukin; glucose-6-phosphate isomeras				9.69E-07	4.41	60.27	17.40	63146.7
AHQ-2-7, 1665	K.HFVALSTNTTK.V	1219.37190	1	1.81E-04	0.64	2.12	-	511.7
AHQ-2-7, 2769	K.NLVTEDVMR.M	1077.23726	1	5.12E-05	0.15	1.91	-	179.1
AHQ-2-7, 2719	K.TFTTQETITNAETAK.E	1656.77283	2	9.69E-07	0.91	3.84	-	939.3
AHQ-2-7, 6021	K.TLAQLNPESSLFIIASK.T	1833.11828	2	3.21E-06	0.86	3.75	-	550.3
AHQ-2-7, 5400 - 5479	R.VDHQTGPIVWGEPGTNGQHAFYQLIHQGTK.M	3317.61777	3	1.33E-04	0.95	5.35	-	1241.5
AHQ-2-7, 2968	K.VFEGNRPTNSIVFTK.L	1709.92693	2	1.12E-06	0.90	3.91	-	956.3
gi|17450333|ref|XP_060887.1| similar to peptidyl-Pro cis trans isomerase [Homo sapiens]				1.01E-06	0.95	10.22	11.00	17503.9
AHQ-2-12, 6787 - 6854	-.M*VNPTVFDIAVDGKPLGR.V	1946.26009	2	1.01E-06	0.95	4.32	-	1307.7
AHQ-2-12, 6915	-.M*VNPTVFDIAVDGKPLGR.V	1946.26009	2	1.34E-05	0.84	3.54	-	862.8
AHQ-2-12, 6569 - 6633	-.M*VNPTVFDIAVDGKPLGR.V	1946.26009	2	7.53E-06	0.92	4.13	-	931.3
gi|21361565|ref|NP_001679.2| ATP synthase, H+ transporting, mitochondrial F0 complex, subunit b, is				1.02E-06	1.75	20.21	9.00	28908.4
AHQ-2-11, 2829	R.HYLFDVQR.N	1078.20513	2	1.02E-06	0.83	2.84	-	854.7
AHQ-2-13-, 7083	R.YGLIPEEFFQFLYPK.T	1892.18434	2	3.05E-04	0.71	2.91	-	338.2
AHQ-2-11, 6872	R.YGLIPEEFFQFLYPK.T	1892.18434	2	3.69E-05	0.92	4.16	-	430.3
gi|4506365|ref|NP_002856.1| RAB2, member RAS oncogene family [Homo sapiens]				1.03E-06	2.12	30.22	16.50	23545.4
AHQ-2-11, 4274 - 4293	K.LQIWDTAGQESFR.S	1551.68478	2	1.03E-06	0.96	4.34	-	1588.8
AHQ-2-11, 4184	K.SCLLLQFTDKR.F	1382.61075	2	3.38E-04	0.71	3.02	-	661.7
AHQ-2-11, 3376	K.YIIIGDTGVGK.S	1136.32280	1	2.51E-04	0.45	2.46	-	499.0
gi|21361122|ref|NP_001440.2| four and a half LIM domains 1; Four-and-a-half LIM domains 1 [Homo sap				1.13E-06	4.09	50.23	21.80	31894.7
AHQ-2-12, 2427	K.AIVAGDQNVEYK.G	1307.43431	2	7.11E-05	0.79	2.98	-	633.5
AHQ-2-11, 2390	K.AIVAGDQNVEYK.G	1307.43431	2	2.84E-04	0.89	3.59	-	876.6
AHQ-2-9, 2996 - 3067	K.CLHPLANETFVAK.D	1501.73210	2	9.24E-06	0.91	3.96	-	524.1
AHQ-2-9, 3620 - 3695	R.FVFHQEQVYCPDCAK.K	1931.13758	2	1.29E-05	0.96	4.64	-	988.9
AHQ-2-13-, 3874	R.FVFHQEQVYCPDCAK.K	1931.13758	2	9.47E-06	0.96	4.00	-	1583.1
AHQ-2-10, 2800 - 2879	K.GEDFYCVTCHETK.F	1648.75260	2	6.71E-06	0.86	3.32	-	713.6
AHQ-2-9, 2926	K.GEDFYCVTCHETK.F	1648.75260	2	1.13E-06	0.91	3.33	-	912.1
AHQ-2-9, 2131	K.NPITGFGK.G	833.95467	1	2.26E-06	0.53	2.33	-	292.7
gi|4506677|ref|NP_002942.1| ribophorin II [Homo sapiens]				1.20E-06	1.77	20.21	5.50	69301.6
AHQ-2-1, 5124	R.LQVTNVLSQPLTQATVK.L	1841.14195	2	1.20E-06	0.92	4.26	-	761.7
AHQ-2-1, 6773	K.TSFTPVGDVFELNFM*NVK.F	2062.33118	2	9.67E-05	0.84	3.66	-	547.4
gi|28395033|ref|NP_786886.1| ras homolog gene family, member C; Aplysia RAS-related homolog 9 (onco				1.21E-06	4.47	50.23	29.00	22006.2
AHQ-2-10, 4238 - 4258	K.HFCPNVPIILVGNK.K	1609.91693	2	1.21E-06	0.97	4.67	-	1913.1
AHQ-2-10, 3762 - 3764	K.HFCPNVPIILVGNKK.D	1738.08985	2	6.95E-06	0.97	4.64	-	1427.2
AHQ-2-10, 6586	R.LRPLSYPDTDVILM*CFSIDSPDSLENIPEK.W	3483.90759	3	4.95E-04	0.92	4.59	-	961.1
AHQ-2-10, 6678	R.LRPLSYPDTDVILMCFSIDSPDSLENIPEK.W	3467.90819	3	5.70E-05	0.84	4.05	-	655.9
AHQ-2-10, 2498	K.LVIVGDGACGK.T	1090.27572	2	2.30E-05	0.76	2.98	-	787.4
AHQ-2-10, 2495	K.LVIVGDGACGK.T	1090.27572	1	7.45E-04	0.62	2.11	-	772.9
gi|4504085|ref|NP_000399.1| glycerol-3-phosphate dehydrogenase 2 (mitochondrial) [Homo sapiens] [MA				1.25E-06	1.81	20.22	4.00	80814.1
AHQ-2-6, 3458	R.CKDVLTGQEFDVR.A	1568.73370	2	1.25E-06	0.95	4.28	-	1408.1
AHQ-2-6, 6170 - 6243	R.YGAATANYMEVVSLLK.K	1731.00632	2	2.18E-04	0.85	3.57	-	968.0
gi|15306356|ref|XP_035638.2| similar to interleukin 25; lymphocyte antigen 6 complex, locus E ligan				1.28E-06	1.79	20.18	13.90	18795.1
AHQ-2-13-, 3473	R.ESDVPLKTEEFEVTK.T	1751.91253	2	1.28E-06	0.86	3.50	-	619.8
AHQ-2-13, 3475	R.ESDVPLKTEEFEVTK.T	1751.91253	2	1.43E-06	0.77	3.08	-	629.1
AHQ-2-12, 3389	R.ESDVPLKTEEFEVTK.T	1751.91253	2	1.94E-06	0.89	3.62	-	707.9
AHQ-2-12, 4610	K.SYLYFTQFK.A	1197.36315	2	4.35E-06	0.93	3.21	-	1258.8

gi|7657649|ref|NP_055362.1| tropomodulin 3 (ubiquitous) [Homo sapiens]				1.31E-06	0.96	10.24	5.40	39594.5
AHQ-2-11, 5052	K.YKDLDEDELLGNLSETELK.Q	2225.39217	2	1.31E-06	0.96	4.70	-	1210.2
gi|4503291|ref|NP_001346.1| D-dopachrome tautomerase [Homo sapiens]				1.32E-06	0.88	10.18	9.30	12711.7
AHQ-2-14-, 5639	R.FFPLESWQIGK.I	1352.56151	2	1.32E-06	0.88	3.40	-	558.1
gi|4557871|ref|NP_001054.1| transferrin [Homo sapiens]				1.33E-06	0.86	10.22	2.70	77049.5
AHQ-2-5, 7344 - 7419	R.SAGWNIPIGLLYCDLPEPR.K	2173.47658	2	1.33E-06	0.86	4.37	-	441.8
gi|20070272|ref|NP_057110.2| androgen-regulated short-chain dehydrogenase/reductase 1; prostate sho				1.36E-06	2.12	30.19	15.40	35414.1
AHQ-2-13, 3735	K.GSGVTTYSVHPGTVQSELVR.H	2075.26811	2	1.36E-06	0.74	3.10	-	490.1
AHQ-2-9, 3272	K.GSGVTTYSVHPGTVQSELVR.H	2075.26811	2	7.31E-06	0.90	3.86	-	744.6
AHQ-2-9, 3779	K.MLSSGVCTSTVQLPGK.V	1666.94223	2	1.76E-04	0.80	3.33	-	657.2
AHQ-2-13-, 2633 - 2643	K.VVVVTGANTGIGK.E	1215.42469	2	2.23E-04	0.58	3.04	-	495.3
AHQ-2-13, 2712	K.VVVVTGANTGIGK.E	1215.42469	2	3.83E-04	0.86	3.01	-	947.8
gi|4758030|ref|NP_004362.1| coatomer protein complex, subunit alpha; alpha coat protein; xenin [Hom				1.40E-06	0.50	10.13	1.20	138330.6
AHQ-2-14, 6025	R.VTTVTEIGKDVIGLR.I	1601.86982	2	1.40E-06	0.50	2.63	-	318.2
gi|30148492|ref|XP_293610.2| similar to Heat shock cognate 71 kDa protein [Homo sapiens]				1.40E-06	2.68	30.22	4.40	72113.4
AHQ-2-6, 4185	K.DAGTIAGLNVLR.I	1200.37012	2	1.29E-04	0.94	3.29	-	1489.0
AHQ-2-7, 4541	R.IINEPTAAAIAYGLDK.K	1660.89259	2	1.01E-05	0.81	3.79	-	885.5
AHQ-2-9, 4618	R.IINEPTAAAIAYGLDK.K	1660.89259	2	1.49E-04	0.47	2.99	-	361.4
AHQ-2-10, 4376 - 4384	R.IINEPTAAAIAYGLDK.K	1660.89259	2	1.29E-05	0.89	3.97	-	629.9
AHQ-2-6, 4023	R.IINEPTAAAIAYGLDKK.V	1789.06550	2	1.40E-06	0.93	4.36	-	758.6
gi|4502905|ref|NP_001822.1| clusterin; complement-associated protein SP-40 [Homo sapiens]				1.44E-06	2.64	40.28	15.10	52494.3
AHQ-2-9, 5871	R.ASSIIDELFQDR.F	1394.51197	1	2.51E-05	0.07	2.37	-	165.0
AHQ-2-9, 2875	R.EILSVDCSTNNPSQAK.L	1764.89299	2	4.43E-04	0.77	3.21	-	634.3
AHQ-2-8, 2792	R.EILSVDCSTNNPSQAK.L	1764.89299	2	6.19E-04	0.49	2.66	-	443.4
AHQ-2-11, 5276	K.LFDSDPITVTVPVEVSR.K	1875.11203	2	4.97E-05	0.84	3.56	-	922.2
AHQ-2-11, 3392	R.VTTVASHTSDSDVPSGVTEVVVK.L	2315.52008	2	1.44E-06	0.96	5.70	-	912.8
AHQ-2-9, 3234	R.VTTVASHTSDSDVPSGVTEVVVK.L	2315.52008	2	3.40E-05	0.95	4.79	-	1123.8
gi|21361619|ref|NP_061882.2| TOLLIP protein; Toll-interacting protein [Homo sapiens]				1.50E-06	1.76	20.20	9.90	30281.6
AHQ-2-11, 5554 - 5629	R.GPVYIGELPQDFLR.I	1604.83072	2	1.50E-06	0.92	4.08	-	959.0
AHQ-2-11, 5637	R.GPVYIGELPQDFLR.I	1604.83072	2	1.87E-06	0.95	4.04	-	1409.4
AHQ-2-11, 4740 - 4745	R.IAWTHITIPESLR.Q	1537.78752	2	5.90E-05	0.83	3.47	-	626.3
gi|4505145|ref|NP_002387.1| malic enzyme 2, NAD(+)-dependent, mitochondrial; Malic enzyme, mitochon				1.59E-06	2.64	30.25	8.90	65443.3
AHQ-2-6, 2967	R.HISDSVFLEAAK.A	1317.47234	2	1.88E-05	0.84	3.43	-	479.3
AHQ-2-6, 2799	K.IETQDIQALR.F	1187.32821	2	1.59E-06	0.91	3.59	-	1006.2
AHQ-2-6, 7217 - 7287	R.ILQDDIESLMPIVYTPTVGLACSQYGHIFR.R	3439.94604	3	3.71E-06	0.90	5.07	-	493.7
gi|17444699|ref|XP_017966.4| similar to Reticulon protein 3 (Neuroendocrine-specific protein-like 2				1.60E-06	0.90	10.16	4.70	25522.7
AHQ-2-1, 2901 - 2905	K.TQIDHYVGIAR.H	1273.42279	2	1.60E-06	0.90	3.25	-	1055.7
gi|30149460|ref|XP_210540.2| similar to Heat shock protein HSP 90-alpha (HSP 86) [Homo sapiens] [MA				1.61E-06	1.70	20.17	11.10	29287.8
AHQ-2-5, 1979	K.EGLELPEDEEEKKKQEEK.K	2188.33193	2	1.30E-04	0.83	3.46	-	712.0
AHQ-2-5, 1996	K.HIYYITGETK.D	1225.37490	2	1.61E-06	0.87	3.08	-	673.3
gi|5729873|ref|NP_006656.1| heparanase; heparanase-1 [Homo sapiens]				1.62E-06	1.78	20.19	4.20	61176.4
AHQ-2-14-, 4999	K.TDFLIFDPK.K	1096.25713	1	1.29E-04	0.37	2.20	-	468.3
AHQ-2-14-, 5005	K.TDFLIFDPK.K	1096.25713	2	1.62E-06	0.94	3.81	-	1369.8
AHQ-2-7, 3296 - 3301	K.YLLRPLGPHGLLSK.S	1564.89924	2	9.10E-04	0.83	2.89	-	931.1
gi|29736026|ref|XP_085123.3| similar to Ras-related protein Rab-15 [Homo sapiens]				1.67E-06	0.35	10.18	5.30	23517.8
AHQ-2-13-, 3470 - 3489	R.IQIWDTAGQER.Y	1317.43243	2	1.53E-05	0.92	3.61	-	952.8
AHQ-2-10, 3160	R.IQIWDTAGQER.Y	1317.43243	1	1.67E-06	0.35	2.83	-	234.8
gi|4505775|ref|NP_002626.1| phosphate carrier precursor isoform 1b; phosphate carrier, mitochondria				1.73E-06	0.81	10.18	7.50	39958.5
AHQ-2-9, 7403 - 7404	K.YYALCGFGGVLSCGLTHTAVVPLDLVK.C	2914.38804	2	1.73E-06	0.81	3.65	-	605.2
gi|5803013|ref|NP_006808.1| endoplasmic reticulum protein 29 precursor; endoplasmic reticulum lumen				1.74E-06	2.19	30.20	15.30	28993.2
AHQ-2-10, 3864 - 3868	R.DGDFENPVPYTGAVK.V	1609.71786	2	7.48E-04	0.32	2.75	-	437.6
AHQ-2-10, 3727	K.ILDQGEDFPASEMTR.I	1709.85924	2	3.98E-05	0.94	4.06	-	980.0
AHQ-2-10, 4476	K.SLNILTAFQK.K	1135.33804	2	1.74E-06	0.92	3.33	-	1321.6
gi|29742410|ref|XP_291183.1| similar to TREM-like transcript 1 [Homo sapiens]				1.79E-06	0.72	10.21	5.10	32678.3
AHQ-2-10, 4027	R.FLPEGCQPLVSSAVDR.R	1776.99174	2	8.45E-06	0.74	3.21	-	348.7
AHQ-2-11, 4205	R.FLPEGCQPLVSSAVDR.R	1776.99174	2	1.79E-06	0.72	2.94	-	384.9
AHQ-2-13-, 4230 - 4238	R.FLPEGCQPLVSSAVDR.R	1776.99174	2	5.87E-06	0.89	4.22	-	472.5
AHQ-2-9, 4288	R.FLPEGCQPLVSSAVDR.R	1776.99174	2	2.21E-04	0.51	3.10	-	135.1
gi|5803135|ref|NP_006852.1| RAB35, member RAS oncogene family; ras-related protein rab-1c (GTP-bind				1.81E-06	0.88	10.18	5.50	23025.1
AHQ-2-13-, 3814 - 3829	K.LLIIGDSGVGK.S	1072.28025	2	5.65E-05	0.89	3.49	-	1052.4
AHQ-2-10, 3406	K.LLIIGDSGVGK.S	1072.28025	2	1.81E-06	0.88	3.38	-	902.6
gi|7706244|ref|NP_057005.1| divalent cation tolerant protein CUTA [Homo sapiens]				1.82E-06	0.97	10.34	17.30	16832.5
AHQ-2-13, 6773	R.SVHPYEVAEVIALPVEQGNFPYLQWVR.Q	3142.55428	3	1.82E-06	0.97	6.78	-	1780.9
gi|18375652|ref|NP_002826.2| protein tyrosine phosphatase, non-receptor type 12; protein-tyrosine p				1.87E-06	0.93	10.22	2.10	88119.9
AHQ-2-9, 7195	R.YWPLYGEDPITFAPFK.I	1945.20411	2	4.27E-04	0.76	3.46	-	329.3
AHQ-2-7, 7128 - 7192	R.YWPLYGEDPITFAPFK.I	1945.20411	2	1.87E-06	0.93	4.37	-	661.7
gi|7019485|ref|NP_037364.1| programmed cell death 6; apoptosis-linked gene 2 [Homo sapiens]				1.89E-06	1.68	20.18	20.90	21868.3
AHQ-2-11, 5445	K.AGVNFSEFTGVWK.Y	1442.59992	2	1.89E-06	0.90	3.56	-	1055.0
AHQ-2-11, 6524	R.SGVISDTELQQALSNGTWTPFNPVTVR.S	2919.19515	2	2.38E-06	0.78	3.52	-	541.8
gi|5174613|ref|NP_005960.1| nucleosome assembly protein 1-like 4; nucleosome assembly protein 2 [Ho				1.89E-06	0.95	10.17	2.70	42823.1
AHQ-2-7, 2619	K.FYEEVHDLER.K	1337.41897	2	1.89E-06	0.95	3.40	-	1659.1
gi|4506205|ref|NP_002791.1| proteasome beta 9 subunit isoform 1 proprotein; proteasome subunit, bet				1.96E-06	0.93	10.16	5.50	23264.2
AHQ-2-11, 4417	R.FTTDAIALAMSR.D	1297.50607	2	1.96E-06	0.93	3.30	-	1431.5
gi|18201911|ref|NP_000629.2| vitronectin precursor; serum spreading factor; somatomedin B; compleme				2.06E-06	0.94	10.21	3.10	54335.4
AHQ-2-13-, 5713 - 5715	R.SIAQYWLGCPAPGHL	1671.90045	2	2.06E-06	0.94	4.17	-	968.9
AHQ-2-13, 5603	R.SIAQYWLGCPAPGHL	1671.90045	2	1.97E-04	0.77	2.88	-	616.2
gi|22748619|ref|NP_689476.1| tropomyosin 3 [Homo sapiens]				2.07E-06	1.82	20.25	11.10	28222.6
AHQ-2-9, 3634	R.ALKDEEKMELQEIQLK.E	1946.25520	2	2.07E-06	0.95	4.92	-	1238.0
AHQ-2-9, 3011 - 3078	R.KLVIIEGDLER.T	1285.51492	2	1.47E-04	0.87	3.40	-	873.6
gi|27413156|ref|NP_000338.2| spectrin, beta, erythrocytic (includes spherocytosis, clinical type I)				2.09E-06	0.90	10.21	1.40	246320.0
AHQ-2-2, 7465	R.DASVAEAWLIAQEPYLASGDFGHTVDSVEK.L	3207.44880	3	2.09E-06	0.90	4.12	-	1098.5
gi|4506363|ref|NP_002861.1| RAB13, member RAS oncogene family; RAS-associated protein RAB13 [Homo s				2.12E-06	1.74	20.19	10.80	22774.0
AHQ-2-11, 3550	K.LLLIGDSGVGK.T	1072.28025	1	2.90E-05	0.82	3.19	-	626.9
AHQ-2-13, 3792	K.LLLIGDSGVGK.T	1072.28025	2	3.69E-04	0.89	3.35	-	964.8
AHQ-2-14-, 3755	K.LLLIGDSGVGK.T	1072.28025	2	2.12E-06	0.93	3.75	-	1259.1
AHQ-2-11, 2930	K.LQVWDTAGQER.F	1303.40576	2	5.05E-05	0.81	3.15	-	812.2
gi|4503359|ref|NP_003965.1| docking protein 2, 56kD [Homo sapiens]				2.13E-06	1.52	20.14	6.30	45547.8
AHQ-2-13, 4000	R.FGASLYGGSDCALAR.L	1546.68659	2	2.13E-06	0.62	2.73	-	621.7
AHQ-2-13-, 3915	R.LYLLAAPAAER.G	1188.40094	2	5.08E-04	0.90	2.89	-	1186.2
gi|4759342|ref|NP_004808.1| tight junction protein 2 (zona occludens 2); Friedreich ataxia region g				2.15E-06	0.76	10.19	2.90	124661.6
AHQ-2-13, 6296	R.DNPHFENGETSIVISDVLPGGPADGLLQENDR.V	3407.60319	3	2.15E-06	0.76	3.89	-	663.7
gi|7706675|ref|NP_057661.1| RAB6B, member RAS oncogene family; small GTPase RAB6B [Homo sapiens] [M				2.15E-06	2.74	40.24	23.10	23461.5
AHQ-2-11, 4600	R.GSDVIIMLVGNK.T	1246.50220	2	9.59E-06	0.96	4.15	-	1961.4
AHQ-2-11, 3425	R.LQLWDTAGQER.F	1317.43243	1	8.52E-05	0.20	2.55	-	217.9
AHQ-2-12, 3315 - 3394	R.LQLWDTAGQER.F	1317.43243	2	2.45E-04	0.62	2.54	-	595.2
AHQ-2-11, 3489	K.LVFLGEQSVGK.T	1177.37492	2	8.48E-05	0.94	3.60	-	1242.9
AHQ-2-11, 3000	R.VASALPGMENVQEK.S	1473.67727	1	2.15E-06	0.64	3.56	-	307.5
gi|30154095|ref|XP_293967.2| similar to gamma actin-like protein [Homo sapiens]				2.15E-06	1.41	20.20	12.20	19259.0
AHQ-2-8, 5073	K.DLYTSTVLSGGTTM*YPGIADR.M	2235.45690	2	2.13E-04	0.37	2.80	-	334.7
AHQ-2-8, 5208	K.DLYTSTVLSGGTTM*YPGIADR.M	2235.45690	2	3.31E-04	0.31	2.72	-	257.0
AHQ-2-10, 4680 - 4694	K.DLYTSTVLSGGTTM*YPGIADR.M	2235.45690	2	5.29E-05	0.37	2.75	-	376.9
AHQ-2-13, 4911 - 4919	K.DLYTSTVLSGGTTM*YPGIADR.M	2235.45690	2	3.62E-05	0.33	2.73	-	306.8
AHQ-2-13, 5213	K.DLYTSTVLSGGTTMYPGIADR.M	2219.45750	2	6.74E-05	0.63	2.70	-	572.8
AHQ-2-14, 5700	K.DLYTSTVLSGGTTM*YPGIADR.M	2235.45690	2	3.66E-05	0.74	3.36	-	526.8
AHQ-2-14, 5824 - 5885	K.DLYTSTVLSGGTTM*YPGIADR.M	2235.45690	2	2.00E-05	0.64	3.31	-	441.7
AHQ-2-14, 6076	K.DLYTSTVLSGGTTM*YPGIADR.M	2235.45690	2	6.86E-05	0.85	3.74	-	725.2
AHQ-2-14, 6196	K.DLYTSTVLSGGTTMYPGIADR.M	2219.45750	2	3.40E-05	0.84	4.08	-	488.9

AHQ-2-13-, 4847	K.DLYTSTVLSGGTTM*YPGIADR.M	2235.45690	2	7.68E-06	0.80	3.53	-	602.6
AHQ-2-13-, 4950 - 5018	K.DLYTSTVLSGGTTM*YPGIADR.M	2235.45690	2	3.87E-04	0.69	3.21	-	484.1
AHQ-2-13-, 5177	K.DLYTSTVLSGGTTM*YPGIADR.M	2235.45690	2	2.25E-05	0.82	3.11	-	886.2
AHQ-2-13-, 5273	K.DLYTSTVLSGGTTMYPGIADR.M	2219.45750	2	1.79E-04	0.75	3.59	-	420.8
AHQ-2-14-, 4775 - 4843	K.DLYTSTVLSGGTTM*YPGIADR.M	2235.45690	2	1.00E-04	0.74	3.20	-	730.6
AHQ-2-14-, 4907 - 4979	K.DLYTSTVLSGGTTM*YPGIADR.M	2235.45690	2	1.21E-04	0.35	3.02	-	363.5
AHQ-2-14-, 5096 - 5111	K.DLYTSTVLSGGTTM*YPGIADR.M	2235.45690	2	2.15E-06	0.56	3.05	-	400.1
gi|19923437|ref|NP_057366.2| adenylate kinase 3 alpha like [Homo sapiens]				2.15E-06	1.79	20.22	12.80	25565.2
AHQ-2-10, 3038	K.AYEDQTKPVLEYYQK.K	1876.05535	2	1.09E-04	0.92	4.36	-	613.9
AHQ-2-10, 6246 - 6286	K.NLTQYSWLLDGFPR.T	1710.91339	2	2.15E-06	0.87	3.20	-	1180.3
gi|5453603|ref|NP_006422.1| chaperonin containing TCP1, subunit 2 (beta); chaperonin containing t-c				2.18E-06	3.63	40.20	11.40	57487.9
AHQ-2-7, 2856	R.EALLSSAVDHGSDEVK.F	1657.76083	2	8.35E-06	0.90	4.03	-	886.8
AHQ-2-7, 2919	R.GATQQILDEAER.S	1331.41427	2	2.18E-06	0.91	3.77	-	1136.4
AHQ-2-7, 5433	K.LGGSLADSYLDEGFLLDKK.I	2042.27480	2	9.27E-05	0.92	3.69	-	1091.2
AHQ-2-7, 5481 - 5545	R.SLHDALCVLAQTVK.D	1556.80920	2	3.87E-06	0.90	3.27	-	1047.5
gi|4759270|ref|NP_004613.1| translin; recombination hotspot associated factor; recombination hotspo				2.19E-06	1.84	20.23	15.80	26182.8
AHQ-2-10, 5059 - 5134	R.EILTLLQGVHQGAGFQDIPK.R	2165.47753	2	2.76E-05	0.87	4.10	-	540.5
AHQ-2-10, 6079	R.PLHISTFINELDSGFR.L	1847.06360	2	2.19E-06	0.97	4.61	-	1797.2
gi|4503481|ref|NP_001395.1| eukaryotic translation elongation factor 1 gamma; elongation factor 1-g				2.25E-06	1.68	20.20	8.20	50118.5
AHQ-2-12, 7026	R.GQELAFPLSPDWQVDYESYTWR.K	2688.88693	2	1.26E-04	0.79	3.33	-	383.5
AHQ-2-12, 2571	R.KLDPGSEETQTLVR.E	1573.73020	2	2.25E-06	0.89	4.00	-	546.1
gi|4504351|ref|NP_000510.1| delta globin [Homo sapiens]				2.27E-06	0.93	10.19	12.90	16055.4
AHQ-2-13-, 5626	R.FFESFGDLSSPDAVMGNPK.V	2046.24551	2	2.27E-06	0.93	3.83	-	1245.9
AHQ-2-13, 5449 - 5528	R.FFESFGDLSSPDAVMGNPK.V	2046.24551	2	8.73E-04	0.85	3.32	-	1030.6
gi|24308263|ref|NP_067045.1| TcD37 homolog; prune [Homo sapiens]				2.33E-06	0.97	10.25	3.80	50199.3
AHQ-2-7, 7660	K.LLPLLQEALSAYFDSMK.I	1940.29226	2	2.33E-06	0.97	5.02	-	1760.3
gi|4504045|ref|NP_002063.1| guanine nucleotide binding protein (G protein), q polypeptide [Homo sap				2.39E-06	1.66	20.24	9.50	42142.8
AHQ-2-8, 3850	R.VADPAYLPTQQDVLR.V	1686.89014	2	2.39E-06	0.71	3.05	-	504.6
AHQ-2-9, 7386 - 7390	R.VPTTGIIEYPFDLQSVIFR.M	2196.53000	2	7.66E-04	0.94	4.77	-	691.4
AHQ-2-8, 7355	R.VPTTGIIEYPFDLQSVIFR.M	2196.53000	2	1.89E-05	0.95	4.72	-	980.9
gi|4758792|ref|NP_004544.1| NADH dehydrogenase (ubiquinone) Fe-S protein 6, 13kDa (NADH-coenzyme Q				2.44E-06	0.59	10.13	9.70	13711.5
AHQ-2-13-, 3931	K.TGTCGYCGLQFR.Q	1422.56875	2	2.44E-06	0.59	2.62	-	665.0
gi|6680033|ref|NP_031379.1| guanine nucleotide binding protein (G protein) alpha 12 [Homo sapiens]				2.45E-06	0.61	10.13	2.90	44250.9
AHQ-2-9, 2971	K.ILLLGAGESGK.S	1058.25357	1	2.95E-04	0.66	2.57	-	622.1
AHQ-2-8, 2807	K.ILLLGAGESGK.S	1058.25357	1	2.45E-06	0.61	2.60	-	503.6
gi|30149013|ref|XP_302138.1| similar to alpha tubulin [Homo sapiens]				2.46E-06	0.85	10.14	4.60	22444.5
AHQ-2-13, 4989	R.EIINLVLDR.I	1085.27902	1	1.63E-04	0.13	2.08	-	134.3
AHQ-2-14-, 5011	R.EIINLVLDR.I	1085.27902	2	3.82E-04	0.76	2.66	-	852.9
AHQ-2-7, 4932 - 4936	R.EIINLVLDR.I	1085.27902	2	2.46E-06	0.85	2.89	-	1050.7
gi|4502011|ref|NP_000467.1| adenylate kinase 1 [Homo sapiens]				2.61E-06	3.97	50.31	35.10	21634.7
AHQ-2-11, 6554 - 6557	K.GQLVPLETVLDM*LR.D	1600.90530	2	6.61E-04	0.92	4.61	-	486.2
AHQ-2-11, 5644	R.IGQPTLLLYVDAGPETMTQR.L	2204.53234	2	3.00E-06	0.95	4.46	-	1087.9
AHQ-2-11, 5148	R.IGQPTLLLYVDAGPETM*TQR.L	2220.53174	2	2.61E-06	0.86	4.13	-	595.4
AHQ-2-11, 3346	K.IIFVVGGPGSGK.G	1131.34939	1	3.52E-04	0.75	2.46	-	934.2
AHQ-2-11, 6321 - 6390	K.VNAEGSVDSVFSQVCTHLDALK	2378.60116	2	4.40E-04	0.49	2.88	-	381.4
gi|5453714|ref|NP_006448.1| LIM protein (similar to rat protein kinase C-binding enigma) [Homo sapi				2.64E-06	0.79	10.16	2.50	64027.9
AHQ-2-13-, 2829 - 2894	K.EVVKPVPITSPAVSK.V	1551.85241	2	2.64E-06	0.79	3.10	-	371.5
AHQ-2-13, 2888 - 2937	K.EVVKPVPITSPAVSK.V	1551.85241	2	8.52E-05	0.68	3.02	-	262.1
gi|4506181|ref|NP_002778.1| proteasome alpha 2 subunit; proteasome subunit HC3; proteasome componen				2.84E-06	1.18	20.22	15.40	25898.4
AHQ-2-10, 4490	R.GYSFSLTTFSPSGK.L	1479.61533	1	7.54E-04	0.26	2.42	-	284.4
AHQ-2-10, 4812	R.KLAQQYYLVYQEPIPTAQLVQR.V	2651.05593	2	2.84E-06	0.92	4.43	-	674.9
gi|4506359|ref|NP_000311.1| quinoid dihydropteridine reductase; dihydropteridine reductase [Homo sa				2.87E-06	0.98	10.26	7.00	25803.4
AHQ-2-10, 3280	K.NRPSSGSLIQVVTTEGR.T	1801.98277	2	2.87E-06	0.98	5.12	-	1874.6
gi|4757714|ref|NP_004291.1| acid phosphatase 1 isoform c; acid phosphatase of erythrocyte; red cell				3.29E-06	0.64	10.18	5.70	18042.4
AHQ-2-11, 3112	R.SPIAEAVFR.K	990.13779	1	3.29E-06	0.64	2.29	-	413.1
gi|21361456|ref|NP_055425.2| alpha glucosidase II alpha subunit; likely ortholog of mouse G2an alph				3.37E-06	3.33	40.20	7.20	109437.2
AHQ-2-5, 5947	K.HHGPQTLYLPVTLSSIPVFQR.G	2391.75581	2	4.49E-06	0.76	3.03	-	646.8
AHQ-2-5, 4280	R.LSFQHDPETSVLVLR.K	1741.96893	2	4.91E-04	0.84	3.72	-	519.6
AHQ-2-5, 3629	K.MMDYLQGSGETPQTDVR.W	1929.12196	2	3.37E-06	0.94	4.10	-	1097.2
AHQ-2-4, 3893	R.VVIIGAGKPAAVVLQTK.G	1665.05702	2	6.35E-04	0.79	3.35	-	652.3
gi|4506285|ref|NP_003470.1| protein tyrosine phosphatase type IVA, member 2 isoform 1; protein tyro				3.41E-06	0.88	10.17	8.40	19127.1
AHQ-2-11, 2837 - 2897	R.FLITHNPTNATLNK.F	1584.80104	2	3.41E-06	0.88	3.30	-	1159.4
gi|20127408|ref|NP_000173.2| hydroxyacyl dehydrogenase, subunit A; trifunctional protein, alpha sub				3.56E-06	3.63	50.21	12.70	82999.1
AHQ-2-6, 6475	R.DSIFSNLTGQLDYQGFEK.A	2063.20943	2	1.10E-04	0.92	4.15	-	915.8
AHQ-2-6, 2481	K.DTSASAVAVGLK.Q	1119.25064	1	5.72E-05	0.18	2.21	-	367.7
AHQ-2-6, 6621 - 6629	K.EVEAVIPDHCIFASNTSALPISEIAAVSK.R	3071.44790	3	3.10E-04	0.79	3.91	-	440.7
AHQ-2-6, 2975	K.TGIEQGSDAGYLCESQK.F	1844.93497	2	3.56E-06	0.93	4.15	-	880.0
AHQ-2-6, 6045	K.TVLGTPEVLLGALPGAGGTQR.L	2008.30795	3	1.49E-04	0.81	3.45	-	948.4
gi|9845511|ref|NP_008839.2| ras-related C3 botulinum toxin substrate 1 isoform Rac1; rho family, sm				3.95E-06	0.96	10.21	5.20	21450.0
AHQ-2-11, 3052	K.YLECSALTQR.G	1242.38392	2	3.95E-06	0.96	4.18	-	1668.5
gi|8923579|ref|NP_060377.1| hypothetical protein FLJ20625 [Homo sapiens]				4.01E-06	1.84	20.27	26.70	17744.7
AHQ-2-13, 6301	K.LPPLPSLTSQPHQVLASEPIPFSDLQQVSR.I	3283.72259	3	7.24E-04	0.87	4.25	-	626.1
AHQ-2-14-, 6339	K.LPPLPSLTSQPHQVLASEPIPFSDLQQVSR.I	3283.72259	3	6.89E-04	0.93	5.38	-	718.7
AHQ-2-12, 5445	R.TDEQALLSSILAK.T	1389.57677	2	4.01E-06	0.91	3.19	-	1195.8
gi|29736622|ref|XP_293924.1| similar to cytoplasmic beta-actin [Homo sapiens]				4.02E-06	1.87	20.24	13.60	42002.9
AHQ-2-8, 7187	R.FRCPEAIFQPSFLGIESSGIHETTFNSIMK.C	3446.89713	3	5.02E-05	0.93	4.78	-	1168.7
AHQ-2-8, 3762	R.VAPDEHPILLTEAPLNPKINR.E	2338.69122	3	4.02E-06	0.94	4.71	-	1399.8
gi|22063113|ref|XP_171447.1| similar to elongin B; transcription elongation factor B (SIII), polype				4.36E-06	1.30	20.16	21.20	13156.8
AHQ-2-12, 3470	R.PQAPATVGLAFR.A	1228.42516	2	4.36E-06	0.88	3.20	-	742.6
AHQ-2-12, 4897	K.TLGECGFTSQTARPQAPATVGLAFR.A	2638.94056	2	6.63E-05	0.41	2.82	-	241.3
gi|27482573|ref|XP_208709.1| similar to Ubiquitin-conjugating enzyme E2-18 kDa UbcH7 (Ubiquitin-pro				4.49E-06	0.96	10.27	11.70	17875.4
AHQ-2-12, 4917	K.GQVCLPVISAENWKPATK.T	2000.30757	2	4.49E-06	0.96	5.38	-	878.0
gi|25306267|ref|NP_001700.2| brain-derived neurotrophic factor isoform a preproprotein [Homo sapien				4.58E-06	1.68	20.20	6.10	27817.8
AHQ-2-13-, 2858	K.TAVDM*SGGTVTVLEK.V	1524.71933	2	4.58E-06	0.73	2.59	-	1013.9
AHQ-2-13-, 3754 - 3758	K.TAVDMSGGTVTVLEK.V	1508.71993	2	7.97E-04	0.80	3.34	-	841.4
AHQ-2-13, 3740 - 3743	K.TAVDMSGGTVTVLEK.V	1508.71993	2	8.34E-05	0.95	3.91	-	1659.7
gi|4505395|ref|NP_002499.1| nidogen (enactin); Nidogen; nidogen (entactin) [Homo sapiens]				4.59E-06	0.95	10.18	0.90	136488.3
AHQ-2-3, 4536	R.VLFETDLVNPR.G	1303.48880	2	4.59E-06	0.95	3.56	-	1467.7
gi|5031569|ref|NP_005727.1| ARP1 actin-related protein 1 homolog A, centractin alpha; ARP1 (actin-r				4.59E-06	0.93	10.23	5.90	42613.4
AHQ-2-8, 5052	K.DQLQTFSEEHPVLLTEAPLNPR.K	2535.79382	2	4.59E-06	0.93	4.42	-	748.7
gi|19923262|ref|NP_004153.2| RAB5A, member RAS oncogene family; RAS-associated protein RAB5A [Homo				4.76E-06	1.84	20.19	10.20	23658.5
AHQ-2-10, 3691	K.FEIWDTAGQER.Y	1352.43364	2	4.37E-05	0.93	3.80	-	1250.9
AHQ-2-10, 3204 - 3206	K.LVLLGESAVGK.S	1086.30692	2	4.76E-06	0.90	3.53	-	1143.8
gi|8393638|ref|NP_058642.1| F11 receptor isoform a precursor; platelet F11 receptor; platelet adhes				5.09E-06	0.68	10.13	3.70	32582.9
AHQ-2-12, 5326	R.VTFLPTGITFK.S	1224.47318	2	5.09E-06	0.68	2.55	-	374.5
gi|4557251|ref|NP_001101.1| a disintegrin and metalloprotease domain 10 [Homo sapiens]				5.12E-06	0.87	10.17	2.00	84141.7
AHQ-2-5, 4676	K.AIDTIYQTTDFSGIR.N	1701.85844	2	5.12E-06	0.87	3.31	-	869.0
gi|20149570|ref|NP_005816.2| RAS guanyl releasing protein 2 isoform 1; calcium and diacylglycerol-r				5.28E-06	0.80	10.14	2.80	75547.0
AHQ-2-7, 6844	K.LWEGLTELVTATGNYGNYR.R	2158.35558	2	5.28E-06	0.80	2.76	-	1014.2
gi|6005786|ref|NP_009214.1| monoglyceride lipase; lysophospholipase-like [Homo sapiens]				5.49E-06	3.47	40.26	19.20	34292.3
AHQ-2-9, 1966	K.ALIFVSHGAGEHSGR.Y	1538.69246	2	5.49E-06	0.95	4.20	-	1159.4
AHQ-2-14-, 6103	K.LTVPFLLLQGSADR.L	1530.79329	2	5.48E-04	0.70	2.88	-	491.7
AHQ-2-9, 6430	R.TPQSIPYQDLPHLVNADGQYLFCR.Y	2835.14314	3	4.62E-04	0.87	4.46	-	400.1
AHQ-2-9, 7346	K.VLNLVLPNLSLGPIDSSVLSR.N	2207.59900	2	5.03E-04	0.95	5.20	-	718.2
gi|8922601|ref|NP_060654.1| hypothetical protein FLJ10702 [Homo sapiens]				6.17E-06	1.04	20.18	13.40	21538.9
AHQ-2-11, 2597 - 2629	R.DLPNALDEK.Q	1015.09928	1	3.53E-04	0.20	1.83	-	463.5
AHQ-2-11, 5433	R.GVNAIVYMIDAADREK.I	1766.01230	2	6.17E-06	0.83	3.56	-	677.3

gi|4826657|ref|NP_004333.1| caldesmon 1 isoform 3; H-CAD; L-CAD [Homo sapiens]				6.37E-06	3.55	40.20	13.00	62662.9
AHQ-2-10, 4634 - 4640	K.EFDPTITDASLSLPSR.R	1749.89986	2	2.63E-05	0.87	3.52	-	786.4
AHQ-2-12, 2503	K.GNVFSSPTAAGTPNK.E	1448.56286	2	5.22E-04	0.71	2.67	-	884.8
AHQ-2-13, 2668	K.GNVFSSPTAAGTPNK.E	1448.56286	2	7.40E-04	0.72	2.85	-	553.1
AHQ-2-13-, 2601	K.GNVFSSPTAAGTPNK.E	1448.56286	2	6.37E-06	0.92	3.81	-	918.8
AHQ-2-12, 2694	K.SAKPTKPAASDLPVPAEGVR.N	1992.26539	2	2.46E-04	0.88	4.10	-	593.8
AHQ-2-10, 3516	K.TTTTNTQVEGDDEAAFLER.L	2099.15534	2	4.98E-05	0.87	3.06	-	980.4
gi|4505329|ref|NP_003818.1| N-ethylmaleimide-sensitive factor attachment protein, alpha; alpha-SNAP				6.55E-06	1.94	20.24	11.20	33246.6
AHQ-2-9, 3974	K.HDAATCFVDAGNAFK.K	1625.74381	2	6.55E-06	0.97	4.58	-	2068.3
AHQ-2-9, 3118	K.LLEAHEEQNVDSYTESVK.E	2092.20594	2	9.22E-05	0.97	4.83	-	1535.2
gi|4759086|ref|NP_004883.1| vesicle trafficking protein sec22b [Homo sapiens]				6.69E-06	2.65	30.20	20.00	24740.4
AHQ-2-12, 1987	R.DLQQYQSQAK.Q	1209.29073	2	3.08E-05	0.83	2.97	-	763.3
AHQ-2-12, 3970	R.NLGSINTELQDVQR.I	1587.71696	2	6.69E-06	0.91	3.48	-	1333.7
AHQ-2-13-, 4089	R.VADGLPLAASMQEDEQSGR.D	1975.12722	2	2.58E-04	0.63	2.94	-	554.4
AHQ-2-12, 4041	R.VADGLPLAASMQEDEQSGR.D	1975.12722	2	4.81E-05	0.91	4.04	-	639.7
gi|4502211|ref|NP_001654.1| ADP-ribosylation factor 6 [Homo sapiens]				6.72E-06	0.81	10.17	6.30	20082.0
AHQ-2-12, 4079	K.FNVWDVGGQDK.I	1265.35598	2	6.72E-06	0.81	2.77	-	1144.5
gi|20270343|ref|NP_620150.1| hypothetical protein BC015408 [Homo sapiens]				6.72E-06	1.88	20.32	17.20	21415.8
AHQ-2-11, 2709	K.MNLSAIQDR.E	1048.19914	2	4.76E-04	0.92	3.23	-	1348.4
AHQ-2-11, 5833 - 5836	K.NELHNLLDKPQLQGIPVLVLGNK.R	2553.98492	2	6.72E-06	0.96	5.28	-	834.7
AHQ-2-11, 5832 - 5897	K.NELHNLLDKPQLQGIPVLVLGNK.R	2553.98492	3	1.16E-05	0.97	6.36	-	1323.7
gi|30148978|ref|XP_291520.2| similar to Keratin, type I cytoskeletal 18 (Cytokeratin 18) (K18) (CK				6.80E-06	0.72	10.16	2.20	68413.1
AHQ-2-11, 4888	R.DWSHYFKTIKDLR.A	1709.92863	2	2.24E-04	0.37	2.59	-	379.0
AHQ-2-13-, 4963	R.DWSHYFKTIKDLR.A	1709.92863	2	1.05E-04	0.63	3.14	-	663.3
AHQ-2-11, 4754 - 4830	R.DWSHYFKTIKDLR.A	1709.92863	2	6.80E-06	0.72	3.10	-	663.6
gi|22129786|ref|NP_059980.1| gp25L2 protein [Homo sapiens]				6.83E-06	0.76	10.19	5.10	25104.5
AHQ-2-10, 2875	R.QLVEQVEQIQK.E	1342.52334	2	6.83E-06	0.76	3.39	-	653.5
gi|4504221|ref|NP_000849.1| guanylate kinase 1 [Homo sapiens]				7.08E-06	0.97	10.21	6.10	21725.4
AHQ-2-11, 5065	R.ICVLDVDLQGVR.N	1388.61530	2	7.08E-06	0.97	4.10	-	2374.2
gi|6996010|ref|NP_002038.1| glycyl-tRNA synthetase; GlyRS; glycine tRNA ligase [Homo sapiens]				7.13E-06	1.57	20.17	4.50	77530.3
AHQ-2-6, 6805	K.TLYVEEVVPNVIEPSFGLGR.I	2219.52197	2	7.13E-06	0.85	3.47	-	757.3
AHQ-2-6, 2991 - 3010	K.TVNVVQFEPSK.G	1248.41001	2	6.57E-05	0.72	2.54	-	837.4
gi|27480842|ref|XP_208238.1| similar to bA92K2.2 (similar to ubiquitin) [Homo sapiens]				7.23E-06	0.88	10.14	5.80	17966.9
AHQ-2-14-, 2283	R.TLSDYNIQK.E	1082.18886	2	7.23E-06	0.88	2.83	-	642.7
gi|4502297|ref|NP_001678.1| ATP synthase, H+ transporting, mitochondrial F1 complex, delta subunit				7.35E-06	0.65	10.21	8.30	17489.8
AHQ-2-13-, 2515	K.AQAELVGTADEATR.A	1432.51860	2	8.18E-04	0.94	4.11	-	1171.8
AHQ-2-13-, 2535	K.AQAELVGTADEATR.A	1432.51860	1	7.35E-06	0.65	3.20	-	204.4
AHQ-2-12, 2471	K.AQAELVGTADEATR.A	1432.51860	2	6.00E-04	0.81	3.06	-	569.9
gi|4503531|ref|NP_001958.1| eukaryotic translation initiation factor 4A, isoform 2 [Homo sapiens] [				7.50E-06	0.38	10.12	3.40	46393.9
AHQ-2-8, 2576	K.GYDVIAQAQSGTGK.T	1395.49996	1	7.50E-06	0.38	2.47	-	550.3
gi|5803217|ref|NP_006821.1| ubiquinol-cytochrome c reductase (6.4kD) subunit [Homo sapiens]				7.63E-06	0.90	10.14	21.40	6569.6
AHQ-2-14-, 6288	R.LILDWVPYINGK.F	1431.70332	2	7.63E-06	0.90	2.89	-	895.3
gi|19743813|ref|NP_002202.2| integrin beta 1 isoform 1A precursor; integrin VLA-4 beta subunit; fib				7.67E-06	0.66	10.16	2.10	88414.9
AHQ-2-5, 5124	R.LKPEDITQIQPQQLVLR.L	2020.36187	2	7.67E-06	0.66	3.02	-	680.1
gi|7706371|ref|NP_057190.1| vesicle transport-related protein [Homo sapiens]				7.69E-06	0.89	10.19	3.00	72479.5
AHQ-2-6, 6641	K.GSPFPEVAESVQQELESYR.A	2153.29097	2	7.69E-06	0.89	3.87	-	779.2
gi|29729950|ref|XP_293759.1| similar to Heat shock protein HSP 90-alpha (HSP 86) [Homo sapiens] [MA				7.75E-06	0.95	10.21	5.00	39776.4
AHQ-2-5, 4823	K.CLELFTELAEDKENYKK.F	2132.37640	2	7.75E-06	0.95	4.16	-	1482.2
gi|29730354|ref|XP_298663.1| hypothetical protein XP_298663 [Homo sapiens]				7.81E-06	0.34	10.15	15.90	19365.2
[truncated: 6,203,893 more chars]
