# Supplementary material for: Proteomic and Phospho-Proteomic Profile of Human Platelets in Basal, Resting State: Insights into Integrin Signaling
Source: PLoS One. 2009 Oct 27;4(10):e7627. doi: 10.1371/journal.pone.0007627 (PMC2762604; doi:10.1371/journal.pone.0007627)
Supplement: Table S7 — A combined list of 1507 platelet proteins identified in this study. Protein uniprot name, Gene name, Uniprot accession number, Protein name, Gene ontology classification, Predicted sub-cellular localization and Protein family are shown based on its descrition in the Uniprot database (www.uniprot.org). Comments column describes whether the protein was identified as associated with the membrane fraction or as a phospho-protein in this study. Additional columns describe if the protein is known to be phosphorylated in literature databases. The function column tags known protein kinases and phosphatases in our dataset. (1.13 MB PDF) [file pone.0007627.s012.pdf]

	Protein Name	Uniprot Name	Other Names	Uniprot IDs	Refseq IDs	Protein Description	GO ID	GO terms	Subcellular location	Comments	Comments	Comments - Database Search	Comments - Database Search	Function
1	ACTN1	ACTN1_HUMAN	ACTN1	P12814; Q9BTN1	NP_001093	Alpha-actinin-1 (Alpha-actinin cytoskeletal isoform) (Non-muscle alpha-actinin-1) (F-actin cross-linking protein)	GO:0003779; GO:0005509; GO:0005737; GO:0005856; GO:0005925; GO:0048041; GO:0005178;	actin binding; calcium ion binding; cytoplasm; cytoskeleton; focal adhesion; focal adhesion formation; integrin binding; negative regulation of cell motion; nucleolus; pseudopodium; regulation of apoptosis; vinculin binding	Cytoplasmcytoskeleton Cytoplasmmyofibrilsarc omereZdisk	Phophorylated - from this Study	Membrane-Fraction - this study	Known in Uniprot to be phosphorylated	Known in HPRD to be phosphorylated	
2	F13A	F13A_HUMAN	F13A; Q59HA7; Q9BX29	P00488; Q59HA7; Q8N6X2; Q96P24; Q9BX29	NP_000120	Coagulation factor XIII A chain (Coagulation factor XIIIa) (EC 2.3.2.13) (Protein-glutamine gamma-glutamyltransferase A chain) (Transglutaminase A chain)	GO:0008415; GO:0007596; GO:0005509; GO:0005737; GO:0005576; GO:0018149; GO:0003810	acyltransferase activity; blood coagulation; calcium ion binding; cytoplasm; extracellular region; peptide cross-linking; protein-glutamine gamma-glutamyltransferase activity	CytoplasmSecreted		Membrane-Fraction - this study			
3	FIBA	FIBA_HUMAN	FIBA	P02671; Q9BX62; Q9UCH2	NP_000499; NP_068657	Fibrinogen alpha chain [Cleaved into: Fibrinopeptide A]	GO:0043499; GO:0009897; GO:0005577; GO:0030168; GO:0031093; GO:0030674; GO:0051258; GO:0005102;	eukaryotic cell surface binding; external side of plasma membrane; fibrinogen complex; platelet activation; platelet alpha granule lumen; protein binding, bridging; protein polymerization; receptor binding; response to calcium ion; signal transduction	Secreted	Phophorylated - from this Study	Membrane-Fraction - this study	Known in Uniprot to be phosphorylated	Known in HPRD to be phosphorylated	
4	FLNA	FLNA_HUMAN	Q60FE6; Q5HY53; Q8NF52; FLNA	P21333; Q60FE6; Q8NF52; Q5HY53	NP_001447; NP_001104 026	Filamin-A (Alpha-filamin) (Filamin-1) (Endothelial actin-binding protein) (Actin-binding protein 280) (ABP-280) (Non-muscle filamin); Filamin A, alpha (Actin binding protein 280) (Filamin A, alpha (Actin binding protein 280), isoform CRA_c); Filamin A	GO:0034988; GO:0034989; GO:0048365; GO:0051764; GO:0015629; GO:0031532; GO:0051015; GO:0005737; GO:0051220; GO:0001948; GO:0007195; GO:0042177; GO:0043433; GO:0005634; GO:0005886; GO:0043123; GO:0042993; GO:0042803; GO:0034394; GO:0050821; GO:0043113; GO:0004871; [illegible]	Fc-gamma receptor I complex binding; GTP-Ral binding; Rac GTPase binding; actin crosslink formation; actin cytoskeleton; actin cytoskeleton reorganization; actin filament binding; cytoplasm; cytoplasmic sequestering of protein; glycoprotein binding; inhibition of adenylate cyclase activity by dopamine receptor signaling pathway; negative regulation of protein catabolic process; negative regulation of transcription factor activity; nucleus; plasma membrane; positive regulation of I-kappaB kinase/NF-kappaB cascade; positive regulation of transcription factor import into nucleus; protein homodimerization activity; protein localization at cell surface; protein stabilization; receptor clustering; signal transducer activity; transcription factor binding; actin binding; [illegible]	CytoplasmcellcortexCy toplasmcytoskeleton;	Phophorylated - from this Study	Membrane-Fraction - this study	Known in Uniprot to be phosphorylated	Known in HPRD to be phosphorylated	
5	GP1BB	GP1BB_HUMAN	GP1BB	P13224; Q14422; Q8NG40	NP_000398	Platelet glycoprotein Ib beta chain (GP-Ib beta) (GPIb-beta) (GPIbB) (Antigen CD42b-beta) (CD antigen CD42c)	GO:0007155; GO:0007166; GO:0005887; GO:0030168; GO:0031092; GO:0005515; GO:0004888	cell adhesion; cell surface receptor linked signal transduction; integral to plasma membrane; platelet activation; platelet alpha granule membrane; protein binding; transmembrane receptor activity	MembraneSinglepassty peImembraneprotein	Phophorylated - from this Study	Membrane-Fraction - this study	Known in Uniprot to be phosphorylated	Known in HPRD to be phosphorylated	
6	HSPB1	HSPB1_HUMAN	HSPB1; B2R4N8	P04792; B2R4N8; Q6FI47; Q96C20; Q96EI7; Q9UC31; Q9UC34; Q9UC35;	NP_001531; XP_293276	Heat shock protein beta-1 (HspB1) (Heat shock 27 kDa protein) (HSP 27) (Stress-responsive protein 27) (SRP27) (Estrogen-regulated 24 kDa protein) (28 kDa heat shock protein)	GO:0006916; GO:0006928; GO:0009986; GO:0005737; GO:0042802; GO:0005634; GO:0006446; GO:0009408;	anti-apoptosis; cell motion; cell surface; cytoplasm; identical protein binding; nucleus; regulation of translational initiation; response to heat; response to unfolded protein; spindle	CytoplasmNucleusSpin dle	Phophorylated - from this Study	Membrane-Fraction - this study	Known in Uniprot to be phosphorylated	Known in HPRD to be phosphorylated	
7	PLEK	PLEK_HUMAN	PLEK; B2R9E8	P08567; Q53SU8; Q6FGM8; Q6FGQ1; Q8WV81; B2R9E8	NP_002655	Pleckstrin (Platelet p47 protein)	GO:0031532; GO:0030866; GO:0005737; GO:0002244; GO:0030845; GO:0007229; GO:0005624; GO:0045744; GO:0050849; GO:0010920; GO:0042301; GO:0043325; GO:0030384; GO:0002576; GO:0032233; GO:0030836; GO:0010925; GO:0033625; GO:0010572; GO:0042803; GO:0005080; GO:0060305; GO:0032587; GO:0031529; GO:0005625; GO:0070493; GO:0006904	actin cytoskeleton reorganization; cortical actin cytoskeleton organization; cytoplasm; hemopoietic progenitor cell differentiation; inhibition of phospholipase C activity involved in G-protein coupled receptor signaling pathway; integrin-mediated signaling pathway; membrane fraction; negative regulation of G-protein coupled receptor protein signaling pathway; negative regulation of calcium-mediated signaling; negative regulation of inositol phosphate biosynthetic process; phosphate binding; phosphatidylinositol-3,4-bisphosphate binding; phosphoinositide metabolic process; platelet degranulation; positive regulation of actin filament bundle formation; positive regulation of actin filament depolymerization; positive regulation of inositol-polyphosphate 5-phosphatase activity; positive regulation of integrin activation; positive regulation of platelet activation; protein homodimerization activity; protein kinase C binding; regulation of cell diameter; ruffle membrane; ruffle organization; soluble fraction; thrombin receptor signaling pathway; vesicle docking during exocytosis		Phophorylated - from this Study	Membrane-Fraction - this study	Known in Uniprot to be phosphorylated	Known in HPRD to be phosphorylated	
8	SCYB7	SCYB7_HUMAN	SCYB7; CXCL7; B2R5F3	P02775; Q6IBJ8; B2R5F3	NP_002695	Platelet basic protein (PBP) (C-X-C motif chemokine 7) (Small-inducible cytokine B7) (Leukocyte-derived growth factor) (LDGF) (Macrophage-derived growth factor) (MDGF) [Cleaved into: Connective tissue-activating peptide III (CTAP-III) (Low-affinity platelet factor IV) (LA-PF4); TC-2; Connective tissue-activating peptide III(1-81) (CTAP-III(1-81)); Beta-thromboglobulin (Beta-TG); Neutrophil-activating peptide 2(74) (NAP-2(74)); Neutrophil-activating peptide 2(73) (NAP-2(73)); Neutrophil-activating peptide 2 (NAP-2); TC-1; Neutrophil-activating peptide 2(1-66) (NAP-2(1-66)); Neutrophil-activating peptide 2(1-63) (NAP-2(1-63))]	GO:0008283; GO:0008009; GO:0006935; GO:0042742; GO:0005615; GO:0005355; GO:0015758; GO:0008083; GO:0006955; GO:0031093	cell proliferation; chemokine activity; chemotaxis; defense response to bacterium; extracellular space; glucose transmembrane transporter activity; glucose transport; growth factor activity; immune response; platelet alpha granule lumen	Secreted	Phophorylated - from this Study	Membrane-Fraction - this study			
9	TAGL2	TAGL2_HUMAN	TAGL2; Q6FGI1	P37802; Q6FGI1; Q5JRQ6; Q5JRQ7; Q9BUH5; Q9H4P0	NP_003555	Transgelin-2 (SM22-alpha homolog)	GO:0005856; GO:0007517; GO:0031965; GO:0005886; GO:0005515	cytoskeleton; muscle organ development; nuclear membrane; plasma membrane; protein binding		Phophorylated - from this Study	Membrane-Fraction - this study	Known in Uniprot to be phosphorylated		
10	CSRP1	CSRP1_HUMAN	CSRP1; Q5U0J2	P21291; Q5U0J2	NP_004069	Cysteine and glycine-rich protein 1 (Cysteine-rich protein 1) (CRP1) (CRP); Cysteine and glycine-rich protein 1 (cDNA FLJ75177, highly similar to Homo sapiens cysteine and glycine-rich protein 1 (CSRP1), mRNA) (Cysteine and glycine-rich protein 1, isoform CRA_b)	GO:0005634; GO:0008270; GO:0005515	nucleus; zinc ion binding; protein binding	Nucleus;	Phophorylated - from this Study	Membrane-Fraction - this study	Known in Uniprot to be phosphorylated		
11	SDPR	SDPR_HUMAN	SDPR	O95810	NP_004648	Serum deprivation-response protein (Phosphatidylserine-binding protein) (PS-p68)	GO:0005829; GO:0016020; GO:0001786; GO:0005515	cytosol; membrane; phosphatidylserine binding; protein binding	CytoplasmcytosolMem branecaveola	Phophorylated - from this Study	Membrane-Fraction - this study	Known in Uniprot to be phosphorylated		
12	PURA	PURA_HUMAN	PURA; Q56A79	Q00577; Q56A79	NP_005850	Transcriptional activator protein Pur-alpha (Purine-rich single-stranded DNA-binding protein alpha); Purine-rich element binding protein A	GO:0005662; GO:0006270; GO:0006268; GO:0003705; GO:0003691; GO:0000784; GO:0003697; GO:0008134; GO:0046332; GO:0005737; GO:0045892; GO:0007399; GO:0008284; GO:0003700	DNA replication factor A complex; DNA replication initiation; DNA unwinding during replication; RNA polymerase II transcription factor activity, enhancer binding; double-stranded telomeric DNA binding; nuclear chromosome, telomeric region; single-stranded DNA binding; transcription factor binding; SMAD binding; cytoplasm; negative regulation of transcription, DNA-dependent; nervous system development; positive regulation of cell proliferation; transcription factor activity	Nucleus;	Phophorylated - from this Study	Membrane-Fraction - this study	Known in Uniprot to be phosphorylated		
13	CAZA1	CAZA1_HUMAN	CAZA1	P52907; Q53FQ6; Q6FHD5	NP_006126	F-actin-capping protein subunit alpha-1 (CapZ alpha-1)	GO:0008290; GO:0003779; GO:0051016; GO:0006928	F-actin capping protein complex; actin binding; barbed-end actin filament capping; cell motion			Membrane-Fraction - this study			
14	IQGA2	IQGA2_HUMAN	IQGA2; Q59HA3	Q13576; Q59HA3	NP_006624	Ras GTPase-activating-like protein IQGAP2; IQ motif containing GTPase activating protein 2 variant (Fragment)	GO:0005095; GO:0005099; GO:0003779; GO:0015629; GO:0005516; GO:0051056; GO:0005099; GO:0005622; GO:0005515	GTPase inhibitor activity; Ras GTPase activator activity; actin binding; actin cytoskeleton; calmodulin binding; regulation of small GTPase mediated signal transduction; Ras GTPase activator activity; intracellular; protein binding		Phophorylated - from this Study	Membrane-Fraction - this study	Known in Uniprot to be phosphorylated		
15	SCAM2	SCAM2_HUMAN	SCAM2; B2RDF0	O15127; Q9BQE8; B2RDF0	NP_005688	Secretory carrier-associated membrane protein 2 (Secretory carrier membrane protein 2)	GO:0016021; GO:0006892; GO:0005515; GO:0015031; GO:0055038; GO:0032588	integral to membrane; post-Golgi vesicle-mediated transport; protein binding; protein transport; recycling endosome membrane; trans-Golgi network membrane	GolgiapparatustransGo lginetworkmembraneM ultipassmembraneprot einRecyclingendosome membraneMultipassme mbraneprotein	Phophorylated - from this Study	Membrane-Fraction - this study	Known in Uniprot to be phosphorylated		
16	GTR3	GTR3_HUMAN	GTR3; B2R606	P11169; Q6I9U2; Q9UG15; B2R606	NP_008862	Solute carrier family 2, facilitated glucose transporter member 3 (Glucose transporter type 3, brain) (GLUT-3)	GO:0005975; GO:0005737; GO:0005355; GO:0015758; GO:0016021; GO:0005886; GO:0005351; GO:0055085	carbohydrate metabolic process; cytoplasm; glucose transmembrane transporter activity; glucose transport; integral to membrane; plasma membrane; sugar:hydrogen symporter activity; transmembrane transport	MembraneMultipassme mbraneprotein	Phophorylated - from this Study	Membrane-Fraction - this study			
17	G3P	G3P_HUMAN	G3P; Q53X65	P04406; Q53X65; P00354	NP_002037; XP_294070; XP_294015; XP_291603; XP_210460	Glyceraldehyde-3-phosphate dehydrogenase (GAPDH) (EC 1.2.1.12); Glyceraldehyde 3-phosphate dehydrogenase (EC 1.2.1.12) (Fragment)	GO:0051287; GO:0005737; GO:0004365; GO:0006096; GO:0016020; GO:0055114; GO:0005515; GO:0006006	NAD or NADH binding; cytoplasm; glyceraldehyde-3-phosphate dehydrogenase (phosphorylating) activity; glycolysis; membrane; oxidation reduction; protein binding; glucose metabolic process	CytoplasmCytoplasmp erinuclearregionMembr ane; Cytoplasm	Phophorylated - from this Study	Membrane-Fraction - this study	Known in Uniprot to be phosphorylated		

	Protein Name	Uniprot Name	Other Names	Uniprot IDs	Refseq IDs	Protein Description	GO ID	GO terms	Subcellular location	Comments	Comments	Comments - Database Search	Comments - Database Search	Function
**18**	MYH9	MYH9_HUMAN	MYH9; Q86XU5; Q60FE2	P35579; Q60FE2; O60805; Q86T83; Q86XU5	NP_002464	Myosin-9 (Myosin heavy chain 9) (Myosin heavy chain, non-muscle IIa) (Non-muscle myosin heavy chain IIa) (NMMHC II-a) (NMMHC-IIA) (Cellular myosin heavy chain, type A) (Non-muscle myosin heavy chain A) (NMMHC-A); Non-muscle myosin heavy polypeptide 9 (MYH9 variant protein) (Myosin, heavy polypeptide 9, non-muscle, isoform CRA_a); MYH9 protein (Fragment)	GO:0043531; GO:0005524; GO:0031532; GO:0051015; GO:0030048; GO:0030898; GO:0001525; GO:0043534; GO:0005516; GO:0032154; GO:0005826; GO:0000910; GO:0005829; GO:0007229; GO:0050900; GO:0006509; GO:0000146; GO:0030224; GO:0016459; GO:0005634; GO:0030220; GO:0043495; GO:0042803; GO:0015031; GO:0008360; GO:0001726; GO:0007605; GO:0001725; GO:0001931; GO:0005524; GO:0003779; GO:0000904; GO:0006928; GO:0005913; GO:0016337;	ADP binding; ATP binding; actin cytoskeleton reorganization; actin filament binding; actin filament-based movement; actin-dependent ATPase activity; angiogenesis; blood vessel endothelial cell migration; calmodulin binding; cleavage furrow; contractile ring; cytokinesis; cytosol; integrin-mediated signaling pathway; leukocyte migration; membrane protein ectodomain proteolysis; microfilament motor activity; monocyte differentiation; myosin complex; nucleus; platelet formation; protein anchor; protein homodimerization activity; protein transport; regulation of cell shape; ruffle; sensory perception of sound; stress fiber; uropod; ATP binding; actin binding; cell morphogenesis involved in differentiation; cell motion; cell-cell adherens junction; cell-cell adhesion; cortical cytoskeleton; establishment of T cell polarity; establishment of meiotic spindle localization; immunological synapse; in utero embryonic development; meiotic metaphase I; meiotic spindle organization; motor activity; myoblast fusion; neuromuscular junction; spindle; uropod organization		Phophorylated - from this Study	Membrane-Fraction - this study	Known in Uniprot to be phosphorylated	Known in HPRD to be phosphorylated	
**19**	TBB1	TBB1_HUMAN	TBB1	Q9H4B7	NP_110400	Tubulin beta-1 chain	GO:0005525; GO:0003924; GO:0005874; GO:0007018; [illegible]	GTP binding; GTPase activity; microtubule; microtubule-based movement; protein complex; protein polymerization		Phophorylated - from this Study	Membrane-Fraction - this study			
**20**	TLN1	TLN1_HUMAN	TLN1; Q9H3E8	Q9Y490; A6NMY0; Q86YD0; Q9NZQ2; Q9UHH8; Q9UPX3; Q9H3E8	NP_006280	Talin-1; MSTP037	GO:0030274; GO:0003779; GO:0006928; GO:0005911; GO:0007043; GO:0005737; GO:0007016; GO:0005856; GO:0005925;	LIM domain binding; actin binding; cell motion; cell-cell junction; cell-cell junction assembly; cytoplasm; cytoskeletal anchoring at plasma membrane; cytoskeleton; focal adhesion; intracellular membrane-bounded organelle; ruffle; structural constituent of cytoskeleton; vinculin binding;	Cellprojectionruffle membranePeripheralmem braneproteinCytoplasm icsideCytoplasmcytosk eleton;	Phophorylated - from this Study	Membrane-Fraction - this study	Known in Uniprot to be phosphorylated		
**21**	TBA4A	TBA4A_HUMAN	TBA1; B3KNQ6; TBA4A	P68366; B3KNQ6; P05215	NP_005991; XP_301321	Tubulin alpha-4A chain (Tubulin alpha-1 chain) (Alpha-tubulin 1) (Testis-specific alpha-tubulin) (Tubulin H2-alpha)	GO:0005525; GO:0003924; GO:0005874; GO:0007018; GO:0005515; GO:0043234; GO:0051258; GO:0005198	GTP binding; GTPase activity; microtubule; microtubule-based movement; protein binding; protein complex; protein polymerization; structural molecule activity		Phophorylated - from this Study	Membrane-Fraction - this study	Known in Uniprot to be phosphorylated	Known in HPRD to be phosphorylated	
**22**	RGS18	RGS18_HUMAN	RGS18; B2RD23	Q9NS28; B2RD23	NP_570138	Regulator of G-protein signaling 18 (RGS18)	GO:0005737; GO:0009968; GO:0004871	cytoplasm; negative regulation of signal transduction; signal transducer activity	Cytoplasm	Phophorylated - from this Study	Membrane-Fraction - this study			
**23**	PARVB	PARVB_HUMAN	PARVB	Q9HBI1; B2R9X6; Q5TGJ5; Q86X93; Q9NSP7; Q9UGT3; Q9Y368; Q9Y3L6;	NP_037459	Beta-parvin (Affixin)	GO:0003779; GO:0007155; GO:0030054; GO:0005737; GO:0005856	actin binding; cell adhesion; cell junction; cytoplasm; cytoskeleton	Celljunctionfocaladhesi onCellmembranePeriph eralmembraneproteinC ytoplasmicsideCytoplas mcytoskeleton	Phophorylated - from this Study	Membrane-Fraction - this study	Known in Uniprot to be phosphorylated		
**24**	ESAM	ESAM_HUMAN	ESAM	Q96AP7; Q96T50	NP_620411	Endothelial cell-selective adhesion molecule	GO:0016021; GO:0005515; GO:0005923	integral to membrane; protein binding; tight junction	Celljunctionadherensju nctionCelljunctiontightj unctionCellmembraneS inglepasstypeImembra neprotein	Phophorylated - from this Study	Membrane-Fraction - this study			
**25**	RTN4	RTN4_HUMAN	RTN4; Q7L7Q8; Q8IUA4; Q96B16; Q7L7Q5	Q9NQC3; Q7L7Q8; O94962; Q96B16; Q9BXG5; Q9H212; Q9H3I3; Q9UQ42; Q9Y293; Q9Y2Y7; Q9Y5U6; Q8IUA4; Q7L7Q5	NP_065393; NP_997404; NP_722550; NP_997403; NP_008939	Reticulon-4 (Neurite outgrowth inhibitor) (Nogo protein) (Foocen) (Neuroendocrine-specific protein) (NSP) (Neuroendocrine-specific protein C homolog) (RTN-x) (Reticulon-5); RTN4 isoform C (RTN4) (Reticulon 4, isoform CRA_d); RTN4 isoform Aa (RTN4 isoform Ab) (RTN4 isoform D) (RTN4 isoform E) (RTN4 isoform F) (RTN4 isoform G) (RNT4) (Reticulon 4, isoform CRA_c) (Reticulon 4, isoform CRA_f)	GO:0030176; GO:0019987; GO:0030517; GO:0005635; GO:0005515; GO:0030334; GO:0005783	integral to endoplasmic reticulum membrane; negative regulation of anti-apoptosis; negative regulation of axon extension; nuclear envelope; protein binding; regulation of cell migration; endoplasmic reticulum	Endoplasmicreticulum membraneMultipassme mbraneprotein;	Phophorylated - from this Study	Membrane-Fraction - this study	Known in Uniprot to be phosphorylated	Known in HPRD to be phosphorylated	
**26**	TSP1	TSP1_HUMAN	TSP1; B9EGH6	P07996; Q15667; B9EGH6	NP_003237	Thrombospondin-1	GO:0000187; GO:0006916; GO:0005509; GO:0007050; GO:0034605; GO:0002544; GO:0070052; GO:0043652; GO:0043499; GO:0009897; GO:0031012; GO:0070051; GO:0005577; GO:0048603; GO:0001968; GO:0008201; GO:0042802; GO:0006955; GO:0005178; GO:0043236; GO:0030169; GO:0016525; GO:0002581; GO:0043537; GO:0010754; GO:0002605; GO:0001937; GO:0051918; GO:0040037; GO:0051895; GO:0032695; GO:0010751; GO:0010748; GO:0010757; GO:0018149;	activation of MAPK activity; anti-apoptosis; calcium ion binding; cell cycle arrest; cellular response to heat; chronic inflammatory response; collagen V binding; engulfment of apoptotic cell; eukaryotic cell surface binding; external side of plasma membrane; extracellular matrix; fibrinogen binding; fibrinogen complex; fibroblast growth factor 2 binding; fibronectin binding; heparin binding; identical protein binding; immune response; integrin binding; laminin binding; low-density lipoprotein binding; negative regulation of angiogenesis; negative regulation of antigen processing and presentation of peptide or polysaccharide antigen via MHC class II; negative regulation of blood vessel endothelial cell migration; negative regulation of cGMP-mediated signaling; negative regulation of dendritic cell antigen processing and presentation; negative regulation of endothelial cell proliferation; negative regulation of fibrinolysis; negative regulation of fibroblast growth factor receptor signaling pathway; negative regulation of focal adhesion formation; negative regulation of interleukin-12 production; negative regulation of nitric oxide mediated signal transduction; negative regulation of plasma membrane long-chain fatty acid		Phophorylated - from this Study	Membrane-Fraction - this study			
**27**	ITB3	ITB3_HUMAN	ITB3	P05106; A0PJW2; O15495; Q12806; Q13413; Q14648; Q16499	NP_000203	Integrin beta-3 (Platelet membrane glycoprotein IIIa) (GPIIIa) (CD antigen CD61)	GO:0007596; GO:0007160; GO:0042802; GO:0008305; GO:0007229; GO:0044419; GO:0010745; GO:0032369; GO:0050748; GO:0045715; GO:0031092; GO:0004872	blood coagulation; cell-matrix adhesion; identical protein binding; integrin complex; integrin-mediated signaling pathway; interspecies interaction between organisms; negative regulation of foam cell differentiation; negative regulation of lipid transport; negative regulation of lipoprotein metabolic process; negative regulation of low-density lipoprotein receptor biosynthetic process; platelet alpha granule membrane; receptor activity	MembraneSinglepassty peImembraneprotein	Phophorylated - from this Study	Membrane-Fraction - this study	Known in Uniprot to be phosphorylated	Known in HPRD to be phosphorylated	
**28**	CALX	CALX_HUMAN	CALX; B2R5V8	P27824; B2R5V8	NP_001737; NP_001019 820	Calnexin (Major histocompatibility complex class I antigen-binding protein p88) (p90) (IP90)	GO:0005509; GO:0005789; GO:0016021; GO:0042470; GO:0006457; GO:0009306; GO:0005529; GO:0051082	calcium ion binding; endoplasmic reticulum membrane; integral to membrane; melanosome; protein folding; protein secretion; sugar binding; unfolded protein binding	Endoplasmicreticulum membraneSinglepassty peImembraneproteinM elanosome	Phophorylated - from this Study	Membrane-Fraction - this study	Known in Uniprot to be phosphorylated	Known in HPRD to be phosphorylated	
**29**	TPM1	TPM1_HUMAN	TPM1; O15513; Q7Z6L8	P09493; O15513; Q7Z6L8; P09494; P10469; Q6DV89; Q6DV90; Q86W64; Q96IK2; Q9UCI1; Q9UCI2; Q9UCY9	NP_000357; NP_001018 006; NP_0010180 04; NP_0010180 20; NP_001018 008; NP_0010180 05	Tropomyosin alpha-1 chain (Tropomyosin-1) (Alpha-tropomyosin); Alpha-tropomyosin (Fragment); Tropomyosin 1 (Alpha) (Tropomyosin 1 (Alpha), isoform CRA_e)	GO:0003779; GO:0032059; GO:0060048; GO:0034614; GO:0030049; GO:0005862; GO:0030336; GO:0032781; GO:0045785; GO:0003065; GO:0051496; GO:0006937; GO:0032587; GO:0031529; GO:0045214; GO:0001725; GO:0005200; GO:0008307; GO:0055010; GO:0042060; GO:0031941; GO:0001701; GO:0030016; GO:0005737;	actin binding; bleb; cardiac muscle contraction; cellular response to reactive oxygen species; muscle filament sliding; muscle thin filament tropomyosin; negative regulation of cell migration; positive regulation of ATPase activity; positive regulation of cell adhesion; positive regulation of heart rate by epinephrine; positive regulation of stress fiber formation; regulation of muscle contraction; ruffle membrane; ruffle organization; sarcomere organization; stress fiber; structural constituent of cytoskeleton; structural constituent of muscle; ventricular cardiac muscle morphogenesis; wound healing; filamentous actin; in utero embryonic development; myofibril; cytoplasm; cytoskeleton; nucleus	Cytoplasmcytoskeleton ;	Phophorylated - from this Study	Membrane-Fraction - this study		Known in HPRD to be phosphorylated	
**30**	ALDOA	ALDOA_HUMAN	ALDOA; Q6FI10	P04075; Q6FI10; B4DXI7; Q6FH76; Q96B15; Q9BWD9; Q9UCN2	NP_000025; NP_001121 089; NP_908930; NP_908932	Fructose-bisphosphate aldolase A (EC 4.1.2.13) (Muscle-type aldolase) (Lung cancer antigen NY-LU 1)	GO:0006754; GO:0031674; GO:0003779; GO:0015629; GO:0007015; GO:0070062; GO:0030388; GO:0070061; GO:0004332; GO:0006096;	ATP biosynthetic process; I band; actin binding; actin cytoskeleton; actin filament organization; extracellular vesicular exosome; fructose 1,6-bisphosphate metabolic process; fructose binding; fructose-bisphosphate aldolase activity; glycolysis; identical protein binding; muscle maintenance; nucleus; regulation of cell shape; striated muscle contraction; tubulin binding		Phophorylated - from this Study	Membrane-Fraction - this study	Known in Uniprot to be phosphorylated		

	Protein Name	Uniprot Name	Other Names	Uniprot IDs	Refseq IDs	Protein Description	GO ID	GO terms	Subcellular location	Comments	Comments	Comments - Database Search	Comments - Database Search	Function
**31**	VINC	VINC_HUMAN	VINC	Q5SWX2; P18206; Q16450; Q7Z3B8; Q8IXU7	NP_003364; NP_054706	Vinculin (Metavinculin)	GO:0003779; GO:0015629; GO:0045294; GO:0043297; GO:0007155; GO:0005911; GO:0043034; GO:0005925; GO:0030032;	actin binding; actin cytoskeleton; alpha-catenin binding; apical junction assembly; cell adhesion; cell-cell junction; costamere; focal adhesion; lamellipodium assembly; negative regulation of cell migration; oxidoreductase activity; protein complex; structural molecule activity	CytoplasmcytoskeletonCelljunctionadherensjunctionCellmembranePeripheralmembraneproteinCytoplasmicside	Phophorylated - from this Study	Membrane-Fraction - this study	Known in Uniprot to be phosphorylated	Known in HPRD to be phosphorylated	
**32**	ACTG	ACTG_HUMAN	ACTG; Q5U032	P63261; Q5U032; A8K7C2; P02571; P14104; P99022; Q96E67	NP_001605; XP_301187; XP_293967; XP_302052	Actin, cytoplasmic 2 (Gamma-actin) [Cleaved into: Actin, cytoplasmic 2, N-terminally processed]	GO:0005524; GO:0006928; GO:0005737; GO:0005856; GO:0042802; GO:0007605; GO:0005200	ATP binding; cell motion; cytoplasm; cytoskeleton; identical protein binding; sensory perception of sound; structural constituent of cytoskeleton	Cytoplasmcytoskeleton	Phophorylated - from this Study		Known in Uniprot to be phosphorylated		
**33**	ACTH	ACTH_HUMAN	ACTH	P63267; Q504R1; B2R7E7; P12718; Q6FI22	NP_001606	Actin, gamma-enteric smooth muscle (Smooth muscle gamma-actin) (Gamma-2-actin) (Alpha-actin-3)	GO:0005524; GO:0005737; GO:0005856; GO:0005515	ATP binding; cytoplasm; cytoskeleton; protein binding	Cytoplasmcytoskeleton	Phophorylated - from this Study				
**34**	ADAM9	ADAM9_HUMAN	ADAM9	Q13443; Q10718; Q8NFM6	NP_003807; NP_001005845	Disintegrin and metalloproteinase domain-containing protein 9 (ADAM 9) (EC 3.4.24.-) (Metalloprotease/disintegrin/cysteine-rich protein 9) (Myeloma cell metalloproteinase) (Meltrin-gamma) (Cellular disintegrin-related protein)	GO:0051088; GO:0017124; GO:0000186; GO:0033631; GO:0007160; GO:0005518; GO:0005615; GO:0016021; GO:0005178; GO:0007229; GO:0031233; GO:0030216; GO:0043236; GO:0004222; GO:0042117; GO:0042301; GO:0033630; GO:0051549; GO:0034241; GO:0051044; GO:0050714; GO:0005080; GO:0051592; GO:0051384; GO:0042542; GO:0010042; GO:0034612; GO:0007179; GO:0008270	PMA-inducible membrane protein ectodomain proteolysis; SH3 domain binding; activation of MAPKK activity; cell-cell adhesion mediated by integrin; cell-matrix adhesion; collagen binding; extracellular space; integral to membrane; integrin binding; integrin-mediated signaling pathway; intrinsic to external side of plasma membrane; keratinocyte differentiation; laminin binding; metalloendopeptidase activity; monocyte activation; phosphate binding; positive regulation of cell adhesion mediated by integrin; positive regulation of keratinocyte migration; positive regulation of macrophage fusion; positive regulation of membrane protein ectodomain proteolysis; positive regulation of protein secretion; protein kinase C binding; response to calcium ion; response to glucocorticoid stimulus; response to hydrogen peroxide; response to manganese ion; response to tumor necrosis factor; transforming growth factor beta receptor signaling pathway; zinc ion binding	CellmembraneSinglepasstypeImembraneproteinSecreted	Phophorylated - from this Study		Known in Uniprot to be phosphorylated		
**35**	FETUA	FETUA_HUMAN	FETUA	P02765; A8K9N6; O14961; O14962; Q9P152	NP_001613	Alpha-2-HS-glycoprotein (Ba-alpha-2-glycoprotein) (Alpha-2-Z-globulin) (Fetuin-A) [Cleaved into: Alpha-2-HS-glycoprotein chain A; Alpha-2-HS-glycoprotein chain B]	GO:0006953; GO:0004869; GO:0005615; GO:0030502; GO:0046627; GO:0006907; GO:0050766; GO:0005515; GO:0050727	acute-phase response; cysteine-type endopeptidase inhibitor activity; extracellular space; negative regulation of bone mineralization; negative regulation of insulin receptor signaling pathway; pinocytosis; positive regulation of phagocytosis; protein binding; regulation of inflammatory response	Secreted	Phophorylated - from this Study		Known in Uniprot to be phosphorylated	Known in HPRD to be phosphorylated	
**36**	DDEF2	DDEF2_HUMAN	DDEF2; ASAP2	O43150	NP_003878; NP_001128663	Arf-GAP with SH3 domain, ANK repeat and PH domain-containing protein 2 (Development and differentiation-enhancing factor 2) (Pyk2 C-terminus-associated protein) (PAP) (Paxillin-associated protein with ARFGAP activity 3) (PAG3)	GO:0008060; GO:0005794; GO:0005886; GO:0005515; GO:0032312; GO:0008270	ARF GTPase activator activity; Golgi apparatus; plasma membrane; protein binding; regulation of ARF GTPase activity; zinc ion binding	CytoplasmGolgiapparatusGolgistackmembranePeripheralmembraneproteinCellmembranePeripheralmembraneprotein	Phophorylated - from this Study		Known in Uniprot to be phosphorylated		
**37**	DEMA	DEMA_HUMAN	DEMA	Q08495; A8K0T5; B3KP70; Q13215; Q9BRE3	NP_001969; NP_001107607; NP_001107608; NP_001107609; NP_001107610	Dematin (Erythrocyte membrane protein band 4.9)	GO:0003779; GO:0015629; GO:0051017; GO:0051016; GO:0005737	actin binding; actin cytoskeleton; actin filament bundle formation; barbed-end actin filament capping; cytoplasm	Cytoplasm	Phophorylated - from this Study		Known in Uniprot to be phosphorylated	Known in HPRD to be phosphorylated	
**38**	EP15	EP15_HUMAN	EP15; EPS15	P42566; Q5SRH4; B2R8J7	NP_001972	Epidermal growth factor receptor substrate 15 (Protein Eps15) (Protein AF-1p)	GO:0017124; GO:0005509; GO:0008283; GO:0005905; GO:0005829; GO:0031901; GO:0007173; GO:0015031; GO:0016050	SH3 domain binding; calcium ion binding; cell proliferation; coated pit; cytosol; early endosome membrane; epidermal growth factor receptor signaling pathway; protein transport; vesicle organization	CytoplasmCellmembranePeripheralmembraneproteinCytoplasmicsideEarlyendosomemembranePeripheralmembraneproteinCytoplasmicside	Phophorylated - from this Study		Known in Uniprot to be phosphorylated	Known in HPRD to be phosphorylated	
**39**	KNG1	KNG1_HUMAN	KNG1; Q05CF8	P01042; A8K474; B2RCR2; P01043; Q53EQ0; Q6PAU9; Q7M4P1; Q05CF8	NP_000884; NP_001095886	Kininogen-1 (High molecular weight kininogen) (HMWK) (Williams-Fitzgerald-Flaujeac factor) (Fitzgerald factor) (Alpha-2-thiol proteinase inhibitor) [Cleaved into: Kininogen-1 heavy chain; T-kinin (Ile-Ser-Bradykinin); Bradykinin (Kallidin I); Lysyl-bradykinin (Kallidin II); Kininogen-1 light chain; Low molecular weight growth-promoting factor]; KNG1 protein	GO:0005813; GO:0004869; GO:0030146; GO:0007204; GO:0005576; GO:0008201; GO:0006954; GO:0030147; GO:0030195; GO:0007162; GO:0005634; GO:0043065; [illegible]	centrosome; cysteine-type endopeptidase inhibitor activity; diuresis; elevation of cytosolic calcium ion concentration; extracellular region; heparin binding; inflammatory response; natriuresis; negative regulation of blood coagulation; negative regulation of cell adhesion; nucleus; positive regulation of apoptosis; receptor binding; smooth muscle contraction; vasodilation; zinc ion binding; cysteine-type endopeptidase inhibitor activity	Secretedextracellularspace;	Phophorylated - from this Study				
**40**	LYN	LYN_HUMAN	LYN	P07948; A0AVQ5	NP_002341	Tyrosine-protein kinase Lyn (EC 2.7.10.2)	GO:0005524; GO:0005794; GO:0030218; GO:0044419; GO:0045121; GO:0004715; GO:0005634; GO:0005886; GO:0008284; GO:0042531; GO:0005515; GO:0004716; GO:0009725	ATP binding; Golgi apparatus; erythrocyte differentiation; interspecies interaction between organisms; membrane raft; non-membrane spanning protein tyrosine kinase activity; nucleus; plasma membrane; positive regulation of cell proliferation; positive regulation of tyrosine phosphorylation of STAT protein; protein binding; receptor signaling protein tyrosine kinase activity; response to hormone stimulus		Phophorylated - from this Study		Known in Uniprot to be phosphorylated	Known in HPRD to be phosphorylated	Kinase
**41**	MOES	MOES_HUMAN	MOES	P26038	NP_002435; XP_171113	Moesin (Membrane-organizing extension spike protein)	GO:0016324; GO:0050839; GO:0005737; GO:0008092; GO:0005856; GO:0019898; GO:0030175; GO:0007159; GO:0050900; GO:0022614; [illegible]	apical plasma membrane; cell adhesion molecule binding; cytoplasm; cytoskeletal protein binding; cytoskeleton; extrinsic to membrane; filopodium; leukocyte adhesion; leukocyte migration; membrane to membrane docking; microvillus; receptor binding; structural constituent of cytoskeleton	CellmembranePeripheralmembraneproteinCytoplasmicsideCytoplasmcytoskeletonApicalcellmembranePeripheralmembraneproteinCytoplasmicsideCellprojectionmicrovillusmembranePeripheralmembraneproteinCytoplasmicside	Phophorylated - from this Study		Known in Uniprot to be phosphorylated	Known in HPRD to be phosphorylated	
**42**	MYPT1	MYPT1_HUMAN	MYPT1; B2RAH5	O14974; Q2NKL4; Q569H0; Q86WU3; Q8NFR6; Q9BYH0; B2RAH5	NP_002471; NP_001137357	Protein phosphatase 1 regulatory subunit 12A (Myosin phosphatase-targeting subunit 1) (Myosin phosphatase target subunit 1) (Protein phosphatase myosin-binding subunit)	GO:0005737; GO:0005515; GO:0004871	cytoplasm; protein binding; signal transducer activity	Cytoplasm	Phophorylated - from this Study		Known in Uniprot to be phosphorylated	Known in HPRD to be phosphorylated	Phosphatase
**43**	ODPA	ODPA_HUMAN	ODPA; Q5JPT8	P08559; Q5JPT8; Q53H41; Q9NP12; Q9UBJ8; Q9UBU0; Q9UNG4; Q9UNG5	NP_000275	Pyruvate dehydrogenase E1 component subunit alpha, somatic form, mitochondrial (EC 1.2.4.1) (PDHE1-A type I); Pyruvate dehydrogenase (Lipoamide) alpha 1 (Pyruvate dehydrogenase (Lipoamide) alpha 1, isoform CRA_a)	GO:0006096; GO:0005759; GO:0055114; GO:0005515; GO:0004739; GO:0008152; GO:0005739; GO:0016624	glycolysis; mitochondrial matrix; oxidation reduction; protein binding; pyruvate dehydrogenase (acetyl-transferring) activity; metabolic process; mitochondrion; oxidoreductase activity, acting on the aldehyde or oxo group of donors, disulfide as acceptor	Mitochondrionmatrix;	Phophorylated - from this Study		Known in Uniprot to be phosphorylated	Known in HPRD to be phosphorylated	
**44**	PGK1	PGK1_HUMAN	PGK1; A8K4W6	P00558; Q5J7W1; Q6IBT6; Q8NI87; A8K4W6	NP_000282	Phosphoglycerate kinase 1 (EC 2.7.2.3) (Primer recognition protein 2) (PRP 2) (Cell migration-inducing gene 10 protein)	GO:0005524; GO:0005737; GO:0006096; GO:0004618; GO:0016310	ATP binding; cytoplasm; glycolysis; phosphoglycerate kinase activity; phosphorylation	Cytoplasm	Phophorylated - from this Study		Known in Uniprot to be phosphorylated		Kinase
**45**	KPCA	KPCA_HUMAN	KPCA; Q7Z727; B5BU22	P17252; Q32M72; Q15137; Q96RE4; B5BU22; Q7Z727	NP_002728	Protein kinase C alpha type (PKC-alpha) (PKC-A) (EC 2.7.11.13); PRKCA protein (Fragment)	GO:0005524; GO:0005509; GO:0005829; GO:0019992; GO:0008624; GO:0005624; GO:0005886; [illegible]	ATP binding; calcium ion binding; cytosol; diacylglycerol binding; induction of apoptosis by extracellular signals; membrane fraction; plasma membrane; protein binding; zinc ion binding; diacylglycerol binding; intracellular signaling cascade		Phophorylated - from this Study		Known in Uniprot to be phosphorylated	Known in HPRD to be phosphorylated	Kinase
**46**	PSA3	PSA3_HUMAN	PSA3; B2RCK6	P25788; Q86U83; Q8N1D8; Q9BS70; B2RCK6	NP_002779; NP_687033	Proteasome subunit alpha type-3 (EC 3.4.25.1) (Proteasome component C8) (Macropain subunit C8) (Multicatalytic endopeptidase complex subunit C8)	GO:0031145; GO:0005856; GO:0005829; GO:0051436; GO:0005634; GO:0051437; GO:0005839; GO:0005515; GO:0004298	anaphase-promoting complex-dependent proteasomal ubiquitin-dependent protein catabolic process; cytoskeleton; cytosol; negative regulation of ubiquitin-protein ligase activity during mitotic cell cycle; nucleus; positive regulation of ubiquitin-protein ligase activity during mitotic cell cycle; proteasome core complex; protein binding; threonine-type endopeptidase activity	CytoplasmNucleus	Phophorylated - from this Study		Known in Uniprot to be phosphorylated	Known in HPRD to be phosphorylated	
**47**	PTPRA	PTPRA_HUMAN	PTPRA	P18433; Q14513; Q7Z2I2; Q96TD9	NP_002827; NP_543030; NP_543031	Receptor-type tyrosine-protein phosphatase alpha (Protein-tyrosine phosphatase alpha) (R-PTP-alpha) (EC 3.1.3.48)	GO:0005887; GO:0006470; GO:0006468; GO:0005001	integral to plasma membrane; protein amino acid dephosphorylation; protein amino acid phosphorylation; transmembrane receptor protein tyrosine phosphatase activity	MembraneSinglepasstypeImembraneprotein	Phophorylated - from this Study		Known in Uniprot to be phosphorylated	Known in HPRD to be phosphorylated	Phosphatase
**48**	SPTB2	SPTB2_HUMAN	SPTB2; Q59ER3; Q53R99; B2ZZ89	Q01082; O60837; Q16057; Q53R99; Q59ER3; Q8IX99; B2ZZ89	NP_003119; NP_842565	Spectrin beta chain, brain 1 (Spectrin, non-erythroid beta chain 1) (Beta-II spectrin) (Fodrin beta chain)	GO:0003779; GO:0051016; GO:0005516; GO:0005730; GO:0005886; GO:0008091; GO:0005200	actin binding; barbed-end actin filament capping; calmodulin binding; nucleolus; plasma membrane; spectrin; structural constituent of cytoskeleton	CytoplasmcytoskeletonCytoplasmmyofibrilsarcomereMbandCellmembranePeripheralmembraneproteinCytoplasmicside	Phophorylated - from this Study		Known in Uniprot to be phosphorylated	Known in HPRD to be phosphorylated	

	Protein Name	Uniprot Name	Other Names	Uniprot IDs	Refseq IDs	Protein Description	GO ID	GO terms	Subcellular location	Comments	Comments	Comments - Database Search	Comments - Database Search	Function
**49**	TA2R	TA2R_HUMAN	TA2R; Q05C92; Q0VAB0	P21731; O75228; Q6DK52; Q9UCY1; Q9UCY2; Q05C92; Q0VAB0	NP_001051; NP_963998	Thromboxane A2 receptor (TXA2-R) (Prostanoid TP receptor); TBXA2R protein (Fragment); TBXA2R protein	GO:0007186; GO:0005887; GO:0004961; GO:0016021; GO:0045987; GO:0019229; GO:0019932; GO:0004960	G-protein coupled receptor protein signaling pathway; integral to plasma membrane; thromboxane A2 receptor activity; integral to membrane; positive regulation of smooth muscle contraction; regulation of vasoconstriction; second-messenger-mediated signaling; thromboxane receptor activity	CellmembraneMultipassmembraneprotein;	Phophorylated - from this Study				
**50**	VASP	VASP_HUMAN	VASP; B2RBT9	P50552; Q6PIZ1; Q93035; B2RBT9	NP_003361	Vasodilator-stimulated phosphoprotein (VASP)	GO:0017124; GO:0003779; GO:0015629; GO:0042995; GO:0005737; GO:0005925	SH3 domain binding; actin binding; actin cytoskeleton; cell projection; cytoplasm; focal adhesion	CytoplasmCytoplasmcytoskeletonCelljunctionfocaladhesionCellprojectionlamellipodiummembraneCellprojectionfilopodiummembrane	Phophorylated - from this Study		Known in Uniprot to be phosphorylated	Known in HPRD to be phosphorylated	
**51**	ACTN3	ACTN3_HUMAN	ACTN3	Q08043; Q4KKV2; A6NP77	NP_001095	Alpha-actinin-3 (Alpha-actinin skeletal muscle isoform 3) (F-actin cross-linking protein)	GO:0003779; GO:0005884; GO:0005509; GO:0005925; GO:0048041; GO:0005178; [illegible]	actin binding; actin filament; calcium ion binding; focal adhesion; focal adhesion formation; integrin binding; protein homodimerization activity; pseudopodium; regulation of apoptosis; structural constituent of muscle		Phophorylated - from this Study				
**52**	MLH1	MLH1_HUMAN	MLH1; Q5GJ64	P40692; Q5GJ64	NP_000240	DNA mismatch repair protein Mlh1 (MutL protein homolog 1); Hypothetical rhabdomyosarcoma antigen MU-RMS-40.5 (Fragment)	GO:0005524; GO:0006298; GO:0030983; GO:0045786; GO:0005634; GO:0005515	ATP binding; mismatch repair; mismatched DNA binding; negative regulation of cell cycle; nucleus; protein binding	Nucleus;			Known in Uniprot to be phosphorylated		
**53**	RHG01	RHG01_HUMAN	RHG01	Q07960	NP_004299	Rho GTPase-activating protein 1 (Rho-type GTPase-activating protein 1) (Rho-related small GTPase protein activator) (GTPase-activating protein rhoOGAP) (p50-RhoGAP) (CDC42 GTPase-activating protein)	GO:0007266; GO:0017124; GO:0005070; GO:0005737; GO:0043231	Rho protein signal transduction; SH3 domain binding; SH3/SH2 adaptor activity; cytoplasm; intracellular membrane-bounded organelle	Cytoplasm	Phophorylated - from this Study		Known in Uniprot to be phosphorylated		
**54**	BNIP2	BNIP2_HUMAN	BNIP2	Q12982	NP_004321	BCL2/adenovirus E1B 19 kDa protein-interacting protein 2	GO:0005096; GO:0006916; GO:0005509; GO:0005737; GO:0005635; GO:0005515	GTPase activator activity; anti-apoptosis; calcium ion binding; cytoplasm; nuclear envelope; protein binding	CytoplasmCytoplasmperinuclearregion	Phophorylated - from this Study		Known in Uniprot to be phosphorylated		
**55**	CSP9	CSP9_HUMAN	CSP9; Q6IAZ5; MED7	O43513; Q6IAZ5	NP_004261; NP_001094286	Mediator of RNA polymerase II transcription subunit 7 (Mediator complex subunit 7) (Cofactor required for Sp1 transcriptional activation subunit 9) (CRSP complex subunit 9) (Transcriptional coactivator CRSP33) (RNA polymerase transcriptional regulation mediator subunit 7 homolog) (hMED7) (Activator-recruited cofactor 34 kDa component) (ARC34); CRSP9 protein (Cofactor required for Sp1 transcriptional activation, subunit 9, 33kDa) (Cofactor required for Sp1 transcriptional activation, subunit 9, 33kDa, isoform CRA_a)	GO:0045941; GO:0006357; GO:0003713; GO:0005667; GO:0006367; GO:0005634; GO:0045941	positive regulation of transcription; regulation of transcription from RNA polymerase II promoter; transcription coactivator activity; transcription factor complex; transcription initiation from RNA polymerase II promoter; nucleus; positive regulation of transcription	Nucleus;	Phophorylated - from this Study				
**56**	DAP1	DAP1_HUMAN	DAP1; Q6FGC3	P51397; Q6FGC3; Q9BUC9	NP_004385	Death-associated protein 1 (DAP-1)	GO:0008624	induction of apoptosis by extracellular signals		Phophorylated - from this Study		Known in Uniprot to be phosphorylated		
**57**	FCERG	FCERG_HUMAN	FCERG	P30273; Q5VTW4	NP_004097	High affinity immunoglobulin epsilon receptor subunit gamma (IgE Fc receptor subunit gamma) (FceRI gamma) (Fc-epsilon RI-gamma)	GO:0005887	integral to plasma membrane	CellmembraneSinglepasstypeImembraneprotein	Phophorylated - from this Study		Known in Uniprot to be phosphorylated		
**58**	GRAP2	GRAP2_HUMAN	GRAP2; Q6FI14	O75791; Q6FI14; O43726; Q9NRB7	NP_004801	GRB2-related adapter protein 2 (Protein GADS) (Growth factor receptor-binding protein) (GRBLG) (Grf40 adapter protein) (Grf-40) (GRB-2-like protein) (GRB2L) (GRBX) (P38) (Hematopoietic cell-associated adapter protein GrpL) (Adapter protein GRID) (SH3-SH2-SH3 adapter Mona); GRAP2 protein (GRB2-related adaptor protein 2, isoform CRA_a) (cDNA, FLJ93267, Homo sapiens GRB2-related adaptor protein 2 (GRAP2), mRNA)	GO:0007265; GO:0005070; GO:0007267; GO:0005829; GO:0005634; GO:0005515	Ras protein signal transduction; SH3/SH2 adaptor activity; cell-cell signaling; cytosol; nucleus; protein binding		Phophorylated - from this Study		Known in Uniprot to be phosphorylated	Known in HPRD to be phosphorylated	
**59**	KAP2	KAP2_HUMAN	KAP2; Q9BUB1	P13861; Q16823; Q9BUB1	NP_004148	cAMP-dependent protein kinase type II-alpha regulatory subunit; PRKAR2A protein (Protein kinase, cAMP-dependent, regulatory, type II, alpha) (Protein kinase, cAMP-dependent, regulatory, type II, alpha, isoform CRA_b)	GO:0030552; GO:0005952; GO:0008603; GO:0005737; GO:0009755; GO:0005624; GO:0005886; GO:0001932; GO:0005952; GO:0016301; GO:0000166; GO:0007165	cAMP binding; cAMP-dependent protein kinase complex; cAMP-dependent protein kinase regulator activity; cytoplasm; hormone-mediated signaling; membrane fraction; plasma membrane; regulation of protein amino acid phosphorylation; cAMP-dependent protein kinase complex; kinase activity; nucleotide binding; signal transduction		Phophorylated - from this Study		Known in Uniprot to be phosphorylated	Known in HPRD to be phosphorylated	Kinase
**60**	NHERF	NHERF_HUMAN	NHERF; NHRF1	O14745; O43552; Q86WQ5	NP_004243	Na(+)/H(+) exchange regulatory cofactor NHE-RF1 (NHERF-1) (Ezrin-radixin-moesin-binding phosphoprotein 50) (EBP50) (Regulatory cofactor of Na(+)/H(+) exchanger) (Sodium-hydrogen exchanger regulatory factor 1) (Solute carrier family 9 isoform A3 regulatory factor 1)	GO:0030165; GO:0016055; GO:0015629; GO:0031698; GO:0042995; GO:0012505; GO:0006461; GO:0032415	PDZ domain binding; Wnt receptor signaling pathway; actin cytoskeleton; beta-2 adrenergic receptor binding; cell projection; endomembrane system; protein complex assembly; regulation of sodium:hydrogen antiporter activity	IntracytoplasmicmembranePeripheralmembraneproteinCellprojectionfilopodiumCellprojectionruffleCellprojectionmicrovillus	Phophorylated - from this Study		Known in Uniprot to be phosphorylated	Known in HPRD to be phosphorylated	
**61**	BCL3	BCL3_HUMAN	BCL3	P20749	NP_005169	B-cell lymphoma 3-encoded protein (Protein Bcl-3)	GO:0032996; GO:0033257; GO:0042771; GO:0007249; GO:0005737; GO:0051457; GO:0043066; GO:0045415; GO:0016481; GO:0005634; GO:0045727; GO:0030674; GO:0000060; GO:0051101; GO:0042345; GO:0010225; GO:0009615; GO:0008134	Bcl3-Bcl10 complex; Bcl3/NF-kappaB2 complex; DNA damage response, signal transduction by p53 class mediator resulting in induction of apoptosis; I-kappaB kinase/NF-kappaB cascade; cytoplasm; maintenance of protein location in nucleus; negative regulation of apoptosis; negative regulation of interleukin-8 biosynthetic process; negative regulation of transcription; nucleus; positive regulation of translation; protein binding, bridging; protein import into nucleus, translocation; regulation of DNA binding; regulation of NF-kappaB import into nucleus; response to UV-C; response to virus; transcription factor binding	Nucleus	Phophorylated - from this Study		Known in Uniprot to be phosphorylated		
**62**	HSP71	HSP71_HUMAN	HSP71; A8K5I0; B3KTT5	Q5SP17; P08107; P19790; Q5JQI4; Q9UQL9; Q9UQM0; A8K5I0; B3KTT5	NP_005336; NP_005337	Heat shock 70 kDa protein 1 (HSP70.1) (HSP70-1/HSP70-2)	GO:0005524; GO:0006916; GO:0005783; GO:0006402; GO:0005739; GO:0005634; GO:0006986; GO:0051082	ATP binding; anti-apoptosis; endoplasmic reticulum; mRNA catabolic process; mitochondrion; nucleus; response to unfolded protein; unfolded protein binding		Phophorylated - from this Study		Known in Uniprot to be phosphorylated		
**63**	NP1L4	NP1L4_HUMAN	NP1L4	Q99733	NP_005960	Nucleosome assembly protein 1-like 4 (Nucleosome assembly protein 2) (NAP2)	GO:0005678; GO:0005737; GO:0006334; GO:0051082	chromatin assembly complex; cytoplasm; nucleosome assembly; unfolded protein binding	Nucleus	Phophorylated - from this Study		Known in Uniprot to be phosphorylated	Known in HPRD to be phosphorylated	
**64**	CAP1	CAP1_HUMAN	CAP1	Q01518; Q53HR7; Q5T0S1; Q5T0S2; Q6I9U6	NP_006358; NP_001099000	Adenylyl cyclase-associated protein 1 (CAP 1)	GO:0003779; GO:0007190; GO:0007163; GO:0005886; GO:0007165	actin binding; activation of adenylate cyclase activity; establishment or maintenance of cell polarity; plasma membrane; signal transduction	CellmembranePeripheralmembraneprotein	Phophorylated - from this Study		Known in Uniprot to be phosphorylated		
**65**	LRRF2	LRRF2_HUMAN	LRRF2	Q9Y608; Q68CV3; Q9NXH5	NP_006300; NP_060194	Leucine-rich repeat flightless-interacting protein 2 (LRR FLII-interacting protein 2)	GO:0030275; GO:0016055	LRR domain binding; Wnt receptor signaling pathway		Phophorylated - from this Study		Known in Uniprot to be phosphorylated		
**66**	PGRC2	PGRC2_HUMAN	PGRC2	O15173; Q569H1	NP_006311	Membrane-associated progesterone receptor component 2 (Progesterone membrane-binding protein) (Steroid receptor protein DG6)	GO:0020037; GO:0016021; GO:0005496; GO:0003707	heme binding; integral to membrane; steroid binding; steroid hormone receptor activity	MembraneSinglepassmembraneprotein	Phophorylated - from this Study		Known in Uniprot to be phosphorylated		
**67**	IPP2	IPP2_HUMAN	IPP2; Q6NXS2	P41236; Q6NXS2	NP_006232	Protein phosphatase inhibitor 2 (IPP-2)	GO:0005977; GO:0005515; GO:0004865; GO:0043666; GO:0009966	glycogen metabolic process; protein binding; protein serine/threonine phosphatase inhibitor activity; regulation of phosphoprotein phosphatase activity; regulation of signal transduction		Phophorylated - from this Study		Known in Uniprot to be phosphorylated	Known in HPRD to be phosphorylated	Phosphatase
**68**	KPCT	KPCT_HUMAN	KPCT	Q04759; Q14DH6; Q3MJF1; Q64FY5; Q9H508; Q9H549	NP_006248	Protein kinase C theta type (EC 2.7.11.13) (nPKC-theta)	GO:0005524; GO:0019992; GO:0005622; GO:0007242; GO:0000287; GO:0006509; GO:0006468; GO:0005515; [illegible]	ATP binding; diacylglycerol binding; intracellular; intracellular signaling cascade; magnesium ion binding; membrane protein ectodomain proteolysis; protein amino acid phosphorylation; protein binding; protein kinase C activity; regulation of cell growth; zinc ion binding		Phophorylated - from this Study		Known in Uniprot to be phosphorylated	Known in HPRD to be phosphorylated	Kinase
**69**	TRAD1	TRAD1_HUMAN	TRAD1	O14545	NP_006691; NP_001137378	TRAF-type zinc finger domain-containing protein 1 (Protein FLN29)	GO:0005622; GO:0005515; GO:0008270	intracellular; protein binding; zinc ion binding		Phophorylated - from this Study		Known in Uniprot to be phosphorylated	Known in HPRD to be phosphorylated	
**70**	PGRC1	PGRC1_HUMAN	PGRC1; Q6IB11	O00264; Q6IB11; Q9UGJ9	NP_006658	Membrane-associated progesterone receptor component 1 (mPR); PGRMC1 protein (Progesterone receptor membrane component 1) (Progesterone receptor membrane component 1, isoform CRA_a) (cDNA, FLJ94602, Homo sapiens progesterone receptor membrane component 1 (PGRMC1),mRNA)	GO:0009986; GO:0005789; GO:0020037; GO:0005887; GO:0005792; GO:0005730; GO:0005515; [illegible]	cell surface; endoplasmic reticulum membrane; heme binding; integral to plasma membrane; microsome; nucleolus; protein binding; receptor activity; steroid binding; axon guidance; transition metal ion binding	MicrosomemembraneSinglepassmembraneproteinEndoplasmicreticulummembraneSinglepassmembraneprotein;	Phophorylated - from this Study		Known in Uniprot to be phosphorylated	Known in HPRD to be phosphorylated	
**71**	TRPC6	TRPC6_HUMAN	TRPC6	Q9Y210; Q52M59; Q9HCW3; Q9NQA8; Q9NQA9	NP_004612	Short transient receptor potential channel 6 (TrpC6)	GO:0005509; GO:0006816; GO:0016021; GO:0005886; GO:0005515	calcium ion binding; calcium ion transport; integral to membrane; plasma membrane; protein binding	MembraneMultipassmembraneprotein	Phophorylated - from this Study		Known in Uniprot to be phosphorylated		
**72**	ZN185	ZN185_HUMAN	ZN185; A4FTV3	O15231; A6NME5; O00345; Q9NSD2; A4FTV3	NP_009081	Zinc finger protein 185 (LIM domain protein ZNF185) (P1-A)	GO:0005737; GO:0005634; GO:0005886; GO:0008270	cytoplasm; nucleus; plasma membrane; zinc ion binding	Nucleus	Phophorylated - from this Study		Known in Uniprot to be phosphorylated		
**73**	FHOD1	FHOD1_HUMAN	FHOD1	Q9Y613; Q59F76; Q6Y1F2; Q76MS8; Q8N521	NP_037373	FH1/FH2 domain-containing protein 1 (Formin homolog overexpressed in spleen 1) (FHOS) (Formin homology 2 domain-containing protein 1)	GO:0003779; GO:0030036; GO:0005737; GO:0005856; GO:0005634	actin binding; actin cytoskeleton organization; cytoplasm; cytoskeleton; nucleus	CytoplasmCytoplasmcytoskeleton	Phophorylated - from this Study		Known in Uniprot to be phosphorylated	Known in HPRD to be phosphorylated	
**74**	DAPP1	DAPP1_HUMAN	DAPP1	Q9UN19; Q8TCK5; Q9UHF2	NP_055210	Dual adapter for phosphotyrosine and 3-phosphotyrosine and 3-phosphoinositide (hDAPP1) (B-cell adapter molecule of 32 kDa) (B lymphocyte adapter protein Bam32)	GO:0005737; GO:0016020; GO:0005543; GO:0006470; GO:0005515; GO:0004725; GO:0007165	cytoplasm; membrane; phospholipid binding; protein amino acid dephosphorylation; protein binding; protein tyrosine phosphatase activity; signal transduction	CytoplasmMembranePeripheralmembraneprotein	Phophorylated - from this Study		Known in Uniprot to be phosphorylated	Known in HPRD to be phosphorylated	

	Protein Name	Uniprot Name	Other Names	Uniprot IDs	Refseq IDs	Protein Description	GO ID	GO terms	Subcellular location	Comments	Comments	Comments - Database Search	Comments - Database Search	Function
**75**	TMOD3	TMOD3_HUMAN	TMOD3	Q9NYL9; B2R6G7; Q9NT43; Q9NZR0	NP_055362	Tropomodulin-3 (Ubiquitous tropomodulin) (U-Tmod)	GO:0003779; GO:0005737; GO:0005856; GO:0005523	actin binding; cytoplasm; cytoskeleton; tropomyosin binding	Cytoplasmcytoskeleton	Phophorylated - from this Study		Known in Uniprot to be phosphorylated		
**76**	LEGL	LEGL_HUMAN	Q9P005; LEGL	Q9P005; Q3ZCW2; B2RBG8; Q6P5T6	NP_054900	Galectin-related protein	GO:0005622; GO:0005529	intracellular; sugar binding		Phophorylated - from this Study		Known in Uniprot to be phosphorylated		
**77**	FARP2	FARP2_HUMAN	FARP2	O94887; Q53QM5; Q8WU27; Q9UFE7	NP_055623	FERM, RhoGEF and pleckstrin domain-containing protein 2 (FERM domain including RhoGEF) (FIR)	GO:0016601; GO:0005089; GO:0005737; GO:0008092; GO:0005856; GO:0019898; GO:0016322; GO:0035023	Rac protein signal transduction; Rho guanyl-nucleotide exchange factor activity; cytoplasm; cytoskeletal protein binding; cytoskeleton; extrinsic to membrane; neuron remodeling; regulation of Rho protein signal transduction		Phophorylated - from this Study		Known in Uniprot to be phosphorylated		
**78**	ABLM3	ABLM3_HUMAN	ABLM3	O94929; A8K121; Q19VH3; Q658S1; Q68CI5; Q9BV32	NP_055760	Actin-binding LIM protein 3 (Actin-binding LIM protein family member 3) (abLIM-3)	GO:0003779; GO:0005737; GO:0007010; GO:0008270	actin binding; cytoplasm; cytoskeleton organization; zinc ion binding	Cytoplasm	Phophorylated - from this Study		Known in Uniprot to be phosphorylated		
**79**	RGC32	RGC32_HUMAN	RGC32	Q9UL69; Q9H4X1; Q6NZ48	NP_054778	Response gene to complement 32 protein (RGC-32)	GO:0005813; GO:0005634; GO:0000079	centrosome; nucleus; regulation of cyclin-dependent protein kinase activity	CytoplasmNucleusCentrosome	Phophorylated - from this Study		Known in Uniprot to be phosphorylated	Known in HPRD to be phosphorylated	
**80**	DC1L1	DC1L1_HUMAN	DC1L1; A2RRG7	Q9Y6G9; Q53HC8; Q53HK7; A2RRG7	NP_057225	Cytoplasmic dynein 1 light intermediate chain 1 (Dynein light intermediate chain 1, cytosolic) (Dynein light chain A) (DLC-A)	GO:0005524; GO:0030286; GO:0005874; GO:0003774	ATP binding; dynein complex; microtubule; motor activity		Phophorylated - from this Study		Known in Uniprot to be phosphorylated	Known in HPRD to be phosphorylated	
**81**	MOL1B	MOL1B_HUMAN	MOL1B	Q9H8S9; Q53S34; Q9H3T5; Q9HAI0; Q9NVE2	NP_060691	Mps one binder kinase activator-like 1B (Mob1 homolog 1B) (Mob1 alpha) (Mob1A) (Protein Mob4B)	GO:0005515; GO:0008270	protein binding; zinc ion binding		Phophorylated - from this Study		Known in Uniprot to be phosphorylated		
**82**	CI167	CI167_HUMAN	A2BFA4; Q9NXH8; CI167	Q9NXH8; A2BFA4	NP_060193	Torsin family protein C9orf167	GO:0005524; GO:0051085; GO:0005783; GO:0016021; GO:0017111	ATP binding; chaperone mediated protein folding requiring cofactor; endoplasmic reticulum; integral to membrane; nucleoside-triphosphatase activity	MembraneSinglepassmembraneprotein	Phophorylated - from this Study		Known in Uniprot to be phosphorylated		
**83**	CKLF6	CKLF6_HUMAN	CKLF6; Q6IAC4	Q9NX76; Q6IAC4	NP_060271	CKLF-like MARVEL transmembrane domain-containing protein 6 (Chemokine-like factor superfamily member 6); CKLF-like MARVEL transmembrane domain containing 6, isoform CRA_a (CKLFSF6 protein)	GO:0006935; GO:0005125; GO:0005615; GO:0016021; GO:0016021	chemotaxis; cytokine activity; extracellular space; integral to membrane; integral to membrane	MembraneMultipassmembraneprotein;	Phophorylated - from this Study				
**84**	WDR1	WDR1_HUMAN	WDR1	O75083; A8K6E9; O75313; Q8N6E5; Q9UG05; Q9UG78; Q9UQE0	NP_059830; NP_005103	WD repeat-containing protein 1 (Actin-interacting protein 1) (AIP1) (NORI-1)	GO:0003779; GO:0005737; GO:0005856; GO:0007605	actin binding; cytoplasm; cytoskeleton; sensory perception of sound	Cytoplasmcytoskeleton	Phophorylated - from this Study		Known in Uniprot to be phosphorylated		
**85**	SEPT5	SEPT5_HUMAN	SEPT5	Q99719; O15251	NP_002679	Septin-5 (Peanut-like protein 1) (Cell division control-related protein 1) (CDCrel-1)	GO:0005525; GO:0003924; GO:0007049; GO:0000910; GO:0005886; GO:0031105; GO:0005198; GO:0008021; GO:0016080	GTP binding; GTPase activity; cell cycle; cytokinesis; plasma membrane; septin complex; structural molecule activity; synaptic vesicle; synaptic vesicle targeting		Phophorylated - from this Study		Known in Uniprot to be phosphorylated	Known in HPRD to be phosphorylated	
**86**	ITPR1	ITPR1_HUMAN	ITPR1	Q14643; Q14660; Q99897	NP_002213; NP_001093422	Inositol 1,4,5-trisphosphate receptor type 1 (Type 1 inositol 1,4,5-trisphosphate receptor) (Type 1 InsP3 receptor) (IP3 receptor isoform 1) (InsP3R1) (IP3R)	GO:0005509; GO:0015085; GO:0006816; GO:0008219; GO:0005789; GO:0005220; GO:0008095; GO:0016021; GO:0031095; GO:0005515; GO:0001666; GO:0007165	calcium ion binding; calcium ion transmembrane transporter activity; calcium ion transport; cell death; endoplasmic reticulum membrane; inositol 1,4,5-triphosphate-sensitive calcium-release channel activity; inositol-1,4,5-triphosphate receptor activity; integral to membrane; platelet dense tubular network membrane; protein binding; response to hypoxia; signal transduction	EndoplasmicreticulummembraneMultipassmembraneprotein	Phophorylated - from this Study		Known in Uniprot to be phosphorylated	Known in HPRD to be phosphorylated	
**87**	KGP1B	KGP1B_HUMAN	KGP1B	P14619; Q5JSJ6	NP_006249	cGMP-dependent protein kinase 1, beta isozyme (cGK 1 beta) (cGKI-beta) (EC 2.7.11.12)	GO:0005524; GO:0030036; GO:0030553; GO:0004692; GO:0005737; GO:0005634; GO:0006468; GO:0007165	ATP binding; actin cytoskeleton organization; cGMP binding; cGMP-dependent protein kinase activity; cytoplasm; nucleus; protein amino acid phosphorylation; signal transduction		Phophorylated - from this Study		Kinase	Known in HPRD to be phosphorylated	
**88**	RTN1	RTN1_HUMAN	RTN1	Q16799; Q5BKZ4; Q16800; Q16801	NP_066959; NP_996739	Reticulon-1 (Neuroendocrine-specific protein)	GO:0030176; GO:0030182; GO:0004871; GO:0007165	integral to endoplasmic reticulum membrane; neuron differentiation; signal transducer activity; signal transduction	EndoplasmicreticulummembraneMultipassmembraneprotein	Phophorylated - from this Study		Known in Uniprot to be phosphorylated		
**89**	TTL12	TTL12_HUMAN	TTL12; Q20WK5	Q14166; Q20WK5; Q9UGU3	NP_055955	Tubulin--tyrosine ligase-like protein 12	GO:0006464; GO:0004835	protein modification process; tubulin-tyrosine ligase activity		Phophorylated - from this Study		Known in Uniprot to be phosphorylated		
**90**	CD2AP	CD2AP_HUMAN	CD2AP; Q5VYA3	Q9Y5K6; Q5VYA3; A6NL34; Q9UG97	NP_036252	CD2-associated protein (Cas ligand with multiple SH3 domains) (Adapter protein CMS)	GO:0017124; GO:0051301; GO:0031941; GO:0007067; GO:0005730; GO:0005886; GO:0006461; GO:0001726; GO:0007165; GO:0005200; GO:0006930	SH3 domain binding; cell division; filamentous actin; mitosis; nucleolus; plasma membrane; protein complex assembly; ruffle; signal transduction; structural constituent of cytoskeleton; substrate-bound cell migration, cell extension	Cytoplasmcytoskeleton Cellprojectionruffle	Phophorylated - from this Study		Known in Uniprot to be phosphorylated		
**91**	SNTB1	SNTB1_HUMAN	SNTB1	Q13884; A8K9E0; O14912; Q4KMG8	NP_066301	Beta-1-syntrophin (59 kDa dystrophin-associated protein A1 basic component 1) (DAPA1B) (Tax interaction protein 43) (TIP-43) (Syntrophin-2) (BSYN2)	GO:0003779; GO:0005509; GO:0005516; GO:0030054; GO:0005737; GO:0005856; GO:0016010; GO:0006936	actin binding; calcium ion binding; calmodulin binding; cell junction; cytoplasm; cytoskeleton; dystrophin-associated glycoprotein complex; muscle contraction	CellmembranesarcolemmaPeripheralmembraneproteinCytoplasmicsideCelljunctionCytoplasmcytoskeleton	Phophorylated - from this Study		Known in Uniprot to be phosphorylated		
**92**	CIP4	CIP4_HUMAN	CIP4; B2R8A6	Q15642; O15184; Q53G22; Q5TZN1; Q6FI24; Q8NFL1; Q8TCY1; Q8TDX3; Q96RJ1; B2R8A6	NP_004231	Cdc42-interacting protein 4 (Thyroid receptor-interacting protein 10) (TRIP-10) (Protein Felic) (Salt tolerant protein) (hSTP)	GO:0005794; GO:0030036; GO:0005856; GO:0006897; GO:0042802; GO:0008289; GO:0005764; GO:0005886; GO:0007165	Golgi apparatus; actin cytoskeleton organization; cytoskeleton; endocytosis; identical protein binding; lipid binding; lysosome; plasma membrane; signal transduction	Cytoplasmcytoskeleton CytoplasmcellcortexLysosomeGolgiapparatusCellmembraneCytoplasmperinuclearregion	Phophorylated - from this Study		Known in Uniprot to be phosphorylated		
**93**	GPSM3	GPSM3_HUMAN	GPSM3; A2BFJ3	Q9Y4H4; A2BFJ3	NP_071390	G-protein-signaling modulator 3 (Activator of G-protein signaling 4) (Protein G18) (G18.1b); G-protein signalling modulator 3 (AGS3-like, C. elegans) (HCG2001537, isoform CRA_h (Hcg2044638)) (cDNA, FLJ94561, Homo sapiens G-protein signalling modulator 3 (AGS3-like, C.elegans) (GPSM3), mRNA)	GO:0005096; GO:0005737; GO:0005515; GO:0007165	GTPase activator activity; cytoplasm; protein binding; signal transduction	Cytoplasm;	Phophorylated - from this Study		Known in Uniprot to be phosphorylated		
**94**	TBAL3	TBAL3_HUMAN	Q9H6Z0; TBAL3	Q9H6Z0; A6NHL2; B4DKL2; Q4QQJ5	NP_079079; XP_302138	Tubulin alpha chain-like 3	GO:0005525; GO:0003924; GO:0005874; GO:0007018; GO:0043234; GO:0051258; GO:0005198	GTP binding; GTPase activity; microtubule; microtubule-based movement; protein complex; protein polymerization; structural molecule activity				Known in Uniprot to be phosphorylated		
**95**	DOK3	DOK3_HUMAN	DOK3	Q7L591; Q8N864; Q9BQB3; Q9H666	NP_079148; NP_001138347; NP_001138348	Docking protein 3 (Downstream of tyrosine kinase 3)	GO:0005737; GO:0005158	cytoplasm; insulin receptor binding	CytoplasmCellmembranePeripheralmembraneproteinCytoplasmicside	Phophorylated - from this Study		Known in Uniprot to be phosphorylated		
**96**	MYCT1	MYCT1_HUMAN	Q9H763; MYCT1	Q9H763; Q8N699; Q8N396; Q8TBE8	NP_079383	Myc target protein 1 (Myc target in myeloid cells protein 1)	GO:0005634	nucleus	Nucleus	Phophorylated - from this Study		Known in Uniprot to be phosphorylated		
**97**	T22D4	T22D4_HUMAN	T22D4; A4D2C3	Q9Y3Q8; A4D2C3	NP_112197	TSC22 domain family protein 4 (TSC22-related-inducible leucine zipper protein 2) (Tsc-22-like protein THG-1); HCG1770615, isoform CRA_a (TSC-22-like)	GO:0005737; GO:0005634; GO:0005515; GO:0006355; GO:0003700; GO:0016564; GO:0006355; GO:0006970	cytoplasm; nucleus; protein binding; regulation of transcription, DNA-dependent; transcription factor activity; transcription repressor activity; regulation of transcription, DNA-dependent; response to osmotic stress	Nucleus;	Phophorylated - from this Study		Known in Uniprot to be phosphorylated		
**98**	KIF11	KIF11_HUMAN	KIF11	Q5VWX0; P52732; A0AV49; B2RMV3; Q15716	NP_004514	Kinesin-like protein KIF11 (Kinesin-related motor protein Eg5) (Kinesin-like spindle protein HKSP) (Thyroid receptor-interacting protein 5) (TRIP-5) (Kinesin-like protein 1)	GO:0005524; GO:0051301; GO:0005737; GO:0005871; GO:0005874; GO:0003777; GO:0007018; GO:0007067; GO:0007052; GO:0005634; GO:0005819; GO:0051300	ATP binding; cell division; cytoplasm; kinesin complex; microtubule; microtubule motor activity; microtubule-based movement; mitosis; mitotic spindle organization; nucleus; spindle; spindle pole body organization		Phophorylated - from this Study		Known in Uniprot to be phosphorylated	Known in HPRD to be phosphorylated	
**99**	CCM2	CCM2_HUMAN	CCM2; A4D2L4	Q9BSQ5; Q71RE5; Q8TAT4; A4D2L4	NP_113631; NP_001025006	Malcavernin (Cerebral cavernous malformations 2 protein); Cerebral cavernous malformation 2 (Cerebral cavernous malformation 2, isoform CRA_d)	GO:0005737; GO:0007229; GO:0005515; GO:0051403; GO:0001570;	cytoplasm; integrin-mediated signaling pathway; protein binding; stress-activated MAPK cascade; vasculogenesis;	Cytoplasm;	Phophorylated - from this Study		Known in Uniprot to be phosphorylated		
**100**	PDLI1	PDLI1_HUMAN	PDLI1	O00151; B2RBS6; Q5VZH5; Q9BPZ9	NP_066272	PDZ and LIM domain protein 1 (Elfin) (LIM domain protein CLP-36) (C-terminal LIM domain protein 1)	GO:0005737; GO:0005856; GO:0006979; GO:0008270	cytoplasm; cytoskeleton; response to oxidative stress; zinc ion binding	CytoplasmCytoplasmcytoskeleton	Phophorylated - from this Study		Known in Uniprot to be phosphorylated	Known in HPRD to be phosphorylated	

	Protein Name	Uniprot Name	Other Names	Uniprot IDs	Refseq IDs	Protein Description	GO ID	GO terms	Subcellular location	Comments	Comments	Comments - Database Search	Comments - Database Search	Function
**101**	PP12C	PP12C_HUMAN	Q9BZL4; PP12C	Q9BZL4; Q59FK8; Q6ZPD1; Q7L8F7; Q96HW1; Q9H5H5; Q9NT00	NP_060077	Protein phosphatase 1 regulatory subunit 12C (Protein phosphatase 1 myosin-binding subunit of 85 kDa) (Protein phosphatase 1 myosin-binding subunit p85)	GO:0005737	cytoplasm	Cytoplasm	Phophorylated - from this Study		Known in Uniprot to be phosphorylated	Known in HPRD to be phosphorylated	Phosphatase
**102**	TBB6	TBB6_HUMAN	TBB6	Q9BUF5; B3KM76; Q9HA42	XP_001131046; NP_115914	Tubulin beta-6 chain	GO:0005525; GO:0003924; GO:0005874; GO:0007018; GO:0043234; GO:0051258; GO:0005198	GTP binding; GTPase activity; microtubule; microtubule-based movement; protein complex; protein polymerization; structural molecule activity		Phophorylated - from this Study		Known in Uniprot to be phosphorylated		
**103**	PURB	PURB_HUMAN	PURB; A4D2L7	Q96QR8; A4D2L7	NP_150093	Transcriptional activator protein Pur-beta (Purine-rich element-binding protein B); Purine-rich element binding protein B	GO:0005662; GO:0003729; GO:0045637; GO:0003697; GO:0008134; GO:0046332; GO:0003690; GO:0045892; GO:0032422; GO:0016566; GO:0003700; GO:0000900	DNA replication factor A complex; mRNA binding; regulation of myeloid cell differentiation; single-stranded DNA binding; transcription factor binding; SMAD binding; double-stranded DNA binding; negative regulation of transcription, DNA-dependent; purine-rich negative regulatory element binding; specific transcriptional repressor activity; transcription factor activity; translation repressor activity, nucleic acid binding	Nucleus;	Phophorylated - from this Study		Known in Uniprot to be phosphorylated	Known in HPRD to be phosphorylated	
**104**	PP14A	PP14A_HUMAN	PP14A	Q96A00; Q7Z4X7; Q96S54	NP_150281	Protein phosphatase 1 regulatory subunit 14A (17 kDa PKC-potentiated inhibitory protein of PP1) (CPI-17)	GO:0005737; GO:0004864; GO:0005515; GO:0042325	cytoplasm; phosphoprotein phosphatase inhibitor activity; protein binding; regulation of phosphorylation	Cytoplasm	Phophorylated - from this Study		Known in Uniprot to be phosphorylated	Known in HPRD to be phosphorylated	Phosphatase
**105**	CALD1	CALD1_HUMAN	CALD1; Q5HY93; Q7Z2Y9; Q6PJM5	Q05682; Q6PJM5; Q13978; Q13979; Q14741; Q14742; Q5HY93; Q7Z2Y9	NP_149131; NP_149130; NP_004333	Caldesmon (CDM); Putative uncharacterized protein DKFZp779D0769 (Fragment); CALD1 protein (Fragment); Putative uncharacterized protein DKFZp686P03208 (Fragment)	GO:0003779; GO:0005516; GO:0006928; GO:0005856; GO:0006936; GO:0017022; GO:0005523; ; GO:0030478; GO:0005624	actin binding; calmodulin binding; cell motion; cytoskeleton; muscle contraction; myosin binding; tropomyosin binding; ; actin cap; membrane fraction		Phophorylated - from this Study		Known in Uniprot to be phosphorylated	Known in HPRD to be phosphorylated	
**106**	KAPCG	KAPCG_HUMAN	KAPCG; Q5VZ02	P22612; Q5VZ02; O60850; Q86YI1	NP_002723	cAMP-dependent protein kinase catalytic subunit gamma (PKA C-gamma) (EC 2.7.11.11)	GO:0005524; GO:0004691; GO:0009755; GO:0008584; GO:0006468; GO:0007243; GO:0007283	ATP binding; cAMP-dependent protein kinase activity; hormone-mediated signaling; male gonad development; protein amino acid phosphorylation; protein kinase cascade; spermatogenesis		Phophorylated - from this Study		Known in Uniprot to be phosphorylated		Kinase
**107**	ML12B	ML12B_HUMAN	Q13182; ML12B	Q13182; O14950; Q7Z5Z4	NP_291024; XP_294099; XP_095590; NP_001138416; NP_001138417	Myosin regulatory light chain MRLC2 (Myosin regulatory light chain 2-B, smooth muscle isoform) (MLC-2A) (MLC-2) (SHUJUN-1)	GO:0005509; GO:0003774; GO:0016459	calcium ion binding; motor activity; myosin complex		Phophorylated - from this Study		Known in Uniprot to be phosphorylated		
**108**	SKAP2	SKAP2_HUMAN	SKAP2; A4D173	O75563; Q53GP6; Q75MK6; Q75MZ4; Q9UBZ3; Q9UED8; A4D173	NP_003921	Src kinase-associated phosphoprotein 2 (Src family-associated phosphoprotein 2) (Src kinase-associated phosphoprotein 55-related protein) (SKAP55 homolog) (SKAP-55HOM) (SKAP-HOM) (Src-associated adapter protein with PH and SH3 domains) (Pyk2/RAFTK-associated protein) (Retinoic acid-induced protein 70); Src family associated phosphoprotein 2 (Src family associated phosphoprotein 2, isoform CRA_b) (cDNA, FLJ93710, Homo sapiens src family associated phosphoprotein 2 (SCAP2), mRNA)	GO:0042113; GO:0005070; GO:0005737; GO:0005886; GO:0006461; GO:0007165; GO:0005737; GO:0008285	B cell activation; SH3/SH2 adaptor activity; cytoplasm; plasma membrane; protein complex assembly; signal transduction; cytoplasm; negative regulation of cell proliferation	Cytoplasm;	Phophorylated - from this Study		Known in Uniprot to be phosphorylated		
**109**	Q9UBY6	Q9UBY6_HUMAN	Q9UBY6	Q9UBY6; Q3ZCP9	NP_444260	Kinase-related protein (Kinase-related protein isoform 2) (Myosin light chain kinase) (Myosin, light polypeptide kinase, isoform CRA_e) (cDNA, FLJ95241, Homo sapiens myosin, light polypeptide kinase (MYLK), transcriptvariant 8, mRNA)	GO:0016301	kinase activity		Phophorylated - from this Study				Kinase
**110**	TJAP1	TJAP1_HUMAN	TJAP1	Q5JTD0; Q05BH9; Q5JTD1; Q5JWW1; Q68DB2; Q6P2P3; Q9H7V7	NP_542171; NP_001139488; NP_001139489	Tight junction-associated protein 1 (Tight junction protein 4) (Protein incorporated later into tight junctions)	GO:0005794; GO:0005515; GO:0005923	Golgi apparatus; protein binding; tight junction	GolgiapparatusCelljunctiontightjunction	Phophorylated - from this Study		Known in Uniprot to be phosphorylated		
**111**	ZCCHL	ZCCHL_HUMAN	Q96H79; ZCCHL	Q96H79; Q8WUD9	NP_542391	Zinc finger CCCH-type antiviral protein 1-like				Phophorylated - from this Study		Known in Uniprot to be phosphorylated		
**112**	PTN3	PTN3_HUMAN	PTN3; B7Z9V1	P26045; A0AUW9; B7Z9V1	NP_002820; NP_001138840	Tyrosine-protein phosphatase non-receptor type 3 (EC 3.1.3.48) (Protein-tyrosine phosphatase H1) (PTP-H1)	GO:0005737; GO:0008092; GO:0005856; GO:0019898; GO:0051045; GO:0005886; GO:0006470; GO:0004725	cytoplasm; cytoskeletal protein binding; cytoskeleton; extrinsic to membrane; negative regulation of membrane protein ectodomain proteolysis; plasma membrane; protein amino acid dephosphorylation; protein tyrosine phosphatase activity	CellmembranePeripheralmembraneproteinCytoplasmicsideCytoplasmcytoskeleton			Known in Uniprot to be phosphorylated		Phosphatase
**113**	PTN6	PTN6_HUMAN	PTN6; Q53EL0; A8K306	P29350; Q969V8; A8K306; Q53EL0	NP_002822; NP_536858; NP_536859	Tyrosine-protein phosphatase non-receptor type 6 (EC 3.1.3.48) (Protein-tyrosine phosphatase 1C) (PTP-1C) (Hematopoietic cell protein-tyrosine phosphatase) (SH-PTP1) (Protein-tyrosine phosphatase SHP-1); Protein tyrosine phosphatase, non-receptor type 6 isoform 1 variant (Fragment)	GO:0007186; GO:0006915; GO:0005829; GO:0016020; GO:0005634; GO:0006470; GO:0005515; GO:0004725; GO:0006470; GO:0004872	G-protein coupled receptor protein signaling pathway; apoptosis; cytosol; membrane; nucleus; protein amino acid dephosphorylation; protein binding; protein tyrosine phosphatase activity; protein amino acid dephosphorylation; receptor activity	CytoplasmNucleus;	Phophorylated - from this Study		Known in Uniprot to be phosphorylated	Known in HPRD to be phosphorylated	Phosphatase
**114**	SYTL4	SYTL4_HUMAN	SYTL4	Q96C24; Q5H9J3; Q5JPG8; Q8N9P4; Q9H4R0; Q9H4R1	NP_542775	Synaptotagmin-like protein 4 (Exophilin-2) (Granuphilin)	GO:0017137; GO:0006887; GO:0006886; GO:0042043; GO:0005543; GO:0008021; GO:0005215; GO:0008270	Rab GTPase binding; exocytosis; intracellular protein transport; neurexin binding; phospholipid binding; synaptic vesicle; transporter activity; zinc ion binding	MembranePeripheralmembraneproteinCellmembranePeripheralmembraneproteinCytoplasmicvesiclesecretoryvesiclemembranePeripheralmembraneprotein	Phophorylated - from this Study		Known in Uniprot to be phosphorylated		
**115**	PTN12	PTN12_HUMAN	PTN12; A4D1C5	Q05209; Q16130; Q59FD6; Q75MN8; Q86XU4; A4D1C5	NP_002826	Tyrosine-protein phosphatase non-receptor type 12 (EC 3.1.3.48) (Protein-tyrosine phosphatase G1) (PTPG1) (PTP-PEST)	GO:0017124; GO:0004726; GO:0006470; GO:0005625	SH3 domain binding; non-membrane spanning protein tyrosine phosphatase activity; protein amino acid dephosphorylation; soluble fraction	Cytoplasm	Phophorylated - from this Study		Known in Uniprot to be phosphorylated	Known in HPRD to be phosphorylated	Phosphatase
**116**	DREB	DREB_HUMAN	DREB	Q16643; B2RBG0; Q9UFZ5	NP_004386; NP_543157	Drebrin (Developmentally-regulated brain protein)	GO:0003779; GO:0007015; GO:0042641; GO:0005737; GO:0030425; GO:0005522; GO:0050773; GO:0048168	actin binding; actin filament organization; actomyosin; cytoplasm; dendrite; profilin binding; regulation of dendrite development; regulation of neuronal synaptic plasticity	Cytoplasm	Phophorylated - from this Study		Known in Uniprot to be phosphorylated	Known in HPRD to be phosphorylated	
**117**	DYR1A	DYR1A_HUMAN	DYR1A	Q13627; O60769; Q92582; Q92810; Q9UNM5	NP_001387; NP_567824; NP_569120; NP_569121; NP_569122	Dual specificity tyrosine-phosphorylation-regulated kinase 1A (EC 2.7.12.1) (Protein kinase minibrain homolog) (MNBH) (hMNB) (HP86) (Dual specificity YAK1-related kinase)	GO:0005524; GO:0007399; GO:0004715; GO:0016607; GO:0018108; GO:0046777; GO:0043621; GO:0004674	ATP binding; nervous system development; non-membrane spanning protein tyrosine kinase activity; nuclear speck; peptidyl-tyrosine phosphorylation; protein amino acid autophosphorylation; protein self-association; protein serine/threonine kinase activity	Nucleusspeckle	Phophorylated - from this Study		Known in Uniprot to be phosphorylated	Known in HPRD to be phosphorylated	Kinase
**118**	G6B	G6B_HUMAN	G6B; A2BEZ3; B0V026; A2BEZ2; B0V023	O95866; A2BEZ1; A2BEZ2; A2BEZ3; A2BEZ4; A2BEZ5; B0UXC8; Q14CK2; Q96A86; Q96A87; Q96A88; Q96A89; Q96A90; Q96A91; B0V026; B0V023	NP_612116; NP_079536; NP_612117; NP_612118; NP_612119; NP_612121	Protein G6b	GO:0005794; GO:0005783; GO:0008201; GO:0016021; GO:0005886; GO:0004872	Golgi apparatus; endoplasmic reticulum; heparin binding; integral to membrane; plasma membrane; receptor activity	EndoplasmicreticulumGolgiapparatusEndoplasmicreticulumGolgiapparatusCellmembraneSinglepasstypeImembraneproteinCellmembraneSinglepasstypeImembraneprotein	Phophorylated - from this Study	Membrane-Fraction - this study	Known in Uniprot to be phosphorylated	Known in HPRD to be phosphorylated	
**119**	F10A1	F10A1_HUMAN	F10A1; Q2TU77	P50502; Q2TU77; O14999	NP_003923; XP_015334	Hsc70-interacting protein (Hip) (Suppression of tumorigenicity protein 13) (Putative tumor suppressor ST13) (Protein FAM10A1) (Progesterone receptor-associated p48 protein) (Renal carcinoma antigen NY-REN-33); Aging-associated protein 2 (Suppression of tumorigenicity 13 (Colon carcinoma) (Hsp70 interacting protein)) (Suppression of tumorigenicity 13 (Colon carcinoma) (Hsp70 interacting protein), isoform CRA_a)	GO:0005737; GO:0030674; GO:0006457; GO:0005488	cytoplasm; protein binding, bridging; protein folding; binding	Cytoplasm;	Phophorylated - from this Study		Known in Uniprot to be phosphorylated		
**120**	AAKB1	AAKB1_HUMAN	AAKB1	Q9Y478; Q9UBV0; Q9UE20; Q9UEX2; Q9Y6V8	NP_006244	5'-AMP-activated protein kinase subunit beta-1 (AMPK beta-1 chain) (AMPKb)	GO:0005737; GO:0006633; GO:0005634; GO:0005515; GO:0007165	cytoplasm; fatty acid biosynthetic process; nucleus; protein binding; signal transduction		Phophorylated - from this Study		Known in Uniprot to be phosphorylated	Known in HPRD to be phosphorylated	
**121**	GA2L1	GA2L1_HUMAN	GA2L1; A0A5E8	Q99501; Q53EN7; Q92640; Q9BUY9; A0A5E8	NP_006469; NP_689423; NP_689422	GAS2-like protein 1 (Growth arrest-specific protein 2-like 1) (GAS2-related protein on chromosome 22); cDNA FLJ16435 fis, clone BRACE3020671, highly similar to GAS2-like protein 1 (cDNA FLJ36067 fis, clone TESTI2019308, highly similar to GAR22 PROTEIN) (Growth arrest-specific 2 like 1, isoform CRA_a)	GO:0007050; GO:0005737; GO:0005856	cell cycle arrest; cytoplasm; cytoskeleton	Cytoplasmcytoskeleton;	Phophorylated - from this Study		Known in Uniprot to be phosphorylated		
**122**	M6PBP	M6PBP_HUMAN	M6PBP	O60664; A8K4Y9; Q53G77; Q9BS03; Q9UBD7; Q9UP92	NP_005808	Mannose-6-phosphate receptor-binding protein 1 (Cargo selection protein TIP47) (47 kDa mannose 6-phosphate receptor-binding protein) (47 kDa MPR-binding protein) (Placental protein 17) (PP17)	GO:0005794; GO:0010008; GO:0034430; GO:0016192	Golgi apparatus; endosome membrane; monolayer-surrounded lipid storage body outer lipid monolayer; vesicle-mediated transport	CytoplasmEndosomemembranePeripheralmembraneproteinCytoplasmicsideLipiddropletmembranePeripheralmembraneproteinCytoplasmicside	Phophorylated - from this Study		Known in Uniprot to be phosphorylated		

	Protein Name	Uniprot Name	Other Names	Uniprot IDs	Refseq IDs	Protein Description	GO ID	GO terms	Subcellular location	Comments	Comments	Comments - Database Search	Comments - Database Search	Function
**123**	HS90B	HS90B_HUMAN	HS90B; B2R5P0	P08238; B2R5P0; Q5T9W7; Q9NQW0; Q9NTK6	NP_031381; XP_293672; XP_292964	Heat shock protein HSP 90-beta (HSP 90) (HSP 84)	GO:0005524; GO:0030911; GO:0042470; GO:0032435; GO:0030235; GO:0045429; GO:0006457; GO:0060334; GO:0060338; GO:0006986; GO:0051082	ATP binding; TPR domain binding; melanosome; negative regulation of proteasomal ubiquitin-dependent protein catabolic process; nitric-oxide synthase regulator activity; positive regulation of nitric oxide biosynthetic process; protein folding; regulation of interferon-gamma-mediated signaling pathway; regulation of type I interferon-mediated signaling pathway; response to unfolded protein; unfolded protein binding	CytoplasmMelanosome	Phophorylated - from this Study		Known in Uniprot to be phosphorylated	Known in HPRD to be phosphorylated	
**124**	NSF1C	NSF1C_HUMAN	NSF1C; B2RD74	Q9UNZ2; Q5JXA5; Q7Z533; Q9H102; Q9NVL9; Q9UI06; B2RD74	NP_057227; NP_061327; NP_872289	NSFL1 cofactor p47 (p97 cofactor p47) (UBX domain-containing protein 2C)	GO:0005794; GO:0008289; GO:0005634; GO:0005515	Golgi apparatus; lipid binding; nucleus; protein binding	NucleusGolgiapparatus Golgistack	Phophorylated - from this Study		Known in Uniprot to be phosphorylated	Known in HPRD to be phosphorylated	
**125**	Q96H99	Q96H99_HUMAN	Q96H99	Q96H99	NP_612632	Cortactin (cDNA FLJ77423, highly similar to Homo sapiens cortactin (CTTN), transcript variant 2, mRNA) (Cortactin, isoform CRA_c)				Phophorylated - from this Study				
**126**	STIM1	STIM1_HUMAN	STIM1; Q8N382	Q8N382; Q13586	NP_003147	Stromal interaction molecule 1	GO:0032237; GO:0005509; GO:0005513; GO:0030176; GO:0005887; GO:0005515	activation of store-operated calcium channel activity; calcium ion binding; detection of calcium ion; integral to endoplasmic reticulum membrane; integral to plasma membrane; protein binding	CellmembraneSinglepasstypeImembraneproteinEndoplasmicreticulummembraneSinglepasstypeImembraneprotein	Phophorylated - from this Study		Known in Uniprot to be phosphorylated		
**127**	AMPD2	AMPD2_HUMAN	AMPD2	Q01433; Q14856; Q14857; Q16686; Q16687; Q16688; Q16729; Q96IA1; Q9UDX8; Q9UDX9; Q9UMU4	NP_004028; NP_631895; NP_981949	AMP deaminase 2 (EC 3.5.4.6) (AMP deaminase isoform L)	GO:0003876; GO:0006144; GO:0009168	AMP deaminase activity; purine base metabolic process; purine ribonucleoside monophosphate biosynthetic process		Phophorylated - from this Study		Known in Uniprot to be phosphorylated		
**128**	TBB4	TBB4_HUMAN	TBB4; B3KQP4	P04350; Q969E5; B3KQP4	NP_006078	Tubulin beta-4 chain (Tubulin 5 beta)	GO:0005525; GO:0003924; GO:0005874; GO:0007018; GO:0043234; GO:0051258; GO:0005198	GTP binding; GTPase activity; microtubule; microtubule-based movement; protein complex; protein polymerization; structural molecule activity		Phophorylated - from this Study		Known in Uniprot to be phosphorylated	Known in HPRD to be phosphorylated	
**129**	GSK3B	GSK3B_HUMAN	GSK3B	P49841; Q9BWH3; Q9UL47	NP_002084; NP_001139628	Glycogen synthase kinase-3 beta (GSK-3 beta) (EC 2.7.11.26)	GO:0005524; GO:0034747; GO:0006983; GO:0051059; GO:0060070; GO:0008013; GO:0030877; GO:0005829; GO:0005977; GO:0004696; GO:0005634; GO:0002039; GO:0018105; GO:0031334; GO:0046827; GO:0034236; GO:0050321	ATP binding; Axin-APC-beta-catenin-GSK3B complex; ER overload response; NF-kappaB binding; Wnt receptor signaling pathway through beta-catenin; beta-catenin binding; beta-catenin destruction complex; cytosol; glycogen metabolic process; glycogen synthase kinase 3 activity; nucleus; p53 binding; peptidyl-serine phosphorylation; positive regulation of protein complex assembly; positive regulation of protein export from nucleus; protein kinase A catalytic subunit binding; tau-protein kinase activity		Phophorylated - from this Study		Known in Uniprot to be phosphorylated	Known in HPRD to be phosphorylated	Kinase
**130**	PARD3	PARD3_HUMAN	PARD3; Q5VWV1	Q8TEW0; Q5VWV1; Q8TEW1; Q8TEW2; Q8TEW3; Q96K28; Q96RM6; Q96RM7; Q9BY57; Q9BY58; Q9HC48; Q9NWL4; Q9NYE6	NP_062565	Partitioning defective 3 homolog (PARD-3) (PAR-3) (Atypical PKC isotype-specific-interacting protein) (ASIP) (CTCL tumor antigen se2-5) (PAR3-alpha); Par-3 partitioning defective 3 homolog (C. elegans) (Par-3 partitioning defective 3 homolog (C. elegans), isoform CRA_c)	GO:0007205; GO:0008356; GO:0007409; GO:0007049; GO:0005737; GO:0012505; GO:0007163; GO:0005515; GO:0006461; GO:0005923; GO:0005515	activation of protein kinase C activity by G protein coupled receptor protein signaling pathway; asymmetric cell division; axonogenesis; cell cycle; cytoplasm; endomembrane system; establishment or maintenance of cell polarity; protein binding; protein complex assembly; tight junction; protein binding	IntracytoplasmicmembraneCelljunctionCelljunctiontightjunctionCellmembraneCytoplasmcell cortex;	Phophorylated - from this Study		Known in Uniprot to be phosphorylated	Known in HPRD to be phosphorylated	
**131**	NEXN	NEXN_HUMAN	Q96DL0; Q14CC3; NEXN	Q96DL0; Q0ZGT2; A0PJ84; Q0D2H2; Q14CC2; Q14CC3; Q16081; Q7Z2X0; Q9Y2V1	NP_653174	Nexilin (F-actin binding protein) (Nelin)	GO:0051015; GO:0005924; GO:0005737; GO:0005856; GO:0030334; GO:0051493	actin filament binding; cell-substrate adherens junction; cytoplasm; cytoskeleton; regulation of cell migration; regulation of cytoskeleton organization	Cytoplasmcytoskeleton Celljunctionadherensjunction	Phophorylated - from this Study		Known in Uniprot to be phosphorylated		
**132**	APOL1	APOL1_HUMAN	APOL1; Q2KHQ6	O14791; A5PLQ4; O60804; Q5R3P7; Q5R3P8; Q96AB8; Q96PM4; Q9BQ03; Q2KHQ6	NP_003652; NP_001130012	Apolipoprotein L1 (Apolipoprotein L-I) (ApoL-I) (Apolipoprotein L) (Apo-L) (ApoL)	GO:0005254; GO:0006821; GO:0008203; GO:0019835; GO:0034364; GO:0045087; GO:0031224; GO:0031640; GO:0008289; GO:0006869; GO:0042157; GO:0005515	chloride channel activity; chloride transport; cholesterol metabolic process; cytolysis; high-density lipoprotein particle; innate immune response; intrinsic to membrane; killing of cells of another organism; lipid binding; lipid transport; lipoprotein metabolic process; protein binding	Secreted	Phophorylated - from this Study				
**133**	MTPN	MTPN_HUMAN	MTPN; Q69YG1	P58546; Q69YG1	NP_665807; NP_001122091	Myotrophin (Protein V-1); Myotrophin, isoform CRA_a (V-1 protein) (cDNA FLJ31663 fis, clone NT2RI2004504, highly similar to MYOTROPHIN) (Myotrophin) (Putative uncharacterized protein DKFZp761E1322)	GO:0016049; GO:0005737; GO:0005515; GO:0016202; GO:0006417; GO:0030424; GO:0006584; GO:0021707; GO:0043403; GO:0051146	cell growth; cytoplasm; protein binding; regulation of striated muscle development; regulation of translation; axon; catecholamine metabolic process; cerebellar granule cell differentiation; skeletal muscle regeneration; striated muscle cell differentiation	Cytoplasm;	Phophorylated - from this Study				
**134**	ARHG6	ARHG6_HUMAN	ARHG6; Q8N4Q3	Q15052; A6NMW9; A8K6S7; Q15396; Q5JQ66; Q7Z3W1; Q86XH0; Q8N4Q3	NP_004831	Rho guanine nucleotide exchange factor 6 (Rac/Cdc42 guanine nucleotide exchange factor 6) (PAK-interacting exchange factor alpha) (Alpha-Pix) (COOL-2); Similar to Rac/Cdc42 guanine nucleotide exchange factor (GEF) 6 (Fragment)	GO:0005096; GO:0007254; GO:0005089; GO:0006915; GO:0005737; GO:0035023;	GTPase activator activity; JNK cascade; Rho guanyl-nucleotide exchange factor activity; apoptosis; cytoplasm; regulation of Rho protein signal transduction;		Phophorylated - from this Study		Known in Uniprot to be phosphorylated	Known in HPRD to be phosphorylated	
**135**	TAC2N	TAC2N_HUMAN	TAC2N	Q8N9U0	NP_689545; NP_001122067; NP_001122068	Tandem C2 domains nuclear protein (Membrane targeting tandem C2 domain-containing protein 1) (Tandem C2 protein in nucleus) (Tac2-N)	GO:0005634	nucleus	Nucleus	Phophorylated - from this Study		Known in Uniprot to be phosphorylated		
**136**	TEBP	TEBP_HUMAN	TEBP; A8K7D0	Q15185; A8K7D0; Q8WU70	NP_006592; XP_208234	Prostaglandin E synthase 3 (EC 5.3.99.3) (Cytosolic prostaglandin E2 synthase) (cPGES) (Telomerase-binding protein p23) (Hsp90 co-chaperone) (Progesterone receptor complex p23)	GO:0000781; GO:0005737; GO:0001516; GO:0050220; GO:0007165; GO:0003720; GO:0005697; GO:0000723; GO:0051082	chromosome, telomeric region; cytoplasm; prostaglandin biosynthetic process; prostaglandin-E synthase activity; signal transduction; telomerase activity; telomerase holoenzyme complex; telomere maintenance; unfolded protein binding	Cytoplasm	Phophorylated - from this Study		Known in Uniprot to be phosphorylated	Known in HPRD to be phosphorylated	
**137**	PA24A	PA24A_HUMAN	PA24A	P47712; B1AKG4; Q29R80	NP_077734	Cytosolic phospholipase A2 (cPLA2) (Phospholipase A2 group IVA) [Includes: Phospholipase A2 (EC 3.1.1.4) (Phosphatidylcholine 2-acylhydrolase); Lysophospholipase (EC 3.1.1.5)]	GO:0005509; GO:0031410; GO:0005829; GO:0006690; GO:0016021; GO:0004622; GO:0004623; GO:0009395; GO:0006663	calcium ion binding; cytoplasmic vesicle; cytosol; icosanoid metabolic process; integral to membrane; lysophospholipase activity; phospholipase A2 activity; phospholipid catabolic process; platelet activating factor biosynthetic process	CytoplasmCytoplasmic vesicle	Phophorylated - from this Study		Known in Uniprot to be phosphorylated	Known in HPRD to be phosphorylated	
**138**	LRC47	LRC47_HUMAN	LRC47	Q8N1G4; Q9ULN5	NP_065761	Leucine-rich repeat-containing protein 47	GO:0003723; GO:0004826; GO:0005515; GO:0006412	RNA binding; phenylalanine-tRNA ligase activity; protein binding; translation		Phophorylated - from this Study		Known in Uniprot to be phosphorylated		
**139**	AT2A2	AT2A2_HUMAN	AT2A2	P16615; P16614	NP_733765; NP_001672; NP_001129237	Sarcoplasmic/endoplasmic reticulum calcium ATPase 2 (SERCA2) (EC 3.6.3.8) (Calcium pump 2) (Calcium-transporting ATPase sarcoplasmic reticulum type, slow twitch skeletal muscle isoform) (SR Ca(2+)-ATPase 2) (Endoplasmic reticulum class 1/2 Ca(2+) ATPase)	GO:0005524; GO:0006754; GO:0048155; GO:0005509; GO:0005388; GO:0007155; GO:0008544; GO:0005887; GO:0000287; GO:0005792; GO:0070296; GO:0033017	ATP binding; ATP biosynthetic process; S100 alpha binding; calcium ion binding; calcium-transporting ATPase activity; cell adhesion; epidermis development; integral to plasma membrane; magnesium ion binding; microsome; sarcoplasmic reticulum calcium ion transport; sarcoplasmic reticulum membrane	EndoplasmicreticulummembraneMultipassmembraneproteinSarcoplasmicreticulummembraneMultipassmembraneprotein	Phophorylated - from this Study	Membrane-Fraction - this study	Known in Uniprot to be phosphorylated	Known in HPRD to be phosphorylated	
**140**	GRP2	GRP2_HUMAN	Q7LDG7; Q9UL65; GRP2	Q7LDG7; Q9UL65; O00538	NP_722541; NP_005816; NP_001092140; NP_001092141	RAS guanyl-releasing protein 2 (Calcium and DAG-regulated guanine nucleotide exchange factor I) (CalDAG-GEFI) (F25B3.3 kinase-like protein) (Cdc25-like protein) (HCDC25L)	GO:0007265; GO:0005509; GO:0030054; GO:0042995; GO:0005829; GO:0019992; GO:0005085; GO:0001558; GO:0051056; GO:0045202; GO:0019717; GO:0008270	Ras protein signal transduction; calcium ion binding; cell junction; cell projection; cytosol; diacylglycerol binding; guanyl-nucleotide exchange factor activity; regulation of cell growth; regulation of small GTPase mediated signal transduction; synapse; synaptosome; zinc ion binding	CytoplasmcytosolCellmembranePeripheralmembraneproteinCelljunctionsynapsesynaptosomeCellprojectionrufflemembranePeripheralmembraneprotein	Phophorylated - from this Study		Known in Uniprot to be phosphorylated		
**141**	ABCA5	ABCA5_HUMAN	ABCA5	Q8WWZ7; Q8IVJ2; Q96LJ1; Q96MS4; Q96PZ9; Q9NY14	NP_061142; NP_758424	ATP-binding cassette sub-family A member 5	GO:0005524; GO:0016887; GO:0000139; GO:0016021; GO:0031902; GO:0005765; GO:0006810	ATP binding; ATPase activity; Golgi membrane; integral to membrane; late endosome membrane; lysosomal membrane; transport	LysosomemembraneMultipassmembraneproteinLateendosomemembraneMultipassmembraneproteinGolgiapparatusmembraneMultipassmembraneprotein	Phophorylated - from this Study				

	Protein Name	Uniprot Name	Other Names	Uniprot IDs	Refseq IDs	Protein Description	GO ID	GO terms	Subcellular location	Comments	Comments	Comments - Database Search	Comments - Database Search	Function
**142**	SPAG1	SPAG1_HUMAN	SPAG1; A6NP70	Q07617; Q7Z5G1; A6NP70	NP_757367; NP_003105	Sperm-associated antigen 1 (Infertility-related sperm protein Spag-1) (HSD-3.8)	GO:0005525; GO:0005737; GO:0016787; GO:0007338	GTP binding; cytoplasm; hydrolase activity; single fertilization	Cytoplasm	Phophorylated - from this Study		Known in Uniprot to be phosphorylated	Known in HPRD to be phosphorylated	
**143**	SL9A9	SL9A9_HUMAN	SL9A9; Q5WA58	Q8IVB4; Q5WA58; A6NMQ9; Q3LIC2; Q5JPI6; Q8NAB9	NP_775924	Sodium/hydrogen exchanger 9 (Na(+)/H(+) exchanger 9) (NHE-9) (Solute carrier family 9 member 9)	GO:0016021; GO:0031902; GO:0055037; GO:0006885; GO:0031402; GO:0006814; GO:0015385	integral to membrane; late endosome membrane; recycling endosome; regulation of pH; sodium ion binding; sodium ion transport; sodium:hydrogen antiporter activity	LateendosomemembraneMultipassmembrane protein	Phophorylated - from this Study				
**144**	SL9A1	SL9A1_HUMAN	SL9A1; Q5VU07; B1ALD6; B2RAH2	P19634; Q5VU07; Q96EM2; B1ALD6; B2RAH2	NP_003038	Sodium/hydrogen exchanger 1 (Na(+)/H(+) exchanger 1) (NHE-1) (Solute carrier family 9 member 1) (Na(+)/H(+) antiporter, amiloride-sensitive) (APNH)	GO:0016021; GO:0006885; GO:0031402; GO:0006814; GO:0015385	integral to membrane; regulation of pH; sodium ion binding; sodium ion transport; sodium:hydrogen antiporter activity	MembraneMultipassmembraneprotein	Phophorylated - from this Study		Known in Uniprot to be phosphorylated	Known in HPRD to be phosphorylated	
**145**	MY18A	MY18A_HUMAN	MY18A	Q92614; Q5H9U3; Q5W9F9; Q5W9G1; Q8IXP8	NP_510880; NP_976063	Myosin-XVIIIa (Myosin containing a PDZ domain) (Molecule associated with JAK3 N-terminus) (MAJN)	GO:0005524; GO:0005793; GO:0006916; GO:0042802; GO:0003774; GO:0016459	ATP binding; ER-Golgi intermediate compartment; anti-apoptosis; identical protein binding; motor activity; myosin complex	EndoplasmicreticulumGolgiintermediatecompartmentCytoplasmcytoskeletonCytoplasm	Phophorylated - from this Study		Known in Uniprot to be phosphorylated	Known in HPRD to be phosphorylated	
**146**	SAM14	SAM14_HUMAN	SAM14	Q8IZD0; Q8N2X0	NP_777580	Sterile alpha motif domain-containing protein 14				Phophorylated - from this Study		Known in Uniprot to be phosphorylated		
**147**	Q5JV32	Q5JV32_HUMAN	Q5JV32; B1ALI0	Q5JV32; B1ALI0	NP_803171					Phophorylated - from this Study				
**148**	RICS	RICS_HUMAN	Q86YL6; RICS	Q86YL6; A7KAX9; O94820; Q8IUG4; Q9BWG3	NP_055530; NP_001136 157	Rho/Cdc42/Rac GTPase-activating protein RICS (p200RhoGAP) (p250GAP) (RhoGAP involved in the beta-catenin-N-cadherin and NMDA receptor signaling) (Brain-specific Rho GTPase-activating protein) (GAB-associated Cdc42/Rac GTPase-activating protein) (GC-GAP) (GTPase regulator interacting with TrkA)	GO:0005096; GO:0000139; GO:0030054; GO:0042995; GO:0005789; GO:0010008; GO:0035091; GO:0045211; GO:0005515; GO:0007165	GTPase activator activity; Golgi membrane; cell junction; cell projection; endoplasmic reticulum membrane; endosome membrane; phosphoinositide binding; postsynaptic membrane; protein binding; signal transduction	CelljunctionsynapsepostsynapticcellmembranepostsynapticdensityCellprojectiondendriticspineCytoplasmcellcortexEndosomemembraneGolgiapparatusmembraneEndoplasmicreticulum membraneMembrane	Phophorylated - from this Study		Known in Uniprot to be phosphorylated		
**149**	TPC1	TPC1_HUMAN	TPC1	Q86XS9; Q9ULQ1; A7E258; Q8NC20	NP_060371; NP_001137 291	Two pore calcium channel protein 1 (Voltage-dependent calcium channel protein TPC1)	GO:0005262; GO:0005509; GO:0006816; GO:0016021; GO:0005244	calcium channel activity; calcium ion binding; calcium ion transport; integral to membrane; voltage-gated ion channel activity	MembraneMultipassmembraneprotein	Phophorylated - from this Study				
**150**	TBB5	TBB5_HUMAN	TBB5; Q5SU16	P07437; Q5SU16; P05218; Q8WUC1; Q9CY33	NP_821133; XP_114617; XP_935073; XP_212565; XP_087939	Tubulin beta chain (Tubulin beta-5 chain); Tubulin beta polypeptide (Tubulin, beta) (Beta 5-tubulin) (Beta-tubulin protein) (Tubulin, beta, isoform CRA_a)	GO:0005525; GO:0003924; GO:0042288; GO:0006928; GO:0005874; GO:0007018; GO:0042267; GO:0051258; GO:0005737; GO:0016600; GO:0043234; GO:0051225; GO:0005200; GO:0005198	GTP binding; GTPase activity; MHC class I protein binding; cell motion; microtubule; microtubule-based movement; natural killer cell mediated cytotoxicity; protein polymerization; cytoplasm; flotillin complex; protein complex; spindle assembly; structural constituent of cytoskeleton; structural molecule activity		Phophorylated - from this Study		Known in Uniprot to be phosphorylated		
**151**	DTD1	DTD1_HUMAN	DTD1; A8K5X5	Q8TEA8; Q496D1; Q5W184; Q8WXU8; Q9BW67; Q9H464; Q9H474; A8K5X5	NP_543010	D-tyrosyl-tRNA(Tyr) deacylase 1 (EC 3.1.-.-) (Histidyl-tRNA synthetase-related)	GO:0019478; GO:0005737; GO:0016788	D-amino acid catabolic process; cytoplasm; hydrolase activity, acting on ester bonds	Cytoplasm	Phophorylated - from this Study		Known in Uniprot to be phosphorylated		
**152**	PGM2L	PGM2L_HUMAN	PGM2L	Q6PCE3; Q96MQ7; Q9UIK3	NP_775853	Glucose 1,6-bisphosphate synthase (EC 2.7.1.106) (Phosphoglucomutase-2-like 1) (PMMLP)	GO:0006006; GO:0047933; GO:0016868	glucose metabolic process; glucose-1,6-bisphosphate synthase activity; intramolecular transferase activity, phosphotransferases		Phophorylated - from this Study		Known in Uniprot to be phosphorylated		
**153**	TXND1	TXND1_HUMAN	TXND1; B2R7A4	Q9H3N1; Q8N487; Q8NBN5; Q9Y4T6; B2R7A4	NP_110382	Thioredoxin domain-containing protein 1 (Transmembrane Trx-related protein) (Thioredoxin-related transmembrane protein)	GO:0006260; GO:0006888; GO:0006916; GO:0030612; GO:0008283; GO:0045454; GO:0015036; GO:0022900; GO:0005789; GO:0016021; GO:0045321; GO:0005624; GO:0005634; GO:0045927; GO:0045893; GO:0006950; GO:0007165	DNA replication; ER to Golgi vesicle-mediated transport; anti-apoptosis; arsenate reductase (thioredoxin) activity; cell proliferation; cell redox homeostasis; disulfide oxidoreductase activity; electron transport chain; endoplasmic reticulum membrane; integral to membrane; leukocyte activation; membrane fraction; nucleus; positive regulation of growth; positive regulation of transcription, DNA-dependent; response to stress; signal transduction	MembraneSinglepasstypeImembraneproteinEndoplasmicreticulummembraneSinglepasstypeImembraneprotein	Phophorylated - from this Study		Known in Uniprot to be phosphorylated	Known in HPRD to be phosphorylated	
**154**	FA63A	FA63A_HUMAN	Q9P2F7; Q8N5J2; FA63A	Q8N5J2; Q9P2F7; B3KWP4; B3KWV8; B4DXF2; B4E1S4; Q5SZF0; Q9NUL9	NP_060849	Protein FAM63A	GO:0005515	protein binding		Phophorylated - from this Study		Known in Uniprot to be phosphorylated		
**155**	TMM40	TMM40_HUMAN	TMM40	Q8NAL4; Q8WWA1; Q9NUZ4	NP_060776	Transmembrane protein 40	GO:0016021	integral to membrane	MembraneMultipassmembraneprotein	Phophorylated - from this Study				
**156**	ANLN	ANLN_HUMAN	ANLN	Q9NQW6; Q5CZ78; Q6NSK5; Q9H8Y4; Q9NVN9; Q9NVP0	NP_061155	Actin-binding protein anillin	GO:0003779; GO:0005826; GO:0000910; GO:0005634; GO:0007096; GO:0000921	actin binding; contractile ring; cytokinesis; nucleus; regulation of exit from mitosis; septin ring assembly	NucleusCytoplasmcytoskeletonCytoplasmcellcortex	Phophorylated - from this Study		Known in Uniprot to be phosphorylated		
**157**	CFP1	CFP1_HUMAN	CFP1; CCNY	Q8ND76; Q2M3V4; Q2TU96; Q6NT86; Q7Z4U7; Q8TEX2; Q8TEX3; Q96M99; Q96P45	NP_659449; NP_859049	Cyclin-Y (Cyclin fold protein 1) (Cyclin box protein 1)	GO:0005634	nucleus		Phophorylated - from this Study		Known in Uniprot to be phosphorylated	Known in HPRD to be phosphorylated	
**158**	BET1L	BET1L_HUMAN	BET1L; A6NI60	Q9NYM9; B3KMY0; A6NI60	NP_057610; NP_001092 257	BET1-like protein (Vesicle transport protein GOS15) (Golgi SNARE with a size of 15 kDa) (GOS-15) (GS15)	GO:0000139; GO:0005484; GO:0031201; GO:0005768; GO:0016021; GO:0015031; GO:0042147	Golgi membrane; SNAP receptor activity; SNARE complex; endosome; integral to membrane; protein transport; retrograde transport, endosome to Golgi	GolgiapparatusmembraneSinglepasstypeIVmembraneprotein	Phophorylated - from this Study		Known in Uniprot to be phosphorylated		
**159**	PAK2	PAK2_HUMAN	PAK2; A8K5M4	Q13177; A8K5M4; Q13154; Q6ISC3	NP_002568; XP_0011261 10	Serine/threonine-protein kinase PAK 2 (EC 2.7.11.1) (p21-activated kinase 2) (PAK-2) (Gamma-PAK) (PAK65) (S6/H4 kinase) (p58) [Cleaved into: PAK-2p27 (p27); PAK-2p34 (p34) (C-t-PAK2)]	GO:0005524; GO:0006915; GO:0005829; GO:0042802; GO:0044419; GO:0016020; GO:0006469; GO:0005634; GO:0046777; GO:0004674; GO:0040008; GO:0007165	ATP binding; apoptosis; cytosol; identical protein binding; interspecies interaction between organisms; membrane; negative regulation of protein kinase activity; nucleus; protein amino acid autophosphorylation; protein serine/threonine kinase activity; regulation of growth; signal transduction	CytoplasmNucleusCytoplasmperinuclearregionMembraneLipidanchor	Phophorylated - from this Study		Known in Uniprot to be phosphorylated	Known in HPRD to be phosphorylated	Kinase
**160**	FGD3	FGD3_HUMAN	FGD3	Q4VX85; Q5JSP0; Q4VX84; Q7Z7D9; Q8N5G1	NP_149077; NP_001077 005	FYVE, RhoGEF and PH domain-containing protein 3 (Zinc finger FYVE domain-containing protein 5)	GO:0005794; GO:0005089; GO:0030036; GO:0005856; GO:0046847; GO:0030027; GO:0043088; GO:0008360; GO:0001726; GO:0031267; GO:0008270	Golgi apparatus; Rho guanyl-nucleotide exchange factor activity; actin cytoskeleton organization; cytoskeleton; filopodium assembly; lamellipodium; regulation of Cdc42 GTPase activity; regulation of cell shape; ruffle; small GTPase binding; zinc ion binding	CytoplasmCytoplasmcytoskeleton	Phophorylated - from this Study		Known in Uniprot to be phosphorylated		
**161**	SKA2	SKA2_HUMAN	SKA2	Q8WVK7; A6NIL3; B3KPL3	NP_872426	Spindle and kinetochore-associated protein 2 (Protein FAM33A)	GO:0051301; GO:0000777; GO:0007067; GO:0005819	cell division; condensed chromosome kinetochore; mitosis; spindle	SpindleKinetochore	Phophorylated - from this Study		Known in Uniprot to be phosphorylated		
**162**	PKHO2	PKHO2_HUMAN	Q8TD55; PKHO2	Q8TD55; Q7L4H4; O8WYS8	NP_079477	Pleckstrin homology domain-containing family O member 2 (Pleckstrin homology domain-containing family O member 1)				Phophorylated - from this Study		Known in Uniprot to be phosphorylated		
**163**	SREC	SREC_HUMAN	SREC; Q8NHD2; A8MQ05	Q14162; O43701; A8MQ05; Q8NHD2	NP_663327; NP_003684	Endothelial cells scavenger receptor (Acetyl LDL receptor) (Scavenger receptor class F member 1); SREC-5	GO:0007155; GO:0006707; GO:0016021; GO:0030169; GO:0006898; GO:0005044; GO:0016358; GO:0005515	cell adhesion; cholesterol catabolic process; integral to membrane; low-density lipoprotein binding; receptor-mediated endocytosis; scavenger receptor activity; dendrite development; protein binding	MembraneSinglepasstypeImembraneprotein;	Phophorylated - from this Study		Known in Uniprot to be phosphorylated		
**164**	UBE2O	UBE2O_HUMAN	UBE2O	Q9C0C9; A6NDU5; Q69YP4; Q6PIZ2; Q86UA4; Q8N425; Q8TBN1; Q9BSW1; Q9H6E6; Q9H7E4; Q9H9B2	NP_071349	Ubiquitin-conjugating enzyme E2 O (EC 6.3.2.19) (Ubiquitin-protein ligase O) (Ubiquitin carrier protein O) (Ubiquitin-conjugating enzyme E2 of 230 kDa) (E2-230K)	GO:0019941; GO:0043687; GO:0051246; GO:0004842	modification-dependent protein catabolic process; post-translational protein modification; regulation of protein metabolic process; ubiquitin-protein ligase activity		Phophorylated - from this Study		Known in Uniprot to be phosphorylated		

	Protein Name	Uniprot Name	Other Names	Uniprot IDs	Refseq IDs	Protein Description	GO ID	GO terms	Subcellular location	Comments	Comments	Comments - Database Search	Comments - Database Search	Function
**165**	RAB7	RAB7_HUMAN	RAB7; RAB7A	P51149; A8K3V6; Q9NWJ0; Q9UPB0	NP_004628	Ras-related protein Rab-7a	GO:0005525; GO:0003924; GO:0005794; GO:0006897; GO:0005770; GO:0005764; GO:0042470; GO:0005515; GO:0015031; GO:0007264	GTP binding; GTPase activity; Golgi apparatus; endocytosis; late endosome; lysosome; melanosome; protein binding; protein transport; small GTPase mediated signal transduction	LateendosomeLysosomeCytoplasmicvesiclephagosomeMelanosome	Phophorylated - from this Study		Known in Uniprot to be phosphorylated		
**166**	GPSM1	GPSM1_HUMAN	GPSM1	Q86YR5; A9Z1X4; B1B0W3; Q86SR5; Q969T1; Q9UFS8	NP_056412; NP_001139110; NP_001139111	G-protein-signaling modulator 1 (Activator of G-protein signaling 3)	GO:0005096; GO:0000139; GO:0005488; GO:0030154; GO:0005789; GO:0007399; GO:0005886; GO:0007165	GTPase activator activity; Golgi membrane; binding; cell differentiation; endoplasmic reticulum membrane; nervous system development; plasma membrane; signal transduction	CytoplasmcytosolEndoplasmicreticulummembranePeripheralmembraneproteinCytoplasmicsideGolgiapparatusmembranePeripheralmembraneproteinCytoplasmicsideCellmembranePeripheralmembraneprotein Cytoplasmicside			Known in Uniprot to be phosphorylated		
**167**	BCAT1	BCAT1_HUMAN	BCAT1	P54687; Q96MY9	NP_005495	Branched-chain-amino-acid aminotransferase, cytosolic (BCAT(c)) (EC 2.6.1.42) (Protein ECA39)	GO:0000082; GO:0009082; GO:0004084; GO:0008283; GO:0005737	G1/S transition of mitotic cell cycle; branched chain family amino acid biosynthetic process; branched-chain-amino-acid transaminase activity; cell proliferation; cytoplasm	Cytoplasm	Phophorylated - from this Study				
**168**	OSTF1	OSTF1_HUMAN	OSTF1	Q92882; Q5W126; Q96IJ4	NP_036515	Osteoclast-stimulating factor 1	GO:0005737; GO:0042802; GO:0001503; GO:0007165	cytoplasm; identical protein binding; ossification; signal transduction	Cytoplasm	Phophorylated - from this Study		Known in Uniprot to be phosphorylated		
**169**	MFSD6	MFSD6_HUMAN	Q86TH2; Q6ZSS7; MFSD6	Q6ZSS7; Q86TH2; Q9NXM3	NP_060164	Major facilitator superfamily domain-containing protein 6 (Macrophage MHC class I receptor 2 homolog)	GO:0016021	integral to membrane	MembraneMultipassmembraneprotein	Phophorylated - from this Study		Known in Uniprot to be phosphorylated		
**170**	MYCPP	MYCPP_HUMAN	MYCPP	Q7Z401; Q14655; Q86T77; Q8IVX2; Q8NB93	NP_005839	C-myc promoter-binding protein (DENN domain-containing protein 4A)	GO:0003677; GO:0005634; GO:0005515; GO:0006355	DNA binding; nucleus; protein binding; regulation of transcription, DNA-dependent	Nucleus	Phophorylated - from this Study		Known in Uniprot to be phosphorylated		
**171**	RHG18	RHG18_HUMAN	RHG18	Q8N392; Q58EZ3; Q6P679; Q6PJD7; Q96S64	NP_277050	Rho GTPase-activating protein 18 (Rho-type GTPase-activating protein 18) (MacGAP)	GO:0005096; GO:0005622; GO:0005515; GO:0007165	GTPase activator activity; intracellular; protein binding; signal transduction		Phophorylated - from this Study				
**172**	XRN1	XRN1_HUMAN	XRN1	Q8IZH2; Q4G0S3; Q68D88; Q6AI24; Q6MZS8; Q86WS7; Q8N8U4; Q9UF39	NP_061874; NP_001036069	5'-3' exoribonuclease 1 (EC 3.1.11.-) (Strand-exchange protein 1 homolog)	GO:0008409; GO:0003677; GO:0003723; GO:0005737; GO:0045786; GO:0006139; GO:0005515	5'-3' exonuclease activity; DNA binding; RNA binding; cytoplasm; negative regulation of cell cycle; nucleobase, nucleoside, nucleotide and nucleic acid metabolic process; protein binding	Cytoplasm	Phophorylated - from this Study		Known in Uniprot to be phosphorylated		
**173**	PXDC2	PXDC2_HUMAN	PXDC2	Q6UX71; Q96E59; Q96PD9; Q96SU9	NP_116201	Plexin domain-containing protein 2 (Tumor endothelial marker 7-related protein)	GO:0016021	integral to membrane	MembraneSinglepasstypeImembraneprotein	Phophorylated - from this Study				
**174**	FYB	FYB_HUMAN	FYB	O15117; O00359	NP_955367	FYN-binding protein (FYN-T-binding protein) (FYB-120/130) (p120/p130) (SLP-76-associated phosphoprotein) (SLAP-130) (Adhesion and degranulation promoting adaptor protein) (ADAP)	GO:0006607; GO:0005737; GO:0006955; GO:0005634; GO:0006468; GO:0005515; GO:0007243	NLS-bearing substrate import into nucleus; cytoplasm; immune response; nucleus; protein amino acid phosphorylation; protein binding; protein kinase cascade	CytoplasmNucleus	Phophorylated - from this Study		Known in Uniprot to be phosphorylated	Known in HPRD to be phosphorylated	
**175**	AFAP1	AFAP1_HUMAN	Q9HBY1; Q8N556; AFAP1	Q9HBY1; Q8N556; A8K442; Q59EY5	NP_940997; NP_001128119	Actin filament-associated protein 1 (110 kDa actin filament-associated protein) (AFAP-110)	GO:0003779; GO:0005737; GO:0005856	actin binding; cytoplasm; cytoskeleton	Cytoplasmcytoskeleton	Phophorylated - from this Study		Known in Uniprot to be phosphorylated	Known in HPRD to be phosphorylated	
**176**	SLAI2	SLAI2_HUMAN	Q9P270; SLAI2	Q9P270; A8K4P1; Q8N5R3	XP_044434; NP_065897	SLAIN motif-containing protein 2				Phophorylated - from this Study		Known in Uniprot to be phosphorylated		
**177**	SLK	SLK_HUMAN	SLK	Q9H2G2; O00211; Q6P1Z4; Q86WU7; Q86WW1; Q92603; Q9NQL0; Q9NQL1	NP_055535	STE20-like serine/threonine-protein kinase (STE20-like kinase) (hSLK) (EC 2.7.11.1) (STE20-related serine/threonine-protein kinase) (STE20-related kinase) (Serine/threonine-protein kinase 2) (CTCL tumor antigen se20-9)	GO:0005524; GO:0003677; GO:0006915; GO:0005737; GO:0004518; GO:0006289; GO:0006468; GO:0004674	ATP binding; DNA binding; apoptosis; cytoplasm; nuclease activity; nucleotide-excision repair; protein amino acid phosphorylation; protein serine/threonine kinase activity	Cytoplasm	Phophorylated - from this Study		Known in Uniprot to be phosphorylated		Kinase
**178**	URP2	URP2_HUMAN	URP2	Q86UX7; Q8IUA1; Q8N207; Q9BT48	NP_848537; NP_113659	Fermitin family homolog 3 (Unc-112-related protein 2) (Kindlin-3) (MIG2-like protein)	GO:0007155; GO:0005737; GO:0005886; GO:0005515	cell adhesion; cytoplasm; plasma membrane; protein binding	CytoplasmCellmembranePeripheralmembraneproteinCytoplasmicside	Phophorylated - from this Study	Membrane-Fraction - this study	Known in Uniprot to be phosphorylated		
**179**	PLEC1	PLEC1_HUMAN	PLEC1; Q6S383	Q6S376; Q15149; Q6S383; Q15148; Q16640; Q6S377; Q6S378; Q6S379; Q6S380; Q6S381; Q6S382	NP_958786; NP_000436; NP_958780; NP_958781; NP_958782; NP_958783; NP_958784; NP_958785	Plectin-1 (PLTN) (PCN) (Hemidesmosomal protein 1) (HD1)	GO:0003779; GO:0005737; GO:0005856; GO:0005886; GO:0008307	actin binding; cytoplasm; cytoskeleton; plasma membrane; structural constituent of muscle	Cytoplasmcytoskeleton	Phophorylated - from this Study		Known in Uniprot to be phosphorylated	Known in HPRD to be phosphorylated	
**180**	FBP1L	FBP1L_HUMAN	FBP1L	Q6R4Q4; Q5T0N5; Q5T0N6; Q6B097; Q6P653; Q9NXG1	NP_060207; NP_001020119	Formin-binding protein 1-like (Transducer of Cdc42 dependent actin assembly protein 1) (Toca-1)	GO:0031410; GO:0005856; GO:0006897; GO:0008289; GO:0005886	cytoplasmic vesicle; cytoskeleton; endocytosis; lipid binding; plasma membrane	CytoplasmCytoplasmcytoskeletonCytoplasmcellcortexCytoplasmicvesicleCellmembranePeripheralmembraneprotein Cytoplasmicside	Phophorylated - from this Study		Known in Uniprot to be phosphorylated		
**181**	ZO2	ZO2_HUMAN	ZO2; A2A3H9	Q9UDY2; A2A3H9; Q5VXL0; Q15883; Q8N756; Q8NI14; Q99839; Q9UDY0; Q9UDY1	NP_963923; NP_004808	Tight junction protein ZO-2 (Zonula occludens protein 2) (Zona occludens protein 2) (Tight junction protein 2); Tight junction protein 2 (Zona occludens 2)	GO:0005737; GO:0004385; GO:0005634; GO:0005515; GO:0005923	cytoplasm; guanylate kinase activity; nucleus; protein binding; tight junction	CelljunctionadherensjunctionCellmembranePeripheralmembraneproteinCytoplasmicsideCelljunctiontightjunctionNucleus;	Phophorylated - from this Study		Known in Uniprot to be phosphorylated	Known in HPRD to be phosphorylated	
**182**	TBB8	TBB8_HUMAN	Q8WZ78; TBB8	Q8WZ78; Q3ZCM7; Q5SQX9	NP_817124	Tubulin beta-8 chain	GO:0005525; GO:0003924; GO:0005874; GO:0007018; GO:0043234; GO:0051258; GO:0005198	GTP binding; GTPase activity; microtubule; microtubule-based movement; protein complex; protein polymerization; structural molecule activity		Phophorylated - from this Study				
**183**	TMM24	TMM24_HUMAN	TMM24; C2C2L	O14523; Q86UT7; Q86V04; Q8N522; Q8TBN4; Q96G10	NP_055622	C2 domain-containing protein 2-like (Transmembrane protein 24)	GO:0016021	integral to membrane	MembraneSinglepassmembraneprotein	Phophorylated - from this Study		Known in Uniprot to be phosphorylated		
**184**	LRMP	LRMP_HUMAN	LRMP	Q12912; A0AVM2; B4E077; Q8N301	NP_006143	Lymphoid-restricted membrane protein (Protein Jaw1) [Cleaved into: Processed lymphoid-restricted membrane protein]	GO:0005789; GO:0005887; GO:0005634; GO:0006906; GO:0006903	endoplasmic reticulum membrane; integral to plasma membrane; nucleus; vesicle fusion; vesicle targeting	CytoplasmEndoplasmicreticulummembraneSinglepasstypeIVmembraneprotein	Phophorylated - from this Study		Known in Uniprot to be phosphorylated		
**185**	WDR44	WDR44_HUMAN	WDR44	Q5JSH3; Q0JS52; Q0JTF3; Q5JSH2; Q6ZSC1; Q7Z365; Q7Z3P6; Q8NAU8; Q8NHU5; Q9NUV4	NP_061918	WD repeat-containing protein 44 (Rabphilin-11)	GO:0005794; GO:0010008	Golgi apparatus; endosome membrane	CytoplasmcytosolCytoplasmperinuclearregionEndosomemembraneGolgiapparatustransGolginetwork	Phophorylated - from this Study		Known in Uniprot to be phosphorylated	Known in HPRD to be phosphorylated	
**186**	DEN2C	DEN2C_HUMAN	DEN2C	Q68D51; B1AL26; Q5TCX6; Q6P3R3	NP_940861	DENN domain-containing protein 2C				Phophorylated - from this Study				
**187**	FA62B	FA62B_HUMAN	FA62B; A4D229; ESYT2	Q6UKI4; A0FGR8; A4D229; Q69YJ2; Q6ZTU0; Q6ZVU1; Q9BQS0; Q9NW47; Q9ULJ2	NP_065779; XP_036408	Extended synaptotagmin-2 (E-Syt2) (Chr2Syt) (Protein FAM62B)	GO:0016021; GO:0005886	integral to membrane; plasma membrane	CellmembraneMultipassmembraneprotein	Phophorylated - from this Study		Known in Uniprot to be phosphorylated	Known in HPRD to be phosphorylated	
**188**	DNJC5	DNJC5_HUMAN	DNJC5; A8K0M0	Q9H3Z4; A8K0M0; B3KY68; Q9H3Z5; Q9H7H2	NP_079495; XP_028966	DnaJ homolog subfamily C member 5 (Cysteine string protein) (CSP)	GO:0031072; GO:0042470; GO:0016020; GO:0006457; GO:0051082	heat shock protein binding; melanosome; membrane; protein folding; unfolded protein binding	MembraneLipidanchor Melanosome	Phophorylated - from this Study		Known in Uniprot to be phosphorylated	Known in HPRD to be phosphorylated	
**189**	DDEF1	DDEF1_HUMAN	DDEF1; ASAP1; B2RNV3	Q9ULH1; B2RNV3	NP_060952	Arf-GAP with SH3 domain, ANK repeat and PH domain-containing protein 1 (130 kDa phosphatidylinositol 4,5-biphosphate-dependent ARF1 GTPase-activating protein) (PIP2-dependent ARF1 GAP) (ADP-ribosylation factor-directed GTPase-activating protein 1) (ARF GTPase-activating protein 1) (Development and differentiation-enhancing factor 1) (Differentiation-enhancing factor 1) (DEF-1)	GO:0008060; GO:0005737; GO:0016020; GO:0005515; GO:0032312; GO:0008270	ARF GTPase activator activity; cytoplasm; membrane; protein binding; regulation of ARF GTPase activity; zinc ion binding	CytoplasmMembrane	Phophorylated - from this Study		Known in Uniprot to be phosphorylated		
**190**	UN13D	UN13D_HUMAN	UN13D	Q70J99; Q9H7K5	NP_954712	Protein unc-13 homolog D (Munc13-4)	GO:0005737; GO:0016020	cytoplasm; membrane	CytoplasmMembranePeripheralmembraneprotein	Phophorylated - from this Study		Known in Uniprot to be phosphorylated		

	Protein Name	Uniprot Name	Other Names	Uniprot IDs	Refseq IDs	Protein Description	GO ID	GO terms	Subcellular location	Comments	Comments	Comments - Database Search	Comments - Database Search	Function
**191**	FINC	FINC_HUMAN	FINC; Q9UQS6; Q6N0A6; Q6MZF4; Q6N084; Q6N025	P02751; O95609; O95610; Q14312; Q14325; Q14326; Q17RV7; Q585T2; Q59EH1; Q60FE4; Q68DP8; Q68DP9; Q68DT4; Q6LDP6; Q6MZU5; Q6N025; Q6N0A6; Q86T27; Q8IVI8; Q96KP7; Q96KP8; Q96KP9; Q9H1B8; Q9HAP3; Q9UMK2; Q9UQS6; Q6MZF4; Q6N084	NP_997640; NP_002017; NP_473375; NP_997639; NP_997641; NP_997647; NP_997643	Fibronectin (FN) (Cold-insoluble globulin) (CIG) [Cleaved into: Ugl-Y1; Ugl-Y2; Ugl-Y3]; Putative uncharacterized protein DKFZp686F219 (Putative uncharacterized protein DKFZp686K139) (Fragment); Putative uncharacterized protein DKFZp686L11144 (Fragment); Fibronectin (Fragment)	GO:0005793; GO:0006953; GO:0007155; GO:0016477; GO:0005518; GO:0005201; GO:0005577; GO:0008201; GO:0018149; GO:0031093; GO:0005578; GO:0008360; GO:0007169; GO:0016324; GO:0005604; GO:0007160; GO:0007044; GO:0016504; GO:0005515; GO:0042060; GO:0005576;	ER-Golgi intermediate compartment; acute-phase response; cell adhesion; cell migration; collagen binding; extracellular matrix structural constituent; fibrinogen complex; heparin binding; peptide cross-linking; platelet alpha granule lumen; proteinaceous extracellular matrix; regulation of cell shape; transmembrane receptor protein tyrosine kinase signaling pathway; apical plasma membrane; basement membrane; cell-matrix adhesion; cell-substrate junction assembly; peptidase activator activity; protein binding; wound healing; extracellular region;	Secretedextracellularspaceextracellularmatrix;	Phophorylated - from this Study	Membrane-Fraction - this study	Known in Uniprot to be phosphorylated		
**192**	MYLK	MYLK_HUMAN	MYLK; Q6P2N0	Q15746; Q7Z4J0; O95796; O95797; O95798; O95799; Q14844; Q16794; Q5MY99; Q5MYA0; Q9C0L5; Q9UBG5; Q9UIT9; Q6P2N0; Q17S15	NP_444254; NP_005956; NP_444253; NP_444255; NP_444256; NP_444259	Myosin light chain kinase, smooth muscle (MLCK) (EC 2.7.11.18) (Telokin) (Kinase-related protein) (KRP); Myosin light chain kinase (Myosin, light polypeptide kinase, isoform CRA_f) (cDNA, FLJ95192, Homo sapiens myosin, light polypeptide kinase (MYLK), transcriptvariant 7, mRNA)	GO:0005524; GO:0005509; GO:0005516; GO:0000287; GO:0004687; GO:0006468; GO:0016301	ATP binding; calcium ion binding; calmodulin binding; magnesium ion binding; myosin light chain kinase activity; protein amino acid phosphorylation; kinase activity		Phophorylated - from this Study		Known in Uniprot to be phosphorylated	Known in HPRD to be phosphorylated	Kinase
**193**	KAP3	KAP3_HUMAN	KAP3; A4D0R9	P31323; A4D0R9	NP_002727	cAMP-dependent protein kinase type II-beta regulatory subunit	GO:0005794; GO:0030552; GO:0005952; GO:0008603; GO:0005813; GO:0009755; GO:0005739; GO:0001932	Golgi apparatus; cAMP binding; cAMP-dependent protein kinase complex; cAMP-dependent protein kinase regulator activity; centrosome; hormone-mediated signaling; mitochondrion; regulation of protein amino acid phosphorylation		Phophorylated - from this Study		Known in Uniprot to be phosphorylated	Known in HPRD to be phosphorylated	
**194**	KPCB	KPCB_HUMAN	KPCB	P05771; O43744; P05127; Q15138; Q93060; Q9UE49; Q9UE50; Q9UEH8; Q9UJ30; Q9UJ33	NP_997700; NP_002729	Protein kinase C beta type (PKC-beta) (PKC-B) (EC 2.7.11.13)	GO:0005524; GO:0005509; GO:0005829; GO:0019992; GO:0007242; GO:0042953; GO:0005886; GO:0006468; GO:0005515; GO:0004697; GO:0008270	ATP binding; calcium ion binding; cytosol; diacylglycerol binding; intracellular signaling cascade; lipoprotein transport; plasma membrane; protein amino acid phosphorylation; protein binding; protein kinase C activity; zinc ion binding	CytoplasmMembranePeripheralmembraneprotein	Phophorylated - from this Study		Known in Uniprot to be phosphorylated	Known in HPRD to be phosphorylated	Kinase
**195**	KPCD	KPCD_HUMAN	KPCD; Q86XJ6	Q86XJ6; Q05655; B2R834; Q15144	NP_997704; NP_006245	Protein kinase C delta type (EC 2.7.11.13) (nPKC-delta)	GO:0005524; GO:0004699; GO:0005829; GO:0019992; GO:0008047; GO:0019899; GO:0043560; GO:0007242; GO:0016020; GO:0046627; GO:0050732; GO:0032091; GO:0005634; GO:0008022; GO:0050821; GO:0010469; GO:0010149; GO:0008270	ATP binding; calcium-independent protein kinase C activity; cytosol; diacylglycerol binding; enzyme activator activity; enzyme binding; insulin receptor substrate binding; intracellular signaling cascade; membrane; negative regulation of insulin receptor signaling pathway; negative regulation of peptidyl-tyrosine phosphorylation; negative regulation of protein binding; nucleus; protein C-terminus binding; protein stabilization; regulation of receptor activity; senescence; zinc ion binding	CytoplasmMembranePeripheralmembraneprotein	Phophorylated - from this Study		Known in Uniprot to be phosphorylated	Known in HPRD to be phosphorylated	Kinase
**196**	GP1BA	GP1BA_HUMAN	GP1BA; A5CKE2	P07359; Q14441; Q16469; Q8N1F3; Q8NG39; Q9HDC7; Q9UEK1; Q9UQS4; A5CKE2	NP_000164	Platelet glycoprotein Ib alpha chain (Glycoprotein Ibalpha) (GP-Ib alpha) (GPIb-alpha) (GPIbA) (Antigen CD42b-alpha) (CD antigen CD42b) [Cleaved into: Glycocalicin]	GO:0007155; GO:0007166; GO:0042730; GO:0005887; GO:0005624; GO:0030168; GO:0031092; GO:0005515; GO:0015057	cell adhesion; cell surface receptor linked signal transduction; fibrinolysis; integral to plasma membrane; membrane fraction; platelet activation; platelet alpha granule membrane; protein binding; thrombin receptor activity	MembraneSinglepasstypeImembraneprotein	Phophorylated - from this Study		Known in Uniprot to be phosphorylated		
**197**	HMHA1	HMHA1_HUMAN	Q6P189; Q92619; Q8HX84; HMHA1	Q8HX84; Q6P189; Q92619; Q7LE26; Q86WS1; Q9GJN9; Q9GJP0; Q9GJP1; Q9MY24	NP_036424	Minor histocompatibility protein HA-1 [Cleaved into: Minor histocompatibility antigen HA-1 (mHag HA-1)]	GO:0005096; GO:0019992; GO:0005622; GO:0007242; GO:0008270	GTPase activator activity; diacylglycerol binding; intracellular; intracellular signaling cascade; zinc ion binding		Phophorylated - from this Study		Known in Uniprot to be phosphorylated		
**198**	BIN2	BIN2_HUMAN	BIN2	Q9UBW5; Q86VV0; Q9NWK4; Q9UKN4	NP_057377; XP_293762	Bridging integrator 2 (Breast cancer-associated protein 1)	GO:0005737; GO:0005515	cytoplasm; protein binding	Cytoplasm	Phophorylated - from this Study				
**199**	TBB3	TBB3_HUMAN	TBB3	Q13509; Q9BTZ0; Q9BW10	NP_006077	Tubulin beta-3 chain (Tubulin beta-III) (Tubulin beta-4)	GO:0005525; GO:0003924; GO:0005874; GO:0007018; GO:0043234; GO:0051258; GO:0005198	GTP binding; GTPase activity; microtubule; microtubule-based movement; protein complex; protein polymerization; structural molecule activity		Phophorylated - from this Study			Known in HPRD to be phosphorylated	
**200**	TMBI1	TMBI1_HUMAN	TMBI1; B3KQY6	Q969X1; Q8N1R3; Q8TAM3; Q96K13; B3KQY6	NP_071435	Transmembrane BAX inhibitor motif-containing protein 1 (Protein RECS1 homolog)	GO:0016021	integral to membrane	MembraneMultipassmembraneprotein	Phophorylated - from this Study		Known in Uniprot to be phosphorylated		
**201**	LY66F	LY66F_HUMAN	Q7Z5H2; LY66F; B0UXB7	Q7Z5H2; Q5SQ64; O95869; Q96QC7; B0UXB7	NP_0010036 93	Lymphocyte antigen 6 complex locus protein G6f	GO:0016021; GO:0005886	integral to membrane; plasma membrane	CellmembraneSinglepasstypeImembraneprotein	Phophorylated - from this Study		Known in Uniprot to be phosphorylated	Known in HPRD to be phosphorylated	
**202**	FKB15	FKB15_HUMAN	Q5T1M5; FKB15	Q5T1M5; Q05DK8; Q5T1M2; Q6DD85; Q9Y4D0	XP_376903; NP_056073	FK506-binding protein 15	GO:0005737; GO:0006457	cytoplasm; protein folding	CytoplasmCellprojectionaxon	Phophorylated - from this Study		Known in Uniprot to be phosphorylated		
**203**	RPGP2	RPGP2_HUMAN	Q684P5; Q684P4; RPGP2	Q684P5; Q684P4; B2RTY5; Q6AI00; Q6ZVF0; Q9UPW2	NP_055900; XP_085748; NP_001093 868	Rap1 GTPase-activating protein 2 (Rap1GAP2) (GTPase-activating Rap/Ran-GAP domain-like protein 4)	GO:0005096; GO:0005737; GO:0051056	GTPase activator activity; cytoplasm; regulation of small GTPase mediated signal transduction	CytoplasmCytoplasmperinuclearregion	Phophorylated - from this Study		Known in Uniprot to be phosphorylated		
**204**	RHG17	RHG17_HUMAN	RHG17	Q68EM7; A8K6M6; Q6ZUS4; Q7Z2F2; Q8NDG2; Q96KS2; Q96KS3; Q96SS8; Q9BVF6; Q9H8U5; Q9NW54	NP_0010066 35; NP_060524	Rho GTPase-activating protein 17 (Rho-type GTPase-activating protein 17) (RhoGAP interacting with CIP4 homologs protein 1) (RICH-1)	GO:0005096; GO:0017124; GO:0005737; GO:0007165; GO:0005923	GTPase activator activity; SH3 domain binding; cytoplasm; signal transduction; tight junction	MembranePeripheralmembraneproteinCytoplasmCelljunctiontightjunction	Phophorylated - from this Study		Known in Uniprot to be phosphorylated		
**205**	TNIK	TNIK_HUMAN	TNIK; A7E2A3	Q9UKE5; O60298; Q8WUY7; Q9UKD8; Q9UKD9; Q9UKE0; Q9UKE1; Q9UKE2; Q9UKE3; Q9UKE4; A7E2A3	NP_055843	TRAF2 and NCK-interacting protein kinase (EC 2.7.11.1)	GO:0005524; GO:0007254; GO:0006468; GO:0005515; GO:0004674; GO:0005083	ATP binding; JNK cascade; protein amino acid phosphorylation; protein binding; protein serine/threonine kinase activity; small GTPase regulator activity		Phophorylated - from this Study		Known in Uniprot to be phosphorylated	Known in HPRD to be phosphorylated	Kinase
**206**	CT032	CT032_HUMAN	CT032; CASS4	Q9NQ75; Q96K09; Q9BYL5	NP_065089	Cas scaffolding protein family member 4 (HEF1-EFS-p130Cas-like protein) (HEPL) (HEF-like protein)	GO:0007155; GO:0030054; GO:0005737; GO:0005856; GO:0005515; GO:0007165; GO:0000155	cell adhesion; cell junction; cytoplasm; cytoskeleton; protein binding; signal transduction; two-component sensor activity	CytoplasmcytoskeletonCelljunctionfocaladhesion	Phophorylated - from this Study		Known in Uniprot to be phosphorylated		
**207**	PIWL3	PIWL3_HUMAN	PIWL3	Q7Z3Z3	NP_0010084 96	Piwi-like protein 3	GO:0007275; GO:0003676	multicellular organismal development; nucleic acid binding	Cytoplasm	Phophorylated - from this Study				

	Protein Name	Uniprot Name	Other Names	Uniprot IDs	Refseq IDs	Protein Description	GO ID	GO terms	Subcellular location	Comments	Comments	Comments - Database Search	Comments - Database Search	Function
**208**	FRIH	FRIH_HUMAN	FRIH; B3KNR5	P02794; Q3KRA8; Q3SWW1; B3KNR5	NP_002023	Ferritin heavy chain (Ferritin H subunit) (EC 1.16.3.1) (Cell proliferation-inducing gene 15 protein)	GO:0008199; GO:0004322; GO:0006955; GO:0008043; GO:0006880; GO:0006826; GO:0008285; GO:0055114; GO:0005515	ferric iron binding; ferroxidase activity; immune response; intracellular ferritin complex; intracellular sequestering of iron ion; iron ion transport; negative regulation of cell proliferation; oxidation reduction; protein binding		Phophorylated - from this Study		Known in Uniprot to be phosphorylated	Known in HPRD to be phosphorylated	
**209**	PDLI5	PDLI5_HUMAN	PDLI5; Q8WVK0; Q5UW38	Q96HC4; A8K6F9; O60705; Q5UW38; Q8WVK0	NP_006448; NP_001011513; NP_001011515; NP_001011516	PDZ and LIM domain protein 5 (Enigma homolog) (Enigma-like PDZ and LIM domains protein); PDZ and LIM domain 5 (cDNA FLJ77159, highly similar to Homo sapiens PDZ and LIM domain 5 (PDLIM5), transcript variant 5, mRNA) (PDZ and LIM domain 5, isoform CRA_b)	GO:0003779; GO:0015629; GO:0042805; GO:0005829; GO:0005624; GO:0005080; GO:0008270	actin binding; actin cytoskeleton; actinin binding; cytosol; membrane fraction; protein kinase C binding; zinc ion binding		Phophorylated - from this Study		Known in Uniprot to be phosphorylated		
**210**	SEPT7	SEPT7_HUMAN	SEPT7; Q3B7A3	Q16181; Q52M76; Q6NX50; Q3B7A3	NP_001779; NP_001011553	Septin-7 (CDC10 protein homolog)	GO:0005525; GO:0000777; GO:0000910; GO:0007067; GO:0005634; GO:0005515; GO:0051291; GO:0031105; GO:0005819; GO:0001725; GO:0005198	GTP binding; condensed chromosome kinetochore; cytokinesis; mitosis; nucleus; protein binding; protein heterooligomerization; septin complex; spindle; stress fiber; structural molecule activity	CytoplasmKinetochore Spindle	Phophorylated - from this Study		Known in Uniprot to be phosphorylated		
**211**	TTC7B	TTC7B_HUMAN	TTC7B; Q6PIF1	Q86TV6; Q86U24; Q86VT3; Q6PIF1	NP_001010854	Tetratricopeptide repeat protein 7B (TPR repeat protein 7B) (Tetratricopeptide repeat protein 7-like-1); TTC7B protein (Fragment)	GO:0005488	binding		Phophorylated - from this Study		Known in Uniprot to be phosphorylated		
**212**	HUWE1	HUWE1_HUMAN	HUWE1	Q7Z6Z7; O15029; Q4G2Z2; Q5H961; Q6P4D0; Q8NG67; Q9BUI0; Q9HCJ4; Q9NSL6; Q9P0A9	NP_113584	E3 ubiquitin-protein ligase HUWE1 (EC 6.3.2.-) (HECT, UBA and WWE domain-containing protein 1) (Upstream regulatory element-binding protein 1) (URE-binding protein 1) (URE-B1) (Mcl-1 ubiquitin ligase E3) (Mule) (ARF-binding protein 1) (ARF-BP1) (Large structure of UREB1) (LASU1) (Homologous to E6AP carboxyl terminus homologous protein 9) (HectH9)	GO:0003677; GO:0030154; GO:0005737; GO:0016574; GO:0019941; GO:0005634; GO:0005515; GO:0000209; GO:0004842	DNA binding; cell differentiation; cytoplasm; histone ubiquitination; modification-dependent protein catabolic process; nucleus; protein binding; protein polyubiquitination; ubiquitin-protein ligase activity	CytoplasmNucleus	Phophorylated - from this Study		Known in Uniprot to be phosphorylated	Known in HPRD to be phosphorylated	
**213**	DBNL	DBNL_HUMAN	DBNL; A4D2I9	Q9UJU6; A4D2I9; P84070; Q6IAI8; Q96F30; Q96K74; Q9HBN8; Q9NR72	NP_001014436; NP_054782; NP_001116428	Drebrin-like protein (Drebrin-F) (SH3 domain-containing protein 7) (Cervical SH3P7) (HPK1-interacting protein of 55 kDa) (HIP-55) (Cervical mucin-associated protein)	GO:0016601; GO:0007257; GO:0005938; GO:0005856; GO:0006897; GO:0008047; GO:0042802; GO:0006955; GO:0030027	Rac protein signal transduction; activation of JUN kinase activity; cell cortex; cytoskeleton; endocytosis; enzyme activator activity; identical protein binding; immune response; lamellipodium	Cytoplasmcytoskeleton Cellprojectionlamellipodium	Phophorylated - from this Study		Known in Uniprot to be phosphorylated	Known in HPRD to be phosphorylated	
**214**	EXOC7	EXOC7_HUMAN	EXOC7; Q63HP7	Q9UPT5; Q8ND93; Q8WV91; Q96FF0; Q9H8C3; Q9H9X3; Q9HA32; Q63HP7	NP_001013861; NP_056034; NP_001138769	Exocyst complex component 7 (Exocyst complex component Exo70); Putative uncharacterized protein DKFZp686P1551	GO:0034451; GO:0000145; GO:0006887; GO:0005886; GO:0015031; GO:0000145	centriolar satellite; exocyst; exocytosis; plasma membrane; protein transport; exocyst	CytoplasmcytosolCellmembranePeripheralmembraneprotein;	Phophorylated - from this Study		Known in Uniprot to be phosphorylated		
**215**	GI24	GI24_HUMAN	GI24	Q9H7M9; Q6UXF3; Q8WUG3; Q8WYZ8	NP_071436	Platelet receptor Gi24	GO:0016021; GO:0004872	integral to membrane; receptor activity	MembraneSinglepasstypeImembraneprotein	Phophorylated - from this Study		Known in Uniprot to be phosphorylated		
**216**	ILK	ILK_HUMAN	ILK	Q13418; Q68DZ3; P57043	NP_001014795; NP_004508; NP_001014794	Integrin-linked protein kinase (EC 2.7.11.1) (ILK-1) (ILK-2) (59 kDa serine/threonine-protein kinase) (p59ILK)	GO:0005524; GO:0007160; GO:0005737; GO:0007229; GO:0006468; GO:0005515; GO:0004674; GO:0004713; GO:0009966	ATP binding; cell-matrix adhesion; cytoplasm; integrin-mediated signaling pathway; protein amino acid phosphorylation; protein binding; protein serine/threonine kinase activity; protein tyrosine kinase activity; regulation of signal transduction	CelljunctionfocaladhesionCellmembranePeripheralmembraneproteinCytoplasmicside	Phophorylated - from this Study		Known in Uniprot to be phosphorylated	Known in HPRD to be phosphorylated	Kinase
**217**	HS90A	HS90A_HUMAN	Q5CAQ7; HS90A; Q86SX1; A8K500	Q5CAQ7; P07900; Q2PP14; Q5CAQ6; Q9BVQ5; Q86SX1; A8K500	NP_001017963; NP_005339; XP_301154; XP_293759; XP_291550; XP_210540; NP_001035231	Heat shock protein HSP 90-alpha (HSP 86) (Renal carcinoma antigen NY-REN-38)	GO:0005524; GO:0030911; GO:0034619; GO:0005829; GO:0042470; GO:0070096; GO:0006839; GO:0030235; GO:0045429; GO:0042803; GO:0042026; GO:0006986; GO:0007165; GO:0051082	ATP binding; TPR domain binding; cellular chaperone-mediated protein complex assembly; cytosol; melanosome; mitochondrial outer membrane translocase complex assembly; mitochondrial transport; nitric-oxide synthase regulator activity; positive regulation of nitric oxide biosynthetic process; protein homodimerization activity; protein refolding; response to unfolded protein; signal transduction; unfolded protein binding	CytoplasmMelanosome	Phophorylated - from this Study		Known in Uniprot to be phosphorylated	Known in HPRD to be phosphorylated	
**218**	RHG08	RHG08_HUMAN	RHG08; Q6PJW1; PTOR1	Q9NSG0; P85299; O75983; O95695; Q5BIW2; Q5EAJ8; Q5EAJ9; Q5XKJ6; Q96RW1; Q96RW2; Q9HA49; Q9HC46; Q9NVX8; Q9NXL1; Q9UH20; P85298; Q6PJW1	NP_001017526; NP_001017528; NP_001017529; NP_001017530; NP_056181; NP_851850; NP_851852	Rho GTPase-activating protein 8 (Rho-type GTPase-activating protein 8); ARHGAP8 protein (Fragment)	GO:0005096; GO:0005622; GO:0007165; GO:0005622	GTPase activator activity; intracellular; signal transduction; intracellular				Known in Uniprot to be phosphorylated		
**219**	TENS1	TENS1_HUMAN	TENS1; Q9UFN8; Q4ZG71; Q86VB0; Q59G71	Q4ZG71; Q9HBL0; Q59G71; Q86VB0; Q9UFN8	NP_072174	Tensin-1; Tensin variant (Fragment); TNS1 protein; Putative uncharacterized protein DKFZp434G162 (Fragment)	GO:0003779; GO:0030054; GO:0005737; GO:0005856; GO:0005515; GO:0016477; GO:0007044; GO:0005925	actin binding; cell junction; cytoplasm; cytoskeleton; protein binding; cell migration; cell-substrate junction assembly; focal adhesion	CelljunctionfocaladhesionCytoplasmcytoskeleton;	Phophorylated - from this Study		Known in Uniprot to be phosphorylated		
**220**	SKIV2	SKIV2_HUMAN	SKIV2; Q9NPK3; Q5ST66	Q5ST66; Q15477; O15005; Q12902; Q15476; Q9NPK3	NP_008860	Helicase SKI2W (EC 3.6.1.-) (Helicase-like protein) (HLP); Superkiller viralicidic activity 2-like (S. cerevisiae); DJ34F7.7 (Superkiller viralicidic activity 2 (S. cerevisiae homolog)-like (SKI2W))	GO:0005524; GO:0004004; GO:0003723; GO:0005634; GO:0005515; GO:0008026; GO:0051059; GO:0005829; GO:0006952; GO:0003676; GO:0043234; GO:0007584; GO:0006986	ATP binding; ATP-dependent RNA helicase activity; RNA binding; nucleus; protein binding; ATP-dependent helicase activity; NF-kappaB binding; cytosol; defense response; nucleic acid binding; protein complex; response to nutrient; response to unfolded protein	Nucleus;	Phophorylated - from this Study		Known in Uniprot to be phosphorylated	Known in HPRD to be phosphorylated	
**221**	DUET	DUET_HUMAN	DUET; KALRN	Q9Y2A5; O60229; A8MSI4; Q6ZN45; Q8TBQ5; Q9NSZ4	NP_008995; XP_293563; NP_001019831; NP_003938	Kalirin (EC 2.7.11.1) (Huntingtin-associated protein-interacting protein) (Protein Duo) (Serine/threonine kinase with Dbl- and pleckstrin homology domain)	GO:0005524; GO:0005089; GO:0015629; GO:0005737; GO:0000287; GO:0007399; GO:0006468; GO:0005515; GO:0004674; GO:0035023; GO:0016192	ATP binding; Rho guanyl-nucleotide exchange factor activity; actin cytoskeleton; cytoplasm; magnesium ion binding; nervous system development; protein amino acid phosphorylation; protein binding; protein serine/threonine kinase activity; regulation of Rho protein signal transduction; vesicle-mediated transport	CytoplasmCytoplasmcytoskeleton	Phophorylated - from this Study		Known in Uniprot to be phosphorylated		Kinase
**222**	H1BP3	H1BP3_HUMAN	Q86VC2; Q9H7U7; Q8N367; Q53T59; H1BP3	Q53T59; Q86VC2; B2RAW2; Q8N367; Q9H7U7	NP_071905	HCLS1-binding protein 3 (HS1-binding protein 3) (HSP1BP-3); cDNA FLJ14249 fis, clone OVARC1001200, weakly similar to Mus musculus HS1 binding protein 3	GO:0007154; GO:0035091; GO:0005515	cell communication; phosphoinositide binding; protein binding		Phophorylated - from this Study		Known in Uniprot to be phosphorylated		
**223**	ABCF1	ABCF1_HUMAN	ABCF1; A2BEP8; A2BF75	Q8NE71; O14897; Q69YP6; A2BF75	NP_001020262; NP_001081	ATP-binding cassette sub-family F member 1 (ATP-binding cassette 50) (TNF-alpha-stimulated ABC protein)	GO:0005524; GO:0042626; GO:0006954; GO:0006412; GO:0008135	ATP binding; ATPase activity, coupled to transmembrane movement of substances; inflammatory response; translation; translation factor activity, nucleic acid binding		Phophorylated - from this Study		Known in Uniprot to be phosphorylated	Known in HPRD to be phosphorylated	
**224**	KSR1	KSR1_HUMAN	KSR1; A8MY87	Q8IVT5; Q13476; A8MY87	NP_055053	Kinase suppressor of Ras 1	GO:0005524; GO:0007265; GO:0005737; GO:0019992; GO:0016020; GO:0006468; GO:0005515; GO:0004674; GO:0004713; GO:0008270	ATP binding; Ras protein signal transduction; cytoplasm; diacylglycerol binding; membrane; protein amino acid phosphorylation; protein binding; protein serine/threonine kinase activity; protein tyrosine kinase activity; zinc ion binding	CytoplasmMembranePeripheralmembraneprotein	Phophorylated - from this Study		Known in Uniprot to be phosphorylated		Kinase
**225**	MAVS	MAVS_HUMAN	MAVS; Q2HWT5	Q7Z434; Q2HWT5; Q3I0Y2; Q5T7I6; Q86VY7; Q9H1H3; Q9H4Y1; Q9H8D3; Q9ULE9	NP_065797	Mitochondrial antiviral-signaling protein (Interferon-beta promoter stimulator protein 1) (IPS-1) (Virus-induced-signaling adapter) (CARD adapter inducing interferon-beta) (Cardif) (Putative NF-kappa-B-activating protein 031N)	GO:0045087; GO:0016021; GO:0044419; GO:0005741; GO:0043123; GO:0005515; GO:0009615; GO:0004871	innate immune response; integral to membrane; interspecies interaction between organisms; mitochondrial outer membrane; positive regulation of I-kappaB kinase/NF-kappaB cascade; protein binding; response to virus; signal transducer activity	Mitochondrionoutermembrane	Phophorylated - from this Study		Known in Uniprot to be phosphorylated		

	Protein Name	Uniprot Name	Other Names	Uniprot IDs	Refseq IDs	Protein Description	GO ID	GO terms	Subcellular location	Comments	Comments	Comments - Database Search	Comments - Database Search	Function
**226**	NEB2	NEB2_HUMAN	NEB2	Q96SB3; Q8TCR9	NP_115984	Neurabin-2 (Neurabin-II) (Spinophilin) (Protein phosphatase 1 regulatory subunit 9B)	GO:0008380; GO:0003779; GO:0007050; GO:0030154; GO:0030054; GO:0042995; GO:0005856; GO:0030308; GO:0007399; GO:0005654; GO:0004864; GO:0008157; GO:0000164; GO:0001560; GO:0042127; GO:0007096; GO:0045202	RNA splicing; actin binding; cell cycle arrest; cell differentiation; cell junction; cell projection; cytoskeleton; negative regulation of cell growth; nervous system development; nucleoplasm; phosphoprotein phosphatase inhibitor activity; protein phosphatase 1 binding; protein phosphatase type 1 complex; regulation of cell growth by extracellular stimulus; regulation of cell proliferation; regulation of exit from mitosis; synapse	Cytoplasmcytoskeleton NucleusCellprojectiondendriticspineCelljunctionsynapse	Phophorylated - from this Study		Known in Uniprot to be phosphorylated	Known in HPRD to be phosphorylated	Phosphatase
**227**	IF2P	IF2P_HUMAN	IF2P; Q8N5A0	O60841; O95805; Q9UF81; Q9UMN7; Q8N5A0	NP_056988	Eukaryotic translation initiation factor 5B (eIF-5B) (Translation initiation factor IF-2); Eukaryotic translation initiation factor 5B	GO:0005525; GO:0003924; GO:0005737; GO:0005515; GO:0006446; GO:0003743	GTP binding; GTPase activity; cytoplasm; protein binding; regulation of translational initiation; translation initiation factor activity	Cytoplasm;	Phophorylated - from this Study		Known in Uniprot to be phosphorylated	Known in HPRD to be phosphorylated	
**228**	S39A9	S39A9_HUMAN	Q5MJQ0; S39A9	Q5MJQ0; Q9NUM3; Q53HN3; Q6P2Q1; Q86WY2	XP_937983; NP_060845	Zinc transporter ZIP9 (Zrt- and Irt-like protein 9) (ZIP-9) (Solute carrier family 39 member 9)	GO:0005794; GO:0016021; GO:0046873; GO:0005730; GO:0008270; GO:0006829	Golgi apparatus; integral to membrane; metal ion transmembrane transporter activity; nucleolus; zinc ion binding; zinc ion transport	MembraneMultipassmembraneprotein	Phophorylated - from this Study				
**229**	UBP24	UBP24_HUMAN	UBP24	Q9UPU5; Q6ZSY2; Q8N2Y4; Q9NXD1	XP_371254; NP_056121	Ubiquitin carboxyl-terminal hydrolase 24 (EC 3.1.2.15) (Ubiquitin thioesterase 24) (Ubiquitin-specific-processing protease 24) (Deubiquitinating enzyme 24)	GO:0008234; GO:0004221; GO:0006511	cysteine-type peptidase activity; ubiquitin thiolesterase activity; ubiquitin-dependent protein catabolic process		Phophorylated - from this Study		Known in Uniprot to be phosphorylated		
**230**	FRM4B	FRM4B_HUMAN	FRM4B	Q9Y2L6; Q8TAI3	XP_114303; NP_055938	FERM domain-containing protein 4B (GRP1-binding protein GRSP1)	GO:0005488; GO:0005737; GO:0005856	binding; cytoplasm; cytoskeleton	Cytoplasmcytoskeleton	Phophorylated - from this Study		Known in Uniprot to be phosphorylated		
**231**	NBEL2	NBEL2_HUMAN	Q6ZNJ1; NBEL2	Q6ZNJ1; O60288; Q6P994; Q6UX91; Q8NAC9	XP_946304; XP_291064; NP_055990	Neurobeachin-like protein 2	GO:0005488	binding		Phophorylated - from this Study		Known in Uniprot to be phosphorylated		
**232**	Q6NXR8	Q6NXR8_HUMAN	Q6NXR8	Q6NXR8	XP_931621	Ribosomal protein S3A	GO:0005840; GO:0003735; GO:0006412	ribosome; structural constituent of ribosome; translation		Phophorylated - from this Study				
**233**	PPR3E	PPR3E_HUMAN	Q9H7J1; PPR3E	Q9H7J1	XP_932122	Protein phosphatase 1 regulatory subunit 3E	GO:0005977	glycogen metabolic process		Phophorylated - from this Study				Phosphatase
**234**	CO4A3	CO4A3_HUMAN	CO4A3	Q01955; Q53QQ1; Q53R14; Q53RW8; Q9BQT2; Q9NYC4	XP_001131951; NP_000082; NP_112730; NP_112733; NP_112734	Collagen alpha-3(IV) chain (Goodpasture antigen) [Cleaved into: Tumstatin]	GO:0006919; GO:0008015; GO:0007155; GO:0007166; GO:0005587; GO:0005201; GO:0006917; GO:0005178; GO:0008191; GO:0016525; GO:0008285; GO:0007605	activation of caspase activity; blood circulation; cell adhesion; cell surface receptor linked signal transduction; collagen type IV; extracellular matrix structural constituent; induction of apoptosis; integrin binding; metalloendopeptidase inhibitor activity; negative regulation of angiogenesis; negative regulation of cell proliferation; sensory perception of sound	Secretedextracellularspaceextracellularmatrixbasementmembrane	Phophorylated - from this Study		Known in Uniprot to be phosphorylated	Known in HPRD to be phosphorylated	
**235**	INF2	INF2_HUMAN	Q27J81; INF2	Q9H6N1; Q27J81; Q27J83; Q69YL8; Q6P1X7; Q6PK22; Q86TR7; Q9BRM1	NP_071934; NP_001026884; NP_116103	Inverted formin-2 (HBEBP2-binding protein C)	GO:0017048; GO:0003779; GO:0030036; GO:0005783; GO:0005634	Rho GTPase binding; actin binding; actin cytoskeleton organization; endoplasmic reticulum; nucleus		Phophorylated - from this Study		Known in Uniprot to be phosphorylated		
**236**	CA062	CA062_HUMAN	Q5T1N1; CA062	Q8NCN9; Q5T1N1; Q5T1N0; Q8N990	NP_689976	Uncharacterized protein C1orf62				Phophorylated - from this Study				
**237**	RHDF2	RHDF2_HUMAN	Q5YGQ8; RHDF2	Q5YGQ8; Q6PJF5; A6NEM3; A8K801; Q5U607; Q9H6E9	NP_001005498; NP_078875	Rhomboid family member 2 (Rhomboid 5 homolog 2) (Rhomboid veinlet-like protein 5) (Rhomboid veinlet-like protein 6)	GO:0005789; GO:0016021	endoplasmic reticulum membrane; integral to membrane	EndoplasmicreticulummembraneMultipassmembraneprotein	Phophorylated - from this Study		Known in Uniprot to be phosphorylated		
**238**	RHG06	RHG06_HUMAN	RHG06	O43182; B2RWQ0; O43437; Q9P1B3; Q9UK81; Q9UK82	NP_038286; NP_006116; NP_038267	Rho GTPase-activating protein 6 (Rho-type GTPase-activating protein 6) (Rho-type GTPase-activating protein RhoGAPX-1)	GO:0005100; GO:0007266; GO:0017124; GO:0005070; GO:0005884; GO:0030041; GO:0005737; GO:0051895; GO:0051497; GO:0043087	Rho GTPase activator activity; Rho protein signal transduction; SH3 domain binding; SH3/SH2 adaptor activity; actin filament; actin filament polymerization; cytoplasm; negative regulation of focal adhesion formation; negative regulation of stress fiber formation; regulation of GTPase activity	Cytoplasm	Phophorylated - from this Study		Known in Uniprot to be phosphorylated		
**239**	FA5	FA5_HUMAN	FA5	P12259; A8K6E8; Q14285; Q2EHR5; Q5R346; Q5R347; Q6UPU6; Q8WWQ6	NP_000121	Coagulation factor V (Activated protein C cofactor) (Proaccelerin, labile factor) [Cleaved into: Coagulation factor V heavy chain; Coagulation factor V light chain]	GO:0007596; GO:0005509; GO:0007155; GO:0005507; GO:0016491; GO:0005886; GO:0031093	blood coagulation; calcium ion binding; cell adhesion; copper ion binding; oxidoreductase activity; plasma membrane; platelet alpha granule lumen	Secreted	Phophorylated - from this Study		Known in Uniprot to be phosphorylated	Known in HPRD to be phosphorylated	
**240**	ASAHL	ASAHL_HUMAN	ASAHL; NAAA	Q02083; Q5KTF2; Q96EY2; Q9BRA8	NP_001035861; NP_055250	N-acylethanolamine-hydrolyzing acid amidase (EC 3.5.1.-) (N-acylsphingosine amidohydrolase-like) (ASAH-like protein) (Acid ceramidase-like protein)	GO:0016787; GO:0006629; GO:0005764	hydrolase activity; lipid metabolic process; lysosome	Lysosome	Phophorylated - from this Study				
**241**	CE170	CE170_HUMAN	CE170	Q5SW79; O75058; Q5SW77; Q5SW78; Q7LGA9; Q86W31; Q9UQ08; Q9UQ09	NP_001035863; NP_001035864; NP_055627	Centrosomal protein of 170 kDa (Cep170) (KARP-1 binding protein) (KARP1-binding protein)	GO:0005737; GO:0005874	cytoplasm; microtubule	CentrosomecentrioleCytoplasmcytoskeleton	Phophorylated - from this Study		Known in Uniprot to be phosphorylated	Known in HPRD to be phosphorylated	
**242**	TGFI1	TGFI1_HUMAN	O43294; Q9BPW3; TGFI1	O43294; Q9BPW3; Q9Y2V5	NP_001035919; NP_057011	Transforming growth factor beta-1-induced transcript 1 protein (Hydrogen peroxide-inducible clone 5 protein) (Hic-5) (Androgen receptor-associated protein of 55 kDa)	GO:0070411; GO:0016055; GO:0050681; GO:0030521; GO:0007155; GO:0030054; GO:0005737; GO:0005856; GO:0008285; GO:0030512; GO:0016363; GO:0010718; GO:0045893; GO:0030511; GO:0003713; GO:0006366; GO:0030579; GO:0008270	I-SMAD binding; Wnt receptor signaling pathway; androgen receptor binding; androgen receptor signaling pathway; cell adhesion; cell junction; cytoplasm; cytoskeleton; negative regulation of cell proliferation; negative regulation of transforming growth factor beta receptor signaling pathway; nuclear matrix; positive regulation of epithelial to mesenchymal transition; positive regulation of transcription, DNA-dependent; positive regulation of transforming growth factor beta receptor signaling pathway; transcription coactivator activity; transcription from RNA polymerase II promoter; ubiquitin-dependent SMAD protein catabolic process; zinc ion binding	CelljunctionfocaladhesionNucleusmatrixCytoplasmcytoskeleton	Phophorylated - from this Study		Known in Uniprot to be phosphorylated		
**243**	FA65C	FA65C_HUMAN	CT175; FA65C	Q96MK2; Q5QPB6; Q9NQQ2	NP_543019	Protein FAM65C				Phophorylated - from this Study				
**244**	SYTL3	SYTL3_HUMAN	Q5U3B9; SYTL3	Q5U3B9; Q4VX76; Q496J4; Q496J6	NP_001009991	Synaptotagmin-like protein 3 (Exophilin-6)	GO:0017137; GO:0012505; GO:0006886; GO:0016020; GO:0008270	Rab GTPase binding; endomembrane system; intracellular protein transport; membrane; zinc ion binding	IntracytoplasmicmembranePeripheralmembraneprotein	Phophorylated - from this Study				
**245**	ARRB1	ARRB1_HUMAN	ARRB1; B6V9G8	P49407; B6V9G8; O75625; O75630; Q2PP20; Q9BTK8	NP_064647; NP_004032	Beta-arrestin-1 (Arrestin beta-1)	GO:0005737; GO:0004857; GO:0005624; GO:0005886; GO:0005515; GO:0005625	cytoplasm; enzyme inhibitor activity; membrane fraction; plasma membrane; protein binding; soluble fraction		Phophorylated - from this Study		Known in Uniprot to be phosphorylated	Known in HPRD to be phosphorylated	
**246**	ADCY6	ADCY6_HUMAN	ADCY6	O43306; Q9NR75	NP_066193; NP_056085	Adenylate cyclase type 6 (EC 4.6.1.1) (Adenylate cyclase type VI) (ATP pyrophosphate-lyase 6) (Adenylyl cyclase 6) (Ca(2+)-inhibitable adenylyl cyclase)	GO:0004016; GO:0006171; GO:0016021; GO:0007242; GO:0000287	adenylate cyclase activity; cAMP biosynthetic process; integral to membrane; intracellular signaling cascade; magnesium ion binding	MembraneMultipassmembraneprotein	Phophorylated - from this Study		Known in Uniprot to be phosphorylated	Known in HPRD to be phosphorylated	
**247**	CEND2	CEND2_HUMAN	CEND2; A3KLL7; ARAP1	Q96P48; A3KLL7; O94879; Q4LDD5; Q59FI7; Q6PHS3; Q8WU51; Q96HP6; Q96L71	NP_001035207; NP_056057	Arf-GAP, Rho-GAP domain, ANK repeat and PH domain-containing protein 1 (Centaurin-delta-2) (Cnt-d2); ARAP1b protein	GO:0008060; GO:0005794; GO:0005100; GO:0030037; GO:0016020; GO:0051497; GO:0005547; GO:0043089; GO:0051491; GO:0005515; GO:0032312; GO:0051270; GO:0008360; GO:0008270; GO:0005622	ARF GTPase activator activity; Golgi apparatus; Rho GTPase activator activity; actin filament reorganization during cell cycle; membrane; negative regulation of stress fiber formation; phosphatidylinositol-3,4,5-triphosphate binding; positive regulation of Cdc42 GTPase activity; positive regulation of filopodium assembly; protein binding; regulation of ARF GTPase activity; regulation of cell motion; regulation of cell shape; zinc ion binding; intracellular	CytoplasmGolgiapparatusGolgistackmembranePeripheralmembraneproteinCellmembrane;	Phophorylated - from this Study		Known in Uniprot to be phosphorylated		
**248**	TBA2	TBA2_HUMAN	TBA2; TBA3C	Q13748; A6NJQ0; Q5W099; Q6PEY3; Q96F18	NP_524575; NP_005992; NP_525125	Tubulin alpha-3C/D chain (Alpha-tubulin 3C/D) (Tubulin alpha-2 chain) (Alpha-tubulin 2)	GO:0005525; GO:0003924; GO:0005874; GO:0007018; GO:0005515; GO:0043234; GO:0051258; GO:0005198	GTP binding; GTPase activity; microtubule; microtubule-based movement; protein binding; protein complex; protein polymerization; structural molecule activity		Phophorylated - from this Study		Known in Uniprot to be phosphorylated		

	Protein Name	Uniprot Name	Other Names	Uniprot IDs	Refseq IDs	Protein Description	GO ID	GO terms	Subcellular location	Comments	Comments	Comments - Database Search	Comments - Database Search	Function
**249**	1433Z	1433Z_HUMAN	1433Z; A8K1N0	P63104; A8K1N0; P29213; P29312; Q32P43; Q5XJ08; Q6GPI2; Q6IN74; Q6NUR9; Q6P3U9; Q86V33	NP_663723; NP_001129171; NP_001129172; NP_001129173; NP_001129174; NP_003397; XP_209737	14-3-3 protein zeta/delta (Protein kinase C inhibitor protein 1) (KCIP-1)	GO:0006916; GO:0042470; GO:0019904; GO:0007165; GO:0008134	anti-apoptosis; melanosome; protein domain specific binding; signal transduction; transcription factor binding	CytoplasmMelanosome		Membrane-Fraction - this study	Known in Uniprot to be phosphorylated	Known in HPRD to be phosphorylated	
**250**	SEPT6	SEPT6_HUMAN	SEPT6; Q5JTK3; Q541S4	Q5JTK3; Q14141; Q541S4; Q5JTK0; Q969W5; Q96A13; Q96GR1; Q96P86; Q96P87	NP_665798; NP_665799; NP_055944	Septin-6	GO:0005525; GO:0007049; GO:0000910; GO:0005634; GO:0005515; GO:0031105	GTP binding; cell cycle; cytokinesis; nucleus; protein binding; septin complex		Phophorylated - from this Study	Membrane-Fraction - this study	Known in Uniprot to be phosphorylated		
**251**	ADDA	ADDA_HUMAN	ADDA; A2A2Q0; A2A3P0	P35611; Q13734; Q14729; Q16156; Q9UJB6; A2A3P0	NP_054908; NP_001110; NP_054909	Alpha-adducin (Erythrocyte adducin subunit alpha)	GO:0008290; GO:0051015; GO:0051017; GO:0051016; GO:0005516; GO:0005829; GO:0046872; GO:0005634; GO:0005886; GO:0032092; GO:0046982; GO:0042803; GO:0030507; GO:0008134	F-actin capping protein complex; actin filament binding; actin filament bundle formation; barbed-end actin filament capping; calmodulin binding; cytosol; metal ion binding; nucleus; plasma membrane; positive regulation of protein binding; protein heterodimerization activity; protein homodimerization activity; spectrin binding; transcription factor binding	CytoplasmcytoskeletonCellmembranePeripheralmembraneproteinCytoplasmicside	Phophorylated - from this Study		Known in Uniprot to be phosphorylated	Known in HPRD to be phosphorylated	
**252**	KPYM	KPYM_HUMAN	KPYM	P14618; A6NFK3; B2R5N8; B3KRY0; B4DFX8; P14786; Q53GK4; Q96E76; Q9BWB5; Q9UCV6; Q9UPF2	NP_872270; NP_002645; XP_294035; XP_290872	Pyruvate kinase isozymes M1/M2 (EC 2.7.1.40) (Pyruvate kinase muscle isozyme) (Pyruvate kinase 2/3) (Cytosolic thyroid hormone-binding protein) (CTHBP) (THBP1)	GO:0005829; GO:0006096; GO:0000287; GO:0030955; GO:0005515; GO:0004743	cytosol; glycolysis; magnesium ion binding; potassium ion binding; protein binding; pyruvate kinase activity		Phophorylated - from this Study		Known in Uniprot to be phosphorylated		Kinase
**253**	RLA1	RLA1_HUMAN	RLA1; A6NIB2	P05386; A6NIB2	NP_998890; XP_087062; NP_000994	60S acidic ribosomal protein P1	GO:0003723; GO:0022625; GO:0005515; GO:0003735; GO:0006414	RNA binding; cytosolic large ribosomal subunit; protein binding; structural constituent of ribosome; translational elongation		Phophorylated - from this Study		Known in Uniprot to be phosphorylated	Known in HPRD to be phosphorylated	
**254**	ZYX	ZYX_HUMAN	ZYX; Q96AF9; A4D2G6	Q15942; Q6I9S4; A4D2G6; Q96AF9	NP_001010972; NP_003452	Zyxin (Zyxin-2); ZYX protein (Fragment)	GO:0007155; GO:0005913; GO:0007267; GO:0005737; GO:0005925; GO:0005887; GO:0044419; GO:0005634; GO:0005515; GO:0007165; GO:0001725; GO:0008270; GO:0005737	cell adhesion; cell-cell adherens junction; cell-cell signaling; cytoplasm; focal adhesion; integral to plasma membrane; interspecies interaction between organisms; nucleus; protein binding; signal transduction; stress fiber; zinc ion binding; cytoplasm	CytoplasmCytoplasmcytoskeletonNucleus;	Phophorylated - from this Study		Known in Uniprot to be phosphorylated	Known in HPRD to be phosphorylated	
**255**	NP1L1	NP1L1_HUMAN	NP1L1	P55209	NP_004528; NP_631946	Nucleosome assembly protein 1-like 1 (NAP-1-related protein) (hNRP)	GO:0006260; GO:0005678; GO:0042470; GO:0006334; GO:0008284; GO:0005515	DNA replication; chromatin assembly complex; melanosome; nucleosome assembly; positive regulation of cell proliferation; protein binding	NucleusMelanosome	Phophorylated - from this Study		Known in Uniprot to be phosphorylated	Known in HPRD to be phosphorylated	
**256**	GRK6	GRK6_HUMAN	GRK6; Q96AD6	P43250; O60541; Q13652; Q96AD6	NP_002073; NP_001004105; NP_001004106	G protein-coupled receptor kinase 6 (EC 2.7.11.16) (G protein-coupled receptor kinase GRK6); G protein-coupled receptor kinase 6	GO:0005524; GO:0004703; GO:0016020; GO:0006468; GO:0008277; GO:0004871; GO:0004872; GO:0007165	ATP binding; G-protein coupled receptor kinase activity; membrane; protein amino acid phosphorylation; regulation of G-protein coupled receptor protein signaling pathway; signal transducer activity; receptor activity; signal transduction	MembraneLipidanchor;	Phophorylated - from this Study		Known in Uniprot to be phosphorylated	Known in HPRD to be phosphorylated	Kinase
**257**	SEPT2	SEPT2_HUMAN	SEPT2	Q15019; Q14132; Q8IUK9; Q96CB0	NP_006146; NP_004395; NP_001008491; NP_001008492	Septin-2 (Neural precursor cell expressed developmentally down-regulated protein 5) (NEDD-5)	GO:0005525; GO:0051301; GO:0007067; GO:0005634; GO:0005515; GO:0031105; GO:0005819	GTP binding; cell division; mitosis; nucleus; protein binding; septin complex; spindle	CytoplasmSpindle	Phophorylated - from this Study		Known in Uniprot to be phosphorylated	Known in HPRD to be phosphorylated	
**258**	ADDG	ADDG_HUMAN	ADDG; Q5VU09	Q5VU09; Q9UEY8; O43243; Q92773; Q9UEY7	NP_001112; NP_058432; NP_063968	Gamma-adducin (Adducin-like protein 70); Adducin 3 (Gamma) (Putative uncharacterized protein DKFZp686O0853) (Adducin 3 (Gamma), isoform CRA_b)	GO:0003779; GO:0005516; GO:0005737; GO:0005856; GO:0046872; GO:0005886; GO:0005200; GO:0005856; GO:0016020; GO:0005198	actin binding; calmodulin binding; cytoplasm; cytoskeleton; metal ion binding; plasma membrane; structural constituent of cytoskeleton; cytoskeleton; membrane; structural molecule activity	CytoplasmcytoskeletonCellmembranePeripheralmembraneproteinCytoplasmicside;	Phophorylated - from this Study		Known in Uniprot to be phosphorylated	Known in HPRD to be phosphorylated	
**259**	LAT	LAT_HUMAN	LAT	O43561; O43919	NP_001014988; NP_001014987; NP_001014989; NP_055202	Linker for activation of T-cells family member 1 (36 kDa phospho-tyrosine adapter protein) (pp36) (p36-38)	GO:0007265; GO:0005070; GO:0019722; GO:0001772; GO:0016021; GO:0007229; GO:0043303; GO:0045121; GO:0050863	Ras protein signal transduction; SH3/SH2 adaptor activity; calcium-mediated signaling; immunological synapse; integral to membrane; integrin-mediated signaling pathway; mast cell degranulation; membrane raft; regulation of T cell activation	CellmembraneSinglepasstypeIIImembraneprotein	Phophorylated - from this Study		Known in Uniprot to be phosphorylated	Known in HPRD to be phosphorylated	
**260**	JAM1	JAM1_HUMAN	JAM1; Q9Y5B2; Q6FIB4	Q9Y624; Q9Y5B2; Q6FIB4	NP_653085; NP_058642	Junctional adhesion molecule A (JAM-A) (Junctional adhesion molecule 1) (JAM-1) (Platelet adhesion molecule 1) (PAM-1) (Platelet F11 receptor) (CD antigen CD321); F11 receptor (F11 receptor protein) (F11R protein) (cDNA FLJ90671 fis, clone PLACE1005544, highly similar to Junctional adhesion molecule 1) (F11 receptor, isoform CRA_c) (JAM1); Junction adhesion molecule	GO:0006954; GO:0016021; GO:0044419; GO:0005923; GO:0007155; GO:0030855; GO:0005515; GO:0004872	inflammatory response; integral to membrane; interspecies interaction between organisms; tight junction; cell adhesion; epithelial cell differentiation; protein binding; receptor activity	CelljunctiontightjunctionCellmembraneSinglepasstypeImembraneprotein;	Phophorylated - from this Study	Membrane-Fraction - this study	Known in Uniprot to be phosphorylated	Known in HPRD to be phosphorylated	
**261**	EF1B	EF1B_HUMAN	EF1B; A8K795	P24534; Q6IBH9; A8K795	XP_935011; NP_001950; NP_001032752; NP_066944	Elongation factor 1-beta (EF-1-beta)	GO:0005829; GO:0005853; GO:0005515; GO:0003746; GO:0006414	cytosol; eukaryotic translation elongation factor 1 complex; protein binding; translation elongation factor activity; translational elongation		Phophorylated - from this Study		Known in Uniprot to be phosphorylated	Known in HPRD to be phosphorylated	
**262**	SSF1	SSF1_HUMAN	SSF1	Q9NQ55; Q9BW97; Q9H170	NP_001035754; NP_064615	Suppressor of SWI4 1 homolog (Ssf-1) (Peter Pan homolog)	GO:0008380; GO:0005634; GO:0005515	RNA splicing; nucleus; protein binding	Nucleusnucleolus			Known in Uniprot to be phosphorylated	Known in HPRD to be phosphorylated	
**263**	Q2NKW9	Q2NKW9_HUMAN	Q2NKW9	Q2NKW9	NP_001036024	Eukaryotic translation initiation factor 4 gamma, 2	GO:0016070; GO:0005515; GO:0003743	RNA metabolic process; protein binding; translation initiation factor activity		Phophorylated - from this Study				
**264**	Q8N1V6	Q8N1V6_HUMAN	Q8N1V6	Q8N1V6	XP_001128823	cDNA FLJ37514 fis, clone BRCAN2000639 (HCG2032358)				Phophorylated - from this Study				
**265**	SFR19	SFR19_HUMAN	Q9NR59; SFR19	Q9NR59; Q9H7N4; Q7Z5V7; Q8WVA1	XP_001130535	Splicing factor, arginine/serine-rich 19 (Serine arginine-rich pre-mRNA splicing factor SR-A1) (SR-A1) (SR-related-CTD-associated factor) (SCAF)	GO:0003723; GO:0008380; GO:0006397; GO:0005634	RNA binding; RNA splicing; mRNA processing; nucleus	Nucleus	Phophorylated - from this Study		Known in Uniprot to be phosphorylated		
**266**	Q9NWG2	Q9NWG2_HUMAN	Q9NWG2	Q9NWG2	XP_001129158	cDNA FLJ10044 fis, clone HEMBA1001088, moderately similar to PINCH PROTEIN	GO:0008270	zinc ion binding		Phophorylated - from this Study				
**267**	ACTS	ACTS_HUMAN	ACTS; Q5T8M9	P68133; Q5T8M9; P02568; P99020	NP_001091	Actin, alpha skeletal muscle (Alpha-actin-1); Actin, alpha 1, skeletal muscle	GO:0043531; GO:0005524; GO:0005884; GO:0006936; GO:0030240; GO:0017022; GO:0048741; GO:0001725; GO:0005865; GO:0005200; GO:0005524; GO:0005515	ADP binding; ATP binding; actin filament; muscle contraction; muscle thin filament assembly; myosin binding; skeletal muscle fiber development; stress fiber; striated muscle thin filament; structural constituent of cytoskeleton; ATP binding; protein binding	Cytoplasmcytoskeleton;		Membrane-Fraction - this study	Known in Uniprot to be phosphorylated		
**268**	ACTB	ACTB_HUMAN	ACTB; Q1KLZ0; ACTBL; B2RPJ1	P60709; Q1KLZ0; Q562R1; Q562R2; Q562S9; Q562X8; P02570; P70514; P99021; Q11211; Q64316; Q75MN2; Q96B34; Q96HG5; B2RPJ1	NP_001092; NP_001017992	Actin, cytoplasmic 1 (Beta-actin) [Cleaved into: Actin, cytoplasmic 1, N-terminally processed]; PS1TP5-binding protein 1 (HCG15971, isoform CRA_a)	GO:0005524; GO:0035267; GO:0006928; GO:0005856; GO:0050998; GO:0007605; GO:0005200; GO:0005829; GO:0005515; GO:0005625	ATP binding; NuA4 histone acetyltransferase complex; cell motion; cytoskeleton; nitric-oxide synthase binding; sensory perception of sound; structural constituent of cytoskeleton; cytosol; protein binding; soluble fraction	Cytoplasmcytoskeleton;		Membrane-Fraction - this study	Known in Uniprot to be phosphorylated		

	Protein Name	Uniprot Name	Other Names	Uniprot IDs	Refseq IDs	Protein Description	GO ID	GO terms	Subcellular location	Comments	Comments	Comments - Database Search	Comments - Database Search	Function
**269**	ACTN2	ACTN2_HUMAN	ACTN2; Q5VXF1; B1ANE4	P35609; Q5VXF1; Q86TF4; Q86TI8; B1ANE4	NP_001094	Alpha-actinin-2 (Alpha-actinin skeletal muscle isoform 2) (F-actin cross-linking protein)	GO:0051374; GO:0051370; GO:0003779; GO:0005884; GO:0005509; GO:0043197; GO:0030175; GO:0005925; GO:0048041; GO:0042802; GO:0005178; GO:0030035; GO:0005730; GO:0046983; GO:0051289; GO:0031143; GO:0042981; GO:0008307; GO:0070080; GO:0031432	FATZ 1 binding; ZASP binding; actin binding; actin filament; calcium ion binding; dendritic spine; filopodium; focal adhesion; focal adhesion formation; identical protein binding; integrin binding; microspike assembly; nucleolus; protein dimerization activity; protein homotetramerization; pseudopodium; regulation of apoptosis; structural constituent of muscle; titin Z domain binding; titin binding	CytoplasmmyofibrilsarcomereZdisk		Membrane-Fraction - this study			
**270**	LOX12	LOX12_HUMAN	LOX12	P18054; O95569; Q6ISF8; Q9UQM4	NP_000688	Arachidonate 12-lipoxygenase, 12S-type (12-LOX) (EC 1.13.11.31) (Platelet-type lipoxygenase 12)	GO:0006916; GO:0004052; GO:0006928; GO:0005829; GO:0019395; GO:0047977; GO:0005506; GO:0019370; GO:0016165; GO:0055114; GO:0045785; GO:0030307; GO:0008284; GO:0005515; GO:0042383; GO:0042554	anti-apoptosis; arachidonate 12-lipoxygenase activity; cell motion; cytosol; fatty acid oxidation; hepoxilin-epoxide hydrolase activity; iron ion binding; leukotriene biosynthetic process; lipoxygenase activity; oxidation reduction; positive regulation of cell adhesion; positive regulation of cell growth; positive regulation of cell proliferation; protein binding; sarcolemma; superoxide release	Cytoplasm		Membrane-Fraction - this study			
**271**	ANXA5	ANXA5_HUMAN	ANXA5; Q6FHB3; A8MTE3	P08758; Q6FHB3; Q6FI16; Q8WV69; A8MTE3	NP_001145	Annexin A5 (Annexin-5) (Annexin V) (Lipocortin V) (Endonexin II) (Calphobindin I) (CBP-I) (Placental anticoagulant protein I) (PAP-I) (Placental anticoagulant protein 4) (PP4) (Thromboplastin inhibitor) (Vascular anticoagulant-alpha) (VAC-alpha) (Anchorin CII); ANXA5 protein (Annexin A5, isoform CRA_b) (Putative uncharacterized protein ANXA5) (cDNA, FLJ93030, Homo sapiens annexin A5 (ANXA5), mRNA) (Fragment)	GO:0006916; GO:0007596; GO:0005509; GO:0005544; GO:0005737; GO:0050819; GO:0004859; GO:0005515; GO:0007165; GO:0005509; GO:0043499; GO:0051260; GO:0010033	anti-apoptosis; blood coagulation; calcium ion binding; calcium-dependent phospholipid binding; cytoplasm; negative regulation of coagulation; phospholipase inhibitor activity; protein binding; signal transduction; calcium ion binding; eukaryotic cell surface binding; protein homooligomerization; response to organic substance			Membrane-Fraction - this study	Known in Uniprot to be phosphorylated		
**272**	A4	A4_HUMAN	A4; B2R5V1	P05067; P09000; P78438; Q13764; Q13778; Q13793; Q16011; Q16014; Q16019; Q16020; Q6GSC0; Q8WZ99; Q9BT38; Q9UC33; Q9UCA9; Q9UCB6; Q9UCC8; Q9UCD1; Q9UQ58; B2R5V1	NP_000475; NP_001129601; NP_958816; NP_958817	Amyloid beta A4 protein (Alzheimer disease amyloid protein) (ABPP) (APPI) (APP) (PreA4) (Cerebral vascular amyloid peptide) (CVAP) (Protease nexin-II) (PN-II) [Cleaved into: Soluble APP-alpha (S-APP-alpha); Soluble APP-beta (S-APP beta); C99; Beta-amyloid protein 42 (Beta-APP42); Beta-amyloid protein 40 (Beta-APP40); C83; P3(42); P3(40); Gamma-secretase C-terminal fragment 59 (Gamma-CTF(59)) (Amyloid intracellular domain 59) (AICD-59) (AID(59)); Gamma-secretase C-terminal fragment 57 (Gamma-CTF(57)) (Amyloid intracellular domain 57) (AICD-57) (AID(57)); Gamma-secretase C-terminal fragment 50 (Gamma-CTF(50)) (Amyloid intracellular domain 50) (AICD-50) (AID(50)); C31]	GO:0005794; GO:0007219; GO:0033130; GO:0006915; GO:0007155; GO:0009986; GO:0006878; GO:0005905; GO:0005507; GO:0006897; GO:0005576; GO:0008201; GO:0042802; GO:0005887; GO:0005506; GO:0050905; GO:0031093; GO:0004867; GO:0008270	Golgi apparatus; Notch signaling pathway; acetylcholine receptor binding; apoptosis; cell adhesion; cell surface; cellular copper ion homeostasis; coated pit; copper ion binding; endocytosis; extracellular region; heparin binding; identical protein binding; integral to plasma membrane; iron ion binding; neuromuscular process; platelet alpha granule lumen; serine-type endopeptidase inhibitor activity; zinc ion binding	MembraneSinglepasstypeImembraneproteinMembraneclathrincoatedpit		Membrane-Fraction - this study	Known in Uniprot to be phosphorylated	Known in HPRD to be phosphorylated	
**273**	ATPO	ATPO_HUMAN	ATPO; B2R4E2	P48047; Q5U042; Q6IBI2; B2R4E2	NP_001688	ATP synthase subunit O, mitochondrial (Oligomycin sensitivity conferral protein) (OSCP)	GO:0006200; GO:0008144; GO:0046933; GO:0042776; GO:0005753; GO:0005886; GO:0045261; GO:0046961	ATP catabolic process; drug binding; hydrogen ion transporting ATP synthase activity, rotational mechanism; mitochondrial ATP synthesis coupled proton transport; mitochondrial proton-transporting ATP synthase complex; plasma membrane; proton-transporting ATP synthase complex, catalytic core F(1); proton-transporting ATPase activity, rotational mechanism	MitochondrionMitochondrioninnermembrane		Membrane-Fraction - this study			
**274**	CD9	CD9_HUMAN	CD9; Q5J7W6	P21926; Q5J7W6; Q96ES4	NP_001760	CD9 antigen (p24) (Leukocyte antigen MIC3) (Motility-related protein) (MRP-1) (Tetraspanin-29) (Tspan-29) (5H9 antigen) (Cell growth-inhibiting gene 2 protein) (CD antigen CD9)	GO:0007155; GO:0006928; GO:0007342; GO:0005887; GO:0030913; GO:0030168; GO:0031092; GO:0005515	cell adhesion; cell motion; fusion of sperm to egg plasma membrane; integral to plasma membrane; paranodal junction assembly; platelet activation; platelet alpha granule membrane; protein binding	MembraneMultipassmembraneprotein		Membrane-Fraction - this study			
**275**	CX7A2	CX7A2_HUMAN	CX7A2	P14406; Q3MIH5; B2R5E1; Q5TF59; Q6FGI2	NP_001856	Cytochrome c oxidase polypeptide 7A2, mitochondrial (Cytochrome c oxidase polypeptide VIIa-liver/heart) (Cytochrome c oxidase subunit VIIa-L) (VIIaL)	GO:0004129; GO:0009055; GO:0005746	cytochrome-c oxidase activity; electron carrier activity; mitochondrial respiratory chain	Mitochondrioninnermembrane		Membrane-Fraction - this study			
**276**	DECR	DECR_HUMAN	DECR	Q16698; Q2M304; Q93085	NP_001350	2,4-dienoyl-CoA reductase, mitochondrial (EC 1.3.1.34) (2,4-dienoyl-CoA reductase [NADPH]) (4-enoyl-CoA reductase [NADPH])	GO:0008670; GO:0070402; GO:0006635; GO:0005739; GO:0055114; GO:0051289	2,4-dienoyl-CoA reductase (NADPH) activity; NADPH binding; fatty acid beta-oxidation; mitochondrion; oxidation reduction; protein homotetramerization	Mitochondrion		Membrane-Fraction - this study			
**277**	NCB5R	NCB5R_HUMAN	NCB5R; NB5R3	P00387; B1AHF2; O75675; Q8TDL8; Q8WTS8; Q9UEN4; Q9UEN5; Q9UL55; Q9UL56	NP_000389; NP_001123291; NP_015565	NADH-cytochrome b5 reductase 3 (Cytochrome b5 reductase) (B5R) (EC 1.6.2.2) (Diaphorase-1) [Cleaved into: NADH-cytochrome b5 reductase 3 membrane-bound form; NADH-cytochrome b5 reductase 3 soluble form]	GO:0008015; GO:0006695; GO:0004128; GO:0009055; GO:0005789; GO:0005833; GO:0005741; GO:0055114	blood circulation; cholesterol biosynthetic process; cytochrome-b5 reductase activity; electron carrier activity; endoplasmic reticulum membrane; hemoglobin complex; mitochondrial outer membrane; oxidation reduction	EndoplasmicreticulummembraneLipidanchorCytoplasmicsideMitochondrionoutermembraneLipidanchorCytoplasmicsideCytoplasm		Membrane-Fraction - this study	Known in Uniprot to be phosphorylated		
**278**	IF5A1	IF5A1_HUMAN	IF5A1; A8K9A0	Q7L7L3; P63241; P10159; Q16182; Q7Z4L1; Q9D0G2; A8K9A0	NP_001961; NP_001137232; NP_001137233; NP_001137234	Eukaryotic translation initiation factor 5A-1 (eIF-5A-1) (eIF-5A1) (Eukaryotic initiation factor 5A isoform 1) (eIF-5A) (eIF-4D) (Rev-binding factor)	GO:0017070; GO:0005642; GO:0008283; GO:0005829; GO:0006917; GO:0006406; GO:0005643; GO:0008612; GO:0045948; GO:0047485; GO:0006611; GO:0003743; GO:0019079	U6 snRNA binding; annulate lamellae; cell proliferation; cytosol; induction of apoptosis; mRNA export from nucleus; nuclear pore; peptidyl-lysine modification to hypusine; positive regulation of translational initiation; protein N-terminus binding; protein export from nucleus; translation initiation factor activity; viral genome replication	Nucleus		Membrane-Fraction - this study			
**279**	GSH0	GSH0_HUMAN	GSH0; A8K334	P48507; Q6FHC1; Q9NPX9; Q9NU74; A8K334	NP_002052	Glutamate--cysteine ligase regulatory subunit (Gamma-glutamylcysteine synthetase regulatory subunit) (Gamma-ECS regulatory subunit) (GCS light chain) (Glutamate--cysteine ligase modifier subunit)	GO:0005829; GO:0006536; GO:0035226; GO:0006750; GO:0055114; GO:0016491; GO:0050880; GO:0042493; GO:0006979; GO:0005625	cytosol; glutamate metabolic process; glutamate-cysteine ligase catalytic subunit binding; glutathione biosynthetic process; oxidation reduction; oxidoreductase activity; regulation of blood vessel size; response to drug; response to oxidative stress; soluble fraction			Membrane-Fraction - this study			
**280**	GNAI2	GNAI2_HUMAN	GNAI2	P04899; Q8IZ71	NP_002061	Guanine nucleotide-binding protein G(i), alpha-2 subunit (Adenylate cyclase-inhibiting G alpha protein)	GO:0005525; GO:0003924; GO:0005515; GO:0007584; GO:0004871	GTP binding; GTPase activity; protein binding; response to nutrient; signal transducer activity			Membrane-Fraction - this study	Known in Uniprot to be phosphorylated		
**281**	GPDM	GPDM_HUMAN	GPDM; A8K4V0	P43304; B3KSA9; Q59FR1; Q9HAP9; A8K4V0	NP_000399; NP_001076581	Glycerol-3-phosphate dehydrogenase, mitochondrial (GPDH-M) (GPD-M) (EC 1.1.5.3) (mtGPD)	GO:0005509; GO:0004368; GO:0009331; GO:0006072; GO:0055114	calcium ion binding; glycerol-3-phosphate dehydrogenase activity; glycerol-3-phosphate dehydrogenase complex; glycerol-3-phosphate metabolic process; oxidation reduction	Mitochondrion		Membrane-Fraction - this study	Known in Uniprot to be phosphorylated		
**282**	GELS	GELS_HUMAN	GELS; Q5T0I2; A2A418	P06396; Q5T0I2; Q8WVV7; A2A418	NP_000168; NP_937895; NP_001121134; NP_001121135; NP_001121136; NP_001121137; NP_001121138; NP_001121139	Gelsolin (Actin-depolymerizing factor) (ADF) (Brevin) (AGEL); Gelsolin (Amyloidosis, Finnish type) (cDNA FLJ38961 fis, clone NT2RI2000276, highly similar to Gelsolin) (cDNA FLJ43831 fis, clone TESTI4005534, highly similar to Gelsolin) (Gelsolin (Amyloidosis, Finnish type), isoform CRA_a); Gelsolin (Amyloidosis, Finnish type) (Gelsolin (Amyloidosis, Finnish type), isoform CRA_c) (cDNA, FLJ96557, Homo sapiens gelsolin (amyloidosis, Finnish type) (GSN), mRNA)	GO:0003779; GO:0015629; GO:0051014; GO:0051016; GO:0005509; GO:0005829; GO:0005576; GO:0030027; GO:0016192; GO:0030041	actin binding; actin cytoskeleton; actin filament severing; barbed-end actin filament capping; calcium ion binding; cytosol; extracellular region; lamellipodium; vesicle-mediated transport; actin filament polymerization	Cytoplasmcytoskeleton Secreted;		Membrane-Fraction - this study	Known in Uniprot to be phosphorylated	Known in HPRD to be phosphorylated	
**283**	HXK1	HXK1_HUMAN	HXK1; A8K7J7	P19367; O43443; O43444; O75574; Q5VTC3; Q96HC8; Q9NNZ4; Q9NNZ5; A8K7J7	NP_000179; NP_277031; NP_277032	Hexokinase-1 (EC 2.7.1.1) (Hexokinase type I) (HK I) (Brain form hexokinase)	GO:0005524; GO:0005829; GO:0006096; GO:0004396; GO:0005741	ATP binding; cytosol; glycolysis; hexokinase activity; mitochondrial outer membrane	Mitochondrionoutermembrane		Membrane-Fraction - this study	Known in Uniprot to be phosphorylated		Kinase
**284**	ITA2	ITA2_HUMAN	ITA2	P17301; Q14595	NP_002194	Integrin alpha-2 (Platelet membrane glycoprotein Ia) (GPIa) (Collagen receptor) (VLA-2 alpha chain) (CD49 antigen-like family member B) (CD antigen CD49b)	GO:0007596; GO:0005509; GO:0007160; GO:0005518; GO:0042802; GO:0008305; GO:0007229; GO:0000287; GO:0009887; GO:0004872	blood coagulation; calcium ion binding; cell-matrix adhesion; collagen binding; identical protein binding; integrin complex; integrin-mediated signaling pathway; magnesium ion binding; organ morphogenesis; receptor activity	MembraneSinglepasstypeImembraneprotein		Membrane-Fraction - this study	Known in Uniprot to be phosphorylated		

	Protein Name	Uniprot Name	Other Names	Uniprot IDs	Refseq IDs	Protein Description	GO ID	GO terms	Subcellular location	Comments	Comments	Comments - Database Search	Comments - Database Search	Function
**285**	MYL6B	MYL6B_HUMAN	MYL6B	P14649	NP_002466	Myosin light chain 6B (Smooth muscle and nonmuscle myosin light chain alkali 6B) (Myosin light chain 1 slow-twitch muscle A isoform) (MLC1sa)	GO:0005509; GO:0003774; GO:0030049; GO:0005859; GO:0005515; GO:0007519; GO:0008307; GO:0016461	calcium ion binding; motor activity; muscle filament sliding; muscle myosin complex; protein binding; skeletal muscle tissue development; structural constituent of muscle; unconventional myosin complex			Membrane-Fraction - this study			
**286**	PLF4	PLF4_HUMAN	PLF4	P02776; Q53X61; Q9UC64; Q9UC65	NP_002610	Platelet factor 4 (PF-4) (C-X-C motif chemokine 4) (Oncostatin-A) (Iroplact) [Cleaved into: Platelet factor 4, short form]	GO:0008009; GO:0019221; GO:0005615; GO:0008201; GO:0006955; GO:0030595; GO:0045347; GO:0016525; GO:0043066; GO:0045918; GO:0045653; GO:0030168; GO:0031093; GO:0010744; GO:0010628; GO:0032760	chemokine activity; cytokine-mediated signaling pathway; extracellular space; heparin binding; immune response; leukocyte chemotaxis; negative regulation of MHC class II biosynthetic process; negative regulation of angiogenesis; negative regulation of apoptosis; negative regulation of cytolysis; negative regulation of megakaryocyte differentiation; platelet activation; platelet alpha granule lumen; positive regulation of foam cell differentiation; positive regulation of gene expression; positive regulation of tumor necrosis factor production	Secreted		Membrane-Fraction - this study			
**287**	RLA2	RLA2_HUMAN	RLA2; Q6FG96	P05387; Q6FG96	NP_000995	60S acidic ribosomal protein P2 (Renal carcinoma antigen NY-REN-44); RPLP2 protein (Ribosomal protein, large, P2, isoform CRA_a) (cDNA, FLJ92222, Homo sapiens ribosomal protein, large P2 (RPLP2), mRNA)	GO:0003723; GO:0022625; GO:0003735; GO:0006414; GO:0005840	RNA binding; cytosolic large ribosomal subunit; structural constituent of ribosome; translational elongation; ribosome			Membrane-Fraction - this study	Known in Uniprot to be phosphorylated	Known in HPRD to be phosphorylated	
**288**	RIB1	RIB1_HUMAN	RIB1; Q68DT1; RPN1; B2R5Z0	P04843; Q68DT1; B2R5Z0	NP_002941	Dolichyl-diphosphooligosaccharide--protein glycosyltransferase subunit 1 (EC 2.4.1.119) (Dolichyl-diphosphooligosaccharide--protein glycosyltransferase 67 kDa subunit) (Ribophorin-1) (Ribophorin I) (RPN-I)	GO:0004579; GO:0016021; GO:0042470; GO:0008250; GO:0018279; GO:0005515; GO:0030867	dolichyl-diphosphooligosaccharide-protein glycotransferase activity; integral to membrane; melanosome; oligosaccharyltransferase complex; protein amino acid N-linked glycosylation via asparagine; protein binding; rough endoplasmic reticulum membrane	EndoplasmicreticulummembraneSinglepasstypeImembraneproteinMelanosome		Membrane-Fraction - this study			
**289**	LYAM3	LYAM3_HUMAN	LYAM3; Q5R349; Q6NUL9	P16109; Q5R344; Q8IVD1; Q5R349; Q6NUL9	NP_002996	P-selectin (Granule membrane protein 140) (GMP-140) (PADGEM) (Leukocyte-endothelial cell adhesion molecule 3) (LECAM3) (CD62 antigen-like family member P) (CD antigen CD62P)	GO:0050829; GO:0009897; GO:0005615; GO:0042806; GO:0043208; GO:0008201; GO:0005887; GO:0007159; GO:0001530; GO:0005624; GO:0031092; GO:0010572; GO:0005515; GO:0032496; GO:0033691; GO:0005625	defense response to Gram-negative bacterium; external side of plasma membrane; extracellular space; fucose binding; glycosphingolipid binding; heparin binding; integral to plasma membrane; leukocyte adhesion; lipopolysaccharide binding; membrane fraction; platelet alpha granule membrane; positive regulation of platelet activation; protein binding; response to lipopolysaccharide; sialic acid binding; soluble fraction	MembraneSinglepasstypeImembraneprotein		Membrane-Fraction - this study			
**290**	SSB	SSB_HUMAN	SSB; A4D1U3; SSBP	Q04837; A4D1U3	NP_003134	Single-stranded DNA-binding protein, mitochondrial (Mt-SSB) (MtSSB) (PWP1-interacting protein 17); Single-stranded DNA binding protein 1, isoform CRA_a (Single-stranded DNA binding protein 1) (cDNA, FLJ93504, Homo sapiens single-stranded DNA binding protein 1 (SSBP1), mRNA)	GO:0006260; GO:0042645; GO:0003697	DNA replication; mitochondrial nucleoid; single-stranded DNA binding	Mitochondrion;		Membrane-Fraction - this study	Known in Uniprot to be phosphorylated	Known in HPRD to be phosphorylated	
**291**	TPM4	TPM4_HUMAN	TPM4; Q5U0D9	P67936; Q5U0D9; P07226; Q15659; Q9BU85; Q9H8Q3; Q9UCS1; Q9UCS2; Q9UCS3; Q9UCS4	XP_088391; NP_003281; NP_001138632; XP_208313	Tropomyosin alpha-4 chain (Tropomyosin-4) (TM30p1)	GO:0003779; GO:0006928; GO:0005862; GO:0008307	actin binding; cell motion; muscle thin filament tropomyosin; structural constituent of muscle	Cytoplasmcytoskeleton		Membrane-Fraction - this study	Known in Uniprot to be phosphorylated		
**292**	TBB2A	TBB2A_HUMAN	TBB2A	Q13885; Q6FGZ8; Q8IWR2	NP_001060	Tubulin beta-2A chain	GO:0005525; GO:0003924; GO:0005874; GO:0007018; GO:0005515; GO:0043234; GO:0051258; GO:0005198	GTP binding; GTPase activity; microtubule; microtubule-based movement; protein binding; protein complex; protein polymerization; structural molecule activity			Membrane-Fraction - this study		Known in HPRD to be phosphorylated	
**293**	UBP7	UBP7_HUMAN	UBP7	Q93009; A6NMY8	NP_003461	Ubiquitin carboxyl-terminal hydrolase 7 (EC 3.1.2.15) (Ubiquitin thioesterase 7) (Ubiquitin-specific-processing protease 7) (Deubiquitinating enzyme 7) (Herpesvirus-associated ubiquitin-specific protease)	GO:0004197; GO:0044419; GO:0005634; GO:0008022; GO:0016579; GO:0004221; GO:0006511; GO:0004843	cysteine-type endopeptidase activity; interspecies interaction between organisms; nucleus; protein C-terminus binding; protein deubiquitination; ubiquitin thiolesterase activity; ubiquitin-dependent protein catabolic process; ubiquitin-specific protease activity	Nucleus		Membrane-Fraction - this study	Known in Uniprot to be phosphorylated	Known in HPRD to be phosphorylated	
**294**	VDAC1	VDAC1_HUMAN	VDAC1; B3KVK4	P21796; Q5FVE7; Q9UIQ5; Q9UPL0; B3KVK4	NP_003365	Voltage-dependent anion-selective channel protein 1 (VDAC-1) (hVDAC1) (Outer mitochondrial membrane protein porin 1) (Plasmalemmal porin) (Porin 31HL) (Porin 31HM)	GO:0006820; GO:0006915; GO:0044419; GO:0042645; GO:0005741; GO:0005886; GO:0046930; GO:0005515; GO:0008308	anion transport; apoptosis; interspecies interaction between organisms; mitochondrial nucleoid; mitochondrial outer membrane; plasma membrane; pore complex; protein binding; voltage-gated anion channel activity	MitochondrionoutermembraneCellmembrane		Membrane-Fraction - this study	Known in Uniprot to be phosphorylated	Known in HPRD to be phosphorylated	
**295**	ADA10	ADA10_HUMAN	ADA10	O14672; Q10742; Q92650	NP_001101	Disintegrin and metalloproteinase domain-containing protein 10 (ADAM 10) (EC 3.4.24.81) (Mammalian disintegrin-metalloprotease) (Kuzbanian protein homolog) (CDw156) (CD antigen CD156c)	GO:0005798; GO:0007219; GO:0051088; GO:0017124; GO:0009986; GO:0007267; GO:0051089; GO:0012505; GO:0001701; GO:0016021; GO:0005178; GO:0007229; GO:0004222; GO:0042117; GO:0007162; GO:0005634; GO:0005886; GO:0010820; GO:0030307; GO:0008284; GO:0006468; GO:0042803; GO:0019901; GO:0034612; GO:0008270	Golgi-associated vesicle; Notch signaling pathway; PMA-inducible membrane protein ectodomain proteolysis; SH3 domain binding; cell surface; cell-cell signaling; constitutive protein ectodomain proteolysis; endomembrane system; in utero embryonic development; integral to membrane; integrin binding; integrin-mediated signaling pathway; metalloendopeptidase activity; monocyte activation; negative regulation of cell adhesion; nucleus; plasma membrane; positive regulation of T cell chemotaxis; positive regulation of cell growth; positive regulation of cell proliferation; protein amino acid phosphorylation; protein homodimerization activity; protein kinase binding; response to tumor necrosis factor; zinc ion binding	CellmembraneSinglepasstypeImembraneproteinIntracytoplasmicmembraneSinglepasstypeImembraneprotein		Membrane-Fraction - this study			
**296**	ATPK	ATPK_HUMAN	ATPK; Q6IBB3	P56134; Q6IBB3; O76079; Q96L83; Q9BTI8	NP_004880; NP_001003713; NP_001003714	ATP synthase subunit f, mitochondrial; ATP synthase, H+ transporting, mitochondrial F0 complex, subunit F2 (ATP synthase, H+ transporting, mitochondrial F0 complex, subunit f, isoform 2) (ATP5J2 protein)	GO:0006754; GO:0015078; GO:0005753; GO:0015992; GO:0045263;	ATP biosynthetic process; hydrogen ion transmembrane transporter activity; mitochondrial proton-transporting ATP synthase complex; proton transport; proton-transporting ATP synthase complex, coupling factor F(o);	MitochondrionMitochondrioninnermembrane;		Membrane-Fraction - this study			
**297**	B2MG	B2MG_HUMAN	B2MG; Q540F8	P61769; Q540F8; P01884; Q6IAT8; Q9UCK0; Q9UDF4	NP_004039	Beta-2-microglobulin [Cleaved into: Beta-2-microglobulin form pI 5.3]	GO:0000139; GO:0042612; GO:0002474; GO:0031901; GO:0005576; GO:0006955; GO:0005515	Golgi membrane; MHC class I protein complex; antigen processing and presentation of peptide antigen via MHC class I; early endosome membrane; extracellular region; immune response; protein binding	Secreted		Membrane-Fraction - this study			
**298**	CLH1	CLH1_HUMAN	CLH1	Q00610; Q6N0A0; Q86TF2	NP_004850	Clathrin heavy chain 1 (CLH-17)	GO:0030132; GO:0030130; GO:0006886; GO:0042470; GO:0005515; GO:0005198; GO:0016192	clathrin coat of coated pit; clathrin coat of trans-Golgi network vesicle; intracellular protein transport; melanosome; protein binding; structural molecule activity; vesicle-mediated transport	CytoplasmicvesiclemembranePeripheralmembraneproteinCytoplasmicsideMembranecoatedpitPeripheralmembraneproteinCytoplasmicsideMelanosome		Membrane-Fraction - this study	Known in Uniprot to be phosphorylated	Known in HPRD to be phosphorylated	
**299**	DCAK1	DCAK1_HUMAN	DCAK1; Q5VZZ0; DCLK1	Q5VZZ0; O15075; Q5VZY8; Q5VZZ1	NP_004725	Serine/threonine-protein kinase DCLK1 (EC 2.7.11.1) (Doublecortin-like and CAM kinase-like 1) (Doublecortin-like kinase 1)	GO:0005524; GO:0030154; GO:0007417; GO:0016197; GO:0005887; GO:0007242; GO:0006468; GO:0005515; GO:0004674; GO:0005057	ATP binding; cell differentiation; central nervous system development; endosome transport; integral to plasma membrane; intracellular signaling cascade; protein amino acid phosphorylation; protein binding; protein serine/threonine kinase activity; receptor signaling protein activity			Membrane-Fraction - this study	Known in Uniprot to be phosphorylated	Known in HPRD to be phosphorylated	Kinase
**300**	GPV	GPV_HUMAN	GPV	P40197	NP_004479	Platelet glycoprotein V (GPV) (CD antigen CD42D)	GO:0007596; GO:0007155; GO:0005887; GO:0031092; GO:0005515	blood coagulation; cell adhesion; integral to plasma membrane; platelet alpha granule membrane; protein binding	MembraneSinglepasstypeImembraneprotein		Membrane-Fraction - this study			
**301**	PPIB	PPIB_HUMAN	PPIB	P23284; A8K534; Q6IBH5; Q9BVK5	NP_000933	Peptidyl-prolyl cis-trans isomerase B (PPIase) (Rotamase) (EC 5.2.1.8) (Cyclophilin B) (S-cyclophilin) (SCYLP) (CYP-S1)	GO:0005783; GO:0005788; GO:0042470; GO:0042277; GO:0003755; GO:0006457; GO:0051082	endoplasmic reticulum; endoplasmic reticulum lumen; melanosome; peptide binding; peptidyl-prolyl cis-trans isomerase activity; protein folding; unfolded protein binding	EndoplasmicreticulumlumenMelanosome		Membrane-Fraction - this study			
**302**	RB11A	RB11A_HUMAN	RB11A; B2R4B6	P62491; P24410; Q5TZN9; Q9JLX1; B2R4B6	NP_004654	Ras-related protein Rab-11A (Rab-11) (YL8)	GO:0005525; GO:0003924; GO:0005886; GO:0048227; GO:0055038; GO:0007264; GO:0019905; GO:0005802; GO:0005215	GTP binding; GTPase activity; plasma membrane; plasma membrane to endosome transport; recycling endosome membrane; small GTPase mediated signal transduction; syntaxin binding; trans-Golgi network; transporter activity	CellmembranePeripheralmembraneproteinRecyclingendosomemembranePeripheralmembraneprotein		Membrane-Fraction - this study			

	Protein Name	Uniprot Name	Other Names	Uniprot IDs	Refseq IDs	Protein Description	GO ID	GO terms	Subcellular location	Comments	Comments	Comments - Database Search	Comments - Database Search	Function
**303**	DHSA	DHSA_HUMAN	DHSA; A8K5J6	P31040; A8K5J6; Q16395; Q9UMY5	NP_004159; XP_291117	Succinate dehydrogenase [ubiquinone] flavoprotein subunit, mitochondrial (EC 1.3.5.1) (Flavoprotein subunit of complex II) (Fp)	GO:0050660; GO:0009055; GO:0005749; GO:0007399; GO:0005515; GO:0022904; GO:0008177; GO:0006105; GO:0006810; GO:0006099	FAD binding; electron carrier activity; mitochondrial respiratory chain complex II; nervous system development; protein binding; respiratory electron transport chain; succinate dehydrogenase (ubiquinone) activity; succinate metabolic process; transport; tricarboxylic acid cycle	MitochondrioninnermembranePeripheralmembraneproteinMatrixside		Membrane-Fraction - this study	Known in Uniprot to be phosphorylated		
**304**	SNP29	SNP29_HUMAN	SNP29	O95721	NP_004773	Synaptosomal-associated protein 29 (SNAP-29) (Vesicle-membrane fusion protein SNAP-29) (Soluble 29 kDa NSF attachment protein)	GO:0005484; GO:0030054; GO:0005737; GO:0006887; GO:0006944; GO:0015031; GO:0045202; GO:0019717; GO:0006903	SNAP receptor activity; cell junction; cytoplasm; exocytosis; membrane fusion; protein transport; synapse; synaptosome; vesicle targeting	CytoplasmMembranePeripheralmembraneproteinCelljunctionsynapse synaptosome		Membrane-Fraction - this study			
**305**	NDUB8	NDUB8_HUMAN	NDUB8; A8K0L4	O95169; Q5W144; Q9UG53; Q9UJR4; Q9UQF3; A8K0L4	NP_004995	NADH dehydrogenase [ubiquinone] 1 beta subcomplex subunit 8, mitochondrial (NADH-ubiquinone oxidoreductase ASHI subunit) (Complex I-ASHI) (CI-ASHI)	GO:0008137; GO:0005783; GO:0016021; GO:0006120; GO:0005747; GO:0006810	NADH dehydrogenase (ubiquinone) activity; endoplasmic reticulum; integral to membrane; mitochondrial electron transport, NADH to ubiquinone; mitochondrial respiratory chain complex I; transport	MitochondrioninnermembraneSinglepassmembraneproteinMatrixside		Membrane-Fraction - this study			
**306**	PROF1	PROF1_HUMAN	PROF1; Q53Y44	P07737; Q53Y44	NP_005013	Profilin-1 (Profilin I); Profilin	GO:0003779; GO:0015629; GO:0030036; GO:0005737; GO:0007010; GO:0001843; GO:0005634; GO:0006357	actin binding; actin cytoskeleton; actin cytoskeleton organization; cytoplasm; cytoskeleton organization; neural tube closure; nucleus; regulation of transcription from RNA polymerase II promoter	Cytoplasmcytoskeleton ;		Membrane-Fraction - this study	Known in Uniprot to be phosphorylated		
**307**	ACTZ	ACTZ_HUMAN	ACTZ; B2R6B0	P61163; P42024; B2R6B0	NP_005727	Alpha-centractin (Centractin) (Centrosome-associated actin homolog) (Actin-RPV) (ARP1)	GO:0005524; GO:0005813; GO:0005869; GO:0005515; GO:0016192	ATP binding; centrosome; dynactin complex; protein binding; vesicle-mediated transport	Cytoplasmcytoskeleton Centrosome		Membrane-Fraction - this study			
**308**	ARP2	ARP2_HUMAN	ARP2; B2RCP5	P61160; O15142; Q96C82; B2RCP5	NP_005713; NP_001005386	Actin-related protein 2 (Actin-like protein 2)	GO:0005524; GO:0005885; GO:0003779; GO:0006928; GO:0042995; GO:0005737	ATP binding; Arp2/3 protein complex; actin binding; cell motion; cell projection; cytoplasm	Cytoplasmcytoskeleton Cellprojection		Membrane-Fraction - this study			
**309**	ARP3	ARP3_HUMAN	ARP3; Q53QM2	P61158; Q53QM2; P32391	NP_005712	Actin-related protein 3 (Actin-like protein 3); ARP3 actin-related protein 3 homolog (Yeast), isoform CRA_a (Putative uncharacterized protein ACTR3) (cDNA, FLJ93049, Homo sapiens ARP3 actin-related protein 3 homolog (yeast) (ACTR3), mRNA)	GO:0005524; GO:0005885; GO:0003779; GO:0006928; GO:0005737; GO:0030833; GO:0005856; GO:0030027; GO:0005515	ATP binding; Arp2/3 protein complex; actin binding; cell motion; cytoplasm; regulation of actin filament polymerization; cytoskeleton; lamellipodium; protein binding	Cytoplasmcytoskeleton Cellprojection;		Membrane-Fraction - this study	Known in Uniprot to be phosphorylated		
**310**	ARPC5	ARPC5_HUMAN	ARPC5	O15511; A6NEC4; Q6PG42	NP_005708	Actin-related protein 2/3 complex subunit 5 (Arp2/3 complex 16 kDa subunit) (p16-ARC)	GO:0005885; GO:0003779; GO:0006928; GO:0042995; GO:0005737; GO:0030833; GO:0005200	Arp2/3 protein complex; actin binding; cell motion; cell projection; cytoplasm; regulation of actin filament polymerization; structural constituent of cytoskeleton	Cytoplasmcytoskeleton Cellprojection		Membrane-Fraction - this study		Known in HPRD to be phosphorylated	
**311**	BET1	BET1_HUMAN	BET1; Q53XK0	O15155; Q53XK0	NP_005859	BET1 homolog (hBET1) (Golgi vesicular membrane-trafficking protein p18); BET1 homolog (S. cerevisiae) (BET1 homolog (S. cerevisiae), isoform CRA_d) (cDNA, FLJ94635, Homo sapiens BET1 homolog (S. cerevisiae) (BET1), mRNA)	GO:0006888; GO:0000139; GO:0005789; GO:0016021; GO:0005515; GO:0015031;	ER to Golgi vesicle-mediated transport; Golgi membrane; endoplasmic reticulum membrane; integral to membrane; protein binding; protein transport;	GolgiapparatusmembraneSinglepasstypeIVmembraneproteinEndoplasmicreticulummembraneSinglepasstypeIVmembraneprotein;		Membrane-Fraction - this study	Known in Uniprot to be phosphorylated		
**312**	COF1	COF1_HUMAN	COF1; B3KUQ1	P23528; B3KUQ1; Q53Y87; Q9UCA2	NP_005498; XP_291459	Cofilin-1 (Cofilin, non-muscle isoform) (18 kDa phosphoprotein) (p18)	GO:0007266; GO:0003779; GO:0006916; GO:0016363	Rho protein signal transduction; actin binding; anti-apoptosis; nuclear matrix	NucleusmatrixCytoplasmcytoskeleton		Membrane-Fraction - this study	Known in Uniprot to be phosphorylated	Known in HPRD to be phosphorylated	
**313**	PDIA6	PDIA6_HUMAN	PDIA6; Q53RC7	Q15084; Q53RC7; Q6ZSH5; Q99778	NP_005733	Protein disulfide-isomerase A6 (EC 5.3.4.1) (Protein disulfide isomerase P5) (Thioredoxin domain-containing protein 7); cDNA FLJ76201, highly similar to Homo sapiens protein disulfide isomerase family A, member 6 (PDIA6), mRNA (Protein disulfide isomerase family A, member 6, isoform CRA_b) (Putative uncharacterized protein P5)	GO:0005793; GO:0045454; GO:0005783; GO:0005788; GO:0042470; GO:0005515; GO:0003756; GO:0006457; GO:0045454; GO:0016853	ER-Golgi intermediate compartment; cell redox homeostasis; endoplasmic reticulum; endoplasmic reticulum lumen; melanosome; protein binding; protein disulfide isomerase activity; protein folding; cell redox homeostasis; isomerase activity	EndoplasmicreticulumlumenMelanosome;		Membrane-Fraction - this study	Known in Uniprot to be phosphorylated	Known in HPRD to be phosphorylated	
**314**	PPIF	PPIF_HUMAN	PPIF	P30405; Q5W131	NP_005720	Peptidyl-prolyl cis-trans isomerase, mitochondrial (PPIase) (Rotamase) (EC 5.2.1.8) (Cyclophilin F)	GO:0005624; GO:0005759; GO:0042277; GO:0003755; GO:0006457	membrane fraction; mitochondrial matrix; peptide binding; peptidyl-prolyl cis-trans isomerase activity; protein folding	Mitochondrionmatrix		Membrane-Fraction - this study			
**315**	ATP5H	ATP5H_HUMAN	ATP5H; A0PJH2; B2R5L6	O75947; A0PJH2; B2R5L6; Q9H3J4	NP_006347; XP_210750; XP_071133; NP_001003785	ATP synthase subunit d, mitochondrial; ATP5H protein (Fragment)	GO:0015986; GO:0015078; GO:0000276	ATP synthesis coupled proton transport; hydrogen ion transmembrane transporter activity; mitochondrial proton-transporting ATP synthase complex, coupling factor F(o)	MitochondrionMitochondrioninnermembrane;		Membrane-Fraction - this study			
**316**	PRAF3	PRAF3_HUMAN	PRAF3; B2R6V5	O75915; Q53ES3; Q5KU08; B2R6V5	NP_006398	PRA1 family protein 3 (ADP-ribosylation factor-like protein 6-interacting protein 5) (ARL-6-interacting protein 5) (Aip-5) (Glutamate transporter EAAC1-interacting protein) (GTRAP3-18) (Prenylated Rab acceptor protein 2) (Protein JWa) (Dermal papilla-derived protein 11) (JM5) (Putative MAPK-activating protein PM27) (Cytoskeleton-related vitamin A-responsive protein)	GO:0015813; GO:0005789; GO:0016021; GO:0005515	L-glutamate transport; endoplasmic reticulum membrane; integral to membrane; protein binding	EndoplasmicreticulummembraneMultipassmembraneproteinCytoplasm		Membrane-Fraction - this study			
**317**	MLRM	MLRM_HUMAN	MLRM; Q53X45; ML12A	P19105; Q53X45	NP_006462	Myosin regulatory light chain MRLC3 (Myosin regulatory light chain 2, nonsarcomeric) (Myosin RLC) (MLC-2B); Myosin regulatory light chain (cDNA FLJ76400, highly similar to Homo sapiens myosin regulatory light chain MRCL3 (MRCL3), mRNA) (Myosin regulatory light chain MRCL3, isoform CRA_a)	GO:0005509; GO:0003774; GO:0016459; GO:0005515	calcium ion binding; motor activity; myosin complex; protein binding			Membrane-Fraction - this study	Known in Uniprot to be phosphorylated		
**318**	HSP7C	HSP7C_HUMAN	HSP7C	P11142; Q9H3R6	NP_006588; XP_293610; XP_291446; XP_166540; NP_694881	Heat shock cognate 71 kDa protein (Heat shock 70 kDa protein 8)	GO:0005524; GO:0042623; GO:0009986; GO:0042470; GO:0005515; GO:0006457; GO:0006986	ATP binding; ATPase activity, coupled; cell surface; melanosome; protein binding; protein folding; response to unfolded protein	CytoplasmMelanosome		Membrane-Fraction - this study	Known in Uniprot to be phosphorylated	Known in HPRD to be phosphorylated	
**319**	RB27B	RB27B_HUMAN	RB27B; B2RAB0	O00194; Q9BZB6; B2RAB0	NP_004154	Ras-related protein Rab-27B (C25KG)	GO:0005525; GO:0003924; GO:0016020; GO:0015031; GO:0007264	GTP binding; GTPase activity; membrane; protein transport; small GTPase mediated signal transduction	MembraneLipidanchor		Membrane-Fraction - this study			
**320**	1433E	1433E_HUMAN	1433E; Q53XZ5; Q4VJB6	P62258; Q53XZ5; B3KY71; P29360; P42655; Q63631; Q7M4R4; Q4VJB6	NP_006752; XP_086931; XP_016144	14-3-3 protein epsilon (14-3-3E)	GO:0019899; GO:0044419; GO:0007242; GO:0042470; GO:0019904	enzyme binding; interspecies interaction between organisms; intracellular signaling cascade; melanosome; protein domain specific binding	CytoplasmMelanosome		Membrane-Fraction - this study	Known in Uniprot to be phosphorylated		
**321**	STXB2	STXB2_HUMAN	STXB2; Q53GF4	Q15833; Q53GF4	NP_008880	Syntaxin-binding protein 2 (Unc-18 homolog 2) (Unc18-2) (Unc-18B)	GO:0006886; GO:0030347; GO:0030348; GO:0006904	intracellular protein transport; syntaxin-2 binding; syntaxin-3 binding; vesicle docking during exocytosis			Membrane-Fraction - this study			
**322**	TWF2	TWF2_HUMAN	TWF2	Q6IBS0; Q9Y3F5	NP_009215	Twinfilin-2 (Twinfilin-1-like protein) (A6-related protein) (hA6RP) (Protein tyrosine kinase 9-like)	GO:0005524; GO:0003779; GO:0005737; GO:0005856	ATP binding; actin binding; cytoplasm; cytoskeleton	Cytoplasmcytoskeleton Cytoplasmperinuclearregion		Membrane-Fraction - this study	Known in Uniprot to be phosphorylated		
**323**	TSP3	TSP3_HUMAN	TSP3; Q4VX26; B1AVR8	P49746; Q4VX26; Q8WV34; B1AVR8	NP_009043	Thrombospondin-3	GO:0005509; GO:0007160; GO:0005576; GO:0031093; GO:0005515; GO:0005198	calcium ion binding; cell-matrix adhesion; extracellular region; platelet alpha granule lumen; protein binding; structural molecule activity			Membrane-Fraction - this study	Known in Uniprot to be phosphorylated		
**324**	RSU1	RSU1_HUMAN	RSU1; Q6FI17; Q96ND5; Q5VWS1; A8KA46	Q15404; Q6FI17; Q5VWS1; A8KA46	NP_036557; XP_291710; NP_689937	Ras suppressor protein 1 (Rsu-1) (RSP-1)	GO:0005515; GO:0007165	protein binding; signal transduction			Membrane-Fraction - this study			
**325**	EHD3	EHD3_HUMAN	EHD3	Q9NZN3; Q8N514; Q9NZB3	NP_055415	EH domain-containing protein 3	GO:0005524; GO:0005525; GO:0003924; GO:0005509; GO:0003676; GO:0005634; GO:0005886; GO:0055038	ATP binding; GTP binding; GTPase activity; calcium ion binding; nucleic acid binding; nucleus; plasma membrane; recycling endosome membrane	CellmembranePeripheralmembraneproteinCytoplasmicsideEndosomemembranePeripheralmembraneproteinRecyclingendosomemembranePeripheralmembraneprotein		Membrane-Fraction - this study			
**326**	CMC2	CMC2_HUMAN	CMC2	Q9UJS0; O14566; O14575; Q9NZW1; Q9UNI7	NP_055066	Calcium-binding mitochondrial carrier protein Aralar2 (Mitochondrial aspartate glutamate carrier 2) (Solute carrier family 25 member 13) (Citrin)	GO:0006754; GO:0015183; GO:0005313; GO:0015813; GO:0015810; GO:0005509; GO:0045333; GO:0005887; GO:0043490; GO:0005743; GO:0051592	ATP biosynthetic process; L-aspartate transmembrane transporter activity; L-glutamate transmembrane transporter activity; L-glutamate transport; aspartate transport; calcium ion binding; cellular respiration; integral to plasma membrane; malate-aspartate shuttle; mitochondrial inner membrane; response to calcium ion	MitochondrioninnermembraneMultipassmembraneprotein		Membrane-Fraction - this study			
**327**	DHB12	DHB12_HUMAN	DHB12; A8K9B0	Q9Y6G8; Q53GQ0; Q96JU2; A8K9B0	NP_057226	Estradiol 17-beta-dehydrogenase 12 (EC 1.1.1.62) (17-beta-hydroxysteroid dehydrogenase 12) (17-beta-HSD 12) (3-ketoacyl-CoA reductase) (KAR) (EC 1.3.1.-)	GO:0005488; GO:0005789; GO:0004303; GO:0016021; GO:0055114; GO:0006694	binding; endoplasmic reticulum membrane; estradiol 17-beta-dehydrogenase activity; integral to membrane; oxidation reduction; steroid biosynthetic process	EndoplasmicreticulummembraneMultipassmembraneprotein		Membrane-Fraction - this study			
**328**	PARVA	PARVA_HUMAN	PARVA	Q9NVD7; Q96C85; Q9HA48	NP_060692	Alpha-parvin (Calponin-like integrin-linked kinase-binding protein) (CH-ILKBP) (Matrix-remodeling-associated protein 2) (Actopaxin)	GO:0003779; GO:0007155; GO:0005737; GO:0005856	actin binding; cell adhesion; cytoplasm; cytoskeleton	CelljunctionfocaladhesionCellmembranePeripheralmembraneproteinCytoplasmicsideCytoplasmcytoskeleton		Membrane-Fraction - this study	Known in Uniprot to be phosphorylated	Known in HPRD to be phosphorylated	
**329**	SEP11	SEP11_HUMAN	SEP11; Q96SP1	Q9NVA2; Q7L4N1; Q96SP1	NP_060713	Septin-11	GO:0005525; GO:0007049; GO:0051301; GO:0005634; GO:0005515; GO:0051291; GO:0031105; GO:0001725	GTP binding; cell cycle; cell division; nucleus; protein binding; protein heterooligomerization; septin complex; stress fiber			Membrane-Fraction - this study			

	Protein Name	Uniprot Name	Other Names	Uniprot IDs	Refseq IDs	Protein Description	GO ID	GO terms	Subcellular location	Comments	Comments	Comments - Database Search	Comments - Database Search	Function
**330**	CK059	CK059_HUMAN	CK059	Q9NWT0; Q6IAA8; Q8WZ09	NP_060377	UPF0404 protein C11orf59					Membrane-Fraction - this study	Known in Uniprot to be phosphorylated		
**331**	MARE2	MARE2_HUMAN	MARE2; B2RE21	Q15555; Q9UQ33; B2RE21	NP_055083; NP_001137298; NP_001137299	Microtubule-associated protein RP/EB family member 2 (APC-binding protein EB2) (End-binding protein 2) (EB2)	GO:0051301; GO:0008283; GO:0005737; GO:0005874; GO:0008017; GO:0007067; GO:0007165	cell division; cell proliferation; cytoplasm; microtubule; microtubule binding; mitosis; signal transduction	Cytoplasm		Membrane-Fraction - this study	Known in Uniprot to be phosphorylated		
**332**	RHOA	RHOA_HUMAN	RHOA; Q5U024; Q9BVT0	P61586; Q5U024; P06749; Q9UEJ4; Q9BVT0	NP_001655	Transforming protein RhoA (H12); ARHA protein (Fragment)	GO:0005525; GO:0003924; GO:0007266; GO:0005737; GO:0005856; GO:0000287; GO:0005886; GO:0043123; GO:0042346; GO:0005515; GO:0030154; GO:0000902; GO:0007160; GO:0043524; GO:0005634; GO:0007519; GO:0007264; GO:0043149	GTP binding; GTPase activity; Rho protein signal transduction; cytoplasm; cytoskeleton; magnesium ion binding; plasma membrane; positive regulation of I-kappaB kinase/NF-kappaB cascade; positive regulation of NF-kappaB import into nucleus; protein binding; cell differentiation; cell morphogenesis; cell-matrix adhesion; negative regulation of neuron apoptosis; nucleus; skeletal muscle tissue development; small GTPase mediated signal transduction; stress fiber formation	CellmembraneLipidanchorCytoplasmicsideCytoplasmcytoskeleton;		Membrane-Fraction - this study		Known in HPRD to be phosphorylated	
**333**	PPIA	PPIA_HUMAN	PPIA; A8K220; B5MD27	P62937; Q3KQW3; P05092; Q6IBU5; Q96IX3; Q9BRU4; Q9BTY9; Q9UC61; A8K220; B5MD27	NP_066953; XP_060887; XP_292963; XP_001130327; XP_060398; XP_067503; XP_293007; XP_293023; XP_067176; XP_116396	Peptidyl-prolyl cis-trans isomerase A (PPIase A) (Rotamase A) (EC 5.2.1.8) (Cyclophilin A) (Cyclosporin A-binding protein)	GO:0005829; GO:0005576; GO:0019059; GO:0044419; GO:0005634; GO:0042277; GO:0003755; GO:0006457; GO:0019047; GO:0045069; GO:0051082; GO:0046790	cytosol; extracellular region; initiation of viral infection; interspecies interaction between organisms; nucleus; peptide binding; peptidyl-prolyl cis-trans isomerase activity; protein folding; provirus integration; regulation of viral genome replication; unfolded protein binding; virion binding	Cytoplasm		Membrane-Fraction - this study	Known in Uniprot to be phosphorylated		
**334**	GBB1	GBB1_HUMAN	GBB1; Q5QPR5	P62873; Q5QPR5; B1AJZ7; P04697; P04901	NP_002065	Guanine nucleotide-binding protein G(I)/G(S)/G(T) subunit beta-1 (Transducin beta chain 1)	GO:0003924; GO:0007265; GO:0009755; GO:0007213; GO:0004871	GTPase activity; Ras protein signal transduction; hormone-mediated signaling; muscarinic acetylcholine receptor signaling pathway; signal transducer activity			Membrane-Fraction - this study			
**335**	K6PP	K6PP_HUMAN	K6PP; Q5VSR8	Q01813; Q5VSR8	NP_002618	6-phosphofructokinase type C (EC 2.7.1.11) (Phosphofructokinase 1) (Phosphohexokinase) (Phosphofructo-1-kinase isozyme C) (PFK-C) (6-phosphofructokinase, platelet type); Phosphofructokinase, platelet (cDNA FLJ78683, highly similar to Homo sapiens phosphofructokinase, platelet (PFKP), mRNA)	GO:0003872; GO:0005945; GO:0005524; GO:0006096; GO:0000287; GO:0005515	6-phosphofructokinase activity; 6-phosphofructokinase complex; ATP binding; glycolysis; magnesium ion binding; protein binding			Membrane-Fraction - this study	Known in Uniprot to be phosphorylated	Known in HPRD to be phosphorylated	Kinase
**336**	WASF2	WASF2_HUMAN	WASF2; Q8NBU0	Q9Y6W5; O60794; Q9UDY7; Q8NBU0	NP_008921	Wiskott-Aldrich syndrome protein family member 2 (WASP family protein member 2) (Protein WAVE-2) (Verprolin homology domain-containing protein 2); cDNA FLJ90750 fis, clone PLACE2000118, weakly similar to WISKOTT-ALDRICH SYNDROME PROTEIN HOMOLOG	GO:0007188; GO:0003779; GO:0015629; GO:0030036; GO:0005737; GO:0030027; GO:0007186; GO:0004969; GO:0016021	G-protein signaling, coupled to cAMP nucleotide second messenger; actin binding; actin cytoskeleton; actin cytoskeleton organization; cytoplasm; lamellipodium; G-protein coupled receptor protein signaling pathway; histamine receptor activity; integral to membrane	Cytoplasmcytoskeleton Cellprojectionlamellipodium;		Membrane-Fraction - this study	Known in Uniprot to be phosphorylated		
**337**	MK1I1	MK1I1_HUMAN	MK1I1; MPKS1	Q9UHA4; B2R4A1; Q9H364	NP_068805	Mitogen-activated protein kinase scaffold protein 1 (Mitogen-activated protein kinase kinase 1-interacting protein 1) (MEK-binding partner 1) (Mp1)	GO:0031902; GO:0005515	late endosome membrane; protein binding	LateendosomemembranePeripheralmembraneproteinCytoplasmicside		Membrane-Fraction - this study			
**338**	ACTN4	ACTN4_HUMAN	ACTN4	O43707; O76048	NP_004915; XP_293669	Alpha-actinin-4 (Non-muscle alpha-actinin 4) (F-actin cross-linking protein)	GO:0051015; GO:0005509; GO:0005178; GO:0005730; GO:0001882; GO:0048471; GO:0051272; GO:0032417; GO:0043234; GO:0042803; GO:0031143; GO:0042981	actin filament binding; calcium ion binding; integrin binding; nucleolus; nucleoside binding; perinuclear region of cytoplasm; positive regulation of cell motion; positive regulation of sodium:hydrogen antiporter activity; protein complex; protein homodimerization activity; pseudopodium; regulation of apoptosis	NucleusCytoplasm		Membrane-Fraction - this study	Known in Uniprot to be phosphorylated		
**339**	CAN1	CAN1_HUMAN	CAN1; Q2TTR0	P07384; Q2TTR0; Q6DHV4	NP_005177	Calpain-1 catalytic subunit (EC 3.4.22.52) (Calpain 1 large subunit) (Calcium-activated neutral proteinase 1) (CANP 1) (Calpain mu-type) (muCANP) (Micromolar-calpain) (Cell proliferation-inducing gene 30 protein)	GO:0005509; GO:0004198; GO:0005737; GO:0005886; GO:0008284; GO:0005515; GO:0006508	calcium ion binding; calcium-dependent cysteine-type endopeptidase activity; cytoplasm; plasma membrane; positive regulation of cell proliferation; protein binding; proteolysis	CytoplasmCellmembrane		Membrane-Fraction - this study			
**340**	MYH11	MYH11_HUMAN	MYH11; Q3MIV8; Q3MNF0	P35749; O00396; O94944; P78422; Q3MIV8; Q3MNF0	NP_002465; NP_074035; NP_001035202; NP_001035203	Myosin-11 (Myosin heavy chain 11) (Myosin heavy chain, smooth muscle isoform) (SMMHC); Myosin, heavy chain 11, smooth muscle (Myosin, heavy polypeptide 11, smooth muscle, isoform CRA_b); Myosin, heavy chain 11, smooth muscle (Smooth muscle myosin heavy chain isoform SM2)	GO:0005524; GO:0003779; GO:0005516; GO:0048739; GO:0048251; GO:0042470; GO:0003774; GO:0030241; GO:0006939; GO:0006941; GO:0005863; GO:0008307; GO:0016459	ATP binding; actin binding; calmodulin binding; cardiac muscle fiber development; elastic fiber assembly; melanosome; motor activity; muscle thick filament assembly; smooth muscle contraction; striated muscle contraction; striated muscle thick filament; structural constituent of muscle; myosin complex	Melanosome;		Membrane-Fraction - this study	Known in Uniprot to be phosphorylated		
**341**	LIMS1	LIMS1_HUMAN	LIMS1	P48059; B2RAJ4; Q53TE0; Q9BS44	NP_004978	LIM and senescent cell antigen-like-containing domain protein 1 (Particularly interesting new Cys-His protein 1) (PINCH-1) (Renal carcinoma antigen NY-REN-48)	GO:0007569; GO:0030054; GO:0008270	cell aging; cell junction; zinc ion binding	CelljunctionfocaladhesionCellmembranePeripheralmembraneproteinCytoplasmicside		Membrane-Fraction - this study			
**342**	HXK2	HXK2_HUMAN	HXK2	P52789; Q8WU87; Q9UN82	NP_000180	Hexokinase-2 (EC 2.7.1.1) (Hexokinase type II) (HK II) (Muscle form hexokinase)	GO:0005524; GO:0005829; GO:0006096; GO:0004396; GO:0005741	ATP binding; cytosol; glycolysis; hexokinase activity; mitochondrial outer membrane	Mitochondrionoutermembrane		Membrane-Fraction - this study	Known in Uniprot to be phosphorylated		Kinase
**343**	LRBA	LRBA_HUMAN	LRBA	P50851; Q9H2U3; Q9H2U4	NP_006717	Lipopolysaccharide-responsive and beige-like anchor protein (CDC4-like protein) (Beige-like protein)	GO:0005515	protein binding			Membrane-Fraction - this study	Known in Uniprot to be phosphorylated		
**344**	PI4KA	PI4KA_HUMAN	PI4KA; Q4LE69	P42356; Q7Z625; Q9UPG2; Q4LE69	NP_477352; NP_002641	Phosphatidylinositol 4-kinase alpha (PI4-kinase alpha) (PtdIns-4-kinase alpha) (PI4K-alpha) (EC 2.7.1.67)	GO:0004430; GO:0005798; GO:0006661; GO:0046854; GO:0048015; GO:0005515; GO:0007268	1-phosphatidylinositol 4-kinase activity; Golgi-associated vesicle; phosphatidylinositol biosynthetic process; phosphoinositide phosphorylation; phosphoinositide-mediated signaling; protein binding; synaptic transmission			Membrane-Fraction - this study	Known in Uniprot to be phosphorylated		Kinase
**345**	MYL6	MYL6_HUMAN	MYL6; Q561V9; Q6IBG5	P60660; Q6IBG5; P16475; P24572; P24573; Q12790; Q561V9; Q6IAZ0; Q6IPY5	NP_066299; NP_524147; XP_945628	Myosin light polypeptide 6 (Smooth muscle and nonmuscle myosin light chain alkali 6) (Myosin light chain alkali 3) (Myosin light chain 3) (MLC-3) (LC17)	GO:0030898; GO:0005509; GO:0003774; GO:0030049; GO:0007519; GO:0008307; GO:0016461	actin-dependent ATPase activity; calcium ion binding; motor activity; muscle filament sliding; skeletal muscle tissue development; structural constituent of muscle; unconventional myosin complex			Membrane-Fraction - this study	Known in Uniprot to be phosphorylated		
**346**	MLE1	MLE1_HUMAN	MLE1; B2R4T6	P05976; Q6IBD5; B2R4T6	NP_524144	Myosin light chain 1, skeletal muscle isoform (MLC1F) (A1 catalytic) (Alkali myosin light chain 1)	GO:0005509; GO:0003774; GO:0030049; GO:0005859; GO:0007517; GO:0030017; GO:0008307	calcium ion binding; motor activity; muscle filament sliding; muscle myosin complex; muscle organ development; sarcomere; structural constituent of muscle			Membrane-Fraction - this study			
**347**	PGH1	PGH1_HUMAN	PGH1; Q5T7T7	Q5T7T7; P23219; Q15122; Q5T7T6; Q5T7T8	NP_000953; NP_542158	Prostaglandin G/H synthase 1 (EC 1.14.99.1) (Cyclooxygenase-1) (COX-1) (Prostaglandin-endoperoxide synthase 1) (Prostaglandin H2 synthase 1) (PGH synthase 1) (PGHS-1) (PHS 1)	GO:0005794; GO:0005789; GO:0020037; GO:0005792; GO:0005634; GO:0055114; GO:0016702; GO:0004601; GO:0005886; GO:0001516; GO:0004666; GO:0008217; GO:0006979	Golgi apparatus; endoplasmic reticulum membrane; heme binding; microsome; nucleus; oxidation reduction; oxidoreductase activity, acting on single donors with incorporation of molecular oxygen, incorporation of two atoms of oxygen; peroxidase activity; plasma membrane; prostaglandin biosynthetic process; prostaglandin-endoperoxide synthase activity; regulation of blood pressure; response to oxidative stress	MicrosomemembranePeripheralmembraneproteinEndoplasmicreticulummembranePeripheralmembraneprotein		Membrane-Fraction - this study			
**348**	ITB1	ITB1_HUMAN	ITB1; Q8WUM6	Q8WUM6; P05556; A8K6N2; P78466; P78467; Q13089; Q13090; Q13091; Q13212; Q14622; Q14647; Q29RW2; Q7Z3V1	NP_002202; NP_389647; NP_391987; NP_391988; NP_391989; NP_596867	Integrin beta-1 (Fibronectin receptor subunit beta) (Integrin VLA-4 subunit beta) (CD antigen CD29)	GO:0030183; GO:0009986; GO:0033631; GO:0007160; GO:0006968; GO:0007156; GO:0042802; GO:0008305; GO:0007229; GO:0044419; GO:0007159; GO:0042470; GO:0031594; GO:0046982; GO:0004872; GO:0001726; GO:0042383	B cell differentiation; cell surface; cell-cell adhesion mediated by integrin; cell-matrix adhesion; cellular defense response; homophilic cell adhesion; identical protein binding; integrin complex; integrin-mediated signaling pathway; interspecies interaction between organisms; leukocyte adhesion; melanosome; neuromuscular junction; protein heterodimerization activity; receptor activity; ruffle; sarcolemma	CellmembraneSinglepasstypeImembraneproteinMelanosome		Membrane-Fraction - this study	Known in Uniprot to be phosphorylated		
**349**	HPCA	HPCA_HUMAN	HPCA; B2R9T3	P84074; P32076; P41211; P70510; B2R9T3	NP_002134	Neuron-specific calcium-binding protein hippocalcin (Calcium-binding protein BDR-2)	GO:0003779; GO:0005509	actin binding; calcium ion binding			Membrane-Fraction - this study			

	Protein Name	Uniprot Name	Other Names	Uniprot IDs	Refseq IDs	Protein Description	GO ID	GO terms	Subcellular location	Comments	Comments	Comments - Database Search	Comments - Database Search	Function
**350**	PECA1	PECA1_HUMAN	PECA1	P16284; Q6LDA9; Q8TBH1; Q96RF5; Q96RF6; Q9NP65; Q9NPB7; Q9NPG9; Q9NQS9; Q9NQT0; Q9NQT1; Q9NQT2	NP_000433	Platelet endothelial cell adhesion molecule (PECAM-1) (EndoCAM) (GPIIA') (CD antigen CD31)	GO:0007155; GO:0008037; GO:0005615; GO:0016021; GO:0005886; GO:0031092; GO:0005515; GO:0007165	cell adhesion; cell recognition; extracellular space; integral to membrane; plasma membrane; platelet alpha granule membrane; protein binding; signal transduction	MembraneSinglepasstypeImembraneprotein		Membrane-Fraction - this study	Known in Uniprot to be phosphorylated	Known in HPRD to be phosphorylated	
**351**	FHL1	FHL1_HUMAN	FHL1; Q6IB30	Q6IB30; Q13642; O95212; Q13230; Q13645; Q5JXI7; Q5M7Y6; Q9NZ40; Q9UKZ8; Q9Y630	NP_001440	Four and a half LIM domains protein 1 (FHL-1) (Skeletal muscle LIM-protein 1) (SLIM 1) (SLIM); cDNA FLJ16189 fis, clone BRTHA2012714, highly similar to Four and a half LIM domains protein 1 (cDNA FLJ78310, highly similar to Homo sapiens four and a half LIM domains 1 (FHL1), mRNA) (FHL1 protein) (Four and a half LIM domains 1, isoform CRA_a)	GO:0030154; GO:0016049; GO:0005737; GO:0007517; GO:0005634; GO:0009887; GO:0008270; GO:0008270	cell differentiation; cell growth; cytoplasm; muscle organ development; nucleus; organ morphogenesis; zinc ion binding; zinc ion binding	CytoplasmCytoplasmNucleusNucleusCytoplasmcytosol;		Membrane-Fraction - this study			
**352**	PDIA3	PDIA3_HUMAN	PDIA3	P30101; Q13453; Q14255; Q8IYF8; Q9UMU7	NP_005304	Protein disulfide-isomerase A3 (EC 5.3.4.1) (Disulfide isomerase ER-60) (ERp60) (58 kDa microsomal protein) (p58) (ERp57) (58 kDa glucose-regulated protein)	GO:0045454; GO:0004197; GO:0005783; GO:0005788; GO:0042470; GO:0004629; GO:0005515; GO:0003756; GO:0006606; GO:0006621; GO:0007165	cell redox homeostasis; cysteine-type endopeptidase activity; endoplasmic reticulum; endoplasmic reticulum lumen; melanosome; phospholipase C activity; protein binding; protein disulfide isomerase activity; protein import into nucleus; protein retention in ER lumen; signal transduction	EndoplasmicreticulumlumenMelanosome		Membrane-Fraction - this study			
**353**	JAM3	JAM3_HUMAN	JAM3	Q9BX67; Q8WWL8; Q96FL1	NP_116190	Junctional adhesion molecule C (JAM-C) (Junctional adhesion molecule 3) (JAM-3) (JAM-2)	GO:0016021; GO:0005886	integral to membrane; plasma membrane	CellmembraneSinglepasstypeImembraneprotein		Membrane-Fraction - this study			
**354**	1433G	1433G_HUMAN	1433G	P61981; O70457; P35214; Q6FH52; Q9UDP2; Q9UN99	NP_036611	14-3-3 protein gamma (Protein kinase C inhibitor protein 1) (KCIP-1) [Cleaved into: 14-3-3 protein gamma, N-terminally processed]	GO:0005737; GO:0005159; GO:0006469; GO:0019904; GO:0005080; GO:0008426; GO:0045664; GO:0009966; GO:0048167	cytoplasm; insulin-like growth factor receptor binding; negative regulation of protein kinase activity; protein domain specific binding; protein kinase C binding; protein kinase C inhibitor activity; regulation of neuron differentiation; regulation of signal transduction; regulation of synaptic	Cytoplasm		Membrane-Fraction - this study	Known in Uniprot to be phosphorylated		
**355**	MDHM	MDHM_HUMAN	MDHM	P40926; A8K414; B2RE78; O43682	NP_005909	Malate dehydrogenase, mitochondrial (EC 1.1.1.37)	GO:0030060; GO:0005488; GO:0006096; GO:0006108; GO:0005759; GO:0055114; GO:0006099	L-malate dehydrogenase activity; binding; glycolysis; malate metabolic process; mitochondrial matrix; oxidation reduction; tricarboxylic acid cycle	Mitochondrionmatrix		Membrane-Fraction - this study	Known in Uniprot to be phosphorylated		
**356**	TPM3	TPM3_HUMAN	TPM3; Q5VU71; Q8NAH0	P06753; Q8NAH0; P12324; Q2QD06; Q5VU71; Q969Q2; Q9NQH8	NP_705935; NP_689476; NP_001036816; NP_001036817; XP_208872	Tropomyosin alpha-3 chain (Tropomyosin-3) (Gamma-tropomyosin) (Tropomyosin-5) (hTM5)	GO:0003779; GO:0006928; GO:0005862; GO:0006937	actin binding; cell motion; muscle thin filament tropomyosin; regulation of muscle contraction	Cytoplasmcytoskeleton		Membrane-Fraction - this study	Known in Uniprot to be phosphorylated	Known in HPRD to be phosphorylated	
**357**	CYFP1	CYFP1_HUMAN	CYFP1; A8K6D9	Q7L576; Q14467; Q5IED0; Q6ZSX1; Q9BSD9; Q9BVC7; A8K6D9	NP_055423; NP_001028200	Cytoplasmic FMR1-interacting protein 1 (Specifically Rac1-associated protein 1) (Sra-1) (p140sra-1)	GO:0048365; GO:0051015; GO:0048675; GO:0030054; GO:0030027; GO:0030032; GO:0048471; GO:0008360; GO:0001726; GO:0031529; GO:0045202; GO:0019717	Rac GTPase binding; actin filament binding; axon extension; cell junction; lamellipodium; lamellipodium assembly; perinuclear region of cytoplasm; regulation of cell shape; ruffle; ruffle organization; synapse; synaptosome	CytoplasmperinuclearregionCellprojectionlamellipodiumCellprojectionruffleCelljunctionsynapsesynaptosome		Membrane-Fraction - this study	Known in Uniprot to be phosphorylated		
**358**	C109A	C109A_HUMAN	C109A	Q8NE86; Q96FL3	NP_612366	Coiled-coil domain-containing protein 109A	GO:0016021	integral to membrane	MembraneMultipassmembraneprotein		Membrane-Fraction - this study			
**359**	STS1	STS1_HUMAN	STS1; UBS3B	Q8TF42; Q53GT5; Q53GT8; Q8NBV7; Q96IG9; Q96NZ2	NP_116262	Ubiquitin-associated and SH3 domain-containing protein B (Suppressor of T-cell receptor signaling 1) (STS-1) (Cbl-interacting protein p70)	GO:0005737; GO:0005634	cytoplasm; nucleus	CytoplasmNucleus		Membrane-Fraction - this study	Known in Uniprot to be phosphorylated		
**360**	1A03	1A03_HUMAN	1A03; Q8WUX3; B1PKY1	P04439; Q65A82; O19546; O19756; Q9GJE6; Q9GJE7; Q9GJE8; Q9MYE6; Q9MYG4; Q9TPR8; B1PKY1; Q8WUX3	NP_002107	HLA class I histocompatibility antigen, A-3 alpha chain (MHC class I antigen A*3); HLA-A protein	GO:0042612; GO:0032393; GO:0002474; GO:0006955; GO:0005887; GO:0044419; GO:0005515; GO:0016021	MHC class I protein complex; MHC class I receptor activity; antigen processing and presentation of peptide antigen via MHC class I; immune response; integral to plasma membrane; interspecies interaction between organisms; protein binding; integral to membrane	MembraneSinglepasstypeImembraneprotein;		Membrane-Fraction - this study		Known in HPRD to be phosphorylated	
**361**	PHF2	PHF2_HUMAN	PHF2; Q4VXB9	O75151; Q4VXG0; Q8N3K2; Q9Y6N4	NP_005383	PHD finger protein 2 (GRC5)	GO:0005634; GO:0005515; GO:0008270	nucleus; protein binding; zinc ion binding	Nucleus		Membrane-Fraction - this study	Known in Uniprot to be phosphorylated		
**362**	VDAC3	VDAC3_HUMAN	VDAC3	Q9Y277; Q9UIS0	NP_005653; NP_001129166	Voltage-dependent anion-selective channel protein 3 (VDAC-3) (hVDAC3) (Outer mitochondrial membrane protein porin 3)	GO:0015853; GO:0006820; GO:0016021; GO:0005741; GO:0000166; GO:0005515; GO:0008308	adenine transport; anion transport; integral to membrane; mitochondrial outer membrane; nucleotide binding; protein binding; voltage-gated anion channel activity	Mitochondrionoutermembrane		Membrane-Fraction - this study	Known in Uniprot to be phosphorylated		
**363**	RHOC	RHOC_HUMAN	RHOC; B3KSW1	P08134; Q6ICN3; B3KSW1	NP_786886; NP_001036143; NP_001036144	Rho-related GTP-binding protein RhoC (H9)	GO:0005525; GO:0003924; GO:0005622; GO:0005886; GO:0043123; GO:0005515; GO:0004871; GO:0007264	GTP binding; GTPase activity; intracellular; plasma membrane; positive regulation of I-kappaB kinase/NF-kappaB cascade; protein binding; signal transducer activity; small GTPase mediated signal transduction	CellmembraneLipidanchorCytoplasmicside		Membrane-Fraction - this study	Known in Uniprot to be phosphorylated		
**364**	MLRN	MLRN_HUMAN	MLRN; Q9BUF9; MYL9	P24844; Q9H136; Q9BUF9; Q9BQL9	NP_006088; NP_852667	Myosin regulatory light polypeptide 9 (Myosin regulatory light chain 9) (Myosin regulatory light chain MRLC1) (Myosin regulatory light chain 2, smooth muscle isoform) (Myosin RLC) (MLC-2C) (LC20); Myosin, light chain 9, regulatory (Regulatory myosin light chain short version) (Myosin, light polypeptide 9, regulatory, isoform CRA_c)	GO:0005509; GO:0003774; GO:0005859; GO:0006937; GO:0008307	calcium ion binding; motor activity; muscle myosin complex; regulation of muscle contraction; structural constituent of muscle			Membrane-Fraction - this study	Known in Uniprot to be phosphorylated		
**365**	ACOX1	ACOX1_HUMAN	ACOX1	Q15067; Q12863; Q15068; Q15101; Q16131; Q9UD31	NP_004026; NP_009223	Peroxisomal acyl-coenzyme A oxidase 1 (AOX) (EC 1.3.3.6) (Palmitoyl-CoA oxidase) (Straight-chain acyl-CoA oxidase) (SCOX)	GO:0050660; GO:0003995; GO:0003997; GO:0009055; GO:0006635; GO:0006091; GO:0055114; GO:0006693; GO:0047485	FAD binding; acyl-CoA dehydrogenase activity; acyl-CoA oxidase activity; electron carrier activity; fatty acid beta-oxidation; generation of precursor metabolites and energy; oxidation reduction; prostaglandin metabolic process; protein N-terminus binding	Peroxisome		Membrane-Fraction - this study	Known in Uniprot to be phosphorylated		
**366**	PCYXL	PCYXL_HUMAN	PCYXL	Q8NBM8; Q7Z4S2; Q8NCY5; Q8NF69; Q9BTE8; Q9BWS3	NP_076933	Prenylcysteine oxidase-like (EC 1.8.3.-)	GO:0005576; GO:0055114; GO:0016670; GO:0030328	extracellular region; oxidation reduction; oxidoreductase activity, acting on sulfur group of donors, oxygen as acceptor; prenylcysteine catabolic process	Secreted		Membrane-Fraction - this study			
**367**	ATPB	ATPB_HUMAN	ATPB; A8K4X0	P06576; Q14283; A8K4X0	NP_001677	ATP synthase subunit beta, mitochondrial (EC 3.6.3.14)	GO:0005524; GO:0015986; GO:0042288; GO:0001525; GO:0009986; GO:0043499; GO:0046933; GO:0008553; GO:0042645; GO:0005754; GO:0005886; GO:0046961; GO:0051453	ATP binding; ATP synthesis coupled proton transport; MHC class I protein binding; angiogenesis; cell surface; eukaryotic cell surface binding; hydrogen ion transporting ATP synthase activity, rotational mechanism; hydrogen-exporting ATPase activity, phosphorylative mechanism; mitochondrial nucleoid; mitochondrial proton-transporting ATP synthase, catalytic core; plasma membrane; proton-transporting ATPase activity, rotational mechanism; regulation of intracellular pH	MitochondrionMitochondrioninnermembrane		Membrane-Fraction - this study	Known in Uniprot to be phosphorylated		
**368**	PCF11	PCF11_HUMAN	PCF11; A6H8W7	O94913; O43671; Q6P0X8; A6H8W7	NP_056969	Pre-mRNA cleavage complex 2 protein Pcf11 (Pre-mRNA cleavage complex II protein Pcf11)	GO:0006379; GO:0005849; GO:0000398	mRNA cleavage; mRNA cleavage factor complex; nuclear mRNA splicing, via spliceosome	Nucleus		Membrane-Fraction - this study	Known in Uniprot to be phosphorylated	Known in HPRD to be phosphorylated	
**369**	NNTM	NNTM_HUMAN	NNTM	Q13423; Q16796; Q2TB60; Q8N3V4	NP_036475; NP_892022	NAD(P) transhydrogenase, mitochondrial (EC 1.6.1.2) (Nicotinamide nucleotide transhydrogenase) (Pyridine nucleotide transhydrogenase)	GO:0051287; GO:0008750; GO:0003957; GO:0050661; GO:0009055; GO:0016021; GO:0005746; GO:0055114; GO:0015992; GO:0006099	NAD or NADH binding; NAD(P)+ transhydrogenase (AB-specific) activity; NAD(P)+ transhydrogenase (B-specific) activity; NADP or NADPH binding; electron carrier activity; integral to membrane; mitochondrial respiratory chain; oxidation reduction; proton transport; tricarboxylic acid cycle	MitochondrioninnermembraneMultipassmembraneproteinMatrixside		Membrane-Fraction - this study			
**370**	RIB2	RIB2_HUMAN	RIB2; RPN2	P04844; Q6IBA5; Q96E21; Q9BUQ3; Q9UBE1	NP_002942	Dolichyl-diphosphooligosaccharide--protein glycosyltransferase subunit 2 (EC 2.4.1.119) (Dolichyl-diphosphooligosaccharide--protein glycosyltransferase 63 kDa subunit) (Ribophorin-2) (Ribophorin II) (RPN-II) (RIBIIR)	GO:0004579; GO:0016021; GO:0008250; GO:0018279; GO:0005515	dolichyl-diphosphooligosaccharide-protein glycotransferase activity; integral to membrane; oligosaccharyltransferase complex; protein amino acid N-linked glycosylation via asparagine; protein binding	EndoplasmicreticulummembraneMultipassmembraneprotein		Membrane-Fraction - this study	Known in Uniprot to be phosphorylated		

	Protein Name	Uniprot Name	Other Names	Uniprot IDs	Refseq IDs	Protein Description	GO ID	GO terms	Subcellular location	Comments	Comments	Comments - Database Search	Comments - Database Search	Function
**371**	STOM	STOM_HUMAN	STOM; Q9H376	P27105; Q14087; Q15609; Q5VX96; Q96FK4; Q9H376	NP_004090; NP_937837	Erythrocyte band 7 integral membrane protein (Stomatin) (Protein 7.2b); PRO0720	GO:0005856; GO:0005887; GO:0042470; GO:0045121; GO:0005515; GO:0051260;	cytoskeleton; integral to plasma membrane; melanosome; membrane raft; protein binding; protein homooligomerization;	CellmembraneSinglepassmembraneproteinCytoplasmicsideCellmembraneLipidanchorCytoplasmicsideMelanosome;		Membrane-Fraction - this study	Known in Uniprot to be phosphorylated	Known in HPRD to be phosphorylated	
**372**	RASA3	RASA3_HUMAN	Q8IUY2; RASA3	Q8IUY2; Q14644; A6NL15	NP_031394	Ras GTPase-activating protein 3 (GAP1(IP4BP)) (Ins P4-binding protein)	GO:0005096; GO:0005622; GO:0005886; GO:0051056; GO:0008270	GTPase activator activity; intracellular; plasma membrane; regulation of small GTPase mediated signal transduction; zinc ion binding	Cellmembrane		Membrane-Fraction - this study	Known in Uniprot to be phosphorylated		
**373**	GANAB	GANAB_HUMAN	GANAB; Q9BS14	Q14697; A6NC20; Q8WTS9; Q9P0X0; Q9BS14	NP_055425; NP_938148; NP_938149	Neutral alpha-glucosidase AB (EC 3.2.1.84) (Glucosidase II subunit alpha) (Alpha-glucosidase 2); GANAB protein (Fragment)	GO:0005794; GO:0005975; GO:0005783; GO:0033919; GO:0042470; GO:0005515; GO:0005975; GO:0004553	Golgi apparatus; carbohydrate metabolic process; endoplasmic reticulum; glucan 1,3-alpha-glucosidase activity; melanosome; protein binding; carbohydrate metabolic process; hydrolase activity, hydrolyzing O-glycosyl compounds	Endoplasmicreticulum GolgiapparatusMelanosome;					
**374**	ATD3A	ATD3A_HUMAN	ATD3A	Q9NVI7; Q5SV23; Q8N275; Q96A50	NP_060658	ATPase family AAA domain-containing protein 3A	GO:0005524; GO:0017111; GO:0005515	ATP binding; nucleoside-triphosphatase activity; protein binding			Membrane-Fraction - this study	Known in Uniprot to be phosphorylated		
**375**	GNA12	GNA12_HUMAN	GNA12; Q6ZQV4; A4D204; B3KXS2	Q03113; A4D204; Q2T9L1; Q86UM8; Q8TD71; Q9UDU9; B3KXS2; Q6ZQV4	NP_031379	Guanine nucleotide-binding protein subunit alpha-12 (G-protein subunit alpha-12) (G alpha-12); cDNA FLJ46868 fis, clone UTERU3012414, highly similar to Guanine nucleotide-binding protein, alpha-12 subunit	GO:0007186; GO:0005525; GO:0003924; GO:0007596; GO:0005886; GO:0005515; GO:0004871; GO:0019001	G-protein coupled receptor protein signaling pathway; GTP binding; GTPase activity; blood coagulation; plasma membrane; protein binding; signal transducer activity; guanyl nucleotide binding	MembraneLipidanchor;		Membrane-Fraction - this study			
**376**	MMRN1	MMRN1_HUMAN	MMRN1; Q6ZUL9; Q4W5L1	Q4W5L1; Q13201; Q6P3T8; Q6ZUL9	NP_031377	Multimerin-1 (Endothelial cell multimerin) (EMILIN-4) (Elastin microfibril interface located protein 4) (Elastin microfibril interfacer 4) [Cleaved into: Platelet glycoprotein Ia*; 155 kDa platelet multimerin (p-155) (p155)]	GO:0007596; GO:0007155; GO:0005576; GO:0031093	blood coagulation; cell adhesion; extracellular region; platelet alpha granule lumen	Secreted		Membrane-Fraction - this study			
**377**	ERIC1	ERIC1_HUMAN	ERIC1	Q86X53; Q9P063	XP_291282; NP_997215	Glutamate-rich protein 1	GO:0005739; GO:0005634	mitochondrion; nucleus				Known in Uniprot to be phosphorylated		
**378**	UQCR1	UQCR1_HUMAN	UQCR1; QCR1; B2R7R8	P31930; Q96DD2; B2R7R8	NP_003356	Cytochrome b-c1 complex subunit 1, mitochondrial (Ubiquinol-cytochrome-c reductase complex core protein 1) (Core protein I) (Complex III subunit 1)	GO:0009060; GO:0004222; GO:0005746; GO:0005515; GO:0006508; GO:0006810; GO:0008121; GO:0008270	aerobic respiration; metalloendopeptidase activity; mitochondrial respiratory chain; protein binding; proteolysis; transport; ubiquinol-cytochrome-c reductase activity; zinc ion binding	Mitochondrioninnermembrane		Membrane-Fraction - this study	Known in Uniprot to be phosphorylated		
**379**	K6PL	K6PL_HUMAN	K6PL; Q7L2M7	P17858; Q96A64; Q96IH4; Q9BR91; Q7L2M7	NP_002617	6-phosphofructokinase, liver type (EC 2.7.1.11) (Phosphofructokinase 1) (Phosphohexokinase) (Phosphofructo-1-kinase isozyme B) (PFK-B); PFKL protein (Fragment)	GO:0003872; GO:0005945; GO:0005524; GO:0006002; GO:0070095; GO:0006096; GO:0042802; GO:0019900; GO:0000287; GO:0051259; GO:0046676; GO:0009749; GO:0005625	6-phosphofructokinase activity; 6-phosphofructokinase complex; ATP binding; fructose 6-phosphate metabolic process; fructose-6-phosphate binding; glycolysis; identical protein binding; kinase binding; magnesium ion binding; protein oligomerization; negative regulation of insulin secretion; response to glucose stimulus; soluble fraction			Membrane-Fraction - this study	Known in Uniprot to be phosphorylated		Kinase
**380**	UQCR2	UQCR2_HUMAN	UQCR2; QCR2; B3KSN4	P22695; Q9BQ05; B3KSN4	NP_003357	Cytochrome b-c1 complex subunit 2, mitochondrial (Ubiquinol-cytochrome-c reductase complex core protein 2) (Core protein II) (Complex III subunit 2)	GO:0009060; GO:0022900; GO:0004222; GO:0005743; GO:0005634; GO:0006119; GO:0005515; GO:0006508; GO:0006810; GO:0008270	aerobic respiration; electron transport chain; metalloendopeptidase activity; mitochondrial inner membrane; nucleus; oxidative phosphorylation; protein binding; proteolysis; transport; zinc ion binding	MitochondrioninnermembranePeripheralmembraneproteinMatrixside		Membrane-Fraction - this study	Known in Uniprot to be phosphorylated		
**381**	1C07	1C07_HUMAN	1C07; Q95HC2; Q6R739	Q6R739; Q95HC2; P10321; O78061; O78083; Q29631; Q29652; Q29867; Q29990; Q95463; Q95603; Q9MY31; Q9TQP9	NP_002108	HLA class I histocompatibility antigen, Cw-7 alpha chain (MHC class I antigen Cw*7); Major histocompatibility complex, class I, C (MHC class I antigen); HLA-C protein (Fragment)	GO:0042612; GO:0002474; GO:0006955; GO:0016021; GO:0044419	MHC class I protein complex; antigen processing and presentation of peptide antigen via MHC class I; immune response; integral to membrane; interspecies interaction between organisms	MembraneSinglepasstypeImembraneprotein;		Membrane-Fraction - this study			
**382**	ARMET	ARMET_HUMAN	ARMET; A8K878	P55145; Q14CX4; Q86U67; Q96IS4; A8K878	NP_006001	Protein ARMET (Mesencephalic astrocyte-derived neurotrophic factor) (MANF) (Arginine-rich protein)	GO:0005576; GO:0008083	extracellular region; growth factor activity	Secreted		Membrane-Fraction - this study	Known in Uniprot to be phosphorylated		
**383**	ADT1	ADT1_HUMAN	ADT1	P12235	NP_001142	ADP/ATP translocase 1 (Adenine nucleotide translocator 1) (ANT 1) (ADP,ATP carrier protein 1) (Solute carrier family 25 member 4) (ADP,ATP carrier protein, heart/skeletal muscle isoform T1)	GO:0015207; GO:0006091; GO:0005887; GO:0044419; GO:0000002; GO:0005743; GO:0005515	adenine transmembrane transporter activity; generation of precursor metabolites and energy; integral to plasma membrane; interspecies interaction between organisms; mitochondrial genome maintenance; mitochondrial inner membrane; protein binding	MitochondrioninnermembraneMultipassmembraneprotein		Membrane-Fraction - this study	Known in Uniprot to be phosphorylated		
**384**	SCPDH	SCPDH_HUMAN	SCPDH	Q8NBX0; Q8TAR0; Q9Y363	NP_057086	Probable saccharopine dehydrogenase (EC 1.5.1.9)	GO:0005488; GO:0055114; GO:0047131	binding; oxidation reduction; saccharopine dehydrogenase (NAD+, L-glutamate-forming) activity			Membrane-Fraction - this study			
**385**	ABI1	ABI1_HUMAN	ABI1; Q5W068; Q5T2R6	Q8IZP0; Q5W068; O15147; O76049; O95060; Q5W070; Q5W072; Q8TB63; Q96S81; Q9NXZ9; Q9NYB8; Q5T2R6	NP_005461; NP_001012768; NP_001012769; NP_001012770	Abl interactor 1 (Abelson interactor 1) (Abi-1) (Spectrin SH3 domain-binding protein 1) (Eps8 SH3 domain-binding protein) (Eps8-binding protein) (e3B1) (Nap1-binding protein) (Nap1BP) (Abl-binding protein 4) (AblBP4)	GO:0030054; GO:0008092; GO:0005856; GO:0005829; GO:0005783; GO:0030027; GO:0008285; GO:0005634; GO:0018108; GO:0005625; GO:0045202; GO:0019717; GO:0007169	cell junction; cytoskeletal protein binding; cytoskeleton; cytosol; endoplasmic reticulum; lamellipodium; negative regulation of cell proliferation; nucleus; peptidyl-tyrosine phosphorylation; soluble fraction; synapse; synaptosome; transmembrane receptor protein tyrosine kinase signaling pathway	CytoplasmNucleusCellprojectionlamellipodiumCellprojectionfilopodiumCellprojectiongrowthconeCelljunctionsynapsesynaptosomeCytoplasmcytoskeleton		Membrane-Fraction - this study	Known in Uniprot to be phosphorylated		
**386**	ACTBM	ACTBM_HUMAN	Q9BYX7; ACTBM	Q9BYX7; Q562N5	XP_065237; NP_001017421; XP_292982	Beta-actin-like protein 3 (Kappa-actin)	GO:0005524; GO:0005737; GO:0005856; GO:0005515	ATP binding; cytoplasm; cytoskeleton; protein binding	Cytoplasmcytoskeleton					
**387**	TCPG	TCPG_HUMAN	TCPG	P49368; Q5SZY1; Q9BR64	NP_005989; NP_001008800; NP_001008883	T-complex protein 1 subunit gamma (TCP-1-gamma) (CCT-gamma) (hTRiC5)	GO:0005524; GO:0005737; GO:0005856; GO:0005634; GO:0005886; GO:0006457; GO:0051082	ATP binding; cytoplasm; cytoskeleton; nucleus; plasma membrane; protein folding; unfolded protein binding	Cytoplasm		Membrane-Fraction - this study	Known in Uniprot to be phosphorylated		
**388**	SPCS1	SPCS1_HUMAN	SPCS1	Q9Y6A9; B3KNF8; Q9BVW1	NP_054760	Signal peptidase complex subunit 1 (EC 3.4.-.-) (Microsomal signal peptidase 12 kDa subunit) (SPase 12 kDa subunit)	GO:0030176; GO:0005792; GO:0008233; GO:0006508; GO:0005787; GO:0006465	integral to endoplasmic reticulum membrane; microsome; peptidase activity; proteolysis; signal peptidase complex; signal peptide processing	MicrosomemembraneMultipassmembraneproteinEndoplasmicreticulummembraneMultipassmembraneprotein		Membrane-Fraction - this study			
**389**	FIBB	FIBB_HUMAN	FIBB; B2R7G3	P02675; Q3KPF2; B2R7G3	NP_005132	Fibrinogen beta chain [Cleaved into: Fibrinopeptide B]	GO:0043499; GO:0009897; GO:0005577; GO:0005634; GO:0030168; GO:0031093; GO:0030674; GO:0051258; GO:0005102; GO:0051592; GO:0007165; GO:0005625	eukaryotic cell surface binding; external side of plasma membrane; fibrinogen complex; nucleus; platelet activation; platelet alpha granule lumen; protein binding, bridging; protein polymerization; receptor binding; response to calcium ion; signal transduction; soluble fraction	Secreted		Membrane-Fraction - this study			
**390**	AP1G1	AP1G1_HUMAN	AP1G1; Q8IY97	O43747; O75709; O75842; Q9UG09; Q9Y3U4; Q8IY97	NP_001119; NP_001025178	AP-1 complex subunit gamma-1 (Adapter-related protein complex 1 subunit gamma-1) (Adaptor protein complex AP-1 subunit gamma-1) (Golgi adaptor HA1/AP1 adaptin subunit gamma-1) (Gamma1-adaptin) (Clathrin assembly protein complex 1 gamma-1 large chain); Adaptor-related protein complex 1, gamma 1 subunit	GO:0044431; GO:0030131; GO:0030136; GO:0005829; GO:0006897; GO:0006886; GO:0005515; GO:0008565; GO:0016192	Golgi apparatus part; clathrin adaptor complex; clathrin-coated vesicle; cytosol; endocytosis; intracellular protein transport; protein binding; protein transporter activity; vesicle-mediated transport	GolgiapparatusCytoplasmicvesicleclathrincoatedvesiclemembranePeripheralmembraneproteinCytoplasmicside;		Membrane-Fraction - this study			
**391**	SND1	SND1_HUMAN	SND1	Q7KZF4; Q13122; Q96AG0	NP_055205	Staphylococcal nuclease domain-containing protein 1 (p100 co-activator) (100 kDa coactivator) (EBNA2 coactivator p100) (Tudor domain-containing protein 11)	GO:0005794; GO:0016246; GO:0016442; GO:0044419; GO:0042470; GO:0004518; GO:0003676; GO:0005634; GO:0006355; GO:0003712	Golgi apparatus; RNA interference; RNA-induced silencing complex; interspecies interaction between organisms; melanosome; nuclease activity; nucleic acid binding; nucleus; regulation of transcription, DNA-dependent; transcription cofactor activity	CytoplasmNucleusMelanosome		Membrane-Fraction - this study	Known in Uniprot to be phosphorylated		
**392**	ITA2B	ITA2B_HUMAN	ITA2B; Q17R67	P08514; B2RCY8; O95366; Q14443; Q17R67	NP_000410	Integrin alpha-IIb (Platelet membrane glycoprotein IIb) (GPalpha IIb) (GPIIb) (CD antigen CD41) [Cleaved into: Integrin alpha-IIb heavy chain; Integrin alpha-IIb light chain, form 1; Integrin alpha-IIb light chain, form 2]	GO:0005509; GO:0007155; GO:0042802; GO:0008305; GO:0007229; GO:0031092; GO:0004872	calcium ion binding; cell adhesion; identical protein binding; integrin complex; integrin-mediated signaling pathway; platelet alpha granule membrane; receptor activity	MembraneSinglepasstypeImembraneprotein		Membrane-Fraction - this study			

	Protein Name	Uniprot Name	Other Names	Uniprot IDs	Refseq IDs	Protein Description	GO ID	GO terms	Subcellular location	Comments	Comments	Comments - Database Search	Comments - Database Search	Function
**393**	VWF	VWF_HUMAN	VWF	P04275; Q99806	NP_000543	von Willebrand factor (vWF) [Cleaved into: von Willebrand antigen 2 (von Willebrand antigen II)]	GO:0033093; GO:0031589; GO:0051087; GO:0005518; GO:0005783; GO:0001948; GO:0019865; GO:0005178; GO:0005730; GO:0030168; GO:0031091; GO:0002020; GO:0047485; GO:0042803; GO:0051260; GO:0005578	Weibel-Palade body; cell-substrate adhesion; chaperone binding; collagen binding; endoplasmic reticulum; glycoprotein binding; immunoglobulin binding; integrin binding; nucleolus; platelet activation; platelet alpha granule; protease binding; protein N-terminus binding; protein homodimerization activity; protein homooligomerization; proteinaceous extracellular matrix	SecretedSecretedextracellularspaceextracellularmatrix		Membrane-Fraction - this study			
**394**	CD68	CD68_HUMAN	CD68; Q53XI3	P34810; Q96BI7; Q53XI3	NP_001242; NP_001035148	Macrosialin (Gp110) (CD antigen CD68); CD68 antigen (CD68 antigen variant) (cDNA FLJ75624, highly similar to Homo sapiens CD68 antigen (CD68), mRNA)	GO:0005768; GO:0016021; GO:0005765; GO:0005624; GO:0005886; GO:0016020	endosome; integral to membrane; lysosomal membrane; membrane fraction; plasma membrane; membrane	CellmembraneSinglepasstypeImembraneproteinEndosomemembraneSinglepasstypeImembraneproteinLysosomemembraneSinglepasstypeImembraneprotein;		Membrane-Fraction - this study			
**395**	SC22B	SC22B_HUMAN	SC22B	O75396; A8K1G0	NP_004883	Vesicle-trafficking protein SEC22b (SEC22 vesicle-trafficking protein homolog B) (SEC22 vesicle-trafficking protein-like 1) (ERS24) (ERS-24)	GO:0006888; GO:0033116; GO:0000139; GO:0005789; GO:0016021; GO:0042470; GO:0005515; GO:0015031	ER to Golgi vesicle-mediated transport; ER-Golgi intermediate compartment membrane; Golgi membrane; endoplasmic reticulum membrane; integral to membrane; melanosome; protein binding; protein transport	EndoplasmicreticulumGolgiintermediatecompartmentmembraneSinglepasstypeIVmembraneproteinGolgiapparatusmembraneSinglepasstypeIVmembraneproteinEndoplasmicreticulummembraneSinglepasstypeIVmembraneproteinMelanosome		Membrane-Fraction - this study	Known in Uniprot to be phosphorylated		
**396**	TMEDA	TMEDA_HUMAN	TMEDA; B2R605	P49755; Q15602; Q16536; Q86TC2; Q86TS5; B2R605	NP_006818	Transmembrane emp24 domain-containing protein 10 (21 kDa transmembrane-trafficking protein) (Transmembrane protein Tmp21) (Tmp-21-I) (p24delta) (S31III125) (S31I125)	GO:0005793; GO:0000139; GO:0005801; GO:0016021; GO:0042470; GO:0005792; GO:0005515; GO:0015031; GO:0045055; GO:0048199; GO:0042589	ER-Golgi intermediate compartment; Golgi membrane; cis-Golgi network; integral to membrane; melanosome; microsome; protein binding; protein transport; regulated secretory pathway; vesicle targeting, to, from or within Golgi; zymogen granule membrane	GolgiapparatusmembraneSinglepasstypeImembraneproteinLumenalsideMelanosome		Membrane-Fraction - this study			
**397**	G6PD	G6PD_HUMAN	G6PD	P11413; Q16000; Q16765; Q8IU70; Q8IU88; Q8IUA6; Q96PQ2	NP_000393; NP_001035810	Glucose-6-phosphate 1-dehydrogenase (G6PD) (EC 1.1.1.49)	GO:0050661; GO:0034599; GO:0005813; GO:0006695; GO:0001816; GO:0005829; GO:0043249; GO:0006010; GO:0005536; GO:0004345; GO:0006749; GO:0009898; GO:0043231; GO:0010734; GO:0055114; GO:0009051; GO:0042803; GO:0046390	NADP or NADPH binding; cellular response to oxidative stress; centrosome; cholesterol biosynthetic process; cytokine production; cytosol; erythrocyte maturation; glucose 6-phosphate utilization; glucose binding; glucose-6-phosphate dehydrogenase activity; glutathione metabolic process; internal side of plasma membrane; intracellular membrane-bounded organelle; negative regulation of protein amino acid glutathionylation; oxidation reduction; pentose-phosphate shunt, oxidative branch; protein homodimerization activity; ribose phosphate biosynthetic process			Membrane-Fraction - this study	Known in Uniprot to be phosphorylated		
**398**	AT2A1	AT2A1_HUMAN	AT2A1; Q7Z675; B3KY17	O14983; A8K5J9; O14984; B3KY17; Q7Z675	NP_004311; NP_775293	Sarcoplasmic/endoplasmic reticulum calcium ATPase 1 (SERCA1) (EC 3.6.3.8) (Calcium pump 1) (Calcium-transporting ATPase sarcoplasmic reticulum type, fast twitch skeletal muscle isoform) (SR Ca(2+)-ATPase 1) (Endoplasmic reticulum class 1/2 Ca(2+) ATPase); Putative uncharacterized protein DKFZp779O2152	GO:0005524; GO:0006754; GO:0005509; GO:0006816; GO:0005388; GO:0016021; GO:0000287; GO:0031448; GO:0005515; GO:0033017; GO:0016529	ATP binding; ATP biosynthetic process; calcium ion binding; calcium ion transport; calcium-transporting ATPase activity; integral to membrane; magnesium ion binding; positive regulation of fast-twitch skeletal muscle fiber contraction; protein binding; sarcoplasmic reticulum membrane; sarcoplasmic reticulum	EndoplasmicreticulummembraneMultipassmembraneproteinSarcoplasmicreticulummembraneMultipassmembraneprotein;		Membrane-Fraction - this study	Known in Uniprot to be phosphorylated		
**399**	ILVBL	ILVBL_HUMAN	Q9BWN5; O43341; ILVBL	O43341; Q9BWN5; A1L0T0; Q96F08; Q99651; Q9UEB2	NP_006835	Acetolactate synthase-like protein (EC 2.2.1.-) (IlvB-like protein)	GO:0016021; GO:0000287; GO:0030976; GO:0016740	integral to membrane; magnesium ion binding; thiamin pyrophosphate binding; transferase activity	MembraneSinglepassmembraneprotein		Membrane-Fraction - this study			
**400**	FYN	FYN_HUMAN	FYN; B5BU57	Q5R3A6; P06241; Q5R3A7; Q16248; Q8N5D7; B5BU57	NP_694592; NP_002028; NP_694593	Proto-oncogene tyrosine-protein kinase Fyn (EC 2.7.10.2) (p59-Fyn) (Protooncogene Syn) (SLK)	GO:0005524; GO:0050852; GO:0006816; GO:0005829; GO:0005768; GO:0007631; GO:0001948; GO:0042802; GO:0044419; GO:0007612; GO:0030145; GO:0007275; GO:0004715; GO:0005886; GO:0006468; GO:0007243	ATP binding; T cell receptor signaling pathway; calcium ion transport; cytosol; endosome; feeding behavior; glycoprotein binding; identical protein binding; interspecies interaction between organisms; learning; manganese ion binding; multicellular organismal development; non-membrane spanning protein tyrosine kinase activity; plasma membrane; protein amino acid phosphorylation; protein kinase cascade	Cellmembrane		Membrane-Fraction - this study	Known in Uniprot to be phosphorylated	Known in HPRD to be phosphorylated	Kinase
**401**	AT2A3	AT2A3_HUMAN	AT2A3	Q93084; O60900; O60901; O75501; O75502; Q16115; Q8TEX5; Q8TEX6	NP_005164; NP_777613; NP_777614; NP_777615; NP_777616; NP_777617; NP_777618	Sarcoplasmic/endoplasmic reticulum calcium ATPase 3 (SERCA3) (EC 3.6.3.8) (Calcium pump 3) (SR Ca(2+)-ATPase 3)	GO:0005524; GO:0006754; GO:0005509; GO:0006816; GO:0005388; GO:0016021; GO:0000287; GO:0031965; GO:0005515; GO:0033017	ATP binding; ATP biosynthetic process; calcium ion binding; calcium ion transport; calcium-transporting ATPase activity; integral to membrane; magnesium ion binding; nuclear membrane; protein binding; sarcoplasmic reticulum membrane	NucleusmembraneMultipassmembraneproteinEndoplasmicreticulummembraneMultipassmembraneproteinSarcoplasmicreticulummembraneMultipassmembraneprotein		Membrane-Fraction - this study	Known in Uniprot to be phosphorylated		
**402**	CLUS	CLUS_HUMAN	CLUS; Q2TU75	P10909; B2R9Q1; P11380; P11381; Q2TU75; Q5HYC1; Q7Z5B9	NP_001822; NP_976084	Clusterin (Complement-associated protein SP-40,40) (Complement cytolysis inhibitor) (CLI) (NA1/NA2) (Apolipoprotein J) (Apo-J) (Testosterone-repressed prostate message 2) (TRPM-2) (Ku70-binding protein 1) (Aging-associated gene 4 protein) [Cleaved into: Clusterin beta chain (ApoJalpha) (Complement cytolysis inhibitor a chain); Clusterin alpha chain (ApoJbeta) (Complement cytolysis inhibitor b chain)]	GO:0006958; GO:0005615; GO:0045087; GO:0006629; GO:0005515	complement activation, classical pathway; extracellular space; innate immune response; lipid metabolic process; protein binding	Secreted		Membrane-Fraction - this study			
**403**	CALM	CALM_HUMAN	CALM; B4DJ51	P62158; P02593; P70667; P99014; Q13942; Q53S29; Q61379; Q61380; Q96HK3; B4DJ51	NP_001734; NP_005175; NP_008819	Calmodulin (CaM)	GO:0007186; GO:0031997; GO:0005509; GO:0005813; GO:0005829; GO:0060315; GO:0005886; GO:0060316; GO:0019904; GO:0032465; GO:0010880; GO:0051592; GO:0005876; GO:0000922; GO:0031432	G-protein coupled receptor protein signaling pathway; N-terminal myristoylation domain binding; calcium ion binding; centrosome; cytosol; negative regulation of ryanodine-sensitive calcium-release channel activity; plasma membrane; positive regulation of ryanodine-sensitive calcium-release channel activity; protein domain specific binding; regulation of cytokinesis; regulation of release of sequestered calcium ion into cytosol by sarcoplasmic reticulum; response to calcium ion; spindle microtubule; spindle pole; titin binding	Spindle		Membrane-Fraction - this study	Known in Uniprot to be phosphorylated	Known in HPRD to be phosphorylated	Kinase
**404**	CPNS1	CPNS1_HUMAN	CPNS1	P04632; A8K0P1; Q8WTX3; Q96EW0	NP_001740; NP_001003962	Calpain small subunit 1 (CSS1) (Calcium-dependent protease small subunit 1) (Calcium-dependent protease small subunit) (CDPS) (Calpain regulatory subunit) (Calcium-activated neutral proteinase small subunit) (CANP small subunit)	GO:0005509; GO:0004198; GO:0005737; GO:0005634; GO:0005886; GO:0008284; GO:0005515	calcium ion binding; calcium-dependent cysteine-type endopeptidase activity; cytoplasm; nucleus; plasma membrane; positive regulation of cell proliferation; protein binding	CytoplasmCellmembrane		Membrane-Fraction - this study			
**405**	PP1B	PP1B_HUMAN	PP1B; B2R5V4	P62140; P37140; Q5U087; Q6FG45; B2R5V4	NP_002700; NP_996759	Serine/threonine-protein phosphatase PP1-beta catalytic subunit (PP-1B) (EC 3.1.3.16)	GO:0007049; GO:0051301; GO:0005737; GO:0005977; GO:0005506; GO:0030145; GO:0005515	cell cycle; cell division; cytoplasm; glycogen metabolic process; iron ion binding; manganese ion binding; protein binding	Cytoplasm		Membrane-Fraction - this study	Known in Uniprot to be phosphorylated	Known in HPRD to be phosphorylated	Phosphatase
**406**	1433B	1433B_HUMAN	1433B; A8K9K2	P31946; A8K9K2	NP_003395; NP_647539	14-3-3 protein beta/alpha (Protein kinase C inhibitor protein 1) (KCIP-1) (Protein 1054) [Cleaved into: 14-3-3 protein beta/alpha, N-terminally processed]	GO:0007265; GO:0008633; GO:0005813; GO:0005829; GO:0019899; GO:0042470; GO:0035308; GO:0005634; GO:0048471; GO:0043085; GO:0019904	Ras protein signal transduction; activation of pro-apoptotic gene products; centrosome; cytosol; enzyme binding; melanosome; negative regulation of protein amino acid dephosphorylation; nucleus; perinuclear region of cytoplasm; positive regulation of catalytic activity; protein domain specific binding	CytoplasmMelanosome		Membrane-Fraction - this study	Known in Uniprot to be phosphorylated	Known in HPRD to be phosphorylated	

	Protein Name	Uniprot Name	Other Names	Uniprot IDs	Refseq IDs	Protein Description	GO ID	GO terms	Subcellular location	Comments	Comments	Comments - Database Search	Comments - Database Search	Function
**407**	CD36	CD36_HUMAN	CD36; A4D1B1	P16671; Q13966; Q16093; Q8TCV7; Q9BPZ8; Q9BQC2; Q9BZM8; Q9BZN3; Q9BZN4; Q9BZN5; A4D1B1	NP_000063; NP_001001547; NP_001001548; NP_001120915; NP_001120916	Platelet glycoprotein 4 (Platelet glycoprotein IV) (GPIV) (Glycoprotein IIIb) (GPIIIB) (Leukocyte differentiation antigen CD36) (PAS IV) (PAS-4) (Platelet collagen receptor) (Fatty acid translocase) (FAT) (Thrombospondin receptor) (CD antigen CD36); CD36 antigen (Collagen type I receptor, thrombospondin receptor) (CD36 antigen (Collagen type I receptor, thrombospondin receptor), isoform CRA_a) (cDNA, FLJ93369, highly similar to Homo sapiens CD36 antigen (collagen type I receptor, thrombospondinreceptor) (CD36), mRNA)	GO:0005794; GO:0007596; GO:0019934; GO:0009986; GO:0030301; GO:0005887; GO:0008289; GO:0006629; GO:0019915; GO:0042159; GO:0034381; GO:0042953; GO:0005041; GO:0005624; GO:0007263; GO:0015911; GO:0031092; GO:0001954; GO:0010744; GO:0070053; GO:0050431; GO:0043277; GO:0007155; GO:0008035; GO:0016020; GO:0004872	Golgi apparatus; blood coagulation; cGMP-mediated signaling; cell surface; cholesterol transport; integral to plasma membrane; lipid binding; lipid metabolic process; lipid storage; lipoprotein catabolic process; lipoprotein particle clearance; lipoprotein transport; low-density lipoprotein receptor activity; membrane fraction; nitric oxide mediated signal transduction; plasma membrane long-chain fatty acid transport; platelet alpha granule membrane; positive regulation of cell-matrix adhesion; positive regulation of foam cell differentiation; thrombospondin receptor activity; transforming growth factor beta binding; apoptotic cell clearance; cell adhesion; high-density lipoprotein binding; membrane; receptor activity	MembraneMultipassmembraneprotein;		Membrane-Fraction - this study		Known in HPRD to be phosphorylated	
**408**	ARPC2	ARPC2_HUMAN	ARPC2; Q53R19	O15144; Q53R19; Q92801; Q9P1D4	NP_005722; NP_690601	Actin-related protein 2/3 complex subunit 2 (Arp2/3 complex 34 kDa subunit) (p34-ARC); cDNA FLJ76076, highly similar to Homo sapiens actin related protein 2/3 complex, subunit 2, 34kDa (ARPC2), transcript variant 2, mRNA (Actin related protein 2/3 complex, subunit 2, 34kDa, isoform CRA_a) (Putative uncharacterized protein ARPC2)	GO:0005885; GO:0003779; GO:0006928; GO:0042995; GO:0005737; GO:0030833; GO:0005200; GO:0005856; GO:0005925	Arp2/3 protein complex; actin binding; cell motion; cell projection; cytoplasm; regulation of actin filament polymerization; structural constituent of cytoskeleton; cytoskeleton; focal adhesion	Cytoplasmcytoskeleton Cellprojection;		Membrane-Fraction - this study			
**409**	MGLL	MGLL_HUMAN	MGLL; Q6IBG9; B3KRC2	Q6IBG9; Q99685; Q96AA5; B3KRC2	NP_009214; NP_001003794	Monoglyceride lipase (MGL) (EC 3.1.1.23) (Lysophospholipase homolog) (Lysophospholipase-like) (HU-K5); MGLL protein (Monoglyceride lipase, isoform CRA_b) (cDNA, FLJ96595, Homo sapiens monoglyceride lipase (MGLL), mRNA)	GO:0047372; GO:0006954; GO:0006629; GO:0004622; GO:0016787	acylglycerol lipase activity; inflammatory response; lipid metabolic process; lysophospholipase activity; hydrolase activity			Membrane-Fraction - this study			
**410**	RAP1A	RAP1A_HUMAN	RAP1A; Q5QPH6; A8KAH9	P62834; Q5QPH6; P10113; A8KAH9	NP_002875; NP_001010935	Ras-related protein Rap-1A (GTP-binding protein smg-p21A) (Ras-related protein Krev-1) (C21KG) (G-22K)	GO:0005525; GO:0003924; GO:0005829; GO:0045786; GO:0005886; GO:0007264	GTP binding; GTPase activity; cytosol; negative regulation of cell cycle; plasma membrane; small GTPase mediated signal transduction	CellmembraneLipidanchor		Membrane-Fraction - this study		Known in HPRD to be phosphorylated	
**411**	COR1B	COR1B_HUMAN	COR1B; B2RD45	Q9BR76; B2RD45	NP_065174; NP_001018080	Coronin-1B (Coronin-2)	GO:0003779; GO:0005737; GO:0005856	actin binding; cytoplasm; cytoskeleton	Cytoplasmcytoskeleton		Membrane-Fraction - this study	Known in Uniprot to be phosphorylated	Known in HPRD to be phosphorylated	
**412**	ARF1	ARF1_HUMAN	ARF1	P84077; P10947; P32889	NP_001649; NP_001019397; NP_001019398; NP_001019399	ADP-ribosylation factor 1	GO:0048205; GO:0005525; GO:0003924; GO:0000139; GO:0005829; GO:0005886; GO:0005515; GO:0015031; GO:0005057; GO:0006890; GO:0007264	COPI coating of Golgi vesicle; GTP binding; GTPase activity; Golgi membrane; cytosol; plasma membrane; protein binding; protein transport; receptor signaling protein activity; retrograde vesicle-mediated transport, Golgi to ER; small GTPase mediated signal transduction	Golgiapparatus		Membrane-Fraction - this study	Known in Uniprot to be phosphorylated		
**413**	FIBG	FIBG_HUMAN	FIBG; Q53Y18	Q53Y18; P02679; A8K057; P04469; P04470; Q96A14; Q96KJ3; Q9UC62; Q9UC63	NP_000500; NP_068656	Fibrinogen gamma chain	GO:0005509; GO:0043499; GO:0009897; GO:0005577; GO:0030168; GO:0031093; GO:0030674; GO:0051258; GO:0005102; GO:0051592; GO:0007165	calcium ion binding; eukaryotic cell surface binding; external side of plasma membrane; fibrinogen complex; platelet activation; platelet alpha granule lumen; protein binding, bridging; protein polymerization; receptor binding; response to calcium ion; signal transduction	Secreted		Membrane-Fraction - this study			
**414**	VDAC2	VDAC2_HUMAN	VDAC2; A6NLE7	P45880; Q5VWK1; Q5VWK3; Q6IB40; Q7L3J5; Q9BWK8; Q9Y5I6; A6NLE7	XP_016625; XP_001133585; NP_003366	Voltage-dependent anion-selective channel protein 2 (VDAC-2) (hVDAC2) (Outer mitochondrial membrane protein porin 2)	GO:0006820; GO:0016021; GO:0042645; GO:0005741; GO:0000166; GO:0005515; GO:0008308	anion transport; integral to membrane; mitochondrial nucleoid; mitochondrial outer membrane; nucleotide binding; protein binding; voltage-gated anion channel activity	Mitochondrionoutermembrane			Known in Uniprot to be phosphorylated		
**415**	Q5SQY0	Q5SQY0_HUMAN	Q5SQY0	Q5SQY0	XP_300862	Novel protein similar to beta-tubulin 4Q (TUBB4Q) (LOC253936) (Tubulin, beta 8, isoform CRA_c)	GO:0005525; GO:0003924; GO:0005737; GO:0005874; GO:0007018; GO:0043234; GO:0051258; GO:0005198	GTP binding; GTPase activity; cytoplasm; microtubule; microtubule-based movement; protein complex; protein polymerization; structural molecule activity						
**416**	Q5T8M7	Q5T8M7_HUMAN	Q5T8M7	Q5T8M7	XP_301899	Actin, alpha 1, skeletal muscle	GO:0005524; GO:0005856; GO:0005515	ATP binding; cytoskeleton; protein binding						
**417**	K1C39	K1C39_HUMAN	Q6IFU6; K1C39	Q6IFU6; Q6A163; B2RXK6	XP_292615; NP_998821; XP_001722656	Keratin, type I cytoskeletal 39 (Cytokeratin-39) (CK-39) (Keratin-39) (K39) (Type I hair keratin Ka35)	GO:0005882; GO:0005198	intermediate filament; structural molecule activity						
**418**	O86WF2	O86WF2_HUMAN	O86WF2	O86WF2	XP_299768	Sarcoma antigen NY-SAR-29 (Fragment)								
**419**	BICC1	BICC1_HUMAN	BICC1	Q9H694	XP_166699; NP_001073981	Protein bicaudal C homolog 1 (Bic-C)	GO:0003723; GO:0007275	RNA binding; multicellular organismal development						
**420**	SPOPL	SPOPL_HUMAN	SPOPL	Q6IQ16	XP_290995; NP_001001664	Speckle-type POZ protein-like (HIB homolog 2) (Roadkill homolog 2)	GO:0019941; GO:0005634; GO:0005515	modification-dependent protein catabolic process; nucleus; protein binding	Nucleus					
**421**	TRMT5	TRMT5_HUMAN	TRMT5	Q32P41; Q9P2F4	XP_050793; NP_065861	tRNA (guanine-N(1)-)-methyltransferase (EC 2.1.1.31) (M1G-methyltransferase) (tRNA [GM37] methyltransferase) (tRNA methyltransferase 5) (TRM5)	GO:0005737; GO:0005730; GO:0009019; GO:0008033	cytoplasm; nucleolus; tRNA (guanine-N1-)-methyltransferase activity; tRNA processing	Cytoplasm					
**422**	ACDSB	ACDSB_HUMAN	ACDSB; Q5SQN6	P45954; Q5SQN6; Q96CX7	NP_001600	Short/branched chain specific acyl-CoA dehydrogenase, mitochondrial (SBCAD) (EC 1.3.99.-) (2-methyl branched chain acyl-CoA dehydrogenase) (2-MEBCAD) (2-methylbutyryl-coenzyme A dehydrogenase) (2-methylbutyryl-CoA dehydrogenase); Acyl-Coenzyme A dehydrogenase, short/branched chain (cDNA, FLJ94986, Homo sapiens acyl-Coenzyme A dehydrogenase, short/branched chain(ACADSB), nuclear gene encoding mitochondrial protein, mRNA)	GO:0050660; GO:0003995; GO:0009055; GO:0006631; GO:0005759; GO:0055114; GO:0008152	FAD binding; acyl-CoA dehydrogenase activity; electron carrier activity; fatty acid metabolic process; mitochondrial matrix; oxidation reduction; metabolic process	Mitochondrionmatrix;					
**423**	ACON	ACON_HUMAN	ACON; Q5JZ41; Q8TAQ6	Q99798; Q5JZ41; O75809; Q6FHX0; Q8TAQ6	NP_001089	Aconitate hydratase, mitochondrial (Aconitase) (EC 4.2.1.3) (Citrate hydro-lyase)	GO:0051539; GO:0003994; GO:0006101; GO:0005506; GO:0005759; GO:0005634; GO:0006099	4 iron, 4 sulfur cluster binding; aconitate hydratase activity; citrate metabolic process; iron ion binding; mitochondrial matrix; nucleus; tricarboxylic acid cycle	Mitochondrion					
**424**	ADAS	ADAS_HUMAN	ADAS; A5D8U9	O00116; Q2TU35; A5D8U9	NP_003650	Alkyldihydroxyacetonephosphate synthase, peroxisomal (Alkyl-DHAP synthase) (EC 2.5.1.26) (Alkylglycerone-phosphate synthase) (Aging-associated gene 5 protein)	GO:0050660; GO:0008609; GO:0005829; GO:0008610; GO:0016491; GO:0005778	FAD binding; alkylglycerone-phosphate synthase activity; cytosol; lipid biosynthetic process; oxidoreductase activity; peroxisomal membrane	Peroxisomemembrane			Known in Uniprot to be phosphorylated		
**425**	KAD1	KAD1_HUMAN	KAD1; Q6FGX9	P00568; Q6FGX9; Q9BVK9; Q9UQC7	NP_000467	Adenylate kinase isoenzyme 1 (AK 1) (EC 2.7.4.3) (ATP-AMP transphosphorylase 1) (Myokinase); Adenylate kinase 1 (AK1 protein) (Adenylate kinase 1, isoform CRA_b) (cDNA, FLJ94359, Homo sapiens adenylate kinase 1 (AK1), mRNA)	GO:0005524; GO:0046034; GO:0004017; GO:0005829; GO:0019206; GO:0005634; GO:0005515; GO:0007050; GO:0005737; GO:0005739; GO:0005886	ATP binding; ATP metabolic process; adenylate kinase activity; cytosol; nucleoside kinase activity; nucleus; protein binding; cell cycle arrest; cytoplasm; mitochondrion; plasma membrane	Cytoplasm;					Kinase
**426**	KAD2	KAD2_HUMAN	KAD2	P54819; A8K6L1; B4DHH7; B4DL64; Q16856; Q5EB54; Q5TIF7; Q8TCY2; Q8TCY3	NP_001616	Adenylate kinase 2, mitochondrial (AK 2) (EC 2.7.4.3) (ATP-AMP transphosphorylase 2)	GO:0005524; GO:0004017; GO:0005758; GO:0006139; GO:0019206	ATP binding; adenylate kinase activity; mitochondrial intermembrane space; nucleobase, nucleoside, nucleotide and nucleic acid metabolic process; nucleoside kinase activity	Mitochondrionintermembranespace					Kinase
**427**	ALBU	ALBU_HUMAN	ALBU; Q645G4	P02768; Q645G4; O95574; P04277; Q13140; Q68DN5; Q6UXK4; Q86YG0; Q9P157; Q9P1I7; Q9UHS3; Q9UJZ0	NP_000468	Serum albumin	GO:0003677; GO:0016209; GO:0009267; GO:0005507; GO:0008144; GO:0005615; GO:0005504; GO:0019836; GO:0051659; GO:0043066; GO:0031093; GO:0005515; GO:0043234; GO:0030170; GO:0015643; GO:0006810	DNA binding; antioxidant activity; cellular response to starvation; copper ion binding; drug binding; extracellular space; fatty acid binding; hemolysis by symbiont of host erythrocytes; maintenance of mitochondrion location; negative regulation of apoptosis; platelet alpha granule lumen; protein binding; protein complex; pyridoxal phosphate binding; toxin binding; transport	Secreted			Known in Uniprot to be phosphorylated		
**428**	ALDR	ALDR_HUMAN	ALDR	P15121; Q5U031; Q9BS21; Q9UCI9	XP_210550; NP_001619	Aldose reductase (AR) (EC 1.1.1.21) (Aldehyde reductase)	GO:0004032; GO:0005975; GO:0005829; GO:0009055; GO:0005615; GO:0055114; GO:0005515; GO:0006950	aldehyde reductase activity; carbohydrate metabolic process; cytosol; electron carrier activity; extracellular space; oxidation reduction; protein binding; response to stress	Cytoplasm			Known in Uniprot to be phosphorylated		

	Protein Name	Uniprot Name	Other Names	Uniprot IDs	Refseq IDs	Protein Description	GO ID	GO terms	Subcellular location	Comments	Comments	Comments - Database Search	Comments - Database Search	Function
**429**	ADT2	ADT2_HUMAN	ADT2	P05141; O43350	NP_001143; XP_170195; XP_293027	ADP/ATP translocase 2 (Adenine nucleotide translocator 2) (ANT 2) (ADP,ATP carrier protein 2) (Solute carrier family 25 member 5) (ADP,ATP carrier protein, fibroblast isoform)	GO:0015207; GO:0005488; GO:0005887; GO:0044419; GO:0005743; GO:0042645; GO:0006810	adenine transmembrane transporter activity; binding; integral to plasma membrane; interspecies interaction between organisms; mitochondrial inner membrane; mitochondrial nucleoid; transport	MitochondrioninnermembraneMultipassmembraneprotein			Known in Uniprot to be phosphorylated		
**430**	ANXA1	ANXA1_HUMAN	ANXA1; Q5TZZ9	P04083; Q5TZZ9	NP_000691	Annexin A1 (Annexin-1) (Annexin I) (Lipocortin I) (Calpactin II) (Chromobindin-9) (p35) (Phospholipase A2 inhibitory protein); ANXA1 protein (Annexin A1) (Annexin A1, isoform CRA_a)	GO:0006916; GO:0016323; GO:0005509; GO:0005544; GO:0006928; GO:0007166; GO:0005929; GO:0001533; GO:0005737; GO:0006954; GO:0030216; GO:0006629; GO:0005634; GO:0018149; GO:0019834; GO:0030674; GO:0005102; GO:0005198; GO:0050482; GO:0007049; GO:0004859; GO:0005515; GO:0042127; GO:0042383; GO:0007165	anti-apoptosis; basolateral plasma membrane; calcium ion binding; calcium-dependent phospholipid binding; cell motion; cell surface receptor linked signal transduction; cilium; cornified envelope; cytoplasm; inflammatory response; keratinocyte differentiation; lipid metabolic process; nucleus; peptide cross-linking; phospholipase A2 inhibitor activity; protein binding, bridging; receptor binding; structural molecule activity; arachidonic acid secretion; cell cycle; phospholipase inhibitor activity; protein binding; regulation of cell proliferation; sarcolemma; signal transduction	NucleusCytoplasmCellprojectionciliumBasolateralcellmembrane;			Known in Uniprot to be phosphorylated	Known in HPRD to be phosphorylated	
**431**	APT	APT_HUMAN	APT	P07741; Q3KP55; Q68DF9	NP_000476	Adenine phosphoribosyltransferase (APRT) (EC 2.4.2.7)	GO:0016208; GO:0003999; GO:0006168; GO:0005829; GO:0005515; GO:0006166	AMP binding; adenine phosphoribosyltransferase activity; adenine salvage; cytosol; protein binding; purine ribonucleoside salvage	Cytoplasm					
**432**	APXL	APXL_HUMAN	APXL; SHRM2	Q13796	XP_296859; NP_001640	Protein Shroom2 (Apical-like protein) (Protein APXL)	GO:0051015; GO:0015280; GO:0016324; GO:0045176; GO:0008013; GO:0007420; GO:0016477; GO:0005913; GO:0043482; GO:0043583; GO:0032401; GO:0008057; GO:0002089; GO:0032438; GO:0005874; GO:0005923	actin filament binding; amiloride-sensitive sodium channel activity; apical plasma membrane; apical protein localization; beta-catenin binding; brain development; cell migration; cell-cell adherens junction; cellular pigment accumulation; ear development; establishment of melanosome localization; eye pigment granule organization; lens morphogenesis in camera-type eye; melanosome organization; microtubule; tight junction	ApicalcellmembraneCelljunctiontightjunctionCytoplasmcytoskeleton			Known in Uniprot to be phosphorylated		
**433**	ARF4	ARF4_HUMAN	ARF4	P18085; B2R7J7; P21371	NP_001651	ADP-ribosylation factor 4	GO:0005525; GO:0003924; GO:0005794; GO:0015031; GO:0007264; GO:0016192	GTP binding; GTPase activity; Golgi apparatus; protein transport; small GTPase mediated signal transduction; vesicle-mediated transport	Golgiapparatus					
**434**	ARF5	ARF5_HUMAN	ARF5; A4D0Z3	P84085; P26437; A4D0Z3	NP_001653	ADP-ribosylation factor 5; ADP-ribosylation factor 5 (ADP-ribosylation factor 5, isoform CRA_a) (cDNA, FLJ92389, Homo sapiens ADP-ribosylation factor 5 (ARF5), mRNA)	GO:0005525; GO:0003924; GO:0005794; GO:0015031; GO:0007264; GO:0016192; GO:0005737; GO:0005622; GO:0005886	GTP binding; GTPase activity; Golgi apparatus; protein transport; small GTPase mediated signal transduction; vesicle-mediated transport; cytoplasm; intracellular; plasma membrane	Golgiapparatus;					
**435**	ARF6	ARF6_HUMAN	ARF6; Q6FGZ2	P62330; Q6FGZ2; P26438	NP_001654	ADP-ribosylation factor 6; ADP-ribosylation factor 6 (ARF6 protein) (cDNA, FLJ94403, Homo sapiens ADP-ribosylation factor 6 (ARF6), mRNA) (Fragment)	GO:0005525; GO:0003924; GO:0005794; GO:0007155; GO:0005938; GO:0006928; GO:0030866; GO:0005768; GO:0005624; GO:0048261; GO:0005886; GO:0030838; GO:0005515; GO:0015031; GO:0035020; GO:0001726; GO:0031529; GO:0006915; GO:0005737; GO:0005622; GO:0001889; GO:0007264	GTP binding; GTPase activity; Golgi apparatus; cell adhesion; cell cortex; cell motion; cortical actin cytoskeleton organization; endosome; membrane fraction; negative regulation of receptor-mediated endocytosis; plasma membrane; positive regulation of actin filament polymerization; protein binding; protein transport; regulation of Rac protein signal transduction; ruffle; ruffle organization; apoptosis; cytoplasm; intracellular; liver development; small GTPase mediated signal transduction	Golgiapparatus;					
**436**	ARL1	ARL1_HUMAN	ARL1; Q53XB1	P40616; Q53XB1; P80417	NP_001168	ADP-ribosylation factor-like protein 1; Putative uncharacterized protein DKFZp686O2082 (ADP-ribosylation factor-like 1) (ADP-ribosylation factor-like 1, isoform CRA_b) (cDNA, FLJ92054, Homo sapiens ADP-ribosylation factor-like 1 (ARL1), mRNA)	GO:0005525; GO:0003924; GO:0000139; GO:0008047; GO:0046872; GO:0005515; GO:0007264; GO:0005622	GTP binding; GTPase activity; Golgi membrane; enzyme activator activity; metal ion binding; protein binding; small GTPase mediated signal transduction; intracellular	GolgiapparatusmembranePeripheralmembraneproteinCytoplasmicside;					
**437**	ANT3	ANT3_HUMAN	ANT3; Q5TC78; Q7KZ97; Q8TCE1	P01008; Q5TC78; B2R6P0; P78439; P78447; Q13815; Q7KZ43; Q7KZ97; Q9UC78; Q8TCE1	NP_000479	Antithrombin-III (ATIII); SERPINC1 protein	GO:0007596; GO:0005576; GO:0008201; GO:0005886; GO:0002020; GO:0004867; GO:0004867	blood coagulation; extracellular region; heparin binding; plasma membrane; protease binding; serine-type endopeptidase inhibitor activity; serine-type endopeptidase inhibitor activity	Secretedextracellularspace;					
**438**	BAF	BAF_HUMAN	BAF	O75531; O60558; Q6FGG7	NP_003851; NP_001137457	Barrier-to-autointegration factor (Breakpoint cluster region protein 1)	GO:0003677; GO:0005694; GO:0005829; GO:0019059; GO:0044419; GO:0005634; GO:0019047; GO:0009615	DNA binding; chromosome; cytosol; initiation of viral infection; interspecies interaction between organisms; nucleus; provirus integration; response to virus	NucleusCytoplasm			Known in Uniprot to be phosphorylated		
**439**	BLVRB	BLVRB_HUMAN	BLVRB	P30043; Q32LZ2; A6NKD8; B2R5C6; P32078; P53005	NP_000704	Flavin reductase (FR) (EC 1.5.1.30) (NADPH-dependent diaphorase) (NADPH-flavin reductase) (FLR) (Biliverdin reductase B) (BVR-B) (EC 1.3.1.24) (Biliverdin-IX beta-reductase) (Green heme-binding protein) (GHBP)	GO:0004074; GO:0044237; GO:0050662; GO:0005737; GO:0042602; GO:0055114	biliverdin reductase activity; cellular metabolic process; coenzyme binding; cytoplasm; flavin reductase activity; oxidation reduction	Cytoplasm					
**440**	BT1A1	BT1A1_HUMAN	BT1A1; Q4VAN3	Q4VAN3; Q13410; Q4VAN4; Q9H458	NP_001723	Butyrophilin subfamily 1 member A1 (BT); Butyrophilin, subfamily 1, member A1	GO:0005576; GO:0005887; GO:0004872; GO:0016021	extracellular region; integral to plasma membrane; receptor activity; integral to membrane	MembraneSinglepasstypeImembraneproteinSecreted;					
**441**	C1QBP	C1QBP_HUMAN	C1QBP	Q07021; Q2HXR8; Q9NNY8	NP_001203	Complement component 1 Q subcomponent-binding protein, mitochondrial (Glycoprotein gC1qBP) (C1qBP) (GC1q-R protein) (Hyaluronan-binding protein 1) (Mitochondrial matrix protein p32) (p33)	GO:0006955; GO:0044419; GO:0005759; GO:0005886	immune response; interspecies interaction between organisms; mitochondrial matrix; plasma membrane	Mitochondrionmatrix			Known in Uniprot to be phosphorylated		
**442**	KCC1A	KCC1A_HUMAN	KCC1A; B0YIY3	Q14012; Q3KPF6; B0YIY3	NP_003647	Calcium/calmodulin-dependent protein kinase type 1 (EC 2.7.11.17) (CaM kinase I) (CaM-KI) (CaM kinase I alpha) (CaMKI-alpha)	GO:0005524; GO:0005516; GO:0004683; GO:0030154; GO:0005737; GO:0007399; GO:0005634; GO:0006468; GO:0007165	ATP binding; calmodulin binding; calmodulin-dependent protein kinase activity; cell differentiation; cytoplasm; nervous system development; nucleus; protein amino acid phosphorylation; signal transduction	CytoplasmNucleus			Known in Uniprot to be phosphorylated	Known in HPRD to be phosphorylated	Kinase
**443**	DHCA	DHCA_HUMAN	DHCA; Q3LHW8; CBR1; B2RBZ7	P16152; Q3LHW8; B2RBZ7	NP_001748	Carbonyl reductase [NADPH] 1 (EC 1.1.1.184) (NADPH-dependent carbonyl reductase 1) (Prostaglandin-E(2) 9-reductase) (EC 1.1.1.189) (Prostaglandin 9-ketoreductase) (15-hydroxyprostaglandin dehydrogenase [NADP+]) (EC 1.1.1.197)	GO:0047021; GO:0004090; GO:0005737; GO:0055114; GO:0050221; GO:0005515	15-hydroxyprostaglandin dehydrogenase (NADP+) activity; carbonyl reductase (NADPH) activity; cytoplasm; oxidation reduction; prostaglandin-E2 9-reductase activity; protein binding	Cytoplasm			Known in Uniprot to be phosphorylated		
**444**	DHC3	DHC3_HUMAN	DHC3; Q6FHP2; CBR3	O75828; Q6FHP2	NP_001227	Carbonyl reductase [NADPH] 3 (EC 1.1.1.184) (NADPH-dependent carbonyl reductase 3)	GO:0005488; GO:0004090; GO:0005737; GO:0055114	binding; carbonyl reductase (NADPH) activity; cytoplasm; oxidation reduction	Cytoplasm					
**445**	CCND3	CCND3_HUMAN	CCND3	P30281; B2RD63; Q5T8J0; Q96F49	XP_301676; NP_001129597; NP_001751	G1/S-specific cyclin-D3	GO:0051301; GO:0000307; GO:0005737; GO:0005634; GO:0045737; GO:0001934; GO:0019901	cell division; cyclin-dependent protein kinase holoenzyme complex; cytoplasm; nucleus; positive regulation of cyclin-dependent protein kinase activity; positive regulation of protein amino acid phosphorylation; protein kinase binding	CytoplasmNucleus					
**446**	TCPZ	TCPZ_HUMAN	TCPZ	P40227; Q3KP28; Q75LP4; Q96S46	NP_001753	T-complex protein 1 subunit zeta (TCP-1-zeta) (CCT-zeta) (CCT-zeta-1) (Tcp20) (HTR3) (Acute morphine dependence-related protein 2)	GO:0005524; GO:0006457; GO:0051082	ATP binding; protein folding; unfolded protein binding	Cytoplasm					
**447**	SLAF5	SLAF5_HUMAN	SLAF5; B2R8T1	O15430; Q9UIB8; O95266; O95660; Q5H9R1; Q6FHA8; Q8WLP1; Q8WWI8; Q9UF04; Q9UIB6; Q9UIB7; Q9UIT7; B2R8T1	NP_003865	SLAM family member 5 (Signaling lymphocytic activation molecule 5) (Leukocyte differentiation antigen CD84) (Cell surface antigen MAX.3) (Hly9-beta) (CD antigen CD84)	GO:0006952; GO:0007156; GO:0005887; GO:0005515; GO:0004872	defense response; homophilic cell adhesion; integral to plasma membrane; protein binding; receptor activity	CellmembraneSinglepasstypeImembraneprotein			Known in Uniprot to be phosphorylated		

	Protein Name	Uniprot Name	Other Names	Uniprot IDs	Refseq IDs	Protein Description	GO ID	GO terms	Subcellular location	Comments	Comments	Comments - Database Search	Comments - Database Search	Function
**448**	AP3S1	AP3S1_HUMAN	AP3S1; Q53XL4	Q92572; Q53XL4; O00647; O00676; O00721; O00727; Q6ICQ2	NP_001275	AP-3 complex subunit sigma-1 (Adapter-related protein complex 3 sigma-1 subunit) (AP-3 complex sigma-3A subunit) (Sigma-adaptin 3a) (Sigma-3A-adaptin) (Sigma3A-adaptin) (Clathrin-associated/assembly/adapter protein, small 3)	GO:0030119; GO:0005794; GO:0030659; GO:0008286; GO:0006886; GO:0005515; GO:0008565; GO:0030133; GO:0016192	AP-type membrane coat adaptor complex; Golgi apparatus; cytoplasmic vesicle membrane; insulin receptor signaling pathway; intracellular protein transport; protein binding; protein transporter activity; transport vesicle; vesicle-mediated transport	GolgiapparatusCytoplasmicvesiclemembranePeripheralmembraneproteinCytoplasmicside					
**449**	CLD5	CLD5_HUMAN	CLD5	O00501; B3KS11; Q53XW2; Q8WUW3	NP_003268; NP_001124333	Claudin-5 (Transmembrane protein deleted in VCFS) (TMDVCF)	GO:0016338; GO:0042802; GO:0016021; GO:0005198; GO:0005923	calcium-independent cell-cell adhesion; identical protein binding; integral to membrane; structural molecule activity; tight junction	CelljunctiontightjunctionCellmembraneMultipassmembraneprotein			Known in Uniprot to be phosphorylated		
**450**	CNN3	CNN3_HUMAN	CNN3; Q6FHA7	Q15417; Q6FHA7	NP_001830	Calponin-3 (Calponin, acidic isoform); CNN3 protein (Calponin 3, acidic) (cDNA, FLJ92692, Homo sapiens calponin 3, acidic (CNN3), mRNA) (Fragment)	GO:0003779; GO:0031032; GO:0005516; GO:0006939; GO:0005523; GO:0030172	actin binding; actomyosin structure organization; calmodulin binding; smooth muscle contraction; tropomyosin binding; troponin C binding				Known in Uniprot to be phosphorylated	Known in HPRD to be phosphorylated	
**451**	COMT	COMT_HUMAN	COMT; Q6IB07	P21964; Q6IB07; Q6ICE6	NP_000745; NP_001128633; NP_001128634; NP_009294	Catechol O-methyltransferase (EC 2.1.1.6)	GO:0016206; GO:0005829; GO:0016021; GO:0043231; GO:0000287; GO:0005792; GO:0042135; GO:0005886; GO:0005515; GO:0005625	catechol O-methyltransferase activity; cytosol; integral to membrane; intracellular membrane-bounded organelle; magnesium ion binding; microsome; neurotransmitter catabolic process; plasma membrane; protein binding; soluble fraction	CytoplasmCellmembraneSinglepasstypeIImembraneproteinExtracellularside			Known in Uniprot to be phosphorylated		
**452**	COX41	COX41_HUMAN	COX41; B2R4J2	P13073; B2R4J2	NP_001852	Cytochrome c oxidase subunit 4 isoform 1, mitochondrial (Cytochrome c oxidase subunit IV isoform 1) (COX IV-1) (Cytochrome c oxidase polypeptide IV)	GO:0004129; GO:0006091; GO:0005743; GO:0005634; GO:0005515	cytochrome-c oxidase activity; generation of precursor metabolites and energy; mitochondrial inner membrane; nucleus; protein binding	Mitochondrioninnermembrane					
**453**	CX6B1	CX6B1_HUMAN	CX6B1	P14854; B2R5C9; Q6IBL4	NP_001854	Cytochrome c oxidase subunit 6B1 (Cytochrome c oxidase subunit VIb isoform 1) (COX VIb-1)	GO:0004129; GO:0005758; GO:0055114	cytochrome-c oxidase activity; mitochondrial intermembrane space; oxidation reduction	Mitochondrionintermembranespace					
**454**	COX7C	COX7C_HUMAN	COX7C	P15954; Q6NR81	NP_001858	Cytochrome c oxidase subunit 7C, mitochondrial (Cytochrome c oxidase polypeptide VIIc)	GO:0004129; GO:0006091; GO:0016021; GO:0005743	cytochrome-c oxidase activity; generation of precursor metabolites and energy; integral to membrane; mitochondrial inner membrane	Mitochondrioninnermembrane					
**455**	CSRP2	CSRP2_HUMAN	CSRP2	Q16527; Q93030	NP_001312	Cysteine and glycine-rich protein 2 (Cysteine-rich protein 2) (CRP2) (Smooth muscle cell LIM protein) (SmLIM) (LIM domain only protein 5)	GO:0016049; GO:0008283; GO:0005634; GO:0009887; GO:0008270	cell growth; cell proliferation; nucleus; organ morphogenesis; zinc ion binding	Nucleus					
**456**	CYTS	CYTS_HUMAN	CYTS	P01036; Q9UBI5; Q9UCS9	NP_001890	Cystatin-S (Cystatin-4) (Salivary acidic protein 1) (Cystatin-SA-III)	GO:0004869; GO:0005576	cysteine-type endopeptidase inhibitor activity; extracellular region	Secreted			Known in Uniprot to be phosphorylated	Known in HPRD to be phosphorylated	
**457**	CYTB	CYTB_HUMAN	CYTB; Q76LA1	P04080; Q76LA1	NP_000091	Cystatin-B (Stefin-B) (Liver thiol proteinase inhibitor) (CPI-B); CSTB protein (Cystatin B (Stefin B)) (Cystatin B) (Cystatin B (Stefin B), isoform CRA_a) (cDNA, FLJ92415, Homo sapiens cystatin B (stefin B) (CSTB), mRNA)	GO:0004869; GO:0005737; GO:0010466; GO:0005634; GO:0002020; GO:0008344; GO:0004869; GO:0005622; GO:0005515	cysteine-type endopeptidase inhibitor activity; cytoplasm; negative regulation of peptidase activity; nucleus; protease binding; adult locomotory behavior; cysteine-type endopeptidase inhibitor activity; intracellular; protein binding	CytoplasmNucleus;					
**458**	CATC	CATC_HUMAN	CATC	P53634; A8K7V2; Q53G93; Q71E75; Q71E76; Q7M4N9; Q8WY99; Q8WYA7; Q8WYA8	NP_001805	Dipeptidyl-peptidase 1 (EC 3.4.14.1) (Dipeptidyl-peptidase I) (DPP-I) (DPPI) (Cathepsin C) (Cathepsin J) (Dipeptidyl transferase) [Cleaved into: Dipeptidyl-peptidase 1 exclusion domain chain (Dipeptidyl-peptidase I exclusion domain chain); Dipeptidyl-peptidase 1 heavy chain (Dipeptidyl-peptidase I heavy chain); Dipeptidyl-peptidase 1 light chain (Dipeptidyl-peptidase I light chain)]	GO:0031404; GO:0004197; GO:0006955; GO:0005764; GO:0006508	chloride ion binding; cysteine-type endopeptidase activity; immune response; lysosome; proteolysis	Lysosome					
**459**	CXAR	CXAR_HUMAN	CXAR; B2R8V8	P78310; O00694; Q8WWT6; Q8WWT7; Q8WWT8; Q9UKV4; B2R8V8	NP_001329	Coxsackievirus and adenovirus receptor (Coxsackievirus B-adenovirus receptor) (HCVADR) (hCAR) (CVB3-binding protein)	GO:0005794; GO:0016323; GO:0007155; GO:0005576; GO:0005887; GO:0044419; GO:0005515; GO:0004872; GO:0005923	Golgi apparatus; basolateral plasma membrane; cell adhesion; extracellular region; integral to plasma membrane; interspecies interaction between organisms; protein binding; receptor activity; tight junction	CellmembraneSinglepasstypeImembraneproteinCelljunctiontightjunctionCelljunctionadherensjunctionBasolateralcellmembraneSinglepasstypeImembraneproteinCellmembraneSinglepasstypeImembraneproteinCelljunctiontightjunctionCelljunctionadherensjunctionBasolateralcellmembraneSinglepassmembraneproteinSecretedSecretedSecreted			Known in Uniprot to be phosphorylated	Known in HPRD to be phosphorylated	
**460**	OFD1	OFD1_HUMAN	OFD1	O75665; O75666; Q4VAK4	NP_003602	Oral-facial-digital syndrome 1 protein (Protein 71-7A)	GO:0005813	centrosome	CytoplasmCentrosome			Known in Uniprot to be phosphorylated		
**461**	DAD1	DAD1_HUMAN	DAD1; Q6FGA3; Q53G02	P61803; Q6FGA3; O08552; O70364; P46966; P46968; Q96GB7; Q53G02	NP_001335	Dolichyl-diphosphooligosaccharide--protein glycosyltransferase subunit DAD1 (Oligosaccharyl transferase subunit DAD1) (EC 2.4.1.119) (Defender against cell death 1) (DAD-1)	GO:0006916; GO:0004579; GO:0016021; GO:0008250; GO:0018279	anti-apoptosis; dolichyl-diphosphooligosaccharide-protein glycotransferase activity; integral to membrane; oligosaccharyltransferase complex; protein amino acid N-linked glycosylation via asparagine	MembraneMultipassmembraneprotein					
**462**	DOPD	DOPD_HUMAN	DOPD; Q53Y51	P30046; Q53Y51; O00774; O60787; Q13534	NP_001346; NP_001077861	D-dopachrome decarboxylase (EC 4.1.1.84) (D-dopachrome tautomerase) (Phenylpyruvate tautomerase II); D-dopachrome tautomerase (D-dopachrome tautomerase, isoform CRA_b) (cDNA FLJ76419, highly similar to Homo sapiens D-dopachrome tautomerase (DDT), mRNA)	GO:0033981; GO:0005737; GO:0004167; GO:0006583; GO:0005515;	D-dopachrome decarboxylase activity; cytoplasm; dopachrome isomerase activity; melanin biosynthetic process from tyrosine; protein binding;	Cytoplasm;					
**463**	DPYL2	DPYL2_HUMAN	DPYL2; A8K5H2	Q16555; O00424; A8K5H2	NP_001377	Dihydropyrimidinase-related protein 2 (DRP-2) (Collapsin response mediator protein 2) (CRMP-2) (N2A3)	GO:0030154; GO:0005856; GO:0004157; GO:0007399; GO:0006139; GO:0005515; GO:0007165	cell differentiation; cytoskeleton; dihydropyrimidinase activity; nervous system development; nucleobase, nucleoside, nucleotide and nucleic acid metabolic process; protein binding; signal transduction	Cytoplasm			Known in Uniprot to be phosphorylated	Known in HPRD to be phosphorylated	
**464**	DUT	DUT_HUMAN	DUT; Q6FHN1	Q6FHN1; P33316; O14785; Q16708; Q16860; Q6NSA3; Q96Q81	NP_001939; NP_001020419	Deoxyuridine 5'-triphosphate nucleotidohydrolase, mitochondrial (dUTPase) (EC 3.6.1.23) (dUTP pyrophosphatase); DUT protein (cDNA, FLJ92404, Homo sapiens dUTP pyrophosphatase (DUT), mRNA)	GO:0006260; GO:0005829; GO:0004170; GO:0046080; GO:0000287; GO:0005739; GO:0005634; GO:0004170	DNA replication; cytosol; dUTP diphosphatase activity; dUTP metabolic process; magnesium ion binding; mitochondrion; nucleus; dUTP diphosphatase activity	MitochondrionNucleus;			Known in Uniprot to be phosphorylated	Known in HPRD to be phosphorylated	Phosphatase
**465**	ECE1	ECE1_HUMAN	ECE1	P42892; A8K3P1; Q14217; Q17RN5; Q58GE7; Q5THM5; Q5THM7; Q5THM8; Q9UJQ6; Q9UPF4; Q9UPM4; Q9Y501	NP_001388; NP_001106818; NP_001106820	Endothelin-converting enzyme 1 (ECE-1) (EC 3.4.24.71)	GO:0005794; GO:0033093; GO:0043583; GO:0042733; GO:0034959; GO:0009897; GO:0007507; GO:0042447; GO:0016021; GO:0031302; GO:0005624; GO:0004222; GO:0017046; GO:0048471; GO:0001921; GO:0042803; GO:0003100; GO:0019229; GO:0008270	Golgi apparatus; Weibel-Palade body; ear development; embryonic digit morphogenesis; endothelin maturation; external side of plasma membrane; heart development; hormone catabolic process; integral to membrane; intrinsic to endosome membrane; membrane fraction; metalloendopeptidase activity; peptide hormone binding; perinuclear region of cytoplasm; positive regulation of receptor recycling; protein homodimerization activity; regulation of systemic arterial blood pressure by endothelin; regulation of vasoconstriction; zinc ion binding	CellmembraneSinglepasstypeIImembraneprotein			Known in Uniprot to be phosphorylated		
**466**	TYPH	TYPH_HUMAN	TYPH	P19971; A8MW15; Q13390; Q8WVB7	NP_001944; NP_001107227; NP_001107228	Thymidine phosphorylase (EC 2.4.2.4) (TdRPase) (TP) (Platelet-derived endothelial cell growth factor) (PD-ECGF) (Gliostatin)	GO:0006260; GO:0001525; GO:0030154; GO:0006935; GO:0005829; GO:0008083; GO:0043231; GO:0000002; GO:0005161; GO:0006206; GO:0006213; GO:0006220; GO:0009032	DNA replication; angiogenesis; cell differentiation; chemotaxis; cytosol; growth factor activity; intracellular membrane-bounded organelle; mitochondrial genome maintenance; platelet-derived growth factor receptor binding; pyrimidine base metabolic process; pyrimidine nucleoside metabolic process; pyrimidine nucleotide metabolic process; thymidine phosphorylase activity						
**467**	EF1A1	EF1A1_HUMAN	EF1A1	P68104; P04719; P04720	XP_208217; XP_166506; NP_001393	Elongation factor 1-alpha 1 (EF-1-alpha-1) (Elongation factor 1 A-1) (eEF1A-1) (Elongation factor Tu) (EF-Tu) (Leukocyte receptor cluster member 7)	GO:0005525; GO:0003924; GO:0005829; GO:0005853; GO:0005515; GO:0003746; GO:0006414	GTP binding; GTPase activity; cytosol; eukaryotic translation elongation factor 1 complex; protein binding; translation elongation factor activity; translational elongation	Cytoplasm			Known in Uniprot to be phosphorylated	Known in HPRD to be phosphorylated	
**468**	EF1G	EF1G_HUMAN	EF1G; Q53YD7	P26641; Q53YD7; Q6PJ62; Q6PK31; Q96CU2; Q9P196	NP_001395	Elongation factor 1-gamma (EF-1-gamma) (eEF-1B gamma); GIG35 (EEF1G protein) (Eukaryotic translation elongation factor 1 gamma) (Eukaryotic translation elongation factor 1 gamma, isoform CRA_c)	GO:0005829; GO:0005853; GO:0005515; GO:0003746; GO:0006414; GO:0005853	cytosol; eukaryotic translation elongation factor 1 complex; protein binding; translation elongation factor activity; translational elongation; eukaryotic translation elongation factor 1 complex				Known in Uniprot to be phosphorylated		
**469**	IF2G	IF2G_HUMAN	IF2G; B5BTZ4	P41091; B5BTZ4	NP_001406	Eukaryotic translation initiation factor 2 subunit 3 (Eukaryotic translation initiation factor 2 subunit gamma) (eIF-2-gamma)	GO:0005525; GO:0003924; GO:0005829; GO:0005515; GO:0003743; GO:0006413	GTP binding; GTPase activity; cytosol; protein binding; translation initiation factor activity; translational initiation				Known in Uniprot to be phosphorylated		
**470**	IF4A1	IF4A1_HUMAN	IF4A1; B2R6L8	P60842; B2R6L8; P04765; Q5U018; Q61516	NP_001407; XP_301443	Eukaryotic initiation factor 4A-I (eIF-4A-I) (eIF4A-I) (EC 3.6.1.-) (ATP-dependent RNA helicase eIF4A-1)	GO:0005524; GO:0008026; GO:0000339; GO:0005829; GO:0016281; GO:0003729; GO:0005515; GO:0006412; GO:0003743	ATP binding; ATP-dependent helicase activity; RNA cap binding; cytosol; eukaryotic translation initiation factor 4F complex; mRNA binding; protein binding; translation; translation initiation factor activity				Known in Uniprot to be phosphorylated		

	Protein Name	Uniprot Name	Other Names	Uniprot IDs	Refseq IDs	Protein Description	GO ID	GO terms	Subcellular location	Comments	Comments	Comments - Database Search	Comments - Database Search	Function
**471**	IF4G2	IF4G2_HUMAN	IF4G2	P78344; O60877; P78404; Q0VH00; Q53EU1; Q58EZ2; Q8NI71; Q96C16	NP_001409	Eukaryotic translation initiation factor 4 gamma 2 (eIF-4-gamma 2) (eIF-4G 2) (eIF4G 2) (p97) (Death-associated protein 5) (DAP-5)	GO:0016070; GO:0007050; GO:0008219; GO:0016281; GO:0005515; GO:0006446; GO:0003743	RNA metabolic process; cell cycle arrest; cell death; eukaryotic translation initiation factor 4F complex; protein binding; regulation of translational initiation; translation initiation factor activity				Known in Uniprot to be phosphorylated	Known in HPRD to be phosphorylated	
**472**	ENOA	ENOA_HUMAN	ENOA	P06733; B2RD59; P22712; Q16704; Q4TUS4; Q53FT9; Q53HR3; Q658M5; Q6GMP2; Q71V37; Q7Z3V6; Q8WU71; Q9UCH6; Q9UM55	NP_001419	Alpha-enolase (EC 4.2.1.11) (2-phospho-D-glycerate hydro-lyase) (Non-neural enolase) (NNE) (Enolase 1) (Phosphopyruvate hydratase) (C-myc promoter-binding protein) (MBP-1) (MPB-1) (Plasminogen-binding protein)	GO:0006096; GO:0000287; GO:0030308; GO:0000122; GO:0005634; GO:0004634; GO:0000015; GO:0005886; GO:0004252; GO:0003714; GO:0003700	glycolysis; magnesium ion binding; negative regulation of cell growth; negative regulation of transcription from RNA polymerase II promoter; nucleus; phosphopyruvate hydratase activity; phosphopyruvate hydratase complex; plasma membrane; serine-type endopeptidase activity; transcription corepressor activity; transcription factor activity	CytoplasmCellmembraneCytoplasmmyofibrilsarcomereMbandNucleus			Known in Uniprot to be phosphorylated	Known in HPRD to be phosphorylated	
**473**	ENOB	ENOB_HUMAN	ENOB	P13929; Q96AE2	NP_001967; NP_443739	Beta-enolase (EC 4.2.1.11) (2-phospho-D-glycerate hydro-lyase) (Muscle-specific enolase) (MSE) (Skeletal muscle enolase) (Enolase 3)	GO:0006096; GO:0000287; GO:0004634; GO:0000015	glycolysis; magnesium ion binding; phosphopyruvate hydratase activity; phosphopyruvate hydratase complex	Cytoplasm					
**474**	ETFA	ETFA_HUMAN	ETFA; Q53XN3	P13804; Q53XN3	NP_000117; NP_001121188	Electron transfer flavoprotein subunit alpha, mitochondrial (Alpha-ETF); Electron-transfer-flavoprotein, alpha polypeptide (Electron-transfer-flavoprotein, alpha polypeptide (Glutaric aciduria II)) (cDNA FLJ76486, highly similar to Homo sapiens electron-transfer-flavoprotein, alpha polypeptide (glutaric aciduria II) (ETFA), nuclear gene encoding mitochondrial protein, mRNA) (Electron transfer flavoprotein alpha-subunit) (Electron-transfer-flavoprotein, alpha polypeptide (Glutaric aciduria II), isoform CRA_a)	GO:0050660; GO:0009055; GO:0022900; GO:0005759; GO:0006810; GO:0005739	FAD binding; electron carrier activity; electron transport chain; mitochondrial matrix; transport; mitochondrion	Mitochondrionmatrix;			Known in Uniprot to be phosphorylated		
**475**	ETFB	ETFB_HUMAN	ETFB	P38117; B3KNY2; Q6IBH7; Q71RF6; Q9Y3S7	NP_001976; NP_001014763	Electron transfer flavoprotein subunit beta (Beta-ETF)	GO:0009055; GO:0022900; GO:0005759; GO:0006810	electron carrier activity; electron transport chain; mitochondrial matrix; transport	Mitochondrionmatrix					
**476**	THRB	THRB_HUMAN	THRB	P00734; B2R7F7; B4E1A7; Q4QZ40; Q53H04; Q53H06; Q7Z7P3; Q9UCA1	NP_000497	Prothrombin (EC 3.4.21.5) (Coagulation factor II) [Cleaved into: Activation peptide fragment 1; Activation peptide fragment 2; Thrombin light chain; Thrombin heavy chain]	GO:0007262; GO:0006919; GO:0006953; GO:0005509; GO:0007166; GO:0005615; GO:0042730; GO:0007275; GO:0005886; GO:0030168; GO:0030194; GO:0032967; GO:0001934; GO:0006508; GO:0005102; GO:0051209; GO:0004252; GO:0005625; GO:0003809; GO:0070053; GO:0007260	STAT protein nuclear translocation; activation of caspase activity; acute-phase response; calcium ion binding; cell surface receptor linked signal transduction; extracellular space; fibrinolysis; multicellular organismal development; plasma membrane; platelet activation; positive regulation of blood coagulation; positive regulation of collagen biosynthetic process; positive regulation of protein amino acid phosphorylation; proteolysis; receptor binding; release of sequestered calcium ion into cytosol; serine-type endopeptidase activity; soluble fraction; thrombin activity; thrombospondin receptor activity; tyrosine phosphorylation of STAT protein	Secretedextracellularspace					
**477**	FLII	FLII_HUMAN	FLII	Q13045	NP_002009	Protein flightless-1 homolog	GO:0003779; GO:0005813; GO:0007275; GO:0006936; GO:0005634; GO:0006355	actin binding; centrosome; multicellular organismal development; muscle contraction; nucleus; regulation of transcription, DNA-dependent	NucleusCytoplasmcytoskeletonCentrosome			Known in Uniprot to be phosphorylated	Known in HPRD to be phosphorylated	
**478**	KU70	KU70_HUMAN	KU70	P12956; Q6FG89; Q9UCQ2; Q9UCQ3	XP_060320; NP_001460	ATP-dependent DNA helicase 2 subunit 1 (ATP-dependent DNA helicase II 70 kDa subunit) (Lupus Ku autoantigen protein p70) (Ku70) (70 kDa subunit of Ku antigen) (Thyroid-lupus autoantigen) (TLAA) (CTC box-binding factor 75 kDa subunit) (CTCBF) (CTC75) (DNA repair protein XRCC6)	GO:0004003; GO:0006266; GO:0005958; GO:0006303; GO:0003690; GO:0019059; GO:0005624; GO:0000783; GO:0010552; GO:0010843; GO:0008022; GO:0019047; GO:0000723; GO:0016563; GO:0005667	ATP-dependent DNA helicase activity; DNA ligation; DNA-dependent protein kinase-DNA ligase 4 complex; double-strand break repair via nonhomologous end joining; double-stranded DNA binding; initiation of viral infection; membrane fraction; nuclear telomere cap complex; positive regulation of specific transcription from RNA polymerase II promoter; promoter binding; protein C-terminus binding; provirus integration; telomere maintenance; transcription activator activity; transcription factor complex	Nucleus			Known in Uniprot to be phosphorylated	Known in HPRD to be phosphorylated	
**479**	GDIA	GDIA_HUMAN	GDIA; Q6FG50	P31150; Q6FG50; P50394; Q7Z2G6; Q7Z2G9; Q7Z2H5; Q7Z2I6	XP_069750; NP_001484	Rab GDP dissociation inhibitor alpha (Rab GDI alpha) (Guanosine diphosphate dissociation inhibitor 1) (GDI-1) (Oligophrenin-2) (Protein XAP-4)	GO:0005096; GO:0005093; GO:0005737; GO:0005515; GO:0015031; GO:0043087	GTPase activator activity; Rab GDP-dissociation inhibitor activity; cytoplasm; protein binding; protein transport; regulation of GTPase activity	Cytoplasm			Known in Uniprot to be phosphorylated		
**480**	GET1	GET1_HUMAN	GET1; STBD1	O95210	XP_292133; NP_003934	Starch-binding domain-containing protein 1 (Genethonin-1)	GO:0030246; GO:0005975; GO:0003824; GO:0005887; GO:0005624; GO:0006936	carbohydrate binding; carbohydrate metabolic process; catalytic activity; integral to plasma membrane; membrane fraction; muscle contraction	MembraneSinglepasstypeIIImembraneprotein			Known in Uniprot to be phosphorylated		
**481**	GFAP	GFAP_HUMAN	GFAP; B2RD44	P14136; Q53H98; Q5D055; Q6ZQS3; Q7Z5J6; Q7Z5J7; Q96KS4; Q96P18; Q9UFD0; B2RD44	NP_002046; NP_001124491	Glial fibrillary acidic protein (GFAP)	GO:0005737; GO:0005882; GO:0005515; GO:0005200	cytoplasm; intermediate filament; protein binding; structural constituent of cytoskeleton	Cytoplasm			Known in Uniprot to be phosphorylated	Known in HPRD to be phosphorylated	
**482**	GGH	GGH_HUMAN	GGH	Q92820	NP_003869	Gamma-glutamyl hydrolase (EC 3.4.19.9) (Gamma-Glu-X carboxypeptidase) (Conjugase) (GH)	GO:0005576; GO:0034722; GO:0006541; GO:0005764; GO:0042470	extracellular region; gamma-glutamyl-peptidase activity; glutamine metabolic process; lysosome; melanosome	SecretedextracellularspaceLysosomeMelanosome					
**483**	GMDS	GMDS_HUMAN	GMDS	O60547; O75357; Q5T954; Q6FH09; Q9UGZ3; Q9UJK9	XP_304825; NP_001491	GDP-mannose 4,6 dehydratase (EC 4.2.1.47) (GDP-D-mannose dehydratase) (GMD)	GO:0042351; GO:0008446; GO:0019673; GO:0050662; GO:0005737; GO:0007159	'de novo' GDP-L-fucose biosynthetic process; GDP-mannose 4,6-dehydratase activity; GDP-mannose metabolic process; coenzyme binding; cytoplasm; leukocyte adhesion						
**484**	GNAZ	GNAZ_HUMAN	GNAZ; Q8N652; Q8IY73; B2R6C1	P19086; Q4QRJ6; B2R6C1; Q8IY73; Q8N652	NP_002064	Guanine nucleotide-binding protein G(z) subunit alpha (G(x) alpha chain) (Gz-alpha); Guanine nucleotide binding protein (G protein), alpha z polypeptide	GO:0007186; GO:0005525; GO:0003924; GO:0005783; GO:0005635; GO:0005886; GO:0005057; GO:0004871	G-protein coupled receptor protein signaling pathway; GTP binding; GTPase activity; endoplasmic reticulum; nuclear envelope; plasma membrane; receptor signaling protein activity; signal transducer activity	MembraneLipidanchor;				known in HPRD to be phosphorylated	
**485**	GNS	GNS_HUMAN	GNS; Q7Z3X3	P15586; Q53F05; Q7Z3X3	NP_002067	N-acetylglucosamine-6-sulfatase (G6S) (Glucosamine-6-sulfatase) (EC 3.1.6.14); Putative uncharacterized protein DKFZp686E12166	GO:0008449; GO:0005509; GO:0006027; GO:0005764; GO:0005515; GO:0030203	N-acetylglucosamine-6-sulfatase activity; calcium ion binding; glycosaminoglycan catabolic process; lysosome; protein binding; glycosaminoglycan metabolic process	Lysosome;					
**486**	GPIX	GPIX_HUMAN	GPIX; Q8N1D1	P14770; Q14445; Q92525; Q8N1D1	NP_000165	Platelet glycoprotein IX (GPIX) (CD antigen CD42a); Glycoprotein IX (Platelet)	GO:0007596; GO:0007155; GO:0005887; GO:0031092; GO:0005515;	blood coagulation; cell adhesion; integral to plasma membrane; platelet alpha granule membrane; protein binding;	MembraneSinglepasstypeImembraneprotein;					
**487**	GRB2	GRB2_HUMAN	GRB2; Q9H2Q4; B0LPF3	P62993; P29354; Q14450; Q63057; Q63059; B0LPF3	NP_002077; NP_987102	Growth factor receptor-bound protein 2 (Adapter protein GRB2) (SH2/SH3 adapter GRB2) (Protein Ash)	GO:0005794; GO:0007265; GO:0005070; GO:0007267; GO:0005829; GO:0005154; GO:0007173; GO:0008286; GO:0043560; GO:0044419	Golgi apparatus; Ras protein signal transduction; SH3/SH2 adaptor activity; cell-cell signaling; cytosol; epidermal growth factor receptor binding; epidermal growth factor receptor signaling pathway; insulin receptor signaling pathway; insulin receptor substrate binding; interspecies interaction between organisms	Golgiapparatus			Known in Uniprot to be phosphorylated	Known in HPRD to be phosphorylated	
**488**	GSTM2	GSTM2_HUMAN	GSTM2	P28161; Q2M318; Q5TZY5; Q8WWE1	NP_000839	Glutathione S-transferase Mu 2 (EC 2.5.1.18) (GSTM2-2) (GST class-mu 2)	GO:0005737; GO:0004364; GO:0008152	cytoplasm; glutathione transferase activity; metabolic process	Cytoplasm					
**489**	GSTP1	GSTP1_HUMAN	GSTP1	P09211; Q5TZY3; O00460; Q15690	NP_000843	Glutathione S-transferase P (EC 2.5.1.18) (GST class-pi) (GSTP1-1)	GO:0006916; GO:0007417; GO:0005737; GO:0004364; GO:0008152; GO:0005515	anti-apoptosis; central nervous system development; cytoplasm; glutathione transferase activity; metabolic process; protein binding				Known in Uniprot to be phosphorylated		
**490**	GCYB1	GCYB1_HUMAN	GCYB1	Q02153; Q86WY5	NP_000848	Guanylate cyclase soluble subunit beta-1 (GCS-beta-1) (EC 4.6.1.2) (Soluble guanylate cyclase small subunit) (GCS-beta-3)	GO:0008015; GO:0006182; GO:0004383; GO:0008074; GO:0020037; GO:0007263; GO:0004872	blood circulation; cGMP biosynthetic process; guanylate cyclase activity; guanylate cyclase complex, soluble; heme binding; nitric oxide mediated signal transduction; receptor activity	Cytoplasm					
**491**	KGUA	KGUA_HUMAN	KGUA; Q6IBG8; Q658U3	Q16774; Q6IBG8; Q658U3	NP_000849; NP_001152863	Guanylate kinase (EC 2.7.4.8) (GMP kinase); Putative uncharacterized protein DKFZp666D023 (Fragment); GUK1 protein (Guanylate kinase 1) (Guanylate kinase 1, isoform CRA_a)	GO:0005524; GO:0005829; GO:0004385; GO:0019206; GO:0006163; GO:0004385; GO:0005737; GO:0016301	ATP binding; cytosol; guanylate kinase activity; nucleoside kinase activity; purine nucleotide metabolic process; guanylate kinase activity; cytoplasm; kinase activity				Known in Uniprot to be phosphorylated		Kinase

	Protein Name	Uniprot Name	Other Names	Uniprot IDs	Refseq IDs	Protein Description	GO ID	GO terms	Subcellular location	Comments	Comments	Comments - Database Search	Comments - Database Search	Function
**492**	HBB	HBB_HUMAN	HBB	P68871; A4GX73; B2ZUE0; P02023; Q13852; Q14481; Q14510; Q45KT0; Q549N7; Q6FI08; Q6R7N2; Q8IZI1; Q9BX96; Q9UCD6; Q9UCP8; Q9UCP9	NP_000509	Hemoglobin subunit beta (Hemoglobin beta chain) (Beta-globin) [Cleaved into: LVV-hemorphin-7]	GO:0020037; GO:0030492; GO:0005833; GO:0030185; GO:0019825; GO:0015671; GO:0005344; GO:0045429; GO:0008217; GO:0050880	heme binding; hemoglobin binding; hemoglobin complex; nitric oxide transport; oxygen binding; oxygen transport; oxygen transporter activity; positive regulation of nitric oxide biosynthetic process; regulation of blood pressure; regulation of blood vessel size				Known in Uniprot to be phosphorylated		
**493**	HBD	HBD_HUMAN	HBD; A0N071	P02042; Q3Y5H3; Q8WXT7; A0N071	NP_000510	Hemoglobin subunit delta (Hemoglobin delta chain) (Delta-globin); Delta-globin chain (Hemoglobin delta) (Hemoglobin, delta)	GO:0020037; GO:0005833; GO:0019825; GO:0015671; GO:0005344; GO:0005506	heme binding; hemoglobin complex; oxygen binding; oxygen transport; oxygen transporter activity; iron ion binding				Known in Uniprot to be phosphorylated		
**494**	HLAG	HLAG_HUMAN	HLAG; Q6DU14	P17693; Q6DU14	NP_002118	HLA class I histocompatibility antigen, alpha chain G (HLA G antigen); HLA-G histocompatibility antigen, class I, G (Major histocompatibility complex, class I, G) (HLA-G histocompatibility antigen, class I, G, isoform CRA_a) (MHC class Ib antigen)	GO:0042612; GO:0032393; GO:0002474; GO:0006968; GO:0006955; GO:0016021; GO:0019882	MHC class I protein complex; MHC class I receptor activity; antigen processing and presentation of peptide antigen via MHC class I; cellular defense response; immune response; integral to membrane; antigen processing and presentation	MembraneSinglepasstypeImembraneprotein;					
**495**	HPRT	HPRT_HUMAN	HPRT	P00492; A6NHF0; B2R8M9	NP_000185	Hypoxanthine-guanine phosphoribosyltransferase (HGPRTase) (HGPRT) (EC 2.4.2.8)	GO:0005829; GO:0046100; GO:0004422; GO:0000287; GO:0045964; GO:0042803; GO:0051289; GO:0006164; GO:0006166	cytosol; hypoxanthine metabolic process; hypoxanthine phosphoribosyltransferase activity; magnesium ion binding; positive regulation of dopamine metabolic process; protein homodimerization activity; protein homotetramerization; purine nucleotide biosynthetic process; purine ribonucleoside salvage	Cytoplasm					
**496**	SRCH	SRCH_HUMAN	SRCH	P23327; Q504Y6	NP_002143	Sarcoplasmic reticulum histidine-rich calcium-binding protein	GO:0005509; GO:0006936; GO:0005634; GO:0033018	calcium ion binding; muscle contraction; nucleus; sarcoplasmic reticulum lumen	Sarcoplasmicreticulumlumen					
**497**	HRG	HRG_HUMAN	HRG; B9EK35	P04196; B9EK35	NP_000403	Histidine-rich glycoprotein (Histidine-proline-rich glycoprotein) (HPRG)	GO:0004869; GO:0005576; GO:0008201; GO:0031093	cysteine-type endopeptidase inhibitor activity; extracellular region; heparin binding; platelet alpha granule lumen	Secreted					
**498**	DHB4	DHB4_HUMAN	DHB4	P51659	NP_000405	Peroxisomal multifunctional enzyme type 2 (MFE-2) (D-bifunctional protein) (DBP) (17-beta-hydroxysteroid dehydrogenase 4) (17-beta-HSD 4) [Includes: 3-hydroxyacyl-CoA dehydrogenase (EC 1.1.1.35); 3-alpha,7-alpha,12-alpha-trihydroxy-5-beta-cholest-24-enoyl-CoA hydratase (EC 4.2.1.107)]	GO:0003857; GO:0033989; GO:0004303; GO:0016853; GO:0055114; GO:0005782; GO:0005515; GO:0005498; GO:0015248	3-hydroxyacyl-CoA dehydrogenase activity; 3alpha,7alpha,12alpha-trihydroxy-5beta-cholest-24-enoyl-CoA hydratase activity; estradiol 17-beta-dehydrogenase activity; isomerase activity; oxidation reduction; peroxisomal matrix; protein binding; sterol carrier activity; sterol transporter activity	Peroxisome			Known in Uniprot to be phosphorylated		
**499**	CH10	CH10_HUMAN	CH10; Q53X54	P61604; Q53X54; O95421; Q04984	NP_002148	10 kDa heat shock protein, mitochondrial (Hsp10) (10 kDa chaperonin) (CPN10) (Early-pregnancy factor) (EPF); HSPE1 protein (Heat shock 10kDa protein 1 (Chaperonin 10), isoform CRA_i) (Putative uncharacterized protein HSPE1) (cDNA, FLJ92386, Homo sapiens heat shock 10kDa protein 1 (chaperonin 10) (HSPE1),mRNA) (Fragment)	GO:0005524; GO:0006919; GO:0051087; GO:0005759; GO:0006457; GO:0006986; GO:0051082; GO:0005739; GO:0006950	ATP binding; activation of caspase activity; chaperone binding; mitochondrial matrix; protein folding; response to unfolded protein; unfolded protein binding; mitochondrion; response to stress	Mitochondrionmatrix;			Known in Uniprot to be phosphorylated		
**500**	ICAM2	ICAM2_HUMAN	ICAM2; Q6FHE2	P13598; Q14600; Q6FHE2	NP_000864; NP_001093256; NP_001093257; NP_001093258; NP_001093259	Intercellular adhesion molecule 2 (ICAM-2) (CD antigen CD102)	GO:0016337; GO:0005887; GO:0005178	cell-cell adhesion; integral to plasma membrane; integrin binding	MembraneSinglepasstypeImembraneprotein					
**501**	ITPR3	ITPR3_HUMAN	ITPR3	Q14573; Q14649; Q5TAQ2	NP_002215	Inositol 1,4,5-trisphosphate receptor type 3 (Type 3 inositol 1,4,5-trisphosphate receptor) (Type 3 InsP3 receptor) (IP3 receptor isoform 3) (InsP3R3)	GO:0007186; GO:0005903; GO:0005509; GO:0015085; GO:0006816; GO:0043025; GO:0007204; GO:0005789; GO:0043533; GO:0005220; GO:0000822; GO:0008095; GO:0016021; GO:0043209; GO:0005640; GO:0005515; GO:0051291; GO:0051260	G-protein coupled receptor protein signaling pathway; brush border; calcium ion binding; calcium ion transmembrane transporter activity; calcium ion transport; cell soma; elevation of cytosolic calcium ion concentration; endoplasmic reticulum membrane; inositol 1,3,4,5 tetrakisphosphate binding; inositol 1,4,5-triphosphate-sensitive calcium-release channel activity; inositol hexakisphosphate binding; inositol-1,4,5-triphosphate receptor activity; integral to membrane; myelin sheath; nuclear outer membrane; protein binding; protein heterooligomerization; protein homooligomerization	EndoplasmicreticulummembraneMultipassmembraneprotein			Known in Uniprot to be phosphorylated		
**502**	KRT81	KRT81_HUMAN	KRT81; Q9NSB5	Q14533; Q14846; Q16274; Q17R48; Q8WU52; Q9BR74; Q9NSB5	NP_002272	Keratin, type II cuticular Hb1 (Type II hair keratin Hb1) (Keratin, hair, basic, 1) (ghHKb1) (ghHb1) (Keratin-81) (K81) (K2.9) (MLN 137); Type II hair keratin 1 (Fragment)	GO:0045095; GO:0005515; GO:0005198	keratin filament; protein binding; structural molecule activity						
**503**	KYNU	KYNU_HUMAN	KYNU; Q6I9T2	Q16719; Q6I9T2; B2RCZ5	NP_003928	Kynureninase (EC 3.7.1.3) (L-kynurenine hydrolase); KYNU protein (Kynureninase (L-kynurenine hydrolase), isoform CRA_b)	GO:0009435; GO:0043420; GO:0005829; GO:0030429; GO:0005739; GO:0042803; GO:0030170; GO:0019805; GO:0034341; GO:0034516; GO:0005625; GO:0006569; GO:0005737	NAD biosynthetic process; anthranilate metabolic process; cytosol; kynureninase activity; mitochondrion; protein homodimerization activity; pyridoxal phosphate binding; quinolinate biosynthetic process; response to interferon-gamma; response to vitamin B6; soluble fraction; tryptophan catabolic process; cytoplasm	Cytoplasm;					
**504**	LEG1	LEG1_HUMAN	LEG1; B2R5E8	P09382; B2R5E8	NP_002296	Galectin-1 (Lectin galactoside-binding soluble 1) (Beta-galactoside-binding lectin L-14-I) (Lactose-binding lectin 1) (S-Lac lectin 1) (Galaptin) (14 kDa lectin) (HPL) (HBL) (Putative MAPK-activating protein PM12)	GO:0005737; GO:0016936; GO:0043123; GO:0005515; GO:0042981; GO:0004871	cytoplasm; galactoside binding; positive regulation of I-kappaB kinase/NF-kappaB cascade; protein binding; regulation of apoptosis; signal transducer activity	Secretedextracellularspaceextracellularmatrix					
**505**	MPRD	MPRD_HUMAN	MPRD; A8K528	P20645; A8K528	NP_002346	Cation-dependent mannose-6-phosphate receptor (CD Man-6-P receptor) (CD-MPR) (46 kDa mannose 6-phosphate receptor) (MPR 46)	GO:0009986; GO:0008333; GO:0005887; GO:0005765; GO:0005537; GO:0015578; GO:0015761; GO:0005515; GO:0006898; GO:0004888	cell surface; endosome to lysosome transport; integral to plasma membrane; lysosomal membrane; mannose binding; mannose transmembrane transporter activity; mannose transport; protein binding; receptor-mediated endocytosis; transmembrane receptor activity	LysosomemembraneSinglepasstypeImembraneprotein			Known in Uniprot to be phosphorylated		
**506**	ACTHR	ACTHR_HUMAN	ACTHR; A8K016	Q01718; Q3MI45; Q504X6; A8K016	NP_000520	Adrenocorticotropic hormone receptor (ACTH receptor) (ACTH-R) (Adrenocorticotropin receptor) (Melanocortin receptor 2) (MC2-R)	GO:0007187; GO:0004978; GO:0005887	G-protein signaling, coupled to cyclic nucleotide second messenger; adrenocorticotropin receptor activity; integral to plasma membrane	CellmembraneMultipassmembraneprotein			Known in Uniprot to be phosphorylated		
**507**	MAOM	MAOM_HUMAN	MAOM; Q9BWL6	P23368; Q9BYG1; Q9H4B2; Q9BWL6	NP_002387	NAD-dependent malic enzyme, mitochondrial (NAD ME) (EC 1.1.1.38) (Malic enzyme 2); Malic enzyme	GO:0051287; GO:0009055; GO:0004471; GO:0016619; GO:0006108; GO:0046872; GO:0005759; GO:0055114; GO:0004470; GO:0005739; GO:0016616	NAD or NADH binding; electron carrier activity; malate dehydrogenase (decarboxylating) activity; malate dehydrogenase (oxaloacetate-decarboxylating) activity; malate metabolic process; metal ion binding; mitochondrial matrix; oxidation reduction; malic enzyme activity; mitochondrion; oxidoreductase activity, acting on the CH-OH group of donors, NAD or NADP as acceptor	Mitochondrionmatrix;					
**508**	MIF	MIF_HUMAN	MIF; B2R4S3	P14174; Q2V4Y5; B2R4S3	NP_002406	Macrophage migration inhibitory factor (MIF) (EC 5.3.2.1) (Phenylpyruvate tautomerase) (Glycosylation-inhibiting factor) (GIF)	GO:0008283; GO:0007166; GO:0005125; GO:0005615; GO:0006954; GO:0043066; GO:0050178; GO:0001516; GO:0043030	cell proliferation; cell surface receptor linked signal transduction; cytokine activity; extracellular space; inflammatory response; negative regulation of apoptosis; phenylpyruvate tautomerase activity; prostaglandin biosynthetic process; regulation of macrophage activation				Known in Uniprot to be phosphorylated		
**509**	MLL2	MLL2_HUMAN	Q6PIA1; MLL2	O14686; Q6PIA1; O14687	NP_003473	MLL2 protein (Fragment)	GO:0003677; GO:0001701; GO:0008168; GO:0005634	DNA binding; in utero embryonic development; methyltransferase activity; nucleus				Known in Uniprot to be phosphorylated	Known in HPRD to be phosphorylated	
**510**	EM55	EM55_HUMAN	EM55; Q5J7V5	Q00013; Q5J7V5	NP_002427	55 kDa erythrocyte membrane protein (p55) (Membrane protein, palmitoylated 1); Migration-related gene 1 protein (cDNA FLJ78470, highly similar to Homo sapiens membrane protein, palmitoylated 1, 55kDa (MPP1), mRNA) (Membrane protein, palmitoylated 1, 55kDa, isoform CRA_b) (cDNA, FLJ92600, Homo sapiens membrane protein, palmitoylated 1, 55kDa (MPP1), mRNA)	GO:0042995; GO:0004385; GO:0005887; GO:0005622; GO:0005624; GO:0005515; GO:0007165; GO:0005515	cell projection; guanylate kinase activity; integral to plasma membrane; intracellular; membrane fraction; protein binding; signal transduction; protein binding	MembranePeripheralmembraneproteinCellprojectionstereocilium;			Known in Uniprot to be phosphorylated		
**511**	MUC2	MUC2_HUMAN	MUC2	Q02817; Q14878	XP_292125; NP_002448	Mucin-2 (MUC-2) (Intestinal mucin-2)	GO:0005576	extracellular region	Secreted			Known in Uniprot to be phosphorylated		
**512**	MYH8	MYH8_HUMAN	MYH8	P13535; Q14910	NP_002463	Myosin-8 (Myosin heavy chain 8) (Myosin heavy chain, skeletal muscle, perinatal) (MyHC-perinatal)	GO:0005524; GO:0003779; GO:0005516; GO:0003774; GO:0006941; GO:0005863; GO:0008307	ATP binding; actin binding; calmodulin binding; motor activity; striated muscle contraction; striated muscle thick filament; structural constituent of muscle	Cytoplasmmyofibril					

	Protein Name	Uniprot Name	Other Names	Uniprot IDs	Refseq IDs	Protein Description	GO ID	GO terms	Subcellular location	Comments	Comments	Comments - Database Search	Comments - Database Search	Function
**513**	NDUA4	NDUA4_HUMAN	NDUA4; A4D109	O00483; A4D109; Q6FHN5	NP_002480; XP_302059	NADH dehydrogenase [ubiquinone] 1 alpha subcomplex subunit 4 (NADH-ubiquinone oxidoreductase MLRQ subunit) (Complex I-MLRQ) (CI-MLRQ)	GO:0008137; GO:0006120; GO:0005747; GO:0006810	NADH dehydrogenase (ubiquinone) activity; mitochondrial electron transport, NADH to ubiquinone; mitochondrial respiratory chain complex I; transport	MitochondrioninnermembranePeripheralmembraneproteinMatrixside					
**514**	NDUS4	NDUS4_HUMAN	NDUS4	O43181; Q9BS69	NP_002486	NADH dehydrogenase [ubiquinone] iron-sulfur protein 4, mitochondrial (NADH-ubiquinone oxidoreductase 18 kDa subunit) (Complex I-18 kDa) (CI-18 kDa) (Complex I-AQDQ) (CI-AQDQ)	GO:0008137; GO:0007420; GO:0019933; GO:0045333; GO:0006120; GO:0005747; GO:0032981; GO:0006800; GO:0048146; GO:0001932; GO:0051591; GO:0006810	NADH dehydrogenase (ubiquinone) activity; brain development; cAMP-mediated signaling; cellular respiration; mitochondrial electron transport, NADH to ubiquinone; mitochondrial respiratory chain complex I; mitochondrial respiratory chain complex I assembly; oxygen and reactive oxygen species metabolic process; positive regulation of fibroblast proliferation; regulation of protein amino acid phosphorylation; response to cAMP; transport	MitochondrioninnermembranePeripheralmembraneproteinMatrixside			Known in Uniprot to be phosphorylated	Known in HPRD to be phosphorylated	
**515**	NID1	NID1_HUMAN	NID1	P14543; Q14942; Q59FL2; Q5TAF2; Q5TAF3; Q86XD7	NP_002499	Nidogen-1 (NID-1) (Entactin)	GO:0005604; GO:0008218; GO:0005509; GO:0007160; GO:0016020; GO:0005515; GO:0018298	basement membrane; bioluminescence; calcium ion binding; cell-matrix adhesion; membrane; protein binding; protein-chromophore linkage	Secretedextracellularspaceextracellularmatrixbasementmembrane					
**516**	NQO2	NQO2_HUMAN	NQO2	P16083; Q5TD04	NP_000895	Ribosyldihydronicotinamide dehydrogenase [quinone] (EC 1.10.99.2) (NRH dehydrogenase [quinone] 2) (NRH:quinone oxidoreductase 2) (Quinone reductase 2) (QR2)	GO:0008753; GO:0050662; GO:0005737; GO:0001512; GO:0009055; GO:0055114; GO:0008270	NADPH dehydrogenase (quinone) activity; coenzyme binding; cytoplasm; dihydronicotinamide riboside quinone reductase activity; electron carrier activity; oxidation reduction; zinc ion binding	Cytoplasm			Known in Uniprot to be phosphorylated		
**517**	RASN	RASN_HUMAN	RASN; Q5U091	P01111; Q5U091; Q14971; Q15104; Q15282	NP_002515	GTPase NRas (Transforming protein N-Ras); Neuroblastoma RAS viral (V-ras) oncogene homolog (Neuroblastoma RAS viral (V-ras) oncogene homolog, isoform CRA_a)	GO:0005525; GO:0000139; GO:0005886; GO:0030036; GO:0007569; GO:0006897; GO:0005622; GO:0005624; GO:0043524; GO:0035022; GO:0008284; GO:0005515; GO:0048169; GO:0032228; GO:0007264; GO:0051146; GO:0008542	GTP binding; Golgi membrane; plasma membrane; actin cytoskeleton organization; cell aging; endocytosis; intracellular; membrane fraction; negative regulation of neuron apoptosis; positive regulation of Rac protein signal transduction; positive regulation of cell proliferation; protein binding; regulation of long-term neuronal synaptic plasticity; regulation of synaptic transmission, GABAergic; small GTPase mediated signal transduction; striated muscle cell differentiation; visual learning	CellmembraneLipidanchorCytoplasmicsideGolgiapparatusmembraneLipidanchor;					
**518**	PA1B2	PA1B2_HUMAN	PA1B2; Q6IBR6	P68402; Q6IBR6; O00687; Q29459	NP_002563	Platelet-activating factor acetylhydrolase IB subunit beta (EC 3.1.1.47) (PAF acetylhydrolase 30 kDa subunit) (PAF-AH 30 kDa subunit) (PAF-AH subunit beta) (PAFAH subunit beta); Intracellular platelet-activating factor acetylhydrolase alpha 2 subunit (PAFAH1B2 protein) (cDNA FLJ75836, highly similar to Homo sapiens platelet-activating factor acetylhydrolase, isoform Ib, beta subunit 30kDa (PAFAH1B2), mRNA) (Platelet-activating factor acetylhydrolase IB subunit beta) (Platelet-activating factor acetylhydrolase, isoform Ib, beta subunit 30kDa, isoform CRA_a)	GO:0003847; GO:0005737; GO:0016042; GO:0005737; GO:0016788; GO:0006629; GO:0005515; GO:0007283	1-alkyl-2-acetylglycerophosphocholine esterase activity; cytoplasm; lipid catabolic process; cytoplasm; hydrolase activity, acting on ester bonds; lipid metabolic process; protein binding; spermatogenesis	Cytoplasm;					
**519**	PEBP1	PEBP1_HUMAN	PEBP1; B2R4S1	P30086; B2R4S1	NP_002558; XP_086916	Phosphatidylethanolamine-binding protein 1 (PEBP-1) (Prostatic-binding protein) (HCNPpp) (Neuropolypeptide h3) (Raf kinase inhibitor protein) (RKIP) [Cleaved into: Hippocampal cholinergic neurostimulating peptide (HCNP)]	GO:0005524; GO:0005737; GO:0008429; GO:0005515; GO:0004867	ATP binding; cytoplasm; phosphatidylethanolamine binding; protein binding; serine-type endopeptidase inhibitor activity	Cytoplasm			Known in Uniprot to be phosphorylated	Known in HPRD to be phosphorylated	
**520**	PDE6D	PDE6D_HUMAN	PDE6D; Q6PKG8; Q6IB24	O43924; Q6IB24; O43250; Q6PKG8	NP_002592	Retinal rod rhodopsin-sensitive cGMP 3',5'-cyclic phosphodiesterase subunit delta (GMP-PDE delta) (Protein p17); PDE6D protein (Phosphodiesterase 6D, cGMP-specific, rod, delta, isoform CRA_b) (Putative uncharacterized protein tmp_locus_12); PDE6D protein	GO:0004114; GO:0005515; GO:0050896; GO:0007601;	3',5'-cyclic-nucleotide phosphodiesterase activity; protein binding; response to stimulus; visual perception;						
**521**	PDPK1	PDPK1_HUMAN	PDPK1; Q9BRD5; Q6FI20	O15530; Q6FI20; Q9BRD5; Q8IV52	NP_002604; NP_112558	3-phosphoinositide-dependent protein kinase 1 (hPDK1) (EC 2.7.11.1); 3-phosphoinositide dependent protein kinase-1	GO:0004676; GO:0005524; GO:0030036; GO:0032148; GO:0032869; GO:0005829; GO:0007242; GO:0006469; GO:0018107; GO:0005886; GO:0005515; GO:0070201; GO:0005524; GO:0006468; GO:0004674	3-phosphoinositide-dependent protein kinase activity; ATP binding; actin cytoskeleton organization; activation of protein kinase B activity; cellular response to insulin stimulus; cytosol; intracellular signaling cascade; negative regulation of protein kinase activity; peptidyl-threonine phosphorylation; plasma membrane; protein binding; regulation of establishment of protein localization; ATP binding; protein amino acid phosphorylation; protein serine/threonine kinase activity	CytoplasmMembranePeripheralmembraneprotein;			Known in Uniprot to be phosphorylated	Known in HPRD to be phosphorylated	Kinase
**522**	PEA15	PEA15_HUMAN	PEA15; Q5JW80; B1AKZ4	Q15121; Q5JW80; O00511; B1AKZ4	NP_003759	Astrocytic phosphoprotein PEA-15 (Phosphoprotein enriched in diabetes) (PED)	GO:0006916; GO:0005737; GO:0005875; GO:0046325; GO:0005515; GO:0005351	anti-apoptosis; cytoplasm; microtubule associated complex; negative regulation of glucose import; protein binding; sugar:hydrogen symporter activity	Cytoplasm			Known in Uniprot to be phosphorylated	Known in HPRD to be phosphorylated	
**523**	PF4V	PF4V_HUMAN	PF4V; A1L4S0	P10720; A1L4S0	NP_002611	Platelet factor 4 variant (PF4var1) (PF4alt) (C-X-C motif chemokine 4 variant) (CXCL4L1) [Cleaved into: Platelet factor 4 variant(4-74); Platelet factor 4 variant(5-74); Platelet factor 4 variant(6-74)]; Platelet factor 4 variant 1	GO:0008009; GO:0005576; GO:0008201; GO:0006955	chemokine activity; extracellular region; heparin binding; immune response	Secreted					
**524**	PGAM1	PGAM1_HUMAN	PGAM1; Q6FHU2	P18669; Q6FHU2; Q9BWC0	NP_002620	Phosphoglycerate mutase 1 (EC 5.4.2.1) (EC 5.4.2.4) (EC 3.1.3.13) (Phosphoglycerate mutase isozyme B) (PGAM-B) (BPG-dependent PGAM 1); Phosphoglycerate mutase (Fragment)	GO:0004083; GO:0004082; GO:0005829; GO:0004619; GO:0019901; GO:0006110; GO:0043456; GO:0045730; GO:0006096; GO:0016868	bisphosphoglycerate 2-phosphatase activity; bisphosphoglycerate mutase activity; cytosol; phosphoglycerate mutase activity; protein kinase binding; regulation of glycolysis; regulation of pentose-phosphate shunt; respiratory burst; glycolysis; intramolecular transferase activity, phosphotransferases				Known in Uniprot to be phosphorylated		
**525**	PHB	PHB_HUMAN	PHB; Q13022; A8K401	P35232; A8K401; Q13022; Q4VBQ0	NP_002625; XP_069253	Prohibitin; Prohibitin (Fragment)	GO:0006260; GO:0016575; GO:0005887; GO:0005743; GO:0008285; GO:0016481; GO:0005654; GO:0005515; GO:0042981; GO:0007165; GO:0016563; GO:0016564;	DNA replication; histone deacetylation; integral to plasma membrane; mitochondrial inner membrane; negative regulation of cell proliferation; negative regulation of transcription; nucleoplasm; protein binding; regulation of apoptosis; signal transduction; transcription activator activity; transcription repressor activity;	Mitochondrioninnermembrane;			Known in Uniprot to be phosphorylated		
**526**	DYL1	DYL1_HUMAN	DYL1; Q6FGH9	P63167; Q6FGH9; Q15701	NP_003737; NP_001032583; NP_001032584	Dynein light chain 1, cytoplasmic (Dynein light chain LC8-type 1) (8 kDa dynein light chain) (DLC8) (Protein inhibitor of neuronal nitric oxide synthase) (PIN); Dynein, light chain, LC8-type 1 (DNCL1 protein) (Dynein, light chain, LC8-type 1, isoform CRA_a) (cDNA, FLJ92051, Homo sapiens dynein, cytoplasmic, light polypeptide 1 (DNCL1),mRNA)	GO:0030036; GO:0008633; GO:0009653; GO:0005868; GO:0005829; GO:0007292; GO:0005874; GO:0003777; GO:0007017; GO:0042326; GO:0005886; GO:0005829; GO:0005624; GO:0005875; GO:0008022; GO:0019904	actin cytoskeleton organization; activation of pro-apoptotic gene products; anatomical structure morphogenesis; cytoplasmic dynein complex; cytosol; female gamete generation; microtubule; microtubule motor activity; microtubule-based process; negative regulation of phosphorylation; plasma membrane; cytosol; membrane fraction; microtubule associated complex; protein C-terminus binding; protein domain specific binding	Cytoplasm;			known in HPRD to be phosphorylated		
**527**	PIP	PIP_HUMAN	PIP; A4D2I1	P12273; A4D2I1	NP_002643	Prolactin-inducible protein (Prolactin-induced protein) (Secretory actin-binding protein) (SABP) (Gross cystic disease fluid protein 15) (GCDFP-15) (gp17); Prolactin-induced protein (cDNA, FLJ92518, Homo sapiens prolactin-induced protein (PIP), mRNA)	GO:0003779; GO:0005576; GO:0005576	actin binding; extracellular region; extracellular region	Secreted;					
**528**	PLMN	PLMN_HUMAN	PLMN; B2R7F8	P00747; Q5TEH4; Q15146; Q6PA00; B2R7F8	NP_000292	Plasminogen (EC 3.4.21.7) [Cleaved into: Plasmin heavy chain A; Activation peptide; Angiostatin; Plasmin heavy chain A, short form; Plasmin light chain B]	GO:0034185; GO:0005509; GO:0005615; GO:0006917; GO:0016525; GO:0043537; GO:0008285; GO:0051918; GO:0004283; GO:0051919; GO:0006508; GO:0004252; GO:0048771	apolipoprotein binding; calcium ion binding; extracellular space; induction of apoptosis; negative regulation of angiogenesis; negative regulation of blood vessel endothelial cell migration; negative regulation of cell proliferation; negative regulation of fibrinolysis; plasmin activity; positive regulation of fibrinolysis; proteolysis; serine-type endopeptidase activity; tissue remodeling	Secreted			Known in Uniprot to be phosphorylated	Known in HPRD to be phosphorylated	
**529**	PML	PML_HUMAN	PML	P29590; P29591; P29592; P29593; Q00755; Q96S41	NP_002666; NP_150241; NP_150242; NP_150243; NP_150247; NP_150249; NP_150250; NP_150252; NP_150253	Probable transcription factor PML (Tripartite motif-containing protein 19) (RING finger protein 71)	GO:0003677; GO:0006977; GO:0042771; GO:0016605; GO:0032184; GO:0005737; GO:0005626; GO:0044419; GO:0051457; GO:0016525; GO:0030308; GO:0008285; GO:0016481; GO:0031965; GO:0005730; GO:0006461; GO:0042803; GO:0006355; GO:0034097; GO:0001666; GO:0008270	DNA binding; DNA damage response, signal transduction by p53 class mediator resulting in cell cycle arrest; DNA damage response, signal transduction by p53 class mediator resulting in induction of apoptosis; PML body; SUMO polymer binding; cytoplasm; insoluble fraction; interspecies interaction between organisms; maintenance of protein location in nucleus; negative regulation of angiogenesis; negative regulation of cell growth; negative regulation of cell proliferation; negative regulation of transcription; nuclear membrane; nucleolus; protein complex assembly; protein homodimerization activity; regulation of transcription, DNA-dependent; response to cytokine stimulus; response to hypoxia; zinc ion binding	NucleusnucleoplasmCytoplasm			Known in Uniprot to be phosphorylated		

	Protein Name	Uniprot Name	Other Names	Uniprot IDs	Refseq IDs	Protein Description	GO ID	GO terms	Subcellular location	Comments	Comments	Comments - Database Search	Comments - Database Search	Function
**530**	RPB2	RPB2_HUMAN	RPB2; A8K1A8	P30876; Q8IZ61; A8K1A8	NP_000929	DNA-directed RNA polymerase II subunit RPB2 (EC 2.7.7.6) (DNA-directed RNA polymerase II subunit B) (RNA polymerase II subunit B2) (RNA polymerase II subunit 2) (DNA-directed RNA polymerase II 140 kDa polypeptide)	GO:0003677; GO:0005665; GO:0003899; GO:0006368; GO:0000287; GO:0000398; GO:0005515; GO:0032549; GO:0006367; GO:0008270	DNA binding; DNA-directed RNA polymerase II, core complex; DNA-directed RNA polymerase activity; RNA elongation from RNA polymerase II promoter; magnesium ion binding; nuclear mRNA splicing, via spliceosome; protein binding; ribonucleoside binding; transcription initiation from RNA polymerase II promoter; zinc ion binding	Nucleus					
**531**	PPGB	PPGB_HUMAN	PPGB	P10619; B2R798; Q561W6; Q5JZH1; Q96KJ2; Q9BR08; Q9BW68	NP_000299; NP_001121 167	Lysosomal protective protein (EC 3.4.16.5) (Cathepsin A) (Carboxypeptidase C) (Protective protein for beta-galactosidase) [Cleaved into: Lysosomal protective protein 32 kDa chain; Lysosomal protective protein 20 kDa chain]	GO:0005783; GO:0008047; GO:0006886; GO:0005764; GO:0005515; GO:0006508; GO:0004185	endoplasmic reticulum; enzyme activator activity; intracellular protein transport; lysosome; protein binding; proteolysis; serine-type carboxypeptidase activity	Lysosome					
**532**	CANB1	CANB1_HUMAN	CANB1; B2RC10	P63098; Q53SL0; P06705; P15117; Q08044; B2RC10	NP_000936	Calcineurin subunit B type 1 (Protein phosphatase 2B regulatory subunit 1) (Protein phosphatase 3 regulatory subunit B alpha isoform 1)	GO:0008633; GO:0005955; GO:0005509; GO:0004723; GO:0005516; GO:0005829	activation of pro-apoptotic gene products; calcineurin complex; calcium ion binding; calcium-dependent protein serine/threonine phosphatase activity; calmodulin binding; cytosol				Known in Uniprot to be phosphorylated		Phosphatase
**533**	KAPCB	KAPCB_HUMAN	KAPCB; B2RB89	P22694; B4E2Q1; Q14VH1; Q59GC0; Q5BNE9; Q5BNF0; Q5BNF1; Q5BNF2; Q5BNF3; Q5CZ92; Q5T1K3; Q7Z3M1; Q8IYR5; Q8IZQ0; Q96B09; B2RB89	NP_002722; NP_891993; NP_997461	cAMP-dependent protein kinase catalytic subunit beta (PKA C-beta) (EC 2.7.11.11)	GO:0005524; GO:0007188; GO:0004691; GO:0005952; GO:0005737; GO:0009755; GO:0000287; GO:0005634; GO:0006468; GO:0007243	ATP binding; G-protein signaling, coupled to cAMP nucleotide second messenger; cAMP-dependent protein kinase activity; cAMP-dependent protein kinase complex; cytoplasm; hormone-mediated signaling; magnesium ion binding; nucleus; protein amino acid phosphorylation; protein kinase cascade	CytoplasmNucleus			Known in Uniprot to be phosphorylated		Kinase
**534**	PROS	PROS_HUMAN	PROS; A8KAC9	P07225; Q15518; Q7Z715; A8KAC9	NP_000304	Vitamin K-dependent protein S	GO:0007596; GO:0005509; GO:0005737; GO:0004866; GO:0005576; GO:0005634	blood coagulation; calcium ion binding; cytoplasm; endopeptidase inhibitor activity; extracellular region; nucleus	Secreted					
**535**	TRY1	TRY1_HUMAN	TRY1; Q5NV57	P07477; Q5NV57; A1A509; B2R5I5; Q7M4N3; Q7M4N4; Q92955; Q9HAN4; Q9HAN5; Q9HAN6; Q9HAN7	NP_002760	Trypsin-1 (EC 3.4.21.4) (Trypsin I) (Cationic trypsinogen) (Serine protease 1) (Beta-trypsin) [Cleaved into: Alpha-trypsin chain 1; Alpha-trypsin chain 2]	GO:0005509; GO:0007586; GO:0005576; GO:0005515; GO:0006508; GO:0004252	calcium ion binding; digestion; extracellular region; protein binding; proteolysis; serine-type endopeptidase activity	Secretedextracellulars pace				known in HPRD to be phosphorylated	
**536**	PSA2	PSA2_HUMAN	PSA2; Q6ICS6	P25787; Q6ICS6; Q9BU45	NP_002778	Proteasome subunit alpha type-2 (EC 3.4.25.1) (Proteasome component C3) (Macropain subunit C3) (Multicatalytic endopeptidase complex subunit C3)	GO:0031145; GO:0005829; GO:0051436; GO:0005634; GO:0051437; GO:0005839; GO:0005515; GO:0004298	anaphase-promoting complex-dependent proteasomal ubiquitin-dependent protein catabolic process; cytosol; negative regulation of ubiquitin-protein ligase activity during mitotic cell cycle; nucleus; positive regulation of ubiquitin-protein ligase activity during mitotic cell cycle; proteasome core complex; protein binding; threonine-type endopeptidase activity	CytoplasmNucleus			Known in Uniprot to be phosphorylated		
**537**	PSA4	PSA4_HUMAN	PSA4; Q53XP2; Q7Z474	P25789; Q53XP2; Q8TBD1; Q7Z474	NP_002780; NP_001096 137	Proteasome subunit alpha type-4 (EC 3.4.25.1) (Proteasome component C9) (Macropain subunit C9) (Multicatalytic endopeptidase complex subunit C9) (Proteasome subunit L); Proteasome subunit alpha type (EC 3.4.25.1)	GO:0031145; GO:0005829; GO:0042802; GO:0051436; GO:0005634; GO:0051437; GO:0005839; GO:0004298; GO:0005829; GO:0006511	anaphase-promoting complex-dependent proteasomal ubiquitin-dependent protein catabolic process; cytosol; identical protein binding; negative regulation of ubiquitin-protein ligase activity during mitotic cell cycle; nucleus; positive regulation of ubiquitin-protein ligase activity during mitotic cell cycle; proteasome core complex; threonine-type endopeptidase activity; cytosol; ubiquitin-dependent protein catabolic process	CytoplasmNucleus;			Known in Uniprot to be phosphorylated		
**538**	PSA7	PSA7_HUMAN	PSA7; B2R515	O14818; Q9BR53; Q9H4K5; Q9UEU8; B2R515	NP_002783	Proteasome subunit alpha type-7 (EC 3.4.25.1) (Proteasome subunit RC6-1) (Proteasome subunit XAPC7)	GO:0031145; GO:0005829; GO:0042802; GO:0051436; GO:0005634; GO:0051437; GO:0005839; GO:0004298	anaphase-promoting complex-dependent proteasomal ubiquitin-dependent protein catabolic process; cytosol; identical protein binding; negative regulation of ubiquitin-protein ligase activity during mitotic cell cycle; nucleus; positive regulation of ubiquitin-protein ligase activity during mitotic cell cycle; proteasome core complex; threonine-type endopeptidase activity	CytoplasmNucleus					
**539**	PSB10	PSB10_HUMAN	PSB10; B2R5J4	P40306; Q5U098; B2R5J4	NP_002792	Proteasome subunit beta type-10 (EC 3.4.25.1) (Proteasome subunit beta-2i) (Proteasome MECl-1) (Macropain subunit MECl-1) (Multicatalytic endopeptidase complex subunit MECl-1)	GO:0031145; GO:0005829; GO:0006959; GO:0051436; GO:0005634; GO:0051437; GO:0005839; GO:0004298	anaphase-promoting complex-dependent proteasomal ubiquitin-dependent protein catabolic process; cytosol; humoral immune response; negative regulation of ubiquitin-protein ligase activity during mitotic cell cycle; nucleus; positive regulation of ubiquitin-protein ligase activity during mitotic cell cycle; proteasome core complex; threonine-type endopeptidase activity	CytoplasmNucleus					
**540**	PSB1	PSB1_HUMAN	PSB1	P20618; B5BU76; Q9BWA8	NP_002784	Proteasome subunit beta type-1 (EC 3.4.25.1) (Proteasome component C5) (Macropain subunit C5) (Multicatalytic endopeptidase complex subunit C5) (Proteasome gamma chain)	GO:0031145; GO:0005829; GO:0051436; GO:0005634; GO:0051437; GO:0005839; GO:0005515; GO:0004298	anaphase-promoting complex-dependent proteasomal ubiquitin-dependent protein catabolic process; cytosol; negative regulation of ubiquitin-protein ligase activity during mitotic cell cycle; nucleus; positive regulation of ubiquitin-protein ligase activity during mitotic cell cycle; proteasome core complex; protein binding; threonine-type endopeptidase activity	CytoplasmNucleus			Known in Uniprot to be phosphorylated		
**541**	PSB2	PSB2_HUMAN	PSB2	P49721; Q9BWZ9; P31145	NP_002785	Proteasome subunit beta type-2 (EC 3.4.25.1) (Proteasome component C7-I) (Macropain subunit C7-I) (Multicatalytic endopeptidase complex subunit C7-I)	GO:0031145; GO:0005829; GO:0051436; GO:0005634; GO:0051437; GO:0005839; GO:0004298	anaphase-promoting complex-dependent proteasomal ubiquitin-dependent protein catabolic process; cytosol; negative regulation of ubiquitin-protein ligase activity during mitotic cell cycle; nucleus; positive regulation of ubiquitin-protein ligase activity during mitotic cell cycle; proteasome core complex; threonine-type endopeptidase activity	CytoplasmNucleus					
**542**	PSB9	PSB9_HUMAN	PSB9; Q5JNW4	P28065; Q5JNW4; Q16523	NP_002791; NP_683756	Proteasome subunit beta type-9 (EC 3.4.25.1) (Proteasome subunit beta-1i) (Proteasome chain 7) (Macropain chain 7) (Multicatalytic endopeptidase complex chain 7) (RING12 protein) (Low molecular mass protein 2); PSMB9 protein (Proteasome (Prosome, macropain) subunit, beta type, 9 (Large multifunctional peptidase 2)) (Proteasome (Prosome, macropain) subunit, beta type, 9 (Large multifunctional peptidase 2), isoform CRA_b)	GO:0031145; GO:0005829; GO:0006955; GO:0051436; GO:0005634; GO:0051437; GO:0005839; GO:0004298; GO:0005829; GO:0006511	anaphase-promoting complex-dependent proteasomal ubiquitin-dependent protein catabolic process; cytosol; immune response; negative regulation of ubiquitin-protein ligase activity during mitotic cell cycle; nucleus; positive regulation of ubiquitin-protein ligase activity during mitotic cell cycle; proteasome core complex; threonine-type endopeptidase activity; cytosol; ubiquitin-dependent protein catabolic process	CytoplasmNucleus;					
**543**	PGH2	PGH2_HUMAN	PGH2; A8K802	P35354; Q16876; A8K802	NP_000954	Prostaglandin G/H synthase 2 (EC 1.14.99.1) (Cyclooxygenase-2) (COX-2) (Prostaglandin-endoperoxide synthase 2) (Prostaglandin H2 synthase 2) (PGH synthase 2) (PGHS-2) (PHS II)	GO:0006928; GO:0019371; GO:0005788; GO:0005789; GO:0020037; GO:0005792; GO:0005634; GO:0055114; GO:0016702; GO:0004601; GO:0004666; GO:0008217; GO:0050727; GO:0006979	cell motion; cyclooxygenase pathway; endoplasmic reticulum lumen; endoplasmic reticulum membrane; heme binding; microsome; nucleus; oxidation reduction; oxidoreductase activity, acting on single donors with incorporation of molecular oxygen, incorporation of two atoms of oxygen; peroxidase activity; prostaglandin-endoperoxide synthase activity; regulation of blood pressure; regulation of inflammatory response; response to oxidative stress	MicrosomemembranePeripheralmembraneproteinEndoplasmicreticulummembranePeripheralmembraneprotein			Known in Uniprot to be phosphorylated		
**544**	DHPR	DHPR_HUMAN	DHPR; A8K158	P09417; Q53F52; Q9H3M5; A8K158	NP_000311	Dihydropteridine reductase (EC 1.5.1.34) (HDHPR) (Quinoid dihydropteridine reductase)	GO:0004155; GO:0006559; GO:0005488; GO:0005737; GO:0051066; GO:0009055; GO:0055114; GO:0006729	6,7-dihydropteridine reductase activity; L-phenylalanine catabolic process; binding; cytoplasm; dihydrobiopterin metabolic process; electron carrier activity; oxidation reduction; tetrahydrobiopterin biosynthetic process						
**545**	RAB13	RAB13_HUMAN	RAB13	P51153; Q5U0A6; A8K6B5; Q6GPG6; Q96GU4	NP_002861	Ras-related protein Rab-13 (Cell growth-inhibiting gene 4 protein)	GO:0005525; GO:0003924; GO:0007155; GO:0030659; GO:0015031; GO:0007264; GO:0005923; GO:0016192	GTP binding; GTPase activity; cell adhesion; cytoplasmic vesicle membrane; protein transport; small GTPase mediated signal transduction; tight junction; vesicle-mediated transport	CelljunctiontightjunctionCellmembraneLipidanchorCytoplasmicsideCytoplasmicvesiclemembraneLipidanchorCytoplasmicside			Known in Uniprot to be phosphorylated		
**546**	RAB2A	RAB2A_HUMAN	RAB2A; B2R5W8	P61019; P08886; B2R5W8	NP_002856	Ras-related protein Rab-2A	GO:0006888; GO:0033116; GO:0005525; GO:0003924; GO:0000139; GO:0005789; GO:0042470; GO:0015031; GO:0007264	ER to Golgi vesicle-mediated transport; ER-Golgi intermediate compartment membrane; GTP binding; GTPase activity; Golgi membrane; endoplasmic reticulum membrane; melanosome; protein transport; small GTPase mediated signal transduction	EndoplasmicreticulumGolgiintermediatecompartmentmembraneLipidanchorMelanosomeEndoplasmicreticulummembraneLipidanchorGolgiapparatusmembraneLipidanchor					

	Protein Name	Uniprot Name	Other Names	Uniprot IDs	Refseq IDs	Protein Description	GO ID	GO terms	Subcellular location	Comments	Comments	Comments - Database Search	Comments - Database Search	Function
**547**	RAC2	RAC2_HUMAN	RAC2	P15153	NP_002863	Ras-related C3 botulinum toxin substrate 2 (p21-Rac2) (Small G protein) (GX)	GO:0005525; GO:0003924; GO:0005737; GO:0005515; GO:0010310; GO:0060263; GO:0007264	GTP binding; GTPase activity; cytoplasm; protein binding; regulation of hydrogen peroxide metabolic process; regulation of respiratory burst; small GTPase mediated signal transduction	Cytoplasm					
**548**	RALB	RALB_HUMAN	RALB; Q6ZS74; Q53T32	P11234; Q53T32; Q6ZS74	NP_002872	Ras-related protein Ral-B; Putative uncharacterized protein RALB (V-ral simian leukemia viral oncogene homolog B (Ras related; GTP binding protein), isoform CRA_a) (cDNA, FLJ92808, Homo sapiens v-ral simian leukemia viral oncogene homolog B (rasrelated; GTP binding protein) (RALB), mRNA); cDNA FLJ45773 fis, clone NETRP2003448, highly similar to Ras-related protein RAL-B (V-ral simian leukemia viral oncogene homolog B (Ras related; GTP binding protein), isoform CRA_b)	GO:0005525; GO:0007265; GO:0005622; GO:0005886; GO:0005515; GO:0007264	GTP binding; Ras protein signal transduction; intracellular; plasma membrane; protein binding; small GTPase mediated signal transduction	CellmembraneLipidanchorCytoplasmicside;					
**549**	RANG	RANG_HUMAN	RANG	P43487	NP_002873	Ran-specific GTPase-activating protein (Ran-binding protein 1) (RanBP1)	GO:0005092; GO:0005096; GO:0008536; GO:0046907; GO:0005634; GO:0007165	GDP-dissociation inhibitor activity; GTPase activator activity; Ran GTPase binding; intracellular transport; nucleus; signal transduction				Known in Uniprot to be phosphorylated		
**550**	RARR2	RARR2_HUMAN	RARR2; Q7LE02	Q99969; Q7LE02	NP_002880	Retinoic acid receptor responder protein 2 (Tazarotene-induced gene 2 protein) (RAR-responsive protein TIG2); RARRES2 protein (Putative uncharacterized protein RARRES2) (Retinoic acid receptor responder (Tazarotene induced) 2, isoform CRA_a) (cDNA, FLJ92484, Homo sapiens retinoic acid receptor responder (tazarotene induced)2 (RARRES2), mRNA)	GO:0005576; GO:0001523; GO:0004872	extracellular region; retinoid metabolic process; receptor activity	Secreted;					
**551**	RADI	RADI_HUMAN	RADI; Q6PKD3; B0YJ88	P35241; Q86Y61; B0YJ88; Q6PKD3	NP_002897	Radixin; RDX protein (Fragment)	GO:0003779; GO:0051016; GO:0005737; GO:0005856; GO:0019898; GO:0005886; GO:0007605; GO:0045177; GO:0045176; GO:0008092; GO:0030175; GO:0030027; GO:0030033; GO:0001726; GO:0032420	actin binding; barbed-end actin filament capping; cytoplasm; cytoskeleton; extrinsic to membrane; plasma membrane; sensory perception of sound; apical part of cell; apical protein localization; cytoskeletal protein binding; filopodium; lamellipodium; microvillus assembly; ruffle; stereocilium	CellmembranePeripheralmembraneproteinCytoplasmicsideCytoplasm cytoskeleton;			Known in Uniprot to be phosphorylated	Known in HPRD to be phosphorylated	
**552**	RGS10	RGS10_HUMAN	RGS10; Q6IAZ6; Q96GN0	Q6IAZ6; O43665; A8K408; Q96GN0	NP_002916; NP_001005339	Regulator of G-protein signaling 10 (RGS10)	GO:0009968; GO:0005515; GO:0004871	negative regulation of signal transduction; protein binding; signal transducer activity				Known in Uniprot to be phosphorylated	Known in HPRD to be phosphorylated	
**553**	ROBO1	ROBO1_HUMAN	ROBO1	Q9Y6N7; Q7Z300; Q9BUS7	NP_002932	Roundabout homolog 1 (H-Robo-1) (Deleted in U twenty twenty)	GO:0008046; GO:0009986; GO:0006935; GO:0007156; GO:0042802; GO:0005887; GO:0050772	axon guidance receptor activity; cell surface; chemotaxis; homophilic cell adhesion; identical protein binding; integral to plasma membrane; positive regulation of axonogenesis	MembraneSinglepasstypeImembraneprotein			Known in Uniprot to be phosphorylated		
**554**	RL19	RL19_HUMAN	RL19	P84098; B2R4K2; P14118; P22908; Q502Y6; Q7Z6E4	XP_068464; NP_000972	60S ribosomal protein L19	GO:0003723; GO:0022625; GO:0003735; GO:0006414	RNA binding; cytosolic large ribosomal subunit; structural constituent of ribosome; translational elongation				Known in Uniprot to be phosphorylated		
**555**	RL26	RL26_HUMAN	RL26	P61254; Q02877; Q6IPY2	XP_291724; NP_000978	60S ribosomal protein L26	GO:0003723; GO:0022625; GO:0005515; GO:0006364; GO:0042273; GO:0003735; GO:0006414	RNA binding; cytosolic large ribosomal subunit; protein binding; rRNA processing; ribosomal large subunit biogenesis; structural constituent of ribosome; translational elongation				Known in Uniprot to be phosphorylated		
**556**	RL31	RL31_HUMAN	RL31	P62899; P12947; Q53SQ5; Q6LBJ6	XP_292188; NP_000984	60S ribosomal protein L31	GO:0003723; GO:0022625; GO:0005515; GO:0003735; GO:0006414	RNA binding; cytosolic large ribosomal subunit; protein binding; structural constituent of ribosome; translational elongation				Known in Uniprot to be phosphorylated		
**557**	RL7A	RL7A_HUMAN	RL7A; Q5T8U4	P62424; Q5T8U4; P11518	XP_210183; NP_000963	60S ribosomal protein L7a (Surfeit locus protein 3) (PLA-X polypeptide); Ribosomal protein L7a (cDNA FLJ78489, highly similar to Homo sapiens ribosomal protein L7a (RPL7A), mRNA) (Ribosomal protein L7a, isoform CRA_c) (cDNA, FLJ92002, Homo sapiens ribosomal protein L7a (RPL7A), mRNA)	GO:0003723; GO:0022625; GO:0005624; GO:0042788; GO:0042254; GO:0003735; GO:0006414; GO:0005840; GO:0006412	RNA binding; cytosolic large ribosomal subunit; membrane fraction; polysomal ribosome; ribosome biogenesis; structural constituent of ribosome; translational elongation; ribosome; translation						
**558**	RS15	RS15_HUMAN	RS15	P62841; A5D8V9; P11174; Q3KRA1	XP_059465; NP_001009	40S ribosomal protein S15 (RIG protein)	GO:0003677; GO:0022627; GO:0005654; GO:0005515; GO:0006364; GO:0042274; GO:0000056; GO:0003735; GO:0006414	DNA binding; cytosolic small ribosomal subunit; nucleoplasm; protein binding; rRNA processing; ribosomal small subunit biogenesis; ribosomal small subunit export from nucleus; structural constituent of ribosome; translational elongation						
**559**	RS20	RS20_HUMAN	RS20	P60866; P17075; Q5M8S9	XP_299876; NP_001014	40S ribosomal protein S20	GO:0003723; GO:0022627; GO:0005515; GO:0003735; GO:0006414	RNA binding; cytosolic small ribosomal subunit; protein binding; structural constituent of ribosome; translational elongation				Known in Uniprot to be phosphorylated		
**560**	UBIQ	UBIQ_HUMAN	UBIQ; RS27A; B2RDW1	P62988; P62979; P14798; Q6LBL4; Q9BQ77; P02248; P02249; P02250; Q29120; Q6LDU5; Q8WYN8; Q91887; Q91888; Q9BWD6; Q9BX98; Q9UEF2; Q9UEG1; Q9UEK8; Q9UPK7; B2RDW1	NP_002945; NP_061828; NP_066289; NP_001129064	40S ribosomal protein S27a; Ubiquitin	GO:0022627; GO:0003735; GO:0006414; GO:0008270; GO:0030433; GO:0031145; GO:0007411; GO:0005829; GO:0042062; GO:0051436; GO:0005654; GO:0045941; GO:0051437; GO:0005515; GO:0016567; GO:0048167; GO:0030528	cytosolic small ribosomal subunit; structural constituent of ribosome; translational elongation; zinc ion binding; ER-associated protein catabolic process; anaphase-promoting complex-dependent proteasomal ubiquitin-dependent protein catabolic process; axon guidance; cytosol; long-term strengthening of neuromuscular junction; negative regulation of ubiquitin-protein ligase activity during mitotic cell cycle; nucleoplasm; positive regulation of transcription; positive regulation of ubiquitin-protein ligase activity during mitotic cell cycle; protein binding; protein ubiquitination; regulation of synaptic plasticity; transcription regulator activity	; CytoplasmNucleus			Known in Uniprot to be phosphorylated		
**561**	RS3A	RS3A_HUMAN	RS3A; B2R4D4	P61247; P33443; P49241; B2R4D4	NP_000997	40S ribosomal protein S3a	GO:0003723; GO:0022627; GO:0006917; GO:0005730; GO:0005515; GO:0003735; GO:0006414; GO:0006413	RNA binding; cytosolic small ribosomal subunit; induction of apoptosis; nucleolus; protein binding; structural constituent of ribosome; translational elongation; translational initiation				Known in Uniprot to be phosphorylated		
**562**	S10A7	S10A7_HUMAN	Q5SY67; S10A7	P31151; Q6FGE3; Q5SY67; Q9H1E2	NP_002954	Protein S100-A7 (S100 calcium-binding protein A7) (Psoriasin)	GO:0001525; GO:0005509; GO:0005829; GO:0050829; GO:0005783; GO:0008544; GO:0005576; GO:0005925; GO:0045087; GO:0030216; GO:0005634; GO:0005515; GO:0000302; GO:0051238; GO:0008270	angiogenesis; calcium ion binding; cytosol; defense response to Gram-negative bacterium; endoplasmic reticulum; epidermis development; extracellular region; focal adhesion; innate immune response; keratinocyte differentiation; nucleus; protein binding; response to reactive oxygen species; sequestering of metal ion; zinc ion binding	CytoplasmSecreted					
**563**	S10A9	S10A9_HUMAN	S10A9	P06702; Q6FGA1; Q9NYM0; Q9UCJ1	NP_002956	Protein S100-A9 (S100 calcium-binding protein A9) (Calgranulin-B) (Migration inhibitory factor-related protein 14) (MRP-14) (P14) (Leukocyte L1 complex heavy chain) (Calprotectin L1H subunit)	GO:0005509; GO:0007267; GO:0005737; GO:0006954; GO:0005634; GO:0005515; GO:0004871	calcium ion binding; cell-cell signaling; cytoplasm; inflammatory response; nucleus; protein binding; signal transducer activity				Known in Uniprot to be phosphorylated	Known in HPRD to be phosphorylated	
**564**	SH3L1	SH3L1_HUMAN	SH3L1	O75368; Q3SYL1; Q5JT50; Q6FIE8; Q9H0N8	NP_003013	SH3 domain-binding glutamic acid-rich-like protein	GO:0017124; GO:0005070; GO:0005737; GO:0005634	SH3 domain binding; SH3/SH2 adaptor activity; cytoplasm; nucleus						
**565**	SYUA	SYUA_HUMAN	SYUA; A8K2A4	P37840; Q13701; Q4JHI3; Q6IAU6; A8K2A4	NP_000336; NP_001139526; NP_001139527	Alpha-synuclein (Non-A beta component of AD amyloid) (Non-A4 component of amyloid precursor) (NACP)	GO:0005829; GO:0043205; GO:0016020; GO:0032769; GO:0005634; GO:0005515; GO:0014059	cytosol; fibril; membrane; negative regulation of monooxygenase activity; nucleus; protein binding; regulation of dopamine secretion	CytoplasmMembraneNucleus			Known in Uniprot to be phosphorylated	Known in HPRD to be phosphorylated	
**566**	SNX3	SNX3_HUMAN	SNX3; Q4TT31	O60493; Q4TT31; O60718; Q5JXJ7; Q5JXJ8; Q96AP9; Q9C0J5; Q9NU45	NP_003786; NP_690040	Sorting nexin-3 (Protein SDP3)	GO:0007154; GO:0005737; GO:0006897; GO:0035091; GO:0019903; GO:0015031	cell communication; cytoplasm; endocytosis; phosphoinositide binding; protein phosphatase binding; protein transport				Known in Uniprot to be phosphorylated		

	Protein Name	Uniprot Name	Other Names	Uniprot IDs	Refseq IDs	Protein Description	GO ID	GO terms	Subcellular location	Comments	Comments	Comments - Database Search	Comments - Database Search	Function
**567**	SODC	SODC_HUMAN	SODC; Q6NR85	P00441; Q6NR85; A6NHJ0; Q16669; Q16711; Q16838; Q16839; Q16840	NP_000445	Superoxide dismutase [Cu-Zn] (EC 1.15.1.1)	GO:0006309; GO:0000187; GO:0016209; GO:0060088; GO:0007569; GO:0043025; GO:0006879; GO:0051087; GO:0005507; GO:0031410; GO:0005829; GO:0032839; GO:0006302; GO:0007566; GO:0031012; GO:0005615; GO:0006749; GO:0060047; GO:0050665; GO:0007626; GO:0005759; GO:0046716; GO:0032287; GO:0002262; GO:0045541; GO:0043524; GO:0060052; GO:0005634; GO:0001541; GO:0055114; GO:0005777; GO:0001890; GO:0043065; GO:0001819; GO:0043234;	DNA fragmentation involved in apoptosis; activation of MAPK activity; antioxidant activity; auditory receptor cell stereocilium organization; cell aging; cell soma; cellular iron ion homeostasis; chaperone binding; copper ion binding; cytoplasmic vesicle; cytosol; dendrite cytoplasm; double-strand break repair; embryo implantation; extracellular matrix; extracellular space; glutathione metabolic process; heart contraction; hydrogen peroxide biosynthetic process; locomotory behavior; mitochondrial matrix; muscle maintenance; myelin maintenance in the peripheral nervous system; myeloid cell homeostasis; negative regulation of cholesterol biosynthetic process; negative regulation of neuron apoptosis; neurofilament cytoskeleton organization; nucleus; ovarian follicle development; oxidation reduction; peroxisome; placenta development; positive regulation of apoptosis; positive regulation of cytokine production; protein complex; protein homodimerization activity; protein phosphatase 2B binding; regulation of T cell differentiation in the thymus; regulation of blood pressure; regulation of mitochondrial membrane potential; regulation of multicellular organism growth; regulation of organ growth;	Cytoplasm			Known in Uniprot to be phosphorylated		
**568**	SORL	SORL_HUMAN	SORL; B2RNX7	Q92673; Q92856; B2RNX7	NP_003096	Sortilin-related receptor (Sorting protein-related receptor containing LDLR class A repeats) (SorLA) (SorLA-1) (Low-density lipoprotein receptor relative with 11 ligand-binding repeats) (LDLR relative with 11 ligand-binding repeats) (LR11)	GO:0008203; GO:0005887; GO:0006869; GO:0030169; GO:0034362; GO:0006898; GO:0004888	cholesterol metabolic process; integral to plasma membrane; lipid transport; low-density lipoprotein binding; low-density lipoprotein particle; receptor-mediated endocytosis; transmembrane receptor activity	MembraneSinglepasstypeImembraneprotein					
**569**	SPRC	SPRC_HUMAN	SPRC; Q6IBK4	P09486; Q6IBK4	NP_003109	SPARC (Secreted protein acidic and rich in cysteine) (Osteonectin) (ON) (Basement-membrane protein 40) (BM-40); SPARC protein (Secreted protein, acidic, cysteine-rich (Osteonectin), isoform CRA_b)	GO:0005604; GO:0005509; GO:0005518; GO:0005507; GO:0001503; GO:0031093; GO:0007169; GO:0005509; GO:0031012; GO:0042127	basement membrane; calcium ion binding; collagen binding; copper ion binding; ossification; platelet alpha granule lumen; transmembrane receptor protein tyrosine kinase signaling pathway; calcium ion binding; extracellular matrix; regulation of cell proliferation	Secretedextracellularspaceextracellularmatrixbasementmembrane;					
**570**	SPTA2	SPTA2_HUMAN	SPTA2	Q13813; Q13186; Q15324; Q16606; Q59EF1; Q5VXV5; Q5VXV6; Q7Z6M5; Q9P0V0	NP_003118; NP_001123910	Spectrin alpha chain, brain (Spectrin, non-erythroid alpha chain) (Alpha-II spectrin) (Fodrin alpha chain)	GO:0003779; GO:0051016; GO:0005509; GO:0005516; GO:0005829; GO:0005624; GO:0008091; GO:0005200	actin binding; barbed-end actin filament capping; calcium ion binding; calmodulin binding; cytosol; membrane fraction; spectrin; structural constituent of cytoskeleton	Cytoplasmcytoskeleton Cytoplasmcellcortex			Known in Uniprot to be phosphorylated	Known in HPRD to be phosphorylated	
**571**	SORCN	SORCN_HUMAN	SORCN	P30626	NP_003121	Sorcin (22 kDa protein) (CP-22) (V19)	GO:0005246; GO:0005509; GO:0005737; GO:0007507; GO:0006880; GO:0007517; GO:0005102; GO:0001508; GO:0008016; GO:0006942; GO:0007165; GO:0006810	calcium channel regulator activity; calcium ion binding; cytoplasm; heart development; intracellular sequestering of iron ion; muscle organ development; receptor binding; regulation of action potential; regulation of heart contraction; regulation of striated muscle contraction; signal transduction; transport	Cytoplasm					
**572**	STAM1	STAM1_HUMAN	STAM1; B0YJ99	Q92783; Q8N6D9; B0YJ99	NP_003464	Signal transducing adapter molecule 1 (STAM-1)	GO:0005070; GO:0005829; GO:0031901; GO:0006886; GO:0007165	SH3/SH2 adaptor activity; cytosol; early endosome membrane; intracellular protein transport; signal transduction	CytoplasmEarlyendosomemembranePeripheralmembraneproteinCytoplasmicside			Known in Uniprot to be phosphorylated	Known in HPRD to be phosphorylated	
**573**	STX7	STX7_HUMAN	STX7	O15400; Q5SZW2; Q96ES9	NP_003560	Syntaxin-7	GO:0005484; GO:0031901; GO:0016021; GO:0006886; GO:0006892	SNAP receptor activity; early endosome membrane; integral to membrane; intracellular protein transport; post-Golgi vesicle-mediated transport	EarlyendosomemembraneSinglepasstypeIVmembraneprotein			Known in Uniprot to be phosphorylated	Known in HPRD to be phosphorylated	
**574**	ST1A2	ST1A2_HUMAN	ST1A2; Q14CJ7	P50226; A9QY25; P78393; Q14CJ7	NP_001045; NP_803564	Sulfotransferase 1A2 (EC 2.8.2.1) (Aryl sulfotransferase 2) (Phenol sulfotransferase 2) (Phenol-sulfating phenol sulfotransferase 2) (P-PST 2) (ST1A2); Sulfotransferase family, cytosolic, 1A, phenol-preferring, member 2	GO:0009309; GO:0004062; GO:0006584; GO:0005737; GO:0008202; GO:0008146	amine biosynthetic process; aryl sulfotransferase activity; catecholamine metabolic process; cytoplasm; steroid metabolic process; sulfotransferase activity	Cytoplasm;					
**575**	THAS	THAS_HUMAN	THAS; Q53F23	P24557; O14987; Q16844; Q8IUN1; Q9GZW4; Q9HD77; Q9HD78; Q9HD79; Q9HD80; Q9HD81; Q9HD82; Q9HD83; Q9HD84; Q53F23	NP_001052; NP_001124438	Thromboxane-A synthase (TXA synthase) (TXS) (EC 5.3.99.5) (Cytochrome P450 5A1)	GO:0009055; GO:0020037; GO:0016021; GO:0004497; GO:0055114; GO:0001516; GO:0004796	electron carrier activity; heme binding; integral to membrane; monooxygenase activity; oxidation reduction; prostaglandin biosynthetic process; thromboxane-A synthase activity	MembraneMultipassmembraneprotein					
**576**	TIMP1	TIMP1_HUMAN	TIMP1; Q6FGX5	P01033; Q6FGX5; Q14252; Q9UCU1	NP_003245	Metalloproteinase inhibitor 1 (Tissue inhibitor of metalloproteinases) (TIMP-1) (Erythroid-potentiating activity) (EPA) (Fibroblast collagenase inhibitor) (Collagenase inhibitor); TIMP metallopeptidase inhibitor 1 (TIMP1 protein) (TIMP metallopeptidase inhibitor 1, isoform CRA_a) (cDNA, FLJ92204, Homo sapiens tissue inhibitor of metalloproteinase 1 (erythroidpotentiating activity, collagenase inhibitor) (TIMP1), mRNA)	GO:0043249; GO:0008191; GO:0051045; GO:0008284; GO:0005515; GO:0005578; GO:0008191	erythrocyte maturation; metalloendopeptidase inhibitor activity; negative regulation of membrane protein ectodomain proteolysis; positive regulation of cell proliferation; protein binding; proteinaceous extracellular matrix; metalloendopeptidase inhibitor activity	Secreted;					
**577**	TIMP3	TIMP3_HUMAN	TIMP3; B2RBY9	P35625; Q5THV4; Q9UC74; Q9UGS2; B2RBY9	NP_000353	Metalloproteinase inhibitor 3 (Tissue inhibitor of metalloproteinases 3) (TIMP-3) (Protein MIG-5)	GO:0008191; GO:0051045; GO:0005515; GO:0007169; GO:0007601	metalloendopeptidase inhibitor activity; negative regulation of membrane protein ectodomain proteolysis; protein binding; transmembrane receptor protein tyrosine kinase signaling pathway; visual perception	Secretedextracellularspaceextracellularmatrix					
**578**	TKT	TKT_HUMAN	TKT; A8K089	P29401; Q8TBA3; Q96HH3; A8K089	NP_001055; NP_001128527	Transketolase (TK) (EC 2.2.1.1)	GO:0005509; GO:0005829; GO:0008152; GO:0005515; GO:0004802	calcium ion binding; cytosol; metabolic process; protein binding; transketolase activity				Known in Uniprot to be phosphorylated	Known in HPRD to be phosphorylated	
**579**	TPIS	TPIS_HUMAN	TPIS; Q6FHP9	P60174; Q6FHP9; P00938; Q6IS07; Q8WWD0; Q96AG5	NP_000356	Triosephosphate isomerase (TIM) (EC 5.3.1.1) (Triose-phosphate isomerase); Triosephosphate isomerase (EC 5.3.1.1) (Fragment)	GO:0005829; GO:0006633; GO:0006094; GO:0006096; GO:0006098; GO:0004807; GO:0009790; GO:0006006; GO:0019682	cytosol; fatty acid biosynthetic process; gluconeogenesis; glycolysis; pentose-phosphate shunt; triose-phosphate isomerase activity; embryonic development; glucose metabolic process; glyceraldehyde-3-phosphate metabolic process				Known in Uniprot to be phosphorylated		
**580**	TPP2	TPP2_HUMAN	TPP2	P29144; Q5VZU8	NP_003282	Tripeptidyl-peptidase 2 (EC 3.4.14.10) (Tripeptidyl-peptidase II) (TPP-II) (Tripeptidyl aminopeptidase)	GO:0004177; GO:0005737; GO:0006508; GO:0004252; GO:0008240	aminopeptidase activity; cytoplasm; proteolysis; serine-type endopeptidase activity; tripeptidyl-peptidase activity	Cytoplasm					
**581**	TCTP	TCTP_HUMAN	TCTP; B2R7E5	P13693; B2R7E5; Q6YLS2; Q7Z4J4; Q96EE2; Q9UC70	NP_003286; XP_294045	Translationally-controlled tumor protein (TCTP) (p23) (Histamine-releasing factor) (HRF) (Fortilin)	GO:0006916; GO:0005509; GO:0006816; GO:0006874; GO:0005615; GO:0005771; GO:0005515	anti-apoptosis; calcium ion binding; calcium ion transport; cellular calcium ion homeostasis; extracellular space; multivesicular body; protein binding	Cytoplasm			Known in Uniprot to be phosphorylated	Known in HPRD to be phosphorylated	
**582**	ENPL	ENPL_HUMAN	ENPL	P14625; Q96A97	NP_003290	Endoplasmin (Heat shock protein 90 kDa beta member 1) (94 kDa glucose-regulated protein) (GRP94) (gp96 homolog) (Tumor rejection antigen 1)	GO:0005524; GO:0003723; GO:0006916; GO:0005509; GO:0005829; GO:0005788; GO:0005789; GO:0050750; GO:0042470; GO:0005792; GO:0048471; GO:0006457; GO:0015031; GO:0001666; GO:0051208; GO:0051082; GO:0046790	ATP binding; RNA binding; anti-apoptosis; calcium ion binding; cytosol; endoplasmic reticulum lumen; endoplasmic reticulum membrane; low-density lipoprotein receptor binding; melanosome; microsome; perinuclear region of cytoplasm; protein folding; protein transport; response to hypoxia; sequestering of calcium ion; unfolded protein binding; virion binding	EndoplasmicreticulumlumenMelanosome			Known in Uniprot to be phosphorylated		
**583**	TTHY	TTHY_HUMAN	TTHY; Q549C7	P02766; Q549C7; Q6IB96; Q9UBZ6; Q9UCM9	NP_000362	Transthyretin (Prealbumin) (TBPA) (TTR) (ATTR); Transthyretin (Transthyretin (Prealbumin, amyloidosis type I), isoform CRA_a) (cDNA, FLJ92332, Homo sapiens transthyretin (prealbumin, amyloidosis type I) (TTR),mRNA)	GO:0005179; GO:0016918; GO:0019841; GO:0005496; GO:0006590; GO:0006810; GO:0005615; GO:0042562; GO:0015349	hormone activity; retinal binding; retinol binding; steroid binding; thyroid hormone generation; transport; extracellular space; hormone binding; thyroid hormone transmembrane transporter activity	Secreted;					
**584**	UBE2H	UBE2H_HUMAN	UBE2H; A4D1L5	P62256; P37286; A4D1L5	NP_003335	Ubiquitin-conjugating enzyme E2 H (EC 6.3.2.19) (Ubiquitin-protein ligase H) (Ubiquitin carrier protein H) (UbcH2) (E2-20K); Ubiquitin carrier protein (EC 6.3.2.-)	GO:0005739; GO:0016567; GO:0051246; GO:0006511; GO:0004842; GO:0016567; GO:0019787; GO:0006512	mitochondrion; protein ubiquitination; regulation of protein metabolic process; ubiquitin-dependent protein catabolic process; ubiquitin-protein ligase activity; protein ubiquitination; small conjugating protein ligase activity; ubiquitin cycle						
**585**	UB2L3	UB2L3_HUMAN	UB2L3; B2R4A7	P68036; B2R4A7; P51966; P70653; Q9HAV1	NP_003338; XP_208709; NP_937800	Ubiquitin-conjugating enzyme E2 L3 (EC 6.3.2.19) (Ubiquitin-protein ligase L3) (Ubiquitin carrier protein L3) (UbcH7) (E2-F1) (L-UBC)	GO:0005737; GO:0019899; GO:0016567; GO:0051246; GO:0000151; GO:0006511; GO:0004842	cytoplasm; enzyme binding; protein ubiquitination; regulation of protein metabolic process; ubiquitin ligase complex; ubiquitin-dependent protein catabolic process; ubiquitin-protein ligase activity						

	Protein Name	Uniprot Name	Other Names	Uniprot IDs	Refseq IDs	Protein Description	GO ID	GO terms	Subcellular location	Comments	Comments	Comments - Database Search	Comments - Database Search	Function
**586**	UBC12	UBC12_HUMAN	UBC12	P61081; O76069; Q8VC50	XP_208901; NP_003960	NEDD8-conjugating enzyme Ubc12 (EC 6.3.2.-) (Ubiquitin-conjugating enzyme E2 M) (NEDD8 protein ligase) (NEDD8 carrier protein)	GO:0005524; GO:0019941; GO:0043687; GO:0005515; GO:0051246; GO:0018169; GO:0004842	ATP binding; modification-dependent protein catabolic process; post-translational protein modification; protein binding; regulation of protein metabolic process; ribosomal S6-glutamic acid ligase activity; ubiquitin-protein ligase activity				Known in Uniprot to be phosphorylated		
**587**	UBE2N	UBE2N_HUMAN	UBE2N	P61088; Q53Y81; Q16781	XP_301485; NP_003339	Ubiquitin-conjugating enzyme E2 N (EC 6.3.2.19) (Ubiquitin-protein ligase N) (Ubiquitin carrier protein N) (Ubc13) (Bendless-like ubiquitin-conjugating enzyme)	GO:0000729; GO:0050852; GO:0031372; GO:0005737; GO:0000724; GO:0019941; GO:0005634; GO:0045739; GO:0043123; GO:0051092; GO:0031058; GO:0051443; GO:0006301; GO:0033182; GO:0043130; GO:0006512; GO:0004842	DNA double-strand break processing; T cell receptor signaling pathway; UBC13-MMS2 complex; cytoplasm; double-strand break repair via homologous recombination; modification-dependent protein catabolic process; nucleus; positive regulation of DNA repair; positive regulation of I-kappaB kinase/NF-kappaB cascade; positive regulation of NF-kappaB transcription factor activity; positive regulation of histone modification; positive regulation of ubiquitin-protein ligase activity; postreplication repair; regulation of histone ubiquitination; ubiquitin binding; ubiquitin cycle; ubiquitin-protein ligase activity						
**588**	UB2V1	UB2V1_HUMAN	UB2V1	Q13404; Q13403; Q13532; Q96H34; Q9GZT0; Q9GZW1; Q9H4J3; Q9H4J4; Q9UKL1; Q9UM48; Q9UM49; Q9UM50	NP_003340	Ubiquitin-conjugating enzyme E2 variant 1 (UEV-1) (CROC-1) (TRAF6-regulated IKK activator 1 beta Uev1A)	GO:0030154; GO:0005737; GO:0019941; GO:0005634; GO:0043123; GO:0051092; GO:0005515; GO:0000209; GO:0006282; GO:0051246; GO:0006355; GO:0019787; GO:0016563; GO:0031371	cell differentiation; cytoplasm; modification-dependent protein catabolic process; nucleus; positive regulation of I-kappaB kinase/NF-kappaB cascade; positive regulation of NF-kappaB transcription factor activity; protein binding; protein polyubiquitination; regulation of DNA repair; regulation of protein metabolic process; regulation of transcription, DNA-dependent; small conjugating protein ligase activity; transcription activator activity; ubiquitin conjugating enzyme complex	Nucleus					
**589**	UB2V2	UB2V2_HUMAN	UB2V2; A0M8W4	Q15819; A0M8W4	NP_003341	Ubiquitin-conjugating enzyme E2 variant 2 (MMS2) (Enterocyte differentiation-associated factor EDAF-1) (Enterocyte differentiation-promoting factor) (EDPF-1) (Vitamin D3-inducible protein) (DDVit 1); Ubiquitin-conjugating enzyme E2 variant 2 (Ubiquitin-conjugating enzyme E2 variant 2, isoform CRA_b) (cDNA, FLJ93989, Homo sapiens ubiquitin-conjugating enzyme E2 variant 2 (UBE2V2),mRNA)	GO:0000729; GO:0031372; GO:0008283; GO:0005737; GO:0019941; GO:0005634; GO:0005515; GO:0000209; GO:0006282; GO:0051246; GO:0019787; GO:0006512; GO:0043687	DNA double-strand break processing; UBC13-MMS2 complex; cell proliferation; cytoplasm; modification-dependent protein catabolic process; nucleus; protein binding; protein polyubiquitination; regulation of DNA repair; regulation of protein metabolic process; small conjugating protein ligase activity; ubiquitin cycle; post-translational protein modification						
**590**	UBP5	UBP5_HUMAN	UBP5	P45974; Q96J22	NP_003472; NP_001092 006	Ubiquitin carboxyl-terminal hydrolase 5 (EC 3.1.2.15) (Ubiquitin thioesterase 5) (Ubiquitin-specific-processing protease 5) (Deubiquitinating enzyme 5) (Isopeptidase T)	GO:0004197; GO:0005764; GO:0005634; GO:0005515; GO:0004221; GO:0006511; GO:0008270	cysteine-type endopeptidase activity; lysosome; nucleus; protein binding; ubiquitin thiolesterase activity; ubiquitin-dependent protein catabolic process; zinc ion binding				Known in Uniprot to be phosphorylated		
**591**	WASP	WASP_HUMAN	WASP	P42768; Q9BU11; Q9UNJ9	NP_000368	Wiskott-Aldrich syndrome protein (WASp)	GO:0015629; GO:0007596; GO:0005737; GO:0006952; GO:0008544; GO:0042802; GO:0006955; GO:0005634; GO:0005083	actin cytoskeleton; blood coagulation; cytoplasm; defense response; epidermis development; identical protein binding; immune response; nucleus; small GTPase regulator activity	Cytoplasmcytoskeleton			Known in Uniprot to be phosphorylated	Known in HPRD to be phosphorylated	
**592**	WNT8B	WNT8B_HUMAN	WNT8B	Q93098; O00771; Q5VX55; Q8WYK9	NP_003384	Protein Wnt-8b	GO:0007223; GO:0007267; GO:0007399; GO:0005578; GO:0004871	Wnt receptor signaling pathway, calcium modulating pathway; cell-cell signaling; nervous system development; proteinaceous extracellular matrix; signal transducer activity	Secretedextracellularspaceextracellularmatrix					
**593**	SYYC	SYYC_HUMAN	SYYC; B3KWK4	P54577; O43276; Q53EN1; B3KWK4	NP_003671	Tyrosyl-tRNA synthetase, cytoplasmic (EC 6.1.1.1) (Tyrosyl--tRNA ligase) (TyrRS)	GO:0005524; GO:0006915; GO:0005737; GO:0005615; GO:0005153; GO:0004871; GO:0005625; GO:0000049; GO:0004831; GO:0006437	ATP binding; apoptosis; cytoplasm; extracellular space; interleukin-8 receptor binding; signal transducer activity; soluble fraction; tRNA binding; tyrosine-tRNA ligase activity; tyrosyl-tRNA aminoacylation	Cytoplasm					
**594**	1433F	1433F_HUMAN	1433F; Q9H4N8	Q04917; Q9H4N8	NP_003396	14-3-3 protein eta (Protein AS1); Homo sapiens clone CDABP0046 mRNA sequence	GO:0003779; GO:0005737; GO:0019899; GO:0006713; GO:0035259; GO:0042921; GO:0005159; GO:0006886; GO:0050774; GO:0045941; GO:0019904; GO:0048167; GO:0016563	actin binding; cytoplasm; enzyme binding; glucocorticoid catabolic process; glucocorticoid receptor binding; glucocorticoid receptor signaling pathway; insulin-like growth factor receptor binding; intracellular protein transport; negative regulation of dendrite morphogenesis; positive regulation of transcription; protein domain specific binding; regulation of synaptic plasticity; transcription activator activity				Known in Uniprot to be phosphorylated		
**595**	TYY1	TYY1_HUMAN	TYY1	P25490; Q14935	NP_003394	Transcriptional repressor protein YY1 (Yin and yang 1) (YY-1) (Delta transcription factor) (NF-E1) (INO80 complex subunit S)	GO:0005737; GO:0016363; GO:0005886; GO:0006357; GO:0003713; GO:0003714; GO:0003700; GO:0008270	cytoplasm; nuclear matrix; plasma membrane; regulation of transcription from RNA polymerase II promoter; transcription coactivator activity; transcription corepressor activity; transcription factor activity; zinc ion binding	Nucleusmatrix			Known in Uniprot to be phosphorylated	Known in HPRD to be phosphorylated	
**596**	CATA	CATA_HUMAN	CATA; Q8TAK2	P04040; A8K6C0; B2RCZ9; Q2M1U4; Q4VXX5; Q9BWT9; Q9UC85; Q8TAK2	NP_001743	Catalase (EC 1.11.1.6); Similar to catalase (Fragment)	GO:0050661; GO:0009650; GO:0004096; GO:0008283; GO:0008083; GO:0020037; GO:0042744; GO:0043066; GO:0055114; GO:0005778; GO:0042803; GO:0051262; GO:0009060; GO:0004046; GO:0008203; GO:0020027; GO:0005506; GO:0005739; GO:0032088; GO:0051092; GO:0014068; GO:0006641	NADP or NADPH binding; UV protection; catalase activity; cell proliferation; growth factor activity; heme binding; hydrogen peroxide catabolic process; negative regulation of apoptosis; oxidation reduction; peroxisomal membrane; protein homodimerization activity; protein tetramerization; aerobic respiration; aminoacylase activity; cholesterol metabolic process; hemoglobin metabolic process; iron ion binding; mitochondrion; negative regulation of NF-kappaB transcription factor activity; positive regulation of NF-kappaB transcription factor activity; positive regulation of phosphoinositide 3-kinase cascade; triglyceride metabolic process	Peroxisome;			Known in Uniprot to be phosphorylated	Known in HPRD to be phosphorylated	
**597**	LDHB	LDHB_HUMAN	LDHB; Q5U077	P07195; Q5U077	NP_002291	L-lactate dehydrogenase B chain (LDH-B) (EC 1.1.1.27) (LDH heart subunit) (LDH-H) (Renal carcinoma antigen NY-REN-46); L-lactate dehydrogenase (EC 1.1.1.27)	GO:0004459; GO:0019642; GO:0005488; GO:0005737; GO:0055114	L-lactate dehydrogenase activity; anaerobic glycolysis; binding; cytoplasm; oxidation reduction	Cytoplasm;			Known in Uniprot to be phosphorylated		
**598**	ACADV	ACADV_HUMAN	ACADV	P49748; O76056; Q8WUL0	NP_000009; NP_001029 031	Very long-chain specific acyl-CoA dehydrogenase, mitochondrial (VLCAD) (EC 1.3.99.-)	GO:0050660; GO:0009055; GO:0015980; GO:0006635; GO:0004466; GO:0005743; GO:0042645; GO:0055114	FAD binding; electron carrier activity; energy derivation by oxidation of organic compounds; fatty acid beta-oxidation; long-chain-acyl-CoA dehydrogenase activity; mitochondrial inner membrane; mitochondrial nucleoid; oxidation reduction	Mitochondrioninnermembrane					
**599**	APOA1	APOA1_HUMAN	APOA1	P02647; A8K866; Q6LDN9; Q6Q785; Q9UCS8; Q9UCT8	NP_000030	Apolipoprotein A-I (Apo-AI) (ApoA-I) [Cleaved into: Apolipoprotein A-I(1-242)]	GO:0032488; GO:0007186; GO:0034191; GO:0001540; GO:0015485; GO:0033344; GO:0042632; GO:0008203; GO:0017127; GO:0030139; GO:0005788; GO:0019899; GO:0034364; GO:0034380; GO:0042802; GO:0042157; GO:0070325; GO:0002740; GO:0050713; GO:0006656; GO:0060228; GO:0005543; GO:0033700; GO:0010873; GO:0051345; GO:0050821; GO:0043691	Cdc42 protein signal transduction; G-protein coupled receptor protein signaling pathway; apolipoprotein A-I receptor binding; beta-amyloid binding; cholesterol binding; cholesterol efflux; cholesterol homeostasis; cholesterol metabolic process; cholesterol transporter activity; endocytic vesicle; endoplasmic reticulum lumen; enzyme binding; high-density lipoprotein particle; high-density lipoprotein particle assembly; identical protein binding; lipoprotein metabolic process; lipoprotein receptor binding; negative regulation of cytokine secretion during immune response; negative regulation of interleukin-1 beta secretion; phosphatidylcholine biosynthetic process; phosphatidylcholine-sterol O-acyltransferase activator activity; phospholipid binding; phospholipid efflux; positive regulation of cholesterol esterification; positive regulation of hydrolase activity; protein stabilization; reverse cholesterol transport	Secreted					

	Protein Name	Uniprot Name	Other Names	Uniprot IDs	Refseq IDs	Protein Description	GO ID	GO terms	Subcellular location	Comments	Comments	Comments - Database Search	Comments - Database Search	Function
**600**	APOC3	APOC3_HUMAN	APOC3; A3KPE2	P02656; Q08E83; Q6Q786; A3KPE2	NP_000031	Apolipoprotein C-III (Apo-CIII) (ApoC-III); Apolipoprotein C-III (Apolipoprotein C-III variant 2) (Apolipoprotein C-III variant 3) (Apolipoprotein C-III, isoform CRA_a)	GO:0032488; GO:0007186; GO:0015485; GO:0033344; GO:0042627; GO:0034375; GO:0034363; GO:0055102; GO:0042157; GO:0045717; GO:0051005; GO:0048261; GO:0010897; GO:0005543; GO:0033700; GO:0034366; GO:0034361; GO:0034447; GO:0005576; GO:0008289; GO:0006869	Cdc42 protein signal transduction; G-protein coupled receptor protein signaling pathway; cholesterol binding; cholesterol efflux; chylomicron; high-density lipoprotein particle remodeling; intermediate-density lipoprotein particle; lipase inhibitor activity; lipoprotein metabolic process; negative regulation of fatty acid biosynthetic process; negative regulation of lipoprotein lipase activity; negative regulation of receptor-mediated endocytosis; negative regulation of triglyceride catabolic process; phospholipid binding; phospholipid efflux; spherical high-density lipoprotein particle; very-low-density lipoprotein particle; very low-density lipoprotein particle clearance; extracellular region; lipid binding; lipid transport	Secreted;					
**601**	APOH	APOH_HUMAN	APOH	P02749; B2R9M3; Q9UCN7	NP_000033	Beta-2-glycoprotein 1 (Beta-2-glycoprotein I) (Beta(2)GPI) (B2GPI) (Apolipoprotein H) (Apo-H) (Activated protein C-binding protein) (APC inhibitor) (Anticardiolipin cofactor)	GO:0007597; GO:0009986; GO:0042627; GO:0005737; GO:0043499; GO:0001948; GO:0008201; GO:0034364; GO:0060230; GO:0016525; GO:0010596; GO:0001937; GO:0051918; GO:0033033; GO:0034392; GO:0005634; GO:0005543; GO:0031639; GO:0030194; GO:0051006; GO:0006641; GO:0034197; GO:0034361	blood coagulation, intrinsic pathway; cell surface; chylomicron; cytoplasm; eukaryotic cell surface binding; glycoprotein binding; heparin binding; high-density lipoprotein particle; lipoprotein lipase activator activity; negative regulation of angiogenesis; negative regulation of endothelial cell migration; negative regulation of endothelial cell proliferation; negative regulation of fibrinolysis; negative regulation of myeloid cell apoptosis; negative regulation of smooth muscle cell apoptosis; nucleus; phospholipid binding; plasminogen activation; positive regulation of blood coagulation; positive regulation of lipoprotein lipase activity; triglyceride metabolic process; triglyceride transport; very-low-density lipoprotein particle	Secreted					
**602**	BID	BID_HUMAN	BID; A8ASI8	P55957; Q549M7; Q71T04; Q7Z4M9; Q8IY86; A8ASI8	NP_001187; NP_932070; NP_932071	BH3-interacting domain death agonist (p22 BID) (BID) [Cleaved into: BH3-interacting domain death agonist p15 (p15 BID); BH3-interacting domain death agonist p13 (p13 BID); BH3-interacting domain death agonist p11 (p11 BID)]	GO:0008633; GO:0005829; GO:0005123; GO:0008625; GO:0005624; GO:0005741; GO:0051402; GO:0001836	activation of pro-apoptotic gene products; cytosol; death receptor binding; induction of apoptosis via death domain receptors; membrane fraction; mitochondrial outer membrane; neuron apoptosis; release of cytochrome c from mitochondria	CytoplasmMitochondrionmembraneMitochondrionmembraneMitochondrionmembraneCytoplasmCytoplasmMitochondrionmembrane			Known in Uniprot to be phosphorylated	Known in HPRD to be phosphorylated	
**603**	BTK	BTK_HUMAN	BTK	Q06187; Q32ML5	NP_000052	Tyrosine-protein kinase BTK (EC 2.7.10.2) (Bruton tyrosine kinase) (Agammaglobulinaemia tyrosine kinase) (ATK) (B-cell progenitor kinase) (BPK)	GO:0005524; GO:0019722; GO:0005737; GO:0042802; GO:0008624; GO:0045121; GO:0007498; GO:0004715; GO:0005634; GO:0006468; GO:0008270	ATP binding; calcium-mediated signaling; cytoplasm; identical protein binding; induction of apoptosis by extracellular signals; membrane raft; mesoderm development; non-membrane spanning protein tyrosine kinase activity; nucleus; protein amino acid phosphorylation; zinc ion binding	CytoplasmMembranePeripheralmembraneproteinNucleus			Known in Uniprot to be phosphorylated	Known in HPRD to be phosphorylated	Kinase
**604**	CO3	CO3_HUMAN	CO3; A7E236	P01024; A7E236	NP_000055; XP_941319	Complement C3 (C3 and PZP-like alpha-2-macroglobulin domain-containing protein 1) [Cleaved into: Complement C3 beta chain; Complement C3 alpha chain; C3a anaphylatoxin; Complement C3b alpha' chain; Complement C3c alpha' chain fragment 1; Complement C3dg fragment; Complement C3g fragment; Complement C3d fragment; Complement C3f fragment; Complement C3c alpha' chain fragment 2]	GO:0007186; GO:0006957; GO:0006958; GO:0004866; GO:0005615; GO:0005102	G-protein coupled receptor protein signaling pathway; complement activation, alternative pathway; complement activation, classical pathway; endopeptidase inhibitor activity; extracellular space; receptor binding	Secreted			Known in Uniprot to be phosphorylated		
**605**	CAH2	CAH2_HUMAN	CAH2; B2R7G8	P00918; Q6FI12; Q96ET9; B2R7G8	NP_000058	Carbonic anhydrase 2 (EC 4.2.1.1) (Carbonic anhydrase II) (CA-II) (Carbonate dehydratase II) (Carbonic anhydrase C) (CAC)	GO:0004089; GO:0005737; GO:0005634; GO:0006730; GO:0008270	carbonate dehydratase activity; cytoplasm; nucleus; one-carbon compound metabolic process; zinc ion binding	Cytoplasm					
**606**	CBS	CBS_HUMAN	CBS; B2R993	P35520; Q99425; Q9BWC5; B2R993	NP_000062	Cystathionine beta-synthase (EC 4.2.1.22) (Serine sulfhydrase) (Beta-thionase)	GO:0004122; GO:0006535; GO:0019343; GO:0005829; GO:0005506; GO:0005730; GO:0030170	cystathionine beta-synthase activity; cysteine biosynthetic process from serine; cysteine biosynthetic process via cystathionine; cytosol; iron ion binding; nucleolus; pyridoxal phosphate binding	CytoplasmNucleus					
**607**	AP2B1	AP2B1_HUMAN	AP2B1	P63010; A6NJP3; P21851; Q96J19	NP_001273; NP_001025177	AP-2 complex subunit beta-1 (Adapter-related protein complex 2 beta-1 subunit) (Beta2-adaptin) (Beta-adaptin) (Plasma membrane adaptor HA2/AP2 adaptin beta subunit) (Clathrin assembly protein complex 2 beta large chain) (AP105B)	GO:0030131; GO:0005905; GO:0005829; GO:0006886; GO:0005515; GO:0008565; GO:0050690; GO:0016192	clathrin adaptor complex; coated pit; cytosol; intracellular protein transport; protein binding; protein transporter activity; regulation of defense response to virus by virus; vesicle-mediated transport	CellmembraneMembranecoatedpitPeripheralmembraneproteinCytoplasmicside			Known in Uniprot to be phosphorylated		
**608**	AP1S1	AP1S1_HUMAN	AP1S1; B2R5D8	P61966; P82267; Q00382; Q53YA7; Q9BTN4; Q9UDW9; B2R5D8	NP_001274	AP-1 complex subunit sigma-1A (Adapter-related protein complex 1 sigma-1A subunit) (Adaptor protein complex AP-1 sigma-1A subunit) (Sigma-adaptin 1A) (Sigma1A-adaptin) (Golgi adaptor HA1/AP1 adaptin sigma-1A subunit) (HA1 19 kDa subunit) (Clathrin assembly protein complex 1 sigma-1A small chain) (Clathrin coat assembly protein AP19) (Sigma 1a subunit of AP-1 clathrin)	GO:0030121; GO:0005905; GO:0005829; GO:0006886; GO:0005515; GO:0008565; GO:0006898	AP-1 adaptor complex; coated pit; cytosol; intracellular protein transport; protein binding; protein transporter activity; receptor-mediated endocytosis	GolgiapparatusCytoplasmicvesiclemembranePeripheralmembraneproteinCytoplasmicsideMembraneclathrincoatedpit			Known in Uniprot to be phosphorylated		
**609**	CERU	CERU_HUMAN	CERU; A5PL27	P00450; Q14063; Q2PP18; Q9UKS4; A5PL27	NP_000087	Ceruloplasmin (EC 1.16.3.1) (Ferroxidase)	GO:0006879; GO:0005507; GO:0006825; GO:0005615; GO:0004322; GO:0055114	cellular iron ion homeostasis; copper ion binding; copper ion transport; extracellular space; ferroxidase activity; oxidation reduction	Secreted			Known in Uniprot to be phosphorylated		
**610**	EMD	EMD_HUMAN	EMD; Q6FI02	P50402; Q6FI02	NP_000108	Emerin; EMD protein (Emerin) (Emerin (Emery-Dreifuss muscular dystrophy), isoform CRA_a)	GO:0005783; GO:0016021; GO:0006936; GO:0007517; GO:0005637; GO:0005515; GO:0005635; GO:0031468; GO:0005819	endoplasmic reticulum; integral to membrane; muscle contraction; muscle organ development; nuclear inner membrane; protein binding; nuclear envelope; nuclear envelope reassembly; spindle	NucleusinnermembraneSinglepassmembraneproteinNucleoplasmicside;			Known in Uniprot to be phosphorylated	Known in HPRD to be phosphorylated	
**611**	FABPE	FABPE_HUMAN	FABPE; FABP5; B2R4K0	Q01469; B2R4K0	NP_001435; XP_001127657; XP_001128089; XP_001718427	Fatty acid-binding protein, epidermal (E-FABP) (Fatty acid-binding protein 5) (Psoriasis-associated fatty acid-binding protein homolog) (PA-FABP)	GO:0005737; GO:0008544; GO:0005504; GO:0006629; GO:0005515; GO:0006810; GO:0005215	cytoplasm; epidermis development; fatty acid binding; lipid metabolic process; protein binding; transport; transporter activity	Cytoplasm			Known in Uniprot to be phosphorylated		
**612**	GLGB	GLGB_HUMAN	Q59ET0; GLGB; B3KWV3	Q59ET0; Q04446; Q96EN0; B3KWV3	NP_000149	Glucan , branching enzyme 1 variant (Fragment)	GO:0005975; GO:0043169; GO:0004553	carbohydrate metabolic process; cation binding; hydrolase activity, hydrolyzing O-glycosyl compounds				Known in Uniprot to be phosphorylated		
**613**	ITA6	ITA6_HUMAN	ITA6; Q53RX7	P23229; B2RMU9; B4DG69; B4DKB8; Q08443; Q0MRC7; Q14646; Q16508; Q53RX7; Q59HB7; Q86VL6; Q9UN03	NP_000201; NP_001073286	Integrin alpha-6 (VLA-6) (CD49 antigen-like family member F) (CD antigen CD49f) [Cleaved into: Integrin alpha-6 heavy chain; Integrin alpha-6 light chain]	GO:0005509; GO:0007155; GO:0007044; GO:0008305; GO:0007229; GO:0005515; GO:0004872	calcium ion binding; cell adhesion; cell-substrate junction assembly; integrin complex; integrin-mediated signaling pathway; protein binding; receptor activity	MembraneSinglepasstypeImembraneprotein			Known in Uniprot to be phosphorylated		
**614**	K1C17	K1C17_HUMAN	K1C17	Q04695; A5Z1M9; A5Z1N0; A5Z1N1; A5Z1N2; A6NDV6; A6NKQ2; Q6IP98; Q8N1P6	NP_000413	Keratin, type I cytoskeletal 17 (Cytokeratin-17) (CK-17) (Keratin-17) (K17) (39.1)	GO:0005737; GO:0008544; GO:0005882; GO:0005634; GO:0005515; GO:0005200	cytoplasm; epidermis development; intermediate filament; nucleus; protein binding; structural constituent of cytoskeleton	Cytoplasm			Known in Uniprot to be phosphorylated		
**615**	MYH7	MYH7_HUMAN	MYH7; A2TDB6	P12883; Q14836; Q14837; Q14904; Q16579; Q2M1Y6; Q92679; Q9H1D5; Q9UMM8; A2TDB6	NP_000248	Myosin-7 (Myosin heavy chain 7) (Myosin heavy chain, cardiac muscle beta isoform) (MyHC-beta) (Myosin heavy chain slow isoform) (MyHC-slow); Myosin, heavy chain 7, cardiac muscle, beta (Myosin, heavy polypeptide 7, cardiac muscle, beta, isoform CRA_b)	GO:0005524; GO:0006200; GO:0003779; GO:0030898; GO:0007512; GO:0005516; GO:0005925; GO:0000146; GO:0030049; GO:0005730; GO:0002027; GO:0006941; GO:0005863; GO:0008307; GO:0055010; GO:0003774	ATP binding; ATP catabolic process; actin binding; actin-dependent ATPase activity; adult heart development; calmodulin binding; focal adhesion; microfilament motor activity; muscle filament sliding; nucleolus; regulation of heart rate; striated muscle contraction; striated muscle thick filament; structural constituent of muscle; ventricular cardiac muscle morphogenesis; motor activity	Cytoplasmmyofibril;			Known in Uniprot to be phosphorylated		

	Protein Name	Uniprot Name	Other Names	Uniprot IDs	Refseq IDs	Protein Description	GO ID	GO terms	Subcellular location	Comments	Comments	Comments - Database Search	Comments - Database Search	Function
**616**	NDKA	NDKA_HUMAN	NDKA; Q6FGK3; Q86XQ2	P15531; Q6FGK3; Q86XQ2	NP_000260; NP_937818	Nucleoside diphosphate kinase A (NDP kinase A) (NDK A) (EC 2.7.4.6) (Tumor metastatic process-associated protein) (Metastasis inhibition factor nm23) (nm23-H1) (Granzyme A-activated DNase) (GAAD)	GO:0005524; GO:0006241; GO:0003677; GO:0005525; GO:0006183; GO:0006228; GO:0005737; GO:0004536; GO:0042802; GO:0000287; GO:0045786; GO:0008285; GO:0004550; GO:0005634; GO:0043388; GO:0050679; GO:0042981	ATP binding; CTP biosynthetic process; DNA binding; GTP binding; GTP biosynthetic process; UTP biosynthetic process; cytoplasm; deoxyribonuclease activity; identical protein binding; magnesium ion binding; negative regulation of cell cycle; negative regulation of cell proliferation; nucleoside diphosphate kinase activity; nucleus; positive regulation of DNA binding; positive regulation of epithelial cell proliferation; regulation of apoptosis	CytoplasmNucleus			Known in Uniprot to be phosphorylated	Known in HPRD to be phosphorylated	Kinase
**617**	PNPH	PNPH_HUMAN	PNPH	P00491; B2R8S5; Q15160; Q5PZ03	NP_000261	Purine nucleoside phosphorylase (PNP) (EC 2.4.2.1) (Inosine phosphorylase)	GO:0034356; GO:0005829; GO:0008144; GO:0006955; GO:0006148; GO:0006738; GO:0001882; GO:0042301; GO:0042102; GO:0046638; GO:0002060; GO:0004731; GO:0042493; GO:0034418	NAD biosynthesis via nicotinamide riboside salvage pathway; cytosol; drug binding; immune response; inosine catabolic process; nicotinamide riboside catabolic process; nucleoside binding; phosphate binding; positive regulation of T cell proliferation; positive regulation of alpha-beta T cell differentiation; purine binding; purine-nucleoside phosphorylase activity; response to drug; urate biosynthetic process						
**618**	TRFE	TRFE_HUMAN	TRFE; Q1HBA5	P02787; O43890; Q9NQB8; Q9UHV0; Q1HBA5	NP_001054	Serotransferrin (Transferrin) (Siderophilin) (Beta-1 metal-binding globulin); Transferrin (Transferrin, isoform CRA_d)	GO:0006879; GO:0030139; GO:0008199; GO:0005739; GO:0006953; GO:0016324; GO:0005604; GO:0006879; GO:0005905; GO:0016023; GO:0005768; GO:0005615; GO:0015091; GO:0015682; GO:0031643; GO:0001666; GO:0014070	cellular iron ion homeostasis; endocytic vesicle; ferric iron binding; mitochondrion; acute-phase response; apical plasma membrane; basement membrane; cellular iron ion homeostasis; coated pit; cytoplasmic membrane-bounded vesicle; endosome; extracellular space; ferric iron transmembrane transporter activity; ferric iron transport; positive regulation of myelination; response to hypoxia; response to organic cyclic substance	Secreted;			Known in Uniprot to be phosphorylated		
**619**	K2C5	K2C5_HUMAN	K2C5	P13647; Q6PI71; Q6UBJ0; Q8TA91	NP_000415	Keratin, type II cytoskeletal 5 (Cytokeratin-5) (CK-5) (Keratin-5) (K5) (58 kDa cytokeratin)	GO:0008544; GO:0045095; GO:0005515; GO:0005200	epidermis development; keratin filament; protein binding; structural constituent of cytoskeleton				Known in Uniprot to be phosphorylated		
**620**	LYSC	LYSC_HUMAN	LYSC; B2R4C5	P61626; P00695; Q13170; Q9UCF8; B2R4C5	NP_000230	Lysozyme C (EC 3.2.1.17) (1,4-beta-N-acetylmuramidase C)	GO:0016998; GO:0019835; GO:0042742; GO:0005615; GO:0006954; GO:0003796; GO:0005515	cell wall catabolic process; cytolysis; defense response to bacterium; extracellular space; inflammatory response; lysozyme activity; protein binding						
**621**	MTM1	MTM1_HUMAN	MTM1	Q13496; Q8NEL1	NP_000243	Myotubularin (EC 3.1.3.48)	GO:0005737; GO:0031012; GO:0007517; GO:0005886; GO:0006470; GO:0004722; GO:0004725	cytoplasm; extracellular matrix; muscle organ development; plasma membrane; protein amino acid dephosphorylation; protein serine/threonine phosphatase activity; protein tyrosine phosphatase activity				Known in Uniprot to be phosphorylated		
**622**	PPAC	PPAC_HUMAN	PPAC; Q53RU0; Q59EH3	P24666; Q53RU0; P24667; Q16035; Q16036; Q16725; Q3KQX8; Q59EH3	NP_004291; NP_009030	Low molecular weight phosphotyrosine protein phosphatase (LMW-PTPase) (LMW-PTP) (EC 3.1.3.48) (Low molecular weight cytosolic acid phosphatase) (EC 3.1.3.2) (Red cell acid phosphatase 1) (Adipocyte acid phosphatase)	GO:0003993; GO:0005737; GO:0042802; GO:0004726; GO:0006470; GO:0005625	acid phosphatase activity; cytoplasm; identical protein binding; non-membrane spanning protein tyrosine phosphatase activity; protein amino acid dephosphorylation; soluble fraction	Cytoplasm			Known in Uniprot to be phosphorylated	Known in HPRD to be phosphorylated	Phosphatase
**623**	AIFM1	AIFM1_HUMAN	AIFM1; A4QPB4	O95831; B2RB08; Q5JUZ7; Q6I9X6; Q9Y3I3; Q9Y3I4; A4QPB4	NP_004199; NP_665811; NP_665812	Apoptosis-inducing factor 1, mitochondrial (EC 1.-.-.-) (Programmed cell death protein 8)	GO:0003677; GO:0008630; GO:0006309; GO:0050660; GO:0045454; GO:0009055; GO:0005758; GO:0005634; GO:0055114; GO:0016491; GO:0005515	DNA binding; DNA damage response, signal transduction resulting in induction of apoptosis; DNA fragmentation involved in apoptosis; FAD binding; cell redox homeostasis; electron carrier activity; mitochondrial intermembrane space; nucleus; oxidation reduction; oxidoreductase activity; protein binding	MitochondrionintermembranespaceNucleus			Known in Uniprot to be phosphorylated		
**624**	GDIR	GDIR_HUMAN	GDIR; Q6IBM5; GDIR1	P52565; Q6IBM5	NP_004300	Rho GDP-dissociation inhibitor 1 (Rho GDI 1) (Rho-GDI alpha); ARHGDIA protein (Rho GDP dissociation inhibitor (GDI) alpha) (Rho GDP dissociation inhibitor (GDI) alpha, isoform CRA_a)	GO:0005096; GO:0005094; GO:0007266; GO:0006916; GO:0006928; GO:0005737; GO:0005856; GO:0042802; GO:0007162; GO:0005094	GTPase activator activity; Rho GDP-dissociation inhibitor activity; Rho protein signal transduction; anti-apoptosis; cell motion; cytoplasm; cytoskeleton; identical protein binding; negative regulation of cell adhesion; Rho GDP-dissociation inhibitor activity	Cytoplasm;					
**625**	ARRC	ARRC_HUMAN	ARRC	P36575; B5B0B9; Q5JT23; Q5JT24; Q6IBF5; Q96EN2	NP_004303	Arrestin-C (Cone arrestin) (C-arrestin) (cArr) (Retinal cone arrestin-3) (X-arrestin)	GO:0005737; GO:0050896; GO:0007165; GO:0005625; GO:0007601	cytoplasm; response to stimulus; signal transduction; soluble fraction; visual perception						
**626**	VATG1	VATG1_HUMAN	VATG1; Q6IB33	O75348; Q6IB33	NP_004879	V-type proton ATPase subunit G 1 (V-ATPase subunit G 1) (Vacuolar proton pump subunit G 1) (V-ATPase 13 kDa subunit 1) (Vacuolar proton pump subunit M16); ATP6V1G1 protein (ATPase, H+ transporting, lysosomal 13kDa, V1 subunit G1) (ATPase, H+ transporting, lysosomal 13kDa, V1 subunit G1, isoform CRA_a)	GO:0015992; GO:0016471; GO:0008553; GO:0005765; GO:0005515; GO:0015992	proton transport; vacuolar proton-transporting V-type ATPase complex; hydrogen-exporting ATPase activity, phosphorylative mechanism; lysosomal membrane; protein binding; proton transport						
**627**	PTTG	PTTG_HUMAN	PTTG; B2RDP7	P53801; Q9NS09; B2RDP7	NP_004330	Pituitary tumor-transforming gene 1 protein-interacting protein (Pituitary tumor-transforming gene protein-binding factor) (PTTG-binding factor) (PBF)	GO:0005737; GO:0016021; GO:0005634; GO:0006606	cytoplasm; integral to membrane; nucleus; protein import into nucleus	MembraneSinglepasstypeImembraneproteinCytoplasmNucleus			Known in Uniprot to be phosphorylated		
**628**	CALR	CALR_HUMAN	CALR; Q6IAT4	P27797; Q6IAT4	NP_004334	Calreticulin (CRP55) (Calregulin) (HACBP) (ERp60) (grp60); CALR protein (Calreticulin) (Calreticulin, isoform CRA_b)	GO:0003677; GO:0042824; GO:0050681; GO:0005509; GO:0007050; GO:0051087; GO:0001849; GO:0005829; GO:0005788; GO:0042921; GO:0005178; GO:0003729; GO:0045665; GO:0048387; GO:0033144; GO:0000122; GO:0017148; GO:0005634; GO:0002502; GO:0048471; GO:0005844; GO:0045740; GO:0045787; GO:0008284; GO:0050766; GO:0006611; GO:0022417; GO:0050821; GO:0005578; GO:0042981; GO:0010149; GO:0051208; GO:0005529; GO:0016564; GO:0051082;	DNA binding; MHC class I peptide loading complex; androgen receptor binding; calcium ion binding; cell cycle arrest; chaperone binding; complement component C1q binding; cytosol; endoplasmic reticulum lumen; glucocorticoid receptor signaling pathway; integrin binding; mRNA binding; negative regulation of neuron differentiation; negative regulation of retinoic acid receptor signaling pathway; negative regulation of steroid hormone receptor signaling pathway; negative regulation of transcription from RNA polymerase II promoter; negative regulation of translation; nucleus; peptide antigen assembly with MHC class I protein complex; perinuclear region of cytoplasm; polysome; positive regulation of DNA replication; positive regulation of cell cycle; positive regulation of cell proliferation; positive regulation of phagocytosis; protein export from nucleus; protein maturation via protein folding; protein stabilization; proteinaceous extracellular matrix; regulation of apoptosis; senescence; sequestering of calcium ion; sugar binding; transcription repressor activity; unfolded protein binding; zinc ion binding; MHC class I peptide loading complex; cortical actin cytoskeleton	EndoplasmicreticulumlumenCytoplasmcytosolSecretedextracellularspaceextracellularmatrixCellsurface;					
**629**	CDN1B	CDN1B_HUMAN	CDN1B; Q6I9V6	P46527; Q6I9V6; Q16307; Q5U0H2; Q9BUS6	NP_004055	Cyclin-dependent kinase inhibitor 1B (Cyclin-dependent kinase inhibitor p27) (p27Kip1); Cyclin-dependent kinase inhibitor 1B (CDKN1B protein) (Cyclin-dependent kinase inhibitor 1B (P27, Kip1), isoform CRA_a) (cDNA, FLJ92816, Homo sapiens cyclin-dependent kinase inhibitor 1B (p27, Kip1)(CDKN1B), mRNA)	GO:0000082; GO:0048102; GO:0007050; GO:0004861; GO:0005829; GO:0006917; GO:0030308; GO:0008285; GO:0042326; GO:0005634; GO:0005515; GO:0000079; GO:0005072; GO:0007050; GO:0016301	G1/S transition of mitotic cell cycle; autophagic cell death; cell cycle arrest; cyclin-dependent protein kinase inhibitor activity; cytosol; induction of apoptosis; negative regulation of cell growth; negative regulation of cell proliferation; negative regulation of phosphorylation; nucleus; protein binding; regulation of cyclin-dependent protein kinase activity; transforming growth factor beta receptor, cytoplasmic mediator activity; cell cycle arrest; kinase activity	NucleusCytoplasm;			Known in Uniprot to be phosphorylated	Known in HPRD to be phosphorylated	Kinase
**630**	CNN2	CNN2_HUMAN	CNN2; A5D8U8	Q99439; A5D8U8; Q92578	NP_004359; XP_167021; NP_958434	Calponin-2 (Calponin H2, smooth muscle) (Neutral calponin)	GO:0003779; GO:0031032; GO:0005516; GO:0005911; GO:0005856	actin binding; actomyosin structure organization; calmodulin binding; cell-cell junction; cytoskeleton				Known in Uniprot to be phosphorylated		

	Protein Name	Uniprot Name	Other Names	Uniprot IDs	Refseq IDs	Protein Description	GO ID	GO terms	Subcellular location	Comments	Comments	Comments - Database Search	Comments - Database Search	Function
**631**	COPA	COPA_HUMAN	COPA	P53621; Q5T201; Q8IXZ9	NP_004362; NP_001091868	Coatomer subunit alpha (Alpha-coat protein) (Alpha-COP) (HEP-COP) (HEPCOP) [Cleaved into: Xenin (Xenopsin-related peptide); Proxenin]	GO:0048205; GO:0030126; GO:0005509; GO:0005829; GO:0005783; GO:0005615; GO:0005179; GO:0006886; GO:0005792; GO:0030157; GO:0006457; GO:0006890; GO:0005625; GO:0005198; GO:0051082	COPI coating of Golgi vesicle; COPI vesicle coat; calcium ion binding; cytosol; endoplasmic reticulum; extracellular space; hormone activity; intracellular protein transport; microsome; pancreatic juice secretion; protein folding; retrograde vesicle-mediated transport, Golgi to ER; soluble fraction; structural molecule activity; unfolded protein binding	CytoplasmGolgiapparatusmembranePeripheralmembraneproteinCytoplasmicsideCytoplasmicvesicleCOPIcoatedvesiclemembranePeripheralmembraneproteinCytoplasmicsideSecreted			Known in Uniprot to be phosphorylated	Known in HPRD to be phosphorylated	
**632**	COPB2	COPB2_HUMAN	COPB2	P35606	NP_004757	Coatomer subunit beta' (Beta'-coat protein) (Beta'-COP) (p102)	GO:0048205; GO:0030126; GO:0005829; GO:0006891; GO:0006886; GO:0005515; GO:0006890; GO:0005198	COPI coating of Golgi vesicle; COPI vesicle coat; cytosol; intra-Golgi vesicle-mediated transport; intracellular protein transport; protein binding; retrograde vesicle-mediated transport, Golgi to ER; structural molecule activity	CytoplasmGolgiapparatusmembranePeripheralmembraneproteinCytoplasmicsideCytoplasmicvesicleCOPIcoatedvesiclemembranePeripheralmembraneproteinCytoplasmicside			Known in Uniprot to be phosphorylated		
**633**	COX5A	COX5A_HUMAN	COX5A	P20674; P30045; Q8TB65	NP_004246	Cytochrome c oxidase subunit 5A, mitochondrial (Cytochrome c oxidase polypeptide Va)	GO:0004129; GO:0009055; GO:0005506; GO:0005743	cytochrome-c oxidase activity; electron carrier activity; iron ion binding; mitochondrial inner membrane	Mitochondrioninnermembrane					
**634**	COX6C	COX6C_HUMAN	COX6C; B2R4D7	P09669; B2R4D7	NP_004365	Cytochrome c oxidase subunit 6C (Cytochrome c oxidase polypeptide VIc)	GO:0004129; GO:0006091; GO:0016021; GO:0005743	cytochrome-c oxidase activity; generation of precursor metabolites and energy; integral to membrane; mitochondrial inner membrane	Mitochondrioninnermembrane					
**635**	CSK	CSK_HUMAN	CSK; B2R6Q4	P41240; Q2M3N2; Q6FGZ6; B2R6Q4	NP_004374; NP_001120662	Tyrosine-protein kinase CSK (EC 2.7.10.2) (C-SRC kinase) (Protein-tyrosine kinase CYL)	GO:0005524; GO:0005737; GO:0004715; GO:0008022; GO:0006468	ATP binding; cytoplasm; non-membrane spanning protein tyrosine kinase activity; protein C-terminus binding; protein amino acid phosphorylation	CytoplasmCellmembrane			Known in Uniprot to be phosphorylated	Known in HPRD to be phosphorylated	Kinase
**636**	DUS3	DUS3_HUMAN	DUS3; Q5U0J1	P51452; Q5U0J1	XP_301571; NP_004081	Dual specificity protein phosphatase 3 (EC 3.1.3.48) (EC 3.1.3.16) (Dual specificity protein phosphatase VHR); Dual specificity phosphatase 3 (Vaccinia virus phosphatase VH1-related) (Dual specificity phosphatase 3 (Vaccinia virus phosphatase VH1-related), isoform CRA_a)	GO:0005654; GO:0006470; GO:0004725; GO:0008138; GO:0006470	nucleoplasm; protein amino acid dephosphorylation; protein tyrosine phosphatase activity; protein tyrosine/serine/threonine phosphatase activity; protein amino acid dephosphorylation					Known in HPRD to be phosphorylated	Phosphatase
**637**	PDIA4	PDIA4_HUMAN	PDIA4; Q549T6	P13667; Q549T6	NP_004902	Protein disulfide-isomerase A4 (EC 5.3.4.1) (Protein ERp-72) (ERp72); Protein disulfide isomerase family A, member 4, isoform CRA_a (Putative uncharacterized protein ERP70)	GO:0045454; GO:0005783; GO:0005788; GO:0042470; GO:0005515; GO:0003756; GO:0009306; GO:0005509; GO:0045454; GO:0016853	cell redox homeostasis; endoplasmic reticulum; endoplasmic reticulum lumen; melanosome; protein binding; protein disulfide isomerase activity; protein secretion; calcium ion binding; cell redox homeostasis; isomerase activity	EndoplasmicreticulumlumenMelanosome;					
**638**	FKBP5	FKBP5_HUMAN	FKBP5; Q5TGM6	Q13451; Q5TGM6	NP_004108; NP_001139247; NP_001139248	FK506-binding protein 5 (EC 5.2.1.8) (Peptidyl-prolyl cis-trans isomerase) (PPIase) (Rotamase) (51 kDa FK506-binding protein) (FKBP-51) (54 kDa progesterone receptor-associated immunophilin) (FKBP54) (P54) (FF1 antigen) (HSP90-binding immunophilin) (Androgen-regulated protein 6); FK506 binding protein 5 (FK506 binding protein 5, isoform CRA_b) (cDNA, FLJ92761, Homo sapiens FK506 binding protein 5 (FKBP5), mRNA)	GO:0005528; GO:0005737; GO:0005634; GO:0003755; GO:0005515; GO:0006457; GO:0005488	FK506 binding; cytoplasm; nucleus; peptidyl-prolyl cis-trans isomerase activity; protein binding; protein folding; binding	CytoplasmNucleus;			Known in Uniprot to be phosphorylated	Known in HPRD to be phosphorylated	
**639**	GMFG	GMFG_HUMAN	GMFG; Q6IB37	O60234; Q6IB37	NP_004868	Glia maturation factor gamma (GMF-gamma); GMFG protein (Glia maturation factor, gamma)	GO:0003779; GO:0008047; GO:0008083; GO:0005739; GO:0005634; GO:0006468; GO:0004860; GO:0005622	actin binding; enzyme activator activity; growth factor activity; mitochondrion; nucleus; protein amino acid phosphorylation; protein kinase inhibitor activity; intracellular						
**640**	GMFB	GMFB_HUMAN	GMFB; B2R499	P60983; P17774; B2R499	NP_004115	Glia maturation factor beta (GMF-beta)	GO:0003779; GO:0008047; GO:0008083; GO:0005739; GO:0007399; GO:0005634; GO:0006468; GO:0004860; GO:0004871; GO:0007165	actin binding; enzyme activator activity; growth factor activity; mitochondrion; nervous system development; nucleus; protein amino acid phosphorylation; protein kinase inhibitor activity; signal transducer activity; signal transduction				Known in Uniprot to be phosphorylated	Known in HPRD to be phosphorylated	
**641**	GSTO1	GSTO1_HUMAN	GSTO1	P78417; Q5TA03; Q7Z3T2	NP_004823; XP_293602	Glutathione S-transferase omega-1 (EC 2.5.1.18) (GSTO 1-1)	GO:0005737; GO:0004364; GO:0008152; GO:0016656	cytoplasm; glutathione transferase activity; metabolic process; monodehydroascorbate reductase (NADH) activity	Cytoplasm					
**642**	HCD2	HCD2_HUMAN	HCD2; Q6IBS9	Q99714; Q6IBS9; Q8TCV9; Q96HD5	NP_004484; NP_001032900	3-hydroxyacyl-CoA dehydrogenase type-2 (EC 1.1.1.35) (3-hydroxyacyl-CoA dehydrogenase type II) (Type II HADH) (3-hydroxy-2-methylbutyryl-CoA dehydrogenase) (EC 1.1.1.178) (17-beta-hydroxysteroid dehydrogenase 10) (Mitochondrial ribonuclease P protein 2) (Mitochondrial RNase P protein 2) (Endoplasmic reticulum-associated amyloid beta-peptide-binding protein) (Short-chain type dehydrogenase/reductase XH98G2); HADH2 protein (Hydroxysteroid (17-beta) dehydrogenase 10) (Hydroxyacyl-Coenzyme A dehydrogenase, type II, isoform CRA_a)	GO:0047015; GO:0003857; GO:0008709; GO:0006629; GO:0005739; GO:0055114; GO:0005886; GO:0005515; GO:0008033; GO:0005488; GO:0008152; GO:0016491	3-hydroxy-2-methylbutyryl-CoA dehydrogenase activity; 3-hydroxyacyl-CoA dehydrogenase activity; 7-alpha-hydroxysteroid dehydrogenase activity; lipid metabolic process; mitochondrion; oxidation reduction; plasma membrane; protein binding; tRNA processing; binding; metabolic process; oxidoreductase activity	Mitochondrion;					
**643**	K2C75	K2C75_HUMAN	O95678; K2C75	O95678; Q9NSA9	NP_004684	Keratin, type II cytoskeletal 75 (Cytokeratin-75) (CK-75) (Keratin-75) (K75) (Type II keratin-18) (Type II keratin-K6hf) (Keratin-6 hair follicle) (hK6hf)	GO:0045095; GO:0005198	keratin filament; structural molecule activity				Known in Uniprot to be phosphorylated		
**644**	PRDX6	PRDX6_HUMAN	PRDX6; A8JZY7	P30041; P32077; Q5TAH4; Q5ZEZ8; A8JZY7	NP_004896	Peroxiredoxin-6 (EC 1.11.1.15) (Antioxidant protein 2) (1-Cys peroxiredoxin) (1-Cys PRX) (Acidic calcium-independent phospholipase A2) (aiPLA2) (EC 3.1.1.-) (Non-selenium glutathione peroxidase) (NSGPx) (EC 1.11.1.7) (24 kDa protein) (Liver 2D page spot 40) (Red blood cells page spot 12)	GO:0045454; GO:0031410; GO:0005829; GO:0005764; GO:0005634; GO:0055114; GO:0051920; GO:0004623; GO:0009395; GO:0006979	cell redox homeostasis; cytoplasmic vesicle; cytosol; lysosome; nucleus; oxidation reduction; peroxiredoxin activity; phospholipase A2 activity; phospholipid catabolic process; response to oxidative stress	CytoplasmLysosomeCytoplasmicvesicle			Known in Uniprot to be phosphorylated		
**645**	KIF2A	KIF2A_HUMAN	KIF2A	O00139; A5YM42; A5YM54; B4DY54; Q7Z5I3; Q8N5Q7	NP_004511; NP_001091981	Kinesin-like protein KIF2A (Kinesin-2) (HK2)	GO:0005524; GO:0030154; GO:0005813; GO:0005874; GO:0003777; GO:0007018; GO:0007399	ATP binding; cell differentiation; centrosome; microtubule; microtubule motor activity; microtubule-based movement; nervous system development	CytoplasmCentrosome			Known in Uniprot to be phosphorylated	Known in HPRD to be phosphorylated	
**646**	KINH	KINH_HUMAN	KINH; Q6P164	P33176; A0AVB2; Q5VZ85; Q6P164	NP_004512	Kinesin-1 heavy chain (Ubiquitous kinesin heavy chain) (UKHC) (Conventional kinesin heavy chain)	GO:0005524; GO:0005871; GO:0005874; GO:0008017; GO:0003777; GO:0048471; GO:0047496	ATP binding; kinesin complex; microtubule; microtubule binding; microtubule motor activity; perinuclear region of cytoplasm; vesicle transport along microtubule	Cytoplasmcytoskeleton					
**647**	MGST3	MGST3_HUMAN	MGST3; B2R592	O14880; Q6ICN4; B2R592	NP_004519	Microsomal glutathione S-transferase 3 (Microsomal GST-3) (EC 2.5.1.18) (Microsomal GST-III)	GO:0005789; GO:0004364; GO:0016021; GO:0006629; GO:0005792; GO:0004601; GO:0007165	endoplasmic reticulum membrane; glutathione transferase activity; integral to membrane; lipid metabolic process; microsome; peroxidase activity; signal transduction	EndoplasmicreticulummembraneMultipassmembraneproteinMicrosomemembraneMultipassmembraneprotein					
**648**	NDUC2	NDUC2_HUMAN	NDUC2; Q549M5	O95298; Q549M5; Q6FIH8; Q9UBJ9	NP_004540	NADH dehydrogenase [ubiquinone] 1 subunit C2 (NADH-ubiquinone oxidoreductase subunit B14.5b) (Complex I-B14.5b) (CI-B14.5b) (Human lung cancer oncogene 1 protein) (HLC-1); NADH dehydrogenase (Putative uncharacterized protein NDUFC2)	GO:0008137; GO:0016021; GO:0006120; GO:0005747; GO:0006810; GO:0005743	NADH dehydrogenase (ubiquinone) activity; integral to membrane; mitochondrial electron transport, NADH to ubiquinone; mitochondrial respiratory chain complex I; transport; mitochondrial inner membrane	MitochondrioninnermembraneSinglepassmembraneproteinMatrixside;					
**649**	NDUS3	NDUS3_HUMAN	NDUS3; B2R9J1	O75489; Q9UNQ8; B2R9J1	NP_004542	NADH dehydrogenase [ubiquinone] iron-sulfur protein 3, mitochondrial (EC 1.6.5.3) (EC 1.6.99.3) (NADH-ubiquinone oxidoreductase 30 kDa subunit) (Complex I-30kD) (CI-30kD)	GO:0008137; GO:0009055; GO:0006917; GO:0006120; GO:0005747; GO:0030308; GO:0006800; GO:0005515; GO:0006810	NADH dehydrogenase (ubiquinone) activity; electron carrier activity; induction of apoptosis; mitochondrial electron transport, NADH to ubiquinone; mitochondrial respiratory chain complex I; negative regulation of cell growth; oxygen and reactive oxygen species metabolic process; protein binding; transport	Mitochondrioninnermembrane					
**650**	NDUS5	NDUS5_HUMAN	NDUS5; Q6IBA0	O43920; Q6IBA0	NP_004543	NADH dehydrogenase [ubiquinone] iron-sulfur protein 5 (NADH-ubiquinone oxidoreductase 15 kDa subunit) (Complex I-15 kDa) (CI-15 kDa); NADH dehydrogenase (Ubiquinone) Fe-S protein 5, 15kDa (NADH-coenzyme Q reductase) (NADH dehydrogenase (Ubiquinone) Fe-S protein 5, 15kDa (NADH-coenzyme Q reductase), isoform CRA_a) (NDUFS5 protein) (cDNA, FLJ92475, Homo sapiens NADH dehydrogenase (ubiquinone) Fe-S protein 5, 15kDa(NADH-coenzyme Q reductase) (NDUFS5), mRNA)	GO:0008137; GO:0006120; GO:0005747; GO:0032981; GO:0006810;	NADH dehydrogenase (ubiquinone) activity; mitochondrial electron transport, NADH to ubiquinone; mitochondrial respiratory chain complex I; mitochondrial respiratory chain complex I assembly; transport;	MitochondrioninnermembranePeripheralmembraneproteinMatrixside;					
**651**	NDUS6	NDUS6_HUMAN	NDUS6; Q6IBC4	O75380; Q6IBC4	NP_004544	NADH dehydrogenase [ubiquinone] iron-sulfur protein 6, mitochondrial (NADH-ubiquinone oxidoreductase 13 kDa-A subunit) (Complex I-13kD-A) (CI-13kD-A); NDUFS6 protein (cDNA FLJ12441 fis, clone NT2RM1000132, highly similar to NADH-ubiquinone oxidoreductase 13 kDa-A subunit, mitochondrial (EC 1.6.5.3)) (NADH dehydrogenase (Ubiquinone) Fe-S protein 6, 13kDa (NADH-coenzyme Q reductase), isoform CRA_a)	GO:0008137; GO:0009055; GO:0006120; GO:0005747; GO:0006810;	NADH dehydrogenase (ubiquinone) activity; electron carrier activity; mitochondrial electron transport, NADH to ubiquinone; mitochondrial respiratory chain complex I; transport;	MitochondrioninnermembranePeripheralmembraneproteinMatrixside;					
**652**	NOLC1	NOLC1_HUMAN	NOLC1	Q14978; Q15030; Q5VV70; Q9BUV3	XP_293394; NP_004732	Nucleolar phosphoprotein p130 (Nucleolar 130 kDa protein) (140 kDa nucleolar phosphoprotein) (Nopp140) (Nucleolar and coiled-body phosphoprotein 1) (HCV NS5A trans-regulated protein 13) (NS5ATP13)	GO:0005524; GO:0005525; GO:0005737; GO:0007067; GO:0005730; GO:0005515; GO:0006364	ATP binding; GTP binding; cytoplasm; mitosis; nucleolus; protein binding; rRNA processing	NucleusnucleolusCytoplasm			Known in Uniprot to be phosphorylated	Known in HPRD to be phosphorylated	

	Protein Name	Uniprot Name	Other Names	Uniprot IDs	Refseq IDs	Protein Description	GO ID	GO terms	Subcellular location	Comments	Comments	Comments - Database Search	Comments - Database Search	Function
**653**	SPB9	SPB9_HUMAN	SPB9; Q5TD03	P50453; Q5TD03; B2RBW3	NP_004146	Serpin B9 (Cytoplasmic antiproteinase 3) (CAP-3) (CAP3) (Proteinase inhibitor 9)	GO:0006916; GO:0005829; GO:0005515; GO:0004867; GO:0007165	anti-apoptosis; cytosol; protein binding; serine-type endopeptidase inhibitor activity; signal transduction	Cytoplasm					
**654**	PSB8	PSB8_HUMAN	PSB8; Q5JNW6	Q5JNW6; P28062; B0UZC0; Q29824; Q96J48	NP_004150; NP_683720	Proteasome subunit beta type-8 (EC 3.4.25.1) (Proteasome subunit beta-5i) (Proteasome component C13) (Macropain subunit C13) (Multicatalytic endopeptidase complex subunit C13); Proteasome (Prosome, macropain) subunit beta type 8 (Large multifunctional protease 7) (Proteasome (Prosome, macropain) subunit, beta type, 8 (Large multifunctional peptidase 7)) (Proteasome (Prosome, macropain) subunit, beta type, 8 (Large multifunctional peptidase 7), isoform CRA_a)	GO:0031145; GO:0005829; GO:0006955; GO:0051436; GO:0005634; GO:0051437; GO:0005839; GO:0004298; GO:0005829; GO:0006511	anaphase-promoting complex-dependent proteasomal ubiquitin-dependent protein catabolic process; cytosol; immune response; negative regulation of ubiquitin-protein ligase activity during mitotic cell cycle; nucleus; positive regulation of ubiquitin-protein ligase activity during mitotic cell cycle; proteasome core complex; threonine-type endopeptidase activity; cytosol; ubiquitin-dependent protein catabolic process	CytoplasmNucleus;					
**655**	RB11B	RB11B_HUMAN	RB11B	Q15907; B2R7I4; Q2YDT2; Q8NI07	NP_004209	Ras-related protein Rab-11B (GTP-binding protein YPT3)	GO:0005525; GO:0003924; GO:0005886; GO:0015031; GO:0007264	GTP binding; GTPase activity; plasma membrane; protein transport; small GTPase mediated signal transduction	CellmembraneLipidanchorCytoplasmicside					
**656**	RAB1A	RAB1A_HUMAN	RAB1A; Q5U0I6	P62820; Q5U0I6; P11476; Q96N61; Q9Y3T2	NP_004152; NP_056358	Ras-related protein Rab-1A (YPT1-related protein); RAB1A protein (RAB1A, member RAS oncogene family) (RAB1A, member RAS oncogene family, isoform CRA_b) (cDNA, FLJ96558, Homo sapiens RAB1A, member RAS oncogene family (RAB1A), mRNA)	GO:0005525; GO:0003924; GO:0005794; GO:0005783; GO:0015031; GO:0007264; GO:0016192	GTP binding; GTPase activity; Golgi apparatus; endoplasmic reticulum; protein transport; small GTPase mediated signal transduction; vesicle-mediated transport	GolgiapparatusEndoplasmicreticulum;			Known in Uniprot to be phosphorylated		
**657**	SLIT2	SLIT2_HUMAN	SLIT2	O94813; O95710; Q17RU3; Q9Y5Q7	NP_004778	Slit homolog 2 protein (Slit-2) [Cleaved into: Slit homolog 2 protein N-product; Slit homolog 2 protein C-product]	GO:0005509; GO:0005576; GO:0008347; GO:0050929; GO:0007509; GO:0008045; GO:0008038; GO:0050772; GO:0005102; GO:0007608; GO:0001657	calcium ion binding; extracellular region; glial cell migration; induction of negative chemotaxis; mesoderm migration; motor axon guidance; neuron recognition; positive regulation of axonogenesis; receptor binding; sensory perception of smell; ureteric bud development	Secreted					
**658**	TBCA	TBCA_HUMAN	TBCA; Q6FGD7	O75347; Q6FGD7	NP_004598	Tubulin-specific chaperone A (Tubulin-folding cofactor A) (CFA) (TCP1-chaperonin cofactor A); TBCA protein (Tubulin folding cofactor A) (Tubulin-specific chaperone a) (cDNA, FLJ92496, Homo sapiens tubulin-specific chaperone a (TBCA), mRNA)	GO:0051087; GO:0005737; GO:0005874; GO:0007023; GO:0007021; GO:0051082; GO:0005874	chaperone binding; cytoplasm; microtubule; post-chaperonin tubulin folding pathway; tubulin complex assembly; unfolded protein binding; microtubule	Cytoplasmcytoskeleton;					
**659**	TSN	TSN_HUMAN	TSN	Q15631; Q5U0K7	NP_004613	Translin	GO:0006310; GO:0005737; GO:0005634; GO:0043565	DNA recombination; cytoplasm; nucleus; sequence-specific DNA binding	CytoplasmNucleus					
**660**	TXNL1	TXNL1_HUMAN	TXNL1	O43396	NP_004777	Thioredoxin-like protein 1 (32 kDa thioredoxin-related protein)	GO:0045454; GO:0005737; GO:0015036; GO:0022900; GO:0006810	cell redox homeostasis; cytoplasm; disulfide oxidoreductase activity; electron transport chain; transport	Cytoplasm					
**661**	VAMP3	VAMP3_HUMAN	VAMP3; Q6FGG2; Q9BRV4	Q15836; Q6FGG2; Q9BRV4	NP_004772	Vesicle-associated membrane protein 3 (VAMP-3) (Synaptobrevin-3) (Cellubrevin) (CEB); VAMP3 protein (Vesicle-associated membrane protein 3 (Cellubrevin)) (cDNA, FLJ92013, Homo sapiens vesicle-associated membrane protein 3 (cellubrevin)(VAMP3), mRNA); Vesicle-associated membrane protein 3 (Cellubrevin)	GO:0030054; GO:0016021; GO:0006944; GO:0006461; GO:0045202; GO:0019717; GO:0006904; GO:0016021; GO:0016192	cell junction; integral to membrane; membrane fusion; protein complex assembly; synapse; synaptosome; vesicle docking during exocytosis; integral to membrane; vesicle-mediated transport	MembraneSinglepasstypeIVmembraneproteinCelljunctionsynapsesynaptosome;			Known in Uniprot to be phosphorylated		
**662**	VAPB	VAPB_HUMAN	VAPB; Q53XM7; Q6ZSP7	O95292; Q53XM7; A2A2F2; O95293; Q9P0H0; Q6ZSP7	NP_004729	Vesicle-associated membrane protein-associated protein B/C (VAMP-associated protein B/C) (VAMP-B/VAMP-C) (VAP-B/VAP-C); VAMP (Vesicle-associated membrane protein)-associated protein B and C (cDNA FLJ13179 fis, clone NT2RP3003918, highly similar to Vesicle-associated membrane protein-associated protein B/C) (VAMP (Vesicle-associated membrane protein)-associated protein B and C, isoform CRA_b); cDNA FLJ45319 fis, clone BRHIP3005801, highly similar to Homo sapiens VAMP (vesicle-associated membrane protein)-associated protein B and C (VAPB)	GO:0005794; GO:0048487; GO:0005789; GO:0030968; GO:0019899; GO:0016021; GO:0005886; GO:0045070; GO:0046982; GO:0042803; GO:0005198; GO:0019048; GO:0005198;	Golgi apparatus; beta-tubulin binding; endoplasmic reticulum membrane; endoplasmic reticulum unfolded protein response; enzyme binding; integral to membrane; plasma membrane; positive regulation of viral genome replication; protein heterodimerization activity; protein homodimerization activity; structural molecule activity; virus-host interaction; structural molecule activity;	CellmembraneSinglepasstypeIVmembraneproteinIntracytoplasmicmembraneSinglepasstypeIVmembraneprotein;			Known in Uniprot to be phosphorylated	Known in HPRD to be phosphorylated	
**663**	ANXA3	ANXA3_HUMAN	ANXA3; B2R9W6	P12429; Q6LET2; B2R9W6	NP_005130	Annexin A3 (Annexin-3) (Annexin III) (Lipocortin III) (Placental anticoagulant protein III) (PAP-III) (35-alpha calcimedin) (Inositol 1,2-cyclic phosphate 2-phosphohydrolase)	GO:0005509; GO:0005544; GO:0005737; GO:0019834; GO:0007165	calcium ion binding; calcium-dependent phospholipid binding; cytoplasm; phospholipase A2 inhibitor activity; signal transduction						
**664**	CAPZB	CAPZB_HUMAN	CAPZB; Q32Q68	Q32Q68; P47756; Q5U0L4; Q8TB49; Q9NUC4	NP_004921	F-actin-capping protein subunit beta (CapZ beta); Capping protein (Actin filament) muscle Z-line, beta (Capping protein (Actin filament) muscle Z-line, beta, isoform CRA_c)	GO:0008290; GO:0003779; GO:0051016; GO:0006928; GO:0005737; GO:0030036; GO:0030027; GO:0030032	F-actin capping protein complex; actin binding; barbed-end actin filament capping; cell motion; cytoplasm; actin cytoskeleton organization; lamellipodium; lamellipodium assembly	Cytoplasmcytoskeleton;			Known in Uniprot to be phosphorylated		
**665**	CCS	CCS_HUMAN	CCS	O14618; Q2M366	NP_005116	Copper chaperone for superoxide dismutase (Superoxide dismutase copper chaperone)	GO:0005507; GO:0005375; GO:0005737; GO:0015680; GO:0005634; GO:0005515; GO:0005625; GO:0006801; GO:0008270	copper ion binding; copper ion transmembrane transporter activity; cytoplasm; intracellular copper ion transport; nucleus; protein binding; soluble fraction; superoxide metabolic process; zinc ion binding	Cytoplasm			Known in Uniprot to be phosphorylated		
**666**	HPT	HPT_HUMAN	HPT	P00738; B0AZL5; P00737; Q0VAC4; Q2PP15; Q3B7J0; Q6LBY9; Q9UC67	NP_005134; NP_001119574	Haptoglobin [Cleaved into: Haptoglobin alpha chain; Haptoglobin beta chain]	GO:0006879; GO:0006952; GO:0005576; GO:0030492; GO:0006508; GO:0004252	cellular iron ion homeostasis; defense response; extracellular region; hemoglobin binding; proteolysis; serine-type endopeptidase activity	Secreted					
**667**	UCRP	UCRP_HUMAN	UCRP; Q5SVA4	P05161; Q5SVA4; Q7Z2G2; Q96GF0	NP_005092	Interferon-induced 17 kDa protein [Cleaved into: Ubiquitin cross-reactive protein (hUCRP) (Interferon-induced 15 kDa protein)]	GO:0032020; GO:0007267; GO:0005737; GO:0005615; GO:0044419; GO:0019941; GO:0005515; GO:0009615; GO:0006512	ISG15-protein conjugation; cell-cell signaling; cytoplasm; extracellular space; interspecies interaction between organisms; modification-dependent protein catabolic process; protein binding; response to virus; ubiquitin cycle	CytoplasmSecreted					
**668**	NDUA5	NDUA5_HUMAN	NDUA5; B2RD98	Q16718; Q6IRX7; B2RD98	NP_004991	NADH dehydrogenase [ubiquinone] 1 alpha subcomplex subunit 5 (NADH-ubiquinone oxidoreductase 13 kDa-B subunit) (Complex I-13kD-B) (CI-13kD-B) (Complex I subunit B13)	GO:0008137; GO:0006120; GO:0005747; GO:0006810	NADH dehydrogenase (ubiquinone) activity; mitochondrial electron transport, NADH to ubiquinone; mitochondrial respiratory chain complex I; transport	MitochondrioninnermembranePeripheralmembraneproteinMatrixside					
**669**	UBP14	UBP14_HUMAN	UBP14; Q53XY5	P54578; Q53XY5	NP_005142; NP_001032411	Ubiquitin carboxyl-terminal hydrolase 14 (EC 3.1.2.15) (Ubiquitin thioesterase 14) (Ubiquitin-specific-processing protease 14) (Deubiquitinating enzyme 14)	GO:0004197; GO:0005737; GO:0006464; GO:0008193; GO:0004221; GO:0006511; GO:0004843	cysteine-type endopeptidase activity; cytoplasm; protein modification process; tRNA guanylyltransferase activity; ubiquitin thiolesterase activity; ubiquitin-dependent protein catabolic process; ubiquitin-specific protease activity				Known in Uniprot to be phosphorylated		
**670**	APJ	APJ_HUMAN	APJ; B3KQN4	P35414; B3KQN4	NP_005152	Apelin receptor (G-protein coupled receptor APJ) (Angiotensin receptor-like 1) (HG11)	GO:0004930; GO:0007186; GO:0005887	G-protein coupled receptor activity; G-protein coupled receptor protein signaling pathway; integral to plasma membrane	CellmembraneMultipassmembraneprotein					
**671**	ALDOC	ALDOC_HUMAN	ALDOC; Q6FH94; B2R5R3	P09972; Q6FH94; Q3SYL3; Q6P0L5; B2R5R3	NP_005156	Fructose-bisphosphate aldolase C (EC 4.1.2.13) (Brain-type aldolase)	GO:0008092; GO:0005856; GO:0030388; GO:0004332; GO:0006096	cytoskeletal protein binding; cytoskeleton; fructose 1,6-bisphosphate metabolic process; fructose-bisphosphate aldolase activity; glycolysis				Known in Uniprot to be phosphorylated		
**672**	CRKL	CRKL_HUMAN	CRKL; A8KA44	P46109; A8KA44	NP_005198	Crk-like protein	GO:0007254; GO:0007265; GO:0005070; GO:0005737; GO:0005886; GO:0004713	JNK cascade; Ras protein signal transduction; SH3/SH2 adaptor activity; cytoplasm; plasma membrane; protein tyrosine kinase activity				Known in Uniprot to be phosphorylated	Known in HPRD to be phosphorylated	
**673**	CYTA	CYTA_HUMAN	CYTA; Q6IB90	P01040; Q6IB90	NP_005204	Cystatin-A (Cystatin-AS) (Stefin-A); CSTA protein (Cystatin A (Stefin A)) (cDNA FLJ76406, highly similar to Homo sapiens cystatin A (stefin A) (CSTA), mRNA)	GO:0001533; GO:0004869; GO:0005737; GO:0030216; GO:0010466; GO:0005634; GO:0018149; GO:0002020; GO:0030674; GO:0005198; GO:0004869; GO:0005622	cornified envelope; cysteine-type endopeptidase inhibitor activity; cytoplasm; keratinocyte differentiation; negative regulation of peptidase activity; nucleus; peptide cross-linking; protease binding; protein binding, bridging; structural molecule activity; cysteine-type endopeptidase inhibitor activity; intracellular	Cytoplasm;					
**674**	FGR	FGR_HUMAN	FGR	P09769; Q9UIQ3	NP_005239; NP_001036194; NP_001036212	Proto-oncogene tyrosine-protein kinase FGR (C-FGR) (EC 2.7.10.2) (P55-FGR)	GO:0005524; GO:0004715; GO:0006468; GO:0005515; GO:0009615	ATP binding; non-membrane spanning protein tyrosine kinase activity; protein amino acid phosphorylation; protein binding; response to virus				Known in Uniprot to be phosphorylated	Known in HPRD to be phosphorylated	Kinase
**675**	DHE3	DHE3_HUMAN	DHE3	P00367; Q5TBU3	NP_005262	Glutamate dehydrogenase 1, mitochondrial (GDH) (EC 1.4.1.3)	GO:0005524; GO:0005525; GO:0006538; GO:0004353; GO:0004352; GO:0005759; GO:0055114; GO:0007169	ATP binding; GTP binding; glutamate catabolic process; glutamate dehydrogenase [NAD(P)+] activity; glutamate dehydrogenase activity; mitochondrial matrix; oxidation reduction; transmembrane receptor protein tyrosine kinase signaling pathway	Mitochondrionmatrix			Known in Uniprot to be phosphorylated		
**676**	GBG5	GBG5_HUMAN	GBG5; B2R5A0	P63218; P30670; Q5VX54; Q61015; B2R5A0	NP_005265	Guanine nucleotide-binding protein G(I)/G(S)/G(O) subunit gamma-5	GO:0007186; GO:0005834; GO:0009755; GO:0004871	G-protein coupled receptor protein signaling pathway; heterotrimeric G-protein complex; hormone-mediated signaling; signal transducer activity	CellmembraneLipidanchorCytoplasmicside					

	Protein Name	Uniprot Name	Other Names	Uniprot IDs	Refseq IDs	Protein Description	GO ID	GO terms	Subcellular location	Comments	Comments	Comments - Database Search	Comments - Database Search	Function
**677**	HCLS1	HCLS1_HUMAN	HCLS1; Q53Y93	P14317; Q53Y93	NP_005326	Hematopoietic lineage cell-specific protein (Hematopoietic cell-specific LYN substrate 1) (LckBP1) (p75); Hematopoietic cell-specific Lyn substrate 1 (cDNA, FLJ93159, Homo sapiens hematopoietic cell-specific Lyn substrate 1 (HCLS1), mRNA)	GO:0005665; GO:0030218; GO:0016020; GO:0005739; GO:0008284; GO:0042531; GO:0006355; GO:0009725; GO:0003700; GO:0017124; GO:0005737; GO:0005634	DNA-directed RNA polymerase II, core complex; erythrocyte differentiation; membrane; mitochondrion; positive regulation of cell proliferation; positive regulation of tyrosine phosphorylation of STAT protein; regulation of transcription, DNA-dependent; response to hormone stimulus; transcription factor activity; SH3 domain binding; cytoplasm; nucleus	MembranePeripheralmembraneproteinCytoplasmMitochondrion;			Known in Uniprot to be phosphorylated	Known in HPRD to be phosphorylated	
**678**	HINT1	HINT1_HUMAN	HINT1	P49773; Q9H5W8	NP_005331	Histidine triad nucleotide-binding protein 1 (EC 3.-.-.-) (Adenosine 5'-monophosphoramidase) (Protein kinase C inhibitor 1) (Protein kinase C-interacting protein 1) (PKCI-1)	GO:0005737; GO:0005856; GO:0016787; GO:0005634; GO:0005080; GO:0007165	cytoplasm; cytoskeleton; hydrolase activity; nucleus; protein kinase C binding; signal transduction	CytoplasmNucleus					
**679**	UBC1	UBC1_HUMAN	UBC1; UBE2K	P61086; A6NJC1; B2RDF8; O54806; P27924; Q16721; Q9CVV9; Q9Y2D3	NP_005330	Ubiquitin-conjugating enzyme E2 K (EC 6.3.2.19) (Ubiquitin-conjugating enzyme E2-25 kDa) (E2(25K)) (E2-25K) (Ubiquitin-protein ligase) (Ubiquitin carrier protein) (Huntingtin-interacting protein 2) (HIP-2)	GO:0005737; GO:0043687; GO:0051246; GO:0031625; GO:0006511; GO:0004842	cytoplasm; post-translational protein modification; regulation of protein metabolic process; ubiquitin protein ligase binding; ubiquitin-dependent protein catabolic process; ubiquitin-protein ligase activity	Cytoplasm					
**680**	RASH	RASH_HUMAN	RASH; Q9BR65	P01112; B5BUA0; Q14080; Q6FHV9; Q9UCE2; Q9BR65	NP_005334; NP_001123 914; NP_789765	GTPase HRas (Transforming protein p21) (p21ras) (H-Ras-1) (c-H-ras) (Ha-Ras) [Cleaved into: GTPase HRas, N-terminally processed]; V-Ha-ras Harvey rat sarcoma viral oncogene homolog (p19 H-RasIDX protein) (V-Ha-ras Harvey rat sarcoma viral oncogene homolog, isoform CRA_b)	GO:0005525; GO:0000139; GO:0007166; GO:0006935; GO:0009887; GO:0005886; GO:0008022; GO:0005622; GO:0007264	GTP binding; Golgi membrane; cell surface receptor linked signal transduction; chemotaxis; organ morphogenesis; plasma membrane; protein C-terminus binding; intracellular; small GTPase mediated signal transduction	CellmembraneLipidanchorCytoplasmicsideGolgiapparatusmembraneLipidanchor;					
**681**	PIMT	PIMT_HUMAN	PIMT; Q5VYC2	Q5VYC2; P22061; A8K109; Q14661; Q16556; Q5VYC1; Q93061; Q96II9; Q99625; Q9BQV7; Q9BQV8; Q9NP03	NP_005380	Protein-L-isoaspartate(D-aspartate) O-methyltransferase (PIMT) (EC 2.1.1.77) (Protein-beta-aspartate methyltransferase) (Protein L-isoaspartyl/D-aspartyl methyltransferase) (L-isoaspartyl protein carboxyl methyltransferase)	GO:0005783; GO:0042802; GO:0006479; GO:0030091; GO:0004719	endoplasmic reticulum; identical protein binding; protein amino acid methylation; protein repair; protein-L-isoaspartate (D-aspartate) O-methyltransferase activity	Cytoplasm					
**682**	ROCK1	ROCK1_HUMAN	ROCK1	Q13464; B0YJ91; Q2KHM4; Q59GZ4	NP_005397	Rho-associated protein kinase 1 (EC 2.7.11.1) (Rho-associated, coiled-coil-containing protein kinase 1) (p160 ROCK-1) (p160ROCK) (Renal carcinoma antigen NY-REN-35)	GO:0005524; GO:0000139; GO:0007266; GO:0030036; GO:0000910; GO:0005829; GO:0019992; GO:0042802; GO:0050901; GO:0022614; GO:0006468; GO:0004674; GO:0008270	ATP binding; Golgi membrane; Rho protein signal transduction; actin cytoskeleton organization; cytokinesis; cytosol; diacylglycerol binding; identical protein binding; leukocyte tethering or rolling; membrane to membrane docking; protein amino acid phosphorylation; protein serine/threonine kinase activity; zinc ion binding	CytoplasmCentrosomecentrioleGolgiapparatusmembranePeripheralmembraneprotein			Known in Uniprot to be phosphorylated		Kinase
**683**	ARPC4	ARPC4_HUMAN	ARPC4	P59998; O15509; Q6P0W5; Q96QJ3	NP_005709	Actin-related protein 2/3 complex subunit 4 (Arp2/3 complex 20 kDa subunit) (p20-ARC)	GO:0005885; GO:0003779; GO:0030041; GO:0045010; GO:0042995; GO:0005737	Arp2/3 protein complex; actin binding; actin filament polymerization; actin nucleation; cell projection; cytoplasm	Cytoplasmcytoskeleton Cellprojection					
**684**	ARPC3	ARPC3_HUMAN	ARPC3	O15145; O00554	NP_005710; XP_208062	Actin-related protein 2/3 complex subunit 3 (Arp2/3 complex 21 kDa subunit) (p21-ARC)	GO:0005885; GO:0003779; GO:0006928; GO:0042995; GO:0005737; GO:0030833; GO:0005200	Arp2/3 protein complex; actin binding; cell motion; cell projection; cytoplasm; regulation of actin filament polymerization; structural constituent of cytoskeleton	Cytoplasmcytoskeleton Cellprojection			Known in Uniprot to be phosphorylated		
**685**	ARC1B	ARC1B_HUMAN	ARC1B; A4D275	O15143; A4D275; Q9BU00	NP_005711; XP_941344	Actin-related protein 2/3 complex subunit 1B (Arp2/3 complex 41 kDa subunit) (p41-ARC); Actin related protein 2/3 complex, subunit 1B, 41kDa (Actin related protein 2/3 complex, subunit 1B, 41kDa, isoform CRA_a) (cDNA, FLJ95695, Homo sapiens actin related protein 2/3 complex, subunit 1B, 41kDa(ARPC1B), mRNA)	GO:0005885; GO:0003779; GO:0006928; GO:0005737; GO:0030833; GO:0005200; GO:0003779; GO:0005856	Arp2/3 protein complex; actin binding; cell motion; cytoplasm; regulation of actin filament polymerization; structural constituent of cytoskeleton; actin binding; cytoskeleton	Cytoplasmcytoskeleton ;			Known in Uniprot to be phosphorylated	Known in HPRD to be phosphorylated	
**686**	ES1	ES1_HUMAN	ES1	P30042; A6NFJ6; A6NJY7; O00650; O00660; O15011; O15012; P55346; P78474; Q92505; Q92507	NP_004640; NP_937798	ES1 protein homolog, mitochondrial (Protein KNP-I) (Protein GT335)	GO:0005739	mitochondrion	Mitochondrion					
**687**	K2C6A	K2C6A_HUMAN	K2C6A	P02538; A4QPC1; P48667; Q08AR4; Q6NT67; Q96CL4	NP_005545	Keratin, type II cytoskeletal 6A (Cytokeratin-6A) (CK 6A) (K6a keratin) (Cytokeratin-6D) (CK 6D) (allergen Hom s 5)	GO:0030154; GO:0007398; GO:0045095; GO:0008284; GO:0005515; GO:0005200	cell differentiation; ectoderm development; keratin filament; positive regulation of cell proliferation; protein binding; structural constituent of cytoskeleton				Known in Uniprot to be phosphorylated		
**688**	LCP2	LCP2_HUMAN	LCP2; Q53XV4	Q13094; Q53XV4	NP_005556	Lymphocyte cytosolic protein 2 (SH2 domain-containing leukocyte protein of 76 kDa) (SLP-76 tyrosine phosphoprotein) (SLP76); Lymphocyte cytosolic protein 2 (SH2 domain containing leukocyte protein of 76kDa) (Lymphocyte cytosolic protein 2 (SH2 domain containing leukocyte protein of 76kDa), isoform CRA_b)	GO:0005829; GO:0006955; GO:0005515; GO:0007169; GO:0050663; GO:0045576	cytosol; immune response; protein binding; transmembrane receptor protein tyrosine kinase signaling pathway; cytokine secretion; mast cell activation	Cytoplasm;			Known in Uniprot to be phosphorylated	Known in HPRD to be phosphorylated	
**689**	LDHA	LDHA_HUMAN	LDHA	P00338; Q53G53; Q6IBM7; Q6ZNV1	NP_005557; XP_208411	L-lactate dehydrogenase A chain (LDH-A) (EC 1.1.1.27) (LDH muscle subunit) (LDH-M) (Renal carcinoma antigen NY-REN-59) (Cell proliferation-inducing gene 19 protein)	GO:0004459; GO:0019642; GO:0005829; GO:0055114; GO:0005515	L-lactate dehydrogenase activity; anaerobic glycolysis; cytosol; oxidation reduction; protein binding	Cytoplasm			Known in Uniprot to be phosphorylated		
**690**	NACA	NACA_HUMAN	NACA; B2R4P8	Q13765; Q3KQV4; Q53A18; Q53G46; B2R4P8	NP_005585; NP_001106 672; NP_0011066 73; NP_0011066 74	Nascent polypeptide-associated complex subunit alpha (NAC-alpha) (Alpha-NAC) (allergen Hom s 2)	GO:0003677; GO:0044419; GO:0005854; GO:0005634; GO:0015031; GO:0006350; GO:0006412	DNA binding; interspecies interaction between organisms; nascent polypeptide-associated complex; nucleus; protein transport; transcription; translation	CytoplasmNucleus			Known in Uniprot to be phosphorylated	Known in HPRD to be phosphorylated	
**691**	CACO2	CACO2_HUMAN	Q13137; CACO2	Q13137; B2RBT0; Q53FQ5; Q53HB5; Q6IBN9; Q9BTF7	NP_005822	Calcium-binding and coiled-coil domain-containing protein 2 (Nuclear domain 10 protein NDP52) (Nuclear domain 10 protein 52) (Nuclear dot protein 52) (Antigen nuclear dot 52 kDa protein)	GO:0005794; GO:0005856; GO:0005634; GO:0048471; GO:0042803; GO:0034341; GO:0005625; GO:0016032	Golgi apparatus; cytoskeleton; nucleus; perinuclear region of cytoplasm; protein homodimerization activity; response to interferon-gamma; soluble fraction; viral reproduction	CytoplasmperinuclearregionGolgiapparatusCytoplasmcytoskeleton					
**692**	NIT1	NIT1_HUMAN	NIT1; Q5SY59; B1AQP3	Q86X76; Q5SY59; O76091; B1AQP3	NP_005591	Nitrilase homolog 1 (EC 3.5.-.-)	GO:0005737; GO:0000257; GO:0006807; GO:0005634	cytoplasm; nitrilase activity; nitrogen compound metabolic process; nucleus						
**693**	PQBP1	PQBP1_HUMAN	PQBP1	O60828; Q4VY25; Q4VY26; Q4VY27; Q4VY29; Q4VY30; Q4VY34; Q4VY35; Q4VY36; Q4VY37; Q4VY38; Q9GZP2; Q9GZU4; Q9GZZ4	NP_005701; NP_001027 553; NP_0010275 54; NP_0010275 55; NP_001027 556	Polyglutamine-binding protein 1 (Polyglutamine tract-binding protein 1) (PQBP-1) (38 kDa nuclear protein containing a WW domain) (Npw38)	GO:0003677; GO:0005737; GO:0005634; GO:0006355; GO:0003713	DNA binding; cytoplasm; nucleus; regulation of transcription, DNA-dependent; transcription coactivator activity	Nucleus			Known in Uniprot to be phosphorylated		
**694**	NTF2	NTF2_HUMAN	NTF2; B2R4G7	P61970; P13662; Q6IB67; B2R4G7	NP_005787	Nuclear transport factor 2 (NTF-2) (Placental protein 15) (PP15)	GO:0005829; GO:0005643; GO:0005515	cytosol; nuclear pore; protein binding	Cytoplasm					
**695**	PYGM	PYGM_HUMAN	PYGM; PHS2	Q9H0N5; P11217; Q8TD40; A0AVK1	NP_005600; NP_115527	Glycogen phosphorylase, muscle form (EC 2.4.1.1) (Myophosphorylase)	GO:0008184; GO:0000166; GO:0005515; GO:0030170	glycogen phosphorylase activity; nucleotide binding; protein binding; pyridoxal phosphate binding				Known in Uniprot to be phosphorylated	Known in HPRD to be phosphorylated	
**696**	RHEB	RHEB_HUMAN	RHEB	Q15382; Q53Y56; B3KWN6; Q99444	NP_005605	GTP-binding protein Rheb (Ras homolog enriched in brain)	GO:0005525; GO:0003924; GO:0005829; GO:0005886; GO:0007264	GTP binding; GTPase activity; cytosol; plasma membrane; small GTPase mediated signal transduction	CellmembraneLipidanchorCytoplasmicside					
**697**	SYBL1	SYBL1_HUMAN	SYBL1; VAMP7	P51809; Q53GY7; Q7Z409; Q9H4A7	NP_005629; NP_001138 621	Vesicle-associated membrane protein 7 (VAMP-7) (Synaptobrevin-like protein 1) (Tetanus-insensitive VAMP) (Ti-VAMP)	GO:0006888; GO:0005794; GO:0031201; GO:0017156; GO:0005789; GO:0008333; GO:0043308; GO:0016021; GO:0031902; GO:0005765; GO:0043312; GO:0045335; GO:0006911; GO:0005515; GO:0015031; GO:0006906	ER to Golgi vesicle-mediated transport; Golgi apparatus; SNARE complex; calcium ion-dependent exocytosis; endoplasmic reticulum membrane; endosome to lysosome transport; eosinophil degranulation; integral to membrane; late endosome membrane; lysosomal membrane; neutrophil degranulation; phagocytic vesicle; phagocytosis, engulfment; protein binding; protein transport; vesicle fusion	CytoplasmicvesiclesecretoryvesiclemembraneSinglepasstypeIVmembraneproteinGolgiapparatustransGolginetworkmembraneSinglepasstypeIVmembraneprotein LateendosomemembraneSinglepasstypeIVmembraneproteinLysosomemembraneSinglepasstypeIVmembraneproteinEndoplasmicreticulummembraneSinglepasstypeIVmembraneprotein CytoplasmicvesiclephagosomemembraneSinglepasstypeIVmembraneprotein					

	Protein Name	Uniprot Name	Other Names	Uniprot IDs	Refseq IDs	Protein Description	GO ID	GO terms	Subcellular location	Comments	Comments	Comments - Database Search	Comments - Database Search	Function
**698**	ELOC	ELOC_HUMAN	ELOC	Q15369; Q567Q6	NP_005639	Transcription elongation factor B polypeptide 1 (RNA polymerase II transcription factor SIII subunit C) (SIII p15) (Elongin 15 kDa subunit) (Elongin-C) (EloC)	GO:0005829; GO:0005654; GO:0005515; GO:0006357; GO:0006511	cytosol; nucleoplasm; protein binding; regulation of transcription from RNA polymerase II promoter; ubiquitin-dependent protein catabolic process	Nucleus					
**699**	THIC	THIC_HUMAN	THIC	Q9BWD1; Q16146; Q5TCL7; Q8TDM4	NP_005882	Acetyl-CoA acetyltransferase, cytosolic (EC 2.3.1.9) (Cytosolic acetoacetyl-CoA thiolase) (Acetyl-CoA transferase-like protein)	GO:0003985; GO:0005737; GO:0006629; GO:0005515	acetyl-CoA C-acetyltransferase activity; cytoplasm; lipid metabolic process; protein binding	Cytoplasm					
**700**	GBLP	GBLP_HUMAN	GBLP; Q6FH47; Q53HU2	P63244; Q6FH47; B3KTJ0; P25388; P99049; Q53HU2; Q5J8M6; Q5VLR4	NP_006089	Guanine nucleotide-binding protein subunit beta-2-like 1 (Guanine nucleotide-binding protein subunit beta-like protein 12.3) (Receptor of activated protein kinase C 1) (RACK1) (Receptor for activated C kinase) (Cell proliferation-inducing gene 21 protein) (Human lung cancer oncogene 7 protein) (HLC-7)	GO:0005886; GO:0019903; GO:0005102	plasma membrane; protein phosphatase binding; receptor binding	Cellmembrane			Known in Uniprot to be phosphorylated		
**701**	MAP1B	MAP1B_HUMAN	MAP1B; A2BDK5; Q6PJD3	P46821; A2BDK5; Q6PJD3	NP_005900	Microtubule-associated protein 1B (MAP-1B) [Cleaved into: MAP1 light chain LC1]	GO:0005874; GO:0005875; GO:0005198	microtubule; microtubule associated complex; structural molecule activity				Known in Uniprot to be phosphorylated	Known in HPRD to be phosphorylated	
**702**	MDHC	MDHC_HUMAN	MDHC; B2R5V5	P40925; B2R5V5; Q6I9V0	NP_005908; XP_066916	Malate dehydrogenase, cytoplasmic (EC 1.1.1.37) (Cytosolic malate dehydrogenase)	GO:0030060; GO:0005829; GO:0006096; GO:0006108; GO:0004470; GO:0055114; GO:0006099	L-malate dehydrogenase activity; cytosol; glycolysis; malate metabolic process; malic enzyme activity; oxidation reduction; tricarboxylic acid cycle	Cytoplasm			Known in Uniprot to be phosphorylated		
**703**	RTN3	RTN3_HUMAN	RTN3	O95197; B3KQS2; Q147U9; Q496K2; Q53GN3; Q59EP0; Q5UEP2; Q6T930; Q7RTM7; Q7RTM8; Q7RTN3	XP_017966; NP_006045; NP_958831; NP_958832; NP_958833	Reticulon-3 (Neuroendocrine-specific protein-like 2) (NSP-like protein II) (NSPLII)	GO:0000139; GO:0006915; GO:0005789; GO:0005615; GO:0016021; GO:0044419; GO:0006950; GO:0016192	Golgi membrane; apoptosis; endoplasmic reticulum membrane; extracellular space; integral to membrane; interspecies interaction between organisms; response to stress; vesicle-mediated transport	Endoplasmicreticulum membraneMultipassme mbraneproteinGolgiap paratusmembraneMulti passmembraneprotein			Known in Uniprot to be phosphorylated		
**704**	SIA10	SIA10_HUMAN	SIA10; B2RCH2	Q9Y274; B2RCH2	NP_006091	Type 2 lactosamine alpha-2,3-sialyltransferase (EC 2.4.99.-) (CMP-NeuAc:beta-galactoside alpha-2,3-sialyltransferase VI) (ST3Gal VI) (Sialyltransferase 10)	GO:0006040; GO:0006664; GO:0030173; GO:0006486; GO:0009249; GO:0008373	amino sugar metabolic process; glycolipid metabolic process; integral to Golgi membrane; protein amino acid glycosylation; protein lipoylation; sialyltransferase activity	Golgiapparatusmembra neSinglepasstypeIIme mbraneprotein					
**705**	TSNAX	TSNAX_HUMAN	TSNAX; Q5VT41; B1APC6	Q99598; Q5VT41; B1APC6	NP_005990	Translin-associated protein X (Translin-associated factor X)	GO:0030154; GO:0005737; GO:0007275; GO:0005634; GO:0008565; GO:0043565; GO:0007283	cell differentiation; cytoplasm; multicellular organismal development; nucleus; protein transporter activity; sequence-specific DNA binding; spermatogenesis	Cytoplasmperinuclearr egionNucleus			Known in Uniprot to be phosphorylated		
**706**	PRDX4	PRDX4_HUMAN	PRDX4	Q13162; Q6FHT3	NP_006397	Peroxiredoxin-4 (EC 1.11.1.15) (Prx-IV) (Thioredoxin peroxidase AO372) (Thioredoxin-dependent peroxide reductase A0372) (Antioxidant enzyme AOE372) (AOE37-2)	GO:0007252; GO:0045454; GO:0055114; GO:0008379	I-kappaB phosphorylation; cell redox homeostasis; oxidation reduction; thioredoxin peroxidase activity	Cytoplasm					
**707**	RAN	RAN_HUMAN	RAN; A8K3Z8	P62826; A8K3Z8; P17080; P28746; P28747; Q6IPB2; Q86V08; Q8NI90; Q9CSP3; Q9CWI7; Q9CZA2; Q9UDJ5; Q9UEU9	NP_006316; XP_293786	GTP-binding nuclear protein Ran (GTPase Ran) (Ras-related nuclear protein) (Ras-like protein TC4) (Androgen receptor-associated protein 24)	GO:0006259; GO:0005525; GO:0003924; GO:0006405; GO:0050681; GO:0030521; GO:0003682; GO:0005829; GO:0044419; GO:0042470; GO:0007067; GO:0007052; GO:0005643; GO:0005654; GO:0045893; GO:0006611; GO:0003713	DNA metabolic process; GTP binding; GTPase activity; RNA export from nucleus; androgen receptor binding; androgen receptor signaling pathway; chromatin binding; cytosol; interspecies interaction between organisms; melanosome; mitosis; mitotic spindle organization; nuclear pore; nucleoplasm; positive regulation of transcription, DNA-dependent; protein export from nucleus; transcription coactivator activity	NucleusCytoplasmMela nosome			Known in Uniprot to be phosphorylated		
**708**	SMC2	SMC2_HUMAN	SMC2; Q05BV1; Q6IEE0; Q7Z2X1; Q05D74	O95347; Q6IEE0; Q9P1P2; Q05BV1; Q05D74; Q7Z2X1	NP_006435; NP_001036015; NP_001036016	Structural maintenance of chromosomes protein 2 (Chromosome-associated protein E) (hCAP-E) (XCAP-E homolog); SMC2 protein (Fragment)	GO:0005524; GO:0051301; GO:0000796; GO:0005737; GO:0007076; GO:0000228; GO:0046982; GO:0005694; GO:0051276; GO:0005515;	ATP binding; cell division; condensin complex; cytoplasm; mitotic chromosome condensation; nuclear chromosome; protein heterodimerization activity; chromosome; chromosome organization; protein binding;	NucleusCytoplasm;					
**709**	CAZA2	CAZA2_HUMAN	CAZA2; A4D0V4	P47755; A4D0V4	NP_006127	F-actin-capping protein subunit alpha-2 (CapZ alpha-2); Capping protein (Actin filament) muscle Z-line, alpha 2 (Capping protein (Actin filament) muscle Z-line, alpha 2, isoform CRA_b) (cDNA, FLJ95684, Homo sapiens capping protein (actin filament) muscle Z-line, alpha2 (CAPZA2), mRNA)	GO:0008290; GO:0003779; GO:0051016; GO:0006928; GO:0005737; GO:0030036	F-actin capping protein complex; actin binding; barbed-end actin filament capping; cell motion; cytoplasm; actin cytoskeleton organization						
**710**	TCPB	TCPB_HUMAN	TCPB	P78371; Q14D36; Q6IAT3	NP_006422	T-complex protein 1 subunit beta (TCP-1-beta) (CCT-beta)	GO:0005524; GO:0005634; GO:0006457; GO:0051082	ATP binding; nucleus; protein folding; unfolded protein binding	Cytoplasm					
**711**	TCPH	TCPH_HUMAN	TCPH; A8K7E6; B7WNW9	Q99832; Q6FI26; O14871; A8K7E6; B7WNW9	NP_006420; NP_001009570	T-complex protein 1 subunit eta (TCP-1-eta) (CCT-eta) (HIV-1 Nef-interacting protein)	GO:0005524; GO:0005737; GO:0006457; GO:0051082	ATP binding; cytoplasm; protein folding; unfolded protein binding	Cytoplasm					
**712**	DCTN2	DCTN2_HUMAN	DCTN2; B2RBK5	Q13561; Q86YN2; Q9BW17; B2RBK5	NP_006391	Dynactin subunit 2 (Dynactin complex 50 kDa subunit) (DCTN-50) (50 kDa dynein-associated polypeptide) (p50 dynamitin)	GO:0008283; GO:0005813; GO:0005869; GO:0030286; GO:0000776; GO:0016020; GO:0005874; GO:0007017; GO:0007067; GO:0003774; GO:0005515	cell proliferation; centrosome; dynactin complex; dynein complex; kinetochore; membrane; microtubule; microtubule-based process; mitosis; motor activity; protein binding	Cytoplasmcytoskeleton MembranePeripheralm embraneprotein			Known in Uniprot to be phosphorylated	Known in HPRD to be phosphorylated	
**713**	GCP3	GCP3_HUMAN	GCP3	Q96CW5; O43631; O60852; O60853; Q7Z4K1; Q96I79	XP_297109; NP_006313	Gamma-tubulin complex component 3 (GCP-3) (hGCP3) (Spindle pole body protein Spc98 homolog) (hSpc98) (h104p)	GO:0005814; GO:0043015; GO:0007020; GO:0005827; GO:0005200	centriole; gamma-tubulin binding; microtubule nucleation; polar microtubule; structural constituent of cytoskeleton	Centrosome					
**714**	LASP1	LASP1_HUMAN	LASP1	Q14847; Q96ED2; Q96IG0	NP_006139	LIM and SH3 domain protein 1 (LASP-1) (MLN 50)	GO:0005070; GO:0030864; GO:0015075; GO:0006811; GO:0008270	SH3/SH2 adaptor activity; cortical actin cytoskeleton; ion transmembrane transporter activity; ion transport; zinc ion binding	CytoplasmcellcortexCy toplasmcytoskeleton			Known in Uniprot to be phosphorylated	Known in HPRD to be phosphorylated	
**715**	LYPA1	LYPA1_HUMAN	LYPA1; Q6IAQ1	O75608; Q6IAQ1; O43202; Q9UQF9	NP_006321	Acyl-protein thioesterase 1 (EC 3.1.2.-) (Lysophospholipase 1) (Lysophospholipase I); LYPLA1 protein (cDNA FLJ78542, highly similar to Homo sapiens lysophospholipase I (LYPLA1), mRNA) (Lysophospholipase I, isoform CRA_a) (cDNA, FLJ94339, Homo sapiens lysophospholipase I (LYPLA1), mRNA)	GO:0005737; GO:0006631; GO:0004622; GO:0016787	cytoplasm; fatty acid metabolic process; lysophospholipase activity; hydrolase activity	Cytoplasm;					
**716**	NEDD8	NEDD8_HUMAN	NEDD8	Q15843; Q3SXN8; Q6LES6	NP_006147	NEDD8 (Ubiquitin-like protein Nedd8) (Neddylin) (Neural precursor cell expressed developmentally down-regulated protein 8) (NEDD-8)	GO:0009653; GO:0005634; GO:0005515; GO:0045116; GO:0006511	anatomical structure morphogenesis; nucleus; protein binding; protein neddylation; ubiquitin-dependent protein catabolic process	Nucleus					
**717**	OXRP	OXRP_HUMAN	OXRP; Q2I204; HYOU1; A8C1Z0	Q9Y4L1; Q2I204; Q53H25; A8C1Z0	NP_006380; NP_001124463	Hypoxia up-regulated protein 1 (150 kDa oxygen-regulated protein) (Orp150)	GO:0005524; GO:0005783; GO:0005788; GO:0005515	ATP binding; endoplasmic reticulum; endoplasmic reticulum lumen; protein binding	Endoplasmicreticuluml umen					
**718**	PCBP1	PCBP1_HUMAN	PCBP1; Q53SS8	Q15365; Q13157; Q14975; Q53SS8	NP_006187	Poly(rC)-binding protein 1 (Alpha-CP1) (hnRNP-E1) (Nucleic acid-binding protein SUB2.3)	GO:0003723; GO:0005737; GO:0000398; GO:0005634; GO:0005515; GO:0030529; GO:0003697	RNA binding; cytoplasm; nuclear mRNA splicing, via spliceosome; nucleus; protein binding; ribonucleoprotein complex; single-stranded DNA binding	NucleusCytoplasm			Known in Uniprot to be phosphorylated	Known in HPRD to be phosphorylated	
**719**	PIN1	PIN1_HUMAN	PIN1; Q53X75; A8K4V9	Q13526; Q53X75; A8K4V9	NP_006212	Peptidyl-prolyl cis-trans isomerase NIMA-interacting 1 (EC 5.2.1.8) (Rotamase Pin1) (PPIase Pin1)	GO:0070412; GO:0030512; GO:0005634; GO:0003755; GO:0050815; GO:0050816; GO:0051443; GO:0006457; GO:0007088; GO:0060393	R-SMAD binding; negative regulation of transforming growth factor beta receptor signaling pathway; nucleus; peptidyl-prolyl cis-trans isomerase activity; phosphoserine binding; phosphothreonine binding; positive regulation of ubiquitin-protein ligase activity; protein folding; regulation of mitosis; regulation of pathway-restricted SMAD protein phosphorylation	Nucleus			Known in Uniprot to be phosphorylated		
**720**	PKN2	PKN2_HUMAN	PKN2	Q16513; Q9H1W4	NP_006247	Serine/threonine-protein kinase N2 (EC 2.7.11.13) (Protein kinase C-like 2) (Protein-kinase C-related kinase 2)	GO:0005524; GO:0005737; GO:0006468; GO:0004697; GO:0007165	ATP binding; cytoplasm; protein amino acid phosphorylation; protein kinase C activity; signal transduction	Cytoplasm			Known in Uniprot to be phosphorylated	Known in HPRD to be phosphorylated	Kinase
**721**	PSME1	PSME1_HUMAN	PSME1; Q6IBM2	Q06323; Q6IBM2; Q9UEF4	NP_006254; NP_788955	Proteasome activator complex subunit 1 (Proteasome activator 28 subunit alpha) (PA28alpha) (PA28a) (Activator of multicatalytic protease subunit 1) (11S regulator complex subunit alpha) (REG-alpha) (Interferon gamma up-regulated I-5111 protein) (IGUP I-5111); PSME1 protein (Proteasome (Prosome, macropain) activator subunit 1 (PA28 alpha), isoform CRA_a) (cDNA, FLJ92500, Homo sapiens proteasome (prosome, macropain) activator subunit 1 (PA28 alpha) (PSME1), mRNA)	GO:0031145; GO:0005829; GO:0051436; GO:0005634; GO:0051437; GO:0008538; GO:0008537; GO:0005829	anaphase-promoting complex-dependent proteasomal ubiquitin-dependent protein catabolic process; cytosol; negative regulation of ubiquitin-protein ligase activity during mitotic cell cycle; nucleus; positive regulation of ubiquitin-protein ligase activity during mitotic cell cycle; proteasome activator activity; proteasome activator complex; cytosol						

	Protein Name	Uniprot Name	Other Names	Uniprot IDs	Refseq IDs	Protein Description	GO ID	GO terms	Subcellular location	Comments	Comments	Comments - Database Search	Comments - Database Search	Function
**722**	IPO7	IPO7_HUMAN	IPO7	O95373; A6NNM5; B2R786; Q1RMF7; Q9H177; Q9NTE3	NP_006382	Importin-7 (Imp7) (Ran-binding protein 7) (RanBP7)	GO:0008536; GO:0005737; GO:0044419; GO:0005643; GO:0000059; GO:0008565; GO:0007165; GO:0005083; GO:0005625	Ran GTPase binding; cytoplasm; interspecies interaction between organisms; nuclear pore; protein import into nucleus, docking; protein transporter activity; signal transduction; small GTPase regulator activity; soluble fraction	CytoplasmNucleus			Known in Uniprot to be phosphorylated		
**723**	RRAS	RRAS_HUMAN	RRAS; Q6FH12	P10301; Q6FH12	NP_006261	Ras-related protein R-Ras (p23); RRAS protein (Related RAS viral (R-ras) oncogene homolog, isoform CRA_a)	GO:0005525; GO:0003924; GO:0007265; GO:0005622; GO:0005886; GO:0005515; GO:0030336; GO:0007264	GTP binding; GTPase activity; Ras protein signal transduction; intracellular; plasma membrane; protein binding; negative regulation of cell migration; small GTPase mediated signal transduction	CellmembraneLipidanchorCytoplasmicside;				known in HPRD to be phosphorylated	
**724**	1433S	1433S_HUMAN	1433S	P31947; Q6FH30; Q6FH51; Q96DH0	XP_059066; NP_006133	14-3-3 protein sigma (Stratifin) (Epithelial cell marker protein 1)	GO:0008630; GO:0005737; GO:0005615; GO:0043154; GO:0006469; GO:0005634; GO:0019904; GO:0008426; GO:0001836	DNA damage response, signal transduction resulting in induction of apoptosis; cytoplasm; extracellular space; negative regulation of caspase activity; negative regulation of protein kinase activity; nucleus; protein domain specific binding; protein kinase C inhibitor activity; release of cytochrome c from mitochondria	CytoplasmNucleusSecreted			Known in Uniprot to be phosphorylated		
**725**	SSRD	SSRD_HUMAN	SSRD; Q53XY1; A8K378	P51571; Q53XY1; A8K378	NP_006271	Translocon-associated protein subunit delta (TRAP-delta) (Signal sequence receptor subunit delta) (SSR-delta)	GO:0005509; GO:0016021; GO:0006886; GO:0005515; GO:0005784	calcium ion binding; integral to membrane; intracellular protein transport; protein binding; translocon complex	EndoplasmicreticulummembraneSinglepasstypeImembraneprotein					
**726**	UCR6	UCR6_HUMAN	UCR6; Q6FGD1; QCR7	P14927; Q6FGD1	NP_006285	Cytochrome b-c1 complex subunit 7 (Ubiquinol-cytochrome c reductase complex 14 kDa protein) (Complex III subunit 7) (Complex III subunit VII) (QP-C); UQCRB protein (Ubiquinol-cytochrome c reductase binding protein)	GO:0009060; GO:0006122; GO:0005746; GO:0006810; GO:0008121; GO:0006122	aerobic respiration; mitochondrial electron transport, ubiquinol to cytochrome c; mitochondrial respiratory chain; transport; ubiquinol-cytochrome-c reductase activity; mitochondrial electron transport, ubiquinol to cytochrome c	Mitochondrioninnermembrane;					
**727**	TPP1	TPP1_HUMAN	TPP1	O14773; Q53HT1; Q5JAK6; Q6UX56; Q71JP6; Q96C37	NP_000382	Tripeptidyl-peptidase 1 (TPP-1) (EC 3.4.14.9) (Tripeptidyl-peptidase I) (TPP-I) (Tripeptidyl aminopeptidase) (Lysosomal pepstatin-insensitive protease) (LPIC) (Cell growth-inhibiting gene 1 protein)	GO:0045453; GO:0006629; GO:0005764; GO:0042470; GO:0007399; GO:0043171; GO:0005515; GO:0030163; GO:0006508; GO:0004252; GO:0005625; GO:0008240	bone resorption; lipid metabolic process; lysosome; melanosome; nervous system development; peptide catabolic process; protein binding; protein catabolic process; proteolysis; serine-type endopeptidase activity; soluble fraction; tripeptidyl-peptidase activity	LysosomeMelanosome					
**728**	CSN8	CSN8_HUMAN	CSN8; Q53QS9	Q99627; Q53QS9	NP_006701; NP_937832	COP9 signalosome complex subunit 8 (Signalosome subunit 8) (SGN8) (JAB1-containing signalosome subunit 8) (COP9 homolog) (hCOP9); COP9 constitutive photomorphogenic homolog subunit 8 (Arabidopsis), isoform CRA_a (Putative uncharacterized protein COP9)	GO:0005737; GO:0008180;	cytoplasm; signalosome;	CytoplasmNucleus;			Known in Uniprot to be phosphorylated		
**729**	NUDT3	NUDT3_HUMAN	NUDT3; B2R8N4	O95989; B2R8N4	NP_006694	Diphosphoinositol polyphosphate phosphohydrolase 1 (DIPP-1) (EC 3.6.1.52) (Diadenosine 5',5'''-P1,P6-hexaphosphate hydrolase 1) (EC 3.6.1.-) (Nucleoside diphosphate-linked moiety X motif 3) (Nudix motif 3)	GO:0007267; GO:0005737; GO:0015961; GO:0008486; GO:0000287	cell-cell signaling; cytoplasm; diadenosine polyphosphate catabolic process; diphosphoinositol-polyphosphate diphosphatase activity; magnesium ion binding	Cytoplasm					
**730**	CD226	CD226_HUMAN	CD226; B2R818	Q15762; B2R818	NP_006557	CD226 antigen (DNAX accessory molecule 1) (DNAM-1) (CD antigen CD226)	GO:0007155; GO:0050839; GO:0008037; GO:0009986; GO:0005887; GO:0005178; GO:0045121; GO:0060369; GO:0002891; GO:0033005; GO:0002860; GO:0019901; GO:0004872	cell adhesion; cell adhesion molecule binding; cell recognition; cell surface; integral to plasma membrane; integrin binding; membrane raft; positive regulation of Fc receptor mediated stimulatory signaling pathway; positive regulation of immunoglobulin mediated immune response; positive regulation of mast cell activation; positive regulation of natural killer cell mediated cytotoxicity directed against tumor cell target; protein kinase binding; receptor activity	MembraneSinglepasstypeImembraneprotein			Known in Uniprot to be phosphorylated		
**731**	LGUL	LGUL_HUMAN	LGUL	Q04760; B2R6P7; P78375; Q5TZW3; Q96FC0; Q96J41	NP_006699	Lactoylglutathione lyase (EC 4.4.1.5) (Methylglyoxalase) (Aldoketomutase) (Glyoxalase I) (Glx I) (Ketone-aldehyde mutase) (S-D-lactoylglutathione methylglyoxal lyase)	GO:0006916; GO:0005975; GO:0005737; GO:0004462; GO:0008270	anti-apoptosis; carbohydrate metabolic process; cytoplasm; lactoylglutathione lyase activity; zinc ion binding						
**732**	PMVK	PMVK_HUMAN	PMVK; Q6FGV9	Q15126; Q6FGV9; Q5TZW9	NP_006547	Phosphomevalonate kinase (PMKase) (EC 2.7.4.2); PMVK protein (Phosphomevalonate kinase) (Phosphomevalonate kinase, isoform CRA_a) (cDNA, FLJ93228, Homo sapiens phosphomevalonate kinase (PMVK), mRNA) (Fragment)	GO:0006695; GO:0005777; GO:0004631; GO:0006468; GO:0005515; GO:0005737	cholesterol biosynthetic process; peroxisome; phosphomevalonate kinase activity; protein amino acid phosphorylation; protein binding; cytoplasm	Peroxisome;					Kinase
**733**	PRS6B	PRS6B_HUMAN	PRS6B; A8K2M0	P43686; Q96FV5; Q9UBM3; Q9UEX3; A8K2M0	NP_006494; NP_694546	26S protease regulatory subunit 6B (Proteasome 26S subunit ATPase 4) (MB67-interacting protein) (MIP224) (TAT-binding protein 7) (TBP-7)	GO:0005524; GO:0016887; GO:0031145; GO:0005829; GO:0051436; GO:0005634; GO:0051437; GO:0000502; GO:0005515	ATP binding; ATPase activity; anaphase-promoting complex-dependent proteasomal ubiquitin-dependent protein catabolic process; cytosol; negative regulation of ubiquitin-protein ligase activity during mitotic cell cycle; nucleus; positive regulation of ubiquitin-protein ligase activity during mitotic cell cycle; proteasome complex; protein binding	CytoplasmNucleus			Known in Uniprot to be phosphorylated		
**734**	RRAGA	RRAGA_HUMAN	RRAGA; B2R7L1	Q7L523; O00290; Q15347; B2R7L1	NP_006561	Ras-related GTP-binding protein A (Rag A) (RagA) (Adenovirus E3 14.7 kDa-interacting protein 1) (FIP-1)	GO:0005525; GO:0005794; GO:0006915; GO:0007166; GO:0005634; GO:0051219; GO:0045919; GO:0046982; GO:0042803; GO:0019048	GTP binding; Golgi apparatus; apoptosis; cell surface receptor linked signal transduction; nucleus; phosphoprotein binding; positive regulation of cytolysis; protein heterodimerization activity; protein homodimerization activity; virus-host interaction	CytoplasmNucleus					
**735**	SRCAP	SRCAP_HUMAN	Q9Y5L9; SRCAP	Q9Y5L9; Q6ZRS2; O15026; Q7Z744	XP_297088; NP_006653	Helicase SRCAP (EC 3.6.1.-) (Snf2-related CBP activator) (Domino homolog 2)	GO:0005524; GO:0003677; GO:0016568; GO:0004386; GO:0004402; GO:0044419; GO:0005634; GO:0006357; GO:0003713	ATP binding; DNA binding; chromatin modification; helicase activity; histone acetyltransferase activity; interspecies interaction between organisms; nucleus; regulation of transcription from RNA polymerase II promoter; transcription coactivator activity	Nucleus			Known in Uniprot to be phosphorylated		
**736**	DEST	DEST_HUMAN	DEST; B2R6N2	P60981; B2R6N2; P18282; Q5W166; Q6IAW2	NP_006861; XP_088393; XP_067423; NP_001011 546	Destrin (Actin-depolymerizing factor) (ADF)	GO:0003779; GO:0051014	actin binding; actin filament severing				Known in Uniprot to be phosphorylated	Known in HPRD to be phosphorylated	
**737**	PRDX3	PRDX3_HUMAN	PRDX3	P30048; B2R7Z0; P35690; Q0D2H1; Q13776; Q5T5V2; Q96HK4	NP_006784; NP_054817	Thioredoxin-dependent peroxide reductase, mitochondrial (EC 1.11.1.15) (Peroxiredoxin-3) (PRX III) (Antioxidant protein 1) (AOP-1) (Protein MER5 homolog) (HBC189)	GO:0008785; GO:0043027; GO:0045454; GO:0034614; GO:0005769; GO:0042744; GO:0005739; GO:0007005; GO:0030099; GO:0043066; GO:0033673; GO:0055114; GO:0051920; GO:0051092; GO:0008284; GO:0008022; GO:0019901; GO:0051881; GO:0032496	alkyl hydroperoxide reductase activity; caspase inhibitor activity; cell redox homeostasis; cellular response to reactive oxygen species; early endosome; hydrogen peroxide catabolic process; mitochondrion; mitochondrion organization; myeloid cell differentiation; negative regulation of apoptosis; negative regulation of kinase activity; oxidation reduction; peroxiredoxin activity; positive regulation of NF-kappaB transcription factor activity; positive regulation of cell proliferation; protein C-terminus binding; protein kinase binding; regulation of mitochondrial membrane potential; response to lipopolysaccharide	Mitochondrion					
**738**	ABEC2	ABEC2_HUMAN	ABEC2; B2R899	Q9Y235; Q53F28; Q5TGU5; Q5TGU6; B2R899	NP_006780	Probable C->U-editing enzyme APOBEC-2 (EC 3.5.4.-)	GO:0003723; GO:0004126; GO:0006397; GO:0008270	RNA binding; cytidine deaminase activity; mRNA processing; zinc ion binding						
**739**	DIAP2	DIAP2_HUMAN	DIAP2	O60879; A6NG19; O60878; Q8WX06; Q8WX48; Q9UJL2	NP_006720; NP_009293	Protein diaphanous homolog 2 (Diaphanous-related formin-2) (DRF2)	GO:0005794; GO:0017048; GO:0003779; GO:0030036; GO:0030154; GO:0000910; GO:0005769; GO:0007275; GO:0048477; GO:0005102	Golgi apparatus; Rho GTPase binding; actin binding; actin cytoskeleton organization; cell differentiation; cytokinesis; early endosome; multicellular organismal development; oogenesis; receptor binding	CytoplasmcytosolEarly endosome			Known in Uniprot to be phosphorylated		
**740**	ENOG	ENOG_HUMAN	ENOG; Q6FHV6	P09104; Q6FHV6; Q96J33	NP_001966	Gamma-enolase (EC 4.2.1.11) (2-phospho-D-glycerate hydro-lyase) (Neural enolase) (Neuron-specific enolase) (NSE) (Enolase 2); Enolase (EC 4.2.1.11)	GO:0006096; GO:0000287; GO:0004634; GO:0000015; GO:0005886	glycolysis; magnesium ion binding; phosphopyruvate hydratase activity; phosphopyruvate hydratase complex; plasma membrane	CytoplasmCellmembrane;			Known in Uniprot to be phosphorylated		
**741**	ERP29	ERP29_HUMAN	ERP29	P30040; Q3MJC3; Q6FHT4	NP_006808	Endoplasmic reticulum protein ERp29 (ERp31) (ERp28)	GO:0005783; GO:0005788; GO:0006886; GO:0042470; GO:0003756; GO:0006457; GO:0009306	endoplasmic reticulum; endoplasmic reticulum lumen; intracellular protein transport; melanosome; protein disulfide isomerase activity; protein folding; protein secretion	EndoplasmicreticulumlumenMelanosome			Known in Uniprot to be phosphorylated		
**742**	ROA0	ROA0_HUMAN	ROA0; Q6IB18	Q13151; Q6IB18	NP_006796	Heterogeneous nuclear ribonucleoprotein A0 (hnRNP A0); HNRPA0 protein (Heterogeneous nuclear ribonucleoprotein A0)	GO:0003723; GO:0030530; GO:0000398; GO:0000166; GO:0003676; GO:0030529	RNA binding; heterogeneous nuclear ribonucleoprotein complex; nuclear mRNA splicing, via spliceosome; nucleotide binding; nucleic acid binding; ribonucleoprotein complex	Nucleus;			Known in Uniprot to be phosphorylated		

	Protein Name	Uniprot Name	Other Names	Uniprot IDs	Refseq IDs	Protein Description	GO ID	GO terms	Subcellular location	Comments	Comments	Comments - Database Search	Comments - Database Search	Function
**743**	IMMT	IMMT_HUMAN	IMMT; B1H0U5	Q16891; Q14539; Q15092; Q68D41; Q69HW5; Q6IBL0; Q7Z3X1; Q8TAJ5; Q9P0V2; B1H0U5	NP_006830; NP_001093639; NP_001093640	Mitochondrial inner membrane protein (Mitofilin) (p87/89) (Cell proliferation-inducing gene 4 protein)	GO:0031305; GO:0005515	integral to mitochondrial inner membrane; protein binding	Mitochondrioninnermembrane					
**744**	PDIA5	PDIA5_HUMAN	PDIA5	Q14554	NP_006801	Protein disulfide-isomerase A5 (EC 5.3.4.1) (Protein disulfide isomerase-related protein)	GO:0045454; GO:0005783; GO:0005788; GO:0055114; GO:0016491; GO:0003756; GO:0006457; GO:0006950	cell redox homeostasis; endoplasmic reticulum; endoplasmic reticulum lumen; oxidation reduction; oxidoreductase activity; protein disulfide isomerase activity; protein folding; response to stress	Endoplasmicreticulumlumen					
**745**	RAB32	RAB32_HUMAN	RAB32	Q13637	NP_006825	Ras-related protein Rab-32	GO:0005525; GO:0005739; GO:0015031; GO:0007264	GTP binding; mitochondrion; protein transport; small GTPase mediated signal transduction	Mitochondrion			Known in Uniprot to be phosphorylated		
**746**	RAB35	RAB35_HUMAN	RAB35; B2R6E0	Q15286; B2R6E0	NP_006852	Ras-related protein Rab-35 (Rab-1C) (GTP-binding protein RAY)	GO:0005525; GO:0003924; GO:0031253; GO:0045334; GO:0005905; GO:0000910; GO:0016197; GO:0045171; GO:0042470; GO:0005546; GO:0015031; GO:0007264	GTP binding; GTPase activity; cell projection membrane; clathrin-coated endocytic vesicle; coated pit; cytokinesis; endosome transport; intercellular bridge; melanosome; phosphatidylinositol-4,5-bisphosphate binding; protein transport; small GTPase mediated signal transduction	CellmembraneLipidanchorCytoplasmicsideMelanosome					
**747**	TMED2	TMED2_HUMAN	TMED2; Q6FHT8	Q15363; Q6FHT8	XP_067504; NP_006806	Transmembrane emp24 domain-containing protein 2 (Membrane protein p24A); RNP24 protein (Transmembrane emp24 domain trafficking protein 2, isoform CRA_a) (cDNA, FLJ93436, Homo sapiens coated vesicle membrane protein (RNP24), mRNA)	GO:0005793; GO:0000139; GO:0016021; GO:0005792; GO:0005515; GO:0015031; GO:0016192; GO:0042589; GO:0016021; GO:0006810	ER-Golgi intermediate compartment; Golgi membrane; integral to membrane; microsome; protein binding; protein transport; vesicle-mediated transport; zymogen granule membrane; integral to membrane; transport	CytoplasmicvesiclemembraneSinglepasstypeImembraneproteinCytoplasmicvesicleCOPIcoatedvesiclemembraneSinglepasstypeImembraneproteinGolgiapparatusmembraneSinglepasstypeImembraneprotein; MembraneSinglepasstypeImembraneprotein					
**748**	SF3A3	SF3A3_HUMAN	SF3A3; B3KY12	Q12874; Q5VT87; Q15460; B3KY12	NP_006793	Splicing factor 3A subunit 3 (Spliceosome-associated protein 61) (SAP 61) (SF3a60)	GO:0031202; GO:0000389; GO:0016607; GO:0003676; GO:0005515; GO:0005681; GO:0008270	RNA splicing factor activity, transesterification mechanism; nuclear mRNA 3'-splice site recognition; nuclear speck; nucleic acid binding; protein binding; spliceosome; zinc ion binding	Nucleusspeckle			Known in Uniprot to be phosphorylated		
**749**	STIP1	STIP1_HUMAN	STIP1; Q5TZU9	P31948; Q5TZU9	NP_006810	Stress-induced-phosphoprotein 1 (STI1) (Hsc70/Hsp90-organizing protein) (Hop) (Transformation-sensitive protein IEF SSP 3521) (NY-REN-11 antigen)	GO:0005794; GO:0005634; GO:0005515; GO:0006950	Golgi apparatus; nucleus; protein binding; response to stress	CytoplasmNucleus			Known in Uniprot to be phosphorylated		
**750**	TALDO	TALDO_HUMAN	TALDO	P37837; Q8WV32; B2R8M2; O00751	NP_006746	Transaldolase (EC 2.2.1.2)	GO:0005829; GO:0006098; GO:0005515; GO:0004801	cytosol; pentose-phosphate shunt; protein binding; transaldolase activity	Cytoplasm			Known in Uniprot to be phosphorylated		
**751**	UCR11	UCR11_HUMAN	UCR11; QCR10; B2R542	O14957; Q9UEA3; Q9UPK4; B2R542	NP_006821	Cytochrome b-c1 complex subunit 10 (Ubiquinol-cytochrome c reductase complex 6.4 kDa protein) (Complex III subunit XI) (Complex III subunit 10)	GO:0009055; GO:0016021; GO:0006122; GO:0005743; GO:0006810; GO:0008121	electron carrier activity; integral to membrane; mitochondrial electron transport, ubiquinol to cytochrome c; mitochondrial inner membrane; transport; ubiquinol-cytochrome-c reductase activity	Mitochondrioninnermembrane					
**752**	1433T	1433T_HUMAN	1433T	P27348; Q567U5; Q5TZU8; Q9UP48	NP_006817	14-3-3 protein theta (14-3-3 protein tau) (14-3-3 protein T-cell) (Protein HS1)	GO:0005813; GO:0045892; GO:0005634; GO:0047485; GO:0019904	centrosome; negative regulation of transcription, DNA-dependent; nucleus; protein N-terminus binding; protein domain specific binding	Cytoplasm			Known in Uniprot to be phosphorylated	Known in HPRD to be phosphorylated	
**753**	CDC37	CDC37_HUMAN	CDC37; Q53YA2	Q16543; Q53YA2	NP_008996	Hsp90 co-chaperone Cdc37 (Hsp90 chaperone protein kinase-targeting subunit) (p50Cdc37); CDC37 cell division cycle 37 homolog (S. cerevisiae) (CDC37 cell division cycle 37 homolog (S. cerevisiae), isoform CRA_a)	GO:0006605; GO:0000079; GO:0060334; GO:0060338; GO:0051082; GO:0051879; GO:0051301; GO:0005829	protein targeting; regulation of cyclin-dependent protein kinase activity; regulation of interferon-gamma-mediated signaling pathway; regulation of type I interferon-mediated signaling pathway; unfolded protein binding; Hsp90 protein binding; cell division; cytosol	Cytoplasm;			Known in Uniprot to be phosphorylated	Known in HPRD to be phosphorylated	
**754**	EMIL1	EMIL1_HUMAN	EMIL1; A5PL03	Q9Y6C2; Q53SY9; Q96G58; Q9UG76; A5PL03	NP_008977	EMILIN-1 (Elastin microfibril interface-located protein 1) (Elastin microfibril interfacer 1)	GO:0007155; GO:0005813; GO:0005634; GO:0005578	cell adhesion; centrosome; nucleus; proteinaceous extracellular matrix	Secretedextracellularspaceextracellularmatrix					
**755**	COR1A	COR1A_HUMAN	COR1A; B2RBL1	P31146; Q2YD73; B2RBL1	NP_009005	Coronin-1A (Coronin-like protein p57) (Coronin-like protein A) (Clipin-A) (Tryptophan aspartate-containing coat protein) (TACO)	GO:0051015; GO:0031589; GO:0030864; GO:0031941; GO:0045087; GO:0030027; GO:0051126; GO:0001891; GO:0045335; GO:0001845; GO:0043548; GO:0050918; GO:0008022; GO:0042803	actin filament binding; cell-substrate adhesion; cortical actin cytoskeleton; filamentous actin; innate immune response; lamellipodium; negative regulation of actin nucleation; phagocytic cup; phagocytic vesicle; phagolysosome formation; phosphoinositide 3-kinase binding; positive chemotaxis; protein C-terminus binding; protein homodimerization activity	CytoplasmcytoskeletonCytoplasmcellcortexCytoplasmicvesiclephagosomemembrane					
**756**	TBCD8	TBCD8_HUMAN	TBCD8	O95759; A6NDL4; Q53SQ4; Q9UQ32	NP_008994; NP_001095896	TBC1 domain family member 8 (Vascular Rab-GAP/TBC-containing protein) (AD 3)	GO:0005097; GO:0008015; GO:0005622; GO:0016020; GO:0008284; GO:0032313	Rab GTPase activator activity; blood circulation; intracellular; membrane; positive regulation of cell proliferation; regulation of Rab GTPase activity						
**757**	ATP5I	ATP5I_HUMAN	ATP5I	P56385; Q0D2L9	NP_009031	ATP synthase subunit e, mitochondrial	GO:0015986; GO:0015078; GO:0000276	ATP synthesis coupled proton transport; hydrogen ion transmembrane transporter activity; mitochondrial proton-transporting ATP synthase complex, coupling factor F(o)	MitochondrionMitochondrioninnermembrane			Known in Uniprot to be phosphorylated		
**758**	CHP1	CHP1_HUMAN	CHP1; Q96HL9; Q6FHZ9	Q99653; Q6FHZ9; B2R6H9	NP_009167	Calcium-binding protein p22 (Calcium-binding protein CHP) (Calcineurin homologous protein) (Calcineurin B homolog)	GO:0005509; GO:0005737; GO:0043231; GO:0015459; GO:0006813; GO:0007264	calcium ion binding; cytoplasm; intracellular membrane-bounded organelle; potassium channel regulator activity; potassium ion transport; small GTPase mediated signal transduction	Cytoplasm			Known in Uniprot to be phosphorylated		
**759**	DCTN3	DCTN3_HUMAN	DCTN3; B2RBM5	O75935; A6NII7; Q5T1I5; Q5T1I7; Q5T8G3; Q9BPU8; B2RBM5	NP_009165; NP_077324	Dynactin subunit 3 (Dynactin complex subunit 22 kDa subunit) (p22)	GO:0005813; GO:0000777; GO:0000910; GO:0005869; GO:0007067; GO:0048471; GO:0005515; GO:0005819; GO:0005198	centrosome; condensed chromosome kinetochore; cytokinesis; dynactin complex; mitosis; perinuclear region of cytoplasm; protein binding; spindle; structural molecule activity	CytoplasmCentrosomeKinetochoreSpindle					
**760**	GBRL2	GBRL2_HUMAN	GBRL2; Q6FG91	P60520; Q6FG91; O08765; Q9DCP8; Q9UQF7	NP_009216	Gamma-aminobutyric acid receptor-associated protein-like 2 (GABA(A) receptor-associated protein-like 2) (Golgi-associated ATPase enhancer of 16 kDa) (GATE-16) (General protein transport factor p16) (Ganglioside expression factor 2) (GEF-2) (MAP1 light chain 3-related protein); GABARAPL2 protein (cDNA FLJ76520, highly similar to Homo sapiens GABA(A) receptor-associated protein-like 2 (GABARAPL2), mRNA) (GABA(A) receptor-associated protein-like 2, isoform CRA_b)	GO:0051117; GO:0050811; GO:0000139; GO:0000149; GO:0000421; GO:0006914; GO:0048487; GO:0005829; GO:0006891; GO:0005624; GO:0008017; GO:0032781; GO:0015031; GO:0000139; GO:0005515; GO:0004872	ATPase binding; GABA receptor binding; Golgi membrane; SNARE binding; autophagic vacuole membrane; autophagy; beta-tubulin binding; cytosol; intra-Golgi vesicle-mediated transport; membrane fraction; microtubule binding; positive regulation of ATPase activity; protein transport; Golgi membrane; protein binding; receptor activity	Golgiapparatus;			Known in Uniprot to be phosphorylated		
**761**	PROL4	PROL4_HUMAN	PROL4	Q16378; A8KA69; Q8NFB3	NP_009175; NP_001092008	Proline-rich protein 4 (Lacrimal proline-rich protein) (Nasopharyngeal carcinoma-associated proline-rich protein 4)	GO:0005615; GO:0007601	extracellular space; visual perception	Secreted					
**762**	PACN2	PACN2_HUMAN	PACN2	Q9UNF0; O95921; Q96HV9; Q9H0D3; Q9NPN1; Q9Y4V2	NP_009160	Protein kinase C and casein kinase substrate in neurons protein 2	GO:0030036; GO:0031410; GO:0005215	actin cytoskeleton organization; cytoplasmic vesicle; transporter activity	Cytoplasmicvesicle			Known in Uniprot to be phosphorylated		
**763**	POLI	POLI_HUMAN	POLI	Q9UNA4; Q8N590; Q9H0S1; Q9NYH6	NP_009126	DNA polymerase iota (EC 2.7.7.7) (RAD30 homolog B) (Eta2)	GO:0006281; GO:0006260; GO:0003887; GO:0003684; GO:0000287; GO:0005654	DNA repair; DNA replication; DNA-directed DNA polymerase activity; damaged DNA binding; magnesium ion binding; nucleoplasm	Nucleus					
**764**	PHB2	PHB2_HUMAN	PHB2	Q99623; Q2YDA4; Q7KYU3; Q92978	NP_009204; NP_001138303	Prohibitin-2 (B-cell receptor-associated protein BAP37) (Repressor of estrogen receptor activity) (D-prohibitin)	GO:0030331; GO:0005743; GO:0016481; GO:0005634; GO:0004872; GO:0006355; GO:0016566	estrogen receptor binding; mitochondrial inner membrane; negative regulation of transcription; nucleus; receptor activity; regulation of transcription, DNA-dependent; specific transcriptional repressor activity	MitochondrioninnermembraneCytoplasmNucleus			Known in Uniprot to be phosphorylated		
**765**	RL35	RL35_HUMAN	RL35	P42766; A8K4V7; Q4VBY5; Q5JTN5; Q6IBC7; Q96QJ7; Q9BYF4	XP_294103; NP_009140	60S ribosomal protein L35	GO:0022625; GO:0003729; GO:0005730; GO:0005515; GO:0003735; GO:0006414	cytosolic large ribosomal subunit; mRNA binding; nucleolus; protein binding; structural constituent of ribosome; translational elongation						
**766**	STXB3	STXB3_HUMAN	STXB3; Q5VTL6	O00186; A8K269; A8K5K7; Q53FW1; Q86YJ3; Q9UPD7	NP_009200	Syntaxin-binding protein 3 (Unc-18 homolog 3) (Unc-18-3) (Unc-18C) (Platelet Sec1 protein) (PSP)	GO:0005829; GO:0016020; GO:0015031; GO:0006904	cytosol; membrane; protein transport; vesicle docking during exocytosis	CytoplasmcytosolCellmembrane			Known in Uniprot to be phosphorylated		

	Protein Name	Uniprot Name	Other Names	Uniprot IDs	Refseq IDs	Protein Description	GO ID	GO terms	Subcellular location	Comments	Comments	Comments - Database Search	Comments - Database Search	Function
**767**	ELOB	ELOB_HUMAN	ELOB	Q15370	NP_009039; XP_171447	Transcription elongation factor B polypeptide 2 (RNA polymerase II transcription factor SIII subunit B) (SIII p18) (Elongin B) (EloB) (Elongin 18 kDa subunit)	GO:0006368; GO:0005829; GO:0019941; GO:0005654; GO:0005515; GO:0006461; GO:0006464; GO:0006355	RNA elongation from RNA polymerase II promoter; cytosol; modification-dependent protein catabolic process; nucleoplasm; protein binding; protein complex assembly; protein modification process; regulation of transcription, DNA-dependent	Nucleus					
**768**	TERA	TERA_HUMAN	TERA; Q5VYM0; Q9NTC4; Q2TAI5	P55072; Q5VYM0; Q969G7; Q2TAI5; Q9NTC4	NP_009057	Transitional endoplasmic reticulum ATPase (TER ATPase) (15S Mg(2+)-ATPase p97 subunit) (Valosin-containing protein) (VCP); Putative uncharacterized protein DKFZp434K0126 (Fragment)	GO:0005524; GO:0016887; GO:0030433; GO:0006919; GO:0005829; GO:0006302; GO:0005783; GO:0030968; GO:0008289; GO:0005792; GO:0005634; GO:0019904; GO:0016567; GO:0030970; GO:0017111; GO:0005515	ATP binding; ATPase activity; ER-associated protein catabolic process; activation of caspase activity; cytosol; double-strand break repair; endoplasmic reticulum; endoplasmic reticulum unfolded protein response; lipid binding; microsome; nucleus; protein domain specific binding; protein ubiquitination; retrograde protein transport, ER to cytosol; nucleoside-triphosphatase activity; protein binding	CytoplasmcytosolNucleus;			Known in Uniprot to be phosphorylated		
**769**	NDUB4	NDUB4_HUMAN	NDUB4; B9EJC7	O95168; B9EJC7	NP_004538	NADH dehydrogenase [ubiquinone] 1 beta subcomplex subunit 4 (NADH-ubiquinone oxidoreductase B15 subunit) (Complex I-B15) (CI-B15)	GO:0008137; GO:0016021; GO:0006120; GO:0005747; GO:0006979; GO:0006810	NADH dehydrogenase (ubiquinone) activity; integral to membrane; mitochondrial electron transport, NADH to ubiquinone; mitochondrial respiratory chain complex I; response to oxidative stress; transport	MitochondrioninnermembraneSinglepassmembraneproteinMatrixside					
**770**	GDIB	GDIB_HUMAN	GDIB; Q6IAT1	P50395; Q6IAT1; O43928; Q9UQM6	NP_001485; NP_001108628	Rab GDP dissociation inhibitor beta (Rab GDI beta) (Guanosine diphosphate dissociation inhibitor 2) (GDI-2); GDI2 protein (GDP dissociation inhibitor 2) (GDP dissociation inhibitor 2 isoform 1) (GDP dissociation inhibitor 2, isoform CRA_b) (cDNA, FLJ92845, Homo sapiens GDP dissociation inhibitor 2 (GDI2), mRNA)	GO:0005096; GO:0005093; GO:0009986; GO:0005737; GO:0016020; GO:0015031; GO:0043087; GO:0007165; GO:0005093	GTPase activator activity; Rab GDP-dissociation inhibitor activity; cell surface; cytoplasm; membrane; protein transport; regulation of GTPase activity; signal transduction; Rab GDP-dissociation inhibitor activity	CytoplasmMembranePeripheralmembraneprotein;			Known in Uniprot to be phosphorylated		
**771**	NDUA9	NDUA9_HUMAN	NDUA9	Q16795; Q14076; Q2NKX0	NP_004993	NADH dehydrogenase [ubiquinone] 1 alpha subcomplex subunit 9, mitochondrial (NADH-ubiquinone oxidoreductase 39 kDa subunit) (Complex I-39kD) (CI-39kD)	GO:0008137; GO:0050662; GO:0006120; GO:0005759; GO:0005747; GO:0005515; GO:0006814	NADH dehydrogenase (ubiquinone) activity; coenzyme binding; mitochondrial electron transport, NADH to ubiquinone; mitochondrial matrix; mitochondrial respiratory chain complex I; protein binding; sodium ion transport	Mitochondrionmatrix					
**772**	PP2BA	PP2BA_HUMAN	PP2BA; A8W6Z7; B5BUA2	Q08209; Q8TAW9; A8W6Z7; B5BUA2	NP_000935; NP_001124163; NP_001124164	Serine/threonine-protein phosphatase 2B catalytic subunit alpha isoform (EC 3.1.3.16) (Calmodulin-dependent calcineurin A subunit alpha isoform) (CAM-PRP catalytic subunit)	GO:0005955; GO:0005509; GO:0005516; GO:0005506; GO:0005634; GO:0006470; GO:0004722; GO:0008270	calcineurin complex; calcium ion binding; calmodulin binding; iron ion binding; nucleus; protein amino acid dephosphorylation; protein serine/threonine phosphatase activity; zinc ion binding	Nucleus			Known in Uniprot to be phosphorylated		Phosphatase
**773**	GOGA4	GOGA4_HUMAN	GOGA4; Q86W71	Q13439; Q13270; Q13654; Q14436; Q86W71	NP_002069	Golgin subfamily A member 4 (Trans-Golgi p230) (256 kDa golgin) (Golgin-245) (Protein 72.1); GOLGA4 protein	GO:0000139; GO:0005802; GO:0016192; GO:0005794	Golgi membrane; trans-Golgi network; vesicle-mediated transport; Golgi apparatus	CytoplasmGolgiapparatusmembranePeripheralmembraneprotein;			Known in Uniprot to be phosphorylated	Known in HPRD to be phosphorylated	
**774**	HBG2	HBG2_HUMAN	HBG2; Q96FH6	P69892; Q96FH6; P02096; P62027; Q68NH9; Q96FH7	NP_000175	Hemoglobin subunit gamma-2 (Hemoglobin gamma-2 chain) (Gamma-2-globin) (Hemoglobin gamma-G chain) (Hb F Ggamma); Hemoglobin, gamma A (Hemoglobin, gamma G) (cDNA FLJ76540, highly similar to Homo sapiens hemoglobin, gamma G (HBG2), mRNA)	GO:0020037; GO:0005833; GO:0019825; GO:0015671; GO:0005344; GO:0005506	heme binding; hemoglobin complex; oxygen binding; oxygen transport; oxygen transporter activity; iron ion binding				Known in Uniprot to be phosphorylated		
**775**	PI52A	PI52A_HUMAN	PI52A; PI42A; B0YJ66	P48426; P53807; Q5VUX3; B0YJ66	NP_005019	Phosphatidylinositol-5-phosphate 4-kinase type-2 alpha (EC 2.7.1.149) (Phosphatidylinositol-5-phosphate 4-kinase type II alpha) (1-phosphatidylinositol-5-phosphate 4-kinase 2-alpha) (PtdIns(5)P-4-kinase isoform 2-alpha) (PIP4KII-alpha) (Diphosphoinositide kinase 2-alpha) (PtdIns(4)P-5-kinase B isoform) (PIP5KIII) (PtdIns(4)P-5-kinase C isoform)	GO:0016308; GO:0016309; GO:0005524; GO:0046488; GO:0016310	1-phosphatidylinositol-4-phosphate 5-kinase activity; 1-phosphatidylinositol-5-phosphate 4-kinase activity; ATP binding; phosphatidylinositol metabolic process; phosphorylation				Known in Uniprot to be phosphorylated		Kinase
**776**	PRDX5	PRDX5_HUMAN	PRDX5	P30044; Q14CK0; Q6IAF2; Q9UBU5; Q9UJU4; Q9UKX4	NP_036226; NP_857634; NP_857635	Peroxiredoxin-5, mitochondrial (EC 1.11.1.15) (Prx-V) (Peroxisomal antioxidant enzyme) (PLP) (Thioredoxin reductase) (Thioredoxin peroxidase PMP20) (Antioxidant enzyme B166) (AOEB166) (TPx type VI) (Liver tissue 2D-page spot 71B) (Alu corepressor 1)	GO:0043027; GO:0045454; GO:0034614; GO:0044445; GO:0006954; GO:0005739; GO:0043066; GO:0055114; GO:0051920; GO:0005777	caspase inhibitor activity; cell redox homeostasis; cellular response to reactive oxygen species; cytosolic part; inflammatory response; mitochondrion; negative regulation of apoptosis; oxidation reduction; peroxiredoxin activity; peroxisome	MitochondrionCytoplasmPeroxisome					
**777**	CHRD1	CHRD1_HUMAN	Q9UHD1; CHRD1	Q9UHD1; Q6IN49; Q8WVL9; Q9H3D6	NP_036256; NP_001137545	Cysteine and histidine-rich domain-containing protein 1 (CHORD domain-containing protein 1) (CHORD-containing protein 1) (CHP-1)						Known in Uniprot to be phosphorylated		
**778**	LMOD1	LMOD1_HUMAN	Q68EN2; LMOD1; B1APV6	P29536; Q68EN2; B1APV6	NP_036266	Leiomodin-1 (Leiomodin, muscle form) (64 kDa autoantigen D1) (64 kDa autoantigen 1D) (64 kDa autoantigen 1D3) (Thyroid-associated ophthalmopathy autoantigen) (Smooth muscle leiomodin) (SM-Lmod)	GO:0005737; GO:0005856; GO:0005624; GO:0005523	cytoplasm; cytoskeleton; membrane fraction; tropomyosin binding	CytoplasmCytoplasmcytoskeleton					
**779**	GRHPR	GRHPR_HUMAN	GRHPR; Q5T945	Q9UBQ7; Q5T945; Q9H3E9; Q9UKX1	NP_036335	Glyoxylate reductase/hydroxypyruvate reductase (EC 1.1.1.79); Glyoxylate reductase/hydroxypyruvate reductase (Glyoxylate reductase/hydroxypyruvate reductase, isoform CRA_b) (cDNA, FLJ96788, Homo sapiens glyoxylate reductase/hydroxypyruvate reductase(GRHPR), mRNA)	GO:0051287; GO:0005737; GO:0007588; GO:0008465; GO:0030267; GO:0016618; GO:0055114; GO:0005515; GO:0008152; GO:0016616	NAD or NADH binding; cytoplasm; excretion; glycerate dehydrogenase activity; glyoxylate reductase (NADP) activity; hydroxypyruvate reductase activity; oxidation reduction; protein binding; metabolic process; oxidoreductase activity, acting on the CH-OH group of donors, NAD or NADP as acceptor						
**780**	TF3C3	TF3C3_HUMAN	TF3C3; Q4ZG48	Q9Y5Q9; Q4ZG48; Q86XJ8; Q8WX84; Q96B44; Q9H5I8; Q9NT97	NP_036218	General transcription factor 3C polypeptide 3 (Transcription factor IIIC subunit gamma) (TF3C-gamma) (TFIIIC 102 kDa subunit) (TFIIIC102)	GO:0042791; GO:0003677; GO:0042797; GO:0000127	5S class rRNA transcription; DNA binding; tRNA transcription from RNA polymerase III promoter; transcription factor TFIIIC complex	Nucleus			Known in Uniprot to be phosphorylated		
**781**	MARE1	MARE1_HUMAN	MARE1; B2R6I7	Q15691; Q3KQS8; B2R6I7	NP_036457	Microtubule-associated protein RP/EB family member 1 (APC-binding protein EB1) (End-binding protein 1) (EB1)	GO:0051301; GO:0008283; GO:0030981; GO:0005874; GO:0051010; GO:0007067; GO:0031115; GO:0008022	cell division; cell proliferation; cortical microtubule cytoskeleton; microtubule; microtubule plus-end binding; mitosis; negative regulation of microtubule polymerization; protein C-terminus binding	Cytoplasm			Known in Uniprot to be phosphorylated		
**782**	MSRA	MSRA_HUMAN	MSRA; Q549N4	Q9UJ68; Q549N4	NP_036463; NP_001129142	Peptide methionine sulfoxide reductase (EC 1.8.4.11) (Protein-methionine-S-oxide reductase) (PMSR) (Peptide-methionine (S)-S-oxide reductase) (Peptide Met(O) reductase); Methionine sulfoxide reductase (Methionine sulfoxide reductase A, isoform CRA_b) (Methionine sulfoxide reductase A1)	GO:0006555; GO:0055114; GO:0008113; GO:0006464; GO:0006979; GO:0055114; GO:0019538	methionine metabolic process; oxidation reduction; peptide-methionine-(S)-S-oxide reductase activity; protein modification process; response to oxidative stress; oxidation reduction; protein metabolic process						
**783**	NUBP2	NUBP2_HUMAN	NUBP2	Q9Y5Y2; O9NWB2	NP_036357	Nucleotide-binding protein 2 (NBP 2)	GO:0005524; GO:0005634	ATP binding; nucleus	Nucleus					
**784**	6PGL	6PGL_HUMAN	6PGL	O95336	NP_036220	6-phosphogluconolactonase (6PGL) (EC 3.1.1.31)	GO:0017057; GO:0006098	6-phosphogluconolactonase activity; pentose-phosphate shunt						
**785**	SIRT4	SIRT4_HUMAN	SIRT4	Q9Y6E7; Q32M33; O43346	NP_036372	NAD-dependent deacetylase sirtuin-4 (EC 3.5.1.-) (SIR2-like protein 4)	GO:0070403; GO:0003950; GO:0006342; GO:0016811; GO:0006471; GO:0006476; GO:0008270	NAD binding; NAD+ ADP-ribosyltransferase activity; chromatin silencing; hydrolase activity, acting on carbon-nitrogen (but not peptide) bonds, in linear amides; protein amino acid ADP-ribosylation; protein amino acid deacetylation; zinc ion binding				Known in Uniprot to be phosphorylated		
**786**	TIM9	TIM9_HUMAN	TIM9; B2R584	Q9Y5J7; B2R584	NP_036592	Mitochondrial import inner membrane translocase subunit Tim9	GO:0065002; GO:0005744; GO:0042719; GO:0045039; GO:0007605; GO:0008270	intracellular protein transmembrane transport; mitochondrial inner membrane presequence translocase complex; mitochondrial intermembrane space protein transporter complex; protein import into mitochondrial inner membrane; sensory perception of sound; zinc ion binding	MitochondrioninnermembranePeripheralmembraneproteinIntermembraneside					
**787**	SYG	SYG_HUMAN	SYG	P41250; B3KQA2; B4DIA0; Q969Y1	NP_002038	Glycyl-tRNA synthetase (EC 6.1.1.14) (Glycine--tRNA ligase) (GlyRS)	GO:0005524; GO:0004820; GO:0006426; GO:0005625	ATP binding; glycine-tRNA ligase activity; glycyl-tRNA aminoacylation; soluble fraction				Known in Uniprot to be phosphorylated		
**788**	SYHC	SYHC_HUMAN	SYHC	P12081	NP_002100	Histidyl-tRNA synthetase, cytoplasmic (EC 6.1.1.21) (Histidine--tRNA ligase) (HisRS)	GO:0005524; GO:0005737; GO:0004821; GO:0006427	ATP binding; cytoplasm; histidine-tRNA ligase activity; histidyl-tRNA aminoacylation	Cytoplasm					
**789**	PDCD6	PDCD6_HUMAN	PDCD6; Q53FC3	O75340; Q5TZS0; B2RD16; Q53FC3	NP_037364	Programmed cell death protein 6 (Apoptosis-linked gene 2 protein) (Probable calcium-binding protein ALG-2); Programmed cell death 6 variant (Fragment)	GO:0005509; GO:0048306; GO:0005789; GO:0008624; GO:0031965; GO:0051592	calcium ion binding; calcium-dependent protein binding; endoplasmic reticulum membrane; induction of apoptosis by extracellular signals; nuclear membrane; response to calcium ion	NucleusmembranePeripheralmembraneproteinEndoplasmicreticulummembranePeripheralmembraneproteinNucleus;					
**790**	NENF	NENF_HUMAN	NENF; A1KYQ8	Q9UMX5; A1KYQ8; Q53FZ6; Q5TM90	NP_037481	Neudesin (Neuron-derived neurotrophic factor) (Secreted protein of unknown function) (SPUF protein) (Cell immortalization-related protein 2)	GO:0020037	heme binding	Secretedextracellularspace					
**791**	MYOF	MYOF_HUMAN	MYOF	Q9NZM1; B3KQN5; Q5VWW2; Q5VWW3; Q5VWW4; Q5VWW5; Q7Z642; Q8IWH0; Q9HBU3; Q9NZM0; Q9ULL3; Q9Y4U4	NP_038479; NP_579899	Myoferlin (Fer-1-like protein 3)	GO:0008015; GO:0030659; GO:0016021; GO:0006936; GO:0031965; GO:0005886; GO:0005515	blood circulation; cytoplasmic vesicle membrane; integral to membrane; muscle contraction; nuclear membrane; plasma membrane; protein binding	CellmembraneSinglepasstypeIImembraneproteinNucleusmembraneSinglepasstypeIImembraneproteinCytoplasmicvesiclemembraneSinglepasstypeIImembraneprotein			Known in Uniprot to be phosphorylated	Known in HPRD to be phosphorylated	

	Protein Name	Uniprot Name	Other Names	Uniprot IDs	Refseq IDs	Protein Description	GO ID	GO terms	Subcellular location	Comments	Comments	Comments - Database Search	Comments - Database Search	Function
**792**	NCKP1	NCKP1_HUMAN	NCKP1; Q53Y35	Q9Y2A7; Q53Y35; O60329; Q53QN5; Q53S94	NP_038464; NP_995314	Nck-associated protein 1 (NAP 1) (p125Nap1) (Membrane-associated protein HEM-2)	GO:0006915; GO:0042995; GO:0007417; GO:0016021; GO:0005886; GO:0005515	apoptosis; cell projection; central nervous system development; integral to membrane; plasma membrane; protein binding	CellmembraneSinglepassmembraneproteinCytoplasmicsideCellprojectionlamellipodiummembraneSinglepassmembraneproteinCytoplasmicside					
**793**	STML2	STML2_HUMAN	STML2	Q9UJZ1; O60376; Q53G29; Q96FY2; Q9P042	NP_038470	Stomatin-like protein 2 (SLP-2) (EPB72-like 2)	GO:0005856; GO:0005102	cytoskeleton; receptor binding	MembranePeripheralmembraneproteinCytoplasmCytoplasmcytoskeleton			Known in Uniprot to be phosphorylated		
**794**	CLIC4	CLIC4_HUMAN	CLIC4; Q5VSX5; Q6FIC5	Q9Y696; Q5VSX5; Q9UFW9; Q9UQJ6; Q6FIC5	NP_039234	Chloride intracellular channel protein 4 (Intracellular chloride ion channel protein p64H1)	GO:0015629; GO:0030154; GO:0031404; GO:0006821; GO:0031410; GO:0016020; GO:0005902; GO:0030336; GO:0005515; GO:0005625; GO:0005247	actin cytoskeleton; cell differentiation; chloride ion binding; chloride transport; cytoplasmic vesicle; membrane; microvillus; negative regulation of cell migration; protein binding; soluble fraction; voltage-gated chloride channel activity	CytoplasmCytoplasmicvesiclemembraneSinglepassmembraneproteinNucleusCellmembraneSinglepassmembraneprotein					
**795**	DDAH2	DDAH2_HUMAN	DDAH2; A2BEZ7	O95865; A2BEZ7	NP_039268	N(G),N(G)-dimethylarginine dimethylaminohydrolase 2 (Dimethylarginine dimethylaminohydrolase 2) (DDAH-2) (EC 3.5.3.18) (DDAHII) (Dimethylargininase-2) (S-phase protein) (Protein G6a)	GO:0006916; GO:0006527; GO:0016403; GO:0006809; GO:0007263	anti-apoptosis; arginine catabolic process; dimethylargininase activity; nitric oxide biosynthetic process; nitric oxide mediated signal transduction						
**796**	CYBP	CYBP_HUMAN	CYBP; Q5R371	Q9HB71; Q5R371; B2ZWH2; O60666	NP_055227	Calcyclin-binding protein (CacyBP) (hCacyBP) (Siah-interacting protein) (S100A6-binding protein); Calcyclin binding protein (Growth-inhibiting gene 5 protein) (cDNA, FLJ93790, Homo sapiens Siah-interacting protein (SIP), mRNA) (Calcyclin binding protein, isoform CRA_a)	GO:0005737; GO:0019941; GO:0005634; GO:0005515;	cytoplasm; modification-dependent protein catabolic process; nucleus; protein binding;	NucleusCytoplasm;			Known in Uniprot to be phosphorylated		
**797**	COR1C	COR1C_HUMAN	COR1C; B3KU12	Q9ULV4; Q9NSK5; B3KU12	NP_055140	Coronin-1C (Coronin-3) (hCRNN4)	GO:0003779; GO:0015629; GO:0006909; GO:0007165	actin binding; actin cytoskeleton; phagocytosis; signal transduction				Known in Uniprot to be phosphorylated		
**798**	TFIP8	TFIP8_HUMAN	Q9UP47; O95379; TFIP8; B3KMH1	Q9UER5; Q9UP47; O95379; B3KMI2; Q9P1Q1; B3KMH1	NP_055165; NP_001071122	Tumor necrosis factor, alpha-induced protein 8 (Head and neck tumor and metastasis-related protein) (TNF-induced protein GG2-1) (SCC-S2) (NF-kappa-B-inducible DED-containing protein) (NDED) (MDC-3.13)	GO:0043027; GO:0005737; GO:0019987; GO:0005515	caspase inhibitor activity; cytoplasm; negative regulation of anti-apoptosis; protein binding	Cytoplasm					
**799**	MSAP	MSAP_HUMAN	MSAP; CNPY2; B2R7B9	Q9Y2B0; Q9UHE9; B2R7B9	NP_055070	Protein canopy homolog 2 (MIR-interacting saposin like protein) (Transmembrane protein 4) (Putative secreted protein ZSIG9)	GO:0005783; GO:0005887; GO:0005515	endoplasmic reticulum; integral to plasma membrane; protein binding	Endoplasmicreticulum					
**800**	LSM1	LSM1_HUMAN	LSM1; B2R5E6	O15116; B2R5E6	NP_055277	U6 snRNA-associated Sm-like protein LSm1 (Cancer-associated Sm-like) (Small nuclear ribonuclear CaSm)	GO:0003723; GO:0008380; GO:0031202; GO:0005737; GO:0006397; GO:0005634; GO:0005515; GO:0030529	RNA binding; RNA splicing; RNA splicing factor activity, transesterification mechanism; cytoplasm; mRNA processing; nucleus; protein binding; ribonucleoprotein complex	Nucleus					
**801**	ORNT1	ORNT1_HUMAN	ORNT1; Q5VZD8	Q9Y619; Q5VZD8; Q9HC45	NP_055067	Mitochondrial ornithine transporter 1 (Solute carrier family 25 member 15); Solute carrier family 25 (Mitochondrial carrier; ornithine transporter) member 15	GO:0000064; GO:0006520; GO:0005488; GO:0016021; GO:0005743; GO:0000066; GO:0000050; GO:0005488; GO:0006810; GO:0005215	L-ornithine transmembrane transporter activity; amino acid metabolic process; binding; integral to membrane; mitochondrial inner membrane; mitochondrial ornithine transport; urea cycle; binding; transport; transporter activity	MitochondrioninnermembraneMultipassmembraneprotein;					
**802**	TMOD2	TMOD2_HUMAN	TMOD2	Q9NZR1	NP_055363	Tropomodulin-2 (Neuronal tropomodulin) (N-Tmod)	GO:0003779; GO:0005737; GO:0005856; GO:0007399; GO:0005523	actin binding; cytoplasm; cytoskeleton; nervous system development; tropomyosin binding	Cytoplasmcytoskeleton					
**803**	BR44	BR44_HUMAN	BR44	O95563; Q3SXR6; A8K261; Q6FIF3	NP_056230; NP_001137146	Brain protein 44	GO:0005739	mitochondrion						
**804**	HIG1A	HIG1A_HUMAN	HIG1A	Q9Y241; Q9UFZ2	NP_054775; NP_001093138; NP_001093139	HIG1 domain family member 1A (Hypoxia-inducible gene 1 protein)	GO:0016021; GO:0005515; GO:0043234; GO:0006950	integral to membrane; protein binding; protein complex; response to stress	MembraneMultipassmembraneprotein					
**805**	MAPIP	MAPIP_HUMAN	MAPIP	Q9Y2Q5; Q5VY97; Q5VY99	NP_054736	Mitogen-activated protein-binding protein-interacting protein (Late endosomal/lysosomal Mp1-interacting protein) (Roadblock domain-containing protein 3) (p14)	GO:0031902; GO:0005765	late endosome membrane; lysosomal membrane	LateendosomemembranePeripheralmembraneproteinCytoplasmicsideLysosomemembranePeripheralmembraneproteinCytoplasmicside					
**806**	RAB21	RAB21_HUMAN	RAB21	Q9UL25; Q569H3; Q14466	NP_055814	Ras-related protein Rab-21	GO:0005525; GO:0000139; GO:0005768; GO:0015031; GO:0007264	GTP binding; Golgi membrane; endosome; protein transport; small GTPase mediated signal transduction	EndoplasmicreticulummembraneLipidanchorGolgiapparatusmembraneEarlyendosomemembraneCytoplasmicvesiclemembrane					
**807**	K0152	K0152_HUMAN	K0152; MLEC	Q14165	NP_055545	Malectin	GO:0030246; GO:0005975; GO:0005789; GO:0016021	carbohydrate binding; carbohydrate metabolic process; endoplasmic reticulum membrane; integral to	EndoplasmicreticulummembraneSinglepasstypeImembraneprotein					
**808**	MCTS1	MCTS1_HUMAN	Q9ULC4; MCTS1	Q9ULC4; Q502X6	NP_054779	Malignant T cell amplified sequence 1 (MCT-1) (Multiple copies T-cell malignancies)	GO:0003723; GO:0005737; GO:0045786; GO:0008284; GO:0040008; GO:0006355; GO:0006974	RNA binding; cytoplasm; negative regulation of cell cycle; positive regulation of cell proliferation; regulation of growth; regulation of transcription, DNA-dependent; response to DNA damage stimulus	Cytoplasm			Known in Uniprot to be phosphorylated		
**809**	LAMP1	LAMP1_HUMAN	Q8WU33; LAMP1	P11279; Q8WU33; Q96I40; Q9BRD2; Q9NP13	NP_005552	Lysosome-associated membrane glycoprotein 1 (LAMP-1) (CD107 antigen-like family member A) (CD antigen CD107a)	GO:0010008; GO:0005887; GO:0005765; GO:0005624	endosome membrane; integral to plasma membrane; lysosomal membrane; membrane fraction	CellmembraneSinglepasstypeImembraneproteinEndosomemembraneSinglepasstypeImembraneproteinLysosomemembraneSinglepasstypeImembraneprotein					
**810**	SPN90	SPN90_HUMAN	SPN90	Q9NZQ3; Q9UGM8	NP_057537	NCK-interacting protein with SH3 domain (SH3 adapter protein SPIN90) (SH3 protein interacting with Nck, 90 kDa) (VacA-interacting protein, 54 kDa) (VIP54) (AF3p21) (Diaphanous protein-interacting protein) (Dia-interacting protein 1) (DIP-1)	GO:0006607; GO:0017124; GO:0008092; GO:0007010; GO:0005882; GO:0005634; GO:0007165	NLS-bearing substrate import into nucleus; SH3 domain binding; cytoskeletal protein binding; cytoskeleton organization; intermediate filament; nucleus; signal transduction	Nucleus			Known in Uniprot to be phosphorylated		
**811**	UFM1	UFM1_HUMAN	UFM1	P61960; Q14346; Q6IAG6; Q9CPX2; O9NZF2	NP_057701	Ubiquitin-fold modifier 1	GO:0005737; GO:0019941; GO:0005634; GO:0005515	cytoplasm; modification-dependent protein catabolic process; nucleus; protein binding	NucleusCytoplasm					
**812**	COPB	COPB_HUMAN	COPB	Q6GTT7; P53618; Q9NTK2; Q9UNW7	NP_057535; NP_001137533; NP_001137534	Coatomer subunit beta (Beta-coat protein) (Beta-COP)	GO:0048205; GO:0030126; GO:0005829; GO:0044419; GO:0006891; GO:0006886; GO:0005515; GO:0006890; GO:0005198	COPI coating of Golgi vesicle; COPI vesicle coat; cytosol; interspecies interaction between organisms; intra-Golgi vesicle-mediated transport; intracellular protein transport; protein binding; retrograde vesicle-mediated transport, Golgi to ER; structural molecule activity	CytoplasmGolgiapparatusmembranePeripheralmembraneproteinCytoplasmicsideCytoplasmicvesicleCOPIcoatedvesiclemembranePeripheralmembraneproteinCytoplasmicside					
**813**	TM14C	TM14C_HUMAN	TM14C; Q5T4I6	Q9P0S9; Q5T4I6	NP_057546	Transmembrane protein 14C; Transmembrane protein 14C (Transmembrane protein 14C, isoform CRA_a)	GO:0016021; GO:0031966	integral to membrane; mitochondrial membrane	MitochondrionmembraneMultipassmembraneprotein;					
**814**	FIS1	FIS1_HUMAN	FIS1	Q9Y3D6; Q9BTP3	NP_057152	Mitochondrial fission 1 protein (Fis1 homolog) (hFis1) (Tetratricopeptide repeat protein 11) (TPR repeat protein 11)	GO:0006915; GO:0031307; GO:0005779; GO:0000266; GO:0016559; GO:0005515	apoptosis; integral to mitochondrial outer membrane; integral to peroxisomal membrane; mitochondrial fission; peroxisome fission; protein binding	MitochondrionoutermembraneSinglepassmembraneproteinPeroxisomemembraneSinglepassmembraneprotein					
**815**	BOLA1	BOLA1_HUMAN	BOLA1; B2R7K2	Q9Y3E2; Q5QNY0; B2R7K2	NP_057158	BolA-like protein 1	GO:0005737; GO:0005634; GO:0005515	cytoplasm; nucleus; protein binding				Known in Uniprot to be phosphorylated		
**816**	GSTK1	GSTK1_HUMAN	GSTK1; Q6FII1	Q9Y2Q3; Q6FII1; Q7Z520; Q9P1S4	NP_057001	Glutathione S-transferase kappa 1 (EC 2.5.1.18) (Glutathione S-transferase subunit 13) (GST 13-13) (GST class-kappa) (GSTK1-1) (hGSTK1); LOC51064 protein (cDNA FLJ78056) (Glutathione S-transferase kappa 1) (Glutathione S-transferase kappa 1, isoform CRA_d)	GO:0004364; GO:0042802; GO:0030288; GO:0005777; GO:0015035; GO:0030288; GO:0016740	glutathione transferase activity; identical protein binding; outer membrane-bounded periplasmic space; peroxisome; protein disulfide oxidoreductase activity; outer membrane-bounded periplasmic space; transferase activity	Peroxisome;					
**817**	IR3IP	IR3IP_HUMAN	IR3IP	Q9Y5U9	NP_057181	Immediate early response 3-interacting protein 1	GO:0005794; GO:0005789; GO:0016021	Golgi apparatus; endoplasmic reticulum membrane; integral to membrane	EndoplasmicreticulummembraneMultipassmembraneprotein					
**818**	RAB9B	RAB9B_HUMAN	RAB9B	Q9NP90; Q52LX2; B2R8M0	NP_057454	Ras-related protein Rab-9B (Rab-9L) (RAB9-like protein)	GO:0005525; GO:0006895; GO:0005886; GO:0015031; GO:0007264	GTP binding; Golgi to endosome transport; plasma membrane; protein transport; small GTPase mediated signal transduction	CellmembraneLipidanchorCytoplasmicside					
**819**	PTH2	PTH2_HUMAN	PTH2; B3KUY4	Q9Y3E5; Q9NTE5; B3KUY4	NP_057161	Peptidyl-tRNA hydrolase 2, mitochondrial (PTH 2) (EC 3.1.1.29) (Bcl-2 inhibitor of transcription 1)	GO:0004045; GO:0006915; GO:0005739; GO:0006412	aminoacyl-tRNA hydrolase activity; apoptosis; mitochondrion; translation	Mitochondrion			Known in Uniprot to be phosphorylated		
**820**	TPC2L	TPC2L_HUMAN	Q9UL33; TPC2L	Q9UL33; B2R4M9; Q6ZTA7; Q9NZZ4	NP_057293	Trafficking protein particle complex subunit 2-like protein	GO:0006888; GO:0005622	ER to Golgi vesicle-mediated transport; intracellular						
**821**	TRAP1	TRAP1_HUMAN	TRAP1	Q12931; O43642; O75235; Q9UHL5	NP_057376	Heat shock protein 75 kDa, mitochondrial (HSP 75) (Tumor necrosis factor type 1 receptor-associated protein) (TRAP-1) (TNFR-associated protein 1)	GO:0005524; GO:0005739; GO:0006457; GO:0006950; GO:0005164; GO:0051082	ATP binding; mitochondrion; protein folding; response to stress; tumor necrosis factor receptor binding; unfolded protein binding	Mitochondrion			Known in Uniprot to be phosphorylated		
**822**	DJB11	DJB11_HUMAN	DJB11; Q542Y5	Q9UBS4; Q542Y5; Q542Y9; Q6IAQ8; Q96JC6	NP_057390	DnaJ homolog subfamily B member 11 (ER-associated dnaJ protein 3) (ERj3p) (ERdj3) (ER-associated Hsp40 co-chaperone) (ER-associated DNAJ) (HEDJ) (hDj9) (PWP1-interacting protein 4) (APOBEC1-binding protein 2) (ABBP-2)	GO:0005783; GO:0005788; GO:0031072; GO:0006457; GO:0051082	endoplasmic reticulum; endoplasmic reticulum lumen; heat shock protein binding; protein folding; unfolded protein binding	Endoplasmicreticulumlumen					

	Protein Name	Uniprot Name	Other Names	Uniprot IDs	Refseq IDs	Protein Description	GO ID	GO terms	Subcellular location	Comments	Comments	Comments - Database Search	Comments - Database Search	Function
**823**	KCY	KCY_HUMAN	KCY; B2R6S5	P30085; Q53GB7; Q5SVZ0; Q96C07; Q9UBQ8; Q9UIA2; B2R6S5	NP_057392; NP_001129612	UMP-CMP kinase (EC 2.7.4.14) (Cytidylate kinase) (Deoxycytidylate kinase) (Cytidine monophosphate kinase) (Uridine monophosphate kinase) (Uridine monophosphate/cytidine monophosphate kinase) (UMP/CMP kinase) (UMP/CMPK)	GO:0005524; GO:0004127; GO:0005737; GO:0005634; GO:0016776; GO:0009220; GO:0004849	ATP binding; cytidylate kinase activity; cytoplasm; nucleus; phosphotransferase activity, phosphate group as acceptor; pyrimidine ribonucleotide biosynthetic process; uridine kinase activity	NucleusCytoplasm					Kinase
**824**	RAB8B	RAB8B_HUMAN	RAB8B; Q5JPC4	Q92930; Q5JPC4; Q9P293	NP_057614	Ras-related protein Rab-8B; Putative uncharacterized protein DKFZp667F1514 (cDNA FLJ10249 fis, clone HEMBB1000725, highly similar to Ras-related protein Rab-8B)	GO:0005525; GO:0003924; GO:0005886; GO:0005515; GO:0015031; GO:0007264	GTP binding; GTPase activity; plasma membrane; protein binding; protein transport; small GTPase mediated signal transduction	CellmembraneLipidanchorCytoplasmicside;					
**825**	CALL5	CALL5_HUMAN	CALL5; Q53H37	Q9NZT1; Q5SQI3; Q8IXU8; Q53H37	NP_059118	Calmodulin-like protein 5 (Calmodulin-like skin protein)	GO:0005509; GO:0008544; GO:0005515; GO:0007165	calcium ion binding; epidermis development; protein binding; signal transduction						
**826**	RNZ1	RNZ1_HUMAN	RNZ1	Q9H777; Q9NS99	NP_061166	Zinc phosphodiesterase ELAC protein 1 (EC 3.1.26.11) (Ribonuclease Z 1) (RNase Z 1) (tRNase Z 1) (tRNA 3 endonuclease 1) (ElaC homolog protein 1) (Deleted in Ma29)	GO:0004519; GO:0005634; GO:0008033; GO:0008270	endonuclease activity; nucleus; tRNA processing; zinc ion binding	Nucleus					
**827**	NECP2	NECP2_HUMAN	NECP2	Q9NVZ3; Q5VSU4; Q5VSU5; Q9H7L1; Q9H8L1	NP_060560; NP_001138749	Adaptin ear-binding coat-associated protein 2 (NECAP endocytosis-associated protein 2) (NECAP-2)	GO:0030125; GO:0005905; GO:0006897; GO:0015031	clathrin vesicle coat; coated pit; endocytosis; protein transport	CytoplasmicvesicleclathrincoatedvesiclemembraneCellmembrane					
**828**	RT18A	RT18A_HUMAN	RT18A	Q9NVS2	NP_060605	28S ribosomal protein S18a, mitochondrial (MRP-S18-a) (Mrps18a) (MRP-S18-3)	GO:0005763; GO:0003735; GO:0006412	mitochondrial small ribosomal subunit; structural constituent of ribosome; translation	Mitochondrion					
**829**	ARL8B	ARL8B_HUMAN	ARL8B	Q9NVJ2	NP_060654	ADP-ribosylation factor-like protein 8B (ADP-ribosylation factor-like protein 10C) (Novel small G protein indispensable for equal chromosome segregation 1)	GO:0019003; GO:0005525; GO:0003924; GO:0043014; GO:0048487; GO:0007059; GO:0031902; GO:0005765; GO:0030496; GO:0007264; GO:0051233	GDP binding; GTP binding; GTPase activity; alpha-tubulin binding; beta-tubulin binding; chromosome segregation; late endosome membrane; lysosomal membrane; midbody; small GTPase mediated signal transduction; spindle midzone	LateendosomemembraneLysosomemembrane					
**830**	TRI62	TRI62_HUMAN	TRI62; B3KVH5	Q9NVG0; Q9BVG3; B3KVH5	NP_060677	Tripartite motif-containing protein 62	GO:0005622; GO:0005515; GO:0008270	intracellular; protein binding; zinc ion binding						
**831**	CNDP2	CNDP2_HUMAN	CNDP2	Q96KP4; Q9BQ94; Q9NVB4	NP_060705	Cytosolic non-specific dipeptidase (CNDP dipeptidase 2) (Glutamate carboxypeptidase-like protein 1) (Peptidase A)	GO:0004180; GO:0008237; GO:0046983; GO:0006508; GO:0008270	carboxypeptidase activity; metallopeptidase activity; protein dimerization activity; proteolysis; zinc ion binding						
**832**	CA106	CA106_HUMAN	CA106	Q9NV65; Q3KP66; Q9NVI0	NP_060735	Uncharacterized protein C1orf106								
**833**	TXLNG	TXLNG_HUMAN	TXLNG	Q9NUQ3; Q5JNZ7; Q9P0X1	NP_060830	Gamma-taxilin (Lipopolysaccharide-specific response protein 5)	GO:0005737	cytoplasm				Known in Uniprot to be phosphorylated		
**834**	MNS1	MNS1_HUMAN	Q9NUP4; MNS1	Q9NUP4; Q8NEH6; Q8IYT6	NP_060835	Meiosis-specific nuclear structural protein 1	GO:0007126	meiosis	Nucleus			Known in Uniprot to be phosphorylated		
**835**	ZN407	ZN407_HUMAN	ZN407	Q9NXD7; Q9C0G0; Q96MY0; Q9H8A1; Q9NXD4	NP_060227; NP_001139661; NP_001139662	Zinc finger protein 407	GO:0003677; GO:0005634; GO:0006355; GO:0008270	DNA binding; nucleus; regulation of transcription, DNA-dependent; zinc ion binding	Nucleus					
**836**	CHCH3	CHCH3_HUMAN	CHCH3	Q9NX63	XP_208225; NP_060282	Coiled-coil-helix-coiled-coil-helix domain-containing protein 3, mitochondrial	GO:0005515	protein binding	Mitochondrion			Known in Uniprot to be phosphorylated	Known in HPRD to be phosphorylated	
**837**	CA123	CA123_HUMAN	CA123	Q9NWV4	NP_060357	UPF0587 protein C1orf123								
**838**	ZN446	ZN446_HUMAN	ZN446	Q9NWS9	NP_060378	Zinc finger protein 446 (Zinc finger protein with KRAB and SCAN domains 20)	GO:0005634; GO:0006355; GO:0003700; GO:0008270	nucleus; regulation of transcription, DNA-dependent; transcription factor activity; zinc ion binding	Nucleus					
**839**	GTR6	GTR6_HUMAN	GTR6	Q9UGQ3; A6NNU6; Q5SXD7	NP_060055	Solute carrier family 2, facilitated glucose transporter member 6 (Glucose transporter type 6) (GLUT-6) (Glucose transporter type 9) (GLUT-9)	GO:0008643; GO:0005355; GO:0016021; GO:0005886; GO:0005351; GO:0055085	carbohydrate transport; glucose transmembrane transporter activity; integral to membrane; plasma membrane; sugar:hydrogen symporter activity; transmembrane transport	CellmembraneMultipassmembraneprotein			Known in Uniprot to be phosphorylated		
**840**	THEM2	THEM2_HUMAN	THEM2	Q9NPJ3; O95549	NP_060943	Thioesterase superfamily member 2	GO:0016787	hydrolase activity						
**841**	BIN3	BIN3_HUMAN	BIN3	Q9NQY0; Q9BVG2; Q9NVY9	NP_061158	Bridging integrator 3	GO:0007015; GO:0000917; GO:0007049; GO:0005737; GO:0008093; GO:0005856; GO:0008104; GO:0009826	actin filament organization; barrier septum formation; cell cycle; cytoplasm; cytoskeletal adaptor activity; cytoskeleton; protein localization; unidimensional cell growth	Cytoplasmcytoskeleton					
**842**	CI046	CI046_HUMAN	CI046	Q9HBL7; Q9NZ44	NP_060935	Transmembrane protein C9orf46	GO:0016021; GO:0005739	integral to membrane; mitochondrion	MembraneMultipassmembraneprotein					
**843**	A1AG1	A1AG1_HUMAN	A1AG1; Q5U067; Q5T539	P02763; Q5U067; Q8TC16; Q5T539	NP_000598	Alpha-1-acid glycoprotein 1 (AGP 1) (Orosomucoid-1) (OMD 1); Orosomucoid 1 (cDNA, FLJ92312, Homo sapiens orosomucoid 1 (ORM1), mRNA)	GO:0006953; GO:0005615; GO:0005515; GO:0002682; GO:0005488	acute-phase response; extracellular space; protein binding; regulation of immune system process; binding	Secreted;					
**844**	TBA8	TBA8_HUMAN	TBA8; B2RCX2	Q9NY65; Q2M3N4; B2RCX2	NP_061816	Tubulin alpha-8 chain (Alpha-tubulin 8) (Tubulin alpha chain-like 2)	GO:0005525; GO:0003924; GO:0005874; GO:0007018; GO:0043234; GO:0051258; GO:0005198	GTP binding; GTPase activity; microtubule; microtubule-based movement; protein complex; protein polymerization; structural molecule activity				Known in Uniprot to be phosphorylated		
**845**	ITB6	ITB6_HUMAN	ITB6; B2R9W5	P18564; Q0VA95; Q16500; Q53RG5; Q53RR6; B2R9W5	NP_000879	Integrin beta-6	GO:0007160; GO:0008305; GO:0007229; GO:0044419; GO:0005515; GO:0004872	cell-matrix adhesion; integrin complex; integrin-mediated signaling pathway; interspecies interaction between organisms; protein binding; receptor activity	MembraneSinglepasstypeImembraneprotein					
**846**	DBLOH	DBLOH_HUMAN	DBLOH	Q9NR28; B2RDQ0; Q96LV0; Q9BT11; Q9HAV6	NP_063940; NP_620307	Diablo homolog, mitochondrial (Second mitochondria-derived activator of caspase) (Smac protein) (Direct IAP-binding protein with low pI)	GO:0008635; GO:0005829; GO:0008625; GO:0005739; GO:0005515	activation of caspase activity by cytochrome c; cytosol; induction of apoptosis via death domain receptors; mitochondrion; protein binding	Mitochondrion					
**847**	LAMB2	LAMB2_HUMAN	LAMB2	P55268; Q16321	NP_002283	Laminin subunit beta-2 (S-laminin) (Laminin B1s chain)	GO:0005605; GO:0007155; GO:0005783; GO:0005634; GO:0005515; GO:0005198	basal lamina; cell adhesion; endoplasmic reticulum; nucleus; protein binding; structural molecule activity	Secretedextracellularspaceextracellularmatrixbasementmembrane					
**848**	RAC1	RAC1_HUMAN	RAC1; A4D2P1	P63000; Q5JAA8; O95501; P15154; Q3Y4D3; Q9BTB4; A4D2P1	XP_171081; NP_008839	Ras-related C3 botulinum toxin substrate 1 (p21-Rac1) (Ras-like protein TC25) (Cell migration-inducing gene 5 protein); Ras-related C3 botulinum toxin substrate 1 isoform Rac1 (cDNA FLJ30431 fis, clone BRACE2008968, highly similar to Ras-related C3 botulinum toxin substrate 1) (cDNA FLJ75553, highly similar to Homo sapiens ras-related C3 botulinum toxin substrate 1 (rho family, small GTP binding protein Rac1) (RAC1), transcript variant Rac1, mRNA) (Ras-related C3 botulinum toxin substrate 1 (Rho family, small GTP binding protein Rac1)) (Ras-related C3 botulinum toxin substrate 1 (Rho family, small GTP binding protein Rac1), isoform CRA_a)	GO:0005525; GO:0030742; GO:0003924; GO:0030041; GO:0007155; GO:0006928; GO:0005829; GO:0019899; GO:0006954; GO:0051668; GO:0042470; GO:0048261; GO:0005886; GO:0035025; GO:0010592; GO:0010310; GO:0060263; GO:0031529; GO:0005622; GO:0016020; GO:0007264	GTP binding; GTP-dependent protein binding; GTPase activity; actin filament polymerization; cell adhesion; cell motion; cytosol; enzyme binding; inflammatory response; localization within membrane; melanosome; negative regulation of receptor-mediated endocytosis; plasma membrane; positive regulation of Rho protein signal transduction; positive regulation of lamellipodium assembly; regulation of hydrogen peroxide metabolic process; regulation of respiratory burst; ruffle organization; intracellular; membrane; small GTPase mediated signal transduction	CellmembraneLipidanchorCytoplasmicsideMelanosome;			Known in Uniprot to be phosphorylated	Known in HPRD to be phosphorylated	
**849**	OTU7B	OTU7B_HUMAN	OTU7B; Q5SZ60	Q6GQQ9; Q8WWA7; Q9NQ53; Q9UFF4; Q5SZ60	NP_064590	OTU domain-containing protein 7B (EC 3.-.-.-) (Zinc finger protein Cezanne) (Zinc finger A20 domain-containing protein 1) (Cellular zinc finger anti-NF-kappa-B protein); OTU domain containing 7B (Zinc finger, A20 domain containing 1, isoform CRA_a)	GO:0003677; GO:0008234; GO:0005737; GO:0019941; GO:0043124; GO:0005634; GO:0005515; GO:0008270; GO:0016579; GO:0004221	DNA binding; cysteine-type peptidase activity; cytoplasm; modification-dependent protein catabolic process; negative regulation of I-kappaB kinase/NF-kappaB cascade; nucleus; protein binding; zinc ion binding; protein deubiquitination; ubiquitin thiolesterase activity	CytoplasmNucleus;			Known in Uniprot to be phosphorylated		
**850**	OTOR	OTOR_HUMAN	OTOR	Q9NRC9; O3MIU6	NP_064542	Otoraplin (Fibrocyte-derived protein) (Melanoma inhibitory activity-like protein)	GO:0005576; GO:0007605	extracellular region; sensory perception of sound	Secreted					
**851**	SAR1A	SAR1A_HUMAN	SAR1A; Q5SQT9	Q9NR31; Q5SQT9	NP_064535; NP_001136120	GTP-binding protein SAR1a (COPII-associated small GTPase); SAR1 gene homolog A (S. cerevisiae) (Small GTP-binding protein) (SAR1 gene homolog A (S. cerevisiae), isoform CRA_a)	GO:0006888; GO:0005525; GO:0003924; GO:0005794; GO:0006886; GO:0005525; GO:0005783; GO:0016529; GO:0007264; GO:0016192	ER to Golgi vesicle-mediated transport; GTP binding; GTPase activity; Golgi apparatus; intracellular protein transport; GTP binding; endoplasmic reticulum; sarcoplasmic reticulum; small GTPase mediated signal transduction; vesicle-mediated transport	Endoplasmicreticulum Golgiapparatus;			Known in Uniprot to be phosphorylated		
**852**	LYPA2	LYPA2_HUMAN	LYPA2	O95372; O7Z4Z2	NP_009191	Acyl-protein thioesterase 2 (EC 3.1.2.-) (Lysophospholipase II) (LPL-I)	GO:0005737; GO:0006631; GO:0016787	cytoplasm; fatty acid metabolic process; hydrolase activity	Cytoplasm					
**853**	PCNP	PCNP_HUMAN	PCNP	Q8WW12; B2RBE7; Q6AI44; Q96CU3; Q9NS81	NP_065090	PEST proteolytic signal-containing nuclear protein (PEST-containing nuclear protein) (PCNP)	GO:0007049; GO:0005634; GO:0043161; GO:0005515; GO:0016567	cell cycle; nucleus; proteasomal ubiquitin-dependent protein catabolic process; protein binding; protein ubiquitination	Nucleus			Known in Uniprot to be phosphorylated		
**854**	IF5A2	IF5A2_HUMAN	IF5A2; B2R4V5	Q9GZV4; B2R4V5	NP_065123	Eukaryotic translation initiation factor 5A-2 (eIF-5A-2) (eIF-5A2) (Eukaryotic initiation factor 5A isoform 2)	GO:0008283; GO:0005829; GO:0008612; GO:0010509; GO:0005515; GO:0007283; GO:0003743; GO:0006413	cell proliferation; cytosol; peptidyl-lysine modification to hypusine; polyamine homeostasis; protein binding; spermatogenesis; translation initiation factor activity; translational initiation						

	Protein Name	Uniprot Name	Other Names	Uniprot IDs	Refseq IDs	Protein Description	GO ID	GO terms	Subcellular location	Comments	Comments	Comments - Database Search	Comments - Database Search	Function
**855**	ZO3	ZO3_HUMAN	ZO3	O95049; A6NFP3	NP_055243	Tight junction protein ZO-3 (Zonula occludens protein 3) (Zona occludens protein 3) (Tight junction protein 3)	GO:0005515; GO:0005923	protein binding; tight junction	CellmembranePeripheralmembraneproteinCytoplasmicsideCelljunctiontightjunction			Known in Uniprot to be phosphorylated		
**856**	ACBP	ACBP_HUMAN	ACBP; B8ZWD2	P07108; P08869; Q53SQ7; Q6IB48; Q9UCI8; B8ZWD2	NP_065438; NP_001073331	Acyl-CoA-binding protein (ACBP) (Diazepam-binding inhibitor) (DBI) (Endozepine) (EP)	GO:0000062; GO:0030156; GO:0006810	acyl-CoA binding; benzodiazepine receptor binding; transport				Known in Uniprot to be phosphorylated		
**857**	CBX8	CBX8_HUMAN	CBX8	Q9HC52; Q96H39; Q9NR07	XP_297653; NP_065700	Chromobox protein homolog 8 (Polycomb 3 homolog) (Pc3) (hPc3) (Rectachrome 1)	GO:0000785; GO:0006333; GO:0003682; GO:0006355	chromatin; chromatin assembly or disassembly; chromatin binding; regulation of transcription, DNA-dependent	Nucleus			Known in Uniprot to be phosphorylated		
**858**	MARE3	MARE3_HUMAN	MARE3; Q6FHB0	Q9UPY8; Q6FHB0; O00265; Q6FI15; Q9BZP7; Q9BZP8	NP_036458	Microtubule-associated protein RP/EB family member 3 (End-binding protein 3) (EB3) (EB1 protein family member 3) (EBF3) (RP3); MAPRE3 protein (Microtubule-associated protein, RP/EB family, member 3, isoform CRA_c) (Putative uncharacterized protein MAPRE3) (Fragment)	GO:0051301; GO:0005737; GO:0005874; GO:0008017; GO:0030496; GO:0007067; GO:0043193; GO:0005083; GO:0008017	cell division; cytoplasm; microtubule; microtubule binding; midbody; mitosis; positive regulation of gene-specific transcription; small GTPase regulator activity; microtubule binding	Cytoplasm;			Known in Uniprot to be phosphorylated		
**859**	I13R2	I13R2_HUMAN	I13R2	Q14627; O00667	NP_000631	Interleukin-13 receptor alpha-2 chain (Interleukin-13-binding protein) (CD antigen CD213a2)	GO:0004896; GO:0005615; GO:0016021; GO:0005625	cytokine receptor activity; extracellular space; integral to membrane; soluble fraction	MembraneSinglepasstypeImembraneprotein					
**860**	NPM	NPM_HUMAN	NPM; A8K3N7	P06748; A8K3N7; P08693; Q12826; Q13440; Q13441; Q14115; Q5EU94; Q5EU95; Q5EU96; Q5EU97; Q5EU98; Q5EU99; Q6V962; Q8WTW5; Q96AT6; Q96DC4; Q96EA5	NP_002511; XP_059067; NP_954654	Nucleophosmin (NPM) (Nucleolar phosphoprotein B23) (Numatrin) (Nucleolar protein NO38)	GO:0051059; GO:0003723; GO:0030957; GO:0006916; GO:0007569; GO:0042393; GO:0044419; GO:0006886; GO:0008285; GO:0010826; GO:0006913; GO:0005730; GO:0005654; GO:0006334; GO:0051092; GO:0046982; GO:0042803; GO:0051259; GO:0006950; GO:0030529; GO:0043023; GO:0043024; GO:0042255; GO:0007165; GO:0031616; GO:0003713; GO:0051082	NF-kappaB binding; RNA binding; Tat protein binding; anti-apoptosis; cell aging; histone binding; interspecies interaction between organisms; intracellular protein transport; negative regulation of cell proliferation; negative regulation of centrosome duplication; nucleocytoplasmic transport; nucleolus; nucleoplasm; nucleosome assembly; positive regulation of NF-kappaB transcription factor activity; protein heterodimerization activity; protein homodimerization activity; protein oligomerization; response to stress; ribonucleoprotein complex; ribosomal large subunit binding; ribosomal small subunit binding; ribosome assembly; signal transduction; spindle pole centrosome; transcription coactivator activity; unfolded protein binding	Nucleusnucleolus			Known in Uniprot to be phosphorylated	Known in HPRD to be phosphorylated	
**861**	KPYR	KPYR_HUMAN	KPYR	P30613; P11973	NP_000289; NP_870986	Pyruvate kinase isozymes R/L (EC 2.7.1.40) (R-type/L-type pyruvate kinase) (Red cell/liver pyruvate kinase) (Pyruvate kinase 1)	GO:0006096; GO:0000287; GO:0030955; GO:0004743	glycolysis; magnesium ion binding; potassium ion binding; pyruvate kinase activity				Known in Uniprot to be phosphorylated		Kinase
**862**	DAF	DAF_HUMAN	DAF; Q8TD14; Q5VW90; B1AP14	P08174; Q5VW90; P09679; P78361; B1AP14; Q8TD14	NP_000565; NP_001108224	Complement decay-accelerating factor (CD antigen CD55); Decay-accelerating factor 1a (Fragment)	GO:0031225; GO:0006958; GO:0007204; GO:0045087; GO:0005887; GO:0045121; GO:0045730; GO:0005625;	anchored to membrane; complement activation, classical pathway; elevation of cytosolic calcium ion concentration; innate immune response; integral to plasma membrane; membrane raft; respiratory burst; soluble fraction;	MembraneSinglepasstypeImembraneproteinCellmembraneLipidanchorGPIanchor;			Known in Uniprot to be phosphorylated		
**863**	LSM2	LSM2_HUMAN	LSM2; Q6FGG1	Q9Y333; Q6FGG1	NP_067000	U6 snRNA-associated Sm-like protein LSm2 (snRNP core Sm-like protein Sm-x5) (Small nuclear ribonuclear protein D homolog) (Protein G7b); LSM2 homolog, U6 small nuclear RNA associated (S. cerevisiae) (LSM2 protein) (LSM2 homolog, U6 small nuclear RNA associated (S. cerevisiae), isoform CRA_a)	GO:0017070; GO:0000398; GO:0005681; GO:0016071; GO:0019901; GO:0030529	U6 snRNA binding; nuclear mRNA splicing, via spliceosome; spliceosome; mRNA metabolic process; protein kinase binding; ribonucleoprotein complex	Nucleus;			Known in Uniprot to be phosphorylated		
**864**	SQRD	SQRD_HUMAN	SQRD	Q9Y6N5; O9UQM8	NP_067022	Sulfide:quinone oxidoreductase, mitochondrial (EC 1.-.-.-)	GO:0055114; GO:0016491	oxidation reduction; oxidoreductase activity	Mitochondrion					
**865**	TPPC1	TPPC1_HUMAN	TPPC1	Q9Y5R8	NP_067033	Trafficking protein particle complex subunit 1 (BET5 homolog) (Multiple myeloma protein 2) (MUM-2)	GO:0006888; GO:0005801; GO:0005783	ER to Golgi vesicle-mediated transport; cis-Golgi network; endoplasmic reticulum	GolgiapparatuscisGolginetworkEndoplasmicreticulum					
**866**	EP15R	EP15R_HUMAN	EP15R	Q9UBC2	NP_067058	Epidermal growth factor receptor substrate 15-like 1 (Eps15-related protein) (Eps15R)	GO:0005509; GO:0005905; GO:0006897; GO:0005634	calcium ion binding; coated pit; endocytosis; nucleus	CellmembranePeripheralmembraneproteinNucleusMembranecoatedpit			Known in Uniprot to be phosphorylated	Known in HPRD to be phosphorylated	
**867**	RAB18	RAB18_HUMAN	RAB18; B3KMC7	Q9NP72; Q53FX8; Q6FIH1; B3KMC7	NP_067075	Ras-related protein Rab-18	GO:0005525; GO:0003924; GO:0006897; GO:0005622; GO:0005886; GO:0015031; GO:0007264	GTP binding; GTPase activity; endocytosis; intracellular; plasma membrane; protein transport; small GTPase mediated signal transduction	CellmembraneLipidanchorCytoplasmicside			Known in Uniprot to be phosphorylated		
**868**	TYB4	TYB4_HUMAN	TYB4; Q0P5T0; A2VCK8; TMSL3	P62328; A2VCK8; Q0P5T0; A8MW06; P01253; P01254; Q546P5; Q63576; Q9UE55	NP_066932; NP_898870	Thymosin beta-4 (T beta-4) (Fx) [Cleaved into: Hematopoietic system regulatory peptide (Seraspenide)]; Thymosin beta 4, X-linked (Thymosin, beta 4, X-linked, isoform CRA_a) (cDNA, FLJ92006, Homo sapiens thymosin, beta 4, X chromosome (TMSB4X), mRNA); TMSB4X protein (Fragment)	GO:0003779; GO:0005737; GO:0005856; GO:0042989; GO:0007010	actin binding; cytoplasm; cytoskeleton; sequestering of actin monomers; cytoskeleton organization	Cytoplasmcytoskeleton;			Known in Uniprot to be phosphorylated		
**869**	CYC	CYC_HUMAN	CYC; A4D166; B2R4I1	P99999; A4D166; P00001; Q6NUR2; Q6NX69; Q96BV4; B2R4I1	NP_061820; XP_088293; XP_291384	Cytochrome c	GO:0006309; GO:0008635; GO:0045333; GO:0005829; GO:0022900; GO:0045155; GO:0020037; GO:0005758; GO:0005759; GO:0005634; GO:0005515; GO:0000159; GO:0006810	DNA fragmentation involved in apoptosis; activation of caspase activity by cytochrome c; cellular respiration; cytosol; electron transport chain; electron transporter, transferring electrons from CoQH2-cytochrome c reductase complex and cytochrome c oxidase complex activity; heme binding; mitochondrial intermembrane space; mitochondrial matrix; nucleus; protein binding; protein phosphatase type 2A complex; transport	Mitochondrionmatrix					
**870**	RAI2	RAI2_HUMAN	RAI2; B2RBE9	Q9Y5P3; B1B1K2; Q8N6X7; B2RBE9	NP_068557	Retinoic acid-induced protein 2	GO:0009790	embryonic development						
**871**	HEMO	HEMO_HUMAN	HEMO	P02790; B2R957	NP_000604	Hemopexin (Beta-1B-glycoprotein)	GO:0006879; GO:0005615; GO:0015886; GO:0015232; GO:0044419; GO:0005506	cellular iron ion homeostasis; extracellular space; heme transport; heme transporter activity; interspecies interaction between organisms; iron ion binding	Secreted					
**872**	A2AP	A2AP_HUMAN	A2AP; Q8N5U7	P08697; Q8N5U7; Q9UCG2; Q9UCG3	NP_000925	Alpha-2-antiplasmin (Alpha-2-AP) (Alpha-2-plasmin inhibitor) (Alpha-2-PI)	GO:0006953; GO:0005576; GO:0005634; GO:0031093; GO:0002020; GO:0004867	acute-phase response; extracellular region; nucleus; platelet alpha granule lumen; protease binding; serine-type endopeptidase inhibitor activity	Secreted					
**873**	PDLI7	PDLI7_HUMAN	PDLI7	Q9NR12; Q14250; Q5XG82; Q6NVZ5; Q96C91; Q9BXB8; Q9BXB9	NP_005442; NP_976227; NP_998801	PDZ and LIM domain protein 7 (LIM mineralization protein) (LMP) (Protein enigma)	GO:0030154; GO:0005737; GO:0005856; GO:0001503; GO:0005515; GO:0006898; GO:0008270	cell differentiation; cytoplasm; cytoskeleton; ossification; protein binding; receptor-mediated endocytosis; zinc ion binding	CytoplasmCytoplasmcytoskeleton			Known in Uniprot to be phosphorylated		
**874**	FN3K	FN3K_HUMAN	FN3K	Q9H479	NP_071441	Fructosamine-3-kinase (EC 2.7.1.-)	GO:0030387; GO:0030393	fructosamine-3-kinase activity; fructoselysine metabolic process				Known in Uniprot to be phosphorylated		Kinase
**875**	IF4H	IF4H_HUMAN	IF4H	Q15056	NP_071496	Eukaryotic translation initiation factor 4H (eIF-4H) (Williams-Beuren syndrome chromosomal region 1 protein)	GO:0003723; GO:0005829; GO:0016281; GO:0044419; GO:0000166; GO:0006446; GO:0003743	RNA binding; cytosol; eukaryotic translation initiation factor 4F complex; interspecies interaction between organisms; nucleotide binding; regulation of translational initiation; translation initiation factor activity	Cytoplasmperinuclearregion			Known in Uniprot to be phosphorylated	Known in HPRD to be phosphorylated	
**876**	JARD2	JARD2_HUMAN	JARD2; A8K9Z6	Q92833; Q5U5L5; A8K9Z6	NP_004964	Protein Jumonji (Jumonji/ARID domain-containing protein 2)	GO:0003677; GO:0007417; GO:0005634	DNA binding; central nervous system development; nucleus	Nucleus					
**877**	ZF106	ZF106_HUMAN	ZF106	Q9H2Y7; Q6NSD9; Q6PEK1; Q86T43; Q86T45; Q86T50; Q86T58; Q86TA9; Q96M37; Q9H7B8	NP_071918	Zinc finger protein 106 homolog (Zfp-106) (Zinc finger protein 474)	GO:0005634; GO:0008270	nucleus; zinc ion binding	Nucleusnucleolus			Known in Uniprot to be phosphorylated		
**878**	CC029	CC029_HUMAN	CC029; MTMRE	Q9H6C0; Q8NCE2; Q0JTH5; Q0JU83; Q6PIZ4; Q6QE21; Q86VK9; Q8IYK1; Q8TCM7	NP_071930; NP_001070993	Myotubularin-related protein 14 (EC 3.1.3.-) (HCV NS5A-transactivated protein 4 splice variant A-binding protein 1) (NS5ATP4ABP1) (hJumpy)	GO:0048471; GO:0004438; GO:0001726	perinuclear region of cytoplasm; phosphatidylinositol-3-phosphatase activity; ruffle	Cytoplasm			Known in Uniprot to be phosphorylated		
**879**	TX1B3	TX1B3_HUMAN	TX1B3; B2RD53	O14907; Q7LCQ4; B2RD53	NP_055419	Tax1-binding protein 3 (Tax interaction protein 1) (TIP-1) (Glutaminase-interacting protein 3)	GO:0007266; GO:0005737; GO:0030178; GO:0005634; GO:0005515	Rho protein signal transduction; cytoplasm; negative regulation of Wnt receptor signaling pathway; nucleus; protein binding	CytoplasmNucleus			Known in Uniprot to be phosphorylated		

	Protein Name	Uniprot Name	Other Names	Uniprot IDs	Refseq IDs	Protein Description	GO ID	GO terms	Subcellular location	Comments	Comments	Comments - Database Search	Comments - Database Search	Function
**880**	FBRL	FBRL_HUMAN	FBRL; B5BUE8	P22087; Q6IAT5; O75259; Q9UPI6; B5BUE8	NP_001427	rRNA 2'-O-methyltransferase fibrillarin (EC 2.1.1.-) (34 kDa nucleolar scleroderma antigen)	GO:0003723; GO:0008168; GO:0005730; GO:0005515; GO:0006364; GO:0030529	RNA binding; methyltransferase activity; nucleolus; protein binding; rRNA processing; ribonucleoprotein complex	Nucleusnucleolus					
**881**	PFD2	PFD2_HUMAN	PFD2; Q5SY55; B1AQP2	Q9UHV9; Q5SY55; Q9P0P7; Q9UN05; B1AQP2	NP_036526	Prefoldin subunit 2	GO:0016272; GO:0006457; GO:0051082	prefoldin complex; protein folding; unfolded protein binding						
**882**	PFD4	PFD4_HUMAN	PFD4	Q9NQP4; Q5TD11; Q92779	NP_002614	Prefoldin subunit 4 (Protein C-1)	GO:0051087; GO:0016272; GO:0051082	chaperone binding; prefoldin complex; unfolded protein binding				Known in Uniprot to be phosphorylated		
**883**	PER2	PER2_HUMAN	PER2; A2I2P7	O15055; Q4ZG49; Q9UQ45; A2I2P7	NP_073728	Period circadian protein homolog 2 (Circadian clock protein PERIOD 2) (hPER2); Period 2 (Period homolog 2 (Drosophila), isoform CRA_c)	GO:0007623; GO:0005737; GO:0005634; GO:0005515; GO:0006355; GO:0004871; GO:0007165; GO:0006355	circadian rhythm; cytoplasm; nucleus; protein binding; regulation of transcription, DNA-dependent; signal transducer activity; signal transduction; regulation of transcription, DNA-dependent	NucleusCytoplasm;			Known in Uniprot to be phosphorylated	Known in HPRD to be phosphorylated	
**884**	ECHM	ECHM_HUMAN	ECHM	P30084; O00739; Q5VWY1; Q96H54	NP_004083	Enoyl-CoA hydratase, mitochondrial (EC 4.2.1.17) (Short chain enoyl-CoA hydratase) (SCEH) (Enoyl-CoA hydratase 1)	GO:0004300; GO:0006635; GO:0005759; GO:0005515	enoyl-CoA hydratase activity; fatty acid beta-oxidation; mitochondrial matrix; protein binding	Mitochondrionmatrix					
**885**	TRAK2	TRAK2_HUMAN	TRAK2	O60296; Q8WVH7; Q96NS2; Q9C0K5; Q9C0K6	NP_055864	Trafficking kinesin-binding protein 2 (Amyotrophic lateral sclerosis 2 chromosomal region candidate gene 3 protein)	GO:0050811; GO:0005737; GO:0005886	GABA receptor binding; cytoplasm; plasma membrane	Cytoplasm					
**886**	CG024	CG024_HUMAN	CG024; GGCT	O75223; B2RDN0; Q9BS37	NP_076956	Gamma-glutamylcyclotransferase (EC 2.3.2.4) (Cytochrome c-releasing factor 21)	GO:0008415; GO:0005829; GO:0003839; GO:0042803; GO:0001836	acyltransferase activity; cytosol; gamma-glutamylcyclotransferase activity; protein homodimerization activity; release of cytochrome c from mitochondria						
**887**	DYNA	DYNA_HUMAN	DYNA; Q6MZZ3; DCTN1; Q6IQ37	Q14203; Q6MZZ3; O95296; Q9BRM9; Q9UIU1; Q9UIU2; Q6IQ37	NP_004073; NP_075408; NP_001128513	Dynactin subunit 1 (150 kDa dynein-associated polypeptide) (DAP-150) (DP-150) (p150-glued) (p135)	GO:0005737; GO:0030286; GO:0005874; GO:0007067; GO:0003774; GO:0007399; GO:0005515; GO:0000922	cytoplasm; dynein complex; microtubule; mitosis; motor activity; nervous system development; protein binding; spindle pole	CytoplasmCytoplasmcytoskeleton			Known in Uniprot to be phosphorylated		
**888**	PDYN	PDYN_HUMAN	PDYN	P01213	XP_299313; NP_077722	Proenkephalin-B (Beta-neoendorphin-dynorphin) (Preprodynorphin) [Cleaved into: Alpha-neoendorphin; Beta-neoendorphin; Big dynorphin (Big Dyn); Dynorphin A(1-17) (Dynorphin A) (Dyn-A17); Dynorphin A(1-13); Dynorphin A(1-8); Leu-enkephalin; Rimorphin (Dynorphin B) (Dyn-B) (Dynorphin B(1-13)); Leumorphin (Dynorphin B-29)]	GO:0005576; GO:0007218; GO:0001515; GO:0005886; GO:0007268	extracellular region; neuropeptide signaling pathway; opioid peptide activity; plasma membrane; synaptic transmission	Secreted					
**889**	CELR2	CELR2_HUMAN	CELR2; Q5T2Y7	Q9HCU4; Q5T2Y7; Q92566	NP_001399	Cadherin EGF LAG seven-pass G-type receptor 2 (Epidermal growth factor-like 2) (Multiple epidermal growth factor-like domains 3) (Flamingo 1)	GO:0004930; GO:0016055; GO:0005509; GO:0005737; GO:0048813; GO:0007156; GO:0016021; GO:0021999; GO:0007218; GO:0005886; GO:0005515; GO:0022407; GO:0032583	G-protein coupled receptor activity; Wnt receptor signaling pathway; calcium ion binding; cytoplasm; dendrite morphogenesis; homophilic cell adhesion; integral to membrane; neural plate anterioposterior pattern formation; neuropeptide signaling pathway; plasma membrane; protein binding; regulation of cell-cell adhesion; regulation of gene-specific transcription	CellmembraneMultipassmembraneprotein					
**890**	ZFAN1	ZFAN1_HUMAN	ZFAN1	Q8TCF1; Q658R7; Q6IA32; Q6PGQ6; Q9H810	NP_078975	AN1-type zinc finger protein 1	GO:0008270	zinc ion binding						
**891**	DDB1	DDB1_HUMAN	DDB1	Q16531; A6NG77; B2R648; O15176; Q13289; Q58F96	NP_001914	DNA damage-binding protein 1 (Damage-specific DNA-binding protein 1) (UV-damaged DNA-binding factor) (DDB p127 subunit) (DNA damage-binding protein a) (DDBa) (UV-damaged DNA-binding protein 1) (UV-DDB 1) (Xeroderma pigmentosum group E-complementing protein) (XPCe) (XPE-binding factor) (XPE-BF) (HBV X-associated protein 1) (XAP-1)	GO:0000075; GO:0005737; GO:0003684; GO:0044419; GO:0019941; GO:0005654; GO:0000718; GO:0005515	cell cycle checkpoint; cytoplasm; damaged DNA binding; interspecies interaction between organisms; modification-dependent protein catabolic process; nucleoplasm; nucleotide-excision repair, DNA damage removal; protein binding	CytoplasmNucleus			Known in Uniprot to be phosphorylated		
**892**	ILEU	ILEU_HUMAN	ILEU	P30740; Q53FB9; Q5W0E1	NP_109591	Leukocyte elastase inhibitor (LEI) (Monocyte/neutrophil elastase inhibitor) (M/NEI) (EI) (Serpin B1)	GO:0005737; GO:0004867	cytoplasm; serine-type endopeptidase inhibitor activity	Cytoplasm			Known in Uniprot to be phosphorylated		
**893**	RAB1B	RAB1B_HUMAN	RAB1B; A8K7S1	Q9H0U4; A8K7S1	NP_112243	Ras-related protein Rab-1B	GO:0005525; GO:0005794; GO:0016020; GO:0005515; GO:0015031; GO:0007264	GTP binding; Golgi apparatus; membrane; protein binding; protein transport; small GTPase mediated signal transduction	MembraneLipidanchorCytoplasmicsideCytoplasm					
**894**	HYI	HYI_HUMAN	Q9BZR4; HYI	Q9BZR4; Q5T013; Q5Q9A2; Q7Z778; Q96S83; Q9BZR3	NP_112484	Putative hydroxypyruvate isomerase (EC 5.3.1.22) (Endothelial cell apoptosis protein E-CE1)	GO:0008903	hydroxypyruvate isomerase activity						
**895**	C1TC	C1TC_HUMAN	C1TC	P11586; Q86VC9; Q9BVP5	NP_005947	C-1-tetrahydrofolate synthase, cytoplasmic (C1-THF synthase) [Includes: Methylenetetrahydrofolate dehydrogenase (EC 1.5.1.5); Methenyltetrahydrofolate cyclohydrolase (EC 3.5.4.9); Formyltetrahydrofolate synthetase (EC 6.3.4.3)]	GO:0005524; GO:0009396; GO:0004329; GO:0000105; GO:0004477; GO:0009086; GO:0004488; GO:0005739; GO:0006730; GO:0055114; GO:0005515; GO:0006164	ATP binding; folic acid and derivative biosynthetic process; formate-tetrahydrofolate ligase activity; histidine biosynthetic process; methenyltetrahydrofolate cyclohydrolase activity; methionine biosynthetic process; methylenetetrahydrofolate dehydrogenase (NADP+) activity; mitochondrion; one-carbon compound metabolic process; oxidation reduction; protein binding; purine nucleotide biosynthetic process	Cytoplasm			Known in Uniprot to be phosphorylated		
**896**	SH3L3	SH3L3_HUMAN	SH3L3; Q5T122	Q9H299; Q5T122	NP_112576	SH3 domain-binding glutamic acid-rich-like protein 3 (SH3 domain-binding protein 1) (SH3BP-1)	GO:0045454; GO:0005737; GO:0009055; GO:0005634; GO:0015035	cell redox homeostasis; cytoplasm; electron carrier activity; nucleus; protein disulfide oxidoreductase activity	CytoplasmNucleus					
**897**	RB33B	RB33B_HUMAN	RB33B	Q9H082; Q4W5B0; B2R987	NP_112586	Ras-related protein Rab-33B	GO:0005525; GO:0000139; GO:0015031; GO:0007264	GTP binding; Golgi membrane; protein transport; small GTPase mediated signal transduction	GolgiapparatusmembraneLipidanchor					
**898**	UCK1	UCK1_HUMAN	UCK1	Q9HA47; Q5JT12; Q6IA74	NP_113620	Uridine-cytidine kinase 1 (UCK 1) (EC 2.7.1.48) (Uridine monophosphokinase 1) (Cytidine monophosphokinase 1)	GO:0005524; GO:0005829; GO:0008152; GO:0016773; GO:0004849	ATP binding; cytosol; metabolic process; phosphotransferase activity, alcohol group as acceptor; uridine kinase activity						Kinase
**899**	PARP9	PARP9_HUMAN	PARP9	Q8IXQ6; Q8TCP3; Q9BZL8; Q9BZL9	NP_113646; NP_001139574	Poly [ADP-ribose] polymerase 9 (PARP-9) (EC 2.4.2.30) (B aggressive lymphoma protein)	GO:0003950; GO:0016477; GO:0005634	NAD+ ADP-ribosyltransferase activity; cell migration; nucleus	Nucleus					
**900**	SH3L2	SH3L2_HUMAN	SH3L2; Q7Z734	Q9UJC5; Q5VV96; Q9BPY5; Q7Z734; Q2VPC2; Q6P9E8	NP_113657	SH3 domain-binding glutamic acid-rich-like protein 2 (Fovea-associated SH3 domain-binding protein); SH3 domain binding glutamic acid-rich protein like 2 (cDNA, FLJ92017, Homo sapiens SH3 domain binding glutamic acid-rich protein like 2(SH3BGRL2), mRNA)	GO:0017124; GO:0005634;	SH3 domain binding; nucleus;	Nucleus;					
**901**	VAMP8	VAMP8_HUMAN	VAMP8	Q9BV40; Q53SP9; O60625; Q6IB09	NP_003752	Vesicle-associated membrane protein 8 (VAMP-8) (Endobrevin) (EDB)	GO:0005769; GO:0016021; GO:0005624; GO:0016192	early endosome; integral to membrane; membrane fraction; vesicle-mediated transport	MembraneSinglepasstypeIVmembraneprotein			Known in Uniprot to be phosphorylated		
**902**	Q6IPF4	Q6IPF4_HUMAN	Q6IPF4; Q68Y55	Q6IPF4; Q68Y55	NP_005007; NP_001092090; NP_001122383; NP_001122384; NP_001122385; NP_001122386; NP_114366	Poly(RC) binding protein 2 (Poly(RC) binding protein 2, isoform CRA_d)	GO:0003723	RNA binding					known in HPRD to be phosphorylated	
**903**	FA62A	FA62A_HUMAN	FA62A; A0FGR7; ESYT1	Q9BSJ8; A0FGR7; A8K2S2; O94848; Q6PJN4; Q9H6J1; Q9H6W2; Q9Y416	NP_056107	Extended synaptotagmin-1 (E-Syt1) (Membrane-bound C2 domain-containing protein) (Protein FAM62A)	GO:0016021	integral to membrane	MembraneMultipassmembraneprotein			Known in Uniprot to be phosphorylated		
**904**	HDHD2	HDHD2_HUMAN	Q9H0R4; HDHD2; A8K7T3	Q9H0R4; Q96NV4; A8K7T3	NP_115500	Haloacid dehalogenase-like hydrolase domain-containing protein 2	GO:0016787; GO:0008152	hydrolase activity; metabolic process						
**905**	TCHP	TCHP_HUMAN	Q9BT92; TCHP	Q9BT92; Q8NAG0	NP_115676; NP_001137324	Trichoplein keratin filament-binding protein (Protein TCHP) (Tumor suppressor protein)	GO:0045179; GO:0045095; GO:0005515	apical cortex; keratin filament; protein binding	Cytoplasmcytoskeleton					
**906**	SLD5	SLD5_HUMAN	Q9BRT9; SLD5; B2R8H5	Q9BRT9; Q8N648; B2R8H5	NP_115712	DNA replication complex GINS protein SLD5 (GINS complex subunit 4)	GO:0006260; GO:0005737; GO:0005634	DNA replication; cytoplasm; nucleus	CytoplasmNucleus					
**907**	VPS25	VPS25_HUMAN	VPS25; B2R581	Q9BRG1; B2R581	NP_115729	Vacuolar protein-sorting-associated protein 25 (hVps25) (ESCRT-II complex subunit VPS25) (ELL-associated protein of 20 kDa) (Dermal papilla-derived protein 9)	GO:0005737; GO:0005634; GO:0015031; GO:0006355	cytoplasm; nucleus; protein transport; regulation of transcription, DNA-dependent	CytoplasmNucleusnucleoplasm					
**908**	CPNS2	CPNS2_HUMAN	CPNS2	Q96L46; Q9BPV4	NP_115706	Calpain small subunit 2 (CSS2) (Calcium-dependent protease small subunit 2)	GO:0005509; GO:0005737; GO:0005886	calcium ion binding; cytoplasm; plasma membrane	CytoplasmCellmembrane					
**909**	HINT2	HINT2_HUMAN	HINT2; Q5TCW3	Q9BX68; Q5TCW3	NP_115982	Histidine triad nucleotide-binding protein 2 (HINT-2) (EC 3.-.-.-) (HINT-3) (HIT-17kDa) (PKCI-1-related HIT protein); Histidine triad nucleotide binding protein 2 (Histidine triad nucleotide binding protein 2, isoform CRA_b)	GO:0005576; GO:0016787; GO:0005739; GO:0003824	extracellular region; hydrolase activity; mitochondrion; catalytic activity	Secreted;					

	Protein Name	Uniprot Name	Other Names	Uniprot IDs	Refseq IDs	Protein Description	GO ID	GO terms	Subcellular location	Comments	Comments	Comments - Database Search	Comments - Database Search	Function
**910**	LOXL3	LOXL3_HUMAN	LOXL3	P58215; Q96RS1	NP_115992	Lysyl oxidase homolog 3 (EC 1.4.3.-) (Lysyl oxidase-like protein 3)	GO:0005507; GO:0005576; GO:0016020; GO:0055114; GO:0004720; GO:0005044	copper ion binding; extracellular region; membrane; oxidation reduction; protein-lysine 6-oxidase activity; scavenger receptor activity	Secretedextracellularspace					
**911**	TXNL5	TXNL5_HUMAN	TXNL5; TXD17; A8K7E8	Q9BRA2; A8K7E8	NP_116120	Thioredoxin domain-containing protein 17 (Thioredoxin-like protein 5) (14 kDa thioredoxin-related protein) (TRP14) (Protein 42-9-9)	GO:0005829; GO:0009055; GO:0004601; GO:0005515; GO:0047134; GO:0033209	cytosol; electron carrier activity; peroxidase activity; protein binding; protein-disulfide reductase activity; tumor necrosis factor-mediated signaling pathway	Cytoplasm					
**912**	ABHEB	ABHEB_HUMAN	ABHEB	Q96IU4; Q86VK8; Q8N8W5	NP_116139; NP_001139786	Abhydrolase domain-containing protein 14B (EC 3.-.-.-) (CCG1-interacting factor B)	GO:0005737; GO:0016787; GO:0005634	cytoplasm; hydrolase activity; nucleus	CytoplasmNucleus					
**913**	CA198	CA198_HUMAN	CA198	Q9H425	NP_116189	Uncharacterized protein C1orf198						Known in Uniprot to be phosphorylated		
**914**	CLIC1	CLIC1_HUMAN	CLIC1; Q5SRT3	O00299; Q502X1; Q15089; Q5SRT3	NP_001279	Chloride intracellular channel protein 1 (Nuclear chloride ion channel 27) (NCC27) (Chloride channel ABP) (Regulatory nuclear chloride ion channel protein) (hRNCC); Chloride intracellular channel 1 (cDNA, FLJ94671, Homo sapiens chloride intracellular channel 1 (CLIC1), mRNA)	GO:0005903; GO:0031404; GO:0006821; GO:0005624; GO:0031965; GO:0005886; GO:0007165; GO:0005625; GO:0005247; GO:0051059; GO:0005829; GO:0006952; GO:0016020; GO:0043234; GO:0007584; GO:0006986	brush border; chloride ion binding; chloride transport; membrane fraction; nuclear membrane; plasma membrane; signal transduction; soluble fraction; voltage-gated chloride channel activity; NF-kappaB binding; cytosol; defense response; membrane; protein complex; response to nutrient; response to unfolded protein	NucleusNucleusmembraneSinglepassmembraneproteinCytoplasmCellmembraneSinglepass membraneprotein;			Known in Uniprot to be phosphorylated		
**915**	RS12	RS12_HUMAN	Q76M58; RS12	Q76M58; P25398	NP_001007; XP_293042	40S ribosomal protein S12	GO:0022627; GO:0003735; GO:0006414	cytosolic small ribosomal subunit; structural constituent of ribosome; translational elongation	Cytoplasm					
**916**	TBA6	TBA6_HUMAN	TBA6; TBA1C	Q9BQE3	NP_116093	Tubulin alpha-1C chain (Tubulin alpha-6 chain) (Alpha-tubulin 6)	GO:0005525; GO:0003924; GO:0005874; GO:0007018; GO:0005515; GO:0043234; GO:0051258; GO:0005198	GTP binding; GTPase activity; microtubule; microtubule-based movement; protein binding; protein complex; protein polymerization; structural molecule activity				Known in Uniprot to be phosphorylated		
**917**	CADH4	CADH4_HUMAN	CADH4	P55283; Q2M208; Q5VZ44; Q9BZ05	NP_001785	Cadherin-4 (Retinal cadherin) (R-cadherin) (R-CAD)	GO:0005509; GO:0007156; GO:0005515	calcium ion binding; homophilic cell adhesion; protein binding	CellmembraneSinglepasstypeImembraneprotein					
**918**	RL5	RL5_HUMAN	RL5; A2RUM7	P46777; A2RUM7; Q32LZ3; Q53HH6; Q9H3F4	NP_000960; XP_301845; XP_294438	60S ribosomal protein L5; Ribosomal protein L5 (Ribosomal protein L5, isoform CRA_c) (cDNA, FLJ95579, Homo sapiens ribosomal protein L5 (RPL5), mRNA)	GO:0008097; GO:0022625; GO:0005634; GO:0005515; GO:0006364; GO:0042273; GO:0003735; GO:0006414; GO:0005840; GO:0006412	5S rRNA binding; cytosolic large ribosomal subunit; nucleus; protein binding; rRNA processing; ribosomal large subunit biogenesis; structural constituent of ribosome; translational elongation; ribosome; translation	CytoplasmNucleusnucleolus;			Known in Uniprot to be phosphorylated		
**919**	K1H1	K1H1_HUMAN	K1H1; Q9UE12	Q9UE12; Q15323	NP_002268	Keratin, type I cuticular Ha1 (Hair keratin, type I Ha1) (Keratin-31) (K31)	GO:0008544; GO:0005882; GO:0005515; GO:0005200	epidermis development; intermediate filament; protein binding; structural constituent of cytoskeleton						
**920**	RS2	RS2_HUMAN	RS2	P15880; Q3MIB1	XP_042500; NP_002943	40S ribosomal protein S2 (S4) (LLRep3 protein)	GO:0003723; GO:0022627; GO:0048602; GO:0048604; GO:0005654; GO:0003735; GO:0006414	RNA binding; cytosolic small ribosomal subunit; fibroblast growth factor 1 binding; fibroblast growth factor 3 binding; nucleoplasm; structural constituent of ribosome; translational elongation				Known in Uniprot to be phosphorylated		
**921**	SYRC	SYRC_HUMAN	SYRC	P54136; Q9BWA1	NP_002878	Arginyl-tRNA synthetase, cytoplasmic (EC 6.1.1.19) (Arginine--tRNA ligase) (ArgRS)	GO:0005524; GO:0004814; GO:0006420; GO:0005737; GO:0005634; GO:0005515; GO:0005625	ATP binding; arginine-tRNA ligase activity; arginyl-tRNA aminoacylation; cytoplasm; nucleus; protein binding; soluble fraction	Cytoplasm					
**922**	K1C14	K1C14_HUMAN	K1C14	P02533; Q14715; Q53XY3; Q9BUE3; Q9UBN2; Q9UBN3; Q9UCY4	NP_000517	Keratin, type I cytoskeletal 14 (Cytokeratin-14) (CK-14) (Keratin-14) (K14)	GO:0005737; GO:0008544; GO:0045110; GO:0045095; GO:0005634; GO:0005515; GO:0005200	cytoplasm; epidermis development; intermediate filament bundle assembly; keratin filament; nucleus; protein binding; structural constituent of cytoskeleton	CytoplasmNucleus					
**923**	CASP1	CASP1_HUMAN	CASP1	P29466; Q53EY6; Q6DMQ1; Q6GSS3; Q6PI75; Q9UCN3	XP_301313; NP_001214; NP_150634; NP_150635; NP_150636; NP_150637	Caspase-1 (CASP-1) (EC 3.4.22.36) (Interleukin-1 beta convertase) (IL-1BC) (Interleukin-1 beta-converting enzyme) (IL-1 beta-converting enzyme) (ICE) (p45) [Cleaved into: Caspase-1 subunit p20; Caspase-1 subunit p10]	GO:0008656; GO:0004197; GO:0005737; GO:0005634; GO:0043123; GO:0005515; GO:0006508; GO:0042981	caspase activator activity; cysteine-type endopeptidase activity; cytoplasm; nucleus; positive regulation of I-kappaB kinase/NF-kappaB cascade; protein binding; proteolysis; regulation of apoptosis	Cytoplasm				known in HPRD to be phosphorylated	
**924**	PSA	PSA_HUMAN	PSA	P55786; Q6P145; Q9NP16; Q9UEM2	NP_006301	Puromycin-sensitive aminopeptidase (PSA) (EC 3.4.11.-)	GO:0004177; GO:0005829; GO:0008237; GO:0005634; GO:0005515; GO:0006508; GO:0008270	aminopeptidase activity; cytosol; metallopeptidase activity; nucleus; protein binding; proteolysis; zinc ion binding	CytoplasmNucleus					
**925**	BMPR2	BMPR2_HUMAN	BMPR2	Q13873; Q16569	NP_001195	Bone morphogenetic protein receptor type-2 (EC 2.7.11.30) (Bone morphogenetic protein receptor type II) (BMP type II receptor) (BMPR-II)	GO:0005524; GO:0030509; GO:0048286; GO:0009952; GO:0009986; GO:0005887; GO:0000287; GO:0030145; GO:0001707; GO:0003085; GO:0045906; GO:0030501; GO:0045669; GO:0006468; GO:0005515; GO:0042127; GO:0014916; GO:0006366; GO:0005024; GO:0048010	ATP binding; BMP signaling pathway; alveolus development; anterior/posterior pattern formation; cell surface; integral to plasma membrane; magnesium ion binding; manganese ion binding; mesoderm formation; negative regulation of systemic arterial blood pressure; negative regulation of vasoconstriction; positive regulation of bone mineralization; positive regulation of osteoblast differentiation; protein amino acid phosphorylation; protein binding; regulation of cell proliferation; regulation of lung blood pressure; transcription from RNA polymerase II promoter; transforming growth factor beta receptor activity; vascular endothelial growth factor receptor signaling pathway	MembraneSinglepasstypeImembraneprotein			Known in Uniprot to be phosphorylated		Kinase
**926**	K2C71	K2C71_HUMAN	Q96DU2; K2C71	Q96DU2; Q3SY84; B3KVC1; Q3SY85	NP_258259	Keratin, type II cytoskeletal 71 (Cytokeratin-71) (CK-71) (Keratin-71) (K71) (Type II keratin-34) (Type II inner root sheath-specific keratin-K6irs1) (Keratin 6 irs) (hK6irs) (hK6irs1)	GO:0045095; GO:0005198	keratin filament; structural molecule activity						
**927**	ITPA	ITPA_HUMAN	ITPA	Q9BY32; A4UIM5; O14878; Q5JWH4; Q9BYN1; Q9BYX0; Q9H3H8	NP_258412; NP_852470	Inosine triphosphate pyrophosphatase (ITPase) (Inosine triphosphatase) (EC 3.6.1.19) (Putative oncogene protein hlc14-06-p)	GO:0005737; GO:0000287; GO:0047429; GO:0009117	cytoplasm; magnesium ion binding; nucleoside-triphosphate diphosphatase activity; nucleotide metabolic process	Cytoplasm					Phosphatase
**928**	SYSC	SYSC_HUMAN	SYSC; Q5T5C8; B2R6Y9	P49591; Q5T5C8; Q9NSE3; B2R6Y9	NP_006504	Seryl-tRNA synthetase, cytoplasmic (EC 6.1.1.11) (Seryl-tRNA(Ser/Sec) synthetase) (Serine--tRNA ligase) (SerRS)	GO:0005524; GO:0003723; GO:0005737; GO:0005515; GO:0004828; GO:0006434; GO:0008033	ATP binding; RNA binding; cytoplasm; protein binding; serine-tRNA ligase activity; seryl-tRNA aminoacylation; tRNA processing	Cytoplasm			Known in Uniprot to be phosphorylated		
**929**	MRCKB	MRCKB_HUMAN	MRCKB; Q86XZ8	Q9Y5S2; Q2L7A5; Q86TJ1; Q9ULU5; Q86XZ8	NP_006026	Serine/threonine-protein kinase MRCK beta (EC 2.7.11.1) (CDC42-binding protein kinase beta) (Myotonic dystrophy kinase-related CDC42-binding kinase beta) (Myotonic dystrophy protein kinase-like beta) (MRCK beta) (DMPK-like beta); CDC42BPB protein (Fragment)	GO:0005524; GO:0031532; GO:0031252; GO:0005911; GO:0005737; GO:0005856; GO:0019992; GO:0007163; GO:0007242; GO:0000287; GO:0006468; GO:0005515; GO:0004674; GO:0005083; GO:0008270	ATP binding; actin cytoskeleton reorganization; cell leading edge; cell-cell junction; cytoplasm; cytoskeleton; diacylglycerol binding; establishment or maintenance of cell polarity; intracellular signaling cascade; magnesium ion binding; protein amino acid phosphorylation; protein binding; protein serine/threonine kinase activity; small GTPase regulator activity; zinc ion binding	Cytoplasm;			Known in Uniprot to be phosphorylated		Kinase
**930**	ZIM3	ZIM3_HUMAN	ZIM3	Q96PE6; Q14CA6	NP_443114	Zinc finger imprinted 3	GO:0003677; GO:0005634; GO:0006355; GO:0008270	DNA binding; nucleus; regulation of transcription, DNA-dependent; zinc ion binding	Nucleus					
**931**	TOP1M	TOP1M_HUMAN	TOP1M	Q969P6; Q86V82	NP_443195	DNA topoisomerase I, mitochondrial (EC 5.99.1.2) (TOP1mt)	GO:0003918; GO:0003917; GO:0006265; GO:0006268; GO:0005694; GO:0042645	DNA topoisomerase (ATP-hydrolyzing) activity; DNA topoisomerase type I activity; DNA topological change; DNA unwinding during replication; chromosome; mitochondrial nucleoid	Mitochondrion					
**932**	GRP78	GRP78_HUMAN	GRP78; B0QZ61	P11021; B0QZ61; Q2EF78; Q9NPF1; Q9UK02	NP_005338; XP_291482	78 kDa glucose-regulated protein (GRP 78) (Heat shock 70 kDa protein 5) (Immunoglobulin heavy chain-binding protein) (BiP) (Endoplasmic reticulum lumenal Ca(2+)-binding protein grp78)	GO:0005524; GO:0005793; GO:0006916; GO:0005509; GO:0043027; GO:0009986; GO:0042149; GO:0005788; GO:0030176; GO:0042470; GO:0043154; GO:0005634; GO:0048471; GO:0030674; GO:0051082	ATP binding; ER-Golgi intermediate compartment; anti-apoptosis; calcium ion binding; caspase inhibitor activity; cell surface; cellular response to glucose starvation; endoplasmic reticulum lumen; integral to endoplasmic reticulum membrane; melanosome; negative regulation of caspase activity; nucleus; perinuclear region of cytoplasm; protein binding, bridging; unfolded protein binding	EndoplasmicreticulumlumenMelanosome			Known in Uniprot to be phosphorylated		

	Protein Name	Uniprot Name	Other Names	Uniprot IDs	Refseq IDs	Protein Description	GO ID	GO terms	Subcellular location	Comments	Comments	Comments - Database Search	Comments - Database Search	Function
**933**	DCD	DCD_HUMAN	DCD; Q53YJ2	P81605; Q53YJ2; P58461	NP_444513	Dermcidin (Preproteolysin) [Cleaved into: Survival-promoting peptide; DCD-1]; Dermcidin (Proteolysis inducing factor)	GO:0042742; GO:0050832; GO:0005576; GO:0030145; GO:0005515; GO:0006805;	defense response to bacterium; defense response to fungus; extracellular region; manganese ion binding; protein binding; xenobiotic metabolic process;	Secreted;					
**934**	SEPR	SEPR_HUMAN	SEPR; Q53TP5	Q53TP5; Q12884; O00199; Q86Z29; Q99998; Q9UID4	NP_004451	Seprase (EC 3.4.21.-) (Fibroblast activation protein alpha) (Integral membrane serine protease) (170 kDa melanoma membrane-bound gelatinase); Fibroblast activation protein, alpha, isoform CRA_a (Putative uncharacterized protein FAP)	GO:0008239; GO:0016021; GO:0030027; GO:0004222; GO:0005886; GO:0042803; GO:0006508; GO:0004252; GO:0004274; GO:0016020; GO:0004287	dipeptidyl-peptidase activity; integral to membrane; lamellipodium; metalloendopeptidase activity; plasma membrane; protein homodimerization activity; proteolysis; serine-type endopeptidase activity; dipeptidyl-peptidase IV activity; membrane; prolyl oligopeptidase activity	CellmembraneSinglepasstypeIImembraneproteinCellprojectionlamellipodiummembraneSinglepasstypeIImembraneproteinCellprojectioninvadopodiummembraneSinglepasstypeIImembraneprotein;					
**935**	RAB8A	RAB8A_HUMAN	RAB8A; Q6FHV5	P61006; Q6FHV5; P24407	NP_005361	Ras-related protein Rab-8A (Oncogene c-mel)	GO:0005525; GO:0048210; GO:0042384; GO:0031513; GO:0005886; GO:0005515; GO:0015031; GO:0007264; GO:0006904	GTP binding; Golgi vesicle fusion to target membrane; cilium assembly; nonmotile primary cilium; plasma membrane; protein binding; protein transport; small GTPase mediated signal transduction; vesicle docking during exocytosis	CellmembraneLipidanchorCytoplasmicside			Known in Uniprot to be phosphorylated		
**936**	COX5B	COX5B_HUMAN	COX5B; Q53YB7	P10606; Q53YB7; Q96J18; Q99610	NP_001853	Cytochrome c oxidase subunit 5B, mitochondrial (Cytochrome c oxidase polypeptide Vb); Cytochrome c oxidase subunit Vb (Putative uncharacterized protein COX5B)	GO:0004129; GO:0005743; GO:0007585; GO:0008270; GO:0005740	cytochrome-c oxidase activity; mitochondrial inner membrane; respiratory gaseous exchange; zinc ion binding; mitochondrial envelope	Mitochondrioninnermembrane;					
**937**	K2C1	K2C1_HUMAN	K2C1	P04264; B2RA01; Q14720; Q6GSJ0; Q9H298	NP_006112	Keratin, type II cytoskeletal 1 (Cytokeratin-1) (CK-1) (Keratin-1) (K1) (67 kDa cytokeratin) (Hair alpha protein)	GO:0001867; GO:0008544; GO:0042730; GO:0045095; GO:0005886; GO:0005515; GO:0004872; GO:0045765; GO:0006979; GO:0005200; GO:0005529	complement activation, lectin pathway; epidermis development; fibrinolysis; keratin filament; plasma membrane; protein binding; receptor activity; regulation of angiogenesis; response to oxidative stress; structural constituent of cytoskeleton; sugar binding	Cellmembrane			Known in Uniprot to be phosphorylated	Known in HPRD to be phosphorylated	
**938**	THTR	THTR_HUMAN	THTR; B3KRM1	Q16762; Q6IB06; B3KRM1	NP_003303	Thiosulfate sulfurtransferase (EC 2.8.1.1) (Rhodanese)	GO:0009440; GO:0005856; GO:0005759; GO:0005886; GO:0008272; GO:0004792	cyanate catabolic process; cytoskeleton; mitochondrial matrix; plasma membrane; sulfate transport; thiosulfate sulfurtransferase activity	Mitochondrionmatrix			Known in Uniprot to be phosphorylated		
**939**	DYH7	DYH7_HUMAN	Q8WXX0; DYH7	Q8WXX0; O00433; O95492; Q4G152; Q53QX7; Q53S64; Q53T02; Q68CY5; Q8N1Z2; Q9UMS3; Q9Y2F3	NP_061720	Dynein heavy chain 7, axonemal (Axonemal beta dynein heavy chain 7) (Ciliary dynein heavy chain 7) (Dynein heavy chain-like protein 2) (HDHC2)	GO:0005524; GO:0016887; GO:0005858; GO:0001539; GO:0005929; GO:0005874; GO:0003777; GO:0007018	ATP binding; ATPase activity; axonemal dynein complex; ciliary or flagellar motility; cilium; microtubule; microtubule motor activity; microtubule-based movement	Cellprojectionciliumaxoneme					
**940**	1B07	1B07_HUMAN	1B07; Q96DW9; Q5SRJ2	P01889; Q5SRJ2; Q29638; Q29681; Q29854; Q29861; Q31613; Q9GIX1; Q9TP95; Q96DW9	NP_005505	HLA class I histocompatibility antigen, B-7 alpha chain (MHC class I antigen B*7); Major histocompatibility complex, class I, B (cDNA, FLJ94549, Homo sapiens major histocompatibility complex, class I, B (HLA-B),mRNA); HLA-B protein	GO:0042612; GO:0032393; GO:0002474; GO:0006955; GO:0005887; GO:0044419; GO:0016021	MHC class I protein complex; MHC class I receptor activity; antigen processing and presentation of peptide antigen via MHC class I; immune response; integral to plasma membrane; interspecies interaction between organisms; integral to membrane	MembraneSinglepasstypeImembraneprotein;				Known in HPRD to be phosphorylated	
**941**	TBA3	TBA3_HUMAN	TBA3; TBA1A; A8K0B8; TBAK; TBA1B	Q71U36; P04687; P05209; A8K0B8; P68363; Q27I68	NP_006000; XP_292459; NP_006073	Tubulin alpha-1A chain (Tubulin B-alpha-1) (Tubulin alpha-3 chain) (Alpha-tubulin 3); Tubulin alpha-1B chain (Tubulin alpha-ubiquitous chain) (Alpha-tubulin ubiquitous) (Tubulin K-alpha-1)	GO:0005525; GO:0003924; GO:0005874; GO:0007018; GO:0043234; GO:0051258; GO:0005198	GTP binding; GTPase activity; microtubule; microtubule-based movement; protein complex; protein polymerization; structural molecule activity				Known in Uniprot to be phosphorylated		
**942**	VPS35	VPS35_HUMAN	VPS35	Q96QK1; Q561W2; Q9H016; Q9H096; Q9H4P3; Q9H8J0; Q9NRS7; Q9NVG2; Q9NX80; Q9NZK2	NP_060676	Vacuolar protein sorting-associated protein 35 (Vesicle protein sorting 35) (hVPS35) (Maternal-embryonic 3)	GO:0005829; GO:0005768; GO:0016020; GO:0005515; GO:0015031; GO:0042147	cytosol; endosome; membrane; protein binding; protein transport; retrograde transport, endosome to Golgi	CytoplasmMembranePeripheralmembraneprotein			Known in Uniprot to be phosphorylated		
**943**	CSKI1	CSKI1_HUMAN	CSKI1	Q8WXD9; Q9P2P0	NP_065815	Caskin-1 (CASK-interacting protein 1)	GO:0005737; GO:0005515; GO:0007165	cytoplasm; protein binding; signal transduction	Cytoplasm			Known in Uniprot to be phosphorylated		
**944**	DYL2	DYL2_HUMAN	DYL2; B2R5B4	Q96FJ2; B2R5B4	NP_542408	Dynein light chain 2, cytoplasmic (Dynein light chain LC8-type 2)	GO:0005829; GO:0030286; GO:0005874; GO:0003777; GO:0007017; GO:0016459; GO:0005886; GO:0005515	cytosol; dynein complex; microtubule; microtubule motor activity; microtubule-based process; myosin complex; plasma membrane; protein binding	Cytoplasm					
**945**	TP4A2	TP4A2_HUMAN	TP4A2	Q12974; O00649; Q15197; Q15259; Q15260; O15261	NP_003470; NP_536316; NP_536317	Protein tyrosine phosphatase type IVA 2 (EC 3.1.3.48) (Protein-tyrosine phosphatase 4a2) (Protein-tyrosine phosphatase of regenerating liver 2) (PRL-2) (PTP(CAAXII)) (HU-PP-1) (OV-1)	GO:0005769; GO:0005886; GO:0004727; GO:0006470; GO:0005515	early endosome; plasma membrane; prenylated protein tyrosine phosphatase activity; protein amino acid dephosphorylation; protein binding	CellmembraneEarlyendosomeCytoplasm					Phosphatase
**946**	COX7R	COX7R_HUMAN	COX7R; Q6FGA0	O14548; Q6FGA0; Q9P118	NP_004709	Cytochrome c oxidase subunit 7A-related protein, mitochondrial (Cytochrome c oxidase subunit VIIa-related protein) (COX7a-related protein) (EB1); COX7A2L protein (cDNA FLJ35592 fis, clone SPLEN2007695, highly similar to Cytochrome c oxidase subunit VIIa-related protein, mitochondrial) (Cytochrome c oxidase subunit VIIa polypeptide 2 like, isoform CRA_a) (Putative uncharacterized protein COX7A2L)	GO:0004129; GO:0009055; GO:0005746	cytochrome-c oxidase activity; electron carrier activity; mitochondrial respiratory chain	Mitochondrioninnermembrane;					
**947**	VP33B	VP33B_HUMAN	VP33B	Q9H267; Q96K14; Q9NRP6; Q9NSF3	NP_061138	Vacuolar protein sorting-associated protein 33B (hVPS33B)	GO:0031902; GO:0005765; GO:0015031; GO:0006904	late endosome membrane; lysosomal membrane; protein transport; vesicle docking during exocytosis	LateendosomemembranePeripheralmembraneproteinCytoplasmicsideLysosomemembranePeripheralmembraneproteinCytoplasmicside					
**948**	G6PI	G6PI_HUMAN	G6PI	P06744; Q9BRD3; Q9BSK5; Q9UHE6	NP_000166	Glucose-6-phosphate isomerase (GPI) (EC 5.3.1.9) (Phosphoglucose isomerase) (PGI) (Phosphohexose isomerase) (PHI) (Autocrine motility factor) (AMF) (Neuroleukin) (NLK) (Sperm antigen 36) (SA-36)	GO:0005125; GO:0005737; GO:0005615; GO:0006094; GO:0004347; GO:0006096; GO:0008083; GO:0007599; GO:0006959	cytokine activity; cytoplasm; extracellular space; gluconeogenesis; glucose-6-phosphate isomerase activity; glycolysis; growth factor activity; hemostasis; humoral immune response	CytoplasmSecreted			Known in Uniprot to be phosphorylated	Known in HPRD to be phosphorylated	
**949**	DUS19	DUS19_HUMAN	DUS19	Q8WTR2; Q547H4; B2RA79; Q8WYN4	NP_543152; NP_001135786	Dual specificity protein phosphatase 19 (EC 3.1.3.48) (EC 3.1.3.16) (Stress-activated protein kinase pathway-regulating phosphatase 1) (SAPK pathway-regulating phosphatase 1) (Protein phosphatase SKRP1) (Dual-specificity phosphatase TS-DSP1) (LMW-DSP3)	GO:0006470; GO:0004725	protein amino acid dephosphorylation; protein tyrosine phosphatase activity						Phosphatase
**950**	DACH1	DACH1_HUMAN	DACH1	Q9UI36; O75523; O75687; Q5VYY3; Q5VYY4; Q96SG3; Q96SG4; Q9H524; Q9UMH4	NP_542937; NP_004383; NP_542938	Dachshund homolog 1 (Dach1)	GO:0007275; GO:0000166; GO:0005515; GO:0006355	multicellular organismal development; nucleotide binding; protein binding; regulation of transcription, DNA-dependent	Nucleus			Known in Uniprot to be phosphorylated		
**951**	PTN18	PTN18_HUMAN	PTN18; Q53P42	Q53P42; Q99952	NP_055184	Tyrosine-protein phosphatase non-receptor type 18 (EC 3.1.3.48) (Brain-derived phosphatase)	GO:0005737; GO:0004726; GO:0005634; GO:0006470	cytoplasm; non-membrane spanning protein tyrosine phosphatase activity; nucleus; protein amino acid dephosphorylation	NucleusCytoplasm			Known in Uniprot to be phosphorylated	Known in HPRD to be phosphorylated	Phosphatase
**952**	SORC3	SORC3_HUMAN	SORC3	Q9UPU3; Q5VXF9; Q9NQJ2	NP_055793	VPS10 domain-containing receptor SorCS3	GO:0016021; GO:0008188; GO:0007218	integral to membrane; neuropeptide receptor activity; neuropeptide signaling pathway	MembraneSinglepasstypeImembraneprotein					
**953**	DPP3	DPP3_HUMAN	DPP3; Q5JPB8	Q9NY33; B2RDB5; O95748; Q969H2; Q9BV67; Q9HAL6; Q5JPB8	NP_005691; NP_569710	Dipeptidyl-peptidase 3 (EC 3.4.14.4) (Dipeptidyl-peptidase III) (DPP III) (Dipeptidyl aminopeptidase III) (Dipeptidyl arylamidase III); Putative uncharacterized protein DKFZp686O1117	GO:0004177; GO:0005737; GO:0008239; GO:0008237; GO:0006508; GO:0008270; GO:0005737	aminopeptidase activity; cytoplasm; dipeptidyl-peptidase activity; metallopeptidase activity; proteolysis; zinc ion binding; cytoplasm	Cytoplasm;			Known in Uniprot to be phosphorylated		
**954**	DLRB2	DLRB2_HUMAN	DLRB2	Q8TF09	NP_570967	Dynein light chain roadblock-type 2 (Dynein light chain 2B, cytoplasmic) (Roadblock domain-containing protein 2)	GO:0005737; GO:0005868; GO:0005874; GO:0003777; GO:0007018; GO:0005515	cytoplasm; cytoplasmic dynein complex; microtubule; microtubule motor activity; microtubule-based movement; protein binding	Cytoplasm					
**955**	SNP23	SNP23_HUMAN	SNP23; A8K287	O00161; A8K287; O00162; Q13602; Q6IAE3	NP_003816; NP_570710	Synaptosomal-associated protein 23 (SNAP-23) (Vesicle-membrane fusion protein SNAP-23)	GO:0030054; GO:0005737; GO:0006944; GO:0005634; GO:0006892; GO:0005515; GO:0015031; GO:0045202; GO:0019717; GO:0006903	cell junction; cytoplasm; membrane fusion; nucleus; post-Golgi vesicle-mediated transport; protein binding; protein transport; synapse; synaptosome; vesicle targeting	CellmembranePeripheralmembraneproteinCelljunctionsynapsesynaptosome			Known in Uniprot to be phosphorylated	Known in HPRD to be phosphorylated	

	Protein Name	Uniprot Name	Other Names	Uniprot IDs	Refseq IDs	Protein Description	GO ID	GO terms	Subcellular location	Comments	Comments	Comments - Database Search	Comments - Database Search	Function
956	PTPRJ	PTPRJ_HUMAN	PTPRJ; Q9NPR5	Q12913; Q15255; Q8NHM2; Q9NPR5	NP_002834	Receptor-type tyrosine-protein phosphatase eta (Protein-tyrosine phosphatase eta) (R-PTP-eta) (EC 3.1.3.48) (HPTP eta) (Protein-tyrosine phosphatase receptor type J) (Density-enhanced phosphatase 1) (DEP-1) (CD antigen CD148)	GO:0030054; GO:0007267; GO:0005887; GO:0005634; GO:0006470; GO:0007169; GO:0005001	cell junction; cell-cell signaling; integral to plasma membrane; nucleus; protein amino acid dephosphorylation; transmembrane receptor protein tyrosine kinase signaling pathway; transmembrane receptor protein tyrosine phosphatase activity	MembraneSinglepassty peImembraneprotein					Phosphatase
957	DPM3	DPM3_HUMAN	DPM3; Q5SR62	Q5SR62; Q9P2X0; Q5SR63; Q9BXN4; Q9BXN5	NP_061846; NP_714963	Dolichol-phosphate mannosyltransferase subunit 3 (Dolichyl-phosphate beta-D-mannosyltransferase subunit 3) (Dolichol-phosphate mannose synthase subunit 3) (DPM synthase complex subunit 3) (Mannose-P-dolichol synthase subunit 3) (MPD synthase subunit 3) (Prostin-1); Dolichyl-phosphate mannosyltransferase polypeptide 3 (Dolichyl-phosphate mannosyltransferase polypeptide 3, isoform CRA_b)	GO:0006506; GO:0033185; GO:0030176; GO:0018406; GO:0035269; GO:0005515; GO:0016757	GPI anchor biosynthetic process; dolichol-phosphate-mannose synthase complex; integral to endoplasmic reticulum membrane; protein amino acid C-linked glycosylation via 2'-alpha-mannosyl-L-tryptophan; protein amino acid O-linked mannosylation; protein binding; transferase activity, transferring glycosyl groups	Endoplasmicreticulum membraneMultipassme mbraneprotein;					
958	DMXL2	DMXL2_HUMAN	DMXL2	Q8TDJ6; O94938	NP_056078	DmX-like protein 2 (Rabconnectin-3)	GO:0017137; GO:0030054; GO:0008021	Rab GTPase binding; cell junction; synaptic vesicle	Cytoplasmicvesiclesecr etoryvesiclesynapticve siclemembranePeripher almembraneprotein			Known in Uniprot to be phosphorylated	Known in HPRD to be phosphorylated	
959	CYTN	CYTN_HUMAN	CYTN	P01037; Q96LE6; Q9UCQ6	NP_001889	Cystatin-SN (Cystatin-1) (Salivary cystatin-SA-1) (Cystain-SA-I)	GO:0004869; GO:0005576	cysteine-type endopeptidase inhibitor activity; extracellular region	Secreted					
960	AP2A1	AP2A1_HUMAN	AP2A1	O95782; Q96CI7; Q96PP6; Q96PP7; Q9H070	NP_055018	AP-2 complex subunit alpha-1 (Adapter-related protein complex 2 alpha-1 subunit) (Adaptor protein complex AP-2 subunit alpha-1) (Alpha1-adaptin) (Plasma membrane adaptor HA2/AP2 adaptin alpha A subunit) (Alpha-adaptin A) (Clathrin assembly protein complex 2 alpha-A large chain) (100 kDa coated vesicle protein A)	GO:0030122; GO:0006895; GO:0030130; GO:0005829; GO:0006897; GO:0006886; GO:0005515; GO:0008565; GO:0050690	AP-2 adaptor complex; Golgi to endosome transport; clathrin coat of trans-Golgi network vesicle; cytosol; endocytosis; intracellular protein transport; protein binding; protein transporter activity; regulation of defense response to virus by virus	GolgiapparatusCytopla smicvesicleclathrincoat edvesiclemembranePer ipheralmembraneprotei nCytoplasmicside			Known in Uniprot to be phosphorylated		
961	VATA1	VATA1_HUMAN	VATA1; Q53YD9; VATA; B2RBR8	P38606; Q53YD9; Q96DY6; Q9UHY3; B2RBR8	NP_001681	V-type proton ATPase catalytic subunit A (V-ATPase subunit A) (EC 3.6.3.14) (Vacuolar proton pump subunit alpha) (V-ATPase 69 kDa subunit) (Vacuolar ATPase isoform VA68)	GO:0005524; GO:0015986; GO:0046933; GO:0005887; GO:0046961; GO:0033180	ATP binding; ATP synthesis coupled proton transport; hydrogen ion transporting ATP synthase activity, rotational mechanism; integral to plasma membrane; proton-transporting ATPase activity, rotational mechanism; proton-transporting V-type ATPase, V1 domain				Known in Uniprot to be phosphorylated		
962	VATB1	VATB1_HUMAN	Q6P4H6; VATB1	Q6P4H6; P15313; Q53FY0	NP_001683	V-type proton ATPase subunit B, kidney isoform (V-ATPase subunit B 1) (Vacuolar proton pump subunit B 1) (Endomembrane proton pump 58 kDa subunit)	GO:0015986; GO:0012505; GO:0007588; GO:0046933; GO:0001503; GO:0046961; GO:0033180; GO:0006885; GO:0007605; GO:0016471	ATP synthesis coupled proton transport; endomembrane system; excretion; hydrogen ion transporting ATP synthase activity, rotational mechanism; ossification; proton-transporting ATPase activity, rotational mechanism; proton-transporting V-type ATPase, V1 domain; regulation of pH; sensory perception of sound; vacuolar proton-transporting V-type ATPase complex	Intracytoplasmicmemb ranePeripheralmembra neprotein					
963	IMB1	IMB1_HUMAN	IMB1; Q53XN2	Q14974; Q53XN2; Q14637; Q96J27	NP_002256	Importin subunit beta-1 (Karyopherin subunit beta-1) (Nuclear factor P97) (Importin-90); Karyopherin (Importin) beta 1 (Karyopherin (Importin) beta 1, isoform CRA_a)	GO:0006607; GO:0005829; GO:0044419; GO:0008139; GO:0005643; GO:0005654; GO:0019904; GO:0000059; GO:0000060; GO:0008565; GO:0008270; GO:0005737; GO:0005515; GO:0006610	NLS-bearing substrate import into nucleus; cytosol; interspecies interaction between organisms; nuclear localization sequence binding; nuclear pore; nucleoplasm; protein domain specific binding; protein import into nucleus, docking; protein import into nucleus, translocation; protein transporter activity; zinc ion binding; cytoplasm; protein binding; ribosomal protein import into nucleus	CytoplasmNucleusenve lope;			Known in Uniprot to be phosphorylated		
964	NLTP	NLTP_HUMAN	NLTP; Q5VVZ1; Q6NXF4	P22307; Q5VVZ1; Q15432; Q16622; Q99430; Q6NXF4	NP_002970; NP_001007 100; NP_0010071 01; NP_0010072 51; NP_001007 099	Non-specific lipid-transfer protein (EC 2.3.1.176) (Propanoyl-CoA C-acyltransferase) (NSL-TP) (Sterol carrier protein 2) (SCP-2) (Sterol carrier protein X) (SCP-X) (SCP-chi) (SCPX); Sterol carrier protein 2 (Sterol carrier protein 2, isoform CRA_a); Sterol carrier protein 2	GO:0006869; GO:0005739; GO:0016491; GO:0005777; GO:0033814; GO:0005515; GO:0006694; GO:0005498; GO:0008152; GO:0016740; GO:0007031	lipid transport; mitochondrion; oxidoreductase activity; peroxisome; propanoyl-CoA C-acyltransferase activity; protein binding; steroid biosynthetic process; sterol carrier activity; metabolic process; transferase activity; peroxisome organization	CytoplasmMitochondrio nPeroxisome;			Known in Uniprot to be phosphorylated		
965	RAB5A	RAB5A_HUMAN	RAB5A; Q6FI44	P20339; Q6FI44	NP_004153	Ras-related protein Rab-5A; Cervical cancer oncogene 10 protein (RAB5A protein) (RAB5A, member RAS oncogene family, isoform CRA_a) (cDNA, FLJ93000, Homo sapiens RAB5A, member RAS oncogene family (RAB5A), mRNA)	GO:0005525; GO:0003924; GO:0031901; GO:0006897; GO:0042470; GO:0005886; GO:0005515; GO:0015031; GO:0007264; GO:0005769; GO:0030139; GO:0045121; GO:0001726	GTP binding; GTPase activity; early endosome membrane; endocytosis; melanosome; plasma membrane; protein binding; protein transport; small GTPase mediated signal transduction; early endosome; endocytic vesicle; membrane raft; ruffle	CellmembraneLipidanc horCytoplasmicsideEarl yendosomemembraneL ipidanchorMelanosome ;			Known in Uniprot to be phosphorylated		
966	RB27A	RB27A_HUMAN	RB27A; A2RU94	Q6FI40; P51159; O00195; Q9UIR9; Q9Y5U3; A2RU94	NP_004571; NP_899057; NP_899058; NP_899059	Ras-related protein Rab-27A (Rab-27) (GTP-binding protein Ram); RAB27A, member RAS oncogene family (RAB27A, member RAS oncogene family, isoform CRA_a) (cDNA, FLJ93274, Homo sapiens RAB27A, member RAS oncogene family (RAB27A), mRNA)	GO:0005525; GO:0003924; GO:0030425; GO:0042470; GO:0016020; GO:0015031; GO:0007264	GTP binding; GTPase activity; dendrite; melanosome; membrane; protein transport; small GTPase mediated signal transduction	MembraneLipidanchor Melanosome;					
967	KAD3	KAD3_HUMAN	KAD3	Q9UIJ7; Q5VYW6; Q9H576; Q9NPB4	NP_057366	GTP:AMP phosphotransferase mitochondrial (EC 2.7.4.10) (Adenylate kinase 3) (AK 3) (Adenylate kinase 3 alpha-like 1)	GO:0005524; GO:0005525; GO:0004017; GO:0005759; GO:0006139; GO:0046899	ATP binding; GTP binding; adenylate kinase activity; mitochondrial matrix; nucleobase, nucleoside, nucleotide and nucleic acid metabolic process; nucleoside triphosphate adenylate kinase activity	Mitochondrionmatrix					Kinase
968	RAB14	RAB14_HUMAN	RAB14; B3KR31	P61106; P35287; Q5JVD4; Q6Q7K5; Q969L0; Q9UI11; B3KR31	NP_057406	Ras-related protein Rab-14	GO:0005525; GO:0003924; GO:0005795; GO:0006895; GO:0005829; GO:0005769; GO:0005770; GO:0005764; GO:0005624; GO:0007269; GO:0042175; GO:0048471; GO:0005886; GO:0005515; GO:0015031; GO:0005791; GO:0007264; GO:0030140	GTP binding; GTPase activity; Golgi stack; Golgi to endosome transport; cytosol; early endosome; late endosome; lysosome; membrane fraction; neurotransmitter secretion; nuclear envelope-endoplasmic reticulum network; perinuclear region of cytoplasm; plasma membrane; protein binding; protein transport; rough endoplasmic reticulum; small GTPase mediated signal transduction; trans-Golgi network transport vesicle	CellmembraneLipidanc horCytoplasmicside			Known in Uniprot to be phosphorylated		
969	EMSY	EMSY_HUMAN	EMSY	Q7Z589; Q4G109; Q8NBU6; Q8TE50; Q9H8I9; Q9NRH0	NP_064578	Protein EMSY	GO:0006281; GO:0016568; GO:0005634; GO:0006355	DNA repair; chromatin modification; nucleus; regulation of transcription, DNA-dependent	Nucleus			Known in Uniprot to be phosphorylated	Known in HPRD to be phosphorylated	
970	ODO2	ODO2_HUMAN	ODO2; Q7LDY7	Q7LDY7; P36957; Q9BQ32	NP_001924	Dihydrolipoyllysine-residue succinyltransferase component of 2-oxoglutarate dehydrogenase complex, mitochondrial (EC 2.3.1.61) (Dihydrolipoamide succinyltransferase component of 2-oxoglutarate dehydrogenase complex) (E2) (E2K)	GO:0004149; GO:0031405; GO:0005759; GO:0005634; GO:0045252; GO:0005515; GO:0006099	dihydrolipoyllysine-residue succinyltransferase activity; lipoic acid binding; mitochondrial matrix; nucleus; oxoglutarate dehydrogenase complex; protein binding; tricarboxylic acid cycle	Mitochondrion					
971	RAB3B	RAB3B_HUMAN	RAB3B	P20337; Q5VUL2; Q9BSI1	NP_002858	Ras-related protein Rab-3B	GO:0005525; GO:0005525; GO:0003924; GO:0005886; GO:0015031; GO:0007264	GTP binding; GTPase activity; plasma membrane; protein transport; small GTPase mediated signal transduction	CellmembraneLipidanc horCytoplasmicside			Known in Uniprot to be phosphorylated		
972	IQCD	IQCD_HUMAN	IQCD	Q96DY2; Q6ZSU0	NP_612460	IQ domain-containing protein D								
973	CKLF5	CKLF5_HUMAN	CKLF5	Q96DZ9	NP_612469; NP_001032 365	CKLF-like MARVEL transmembrane domain-containing protein 5 (Chemokine-like factor superfamily member 5)	GO:0006935; GO:0005125; GO:0005615; GO:0016021	chemotaxis; cytokine activity; extracellular space; integral to membrane	MembraneMultipassme mbraneprotein					
974	PDIA1	PDIA1_HUMAN	PDIA1; B2RDQ2	P07237; P30037; P32079; Q15205; Q6LDE5; B2RDQ2	NP_000909	Protein disulfide-isomerase (PDI) (EC 5.3.4.1) (Prolyl 4-hydroxylase subunit beta) (Cellular thyroid hormone-binding protein) (p55)	GO:0005793; GO:0045454; GO:0009986; GO:0005783; GO:0005788; GO:0005576; GO:0042470; GO:0018401; GO:0005886; GO:0004656; GO:0005515; GO:0003756	ER-Golgi intermediate compartment; cell redox homeostasis; cell surface; endoplasmic reticulum; endoplasmic reticulum lumen; extracellular region; melanosome; peptidyl-proline hydroxylation to 4-hydroxy-L-proline; plasma membrane; procollagen-proline 4-dioxygenase activity; protein binding; protein disulfide isomerase activity	EndoplasmicreticulumI umenMelanosomeCell membranePeripheralm embraneprotein					
975	STK24	STK24_HUMAN	STK24; Q5T5B3; Q5JV92	Q9Y6E0; Q5T5B3; O14840; Q5JV92	NP_003567	Serine/threonine-protein kinase 24 (EC 2.7.11.1) (STE20-like kinase MST3) (MST-3) (Mammalian STE20-like protein kinase 3)	GO:0005524; GO:0005737; GO:0006468; GO:0004674; GO:0007165	ATP binding; cytoplasm; protein amino acid phosphorylation; protein serine/threonine kinase activity; signal transduction	Cytoplasm			Known in Uniprot to be phosphorylated	Known in HPRD to be phosphorylated	Kinase
976	RDH11	RDH11_HUMAN	RDH11; B2RB26	Q8TC12; A6NDK3; Q6IAH5; Q9NRW0; Q9Y391; B2RB26	NP_057110	Retinol dehydrogenase 11 (EC 1.1.1.-) (Retinal reductase 1) (RalR1) (Prostate short-chain dehydrogenase/reductase 1) (Androgen-regulated short-chain dehydrogenase/reductase 1) (HCV core-binding protein HCBP12)	GO:0005488; GO:0005789; GO:0016021; GO:0055114; GO:0004745; GO:0042572	binding; endoplasmic reticulum membrane; integral to membrane; oxidation reduction; retinol dehydrogenase activity; retinol metabolic process	Endoplasmicreticulum membraneSinglepassty peIImembraneprotein			Known in Uniprot to be phosphorylated		
977	ECHA	ECHA_HUMAN	ECHA; B2R7L4	P40939; Q16679; Q96GT7; B2R7L4	NP_000173	Trifunctional enzyme subunit alpha, mitochondrial (TP-alpha) (78 kDa gastrin-binding protein) [Includes: Long-chain enoyl-CoA hydratase (EC 4.2.1.17); Long chain 3-hydroxyacyl-CoA dehydrogenase (EC 1.1.1.211)]	GO:0003857; GO:0003985; GO:0004300; GO:0006635; GO:0016507; GO:0016509; GO:0042645; GO:0055114	3-hydroxyacyl-CoA dehydrogenase activity; acetyl-CoA C-acetyltransferase activity; enoyl-CoA hydratase activity; fatty acid beta-oxidation; fatty acid beta-oxidation multienzyme complex; long-chain-3-hydroxyacyl-CoA dehydrogenase activity; mitochondrial nucleoid; oxidation reduction	Mitochondrion			Known in Uniprot to be phosphorylated		

	Protein Name	Uniprot Name	Other Names	Uniprot IDs	Refseq IDs	Protein Description	GO ID	GO terms	Subcellular location	Comments	Comments	Comments - Database Search	Comments - Database Search	Function
**978**	PUR9	PUR9_HUMAN	PUR9; Q53S28	P31939; Q53S28; Q13856	NP_004035	Bifunctional purine biosynthesis protein PURH [Includes: Phosphoribosylaminoimidazolecarboxamide formyltransferase (EC 2.1.2.3) (5-aminoimidazole-4-carboxamide ribonucleotide formyltransferase) (AICAR transformylase); IMP cyclohydrolase (EC 3.5.4.10) (Inosinicase) (IMP synthetase) (ATIC)]	GO:0006188; GO:0003937; GO:0004643; GO:0005515	IMP biosynthetic process; IMP cyclohydrolase activity; phosphoribosylaminoimidazolecarboxamide formyltransferase activity; protein binding				Known in Uniprot to be phosphorylated		
**979**	Q4ZG20	Q4ZG20_HUMAN	Q4ZG20	Q4ZG20	NP_596870	Putative uncharacterized protein TTN								
**980**	NMD3A	NMD3A_HUMAN	NMD3A	Q8TCU5; B3DLF9; Q5VTR3; Q8TF29; Q8WXI6	NP_597702	Glutamate [NMDA] receptor subunit 3A (N-methyl-D-aspartate receptor subtype NR3A) (NMDAR-L)	GO:0004972; GO:0017146; GO:0005509; GO:0006816; GO:0030054; GO:0043025; GO:0005234; GO:0016594; GO:0042802; GO:0000287; GO:0043005; GO:0045211; GO:0051721; GO:0045471	N-methyl-D-aspartate selective glutamate receptor activity; N-methyl-D-aspartate selective glutamate receptor complex; calcium ion binding; calcium ion transport; cell junction; cell soma; extracellular-glutamate-gated ion channel activity; glycine binding; identical protein binding; magnesium ion binding; neuron projection; postsynaptic membrane; protein phosphatase 2A binding; response to ethanol	CellmembraneMultipassmembraneproteinCelljunctionsynapsepostsynapticcellmembraneCelljunctionsynapsepostsynapticcellmembranepostsynapticdensity					
**981**	STML1	STML1_HUMAN	STML1; B3KQN0	Q9UBI4; O95675; Q6FGL8; Q8WYI7; Q9UMB9; Q9UMC0; Q9Y6H9; B3KQN0	NP_004800	Stomatin-like protein 1 (SLP-1) (Stomatin-related protein) (STORP) (EPB72-like 1) (UNC24 homolog)	GO:0016021; GO:0005498	integral to membrane; sterol carrier activity	MembraneSinglepasstypeIIImembraneprotein					
**982**	DAK	DAK_HUMAN	DAK; DHAK	Q3LXA3; Q53EQ9; Q9BVA7; Q9H895	NP_056348	Bifunctional ATP-dependent dihydroxyacetone kinase/FAD-AMP lyase (cyclizing) [Includes: ATP-dependent dihydroxyacetone kinase (DHA kinase) (EC 2.7.1.29) (Glycerone kinase); FAD-AMP lyase (cyclizing) (EC 4.6.1.15) (FAD-AMP lyase (cyclic FMN forming)) (FMN cyclase)]	GO:0005524; GO:0006071; GO:0004371; GO:0016829	ATP binding; glycerol metabolic process; glycerone kinase activity; lyase activity						Kinase
**983**	MIRO2	MIRO2_HUMAN	MIRO2; A2IDC2	Q8IXI1; Q8NF53; Q96C13; Q96S17; Q9BT60; Q9H7M8; A2IDC2	NP_620124	Mitochondrial Rho GTPase 2 (hMiro-2) (MIRO-2) (EC 3.6.5.-) (Ras homolog gene family member T2); Ras homolog gene family, member T2 (Ras homolog gene family, member T2, isoform CRA_a)	GO:0005525; GO:0006915; GO:0005509; GO:0019725; GO:0016787; GO:0031307; GO:0047497; GO:0005515; GO:0007264; GO:0005622	GTP binding; apoptosis; calcium ion binding; cellular homeostasis; hydrolase activity; integral to mitochondrial outer membrane; mitochondrion transport along microtubule; protein binding; small GTPase mediated signal transduction; intracellular	MitochondrionoutermembraneSinglepasstypeIVmembraneprotein;					
**984**	ARL8A	ARL8A_HUMAN	ARL8A; B3KXD0	Q96BM9; B3KXD0	NP_620150	ADP-ribosylation factor-like protein 8A (ADP-ribosylation factor-like protein 10B) (Novel small G protein indispensable for equal chromosome segregation 2)	GO:0005525; GO:0003924; GO:0043014; GO:0048487; GO:0007059; GO:0031902; GO:0005765; GO:0030496; GO:0007264; GO:0051233	GTP binding; GTPase activity; alpha-tubulin binding; beta-tubulin binding; chromosome segregation; late endosome membrane; lysosomal membrane; midbody; small GTPase mediated signal transduction; spindle midzone	LateendosomemembraneLysosomemembrane					
**985**	TRI43	TRI43_HUMAN	TRI43	Q96BQ3; Q53TJ7	XP_304295; XP_210184; NP_620155	Tripartite motif-containing protein 43	GO:0005622; GO:0005515; GO:0008270	intracellular; protein binding; zinc ion binding						
**986**	GNAT2	GNAT2_HUMAN	GNAT2; Q5T697	P19087; Q5T697	NP_005263	Guanine nucleotide-binding protein G(t) subunit alpha-2 (Transducin alpha-2 chain); Guanine nucleotide binding protein (G protein), alpha transducing activity polypeptide 2 (Guanine nucleotide binding protein (G protein), alpha transducing activity polypeptide 2, isoform CRA_a)	GO:0008020; GO:0007186; GO:0005525; GO:0005834; GO:0001917; GO:0042622; GO:0007602; GO:0007186; GO:0046549; GO:0004871	G-protein coupled photoreceptor activity; G-protein coupled receptor protein signaling pathway; GTP binding; heterotrimeric G-protein complex; photoreceptor inner segment; photoreceptor outer segment membrane; phototransduction; G-protein coupled receptor protein signaling pathway; retinal cone cell development; signal transducer activity						
**987**	HEBP1	HEBP1_HUMAN	HEBP1; A8K1G2	Q9NRV9; Q9Y5Z5; A8K1G2	NP_057071	Heme-binding protein 1 (p22HBP)	GO:0007623	circadian rhythm	Cytoplasm					
**988**	GBB2	GBB2_HUMAN	GBB2; Q6FHM2	P62879; Q6FHM2; P11016; P54312	NP_005264	Guanine nucleotide-binding protein G(I)/G(S)/G(T) subunit beta-2 (Transducin beta chain 2) (G protein subunit beta-2); GNB2 protein (cDNA FLJ77321, highly similar to Homo sapiens guanine nucleotide binding protein (G protein), beta polypeptide 2 (GNB2), mRNA) (Guanine nucleotide binding protein (G protein), beta polypeptide 2, isoform CRA_a)	GO:0007186; GO:0003924; GO:0009755; GO:0004871; GO:0005246	G-protein coupled receptor protein signaling pathway; GTPase activity; hormone-mediated signaling; signal transducer activity; calcium channel regulator activity				Known in Uniprot to be phosphorylated		
**989**	SRC8	SRC8_HUMAN	SRC8; Q53HG7	Q14247; Q53HG7	NP_005222	Src substrate cortactin (Amplaxin) (Oncogene EMS1)	GO:0005938; GO:0005856; GO:0030027; GO:0005515; GO:0001726; GO:0005625	cell cortex; cytoskeleton; lamellipodium; protein binding; ruffle; soluble fraction	CytoplasmcytoskeletonCellprojectionlamellipodiumCellprojectionruffle			Known in Uniprot to be phosphorylated	Known in HPRD to be phosphorylated	
**990**	LCK	LCK_HUMAN	LCK; Q7RTZ3	P06239; P07100; Q12850; Q13152; Q5TDH8; Q5TDH9; Q7RTZ3; Q96DW4; Q9NYT8	NP_005347; NP_001036236	Proto-oncogene tyrosine-protein kinase LCK (EC 2.7.10.2) (Lymphocyte cell-specific protein-tyrosine kinase) (p56-LCK) (LSK) (T cell-specific protein-tyrosine kinase)	GO:0005524; GO:0051117; GO:0042609; GO:0042610; GO:0005794; GO:0007265; GO:0042169; GO:0006919; GO:0006882; GO:0005829; GO:0001948; GO:0006917; GO:0044419; GO:0045121; GO:0004715; GO:0000242; GO:0043548; GO:0005886; GO:0050862; GO:0008022; GO:0019901; GO:0004722; GO:0051209; GO:0042493	ATP binding; ATPase binding; CD4 receptor binding; CD8 receptor binding; Golgi apparatus; Ras protein signal transduction; SH2 domain binding; activation of caspase activity; cellular zinc ion homeostasis; cytosol; glycoprotein binding; induction of apoptosis; interspecies interaction between organisms; membrane raft; non membrane spanning protein tyrosine kinase activity; pericentriolar material; phosphoinositide 3-kinase binding; plasma membrane; positive regulation of T cell receptor signaling pathway; protein C-terminus binding; protein kinase binding; protein serine/threonine phosphatase activity; release of sequestered calcium ion into cytosol; response to drug	CytoplasmCellmembraneLipidanchorCytoplasmicside			Known in Uniprot to be phosphorylated	Known in HPRD to be phosphorylated	Kinase
**991**	A1BG	A1BG_HUMAN	A1BG; Q68CK0	Q68CK0; P04217; Q8IYJ6; Q96P39	NP_570602	Alpha-1B-glycoprotein (Alpha-1-B glycoprotein)	GO:0005576	extracellular region	Secreted					
**992**	TF2LY	TF2LY_HUMAN	TF2LY; A2VCU1	Q8IUE0; A2VCU1	NP_631960	Homeobox protein TGIF2LY (TGFB-induced factor 2 like protein, Y-linked) (TGF(beta)induced transcription factor 2-like protein) (TGIF-like on the Y); cDNA FLJ75815, highly similar to Homo sapiens TGFB-induced factor 2-like, Y-linked (TGIF2LY), mRNA (TGFB-induced factor homeobox 2-like, Y-linked)	GO:0005634; GO:0006355; GO:0043565; GO:0003700	nucleus; regulation of transcription, DNA-dependent; sequence-specific DNA binding; transcription factor activity	Nucleus					
**993**	ATS17	ATS17_HUMAN	ATS17	Q8TE56	NP_620688	A disintegrin and metalloproteinase with thrombospondin motifs 17 (ADAMTS-17) (ADAM-TS 17) (ADAM-TS17) (EC 3.4.24.-)	GO:0004222; GO:0005578; GO:0006508; GO:0008270	metalloendopeptidase activity; proteinaceous extracellular matrix; proteolysis; zinc ion binding	Secretedextracellularspaceextracellularmatrix					
**994**	CY1	CY1_HUMAN	CY1	P08574; Q5U062; Q6FHS7	NP_001907	Cytochrome c1, heme protein, mitochondrial (Ubiquinol-cytochrome-c reductase complex cytochrome c1 subunit) (Cytochrome c-1) (Cytochrome b-c1 complex subunit 4) (Complex III subunit 4) (Complex III subunit IV)	GO:0022900; GO:0045155; GO:0020037; GO:0016021; GO:0005743; GO:0006810	electron transport chain; electron transporter, transferring electrons from CoQH2-cytochrome c reductase complex and cytochrome c oxidase complex activity; heme binding; integral to membrane; mitochondrial inner membrane; transport	MitochondrioninnermembraneSinglepassmembraneproteinIntermembraneside					
**995**	KLHL2	KLHL2_HUMAN	KLHL2	O95198; A6NCM7; Q8N484; Q8TBH5	NP_009177	Kelch-like protein 2 (Actin-binding protein Mayven)	GO:0003779; GO:0015629; GO:0005737; GO:0006886; GO:0005215	actin binding; actin cytoskeleton; cytoplasm; intracellular protein transport; transporter activity	Cytoplasmcytoskeleton					
**996**	XPP1	XPP1_HUMAN	XPP1	Q9NQW7; O15250; Q53EX6; Q8N3Q0; Q96D23	NP_006514; NP_065116	Xaa-Pro aminopeptidase 1 (EC 3.4.11.9) (X-Pro aminopeptidase 1) (X-prolyl aminopeptidase 1, soluble) (Cytosolic aminopeptidase P) (Soluble aminopeptidase P) (sAmp) (Aminoacylproline aminopeptidase)	GO:0004177; GO:0005737; GO:0030145; GO:0008235; GO:0006508	aminopeptidase activity; cytoplasm; manganese ion binding; metalloexopeptidase activity; proteolysis	Cytoplasm					
**997**	Q9BSS6	Q9BSS6_HUMAN	Q9BSS6	Q9BSS6	NP_005596	Serine/threonine protein phosphatase (EC 3.1.3.16)	GO:0005506; GO:0004721	iron ion binding; phosphoprotein phosphatase activity						Phosphatase
**998**	CGL	CGL_HUMAN	CGL; Q53Y79	P32929; Q53Y79; Q53FB3; Q9H4W7; Q9H4W8	NP_001893; NP_714964	Cystathionine gamma-lyase (EC 4.4.1.1) (Gamma-cystathionase); Cystathionase (Cystathionine gamma-lyase) (Cystathionase (Cystathionine gamma-lyase), isoform CRA_a)	GO:0004123; GO:0019344; GO:0005737; GO:0030170; GO:0004121; GO:0006749; GO:0050667; GO:0030308; GO:0008285	cystathionine gamma-lyase activity; cysteine biosynthetic process; cytoplasm; pyridoxal phosphate binding; cystathionine beta-lyase activity; glutathione metabolic process; homocysteine metabolic process; negative regulation of cell growth; negative regulation of cell proliferation	Cytoplasm;			Known in Uniprot to be phosphorylated		
**999**	STK25	STK25_HUMAN	STK25; A8K7D2	O00506; Q15522; Q5BJF1; A8K7D2	NP_006365	Serine/threonine-protein kinase 25 (EC 2.7.11.1) (Sterile 20/oxidant stress-response kinase 1) (Ste20/oxidant stress response kinase 1) (SOK-1) (Ste20-like kinase)	GO:0005524; GO:0005794; GO:0042802; GO:0000287; GO:0006468; GO:0004674; GO:0006979; GO:0007165	ATP binding; Golgi apparatus; identical protein binding; magnesium ion binding; protein amino acid phosphorylation; protein serine/threonine kinase activity; response to oxidative stress; signal transduction	CytoplasmGolgiapparatus			Known in Uniprot to be phosphorylated		Kinase
**1000**	PYGB	PYGB_HUMAN	PYGB	P11216; Q96AK1; Q9NPX8	NP_002853	Glycogen phosphorylase, brain form (EC 2.4.1.1)	GO:0005737; GO:0005980; GO:0008184; GO:0030170	cytoplasm; glycogen catabolic process; glycogen phosphorylase activity; pyridoxal phosphate binding				Known in Uniprot to be phosphorylated		

	Protein Name	Uniprot Name	Other Names	Uniprot IDs	Refseq IDs	Protein Description	GO ID	GO terms	Subcellular location	Comments	Comments	Comments - Database Search	Comments - Database Search	Function
**1001**	RGAP1	RGAP1_HUMAN	RGAP1	Q9H0H5; Q6PJ26; Q9NWN2; Q9P250; Q9P2W2	NP_037409; NP_001119575; NP_001119576	Rac GTPase-activating protein 1 (Male germ cell RacGap) (MgcRacGAP)	GO:0005096; GO:0001669; GO:0043014; GO:0048487; GO:0000915; GO:0007108; GO:0019992; GO:0009790; GO:0043015; GO:0007242; GO:0005874; GO:0007405; GO:0005634; GO:0008022; GO:0007283; GO:0005819; GO:0008272; GO:0008270	GTPase activator activity; acrosomal vesicle; alpha-tubulin binding; beta-tubulin binding; cytokinesis, contractile ring formation; cytokinesis, initiation of separation; diacylglycerol binding; embryonic development; gamma-tubulin binding; intracellular signaling cascade; microtubule; neuroblast proliferation; nucleus; protein C-terminus binding; spermatogenesis; spindle; sulfate transport; zinc ion binding	NucleusCytoplasmSpindleAcrosome			Known in Uniprot to be phosphorylated	Known in HPRD to be phosphorylated	
**1002**	2AAA	2AAA_HUMAN	2AAA; A8K7B7	Q96DH3; P30153; Q13773; Q6ICQ3; A8K7B7	NP_055040	Serine/threonine-protein phosphatase 2A 65 kDa regulatory subunit A alpha isoform (PP2A, subunit A, PR65-alpha isoform) (PP2A, subunit A, R1-alpha isoform) (Medium tumor antigen-associated 61 kDa protein)	GO:0008380; GO:0003823; GO:0006672; GO:0005829; GO:0000188; GO:0006917; GO:0016020; GO:0015630; GO:0005739; GO:0030308; GO:0042518; GO:0005634; GO:0006470; GO:0006461; GO:0046982; GO:0000159; GO:0008601; GO:0006275; GO:0030111; GO:0030155; GO:0045595; GO:0045449; GO:0010033; GO:0019932; GO:0005625	RNA splicing; antigen binding; ceramide metabolic process; cytosol; inactivation of MAPK activity; induction of apoptosis; membrane; microtubule cytoskeleton; mitochondrion; negative regulation of cell growth; negative regulation of tyrosine phosphorylation of Stat3 protein; nucleus; protein amino acid dephosphorylation; protein complex assembly; protein heterodimerization activity; protein phosphatase type 2A complex; protein phosphatase type 2A regulator activity; regulation of DNA replication; regulation of Wnt receptor signaling pathway; regulation of cell adhesion; regulation of cell differentiation; regulation of transcription; response to organic substance; second-messenger-mediated signaling; soluble fraction						Phosphatase
**1003**	RRAS2	RRAS2_HUMAN	RRAS2; B2R9Z3	P62070; P17082; B2R9Z3	NP_036382; NP_001096139	Ras-related protein R-Ras2 (Ras-like protein TC21) (Teratocarcinoma oncogene)	GO:0005525; GO:0003924; GO:0005783; GO:0005886; GO:0005515	GTP binding; GTPase activity; endoplasmic reticulum; plasma membrane; protein binding	CellmembraneLipidanchorCytoplasmicside			Known in Uniprot to be phosphorylated		
**1004**	CA048	CA048_HUMAN	CA048; Q53FM2; NSL1	Q96IY1; Q5SY75; Q9H2M5; Q9NRN8; Q9Y415; Q53FM2	NP_056286	Kinetochore-associated protein NSL1 homolog; Chromosome 1 open reading frame 48 variant (Chromosome 1 open reading frame 48, isoform CRA_a) (Fragment)	GO:0000444; GO:0051301; GO:0007059; GO:0007067; GO:0005634; GO:0005515	MIS12/MIND type complex; cell division; chromosome segregation; mitosis; nucleus; protein binding;	NucleusKinetochore;			Known in Uniprot to be phosphorylated	Known in HPRD to be phosphorylated	
**1005**	KSYK	KSYK_HUMAN	KSYK; Q5T6N7; A8K4G2	P43405; Q5T6N7; A8K4G2	NP_003168	Tyrosine-protein kinase SYK (EC 2.7.10.2) (Spleen tyrosine kinase)	GO:0005524; GO:0042101; GO:0005737; GO:0005178; GO:0007229; GO:0044419; GO:0007159; GO:0030593; GO:0004715; GO:0005634; GO:0009887; GO:0006461	ATP binding; T cell receptor complex; cytoplasm; integrin binding; integrin-mediated signaling pathway; interspecies interaction between organisms; leukocyte adhesion; neutrophil chemotaxis; non-membrane spanning protein tyrosine kinase activity; nucleus; organ morphogenesis; protein complex assembly				Known in Uniprot to be phosphorylated	Known in HPRD to be phosphorylated	Kinase
**1006**	AT5F1	AT5F1_HUMAN	AT5F1; Q08ET0	P24539; Q9BQ68; Q9BRU8; Q08ET0	NP_001679	ATP synthase subunit b, mitochondrial; Cell proliferation-inducing protein 47 (HCG39985, isoform CRA_a) (cDNA, FLJ94101, Homo sapiens ATP synthase, H+ transporting, mitochondrial F0complex, subunit b, isoform 1 (ATP5F1), mRNA)	GO:0015986; GO:0015078; GO:0005759; GO:0000276; GO:0005515	ATP synthesis coupled proton transport; hydrogen ion transmembrane transporter activity; mitochondrial matrix; mitochondrial proton-transporting ATP synthase complex, coupling factor F(o); protein binding	MitochondrionMitochondrioninnermembrane;					
**1007**	TOLIP	TOLIP_HUMAN	TOLIP; Q6FIE9	Q9H0E2; Q6FIE9; Q9H9E6; Q9UJ69	NP_061882	Toll-interacting protein; TOLLIP protein (Toll interacting protein) (Toll interacting protein, isoform CRA_a) (cDNA, FLJ96670, Homo sapiens toll interacting protein (TOLLIP), mRNA)	GO:0005121; GO:0007267; GO:0005737; GO:0006955; GO:0006954; GO:0045323; GO:0045092; GO:0007242; GO:0045321; GO:0016310; GO:0004871;	Toll binding; cell-cell signaling; cytoplasm; immune response; inflammatory response; interleukin-1 receptor complex; interleukin-18 receptor complex; intracellular signaling cascade; leukocyte activation; phosphorylation; signal transducer activity;	Cytoplasm;					
**1008**	PGM1	PGM1_HUMAN	PGM1	P36871; Q16105; Q5BKZ9; Q6NW22; Q86U74; Q96J40; Q9NTY4	NP_002624	Phosphoglucomutase-1 (PGM 1) (EC 5.4.2.2) (Glucose phosphomutase 1)	GO:0005829; GO:0006006; GO:0000287; GO:0004614	cytosol; glucose metabolic process; magnesium ion binding; phosphoglucomutase activity	Cytoplasm			Known in Uniprot to be phosphorylated		
**1009**	CF055	CF055_HUMAN	CF055; Q5TGM1; VTA1	Q9NP79; Q5TGM1; Q6IAE8; Q9H0R2; Q9H3K9; Q9P0Q0	NP_057569	Vacuolar protein sorting-associated protein VTA1 homolog (Dopamine-responsive protein DRG-1) (SKD1 binding protein 1) (SBP1) (LYST-interacting protein 5) (LIP5)			CytoplasmEndosomemembranePeripheralmembraneprotein					
**1010**	CAND1	CAND1_HUMAN	CAND1; B2RAU3	Q86VP6; O94918; Q6PIY4; Q8NDJ4; Q96JZ9; Q96T19; Q9BTC4; Q9H0G2; Q9P0H7; Q9UF85; B2RAU3	NP_060918	Cullin-associated NEDD8-dissociated protein 1 (Cullin-associated and neddylation-dissociated protein 1) (p120 CAND1) (TBP-interacting protein TIP120A) (TBP-interacting protein of 120 kDa A)	GO:0030154; GO:0019941; GO:0043086; GO:0005634; GO:0005515; GO:0016567; GO:0000151	cell differentiation; modification-dependent protein catabolic process; negative regulation of catalytic activity; nucleus; protein binding; protein ubiquitination; ubiquitin ligase complex	Nucleus			Known in Uniprot to be phosphorylated		
**1011**	NDUAD	NDUAD_HUMAN	NDUAD	Q9P0J0; Q6PKI0; Q9H2L3; Q9Y327	NP_057049	NADH dehydrogenase [ubiquinone] 1 alpha subcomplex subunit 13 (NADH-ubiquinone oxidoreductase B16.6 subunit) (Complex I-B16.6) (CI-B16.6) (Gene associated with retinoic-interferon-induced mortality 19 protein) (GRIM-19) (Cell death regulatory protein GRIM-19)	GO:0005524; GO:0008137; GO:0030262; GO:0022900; GO:0008624; GO:0016021; GO:0005747; GO:0030308; GO:0045892; GO:0017148; GO:0005654; GO:0006800; GO:0005515; GO:0006606	ATP binding; NADH dehydrogenase (ubiquinone) activity; apoptotic nuclear changes; electron transport chain; induction of apoptosis by extracellular signals; integral to membrane; mitochondrial respiratory chain complex I; negative regulation of cell growth; negative regulation of transcription, DNA-dependent; negative regulation of translation; nucleoplasm; oxygen and reactive oxygen species metabolic process; protein binding; protein import into nucleus	MitochondrioninnermembraneSinglepassmembraneproteinMatrixsideNucleus					
**1012**	MTA70	MTA70_HUMAN	MTA70	Q86U44; O14736; Q86V05; Q9HB32	NP_062826	N6-adenosine-methyltransferase 70 kDa subunit (MT-A70) (EC 2.1.1.62) (Methyltransferase-like protein 3)	GO:0003723; GO:0001510; GO:0016422; GO:0016607	RNA binding; RNA methylation; mRNA (2'-O-methyladenosine-N6-)-methyltransferase activity; nuclear speck	Nucleusspeckle			Known in Uniprot to be phosphorylated		
**1013**	RAB2B	RAB2B_HUMAN	RAB2B; B2RD03	Q8WUD1; B2RD03	NP_116235	Ras-related protein Rab-2B	GO:0005525; GO:0000139; GO:0005789; GO:0005886; GO:0015031; GO:0007264; GO:0016192	GTP binding; Golgi membrane; endoplasmic reticulum membrane; plasma membrane; protein transport; small GTPase mediated signal transduction; vesicle-mediated transport	CellmembraneLipidanchorCytoplasmicsideEndoplasmicreticulummembraneGolgiapparatusmembrane					
**1014**	LZIC	LZIC_HUMAN	LZIC; B2R6F0	Q8WZA0; Q96IU1; B2R6F0	NP_115744	Protein LZIC (Leucine zipper and ICAT homologous domain-containing protein) (Leucine zipper and CTNNBIP1 domain-containing protein)	GO:0008013	beta-catenin binding						
**1015**	JKIP1	JKIP1_HUMAN	Q96N16; Q86Y69; JKIP1	Q96N16; A6H2J2; A6H2J3; A6H2J4; A6H2J5; A8MTK6; Q86Y69; Q8N7G3	NP_653321; NP_001092903	Janus kinase and microtubule-interacting protein 1 (GABA-B receptor-binding protein) (Multiple alpha-helices and RNA-linker protein 1) (Marlin-1)	GO:0050811; GO:0003723; GO:0005737; GO:0016020; GO:0005874; GO:0000300; GO:0015031; GO:0030529	GABA receptor binding; RNA binding; cytoplasm; membrane; microtubule; peripheral to membrane of membrane fraction; protein transport; ribonucleoprotein complex	CytoplasmcytoskeletonMembranePeripheralmembraneprotein			Known in Uniprot to be phosphorylated		
**1016**	CC138	CC138_HUMAN	Q96M89; CC138	Q96M89; Q05DF1; Q4ZG07; Q53TE1; Q6ZUY5; Q86VL7	NP_659415	Coiled-coil domain-containing protein 138								
**1017**	ENKUR	ENKUR_HUMAN	ENKUR; A8K8Y0	Q8TC29; A8K8Y0	NP_659447	Enkurin	GO:0017124; GO:0005516; GO:0005929; GO:0019861	SH3 domain binding; calmodulin binding; cilium; flagellum	Cellprojectionciliumflagellum					
**1018**	STAC3	STAC3_HUMAN	STAC3	Q96MF2; Q96HU5	NP_659501	SH3 and cysteine-rich domain-containing protein 3	GO:0019992; GO:0042802; GO:0007242; GO:0008270	diacylglycerol binding; identical protein binding; intracellular signaling cascade; zinc ion binding						
**1019**	PFD1	PFD1_HUMAN	PFD1; B2RD02	O60925; Q53F95; Q96EX6; B2RD02	NP_002613	Prefoldin subunit 1	GO:0007049; GO:0005886; GO:0016272; GO:0006457; GO:0003700; GO:0051082	cell cycle; plasma membrane; prefoldin complex; protein folding; transcription factor activity; unfolded protein binding						
**1020**	TRY3	TRY3_HUMAN	TRY3; Q6ISJ4	Q6ISJ4; P35030; P15951; Q15665; Q5VXV0; Q9UQV3	NP_002762	Trypsin-3 (EC 3.4.21.4) (Trypsin III) (Brain trypsinogen) (Mesotrypsinogen) (Trypsin IV) (Serine protease 3) (Serine protease 4); Protease, serine, 3 (Mesotrypsinogen) (Protease, serine, 3 (Mesotrypsin), isoform CRA_b)	GO:0005509; GO:0007586; GO:0043542; GO:0005615; GO:0005515; GO:0006508; GO:0004252; GO:0031638; GO:0006508	calcium ion binding; digestion; endothelial cell migration; extracellular space; protein binding; proteolysis; serine-type endopeptidase activity; zymogen activation; proteolysis	Secreted;					

	Protein Name	Uniprot Name	Other Names	Uniprot IDs	Refseq IDs	Protein Description	GO ID	GO terms	Subcellular location	Comments	Comments	Comments - Database Search	Comments - Database Search	Function
**1021**	EZRI	EZRI_HUMAN	EZRI	Q4VX75; P15311; P23714; Q96CU8; Q9NSJ4	NP_003370; NP_001104547	Ezrin (p81) (Cytovillin) (Villin-2)	GO:0005884; GO:0051015; GO:0051017; GO:0016324; GO:0050839; GO:0030863; GO:0007016; GO:0005829; GO:0019898; GO:0030175; GO:0007159; GO:0022614; GO:0005902; GO:0043621; GO:0008360; GO:0001726	actin filament; actin filament binding; actin filament bundle formation; apical plasma membrane; cell adhesion molecule binding; cortical cytoskeleton; cytoskeletal anchoring at plasma membrane; cytosol; extrinsic to membrane; filopodium; leukocyte adhesion; membrane to membrane docking; microvillus; protein self-association; regulation of cell shape; ruffle	ApicalcellmembranePeripheralmembraneproteinCytoplasmicsideCellprojectionCellprojection microvillusmembranePeripheralmembraneproteinCytoplasmicsideCellprojectionrufflemembranePeripheralmembraneproteinCytoplasmicsideCytoplasmcellcortexCytoplasmcytoskeleton			Known in Uniprot to be phosphorylated	Known in HPRD to be phosphorylated	
**1022**	S10A8	S10A8_HUMAN	S10A8	P05109; Q5SY70; A8K5L3; Q9UC84; Q9UC92; Q9UCJ0; Q9UCM6	NP_002955	Protein S100-A8 (S100 calcium-binding protein A8) (Calgranulin-A) (Migration inhibitory factor-related protein 8) (MRP-8) (P8) (Cystic fibrosis antigen) (CFAG) (Leukocyte L1 complex light chain) (Calprotectin L1L subunit) (Urinary stone protein band A)	GO:0005509; GO:0006954; GO:0005515	calcium ion binding; inflammatory response; protein binding						
**1023**	CACP	CACP_HUMAN	CACP	P43155; Q5T952; Q9BW16	NP_000746; NP_003994	Carnitine O-acetyltransferase (Carnitine acetylase) (EC 2.3.1.7) (Carnitine acetyltransferase) (CrAT) (CAT)	GO:0004092; GO:0005783; GO:0015980; GO:0006631; GO:0005743; GO:0005777; GO:0006810	carnitine O-acetyltransferase activity; endoplasmic reticulum; energy derivation by oxidation of organic compounds; fatty acid metabolic process; mitochondrial inner membrane; peroxisome; transport	EndoplasmicreticulumPeroxisomeMitochondrioninnermembranePeripheralmembraneproteinMatrixside					
**1024**	STA5A	STA5A_HUMAN	STA5A	P42229	NP_003143	Signal transducer and activator of transcription 5A	GO:0006103; GO:0007259; GO:0000255; GO:0005509; GO:0006101; GO:0006600; GO:0046449; GO:0005737; GO:0006631; GO:0006549; GO:0005634; GO:0006107; GO:0042301; GO:0005515; GO:0004871; GO:0006105; GO:0019530; GO:0003700; GO:0006573	2-oxoglutarate metabolic process; JAK-STAT cascade; allantoin metabolic process; calcium ion binding; citrate metabolic process; creatine metabolic process; creatinine metabolic process; cytoplasm; fatty acid metabolic process; isoleucine metabolic process; nucleus; oxaloacetate metabolic process; phosphate binding; protein binding; signal transducer activity; succinate metabolic process; taurine metabolic process; transcription factor activity; valine metabolic process	CytoplasmNucleus			Known in Uniprot to be phosphorylated	Known in HPRD to be phosphorylated	
**1025**	COTL1	COTL1_HUMAN	COTL1; B2RDU3	Q14019; Q86XM5; B2RDU3	NP_066972	Coactosin-like protein	GO:0003779; GO:0005737; GO:0005856; GO:0019899	actin binding; cytoplasm; cytoskeleton; enzyme binding	Cytoplasmcytoskeleton			Known in Uniprot to be phosphorylated		
**1026**	PDC6I	PDC6I_HUMAN	PDC6I	Q8WUM4; Q9BX86; Q9NUN0; Q9P2H2; Q9UKL5	NP_037506	Programmed cell death 6-interacting protein (PDCD6-interacting protein) (ALG-2-interacting protein 1) (Hp95)	GO:0006915; GO:0044419; GO:0042470; GO:0005515; GO:0015031	apoptosis; interspecies interaction between organisms; melanosome; protein binding; protein transport	CytoplasmcytosolMelanosomeCentrosome			Known in Uniprot to be phosphorylated		
**1027**	AP1B1	AP1B1_HUMAN	AP1B1	Q10567; P78436	NP_001118; NP_663782	AP-1 complex subunit beta-1 (Adapter-related protein complex 1 subunit beta-1) (Adaptor protein complex AP-1 subunit beta-1) (Beta-adaptin 1) (Beta1-adaptin) (Golgi adaptor HA1/AP1 adaptin beta subunit) (Clathrin assembly protein complex 1 beta large chain)	GO:0005794; GO:0030131; GO:0031410; GO:0005829; GO:0006897; GO:0006886; GO:0005515; GO:0008565	Golgi apparatus; clathrin adaptor complex; cytoplasmic vesicle; cytosol; endocytosis; intracellular protein transport; protein binding; protein transporter activity	GolgiapparatusCytoplasmicvesicleclathrincoatedvesiclemembranePeripheralmembraneproteinCytoplasmicside			Known in Uniprot to be phosphorylated		
**1028**	AP1S2	AP1S2_HUMAN	AP1S2; Q549M9	P56377; Q549M9; O95326; Q9H2N6	NP_003907	AP-1 complex subunit sigma-2 (Adapter-related protein complex 1 sigma-1B subunit) (Adaptor protein complex AP-1 sigma-1B subunit) (Sigma-adaptin 1B) (Sigma1B-adaptin) (Golgi adaptor HA1/AP1 adaptin sigma-1B subunit) (Clathrin assembly protein complex 1 sigma-1B small chain) (Sigma 1B subunit of AP-1 clathrin); Adaptor-related protein complex 1, sigma 2 subunit, isoform CRA_e (Clathrin-associated protein 19)	GO:0030131; GO:0030130; GO:0005905; GO:0005829; GO:0006897; GO:0006886; GO:0005515; GO:0008565; GO:0016192	clathrin adaptor complex; clathrin coat of trans-Golgi network vesicle; coated pit; cytosol; endocytosis; intracellular protein transport; protein binding; protein transporter activity; vesicle-mediated transport	GolgiapparatusCytoplasmicvesiclemembranePeripheralmembraneproteinCytoplasmicsideMembraneclathrincoatedpit;					
**1029**	M4K2	M4K2_HUMAN	M4K2	Q12851	NP_004570	Mitogen-activated protein kinase kinase kinase kinase 2 (EC 2.7.11.1) (MAPK/ERK kinase kinase kinase 2) (MEK kinase kinase 2) (MEKKK 2) (Germinal center kinase) (GC kinase) (Rab8-interacting protein) (B lymphocyte serine/threonine-protein kinase)	GO:0005524; GO:0000139; GO:0007254; GO:0016323; GO:0006955; GO:0006468; GO:0005515; GO:0004674; GO:0005083; GO:0005625; GO:0006903	ATP binding; Golgi membrane; JNK cascade; basolateral plasma membrane; immune response; protein amino acid phosphorylation; protein binding; protein serine/threonine kinase activity; small GTPase regulator activity; soluble fraction; vesicle targeting	CytoplasmBasolateralcellmembranePeripheralmembraneproteinGolgiapparatusmembranePeripheralmembraneprotein			Known in Uniprot to be phosphorylated		Kinase
**1030**	TLN2	TLN2_HUMAN	TLN2	Q9Y4G6; A6NLB8	NP_055874	Talin-2	GO:0003779; GO:0015629; GO:0007155; GO:0005911; GO:0007043; GO:0005737; GO:0007016; GO:0005925; GO:0001726; GO:0005200; GO:0045202	actin binding; actin cytoskeleton; cell adhesion; cell-cell junction; cell-cell junction assembly; cytoplasm; cytoskeletal anchoring at plasma membrane; focal adhesion; ruffle; structural constituent of cytoskeleton; synapse	CelljunctionfocaladhesionCelljunctionsynapse CellmembranePeripheralmembraneproteinCytoplasmicsideCytoplasm cytoskeleton			Known in Uniprot to be phosphorylated	Known in HPRD to be phosphorylated	
**1031**	GFRA3	GFRA3_HUMAN	GFRA3; B2RA36	O60609; Q6UW20; Q8IUZ2; B2RA36	NP_001487	GDNF family receptor alpha-3 (GFR-alpha-3)	GO:0031225; GO:0019898; GO:0007422; GO:0005102; GO:0007165	anchored to membrane; extrinsic to membrane; peripheral nervous system development; receptor binding; signal transduction	CellmembraneLipidanchorGPIanchor					
**1032**	PDS5A	PDS5A_HUMAN	Q8NG14; PDS5A	Q8NG14; Q29RF7; Q2TTR5; Q68DF7; Q8N7J4; Q9Y4D4	NP_056015; NP_001093869; NP_001093870	Sister chromatid cohesion protein PDS5 homolog A (Sister chromatid cohesion protein 112) (SCC-112) (Cell proliferation-inducing gene 54 protein)	GO:0051301; GO:0042802; GO:0007067; GO:0005634	cell division; identical protein binding; mitosis; nucleus	Nucleus			Known in Uniprot to be phosphorylated		
**1033**	CREB3	CREB3_HUMAN	CREB3; Q5TCV1	Q5TCV1; O43889; O14671; O14919; Q96GK8; Q9H2W3; Q9UE77	NP_006359	Cyclic AMP-responsive element-binding protein 3 (cAMP-responsive element-binding protein 3) (CREB-3) (Luman) (Transcription factor LZIP-alpha) [Cleaved into: Processed cyclic AMP-responsive element-binding protein 3]	GO:0006935; GO:0005789; GO:0016021; GO:0044419; GO:0005634; GO:0046983; GO:0006355; GO:0043565; GO:0003700	chemotaxis; endoplasmic reticulum membrane; integral to membrane; interspecies interaction between organisms; nucleus; protein dimerization activity; regulation of transcription, DNA-dependent; sequence-specific DNA binding; transcription factor activity	Endoplasmicreticulum membraneSinglepasstypeIImembraneprotein Nucleus					
**1034**	NPS3A	NPS3A_HUMAN	NPS3A; Q5VX32	Q9UFN0; Q5VX32; A6NM55; Q9BRV7; Q9H843; Q9P083	NP_056284	Protein NipSnap homolog 3A (NipSnap3A) (NipSnap4) (Target for Salmonella secreted protein C) (TassC)	GO:0005737	cytoplasm	Cytoplasmcytosol					
**1035**	NCOA1	NCOA1_HUMAN	NCOA1; Q53SX3	Q53SX3; Q15788; O00150; O43792; O43793; Q13071; Q13420; Q2T9G5; Q6GVI5; Q7KYV3	NP_003734; NP_671756; NP_671766	Nuclear receptor coactivator 1 (NCoA-1) (EC 2.3.1.48) (Steroid receptor coactivator 1) (SRC-1) (RIP160) (Protein Hin-2) (Renal carcinoma antigen NY-REN-52)	GO:0050681; GO:0030521; GO:0004402; GO:0005634; GO:0047485; GO:0004871; GO:0003713	androgen receptor binding; androgen receptor signaling pathway; histone acetyltransferase activity; nucleus; protein N-terminus binding; signal transducer activity; transcription coactivator activity	Nucleus			Known in Uniprot to be phosphorylated	Known in HPRD to be phosphorylated	
**1036**	PSB3	PSB3_HUMAN	PSB3	P49720; P31147; Q0P6J7; Q96E27	NP_002786	Proteasome subunit beta type-3 (EC 3.4.25.1) (Proteasome theta chain) (Proteasome chain 13) (Proteasome component C10-II)	GO:0031145; GO:0005829; GO:0051436; GO:0005634; GO:0051437; GO:0005839; GO:0005515; GO:0004298	anaphase-promoting complex-dependent proteasomal ubiquitin-dependent protein catabolic process; cytosol; negative regulation of ubiquitin-protein ligase activity during mitotic cell cycle; nucleus; positive regulation of ubiquitin-protein ligase activity during mitotic cell cycle; proteasome core complex; protein binding; threonine-type endopeptidase activity	CytoplasmNucleus					
**1037**	PSB4	PSB4_HUMAN	PSB4; Q5SZS5	Q5SZS5; P28070; P31148; Q969L6	NP_002787	Proteasome subunit beta type-4 (Proteasome beta chain) (EC 3.4.25.1) (Macropain beta chain) (Multicatalytic endopeptidase complex beta chain) (Proteasome chain 3) (HSN3) (HsBPROS26)	GO:0031145; GO:0005813; GO:0005829; GO:0051436; GO:0005634; GO:0051437; GO:0005839; GO:0004298	anaphase-promoting complex-dependent proteasomal ubiquitin-dependent protein catabolic process; centrosome; cytosol; negative regulation of ubiquitin-protein ligase activity during mitotic cell cycle; nucleus; positive regulation of ubiquitin-protein ligase activity during mitotic cell cycle; proteasome core complex; threonine-type endopeptidase activity	CytoplasmNucleus			Known in Uniprot to be phosphorylated		
**1038**	CCL5	CCL5_HUMAN	CCL5; Q9UBL2	P13501; O43646; Q0QVW8; Q4ZGJ1; Q9NYA2; Q9UBG2; Q9UC99; Q9UBL2	NP_002976	C-C motif chemokine 5 (Small-inducible cytokine A5) (T-cell-specific protein RANTES) (SIS-delta) (T cell-specific protein P228) (TCP228) (Eosinophil-chemotactic cytokine) (EoCP) [Cleaved into: RANTES(3-68); RANTES(4-68)]; RANTES chemokine (Fragment)	GO:0007155; GO:0006928; GO:0007267; GO:0006874; GO:0006968; GO:0042056; GO:0008009; GO:0006935; GO:0006887; GO:0005615; GO:0006955; GO:0006954; GO:0045071; GO:0006979; GO:0009615; GO:0004871; GO:0007165;	cell adhesion; cell motion; cell-cell signaling; cellular calcium ion homeostasis; cellular defense response; chemoattractant activity; chemokine activity; chemotaxis; exocytosis; extracellular space; immune response; inflammatory response; negative regulation of viral genome replication; response to oxidative stress; response to virus; signal transducer activity; signal transduction;	Secreted;					

	Protein Name	Uniprot Name	Other Names	Uniprot IDs	Refseq IDs	Protein Description	GO ID	GO terms	Subcellular location	Comments	Comments	Comments - Database Search	Comments - Database Search	Function
**1039**	GLYC	GLYC_HUMAN	GLYC; Q96HY0	P34896; Q9UMD1; Q9UMD2; Q96HY0	NP_004160; NP_683718	Serine hydroxymethyltransferase, cytosolic (SHMT) (Serine methylase) (EC 2.1.2.1) (Glycine hydroxymethyltransferase); Serine hydroxymethyltransferase (EC 2.1.2.1)	GO:0006565; GO:0005829; GO:0046655; GO:0004372; GO:0006544; GO:0006730; GO:0042803; GO:0051262; GO:0009113; GO:0030170; GO:0006563	L-serine catabolic process; cytosol; folic acid metabolic process; glycine hydroxymethyltransferase activity; glycine metabolic process; one-carbon compound metabolic process; protein homodimerization activity; protein tetramerization; purine base biosynthetic process; pyridoxal phosphate binding; L-serine metabolic process	Cytoplasm;			Known in Uniprot to be phosphorylated		
**1040**	PURA1	PURA1_HUMAN	PURA1	Q8N142; Q86TT6; Q8N714	NP_689541; NP_954634	Adenylosuccinate synthetase isozyme 1 (AMPSase 1) (AdSS 1) (EC 6.3.4.4) (IMP--aspartate ligase 1)	GO:0006167; GO:0005525; GO:0004019; GO:0005737; GO:0002376; GO:0000287; GO:0042301	AMP biosynthetic process; GTP binding; adenylosuccinate synthase activity; cytoplasm; immune system process; magnesium ion binding; phosphate binding	Cytoplasm			Known in Uniprot to be phosphorylated		
**1041**	CA093	CA093_HUMAN	Q8TBF2; CA093; B9DI92	Q8TBF2; Q8N2H0; B9DI92	NP_689584	Uncharacterized protein C1orf93	GO:0005737; GO:0005634; GO:0005886	cytoplasm; nucleus; plasma membrane				Known in Uniprot to be phosphorylated		
**1042**	CA087	CA087_HUMAN	Q8N0U7; CA087	Q8N0U7; Q6ZU07; Q8IVS0	NP_689590	Uncharacterized protein C1orf87								
**1043**	CB063	CB063_HUMAN	Q8NHS4; CB063	Q8NHS4; B2RDV1; Q53R93; Q8N403	NP_689598; NP_001129070	Uncharacterized protein C2orf63	GO:0005488	binding						
**1044**	VP37A	VP37A_HUMAN	Q96DL9; VP37A	Q96DL9; Q8NEZ2; Q336D5; Q6NW27; Q8N3D7; Q8TBL7	NP_689628; NP_001138624	Vacuolar protein sorting-associated protein 37A (hVps37A) (ESCRT-I complex subunit VPS37A) (Hepatocellular carcinoma-related protein 1)	GO:0031902; GO:0005634; GO:0015031	late endosome membrane; nucleus; protein transport	LateendosomemembranePeripheralmembraneproteinNucleus			Known in Uniprot to be phosphorylated		
**1045**	FA81A	FA81A_HUMAN	FA81A	Q8TBF8	NP_689663	Protein FAM81A								
**1046**	FA81B	FA81B_HUMAN	FA81B	Q96LP2	NP_689761	Protein FAM81B								
**1047**	CA071	CA071_HUMAN	Q96MR5; CA071	Q96MR5; Q6PJW8; Q5VSY9; Q5VTM7; Q8IYA9; Q8N3L5; Q8NB09; Q8TEI2	NP_689822; NP_001132931	Uncharacterized protein C1orf71	GO:0016021; GO:0043234	integral to membrane; protein complex	MembraneSinglepassmembraneprotein					
**1048**	CCD89	CCD89_HUMAN	CCD89	Q8N998	NP_689936	Coiled-coil domain-containing protein 89 (Bc8 orange-interacting protein)			CytoplasmNucleus					
**1049**	CQ061	CQ061_HUMAN	Q8N2U0; CQ061	Q8N2U0	NP_689979	UPF0451 protein C17orf61	GO:0016021	integral to membrane	MembraneMultipassmembraneprotein					
**1050**	ABC3C	ABC3C_HUMAN	ABC3C; B2R884	Q9NRW3; Q5JZ92; Q7Z2N7; Q96F12; B2R884	NP_055323	Probable DNA dC->dU-editing enzyme APOBEC-3C (EC 3.5.4.-) (APOBEC1-like) (Phorbolin I protein)	GO:0016814; GO:0005515; GO:0008270	hydrolase activity, acting on carbon-nitrogen (but not peptide) bonds, in cyclic amidines; protein binding; zinc ion binding						
**1051**	HIF3A	HIF3A_HUMAN	Q9Y2N7; Q66K72; HIF3A	Q9Y2N7; Q58A43; Q66K72; Q8WXA1; Q96K34; Q9H7Z9; Q9HAI2	NP_690007; NP_071907; NP_690008	Hypoxia-inducible factor 3 alpha (HIF-3 alpha) (HIF3 alpha 1) (Member of PAS protein 7) (Basic-helix-loop-helix-PAS protein MOP7) (Inhibitory PAS domain protein) (IPAS)	GO:0003677; GO:0005737; GO:0005634; GO:0006355; GO:0004871; GO:0007165; GO:0030528	DNA binding; cytoplasm; nucleus; regulation of transcription, DNA-dependent; signal transducer activity; signal transduction; transcription regulator activity	NucleusCytoplasm					
**1052**	GSTM1	GSTM1_HUMAN	GSTM1	P09488; Q5GHG0; Q6FH88; Q8TC98; Q9UC96	NP_000552; NP_666533	Glutathione S-transferase Mu 1 (EC 2.5.1.18) (GSTM1-1) (GST class-mu 1) (GSTM1a-1a) (GSTM1b-1b) (HB subunit 4) (GTH4)	GO:0005737; GO:0004364; GO:0008152	cytoplasm; glutathione transferase activity; metabolic process	Cytoplasm			Known in Uniprot to be phosphorylated		
**1053**	GSTM3	GSTM3_HUMAN	GSTM3; Q6FGJ9	P21266; Q6FGJ9; O60550; Q96HA3	NP_000840	Glutathione S-transferase Mu 3 (EC 2.5.1.18) (GSTM3-3) (GST class-mu 3) (hGSTM3-3); GSTM3 protein (Glutathione S-transferase M3 (Brain)) (cDNA, FLJ93985, Homo sapiens glutathione S-transferase M3 (brain) (GSTM3), mRNA)	GO:0005737; GO:0008065; GO:0004364; GO:0008152; GO:0005515; GO:0043627; GO:0004364	cytoplasm; establishment of blood-nerve barrier; glutathione transferase activity; metabolic process; protein binding; response to estrogen stimulus; glutathione transferase activity	Cytoplasm;					
**1054**	SYNE1	SYNE1_HUMAN	SYNE1	Q8NF91; O94890; Q5JV19; Q5JV22; Q8N9P7; Q8TCP1; Q8WWW6; Q8WWW7; Q8WXF6; Q96N17; Q9C0A7; Q9H525; Q9H526; Q9NS36; Q9NU50; Q9UJ06; Q9UJ07; Q9ULF8	NP_149062; NP_056108; NP_598411; NP_892006	Nesprin-1 (Nuclear envelope spectrin repeat protein 1) (Synaptic nuclear envelope protein 1) (Syne-1) (Myocyte nuclear envelope protein 1) (Myne-1) (Enaptin)	GO:0005794; GO:0007030; GO:0003779; GO:0008219; GO:0005856; GO:0016021; GO:0005521; GO:0042692; GO:0005640; GO:0006997; GO:0045211	Golgi apparatus; Golgi organization; actin binding; cell death; cytoskeleton; integral to membrane; lamin binding; muscle cell differentiation; nuclear outer membrane; nucleus organization; postsynaptic membrane	NucleusoutermembraneSinglepasstypeIVmembraneproteinCytoplasmicsideCytoplasmcytoskeletonCytoplasmmyofibrilsarcomere			Known in Uniprot to be phosphorylated		
**1055**	PSB6	PSB6_HUMAN	PSB6; Q6IAT9	P28072; Q6IAT9; Q96J55	NP_002789	Proteasome subunit beta type-6 (EC 3.4.25.1) (Proteasome delta chain) (Macropain delta chain) (Multicatalytic endopeptidase complex delta chain) (Proteasome subunit Y); PSMB6 protein (Proteasome (Prosome, macropain) subunit, beta type, 6) (cDNA, FLJ92029, Homo sapiens proteasome (prosome, macropain) subunit, beta type, 6(PSMB6), mRNA)	GO:0031145; GO:0005829; GO:0051436; GO:0005634; GO:0051437; GO:0005839; GO:0004298; GO:0005829; GO:0006511	anaphase-promoting complex-dependent proteasomal ubiquitin-dependent protein catabolic process; cytosol; negative regulation of ubiquitin-protein ligase activity during mitotic cell cycle; nucleus; positive regulation of ubiquitin-protein ligase activity during mitotic cell cycle; proteasome core complex; threonine-type endopeptidase activity; cytosol; ubiquitin-dependent protein catabolic process	CytoplasmNucleus;					
**1056**	PSA5	PSA5_HUMAN	PSA5; B2R8F6	P28066; Q3T1C1; Q6IBF7; B2R8F6	NP_002781	Proteasome subunit alpha type-5 (EC 3.4.25.1) (Proteasome zeta chain) (Macropain zeta chain) (Multicatalytic endopeptidase complex zeta chain)	GO:0031145; GO:0005829; GO:0051436; GO:0005634; GO:0051437; GO:0005839; GO:0005515; GO:0004298	anaphase-promoting complex-dependent proteasomal ubiquitin-dependent protein catabolic process; cytosol; negative regulation of ubiquitin-protein ligase activity during mitotic cell cycle; nucleus; positive regulation of ubiquitin-protein ligase activity during mitotic cell cycle; proteasome core complex; protein binding; threonine-type endopeptidase activity	CytoplasmNucleus			Known in Uniprot to be phosphorylated	Known in HPRD to be phosphorylated	
**1057**	PSA6	PSA6_HUMAN	PSA6; B2R7J9	P60900; B2R7J9; P34062; Q6IB60	NP_002782; XP_062849	Proteasome subunit alpha type-6 (EC 3.4.25.1) (Proteasome iota chain) (Macropain iota chain) (Multicatalytic endopeptidase complex iota chain) (27 kDa prosomal protein) (PROS-27) (p27K)	GO:0003723; GO:0031145; GO:0005829; GO:0005739; GO:0051436; GO:0005634; GO:0051437; GO:0005839; GO:0005515; GO:0004298	RNA binding; anaphase-promoting complex-dependent proteasomal ubiquitin-dependent protein catabolic process; cytosol; mitochondrion; negative regulation of ubiquitin-protein ligase activity during mitotic cell cycle; nucleus; positive regulation of ubiquitin-protein ligase activity during mitotic cell cycle; proteasome core complex; protein binding; threonine-type endopeptidase activity	CytoplasmNucleus			Known in Uniprot to be phosphorylated		
**1058**	SNX12	SNX12_HUMAN	SNX12; Q3SYF1	Q3SYF1; Q9UMY4; Q8WUG9	NP_037478	Sorting nexin-12; Sorting nexin 12 (Sorting nexin 12, isoform CRA_a) (cDNA, FLJ93438, Homo sapiens sorting nexin 12 (SNX12), mRNA)	GO:0007154; GO:0035091; GO:0005515; GO:0015031	cell communication; phosphoinositide binding; protein binding; protein transport				Known in Uniprot to be phosphorylated		
**1059**	ABHDB	ABHDB_HUMAN	ABHDB	Q8NFV3; Q8NFV4; Q6PJU0; Q8N722; Q8N723; Q8NFV2; Q9HBS8	NP_112585; NP_001138835; NP_001138836; NP_683710; NP_683711; NP_683714	Abhydrolase domain-containing protein 11 (EC 3.-.-.-) (Williams-Beuren syndrome chromosomal region 21 protein)	GO:0016787	hydrolase activity						
**1060**	ASGL1	ASGL1_HUMAN	Q8NI34; ASGL1; B2R7Q0	Q8NI34; Q7L266; Q567Q4; Q6P1P0; Q9H6F7; B2R7Q0	NP_079356; NP_001077395	L-asparaginase (EC 3.5.1.1) (L-asparagine amidohydrolase) (Asparaginase-like protein 1)	GO:0003948; GO:0033345; GO:0005737	N4-(beta-N-acetylglucosaminyl)-L-asparaginase activity; asparagine catabolic process via L-aspartate; cytoplasm	Cytoplasm					
**1061**	GIMA7	GIMA7_HUMAN	GIMA7	Q8NHV1	NP_694968	GTPase IMAP family member 7 (Immunity-associated nucleotide 7 protein)	GO:0005525	GTP binding						
**1062**	A16A1	A16A1_HUMAN	Q8IZ83; A16A1	Q8IZ83; Q86YF0; Q8IYL4; Q8TEI8	NP_699160	Aldehyde dehydrogenase family 16 member A1	GO:0008152; GO:0016491; GO:0005515	metabolic process; oxidoreductase activity; protein binding						
**1063**	T161B	T161B_HUMAN	Q8NDZ6; T161B	Q8NDZ6; Q5CZH7; Q6UWQ6	XP_291762; NP_699185	Transmembrane protein 161B	GO:0016021	integral to membrane	MembraneMultipassmembraneprotein					
**1064**	ACPH	ACPH_HUMAN	ACPH	P13798; Q9BQ33; Q9P0Y2	NP_001631	Acylamino-acid-releasing enzyme (AARE) (EC 3.4.19.1) (Acyl-peptide hydrolase) (APH) (Acylaminoacyl-peptidase) (Oxidized protein hydrolase) (OPH)	GO:0005737; GO:0006508; GO:0004252	cytoplasm; proteolysis; serine-type endopeptidase activity	Cytoplasm			Known in Uniprot to be phosphorylated		
**1065**	MDGA1	MDGA1_HUMAN	MDGA1	Q8NFP4; Q8NBE3	NP_705691	MAM domain-containing glycosylphosphatidylinositol anchor protein 1 (MAM domain-containing protein 3) (Glycosylphosphatidylinositol-MAM) (GPI and MAM protein) (GPIM)	GO:0046658; GO:0007420; GO:0001764; GO:0021527	anchored to plasma membrane; brain development; neuron migration; spinal cord association neuron differentiation	CellmembraneLipidanchorGPIanchor					
**1066**	GNA13	GNA13_HUMAN	GNA13; B2R977	Q14344; Q8TD70; B2R977	NP_006563	Guanine nucleotide-binding protein subunit alpha-13 (G-protein subunit alpha-13) (G alpha-13)	GO:0007186; GO:0005525; GO:0003924; GO:0042470; GO:0016020; GO:0005515; GO:0004871	G-protein coupled receptor protein signaling pathway; GTP binding; GTPase activity; melanosome; membrane; protein binding; signal transducer activity	MembraneLipidanchor Melanosome			Known in Uniprot to be phosphorylated	Known in HPRD to be phosphorylated	

	Protein Name	Uniprot Name	Other Names	Uniprot IDs	Refseq IDs	Protein Description	GO ID	GO terms	Subcellular location	Comments	Comments	Comments - Database Search	Comments - Database Search	Function
**1067**	NALCN	NALCN_HUMAN	Q8IZF0; NALCN	Q8IZF0; Q6P2S6; Q6ZMI7; Q8IZZ1; Q8TAH1	NP_443099	Sodium leak channel non-selective protein (Voltage gated channel-like protein 1) (CanIon)	GO:0016021; GO:0005272; GO:0031402; GO:0006814; GO:0005244	integral to membrane; sodium channel activity; sodium ion binding; sodium ion transport; voltage-gated ion channel activity	MembraneMultipassmembraneprotein			Known in Uniprot to be phosphorylated		
**1068**	GRP75	GRP75_HUMAN	GRP75; Q8N1C8; Q1HB43	P38646; B2RCM1; P30036; P31932; Q1HB43; Q53H23; Q6GU03; Q9BWB7; Q9UC56; Q8N1C8	NP_004125; XP_293066	Stress-70 protein, mitochondrial (75 kDa glucose-regulated protein) (GRP 75) (Heat shock 70 kDa protein 9) (Peptide-binding protein 74) (PBP74) (Mortalin) (MOT); HSPA9 protein (Fragment)	GO:0005524; GO:0006916; GO:0009986; GO:0042645; GO:0006457; GO:0051082; GO:0005739; GO:0006611	ATP binding; anti-apoptosis; cell surface; mitochondrial nucleoid; protein folding; unfolded protein binding; mitochondrion; protein export from nucleus	Mitochondrion;			Known in Uniprot to be phosphorylated		
**1069**	GDN	GDN_HUMAN	GDN; Q53S15	P07093; Q53S15	NP_006207	Glia-derived nexin (GDN) (Protease nexin I) (PN-1) (Protease inhibitor 7); Putative uncharacterized protein SERPINE2 (Serpin peptidase inhibitor, clade E (Nexin, plasminogen activator inhibitor type 1), member 2, isoform CRA_a)	GO:0030154; GO:0005576; GO:0008201; GO:0007399; GO:0030334; GO:0030162; GO:0004867; GO:0004867	cell differentiation; extracellular region; heparin binding; nervous system development; regulation of cell migration; regulation of proteolysis; serine-type endopeptidase inhibitor activity; serine-type endopeptidase inhibitor activity	Secretedextracellularspace;					
**1070**	TCPE	TCPE_HUMAN	TCPE; A8JZY8	P48643; A8JZY8	NP_036205	T-complex protein 1 subunit epsilon (TCP-1-epsilon) (CCT-epsilon)	GO:0005524; GO:0005730; GO:0006457; GO:0051082	ATP binding; nucleolus; protein folding; unfolded protein binding	Cytoplasm					
**1071**	STK36	STK36_HUMAN	STK36	Q9NRP7; Q8TC32; Q9H9N9; Q9UF35; Q9ULE2	NP_056505	Serine/threonine-protein kinase 36 (EC 2.7.11.1) (Fused homolog)	GO:0005524; GO:0005737; GO:0000287; GO:0005634; GO:0009791; GO:0006468; GO:0004674; GO:0051090; GO:0008134	ATP binding; cytoplasm; magnesium ion binding; nucleus; post-embryonic development; protein amino acid phosphorylation; protein serine/threonine kinase activity; regulation of transcription factor activity; transcription factor binding	CytoplasmNucleus					Kinase
**1072**	FA20A	FA20A_HUMAN	FA20A	Q96MK3; B2RN49; Q9UF95	NP_060035	Protein FAM20A	GO:0005576	extracellular region	Secreted					
**1073**	CT2NL	CT2NL_HUMAN	CT2NL	Q9P2B4; Q96B40	NP_061174	CTTNBP2 N-terminal-like protein	GO:0015629; GO:0005737; GO:0005730	actin cytoskeleton; cytoplasm; nucleolus				Known in Uniprot to be phosphorylated	Known in HPRD to be phosphorylated	
**1074**	APMAP	APMAP_HUMAN	APMAP	Q9HDC9; A8K514; B4DXG1; Q6UVZ8; Q9GZS8; Q9NUB2	NP_065392	Adipocyte plasma membrane-associated protein (Protein BSCv)	GO:0004064; GO:0009058; GO:0009986; GO:0016021; GO:0016844	arylesterase activity; biosynthetic process; cell surface; integral to membrane; strictosidine synthase activity	MembraneSinglepasstypeIImembraneprotein					
**1075**	PRUNE	PRUNE_HUMAN	Q8N654; PRUNE	Q8N654; Q86TP1; B2RCH8; B4DFL7; Q5SZF9; Q659E5; Q6P4E0; Q96JU5; Q9C071; Q9C072; Q9UIV0	NP_067045	Protein prune homolog (hPrune) (EC 3.6.1.1) (Drosophila-related expressed sequence 17) (DRES-17) (DRES17) (HTcD37)	GO:0030054; GO:0005737; GO:0004427; GO:0030145; GO:0005634; GO:0005515	cell junction; cytoplasm; inorganic diphosphatase activity; manganese ion binding; nucleus; protein binding	CytoplasmNucleusCelljunctionfocaladhesion					
**1076**	TAF1L	TAF1L_HUMAN	TAF1L	Q8IZX4; Q0VG57	NP_722516	Transcription initiation factor TFIID 210 kDa subunit (TBP-associated factor 210 kDa) (TAF(II)210) (TBP-associated factor 1-like)	GO:0003677; GO:0017025; GO:0004402; GO:0007140; GO:0004674; GO:0006357; GO:0016563; GO:0005669; GO:0006352	DNA binding; TATA-binding protein binding; histone acetyltransferase activity; male meiosis; protein serine/threonine kinase activity; regulation of transcription from RNA polymerase II promoter; transcription activator activity; transcription factor TFIID complex; transcription initiation	Nucleus					
**1077**	WBP2	WBP2_HUMAN	WBP2	Q969T9; O95638	NP_036610	WW domain-binding protein 2 (WBP-2)	GO:0005515	protein binding				Known in Uniprot to be phosphorylated	Known in HPRD to be phosphorylated	
**1078**	PRS4	PRS4_HUMAN	PRS4; Q53XL8	P62191; Q53XL8; P49014; Q03527; Q6IAW0; Q96AZ3	XP_115995; NP_002793	26S protease regulatory subunit 4 (P26s4) (Proteasome 26S subunit ATPase 1); Proteasome (Prosome, macropain) 26S subunit, ATPase, 1, isoform CRA_a (Proteasome 26S ATPase subunit 1) (Proteasome (Prosome, macropain) 26S subunit, ATPase, 1) (cDNA, FLJ93843, Homo sapiens proteasome (prosome, macropain) 26S subunit, ATPase, 1(PSMC1), mRNA)	GO:0005524; GO:0016887; GO:0031145; GO:0005829; GO:0051436; GO:0005634; GO:0051437; GO:0000502; GO:0005515; GO:0017111; GO:0030163; GO:0043234	ATP binding; ATPase activity; anaphase-promoting complex-dependent proteasomal ubiquitin-dependent protein catabolic process; cytosol; negative regulation of ubiquitin-protein ligase activity during mitotic cell cycle; nucleus; positive regulation of ubiquitin-protein ligase activity during mitotic cell cycle; proteasome complex; protein binding; nucleoside-triphosphatase activity; protein catabolic process; protein complex	CytoplasmNucleus;			Known in Uniprot to be phosphorylated	Known in HPRD to be phosphorylated	
**1079**	K1C16	K1C16_HUMAN	K1C16; Q16195; A8K488	P08779; P30654; Q16402; Q9UBG8; A8K488; Q16195	NP_005548	Keratin, type I cytoskeletal 16 (Cytokeratin-16) (CK-16) (Keratin-16) (K16); Keratin (Fragment)	GO:0008283; GO:0008544; GO:0005882; GO:0005515; GO:0005200; GO:0005882; GO:0045104	cell proliferation; epidermis development; intermediate filament; protein binding; structural constituent of cytoskeleton; intermediate filament; intermediate filament cytoskeleton organization						
**1080**	ZN668	ZN668_HUMAN	ZN668	Q8N669; Q96K58; Q59EV1; Q9H8L4	NP_078982	Zinc finger protein 668	GO:0003677; GO:0005634; GO:0006355; GO:0008270	DNA binding; nucleus; regulation of transcription, DNA-dependent; zinc ion binding	Nucleus					
**1081**	SPZ1	SPZ1_HUMAN	SPZ1; B2RA21	Q8N4P1; Q9BXG8; Q8N7E9; B2RA21	NP_115956	Spermatogenic leucine zipper protein 1 (Testis-specific protein 1) (Testis-specific protein NYD-TSP1)	GO:0003677; GO:0005737; GO:0005634; GO:0006355	DNA binding; cytoplasm; nucleus; regulation of transcription, DNA-dependent	CytoplasmNucleus			Known in Uniprot to be phosphorylated		
**1082**	PHP14	PHP14_HUMAN	PHP14; Q5T5S3; B1AMX1; Q9P019	Q9NRX4; Q5T5S3; Q9H0Y3; B1AMX1; Q9P019	NP_054891; NP_001129333	14 kDa phosphohistidine phosphatase (EC 3.1.3.-) (Phosphohistidine phosphatase 1) (Protein janus-A homolog)	GO:0005829; GO:0016311; GO:0008969; GO:0004721	cytosol; dephosphorylation; phosphohistidine phosphatase activity; phosphoprotein phosphatase activity	Cytoplasm			Known in Uniprot to be phosphorylated		Phosphatase
**1083**	PRS8	PRS8_HUMAN	PRS8; A8K763	P62195; O35051; O43208; P47210; P52915; P52916; A8K763	NP_002796	26S protease regulatory subunit 8 (Proteasome 26S subunit ATPase 5) (Proteasome subunit p45) (p45/SUG) (Thyroid hormone receptor-interacting protein 1) (TRIP1)	GO:0005524; GO:0016887; GO:0031145; GO:0005829; GO:0043069; GO:0051436; GO:0005634; GO:0043193; GO:0051437; GO:0000502; GO:0031531; GO:0003712; GO:0006366	ATP binding; ATPase activity; anaphase-promoting complex-dependent proteasomal ubiquitin-dependent protein catabolic process; cytosol; negative regulation of programmed cell death; negative regulation of ubiquitin-protein ligase activity during mitotic cell cycle; nucleus; positive regulation of gene-specific transcription; positive regulation of ubiquitin-protein ligase activity during mitotic cell cycle; proteasome complex; thyrotropin-releasing hormone receptor binding; transcription cofactor activity; transcription from RNA polymerase II promoter	CytoplasmNucleus					
**1084**	IL12B	IL12B_HUMAN	IL12B	P29460	NP_002178	Interleukin-12 subunit beta (IL-12B) (IL-12 subunit p40) (Cytotoxic lymphocyte maturation factor 40 kDa subunit) (CLMF p40) (NK cell stimulatory factor chain 2) (NKSF2)	GO:0042088; GO:0042093; GO:0007050; GO:0016477; GO:0005125; GO:0004896; GO:0043514; GO:0005143; GO:0016020; GO:0048662; GO:0042104; GO:0045785; GO:0045078; GO:0032729; GO:0032816; GO:0002860; GO:0034393; GO:0046982; GO:0010224	T-helper 1 type immune response; T-helper cell differentiation; cell cycle arrest; cell migration; cytokine activity; cytokine receptor activity; interleukin-12 complex; interleukin-12 receptor binding; membrane; negative regulation of smooth muscle cell proliferation; positive regulation of activated T cell proliferation; positive regulation of cell adhesion; positive regulation of interferon-gamma biosynthetic process; positive regulation of interferon-gamma production; positive regulation of natural killer cell activation; positive regulation of natural killer cell mediated cytotoxicity directed against tumor cell target; positive regulation of smooth muscle cell apoptosis; protein heterodimerization activity; response to UV-B	Secreted					
**1085**	EF1D	EF1D_HUMAN	EF1D; Q9H7G6; B2RAR6	P29692; Q969J1; B2RAR6; Q9H7G6	NP_001951; NP_001123527; NP_001123529; NP_001123525; NP_001123526; NP_001123528; NP_115754	Elongation factor 1-delta (EF-1-delta) (Antigen NY-CO-4); cDNA: FLJ20897 fis, clone ADKA03573	GO:0005829; GO:0005853; GO:0043123; GO:0005515; GO:0004871; GO:0003746; GO:0006414; GO:0005853	cytosol; eukaryotic translation elongation factor 1 complex; positive regulation of I-kappaB kinase/NF-kappaB cascade; protein binding; signal transducer activity; translation elongation factor activity; translational elongation; eukaryotic translation elongation factor 1 complex				Known in Uniprot to be phosphorylated	Known in HPRD to be phosphorylated	
**1086**	PSMD1	PSMD1_HUMAN	PSMD1	Q99460; Q24JU0; Q53TI2; Q6GMU5; Q6P2P4; Q6PJM7; Q6PKG9; Q86VU1; Q8IV79	NP_002798	26S proteasome non-ATPase regulatory subunit 1 (26S proteasome regulatory subunit RPN2) (26S proteasome regulatory subunit S1) (26S proteasome subunit p112)	GO:0031145; GO:0005829; GO:0030234; GO:0051436; GO:0051437; GO:0005838; GO:0005515; GO:0042176	anaphase-promoting complex-dependent proteasomal ubiquitin-dependent protein catabolic process; cytosol; enzyme regulator activity; negative regulation of ubiquitin-protein ligase activity during mitotic cell cycle; positive regulation of ubiquitin-protein ligase activity during mitotic cell cycle; proteasome regulatory particle; protein binding; regulation of protein catabolic process				Known in Uniprot to be phosphorylated	Known in HPRD to be phosphorylated	
**1087**	KCNQ4	KCNQ4_HUMAN	KCNQ4	P56696; O96025	NP_004691; NP_751895	Potassium voltage-gated channel subfamily KQT member 4 (Voltage-gated potassium channel subunit Kv7.4) (Potassium channel subunit alpha KvLQT4) (KQT-like 4)	GO:0009925; GO:0030955; GO:0006813; GO:0007605; GO:0005249; GO:0008076	basal plasma membrane; potassium ion binding; potassium ion transport; sensory perception of sound; voltage-gated potassium channel activity; voltage-gated potassium channel complex	BasalcellmembraneMultipassmembraneprotein					

	Protein Name	Uniprot Name	Other Names	Uniprot IDs	Refseq IDs	Protein Description	GO ID	GO terms	Subcellular location	Comments	Comments	Comments - Database Search	Comments - Database Search	Function
**1088**	NAV1	NAV1_HUMAN	Q8NEY1; NAV1	Q8NEY1; Q5SVH1; Q5SVH2; Q5SVH3; Q5SVH7; Q5VUY9; Q8IVL2; Q96II1; Q9H7V9; Q9H9S9; Q9H9T5; Q9UGI1; Q9ULK7; Q9ULR9	NP_065176	Neuron navigator 1 (Steerin-1) (Pore membrane and/or filament-interacting-like protein 3) (Unc-53 homolog 1) (unc53H1)	GO:0030154; GO:0005737; GO:0005874; GO:0007399; GO:0017111; GO:0000166	cell differentiation; cytoplasm; microtubule; nervous system development; nucleoside-triphosphatase activity; nucleotide binding	Cytoplasmcytoskeleton			Known in Uniprot to be phosphorylated		
**1089**	TIM16	TIM16_HUMAN	TIM16	Q9Y3D7; Q6I9Z3; Q9H5X3	NP_057153	Mitochondrial import inner membrane translocase subunit Tim16 (Mitochondria-associated granulocyte macrophage CSF-signaling molecule)	GO:0065002; GO:0005743	intracellular protein transmembrane transport; mitochondrial inner membrane	MitochondrioninnermembranePeripheralmembraneprotein					
**1090**	HS70L	HS70L_HUMAN	HS70L; HS71L	P34931; A6NNB0; B0UXW8; O75634; Q2HXR3; Q8NE72; Q96QC9; Q9UQM1	NP_005518	Heat shock 70 kDa protein 1L (Heat shock 70 kDa protein 1-like) (Heat shock 70 kDa protein 1-Hom) (HSP70-Hom)	GO:0005524; GO:0006986	ATP binding; response to unfolded protein						
**1091**	NDF6	NDF6_HUMAN	NDF6; Q548T9	Q96NK8; Q548T9; Q9H3H6	NP_073565	Neurogenic differentiation factor 6 (NeuroD6) (Protein atonal homolog 2); MATH2 protein (Neurogenic differentiation 6) (cDNA FLJ78237, highly similar to Homo sapiens neurogenic differentiation 6 (NEUROD6), mRNA)	GO:0003677; GO:0030154; GO:0007399; GO:0005634; GO:0006355; GO:0030528; GO:0005634; GO:0045449	DNA binding; cell differentiation; nervous system development; nucleus; regulation of transcription, DNA-dependent; transcription regulator activity; nucleus; regulation of transcription	Nucleus;					
**1092**	PO210	PO210_HUMAN	PO210	Q8TEM1; A6NN56; O94980; Q6NXG6; Q8NBJ1; Q9H6C8; Q9UFP3	NP_079199	Nuclear pore membrane glycoprotein 210 (POM210) (Nuclear pore protein gp210)	GO:0005789; GO:0065002; GO:0051028; GO:0031965; GO:0005643; GO:0005515	endoplasmic reticulum membrane; intracellular protein transmembrane transport; mRNA transport; nuclear membrane; nuclear pore; protein binding	NucleusnuclearporecomplexNucleusmembraneSinglepasstypeImembraneproteinEndoplasmicreticulummembraneSinglepasstypeImembraneprotein			Known in Uniprot to be phosphorylated	Known in HPRD to be phosphorylated	
**1093**	DENR	DENR_HUMAN	DENR	O43583; Q9H3U6; Q9UKZ0	XP_113714; NP_003668	Density-regulated protein (DRP) (Protein DRP1) (Smooth muscle cell-associated protein 3) (SMAP-3)	GO:0003743; GO:0006413	translation initiation factor activity; translational initiation				Known in Uniprot to be phosphorylated		
**1094**	BRK1	BRK1_HUMAN	BRK1; B2R5E2	Q8WUW1; Q9P082; B2R5E2	NP_060932	Probable protein BRICK1	GO:0005737; GO:0005856	cytoplasm; cytoskeleton	Cytoplasmcytoskeleton					
**1095**	PCDH1	PCDH1_HUMAN	Q8IUP2; PCDH1	Q8IUP2; Q08174	NP_002578	Protocadherin-1 (Protocadherin-42) (PC42) (Cadherin-like protein 1)	GO:0005509; GO:0005911; GO:0007267; GO:0007156; GO:0005887; GO:0007399; GO:0005515	calcium ion binding; cell-cell junction; cell-cell signaling; homophilic cell adhesion; integral to plasma membrane; nervous system development; protein binding	CelljunctionCellmembraneSinglepasstypeImembraneprotein			Known in Uniprot to be phosphorylated	Known in HPRD to be phosphorylated	
**1096**	CRLF3	CRLF3_HUMAN	Q9Y6M8; CRLF3	Q9Y6M8; Q8IUI8; A6NJF3; B2R8C3; Q2MLY7; Q9UNI3	NP_057070	Cytokine receptor-like factor 3 (Cytokine receptor-related protein 4) (Cytokine receptor-like molecule 9) (CREME-9) (Type I cytokine receptor-like factor p48)	GO:0005737; GO:0005886	cytoplasm; plasma membrane						
**1097**	IDHC	IDHC_HUMAN	IDHC; Q6FHQ6	O75874; Q6FHQ6; Q93090; Q9NTJ9; Q9UKW8	NP_005887	Isocitrate dehydrogenase [NADP] cytoplasmic (IDH) (EC 1.1.1.42) (Cytosolic NADP-isocitrate dehydrogenase) (Oxalosuccinate decarboxylase) (NADP(+)-specific ICDH) (IDP); IDH1 protein (Isocitrate dehydrogenase 1 (NADP+), soluble, isoform CRA_a) (Putative uncharacterized protein IDH1) (Fragment)	GO:0051287; GO:0005829; GO:0006097; GO:0004450; GO:0006102; GO:0000287; GO:0030145; GO:0055114; GO:0005777; GO:0006099; GO:0005829; GO:0006749; GO:0006979	NAD or NADH binding; cytosol; glyoxylate cycle; isocitrate dehydrogenase (NADP+) activity; isocitrate metabolic process; magnesium ion binding; manganese ion binding; oxidation reduction; peroxisome; tricarboxylic acid cycle; cytosol; glutathione metabolic process; response to oxidative stress	CytoplasmPeroxisome;					
**1098**	IDHP	IDHP_HUMAN	IDHP; B2R6L6	P48735; Q96GT3; B2R6L6	NP_002159	Isocitrate dehydrogenase [NADP], mitochondrial (IDH) (EC 1.1.1.42) (Oxalosuccinate decarboxylase) (NADP(+)-specific ICDH) (IDP) (ICD-M)	GO:0051287; GO:0006097; GO:0004450; GO:0006102; GO:0000287; GO:0030145; GO:0005739; GO:0055114; GO:0006099	NAD or NADH binding; glyoxylate cycle; isocitrate dehydrogenase (NADP+) activity; isocitrate metabolic process; magnesium ion binding; manganese ion binding; mitochondrion; oxidation reduction; tricarboxylic acid cycle	Mitochondrion					
**1099**	NDUAB	NDUAB_HUMAN	NDUAB	Q86Y39; Q6ZS66	NP_783313	NADH dehydrogenase [ubiquinone] 1 alpha subcomplex subunit 11 (NADH-ubiquinone oxidoreductase subunit B14.7) (Complex I-B14.7) (CI-B14.7)	GO:0022900; GO:0016021; GO:0005743; GO:0006810	electron transport chain; integral to membrane; mitochondrial inner membrane; transport	MitochondrioninnermembraneMultipassmembraneproteinMatrixside					
**1100**	CH042	CH042_HUMAN	Q86YL5; CH042	Q86YL5	NP_778250	Uncharacterized protein C8orf42 (Protein INM01)								
**1101**	ANUB1	ANUB1_HUMAN	ANUB1; B2RAX2	Q86XD8; A8K8V4; Q5VVY5; B2RAX2	NP_777550; NP_001121796	AN1-type zinc finger and ubiquitin domain-containing protein 1 (AN1-type zinc finger protein 4)	GO:0006464; GO:0008270	protein modification process; zinc ion binding						
**1102**	TTBK2	TTBK2_HUMAN	TTBK2; Q8IWY7	Q8IWY7; Q6IQ55; O94932; Q6ZN52; Q8IVV1	NP_775771; XP_293277	Tau-tubulin kinase 2 (EC 2.7.11.1); Tau-tubulin kinase	GO:0005524; GO:0008219; GO:0004674; GO:0045095; GO:0006468; GO:0004672; GO:0005198	ATP binding; cell death; protein serine/threonine kinase activity; keratin filament; protein amino acid phosphorylation; protein kinase activity; structural molecule activity						Kinase
**1103**	PRPS3	PRPS3_HUMAN	PRPS3	P21108; Q6P5P6	NP_787082	Ribose-phosphate pyrophosphokinase 3 (EC 2.7.6.1) (Phosphoribosyl pyrophosphate synthetase III) (PRS-III) (Phosphoribosyl pyrophosphate synthetase 1-like 1) (PRPS1-like 1)	GO:0044249; GO:0016301; GO:0000287; GO:0009116; GO:0009156; GO:0004749	cellular biosynthetic process; kinase activity; magnesium ion binding; nucleoside metabolic process; ribonucleoside monophosphate biosynthetic process; ribose phosphate diphosphokinase activity						Kinase
**1104**	FUND1	FUND1_HUMAN	FUND1	Q8IVP5	NP_776155	FUN14 domain-containing protein 1						Known in Uniprot to be phosphorylated		
**1105**	PSD13	PSD13_HUMAN	PSD13; Q53XU2	Q9UNM6; Q53XU2; O75831; Q9UNV3	NP_002808	26S proteasome non-ATPase regulatory subunit 13 (26S proteasome regulatory subunit S11) (26S proteasome regulatory subunit p40.5); Proteasome (Prosome, macropain) 26S subunit, non-ATPase, 13 (Proteasome (Prosome, macropain) 26S subunit, non-ATPase, 13, isoform CRA_a)	GO:0031145; GO:0005829; GO:0051436; GO:0051437; GO:0005838; GO:0005515; GO:0005829; GO:0007127	anaphase-promoting complex-dependent proteasomal ubiquitin-dependent protein catabolic process; cytosol; negative regulation of ubiquitin-protein ligase activity during mitotic cell cycle; positive regulation of ubiquitin-protein ligase activity during mitotic cell cycle; proteasome regulatory particle; protein binding; cytosol; meiosis I				Known in Uniprot to be phosphorylated		
**1106**	BA2D1	BA2D1_HUMAN	Q9NSM8; Q9UF29; Q9Y520; BA2D1	Q9Y520; Q9NSM8; Q05DM8; Q49A39; Q6PD54; Q9H2N2; Q9HA05; Q9NXL3; Q9UF29; Q9UPQ6	NP_055987	BAT2 domain-containing protein 1 (HBxAg transactivated protein 2)						Known in Uniprot to be phosphorylated		
**1107**	XPO2	XPO2_HUMAN	XPO2; B2R5T4	P55060; O75432; Q32M40; Q9H5B7; Q9NTS0; Q9UP98; Q9UP99; Q9UPA0; B2R5T4	NP_001307	Exportin-2 (Exp2) (Importin-alpha re-exporter) (Chromosome segregation 1-like protein) (Cellular apoptosis susceptibility protein)	GO:0006915; GO:0008283; GO:0005737; GO:0008262; GO:0005643; GO:0000059	apoptosis; cell proliferation; cytoplasm; importin-alpha export receptor activity; nuclear pore; protein import into nucleus, docking	CytoplasmNucleus					
**1108**	PKHL1	PKHL1_HUMAN	Q86WI1; PKHL1	Q86WI1; Q567P2; Q9UF27	NP_803875	Fibrocystin-L (Polycystic kidney and hepatic disease 1-like protein 1) (PKHD1-like protein 1)	GO:0016021; GO:0004872	integral to membrane; receptor activity	MembraneSinglepassmembraneprotein					
**1109**	UN45A	UN45A_HUMAN	UN45A; A8K6F7	Q9H3U1; Q7L3Y6; Q9H3U8; Q9NSE8; Q9NSE9; A8K6F7	NP_061141; NP_001034764	Protein unc-45 homolog A (UNC-45A) (GCUNC-45) (Smooth muscle cell-associated protein 1) (SMAP-1)	GO:0030154; GO:0005737; GO:0005634; GO:0005515; GO:0007519	cell differentiation; cytoplasm; nucleus; protein binding; skeletal muscle tissue development	CytoplasmCytoplasmperinuclearregionNucleus			Known in Uniprot to be phosphorylated		
**1110**	PTPA	PTPA_HUMAN	PTPA; A9IZU4	Q5TZQ2; Q15257; B4DXM4; Q15258; Q53GZ3; Q9BUK1; Q9NNZ7; Q9NNZ8; Q9NNZ9; A9IZU4	NP_066954; NP_821067; NP_821068; NP_821070	Serine/threonine-protein phosphatase 2A regulatory subunit B' (PP2A, subunit B', PR53 isoform) (Phosphotyrosyl phosphatase activator) (PTPA)	GO:0005524; GO:0034704; GO:0032515; GO:0035308; GO:0005634; GO:0032516; GO:0035307; GO:0046982; GO:0042803; GO:0051721; GO:0000159; GO:0008601; GO:0008160; GO:0005102; GO:0005625	ATP binding; calcium channel complex; negative regulation of phosphoprotein phosphatase activity; negative regulation of protein amino acid dephosphorylation; nucleus; positive regulation of phosphoprotein phosphatase activity; positive regulation of protein amino acid dephosphorylation; protein heterodimerization activity; protein homodimerization activity; protein phosphatase 2A binding; protein phosphatase type 2A complex; protein phosphatase type 2A regulator activity; protein tyrosine phosphatase activator activity; receptor binding; soluble fraction						Phosphatase
**1111**	ZN490	ZN490_HUMAN	ZN490	Q9ULM2	NP_065765	Zinc finger protein 490	GO:0003677; GO:0005634; GO:0006355; GO:0008270	DNA binding; nucleus; regulation of transcription, DNA-dependent; zinc ion binding	Nucleus					
**1112**	Q6W898	Q6W898_HUMAN	Q6W898; A8K0B6	Q6W898; A8K0B6	NP_004306; NP_808592	HSD-33	GO:0006629; GO:0005764	lipid metabolic process; lysosome						
**1113**	GOGA3	GOGA3_HUMAN	GOGA3	Q08378; O43241; Q6P9C7; Q86XW3; Q8TDA9; Q8WZA3	NP_005886	Golgin subfamily A member 3 (Golgin-160) (Golgi complex-associated protein of 170 kDa) (GCP170)	GO:0000139; GO:0017119; GO:0006891; GO:0005515; GO:0005215	Golgi membrane; Golgi transport complex; intra-Golgi vesicle-mediated transport; protein binding; transporter activity	CytoplasmGolgiapparatusGolgistackmembranePeripheralmembraneprotein			Known in Uniprot to be phosphorylated		

	Protein Name	Uniprot Name	Other Names	Uniprot IDs	Refseq IDs	Protein Description	GO ID	GO terms	Subcellular location	Comments	Comments	Comments - Database Search	Comments - Database Search	Function
**1114**	EHD1	EHD1_HUMAN	EHD1; B2R5U3	Q9H4M9; O14611; Q2M3Q4; Q9UNR3; B2R5U3	NP_006786	EH domain-containing protein 1 (Testilin) (hPAST1)	GO:0005524; GO:0005525; GO:0003924; GO:0005509; GO:0031901; GO:0005886; GO:0005515; GO:0055038	ATP binding; GTP binding; GTPase activity; calcium ion binding; early endosome membrane; plasma membrane; protein binding; recycling endosome membrane	CellmembranePeripheralmembraneproteinCytoplasmicsideEarlyendosomemembranePeripheralmembraneproteinRecyclingendosomemembranePeripheralmembraneprotein			Known in Uniprot to be phosphorylated		
**1115**	PSME2	PSME2_HUMAN	PSME2; Q86SZ7	Q86SZ7; Q9UL46; Q15129	NP_002809; XP_291768	Proteasome activator complex subunit 2 (Proteasome activator 28 subunit beta) (PA28beta) (PA28b) (Activator of multicatalytic protease subunit 2) (11S regulator complex subunit beta) (REG-beta); Full-length cDNA clone CS0DJ015YJ12 of T cells (Jurkat cell line) of Homo sapiens (human) (PSME2 protein)	GO:0031145; GO:0005829; GO:0051436; GO:0051437; GO:0008538; GO:0008537; GO:0008538	anaphase-promoting complex-dependent proteasomal ubiquitin-dependent protein catabolic process; cytosol; negative regulation of ubiquitin-protein ligase activity during mitotic cell cycle; positive regulation of ubiquitin-protein ligase activity during mitotic cell cycle; proteasome activator activity; proteasome activator complex; proteasome activator activity						
**1116**	ANO4	ANO4_HUMAN	Q8NB39; ANO4	Q8NB39; Q32M45; Q8NAJ0; Q8NB53	XP_058577; NP_849148	Anoctamin-4 (Transmembrane protein 16D)	GO:0005509; GO:0005254; GO:0031404; GO:0016021; GO:0006811	calcium ion binding; chloride channel activity; chloride ion binding; integral to membrane; ion transport	MembraneMultipassmembraneprotein					
**1117**	HDBP1	HDBP1_HUMAN	Q8IV16; HDBP1	Q8IV16; Q6P3T2; Q86W15	XP_296036; NP_835466	Glycosylphosphatidylinositol-anchored high density lipoprotein-binding protein 1 (GPI-anchored HDL-binding protein 1) (GPI-HBP1) (High density lipoprotein-binding protein 1)	GO:0034364; GO:0016021; GO:0008289	high-density lipoprotein particle; integral to membrane; lipid binding	CellmembraneSinglepassmembraneprotein					
**1118**	FBXW8	FBXW8_HUMAN	FBXW8	Q8N3Y1; Q9UK95	NP_699179; NP_036306	F-box/WD repeat-containing protein 8 (F-box and WD-40 domain-containing protein 8) (F-box only protein 29)	GO:0019941; GO:0005515	modification-dependent protein catabolic process; protein binding				Known in Uniprot to be phosphorylated		
**1119**	CRNL1	CRNL1_HUMAN	CRNL1	Q9BZJ0; A8K9T4; Q5JY64; Q8WYI5; Q9BZI9; Q9BZJ1; Q9BZJ2; Q9GZW7; Q9H8F8; Q9NQH5; Q9NYD8	NP_057736	Crooked neck-like protein 1 (Crooked neck homolog) (hCrn)	GO:0003723; GO:0005737; GO:0016607; GO:0005681; GO:0000245	RNA binding; cytoplasm; nuclear speck; spliceosome; spliceosome assembly	NucleusspeckleCytoplasm					
**1120**	FRDA	FRDA_HUMAN	FRDA; Q5VZ01	Q16595; Q5VZ01; O15545; O95656; Q15294	NP_000135; NP_852090	Frataxin, mitochondrial (Friedreich ataxia protein) (Fxn) [Cleaved into: Frataxin intermediate form; Frataxin(56-210); Frataxin(81-210)]	GO:0051537; GO:0006879; GO:0005829; GO:0008199; GO:0008198; GO:0006783; GO:0034986; GO:0018283; GO:0005759; GO:0030307; GO:0008284; GO:0051349; GO:0048554; GO:0051353; GO:0051347; GO:0016540; GO:0005515; GO:0010722; GO:0010039	2 iron, 2 sulfur cluster binding; cellular iron ion homeostasis; cytosol; ferric iron binding; ferrous iron binding; heme biosynthetic process; iron chaperone activity; iron incorporation into metallo-sulfur cluster; mitochondrial matrix; positive regulation of cell growth; positive regulation of cell proliferation; positive regulation of lyase activity; positive regulation of metalloenzyme activity; positive regulation of oxidoreductase activity; positive regulation of transferase activity; protein autoprocessing; protein binding; regulation of ferrochelatase activity; response to iron ion	Mitochondrion					
**1121**	FSIP1	FSIP1_HUMAN	Q86Y89; FSIP1	Q86Y89; Q8NA03; Q6X2C8	XP_298422; NP_689810	Fibrous sheath-interacting protein 1								
**1122**	DOCK3	DOCK3_HUMAN	DOCK3	Q8IZD9; O15017	XP_039259; NP_004938	Dedicator of cytokinesis protein 3 (Modifier of cell adhesion) (Presenilin-binding protein) (PBP)	GO:0005525; GO:0051020; GO:0017124; GO:0005737; GO:0005085	GTP binding; GTPase binding; SH3 domain binding; cytoplasm; guanyl-nucleotide exchange factor activity	Cytoplasm			Known in Uniprot to be phosphorylated		
**1123**	HS74L	HS74L_HUMAN	HS74L; A2ICT2	O95757; Q4W5M5; Q8IWA2; A2ICT2	NP_055093	Heat shock 70 kDa protein 4L (Osmotic stress protein 94) (Heat shock 70-related protein APG-1); Heat shock 70 kDa protein 4-like protein (Heat shock 70kDa protein 4-like, isoform CRA_b)	GO:0005524; GO:0005737; GO:0005634; GO:0005515; GO:0006457; GO:0006986; GO:0006950	ATP binding; cytoplasm; nucleus; protein binding; protein folding; response to unfolded protein; response to stress	CytoplasmNucleus;			Known in Uniprot to be phosphorylated		
**1124**	COPE	COPE_HUMAN	COPE; Q6IBB8	O14579; Q6IBB8; O76097	NP_009194	Coatomer subunit epsilon (Epsilon-coat protein) (Epsilon-COP); COPE protein (Coatomer protein complex, subunit epsilon, isoform CRA_i)	GO:0048205; GO:0030126; GO:0005829; GO:0006891; GO:0005515; GO:0015031; GO:0006890; GO:0005198; GO:0030126	COPI coating of Golgi vesicle; COPI vesicle coat; cytosol; intra-Golgi vesicle-mediated transport; protein binding; protein transport; retrograde vesicle-mediated transport, Golgi to ER; structural molecule activity; COPI vesicle coat	CytoplasmGolgiapparatusmembranePeripheralmembraneproteinCytoplasmicsideCytoplasmicvesicleCOPIcoatedvesiclemembranePeripheralmembraneproteinCytoplasmicside;			Known in Uniprot to be phosphorylated		
**1125**	ARP5	ARP5_HUMAN	ARP5	Q9H9F9; Q86WF7; Q8IUY5; Q8N724; Q9BRN0; Q9BVB7	NP_079131	Actin-related protein 5 (hARP5) (Sarcoma antigen NY-SAR-16)	GO:0005524; GO:0005515; GO:0006355	ATP binding; protein binding; regulation of transcription, DNA-dependent						
**1126**	GMPR1	GMPR1_HUMAN	GMPR1	P36959; Q96HQ6	NP_006868	GMP reductase 1 (EC 1.7.1.7) (Guanosine 5'-monophosphate oxidoreductase 1) (Guanosine monophosphate reductase 1)	GO:0003920; GO:0009117; GO:0055114; GO:0030955; GO:0009409	GMP reductase activity; nucleotide metabolic process; oxidation reduction; potassium ion binding; response to cold						
**1127**	PARK7	PARK7_HUMAN	PARK7; B2R4Z1	Q99497; O14805; Q6DR95; Q7LFU2; B2R4Z1	NP_009193; NP_001116849	Protein DJ-1 (Oncogene DJ1) (Parkinson disease protein 7)	GO:0007265; GO:0005634; GO:0005515	Ras protein signal transduction; nucleus; protein binding	NucleusCytoplasm			Known in Uniprot to be phosphorylated		
**1128**	PGK2	PGK2_HUMAN	PGK2	P07205; B2R6Y8; Q9H107	NP_620061	Phosphoglycerate kinase 2 (EC 2.7.2.3) (Phosphoglycerate kinase, testis specific)	GO:0005524; GO:0005829; GO:0006096; GO:0004618; GO:0016310	ATP binding; cytosol; glycolysis; phosphoglycerate kinase activity; phosphorylation	Cytoplasm			Known in Uniprot to be phosphorylated		Kinase
**1129**	DPOE3	DPOE3_HUMAN	DPOE3	Q9NRF9; Q5W0U1; Q8N758; Q8NCE5; Q9NR32	NP_059139	DNA polymerase epsilon subunit 3 (DNA polymerase II subunit 3) (EC 2.7.7.7) (DNA polymerase epsilon subunit p17) (Chromatin accessibility complex 17) (HuCHRAC17) (CHRAC-17) (Arsenic-transactivated protein) (AsTP)	GO:0006260; GO:0003887; GO:0005634; GO:0005515; GO:0043565	DNA replication; DNA-directed DNA polymerase activity; nucleus; protein binding; sequence-specific DNA binding	Nucleus			Known in Uniprot to be phosphorylated		
**1130**	NPT2A	NPT2A_HUMAN	Q86VN6; NPT2A; Q7Z725	Q86VN6; Q06495; Q7Z725	NP_003043	Solute carrier family 34 (Sodium phosphate), member 1	GO:0046849; GO:0016020; GO:0006817; GO:0015321	bone remodeling; membrane; phosphate transport; sodium-dependent phosphate transmembrane transporter activity				Known in Uniprot to be phosphorylated		
**1131**	DIAP1	DIAP1_HUMAN	Q59FH8; Q17RN4; DIAP1; B7ZKW2	Q59FH8; O60610; A6NF18; Q9UC76; B7ZKW2; Q17RN4	NP_005210; NP_001073280	Protein diaphanous homolog 1 (Diaphanous-related formin-1) (DRF1); DIAPH1 protein	GO:0017048; GO:0003779; GO:0030036; GO:0042995; GO:0005737; GO:0005856; GO:0005886; GO:0005102; GO:0007605; GO:0016043	Rho GTPase binding; actin binding; actin cytoskeleton organization; cell projection; cytoplasm; cytoskeleton; plasma membrane; receptor binding; sensory perception of sound; cellular component organization	CellmembraneCellprojectionrufflemembraneCytoplasmcytoskeleton;			Known in Uniprot to be phosphorylated		
**1132**	KR132	KR132_HUMAN	KR132	Q52LG2	XP_298663; NP_853652	Keratin-associated protein 13-2	GO:0005882	intermediate filament						
**1133**	PRDX2	PRDX2_HUMAN	PRDX2	P32119; P31945; P32118; P35701; Q6FHG4; Q92763; Q9UC23	NP_005800	Peroxiredoxin-2 (EC 1.11.1.15) (Thioredoxin peroxidase 1) (Thioredoxin-dependent peroxide reductase 1) (Thiol-specific antioxidant protein) (TSA) (PRP) (Natural killer cell-enhancing factor B) (NKEF-B)	GO:0006916; GO:0045454; GO:0005737; GO:0055114; GO:0006979; GO:0008379	anti-apoptosis; cell redox homeostasis; cytoplasm; oxidation reduction; response to oxidative stress; thioredoxin peroxidase activity	Cytoplasm					
**1134**	VTDB	VTDB_HUMAN	VTDB	P02774; Q16309; Q16310	NP_000574	Vitamin D-binding protein (DBP) (VDB) (Group-specific component) (Gc-globulin)	GO:0003779; GO:0005813; GO:0005615; GO:0005499; GO:0051180; GO:0051183	actin binding; centrosome; extracellular space; vitamin D binding; vitamin transport; vitamin transporter activity	Secreted					
**1135**	PDDC1	PDDC1_HUMAN	Q8NB37; PDDC1	Q8NB37; Q2NL76; Q6ZQY0; Q8NAE0	XP_300550; NP_872418	Parkinson disease 7 domain-containing protein 1	GO:0005576	extracellular region	Secreted					
**1136**	RHG07	RHG07_HUMAN	RHG07	Q96QB1; O14868; O43199; Q9C0E0	NP_006085	Rho GTPase-activating protein 7 (Rho-type GTPase-activating protein 7) (Deleted in liver cancer 1 protein) (Dlc-1) (StAR-related lipid transfer protein 12) (START domain-containing protein 12) (StARD12) (HP protein)	GO:0005100; GO:0042169; GO:0006919; GO:0005901; GO:0005737; GO:0005925; GO:0048041; GO:0030900; GO:0003007; GO:0021575; GO:0006917; GO:0035024; GO:0030336; GO:0008285; GO:0051497; GO:0001843; GO:0005634; GO:0035307; GO:0008360	Rho GTPase activator activity; SH2 domain binding; activation of caspase activity; caveola; cytoplasm; focal adhesion; focal adhesion formation; forebrain development; heart morphogenesis; hindbrain morphogenesis; induction of apoptosis; negative regulation of Rho protein signal transduction; negative regulation of cell migration; negative regulation of cell proliferation; negative regulation of stress fiber formation; neural tube closure; nucleus; positive regulation of protein amino acid dephosphorylation; regulation of cell shape	Cytoplasm			Known in Uniprot to be phosphorylated	Known in HPRD to be phosphorylated	
**1137**	MACF1	MACF1_HUMAN	MACF1	Q9UPN3; O75053; Q5VW20; Q8WXY2; Q9H540; Q9UKP0; Q9ULG9	NP_036222	Microtubule-actin cross-linking factor 1, isoforms 1/2/3/5 (Actin cross-linking family protein 7) (Macrophin-1) (Trabeculin-alpha) (620 kDa actin-binding protein) (ABP620)	GO:0051015; GO:0005509; GO:0007050; GO:0005737; GO:0005856; GO:0008017	actin filament binding; calcium ion binding; cell cycle arrest; cytoplasm; cytoskeleton; microtubule binding	Cytoplasmcytoskeleton			Known in Uniprot to be phosphorylated		

	Protein Name	Uniprot Name	Other Names	Uniprot IDs	Refseq IDs	Protein Description	GO ID	GO terms	Subcellular location	Comments	Comments	Comments - Database Search	Comments - Database Search	Function
**1138**	MYO9B	MYO9B_HUMAN	MYO9B; Q8WVD2; B0I1T6	Q13459; O75314; Q9NUJ2; Q9UHN0; B0I1T6; Q8WVD2	NP_004136	Myosin-IXb (Unconventional myosin-9b); MYO9B protein (Fragment)	GO:0043531; GO:0005524; GO:0016887; GO:0005100; GO:0007266; GO:0003779; GO:0030048; GO:0005516; GO:0005938; GO:0019992; GO:0031941; GO:0000146; GO:0016459; GO:0048471; GO:0008270; GO:0005524; GO:0030898; GO:0005622; GO:0007242	ADP binding; ATP binding; ATPase activity; Rho GTPase activator activity; Rho protein signal transduction; actin binding; actin filament-based movement; calmodulin binding; cell cortex; diacylglycerol binding; filamentous actin; microfilament motor activity; myosin complex; perinuclear region of cytoplasm; zinc ion binding; ATP binding; actin-dependent ATPase activity; intracellular; intracellular signaling cascade	CytoplasmcellcortexCytoplasmperinuclearregionCytoplasmcytoskeleton;			Known in Uniprot to be phosphorylated		
**1139**	MCM10	MCM10_HUMAN	MCM10; B7ZKZ8	Q9H3P9; Q7L590; A8K9I6; Q3MIR3; Q7LD55; Q96GX4; Q96NB6; Q9H0D7; Q9P177; B7ZKZ8	NP_060988; NP_877428	Protein MCM10 homolog (HsMCM10)	GO:0006260; GO:0005654; GO:0005515; GO:0008270	DNA replication; nucleoplasm; protein binding; zinc ion binding	Nucleus			Known in Uniprot to be phosphorylated		
**1140**	ESTD	ESTD_HUMAN	ESTD	P10768; Q5TBV0; Q5TBU8; Q5TBV2; Q9BVJ2	XP_166266; NP_001975	S-formylglutathione hydrolase (FGH) (EC 3.1.2.12) (Esterase D)	GO:0018738; GO:0004091; GO:0016023	S-formylglutathione hydrolase activity; carboxylesterase activity; cytoplasmic membrane-bounded vesicle	CytoplasmCytoplasmic vesicle					
**1141**	CS010	CS010_HUMAN	CS010	Q969H8; O75256; O75272; Q9BTK7; Q9NP69	XP_035638; NP_061980	UPF0556 protein C19orf10 (Stromal cell-derived growth factor SF20) (Interleukin-25) (IL-25)	GO:0005793; GO:0005576	ER-Golgi intermediate compartment; extracellular region	SecretedEndoplasmicreticulumGolgiintermediatecompartment					
**1142**	EGFLA	EGFLA_HUMAN	Q8N8N5; EGFLA	Q8N8N5; Q63HQ2; A8K6D7; Q5U643; Q6P3V1; Q8N124; Q8N197; Q8N7Y0; Q8NAL2	NP_689616; NP_877950; NP_877951; NP_877953	Pikachurin (EGF-like, fibronectin type-III and laminin G-like domain-containing protein)	GO:0030054; GO:0005578; GO:0045202	cell junction; proteinaceous extracellular matrix; synapse	SecretedextracellularspaceextracellularmatrixCelljunctionsynapse					
**1143**	SCFD1	SCFD1_HUMAN	SCFD1	Q8WVM8; A8K2Z5; O60754; O94990; Q7Z529; Q9BZI3; Q9UNL3; Q9Y6A8	NP_057190; NP_878255	Sec1 family domain-containing protein 1 (Syntaxin-binding protein 1-like 2) (Sly1p)	GO:0005794; GO:0005789; GO:0005634; GO:0015031; GO:0006904	Golgi apparatus; endoplasmic reticulum membrane; nucleus; protein transport; vesicle docking during exocytosis	CytoplasmEndoplasmicreticulummembranePeripheralmembraneproteinGolgiapparatusGolgistackmembranePeripheralmembraneprotein			Known in Uniprot to be phosphorylated		
**1144**	NID2	NID2_HUMAN	NID2; Q8IV28	Q14112; O43710; Q8IV28	NP_031387	Nidogen-2 (NID-2) (Osteonidogen); NID2 protein	GO:0005604; GO:0008218; GO:0005509; GO:0007160; GO:0005518; GO:0016020; GO:0018298; GO:0008218	basement membrane; bioluminescence; calcium ion binding; cell-matrix adhesion; collagen binding; membrane; protein-chromophore linkage; bioluminescence	Secretedextracellularspaceextracellularmatrixbasementmembrane;					
**1145**	TRXR1	TRXR1_HUMAN	TRXR1	Q16881; Q6FI31; Q6VB40; Q6VB41; Q6VB42; Q6VBP2; Q6VBP3; Q6VBP4; Q6VBP5; Q6VBP9; Q6VBQ0; Q6YNQ1; Q76P53; Q7LA96; Q8WVC8; Q99475; Q9UES8; Q9UH79	NP_003321; NP_001087 240; NP_877393; NP_877419; NP_877420	Thioredoxin reductase 1, cytoplasmic (TR) (EC 1.8.1.9) (Thioredoxin reductase TR1) (KM-102-derived reductase-like factor) (Gene associated with retinoid-IFN-induced mortality 12 protein) (GRIM-12)	GO:0050660; GO:0050661; GO:0045454; GO:0005829; GO:0009055; GO:0022900; GO:0005634; GO:0015035; GO:0008430; GO:0007165; GO:0004791; GO:0006810	FAD binding; NADP or NADPH binding; cell redox homeostasis; cytosol; electron carrier activity; electron transport chain; nucleus; protein disulfide oxidoreductase activity; selenium binding; signal transduction; thioredoxin-disulfide reductase activity; transport	CytoplasmCytoplasmNucleusCytoplasm			Known in Uniprot to be phosphorylated		
**1146**	BIEA	BIEA_HUMAN	BIEA	P53004; A8K747; O95019; Q86UX0; Q96QL4; Q9BRW8	NP_000703	Biliverdin reductase A (BVR A) (EC 1.3.1.24) (Biliverdin-IX alpha-reductase)	GO:0004074; GO:0005737; GO:0042167; GO:0055114; GO:0008270	biliverdin reductase activity; cytoplasm; heme catabolic process; oxidation reduction; zinc ion binding	Cytoplasm			Known in Uniprot to be phosphorylated		
**1147**	SBNO1	SBNO1_HUMAN	Q9H3T8; SBNO1	Q9H3T8; A3KN83; Q05C06; Q3ZTS3; Q9NVB2	NP_060653	Protein strawberry notch homolog 1 (Monocyte protein 3) (MOP-3)						Known in Uniprot to be phosphorylated		
**1148**	STX11	STX11_HUMAN	STX11; Q5TCL6	O75558; Q5TCL6; O75378; O95148	NP_003755	Syntaxin-11	GO:0005794; GO:0005484; GO:0006886; GO:0016020; GO:0006944; GO:0016192	Golgi apparatus; SNAP receptor activity; intracellular protein transport; membrane; membrane fusion; vesicle-mediated transport	MembranePeripheralmembraneproteinGolgiapparatustransGolginetworkmembranePeripheralmembraneprotein					
**1149**	RAB10	RAB10_HUMAN	RAB10	P61026; O88386; Q6IA52; Q9D7X6; Q9H0T3	NP_057215	Ras-related protein Rab-10	GO:0005525; GO:0005886; GO:0005515; GO:0015031; GO:0007264	GTP binding; plasma membrane; protein binding; protein transport; small GTPase mediated signal transduction	CellmembraneLipidanchorCytoplasmicside					
**1150**	RALA	RALA_HUMAN	RALA; A4D1W3	P11233; A4D1W3	NP_005393	Ras-related protein Ral-A; V-ral simian leukemia viral oncogene homolog A (Ras related) (V-ral simian leukemia viral oncogene homolog A (Ras related), isoform CRA_b)	GO:0005525; GO:0007265; GO:0031532; GO:0006935; GO:0044419; GO:0005622; GO:0005886; GO:0051491; GO:0005515; GO:0007264	GTP binding; Ras protein signal transduction; actin cytoskeleton reorganization; chemotaxis; interspecies interaction between organisms; intracellular; plasma membrane; positive regulation of filopodium assembly; protein binding; small GTPase mediated signal transduction	CellmembraneLipidanchorCytoplasmicside;					
**1151**	EFTU	EFTU_HUMAN	EFTU	P49411; O15276	NP_003312	Elongation factor Tu, mitochondrial (EF-Tu) (P43)	GO:0005525; GO:0003924; GO:0042645; GO:0005515; GO:0003746; GO:0006414	GTP binding; GTPase activity; mitochondrial nucleoid; protein binding; translation elongation factor activity; translational elongation	Mitochondrion					
**1152**	HSP76	HSP76_HUMAN	HSP76; Q5QNY2; Q1HBA8	P17066; Q5QNY2; Q8IYK7; Q9BT95; Q1HBA8	NP_002146	Heat shock 70 kDa protein 6 (Heat shock 70 kDa protein B')	GO:0005524; GO:0006986	ATP binding; response to unfolded protein						
**1153**	GALT4	GALT4_HUMAN	GALT4	Q8N4A0; O00208	NP_003765	Polypeptide N-acetylgalactosaminyltransferase 4 (EC 2.4.1.41) (Polypeptide GalNAc transferase 4) (pp-GaNTase 4) (GalNAc-T4) (Protein-UDP acetylgalactosaminyltransferase 4) (UDP-GalNAc:polypeptide N-acetylgalactosaminyltransferase 4)	GO:0000139; GO:0005509; GO:0005975; GO:0016021; GO:0030145; GO:0048471; GO:0004653; GO:0005529	Golgi membrane; calcium ion binding; carbohydrate metabolic process; integral to membrane; manganese ion binding; perinuclear region of cytoplasm; polypeptide N-acetylgalactosaminyltransferase activity; sugar binding	GolgiapparatusmembraneSinglepasstypeIImembraneprotein					
**1154**	PREX1	PREX1_HUMAN	PREX1	Q8TCU6; Q5JS95; Q5JS96; Q69YL2; Q7Z2L9; Q9BQH0; Q9BX55; Q9H4Q6; Q9P2D2; Q9UGQ4	NP_065871	Phosphatidylinositol 3,4,5-trisphosphate-dependent Rac exchanger 1 protein (PtdIns(3,4,5)-dependent Rac exchanger 1) (P-Rex1)	GO:0005100; GO:0005089; GO:0030041; GO:0005829; GO:0019899; GO:0042119; GO:0005543; GO:0005886; GO:0035023; GO:0006801	Rho GTPase activator activity; Rho guanyl nucleotide exchange factor activity; actin filament polymerization; cytosol; enzyme binding; neutrophil activation; phospholipid binding; plasma membrane; regulation of Rho protein signal transduction; superoxide metabolic process	CytoplasmcytosolCellmembrane			Known in Uniprot to be phosphorylated		
**1155**	HEM2	HEM2_HUMAN	HEM2; Q6ZMU0; A8K375	P13716; B2R6F2; Q16870; Q16871; Q9BVQ9; A8K375; Q6ZMU0	NP_000022	Delta-aminolevulinic acid dehydratase (ALADH) (EC 4.2.1.24) (Porphobilinogen synthase); Delta-aminolevulinic acid dehydratase (EC 4.2.1.24)	GO:0005829; GO:0006783; GO:0004655; GO:0008270; GO:0046872; GO:0033014	cytosol; heme biosynthetic process; porphobilinogen synthase activity; zinc ion binding; metal ion binding; tetrapyrrole biosynthetic process						
**1156**	GBRD	GBRD_HUMAN	GBRD	O14764; Q8N4N9	NP_000806	Gamma-aminobutyric acid receptor subunit delta (GABA(A) receptor subunit delta)	GO:0004890; GO:0030054; GO:0005254; GO:0031404; GO:0005230; GO:0005887; GO:0006811; GO:0030594; GO:0045211; GO:0007165	GABA-A receptor activity; cell junction; chloride channel activity; chloride ion binding; extracellular ligand-gated ion channel activity; integral to plasma membrane; ion transport; neurotransmitter receptor activity; postsynaptic membrane; signal transduction	CelljunctionsynapsepostsynapticcellmembraneMultipassmembraneproteinCellmembraneMultipassmembraneprotein					
**1157**	DCNL1	DCNL1_HUMAN	DCNL1; B2RB37	Q96GG9; Q7L3G9; Q8TEX7; Q9H6M1; Q9HCT3; B2RB37	NP_065691	DCN1-like protein 1 (Defective in cullin neddylation protein 1-like protein 1) (DCUN1 domain-containing protein 1) (Squamous cell carcinoma-related oncogene)	GO:0019941	modification-dependent protein catabolic process						
**1158**	GPVI	GPVI_HUMAN	GPVI	Q9HCN6; Q9HCN7; Q9UIF2	NP_057447	Platelet glycoprotein VI	GO:0005518; GO:0007167; GO:0005887; GO:0030168; GO:0004888	collagen binding; enzyme linked receptor protein signaling pathway; integral to plasma membrane; platelet activation; transmembrane receptor activity	CellmembraneSinglepassmembraneprotein					

	Protein Name	Uniprot Name	Other Names	Uniprot IDs	Refseq IDs	Protein Description	GO ID	GO terms	Subcellular location	Comments	Comments	Comments - Database Search	Comments - Database Search	Function
**1159**	UB2J1	UB2J1_HUMAN	UB2J1	Q9Y385; A8K3F9; Q5W0N4; Q9BZ32; Q9NQL3; Q9NY66; Q9P011; Q9P0S0; Q9UF10	NP_057105	Ubiquitin-conjugating enzyme E2 J1 (EC 6.3.2.19) (Non-canonical ubiquitin-conjugating enzyme 1) (NCUBE1) (Yeast ubiquitin-conjugating enzyme UBC6 homolog E) (HSUBC6e)	GO:0005789; GO:0016021; GO:0019941; GO:0043687; GO:0051246; GO:0004842	endoplasmic reticulum membrane; integral to membrane; modification-dependent protein catabolic process; post translational protein modification; regulation of protein metabolic process; ubiquitin-protein ligase activity	EndoplasmicreticulummembraneSinglepasstypeIVmembraneprotein			Known in Uniprot to be phosphorylated		
**1160**	SATB2	SATB2_HUMAN	SATB2; A8K5Z8	Q9UPW6; Q4V763; A8K5Z8	XP_031223; NP_056080	DNA-binding protein SATB2 (Special AT-rich sequence-binding protein 2)	GO:0005634; GO:0006355; GO:0043565; GO:0003700	nucleus; regulation of transcription, DNA-dependent; sequence-specific DNA binding; transcription factor activity	Nucleus			Known in Uniprot to be phosphorylated		
**1161**	RHOF	RHOF_HUMAN	RHOF	Q9HBH0; Q8WVB1; Q9NXH6	NP_061907	Rho-related GTP-binding protein RhoF (Rho family GTPase Rif) (Rho in filopodia)	GO:0005525; GO:0003924; GO:0007015; GO:0005737; GO:0005856; GO:0005886; GO:0007264	GTP binding; GTPase activity; actin filament organization; cytoplasm; cytoskeleton; plasma membrane; small GTPase mediated signal transduction	CellmembraneLipidanchorCytoplasmicsideCytoplasmcytoskeleton					
**1162**	UBA7	UBA7_HUMAN	Q9BRB2; UBA7	Q9BRB2; P41226	NP_003326	Ubiquitin-like modifier-activating enzyme 7 (Ubiquitin-activating enzyme 7) (Ubiquitin-activating enzyme E1 homolog) (D8)	GO:0005524; GO:0019782; GO:0032020; GO:0016874; GO:0019941	ATP binding; ISG15 activating enzyme activity; ISG15-protein conjugation; ligase activity; modification-dependent protein catabolic process						
**1163**	UN13B	UN13B_HUMAN	UN13B; Q4LE73; Q5VYM8	O14795; Q5VYM8; Q4LE73	NP_006368	Protein unc-13 homolog B (Munc13-2) (munc13); UNC13B variant protein (Fragment)	GO:0005794; GO:0030054; GO:0019992; GO:0007588; GO:0006917; GO:0007242; GO:0004872; GO:0045202; GO:0008270; GO:0019992; GO:0016082	Golgi apparatus; cell junction; diacylglycerol binding; excretion; induction of apoptosis; intracellular signaling cascade; receptor activity; synapse; zinc ion binding; diacylglycerol binding; synaptic vesicle priming	CytoplasmMembranePeripheralmembraneproteinCellmembraneCelljunctionsynapse;			Known in Uniprot to be phosphorylated	Known in HPRD to be phosphorylated	
**1164**	RAP2B	RAP2B_HUMAN	RAP2B; Q5JQ44	P61225; P17964; Q96EG5; Q9CXG0; Q5JQ44	NP_002877	Ras-related protein Rap-2b; Putative uncharacterized protein DKFZp547A0616 (Fragment)	GO:0005525; GO:0005622; GO:0005886; GO:0007264; GO:0019904	GTP binding; intracellular; plasma membrane; small GTPase mediated signal transduction; protein domain specific binding	CellmembraneLipidanchorCytoplasmicside;					
**1165**	ELAV1	ELAV1_HUMAN	ELAV1; Q53XN6	Q15717; Q53XN6; Q9BTT1	NP_001410	ELAV-like protein 1 (Hu-antigen R) (HuR); ELAV (Embryonic lethal, abnormal vision, Drosophila)-like 1 (Hu antigen R) (ELAV (Embryonic lethal, abnormal vision, Drosophila)-like 1 (Hu antigen R), isoform CRA_a)	GO:0042802; GO:0003729; GO:0007275; GO:0000166; GO:0003723; GO:0048255; GO:0005634	identical protein binding; mRNA binding; multicellular organismal development; nucleotide binding; RNA binding; mRNA stabilization; nucleus				Known in Uniprot to be phosphorylated		
**1166**	AOFB	AOFB_HUMAN	AOFB; Q8TBI1; B2R6R3	P27338; Q7Z6S2; B2R6R3; Q8TBI1	NP_000889	Amine oxidase [flavin-containing] B (EC 1.4.3.4) (Monoamine oxidase type B) (MAO-B); Monoamine oxidase B	GO:0008131; GO:0009055; GO:0016021; GO:0005741; GO:0055114; GO:0009055; GO:0005743; GO:0016491	amine oxidase activity; electron carrier activity; integral to membrane; mitochondrial outer membrane; oxidation reduction; electron carrier activity; mitochondrial inner membrane; oxidoreductase activity	MitochondrionoutermembraneSinglepasstypeIVmembraneproteinCytoplasmicside;					
**1167**	SYTC	SYTC_HUMAN	SYTC; A8K8I1	P26639; Q96FP5; Q9BWA6; A8K8I1	NP_003182; NP_689508	Threonyl-tRNA synthetase, cytoplasmic (EC 6.1.1.3) (Threonine--tRNA ligase) (ThrRS)	GO:0005524; GO:0005737; GO:0042803; GO:0004829; GO:0006435	ATP binding; cytoplasm; protein homodimerization activity; threonine-tRNA ligase activity; threonyl-tRNA aminoacylation	Cytoplasm			Known in Uniprot to be phosphorylated		
**1168**	CISY	CISY_HUMAN	CISY	O75390; Q71UT9; Q7KZH0; Q96FZ8; Q9BWN8	NP_004068	Citrate synthase, mitochondrial (EC 2.3.3.1)	GO:0044262; GO:0004108; GO:0005759; GO:0006099	cellular carbohydrate metabolic process; citrate (Si)-synthase activity; mitochondrial matrix; tricarboxylic acid cycle	Mitochondrionmatrix					
**1169**	PRR20	PRR20_HUMAN	Q8N7V5; PRR20	Q8N7V5	XP_297707; NP_001123876; NP_001123877; NP_001123878; NP_001123879; NP_940843	Proline-rich protein 20								
**1170**	BASI	BASI_HUMAN	BASI	P35613; Q7Z796; Q8IZL7	NP_001719; NP_940991	Basigin (Leukocyte activation antigen M6) (Collagenase stimulatory factor) (Extracellular matrix metalloproteinase inducer) (EMMPRIN) (5F7) (Tumor cell-derived collagenase stimulatory factor) (TCSF) (OK blood group antigen) (CD antigen CD147)	GO:0000139; GO:0007166; GO:0016021; GO:0005537; GO:0042470; GO:0005886; GO:0005515	Golgi membrane; cell surface receptor linked signal transduction; integral to membrane; mannose binding; melanosome; plasma membrane; protein binding	CellmembraneSinglepasstypeImembraneproteinMelanosome			Known in Uniprot to be phosphorylated		
**1171**	TCPD	TCPD_HUMAN	TCPD	P50991; Q53QP9; B2R6I3; O14870; O96C51	NP_006421	T-complex protein 1 subunit delta (TCP-1-delta) (CCT-delta) (Stimulator of TAR RNA-binding)	GO:0005524; GO:0042470; GO:0006457; GO:0051082	ATP binding; melanosome; protein folding; unfolded protein binding	CytoplasmMelanosome					
**1172**	CV025	CV025_HUMAN	CV025	Q8IW05; Q6ICL3; Q8NAL0; Q8TCS0; Q96M16	NP_690870	Uncharacterized protein C22orf25								
**1173**	F111A	F111A_HUMAN	F111A	Q68DK9; Q96PZ2; A8K5Y8; Q5RKS9; Q5XKM2; Q6IPR7; Q9H5Y1	NP_071357; NP_001135991; NP_001135992; NP_001135993; NP_942144	Protein FAM111A	GO:0003824	catalytic activity						
**1174**	ACLY	ACLY_HUMAN	ACLY	P53396; Q13037; Q9BRL0	NP_001087; NP_942127	ATP-citrate synthase (EC 2.3.3.8) (ATP-citrate (pro[illegible] S-)-lyase) (Citrate cleavage enzyme)	GO:0005524; GO:0006200; GO:0003878; GO:0044262; GO:0008815; GO:0009346; GO:0006101; GO:0015936; GO:0005829; GO:0008610; GO:0000287; GO:0005515; GO:0004775	ATP binding; ATP catabolic process; ATP citrate synthase activity; cellular carbohydrate metabolic process; citrate (pro-3S)-lyase activity; citrate lyase complex; citrate metabolic process; coenzyme A metabolic process; cytosol; lipid biosynthetic process; magnesium ion binding; protein binding; succinate-CoA ligase (ADP-forming) activity	Cytoplasm			Known in Uniprot to be phosphorylated	Known in HPRD to be phosphorylated	
**1175**	ZN792	ZN792_HUMAN	Q8N932; Q495L3; ZN792	Q495L3; Q8N932; Q3KQV3; B4E333; Q495L1	NP_787068	Zinc finger protein 792	GO:0003677; GO:0005634; GO:0006355; GO:0008270	DNA binding; nucleus; regulation of transcription, DNA-dependent; zinc ion binding	Nucleus					
**1176**	PIN4	PIN4_HUMAN	PIN4	Q9Y237; Q5HYW6	NP_006214	Peptidyl-prolyl cis-trans isomerase NIMA-interacting 4 (EC 5.2.1.8) (Rotamase Pin4) (PPIase Pin4) (Parvulin 14) (hPar14) (Par14) (Peptidyl-prolyl cis/trans isomerase EPVH)	GO:0005759; GO:0003755; GO:0006457	mitochondrial matrix; peptidyl-prolyl cis-trans isomerase activity; protein folding				known in HPRD to be phosphorylated		
**1177**	SNX25	SNX25_HUMAN	SNX25; Q3ZT30; B3KTI8	Q3ZT30; Q9H3E2; B3KTI8	NP_114159	Sorting nexin-25	GO:0007154; GO:0035091; GO:0005515; GO:0015031; GO:0004871	cell communication; phosphoinositide binding; protein binding; protein transport; signal transducer activity				Known in Uniprot to be phosphorylated		
**1178**	XIP	XIP_HUMAN	XIP; HBXIP	O43504; Q6IBD8	NP_006393	Hepatitis B virus X-interacting protein (HBX-interacting protein) (HBV X-interacting protein)	GO:0006916; GO:0005829; GO:0043154; GO:0005515; GO:0009615; GO:0019079	anti-apoptosis; cytosol; negative regulation of caspase activity; protein binding; response to virus; viral genome replication	Cytoplasm			Known in Uniprot to be phosphorylated		
**1179**	TMED9	TMED9_HUMAN	TMED9	Q9BVK6; Q14437; Q8WZ61	NP_059980	Transmembrane emp24 domain-containing protein 9 (Glycoprotein 25L2)	GO:0005793; GO:0005789; GO:0016021; GO:0006810	ER-Golgi intermediate compartment; endoplasmic reticulum membrane; integral to membrane; transport	EndoplasmicreticulummembraneSinglepasstypeImembraneprotein					
**1180**	6PGD	6PGD_HUMAN	6PGD; Q4VXH6; A9Z1X1	P52209; Q4VXH6; Q9BWD8; A9Z1X1	NP_002622	6-phosphogluconate dehydrogenase, decarboxylating (EC 1.1.1.44)	GO:0050661; GO:0055114; GO:0009051; GO:0004616; GO:0005515	NADP or NADPH binding; oxidation reduction; pentose-phosphate shunt, oxidative branch; phosphogluconate dehydrogenase (decarboxylating) activity; protein binding						
**1181**	CK063	CK063_HUMAN	CK063; A8K6G0; B3KUM2	Q6NUN7; Q96GB5; Q9H5D6; A8K6G0; B3KUM2	NP_079082; NP_954575	Uncharacterized protein C11orf63								
**1182**	U520	U520_HUMAN	U520; Q8NBE6	O75643; O94884; Q6NZY0; Q6PX59; Q8NBE6; Q96IF2; Q9H7S0	NP_054733	U5 small nuclear ribonucleoprotein 200 kDa helicase (EC 3.6.1.-) (U5 snRNP-specific 200 kDa protein) (U5-200KD) (Activating signal cointegrator 1 complex subunit 3-like 1) (BRR2 homolog)	GO:0005524; GO:0008026; GO:0000354; GO:0003676; GO:0005515; GO:0005682	ATP binding; ATP-dependent helicase activity; cis assembly of pre-catalytic spliceosome; nucleic acid binding; protein binding; snRNP U5	Nucleus			Known in Uniprot to be phosphorylated	Known in HPRD to be phosphorylated	
**1183**	GNAQ	GNAQ_HUMAN	GNAQ; Q6NT27	Q6NT27; P50148; O15108; Q13462; Q92471; Q9BZB9	NP_002063	Guanine nucleotide-binding protein G(q) subunit alpha (Guanine nucleotide-binding protein alpha-q); Guanine nucleotide binding protein (G protein), q polypeptide (Guanine nucleotide binding protein (G protein), q polypeptide, isoform CRA_c)	GO:0005525; GO:0003924; GO:0007596; GO:0005737; GO:0005886; GO:0006471; GO:0004871; GO:0007186; GO:0005525; GO:0007189; GO:0060158; GO:0007610; GO:0042733; GO:0021884; GO:0007215; GO:0007507; GO:0016322; GO:0009791; GO:0001508; GO:0045634; GO:0001501	GTP binding; GTPase activity; blood coagulation; cytoplasm; plasma membrane; protein amino acid ADP-ribosylation; signal transducer activity; G-protein coupled receptor protein signaling pathway; GTP binding; activation of adenylate cyclase activity by G-protein signaling pathway; activation of phospholipase C activity by dopamine receptor signaling pathway; behavior; embryonic digit morphogenesis; forebrain neuron development; glutamate signaling pathway; heart development; neuron remodeling; post-embryonic development; regulation of action potential; regulation of melanocyte differentiation; skeletal system development						
**1184**	OTU6B	OTU6B_HUMAN	OTU6B	Q8N6M0; A8K6I1; Q9NTA4; Q9Y387	NP_057107	OTU domain-containing protein 6B (DUBA-5)								

	Protein Name	Uniprot Name	Other Names	Uniprot IDs	Refseq IDs	Protein Description	GO ID	GO terms	Subcellular location	Comments	Comments	Comments - Database Search	Comments - Database Search	Function
**1185**	PNCB	PNCB_HUMAN	Q6PJL1; PNCB	Q6PJL1; Q6XQN6; A7BFI3; Q6XQN4; Q6XQN5; Q8N5E8; Q9BRG0	NP_660202	Nicotinate phosphoribosyltransferase (NAPRTase) (EC 2.4.2.11) (Nicotinate phosphoribosyltransferase domain-containing protein 1) (FHA-HIT-interacting protein)	GO:0005829; GO:0004516; GO:0019363; GO:0006979	cytosol; nicotinate phosphoribosyltransferase activity; pyridine nucleotide biosynthetic process; response to oxidative stress	Cytoplasmcytosol			Known in Uniprot to be phosphorylated		
**1186**	8ODP	8ODP_HUMAN	8ODP; Q7Z7N6	Q6LES7; P36639; Q6P0Y6; Q8IV95; Q9UBM0; Q9UBM9; Q7Z7N6	NP_002443; NP_945186; NP_945187; NP_945188; NP_945190; NP_945191; NP_945192	7,8-dihydro-8-oxoguanine triphosphatase (EC 3.1.6.-) (8-oxo-dGTPase) (Nucleoside diphosphate-linked moiety X motif 1) (Nudix motif 1); NUDT1 protein (Nudix (Nucleoside diphosphate linked moiety X)-type motif 1) (Nudix (Nucleoside diphosphate linked moiety X)-type motif 1, isoform CRA_a)	GO:0008413; GO:0006281; GO:0003924; GO:0005515; GO:0006979	8-oxo-7,8-dihydroguanine triphosphatase activity; DNA repair; GTPase activity; protein binding; response to oxidative stress						Phosphatase
**1187**	TSP2	TSP2_HUMAN	TSP2; Q5RI52; Q6MZL6	Q5RI52; P35442; Q6MZL6	NP_003238	Thrombospondin-2; Putative uncharacterized protein DKFZp686G02190 (Fragment)	GO:0005509; GO:0007155; GO:0005576; GO:0008201; GO:0031093; GO:0005515; GO:0005198	calcium ion binding; cell adhesion; extracellular region; heparin binding; platelet alpha granule lumen; protein binding; structural molecule activity						
**1188**	K1C10	K1C10_HUMAN	K1C10	Q8N175; P13645; Q14664	NP_000412	Keratin, type I cytoskeletal 10 (Cytokeratin-10) (CK-10) (Keratin-10) (K10)	GO:0008544; GO:0005882; GO:0005515; GO:0030280	epidermis development; intermediate filament; protein binding; structural constituent of epidermis				Known in Uniprot to be phosphorylated		
**1189**	TPD54	TPD54_HUMAN	TPD54; Q6FGS1; O43398	O43399; Q6FGS1; O43398; Q9H3Z6	NP_003279; NP_955392	Tumor protein D54 (hD54) (Tumor protein D52-like 2); TPD52L2 protein (Tumor protein D52-like 2, isoform CRA_a) (cDNA, FLJ95317, highly similar to Homo sapiens tumor protein D52-like 2 (TPD52L2), mRNA)	GO:0005515;	protein binding;				Known in Uniprot to be phosphorylated		
**1190**	SPB6	SPB6_HUMAN	SPB6; Q5TD06	Q5TD06; P35237; Q96J44	NP_004559	Serpin B6 (Placental thrombin inhibitor) (Cytoplasmic antiproteinase) (CAP) (Proteinase inhibitor 6) (PI-6)	GO:0005813; GO:0005829; GO:0005515; GO:0004867	centrosome; cytosol; protein binding; serine-type endopeptidase inhibitor activity	Cytoplasm					
**1191**	CND1	CND1_HUMAN	CND1	Q15021; Q8N6U3	XP_294234	Condensin complex subunit 1 (Non-SMC condensin I complex subunit D2) (Chromosome condensation-related SMC-associated protein 1) (Chromosome-associated protein D2) (hCAP-D2) (XCAP-D2 homolog)	GO:0051301; GO:0000797; GO:0005737; GO:0042393; GO:0007076; GO:0005634	cell division; condensin core heterodimer; cytoplasm; histone binding; mitotic chromosome condensation; nucleus	NucleusCytoplasm			Known in Uniprot to be phosphorylated		
**1192**	SAC1	SAC1_HUMAN	Q9NTJ5; SAC1	Q9NTJ5; A8K527; O94935; Q7LA14; Q7LA22; Q96AX7; Q9NQ46; Q9NQ57	NP_054735	Phosphatidylinositide phosphatase SAC1 (EC 3.1.3.) (Suppressor of actin mutations 1-like protein)	GO:0005794; GO:0005789; GO:0016021; GO:0016791	Golgi apparatus; endoplasmic reticulum membrane; integral to membrane; phosphatase activity	Endoplasmicreticulum membraneMultipassme mbraneprotein					Phosphatase
**1193**	CRUM1	CRUM1_HUMAN	CRUM1	P82279; Q5K3A6; Q5TC28; Q5VUT1; Q6N027; Q8WWY0	NP_036208; NP_957705	Crumbs homolog 1	GO:0016324; GO:0005509; GO:0007267; GO:0007163; GO:0005576; GO:0016021; GO:0050896; GO:0007601	apical plasma membrane; calcium ion binding; cell-cell signaling; establishment or maintenance of cell polarity; extracellular region; integral to membrane; response to stimulus; visual perception	ApicalcellmembraneSin glepasstypeImembran eproteinSecreted					
**1194**	ARK72	ARK72_HUMAN	ARK72	O43488; O75749; Q5TG63	NP_003680	Aflatoxin B1 aldehyde reductase member 2 (EC 1.-.-.-) (AFB1-AR 1) (Aldoketoreductase 7)	GO:0005794; GO:0004032; GO:0005975; GO:0006081; GO:0009055; GO:0055114	Golgi apparatus; aldehyde reductase activity; carbohydrate metabolic process; cellular aldehyde metabolic process; electron carrier activity; oxidation reduction	Golgiapparatus			Known in Uniprot to be phosphorylated		
**1195**	DDX46	DDX46_HUMAN	DDX46	Q7L014; O94894; Q96EI0; Q9Y658	NP_055644	Probable ATP-dependent RNA helicase DDX46 (EC 3.6.1.-) (DEAD box protein 46) (PRP5 homolog)	GO:0005524; GO:0008026; GO:0003723; GO:0008380; GO:0006397; GO:0016607	ATP binding; ATP-dependent helicase activity; RNA binding; RNA splicing; mRNA processing; nuclear speck	NucleusspeckleNucleus Cajalbody			Known in Uniprot to be phosphorylated	Known in HPRD to be phosphorylated	
**1196**	MOB3	MOB3_HUMAN	MOB3; Q53SE0; MOBL3	Q9Y3A3; Q53SE0; Q7Z4Y6; Q9H2P3; Q9H5J1; Q9Y4T8	NP_056202; NP_955776	Mps one binder kinase activator-like 3 (Preimplantation protein 3) (Mob1 homolog 3) (Mob3) (Class II mMOB1) (2C4D); cDNA FLJ78747, highly similar to Homo sapiens preimplantation protein 3 (PREI3), transcript variant 1, mRNA (Preimplantation protein 3, isoform CRA_a) (Putative uncharacterized protein tmp_locus_8)	GO:0005794; GO:0016020; GO:0005515; GO:0006810; GO:0008270; GO:0005515	Golgi apparatus; membrane; protein binding; transport; zinc ion binding; protein binding	Cytoplasmperinuclearr egionMembranePeriphe ralmembraneproteinGo lgiapparatusGolgistack membranePeripheralm embraneprotein;			Known in Uniprot to be phosphorylated		
**1197**	PPCE	PPCE_HUMAN	Q8N6D4; Q9UM02; PPCE	Q8N6D4; P48147; Q9UM02	NP_002717	Prolyl endopeptidase (PE) (EC 3.4.21.26) (Post-proline cleaving enzyme); Prolyl endopeptidase (EC 3.4.21.26) (Prolyl oligopeptidase)	GO:0005737; GO:0006508; GO:0004252; GO:0006508	cytoplasm; proteolysis; serine-type endopeptidase activity; proteolysis	Cytoplasm;					
**1198**	CSMD1	CSMD1_HUMAN	CSMD1	Q96PZ7; Q0H0J5; Q96QU9; Q96RM4	NP_150094	CUB and sushi domain-containing protein 1 (CUB and sushi multiple domains protein 1)	GO:0016021	integral to membrane	MembraneSinglepassty peImembraneprotein					
**1199**	DOK2	DOK2_HUMAN	DOK2	Q8N5A4; O60496	NP_003965	Docking protein 2 (Downstream of tyrosine kinase 2) (p56(dok-2))	GO:0042802; GO:0005158	identical protein binding; insulin receptor binding				Known in Uniprot to be phosphorylated	Known in HPRD to be phosphorylated	
**1200**	MYH10	MYH10_HUMAN	MYH10; Q9BWG0; Q149N3; Q6PK16	P35580; Q12989; Q149N3; Q16087; Q6PK16; Q9BWG0	NP_005955	Myosin-10 (Myosin heavy chain 10) (Myosin heavy chain, non-muscle IIb) (Non-muscle myosin heavy chain IIb) (NMMHC II-b) (NMMHC-IIB) (Cellular myosin heavy chain, type B) (Non-muscle myosin heavy chain B) (NMMHC-B); MYH10 protein (Fragment)	GO:0043531; GO:0005524; GO:0051015; GO:0030048; GO:0030898; GO:0005516; GO:0005938; GO:0032154; GO:0000281; GO:0000146; GO:0030496; GO:0016459; GO:0008360; GO:0001725; GO:0005524; GO:0007512; GO:0030424; GO:0007411; GO:0055003; GO:0008283; GO:0043025; GO:0021680; GO:0043197; GO:0006887; GO:0021592; GO:0030426; GO:0001701; GO:0021670; GO:0003774; GO:0031594; GO:0050885; GO:0001764; GO:0007097; GO:0005886; GO:0001778;	ADP binding; ATP binding; actin filament binding; actin filament-based movement; actin-dependent ATPase activity; calmodulin binding; cell cortex; cleavage furrow; cytokinesis after mitosis; microfilament motor activity; midbody; myosin complex; regulation of cell shape; stress fiber; ATP binding; adult heart development; axon; axon guidance; cardiac myofibril assembly; cell proliferation; cell soma; cerebellar Purkinje cell layer development; dendritic spine; exocytosis; fourth ventricle development; growth cone; in utero embryonic development; lateral ventricle development; motor activity; neuromuscular junction; neuromuscular process controlling balance; neuron migration; nuclear migration; plasma membrane; plasma membrane repair; protein binding; retina development in camera-type eye; spindle; substrate-bound cell migration, cell extension; third ventricle development; ventricular cardiac muscle cell development; adult heart development				Known in Uniprot to be phosphorylated		
**1201**	GPX1	GPX1_HUMAN	GPX1	P07203; Q7Z5H1; Q9BW12	NP_000572; NP_958799	Glutathione peroxidase 1 (EC 1.11.1.9) (GSHPx-1) (GPx-1) (Cellular glutathione peroxidase)	GO:0017124; GO:0009650; GO:0006916; GO:0005829; GO:0043295; GO:0006749; GO:0004602; GO:0060047; GO:0042744; GO:0005739; GO:0043154; GO:0055114; GO:0008539; GO:0040029; GO:0033599; GO:0001836; GO:0010269; GO:0008430	SH3 domain binding; UV protection; anti-apoptosis; cytosol; glutathione binding; glutathione metabolic process; glutathione peroxidase activity; heart contraction; hydrogen peroxide catabolic process; mitochondrion; negative regulation of caspase activity; oxidation reduction; proteasome inhibitor activity; regulation of gene expression, epigenetic; regulation of mammary gland epithelial cell proliferation; release of cytochrome c from mitochondria; response to selenium ion; selenium binding	Cytoplasm				known in HPRD to be phosphorylated	
**1202**	ROCK2	ROCK2_HUMAN	ROCK2	O75116; Q9UQN5	NP_004841	Rho-associated protein kinase 2 (EC 2.7.11.1) (Rho-associated, coiled-coil-containing protein kinase 2) (p164 ROCK-2) (Rho kinase 2)	GO:0005524; GO:0000910; GO:0005737; GO:0019992; GO:0007242; GO:0005886; GO:0006468; GO:0005515; GO:0004674; GO:0005198; GO:0008270	ATP binding; cytokinesis; cytoplasm; diacylglycerol binding; intracellular signaling cascade; plasma membrane; protein amino acid phosphorylation; protein binding; protein serine/threonine kinase activity; structural molecule activity; zinc ion binding	CytoplasmCellmembra nePeripheralmembrane protein			Known in Uniprot to be phosphorylated	Known in HPRD to be phosphorylated	Kinase
**1203**	FAS	FAS_HUMAN	FAS	P49327; Q13479; Q16702; Q6P4U5; Q6SS02; Q969R1; Q96C68; Q96IT0	NP_004095	Fatty acid synthase (EC 2.3.1.85) [Includes: [Acyl-carrier-protein] S-acetyltransferase (EC 2.3.1.38); [Acyl-carrier-protein] S-malonyltransferase (EC 2.3.1.39); 3-oxoacyl-[acyl-carrier-protein] synthase (EC 2.3.1.41); 3-oxoacyl-[acyl-carrier-protein] reductase (EC 1.1.1.100); 3-hydroxypalmitoyl-[acyl-carrier-protein] dehydratase (EC 4.2.1.61); Enoyl-[acyl-carrier-protein] reductase (EC 1.3.1.10); Oleoyl-[acyl-carrier-protein] hydrolase (EC 3.1.2.14)]	GO:0004317; GO:0004316; GO:0004315; GO:0005794; GO:0004313; GO:0004314; GO:0000036; GO:0048037; GO:0005829; GO:0004319; GO:0006633; GO:0042470; GO:0004320; GO:0055114; GO:0031177; GO:0005515; GO:0008270	3-hydroxypalmitoyl-[acyl-carrier-protein] dehydratase activity; 3-oxoacyl-[acyl-carrier-protein] reductase activity; 3-oxoacyl-[acyl-carrier-protein] synthase activity; Golgi apparatus; [acyl-carrier-protein] S-acetyltransferase activity; [acyl-carrier-protein] S-malonyltransferase activity; acyl carrier activity; cofactor binding; cytosol; enoyl-[acyl-carrier-protein] reductase (NADPH, B-specific) activity; fatty acid biosynthetic process; melanosome; oleoyl-[acyl-carrier-protein] hydrolase activity; oxidation reduction; phosphopantetheine binding; protein binding; zinc ion binding	CytoplasmMelanosome			Known in Uniprot to be phosphorylated	Known in HPRD to be phosphorylated	
**1204**	O05DE9	O05DE9_HUMAN	O05DE9	O05DE9	NP_001456	FYB protein (Fragment)								
**1205**	TPM2	TPM2_HUMAN	TPM2	P07951; Q5TCU7; A6NM85; P06468; Q13894; Q53FM4; Q5TCU4; Q9UH67	NP_003280; NP_998839	Tropomyosin beta chain (Tropomyosin-2) (Beta-tropomyosin)	GO:0003779; GO:0005862; GO:0043462; GO:0008307	actin binding; muscle thin filament tropomyosin; regulation of ATPase activity; structural constituent of muscle	Cytoplasmcytoskeleton			Known in Uniprot to be phosphorylated		

	Protein Name	Uniprot Name	Other Names	Uniprot IDs	Refseq IDs	Protein Description	GO ID	GO terms	Subcellular location	Comments	Comments	Comments - Database Search	Comments - Database Search	Function
**1206**	DTYMK	DTYMK_HUMAN	DTYMK; Q6FGU2; KTHY	P23919; Q6FGU2; Q6FGX1; Q9BUX4	NP_036277; XP_001126211	Thymidylate kinase (EC 2.7.4.9) (dTMP kinase); DTYMK protein (Deoxythymidylate kinase (Thymidylate kinase)) (Deoxythymidylate kinase (Thymidylate kinase), isoform CRA_b) (Putative uncharacterized protein tmp_locus_5) (Fragment)	GO:0005524; GO:0007049; GO:0008283; GO:0005829; GO:0006233; GO:0019206; GO:0004798	ATP binding; cell cycle; cell proliferation; cytosol; dTDP biosynthetic process; nucleoside kinase activity; thymidylate kinase activity						Kinase
**1207**	TSSK4	TSSK4_HUMAN	TSSK4	Q6SA08; Q2TA60; Q6ZNM2	NP_777604	Testis-specific serine/threonine-protein kinase 4 (TSSK-4) (Testis-specific kinase 4) (TSK-4) (EC 2.7.11.1) (Serine/threonine-protein kinase 22E)	GO:0005524; GO:0030154; GO:0000287; GO:0007275; GO:0032793; GO:0006468; GO:0005515; GO:0004674; GO:0007283	ATP binding; cell differentiation; magnesium ion binding; multicellular organismal development; positive regulation of CREB transcription factor activity; protein amino acid phosphorylation; protein binding; protein serine/threonine kinase activity; spermatogenesis				Known in Uniprot to be phosphorylated		Kinase
**1208**	SEPT15	SEPT15_HUMAN	SEPT15	O60613; Q4GZG7; Q8WU00; Q9BS64; Q9GZW0; Q9NR01	NP_004252; NP_976086	15 kDa selenoprotein	GO:0051084; GO:0005783; GO:0005788; GO:0005515; GO:0008430	'de novo' posttranslational protein folding; endoplasmic reticulum; endoplasmic reticulum lumen; protein binding; selenium binding	Endoplasmicreticulumlumen					
**1209**	ACSL3	ACSL3_HUMAN	ACSL3	O95573; Q60I92; Q8IUM9	NP_004448; NP_976251	Long-chain-fatty-acid--CoA ligase 3 (EC 6.2.1.3) (Long-chain acyl-CoA synthetase 3) (LACS 3)	GO:0005789; GO:0006631; GO:0004321; GO:0016021; GO:0004467; GO:0000287; GO:0005792; GO:0005741; GO:0005778; GO:0005515	endoplasmic reticulum membrane; fatty acid metabolic process; fatty-acyl-CoA synthase activity; integral to membrane; long-chain-fatty-acid-CoA ligase activity; magnesium ion binding; microsome; mitochondrial outer membrane; peroxisomal membrane; protein binding	MitochondrionoutermembraneSinglepasstypeIIImembraneproteinPeroxisomemembraneSinglepasstypeIIImembraneproteinMicrosomemembraneSinglepasstypeIIImembraneproteinEndoplasmicreticulummembraneSinglepasstypeIIImembraneprotein			Known in Uniprot to be phosphorylated	Known in HPRD to be phosphorylated	
**1210**	MCA1	MCA1_HUMAN	MCA1	Q12904; B3KTR2; Q6FG28; Q96CQ9	NP_004748; NP_001135887	Multisynthetase complex auxiliary component p43 [Cleaved into: Endothelial monocyte-activating polypeptide 2 (EMAP-II) (Small inducible cytokine subfamily E member 1)]	GO:0017101; GO:0043498; GO:0007267; GO:0006935; GO:0005125; GO:0005615; GO:0006954; GO:0050900; GO:0001937; GO:0042803; GO:0007165; GO:0006418; GO:0000049	aminoacyl-tRNA synthetase multienzyme complex; cell surface binding; cell-cell signaling; chemotaxis; cytokine activity; extracellular space; inflammatory response; leukocyte migration; negative regulation of endothelial cell proliferation; protein homodimerization activity; signal transduction; tRNA aminoacylation for protein translation; tRNA binding						
**1211**	OST5	OST5_HUMAN	OST5; HS3S5; A8K1J2	Q8IZT8; Q52LI2; Q8N285; A8K1J2	NP_705840	Heparan sulfate glucosamine 3-O-sulfotransferase 5 (EC 2.8.2.23) (Heparan sulfate D-glucosaminyl 3-O-sulfotransferase 5) (Heparan sulfate 3-O-sulfotransferase 5) (h3-OST-5)	GO:0050656; GO:0000139; GO:0008467; GO:0015015; GO:0016021; GO:0050819; GO:0006477; GO:0005515; GO:0046596	3'-phosphoadenosine 5'-phosphosulfate binding; Golgi membrane; [heparan sulfate]-glucosamine 3-sulfotransferase 1 activity; heparan sulfate proteoglycan biosynthetic process, enzymatic modification; integral to membrane; negative regulation of coagulation; protein amino acid sulfation; protein binding; regulation of virion penetration into host cell	GolgiapparatusmembraneSinglepasstypeIImembraneprotein					
**1212**	QIL1	QIL1_HUMAN	Q5XKP0; QIL1	Q5XKP0; Q86YE5	XP_058957; NP 991330	Protein QIL1 (Protein P117)	GO:0005515	protein binding						
**1213**	PG12B	PG12B_HUMAN	PG12B	Q9BX93; Q52LB2; Q96Q99	NP_115951	Group XIIB secretory phospholipase A2-like protein (Group XIII secretory phospholipase A2-like protein) (GXIII sPLA2-like) (sPLA2-GXIIB) (GXIIB)	GO:0005509; GO:0005576; GO:0016042; GO:0004623	calcium ion binding; extracellular region; lipid catabolic process; phospholipase A2 activity	Secreted					
**1214**	BCAP	BCAP_HUMAN	Q6ZUJ8; Q86YV3; Q8NAC8; BCAP	Q6ZUJ8; Q5TB56; Q5VXJ9; Q8N6J6; Q8NAC8; Q86YV3	NP_689522	Phosphoinositide 3-kinase adapter protein 1 (B-cell phosphoinositide 3-kinase adapter protein 1) (B-cell adapter for phosphoinositide 3-kinase); FLJ00308 protein (Fragment)	GO:0005737; GO:0005886; GO:0005515; GO:0005066	cytoplasm; plasma membrane; protein binding; transmembrane receptor protein tyrosine kinase signaling protein activity	CytoplasmCellmembrane;			Known in Uniprot to be phosphorylated		
**1215**	GTPB2	GTPB2_HUMAN	GTPB2	Q9BX10; Q5T7E8; Q8ND84; Q8TAH7; Q8WUA5; Q9HCS9; Q9NRU4; Q9NX60	NP_061969	GTP-binding protein 2	GO:0005525; GO:0003924	GTP binding; GTPase activity						
**1216**	TLK2	TLK2_HUMAN	TLK2	Q86UE8; Q9UKI7; Q9Y4F7	NP_006843; NP_001106178	Serine/threonine-protein kinase tousled-like 2 (EC 2.7.11.1) (Tousled-like kinase 2) (PKU-alpha)	GO:0005524; GO:0007049; GO:0016568; GO:0007242; GO:0005634; GO:0006468; GO:0005515; GO:0004674; GO:0001672; GO:0006974	ATP binding; cell cycle; chromatin modification; intracellular signaling cascade; nucleus; protein amino acid phosphorylation; protein binding; protein serine/threonine kinase activity; regulation of chromatin assembly or disassembly; response to DNA damage stimulus	Nucleus			Known in Uniprot to be phosphorylated	Known in HPRD to be phosphorylated	Kinase
**1217**	PGSG	PGSG_HUMAN	PGSG; Q5VW06; SRGN	Q5VW06; P10124	NP_002718	Serglycin (Secretory granule proteoglycan core protein) (Platelet proteoglycan core protein) (P.PG) (Hematopoetic proteoglycan core protein)	GO:0005794; GO:0006915; GO:0005615; GO:0033382; GO:0033373; GO:0042629; GO:0030502; GO:0050710; GO:0005515; GO:0051605	Golgi apparatus; apoptosis; extracellular space; maintenance of granzyme B location in T cell secretory granule; maintenance of protease location in mast cell secretory granule; mast cell granule; negative regulation of bone mineralization; negative regulation of cytokine secretion; protein binding; protein maturation via proteolysis	CytoplasmicgranuleSecretedextracellularspaceGolgiapparatus					
**1218**	MPPH1	MPPH1_HUMAN	MPPH1	Q5T9M9; Q96Q89; O43277; Q09471; Q2KQ73; Q32NE1; Q561V3; Q58EX8; Q5T9M8; Q5T9N0; Q5T9N1; Q7KZ68; Q7Z5E0; Q7Z5E1; Q7Z6M9; Q86X82; Q9H3R8; Q9H6Q9; Q9H755; Q9NTC1; Q9UFR5	NP_057279	M-phase phosphoprotein 1 (MPP1) (Kinesin-related motor interacting with PIN1) (Cancer/testis antigen 90) (CT90)	GO:0005524; GO:0016887; GO:0050699; GO:0007050; GO:0051301; GO:0005813; GO:0005874; GO:0003777; GO:0007018; GO:0005730; GO:0005654; GO:0007088	ATP binding; ATPase activity; WW domain binding; cell cycle arrest; cell division; centrosome; microtubule; microtubule motor activity; microtubule-based movement; nucleolus; nucleoplasm; regulation of mitosis	CytoplasmNucleus			Known in Uniprot to be phosphorylated	Known in HPRD to be phosphorylated	
**1219**	SPG17	SPG17_HUMAN	Q6Q759; SPG17	Q6Q759; Q8NAZ1; Q9NT21	XP_117198; NP_996879	Sperm-associated antigen 17 (Projection protein PF6 homolog)	GO:0005929; GO:0005737; GO:0019861; GO:0005874	cilium; cytoplasm; flagellum; microtubule	CytoplasmCellprojectionciliumflagellumaxoneme					
**1220**	RHOG	RHOG_HUMAN	RHOG; Q6ICQ8	P84095; Q6ICQ8; P35238; Q8NI04	NP_001656	Rho-related GTP-binding protein RhoG; ARHG protein (Ras homolog gene family, member G (Rho G)) (Ras homolog gene family, member G (Rho G), isoform CRA_a) (Fragment)	GO:0005525; GO:0003924; GO:0007266; GO:0005622; GO:0005886; GO:0008284; GO:0005515; GO:0016020; GO:0007264	GTP binding; GTPase activity; Rho protein signal transduction; intracellular; plasma membrane; positive regulation of cell proliferation; protein binding; membrane; small GTPase mediated signal transduction	CellmembraneLipidanchorCytoplasmicside;					
**1221**	LTB1S	LTB1S_HUMAN	LTB1S; LTBP1; LTB1L	P22064; Q14766; A1L3V1; Q53SD8; Q53SF3; Q53SG1; Q59HF7; Q8TD95	NP_000618; NP_996826; XP_115812	Latent-transforming growth factor beta-binding protein 1 (LTBP-1) (Transforming growth factor beta-1-binding protein 1) (TGF-beta1-BP-1)	GO:0005509; GO:0005737; GO:0019838; GO:0005634; GO:0005578; GO:0005024	calcium ion binding; cytoplasm; growth factor binding; nucleus; proteinaceous extracellular matrix; transforming growth factor beta receptor activity	Secreted			Known in Uniprot to be phosphorylated		
**1222**	ANR53	ANR53_HUMAN	ANR53	Q8IYP8; Q8N9V6	NP_079209; NP_001108588	Ankyrin repeat domain-containing protein 53								
**1223**	TBA3E	TBA3E_HUMAN	Q6PEY2; TBA3E	Q6PEY2	XP_053177; NP_997195	Tubulin alpha-3E chain (Alpha-tubulin 3E)	GO:0005525; GO:0003924; GO:0005874; GO:0007018; GO:0043234; GO:0051258; GO:0005198	GTP binding; GTPase activity; microtubule; microtubule-based movement; protein complex; protein polymerization; structural molecule activity				Known in Uniprot to be phosphorylated	Known in HPRD to be phosphorylated	
**1224**	NLF1	NLF1_HUMAN	Q8NCU7; NLF1	Q8NCU7	XP_096852; NP 997205	Nuclear-localized factor 1 (Protein FAM148A)	GO:0005634	nucleus	Nucleus					
**1225**	CCDC6	CCDC6_HUMAN	CCDC6; Q05CP8; Q6GSG7	Q16204; Q15250; Q05CP8; Q6GSG7	NP_005427	Coiled-coil domain-containing protein 6 (Protein H4) (Papillary thyroid carcinoma-encoded protein); CCDC6 protein (Fragment); Coiled-coil domain containing 6 (Coiled-coil domain containing 6, isoform CRA_a) (cDNA FLJ75411, highly similar to Homo sapiens coiled-coil domain containing 6, mRNA)	GO:0017124; GO:0005737; GO:0005856; GO:0005200;	SH3 domain binding; cytoplasm; cytoskeleton; structural constituent of cytoskeleton;	CytoplasmCytoplasmcytoskeleton;			Known in Uniprot to be phosphorylated	Known in HPRD to be phosphorylated	
**1226**	ASB18	ASB18_HUMAN	Q6ZVZ8; ASB18	Q6ZVZ8	XP_292989; NP_997721	Ankyrin repeat and SOCS box-containing protein 18 (ASB-18)	GO:0007242; GO:0019941	intracellular signaling cascade; modification-dependent protein catabolic process						
**1227**	TMED8	TMED8_HUMAN	TMED8	Q6PL24; B3KTI6; Q3MJB0; Q9P1V9	XP_208746; NP_998766	Protein TMED8	GO:0005737; GO:0005634	cytoplasm; nucleus						
**1228**	K22E	K22E_HUMAN	K22E	Q4VAQ2; P35908	NP_000414	Keratin, type II cytoskeletal 2 epidermal (Cytokeratin-2e) (CK 2e) (K2e) (keratin-2)	GO:0005813; GO:0045095; GO:0031424; GO:0032980; GO:0051546; GO:0043616; GO:0005515; GO:0005200	centrosome; keratin filament; keratinization; keratinocyte activation; keratinocyte migration; keratinocyte proliferation; protein binding; structural constituent of cytoskeleton				Known in Uniprot to be phosphorylated		

	Protein Name	Uniprot Name	Other Names	Uniprot IDs	Refseq IDs	Protein Description	GO ID	GO terms	Subcellular location	Comments	Comments	Comments - Database Search	Comments - Database Search	Function
**1229**	MTAP	MTAP_HUMAN	MTAP; Q5T3P3	Q5T3P3; Q13126; Q9H010	NP_002442	S-methyl-5'-thioadenosine phosphorylase (EC 2.4.2.28) (5'-methylthioadenosine phosphorylase) (MTA phosphorylase) (MTAPase); Methylthioadenosine phosphorylase (Methylthioadenosine phosphorylase, isoform CRA_b)	GO:0017061; GO:0005737; GO:0009116; GO:0004645; GO:0009116; GO:0016763	S-methyl-5-thioadenosine phosphorylase activity; cytoplasm; nucleoside metabolic process; phosphorylase activity; nucleoside metabolic process; transferase activity, transferring pentosyl groups	Cytoplasm;			Known in Uniprot to be phosphorylated	Known in HPRD to be phosphorylated	
**1230**	SYWC	SYWC_HUMAN	SYWC; Q53XB6	P23381; Q53XB6; P78535; Q9UDL3	NP_004175; NP_776049; NP_998810; NP_998811	Tryptophanyl-tRNA synthetase, cytoplasmic (EC 6.1.1.2) (Tryptophan--tRNA ligase) (TrpRS) (Interferon-induced protein 53) (IFP53) (hWRS); Full-length cDNA clone CS0DM004YH09 of Fetal liver of Homo sapiens (human) (Tryptophanyl-tRNA synthetase, isoform CRA_a) (cDNA FLJ31538 fis, clone NT2RI2000727, highly similar to TRYPTOPHANYL-TRNA SYNTHETASE (EC 6.1.1.2)) (Putative uncharacterized protein WARS)	GO:0005524; GO:0005737; GO:0008285; GO:0005625; GO:0004830; GO:0006436	ATP binding; cytoplasm; negative regulation of cell proliferation; soluble fraction; tryptophan-tRNA ligase activity; tryptophanyl-tRNA aminoacylation	Cytoplasm;			Known in Uniprot to be phosphorylated	Known in HPRD to be phosphorylated	
**1231**	SNAA	SNAA_HUMAN	SNAA; A8K879	Q96IK3; P54920; Q9BVJ3; A8K879	NP_003818	Alpha-soluble NSF attachment protein (SNAP-alpha) (N-ethylmaleimide-sensitive factor attachment protein alpha)	GO:0005794; GO:0005783; GO:0006891; GO:0006886; GO:0016020; GO:0006944	Golgi apparatus; endoplasmic reticulum; intra-Golgi vesicle-mediated transport; intracellular protein transport; membrane; membrane fusion	MembranePeripheralmembraneprotein					
**1232**	GTR9	GTR9_HUMAN	Q8WV30; GTR9	Q8WV30; Q9NRM0; Q0VGC4; Q4W5D1; Q96P00	NP_064425; NP_001001290	Solute carrier family 2, facilitated glucose transporter member 9 (Glucose transporter type 9) (GLUT-9)	GO:0015758; GO:0016021; GO:0005351; GO:0055085	glucose transport; integral to membrane; sugar:hydrogen symporter activity; transmembrane transport	MembraneMultipassmembraneprotein					
**1233**	GLU2B	GLU2B_HUMAN	GLU2B	P14314; Q96BU9; Q96D06; Q9P0W9	NP_002734; NP_001001329	Glucosidase 2 subunit beta (Glucosidase II subunit beta) (Protein kinase C substrate 60.1 kDa protein heavy chain) (PKCSH) (80K-H protein)	GO:0005509; GO:0005783; GO:0005515; GO:0007243	calcium ion binding; endoplasmic reticulum; protein binding; protein kinase cascade	Endoplasmicreticulum			Known in Uniprot to be phosphorylated		
**1234**	UGPA2	UGPA2_HUMAN	UGPA2; UGPA	Q16851; Q07131; Q0P6K2; Q86Y81; Q9BU15	NP_006750; NP_001001521	UTP--glucose-1-phosphate uridylyltransferase (EC 2.7.7.9) (UDP-glucose pyrophosphorylase) (UDPGP) (UGPase)	GO:0006011; GO:0003983; GO:0005829; GO:0016310; GO:0005515	UDP-glucose metabolic process; UTP:glucose-1-phosphate uridylyltransferase activity; cytosol; phosphorylation; protein binding	Cytoplasm					
**1235**	TCPQ	TCPQ_HUMAN	TCPQ	P50990; Q4VBP8; A6NN54	NP_006576	T-complex protein 1 subunit theta (TCP-1-theta) (CCT-theta) (Renal carcinoma antigen NY-REN-15)	GO:0005524; GO:0042623; GO:0005829; GO:0006457; GO:0051082	ATP binding; ATPase activity, coupled; cytosol; protein folding; unfolded protein binding	Cytoplasm			Known in Uniprot to be phosphorylated		
**1236**	E2IG5	E2IG5_HUMAN	E2IG5; Q9H2P1; F162A	Q9NRN6; Q96A26; Q9UJX8; Q9H2P1	NP_055182	UPF0389 protein FAM162A (E2-induced gene 5 protein); DC16	GO:0016021;	integral to membrane;	MembraneSinglepassmembraneprotein;					
**1237**	GSHR	GSHR_HUMAN	GSHR	P00390; Q7Z5C9; Q9NP63	NP_000628	Glutathione reductase, mitochondrial (GRase) (GR) (EC 1.8.1.7)	GO:0050660; GO:0050661; GO:0045454; GO:0005829; GO:0009055; GO:0006749; GO:0004362; GO:0005739; GO:0055114	FAD binding; NADP or NADPH binding; cell redox homeostasis; cytosol; electron carrier activity; glutathione metabolic process; glutathione-disulfide reductase activity; mitochondrion; oxidation reduction	MitochondrionCytoplasm			Known in Uniprot to be phosphorylated		
**1238**	TBCB	TBCB_HUMAN	TBCB	Q99426; O00111; O00674; O14728	NP_001272	Tubulin folding cofactor B (Tubulin-specific chaperone B) (Cytoskeleton-associated protein 1) (Cytoskeleton-associated protein CKAPI)	GO:0030154; GO:0005737; GO:0005874; GO:0007399; GO:0005515	cell differentiation; cytoplasm; microtubule; nervous system development; protein binding	CytoplasmCytoplasmcytoskeleton			Known in Uniprot to be phosphorylated	Known in HPRD to be phosphorylated	
**1239**	THIO	THIO_HUMAN	THIO; Q5T937	P10599; Q5T937; Q53X69; Q96KI3	NP_003320	Thioredoxin (Trx) (ATL-derived factor) (ADF) (Surface-associated sulphydryl protein) (SASP)	GO:0006928; GO:0008283; GO:0045454; GO:0007267; GO:0005829; GO:0022900; GO:0005515; GO:0007165; GO:0006810	cell motion; cell proliferation; cell redox homeostasis; cell-cell signaling; cytosol; electron transport chain; protein binding; signal transduction; transport	Cytoplasm			Known in Uniprot to be phosphorylated		
**1240**	PGAM2	PGAM2_HUMAN	PGAM2	P15259	NP_000281	Phosphoglycerate mutase 2 (EC 5.4.2.1) (EC 5.4.2.4) (EC 3.1.3.13) (Phosphoglycerate mutase isozyme M) (PGAM-M) (BPG-dependent PGAM 2) (Muscle-specific phosphoglycerate mutase)	GO:0004083; GO:0004082; GO:0006096; GO:0004619; GO:0006941	bisphosphoglycerate 2-phosphatase activity; bisphosphoglycerate mutase activity; glycolysis; phosphoglycerate mutase activity; striated muscle contraction						
**1241**	MFAP1	MFAP1_HUMAN	Q86TG6; MFAP1	Q86TG6; P55081	NP_005917	Microfibrillar-associated protein 1	GO:0001527	microfibril	Secretedextracellularspaceextracellularmatrix			Known in Uniprot to be phosphorylated	Known in HPRD to be phosphorylated	
**1242**	CV019	CV019_HUMAN	CV019; THOC5	Q13769; O60839; Q9UPZ5	NP_003669; NP_001002877; NP_001002878; NP_001002879	THO complex subunit 5 homolog (NF2/meningioma region protein pK1.3) (Placental protein 39.2)	GO:0005737; GO:0005634	cytoplasm; nucleus	NucleusCytoplasm			Known in Uniprot to be phosphorylated	Known in HPRD to be phosphorylated	
**1243**	LY66D	LY66D_HUMAN	O95868; Q4VX50; LY66D	O95868; A2BEY8; B0UXC1; B0V019; B0V1Y6; Q4VX50	NP_067069	Lymphocyte antigen 6 complex locus protein G6d (Protein Ly6-D) (Megakaryocyte-enhanced gene transcript 1 protein)	GO:0031225; GO:0005886	anchored to membrane; plasma membrane	CellmembraneLipidanchorGPIanchor					
**1244**	MY18B	MY18B_HUMAN	MY18B	Q8IUG5; Q8NDI8; Q8TE65; Q8WWS0; Q96KH2; Q96KR8; Q96KR9	NP_115997	Myosin-XVIIIb	GO:0005524; GO:0003779; GO:0005737; GO:0003774; GO:0005634; GO:0016461	ATP binding; actin binding; cytoplasm; motor activity; nucleus; unconventional myosin complex	CytoplasmNucleusCytoplasmmyofibrilsarcomere			Known in Uniprot to be phosphorylated		
**1245**	NDUA6	NDUA6_HUMAN	NDUA6	P56556; B2RE54; O43675; Q6FGW0; Q6IBT8; Q6IC39	NP_002481	NADH dehydrogenase [ubiquinone] 1 alpha subcomplex subunit 6 (NADH-ubiquinone oxidoreductase B14 subunit) (Complex I-B14) (CI-B14) (LYR motif-containing protein 6)	GO:0008137; GO:0006120; GO:0005747; GO:0006979; GO:0006810	NADH dehydrogenase (ubiquinone) activity; mitochondrial electron transport, NADH to ubiquinone; mitochondrial respiratory chain complex I; response to oxidative stress; transport	MitochondrioninnermembranePeripheralmembraneproteinMatrixside					
**1246**	ENDD1	ENDD1_HUMAN	ENDD1	O94919; Q8TAQ8	XP_290546; NP_055851	Endonuclease domain-containing 1 protein (EC 3.1.30.-)	GO:0004519; GO:0005576; GO:0046872; GO:0003676	endonuclease activity; extracellular region; metal ion binding; nucleic acid binding	Secreted					
**1247**	ATP5L	ATP5L_HUMAN	ATP5L; A8K0K3	O75964; Q96BV6; Q9UBZ7; A8K0K3	NP_006467	ATP synthase subunit g, mitochondrial (ATPase subunit g)	GO:0015986; GO:0015078; GO:0000276; GO:0005515	ATP synthesis coupled proton transport; hydrogen ion transmembrane transporter activity; mitochondrial proton-transporting ATP synthase complex, coupling factor F(o); protein binding	MitochondrionMitochondrioninnermembrane					
**1248**	OR1J1	OR1J1_HUMAN	OR1J1; A3KFL8	Q8NGS3; Q6IF10; Q96R88; A3KFL8	XP_294531; NP_001004451	Olfactory receptor 1J1 (Olfactory receptor OR9-18); Olfactory receptor, family 1, subfamily J, member 1	GO:0007186; GO:0016021; GO:0004984; GO:0005886; GO:0050896; GO:0007608	G-protein coupled receptor protein signaling pathway; integral to membrane; olfactory receptor activity; plasma membrane; response to stimulus; sensory perception of smell	CellmembraneMultipassmembraneprotein;					
**1249**	TXND4	TXND4_HUMAN	TXND4; ERP44	Q9BS26; O60319; Q4VXC1; Q5VWZ7; Q6UW14; Q8WX67	XP_088476; NP_055866	Thioredoxin domain-containing protein 4 (Endoplasmic reticulum resident protein ERp44)	GO:0005793; GO:0045454; GO:0005788; GO:0005789; GO:0009100; GO:0005515; GO:0003756; GO:0006457; GO:0006986	ER-Golgi intermediate compartment; cell redox homeostasis; endoplasmic reticulum lumen; endoplasmic reticulum membrane; glycoprotein metabolic process; protein binding; protein disulfide isomerase activity; protein folding; response to unfolded protein	Endoplasmicreticulumlumen					
**1250**	LY10	LY10_HUMAN	LY10; Q8IWJ1; B4DVW8	Q13342; Q13341; Q4ZG66; Q53TG1; Q92881; Q96TG3; B4DVW8; Q8IWJ1	NP_009168; NP_001005176	Nuclear body protein SP140 (Nuclear autoantigen Sp-140) (Speckled 140 kDa) (Lymphoid-restricted homolog of Sp100) (LYSp100 protein); Similar to SP140 nuclear body protein (Fragment)	GO:0005737; GO:0006952; GO:0005635; GO:0005654; GO:0005515; GO:0003700; GO:0008270; GO:0005634	cytoplasm; defense response; nuclear envelope; nucleoplasm; protein binding; transcription factor activity; zinc ion binding; nucleus	NucleusCytoplasm;					
**1251**	APC	APC_HUMAN	APC	P25054; Q15162; Q15163; Q93042	NP_000029; NP_001120982; NP_001120983	Adenomatous polyposis coli protein (Protein APC) (Deleted in polyposis 2.5)	GO:0034747; GO:0060070; GO:0008013; GO:0030877; GO:0007155; GO:0007050; GO:0005813; GO:0000281; GO:0005829; GO:0000776; GO:0016328; GO:0008017; GO:0007094; GO:0045736; GO:0007026; GO:0005634; GO:0043065; GO:0030335; GO:0031274; GO:0006461; GO:0008605; GO:0019901; GO:0051988; GO:0006974	Axin-APC-beta-catenin-GSK3B complex; Wnt receptor signaling pathway through beta-catenin; beta-catenin binding; beta-catenin destruction complex; cell adhesion; cell cycle arrest; centrosome; cytokinesis after mitosis; cytosol; kinetochore; lateral plasma membrane; microtubule binding; mitotic cell cycle spindle assembly checkpoint; negative regulation of cyclin-dependent protein kinase activity; negative regulation of microtubule depolymerization; nucleus; positive regulation of apoptosis; positive regulation of cell migration; positive regulation of pseudopodium assembly; protein complex assembly; protein kinase CK2 regulator activity; protein kinase binding; regulation of attachment of spindle microtubules to kinetochore; response to DNA damage stimulus				Known in Uniprot to be phosphorylated	Known in HPRD to be phosphorylated	
**1252**	WDR60	WDR60_HUMAN	WDR60	Q8WVS4; Q9NW58	NP_060521	WD repeat-containing protein 60						Known in Uniprot to be phosphorylated		
**1253**	SUMO2	SUMO2_HUMAN	SUMO2; B2R4I2	P61956; Q32Q42; P55855; Q6IPZ6; Q96HK1; B2R4I2	NP_008868; NP_001005849	Small ubiquitin-related modifier 2 (SUMO-2) (Ubiquitin-like protein SMT3B) (SMT3 homolog 2) (Sentrin-2) (HSMT3) (SUMO-3)	GO:0019941; GO:0005634; GO:0005515; GO:0006464	modification-dependent protein catabolic process; nucleus; protein binding; protein modification process	Nucleus					
**1254**	PK3CA	PK3CA_HUMAN	PK3CA	P42336; Q14CW1; Q99762	NP_006209	Phosphatidylinositol-4,5-bisphosphate 3-kinase catalytic subunit alpha isoform (EC 2.7.1.153) (PI3 kinase p110 subunit alpha) (PtdIns-3-kinase p110) (PI3K)	GO:0016303; GO:0005739; GO:0046934; GO:0005942; GO:0046854; GO:0048015	1-phosphatidylinositol-3-kinase activity; mitochondrion; phosphatidylinositol-4,5-bisphosphate 3-kinase activity; phosphoinositide 3-kinase complex; phosphoinositide phosphorylation; phosphoinositide-mediated signaling						Kinase

	Protein Name	Uniprot Name	Other Names	Uniprot IDs	Refseq IDs	Protein Description	GO ID	GO terms	Subcellular location	Comments	Comments	Comments - Database Search	Comments - Database Search	Function
**1255**	S6A16	S6A16_HUMAN	S6A16	Q9GZN6; Q9Y5I9	NP_054756	Orphan sodium- and chloride-dependent neurotransmitter transporter NTT5 (Solute carrier family 6 member 16)	GO:0005887; GO:0005622; GO:0006836; GO:0005328	integral to plasma membrane; intracellular; neurotransmitter transport; neurotransmitter:sodium symporter activity	MembraneMultipassmembraneprotein			Known in Uniprot to be phosphorylated		
**1256**	CF223	CF223_HUMAN	Q8N575; Q8N319; CF223	Q8N319; Q8N575	NP_694978	Uncharacterized protein C6orf223								
**1257**	ZN382	ZN382_HUMAN	ZN382	Q96SR6; A3KMP6; A8MT55; Q53ZY8; Q5JPJ2	NP_116214	Zinc finger protein 382 (Multiple zinc finger and krueppel-associated box protein KS1) (KRAB/zinc finger suppressor protein 1) (KS1)	GO:0003677; GO:0005634; GO:0006355; GO:0008270	DNA binding; nucleus; regulation of transcription, DNA-dependent; zinc ion binding	Nucleus					
**1258**	CTNA2	CTNA2_HUMAN	CTNA2	Q7Z3Y0; P26232; B3KXE5; Q4ZFW1; Q53R26; Q53R33; Q53T67; Q53T71; Q53TM8	NP_004380	Catenin alpha-2 (Alpha-catenin-related protein) (Alpha N-catenin)	GO:0015629; GO:0045296; GO:0007155; GO:0030054; GO:0005737; GO:0005200	actin cytoskeleton; cadherin binding; cell adhesion; cell junction; cytoplasm; structural constituent of cytoskeleton	CytoplasmcytoskeletonCelljunctionadherensjunctionCellmembranePeripheralmembraneproteinCytoplasmicsideCelljunction			Known in Uniprot to be phosphorylated	Known in HPRD to be phosphorylated	
**1259**	NOG1	NOG1_HUMAN	NOG1; Q5T3R8	Q9BZE4; Q5T3R8; O95446; Q9NVJ8	NP_036473	Nucleolar GTP-binding protein 1 (Chronic renal failure gene protein) (GTP-binding protein NGB); GTP binding protein 4 (GTP binding protein 4, isoform CRA_a)	GO:0005525; GO:0003924; GO:0008156; GO:0030336; GO:0008285; GO:0022408; GO:0033342; GO:0031397; GO:0005634; GO:0048471; GO:0005515; GO:0050821; GO:0000079; GO:0042254; GO:0007264	GTP binding; GTPase activity; negative regulation of DNA replication; negative regulation of cell migration; negative regulation of cell proliferation; negative regulation of cell-cell adhesion; negative regulation of collagen binding; negative regulation of protein ubiquitination; nucleus; perinuclear region of cytoplasm; protein binding; protein stabilization; regulation of cyclin-dependent protein kinase activity; ribosome biogenesis; small GTPase mediated signal transduction	Nucleusnucleolus;			Known in Uniprot to be phosphorylated		
**1260**	K1C9	K1C9_HUMAN	K1C9	P35527; O00109; Q0IJ47; Q14665	NP_000217	Keratin, type I cytoskeletal 9 (Cytokeratin-9) (CK-9) (Keratin-9) (K9)	GO:0005737; GO:0005882; GO:0045109; GO:0005515; GO:0043588; GO:0005200	cytoplasm; intermediate filament; intermediate filament organization; protein binding; skin development; structural constituent of cytoskeleton						
**1261**	ITA5	ITA5_HUMAN	ITA5	P08648; Q96HA5	NP_002196	Integrin alpha-5 (Fibronectin receptor subunit alpha) (Integrin alpha-F) (VLA-5) (CD49 antigen-like family member E) (CD antigen CD49e) [Cleaved into: Integrin alpha-5 heavy chain; Integrin alpha-5 light chain]	GO:0005794; GO:0005509; GO:0008305; GO:0007229; GO:0044419; GO:0005634; GO:0004872; GO:0001726; GO:0035313	Golgi apparatus; calcium ion binding; integrin complex; integrin-mediated signaling pathway; interspecies interaction between organisms; nucleus; receptor activity; ruffle; wound healing, spreading of epidermal cells	MembraneSinglepasstypeImembraneprotein					
**1262**	TCHL1	TCHL1_HUMAN	Q5QJ38; TCHL1	Q5QJ38; B2RPK8; Q5VTJ9	XP_060104; NP_001008536	Trichohyalin-like protein 1 (Basalin) (Protein S100-A17) (S100 calcium-binding protein A17)	GO:0005509	calcium ion binding						
**1263**	GDIS	GDIS_HUMAN	GDIS; GDIR2; B5BU79	P52566; B5BU79	NP_001166	Rho GDP-dissociation inhibitor 2 (Rho GDI 2) (Rho-GDI beta) (Ly-GDI)	GO:0005096; GO:0005094; GO:0007266; GO:0030036; GO:0006928; GO:0016023; GO:0005856; GO:0006955; GO:0007275; GO:0007162	GTPase activator activity; Rho GDP-dissociation inhibitor activity; Rho protein signal transduction; actin cytoskeleton organization; cell motion; cytoplasmic membrane-bounded vesicle; cytoskeleton; immune response; multicellular organismal development; negative regulation of cell adhesion	Cytoplasm			Known in Uniprot to be phosphorylated		
**1264**	K2018	K2018_HUMAN	Q68DE3; K2018	Q68DE3; Q7Z3L9; Q8IVF3; Q9H8T4	XP_305530	Basic helix-loop-helix domain-containing protein KIAA2018	GO:0003677; GO:0005509; GO:0004571; GO:0016020; GO:0005634; GO:0045449; GO:0030528	DNA binding; calcium ion binding; mannosyl-oligosaccharide 1,2-alpha-mannosidase activity; membrane; nucleus; regulation of transcription; transcription regulator activity	Nucleus			Known in Uniprot to be phosphorylated		
**1265**	CK5P2	CK5P2_HUMAN	CK5P2; Q05CZ3; Q5VU02; B3KVI2; Q5JV18	Q5VU02; Q96SN8; Q7Z3L4; Q7Z3U1; Q7Z7I6; Q9BSW0; Q9H6J6; Q9HCD9; Q9NV90; Q9UIW9; B3KVI2; Q05CZ3; Q5JV18	NP_060719; NP_001011649	CDK5 regulatory subunit-associated protein 2 (CDK5 activator-binding protein C48) (Centrosome-associated protein 215); CDK5RAP2 protein (Fragment)	GO:0007420; GO:0005813; GO:0008017; GO:0042808; GO:0045664; GO:0005813	brain development; centrosome; microtubule binding; neuronal Cdc2-like kinase binding; regulation of neuron differentiation; centrosome	Centrosome;			Known in Uniprot to be phosphorylated	Known in HPRD to be phosphorylated	
**1266**	GTPB9	GTPB9_HUMAN	GTPB9; Q53SQ6; OLA1	Q9NTK5; Q5BJD7; Q8NCI8; Q96CU1; Q96SV2; Q9P1D3; Q9UNY9; Q9Y6G4; Q53SQ6	NP_037473; NP_001011708	Obg-like ATPase 1 (EC 3.6.3.-) (GTP-binding protein 9); Putative uncharacterized protein PTD004 (Fragment)	GO:0005524; GO:0006200; GO:0005525; GO:0005737; GO:0016787; GO:0005515;	ATP binding; ATP catabolic process; GTP binding; cytoplasm; hydrolase activity; protein binding;						
**1267**	K0408	K0408_HUMAN	K0408	Q6ZU52; O43158; Q5TF20; Q7L2M2	NP_055517	Uncharacterized protein KIAA0408								
**1268**	DYN1	DYN1_HUMAN	DYN1; Q5SYX2; Q86VD2; Q6P3T6	Q5SYX2; Q05193; A6NLM6; Q5SYX0; Q6P3T6; Q86VD2	NP_004399; NP_001005336	Dynamin-1 (EC 3.6.5.5)	GO:0005525; GO:0003924; GO:0005737; GO:0005874; GO:0003774; GO:0005515; GO:0006898	GTP binding; GTPase activity; cytoplasm; microtubule; motor activity; protein binding; receptor-mediated endocytosis	CytoplasmCytoplasmcytoskeleton			Known in Uniprot to be phosphorylated	Known in HPRD to be phosphorylated	
**1269**	HEMH	HEMH_HUMAN	HEMH; A8KA72	P22830; Q8IXN1; A8KA72	NP_000131	Ferrochelatase, mitochondrial (EC 4.99.1.1) (Protoheme ferro-lyase) (Heme synthetase)	GO:0051537; GO:0004325; GO:0008198; GO:0006091; GO:0006783; GO:0005743; GO:0005759; GO:0005515; GO:0046501	2 iron, 2 sulfur cluster binding; ferrochelatase activity; ferrous iron binding; generation of precursor metabolites and energy; heme biosynthetic process; mitochondrial inner membrane; mitochondrial matrix; protein binding; protoporphyrinogen IX metabolic process	MitochondrioninnermembranePeripheralmembraneproteinMatrixside					
**1270**	Q5KTZ5	Q5KTZ5_HUMAN	Q5KTZ5	Q5KTZ5	XP_035037; NP_002325	Low density lipoprotein receptor-related protein 10	GO:0005509; GO:0009953; GO:0042733; GO:0001942; GO:0001822; GO:0030178; GO:0042475; GO:0005886; GO:0008104; GO:0009954; GO:0004872; GO:0043113; GO:0051124	calcium ion binding; dorsal/ventral pattern formation; embryonic digit morphogenesis; hair follicle development; kidney development; negative regulation of Wnt receptor signaling pathway; odontogenesis of dentine-containing tooth; plasma membrane; protein localization; proximal/distal pattern formation; receptor activity; receptor clustering; synaptic growth at neuromuscular junction						
**1271**	PDE5A	PDE5A_HUMAN	PDE5A; Q4W5L4	O76074; A0AV69; A8K2C4; O75026; O75887; Q86UI0; Q86V66; Q9Y6Z6; Q4W5L4	NP_001074; NP_236914; NP_246273	cGMP-specific 3',5'-cyclic phosphodiesterase (EC 3.1.4.35) (cGMP-binding cGMP-specific phosphodiesterase) (CGB-PDE); Putative uncharacterized protein PDE5A (Fragment)	GO:0047555; GO:0030553; GO:0009187; GO:0005737; GO:0000287; GO:0007165; GO:0008270; GO:0004114; GO:0046069; GO:0007399; GO:0001666; GO:0042311	3',5'-cyclic-GMP phosphodiesterase activity; cGMP binding; cyclic nucleotide metabolic process; cytoplasm; magnesium ion binding; signal transduction; zinc ion binding; 3',5'-cyclic-nucleotide phosphodiesterase activity; cGMP catabolic process; nervous system development; response to hypoxia; vasodilation				Known in Uniprot to be phosphorylated	Known in HPRD to be phosphorylated	
**1272**	YG024	YG024_HUMAN	Q6ZRM9; YG024	Q6ZRM9	XP_294951	Putative uncharacterized protein FLJ46235								
**1273**	IBP3	IBP3_HUMAN	IBP3; A4D2F5	P17936; A4D2F5; Q2V509; Q6P1M6; Q9UCL4	NP_000589; NP_001013416	Insulin-like growth factor-binding protein 3 (IGF-binding protein 3) (IGFBP-3) (IBP-3)	GO:0031994; GO:0016942; GO:0046872; GO:0001933; GO:0009968; GO:0014912; GO:0048662; GO:0005634; GO:0043065; GO:0045663; GO:0008160; GO:0001558	insulin-like growth factor I binding; insulin-like growth factor binding protein complex; metal ion binding; negative regulation of protein amino acid phosphorylation; negative regulation of signal transduction; negative regulation of smooth muscle cell migration; negative regulation of smooth muscle cell proliferation; nucleus; positive regulation of apoptosis; positive regulation of myoblast differentiation; protein tyrosine phosphatase activator activity; regulation of cell growth	Secreted			known in HPRD to be phosphorylated		
**1274**	CFAH	CFAH_HUMAN	CFAH	P08603; A5PL14; P78435; Q14570; Q2TAZ5; Q38G77; Q5TFM3; Q8N708; Q9NU86	NP_000177	Complement factor H (H factor 1)	GO:0006957; GO:0005615	complement activation, alternative pathway; extracellular space	Secreted					
**1275**	LGR6	LGR6_HUMAN	LGR6	Q9HBX8; Q5T509; Q5T512; Q6UY15; Q86VU0; Q96K69; Q9BYD7	XP_303366; NP_001017403; NP_001017404; NP_067649	Leucine-rich repeat-containing G-protein coupled receptor 6	GO:0007186; GO:0016021; GO:0005886; GO:0005515; GO:0016500	G-protein coupled receptor protein signaling pathway; integral to membrane; plasma membrane; protein binding; protein-hormone receptor activity	CellmembraneMultipassmembraneprotein			Known in Uniprot to be phosphorylated		

	Protein Name	Uniprot Name	Other Names	Uniprot IDs	Refseq IDs	Protein Description	GO ID	GO terms	Subcellular location	Comments	Comments	Comments - Database Search	Comments - Database Search	Function
**1276**	TGFB1	TGFB1_HUMAN	TGFB1	P01137; Q9UCG4	NP_000651	Transforming growth factor beta-1 (TGF-beta-1) [Cleaved into: Latency-associated peptide (LAP)]	GO:0006754; GO:0031575; GO:0005796; GO:0007183; GO:0007050; GO:0002248; GO:0007173; GO:0019049; GO:0005615; GO:0010742; GO:0008083; GO:0006917; GO:0048535; GO:0008156; GO:0043537; GO:0030308; GO:0022408; GO:0050680; GO:0045930; GO:0001933; GO:0016481; GO:0005634; GO:0042476; GO:0043932; GO:0031093; GO:0043406; GO:0060391; GO:0043536; GO:0030501; GO:0050921; GO:0032967; GO:0010718; GO:0010763; GO:0032740; GO:0048298;	ATP biosynthetic process; G1/S transition checkpoint; Golgi lumen; SMAD protein complex assembly; cell cycle arrest; connective tissue replacement during inflammatory response; epidermal growth factor receptor signaling pathway; evasion of host defenses by virus; extracellular space; foam cell differentiation; growth factor activity; induction of apoptosis; lymph node development; negative regulation of DNA replication; negative regulation of blood vessel endothelial cell migration; negative regulation of cell growth; negative regulation of cell-cell adhesion; negative regulation of epithelial cell proliferation; negative regulation of mitotic cell cycle; negative regulation of protein amino acid phosphorylation; negative regulation of transcription; nucleus; odontogenesis; ossification involved in bone remodeling; platelet alpha granule lumen; positive regulation of MAP kinase activity; positive regulation of SMAD protein nuclear translocation; positive regulation of blood vessel endothelial cell migration; positive regulation of bone mineralization; positive regulation of chemotaxis; positive regulation of collagen biosynthetic process; positive regulation of epithelial to mesenchymal transition; positive	Secreted					
**1277**	PGM2	PGM2_HUMAN	PGM2	Q96G03; Q53FP5; Q5QTR0; Q9H0P9; Q9NV22	NP_060760	Phosphoglucomutase-2 (PGM 2) (EC 5.4.2.2) (Glucose phosphomutase 2)	GO:0005737; GO:0006006; GO:0000287; GO:0004614	cytoplasm; glucose metabolic process; magnesium ion binding; phosphoglucomutase activity	Cytoplasm			Known in Uniprot to be phosphorylated		
**1278**	FLNC	FLNC_HUMAN	FLNC; Q59H94; B2ZZ88	Q14315; O95303; Q07985; Q9NS12; Q9NYE5; Q9UMR8; Q9Y503; B2ZZ88; Q59H94	NP_001449; NP_001120959	Filamin-C (Gamma-filamin) (Filamin-2) (Protein FLNc) (Actin-binding-like protein) (ABP-L) (ABP-280-like protein); Gamma filamin variant (Fragment)	GO:0003779; GO:0005737; GO:0005856; GO:0005886;	actin binding; cytoplasm; cytoskeleton; plasma membrane;	CytoplasmMembranePeripheralmembraneproteinCytoplasmcytoskeletonCytoplasmmyofibrilsarcomereZdisk;			Known in Uniprot to be phosphorylated		
**1279**	MAGI1	MAGI1_HUMAN	MAGI1	Q96QZ7; O00309; O43863; O75085; Q96QZ8; Q96QZ9	NP_004733; NP_001028229; NP_056335	Membrane-associated guanylate kinase, WW and PDZ domain-containing protein 1 (BAI1-associated protein 1) (BAP-1) (Membrane-associated guanylate kinase inverted 1) (MAGI-1) (Atrophin-1-interacting protein 3) (AIP3) (WW domain-containing protein 3) (WWP3) (Trinucleotide repeat containing gene 19 protein)	GO:0005524; GO:0007155; GO:0007166; GO:0008022; GO:0006461; GO:0005923	ATP binding; cell adhesion; cell surface receptor linked signal transduction; protein C-terminus binding; protein complex assembly; tight junction	CelljunctiontightjunctionCellmembranePeripheralmembraneprotein			Known in Uniprot to be phosphorylated		
**1280**	MGR2	MGR2_HUMAN	MGR2; Q86YG3; GRM2; B0M0K7	Q14416; Q14CU5; Q52MC6; Q9H3N6; B0M0K7; Q86YG3	NP_000830; NP_001123535	Metabotropic glutamate receptor 2 (mGluR2); Similar to glutamate receptor, metabotropic 2 (Fragment)	GO:0004930; GO:0007186; GO:0008066; GO:0005887; GO:0007194; GO:0007268; GO:0030424; GO:0014047; GO:0016021; GO:0005622; GO:0005886; GO:0042734	G-protein coupled receptor activity; G-protein coupled receptor protein signaling pathway; glutamate receptor activity; integral to plasma membrane; negative regulation of adenylate cyclase activity; synaptic transmission; axon; glutamate secretion; integral to membrane; intracellular; plasma membrane; presynaptic membrane	CellmembraneMultipassmembraneprotein					
**1281**	A2MG	A2MG_HUMAN	A2MG	P01023; Q13677; Q59F47; Q5QTS0; Q68DN2; Q6PIY3; Q6PN97	NP_000005	Alpha-2-macroglobulin (Alpha-2-M) (C3 and PZP-like alpha-2-macroglobulin domain-containing protein 5)	GO:0019899; GO:0005615; GO:0019966; GO:0019959; GO:0005634; GO:0031093; GO:0051260; GO:0004867; GO:0043120; GO:0017114	enzyme binding; extracellular space; interleukin-1 binding; interleukin-8 binding; nucleus; platelet alpha granule lumen; protein homooligomerization; serine-type endopeptidase inhibitor activity; tumor necrosis factor binding; wide-spectrum protease inhibitor activity	Secreted					
**1282**	MECR	MECR_HUMAN	MECR	Q9BV79; Q5SYU0; Q5SYU1; Q5SYU2; Q6IBU9; Q9Y373	NP_057095	Trans-2-enoyl-CoA reductase, mitochondrial (HsNrbf-1) (EC 1.3.1.38) (NRBF-1)	GO:0006633; GO:0005739; GO:0055114; GO:0019166; GO:0008270	fatty acid biosynthetic process; mitochondrion; oxidation reduction; trans-2-enoyl-CoA reductase (NADPH) activity; zinc ion binding	Mitochondrion					
**1283**	GCYA3	GCYA3_HUMAN	GCYA3	Q02108; O43843; Q8TAH3	NP_000847; NP_001124154; NP_001124155; NP_001124156	Guanylate cyclase soluble subunit alpha-3 (GCS-alpha-3) (EC 4.6.1.2) (Soluble guanylate cyclase large subunit) (GCS-alpha-1)	GO:0004383; GO:0008074; GO:0007263; GO:0004872	guanylate cyclase activity; guanylate cyclase complex, soluble; nitric oxide mediated signal transduction; receptor activity	Cytoplasm					
**1284**	SODM	SODM_HUMAN	SODM; B2R7R1	P04179; P78434; Q16792; Q5TCM1; Q96EE6; Q9P2Z3; B2R7R1	NP_000627; NP_001019636	Superoxide dismutase [Mn], mitochondrial (EC 1.15.1.1)	GO:0001315; GO:0030145; GO:0005759; GO:0043524; GO:0032364; GO:0005515; GO:0006357; GO:0001836; GO:0019430; GO:0000303; GO:0004784; GO:0003069	age-dependent response to reactive oxygen species; manganese ion binding; mitochondrial matrix; negative regulation of neuron apoptosis; oxygen homeostasis; protein binding; regulation of transcription from RNA polymerase II promoter; release of cytochrome c from mitochondria; removal of superoxide radicals; response to superoxide; superoxide dismutase activity; vasodilation by acetylcholine involved in regulation of systemic arterial blood pressure	Mitochondrionmatrix					
**1285**	SPTB1	SPTB1_HUMAN	Q59FP5; SPTB1	Q59FP5; P11277; Q15510; Q15519	NP_000338; NP_001020029	Spectrin, beta, erythrocytic (Includes spherocytosis, clinical type I) variant (Fragment)	GO:0005794; GO:0003779; GO:0005634; GO:0005886; GO:0008091; GO:0005200	Golgi apparatus; actin binding; nucleus; plasma membrane; spectrin; structural constituent of cytoskeleton				Known in Uniprot to be phosphorylated		
**1286**	SCG2	SCG2_HUMAN	SCG2; B2R662	Q53T11; P13521; Q8TBH3; B2R662	NP_003460	Secretogranin-2 (Secretogranin II) (SgII) (Chromogranin-C) [Cleaved into: Secretoneurin (SN)]	GO:0000165; GO:0001525; GO:0005509; GO:0042056; GO:0005125; GO:0043542; GO:0048245; GO:0005615; GO:0050930; GO:0006954; GO:0043066; GO:0001937; GO:0001938; GO:0009306	MAPKKK cascade; angiogenesis; calcium ion binding; chemoattractant activity; cytokine activity; endothelial cell migration; eosinophil chemotaxis; extracellular space; induction of positive chemotaxis; inflammatory response; negative regulation of apoptosis; negative regulation of endothelial cell proliferation; positive regulation of endothelial cell proliferation; protein secretion	Secreted			Known in Uniprot to be phosphorylated		
**1287**	PSA7L	PSA7L_HUMAN	PSA7L; B0YJ75	Q8TAA3; Q8IVP4; Q8TA98; Q8TAA2; B0YJ75	NP_653263; NP_001020267; NP_001020268	Proteasome subunit alpha type-7-like (EC 3.4.25.1)	GO:0005829; GO:0005634; GO:0005839; GO:0004298; GO:0006511	cytosol; nucleus; proteasome core complex; threonine-type endopeptidase activity; ubiquitin-dependent protein catabolic process	CytoplasmNucleus					
**1288**	ZMYM6	ZMYM6_HUMAN	Q69YL4; Q5SW00; Q32Q23; ZMYM6	Q5SW01; Q69YL4; O95789; B4DRJ6; Q32Q23; Q4G108; Q5SVZ9; Q5SW00; Q96IY0; Q9NWF1; Q9P2J4	NP_009098	Zinc finger MYM-type protein 6 (Zinc finger protein 258)	GO:0003677; GO:0007275; GO:0005634; GO:0008270	DNA binding; multicellular organismal development; nucleus; zinc ion binding	Nucleus			Known in Uniprot to be phosphorylated		
**1289**	TM16F	TM16F_HUMAN	TM16F; ANO6; B3KX12	Q4KMQ2; A6NNM6; Q8N3Q2; B3KX12	XP_113743; NP_001020527; NP_001136150; NP_001136151	Anoctamin-6 (Transmembrane protein 16F)	GO:0005509; GO:0005254; GO:0031404; GO:0016021; GO:0006811	calcium ion binding; chloride channel activity; chloride ion binding; integral to membrane; ion transport	MembraneMultipassmembraneprotein					
**1290**	AP2S1	AP2S1_HUMAN	AP2S1; B2R4Z4	P53680; O75977; Q6PK67; B2R4Z4	NP_004060; NP_067586	AP-2 complex subunit sigma-1 (Adapter-related protein complex 2 sigma-1 subunit) (Sigma2-adaptin) (Plasma membrane adaptor AP-2 17 kDa protein) (HA2 17 kDa subunit) (Clathrin assembly protein 2 small chain) (Clathrin coat assembly protein AP17) (Clathrin coat-associated protein AP17)	GO:0030122; GO:0048268; GO:0005829; GO:0006886; GO:0005515; GO:0008565; GO:0050690; GO:0030100	AP-2 adaptor complex; clathrin coat assembly; cytosol; intracellular protein transport; protein binding; protein transporter activity; regulation of defense response to virus by virus; regulation of endocytosis	CellmembraneMembranecoatedpitPeripheralmembraneproteinCytoplasmicside			Known in Uniprot to be phosphorylated		
**1291**	ECH1	ECH1_HUMAN	ECH1	Q13011; Q8WVX0; Q96EZ9	NP_001389	Delta(3,5)-Delta(2,4)-dienoyl-CoA isomerase, mitochondrial (EC 5.3.3.-)	GO:0004300; GO:0006635; GO:0006091; GO:0016853; GO:0005739; GO:0005777; GO:0005515	enoyl-CoA hydratase activity; fatty acid beta-oxidation; generation of precursor metabolites and energy; isomerase activity; mitochondrion; peroxisome; protein binding	MitochondrionPeroxisome			Known in Uniprot to be phosphorylated		

	Protein Name	Uniprot Name	Other Names	Uniprot IDs	Refseq IDs	Protein Description	GO ID	GO terms	Subcellular location	Comments	Comments	Comments - Database Search	Comments - Database Search	Function
**1292**	PYGL	PYGL_HUMAN	PYGL	P06737; A6NDQ4; O60567; O60752; O60913; Q501V9; Q641R5; Q96G82	NP_002854	Glycogen phosphorylase, liver form (EC 2.4.1.1)	GO:0016208; GO:0005524; GO:0032052; GO:0005737; GO:0008144; GO:0005536; GO:0042593; GO:0005977; GO:0008184; GO:0005634; GO:0005886; GO:0042803; GO:0002060; GO:0030170; GO:0005625	AMP binding; ATP binding; bile acid binding; cytoplasm; drug binding; glucose binding; glucose homeostasis; glycogen metabolic process; glycogen phosphorylase activity; nucleus; plasma membrane; protein homodimerization activity; purine binding; pyridoxal phosphate binding; soluble fraction				Known in Uniprot to be phosphorylated		
**1293**	DCUP	DCUP_HUMAN	DCUP; A8K762	P06132; Q6IB28; Q16863; Q16883; Q9BUZ0; A8K762	NP_000365	Uroporphyrinogen decarboxylase (URO-D) (UPD) (EC 4.1.1.37)	GO:0005737; GO:0006783; GO:0004853	cytoplasm; heme biosynthetic process; uroporphyrinogen decarboxylase activity	Cytoplasm			Known in Uniprot to be phosphorylated		
**1294**	ADHX	ADHX_HUMAN	ADHX; Q6IRT1	P11766; Q6FHR2; Q6IRT1	NP_000662	Alcohol dehydrogenase class-3 (EC 1.1.1.1) (Alcohol dehydrogenase class-III) (Alcohol dehydrogenase 5) (Alcohol dehydrogenase class chi chain) (S-(hydroxymethyl)glutathione dehydrogenase) (EC 1.1.1.284) (Glutathione-dependent formaldehyde dehydrogenase) (GSH-FDH) (FALDH) (FDH) (EC 1.1.1.-)	GO:0051903; GO:0004022; GO:0009055; GO:0006069; GO:0005504; GO:0018467; GO:0055114; GO:0051775; GO:0008270	S-(hydroxymethyl)glutathione dehydrogenase activity; alcohol dehydrogenase activity; electron carrier activity; ethanol oxidation; fatty acid binding; formaldehyde dehydrogenase activity; oxidation reduction; response to redox state; zinc ion binding	Cytoplasm					
**1295**	COAC	COAC_HUMAN	COAC	Q96CD2; Q96SX0; Q9HC17	NP_068595	Phosphopantothenoylcysteine decarboxylase (PPC-DC) (EC 4.1.1.36) (CoaC)	GO:0015937; GO:0004633	coenzyme A biosynthetic process; phosphopantothenoylcysteine decarboxylase activity						
**1296**	SNX32	SNX32_HUMAN	Q86XE0; SNX32	Q86XE0; Q8IW53; Q96NG4	NP_689973	Sorting nexin-32 (Sortin nexin-6B)	GO:0007154; GO:0035091; GO:0005515; GO:0015031	cell communication; phosphoinositide binding; protein binding; protein transport						
**1297**	MESD2	MESD2_HUMAN	MESD2	Q14696; O969U1	XP_051854; NP_055969	Mesoderm development candidate 2 (Renal carcinoma antigen NY-REN-61)	GO:0007498	mesoderm development						
**1298**	GPX4	GPX4_HUMAN	GPX4	P36969; O43381; Q6PJ59; Q9UPK2	NP_002076; NP_001034936; NP_001034937	Phospholipid hydroperoxide glutathione peroxidase, mitochondrial (PHGPx) (EC 1.11.1.12) (GPX-4)	GO:0004602; GO:0007275; GO:0055114; GO:0006644; GO:0047066; GO:0008430	glutathione peroxidase activity; multicellular organismal development; oxidation reduction; phospholipid metabolic process; phospholipid-hydroperoxide glutathione peroxidase activity; selenium binding	MitochondrionCytoplasm					
**1299**	HELC1	HELC1_HUMAN	HELC1; Q4G1A0	Q8N3C0; O43738; Q5VTN2; Q9H1I9; Q9NTR0; Q4G1A0	NP_006819; NP_071374	Activating signal cointegrator 1 complex subunit 3 (EC 3.6.1.-) (ASC-1 complex subunit p200) (Trip4 complex subunit p200) (Helicase, ATP binding 1); ASCC3 protein	GO:0005524; GO:0008026; GO:0005737; GO:0003676; GO:0006355	ATP binding; ATP-dependent helicase activity; cytoplasm; nucleic acid binding; regulation of transcription, DNA-dependent	Cytoplasm;					
**1300**	NRCAM	NRCAM_HUMAN	NRCAM; Q75ML9	Q92823; A4D0S3; O15051; O15179; Q9UHI3; Q9UHI4; Q75ML9	NP_005001; NP_001032209	Neuronal cell adhesion molecule (Nr-CAM) (NgCAM-related cell adhesion molecule) (Ng-CAM-related) (hBravo); Putative uncharacterized protein NRCAM (Fragment)	GO:0030506; GO:0007413; GO:0016337; GO:0007417; GO:0045162; GO:0009897; GO:0005887; GO:0001764; GO:0043005; GO:0045666; GO:0030516; GO:0007416;	ankyrin binding; axonal fasciculation; cell-cell adhesion; central nervous system development; clustering of voltage-gated sodium channels; external side of plasma membrane; integral to plasma membrane; neuron migration; neuron projection; positive regulation of neuron differentiation; regulation of axon extension; synaptogenesis;	CellmembraneSinglepasstypeImembraneprotein;			Known in Uniprot to be phosphorylated		
**1301**	CYFP2	CYFP2_HUMAN	CYFP2	Q96F07; A6NLT2; Q53EN5; Q9NTK4; Q9ULQ2; Q9UN29	NP_055191; NP_001032409; NP_001032410	Cytoplasmic FMR1-interacting protein 2 (p53-inducible protein 121)	GO:0006915; GO:0030054; GO:0016337; GO:0048471; GO:0005515; GO:0045202; GO:0019717	apoptosis; cell junction; cell-cell adhesion; perinuclear region of cytoplasm; protein binding; synapse; synaptosome	CytoplasmCytoplasmperinuclearregionCelljunctionsynapsesynaptosome			Known in Uniprot to be phosphorylated		
**1302**	CLIC5	CLIC5_HUMAN	CLIC5; Q53G01	Q9NZA1; Q5T4Z0; Q96JT5; Q9BWZ0; Q53G01	NP_058625; NP_001107558; XP_001723610	Chloride intracellular channel protein 5; Chloride intracellular channel 5 variant (Chloride intracellular channel 5A) (Chloride intracellular channel 5, isoform CRA_a) (Fragment)	GO:0005794; GO:0015629; GO:0005813; GO:0031404; GO:0006821; GO:0007565; GO:0005626; GO:0016020; GO:0005902; GO:0005515; GO:0005247; GO:0006821	Golgi apparatus; actin cytoskeleton; centrosome; chloride ion binding; chloride transport; female pregnancy; insoluble fraction; membrane; microvillus; protein binding; voltage-gated chloride channel activity; chloride transport	CytoplasmcellcortexCytoplasmcytoskeletonMembraneSinglepassmembraneproteinGolgiapparatusCentrosomeMembraneSinglepassmembraneprotein;					
**1303**	UCRQ	UCRQ_HUMAN	UCRQ; GDF9; QCR8	O14949; O60383; Q4VAW5; Q5FVE2; Q9BV88; Q9T2V7	NP_055217; NP_005251	Growth/differentiation factor 9 (GDF-9); Cytochrome b-c1 complex subunit 8 (Ubiquinol-cytochrome c reductase complex ubiquinone-binding protein QP-C) (Ubiquinol-cytochrome c reductase complex 9.5 kDa protein) (Complex III subunit VIII) (Complex III subunit 8)	GO:0005125; GO:0005615; GO:0008083; GO:0007179; GO:0022900; GO:0005743; GO:0006810; GO:0008121	cytokine activity; extracellular space; growth factor activity; transforming growth factor beta receptor signaling pathway; electron transport chain; mitochondrial inner membrane; transport; ubiquinol-cytochrome-c reductase activity	Secreted; Mitochondrioninnermembrane					
**1304**	UCRH	UCRH_HUMAN	UCRH; QCR6; B2R4V9	P07919; Q5TDF6; Q6LDB8; Q9BQ91; B2R4V9	NP_005995	Cytochrome b-c1 complex subunit 6, mitochondrial (Ubiquinol-cytochrome c reductase complex 11 kDa protein) (Cytochrome c1 non-heme 11 kDa protein) (Mitochondrial hinge protein) (Complex III subunit VIII) (Complex III subunit 6)	GO:0009060; GO:0006122; GO:0005746; GO:0006810; GO:0008121	aerobic respiration; mitochondrial electron transport, ubiquinol to cytochrome c; mitochondrial respiratory chain; transport; ubiquinol-cytochrome-c reductase activity	Mitochondrioninnermembrane			Known in Uniprot to be phosphorylated		
**1305**	IF4A2	IF4A2_HUMAN	IF4A2; Q53XJ6	Q14240; Q96B90; Q96EA8; Q53XJ6	NP_001958	Eukaryotic initiation factor 4A-II (eIF-4A-II) (eIF4A-II) (EC 3.6.1.-) (ATP-dependent RNA helicase eIF4A-2); Eukaryotic translation initiation factor 4A, isoform 2 (Eukaryotic translation initiation factor 4A, isoform 2, isoform CRA_a)	GO:0005524; GO:0008026; GO:0003723; GO:0005829; GO:0016281; GO:0044419; GO:0005515; GO:0006446; GO:0003743	ATP binding; ATP-dependent helicase activity; RNA binding; cytosol; eukaryotic translation initiation factor 4F complex; interspecies interaction between organisms; protein binding; regulation of translational initiation; translation initiation factor activity						
**1306**	ACBG2	ACBG2_HUMAN	Q5FVE4; ACBG2; B3KSF2	Q8WW03; Q5FVE4; Q6UWJ3; Q7Z5A0; Q96M36; Q9BYZ3; Q9H0C4; B3KSF2	NP_112186	Long-chain-fatty-acid--CoA ligase ACSBG2 (EC 6.2.1.3) (Acyl-CoA synthetase bubblegum family member 2) (Bubblegum-related protein) (PRTD-NY3)	GO:0005524; GO:0016291; GO:0030154; GO:0006631; GO:0004467; GO:0016020; GO:0005792; GO:0005739; GO:0007275; GO:0007283	ATP binding; acyl-CoA thioesterase activity; cell differentiation; fatty acid metabolic process; long-chain-fatty-acid-CoA ligase activity; membrane; microsome; mitochondrion; multicellular organismal development; spermatogenesis	CytoplasmMembranePeripheralmembraneprotein					
**1307**	SEBP2	SEBP2_HUMAN	SEBP2; Q5HYY1	Q96T21; Q8IYC0; Q5HYY1	NP_076982	SECIS-binding protein 2 (Selenocysteine insertion sequence-binding protein 2); SECIS binding protein 2 (SECIS binding protein 2, isoform CRA_b)	GO:0003730; GO:0005634; GO:0006412;	mRNA 3'-UTR binding; nucleus; translation;	Nucleus;					
**1308**	CYB5B	CYB5B_HUMAN	CYB5B	O43169; A8K6B1; Q96CC3; Q9BT35	NP_085056	Cytochrome b5 type B (Cytochrome b5 outer mitochondrial membrane isoform)	GO:0022900; GO:0020037; GO:0016021; GO:0005741; GO:0006810	electron transport chain; heme binding; integral to membrane; mitochondrial outer membrane; transport	Mitochondrionoutermembrane			Known in Uniprot to be phosphorylated		
**1309**	VTNC	VTNC_HUMAN	VTNC	P04004; B2R7G0; P01141; Q9BSH7	NP_000629	Vitronectin (Serum-spreading factor) (S-protein) (V75) [Cleaved into: Vitronectin V65 subunit; Vitronectin V10 subunit; Somatomedin-B]	GO:0005615; GO:0008201; GO:0006955; GO:0005515	extracellular space; heparin binding; immune response; protein binding	Secretedextracellularspace			Known in Uniprot to be phosphorylated	Known in HPRD to be phosphorylated	
**1310**	GRLF1	GRLF1_HUMAN	GRLF1; A7E2A4	Q9NRY4; Q14452; Q9C0E1; A7E2A4	NP_004482	Glucocorticoid receptor DNA-binding factor 1 (Glucocorticoid receptor repression factor 1) (GRF-1) (Rho GAP p190A) (p190-A)	GO:0003677; GO:0005096; GO:0005737; GO:0045786; GO:0016481; GO:0005634; GO:0006355; GO:0007165; GO:0003714	DNA binding; GTPase activator activity; cytoplasm; negative regulation of cell cycle; negative regulation of transcription; nucleus; regulation of transcription, DNA-dependent; signal transduction; transcription corepressor activity	CytoplasmNucleus			Known in Uniprot to be phosphorylated	Known in HPRD to be phosphorylated	
**1311**	CA167	CA167_HUMAN	Q5SNV9; CA167	Q5SNV9; Q8NDA9; Q8NDF3	XP_209234	Uncharacterized protein C1orf167								
**1312**	DDX6L	DDX6L_HUMAN	Q5H9U9; DDX6L	Q5H9U9; Q96ND6	XP_037817; NP_001012985	Probable ATP-dependent RNA helicase DDX60-like (EC 3.6.1.-) (DEAD box protein 60-like)	GO:0005524; GO:0008026; GO:0003723	ATP binding; ATP-dependent helicase activity; RNA binding						
**1313**	S14L5	S14L5_HUMAN	O43304; S14L5	O43304	XP_032693; NP_055507	SEC14-like protein 5	GO:0005622; GO:0006810; GO:0005215	intracellular; transport; transporter activity						
**1314**	MET7A	MET7A_HUMAN	MET7A	Q9H7R3; Q9H8H3; Q9UHZ7; Q9Y422	NP_054752	Methyltransferase-like protein 7A (EC 2.1.1.-) (Protein AAM-B)	GO:0008152; GO:0008168	metabolic process; methyltransferase activity						
**1315**	DLDH	DLDH_HUMAN	Q8WTS4; DLDH; B2R5X0	P09622; Q8WTS4; Q14131; Q14167; Q59EV8; B2R5X0	NP_000099	Dihydrolipoyl dehydrogenase, mitochondrial (EC 1.8.1.4) (Dihydrolipoamide dehydrogenase) (Glycine cleavage system L protein)	GO:0050660; GO:0045454; GO:0004148; GO:0005759	FAD binding; cell redox homeostasis; dihydrolipoyl dehydrogenase activity; mitochondrial matrix	Mitochondrionmatrix					
**1316**	LRP1B	LRP1B_HUMAN	LRP1B; Q53TB8; Q53RA0; Q53RG4; Q53S26; Q580W7; Q53QP5	Q9NZR2; Q8WY29; Q8WY30; Q8WY31; Q53QP5; Q53RA0; Q53RG4; Q53S26; Q53TB8; Q580W7	NP_061027	Low-density lipoprotein receptor-related protein 1B (Low-density lipoprotein receptor-related protein-deleted in tumor) (LRP-DIT); Putative uncharacterized protein LRP1B (Fragment)	GO:0005509; GO:0016021; GO:0015031; GO:0006898; GO:0005041; GO:0016020; GO:0005624; GO:0005041	calcium ion binding; integral to membrane; protein transport; receptor-mediated endocytosis; low-density lipoprotein receptor activity; membrane; membrane fraction; low-density lipoprotein receptor activity	MembraneSinglepasstypeImembraneprotein;					
**1317**	SPTCS	SPTCS_HUMAN	Q96JI7; Q4VC11; SPTCS	Q96JI7; A8KAX9; Q4VC11; Q58G86; Q69YG6; Q6NW01; Q8N270; Q8TBU9; Q9H734	NP_079413	Spatacsin (Spastic paraplegia 11 protein) (Colorectal carcinoma-associated protein)	GO:0005737; GO:0016021; GO:0005634	cytoplasm; integral to membrane; nucleus	MembraneMultipassmembraneproteinCytoplasmcytosolNucleus			Known in Uniprot to be phosphorylated		

	Protein Name	Uniprot Name	Other Names	Uniprot IDs	Refseq IDs	Protein Description	GO ID	GO terms	Subcellular location	Comments	Comments	Comments - Database Search	Comments - Database Search	Function
**1318**	MD12L	MD12L_HUMAN	Q86YW9; MD12L	Q86YW9; Q96PC7; Q96PC8; Q9H9M5; Q9HCD7; Q9UI69	NP_443728	Mediator of RNA polymerase II transcription subunit 12-like protein (Mediator complex subunit 12-like protein) (Thyroid hormone receptor-associated-like protein) (Trinucleotide repeat-containing gene 11 protein-like)	GO:0005634; GO:0006355; GO:0030528	nucleus; regulation of transcription, DNA-dependent; transcription regulator activity	Nucleus			Known in Uniprot to be phosphorylated		
**1319**	SCN4A	SCN4A_HUMAN	SCN4A	P35499; Q15478; Q16447; Q7Z6B1	NP_000325	Sodium channel protein type 4 subunit alpha (Sodium channel protein type IV subunit alpha) (Voltage-gated sodium channel subunit alpha Nav1.4) (Sodium channel protein skeletal muscle subunit alpha) (SkM1)	GO:0006936; GO:0031402; GO:0006814; GO:0005248; GO:0001518	muscle contraction; sodium ion binding; sodium ion transport; voltage-gated sodium channel activity; voltage-gated sodium channel complex	MembraneMultipassmembraneprotein			Known in Uniprot to be phosphorylated		
**1320**	TDRD4	TDRD4_HUMAN	TDRD4; Q5T2J9; RNF17	Q9BXT8; Q9NUY9; Q5T2J9; Q6P1W3; Q9BXT7	NP_112567	RING finger protein 17 (Tudor domain-containing protein 4)	GO:0005737; GO:0016788; GO:0007275; GO:0003676; GO:0005634; GO:0008270	cytoplasm; hydrolase activity, acting on ester bonds; multicellular organismal development; nucleic acid binding; nucleus; zinc ion binding	CytoplasmNucleus					
**1321**	RPIA	RPIA_HUMAN	RPIA	P49247; Q541P9; Q96BJ6	NP_653164	Ribose-5-phosphate isomerase (EC 5.3.1.6) (Phosphoriboisomerase)	GO:0005829; GO:0009052; GO:0004751	cytosol; pentose-phosphate shunt, non-oxidative branch; ribose-5-phosphate isomerase activity						
**1322**	U773	U773_HUMAN	U773; ZG16B	Q96DA0; A6NIY1; B2R4F6; Q6UW28	NP_660295	Zymogen granule protein 16 homolog B	GO:0005576; GO:0005529	extracellular region; sugar binding	Secreted					
**1323**	HCDH	HCDH_HUMAN	HCDH	Q16836; O00324; O00397; O00753	NP_005318	Hydroxyacyl-coenzyme A dehydrogenase, mitochondrial (HCDH) (EC 1.1.1.35) (Short chain 3-hydroxyacyl-CoA dehydrogenase) (Medium and short chain L-3-hydroxyacyl-coenzyme A dehydrogenase)	GO:0003857; GO:0050662; GO:0006631; GO:0005759; GO:0055114	3-hydroxyacyl-CoA dehydrogenase activity; coenzyme binding; fatty acid metabolic process; mitochondrial matrix; oxidation reduction	Mitochondrionmatrix					
**1324**	RUFY1	RUFY1_HUMAN	RUFY1	Q96T51; Q59FF3; Q71S93; Q9H6I3	NP_079434; NP_001035541; NP_001035542	RUN and FYVE domain-containing protein 1 (FYVE-finger protein EIP1) (Zinc finger FYVE domain-containing protein 12) (La-binding protein 1) (Rab4-interacting protein)	GO:0031901; GO:0006897; GO:0008289; GO:0015031; GO:0008270	early endosome membrane; endocytosis; lipid binding; protein transport; zinc ion binding	CytoplasmEarlyendosomemembranePeripheralmembraneprotein			Known in Uniprot to be phosphorylated		
**1325**	HPSE	HPSE_HUMAN	HPSE	Q9Y251; Q53GE5; Q9UL39	NP_006656; NP_001092010	Heparanase (EC 3.2.-.-) (Heparanase-1) (Hpa1) (Endo-glucoronidase) [Cleaved into: Heparanase 8 kDa subunit; Heparanase 50 kDa subunit]	GO:0004566; GO:0005509; GO:0005576; GO:0005765; GO:0000287; GO:0006029	beta-glucuronidase activity; calcium ion binding; extracellular region; lysosomal membrane; magnesium ion binding; proteoglycan metabolic process	LysosomemembranePeripheralmembraneproteinSecreted					
**1326**	ARTS1	ARTS1_HUMAN	ARTS1; ERAP1	Q9NZ08; O60278; Q6UWY6; Q8NEL4; Q8TAD0; Q9UHF8; Q9UKY2	NP_057526; NP_001035548	Endoplasmic reticulum aminopeptidase 1 (EC 3.4.11.-) (Adipocyte-derived leucine aminopeptidase) (A-LAP) (ARTS-1) (Aminopeptidase PILS) (Puromycin-insensitive leucyl-specific aminopeptidase) (PILS-AP) (Type 1 tumor necrosis factor receptor shedding aminopeptidase regulator)	GO:0004177; GO:0019885; GO:0005829; GO:0005788; GO:0005789; GO:0005576; GO:0045444; GO:0016021; GO:0005151; GO:0005138; GO:0006509; GO:0008235; GO:0008217; GO:0045088; GO:0009617; GO:0008270	aminopeptidase activity; antigen processing and presentation of endogenous peptide antigen via MHC class I; cytosol; endoplasmic reticulum lumen; endoplasmic reticulum membrane; extracellular region; fat cell differentiation; integral to membrane; interleukin-1, Type II receptor binding; interleukin-6 receptor binding; membrane protein ectodomain proteolysis; metalloexopeptidase activity; regulation of blood pressure; regulation of innate immune response; response to bacterium; zinc ion binding	EndoplasmicreticulummembraneSinglepasstypeIImembraneprotein					
**1327**	ITPR2	ITPR2_HUMAN	ITPR2	Q14571; O94773	NP_002214	Inositol 1,4,5-trisphosphate receptor type 2 (Type 2 inositol 1,4,5-trisphosphate receptor) (Type 2 InsP3 receptor) (IP3 receptor isoform 2) (InsP3R2)	GO:0005509; GO:0015085; GO:0006816; GO:0005789; GO:0005220; GO:0008095; GO:0016021; GO:0005886; GO:0001666; GO:0007165	calcium ion binding; calcium ion transmembrane transporter activity; calcium ion transport; endoplasmic reticulum membrane; inositol 1,4,5-triphosphate-sensitive calcium-release channel activity; inositol-1,4,5-triphosphate receptor activity; integral to membrane; plasma membrane; response to hypoxia; signal transduction	EndoplasmicreticulummembraneMultipassmembraneprotein			Known in Uniprot to be phosphorylated		
**1328**	RAB6B	RAB6B_HUMAN	RAB6B; B2R5Z9; Q6AZ91	Q6AZ91; Q9NRW1; B2R5Z9	NP_057661	Ras-related protein Rab-6B	GO:0005525; GO:0003924; GO:0000139; GO:0015031; GO:0006890; GO:0007264	GTP binding; GTPase activity; Golgi membrane; protein transport; retrograde vesicle-mediated transport, Golgi to ER; small GTPase mediated signal transduction	GolgiapparatusmembraneLipidanchor					
**1329**	ISOC1	ISOC1_HUMAN	ISOC1; Q9Y3B5	Q9Y3B5; Q96CN7; Q7Z770	NP_057132	Isochorismatase domain-containing protein 1	GO:0003824; GO:0008152; GO:0005777	catalytic activity; metabolic process; peroxisome						
**1330**	FLNB	FLNB_HUMAN	FLNB; Q60FE7; B2ZZ85	O75369; Q13706; Q59EC2; Q6MZJ1; Q8WXS9; Q8WXT0; Q8WXT1; Q8WXT2; Q9NRB5; Q9NT26; Q9UEV9; B2ZZ85; Q60FE7	NP_001448	Filamin-B (FLN-B) (Beta-filamin) (Actin-binding-like protein) (Thyroid autoantigen) (Truncated actin-binding protein) (Truncated ABP) (ABP-280 homolog) (ABP-278) (Filamin 3) (Filamin homolog 1) (Fh1); Filamin B	GO:0003779; GO:0015629; GO:0030036; GO:0030154; GO:0005737; GO:0007016; GO:0016021; GO:0005634; GO:0007165; GO:0007519	actin binding; actin cytoskeleton; actin cytoskeleton organization; cell differentiation; cytoplasm; cytoskeletal anchoring at plasma membrane; integral to membrane; nucleus; signal transduction; skeletal muscle tissue development	CytoplasmcellcortexCytoplasmcytoskeletonCytoplasmmyofibrilsarcomereZdiskCytoplasmcytoskeletonCytoplasmcytoskeletonCytoplasmcytoskeleton;			Known in Uniprot to be phosphorylated		
**1331**	APOB	APOB_HUMAN	APOB; Q59HB3; Q7Z7Q0	P04114; O00502; P78479; P78480; P78481; Q13779; Q13785; Q13786; Q13787; Q13788; Q7Z600; Q9UMN0; Q59HB3; Q7Z7Q0	NP_000375	Apolipoprotein B-100 (Apo B-100) [Cleaved into: Apolipoprotein B-48 (Apo B-48)]; Apolipoprotein B variant (Fragment); APOB protein	GO:0042632; GO:0030301; GO:0017127; GO:0034360; GO:0030669; GO:0005788; GO:0031904; GO:0010008; GO:0008201; GO:0034363; GO:0034362; GO:0034383; GO:0034374; GO:0050750; GO:0034359; GO:0005792; GO:0005543; GO:0005886; GO:0010744; GO:0045834; GO:0046982; GO:0034361; GO:0034379; GO:0048844; GO:0042632; GO:0005783; GO:0009566; GO:0001701; GO:0042158; GO:0042159; GO:0042953; GO:0007399; GO:0009791; GO:0045540; GO:0030317; GO:0007283; GO:0019433;	cholesterol homeostasis; cholesterol transport; cholesterol transporter activity; chylomicron remnant; clathrin-coated endocytic vesicle membrane; endoplasmic reticulum lumen; endosome lumen; endosome membrane; heparin binding; intermediate-density lipoprotein particle; low-density lipoprotein particle; low-density lipoprotein particle clearance; low-density lipoprotein particle remodeling; low-density lipoprotein receptor binding; mature chylomicron; microsome; phospholipid binding; plasma membrane; positive regulation of foam cell differentiation; positive regulation of lipid metabolic process; protein heterodimerization activity; very-low-density lipoprotein particle; very-low-density lipoprotein particle assembly; artery morphogenesis; cholesterol homeostasis; endoplasmic reticulum; fertilization; in utero embryonic development; lipoprotein biosynthetic process; lipoprotein catabolic process; lipoprotein transport; nervous system development; post-embryonic development; regulation of cholesterol biosynthetic process; sperm motility; spermatogenesis; triglyceride catabolic process; triglyceride mobilization; lipid transport; lipid transporter activity	Secreted;			Known in Uniprot to be phosphorylated		
**1332**	PDE6B	PDE6B_HUMAN	PDE6B	P35913; Q53XN5; Q9BWH5	NP_000274; NP_001138763	Rod cGMP-specific 3',5'-cyclic phosphodiesterase subunit beta (GMP-PDE beta) (EC 3.1.4.35)	GO:0047555; GO:0016020; GO:0007603; GO:0007165	3',5'-cyclic-GMP phosphodiesterase activity; membrane; phototransduction, visible light; signal transduction	MembraneLipidanchor					
**1333**	UBP44	UBP44_HUMAN	UBP44	Q9H0E7; B2RDW3	NP_115523	Ubiquitin carboxyl-terminal hydrolase 44 (EC 3.1.2.15) (Ubiquitin thioesterase 44) (Ubiquitin-specific-processing protease 44) (Deubiquitinating enzyme 44)	GO:0008234; GO:0005634; GO:0004221; GO:0006511; GO:0008270	cysteine-type peptidase activity; nucleus; ubiquitin thiolesterase activity; ubiquitin-dependent protein catabolic process; zinc ion binding						
**1334**	OTUB1	OTUB1_HUMAN	OTUB1; B3KUV5	Q96FW1; Q32Q78; Q96II3; Q9NXQ4; Q9P0B8; B3KUV5	NP_060140	Ubiquitin thioesterase OTUB1 (EC 3.1.2.-) (Otubain-1) (OTU domain-containing ubiquitin aldehyde-binding protein 1) (Ubiquitin-specific-processing protease OTUB1) (Deubiquitinating enzyme OTUB1)	GO:0008234; GO:0006955; GO:0019941	cysteine-type peptidase activity; immune response; modification-dependent protein catabolic process				Known in Uniprot to be phosphorylated		
**1335**	SYAC	SYAC_HUMAN	SYAC	P49588; A6NF14; Q53GV7; Q96FA0	NP_001596	Alanyl-tRNA synthetase, cytoplasmic (EC 6.1.1.7) (Alanine--tRNA ligase) (AlaRS) (Renal carcinoma antigen NY-REN-42)	GO:0005524; GO:0004813; GO:0006419; GO:0005737; GO:0005625; GO:0000049	ATP binding; alanine-tRNA ligase activity; alanyl-tRNA aminoacylation; cytoplasm; soluble fraction; tRNA binding	Cytoplasm			Known in Uniprot to be phosphorylated		
**1336**	CE290	CE290_HUMAN	CE290; Q05BJ6	Q66GS8; O15078; Q1PSK5; Q9H2G6; Q9H6Q7; Q9H8I0; Q05BJ6	NP_079390	Centrosomal protein of 290 kDa (Cep290) (Nephrocystin-6) (Tumor antigen se2-2) (Cancer/testis antigen 87) (CT87); CEP290 protein	GO:0030030; GO:0009986; GO:0005813; GO:0005829; GO:0042462; GO:0030902; GO:0005634; GO:0030916; GO:0032391; GO:0048793; GO:0005515; GO:0015031; GO:0016563; GO:0005813	cell projection organization; cell surface; centrosome; cytosol; eye photoreceptor cell development; hindbrain development; nucleus; otic vesicle formation; photoreceptor connecting cilium; pronephros development; protein binding; protein transport; transcription activator activity; centrosome	CentrosomeNucleusCellprojectioncilium;			Known in Uniprot to be phosphorylated		
**1337**	SUCA	SUCA_HUMAN	SUCA	P53597; Q9BWB0; Q9UNP6	NP_003840	Succinyl-CoA ligase [GDP-forming] subunit alpha, mitochondrial (EC 6.2.1.4) (Succinyl-CoA synthetase subunit alpha) (SCS-alpha)	GO:0003878; GO:0005525; GO:0005759; GO:0004775; GO:0004776; GO:0006099	ATP citrate synthase activity; GTP binding; mitochondrial matrix; succinate-CoA ligase (ADP-forming) activity; succinate-CoA ligase (GDP-forming) activity; tricarboxylic acid cycle	Mitochondrion			Known in Uniprot to be phosphorylated		
**1338**	PTPRF	PTPRF_HUMAN	PTPRF; Q86WS0; Q5T022	P10586; Q5T021; Q5T022; Q5W9G2; Q86WS0	NP_002831; NP_569707	Receptor-type tyrosine-protein phosphatase F (EC 3.1.3.48) (Leukocyte common antigen related) (LAR)	GO:0007155; GO:0005887; GO:0006470; GO:0005515; GO:0005001; GO:0007185	cell adhesion; integral to plasma membrane; protein amino acid dephosphorylation; protein binding; transmembrane receptor protein tyrosine phosphatase activity; transmembrane receptor protein tyrosine phosphatase signaling pathway	MembraneSinglepasstypeImembraneprotein				Known in HPRD to be phosphorylated	Phosphatase

	Protein Name	Uniprot Name	Other Names	Uniprot IDs	Refseq IDs	Protein Description	GO ID	GO terms	Subcellular location	Comments	Comments	Comments - Database Search	Comments - Database Search	Function
**1339**	BDP1	BDP1_HUMAN	Q9C0H4; BDP1	Q9HCY0; Q9C0H4; A6H8Y1; Q68DS6; Q68DY5; Q6MZL9; Q6PIM7; Q86W98; Q96LR8; Q9H197; Q9H1A1; Q9HAW1; Q9HAW2; Q9ULH9	NP_060899	Transcription factor TFIIIB component B'' homolog (Transcription factor IIIB 150) (TFIIIB150) (Transcription factor-like nuclear regulator)	GO:0003677; GO:0005634; GO:0006355	DNA binding; nucleus; regulation of transcription, DNA-dependent	Nucleus			Known in Uniprot to be phosphorylated		
**1340**	SRP72	SRP72_HUMAN	SRP72; Q7Z3C0; Q71V07	Q71V07; O76094; Q7Z3C0	NP_008878	Signal recognition particle 72 kDa protein (SRP72); Putative uncharacterized protein DKFZp686A04192 (Fragment)	GO:0008312; GO:0006614; GO:0005786; GO:0048500	7S RNA binding; SRP-dependent cotranslational protein targeting to membrane; signal recognition particle, endoplasmic reticulum targeting; signal recognition particle	Cytoplasm;			Known in Uniprot to be phosphorylated		
**1341**	PP2CA	PP2CA_HUMAN	PP2CA; PPM1A; B5BU11	P35813; O75551; B5BU11	NP_066283; NP_808820	Protein phosphatase 1A (EC 3.1.3.16) (Protein phosphatase 2C isoform alpha) (PP2C-alpha) (IA)	GO:0000287; GO:0030145; GO:0043123; GO:0030177; GO:0045893; GO:0006470; GO:0004722; GO:0008287; GO:0004871	magnesium ion binding; manganese ion binding; positive regulation of I-kappaB kinase/NF-kappaB cascade; positive regulation of Wnt receptor signaling pathway; positive regulation of transcription, DNA-dependent; protein amino acid dephosphorylation; protein serine/threonine phosphatase activity; protein serine/threonine phosphatase complex; signal transducer activity				Known in Uniprot to be phosphorylated		Phosphatase
**1342**	HNRDL	HNRDL_HUMAN	O14979; HNRDL	O14979; Q6SPF2; Q7KZ74; Q7KZ75; Q96IM0; Q96S43	NP_005454; NP_112740	Heterogeneous nuclear ribonucleoprotein D-like (hnRPD-like protein) (hnHNRP-DL) (JKT41-binding protein) (AU-rich element RNA-binding factor) (Protein laAUF1)	GO:0006396; GO:0005737; GO:0003690; GO:0030530; GO:0000166; GO:0008143; GO:0005515; GO:0006355; GO:0003697	RNA processing; cytoplasm; double-stranded DNA binding; heterogeneous nuclear ribonucleoprotein complex; nucleotide binding; poly(A) binding; protein binding; regulation of transcription, DNA-dependent; single-stranded DNA binding	NucleusCytoplasm			Known in Uniprot to be phosphorylated	Known in HPRD to be phosphorylated	
**1343**	HNRPK	HNRPK_HUMAN	HNRPK; Q6IBN1	Q6IBN1; P61978; Q07244; Q15671; Q59F98; Q5T6W4; Q60577; Q922Y7; Q96J62	NP_002131; NP_112552; NP_112553	Heterogeneous nuclear ribonucleoprotein K (hnRNP K) (Transformation up-regulated nuclear protein) (TUNP); HNRPK protein (Heterogeneous nuclear ribonucleoprotein K) (Heterogeneous nuclear ribonucleoprotein K isoform a) (cDNA FLJ77137, highly similar to Homo sapiens heterogeneous nuclear ribonucleoprotein K (HNRPK), transcript variant 1, mRNA) (Heterogeneous nuclear ribonucleoprotein K, isoform CRA_c)	GO:0003677; GO:0003723; GO:0005737; GO:0030530; GO:0044419; GO:0000398; GO:0005654; GO:0005515; GO:0007165; GO:0005681; GO:0003723; GO:0005634; GO:0030529	DNA binding; RNA binding; cytoplasm; heterogeneous nuclear ribonucleoprotein complex; interspecies interaction between organisms; nuclear mRNA splicing, via spliceosome; nucleoplasm; protein binding; signal transduction; spliceosome; RNA binding; nucleus; ribonucleoprotein complex	CytoplasmNucleusnucleoplasm;			Known in Uniprot to be phosphorylated	Known in HPRD to be phosphorylated	
**1344**	RASK	RASK_HUMAN	RASK; Q96FS0; A8K8Z5	P01116; P01118; Q96D10; A8K8Z5	NP_004976; NP_203524	GTPase KRas (K-Ras 2) (Ki-Ras) (c-K-ras) (c-Ki-ras) [Cleaved into: GTPase KRas, N-terminally processed]	GO:0005525; GO:0005622; GO:0005886; GO:0005515; GO:0007605	GTP binding; intracellular; plasma membrane; protein binding; sensory perception of sound	CellmembraneLipidanchorCytoplasmicside					
**1345**	RL34	RL34_HUMAN	RL34	P49207; Q6FG66; Q9BUZ2	XP_301350; NP_000986; NP_296374	60S ribosomal protein L34	GO:0003723; GO:0022625; GO:0005515; GO:0003735; GO:0006414	RNA binding; cytosolic large ribosomal subunit; protein binding; structural constituent of ribosome; translational elongation				Known in Uniprot to be phosphorylated		
**1346**	CDC42	CDC42_HUMAN	CDC42	P60953; P21181; P25763; Q7L8R5	NP_001782; NP_001034891; NP_426359	Cell division control protein 42 homolog (G25K GTP-binding protein)	GO:0005525; GO:0003924; GO:0030036; GO:0005829; GO:0007163; GO:0030175; GO:0030225; GO:0031333; GO:0005886; GO:0031274; GO:0005515; GO:0007264	GTP binding; GTPase activity; actin cytoskeleton organization; cytosol; establishment or maintenance of cell polarity; filopodium; macrophage differentiation; negative regulation of protein complex assembly; plasma membrane; positive regulation of pseudopodium assembly; protein binding; small GTPase mediated signal transduction	CellmembraneLipidanchorCytoplasmicside					
**1347**	RL6	RL6_HUMAN	RL6	Q02878; Q2M3Q3; Q8WW97	XP_294006; NP_000961; NP_001019833	60S ribosomal protein L6 (TAX-responsive enhancer element-binding protein 107) (TAXREB107) (Neoplasm-related protein C140)	GO:0003677; GO:0003723; GO:0022625; GO:0006355; GO:0003735; GO:0006414	DNA binding; RNA binding; cytosolic large ribosomal subunit; regulation of transcription, DNA-dependent; structural constituent of ribosome; translational elongation				Known in Uniprot to be phosphorylated		
**1348**	CDK2	CDK2_HUMAN	CDK2; O75100	P24941; O75100	NP_439892; NP_001789	Cell division protein kinase 2 (EC 2.7.11.22) (p33 protein kinase); D-HSCDK2	GO:0005524; GO:0000086; GO:0051301; GO:0004693; GO:0000307; GO:0005829; GO:0042802; GO:0007067; GO:0005654; GO:0008284; GO:0006468; GO:0006275; GO:0007089; GO:0004674	ATP binding; G2/M transition of mitotic cell cycle; cell division; cyclin-dependent protein kinase activity; cyclin-dependent protein kinase holoenzyme complex; cytosol; identical protein binding; mitosis; nucleoplasm; positive regulation of cell proliferation; protein amino acid phosphorylation; regulation of DNA replication; traversing start control point of mitotic cell cycle; protein serine/threonine kinase activity				Known in Uniprot to be phosphorylated	Known in HPRD to be phosphorylated	Kinase
**1349**	PSIP1	PSIP1_HUMAN	PSIP1	O75475; O00256; O95368; Q6P391; Q86YB9; Q9NZI3; Q9UER6	NP_066967; NP_001121689; NP_150091	PC4 and SFRS1-interacting protein (Lens epithelium-derived growth factor) (Transcriptional coactivator p75/p52) (Dense fine speckles 70 kDa protein) (DFS 70) (CLL-associated antigen KW-7)	GO:0003677; GO:0005829; GO:0019059; GO:0044419; GO:0005634; GO:0019047; GO:0006355	DNA binding; cytosol; initiation of viral infection; interspecies interaction between organisms; nucleus; provirus integration; regulation of transcription, DNA-dependent	Nucleus			Known in Uniprot to be phosphorylated	Known in HPRD to be phosphorylated	
**1350**	ASSY	ASSY_HUMAN	ASSY	P00966; Q6LDL2; Q86UZ0; Q96GT4	XP_294215; NP_000041; NP_446464	Argininosuccinate synthase (EC 6.3.4.5) (Citrulline--aspartate ligase)	GO:0005524; GO:0006526; GO:0004055; GO:0005737; GO:0005515; GO:0000050	ATP binding; arginine biosynthetic process; argininosuccinate synthase activity; cytoplasm; protein binding; urea cycle				Known in Uniprot to be phosphorylated		
**1351**	FKB1A	FKB1A_HUMAN	FKB1A; Q4VC47; Q0VDC6	P62942; P20071; Q4VC47; Q6FGD9; Q6LEU3; Q9H103; Q9H566; Q0VDC6	NP_000792; NP_463460; NP_001011510	Peptidyl-prolyl cis-trans isomerase FKBP1A (PPIase FKBP1A) (EC 5.2.1.8) (FK506-binding protein 1A) (FKBP-1A) (Rotamase) (Immunophilin FKBP12) (12 kDa FKBP) (FKBP-12); FKBP1A protein	GO:0006458; GO:0005528; GO:0046332; GO:0007183; GO:0042110; GO:0048185; GO:0034205; GO:0005829; GO:0043206; GO:0060347; GO:0032513; GO:0003755; GO:0043123; GO:0032092; GO:0031398; GO:0022417; GO:0042026; GO:0032925; GO:0050776; GO:0060314; GO:0004871; GO:0014802; GO:0034713; GO:0055010; GO:0003755; GO:0006457	'de novo' protein folding; FK506 binding; SMAD binding; SMAD protein complex assembly; T cell activation; activin binding; beta-amyloid formation; cytosol; fibril organization; heart trabecula formation; negative regulation of protein phosphatase type 2B activity; peptidyl-prolyl cis-trans isomerase activity; positive regulation of I-kappaB kinase/NF-kappaB cascade; positive regulation of protein binding; positive regulation of protein ubiquitination; protein maturation via protein folding; protein refolding; regulation of activin receptor signaling pathway; regulation of immune response; regulation of ryanodine-sensitive calcium-release channel activity; signal transducer activity; terminal cisterna; type I transforming growth factor beta receptor binding; ventricular cardiac muscle morphogenesis; peptidyl-prolyl cis-trans isomerase activity; protein folding	Cytoplasm;					
**1352**	FKBP2	FKBP2_HUMAN	FKBP2; Q53XJ5	P26885; Q53XJ5; Q5BJH9; Q9BTS7	NP_004461; NP_001128680; NP_476433	FK506-binding protein 2 (EC 5.2.1.8) (Peptidyl-prolyl cis-trans isomerase) (PPIase) (Rotamase) (13 kDa FKBP) (FKBP-13); FK506 binding protein 2, 13kDa (FKBP2 protein) (FK506 binding protein 2, 13kDa, isoform CRA_a)	GO:0005528; GO:0005789; GO:0003755; GO:0005515; GO:0006457; GO:0003755	FK506 binding; endoplasmic reticulum membrane; peptidyl-prolyl cis-trans isomerase activity; protein binding; protein folding; peptidyl-prolyl cis-trans isomerase activity	EndoplasmicreticulummembranePeripheralmembraneprotein;					
**1353**	MVP	MVP_HUMAN	MVP	Q14764; Q96BG4; Q9BPW6; Q9BQT1; Q9UBD1	NP_005106; NP_059447	Major vault protein (MVP) (Lung resistance-related protein)	GO:0005737; GO:0065002; GO:0051028; GO:0005643; GO:0042493; GO:0030529	cytoplasm; intracellular protein transmembrane transport; mRNA transport; nuclear pore; response to drug; ribonucleoprotein complex	CytoplasmNucleusnuclearporecomplex			Known in Uniprot to be phosphorylated		
**1354**	HPCL1	HPCL1_HUMAN	HPCL1; Q6FGY1; O75544	P37235; Q6FGY1; Q969S5; O75544	NP_002140; NP_602293	Hippocalcin-like protein 1 (Visinin-like protein 3) (VILIP-3) (Calcium-binding protein BDR-1) (HLP2); BDP-1 protein (Fragment); cDNA FLJ78291, highly similar to Homo sapiens hippocalcin-like 1 (HPCAL1), transcript variant 2, mRNA (HPCAL1 protein) (Hippocalcin-like 1) (Hippocalcin-like 1, isoform CRA_a) (Putative uncharacterized protein HPCAL1)	GO:0005509; GO:0005515	calcium ion binding; protein binding						
**1355**	MK14	MK14_HUMAN	MK14	Q16539; A6ZJ92; A8K6P4; B0LPH0; O60776; Q13083; Q14084; Q8TDX0	NP_001306; NP_620581; NP_620583	Mitogen-activated protein kinase 14 (EC 2.7.11.24) (Mitogen-activated protein kinase p38 alpha) (MAP kinase p38 alpha) (Cytokine suppressive anti-inflammatory drug-binding protein) (CSAID-binding protein) (CSBP) (MAX-interacting protein 2) (MAP kinase MXI2) (SAPK2A)	GO:0005524; GO:0004708; GO:0008339; GO:0007265; GO:0006928; GO:0007166; GO:0006935; GO:0005829; GO:0005654; GO:0006468; GO:0005515; GO:0007243; GO:0006950	ATP binding; MAP kinase kinase activity; MP kinase activity; Ras protein signal transduction; cell motion; cell surface receptor linked signal transduction; chemotaxis; cytosol; nucleoplasm; protein amino acid phosphorylation; protein binding; protein kinase cascade; response to stress	CytoplasmNucleus			Known in Uniprot to be phosphorylated	Known in HPRD to be phosphorylated	Kinase
**1356**	BC11B	BC11B_HUMAN	BC11B	Q9C0K0; Q9H162	NP_075049; NP_612808	B-cell lymphoma/leukemia 11B (B-cell CLL/lymphoma 11B) (Radiation-induced tumor suppressor gene 1 protein) (hRit1) (COUP-TF-interacting protein 2)	GO:0003676; GO:0005634; GO:0006355; GO:0008270	nucleic acid binding; nucleus; regulation of transcription, DNA-dependent; zinc ion binding	Nucleus					

	Protein Name	Uniprot Name	Other Names	Uniprot IDs	Refseq IDs	Protein Description	GO ID	GO terms	Subcellular location	Comments	Comments	Comments - Database Search	Comments - Database Search	Function
**1357**	STAT3	STAT3_HUMAN	STAT3	P40763; O14916; Q9BW54	NP_003141; NP_644805; NP_998827	Signal transducer and activator of transcription 3 (Acute-phase response factor)	GO:0060397; GO:0005509; GO:0006928; GO:0032870; GO:0005737; GO:0005062; GO:0070102; GO:0044419; GO:0000122; GO:0007399; GO:0005634; GO:0019901; GO:0032355; GO:0003700; GO:0008134	JAK-STAT cascade involved in growth hormone signaling pathway; calcium ion binding; cell motion; cellular response to hormone stimulus; cytoplasm; hematopoietin/interferon-class (D200-domain) cytokine receptor signal transducer activity; interleukin-6-mediated signaling pathway; interspecies interaction between organisms; negative regulation of transcription from RNA polymerase II promoter; nervous system development; nucleus; protein kinase binding; response to estradiol stimulus; transcription factor activity; transcription factor binding	CytoplasmNucleus			Known in Uniprot to be phosphorylated	Known in HPRD to be phosphorylated	
**1358**	TNR6A	TNR6A_HUMAN	TNR6A	Q8NDV7; O15408; Q658L5; Q6NVB5; Q8NEZ0; Q8TBT8; Q8TCR0; Q9NV59; Q9P268	NP_055309	Trinucleotide repeat-containing gene 6A protein (CAG repeat protein 26) (Glycine-tryptophan protein of 182 kDa) (GW182 autoantigen) (Protein GW1) (EMSY interactor protein)	GO:0003723; GO:0005737; GO:0000166	RNA binding; cytoplasm; nucleotide binding	CytoplasmPbody			Known in Uniprot to be phosphorylated		
**1359**	AKAP9	AKAP9_HUMAN	AKAP9; A4D1F2; Q6PJH3	Q99996; A4D1F0; A4D1F2; A4D1F4; O14869; O43355; O94895; Q75N20; Q9UQH3; Q9UQQ4; Q9Y6B8; Q9Y6Y2; Q6PJH3	NP_005742; NP_671714	A-kinase anchor protein 9 (Protein kinase A-anchoring protein 9) (PRKA9) (A-kinase anchor protein 450 kDa) (AKAP 450) (A-kinase anchor protein 350 kDa) (AKAP 350) (hgAKAP 350) (AKAP 120-like protein) (Protein hyperion) (Protein yotiao) (Centrosome- and Golgi-localized PKN-associated protein) (CG-NAP)	GO:0005794; GO:0005813; GO:0005102; GO:0007165; GO:0007268; GO:0006810	Golgi apparatus; centrosome; receptor binding; signal transduction; synaptic transmission; transport	CytoplasmCentrosome Golgiapparatus			Known in Uniprot to be phosphorylated		Kinase
**1360**	PDC10	PDC10_HUMAN	PDC10; A8K515	Q9BUL8; O14811; A8K515	NP_009148; NP_665858; NP_665859	Programmed cell death protein 10 (TF-1 cell apoptosis-related protein 15) (Cerebral cavernous malformations 3 protein)	GO:0006915; GO:0005515	apoptosis; protein binding						
**1361**	CPNE1	CPNE1_HUMAN	CPNE1; B0QZ18	Q99829; Q6IBL3; Q9H243; Q9NTZ7; B0QZ18	NP_003906; NP_690902; NP_690903; NP_690904; NP_690905; NP_690906; NP_690907; NP_690908	Copine-1 (Copine I)	GO:0005544; GO:0006629; GO:0001786; GO:0005215; GO:0016192	calcium-dependent phospholipid binding; lipid metabolic process; phosphatidylserine binding; transporter activity; vesicle-mediated transport						
**1362**	UBE1	UBE1_HUMAN	UBE1; Q5JRR8; UBA1	P22314; Q5JRR8; Q96E13	NP_003325; NP_695012	Ubiquitin-like modifier-activating enzyme 1 (Ubiquitin-activating enzyme E1) (Protein A1S9)	GO:0005524; GO:0005737; GO:0016874; GO:0019941; GO:0005634; GO:0005515; GO:0006464; GO:0008641	ATP binding; cytoplasm; ligase activity; modification-dependent protein catabolic process; nucleus; protein binding; protein modification process; small protein activating enzyme activity				Known in Uniprot to be phosphorylated	Known in HPRD to be phosphorylated	
**1363**	RBM12	RBM12_HUMAN	RBM12	Q9NTZ6; O94865; Q8N3B1; Q9H196	XP_305804; NP_006038; NP_690051	RNA-binding protein 12 (RNA-binding motif protein 12) (SH3/WW domain anchor protein in the nucleus) (SWAN)	GO:0003723; GO:0000166; GO:0005634; GO:0005515	RNA binding; nucleotide binding; nucleus; protein binding	Nucleus			Known in Uniprot to be phosphorylated	Known in HPRD to be phosphorylated	
**1364**	BDNF	BDNF_HUMAN	BDNF; Q6YNR3; Q598Q1	P23560; Q598Q1; Q9BYY7; Q9UC24; Q6YNR3	NP_001700; NP_001137277; NP_001137278; NP_001137279; NP_001137280; NP_001137283; NP_001137284; NP_001137285; NP_001137286; NP_001137287; NP_001137288; NP_733928; NP_733929; NP_733931; NP_733927	Brain-derived neurotrophic factor (BDNF) (Abrineurin); Brain derived neurotrophic factor transcript III (Brain derived neurotrophic factor transcript IIa) (Brain derived neurotrophic factor transcript IIb) (Brain derived neurotrophic factor transcript IIc) (Brain derived neurotrophic factor transcript IV) (Brain-derived neurotrophic factor BDNF2A) (Brain-derived neurotrophic factor BDNF2B) (Brain-derived neurotrophic factor BDNF2C) (Brain derived neurotrophic factor variant) (Brain-derived neurotrophic factor); Brain derived neurotrophic factor variant (Brain-derived neurotrophic factor BDNF1) (Brain-derived neurotrophic factor, isoform CRA_c) (cDNA FLJ77015, highly similar to Homo sapiens brain-derived neurotrophic factor (BDNF), transcript variant 3, mRNA)	GO:0005576; GO:0008083; GO:0006916; GO:0007411; GO:0007412; GO:0016023; GO:0016358; GO:0042596; GO:0007631; GO:0014047; GO:0048839; GO:0007611; GO:0042490; GO:0007406; GO:0043524; GO:0021675; GO:0008038; GO:0045666; GO:0019222; GO:0046668; GO:0048167; GO:0042493; GO:0001657	extracellular region; growth factor activity; anti-apoptosis; axon guidance; axon target recognition; cytoplasmic membrane-bounded vesicle; dendrite development; fear response; feeding behavior; glutamate secretion; inner ear development; learning or memory; mechanoreceptor differentiation; negative regulation of neuroblast proliferation; negative regulation of neuron apoptosis; nerve development; neuron recognition; positive regulation of neuron differentiation; regulation of metabolic process; regulation of retinal cell programmed cell death; regulation of synaptic plasticity; response to drug; ureteric bud development	Secreted;					
**1365**	RAB37	RAB37_HUMAN	RAB37; Q8IWA7	Q8IWA7; Q96AX2	NP_783865; NP_001006639	Ras-related protein Rab-37; RAB37, member RAS oncogene family (RAB37, member RAS oncogene family, isoform CRA_d) (cDNA FLJ75617, highly similar to Homo sapiens RAB37, member RAS oncogene family (RAB37), transcript variant 3, mRNA)	GO:0005793; GO:0005525; GO:0031410; GO:0015031; GO:0007264; GO:0005525	ER-Golgi intermediate compartment; GTP binding; cytoplasmic vesicle; protein transport; small GTPase mediated signal transduction; GTP binding	Cytoplasmicvesicle;					
**1366**	ST1A3	ST1A3_HUMAN	ST1A3; Q1ET61	P50224; B4DNV0; O95603; Q6ZWJ5; Q1ET61	NP_003157; NP_001017389; NP_001017390; NP_808220	Sulfotransferase 1A3/1A4 (EC 2.8.2.1) (Monoamine-sulfating phenol sulfotransferase) (Aryl sulfotransferase 1A3/1A4) (Sulfotransferase, monoamine-preferring) (M-PST) (Thermolabile phenol sulfotransferase) (TL-PST) (Placental estrogen sulfotransferase) (Catecholamine-sulfating phenol sulfotransferase) (HAST3); Phenol sulfotransferase 1A5*1A (Phenol sulfotransferase 1A5*1B) (Sulfotransferase family, cytosolic, 1A, phenol-preferring, member 3) (cDNA FLJ16185 fis, clone BRTHA2005864, highly similar to Monoamine sulfating phenol sulfotransferase (EC 2.8.2.1))	GO:0004062; GO:0006584; GO:0005737; GO:0008202; GO:0008146	aryl sulfotransferase activity; catecholamine metabolic process; cytoplasm; steroid metabolic process; sulfotransferase activity	Cytoplasm;					
**1367**	PRDX1	PRDX1_HUMAN	PRDX1; B5BU26	Q06830; P35703; Q2V576; Q5T154; Q5T155; B5BU26	NP_002565; NP_859047; NP_859048	Peroxiredoxin-1 (EC 1.11.1.15) (Thioredoxin peroxidase 2) (Thioredoxin-dependent peroxide reductase 2) (Proliferation-associated gene protein) (PAG) (Natural killer cell-enhancing factor A) (NKEF-A)	GO:0008283; GO:0045454; GO:0042744; GO:0042470; GO:0005634; GO:0055114; GO:0051920; GO:0005515; GO:0001501	cell proliferation; cell redox homeostasis; hydrogen peroxide catabolic process; melanosome; nucleus; oxidation reduction; peroxiredoxin activity; protein binding; skeletal system development	CytoplasmMelanosome			Known in Uniprot to be phosphorylated		
**1368**	GEM	GEM_HUMAN	GEM; B2RA31	P55040; B2RA31	NP_005252; NP_859053	GTP-binding protein GEM (GTP-binding mitogen-induced T-cell protein) (RAS-like protein KIR)	GO:0019003; GO:0005525; GO:0003924; GO:0005516; GO:0007166; GO:0006955; GO:0009898; GO:0000287; GO:0007264	GDP binding; GTP binding; GTPase activity; calmodulin binding; cell surface receptor linked signal transduction; immune response; internal side of plasma membrane; magnesium ion binding; small GTPase mediated signal transduction	CellmembranePeripheralmembraneproteinCytoplasmicside			Known in Uniprot to be phosphorylated	Known in HPRD to be phosphorylated	
**1369**	UB2D3	UB2D3_HUMAN	UB2D3	P61077; A6NM99; P47986; Q6IB88; Q6NXS4	NP_003331; NP_871615; NP_871616; NP_871617; NP_871618; NP_871619; NP_871620; NP_871622	Ubiquitin-conjugating enzyme E2 D3 (EC 6.3.2.19) (Ubiquitin-protein ligase D3) (Ubiquitin carrier protein D3) (Ubiquitin-conjugating enzyme E2-17 kDa 3) (E2(17)KB 3)	GO:0030509; GO:0005515; GO:0016567; GO:0051246; GO:0006511; GO:0004842	BMP signaling pathway; protein binding; protein ubiquitination; regulation of protein metabolic process; ubiquitin-dependent protein catabolic process; ubiquitin-protein ligase activity						
**1370**	PCNA	PCNA_HUMAN	PCNA; B2R897	P12004; B2R897	NP_002583; NP_872590	Proliferating cell nuclear antigen (PCNA) (Cyclin)	GO:0030337; GO:0005663; GO:0032405; GO:0043626; GO:0008283; GO:0032139; GO:0006298; GO:0005654; GO:0006297; GO:0048015; GO:0000701; GO:0006275	DNA polymerase processivity factor activity; DNA replication factor C complex; MutLalpha complex binding; PCNA complex; cell proliferation; dinucleotide insertion or deletion binding; mismatch repair; nucleoplasm; nucleotide-excision repair, DNA gap filling; phosphoinositide-mediated signaling; purine-specific mismatch base pair DNA N-glycosylase activity; regulation of DNA replication	Nucleus					
**1371**	RNF14	RNF14_HUMAN	RNF14; B7ZLV2	Q9UBS8; A0AV26; A6NMR2; B3KN72; O94793; Q6IBV0; B7ZLV2	NP_004281; NP_899646; NP_899647; NP_899648	E3 ubiquitin-protein ligase RNF14 (EC 6.3.2.-) (RING finger protein 14) (Androgen receptor-associated protein 54) (Triad2 protein) (HFB30)	GO:0050681; GO:0030521; GO:0005737; GO:0019941; GO:0005634; GO:0045893; GO:0016567; GO:0006357; GO:0032355; GO:0019787; GO:0003713; GO:0008270	androgen receptor binding; androgen receptor signaling pathway; cytoplasm; modification-dependent protein catabolic process; nucleus; positive regulation of transcription, DNA-dependent; protein ubiquitination; regulation of transcription from RNA polymerase II promoter; response to estradiol stimulus; small conjugating protein ligase activity; transcription coactivator activity; zinc ion binding	CytoplasmNucleus			Known in Uniprot to be phosphorylated		
**1372**	SRC	SRC_HUMAN	SRC	P12931; Q86VB9; Q9H5A8	NP_005408; NP_938033	Proto-oncogene tyrosine-protein kinase Src (EC 2.7.10.2) (pp60c-src) (p60-Src) (c-Src)	GO:0005524; GO:0007265; GO:0042169; GO:0005070; GO:0005901; GO:0005829; GO:0044419; GO:0004715; GO:0007243; GO:0007172	ATP binding; Ras protein signal transduction; SH2 domain binding; SH3/SH2 adaptor activity; caveola; cytosol; interspecies interaction between organisms; non-membrane spanning protein tyrosine kinase activity; protein kinase cascade; signal complex assembly				Known in Uniprot to be phosphorylated	Known in HPRD to be phosphorylated	Kinase
**1373**	RAB6A	RAB6A_HUMAN	RAB6A	P20340; A8K133; Q5U0A8; Q9UBE4	NP_002860; NP_942599	Ras-related protein Rab-6A (Rab-6)	GO:0005525; GO:0003924; GO:0000139; GO:0018125; GO:0005515; GO:0015031; GO:0007264; GO:0016192	GTP binding; GTPase activity; Golgi membrane; peptidyl-cysteine methylation; protein binding; protein transport; small GTPase mediated signal transduction; vesicle-mediated transport	GolgiapparatusmembraneLipidanchor			Known in Uniprot to be phosphorylated		

	Protein Name	Uniprot Name	Other Names	Uniprot IDs	Refseq IDs	Protein Description	GO ID	GO terms	Subcellular location	Comments	Comments	Comments - Database Search	Comments - Database Search	Function
**1374**	K1C18	K1C18_HUMAN	K1C18	P05783; Q53G38; Q5U0N8; Q9BW26	XP_294126; XP_293814; XP_293411; XP_291520; XP_062437; NP_000215; NP_954657	Keratin, type I cytoskeletal 18 (Cytokeratin-18) (CK-18) (Keratin-18) (K18) (Cell proliferation-inducing gene 46 protein)	GO:0043000; GO:0009653; GO:0007049; GO:0034451; GO:0044419; GO:0045095; GO:0043066; GO:0005515; GO:0005198	Golgi to plasma membrane CFTR protein transport; anatomical structure morphogenesis; cell cycle; centriolar satellite; interspecies interaction between organisms; keratin filament; negative regulation of apoptosis; protein binding; structural molecule activity	Cytoplasmperinuclearregion			Known in Uniprot to be phosphorylated	Known in HPRD to be phosphorylated	
**1375**	CH60	CH60_HUMAN	CH60	P10809; B2R5M6; Q38L19; Q9UCR6	NP_002147; XP_293567; XP_116321; NP_955472	60 kDa heat shock protein, mitochondrial (Heat shock protein 60) (HSP-60) (Hsp60) (60 kDa chaperonin) (Chaperonin 60) (CPN60) (Mitochondrial matrix protein P1) (P60 lymphocyte protein) (HuCHA60)	GO:0006458; GO:0005524; GO:0016887; GO:0002368; GO:0042100; GO:0003688; GO:0002755; GO:0006919; GO:0009986; GO:0043498; GO:0051087; GO:0051131; GO:0005905; GO:0030135; GO:0005829; GO:0005769; GO:0005615; GO:0044419; GO:0048291; GO:0001530; GO:0046696; GO:0005743; GO:0005759; GO:0043066; GO:0002039; GO:0050870; GO:0002842; GO:0043065; GO:0032727; GO:0032729; GO:0032733; GO:0032735; GO:0032755; GO:0043032; GO:0051604;	'de novo' protein folding; ATP binding; ATPase activity; B cell cytokine production; B cell proliferation; DNA replication origin binding; MyD88-dependent toll-like receptor signaling pathway; activation of caspase activity; cell surface; cell surface binding; chaperone binding; chaperone-mediated protein complex assembly; coated pit; coated vesicle; cytosol; early endosome; extracellular space; interspecies interaction between organisms; isotype switching to IgG isotypes; lipopolysaccharide binding; lipopolysaccharide receptor complex; mitochondrial inner membrane; mitochondrial matrix; negative regulation of apoptosis; p53 binding; positive regulation of T cell activation; positive regulation of T cell mediated immune response to tumor cell; positive regulation of apoptosis; positive regulation of interferon-alpha production; positive regulation of interferon-gamma production; positive regulation of interleukin-10 production; positive regulation of interleukin-12 production; positive regulation of interleukin-6 production; positive regulation of macrophage activation; protein maturation; protein refolding; protein stabilization; response to unfolded	Mitochondrionmatrix			Known in Uniprot to be phosphorylated		
**1376**	RINI	RINI_HUMAN	RINI	P13489	NP_002930; NP_976317; NP_976318; NP_976319; NP_976320; NP_976321; NP_976322; NP_976323	Ribonuclease inhibitor (Ribonuclease/angiogenin inhibitor 1) (RAI) (Placental ribonuclease inhibitor) (RNase inhibitor) (RI)	GO:0032311; GO:0005737; GO:0006402; GO:0005515; GO:0045765; GO:0008428	angiogenin-PRI complex; cytoplasm; mRNA catabolic process; protein binding; regulation of angiogenesis; ribonuclease inhibitor activity	Cytoplasm			Known in Uniprot to be phosphorylated		
**1377**	AP4A	AP4A_HUMAN	AP4A; Q5T589	P50583; Q5T589	NP_001152; NP_671701; NP_671702	Bis(5'-nucleosyl)-tetraphosphatase [asymmetrical] (EC 3.6.1.17) (Diadenosine 5',5'''-P1,P4-tetraphosphate asymmetrical hydrolase) (Diadenosine tetraphosphatase) (Ap4A hydrolase) (Ap4Aase) (Nucleoside diphosphate-linked moiety X motif 2) (Nudix motif 2)	GO:0004081; GO:0008803; GO:0006917; GO:0006139	bis(5'-nucleosyl)-tetraphosphatase (asymmetrical) activity; bis(5'-nucleosyl)-tetraphosphatase (symmetrical) activity; induction of apoptosis; nucleobase, nucleoside, nucleotide and nucleic acid metabolic process						Phosphatase
**1378**	BIRC3	BIRC3_HUMAN	BIRC3	Q13489; Q16628; Q9HC27; Q9UP46	NP_001156; NP_892007	Baculoviral IAP repeat-containing protein 3 (Inhibitor of apoptosis protein 1) (HIAP-1) (HIAP1) (C-IAP2) (TNFR2-TRAF-signaling complex protein 1) (IAP homolog C) (Apoptosis inhibitor 2) (API2) (RING finger protein 49)	GO:0006916; GO:0007166; GO:0005737; GO:0005634; GO:0005515; GO:0004842; GO:0008270	anti-apoptosis; cell surface receptor linked signal transduction; cytoplasm; nucleus; protein binding; ubiquitin-protein ligase activity; zinc ion binding	Cytoplasm					
**1379**	CYTC	CYTC_HUMAN	CYTC; CYTT	P09228; P01034; B2R5J9; Q6FGW9; Q9UCQ7	NP_001313; NP_000090	Cystatin-C (Cystatin-3) (Neuroendocrine basic polypeptide) (Gamma-trace) (Post-gamma-globulin); Cystatin-SA (Cystatin-2) (Cystatin-S5)	GO:0001540; GO:0004869; GO:0006952; GO:0005615; GO:0043206; GO:0060313; GO:0010711; GO:0060311; GO:0010716; GO:0010703; GO:0010466; GO:0002020; GO:0004869; GO:0005576	beta-amyloid binding; cysteine-type endopeptidase inhibitor activity; defense response; extracellular space; fibril organization; negative regulation of blood vessel remodeling; negative regulation of collagen catabolic process; negative regulation of elastin catabolic process; negative regulation of extracellular matrix disassembly; negative regulation of histolysis; negative regulation of peptidase activity; protease binding; cysteine-type endopeptidase inhibitor activity; extracellular region	Secreted					
**1380**	GBRB2	GBRB2_HUMAN	GBRB2	P47870; Q16323; Q4FZB2	NP_068711; NP_000804	Gamma-aminobutyric acid receptor subunit beta-2 (GABA(A) receptor subunit beta-2)	GO:0004890; GO:0030054; GO:0005254; GO:0031404; GO:0005230; GO:0007214; GO:0005887; GO:0006811; GO:0030594; GO:0045211; GO:0007268	GABA-A receptor activity; cell junction; chloride channel activity; chloride ion binding; extracellular ligand-gated ion channel activity; gamma-aminobutyric acid signaling pathway; integral to plasma membrane; ion transport; neurotransmitter receptor activity; postsynaptic membrane; synaptic transmission	CelljunctionsynapsepostsynapticcellmembraneMultipassmembraneproteinCellmembraneMultipassmembraneprotein			Known in Uniprot to be phosphorylated	Known in HPRD to be phosphorylated	
**1381**	TF3C2	TF3C2_HUMAN	TF3C2	Q8WUA4; Q16632; Q9BWI7	NP_001512; NP_001030598	General transcription factor 3C polypeptide 2 (Transcription factor IIIC subunit beta) (TF3C-beta) (TFIIIC 110 kDa subunit) (TFIIIC110)	GO:0042791; GO:0005515; GO:0042797; GO:0000127	5S class rRNA transcription; protein binding; tRNA transcription from RNA polymerase III promoter; transcription factor TFIIIC complex	Nucleus			Known in Uniprot to be phosphorylated		
**1382**	HBA	HBA_HUMAN	HBA; Q3MIF5	P69905; Q3MIF5; P01922; Q1HDT5; Q53F97; Q96KF1; Q9NYR7; Q9UCM0	NP_000508; NP_000549	Hemoglobin subunit alpha (Hemoglobin alpha chain) (Alpha-globin)	GO:0022627; GO:0020037; GO:0005833; GO:0019825; GO:0015671; GO:0005344; GO:0005515	cytosolic small ribosomal subunit; heme binding; hemoglobin complex; oxygen binding; oxygen transport; oxygen transporter activity; protein binding				Known in Uniprot to be phosphorylated		
**1383**	ROA2	ROA2_HUMAN	ROA2	P22626; A8K064; P22627; Q9UC98	NP_002128; NP_112533	Heterogeneous nuclear ribonucleoproteins A2/B1 (hnRNP A2 / hnRNP B1)	GO:0003723; GO:0050658; GO:0005737; GO:0030530; GO:0000398; GO:0000166; GO:0005515; GO:0043047; GO:0005681	RNA binding; RNA transport; cytoplasm; heterogeneous nuclear ribonucleoprotein complex; nuclear mRNA splicing, via spliceosome; nucleotide binding; protein binding; single-stranded telomeric DNA binding; spliceosome	NucleusnucleoplasmCytoplasm			Known in Uniprot to be phosphorylated	Known in HPRD to be phosphorylated	
**1384**	LDHC	LDHC_HUMAN	LDHC	P07864; Q6GSG8; Q7Z7J4	NP_002292; NP_059144	L-lactate dehydrogenase C chain (LDH-C) (EC 1.1.1.27) (LDH testis subunit) (LDH-X) (Cancer/testis antigen 32) (CT32)	GO:0004459; GO:0019642; GO:0005488; GO:0005737; GO:0055114	L-lactate dehydrogenase activity; anaerobic glycolysis; binding; cytoplasm; oxidation reduction	Cytoplasm					
**1385**	NDKB	NDKB_HUMAN	NDKB; Q6FHN3	P22392; Q6FHN3; A8MWA3; Q1WM23; Q6LCT6	NP_002503; NP_001018146; NP_001018147; NP_001018148; NP_001018149	Nucleoside diphosphate kinase B (NDP kinase B) (NDK B) (EC 2.7.4.6) (nm23-H2) (C-myc purine-binding transcription factor PUF); Nucleoside diphosphate kinase (EC 2.7.4.6)	GO:0005524; GO:0006241; GO:0006183; GO:0006228; GO:0007155; GO:0005737; GO:0030027; GO:0000287; GO:0043066; GO:0045786; GO:0008285; GO:0004550; GO:0005634; GO:0050679; GO:0045618; GO:0005515; GO:0045682; GO:0006355; GO:0001726; GO:0003700	ATP binding; CTP biosynthetic process; GTP biosynthetic process; UTP biosynthetic process; cell adhesion; cytoplasm; lamellipodium; magnesium ion binding; negative regulation of apoptosis; negative regulation of cell cycle; negative regulation of cell proliferation; nucleoside diphosphate kinase activity; nucleus; positive regulation of epithelial cell proliferation; positive regulation of keratinocyte differentiation; protein binding; regulation of epidermis development; regulation of transcription, DNA-dependent; ruffle; transcription factor activity	CytoplasmNucleus;			Known in Uniprot to be phosphorylated	Known in HPRD to be phosphorylated	Kinase
**1386**	KAP0	KAP0_HUMAN	KAP0; B2R5T5	P10644; Q567S7; B2R5T5	NP_002725; NP_997636; NP_997637	cAMP-dependent protein kinase type I-alpha regulatory subunit (Tissue-specific extinguisher 1) (TSE1)	GO:0030552; GO:0005952; GO:0008603; GO:0009755; GO:0005515; GO:0001932; GO:0006357	cAMP binding; cAMP-dependent protein kinase complex; cAMP-dependent protein kinase regulator activity; hormone-mediated signaling; protein binding; regulation of protein amino acid phosphorylation; regulation of transcription from RNA polymerase II promoter				Known in Uniprot to be phosphorylated	Known in HPRD to be phosphorylated	Kinase
**1387**	PTBP1	PTBP1_HUMAN	PTBP1; Q9BUQ0	Q9BUQ0; P26599	NP_002810; NP_114367; NP_114368; NP_787041	Polypyrimidine tract-binding protein 1 (PTB) (Heterogeneous nuclear ribonucleoprotein I) (hnRNP I) (57 kDa RNA-binding protein PPTB-1); Polypyrimidine tract binding protein 1 (Polypyrimidine tract binding protein 1, isoform CRA_c)	GO:0030530; GO:0000398; GO:0005730; GO:0005654; GO:0000166; GO:0008187; GO:0005515; GO:0003723; GO:0006397; GO:0005634	heterogeneous nuclear ribonucleoprotein complex; nuclear mRNA splicing, via spliceosome; nucleolus; nucleoplasm; nucleotide binding; poly-pyrimidine tract binding; protein binding; RNA binding; mRNA processing; nucleus	Nucleus;			Known in Uniprot to be phosphorylated	Known in HPRD to be phosphorylated	
**1388**	S10A4	S10A4_HUMAN	S10A4; A8K7R8	P26447; Q6ICP8; A8K7R8	NP_002952; NP_062427	Protein S100-A4 (S100 calcium-binding protein A4) (Metastasin) (Protein Mts1) (Placental calcium-binding protein) (Calvasculin)	GO:0005509; GO:0005737; GO:0001837; GO:0005634	calcium ion binding; cytoplasm; epithelial to mesenchymal transition; nucleus						
**1389**	ANX11	ANX11_HUMAN	ANX11; Q5T0G8	P50995; Q5T0G8	NP_001148; NP_665875; NP_665876	Annexin A11 (Annexin-11) (Annexin XI) (Calcyclin-associated annexin 50) (CAP-50) (56 kDa autoantigen); Annexin A11 (cDNA FLJ31545 fis, clone NT2RI2000932, highly similar to Annexin A11) (cDNA FLJ44306 fis, clone TRACH2025084, highly similar to Annexin A11) (Annexin A11, isoform CRA_b)	GO:0005509; GO:0005544; GO:0006955; GO:0042470; GO:0005635; GO:0005654; GO:0005515; GO:0008429	calcium ion binding; calcium-dependent phospholipid binding; immune response; melanosome; nuclear envelope; nucleoplasm; protein binding; phosphatidylethanolamine binding	CytoplasmNucleusMelanosome;			Known in Uniprot to be phosphorylated		
**1390**	ANXA2	ANXA2_HUMAN	ANXA2	P07355; Q567R4; Q96DD5	NP_004030; NP_001002857; NP_001129487	Annexin A2 (Annexin-2) (Annexin II) (Lipocortin II) (Calpactin I heavy chain) (Chromobindin-8) (p36) (Protein I) (Placental anticoagulant protein IV) (PAP-IV)	GO:0005604; GO:0005509; GO:0005544; GO:0008092; GO:0042470; GO:0004859; GO:0001501; GO:0005625	basement membrane; calcium ion binding; calcium-dependent phospholipid binding; cytoskeletal protein binding; melanosome; phospholipase inhibitor activity; skeletal system development; soluble fraction	SecretedextracellularspaceextracellularmatrixbasementmembraneMelanosome			Known in Uniprot to be phosphorylated	Known in HPRD to be phosphorylated	

	Protein Name	Uniprot Name	Other Names	Uniprot IDs	Refseq IDs	Protein Description	GO ID	GO terms	Subcellular location	Comments	Comments	Comments - Database Search	Comments - Database Search	Function
**1391**	ATPA	ATPA_HUMAN	ATPA; Q53XX6	P25705; Q53XX6; Q8IXV2; Q96FB4; Q96HW2; Q96IR6; Q9BTV8	NP_004037; NP_001001937	ATP synthase subunit alpha, mitochondrial	GO:0005524; GO:0015986; GO:0042288; GO:0009790; GO:0043499; GO:0046933; GO:0006629; GO:0005759; GO:0005753; GO:0001937; GO:0005886; GO:0045261; GO:0046961	ATP binding; ATP synthesis coupled proton transport; MHC class I protein binding; embryonic development; eukaryotic cell surface binding; hydrogen ion transporting ATP synthase activity, rotational mechanism; lipid metabolic process; mitochondrial matrix; mitochondrial proton-transporting ATP synthase complex; negative regulation of endothelial cell proliferation; plasma membrane; proton-transporting ATP synthase complex, catalytic core F(1); proton-transporting ATPase activity, rotational mechanism	Mitochondrioninnermembrane			Known in Uniprot to be phosphorylated		
**1392**	ATPG	ATPG_HUMAN	ATPG; Q8TAS0; A8KA31	P36542; Q5VYP3; Q96AS8; A8KA31; Q8TAS0	NP_005165; NP_001001973	ATP synthase subunit gamma, mitochondrial (F-ATPase gamma subunit); ATP synthase gamma chain (Fragment)	GO:0015986; GO:0046933; GO:0005759; GO:0000275; GO:0046961; GO:0045261	ATP synthesis coupled proton transport; hydrogen ion transporting ATP synthase activity, rotational mechanism; mitochondrial matrix; mitochondrial proton-transporting ATP synthase complex, catalytic core F(1); proton-transporting ATPase activity, rotational mechanism; proton-transporting ATP synthase complex, catalytic core F(1)	MitochondrionMitochondrioninnermembrane;					
**1393**	DNM1L	DNM1L_HUMAN	DNM1L	O00429; A8K4X9; O14541; O60709; Q7L6B3; Q8TBT7; Q9BWM1; Q9Y5J2	NP_005681; NP_036192; NP_036193	Dynamin-1-like protein (EC 3.6.5.5) (Dynamin-like protein) (Dnm1p/Vps1p-like protein) (DVLP) (Dynamin family member proline-rich carboxyl-terminal domain less) (Dymple) (Dynamin-related protein 1) (Dynamin-like protein 4) (Dynamin-like protein IV) (HdynIV)	GO:0005525; GO:0003924; GO:0007154; GO:0005801; GO:0012505; GO:0005783; GO:0016020; GO:0043653; GO:0007006; GO:0007275; GO:0005515	GTP binding; GTPase activity; cell communication; cis-Golgi network; endomembrane system; endoplasmic reticulum; membrane; mitochondrial fragmentation during apoptosis; mitochondrial membrane organization; multicellular organismal development; protein binding	CytoplasmcytosolIntracytoplasmicmembranePeripheralmembraneprotein			Known in Uniprot to be phosphorylated		
**1394**	ATPD	ATPD_HUMAN	ATPD; Q6FG90	P30049; Q6FG90	NP_001678; NP_001001975	ATP synthase subunit delta, mitochondrial (F-ATPase delta subunit)	GO:0006200; GO:0046933; GO:0042776; GO:0005759; GO:0000275; GO:0046961; GO:0046688	ATP catabolic process; hydrogen ion transporting ATP synthase activity, rotational mechanism; mitochondrial ATP synthesis coupled proton transport; mitochondrial matrix; mitochondrial proton-transporting ATP synthase complex, catalytic core F(1); proton-transporting ATPase activity, rotational mechanism; response to copper ion	MitochondrionMitochondrioninnermembrane					
**1395**	A1AT	A1AT_HUMAN	A1AT; Q53XB8	P01009; Q53XB8; A6PX14; B2RDQ8; Q0PVP5; Q13672; Q5U0M1; Q7M4R2; Q86U18; Q86U19; Q96BF9; Q96ES1; Q9P1P0; Q9UCE6; Q9UCM3	NP_000286; NP_001002235; NP_001002236; NP_001121172; NP_001121173; NP_001121174; NP_001121175; NP_001121176; NP_001121177; NP_001121178; NP_001121179	Alpha-1-antitrypsin (Alpha-1 protease inhibitor) (Alpha-1-antiproteinase) [Cleaved into: Short peptide from AAT (SPAAT)]	GO:0006953; GO:0007596; GO:0005615; GO:0005730; GO:0008233; GO:0031093; GO:0002020; GO:0005578; GO:0004867	acute-phase response; blood coagulation; extracellular space; nucleolus; peptidase activity; platelet alpha granule lumen; protease binding; proteinaceous extracellular matrix; serine-type endopeptidase inhibitor activity	SecretedSecretedextracellularspaceextracellularmatrix					
**1396**	MYL4	MYL4_HUMAN	MYL4	P12829; P11783	NP_002467; NP_001002841	Myosin light chain 4 (Myosin light chain 1, embryonic muscle/atrial isoform) (Myosin light chain alkali, GT-1 isoform)	GO:0031672; GO:0051015; GO:0003785; GO:0005509; GO:0060048; GO:0003774; GO:0005859; GO:0007517; GO:0032038; GO:0032781; GO:0002026; GO:0008307	A band; actin filament binding; actin monomer binding; calcium ion binding; cardiac muscle contraction; motor activity; muscle myosin complex; muscle organ development; myosin II heavy chain binding; positive regulation of ATPase activity; regulation of the force of heart contraction; structural constituent of muscle				known in HPRD to be phosphorylated		
**1397**	DBX2	DBX2_HUMAN	Q6ZNG2; DBX2	Q6ZNG2	NP_001025036; NP_001004329	Homeobox protein DBX2 (Developing brain homeobox protein 2)	GO:0005634; GO:0006355; GO:0043565; GO:0003700	nucleus; regulation of transcription, DNA-dependent; sequence-specific DNA binding; transcription factor activity	Nucleus					
**1398**	STABP	STABP_HUMAN	STABP	O95630; Q3MJE7	NP_006454; NP_964010; NP_998787	STAM-binding protein (EC 3.1.2.15) (Associated molecule with the SH3 domain of STAM)	GO:0007259; GO:0005737; GO:0016020; GO:0008237; GO:0019941; GO:0005634; GO:0008284; GO:0005515; GO:0004221; GO:0008270	JAK-STAT cascade; cytoplasm; membrane; metallopeptidase activity; modification-dependent protein catabolic process; nucleus; positive regulation of cell proliferation; protein binding; ubiquitin thiolesterase activity; zinc ion binding	NucleusMembranePeripheralmembraneproteinCytoplasmEarlyendosome			Known in Uniprot to be phosphorylated	Known in HPRD to be phosphorylated	
**1399**	FRG1	FRG1_HUMAN	FRG1; TBB4Q	Q14331; Q99867; P78353	NP_064424; NP_004468	Protein FRG1 (FSHD region gene 1 protein); Putative tubulin beta-4q chain	GO:0008380; GO:0006397; GO:0016607; GO:0006364; GO:0005681; GO:0005525; GO:0003924; GO:0005874; GO:0007018; GO:0043234; GO:0051258; GO:0005198	RNA splicing; mRNA processing; nuclear speck; rRNA processing; spliceosome; GTP binding; GTPase activity; microtubule; microtubule-based movement; protein complex; protein polymerization; structural molecule activity	NucleusCajalbodyNucleusspeckleNucleusnucleolus;					
**1400**	TAGL3	TAGL3_HUMAN	TAGL3	Q9UI15; Q96A74	NP_037391; NP_001008273; NP_001008274	Transgelin-3 (Neuronal protein NP25) (Neuronal protein 22) (NP22)	GO:0007417; GO:0007517	central nervous system development; muscle organ development				Known in Uniprot to be phosphorylated		
**1401**	LGMN	LGMN_HUMAN	LGMN; Q96CY7; Q53XC6	Q99538; Q53XC6; O00123; Q9BTY1; Q96CY7	NP_005597; NP_001008530	Legumain (EC 3.4.22.34) (Asparaginyl endopeptidase) (Protease, cysteine 1); Full-length cDNA clone CS0DI011YN22 of Placenta of Homo sapiens (human) (Legumain, isoform CRA_a) (cDNA FLJ78453, highly similar to Homo sapiens legumain (LGMN), transcript variant 2, mRNA); Putative uncharacterized protein (Fragment)	GO:0004197; GO:0005764; GO:0004674; GO:0006508; GO:0045177; GO:0004197; GO:0005770; GO:0001509; GO:0040015	cysteine-type endopeptidase activity; lysosome; protein serine/threonine kinase activity; proteolysis; apical part of cell; cysteine-type endopeptidase activity; late endosome; legumain activity; negative regulation of multicellular organism growth	Lysosome;					
**1402**	PP1A	PP1A_HUMAN	PP1A; Q07161	Q07161; P62136; B2R908; P08129; P20653; P22802	NP_001008709; NP_002699; NP_996756	Serine/threonine-protein phosphatase PP1-alpha catalytic subunit (PP-1A) (EC 3.1.3.16); Serine/threonine protein phosphatase (EC 3.1.3.16)	GO:0007049; GO:0051301; GO:0005737; GO:0005977; GO:0005506; GO:0030145; GO:0006470; GO:0005515; GO:0004722; GO:0004721	cell cycle; cell division; cytoplasm; glycogen metabolic process; iron ion binding; manganese ion binding; protein amino acid dephosphorylation; protein binding; protein serine/threonine phosphatase activity; phosphoprotein phosphatase activity	Cytoplasm;			Known in Uniprot to be phosphorylated		Phosphatase
**1403**	RAP1B	RAP1B_HUMAN	RAP1B; Q5TZR4	P61224; Q5TZR4; B2R5Z2; P09526; Q502X3; Q6DCA1; Q6LES0	XP_933978; NP_056461; NP_001010942	Ras-related protein Rap-1b (GTP-binding protein smg p21B)	GO:0005525; GO:0005737; GO:0005886; GO:0007264	GTP binding; cytoplasm; plasma membrane; small GTPase mediated signal transduction	CellmembraneCytoplasmcytosol			Known in Uniprot to be phosphorylated	Known in HPRD to be phosphorylated	
**1404**	RA1L2	RA1L2_HUMAN	Q5TBS2; RA1L2	Q5TBS2; Q32P51	XP_291501; NP_001011724; NP_001011725	Heterogeneous nuclear ribonucleoprotein A1-like protein 2 (hnRNP core protein A1-like protein 2)	GO:0003723; GO:0008380; GO:0005737; GO:0006397; GO:0051028; GO:0000166; GO:0005681	RNA binding; RNA splicing; cytoplasm; mRNA processing; mRNA transport; nucleotide binding; spliceosome	NucleusCytoplasm					
**1405**	MPCP	MPCP_HUMAN	MPCP; Q6MZF9; Q7Z7N7	Q00325; Q96A03; Q6MZF9; Q7Z7N7	NP_002626; NP_005879; NP_998776	Phosphate carrier protein, mitochondrial (Phosphate transport protein) (PTP) (Solute carrier family 25 member 3); Putative uncharacterized protein DKFZp686J11229 (Fragment); Solute carrier family 25 (Mitochondrial carrier; phosphate carrier), member 3 (Solute carrier family 25 (Mitochondrial carrier; phosphate carrier), member 3, isoform CRA_a)	GO:0005488; GO:0006091; GO:0005887; GO:0005743; GO:0015320; GO:0015293; GO:0006810; GO:0005743	binding; generation of precursor metabolites and energy; integral to plasma membrane; mitochondrial inner membrane; phosphate carrier activity; symporter activity; transport; mitochondrial inner membrane	MitochondrioninnermembraneMultipassmembraneprotein;					
**1406**	THTM	THTM_HUMAN	THTM; Q6FHN9	P25325; Q6FHN9; O75750; A8MZ34	NP_066949; NP_001013454; NP_001123989	3-mercaptopyruvate sulfurtransferase (MST) (EC 2.8.1.2); MPST protein (Mercaptopyruvate sulfurtransferase, isoform CRA_a) (Putative uncharacterized protein MPST) (Fragment)	GO:0016784; GO:0009440; GO:0005739; GO:0009636; GO:0008272; GO:0004792; GO:0008272	3-mercaptopyruvate sulfurtransferase activity; cyanate catabolic process; mitochondrion; response to toxin; sulfate transport; thiosulfate sulfurtransferase activity; sulfate transport	Cytoplasm;					
**1407**	KATL1	KATL1_HUMAN	KATL1; A8K5X4	Q9BW62; A8K5X4	NP_115492; NP_001014402	Katanin p60 ATPase-containing subunit A-like 1 (Katanin p60 subunit A-like 1) (EC 3.6.4.3) (p60 katanin-like 1)	GO:0005524; GO:0005874; GO:0008568	ATP binding; microtubule; microtubule-severing ATPase activity						
**1408**	CUTA	CUTA_HUMAN	CUTA	O60888; A2AB26; A2BEL4; Q3B784; Q5JXM9; Q5SU05; Q9NYQ9	NP_057005; NP_001014433; NP_001014837; NP_001014838; NP_001014840	Protein CutA (Brain acetylcholinesterase putative membrane anchor) (Acetylcholinesterase-associated protein)	GO:0019899; GO:0016020; GO:0008104; GO:0010038	enzyme binding; membrane; protein localization; response to metal ion						

	Protein Name	Uniprot Name	Other Names	Uniprot IDs	Refseq IDs	Protein Description	GO ID	GO terms	Subcellular location	Comments	Comments	Comments - Database Search	Comments - Database Search	Function
**1409**	BRCA1	BRCA1_HUMAN	BRCA1; Q3LRJ6; Q6IN79	P38398; Q3LRJ6; O15129; Q7KYU9; Q6IN79	NP_009225; NP_009226; NP_009227; NP_009228; NP_009235; NP_009229; NP_009230; NP_009231; NP_009233; NP_009234; NP_009236	Breast cancer type 1 susceptibility protein (EC 6.3.2.-) (RING finger protein 53); Breast cancer 1 early onset; BRCA1 protein	GO:0031436; GO:0006978; GO:0008630; GO:0050681; GO:0030521; GO:0007059; GO:0000724; GO:0019899; GO:0008274; GO:0042802; GO:0045786; GO:0046600; GO:0045717; GO:0016481; GO:0005654; GO:0045739; GO:0031398; GO:0045893; GO:0006301; GO:0042127; GO:0006357; GO:0006359; GO:0043627; GO:0030529; GO:0003713; GO:0015631; GO:0008270; GO:0006281; GO:0006260; GO:0007098; GO:0043009; GO:0000793; GO:0005737; GO:0003684; GO:0009048; GO:0005634; GO:0005515; GO:0003677; GO:0006281	BRCA1-BARD1 complex; DNA damage response, signal transduction by p53 class mediator resulting in transcription of p21 class mediator; DNA damage response, signal transduction resulting in induction of apoptosis; androgen receptor binding; androgen receptor signaling pathway; chromosome segregation; double-strand break repair via homologous recombination; enzyme binding; gamma-tubulin ring complex; identical protein binding; negative regulation of cell cycle; negative regulation of centriole replication; negative regulation of fatty acid biosynthetic process; negative regulation of transcription; nucleoplasm; positive regulation of DNA repair; positive regulation of protein ubiquitination; positive regulation of transcription, DNA-dependent; postreplication repair; regulation of cell proliferation; regulation of transcription from RNA polymerase II promoter; regulation of transcription from RNA polymerase III promoter; response to estrogen stimulus; ribonucleoprotein complex; transcription coactivator activity; tubulin binding; zinc ion binding; DNA repair; DNA replication; centrosome cycle; chordate embryonic development; condensed chromosome; cytoplasm; damaged DNA binding; dosage compensation, by inactivation of X	NucleusCytoplasmCytoplasm;			Known in Uniprot to be phosphorylated	Known in HPRD to be phosphorylated	
**1410**	WASF1	WASF1_HUMAN	WASF1; Q5SZK7	Q92558; Q5SZK7	NP_003922; NP_001020105; NP_001020106; NP_001020107	Wiskott-Aldrich syndrome protein family member 1 (WASP family protein member 1) (Protein WAVE-1) (Verprolin homology domain-containing protein 1); WAS protein family, member 1 (WAS protein family, member 1, isoform CRA_a)	GO:0003779; GO:0015629; GO:0030041; GO:0006928; GO:0005739;	actin binding; actin cytoskeleton; actin filament polymerization; cell motion; mitochondrion;	Cytoplasmcytoskeleton;					
**1411**	AP3M1	AP3M1_HUMAN	AP3M1; Q9H5L2; Q5JQ12	Q9Y2T2; Q5JQ12; Q9H5L2	NP_036227; NP_996895	AP-3 complex subunit mu-1 (Adapter-related protein complex 3 mu-1 subunit) (AP-3 adapter complex mu3A subunit) (Mu-adaptin 3A) (Mu3A-adaptin)	GO:0005794; GO:0030131; GO:0005764; GO:0005515; GO:0006622; GO:0016192	Golgi apparatus; clathrin adaptor complex; lysosome; protein binding; protein targeting to lysosome; vesicle-mediated transport	Golgiapparatus					
**1412**	PP13	PP13_HUMAN	PP13	Q9UHV8	XP_086001; NP_037400	Galactoside-binding soluble lectin 13 (EC 3.1.1.5) (Placental tissue protein 13) (Placenta protein 13) (PP13) (Galectin-13)	GO:0016042; GO:0004622; GO:0006644; GO:0005529	lipid catabolic process; lysophospholipase activity; phospholipid metabolic process; sugar binding						
**1413**	5NT3	5NT3_HUMAN	5NT3	Q9H0P0; B2RAA5; Q6IPZ1; Q6NXS6; Q7L3G6; Q9P0P5; Q9UC42; Q9UC43; Q9UC44; Q9UC45	XP_208318; NP_001002009; NP_001002010; NP_057573	Cytosolic 5'-nucleotidase 3 (EC 3.1.3.5) (Cytosolic 5'-nucleotidase III) (cN-III) (Pyrimidine 5'-nucleotidase 1) (P5'N-1) (P5N-1) (PN-I) (Uridine 5' monophosphate hydrolase 1) (p36)	GO:0008665; GO:0008253; GO:0005783; GO:0000287; GO:0000166; GO:0009117; GO:0006213	2'-phosphotransferase activity; 5'-nucleotidase activity; endoplasmic reticulum; magnesium ion binding; nucleotide binding; nucleotide metabolic process; pyrimidine nucleoside metabolic process	CytoplasmEndoplasmic reticulum					
**1414**	UGGG1	UGGG1_HUMAN	UGGG1; Q53QP2	Q9NYU2; Q53QP2; Q53SL3; Q8IW30; Q9H8I4	NP_064505; NP_001020948	UDP-glucose:glycoprotein glucosyltransferase 1 (EC 2.4.1.-) (UDP-glucose ceramide glucosyltransferase-like 1) (UDP--Glc:glycoprotein glucosyltransferase) (HUGT1)	GO:0051084; GO:0005793; GO:0003980; GO:0005783; GO:0005788; GO:0006486; GO:0051082	'de novo' posttranslational protein folding; ER-Golgi intermediate compartment; UDP-glucose:glycoprotein glucosyltransferase activity; endoplasmic reticulum; endoplasmic reticulum lumen; protein amino acid glycosylation; unfolded protein binding	EndoplasmicreticulumlumenEndoplasmicreticulumGolgiintermediate compartment			Known in Uniprot to be phosphorylated		
**1415**	TPPC2	TPPC2_HUMAN	TPPC2; Q6IBE5	O14582; Q6IBE5; A6NEG0; Q9HD16	NP_055378; NP_001011658	Trafficking protein particle complex subunit 2 (Sedlin) (MBP-1-interacting protein 2A) (MIP-2A); ZNF547 protein (Trafficking protein particle complex 2, isoform CRA_a) (Trafficking protein particle complex 2, isoform CRA_b) (Zinc finger protein 547, isoform CRA_a) (cDNA, FLJ94336, Homo sapiens spondyloepiphyseal dysplasia, late (SEDL), mRNA)	GO:0006888; GO:0005794; GO:0005783; GO:0006355; GO:0001501; GO:0008134; GO:0005622	ER to Golgi vesicle-mediated transport; Golgi apparatus; endoplasmic reticulum; regulation of transcription, DNA-dependent; skeletal system development; transcription factor binding; intracellular	CytoplasmperinuclearregionEndoplasmicreticulumGolgiapparatus;					
**1416**	SAR1B	SAR1B_HUMAN	SAR1B	Q9Y6B6; Q567T4	NP_057187; NP_001028675	GTP-binding protein SAR1b (GTBPB)	GO:0006888; GO:0005525; GO:0005794; GO:0005789; GO:0006886; GO:0000287; GO:0016044	ER to Golgi vesicle-mediated transport; GTP binding; Golgi apparatus; endoplasmic reticulum membrane; intracellular protein transport; magnesium ion binding; membrane organization	EndoplasmicreticulummembranePeripheralmembraneproteinGolgiapparatusGolgistackmembranePeripheralmembraneprotein					
**1417**	TKN1	TKN1_HUMAN	TKN1; Q549V2	Q549V2; P20366; O60600; O60601; Q00072; Q53GH4	NP_054702; NP_003173; NP_054704	Protachykinin-1 (PPT) [Cleaved into: Substance P; Neurokinin A (NKA) (Substance K) (Neuromedin L); Neuropeptide K (NPK); Neuropeptide gamma; C-terminal-flanking peptide]; Putative uncharacterized protein TAC1 (Tachykinin, 1 (Substance K, substance P, neurokinin 1, neurokinin 2, neuromedin L, neurokinin alpha, neuropeptide K, neuropeptide gamma)) (Tachykinin, 1 (Substance K, substance P, neurokinin 1, neurokinin 2, neuromedin L, neurokinin alpha, neuropeptide K, neuropeptide gamma), isoform CRA_d)	GO:0009582; GO:0007204; GO:0005615; GO:0007320; GO:0007218; GO:0007268; GO:0007217; GO:0005576	detection of abiotic stimulus; elevation of cytosolic calcium ion concentration; extracellular space; insemination; neuropeptide signaling pathway; synaptic transmission; tachykinin receptor signaling pathway; extracellular region	Secreted					
**1418**	LUC7L	LUC7L_HUMAN	LUC7L; Q1W6G4	Q9NQ29; Q96S32; Q9NPH4; Q1W6G4	NP_060502; NP_958815	Putative RNA-binding protein Luc7-like 1 (SR+89) (Putative SR protein LUC7B1); LUC7-like (S. cerevisiae) (LUC7-like (S. cerevisiae), isoform CRA_g) (Putative RNA-binding protein Luc7-like 1)	GO:0008270; GO:0050733; GO:0045843; GO:0005634	zinc ion binding; RS domain binding; negative regulation of striated muscle development; nucleus				Known in Uniprot to be phosphorylated		
**1419**	VAPA	VAPA_HUMAN	VAPA; A6NDZ0	Q9P0L0; O75453; Q5U0E7; Q9UBZ2; A6NDZ0	NP_003565; NP_919415	Vesicle-associated membrane protein-associated protein A (VAMP-associated protein A) (VAMP-A) (VAP-A) (33 kDa Vamp-associated protein) (VAP-33)	GO:0012505; GO:0016021; GO:0006944; GO:0005886; GO:0043123; GO:0046982; GO:0004871; GO:0005198; GO:0031982	endomembrane system; integral to membrane; membrane fusion; plasma membrane; positive regulation of I-kappaB kinase/NF-kappaB cascade; protein heterodimerization activity; signal transducer activity; structural molecule activity; vesicle	CellmembraneSinglepasstypeIVmembraneproteinIntracytoplasmicmembraneSinglepasstypeIVmembraneprotein					
**1420**	RSSA	RSSA_HUMAN	RSSA; Q6NSD1	P08865; Q6IPD2; P11085; P12030; Q16471; Q6IPD1; Q6NSD1; Q6NXQ8; Q86VC0	NP_002286; NP_001012321	40S ribosomal protein SA (Laminin receptor 1) (LamR) (37/67 kDa laminin receptor) (LRP/LR) (Laminin-binding protein precursor p40) (LBP/p40) (37 kDa laminin receptor precursor) (37LRP) (67 kDa laminin receptor) (67LR) (Colon carcinoma laminin-binding protein) (NEM/1CHD4) (Multidrug resistance-associated protein MGr1-Ag)	GO:0007155; GO:0022627; GO:0005634; GO:0043022; GO:0003735; GO:0006414	cell adhesion; cytosolic small ribosomal subunit; nucleus; ribosome binding; structural constituent of ribosome; translational elongation	CellmembraneCytoplasmNucleus			Known in Uniprot to be phosphorylated		
**1421**	CAC1A	CAC1A_HUMAN	CAC1A	O00555; P78510; P78511; Q16290; Q92690; Q99790; Q99791; Q99792; Q99793	XP_212118; NP_001120693; NP_001120694	Voltage-dependent P/Q-type calcium channel subunit alpha-1A (Voltage-gated calcium channel subunit alpha Cav2.1) (Calcium channel, L type, alpha-1 polypeptide isoform 4) (Brain calcium channel I) (BI)	GO:0005509; GO:0006816; GO:0008219; GO:0042995; GO:0005737; GO:0007204; GO:0005634; GO:0019905; GO:0005245; GO:0005891	calcium ion binding; calcium ion transport; cell death; cell projection; cytoplasm; elevation of cytosolic calcium ion concentration; nucleus; syntaxin binding; voltage-gated calcium channel activity; voltage-gated calcium channel complex	MembraneMultipassmembraneprotein			Known in Uniprot to be phosphorylated		
**1422**	PUR4	PUR4_HUMAN	PUR4	O15067; A6H8V8	XP_290742	Phosphoribosylformylglycinamidine synthase (FGAM synthase) (FGAMS) (EC 6.3.5.3) (Formylglycinamide ribotide amidotransferase) (FGARAT) (Formylglycinamide ribotide synthetase)	GO:0006189; GO:0005524; GO:0005829; GO:0006541; GO:0004642; GO:0005515	'de novo' IMP biosynthetic process; ATP binding; cytosol; glutamine metabolic process; phosphoribosylformylglycinamidine synthase activity; protein binding	Cytoplasm			Known in Uniprot to be phosphorylated		
**1423**	MYO1B	MYO1B_HUMAN	MYO1B; B0I1S9	O43795; O43794; Q7Z6L5; B0I1S9	XP_290989; NP_036355; NP_001123630	Myosin-Ib (Myosin I alpha) (MMI-alpha) (MMIa) (MYH-1c)	GO:0005524; GO:0003779; GO:0005516; GO:0003774; GO:0016459	ATP binding; actin binding; calmodulin binding; motor activity; myosin complex						
**1424**	OR2T1	OR2T1_HUMAN	OR2T1	O43869; Q6IEZ9	XP_060316; NP_112166	Olfactory receptor 2T1 (Olfactory receptor OR1-61) (Olfactory receptor 1-25) (OR1-25)	GO:0007186; GO:0016021; GO:0004984; GO:0005886; GO:0050896; GO:0007608	G-protein coupled receptor protein signaling pathway; integral to membrane; olfactory receptor activity; plasma membrane; response to stimulus; sensory perception of smell	CellmembraneMultipassmembraneprotein					
**1425**	NDK8	NDK8_HUMAN	NDK8	O60361	NP_789797	Putative nucleoside diphosphate kinase (NDP kinase) (NDK) (EC 2.7.4.6)	GO:0005524; GO:0006241; GO:0006183; GO:0006228; GO:0000287; GO:0004550	ATP binding; CTP biosynthetic process; GTP biosynthetic process; UTP biosynthetic process; magnesium ion binding; nucleoside diphosphate kinase activity				Known in Uniprot to be phosphorylated		Kinase
**1426**	LIRB3	LIRB3_HUMAN	LIRB3	O75022; O15471; Q86U49	XP_115639	Leukocyte immunoglobulin-like receptor subfamily B member 3 (Leukocyte immunoglobulin-like receptor 3) (LIR-3) (Immunoglobulin-like transcript 5) (ILT-5) (Monocyte inhibitory receptor HL9) (CD85 antigen-like family member A) (CD antigen CD85a)	GO:0007166; GO:0006952; GO:0006955; GO:0005887; GO:0004888	cell surface receptor linked signal transduction; defense response; immune response; integral to plasma membrane; transmembrane receptor activity	MembraneSinglepasstypeImembraneprotein			Known in Uniprot to be phosphorylated		
**1427**	RGS9	RGS9_HUMAN	RGS9	O75916; O75573; Q696R2; Q8TD64; Q8TD65; Q9HC32; Q9HC33	XP_295854; NP_001075424; NP_003826	Regulator of G-protein signaling 9 (RGS9)	GO:0005096; GO:0005834; GO:0007242; GO:0009968; GO:0008277; GO:0050896; GO:0004871; GO:0007601	GTPase activator activity; heterotrimeric G-protein complex; intracellular signaling cascade; negative regulation of signal transduction; regulation of G-protein coupled receptor protein signaling pathway; response to stimulus; signal transducer activity; visual perception	MembranePeripheralmembraneprotein			Known in Uniprot to be phosphorylated		
**1428**	DNJC8	DNJC8_HUMAN	DNJC8	O75937; Q6IBA4; Q8N4Z5; Q9P051; Q9P067	XP_292230; NP_055095	DnaJ homolog subfamily C member 8 (Splicing protein spf31)	GO:0031072; GO:0000398	heat shock protein binding; nuclear mRNA splicing, via spliceosome				Known in Uniprot to be phosphorylated		

	Protein Name	Uniprot Name	Other Names	Uniprot IDs	Refseq IDs	Protein Description	GO ID	GO terms	Subcellular location	Comments	Comments	Comments - Database Search	Comments - Database Search	Function
**1429**	K2C8	K2C8_HUMAN	K2C8	P05787; Q14099; Q14716; Q14717; Q53GJ0; Q6DHW5; Q6GMY0; Q6P4C7; Q96J60	XP_293010; XP_301540; NP_002264	Keratin, type II cytoskeletal 8 (Cytokeratin-8) (CK-8) (Keratin-8) (K8)	GO:0007010; GO:0044419; GO:0045095; GO:0005515; GO:0005198	cytoskeleton organization; interspecies interaction between organisms; keratin filament; protein binding; structural molecule activity	Cytoplasm			Known in Uniprot to be phosphorylated	Known in HPRD to be phosphorylated	
**1430**	ROA1	ROA1_HUMAN	ROA1; Q3MIB7	P09651; Q3MIB7; Q6PJZ7	XP_301830; XP_293689; XP_301750; NP_002127	Heterogeneous nuclear ribonucleoprotein A1 (hnRNP core protein A1) (Helix-destabilizing protein) (Single-strand RNA-binding protein)	GO:0006405; GO:0005737; GO:0030530; GO:0051028; GO:0051170; GO:0000398; GO:0005654; GO:0000166; GO:0005515; GO:0003697; GO:0003727; GO:0005681	RNA export from nucleus; cytoplasm; heterogeneous nuclear ribonucleoprotein complex; mRNA transport; nuclear import; nuclear mRNA splicing, via spliceosome; nucleoplasm; nucleotide binding; protein binding; single-stranded DNA binding; single-stranded RNA binding; spliceosome	NucleusCytoplasm			Known in Uniprot to be phosphorylated	Known in HPRD to be phosphorylated	
**1431**	CO4B	CO4B_HUMAN	CO4B; Q6U2E9; A7E2V2	Q6U2E9; P0C0L5; P01028; P78445; Q13160; Q13906; Q14033; Q14835; Q9NPK5; Q9UIP5; A7E2V2	NP_000583; NP_001002029	Complement C4-B (Basic complement C4) (C3 and PZP-like alpha-2-macroglobulin domain-containing protein 3) [Cleaved into: Complement C4 beta chain; Complement C4-B alpha chain; C4a anaphylatoxin; C4b-B; C4d-B; Complement C4 gamma chain]; Complement component 4B (Childo blood group) (C4B1)	GO:0006958; GO:0004866; GO:0005615; GO:0045087; GO:0005515; GO:0006956	complement activation, classical pathway; endopeptidase inhibitor activity; extracellular space; innate immune response; protein binding; complement activation	Secreted;					
**1432**	IL32	IL32_HUMAN	IL32	P24001; Q5VFH7; Q5VFH8; Q8WV38; Q96GK9	XP_301585; NP_001012649; NP_001012651; NP_001012736; NP_004212	Interleukin-32 (IL-32) (Natural killer cells protein 4) (Tumor necrosis factor alpha-inducing factor)	GO:0007155; GO:0005125; GO:0006952; GO:0005615; GO:0006955	cell adhesion; cytokine activity; defense response; extracellular space; immune response	Secreted					
**1433**	CLAT	CLAT_HUMAN	CLAT	P28329; Q16488; Q9BQ23; Q9BQ35; Q9BQE1	XP_305444; NP_001136401; NP_001136405; NP_001136406; NP_065574; NP_066264	Choline O-acetyltransferase (Choline acetylase) (CHOACTase) (ChAT) (EC 2.3.1.6)	GO:0004102; GO:0005737; GO:0042136; GO:0005634	choline O-acetyltransferase activity; cytoplasm; neurotransmitter biosynthetic process; nucleus					known in HPRD to be phosphorylated	
**1434**	RBM25	RBM25_HUMAN	RBM25	P49756; A0PJL9; B2RNA8; B3KT03; Q2TA72; Q5XJ17; Q6P665; Q9H6A1; Q9UEQ5; Q9UIE9	XP_299177; NP_067062	RNA-binding protein 25 (RNA-binding motif protein 25) (RNA-binding region-containing protein 7) (Arg/Glu/Asp-rich protein of 120 kDa) (RED120) (Protein S164)	GO:0003729; GO:0000398; GO:0000166	mRNA binding; nuclear mRNA splicing, via spliceosome; nucleotide binding	NucleusspeckleCytoplasm			Known in Uniprot to be phosphorylated	Known in HPRD to be phosphorylated	
**1435**	RAB15	RAB15_HUMAN	RAB15	P59190; Q86TX7; Q8IW89	XP_085123; NP_941959	Ras-related protein Rab-15	GO:0005525; GO:0005886; GO:0015031; GO:0007264	GTP binding; plasma membrane; protein transport; small GTPase mediated signal transduction	CellmembraneLipidanchorCytoplasmicside					
**1436**	FOXD4	FOXD4_HUMAN	FOXD4	Q12950; B2RN05; Q5VVK1; Q8WXT6	XP_296644; NP_997188	Forkhead box protein D4 (Forkhead-related protein FKHL9) (Forkhead-related transcription factor 5) (FREAC-5) (Myeloid factor-alpha)	GO:0008301; GO:0007275; GO:0005634; GO:0006355; GO:0043565; GO:0003700	DNA bending activity; multicellular organismal development; nucleus; regulation of transcription, DNA-dependent; sequence-specific DNA binding; transcription factor activity	Nucleus					
**1437**	RRP5	RRP5_HUMAN	RRP5	Q14690; Q2TA92; Q5W093; Q6P2J3; Q86VQ8; Q9H4P1	XP_031992; NP_055791	Protein RRP5 homolog (Programmed cell death protein 11) (NF-kappa-B-binding protein) (NFBP)	GO:0003723; GO:0005634; GO:0006364; GO:0008134	RNA binding; nucleus; rRNA processing; transcription factor binding	Nucleusnucleolus			Known in Uniprot to be phosphorylated		
**1438**	MTF1	MTF1_HUMAN	MTF1	Q14872; Q96CB1	XP_210576; NP_005946	Metal regulatory transcription factor 1 (Transcription factor MTF-1) (MRE-binding transcription factor)	GO:0005634; GO:0003713; GO:0003700; GO:0008270	nucleus; transcription coactivator activity; transcription factor activity; zinc ion binding	Nucleus				known in HPRD to be phosphorylated	
**1439**	Q15206	Q15206_HUMAN	Q15206	Q15206	XP_305873	Profilaggrin (Fragment)	GO:0005198	structural molecule activity						
**1440**	Q2QD09	Q2QD09_HUMAN	Q2QD09	Q2QD09	XP_208215; XP_001133949; XP_001133950	Triosephosphate isomerase (EC 5.3.1.1) (Fragment)	GO:0009790; GO:0006006; GO:0019682; GO:0004807	embryonic development; glucose metabolic process; glyceraldehyde-3-phosphate metabolic process; triose-phosphate isomerase activity						
**1441**	HSP74	HSP74_HUMAN	Q2TAL4; HSP74	Q2TAL4; P34932; O95756	XP_114482; NP_002145	Heat shock 70 kDa protein 4 (Heat shock 70-related protein APG-2) (HSP70RY)	GO:0005524; GO:0034619; GO:0005737; GO:0070096; GO:0006986	ATP binding; cellular chaperone-mediated protein complex assembly; cytoplasm; mitochondrial outer membrane translocase complex assembly; response to unfolded protein	Cytoplasm			Known in Uniprot to be phosphorylated		
**1442**	ZN791	ZN791_HUMAN	Q3KP31; ZN791	Q3KP31; Q8NC99	XP_294548; NP_699189	Zinc finger protein 791	GO:0003677; GO:0005634; GO:0006355; GO:0008270	DNA binding; nucleus; regulation of transcription, DNA-dependent; zinc ion binding	Nucleus					
**1443**	Q58FF2	Q58FF2_HUMAN	Q58FF2	Q58FF2	XP_291587	Heat shock protein 94c	GO:0006457; GO:0006950; GO:0051082	protein folding; response to stress; unfolded protein binding						
**1444**	Q58FF4	Q58FF4_HUMAN	Q58FF4	Q58FF4	XP_301425	Heat shock protein 90Bf	GO:0006457; GO:0006950; GO:0051082	protein folding; response to stress; unfolded protein binding						
**1445**	HS904	HS904_HUMAN	Q58FG1; HS904	Q58FG1	XP_293716	Putative heat shock protein HSP 90-alpha A4 (Heat shock protein 90-alpha D) (Heat shock protein 90Ad) (Heat shock 90 kDa protein 1 alpha-like 2)	GO:0005524; GO:0005737; GO:0006457; GO:0006950; GO:0051082	ATP binding; cytoplasm; protein folding; response to stress; unfolded protein binding	Cytoplasm					
**1446**	Q59FI9	Q59FI9_HUMAN	Q59FI9	Q59FI9	XP_301421	Ribosomal protein L12 variant (Fragment)	GO:0005840; GO:0003735; GO:0006412	ribosome; structural constituent of ribosome; translation						
**1447**	Q5JWW0	Q5JWW0_HUMAN	Q5JWW0	Q5JWW0	XP_301652	Putative uncharacterized protein DKFZp566L241 (Fragment)								
**1448**	K1755	K1755_HUMAN	Q5JYT7; K1755	Q5JYT7; Q9C0A8	XP_028810; NP_001025035	Uncharacterized protein KIAA1755								
**1449**	Q5RKT7	Q5RKT7_HUMAN	Q5RKT7	Q5RKT7	XP_208238	Ribosomal protein S27a	GO:0005515; GO:0006464; GO:0005840; GO:0003735; GO:0006412	protein binding; protein modification process; ribosome; structural constituent of ribosome; translation						
**1450**	CARKD	CARKD_HUMAN	Q5T9X3; CARKD	Q5T9X3; Q8IW45; Q9H7W1; Q9NVF5	XP_305433; NP_060680	Carbohydrate kinase domain-containing protein						Known in Uniprot to be phosphorylated		
**1451**	ZCH11	ZCH11_HUMAN	ZCH11	Q5TAX3; A2RRP0; Q12764; Q5TAX4; Q86XZ3	XP_038288; NP_001009882; NP_001009881; NP_056084	Zinc finger CCHC domain-containing protein 11	GO:0005737; GO:0003676; GO:0016779; GO:0005634; GO:0005515; GO:0008270	cytoplasm; nucleic acid binding; nucleotidyltransferase activity; nucleus; protein binding; zinc ion binding	NucleusCytoplasm			Known in Uniprot to be phosphorylated		
**1452**	EF1A3	EF1A3_HUMAN	Q5VTE0; EF1A3	Q5VTE0	XP_291573	Putative elongation factor 1-alpha-like 3 (EF-1-alpha-like 3) (Elongation factor 1 A-like 3) (eEF1A-like 3)	GO:0005525; GO:0003924; GO:0005737; GO:0003746; GO:0006414	GTP binding; GTPase activity; cytoplasm; translation elongation factor activity; translational elongation	Cytoplasm			Known in Uniprot to be phosphorylated		
**1453**	PEAR1	PEAR1_HUMAN	Q5VY43; PEAR1	Q5VY43; Q8TEK2	XP_371320; NP_001073940	Platelet endothelial aggregation receptor 1 (hPEAR1) (Multiple epidermal growth factor-like domains 12) (Multiple EGF-like-domains 12)	GO:0016021; GO:0005886	integral to membrane; plasma membrane	CellmembraneSinglepassmembraneprotein	Phophorylated - from this Study		Known in Uniprot to be phosphorylated	Known in HPRD to be phosphorylated	
**1454**	Q5W161	Q5W161_HUMAN	Q5W161	Q5W161	XP_087572	Novel protein	GO:0005525; GO:0007049	GTP binding; cell cycle						
**1455**	F104B	F104B_HUMAN	F104B	Q5XKR9; A6NEH1; Q8WVU5; Q9BRA1	XP_033811; NP_612371	Protein FAM104B								
**1456**	IF5AL	IF5AL_HUMAN	Q6IS14; IF5AL	Q6IS14	XP_084467; NP_001093162	Eukaryotic translation initiation factor 5A-1-like (eIF-5A-1-like) (eIF-5A1-like) (Eukaryotic initiation factor 5A isoform 1-like)	GO:0003743; GO:0006413	translation initiation factor activity; translational initiation						
**1457**	TWF1	TWF1_HUMAN	Q6NUK9; TWF1	Q6NUK9; Q12792; A8K5A8; Q59G07; Q5U0B1; Q6FHJ1; Q6FHL6; Q86XL6; Q8TCD3	XP_208162; NP_002813	Twinfilin-1 (Protein A6) (Protein tyrosine kinase 9)	GO:0003779; GO:0015629; GO:0005737; GO:0006468; GO:0004713	actin binding; actin cytoskeleton; cytoplasm; protein amino acid phosphorylation; protein tyrosine kinase activity	CytoplasmCytoplasmcytoskeleton			Known in Uniprot to be phosphorylated		
**1458**	Q6P6D7	Q6P6D7_HUMAN	Q6P6D7	Q6P6D7	XP_293925	Phosphoglycerate mutase	GO:0006096; GO:0016868	glycolysis; intramolecular transferase activity, phosphotransferases						
**1459**	Q6PUJ7	Q6PUJ7_HUMAN	Q6PUJ7	Q6PUJ7	XP_070805	Prohibitin	GO:0016020	membrane						
**1460**	ZN788	ZN788_HUMAN	Q6ZQV5; ZN788	Q6ZQV5; Q6ZRE4	XP_292814	Zinc finger protein 788	GO:0003677; GO:0005634; GO:0006355; GO:0008270	DNA binding; nucleus; regulation of transcription, DNA-dependent; zinc ion binding	Nucleus					
**1461**	K1875	K1875_HUMAN	Q6ZVG2; Q96JF2; K1875	Q6ZVG2; A6NE52; Q96JF2	XP_291269; NP_115918	WD repeat-containing protein KIAA1875; cDNA FLJ42620 fis, clone BRACE3015121								
**1462**	MON2	MON2_HUMAN	Q8N3I5; Q8NHE2; MON2	Q7Z3U7; Q8N3I5; A5D8U7; A7E2Y0; Q86TA2; Q8NAI0; Q8NHE2; Q9UPW1	XP_051091; NP_055841	Protein MON2 homolog (Protein SF21)	GO:0006895; GO:0005488; GO:0015031	Golgi to endosome transport; binding; protein transport				Known in Uniprot to be phosphorylated		
**1463**	PK1L3	PK1L3_HUMAN	Q7Z443; PK1L3	Q7Z443	XP_292513	Polycystic kidney disease protein 1-like 3 (Polycystin-1L3) (PC1-like 3 protein)	GO:0016021; GO:0005216; GO:0006811; GO:0007218; GO:0005529	integral to membrane; ion channel activity; ion transport; neuropeptide signaling pathway; sugar binding	MembraneMultipassmembraneprotein					

	Protein Name	Uniprot Name	Other Names	Uniprot IDs	Refseq IDs	Protein Description	GO ID	GO terms	Subcellular location	Comments	Comments	Comments - Database Search	Comments - Database Search	Function
**1464**	CB010	CB010_HUMAN	CB010; Z804A	Q7Z570; A7E253; Q6ZN26	XP_293011; NP_919226	Zinc finger protein 804A	GO:0005622; GO:0008270	intracellular; zinc ion binding						
**1465**	FLIP1	FLIP1_HUMAN	FLIP1	Q7Z7B0; Q5VUL6; Q86TC3; Q8N8B9; Q96SK6; Q9NVI8; Q9ULE5	XP_029179; NP_056502	Filamin-A-interacting protein 1 (FILIP)						Known in Uniprot to be phosphorylated		
**1466**	VP13B	VP13B_HUMAN	VP13B	Q7Z7G8; Q709C6; Q709C7; Q7Z7G4; Q7Z7G5; Q7Z7G6; Q7Z7G7; Q8NB77; Q9NWV1; Q9Y4E7	XP_047659; NP_056058; NP_060360; NP_689777; NP_858047	Vacuolar protein sorting-associated protein 13B (Cohen syndrome protein 1)	GO:0015031	protein transport				Known in Uniprot to be phosphorylated		
**1467**	Q7Z7J6	Q7Z7J6_HUMAN	Q7Z7J6	Q7Z7J6	XP_301583	Actin alpha 1 skeletal muscle protein	GO:0005524; GO:0005856; GO:0005515	ATP binding; cytoskeleton; protein binding						
**1468**	ACOT1	ACOT1_HUMAN	ACOT1	Q86TX2; A1L173; Q3I5F9	XP_301494; NP_001032238	Acyl-coenzyme A thioesterase 1 (Acyl-CoA thioesterase 1) (EC 3.1.2.2) (Inducible cytosolic acyl-coenzyme A thioester hydrolase) (Long chain acyl-CoA thioester hydrolase) (Long chain acyl-CoA hydrolase) (CTE-I) (CTE-Ib)	GO:0006637; GO:0004091; GO:0005829; GO:0001676; GO:0016290; GO:0000038	acyl-CoA metabolic process; carboxylesterase activity; cytosol; long-chain fatty acid metabolic process; palmitoyl-CoA hydrolase activity; very-long-chain fatty acid metabolic process	Cytoplasm					
**1469**	Q8IZS0	Q8IZS0_HUMAN	Q8IZS0	Q8IZS0	XP_210664	Orofacial cleft 1 candidate gene 1 protein. (Fragment)								
**1470**	NGDN	NGDN_HUMAN	NGDN	Q8NEJ9; Q9Y400	XP_033371; NP_056329; NP_001036100	Neuroguidin (EIF4E-binding protein)	GO:0042995; GO:0005737; GO:0005634; GO:0006417	cell projection; cytoplasm; nucleus; regulation of translation	NucleusCytoplasmCellprojectionaxonCellprojectiondendriteCellprojectionfilopodium			Known in Uniprot to be phosphorylated	Known in HPRD to be phosphorylated	
**1471**	F10A5	F10A5_HUMAN	F10A5	Q8NFI4	NP_695023	Protein FAM10A5	GO:0005488; GO:0005737	binding; cytoplasm	Cytoplasm					
**1472**	Q8TBK7	Q8TBK7_HUMAN	Q8TBK7	Q8TBK7	XP_302053	TPT1 protein (Tumor protein, translationally-controlled 1) (Tumor protein, translationally-controlled 1, isoform CRA_a)	GO:0005737	cytoplasm						
**1473**	Q8WU19	Q8WU19_HUMAN	Q8WU19	Q8WU19	XP_084610	TUBA1B protein	GO:0005525; GO:0003924; GO:0005874; GO:0007018; GO:0043234; GO:0051258; GO:0005198	GTP binding; GTPase activity; microtubule; microtubule-based movement; protein complex; protein polymerization; structural molecule activity						
**1474**	SKT	SKT_HUMAN	Q8WV45; Q9NSR2; SKT	Q8WV45; Q5T5P2; A5LHW9; A6PVQ5; A6PVQ6; A6PVQ7; Q4KMG4; Q5T5P3; Q5T7H3; Q6MZZ6; Q6ZUI4; Q9NSR2; Q9ULK3	XP_166112; NP_062536; NP_001091970; NP_001091971	Sickle tail protein homolog	GO:0005737; GO:0048706	cytoplasm; embryonic skeletal system development	Cytoplasm			Known in Uniprot to be phosphorylated		
**1475**	GCN1L	GCN1L_HUMAN	GCN1L	Q92616; A8KAY1; O95001; O95651; Q6P2S3; Q86X65; Q8N5I5; Q8WU80; Q99736; Q9UE60	XP_045792; NP_006827	Translational activator GCN1 (GCN1-like protein 1) (HsGCN1)	GO:0005515; GO:0006417; GO:0005840; GO:0008135	protein binding; regulation of translation; ribosome; translation factor activity, nucleic acid binding				Known in Uniprot to be phosphorylated		
**1476**	ZN582	ZN582_HUMAN	ZN582	Q96NG8; Q6PJT6	XP_033004; NP_653291	Zinc finger protein 582	GO:0003677; GO:0005634; GO:0006355; GO:0008270	DNA binding; nucleus; regulation of transcription, DNA-dependent; zinc ion binding	Nucleus					
**1477**	Q96RH9	Q96RH9_HUMAN	Q96RH9	Q96RH9	XP_097006; XP_001715069; XP_001725690; XP_001726928	HCG1980276 (MUSP1)								
**1478**	LDH6B	LDH6B_HUMAN	LDH6B	Q9BYZ2; Q6DUY4; Q96LI2	XP_062669; NP_149972	L-lactate dehydrogenase A-like 6B (EC 1.1.1.27)	GO:0004459; GO:0019642; GO:0005737; GO:0055114; GO:0005515	L-lactate dehydrogenase activity; anaerobic glycolysis; cytoplasm; oxidation reduction; protein binding						
**1479**	MTMRC	MTMRC_HUMAN	Q9C0I1; MTMRC	Q9C0I1; Q69YJ4; Q6PFW3; Q96QU2; Q9NX27	NP_061934; NP_001035536	Myotubularin-related protein 12 (Phosphatidylinositol-3 phosphate 3-phosphatase adapter subunit) (3-phosphatase adapter protein) (3-PAP)	GO:0005737; GO:0016311; GO:0016791	cytoplasm; dephosphorylation; phosphatase activity	Cytoplasm			Known in Uniprot to be phosphorylated		Phosphatase
**1480**	K1549	K1549_HUMAN	Q9H0M3; K1549	Q9H0M3; Q9HCM3; Q5BJD6; Q8IY15	XP_297463; NP_065961	UPF0606 protein KIAA1549	GO:0016021	integral to membrane	MembraneMultipassmembraneprotein			Known in Uniprot to be phosphorylated		
**1481**	CK067	CK067_HUMAN	CK067	Q9H7C9; Q96AQ4; Q9Y6B1	XP_166241; NP_078960	UPF0366 protein C11orf67								
**1482**	TBA4B	TBA4B_HUMAN	Q9H853; TBA4B	Q9H853	NP_079295	Putative tubulin-like protein alpha-4B (Alpha-tubulin 4B)	GO:0005525; GO:0003924; GO:0005874; GO:0007018; GO:0043234; GO:0051258; GO:0005198	GTP binding; GTPase activity; microtubule; microtubule-based movement; protein complex; protein polymerization; structural molecule activity						
**1483**	C2D2A	C2D2A_HUMAN	Q9H8A7; C2D2A	Q9H8A7; Q9P2K1; A6ND97; B3FW08; Q3SYP3	XP_291660; NP_001073991; NP_065836	Coiled-coil and C2 domain-containing protein 2A	GO:0005737; GO:0050896; GO:0007601	cytoplasm; response to stimulus; visual perception	CytoplasmCellprojectionciliumbasalbody					
**1484**	Q9H9G2	Q9H9G2_HUMAN	Q9H9G2	Q9H9G2	XP_296484; XP_001720486	cDNA FLJ12781 fis, clone NT2RP2001861								
**1485**	CO002	CO002_HUMAN	CO002	Q9NZP6	XP_294519; NP_061831	Protein C15orf2	GO:0030154; GO:0007275; GO:0007283	cell differentiation; multicellular organismal development; spermatogenesis						
**1486**	CF115	CF115_HUMAN	CF115	Q9P1F3; Q5SZC8; Q9P0A1	XP_302246; NP_067066	UPF0727 protein C6orf115								
**1487**	DYH2	DYH2_HUMAN	Q9P225; DYH2	Q9P225; A8K992; O15434; Q6PIH3; Q6ZR42	XP_305800; NP_065928	Dynein heavy chain 2, axonemal (Axonemal beta dynein heavy chain 2) (Ciliary dynein heavy chain 2) (Dynein heavy chain domain-containing protein 3)	GO:0005524; GO:0016887; GO:0005858; GO:0001539; GO:0005929; GO:0005874; GO:0003777; GO:0007018	ATP binding; ATPase activity; axonemal dynein complex; ciliary or flagellar motility; cilium; microtubule; microtubule motor activity; microtubule-based movement	Cellprojectionciliumaxoneme			Known in Uniprot to be phosphorylated		
**1488**	K1370	K1370_HUMAN	Q9P2H7; K1370	Q9P2H7; Q32MH5; A8KA52; Q32MH6; Q4G0R7; Q5XJ16; Q6PDA3; Q9NV24	XP_035527; NP_062546	Uncharacterized protein KIAA1370								
**1489**	EPHA6	EPHA6_HUMAN	EPHA6; B3KS12	Q9UF33; B3KS12	NP_001073917; XP_114973; NP_775926	Ephrin type-A receptor 6 (EC 2.7.10.1) (Tyrosine-protein kinase receptor EHK-2) (EPH homology kinase 2)	GO:0005524; GO:0005003; GO:0016021; GO:0006468; GO:0007169	ATP binding; ephrin receptor activity; integral to membrane; protein amino acid phosphorylation; transmembrane receptor protein tyrosine kinase signaling pathway	MembraneSinglepasstypeImembraneprotein	Phophorylated - from this Study		Known in Uniprot to be phosphorylated		Kinase
**1490**	K1210	K1210_HUMAN	K1210	Q9ULL0; Q5JPN4	XP_297644	Uncharacterized protein KIAA1210	GO:0005634	nucleus						
**1491**	MRVI1	MRVI1_HUMAN	Q9UNY1; MRVI1	Q9UNY1; Q9Y6F6	NP_006060; NP_569056; NP_001092049	Protein MRVI1 (Inositol 1,4,5-triphosphate receptor-associated cGMP kinase substrate) (JAW1-related protein MRVI1)	GO:0005789; GO:0016021; GO:0005634; GO:0016529	endoplasmic reticulum membrane; integral to membrane; nucleus; sarcoplasmic reticulum	CytoplasmperinuclearregionSarcoplasmicreticulumMembraneSinglepassmembraneprotein	Phophorylated - from this Study		Known in Uniprot to be phosphorylated	Known in HPRD to be phosphorylated	
**1492**	CCD56	CCD56_HUMAN	CCD56	Q9Y2R0	NP_054738; NP_001035521	Coiled-coil domain-containing protein 56	GO:0016021	integral to membrane	MembraneSinglepassmembraneprotein					
**1493**	ZN134	ZN134_HUMAN	Q9Y4B2; ZN134	Q9Y4B2; P52741	XP_301130; NP_003426	Zinc finger protein 134	GO:0005634; GO:0006355; GO:0003700; GO:0008270	nucleus; regulation of transcription, DNA-dependent; transcription factor activity; zinc ion binding	Nucleus					
**1494**	PPAL4	PPAL4_HUMAN	A1L431; PPAL4	A2BFH1; A1L431	XP_089309; NP_001116540	Peptidylprolyl cis-trans isomerase A-like 4 (PPIase A-like 4) (EC 5.2.1.8) (Chromosome 1-amplified sequence 2) (COAS-2)	GO:0005737; GO:0003755; GO:0006457	cytoplasm; peptidyl-prolyl cis-trans isomerase activity; protein folding	Cytoplasm					
**1495**	A1L454	A1L454_HUMAN	A1L454	A1L454	XP_290702	BolA homolog 2 (E. coli)								
**1496**	LSDP5	LSDP5_HUMAN	A2RRC1; LSDP5	Q00G26; A2RRC1; Q6ZS68	XP_292801; NP_001013728	Lipid storage droplet protein 5	GO:0012511	monolayer-surrounded lipid storage body	Lipiddroplet					
**1497**	A4FU88	A4FU88_HUMAN	A4FU88		XP_297123									
**1498**	ARHL1	ARHL1_HUMAN	ARHL1	Q8NDY3; Q5JUG2; Q96GD1	XP_097771; NP_612439; NP_954631	[Protein ADP-ribosylarginine] hydrolase-like protein 1 (ADP-ribosylhydrolase 2)	GO:0003875; GO:0000287; GO:0051725	ADP-ribosylarginine hydrolase activity; magnesium ion binding; protein amino acid de-ADP-ribosylation						

	Protein Name	Uniprot Name	Other Names	Uniprot IDs	Refseq IDs	Protein Description	GO ID	GO terms	Subcellular location	Comments	Comments	Comments - Database Search	Comments - Database Search	Function
**1499**	HMGB1	HMGB1_HUMAN	HMGB1	P09429; A5D8W9; Q14321; Q5T7C3; Q6IBE1	XP_299053; NP_002119	High mobility group protein B1 (High mobility group protein 1) (HMG-1)	GO:0008301; GO:0006310; GO:0006268; GO:0006916; GO:0006288; GO:0000793; GO:0006325; GO:0017055; GO:0005730; GO:0007165; GO:0008134	DNA bending activity; DNA recombination; DNA unwinding during replication; anti-apoptosis; base-excision repair, DNA ligation; condensed chromosome; establishment or maintenance of chromatin architecture; negative regulation of transcriptional preinitiation complex assembly; nucleolus; signal transduction; transcription factor binding	Nucleus			Known in Uniprot to be phosphorylated		
**1500**	NACAK	NACAK_HUMAN	NACAK		XP_304146									
**1501**	NACP1	NACP1_HUMAN	NACP1	Q9BZK3	NP_114420	Putative nascent polypeptide-associated complex subunit alpha-like protein (NAC-alpha pseudogene 1) (Alpha-NAC pseudogene 1)	GO:0005515	protein binding						
**1502**	Q05AH0	Q05AH0_HUMAN	Q05AH0	Q05AH0	XP_298144	FSHR protein	GO:0007186; GO:0004963; GO:0016021; GO:0005515	G-protein coupled receptor protein signaling pathway; follicle-stimulating hormone receptor activity; integral to membrane; protein binding						
**1503**	APC2	APC2_HUMAN	Q05BW4; APC2	O95996; Q05BW4; Q9UBZ1; Q9UEM8; Q9UQJ8; Q9UQJ9; Q9Y632	XP_297980; NP_005874	Adenomatous polyposis coli protein 2 (Adenomatous polyposis coli protein-like) (APC-like)	GO:0000139; GO:0016055; GO:0008013; GO:0016342; GO:0005874; GO:0008017; GO:0006461	Golgi membrane; Wnt receptor signaling pathway; beta-catenin binding; catenin complex; microtubule; microtubule binding; protein complex assembly	CytoplasmcytoskeletonGolgiapparatusmembranePeripheralmembraneproteinCytoplasmicsideCellmembranePeripheralmembraneproteinCytoplasmicside					
**1504**	T183A	T183A_HUMAN	T183A	Q8IXX5; Q6NW15; Q96E06	XP_293932; NP_612400	Transmembrane protein 183A	GO:0016021	integral to membrane	MembraneSinglepassmembraneprotein					
**1505**	TRML1	TRML1_HUMAN	TRML1	Q86YW5; Q496B3; Q8IWY1; Q8IWY2	XP_291183; NP_835468	Trem-like transcript 1 protein (TLT-1) (Triggering receptor expressed on myeloid cells-like protein 1)	GO:0045087; GO:0016021; GO:0005886; GO:0004872	innate immune response; integral to membrane; plasma membrane; receptor activity	CellmembraneSinglepasstypeImembraneproteinCytoplasm			Known in Uniprot to be phosphorylated	Known in HPRD to be phosphorylated	
**1506**	ANGT	ANGT_HUMAN	ANGT; B0ZBE2; B2R5S1	P01019; Q16358; Q16359; Q96F91; B0ZBE2; B2R5S1	NP_000020; 670366A									
**1507**	LOC730184	LOC730184	LOC730184		XP_001132826					Phophorylated - from this Study				
